# Elusive elapids: biogeographic venom variation in Indian kraits and its repercussion on snakebite therapy

**DOI:** 10.3389/fphar.2024.1443073

**Published:** 2024-11-07

**Authors:** U. Rashmi, Siddharth Bhatia, Muralidhar Nayak, Suyog Khochare, Kartik Sunagar

**Affiliations:** Evolutionary Venomics Lab, Centre for Ecological Sciences, Indian Institute of Science, Bangalore, Karnataka, India

**Keywords:** *Bungarus caeruleus*, snake venom, Elapidae, Indian antivenoms, snakebite

## Abstract

Snakebite is a major public health concern in many parts of the world, including India, where over 58,000 deaths occur annually due to snake envenoming. The common krait (*Bungarus caeruleus*) is responsible for the second-highest number of snakebite-related mortalities in the country. However, despite its notoriety, little is known about its venom ecology, functions and compositional variation across bioclimatic zones, partly because these nocturnal snakes are highly elusive, making it difficult to find them in the wild. We aim to address this knowledge gap by characterising the venom composition and toxicity profiles of the pan-Indian populations (n = 8) of *B. caeruleus* using a combination of proteomics, receptor-toxin interaction assays, biochemical experiments, pharmacological tests and preclinical evaluations. We reveal considerable variation in venom composition, functions, and pharmacological activities among the geographically distinct populations of *B. caeruleus*. Furthermore, toxin-receptor interaction assays provide insights into their feeding ecology and prey-predator interactions. Finally, *in vitro* and *in vivo* experiments revealed the poor neutralising potencies of Indian antivenoms towards most populations of the common krait. Our findings highlight the alarming need to develop efficacious snakebite therapy in India to treat bites from this medically most important elapid snake.

## 1 Introduction

Asian kraits are amongst the most enigmatic snakes in the world, well-known for their highly toxic venoms and clinically important envenomations. The common krait (*Bungarus caeruleus*) in India, for example, is considered one of the “big four” snake species that accounts for the second highest number of snakebite-related deaths in the country (Suraweera et al., 2020). Bites from this species frequently occur at night, primarily to individuals who sleep on the ground. Victims remain unmindful of the bite, as krait envenoming is often painless with minimal local symptoms, resulting in delayed diagnosis and treatment (Kularatne, 2002). The venom of *B. caeruleus* is known to be primarily constituted by presynaptic neurotoxins that cause severe neuromuscular paralysis in prey animals and accidental bite victims (Oh et al., 2017; Chen and Lee, 1970; Ranawaka et al., 2013; Silva et al., 2016; Sunagar et al., 2013; Sunagar et al., 2021). Unlike the other “big four” snakes, the common krait is mostly docile during the day and highly active at night, making it very difficult for researchers to capture them from the wild. This is further complicated by constraints associated with sampling venoms in India, including the prohibition on sampling at night, permission to collect venoms from a limited number of snakes and the delays associated with obtaining sampling permits and associated clearances. In addition to being elusive, kraits produce minimal amounts of venom. Consequently, despite being widely distributed across India and causing the second-largest number of human deaths, there have been fewer studies on the common krait (Oh et al., 2017; Sunagar et al., 2021; Senji Laxme et al., 2019; Singh et al., 2001; Choudhury et al., 2017; Patra et al., 2019). Moreover, given their nocturnal nature, we have a limited understanding of their biology, including ecology, specificity and potency of venom.

Several recent studies have highlighted the remarkable venom variation in Indian snakes across their geographically disparate populations (Senji Laxme et al., 2021a; Senji Laxme et al., 2021b; Bhatia and Vasudevan, 2020; Shashidharamurthy et al., 2002; Shashidharamurthy and Kemparaju, 2007; Sharma et al., 2014; Prasad et al., 1999; Jayanthi and Gowda, 1988). The consequent result of this intraspecific venom variation is significantly reduced effectiveness of commercial antivenoms across the Indian subcontinent (Senji Laxme et al., 2021a; Senji Laxme et al., 2021b; Casewell et al., 2010). While a few of these investigations are conducted on *B. caeruleus*, most of them have sourced venoms from the Irula Snake Catchers’ Industrial Cooperative Society in Tamil Nadu (Oh et al., 2017; Choudhury et al., 2017; Patra et al., 2019) or from the captive snakes at Kolkata snake park in West Bengal (Bhattacharya et al., 2013). Only a couple of studies in the past have assessed *B. caeruleus* venoms from the wild (Sunagar et al., 2021; Senji Laxme et al., 2019). Limited sampling efforts and the assessment of venom from geographically-restricted populations have resulted in a lack of understanding of the venom variation in Indian kraits and the impact of this variation on the effectiveness of antivenoms.

To this end, we have characterised *B. caeruleus* venoms from eight geographically distinct populations across India in the northern (Punjab: PB), southern (Karnataka: KA; Tamil Nadu: TN), eastern (West Bengal: WB), southeastern (Andhra Pradesh: AP), western (Maharashtra: MH), southwestern (Goa: GA), and central (Madhya Pradesh: MP) India. These locations belong to various biogeographic regions, including the coasts (TN and AP), the Western Ghats (GA, MH and KA), the Deccan Plateau (MP), the Gangetic plains (WB) and semi-arid regions (PB). Analyses of venom proteome, biochemistry, and pharmacological activity provided insights into venom composition and function. Evaluating the binding affinity of *Bungarus* venom toxins towards the nicotinic acetylcholine receptors (ɑ-1 nAChR) from various prey and predatory species provided fascinating insights into the venom ecology of this elusive elapid. Furthermore, outcomes of *in vivo* experiments reveal the consequences of interpopulation venom variation on the effectiveness of the Indian polyvalent antivenoms in treating bites from *B. caeruleus*.

## 2 Materials and methods

### 2.1 Snake venoms and commercially available antivenoms

Krait venoms were collected from different locations across India with the permission of the respective state forest departments. North: Punjab (#3615; 11/10/12), South: Karnataka (PCCF(WL)/C1(C3)/CR-09/2017-18), Southeast: Andhra Pradesh (#13526/2017/WL-3), West: Maharashtra (Desk-22 (8)/Research/CR-80 (16–17)/943/2017-18), Southwest: Goa (No.2-66-WL-RESEARCH PERMISSIONS-FD-2022-23-Vol.IV/858), East: West Bengal (No.WL/4R-6/2017) and Central: Madhya Pradesh (#/TK-1/48-II/606). Snake venom samples (n = 34) were collected individually or by pooling, flash-frozen, lyophilised, and stored at −80°C. Additionally, lyophilised krait venom was procured from the Irula Snake Catchers’ Industrial Cooperative Society (ISCICS) in Tamil Nadu. Details of venoms and antivenoms investigated in this study have been provided in Supplementary Tables S1, S2, respectively.

### 2.2 Ethical statement

Toxicities of krait venoms and the neutralising potencies of commercial Indian antivenom were analysed in the murine model of venoming, following the standard protocol recommended by the World Health Organization (WHO). Ethical approvals were obtained from (i) the Committee for Control and Supervision of Experiments on Animals (CCSEA), Government of India; and (ii) the Institutional Animal Ethics Committee (IAEC), Indian Institute of Science (IISc), Bangalore (CAF/Ethics/642/2018; 16/10/2020). For investigating the coagulopathic effects of krait venoms on human blood, ethical clearance was obtained from the Institute Human Ethical Committee (IHEC No: 18/20201216; approval date: 16th December 2020), and subsequently, blood was collected from healthy volunteers with informed consent. Male CD-1 mice procured from Hylasco Biotechnology India Pvt. Ltd (Hyderabad, Telangana, India) were maintained at IISc’s Central Animal Facility. After a 7-day quarantine period, 3 to 4-week-old mice weighing 18–20 g were randomly assigned to cages (n = 5). The cages were kept at 18°–24°C with 60%–65% relative humidity and a 12:12 day/night cycle.

### 2.3 Protein concentration and sodium dodecyl sulphate polyacrylamide gel electrophoresis (SDS-PAGE)

The total protein concentrations of krait venoms and Indian antivenoms were assessed with Bovine Serum Albumin (BSA, Sigma-Aldrich, United States) and Bovine Gamma Globulin (BGG, Bio-Rad) as respective controls using the Bradford reagent (de Yebra and Oliva, 1993; Bradford, 1976) (Supplementary Tables S1, S2). Krait venoms (12 μg) were loaded into a 12.5% gel, followed by an electrophoretic separation in the Tris-Glycine-SDS buffer at 80 V (Laemmli and Favre, 1970). The Precision Plus Protein Dual Colour Xtra Standard (Bio-Rad Laboratories, United States) was used as a marker, and the gel was stained with Coomassie Brilliant Blue R-250 (Sisco Research Laboratories Pvt. Ltd., India). The gel was visualised with the iBright CL1000 gel documentation system (Thermo Fisher Scientific, United States).

### 2.4 Reversed-phase high-performance liquid chromatography (RP-HPLC)

A total of 1 mg of krait venoms were separated into different fractions via RP-HPLC using a C18 column (Shim-pack GIST C18, Shimadzu P.No. 227–30017-08) having dimensions (4.6 × 250 mm) with 5 μm particle size in the Shimadzu LC-20AD series HPLC system (Kyoto, Japan) according to the previously described methods (Lomonte and Calvete, 2017). Venom fractions were separated at a flow rate of 1 mL/min using a gradient of buffer A (0.1% Trifluoroacetic acid in water suitable for HPLC), buffer B (0.1% TFA in acetonitrile), and an isocratic phase with 5% buffer B for 5 min. This was followed by a gradient from 5% to 15% buffer B for 10 min, 15%–45% buffer B for 60 min, and subsequently from 45% to 70% buffer B over 10 min. Fractions were collected individually by monitoring absorbance peaks at 215 nm, and their relative abundances were estimated based on the areas under these peaks.

### 2.5 Liquid chromatography-tandem mass spectrometry (LC-MS/MS)

The fractions collected using RP-HPLC were either subjected to in-gel or in-solution digestion. In-gel digestion was carried out for fractions with protein amounts exceeding 10 μg, while in-solution digestion was performed for all other samples following methods employed previously (Jaglan et al., 2023). In the case of in-gel digestion, samples were run on the SDS-PAGE, and bands were excised, destained, and dehydrated using 70% acetonitrile. Samples were reduced using the 10 mM DTT (dithiothreitol, D-5545-Sigma-Aldrich, United States) at 95°C for 5 min (for in-solution) and at 55°C for 45 min (for in-gel) and treated with 100 mM iodoacetamide for 30 min at RT in the dark followed by overnight digestion with the trypsin (10 μg/mL; 1:60) at the 37°C. Formic acid (0.1%) was used to stop the reaction, followed by the desalting of samples in the ZipTip (C18, with Tip Size P10, Cat. No: ZTC18S960, MILLIPORE) with 0.1% formic acid and acetonitrile. The digested peptides were subjected to nano-LC and electrospray ionisation tandem mass spectrometry (ESI-MS/MS). These samples were introduced into a PepMap C18 nano-LC column (50 cm × 75 μm, with 2 μm particle size and 100 Å pore size) installed on the Thermo EASY nLC Ultimate 3,000 series system (Thermo Fisher Scientific, MA, United States). Gradient elution of buffer A (0.1% formic acid in MS grade water) and buffer B (0.1% formic acid in 80% acetonitrile) was carried out at a steady flow rate of 250 nL/min for 90 min. The elution utilised an 8%–35% gradient of buffer B for the first 70 min, followed by 35%–95% over the next 5 min, and finally maintained at 95% for the last 15 min. The fractions from the nano-LC were then directed into a Thermo Orbitrap Fusion Mass Spectrometer (Thermo Fisher Scientific, MA, United States). For MS scans, a scan range (m/z) of 300–2,000 was utilised, with a resolution set at 120,000 and a maximum injection time of 100 ms. Precursor (MS) and fragment (MS/MS) scans were performed using an orbitrap detector employing high collision dissociation (HCD) fragmentation (30%), with scan parameters set at a range of m/z 110–2,000 and a maximum injection time of 50 ms. The raw MS/MS analysis data is uploaded to the ProteomeXchange Consortium via the PRIDE partner repository with data identifier PXD052611. The list of toxins identified through tandem MS and their relative proportions are tabulated in Supplementary Table S5A–E.

The MS/MS sequenced peptides were identified using PEAKS Studio 10 (Bioinformatics Solutions Inc., Ontario, Canada) (Perez-Riverol et al., 2019). Each obtained sequence was searched against the NCBI-NR Serpentes databases (taxid: 8,570; September 2023) using the following parameters: 0.6 Da of fragment mass tolerance, 10 ppm of peptide mass tolerance, and 0.1 of False Discovery Rate (FDR). Peptides with at least one unique match with the peptide were considered for downstream analysis. Proteins found redundant were manually removed from the data. The relative abundance of toxin families was calculated using information from HPLC peak fraction, densitometry of SDS-PAGE bands, and precursor intensity from MS/MS analysis as described in the following equation given below, where N indicates the number of fractions (Calvete et al., 2023).

$$Relative\;abundance\;of\;X\;\left(\%\right)=\sum_{n=1}^{N}\frac{Mean\;spectral\;intesity\;of\;toxin\;X\;in\;fraction\;F_{n\;}}{Total\;mean\;spectral\;intensity\;all\;toxin\;hits\;in\;Fraction\;F_{n}}\times AUC\;of\;Fraction\;F_{n}\times Densitometry$$

### 2.6 Biolayer interferometry (BLI)

The nAChR binding profiles of krait venoms were analysed as described previously (Zdenek et al., 2019). Binding kinetics of krait venoms with mimotope, synthesised with the orthosteric site of the *ɑ-1* nAChR, were analysed using the Octet RED 96 system (ForteBio, Fremont, CA, United States) and Greiner black 96 microtiter well plates at 30°C. Krait venoms (1 mg/mL) were diluted in running buffer (1x Dulbecco’s Phosphate Buffered Saline, 1% BSA and 0.05% Tween 20) to a final 25 μg/mL concentration. Mimotopes were diluted to 1 μg/mL concentration in the running buffer and the final concentration was kept at 0.2 μg per well. Krait venoms (1 mg/mL) were diluted in a running buffer (1X DPBS, 1% BSA, and 0.05% Tween 20) to a final 25 μg/mL concentration. In the microtiter plate setup, mimotopes were placed in the 2nd column, venom was added in the 4th column, and a running buffer was added to the 1st and 3rd columns. The running buffer was used as the negative control. Before starting the assay, the streptavidin biosensor tip was hydrated with the running buffer for 30–60 min while agitated at 2 RPM on a shaker. The orthosteric binding kinetic assay was done with the following program: 60 s for the first baseline, 50 s for loading, 120 s for the second baseline, 400 s for association, and 200 s for dissociation. Data were exported from Octet RED 96 system (ForteBio, Fremont, CA, United States), then imported to GraphPad Prism 8 (GraphPad Software, La Jolla California United States, www.graphpad.com), where the area under the curve (AUC) was calculated and graphs were plotted.

### 2.7 Biochemical characterisation

#### 2.7.1 PLA_2_ assay

The PLA_2_ activities of krait venoms were estimated using the 4-Nitro-3 (octanoyloxy) benzoic acid (NOB; Enzo LifeSciences, New York, NY, United States) substrate (Freitas-de-Sousa et al., 2020). A 5 μg venom sample was added to the 200 μL reaction mixture containing 500 μM NOB in buffer (10 mM Tris-HCl, 10 mM CaCl_2_, 100 mM NaCl, pH 7.8) and incubated for 40 min at 37°C. The absorbance was measured at 425 nm in an Epoch 2 microplate spectrophotometer (BioTek Instruments, Inc., United States) every 10 min. An identical procedure was used for plotting the standard curve using various concentrations of the NOB substrate (4–130 nmol) and 4M NaOH. The amount of the cleaved phospholipid substrate (nmol) was calculated as nmol/ng/min from the standard curve.

#### 2.7.2 Snake venom protease assay

The snake venom protease activity was assessed using previously established protocols (Chowdhury et al., 1990; Rashmi et al., 2021). Briefly, a known amount of the crude venom (10 μg) was incubated with the azocasein substrate at 37 C for 90 min. Post-incubation, trichloroacetic acid (200 μL) was added to the reaction mixture to precipitate any intact protein not cleaved by the venom. This reaction mixture was then centrifuged at 1,000 x g for 5 min. The supernatant was mixed with equal volumes of 0.5 M NaOH, and the absorbance was measured in an Epoch 2 microplate spectrophotometer (BioTek Instruments, Inc., Winooski, VT, United States) at 440 nm. Purified bovine pancreatic protease (Sigma-Aldrich, Burlington, MA, United States) was used as a positive control, and the relative proteolytic activities of krait venoms were calculated.

#### 2.7.3 L-amino acid oxidase (LAAO) assay

LAAO activity was estimated using the colourimetric method described previously (Senji Laxme et al., 2019; Kishimoto and Takahashi, 2001). A total of 0.5 μg venom and 200 μL of reaction mixture containing 5 mM L-leucine as a substrate, 2 mM o-phenylenediamine dihydrochloride (OPD), 5 IU/mL horseradish peroxidase (HRP), and 50 nM Tris-Cl buffer (pH 8) were preincubated separately for 10 min at 37°C. The reaction mixture was mixed with venom and incubated at 37°C for 10 min. Post-incubation, the reaction was terminated by adding 2M H_2_SO_4_, and the release of H_2_O_2_ was assayed by measuring absorbance at 492 nm in an Epoch 2 microplate spectrophotometer (BioTek Instruments, Inc., United States). The specific activity of each sample was calculated by converting the obtained optical density into nanomoles released per minute. This conversion was based on a standard curve generated using the H_2_O_2_ with the same experimental protocol.

#### 2.7.4 Fibrinogenolytic assay

The fibrinogenolytic activity was assessed by incubating the venom (1.5 μg) with the human fibrinogen (15 μg) (Sigma-Aldrich, Burlington, MA, United States) in 1X PBS at 37°C for 60 min as described previously (Senji Laxme et al., 2019; Rashmi et al., 2021; Teng et al., 1985). After incubation, an equal amount of 5X loading dye (50% Glycerol; 0.5% Bromophenol blue; 10% SDS; 1 M Tris-HCl, pH 6.8; 20% β-mercaptoethanol) was added to the reaction mixture, and heated at 70°C for 10 min. Samples were subjected to 15% SDS-PAGE, and densitometry was performed using ImageJ software to quantify the cleavage pattern of fibrinogen.

#### 2.7.5 DNase assay

DNase activity for various krait populations was evaluated by incubating 500 ng of purified calf thymus DNA (Sigma-Aldrich, Burlington, MA, United States) with a known amount of crude venom in PBS (Phosphate-buffered saline; 7.4 pH) at 37°C for 60 min. Samples were run on a 0.8% agarose gel and imaged using an iBright CL1000 (Thermo Fisher Scientific, MA, United States). Intact DNA from the calf thymus and DNase I from the bovine pancreas (15 U) were used as negative and positive controls, respectively. The relative DNAse activity was determined by dividing the DNA band intensity of samples by that of the negative control (Senji Laxme et al., 2019; Gerceker et al., 2009).

#### 2.7.6 Haemolytic assay

To evaluate the haemolytic potential of venoms, red blood cells (RBCs) were isolated from whole blood by centrifugation at 4°C for 10 min at 3,000 x g and then washed with PBS (7.4 pH) five times as described previously (Maisano et al., 2013). The RBC pellet was resuspended in PBS (1:10 ratio) and was incubated for 24 h at 37 C with various concentrations (5, 10, and 20 μg) of venom. After incubation, processed samples were centrifuged at 4°C for 10 min at 3,000 x g, and the absorbance of the supernatant was measured at 540 nm using an Epoch 2 microplate spectrophotometer. Triton X (0.5%) was used as a positive control for calculating the relative haemolytic activity for each sample, and PBS was used as a negative control.

### 2.8 Estimating *in vivo* and *in vitro* efficacy of commercially available antivenom

#### 2.8.1 Indirect enzyme-linked immunosorbent assay (ELISA)

Antivenom efficacy in recognising *B. caeruleus* venoms was estimated via indirect ELISA (Senji Laxme et al., 2019; Peres et al., 2006; Khochare et al., 2024). 100 ng of venom was diluted in the carbonated buffer (pH 9.6), coated on the 96 well plates and incubated overnight at 4°C. Unbound proteins were removed by washing the wells six times with Tris-buffered saline (0.01 M, pH-8.5, 0.15 M NaCl) and 1% Tween 20 (TBST). Blocking was done with a blocking buffer (5% skimmed milk in TBST) for 3 h at room temperature. The plate was incubated overnight at 4°C with various dilutions (1:4 to 1:312,500) of Indian antivenoms (Premium Serums, VINS, Bharat, Haffkine). Unbound primary antibodies were removed by washing the plates with a TBST solution and then incubated for 2 h at room temperature with HRP-conjugated rabbit anti-horse secondary antibody (1:1,000). The plate was washed with TBST to remove the unbound HRP-conjugated rabbit anti-horse secondary antibody (1:1,000). Then 100 µL of ABTS [2,2-azino-bis (3-ethylbenzthiazoline-6-sulphonic acid)] substrate solution (Sigma-Aldrich, United States) was added to the plate post-incubation, followed by six rounds TBST washing. Absorbance was measured at a wavelength of 405 nm for 40 min using an EPOCH2 microplate spectrophotometer (BioTeK, United States). In addition, purified antibodies from naive horses (Bio-Rad Laboratories, United States) were used as a negative control.

#### 2.8.2 Median lethal dose (LD_50_)

Venom potencies of *B. caeruleus* were estimated by calculating the LD_50_, which is defined as the minimum amount of venom required to kill 50% of the test population, using the murine model of envenoming. Five graded concentrations of krait venoms were prepared in physiological saline (0.9% NaCl) and intravenously injected into the caudal vein of CD-1 mice (n = 5). Death and survival patterns were recorded for each venom dose group (n = 5) 24 h post-venom injection. Finally, the LD_50_ values were calculated with 95% confidence intervals using Probit analysis (Finney, 1971; World Health Organization WHO and Others, 2010).

#### 2.8.3 Median effective dose (ED_50_)

The neutralisation potency of antivenom was estimated by calculating the ED_50_ value, which corresponds to the minimum amount of antivenom required to save 50% of the test population of mice (Finney, 1971; World Health Organization WHO and Others, 2010). Four antivenom dilutions were challenged against a fixed dose of venom that was five times the LD_50_. The venom and antivenom mixture was preincubated at 37°C for 30 min and injected into the caudal vein of five CD-1 mice per dilution. The ED_50_ of antivenom was calculated using Probit statistics 24 h post-venom injection. The antivenom’s neutralisation potency was expressed in milligrams of venom neutralised per millilitre of antivenom using the following equation:

$$Neutralisation\;potency\;\left(mg/ml\right)=\frac{\left(n-1\right)\;{\times LD}_{50}\;of\;venom\;\left(mg/mouse\right)}{{ED}_{50}\;\left(mL\right)}$$

### 2.9 Statistical analysis

Statistical comparisons for all biochemical assays were performed using one-way ANOVA, two-way ANOVA or Dunnett’s multiple comparison tests in GraphPad Prism 8 (GraphPad Software, La Jolla California United States, www.graphpad.com). The median doses (LD_50_ and ED_50_) with 95% CI were calculated using Probit analysis. The area under the association and dissociation curve, and ELISA results were compared using two-way ANOVA.

## 3 Results

### 3.1 Proteomic composition of krait venoms

**FIGURE 1:**
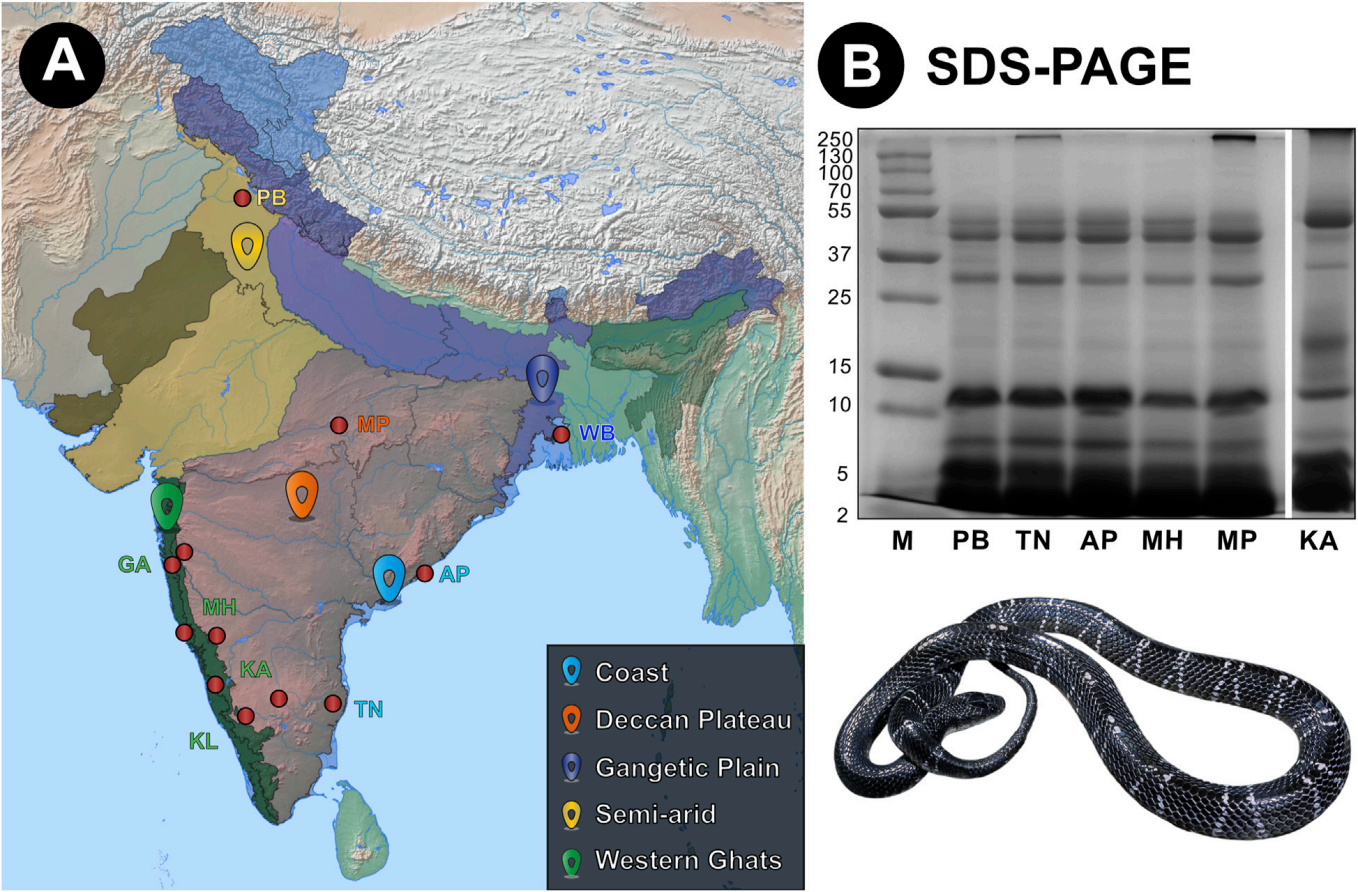
Sampling locations of krait venoms from biogeographically distinct environments across India and their venom profiles. Here, panels **(A)** and **(B)** show sampling locations and the reduced SDS-PAGE profiles of krait venoms, respectively. In panel **(A)**, red dots highlight sampling locations, while uniquely coloured landmarks indicate the respective biogeographic location. Abbreviations: M, Molecular weight marker; PB, Punjab; TN, Tamil Nadu; AP, Andhra Pradesh; MH, Maharashtra; MP, Madra Pradesh; and KA, Karnataka.

In this study, we investigated the venom proteomic compositions of *B. caeruleus* from eight geographically distinct populations across India (Figure 1A). The crude venom profile of each population was generated using reducing sodium dodecyl sulfate-polyacrylamide gel electrophoresis (SDS-PAGE) and reversed-phase high-performance liquid chromatography (RP-HPLC). Overall, the SDS-PAGE venom profiles of *B. caeruleus* populations were highly similar and revealed an abundance of low-molecular-weight toxins (Figure 1B). Only minor differences in band intensities between certain populations were observed. The venom of the southern (KA) krait population was distinct from other populations as it exhibited a band with a molecular weight between 15 and 25 kDa. This population was also typified by a dense band at 55 kDa while possessing a relatively lighter band at 15 kDa (Figure 1B).

**FIGURE 2:**
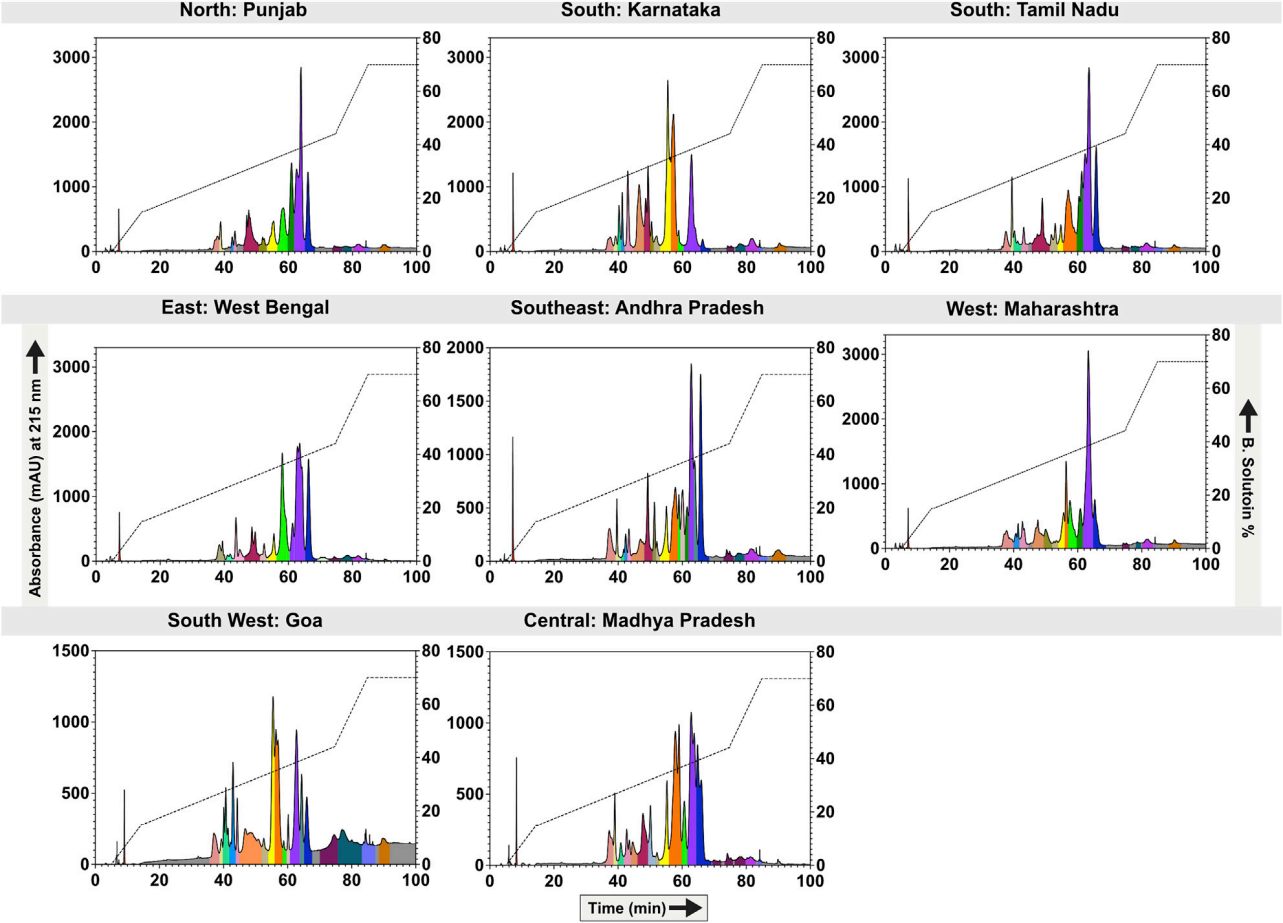
Biogeographical venom variability of krait populations. This figure depicts the RP-HPLC profiles of the eight geographically remote krait populations. The absorbance values (mAU) are indicated on the *y*-axis and plotted against the retention time (min) on the *x*-axis. Each fraction is uniquely colour-coded. For the location information, please refer to Figure 1 and Supplementary Table S1.

In contrast to SDS-PAGE profiles, significant differences among the populations were observed in RP-HPLC analyses. Comparative RP-HPLC profiles of the eight distinct krait populations identified the presence of as many as 23 prominent peaks with stark disparities in the peak intensities and area (Figure 2). The first RP-HPLC peak at the 8th minute exhibited relatively lower intensities in northern (PB) and western (MH) populations. The subsequent peaks between the 35th and 45th minutes exhibited high variability across all populations. Specifically, the peaks around 40th min were prominent in krait venoms from southern (KA and TN), southeast (AP) and southwestern (GA) populations compared to others. The peaks eluting between the 45th and 50th min were characterised by a large area in the southern (KA) and southeastern (AP) krait populations and were negligible in others. Interestingly, peaks between the 50th and 60th minutes displayed higher intensities in the southern (KA), southwestern (GA), eastern (WB) and central (MP) populations. The peaks eluting between 60th – 65th minutes were relatively lower in the southern (KA) and southeastern (AP) populations, whereas it was abundant in the other populations of *B. caeruleus* (Figure 2).

**FIGURE 3:**
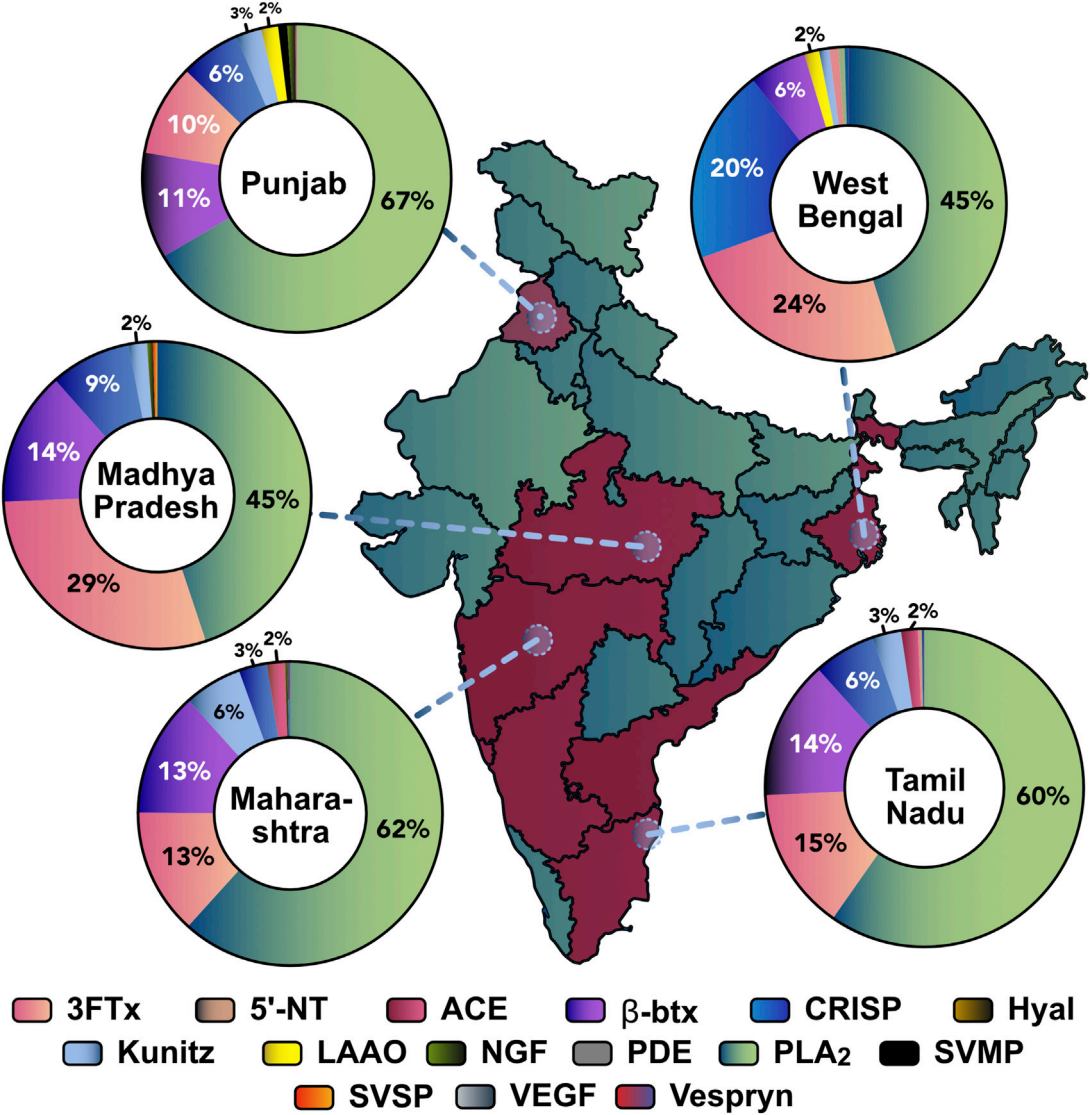
Comparative proteomics profiles of various krait populations across India. States from where the krait venoms were sourced are highlighted in red. The circles denote venom sampling points. The doughnut charts represent the relative abundance of toxin families in each population: 3FTx: three-finger toxins; 5′-NT: 5′-nucleotidase; ACE: acetylcholinesterase; β-btx: beta bungarotoxins; CRISP: cysteine-rich secretory proteins; LAAO: L-amino acid oxidase; NGF: nerve growth factor; PDE: phosphodiesterases; PLA_2_: phospholipase A_2_; SVMP: snake venom metalloproteinases; SVSP: snake venom serine proteinases; VEGF: vascular endothelial growth factors.

Each RP-HPLC fraction from southern (TN), eastern (WB), northern (PB), western (MH), and central (MP) Indian krait populations were subjected to SDS-PAGE, in-gel digestion, and analysed using tandem mass spectrometry (LC-MS/MS) to unravel the proteomic composition of *B. caeruleus* venoms. Mass spectrometry results suggest differences in the abundance of toxin families. The venoms of all populations under investigation were rich in low-molecular-weight phospholipase A_2_ (PLA_2_; 45%–62%) and three-finger toxins (3FTx; 13%–29%). Notably, the abundance of 3FTx subfamilies varied across different locations. In general, type I and II ⍺-neurotoxins and к-bungarotoxins (*κ*-btx) constituted the major portion of the 3FTx subfamilies in the venoms from northern (PB), southern (TN), and eastern (WB) krait populations. Cytotoxic 3FTxs (C-3FTx) were not detected in the eastern (WB) population and were negligible in the southern (TN) and northern (PB) populations while being dominant in others. The β-bungarotoxins (β-btx: 5%–15%) - a unique heteromeric toxin class with PLA_2_ and Kunitz peptide - and CRISPs (3%–20%) were present in moderate abundance across all populations. Numerous toxin families were also identified as minor components (<1%), such as Kunitz, acetylcholinesterases (ACE), L-amino acid oxidase (LAAO), snake venom metalloproteinases (SVMP), hyaluronidase, nerve growth factor (NGF), 5′-nucleotidases (5′-NT), snake venom serine proteinases (SVSP), phosphodiesterases (PDE), Vespryn and vascular endothelial growth factor (VEGF) (Figure 3).

### 3.2 Orthosteric binding profiles of krait venom towards ɑ-1 nAChRs

**FIGURE 4:**
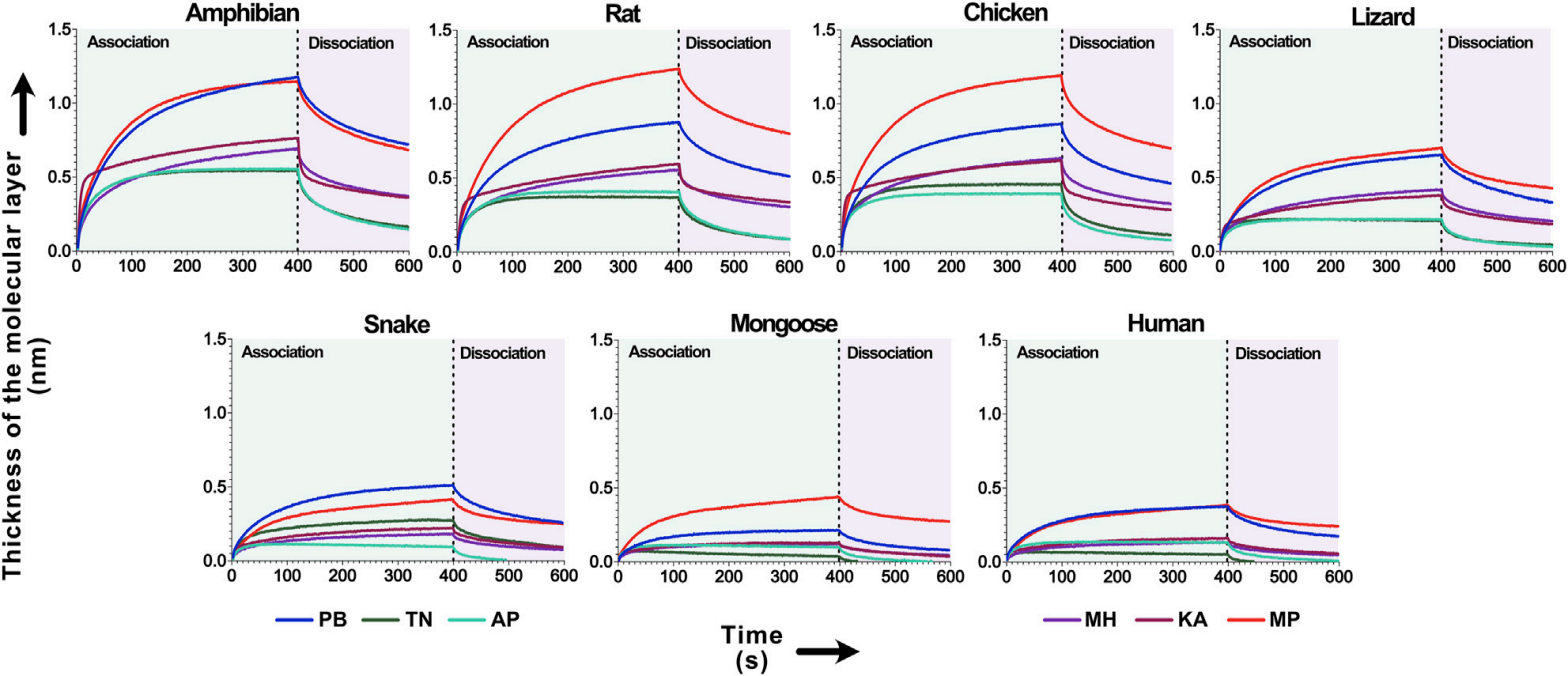
The binding affinity of krait venoms towards α-1 nAChRs of their prey and predatory animals. This figure depicts the binding affinity of 3FTxs from the pan-Indian populations of *B. caeruleus* towards the ɑ-1 nAChRs from various animals. Here, the binding patterns of krait populations are uniquely colour-coded. The time of association and dissociation (seconds) of venom toxins with the ɑ-1 nAChRs are indicated on the *x*-axis, and the thickness of the molecular layer in nm (shifts in the wavelength because of ligand-receptor binding) is shown on the *y*-axis. The dashed line in the plot at 400 s delineates the boundary between the association and dissociation phases.

Mass spectrometry shows that krait venoms are rich in 3FTxs that target ɑ-1 subtype of the nicotinic acetylcholine receptors in muscles (Fry, 2015). The binding patterns of these toxins from geographically distinct krait populations towards ɑ-1 nAChR mimotopes were investigated using biolayer interferometry (Zdenek et al., 2019; Harris et al., 2020). The AUC comparison of association and dissociation curve across all krait populations revealed a varied specificity for ɑ-1 nAChRs of various prey and predatory animals (Figure 4).

Venoms from the central (MP) and northern (PB) Indian krait populations exhibited higher binding towards nAChRs of various animals, including amphibians, rodents, birds and lizards (p < 0.0001; Figure 4). Overall, all the populations showed a similar order of binding pattern, with the highest binding being observed against amphibian, rodent, avian and lizard nAChRs (p < 0.0001). The least binding was observed against the snake, mongoose and human channels (p < 0.0001; Figure 4).

### 3.3 Enzymatic activities of krait venom toxins

Enzymatic activities of *B. caeruleus* venoms were assessed using various biochemical assays described below.

#### 3.3.1 Phospholipase A_2_ assay

The ability of the pan-Indian krait venoms to cleave 4-Nitro-3 (octanoyloxy) benzoic acid or the NOB substrate was assayed to evaluate their PLA_2_ activities. Krait venoms from eastern (WB) and few individuals from southern (KA) and western (MH) India showed significantly higher PLA_2_ activities (238.015–351.148 nmol/mg/min) in comparison to northern (PB), southern (TN), southeastern (AP), southwestern (GA), and central (MP) populations (177.81–205.314 nmol/mg/min; p < 0.0001). Moreover, significant differences in the PLA_2_ activities among the individuals from the same region in southern (KA) and western (MH) India were also observed (p < 0.0001). Interestingly, despite the considerable distance separating them, highly similar activities were observed between a few populations, including southern (TN), northern (PB), and one of the southern (KA) Indian krait populations (Supplementary Figure S1A).

#### 3.3.2 Snake venom protease assay

An azocasein substrate was incubated with a known amount (10 µg) of venom to measure the proteolytic effects of krait venoms. The relative activity was calculated using a purified bovine pancreatic protease as a positive control. All geographically distinct krait populations had negligible proteolytic activity (1.5%–3.34%; p < 0.0001; Supplementary Figure S1C), which was consistent with previous findings (Senji Laxme et al., 2019).

#### 3.3.3 L-amino acid oxidase assay

The abilities of krait venoms in catalysing the cleavage of the L-leucine substrate were estimated by incubating the latter with a known amount of venom (0.5 µg) and measuring the absorbance. Statistically significant differences in LAAO activity were observed among the venoms of krait populations from different geographical regions (p < 0.0001). The maximum LAAO activity was exhibited by the central (MP) population, followed by the southern (KA) Indian kraits, while other populations exhibited relatively lower activity (p < 0.0001; Supplementary Figure S1B).

#### 3.3.4 DNase assay

The DNase activity of krait venoms was determined by incubating *B. caeruleus* venom with the calf thymus DNA for 60 min, followed by gel electrophoresis and densitometric analysis. The venoms of all populations were found to exhibit significant DNase activity (Supplementary Figure S2). The southern (KA) population exhibited highest activity (96.96%), followed by the southern (TN) and southeastern (AP) population (∼70%) and others. The western (MH) population (31%) exhibited relatively lower DNase activity than all others (Supplementary Figures S1D, S2). These observations were based on densitometric analyses of band intensities, and statistical tests were not conducted as this assay was performed without replicates.

#### 3.3.5 Fibrinogenolytic assay

The fibrinogenolytic activity of the venoms of the pan-Indian populations of kraits was assessed by incubating human fibrinogen with venom and subjecting the cleavage products to gel electrophoresis (Supplementary Figure S3). In this experiment, venoms of all krait populations degraded the Aɑ chain of the human fibrinogen with varying intensities, with populations from southern (KA) and central (MP) India showing relatively lower degradation as compared to other populations (Supplementary Figure S3).

### 3.4 Haemolytic assay

**FIGURE 5:**
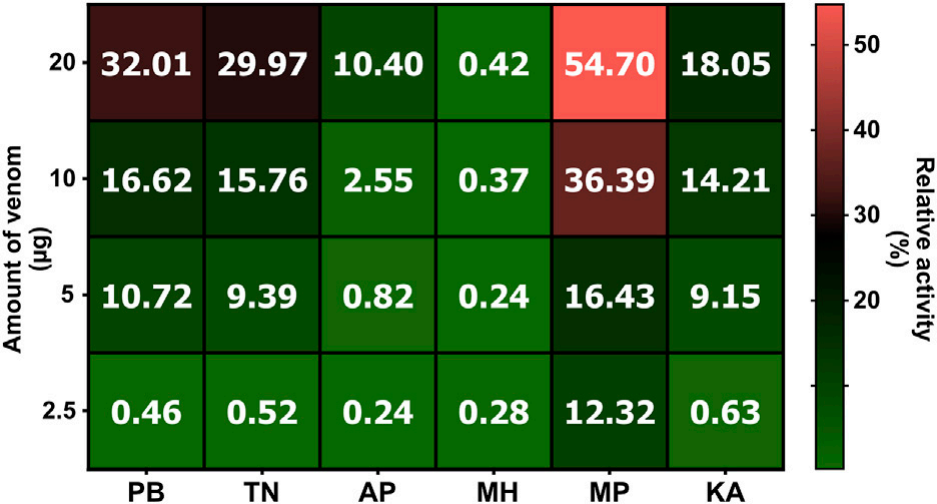
The haemolytic potential *B. caeruleus* venoms. This figure depicts the haemolytic potential of the pan-Indian populations of *B. caeruleus* venoms. Assays were carried out in triplicates. The colour scale and cell numbers represent the percentage of relative activity with respect to the positive control (0.5% Triton X). The amount of venom tested is indicated on the *y*-axis, while sample names are shown on the *x*-axis. Abbreviations: PB, Punjab; TN, Tamil Nadu; AP, Andhra Pradesh; MH, Maharashtra; MP, Madhya Pradesh; and KA, Karnataka.

The venoms of various krait populations were incubated with a 1% RBC solution for 24 h to estimate their haemolytic potential. The outcomes of these experiments suggested that all populations except for the western (MH) population exhibited hemolytic activity that increased with increasing venom concentrations (Figure 5). The central (MP) population induced the highest lysis of RBC, followed by the northern (PB) and southern (TN and KA) Indian populations (p < 0.0001). The southeastern (AP) population was observed to have negligible hemolysis, and the least activity was documented for the western (MH) population of *B. caeruleus*, even at a concentration of 20 µg.

### 3.5 The preclinical efficacy of indian antivenoms against *B. caeruleus*

#### 3.5.1 Venom binding potential via indirect enzyme-linked immunosorbent assay (ELISA)

**FIGURE 6:**
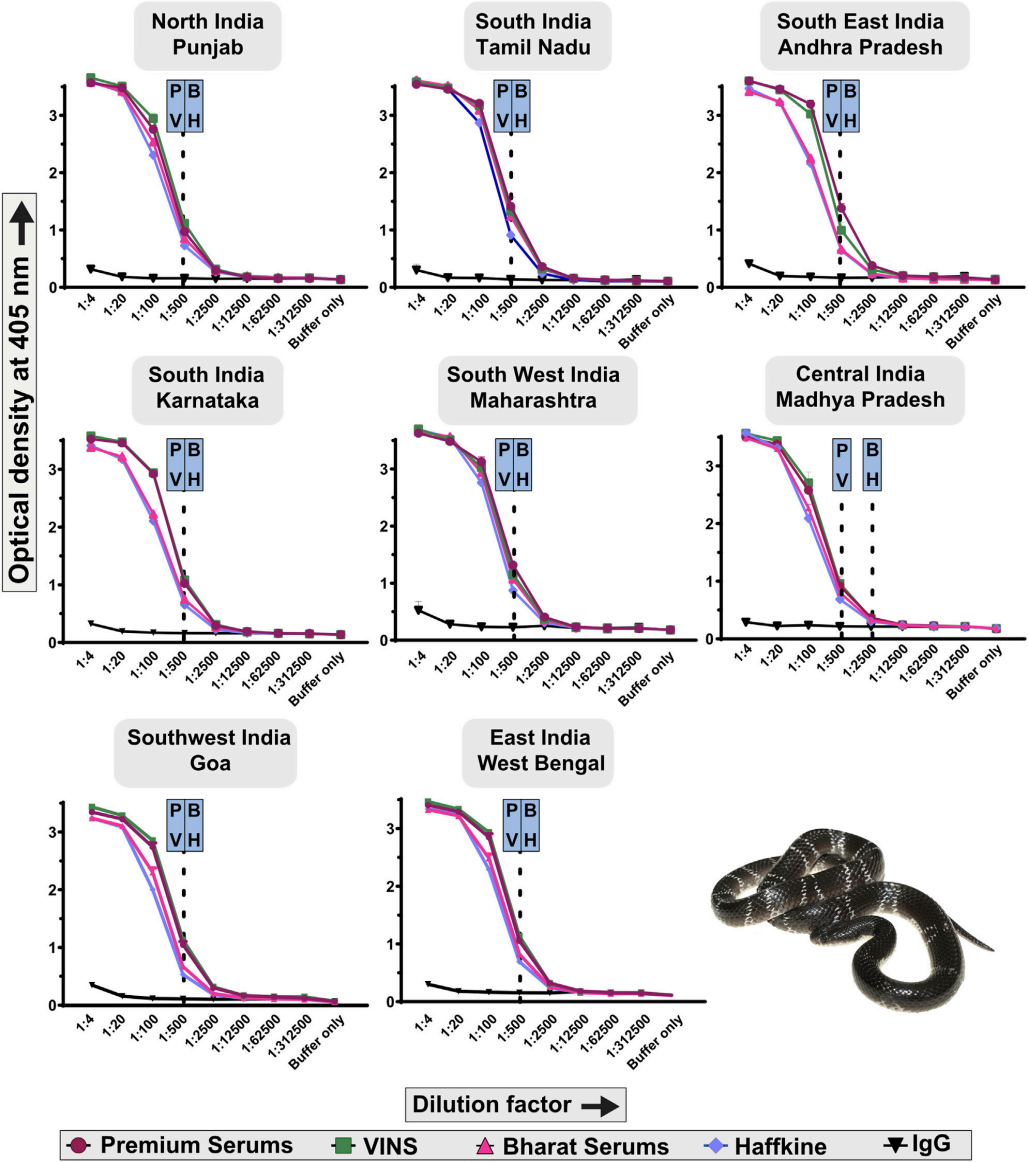
Immunological recognition of commercial Indian antivenoms against the pan-Indian populations of *B. caeruleus* venoms. Various dilutions of antivenoms (1:4 to 1:312,500) are shown on the *x*-axis, while the absorbance values measured at 405 nm, corresponding to the venom-recognition potential of antivenoms, are shown on the *y*-axis. Assays were carried out in triplicates, and the standard deviation is shown as error bars. The dotted lines represent the titers of the respective antivenoms, which were determined using the naive horse IgG at 1:4 dilution. Abbreviation used: P: Premium Serums and Vaccines Pvt. Ltd.; V: VINS Bioproducts Ltd.; B: Bharat Serums and Vaccines Ltd.; and H: Haffkine Institute.

Indirect ELISA was performed to assess the *B. caeruleus* venom-recognition potential of commercial Indian antivenoms. The immunological reactivity of four commercially available polyvalent antivenoms, namely, Premium Serums, VINS, Haffkine, and Bharat Serums, was analysed by indirect ELISA (Figure 6). In these experiments, various dilutions of antivenoms were incubated with the venom. The absorbance obtained at 405 nm was plotted against multiple dilutions of the antivenom, which is directly proportional to the binding of antivenom to venom toxins. Surprisingly, even though the big four polyvalent antivenoms are raised against the common krait from southern India (TN), their venom-recognition potential towards the pan-Indian populations of kraits was very low (endpoint titer of 1:500; Figure 6), relative to the venoms of the other ‘big four’ counterparts [p-value<0.05 (Senji Laxme et al., 2019; Senji Laxme et al., 2021a; Senji Laxme et al., 2021b)]. Among all krait populations, only Premium Serums and VINS exhibited relatively higher binding potential towards the central (MP) Indian population with an endpoint titer of 1:2,500. Overall, the antivenom manufactured by Premium Serums outperformed its competitors w.r.t. *in vitro* binding efficacy (p < 0.05; Figure 6).

#### 3.5.2 Toxicity profiles of the pan-Indian *B. caeruleus*

**FIGURE 7:**
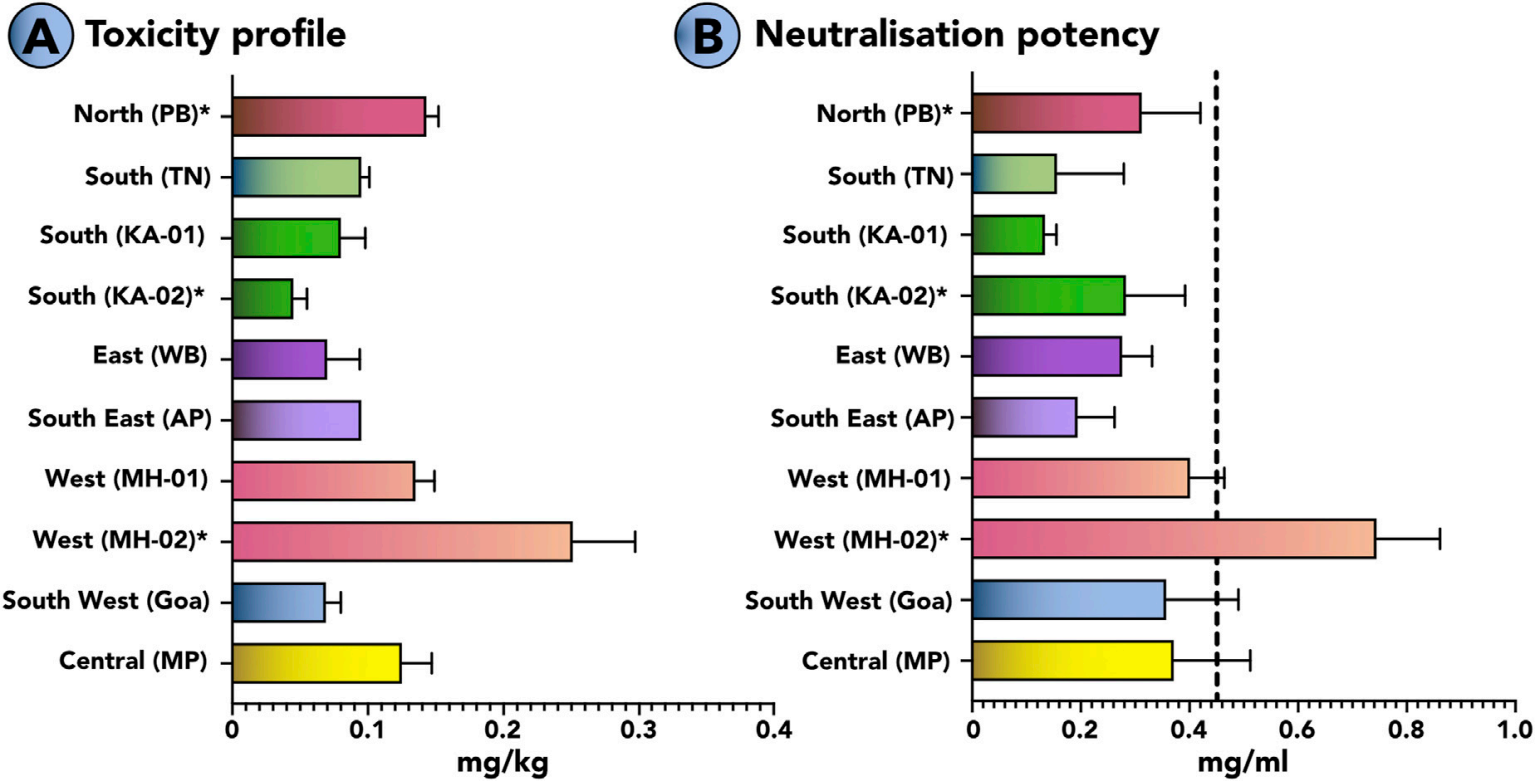
Toxicity profiles of *B* ***.*** *caeruleus* venoms and the neutralisation potencies of commercial antivenom against them. This figure highlights **(A)** the median lethal dose (mg/kg) of various populations of *B. caeruleus* and **(B)** the neutralising potencies (mg/mL) of the Premium Serums antivenom against them. The vertical dotted line in panel B indicates the marketed neutralising potency (0.45 mg/mL) of Indian antivenoms against *B. caeruleus*. The error bars represent 95% of the CI estimated from the probit analysis. Detailed information on venom doses (µg), number of test animals, survival patterns, LD_50_, and ED_50_ with 95% CI are provided in Supplementary Tables S3, S4. *represents values from previous publications (Sunagar et al., 2021; Senji Laxme et al., 2019; Attarde et al., 2022).

The murine model of envenoming was used to estimate the toxicities of krait venoms using WHO-recommended protocols. The LD_50_ (median lethal dose) of the venoms from the pan-Indian populations of *B. caeruleus* ranged between 0.045 and 0.251 mg/kg (Figure 7A; Supplementary Table S3). Among all krait venoms under investigation, the venom of *B. caeruleus* from the southern Indian population of Karnataka in the western ghats was the most toxic to mice (0.045 mg/kg). This was followed by the venoms of kraits from the eastern (WB: 0.069 mg/kg), southwestern (GA: 0.08 mg/kg) and southern (TN: 0.09 mg/kg) Indian populations.

#### 3.5.3 Neutralisation potential of Indian antivenoms against the pan-Indian populations of *B. caeruleus*

The WHO-recommended preclinical assay was employed to estimate the neutralisation potency of the Indian polyvalent antivenom manufactured by Premium Serums, which performed relatively better than other antivenoms under *in vitro* conditions (Figure 7B). In these experiments, the polyvalent product failed to match the marketed label claim of 0.45 mg/mL when tested against a 5x challenge dose of krait venom from seven biogeographic regions, namely, northern (PB), southern (KA and TN), southerneastern (AP), eastern (WB), western (MH), southernwertern (GA) and central MP) India. The antivenom was unable to meet the marketed claim of neutralisation against krait venoms from the pan-Indian populations of *B. caeruleus*, including northern (PB: 0.310 mg/mL), southern (KA: 0.156 mg/mL and 0.134 mg/mL; (Sunagar et al., 2021); TN: 0.283 mg/mL), southeastern (AP: 0.194 mg/mL), southernwestern (GA: 0.357 mg/mL), eastern (WB: 0.276 mg/mL), western (MH: 0.400 mg/mL and 0.744 mg/mL (Sunagar et al., 2021)) and central (MP: 0.371 mg/mL) populations. Surprisingly, it exhibited poor efficacy (0.283 mg/mL) even against the *B. caeruleus* venom from southern (TN) India, using which the commercial antivenoms are manufactured (Figure 7B; Supplementary Table S4). The antivenom exhibited superior neutralisation potency than the marketed potency against one of the western India (MH: 0.744 mg/mL) populations.

## 4 Discussion

### 4.1 Stark variation in venom profiles of the pan-Indian *B. caeruleus*

India’s common krait, *B. caeruleus*, poses a significant medical threat given its potent venom. Accidental bites from this snake lead to severe neurotoxic effects, resulting in paralysis, respiratory distress, and even death. Their venom primarily consists of presynaptic and postsynaptic neurotoxins, such as 3FTxs, neurotoxic PLA_2_s and β-bungarotoxins (Oh et al., 2017; Chen and Lee, 1970; Ranawaka et al., 2013; Silva et al., 2016; Sunagar et al., 2013; Sunagar et al., 2021; Fry, 2015; Barber et al., 2013; Ueno and Rosenberg, 1996; Young et al., 2003; Dixon and Harris, 1999). The presence of β-bungarotoxin, a heterodimeric toxin consisting of enzymatic PLA_2_ and non-enzymatic Kunitz domains, distinguishes krait venoms from the venoms of most other snakes. This toxin exerts its effects presynaptically by interfering with the release of acetylcholine (Rugolo et al., 1986). Different isoforms of 3FTx have been reported in *Bungarus* spp., including α-neurotoxins (Type-I and Type-II), k-bungarotoxins, and unconventional 3FTxs (Ranawaka et al., 2013; Fry, 2015; Young et al., 2003; Nys et al., 2022).

While efforts have been devoted to unravelling the composition of krait venoms in southern India, a notable knowledge gap remains regarding how their venom profiles may vary across geography. Such studies are essential for understanding venom diversity and developing efficient snakebite therapeutics. Our study examines krait venoms across five biogeographic zones of India and reports their venom proteome composition, biochemical activities and toxicity profiles. Venom proteomics identified PLA_2_ as the most abundant toxin family in the pan-Indian populations of *B. caeruleus* (Figure 3; Supplementary Table S5A–E. The populations from northern (PB), western (MH) and southern (TN) Indian regions were found to consist of larger amounts of PLA_2_s (60%–62%), whereas central (MP) and eastern (WB) Indian populations had relatively lower amounts of this enzymatic toxin (45%). 3FTx also exhibited notable variations, being the most abundant in the central (MP) population (29%) and least abundant in the western (MH) Indian population (13%). Additionally, the composition of the 3FTx subfamily varies across populations. For instance, C-3FTx were substantially present in the western (MH) and central (MP) populations (17%–20% of all 3FTxs) but were negligible in others. However, their biological role in krait venoms remains unknown. In the case of the southern (TN) Indian population, type II ⍺-neurotoxin formed a major proportion of the 3FTx subfamily (50%), while in the northern (PB) population, κ-bungarotoxins were predominant (42%). In eastern (WB) and northern (PB) populations, type I and II ⍺-neurotoxins were in equal proportions (38%–42% of all 3FTxs). Other toxin families, including CRISPs, were relatively more abundant in eastern (WB) and northern (PB) populations (12%–20%) than others (6%–9%). In comparison, β-btx was found to be higher in southern (TN), western (MH) and central (MP) Indian populations (13%–14%).

The functional characterisation of *B. caeruleus* venoms remains poorly understood. Hence, we performed various assays to investigate PLA_2_, proteolytic, LAAO, and DNAse activities. The enzymatic activity of PLA_2_ was significantly higher for populations from eastern (WB), southern (KA) and western (MH) regions. Surprisingly, despite the relatively lower abundance of PLA_2_ for eastern (WB) populations, the observed activity was notably high. This inconsistency in proteomic abundance and function has also been reported previously (Senji Laxme et al., 2021a), suggesting a varying proportion of catalytically active PLA_2_s across regions. The venoms from all populations were specific towards the Aα-chain of human fibrinogen, leaving the Bβ-chain and gamma-chains intact. Furthermore, DNAse activity varied among populations, with the southern (KA) population exhibiting higher activity than others. This variation in the functional activity may contribute to differences in symptoms among the bite victims across different regions. A combination of functional variation and the compositional difference in the abundance of 3FTx subfamilies and their affinities for nAChRs of target animals may affect the observed differences in potency of *B. caeruleus* venoms across India. The LD_50_ of venoms in the mouse model of envenoming, indeed, varied across regions with southern (TN and KA) and eastern populations exhibiting relatively higher toxicity in comparison to western (MH) and central (MP) Indian krait populations.

### 4.2 Orthosteric binding profiles of krait venoms provide insights into the feeding ecology of these elusive elapids

Krait envenoming results in prey paralysis, primarily through the binding of 3FTx to the presynaptic α-1 nAChRs at the neuromuscular junction (Ueno and Rosenberg, 1996; Dixon and Harris, 1999). Given their nocturnal nature, field observations of their dietary behaviour are challenging. Moreover, India’s stringent wildlife protection acts rule out the possibility of capturing them to analyse their gut content. Hence, despite being widespread, the venom ecology of these elusive elapids has remained largely unknown. The evolution of 3FTxs and their ability to target specific receptors can shed light on the coevolutionary dynamics between predator-prey systems. Here, we have evaluated the binding patterns of krait venoms to their potential target prey and predators to understand their venom ecology. Overall, the venoms of the pan-Indian krait populations exhibited a varied binding pattern towards nAChRs of various prey and predatory species (Figure 4). The highest binding was observed against rodents, amphibians, and birds. Intermediate affinity was observed towards the lizard nAChRs. In contrast, the least binding affinity was observed towards human, mongoose, and snake nAChRs (Figure 4). Kraits have been documented to feed on frogs and rats (Dixon and Harris, 1999; Captain and Whitaker, 2015). The orthosteric binding patterns of neurotoxins and nAChRs obtained in this study largely support these observations. The reduced binding affinity towards mongoose nAChRs is, perhaps, a result of the evolution of resistance to neurotoxins in these animals that often prey on snakes. Since humans are not the primary target species of the common krait, it is unsurprising that their venom toxins exhibit relatively decreased affinity towards our nAChRs.

When intraspecific variations in binding affinities towards nAChRs were evaluated, the venoms of certain populations exhibited interesting binding profiles. The venom of the central Indian (MP) krait population showed the highest binding towards nAChRs of amphibians, rodents, and lizards (p < 0.0001; Figure 4). Surprisingly, the north Indian (PB) krait venom exhibited the highest binding affinity towards amphibian nAChRs compared to the rodent channels (p < 0.0001; Figure 4). Although the differences in binding affinities were several times greater, the biological relevance of these differences remains unclear. Overall, krait venoms across India exhibited strong binding affinities towards rodent channels. These observations were also corroborated by the outcomes of our *in vivo* experiments in the mouse model of envenoming, where the venoms of the pan-Indian populations of kraits were found to be highly potent: LD_50_ ranged between 0.045 and 0.251 mg/kg (Figure 7A). Thus, our findings provide novel insights into the feeding ecology of *B. caeruleus*. However, it is important to obtain additional lines of evidence supporting these findings, such as field observations of the feeding behavior and toxicity assessment against the target prey species, to gain a better understanding of the prey-predator interactions.

### 4.3 Commercial Indian antivenoms are inefficacious in neutralising the venoms of the pan-Indian populations of kraits

The current therapeutic approach for treating snake bites involves using polyclonal antibodies derived from equines immunised with the venoms of the ‘big four’ snakes (Kaur et al., 2021). However, this approach has several limitations, including reduced antivenom effectiveness in certain parts of the country, poor dose efficacy and a lack of broad effectivity against multiple species/populations (Senji Laxme et al., 2019; Senji Laxme et al., 2021a; Senji Laxme et al., 2021b; Shashidharamurthy and Kemparaju, 2007; Alangode et al., 2020; Casewell et al., 2020; Gutierrez et al., 2007). Previous studies have highlighted the repercussions of venom variation in kraits on the neutralising effect of the Indian polyvalent antivenom (Sunagar et al., 2021; Senji Laxme et al., 2019). While the antivenom was found to match the marketed claim of neutralisation against the venom of the source population in southern India, it exhibited poor neutralisation against the venom of the krait from northern India (Senji Laxme et al., 2019), as well as against the venoms of kraits in the neighbouring region of Karnataka (Sunagar et al., 2021).

*In vivo* experiments in the murine model of envenoming in this study support these findings and reveal a much more alarming outcome. When we tested the best-binding antivenom (*in vitro* conditions) in countering toxicities inflicted by the pan-Indian populations of *B. caeruleus*, the antivenom failed to reach the marketed neutralising potency (0.45 mg/mL) against the northern population (Punjab: 0.310 mg/mL). Surprisingly, this antivenom performed poorly even against the southern populations, including Tamil Nadu (0.283 mg/mL), where venoms are sourced for the commercial production of antivenoms. In addition, a preclinical inefficacy was documented against the common krait venom from Karnataka (0.156 mg/mL) and Andhra Pradesh (0.194 mg/mL) in southern India, as well as against Maharashtra (0.40 mg/mL) and Madhya Pradesh (0.371 mg/mL) in western and central India, respectively (Figure 7B). The consistent failure of antivenom to reach the marketed neutralisation potency of 0.45 mg/mL, which is already far lower than what would be desirable for effective snakebite treatment, is alarming and highlights the urgency to improve the performance of antivenom products in treating snakebites. The absence of sufficient neutralising antibodies in conventional antivenoms against 3FTxs may reflect the inferior immunogenicity of these toxins, emphasising the need for next-generation snakebite solutions, including broadly neutralising antibodies (Casewell et al., 2020; Khalek et al., 2024; Ledsgaard et al., 2023).

## 5 Conclusion

The proteomic profiling, *in vitro* enzymatic assays, and *in vivo* animal experiments in this study revealed variations in the venom composition, functional activities, feeding ecologies, and toxic potencies of the geographically disparate populations of the common Indian krait, *B. caeruleus*. *In vitro* binding assays revealed a poor venom-recognition potential by all Indian antivenoms. Consistently, *in vivo* venom neutralisation assays revealed that even the relatively better binding antivenom fails to reach the marketed potency claimed by the manufacturers against the pan-Indian krait venoms. These findings highlight the urgent need to improve therapeutics for treating krait bites in India.

## Data Availability

The datasets presented in this study can be found in online repositories. The names of the repository/repositories and accession number(s) can be found in the article/Supplementary Material.
