# Supplementary material for: Elusive elapids: biogeographic venom variation in Indian kraits and its repercussion on snakebite therapy
Source: Front Pharmacol. 2024 Nov 7;15:1443073. doi: 10.3389/fphar.2024.1443073 (PMC11579489; doi:10.3389/fphar.2024.1443073)
Supplement: Supplementary file 2 [file DataSheet1.PDF]

Table S1. Details of krait venom samples investigated in this study.

Sample ID	Number of individuals	Protein concentration (µg/µl)	Region	Biogeographical region
BuCaPB01	4	0.166	Nawanshahr, Punjab	Semi-arid
BuCaTN01	Unknown	0.144	Kanchipuram district, Tamil Nadu	Coastal-plains
BuCaMH01	10	0.109	Mahad, Maharashtra	Western ghats
BuCaMH02	5	0.087	Pune, Maharashtra	Western ghats
BuCaMH03	1	0.126	Mahad, Maharashtra	Western ghats
BuCaMH04	1	0.263	Warje, Maharashtra	Western ghats
BuCaKA01	1	0.116	Agumbe, Karnataka	Western ghats
BuCaKA02	1	0.122	Bannerghatta, Karnataka	Western ghats
BuCaKA03	1	0.165	Hunsur, Karnataka	Western ghats
BuCaKA04	1	0.151	Hunsur, Karnataka	Western ghats
BuCaKA05	1	0.141	Hunsur, Karnataka	Western ghats
BuCaKA06	1	0.138	Hunsur, Karnataka	Western ghats

BuCaKA07	1	0.157	Agumbe, Karnataka	Western ghats
BuCaKA08	1	0.146	Banglore, Karnataka	Western ghats
BuCaKA09	1	0.478	Dharwad, Karnataka	Western ghats
BuCaMP02	1	0.124	Jabalpur, Madhya Pradesh	Deccan plateau
BuCaAP01	1	0.107	Visakhapatnam, Andhra Pradesh	Coastal-plains
BuCaGA01	1	0.112	Bicholim, Goa	Western ghats
BuCaWB32	1	0.125	Habra, West Bengal	Gangetic plains

Table S2. Details of antivenom samples investigated in this study.

Manufacturer	Batch	Manufacture (M) and expiry (E) dates	Protein content (mg/ml)	Marketed neutralising efficacy (mg/ml)
**Indian polyvalent antivenoms**				
**Bharat Serums and Vaccines Ltd.**	A05318087	**M:** 10/2018<br>**E:** 09/2022	26.5 ± 0.77	*B. caeruleus*: 0.45
**Haffkine BioPharmaceutical Corporation Ltd.**	AS180611	**M:** 06/2018<br>**E:** 11/2022	24.7± 0.5	
**Premium Serums & Vaccines Pvt. Ltd.**	ASVS(I)-Lyo013	**M:** 12/2018<br>**E:** 11/2022	26.2± 1.2	
**VINS Bioproducts Ltd.**	01AS18067	**M:** 08/2018<br>**E:** 07/2023	31.4 ± 0.54	

Details of the commercial Indian antivenoms tested in this study are provided here. Batch numbers, manufacturing and expiry dates, protein concentrations, and marketed neutralising potencies are shown.

Table S3. The median lethal dose of krait venoms.

Name of sample	Venom Dose (µg)					Number of survivors					LD_{50} (µg/mouse)	LD_{50} (mg/kg)
BuCaPB01*	2.56	3.20	4.0	5.0	6.25	4	1	0	0	0	2.86<br>2.58-3.03	0.143<br>0.13-0.15
BuCaTN01	1.44	1.73	2.08	2.50	3.0	5	4	1	0	0	1.90<br>1.78-2.02	0.09<br>0.09-0.10
BuCaMH01	2.04	2.56	3.20	4.0	5.0	5	3	1	0	0	2.70<br>2.44-2.98	0.13<br>0.12-0.15
BuCaMH02*	2.88	3.6	4.5	5.62	7.03	5	5	3	2	0	5.03<br>4.25–5.95	0.251<br>0.213–0.297
BuCaKA01	0.68	1.02	1.53	2.30	3.44	5	4	3	1	0	1.59<br>1.29-1.97	0.08<br>0.06-0.10
BuCaKA02*	1	1.28	1.6	2	2.5	2	1	0	0	0	0.90<br>0.734–1.10	0.045<br>0.037–0.055
BuCaMP02	1.80	2.25	2.81	3.52	4.39	0	3	2	1	0	2.51<br>2.14-2.93	0.125<br>0.10-0.14
BuCaAP01	1.73	2.08	2.50	3.00	3.60	4	0	0	0	0	1.90	0.095
BuCaGA01	1.13	1.41	1.77	2.21	2.76	5	5	2	4	5	1.60<br>1.38-1.86	0.08<br>0.069- 0.093
BuCaWB32	0.50	0.75	1.13	1.69	2.53	5	5	3	2	0	1.38	0.069

											1.019-1.874	0.051-0.094

The table indicates various dose groups, survival patterns, and toxicities of krait venoms from different biogeographical zones. * Previously published data (Senji Laxme et al. 2019; Sunagar et al. 2021; Attarde et al. 2022).

Table S4. Median effective doses and neutralisation potencies of Premium Serums antivenom against Pan-Indian populations of *B. caeruleus* venom.

Venom	Challenge dose 5X LD_{50} (µg/mouse)	Amount of antivenom injected in the venom-antivenom mixture (µl)				ED_{50} (µl)	ED_{50} (µl antivenom/mg venom)	Potency of antivenom (mg/ml)
BuCaPB01	14.3	32.94	21.97	14.64	9.76	26.17<br>19.35-35.36	1830.07<br>1353.15-2472.73	0.310<br>0.230-0.419
BuCaTN01	9.5	49.40	32.94	21.97	14.64	26.90<br>19.36-37.36	2831.58<br>20380.95-3932.63	0.283<br>0.203-0.392
BuCaMH01	13.5	49.40	32.94	21.97	14.64	26.90<br>23.29-31.06	1992.59<br>1745.9-2300.75	0.40<br>0.35-0.46
BuCaMH02*	25.15	32.93	21.96	14.63	9.75	26.90<br>23.21-31.16	1069.58<br>922.86- 1238.97	0.74<br>0.64- 0.86
BuCaKA01	7.95	32.94	21.97	14.64	9.76	40.69<br>22.78-72.68	5,118.24<br>2,864.15-9,142.14	0.156<br>0.087-0.279
BuCaKA02*	4.5	32.93	21.96	14.63	9.75	26.90<br>23.21-31.16	5977.78<br>5157.78-6924.44	0.13<br>0.11- 0.15

BuCaMP02	12.55	32.94	21.97	14.64	9.76	27.03<br>19.60-37.2<br>7	2,156.97<br>1,561.75-<br>2,969.72	0.371<br>0.269-0.512
BuCaAP01	9.5	49.40	32.94	21.97	14.64	39.21<br>29.03-<br>52.97	4,127.37<br>3,055.79-<br>5,575.79	0.194<br>0.143-0.262
BuCaGA01	8	32.94	21.97	14.64	9.76	17.93<br>12.9-24.93	2,241.25<br>1,612.50-<br>3,116.25	0.357<br>0.321- 0.620
BuCaWB32	6.9	32.93	21.9	14.63	9.76	20.00<br>16.65-24.0<br>13	2898.55<br>2413.0-3478.26	0.276<br>0.23-0.331

The above table shows the toxicity neutralisation potencies of commercial Indian antivenom manufactured by Premium Serum and Vaccines Pvt. Ltd. The neutralisation potencies were estimated against krait venoms from different biogeographical with 5X LD_{50} as 'challenge dose' as noted in the table. * Previously published data (Senji Laxme et al. 2019; Sunagar et al. 2021; Attarde et al. 2022).

Table S5A: Proteomic information of venom from southern (TN) populations of *B. caeruleus* . Assignment of peptides sequenced from MS/MS spectra using Serpentes NCBI database (TaxID: 8570) in PEAKS X (Bioinformatics Solutions Inc., Canada) . Relative abundance of each toxin was estimated by using peak fraction area (obtained from RP-HPLC and SDS PAGE) and spectral intensity of all peptides matched to their respective proteins. -10logP is the matching score given by PEAKS Algorithm, m/z is mass to charge ratio of the peptides.

HPLC Peak no.	Protein information			Peptide Information						Calculation of Toxin abundance				
	NCBI Accession ID	Description	Toxin family	Peptide Sequence	-10LgP	m/z	z	RT	Precursor intensity	HPLC Peak	SDS Densitometry	Relative peak fraction	Relative abundance	% Proportion of Toxin
F2a	1MR6	Chain A neurotoxin	3FTx (Type II NTx)	CPNSETCPDGKNICVKR	43.85	573.6002	3	29.98	930000000	0.0247	1	0.0247	0.4968	0.0122906
				SFYTCPNSETCPDGKN	82.69	838.3397	2	36.79	930000000					
				FYTCPNSETCPDGKNICVKR	54.84	1071.968	2	36.35	930000000					
				MQCKTCSFYTCPNSETCPDGKNICVKR	49.78	1067.783	3	50.04	930000000					
	CAA06887.1	neurotoxin-like protein [Bungarus multicinctus multicinctus]	3FTx (Type I NTx)	RGCVATCPKPKKNEIIQCCAKD	55.95	591.2933	4	30.17	824000000				0.4402	0.0108897
				KYSQANESSKTCPSGQLLC	87.01	935.4362	2	44.07	824000000					
				KKNEIIQCCAKDKC	58.02	502.9184	3	23.49	824000000					
				KWEIGNPSGKE	56.58	494.2478	2	44.83	824000000					
				KRGCVATCPKPKK	45.87	425.2213	3	17.91	824000000					
				KTCPSGQLLCLKKWEIGNPSGKE	46.52	594.0562	4	66.49	824000000					
				KKWEIGNPSGKE	71.37	558.2964	2	79.97	824000000					
	Q8AY42.2	RecName: Full=Kunitz-type serine protease inhibitor B; AltName: Full=Kunitz inhibitor B; Flags: Precursor		EFTYGGCGGNANNFKS	86.88	753.8243	2	42.8	94900000				0.0507	0.0012542
	XP_026579406.1	BPTI/Kunitz domain-containing protein-like isoform X3 [Pseudonaja textilis]	Kunitz	KFTYGGCAGNANNFKKH	41.93	550.256	3	36.56	9870000				0.0053	0.0001304
				GLFEGSCSIFPNDSQRC	49.11	878.9021	2	63.05	9870000					
	KAG8131486.1	putative Kunitz-type serine protease inhibitor spermatin protein [Naja naja]		RCNAYITAFYYNPDSRR	72.57	927.9088	2	68.44	8570000				0.0046	0.0001133
				KGRCNAYITAFYYNPDSRR	59.9	689.9836	3	62.53	8570000					
				GFCELPAAKG	48.05	468.2365	2	37.5	8570000					
	0412250A	toxin A beta1 bungaro	PLA2	RTIICYGAAGGTCRI	67.19	700.3264	2	42.08	4680000				0.0025	0.0000618
F3a	1MR6	Chain A neurotoxin	3FTx (Type II NTx)	FYTCPNSETCPDGKNICVKR	54.84	1071.968	2	36.35	21200000000	0.0349	1	0.0349	0.8678	0.0303111
				CPNSETCPDGKNICVKR	43.85	573.6002	3	29.98	21200000000					
				MQCKTCSFYTCPNSETCPDGKNICVKR	49.78	1067.783	3	50.04	21200000000					
				SFYTCPNSETCPDGKNICVKR	103.15	1145.5	2	42.38	21200000000					
	Q8AY42.2	RecName: Full=Kunitz-type serine protease inhibitor B; AltName: Full=Kunitz inhibitor B; Flags: Precursor	Kunitz	EFTYGGCGGNANNFKS	86.88	753.8243	2	42.8	2580000000				0.1056	0.0036888
	XP_026579406.1	BPTI/Kunitz domain-containing protein-like isoform X3 [Pseudonaja textilis]		KFTYGGCAGNANNFKK	93.7	760.8345	2	42.5	377000000				0.0154	0.0005390
				GLFEGSCSIFPNDSQRC	49.11	878.9021	2	63.05	377000000					
	CAA06887.1	neurotoxin-like protein [Bungarus multicinctus multicinctus]	3FTx (Type I NTx)	KKNEIIQCCAKDKC	58.02	502.9184	3	23.49	241000000				0.0099	0.0003446
				RGCVATCPKPKK	44.97	559.2778	2	18.98	241000000					
				KKWEIGNPSGKE	71.37	558.2964	2	79.97	241000000					
				KWEIGNPSGKE	56.58	494.2478	2	44.83	241000000					
				KRGCVATCPKPKK	45.87	425.2213	3	17.91	241000000					
				KYSQANESSKTCPSGQLLC	87.01	935.4362	2	44.07	241000000					
				KTCPSGQLLCLKKWEIGNPSGKE	46.52	594.0562	4	66.49	241000000					
	AAL30054.1	kappa 1a bungaratoxin [Bungarus candidus]	3FTx (κ-bungarotoxin)	CLISPSSTPQTCPQGQDICFLKA	51.11	793.0555	3	62.66	26900000				0.0011	0.0000385
				RTCLISPSSTPQTCPQGQDICFLKA	100.66	880.0802	3	66.27	26900000					
				TRTCLISPSSTPQTCPQGQDICFLKA	101.81	932.1138	3	60.45	26900000					
	P01388.1	RecName: Full=Long neurotoxin 2; AltName: Full=Neurotoxin B; AltName: Full=Tx-NM4	3FTx (Type II NTx)	VDLGCAATCPTVKPGVNIKC	46.43	634.3239	3	57.43	2010000				0.0001	0.0000029
	ADF50022.1	non-conventional three finger toxin isoform 7 [Bungarus flaviceps]	3FTx	GYTLTCLICPEKYCQKV	42.49	659.6476	3	64.39	1510000				0.0001	0.0000022
				TLTCLICPEKYCQKV	70.27	856.9109	2	49.62	1510000					
	0412250A	toxin A beta1 bungaro	PLA2	RTIICYGAAGGTCRI	67.19	700.3264	2	42.08	923000				0.0000	0.0000013
	KAG8131486.1	putative Kunitz-type serine protease inhibitor spermatin protein [Naja naja]	Kunitz	KGRCNAYITAFYYNPDSRR	59.9	689.9836	3	62.53	495000				0.0000	0.0000007
				RCNAYITAFYYNPDSRR	72.57	927.9088	2	68.44	495000					
				GFCELPAAKG	48.05	468.2365	2	37.5	495000					
	B4ESA4.1	RecName: Full=Kunitz-type serine protease inhibitor PILP-3; AltName: Full=Protease inhibitor-like protein 3; Flags: Precursor		RKCIEFSYGGCGGNANNFKS	58.51	674.9644	3	52.31	410000				0.0000	0.0000006
	Q9W729.1	RecName: Full=Kappa-6-bungarotoxin; Short=K-6 bungarotoxin; Flags: Precursor	3FTx (κ-bungarotoxin)	RGAVIEQGCVATCPEFRS	101.41	897.4203	2	51.79	247000				0.0000	0.0000004
				QGCVATCPEFRS	45.84	598.7631	2	39.55	247000					
				AVIEQGCVATCPEFRS	73.41	833.3901	2	48.13	247000					
				TRTCHISTSSTPQTCPKGQDICFRK	58.96	913.08	3	41.93	247000					
F4	Q8AY42.2	RecName: Full=Kunitz-type serine protease inhibitor B; AltName: Full=Kunitz inhibitor B; Flags: Precursor	Kunitz	EFTYGGCGGNANNFKS	86.88	753.8243	2	42.8	1040000000	0.0161	1	0.0161	0.9741	0.0156438
	UNJ97059.1	kunitz peptide partial [Boiga dendrophila dendrophila]		EFEYGGCGGNANNFKT	43.67	767.8224	2	57.89	12200000				0.0114	0.0001835
	CAA06887.1	neurotoxin-like protein [Bungarus multicinctus multicinctus]	3FTx (Type I NTx)	KWEIGNPSGKE	56.58	494.2478	2	44.83	11600000				0.0109	0.0001745
				KRGCVATCPKPKK	45.87	425.2213	3	17.91	11600000					
				RGCVATCPKPKKNEIIQCCAKD	55.95	591.2933	4	30.17	11600000					
				KKWEIGNPSGKE	71.37	558.2964	2	79.97	11600000					
				KTCPSGQLLCLKKW	44.46	468.9153	3	49.32	11600000					
				KKNEIIQCCAKD	58.97	632.3132	2	25.62	11600000					
				KYSQANESSKTCPSGQLLC	87.01	935.4362	2	44.07	11600000					
	1MR6	Chain A neurotoxin	3FTx (Type II NTx)	SFYTCPNSETCPDGKNICVKR	103.15	1145.5	2	42.38	3870000				0.0036	0.0000582
				FYTCPNSETCPDGKNICVKR	54.84	1071.968	2	36.35	3870000					
				CPNSETCPDGKNICVKR	43.85	573.6002	3	29.98	3870000					
				MQCKTCSFYTCPNSETCPDGKNICVKR	49.78	1067.783	3	50.04	3870000					
F5	ADF50022.1	non-conventional three finger toxin isoform 7 [Bungarus flaviceps]	3FTx	TLTCLICPEKYCQKV	70.27	856.9109	2	49.62	42000000	0.0119	1	0.0119	0.5846	0.0069562
				GYTLTCLICPEKYCQKV	42.49	659.6476	3	64.39	42000000					
	Q8AY42.2	RecName: Full=Kunitz-type serine protease inhibitor B; AltName: Full=Kunitz inhibitor B; Flags: Precursor	Kunitz	EFTYGGCGGNANNFKS	86.88	753.8243	2	42.8	15100000				0.2102	0.0025009
	CAA06887.1	neurotoxin-like protein [Bungarus multicinctus multicinctus]	3FTx (Type I NTx)	KWEIGNPSGKE	56.58	494.2478	2	44.83	6580000				0.0916	0.0010898
				KKNEIIQCCAKD	58.97	632.3132	2	25.62	6580000					
				KKWEIGNPSGKE	71.37	558.2964	2	79.97	6580000					
				KTCPSGQLLCLKKW	44.46	468.9153	3	49.32	6580000					
				KRGCVATCPKPKK	45.87	425.2213	3	17.91	6580000					
				RGCVATCPKPKK	44.97	559.2778	2	18.98	6580000					
				KYSQANESSKTCPSGQLLC	87.01	935.4362	2	44.07	6580000					
	Q8AY45.2	RecName: Full=Kunitz-type serine protease inhibitor homolog beta-bungarotoxin B2a chain; Flags: Precursor	Kunitz	RAFYYKPSAKR	41.37	358.8592	3	33.53	3180000				0.0443	0.0005267
	Q8JFX7.1	RecName: Full=Muscarinic toxin BM14; Flags: Precursor	3FTx	KSYWVNENGKQKK	46.96	451.5582	3	32.73	2950000				0.0411	0.0004886
				RCYKSYWVNENGKQKK	43.98	902.4322	2	35.46	2950000					
	P25668.1	RecName: Full=Long neurotoxin 1; AltName: Full=Toxin A	3FTx (Type II NTx)	RVDLGCAATCPTVRT	72.6	710.3397	2	42.98	2040000				0.0284	0.0003379
				KRVDLGCAATCPTVRT	76.14	525.9288	3	39.07	2040000					
				RGKRVDLGCAATCPTVRT	72.05	440.9781	4	34.58	2040000					
F6a	AAL30054.1	kappa 1a bungaratoxin [Bungarus candidus]	3FTx (κ-bungarotoxin)	RTCLISPSSTPQTCPQGQDICFLKA	100.66	880.0802	3	66.27	1280000000	0.0664	1	0.0664	0.3428	0.0227502
				CLISPSSTPQTCPQGQDICFLKA	51.11	793.0555	3	62.66	1280000000					
	1F94	Chain A BUCANDIN	3FTx	LKITCSAEETFCYKW	52.26	546.2498	3	42.98	899000000				0.2408	0.0159785
				KITCSAEETFCYKWLNKISNERW	70.93	883.7543	3	70	899000000					
				CSAEETFCYKWLNKI	59.35	838.3919	2	59.22	899000000					
				RCGVSGCHLKITCSAEETFCYKW	88.69	836.3639	3	53.55	899000000					
				IDTWNVYNKCCTTNLCNT	43.96	1082.447	2	54.91	899000000					
				KWLNKISNERW	41.68	580.3146	2	36.65	899000000					
	1MR6	Chain A neurotoxin		MQCKTCSFYTCPNSETCPDGKNICVKR	49.78	1067.783	3	50.04	790000000				0.2116	0.0140411
				CPNSETCPDGKNICVKR	43.85	573.6002	3	29.98	790000000					
				SFYTCPNSETCPDGKN	82.69	838.3397	2	36.79	790000000					
				FYTCPNSETCPDGKNICVKR	54.84	1071.968	2	36.35	790000000					
	CAM11302.1	alpha-delta-bungarotoxin-4 partial [Bungarus caeruleus]	3FTx (Type II NTx)	TSTSPISTVTCPSGENLCYTKM	86.24	1101.506	2	54.17	224000000				0.0600	0.0039813
				KVIELGCVATCPQPKPYEEVTCCSTDKC	78.78	1014.463	3	58.23	224000000					
				MWCDAFCSSRG	44.41	594.7312	2	46.96	224000000					
				STSPISTVTCPSGENLCYTKM	63.33	1057.989	2	53.66	224000000					
				SPISTVTCPSGENLCYTKM	41.83	963.9484	2	49.07	224000000					
				HTTSTSPISTVTCPSGENLCYTKM	41.81	1202.553	2	54.92	224000000					
				KMWCDAFCSSRG	57.87	660.2519	2	56.42	224000000					
	Q8AY42.2	RecName: Full=Kunitz-type serine protease inhibitor B; AltName: Full=Kunitz inhibitor B; Flags: Precursor	Kunitz	EFTYGGCGGNANNFKS	86.88	753.8243	2	42.8	144000000				0.0386	0.0025594
	AAL87004.1	beta-bungarotoxin A2 chain precursor [Bungarus caeruleus]	b-BTx	RYTIPCDKTWGHYADYGCYCGAGGSGTPVDALDRC	99.95	1228.527	3	67.23	122000000				0.0327	0.0021684
				RCCYVHDNCYGVAENKH	118.36	944.8733	2	36.88	122000000					
				KTWGHYADYGCYCGAGGSGTPVDALDRC	123.31	936.059	3	63.11	122000000					
				CFGDSEYIGAHKN	51.49	408.5277	3	37.07	122000000					
				RIVCDCDRTAALCFGDSEYIGAHKNI	70.09	693.8093	4	55.91	122000000					
				RTAALCFGDSEYIGAHKNIDTKR	74.86	578.5314	4	53.11	122000000					
				ADYGCYCGAGGSGTPVDALDRC	91.42	1045.929	2	55.35	122000000					
				HYADYGCYCGAGGSGTPVDALDRC	68.53	1162.982	2	58.07	122000000					
				YADYGCYCGAGGSGTPVDALDRC	71	1081.448	2	55.1	122000000					
				WGHYADYGCYCGAGGSGTPVDALDRC	69.79	840.3488	3	53.65	122000000					
				ALCFGDSEYIGAHKN	44.88	499.5666	3	51.25	122000000					
				IRYTIPCDKTWGHYADYGCYCGAGGSGTPVDALDRC	52.95	960.6711	4	64.11	122000000					
				AALCFGDSEYIGAHKN	60.4	523.2436	3	53.39	122000000					
				LCFGDSEYIGAHKN	57.66	461.8716	3	40.75	122000000					
	7ULR	Chain B Kappa-bungarotoxin	3FTx (κ-bungarotoxin)	MRTCLISPSSTPQTCPNGQDICFLKAQ	53.1	951.1222	3	61.02	87500000				0.0234	0.0015552
	AAS20530.1	phospholipase A2 isoform 1 partial [Bungarus caeruleus]	PLA2	ARFVCDCDRTAAICFAKA	78.26	498.2325	4	47.48	40100000				0.0107	0.0007127
				CDCDRTAAICFAKA	60.32	476.5518	3	40.33	40100000					
	ADF50022.1	non-conventional three finger toxin isoform 7 [Bungarus flaviceps]	3FTx	TLTCLICPEKYCQKV	70.27	856.9109	2	49.62	38600000				0.0103	0.0006861
				GYTLTCLICPEKYCQKV	42.49	659.6476	3	64.39	38600000					
	P25668.1	RecName: Full=Long neurotoxin 1; AltName: Full=Toxin A	3FTx (Type II NTx)	KRVDLGCAATCPTVRT	76.14	525.9288	3	39.07	22500000				0.0060	0.0003999
				RGKRVDLGCAATCPTVRT	72.05	440.9781	4	34.58	22500000					
				RVDLGCAATCPTVRT	72.6	710.3397	2	42.98	22500000					
	XP_026579406.1	BPTI/Kunitz domain-containing protein-like isoform X3 [Pseudonaja textilis]	Kunitz	KFTYGGCAGNANNFKK	93.7	760.8345	2	42.5	20900000				0.0056	0.0003715
				GLFEGSCSIFPNDSQRC	49.11	878.9021	2	63.05	20900000					
	CAA06887.1	neurotoxin-like protein [Bungarus multicinctus multicinctus]	3FTx (Type I NTx)	KKWEIGNPSGKE	71.37	558.2964	2	79.97	20500000				0.0055	0.0003644
				KKNEIIQCCAKD	58.97	632.3132	2	25.62	20500000					
				KRGCVATCPKPKK	45.87	425.2213	3	17.91	20500000					
				KTCPSGQLLCLKKWEIGNPSGKE	46.52	594.0562	4	66.49	20500000					
				RGCVATCPKPKKNEIIQCCAKD	55.95	591.2933	4	30.17	20500000					
				KYSQANESSKTCPSGQLLC	87.01	935.4362	2	44.07	20500000					
				KWEIGNPSGKE	56.58	494.2478	2	44.83	20500000					
	Q8QFW4.1	RecName: Full=Basic phospholipase A2 beta-bungarotoxin A1 chain; Short=Beta-BuTX A1 chain; Short=svPLA2; AltName: Full=Phosphatidylcholine 2-acylhydrolase; Flags: Precursor	b-BTx	YTNYGCYCGAGGSGTPIDALDRC	68.28	1102.969	2	58.37	10500000				0.0028	0.0001866
				PLNLINFMNMIRY	47.61	460.2495	3	92.93	10500000					
				RYTIPCEKTWGEYTNYGCYCGAGGSGTPIDALDRC	105.66	1244.874	3	73.77	10500000					
				CEKTWGEYTNYGCYCGAGGSGTPIDALDRC	41.95	1033.446	3	66.75	10500000					
				AANIPPHPLNLINFMNMIRY	58.77	702.3773	3	90.03	10500000					
				EYTNYGCYCGAGGSGTPIDALDRC	66.55	1184.503	2	60.13	10500000					
				KCSPKTQSYSYKLTRR	68.1	430.4687	4	34.43	10500000					
				KTQSYSYKLTRR	52.61	416.2189	3	35.98	10500000					
				KTWGEYTNYGCYCGAGGSGTPIDALDRC	115.46	1421.095	2	71.09	10500000					

				LNLINFMNMIRY	45.64	633.3276	2	85.48	10500000					
				QGCVATCPEFRS	45.84	598.7631	2	39.55	9080000					
	Q9W729.1	RecName: Full=Kappa-6-bungarotoxin; Short=K-6 bungarotoxin; Flags: Precursor	3FTx (κ-bungarotoxin)	RGAVIEQGCVATC	49.46	552.7714	2	40.25	9080000				0.0024	0.0001614
				TRTCHISTSSTPQTCPKGQDICFRK	58.96	913.08	3	41.93	9080000					
				AVIEQGCVATCPEFRS	73.41	833.3901	2	48.13	9080000					
	TIKFBU	beta-1 bungarotoxin chain B major component - many-banded krait	b-BTx	DGDHGNFKTETLCRC	48.7	512.2404	3	32.87	5280000				0.0014	0.0000938
	CAB53358.1	cardiotoxin-like protein [Bungarus multicinctus multicinctus]	3FTx (C-3FTx)	RCYKSYWVNENGKQEA	55.89	902.9018	2	44.12	3810000				0.0010	0.0000677
	P01383.1	RecName: Full=Long neurotoxin 1; AltName: Full=Neurotoxin 3.9.4	3FTx (Type II NTx)	KKWCADWCTSRG	47.31	457.1966	3	47.04	3610000				0.0010	0.0000642
	1G0Z	Chain B PHOSPHOLIPASE A2	PLA2	IGYGCYCGYGGSGTPVDELDRC	100.48	1091.944	2	57.45	3370000				0.0009	0.0000599
				NIPGCNPLIKT	52.95	506.2869	2	41.5	3370000					
	XP_026579252.1	kunitz/BPTI-like toxin isoform X1 [Pseudonaja textilis]	Kunitz	KAHPGTITICPVRC	66.07	441.2395	3	40.88	3160000				0.0008	0.0000562
	P82463.1	RecName: Full=Muscarinic toxin-like protein 2; Short=MTLP-2	3FTx (Type I NTx)	KSIFGVTTEDCPDGQNLCFKR	44.5	1094.494	2	69.31	1900000				0.0005	0.0000338
	CAJ77819.1	alpha-delta-bungarotoxin-1 partial [Bungarus candidus]	3FTx (Type II NTx)	RGKVIELGCAATC	47.4	559.7982	2	41.67	1810000				0.0005	0.0000322
	ADF50023.1	kappa-toxin partial [Bungarus flaviceps]	3FTx (κ-bungarotoxin)	RGPVIEQGCAATCPEFTSR	62.63	912.4055	2	48.71	1200000				0.0003	0.0000213
	AED89576.1	putative phospholipase A2 precursor [Micrurus altirostris]	PLA2	SGGGGTPVDDLDRC	63.05	579.7731	2	40.47	518000				0.0001	0.0000092
	C0HKZ8.2	RecName: Full=Actiflagelin	3FTx	MECYKCGVSGCHLKI	46.61	582.2477	3	29.58	253000				0.0001	0.0000045
	AED89576.1	putative phospholipase A2 precursor [Micrurus altirostris]		SGGGGTPVDDLDRC	63.05	579.7731	2	40.47	627000000				0.7207	0.0091132
	AAS20530.1	phospholipase A2 isoform 1 partial [Bungarus caeruleus]	PLA2	CDCDRTAAICFAKA	60.32	476.5518	3	40.33	169000000				0.1942	0.0024563
				ARFVCDCDRTAAICFAKA	78.26	498.2325	4	47.48	169000000					
				NSYCTTTDTFVKA	52.05	661.8008	2	43.31	36000000					
				KATDIKGNTVTVMVDVNLNNEVYKQ	57.75	846.4348	3	68.45	36000000					
				RHWNSYCTTTDTFVKA	118.89	880.3908	2	49.91	36000000					
	AAB25729.1	nerve growth factor precursor [Bungarus multicinctus]	NGF	WNSYCTTTDTFVKA	42.93	718.8217	2	43.85	36000000				0.0414	0.0005232
				RIDTACVCVISRKTENF	52.36	638.3134	3	56.13	36000000					
				RGIDSRHWNSYCTTTDTFVKA	53.49	572.7655	4	54.55	36000000					
				RGAVIEQGCVATC	49.46	552.7714	2	40.25	9370000					
	Q9W729.1	RecName: Full=Kappa-6-bungarotoxin; Short=K-6 bungarotoxin; Flags: Precursor	3FTx (κ-bungarotoxin)	QGCVATCPEFRS	45.84	598.7631	2	39.55	9370000				0.0108	0.0001362
				AVIEQGCVATCPEFRS	73.41	833.3901	2	48.13	9370000					
				TRTCHISTSSTPQTCPKGQDICFRK	58.96	913.08	3	41.93	9370000					
	XP_058034421.1	beta-nerve growth factor isoform X2 [Ahaetulla prasina]	NGF	RFIRIDTACVCIISRK	42.69	575.3046	3	61.95	4160000				0.0048	0.0000605
				RIDTACVCIISRK	59	654.3257	2	47.77	4160000					
				SFYTCPNSETCPDGKNICVKR	103.15	1145.5	2	42.38	3400000					
	1MR6	Chain A neurotoxin	3FTx (Type II NTx)	MQCKTCSFYTCPNSETCPDGKNICVKR	49.78	1067.783	3	50.04	3400000				0.0039	0.0000494
				FYTCPNSETCPDGKNICVKR	54.84	1071.968	2	36.35	3400000					
				CPNSETCPDGKNICVKR	43.85	573.6002	3	29.98	3400000					
	Q8AY42.2	RecName: Full=Kunitz-type serine protease inhibitor B; AltName: Full=Kunitz inhibitor B; Flags: Precursor	Kunitz	EFTYGGCGGNANNFKS	86.88	753.8243	2	42.8	2040000				0.0023	0.0000297
	BAP39981.1	nerve growth factor partial [Protobothrops flavoviridis]	NGF	KCRNPNPVPTGCRG	51.04	476.5593	3	25.43	1720000				0.0020	0.0000250
				RNPNPVPTGCRG	50.81	556.2693	2	27.71	1720000					
				RTAALCFGDSEYIGAHKNIDTKR	74.86	578.5314	4	53.11	1670000					
				YADYGCYCGAGGSGTPVDALDRC	71	1081.448	2	55.1	1670000					
				KTWGHYADYGCYCGAGGSGTPVDALDRC	123.31	936.059	3	63.11	1670000					
				RIVCDCDRTAALCFGDSEYIGAHKNI	70.09	693.8093	4	55.91	1670000					
				ADYGCYCGAGGSGTPVDALDRC	91.42	1045.929	2	55.35	1670000					
				WGHYADYGCYCGAGGSGTPVDALDRC	69.79	840.3488	3	53.65	1670000					
				CFGDSEYIGAHKN	51.49	408.5277	3	37.07	1670000					
	AAL87004.1	beta-bungarotoxin A2 chain precursor [Bungarus caeruleus]	b-BTx	RCCYVHDNCYGVAENKHKCNPKT	55.99	664.0339	4	30.13	1670000				0.0019	0.0000243
				AALCFGDSEYIGAHKN	60.4	523.2436	3	53.39	1670000					
				ALCFGDSEYIGAHKN	44.88	499.5666	3	51.25	1670000					
				LCFGDSEYIGAHKN	57.66	461.8716	3	40.75	1670000					
				RYTIPCDKTWGHYADYGCYCGAGGSGTPVD ALDRC	99.95	1228.527	3	67.23	1670000					
				HYADYGCYCGAGGSGTPVDALDRC	68.53	1162.982	2	58.07	1670000					
				IRYTIPCDKTWGHYADYGCYCGAGGSGTPVD ALDRC	52.95	960.6711	4	64.11	1670000					
				NGNTVGLAALGSLCSVKY	58.77	773.9169	2	68.86	1580000					
				KCPTLTNQCIALMGPNVKV	55.66	644.9832	3	55.08	1580000					
	ABN72537.1	metalloproteinase [Bungarus multicinctus]	SVMP	GVCNCLILPDDPNYGMVETGTKC	43.93	799.3678	3	67.9	1580000				0.0018	0.0000230
				RKCVKLQTVY	41.46	569.8188	2	37.52	1580000					
				DPNYGMVETGTKC	51.49	598.7869	2	37.39	1580000					
				RTCLISPSSTPQTCPQGQDICFLKA	100.66	880.0802	3	66.27	1440000					
	AAL30054.1	kappa 1a bungaratoxin [Bungarus candidus]	3FTx (κ-bungarotoxin)	TRTCLISPSSTPQTCPQGQDICFLKAQCDKF	61.53	849.9005	4	57.35	1440000				0.0017	0.0000209
				CLISPSSTPQTCPQGQDICFLKA	51.11	793.0555	3	62.66	1440000					
				PLNLINFMNMIRY	47.61	460.2495	3	92.93	1140000					
				CEKTWGEYTNYGCYCGAGGSGTPIDALDRC	41.95	1033.446	3	66.75	1140000					
F7a				RYTIPCEKTWGEYTNYGCYCGAGGSGTPIDAL DRC	105.66	1244.874	3	73.77	1140000		0.4675	0.0126		
	Q8QFW4.1	RecName: Full=Basic phospholipase A2 beta-bungarotoxin A1 chain; Short=Beta-BuTX A1 chain; Short=svPLA2; AltName: Full=Phosphatidylcholine 2-acylhydrolase; Flags: Precursor	b-BTx	AANIPPHPLNLINFMNMIRY	58.77	702.3773	3	90.03	1140000				0.0013	0.0000166
				KCSPKTQSYSYKLTRR	68.1	430.4687	4	34.43	1140000					
				KTQSYSYKLTRR	52.61	416.2189	3	35.98	1140000					
				KTWGEYTNYGCYCGAGGSGTPIDALDRC	115.46	1421.095	2	71.09	1140000					
				EYTNYGCYCGAGGSGTPIDALDRC	66.55	1184.503	2	60.13	1140000					
				LNLINFMNMIRY	45.64	633.3276	2	85.48	1140000					
				IDTWNVYNKCCTTNLCNT	43.96	1082.447	2	54.91	1100000					
				KWLNKISNERW	41.68	580.3146	2	36.65	1100000					
				RCGVSGCHLKITCSAEETFCYKW	88.69	836.3639	3	53.55	1100000					
	1F94	Chain A BUCANDIN	3FTx	KITCSAEETFCYKWLNKISNERW	70.93	883.7543	3	70	1100000				0.0013	0.0000160
				LKITCSAEETFCYKW	52.26	546.2498	3	42.98	1100000					
				CSAEETFCYKWLNKI	59.35	838.3919	2	59.22	1100000					
	1G0Z	Chain B PHOSPHOLIPASE A2	PLA2	NIPGCNPLIKT	52.95	506.2869	2	41.5	830000				0.0010	0.0000121
				IGYGCYCGYGGSGTPVDELDRC	100.48	1091.944	2	57.45	830000					
	XP_026522979.1	vascular endothelial growth factor C [Notechis scutatus]	VEGF	RCDSGFYYSEEVCRC	64.83	836.3251	2	52.61	510000				0.0006	0.0000074
				REVTVDVGKESGATTNIFFKPPCVSVYRC	47.16	750.8829	4	70.61	510000					
	XP_026579406.1	BPTI/Kunitz domain-containing protein-like isoform X3 [Pseudonaja textilis]	Kunitz	KFTYGGCAGNANNFKKH	41.93	550.256	3	36.56	443000				0.0005	0.0000064
				GLFEGSCSIFPNDSQRC	49.11	878.9021	2	63.05	443000					
	KAG8139853.1	hypothetical protein E2320 002630 [Naja naja]	PLA2	KTTAIVGNLPSSRD	53.13	608.3383	2	43.88	393000				0.0005	0.0000057
	BAC77655.1	phospholipase A2I precursor [Bungarus flaviceps]		RPTWHYAHYGCYCGKG	69.3	450.6913	4	43.61	361000				0.0004	0.0000052
	XP_013922516.1	PREDICTED: vascular endothelial growth factor A isoform X3 [Thamnophis sirtalis]	VEGF	KHLYKQDPLTCKC	79.51	468.2427	3	32.91	324000				0.0004	0.0000047
				KHFQSQHIHPMSFQQHSKC	44.08	421.6044	5	37.1	324000					
				TSTSPISTVTCPSGENLCYTKM	86.24	1101.506	2	54.17	181000					
				KMWCDAFCSSRG	57.87	660.2519	2	56.42	181000					
				KVIELGCVATCPQPKPYEEVTCCSTDKCNPHP KQ	52.88	755.7468	5	53.01	181000					
	CAM11302.1	alpha-delta-bungarotoxin-4 partial [Bungarus caeruleus]	3FTx (Type II NTx)	STSPISTVTCPSGENLCYTKM	63.33	1057.989	2	53.66	181000				0.0002	0.0000026
				RGKVIELGCVATCPQPKPYEEVTCCSTDKC	84.43	1076.167	3	54.32	181000					
				MWCDAFCSSRG	44.41	594.7312	2	46.96	181000					
				SPISTVTCPSGENLCYTKM	41.83	963.9484	2	49.07	181000					
				HTTSTSPISTVTCPSGENLCYTKM	41.81	1202.553	2	54.92	181000					
	Q9PVK7.1	RecName: Full=Zinc metalloproteinase-disintegrin-like cobrin; AltName: Full=Snake venom metalloproteinase; Short=SVMP; Flags: Precursor	SVMP	KTSAAVVQDYSKS	55.94	584.7958	2	33.62	66600				0.0001	0.0000010
				RDPSYGMVEPGTKC	50.17	648.7926	2	34.12	66600					
				KRVDLGCAATCPTVRT	76.14	525.9288	3	39.07	59700					
	P25668.1	RecName: Full=Long neurotoxin 1; AltName: Full=Toxin A	3FTx (Type II NTx)	RVDLGCAATCPTVRT	72.6	710.3397	2	42.98	59700				0.0001	0.0000009
				RGKRVDLGCAATCPTVRT	72.05	440.9781	4	34.58	59700					
	XP_026579252.1	kunitz/BPTI-like toxin isoform X1 [Pseudonaja textilis]	Kunitz	KAHPGTITICPVRC	66.07	441.2395	3	40.88	58400				0.0001	0.0000008
				KYSQANESSKTCPSGQLLC	87.01	935.4362	2	44.07	47500					
				RGCVATCPKPKKNEIIQCCAKD	55.95	591.2933	4	30.17	47500					
				KWEIGNPSGKE	56.58	494.2478	2	44.83	47500					
	CAA06887.1	neurotoxin-like protein [Bungarus multicinctus multicinctus]	3FTx (Type I NTx)	KKNEIIQCCAKDKC	58.02	502.9184	3	23.49	47500				0.0001	0.0000007
				KTCPSGQLLCLKKW	44.46	468.9153	3	49.32	47500					
				KRGCVATCPKPKK	45.87	425.2213	3	17.91	47500					
				KKWEIGNPSGKE	71.37	558.2964	2	79.97	47500					
				KTKTYSYTCTKPNLTC	57.97	839.4109	2	38.12	8840					
				AANVPPQHLNLYQFKN	41	556.9668	3	55.58	8840					
	P00606.2	RecName: Full=Acidic phospholipase A2; Short=svPLA2; AltName: Full=Phosphatidylcholine 2-acylhydrolase; Flags: Precursor	PLA2	GYVNYGCYCGAGGSGTPVDELDRC	57.61	1205.508	2	58.75	8840				0.0000	0.0000001
				LNLYQFKNMIVCAGTRPWIGYVNYGCYCGA GGSGTPVDELDRC	50.74	1169.533	4	89.73	8840					
				YVNYGCYCGAGGSGTPVDELDRC	79.29	749.6523	3	55.84	8840	0.0271				
				AVIEQGCVATCPEFRS	73.41	833.3901	2	48.13	659000000					
	Q9W729.1	RecName: Full=Kappa-6-bungarotoxin; Short=K-6 bungarotoxin; Flags: Precursor	3FTx (κ-bungarotoxin)	TRTCHISTSSTPQTCPKGQDICFRK	58.96	913.08	3	41.93	659000000				0.3368	0.0048520
				RGAVIEQGCVATCPEFRS	101.41	897.4203	2	51.79	659000000					
				QGCVATCPEFRS	45.84	598.7631	2	39.55	659000000					
	AAS20530.1	phospholipase A2 isoform 1 partial [Bungarus caeruleus]	PLA2	CDCDRTAAICFAKA	60.32	476.5518	3	40.33	591000000				0.3021	0.0043514
				ARFVCDCDRTAAICFAKA	78.26	498.2325	4	47.48	591000000					
				MQCKTCSFYTCPNSETCPDGKNICVKR	49.78	1067.783	3	50.04	194000000					
	1MR6	Chain A neurotoxin	3FTx (Type II NTx)	FYTCPNSETCPDGKNICVKR	54.84	1071.968	2	36.35	194000000				0.0992	0.0014284
				SFYTCPNSETCPDGKNICVKR	103.15	1145.5	2	42.38	194000000					
				CPNSETCPDGKNICVKR	43.85	573.6002	3	29.98	194000000					
				RCCYVHDNCYGVAENKH	118.36	944.8733	2	36.88	131000000					
				RTAALCFGDSEYIGAHK	44.82	806.3602	2	62.33	131000000					
				KTWGHYADYGCYCGAGGSGTPVDALDRC	123.31	936.059	3	63.11	131000000					
				ADYGCYCGAGGSGTPVDALDRC	91.42	1045.929	2	55.35	131000000					
				RIVCDCDRTAALCFGDSEYIGAHKN	105.04	886.7296	3	57.28	131000000					
				AALCFGDSEYIGAHKN	60.4	523.2436	3	53.39	131000000					
	AAL87004.1	beta-bungarotoxin A2 chain precursor [Bungarus caeruleus]	b-BTx	RYTIPCDKTWGHYADYGCYCGAGGSGTPVD ALDRC	99.95	1228.527	3	67.23	131000000				0.0670	0.0009645
				LCFGDSEYIGAHKN	57.66	461.8716	3	40.75	131000000					
				IRYTIPCDKTWGHYADYGCYCGAGGSGTPVD ALDRC	52.95	960.6711	4	64.11	131000000					
				HYADYGCYCGAGGSGTPVDALDRC	68.53	1162.982	2	58.07	131000000					
				CFGDSEYIGAHKN	51.49	408.5277	3	37.07	131000000					
				WGHYADYGCYCGAGGSGTPVDALDRC	69.79	840.3488	3	53.65	131000000					
				ALCFGDSEYIGAHKN	44.88	499.5666	3	51.25	131000000					
				YADYGCYCGAGGSGTPVDALDRC	71	1081.448	2	55.1	131000000					
	AED89576.1	putative phospholipase A2 precursor [Micrurus altirostris]	PLA2	SGGGGTPVDDLDRC	63.05	579.7731	2	40.47	99200000				0.0507	0.0007304
				EYTNYGCYCGAGGSGTPIDALDRC	66.55	1184.503	2	60.13	78200000					
				KTQSYSYKLTRR	52.61	416.2189	3	35.98	78200000					
				KTWGEYTNYGCYCGAGGSGTPIDALDRCCYV HDNCYGDAEKI	49.16	1153.961	4	69.18	78200000					
				LNLINFMNMIRY	45.64	633.3276	2	85.48	78200000					
	Q8QFW4.1	RecName: Full=Basic phospholipase A2 beta-bungarotoxin A1 chain; Short=Beta-BuTX A1 chain; Short=svPLA2; AltName: Full=Phosphatidylcholine 2-acylhydrolase; Flags: Precursor	b-BTx	PLNLINFMNMIRY	47.61	460.2495	3	92.93	78200000				0.0400	0.0005758
				KCSPKTQSYSYKLTRR	68.1	430.4687	4	34.43	78200000					
				CEKTWGEYTNYGCYCGAGGSGTPIDALDRC	41.95	1033.446	3	66.75	78200000					
				AANIPPHPLNLINFMNMIRY	58.77	702.3773	3	90.03	78200000					
				RYTIPCEKTWGEYTNYGCYCGAGGSGTPIDAL DRC	105.66	1244.874	3	73.77	78200000					
				RTCLISPSSTPQTCPQGQDICFLKAQCDKF	53.44	1080.832	3	61.37	42000000					
	AAL30054.1	kappa 1a bungaratoxin [Bungarus candidus]	3FTx (κ-bungarotoxin)	TRTCLISPSSTPQTCPQGQDICFLKA	101.81	932.1138	3	60.45	42000000				0.0215	0.0003092
				CLISPSSTPQTCPQGQDICFLKA	51.11	793.0555	3	62.66	42000000					

	Q8AY42.2	RecName: Full=Kunitz-type serine protease inhibitor B; AltName: Full=Kunitz inhibitor B; Flags: Precursor	Kunitz	EFTYGGCGGNANNFKS	86.88	753.8243	2	42.8	36800000				0.0188	0.0002709
				KWLNKISNERW	41.68	580.3146	2	36.65	28700000					
				IDTWNVYNKCCTTNLCNT	43.96	1082.447	2	54.91	28700000					
	1F94	Chain A BUCANDIN	3FTx	KITCSAEETFCYKWLNKISNERW	70.93	883.7543	3	70	28700000				0.0147	0.0002113
				CSAEETFCYKWLNKI	59.35	838.3919	2	59.22	28700000					
				LKITCSAEETFCYKW	52.26	546.2498	3	42.98	28700000					
				RCGVSGCHLKITCSAEETFCYKW	88.69	836.3639	3	53.55	28700000					
F7b	1G0Z	Chain B PHOSPHOLIPASE A2	PLA2	NIPGCNPLIKT	52.95	506.2869	2	41.5	27000000		0.5325	0.0144	0.0138	0.0001988
				IGYGCYCGYGGSGTPVDELDRC	100.48	1091.944	2	57.45	27000000					
				KKWEIGNPSGKE	71.37	558.2964	2	79.97	20000000					
				KKNEIIQCCAKD	58.97	632.3132	2	25.62	20000000					
				KRGCVATCPKPKK	45.87	425.2213	3	17.91	20000000					
	CAA06887.1	neurotoxin-like protein [Bungarus multicinctus multicinctus]	3FTx (Type I NTx)	KTCPSGQLLCLKKWEIGNPSGKE	46.52	594.0562	4	66.49	20000000				0.0102	0.0001473
				RGCVATCPKPKKNEIIQCCAKD	55.95	591.2933	4	30.17	20000000					
				KYSQANESSKTCPSGQLLC	87.01	935.4362	2	44.07	20000000					
				KWEIGNPSGKE	56.58	494.2478	2	44.83	20000000					
				WNSYCTTTDTFVKA	42.93	718.8217	2	43.85	15400000					
				RIDTACVCVISRKTENF	52.36	638.3134	3	56.13	15400000					
	AAB25729.1	nerve growth factor precursor [Bungarus multicinctus]	NGF	KATDIKGNTVTVMVDVNLNNEVYKQ	57.75	846.4348	3	68.45	15400000				0.0079	0.0001134
				NSYCTTTDTFVKA	52.05	661.8008	2	43.31	15400000					
				RHWNSYCTTTDTFVKALTMEGNRA	63.84	878.068	3	74.6	15400000					
				RGIDSRHWNSYCTTTDTFVKA	53.49	572.7655	4	54.55	15400000					
				RGKVIELGCVATCPQPKPYEEVTC	48.69	825.7491	3	56.58	10400000					
				HTTSTSPISTVTCPSGENLCYTKM	41.81	1202.553	2	54.92	10400000					
				STSPISTVTCPSGENLCYTKM	63.33	1057.989	2	53.66	10400000					
	CAM11302.1	alpha-delta-bungarotoxin-4 partial [Bungarus caeruleus]	3FTx (Type II NTx)	KVIELGCVATCPQPKPYEEVTC	43.75	1145.563	2	61.95	10400000				0.0053	0.0000766
				KMWCDAFCSSRG	57.87	660.2519	2	56.42	10400000					
				SPISTVTCPSGENLCYTKM	41.83	963.9484	2	49.07	10400000					
				TSTSPISTVTCPSGENLCYTKM	86.24	1101.506	2	54.17	10400000					
				MWCDAFCSSRG	44.41	594.7312	2	46.96	10400000					
	XP_026579406.1	BPTI/Kunitz domain-containing protein-like isoform X3 [Pseudonaja textilis]	Kunitz	GLFEGSCSIFPNDSQRC	49.11	878.9021	2	63.05	3980000				0.0020	0.0000293
				KFTYGGCAGNANNFKKH	41.93	550.256	3	36.56	3980000					
	Q75S49.1	RecName: Full=Kunitz-type serine protease inhibitor homolog beta-bungarotoxin B4 chain; Flags: Precursor		NGNGNHFKTETLCRC	47.23	511.9124	3	29.79	3530000				0.0018	0.0000260
	Q7ZT13.1	RecName: Full=Neurotoxin-like protein pMD18-NTL1/2/4/5; Flags: Precursor	3FTx (Type I NTx)	RGCSLTCPDIKY	52.39	575.7656	2	40.22	3210000				0.0016	0.0000236
				RIERGCSLTCPDIKY	43.69	516.9222	3	39.13	3210000					
	Q75S48.1	RecName: Full=Acidic phospholipase A2 1; Short=svPLA2; AltName: Full=PA2-I; AltName: Full=Phosphatidylcholine 2-acylhydrolase; Flags:	PLA2	RGTPVDELDRCCQTHDNCYAEAEEHPKC	57.32	783.5762	4	45.17	2210000				0.0011	0.0000163
				RGSLTYLDYMDYGCYCGTGSRG	60.09	1169.975	2	74.07	2210000					
				KRVDLGCAATCPTVRT	76.14	525.9288	3	39.07	1940000					
	P25668.1	RecName: Full=Long neurotoxin 1; AltName: Full=Toxin A	3FTx (Type II NTx)	RVDLGCAATCPTVRT	72.6	710.3397	2	42.98	1940000				0.0010	0.0000143
				RGKRVDLGCAATCPTVRT	72.05	440.9781	4	34.58	1940000					
	BAC77655.1	phospholipase A2I precursor [Bungarus flaviceps]	PLA2	RPTWHYAHYGCYCGKG	69.3	450.6913	4	43.61	1860000				0.0010	0.0000137
	XP_026579252.1	kunitz/BPTI-like toxin isoform X1 [Pseudonaja textilis]	Kunitz	KAHPGTITICPVRC	66.07	441.2395	3	40.88	1800000				0.0009	0.0000133
	ABU63166.1	phospholipase A2 precursor BF-42 [Bungarus fasciatus]	PLA2	YVSYGCYCGYGGSGTPVDELDRC	60.66	1156.483	2	60.24	1170000				0.0006	0.0000086
	ADF50022.1	non-conventional three finger toxin isoform 7 [Bungarus flaviceps]	3FTx	TLTCLICPEKY	58.09	567.2889	2	45.4	1110000				0.0006	0.0000082
				GYTLTCLICPEKYCQKV	42.49	659.6476	3	64.39	1110000					
	CAA72434.1	kappa3-bungarotoxin [Bungarus multicinctus]	3FTx (κ-bungarotoxin)	SSTPQTCPNGQDICFRK	48.47	890.8908	2	45.52	900000				0.0005	0.0000066
	Q3HXY1.1	RecName: Full=Venom nerve growth factor 3; Short=v-NGF-3; Short=vNGF-3; Flags: Precursor	NGF	RFIRIDTACVCVITKKT	52.21	456.7515	4	56.15	467000				0.0002	0.0000034
	AAO84769.1	phospholipase A2 partial [Bungarus candidus]	PLA2	RTAALCFAEAPYKRR	71.55	499.9215	3	50.04	460000				0.0002	0.0000034
	CAB42053.1	cardiotoxin-31 [Naja atra]	3FTx (C-3FTx)	KLVPLFYKTCPAGKN	43.41	498.611	3	54.37	339000				0.0002	0.0000025
	1CDT	Chain B CARDIOTOXIN VII4		KLIPIAYKTCPEGKN	42.68	497.2742	3	44.28	291000				0.0001	0.0000021
				GYVNYGCYCGAGGSGTPVDELDRC	57.61	1205.508	2	58.75	281000					
	P00606.2	RecName: Full=Acidic phospholipase A2; Short=svPLA2; AltName: Full=Phosphatidylcholine 2-acylhydrolase; Flags: Precursor	PLA2	LNLYQFKNMIVCAGTRPWIGYVNYGCYCGAGGSGTPVDELDRC	50.74	1169.533	4	89.73	281000				0.0001	0.0000021
				AANVPPQHLNLYQFKN	41	556.9668	3	55.58	281000					
				KTKTYSYTCTKPNLTC	57.97	839.4109	2	38.12	281000					
				YVNYGCYCGAGGSGTPVDELDRC	79.29	749.6523	3	55.84	281000					
	C0HKZ8.2	RecName: Full=Actiflagelin	3FTx	MECYKCGVSGCHLKI	46.61	582.2477	3	29.58	143000				0.0001	0.0000011
	1DPY	Chain A PHOSPHOLIPASE A2	PLA2	RIWTAYVAYGCYCGKGGSGTPVDELDRC	84.92	965.7628	3	76.75	56700000				0.6465	0.0134721
				NLIQFKNMIQCAGTRIWTAYV	41.7	815.4131	3	84.36	56700000					
				ADYGCYCGAGGSGTPVDALDRC	91.42	1045.929	2	55.35	9970000					
				HYADYGCYCGAGGSGTPVDALDRC	68.53	1162.982	2	58.07	9970000					
				RYTIPCDKTWGHYADYGCYCGAGGSGTPVDALDRC	99.95	1228.527	3	67.23	9970000					
				RTAALCFGDSEYIGAHKNI	69.1	618.621	3	53.97	9970000					
				RIVCDCDRTAALCFGDSEYIGAHKN	105.04	886.7296	3	57.28	9970000					
				ALCFGDSEYIGAHKN	44.88	499.5666	3	51.25	9970000					
	AAL87004.1	beta-bungarotoxin A2 chain precursor [Bungarus caeruleus]	b-BTx	KTWGHYADYGCYCGAGGSGTPVDALDRC	123.31	936.059	3	63.11	9970000				0.1137	0.0023689
				LCFGDSEYIGAHKN	57.66	461.8716	3	40.75	9970000					
				AALCFGDSEYIGAHKN	60.4	523.2436	3	53.39	9970000					
				IRYTIPCDKTWGHYADYGCYCGAGGSGTPVDALDRC	52.95	960.6711	4	64.11	9970000					
				RCCYVHDNCYGVAENKHKCNPKT	55.99	664.0339	4	30.13	9970000					
				WGHYADYGCYCGAGGSGTPVDALDRC	69.79	840.3488	3	53.65	9970000					
				CFGDSEYIGAHKN	51.49	408.5277	3	37.07	9970000					
				YADYGCYCGAGGSGTPVDALDRC	71	1081.448	2	55.1	9970000					
	Q7LZQ6.1	RecName: Full=Basic phospholipase A2 13; Short=svPLA2; AltName: Full=Cytotoxin XIII; AltName: Full=Phosphatidylcholine 2-acylhydrolase; AltName: Full=Phospholipase A2 XIII	PLA2	NLYQFKNMIECAGTRT	62.3	615.6313	3	65.95	5980000				0.0682	0.0014209
				RGAVIEQGCVATC	49.46	552.7714	2	40.25	5960000					
	Q9W729.1	RecName: Full=Kappa-6-bungarotoxin; Short=K-6 bungarotoxin; Flags: Precursor	3FTx (κ-bungarotoxin)	QGCVATCPEFRS	45.84	598.7631	2	39.55	5960000				0.0680	0.0014161
				AVIEQGCVATCPEFRS	73.41	833.3901	2	48.13	5960000					
				TRTCHISTSSTPQTCPKGQDICFRK	58.96	913.08	3	41.93	5960000					
	AED89576.1	putative phospholipase A2 precursor [Micrurus altirostris]	PLA2	SGGGGTPVDDLDRC	63.05	579.7731	2	40.47	2060000				0.0235	0.0004895
	LAB21297.1	hypothetical protein partial [Micrurus spixii]		LNLYQFKNMIQCTNTRS	60.18	644.3137	3	64.12	1770000				0.0202	0.0004206
	0412250A	toxin A beta1 bungaro		RTIICYGAAGGTCRI	67.19	700.3264	2	42.08	1360000				0.0155	0.0003231
				KWLNKISNERW	41.68	580.3146	2	36.65	1290000					
F8				CSAEETFCYKWLNKI	59.35	838.3919	2	59.22	1290000	0.0208		0.0208		
	1F94	Chain A BUCANDIN	3FTx	KITCSAEETFCYKWLNKI	72.94	683.9857	3	63.58	1290000				0.0147	0.0003065
				LKITCSAEETFCYKW	52.26	546.2498	3	42.98	1290000					
				RCGVSGCHLKITCSAEETFCYKW	88.69	836.3639	3	53.55	1290000					
				IDTWNVYNKCCTTNLCNT	43.96	1082.447	2	54.91	1290000					
				PLNLINFMNMIRY	43.01	697.8675	2	86.19	1070000					
				KTQSYSYKLTRR	52.61	416.2189	3	35.98	1070000					
				CEKTWGEYTNYGCYCGAGGSGTPIDALDRC	41.95	1033.446	3	66.75	1070000					
				YTNYGCYCGAGGSGTPIDALDRC	68.28	1102.969	2	58.37	1070000					
	Q8QFW4.1	RecName: Full=Basic phospholipase A2 beta-bungarotoxin A1 chain; Short=Beta-BuTX A1 chain; Short=svPLA2; AltName: Full=Phosphatidylcholine 2-acylhydrolase; Flags: Precursor	b-BTx	KTWGEYTNYGCYCGAGGSGTPIDALDRCCYVHDNCYGDAEKI	49.16	1153.961	4	69.18	1070000				0.0122	0.0002542
				EYTNYGCYCGAGGSGTPIDALDRC	66.55	1184.503	2	60.13	1070000					
				AANIPPHPLNLINFMNMIRY	58.77	702.3773	3	90.03	1070000					
				RYTIPCEKTWGEYTNYGCYCGAGGSGTPIDALDRC	105.66	1244.874	3	73.77	1070000					
				KCSPKTQSYSYKLTRR	68.1	430.4687	4	34.43	1070000					
				LNLINFMNMIRY	45.64	633.3276	2	85.48	1070000					
				KATDIKGNTVTVMVDVNLNNEVYKQ	57.75	846.4348	3	68.45	598000					
				RHWNSYCTTTDTFVKA	118.89	880.3908	2	49.91	598000					
	AAB25729.1	nerve growth factor precursor [Bungarus multicinctus]	NGF	RGIDSRHWNSYCTTTDTFVKA	53.49	572.7655	4	54.55	598000				0.0068	0.0001421
				NSYCTTTDTFVKA	52.05	661.8008	2	43.31	598000					
				RIDTACVCVISRKTENF	52.36	638.3134	3	56.13	598000					
				WNSYCTTTDTFVKA	42.93	718.8217	2	43.85	598000					
	P82464.1	RecName: Full=Muscarinic toxin-like protein 3; Short=MTLP-3	3FTx (Type I NTx)	TICYNHLTRT	42.32	393.1976	3	35.2	315000				0.0036	0.0000748
	Q9DF52.1	RecName: Full=Basic phospholipase A2 KPA2; Short=svPLA2; AltName: Full=Phosphatidylcholine 2-acylhydrolase; Flags: Precursor	PLA2	KNMIQCAGTRPWTAYVNYGCYCGKGGSGTPVDELDRC	45.08	993.1912	4	71.41	299000				0.0034	0.0000710
	XP_034282052.1	phospholipase A2 GL16-1-like [Pantherophis guttatus]		CGYGGSGTPVDQLDRC	42.21	711.334	2	47.12	278000				0.0032	0.0000661
				DYGCYCGYGGSGTPVDQLDRC	41.23	1062.929	2	57.85	278000					
	Q9PVK7.1	RecName: Full=Zinc metalloproteinase-disintegrin-like cobrin; AltName: Full=Snake venom metalloproteinase; Short=SVMP; Flags: Precursor	SVMP	KTSAAVVQDYSKSTRM	59.58	504.9269	3	33.48	59100				0.0007	0.0000140
				RDPSYGMVEPGTKC	50.17	648.7926	2	34.12	59100					
	AAS20530.1	phospholipase A2 isoform 1 partial [Bungarus caeruleus]	PLA2	ARFVCDCDRTAAICFAKA	78.26	498.2325	4	47.48	2630000000				0.3398	0.0537326
				CDCDRTAAICFAKA	60.32	476.5518	3	40.33	2630000000					
				YADYGCYCGAGGSGTPVDALDRC	71	1081.448	2	55.1	2370000000					
				WGHYADYGCYCGAGGSGTPVDALDRC	69.79	840.3488	3	53.65	2370000000					
				RIVCDCDRTAALCFGDSEYIGAHKNIDTKR	72.1	808.1238	4	55.94	2370000000					
				ADYGCYCGAGGSGTPVDALDRC	91.42	1045.929	2	55.35	2370000000					
				RCCYVHDNCYGVAENKH	118.36	944.8733	2	36.88	2370000000					
				AALCFGDSEYIGAHKN	60.4	523.2436	3	53.39	2370000000					
				RTAALCFGDSEYIGAHKNIDTKR	74.86	578.5314	4	53.11	2370000000					
	AAL87004.1	beta-bungarotoxin A2 chain precursor [Bungarus caeruleus]		KTWGHYADYGCYCGAGGSGTPVDALDRC	123.31	936.059	3	63.11	2370000000				0.3062	0.0484206
				ALCFGDSEYIGAHKN	44.88	499.5666	3	51.25	2370000000					
				HYADYGCYCGAGGSGTPVDALDRC	68.53	1162.982	2	58.07	2370000000					
				CFGDSEYIGAHKN	51.49	408.5277	3	37.07	2370000000					
				IRYTIPCDKTWGHYADYGCYCGAGGSGTPVDALDRC	52.95	960.6711	4	64.11	2370000000					
			b-BTx	RYTIPCDKTWGHYADYGCYCGAGGSGTPVDALDRC	99.95	1228.527	3	67.23	2370000000					
				LCFGDSEYIGAHKN	57.66	461.8716	3	40.75	2370000000					
				KTWGEYTNYGCYCGAGGSGTPIDALDRC	115.46	1421.095	2	71.09	2210000000					
				EYTNYGCYCGAGGSGTPIDALDRC	66.55	1184.503	2	60.13	2210000000					
				KTQSYSYKLTRR	52.61	416.2189	3	35.98	2210000000					
	Q8QFW4.1	RecName: Full=Basic phospholipase A2 beta-bungarotoxin A1 chain; Short=Beta-BuTX A1 chain; Short=svPLA2; AltName: Full=Phosphatidylcholine 2-acylhydrolase; Flags: Precursor		CEKTWGEYTNYGCYCGAGGSGTPIDALDRC	41.95	1033.446	3	66.75	2210000000				0.2856	0.0451517
				PLNLINFMNMIRY	47.61	460.2495	3	92.93	2210000000					
				RYTIPCEKTWGEYTNYGCYCGAGGSGTPIDALDRC	105.66	1244.874	3	73.77	2210000000					
				AANIPPHPLNLINFMNMIRY	58.77	702.3773	3	90.03	2210000000					
				KCSPKTQSYSYKLTRR	68.1	430.4687	4	34.43	2210000000					
				LNLINFMNMIRY	45.64	633.3276	2	85.48	2210000000					
				KVIELGCVATCPQPKP	75.48	786.3998	2	51.11	241000000					
				KMWCDAFCSSRG	57.87	660.2519	2	56.42	241000000					
				STSPISTVTCPSGENLCYTKM	63.33	1057.989	2	53.66	241000000					
	CAM11302.1	alpha-delta-bungarotoxin-4 partial [Bungarus caeruleus]	3FTx (Type II NTx)	HTTSTSPISTVTCPSGENLCYTKM	41.81	1202.553	2	54.92	241000000				0.0311	0.0049238
				TSTSPISTVTCPSGENLCYTKM	86.24	1101.506	2	54.17	241000000					
				MWCDAFCSSRG	44.41	594.7312	2	46.96	241000000					
				SPISTVTCPSGENLCYTKM	41.83	963.9484	2	49.07	241000000					
	1G0Z	Chain B PHOSPHOLIPASE A2	PLA2	NIPGCNPLIKT	52.95	506.2869	2	41.5	95600000				0.0124	0.0019532
				IGYGCYCGYGGSGTPVDELDRC	100.48	1091.944	2	57.45	95600000					
	AED89576.1	putative phospholipase A2 precursor [Micrurus altirostris]		SGGGGTPVDDLDRC	63.05	579.7731	2	40.47	44900000				0.0058	0.0009173
	UNW14928.1	phospholipase A2 protein 2-3 partial [Pseudonaja affinis]		RDVLDYANYGCYCGKGGSGTPVDELDRC	50.95	961.0906	3	76.66	43400000				0.0056	0.0008867
				SFYTCPNSETCPDGKN	82.69	838.3397	2	36.79	42900000					

F9a	1MR6	Chain A neurotoxin	3FTx (Type II NTx)	CPNSETCPDGKNICVKR	43.85	573.6002	3	29.98	42900000	0.1581	1.0000	0.1581	0.0055	0.0008765
				MQCKTCSFYTCPNSETCPDGKNICVKR	49.78	1067.783	3	50.04	42900000					
				FYTCPNSETCPDGKNICVKR	54.84	1071.968	2	36.35	42900000					
	Q8AY42.2	RecName: Full=Kunitz-type serine protease inhibitor B; AltName: Full=Kunitz inhibitor B; Flags: Precursor	Kunitz	EFTYGGCGGNANNFKS	86.88	753.8243	2	42.8	8760000				0.0011	0.0001790
	AAL55556.1	phospholipase A2 partial [Hydrophis hardwickii]	PLA2	RRSSLDYADYGCYCGAGGSGTPVDELDRC	54.51	981.0878	3	76.69	7810000				0.0010	0.0001596
	Q9W729.1	RecName: Full=Kappa-6-bungarotoxin; Short=K-6 bungarotoxin; Flags: Precursor	3FTx (κ-bungarotoxin)	TRTCHISTSSTPQTCPKGQDICFRK	58.96	913.08	3	41.93	6650000				0.0009	0.0001359
				QGCVATCPEFRS	45.84	598.7631	2	39.55	6650000					
				RGAVIEQGCVATCPEFRSNYRS	52.37	772.0299	3	50.84	6650000					
				AVIEQGCVATCPEFRS	73.41	833.3901	2	48.13	6650000					
	CAA06887.1	neurotoxin-like protein [Bungarus multicinctus multicinctus]	3FTx (Type I NTx)	KRGCVATCPKPKK	45.87	425.2213	3	17.91	6440000				0.0008	0.0001316
				KTCPSGQLLCLKKWEIGNPSGKE	46.52	594.0562	4	66.49	6440000					
				KYSQANESSKTCPSGQLLC	87.01	935.4362	2	44.07	6440000					
				KKNEIIQCCAKDKC	58.02	502.9184	3	23.49	6440000					
				KWEIGNPSGKE	56.58	494.2478	2	44.83	6440000					
				RGCVATCPKPKKNEIIQCCAKD	55.95	591.2933	4	30.17	6440000					
				KKWEIGNPSGKE	71.37	558.2964	2	79.97	6440000					
	1POB	Chain B PHOSPHOLIPASE A2	PLA2	RLAAICFAGAPYNDNDYNINLKA	55.21	1179.065	2	73.58	5850000				0.0008	0.0001195
	ABU63166.1	phospholipase A2 precursor BF-42 [Bungarus fasciatus]		YVSYGCYCGYGGSGTPVDELDRC	60.66	1156.483	2	60.24	5700000				0.0007	0.0001165
	AAO84769.1	phospholipase A2 partial [Bungarus candidus]		RTAALCFAEAPYKRR	71.55	499.9215	3	50.04	5090000				0.0007	0.0001040
	AAL30054.1	kappa 1a bungaratoxin [Bungarus candidus]	3FTx (κ-bungarotoxin)	RTCLISPSSTPQTCPQGQDICFLKAQCDKF	53.44	1080.832	3	61.37	3890000				0.0005	0.0000795
				TRTCLISPSSTPQTCPQGQDICFLKAQCDKF	61.53	849.9005	4	57.35	3890000					
				CLISPSSTPQTCPQGQDICFLKA	51.11	793.0555	3	62.66	3890000					
	BAC77655.1	phospholipase A2I precursor [Bungarus flaviceps]	PLA2	RPTWHYAHYGCYCGKG	69.3	450.6913	4	43.61	2590000				0.0003	0.0000529
	1F94	Chain A BUCANDIN	3FTx	CSAEETFCYKWLNKI	59.35	838.3919	2	59.22	2030000				0.0003	0.0000415
				KITCSAEETFCYKWLNKI	72.94	683.9857	3	63.58	2030000					
				LKITCSAEETFCYKW	52.26	546.2498	3	42.98	2030000					
				RCGVSGCHLKITCSAEETFCYKW	88.69	836.3639	3	53.55	2030000					
				IDTWNVYNKCCTTNLCNT	43.96	1082.447	2	54.91	2030000					
				KWLNKISNERW	41.68	580.3146	2	36.65	2030000					
	P00600.1	RecName: Full=Acidic phospholipase A2 DE-II; Short=svPLA2; AltName: Full=Phosphatidylcholine 2-acylhydrolase	PLA2	KISGCWPYIKT	48.44	562.284	2	62.17	1930000				0.0002	0.0000394
	CAD24463.1	beta-bungarotoxin A8 chain [Bungarus multicinctus]	b-BTx	RTAALCFGNSEYIGAHKN	45.29	580.2771	3	66.1	984000				0.0001	0.0000201
	P00602.1	RecName: Full=Acidic phospholipase A2 CM-I; Short=svPLA2; AltName: Full=Phosphatidylcholine 2-acylhydrolase	PLA2	PYIDANYNVNLKE	42.85	663.8372	2	50.28	788000				0.0001	0.0000161
	P00606.2	RecName: Full=Acidic phospholipase A2; Short=svPLA2; AltName: Full=Phosphatidylcholine 2-acylhydrolase; Flags: Precursor		AANVPPQHLNLYQFKN	41	556.9668	3	55.58	713000				0.0001	0.0000146
				YVNYGCYCGAGGSGTPVDELDRC	79.29	749.6523	3	55.84	713000					
				GYVNYGCYCGAGGSGTPVDELDRC	57.61	1205.508	2	58.75	713000					
				KTKTYSYTCTKPNLTC	57.97	839.4109	2	38.12	713000					
				LNLYQFKNMIVCAGTRPWIGYVNYGCYCGAGGSGTPVDELDRC	50.74	1169.533	4	89.73	713000					
	TIKFBU	beta-1 bungarotoxin chain B major component - many-banded krait	b-BTx	DGDHGNFKTETLCRC	48.7	512.2404	3	32.87	630000				0.0001	0.0000129
	CAJ77819.1	alpha-delta-bungarotoxin-1 partial [Bungarus candidus]	3FTx (Type II NTx)	RGKVIELGCAATC	47.4	559.7982	2	41.67	382000				0.0000	0.0000078
	AAB25729.1	nerve growth factor precursor [Bungarus multicinctus]	NGF	RIDTACVCVISRKTENF	52.36	638.3134	3	56.13	338000				0.0000	0.0000069
				NSYCTTTDTFVKA	52.05	661.8008	2	43.31	338000					
				WNSYCTTTDTFVKA	42.93	718.8217	2	43.85	338000					
				RHWNSYCTTTDTFVKALTMEGNRA	63.84	878.068	3	74.6	338000					
				KATDIKGNTVTVMVDVNLNNEVYKQYFFETKC	42.12	870.9398	4	82.05	338000					
				RGIDSRHWNSYCTTTDTFVKA	53.49	572.7655	4	54.55	338000					
	0412250A	toxin A beta1 bungaro	PLA2	RTIICYGAAGGTCRI	67.19	700.3264	2	42.08	218000				0.0000	0.0000045
	P01383.1	RecName: Full=Long neurotoxin 1; AltName: Full=Neurotoxin 3.9.4	3FTx (Type II NTx)	KKWCADWCTSRG	47.31	457.1966	3	47.04	158000				0.0000	0.0000032
	1ZM6	Chain A Phospholipase A2 isoform 3	PLA2	KTYSYECTQGTLTCKG	50.11	856.3701	2	41.67	92300				0.0000	0.0000019
	XP_013922516.1	PREDICTED: vascular endothelial growth factor A isoform X3 [Thamnophis sirtalis]	VEGF	KHFQSQHIHPMSFQQHSKC	44.08	421.6044	5	37.1	25200				0.0000	0.0000005
				KHLYKQDPLTCKC	79.51	468.2427	3	32.91	25200					
F10a	AAL87004.1	beta-bungarotoxin A2 chain precursor [Bungarus caeruleus]	b-BTx	RTAALCFGDSEYIGAHKNI	69.1	618.621	3	53.97	174000000		0.0693	0.0088	0.4303	0.0037661
				LCFGDSEYIGAHKN	57.66	461.8716	3	40.75	174000000					
				HYADYGCYCGAGGSGTPVDALDRC	68.53	1162.982	2	58.07	174000000					
				ADYGCYCGAGGSGTPVDALDRC	91.42	1045.929	2	55.35	174000000					
				RIVCDCDRTAALCFGDSEYIGAHKN	105.04	886.7296	3	57.28	174000000					
				WGHYADYGCYCGAGGSGTPVDALDRC	69.79	840.3488	3	53.65	174000000					
				RCCYVHDNCYGVAENKHKCNPKT	55.99	664.0339	4	30.13	174000000					
				IRYTIPCDKTWGHYADYGCYCGAGGSGTPVDALDRC	52.95	960.6711	4	64.11	174000000					
				RYTIPCDKTWGHYADYGCYCGAGGSGTPVDALDRC	99.95	1228.527	3	67.23	174000000					
				ALCFGDSEYIGAHKN	44.88	499.5666	3	51.25	174000000					
				CFGDSEYIGAHKN	51.49	408.5277	3	37.07	174000000					
				YADYGCYCGAGGSGTPVDALDRC	71	1081.448	2	55.1	174000000					
				KTWGHYADYGCYCGAGGSGTPVDALDRC	123.31	936.059	3	63.11	174000000					
				AALCFGDSEYIGAHKN	60.4	523.2436	3	53.39	174000000					
	Q8QFW4.1	RecName: Full=Basic phospholipase A2 beta-bungarotoxin A1 chain; Short=Beta-BuTX A1 chain; Short=svPLA2; AltName: Full=Phosphatidylcholine 2-acylhydrolase; Flags: Precursor		CEKTWGEYTNYGCYCGAGGSGTPIDALDRC	41.95	1033.446	3	66.75	116000000				0.2869	0.0025108
				PLNLINFMNMIRY	47.61	460.2495	3	92.93	116000000					
				KTWGEYTNYGCYCGAGGSGTPIDALDRCCYVHDNCYGDAEKI	49.16	1153.961	4	69.18	116000000					
				KTQSYSYKLTRR	52.61	416.2189	3	35.98	116000000					
				EYTNYGCYCGAGGSGTPIDALDRC	66.55	1184.503	2	60.13	116000000					
				AANIPPHPLNLINFMNMIRY	58.77	702.3773	3	90.03	116000000					
				KCSPKTQSYSYKLTRR	68.1	430.4687	4	34.43	116000000					
				LNLINFMNMIRY	45.64	633.3276	2	85.48	116000000					
				RYTIPCEKTWGEYTNYGCYCGAGGSGTPIDALDRC	105.66	1244.874	3	73.77	116000000					
	AAS20530.1	phospholipase A2 isoform 1 partial [Bungarus caeruleus]	PLA2	CDCDRTAAICFAKA	60.32	476.5518	3	40.33	53100000				0.1313	0.0011493
				ARFVCDCDRTAAICFAKA	78.26	498.2325	4	47.48	53100000					
	ACE73577.1	cysteine-rich seceretory protein Bc-CRPb [Bungarus candidus]	CRISP	KSGPPCGDCPSACVNGLCTNPCKYNDDYTNCKS	82.55	1194.466	3	51.22	40300000				0.0997	0.0008723
				KYNDDYTNCKS	52.62	596.7331	2	28.31	40300000					
	UNW14927.1	phospholipase A2 protein 2-4 partial [Pseudonaja affinis]	PLA2	RNVLDYADYGCYCGKGGSGTPVDELDRC	47.7	961.0912	3	76.75	10300000				0.0255	0.0002229
	AED89576.1	putative phospholipase A2 precursor [Micrurus altirostris]		SGGGGTPVDDLDRC	63.05	579.7731	2	40.47	3440000				0.0085	0.0000745
	Q3SB03.1	RecName: Full=Cysteine-rich venom protein pseudechetoxin-like; Short=CRVP; Flags: Precursor	CRISP	KSGPPCADCPSACVNGLCTNPCKH	98.41	807.9952	3	47.34	3060000				0.0076	0.0000662
				RGSIATPYKSGPPCADCPSACVNGLCTNPCKH	62.79	1080.474	3	52.03	3060000					
	1G0Z	Chain B PHOSPHOLIPASE A2	PLA2	IGYGCYCGYGGSGTPVDELDRC	100.48	1091.944	2	57.45	1810000				0.0045	0.0000392
				NIPGCNPLIKT	52.95	506.2869	2	41.5	1810000					
	Q9DF52.1	RecName: Full=Basic phospholipase A2 KPA2; Short=svPLA2; AltName: Full=Phosphatidylcholine 2-acylhydrolase; Flags: Precursor		KNMIQCAGTRPWTAYVNYGCYCGKGGSGTPVDELDRC	45.08	993.1912	4	71.41	1310000				0.0032	0.0000284
	XP_013922516.1	PREDICTED: vascular endothelial growth factor A isoform X3 [Thamnophis sirtalis]	VEGF	KHFQSQHIHPMSFQQHSKC	44.08	421.6044	5	37.1	434000				0.0011	0.0000094
				KHLYKQDPLTCKC	79.51	468.2427	3	32.91	434000					
	AAB20783.1	notechis 11'2=non-toxic phospholipase A2 [Notechis scutatus=Australian tiger snakes ssp. scutatus venom Peptide 118 aa]	PLA2	NLYQFGNMIQCANHGRRPTLAYADYGCYCGAGGSGTPVDELDRC	51.08	965.8414	5	78.59	408000				0.0010	0.0000088
	P00606.2	RecName: Full=Acidic phospholipase A2; Short=svPLA2; AltName: Full=Phosphatidylcholine 2-acylhydrolase; Flags: Precursor		KTKTYSYTCTKPNLTC	57.97	839.4109	2	38.12	208000				0.0005	0.0000045
				YVNYGCYCGAGGSGTPVDELDRC	79.29	749.6523	3	55.84	208000					
				AANVPPQHLNLYQFKN	41	556.9668	3	55.58	208000					
				LNLYQFKNMIVCAGTRPWIGYVNYGCYCGAGGSGTPVDELDRC	50.74	1169.533	4	89.73	208000					
				GYVNYGCYCGAGGSGTPVDELDRC	57.61	1205.508	2	58.75	208000					
	Q9W729.1	RecName: Full=Kappa-6-bungarotoxin; Short=K-6 bungarotoxin; Flags: Precursor	3FTx (κ-bungarotoxin)	AVIEQGCVATCPEFRS	73.41	833.3901	2	48.13	17200				0.0000	0.0000004
				TRTCHISTSSTPQTCPKGQDICFRK	58.96	913.08	3	41.93	17200					
				RGAVIEQGCVATCPEFRS	101.41	897.4203	2	51.79	17200					
				QGCVATCPEFRS	45.84	598.7631	2	39.55	17200					
	AAS20530.1	phospholipase A2 isoform 1 partial [Bungarus caeruleus]	PLA2	ARFVCDCDRTAAICFAKA	78.26	498.2325	4	47.48	6680000000				0.8522	0.1001784
				CDCDRTAAICFAKA	60.32	476.5518	3	40.33	6680000000					
	1G0Z	Chain B PHOSPHOLIPASE A2		IGYGCYCGYGGSGTPVDELDRC	100.48	1091.944	2	57.45	411000000				0.0524	0.0061637
				NIPGCNPLIKT	52.95	506.2869	2	41.5	411000000					
	Q8QFW4.1	RecName: Full=Basic phospholipase A2 beta-bungarotoxin A1 chain; Short=Beta-BuTX A1 chain; Short=svPLA2; AltName: Full=Phosphatidylcholine 2-acylhydrolase; Flags: Precursor	b-BTx	RYTIPCEKTWGEYTNYGCYCGAGGSGTPIDALDRC	105.66	1244.874	3	73.77	241000000				0.0307	0.0036142
				KTWGEYTNYGCYCGAGGSGTPIDALDRC	115.46	1421.095	2	71.09	241000000					
				AANIPPHPLNLINFMNMIRY	58.77	702.3773	3	90.03	241000000					
				KCSPKTQSYSYKLTRR	68.1	430.4687	4	34.43	241000000					
				PLNLINFMNMIRY	47.61	460.2495	3	92.93	241000000					
				EYTNYGCYCGAGGSGTPIDALDRC	66.55	1184.503	2	60.13	241000000	0.1263				
				CEKTWGEYTNYGCYCGAGGSGTPIDALDRC	41.95	1033.446	3	66.75	241000000					
				LNLINFMNMIRY	45.64	633.3276	2	85.48	241000000					
				KTQSYSYKLTRR	52.61	416.2189	3	35.98	241000000					
	AAL87004.1	beta-bungarotoxin A2 chain precursor [Bungarus caeruleus]		RIVCDCDRTAALCFGDSEYIGAHKNIDTKR	72.1	808.1238	4	55.94	224000000				0.0286	0.0033593
				YADYGCYCGAGGSGTPVDALDRC	71	1081.448	2	55.1	224000000					
				WGHYADYGCYCGAGGSGTPVDALDRC	69.79	840.3488	3	53.65	224000000					
				RCCYVHDNCYGVAENKH	118.36	944.8733	2	36.88	224000000					
				RTAALCFGDSEYIGAHKNIDTKR	74.86	578.5314	4	53.11	224000000					
				CFGDSEYIGAHKN	51.49	408.5277	3	37.07	224000000					
				IRYTIPCDKTWGHYADYGCYCGAGGSGTPVDALDRC	52.95	960.6711	4	64.11	224000000					
				HYADYGCYCGAGGSGTPVDALDRC	68.53	1162.982	2	58.07	224000000					
				LCFGDSEYIGAHKN	57.66	461.8716	3	40.75	224000000					
				ADYGCYCGAGGSGTPVDALDRC	91.42	1045.929	2	55.35	224000000					
				KTWGHYADYGCYCGAGGSGTPVDALDRC	123.31	936.059	3	63.11	224000000					
				RYTIPCDKTWGHYADYGCYCGAGGSGTPVDALDRC	99.95	1228.527	3	67.23	224000000					
				ALCFGDSEYIGAHKN	44.88	499.5666	3	51.25	224000000					
				AALCFGDSEYIGAHKN	60.4	523.2436	3	53.39	224000000					
	UNW14928.1	phospholipase A2 protein 2-3 partial [Pseudonaja affinis]	PLA2	RDVLDYANYGCYCGKGGSGTPVDELDRC	50.95	961.0906	3	76.66	136000000				0.0173	0.0020396
	1MR6	Chain A neurotoxin	3FTx (Type II NTx)	CPNSETCPDGKNICVKR	43.85	573.6002	3	29.98	48600000				0.0062	0.0007288
				SFYTCPNSETCPDGKNICVKRS	43.09	612.2789	4	39.69	48600000					
				MQCKTCSFYTCPNSETCPDGKNICVKR	49.78	1067.783	3	50.04	48600000					
				FYTCPNSETCPDGKNICVKR	54.84	1071.968	2	36.35	48600000					
F10b	Q9DF52.1	RecName: Full=Basic phospholipase A2 KPA2; Short=svPLA2; AltName: Full=Phosphatidylcholine 2-acylhydrolase; Flags: Precursor	PLA2	KNMIQCAGTRPW	41.67	574.2716	2	36.79	22000000		0.9307	0.1176	0.0028	0.0003299
	CAA06887.1	neurotoxin-like protein [Bungarus multicinctus multicinctus]	3FTx (Type I NTx)	RGCVATCPKPKK	44.97	559.2778	2	18.98	14100000				0.0018	0.0002115
				KRGCVATCPKPKK	45.87	425.2213	3	17.91	14100000					
				KWEIGNPSGKE	56.58	494.2478	2	44.83	14100000					
				KYSQANESSKTCPSGQLLC	87.01	935.4362	2	44.07	14100000					
				KTCPSGQLLCLKKW	44.46	468.9153	3	49.32	14100000					
				KKNEIIQCCAKDKC	58.02	502.9184	3	23.49	14100000					
				KKWEIGNPSGKE	71.37	558.2964	2	79.97	14100000					
	1DPY	Chain A PHOSPHOLIPASE A2	PLA2	NLIQFKNMIQCAGTRIWTAYV	41.7	815.4131	3	84.36	11200000				0.0014	0.0001680
				RIWTAYVAYGCYCGKGGSGTPVDELDRC	84.92	965.7628	3	76.75	11200000					
				KVIELGCVATCPQPKPYEEVTCCSTDKC	78.78	1014.463	3	58.23	10600000					
				HTTSTSPISTVTCPSGENLCYTKM	41.81	1202.553	2	54.92	10600000					

				RGKVIELGCVATCPQPKPYEEVTC	48.69	825.7491	3	56.58	10600000					
	CAM11302.1	alpha-delta-bungarotoxin-4 partial [Bungarus caeruleus]	3FTx (Type II NTx)	KMWCDAFCSSRG	50.69	668.2499	2	51.87	10600000				0.0014	0.0001590
				MWCDAFCSSRG	44.41	594.7312	2	46.96	10600000					
				SPISTVTCPSGENLCYTKM	41.83	963.9484	2	49.07	10600000					
				TSTSPISTVTCPSGENLCYTKM	86.24	1101.506	2	54.17	10600000					
				STSPISTVTCPSGENLCYTKM	63.33	1057.989	2	53.66	10600000					
	Q8AY42.2	RecName: Full=Kunitz-type serine protease inhibitor B; AltName: Full=Kunitz inhibitor B; Flags: Precursor	Kunitz	EFTYGGCGGNANNFKS	86.88	753.8243	2	42.8	9950000				0.0013	0.0001492
				GYVNYGCYCGAGGSGTPVDELDRC	57.61	1205.508	2	58.75	7700000					
				KTKTYSYTCTKPNLTC	57.97	839.4109	2	38.12	7700000					
	P00606.2	RecName: Full=Acidic phospholipase A2; Short=svPLA2; AltName: Full=Phosphatidylcholine 2-acylhydrolase; Flags: Precursor	PLA2	AANVPPQHLNLYQFKN	41	556.9668	3	55.58	7700000				0.0010	0.0001155
				YVNYGCYCGAGGSGTPVDELDRC	79.29	749.6523	3	55.84	7700000					
				LNLYQFKNMIVCAGTRPWIGYVNYGCYCGAGGSGTPVDELDRC	50.74	1169.533	4	89.73	7700000					
				CLISPSSTPQTCPQGQDICFLKA	51.11	793.0555	3	62.66	7410000					
	AAL30054.1	kappa 1a bungaratoxin [Bungarus candidus]	3FTx (κ-bungarotoxin)	TRTCLISPSSTPQTCPQGQDICFLKAQCDKF	61.53	849.9005	4	57.35	7410000				0.0009	0.0001111
				RTCLISPSSTPQTCPQGQDICFLKA	100.66	880.0802	3	66.27	7410000					
	ABW24180.1	PLA-18 precursor [Austrelaps superbus]	PLA2	SGGTGTPVDELDRC	43.22	608.7935	2	43.43	6300000				0.0008	0.0000945
				DYGCYCGSGGTGTPVDELDRC	46.48	1032.417	2	62.52	6300000					
	AAO84769.1	phospholipase A2 partial [Bungarus candidus]		RTAALCFAEAPYKR	79.51	671.3285	2	56.61	3390000				0.0004	0.0000508
				RGAVIEQGCVATCPEFRS	101.41	897.4203	2	51.79	3120000					
	Q9W729.1	RecName: Full=Kappa-6-bungarotoxin; Short=K-6 bungarotoxin; Flags: Precursor	3FTx (κ-bungarotoxin)	TRTCHISTSSTPQTCPKGQDICFRK	58.96	913.08	3	41.93	3120000				0.0004	0.0000468
				QGCVATCPEFRS	45.84	598.7631	2	39.55	3120000					
				AVIEQGCVATCPEFRS	73.41	833.3901	2	48.13	3120000					
	TIKFBU	beta-1 bungarotoxin chain B major component - many-banded krait	b-BTx	DGDHGNFKTETLCRC	48.7	512.2404	3	32.87	2180000				0.0003	0.0000327
	C0HLB8.1	RecName: Full=Phospholipase A2; Short=MIPLA2-1; Short=svPLA2; AltName: Full=Phosphatidylcholine 2-acylhydrolase		DYGCYCGYGGSGTPVDELDRC	62.53	1143.45	2	58.45	155000				0.0000	0.0000023
	P20255.1	RecName: Full=Basic phospholipase A2 PA-12A; Short=svPLA2; AltName: Full=Phosphatidylcholine 2-acylhydrolase	PLA2	NLIQFGNMIQCANKGSRPSLNYADYGCYCGWGGSGTPVDELDRC	43.47	1211.045	4	78.19	113000				0.0000	0.0000017
	JAA74725.1	PLA-2-Den-2 [Denisonia devisi]		SRGALDYADYGCYCGKGGSGTPVDELDRC	44.99	984.7706	3	76.45	24700				0.0000	0.0000004
	ACE73577.1	cysteine-rich seceretory protein Bc-CRPb [Bungarus candidus]	CRISP	KSGPPCGDCPSACVNGLCTNPCKYNDDYTNCKSLAKQ	54.24	995.9156	4	53.01	47800000				0.2313	0.0229463
				KYNDDYTNCKS	52.62	596.7331	2	28.31	47800000					
	P81993.1	RecName: Full=Cysteine-rich venom protein bucarin		KRFADRCTFAHSPPHLRT	68.18	492.745	4	80.99	47400000				0.2293	0.0227543
	1G0Z	Chain B PHOSPHOLIPASE A2	PLA2	NIPGCNPLIKT	52.95	506.2869	2	41.5	42500000				0.2056	0.0204021
				IGYGCYCGYGGSGTPVDELDRC	100.48	1091.944	2	57.45	42500000					
	Q3SB03.1	RecName: Full=Cysteine-rich venom protein pseudechetoxin-like; Short=CRVP; Flags: Precursor	CRISP	KSGPPCADCPSACVNGLC	49.43	831.3409	2	52.23	28100000				0.1359	0.0134894
				RGSIATPYKSGPPCADCPSACVNGLCTNPCKH	62.79	1080.474	3	52.03	28100000					
	AAS20530.1	phospholipase A2 isoform 1 partial [Bungarus caeruleus]	PLA2	ARFVCDCDRTAAICFAKA	78.26	498.2325	4	47.48	17200000				0.0832	0.0082568
				CDCDRTAAICFAKA	60.32	476.5518	3	40.33	17200000					
				CFGDSEYIGAHKN	51.49	408.5277	3	37.07	13800000					
				RCCYVHDNCYGVAENKHKC	96.11	718.6368	3	30.97	13800000					
				ADYGCYCGAGGSGTPVDALDRC	91.42	1045.929	2	55.35	13800000					
				RYTIPCDKTWGHYADYGCYCGAGGSGTPVDALDRC	99.95	1228.527	3	67.23	13800000					
				RTAALCFGDSEYIGAHKN	115.33	870.407	2	55.64	13800000					
				AALCFGDSEYIGAHKN	60.4	523.2436	3	53.39	13800000					
	AAL87004.1	beta-bungarotoxin A2 chain precursor [Bungarus caeruleus]		LCFGDSEYIGAHKN	57.66	461.8716	3	40.75	13800000				0.0668	0.0066247
				RIVCDCDRTAALCFGDSEYIGAHKNI	70.09	693.8093	4	55.91	13800000					
				YADYGCYCGAGGSGTPVDALDRC	71	1081.448	2	55.1	13800000					
				ALCFGDSEYIGAHKN	44.88	499.5666	3	51.25	13800000					
F11a				IRYTIPCDKTWGHYADYGCYCGAGGSGTPVDALDRC	52.95	960.6711	4	64.11	13800000		0.2908	0.0992		
			b-BTx	KTWGHYADYGCYCGAGGSGTPVDALDRC	123.31	936.059	3	63.11	13800000					
				HYADYGCYCGAGGSGTPVDALDRC	68.53	1162.982	2	58.07	13800000					
				WGHYADYGCYCGAGGSGTPVDALDRC	69.79	840.3488	3	53.65	13800000					
				KTWGEYTNYGCYCGAGGSGTPIDALDRC	115.46	1421.095	2	71.09	6560000					
				KCSPKTQSYSYKLTRR	68.1	430.4687	4	34.43	6560000					
				PLNLINFMNMIRY	43.01	697.8675	2	86.19	6560000					
				LNLINFMNMIRY	45.64	633.3276	2	85.48	6560000					
				AANIPPHPLNLINFMNMIRY	58.77	702.3773	3	90.03	6560000					
	Q8QFW4.1	RecName: Full=Basic phospholipase A2 beta-bungarotoxin A1 chain; Short=Beta-BuTX A1 chain; Short=svPLA2; AltName: Full=Phosphatidylcholine 2-acylhydrolase; Flags: Precursor		EYTNYGCYCGAGGSGTPIDALDRC	66.55	1184.503	2	60.13	6560000				0.0317	0.0031491
				CEKTWGEYTNYGCYCGAGGSGTPIDALDRC	41.95	1033.446	3	66.75	6560000					
				RYTIPCEKTWGEYTNYGCYCGAGGSGTPIDALDRC	105.66	1244.874	3	73.77	6560000					
				KTQSYSYKLTRR	52.61	416.2189	3	35.98	6560000					
				YTNYGCYCGAGGSGTPIDALDRC	68.28	1102.969	2	58.37	6560000					
	XP_015678372.1	serotriflin [Protobothrops mucrosquamatus]	CRISP	KSGPPCEDCPSACVNGLCTNPCKH	50.54	827.3306	3	39.81	2570000				0.0124	0.0012337
				RFLTEHNPECIINPPLRT	74.47	650.6686	3	60.7	322000					
	AAZ39880.1	hemorrhagic metalloproteinase russelysin [Daboia russelii]	SVMP	RVAVTMAHEIGHNLGLTHDG	41.87	638.9882	3	60.1	322000				0.0016	0.0001546
				RLGVYYAYCRK	55.43	582.779	2	51.02	322000					
	AAO84769.1	phospholipase A2 partial [Bungarus candidus]		RTAALCFAEAPYKRR	71.55	499.9215	3	50.04	319000				0.0015	0.0001531
	JAA74725.1	PLA-2-Den-2 [Denisonia devisi]		SRGALDYADYGCYCGKGGSGTPVDELDRC	44.99	984.7706	3	76.45	101000				0.0005	0.0000485
	1G0Z	Chain B PHOSPHOLIPASE A2		NIPGCNPLIKT	52.95	506.2869	2	41.5	1750000000				0.4418	0.1068847
			PLA2	IGYGCYCGYGGSGTPVDELDRC	100.48	1091.944	2	57.45	1750000000					
	AAS20530.1	phospholipase A2 isoform 1 partial [Bungarus caeruleus]		ARFVCDCDRTAAICFAKA	78.26	498.2325	4	47.48	1740000000				0.4392	0.1062739
				CDCDRTAAICFAKA	60.32	476.5518	3	40.33	1740000000					
	AAO84769.1	phospholipase A2 partial [Bungarus candidus]		RTAALCFAEAPYKR	79.51	671.3285	2	56.61	144000000				0.0364	0.0087951
				ALCFGDSEYIGAHKN	44.88	499.5666	3	51.25	88000000					
				RTAALCFGDSEYIGAHKNI	69.1	618.621	3	53.97	88000000					
				HYADYGCYCGAGGSGTPVDALDRC	68.53	1162.982	2	58.07	88000000					
				KTWGHYADYGCYCGAGGSGTPVDALDRC	123.31	936.059	3	63.11	88000000					
				IRYTIPCDKTWGHYADYGCYCGAGGSGTPVDALDRC	52.95	960.6711	4	64.11	88000000					
				YADYGCYCGAGGSGTPVDALDRC	71	1081.448	2	55.1	88000000					
	AAL87004.1	beta-bungarotoxin A2 chain precursor [Bungarus caeruleus]	b-BTx	RIVCDCDRTAALCFGDSEYIGAHKNIDTKR	72.1	808.1238	4	55.94	88000000				0.0222	0.0053748
				ADYGCYCGAGGSGTPVDALDRC	91.42	1045.929	2	55.35	88000000					
				WGHYADYGCYCGAGGSGTPVDALDRC	69.79	840.3488	3	53.65	88000000					
				LCFGDSEYIGAHKN	57.66	461.8716	3	40.75	88000000					
				RCCYVHDNCYGVAENKHKCNPKT	55.99	664.0339	4	30.13	88000000					
				CFGDSEYIGAHKN	51.49	408.5277	3	37.07	88000000					
				RYTIPCDKTWGHYADYGCYCGAGGSGTPVDALDRC	99.95	1228.527	3	67.23	88000000	0.3412				
				AALCFGDSEYIGAHKN	60.4	523.2436	3	53.39	88000000					
	0412250A	toxin A beta1 bungaro	PLA2	RTIICYGAAGGTCRI	67.19	700.3264	2	42.08	57100000				0.0144	0.0034875
	QSI84030.1	phospholipase A2 IB [Calliophis bivirgatus]		SWRSFVDYGCYCGKGGSGTPVDELDRC	50.71	961.0908	3	76.53	45800000				0.0116	0.0027973
				PLNLINFMNMIRY	47.61	460.2495	3	92.93	44800000					
				RYTIPCEKTWGEYTNYGCYCGAGGSGTPIDALDRC	105.66	1244.874	3	73.77	44800000					
				KTWGEYTNYGCYCGAGGSGTPIDALDRC	115.46	1421.095	2	71.09	44800000					
	Q8QFW4.1	RecName: Full=Basic phospholipase A2 beta-bungarotoxin A1 chain; Short=Beta-BuTX A1 chain; Short=svPLA2; AltName: Full=Phosphatidylcholine 2-acylhydrolase; Flags: Precursor	b-BTx	LNLINFMNMIRY	45.64	633.3276	2	85.48	44800000				0.0113	0.0027362
				CEKTWGEYTNYGCYCGAGGSGTPIDALDRC	41.95	1033.446	3	66.75	44800000					
				AANIPPHPLNLINFMNMIRY	58.77	702.3773	3	90.03	44800000					
				KTQSYSYKLTRR	52.61	416.2189	3	35.98	44800000					
				KCSPKTQSYSYKLTRR	68.1	430.4687	4	34.43	44800000					
				EYTNYGCYCGAGGSGTPIDALDRC	66.55	1184.503	2	60.13	44800000					
	1DPY	Chain A PHOSPHOLIPASE A2	PLA2	RIWTAYVAYGCYCGKGGSGTPVDELDRC	84.92	965.7628	3	76.75	22100000				0.0056	0.0013498
				NLIQFKNMIQCAGTRIWTAYV	41.7	815.4131	3	84.36	22100000					
				CPNSETCPDGKNICVKR	43.85	573.6002	3	29.98	16800000					
	1MR6	Chain A neurotoxin	3FTx (Type II NTx)	SFYTCPNSETCPDGKNICVKRS	43.09	612.2789	4	39.69	16800000				0.0042	0.0010261
				MQCKTCSFYTCPNSETCPDGKNICVKR	49.78	1067.783	3	50.04	16800000					
				FYTCPNSETCPDGKNICVKR	54.84	1071.968	2	36.35	16800000					
				LNLYQFKNMIVCAGTRPWIGYVNYGCYCGAGGSGTPVDELDRC	50.74	1169.533	4	89.73	13100000					
F11b	P00606.2	RecName: Full=Acidic phospholipase A2; Short=svPLA2; AltName: Full=Phosphatidylcholine 2-acylhydrolase; Flags: Precursor	PLA2	GYVNYGCYCGAGGSGTPVDELDRC	57.61	1205.508	2	58.75	13100000		0.7092	0.2419	0.0033	0.0008001
				YVNYGCYCGAGGSGTPVDELDRC	79.29	749.6523	3	55.84	13100000					
				KTKTYSYTCTKPNLTC	57.97	839.4109	2	38.12	13100000					
				AANVPPQHLNLYQFKN	41	556.9668	3	55.58	13100000					
	P81993.1	RecName: Full=Cysteine-rich venom protein bucarin	CRISP	KRFADRCTFAHSPPHLRT	68.18	492.745	4	80.99	8300000				0.0021	0.0005069
				CLISPSSTPQTCPQGQDICFLKA	51.11	793.0555	3	62.66	7030000					
	AAL30054.1	kappa 1a bungaratoxin [Bungarus candidus]	3FTx (κ-bungarotoxin)	TRTCLISPSSTPQTCPQGQDICFLKAQCDKF	61.53	849.9005	4	57.35	7030000				0.0018	0.0004294
				RTCLISPSSTPQTCPQGQDICFLKA	100.66	880.0802	3	66.27	7030000					
	Q8AY42.2	RecName: Full=Kunitz-type serine protease inhibitor B; AltName: Full=Kunitz inhibitor B; Flags: Precursor	Kunitz	EFTYGGCGGNANNFKS	86.88	753.8243	2	42.8	6900000				0.0017	0.0004214
				RGCVATCPKPKK	44.97	559.2778	2	18.98	6790000					
				KWEIGNPSGKE	56.58	494.2478	2	44.83	6790000					
				KRGCVATCPKPKK	45.87	425.2213	3	17.91	6790000					
	CAA06887.1	neurotoxin-like protein [Bungarus multicinctus multicinctus]	3FTx (Type I NTx)	KYSQANESSKTCPSGQLLC	87.01	935.4362	2	44.07	6790000				0.0017	0.0004147
				KKNEIIQCCAKD	58.97	632.3132	2	25.62	6790000					
				KTCPSGQLLCLKKW	44.46	468.9153	3	49.32	6790000					
				KKWEIGNPSGKE	71.37	558.2964	2	79.97	6790000					
				KVIELGCVATCPQPKPYEEVTCCSTDKC	78.78	1014.463	3	58.23	3790000					
				RGKVIELGCVATCPQPKP	64.32	878.9581	2	45.61	3790000					
				KMWCDAFCSSRG	57.87	660.2519	2	56.42	3790000					
	CAM11302.1	alpha-delta-bungarotoxin-4 partial [Bungarus caeruleus]	3FTx (Type II NTx)	SPISTVTCPSGENLCYTKM	41.83	963.9484	2	49.07	3790000				0.0010	0.0002315
				HTTSTSPISTVTCPSGENLCYTKM	41.81	1202.553	2	54.92	3790000					
				STSPISTVTCPSGENLCYTKM	63.33	1057.989	2	53.66	3790000					
				TSTSPISTVTCPSGENLCYTKM	86.24	1101.506	2	54.17	3790000					
				MWCDAFCSSRG	44.41	594.7312	2	46.96	3790000					
				RGAVIEQGCVATCPEFRSNYRS	52.37	772.0299	3	50.84	3550000					
	Q9W729.1	RecName: Full=Kappa-6-bungarotoxin; Short=K-6 bungarotoxin; Flags: Precursor	3FTx (κ-bungarotoxin)	QGCVATCPEFRS	45.84	598.7631	2	39.55	3550000				0.0009	0.0002168
				TRTCHISTSSTPQTCPKGQDICFRK	58.96	913.08	3	41.93	3550000					
				AVIEQGCVATCPEFRS	73.41	833.3901	2	48.13	3550000					
	C0HLB8.1	RecName: Full=Phospholipase A2; Short=MIPLA2-1; Short=svPLA2; AltName: Full=Phosphatidylcholine 2-acylhydrolase	PLA2	DYGCYCGYGGSGTPVDELDRC	62.53	1143.45	2	58.45	1100000				0.0003	0.0000672
	ABU63166.1	phospholipase A2 precursor BF-42 [Bungarus fasciatus]		YVSYGCYCGYGGSGTPVDELDRC	60.66	1156.483	2	60.24	899000				0.0002	0.0000549
	XP_026579406.1	BPTI/Kunitz domain-containing protein-like isoform X3 [Pseudonaja textilis]	Kunitz	KFTYGGCAGNANNFKKH	41.93	550.256	3	36.56	606000				0.0002	0.0000370
				GLFEGSCSIFPNDSQRC	49.11	878.9021	2	63.05	606000					
	Q3SB03.1	RecName: Full=Cysteine-rich venom protein pseudechetoxin-like; Short=CRVP; Flags: Precursor	CRISP	KSGPPCADCPSACVNGLC	49.43	831.3409	2	52.23	383000				0.0001	0.0000234
				RGSIATPYKSGPPCADCPSACVNGLCTNPCKH	62.79	1080.474	3	52.03	383000					
				RKCVKLQTVY	41.46	569.8188	2	37.52	177000					
				NGNTVGLAALGSLCSVKY	58.77	773.9169	2	68.86	177000					
	ABN72537.1	metalloproteinase [Bungarus multicinctus]	SVMP	DPNYGMVETGTKC	51.49	598.7869	2	37.39	177000				0.0000	0.0000108
				GVCNCLILPDDPNYGMVETGTKC	43.93	799.3678	3	67.9	177000					
				KCPTLTNQCIALMGPNVKV	75.87	958.973	2	61.86	177000					
	JAA75026.1	PLA2-Sut-16 [Suta fasciata]		YCGPGGSGTPVDELDRC	44.56	758.8375	2	50.8	104000				0.0000	0.0000064
	AAS20530.1	phospholipase A2 isoform 1 partial [Bungarus caeruleus]		CDCDRTAAICFAKA	60.32	476.5518	3	40.33	4020000000				0.9044	0.1154223

	[illegible]	phospholipase A2 isoform 1 partial [Bungarus caeruleus]	PLA2	ARFVCDCDRTAAICFAKA	78.26	498.2325	4	47.48	4020000000				[illegible]	[illegible]
	1G0Z	Chain B PHOSPHOLIPASE A2		NIPGCNPLIKT	52.95	506.2869	2	41.5	159000000				0.0358	0.0045652
				IGYGCYCGYGGSGTPVDELDRC	100.48	1091.944	2	57.45	159000000					
				RIVCDCDRTAALCFGDSEYIGAHKNI	70.09	693.8093	4	55.91	80700000					
				WGHYADYGCYCGAGGSGTPVDALDRC	69.79	840.3488	3	53.65	80700000					
				RTAALCFGDSEYIGAHKNI	69.1	618.621	3	53.97	80700000					
				YADYGCYCGAGGSGTPVDALDRC	71	1081.448	2	55.1	80700000					
				ADYGCYCGAGGSGTPVDALDRC	91.42	1045.929	2	55.35	80700000					
				LCFGDSEYIGAHKN	57.66	461.8716	3	40.75	80700000					
				AALCFGDSEYIGAHKN	60.4	523.2436	3	53.39	80700000					
	AAL87004.1	beta-bungarotoxin A2 chain precursor [Bungarus caeruleus]		HYADYGCYCGAGGSGTPVDALDRC	68.53	1162.982	2	58.07	80700000				0.0182	0.0023171
				KTWGHYADYGCYCGAGGSGTPVDALDRC	123.31	936.059	3	63.11	80700000					
				ALCFGDSEYIGAHKN	44.88	499.5666	3	51.25	80700000					
				IRYTIPCDKTWGHYADYGCYCGAGGSGTPVD ALDRC	52.95	960.6711	4	64.11	80700000					
				RCCYVHDNCYGVAENKH	118.36	944.8733	2	36.88	80700000					
			b-BTx	CFGDSEYIGAHKN	51.49	408.5277	3	37.07	80700000					
				RYTIPCDKTWGHYADYGCYCGAGGSGTPVD ALDRC	99.95	1228.527	3	67.23	80700000					
				PLNLINFMNMIRY	41.43	697.868	2	80.66	74200000					
				LNLINFMNMIRY	45.64	633.3276	2	85.48	74200000					
				RYTIPCEKTWGEYTNYGCYCGAGGSGTPIDAL DRC	105.66	1244.874	3	73.77	74200000					
	Q8QFW4.1	RecName: Full=Basic phospholipase A2 beta-bungarotoxin A1 chain; Short=Beta-BuTX A1 chain; Short=svPLA2; AltName: Full=Phosphatidylcholine 2-acylhydrolase; Flags: Precursor		CEKTWGEYTNYGCYCGAGGSGTPIDALDRC	41.95	1033.446	3	66.75	74200000				0.0167	0.0021304
				EYTNYGCYCGAGGSGTPIDALDRC	66.55	1184.503	2	60.13	74200000					
				YTNYGCYCGAGGSGTPIDALDRC	68.28	1102.969	2	58.37	74200000					
				KTQSYSYKLTRR	52.61	416.2189	3	35.98	74200000					
				AANIPPHPLNLINFMNMIRY	58.77	702.3773	3	90.03	74200000					
				KCSPKTQSYSYKLTRR	68.1	430.4687	4	34.43	74200000					
F12a				KTWGEYTNYGCYCGAGGSGTPIDALDRC	115.46	1421.095	2	71.09	74200000	0.1276	1.0000	0.1276		
	ABW24180.1	PLA-18 precursor [Austrelaps superbus]		DYGCYCGSGGTGTPVDELDRC	46.48	1032.417	2	62.52	48000000				0.0108	0.0013782
			PLA2	SGGTGTPVDELDRC	43.22	608.7935	2	43.43	48000000					
	Q75S48.1	RecName: Full=Acidic phospholipase A2 1; Short=svPLA2; AltName: Full=PA2-I; AltName: Full=Phosphatidylcholine 2-acylhydrolase; Flags:		RGSLTYLDYMDYGCYCGTGSRG	60.09	1169.975	2	74.07	41300000				0.0093	0.0011858
				RGTPVDELDRCCQTHDNCYAEAEEHPKC	57.32	783.5762	4	45.17	41300000					
				FYTCPNSETCPDGKNICVKR	54.84	1071.968	2	36.35	11200000					
	1MR6	Chain A neurotoxin	3FTx (Type II NTx)	MQCKTCSFYTCPNSETCPDGKNICVKR	49.78	1067.783	3	50.04	11200000				0.0025	0.0003216
				SFYTCPNSETCPDGKNICVKR	103.15	1145.5	2	42.38	11200000					
				CPNSETCPDGKNICVKR	43.85	573.6002	3	29.98	11200000					
	AAO84769.1	phospholipase A2 partial [Bungarus candidus]	PLA2	RTAALCFAEAPYKR	79.51	671.3285	2	56.61	5350000				0.0012	0.0001536
				MWCDAFCSSRG	44.41	594.7312	2	46.96	1940000					
				KVIELGCVATCPQPKPYEEVTCCSTDKC	78.78	1014.463	3	58.23	1940000					
				HTTSTSPISTVTCPSGENLCYTKM	41.81	1202.553	2	54.92	1940000					
	CAM11302.1	alpha-delta-bungarotoxin-4 partial [Bungarus caeruleus]	3FTx (Type II NTx)	TSTSPISTVTCPSGENLCYTKM	86.24	1101.506	2	54.17	1940000				0.0004	0.0000557
				RGKVIELGCVATCPQPKPYEEVTC	48.69	825.7491	3	56.58	1940000					
				SPISTVTCPSGENLCYTKM	41.83	963.9484	2	49.07	1940000					
				KMWCDAFCSSRG	50.69	668.2499	2	51.87	1940000					
				STSPISTVTCPSGENLCYTKM	63.33	1057.989	2	53.66	1940000					
	Q8AY42.2	RecName: Full=Kunitz-type serine protease inhibitor B; AltName: Full=Kunitz inhibitor B; Flags: Precursor	Kunitz	EFTYGGCGGNANNFKS	86.88	753.8243	2	42.8	1400000				0.0003	0.0000402
	1DPY	Chain A PHOSPHOLIPASE A2	PLA2	NLIQFKNMIQCAGTRIWTAYV	41.7	815.4131	3	84.36	1220000				0.0003	0.0000350
				RIWTAYVAYGCYCGKGGSGTPVDELDRC	84.92	965.7628	3	76.75	1220000					
				KWEIGNPSGKE	56.58	494.2478	2	44.83	264000					
				RGCVATCPKPKK	44.97	559.2778	2	18.98	264000					
				KYSQANESSKTCPSGQLLC	87.01	935.4362	2	44.07	264000					
	CAA06887.1	neurotoxin-like protein [Bungarus multicinctus multicinctus]	3FTx (Type I NTx)	KTCPSGQLLCLKKW	44.46	468.9153	3	49.32	264000				0.0001	0.0000076
				KRGCVATCPKPKK	45.87	425.2213	3	17.91	264000					
				KKWEIGNPSGKE	71.37	558.2964	2	79.97	264000					
				KKNEIIQCCAKD	58.97	632.3132	2	25.62	264000					
	AAL55556.1	phospholipase A2 partial [Hydrophis hardwickii]	PLA2	RRSSLDYADYGCYCGAGGSGTPVDELDRC	54.51	981.0878	3	76.69	130000				0.0000	0.0000037
	Q4JHE1.1	RecName: Full=L-amino-acid oxidase; Short=LAAO; Short=LAO; Flags: Precursor	LAAO	KIFLTCSQKFWEADGIHGGKS	45.26	549.2733	4	65.32	127000				0.0000	0.0000036
				GRAGELKVSTQTGSVRG	64.81	549.3041	3	30.39	100000000					
				KSGAWPTYTASQPQYVQLNTQPLATQPSLRA	90.63	1068.543	3	72.87	100000000					
				RLALQWIQNNIHPFGGNPRA	61.8	692.3699	3	78.32	100000000					
	4QWW	Chain B Acetylcholinesterase	ACHE	RSKNPQELIDEEWSVLPYKS	100.39	725.7046	3	78.13	100000000				0.6812	0.0167023
				RAGELKVSTQTGSVRG	85.19	478.2635	3	32.16	100000000					
				RTGNPTDPADKSGAWPTYTAS	64.47	975.4483	2	54.6	100000000					
				KNPQELIDEEWSVLPYKS	56.05	980.4882	2	83.04	100000000					
				RTLFQRAILQSGGPNAPWATVTPAESRG	67.34	923.4873	3	74.2	100000000					
				RYTIPCDKTWGHYADYGCYCGAGGSGTPVD ALDRC	99.95	1228.527	3	67.23	9330000					
				RIVCDCDRTAALCFGDSEYIGAHKN	105.04	886.7296	3	57.28	9330000					
				AALCFGDSEYIGAHKN	60.4	523.2436	3	53.39	9330000					
				ADYGCYCGAGGSGTPVDALDRC	91.42	1045.929	2	55.35	9330000					
				RCCYVHDNCYGVAENKH	118.36	944.8733	2	36.88	9330000					
				RTAALCFGDSEYIGAHKNIDTKRH	66.98	617.5566	4	51.24	9330000					
	AAL87004.1	beta-bungarotoxin A2 chain precursor [Bungarus caeruleus]	b-BTx	HYADYGCYCGAGGSGTPVDALDRC	68.53	1162.982	2	58.07	9330000				0.0636	0.0015583
				KTWGHYADYGCYCGAGGSGTPVDALDRC	123.31	936.059	3	63.11	9330000					
				LCFGDSEYIGAHKN	57.66	461.8716	3	40.75	9330000					
				CFGDSEYIGAHKN	51.49	408.5277	3	37.07	9330000					
				YADYGCYCGAGGSGTPVDALDRC	71	1081.448	2	55.1	9330000					
				WGHYADYGCYCGAGGSGTPVDALDRC	69.79	840.3488	3	53.65	9330000					
				IRYTIPCDKTWGHYADYGCYCGAGGSGTPVD ALDRC	52.95	960.6711	4	64.11	9330000					
				ALCFGDSEYIGAHKN	44.88	499.5666	3	51.25	9330000					
				RFDEISGGFDQLPKS	56.72	726.8532	2	63.8	6710000					
	ABN72539.1	L-amino acid oxidase [Bungarus multicinctus]	LAAO	KHVVVVGAGMAGLSAAYVLEKA	64.42	657.6966	3	72.52	6710000				0.0457	0.0011207
				KRFDEISGGFDQLPKS	71.5	536.9388	3	60.82	6710000					
				YQFQDYSETVAAPVGRI	42.19	834.4025	2	51.1	6710000					
				PLNLINFMNMIRY	43.01	697.8675	2	86.19	5410000					
				RYTIPCEKTWGEYTNYGCYCGAGGSGTPIDAL DRC	105.66	1244.874	3	73.77	5410000					
				YTNYGCYCGAGGSGTPIDALDRC	68.28	1102.969	2	58.37	5410000					
	Q8QFW4.1	RecName: Full=Basic phospholipase A2 beta-bungarotoxin A1 chain; Short=Beta-BuTX A1 chain; Short=svPLA2; AltName: Full=Phosphatidylcholine 2-acylhydrolase; Flags: Precursor	b-BTx	CEKTWGEYTNYGCYCGAGGSGTPIDALDRC	41.95	1033.446	3	66.75	5410000				0.0369	0.0009036
F13				KTWGEYTNYGCYCGAGGSGTPIDALDRC	115.46	1421.095	2	71.09	5410000	0.0245	1.0000	0.0245		
				LNLINFMNMIRY	45.64	633.3276	2	85.48	5410000					
				KTQSYSYKLTRR	52.61	416.2189	3	35.98	5410000					
				AANIPPHPLNLINFMNMIRY	58.77	702.3773	3	90.03	5410000					
				EYTNYGCYCGAGGSGTPIDALDRC	66.55	1184.503	2	60.13	5410000					
				KCSPKTQSYSYKLTRR	68.1	430.4687	4	34.43	5410000					
				KTSNPKHIVIVGAGMSGLSAAYVLAGAGHKV	44.82	565.3079	5	64.36	5330000					
	ACF70483.1	secreted L-amino acid oxidase precursor [Daboia russelii]	LAAO	RNVKEGWYANLGPMRV	43.06	545.608	3	59.55	5330000				0.0363	0.0008902
				KHIVIVGAGMSGLSAAYVLAGAGHKV	78.91	570.5635	4	74.97	5330000					
				RITFKPPLPPKK	45.34	379.906	3	49.85	5330000					
				KVTVLEASERPGG	45.59	579.3118	2	39.81	5330000					
	AAM51550.1	mocarhagin 1 [Naja mossambica]	SVMP	DPNYGMVAPGTKC	52.68	575.7822	2	30.43	5080000				0.0346	0.0008485
	JAS05169.1	Hyaluronidase [Micrurus tener]	Hyaluronidase	KLELENLKYLRK	42.76	430.9187	3	61.68	3810000				0.0260	0.0006364
	KAG8142835.1	hypothetical protein E2320_006056 partial [Naja naja]		KQLHPELSEAAIKR	69.94	445.914	3	43.75	3530000				0.0240	0.0005896
	Q9PVK7.1	RecName: Full=Zinc metalloproteinase-disintegrin-like cobrin; AltName: Full=Snake venom metalloproteinase; Short=SVMP; Flags: Precursor	SVMP	KTSAAVVQDYSKSTRM	59.58	504.9269	3	33.48	1640000				0.0112	0.0002739
				RDPSYGMVEPGTKC	50.17	648.7926	2	34.12	1640000					
	JAS05143.1	ecto-5'-nucleotidase [Micrurus tener]	5'-NT	KSIQEDPAVKAEVSRM	61.28	510.2708	3	36.48	1280000				0.0087	0.0002138
				RFLTEHNPECIINPPLRT	74.47	650.6686	3	60.7	1250000					
	AAZ39880.1	hemorrhagic metalloproteinase russelysin [Daboia russelii]	SVMP	RVAVTMAHEIGHNLGLTHDG	41.87	638.9882	3	60.1	1250000				0.0085	0.0002088
				RLGVYYAYCRK	55.43	582.779	2	51.02	1250000					
	P81383.3	RecName: Full=L-amino-acid oxidase; Short=LAO; Short=Oh-LAAO; Flags: Precursor	LAAO	REAGHEVVILEASDRV	47.75	508.9266	3	45.03	1110000				0.0076	0.0001854
				KIVIVGAGISGLTAAKL	43.78	685.4247	2	65	1110000					
	E5L0E4.1	RecName: Full=Beta-fibrinogenase-like; Short=RVBF; AltName: Full=Snake venom serine protease; Short=SVSP; Flags: Precursor	SVSP	KTSTYIAPLSLPSSPPRV	70.94	843.9573	2	63.72	949000				0.0065	0.0001585
				RAAYKGLPAQSRT	47.16	387.8849	3	28.26	949000					
				RIHFAGEYTGRF	49.04	384.1932	3	40.04	612000					
	AVX27607.1	L-amino acid oxidase partial [Naja atra]	LAAO	DPSLLKYPVKPSEEGKS	41.7	418.7384	4	42.14	612000				0.0042	0.0001022
				KSASQLYQESLRK	48.08	641.3243	2	45.12	612000					
	W8E7D1.1	RecName: Full=Venom phosphodiesterase; Short=PDE; Short=VLPDE; Flags: Precursor	VLPDE	KRLHYVNNIRI	56.01	395.5595	3	32.3	302000				0.0021	0.0000504
	QBF53421.1	phospholipase B [Vipera ammodytes ammodytes]	PLA2	KFTAYAINGPPVEKD	57.42	703.8698	2	52.52	215000				0.0015	0.0000359
				DPNYGMVETGTKC	51.49	598.7869	2	37.39	210000					
				RKCVKLQTVY	41.46	569.8188	2	37.52	210000					
	ABN72537.1	metalloproteinase [Bungarus multicinctus]	SVMP	NGNTVGLAALGSLCSVKY	58.77	773.9169	2	68.86	210000				0.0014	0.0000351
				KCPTLTNQCIALMGPNVKV	75.87	958.973	2	61.86	210000					
				GVCNCLILPDDPNYGMVETGTKC	43.93	799.3678	3	67.9	210000					
	AUF41653.1	RVV-X heavy chain [Daboia siamensis]		KSHDNALLFTDMRF	49.84	473.8941	3	58.17	17400				0.0001	0.0000029

Table S5B: Proteomic information of venom from western (MH) population of *B. caeruleus* . Assignment of peptides sequenced from MS/MS spectra using Serpentes NCBI database (TaxID: 8570) in PEAKS X (Bioinformatics Solutions Inc., Canada). Relative abundance of each toxin was estimated by using peak fraction area (obtained from RP-HPLC and SDS PAGE) and spectral intensity of all peptides matched to their respective proteins. -10logP is the matching score given by PEAKS Algorithm, m/z is mass to charge ratio of the peptides.

HPLC Peak no.	Protein information			Peptide Information						Calculation of Toxin abundance				
	NCBI Accession ID	Description	Toxin family	Peptide Sequence	-10LgP	m/z	z	RT	Precursor intensity	HPLC Peak Area	SDS Densitometry	Relative peak fraction	Relative abundance	% Proportion of Toxin
				KRGCVATCPKPKK	56.8	425.2221	3	17.2	1280000000					
				KKNEIIQCCAKDKC	62.55	502.9182	3	22.84	1280000000					
				KYSQANESSKTCPSGQ	39.77	758.3225	2	22.76	1280000000					
	CAA06887.1	neurotoxin-like protein [Bungarus multicinctus multicinctus]	3FTx (Type I NTx)	KWEIGNPSGKE	42.38	494.2483	2	46.47	1280000000				0.8113	0.006285
				KNEIIQCCAKD	49.59	568.2655	2	33.07	1280000000					
				KKWEIGNPSGKE	61.7	558.2943	2	67.29	1280000000					
				RGCVATCPKPKK	55.65	559.2771	2	19.57	1280000000					
	Q8AY42.2	RecName: Full=Kunitz-type serine protease inhibitor B; AltName: Full=Kunitz inhibitor B; Flags: Precursor	Kunitz	FTYGGCGGNANNFKS	70.31	680.2905	2	32.54	117000000				0.0742	0.000574
				EFTYGGCGGNANNFKS	83.76	753.8247	2	44.42	117000000					
				NSETCPDGKNICVKR	52.09	754.3478	2	31.46	65100000					
	O12963.1	RecName: Full=Long neurotoxin homolog TA-bm16; AltName: Full=Delta-protein; Flags: Precursor	3FTx (Type-II NTx)	SFYTCPNSETCPDGKNICVKR	42.93	1145.503	2	45	65100000				0.0413	0.000320
				ETCPDGKNICVKR	41.3	646.3103	2	28.49	65100000					
				FYTCPNSETCPDGKN	46.19	764.8057	2	30.26	65100000					
	CAB53358.1	cardiotoxin-like protein [Bungarus multicinctus multicinctus]	3FTx (C-3FTx)	EAYHGKYPVILERG	63.36	482.6015	3	44.53	62900000				0.0399	0.000309
				RFSYGGCGGNGNHFKT	83.88	751.3175	2	38.02	38300000					
	ABG90493.1	beta-bungarotoxin B5 chain partial [Bungarus multicinctus]	beta-BTx	RQRHPDCDKPPDTKR	40.18	350.7746	5	16.57	38300000				0.0243	0.000188
				KNCERFSYGGCGGNGNHFKTKQ	38.74	573.2523	4	34.76	38300000					
				RHPDCDKPPDTKRC	62.44	489.2336	3	16.8	38300000					
	Q75S50.1	RecName: Full=Kunitz-type serine protease inhibitor homolog beta-bungarotoxin B3 chain; Flags: Precursor		KRHPDCDKPPDTRI	52.11	374.1784	4	18.08	6440000				0.0041	0.000032
			Kunitz	RYGGCKGNGNHFKS	42.94	669.8041	2	20.12	6440000					
	XP_026579406.1	BPTI/Kunitz domain-containing protein-like isoform X3 [Pseudonaja textilis]		KFTYGGCAGNANNFKK	63.57	760.8326	2	45.1	5290000				0.0034	0.000026
				RTAALCFGDSEYIGAHKN	107.41	870.4016	2	56.54	1520000					
				CYVHDNCYGVAENKH	62.88	784.8428	2	31.8	1520000					
				RYTIPCDKTWGHYADYGCYCGAGGSGTPVDA	91.58	1228.526	3	67.4	1520000					
				CFGDSEYIGAHKN	54.06	408.5278	3	38.1	1520000					
				FGDSEYIGAHKN	48.36	538.754	2	25.63	1520000					
				WGHYADYGCYCGAGGSGTPVDALDRC	89.97	1260.015	2	55.61	1520000					
F2a				KTWGHYADYGCYCGAGGSGTPVDA	55.81	1175.957	2	60.44	1520000		0.1638	0.0077		
				DSEYIGAHKNIDTKR	61.09	738.3781	2	27.75	1520000					
				ALCFGDSEYIGAHKNIDTKR	39.52	517.7491	4	51.19	1520000					
				GHYADYGCYCGAGGSGTPVDALDRC	93.45	1231.504	2	55.39	1520000					
				DRTAALCFGDSEYIGAHKN	71.32	474.732	4	49.45	1520000					
				TAALCFGDSEYIGAHKN	59.48	546.9218	3	56.05	1520000					
				ADYGCYCGAGGSGTPVDALDRC	92	1045.926	2	58.4	1520000					
	AAL87004.1	beta-bungarotoxin A2 chain precursor [Bungarus caeruleus]		LCFGDSEYIGAHKN	52.42	461.8709	3	41.83	1520000				0.0010	0.000007
				RIVCDCDRTAALCFGDSEYIGAHKNIDTKRH	43.33	565.0986	6	53.05	1520000					
				YVHDNCYGVAENKH	57.8	703.3104	2	27.05	1520000					
				VCDCDRTAALCFGDSEYIGAHKN	49.99	816.0133	3	57.63	1520000					
				AALCFGDSEYIGAHKN	59.82	523.243	3	54.07	1520000					
			beta-BTx	TWGHYADYGCYCGAGGSGTPVDALDRC	40.69	902.3782	3	65.23	1520000					
				RCCYVHDNCYGVAENKH	93.27	944.8749	2	37.7	1520000					
				GDSEYIGAHKNIDTKR	75.27	795.8921	2	31.67	1520000					
				HYADYGCYCGAGGSGTPVDALDRC	85.47	1162.981	2	59.65	1520000					
				AANIPPYPLNLINFMEMIRY	48.37	1089.558	2	87.52	1520000					
				DKTWGHYADYGCYCGAGGSGTPVDALDRC	48.65	734.3186	4	58.89	1520000					
				CDRTAALCFGDSEYIGAHKN	46.97	670.9794	3	54.67	1520000					
				YADYGCYCGAGGSGTPVDALDRC	107.13	1081.444	2	57.32	1520000					
	TIKFBU	beta-1 bungarotoxin chain B major component - many-banded krait		DGDHGNFKTETLCRC	48.32	512.2404	3	35.16	1010000				0.0006	0.000005
				WGEYTNYGCYCGAGGSGTPIDALDRC	88.47	1277.534	2	64.71	62800	0.0473				
				CFGDSEYIEGHKRI	38.53	360.1743	4	34.62	62800					
				YTNYGCYCGAGGSGTPIDALDRC	100.21	1102.969	2	59.88	62800					
				KTWGEYTNYGCYCGAGGSGTPIDALDRC	109.61	1421.096	2	74.13	62800					
	Q8QFW4.1	RecName: Full=Basic phospholipase A2 beta-bungarotoxin A1 chain; Short=Beta-BuTX A1 chain; Short=svPLA2; AltName: Full=Phosphatidylcholine 2-acylhydrolase; Flags: Precursor		KTQSYSYKLTRR	48.03	416.2192	3	36.77	62800				0.0000	0.000000
				EYTNYGCYCGAGGSGTPIDALDRC	108.7	1184.501	2	62.95	62800					
				GEYTNYGCYCGAGGSGTPIDALDRC	95.53	1249.022	2	64.78	62800					
				RYTIPCEKTWGEYTNYGCYCGAGGSGTPIDAL	94.59	1244.866	3	77.12	62800					
				EKTWGEYTNYGCYCGAGGSGTPIDALDRC	43.32	990.4321	3	68.9	62800					
				PLNLINFMNMIRY	38.8	697.868	2	87.72	62800					
				KYSQANESSKTC	45.41	557.7542	2	18.14	45600000					
				KRGCVATCPKPKK	56.8	425.2221	3	17.2	45600000					
				KKNEIIQCCAKDKCNK	57.67	636.9746	3	20.68	45600000					
	CAA06887.1	neurotoxin-like protein [Bungarus multicinctus multicinctus]	3FTx (Type I NTx)	KWEIGNPSGKE	42.38	494.2483	2	46.47	45600000				0.6161	0.024369
				RGCVATCPKPKK	55.65	559.2771	2	19.57	45600000					
				KNEIIQCCAKDKC	51.26	689.828	2	27.46	45600000					
				KKWEIGNPSGKE	61.7	558.2943	2	67.29	45600000					
	Q8AY42.2	RecName: Full=Kunitz-type serine protease inhibitor B; AltName: Full=Kunitz inhibitor B; Flags: Precursor		FTYGGCGGNANNFKS	70.31	680.2905	2	32.54	23800000				0.3216	0.012719
			Kunitz	EFTYGGCGGNANNFKS	83.76	753.8247	2	44.42	23800000					
	Q75S50.1	RecName: Full=Kunitz-type serine protease inhibitor homolog beta-bungarotoxin B3 chain; Flags: Precursor		RYGGCKGNGNHFKS	42.94	669.8041	2	20.12	2680000				0.0362	0.001432
				KRHPDCDKPPDTRI	52.11	374.1784	4	18.08	2680000					
				RQRHPDCDKPPDTKR	37.72	399.1914	4	18.01	1900000					
	ABG90493.1	beta-bungarotoxin B5 chain partial [Bungarus multicinctus]		RFSYGGCGGNGNHFKTKQ	77.03	865.8912	2	34.6	1900000				0.0257	0.001015
				RHPDCDKPPDTKRC	62.44	489.2336	3	16.8	1900000					
				KNCERFSYGGCGGNGNHFKTKQ	38.74	573.2523	4	34.76	1900000					
				RIVCDCDRTAALCFGDSEYIGA	49.96	1161.493	2	69.69	34100					
				RTAALCFGDSEYIGAHKNIDTKR	118.1	1156.051	2	54.34	34100					
				HYADYGCYCGAGGSGTPVDALDRC	85.47	1162.981	2	59.65	34100					
				FGDSEYIGAHKNIDTKR	72.7	824.397	2	31.66	34100					
				CFGDSEYIGAHKN	54.06	408.5278	3	38.1	34100					
F2b				ALCFGDSEYIGAHKN	67.7	499.5642	3	52.66	34100		0.8362	0.0396		
				ADYGCYCGAGGSGTPVDALDRC	92	1045.926	2	58.4	34100					
				GDSEYIGAHKNIDTKR	75.27	795.8921	2	31.67	34100					
				CDRTAALCFGDSEYIGAHKN	46.97	670.9794	3	54.67	34100					
				LCFGDSEYIGAHKN	52.42	461.8709	3	41.83	34100					
			beta-BTx	YADYGCYCGAGGSGTPVDALDRC	107.13	1081.444	2	57.32	34100					
				AANIPPYPLNLINFMEMIRY	48.37	1089.558	2	87.52	34100					
	AAL87004.1	beta-bungarotoxin A2 chain precursor [Bungarus caeruleus]		CYVHDNCYGVAENKHKC	40.45	611.9497	3	25.3	34100				0.0005	0.000018
				YVHDNCYGVAENKH	57.8	703.3104	2	27.05	34100					
				WGHYADYGCYCGAGGSGTPVDALDRC	89.97	1260.015	2	55.61	34100					
				DSEYIGAHKNIDTKRH	40.7	544.6212	3	26.69	34100					
				KTWGHYADYGCYCGAGGSGTPVDALDRC	110.72	1403.582	2	65.03	34100					
				GHYADYGCYCGAGGSGTPVDALDRC	93.45	1231.504	2	55.39	34100					
				RCCYVHDNCYGVAENKH	93.27	944.8749	2	37.7	34100					
				RYTIPCDKTWGHYADYGCYCGAGGSGTPVDA	91.58	1228.526	3	67.4	34100					
				VCDCDRTAALCFGDSEYIGAHKN	49.99	816.0133	3	57.63	34100					
				AALCFGDSEYIGAHKN	59.82	523.243	3	54.07	34100					
				TWGHYADYGCYCGAGGSGTPVDALDRC	40.69	902.3782	3	65.23	34100					
				DKTWGHYADYGCYCGAGGSGTPVDALDRC	48.65	734.3186	4	58.89	34100					
				DRTAALCFGDSEYIGAHKN	71.32	474.732	4	49.45	34100					
				TAALCFGDSEYIGAHKN	59.48	546.9218	3	56.05	34100					
	CAB53358.1	cardiotoxin-like protein [Bungarus multicinctus multicinctus]	3FTx (C-3FTx)	EAYHGKYPVILERG	63.36	482.6015	3	44.53	191000000				0.5041	0.001590
				SFYTCPNSETCPDGKNICVKR	42.93	1145.503	2	45	151000000					
	O12963.1	RecName: Full=Long neurotoxin homolog TA-bm16; AltName: Full=Delta-protein; Flags: Precursor	3FTx (Type-II NTx)	ETCPDGKNICVKR	41.3	646.3103	2	28.49	151000000				0.3986	0.001257
				FYTCPNSETCPDGKN	46.19	764.8057	2	30.26	151000000					
				NSETCPDGKNICVKR	52.09	754.3478	2	31.46	151000000					
	Q8AY42.2	RecName: Full=Kunitz-type serine protease inhibitor B; AltName: Full=Kunitz inhibitor B; Flags: Precursor	Kunitz	EFTYGGCGGNANNFKS	83.76	753.8247	2	44.42	29100000				0.0768	0.000242
				FTYGGCGGNANNFKS	70.31	680.2905	2	32.54	29100000					
				KYSQANESSKTCPSGQ	39.77	758.3225	2	22.76	4960000					
				KKNEIIQCCAKDKCNK	57.67	636.9746	3	20.68	4960000					
				KWEIGNPSGKE	42.38	494.2483	2	46.47	4960000					
	CAA06887.1	neurotoxin-like protein [Bungarus multicinctus multicinctus]	3FTx (Type I NTx)	KRGCVATCPKPKK	56.8	425.2221	3	17.2	4960000				0.0131	0.000041
				KNEIIQCCAKD	49.59	568.2655	2	33.07	4960000					
				KKWEIGNPSGKE	61.7	558.2943	2	67.29	4960000					
				RGCVATCPKPKK	55.65	559.2771	2	19.57	4960000					
	XP_026579406.1	BPTI/Kunitz domain-containing protein-like isoform X3 [Pseudonaja textilis]	Kunitz	KFTYGGCAGNANNFKK	63.57	760.8326	2	45.1	1220000				0.0032	0.000010
				RFSYGGCGGNGNHFKT	83.88	751.3175	2	38.02	586000					
	ABG90493.1	beta-bungarotoxin B5 chain partial [Bungarus multicinctus]	beta-BTx	RHPDCDKPPDTKRC	62.44	489.2336	3	16.8	586000				0.0015	0.000005
				RQRHPDCDKPPDTKR	37.72	399.1914	4	18.01	586000					
				KNCERFSYGGCGGNGNHFKTKQ	38.74	573.2523	4	34.76	586000					
	Q75S50.1	RecName: Full=Kunitz-type serine protease inhibitor homolog beta-bungarotoxin B3 chain; Flags: Precursor	Kunitz	RYGGCKGNGNHFKS	42.94	669.8041	2	20.12	573000				0.0015	0.000005
				KRHPDCDKPPDTRI	52.11	374.1784	4	18.08	573000					
				WGHYADYGCYCGAGGSGTPVDALDRC	89.97	1260.015	2	55.61	389000					
				FGDSEYIGAHKNIDTKR	72.7	824.397	2	31.66	389000					
F3a				RTAALCFGDSEYIGAHKNIDTKRH	70.08	617.5548	4	50.92	389000		0.1101	0.0032		
				CYVHDNCYGVAENKH	62.88	784.8428	2	31.8	389000					
				ALCFGDSEYIGAHKNIDTKR	39.52	517.7491	4	51.19	389000					
				RIVCDCDRTAALCFGDSEYIGAHK	58.56	844.0308	3	63.7	389000					
				DSEYIGAHKNIDTKRH	40.7	544.6212	3	26.69	389000					
				KTWGHYADYGCYCGAGGSGTPVDALDRC	110.72	1403.582	2	65.03	389000					
				GHYADYGCYCGAGGSGTPVDALDRC	93.45	1231.504	2	55.39	389000	0.0286				
				DRTAALCFGDSEYIGAHKN	71.32	474.732	4	49.45	389000					
				RYTIPCDKTWGHYADYGCYCGAGGSGTPVDA	91.58	1228.526	3	67.4	389000					
				VCDCDRTAALCFGDSEYIGAHKN	49.99	816.0133	3	57.63	389000					
	AAL87004.1	beta-bungarotoxin A2 chain precursor [Bungarus caeruleus]	beta-BTx	ADYGCYCGAGGSGTPVDALDRC	92	1045.926	2	58.4	389000				0.0010	0.000003
				LCFGDSEYIGAHKN	52.42	461.8709	3	41.83	389000					
				CFGDSEYIGAHKN	54.06	408.5278	3	38.1	389000					
				GDSEYIGAHKNIDTKR	75.27	795.8921	2	31.67	389000					
				AALCFGDSEYIGAHKN	59.82	523.243	3	54.07	389000					
				CDRTAALCFGDSEYIGAHKN	46.97	670.9794	3	54.67	389000					
				AANIPPYPLNLINFMEMIRY	48.37	1089.558	2	87.52	389000					
				TAALCFGDSEYIGAHKN	59.48	546.9218	3	56.05	389000					
				YVHDNCYGVAENKH	57.8	703.3104	2	27.05	389000					
				HYADYGCYCGAGGSGTPVDALDRC	85.47	1162.981	2	59.65	389000					

				RCCYVHDNCYGVAENKH	93.27	944.8749	2	37.7	389000					
				DKTWGHYADYGCYCGAGGSGTPVDALDRC	48.65	734.3186	4	58.89	389000					
				TWGHYADYGCYCGAGGSGTPVDALDRC	40.69	902.3782	3	65.23	389000					
				YADYGCYCGAGGSGTPVDALDRC	107.13	1081.444	2	57.32	389000					
	Q7T2I3.1	RecName: Full=Long neurotoxin LlLong; Flags: Precursor	3FTx (Type-II NTx)	KVIELGCTATCPRA	80.78	688.8364	2	45.77	43200				0.0001	0.000000
	CAB53358.1	cardiotoxin-like protein [Bungarus multicinctus multicinctus]	3FTx (C-3FTx)	EAYHGKYPVILERG	63.36	482.6015	3	44.53	164000000				0.5258	0.013402
				RQRHPDCDKPPDTKR	40.18	350.7746	5	16.57	66600000					
	ABG90493.1	beta-bungarotoxin B5 chain partial [Bungarus multicinctus]	beta-BTx	KNCERFSYGGCGGNGNHFKTKQ	38.74	573.2523	4	34.76	66600000				0.2135	0.005442
				RFSYGGCGGNGNHFKTKQ	77.03	865.8912	2	34.6	66600000					
				RHPDCDKPPDTKRC	62.44	489.2336	3	16.8	66600000					
	Q75S50.1	RecName: Full=Kunitz-type serine protease inhibitor homolog beta-bungarotoxin B3 chain; Flags: Precursor		RYGGCKGNGNHFKS	42.94	669.8041	2	20.12	49500000				0.1587	0.004045
F3b			Kunitz	KRHPDCDKPPDTRI	52.11	374.1784	4	18.08	49500000		0.8899	0.0255		
	Q8AY42.2	RecName: Full=Kunitz-type serine protease inhibitor B; AltName: Full=Kunitz inhibitor B; Flags: Precursor		FTYGGCGGNANNFKS	70.31	680.2905	2	32.54	23700000				0.0760	0.001937
				EFTYGGCGGNANNFKS	83.76	753.8247	2	44.42	23700000					
				SFYTCPNSETCPDGKN	77.35	838.3411	2	38.55	8090000					
	O12963.1	RecName: Full=Long neurotoxin homolog TA-bm16; AltName: Full=Delta-protein; Flags: Precursor	3FTx (Type-II NTx)	NSETCPDGKNICVKR	52.09	754.3478	2	31.46	8090000				0.0259	0.000661
				FYTCPNSETCPDGKN	46.19	764.8057	2	30.26	8090000					
				ETCPDGKNICVKR	41.3	646.3103	2	28.49	8090000					
	Q75S50.1	RecName: Full=Kunitz-type serine protease inhibitor homolog beta-bungarotoxin B3 chain; Flags: Precursor	Kunitz	KRHPDCDKPPDTRI	52.11	374.1784	4	18.08	26200000				0.8517	0.030541
				RYGGCKGNGNHFKS	42.94	669.8041	2	20.12	26200000					
				RHPDCDKPPDTKRC	62.44	489.2336	3	16.8	3060000					
	ABG90493.1	beta-bungarotoxin B5 chain partial [Bungarus multicinctus]	beta-BTx	RFSYGGCGGNGNHFKTKQ	77.03	865.8912	2	34.6	3060000				0.0995	0.003567
				RQRHPDCDKPPDTKR	40.18	350.7746	5	16.57	3060000					
F4a				KNCERFSYGGCGGNGNHFKTKQ	38.74	573.2523	4	34.76	3060000	0.0359	1.0000	0.0359		
	CAB53358.1	cardiotoxin-like protein [Bungarus multicinctus multicinctus]	3FTx (C-3FTx)	EAYHGKYPVILERG	63.36	482.6015	3	44.53	1130000				0.0367	0.001317
				QGCAATCPEFRS	54.04	584.7479	2	36.63	373000					
				RGPVIEQGCAATCPEFRS	127.31	896.4115	2	52.12	373000					
	O12962.1	RecName: Full=Kappa-5-bungarotoxin; Flags: Precursor	3FTx (k-bungarotoxin)	GCAATCPEFRS	43.96	556.2367	2	36.4	373000				0.0121	0.000435
				GPVIEQGCAATCPEFRS	66.43	867.8996	2	51.43	373000					
				KTCLISPSSTPQTCPQGQDTCFLKA	59.64	1313.599	2	71.79	373000					
	1F94	Chain A BUCANDIN	3FTx	KITCSAEETFCYKW	90.62	754.8226	2	49.56	12400000				0.2414	0.001415
				QPKPYEEVTCCSTDKC	40.5	571.9202	3	34.23	12000000					
				KVIELGCVATCPQPKPYE	48.11	916.4513	2	61.8	12000000					
				ELGCVATCPQPKPYEEVTCCSTDKC	50.75	900.73	3	49.9	12000000					
				KMWCDAFCSSRG	46.58	668.2497	2	53.85	12000000					
				RGKVIELGCVATCPQPKPYEEVTCC	67.27	879.09	3	61.19	12000000					
				CPSGENLCYTKM	57.02	584.7686	2	33.57	12000000					
	CAM11302.1	alpha-delta-bungarotoxin-4 partial [Bungarus caeruleus]	3FTx (Type II NTx)	VTCPSGENLCYTKM	40.96	715.3087	2	36.56	12000000				0.2336	0.001370
				CVATCPQPKPYEEVTCCSTDKC	64.24	790.6856	3	41.8	12000000					
				TCPSGENLCYTKM	59.41	664.7845	2	35.32	12000000					
				PSGENLCYTKM	47.53	536.2424	2	31.4	12000000					
				CPQPKPYEEVTCCSTDKC	43.69	646.9566	3	37.38	12000000					
				LGCVATCPQPKPYEEVTCCSTDKC	43.12	863.0353	3	45.16	12000000					
				TVTCPSGENLCYTKM	57.59	764.8428	2	41.53	12000000					
	CAB53358.1	cardiotoxin-like protein [Bungarus multicinctus multicinctus]	3FTx (C-3FTx)	EAYHGKYPVILERG	63.36	482.6015	3	44.53	8890000				0.1730	0.001015
				DDPNYGMVETGTKC	61.2	664.296	2	35.47	5320000					
				GVCNCLILPDDPNYGMVETGTKC	40.49	804.7002	3	65.02	5320000					
	ABN72537.1	metalloproteinase [Bungarus multicinctus]	SVMP	RMQNGAKIPCAAKD	41.63	435.5528	3	25.55	5320000				0.1036	0.000607
				PTLTNQCIALMGPNVKV	41.35	838.4294	2	53.34	5320000					
				DPNYGMVETGTKC	51.83	606.782	2	31.66	5320000					
				FNGNTVGLAALGSLCSVKY	76.33	830.9376	2	83.01	5320000					
				RGPVIEQGCAATCPEFRS	127.31	896.4115	2	52.12	4260000					
	O12962.1	RecName: Full=Kappa-5-bungarotoxin; Flags: Precursor	3FTx (k-bungarotoxin)	GPVIEQGCAATCPEFRS	66.43	867.8996	2	51.43	4260000				0.0829	0.000486
				GCAATCPEFRS	43.96	556.2367	2	36.4	4260000					
				QGCAATCPEFRS	54.04	584.7479	2	36.63	4260000					
	P0C554.1	RecName: Full=Neurotoxin 3FTx-RK; Flags: Precursor	3FTx	GCVATCPQPKK	38.81	530.7493	2	24.03	2520000				0.0491	0.000288
	SMD28268.1	vascular endothelial growth factor CdtVEGF3 [Crotalus durissus terrificus]	VEGF	KHQPLNPTKC	43.12	467.7589	2	20.33	2350000				0.0457	0.000268
				RTAALCFGDSEYIGAHKNIDTKR	118.1	1156.051	2	54.34	1240000					
				YADYGCYCGAGGSGTPVDALDRC	107.13	1081.444	2	57.32	1240000					
				LCFGDSEYIGAHKNIDTKR	39.77	489.4792	4	45.57	1240000					
				CDRTAALCFGDSEYIGAHKN	46.97	670.9794	3	54.67	1240000					
F5a				GDSEYIGAHKNIDTKR	75.27	795.8921	2	31.67	1240000		0.1047	0.0059		
				YVHDNCYGVAENKH	57.8	703.3104	2	27.05	1240000					
				RIVCDCDRTAALCFGDSEYIGAHK	58.56	844.0308	3	63.7	1240000					
				AALCFGDSEYIGAHKN	59.82	523.243	3	54.07	1240000					
				AANIPPYPLNLINFMEMIRY	48.37	1089.558	2	87.52	1240000					
				ALCFGDSEYIGAHKNIDTKR	39.52	517.7491	4	51.19	1240000					
				KTWGHYADYGCYCGAGGSGTPVDA	55.81	1175.957	2	60.44	1240000					
				RCCYVHDNCYGVAENKH	93.27	944.8749	2	37.7	1240000					
	AAL87004.1	beta-bungarotoxin A2 chain precursor [Bungarus caeruleus]	beta-BTx	CYVHDNCYGVAENKH	62.88	784.8428	2	31.8	1240000				0.0241	0.000142
				HYADYGCYCGAGGSGTPVDALDRC	85.47	1162.981	2	59.65	1240000					
				FGDSEYIGAHKN	48.36	538.754	2	25.63	1240000					
				DKTWGHYADYGCYCGAGGSGTPVDALDRC	48.65	734.3186	4	58.89	1240000					
				DSEYIGAHKNIDTKR	61.09	738.3781	2	27.75	1240000					
				TAALCFGDSEYIGAHKN	59.48	546.9218	3	56.05	1240000					
				DRTAALCFGDSEYIGAHKNIDTKR	54.14	494.2419	5	50.48	1240000					
				TWGHYADYGCYCGAGGSGTPVDALDRC	40.69	902.3782	3	65.23	1240000					
				VCDCDRTAALCFGDSEYIGAHKN	49.99	816.0133	3	57.63	1240000					
				GHYADYGCYCGAGGSGTPVDALDRC	93.45	1231.504	2	55.39	1240000					
				ADYGCYCGAGGSGTPVDALDRC	92	1045.926	2	58.4	1240000					
				WGHYADYGCYCGAGGSGTPVDALDRC	89.97	1260.015	2	55.61	1240000					
				RYTIPCDKTWGHYADYGCYCGAGGSGTPVDA	91.58	1228.526	3	67.4	1240000					
				CFGDSEYIGAHKN	54.06	408.5278	3	38.1	1240000					
	7ULR	Chain B Kappa-bungarotoxin	3FTx (k-bungarotoxin)	MRTCLISPSSTPQTCPNGQDICFLKA	117.42	927.4427	3	63.83	833000				0.0162	0.000095
				RTCLISPSSTPQTCPNGQDICFLKA	72.47	875.4102	3	69.47	833000					
	ADF50020.1	non-conventional three finger toxin isoform 5 [Bungarus flaviceps]	3FTx	TLTCLICPEKYCQKV	48.59	856.9116	2	51.61	794000				0.0155	0.000091
				KRHPDCDKPPDTRI	52.11	374.1784	4	18.08	621000					
	Q75S50.1	RecName: Full=Kunitz-type serine protease inhibitor homolog beta-bungarotoxin B3 chain; Flags: Precursor	Kunitz	RYGGCKGNGNHFKS	42.94	669.8041	2	20.12	621000				0.0121	0.000071
				RKRHPDCDKPPDTRI	54.17	406.2005	4	16.23	621000					
				RHPDCDKPPDTRI	66.87	446.535	3	18.44	621000					
	Q7T2I3.1	RecName: Full=Long neurotoxin LlLong; Flags: Precursor	3FTx (Type-II NTx)	KVIELGCTATCPRA	80.78	688.8364	2	45.77	90000				0.0018	0.000010
	ABK63559.1	metalloproteinase precursor [Demansia vestigiata]	SVMP	KDDCDLPETCTGRS	39.32	719.7827	2	37.56	49900				0.0010	0.000006
				RAAKDDCDLPETCTGRS	50.44	854.8654	2	32.39	49900					
	AAL30054.1	kappa 1a bungaratoxin [Bungarus candidus]	3FTx (k-bungarotoxin)	TCLISPSSTPQTCPQGQDICFLKA	38.98	846.3981	3	68.74	5760				0.0001	0.000001
	1F94	Chain A BUCANDIN	3FTx	KITCSAEETFCYKW	90.62	754.8226	2	49.56	428000000				0.5756	0.028872
				GPVIEQGCAATCPEFRS	66.43	867.8996	2	51.43	93600000	0.0560				
	O12962.1	RecName: Full=Kappa-5-bungarotoxin; Flags: Precursor	3FTx (k-bungarotoxin)	GCAATCPEFRS	43.96	556.2367	2	36.4	93600000				0.1259	0.006314
				QGCAATCPEFRS	54.04	584.7479	2	36.63	93600000					
				RGPVIEQGCAATCPEFRS	127.31	896.4115	2	52.12	93600000					
	TIKFBU	beta-1 bungarotoxin chain B major component - many-banded krait	beta-BTx	DGDHGNFKTETLCRC	48.32	512.2404	3	35.16	34000000				0.0457	0.002294
	CAB53358.1	cardiotoxin-like protein [Bungarus multicinctus multicinctus]	3FTx (C-3FTx)	EAYHGKYPVILERG	63.36	482.6015	3	44.53	30900000				0.0416	0.002084
	CAD56381.1	BM10-1-like neurotoxin precursor [Bungarus multicinctus]	3FTx	KIIRGCSSSCPEKK	54.58	465.2236	3	21.96	30800000				0.0414	0.002078
	Q75S49.1	RecName: Full=Kunitz-type serine protease inhibitor homolog beta-bungarotoxin B4 chain; Flags: Precursor	Kunitz	NGNHFKTETLCRC	39.19	454.8912	3	30.15	23000000				0.0309	0.001552
				NGNGNHFKTETLCRC	54.41	767.3657	2	30.48	23000000					
				FGDSEYIGAHKN	48.36	538.754	2	25.63	21600000					
				DSEYIGAHKNIDTKR	61.09	738.3781	2	27.75	21600000					
				LCFGDSEYIGAHKNIDTKR	39.77	489.4792	4	45.57	21600000					
				GDSEYIGAHKNIDTKR	75.27	795.8921	2	31.67	21600000					
				TAALCFGDSEYIGAHKN	59.48	546.9218	3	56.05	21600000					
				RTAALCFGDSEYIGAHKNIDT	70.4	1041.482	2	60.39	21600000					
				AALCFGDSEYIGAHKN	59.82	523.243	3	54.07	21600000					
				CYVHDNCYGVAENKHKC	40.45	611.9497	3	25.3	21600000					
				CDRTAALCFGDSEYIGAHKN	46.97	670.9794	3	54.67	21600000					
				YADYGCYCGAGGSGTPVDALDRC	107.13	1081.444	2	57.32	21600000					
				GHYADYGCYCGAGGSGTPVDALDRC	93.45	1231.504	2	55.39	21600000					
	AAL87004.1	beta-bungarotoxin A2 chain precursor [Bungarus caeruleus]	beta-BTx	AANIPPYPLNLINFMEMIRY	48.37	1089.558	2	87.52	21600000				0.0291	0.001457
				RYTIPCDKTWGHYADYGCYCGAGGSGTPVDA	91.58	1228.526	3	67.4	21600000					
				ADYGCYCGAGGSGTPVDALDRC	92	1045.926	2	58.4	21600000					
				HYADYGCYCGAGGSGTPVDALDRC	85.47	1162.981	2	59.65	21600000					
				KTWGHYADYGCYCGAGGSGTPVDA	55.81	1175.957	2	60.44	21600000					
				YVHDNCYGVAENKH	57.8	703.3104	2	27.05	21600000					
				RCCYVHDNCYGVAENKH	93.27	944.8749	2	37.7	21600000					
				DRTAALCFGDSEYIGAHKN	71.32	474.732	4	49.45	21600000					
				CFGDSEYIGAHKN	54.06	408.5278	3	38.1	21600000					
				ALCFGDSEYIGAHKN	67.7	499.5642	3	52.66	21600000					
				TWGHYADYGCYCGAGGSGTPVDALDRC	40.69	902.3782	3	65.23	21600000					
				DKTWGHYADYGCYCGAGGSGTPVDALDRC	48.65	734.3186	4	58.89	21600000					
				WGHYADYGCYCGAGGSGTPVDALDRC	89.97	1260.015	2	55.61	21600000					
F5b				RGKVIELGCVATCPQPKPYE	50.23	673.0126	3	57.77	21500000		0.8953	0.0502		
				KVIELGCVATCPQPKPYEEV	79.68	1045.494	2	61.16	21500000					
				QPKPYEEVTCCSTDKC	40.5	571.9202	3	34.23	21500000					
				CPSGENLCYTKM	57.02	584.7686	2	33.57	21500000					
				CPQPKPYEEVTCCSTDKC	43.69	646.9566	3	37.38	21500000					
				TCPSGENLCYTKM	59.41	664.7845	2	35.32	21500000					
	CAM11302.1	alpha-delta-bungarotoxin-4 partial [Bungarus caeruleus]	3FTx (Type II NTx)	LGCVATCPQPKPYEEVTCCSTDKC	43.12	863.0353	3	45.16	21500000				0.0289	0.001450
				TVTCPSGENLCYTKM	57.59	764.8428	2	41.53	21500000					
				KMWCDAFCSSRG	46.58	668.2497	2	53.85	21500000					
				PSGENLCYTKM	47.53	536.2424	2	31.4	21500000					
				ELGCVATCPQPKPYEEVTCCSTDKC	50.75	900.73	3	49.9	21500000					
				CVATCPQPKPYEEVTCCSTDKC	64.24	790.6856	3	41.8	21500000					
				VTCPSGENLCYTKM	40.96	715.3087	2	36.56	21500000					
	7ULR	Chain B Kappa-bungarotoxin	3FTx (k-bungarotoxin)	MRTCLISPSSTPQTCPNGQDICFLKA	117.42	927.4427	3	63.83	19000000				0.0256	0.001282
				RTCLISPSSTPQTCPNGQDICFLKA	72.47	875.4102	3	69.47	19000000					
	Q75S50.1	RecName: Full=Kunitz-type serine protease inhibitor homolog beta-bungarotoxin B3 chain; Flags: Precursor	Kunitz	RYGGCKGNGNHFKS	42.94	669.8041	2	20.12	14500000				0.0195	0.000978
				KRHPDCDKPPDTRI	52.11	374.1784	4	18.08	14500000					
	AAL30054.1	kappa 1a bungaratoxin [Bungarus candidus]	3FTx (k-bungarotoxin)	TCLISPSSTPQTCPQGQDICFLKA	38.98	846.3981	3	68.74	14000000				0.0188	0.000944
	Q9W729.1	RecName: Full=Kappa-6-bungarotoxin; Short=K-6 bungarotoxin; Flags: Precursor		AVIEQGCVATCPEFRS	47.32	833.3891	2	50.55	4440000				0.0060	0.000300
				QGCVATCPEFRS	37.81	598.7631	2	41.83	4440000					
	Q8AY42.2	RecName: Full=Kunitz-type serine protease inhibitor B; AltName: Full=Kunitz inhibitor B; Flags: Precursor	Kunitz	EFTYGGCGGNANNFKS	83.76	753.8247	2	44.42	2740000				0.0037	0.000185
				FTYGGCGGNANNFKS	70.31	680.2905	2	32.54	2740000					
	P0C554.1	RecName: Full=Neurotoxin 3FTx-RK; Flags: Precursor	3FTx	GCVATCPQPKK	38.81	530.7493	2	24.03	2180000				0.0029	0.000147
				RFVCDCDRTAAICFAKAPYN	40.96	722.3257	3	61.05	1140000					
				FVCDCDRTAAICFAKA	41.1	562.9182	3	48.6	1140000					
	1TC8	Chain A phospholipase A2 isoform 1	PLA2	VCDCDRTAAICFAKA	51.37	794.3394	2	42.3	1140000				0.0015	0.000077
				CDCDRTAAICFAKA	62	476.5507	3	41.93	1140000					
				ARFVCDCDRTAAICFAKA	60.48	498.2314	4	46.87	1140000					
	ADF50020.1	non-conventional three finger toxin isoform 5 [Bungarus flaviceps]	3FTx	TLTCLICPEKYCQKV	48.59	856.9116	2	51.61	1040000				0.0014	0.000070
	C0HKZ8.2	RecName: Full=Actiflagelin		MECYKCGVSGCHLKI	50.01	582.2449	3	30.68	530000				0.0007	0.000036
	0412250A	toxin A beta1 bungaro	PLA2	RTIICYGAAGGTCRI	58.71	700.3253	2	46.34	139000				0.0002	0.000009

				RHPDCDKPPDTKRC	62.44	489.2336	3	16.8	56500					
	ABG90493.1	beta-bungarotoxin B5 chain partial [Bungarus multicinctus]	beta-BTx	RFSYGGCGGNGNHFKT	83.88	751.3175	2	38.02	56500				0.0001	0.000004
				KNCERFSYGGCGGNGNHFKTKQ	38.74	573.2523	4	34.76	56500					
				RQRHPDCDKPPDTKR	40.18	350.7746	5	16.57	56500					
				RGKVIELGCVATCPQPKP	73.17	878.9552	2	47.65	45000000					
				KMWCDAFCSSRG	46.58	668.2497	2	53.85	45000000					
				CVATCPQPKPYEEVTCCSTDKC	64.24	790.6856	3	41.8	45000000					
				KVIELGCVATCPQPKPYEEV	79.68	1045.494	2	61.16	45000000					
				VTCPSGENLCYTKM	40.96	715.3087	2	36.56	45000000					
				TVTCPSGENLCYTKM	57.59	764.8428	2	41.53	45000000					
	CAM11302.1	alpha-delta-bungarotoxin-4 partial [Bungarus caeruleus]	3FTx (Type II NTx)	CPQPKPYEEVTCCSTDKC	43.69	646.9566	3	37.38	45000000				0.8899	0.002342
				QPKPYEEVTCCSTDKC	40.5	571.9202	3	34.23	45000000					
				CPSGENLCYTKM	57.02	584.7686	2	33.57	45000000					
				ELGCVATCPQPKPYEEVTCCSTDKC	50.75	900.73	3	49.9	45000000					
				PSGENLCYTKM	47.53	536.2424	2	31.4	45000000					
				TCPSGENLCYTKM	59.41	664.7845	2	35.32	45000000					
				LGCVATCPQPKPYEEVTCCSTDKC	43.12	863.0353	3	45.16	45000000					
	P0C554.1	RecName: Full=Neurotoxin 3FTx-RK; Flags: Precursor	3FTx	GCVATCPQPKK	38.81	530.7493	2	24.03	4430000				0.0876	0.000231
	AXL96578.1	snake venom metalloproteinase 28 partial [Borikenophis portoricensis]	SVMP	DPDNGMVEPGTKC	48.44	580.7662	2	21.53	992000				0.0196	0.000052
				LCFGDSEYIGAHKN	52.42	461.8709	3	41.83	144000					
				GHYADYGCYCGAGGSGTPVDALDRC	93.45	1231.504	2	55.39	144000					
				KTWGHYADYGCYC	41.44	696.77	2	60.8	144000					
				CFGDSEYIGAHKN	54.06	408.5278	3	38.1	144000					
F6a				ALCFGDSEYIGAHKN	67.7	499.5642	3	52.66	144000		0.0936	0.0026		
				DRTAALCFGDSEYIGAHKNIDTKR	54.14	494.2419	5	50.48	144000					
				CYVHDNCYGVAENKHKC	40.45	611.9497	3	25.3	144000					
				DSEYIGAHKNIDTKRH	40.7	544.6212	3	26.69	144000					
				RCCYVHDNCYGVAENKH	93.27	944.8749	2	37.7	144000					
				ADYGCYCGAGGSGTPVDALDRC	92	1045.926	2	58.4	144000					
				AALCFGDSEYIGAHKN	59.82	523.243	3	54.07	144000					
				YVHDNCYGVAENKH	57.8	703.3104	2	27.05	144000					
	AAL87004.1	beta-bungarotoxin A2 chain precursor [Bungarus caeruleus]	beta-BTx	RIVCDCDRTAALCFGDSEYIGAHKNIDT	74.24	1000.777	3	60.91	144000				0.0028	0.000007
				RTAALCFGDSEYIGAHK	43.8	806.3585	2	65.08	144000					
				VCDCDRTAALCFGDSEYIGAHKN	49.99	816.0133	3	57.63	144000					
				RYTIPCDKTWGHYADYGCYCGAGGSGTPVDA	91.58	1228.526	3	67.4	144000					
				WGHYADYGCYCGAGGSGTPVDALDRC	89.97	1260.015	2	55.61	144000					
				FGDSEYIGAHKN	48.36	538.754	2	25.63	144000					
				HYADYGCYCGAGGSGTPVDALDRC	85.47	1162.981	2	59.65	144000					
				TWGHYADYGCYCGAGGSGTPVDALDRC	40.69	902.3782	3	65.23	144000					
				AANIPPYPLNLINFMEMIRY	48.37	1089.558	2	87.52	144000					
				TAALCFGDSEYIGAHKN	59.48	546.9218	3	56.05	144000					
				GDSEYIGAHKNIDTKR	75.27	795.8921	2	31.67	144000					
				CDRTAALCFGDSEYIGAHKN	46.97	670.9794	3	54.67	144000					
				DKTWGHYADYGCYCGAGGSGTPVDALDRC	48.65	734.3186	4	58.89	144000					
				YADYGCYCGAGGSGTPVDALDRC	107.13	1081.444	2	57.32	144000					
				KMWCDAFCSSRG	46.58	668.2497	2	53.85	128000000					
				RGKVIELGCVATCPQPKPYEEVTCCSTDK	48.39	1033.459	3	60.43	128000000					
				KVIELGCVATCPQPKPYEEVTCCS	71.11	1305.592	2	65.48	128000000					
				VTCPSGENLCYTKM	40.96	715.3087	2	36.56	128000000					
				TCPSGENLCYTKM	59.41	664.7845	2	35.32	128000000					
	CAM11302.1	alpha-delta-bungarotoxin-4 partial [Bungarus caeruleus]	3FTx (Type II NTx)	TVTCPSGENLCYTKM	57.59	764.8428	2	41.53	128000000				0.6579	0.003034
				CVATCPQPKPYEEVTCCSTDKC	64.24	790.6856	3	41.8	128000000					
				CPSGENLCYTKM	57.02	584.7686	2	33.57	128000000					
				QPKPYEEVTCCSTDKC	40.5	571.9202	3	34.23	128000000					
				CPQPKPYEEVTCCSTDKC	43.69	646.9566	3	37.38	128000000					
				LGCVATCPQPKPYEEVTCCSTDKC	43.12	863.0353	3	45.16	128000000					
				PSGENLCYTKM	47.53	536.2424	2	31.4	128000000					
				RMQNGAKIPCAAKD	41.63	435.5528	3	25.55	43700000					
				DPNYGMVETGTKC	51.83	606.782	2	31.66	43700000					
	ABN72537.1	metalloproteinase [Bungarus multicinctus]		DDPNYGMVETGTKC	61.2	664.296	2	35.47	43700000				0.2246	0.001036
			SVMP	FNGNTVGLAALGSLCSVKY	76.33	830.9376	2	83.01	43700000					
				PTLTNQCIALMGPNVKV	41.35	838.4294	2	53.34	43700000					
				GVCNCLILPDDPNYGMVETGTKC	40.49	804.7002	3	65.02	43700000					
	ABK63559.1	metalloproteinase precursor [Demansia vestigiata]		RAAKDDCDLPETCTGRS	50.44	854.8654	2	32.39	6700000				0.0344	0.000159
				KDDCDLPETCTGRS	39.32	719.7827	2	37.56	6700000					
	0412250A	toxin A beta1 bungaro	PLA2	RTIICYGAAGGTCRI	58.71	700.3253	2	46.34	6040000				0.0310	0.000143
	CAB53358.1	cardiotoxin-like protein [Bungarus multicinctus multicinctus]	3FTx (C-3FTx)	EAYHGKYPVILERG	63.36	482.6015	3	44.53	1950000				0.0100	0.000046
	AAB25729.1	nerve growth factor precursor [Bungarus multicinctus]	NGF	RHWNSYCTTTDTFVKA	49.13	587.2627	3	51.15	1710000				0.0088	0.000041
				RNPNPVPSGCRG	49.85	549.2616	2	28.03	1710000					
	P0C554.1	RecName: Full=Neurotoxin 3FTx-RK; Flags: Precursor	3FTx	GCVATCPQPKK	38.81	530.7493	2	24.03	1670000				0.0086	0.000040
	AED89576.1	putative phospholipase A2 precursor [Micrurus altirostris]	PLA2	SGGGGTPVDDLDRC	48.75	579.7731	2	37.13	1060000				0.0054	0.000025
				GGGGTPVDDLDRC	46.01	551.2618	2	39.85	1060000					
	JAA74864.1	SVMP-Hop-23 partial [Hoplocephalus bungaroides]	SVMP	RMENGAKIPCAAKD	39.07	653.3184	2	27.83	918000				0.0047	0.000022
	Q8AY42.2	RecName: Full=Kunitz-type serine protease inhibitor B; AltName: Full=Kunitz inhibitor B; Flags: Precursor	Kunitz	EFTYGGCGGNANNFKS	83.76	753.8247	2	44.42	814000				0.0042	0.000019
				FTYGGCGGNANNFKS	70.31	680.2905	2	32.54	814000					
				DSEYIGAHKNIDTKRH	40.7	544.6212	3	26.69	643000					
				KTWGHYADYGCYCGAGGSGTPVDALDR	54.01	1325.538	2	69.52	643000					
				RTAALCFGDSEYIGAHKNIDT	70.4	1041.482	2	60.39	643000					
				RCCYVHDNCYGVAENKH	93.27	944.8749	2	37.7	643000					
F6b				RIVCDCDRTAALCFGDSEYIGAHKNIDTKRH	43.33	565.0986	6	53.05	643000		0.1640	0.0046		
				TAALCFGDSEYIGAHKN	59.48	546.9218	3	56.05	643000					
				ALCFGDSEYIGAHKNIDTKR	39.52	517.7491	4	51.19	643000					
				DRTAALCFGDSEYIGAHKNIDTKR	54.14	494.2419	5	50.48	643000					
				WGHYADYGCYCGAGGSGTPVDALDRC	89.97	1260.015	2	55.61	643000					
				LCFGDSEYIGAHKNIDTKR	39.77	489.4792	4	45.57	643000					
				RYTIPCDKTWGHYADYGCYCGAGGSGTPVDA	91.58	1228.526	3	67.4	643000					
				FGDSEYIGAHKNIDTKR	72.7	824.397	2	31.66	643000					
	AAL87004.1	beta-bungarotoxin A2 chain precursor [Bungarus caeruleus]	beta-BTx	TWGHYADYGCYCGAGGSGTPVDALDRC	40.69	902.3782	3	65.23	643000				0.0033	0.000015
				CYVHDNCYGVAENKH	62.88	784.8428	2	31.8	643000					
				GHYADYGCYCGAGGSGTPVDALDRC	93.45	1231.504	2	55.39	643000					
				YADYGCYCGAGGSGTPVDALDRC	107.13	1081.444	2	57.32	643000					
				HYADYGCYCGAGGSGTPVDALDRC	85.47	1162.981	2	59.65	643000	0.0281				
				DKTWGHYADYGCYCGAGGSGTPVDALDRC	48.65	734.3186	4	58.89	643000					
				CDRTAALCFGDSEYIGAHKN	46.97	670.9794	3	54.67	643000					
				CFGDSEYIGAHKN	54.06	408.5278	3	38.1	643000					
				ADYGCYCGAGGSGTPVDALDRC	92	1045.926	2	58.4	643000					
				YVHDNCYGVAENKH	57.8	703.3104	2	27.05	643000					
				GDSEYIGAHKNIDTKR	75.27	795.8921	2	31.67	643000					
				AALCFGDSEYIGAHKN	59.82	523.243	3	54.07	643000					
				AANIPPYPLNLINFMEMIRY	48.37	1089.558	2	87.52	643000					
				VCDCDRTAALCFGDSEYIGAHKN	49.99	816.0133	3	57.63	643000					
				KNEIIQCCAKDKC	51.26	689.828	2	27.46	454000					
				KKNEIIQCCAKDKC	62.55	502.9182	3	22.84	454000					
				KYSQANESSKTC	45.41	557.7542	2	18.14	454000					
	CAA06887.1	neurotoxin-like protein [Bungarus multicinctus multicinctus]	3FTx (Type I NTx)	KRGCVATCPKPKK	56.8	425.2221	3	17.2	454000				0.0023	0.000011
				KWEIGNPSGKE	42.38	494.2483	2	46.47	454000					
				RGCVATCPKPKK	55.65	559.2771	2	19.57	454000					
				KKWEIGNPSGKE	61.7	558.2943	2	67.29	454000					
				RFSYGGCGGNGNHFKT	83.88	751.3175	2	38.02	414000					
	ABG90493.1	beta-bungarotoxin B5 chain partial [Bungarus multicinctus]	beta-BTx	RHPDCDKPPDTKRC	62.44	489.2336	3	16.8	414000				0.0021	0.000010
				KNCERFSYGGCGGNGNHFKTKQ	38.74	573.2523	4	34.76	414000					
				RQRHPDCDKPPDTKR	40.18	350.7746	5	16.57	414000					
	CAD56381.1	BM10-1-like neurotoxin precursor [Bungarus multicinctus]	3FTx	KIIRGCSSSCPEKKN	45.52	507.9211	3	18.67	329000				0.0017	0.000008
	7ULR	Chain B Kappa-bungarotoxin	3FTx (k-bungarotoxin)	MRTCLISPSSTPQTCPNG	69.71	909.9255	2	44.06	152000				0.0008	0.000004
				RTCLISPSSTPQTCPNGQDICFLKA	72.47	875.4102	3	69.47	152000					
				KVIELGCVATCPQPKPYEEVTC	76.58	1145.557	2	64.96	89800000					
				LGCVATCPQPKPYEEVTCCSTDKC	43.12	863.0353	3	45.16	89800000					
				CPQPKPYEEVTCCSTDKC	43.69	646.9566	3	37.38	89800000					
				CVATCPQPKPYEEVTCCSTDKC	64.24	790.6856	3	41.8	89800000					
				KMWCDAFCSSRG	46.58	668.2497	2	53.85	89800000					
				VTCPSGENLCYTKM	40.96	715.3087	2	36.56	89800000					
	CAM11302.1	alpha-delta-bungarotoxin-4 partial [Bungarus caeruleus]	3FTx (Type II NTx)	RGKVIELGCVATCPQPKPYEEVTCC	67.27	879.09	3	61.19	89800000				0.4208	0.008786
				PSGENLCYTKM	47.53	536.2424	2	31.4	89800000					
				CPSGENLCYTKM	57.02	584.7686	2	33.57	89800000					
				TVTCPSGENLCYTKM	57.59	764.8428	2	41.53	89800000					
				QPKPYEEVTCCSTDKC	40.5	571.9202	3	34.23	89800000					
				ELGCVATCPQPKPYEEVTCCSTDKC	50.75	900.73	3	49.9	89800000					
				TCPSGENLCYTKM	59.41	664.7845	2	35.32	89800000					
	P0C554.1	RecName: Full=Neurotoxin 3FTx-RK; Flags: Precursor	3FTx	GCVATCPQPKK	38.81	530.7493	2	24.03	74100000				0.3473	0.007250
	Q75S50.1	RecName: Full=Kunitz-type serine protease inhibitor homolog beta-bungarotoxin B3 chain; Flags: Precursor	Kunitz	RYGGCKGNGNHFKS	42.94	669.8041	2	20.12	27900000				0.1308	0.002730
				KRHPDCDKPPDTRI	52.11	374.1784	4	18.08	27900000					
				RHPDCDKPPDTKRC	62.44	489.2336	3	16.8	12200000					
	ABG90493.1	beta-bungarotoxin B5 chain partial [Bungarus multicinctus]		RFSYGGCGGNGNHFKTKQ	77.03	865.8912	2	34.6	12200000				0.0572	0.001194
				RQRHPDCDKPPDTKR	40.18	350.7746	5	16.57	12200000					
				KNCERFSYGGCGGNGNHFKTKQ	38.74	573.2523	4	34.76	12200000					
				GHYADYGCYCGAGGSGTPVDALDRC	93.45	1231.504	2	55.39	6470000					
				TAALCFGDSEYIGAHKN	59.48	546.9218	3	56.05	6470000					
				CFGDSEYIGAHKN	54.06	408.5278	3	38.1	6470000					
				RYTIPCDKTWGHYADYGCYCGAGGSGTPVDA	91.58	1228.526	3	67.4	6470000					
				RTAALCFGDSEYIGAHK	43.8	806.3585	2	65.08	6470000					
				RCCYVHDNCYGVAENKHKC	42.54	431.5845	5	31.64	6470000					
				DSEYIGAHKNIDTKR	61.09	738.3781	2	27.75	6470000					
				KTWGHYADYGCYCGAGGSGTPVDALDR	54.01	1325.538	2	69.52	6470000					
				CYVHDNCYGVAENKH	62.88	784.8428	2	31.8	6470000					
			beta-BTx	FGDSEYIGAHKN	48.36	538.754	2	25.63	6470000					
				DRTAALCFGDSEYIGAHKN	71.32	474.732	4	49.45	6470000					
	AAL87004.1	beta-bungarotoxin A2 chain precursor [Bungarus caeruleus]		LCFGDSEYIGAHKN	52.42	461.8709	3	41.83	6470000				0.0303	0.000633
F6c				ADYGCYCGAGGSGTPVDALDRC	92	1045.926	2	58.4	6470000		0.7424	0.0209		
				ALCFGDSEYIGAHKN	67.7	499.5642	3	52.66	6470000					
				YADYGCYCGAGGSGTPVDALDRC	107.13	1081.444	2	57.32	6470000					
				YVHDNCYGVAENKH	57.8	703.3104	2	27.05	6470000					
				DKTWGHYADYGCYCGAGGSGTPVDALDRC	48.65	734.3186	4	58.89	6470000					
				AALCFGDSEYIGAHKN	59.82	523.243	3	54.07	6470000					
				WGHYADYGCYCGAGGSGTPVDALDRC	89.97	1260.015	2	55.61	6470000					
				HYADYGCYCGAGGSGTPVDALDRC	85.47	1162.981	2	59.65	6470000					
				AANIPPYPLNLINFMEMIRY	48.37	1089.558	2	87.52	6470000					
				CDRTAALCFGDSEYIGAHKN	46.97	670.9794	3	54.67	6470000					
				GDSEYIGAHKNIDTKR	75.27	795.8921	2	31.67	6470000					
				TWGHYADYGCYCGAGGSGTPVDALDRC	40.69	902.3782	3	65.23	6470000					

				RFVCDCDRTAAICFAKAP	55.19	952.9258	2	52.54	2370000					
				ARFVCDCDRTAAICFAKA	60.48	498.2314	4	46.87	2370000					
	1TC8	Chain A phospholipase A2 isoform 1		VCDCDRTAAICFAKA	51.37	794.3394	2	42.3	2370000				0.0111	0.000232
				CDCDRTAAICFAKA	62	476.5507	3	41.93	2370000					
			PLA2	FVCDCDRTAAICFAKA	41.1	562.9182	3	48.6	2370000					
				VSYGCYCGYGGSGTPVDELDRC	89.7	1106.945	2	60.06	282000					
	ABU63166.1	phospholipase A2 precursor BF-42 [Bungarus fasciatus]		HYVSYGCYCGYGGSGTPVDELDRC	73.89	1238.014	2	65.19	282000				0.0013	0.000028
				YVSYGCYCGYGGSGTPVDELDRC	102.32	1156.48	2	62.51	282000					
				ENMIECAGTRSWTHYVSYGCYCGYGGSGTPV	57.16	1345.55	3	58.41	282000					
				TCLISPSSTPQTCPQGQDICFLKA	38.98	846.3981	3	68.74	197000					
	AAL30054.1	kappa 1a bungaratoxin [Bungarus candidus]	3FTx (k-bungarotoxin)	RTCLISPSSTPQTCPQGQDICFLKA	53.51	1319.619	2	69.21	197000				0.0009	0.000019
				TRTCLISPSSTPQTCPQGQDICFLKA	77.16	932.1146	3	63.54	197000					
				CFGDSEYIEGHKRI	38.53	360.1743	4	34.62	61300					
				GEYTNYGCYCGAGGSGTPIDALDRC	95.53	1249.022	2	64.78	61300					
				EYTNYGCYCGAGGSGTPIDALDRC	108.7	1184.501	2	62.95	61300					
				YTNYGCYCGAGGSGTPIDALDRC	100.21	1102.969	2	59.88	61300					
	Q8QFW4.1	RecName: Full=Basic phospholipase A2 beta-bungarotoxin A1 chain; Short=Beta-BuTX A1 chain; Short=svPLA2; AltName: Full=Phosphatidylcholine 2-acylhydrolase; Flags: Precursor	beta-BTx	PLNLINFMNMIRY	38.8	697.868	2	87.72	61300				0.0003	0.000006
				KTWGEYTNYGCYCGAGGSGTPIDALDRC	109.61	1421.096	2	74.13	61300					
				KTQSYSYKLTRR	48.03	416.2192	3	36.77	61300					
				EKTWGEYTNYGCYCGAGGSGTPIDALDRC	43.32	990.4321	3	68.9	61300					
				WGEYTNYGCYCGAGGSGTPIDALDRC	88.47	1277.534	2	64.71	61300					
				RYTIPCEKTWGEYTNYGCYCGAGGSGTPIDAL	94.59	1244.866	3	77.12	61300					
				RTAALCFGDSEYIGAHKN	107.41	870.4016	2	56.54	147000					
				KTWGHYADYGCYCGAGGSGTPVDA	55.81	1175.957	2	60.44	147000					
				CYVHDNCYGVAENKH	62.88	784.8428	2	31.8	147000					
				RCCYVHDNCYGVAENKHKC	42.54	431.5845	5	31.64	147000					
				FGDSEYIGAHKNIDTKR	72.7	824.397	2	31.66	147000					
				WGHYADYGCYCGAGGSGTPVDALDRC	89.97	1260.015	2	55.61	147000					
				ALCFGDSEYIGAHKNIDTKR	39.52	517.7491	4	51.19	147000					
				RIVCDCDRTAALCFGDSEYIGAHKNIDTKRH	43.33	565.0986	6	53.05	147000					
				DKTWGHYADYGCYCGAGGSGTPVDALDRC	48.65	734.3186	4	58.89	147000					
				TWGHYADYGCYCGAGGSGTPVDALDRC	40.69	902.3782	3	65.23	147000					
				AANIPPYPLNLINFMEMIRY	48.37	1089.558	2	87.52	147000					
				GDSEYIGAHKNIDTKR	75.27	795.8921	2	31.67	147000					
	AAL87004.1	beta-bungarotoxin A2 chain precursor [Bungarus caeruleus]	beta-BTx	CDRTAALCFGDSEYIGAHKN	46.97	670.9794	3	54.67	147000				0.5589	0.003124
F7				LCFGDSEYIGAHKNIDTKR	39.77	489.4792	4	45.57	147000	0.0056	1.0000	0.0056		
				YADYGCYCGAGGSGTPVDALDRC	107.13	1081.444	2	57.32	147000					
				GHYADYGCYCGAGGSGTPVDALDRC	93.45	1231.504	2	55.39	147000					
				DRTAALCFGDSEYIGAHKN	71.32	474.732	4	49.45	147000					
				VCDCDRTAALCFGDSEYIGAHKN	49.99	816.0133	3	57.63	147000					
				AALCFGDSEYIGAHKN	59.82	523.243	3	54.07	147000					
				ADYGCYCGAGGSGTPVDALDRC	92	1045.926	2	58.4	147000					
				CFGDSEYIGAHKN	54.06	408.5278	3	38.1	147000					
				DSEYIGAHKNIDTKRH	40.7	544.6212	3	26.69	147000					
				HYADYGCYCGAGGSGTPVDALDRC	85.47	1162.981	2	59.65	147000					
				RYTIPCDKTWGHYADYGCYCGAGGSGTPVDA	91.58	1228.526	3	67.4	147000					
				TAALCFGDSEYIGAHKN	59.48	546.9218	3	56.05	147000					
				YVHDNCYGVAENKH	57.8	703.3104	2	27.05	147000					
	AAB25729.1	nerve growth factor precursor [Bungarus multicinctus]	NGF	RHWNSYCTTTDTFVKA	49.13	587.2627	3	51.15	116000				0.4411	0.002466
				RNPNPVPSGCRG	49.85	549.2616	2	28.03	116000					
	AED89576.1	putative phospholipase A2 precursor [Micrurus altirostris]	PLA2	SGGGGTPVDDLDRC	48.75	579.7731	2	37.13	894000000				0.9876	0.015370
				GGGGTPVDDLDRC	46.01	551.2618	2	39.85	894000000					
	AAB25729.1	nerve growth factor precursor [Bungarus multicinctus]	NGF	RHWNSYCTTTDTFVKA	49.13	587.2627	3	51.15	8840000				0.0098	0.000152
				RNPNPVPSGCRG	49.85	549.2616	2	28.03	8840000					
F8a	Q75S50.1	RecName: Full=Kunitz-type serine protease inhibitor homolog beta-bungarotoxin B3 chain; Flags: Precursor	Kunitz	RYGGCKGNGNHFKS	42.94	669.8041	2	20.12	1750000		0.3475	0.0156	0.0019	0.000030
				KRHPDCDKPPDTRI	52.11	374.1784	4	18.08	1750000					
				RQRHPDCDKPPDTKR	40.18	350.7746	5	16.57	668000					
	ABG90493.1	beta-bungarotoxin B5 chain partial [Bungarus multicinctus]	beta-BTx	RHPDCDKPPDTKRC	62.44	489.2336	3	16.8	668000				0.0007	0.000011
				RFSYGGCGGNGNHFKTKQ	77.03	865.8912	2	34.6	668000					
				KNCERFSYGGCGGNGNHFKTKQ	38.74	573.2523	4	34.76	668000					
				VCDCDRTAAICFAKA	51.37	794.3394	2	42.3	613000000					
				RFVCDCDRTAAICFAK	50.7	853.3602	2	62.15	613000000					
	1TC8	Chain A phospholipase A2 isoform 1	PLA2	ARFVCDCDRTAAICFAKA	60.48	498.2314	4	46.87	613000000				0.8041	0.023502
				CDCDRTAAICFAKA	62	476.5507	3	41.93	613000000					
				FVCDCDRTAAICFAKA	41.1	562.9182	3	48.6	613000000					
				YVHDNCYGVAENKH	57.8	703.3104	2	27.05	106000000					
				DKTWGHYADYGCYCGAGGSGTPVDALDRC	48.65	734.3186	4	58.89	106000000					
				HYADYGCYCGAGGSGTPVDALDRC	85.47	1162.981	2	59.65	106000000					
				TWGHYADYGCYCGAGGSGTPVDALDRC	40.69	902.3782	3	65.23	106000000					
				DRTAALCFGDSEYIGAHKN	71.32	474.732	4	49.45	106000000					
				RCCYVHDNCYGVAENKHKC	42.54	431.5845	5	31.64	106000000					
				CFGDSEYIGAHKN	54.06	408.5278	3	38.1	106000000					
				KTWGHYADYGCYC	41.44	696.77	2	60.8	106000000					
				DSEYIGAHKNIDTKRH	40.7	544.6212	3	26.69	106000000					
				TAALCFGDSEYIGAHKN	59.48	546.9218	3	56.05	106000000					
				AANIPPYPLNLINFMEMIRY	48.37	1089.558	2	87.52	106000000					
	AAL87004.1	beta-bungarotoxin A2 chain precursor [Bungarus caeruleus]	beta-BTx	CDRTAALCFGDSEYIGAHKN	46.97	670.9794	3	54.67	106000000				0.1391	0.004064
				RTAALCFGDSEYIGAHKNIDTKR	118.1	1156.051	2	54.34	106000000					
				GHYADYGCYCGAGGSGTPVDALDRC	93.45	1231.504	2	55.39	106000000					
				LCFGDSEYIGAHKN	52.42	461.8709	3	41.83	106000000					
				FGDSEYIGAHKNIDTKR	72.7	824.397	2	31.66	106000000					
				WGHYADYGCYCGAGGSGTPVDALDRC	89.97	1260.015	2	55.61	106000000					
				ALCFGDSEYIGAHKNIDTKR	39.52	517.7491	4	51.19	106000000					
				YADYGCYCGAGGSGTPVDALDRC	107.13	1081.444	2	57.32	106000000					
				GDSEYIGAHKNIDTKR	75.27	795.8921	2	31.67	106000000					
				AALCFGDSEYIGAHKN	59.82	523.243	3	54.07	106000000					
				CYVHDNCYGVAENKHKC	40.45	611.9497	3	25.3	106000000					
				ADYGCYCGAGGSGTPVDALDRC	92	1045.926	2	58.4	106000000					
				RYTIPCDKTWGHYADYGCYCGAGGSGTPVDA	91.58	1228.526	3	67.4	106000000					
	Q75S50.1	RecName: Full=Kunitz-type serine protease inhibitor homolog beta-bungarotoxin B3 chain; Flags: Precursor	Kunitz	KRHPDCDKPPDTRI	52.11	374.1784	4	18.08	9680000				0.0127	0.000371
				RYGGCKGNGNHFKS	42.94	669.8041	2	20.12	9680000					
				PLNLINFMNMIRY	38.8	697.868	2	87.72	7100000					
				CFGDSEYIEGHKRI	38.53	360.1743	4	34.62	7100000					
				RYTIPCEKTWGEYTNYGCYCGAGGSGTPIDAL	94.59	1244.866	3	77.12	7100000					
				GEYTNYGCYCGAGGSGTPIDALDRC	95.53	1249.022	2	64.78	7100000					
	Q8QFW4.1	RecName: Full=Basic phospholipase A2 beta-bungarotoxin A1 chain; Short=Beta-BuTX A1 chain; Short=svPLA2; AltName: Full=Phosphatidylcholine 2-acylhydrolase; Flags: Precursor		EYTNYGCYCGAGGSGTPIDALDRC	108.7	1184.501	2	62.95	7100000	0.0448			0.0093	0.000272
				EKTWGEYTNYGCYCGAGGSGTPIDALDRC	43.32	990.4321	3	68.9	7100000					
			beta-BTx	WGEYTNYGCYCGAGGSGTPIDALDRC	88.47	1277.534	2	64.71	7100000					
				KTWGEYTNYGCYCGAGGSGTPIDALDRC	109.61	1421.096	2	74.13	7100000					
				KTQSYSYKLTRR	48.03	416.2192	3	36.77	7100000					
F8b				KNCERFSYGGCGGNGNHFKTKQ	38.74	573.2523	4	34.76	7050000		0.6525	0.0292		
	ABG90493.1	beta-bungarotoxin B5 chain partial [Bungarus multicinctus]		RQRHPDCDKPPDTKR	37.72	399.1914	4	18.01	7050000				0.0092	0.000270
				RFSYGGCGGNGNHFKTKQ	77.03	865.8912	2	34.6	7050000					
				RHPDCDKPPDTKRC	62.44	489.2336	3	16.8	7050000					
				YVSYGCYCGYGGSGTPVDELDRC	102.32	1156.48	2	62.51	5870000					
	ABU63166.1	phospholipase A2 precursor BF-42 [Bungarus fasciatus]		ENMIECAGTRSWTHYVSYGCYCGYGGSGTPV	57.16	1345.55	3	58.41	5870000				0.0077	0.000225
				VSYGCYCGYGGSGTPVDELDRC	89.7	1106.945	2	60.06	5870000					
			PLA2	HYVSYGCYCGYGGSGTPVDELDRC	73.89	1238.014	2	65.19	5870000					
	0412250A	toxin A beta1 bungaro		RTIICYGAAGGTCRI	58.71	700.3253	2	46.34	5260000				0.0069	0.000202
				NYGCYCGAGGSGTPVGELDRC	48.86	988.4166	2	54.16	2190000					
	JAS05103.1	Phospholipase A2 8i [Micrurus tener]		YGCYCGAGGSGTPVGELDRC	52.62	906.8846	2	52.68	2190000				0.0029	0.000084
				CYCGAGGSGTPVGELDRC	48.09	798.3584	2	48.13	2190000					
	ABK63544.1	LNTX-1 precursor [Demansia vestigiata]	3FTx	RGKVIELGCAATCPRQEPGKE	50.43	691.0186	3	41.69	1240000				0.0016	0.000048
	AAO84769.1	phospholipase A2 partial [Bungarus candidus]	PLA2	RTAALCFAEAPYKRR	55.35	499.9223	3	50.33	931000				0.0012	0.000036
	ABX58164.1	putative long neurotoxin [Austrelaps labialis]		KVIELGCAATCPPAEPKKGI	38.86	633.3218	3	50.79	438000				0.0006	0.000017
				SFYTCPNSETCPDGKN	77.35	838.3411	2	38.55	305000					
	O12963.1	RecName: Full=Long neurotoxin homolog TA-bm16; AltName: Full=Delta-protein; Flags: Precursor	3FTx (Type-II NTx)	ETCPDGKNICVKR	41.3	646.3103	2	28.49	305000				0.0004	0.000012
				FYTCPNSETCPDGKN	46.19	764.8057	2	30.26	305000					
				NSETCPDGKNICVKR	52.09	754.3478	2	31.46	305000					
				KTYSYTCTKPNLTCTDA	83.76	912.8915	2	46.31	156000					
				KTKTYSYTCTKPNLTC	59.92	839.4105	2	42.15	156000					
	0702209A	phospholipase A	PLA2	YVNYGCYCGAGGSGTPVDELDRC	105.56	1123.968	2	56.53	156000				0.0002	0.000006
				GYVNYGCYCGAGGSGTPVDELDRC	97.63	1205.504	2	60.35	156000					
				NLTCTDAAGTCARI	44.87	648.787	2	27.9	156000					
				PSGENLCYTKM	47.53	536.2424	2	31.4	128000					
				ELGCVATCPQPKPYEEVTCCSTDKC	50.75	900.73	3	49.9	128000					
				KVIELGCVATCPQPKPYE	48.11	916.4513	2	61.8	128000					
				CPSGENLCYTKM	57.02	584.7686	2	33.57	128000					
				TCPSGENLCYTKM	59.41	664.7845	2	35.32	128000					
	CAM11302.1	alpha-delta-bungarotoxin-4 partial [Bungarus caeruleus]	3FTx (Type II NTx)	VTCPSGENLCYTKM	40.96	715.3087	2	36.56	128000				0.0002	0.000005
				TVTCPSGENLCYTKM	57.59	764.8428	2	41.53	128000					
				CPQPKPYEEVTCCSTDKC	43.69	646.9566	3	37.38	128000					
				CVATCPQPKPYEEVTCCSTDKC	64.24	790.6856	3	41.8	128000					
				LGCVATCPQPKPYEEVTCCSTDKC	43.12	863.0353	3	45.16	128000					
				KMWCDAFCSSRG	46.58	668.2497	2	53.85	128000					
				QPKPYEEVTCCSTDKC	40.5	571.9202	3	34.23	128000					
				GCAATCPEFRS	43.96	556.2367	2	36.4	12000					
				QGCAATCPEFRS	54.04	584.7479	2	36.63	12000					
	O12962.1	RecName: Full=Kappa-5-bungarotoxin; Flags: Precursor	3FTx (k-bungarotoxin)	RGPVIEQGCAATCPEFRS	127.31	896.4115	2	52.12	12000				0.0000	0.000000
				KTCLISPSSTPQTCPQGQDTCFLKA	59.64	1313.599	2	71.79	12000					
				GPVIEQGCAATCPEFRS	66.43	867.8996	2	51.43	12000					
				RFVCDCDRTAAICFAKAP	55.19	952.9258	2	52.54	528000000					
				FVCDCDRTAAICFAKA	41.1	562.9182	3	48.6	528000000					
	1TC8	Chain A phospholipase A2 isoform 1	PLA2	CDCDRTAAICFAKA	62	476.5507	3	41.93	528000000				0.4375	0.004096
				ARFVCDCDRTAAICFAKA	60.48	498.2314	4	46.87	528000000					
				VCDCDRTAAICFAKA	51.37	794.3394	2	42.3	528000000					
				RCCYVHDNCYGVAENKHKC	42.54	431.5845	5	31.64	460000000					
				DKTWGHYADYGCYCGAGGSGTPVDALDRC	48.65	734.3186	4	58.89	460000000					
				YVHDNCYGVAENKH	57.8	703.3104	2	27.05	460000000					
				TWGHYADYGCYCGAGGSGTPVDALDRC	40.69	902.3782	3	65.23	460000000					
				CDRTAALCFGDSEYIGAHKN	46.97	670.9794	3	54.67	460000000					
				KTWGHYADYGCYCGAGGSGTPVDALDR	54.01	1325.538	2	69.52	460000000					
				FGDSEYIGAHKN	48.36	538.754	2	25.63	460000000					
				LCFGDSEYIGAHKNIDTKR	39.77	489.4792	4	45.57	460000000					
				HYADYGCYCGAGGSGTPVDALDRC	85.47	1162.981	2	59.65	460000000					
				WGHYADYGCYCGAGGSGTPVDALDRC	89.97	1260.015	2	55.61	460000000					
				DRTAALCFGDSEYIGAHKN	71.32	474.732	4	49.45	460000000					
	AAL87004.1	beta-bungarotoxin A2 chain precursor [Bungarus caeruleus]	beta-BTx	DSEYIGAHKNIDTKR	61.09	738.3781	2	27.75	460000000				0.3811	0.003568
				CFGDSEYIGAHKN	54.06	408.5278	3	38.1	460000000					
				RTAALCFGDSEYIGAHKNIDTKRH	70.08	617.5548	4	50.92	460000000					

				AANIPPYPLNLINFMEMIRY	48.37	1089.558	2	87.52	460000000					
				RYTIPCDKTWGHYADYGCYCGAGGSGTPVDA	91.58	1228.526	3	67.4	460000000					
				GHYADYGCYCGAGGSGTPVDALDRC	93.45	1231.504	2	55.39	460000000					
				TAALCFGDSEYIGAHKN	59.48	546.9218	3	56.05	460000000					
				YADYGCYCGAGGSGTPVDALDRC	107.13	1081.444	2	57.32	460000000					
				ALCFGDSEYIGAHKNIDTKR	39.52	517.7491	4	51.19	460000000					
				CYVHDNCYGVAENKH	62.88	784.8428	2	31.8	460000000					
				GDSEYIGAHKNIDTKR	75.27	795.8921	2	31.67	460000000					
				ADYGCYCGAGGSGTPVDALDRC	92	1045.926	2	58.4	460000000					
				AALCFGDSEYIGAHKN	59.82	523.243	3	54.07	460000000					
				CPQPKPYEEVTCCSTDKC	43.69	646.9566	3	37.38	78000000					
				QPKPYEEVTCCSTDKC	40.5	571.9202	3	34.23	78000000					
				CPSGENLCYTKM	57.02	584.7686	2	33.57	78000000					
				KVIELGCVATCPQPKPYEEVTCCSTDK	45.29	1457.142	2	66.53	78000000					
				PSGENLCYTKM	47.53	536.2424	2	31.4	78000000					
	CAM11302.1	alpha-delta-bungarotoxin-4 partial [Bungarus caeruleus]	3FTx (Type II NTx)	LGCVATCPQPKPYEEVTCCSTDKC	43.12	863.0353	3	45.16	78000000				0.0646	0.000605
				TVTCPSGENLCYTKM	57.59	764.8428	2	41.53	78000000					
				ELGCVATCPQPKPYEEVTCCSTDKC	50.75	900.73	3	49.9	78000000					
				KMWCDAFCSSRG	46.58	668.2497	2	53.85	78000000					
				VTCPSGENLCYTKM	40.96	715.3087	2	36.56	78000000					
				TCPSGENLCYTKM	59.41	664.7845	2	35.32	78000000					
				CVATCPQPKPYEEVTCCSTDKC	64.24	790.6856	3	41.8	78000000					
				RYTIPCEKTWGEYTNYGCYCGAGGSGTPIDAL	94.59	1244.866	3	77.12	67500000					
				CFGDSEYIEGHKRI	38.53	360.1743	4	34.62	67500000					
				PLNLINFMNMIRY	38.8	697.868	2	87.72	67500000					
F9a				EYTNYGCYCGAGGSGTPIDALDRC	108.7	1184.501	2	62.95	67500000		0.1242	0.0094		
	Q8QFW4.1	RecName: Full=Basic phospholipase A2 beta-bungarotoxin A1 chain; Short=Beta-BuTX A1 chain; Short=svPLA2; AltName: Full=Phosphatidylcholine 2-acylhydrolase; Flags: Precursor	beta-BTx	GEYTNYGCYCGAGGSGTPIDALDRC	95.53	1249.022	2	64.78	67500000				0.0559	0.000524
				WGEYTNYGCYCGAGGSGTPIDALDRC	88.47	1277.534	2	64.71	67500000					
				EKTWGEYTNYGCYCGAGGSGTPIDALDRC	43.32	990.4321	3	68.9	67500000					
				KTWGEYTNYGCYCGAGGSGTPIDALDRC	109.61	1421.096	2	74.13	67500000					
				KTQSYSYKLTRR	48.03	416.2192	3	36.77	67500000					
				ENMIECAGTRSWTHYVSYGCYCGYGGSGTPV	57.16	1345.55	3	58.41	41300000					
	ABU63166.1	phospholipase A2 precursor BF-42 [Bungarus fasciatus]	PLA2	HYVSYGCYCGYGGSGTPVDELDRC	73.89	1238.014	2	65.19	41300000				0.0342	0.000320
				YVSYGCYCGYGGSGTPVDELDRC	102.32	1156.48	2	62.51	41300000					
				RFSYGGCGGNGNHFKTKQ	77.03	865.8912	2	34.6	19900000					
	ABG90493.1	beta-bungarotoxin B5 chain partial [Bungarus multicinctus]	beta-BTx	RQRHPDCDKPPDTKR	40.18	350.7746	5	16.57	19900000				0.0165	0.000154
				KNCERFSYGGCGGNGNHFKTKQ	38.74	573.2523	4	34.76	19900000					
				RHPDCDKPPDTKRC	62.44	489.2336	3	16.8	19900000					
	TIKFBU	beta-1 bungarotoxin chain B major component - many-banded krait		DGDHGNFKTETLCRC	48.32	512.2404	3	35.16	4880000				0.0040	0.000038
	JAB52755.1	vascular endothelial growth factor 3 [Micrurus fulvius]	VEGF	KHFQSQHIHPMSFQQHSKC	42.48	530.7524	4	26.48	2160000				0.0018	0.000017
				KHLYKQDPLTCKC	69.75	468.2431	3	31.6	2160000					
	P00610.1	RecName: Full=Basic phospholipase A2; Short=svPLA2; AltName: Full=Myotoxin; AltName: Full=Phosphatidylcholine 2-acylhydrolase; AltName: Full=Toxin VI-5; AltName: Full=Toxin VI:5b	PLA2	RRSSLDYADYGCYCGAGGSGTPVDELDRC	67.89	1471.124	2	79.63	1520000				0.0013	0.000012
	Q8AY42.2	RecName: Full=Kunitz-type serine protease inhibitor B; AltName: Full=Kunitz inhibitor B; Flags: Precursor	Kunitz	FTYGGCGGNANNFKS	70.31	680.2905	2	32.54	1010000				0.0008	0.000008
				EFTYGGCGGNANNFKS	83.76	753.8247	2	44.42	1010000					
				YVNYGCYCGAGGSGTPVDELDRC	105.56	1123.968	2	56.53	535000					
				GYVNYGCYCGAGGSGTPVDELDRC	97.63	1205.504	2	60.35	535000					
	0702209A	phospholipase A	PLA2	KTYSYTCTKPNLTCTDA	83.76	912.8915	2	46.31	535000				0.0004	0.000004
				KTKTYSYTCTKPNLTC	59.92	839.4105	2	42.15	535000					
				NLTCTDAAGTCARI	44.87	648.787	2	27.9	535000					
	1F94	Chain A BUCANDIN	3FTx	KITCSAEETFCYKW	90.62	754.8226	2	49.56	436000				0.0004	0.000003
	AAO84769.1	phospholipase A2 partial [Bungarus candidus]		RTAALCFAEAPYKR	75.14	671.3286	2	56.71	403000				0.0003	0.000003
				KSPYMNLYSYTCSGGTITCNADNDECGAFICN	48.41	1622.332	3	75.09	387000					
				RGSLTYLDYMDYGCYCGTGSRG	82.36	1169.977	2	74.6	387000					
	Q75S48.1	RecName: Full=Acidic phospholipase A2 1; Short=svPLA2; AltName: Full=PA2-I; AltName: Full=Phosphatidylcholine 2-acylhydrolase; Flags: Precursor	PLA2	RGTPVDELDRCCQTHDNCYAEAEEHPKC	51.58	1044.431	3	43.94	387000				0.0003	0.000003
				GSLTYLDYMDYGCYCGTGSRG	44.95	1149.455	2	66.5	387000					
				DNDECGAFICNCDRTAALCFAKA	45.36	831.6834	3	61.3	387000					
				RYTEMIQCTIRG	51.59	665.8098	2	41.6	387000					
	0412250A	toxin A beta1 bungaro		RTIICYGAAGGTCRI	58.71	700.3253	2	46.34	346000				0.0003	0.000003
				GCAATCPEFRS	43.96	556.2367	2	36.4	283000					
				RGPVIEQGCAATCPEFRS	127.31	896.4115	2	52.12	283000					
	O12962.1	RecName: Full=Kappa-5-bungarotoxin; Flags: Precursor	3FTx (k-bungarotoxin)	GPVIEQGCAATCPEFRS	66.43	867.8996	2	51.43	283000				0.0002	0.000002
				QGCAATCPEFRS	54.04	584.7479	2	36.63	283000					
				KTCLISPSSTPQTCPQGQDTCFLKA	59.64	1313.599	2	71.79	283000					
				FKNMIQCAGTRTWTSYIGYGCYCGYGGSGTP	44.45	1370.587	3	78.66	200000					
	1G0Z	Chain B PHOSPHOLIPASE A2	PLA2	IGYGCYCGYGGSGTPVDELDRC	97.16	1091.943	2	57.98	200000				0.0002	0.000002
				NIPGCNPLIKT	47.8	506.2864	2	42.68	200000					
	ABX58164.1	putative long neurotoxin [Austrelaps labialis]	3FTx (Type-II NTx)	RGKVIELGCAATCPPAEPKKGI	46.49	695.0282	3	41.33	44100				0.0000	0.000000
				KVIELGCAATCPPAEPKKGI	38.86	633.3218	3	50.79	44100					
				DRTAALCFGDSEYIGAHKN	71.32	474.732	4	49.45	14400000000					
				AALCFGDSEYIGAHKN	59.82	523.243	3	54.07	14400000000	0.0754				
				RTAALCFGDSEYIGAHK	43.8	806.3585	2	65.08	14400000000					
				ALCFGDSEYIGAHKN	67.7	499.5642	3	52.66	14400000000					
				GHYADYGCYCGAGGSGTPVDALDRC	93.45	1231.504	2	55.39	14400000000					
				KTWGHYADYGCYCGAGGSGTPVDALDR	54.01	1325.538	2	69.52	14400000000					
				TWGHYADYGCYCGAGGSGTPVDALDRC	40.69	902.3782	3	65.23	14400000000					
				RCCYVHDNCYGVAENKH	93.27	944.8749	2	37.7	14400000000					
				CYVHDNCYGVAENKHKC	40.45	611.9497	3	25.3	14400000000					
				YADYGCYCGAGGSGTPVDALDRC	107.13	1081.444	2	57.32	14400000000					
				DSEYIGAHKNIDTKR	61.09	738.3781	2	27.75	14400000000					
	AAL87004.1	beta-bungarotoxin A2 chain precursor [Bungarus caeruleus]	beta-BTx	RYTIPCDKTWGHYADYGCYCGAGGSGTPVDA	91.58	1228.526	3	67.4	14400000000				0.4671	0.030843
				WGHYADYGCYCGAGGSGTPVDALDRC	89.97	1260.015	2	55.61	14400000000					
				CFGDSEYIGAHKN	54.06	408.5278	3	38.1	14400000000					
				DKTWGHYADYGCYCGAGGSGTPVDALDRC	48.65	734.3186	4	58.89	14400000000					
				YVHDNCYGVAENKH	57.8	703.3104	2	27.05	14400000000					
				LCFGDSEYIGAHKNIDTKR	39.77	489.4792	4	45.57	14400000000					
				AANIPPYPLNLINFMEMIRY	48.37	1089.558	2	87.52	14400000000					
				CDRTAALCFGDSEYIGAHKN	46.97	670.9794	3	54.67	14400000000					
				GDSEYIGAHKNIDTKR	75.27	795.8921	2	31.67	14400000000					
				HYADYGCYCGAGGSGTPVDALDRC	85.47	1162.981	2	59.65	14400000000					
				FGDSEYIGAHKN	48.36	538.754	2	25.63	14400000000					
				TAALCFGDSEYIGAHKN	59.48	546.9218	3	56.05	14400000000					
				ADYGCYCGAGGSGTPVDALDRC	92	1045.926	2	58.4	14400000000					
				RFVCDCDRTAAICFAKAPYN	40.96	722.3257	3	61.05	13500000000					
				CDCDRTAAICFAKA	62	476.5507	3	41.93	13500000000					
	1TC8	Chain A phospholipase A2 isoform 1	PLA2	ARFVCDCDRTAAICFAKA	60.48	498.2314	4	46.87	13500000000				0.4379	0.028916
				FVCDCDRTAAICFAKA	41.1	562.9182	3	48.6	13500000000					
				VCDCDRTAAICFAKA	51.37	794.3394	2	42.3	13500000000					
				EYTNYGCYCGAGGSGTPIDALDRC	108.7	1184.501	2	62.95	1710000000					
				WGEYTNYGCYCGAGGSGTPIDALDRC	88.47	1277.534	2	64.71	1710000000					
				EKTWGEYTNYGCYCGAGGSGTPIDALDRC	43.32	990.4321	3	68.9	1710000000					
				GEYTNYGCYCGAGGSGTPIDALDRC	95.53	1249.022	2	64.78	1710000000					
	Q8QFW4.1	RecName: Full=Basic phospholipase A2 beta-bungarotoxin A1 chain; Short=Beta-BuTX A1 chain; Short=svPLA2; AltName: Full=Phosphatidylcholine 2-acylhydrolase; Flags: Precursor	beta-BTx	RYTIPCEKTWGEYTNYGCYCGAGGSGTPIDAL	94.59	1244.866	3	77.12	1710000000				0.0555	0.003663
				KTQSYSYKLTRR	48.03	416.2192	3	36.77	1710000000					
				PLNLINFMNMIRY	38.8	697.868	2	87.72	1710000000					
				KTWGEYTNYGCYCGAGGSGTPIDALDRC	109.61	1421.096	2	74.13	1710000000					
				CFGDSEYIEGHKRI	38.53	360.1743	4	34.62	1710000000					
				CPQPKPYEEVTCCSTDKC	43.69	646.9566	3	37.38	356000000					
				KVIELGCVATCPQPKP	79.53	786.3962	2	53.12	356000000					
				LGCVATCPQPKPYEEVTCCSTDKC	43.12	863.0353	3	45.16	356000000					
				RGKVIELGCVATCPQPKPYEE	42.3	716.0275	3	56.93	356000000					
				VTCPSGENLCYTKM	40.96	715.3087	2	36.56	356000000					
				PSGENLCYTKM	47.53	536.2424	2	31.4	356000000					
	CAM11302.1	alpha-delta-bungarotoxin-4 partial [Bungarus caeruleus]	3FTx (Type II NTx)	QPKPYEEVTCCSTDKC	40.5	571.9202	3	34.23	356000000				0.0115	0.000763
F9b				CPSGENLCYTKM	57.02	584.7686	2	33.57	356000000		0.8758	0.0660		
				TCPSGENLCYTKM	59.41	664.7845	2	35.32	356000000					
				TVTCPSGENLCYTKM	57.59	764.8428	2	41.53	356000000					
				KMWCDAFCSSRG	46.58	668.2497	2	53.85	356000000					
				ELGCVATCPQPKPYEEVTCCSTDKC	50.75	900.73	3	49.9	356000000					
				CVATCPQPKPYEEVTCCSTDKC	64.24	790.6856	3	41.8	356000000					
	Q75S49.1	RecName: Full=Kunitz-type serine protease inhibitor homolog beta-bungarotoxin B4 chain; Flags: Precursor	Kunitz	NGNGNHFKTETLCRC	54.41	767.3657	2	30.48	280000000				0.0091	0.000600
				NGNHFKTETLCRC	39.19	454.8912	3	30.15	280000000					
				ENMIECAGTRSWTHYVSYGCYCGYGGSGTPV	57.16	1345.55	3	58.41	192000000					
	ABU63166.1	phospholipase A2 precursor BF-42 [Bungarus fasciatus]	PLA2	YVSYGCYCGYGGSGTPVDELDRC	102.32	1156.48	2	62.51	192000000				0.0062	0.000411
				HYVSYGCYCGYGGSGTPVDELDRC	73.89	1238.014	2	65.19	192000000					
				RFSYGGCGGNGNHFKT	83.88	751.3175	2	38.02	123000000					
	ABG90493.1	beta-bungarotoxin B5 chain partial [Bungarus multicinctus]	beta-BTx	RQRHPDCDKPPDTKR	37.72	399.1914	4	18.01	123000000				0.0040	0.000263
				KNCERFSYGGCGGNGNHFKTKQ	38.74	573.2523	4	34.76	123000000					
				RHPDCDKPPDTKRC	62.44	489.2336	3	16.8	123000000					
	Q75S50.1	RecName: Full=Kunitz-type serine protease inhibitor homolog beta-bungarotoxin B3 chain; Flags: Precursor	Kunitz	RYGGCKGNGNHFKS	42.94	669.8041	2	20.12	45200000				0.0015	0.000097
				KRHPDCDKPPDTRI	52.11	374.1784	4	18.08	45200000					
	JAS05119.1	Phospholipase A2 6c [Micrurus tener]		RTAALCFAKAPYNNNNHKIDPSRCQ	44.83	930.7732	3	75.13	36400000				0.0012	0.000078
	P00610.1	RecName: Full=Basic phospholipase A2; Short=svPLA2; AltName: Full=Myotoxin; AltName: Full=Phosphatidylcholine 2-acylhydrolase; AltName: Full=Toxin VI-5; AltName: Full=Toxin VI:5b	PLA2	RRSSLDYADYGCYCGAGGSGTPVDELDRC	67.89	1471.124	2	79.63	30000000				0.0010	0.000064
	Q7T2I3.1	RecName: Full=Long neurotoxin LlLong; Flags: Precursor	3FTx (Type-II NTx)	KVIELGCTATCPRA	80.78	688.8364	2	45.77	26500000				0.0009	0.000057
	TIKFBU	beta-1 bungarotoxin chain B major component - many-banded krait	beta-BTx	DGDHGNFKTETLCRC	48.32	512.2404	3	35.16	11900000				0.0004	0.000025
	AAO84769.1	phospholipase A2 partial [Bungarus candidus]	PLA2	RTAALCFAEAPYKR	75.14	671.3286	2	56.71	8390000				0.0003	0.000018
	CAB62501.1	beta-bungarotoxin A chain partial [Bungarus multicinctus]	beta-BTx	RTIICYGAAGTCERI	62.76	736.335	2	47.63	5340000				0.0002	0.000011
	UNW14933.1	phospholipase A2 protein 2-1 partial [Pseudonaja nuchalis]		AHYGCFCGSGGSGTPVDELDRC	42.16	1085.938	2	61.56	1030000				0.0000	0.000002
				LITCANRGSRNWWDYAHYGCFCGSGGSGTPV	38.45	1341.251	3	80.15	1030000					
	P20255.1	RecName: Full=Basic phospholipase A2 PA-12A; Short=svPLA2; AltName: Full=Phosphatidylcholine 2-acylhydrolase	PLA2	NLIQFGNMIQCANKGSRPSLNYADYGCYCGW	48.32	1211.048	4	84.8	1010000				0.0000	0.000002
	JAA74725.1	PLA-2-Den-2 [Denisonia devisi]		KDMIECANHGSRGALDYADYGCYCGKGGSGT	38.01	1361.576	3	79.92	655000				0.0000	0.000001
	AAB25587.1	alpha-neurotoxin Oh-4 [Ophiophagus hannah=King cobras venom Peptide 72 aa]	3FTx (Type-II NTx)	RIISEACPPGQDLCYMKT	41.86	949.4233	2	50.67	486000				0.0000	0.000001
				TVKPHEQITCCSTDNCNPHPKM	47.34	606.2728	4	26.5	486000					
	P59069.1	RecName: Full=Phospholipase A2 superbin c; Short=svPLA2; AltName: Full=Phosphatidylcholine 2-acylhydrolase		IPGSRPLLHYTDYGCYCGKGGSGTPVDELDRC	38.79	1109.831	3	81.43	477000				0.0000	0.000001
				KTKTYSYTCTKPNLTC	59.92	839.4105	2	42.15	270000					
				NLTCTDAAGTCARI	44.87	648.787	2	27.9	270000					
	0702209A	phospholipase A		KTYSYTCTKPNLTCTD	57.48	855.3762	2	46.26	270000				0.0000	0.000001
				YVNYGCYCGAGGSGTPVDELDRC	105.56	1123.968	2	56.53	270000					
				GYVNYGCYCGAGGSGTPVDELDRC	97.63	1205.504	2	60.35	270000					
	AHZ08805.1	phospholipase A2 [Micropechis ikaheca]	PLA2	RNMIICTIPDREPLLAFSNYGCYCGKGGSGTPV	38.22	1392.632	3	95.15	136000				0.0000	0.000000
	P20251.1	RecName: Full=Acidic phospholipase A2 PA-3; Short=svPLA2; AltName: Full=Phosphatidylcholine 2-acylhydrolase		MIQCANKGSRPTRHYMDYGCYCGWGGSGTP	37.97	1360.586	3	78.52	101000				0.0000	0.000000
	1G0Z	Chain B PHOSPHOLIPASE A2		IGYGCYCGYGGSGTPVDELDRC	97.16	1091.943	2	57.98	83400				0.0000	0.000000
				NIPGCNPLIKT	47.8	506.2864	2	42.68	83400					
				FKNMIQCAGTRTWTSYIGYGCYCGYGGSGTP	44.45	1370.587	3	78.66	83400					

	JAB52811.1	phospholipase A2 12 [Micrurus fulvius]		KRSWWHFADYGCYCGSGGSGTPVDELDRC	49.97	1045.763	3	73.79	12100				0.0000	0.000000
				KNMIECTTKRSWWHFADYGCYCGSGGSGTP	39.12	1371.574	3	80.4	12100					
	Q8QFW4.1	RecName: Full=Basic phospholipase A2 beta-bungarotoxin A1 chain; Short=Beta-BuTX A1 chain; Short=svPLA2; AltName: Full=Phosphatidylcholine 2-acylhydrolase; Flags: Precursor	beta-BTx	WGEYTNYGCYCGAGGSGTPIDALDRC	88.47	1277.534	2	64.71	447000000				0.5146	0.047495
				EKTWGEYTNYGCYCGAGGSGTPIDALDRC	43.32	990.4321	3	68.9	447000000					
				KTWGEYTNYGCYCGAGGSGTPIDALDRC	109.61	1421.096	2	74.13	447000000					
				KTQSYSYKLTRR	48.03	416.2192	3	36.77	447000000					
				CFGDSEYIEGHKRI	38.53	360.1743	4	34.62	447000000					
				PLNLINFMNMIRY	38.8	697.868	2	87.72	447000000					
				RYTIPCEKTWGEYTNYGCYCGAGGSGTPIDAL	94.59	1244.866	3	77.12	447000000					
				GEYTNYGCYCGAGGSGTPIDALDRC	95.53	1249.022	2	64.78	447000000					
	1TC8	Chain A phospholipase A2 isoform 1	PLA2	RFVCDCDRTAAICFAKAPYNT	58.51	760.3383	3	58.33	140000000				0.1612	0.014875
				CDCDRTAAICFAKA	62	476.5507	3	41.93	140000000					
				FVCDCDRTAAICFAKA	41.1	562.9182	3	48.6	140000000					
				VCDCDRTAAICFAKA	51.37	794.3394	2	42.3	140000000					
				ARFVCDCDRTAAICFAKA	60.48	498.2314	4	46.87	140000000					
	AAL87004.1	beta-bungarotoxin A2 chain precursor [Bungarus caeruleus]	beta-BTx	FGDSEYIGAHKNIDTKR	72.7	824.397	2	31.66	92400000				0.1064	0.009818
				WGHYADYGCYCGAGGSGTPVDALDRC	89.97	1260.015	2	55.61	92400000					
				ALCFGDSEYIGAHKNIDTKR	39.52	517.7491	4	51.19	92400000					
				KTWGHYADYGCYCGAGGSGTPVDALDRC	110.72	1403.582	2	65.03	92400000					
				RTAALCFGDSEYIGAHKN	107.41	870.4016	2	56.54	92400000					
				DKTWGHYADYGCYCGAGGSGTPVDALDRC	48.65	734.3186	4	58.89	92400000					
				RCCYVHDNCYGVAENKHKC	42.54	431.5845	5	31.64	92400000					
				TWGHYADYGCYCGAGGSGTPVDALDRC	40.69	902.3782	3	65.23	92400000					
				AANIPPYPLNLINFMEMIRY	48.37	1089.558	2	87.52	92400000					
				GDSEYIGAHKNIDTKR	75.27	795.8921	2	31.67	92400000					
				LCFGDSEYIGAHKNIDTKR	39.77	489.4792	4	45.57	92400000					
				YADYGCYCGAGGSGTPVDALDRC	107.13	1081.444	2	57.32	92400000					
				GHYADYGCYCGAGGSGTPVDALDRC	93.45	1231.504	2	55.39	92400000					
				DRTAALCFGDSEYIGAHKN	71.32	474.732	4	49.45	92400000					
				AALCFGDSEYIGAHKN	59.82	523.243	3	54.07	92400000					
				CYVHDNCYGVAENKHKC	40.45	611.9497	3	25.3	92400000					
				ADYGCYCGAGGSGTPVDALDRC	92	1045.926	2	58.4	92400000					
				DSEYIGAHKNIDTKRH	40.7	544.6212	3	26.69	92400000					
				TAALCFGDSEYIGAHKN	59.48	546.9218	3	56.05	92400000					
				HYADYGCYCGAGGSGTPVDALDRC	85.47	1162.981	2	59.65	92400000					
				YVHDNCYGVAENKH	57.8	703.3104	2	27.05	92400000					
				CDRTAALCFGDSEYIGAHKN	46.97	670.9794	3	54.67	92400000					
				CFGDSEYIGAHKN	54.06	408.5278	3	38.1	92400000					
				RYTIPCDKTWGHYADYGCYCGAGGSGTPVDA	91.58	1228.526	3	67.4	92400000					
	CAM11302.1	alpha-delta-bungarotoxin-4 partial [Bungarus caeruleus]	3FTx (Type II NTx)	RGKVIELGCVATCPQPKPYEEVTCCSTDKCNPH	51.23	792.7693	5	50.64	91600000				0.1054	0.009733
				KVIELGCVATCPQPKP	79.53	786.3962	2	53.12	91600000					
				LGCVATCPQPKPYEEVTCCSTDKC	43.12	863.0353	3	45.16	91600000					
				CPQPKPYEEVTCCSTDKC	43.69	646.9566	3	37.38	91600000					
				CVATCPQPKPYEEVTCCSTDKC	64.24	790.6856	3	41.8	91600000					
				TVTCPSGENLCYTKM	57.59	764.8428	2	41.53	91600000					
F10a				PSGENLCYTKM	47.53	536.2424	2	31.4	91600000	0.0923	1.0000	0.0923		
				CPSGENLCYTKM	57.02	584.7686	2	33.57	91600000					
				QPKPYEEVTCCSTDKC	40.5	571.9202	3	34.23	91600000					
				TCPSGENLCYTKM	59.41	664.7845	2	35.32	91600000					
				ELGCVATCPQPKPYEEVTCCSTDKC	50.75	900.73	3	49.9	91600000					
				VTCPSGENLCYTKM	40.96	715.3087	2	36.56	91600000					
				KMWCDAFCSSRG	46.58	668.2497	2	53.85	91600000					
	TIKFBU	beta-1 bungarotoxin chain B major component - many-banded krait	beta-BTx	DGDHGNFKTETLCRC	48.32	512.2404	3	35.16	23700000				0.0273	0.002518
	Q75S49.1	RecName: Full=Kunitz-type serine protease inhibitor homolog beta-bungarotoxin B4 chain; Flags: Precursor	Kunitz	NGNGNHFKTETLCRC	54.41	767.3657	2	30.48	21400000				0.0246	0.002274
				NGNHFKTETLCRC	39.19	454.8912	3	30.15	21400000					
	Q7LZQ6.1	RecName: Full=Basic phospholipase A2 13; Short=svPLA2; AltName: Full=Cytotoxin XIII; AltName: Full=Phosphatidylcholine 2-acylhydrolase; AltName: Full=Phospholipase A2 XIII	PLA2	NLYQFKNMIECAGTRT	79.46	930.9393	2	55.02	14100000				0.0162	0.001498
	ABW24180.1	PLA-18 precursor [Austrelaps superbus]		DYGCYCGSGGTGTPVDELDRC	53.37	1032.413	2	64.81	10400000				0.0120	0.001105
				SGGTGTPVDELDRC	41.12	608.7931	2	46.39	10400000					
	LAB21274.1	hypothetical protein partial [Micrurus spixii]		DYGCYCGYGGSGKPVDELDRC	41.54	1076.943	2	64.71	10200000				0.0117	0.001084
	ABG90493.1	beta-bungarotoxin B5 chain partial [Bungarus multicinctus]	beta-BTx	RFSYGGCGGNGNHFKTKQ	77.03	865.8912	2	34.6	8860000				0.0102	0.000941
				RHPDCDKPPDTKRC	62.44	489.2336	3	16.8	8860000					
				RQRHPDCDKPPDTKR	37.72	399.1914	4	18.01	8860000					
				KNCERFSYGGCGGNGNHFKTKQ	38.74	573.2523	4	34.76	8860000					
	ABU63166.1	phospholipase A2 precursor BF-42 [Bungarus fasciatus]	PLA2	ENMIECAGTRSWTHYVSYGCYCGYGGSGTPV	38.89	1340.242	3	81.25	2510000				0.0029	0.000267
				YVSYGCYCGYGGSGTPVDELDRC	102.32	1156.48	2	62.51	2510000					
				HYVSYGCYCGYGGSGTPVDELDRC	73.89	1238.014	2	65.19	2510000					
	Q75S50.1	RecName: Full=Kunitz-type serine protease inhibitor homolog beta-bungarotoxin B3 chain; Flags: Precursor	Kunitz	KRHPDCDKPPDTRI	52.11	374.1784	4	18.08	2150000				0.0025	0.000228
				RYGGCKGNGNHFKS	42.94	669.8041	2	20.12	2150000					
	0702209A	phospholipase A		YVNYGCYCGAGGSGTPVDELDRC	105.56	1123.968	2	56.53	2000000				0.0023	0.000213
				KTKTYSYTCTKPNLTC	59.92	839.4105	2	42.15	2000000					
				GYVNYGCYCGAGGSGTPVDELDRC	97.63	1205.504	2	60.35	2000000					
				NLTCTDAAGTCARI	44.87	648.787	2	27.9	2000000					
				KTYSYTCTKPNLTCTDAAGTCARI	56.61	838.0289	3	46.85	2000000					
	P00610.1	RecName: Full=Basic phospholipase A2; Short=svPLA2; AltName: Full=Myotoxin; AltName: Full=Phosphatidylcholine 2-acylhydrolase; AltName: Full=Toxin VI-5; AltName: Full=Toxin VI:5b	PLA2	RRSSLDYADYGCYCGAGGSGTPVDELDRC	67.89	1471.124	2	79.63	1150000				0.0013	0.000122
	1G0Z	Chain B PHOSPHOLIPASE A2		FKNMIQCAGTRTWTSYIGYGCYCGYGGSGTP	44.45	1370.587	3	78.66	870000				0.0010	0.000092
				NIPGCNPLIKT	47.8	506.2864	2	42.68	870000					
				IGYGCYCGYGGSGTPVDELDRC	97.16	1091.943	2	57.98	870000					
	O12963.1	RecName: Full=Long neurotoxin homolog TA-bm16; AltName: Full=Delta-protein; Flags: Precursor	3FTx (Type-II NTx)	ETCPDGKNICVKR	41.3	646.3103	2	28.49	106000				0.0001	0.000011
				FYTCPNSETCPDGKN	46.19	764.8057	2	30.26	106000					
				SFYTCPNSETCPDGKN	77.35	838.3411	2	38.55	106000					
				NSETCPDGKNICVKR	52.09	754.3478	2	31.46	106000					
	P59068.1	RecName: Full=Phospholipase A2 superbin b; Short=svPLA2; AltName: Full=Phosphatidylcholine 2-acylhydrolase	PLA2	HWLAYADYGCYCGWGGSGTPVDELDRC	41.35	956.7444	3	81.16	91300				0.0001	0.000010
	JAB52760.1	phospholipase A2 9 [Micrurus fulvius]		DFTNYGCYCGSGGSGTPVDELDRC	39.35	1206.497	2	61.6	90700				0.0001	0.000010
	ABB83636.1	weak neurotoxin precursor [Ophiophagus hannah]	3FTx	RVNPGISIICCKT	57.16	630.8262	2	54.19	58700				0.0001	0.000006
	1TC8	Chain A phospholipase A2 isoform 1	PLA2	ARFVCDCDRTAAICFAKA	60.48	498.2314	4	46.87	537000000				0.8378	0.003609
				RFVCDCDRTAAICFAKA	99.44	917.4034	2	51.68	537000000					
				FVCDCDRTAAICFAKA	41.1	562.9182	3	48.6	537000000					
				CDCDRTAAICFAKA	62	476.5507	3	41.93	537000000					
				VCDCDRTAAICFAKA	51.37	794.3394	2	42.3	537000000					
	AAL87004.1	beta-bungarotoxin A2 chain precursor [Bungarus caeruleus]	beta-BTx	DSEYIGAHKNIDTKR	61.09	738.3781	2	27.75	23600000				0.0368	0.000159
				FGDSEYIGAHKN	48.36	538.754	2	25.63	23600000					
				RYTIPCDKTWGHYADYGCYCGAGGSGTPVDA	91.58	1228.526	3	67.4	23600000					
				RTAALCFGDSEYIGAHKN	107.41	870.4016	2	56.54	23600000					
				TAALCFGDSEYIGAHKN	59.48	546.9218	3	56.05	23600000					
				GHYADYGCYCGAGGSGTPVDALDRC	93.45	1231.504	2	55.39	23600000					
				KTWGHYADYGCYCGAGGSGTPVDALDR	54.01	1325.538	2	69.52	23600000					
				DRTAALCFGDSEYIGAHKN	71.32	474.732	4	49.45	23600000					
				CYVHDNCYGVAENKH	62.88	784.8428	2	31.8	23600000					
				TWGHYADYGCYCGAGGSGTPVDALDRC	40.69	902.3782	3	65.23	23600000					
				ALCFGDSEYIGAHKNIDTKR	39.52	517.7491	4	51.19	23600000					
				YADYGCYCGAGGSGTPVDALDRC	107.13	1081.444	2	57.32	23600000					
				WGHYADYGCYCGAGGSGTPVDALDRC	89.97	1260.015	2	55.61	23600000					
				ADYGCYCGAGGSGTPVDALDRC	92	1045.926	2	58.4	23600000					
				DKTWGHYADYGCYCGAGGSGTPVDALDRC	48.65	734.3186	4	58.89	23600000					
				RCCYVHDNCYGVAENKHKC	42.54	431.5845	5	31.64	23600000					
				CDRTAALCFGDSEYIGAHKN	46.97	670.9794	3	54.67	23600000					
				LCFGDSEYIGAHKNIDTKR	39.77	489.4792	4	45.57	23600000					
				AANIPPYPLNLINFMEMIRY	48.37	1089.558	2	87.52	23600000					
				HYADYGCYCGAGGSGTPVDALDRC	85.47	1162.981	2	59.65	23600000					
				YVHDNCYGVAENKH	57.8	703.3104	2	27.05	23600000					
				AALCFGDSEYIGAHKN	59.82	523.243	3	54.07	23600000					
				CFGDSEYIGAHKN	54.06	408.5278	3	38.1	23600000					
				GDSEYIGAHKNIDTKR	75.27	795.8921	2	31.67	23600000					
	Q7LZQ6.1	RecName: Full=Basic phospholipase A2 13; Short=svPLA2; AltName: Full=Cytotoxin XIII; AltName: Full=Phosphatidylcholine 2-acylhydrolase; AltName: Full=Phospholipase A2 XIII	PLA2	NLYQFKNMIECAGTRT	79.46	930.9393	2	55.02	21100000				0.0329	0.000142
	1G0Z	Chain B PHOSPHOLIPASE A2		IGYGCYCGYGGSGTPVDELDRC	97.16	1091.943	2	57.98	19000000				0.0296	0.000128
				FKNMIQCAGTRTWTSYIGYGCYCGYGGSGTP	44.45	1370.587	3	78.66	19000000					
				NIPGCNPLIKT	47.8	506.2864	2	42.68	19000000					
	Q8QFW4.1	RecName: Full=Basic phospholipase A2 beta-bungarotoxin A1 chain; Short=Beta-BuTX A1 chain; Short=svPLA2; AltName: Full=Phosphatidylcholine 2-acylhydrolase; Flags: Precursor	beta-BTx	PLNLINFMNMIRY	38.8	697.868	2	87.72	9440000				0.0147	0.000063
				KTQSYSYKLTRR	48.03	416.2192	3	36.77	9440000					
				EYTNYGCYCGAGGSGTPIDALDRC	108.7	1184.501	2	62.95	9440000					
				WGEYTNYGCYCGAGGSGTPIDALDRC	88.47	1277.534	2	64.71	9440000					
				EKTWGEYTNYGCYCGAGGSGTPIDALDRC	43.32	990.4321	3	68.9	9440000					
				KTWGEYTNYGCYCGAGGSGTPIDALDRC	109.61	1421.096	2	74.13	9440000					
				CFGDSEYIEGHKRI	38.53	360.1743	4	34.62	9440000					
				RYTIPCEKTWGEYTNYGCYCGAGGSGTPIDAL	94.59	1244.866	3	77.12	9440000					
				GEYTNYGCYCGAGGSGTPIDALDRC	95.53	1249.022	2	64.78	9440000					
F11a	JAB52755.1	vascular endothelial growth factor 3 [Micrurus fulvius]	VEGF	KHFQSQHIHPMSFQQHSKC	42.48	530.7524	4	26.48	8850000		0.0674	0.0043	0.0138	0.000059
				KHLYKQDPLTCKC	69.75	468.2431	3	31.6	8850000					
	0702209A	phospholipase A	PLA2	KTYSYTCTKPNLTCTDAAGTCARI	56.61	838.0289	3	46.85	5640000				0.0088	0.000038
				YVNYGCYCGAGGSGTPVDELDRC	105.56	1123.968	2	56.53	5640000					
				KTKTYSYTCTKPNLTC	59.92	839.4105	2	42.15	5640000					
				GYVNYGCYCGAGGSGTPVDELDRC	97.63	1205.504	2	60.35	5640000					
				NLTCTDAAGTCARI	44.87	648.787	2	27.9	5640000					
	ACE73577.1	cysteine-rich seceretory protein Bc-CRPb [Bungarus candidus]	CRISP	KYNDDYTNCKSLAKQ	60.51	796.3566	2	34.23	4950000				0.0077	0.000033
				YNDDYTNCKSLAKQ	49.97	714.825	2	28.91	4950000					
				NGLCTNPCKYNDDYTNCKS	53.04	1061.934	2	41.89	4950000					
				KSGPPCGDCPSACVNGLCTNPCKYNDDYTNC	67.79	1194.464	3	54.86	4950000					
	XP_032083329.1	phospholipase A2 inhibitor subunit gamma A-like [Thamnophis elegans]	PLA2	RYPGDIAYNIKG	48.55	577.2981	2	53.07	3440000				0.0054	0.000023
				KERYPGDIAYNIKG	54.85	480.2493	3	49.38	3440000					
	CAB53358.1	cardiotoxin-like protein [Bungarus multicinctus multicinctus]	3FTx (C-3FTx)	EAYHGKYPVILERG	63.36	482.6015	3	44.53	2940000				0.0046	0.000020
	CAM11302.1	alpha-delta-bungarotoxin-4 partial [Bungarus caeruleus]	3FTx (Type II NTx)	CPSGENLCYTKM	57.02	584.7686	2	33.57	1930000				0.0030	0.000013
				RGKVIELGCVATCPQPKPYEEVTCCSTDK	48.39	1033.459	3	60.43	1930000					
				KVIELGCVATCPQPKPYEEVTCCSTDKC	69.81	1014.462	3	60.65	1930000					
				PSGENLCYTKM	47.53	536.2424	2	31.4	1930000					
				TCPSGENLCYTKM	59.41	664.7845	2	35.32	1930000					
				QPKPYEEVTCCSTDKC	40.5	571.9202	3	34.23	1930000					
				TVTCPSGENLCYTKM	57.59	764.8428	2	41.53	1930000					
				LGCVATCPQPKPYEEVTCCSTDKC	43.12	863.0353	3	45.16	1930000					
				CPQPKPYEEVTCCSTDKC	43.69	646.9566	3	37.38	1930000					
				CVATCPQPKPYEEVTCCSTDKC	64.24	790.6856	3	41.8	1930000					
				KMWCDAFCSSRG	46.58	668.2497	2	53.85	1930000					
				ELGCVATCPQPKPYEEVTCCSTDKC	50.75	900.73	3	49.9	1930000					
				VTCPSGENLCYTKM	40.96	715.3087	2	36.56	1930000					

	Q8AY42.2	RecName: Full=Kunitz-type serine protease inhibitor B; AltName: Full=Kunitz inhibitor B; Flags: Precursor	Kunitz	FTYGGCGGNANNFKS	70.31	680.2905	2	32.54	1170000				0.0018	0.000008
				EFTYGGCGGNANNFKS	83.76	753.8247	2	44.42	1170000					
	AOS50879.1	vespryn [Pseudechis papuanus]	Vespryn	RSGRHFFEVKY	44.15	369.529	3	35.21	718000				0.0011	0.000005
	JAC94987.1	Vespryn [Opheodrys aestivus]		KADVTFDSNTAFESLVVSPDKKT	39.71	757.3773	3	72.4	393000				0.0006	0.000003
	1F94	Chain A BUCANDIN	3FTx	KITCSAEETFCYKW	90.62	754.8226	2	49.56	279000				0.0004	0.000002
				ENMIECAGTRSWTHYVSYGCYCGYGGSGTPV	57.16	1345.55	3	58.41	165000					
	ABU63166.1	phospholipase A2 precursor BF-42 [Bungarus fasciatus]	PLA2	HYVSYGCYCGYGGSGTPVDELDRC	73.89	1238.014	2	65.19	165000				0.0003	0.000001
				YVSYGCYCGYGGSGTPVDELDRC	102.32	1156.48	2	62.51	165000					
	AAO84769.1	phospholipase A2 partial [Bungarus candidus]		RTAALCFAEAPYKR	75.14	671.3286	2	56.71	150000				0.0002	0.000001
	ABB83636.1	weak neurotoxin precursor [Ophiophagus hannah]	3FTx	RVNPGISIICCKT	57.16	630.8262	2	54.19	113000				0.0002	0.000001
				ETCPDGKNICVKR	41.3	646.3103	2	28.49	61400					
	O12963.1	RecName: Full=Long neurotoxin homolog TA-bm16; AltName: Full=Delta-protein; Flags: Precursor	3FTx (Type-II NTx)	FYTCPNSETCPDGKN	46.19	764.8057	2	30.26	61400				0.0001	0.000000
				SFYTCPNSETCPDGKNICVKR	42.93	1145.503	2	45	61400					
				NSETCPDGKNICVKR	52.09	754.3478	2	31.46	61400					
				RHPDCDKPPDTKRC	62.44	489.2336	3	16.8	28300					
	ABG90493.1	beta-bungarotoxin B5 chain partial [Bungarus multicinctus]	beta-BTx	RFSYGGCGGNGNHFKTKQ	77.03	865.8912	2	34.6	28300				0.0000	0.000000
				RQRHPDCDKPPDTKR	40.18	350.7746	5	16.57	28300					
				KNCERFSYGGCGGNGNHFKTKQ	38.74	573.2523	4	34.76	28300					
	Q9DF52.1	RecName: Full=Basic phospholipase A2 KPA2; Short=svPLA2; AltName: Full=Phosphatidylcholine 2-acylhydrolase; Flags: Precursor		PWTAYVNYGCYCGKGGSGTPVDELDRC	50.11	942.4145	3	77.27	10100				0.0000	0.000000
				KNMIQCAGTRPWTAYVNYGCYCGKGGSGTP	47.09	993.1893	4	70.95	10100					
				CDCDRTAAICFAKA	62	476.5507	3	41.93	25400000000					
				RFVCDCDRTAAICFAKAPYN	40.96	722.3257	3	61.05	25400000000					
	1TC8	Chain A phospholipase A2 isoform 1		FVCDCDRTAAICFAKA	41.1	562.9182	3	48.6	25400000000				0.8979	0.053510
				ARFVCDCDRTAAICFAKA	60.48	498.2314	4	46.87	25400000000					
			PLA2	VCDCDRTAAICFAKA	51.37	794.3394	2	42.3	25400000000	0.0639				
	Q7LZQ6.1	RecName: Full=Basic phospholipase A2 13; Short=svPLA2; AltName: Full=Cytotoxin XIII; AltName: Full=Phosphatidylcholine 2-acylhydrolase; AltName: Full=Phospholipase A2 XIII		NLYQFKNMIECAGTRT	79.46	930.9393	2	55.02	703000000				0.0249	0.001481
	UNW14927.1	phospholipase A2 protein 2-4 partial [Pseudonaja affinis]		RNVLDYADYGCYCGKGGSGTPVDELDRC	44.64	961.0893	3	78.24	584000000				0.0206	0.001230
				IGYGCYCGYGGSGTPVDELDRC	97.16	1091.943	2	57.98	524000000					
	1G0Z	Chain B PHOSPHOLIPASE A2		FKNMIQCAGTRTWTSYIGYGCYCGYGGSGTP	44.45	1370.587	3	78.66	524000000				0.0185	0.001104
				NIPGCNPLIKT	47.8	506.2864	2	42.68	524000000					
				LCFGDSEYIGAHKN	52.42	461.8709	3	41.83	502000000					
				CFGDSEYIGAHKN	54.06	408.5278	3	38.1	502000000					
				ADYGCYCGAGGSGTPVDALDRC	92	1045.926	2	58.4	502000000					
				CYVHDNCYGVAENKHKC	40.45	611.9497	3	25.3	502000000					
				RYTIPCDKTWGHYADYGCYCGAGGSGTPVDA	91.58	1228.526	3	67.4	502000000					
				TAALCFGDSEYIGAHKN	59.48	546.9218	3	56.05	502000000					
				DRTAALCFGDSEYIGAHKN	71.32	474.732	4	49.45	502000000					
				AALCFGDSEYIGAHKN	59.82	523.243	3	54.07	502000000					
				RTAALCFGDSEYIGAHKNIDTKRH	70.08	617.5548	4	50.92	502000000					
				HYADYGCYCGAGGSGTPVDALDRC	85.47	1162.981	2	59.65	502000000					
				RCCYVHDNCYGVAENKHKC	42.54	431.5845	5	31.64	502000000					
	AAL87004.1	beta-bungarotoxin A2 chain precursor [Bungarus caeruleus]		ALCFGDSEYIGAHKN	67.7	499.5642	3	52.66	502000000				0.0177	0.001058
				DKTWGHYADYGCYCGAGGSGTPVDALDRC	48.65	734.3186	4	58.89	502000000					
				KTWGHYADYGCYCGAGGSGTPVDALDRC	110.72	1403.582	2	65.03	502000000					
				GHYADYGCYCGAGGSGTPVDALDRC	93.45	1231.504	2	55.39	502000000					
				WGHYADYGCYCGAGGSGTPVDALDRC	89.97	1260.015	2	55.61	502000000					
			beta-BTx	TWGHYADYGCYCGAGGSGTPVDALDRC	40.69	902.3782	3	65.23	502000000					
				FGDSEYIGAHKNIDTKR	72.7	824.397	2	31.66	502000000					
				YADYGCYCGAGGSGTPVDALDRC	107.13	1081.444	2	57.32	502000000					
				CDRTAALCFGDSEYIGAHKN	46.97	670.9794	3	54.67	502000000					
				DSEYIGAHKNIDTKRH	40.7	544.6212	3	26.69	502000000					
				YVHDNCYGVAENKH	57.8	703.3104	2	27.05	502000000					
				AANIPPYPLNLINFMEMIRY	48.37	1089.558	2	87.52	502000000					
				GDSEYIGAHKNIDTKR	75.27	795.8921	2	31.67	502000000					
				PLNLINFMNMIRY	38.8	697.868	2	87.72	292000000					
				KTWGEYTNYGCYCGAGGSGTPIDALDRC	109.61	1421.096	2	74.13	292000000					
				CFGDSEYIEGHKRI	38.53	360.1743	4	34.62	292000000					
				EYTNYGCYCGAGGSGTPIDALDRC	108.7	1184.501	2	62.95	292000000					
	Q8QFW4.1	RecName: Full=Basic phospholipase A2 beta-bungarotoxin A1 chain; Short=Beta-BuTX A1 chain; Short=svPLA2; AltName: Full=Phosphatidylcholine 2-acylhydrolase; Flags: Precursor		EKTWGEYTNYGCYCGAGGSGTPIDALDRC	43.32	990.4321	3	68.9	292000000				0.0103	0.000615
				RYTIPCEKTWGEYTNYGCYCGAGGSGTPIDAL	94.59	1244.866	3	77.12	292000000					
				GEYTNYGCYCGAGGSGTPIDALDRC	95.53	1249.022	2	64.78	292000000					
				WGEYTNYGCYCGAGGSGTPIDALDRC	88.47	1277.534	2	64.71	292000000					
				KTQSYSYKLTRR	48.03	416.2192	3	36.77	292000000					
				KTYSYTCTKPNLTCTDAAGTCARI	56.61	838.0289	3	46.85	118000000					
				NLTCTDAAGTCARI	44.87	648.787	2	27.9	118000000					
	0702209A	phospholipase A		GYVNYGCYCGAGGSGTPVDELDRC	97.63	1205.504	2	60.35	118000000				0.0042	0.000249
				KTKTYSYTCTKPNLTC	59.92	839.4105	2	42.15	118000000					
				YVNYGCYCGAGGSGTPVDELDRC	105.56	1123.968	2	56.53	118000000					
F11b				AYVAYGCYCGKGGSGTPVDELDRC	41.56	1212.53	2	56.2	100000000		0.9326	0.0596		
				IWTAYVAYGCYCGKGGSGTPVDELDRC	53.76	928.0782	3	80.32	100000000					
	1DPY	Chain A PHOSPHOLIPASE A2		KNMIQCAGTRIWTAYVAYGCYCGKGGSGTPV	53.41	986.4463	4	78.23	100000000				0.0035	0.000211
			PLA2	TRIWTAYVAYGCYCGKGGSGTPVDELDRC	38.85	1017.796	3	91.73	100000000					
				WTAYVAYGCYCGKGGSGTPVDELDRC	38.7	1298.561	2	67.33	100000000					
				RIWTAYVAYGCYCGKGGSGTPVDELDRC	80.45	965.7621	3	78.48	100000000					
	QSI84030.1	phospholipase A2 IB [Calliophis bivirgatus]		SWRSFVDYGCYCGKGGSGTPVDELDRC	44.05	961.0906	3	87.24	21600000				0.0008	0.000046
	UNW14928.1	phospholipase A2 protein 2-3 partial [Pseudonaja affinis]		RDVLDYANYGCYCGKGGSGTPVDELDRC	41.43	1441.127	2	76.27	14500000				0.0005	0.000031
				YVSYGCYCGYGGSGTPVDELDRC	102.32	1156.48	2	62.51	7700000					
	ABU63166.1	phospholipase A2 precursor BF-42 [Bungarus fasciatus]		HYVSYGCYCGYGGSGTPVDELDRC	73.89	1238.014	2	65.19	7700000				0.0003	0.000016
				ENMIECAGTRSWTHYVSYGCYCGYGGSGTPV	38.89	1340.242	3	81.25	7700000					
	AAO84769.1	phospholipase A2 partial [Bungarus candidus]		RTAALCFAEAPYKR	75.14	671.3286	2	56.71	4860000				0.0002	0.000010
				LGCVATCPQPKPYEEVTCCSTDKC	43.12	863.0353	3	45.16	3930000					
				KVIELGCVATCPQPKP	79.53	786.3962	2	53.12	3930000					
				RGKVIELGCVATCPQPKPYEEV	72.59	1138.057	2	57.22	3930000					
				TCPSGENLCYTKM	59.41	664.7845	2	35.32	3930000					
				CPQPKPYEEVTCCSTDKC	43.69	646.9566	3	37.38	3930000					
				CPSGENLCYTKM	57.02	584.7686	2	33.57	3930000					
	CAM11302.1	alpha-delta-bungarotoxin-4 partial [Bungarus caeruleus]	3FTx (Type II NTx)	PSGENLCYTKM	47.53	536.2424	2	31.4	3930000				0.0001	0.000008
				VTCPSGENLCYTKM	40.96	715.3087	2	36.56	3930000					
				ELGCVATCPQPKPYEEVTCCSTDKC	50.75	900.73	3	49.9	3930000					
				CVATCPQPKPYEEVTCCSTDKC	64.24	790.6856	3	41.8	3930000					
				QPKPYEEVTCCSTDKC	40.5	571.9202	3	34.23	3930000					
				TVTCPSGENLCYTKM	57.59	764.8428	2	41.53	3930000					
				KMWCDAFCSSRG	46.58	668.2497	2	53.85	3930000					
				RHPDCDKPPDTKRC	62.44	489.2336	3	16.8	2490000					
	ABG90493.1	beta-bungarotoxin B5 chain partial [Bungarus multicinctus]	beta-BTx	RFSYGGCGGNGNHFKTKQ	77.03	865.8912	2	34.6	2490000				0.0001	0.000005
				RQRHPDCDKPPDTKR	40.18	350.7746	5	16.57	2490000					
				KNCERFSYGGCGGNGNHFKTKQ	38.74	573.2523	4	34.76	2490000					
	AAZ22652.1	PLA-2 precursor [Notechis scutatus]		RRPARHYMDYGCYCGKGGSGTPVDELDRC	38.8	1039.799	3	77.79	1310000				0.0000	0.000003
				IQCANHGRRPARHYMDYGCYCGKGGSGTPVI	39.86	985.9385	4	78.11	1310000					
			PLA2	NLYQFGNMIQCANHGRRPTLAYADYGCYCGA	40	1207.046	4	85.32	1220000					
	AAB20783.1	notechis 11'2=non-toxic phospholipase A2 [Notechis scutatus=Australian tiger snakes ssp. scutatus venom Peptide 118 aa]		MIQCANHGRRPTLAYADYGCYCGAGGSGTPV	53.38	1286.561	3	76.51	1220000				0.0000	0.000003
				RPTLAYADYGCYCGAGGSGTPVDELDRC	40.3	1383.099	2	80.71	1220000					
	Q75S50.1	RecName: Full=Kunitz-type serine protease inhibitor homolog beta-bungarotoxin B3 chain; Flags: Precursor	Kunitz	RYGGCKGNGNHFKS	42.94	669.8041	2	20.12	739000				0.0000	0.000002
				KRHPDCDKPPDTRI	52.11	374.1784	4	18.08	739000					
	4E4C	Chain A Phospholipase A2		KRPTWHYMDYGCYCGAGGSGTPVDELDRC	53.57	1046.09	3	75.09	611000				0.0000	0.000001
	JAB52811.1	phospholipase A2 12 [Micrurus fulvius]		KRSWWHFADYGCYCGSGGSGTPVDELDRC	49.97	1045.763	3	73.79	406000				0.0000	0.000001
				KNMIECTTKRSWWHFADYGCYCGSGGSGTP	39.12	1371.574	3	80.4	406000					
				YADYGCYCSAGGSGTPVDELDRC	43.4	1125.46	2	60.86	336000					
	P00607.1	RecName: Full=Basic phospholipase A2 homolog 1; Short=svPLA2 homolog; AltName: Full=Notechis II-1		GSRPSLAYADYGCYCSAGGSGTPVDELDRC	44.1	1008.775	3	91.13	336000				0.0000	0.000001
				SNMIQCANHGSRPSLAYADYGCYCSAGGSGT	40.18	1355.917	3	78.38	336000					
				SLAYADYGCYCSAGGSGTPVDELDRC	46.35	1299.056	2	67.93	336000					
	LAB37300.1	hypothetical protein partial [Micrurus spixii]	PLA2	RYAWDFTNYGCYCGAGGSGTPVDELDRC	41.3	977.7333	3	71.42	269000				0.0000	0.000001
	P81237.1	RecName: Full=Basic phospholipase A2 acanthin-2; Short=svPLA2; AltName: Full=Acanthin II; AltName: Full=Phosphatidylcholine 2-acylhydrolase		SWLSYVNYGCYCGWGGSGTPVDELDRC	37.97	1456.13	2	78.3	231000				0.0000	0.000000
	QSI84023.1	phospholipase A2 IB [Calliophis bivirgatus]		RLSWTSFVDYGCYCGKGGSGTPVDELDRC	43.27	1009.448	3	73.5	195000				0.0000	0.000000
				SWTSFVDYGCYCGKGGSGTPVDELDRC	39.99	942.7385	3	82.25	195000					
	JAA74871.1	PLA2-Hop-15 [Hoplocephalus bungaroides]		RTWWAYAHYGCYCGKGGSGTPVDELDRC	37.97	1002.774	3	72.86	44800				0.0000	0.000000
	JAA74902.1	PLA2-Pse-8 [Pseudonaja modesta]		RNVYDYVDYGCYCGKGGSGTPVDELDRC	38.86	987.0883	3	74.32	26500				0.0000	0.000000
				KSGPPCGDCPSACVNGLCTNPCKYNDDYTNCI	67.79	1194.464	3	54.86	30100000					
	ACE73577.1	cysteine-rich seceretory protein Bc-CRPb [Bungarus candidus]	CRISP	NGLCTNPCKYNDDYTNCKS	53.04	1061.934	2	41.89	30100000				0.3068	0.005619
				YNDDYTNCKSLAKQ	49.97	714.825	2	28.91	30100000					
				KYNDDYTNCKS	57.64	596.733	2	28.75	30100000					
				ARFVCDCDRTAAICFAKA	60.48	498.2314	4	46.87	25400000					
				VCDCDRTAAICFAKA	51.37	794.3394	2	42.3	25400000					
	1TC8	Chain A phospholipase A2 isoform 1		RFVCDCDRTAAICFAK	50.7	853.3602	2	62.15	25400000				0.2589	0.004741
			PLA2	FVCDCDRTAAICFAKA	41.1	562.9182	3	48.6	25400000					
				CDCDRTAAICFAKA	62	476.5507	3	41.93	25400000					
				FKNMIQCAGTRTWTSYIGYGCYCGYGGSGTP	44.45	1370.587	3	78.66	25200000					
	1G0Z	Chain B PHOSPHOLIPASE A2		NIPGCNPLIKT	47.8	506.2864	2	42.68	25200000				0.2569	0.004704
				IGYGCYCGYGGSGTPVDELDRC	97.16	1091.943	2	57.98	25200000					
				KTWGHYADYGCYC	41.44	696.77	2	60.8	7560000					
				DSEYIGAHKNIDTKRH	40.7	544.6212	3	26.69	7560000					
				DRTAALCFGDSEYIGAHKNIDTKR	54.14	494.2419	5	50.48	7560000					
				CFGDSEYIGAHKN	54.06	408.5278	3	38.1	7560000					
				LCFGDSEYIGAHKN	52.42	461.8709	3	41.83	7560000					
				RTAALCFGDSEYIGAHKN	107.41	870.4016	2	56.54	7560000					
				FGDSEYIGAHKNIDTKR	72.7	824.397	2	31.66	7560000					
				YVHDNCYGVAENKH	57.8	703.3104	2	27.05	7560000					
				CYVHDNCYGVAENKH	62.88	784.8428	2	31.8	7560000					
				ALCFGDSEYIGAHKNIDTKR	39.52	517.7491	4	51.19	7560000					
				AANIPPYPLNLINFMEMIRY	48.37	1089.558	2	87.52	7560000					
	AAL87004.1	beta-bungarotoxin A2 chain precursor [Bungarus caeruleus]	beta-BTx	ADYGCYCGAGGSGTPVDALDRC	92	1045.926	2	58.4	7560000				0.0771	0.001411
				WGHYADYGCYCGAGGSGTPVDALDRC	89.97	1260.015	2	55.61	7560000					
				RCCYVHDNCYGVAENKHKC	42.54	431.5845	5	31.64	7560000					
				AALCFGDSEYIGAHKN	59.82	523.243	3	54.07	7560000					
				GDSEYIGAHKNIDTKR	75.27	795.8921	2	31.67	7560000					
				CDRTAALCFGDSEYIGAHKN	46.97	670.9794	3	54.67	7560000					
				HYADYGCYCGAGGSGTPVDALDRC	85.47	1162.981	2	59.65	7560000					
				DKTWGHYADYGCYCGAGGSGTPVDALDRC	48.65	734.3186	4	58.89	7560000					
				RYTIPCDKTWGHYADYGCYCGAGGSGTPVDA	91.58	1228.526	3	67.4	7560000					
				TAALCFGDSEYIGAHKN	59.48	546.9218	3	56.05	7560000					
				YADYGCYCGAGGSGTPVDALDRC	107.13	1081.444	2	57.32	7560000					
				TWGHYADYGCYCGAGGSGTPVDALDRC	40.69	902.3782	3	65.23	7560000					
				GHYADYGCYCGAGGSGTPVDALDRC	93.45	1231.504	2	55.39	7560000					
	0412250A	toxin A beta1 bungaro	PLA2	RTIICYGAAGGTCRI	58.71	700.3253	2	46.34	3570000				0.0364	0.000666
	CAB53358.1	cardiotoxin-like protein [Bungarus multicinctus multicinctus]	3FTx (C-3FTx)	EAYHGKYPVILERG	63.36	482.6015	3	44.53	3390000				0.0346	0.000633
				KTYSYTCTKPNLTCTD	57.48	855.3762	2	46.26	1360000					

F12a	0702209A	phospholipase A	PLA2	KTKTYSYTCTKPNLTC	59.92	839.4105	2	42.15	1360000		0.0444	0.0183	0.0139	0.000254
				YVNYGCYCGAGGSGTPVDELDRC	105.56	1123.968	2	56.53	1360000					
				NLTCTDAAGTCARI	44.87	648.787	2	27.9	1360000					
				GYVNYGCYCGAGGSGTPVDELDRC	97.63	1205.504	2	60.35	1360000					
	Q8AY42.2	RecName: Full=Kunitz-type serine protease inhibitor B; AltName: Full=Kunitz inhibitor B; Flags: Precursor	Kunitz	EFTYGGCGGNANNFKS	83.76	753.8247	2	44.42	556000				0.0057	0.000104
				FTYGGCGGNANNFKS	70.31	680.2905	2	32.54	556000					
	Q8QFW4.1	RecName: Full=Basic phospholipase A2 beta-bungarotoxin A1 chain; Short=Beta-BuTX A1 chain; Short=svPLA2; AltName: Full=Phosphatidylcholine 2-acylhydrolase; Flags: Precursor	beta-BTx	EYTNYGCYCGAGGSGTPIDALDRC	108.7	1184.501	2	62.95	352000				0.0036	0.000066
				EKTWGEYTNYGCYCGAGGSGTPIDALDRC	43.32	990.4321	3	68.9	352000					
				WGEYTNYGCYCGAGGSGTPIDALDRC	88.47	1277.534	2	64.71	352000					
				KTQSYSYKLTRR	48.03	416.2192	3	36.77	352000					
				KTWGEYTNYGCYCGAGGSGTPIDALDRC	109.61	1421.096	2	74.13	352000					
				GEYTNYGCYCGAGGSGTPIDALDRC	95.53	1249.022	2	64.78	352000					
				CFGDSEYIEGHKRI	38.53	360.1743	4	34.62	352000					
				RYTIPCEKTWGEYTNYGCYCGAGGSGTPIDAL	94.59	1244.866	3	77.12	352000					
				PLNLINFMNMIRY	38.8	697.868	2	87.72	352000					
	CAA06887.1	neurotoxin-like protein [Bungarus multicinctus multicinctus]	3FTx (Type I NTx)	RGCVATCPKPKK	55.65	559.2771	2	19.57	213000				0.0022	0.000040
				KNEIIQCCAKDKC	51.26	689.828	2	27.46	213000					
				KYSQANESSKTC	45.41	557.7542	2	18.14	213000					
				KKNEIIQCCAKD	71.4	632.3129	2	26.01	213000					
				KKWEIGNPSGKE	61.7	558.2943	2	67.29	213000					
				KWEIGNPSGKE	42.38	494.2483	2	46.47	213000					
				KRGCVATCPKPKK	56.8	425.2221	3	17.2	213000					
	CAM11302.1	alpha-delta-bungarotoxin-4 partial [Bungarus caeruleus]	3FTx (Type II NTx)	RGKVIELGCVATCPQPKPYEEVTCCSTDKCNPH	51.23	792.7693	5	50.64	126000				0.0013	0.000024
				CVATCPQPKPYEEVTCCSTDKC	64.24	790.6856	3	41.8	126000					
				KVIELGCVATCPQPKPYEEV	79.68	1045.494	2	61.16	126000					
				LGCVATCPQPKPYEEVTCCSTDKC	43.12	863.0353	3	45.16	126000					
				TCPSGENLCYTKM	59.41	664.7845	2	35.32	126000					
				TVTCPSGENLCYTKM	57.59	764.8428	2	41.53	126000					
				PSGENLCYTKM	47.53	536.2424	2	31.4	126000					
				CPSGENLCYTKM	57.02	584.7686	2	33.57	126000					
				KMWCDAFCSSRG	46.58	668.2497	2	53.85	126000					
				QPKPYEEVTCCSTDKC	40.5	571.9202	3	34.23	126000					
				CPQPKPYEEVTCCSTDKC	43.69	646.9566	3	37.38	126000					
				VTCPSGENLCYTKM	40.96	715.3087	2	36.56	126000					
	Q7T2I3.1	RecName: Full=Long neurotoxin LlLong; Flags: Precursor	3FTx (Type-II NTx)	KVIELGCTATCPRA	80.78	688.8364	2	45.77	116000				0.0012	0.000022
	ABB83636.1	weak neurotoxin precursor [Ophiophagus hannah]	3FTx	RVNPGISIICCKT	57.16	630.8262	2	54.19	110000				0.0011	0.000021
	O12963.1	RecName: Full=Long neurotoxin homolog TA-bm16; AltName: Full=Delta-protein; Flags: Precursor	3FTx (Type-II NTx)	SFYTCPNSETCPDGKNICVKR	42.93	1145.503	2	45	44700				0.0005	0.000008
				ETCPDGKNICVKR	41.3	646.3103	2	28.49	44700					
				FYTCPNSETCPDGKN	46.19	764.8057	2	30.26	44700					
				NSETCPDGKNICVKR	52.09	754.3478	2	31.46	44700					
	1TC8	Chain A phospholipase A2 isoform 1	PLA2	RFVCDCDRTAAICFAKAPYNT	58.51	760.3383	3	58.33	210000000				0.4106	0.026808
				VCDCDRTAAICFAKA	51.37	794.3394	2	42.3	210000000					
				ARFVCDCDRTAAICFAKA	60.48	498.2314	4	46.87	210000000					
				FVCDCDRTAAICFAKA	41.1	562.9182	3	48.6	210000000					
				CDCDRTAAICFAKA	62	476.5507	3	41.93	210000000					
	ACE73577.1	cysteine-rich seceretory protein Bc-CRPb [Bungarus candidus]	CRISP	NGLCTNPCKYNDDYTNCKS	53.04	1061.934	2	41.89	163000000				0.3187	0.020808
				KSGPPCGDCPSACVNGLCTNPCKYNDDYTNCI	67.79	1194.464	3	54.86	163000000					
				YNDDYTNCKSLAKQ	49.97	714.825	2	28.91	163000000					
				KYNDDYTNCKSLAKQ	60.51	796.3566	2	34.23	163000000					
	1G0Z	Chain B PHOSPHOLIPASE A2	PLA2	IGYGCYCGYGGSGTPVDELDRC	97.16	1091.943	2	57.98	105000000				0.2053	0.013404
				FKNMIQCAGTRTWTSYIGYGCYCGYGGSGTP	44.45	1370.587	3	78.66	105000000					
				NIPGCNPLIKT	47.8	506.2864	2	42.68	105000000					
	JAA74998.1	CRiSP-Ver-3 [Vermicella annulata]	CRISP	ASCFCHTEII	38.82	583.7526	2	52.1	19900000				0.0389	0.002540
	AAL87004.1	beta-bungarotoxin A2 chain precursor [Bungarus caeruleus]	beta-BTx	GHYADYGCYCGAGGSGTPVDALDRC	93.45	1231.504	2	55.39	3710000				0.0073	0.000474
				KTWGHYADYGCYCGAGGSGTPVDALDR	54.01	1325.538	2	69.52	3710000					
				ALCFGDSEYIGAHKN	67.7	499.5642	3	52.66	3710000					
				AANIPPYPLNLINFMEMIRY	48.37	1089.558	2	87.52	3710000					
				CYVHDNCYGVAENKHKC	40.45	611.9497	3	25.3	3710000					
				RTAALCFGDSEYIGAHKNIDT	70.4	1041.482	2	60.39	3710000					
				CDRTAALCFGDSEYIGAHKN	46.97	670.9794	3	54.67	3710000					
				AALCFGDSEYIGAHKN	59.82	523.243	3	54.07	3710000					
				YADYGCYCGAGGSGTPVDALDRC	107.13	1081.444	2	57.32	3710000					
				LCFGDSEYIGAHKNIDTKR	39.77	489.4792	4	45.57	3710000					
				RYTIPCDKTWGHYADYGCYCGAGGSGTPVDA	91.58	1228.526	3	67.4	3710000					
				TAALCFGDSEYIGAHKN	59.48	546.9218	3	56.05	3710000					
				CFGDSEYIGAHKN	54.06	408.5278	3	38.1	3710000	0.4127				
				GDSEYIGAHKNIDTKR	75.27	795.8921	2	31.67	3710000					
F12b				RCCYVHDNCYGVAENKH	93.27	944.8749	2	37.7	3710000		0.1582	0.0653		
				DRTAALCFGDSEYIGAHKN	71.32	474.732	4	49.45	3710000					
				ADYGCYCGAGGSGTPVDALDRC	92	1045.926	2	58.4	3710000					
				HYADYGCYCGAGGSGTPVDALDRC	85.47	1162.981	2	59.65	3710000					
				YVHDNCYGVAENKH	57.8	703.3104	2	27.05	3710000					
				FGDSEYIGAHKNIDTKR	72.7	824.397	2	31.66	3710000					
				DSEYIGAHKNIDTKRH	40.7	544.6212	3	26.69	3710000					
				TWGHYADYGCYCGAGGSGTPVDALDRC	40.69	902.3782	3	65.23	3710000					
				DKTWGHYADYGCYCGAGGSGTPVDALDRC	48.65	734.3186	4	58.89	3710000					
				WGHYADYGCYCGAGGSGTPVDALDRC	89.97	1260.015	2	55.61	3710000					
	Q3SB03.1	RecName: Full=Cysteine-rich venom protein pseudechetoxin-like; Short=CRVP; Flags: Precursor	CRISP	RGSIATPYKSGPPCADCPSACVNGLCTNPCKH	44.88	1080.466	3	52	3670000				0.0072	0.000468
				KSGPPCADCPSACVNGLCTNPCKH	90.42	807.9963	3	48.72	3670000					
	0702209A	phospholipase A	PLA2	NLTCTDAAGTCARI	44.87	648.787	2	27.9	3520000				0.0069	0.000449
				YVNYGCYCGAGGSGTPVDELDRC	105.56	1123.968	2	56.53	3520000					
				KTYSYTCTKPNLTCTD	57.48	855.3762	2	46.26	3520000					
				KTKTYSYTCTKPNLTC	59.92	839.4105	2	42.15	3520000					
				GYVNYGCYCGAGGSGTPVDELDRC	97.63	1205.504	2	60.35	3520000					
	AAO84769.1	phospholipase A2 partial [Bungarus candidus]		RTAALCFAEAPYKRR	55.35	499.9223	3	50.33	989000				0.0019	0.000126
	ABB83636.1	weak neurotoxin precursor [Ophiophagus hannah]	3FTx	RVNPGISIICCKT	57.16	630.8262	2	54.19	180000				0.0004	0.000023
	Q7T2I3.1	RecName: Full=Long neurotoxin LlLong; Flags: Precursor	3FTx (Type-II NTx)	KVIELGCTATCPRA	80.78	688.8364	2	45.77	84500				0.0002	0.000011
	Q8QFW4.1	RecName: Full=Basic phospholipase A2 beta-bungarotoxin A1 chain; Short=Beta-BuTX A1 chain; Short=svPLA2; AltName: Full=Phosphatidylcholine 2-acylhydrolase; Flags: Precursor	beta-BTx	EYTNYGCYCGAGGSGTPIDALDRC	108.7	1184.501	2	62.95	61500				0.0001	0.000008
				CFGDSEYIEGHKRI	38.53	360.1743	4	34.62	61500					
				EKTWGEYTNYGCYCGAGGSGTPIDALDRC	43.32	990.4321	3	68.9	61500					
				PLNLINFMNMIRY	38.8	697.868	2	87.72	61500					
				KTQSYSYKLTRR	48.03	416.2192	3	36.77	61500					
				WGEYTNYGCYCGAGGSGTPIDALDRC	88.47	1277.534	2	64.71	61500					
				KTWGEYTNYGCYCGAGGSGTPIDALDRC	109.61	1421.096	2	74.13	61500					
				RYTIPCEKTWGEYTNYGCYCGAGGSGTPIDAL	94.59	1244.866	3	77.12	61500					
				GEYTNYGCYCGAGGSGTPIDALDRC	95.53	1249.022	2	64.78	61500					
	1TC8	Chain A phospholipase A2 isoform 1	PLA2	ARFVCDCDRTAAICFAKA	60.48	498.2314	4	46.87	11100000000				0.7168	0.235867
				RFVCDCDRTAAICFAKAP	55.19	952.9258	2	52.54	11100000000					
				VCDCDRTAAICFAKA	51.37	794.3394	2	42.3	11100000000					
				CDCDRTAAICFAKA	62	476.5507	3	41.93	11100000000					
				FVCDCDRTAAICFAKA	41.1	562.9182	3	48.6	11100000000					
	1G0Z	Chain B PHOSPHOLIPASE A2		FKNMIQCAGTRTWTSYIGYGCYCGYGGSGTP	44.45	1370.587	3	78.66	3920000000				0.2531	0.083297
				IGYGCYCGYGGSGTPVDELDRC	97.16	1091.943	2	57.98	3920000000					
				NIPGCNPLIKT	47.8	506.2864	2	42.68	3920000000					
	AAO84769.1	phospholipase A2 partial [Bungarus candidus]		RTAALCFAEAPYKR	75.14	671.3286	2	56.71	167000000				0.0108	0.003549
	AAL87004.1	beta-bungarotoxin A2 chain precursor [Bungarus caeruleus]	beta-BTx	LCFGDSEYIGAHKNIDTKR	39.77	489.4792	4	45.57	93000000				0.0060	0.001976
				GDSEYIGAHKNIDTKR	75.27	795.8921	2	31.67	93000000					
				RTAALCFGDSEYIGAHKNIDTKR	118.1	1156.051	2	54.34	93000000					
				CDRTAALCFGDSEYIGAHKN	46.97	670.9794	3	54.67	93000000					
				CYVHDNCYGVAENKH	62.88	784.8428	2	31.8	93000000					
				KTWGHYADYGCYCGAGGSGTPVDA	55.81	1175.957	2	60.44	93000000					
				AANIPPYPLNLINFMEMIRY	48.37	1089.558	2	87.52	93000000					
				ADYGCYCGAGGSGTPVDALDRC	92	1045.926	2	58.4	93000000					
				RCCYVHDNCYGVAENKH	93.27	944.8749	2	37.7	93000000					
				RYTIPCDKTWGHYADYGCYCGAGGSGTPVDA	91.58	1228.526	3	67.4	93000000					
				TAALCFGDSEYIGAHKN	59.48	546.9218	3	56.05	93000000					
				AALCFGDSEYIGAHKN	59.82	523.243	3	54.07	93000000					
				CFGDSEYIGAHKN	54.06	408.5278	3	38.1	93000000					
				DRTAALCFGDSEYIGAHKNIDTKR	54.14	494.2419	5	50.48	93000000					
				WGHYADYGCYCGAGGSGTPVDALDRC	89.97	1260.015	2	55.61	93000000					
				GHYADYGCYCGAGGSGTPVDALDRC	93.45	1231.504	2	55.39	93000000					
				YADYGCYCGAGGSGTPVDALDRC	107.13	1081.444	2	57.32	93000000					
				ALCFGDSEYIGAHKN	67.7	499.5642	3	52.66	93000000					
				HYADYGCYCGAGGSGTPVDALDRC	85.47	1162.981	2	59.65	93000000					
				DKTWGHYADYGCYCGAGGSGTPVDALDRC	48.65	734.3186	4	58.89	93000000					
				TWGHYADYGCYCGAGGSGTPVDALDRC	40.69	902.3782	3	65.23	93000000					
				DSEYIGAHKNIDTKRH	40.7	544.6212	3	26.69	93000000					
				YVHDNCYGVAENKH	57.8	703.3104	2	27.05	93000000					
				FGDSEYIGAHKNIDTKR	72.7	824.397	2	31.66	93000000					
	Q7LZQ6.1	RecName: Full=Basic phospholipase A2 13; Short=svPLA2; AltName: Full=Cytotoxin XIII; AltName: Full=Phosphatidylcholine 2-acylhydrolase; AltName: Full=Phospholipase A2 XIII		NLYQFKNMIECAGTRT	79.46	930.9393	2	55.02	77400000				0.0050	0.001645
F12c	0702209A	phospholipase A	PLA2	KTYSYTCTKPNLTCTDAAGTCARI	56.61	838.0289	3	46.85	74900000		0.7974	0.3290	0.0048	0.001592
				NLTCTDAAGTCARI	44.87	648.787	2	27.9	74900000					
				GYVNYGCYCGAGGSGTPVDELDRC	97.63	1205.504	2	60.35	74900000					
				KTKTYSYTCTKPNLTC	59.92	839.4105	2	42.15	74900000					
				YVNYGCYCGAGGSGTPVDELDRC	105.56	1123.968	2	56.53	74900000					
	XP_034282052.1	phospholipase A2 GL16-1-like [Pantherophis guttatus]		DYGCYCGYGGSGTPVDQLDRC	69.11	1062.939	2	57.27	24200000				0.0016	0.000514
	Q8QFW4.1	RecName: Full=Basic phospholipase A2 beta-bungarotoxin A1 chain; Short=Beta-BuTX A1 chain; Short=svPLA2; AltName: Full=Phosphatidylcholine 2-acylhydrolase; Flags: Precursor	beta-BTx	EYTNYGCYCGAGGSGTPIDALDRC	108.7	1184.501	2	62.95	11200000				0.0007	0.000238
				WGEYTNYGCYCGAGGSGTPIDALDRC	88.47	1277.534	2	64.71	11200000					
				EKTWGEYTNYGCYCGAGGSGTPIDALDRC	43.32	990.4321	3	68.9	11200000					
				GEYTNYGCYCGAGGSGTPIDALDRC	95.53	1249.022	2	64.78	11200000					
				CFGDSEYIEGHKRI	38.53	360.1743	4	34.62	11200000					
				KTQSYSYKLTRR	48.03	416.2192	3	36.77	11200000					
				KTWGEYTNYGCYCGAGGSGTPIDALDRC	109.61	1421.096	2	74.13	11200000					
				RYTIPCEKTWGEYTNYGCYCGAGGSGTPIDAL	94.59	1244.866	3	77.12	11200000					
				PLNLINFMNMIRY	38.8	697.868	2	87.72	11200000					
	1DPY	Chain A PHOSPHOLIPASE A2		IWTAYVAYGCYCGKGGSGTPVDELDRC	53.76	928.0782	3	80.32	5230000				0.0003	0.000111
				KNMIQCAGTRIWTAYVAYGCYCGKGGSGTPV	53.41	986.4463	4	78.23	5230000					
				WTAYVAYGCYCGKGGSGTPVDELDRC	38.7	1298.561	2	67.33	5230000					
				RIWTAYVAYGCYCGKGGSGTPVDELDRC	80.45	965.7621	3	78.48	5230000					
				AYVAYGCYCGKGGSGTPVDELDRC	41.56	1212.53	2	56.2	5230000					
				TRIWTAYVAYGCYCGKGGSGTPVDELDRC	38.85	1017.796	3	91.73	5230000					
	UNW14928.1	phospholipase A2 protein 2-3 partial [Pseudonaja affinis]	PLA2	RDVLDYANYGCYCGKGGSGTPVDELDRC	41.43	1441.127	2	76.27	3890000				0.0003	0.000083
	P00610.1	RecName: Full=Basic phospholipase A2; Short=svPLA2; AltName: Full=Myotoxin; AltName: Full=Phosphatidylcholine 2-acylhydrolase; AltName: Full=Toxin VI-5; AltName: Full=Toxin VI:5b		RRSSLDYADYGCYCGAGGSGTPVDELDRC	67.89	1471.124	2	79.63	3450000				0.0002	0.000073
	P0C551.1	RecName: Full=Acidic phospholipase A2 KBf-grIB; Short=svPLA2; AltName: Full=Phosphatidylcholine 2-acylhydrolase; Flags: Precursor		KEHDDCYAQIKENPKC	69.02	582.9313	3	28.55	1950000				0.0001	0.000041
	JAB52755.1	vascular endothelial growth factor 3 [Micrurus fulvius]	VEGF	KHLYKQDPLTCKC	69.75	468.2431	3	31.6	1400000				0.0001	0.000030

	[illegible]	[illegible]	[illegible]	KHFQSQHIHPMSFQQHSKC	42.48	530.7524	4	26.48	1400000				[illegible]	[illegible]
	ABU63166.1	phospholipase A2 precursor BF-42 [Bungarus fasciatus]		YVSYGCYCGYGGSGTPVDELDRC	102.32	1156.48	2	62.51	669000				0.0000	0.000014
				ENMIECAGTRSWTHYVSYGCYCGYGGSGTPV	57.16	1345.55	3	58.41	669000					
				HYVSYGCYCGYGGSGTPVDELDRC	73.89	1238.014	2	65.19	669000					
	P59067.1	RecName: Full=Phospholipase A2 superbin a; Short=svPLA2; AltName: Full=Phosphatidylcholine 2-acylhydrolase	PLA2	KNMIQCANHGRRATWHYLDYGCYCGPGGLG	38.08	1082.223	4	73.33	445000				0.0000	0.000009
	UNW14933.1	phospholipase A2 protein 2-1 partial [Pseudonaja nuchalis]		AHYGCFCGSGGSGTPVDELDRC	42.16	1085.938	2	61.56	260000				0.0000	0.000006
				LITCANRGSRNWWDYAHYGCFCGSGGSGTPV	38.45	1341.251	3	80.15	260000					
	ABW24180.1	PLA-18 precursor [Austrelaps superbus]		DYGCYCGSGGTGTPVDELDRC	53.37	1032.413	2	64.81	55500				0.0000	0.000001
				SGGTGTPVDELDRC	41.12	608.7931	2	46.39	55500					
	1TC8	Chain A phospholipase A2 isoform 1	PLA2	RFVCDCDRTAAICFAKAPYNT	58.51	760.3383	3	58.33	614000000				0.8742	0.006172
				ARFVCDCDRTAAICFAKA	60.48	498.2314	4	46.87	614000000					
				FVCDCDRTAAICFAKA	41.1	562.9182	3	48.6	614000000					
				VCDCDRTAAICFAKA	51.37	794.3394	2	42.3	614000000					
				CDCDRTAAICFAKA	62	476.5507	3	41.93	614000000					
	1G0Z	Chain B PHOSPHOLIPASE A2		FKNMIQCAGTRTWTSYIGYGCYCGYGGSGTP	44.45	1370.587	3	78.66	59600000				0.0849	0.000599
				NIPGCNPLIKT	47.8	506.2864	2	42.68	59600000					
				IGYGCYCGYGGSGTPVDELDRC	97.16	1091.943	2	57.98	59600000					
	AAL87004.1	beta-bungarotoxin A2 chain precursor [Bungarus caeruleus]	beta-BTx	RTAALCFGDSEYIGAHKN	107.41	870.4016	2	56.54	11600000				0.0165	0.000117
				AANIPPYPLNLINFMEMIRY	48.37	1089.558	2	87.52	11600000					
				DRTAALCFGDSEYIGAHKNIDTKR	54.14	494.2419	5	50.48	11600000					
				CYVHDNCYGVAENKH	62.88	784.8428	2	31.8	11600000					
				ALCFGDSEYIGAHKN	67.7	499.5642	3	52.66	11600000					
				GHYADYGCYCGAGGSGTPVDALDRC	93.45	1231.504	2	55.39	11600000					
				CDRTAALCFGDSEYIGAHKN	46.97	670.9794	3	54.67	11600000					
				GDSEYIGAHKNIDTKR	75.27	795.8921	2	31.67	11600000					
				KTWGHYADYGCYCGAGGSGTPVDA	55.81	1175.957	2	60.44	11600000					
				LCFGDSEYIGAHKNIDTKR	39.77	489.4792	4	45.57	11600000					
				RCCYVHDNCYGVAENKH	93.27	944.8749	2	37.7	11600000					
				TWGHYADYGCYCGAGGSGTPVDALDRC	40.69	902.3782	3	65.23	11600000					
				YADYGCYCGAGGSGTPVDALDRC	107.13	1081.444	2	57.32	11600000					
				DSEYIGAHKNIDTKRH	40.7	544.6212	3	26.69	11600000					
				YVHDNCYGVAENKH	57.8	703.3104	2	27.05	11600000					
				FGDSEYIGAHKNIDTKR	72.7	824.397	2	31.66	11600000					
				WGHYADYGCYCGAGGSGTPVDALDRC	89.97	1260.015	2	55.61	11600000					
				RYTIPCDKTWGHYADYGCYCGAGGSGTPVDA	91.58	1228.526	3	67.4	11600000					
				ADYGCYCGAGGSGTPVDALDRC	92	1045.926	2	58.4	11600000					
				TAALCFGDSEYIGAHKN	59.48	546.9218	3	56.05	11600000					
				AALCFGDSEYIGAHKN	59.82	523.243	3	54.07	11600000					
				DKTWGHYADYGCYCGAGGSGTPVDALDRC	48.65	734.3186	4	58.89	11600000					
				CFGDSEYIGAHKN	54.06	408.5278	3	38.1	11600000					
				HYADYGCYCGAGGSGTPVDALDRC	85.47	1162.981	2	59.65	11600000					
	ACE73577.1	cysteine-rich seceretory protein Bc-CRPb [Bungarus candidus]	CRISP	KSGPPCGDCPSACVNGLCTNPCKYNDDYTNCI	67.79	1194.464	3	54.86	5800000				0.0083	0.000058
				NGLCTNPCKYNDDYTNCKS	53.04	1061.934	2	41.89	5800000					
				KYNDDYTNCKS	57.64	596.733	2	28.75	5800000					
				YNDDYTNCKSLAKQ	49.97	714.825	2	28.91	5800000					
F13a	Q75S48.1	RecName: Full=Acidic phospholipase A2 1; Short=svPLA2; AltName: Full=PA2-I; AltName: Full=Phosphatidylcholine 2-acylhydrolase; Flags: Precursor	PLA2	RGSLTYLDYMDYGCYCGTGSRG	101.53	1177.97	2	66.7	4010000		0.0905	0.0071	0.0057	0.000040
				DNDECGAFICNCDRTAALCFAKA	45.36	831.6834	3	61.3	4010000					
				GSLTYLDYMDYGCYCGTGSRG	44.95	1149.455	2	66.5	4010000					
				KSPYMNLYSYTCSGGTITCNADNDECGAFICN	71.11	1334.853	3	70.67	4010000					
				RYTEMIQCTIRG	51.59	665.8098	2	41.6	4010000					
				RGTPVDELDRCCQTHDNCYAEAEEHPKC	51.58	1044.431	3	43.94	4010000					
	CAB53358.1	cardiotoxin-like protein [Bungarus multicinctus multicinctus]	3FTx (C-3FTx)	EAYHGKYPVILERG	63.36	482.6015	3	44.53	1940000				0.0028	0.000020
	AAO84769.1	phospholipase A2 partial [Bungarus candidus]	PLA2	RTAALCFAEAPYKR	75.14	671.3286	2	56.71	1430000					
	Q8QFW4.1	RecName: Full=Basic phospholipase A2 beta-bungarotoxin A1 chain; Short=Beta-BuTX A1 chain; Short=svPLA2; AltName: Full=Phosphatidylcholine 2-acylhydrolase; Flags: Precursor	beta-BTx	WGEYTNYGCYCGAGGSGTPIDALDRC	88.47	1277.534	2	64.71	1430000				0.0020	0.000014
				KTQSYSYKLTRR	48.03	416.2192	3	36.77	1430000					
				PLNLINFMNMIRY	38.8	697.868	2	87.72	1430000					
				CFGDSEYIEGHKRI	38.53	360.1743	4	34.62	1430000					
				KTWGEYTNYGCYCGAGGSGTPIDALDRC	109.61	1421.096	2	74.13	1430000					
				EKTWGEYTNYGCYCGAGGSGTPIDALDRC	43.32	990.4321	3	68.9	1430000					
				EYTNYGCYCGAGGSGTPIDALDRC	108.7	1184.501	2	62.95	1430000					
				GEYTNYGCYCGAGGSGTPIDALDRC	95.53	1249.022	2	64.78	1430000					
				RYTIPCEKTWGEYTNYGCYCGAGGSGTPIDAL	94.59	1244.866	3	77.12	1430000					
	0702209A	phospholipase A	PLA2	GYVNYGCYCGAGGSGTPVDELDRC	97.63	1205.504	2	60.35	1320000				0.0019	0.000013
				NLTCTDAAGTCARI	44.87	648.787	2	27.9	1320000					
				YVNYGCYCGAGGSGTPVDELDRC	105.56	1123.968	2	56.53	1320000					
				KTYSYTCTKPNLTCTDAAGTCARI	56.61	838.0289	3	46.85	1320000					
				KTKTYSYTCTKPNLTC	59.92	839.4105	2	42.15	1320000					
	Q8AY42.2	RecName: Full=Kunitz-type serine protease inhibitor B; AltName: Full=Kunitz inhibitor B; Flags: Precursor	Kunitz	FTYGGCGGNANNFKS	70.31	680.2905	2	32.54	617000				0.0009	0.000006
				EFTYGGCGGNANNFKS	83.76	753.8247	2	44.42	617000					
	ABB83636.1	weak neurotoxin precursor [Ophiophagus hannah]	3FTx	RVNPGISIICCKT	57.16	630.8262	2	54.19	220000				0.0003	0.000002
	0412250A	toxin A beta1 bungaro	PLA2	RTIICYGAAGGTCRI	58.71	700.3253	2	46.34	157000				0.0002	0.000002
	O12963.1	RecName: Full=Long neurotoxin homolog TA-bm16; AltName: Full=Delta-protein; Flags: Precursor	3FTx (Type-II NTx)	FYTCPNSETCPDGKN	46.19	764.8057	2	30.26	117000				0.0002	0.000001
				NSETCPDGKNICVKR	52.09	754.3478	2	31.46	117000					
				SFYTCPNSETCPDGKN	77.35	838.3411	2	38.55	117000					
				ETCPDGKNICVKR	41.3	646.3103	2	28.49	117000	0.0781				
	CAA06887.1	neurotoxin-like protein [Bungarus multicinctus multicinctus]	3FTx (Type I NTx)	KKNEIIQCCAKDKCNK	57.67	636.9746	3	20.68	83000				0.0001	0.000001
				KKWEIGNPSGKE	61.7	558.2943	2	67.29	83000					
				KYSQANESSKTC	45.41	557.7542	2	18.14	83000					
				KWEIGNPSGKE	42.38	494.2483	2	46.47	83000					
				RGCVATCPKPKK	55.65	559.2771	2	19.57	83000					
				KNEIIQCCAKD	49.59	568.2655	2	33.07	83000					
				KRGCVATCPKPKK	56.8	425.2221	3	17.2	83000					
	1TC8	Chain A phospholipase A2 isoform 1	PLA2	RFVCDCDRTAAICFAKA	99.44	917.4034	2	51.68	19900000000				0.9732	0.069087
				FVCDCDRTAAICFAKA	41.1	562.9182	3	48.6	19900000000					
				VCDCDRTAAICFAKA	51.37	794.3394	2	42.3	19900000000					
				CDCDRTAAICFAKA	62	476.5507	3	41.93	19900000000					
				ARFVCDCDRTAAICFAKA	60.48	498.2314	4	46.87	19900000000					
	AAL87004.1	beta-bungarotoxin A2 chain precursor [Bungarus caeruleus]	beta-BTx	RTAALCFGDSEYIGAHKN	107.41	870.4016	2	56.54	255000000				0.0125	0.000885
				CYVHDNCYGVAENKH	62.88	784.8428	2	31.8	255000000					
				KTWGHYADYGCYCGAGGSGTPVDALDRC	110.72	1403.582	2	65.03	255000000					
				DSEYIGAHKNIDTKRH	40.7	544.6212	3	26.69	255000000					
				DRTAALCFGDSEYIGAHKNIDTKR	54.14	494.2419	5	50.48	255000000					
				WGHYADYGCYCGAGGSGTPVDALDRC	89.97	1260.015	2	55.61	255000000					
				ALCFGDSEYIGAHKNIDTKR	39.52	517.7491	4	51.19	255000000					
				CDRTAALCFGDSEYIGAHKN	46.97	670.9794	3	54.67	255000000					
				YVHDNCYGVAENKH	57.8	703.3104	2	27.05	255000000					
				YADYGCYCGAGGSGTPVDALDRC	107.13	1081.444	2	57.32	255000000					
				LCFGDSEYIGAHKN	52.42	461.8709	3	41.83	255000000					
				CFGDSEYIGAHKN	54.06	408.5278	3	38.1	255000000					
				GHYADYGCYCGAGGSGTPVDALDRC	93.45	1231.504	2	55.39	255000000					
				FGDSEYIGAHKNIDTKR	72.7	824.397	2	31.66	255000000					
				ADYGCYCGAGGSGTPVDALDRC	92	1045.926	2	58.4	255000000					
				RCCYVHDNCYGVAENKHKC	42.54	431.5845	5	31.64	255000000					
				AALCFGDSEYIGAHKN	59.82	523.243	3	54.07	255000000					
				DKTWGHYADYGCYCGAGGSGTPVDALDRC	48.65	734.3186	4	58.89	255000000					
				RYTIPCDKTWGHYADYGCYCGAGGSGTPVDA	91.58	1228.526	3	67.4	255000000					
				HYADYGCYCGAGGSGTPVDALDRC	85.47	1162.981	2	59.65	255000000					
				GDSEYIGAHKNIDTKR	75.27	795.8921	2	31.67	255000000					
				AANIPPYPLNLINFMEMIRY	48.37	1089.558	2	87.52	255000000					
				TAALCFGDSEYIGAHKN	59.48	546.9218	3	56.05	255000000					
F13b				TWGHYADYGCYCGAGGSGTPVDALDRC	40.69	902.3782	3	65.23	255000000		0.9095	0.0710		
	Q75S48.1	RecName: Full=Acidic phospholipase A2 1; Short=svPLA2; AltName: Full=PA2-I; AltName: Full=Phosphatidylcholine 2-acylhydrolase; Flags: Precursor	PLA2	KSPYMNLYSYTCSGGTITCNADNDECGAFICN	48.41	1622.332	3	75.09	212000000				0.0104	0.000736
				RGSLTYLDYMDYGCYCGTGSRG	82.36	1169.977	2	74.6	212000000					
				RYTEMIQCTIRG	51.59	665.8098	2	41.6	212000000					
				GSLTYLDYMDYGCYCGTGSRG	44.95	1149.455	2	66.5	212000000					
				RGTPVDELDRCCQTHDNCYAEAEEHPKC	51.58	1044.431	3	43.94	212000000					
				DNDECGAFICNCDRTAALCFAKA	45.36	831.6834	3	61.3	212000000					
	Q8QFW4.1	RecName: Full=Basic phospholipase A2 beta-bungarotoxin A1 chain; Short=Beta-BuTX A1 chain; Short=svPLA2; AltName: Full=Phosphatidylcholine 2-acylhydrolase; Flags: Precursor	beta-BTx	PLNLINFMNMIRY	38.8	697.868	2	87.72	72600000				0.0036	0.000252
				GEYTNYGCYCGAGGSGTPIDALDRC	95.53	1249.022	2	64.78	72600000					
				RYTIPCEKTWGEYTNYGCYCGAGGSGTPIDAL	94.59	1244.866	3	77.12	72600000					
				WGEYTNYGCYCGAGGSGTPIDALDRC	88.47	1277.534	2	64.71	72600000					
				EKTWGEYTNYGCYCGAGGSGTPIDALDRC	43.32	990.4321	3	68.9	72600000					
				KTWGEYTNYGCYCGAGGSGTPIDALDRC	109.61	1421.096	2	74.13	72600000					
				CFGDSEYIEGHKRI	38.53	360.1743	4	34.62	72600000					
				KTQSYSYKLTRR	48.03	416.2192	3	36.77	72600000					
				EYTNYGCYCGAGGSGTPIDALDRC	108.7	1184.501	2	62.95	72600000					
	0702209A	phospholipase A	PLA2	KTYSYTCTKPNLTCTDAAGTCARI	56.61	838.0289	3	46.85	3690000				0.0002	0.000013
				KTKTYSYTCTKPNLTC	59.92	839.4105	2	42.15	3690000					
				YVNYGCYCGAGGSGTPVDELDRC	105.56	1123.968	2	56.53	3690000					
				GYVNYGCYCGAGGSGTPVDELDRC	97.63	1205.504	2	60.35	3690000					
				NLTCTDAAGTCARI	44.87	648.787	2	27.9	3690000					
	AAO84769.1	phospholipase A2 partial [Bungarus candidus]		RTAALCFAEAPYKRR	55.35	499.9223	3	50.33	3390000				0.0002	0.000012
	Q7LZQ6.1	RecName: Full=Basic phospholipase A2 13; Short=svPLA2; AltName: Full=Cytotoxin XIII; AltName: Full=Phosphatidylcholine 2-acylhydrolase; AltName: Full=Phospholipase A2 XIII		NLYQFKNMIECAGTRT	79.46	930.9393	2	55.02	1120000				0.0001	0.000004
	Q8AY42.2	RecName: Full=Kunitz-type serine protease inhibitor B; AltName: Full=Kunitz inhibitor B; Flags: Precursor	Kunitz	EFTYGGCGGNANNFKS	83.76	753.8247	2	44.42	234000				0.0000	0.000001
				FTYGGCGGNANNFKS	70.31	680.2905	2	32.54	234000					
	P00610.1	RecName: Full=Basic phospholipase A2; Short=svPLA2; AltName: Full=Myotoxin; AltName: Full=Phosphatidylcholine 2-acylhydrolase; AltName: Full=Toxin VI-5; AltName: Full=Toxin VI:5b	PLA2	RRSSLDYADYGCYCGAGGSGTPVDELDRC	67.89	1471.124	2	79.63	74800				0.0000	0.000000
	AHY20009.1	acetylcholinesterase [Echis coloratus]	ACE	RQDFLEGVRM	40.73	482.2484	2	55.66	35700000				0.9454	0.018474
	ABN72539.1	L-amino acid oxidase [Bungarus multicinctus]	LAAO	DYSETVAAPVGRI	43.81	575.2986	2	38.68	1540000				0.0408	0.000797
				QDYSETVAAPVGRI	47.14	632.8118	2	44.18	1540000					
F14				RFDEISGGFDQLPKS	68.66	726.8542	2	77.98	1540000	0.0195	1.0000	0.0195		
	4QWW	Chain B Acetylcholinesterase	ACE	QYTDWQDQDNRE	39.43	670.7791	2	36.7	336000				0.0089	0.000174
				RSKNPQELIDEEWSVLPYKS	70.24	725.7029	3	93.83	336000					
				KSGAWPTYTASQPQYVQLNTQPLATQPSLRA	76.03	1068.542	3	84.22	336000					
	ABN72540.1	L-amino acid oxidase [Bungarus fasciatus]	LAAO	RISFEPPLPPKK	50.8	562.8209	2	79.03	184000				0.0049	0.000095

Table S5C: Proteomic information of venom from eastern (WB) population of *B. caeruleus* . Assignment of peptides sequenced from MS/MS spectra using Serpentes NCBI database (TaxID: 8570) in PEAKS X (Bioinformatics Solutions Inc., Canada) . Relative abundance of each toxin was estimated by using peak fraction area (obtained from RP-HPLC and SDS PAGE) and spectral intensity of all peptides matched to their respective proteins. -10logP is the matching score given by PEAKS Algorithm, m/z is mass to charge ratio of the peptides.

HPLC Peak no.	Protein information			Peptide Information						Calculation of Toxin abundance				
	NCBI Accession ID	Description	Toxin family	Peptide Sequence	-10LgP	m/z	z	RT	Precursor intensity	HPLC Peak Area	SDS Densitometry	Relative peak fraction	Relative abundance	% Proportion of Toxin
F2	CAA06887.1	neurotoxin-like protein [Bungarus multicinctus multicinctus]	3FTx (Type I NTx)	KKWEIGNPSGKE	69.67	558.2956	2	43.41	35829000	0.0352	1.0000	0.0352	1.0000	0.03519
				KWEIGNPSGKE	56.5	494.2484	2	51.44	35829000					
F3	Q8AY42.2	RecName: Full=Kunitz-type serine protease inhibitor B; AltName: Full=Kunitz inhibitor B; Flags: Precursor	Kunitz	EFTYGGCGGNANNFKS	63.42	753.8246	2	50.14	31596000	0.0101	1.0000	0.0101	1.0000	0.01009
F4	Q8JFX7.1	RecName: Full=Muscarinic toxin BM14; Flags: Precursor	3FTx	KSYWVNENGKQKK	55.82	676.8336	2	39.35	95158000	0.0308	1.0000	0.0308	0.6407	0.019757715
	CAA06887.1	neurotoxin-like protein [Bungarus multicinctus multicinctus]	3FTx (Type I NTx)	KKWEIGNPSGKE	69.67	558.2956	2	43.41	53375000				0.3593	0.011082285
				KWEIGNPSGKE	56.5	494.2484	2	51.44	53375000					
F5	Q8JFX7.1	RecName: Full=Muscarinic toxin BM14; Flags: Precursor	3FTx	KSYWVNENGKQKK	55.82	676.8336	2	39.35	94120000	0.0157	1.0000	0.0157	0.9894	0.015562699
	CAA06887.1	neurotoxin-like protein [Bungarus multicinctus multicinctus]	3FTx (Type I NTx)	KKWEIGNPSGKE	69.67	558.2956	2	43.41	1011800				0.0106	0.000167301
				KWEIGNPSGKE	56.5	494.2484	2	51.44	1011800					
F6a	P15816.1	RecName: Full=Kappa-2-bungarotoxin; AltName: Full=Kappa-neurotoxin CB1; Flags: Precursor	3FTx (k-bungarotoxin)	IRGPVIEQGCAATCPEFRS	92.27	649.9771	3	51.77	1183200	0.0411	0.0129	0.0005	0.9675	0.000512998
				EQGCAATCPEFRS	71.32	648.7755	2	41.59	1183200					
				PIRGPVIEQGCAATCPEFRS	48.89	687.6708	3	57.44	1183200					
				QGCAATCPEFRS	56.71	584.7461	2	41.28	1183200					
				RGPVIEQGCAATCPEFRS	124.92	896.4117	2	57.46	1183200					
	1KBA	Chain B KAPPA-BUNGAROTOXIN		RTCLISPSSTPQTCPNGQDICFLKA	106.77	875.4095	3	75.79	39739				0.0325	1.72296E-05
F6b	P15816.1	RecName: Full=Kappa-2-bungarotoxin; AltName: Full=Kappa-neurotoxin CB1; Flags: Precursor	3FTx (k-bungarotoxin)	PIRGPVIEQGCAATCPEFRS	48.89	687.6708	3	57.44	113200000		0.9871	0.0405	0.4963	0.020120628
				IRGPVIEQGCAATCPEFRS	92.27	649.9771	3	51.77	113200000					
				QGCAATCPEFRS	56.71	584.7461	2	41.28	113200000					
				EQGCAATCPEFRS	71.32	648.7755	2	41.59	113200000					
	1KBA	Chain B KAPPA-BUNGAROTOXIN		RTCLISPSSTPQTCPNGQDICFLKA	106.77	875.4095	3	75.79	35319000				0.1549	0.006277743
	1F94	Chain A BUCANDIN	3FTx	TCSAEETFCYKW	51.29	647.7562	2	49.39	24226000				0.1062	0.004306028
				KWLNKISNERW	56.67	580.3152	2	42.86	24226000					
				KITCSAEETFCYKW	69.52	754.8231	2	55.58	24226000					
	AAL30054.1	kappa 1a bungarotoxin [Bungarus candidus]	3FTx (k-bungarotoxin)	TRTCLISPSSTPQTCPQGQDICFLKA	69.89	932.116	3	69.74	17693000				0.0776	0.003144826
	Q8JFX7.1	RecName: Full=Muscarinic toxin BM14; Flags: Precursor	3FTx	KSYWVNENGKQ	60.4	548.7562	2	46.87	14925000				0.0654	0.00265283
	P15815.1	RecName: Full=Kappa-flavitoxin; Short=Kappa-ftx; Short=Kappa-fvt; AltName: Full=Long neurotoxin 2	3FTx (k-bungarotoxin)	RGPVIEQGCAATCPEFTSR	82.34	912.3972	2	59.79	11730000				0.0514	0.002084938
	P01383.1	RecName: Full=Long neurotoxin 1; AltName: Full=Neurotoxin 3.9.4	3FTx (Type II NTx)	KKWCADWCTSRG	58.56	457.1972	3	54.13	10733000				0.0471	0.001907727
	CAA06887.1	neurotoxin-like protein [Bungarus multicinctus multicinctus]	3FTx (Type I NTx)	KWEIGNPSGKE	56.5	494.2484	2	51.44	253470				0.0011	4.50528E-05
				KKWEIGNPSGKE	69.67	558.2956	2	43.41	253470					
F7	Q8QFW4.1	RecName: Full=Basic phospholipase A2 beta-bungarotoxin A1 chain; Short=Beta-BuTX A1 chain; Short=svPLA2; AltName: Full=Phosphatidylcholine 2-acylhydrolase; Flags: Precursor	beta-BTx	RYTIPCEKTWGEYTNYGCYCGAGGSGTPIDALDRC	48.61	1244.873	3	82.32	591780	0.0339	1.0000	0.0339	1.0000	0.03388
				EKTWGEYTNYGCYCGAGGSGTPIDALDRC	86.84	990.4312	3	74.1	591780					
				KTWGEYTNYGCYCGAGGSGTPIDALDRC	95.94	1421.097	2	80.43	591780					
				KTQSYSYKLTRR	52.06	416.2185	3	41.21	591780					
F9	AED89576.1	putative phospholipase A2 precursor [Micrurus altirostris]	PLA2	SGGGGTPVDDLDRC	52.05	579.7733	2	44.9	9284800	0.0332	1.0000	0.0332	0.8829	0.029322702
	P34128.1	RecName: Full=Venom nerve growth factor; Short=v-NGF; Short=vNGF; Flags: Precursor	NGF	RHWNSYCTTTDTFVKA	64.25	587.2623	3	56.63	1164500				0.1107	0.003677654
				RNPNPVPSGCRG	44.98	549.2618	2	32.27	1164500					
	Q9PVK7.1	RecName: Full=Zinc metalloproteinase-disintegrin-like cobrin; AltName: Full=Snake venom metalloproteinase; Short=SVMP; Flags: Precursor	SVMP	KTSAAVVQDYSKS	70.46	584.7961	2	40.22	66382				0.0063	0.000209644
				NPKTSAAVVQDYSKS	44.12	465.248	3	36.88	66382					
F10a	1PO8	Chain A Phospholipase A2	PLA2	RFVCDCDRTAAICFAKA	82.63	917.4072	2	57.2	198970000	0.2044	1.0000	0.2044	0.3282	0.067093972
				VCDCDRTAAICFAKA	57.3	529.8957	3	46.76	198970000					
	0512217A	neurotoxin	3FTx	KTWCDAFCSSRG	62.88	645.2549	2	62.05	187550000				0.3094	0.063243074
	CAM11302.1	alpha-delta-bungarotoxin-4 partial [Bungarus caeruleus]	3FTx (Type II NTx)	RGKVIELGCVATCPQPKP	63.9	586.3066	3	52.42	124490000				0.2054	0.041978834
				KVIELGCVATCPQPKP	76.37	786.3978	2	58.94	124490000					
	Q8QFW3.1	RecName: Full=Basic phospholipase A2 beta-bungarotoxin A2 chain; Short=Beta-BuTX A2 chain; Short=svPLA2; AltName: Full=Phosphatidylcholine 2-acylhydrolase; Flags: Precursor	beta-BTx	YADYGCYCGAGGSGTPVDALDRC	55.54	1081.451	2	63.89	46647000				0.0770	0.01572967
				KTWGHYADYGCYCGAGGSGTPVDALDRC	56.92	936.0579	3	70.88	46647000					
				RCCYVHDNCYGVAENKH	60.12	630.2524	3	43.3	46647000					
	JAA74769.1	3FTx-Ech-25 [Echiopsis curta]	3FTx	KTWCDAFCTSRG	53.77	652.2639	2	62.84	19504000				0.0322	0.006576875
	AED89576.1	putative phospholipase A2 precursor [Micrurus altirostris]	PLA2	SGGGGTPVDDLDRC	52.05	579.7733	2	44.9	12389000				0.0204	0.004177651
	Q8QFW4.1	RecName: Full=Basic phospholipase A2 beta-bungarotoxin A1 chain; Short=Beta-BuTX A1 chain; Short=svPLA2; AltName: Full=Phosphatidylcholine 2-acylhydrolase; Flags: Precursor	beta-BTx	EKTWGEYTNYGCYCGAGGSGTPIDALDRC	86.84	990.4312	3	74.1	8932500				0.0147	0.003012097
				KTQSYSYKLTRR	52.06	416.2185	3	41.21	8932500					
				KTWGEYTNYGCYCGAGGSGTPIDALDRC	95.94	1421.097	2	80.43	8932500					
				RYTIPCEKTWGEYTNYGCYCGAGGSGTPIDALDRC	48.61	1244.873	3	82.32	8932500					
	CAB62385.1	beta-bungarotoxin A3 chain partial [Bungarus multicinctus]		RTAALCFGDSEYIEGHKNID	58.13	506.9928	4	56.97	5347600				0.0088	0.001803245
	AAO84769.1	phospholipase A2 partial [Bungarus candidus]		RTAALCFAEAPYKRR	58.63	499.9219	3	57.53	1266000				0.0021	0.000426903
			PLA2	RGTPVDMLDRC	46.75	502.2476	2	56.18	1266000					
	ABU63164.1	phospholipase A2 precursor BF-40 [Bungarus fasciatus]		YVSYGCYCGYGGSGTPVDELDRC	58.03	1156.483	2	68.94	763250				0.0013	0.000257373
	AAA66029.1	phospholipase A2 [Naja naja]		RLAAICFAGAPYNDNNYNIDLKA	44.33	786.3793	3	82.41	297460				0.0005	0.000100305
F11a	Q8QFW3.1	RecName: Full=Basic phospholipase A2 beta-bungarotoxin A2 chain; Short=Beta-BuTX A2 chain; Short=svPLA2; AltName: Full=Phosphatidylcholine 2-acylhydrolase; Flags: Precursor	beta-BTx	RCCYVHDNCYGVAENKH	60.12	630.2524	3	43.3	2266800	0.0453	0.0278	0.0013	1.0000	0.001261228
				RTAALCFGDSEYIGAHKN	95.58	580.6065	3	62.88	2266800					
				YADYGCYCGAGGSGTPVDALDRC	55.54	1081.451	2	63.89	2266800					
				KTWGHYADYGCYCGAGGSGTPVDALDRC	56.92	936.0579	3	70.88	2266800					
				RIVCDCDRTAALCFGDSEYIGAHKNIDTKR	44.36	646.7003	5	62.78	2266800					
F11b	1PO8	Chain A Phospholipase A2		VCDCDRTAAICFAKA	57.3	529.8957	3	46.76	216750000		0.9722	0.0441	0.6298	0.027748191
				RFVCDCDRTAAICFAKA	82.63	917.4072	2	57.2	216750000					
	1G0Z	Chain B PHOSPHOLIPASE A2		NIPGCNPLIKT	51.26	506.2859	2	47.82	42529000				0.1236	0.005444534
				IGYGCYCGYGGSGTPVDELDRC	80.25	1091.944	2	65.99	42529000					
	UNW14922.1	phospholipase A2 protein 2-3 partial [Pseudonaja textilis]	PLA2	RNVLDYADYGCYCGKGGSGTPVDELDRC	48.03	961.0908	3	84.78	27641000				0.0803	0.003538582
	UNW14928.1	phospholipase A2 protein 2-3 partial [Pseudonaja affinis]		RDVLDYANYGCYCGKGGSGTPVDELDRC	45.13	961.0908	3	84.78	27641000					
	1DPY	Chain A PHOSPHOLIPASE A2		RIWTAYVAYGCYCGKGGSGTPVDELDRC	70.99	965.7622	3	84.93	15419000				0.0448	0.00197393
	P00606.2	RecName: Full=Acidic phospholipase A2; Short=svPLA2; AltName: Full=Phosphatidylcholine 2-acylhydrolase; Flags: Precursor		YVNYGCYCGAGGSGTPVDELDRC	59.21	749.6531	3	64.45	6107200				0.0177	0.00078184
				GYVNYGCYCGAGGSGTPVDELDRC	60.25	1205.506	2	67.35	6107200					
	Q8QFW3.1	RecName: Full=Basic phospholipase A2 beta-bungarotoxin A2 chain; Short=Beta-BuTX A2 chain; Short=svPLA2; AltName: Full=Phosphatidylcholine 2-acylhydrolase; Flags: Precursor		KTWGHYADYGCYCGAGGSGTPVDALDRC	56.92	936.0579	3	70.88	4372500				0.0127	0.000559765
				RCCYVHDNCYGVAENKH	60.12	630.2524	3	43.3	4372500					
				RTAALCFGDSEYIGAHKNI	60.45	618.6208	3	61.23	4372500					
				YADYGCYCGAGGSGTPVDALDRC	55.54	1081.451	2	63.89	4372500					
	Q8QFW4.1	RecName: Full=Basic phospholipase A2 beta-bungarotoxin A1 chain; Short=Beta-BuTX A1 chain; Short=svPLA2; AltName: Full=Phosphatidylcholine 2-acylhydrolase; Flags: Precursor	beta-BTx	EKTWGEYTNYGCYCGAGGSGTPIDALDRC	86.84	990.4312	3	74.1	2261200				0.0066	0.000289477
				KTWGEYTNYGCYCGAGGSGTPIDALDRC	95.94	1421.097	2	80.43	2261200					
				KTQSYSYKLTRR	52.06	416.2185	3	41.21	2261200					
				RYTIPCEKTWGEYTNYGCYCGAGGSGTPIDALDRC	48.61	1244.873	3	82.32	2261200					
	AAO84769.1	phospholipase A2 partial [Bungarus candidus]	PLA2	RTAALCFAEAPYKR	67.49	671.3296	2	64.86	1386800				0.0040	0.000177537
				RGTPVDMLDRC	46.75	502.2476	2	56.18	1386800					
	P34128.1	RecName: Full=Venom nerve growth factor; Short=v-NGF; Short=vNGF; Flags: Precursor	NGF	RNPNPVPSGCRG	44.98	549.2618	2	32.27	49465				0.0001	6.33248E-06
				RHWNSYCTTTDTFVKA	64.25	587.2623	3	56.63	49465					
F12a	1G0Z	Chain B PHOSPHOLIPASE A2	PLA2	NIPGCNPLIKT	51.26	506.2859	2	47.82	109910000	0.3490	0.0728	0.0254	0.9370	0.023818159
				IGYGCYCGYGGSGTPVDELDRC	80.25	1091.944	2	65.99	109910000					
	JAC94920.1	Cysteine-rich secretory protein A [Python regius]	CRISP	INSIATPYKS	47.99	447.2398	2	37.37	6363400				0.0542	0.001378987
	ACE73577.1	cysteine-rich seceretory protein Bc-CRPb [Bungarus candidus]		RWADRCTFAHSPPHLRT	65.05	463.4768	4	57.39	912890				0.0078	0.000197829
				KYNDDYTNCKS	58.92	596.733	2	34.97	912890					
	Q8QFW3.1	RecName: Full=Basic phospholipase A2 beta-bungarotoxin A2 chain; Short=Beta-BuTX A2 chain; Short=svPLA2; AltName: Full=Phosphatidylcholine 2-acylhydrolase; Flags: Precursor	beta-BTx	RIVCDCDRTAALCFGDSEYIGAHKN	82.36	886.7293	3	64.14	118760				0.0010	2.5736E-05
				RTAALCFGDSEYIGAHKN	95.58	580.6065	3	62.88	118760					
				YADYGCYCGAGGSGTPVDALDRC	55.54	1081.451	2	63.89	118760					
				KTWGHYADYGCYCGAGGSGTPVDALDRC	56.92	936.0579	3	70.88	118760					
				RCCYVHDNCYGVAENKH	60.12	630.2524	3	43.3	118760					
F12b	JAC94920.1	Cysteine-rich secretory protein A [Python regius]	CRISP	INSIATPYKS	47.99	447.2398	2	37.37	23634000		0.2169	0.0757	0.9555	0.07232106
	ACE73577.1	cysteine-rich seceretory protein Bc-CRPb [Bungarus candidus]		KYNDDYTNCKS	58.92	596.733	2	34.97	1101600				0.0445	0.003370944
				RWADRCTFAHSPPHLRT	65.05	463.4768	4	57.39	1101600					
F12c	1G0Z	Chain B PHOSPHOLIPASE A2	PLA2	NIPGCNPLIKT	51.26	506.2859	2	47.82	19357000		0.7103	0.2479	0.9101	0.225627308
				IGYGCYCGYGGSGTPVDELDRC	80.25	1091.944	2	65.99	19357000					
	AAO84769.1	phospholipase A2 partial [Bungarus candidus]		RTAALCFAEAPYKRR	58.63	499.9219	3	57.53	1912300				0.0899	0.022289978
				RGTPVDMLDRC	46.75	502.2476	2	56.18	1912300					
F13a	1G0Z	Chain B PHOSPHOLIPASE A2	PLA2	NIPGCNPLIKT	51.26	506.2859	2	47.82	42333000	0.1361	0.0434	0.0059	0.7685	0.004534996
				IGYGCYCGYGGSGTPVDELDRC	80.25	1091.944	2	65.99	42333000					
	JAC94920.1	Cysteine-rich secretory protein A [Python regius]	CRISP	INSIATPYKS	47.99	447.2398	2	37.37	6992100				0.1269	0.000749041
	ACE73577.1	cysteine-rich seceretory protein Bc-CRPb [Bungarus candidus]		RWADRCTFAHSPPHLRT	65.05	463.4768	4	57.39	5345000				0.0970	0.000572592
				KYNDDYTNCKS	58.92	596.733	2	34.97	5345000					
	AAO84769.1	phospholipase A2 partial [Bungarus candidus]	PLA2	RTAALCFAEAPYKR	67.49	671.3296	2	64.86	414120				0.0075	4.43633E-05
				RGTPVDMLDRC	46.75	502.2476	2	56.18	414120					
F13b	JAC94920.1	Cysteine-rich secretory protein A [Python regius]	CRISP	INSIATPYKS	47.99	447.2398	2	37.37	34530000		0.9566	0.1301	0.7707	0.100302315
	ACE73577.1	cysteine-rich seceretory protein Bc-CRPb [Bungarus candidus]		KYNDDYTNCKS	58.92	596.733	2	34.97	5220400				0.1165	0.015164153
				RWADRCTFAHSPPHLRT	65.05	463.4768	4	57.39	5220400					
	1G0Z	Chain B PHOSPHOLIPASE A2	PLA2	NIPGCNPLIKT	51.26	506.2859	2	47.82	5054600				0.1128	0.014682539
				IGYGCYCGYGGSGTPVDELDRC	80.25	1091.944	2	65.99	5054600					
F14	4QWW	Chain B Acetylcholinesterase	ACE	RAGELKVSTQTGSVRG	77.62	478.2633	3	39.01	11010000	0.0303	1.0000	0.0303	0.2952	0.008948318
				RTGNPTDPADKS	60.16	508.238	2	24.56	11010000					
				RAILQSGGPNAPWATVTPAESRG	68.43	708.368	3	78.59	11010000					
	G8XQX1.1	RecName: Full=L-amino-acid oxidase; Short=DrLAO; Short=LAAO; Flags: Precursor	LAAO	KSAGQLYQESLGKA	46.6	640.8277	2	51.32	10993000				0.2948	0.008934501
				RDVNRASEL	44.35	452.2299	2	37.75	10993000					
	ABN72539.1	L-amino acid oxidase [Bungarus multicinctus]		RFDEISGGFDQLPKS	66.21	726.8545	2	73.14	7244100				0.1942	0.005887603
				RIYFAGEYTARV	49.93	595.7959	2	60.13	7244100					
				KRFDEISGGFDQLPKS	59.12	536.9387	3	81.38	7244100					
				RVAYQTPAKT	49.9	439.2426	2	30.8	7244100					
	Q9PVK7.1	RecName: Full=Zinc metalloproteinase-disintegrin-like cobrin; AltName: Full=Snake venom metalloproteinase; Short=SVMP; Flags: Precursor	SVMP	KTSAAVVQDYSKS	70.46	584.7961	2	40.22	7156900				0.1919	0.005816732
				NPKTSAAVVQDYSKS	44.12	465.248	3	36.88	7156900					
	XP_034264823.1	5'-nucleotidase [Pantherophis guttatus]	5'-NT	NASGNPILLNKD	51.62	513.8008	2	47.39	671410				0.0180	0.000545685
	KAG8141327.1	putative Snake venom metalloprotein partial [Naja naja]	SVMP	KSVAVVQDHSKSTRM	45.81	471.9194	3	23.98	217980				0.0058	0.000177162

Table S5D: Proteomic information of venom from central (MP) population of *B. caeruleus* . Assignment of peptides sequenced from MS/MS spectra using Serpentes NCBI database (TaxID: 8570) in PEAKS X (Bioinformatics Solutions Inc., Canada). Relative abundance of each toxin was estimated by using peak fraction area (obtained from RP-HPLC and SDS PAGE) and spectral intensity of all peptides matched to their respective proteins. -10logP is the matching score given by PEAKS Algorithm, m/z is mass to charge ratio of the peptides.

HPLC Peak no.	Protein information			Peptide Information						Calculation of Toxin abundance				
	NCBI Accession ID	Description	Toxin family	Peptide Sequence	-10LgP	m/z	z	RT	Precursor intensity	HPLC Peak Area	SDS Densitometry	Relative peak fraction	Relative abundance	% Proportion of Toxin
F2	CAA06887.1	neurotoxin-like protein [Bungarus multicinctus multicinctus]	3FTx (Type I NTx)	KKNEIIQCCAKDKC	42.66	502.9181	3	25.99	137820000	0.0374	1.0000	0.0374	0.9335	0.03495131
				KYSQANESSKTCPSGQLLCL	50.07	1015.451	2	45.87	137820000					
				RGCVATCPKPKK	45.14	559.2786	2	21.65	137820000					
				KWEIGNPSGKE	50.86	494.2477	2	49.72	137820000					
				KKWEIGNPSGKE	71.07	558.2958	2	85.44	137820000					
				KRGCVATCPKPKK	48.16	425.2216	3	19.59	137820000					
	Q8AY42.2	RecName: Full=Kunitz-type serine protease inhibitor B; AltName: Full=Kunitz inhibitor B; Flags: Precursor	Kunitz	FTYGGCGGNANNFKS	47.41	680.2912	2	33.94	9813400				0.0665	0.00248869
				EFTYGGCGGNANNFKS	63.19	753.8245	2	60.28	9813400					
F3	CAA06887.1	neurotoxin-like protein [Bungarus multicinctus multicinctus]	3FTx (Type I NTx)	RGCVATCPKPKK	45.14	559.2786	2	21.65	304160000	0.0262	1.0000	0.0262	1.0000	0.02615
				KKWEIGNPSGKE	71.07	558.2958	2	85.44	304160000					
				KRGCVATCPKPKK	48.16	425.2216	3	19.59	304160000					
				KKNEIIQCCAKDKC	42.66	502.9181	3	25.99	304160000					
				KYSQANESSKTCPSGQLLCL	50.07	1015.451	2	45.87	304160000					
				KWEIGNPSGKE	50.86	494.2477	2	49.72	304160000					
F4	Q8AY42.2	RecName: Full=Kunitz-type serine protease inhibitor B; AltName: Full=Kunitz inhibitor B; Flags: Precursor	Kunitz	FTYGGCGGNANNFKS	47.41	680.2912	2	33.94	9020000	0.0135	1.0000	0.0135	1.0000	0.01349
				EFTYGGCGGNANNFKS	63.19	753.8245	2	60.28	9020000					
F5	AAB19289.1	miscellaneous type neurotoxin [Naja naja=cobra ssp. naja Peptide 65 aa]	3FTx	LTCLICPEKY	44.36	567.2886	2	56.16	1948700	0.0188	1.0000	0.0188	0.6394	0.012021197
	764177A	toxin B	3FTx	KRVDLGCAATCPTVRT	70.15	525.9294	3	40.13	970590				0.3185	0.005987404
				KTWCDGFCSSRG	54.04	638.2455	2	45.76	970590					
				RGKRVDLGCAATCPTVRT	68.14	587.6337	3	35.05	970590					
				RVDLGCAATCPTVRT	80.67	710.3403	2	44.73	970590					
	Q8AY42.2	RecName: Full=Kunitz-type serine protease inhibitor B; AltName: Full=Kunitz inhibitor B; Flags: Precursor	Kunitz	FTYGGCGGNANNFKS	47.41	680.2912	2	33.94	128290				0.0421	0.000791399
				EFTYGGCGGNANNFKS	63.19	753.8245	2	60.28	128290					
F7	764177A	toxin B	3FTx	RVDLGCAATCPTVRT	80.67	710.3403	2	44.73	29071	0.0214	1.0000	0.0214	1.0000	0.02136
				RGKRVDLGCAATCPTVRT	68.14	587.6337	3	35.05	29071					
				KRVDLGCAATCPTVRT	70.15	525.9294	3	40.13	29071					
				KTWCDGFCSSRG	54.04	638.2455	2	45.76	29071					
F8a	764177A	toxin B	3FTx	RGKRVDLGCAATCPTVRT	68.14	587.6337	3	35.05	107180000	0.0676	1.0000	0.0676	0.2962	0.020008661
				KTWCDGFCSSRG	54.04	638.2455	2	45.76	107180000					
				KRVDLGCAATCPTVRT	70.15	525.9294	3	40.13	107180000					
				RVDLGCAATCPTVRT	80.67	710.3403	2	44.73	107180000					
	AAL30054.1	kappa 1a bungaratoxin [Bungarus candidus]	3FTx (k-bungarotoxin)	CLISPSSTPQTCPQGQDICFLKA	40.02	793.0553	3	63.84	103100000				0.2849	0.019246995
				TRTCLISPSSTPQTCPQGQDICFLKA	75.77	932.1163	3	61.54	103100000					
	P01391.1	RecName: Full=Alpha-cobratoxin; Short=Alpha-CT; Short=Alpha-CbT; Short=Alpha-Cbtx; Short=Alpha-Ctx; AltName: Full=Alpha-elapitoxin-Nk2a;	3FTx	KTGVDIQCCSTDNCNPFPTRK	119.6	1121.468	2	54.75	69185000				0.1912	0.012915649
				CSTDNCNPFPTRK	56.03	654.7863	2	38.76	69185000					
	CAB53358.1	cardiotoxin-like protein [Bungarus multicinctus multicinctus]	3FTx (C-3FTx)	RCYKSYWVNENGKQEA	57.16	902.8994	2	44.51	36687000				0.1014	0.006848831
	1F94	Chain A BUCANDIN	3FTx	KITCSAEETFCYKWLNKISNERW	42.8	663.0667	4	69.14	24908000				0.0688	0.004649895
				IDTWNVYNKC	40.08	520.2455	2	45.13	24908000					
				RCGVSGCHLKI	44.56	509.2338	2	21.15	24908000					
	BAU24673.1	cytotoxin 14 partial [Naja naja]	3FTx	KLIPLAYKTCPAGKN	65.07	716.4058	2	44.6	5792700				0.0160	0.001081397
				KCNKLIPLAYKT	63.54	610.3472	2	47.5	5792700					
	AAL87004.1	beta-bungarotoxin A2 chain precursor [Bungarus caeruleus]	beta-BTx	KTWGHYADYGCYCGAGGSGTPVDALDRC	88.42	1403.583	2	63.03	1613000				0.0045	0.000301119
				VCDCDRTAALCFGDSEYIGAHKNIDTKR	41.46	604.267	5	52.2	1613000					
				RYTIPCDKTWGHYADYGCYCGAGGSGTPVDA	70.47	921.6452	4	66.83	1613000					
				RTAALCFGDSEYIGAHKNIDTKR	88.59	1156.054	2	52.44	1613000					
				ADYGCYCGAGGSGTPVDALDRC	77.71	1045.931	2	57.53	1613000					
				FGDSEYIGAHKNIDTKR	61.08	824.4026	2	29.07	1613000					
				RIVCDCDRTAALCFGDSEYIGAHKNIDTKRH	67.9	1129.196	3	51.64	1613000					
				YADYGCYCGAGGSGTPVDALDRC	107.82	1081.45	2	56.11	1613000					
				AALCFGDSEYIGAHKN	58.24	523.2444	3	52.74	1613000					
				RCCYVHDNCYGVAENKH	83.59	944.8713	2	38.15	1613000					
				LCFGDSEYIGAHKN	51.44	461.8715	3	42.29	1613000					
				TWGHYADYGCYCGAGGSGTPVDALDRC	61.6	902.3785	3	61.68	1613000					
	Q8AY42.2	RecName: Full=Kunitz-type serine protease inhibitor B; AltName: Full=Kunitz inhibitor B; Flags: Precursor	Kunitz	FTYGGCGGNANNFKS	47.41	680.2912	2	33.94	512090				0.0014	9.55984E-05
				EFTYGGCGGNANNFKS	63.19	753.8245	2	60.28	512090					
	AAB19289.1	miscellaneous type neurotoxin [Naja naja=cobra ssp. naja Peptide 65 aa]	3FTx	LTCLICPEKY	44.36	567.2886	2	56.16	313910				0.0009	5.86016E-05
	P25672.1	RecName: Full=Long neurotoxin 4; AltName: Full=Toxin D	3FTx	RGERVDLGCAATCPTVKT	67.52	578.6154	3	41.16	294480				0.0008	5.49743E-05
	CAA06887.1	neurotoxin-like protein [Bungarus multicinctus multicinctus]	3FTx (Type I NTx)	KRGCVATCPKPKK	48.16	425.2216	3	19.59	156580				0.0004	2.92308E-05
				KKWEIGNPSGKE	71.07	558.2958	2	85.44	156580					
				KKNEIIQCCAKDKCNK	58.26	477.9827	4	23.71	156580					
				KYSQANESSKTCPSGQLLCL	50.07	1015.451	2	45.87	156580					
				RGCVATCPKPKK	45.14	559.2786	2	21.65	156580					
				KWEIGNPSGKE	50.86	494.2477	2	49.72	156580					
	1KBA	Chain B KAPPA-BUNGAROTOXIN	3FTx (k-bungarotoxin)	RTCLISPSSTPQTCPNGQDICFLKAQ	53.49	951.1231	3	61.5	133640				0.0004	2.49483E-05
	P29181.1	RecName: Full=Weak neurotoxin 7	3FTx	LTCLNCPEVYCRR	47.13	792.8533	2	49.9	32124				0.0001	5.997E-06
	1POB	Chain B PHOSPHOLIPASE A2	PLA2	RLAAICFAGAPYNDNDYNINLKA	49.62	1179.06	2	74.32	20229				0.0001	3.77641E-06
F11a	AED89576.1	putative phospholipase A2 precursor [Micrurus altirostris]	PLA2	SGGGGTPVDDLDRC	59.71	579.7737	2	39.03	61966000	0.0517	0.0478	0.0025	0.9119	0.002251439
	Q7ZT13.1	RecName: Full=Neurotoxin-like protein pMD18-NTL1/2/4/5; Flags: Precursor	3FTx (Type I NTx)	RGCSLTCPDIKYNGKY	62.43	806.876	2	43.32	3916300				0.0576	0.000142293
				RIERGCSLTCPDIKY	43.44	516.9218	3	39.64	3916300					
				RHSMYFCFKN	59.2	568.2388	2	45.58	3916300					
	ABN72537.1	metalloproteinase [Bungarus multicinctus]	SVMP	KYSVAVIQDYSKR	57.82	636.8276	2	54.63	1161600				0.0171	4.22049E-05
				NGNTVGLAALGSLCSVKY	45.47	773.9166	2	71.76	1161600					
	P82464.1	RecName: Full=Muscarinic toxin-like protein 3; Short=MTLP-3	3FTx (Type I NTx)	KISLADGNDVRI	54.49	530.2751	2	40.34	426230				0.0063	1.54864E-05
				TICYNHLTRT	43.82	393.1979	3	34.72	426230					
	AAL87004.1	beta-bungarotoxin A2 chain precursor [Bungarus caeruleus]	beta-BTx	RIVCDCDRTAALCFGDSEYIGAHKNIDTKR	78.18	1077.161	3	54.61	324690				0.0048	1.17971E-05
				RCCYVHDNCYGVAENKHKC	66.85	539.2285	4	31.61	324690					
				ADYGCYCGAGGSGTPVDALDRC	77.71	1045.931	2	57.53	324690					
				RYTIPCDKTWGHYADYGCYCGAGGSGTPVDA	70.47	921.6452	4	66.83	324690					
				RTAALCFGDSEYIGAHKNIDTKRH	51.79	494.2465	5	48.81	324690					
				LCFGDSEYIGAHKNIDTKR	56.27	977.953	2	41.13	324690					
				TWGHYADYGCYCGAGGSGTPVDALDRC	61.6	902.3785	3	61.68	324690					
				AALCFGDSEYIGAHKN	58.24	523.2444	3	52.74	324690					
				YADYGCYCGAGGSGTPVDALDRC	107.82	1081.45	2	56.11	324690					
				VCDCDRTAALCFGDSEYIGAHKN	61.73	612.2606	4	53.6	324690					
				FGDSEYIGAHKNIDTKR	61.08	824.4026	2	29.07	324690					
				KTWGHYADYGCYCGAGGSGTPVDALDRC	88.42	1403.583	2	63.03	324690					
	0412250A	toxin A beta1 bungaro	PLA2	RTIICYGAAGGTCRI	45.47	700.3263	2	43.56	143320				0.0021	5.20731E-06
	764177A	toxin B	3FTx	KTWCDGFCSSRG	54.04	638.2455	2	45.76	13977				0.0002	5.07833E-07
				RGKRVDLGCAATCPTVRT	68.14	587.6337	3	35.05	13977					
				RVDLGCAATCPTVRT	80.67	710.3403	2	44.73	13977					
				KRVDLGCAATCPTVRT	70.15	525.9294	3	40.13	13977					
F11b	AED89576.1	putative phospholipase A2 precursor [Micrurus altirostris]	PLA2	SGGGGTPVDDLDRC	59.71	579.7737	2	39.03	6175900		0.0233	0.0012	0.6046	0.000728366
	Q7ZT13.1	RecName: Full=Neurotoxin-like protein pMD18-NTL1/2/4/5; Flags: Precursor	3FTx (Type I NTx)	RHSMYFCFKN	59.2	568.2388	2	45.58	3374700				0.3304	0.000398001
				RGCSLTCPDIKYNGKY	62.43	806.876	2	43.32	3374700					
				RIERGCSLTCPDIKY	43.44	516.9218	3	39.64	3374700					
	P82464.1	RecName: Full=Muscarinic toxin-like protein 3; Short=MTLP-3	3FTx (Type I NTx)	KISLADGNDVRI	54.49	530.2751	2	40.34	387760				0.0380	4.57312E-05
				TICYNHLTRT	43.82	393.1979	3	34.72	387760					
	AAL87004.1	beta-bungarotoxin A2 chain precursor [Bungarus caeruleus]	beta-BTx	VCDCDRTAALCFGDSEYIGAHKN	61.73	612.2606	4	53.6	167840				0.0164	1.97945E-05
				KTWGHYADYGCYCGAGGSGTPVDALDRC	88.42	1403.583	2	63.03	167840					
				YADYGCYCGAGGSGTPVDALDRC	107.82	1081.45	2	56.11	167840					
				AALCFGDSEYIGAHKN	58.24	523.2444	3	52.74	167840					
				RYTIPCDKTWGHYADYGCYCGAGGSGTPVDA	70.47	921.6452	4	66.83	167840					
				RIVCDCDRTAALCFGDSEYIGAHKNIDTKRH	67.9	1129.196	3	51.64	167840					
				RCCYVHDNCYGVAENKH	83.59	944.8713	2	38.15	167840					
				RTAALCFGDSEYIGAHKNIDTKRH	51.79	494.2465	5	48.81	167840					
				FGDSEYIGAHKNIDTKR	61.08	824.4026	2	29.07	167840					
				LCFGDSEYIGAHKN	51.44	461.8715	3	42.29	167840					
				ADYGCYCGAGGSGTPVDALDRC	77.71	1045.931	2	57.53	167840					
				TWGHYADYGCYCGAGGSGTPVDALDRC	61.6	902.3785	3	61.68	167840					
	CAM11302.1	alpha-delta-bungarotoxin-4 partial [Bungarus caeruleus]	3FTx (Type II NTx)	KVIELGCVATCPQPKPYEEVTCC	60.55	1225.571	2	65.3	109000				0.0107	1.28551E-05
				RGKVIELGCVATCPQPKPYEEVTCCSTDKC	76.38	807.3763	4	54.1	109000					
				KPYEEVTCCSTDKC	48.07	744.8025	2	34.26	109000					
				ELGCVATCPQPKPYEEVTCCSTDKC	41	900.7305	3	46.5	109000					
F11c	AED89576.1	putative phospholipase A2 precursor [Micrurus altirostris]	PLA2	SGGGGTPVDDLDRC	59.71	579.7737	2	39.03	53231000		0.3705	0.0191	0.8575	0.016408245
	Q7ZT13.1	RecName: Full=Neurotoxin-like protein pMD18-NTL1/2/4/5; Flags: Precursor	3FTx (Type I NTx)	RIERGCSLTCPDIKY	43.44	516.9218	3	39.64	4666800				0.0752	0.001438523
				RHSMYFCFKN	59.2	568.2388	2	45.58	4666800					
				RGCSLTCPDIKY	56.97	575.765	2	41.17	4666800					
	AAL87004.1	beta-bungarotoxin A2 chain precursor [Bungarus caeruleus]	beta-BTx	RTAALCFGDSEYIGAHKN	98.16	580.6066	3	55.17	1854500				0.0299	0.000571642
				KTWGHYADYGCYCGAGGSGTPVDALDRC	88.42	1403.583	2	63.03	1854500					
				RIVCDCDRTAALCFGDSEYIGAHKNI	57.94	924.7433	3	54.95	1854500					
				RCCYVHDNCYGVAENKH	83.59	944.8713	2	38.15	1854500					
				AALCFGDSEYIGAHKN	58.24	523.2444	3	52.74	1854500					
				VCDCDRTAALCFGDSEYIGAHKNIDTKR	41.46	604.267	5	52.2	1854500					
				FGDSEYIGAHKNIDTKR	61.08	824.4026	2	29.07	1854500					
				RYTIPCDKTWGHYADYGCYCGAGGSGTPVDA	70.47	921.6452	4	66.83	1854500					
				TWGHYADYGCYCGAGGSGTPVDALDRC	61.6	902.3785	3	61.68	1854500					
				LCFGDSEYIGAHKN	51.44	461.8715	3	42.29	1854500					
				YADYGCYCGAGGSGTPVDALDRC	107.82	1081.45	2	56.11	1854500					
				ADYGCYCGAGGSGTPVDALDRC	77.71	1045.931	2	57.53	1854500					
	XP_058034421.1	beta-nerve growth factor isoform X2 [Ahaetulla prasina]	NGF	RIDTACVCIISRK	53.23	654.3257	2	49.09	790310				0.0127	0.00024361
	1G0Z	Chain B PHOSPHOLIPASE A2	PLA2	IGYGCYCGYGGSGTPVDELDRC	95.24	1091.944	2	58.52	480860				0.0077	0.000148223
	CAM11302.1	alpha-delta-bungarotoxin-4 partial [Bungarus caeruleus]	3FTx (Type II NTx)	KVIELGCVATCPQPKP	78.19	786.3979	2	54.94	233790				0.0038	7.20648E-05
				RGKVIELGCVATCPQPKP	85.71	878.9556	2	48.13	233790					
				KPYEEVTCCSTDKC	48.07	744.8025	2	34.26	233790					
				ELGCVATCPQPKPYEEVTCCSTDKC	41	900.7305	3	46.5	233790					
	AAB25729.1	nerve growth factor precursor [Bungarus multicinctus]	NGF	RHWNSYCTTTDTFVKA	68.1	587.2635	3	51.56	227940				0.0037	7.02616E-05
	CAB62501.1	beta-bungarotoxin A chain partial [Bungarus multicinctus]	beta-BTx	KRTIICYGAAGTCERI	42.23	543.2616	3	39.62	130320				0.0021	4.01706E-05
				RTIICYGAAGTCERI	50.96	736.3374	2	48.32	130320					
	BAU24673.1	cytotoxin 14 partial [Naja naja]	3FTx	KLIPLAYKTCPAGKN	65.07	716.4058	2	44.6	114780				0.0018	3.53805E-05
				KCNKLIPLAYKT	63.54	610.3472	2	47.5	114780					
	764177A	toxin B	3FTx	RVDLGCAATCPTVRT	80.67	710.3403	2	44.73	110010				0.0018	3.39101E-05
				RGKRVDLGCAATCPTVRT	68.14	587.6337	3	35.05	110010					
				KTWCDGFCSSRG	54.04	638.2455	2	45.76	110010					
				KRVDLGCAATCPTVRT	70.15	525.9294	3	40.13	110010					
	P82464.1	RecName: Full=Muscarinic toxin-like protein 3; Short=MTLP-3	3FTx (Type I NTx)	KISLADGNDVRI	54.49	530.2751	2	40.34	95705				0.0015	2.95007E-05

F11d	[illegible]	[illegible]	[illegible]	TICYNHLTRT	43.82	393.1979	3	34.72	95705		0.5584	0.0288	[illegible]	[illegible]
	ABU63164.1	phospholipase A2 precursor BF-40 [Bungarus fasciatus]	PLA2	HYVSYGCYCGYGGSGTPVDELDRC	70.24	1238.016	2	63.7	65205				0.0011	2.00992E-05
				YVSYGCYCGYGGSGTPVDELDRC	108.32	1156.482	2	62.54	65205					
	ABN72537.1	metalloproteinase [Bungarus multicinctus]	SVMP	KYSVAVIQDYSKRT	48.4	476.9205	3	49.98	54054				0.0009	1.66619E-05
				NGNTVGLAALGSLCSVKY	45.47	773.9166	2	71.76	54054					
	Q8AY42.2	RecName: Full=Kunitz-type serine protease inhibitor B; AltName: Full=Kunitz inhibitor B; Flags: Precursor	Kunitz	FTYGGCGGNANNFKS	47.41	680.2912	2	33.94	22850				0.0004	7.04342E-06
				EFTYGGCGGNANNFKS	63.19	753.8245	2	60.28	22850					
	Q7ZT13.1	RecName: Full=Neurotoxin-like protein pMD18-NTL1/2/4/5; Flags: Precursor	3FTx (Type I NTx)	RGCSLTCPDIKYNGKY	62.43	806.876	2	43.32	61533000				0.6146	0.017725406
				RHSMYFCFKN	59.2	568.2388	2	45.58	61533000					
				RIERGCSLTCPDIKY	43.44	516.9218	3	39.64	61533000					
	1CB9	Chain A PROTEIN (CYTOTOXIN 2)	3FTx	KMFMVAAPHVPVKR	64.83	679.8519	2	51.04	9935000				0.0992	0.00286191
				FMVAAPHVPVKRG	51.01	407.569	3	30.79	9935000					
				KYVCCNTDKCN	40.6	667.2523	2	22.05	9935000					
				MVAAPHVPVKR	61	459.2823	2	32.83	9935000					
				VAAPHVPVKR	48.02	409.748	2	33.25	9935000					
				KKLVPLFSKT	42.91	466.3023	2	53.01	9935000					
	P82464.1	RecName: Full=Muscarinic toxin-like protein 3; Short=MTLP-3	3FTx (Type I NTx)	TICYNHLTRT	43.82	393.1979	3	34.72	8194200				0.0818	0.002360449
				KISLADGNDVRI	54.49	530.2751	2	40.34	8194200					
	AED89576.1	putative phospholipase A2 precursor [Micrurus altirostris]	PLA2	SGGGGTPVDDLDRC	59.71	579.7737	2	39.03	7152100				0.0714	0.002060258
	CAM11302.1	alpha-delta-bungarotoxin-4 partial [Bungarus caeruleus]	3FTx (Type II NTx)	RGKVIELGCVATCPQPKPYEEVTCCS	45.68	932.4345	3	60.19	4633500				0.0463	0.001334742
				KVIELGCVATCPQPKPYEEV	87.48	1045.5	2	59.29	4633500					
				ELGCVATCPQPKPYEEVTCCSTDKC	41	900.7305	3	46.5	4633500					
				KPYEEVTCCSTDKC	48.07	744.8025	2	34.26	4633500					
	BAU24673.1	cytotoxin 14 partial [Naja naja]	3FTx	KLIPLAYKTCPAGKN	65.07	716.4058	2	44.6	3926500				0.0392	0.001131081
				KCNKLIPLAYKT	63.54	610.3472	2	47.5	3926500					
	AAL87004.1	beta-bungarotoxin A2 chain precursor [Bungarus caeruleus]	beta-BTx	YADYGCYCGAGGSGTPVDALDRC	107.82	1081.45	2	56.11	3281300				0.0328	0.000945222
				VCDCDRTAALCFGDSEYIGAHKN	61.73	612.2606	4	53.6	3281300					
				LCFGDSEYIGAHKNIDTKR	56.27	977.953	2	41.13	3281300					
				RIVCDCDRTAALCFGDSEYIGAHKNIDTKR	78.18	1077.161	3	54.61	3281300					
				RYTIPCDKTWGHYADYGCYCGAGGSGTPVDA	70.47	921.6452	4	66.83	3281300					
				ADYGCYCGAGGSGTPVDALDRC	77.71	1045.931	2	57.53	3281300					
				RCCYVHDNCYGVAENKHKC	66.85	539.2285	4	31.61	3281300					
				FGDSEYIGAHKNIDTKR	61.08	824.4026	2	29.07	3281300					
				RTAALCFGDSEYIGAHKN	98.16	580.6066	3	55.17	3281300					
				AALCFGDSEYIGAHKN	58.24	523.2444	3	52.74	3281300					
				TWGHYADYGCYCGAGGSGTPVDALDRC	61.6	902.3785	3	61.68	3281300					
				KTWGHYADYGCYCGAGGSGTPVDALDRC	88.42	1403.583	2	63.03	3281300					
	0412250A	toxin A beta1 bungaro	PLA2	RTIICYGAAGGTCRI	45.47	700.3263	2	43.56	779280				0.0078	0.000224482
	764177A	toxin B	3FTx	KTWCDGFCSSRG	54.04	638.2455	2	45.76	309980				0.0031	8.92939E-05
				RGKRVDLGCAATCPTVRT	68.14	587.6337	3	35.05	309980					
				RVDLGCAATCPTVRT	80.67	710.3403	2	44.73	309980					
				KRVDLGCAATCPTVRT	70.15	525.9294	3	40.13	309980					
	1G0Z	Chain B PHOSPHOLIPASE A2	PLA2	IGYGCYCGYGGSGTPVDELDRC	95.24	1091.944	2	58.52	179590				0.0018	5.17333E-05
	ABN72537.1	metalloproteinase [Bungarus multicinctus]	SVMP	NGNTVGLAALGSLCSVKY	45.47	773.9166	2	71.76	106360				0.0011	3.06384E-05
				KYSVAVIQDYSKR	57.82	636.8276	2	54.63	106360					
	AAO84769.1	phospholipase A2 partial [Bungarus candidus]	PLA2	RTAALCFAEAPYKR	69.81	671.3284	2	58.29	89439				0.0009	2.57641E-05
				RGTPVDMLDRC	53.42	502.247	2	50.85	89439					
F12a	AED89576.1	putative phospholipase A2 precursor [Micrurus altirostris]	PLA2	SGGGGTPVDDLDRC	59.71	579.7737	2	39.03	1827300		0.1347	0.0211	0.6999	0.014740348
	AAL87004.1	beta-bungarotoxin A2 chain precursor [Bungarus caeruleus]	beta-BTx	AALCFGDSEYIGAHKN	58.24	523.2444	3	52.74	649140				0.2486	0.005236441
				VCDCDRTAALCFGDSEYIGAHKN	61.73	612.2606	4	53.6	649140					
				RIVCDCDRTAALCFGDSEYIGAHKNIDTKR	78.18	1077.161	3	54.61	649140					
				RTAALCFGDSEYIGAHKN	98.16	580.6066	3	55.17	649140					
				RCCYVHDNCYGVAENKH	83.59	944.8713	2	38.15	649140					
				YADYGCYCGAGGSGTPVDALDRC	107.82	1081.45	2	56.11	649140					
				LCFGDSEYIGAHKN	51.44	461.8715	3	42.29	649140					
				FGDSEYIGAHKNIDTKR	61.08	824.4026	2	29.07	649140					
				RYTIPCDKTWGHYADYGCYCGAGGSGTPVDA	70.47	921.6452	4	66.83	649140					
				KTWGHYADYGCYCGAGGSGTPVDALDRC	88.42	1403.583	2	63.03	649140					
				TWGHYADYGCYCGAGGSGTPVDALDRC	61.6	902.3785	3	61.68	649140					
				ADYGCYCGAGGSGTPVDALDRC	77.71	1045.931	2	57.53	649140					
	CAM11302.1	alpha-delta-bungarotoxin-4 partial [Bungarus caeruleus]	3FTx (Type II NTx)	KVIELGCVATCPQPKPYEEV	87.48	1045.5	2	59.29	134400				0.0515	0.001084169
				RGKVIELGCVATCPQPKPYEE	82.39	1073.534	2	52.66	134400					
				ELGCVATCPQPKPYEEVTCCSTDKC	41	900.7305	3	46.5	134400					
				KPYEEVTCCSTDKC	48.07	744.8025	2	34.26	134400					
F12b	1TC8	Chain A phospholipase A2 isoform 1	PLA2	RFVCDCDRTAAICFAKAPYNT	57.06	760.3405	3	57.65	511710000		0.8653	0.1353	0.3481	0.047081439
	AAL87004.1	beta-bungarotoxin A2 chain precursor [Bungarus caeruleus]	beta-BTx	LCFGDSEYIGAHKN	51.44	461.8715	3	42.29	492640000				0.3351	0.045326845
				VCDCDRTAALCFGDSEYIGAHKN	61.73	612.2606	4	53.6	492640000					
				FGDSEYIGAHKNIDTKR	61.08	824.4026	2	29.07	492640000					
				AALCFGDSEYIGAHKN	58.24	523.2444	3	52.74	492640000					
				RCCYVHDNCYGVAENKHKC	66.85	539.2285	4	31.61	492640000					
				KTWGHYADYGCYCGAGGSGTPVDALDRC	88.42	1403.583	2	63.03	492640000					
				RTAALCFGDSEYIGAHKNIDTKRH	51.79	494.2465	5	48.81	492640000					
				YADYGCYCGAGGSGTPVDALDRC	107.82	1081.45	2	56.11	492640000					
				ADYGCYCGAGGSGTPVDALDRC	77.71	1045.931	2	57.53	492640000					
				TWGHYADYGCYCGAGGSGTPVDALDRC	61.6	902.3785	3	61.68	492640000					
				RYTIPCDKTWGHYADYGCYCGAGGSGTPVDA	70.47	921.6452	4	66.83	492640000	0.1563				
	CAM11302.1	alpha-delta-bungarotoxin-4 partial [Bungarus caeruleus]	3FTx (Type II NTx)	RGKVIELGCVATCPQPKPYE	62.54	673.0121	3	53.16	299130000				0.2035	0.027522368
				KVIELGCVATCPQPKPYEEV	87.48	1045.5	2	59.29	299130000					
				KPYEEVTCCSTDKC	48.07	744.8025	2	34.26	299130000					
				ELGCVATCPQPKPYEEVTCCSTDKC	41	900.7305	3	46.5	299130000					
	Q8QFW4.1	RecName: Full=Basic phospholipase A2 beta-bungarotoxin A1 chain; Short=Beta-BuTX A1 chain; Short=svPLA2; AltName: Full=Phosphatidylcholine 2-acylhydrolase; Flags: Precursor	beta-BTx	RYTIPCEKTWGEYTNYGCYCGAGGSGTPIDAL	87.58	1244.872	3	76.01	91034000				0.0619	0.008375861
				TWGEYTNYGCYCGAGGSGTPIDALDRC	44.95	1370.573	2	71.12	91034000					
				KTWGEYTNYGCYCGAGGSGTPIDALDRC	114.9	1421.097	2	74.06	91034000					
				EKTWGEYTNYGCYCGAGGSGTPIDALDRC	95.55	990.4334	3	67.63	91034000					
				EYTNYGCYCGAGGSGTPIDALDRC	96.63	1184.501	2	63.29	91034000					
	BAU24673.1	cytotoxin 14 partial [Naja naja]	3FTx	KLIPLAYKTCPAGKN	65.07	716.4058	2	44.6	22648000				0.0154	0.002083798
				KCNKLIPLAYKT	63.54	610.3472	2	47.5	22648000					
	CAB62501.1	beta-bungarotoxin A chain partial [Bungarus multicinctus]	beta-BTx	RTIICYGAAGTCERI	50.96	736.3374	2	48.32	15450000				0.0105	0.001421524
				KRTIICYGAAGTCERI	42.23	543.2616	3	39.62	15450000					
	P00606.2	RecName: Full=Acidic phospholipase A2; Short=svPLA2; AltName: Full=Phosphatidylcholine 2-acylhydrolase; Flags: Precursor		GYVNYGCYCGAGGSGTPVDELDRC	56.62	1205.507	2	61.14	13788000				0.0094	0.001268607
				YVNYGCYCGAGGSGTPVDELDRC	80.33	1123.973	2	58.44	13788000					
				KTKTYSYTCTKPNLTC	53.86	839.4119	2	38	13788000					
	ABU63164.1	phospholipase A2 precursor BF-40 [Bungarus fasciatus]		HYVSYGCYCGYGGSGTPVDELDRC	70.24	1238.016	2	63.7	10150000				0.0069	0.000933882
				YVSYGCYCGYGGSGTPVDELDRC	108.32	1156.482	2	62.54	10150000					
	1ZM6	Chain A Phospholipase A2 isoform 3	PLA2	ECTQGTLTCKGDNNACAASVCDCDRL	41.93	912.0278	3	36.47	7113800				0.0048	0.000654527
				FAGAPYNDDNYNIDLKA	50.59	841.888	2	53.88	7113800					
				KTYSYECTQGTLTCKG	54.58	856.3684	2	43.43	7113800					
				AAICFAGAPYNDDNYNIDLKA	74.49	1087.498	2	70.18	7113800					
				RLAAICFAGAPYNDDNYNIDLKA	76.38	1179.565	2	77.17	7113800					
	1G0Z	Chain B PHOSPHOLIPASE A2		IGYGCYCGYGGSGTPVDELDRC	95.24	1091.944	2	58.52	4477900				0.0030	0.000412003
	1A3D	Chain A PHOSPHOLIPASE A2		GDNNACAASVCDCDRLAAICFAGAPYNDNNY	68.23	1322.57	3	76.54	1843700				0.0013	0.000169635
				KGDNNACAASVCDCDRLAAICFAGAPYNDNN	69.75	1341.578	3	76.49	1843700					
				ECSQGTLTCKGDNNACAASVCDCDRL	54.77	907.3585	3	36.27	1843700					
	AAO84769.1	phospholipase A2 partial [Bungarus candidus]		RTAALCFAEAPYKR	69.81	671.3284	2	58.29	81322				0.0001	7.48228E-06
				RGTPVDMLDRC	53.42	502.247	2	50.85	81322					
F13a	1TC8	Chain A phospholipase A2 isoform 1	PLA2	RFVCDCDRTAAICFAKAPYNT	57.06	760.3405	3	57.65	4274100000	0.0842	1.0000	0.0842	0.6259	0.052684499
	CAM11302.1	alpha-delta-bungarotoxin-4 partial [Bungarus caeruleus]	3FTx (Type II NTx)	KVIELGCVATCPQPKPYEEVTCC	60.55	1225.571	2	65.3	892680000				0.1307	0.011003579
				RGKVIELGCVATCPQPKPYEEVTCC	55.29	879.0901	3	57.59	892680000					
				ELGCVATCPQPKPYEEVTCCSTDKC	41	900.7305	3	46.5	892680000					
				KPYEEVTCCSTDKC	48.07	744.8025	2	34.26	892680000					
	Q8QFW4.1	RecName: Full=Basic phospholipase A2 beta-bungarotoxin A1 chain; Short=Beta-BuTX A1 chain; Short=svPLA2; AltName: Full=Phosphatidylcholine 2-acylhydrolase; Flags: Precursor	beta-BTx	KTWGEYTNYGCYCGAGGSGTPIDALDRC	114.9	1421.097	2	74.06	584360000				0.0856	0.007203087
				EKTWGEYTNYGCYCGAGGSGTPIDALDRC	95.55	990.4334	3	67.63	584360000					
				RYTIPCEKTWGEYTNYGCYCGAGGSGTPIDAL	87.58	1244.872	3	76.01	584360000					
				TWGEYTNYGCYCGAGGSGTPIDALDRC	44.95	1370.573	2	71.12	584360000					
				EYTNYGCYCGAGGSGTPIDALDRC	96.63	1184.501	2	63.29	584360000					
	1CB9	Chain A PROTEIN (CYTOTOXIN 2)	3FTx	KMFMVAAPHVPVKR	53.24	671.8546	2	55.44	584220000				0.0856	0.007201361
				FMVAAPHVPVKRG	51.01	407.569	3	30.79	584220000					
				KYVCCNTDKCN	40.6	667.2523	2	22.05	584220000					
				MVAAPHVPVKR	61	459.2823	2	32.83	584220000					
				VAAPHVPVKR	48.02	409.748	2	33.25	584220000					
				KKLVPLFSKT	42.91	466.3023	2	53.01	584220000					
	AAL87004.1	beta-bungarotoxin A2 chain precursor [Bungarus caeruleus]	beta-BTx	RCCYVHDNCYGVAENKHKC	66.85	539.2285	4	31.61	237300000				0.0348	0.002925068
				RTAALCFGDSEYIGAHKN	98.16	580.6066	3	55.17	237300000					
				LCFGDSEYIGAHKNIDTKR	56.27	977.953	2	41.13	237300000					
				VCDCDRTAALCFGDSEYIGAHKN	61.73	612.2606	4	53.6	237300000					
				RYTIPCDKTWGHYADYGCYCGAGGSGTPVDA	70.47	921.6452	4	66.83	237300000					
				YADYGCYCGAGGSGTPVDALDRC	107.82	1081.45	2	56.11	237300000					
				TWGHYADYGCYCGAGGSGTPVDALDRC	61.6	902.3785	3	61.68	237300000					
				AALCFGDSEYIGAHKN	58.24	523.2444	3	52.74	237300000					
				FGDSEYIGAHKNIDTKR	61.08	824.4026	2	29.07	237300000					
				KTWGHYADYGCYCGAGGSGTPVDALDRC	88.42	1403.583	2	63.03	237300000					
				ADYGCYCGAGGSGTPVDALDRC	77.71	1045.931	2	57.53	237300000					
	ABU63164.1	phospholipase A2 precursor BF-40 [Bungarus fasciatus]	PLA2	YVSYGCYCGYGGSGTPVDELDRC	108.32	1156.482	2	62.54	123480000				0.0181	0.001522071
				HYVSYGCYCGYGGSGTPVDELDRC	70.24	1238.016	2	63.7	123480000					
	P62377.1	RecName: Full=Cytotoxin-like basic protein; Short=CLBP; AltName: Full=Less-cytotoxic basic polypeptide; Short=LCBP	3FTx	KKFPLKFPVKR	49.18	368.5708	3	52.91	45250000				0.0066	0.000557772
				KCHNTQLPFIYKT	65.89	710.8536	2	55.66	45250000					
				LKCHNTQLPFIYKT	59.67	831.444	2	51.5	45250000					
	UNW14927.1	phospholipase A2 protein 2-4 partial [Pseudonaja affinis]		RNVLDYADYGCYCGKGGSGTPVDELDRC	41.55	961.0897	3	79.32	19737000				0.0029	0.000243287
	AAL55556.1	phospholipase A2 partial [Hydrophis hardwickii]		RRSSLDYADYGCYCGAGGSGTPVDELDRC	67.53	1471.126	2	79.89	15386000				0.0023	0.000189655
	1G0Z	Chain B PHOSPHOLIPASE A2	PLA2	IGYGCYCGYGGSGTPVDELDRC	95.24	1091.944	2	58.52	11993000				0.0018	0.000147831
	1ZM6	Chain A Phospholipase A2 isoform 3		KTYSYECTQGTLTCKG	54.58	856.3684	2	43.43	10722000				0.0016	0.000132164
				ECTQGTLTCKGDNNACAASVCDCDRL	41.93	912.0278	3	36.47	10722000					
				FAGAPYNDDNYNIDLKA	50.59	841.888	2	53.88	10722000					
				RLAAICFAGAPYNDDNYNIDLKA	76.38	1179.565	2	77.17	10722000					
				AAICFAGAPYNDDNYNIDLKA	74.49	1087.498	2	70.18	10722000					
	Q75S49.1	RecName: Full=Kunitz-type serine protease inhibitor homolog beta-bungarotoxin B4 chain; Flags: Precursor	Kunitz	NGNGNHFKTETLCRC	50.12	511.9126	3	30.4	9373400				0.0014	0.000115541
	1A3D	Chain A PHOSPHOLIPASE A2	PLA2	GDNNACAASVCDCDRLAAICFAGAPYNDNNY	68.23	1322.57	3	76.54	7584700				0.0011	9.34925E-05
				ECSQGTLTCKGDNNACAASVCDCDRL	54.77	907.3585	3	36.27	7584700					
				KGDNNACAASVCDCDRLAAICFAGAPYNDNN	69.75	1341.578	3	76.49	7584700					
	CAB62501.1	beta-bungarotoxin A chain partial [Bungarus multicinctus]	beta-BTx	KRTIICYGAAGTCERI	42.23	543.2616	3	39.62	6608100				0.0010	8.14544E-05
				RTIICYGAAGTCERI	50.96	736.3374	2	48.32	6608100					
	P00606.2	RecName: Full=Acidic phospholipase A2; Short=svPLA2; AltName: Full=Phosphatidylcholine 2-acylhydrolase; Flags: Precursor	PLA2	KTKTYSYTCTKPNLTC	53.86	839.4119	2	38	2807800				0.0004	3.46102E-05
				YVNYGCYCGAGGSGTPVDELDRC	80.33	1123.973	2	58.44	2807800					
				GYVNYGCYCGAGGSGTPVDELDRC	56.62	1205.507	2	61.14	2807800					
	AAB25729.1	nerve growth factor precursor [Bungarus multicinctus]	NGF	RHWNSYCTTTDTFVKA	68.1	587.2635	3	51.56	1266200				0.0002	1.56078E-05
	AAO84769.1	phospholipase A2 partial [Bungarus candidus]	PLA2	RTAALCFAEAPYKR	69.81	671.3284	2	58.29	1106300				0.0002	1.36368E-05
				RGTPVDMLDRC	53.42	502.247	2	50.85	1106300					
	ABN72537.1	metalloproteinase [Bungarus multicinctus]	SVMP	KYSVAVIQDYSKRT	48.4	476.9205	3	49.98	428600				0.0001	5.28312E-06
				NGNTVGLAALGSLCSVKY	45.47	773.9166	2	71.76	428600					

	1TC8	Chain A phospholipase A2 isoform 1	PLA2	RFVCDCDRTAAICFAKA	98.02	917.4055	2	52.9	15553000				0.6310	0.001125696
				KMFMVAAPHVPVKRG	64.83	679.8519	2	51.04	3796200					
				FMVAAPHVPVKRG	51.01	407.569	3	30.79	3796200					
	1CB9	Chain A PROTEIN (CYTOTOXIN 2)	3FTx	KYVCCNTDKCN	40.6	667.2523	2	22.05	3796200				0.1540	0.000274762
				MVAAPHVPVKR	61	459.2823	2	32.83	3796200					
				VAAPHVPVKR	48.02	409.748	2	33.25	3796200					
				KKLVPLFSKT	42.91	466.3023	2	53.01	3796200					
	UNW14927.1	phospholipase A2 protein 2-4 partial [Pseudonaja affinis]	PLA2	RNVLDYADYGCYCGKGGSGTPVDELDRC	41.55	961.0897	3	79.32	3230100				0.1311	0.000233788
	P62377.1	RecName: Full=Cytotoxin-like basic protein; Short=CLBP; AltName: Full=Less-cytotoxic basic polypeptide; Short=LCBP	3FTx	KKFPLKFPVKR	49.18	368.5708	3	52.91	869540				0.0353	6.29356E-05
				LKCHNTQLPFIYKT	59.67	831.444	2	51.5	869540					
				KCHNTQLPFIYKT	65.89	710.8536	2	55.66	869540					
				YADYGCYCGAGGSGTPVDALDRC	107.82	1081.45	2	56.11	477150					
				VCDCDRTAALCFGDSEYIGAHKN	61.73	612.2606	4	53.6	477150					
				LCFGDSEYIGAHKN	51.44	461.8715	3	42.29	477150					
				KTWGHYADYGCYCGAGGSGTPVDALDRC	88.42	1403.583	2	63.03	477150					
				RCCYVHDNCYGVAENKHKC	66.85	539.2285	4	31.61	477150					
	AAL87004.1	beta-bungarotoxin A2 chain precursor [Bungarus caeruleus]	beta-BTx	TWGHYADYGCYCGAGGSGTPVDALDRC	61.6	902.3785	3	61.68	477150				0.0194	3.45352E-05
				RTAALCFGDSEYIGAHKN	98.16	580.6066	3	55.17	477150					
F14a				AALCFGDSEYIGAHKN	58.24	523.2444	3	52.74	477150		0.0338	0.0018		
				ADYGCYCGAGGSGTPVDALDRC	77.71	1045.931	2	57.53	477150					
				RYTIPCDKTWGHYADYGCYCGAGGSGTPVDA	70.47	921.6452	4	66.83	477150					
				FGDSEYIGAHKNIDTKR	61.08	824.4026	2	29.07	477150					
				RGIDSSHWNSYCTETDTFIKA	43.65	754.3318	3	64.82	207960					
				EHSVCDSVSAWVTKT	40.24	492.5703	3	57.21	207960					
	AAS94269.1	nerve growth factor II [Naja sputatrix]	NGF	KTTATDIKGNTVTVMENVNLDNKVYKQ	56.4	928.8076	3	53.31	207960				0.0084	1.50517E-05
				REDHPVHNLGEHSVCDSVSAWVTKT	62.83	651.5536	4	58.95	207960					
				KGNTVTVMENVNLDNKVYKQ	41.51	685.3459	3	49.64	207960					
	E5L0E4.1	RecName: Full=Beta-fibrinogenase-like; Short=RVBF; AltName: Full=Snake venom serine protease; Short=SVSP; Flags: Precursor	SVSP	KTSTYIAPLSLPSSPPRV	65.11	843.9543	2	66.36	147640				0.0060	1.06859E-05
	AED89576.1	putative phospholipase A2 precursor [Micrurus altirostris]	PLA2	SGGGGTPVDDLDRC	59.71	579.7737	2	39.03	135280				0.0055	9.79131E-06
				TWGEYTNYGCYCGAGGSGTPIDALDRC	44.95	1370.573	2	71.12	95620					
				EYTNYGCYCGAGGSGTPIDALDRC	96.63	1184.501	2	63.29	95620					
	Q8QFW4.1	RecName: Full=Basic phospholipase A2 beta-bungarotoxin A1 chain; Short=Beta-BuTX A1 chain; Short=svPLA2; AltName: Full=Phosphatidylcholine 2-acylhydrolase; Flags: Precursor	beta-BTx	KTWGEYTNYGCYCGAGGSGTPIDALDRC	114.9	1421.097	2	74.06	95620				0.0039	6.92079E-06
				RYTIPCEKTWGEYTNYGCYCGAGGSGTPIDAL	87.58	1244.872	3	76.01	95620					
				EKTWGEYTNYGCYCGAGGSGTPIDALDRC	95.55	990.4334	3	67.63	95620					
	0412250A	toxin A beta1 bungaro		RTIICYGAAGGTCRI	45.47	700.3263	2	43.56	93747				0.0038	6.78523E-06
				RIWTAYVAYGCYCGKGGSGTPVDELDRC	85.05	965.7619	3	79.45	40066				0.0016	2.8999E-06
			PLA2	IWTAYVAYGCYCGKGGSGTPVDELDRC	50.21	928.0788	3	81.32	40066					
	1DPY	Chain A PHOSPHOLIPASE A2		RIWTAYVAYGCYCGKGGSGTPVDELDRC	85.05	965.7619	3	79.45	4640600				0.5733	0.021843427
				IWTAYVAYGCYCGKGGSGTPVDELDRC	50.21	928.0788	3	81.32	4640600					
				EHSVCDSVSAWVTKT	40.24	492.5703	3	57.21	1092500					
				RGIDSSHWNSYCTETDTFIKA	43.65	754.3318	3	64.82	1092500					
	AAS94269.1	nerve growth factor II [Naja sputatrix]	NGF	REDHPVHNLGEHSVCDSVSAWVTKT	62.83	651.5536	4	58.95	1092500				0.1350	0.005142426
				KTTATDIKGNTVTVMENVNLDNKVYKQ	56.4	928.8076	3	53.31	1092500					
				KGNTVTVMENVNLDNKVYKQ	41.51	685.3459	3	49.64	1092500					
				KMFMVAAPHVPVKR	56.9	448.2385	3	55.59	886920					
				FMVAAPHVPVKRG	51.01	407.569	3	30.79	886920					
				MVAAPHVPVKR	61	459.2823	2	32.83	886920	0.0528				
	1CB9	Chain A PROTEIN (CYTOTOXIN 2)	3FTx	KKLVPLFSKT	42.91	466.3023	2	53.01	886920				0.1096	0.004174756
				VAAPHVPVKR	48.02	409.748	2	33.25	886920					
				KYVCCNTDKCN	40.6	667.2523	2	22.05	886920					
				RIVCDCDRTAALCFGDSEYIEGHKRI	87.78	958.0972	3	51.93	783830					
				RCCYVHDNCYGDAEKIHKC	63.3	723.636	3	35.64	783830					
F14b				KTWGEYTNYGCYCGAGGSGTPIDALDRC	114.9	1421.097	2	74.06	783830		0.7223	0.0381		
				TWGEYTNYGCYCGAGGSGTPIDALDRC	44.95	1370.573	2	71.12	783830					
	Q8QFW4.1	RecName: Full=Basic phospholipase A2 beta-bungarotoxin A1 chain; Short=Beta-BuTX A1 chain; Short=svPLA2; AltName: Full=Phosphatidylcholine 2-acylhydrolase; Flags: Precursor	beta-BTx	VCDCDRTAALCFGDSEYIEGHKRI	54.87	887.3819	3	51.52	783830				0.0968	0.003689509
				RYTIPCEKTWGEYTNYGCYCGAGGSGTPIDAL	87.58	1244.872	3	76.01	783830					
				EYTNYGCYCGAGGSGTPIDALDRC	96.63	1184.501	2	63.29	783830					
				RTAALCFGDSEYIEGHKRI	80.88	977.4579	2	51.77	783830					
				EKTWGEYTNYGCYCGAGGSGTPIDALDRC	95.55	990.4334	3	67.63	783830					
				RGKVIELGCVATCPQPKPYE	62.54	673.0121	3	53.16	486890					
	CAM11302.1	alpha-delta-bungarotoxin-4 partial [Bungarus caeruleus]	3FTx (Type II NTx)	KVIELGCVATCPQPKPYEEV	87.48	1045.5	2	59.29	486890				0.0602	0.002291804
				KPYEEVTCCSTDKC	48.07	744.8025	2	34.26	486890					
				ELGCVATCPQPKPYEEVTCCSTDKC	41	900.7305	3	46.5	486890					
				KTKTYSYTCTKPNLTC	53.86	839.4119	2	38	130360					
	P00606.2	RecName: Full=Acidic phospholipase A2; Short=svPLA2; AltName: Full=Phosphatidylcholine 2-acylhydrolase; Flags: Precursor	PLA2	YVNYGCYCGAGGSGTPVDELDRC	80.33	1123.973	2	58.44	130360				0.0161	0.000613608
				GYVNYGCYCGAGGSGTPVDELDRC	56.62	1205.507	2	61.14	130360					
	JAA74875.1	PLA2-Hop-6 [Hoplocephalus bungaroides]		YGCYCSSGGSGTPVDELDRC	41.01	958.882	2	52.9	73381				0.0091	0.000345406
				KMFMVAAPHVPVKRG	53.24	671.8546	2	55.44	27603000					
				VAAPHVPVKR	48.02	409.748	2	33.25	27603000					
	1CB9	Chain A PROTEIN (CYTOTOXIN 2)	3FTx	MVAAPHVPVKR	61	459.2823	2	32.83	27603000				0.5914	0.007607958
				KYVCCNTDKCN	40.6	667.2523	2	22.05	27603000					
				KKLVPLFSKT	42.91	466.3023	2	53.01	27603000					
				FMVAAPHVPVKRG	51.01	407.569	3	30.79	27603000					
	1TC8	Chain A phospholipase A2 isoform 1	PLA2	RFVCDCDRTAAICFAK	49.7	853.3586	2	62.05	18102000				0.3878	0.004989286
				KVIELGCVATCPQPKPYEEV	87.48	1045.5	2	59.29	163890					
	CAM11302.1	alpha-delta-bungarotoxin-4 partial [Bungarus caeruleus]	3FTx (Type II NTx)	RGKVIELGCVATCPQPKPYE	62.54	673.0121	3	53.16	163890				0.0035	4.51715E-05
				ELGCVATCPQPKPYEEVTCCSTDKC	41	900.7305	3	46.5	163890					
				KPYEEVTCCSTDKC	48.07	744.8025	2	34.26	163890					
	P86538.2	RecName: Full=Cytotoxin 2a; Short=CTX2a; AltName: Full=Cytotoxin 2; Short=CTX2	3FTx	KMFMVSDLTIPVKRG	53.31	523.6117	3	63.32	119050				0.0026	3.28126E-05
F14c				EKTWGEYTNYGCYCGAGGSGTPIDALDRC	95.55	990.4334	3	67.63	59498		0.2439	0.0129		
				RCCYVHDNCYGDAEKIHKC	63.3	723.636	3	35.64	59498					
				RYTIPCEKTWGEYTNYGCYCGAGGSGTPIDAL	87.58	1244.872	3	76.01	59498					
				EYTNYGCYCGAGGSGTPIDALDRC	96.63	1184.501	2	63.29	59498					
	Q8QFW4.1	RecName: Full=Basic phospholipase A2 beta-bungarotoxin A1 chain; Short=Beta-BuTX A1 chain; Short=svPLA2; AltName: Full=Phosphatidylcholine 2-acylhydrolase; Flags: Precursor	beta-BTx	RTAALCFGDSEYIEGHKRI	80.88	977.4579	2	51.77	59498				0.0013	1.63989E-05
				KTWGEYTNYGCYCGAGGSGTPIDALDRC	114.9	1421.097	2	74.06	59498					
				TWGEYTNYGCYCGAGGSGTPIDALDRC	44.95	1370.573	2	71.12	59498					
				VCDCDRTAALCFGDSEYIEGHKRI	54.87	887.3819	3	51.52	59498					
	AAO84769.1	phospholipase A2 partial [Bungarus candidus]	PLA2	RTAALCFAEAPYKRR	47.19	749.3792	2	51.04	55691				0.0012	1.53496E-05
				RGTPVDMLDRC	53.42	502.247	2	50.85	55691					
				KTWCDGFCSSRG	54.04	638.2455	2	45.76	22808					
	764177A	toxin B	3FTx	RVDLGCAATCPTVRT	80.67	710.3403	2	44.73	22808				0.0005	6.28636E-06
				RGKRVDLGCAATCPTVRT	68.14	587.6337	3	35.05	22808					
				KRVDLGCAATCPTVRT	70.15	525.9294	3	40.13	22808					
F15a	E5L0E4.1	RecName: Full=Beta-fibrinogenase-like; Short=RVBF; AltName: Full=Snake venom serine protease; Short=SVSP; Flags: Precursor	SVSP	KTSTYIAPLSLPSSPPRV	65.11	843.9543	2	66.36	51528		0.0176	0.0039	1.0000	0.003912015
	JAC94920.1	Cysteine-rich secretory protein A [Python regius]		INSIATPYKS	41.95	447.2403	2	32.97	15117000				0.9694	0.004133438
	ACE73577.1	cysteine-rich secretory protein Bc-CRPb [Bungarus candidus]	CRISP	KYNDDYTNCKS	40.57	596.7323	2	29.12	228320				0.0146	6.24295E-05
F15b				RWADRCTFAHSPPHLRT	78.11	463.4775	4	50.85	228320		0.0192	0.0043		
	AAO84769.1	phospholipase A2 partial [Bungarus candidus]	PLA2	RTAALCFAEAPYKRR	69.81	671.3284	2	58.29	220680				0.0142	6.03405E-05
				RGTPVDMLDRC	53.42	502.247	2	50.85	220680					
	1TC8	Chain A phospholipase A2 isoform 1		RFVCDCDRTAAICFAKA	98.02	917.4055	2	52.9	27767				0.0018	7.59232E-06
	ACE73577.1	cysteine-rich secretory protein Bc-CRPb [Bungarus candidus]		KYNDDYTNCKS	40.57	596.7323	2	29.12	9611300				0.8243	0.049994736
			CRISP	RWADRCTFAHSPPHLRT	78.11	463.4775	4	50.85	9611300					
	Q3SB03.1	RecName: Full=Cysteine-rich venom protein pseudechetoxin-like; Short=CRVP; Flags: Precursor		KSGPPCADCPSACVNGLCTNPCKH	94.46	1211.49	2	49.58	1454200				0.1247	0.007564257
F15c	AAO84769.1	phospholipase A2 partial [Bungarus candidus]	PLA2	RTAALCFAEAPYKRR	47.19	749.3792	2	51.04	453490		0.2735	0.0607	0.0389	0.002358902
				RGTPVDMLDRC	53.42	502.247	2	50.85	453490					
	ACY68723.1	CRISP isoform 2 [Parasuta nigriceps]	CRISP	KSGPPCGDCPSACVKGLCTNPCKH	61.13	807.9966	3	49.93	110330				0.0095	0.000573899
	AAB32582.1	phospholipase A2 31 kda subunit PLA2 31 kda subunit=urokinase-type plasminogen activator receptor homolog [Naja naja kaouthia=Thailand cobras blood Peptide 188 aa]		KLGSFDVNVGQNTYVRG	64.29	834.9223	2	62.97	31072				0.0027	0.000161626
	AAO84769.1	phospholipase A2 partial [Bungarus candidus]	PLA2	RGTPVDMLDRC	53.42	502.247	2	50.85	6518600	0.2218			0.8221	0.105176091
				RTAALCFAEAPYKRR	47.19	749.3792	2	51.04	6518600					
	P0C551.1	RecName: Full=Acidic phospholipase A2 KBf-grIB; Short=svPLA2; AltName: Full=Phosphatidylcholine 2-acylhydrolase; Flags: Precursor		KENPKCSSLLNVPYVKQ	52.4	583.3048	3	57.09	701190				0.0884	0.011313537
				RTAALCFGDSEYIGAHKNIDTKRH	51.79	494.2465	5	48.81	664200					
				RIVCDCDRTAALCFGDSEYIGAHKN	90.22	886.7267	3	56.51	664200					
				TWGHYADYGCYCGAGGSGTPVDALDRC	61.6	902.3785	3	61.68	664200					
				RCCYVHDNCYGVAENKHKC	66.85	539.2285	4	31.61	664200					
				RYTIPCDKTWGHYADYGCYCGAGGSGTPVDA	70.47	921.6452	4	66.83	664200					
F15d	AAL87004.1	beta-bungarotoxin A2 chain precursor [Bungarus caeruleus]	beta-BTx	LCFGDSEYIGAHKN	51.44	461.8715	3	42.29	664200		0.5769	0.1279	0.0838	0.010716712
				ADYGCYCGAGGSGTPVDALDRC	77.71	1045.931	2	57.53	664200					
				FGDSEYIGAHKNIDTKR	61.08	824.4026	2	29.07	664200					
				YADYGCYCGAGGSGTPVDALDRC	107.82	1081.45	2	56.11	664200					
				VCDCDRTAALCFGDSEYIGAHKN	61.73	612.2606	4	53.6	664200					
				AALCFGDSEYIGAHKN	58.24	523.2444	3	52.74	664200					
				KTWGHYADYGCYCGAGGSGTPVDALDRC	88.42	1403.583	2	63.03	664200					
				KTKTYSYTCTKPNLTC	53.86	839.4119	2	38	45592					
	P00606.2	RecName: Full=Acidic phospholipase A2; Short=svPLA2; AltName: Full=Phosphatidylcholine 2-acylhydrolase; Flags: Precursor	PLA2	GYVNYGCYCGAGGSGTPVDELDRC	56.62	1205.507	2	61.14	45592				0.0057	0.000735616
				YVNYGCYCGAGGSGTPVDELDRC	80.33	1123.973	2	58.44	45592					
	KAG8124481.1	putative Cysteine-rich venom protein [Naja naja]	CRISP	KMEWYPEAASNAERW	60.77	785.3354	2	50.51	359610				0.7191	0.011810792
				KTWGHYADYGCYCGAGGSGTPVDALDRC	88.42	1403.583	2	63.03	109940					
				VCDCDRTAALCFGDSEYIGAHKN	61.73	612.2606	4	53.6	109940					
				YADYGCYCGAGGSGTPVDALDRC	107.82	1081.45	2	56.11	109940					
				LCFGDSEYIGAHKN	51.44	461.8715	3	42.29	109940					
				RCCYVHDNCYGVAENKH	83.59	944.8713	2	38.15	109940					
F16a	AAL87004.1	beta-bungarotoxin A2 chain precursor [Bungarus caeruleus]	beta-BTx	RTAALCFGDSEYIGAHKNIDTKR	88.59	1156.054	2	52.44	109940		0.1054	0.0164	0.2198	0.003610796
				RYTIPCDKTWGHYADYGCYCGAGGSGTPVDA	70.47	921.6452	4	66.83	109940					
				RIVCDCDRTAALCFGDSEYIGAHKNIDTKR	78.18	1077.161	3	54.61	109940					
				AALCFGDSEYIGAHKN	58.24	523.2444	3	52.74	109940					
				FGDSEYIGAHKNIDTKR	61.08	824.4026	2	29.07	109940					
				TWGHYADYGCYCGAGGSGTPVDALDRC	61.6	902.3785	3	61.68	109940					
				ADYGCYCGAGGSGTPVDALDRC	77.71	1045.931	2	57.53	109940					
	KAG8124483.1	putative Cysteine-rich venom protein [Naja naja]	CRISP	KIGCGENLFMSSQPYAWSRV	48.13	1059.973	2	70.97	30557				0.0611	0.001003594
	1TC8	Chain A phospholipase A2 isoform 1	PLA2	RFVCDCDRTAAICFAK	49.7	853.3586	2	62.05	201740000				0.8849	0.099418871
				RCCYVHDNCYGVAENKH	83.59	944.8713	2	38.15	13874000					
				VCDCDRTAALCFGDSEYIGAHKNIDTKR	41.46	604.267	5	52.2	13874000					
				AALCFGDSEYIGAHKN	58.24	523.2444	3	52.74	13874000					
				RYTIPCDKTWGHYADYGCYCGAGGSGTPVDA	70.47	921.6452	4	66.83	13874000					
				RTAALCFGDSEYIGAHKN	98.16	580.6066	3	55.17	13874000					
	AAL87004.1	beta-bungarotoxin A2 chain precursor [Bungarus caeruleus]	beta-BTx	TWGHYADYGCYCGAGGSGTPVDALDRC	61.6	902.3785	3	61.68	13874000				0.0609	0.006837203
				YADYGCYCGAGGSGTPVDALDRC	107.82	1081.45	2	56.11	13874000					
				LCFGDSEYIGAHKN	51.44	461.8715	3	42.29	13874000					
				FGDSEYIGAHKNIDTKR	61.08	824.4026	2	29.07	13874000					
				KTWGHYADYGCYCGAGGSGTPVDALDRC	88.42	1403.583	2	63.03	13874000					
				ADYGCYCGAGGSGTPVDALDRC	77.71	1045.931	2	57.53	13874000					
	ABW24180.1	PLA-18 precursor [Austrelaps superbus]		SGGTGTPVDELDRC	46.82	608.7946	2	45.83	5687000				0.0249	0.002802593
	AAO84769.1	phospholipase A2 partial [Bungarus candidus]	PLA2	RTAALCFAEAPYKRR	69.81	671.3284	2	58.29	5236400	0.1559			0.0230	0.002580534

F16b	[illegible]	phospholipase A2 partial [Bungarus candidus]		RGTPVDMLDRC	53.42	502.247	2	50.85	5236400		0.7208	0.1124	[illegible]	[illegible]
				KTWGEYTNYGCYCGAGGSGTPIDALDRC	114.9	1421.097	2	74.06	843220					
		RecName: Full=Basic phospholipase A2 beta-bungarotoxin A1 chain;		EYTNYGCYCGAGGSGTPIDALDRC	96.63	1184.501	2	63.29	843220					
	Q8QFW4.1	Short=Beta-BuTX A1 chain; Short=svPLA2; AltName:	beta-BTx	EKTWGEYTNYGCYCGAGGSGTPIDALDRC	95.55	990.4334	3	67.63	843220				0.0037	0.000415545
		Full=Phosphatidylcholine 2-acylhydrolase; Flags: Precursor		RYTIPCEKTWGEYTNYGCYCGAGGSGTPIDAL	87.58	1244.872	3	76.01	843220					
				TWGEYTNYGCYCGAGGSGTPIDALDRC	44.95	1370.573	2	71.12	843220					
				ELGCVATCPQPKPYEEVTCCSTDKC	41	900.7305	3	46.5	559330					
	CAM11302.1	alpha-delta-bungarotoxin-4 partial [Bungarus caeruleus]	3FTx (Type II NTx)	RGKVIELGCVATCPQPKPYEEVTCCSTDKC	76.38	807.3763	4	54.1	559330				0.0025	0.000275642
				KVIELGCVATCPQPKPYEE	77.7	980.9762	2	59.09	559330					
				KPYEEVTCCSTDKC	48.07	744.8025	2	34.26	559330					
		RecName: Full=Acidic phospholipase A2 1; Short=svPLA2; AltName:												
	Q75S48.1	Full=PA2-I; AltName: Full=Phosphatidylcholine 2-acylhydrolase; Flags:	PLA2										0.0001	1.4319E-05
		Precursor		RGSLTYLDYMDYGCYCGTGSRG	79.64	1169.974	2	75.82	29056					
	CAB62501.1	beta-bungarotoxin A chain partial [Bungarus multicinctus]		RTIICYGAAGTCERI	50.96	736.3374	2	48.32	15364				0.0001	7.57149E-06
				KRTIICYGAAGTCERI	42.23	543.2616	3	39.62	15364					
				RTAALCFGDSEYIGAHKNI	66.89	927.4256	2	53.35	221790					
				RCCYVHDNCYGVAENKH	83.59	944.8713	2	38.15	221790					
				RIVCDCDRTAALCFGDSEYIGAHKNIDTKR	78.18	1077.161	3	54.61	221790					
				LCFGDSEYIGAHKNIDTKR	56.27	977.953	2	41.13	221790					
			beta-BTx	TWGHYADYGCYCGAGGSGTPVDALDRC	61.6	902.3785	3	61.68	221790					
F16c	AAL87004.1	beta-bungarotoxin A2 chain precursor [Bungarus caeruleus]		KTWGHYADYGCYCGAGGSGTPVDALDRC	88.42	1403.583	2	63.03	221790		0.1738	0.0271	1.0000	0.02709254
				VCDCDRTAALCFGDSEYIGAHKN	61.73	612.2606	4	53.6	221790					
				FGDSEYIGAHKNIDTKR	61.08	824.4026	2	29.07	221790					
				AALCFGDSEYIGAHKN	58.24	523.2444	3	52.74	221790					
				ADYGCYCGAGGSGTPVDALDRC	77.71	1045.931	2	57.53	221790					
				YADYGCYCGAGGSGTPVDALDRC	107.82	1081.45	2	56.11	221790					
				RYTIPCDKTWGHYADYGCYCGAGGSGTPVDA	70.47	921.6452	4	66.83	221790					

Table S5E: Proteomic information of venom from *B. caeruleus* from northern (PB). Assignment of peptides sequenced from MS/MS spectra using Serpentes NCBI database (TaxID: 8570) in PEAKS X (Bioinformatics Solutions Inc., Canada) . Relative abundance of each toxin was estimated by using peak fraction area (obtained from RP-HPLC and SDS PAGE) and spectral intensity of all peptides matched to their respective proteins. -10logP is the matching score given by PEAKS Algorithm, m/z is mass to charge ratio of the peptides.

HPLC Peak no.	Protein information			Peptide Information						Calculation of Toxin abundance				
	NCBI Accession ID	Description	Toxin family	Peptide Sequence	-10LgP	m/z	z	RT	Precursor intensity	HPLC Peak Area	SDS Densitometry	Relative peak fraction	Relative abundance	% Proportion of Toxin
F2a	CAA06887.1	neurotoxin-like protein [Bungarus multicinctus multicinctus]	3FTx (Type I NTx)	KRGCVATCPKPKK	53.36	425.2242	3	6.06	3678600	0.0281	1.0000	0.0281	0.8200	0.023016223
				KWEIGNPSGKE	73.78	494.2521	2	28.55	3678600					
				KNEIIQCCAKD	47.82	568.2705	2	25.5	3678600					
				KKNEIIQCCAKDKCNK	88.18	636.9801	3	9.62	3678600					
				KYSQANESSKTCPSGQLLC	83.99	935.4375	2	28.46	3678600					
				KKWEIGNPSGKE	70.82	558.2988	2	27.09	3678600					
				RGCVATCPKPKK	66.47	559.2834	2	9.62	3678600					
	Q8AY43.2	RecName: Full=Kunitz-type serine protease inhibitor A; AltName: Full=Kunitz inhibitor A; Flags: Precursor	Kunitz	EFTYGGCGGNANNFKS	91.64	753.827	2	27.01	611730				0.1364	0.003827465
	1MR6	Chain A neurotoxin	3FTx (Type II NTx)	SFYTCPNSETCPDGKNICVKR	106.13	764.0095	3	26.32	154590				0.0345	0.000967237
	XP_026579406.1	BPTI/Kunitz domain-containing protein-like isoform X3 [Pseudonaja textilis]	Kunitz	KFTYGGCAGNANNFKK	69.09	760.8354	2	27.18	41407				0.0092	0.000259075
F3a	1MR6	Chain A neurotoxin	3FTx (Type II NTx)	SFYTCPNSETCPDGKN	78.6	838.3473	2	25.8	1755000	0.0229	1.0000	0.0229	0.6833	0.015667813
	CAA06887.1	neurotoxin-like protein [Bungarus multicinctus multicinctus]	3FTx (Type I NTx)	KNEIIQCCAKD	47.82	568.2705	2	25.5	813460				0.3167	0.007262187
				KWEIGNPSGKE	73.78	494.2521	2	28.55	813460					
				KKNEIIQCCAKDKCNK	88.18	636.9801	3	9.62	813460					
				KKWEIGNPSGKE	70.82	558.2988	2	27.09	813460					
				KRGCVATCPKPKK	53.36	425.2242	3	6.06	813460					
				RGCVATCPKPKK	66.47	559.2834	2	9.62	813460					
				KYSQANESSKTCPSGQLLC	83.99	935.4375	2	28.46	813460					
F4	AED89558.1	putative three finger toxin precursor [Micrurus altirostris]	3FTx	QTCLAGQNICFKK	53.81	656.3127	2	30.5	3298200	0.0080	1.0000	0.0080	0.5780	0.004624064
				RGCAVTCPKP	54.12	446.7078	2	9.65	3298200					
	ADF50022.1	non-conventional three finger toxin isoform 7 [Bungarus flaviceps]		CLICPEKYCQKV	46.14	446.8907	3	25.45	1405800				0.2464	0.001970926
				TLTCLICPEKYCQKV	97.84	856.9169	2	27.92	1405800					
	P85520.2	RecName: Full=Oxiana weak toxin; Short=OWT		LTCLICPEKYC	59.67	648.8256	2	29.84	433880				0.0760	0.000608298
	Q8AY43.2	RecName: Full=Kunitz-type serine protease inhibitor A; AltName: Full=Kunitz inhibitor A; Flags: Precursor	Kunitz	EFTYGGCGGNANNFKS	91.64	753.827	2	27.01	377630				0.0662	0.000529436
	CAA06887.1	neurotoxin-like protein [Bungarus multicinctus multicinctus]	3FTx (Type I NTx)	KNEIIQCCAKD	47.82	568.2705	2	25.5	68215				0.0120	9.56372E-05
				KKWEIGNPSGKE	70.82	558.2988	2	27.09	68215					
				RGCVATCPKPKK	66.47	559.2834	2	9.62	68215					
				KWEIGNPSGKE	73.78	494.2521	2	28.55	68215					
				KKNEIIQCCAKDKC	59.23	502.9199	3	10.37	68215					
				KYSQANESSKTCPSGQLLC	83.99	935.4375	2	28.46	68215					
				KRGCVATCPKPKK	53.36	425.2242	3	6.06	68215					
	Q8AY46.2	RecName: Full=Kunitz-type serine protease inhibitor homolog beta-bungarotoxin B1 chain; Flags: Precursor	Kunitz	NGNGNHFKTETLCRC	75.4	511.9108	3	24.25	52600				0.0092	7.3745E-05
	AAL30054.1	kappa 1a bungaratoxin [Bungarus candidus]	3FTx (k-bungarotoxin)	RTCLISPSSTPQTCPQGQDICFLKA	69.27	880.085	3	30.31	20188				0.0035	2.83035E-05
				RSLLCCTTDNCNH	81.68	747.7958	2	27.01	20188					
				TRTCLISPSSTPQTCPQGQDICFLKA	101.95	932.1204	3	29.3	20188					
	ABI33870.1	three finger toxin [Bungarus fasciatus]	3FTx	KETVECCSTDKCNK	74.95	544.2265	3	7.3	15463				0.0027	2.16791E-05
	KAG8142834.1	hypothetical protein E2320_006056 partial [Naja naja]	Hyaluronidase	KQLHPELSEAAIKR	50.09	445.9182	3	27.16	12420				0.0022	1.74128E-05
				KNFMCQCYQGWKG	62.42	761.3064	2	29.76	12420					
	CAB53358.1	cardiotoxin-like protein [Bungarus multicinctus multicinctus]	3FTx (C-3FTx)	KSYWVNENGKQ	70.88	548.7592	2	27.46	7441.6				0.0013	1.04331E-05
	0412250A	toxin A beta1 bungaro	PLA2	RTIICYGAAGGTCRI	52.36	700.3334	2	27.18	7178.8				0.0013	1.00647E-05
	ABG90493.1	beta-bungarotoxin B5 chain partial [Bungarus multicinctus]	beta-BTx	RHPDCDKPPDTKRC	63.57	367.1754	4	5.65	7132.8				0.0013	1.00002E-05
F5	AED89558.1	putative three finger toxin precursor [Micrurus altirostris]	3FTx	QTCLAGQNICFKK	53.81	656.3127	2	30.5	3889200	0.0117	1.0000	0.0117	0.4312	0.005044484
				RGCAVTCPKP	54.12	446.7078	2	9.65	3889200					
	ADF50022.1	non-conventional three finger toxin isoform 7 [Bungarus flaviceps]		CLICPEKYCQKV	46.14	446.8907	3	25.45	3372200				0.3738	0.00437391
				TLTCLICPEKYCQKV	97.84	856.9169	2	27.92	3372200					
	P85520.2	RecName: Full=Oxiana weak toxin; Short=OWT		LTCLICPEKYCN	54.81	728.835	2	29.49	733600				0.0813	0.000951515
	CAB53358.1	cardiotoxin-like protein [Bungarus multicinctus multicinctus]	3FTx (C-3FTx)	KSYWVNENGKQ	70.88	548.7592	2	27.46	435980				0.0483	0.000565488
	Q8AY43.2	RecName: Full=Kunitz-type serine protease inhibitor A; AltName: Full=Kunitz inhibitor A; Flags: Precursor	Kunitz	EFTYGGCGGNANNFKS	91.64	753.827	2	27.01	189020				0.0210	0.000245168
	AAL30054.1	kappa 1a bungaratoxin [Bungarus candidus]	3FTx (k-bungarotoxin)	TRTCLISPSSTPQTCPQGQDICFLKA	101.95	932.1204	3	29.3	165510				0.0183	0.000214675
				RSLLCCTTDNCNH	81.68	747.7958	2	27.01	165510					
				RTCLISPSSTPQTCPQGQDICFLKA	69.27	880.085	3	30.31	165510					
	TIKFBU	beta-1 bungarotoxin chain B major component - many-banded krait	beta-BTx	DGDHGNFKTETLCRC	75.8	512.239	3	24.99	143440				0.0159	0.000186049
	Q8AY46.2	RecName: Full=Kunitz-type serine protease inhibitor homolog beta-bungarotoxin B1 chain; Flags: Precursor	Kunitz	NGNGNHFKTETLCRC	75.4	511.9108	3	24.25	44586				0.0049	5.78302E-05
	KAG8142834.1	hypothetical protein E2320_006056 partial [Naja naja]	Hyaluronidase	KQLHPELSEAAIKR	50.09	445.9182	3	27.16	23913				0.0027	3.10163E-05
				KNFMCQCYQGWKG	62.42	761.3064	2	29.76	23913					
	CAA06887.1	neurotoxin-like protein [Bungarus multicinctus multicinctus]	3FTx (Type I NTx)	KYSQANESSKTCPSGQLLC	83.99	935.4375	2	28.46	16366				0.0018	2.12275E-05
				KKNEIIQCCAKD	74.38	632.3169	2	24.21	16366					
				KRGCVATCPKPKK	53.36	425.2242	3	6.06	16366					
				KNEIIQCCAKD	47.82	568.2705	2	25.5	16366					
				KKWEIGNPSGKE	70.82	558.2988	2	27.09	16366					
				KWEIGNPSGKE	73.78	494.2521	2	28.55	16366					
				RGCVATCPKPKK	66.47	559.2834	2	9.62	16366					
	Q8AY45.2	RecName: Full=Kunitz-type serine protease inhibitor homolog beta-bungarotoxin B2a chain; Flags: Precursor	Kunitz	RAFYYKPSAKR	46.46	358.8605	3	25.72	6659.2				0.0007	8.63731E-06
F6a	AAL30054.1	kappa 1a bungaratoxin [Bungarus candidus]	3FTx (k-bungarotoxin)	RTCLISPSSTPQTCPQGQDICFLKA	69.27	880.085	3	30.31	6424.5	0.0953	0.1048	0.0100	0.6323	0.006313787
				TRTCLISPSSTPQTCPQGQDICFLKA	101.95	932.1204	3	29.3	6424.5					
				RSLLCCTTDNCNH	81.68	747.7958	2	27.01	6424.5					
	AED89576.1	putative phospholipase A2 precursor [Micrurus altirostris]	PLA2	SGGGGTPVDDLDRC	59.5	579.7812	2	26.82	3736.2				0.3677	0.003671814
F6b	AAL30054.1	kappa 1a bungaratoxin [Bungarus candidus]	3FTx (k-bungarotoxin)	RTCLISPSSTPQTCPQGQDICFLKA	69.27	880.085	3	30.31	404110		0.8952	0.0853	0.6639	0.056643493
				TRTCLISPSSTPQTCPQGQDICFLKA	101.95	932.1204	3	29.3	404110					
				RSLLCCTTDNCNH	81.68	747.7958	2	27.01	404110					
	P01383.1	RecName: Full=Long neurotoxin 1; AltName: Full=Neurotoxin 3.9.4	3FTx (Type II NTx)	KWCADWCTSRG	51.23	621.2437	2	29.12	128210				0.2106	0.017971003
				KKWCADWCTSRG	56.48	457.1992	3	28.02	128210					
	CAD56381.1	BM10-1-like neurotoxin precursor [Bungarus multicinctus]	3FTx	KIIRGCSSSCPEKKN	48.12	507.9249	3	9.75	48324				0.0794	0.006773503
	CAJ77819.1	alpha-delta-bungarotoxin-1 partial [Bungarus candidus]	3FTx (Type II NTx)	MWCDAWCSSRG	49.71	614.2375	2	28.84	28012				0.0460	0.0039264
F7	Q9W729.1	RecName: Full=Kappa-6-bungarotoxin; Short=K-6 bungarotoxin; Flags: Precursor	3FTx (k-bungarotoxin)	QTCPKGQDICFRK	56.88	691.3148	2	27.49	79428	0.0106	1.0000	0.0106	0.5001	0.005276162
				SSTPQTCPKGQDICFRK	77.1	897.9149	2	27.62	79428					
	P34128.1	RecName: Full=Venom nerve growth factor; Short=v-NGF; Short=vNGF; Flags: Precursor	NGF	KQYFFETKC	51.91	481.7411	2	30.04	50046				0.3151	0.003324405
				KALTMEGNRA	59.62	446.2254	2	25.65	50046					
				RNPNPVPSGCRG	66.55	549.2611	2	24.06	50046					
				RHWNSYCTTTDTFVKA	85.59	880.395	2	27.99	50046					
				KCRNPNPVPSGCRG	47.57	471.8936	3	9.87	50046					
				RIDTACVCVISRK	67.31	647.3271	2	28	50046					
	ABM88802.1	PLA2 Hs-3 precursor [Hoplocephalus stephensii]	PLA2	AYAHYGCYCGKG	59.67	639.7593	2	24.37	16749				0.1055	0.001112586
	P01385.1	RecName: Full=Alpha-elapitoxin-Aa2b; Short=Alpha-EPTX-Aa2b; AltName: Full=Long neurotoxin 1	Kunitz	RTWCDAFCSSRG	63.11	645.2618	2	29.5	12598				0.0793	0.000836847
F8a	P34128.1	RecName: Full=Venom nerve growth factor; Short=v-NGF; Short=vNGF; Flags: Precursor	NGF	KQYFFETKC	51.91	481.7411	2	30.04	92990	0.0484	0.0521	0.0025	0.6185	0.001558336
				RHWNSYCTTTDTFVKA	85.59	880.395	2	27.99	92990					
				KALTMEGNRA	59.62	446.2254	2	25.65	92990					
				RNPNPVPSGCRG	66.55	549.2611	2	24.06	92990					
				KCRNPNPVPSGCRG	47.57	471.8936	3	9.87	92990					
				RIDTACVCVISRK	67.31	647.3271	2	28	92990					
	AED89576.1	putative phospholipase A2 precursor [Micrurus altirostris]	PLA2	SGGGGTPVDDLDRC	59.5	579.7812	2	26.82	57367				0.3815	0.000961362
F8b	P34128.1	RecName: Full=Venom nerve growth factor; Short=v-NGF; Short=vNGF; Flags: Precursor	NGF	RNPNPVPSGCRG	66.55	549.2611	2	24.06	21666		0.3862	0.0187	0.6834	0.012760984
				KQYFFETKC	51.91	481.7411	2	30.04	21666					
				KALTMEGNRA	59.62	446.2254	2	25.65	21666					
				RIDTACVCVISRK	67.31	647.3271	2	28	21666					
				RHWNSYCTTTDTFVKA	85.59	880.395	2	27.99	21666					
				KCRNPNPVPSGCRG	47.57	471.8936	3	9.87	21666					
	Q8QFW3.1	RecName: Full=Basic phospholipase A2 beta-bungarotoxin A2 chain; Short=Beta-BuTX A2 chain; Short=svPLA2; AltName: Full=Phosphatidylcholine 2-acylhydrolase; Flags: Precursor	beta-BTx	RIVCDCDRTAALCFGDSEYIGAHKN	87.93	665.3049	4	28.72	10036				0.3166	0.005911069
				RTAALCFGDSEYIGAHKN	105.26	580.6064	3	28.83	10036					
				RYTIPCDKT	44.54	448.7123	2	26.15	10036					
F8c				RYTIPCDKT	44.54	448.7123	2	26.15	118320		0.5617	0.0272	0.6863	0.018637813
				RIVCDCDRTAALCFGDSEYIGAHKN	87.93	665.3049	4	28.72	118320					
				RTAALCFGDSEYIGAHKN	105.26	580.6064	3	28.83	118320					
	Q8QFW4.1	RecName: Full=Basic phospholipase A2 beta-bungarotoxin A1 chain; Short=Beta-BuTX A1 chain; Short=svPLA2; AltName: Full=Phosphatidylcholine 2-acylhydrolase; Flags: Precursor		KTQSYSYKLTRR	69.06	416.219	3	25.62	28242				0.1638	0.004448691
				KTWGEYTNYGCYCGAGGSGTPIDALDRC	112.89	947.7322	3	30.76	28242					
				PLNLINFMNMIRY	50.56	689.8645	2	35.87	28242					
				LNLINFMNMIRY	63.06	633.327	2	34.52	28242					
	JAA75031.1	PLA2-Sut-23 [Suta fasciata]	PLA2	RFVCACDAVAAKCFAKA	47.39	573.2651	3	29.09	25849				0.1499	0.004071745
F9a	1PO8	Chain A Phospholipase A2	PLA2	ARFVCDCDRTAAICFAKA	53.51	498.2289	4	27.43	514840	0.1073	1.0000	0.1073	0.5042	0.054077142
				RFVCDCDRTAAICFAK	52.69	853.36	2	30.35	514840					
	Q8QFW4.1	RecName: Full=Basic phospholipase A2 beta-bungarotoxin A1 chain; Short=Beta-BuTX A1 chain; Short=svPLA2; AltName: Full=Phosphatidylcholine 2-acylhydrolase; Flags: Precursor	beta-BTx	KTQSYSYKLTRR	69.06	416.219	3	25.62	168860				0.1654	0.017736513
				PLNLINFMNMIRY	50.56	689.8645	2	35.87	168860					
				KTWGEYTNYGCYCGAGGSGTPIDALDRC	112.89	947.7322	3	30.76	168860					
				LNLINFMNMIRY	63.06	633.327	2	34.52	168860					
	1G0Z	Chain B Phospholipase A2	PLA2	IGYGCYCGYGGSGTPVDELDRC	113.03	1091.943	2	28.96	145300				0.1423	0.015261846
	Q6YIJ3.1	RecName: Full=Acidic phospholipase A2 beta-bungarotoxin A chain; Short=Beta-BuTX A chain; Short=svPLA2; AltName: Full=Phosphatidylcholine 2-acylhydrolase; Flags: Precursor	beta-BTx	RTAALCFGDSEYIEGHKNID	51.4	506.9896	4	27.92	116260				0.1139	0.012211577
	Q8QFW3.1	RecName: Full=Basic phospholipase A2 beta-bungarotoxin A2 chain; Short=Beta-BuTX A2 chain; Short=svPLA2; AltName: Full=Phosphatidylcholine 2-acylhydrolase; Flags: Precursor		RYTIPCDKT	44.54	448.7123	2	26.15	75906				0.0743	0.007972923
F10a	1PO8	Chain A Phospholipase A2	PLA2	RFVCDCDRTAAICFAK	52.69	853.36	2	30.35	2922300	0.1476	1.0000	0.1476	0.7796	0.115068891
				ARFVCDCDRTAAICFAKA	53.51	498.2289	4	27.43	2922300					
	UNW14922.1	phospholipase A2 protein 2-3 partial [Pseudonaja textilis]		RNVLDYADYGCYCGKGGSGTPVDELDRC	47.18	961.0878	3	31.85	592710				0.1581	0.023338631
	1G0Z	Chain B Phospholipase A2		IGYGCYCGYGGSGTPVDELDRC	113.03	1091.943	2	28.96	206640				0.0551	0.008136685
	Q8QFW4.1	RecName: Full=Basic phospholipase A2 beta-bungarotoxin A1 chain; Short=Beta-BuTX A1 chain; Short=svPLA2; AltName: Full=Phosphatidylcholine 2-acylhydrolase; Flags: Precursor	beta-BTx	KTQSYSYKLTRR	69.06	416.219	3	25.62	26813				0.0072	0.001055792
				PLNLINFMNMIRY	50.56	689.8645	2	35.87	26813					
				LNLINFMNMIRY	63.06	633.327	2	34.52	26813					
				KTWGEYTNYGCYCGAGGSGTPIDALDRC	112.89	947.7322	3	30.76	26813					
F11a	ETE62137.1	hypothetical protein L345 12110 partial [Ophiophagus hannah]	CRISP	KYLYVCQYCPAGNIRG	75.67	888.9114	2	29.95	48524		0.0798	0.0303	0.4841	0.014652876
	1PO8	Chain A Phospholipase A2	PLA2	RFVCDCDRTAAICFAK	52.69	853.36	2	30.35	24657				0.2460	0.007445717
				ARFVCDCDRTAAICFAKA	53.51	498.2289	4	27.43	24657					
	ACE73577.1	cysteine-rich seceretory protein Bc-CRPb [Bungarus candidus]	CRISP	KYNDDYTNCKS	56.45	596.7271	2	24.35	14921				0.1489	0.00450572
	1G0Z	Chain B Phospholipase A2	PLA2	IGYGCYCGYGGSGTPVDELDRC	113.03	1091.943	2	28.96	12129				0.1210	0.003662615
F11b	ACE73577.1	cysteine-rich seceretory protein Bc-CRPb [Bungarus candidus]	CRISP	KYNDDYTNCKS	56.45	596.7271	2	24.35	43940		0.2860	0.1084	0.5189	0.056272081
	ETE62137.1	hypothetical protein L345_12110 partial [Ophiophagus hannah]		KYLYVCQYCPAGNIRG	75.67	888.9114	2	29.95	18899				0.2232	0.024203142
	1G0Z	Chain B Phospholipase A2		IGYGCYCGYGGSGTPVDELDRC	113.03	1091.943	2	28.96	5655.2				0.0668	0.007242373
	1PO8	Chain A Phospholipase A2		RFVCDCDRTAAICFAKA	111.06	917.4059	2	28.15	966360				0.5503	0.132301716

F11c	[illegible]	Chain A Phospholipase A2		ARFVCDCDRTAAICFAKA	53.51	498.2289	4	27.43	966360	0.3791	0.6341	0.2404	[illegible]	[illegible]
	1G0Z	Chain B Phospholipase A2	PLA2	IGYGCYCGYGGSGTPVDELDRC	113.03	1091.943	2	28.96	525670				0.2993	0.071968048
	AAO84769.1	phospholipase A2 partial [Bungarus candidus]		RTAALCFAEAPYKRR	71.54	499.9221	3	28.4	113790				0.0648	0.015578679
				KCSSLLNVPYVKQ	72.1	640.3353	2	30.7	113790					
	UNW14922.1	phospholipase A2 protein 2-3 partial [Pseudonaja textilis]		RNVLDYADYGCYCGKGGSGTPVDELDRC	47.18	961.0878	3	31.85	107170				0.0610	0.014672353
	Q8QFW4.1	RecName: Full=Basic phospholipase A2 beta-bungarotoxin A1 chain; Short=Beta-BuTX A1 chain; Short=svPLA2; AltName: Full=Phosphatidylcholine 2-acylhydrolase; Flags: Precursor	beta-BTx	KTWGEYTNYGCYCGAGGSGTPIDALDRC	112.89	947.7322	3	30.76	26766				0.0152	0.00366446
				KTQSYSYKLTRR	69.06	416.219	3	25.62	26766					
				LNLINFMNMIRY	63.06	633.327	2	34.52	26766					
				PLNLINFMNMIRY	50.56	689.8645	2	35.87	26766					
	0412250A	toxin A beta1 bungaro	PLA2	RTIICYGAAGGTCRI	52.36	700.3334	2	27.18	16290				0.0093	0.00223022
F12a	1PO8	Chain A Phospholipase A2	PLA2	ARFVCDCDRTAAICFAKA	53.51	498.2289	4	27.43	554520	0.1106	0.2565	0.0284	0.8696	0.024664248
				RFVCDCDRTAAICFAKA	111.06	917.4059	2	28.15	554520					
	ABW24180.1	PLA-18 precursor [Austrelaps superbus]		SGGTGTPVDELDRC	57.51	608.7959	2	27.41	52709				0.0827	0.00234442
	ETE62137.1	hypothetical protein L345_12110 partial [Ophiophagus hannah]	CRISP	KYLYVCQYCPAGNIRG	75.67	888.9114	2	29.95	30478				0.0478	0.001355617
F12b	1PO8	Chain A Phospholipase A2		RFVCDCDRTAAICFAK	52.69	853.36	2	30.35	3421400		0.7435	0.0822	0.9625	0.079128959
				ARFVCDCDRTAAICFAKA	53.51	498.2289	4	27.43	3421400					
	ABW24180.1	PLA-18 precursor [Austrelaps superbus]	PLA2	SGGTGTPVDELDRC	57.51	608.7959	2	27.41	63248				0.0178	0.001462778
	Q75S48.1	RecName: Full=Acidic phospholipase A2 1; Short=svPLA2; AltName: Full=PA2-I; AltName: Full=Phosphatidylcholine 2-acylhydrolase; Flags: Precursor		RGSLTYLDYMDYGCYCGTGSRG	114.78	1169.962	2	31.73	53464				0.0150	0.001236497
	Q8QFW3.1	RecName: Full=Basic phospholipase A2 beta-bungarotoxin A2 chain; Short=Beta-BuTX A2 chain; Short=svPLA2; AltName:	beta-BTx	RYTIPCDKT	44.54	448.7123	2	26.15	16754				0.0047	0.000387481
				RTAALCFGDSEYIGAHKN	105.26	580.6064	3	28.83	16754					
F13	ABN72539.1	L-amino acid oxidase [Bungarus multicinctus]	LAAO	YQFQDYSETVAAPVGRI	67.96	834.415	2	28.15	147890	0.0046	1.0000	0.0046	0.4894	0.002241331
				RFDEISGGFDQLPKS	84.73	484.9081	3	30.54	147890					
				KHVVVVGAGMAGLSAAYVLEKA	88.78	657.6938	3	31.38	147890					
				KRFDEISGGFDQLPKS	82.89	536.9412	3	29.71	147890					
	ABN72537.1	metalloproteinase [Bungarus multicinctus]	SVMP	GVCNCLILPDDPNYGMVETGTKC	55.23	799.3771	3	30.83	93991				0.3110	0.00142447
				NGNTVGLAALGSLCSVKY	64.92	773.9238	2	31.57	93991					
				RDKINVQSDVKA	60.32	573.3164	2	24.77	93991					
	4QWW	Chain B Acetylcholinesterase	ACE	KNPQELIDEEWSVLPYKS	104.26	980.4996	2	33.3	27076				0.0896	0.000410347
				RLALQWIQNNIHPFGGNPRA	104.39	692.3798	3	32.2	27076					
				RSKNPQELIDEEWSVLPYKS	99.27	725.7144	3	32.11	27076					
				KSGAWPTYTASQPQYVQLNTQPLATQPSLRA	126.54	1068.553	3	30.92	27076					
				RAGELKVSTQTGSVRG	56.35	478.2693	3	25.16	27076					
				YTASQPQYVQLNTQPLATQPSLRA	62.45	814.4396	3	29.45	27076					
				KVSTQTGSVRG	54.14	467.7521	2	23.22	27076					
	AXL95288.1	L-amino acid oxidase 2 partial [Spilotes sulphureus]	LAAO	KSTADIVINDLSLIHQLPKK	76.92	659.7126	3	32.83	13118				0.0434	0.000198808
	AED89558.1	putative three finger toxin precursor [Micrurus altirostris]	3FTx	QTCLAGQNICFKK	53.81	656.3127	2	30.5	11841				0.0392	0.000179455
				RGCAVTCPKP	54.12	446.7078	2	9.65	11841					
	JAS05143.1	ecto-5'-nucleotidase [Micrurus tener]	5'-NT	KSIQEDPAVKAEVSRM	63.14	510.2727	3	25.62	6465.7				0.0214	9.79902E-05
	XP_026582322.1	5'-nucleotidase [Pseudonaja textilis]		RHGQGSGELLQVSGIKV	64.2	503.9454	3	28.49	1821				0.0060	2.7598E-05
F14	ABN72539.1	L-amino acid oxidase [Bungarus multicinctus]	LAAO	RFDEISGGFDQLPKS	84.73	484.9081	3	30.54	592840	0.0033	1.0000	0.0033	0.7618	0.002475811
				KHVVVVGAGMAGLSAAYVLEKA	88.78	657.6938	3	31.38	592840					
				YQFQDYSETVAAPVGRI	67.96	834.415	2	28.15	592840					
				KRFDEISGGFDQLPKS	82.89	536.9412	3	29.71	592840					
	AXL95288.1	L-amino acid oxidase 2 partial [Spilotes sulphureus]		KSTADIVINDLSLIHQLPKK	76.92	659.7126	3	32.83	138020				0.1774	0.000576397
	ABN72537.1	metalloproteinase [Bungarus multicinctus]	SVMP	GVCNCLILPDDPNYGMVETGTKC	55.23	799.3771	3	30.83	33459				0.0430	0.000139731
				RDKINVQSDVKA	60.32	573.3164	2	24.77	33459					
				NGNTVGLAALGSLCSVKY	64.92	773.9238	2	31.57	33459					
	XP_026582322.1	5'-nucleotidase [Pseudonaja textilis]	5'-NT	RHGQGSGELLQVSGIKV	64.2	503.9454	3	28.49	9106.6				0.0117	3.80309E-05
	AED89558.1	putative three finger toxin precursor [Micrurus altirostris]	3FTx	RGCAVTCPKP	54.12	446.7078	2	9.65	4796.1				0.0062	2.00294E-05
				QTCLAGQNICFKK	53.81	656.3127	2	30.5	4796.1					
F15	4QWW	Chain B Acetylcholinesterase	ACE	YTASQPQYVQLNTQPLATQPSLRA	62.45	814.4396	3	29.45	1303100	0.0082	1.0000	0.0082	0.8152	0.006684839
				RLALQWIQNNIHPFGGNPRA	104.39	692.3798	3	32.2	1303100					
				RAGELKVSTQTGSVRG	56.35	478.2693	3	25.16	1303100					
				KSGAWPTYTASQPQYVQLNTQPLATQPSLRA	126.54	1068.553	3	30.92	1303100					
				KNPQELIDEEWSVLPYKS	104.26	980.4996	2	33.3	1303100					
				RSKNPQELIDEEWSVLPYKS	99.27	725.7144	3	32.11	1303100					
				KVSTQTGSVRG	54.14	467.7521	2	23.22	1303100					
	ABN72539.1	L-amino acid oxidase [Bungarus multicinctus]	LAAO	YQFQDYSETVAAPVGRI	67.96	834.415	2	28.15	148930				0.0932	0.000764004
				KHVVVVGAGMAGLSAAYVLEKA	88.78	657.6938	3	31.38	148930					
				KRFDEISGGFDQLPKS	82.89	536.9412	3	29.71	148930					
				RFDEISGGFDQLPKS	84.73	484.9081	3	30.54	148930					
	AXL95288.1	L-amino acid oxidase 2 partial [Spilotes sulphureus]		KSTADIVINDLSLIHQLPKK	76.92	659.7126	3	32.83	87279				0.0546	0.000447737
	ASU45022.1	acetylcholinesterase partial [Daboia russelii]	ACE	RQDFLEGVRM	49.76	482.2523	2	30	42512				0.0266	0.000218084
	KAG8142834.1	hypothetical protein E2320_006056 partial [Naja naja]	Hyaluronidase	KQLHPELSEAAIKR	50.09	445.9182	3	27.16	15484				0.0097	7.94322E-05
				KNFMCQCYQGWKG	62.42	761.3064	2	29.76	15484					
	JAS05143.1	ecto-5'-nucleotidase [Micrurus tener]	5'-NT	KSIQEDPAVKAEVSRM	63.14	510.2727	3	25.62	1150.8				0.0007	5.90355E-06
F16	4QWW	Chain B Acetylcholinesterase	ACE	YTASQPQYVQLNTQPLATQPSLRA	62.45	814.4396	3	29.45	16385	0.0013	1.0000	0.0013	0.5080	0.000675639
				RSKNPQELIDEEWSVLPYKS	99.27	725.7144	3	32.11	16385					
				KVSTQTGSVRG	54.14	467.7521	2	23.22	16385					
				KNPQELIDEEWSVLPYKS	104.26	980.4996	2	33.3	16385					
				RLALQWIQNNIHPFGGNPRA	104.39	692.3798	3	32.2	16385					
				RAGELKVSTQTGSVRG	56.35	478.2693	3	25.16	16385					
				KSGAWPTYTASQPQYVQLNTQPLATQPSLRA	126.54	1068.553	3	30.92	16385					
	AED89558.1	putative three finger toxin precursor [Micrurus altirostris]	3FTx	RGCAVTCPKP	54.12	446.7078	2	9.65	15869				0.4920	0.000654361
				QTCLAGQNICFKK	53.81	656.3127	2	30.5	15869					

Figure S1. Biochemical characterisation of different populations of krait venoms.

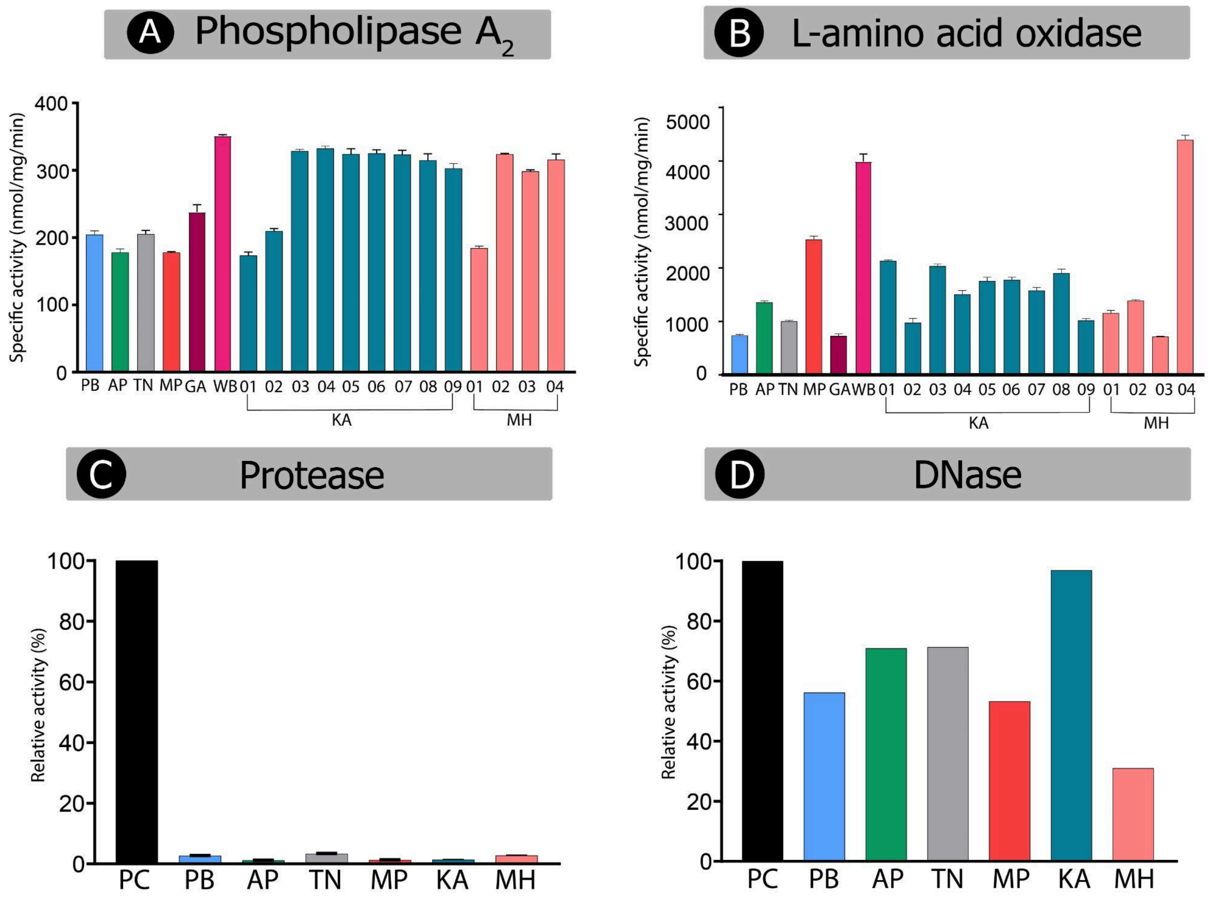

Biochemical activity profiles of krait venom (A) Specific Phospholipase A_2 activity (nmol/mg/min); (B) Specific LAAO activity (nmol/mg/min); (C) Relative protease activity (%); and (D) Relative DNase activity (%) of krait venom. **PC**: positive control; **NC**: negative control; **PB**: Punjab; **AP**: Andhra Pradesh; **TN**: Tamil Nadu; **KA**: Karnataka; **MH**: Maharashtra; and **MP**: Madhya Pradesh; **GA**: Goa. For each assay, three replicates were used, and the standard deviation is represented by error bars.

Figure S2. DNase activities of *Bungarus caeruleus* venoms demonstrated through agarose gel electrophoresis.

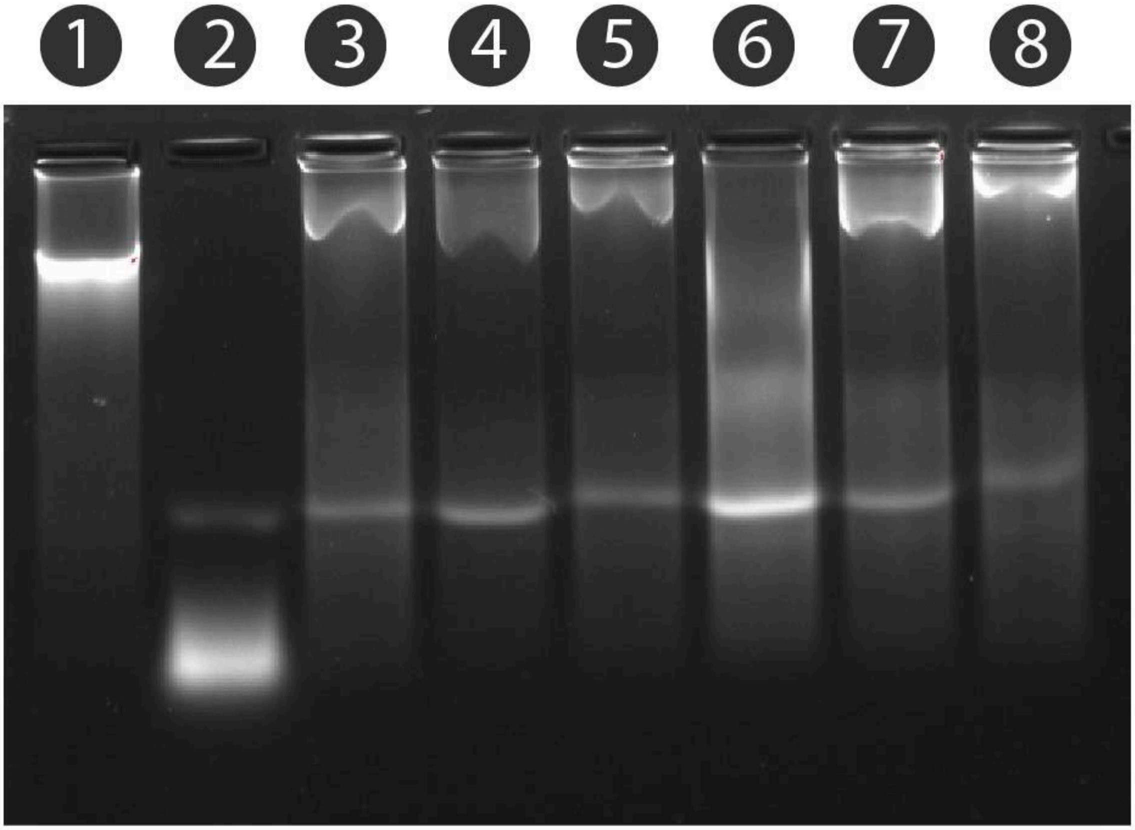

Agarose gel displaying DNase activities of geographically distinct *Bungarus caeruleus* venoms. Lane **1**. DNA only (negative control); **2**. DNA + 15 U DNase (positive control); **3.** PB: Punjab; **4.** AP: Andhra Pradesh; **5.** TN: Tamil Nadu; **6.** KA: Karnataka; **7.** MH: Maharashtra; and **8.** MP: Madhya Pradesh.

Figure S3. Fibrinogenolytic activities of *Bungarus caeruleus* venoms from distinct biogeographical locations across India.

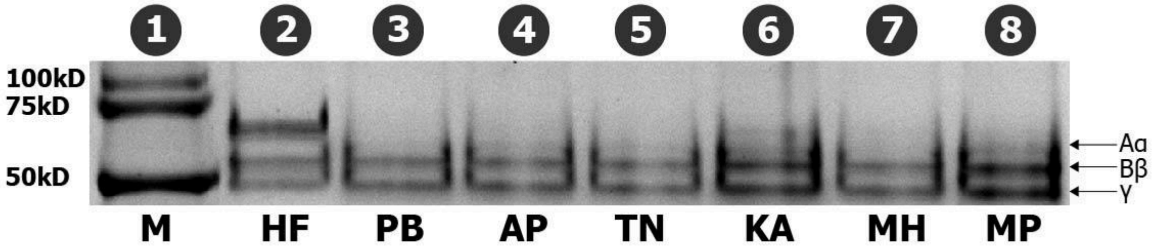

Fibrinogen degrading abilities of *Bungarus caeruleus* venoms are depicted here. **M**: Pre-stained protein marker; **HF**: human fibrinogen; **PB**: Punjab; **AP**: Andhra Pradesh; **TN**: Tamil Nadu; **KA**: Karnataka; **MH**: Maharashtra; and **MP**: Madhya Pradesh.
