# Supplementary material for: Elusive elapids: biogeographic venom variation in Indian kraits and its repercussion on snakebite therapy
Source: Front Pharmacol. 2024 Nov 7;15:1443073. doi: 10.3389/fphar.2024.1443073 (PMC11579489; doi:10.3389/fphar.2024.1443073)
Supplement: Supplementary file 1 [file DataSheet2.PDF]

## Venom protein list- Southern (TN) population of *B. caeruleus*

Protein Accession Contains:
Protein Description Contains:
Protein Sample Area >=
Protein PTM Contains:

| Protein Group | Protein ID | Accession | -10lgP | Coverage (%) | Coverage (%) F1 | Coverage (%) F2a | Coverage (%) F3a | Coverage (%) F4 | Coverage (%) F5 | Coverage (%) F6a | Coverage (%) F7a | Coverage (%) F7b | Coverage (%) F8 | Coverage (%) F9a | Coverage (%) Sample 1F10a | Coverage (%) F10b | Coverage (%) F11a | Coverage (%) F11b | Coverage (%) F12a | Coverage (%) F13 | Area F1 | Area F2a | Area F3a | Area F4 | Area F5 | Area F6a | Area F7a | Area F7b | Area F8 | Area F9a | Area Sample 1F10a | Area F10b | Area F11a | Area F11b | Area F12a | Area F13 | #Peptides | #Unique | #Spec F1 | #Spec F2a | #Spec F3a | #Spec F4 | #Spec F5 | #Spec F6a | #Spec F7a | #Spec F7b | #Spec F8 | #Spec F9a | #Spec Sample 1F10a | #Spec F10b | #Spec F11a | #Spec F11b | #Spec F12a | #Spec F13 | PTM | Avg. Mass | Description |
|---|---|---|---|---|---|---|---|---|---|---|---|---|---|---|---|---|---|---|---|---|---|---|---|---|---|---|---|---|---|---|---|---|---|---|---|---|---|---|---|---|---|---|---|---|---|---|---|---|---|---|---|---|---|---|---|---|---|
| **2** | 41 | AAL87004.1 | 407.22 | 73 | 0 | 9 | 9 | 0 | 9 | 64 | 51 | 70 | 26 | 73 | 59 | 70 | 29 | 61 | 69 | 19 | | | | | | 1.2177E8 | 1.6678E6 | 1.3088E8 | 9.9682E6 | 2.3742E9 | 1.7445E8 | 2.237E8 | 1.3823E7 | 8.8023E7 | 8.0736E7 | 9.3332E6 | 34 | 23 | 0 | 2 | 1 | 0 | 1 | 32 | 7 | 65 | 13 | 418 | 167 | 107 | 11 | 27 | 35 | 10 | Y | 16120 | beta-bungarotoxin A2 chain precursor [Bungarus caeruleus] |
| **2** | 42 | Q8QFW3.1 | 407.22 | 73 | 0 | 9 | 9 | 0 | 9 | 64 | 51 | 70 | 26 | 73 | 59 | 70 | 29 | 61 | 69 | 19 | | | | | | 1.2177E8 | 1.6678E6 | 1.3088E8 | 9.9682E6 | 2.3742E9 | 1.7445E8 | 2.237E8 | 1.3823E7 | 8.8023E7 | 8.0736E7 | 9.3332E6 | 34 | 23 | 0 | 2 | 1 | 0 | 1 | 32 | 7 | 65 | 13 | 418 | 167 | 107 | 11 | 27 | 35 | 10 | Y | 16120 | RecName: Full=Basic phospholipase A2 beta-bungarotoxin A2 chain; Short=Beta-BuTX A2 chain; Short=svPLA2; AltName: Full=Phosphatidylcholine 2-acylhydrolase; Flags: Precursor |
| **3** | 26 | Q8QFW4.1 | 392.63 | 84 | 0 | 0 | 0 | 0 | 0 | 61 | 33 | 67 | 21 | 84 | 58 | 69 | 26 | 55 | 60 | 16 | | | | | | 1.0465E7 | 1.1441E6 | 7.8211E7 | 1.0674E6 | 2.2098E9 | 1.1606E8 | 2.406E8 | 6.5634E6 | 4.4766E7 | 7.4154E7 | 5.41E6 | 36 | 18 | 0 | 0 | 0 | 0 | 0 | 13 | 6 | 46 | 5 | 328 | 57 | 53 | 9 | 25 | 41 | 7 | Y | 16358 | RecName: Full=Basic phospholipase A2 beta-bungarotoxin A1 chain; Short=Beta-BuTX A1 chain; Short=svPLA2; AltName: Full=Phosphatidylcholine 2-acylhydrolase; Flags: Precursor |
| **3** | 27 | AAL87003.1 | 392.63 | 84 | 0 | 0 | 0 | 0 | 0 | 61 | 33 | 67 | 21 | 84 | 58 | 69 | 26 | 55 | 60 | 16 | | | | | | 1.0465E7 | 1.1441E6 | 7.8211E7 | 1.0674E6 | 2.2098E9 | 1.1606E8 | 2.406E8 | 6.5634E6 | 4.4766E7 | 7.4154E7 | 5.41E6 | 36 | 18 | 0 | 0 | 0 | 0 | 0 | 13 | 6 | 46 | 5 | 328 | 57 | 53 | 9 | 25 | 41 | 7 | Y | 16358 | beta-bungarotoxin A chain precursor [Bungarus caeruleus] |
| **56** | 7 | 4QWW | 328.17 | 33 | 0 | 0 | 0 | 0 | 0 | 0 | 0 | 0 | 4 | 0 | 0 | 0 | 0 | 0 | 0 | 33 | | | | | | | | | | | | | | | | 1.0019E8 | 20 | 9 | 0 | 0 | 0 | 0 | 0 | 0 | 0 | 0 | 2 | 0 | 0 | 0 | 0 | 0 | 0 | 52 | Y | 60267 | Chain B, Acetylcholinesterase |
| **56** | 8 | AAC59905.1 | 328.17 | 31 | 0 | 0 | 0 | 0 | 0 | 0 | 0 | 0 | 4 | 0 | 0 | 0 | 0 | 0 | 0 | 31 | | | | | | | | | | | | | | | | 1.0019E8 | 20 | 9 | 0 | 0 | 0 | 0 | 0 | 0 | 0 | 0 | 2 | 0 | 0 | 0 | 0 | 0 | 0 | 52 | Y | 64723 | acetylcholinesterase [Bungarus fasciatus] |
| **56** | 9 | Q92035.2 | 328.17 | 30 | 0 | 0 | 0 | 0 | 0 | 0 | 0 | 0 | 3 | 0 | 0 | 0 | 0 | 0 | 0 | 30 | | | | | | | | | | | | | | | | 1.0019E8 | 20 | 9 | 0 | 0 | 0 | 0 | 0 | 0 | 0 | 0 | 2 | 0 | 0 | 0 | 0 | 0 | 0 | 52 | Y | 68074 | RecName: Full=Acetylcholinesterase; Short=BfAChE; Flags: Precursor |
| **6** | 258 | AAZ29513.1 | 315.99 | 51 | 0 | 0 | 14 | 0 | 0 | 23 | 29 | 34 | 6 | 50 | 32 | 29 | 21 | 25 | 22 | 0 | | | | | | | | | | 0 | | | | | | | 22 | 1 | 0 | 0 | 1 | 0 | 0 | 4 | 19 | 21 | 1 | 23 | 8 | 32 | 10 | 136 | 23 | 0 | Y | 15328 | phospholipase A2, partial [Micrurus fulvius] |
| **6** | 259 | JAI09008.1 | 315.99 | 48 | 0 | 0 | 13 | 0 | 0 | 22 | 27 | 32 | 6 | 47 | 31 | 28 | 19 | 24 | 21 | 0 | | | | | | | | | | 0 | | | | | | | 22 | 1 | 0 | 0 | 1 | 0 | 0 | 4 | 19 | 21 | 1 | 23 | 8 | 32 | 10 | 136 | 23 | 0 | Y | 16161 | Phospholipase A2 4c [Micrurus fulvius] |
| **6** | 260 | JAS04997.1 | 315.99 | 48 | 0 | 0 | 13 | 0 | 0 | 22 | 27 | 32 | 6 | 47 | 31 | 28 | 19 | 24 | 21 | 0 | | | | | | | | | | 0 | | | | | | | 22 | 1 | 0 | 0 | 1 | 0 | 0 | 4 | 19 | 21 | 1 | 23 | 8 | 32 | 10 | 136 | 23 | 0 | Y | 16161 | Phospholipase A2 4c [Micrurus fulvius] |
| **6** | 261 | JAB52790.1 | 315.99 | 49 | 0 | 0 | 13 | 0 | 0 | 22 | 27 | 32 | 6 | 48 | 31 | 28 | 20 | 24 | 21 | 0 | | | | | | | | | | 0 | | | | | | | 22 | 1 | 0 | 0 | 1 | 0 | 0 | 4 | 19 | 21 | 1 | 23 | 8 | 32 | 10 | 136 | 23 | 0 | Y | 15975 | phospholipase A2 29 [Micrurus fulvius] |
| **6** | 262 | JAB52809.1 | 315.99 | 49 | 0 | 0 | 13 | 0 | 0 | 22 | 27 | 32 | 6 | 48 | 31 | 28 | 20 | 24 | 21 | 0 | | | | | | | | | | 0 | | | | | | | 22 | 1 | 0 | 0 | 1 | 0 | 0 | 4 | 19 | 21 | 1 | 23 | 8 | 32 | 10 | 136 | 23 | 0 | Y | 16079 | phospholipase A2 14 [Micrurus fulvius] |
| **6** | 263 | JAS04998.1 | 315.99 | 48 | 0 | 0 | 13 | 0 | 0 | 22 | 27 | 32 | 6 | 47 | 31 | 28 | 19 | 24 | 21 | 0 | | | | | | | | | | 0 | | | | | | | 22 | 1 | 0 | 0 | 1 | 0 | 0 | 4 | 19 | 21 | 1 | 23 | 8 | 32 | 10 | 136 | 23 | 0 | Y | 16157 | Phospholipase A2 4b [Micrurus fulvius] |
| **6** | 264 | JAB52772.1 | 315.99 | 48 | 0 | 0 | 13 | 0 | 0 | 22 | 27 | 32 | 6 | 47 | 31 | 28 | 19 | 24 | 21 | 0 | | | | | | | | | | 0 | | | | | | | 22 | 1 | 0 | 0 | 1 | 0 | 0 | 4 | 19 | 21 | 1 | 23 | 8 | 32 | 10 | 136 | 23 | 0 | Y | 16191 | phospholipase A2 4c [Micrurus fulvius] |
| **6** | 265 | JAS04995.1 | 315.99 | 48 | 0 | 0 | 13 | 0 | 0 | 22 | 27 | 32 | 6 | 47 | 31 | 28 | 19 | 24 | 21 | 0 | | | | | | | | | | 0 | | | | | | | 22 | 1 | 0 | 0 | 1 | 0 | 0 | 4 | 19 | 21 | 1 | 23 | 8 | 32 | 10 | 136 | 23 | 0 | Y | 16176 | Phospholipase A2 4e [Micrurus fulvius] |
| **6** | 266 | JAS04999.1 | 315.99 | 48 | 0 | 0 | 13 | 0 | 0 | 22 | 27 | 32 | 6 | 47 | 31 | 28 | 19 | 24 | 21 | 0 | | | | | | | | | | 0 | | | | | | | 22 | 1 | 0 | 0 | 1 | 0 | 0 | 4 | 19 | 21 | 1 | 23 | 8 | 32 | 10 | 136 | 23 | 0 | Y | 16176 | Phospholipase A2 4a [Micrurus fulvius] |
| **6** | 267 | JAB52770.1 | 315.99 | 48 | 0 | 0 | 13 | 0 | 0 | 22 | 27 | 32 | 6 | 47 | 31 | 28 | 19 | 24 | 21 | 0 | | | | | | | | | | 0 | | | | | | | 22 | 1 | 0 | 0 | 1 | 0 | 0 | 4 | 19 | 21 | 1 | 23 | 8 | 32 | 10 | 136 | 23 | 0 | Y | 16157 | phospholipase A2 4e [Micrurus fulvius] |
| **6** | 268 | JAB52774.1 | 315.99 | 48 | 0 | 0 | 13 | 0 | 0 | 22 | 27 | 32 | 6 | 47 | 31 | 28 | 19 | 24 | 21 | 0 | | | | | | | | | | 0 | | | | | | | 22 | 1 | 0 | 0 | 1 | 0 | 0 | 4 | 19 | 21 | 1 | 23 | 8 | 32 | 10 | 136 | 23 | 0 | Y | 16176 | phospholipase A2 4a [Micrurus fulvius] |
| **6** | 269 | JAI09006.1 | 315.99 | 48 | 0 | 0 | 13 | 0 | 0 | 22 | 27 | 32 | 6 | 47 | 31 | 28 | 19 | 24 | 21 | 0 | | | | | | | | | | 0 | | | | | | | 22 | 1 | 0 | 0 | 1 | 0 | 0 | 4 | 19 | 21 | 1 | 23 | 8 | 32 | 10 | 136 | 23 | 0 | Y | 16176 | Phospholipase A2 4e [Micrurus fulvius] |
| **6** | 270 | JAI09009.1 | 315.99 | 48 | 0 | 0 | 13 | 0 | 0 | 22 | 27 | 32 | 6 | 47 | 31 | 28 | 19 | 24 | 21 | 0 | | | | | | | | | | 0 | | | | | | | 22 | 1 | 0 | 0 | 1 | 0 | 0 | 4 | 19 | 21 | 1 | 23 | 8 | 32 | 10 | 136 | 23 | 0 | Y | 16157 | Phospholipase A2 4b [Micrurus fulvius] |
| **6** | 271 | JAB52771.1 | 315.99 | 48 | 0 | 0 | 13 | 0 | 0 | 22 | 27 | 32 | 6 | 47 | 31 | 28 | 19 | 24 | 21 | 0 | | | | | | | | | | 0 | | | | | | | 22 | 1 | 0 | 0 | 1 | 0 | 0 | 4 | 19 | 21 | 1 | 23 | 8 | 32 | 10 | 136 | 23 | 0 | Y | 16208 | phospholipase A2 4d [Micrurus fulvius] |
| **6** | 272 | JAB52773.1 | 315.99 | 48 | 0 | 0 | 13 | 0 | 0 | 22 | 27 | 32 | 6 | 47 | 31 | 28 | 19 | 24 | 21 | 0 | | | | | | | | | | 0 | | | | | | | 22 | 1 | 0 | 0 | 1 | 0 | 0 | 4 | 19 | 21 | 1 | 23 | 8 | 32 | 10 | 136 | 23 | 0 | Y | 16217 | phospholipase A2 4b [Micrurus fulvius] |
| **6** | 273 | JAI09010.1 | 315.99 | 48 | 0 | 0 | 13 | 0 | 0 | 22 | 27 | 32 | 6 | 47 | 31 | 28 | 19 | 24 | 21 | 0 | | | | | | | | | | 0 | | | | | | | 22 | 1 | 0 | 0 | 1 | 0 | 0 | 4 | 19 | 21 | 1 | 23 | 8 | 32 | 10 | 136 | 23 | 0 | Y | 16176 | Phospholipase A2 4a [Micrurus fulvius] |
| **6** | 274 | JAB52795.1 | 315.99 | 48 | 0 | 0 | 13 | 0 | 0 | 22 | 27 | 32 | 6 | 47 | 31 | 28 | 19 | 24 | 21 | 0 | | | | | | | | | | 0 | | | | | | | 22 | 1 | 0 | 0 | 1 | 0 | 0 | 4 | 19 | 21 | 1 | 23 | 8 | 32 | 10 | 136 | 23 | 0 | Y | 16236 | phospholipase A2 24 [Micrurus fulvius] |
| **6** | 275 | JAI09004.1 | 315.99 | 48 | 0 | 0 | 13 | 0 | 0 | 22 | 27 | 32 | 6 | 47 | 31 | 28 | 19 | 24 | 21 | 0 | | | | | | | | | | 0 | | | | | | | 22 | 1 | 0 | 0 | 1 | 0 | 0 | 4 | 19 | 21 | 1 | 23 | 8 | 32 | 10 | 136 | 23 | 0 | Y | 16300 | Phospholipase A2 5b [Micrurus fulvius] |
| **6** | 276 | JAS04994.1 | 315.99 | 48 | 0 | 0 | 13 | 0 | 0 | 22 | 27 | 32 | 6 | 47 | 31 | 28 | 19 | 24 | 21 | 0 | | | | | | | | | | 0 | | | | | | | 22 | 1 | 0 | 0 | 1 | 0 | 0 | 4 | 19 | 21 | 1 | 23 | 8 | 32 | 10 | 136 | 23 | 0 | Y | 16236 | Phospholipase A2 5a [Micrurus fulvius] |
| **6** | 277 | JAI09005.1 | 315.99 | 48 | 0 | 0 | 13 | 0 | 0 | 22 | 27 | 32 | 6 | 47 | 31 | 28 | 19 | 24 | 21 | 0 | | | | | | | | | | 0 | | | | | | | 22 | 1 | 0 | 0 | 1 | 0 | 0 | 4 | 19 | 21 | 1 | 23 | 8 | 32 | 10 | 136 | 23 | 0 | Y | 16236 | Phospholipase A2 5a [Micrurus fulvius] |
| **6** | 278 | JAS04993.1 | 315.99 | 48 | 0 | 0 | 13 | 0 | 0 | 22 | 27 | 32 | 6 | 47 | 31 | 28 | 19 | 24 | 21 | 0 | | | | | | | | | | 0 | | | | | | | 22 | 1 | 0 | 0 | 1 | 0 | 0 | 4 | 19 | 21 | 1 | 23 | 8 | 32 | 10 | 136 | 23 | 0 | Y | 16300 | Phospholipase A2 5b [Micrurus fulvius] |
| **6** | 366 | JAB52794.1 | 315.99 | 49 | 0 | 0 | 13 | 0 | 0 | 22 | 27 | 32 | 6 | 48 | 31 | 28 | 20 | 24 | 21 | 0 | | | | | | | | | | 0 | | | | | | | 22 | 1 | 0 | 0 | 1 | 0 | 0 | 4 | 19 | 21 | 1 | 23 | 8 | 32 | 10 | 136 | 23 | 0 | Y | 16031 | phospholipase A2 25 [Micrurus fulvius] |
| **7** | 90 | 1G0Z | 307.61 | 47 | 0 | 0 | 16 | 0 | 0 | 24 | 25 | 45 | 0 | 25 | 25 | 35 | 25 | 46 | 36 | 0 | | | | | | 3.3656E6 | 8.2953E5 | 2.7046E7 | | 9.5567E7 | 1.8089E6 | 4.1061E8 | 4.2494E7 | 1.7508E9 | 1.5914E8 | | 16 | 2 | 0 | 0 | 1 | 0 | 0 | 2 | 5 | 16 | 0 | 19 | 5 | 29 | 15 | 146 | 26 | 0 | Y | 12970 | Chain B, PHOSPHOLIPASE A2 |
| **7** | 91 | 1U4J | 307.61 | 47 | 0 | 0 | 16 | 0 | 0 | 24 | 25 | 45 | 0 | 25 | 25 | 35 | 25 | 46 | 36 | 0 | | | | | | 3.3656E6 | 8.2953E5 | 2.7046E7 | | 9.5567E7 | 1.8089E6 | 4.1061E8 | 4.2494E7 | 1.7508E9 | 1.5914E8 | | 16 | 2 | 0 | 0 | 1 | 0 | 0 | 2 | 5 | 16 | 0 | 19 | 5 | 29 | 15 | 146 | 26 | 0 | Y | 12970 | Chain B, phospholipase A2 isoform 2 |
| **7** | 92 | Q6SLM1.1 | 307.61 | 40 | 0 | 0 | 14 | 0 | 0 | 20 | 21 | 39 | 0 | 21 | 21 | 30 | 21 | 39 | 31 | 0 | | | | | | 3.3656E6 | 8.2953E5 | 2.7046E7 | | 9.5567E7 | 1.8089E6 | 4.1061E8 | 4.2494E7 | 1.7508E9 | 1.5914E8 | | 16 | 2 | 0 | 0 | 1 | 0 | 0 | 2 | 5 | 16 | 0 | 19 | 5 | 29 | 15 | 146 | 26 | 0 | Y | 14827 | RecName: Full=Basic phospholipase A2 2; Short=svPLA2; AltName: Full=Phosphatidylcholine 2-acylhydrolase; Flags: Precursor |
| **7** | 93 | AAR19228.1 | 307.61 | 40 | 0 | 0 | 14 | 0 | 0 | 20 | 21 | 39 | 0 | 21 | 21 | 30 | 21 | 39 | 31 | 0 | | | | | | 3.3656E6 | 8.2953E5 | 2.7046E7 | | 9.5567E7 | 1.8089E6 | 4.1061E8 | 4.2494E7 | 1.7508E9 | 1.5914E8 | | 16 | 2 | 0 | 0 | 1 | 0 | 0 | 2 | 5 | 16 | 0 | 19 | 5 | 29 | 15 | 146 | 26 | 0 | Y | 14827 | phospholipase A2 isoform 2, partial [Bungarus caeruleus] |
| **40** | 30 | AAB25729.1 | 291.81 | 36 | 0 | 0 | 0 | 0 | 0 | 5 | 36 | 24 | 12 | 12 | 0 | 0 | 0 | 6 | 0 | 0 | | | | | | | 3.5964E7 | 1.5394E7 | 5.9762E5 | 3.3814E5 | | | | | | | 19 | 8 | 0 | 0 | 0 | 0 | 0 | 1 | 69 | 17 | 3 | 3 | 0 | 0 | 0 | 2 | 0 | 0 | Y | 27514 | nerve growth factor precursor [Bungarus multicinctus] |
| **40** | 31 | P34128.1 | 291.81 | 36 | 0 | 0 | 0 | 0 | 0 | 5 | 36 | 24 | 12 | 12 | 0 | 0 | 0 | 6 | 0 | 0 | | | | | | | 3.5964E7 | 1.5394E7 | 5.9762E5 | 3.3814E5 | | | | | | | 19 | 8 | 0 | 0 | 0 | 0 | 0 | 1 | 69 | 17 | 3 | 3 | 0 | 0 | 0 | 2 | 0 | 0 | Y | 27514 | RecName: Full=Venom nerve growth factor; Short=v-NGF; Short=vNGF; Flags: Precursor |
| **39** | 47 | ACE73577.1 | 288.93 | 39 | 0 | 0 | 0 | 0 | 0 | 0 | 0 | 0 | 0 | 0 | 26 | 0 | 33 | 21 | 0 | 7 | | | | | | | | | | | 4.025E7 | | 4.7764E7 | | | | 14 | 3 | 0 | 0 | 0 | 0 | 0 | 0 | 0 | 0 | 0 | 0 | 37 | 0 | 44 | 7 | 0 | 6 | Y | 26458 | cysteine-rich secretory protein Bc-CRPb [Bungarus candidus] |
| **39** | 50 | ACE73578.1 | 288.93 | 39 | 0 | 0 | 0 | 0 | 0 | 0 | 0 | 0 | 0 | 0 | 26 | 0 | 33 | 21 | 0 | 7 | | | | | | | | | | | 4.025E7 | | 4.7764E7 | | | | 14 | 3 | 0 | 0 | 0 | 0 | 0 | 0 | 0 | 0 | 0 | 0 | 37 | 0 | 44 | 7 | 0 | 6 | Y | 26443 | cysteine-rich secretory protein Bc-CRPa [Bungarus candidus] |
| **33** | 248 | AAL30054.1 | 286.43 | 70 | 0 | 0 | 41 | 0 | 0 | 70 | 41 | 62 | 0 | 41 | 0 | 41 | 0 | 60 | 14 | 0 | | | 2.6909E7 | | | 1.2825E9 | 1.4449E6 | 4.1971E7 | | 3.893E6 | | 7.4117E6 | | 7.0334E6 | | | 15 | 5 | 0 | 0 | 5 | 0 | 0 | 65 | 3 | 12 | 0 | 3 | 0 | 2 | 0 | 4 | 1 | 0 | Y | 9580 | kappa 1a bungarotoxin [Bungarus candidus] |
| **33** | 249 | Q8AY56.1 | 286.43 | 70 | 0 | 0 | 41 | 0 | 0 | 70 | 41 | 62 | 0 | 41 | 0 | 41 | 0 | 60 | 14 | 0 | | | 2.6909E7 | | | 1.2825E9 | 1.4449E6 | 4.1971E7 | | 3.893E6 | | 7.4117E6 | | 7.0334E6 | | | 15 | 5 | 0 | 0 | 5 | 0 | 0 | 65 | 3 | 12 | 0 | 3 | 0 | 2 | 0 | 4 | 1 | 0 | Y | 9580 | RecName: Full=Kappa 1a-bungarotoxin; Flags: Precursor |
| **9** | 467 | AAS20530.1 | 281.85 | 23 | 0 | 0 | 0 | 0 | 0 | 14 | 20 | 15 | 6 | 23 | 14 | 15 | 11 | 14 | 14 | 0 | | | | | | 4.0111E7 | 1.6917E8 | 5.9068E8 | | 2.632E9 | 5.3133E7 | 6.6832E9 | 1.7192E7 | 1.7383E9 | 4.0162E9 | | 17 | 6 | 0 | 0 | 0 | 0 | 0 | 8 | 18 | 17 | 1 | 27 | 10 | 63 | 5 | 26 | 35 | 0 | Y | 15058 | phospholipase A2 isoform 1, partial [Bungarus caeruleus] |
| **9** | 468 | Q6SLM2.1 | 281.85 | 23 | 0 | 0 | 0 | 0 | 0 | 14 | 20 | 15 | 6 | 23 | 14 | 15 | 11 | 14 | 14 | 0 | | | | | | 4.0111E7 | 1.6917E8 | 5.9068E8 | | 2.632E9 | 5.3133E7 | 6.6832E9 | 1.7192E7 | 1.7383E9 | 4.0162E9 | | 17 | 6 | 0 | 0 | 0 | 0 | 0 | 8 | 18 | 17 | 1 | 27 | 10 | 63 | 5 | 26 | 35 | 0 | Y | 15058 | RecName: Full=Acidic phospholipase A2 1; Short=svPLA2; AltName: Full=Phosphatidylcholine 2-acylhydrolase; Flags: Precursor |
| **9** | 471 | 1TC8 | 281.85 | 27 | 0 | 0 | 0 | 0 | 0 | 16 | 24 | 17 | 7 | 26 | 16 | 17 | 13 | 16 | 16 | 0 | | | | | | 4.0111E7 | 1.6917E8 | 5.9068E8 | | 2.632E9 | 5.3133E7 | 6.6832E9 | 1.7192E7 | 1.7383E9 | 4.0162E9 | | 17 | 6 | 0 | 0 | 0 | 0 | 0 | 8 | 18 | 17 | 1 | 27 | 10 | 63 | 5 | 26 | 35 | 0 | Y | 13201 | Chain A, phospholipase A2 isoform 1 |
| **9** | 472 | 1PO8 | 281.85 | 27 | 0 | 0 | 0 | 0 | 0 | 16 | 24 | 17 | 7 | 26 | 16 | 17 | 13 | 16 | 16 | 0 | | | | | | 4.0111E7 | 1.6917E8 | 5.9068E8 | | 2.632E9 | 5.3133E7 | 6.6832E9 | 1.7192E7 | 1.7383E9 | 4.0162E9 | | 17 | 6 | 0 | 0 | 0 | 0 | 0 | 8 | 18 | 17 | 1 | 27 | 10 | 63 | 5 | 26 | 35 | 0 | Y | 13201 | Chain A, Phospholipase A2 |
| | | | | | | | | | | | | | | | | | | | | | | | | | | | | | | | | | | | | | | | | | | | | | | | | | | | | | | | | | phospholipase A2 precursor BF- |

| | | | | | | | | | | | | | | | | | | | | | | | | | | | | | | | | | | | | | | | | | | | | | | | | | | | | | | | | | |
|---|---|---|---|---|---|---|---|---|---|---|---|---|---|---|---|---|---|---|---|---|---|---|---|---|---|---|---|---|---|---|---|---|---|---|---|---|---|---|---|---|---|---|---|---|---|---|---|---|---|---|---|---|---|---|---|---|---|
| **16** | 1140 | ABU63166.1 | 272.09 | 15 | 0 | 0 | 13 | 0 | 0 | 13 | 10 | 15 | 0 | 15 | 13 | 13 | 13 | 14 | 13 | 0 | | | | | | | | 1.1687E6 | | 5.7042E6 | | | | 8.9925E5 | | | 11 | 2 | 0 | 0 | 1 | 0 | 0 | 1 | 3 | 12 | 0 | 15 | 2 | 18 | 7 | 124 | 12 | 0 | Y | 16004 | 42 [Bungarus fasciatus] |
| **16** | 1175 | ABU63164.1 | 272.09 | 15 | 0 | 0 | 13 | 0 | 0 | 13 | 10 | 15 | 0 | 15 | 13 | 13 | 13 | 14 | 13 | 0 | | | | | | | | 1.1687E6 | | 5.7042E6 | | | | 8.9925E5 | | | 11 | 2 | 0 | 0 | 1 | 0 | 0 | 1 | 3 | 12 | 0 | 15 | 2 | 18 | 7 | 124 | 12 | 0 | Y | 15950 | phospholipase A2 precursor BF-40 [Bungarus fasciatus] |
| **18** | 2388 | C0HLB8.1 | 267.40 | 62 | 0 | 0 | 59 | 0 | 0 | 59 | 44 | 59 | 0 | 59 | 59 | 62 | 59 | 62 | 59 | 0 | | | | | | | | | | | | 1.5544E5 | | 1.0963E6 | | | 10 | 1 | 0 | 0 | 1 | 0 | 0 | 1 | 3 | 11 | 0 | 13 | 2 | 20 | 7 | 124 | 12 | 0 | Y | 3728 | RecName: Full=Phospholipase A2; Short=MIPLA2-1; Short=svPLA2; AltName: Full=Phosphatidylcholine 2-acylhydrolase |
| **50** | 114 | P00606.2 | 259.76 | 61 | 0 | 0 | 0 | 0 | 0 | 0 | 14 | 30 | 0 | 22 | 14 | 55 | 8 | 50 | 17 | 0 | | | | | | | 8.8358E3 | 2.8116E5 | | 7.1274E5 | 2.079E5 | 7.6973E6 | | 1.3127E7 | | | 21 | 5 | 0 | 0 | 0 | 0 | 0 | 0 | 2 | 8 | 0 | 6 | 4 | 23 | 1 | 19 | 5 | 0 | Y | 15593 | RecName: Full=Acidic phospholipase A2; Short=svPLA2; AltName: Full=Phosphatidylcholine 2-acylhydrolase; Flags: Precursor |
| **50** | 115 | CAA37482.1 | 259.76 | 61 | 0 | 0 | 0 | 0 | 0 | 0 | 14 | 30 | 0 | 22 | 14 | 55 | 8 | 50 | 17 | 0 | | | | | | | 8.8358E3 | 2.8116E5 | | 7.1274E5 | 2.079E5 | 7.6973E6 | | 1.3127E7 | | | 21 | 5 | 0 | 0 | 0 | 0 | 0 | 0 | 2 | 8 | 0 | 6 | 4 | 23 | 1 | 19 | 5 | 0 | Y | 15593 | precursor polypeptide (AA-27 to 118) [Bungarus multicinctus] |
| **30** | 464 | Q3SB03.1 | 253.80 | 21 | 0 | 0 | 0 | 0 | 0 | 0 | 6 | 6 | 0 | 0 | 21 | 6 | 21 | 18 | 6 | 0 | | | | | | | | | | | 3.0597E6 | | 2.8062E7 | 3.8278E5 | | | 12 | 3 | 0 | 0 | 0 | 0 | 0 | 0 | 1 | 1 | 0 | 0 | 13 | 5 | 78 | 9 | 2 | 0 | Y | 26301 | RecName: Full=Cysteine-rich venom protein pseudechetoxin-like; Short=CRVP; Flags: Precursor |
| **30** | 465 | AAZ38984.1 | 253.80 | 21 | 0 | 0 | 0 | 0 | 0 | 0 | 6 | 6 | 0 | 0 | 21 | 6 | 21 | 18 | 6 | 0 | | | | | | | | | | | 3.0597E6 | | 2.8062E7 | 3.8278E5 | | | 12 | 3 | 0 | 0 | 0 | 0 | 0 | 0 | 1 | 1 | 0 | 0 | 13 | 5 | 78 | 9 | 2 | 0 | Y | 26301 | pseudechetoxin-like protein precursor [Hoplocephalus stephensii] |
| **53** | 281 | CAM11302.1 | 247.36 | 87 | 0 | 0 | 0 | 0 | 0 | 84 | 18 | 67 | 0 | 45 | 0 | 37 | 0 | 18 | 18 | 0 | | | | | | 2.2415E8 | 1.8122E5 | 1.0395E7 | | 2.4108E8 | | 1.0595E7 | | 3.7932E6 | 1.9368E6 | | 15 | 14 | 0 | 0 | 0 | 0 | 0 | 12 | 1 | 8 | 0 | 16 | 0 | 8 | 0 | 2 | 4 | 0 | Y | 8306 | alpha-delta-bungarotoxin-4, partial [Bungarus caeruleus] |
| **53** | 282 | D2N116.1 | 247.36 | 87 | 0 | 0 | 0 | 0 | 0 | 84 | 18 | 67 | 0 | 45 | 0 | 37 | 0 | 18 | 18 | 0 | | | | | | 2.2415E8 | 1.8122E5 | 1.0395E7 | | 2.4108E8 | | 1.0595E7 | | 3.7932E6 | 1.9368E6 | | 15 | 14 | 0 | 0 | 0 | 0 | 0 | 12 | 1 | 8 | 0 | 16 | 0 | 8 | 0 | 2 | 4 | 0 | Y | 8306 | RecName: Full=Alpha-delta-bungarotoxin-4; Short=Alpha-delta-Bgt-4; Flags: Precursor |
| **89** | 12 | ABN72539.1 | 240.54 | 22 | 0 | 0 | 0 | 0 | 0 | 0 | 0 | 0 | 0 | 0 | 0 | 0 | 0 | 0 | 0 | 22 | | | | | | | | | | | | | | | | 6.7089E6 | 11 | 5 | 0 | 0 | 0 | 0 | 0 | 0 | 0 | 0 | 0 | 0 | 0 | 0 | 0 | 0 | 0 | 18 | N | 58811 | L-amino acid oxidase [Bungarus multicinctus] |
| **89** | 13 | A8QL51.1 | 240.54 | 22 | 0 | 0 | 0 | 0 | 0 | 0 | 0 | 0 | 0 | 0 | 0 | 0 | 0 | 0 | 0 | 22 | | | | | | | | | | | | | | | | 6.7089E6 | 11 | 5 | 0 | 0 | 0 | 0 | 0 | 0 | 0 | 0 | 0 | 0 | 0 | 0 | 0 | 0 | 0 | 18 | N | 58811 | RecName: Full=L-amino-acid oxidase; Short=Bm-LAAO; Short=LAO; Flags: Precursor |
| **76** | 10 | ABN72537.1 | 231.01 | 19 | 0 | 0 | 0 | 0 | 0 | 5 | 13 | 2 | 2 | 0 | 0 | 0 | 0 | 3 | 0 | 8 | | | | | | | 1.5828E6 | | | | | | | 1.7694E5 | | 2.0955E5 | 14 | 5 | 0 | 0 | 0 | 0 | 0 | 4 | 10 | 1 | 4 | 0 | 0 | 0 | 0 | 1 | 0 | 7 | Y | 68988 | metalloproteinase [Bungarus multicinctus] |
| **76** | 11 | A8QL49.1 | 231.01 | 19 | 0 | 0 | 0 | 0 | 0 | 5 | 13 | 2 | 2 | 0 | 0 | 0 | 0 | 3 | 0 | 8 | | | | | | | 1.5828E6 | | | | | | | 1.7694E5 | | 2.0955E5 | 14 | 5 | 0 | 0 | 0 | 0 | 0 | 4 | 10 | 1 | 4 | 0 | 0 | 0 | 0 | 1 | 0 | 7 | Y | 68988 | RecName: Full=Zinc metalloproteinase-disintegrin-like BmMP; AltName: Full=Snake venom metalloproteinase; Short=SVMP; Flags: Precursor |
| **64** | 432 | 7ULR | 230.07 | 85 | 0 | 0 | 18 | 0 | 0 | 85 | 18 | 45 | 0 | 18 | 0 | 18 | 0 | 42 | 18 | 0 | | | | | | 8.7481E7 | | | | | | | | | | | 9 | 2 | 0 | 0 | 1 | 0 | 0 | 15 | 2 | 7 | 0 | 1 | 0 | 1 | 0 | 2 | 1 | 0 | Y | 7407 | Chain B, Kappa-bungarotoxin |
| **64** | 452 | 1KBA | 230.07 | 86 | 0 | 0 | 18 | 0 | 0 | 86 | 18 | 45 | 0 | 18 | 0 | 18 | 0 | 42 | 18 | 0 | | | | | | 8.7481E7 | | | | | | | | | | | 9 | 2 | 0 | 0 | 1 | 0 | 0 | 15 | 2 | 7 | 0 | 1 | 0 | 1 | 0 | 2 | 1 | 0 | Y | 7275 | Chain B, KAPPA-BUNGAROTOXIN |
| **64** | 453 | 2NBT | 230.07 | 86 | 0 | 0 | 18 | 0 | 0 | 86 | 18 | 45 | 0 | 18 | 0 | 18 | 0 | 42 | 18 | 0 | | | | | | 8.7481E7 | | | | | | | | | | | 9 | 2 | 0 | 0 | 1 | 0 | 0 | 15 | 2 | 7 | 0 | 1 | 0 | 1 | 0 | 2 | 1 | 0 | Y | 7275 | Chain B, NEURONAL BUNGAROTOXIN |
| **93** | 44 | ACF70483.1 | 227.30 | 16 | 0 | 0 | 0 | 0 | 0 | 0 | 0 | 0 | 0 | 0 | 0 | 0 | 0 | 0 | 0 | 16 | | | | | | | | | | | | | | | | 5.3314E6 | 10 | 6 | 0 | 0 | 0 | 0 | 0 | 0 | 0 | 0 | 0 | 0 | 0 | 0 | 0 | 0 | 0 | 18 | Y | 56888 | secreted L-amino acid oxidase precursor [Daboia russelii] |
| **93** | 45 | G8XQX1.1 | 227.30 | 16 | 0 | 0 | 0 | 0 | 0 | 0 | 0 | 0 | 0 | 0 | 0 | 0 | 0 | 0 | 0 | 16 | | | | | | | | | | | | | | | | 5.3314E6 | 10 | 6 | 0 | 0 | 0 | 0 | 0 | 0 | 0 | 0 | 0 | 0 | 0 | 0 | 0 | 0 | 0 | 18 | Y | 56888 | RecName: Full=L-amino-acid oxidase; Short=DrLAO; Short=LAAO; Flags: Precursor |
| **32** | 391 | 1F94 | 208.62 | 75 | 0 | 0 | 0 | 0 | 0 | 75 | 19 | 19 | 19 | 19 | 0 | 19 | 0 | 0 | 0 | 0 | | | | | | 8.9885E8 | 1.1028E6 | 2.8689E7 | 1.2868E6 | 2.0316E6 | | 0 | | | | | 8 | 8 | 0 | 0 | 0 | 0 | 0 | 90 | 2 | 3 | 1 | 2 | 0 | 1 | 0 | 0 | 0 | 0 | Y | 7285 | Chain A, BUCANDIN |
| **32** | 392 | 1IJC | 208.62 | 75 | 0 | 0 | 0 | 0 | 0 | 75 | 19 | 19 | 19 | 19 | 0 | 19 | 0 | 0 | 0 | 0 | | | | | | 8.9885E8 | 1.1028E6 | 2.8689E7 | 1.2868E6 | 2.0316E6 | | 0 | | | | | 8 | 8 | 0 | 0 | 0 | 0 | 0 | 90 | 2 | 3 | 1 | 2 | 0 | 1 | 0 | 0 | 0 | 0 | Y | 7285 | Chain A, bucandin |
| **32** | 393 | P81782.1 | 208.62 | 75 | 0 | 0 | 0 | 0 | 0 | 75 | 19 | 19 | 19 | 19 | 0 | 19 | 0 | 0 | 0 | 0 | | | | | | 8.9885E8 | 1.1028E6 | 2.8689E7 | 1.2868E6 | 2.0316E6 | | 0 | | | | | 8 | 8 | 0 | 0 | 0 | 0 | 0 | 90 | 2 | 3 | 1 | 2 | 0 | 1 | 0 | 0 | 0 | 0 | Y | 7285 | RecName: Full=Bucandin |
| **67** | 2286 | AAL55556.1 | 204.25 | 23 | 0 | 0 | 0 | 0 | 0 | 0 | 10 | 16 | 0 | 23 | 13 | 16 | 10 | 16 | 23 | 0 | | | | | | | | | | 7.812E6 | | | | | 1.299E5 | | 10 | 1 | 0 | 0 | 0 | 0 | 0 | 0 | 1 | 5 | 0 | 4 | 3 | 11 | 1 | 7 | 1 | 0 | Y | 13210 | phospholipase A2, partial [Hydrophis hardwickii] |
| **67** | 2287 | ABN54807.1 | 204.25 | 18 | 0 | 0 | 0 | 0 | 0 | 0 | 8 | 13 | 0 | 18 | 10 | 13 | 8 | 13 | 18 | 0 | | | | | | | | | | 7.812E6 | | | | | 1.299E5 | | 10 | 1 | 0 | 0 | 0 | 0 | 0 | 0 | 1 | 5 | 0 | 4 | 3 | 11 | 1 | 7 | 1 | 0 | Y | 16078 | phospholipase A2 73 [Hydrophis hardwickii] |
| **67** | 2288 | Q8UW30.2 | 204.25 | 18 | 0 | 0 | 0 | 0 | 0 | 0 | 8 | 13 | 0 | 18 | 10 | 13 | 8 | 13 | 18 | 0 | | | | | | | | | | 7.812E6 | | | | | 1.299E5 | | 10 | 1 | 0 | 0 | 0 | 0 | 0 | 0 | 1 | 5 | 0 | 4 | 3 | 11 | 1 | 7 | 1 | 0 | Y | 16078 | RecName: Full=Basic phospholipase A2 73; Short=svPLA2; AltName: Full=Phosphatidylcholine 2-acylhydrolase; Flags: Precursor |
| **67** | 3068 | P00610.1 | 204.25 | 23 | 0 | 0 | 0 | 0 | 0 | 0 | 10 | 16 | 0 | 23 | 13 | 16 | 10 | 16 | 23 | 0 | | | | | | | | | | 7.812E6 | | | | | 1.299E5 | | 10 | 1 | 0 | 0 | 0 | 0 | 0 | 0 | 1 | 5 | 0 | 4 | 3 | 11 | 1 | 7 | 1 | 0 | Y | 13446 | RecName: Full=Basic phospholipase A2; Short=svPLA2; AltName: Full=Myotoxin; AltName: Full=Phosphatidylcholine 2-acylhydrolase; AltName: Full=Toxin VI-5; AltName: Full=Toxin VI:5b |
| **63** | 3076 | AAB20783.1 | 203.53 | 36 | 0 | 0 | 0 | 0 | 0 | 0 | 10 | 16 | 0 | 16 | 36 | 16 | 10 | 16 | 0 | 0 | | | | | | | | | | | 4.0825E5 | | | | | | 10 | 1 | 0 | 0 | 0 | 0 | 0 | 0 | 1 | 5 | 0 | 3 | 5 | 11 | 1 | 7 | 0 | 0 | Y | 13014 | notechis 11'2=non-toxic phospholipase A2 [Notechis scutatus=Australian tiger snakes, ssp. scutatus, venom, Peptide, 118 aa] |
| **63** | 3077 | P08873.1 | 203.53 | 30 | 0 | 0 | 0 | 0 | 0 | 0 | 8 | 13 | 0 | 13 | 30 | 13 | 8 | 13 | 0 | 0 | | | | | | | | | | | 4.0825E5 | | | | | | 10 | 1 | 0 | 0 | 0 | 0 | 0 | 0 | 1 | 5 | 0 | 3 | 5 | 11 | 1 | 7 | 0 | 0 | Y | 15903 | RecName: Full=Basic phospholipase A2 notechis 11'2; Short=svPLA2; AltName: Full=Phosphatidylcholine 2-acylhydrolase; Flags: Precursor |
| **63** | 3078 | CAA31125.1 | 203.53 | 30 | 0 | 0 | 0 | 0 | 0 | 0 | 8 | 13 | 0 | 13 | 30 | 13 | 8 | 13 | 0 | 0 | | | | | | | | | | | 4.0825E5 | | | | | | 10 | 1 | 0 | 0 | 0 | 0 | 0 | 0 | 1 | 5 | 0 | 3 | 5 | 11 | 1 | 7 | 0 | 0 | Y | 15903 | PLA2 preprotein (AA-27 to 118) [Notechis scutatus] |
| **51** | 160 | BAP39981.1 | 202.57 | 13 | 0 | 0 | 0 | 0 | 0 | 4 | 12 | 9 | 5 | 5 | 0 | 0 | 0 | 5 | 0 | 0 | | | | | | | 1.7175E6 | | | | | | | | | | 10 | 2 | 0 | 0 | 0 | 0 | 0 | 1 | 55 | 12 | 2 | 2 | 0 | 0 | 0 | 2 | 0 | 0 | Y | 32925 | nerve growth factor, partial [Protobothrops flavoviridis] |
| **51** | 161 | BAN82015.1 | 202.57 | 15 | 0 | 0 | 0 | 0 | 0 | 5 | 14 | 10 | 6 | 6 | 0 | 0 | 0 | 6 | 0 | 0 | | | | | | | 1.7175E6 | | | | | | | | | | 10 | 2 | 0 | 0 | 0 | 0 | 0 | 1 | 55 | 12 | 2 | 2 | 0 | 0 | 0 | 2 | 0 | 0 | Y | 27163 | nerve growth factor [Protobothrops flavoviridis] |
| **51** | 208 | AAG12169.1 | 202.57 | 15 | 0 | 0 | 0 | 0 | 0 | 5 | 14 | 10 | 6 | 6 | 0 | 0 | 0 | 6 | 0 | 0 | | | | | | | 1.7175E6 | | | | | | | | | | 10 | 2 | 0 | 0 | 0 | 0 | 0 | 1 | 55 | 12 | 2 | 2 | 0 | 0 | 0 | 2 | 0 | 0 | Y | 27162 | putative neurotrophic growth factor precursor [Bothrops jararacussu] |
| **23** | 1358 | 1MR6 | 201.96 | 40 | 0 | 28 | 40 | 28 | 0 | 28 | 28 | 28 | 0 | 28 | 0 | 28 | 0 | 21 | 21 | 0 | | 9.2952E8 | 2.1175E10 | 3.8699E6 | | 7.8995E8 | 3.3952E6 | 1.9356E8 | | 4.2921E7 | | 4.8584E7 | | 1.6806E7 | 1.1184E7 | | 6 | 6 | 0 | 18 | 124 | 2 | 0 | 9 | 3 | 5 | 0 | 5 | 0 | 4 | 0 | 1 | 3 | 0 | Y | 7535 | Chain A, neurotoxin |
| **23** | 1359 | CAA06885.1 | 201.96 | 30 | 0 | 21 | 30 | 21 | 0 | 21 | 21 | 21 | 0 | 21 | 0 | 21 | 0 | 16 | 16 | 0 | | 9.2952E8 | 2.1175E10 | 3.8699E6 | | 7.8995E8 | 3.3952E6 | 1.9356E8 | | 4.2921E7 | | 4.8584E7 | | 1.6806E7 | 1.1184E7 | | 6 | 6 | 0 | 18 | 124 | 2 | 0 | 9 | 3 | 5 | 0 | 5 | 0 | 4 | 0 | 1 | 3 | 0 | Y | 9826 | neurotoxin-like protein [Bungarus multicinctus multicinctus] |
| **23** | 1360 | Q9YGJ0.1 | 201.96 | 30 | 0 | 21 | 30 | 21 | 0 | 21 | 21 | 21 | 0 | 21 | 0 | 21 | 0 | 16 | 16 | 0 | | 9.2952E8 | 2.1175E10 | 3.8699E6 | | 7.8995E8 | 3.3952E6 | 1.9356E8 | | 4.2921E7 | | 4.8584E7 | | 1.6806E7 | 1.1184E7 | | 6 | 6 | 0 | 18 | 124 | 2 | 0 | 9 | 3 | 5 | 0 | 5 | 0 | 4 | 0 | 1 | 3 | 0 | Y | 9826 | RecName: Full=Gamma-bungarotoxin; AltName: Full=Long neurotoxin homolog NTL2I; Flags: Precursor |
| **23** | 1361 | CAD01082.1 | 201.96 | 30 | 0 | 21 | 30 | 21 | 0 | 21 | 21 | 21 | 0 | 21 | 0 | 21 | 0 | 16 | 16 | 0 | | 9.2952E8 | 2.1175E10 | 3.8699E6 | | 7.8995E8 | 3.3952E6 | 1.9356E8 | | 4.2921E7 | | 4.8584E7 | | 1.6806E7 | 1.1184E7 | | 6 | 6 | 0 | 18 | 124 | 2 | 0 | 9 | 3 | 5 | 0 | 5 | 0 | 4 | 0 | 1 | 3 | 0 | Y | 9826 | gamma-bungarotoxin [Bungarus multicinctus] |
| **23** | 1362 | O12963.1 | 201.96 | 30 | 0 | 21 | 30 | 21 | 0 | 21 | 21 | 21 | 0 | 21 | 0 | 21 | 0 | 16 | 16 | 0 | | 9.2952E8 | 2.1175E10 | 3.8699E6 | | 7.8995E8 | 3.3952E6 | 1.9356E8 | | 4.2921E7 | | 4.8584E7 | | 1.6806E7 | 1.1184E7 | | 6 | 6 | 0 | 18 | 124 | 2 | 0 | 9 | 3 | 5 | 0 | 5 | 0 | 4 | 0 | 1 | 3 | 0 | Y | 9756 | RecName: Full=Long neurotoxin homolog TA-bm16; AltName: Full=Delta-protein; Flags: Precursor |
| **23** | 1363 | AAD41806.1 | 201.96 | 30 | 0 | 21 | 30 | 21 | 0 | 21 | 21 | 21 | 0 | 21 | 0 | 21 | 0 | 16 | 16 | 0 | | 9.2952E8 | 2.1175E10 | 3.8699E6 | | 7.8995E8 | 3.3952E6 | 1.9356E8 | | 4.2921E7 | | 4.8584E7 | | 1.6806E7 | 1.1184E7 | | 6 | 6 | 0 | 18 | 124 | 2 | 0 | 9 | 3 | 5 | 0 | 5 | 0 | 4 | 0 | 1 | 3 | 0 | Y | 9826 | neurotoxin precursor [Bungarus multicinctus] |
| **23** | 1364 | CAA72941.1 | 201.96 | 30 | 0 | 21 | 30 | 21 | 0 | 21 | 21 | 21 | 0 | 21 | 0 | 21 | 0 | 16 | 16 | 0 | | 9.2952E8 | 2.1175E10 | 3.8699E6 | | 7.8995E8 | 3.3952E6 | 1.9356E8 | | 4.2921E7 | | 4.8584E7 | | 1.6806E7 | 1.1184E7 | | 6 | 6 | 0 | 18 | 124 | 2 | 0 | 9 | 3 | 5 | 0 | 5 | 0 | 4 | 0 | 1 | 3 | 0 | Y | 9756 | neurotoxin [Bungarus multicinctus] |
| **23** | 2146 | CAA11214.1 | 201.96 | 30 | 0 | 21 | 30 | 21 | 0 | 21 | 21 | 21 | 0 | 21 | 0 | 21 | 0 | 16 | 16 | 0 | | 9.2952E8 | 2.1175E10 | 3.8699E6 | | 7.8995E8 | 3.3952E6 | 1.9356E8 | | 4.2921E7 | | 4.8584E7 | | 1.6806E7 | 1.1184E7 | | 6 | 6 | 0 | 18 | 124 | 2 | 0 | 9 | 3 | 5 | 0 | 5 | 0 | 4 | 0 | 1 | 3 | 0 | Y | 9873 | neurotoxin-like protein [Bungarus multicinctus multicinctus] |

| | | | | | | | | | | | | | | | | | | | | | | | | | | | | | | | | | | | | | | | | | | | | | | | | | | | | | | | | | |
|---|---|---|---|---|---|---|---|---|---|---|---|---|---|---|---|---|---|---|---|---|---|---|---|---|---|---|---|---|---|---|---|---|---|---|---|---|---|---|---|---|---|---|---|---|---|---|---|---|---|---|---|---|---|---|---|---|---|
| **23** | 2147 | Q9YGH9.1 | 201.96 | 30 | 0 | 21 | 30 | 21 | 0 | 21 | 21 | 21 | 0 | 21 | 0 | 21 | 0 | 16 | 16 | 0 | | 9.2952E8 | 2.1175E10 | 3.8699E6 | | 7.8995E8 | 3.3952E6 | 1.9356E8 | | 4.2921E7 | | 4.8584E7 | | 1.6806E7 | 1.1184E7 | | 6 | 6 | 0 | 18 | 124 | 2 | 0 | 9 | 3 | 5 | 0 | 5 | 0 | 4 | 0 | 1 | 3 | 0 | Y | 9873 | RecName: Full=Long neurotoxin homolog NTL2; Flags: Precursor |
| **107** | 14 | JAS05143.1 | 199.42 | 18 | 0 | 0 | 0 | 0 | 0 | 0 | 0 | 2 | 0 | 0 | 0 | 0 | 0 | 0 | 0 | 16 | | | | | | | | | | | | | | | | 1.2835E6 | 12 | 1 | 0 | 0 | 0 | 0 | 0 | 0 | 0 | 1 | 0 | 0 | 0 | 0 | 0 | 0 | 0 | 14 | Y | 63012 | ecto-5'-nucleotidase [Micrurus tener] |
| **107** | 15 | JAI09047.1 | 199.42 | 18 | 0 | 0 | 0 | 0 | 0 | 0 | 0 | 2 | 0 | 0 | 0 | 0 | 0 | 0 | 0 | 16 | | | | | | | | | | | | | | | | 1.2835E6 | 12 | 1 | 0 | 0 | 0 | 0 | 0 | 0 | 0 | 1 | 0 | 0 | 0 | 0 | 0 | 0 | 0 | 14 | Y | 62983 | Ecto-5'-nucleotidase 1c [Micrurus fulvius] |
| **107** | 16 | JAB52815.1 | 199.42 | 18 | 0 | 0 | 0 | 0 | 0 | 0 | 0 | 2 | 0 | 0 | 0 | 0 | 0 | 0 | 0 | 16 | | | | | | | | | | | | | | | | 1.2835E6 | 12 | 1 | 0 | 0 | 0 | 0 | 0 | 0 | 0 | 1 | 0 | 0 | 0 | 0 | 0 | 0 | 0 | 14 | Y | 62983 | Ecto-5'-nucleotidase 1 [Micrurus fulvius] |
| **107** | 17 | JAI09048.1 | 199.42 | 18 | 0 | 0 | 0 | 0 | 0 | 0 | 0 | 2 | 0 | 0 | 0 | 0 | 0 | 0 | 0 | 16 | | | | | | | | | | | | | | | | 1.2835E6 | 12 | 1 | 0 | 0 | 0 | 0 | 0 | 0 | 0 | 1 | 0 | 0 | 0 | 0 | 0 | 0 | 0 | 14 | Y | 63025 | Ecto-5'-nucleotidase 1b [Micrurus fulvius] |
| **107** | 18 | JAS05038.1 | 199.42 | 18 | 0 | 0 | 0 | 0 | 0 | 0 | 0 | 2 | 0 | 0 | 0 | 0 | 0 | 0 | 0 | 16 | | | | | | | | | | | | | | | | 1.2835E6 | 12 | 1 | 0 | 0 | 0 | 0 | 0 | 0 | 0 | 1 | 0 | 0 | 0 | 0 | 0 | 0 | 0 | 14 | Y | 63025 | Ecto-5'-nucleotidase 1a [Micrurus fulvius] |
| **107** | 19 | JAS05036.1 | 199.42 | 18 | 0 | 0 | 0 | 0 | 0 | 0 | 0 | 2 | 0 | 0 | 0 | 0 | 0 | 0 | 0 | 16 | | | | | | | | | | | | | | | | 1.2835E6 | 12 | 1 | 0 | 0 | 0 | 0 | 0 | 0 | 0 | 1 | 0 | 0 | 0 | 0 | 0 | 0 | 0 | 14 | Y | 62983 | Ecto-5'-nucleotidase 1c [Micrurus fulvius] |
| **107** | 20 | JAS05037.1 | 199.42 | 18 | 0 | 0 | 0 | 0 | 0 | 0 | 0 | 2 | 0 | 0 | 0 | 0 | 0 | 0 | 0 | 16 | | | | | | | | | | | | | | | | 1.2835E6 | 12 | 1 | 0 | 0 | 0 | 0 | 0 | 0 | 0 | 1 | 0 | 0 | 0 | 0 | 0 | 0 | 0 | 14 | Y | 63025 | Ecto-5'-nucleotidase 1b [Micrurus fulvius] |
| **107** | 21 | JAI09049.1 | 199.42 | 18 | 0 | 0 | 0 | 0 | 0 | 0 | 0 | 2 | 0 | 0 | 0 | 0 | 0 | 0 | 0 | 16 | | | | | | | | | | | | | | | | 1.2835E6 | 12 | 1 | 0 | 0 | 0 | 0 | 0 | 0 | 0 | 1 | 0 | 0 | 0 | 0 | 0 | 0 | 0 | 14 | Y | 63025 | Ecto-5'-nucleotidase 1a [Micrurus fulvius] |
| **10** | 189 | CAA06887.1 | 199.33 | 62 | 0 | 62 | 38 | 31 | 12 | 26 | 10 | 35 | 0 | 12 | 0 | 12 | 0 | 12 | 12 | 0 | | 8.2362E8 | 2.4129E8 | 1.156E7 | 6.5847E6 | 2.053E7 | 4.752E4 | 1.9998E7 | | 6.4441E6 | | 1.4126E7 | | 6.7916E6 | 2.6445E5 | | 10 | 10 | 0 | 122 | 58 | 5 | 4 | 6 | 1 | 11 | 0 | 4 | 0 | 7 | 0 | 4 | 1 | 0 | Y | 9519 | neurotoxin-like protein [Bungarus multicinctus multicinctus] |
| **10** | 190 | Q9YGI8.1 | 199.33 | 62 | 0 | 62 | 38 | 31 | 12 | 26 | 10 | 35 | 0 | 12 | 0 | 12 | 0 | 12 | 12 | 0 | | 8.2362E8 | 2.4129E8 | 1.156E7 | 6.5847E6 | 2.053E7 | 4.752E4 | 1.9998E7 | | 6.4441E6 | | 1.4126E7 | | 6.7916E6 | 2.6445E5 | | 10 | 10 | 0 | 122 | 58 | 5 | 4 | 6 | 1 | 11 | 0 | 4 | 0 | 7 | 0 | 4 | 1 | 0 | Y | 9519 | RecName: Full=Short neurotoxin homolog NTL4; Flags: Precursor |
| **70** | 726 | Q9W729.1 | 198.58 | 49 | 0 | 0 | 18 | 0 | 0 | 18 | 18 | 49 | 23 | 18 | 18 | 18 | 0 | 18 | 0 | 0 | | | 2.4705E5 | | | 9.083E6 | 9.3683E6 | 6.5868E8 | 5.9609E6 | 6.6479E6 | 1.7237E4 | 3.1182E6 | | 3.5463E6 | | | 6 | 6 | 0 | 0 | 2 | 0 | 0 | 4 | 5 | 13 | 4 | 3 | 1 | 2 | 0 | 2 | 0 | 0 | Y | 9657 | RecName: Full=Kappa-6-bungarotoxin; Short=K-6 bungarotoxin; Flags: Precursor |
| **70** | 727 | CAB46659.1 | 198.58 | 49 | 0 | 0 | 18 | 0 | 0 | 18 | 18 | 49 | 23 | 18 | 18 | 18 | 0 | 18 | 0 | 0 | | | 2.4705E5 | | | 9.083E6 | 9.3683E6 | 6.5868E8 | 5.9609E6 | 6.6479E6 | 1.7237E4 | 3.1182E6 | | 3.5463E6 | | | 6 | 6 | 0 | 0 | 2 | 0 | 0 | 4 | 5 | 13 | 4 | 3 | 1 | 2 | 0 | 2 | 0 | 0 | Y | 9657 | k-6 bungarotoxin [Bungarus multicinctus] |
| **138** | 48 | 1POB | 192.45 | 72 | 0 | 0 | 0 | 0 | 0 | 0 | 8 | 0 | 13 | 59 | 0 | 0 | 0 | 0 | 0 | 0 | | | | | | | | | | 5.8457E6 | | | | | | | 8 | 1 | 0 | 0 | 0 | 0 | 0 | 0 | 3 | 0 | 1 | 6 | 0 | 0 | 0 | 0 | 0 | 0 | Y | 13144 | Chain B, PHOSPHOLIPASE A2 |
| **138** | 49 | 1POA | 192.45 | 72 | 0 | 0 | 0 | 0 | 0 | 0 | 8 | 0 | 13 | 59 | 0 | 0 | 0 | 0 | 0 | 0 | | | | | | | | | | 5.8457E6 | | | | | | | 8 | 1 | 0 | 0 | 0 | 0 | 0 | 0 | 3 | 0 | 1 | 6 | 0 | 0 | 0 | 0 | 0 | 0 | Y | 13144 | Chain A, PHOSPHOLIPASE A2 |
| **140** | 722 | CAA72434.1 | 191.07 | 36 | 0 | 0 | 0 | 0 | 0 | 18 | 0 | 33 | 0 | 0 | 0 | 0 | 0 | 18 | 0 | 0 | | | | | | | | 8.9982E5 | | | | | | | | | 6 | 1 | 0 | 0 | 0 | 0 | 0 | 4 | 0 | 3 | 0 | 0 | 0 | 0 | 0 | 1 | 0 | 0 | Y | 9674 | kappa3-bungarotoxin [Bungarus multicinctus] |
| **140** | 723 | CAA35775.1 | 191.07 | 36 | 0 | 0 | 0 | 0 | 0 | 18 | 0 | 33 | 0 | 0 | 0 | 0 | 0 | 18 | 0 | 0 | | | | | | | | 8.9982E5 | | | | | | | | | 6 | 1 | 0 | 0 | 0 | 0 | 0 | 4 | 0 | 3 | 0 | 0 | 0 | 0 | 0 | 1 | 0 | 0 | Y | 9660 | unnamed protein product [Bungarus multicinctus] |
| **140** | 724 | P15817.1 | 191.07 | 36 | 0 | 0 | 0 | 0 | 0 | 18 | 0 | 33 | 0 | 0 | 0 | 0 | 0 | 18 | 0 | 0 | | | | | | | | 8.9982E5 | | | | | | | | | 6 | 1 | 0 | 0 | 0 | 0 | 0 | 4 | 0 | 3 | 0 | 0 | 0 | 0 | 0 | 1 | 0 | 0 | Y | 9660 | RecName: Full=Kappa-3-bungarotoxin; Short=K3Bgt; AltName: Full=Kappa-neurotoxin; AltName: Full=Long neurotoxin CR1; Flags: Precursor |
| **34** | 216 | CAD24463.1 | 190.56 | 47 | 0 | 0 | 0 | 0 | 0 | 0 | 7 | 36 | 7 | 42 | 7 | 40 | 0 | 17 | 40 | 0 | | | | | | | | | | 9.8379E5 | | 0 | | | 0 | | 10 | 2 | 0 | 0 | 0 | 0 | 0 | 0 | 1 | 13 | 6 | 41 | 2 | 13 | 0 | 4 | 16 | 0 | Y | 16122 | beta-bungarotoxin A8 chain [Bungarus multicinctus] |
| **28** | 150 | 0412250A | 185.70 | 57 | 0 | 11 | 11 | 0 | 0 | 0 | 8 | 35 | 19 | 50 | 22 | 51 | 0 | 32 | 30 | 0 | | 4.6764E6 | 9.2303E5 | | | | | | 1.3558E6 | 2.1784E5 | | 0 | | 5.708E7 | | | 8 | 1 | 0 | 1 | 2 | 0 | 0 | 0 | 1 | 11 | 7 | 50 | 6 | 13 | 0 | 5 | 14 | 0 | Y | 13489 | toxin A,beta1 bungaro |
| **49** | 226 | Q75S48.1 | 184.72 | 40 | 0 | 0 | 0 | 0 | 0 | 8 | 7 | 25 | 5 | 21 | 7 | 10 | 5 | 9 | 38 | 0 | | | | | | | | 2.2127E6 | | 0 | | | | | 4.1281E7 | | 7 | 2 | 0 | 0 | 0 | 0 | 0 | 3 | 6 | 7 | 1 | 7 | 5 | 11 | 2 | 10 | 15 | 0 | Y | 16855 | RecName: Full=Acidic phospholipase A2 1; Short=svPLA2; AltName: Full=PA2-I; AltName: Full=Phosphatidylcholine 2-acylhydrolase; Flags: Precursor |
| **49** | 227 | BAD06270.1 | 184.72 | 40 | 0 | 0 | 0 | 0 | 0 | 8 | 7 | 25 | 5 | 21 | 7 | 10 | 5 | 9 | 38 | 0 | | | | | | | | 2.2127E6 | | 0 | | | | | 4.1281E7 | | 7 | 2 | 0 | 0 | 0 | 0 | 0 | 3 | 6 | 7 | 1 | 7 | 5 | 11 | 2 | 10 | 15 | 0 | Y | 16855 | phospholipase A2-I precursor [Bungarus candidus] |
| **55** | 649 | P81993.1 | 184.36 | 63 | 0 | 0 | 0 | 0 | 0 | 0 | 0 | 0 | 0 | 0 | 40 | 24 | 63 | 24 | 0 | 25 | | | | | | | | | | | | 0 | 4.7351E7 | 8.3018E6 | | | 4 | 1 | 0 | 0 | 0 | 0 | 0 | 0 | 0 | 0 | 0 | 0 | 18 | 1 | 30 | 2 | 0 | 6 | Y | 7752 | RecName: Full=Cysteine-rich venom protein bucarin |
| **97** | 577 | P25668.1 | 183.25 | 39 | 0 | 0 | 0 | 0 | 23 | 39 | 35 | 37 | 0 | 0 | 0 | 0 | 0 | 0 | 0 | 0 | | | | | 2.0409E6 | 2.2467E7 | 5.9731E4 | 1.9376E6 | | | | | | | | | 5 | 3 | 0 | 0 | 0 | 0 | 3 | 10 | 2 | 2 | 0 | 0 | 0 | 0 | 0 | 0 | 0 | 0 | Y | 7847 | RecName: Full=Long neurotoxin 1; AltName: Full=Toxin A |
| **113** | 210 | Q3HXY1.1 | 182.60 | 17 | 0 | 0 | 0 | 0 | 0 | 0 | 8 | 17 | 0 | 0 | 0 | 0 | 0 | 0 | 0 | 0 | | | | | | | | 4.6715E5 | | | | | | | | | 9 | 2 | 0 | 0 | 0 | 0 | 0 | 0 | 6 | 7 | 0 | 0 | 0 | 0 | 0 | 0 | 0 | 0 | Y | 27138 | RecName: Full=Venom nerve growth factor 3; Short=v-NGF-3; Short=vNGF-3; Flags: Precursor |
| **113** | 211 | ABA60126.1 | 182.60 | 17 | 0 | 0 | 0 | 0 | 0 | 0 | 8 | 17 | 0 | 0 | 0 | 0 | 0 | 0 | 0 | 0 | | | | | | | | 4.6715E5 | | | | | | | | | 9 | 2 | 0 | 0 | 0 | 0 | 0 | 0 | 6 | 7 | 0 | 0 | 0 | 0 | 0 | 0 | 0 | 0 | Y | 27138 | venom nerve growth factor precursor [Pseudechis australis] |
| **113** | 212 | Q3HXY3.1 | 182.60 | 17 | 0 | 0 | 0 | 0 | 0 | 0 | 8 | 17 | 0 | 0 | 0 | 0 | 0 | 0 | 0 | 0 | | | | | | | | 4.6715E5 | | | | | | | | | 9 | 2 | 0 | 0 | 0 | 0 | 0 | 0 | 6 | 7 | 0 | 0 | 0 | 0 | 0 | 0 | 0 | 0 | Y | 27156 | RecName: Full=Venom nerve growth factor 1; Short=v-NGF-1; Short=vNGF-1; Flags: Precursor |
| **113** | 213 | ABA60124.1 | 182.60 | 17 | 0 | 0 | 0 | 0 | 0 | 0 | 8 | 17 | 0 | 0 | 0 | 0 | 0 | 0 | 0 | 0 | | | | | | | | 4.6715E5 | | | | | | | | | 9 | 2 | 0 | 0 | 0 | 0 | 0 | 0 | 6 | 7 | 0 | 0 | 0 | 0 | 0 | 0 | 0 | 0 | Y | 27156 | venom nerve growth factor precursor [Pseudechis australis] |
| **84** | 458 | AAO84769.1 | 178.07 | 22 | 0 | 0 | 0 | 0 | 0 | 0 | 0 | 21 | 0 | 22 | 8 | 22 | 16 | 22 | 22 | 0 | | | | | | | | 4.5975E5 | | 5.0928E6 | | 3.3872E6 | 3.1907E5 | 1.4405E8 | 5.3528E6 | | 5 | 2 | 0 | 0 | 0 | 0 | 0 | 0 | 0 | 3 | 0 | 4 | 1 | 4 | 2 | 12 | 4 | 0 | Y | 15641 | phospholipase A2, partial [Bungarus candidus] |
| **84** | 459 | Q802I1.1 | 178.07 | 22 | 0 | 0 | 0 | 0 | 0 | 0 | 0 | 21 | 0 | 22 | 8 | 22 | 16 | 22 | 22 | 0 | | | | | | | | 4.5975E5 | | 5.0928E6 | | 3.3872E6 | 3.1907E5 | 1.4405E8 | 5.3528E6 | | 5 | 2 | 0 | 0 | 0 | 0 | 0 | 0 | 0 | 3 | 0 | 4 | 1 | 4 | 2 | 12 | 4 | 0 | Y | 15641 | RecName: Full=Acidic phospholipase A2 Bc-PL; Short=svPLA2; AltName: Full=Phosphatidylcholine 2-acylhydrolase; Flags: Precursor |
| **139** | 46 | XP_026582322.1 | 176.21 | 16 | 0 | 0 | 0 | 0 | 0 | 0 | 0 | 2 | 0 | 0 | 0 | 0 | 0 | 0 | 0 | 13 | | | | | | | | | | | | | | | | 0 | 9 | 1 | 0 | 0 | 0 | 0 | 0 | 0 | 0 | 1 | 0 | 0 | 0 | 0 | 0 | 0 | 0 | 10 | Y | 55369 | 5'-nucleotidase [Pseudonaja textilis] |
| **137** | 229 | Q4JHE1.1 | 174.61 | 15 | 0 | 0 | 0 | 0 | 0 | 0 | 0 | 0 | 0 | 0 | 0 | 0 | 0 | 0 | 4 | 12 | | | | | | | | | | | | | | | 1.2745E5 | | 6 | 1 | 0 | 0 | 0 | 0 | 0 | 0 | 0 | 0 | 0 | 0 | 0 | 0 | 0 | 0 | 1 | 8 | Y | 58744 | RecName: Full=L-amino-acid oxidase; Short=LAAO; Short=LAO; Flags: Precursor |
| **137** | 230 | AAY89682.1 | 174.61 | 15 | 0 | 0 | 0 | 0 | 0 | 0 | 0 | 0 | 0 | 0 | 0 | 0 | 0 | 0 | 4 | 12 | | | | | | | | | | | | | | | 1.2745E5 | | 6 | 1 | 0 | 0 | 0 | 0 | 0 | 0 | 0 | 0 | 0 | 0 | 0 | 0 | 0 | 0 | 1 | 8 | Y | 58744 | L-amino acid oxidase precursor [Pseudechis australis] |
| **102** | 112 | Q9PVK7.1 | 161.20 | 12 | 0 | 0 | 0 | 0 | 0 | 2 | 10 | 2 | 2 | 0 | 0 | 0 | 0 | 0 | 0 | 8 | | | | | | | 6.6604E4 | | 5.9107E4 | | | | | | | 1.6367E6 | 7 | 3 | 0 | 0 | 0 | 0 | 0 | 1 | 5 | 2 | 1 | 0 | 0 | 0 | 0 | 0 | 0 | 7 | Y | 67662 | RecName: Full=Zinc metalloproteinase-disintegrin-like cobrin; AltName: Full=Snake venom metalloproteinase; Short=SVMP; Flags: Precursor |
| **102** | 113 | AAF00693.1 | 161.20 | 12 | 0 | 0 | 0 | 0 | 0 | 2 | 10 | 2 | 2 | 0 | 0 | 0 | 0 | 0 | 0 | 8 | | | | | | | 6.6604E4 | | 5.9107E4 | | | | | | | 1.6367E6 | 7 | 3 | 0 | 0 | 0 | 0 | 0 | 1 | 5 | 2 | 1 | 0 | 0 | 0 | 0 | 0 | 0 | 7 | Y | 67662 | cobrin precursor [Naja naja] |
| **96** | 1007 | 1DPY | 158.31 | 35 | 0 | 0 | 0 | 0 | 0 | 0 | 10 | 10 | 17 | 10 | 10 | 22 | 10 | 22 | 22 | 0 | | | | | | | | | 5.6665E7 | | | 1.1216E7 | | 2.2097E7 | 1.2222E6 | | 4 | 2 | 0 | 0 | 0 | 0 | 0 | 0 | 1 | 3 | 1 | 1 | 1 | 4 | 1 | 6 | 2 | 0 | Y | 12936 | Chain A, PHOSPHOLIPASE A2 |
| **155** | 142 | 1ZM6 | 150.24 | 34 | 0 | 0 | 0 | 0 | 0 | 0 | 8 | 0 | 13 | 22 | 0 | 0 | 0 | 0 | 0 | 0 | | | | | | | | | | 9.234E4 | | | | | | | 5 | 1 | 0 | 0 | 0 | 0 | 0 | 0 | 3 | 0 | 1 | 2 | 0 | 0 | 0 | 0 | 0 | 0 | Y | 13162 | Chain A, Phospholipase A2 isoform 3 |
| **100** | 454 | Q9DF52.1 | 144.38 | 33 | 0 | 0 | 0 | 0 | 0 | 0 | 8 | 17 | 7 | 8 | 24 | 24 | 8 | 17 | 0 | 0 | | | | | | | | | 2.992E5 | | 1.3115E6 | 2.2032E7 | | | | | 6 | 2 | 0 | 0 | 0 | 0 | 0 | 0 | 1 | 4 | 1 | 1 | 2 | 1 | 1 | 2 | 0 | 0 | Y | 15765 | RecName: Full=Basic phospholipase A2 KPA2; Short=svPLA2; AltName: Full=Phosphatidylcholine 2-acylhydrolase; Flags: Precursor |
| **100** | 455 | AAG13412.1 | 144.38 | 33 | 0 | 0 | 0 | 0 | 0 | 0 | 8 | 17 | 7 | 8 | 24 | 24 | 8 | 17 | 0 | 0 | | | | | | | | | 2.992E5 | | 1.3115E6 | 2.2032E7 | | | | | 6 | 2 | 0 | 0 | 0 | 0 | 0 | 0 | 1 | 4 | 1 | 1 | 2 | 1 | 1 | 2 | 0 | 0 | Y | 15765 | phospholipase A2 [Bungarus caeruleus] |
| **175** | 174 | AAZ39880.1 | 139.46 | 7 | 0 | 0 | 0 | 0 | 0 | 0 | 0 | 0 | 0 | 0 | 0 | 0 | 1 | 0 | 0 | 6 | | | | | | | | | | | | | 3.2243E5 | | | 1.2523E6 | 3 | 3 | 0 | 0 | 0 | 0 | 0 | 0 | 0 | 0 | 0 | 0 | 0 | 0 | 1 | 0 | 0 | 3 | Y | 69555 | hemorrhagic metalloproteinase russelysin [Daboia russelii] |
| **175** | 175 | B8K1W0.1 | 139.46 | 7 | 0 | 0 | 0 | 0 | 0 | 0 | 0 | 0 | 0 | 0 | 0 | 0 | 1 | 0 | 0 | 6 | | | | | | | | | | | | | 3.2243E5 | | | 1.2523E6 | 3 | 3 | 0 | 0 | 0 | 0 | 0 | 0 | 0 | 0 | 0 | 0 | 0 | 0 | 1 | 0 | 0 | 3 | Y | 69555 | RecName: Full=Zinc metalloproteinase-disintegrin-like daborhagin-K; AltName: Full=Haemorrhagic metalloproteinase russelysin; AltName: Full=Snake venom metalloproteinase; Short=SVMP; Flags: Precursor |
| | | | | | | | | | | | | | | | | | | | | | | | | | | | | | | | | | | | | | | | | | | | | | | | | | | | | | | | | | beta-nerve gro |

| | | | | | | | | | | | | | | | | | | | | | | | | | | | | | | | | | | | | | | | | | | | | | | | | | | | | | | | | | |
|---|---|---|---|---|---|---|---|---|---|---|---|---|---|---|---|---|---|---|---|---|---|---|---|---|---|---|---|---|---|---|---|---|---|---|---|---|---|---|---|---|---|---|---|---|---|---|---|---|---|---|---|---|---|---|---|---|---|
| 151 | 356 | XP_058034421.1 | 137.59 | 11 | 0 | 0 | 0 | 0 | 0 | 0 | 10 | 8 | 0 | 0 | 0 | 0 | 0 | 0 | 0 | 0 |  |  |  |  |  |  | 4.1586E6 |  |  |  |  |  |  |  |  |  | 6 | 2 | 0 | 0 | 0 | 0 | 0 | 0 | 4 | 3 | 0 | 0 | 0 | 0 | 0 | 0 | 0 | 0 | Y | 27021 | wth factor isoform X2 [Ahaetulla prasina] |
| 151 | 357 | XP_058034419.1 | 137.59 | 11 | 0 | 0 | 0 | 0 | 0 | 0 | 10 | 8 | 0 | 0 | 0 | 0 | 0 | 0 | 0 | 0 |  |  |  |  |  |  | 4.1586E6 |  |  |  |  |  |  |  |  |  | 6 | 2 | 0 | 0 | 0 | 0 | 0 | 0 | 4 | 3 | 0 | 0 | 0 | 0 | 0 | 0 | 0 | 0 | Y | 27021 | beta-nerve growth factor isoform X2 [Ahaetulla prasina] |
| 151 | 358 | XP_058034418.1 | 137.59 | 11 | 0 | 0 | 0 | 0 | 0 | 0 | 10 | 8 | 0 | 0 | 0 | 0 | 0 | 0 | 0 | 0 |  |  |  |  |  |  | 4.1586E6 |  |  |  |  |  |  |  |  |  | 6 | 2 | 0 | 0 | 0 | 0 | 0 | 0 | 4 | 3 | 0 | 0 | 0 | 0 | 0 | 0 | 0 | 0 | Y | 27021 | beta-nerve growth factor isoform X2 [Ahaetulla prasina] |
| 151 | 359 | XP_058034420.1 | 137.59 | 11 | 0 | 0 | 0 | 0 | 0 | 0 | 10 | 8 | 0 | 0 | 0 | 0 | 0 | 0 | 0 | 0 |  |  |  |  |  |  | 4.1586E6 |  |  |  |  |  |  |  |  |  | 6 | 2 | 0 | 0 | 0 | 0 | 0 | 0 | 4 | 3 | 0 | 0 | 0 | 0 | 0 | 0 | 0 | 0 | Y | 27021 | beta-nerve growth factor isoform X2 [Ahaetulla prasina] |
| 151 | 360 | XP_058034417.1 | 137.59 | 11 | 0 | 0 | 0 | 0 | 0 | 0 | 9 | 8 | 0 | 0 | 0 | 0 | 0 | 0 | 0 | 0 |  |  |  |  |  |  | 4.1586E6 |  |  |  |  |  |  |  |  |  | 6 | 2 | 0 | 0 | 0 | 0 | 0 | 0 | 4 | 3 | 0 | 0 | 0 | 0 | 0 | 0 | 0 | 0 | Y | 27702 | beta-nerve growth factor isoform X1 [Ahaetulla prasina] |
| 174 | 147 | AVX27607.1 | 133.19 | 14 | 0 | 0 | 0 | 0 | 0 | 0 | 0 | 0 | 0 | 0 | 0 | 0 | 0 | 0 | 0 | 14 |  |  |  |  |  |  |  |  |  |  |  |  |  |  |  | 6.124E5 | 6 | 3 | 0 | 0 | 0 | 0 | 0 | 0 | 0 | 0 | 0 | 0 | 0 | 0 | 0 | 0 | 0 | 7 | Y | 57963 | L-amino acid oxidase, partial [Naja atra] |
| 174 | 148 | 5Z2G | 133.19 | 14 | 0 | 0 | 0 | 0 | 0 | 0 | 0 | 0 | 0 | 0 | 0 | 0 | 0 | 0 | 0 | 14 |  |  |  |  |  |  |  |  |  |  |  |  |  |  |  | 6.124E5 | 6 | 3 | 0 | 0 | 0 | 0 | 0 | 0 | 0 | 0 | 0 | 0 | 0 | 0 | 0 | 0 | 0 | 7 | Y | 57963 | Chain B, L-amino acid oxidase |
| 174 | 149 | A8QL58.2 | 133.19 | 14 | 0 | 0 | 0 | 0 | 0 | 0 | 0 | 0 | 0 | 0 | 0 | 0 | 0 | 0 | 0 | 14 |  |  |  |  |  |  |  |  |  |  |  |  |  |  |  | 6.124E5 | 6 | 3 | 0 | 0 | 0 | 0 | 0 | 0 | 0 | 0 | 0 | 0 | 0 | 0 | 0 | 0 | 0 | 7 | Y | 57963 | RecName: Full=L-amino-acid oxidase; Short=LAO; Short=NA-LAAO; Flags: Precursor |
| 99 | 966 | XP_026579406.1 | 132.23 | 15 | 0 | 7 | 8 | 0 | 0 | 7 | 15 | 7 | 0 | 0 | 0 | 7 | 0 | 7 | 0 | 0 |  | 9.87E6 | 3.7739E8 |  |  | 2.0917E7 | 4.4269E5 | 3.983E6 |  |  |  | 0 |  | 6.0577E5 |  |  | 3 | 3 | 0 | 3 | 9 | 0 | 0 | 2 | 3 | 1 | 0 | 0 | 0 | 1 | 0 | 2 | 0 | 0 | Y | 22428 | BPTI/Kunitz domain-containing protein-like isoform X3 [Pseudonaja textilis] |
| 99 | 967 | XP_026579408.1 | 132.23 | 15 | 0 | 7 | 8 | 0 | 0 | 7 | 15 | 7 | 0 | 0 | 0 | 7 | 0 | 7 | 0 | 0 |  | 9.87E6 | 3.7739E8 |  |  | 2.0917E7 | 4.4269E5 | 3.983E6 |  |  |  | 0 |  | 6.0577E5 |  |  | 3 | 3 | 0 | 3 | 9 | 0 | 0 | 2 | 3 | 1 | 0 | 0 | 0 | 1 | 0 | 2 | 0 | 0 | Y | 22376 | BPTI/Kunitz domain-containing protein-like isoform X1 [Pseudonaja textilis] |
| 99 | 968 | XP_026579405.1 | 132.23 | 15 | 0 | 7 | 8 | 0 | 0 | 7 | 15 | 7 | 0 | 0 | 0 | 7 | 0 | 7 | 0 | 0 |  | 9.87E6 | 3.7739E8 |  |  | 2.0917E7 | 4.4269E5 | 3.983E6 |  |  |  | 0 |  | 6.0577E5 |  |  | 3 | 3 | 0 | 3 | 9 | 0 | 0 | 2 | 3 | 1 | 0 | 0 | 0 | 1 | 0 | 2 | 0 | 0 | Y | 22372 | BPTI/Kunitz domain-containing protein-like isoform X2 [Pseudonaja textilis] |
| 99 | 969 | XP_026579404.1 | 132.23 | 15 | 0 | 7 | 8 | 0 | 0 | 7 | 15 | 7 | 0 | 0 | 0 | 7 | 0 | 7 | 0 | 0 |  | 9.87E6 | 3.7739E8 |  |  | 2.0917E7 | 4.4269E5 | 3.983E6 |  |  |  | 0 |  | 6.0577E5 |  |  | 3 | 3 | 0 | 3 | 9 | 0 | 0 | 2 | 3 | 1 | 0 | 0 | 0 | 1 | 0 | 2 | 0 | 0 | Y | 22376 | BPTI/Kunitz domain-containing protein-like isoform X1 [Pseudonaja textilis] |
| 99 | 970 | XP_026579409.1 | 132.23 | 15 | 0 | 7 | 8 | 0 | 0 | 7 | 15 | 7 | 0 | 0 | 0 | 7 | 0 | 7 | 0 | 0 |  | 9.87E6 | 3.7739E8 |  |  | 2.0917E7 | 4.4269E5 | 3.983E6 |  |  |  | 0 |  | 6.0577E5 |  |  | 3 | 3 | 0 | 3 | 9 | 0 | 0 | 2 | 3 | 1 | 0 | 0 | 0 | 1 | 0 | 2 | 0 | 0 | Y | 22372 | BPTI/Kunitz domain-containing protein-like isoform X2 [Pseudonaja textilis] |
| 169 | 774 | P00600.1 | 130.57 | 18 | 0 | 0 | 0 | 0 | 0 | 0 | 8 | 0 | 0 | 18 | 0 | 0 | 0 | 0 | 0 | 0 |  |  |  |  |  |  |  |  |  | 1.9309E6 |  |  |  |  |  |  | 4 | 1 | 0 | 0 | 0 | 0 | 0 | 0 | 3 | 0 | 0 | 3 | 0 | 0 | 0 | 0 | 0 | 0 | Y | 13427 | RecName: Full=Acidic phospholipase A2 DE-II; Short=svPLA2; AltName: Full=Phosphatidylcholine 2-acylhydrolase |
| 82 | 89 | ETE67131.1 | 129.51 | 3 | 0 | 1 | 0 | 0 | 0 | 0 | 1 | 1 | 0 | 0 | 0 | 1 | 2 | 0 | 0 | 0 |  |  |  |  |  |  |  |  |  |  |  |  | 0 |  |  |  | 6 | 1 | 1 | 2 | 0 | 0 | 0 | 1 | 1 | 5 | 0 | 1 | 1 | 2 | 6 | 0 | 2 | 1 | N | 240642 | Keratin, type II cytoskeletal 1, partial [Ophiophagus hannah] |
| 158 | 1249 | Q8JFX7.1 | 128.50 | 14 | 0 | 0 | 0 | 14 | 14 | 12 | 0 | 0 | 0 | 0 | 0 | 0 | 0 | 0 | 0 | 0 |  |  |  | 0 | 2.945E6 |  |  |  |  |  |  |  |  |  |  |  | 4 | 2 | 0 | 0 | 0 | 2 | 4 | 2 | 0 | 0 | 0 | 0 | 0 | 0 | 0 | 0 | 0 | 0 | Y | 11390 | RecName: Full=Muscarinic toxin BM14; Flags: Precursor |
| 158 | 1250 | CAD18848.1 | 128.50 | 14 | 0 | 0 | 0 | 14 | 14 | 12 | 0 | 0 | 0 | 0 | 0 | 0 | 0 | 0 | 0 | 0 |  |  |  | 0 | 2.945E6 |  |  |  |  |  |  |  |  |  |  |  | 4 | 2 | 0 | 0 | 0 | 2 | 4 | 2 | 0 | 0 | 0 | 0 | 0 | 0 | 0 | 0 | 0 | 0 | Y | 11390 | muscarinic toxin-like protein [Bungarus multicinctus] |
| 122 | 2197 | P59069.1 | 120.87 | 57 | 0 | 0 | 0 | 0 | 0 | 0 | 26 | 26 | 0 | 26 | 26 | 57 | 26 | 26 | 0 | 0 |  |  |  |  |  |  |  |  |  |  |  | 0 |  |  |  |  | 3 | 1 | 0 | 0 | 0 | 0 | 0 | 0 | 1 | 3 | 0 | 1 | 1 | 1 | 1 | 1 | 0 | 0 | Y | 4992 | RecName: Full=Phospholipase A2 superbin c; Short=svPLA2; AltName: Full=Phosphatidylcholine 2-acylhydrolase |
| 171 | 2463 | CAB53358.1 | 120.48 | 14 | 0 | 0 | 0 | 12 | 12 | 14 | 0 | 0 | 0 | 0 | 0 | 0 | 0 | 0 | 0 | 0 |  |  |  |  |  | 3.8075E6 |  |  |  |  |  |  |  |  |  |  | 3 | 1 | 0 | 0 | 0 | 1 | 3 | 3 | 0 | 0 | 0 | 0 | 0 | 0 | 0 | 0 | 0 | 0 | Y | 11334 | cardiotoxin-like protein [Bungarus multicinctus multicinctus] |
| 171 | 2464 | Q9PW19.1 | 120.48 | 14 | 0 | 0 | 0 | 12 | 12 | 14 | 0 | 0 | 0 | 0 | 0 | 0 | 0 | 0 | 0 | 0 |  |  |  |  |  | 3.8075E6 |  |  |  |  |  |  |  |  |  |  | 3 | 1 | 0 | 0 | 0 | 1 | 3 | 3 | 0 | 0 | 0 | 0 | 0 | 0 | 0 | 0 | 0 | 0 | Y | 11334 | RecName: Full=Toxin BMLCL; AltName: Full=BM8; Flags: Precursor |
| 95 | 390 | XP_025030548.1 | 120.34 | 8 | 1 | 4 | 0 | 0 | 0 | 1 | 2 | 1 | 0 | 1 | 0 | 2 | 3 | 3 | 1 | 2 |  |  |  |  |  |  |  |  |  |  |  |  |  | 0 |  |  | 5 | 1 | 1 | 2 | 0 | 0 | 0 | 1 | 1 | 3 | 0 | 1 | 0 | 2 | 2 | 1 | 2 | 1 | N | 64622 | keratin, type II cytoskeletal 5-like [Python bivittatus] |
| 145 | 1010 | BAC77656.1 | 120.32 | 18 | 0 | 0 | 0 | 0 | 0 | 0 | 18 | 0 | 8 | 0 | 0 | 0 | 0 | 0 | 0 | 0 |  |  |  |  |  |  | 7.1389E6 |  |  |  |  |  |  |  |  |  | 3 | 1 | 0 | 0 | 0 | 0 | 0 | 0 | 7 | 0 | 1 | 0 | 0 | 0 | 0 | 0 | 0 | 0 | Y | 15875 | phospholipase A2II precursor [Bungarus flaviceps] |
| 145 | 1011 | Q7T2Q4.1 | 120.32 | 18 | 0 | 0 | 0 | 0 | 0 | 0 | 18 | 0 | 8 | 0 | 0 | 0 | 0 | 0 | 0 | 0 |  |  |  |  |  |  | 7.1389E6 |  |  |  |  |  |  |  |  |  | 3 | 1 | 0 | 0 | 0 | 0 | 0 | 0 | 7 | 0 | 1 | 0 | 0 | 0 | 0 | 0 | 0 | 0 | Y | 15875 | RecName: Full=Acidic phospholipase A2 2; Short=svPLA2; AltName: Full=PA2-II; AltName: Full=Phosphatidylcholine 2-acylhydrolase; Flags: Precursor |
| 145 | 1024 | ADF50035.1 | 120.32 | 18 | 0 | 0 | 0 | 0 | 0 | 0 | 18 | 0 | 8 | 0 | 0 | 0 | 0 | 0 | 0 | 0 |  |  |  |  |  |  | 7.1389E6 |  |  |  |  |  |  |  |  |  | 3 | 1 | 0 | 0 | 0 | 0 | 0 | 0 | 7 | 0 | 1 | 0 | 0 | 0 | 0 | 0 | 0 | 0 | Y | 15845 | phospholipase a2 isoform 1 [Bungarus flaviceps] |
| 202 | 1123 | P01388.1 | 119.34 | 27 | 0 | 0 | 25 | 0 | 0 | 18 | 0 | 0 | 0 | 0 | 0 | 0 | 0 | 0 | 0 | 0 |  |  | 2.007E6 |  |  |  |  |  |  |  |  |  |  |  |  |  | 2 | 1 | 0 | 0 | 2 | 0 | 0 | 1 | 0 | 0 | 0 | 0 | 0 | 0 | 0 | 0 | 0 | 0 | Y | 7772 | RecName: Full=Long neurotoxin 2; AltName: Full=Neurotoxin B; AltName: Full=Tx-NM4 |
| 180 | 823 | KAG8131486.1 | 118.55 | 31 | 0 | 31 | 19 | 0 | 0 | 0 | 0 | 0 | 0 | 0 | 0 | 0 | 0 | 0 | 0 | 0 |  | 8.5655E6 | 4.9528E5 |  |  |  |  |  |  |  |  |  |  |  |  |  | 3 | 3 | 0 | 5 | 1 | 0 | 0 | 0 | 0 | 0 | 0 | 0 | 0 | 0 | 0 | 0 | 0 | 0 | Y | 8923 | putative Kunitz-type serine protease inhibitor spermatin protein [Naja naja] |
| 162 | 1218 | BAC77655.1 | 117.56 | 20 | 0 | 0 | 0 | 0 | 0 | 0 | 20 | 10 | 0 | 10 | 0 | 0 | 0 | 0 | 0 | 0 |  |  |  |  |  |  | 3.6093E5 | 1.8578E6 |  | 2.5918E6 |  |  |  |  |  |  | 2 | 1 | 0 | 0 | 0 | 0 | 0 | 0 | 2 | 1 | 0 | 2 | 0 | 0 | 0 | 0 | 0 | 0 | Y | 16169 | phospholipase A2I precursor [Bungarus flaviceps] |
| 162 | 1219 | Q7T2Q5.1 | 117.56 | 20 | 0 | 0 | 0 | 0 | 0 | 0 | 20 | 10 | 0 | 10 | 0 | 0 | 0 | 0 | 0 | 0 |  |  |  |  |  |  | 3.6093E5 | 1.8578E6 |  | 2.5918E6 |  |  |  |  |  |  | 2 | 1 | 0 | 0 | 0 | 0 | 0 | 0 | 2 | 1 | 0 | 2 | 0 | 0 | 0 | 0 | 0 | 0 | Y | 16169 | RecName: Full=Acidic phospholipase A2 1; Short=svPLA2; AltName: Full=PA2-I; AltName: Full=Phosphatidylcholine 2-acylhydrolase; Flags: Precursor |
| 46 | 3214 | Q8AY42.2 | 117.12 | 17 | 0 | 17 | 17 | 17 | 17 | 17 | 17 | 17 | 0 | 17 | 0 | 17 | 0 | 17 | 17 | 0 |  | 9.4887E7 | 2.5801E9 | 1.0399E9 | 1.5114E7 | 1.4385E8 | 2.0429E6 | 3.6801E7 |  | 8.7639E6 |  | 9.9524E6 |  | 6.8982E6 | 1.398E6 |  | 1 | 1 | 0 | 3 | 52 | 5 | 2 | 2 | 1 | 3 | 0 | 3 | 0 | 1 | 0 | 1 | 1 | 0 | Y | 8919 | RecName: Full=Kunitz-type serine protease inhibitor B; AltName: Full=Kunitz inhibitor B; Flags: Precursor |
| 46 | 3215 | AAL30069.1 | 117.12 | 16 | 0 | 16 | 16 | 16 | 16 | 16 | 16 | 16 | 0 | 16 | 0 | 16 | 0 | 16 | 16 | 0 |  | 9.4887E7 | 2.5801E9 | 1.0399E9 | 1.5114E7 | 1.4385E8 | 2.0429E6 | 3.6801E7 |  | 8.7639E6 |  | 9.9524E6 |  | 6.8982E6 | 1.398E6 |  | 1 | 1 | 0 | 3 | 52 | 5 | 2 | 2 | 1 | 3 | 0 | 3 | 0 | 1 | 0 | 1 | 1 | 0 | Y | 9391 | Kunitz inhibitor b, partial [Bungarus candidus] |
| 46 | 4003 | Q8AY43.2 | 117.12 | 17 | 0 | 17 | 17 | 17 | 17 | 17 | 17 | 17 | 0 | 17 | 0 | 17 | 0 | 17 | 17 | 0 |  | 9.4887E7 | 2.5801E9 | 1.0399E9 | 1.5114E7 | 1.4385E8 | 2.0429E6 | 3.6801E7 |  | 8.7639E6 |  | 9.9524E6 |  | 6.8982E6 | 1.398E6 |  | 1 | 1 | 0 | 3 | 52 | 5 | 2 | 2 | 1 | 3 | 0 | 3 | 0 | 1 | 0 | 1 | 1 | 0 | Y | 9077 | RecName: Full=Kunitz-type serine protease inhibitor A; AltName: Full=Kunitz inhibitor A; Flags: Precursor |
| 46 | 4004 | AAL30068.1 | 117.12 | 16 | 0 | 16 | 16 | 16 | 16 | 16 | 16 | 16 | 0 | 16 | 0 | 16 | 0 | 16 | 16 | 0 |  | 9.4887E7 | 2.5801E9 | 1.0399E9 | 1.5114E7 | 1.4385E8 | 2.0429E6 | 3.6801E7 |  | 8.7639E6 |  | 9.9524E6 |  | 6.8982E6 | 1.398E6 |  | 1 | 1 | 0 | 3 | 52 | 5 | 2 | 2 | 1 | 3 | 0 | 3 | 0 | 1 | 0 | 1 | 1 | 0 | Y | 9606 | Kunitz inhibitor a, partial [Bungarus candidus] |
| 200 | 373 | P0DI84.1 | 114.89 | 5 | 0 | 0 | 0 | 0 | 0 | 0 | 0 | 0 | 0 | 0 | 0 | 0 | 0 | 0 | 0 | 5 |  |  |  |  |  |  |  |  |  |  |  |  |  |  |  | 2.0715E4 | 2 | 1 | 0 | 0 | 0 | 0 | 0 | 0 | 0 | 0 | 0 | 0 | 0 | 0 | 0 | 0 | 0 | 2 | Y | 54748 | RecName: Full=L-amino-acid oxidase; Short=LAO; Short=VAA-LAAO I |
| 200 | 374 | 3KVE | 114.89 | 5 | 0 | 0 | 0 | 0 | 0 | 0 | 0 | 0 | 0 | 0 | 0 | 0 | 0 | 0 | 0 | 5 |  |  |  |  |  |  |  |  |  |  |  |  |  |  |  | 2.0715E4 | 2 | 1 | 0 | 0 | 0 | 0 | 0 | 0 | 0 | 0 | 0 | 0 | 0 | 0 | 0 | 0 | 0 | 2 | Y | 54933 | Chain D, L-amino acid oxidase |
| 212 | 672 | CAB42053.1 | 114.63 | 46 | 0 | 0 | 0 | 0 | 0 | 0 | 0 | 46 | 0 | 0 | 0 | 0 | 0 | 0 | 0 | 0 |  |  |  |  |  |  |  | 3.3915E5 |  |  |  |  |  |  |  |  | 3 | 1 | 0 | 0 | 0 | 0 | 0 | 0 | 0 | 3 | 0 | 0 | 0 | 0 | 0 | 0 | 0 | 0 | Y | 9065 | cardiotoxin-31 [Naja atra] |
| 212 | 673 | AAB18383.1 | 114.63 | 46 | 0 | 0 | 0 | 0 | 0 | 0 | 0 | 46 | 0 | 0 | 0 | 0 | 0 | 0 | 0 | 0 |  |  |  |  |  |  |  | 3.3915E5 |  |  |  |  |  |  |  |  | 3 | 1 | 0 | 0 | 0 | 0 | 0 | 0 | 0 | 3 | 0 | 0 | 0 | 0 | 0 | 0 | 0 | 0 | Y | 9065 | cardiotoxin 3a [Naja atra] |
| 111 | 886 | JAA74725.1 | 114.04 | 27 | 0 | 0 | 0 | 0 | 0 | 0 | 17 | 8 | 0 | 8 | 8 | 18 | 18 | 8 | 0 | 0 |  |  |  |  |  |  |  |  |  |  |  | 2.4671E4 | 1.0132E5 |  |  |  | 4 | 1 | 0 | 0 | 0 | 0 | 0 | 0 | 2 | 3 | 0 | 1 | 1 | 1 | 2 | 1 | 0 | 0 | Y | 16896 | PLA-2-Den-2 [Denisonia devisi] |
| 58 | 2559 | AED89576.1 | 114.01 | 8 | 0 | 0 | 0 | 0 | 0 | 8 | 8 | 8 | 8 | 8 | 8 | 0 | 0 | 0 | 0 | 0 |  |  |  |  |  | 5.1797E5 | 6.2655E8 | 9.9245E7 | 2.0593E6 | 4.4931E7 | 3.4374E6 |  |  |  |  |  | 3 | 1 | 0 | 0 | 0 | 0 | 0 | 1 | 27 | 1 | 1 | 1 | 1 | 0 | 0 | 0 | 0 | 0 | N | 15957 | putative phospholipase A2 precursor [Micrurus altirostris] |
| 77 | 1056 | XP_058026748.1 | 113.58 | 7 | 2 | 7 | 4 | 0 | 2 | 2 | 2 | 5 | 2 | 2 | 5 | 2 | 5 | 2 | 2 | 2 |  | 1.0416E6 | 2.6124E5 |  |  |  |  |  |  |  |  |  |  |  |  |  | 3 | 1 | 3 | 6 | 3 | 0 | 1 | 1 | 1 | 3 | 1 | 3 | 2 | 2 | 3 | 1 | 1 | 1 | N | 53316 | keratin, type II cytoskeletal cochleal-like [Ahaetulla prasina] |
| 126 | 2875 | ADF50022.1 | 113.48 | 17 | 0 | 0 | 10 | 0 | 15 | 17 | 10 | 15 | 0 | 0 | 0 | 0 | 0 | 0 | 0 | 0 |  |  | 1.5108E6 |  | 4.2041E7 | 3.8613E7 | 0 | 1.1097E6 |  |  |  |  |  |  |  |  | 3 | 3 | 0 | 0 | 2 | 0 | 4 | 4 | 1 | 3 | 0 | 0 | 0 | 0 | 0 | 0 | 0 | 0 | Y | 9755 | non-conventional three finger toxin isoform 7 [Bungarus flaviceps] |
|  |  |  |  |  |  |  |  |  |  |  |  |  |  |  |  |  |  |  |  |  |  |  |  |  |  |  |  |  |  |  |  |  |  |  |  |  |  |  |  |  |  |  |  |  |  |  |  |  |  |  |  |  |  |  |  |  | RecName: Full= |

| | | | | | | | | | | | | | | | | | | | | | | | | | | | | | | | | | | | | | | | | | | | | | | | | | | | | | | | | | |
|---|---|---|---|---|---|---|---|---|---|---|---|---|---|---|---|---|---|---|---|---|---|---|---|---|---|---|---|---|---|---|---|---|---|---|---|---|---|---|---|---|---|---|---|---|---|---|---|---|---|---|---|---|---|---|---|---|---|
| **126** | 2876 | Q6IZ95.1 | 113.48 | 17 | 0 | 0 | 10 | 0 | 15 | 17 | 10 | 15 | 0 | 0 | 0 | 0 | 0 | 0 | 0 | 0 | | | 1.5108E6 | | 4.2041E7 | 3.8613E7 | 0 | 1.1097E6 | | | | | | | | | 3 | 3 | 0 | 0 | 2 | 0 | 4 | 4 | 1 | 3 | 0 | 0 | 0 | 0 | 0 | 0 | 0 | 0 | Y | 9723 | Neurotoxin Ntx4; Flags: Precursor |
| **126** | 2877 | ADF50021.1 | 113.48 | 17 | 0 | 0 | 10 | 0 | 15 | 17 | 10 | 15 | 0 | 0 | 0 | 0 | 0 | 0 | 0 | 0 | | | 1.5108E6 | | 4.2041E7 | 3.8613E7 | 0 | 1.1097E6 | | | | | | | | | 3 | 3 | 0 | 0 | 2 | 0 | 4 | 4 | 1 | 3 | 0 | 0 | 0 | 0 | 0 | 0 | 0 | 0 | Y | 9740 | non-conventional three finger toxin isoform 6 [Bungarus flaviceps] |
| **126** | 2878 | AAT38875.1 | 113.48 | 17 | 0 | 0 | 10 | 0 | 15 | 17 | 10 | 15 | 0 | 0 | 0 | 0 | 0 | 0 | 0 | 0 | | | 1.5108E6 | | 4.2041E7 | 3.8613E7 | 0 | 1.1097E6 | | | | | | | | | 3 | 3 | 0 | 0 | 2 | 0 | 4 | 4 | 1 | 3 | 0 | 0 | 0 | 0 | 0 | 0 | 0 | 0 | Y | 9723 | Ntx4 [Bungarus candidus] |
| **126** | 2879 | A2CKF7.1 | 113.48 | 17 | 0 | 0 | 10 | 0 | 15 | 17 | 10 | 15 | 0 | 0 | 0 | 0 | 0 | 0 | 0 | 0 | | | 1.5108E6 | | 4.2041E7 | 3.8613E7 | 0 | 1.1097E6 | | | | | | | | | 3 | 3 | 0 | 0 | 2 | 0 | 4 | 4 | 1 | 3 | 0 | 0 | 0 | 0 | 0 | 0 | 0 | 0 | Y | 9723 | RecName: Full=Neurotoxin 3FTx-LT; Flags: Precursor |
| **126** | 2880 | ABI33871.1 | 113.48 | 17 | 0 | 0 | 10 | 0 | 15 | 17 | 10 | 15 | 0 | 0 | 0 | 0 | 0 | 0 | 0 | 0 | | | 1.5108E6 | | 4.2041E7 | 3.8613E7 | 0 | 1.1097E6 | | | | | | | | | 3 | 3 | 0 | 0 | 2 | 0 | 4 | 4 | 1 | 3 | 0 | 0 | 0 | 0 | 0 | 0 | 0 | 0 | Y | 9723 | three finger toxin [Bungarus fasciatus] |
| **126** | 2881 | ADF50020.1 | 113.48 | 17 | 0 | 0 | 10 | 0 | 15 | 17 | 10 | 15 | 0 | 0 | 0 | 0 | 0 | 0 | 0 | 0 | | | 1.5108E6 | | 4.2041E7 | 3.8613E7 | 0 | 1.1097E6 | | | | | | | | | 3 | 3 | 0 | 0 | 2 | 0 | 4 | 4 | 1 | 3 | 0 | 0 | 0 | 0 | 0 | 0 | 0 | 0 | Y | 9679 | non-conventional three finger toxin isoform 5 [Bungarus flaviceps] |
| **131** | 3224 | P20255.1 | 112.18 | 36 | 0 | 0 | 0 | 0 | 0 | 0 | 10 | 10 | 0 | 10 | 10 | 36 | 10 | 10 | 0 | 0 | | | | | | | | | | | | 1.126E5 | | | | | 3 | 1 | 0 | 0 | 0 | 0 | 0 | 0 | 1 | 3 | 0 | 1 | 1 | 1 | 1 | 1 | 0 | 0 | Y | 12969 | RecName: Full=Basic phospholipase A2 PA-12A; Short=svPLA2; AltName: Full=Phosphatidylcholine 2-acylhydrolase |
| **98** | 2081 | ADF50023.1 | 111.13 | 46 | 0 | 0 | 19 | 0 | 0 | 46 | 19 | 19 | 0 | 19 | 0 | 19 | 0 | 19 | 19 | 0 | | | | | | 1.2004E6 | | | | | | | | | | | 2 | 1 | 0 | 0 | 1 | 0 | 0 | 8 | 2 | 4 | 0 | 1 | 0 | 1 | 0 | 1 | 1 | 0 | Y | 6899 | kappa-toxin, partial [Bungarus flaviceps] |
| **98** | 2082 | P15815.1 | 111.13 | 44 | 0 | 0 | 18 | 0 | 0 | 44 | 18 | 18 | 0 | 18 | 0 | 18 | 0 | 18 | 18 | 0 | | | | | | 1.2004E6 | | | | | | | | | | | 2 | 1 | 0 | 0 | 1 | 0 | 0 | 8 | 2 | 4 | 0 | 1 | 0 | 1 | 0 | 1 | 1 | 0 | Y | 7259 | RecName: Full=Kappa-flavitoxin; Short=Kappa-ftx; Short=Kappa-fvt; AltName: Full=Long neurotoxin 2 |
| **177** | 1233 | P01383.1 | 108.49 | 62 | 0 | 0 | 0 | 0 | 0 | 14 | 0 | 45 | 0 | 48 | 0 | 0 | 0 | 0 | 0 | 0 | | | | | | 3.6091E6 | | 0 | | 1.5789E5 | | | | | | | 4 | 4 | 0 | 0 | 0 | 0 | 0 | 1 | 0 | 1 | 0 | 3 | 0 | 0 | 0 | 0 | 0 | 0 | Y | 8056 | RecName: Full=Long neurotoxin 1; AltName: Full=Neurotoxin 3.9.4 |
| **194** | 1016 | P00605.1 | 107.14 | 17 | 0 | 0 | 0 | 0 | 0 | 0 | 8 | 0 | 0 | 9 | 0 | 0 | 0 | 0 | 0 | 0 | | | | | | | | | | 0 | | | | | | | 3 | 1 | 0 | 0 | 0 | 0 | 0 | 0 | 3 | 0 | 0 | 1 | 0 | 0 | 0 | 0 | 0 | 0 | Y | 13268 | RecName: Full=Phospholipase A2 'basic'; Short=svPLA2; AltName: Full=Phosphatidylcholine 2-acylhydrolase; AltName: Full=Phospholipase A2 isozyme III/IV; Short=CM-III/CM-IV |
| **194** | 1017 | PSNJ3B | 107.14 | 17 | 0 | 0 | 0 | 0 | 0 | 0 | 8 | 0 | 0 | 9 | 0 | 0 | 0 | 0 | 0 | 0 | | | | | | | | | | 0 | | | | | | | 3 | 1 | 0 | 0 | 0 | 0 | 0 | 0 | 3 | 0 | 0 | 1 | 0 | 0 | 0 | 0 | 0 | 0 | Y | 13268 | phospholipase A2 (EC 3.1.1.4) III - cobra (Naja mossambica pallida) |
| **104** | 2649 | UNW14928.1 | 106.85 | 18 | 0 | 0 | 0 | 0 | 0 | 0 | 8 | 8 | 0 | 18 | 8 | 18 | 8 | 8 | 0 | 0 | | | | | | | | | | 4.3389E7 | | 1.3553E8 | | | | | 3 | 1 | 0 | 0 | 0 | 0 | 0 | 0 | 1 | 3 | 0 | 3 | 1 | 6 | 1 | 1 | 0 | 0 | Y | 15587 | phospholipase A2 protein 2-3, partial [Pseudonaja affinis] |
| **128** | 1568 | QSI84030.1 | 106.73 | 16 | 0 | 0 | 0 | 0 | 0 | 0 | 8 | 8 | 0 | 8 | 8 | 0 | 8 | 16 | 0 | 0 | | | | | | | | | | | | | | 4.582E7 | | | 3 | 1 | 0 | 0 | 0 | 0 | 0 | 0 | 1 | 3 | 0 | 1 | 1 | 0 | 1 | 2 | 0 | 0 | Y | 16765 | phospholipase A2 IB [Calliophis bivirgatus] |
| **128** | 1611 | QSI84025.1 | 106.73 | 16 | 0 | 0 | 0 | 0 | 0 | 0 | 8 | 8 | 0 | 8 | 8 | 0 | 8 | 16 | 0 | 0 | | | | | | | | | | | | | | 4.582E7 | | | 3 | 1 | 0 | 0 | 0 | 0 | 0 | 0 | 1 | 3 | 0 | 1 | 1 | 0 | 1 | 2 | 0 | 0 | Y | 16885 | phospholipase A2 IB [Calliophis bivirgatus] |
| **128** | 1632 | QSI84033.1 | 106.73 | 16 | 0 | 0 | 0 | 0 | 0 | 0 | 8 | 8 | 0 | 8 | 8 | 0 | 8 | 16 | 0 | 0 | | | | | | | | | | | | | | 4.582E7 | | | 3 | 1 | 0 | 0 | 0 | 0 | 0 | 0 | 1 | 3 | 0 | 1 | 1 | 0 | 1 | 2 | 0 | 0 | Y | 16790 | phospholipase A2 IB [Calliophis bivirgatus] |
| **128** | 1640 | QSI84029.1 | 106.73 | 16 | 0 | 0 | 0 | 0 | 0 | 0 | 8 | 8 | 0 | 8 | 8 | 0 | 8 | 16 | 0 | 0 | | | | | | | | | | | | | | 4.582E7 | | | 3 | 1 | 0 | 0 | 0 | 0 | 0 | 0 | 1 | 3 | 0 | 1 | 1 | 0 | 1 | 2 | 0 | 0 | Y | 16884 | phospholipase A2 IB [Calliophis bivirgatus] |
| **128** | 1642 | QSI84028.1 | 106.73 | 16 | 0 | 0 | 0 | 0 | 0 | 0 | 8 | 8 | 0 | 8 | 8 | 0 | 8 | 16 | 0 | 0 | | | | | | | | | | | | | | 4.582E7 | | | 3 | 1 | 0 | 0 | 0 | 0 | 0 | 0 | 1 | 3 | 0 | 1 | 1 | 0 | 1 | 2 | 0 | 0 | Y | 16871 | phospholipase A2 IB [Calliophis bivirgatus] |
| **128** | 1890 | QSI84035.1 | 106.73 | 16 | 0 | 0 | 0 | 0 | 0 | 0 | 8 | 8 | 0 | 8 | 8 | 0 | 8 | 16 | 0 | 0 | | | | | | | | | | | | | | 4.582E7 | | | 3 | 1 | 0 | 0 | 0 | 0 | 0 | 0 | 1 | 3 | 0 | 1 | 1 | 0 | 1 | 2 | 0 | 0 | Y | 16745 | phospholipase A2 IB [Calliophis bivirgatus] |
| **128** | 2506 | QSI84034.1 | 106.73 | 16 | 0 | 0 | 0 | 0 | 0 | 0 | 8 | 8 | 0 | 8 | 8 | 0 | 8 | 16 | 0 | 0 | | | | | | | | | | | | | | 4.582E7 | | | 3 | 1 | 0 | 0 | 0 | 0 | 0 | 0 | 1 | 3 | 0 | 1 | 1 | 0 | 1 | 2 | 0 | 0 | Y | 16767 | phospholipase A2 IB [Calliophis bivirgatus] |
| **144** | 2221 | LAB37309.1 | 106.24 | 16 | 0 | 0 | 0 | 0 | 0 | 0 | 10 | 10 | 0 | 10 | 10 | 0 | 10 | 16 | 0 | 0 | | | | | | | | | | | | | | 0 | | | 3 | 1 | 0 | 0 | 0 | 0 | 0 | 0 | 1 | 3 | 0 | 1 | 1 | 0 | 1 | 2 | 0 | 0 | Y | 12824 | hypothetical protein, partial [Micrurus spixii] |
| **141** | 285 | XP_034272439.1 | 106.19 | 3 | 1 | 0 | 0 | 0 | 0 | 0 | 2 | 2 | 0 | 0 | 0 | 0 | 0 | 0 | 0 | 0 | | | | | | | 4.9848E4 | 5.0465E5 | | | | | | | | | 4 | 2 | 1 | 0 | 0 | 0 | 0 | 0 | 2 | 2 | 0 | 0 | 0 | 0 | 0 | 0 | 0 | 0 | N | 112679 | keratin, type I cytoskeletal 9-like [Pantherophis guttatus] |
| **141** | 286 | XP_039209904.1 | 106.19 | 4 | 1 | 0 | 0 | 0 | 0 | 0 | 2 | 2 | 0 | 0 | 0 | 0 | 0 | 0 | 0 | 0 | | | | | | | 4.9848E4 | 5.0465E5 | | | | | | | | | 4 | 2 | 1 | 0 | 0 | 0 | 0 | 0 | 2 | 2 | 0 | 0 | 0 | 0 | 0 | 0 | 0 | 0 | N | 101157 | keratin, type I cytoskeletal 10-like [Crotalus tigris] |
| **105** | 2054 | UNW14927.1 | 105.45 | 17 | 0 | 0 | 0 | 0 | 0 | 0 | 8 | 8 | 0 | 8 | 17 | 17 | 8 | 8 | 0 | 0 | | | | | | | | | | | 1.0327E7 | 0 | | | | | 3 | 1 | 0 | 0 | 0 | 0 | 0 | 0 | 1 | 3 | 0 | 1 | 2 | 2 | 1 | 1 | 0 | 0 | Y | 16612 | phospholipase A2 protein 2-4, partial [Pseudonaja affinis] |
| **105** | 2329 | UNW14922.1 | 105.45 | 17 | 0 | 0 | 0 | 0 | 0 | 0 | 8 | 8 | 0 | 8 | 17 | 17 | 8 | 8 | 0 | 0 | | | | | | | | | | | 1.0327E7 | 0 | | | | | 3 | 1 | 0 | 0 | 0 | 0 | 0 | 0 | 1 | 3 | 0 | 1 | 2 | 2 | 1 | 1 | 0 | 0 | Y | 17011 | phospholipase A2 protein 2-3, partial [Pseudonaja textilis] |
| **105** | 3370 | AAD40976.1 | 105.45 | 17 | 0 | 0 | 0 | 0 | 0 | 0 | 8 | 8 | 0 | 8 | 17 | 17 | 8 | 8 | 0 | 0 | | | | | | | | | | | 1.0327E7 | 0 | | | | | 3 | 1 | 0 | 0 | 0 | 0 | 0 | 0 | 1 | 3 | 0 | 1 | 2 | 2 | 1 | 1 | 0 | 0 | Y | 17140 | phospholipase A2 precursor [Pseudonaja textilis] |
| **105** | 3371 | AAK15776.1 | 105.45 | 17 | 0 | 0 | 0 | 0 | 0 | 0 | 8 | 8 | 0 | 8 | 17 | 17 | 8 | 8 | 0 | 0 | | | | | | | | | | | 1.0327E7 | 0 | | | | | 3 | 1 | 0 | 0 | 0 | 0 | 0 | 0 | 1 | 3 | 0 | 1 | 2 | 2 | 1 | 1 | 0 | 0 | Y | 17140 | class 1B phospholipase A2 Pt-PLA2 [Pseudonaja textilis] |
| **105** | 3372 | Q9W7J3.1 | 105.45 | 17 | 0 | 0 | 0 | 0 | 0 | 0 | 8 | 8 | 0 | 8 | 17 | 17 | 8 | 8 | 0 | 0 | | | | | | | | | | | 1.0327E7 | 0 | | | | | 3 | 1 | 0 | 0 | 0 | 0 | 0 | 0 | 1 | 3 | 0 | 1 | 2 | 2 | 1 | 1 | 0 | 0 | Y | 17140 | RecName: Full=Acidic phospholipase A2 2; Short=svPLA2; AltName: Full=Phosphatidylcholine 2-acylhydrolase; AltName: Full=Pt-PLA2; Flags: Precursor |
| **147** | 1627 | JAA75026.1 | 104.41 | 10 | 0 | 0 | 0 | 0 | 0 | 0 | 8 | 8 | 0 | 8 | 8 | 0 | 8 | 10 | 0 | 0 | | | | | | | | | | | | | | 1.0361E5 | | | 3 | 1 | 0 | 0 | 0 | 0 | 0 | 0 | 1 | 3 | 0 | 1 | 1 | 0 | 1 | 2 | 0 | 0 | Y | 15912 | PLA2-Sut-16 [Suta fasciata] |
| **147** | 3378 | CAA68449.1 | 104.41 | 10 | 0 | 0 | 0 | 0 | 0 | 0 | 8 | 8 | 0 | 8 | 8 | 0 | 8 | 10 | 0 | 0 | | | | | | | | | | | | | | 1.0361E5 | | | 3 | 1 | 0 | 0 | 0 | 0 | 0 | 0 | 1 | 3 | 0 | 1 | 1 | 0 | 1 | 2 | 0 | 0 | Y | 16242 | unnamed protein product [Laticauda laticaudata] |
| **147** | 3379 | P19000.1 | 104.41 | 10 | 0 | 0 | 0 | 0 | 0 | 0 | 8 | 8 | 0 | 8 | 8 | 0 | 8 | 10 | 0 | 0 | | | | | | | | | | | | | | 1.0361E5 | | | 3 | 1 | 0 | 0 | 0 | 0 | 0 | 0 | 1 | 3 | 0 | 1 | 1 | 0 | 1 | 2 | 0 | 0 | Y | 16242 | RecName: Full=Basic phospholipase A2 P'513; Short=svPLA2; AltName: Full=Phosphatidylcholine 2-acylhydrolase; Flags: Precursor |
| **147** | 3380 | Q8UUI4.1 | 104.41 | 10 | 0 | 0 | 0 | 0 | 0 | 0 | 8 | 8 | 0 | 8 | 8 | 0 | 8 | 10 | 0 | 0 | | | | | | | | | | | | | | 1.0361E5 | | | 3 | 1 | 0 | 0 | 0 | 0 | 0 | 0 | 1 | 3 | 0 | 1 | 1 | 0 | 1 | 2 | 0 | 0 | Y | 16244 | RecName: Full=Basic phospholipase A2 PC10; Short=svPLA2; AltName: Full=Phosphatidylcholine 2-acylhydrolase; Flags: Precursor |
| **147** | 3381 | BAB72248.1 | 104.41 | 10 | 0 | 0 | 0 | 0 | 0 | 0 | 8 | 8 | 0 | 8 | 8 | 0 | 8 | 10 | 0 | 0 | | | | | | | | | | | | | | 1.0361E5 | | | 3 | 1 | 0 | 0 | 0 | 0 | 0 | 0 | 1 | 3 | 0 | 1 | 1 | 0 | 1 | 2 | 0 | 0 | Y | 16244 | phospholipase A2 [Laticauda laticaudata] |
| **150** | 3169 | Q7LZQ6.1 | 103.65 | 48 | 0 | 0 | 0 | 0 | 0 | 0 | 0 | 0 | 48 | 0 | 0 | 48 | 0 | 0 | 0 | 0 | | | | | | | | | 5.9813E6 | | | 0 | | | | | 2 | 2 | 0 | 0 | 0 | 0 | 0 | 0 | 0 | 0 | 6 | 0 | 0 | 1 | 0 | 0 | 0 | 0 | Y | 3704 | RecName: Full=Basic phospholipase A2 13; Short=svPLA2; AltName: Full=Cytotoxin XIII; AltName: Full=Phosphatidylcholine 2-acylhydrolase; AltName: Full=Phospholipase A2 XIII |
| **191** | 395 | P81383.3 | 102.58 | 6 | 0 | 0 | 0 | 0 | 0 | 0 | 0 | 0 | 0 | 0 | 0 | 0 | 0 | 0 | 0 | 6 | | | | | | | | | | | | | | | | 1.1105E6 | 3 | 3 | 0 | 0 | 0 | 0 | 0 | 0 | 0 | 0 | 0 | 0 | 0 | 0 | 0 | 0 | 0 | 4 | N | 55977 | RecName: Full=L-amino-acid oxidase; Short=LAO; Short=Oh-LAAO; Flags: Precursor |
| **191** | 396 | ABN72538.1 | 102.58 | 6 | 0 | 0 | 0 | 0 | 0 | 0 | 0 | 0 | 0 | 0 | 0 | 0 | 0 | 0 | 0 | 6 | | | | | | | | | | | | | | | | 1.1105E6 | 3 | 3 | 0 | 0 | 0 | 0 | 0 | 0 | 0 | 0 | 0 | 0 | 0 | 0 | 0 | 0 | 0 | 4 | N | 55977 | L-amino acid oxidase [Ophiophagus hannah] |
| **191** | 401 | ETE56914.1 | 102.58 | 6 | 0 | 0 | 0 | 0 | 0 | 0 | 0 | 0 | 0 | 0 | 0 | 0 | 0 | 0 | 0 | 6 | | | | | | | | | | | | | | | | 1.1105E6 | 3 | 3 | 0 | 0 | 0 | 0 | 0 | 0 | 0 | 0 | 0 | 0 | 0 | 0 | 0 | 0 | 0 | 4 | N | 55593 | hypothetical protein L345_17374, partial [Ophiophagus hannah] |
| **181** | 1196 | XP_013922516.1 | 101.55 | 15 | 0 | 0 | 0 | 0 | 0 | 0 | 6 | 0 | 0 | 9 | 6 | 0 | 0 | 0 | 0 | 0 | | | | | | | 3.2403E5 | | | 2.5195E4 | 4.3374E5 | | | | | | 2 | 2 | 0 | 0 | 0 | 0 | 0 | 0 | 1 | 0 | 0 | 1 | 1 | 0 | 0 | 0 | 0 | 0 | Y | 22563 | PREDICTED: vascular endothelial growth factor A isoform X3 [Thamnophis sirtalis] |
| **181** | 1208 | JAB52756.1 | 101.55 | 13 | 0 | 0 | 0 | 0 | 0 | 0 | 5 | 0 | 0 | 8 | 5 | 0 | 0 | 0 | 0 | 0 | | | | | | | 3.2403E5 | | | 2.5195E4 | 4.3374E5 | | | | | | 2 | 2 | 0 | 0 | 0 | 0 | 0 | 0 | 1 | 0 | 0 | 1 | 1 | 0 | 0 | 0 | 0 | 0 | Y | 24611 | vascular endothelial growth factor 2 [Micrurus fulvius] |

| | | | | | | | | | | | | | | | | | | | | | | | | | | | | | | | | | | | | | | | | | | | | | | | | | | | | | | | | | |
|---|---|---|---|---|---|---|---|---|---|---|---|---|---|---|---|---|---|---|---|---|---|---|---|---|---|---|---|---|---|---|---|---|---|---|---|---|---|---|---|---|---|---|---|---|---|---|---|---|---|---|---|---|---|---|---|---|---|
| **181** | 1227 | AXL96619.1 | 101.55 | 15 | 0 | 0 | 0 | 0 | 0 | 0 | 6 | 0 | 0 | 9 | 6 | 0 | 0 | 0 | 0 | 0 | | | | | | | 3.2403E5 | | | 2.5195E4 | 4.3374E5 | | | | | | 2 | 2 | 0 | 0 | 0 | 0 | 0 | 0 | 1 | 0 | 0 | 1 | 1 | 0 | 0 | 0 | 0 | 0 | Y | 22632 | vascular endothelial growth factor, partial [Ahaetulla prasina] |
| **181** | 1228 | XP_058018264.1 | 101.55 | 15 | 0 | 0 | 0 | 0 | 0 | 0 | 6 | 0 | 0 | 9 | 6 | 0 | 0 | 0 | 0 | 0 | | | | | | | 3.2403E5 | | | 2.5195E4 | 4.3374E5 | | | | | | 2 | 2 | 0 | 0 | 0 | 0 | 0 | 0 | 1 | 0 | 0 | 1 | 1 | 0 | 0 | 0 | 0 | 0 | Y | 22632 | vascular endothelial growth factor A, long form isoform X5 [Ahaetulla prasina] |
| **181** | 1229 | XP_058018253.1 | 101.55 | 14 | 0 | 0 | 0 | 0 | 0 | 0 | 5 | 0 | 0 | 8 | 5 | 0 | 0 | 0 | 0 | 0 | | | | | | | 3.2403E5 | | | 2.5195E4 | 4.3374E5 | | | | | | 2 | 2 | 0 | 0 | 0 | 0 | 0 | 0 | 1 | 0 | 0 | 1 | 1 | 0 | 0 | 0 | 0 | 0 | Y | 23887 | vascular endothelial growth factor A, long form isoform X4 [Ahaetulla prasina] |
| **181** | 1230 | XP_058018245.1 | 101.55 | 13 | 0 | 0 | 0 | 0 | 0 | 0 | 5 | 0 | 0 | 8 | 5 | 0 | 0 | 0 | 0 | 0 | | | | | | | 3.2403E5 | | | 2.5195E4 | 4.3374E5 | | | | | | 2 | 2 | 0 | 0 | 0 | 0 | 0 | 0 | 1 | 0 | 0 | 1 | 1 | 0 | 0 | 0 | 0 | 0 | Y | 25546 | vascular endothelial growth factor A, long form isoform X3 [Ahaetulla prasina] |
| **181** | 1231 | XP_058018236.1 | 101.55 | 13 | 0 | 0 | 0 | 0 | 0 | 0 | 5 | 0 | 0 | 8 | 5 | 0 | 0 | 0 | 0 | 0 | | | | | | | 3.2403E5 | | | 2.5195E4 | 4.3374E5 | | | | | | 2 | 2 | 0 | 0 | 0 | 0 | 0 | 0 | 1 | 0 | 0 | 1 | 1 | 0 | 0 | 0 | 0 | 0 | Y | 26022 | vascular endothelial growth factor A, long form isoform X2 [Ahaetulla prasina] |
| **181** | 1232 | XP_058018228.1 | 101.55 | 12 | 0 | 0 | 0 | 0 | 0 | 0 | 5 | 0 | 0 | 7 | 5 | 0 | 0 | 0 | 0 | 0 | | | | | | | 3.2403E5 | | | 2.5195E4 | 4.3374E5 | | | | | | 2 | 2 | 0 | 0 | 0 | 0 | 0 | 0 | 1 | 0 | 0 | 1 | 1 | 0 | 0 | 0 | 0 | 0 | Y | 26800 | vascular endothelial growth factor A, long form isoform X1 [Ahaetulla prasina] |
| **181** | 1237 | AWX67617.1 | 101.55 | 15 | 0 | 0 | 0 | 0 | 0 | 0 | 6 | 0 | 0 | 9 | 6 | 0 | 0 | 0 | 0 | 0 | | | | | | | 3.2403E5 | | | 2.5195E4 | 4.3374E5 | | | | | | 2 | 2 | 0 | 0 | 0 | 0 | 0 | 0 | 1 | 0 | 0 | 1 | 1 | 0 | 0 | 0 | 0 | 0 | Y | 22548 | vascular endothelial growth factor, partial [Boiga irregularis] |
| **181** | 1238 | JAS04556.1 | 101.55 | 15 | 0 | 0 | 0 | 0 | 0 | 0 | 6 | 0 | 0 | 9 | 6 | 0 | 0 | 0 | 0 | 0 | | | | | | | 3.2403E5 | | | 2.5195E4 | 4.3374E5 | | | | | | 2 | 2 | 0 | 0 | 0 | 0 | 0 | 0 | 1 | 0 | 0 | 1 | 1 | 0 | 0 | 0 | 0 | 0 | Y | 22548 | Vascular endothelial growth factor A [Boiga irregularis] |
| **181** | 1239 | XP_032072241.1 | 101.55 | 15 | 0 | 0 | 0 | 0 | 0 | 0 | 6 | 0 | 0 | 9 | 6 | 0 | 0 | 0 | 0 | 0 | | | | | | | 3.2403E5 | | | 2.5195E4 | 4.3374E5 | | | | | | 2 | 2 | 0 | 0 | 0 | 0 | 0 | 0 | 1 | 0 | 0 | 1 | 1 | 0 | 0 | 0 | 0 | 0 | Y | 22618 | vascular endothelial growth factor A isoform X2 [Thamnophis elegans] |
| **181** | 1240 | JAG68919.1 | 101.55 | 15 | 0 | 0 | 0 | 0 | 0 | 0 | 6 | 0 | 0 | 9 | 6 | 0 | 0 | 0 | 0 | 0 | | | | | | | 3.2403E5 | | | 2.5195E4 | 4.3374E5 | | | | | | 2 | 2 | 0 | 0 | 0 | 0 | 0 | 0 | 1 | 0 | 0 | 1 | 1 | 0 | 0 | 0 | 0 | 0 | Y | 22548 | Vascular endothelial growth factor A [Boiga irregularis] |
| **181** | 1241 | XP_013922515.1 | 101.55 | 13 | 0 | 0 | 0 | 0 | 0 | 0 | 5 | 0 | 0 | 8 | 5 | 0 | 0 | 0 | 0 | 0 | | | | | | | 3.2403E5 | | | 2.5195E4 | 4.3374E5 | | | | | | 2 | 2 | 0 | 0 | 0 | 0 | 0 | 0 | 1 | 0 | 0 | 1 | 1 | 0 | 0 | 0 | 0 | 0 | Y | 24727 | PREDICTED: vascular endothelial growth factor A isoform X2 [Thamnophis sirtalis] |
| **181** | 1242 | XP_032072240.1 | 101.55 | 13 | 0 | 0 | 0 | 0 | 0 | 0 | 5 | 0 | 0 | 8 | 5 | 0 | 0 | 0 | 0 | 0 | | | | | | | 3.2403E5 | | | 2.5195E4 | 4.3374E5 | | | | | | 2 | 2 | 0 | 0 | 0 | 0 | 0 | 0 | 1 | 0 | 0 | 1 | 1 | 0 | 0 | 0 | 0 | 0 | Y | 25560 | vascular endothelial growth factor A isoform X1 [Thamnophis elegans] |
| **181** | 1243 | XP_013922514.1 | 101.55 | 13 | 0 | 0 | 0 | 0 | 0 | 0 | 5 | 0 | 0 | 8 | 5 | 0 | 0 | 0 | 0 | 0 | | | | | | | 3.2403E5 | | | 2.5195E4 | 4.3374E5 | | | | | | 2 | 2 | 0 | 0 | 0 | 0 | 0 | 0 | 1 | 0 | 0 | 1 | 1 | 0 | 0 | 0 | 0 | 0 | Y | 25505 | PREDICTED: vascular endothelial growth factor A isoform X1 [Thamnophis sirtalis] |
| **181** | 1244 | AWX67680.1 | 101.55 | 13 | 0 | 0 | 0 | 0 | 0 | 0 | 5 | 0 | 0 | 8 | 5 | 0 | 0 | 0 | 0 | 0 | | | | | | | 3.2403E5 | | | 2.5195E4 | 4.3374E5 | | | | | | 2 | 2 | 0 | 0 | 0 | 0 | 0 | 0 | 1 | 0 | 0 | 1 | 1 | 0 | 0 | 0 | 0 | 0 | Y | 25462 | vascular endothelial growth factor, partial [Boiga irregularis] |
| **181** | 1245 | JAC94979.1 | 101.55 | 13 | 0 | 0 | 0 | 0 | 0 | 0 | 5 | 0 | 0 | 8 | 5 | 0 | 0 | 0 | 0 | 0 | | | | | | | 3.2403E5 | | | 2.5195E4 | 4.3374E5 | | | | | | 2 | 2 | 0 | 0 | 0 | 0 | 0 | 0 | 1 | 0 | 0 | 1 | 1 | 0 | 0 | 0 | 0 | 0 | Y | 25532 | Vascular endothelial growth factor A [Opheodrys aestivus] |
| **181** | 1309 | XP_034283975.1 | 101.55 | 15 | 0 | 0 | 0 | 0 | 0 | 0 | 6 | 0 | 0 | 9 | 6 | 0 | 0 | 0 | 0 | 0 | | | | | | | 3.2403E5 | | | 2.5195E4 | 4.3374E5 | | | | | | 2 | 2 | 0 | 0 | 0 | 0 | 0 | 0 | 1 | 0 | 0 | 1 | 1 | 0 | 0 | 0 | 0 | 0 | Y | 22648 | vascular endothelial growth factor A isoform X3 [Pantherophis guttatus] |
| **181** | 1310 | JAC95039.1 | 101.55 | 15 | 0 | 0 | 0 | 0 | 0 | 0 | 6 | 0 | 0 | 9 | 6 | 0 | 0 | 0 | 0 | 0 | | | | | | | 3.2403E5 | | | 2.5195E4 | 4.3374E5 | | | | | | 2 | 2 | 0 | 0 | 0 | 0 | 0 | 0 | 1 | 0 | 0 | 1 | 1 | 0 | 0 | 0 | 0 | 0 | Y | 22648 | Vascular endothelial growth factor A [Pantherophis guttatus] |
| **181** | 1311 | XP_034283974.1 | 101.55 | 13 | 0 | 0 | 0 | 0 | 0 | 0 | 5 | 0 | 0 | 8 | 5 | 0 | 0 | 0 | 0 | 0 | | | | | | | 3.2403E5 | | | 2.5195E4 | 4.3374E5 | | | | | | 2 | 2 | 0 | 0 | 0 | 0 | 0 | 0 | 1 | 0 | 0 | 1 | 1 | 0 | 0 | 0 | 0 | 0 | Y | 24784 | vascular endothelial growth factor A isoform X2 [Pantherophis guttatus] |
| **181** | 1312 | XP_034283973.1 | 101.55 | 13 | 0 | 0 | 0 | 0 | 0 | 0 | 5 | 0 | 0 | 8 | 5 | 0 | 0 | 0 | 0 | 0 | | | | | | | 3.2403E5 | | | 2.5195E4 | 4.3374E5 | | | | | | 2 | 2 | 0 | 0 | 0 | 0 | 0 | 0 | 1 | 0 | 0 | 1 | 1 | 0 | 0 | 0 | 0 | 0 | Y | 25562 | vascular endothelial growth factor A isoform X1 [Pantherophis guttatus] |
| **181** | 1338 | JAC96418.1 | 101.55 | 15 | 0 | 0 | 0 | 0 | 0 | 0 | 6 | 0 | 0 | 9 | 6 | 0 | 0 | 0 | 0 | 0 | | | | | | | 3.2403E5 | | | 2.5195E4 | 4.3374E5 | | | | | | 2 | 2 | 0 | 0 | 0 | 0 | 0 | 0 | 1 | 0 | 0 | 1 | 1 | 0 | 0 | 0 | 0 | 0 | Y | 22534 | Vascular endothelial growth factor [Hypsiglena sp. JMG-2014] |
| **181** | 1449 | JAB52755.1 | 101.55 | 15 | 0 | 0 | 0 | 0 | 0 | 0 | 6 | 0 | 0 | 9 | 6 | 0 | 0 | 0 | 0 | 0 | | | | | | | 3.2403E5 | | | 2.5195E4 | 4.3374E5 | | | | | | 2 | 2 | 0 | 0 | 0 | 0 | 0 | 0 | 1 | 0 | 0 | 1 | 1 | 0 | 0 | 0 | 0 | 0 | Y | 22516 | vascular endothelial growth factor 3 [Micrurus fulvius] |
| **181** | 1450 | JAI08989.1 | 101.55 | 15 | 0 | 0 | 0 | 0 | 0 | 0 | 6 | 0 | 0 | 9 | 6 | 0 | 0 | 0 | 0 | 0 | | | | | | | 3.2403E5 | | | 2.5195E4 | 4.3374E5 | | | | | | 2 | 2 | 0 | 0 | 0 | 0 | 0 | 0 | 1 | 0 | 0 | 1 | 1 | 0 | 0 | 0 | 0 | 0 | Y | 22516 | Vascular endothelial growth factor 1 [Micrurus fulvius] |
| **181** | 1451 | ETE70050.1 | 101.55 | 12 | 0 | 0 | 0 | 0 | 0 | 0 | 5 | 0 | 0 | 7 | 5 | 0 | 0 | 0 | 0 | 0 | | | | | | | 3.2403E5 | | | 2.5195E4 | 4.3374E5 | | | | | | 2 | 2 | 0 | 0 | 0 | 0 | 0 | 0 | 1 | 0 | 0 | 1 | 1 | 0 | 0 | 0 | 0 | 0 | Y | 27426 | hypothetical protein L345_04144, partial [Ophiophagus hannah] |
| **192** | 183 | XP_015681610.1 | 101.25 | 6 | 0 | 0 | 0 | 0 | 0 | 0 | 0 | 0 | 0 | 0 | 0 | 0 | 0 | 0 | 0 | 6 | | | | | | | | | | | | | | | | 4.389E5 | 3 | 3 | 0 | 0 | 0 | 0 | 0 | 0 | 0 | 0 | 0 | 0 | 0 | 0 | 0 | 0 | 0 | 3 | N | 72155 | endoplasmic reticulum chaperone BiP [Protobothrops mucrosquamatus] |
| **192** | 185 | XP_026568326.1 | 101.25 | 6 | 0 | 0 | 0 | 0 | 0 | 0 | 0 | 0 | 0 | 0 | 0 | 0 | 0 | 0 | 0 | 6 | | | | | | | | | | | | | | | | 4.389E5 | 3 | 3 | 0 | 0 | 0 | 0 | 0 | 0 | 0 | 0 | 0 | 0 | 0 | 0 | 0 | 0 | 0 | 3 | N | 72187 | endoplasmic reticulum chaperone BiP [Pseudonaja textilis] |
| **192** | 186 | XP_058015571.1 | 101.25 | 6 | 0 | 0 | 0 | 0 | 0 | 0 | 0 | 0 | 0 | 0 | 0 | 0 | 0 | 0 | 0 | 6 | | | | | | | | | | | | | | | | 4.389E5 | 3 | 3 | 0 | 0 | 0 | 0 | 0 | 0 | 0 | 0 | 0 | 0 | 0 | 0 | 0 | 0 | 0 | 3 | N | 72019 | endoplasmic reticulum chaperone BiP [Ahaetulla prasina] |
| **192** | 187 | JAC95528.1 | 101.25 | 6 | 0 | 0 | 0 | 0 | 0 | 0 | 0 | 0 | 0 | 0 | 0 | 0 | 0 | 0 | 0 | 6 | | | | | | | | | | | | | | | | 4.389E5 | 3 | 3 | 0 | 0 | 0 | 0 | 0 | 0 | 0 | 0 | 0 | 0 | 0 | 0 | 0 | 0 | 0 | 3 | N | 72299 | 78 kDa glucose-regulated protein-like [Hypsiglena sp. JMG-2014] |
| **192** | 188 | XP_025022112.1 | 101.25 | 6 | 0 | 0 | 0 | 0 | 0 | 0 | 0 | 0 | 0 | 0 | 0 | 0 | 0 | 0 | 0 | 6 | | | | | | | | | | | | | | | | 4.389E5 | 3 | 3 | 0 | 0 | 0 | 0 | 0 | 0 | 0 | 0 | 0 | 0 | 0 | 0 | 0 | 0 | 0 | 3 | N | 71864 | endoplasmic reticulum chaperone BiP [Python bivittatus] |
| **192** | 191 | JAB54174.1 | 101.25 | 6 | 0 | 0 | 0 | 0 | 0 | 0 | 0 | 0 | 0 | 0 | 0 | 0 | 0 | 0 | 0 | 6 | | | | | | | | | | | | | | | | 4.389E5 | 3 | 3 | 0 | 0 | 0 | 0 | 0 | 0 | 0 | 0 | 0 | 0 | 0 | 0 | 0 | 0 | 0 | 3 | N | 72106 | glucose-regulated protein [Micrurus fulvius] |
| **192** | 192 | JAG67806.1 | 101.25 | 6 | 0 | 0 | 0 | 0 | 0 | 0 | 0 | 0 | 0 | 0 | 0 | 0 | 0 | 0 | 0 | 6 | | | | | | | | | | | | | | | | 4.389E5 | 3 | 3 | 0 | 0 | 0 | 0 | 0 | 0 | 0 | 0 | 0 | 0 | 0 | 0 | 0 | 0 | 0 | 3 | N | 71941 | 78 kDa glucose-regulated protein [Boiga irregularis] |
| **192** | 193 | XP_026542482.1 | 101.25 | 6 | 0 | 0 | 0 | 0 | 0 | 0 | 0 | 0 | 0 | 0 | 0 | 0 | 0 | 0 | 0 | 6 | | | | | | | | | | | | | | | | 4.389E5 | 3 | 3 | 0 | 0 | 0 | 0 | 0 | 0 | 0 | 0 | 0 | 0 | 0 | 0 | 0 | 0 | 0 | 3 | N | 72286 | endoplasmic reticulum chaperone BiP [Notechis scutatus] |
| **192** | 194 | XP_034262421.1 | 101.25 | 7 | 0 | 0 | 0 | 0 | 0 | 0 | 0 | 0 | 0 | 0 | 0 | 0 | 0 | 0 | 0 | 7 | | | | | | | | | | | | | | | | 4.389E5 | 3 | 3 | 0 | 0 | 0 | 0 | 0 | 0 | 0 | 0 | 0 | 0 | 0 | 0 | 0 | 0 | 0 | 3 | N | 68299 | endoplasmic reticulum chaperone BiP, partial [Pantherophis guttatus] |
| **192** | 195 | XP_032088936.1 | 101.25 | 6 | 0 | 0 | 0 | 0 | 0 | 0 | 0 | 0 | 0 | 0 | 0 | 0 | 0 | 0 | 0 | 6 | | | | | | | | | | | | | | | | 4.389E5 | 3 | 3 | 0 | 0 | 0 | 0 | 0 | 0 | 0 | 0 | 0 | 0 | 0 | 0 | 0 | 0 | 0 | 3 | N | 72006 | endoplasmic reticulum chaperone BiP [Thamnophis elegans] |
| **192** | 196 | XP_013910585.1 | 101.25 | 6 | 0 | 0 | 0 | 0 | 0 | 0 | 0 | 0 | 0 | 0 | 0 | 0 | 0 | 0 | 0 | 6 | | | | | | | | | | | | | | | | 4.389E5 | 3 | 3 | 0 | 0 | 0 | 0 | 0 | 0 | 0 | 0 | 0 | 0 | 0 | 0 | 0 | 0 | 0 | 3 | N | 72006 | PREDICTED: 78 kDa glucose-regulated protein [Thamnophis sirtalis] |
| **192** | 197 | JAV50419.1 | 101.25 | 6 | 0 | 0 | 0 | 0 | 0 | 0 | 0 | 0 | 0 | 0 | 0 | 0 | 0 | 0 | 0 | 6 | | | | | | | | | | | | | | | | 4.389E5 | 3 | 3 | 0 | 0 | 0 | 0 | 0 | 0 | 0 | 0 | 0 | 0 | 0 | 0 | 0 | 0 | 0 | 3 | N | 72067 | 78 kDa glucose-regulated protein [Agkistrodon contortrix contortrix] |
| **192** | 198 | XP_039178976.1 | 101.25 | 6 | 0 | 0 | 0 | 0 | 0 | 0 | 0 | 0 | 0 | 0 | 0 | 0 | 0 | 0 | 0 | 6 | | | | | | | | | | | | | | | | 4.389E5 | 3 | 3 | 0 | 0 | 0 | 0 | 0 | 0 | 0 | 0 | 0 | 0 | 0 | 0 | 0 | 0 | 0 | 3 | N | 72105 | endoplasmic reticulum chaperone BiP [Crotalus tigris] |
| **192** | 199 | JAA96909.1 | 101.25 | 6 | 0 | 0 | 0 | 0 | 0 | 0 | 0 | 0 | 0 | 0 | 0 | 0 | 0 | 0 | 0 | 6 | | | | | | | | | | | | | | | | 4.389E5 | 3 | 3 | 0 | 0 | 0 | 0 | 0 | 0 | 0 | 0 | 0 | 0 | 0 | 0 | 0 | 0 | 0 | 3 | N | 72105 | 78 kDa glucose-regulated protein [Crotalus horridus] |
| **192** | 200 | JAG44540.1 | 101.25 | 6 | 0 | 0 | 0 | 0 | 0 | 0 | 0 | 0 | 0 | 0 | 0 | 0 | 0 | 0 | 0 | 6 | | | | | | | | | | | | | | | | 4.389E5 | 3 | 3 | 0 | 0 | 0 | 0 | 0 | 0 | 0 | 0 | 0 | 0 | 0 | 0 | 0 | 0 | 0 | 3 | N | 72105 | 78 kDa glucose-regulated protein precursor [Crotalus horridus] |
| **192** | 201 | JAI13107.1 | 101.25 | 6 | 0 | 0 | 0 | 0 | 0 | 0 | 0 | 0 | 0 | 0 | 0 | 0 | 0 | 0 | 0 | 6 | | | | | | | | | | | | | | | | 4.389E5 | 3 | 3 | 0 | 0 | 0 | 0 | 0 | 0 | 0 | 0 | 0 | 0 | 0 | 0 | 0 | 0 | 0 | 3 | N | 72105 | 78 kDa glucose-regulated protein [Crotalus adamanteus] |
| **192** | 202 | JAG46692.1 | 101.25 | 6 | 0 | 0 | 0 | 0 | 0 | 0 | 0 | 0 | 0 | 0 | 0 | 0 | 0 | 0 | 0 | 6 | | | | | | | | | | | | | | | | 4.389E5 | 3 | 3 | 0 | 0 | 0 | 0 | 0 | 0 | 0 | 0 | 0 | 0 | 0 | 0 | 0 | 0 | 0 | 3 | N | 72105 | 78 kDa glucose-regulated protein [Crotalus horridus] |
| **192** | 203 | AFJ50327.1 | 101.25 | 6 | 0 | 0 | 0 | 0 | 0 | 0 | 0 | 0 | 0 | 0 | 0 | 0 | 0 | 0 | 0 | 6 | | | | | | | | | | | | | | | | 4.389E5 | 3 | 3 | 0 | 0 | 0 | 0 | 0 | 0 | 0 | 0 | 0 | 0 | 0 | 0 | 0 | 0 | 0 | 3 | N | 72105 | Heat shock protein 5 [Crotalus adamanteus] |
| **192** | 204 | AFJ50239.1 | 101.25 | 6 | 0 | 0 | 0 | 0 | 0 | 0 | 0 | 0 | 0 | 0 | 0 | 0 | 0 | 0 | 0 | 6 | | | | | | | | | | | | | | | | 4.389E5 | 3 | 3 | 0 | 0 | 0 | 0 | 0 | 0 | 0 | 0 | 0 | 0 | 0 | 0 | 0 | 0 | 0 | 3 | N | 75480 | 78 kDa glucose-regulated protein precursor [Crotalus adamant |

| | | | | | | | | | | | | | | | | | | | | | | | | | | | | | | | | | | | | | | | | | | | | | | | | | | | | | | | | | |
|---|---|---|---|---|---|---|---|---|---|---|---|---|---|---|---|---|---|---|---|---|---|---|---|---|---|---|---|---|---|---|---|---|---|---|---|---|---|---|---|---|---|---|---|---|---|---|---|---|---|---|---|---|---|---|---|---|---|
| | | | | | | | | | | | | | | | | | | | | | | | | | | | | | | | | | | | | | | | | | | | | | | | | | | | | | | | | eus] |
| **192** | 279 | ETE59912.1 | 101.25 | 7 | 0 | 0 | 0 | 0 | 0 | 0 | 0 | 0 | 0 | 0 | 0 | 0 | 0 | 0 | 0 | 7 | | | | | | | | | | | | | | | | 4.389E5 | 3 | 3 | 0 | 0 | 0 | 0 | 0 | 0 | 0 | 0 | 0 | 0 | 0 | 0 | 0 | 0 | 0 | 3 | N | 67365 | 78 kDa glucose-regulated protein [Ophiophagus hannah] |
| **168** | 566 | QSI83986.1 | 100.63 | 18 | 0 | 0 | 0 | 0 | 0 | 0 | 0 | 0 | 0 | 0 | 3 | 0 | 18 | 3 | 0 | 0 | | | | | | | | | | | | | 2.3815E4 | | | | 3 | 1 | 0 | 0 | 0 | 0 | 0 | 0 | 0 | 0 | 0 | 0 | 1 | 0 | 5 | 1 | 0 | 0 | Y | 26492 | cysteine-rich secretory protein [Calliophis bivirgatus] |
| **94** | 1126 | XP_026539628.1 | 99.76 | 6 | 2 | 0 | 0 | 0 | 0 | 2 | 0 | 2 | 0 | 6 | 0 | 2 | 2 | 4 | 6 | 0 | | | | | | | | | | 1.3253E8 | | | | 1.1967E8 | 2.7284E8 | | 2 | 1 | 1 | 0 | 0 | 0 | 0 | 1 | 0 | 3 | 0 | 2 | 0 | 1 | 1 | 1 | 4 | 0 | N | 62299 | keratin, type II cytoskeletal 5-like [Notechis scutatus] |
| **188** | 296 | XP_026531043.1 | 98.70 | 4 | 0 | 0 | 0 | 0 | 0 | 0 | 0 | 0 | 0 | 0 | 0 | 0 | 0 | 0 | 0 | 4 | | | | | | | | | | | | | | | | 9.9004E5 | 2 | 2 | 0 | 0 | 0 | 0 | 0 | 0 | 0 | 0 | 0 | 0 | 0 | 0 | 0 | 0 | 0 | 3 | N | 69439 | ezrin [Notechis scutatus] |
| **188** | 297 | XP_026531042.1 | 98.70 | 4 | 0 | 0 | 0 | 0 | 0 | 0 | 0 | 0 | 0 | 0 | 0 | 0 | 0 | 0 | 0 | 4 | | | | | | | | | | | | | | | | 9.9004E5 | 2 | 2 | 0 | 0 | 0 | 0 | 0 | 0 | 0 | 0 | 0 | 0 | 0 | 0 | 0 | 0 | 0 | 3 | N | 69439 | ezrin [Notechis scutatus] |
| **188** | 298 | XP_026560859.1 | 98.70 | 4 | 0 | 0 | 0 | 0 | 0 | 0 | 0 | 0 | 0 | 0 | 0 | 0 | 0 | 0 | 0 | 4 | | | | | | | | | | | | | | | | 9.9004E5 | 2 | 2 | 0 | 0 | 0 | 0 | 0 | 0 | 0 | 0 | 0 | 0 | 0 | 0 | 0 | 0 | 0 | 3 | N | 69465 | ezrin isoform X1 [Pseudonaja textilis] |
| **188** | 299 | XP_026560860.1 | 98.70 | 4 | 0 | 0 | 0 | 0 | 0 | 0 | 0 | 0 | 0 | 0 | 0 | 0 | 0 | 0 | 0 | 4 | | | | | | | | | | | | | | | | 9.9004E5 | 2 | 2 | 0 | 0 | 0 | 0 | 0 | 0 | 0 | 0 | 0 | 0 | 0 | 0 | 0 | 0 | 0 | 3 | N | 69497 | ezrin isoform X2 [Pseudonaja textilis] |
| **188** | 300 | XP_026560858.1 | 98.70 | 4 | 0 | 0 | 0 | 0 | 0 | 0 | 0 | 0 | 0 | 0 | 0 | 0 | 0 | 0 | 0 | 4 | | | | | | | | | | | | | | | | 9.9004E5 | 2 | 2 | 0 | 0 | 0 | 0 | 0 | 0 | 0 | 0 | 0 | 0 | 0 | 0 | 0 | 0 | 0 | 3 | N | 69465 | ezrin isoform X1 [Pseudonaja textilis] |
| **188** | 311 | JAC95627.1 | 98.70 | 4 | 0 | 0 | 0 | 0 | 0 | 0 | 0 | 0 | 0 | 0 | 0 | 0 | 0 | 0 | 0 | 4 | | | | | | | | | | | | | | | | 9.9004E5 | 2 | 2 | 0 | 0 | 0 | 0 | 0 | 0 | 0 | 0 | 0 | 0 | 0 | 0 | 0 | 0 | 0 | 3 | N | 69440 | Ezrin-like protein [Hypsiglena sp. JMG-2014] |
| **187** | 814 | UNW14903.1 | 97.05 | 14 | 0 | 0 | 0 | 0 | 0 | 14 | 0 | 0 | 0 | 0 | 0 | 0 | 0 | 0 | 0 | 0 | | | | | | 3.7657E4 | | | | | | | | | | | 1 | 1 | 0 | 0 | 0 | 0 | 0 | 1 | 0 | 0 | 0 | 0 | 0 | 0 | 0 | 0 | 0 | 0 | Y | 11126 | three finger toxin Intx-3, partial [Pseudonaja aspidorhyncha] |
| **204** | 2263 | Q7ZT13.1 | 96.19 | 15 | 0 | 0 | 0 | 0 | 0 | 0 | 0 | 15 | 0 | 0 | 0 | 0 | 0 | 0 | 0 | 0 | | | | | | | | 3.2137E6 | | | | | | | | | 2 | 2 | 0 | 0 | 0 | 0 | 0 | 0 | 0 | 2 | 0 | 0 | 0 | 0 | 0 | 0 | 0 | 0 | Y | 9790 | RecName: Full=Neurotoxin-like protein pMD18-NTL1/2/4/5; Flags: Precursor |
| **204** | 2264 | AAL30058.1 | 96.19 | 15 | 0 | 0 | 0 | 0 | 0 | 0 | 0 | 15 | 0 | 0 | 0 | 0 | 0 | 0 | 0 | 0 | | | | | | | | 3.2137E6 | | | | | | | | | 2 | 2 | 0 | 0 | 0 | 0 | 0 | 0 | 0 | 2 | 0 | 0 | 0 | 0 | 0 | 0 | 0 | 0 | Y | 9759 | candiduxin 2 [Bungarus candidus] |
| **204** | 2265 | AAO47840.1 | 96.19 | 15 | 0 | 0 | 0 | 0 | 0 | 0 | 0 | 15 | 0 | 0 | 0 | 0 | 0 | 0 | 0 | 0 | | | | | | | | 3.2137E6 | | | | | | | | | 2 | 2 | 0 | 0 | 0 | 0 | 0 | 0 | 0 | 2 | 0 | 0 | 0 | 0 | 0 | 0 | 0 | 0 | Y | 9790 | neurotoxin-like protein [Bungarus multicinctus] |
| **204** | 2266 | AAO47842.1 | 96.19 | 15 | 0 | 0 | 0 | 0 | 0 | 0 | 0 | 15 | 0 | 0 | 0 | 0 | 0 | 0 | 0 | 0 | | | | | | | | 3.2137E6 | | | | | | | | | 2 | 2 | 0 | 0 | 0 | 0 | 0 | 0 | 0 | 2 | 0 | 0 | 0 | 0 | 0 | 0 | 0 | 0 | Y | 9790 | neurotoxin-like protein [Bungarus multicinctus] |
| **204** | 2267 | Q8AY53.1 | 96.19 | 15 | 0 | 0 | 0 | 0 | 0 | 0 | 0 | 15 | 0 | 0 | 0 | 0 | 0 | 0 | 0 | 0 | | | | | | | | 3.2137E6 | | | | | | | | | 2 | 2 | 0 | 0 | 0 | 0 | 0 | 0 | 0 | 2 | 0 | 0 | 0 | 0 | 0 | 0 | 0 | 0 | Y | 9760 | RecName: Full=Candiduxin-1; Flags: Precursor |
| **204** | 2268 | AAO47839.1 | 96.19 | 15 | 0 | 0 | 0 | 0 | 0 | 0 | 0 | 15 | 0 | 0 | 0 | 0 | 0 | 0 | 0 | 0 | | | | | | | | 3.2137E6 | | | | | | | | | 2 | 2 | 0 | 0 | 0 | 0 | 0 | 0 | 0 | 2 | 0 | 0 | 0 | 0 | 0 | 0 | 0 | 0 | Y | 9790 | neurotoxin-like protein [Bungarus multicinctus] |
| **204** | 2269 | CAA11213.1 | 96.19 | 15 | 0 | 0 | 0 | 0 | 0 | 0 | 0 | 15 | 0 | 0 | 0 | 0 | 0 | 0 | 0 | 0 | | | | | | | | 3.2137E6 | | | | | | | | | 2 | 2 | 0 | 0 | 0 | 0 | 0 | 0 | 0 | 2 | 0 | 0 | 0 | 0 | 0 | 0 | 0 | 0 | Y | 9776 | neurotoxin-like protein [Bungarus multicinctus multicinctus] |
| **204** | 2270 | Q8AY52.1 | 96.19 | 15 | 0 | 0 | 0 | 0 | 0 | 0 | 0 | 15 | 0 | 0 | 0 | 0 | 0 | 0 | 0 | 0 | | | | | | | | 3.2137E6 | | | | | | | | | 2 | 2 | 0 | 0 | 0 | 0 | 0 | 0 | 0 | 2 | 0 | 0 | 0 | 0 | 0 | 0 | 0 | 0 | Y | 9759 | RecName: Full=Candiduxin-2; Flags: Precursor |
| **204** | 2271 | Q9YGI0.1 | 96.19 | 15 | 0 | 0 | 0 | 0 | 0 | 0 | 0 | 15 | 0 | 0 | 0 | 0 | 0 | 0 | 0 | 0 | | | | | | | | 3.2137E6 | | | | | | | | | 2 | 2 | 0 | 0 | 0 | 0 | 0 | 0 | 0 | 2 | 0 | 0 | 0 | 0 | 0 | 0 | 0 | 0 | Y | 9776 | RecName: Full=Short neurotoxin homolog NTL1; AltName: Full=BM10-2; Flags: Precursor |
| **204** | 2272 | AAL30057.1 | 96.19 | 15 | 0 | 0 | 0 | 0 | 0 | 0 | 0 | 15 | 0 | 0 | 0 | 0 | 0 | 0 | 0 | 0 | | | | | | | | 3.2137E6 | | | | | | | | | 2 | 2 | 0 | 0 | 0 | 0 | 0 | 0 | 0 | 2 | 0 | 0 | 0 | 0 | 0 | 0 | 0 | 0 | Y | 9760 | candiduxin 1 [Bungarus candidus] |
| **204** | 2273 | AAO47843.1 | 96.19 | 15 | 0 | 0 | 0 | 0 | 0 | 0 | 0 | 15 | 0 | 0 | 0 | 0 | 0 | 0 | 0 | 0 | | | | | | | | 3.2137E6 | | | | | | | | | 2 | 2 | 0 | 0 | 0 | 0 | 0 | 0 | 0 | 2 | 0 | 0 | 0 | 0 | 0 | 0 | 0 | 0 | Y | 9790 | neurotoxin-like protein [Bungarus multicinctus] |
| **193** | 973 | E5L0E4.1 | 94.53 | 11 | 0 | 0 | 0 | 0 | 0 | 0 | 0 | 0 | 0 | 0 | 6 | 0 | 0 | 0 | 0 | 11 | | | | | | | | | | | 0 | | | | | 9.4866E5 | 2 | 2 | 0 | 0 | 0 | 0 | 0 | 0 | 0 | 0 | 0 | 0 | 1 | 0 | 0 | 0 | 0 | 3 | N | 28035 | RecName: Full=Beta-fibrinogenase-like; Short=RVBF; AltName: Full=Snake venom serine protease; Short=SVSP; Flags: Precursor |
| **193** | 974 | ADP88560.1 | 94.53 | 11 | 0 | 0 | 0 | 0 | 0 | 0 | 0 | 0 | 0 | 0 | 6 | 0 | 0 | 0 | 0 | 11 | | | | | | | | | | | 0 | | | | | 9.4866E5 | 2 | 2 | 0 | 0 | 0 | 0 | 0 | 0 | 0 | 0 | 0 | 0 | 1 | 0 | 0 | 0 | 0 | 3 | N | 28035 | serine beta-fibrinogenase-like protein precursor [Daboia siamensis] |
| **157** | 1652 | CAJ77819.1 | 93.05 | 39 | 0 | 0 | 0 | 0 | 0 | 39 | 0 | 0 | 0 | 15 | 0 | 0 | 0 | 0 | 0 | 0 | | | | | | 1.8135E6 | | | | 3.8172E5 | | | | | | | 2 | 1 | 0 | 0 | 0 | 0 | 0 | 3 | 0 | 0 | 0 | 1 | 0 | 0 | 0 | 0 | 0 | 0 | Y | 8306 | alpha-delta-bungarotoxin-1, partial [Bungarus candidus] |
| **157** | 1653 | A1IVR8.1 | 93.05 | 39 | 0 | 0 | 0 | 0 | 0 | 39 | 0 | 0 | 0 | 15 | 0 | 0 | 0 | 0 | 0 | 0 | | | | | | 1.8135E6 | | | | 3.8172E5 | | | | | | | 2 | 1 | 0 | 0 | 0 | 0 | 0 | 3 | 0 | 0 | 0 | 1 | 0 | 0 | 0 | 0 | 0 | 0 | Y | 8306 | RecName: Full=Alpha-elapitoxin-Bc2a; Short=Alpha-EPTX-Bc2a; AltName: Full=Alpha-delta-Bgt-1; AltName: Full=Alpha/delta-bungarotoxin-1; Short=Alpha/delta-BgTx-1; Flags: Precursor |
| **214** | 1319 | XP_026541740.1 | 89.79 | 6 | 0 | 0 | 0 | 0 | 0 | 0 | 6 | 0 | 0 | 0 | 0 | 0 | 0 | 0 | 0 | 0 | | | | | | | 3.3246E5 | | | | | | | | | | 2 | 2 | 0 | 0 | 0 | 0 | 0 | 0 | 2 | 0 | 0 | 0 | 0 | 0 | 0 | 0 | 0 | 0 | Y | 39954 | extracellular matrix protein 1 isoform X2 [Notechis scutatus] |
| **214** | 1320 | XP_026541739.1 | 89.79 | 6 | 0 | 0 | 0 | 0 | 0 | 0 | 6 | 0 | 0 | 0 | 0 | 0 | 0 | 0 | 0 | 0 | | | | | | | 3.3246E5 | | | | | | | | | | 2 | 2 | 0 | 0 | 0 | 0 | 0 | 0 | 2 | 0 | 0 | 0 | 0 | 0 | 0 | 0 | 0 | 0 | Y | 40082 | extracellular matrix protein 1 isoform X1 [Notechis scutatus] |
| **236** | 818 | KAG8142835.1 | 89.76 | 4 | 0 | 0 | 0 | 0 | 0 | 0 | 0 | 0 | 0 | 0 | 0 | 0 | 0 | 0 | 0 | 4 | | | | | | | | | | | | | | | | 3.5323E6 | 1 | 1 | 0 | 0 | 0 | 0 | 0 | 0 | 0 | 0 | 0 | 0 | 0 | 0 | 0 | 0 | 0 | 1 | N | 38182 | hypothetical protein E2320_006056, partial [Naja naja] |
| **236** | 819 | KAG8142834.1 | 89.76 | 3 | 0 | 0 | 0 | 0 | 0 | 0 | 0 | 0 | 0 | 0 | 0 | 0 | 0 | 0 | 0 | 3 | | | | | | | | | | | | | | | | 3.5323E6 | 1 | 1 | 0 | 0 | 0 | 0 | 0 | 0 | 0 | 0 | 0 | 0 | 0 | 0 | 0 | 0 | 0 | 1 | N | 53877 | hypothetical protein E2320_006056, partial [Naja naja] |
| **233** | 863 | XP_026522979.1 | 88.41 | 10 | 0 | 0 | 0 | 0 | 0 | 0 | 10 | 0 | 0 | 0 | 0 | 0 | 0 | 0 | 0 | 0 | | | | | | | 5.0971E5 | | | | | | | | | | 2 | 2 | 0 | 0 | 0 | 0 | 0 | 0 | 2 | 0 | 0 | 0 | 0 | 0 | 0 | 0 | 0 | 0 | Y | 47528 | vascular endothelial growth factor C [Notechis scutatus] |
| **233** | 864 | LAB17921.1 | 88.41 | 8 | 0 | 0 | 0 | 0 | 0 | 0 | 8 | 0 | 0 | 0 | 0 | 0 | 0 | 0 | 0 | 0 | | | | | | | 5.0971E5 | | | | | | | | | | 2 | 2 | 0 | 0 | 0 | 0 | 0 | 0 | 2 | 0 | 0 | 0 | 0 | 0 | 0 | 0 | 0 | 0 | Y | 53456 | hypothetical protein, partial [Micrurus spixii] |
| **233** | 866 | LAA17506.1 | 88.41 | 8 | 0 | 0 | 0 | 0 | 0 | 0 | 8 | 0 | 0 | 0 | 0 | 0 | 0 | 0 | 0 | 0 | | | | | | | 5.0971E5 | | | | | | | | | | 2 | 2 | 0 | 0 | 0 | 0 | 0 | 0 | 2 | 0 | 0 | 0 | 0 | 0 | 0 | 0 | 0 | 0 | Y | 53369 | hypothetical protein, partial [Micrurus lemniscatus carvalhoi] |
| **233** | 867 | ETE60014.1 | 88.41 | 10 | 0 | 0 | 0 | 0 | 0 | 0 | 10 | 0 | 0 | 0 | 0 | 0 | 0 | 0 | 0 | 0 | | | | | | | 5.0971E5 | | | | | | | | | | 2 | 2 | 0 | 0 | 0 | 0 | 0 | 0 | 2 | 0 | 0 | 0 | 0 | 0 | 0 | 0 | 0 | 0 | Y | 47557 | Vascular endothelial growth factor C [Ophiophagus hannah] |
| **233** | 868 | XP_026555459.1 | 88.41 | 10 | 0 | 0 | 0 | 0 | 0 | 0 | 10 | 0 | 0 | 0 | 0 | 0 | 0 | 0 | 0 | 0 | | | | | | | 5.0971E5 | | | | | | | | | | 2 | 2 | 0 | 0 | 0 | 0 | 0 | 0 | 2 | 0 | 0 | 0 | 0 | 0 | 0 | 0 | 0 | 0 | Y | 47615 | vascular endothelial growth factor C [Pseudonaja textilis] |
| **233** | 869 | LAB17920.1 | 88.41 | 14 | 0 | 0 | 0 | 0 | 0 | 0 | 14 | 0 | 0 | 0 | 0 | 0 | 0 | 0 | 0 | 0 | | | | | | | 5.0971E5 | | | | | | | | | | 2 | 2 | 0 | 0 | 0 | 0 | 0 | 0 | 2 | 0 | 0 | 0 | 0 | 0 | 0 | 0 | 0 | 0 | Y | 32584 | hypothetical protein [Micrurus spixii] |
| **240** | 1566 | KAG8131487.1 | 86.01 | 14 | 0 | 0 | 0 | 0 | 0 | 14 | 0 | 0 | 0 | 0 | 0 | 0 | 0 | 0 | 0 | 0 | | | | | | 0 | | | | | | | | | | | 1 | 1 | 0 | 0 | 0 | 0 | 0 | 1 | 0 | 0 | 0 | 0 | 0 | 0 | 0 | 0 | 0 | 0 | Y | 14770 | putative Kunitz-type serine protease inhibitor NACI protein [Naja naja] |
| **215** | 742 | ETE70041.1 | 85.64 | 2 | 0 | 0 | 0 | 0 | 0 | 0 | 0 | 0 | 0 | 0 | 0 | 0 | 0 | 0 | 0 | 2 | | | | | | | | | | | | | | | | 2.3744E4 | 1 | 1 | 0 | 0 | 0 | 0 | 0 | 0 | 0 | 0 | 0 | 0 | 0 | 0 | 0 | 0 | 0 | 1 | N | 73994 | Sulfhydryl oxidase 1 [Ophiophagus hannah] |
| **215** | 746 | XP_026559083.1 | 85.64 | 1 | 0 | 0 | 0 | 0 | 0 | 0 | 0 | 0 | 0 | 0 | 0 | 0 | 0 | 0 | 0 | 1 | | | | | | | | | | | | | | | | 2.3744E4 | 1 | 1 | 0 | 0 | 0 | 0 | 0 | 0 | 0 | 0 | 0 | 0 | 0 | 0 | 0 | 0 | 0 | 1 | N | 88597 | sulfhydryl oxidase 1 [Pseudonaja textilis] |
| **208** | 533 | JAG68843.1 | 82.22 | 12 | 12 | 0 | 0 | 0 | 0 | 0 | 0 | 0 | 0 | 0 | 0 | 0 | 0 | 0 | 0 | 0 | 1.4922E5 | | | | | | | | | | | | | | | | 2 | 2 | 3 | 0 | 0 | 0 | 0 | 0 | 0 | 0 | 0 | 0 | 0 | 0 | 0 | 0 | 0 | 0 | N | 41793 | Actin, cytoplasmic 2 [Boiga irregularis] |
| **208** | 534 | XP_013921350.1 | 82.22 | 12 | 12 | 0 | 0 | 0 | 0 | 0 | 0 | 0 | 0 | 0 | 0 | 0 | 0 | 0 | 0 | 0 | 1.4922E5 | | | | | | | | | | | | | | | | 2 | 2 | 3 | 0 | 0 | 0 | 0 | 0 | 0 | 0 | 0 | 0 | 0 | 0 | 0 | 0 | 0 | 0 | N | 41793 | PREDICTED: actin, cytoplasmic 2 [Thamnophis sirtalis] |
| **208** | 535 | JAV51372.1 | 82.22 | 12 | 12 | 0 | 0 | 0 | 0 | 0 | 0 | 0 | 0 | 0 | 0 | 0 | 0 | 0 | 0 | 0 | 1.4922E5 | | | | | | | | | | | | | | | | 2 | 2 | 3 | 0 | 0 | 0 | 0 | 0 | 0 | 0 | 0 | 0 | 0 | 0 | 0 | 0 | 0 | 0 | N | 41793 | Actin, cytoplasmic 2 [Agkistrodon contortrix contortrix] |
| **208** | 536 | JAB54395.1 | 82.22 | 12 | 12 | 0 | 0 | 0 | 0 | 0 | 0 | 0 | 0 | 0 | 0 | 0 | 0 | 0 | 0 | 0 | 1.4922E5 | | | | | | | | | | | | | | | | 2 | 2 | 3 | 0 | 0 | 0 | 0 | 0 | 0 | 0 | 0 | 0 | 0 | 0 | 0 | 0 | 0 | 0 | N | 41793 | cytoplasmic 2 [Micrurus fulvius] |
| **208** | 537 | JAC96363.1 | 82.22 | 12 | 12 | 0 | 0 | 0 | 0 | 0 | 0 | 0 | 0 | 0 | 0 | 0 | 0 | 0 | 0 | 0 | 1.4922E5 | | | | | | | | | | | | | | | | 2 | 2 | 3 | 0 | 0 | 0 | 0 | 0 | 0 | 0 | 0 | 0 | 0 | 0 | 0 | 0 | 0 | 0 | N | 41793 | Actin, cytoplasmic 2 [Hypsiglena sp. JMG-2014] |
| **208** | 538 | JAA97900.1 | 82.22 | 12 | 12 | 0 | 0 | 0 | 0 | 0 | 0 | 0 | 0 | 0 | 0 | 0 | 0 | 0 | 0 | 0 | 1.4922E5 | | | | | | | | | | | | | | | | 2 | 2 | 3 | 0 | 0 | 0 | 0 | 0 | 0 | 0 | 0 | 0 | 0 | 0 | 0 | 0 | 0 | 0 | N | 41793 | Actin, cytoplasmic 2 [Crotalus horridus] |
| **208** | 539 | LAA21099.1 | 82.22 | 12 | 12 | 0 | 0 | 0 | 0 | 0 | 0 | 0 | 0 | 0 | 0 | 0 | 0 | 0 | 0 | 0 | 1.4922E5 | | | | | | | | | | | | | | | | 2 | 2 | 3 | 0 | 0 | 0 | 0 | 0 | 0 | 0 | 0 | 0 | 0 | 0 | 0 | 0 | 0 | 0 | N | 41793 | hypothetical protein [Micrurus lemniscatus carvalhoi] |
| **208** | 540 | XP_026531551.1 | 82.22 | 12 | 12 | 0 | 0 | 0 | 0 | 0 | 0 | 0 | 0 | 0 | 0 | 0 | 0 | 0 | 0 | 0 | 1.4922E5 | | | | | | | | | | | | | | | | 2 | 2 | 3 | 0 | 0 | 0 | 0 | 0 | 0 | 0 | 0 | 0 | 0 | 0 | 0 | 0 | 0 | 0 | N | 41793 | actin, cytoplasmic 2 [Notechis scutatus] |
| **208** | 541 | LAA21098.1 | 82.22 | 12 | 12 | 0 | 0 | 0 | 0 | 0 | 0 | 0 | 0 | 0 | 0 | 0 | 0 | 0 | 0 | 0 | 1.4922E5 | | | | | | | | | | | | | | | | 2 | 2 | 3 | 0 | 0 | 0 | 0 | 0 | 0 | 0 | 0 | 0 | 0 | 0 | 0 | 0 | 0 | 0 | N | 41793 | hypothetical protein [Micrurus lemniscatus carvalhoi] |

| | | | | | | | | | | | | | | | | | | | | | | | | | | | | | | | | | | | | | | | | | | | | | | | | | | | | | | | | | |
|---|---|---|---|---|---|---|---|---|---|---|---|---|---|---|---|---|---|---|---|---|---|---|---|---|---|---|---|---|---|---|---|---|---|---|---|---|---|---|---|---|---|---|---|---|---|---|---|---|---|---|---|---|---|---|---|---|---|
| **208** | 542 | AFJ49303.1 | 82.22 | 12 | 12 | 0 | 0 | 0 | 0 | 0 | 0 | 0 | 0 | 0 | 0 | 0 | 0 | 0 | 0 | 0 | 1.4922E5 | | | | | | | | | | | | | | | | 2 | 2 | 3 | 0 | 0 | 0 | 0 | 0 | 0 | 0 | 0 | 0 | 0 | 0 | 0 | 0 | 0 | 0 | N | 41793 | Actin, cytoplasmic 2 [Crotalus adamanteus] |
| **208** | 543 | JAI10341.1 | 82.22 | 12 | 12 | 0 | 0 | 0 | 0 | 0 | 0 | 0 | 0 | 0 | 0 | 0 | 0 | 0 | 0 | 0 | 1.4922E5 | | | | | | | | | | | | | | | | 2 | 2 | 3 | 0 | 0 | 0 | 0 | 0 | 0 | 0 | 0 | 0 | 0 | 0 | 0 | 0 | 0 | 0 | N | 41793 | Actin, cytoplasmic 2 [Micrurus fulvius] |
| **208** | 544 | JAI14495.1 | 82.22 | 12 | 12 | 0 | 0 | 0 | 0 | 0 | 0 | 0 | 0 | 0 | 0 | 0 | 0 | 0 | 0 | 0 | 1.4922E5 | | | | | | | | | | | | | | | | 2 | 2 | 3 | 0 | 0 | 0 | 0 | 0 | 0 | 0 | 0 | 0 | 0 | 0 | 0 | 0 | 0 | 0 | N | 41793 | Actin, cytoplasmic 2 [Crotalus adamanteus] |
| **208** | 545 | XP_007419915.2 | 82.22 | 12 | 12 | 0 | 0 | 0 | 0 | 0 | 0 | 0 | 0 | 0 | 0 | 0 | 0 | 0 | 0 | 0 | 1.4922E5 | | | | | | | | | | | | | | | | 2 | 2 | 3 | 0 | 0 | 0 | 0 | 0 | 0 | 0 | 0 | 0 | 0 | 0 | 0 | 0 | 0 | 0 | N | 41793 | actin, cytoplasmic 2 [Python bivittatus] |
| **208** | 546 | JAG47579.1 | 82.22 | 12 | 12 | 0 | 0 | 0 | 0 | 0 | 0 | 0 | 0 | 0 | 0 | 0 | 0 | 0 | 0 | 0 | 1.4922E5 | | | | | | | | | | | | | | | | 2 | 2 | 3 | 0 | 0 | 0 | 0 | 0 | 0 | 0 | 0 | 0 | 0 | 0 | 0 | 0 | 0 | 0 | N | 41793 | Actin, cytoplasmic 2 [Crotalus horridus] |
| **208** | 547 | JAG45996.1 | 82.22 | 12 | 12 | 0 | 0 | 0 | 0 | 0 | 0 | 0 | 0 | 0 | 0 | 0 | 0 | 0 | 0 | 0 | 1.4922E5 | | | | | | | | | | | | | | | | 2 | 2 | 3 | 0 | 0 | 0 | 0 | 0 | 0 | 0 | 0 | 0 | 0 | 0 | 0 | 0 | 0 | 0 | N | 41793 | Actin, cytoplasmic 2 [Crotalus horridus] |
| **208** | 548 | ETE74025.1 | 82.22 | 12 | 12 | 0 | 0 | 0 | 0 | 0 | 0 | 0 | 0 | 0 | 0 | 0 | 0 | 0 | 0 | 0 | 1.4922E5 | | | | | | | | | | | | | | | | 2 | 2 | 3 | 0 | 0 | 0 | 0 | 0 | 0 | 0 | 0 | 0 | 0 | 0 | 0 | 0 | 0 | 0 | N | 41793 | Actin, cytoplasmic 2 [Ophiophagus hannah] |
| **208** | 550 | LAA58652.1 | 82.22 | 12 | 12 | 0 | 0 | 0 | 0 | 0 | 0 | 0 | 0 | 0 | 0 | 0 | 0 | 0 | 0 | 0 | 1.4922E5 | | | | | | | | | | | | | | | | 2 | 2 | 3 | 0 | 0 | 0 | 0 | 0 | 0 | 0 | 0 | 0 | 0 | 0 | 0 | 0 | 0 | 0 | N | 41737 | hypothetical protein [Micrurus corallinus] |
| **208** | 551 | JAA97901.1 | 82.22 | 12 | 12 | 0 | 0 | 0 | 0 | 0 | 0 | 0 | 0 | 0 | 0 | 0 | 0 | 0 | 0 | 0 | 1.4922E5 | | | | | | | | | | | | | | | | 2 | 2 | 3 | 0 | 0 | 0 | 0 | 0 | 0 | 0 | 0 | 0 | 0 | 0 | 0 | 0 | 0 | 0 | N | 41737 | Actin, cytoplasmic 1 [Crotalus horridus] |
| **208** | 552 | JAG47580.1 | 82.22 | 12 | 12 | 0 | 0 | 0 | 0 | 0 | 0 | 0 | 0 | 0 | 0 | 0 | 0 | 0 | 0 | 0 | 1.4922E5 | | | | | | | | | | | | | | | | 2 | 2 | 3 | 0 | 0 | 0 | 0 | 0 | 0 | 0 | 0 | 0 | 0 | 0 | 0 | 0 | 0 | 0 | N | 41737 | Actin, cytoplasmic 1 [Crotalus horridus] |
| **208** | 553 | JAI14496.1 | 82.22 | 12 | 12 | 0 | 0 | 0 | 0 | 0 | 0 | 0 | 0 | 0 | 0 | 0 | 0 | 0 | 0 | 0 | 1.4922E5 | | | | | | | | | | | | | | | | 2 | 2 | 3 | 0 | 0 | 0 | 0 | 0 | 0 | 0 | 0 | 0 | 0 | 0 | 0 | 0 | 0 | 0 | N | 41737 | Actin, cytoplasmic 1 [Crotalus adamanteus] |
| **208** | 554 | XP_007436312.1 | 82.22 | 12 | 12 | 0 | 0 | 0 | 0 | 0 | 0 | 0 | 0 | 0 | 0 | 0 | 0 | 0 | 0 | 0 | 1.4922E5 | | | | | | | | | | | | | | | | 2 | 2 | 3 | 0 | 0 | 0 | 0 | 0 | 0 | 0 | 0 | 0 | 0 | 0 | 0 | 0 | 0 | 0 | N | 41737 | actin-like protein 8 [Python bivittatus] |
| **208** | 555 | JAC96364.1 | 82.22 | 12 | 12 | 0 | 0 | 0 | 0 | 0 | 0 | 0 | 0 | 0 | 0 | 0 | 0 | 0 | 0 | 0 | 1.4922E5 | | | | | | | | | | | | | | | | 2 | 2 | 3 | 0 | 0 | 0 | 0 | 0 | 0 | 0 | 0 | 0 | 0 | 0 | 0 | 0 | 0 | 0 | N | 41737 | Actin, cytoplasmic 1 [Hypsiglena sp. JMG-2014] |
| **208** | 556 | BAQ22177.1 | 82.22 | 12 | 12 | 0 | 0 | 0 | 0 | 0 | 0 | 0 | 0 | 0 | 0 | 0 | 0 | 0 | 0 | 0 | 1.4922E5 | | | | | | | | | | | | | | | | 2 | 2 | 3 | 0 | 0 | 0 | 0 | 0 | 0 | 0 | 0 | 0 | 0 | 0 | 0 | 0 | 0 | 0 | N | 41737 | beta-actin [Protobothrops flavoviridis] |
| **208** | 557 | JAG45999.1 | 82.22 | 12 | 12 | 0 | 0 | 0 | 0 | 0 | 0 | 0 | 0 | 0 | 0 | 0 | 0 | 0 | 0 | 0 | 1.4922E5 | | | | | | | | | | | | | | | | 2 | 2 | 3 | 0 | 0 | 0 | 0 | 0 | 0 | 0 | 0 | 0 | 0 | 0 | 0 | 0 | 0 | 0 | N | 41737 | actin, cytoplasmic 1 [Crotalus horridus] |
| **208** | 558 | JAI10342.1 | 82.22 | 12 | 12 | 0 | 0 | 0 | 0 | 0 | 0 | 0 | 0 | 0 | 0 | 0 | 0 | 0 | 0 | 0 | 1.4922E5 | | | | | | | | | | | | | | | | 2 | 2 | 3 | 0 | 0 | 0 | 0 | 0 | 0 | 0 | 0 | 0 | 0 | 0 | 0 | 0 | 0 | 0 | N | 41737 | Actin, cytoplasmic 1 [Micrurus fulvius] |
| **208** | 559 | JAB54771.1 | 82.22 | 12 | 12 | 0 | 0 | 0 | 0 | 0 | 0 | 0 | 0 | 0 | 0 | 0 | 0 | 0 | 0 | 0 | 1.4922E5 | | | | | | | | | | | | | | | | 2 | 2 | 3 | 0 | 0 | 0 | 0 | 0 | 0 | 0 | 0 | 0 | 0 | 0 | 0 | 0 | 0 | 0 | N | 41737 | Actin [Micrurus fulvius] |
| **208** | 560 | JAG68844.1 | 82.22 | 12 | 12 | 0 | 0 | 0 | 0 | 0 | 0 | 0 | 0 | 0 | 0 | 0 | 0 | 0 | 0 | 0 | 1.4922E5 | | | | | | | | | | | | | | | | 2 | 2 | 3 | 0 | 0 | 0 | 0 | 0 | 0 | 0 | 0 | 0 | 0 | 0 | 0 | 0 | 0 | 0 | N | 41737 | Actin, cytoplasmic 1 [Boiga irregularis] |
| **208** | 561 | JAV51374.1 | 82.22 | 12 | 12 | 0 | 0 | 0 | 0 | 0 | 0 | 0 | 0 | 0 | 0 | 0 | 0 | 0 | 0 | 0 | 1.4922E5 | | | | | | | | | | | | | | | | 2 | 2 | 3 | 0 | 0 | 0 | 0 | 0 | 0 | 0 | 0 | 0 | 0 | 0 | 0 | 0 | 0 | 0 | N | 41737 | Actin, cytoplasmic 1 [Agkistrodon contortrix contortrix] |
| **208** | 562 | AFJ49302.1 | 82.22 | 12 | 12 | 0 | 0 | 0 | 0 | 0 | 0 | 0 | 0 | 0 | 0 | 0 | 0 | 0 | 0 | 0 | 1.4922E5 | | | | | | | | | | | | | | | | 2 | 2 | 3 | 0 | 0 | 0 | 0 | 0 | 0 | 0 | 0 | 0 | 0 | 0 | 0 | 0 | 0 | 0 | N | 41737 | Actin, cytoplasmic 1 [Crotalus adamanteus] |
| **208** | 564 | ETE57948.1 | 82.22 | 12 | 12 | 0 | 0 | 0 | 0 | 0 | 0 | 0 | 0 | 0 | 0 | 0 | 0 | 0 | 0 | 0 | 1.4922E5 | | | | | | | | | | | | | | | | 2 | 2 | 3 | 0 | 0 | 0 | 0 | 0 | 0 | 0 | 0 | 0 | 0 | 0 | 0 | 0 | 0 | 0 | N | 40263 | hypothetical protein L345_16333, partial [Ophiophagus hannah] |
| **154** | 3202 | ABW24180.1 | 79.62 | 13 | 0 | 0 | 0 | 0 | 0 | 0 | 0 | 0 | 0 | 0 | 0 | 8 | 13 | 0 | 13 | 0 | | | | | | | | | | | | 6.303E6 | 0 | | 4.7967E7 | | 2 | 2 | 0 | 0 | 0 | 0 | 0 | 0 | 0 | 0 | 0 | 0 | 0 | 1 | 1 | 0 | 2 | 0 | Y | 16353 | PLA-18 precursor [Austrelaps superbus] |
| **209** | 954 | QBF53421.1 | 77.88 | 2 | 0 | 0 | 0 | 0 | 0 | 0 | 0 | 0 | 0 | 0 | 0 | 0 | 0 | 0 | 0 | 2 | | | | | | | | | | | | | | | | 2.1472E5 | 1 | 1 | 0 | 0 | 0 | 0 | 0 | 0 | 0 | 0 | 0 | 0 | 0 | 0 | 0 | 0 | 0 | 1 | N | 64310 | phospholipase B [Vipera ammodytes ammodytes] |
| **209** | 1051 | XP_026550416.1 | 77.88 | 2 | 0 | 0 | 0 | 0 | 0 | 0 | 0 | 0 | 0 | 0 | 0 | 0 | 0 | 0 | 0 | 2 | | | | | | | | | | | | | | | | 2.1472E5 | 1 | 1 | 0 | 0 | 0 | 0 | 0 | 0 | 0 | 0 | 0 | 0 | 0 | 0 | 0 | 0 | 0 | 1 | N | 64214 | phospholipase B-like 1 [Notechis scutatus] |
| **209** | 1054 | ACR78473.1 | 77.88 | 2 | 0 | 0 | 0 | 0 | 0 | 0 | 0 | 0 | 0 | 0 | 0 | 0 | 0 | 0 | 0 | 2 | | | | | | | | | | | | | | | | 2.1472E5 | 1 | 1 | 0 | 0 | 0 | 0 | 0 | 0 | 0 | 0 | 0 | 0 | 0 | 0 | 0 | 0 | 0 | 1 | N | 64103 | putative phospholipase B 81b [Drysdalia coronoides] |
| **209** | 1055 | F8J2D3.1 | 77.88 | 2 | 0 | 0 | 0 | 0 | 0 | 0 | 0 | 0 | 0 | 0 | 0 | 0 | 0 | 0 | 0 | 2 | | | | | | | | | | | | | | | | 2.1472E5 | 1 | 1 | 0 | 0 | 0 | 0 | 0 | 0 | 0 | 0 | 0 | 0 | 0 | 0 | 0 | 0 | 0 | 1 | N | 64103 | RecName: Full=Phospholipase-B 81; Short=PLB; Flags: Precursor |
| **209** | 1057 | AWX67672.1 | 77.88 | 2 | 0 | 0 | 0 | 0 | 0 | 0 | 0 | 0 | 0 | 0 | 0 | 0 | 0 | 0 | 0 | 2 | | | | | | | | | | | | | | | | 2.1472E5 | 1 | 1 | 0 | 0 | 0 | 0 | 0 | 0 | 0 | 0 | 0 | 0 | 0 | 0 | 0 | 0 | 0 | 1 | N | 64164 | phospholipase B, partial [Boiga irregularis] |
| **209** | 1059 | JAG67025.1 | 77.88 | 2 | 0 | 0 | 0 | 0 | 0 | 0 | 0 | 0 | 0 | 0 | 0 | 0 | 0 | 0 | 0 | 2 | | | | | | | | | | | | | | | | 2.1472E5 | 1 | 1 | 0 | 0 | 0 | 0 | 0 | 0 | 0 | 0 | 0 | 0 | 0 | 0 | 0 | 0 | 0 | 1 | N | 64088 | Phospholipase B [Boiga irregularis] |
| **209** | 1086 | BAN82155.1 | 77.88 | 2 | 0 | 0 | 0 | 0 | 0 | 0 | 0 | 0 | 0 | 0 | 0 | 0 | 0 | 0 | 0 | 2 | | | | | | | | | | | | | | | | 2.1472E5 | 1 | 1 | 0 | 0 | 0 | 0 | 0 | 0 | 0 | 0 | 0 | 0 | 0 | 0 | 0 | 0 | 0 | 1 | N | 64133 | phospholipase b [Ovophis okinavensis] |
| **209** | 1087 | BAP39922.1 | 77.88 | 2 | 0 | 0 | 0 | 0 | 0 | 0 | 0 | 0 | 0 | 0 | 0 | 0 | 0 | 0 | 0 | 2 | | | | | | | | | | | | | | | | 2.1472E5 | 1 | 1 | 0 | 0 | 0 | 0 | 0 | 0 | 0 | 0 | 0 | 0 | 0 | 0 | 0 | 0 | 0 | 1 | N | 64115 | phospholipase B [Protobothrops elegans] |
| **209** | 1089 | BAN82026.1 | 77.88 | 2 | 0 | 0 | 0 | 0 | 0 | 0 | 0 | 0 | 0 | 0 | 0 | 0 | 0 | 0 | 0 | 2 | | | | | | | | | | | | | | | | 2.1472E5 | 1 | 1 | 0 | 0 | 0 | 0 | 0 | 0 | 0 | 0 | 0 | 0 | 0 | 0 | 0 | 0 | 0 | 1 | N | 64233 | phospholipase b [Protobothrops flavoviridis] |
| **209** | 1090 | BAP39973.1 | 77.88 | 2 | 0 | 0 | 0 | 0 | 0 | 0 | 0 | 0 | 0 | 0 | 0 | 0 | 0 | 0 | 0 | 2 | | | | | | | | | | | | | | | | 2.1472E5 | 1 | 1 | 0 | 0 | 0 | 0 | 0 | 0 | 0 | 0 | 0 | 0 | 0 | 0 | 0 | 0 | 0 | 1 | N | 64233 | phospholipase B [Protobothrops flavoviridis] |
| **209** | 1092 | JAI12031.1 | 77.88 | 2 | 0 | 0 | 0 | 0 | 0 | 0 | 0 | 0 | 0 | 0 | 0 | 0 | 0 | 0 | 0 | 2 | | | | | | | | | | | | | | | | 2.1472E5 | 1 | 1 | 0 | 0 | 0 | 0 | 0 | 0 | 0 | 0 | 0 | 0 | 0 | 0 | 0 | 0 | 0 | 1 | N | 64091 | Phospholipase B [Crotalus adamanteus] |
| **209** | 1093 | F8S101.1 | 77.88 | 2 | 0 | 0 | 0 | 0 | 0 | 0 | 0 | 0 | 0 | 0 | 0 | 0 | 0 | 0 | 0 | 2 | | | | | | | | | | | | | | | | 2.1472E5 | 1 | 1 | 0 | 0 | 0 | 0 | 0 | 0 | 0 | 0 | 0 | 0 | 0 | 0 | 0 | 0 | 0 | 1 | N | 64049 | RecName: Full=Phospholipase B; Short=PLB; Flags: Precursor |
| **209** | 1094 | AFJ50957.1 | 77.88 | 2 | 0 | 0 | 0 | 0 | 0 | 0 | 0 | 0 | 0 | 0 | 0 | 0 | 0 | 0 | 0 | 2 | | | | | | | | | | | | | | | | 2.1472E5 | 1 | 1 | 0 | 0 | 0 | 0 | 0 | 0 | 0 | 0 | 0 | 0 | 0 | 0 | 0 | 0 | 0 | 1 | N | 64091 | Phospholipase B [Crotalus adamanteus] |
| **209** | 1095 | AEJ31983.1 | 77.88 | 2 | 0 | 0 | 0 | 0 | 0 | 0 | 0 | 0 | 0 | 0 | 0 | 0 | 0 | 0 | 0 | 2 | | | | | | | | | | | | | | | | 2.1472E5 | 1 | 1 | 0 | 0 | 0 | 0 | 0 | 0 | 0 | 0 | 0 | 0 | 0 | 0 | 0 | 0 | 0 | 1 | N | 64049 | phospholipase B [Crotalus adamanteus] |
| **209** | 1096 | JAA96126.1 | 77.88 | 2 | 0 | 0 | 0 | 0 | 0 | 0 | 0 | 0 | 0 | 0 | 0 | 0 | 0 | 0 | 0 | 2 | | | | | | | | | | | | | | | | 2.1472E5 | 1 | 1 | 0 | 0 | 0 | 0 | 0 | 0 | 0 | 0 | 0 | 0 | 0 | 0 | 0 | 0 | 0 | 1 | N | 64180 | Phospholipase B [Crotalus horridus] |
| **209** | 1097 | JAC94989.1 | 77.88 | 2 | 0 | 0 | 0 | 0 | 0 | 0 | 0 | 0 | 0 | 0 | 0 | 0 | 0 | 0 | 0 | 2 | | | | | | | | | | | | | | | | 2.1472E5 | 1 | 1 | 0 | 0 | 0 | 0 | 0 | 0 | 0 | 0 | 0 | 0 | 0 | 0 | 0 | 0 | 0 | 1 | N | 63907 | Phospholipase B [Opheodrys aestivus] |
| **209** | 1098 | SMD28241.1 | 77.88 | 2 | 0 | 0 | 0 | 0 | 0 | 0 | 0 | 0 | 0 | 0 | 0 | 0 | 0 | 0 | 0 | 2 | | | | | | | | | | | | | | | | 2.1472E5 | 1 | 1 | 0 | 0 | 0 | 0 | 0 | 0 | 0 | 0 | 0 | 0 | 0 | 0 | 0 | 0 | 0 | 1 | N | 60434 | phospholipase B CdtPLB2 [Crotalus durissus terrificus] |
| **209** | 1099 | SMD28240.1 | 77.88 | 2 | 0 | 0 | 0 | 0 | 0 | 0 | 0 | 0 | 0 | 0 | 0 | 0 | 0 | 0 | 0 | 2 | | | | | | | | | | | | | | | | 2.1472E5 | 1 | 1 | 0 | 0 | 0 | 0 | 0 | 0 | 0 | 0 | 0 | 0 | 0 | 0 | 0 | 0 | 0 | 1 | N | 64104 | phospholipase B CdtPLB1 [Crotalus durissus terrificus] |
| **209** | 1100 | JAV49636.1 | 77.88 | 2 | 0 | 0 | 0 | 0 | 0 | 0 | 0 | 0 | 0 | 0 | 0 | 0 | 0 | 0 | 0 | 2 | | | | | | | | | | | | | | | | 2.1472E5 | 1 | 1 | 0 | 0 | 0 | 0 | 0 | 0 | 0 | 0 | 0 | 0 | 0 | 0 | 0 | 0 | 0 | 1 | N | 64129 | Phospholipase B [Agkistrodon contortrix contortrix] |
| **209** | 1101 | XP_039191573.1 | 77.88 | 2 | 0 | 0 | 0 | 0 | 0 | 0 | 0 | 0 | 0 | 0 | 0 | 0 | 0 | 0 | 0 | 2 | | | | | | | | | | | | | | | | 2.1472E5 | 1 | 1 | 0 | 0 | 0 | 0 | 0 | 0 | 0 | 0 | 0 | 0 | 0 | 0 | 0 | 0 | 0 | 1 | N | 64098 | phospholipase B-like 1 [Crotalus tigris] |
| **209** | 1102 | JAV48419.1 | 77.88 | 2 | 0 | 0 | 0 | 0 | 0 | 0 | 0 | 0 | 0 | 0 | 0 | 0 | 0 | 0 | 0 | 2 | | | | | | | | | | | | | | | | 2.1472E5 | 1 | 1 | 0 | 0 | 0 | 0 | 0 | 0 | 0 | 0 | 0 | 0 | 0 | 0 | 0 | 0 | 0 | 1 | N | 64110 | Cadam10_PLB-1 [Crotalus adamanteus] |
| **209** | 1110 | ETE59578.1 | 77.88 | 3 | 0 | 0 | 0 | 0 | 0 | 0 | 0 | 0 | 0 | 0 | 0 | 0 | 0 | 0 | 0 | 3 | | | | | | | | | | | | | | | | 2.1472E5 | 1 | 1 | 0 | 0 | 0 | 0 | 0 | 0 | 0 | 0 | 0 | 0 | 0 | 0 | 0 | 0 | 0 | 1 | N | 58279 | Phospholipase B-like 1, partial [Ophiophagus hannah] |
| **209** | 1111 | XP_015687635.1 | 77.88 | 2 | 0 | 0 | 0 | 0 | 0 | 0 | 0 | 0 | 0 | 0 | 0 | 0 | 0 | 0 | 0 | 2 | | | | | | | | | | | | | | | | 2.1472E5 | 1 | 1 | 0 | 0 | 0 | 0 | 0 | 0 | 0 | 0 | 0 | 0 | 0 | 0 | 0 | 0 | 0 | 1 | N | 63708 | phospholipase B-like 1 [Protobothrops mucrosquamatus] |
| **209** | 1132 | JAI09658.1 | 77.88 | 2 | 0 | 0 | 0 | 0 | 0 | 0 | 0 | 0 | 0 | 0 | 0 | 0 | 0 | 0 | 0 | 2 | | | | | | | | | | | | | | | | 2.1472E5 | 1 | 1 | 0 | 0 | 0 | 0 | 0 | 0 | 0 | 0 | 0 | 0 | 0 | 0 | 0 | 0 | 0 | 1 | N | 63500 | phospholipase B [Micrurus fulvius] |
| **209** | 1133 | JAB52759.1 | 77.88 | 2 | 0 | 0 | 0 | 0 | 0 | 0 | 0 | 0 | 0 | 0 | 0 | 0 | 0 | 0 | 0 | 2 | | | | | | | | | | | | | | | | 2.1472E5 | 1 | 1 | 0 | 0 | 0 | 0 | 0 | 0 | 0 | 0 | 0 | 0 | 0 | 0 | 0 | 0 | 0 | 1 | N | 63500 | phospholipase B 1 [Micrurus fulvius] |
| **238** | 829 | XP_039177250.1 | 77.43 | 7 | 0 | 0 | 0 | 0 | 0 | 0 | 0 | 0 | 0 | 0 | 0 | 7 | 0 | 0 | 0 | 0 | | | | | | | | | | | | 2.871E5 | | | | | 2 | 2 | 0 | 0 | 0 | 0 | 0 | 0 | 0 | 0 | 0 | 0 | 0 | 2 | 0 | 0 | 0 | 0 | N | 51399 | polyubiquitin-C [Crotalus tigris] |
| **238** | 830 | JAG66642.1 | 77.43 | 22 | 0 | 0 | 0 | 0 | 0 | 0 | 0 | 0 | 0 | 0 | 0 | 22 | 0 | 0 | 0 | 0 | | | | | | | | | | | | 2.871E5 | | | | | 2 | 2 | 0 | 0 | 0 | 0 | 0 | 0 | 0 | 0 | 0 | 0 | 0 | 2 | 0 | 0 | 0 | 0 | N | 17979 | ubiquitin-40S ribosomal protein S27a [Boiga irregularis] |
| **238** | 831 | JAB53407.1 | 77.43 | 22 | 0 | 0 | 0 | 0 | 0 | 0 | 0 | 0 | 0 | 0 | 0 | 22 | 0 | 0 | 0 | 0 | | | | | | | | | | | | 2.871E5 | | | | | 2 | 2 | 0 | 0 | 0 | 0 | 0 | 0 | 0 | 0 | 0 | 0 | 0 | 2 | 0 | 0 | 0 | 0 | N | 17979 | Ubiquitin-40S ribosomal protein S27a [Micrurus fulvius] |
| **238** | 832 | JAI11580.1 | 77.43 | 22 | 0 | 0 | 0 | 0 | 0 | 0 | 0 | 0 | 0 | 0 | 0 | 22 | 0 | 0 | 0 | 0 | | | | | | | | | | | | 2.871E5 | | | | | 2 | 2 | 0 | 0 | 0 | 0 | 0 | 0 | 0 | 0 | 0 | 0 | 0 | 2 | 0 | 0 | 0 | 0 | N | 17979 | ubiquitin-40S ribosomal protein S27a [Crotalus adamanteus] |
| **238** | 833 | LAA53283.1 | 77.43 | 22 | 0 | 0 | 0 | 0 | 0 | 0 | 0 | 0 | 0 | 0 | 0 | 22 | 0 | 0 | 0 | 0 | | | | | | | | | | | | 2.871E5 | | | | | 2 | 2 | 0 | 0 | 0 | 0 | 0 | 0 | 0 | 0 | 0 | 0 | 0 | 2 | 0 | 0 | 0 | 0 | N | 17979 | hypothetical protein [Micrurus corallinus] |
| **238** | 834 | AFJ51304.1 | 77.43 | 22 | 0 | 0 | 0 | 0 | 0 | 0 | 0 | 0 | 0 | 0 | 0 | 22 | 0 | 0 | 0 | 0 | | | | | | | | | | | | 2.871E5 | | | | | 2 | 2 | 0 | 0 | 0 | 0 | 0 | 0 | 0 | 0 | 0 | 0 | 0 | 2 | 0 | 0 | 0 | 0 | N | 17979 | Ubiquitin-40S ribosomal protein S27a-like [Crotalus adamanteus] |
| **238** | 835 | JAA95751.1 | 77.43 | 22 | 0 | 0 | 0 | 0 | 0 | 0 | 0 | 0 | 0 | 0 | 0 | 22 | 0 | 0 | 0 | 0 | | | | | | | | | | | | 2.871E5 | | | | | 2 | 2 | 0 | 0 | 0 | 0 | 0 | 0 | 0 | 0 | 0 | 0 | 0 | 2 | 0 | 0 | 0 | 0 | N | 17979 | ubiquitin-40S ribosomal protein S27a [Crotalus horridus] |

| | | | | | | | | | | | | | | | | | | | | | | | | | | | | | | | | | | | | | | | | | | | | | | | | | | | | | | | | | |
|---|---|---|---|---|---|---|---|---|---|---|---|---|---|---|---|---|---|---|---|---|---|---|---|---|---|---|---|---|---|---|---|---|---|---|---|---|---|---|---|---|---|---|---|---|---|---|---|---|---|---|---|---|---|---|---|---|---|
| **238** | 836 | JAI09470.1 | 77.43 | 22 | 0 | 0 | 0 | 0 | 0 | 0 | 0 | 0 | 0 | 0 | 0 | 22 | 0 | 0 | 0 | 0 | | | | | | | | | | | | 2.871E5 | | | | | 2 | 2 | 0 | 0 | 0 | 0 | 0 | 0 | 0 | 0 | 0 | 0 | 0 | 2 | 0 | 0 | 0 | 0 | N | 17979 | ubiquitin-40S ribosomal protein S27a [Micrurus fulvius] |
| **238** | 845 | JAI10738.1 | 77.43 | 35 | 0 | 0 | 0 | 0 | 0 | 0 | 0 | 0 | 0 | 0 | 0 | 35 | 0 | 0 | 0 | 0 | | | | | | | | | | | | 2.871E5 | | | | | 2 | 2 | 0 | 0 | 0 | 0 | 0 | 0 | 0 | 0 | 0 | 0 | 0 | 2 | 0 | 0 | 0 | 0 | N | 10986 | ubiquitin C [Crotalus adamanteus] |
| **238** | 846 | LAA24946.1 | 77.43 | 31 | 0 | 0 | 0 | 0 | 0 | 0 | 0 | 0 | 0 | 0 | 0 | 31 | 0 | 0 | 0 | 0 | | | | | | | | | | | | 2.871E5 | | | | | 2 | 2 | 0 | 0 | 0 | 0 | 0 | 0 | 0 | 0 | 0 | 0 | 0 | 2 | 0 | 0 | 0 | 0 | N | 12219 | hypothetical protein, partial [Micrurus lemniscatus carvalhoi] |
| **238** | 847 | XP_013929631.1 | 77.43 | 27 | 0 | 0 | 0 | 0 | 0 | 0 | 0 | 0 | 0 | 0 | 0 | 27 | 0 | 0 | 0 | 0 | | | | | | | | | | | | 2.871E5 | | | | | 2 | 2 | 0 | 0 | 0 | 0 | 0 | 0 | 0 | 0 | 0 | 0 | 0 | 2 | 0 | 0 | 0 | 0 | N | 14674 | PREDICTED: ubiquitin-60S ribosomal protein L40 [Thamnophis sirtalis] |
| **238** | 848 | JAI09497.1 | 77.43 | 27 | 0 | 0 | 0 | 0 | 0 | 0 | 0 | 0 | 0 | 0 | 0 | 27 | 0 | 0 | 0 | 0 | | | | | | | | | | | | 2.871E5 | | | | | 2 | 2 | 0 | 0 | 0 | 0 | 0 | 0 | 0 | 0 | 0 | 0 | 0 | 2 | 0 | 0 | 0 | 0 | N | 14728 | 60S ribosomal protein L40 [Micrurus fulvius] |
| **238** | 849 | JAB53439.1 | 77.43 | 27 | 0 | 0 | 0 | 0 | 0 | 0 | 0 | 0 | 0 | 0 | 0 | 27 | 0 | 0 | 0 | 0 | | | | | | | | | | | | 2.871E5 | | | | | 2 | 2 | 0 | 0 | 0 | 0 | 0 | 0 | 0 | 0 | 0 | 0 | 0 | 2 | 0 | 0 | 0 | 0 | N | 14728 | Ubiquitin-60S ribosomal protein L40 [Micrurus fulvius] |
| **238** | 850 | JAI11613.1 | 77.43 | 27 | 0 | 0 | 0 | 0 | 0 | 0 | 0 | 0 | 0 | 0 | 0 | 27 | 0 | 0 | 0 | 0 | | | | | | | | | | | | 2.871E5 | | | | | 2 | 2 | 0 | 0 | 0 | 0 | 0 | 0 | 0 | 0 | 0 | 0 | 0 | 2 | 0 | 0 | 0 | 0 | N | 14728 | 60S ribosomal protein L40 [Crotalus adamanteus] |
| **238** | 851 | XP_013929630.1 | 77.43 | 27 | 0 | 0 | 0 | 0 | 0 | 0 | 0 | 0 | 0 | 0 | 0 | 27 | 0 | 0 | 0 | 0 | | | | | | | | | | | | 2.871E5 | | | | | 2 | 2 | 0 | 0 | 0 | 0 | 0 | 0 | 0 | 0 | 0 | 0 | 0 | 2 | 0 | 0 | 0 | 0 | N | 14674 | PREDICTED: ubiquitin-60S ribosomal protein L40 [Thamnophis sirtalis] |
| **238** | 852 | AFJ51273.1 | 77.43 | 27 | 0 | 0 | 0 | 0 | 0 | 0 | 0 | 0 | 0 | 0 | 0 | 27 | 0 | 0 | 0 | 0 | | | | | | | | | | | | 2.871E5 | | | | | 2 | 2 | 0 | 0 | 0 | 0 | 0 | 0 | 0 | 0 | 0 | 0 | 0 | 2 | 0 | 0 | 0 | 0 | N | 14728 | Ubiquitin-60S ribosomal protein L40 [Crotalus adamanteus] |
| **238** | 853 | JAG66673.1 | 77.43 | 27 | 0 | 0 | 0 | 0 | 0 | 0 | 0 | 0 | 0 | 0 | 0 | 27 | 0 | 0 | 0 | 0 | | | | | | | | | | | | 2.871E5 | | | | | 2 | 2 | 0 | 0 | 0 | 0 | 0 | 0 | 0 | 0 | 0 | 0 | 0 | 2 | 0 | 0 | 0 | 0 | N | 14728 | 60S ribosomal protein L40 [Boiga irregularis] |
| **238** | 854 | JAA95781.1 | 77.43 | 27 | 0 | 0 | 0 | 0 | 0 | 0 | 0 | 0 | 0 | 0 | 0 | 27 | 0 | 0 | 0 | 0 | | | | | | | | | | | | 2.871E5 | | | | | 2 | 2 | 0 | 0 | 0 | 0 | 0 | 0 | 0 | 0 | 0 | 0 | 0 | 2 | 0 | 0 | 0 | 0 | N | 14728 | 60S ribosomal protein L40 [Crotalus horridus] |
| **238** | 855 | JAB53440.1 | 77.43 | 27 | 0 | 0 | 0 | 0 | 0 | 0 | 0 | 0 | 0 | 0 | 0 | 27 | 0 | 0 | 0 | 0 | | | | | | | | | | | | 2.871E5 | | | | | 2 | 2 | 0 | 0 | 0 | 0 | 0 | 0 | 0 | 0 | 0 | 0 | 0 | 2 | 0 | 0 | 0 | 0 | N | 14716 | ubiquitin-60S ribosomal protein L40 [Micrurus fulvius] |
| **238** | 856 | AAG17445.1 | 77.43 | 27 | 0 | 0 | 0 | 0 | 0 | 0 | 0 | 0 | 0 | 0 | 0 | 27 | 0 | 0 | 0 | 0 | | | | | | | | | | | | 2.871E5 | | | | | 2 | 2 | 0 | 0 | 0 | 0 | 0 | 0 | 0 | 0 | 0 | 0 | 0 | 2 | 0 | 0 | 0 | 0 | N | 14728 | ubiquitin fusion protein [Ophiophagus hannah] |
| **238** | 857 | XP_013928637.1 | 77.43 | 15 | 0 | 0 | 0 | 0 | 0 | 0 | 0 | 0 | 0 | 0 | 0 | 15 | 0 | 0 | 0 | 0 | | | | | | | | | | | | 2.871E5 | | | | | 2 | 2 | 0 | 0 | 0 | 0 | 0 | 0 | 0 | 0 | 0 | 0 | 0 | 2 | 0 | 0 | 0 | 0 | N | 25822 | PREDICTED: polyubiquitin-B [Thamnophis sirtalis] |
| **238** | 858 | AFJ51941.1 | 77.43 | 15 | 0 | 0 | 0 | 0 | 0 | 0 | 0 | 0 | 0 | 0 | 0 | 15 | 0 | 0 | 0 | 0 | | | | | | | | | | | | 2.871E5 | | | | | 2 | 2 | 0 | 0 | 0 | 0 | 0 | 0 | 0 | 0 | 0 | 0 | 0 | 2 | 0 | 0 | 0 | 0 | N | 25806 | putative ubiquitin B [Crotalus adamanteus] |
| **238** | 859 | JAB53023.1 | 77.43 | 9 | 0 | 0 | 0 | 0 | 0 | 0 | 0 | 0 | 0 | 0 | 0 | 9 | 0 | 0 | 0 | 0 | | | | | | | | | | | | 2.871E5 | | | | | 2 | 2 | 0 | 0 | 0 | 0 | 0 | 0 | 0 | 0 | 0 | 0 | 0 | 2 | 0 | 0 | 0 | 0 | N | 42865 | polyubiquitin-C isoform 2 [Micrurus fulvius] |
| **238** | 860 | XP_015684186.1 | 77.43 | 6 | 0 | 0 | 0 | 0 | 0 | 0 | 0 | 0 | 0 | 0 | 0 | 6 | 0 | 0 | 0 | 0 | | | | | | | | | | | | 2.871E5 | | | | | 2 | 2 | 0 | 0 | 0 | 0 | 0 | 0 | 0 | 0 | 0 | 0 | 0 | 2 | 0 | 0 | 0 | 0 | N | 68506 | polyubiquitin-C [Protobothrops mucrosquamatus] |
| **238** | 861 | XP_015666146.1 | 77.43 | 4 | 0 | 0 | 0 | 0 | 0 | 0 | 0 | 0 | 0 | 0 | 0 | 4 | 0 | 0 | 0 | 0 | | | | | | | | | | | | 2.871E5 | | | | | 2 | 2 | 0 | 0 | 0 | 0 | 0 | 0 | 0 | 0 | 0 | 0 | 0 | 2 | 0 | 0 | 0 | 0 | N | 85633 | polyubiquitin-C [Protobothrops mucrosquamatus] |
| **153** | 1046 | TIKFBU | 77.32 | 32 | 0 | 0 | 0 | 0 | 0 | 22 | 0 | 32 | 0 | 22 | 0 | 22 | 0 | 0 | 0 | 0 | | | | | | 5.284E6 | | 0 | | 6.3042E5 | | 2.1794E6 | | | | | 2 | 1 | 0 | 0 | 0 | 0 | 0 | 2 | 0 | 2 | 0 | 1 | 0 | 1 | 0 | 0 | 0 | 0 | Y | 7034 | beta-1 bungarotoxin chain B, major component - many-banded krait |
| **153** | 1047 | 0402253B | 77.32 | 32 | 0 | 0 | 0 | 0 | 0 | 22 | 0 | 32 | 0 | 22 | 0 | 22 | 0 | 0 | 0 | 0 | | | | | | 5.284E6 | | 0 | | 6.3042E5 | | 2.1794E6 | | | | | 2 | 1 | 0 | 0 | 0 | 0 | 0 | 2 | 0 | 2 | 0 | 1 | 0 | 1 | 0 | 0 | 0 | 0 | Y | 7034 | toxin RCM B,beta1 bungaro |
| **235** | 3265 | B4ESA4.1 | 77.03 | 22 | 0 | 0 | 22 | 0 | 0 | 0 | 0 | 0 | 0 | 0 | 0 | 0 | 0 | 0 | 0 | 0 | | | 4.0964E5 | | | | | | | | | | | | | | 1 | 1 | 0 | 0 | 1 | 0 | 0 | 0 | 0 | 0 | 0 | 0 | 0 | 0 | 0 | 0 | 0 | 0 | Y | 9227 | RecName: Full=Kunitz-type serine protease inhibitor PILP-3; AltName: Full=Protease inhibitor-like protein 3; Flags: Precursor |
| **235** | 3266 | CAP74383.1 | 77.03 | 22 | 0 | 0 | 22 | 0 | 0 | 0 | 0 | 0 | 0 | 0 | 0 | 0 | 0 | 0 | 0 | 0 | | | 4.0964E5 | | | | | | | | | | | | | | 1 | 1 | 0 | 0 | 1 | 0 | 0 | 0 | 0 | 0 | 0 | 0 | 0 | 0 | 0 | 0 | 0 | 0 | Y | 9227 | protease inhibitor-like protein 3 precursor [Bungarus multicinctus] |
| **197** | 1039 | Q75S49.1 | 76.59 | 23 | 0 | 0 | 0 | 0 | 0 | 0 | 0 | 23 | 0 | 0 | 0 | 0 | 0 | 0 | 0 | 0 | | | | | | | | 3.5322E6 | | | | | | | | | 2 | 1 | 0 | 0 | 0 | 0 | 0 | 0 | 0 | 2 | 0 | 0 | 0 | 0 | 0 | 0 | 0 | 0 | Y | 9399 | RecName: Full=Kunitz-type serine protease inhibitor homolog beta-bungarotoxin B4 chain; Flags: Precursor |
| **197** | 1040 | BAD06269.1 | 76.59 | 23 | 0 | 0 | 0 | 0 | 0 | 0 | 0 | 23 | 0 | 0 | 0 | 0 | 0 | 0 | 0 | 0 | | | | | | | | 3.5322E6 | | | | | | | | | 2 | 1 | 0 | 0 | 0 | 0 | 0 | 0 | 0 | 2 | 0 | 0 | 0 | 0 | 0 | 0 | 0 | 0 | Y | 9399 | beta-bungarotoxin B4 chain precursor, partial [Bungarus candidus] |
| **197** | 1041 | Q8AY46.2 | 76.59 | 22 | 0 | 0 | 0 | 0 | 0 | 0 | 0 | 22 | 0 | 0 | 0 | 0 | 0 | 0 | 0 | 0 | | | | | | | | 3.5322E6 | | | | | | | | | 2 | 1 | 0 | 0 | 0 | 0 | 0 | 0 | 0 | 2 | 0 | 0 | 0 | 0 | 0 | 0 | 0 | 0 | Y | 9518 | RecName: Full=Kunitz-type serine protease inhibitor homolog beta-bungarotoxin B1 chain; Flags: Precursor |
| **197** | 1042 | AAL30065.1 | 76.59 | 21 | 0 | 0 | 0 | 0 | 0 | 0 | 0 | 21 | 0 | 0 | 0 | 0 | 0 | 0 | 0 | 0 | | | | | | | | 3.5322E6 | | | | | | | | | 2 | 1 | 0 | 0 | 0 | 0 | 0 | 0 | 0 | 2 | 0 | 0 | 0 | 0 | 0 | 0 | 0 | 0 | Y | 10048 | beta bungaratoxin B1 chain, partial [Bungarus candidus] |
| **197** | 1048 | CAB62503.1 | 76.59 | 22 | 0 | 0 | 0 | 0 | 0 | 0 | 0 | 22 | 0 | 0 | 0 | 0 | 0 | 0 | 0 | 0 | | | | | | | | 3.5322E6 | | | | | | | | | 2 | 1 | 0 | 0 | 0 | 0 | 0 | 0 | 0 | 2 | 0 | 0 | 0 | 0 | 0 | 0 | 0 | 0 | Y | 9555 | beta-bungarotoxin B1 [Bungarus multicinctus] |
| **197** | 1049 | CAA72809.1 | 76.59 | 22 | 0 | 0 | 0 | 0 | 0 | 0 | 0 | 22 | 0 | 0 | 0 | 0 | 0 | 0 | 0 | 0 | | | | | | | | 3.5322E6 | | | | | | | | | 2 | 1 | 0 | 0 | 0 | 0 | 0 | 0 | 0 | 2 | 0 | 0 | 0 | 0 | 0 | 0 | 0 | 0 | Y | 9571 | bungarotoxin, B1 chain [Bungarus multicinctus] |
| **197** | 1050 | P00987.2 | 76.59 | 22 | 0 | 0 | 0 | 0 | 0 | 0 | 0 | 22 | 0 | 0 | 0 | 0 | 0 | 0 | 0 | 0 | | | | | | | | 3.5322E6 | | | | | | | | | 2 | 1 | 0 | 0 | 0 | 0 | 0 | 0 | 0 | 2 | 0 | 0 | 0 | 0 | 0 | 0 | 0 | 0 | Y | 9571 | RecName: Full=Kunitz-type serine protease inhibitor homolog beta-bungarotoxin B1 chain, major component; AltName: Full=Beta-1-bungarotoxin; Flags: Precursor |
| **227** | 515 | W8E7D1.1 | 76.50 | 1 | 0 | 0 | 0 | 0 | 0 | 0 | 0 | 0 | 0 | 0 | 0 | 0 | 0 | 0 | 0 | 1 | | | | | | | | | | | | | | | | 3.0155E5 | 1 | 1 | 0 | 0 | 0 | 0 | 0 | 0 | 0 | 0 | 0 | 0 | 0 | 0 | 0 | 0 | 0 | 1 | N | 96181 | RecName: Full=Venom phosphodiesterase; Short=PDE; Short=VLPDE; Flags: Precursor |
| **227** | 516 | AHJ80885.1 | 76.50 | 1 | 0 | 0 | 0 | 0 | 0 | 0 | 0 | 0 | 0 | 0 | 0 | 0 | 0 | 0 | 0 | 1 | | | | | | | | | | | | | | | | 3.0155E5 | 1 | 1 | 0 | 0 | 0 | 0 | 0 | 0 | 0 | 0 | 0 | 0 | 0 | 0 | 0 | 0 | 0 | 1 | N | 96181 | phosphodiesterase [Macrovipera lebetina] |
| **189** | 824 | JAS05169.1 | 75.02 | 2 | 0 | 0 | 0 | 0 | 0 | 0 | 0 | 0 | 0 | 0 | 0 | 0 | 0 | 0 | 0 | 2 | | | | | | | | | | | | | | | | 3.8116E6 | 1 | 1 | 0 | 0 | 0 | 0 | 0 | 0 | 0 | 0 | 0 | 0 | 0 | 0 | 0 | 0 | 0 | 1 | N | 52018 | Hyaluronidase [Micrurus tener] |
| **189** | 826 | JAB52840.1 | 75.02 | 2 | 0 | 0 | 0 | 0 | 0 | 0 | 0 | 0 | 0 | 0 | 0 | 0 | 0 | 0 | 0 | 2 | | | | | | | | | | | | | | | | 3.8116E6 | 1 | 1 | 0 | 0 | 0 | 0 | 0 | 0 | 0 | 0 | 0 | 0 | 0 | 0 | 0 | 0 | 0 | 1 | N | 52078 | Hyaluronidase 1 [Micrurus fulvius] |
| **189** | 827 | JAS05059.1 | 75.02 | 2 | 0 | 0 | 0 | 0 | 0 | 0 | 0 | 0 | 0 | 0 | 0 | 0 | 0 | 0 | 0 | 2 | | | | | | | | | | | | | | | | 3.8116E6 | 1 | 1 | 0 | 0 | 0 | 0 | 0 | 0 | 0 | 0 | 0 | 0 | 0 | 0 | 0 | 0 | 0 | 1 | N | 52078 | Hyaluronidase [Micrurus fulvius] |
| **189** | 828 | JAI09070.1 | 75.02 | 2 | 0 | 0 | 0 | 0 | 0 | 0 | 0 | 0 | 0 | 0 | 0 | 0 | 0 | 0 | 0 | 2 | | | | | | | | | | | | | | | | 3.8116E6 | 1 | 1 | 0 | 0 | 0 | 0 | 0 | 0 | 0 | 0 | 0 | 0 | 0 | 0 | 0 | 0 | 0 | 1 | N | 52078 | Hyaluronidase [Micrurus fulvius] |
| **189** | 1429 | LAA86767.1 | 75.02 | 8 | 0 | 0 | 0 | 0 | 0 | 0 | 0 | 0 | 0 | 0 | 0 | 0 | 0 | 0 | 0 | 8 | | | | | | | | | | | | | | | | 3.8116E6 | 1 | 1 | 0 | 0 | 0 | 0 | 0 | 0 | 0 | 0 | 0 | 0 | 0 | 0 | 0 | 0 | 0 | 1 | N | 14521 | hypothetical protein [Micrurus lemniscatus lemniscatus] |
| **189** | 1430 | LAA86741.1 | 75.02 | 8 | 0 | 0 | 0 | 0 | 0 | 0 | 0 | 0 | 0 | 0 | 0 | 0 | 0 | 0 | 0 | 8 | | | | | | | | | | | | | | | | 3.8116E6 | 1 | 1 | 0 | 0 | 0 | 0 | 0 | 0 | 0 | 0 | 0 | 0 | 0 | 0 | 0 | 0 | 0 | 1 | N | 14521 | hypothetical protein [Micrurus lemniscatus lemniscatus] |
| **189** | 1431 | LAA86748.1 | 75.02 | 8 | 0 | 0 | 0 | 0 | 0 | 0 | 0 | 0 | 0 | 0 | 0 | 0 | 0 | 0 | 0 | 8 | | | | | | | | | | | | | | | | 3.8116E6 | 1 | 1 | 0 | 0 | 0 | 0 | 0 | 0 | 0 | 0 | 0 | 0 | 0 | 0 | 0 | 0 | 0 | 1 | N | 14521 | hypothetical protein [Micrurus lemniscatus lemniscatus] |
| **189** | 1432 | LAA86751.1 | 75.02 | 8 | 0 | 0 | 0 | 0 | 0 | 0 | 0 | 0 | 0 | 0 | 0 | 0 | 0 | 0 | 0 | 8 | | | | | | | | | | | | | | | | 3.8116E6 | 1 | 1 | 0 | 0 | 0 | 0 | 0 | 0 | 0 | 0 | 0 | 0 | 0 | 0 | 0 | 0 | 0 | 1 | N | 14521 | hypothetical protein [Micrurus lemniscatus lemniscatus] |
| **189** | 1433 | LAA86759.1 | 75.02 | 8 | 0 | 0 | 0 | 0 | 0 | 0 | 0 | 0 | 0 | 0 | 0 | 0 | 0 | 0 | 0 | 8 | | | | | | | | | | | | | | | | 3.8116E6 | 1 | 1 | 0 | 0 | 0 | 0 | 0 | 0 | 0 | 0 | 0 | 0 | 0 | 0 | 0 | 0 | 0 | 1 | N | 14521 | hypothetical protein [Micrurus lemniscatus lemniscatus] |
| **189** | 1434 | LAA86757.1 | 75.02 | 8 | 0 | 0 | 0 | 0 | 0 | 0 | 0 | 0 | 0 | 0 | 0 | 0 | 0 | 0 | 0 | 8 | | | | | | | | | | | | | | | | 3.8116E6 | 1 | 1 | 0 | 0 | 0 | 0 | 0 | 0 | 0 | 0 | 0 | 0 | 0 | 0 | 0 | 0 | 0 | 1 | N | 14521 | hypothetical protein [Micrurus lemniscatus lemniscatus] |
| **189** | 1435 | LAA86750.1 | 75.02 | 8 | 0 | 0 | 0 | 0 | 0 | 0 | 0 | 0 | 0 | 0 | 0 | 0 | 0 | 0 | 0 | 8 | | | | | | | | | | | | | | | | 3.8116E6 | 1 | 1 | 0 | 0 | 0 | 0 | 0 | 0 | 0 | 0 | 0 | 0 | 0 | 0 | 0 | 0 | 0 | 1 | N | 14521 | hypothetical protein [Micrurus lemniscatus lemniscatus] |
| **189** | 1436 | LAA86749.1 | 75.02 | 8 | 0 | 0 | 0 | 0 | 0 | 0 | 0 | 0 | 0 | 0 | 0 | 0 | 0 | 0 | 0 | 8 | | | | | | | | | | | | | | | | 3.8116E6 | 1 | 1 | 0 | 0 | 0 | 0 | 0 | 0 | 0 | 0 | 0 | 0 | 0 | 0 | 0 | 0 | 0 | 1 | N | 14521 | hypothetical protein [Micrurus lemniscatus lemniscatus] |
| **189** | 1437 | LAA86742.1 | 75.02 | 8 | 0 | 0 | 0 | 0 | 0 | 0 | 0 | 0 | 0 | 0 | 0 | 0 | 0 | 0 | 0 | 8 | | | | | | | | | | | | | | | | 3.8116E6 | 1 | 1 | 0 | 0 | 0 | 0 | 0 | 0 | 0 | 0 | 0 | 0 | 0 | 0 | 0 | 0 | 0 | 1 | N | 14521 | hypothetical protein [Micrurus lemniscatus lemniscatus] |
| | | | | | | | | | | | | | | | | | | | | | | | | | | | | | | | | | | | | | | | | | | | | | | | | | | | | | | | | | hypothetical pro |

| | | | | | | | | | | | | | | | | | | | | | | | | | | | | | | | | | | | | | | | | | | | | | | | | | | | | | | | | | | |
|---|---|---|---|---|---|---|---|---|---|---|---|---|---|---|---|---|---|---|---|---|---|---|---|---|---|---|---|---|---|---|---|---|---|---|---|---|---|---|---|---|---|---|---|---|---|---|---|---|---|---|---|---|---|---|---|---|---|---|
| **189** | 1438 | LAA86765.1 | 75.02 | 8 | 0 | 0 | 0 | 0 | 0 | 0 | 0 | 0 | 0 | 0 | 0 | 0 | 0 | 0 | 0 | 8 |  |  |  |  |  |  |  |  |  |  |  |  |  |  |  |  | 3.8116E6 | 1 | 1 | 0 | 0 | 0 | 0 | 0 | 0 | 0 | 0 | 0 | 0 | 0 | 0 | 0 | 0 | 0 | 1 | N | 14521 | tein [Micrurus lemniscatus lemniscatus] |
| **189** | 1439 | LAA86747.1 | 75.02 | 8 | 0 | 0 | 0 | 0 | 0 | 0 | 0 | 0 | 0 | 0 | 0 | 0 | 0 | 0 | 0 | 8 |  |  |  |  |  |  |  |  |  |  |  |  |  |  |  |  | 3.8116E6 | 1 | 1 | 0 | 0 | 0 | 0 | 0 | 0 | 0 | 0 | 0 | 0 | 0 | 0 | 0 | 0 | 0 | 1 | N | 14521 | hypothetical protein [Micrurus lemniscatus lemniscatus] |
| **189** | 1440 | LAA86770.1 | 75.02 | 8 | 0 | 0 | 0 | 0 | 0 | 0 | 0 | 0 | 0 | 0 | 0 | 0 | 0 | 0 | 0 | 8 |  |  |  |  |  |  |  |  |  |  |  |  |  |  |  |  | 3.8116E6 | 1 | 1 | 0 | 0 | 0 | 0 | 0 | 0 | 0 | 0 | 0 | 0 | 0 | 0 | 0 | 0 | 0 | 1 | N | 14521 | hypothetical protein [Micrurus lemniscatus lemniscatus] |
| **189** | 1441 | LAA86752.1 | 75.02 | 8 | 0 | 0 | 0 | 0 | 0 | 0 | 0 | 0 | 0 | 0 | 0 | 0 | 0 | 0 | 0 | 8 |  |  |  |  |  |  |  |  |  |  |  |  |  |  |  |  | 3.8116E6 | 1 | 1 | 0 | 0 | 0 | 0 | 0 | 0 | 0 | 0 | 0 | 0 | 0 | 0 | 0 | 0 | 0 | 1 | N | 14521 | hypothetical protein [Micrurus lemniscatus lemniscatus] |
| **189** | 1442 | LAA86761.1 | 75.02 | 8 | 0 | 0 | 0 | 0 | 0 | 0 | 0 | 0 | 0 | 0 | 0 | 0 | 0 | 0 | 0 | 8 |  |  |  |  |  |  |  |  |  |  |  |  |  |  |  |  | 3.8116E6 | 1 | 1 | 0 | 0 | 0 | 0 | 0 | 0 | 0 | 0 | 0 | 0 | 0 | 0 | 0 | 0 | 0 | 1 | N | 14521 | hypothetical protein [Micrurus lemniscatus lemniscatus] |
| **189** | 1443 | LAA86756.1 | 75.02 | 8 | 0 | 0 | 0 | 0 | 0 | 0 | 0 | 0 | 0 | 0 | 0 | 0 | 0 | 0 | 0 | 8 |  |  |  |  |  |  |  |  |  |  |  |  |  |  |  |  | 3.8116E6 | 1 | 1 | 0 | 0 | 0 | 0 | 0 | 0 | 0 | 0 | 0 | 0 | 0 | 0 | 0 | 0 | 0 | 1 | N | 14521 | hypothetical protein [Micrurus lemniscatus lemniscatus] |
| **189** | 1444 | LAA86746.1 | 75.02 | 8 | 0 | 0 | 0 | 0 | 0 | 0 | 0 | 0 | 0 | 0 | 0 | 0 | 0 | 0 | 0 | 8 |  |  |  |  |  |  |  |  |  |  |  |  |  |  |  |  | 3.8116E6 | 1 | 1 | 0 | 0 | 0 | 0 | 0 | 0 | 0 | 0 | 0 | 0 | 0 | 0 | 0 | 0 | 0 | 1 | N | 14521 | hypothetical protein [Micrurus lemniscatus lemniscatus] |
| **189** | 1445 | LAA86771.1 | 75.02 | 8 | 0 | 0 | 0 | 0 | 0 | 0 | 0 | 0 | 0 | 0 | 0 | 0 | 0 | 0 | 0 | 8 |  |  |  |  |  |  |  |  |  |  |  |  |  |  |  |  | 3.8116E6 | 1 | 1 | 0 | 0 | 0 | 0 | 0 | 0 | 0 | 0 | 0 | 0 | 0 | 0 | 0 | 0 | 0 | 1 | N | 14521 | hypothetical protein [Micrurus lemniscatus lemniscatus] |
| **189** | 1446 | LAA86763.1 | 75.02 | 8 | 0 | 0 | 0 | 0 | 0 | 0 | 0 | 0 | 0 | 0 | 0 | 0 | 0 | 0 | 0 | 8 |  |  |  |  |  |  |  |  |  |  |  |  |  |  |  |  | 3.8116E6 | 1 | 1 | 0 | 0 | 0 | 0 | 0 | 0 | 0 | 0 | 0 | 0 | 0 | 0 | 0 | 0 | 0 | 1 | N | 14521 | hypothetical protein [Micrurus lemniscatus lemniscatus] |
| **189** | 1447 | LAA86754.1 | 75.02 | 8 | 0 | 0 | 0 | 0 | 0 | 0 | 0 | 0 | 0 | 0 | 0 | 0 | 0 | 0 | 0 | 8 |  |  |  |  |  |  |  |  |  |  |  |  |  |  |  |  | 3.8116E6 | 1 | 1 | 0 | 0 | 0 | 0 | 0 | 0 | 0 | 0 | 0 | 0 | 0 | 0 | 0 | 0 | 0 | 1 | N | 14521 | hypothetical protein [Micrurus lemniscatus lemniscatus] |
| **196** | 2026 | XP_015678372.1 | 72.18 | 9 | 0 | 0 | 0 | 0 | 0 | 0 | 0 | 0 | 0 | 0 | 0 | 0 | 9 | 0 | 0 | 0 |  |  |  |  |  |  |  |  |  |  |  |  |  | 2.5656E6 |  |  |  | 2 | 1 | 0 | 0 | 0 | 0 | 0 | 0 | 0 | 0 | 0 | 0 | 0 | 0 | 3 | 0 | 0 | 0 | Y | 27088 | serotriflin [Protobothrops mucrosquamatus] |
| **243** | 3748 | P82463.1 | 70.23 | 31 | 0 | 0 | 0 | 0 | 0 | 31 | 0 | 0 | 0 | 0 | 0 | 0 | 0 | 0 | 0 | 0 |  |  |  |  |  |  | 1.9042E6 |  |  |  |  |  |  |  |  |  |  | 2 | 2 | 0 | 0 | 0 | 0 | 0 | 2 | 0 | 0 | 0 | 0 | 0 | 0 | 0 | 0 | 0 | 0 | Y | 7298 | RecName: Full=Muscarinic toxin-like protein 2; Short=MTLP-2 |
| **210** | 728 | AUF41653.1 | 69.98 | 2 | 0 | 0 | 0 | 0 | 0 | 0 | 0 | 0 | 0 | 0 | 0 | 0 | 0 | 0 | 0 | 2 |  |  |  |  |  |  |  |  |  |  |  |  |  |  |  |  | 1.7363E4 | 1 | 1 | 0 | 0 | 0 | 0 | 0 | 0 | 0 | 0 | 0 | 0 | 0 | 0 | 0 | 0 | 0 | 1 | N | 69358 | RVV-X heavy chain [Daboia siamensis] |
| **210** | 729 | AUF41652.1 | 69.98 | 2 | 0 | 0 | 0 | 0 | 0 | 0 | 0 | 0 | 0 | 0 | 0 | 0 | 0 | 0 | 0 | 2 |  |  |  |  |  |  |  |  |  |  |  |  |  |  |  |  | 1.7363E4 | 1 | 1 | 0 | 0 | 0 | 0 | 0 | 0 | 0 | 0 | 0 | 0 | 0 | 0 | 0 | 0 | 0 | 1 | N | 69358 | RVV-X heavy chain [Daboia siamensis] |
| **210** | 732 | ADJ67475.1 | 69.98 | 2 | 0 | 0 | 0 | 0 | 0 | 0 | 0 | 0 | 0 | 0 | 0 | 0 | 0 | 0 | 0 | 2 |  |  |  |  |  |  |  |  |  |  |  |  |  |  |  |  | 1.7363E4 | 1 | 1 | 0 | 0 | 0 | 0 | 0 | 0 | 0 | 0 | 0 | 0 | 0 | 0 | 0 | 0 | 0 | 1 | N | 69521 | factor X activator heavy chain [Daboia russelii russelii] |
| **210** | 776 | AAZ39881.1 | 69.98 | 2 | 0 | 0 | 0 | 0 | 0 | 0 | 0 | 0 | 0 | 0 | 0 | 0 | 0 | 0 | 0 | 2 |  |  |  |  |  |  |  |  |  |  |  |  |  |  |  |  | 1.7363E4 | 1 | 1 | 0 | 0 | 0 | 0 | 0 | 0 | 0 | 0 | 0 | 0 | 0 | 0 | 0 | 0 | 0 | 1 | N | 69648 | RVV-X-heavy chain [Daboia russelii] |
| **210** | 777 | Q7LZ61.2 | 69.98 | 2 | 0 | 0 | 0 | 0 | 0 | 0 | 0 | 0 | 0 | 0 | 0 | 0 | 0 | 0 | 0 | 2 |  |  |  |  |  |  |  |  |  |  |  |  |  |  |  |  | 1.7363E4 | 1 | 1 | 0 | 0 | 0 | 0 | 0 | 0 | 0 | 0 | 0 | 0 | 0 | 0 | 0 | 0 | 0 | 1 | N | 69648 | RecName: Full=Coagulation factor X-activating enzyme heavy chain; AltName: Full=Coagulation factor X-activating enzyme chain alpha; AltName: Full=RVV-X heavy chain; AltName: Full=Russellysin; AltName: Full=Snake venom metalloprote... |
| **210** | 781 | AAB22477.1 | 69.98 | 3 | 0 | 0 | 0 | 0 | 0 | 0 | 0 | 0 | 0 | 0 | 0 | 0 | 0 | 0 | 0 | 3 |  |  |  |  |  |  |  |  |  |  |  |  |  |  |  |  | 1.7363E4 | 1 | 1 | 0 | 0 | 0 | 0 | 0 | 0 | 0 | 0 | 0 | 0 | 0 | 0 | 0 | 0 | 0 | 1 | N | 47975 | coagulation factor X activating enzyme heavy chain, RVV-X-heavy chain=metalloproteinase with disintegrin (platelet aggregation inhibitor)-like and C-type lectin-like domains [Vipera russelli=Russell's viper, Siamensis, venom, Peptide, 429 aa] |
| **201** | 1115 | AAM51550.1 | 69.34 | 2 | 0 | 0 | 0 | 0 | 0 | 0 | 0 | 0 | 0 | 0 | 0 | 0 | 0 | 0 | 0 | 2 |  |  |  |  |  |  |  |  |  |  |  |  |  |  |  |  | 5.0763E6 | 1 | 1 | 0 | 0 | 0 | 0 | 0 | 0 | 0 | 0 | 0 | 0 | 0 | 0 | 0 | 0 | 0 | 3 | Y | 68176 | mocarhagin 1 [Naja mossambica] |
| **201** | 1116 | Q10749.3 | 69.34 | 2 | 0 | 0 | 0 | 0 | 0 | 0 | 0 | 0 | 0 | 0 | 0 | 0 | 0 | 0 | 0 | 2 |  |  |  |  |  |  |  |  |  |  |  |  |  |  |  |  | 5.0763E6 | 1 | 1 | 0 | 0 | 0 | 0 | 0 | 0 | 0 | 0 | 0 | 0 | 0 | 0 | 0 | 0 | 0 | 3 | Y | 68176 | RecName: Full=Snake venom metalloproteinase-disintegrin-like mocarhagin; Short=MOC; Short=Mocarhagin-1; Short=SVMP; AltName: Full=Zinc metalloproteinase mocarhagin; Flags: Precursor |
| **199** | 2324 | 1CDT | 66.57 | 30 | 0 | 0 | 0 | 0 | 0 | 0 | 0 | 30 | 0 | 0 | 0 | 0 | 0 | 0 | 0 | 0 |  |  |  |  |  |  |  |  | 2.9139E5 |  |  |  |  |  |  |  |  | 2 | 1 | 0 | 0 | 0 | 0 | 0 | 0 | 0 | 2 | 0 | 0 | 0 | 0 | 0 | 0 | 0 | 0 | Y | 6715 | Chain B, CARDIOTOXIN VII4 |
| **199** | 2325 | P01452.1 | 66.57 | 30 | 0 | 0 | 0 | 0 | 0 | 0 | 0 | 30 | 0 | 0 | 0 | 0 | 0 | 0 | 0 | 0 |  |  |  |  |  |  |  |  | 2.9139E5 |  |  |  |  |  |  |  |  | 2 | 1 | 0 | 0 | 0 | 0 | 0 | 0 | 0 | 2 | 0 | 0 | 0 | 0 | 0 | 0 | 0 | 0 | Y | 6715 | RecName: Full=Cytotoxin 4; AltName: Full=CTX M3; AltName: Full=Cardiotoxin VII4; AltName: Full=Cytotoxin V(II)4 |
| **216** | 2779 | XP_026579252.1 | 66.07 | 9 | 0 | 0 | 0 | 0 | 0 | 9 | 9 | 9 | 0 | 0 | 0 | 0 | 0 | 0 | 0 | 0 |  |  |  |  |  |  | 3.1615E6 | 5.8354E4 | 1.8042E6 |  |  |  |  |  |  |  |  | 1 | 1 | 0 | 0 | 0 | 0 | 0 | 1 | 1 | 1 | 0 | 0 | 0 | 0 | 0 | 0 | 0 | 0 | Y | 14264 | kunitz/BPTI-like toxin isoform X1 [Pseudonaja textilis] |
| **207** | 680 | XP_034285002.1 | 65.12 | 0 | 0 | 0 | 0 | 0 | 0 | 0 | 0 | 0 | 0 | 0 | 0 | 0 | 0 | 0 | 0 | 0 |  |  |  |  |  |  |  |  | 0 | 9.2313E4 |  |  |  |  |  |  |  | 1 | 1 | 0 | 0 | 0 | 0 | 0 | 0 | 0 | 1 | 1 | 0 | 0 | 0 | 0 | 0 | 0 | 0 | N | 258226 | desmoplakin isoform X3 [Pantherophis guttatus] |
| **207** | 681 | XP_034285001.1 | 65.12 | 0 | 0 | 0 | 0 | 0 | 0 | 0 | 0 | 0 | 0 | 0 | 0 | 0 | 0 | 0 | 0 | 0 |  |  |  |  |  |  |  |  | 0 | 9.2313E4 |  |  |  |  |  |  |  | 1 | 1 | 0 | 0 | 0 | 0 | 0 | 0 | 0 | 1 | 1 | 0 | 0 | 0 | 0 | 0 | 0 | 0 | N | 276847 | desmoplakin isoform X2 [Pantherophis guttatus] |
| **207** | 682 | XP_034285000.1 | 65.12 | 0 | 0 | 0 | 0 | 0 | 0 | 0 | 0 | 0 | 0 | 0 | 0 | 0 | 0 | 0 | 0 | 0 |  |  |  |  |  |  |  |  | 0 | 9.2313E4 |  |  |  |  |  |  |  | 1 | 1 | 0 | 0 | 0 | 0 | 0 | 0 | 0 | 1 | 1 | 0 | 0 | 0 | 0 | 0 | 0 | 0 | N | 329611 | desmoplakin isoform X1 [Pantherophis guttatus] |
| **207** | 684 | JAC95787.1 | 65.12 | 0 | 0 | 0 | 0 | 0 | 0 | 0 | 0 | 0 | 0 | 0 | 0 | 0 | 0 | 0 | 0 | 0 |  |  |  |  |  |  |  |  | 0 | 9.2313E4 |  |  |  |  |  |  |  | 1 | 1 | 0 | 0 | 0 | 0 | 0 | 0 | 0 | 1 | 1 | 0 | 0 | 0 | 0 | 0 | 0 | 0 | N | 257947 | desmoplakin isoform II [Hypsiglena sp. JMG-2014] |
| **207** | 691 | XP_007422203.1 | 65.12 | 0 | 0 | 0 | 0 | 0 | 0 | 0 | 0 | 0 | 0 | 0 | 0 | 0 | 0 | 0 | 0 | 0 |  |  |  |  |  |  |  |  | 0 | 9.2313E4 |  |  |  |  |  |  |  | 1 | 1 | 0 | 0 | 0 | 0 | 0 | 0 | 0 | 1 | 1 | 0 | 0 | 0 | 0 | 0 | 0 | 0 | N | 258600 | desmoplakin isoform X3 [Python bivittatus] |
| **207** | 692 | XP_007422202.1 | 65.12 | 0 | 0 | 0 | 0 | 0 | 0 | 0 | 0 | 0 | 0 | 0 | 0 | 0 | 0 | 0 | 0 | 0 |  |  |  |  |  |  |  |  | 0 | 9.2313E4 |  |  |  |  |  |  |  | 1 | 1 | 0 | 0 | 0 | 0 | 0 | 0 | 0 | 1 | 1 | 0 | 0 | 0 | 0 | 0 | 0 | 0 | N | 277098 | desmoplakin isoform X2 [Python bivittatus] |
| **207** | 693 | XP_026570833.1 | 65.12 | 0 | 0 | 0 | 0 | 0 | 0 | 0 | 0 | 0 | 0 | 0 | 0 | 0 | 0 | 0 | 0 | 0 |  |  |  |  |  |  |  |  | 0 | 9.2313E4 |  |  |  |  |  |  |  | 1 | 1 | 0 | 0 | 0 | 0 | 0 | 0 | 0 | 1 | 1 | 0 | 0 | 0 | 0 | 0 | 0 | 0 | N | 327299 | desmoplakin [Pseudonaja textilis] |
| **207** | 694 | XP_007422201.1 | 65.12 | 0 | 0 | 0 | 0 | 0 | 0 | 0 | 0 | 0 | 0 | 0 | 0 | 0 | 0 | 0 | 0 | 0 |  |  |  |  |  |  |  |  | 0 | 9.2313E4 |  |  |  |  |  |  |  | 1 | 1 | 0 | 0 | 0 | 0 | 0 | 0 | 0 | 1 | 1 | 0 | 0 | 0 | 0 | 0 | 0 | 0 | N | 329911 | desmoplakin isoform X1 [Python bivittatus] |
| **207** | 695 | ETE64922.1 | 65.12 | 1 | 0 | 0 | 0 | 0 | 0 | 0 | 0 | 1 | 1 | 0 | 0 | 0 | 0 | 0 | 0 | 0 |  |  |  |  |  |  |  |  | 0 | 9.2313E4 |  |  |  |  |  |  |  | 1 | 1 | 0 | 0 | 0 | 0 | 0 | 0 | 0 | 1 | 1 | 0 | 0 | 0 | 0 | 0 | 0 | 0 | N | 223095 | Desmoplakin [Ophiophagus hannah] |
| **207** | 696 | XP_026532454.1 | 65.12 | 0 | 0 | 0 | 0 | 0 | 0 | 0 | 0 | 0 | 0 | 0 | 0 | 0 | 0 | 0 | 0 | 0 |  |  |  |  |  |  |  |  | 0 | 9.2313E4 |  |  |  |  |  |  |  | 1 | 1 | 0 | 0 | 0 | 0 | 0 | 0 | 0 | 1 | 1 | 0 | 0 | 0 | 0 | 0 | 0 | 0 | N | 248135 | desmoplakin isoform X3 [Notechis scutatus] |
| **207** | 698 | XP_026532453.1 | 65.12 | 0 | 0 | 0 | 0 | 0 | 0 | 0 | 0 | 0 | 0 | 0 | 0 | 0 | 0 | 0 | 0 | 0 |  |  |  |  |  |  |  |  | 0 | 9.2313E4 |  |  |  |  |  |  |  | 1 | 1 | 0 | 0 | 0 | 0 | 0 | 0 | 0 | 1 | 1 | 0 | 0 | 0 | 0 | 0 | 0 | 0 | N | 266816 | desmoplakin isoform X2 [Notechis scutatus] |
| **207** | 699 | XP_026532452.1 | 65.12 | 0 | 0 | 0 | 0 | 0 | 0 | 0 | 0 | 0 | 0 | 0 | 0 | 0 | 0 | 0 | 0 | 0 |  |  |  |  |  |  |  |  | 0 | 9.2313E4 |  |  |  |  |  |  |  | 1 | 1 | 0 | 0 | 0 | 0 | 0 | 0 | 0 | 1 | 1 | 0 | 0 | 0 | 0 | 0 | 0 | 0 | N | 319556 | desmoplakin isoform X1 [Notechis scutatus] |
| **207** | 700 | LAA69945.1 | 65.12 | 0 | 0 | 0 | 0 | 0 | 0 | 0 | 0 | 0 | 0 | 0 | 0 | 0 | 0 | 0 | 0 | 0 |  |  |  |  |  |  |  |  | 0 | 9.2313E4 |  |  |  |  |  |  |  | 1 | 1 | 0 | 0 | 0 | 0 | 0 | 0 | 0 | 1 | 1 | 0 | 0 | 0 | 0 | 0 | 0 | 0 | N | 329375 | hypothetical protein [Micrurus lemniscatus lemniscatus] |
| **207** | 701 | XP_058034978.1 | 65.12 | 0 | 0 | 0 | 0 | 0 | 0 | 0 | 0 | 0 | 0 | 0 | 0 | 0 | 0 | 0 | 0 | 0 |  |  |  |  |  |  |  |  | 0 | 9.2313E4 |  |  |  |  |  |  |  | 1 | 1 | 0 | 0 | 0 | 0 | 0 | 0 | 0 | 1 | 1 | 0 | 0 | 0 | 0 | 0 | 0 | 0 | N | 258087 | desmoplakin isoform X3 [Ahaetulla prasina] |
| **207** | 702 | XP_058034977.1 | 65.12 | 0 | 0 | 0 | 0 | 0 | 0 | 0 | 0 | 0 | 0 | 0 | 0 | 0 | 0 | 0 | 0 | 0 |  |  |  |  |  |  |  |  | 0 | 9.2313E4 |  |  |  |  |  |  |  | 1 | 1 | 0 | 0 | 0 | 0 | 0 | 0 | 0 | 1 | 1 | 0 | 0 | 0 | 0 | 0 | 0 | 0 | N | 276824 | desmoplakin isoform X2 [Ahaetulla prasina] |
| **207** | 703 | XP_058034976.1 | 65.12 | 0 | 0 | 0 | 0 | 0 | 0 | 0 | 0 | 0 | 0 | 0 | 0 | 0 | 0 | 0 | 0 | 0 |  |  |  |  |  |  |  |  | 0 | 9.2313E4 |  |  |  |  |  |  |  | 1 | 1 | 0 | 0 | 0 | 0 | 0 | 0 | 0 | 1 | 1 | 0 | 0 | 0 | 0 | 0 | 0 | 0 | N | 329614 | desmoplakin isoform X1 [Ahaetulla prasina] |
| **207** | 736 | XP_032079163.1 | 65.12 | 0 | 0 | 0 | 0 | 0 | 0 | 0 | 0 | 0 | 0 | 0 | 0 | 0 | 0 | 0 | 0 | 0 |  |  |  |  |  |  |  |  | 0 | 9.2313E4 |  |  |  |  |  |  |  | 1 | 1 | 0 | 0 | 0 | 0 | 0 | 0 | 0 | 1 | 1 | 0 | 0 | 0 | 0 | 0 | 0 | 0 | N | 258448 | desmoplakin isoform X3 [Thamnophis elegans] |
| **207** | 737 | XP_032079162.1 | 65.12 | 0 | 0 | 0 | 0 | 0 | 0 | 0 | 0 | 0 | 0 | 0 | 0 | 0 | 0 | 0 | 0 | 0 |  |  |  |  |  |  |  |  | 0 | 9.2313E4 |  |  |  |  |  |  |  | 1 | 1 | 0 | 0 | 0 | 0 | 0 | 0 | 0 | 1 | 1 | 0 | 0 | 0 | 0 | 0 | 0 | 0 | N | 277026 | desmoplakin isoform X2 [Thamnophis elegans] |
| **207** | 743 | XP_013912202.1 | 65.12 | 0 | 0 | 0 | 0 | 0 | 0 | 0 | 0 | 0 | 0 | 0 | 0 | 0 | 0 | 0 | 0 | 0 |  |  |  |  |  |  |  |  | 0 | 9.2313E4 |  |  |  |  |  |  |  | 1 | 1 | 0 | 0 | 0 | 0 | 0 | 0 | 0 | 1 | 1 | 0 | 0 | 0 | 0 | 0 | 0 | 0 | N | 314164 | PREDICTED: desmoplakin [Thamnophis sirtalis] |
|  |  |  |  |  |  |  |  |  |  |  |  |  |  |  |  |  |  |  |  |  |  |  |  |  |  |  |  |  |  |  |  |  |  |  |  |  |  |  |  |  |  |  |  |  |  |  |  |  |  |  |  |  |  |  |  |  |  | desmoplakin iso |

| | | | | | | | | | | | | | | | | | | | | | | | | | | | | | | | | | | | | | | | | | | | | | | | | | | | | | | | | | |
|---|---|---|---|---|---|---|---|---|---|---|---|---|---|---|---|---|---|---|---|---|---|---|---|---|---|---|---|---|---|---|---|---|---|---|---|---|---|---|---|---|---|---|---|---|---|---|---|---|---|---|---|---|---|---|---|---|---|
| **207** | 744 | XP_032079161.1 | 65.12 | 0 | 0 | 0 | 0 | 0 | 0 | 0 | 0 | 0 | 0 | 0 | 0 | 0 | 0 | 0 | 0 | 0 | | | | | | | | 0 | 9.2313E4 | | | | | | | | 1 | 1 | 0 | 0 | 0 | 0 | 0 | 0 | 0 | 1 | 1 | 0 | 0 | 0 | 0 | 0 | 0 | 0 | N | 329798 | form X1 [Thamnophis elegans] |
| **207** | 747 | XP_015666325.1 | 65.12 | 0 | 0 | 0 | 0 | 0 | 0 | 0 | 0 | 0 | 0 | 0 | 0 | 0 | 0 | 0 | 0 | 0 | | | | | | | | 0 | 9.2313E4 | | | | | | | | 1 | 1 | 0 | 0 | 0 | 0 | 0 | 0 | 0 | 1 | 1 | 0 | 0 | 0 | 0 | 0 | 0 | 0 | N | 329546 | desmoplakin [Protobothrops mucrosquamatus] |
| **207** | 748 | XP_039206270.1 | 65.12 | 0 | 0 | 0 | 0 | 0 | 0 | 0 | 0 | 0 | 0 | 0 | 0 | 0 | 0 | 0 | 0 | 0 | | | | | | | | 0 | 9.2313E4 | | | | | | | | 1 | 1 | 0 | 0 | 0 | 0 | 0 | 0 | 0 | 1 | 1 | 0 | 0 | 0 | 0 | 0 | 0 | 0 | N | 258525 | desmoplakin isoform X4 [Crotalus tigris] |
| **207** | 749 | XP_039206261.1 | 65.12 | 0 | 0 | 0 | 0 | 0 | 0 | 0 | 0 | 0 | 0 | 0 | 0 | 0 | 0 | 0 | 0 | 0 | | | | | | | | 0 | 9.2313E4 | | | | | | | | 1 | 1 | 0 | 0 | 0 | 0 | 0 | 0 | 0 | 1 | 1 | 0 | 0 | 0 | 0 | 0 | 0 | 0 | N | 277094 | desmoplakin isoform X3 [Crotalus tigris] |
| **207** | 750 | XP_039206254.1 | 65.12 | 0 | 0 | 0 | 0 | 0 | 0 | 0 | 0 | 0 | 0 | 0 | 0 | 0 | 0 | 0 | 0 | 0 | | | | | | | | 0 | 9.2313E4 | | | | | | | | 1 | 1 | 0 | 0 | 0 | 0 | 0 | 0 | 0 | 1 | 1 | 0 | 0 | 0 | 0 | 0 | 0 | 0 | N | 319610 | desmoplakin isoform X2 [Crotalus tigris] |
| **207** | 751 | XP_039206245.1 | 65.12 | 0 | 0 | 0 | 0 | 0 | 0 | 0 | 0 | 0 | 0 | 0 | 0 | 0 | 0 | 0 | 0 | 0 | | | | | | | | 0 | 9.2313E4 | | | | | | | | 1 | 1 | 0 | 0 | 0 | 0 | 0 | 0 | 0 | 1 | 1 | 0 | 0 | 0 | 0 | 0 | 0 | 0 | N | 329886 | desmoplakin isoform X1 [Crotalus tigris] |
| **207** | 773 | JAI13616.1 | 65.12 | 0 | 0 | 0 | 0 | 0 | 0 | 0 | 0 | 0 | 0 | 0 | 0 | 0 | 0 | 0 | 0 | 0 | | | | | | | | 0 | 9.2313E4 | | | | | | | | 1 | 1 | 0 | 0 | 0 | 0 | 0 | 0 | 0 | 1 | 1 | 0 | 0 | 0 | 0 | 0 | 0 | 0 | N | 324549 | desmoplakin [Crotalus adamanteus] |
| **211** | 2715 | XP_034282052.1 | 62.83 | 12 | 0 | 0 | 0 | 0 | 0 | 0 | 0 | 0 | 9 | 0 | 0 | 0 | 0 | 12 | 0 | 0 | | | | | | | | | 2.7841E5 | | | | | 0 | | | 2 | 2 | 0 | 0 | 0 | 0 | 0 | 0 | 0 | 0 | 1 | 0 | 0 | 0 | 0 | 1 | 0 | 0 | Y | 16591 | phospholipase A2 GL16-1-like [Pantherophis guttatus] |
| **211** | 2770 | C1IC46.1 | 62.83 | 14 | 0 | 0 | 0 | 0 | 0 | 0 | 0 | 0 | 10 | 0 | 0 | 0 | 0 | 14 | 0 | 0 | | | | | | | | | 2.7841E5 | | | | | 0 | | | 2 | 2 | 0 | 0 | 0 | 0 | 0 | 0 | 0 | 0 | 1 | 0 | 0 | 0 | 0 | 1 | 0 | 0 | Y | 15034 | RecName: Full=Acidic phospholipase A2 PL-I; Short=svPLA2; AltName: Full=Phosphatidylcholine 2-acylhydrolase; Flags: Precursor |
| **211** | 2771 | ABX82863.1 | 62.83 | 14 | 0 | 0 | 0 | 0 | 0 | 0 | 0 | 0 | 10 | 0 | 0 | 0 | 0 | 14 | 0 | 0 | | | | | | | | | 2.7841E5 | | | | | 0 | | | 2 | 2 | 0 | 0 | 0 | 0 | 0 | 0 | 0 | 0 | 1 | 0 | 0 | 0 | 0 | 1 | 0 | 0 | Y | 15034 | acidic phospholipase A2 I precursor, partial [Walterinnesia aegyptia] |
| **211** | 3798 | ABX82862.1 | 62.83 | 14 | 0 | 0 | 0 | 0 | 0 | 0 | 0 | 0 | 10 | 0 | 0 | 0 | 0 | 14 | 0 | 0 | | | | | | | | | 2.7841E5 | | | | | 0 | | | 2 | 2 | 0 | 0 | 0 | 0 | 0 | 0 | 0 | 0 | 1 | 0 | 0 | 0 | 0 | 1 | 0 | 0 | Y | 15035 | acidic phospholipase A2 II precursor, partial [Walterinnesia aegyptia] |
| **211** | 3799 | C1IC45.1 | 62.83 | 14 | 0 | 0 | 0 | 0 | 0 | 0 | 0 | 0 | 10 | 0 | 0 | 0 | 0 | 14 | 0 | 0 | | | | | | | | | 2.7841E5 | | | | | 0 | | | 2 | 2 | 0 | 0 | 0 | 0 | 0 | 0 | 0 | 0 | 1 | 0 | 0 | 0 | 0 | 1 | 0 | 0 | Y | 15035 | RecName: Full=Acidic phospholipase A2 PL-II; Short=svPLA2; AltName: Full=Phosphatidylcholine 2-acylhydrolase; Flags: Precursor |
| **221** | 1470 | XP_015671648.1 | 62.69 | 8 | 0 | 0 | 0 | 0 | 0 | 0 | 0 | 0 | 0 | 0 | 0 | 8 | 0 | 0 | 0 | 0 | | | | | | | | | | | | 8.2966E4 | | | | | 1 | 1 | 0 | 0 | 0 | 0 | 0 | 0 | 0 | 0 | 0 | 0 | 0 | 1 | 0 | 0 | 0 | 0 | N | 33015 | short stature homeobox protein isoform X1 [Protobothrops mucrosquamatus] |
| **221** | 1471 | XP_029140011.1 | 62.69 | 8 | 0 | 0 | 0 | 0 | 0 | 0 | 0 | 0 | 0 | 0 | 0 | 8 | 0 | 0 | 0 | 0 | | | | | | | | | | | | 8.2966E4 | | | | | 1 | 1 | 0 | 0 | 0 | 0 | 0 | 0 | 0 | 0 | 0 | 0 | 0 | 1 | 0 | 0 | 0 | 0 | N | 33114 | short stature homeobox protein isoform X2 [Protobothrops mucrosquamatus] |
| **182** | 1996 | P00602.1 | 62.49 | 9 | 0 | 0 | 0 | 0 | 0 | 0 | 0 | 0 | 0 | 9 | 0 | 0 | 0 | 0 | 0 | 0 | | | | | | | | | | 7.878E5 | | | | | | | 1 | 1 | 0 | 0 | 0 | 0 | 0 | 0 | 0 | 0 | 0 | 1 | 0 | 0 | 0 | 0 | 0 | 0 | N | 13219 | RecName: Full=Acidic phospholipase A2 CM-I; Short=svPLA2; AltName: Full=Phosphatidylcholine 2-acylhydrolase |
| **213** | 3765 | UNJ97059.1 | 62.19 | 10 | 0 | 0 | 0 | 10 | 0 | 0 | 0 | 0 | 0 | 0 | 0 | 0 | 0 | 0 | 0 | 0 | | | | 1.2175E7 | | | | | | | | | | | | | 1 | 1 | 0 | 0 | 0 | 1 | 0 | 0 | 0 | 0 | 0 | 0 | 0 | 0 | 0 | 0 | 0 | 0 | Y | 16412 | kunitz peptide, partial [Boiga dendrophila dendrophila] |
| **213** | 4416 | UNJ97095.1 | 62.19 | 11 | 0 | 0 | 0 | 11 | 0 | 0 | 0 | 0 | 0 | 0 | 0 | 0 | 0 | 0 | 0 | 0 | | | | 1.2175E7 | | | | | | | | | | | | | 1 | 1 | 0 | 0 | 0 | 1 | 0 | 0 | 0 | 0 | 0 | 0 | 0 | 0 | 0 | 0 | 0 | 0 | Y | 14274 | kunitz peptide, partial [Boiga nigriceps] |
| **213** | 4417 | UNJ97090.1 | 62.19 | 11 | 0 | 0 | 0 | 11 | 0 | 0 | 0 | 0 | 0 | 0 | 0 | 0 | 0 | 0 | 0 | 0 | | | | 1.2175E7 | | | | | | | | | | | | | 1 | 1 | 0 | 0 | 0 | 1 | 0 | 0 | 0 | 0 | 0 | 0 | 0 | 0 | 0 | 0 | 0 | 0 | Y | 14306 | kunitz peptide, partial [Boiga irregularis] |
| **213** | 4418 | UNJ97082.1 | 62.19 | 10 | 0 | 0 | 0 | 10 | 0 | 0 | 0 | 0 | 0 | 0 | 0 | 0 | 0 | 0 | 0 | 0 | | | | 1.2175E7 | | | | | | | | | | | | | 1 | 1 | 0 | 0 | 0 | 1 | 0 | 0 | 0 | 0 | 0 | 0 | 0 | 0 | 0 | 0 | 0 | 0 | Y | 16263 | kunitz peptide, partial [Boiga irregularis] |
| **213** | 4419 | UNJ97094.1 | 62.19 | 10 | 0 | 0 | 0 | 10 | 0 | 0 | 0 | 0 | 0 | 0 | 0 | 0 | 0 | 0 | 0 | 0 | | | | 1.2175E7 | | | | | | | | | | | | | 1 | 1 | 0 | 0 | 0 | 1 | 0 | 0 | 0 | 0 | 0 | 0 | 0 | 0 | 0 | 0 | 0 | 0 | Y | 16197 | kunitz peptide, partial [Boiga nigriceps] |
| **203** | 1523 | P62377.1 | 62.14 | 15 | 0 | 0 | 0 | 0 | 0 | 0 | 0 | 0 | 0 | 15 | 0 | 0 | 0 | 0 | 0 | 0 | | | | | | | | | | 0 | | | | | | | 1 | 1 | 0 | 0 | 0 | 0 | 0 | 0 | 0 | 0 | 0 | 1 | 0 | 0 | 0 | 0 | 0 | 0 | N | 7014 | RecName: Full=Cytotoxin-like basic protein; Short=CLBP; AltName: Full=Less-cytotoxic basic polypeptide; Short=LCBP |
| **203** | 1524 | 1CVO | 62.14 | 15 | 0 | 0 | 0 | 0 | 0 | 0 | 0 | 0 | 0 | 15 | 0 | 0 | 0 | 0 | 0 | 0 | | | | | | | | | | 0 | | | | | | | 1 | 1 | 0 | 0 | 0 | 0 | 0 | 0 | 0 | 0 | 0 | 1 | 0 | 0 | 0 | 0 | 0 | 0 | N | 7014 | Chain A, CARDIOTOXIN V |
| **203** | 1525 | 1KXI | 62.14 | 15 | 0 | 0 | 0 | 0 | 0 | 0 | 0 | 0 | 0 | 15 | 0 | 0 | 0 | 0 | 0 | 0 | | | | | | | | | | 0 | | | | | | | 1 | 1 | 0 | 0 | 0 | 0 | 0 | 0 | 0 | 0 | 0 | 1 | 0 | 0 | 0 | 0 | 0 | 0 | N | 7014 | Chain B, CARDIOTOXIN V |
| **203** | 1526 | Q9W716.1 | 62.14 | 11 | 0 | 0 | 0 | 0 | 0 | 0 | 0 | 0 | 0 | 11 | 0 | 0 | 0 | 0 | 0 | 0 | | | | | | | | | | 0 | | | | | | | 1 | 1 | 0 | 0 | 0 | 0 | 0 | 0 | 0 | 0 | 0 | 1 | 0 | 0 | 0 | 0 | 0 | 0 | N | 9333 | RecName: Full=Cytotoxin homolog 5V; AltName: Full=Cardiotoxin-5V; Short=CTX5V; Flags: Precursor |
| **203** | 1527 | AAB01540.1 | 62.14 | 11 | 0 | 0 | 0 | 0 | 0 | 0 | 0 | 0 | 0 | 11 | 0 | 0 | 0 | 0 | 0 | 0 | | | | | | | | | | 0 | | | | | | | 1 | 1 | 0 | 0 | 0 | 0 | 0 | 0 | 0 | 0 | 0 | 1 | 0 | 0 | 0 | 0 | 0 | 0 | N | 9305 | cardiotoxin VII [Naja atra] |
| **203** | 1528 | CAB50697.1 | 62.14 | 11 | 0 | 0 | 0 | 0 | 0 | 0 | 0 | 0 | 0 | 11 | 0 | 0 | 0 | 0 | 0 | 0 | | | | | | | | | | 0 | | | | | | | 1 | 1 | 0 | 0 | 0 | 0 | 0 | 0 | 0 | 0 | 0 | 1 | 0 | 0 | 0 | 0 | 0 | 0 | N | 9333 | snake venom [Naja atra] |
| **203** | 1529 | AAB36928.1 | 62.14 | 11 | 0 | 0 | 0 | 0 | 0 | 0 | 0 | 0 | 0 | 11 | 0 | 0 | 0 | 0 | 0 | 0 | | | | | | | | | | 0 | | | | | | | 1 | 1 | 0 | 0 | 0 | 0 | 0 | 0 | 0 | 0 | 0 | 1 | 0 | 0 | 0 | 0 | 0 | 0 | N | 9305 | cardiotoxin 7'' [Naja atra] |
| **203** | 1530 | Q91996.1 | 62.14 | 11 | 0 | 0 | 0 | 0 | 0 | 0 | 0 | 0 | 0 | 11 | 0 | 0 | 0 | 0 | 0 | 0 | | | | | | | | | | 0 | | | | | | | 1 | 1 | 0 | 0 | 0 | 0 | 0 | 0 | 0 | 0 | 0 | 1 | 0 | 0 | 0 | 0 | 0 | 0 | N | 9305 | RecName: Full=Cardiotoxin 7''; AltName: Full=Cardiotoxin 7'; AltName: Full=Cardiotoxin VII; Flags: Precursor |
| **203** | 1531 | AAB36929.1 | 62.14 | 11 | 0 | 0 | 0 | 0 | 0 | 0 | 0 | 0 | 0 | 11 | 0 | 0 | 0 | 0 | 0 | 0 | | | | | | | | | | 0 | | | | | | | 1 | 1 | 0 | 0 | 0 | 0 | 0 | 0 | 0 | 0 | 0 | 1 | 0 | 0 | 0 | 0 | 0 | 0 | N | 9319 | cardiotoxin 7a [Naja atra] |
| **203** | 1532 | AAB03219.1 | 62.14 | 11 | 0 | 0 | 0 | 0 | 0 | 0 | 0 | 0 | 0 | 11 | 0 | 0 | 0 | 0 | 0 | 0 | | | | | | | | | | 0 | | | | | | | 1 | 1 | 0 | 0 | 0 | 0 | 0 | 0 | 0 | 0 | 0 | 1 | 0 | 0 | 0 | 0 | 0 | 0 | N | 9305 | cardiotoxin 7'' [Naja atra] |
| **203** | 1533 | CAA90964.1 | 62.14 | 11 | 0 | 0 | 0 | 0 | 0 | 0 | 0 | 0 | 0 | 11 | 0 | 0 | 0 | 0 | 0 | 0 | | | | | | | | | | 0 | | | | | | | 1 | 1 | 0 | 0 | 0 | 0 | 0 | 0 | 0 | 0 | 0 | 1 | 0 | 0 | 0 | 0 | 0 | 0 | N | 9323 | cardiotoxin V [Naja naja] |
| **203** | 1534 | CAA63979.1 | 62.14 | 11 | 0 | 0 | 0 | 0 | 0 | 0 | 0 | 0 | 0 | 11 | 0 | 0 | 0 | 0 | 0 | 0 | | | | | | | | | | 0 | | | | | | | 1 | 1 | 0 | 0 | 0 | 0 | 0 | 0 | 0 | 0 | 0 | 1 | 0 | 0 | 0 | 0 | 0 | 0 | N | 9309 | cardiotoxin V-like protein [Naja atra] |
| **203** | 1535 | AAB03220.1 | 62.14 | 11 | 0 | 0 | 0 | 0 | 0 | 0 | 0 | 0 | 0 | 11 | 0 | 0 | 0 | 0 | 0 | 0 | | | | | | | | | | 0 | | | | | | | 1 | 1 | 0 | 0 | 0 | 0 | 0 | 0 | 0 | 0 | 0 | 1 | 0 | 0 | 0 | 0 | 0 | 0 | N | 9319 | cardiotoxin 7a [Naja atra] |
| **203** | 1536 | AAB18387.1 | 62.14 | 11 | 0 | 0 | 0 | 0 | 0 | 0 | 0 | 0 | 0 | 11 | 0 | 0 | 0 | 0 | 0 | 0 | | | | | | | | | | 0 | | | | | | | 1 | 1 | 0 | 0 | 0 | 0 | 0 | 0 | 0 | 0 | 0 | 1 | 0 | 0 | 0 | 0 | 0 | 0 | N | 9305 | cardiotoxin 7' [Naja atra] |
| **203** | 1537 | Q91137.1 | 62.14 | 11 | 0 | 0 | 0 | 0 | 0 | 0 | 0 | 0 | 0 | 11 | 0 | 0 | 0 | 0 | 0 | 0 | | | | | | | | | | 0 | | | | | | | 1 | 1 | 0 | 0 | 0 | 0 | 0 | 0 | 0 | 0 | 0 | 1 | 0 | 0 | 0 | 0 | 0 | 0 | N | 9309 | RecName: Full=Cytotoxin homolog 5; AltName: Full=Cardiotoxin V-like; Flags: Precursor |
| **203** | 1538 | P62375.1 | 62.14 | 11 | 0 | 0 | 0 | 0 | 0 | 0 | 0 | 0 | 0 | 11 | 0 | 0 | 0 | 0 | 0 | 0 | | | | | | | | | | 0 | | | | | | | 1 | 1 | 0 | 0 | 0 | 0 | 0 | 0 | 0 | 0 | 0 | 1 | 0 | 0 | 0 | 0 | 0 | 0 | N | 9323 | RecName: Full=Cytotoxin A5; AltName: Full=CTX A5; Short=CTX A5; AltName: Full=Cardiotoxin A5; AltName: Full=Cardiotoxin V; Short=CTX V; Short=CTXV; AltName: Full=Cardiotoxin-like basic polypeptide; Short=CLBP; Flags: Precursor |
| **203** | 1539 | Q91126.1 | 62.14 | 11 | 0 | 0 | 0 | 0 | 0 | 0 | 0 | 0 | 0 | 11 | 0 | 0 | 0 | 0 | 0 | 0 | | | | | | | | | | 0 | | | | | | | 1 | 1 | 0 | 0 | 0 | 0 | 0 | 0 | 0 | 0 | 0 | 1 | 0 | 0 | 0 | 0 | 0 | 0 | N | 9319 | RecName: Full=Cardiotoxin 7a; Flags: Precursor |
| **382** | 1188 | XP_026579206.1 | 60.38 | 5 | 5 | 0 | 0 | 0 | 0 | 0 | 0 | 0 | 0 | 0 | 0 | 0 | 0 | 0 | 0 | 0 | 2.6276E5 | | | | | | | | | | | | | | | | 1 | 1 | 1 | 0 | 0 | 0 | 0 | 0 | 0 | 0 | 0 | 0 | 0 | 0 | 0 | 0 | 0 | 0 | N | 31175 | endonuclease domain-containing 1 protein-like [Pseudonaja textilis] |
| **382** | 1189 | XP_026579207.1 | 60.38 | 5 | 5 | 0 | 0 | 0 | 0 | 0 | 0 | 0 | 0 | 0 | 0 | 0 | 0 | 0 | 0 | 0 | 2.6276E5 | | | | | | | | | | | | | | | | 1 | 1 | 1 | 0 | 0 | 0 | 0 | 0 | 0 | 0 | 0 | 0 | 0 | 0 | 0 | 0 | 0 | 0 | N | 31175 | endonuclease domain-containing 1 protein-like [Pseudonaja textilis] |
| **382** | 1402 | JAB54304.1 | 60.38 | 5 | 5 | 0 | 0 | 0 | 0 | 0 | 0 | 0 | 0 | 0 | 0 | 0 | 0 | 0 | 0 | 0 | 2.6276E5 | | | | | | | | | | | | | | | | 1 | 1 | 1 | 0 | 0 | 0 | 0 | 0 | 0 | 0 | 0 | 0 | 0 | 0 | 0 | 0 | 0 | 0 | N | 31577 | endonuclease domain-containing 1 protein [Micrurus fulvius] |
| **382** | 2399 | ETE56723.1 | 60.38 | 9 | 9 | 0 | 0 | 0 | 0 | 0 | 0 | 0 | 0 | 0 | 0 | 0 | 0 | 0 | 0 | 0 | 2.6276E5 | | | | | | | | | | | | | | | | 1 | 1 | 1 | 0 | 0 | 0 | 0 | 0 | 0 | 0 | 0 | 0 | 0 | 0 | 0 | 0 | 0 | 0 | N | 18083 | Endonuclease domain-containing 1 protein [Ophiophagus hannah] |
| **382** | 2424 | XP_058047681.1 | 60.38 | 3 | 3 | 0 | 0 | 0 | 0 | 0 | 0 | 0 | 0 | 0 | 0 | 0 | 0 | 0 | 0 | 0 | 2.6276E5 | | | | | | | | | | | | | | | | 1 | 1 | 1 | 0 | 0 | 0 | 0 | 0 | 0 | 0 | 0 | 0 | 0 | 0 | 0 | 0 | 0 | 0 | N | 55643 | uncharacterized protein LOC131202596 [Ahaetulla prasina] |
| **382** | 2643 | ETE59939.1 | 60.38 | 3 | 3 | 0 | 0 | 0 | 0 | 0 | 0 | 0 | 0 | 0 | 0 | 0 | 0 | 0 | 0 | 0 | 2.6276E5 | | | | | | | | | | | | | | | | 1 | 1 | 1 | 0 | 0 | 0 | 0 | 0 | 0 | 0 | 0 | 0 | 0 | 0 | 0 | 0 | 0 | 0 | N | 51067 | Endonuclease domain-containing 1 protein, partial [Ophiophagus hannah] |
| | | | | | | | | | | | | | | | | | | | | | | | | | | | | | | | | | | | | | | | | | | | | | | | | | | | | | | | | endonuclease domain-containin |

| | | | | | | | | | | | | | | | | | | | | | | | | | | | | | | | | | | | | | | | | | | | | | | | | | | | | | | | | | |
|---|---|---|---|---|---|---|---|---|---|---|---|---|---|---|---|---|---|---|---|---|---|---|---|---|---|---|---|---|---|---|---|---|---|---|---|---|---|---|---|---|---|---|---|---|---|---|---|---|---|---|---|---|---|---|---|---|---|
| **382** | 2913 | XP_039225468.1 | 60.38 | 5 | 5 | 0 | 0 | 0 | 0 | 0 | 0 | 0 | 0 | 0 | 0 | 0 | 0 | 0 | 0 | 0 | 2.6276E5 | | | | | | | | | | | | | | | | 1 | 1 | 1 | 0 | 0 | 0 | 0 | 0 | 0 | 0 | 0 | 0 | 0 | 0 | 0 | 0 | 0 | 0 | N | 31299 | g 1 protein-like [Crotalus tigris] |
| **382** | 2914 | XP_039225467.1 | 60.38 | 5 | 5 | 0 | 0 | 0 | 0 | 0 | 0 | 0 | 0 | 0 | 0 | 0 | 0 | 0 | 0 | 0 | 2.6276E5 | | | | | | | | | | | | | | | | 1 | 1 | 1 | 0 | 0 | 0 | 0 | 0 | 0 | 0 | 0 | 0 | 0 | 0 | 0 | 0 | 0 | 0 | N | 31299 | endonuclease domain-containing 1 protein-like [Crotalus tigris] |
| **382** | 3167 | XP_032076462.1 | 60.38 | 5 | 5 | 0 | 0 | 0 | 0 | 0 | 0 | 0 | 0 | 0 | 0 | 0 | 0 | 0 | 0 | 0 | 2.6276E5 | | | | | | | | | | | | | | | | 1 | 1 | 1 | 0 | 0 | 0 | 0 | 0 | 0 | 0 | 0 | 0 | 0 | 0 | 0 | 0 | 0 | 0 | N | 31282 | endonuclease domain-containing 1 protein-like isoform X2 [Thamnophis elegans] |
| **382** | 3168 | XP_032076461.1 | 60.38 | 5 | 5 | 0 | 0 | 0 | 0 | 0 | 0 | 0 | 0 | 0 | 0 | 0 | 0 | 0 | 0 | 0 | 2.6276E5 | | | | | | | | | | | | | | | | 1 | 1 | 1 | 0 | 0 | 0 | 0 | 0 | 0 | 0 | 0 | 0 | 0 | 0 | 0 | 0 | 0 | 0 | N | 34799 | endonuclease domain-containing 1 protein-like isoform X1 [Thamnophis elegans] |
| **328** | 1373 | LAB21297.1 | 60.18 | 11 | 0 | 0 | 0 | 0 | 0 | 0 | 0 | 0 | 11 | 0 | 0 | 0 | 0 | 0 | 0 | 0 | | | | | | | | | 1.7685E6 | | | | | | | | 1 | 1 | 0 | 0 | 0 | 0 | 0 | 0 | 0 | 0 | 1 | 0 | 0 | 0 | 0 | 0 | 0 | 0 | Y | 14576 | hypothetical protein, partial [Micrurus spixii] |
| **393** | 1556 | XP_026545503.1 | 58.63 | 11 | 0 | 0 | 0 | 0 | 0 | 0 | 0 | 0 | 0 | 0 | 0 | 0 | 0 | 0 | 0 | 11 | | | | | | | | | | | | | | | | 1.0855E5 | 1 | 1 | 0 | 0 | 0 | 0 | 0 | 0 | 0 | 0 | 0 | 0 | 0 | 0 | 0 | 0 | 0 | 1 | N | 11877 | protein S100-A11-like [Notechis scutatus] |
| **393** | 2119 | XP_034291543.1 | 58.63 | 5 | 0 | 0 | 0 | 0 | 0 | 0 | 0 | 0 | 0 | 0 | 0 | 0 | 0 | 0 | 0 | 5 | | | | | | | | | | | | | | | | 1.0855E5 | 1 | 1 | 0 | 0 | 0 | 0 | 0 | 0 | 0 | 0 | 0 | 0 | 0 | 0 | 0 | 0 | 0 | 1 | N | 28872 | uncharacterized protein LOC117676273 [Pantherophis guttatus] |
| **393** | 2243 | XP_026580631.1 | 58.63 | 11 | 0 | 0 | 0 | 0 | 0 | 0 | 0 | 0 | 0 | 0 | 0 | 0 | 0 | 0 | 0 | 11 | | | | | | | | | | | | | | | | 1.0855E5 | 1 | 1 | 0 | 0 | 0 | 0 | 0 | 0 | 0 | 0 | 0 | 0 | 0 | 0 | 0 | 0 | 0 | 1 | N | 11919 | protein S100-A11-like [Pseudonaja textilis] |
| **393** | 2244 | XP_026580634.1 | 58.63 | 11 | 0 | 0 | 0 | 0 | 0 | 0 | 0 | 0 | 0 | 0 | 0 | 0 | 0 | 0 | 0 | 11 | | | | | | | | | | | | | | | | 1.0855E5 | 1 | 1 | 0 | 0 | 0 | 0 | 0 | 0 | 0 | 0 | 0 | 0 | 0 | 0 | 0 | 0 | 0 | 1 | N | 11919 | protein S100-A11-like [Pseudonaja textilis] |
| **393** | 2245 | XP_026580630.1 | 58.63 | 11 | 0 | 0 | 0 | 0 | 0 | 0 | 0 | 0 | 0 | 0 | 0 | 0 | 0 | 0 | 0 | 11 | | | | | | | | | | | | | | | | 1.0855E5 | 1 | 1 | 0 | 0 | 0 | 0 | 0 | 0 | 0 | 0 | 0 | 0 | 0 | 0 | 0 | 0 | 0 | 1 | N | 11919 | protein S100-A11-like [Pseudonaja textilis] |
| **393** | 2246 | XP_026580635.1 | 58.63 | 11 | 0 | 0 | 0 | 0 | 0 | 0 | 0 | 0 | 0 | 0 | 0 | 0 | 0 | 0 | 0 | 11 | | | | | | | | | | | | | | | | 1.0855E5 | 1 | 1 | 0 | 0 | 0 | 0 | 0 | 0 | 0 | 0 | 0 | 0 | 0 | 0 | 0 | 0 | 0 | 1 | N | 11919 | protein S100-A11-like [Pseudonaja textilis] |
| **393** | 3360 | XP_015668861.1 | 58.63 | 11 | 0 | 0 | 0 | 0 | 0 | 0 | 0 | 0 | 0 | 0 | 0 | 0 | 0 | 0 | 0 | 11 | | | | | | | | | | | | | | | | 1.0855E5 | 1 | 1 | 0 | 0 | 0 | 0 | 0 | 0 | 0 | 0 | 0 | 0 | 0 | 0 | 0 | 0 | 0 | 1 | N | 12106 | protein S100-A11 [Protobothrops mucrosquamatus] |
| **244** | 4023 | BAK64382.1 | 58.41 | 13 | 0 | 0 | 0 | 0 | 0 | 13 | 0 | 13 | 0 | 0 | 0 | 0 | 0 | 0 | 0 | 0 | | | | | | 7.3485E3 | | 0 | | | | | | | | | 1 | 1 | 0 | 0 | 0 | 0 | 0 | 1 | 0 | 1 | 0 | 0 | 0 | 0 | 0 | 0 | 0 | 0 | Y | 12351 | small serum protein [Laticauda semifasciata] |
| **244** | 4024 | BAJ14707.1 | 58.41 | 13 | 0 | 0 | 0 | 0 | 0 | 13 | 0 | 13 | 0 | 0 | 0 | 0 | 0 | 0 | 0 | 0 | | | | | | 7.3485E3 | | 0 | | | | | | | | | 1 | 1 | 0 | 0 | 0 | 0 | 0 | 1 | 0 | 1 | 0 | 0 | 0 | 0 | 0 | 0 | 0 | 0 | Y | 12412 | small serum protein-2 [Gloydius blomhoffii blomhoffii] |
| **244** | 4025 | BBV14780.1 | 58.41 | 13 | 0 | 0 | 0 | 0 | 0 | 13 | 0 | 13 | 0 | 0 | 0 | 0 | 0 | 0 | 0 | 0 | | | | | | 7.3485E3 | | 0 | | | | | | | | | 1 | 1 | 0 | 0 | 0 | 0 | 0 | 1 | 0 | 1 | 0 | 0 | 0 | 0 | 0 | 0 | 0 | 0 | Y | 12507 | small serum protein-5a [Ophiophagus hannah] |
| **244** | 4026 | XP_026571031.1 | 58.41 | 13 | 0 | 0 | 0 | 0 | 0 | 13 | 0 | 13 | 0 | 0 | 0 | 0 | 0 | 0 | 0 | 0 | | | | | | 7.3485E3 | | 0 | | | | | | | | | 1 | 1 | 0 | 0 | 0 | 0 | 0 | 1 | 0 | 1 | 0 | 0 | 0 | 0 | 0 | 0 | 0 | 0 | Y | 12205 | small serum protein 5-like [Pseudonaja textilis] |
| **244** | 4027 | BBV14781.1 | 58.41 | 13 | 0 | 0 | 0 | 0 | 0 | 13 | 0 | 13 | 0 | 0 | 0 | 0 | 0 | 0 | 0 | 0 | | | | | | 7.3485E3 | | 0 | | | | | | | | | 1 | 1 | 0 | 0 | 0 | 0 | 0 | 1 | 0 | 1 | 0 | 0 | 0 | 0 | 0 | 0 | 0 | 0 | Y | 12410 | small serum protein-5b [Ophiophagus hannah] |
| **244** | 4028 | XP_058050813.1 | 58.41 | 12 | 0 | 0 | 0 | 0 | 0 | 12 | 0 | 12 | 0 | 0 | 0 | 0 | 0 | 0 | 0 | 0 | | | | | | 7.3485E3 | | 0 | | | | | | | | | 1 | 1 | 0 | 0 | 0 | 0 | 0 | 1 | 0 | 1 | 0 | 0 | 0 | 0 | 0 | 0 | 0 | 0 | Y | 13436 | small serum protein 5-like [Ahaetulla prasina] |
| **339** | 1012 | KAG8139853.1 | 53.13 | 2 | 0 | 0 | 0 | 0 | 0 | 0 | 2 | 0 | 0 | 0 | 0 | 0 | 0 | 0 | 0 | 0 | | | | | | | 3.9332E5 | | | | | | | | | | 1 | 1 | 0 | 0 | 0 | 0 | 0 | 0 | 1 | 0 | 0 | 0 | 0 | 0 | 0 | 0 | 0 | 0 | N | 80626 | hypothetical protein E2320_002630 [Naja naja] |
| **339** | 1018 | XP_026573999.1 | 53.13 | 2 | 0 | 0 | 0 | 0 | 0 | 0 | 2 | 0 | 0 | 0 | 0 | 0 | 0 | 0 | 0 | 0 | | | | | | | 3.9332E5 | | | | | | | | | | 1 | 1 | 0 | 0 | 0 | 0 | 0 | 0 | 1 | 0 | 0 | 0 | 0 | 0 | 0 | 0 | 0 | 0 | N | 66924 | granulins isoform X2 [Pseudonaja textilis] |
| **339** | 1019 | XP_026573998.1 | 53.13 | 2 | 0 | 0 | 0 | 0 | 0 | 0 | 2 | 0 | 0 | 0 | 0 | 0 | 0 | 0 | 0 | 0 | | | | | | | 3.9332E5 | | | | | | | | | | 1 | 1 | 0 | 0 | 0 | 0 | 0 | 0 | 1 | 0 | 0 | 0 | 0 | 0 | 0 | 0 | 0 | 0 | N | 67564 | granulins isoform X1 [Pseudonaja textilis] |
| **339** | 1020 | XP_026539436.1 | 53.13 | 2 | 0 | 0 | 0 | 0 | 0 | 0 | 2 | 0 | 0 | 0 | 0 | 0 | 0 | 0 | 0 | 0 | | | | | | | 3.9332E5 | | | | | | | | | | 1 | 1 | 0 | 0 | 0 | 0 | 0 | 0 | 1 | 0 | 0 | 0 | 0 | 0 | 0 | 0 | 0 | 0 | N | 67643 | granulins isoform X1 [Notechis scutatus] |
| **339** | 1021 | XP_026539435.1 | 53.13 | 2 | 0 | 0 | 0 | 0 | 0 | 0 | 2 | 0 | 0 | 0 | 0 | 0 | 0 | 0 | 0 | 0 | | | | | | | 3.9332E5 | | | | | | | | | | 1 | 1 | 0 | 0 | 0 | 0 | 0 | 0 | 1 | 0 | 0 | 0 | 0 | 0 | 0 | 0 | 0 | 0 | N | 67643 | granulins isoform X1 [Notechis scutatus] |
| **339** | 1022 | XP_026539437.1 | 53.13 | 2 | 0 | 0 | 0 | 0 | 0 | 0 | 2 | 0 | 0 | 0 | 0 | 0 | 0 | 0 | 0 | 0 | | | | | | | 3.9332E5 | | | | | | | | | | 1 | 1 | 0 | 0 | 0 | 0 | 0 | 0 | 1 | 0 | 0 | 0 | 0 | 0 | 0 | 0 | 0 | 0 | N | 67003 | granulins isoform X2 [Notechis scutatus] |
| **339** | 1023 | ETE73811.1 | 53.13 | 1 | 0 | 0 | 0 | 0 | 0 | 0 | 1 | 0 | 0 | 0 | 0 | 0 | 0 | 0 | 0 | 0 | | | | | | | 3.9332E5 | | | | | | | | | | 1 | 1 | 0 | 0 | 0 | 0 | 0 | 0 | 1 | 0 | 0 | 0 | 0 | 0 | 0 | 0 | 0 | 0 | N | 86238 | Granulin [Ophiophagus hannah] |
| **339** | 1460 | LAA86163.1 | 53.13 | 2 | 0 | 0 | 0 | 0 | 0 | 0 | 2 | 0 | 0 | 0 | 0 | 0 | 0 | 0 | 0 | 0 | | | | | | | 3.9332E5 | | | | | | | | | | 1 | 1 | 0 | 0 | 0 | 0 | 0 | 0 | 1 | 0 | 0 | 0 | 0 | 0 | 0 | 0 | 0 | 0 | N | 67294 | hypothetical protein [Micrurus lemniscatus lemniscatus] |
| **339** | 1472 | XP_026574000.1 | 53.13 | 2 | 0 | 0 | 0 | 0 | 0 | 0 | 2 | 0 | 0 | 0 | 0 | 0 | 0 | 0 | 0 | 0 | | | | | | | 3.9332E5 | | | | | | | | | | 1 | 1 | 0 | 0 | 0 | 0 | 0 | 0 | 1 | 0 | 0 | 0 | 0 | 0 | 0 | 0 | 0 | 0 | N | 58279 | granulins isoform X3 [Pseudonaja textilis] |
| **339** | 1473 | XP_026539438.1 | 53.13 | 2 | 0 | 0 | 0 | 0 | 0 | 0 | 2 | 0 | 0 | 0 | 0 | 0 | 0 | 0 | 0 | 0 | | | | | | | 3.9332E5 | | | | | | | | | | 1 | 1 | 0 | 0 | 0 | 0 | 0 | 0 | 1 | 0 | 0 | 0 | 0 | 0 | 0 | 0 | 0 | 0 | N | 58322 | granulins isoform X3 [Notechis scutatus] |
| **339** | 1509 | LAA38501.1 | 53.13 | 2 | 0 | 0 | 0 | 0 | 0 | 0 | 2 | 0 | 0 | 0 | 0 | 0 | 0 | 0 | 0 | 0 | | | | | | | 3.9332E5 | | | | | | | | | | 1 | 1 | 0 | 0 | 0 | 0 | 0 | 0 | 1 | 0 | 0 | 0 | 0 | 0 | 0 | 0 | 0 | 0 | N | 58521 | hypothetical protein [Micrurus corallinus] |
| **339** | 1549 | LAB57775.1 | 53.13 | 4 | 0 | 0 | 0 | 0 | 0 | 0 | 4 | 0 | 0 | 0 | 0 | 0 | 0 | 0 | 0 | 0 | | | | | | | 3.9332E5 | | | | | | | | | | 1 | 1 | 0 | 0 | 0 | 0 | 0 | 0 | 1 | 0 | 0 | 0 | 0 | 0 | 0 | 0 | 0 | 0 | N | 30373 | hypothetical protein, partial [Micrurus surinamensis] |
| **339** | 1584 | LAA20302.1 | 53.13 | 3 | 0 | 0 | 0 | 0 | 0 | 0 | 3 | 0 | 0 | 0 | 0 | 0 | 0 | 0 | 0 | 0 | | | | | | | 3.9332E5 | | | | | | | | | | 1 | 1 | 0 | 0 | 0 | 0 | 0 | 0 | 1 | 0 | 0 | 0 | 0 | 0 | 0 | 0 | 0 | 0 | N | 40689 | hypothetical protein, partial [Micrurus lemniscatus carvalhoi] |
| **339** | 3231 | LAA38500.1 | 53.13 | 5 | 0 | 0 | 0 | 0 | 0 | 0 | 5 | 0 | 0 | 0 | 0 | 0 | 0 | 0 | 0 | 0 | | | | | | | 3.9332E5 | | | | | | | | | | 1 | 1 | 0 | 0 | 0 | 0 | 0 | 0 | 1 | 0 | 0 | 0 | 0 | 0 | 0 | 0 | 0 | 0 | N | 26065 | hypothetical protein [Micrurus corallinus] |
| **339** | 3232 | LAB57770.1 | 53.13 | 3 | 0 | 0 | 0 | 0 | 0 | 0 | 3 | 0 | 0 | 0 | 0 | 0 | 0 | 0 | 0 | 0 | | | | | | | 3.9332E5 | | | | | | | | | | 1 | 1 | 0 | 0 | 0 | 0 | 0 | 0 | 1 | 0 | 0 | 0 | 0 | 0 | 0 | 0 | 0 | 0 | N | 47224 | hypothetical protein, partial [Micrurus surinamensis] |
| **190** | 1851 | C0HKZ8.2 | 49.41 | 22 | 0 | 0 | 0 | 0 | 0 | 22 | 0 | 22 | 0 | 0 | 0 | 0 | 0 | 0 | 0 | 0 | | | | | | 2.5296E5 | | 1.4348E5 | | | | | | | | | 1 | 1 | 0 | 0 | 0 | 0 | 0 | 1 | 0 | 1 | 0 | 0 | 0 | 0 | 0 | 0 | 0 | 0 | Y | 7340 | RecName: Full=Actiflagelin |
| **251** | 1597 | XP_039188891.1 | 48.76 | 2 | 0 | 0 | 2 | 0 | 0 | 0 | 0 | 0 | 0 | 0 | 0 | 0 | 0 | 0 | 0 | 0 | | | 7.2678E4 | | | | | | | | | | | | | | 1 | 1 | 0 | 0 | 1 | 0 | 0 | 0 | 0 | 0 | 0 | 0 | 0 | 0 | 0 | 0 | 0 | 0 | N | 69856 | zinc finger protein 703 [Crotalus tigris] |
| **318** | 1639 | KAG8122958.1 | 47.27 | 3 | 3 | 0 | 0 | 0 | 0 | 0 | 0 | 0 | 0 | 0 | 0 | 0 | 0 | 0 | 0 | 0 | 4.5257E5 | | | | | | | | | | | | | | | | 1 | 1 | 1 | 0 | 0 | 0 | 0 | 0 | 0 | 0 | 0 | 0 | 0 | 0 | 0 | 0 | 0 | 0 | N | 91720 | hypothetical protein E2320_018454 [Naja naja] |
| **281** | 1550 | KAG8137413.1 | 46.68 | 8 | 0 | 0 | 0 | 0 | 0 | 0 | 0 | 0 | 0 | 0 | 0 | 0 | 0 | 8 | 0 | 0 | | | | | | | | | | | | | | 0 | | | 1 | 1 | 0 | 0 | 0 | 0 | 0 | 0 | 0 | 0 | 0 | 0 | 0 | 0 | 0 | 1 | 0 | 0 | N | 35528 | hypothetical protein E2320_004651 [Naja naja] |
| **234** | 1182 | KAG8138472.1 | 46.62 | 10 | 10 | 0 | 0 | 0 | 0 | 0 | 0 | 0 | 0 | 0 | 0 | 0 | 0 | 0 | 0 | 0 | 3.8789E5 | | | | | | | | | | | | | | | | 1 | 1 | 1 | 0 | 0 | 0 | 0 | 0 | 0 | 0 | 0 | 0 | 0 | 0 | 0 | 0 | 0 | 0 | N | 16307 | putative Endonuclease domain-containing 1 protein [Naja naja] |
| **392** | 1819 | XP_026569519.1 | 43.98 | 13 | 0 | 0 | 0 | 0 | 0 | 0 | 0 | 0 | 0 | 0 | 0 | 0 | 0 | 0 | 0 | 13 | | | | | | | | | | | | | | | | 1.7557E4 | 1 | 1 | 0 | 0 | 0 | 0 | 0 | 0 | 0 | 0 | 0 | 0 | 0 | 0 | 0 | 0 | 0 | 1 | N | 13980 | histone H2B 5 [Pseudonaja textilis] |
| **392** | 1820 | XP_026576366.1 | 43.98 | 13 | 0 | 0 | 0 | 0 | 0 | 0 | 0 | 0 | 0 | 0 | 0 | 0 | 0 | 0 | 0 | 13 | | | | | | | | | | | | | | | | 1.7557E4 | 1 | 1 | 0 | 0 | 0 | 0 | 0 | 0 | 0 | 0 | 0 | 0 | 0 | 0 | 0 | 0 | 0 | 1 | N | 13952 | histone H2B 1/2/3/4/6 [Pseudonaja textilis] |
| **392** | 1821 | XP_026569640.1 | 43.98 | 13 | 0 | 0 | 0 | 0 | 0 | 0 | 0 | 0 | 0 | 0 | 0 | 0 | 0 | 0 | 0 | 13 | | | | | | | | | | | | | | | | 1.7557E4 | 1 | 1 | 0 | 0 | 0 | 0 | 0 | 0 | 0 | 0 | 0 | 0 | 0 | 0 | 0 | 0 | 0 | 1 | N | 13980 | histone H2B 5 [Pseudonaja textilis] |
| **392** | 1822 | XP_015678747.1 | 43.98 | 13 | 0 | 0 | 0 | 0 | 0 | 0 | 0 | 0 | 0 | 0 | 0 | 0 | 0 | 0 | 0 | 13 | | | | | | | | | | | | | | | | 1.7557E4 | 1 | 1 | 0 | 0 | 0 | 0 | 0 | 0 | 0 | 0 | 0 | 0 | 0 | 0 | 0 | 0 | 0 | 1 | N | 13994 | histone H2B 8 [Protobothrops mucrosquamatus] |
| **392** | 1823 | XP_026529644.1 | 43.98 | 13 | 0 | 0 | 0 | 0 | 0 | 0 | 0 | 0 | 0 | 0 | 0 | 0 | 0 | 0 | 0 | 13 | | | | | | | | | | | | | | | | 1.7557E4 | 1 | 1 | 0 | 0 | 0 | 0 | 0 | 0 | 0 | 0 | 0 | 0 | 0 | 0 | 0 | 0 | 0 | 1 | N | 13980 | histone H2B 5 [Notechis scutatus] |
| **392** | 1824 | XP_026539722.1 | 43.98 | 13 | 0 | 0 | 0 | 0 | 0 | 0 | 0 | 0 | 0 | 0 | 0 | 0 | 0 | 0 | 0 | 13 | | | | | | | | | | | | | | | | 1.7557E4 | 1 | 1 | 0 | 0 | 0 | 0 | 0 | 0 | 0 | 0 | 0 | 0 | 0 | 0 | 0 | 0 | 0 | 1 | N | 13952 | histone H2B 1/2/3/4/6 [Notechis scutatus] |
| **392** | 1825 | LAA79020.1 | 43.98 | 13 | 0 | 0 | 0 | 0 | 0 | 0 | 0 | 0 | 0 | 0 | 0 | 0 | 0 | 0 | 0 | 13 | | | | | | | | | | | | | | | | 1.7557E4 | 1 | 1 | 0 | 0 | 0 | 0 | 0 | 0 | 0 | 0 | 0 | 0 | 0 | 0 | 0 | 0 | 0 | 1 | N | 13980 | hypothetical protein [Micrurus lemniscatus lemniscatus] |
| **392** | 1826 | XP_015678744.1 | 43.98 | 13 | 0 | 0 | 0 | 0 | 0 | 0 | 0 | 0 | 0 | 0 | 0 | 0 | 0 | 0 | 0 | 13 | | | | | | | | | | | | | | | | 1.7557E4 | 1 | 1 | 0 | 0 | 0 | 0 | 0 | 0 | 0 | 0 | 0 | 0 | 0 | 0 | 0 | 0 | 0 | 1 | N | 13980 | histone H2B 5 [Protobothrops mucrosquamatus] |
| **392** | 1827 | XP_026529646.1 | 43.98 | 13 | 0 | 0 | 0 | 0 | 0 | 0 | 0 | 0 | 0 | 0 | 0 | 0 | 0 | 0 | 0 | 13 | | | | | | | | | | | | | | | | 1.7557E4 | 1 | 1 | 0 | 0 | 0 | 0 | 0 | 0 | 0 | 0 | 0 | 0 | 0 | 0 | 0 | 0 | 0 | 1 | N | 13980 | histone H2B 5 [Notechis scutatus] |
| **392** | 1828 | XP_025022125.1 | 43.98 | 13 | 0 | 0 | 0 | 0 | 0 | 0 | 0 | 0 | 0 | 0 | 0 | 0 | 0 | 0 | 0 | 13 | | | | | | | | | | | | | | | | 1.7557E4 | 1 | 1 | 0 | 0 | 0 | 0 | 0 | 0 | 0 | 0 | 0 | 0 | 0 | 0 | 0 | 0 | 0 | 1 | N | 13980 | histone H2B 5 [Python bivittatus] |
| **392** | 1902 | XP_007429384.1 | 43.98 | 13 | 0 | 0 | 0 | 0 | 0 | 0 | 0 | 0 | 0 | 0 | 0 | 0 | 0 | 0 | 0 | 13 | | | | | | | | | | | | | | | | 1.7557E4 | 1 | 1 | 0 | 0 | 0 | 0 | 0 | 0 | 0 | 0 | 0 | 0 | 0 | 0 | 0 | 0 | 0 | 1 | N | 14137 | histone H2B 1/2/3/4/6-like [Python bivittatus] |
| **392** | 1903 | XP_015672062.1 | 43.98 | 13 | 0 | 0 | 0 | 0 | 0 | 0 | 0 | 0 | 0 | 0 | 0 | 0 | 0 | 0 | 0 | 13 | | | | | | | | | | | | | | | | 1.7557E4 | 1 | 1 | 0 | 0 | 0 | 0 | 0 | 0 | 0 | 0 | 0 | 0 | 0 | 0 | 0 | 0 | 0 | 1 | N | 14036 | histone H2B 8-like [Protobothrops mucrosquamatus] |
| **392** | 1904 | XP_026545426.1 | 43.98 | 13 | 0 | 0 | 0 | 0 | 0 | 0 | 0 | 0 | 0 | 0 | 0 | 0 | 0 | 0 | 0 | 13 | | | | | | | | | | | | | | | | 1.7557E4 | 1 | 1 | 0 | 0 | 0 | 0 | 0 | 0 | 0 | 0 | 0 | 0 | 0 | 0 | 0 | 0 | 0 | 1 | N | 14050 | histone H2B 8-like [Notechis scutatus] |
| **392** | 1905 | XP_026547833.1 | 43.98 | 13 | 0 | 0 | 0 | 0 | 0 | 0 | 0 | 0 | 0 | 0 | 0 | 0 | 0 | 0 | 0 | 13 | | | | | | | | | | | | | | | | 1.7557E4 | 1 | 1 | 0 | 0 | 0 | 0 | 0 | 0 | 0 | 0 | 0 | 0 | 0 | 0 | 0 | 0 | 0 | 1 | N | 14008 | histone H2B 1/2/3/4/6-like [Notechis scutatus] |
| **392** | 1906 | XP_015672174.1 | 43.98 | 13 | 0 | 0 | 0 | 0 | 0 | 0 | 0 | 0 | 0 | 0 | 0 | 0 | 0 | 0 | 0 | 13 | | | | | | | | | | | | | | | | 1.7557E4 | 1 | 1 | 0 | 0 | 0 | 0 | 0 | 0 | 0 | 0 | 0 | 0 | 0 | 0 | 0 | 0 | 0 | 1 | N | 13996 | histone H2B 8-like [Protobothrops mucrosquamatus] |
| **392** | 1907 | XP_026576334.1 | 43.98 | 13 | 0 | 0 | 0 | 0 | 0 | 0 | 0 | 0 | 0 | 0 | 0 | 0 | 0 | 0 | 0 | 13 | | | | | | | | | | | | | | | | 1.7557E4 | 1 | 1 | 0 | 0 | 0 | 0 | 0 | 0 | 0 | 0 | 0 | 0 | 0 | 0 | 0 | 0 | 0 | 1 | N | 14008 | histone H2B 1/2/3/4/6-like [Pseudonaja textilis] |

| | | | | | | | | | | | | | | | | | | | | | | | | | | | | | | | | | | | | | | | | | | | | | | | | | | | | | | | | | |
|---|---|---|---|---|---|---|---|---|---|---|---|---|---|---|---|---|---|---|---|---|---|---|---|---|---|---|---|---|---|---|---|---|---|---|---|---|---|---|---|---|---|---|---|---|---|---|---|---|---|---|---|---|---|---|---|---|---|
| **392** | 1908 | ETE56555.1 | 43.98 | 13 | 0 | 0 | 0 | 0 | 0 | 0 | 0 | 0 | 0 | 0 | 0 | 0 | 0 | 0 | 0 | 13 | | | | | | | | | | | | | | | | 1.7557E4 | 1 | 1 | 0 | 0 | 0 | 0 | 0 | 0 | 0 | 0 | 0 | 0 | 0 | 0 | 0 | 0 | 0 | 1 | N | 14008 | Histone H2B 1/2/3/4/6, partial [Ophiophagus hannah] |
| **392** | 1909 | XP_013925621.1 | 43.98 | 13 | 0 | 0 | 0 | 0 | 0 | 0 | 0 | 0 | 0 | 0 | 0 | 0 | 0 | 0 | 0 | 13 | | | | | | | | | | | | | | | | 1.7557E4 | 1 | 1 | 0 | 0 | 0 | 0 | 0 | 0 | 0 | 0 | 0 | 0 | 0 | 0 | 0 | 0 | 0 | 1 | N | 14036 | PREDICTED: histone H2B 5 [Thamnophis sirtalis] |
| **392** | 1910 | XP_007429385.1 | 43.98 | 13 | 0 | 0 | 0 | 0 | 0 | 0 | 0 | 0 | 0 | 0 | 0 | 0 | 0 | 0 | 0 | 13 | | | | | | | | | | | | | | | | 1.7557E4 | 1 | 1 | 0 | 0 | 0 | 0 | 0 | 0 | 0 | 0 | 0 | 0 | 0 | 0 | 0 | 0 | 0 | 1 | N | 13994 | histone H2B 1/2/3/4/6-like [Python bivittatus] |
| **392** | 1911 | XP_026580816.1 | 43.98 | 13 | 0 | 0 | 0 | 0 | 0 | 0 | 0 | 0 | 0 | 0 | 0 | 0 | 0 | 0 | 0 | 13 | | | | | | | | | | | | | | | | 1.7557E4 | 1 | 1 | 0 | 0 | 0 | 0 | 0 | 0 | 0 | 0 | 0 | 0 | 0 | 0 | 0 | 0 | 0 | 1 | N | 14050 | histone H2B 8-like [Pseudonaja textilis] |
| **392** | 1912 | XP_026548170.1 | 43.98 | 13 | 0 | 0 | 0 | 0 | 0 | 0 | 0 | 0 | 0 | 0 | 0 | 0 | 0 | 0 | 0 | 13 | | | | | | | | | | | | | | | | 1.7557E4 | 1 | 1 | 0 | 0 | 0 | 0 | 0 | 0 | 0 | 0 | 0 | 0 | 0 | 0 | 0 | 0 | 0 | 1 | N | 13994 | histone H2B 1/2/3/4/6-like [Notechis scutatus] |
| **392** | 1913 | XP_026576364.1 | 43.98 | 13 | 0 | 0 | 0 | 0 | 0 | 0 | 0 | 0 | 0 | 0 | 0 | 0 | 0 | 0 | 0 | 13 | | | | | | | | | | | | | | | | 1.7557E4 | 1 | 1 | 0 | 0 | 0 | 0 | 0 | 0 | 0 | 0 | 0 | 0 | 0 | 0 | 0 | 0 | 0 | 1 | N | 13980 | histone H2B 1/2/3/4/6-like [Pseudonaja textilis] |
| **392** | 1914 | XP_026545427.1 | 43.98 | 13 | 0 | 0 | 0 | 0 | 0 | 0 | 0 | 0 | 0 | 0 | 0 | 0 | 0 | 0 | 0 | 13 | | | | | | | | | | | | | | | | 1.7557E4 | 1 | 1 | 0 | 0 | 0 | 0 | 0 | 0 | 0 | 0 | 0 | 0 | 0 | 0 | 0 | 0 | 0 | 1 | N | 14050 | histone H2B 8-like [Notechis scutatus] |
| **392** | 1915 | XP_026576365.1 | 43.98 | 13 | 0 | 0 | 0 | 0 | 0 | 0 | 0 | 0 | 0 | 0 | 0 | 0 | 0 | 0 | 0 | 13 | | | | | | | | | | | | | | | | 1.7557E4 | 1 | 1 | 0 | 0 | 0 | 0 | 0 | 0 | 0 | 0 | 0 | 0 | 0 | 0 | 0 | 0 | 0 | 1 | N | 13994 | histone H2B 1/2/3/4/6-like [Pseudonaja textilis] |
| **392** | 1916 | XP_013930808.1 | 43.98 | 13 | 0 | 0 | 0 | 0 | 0 | 0 | 0 | 0 | 0 | 0 | 0 | 0 | 0 | 0 | 0 | 13 | | | | | | | | | | | | | | | | 1.7557E4 | 1 | 1 | 0 | 0 | 0 | 0 | 0 | 0 | 0 | 0 | 0 | 0 | 0 | 0 | 0 | 0 | 0 | 1 | N | 13931 | PREDICTED: histone H2B 1/2/3/4/6-like [Thamnophis sirtalis] |
| **392** | 1917 | XP_026539701.1 | 43.98 | 13 | 0 | 0 | 0 | 0 | 0 | 0 | 0 | 0 | 0 | 0 | 0 | 0 | 0 | 0 | 0 | 13 | | | | | | | | | | | | | | | | 1.7557E4 | 1 | 1 | 0 | 0 | 0 | 0 | 0 | 0 | 0 | 0 | 0 | 0 | 0 | 0 | 0 | 0 | 0 | 1 | N | 14008 | histone H2B 1/2/3/4/6-like [Notechis scutatus] |
| **392** | 3252 | XP_015675791.1 | 43.98 | 22 | 0 | 0 | 0 | 0 | 0 | 0 | 0 | 0 | 0 | 0 | 0 | 0 | 0 | 0 | 0 | 22 | | | | | | | | | | | | | | | | 1.7557E4 | 1 | 1 | 0 | 0 | 0 | 0 | 0 | 0 | 0 | 0 | 0 | 0 | 0 | 0 | 0 | 0 | 0 | 1 | N | 8685 | histone H2B-like [Protobothrops mucrosquamatus] |
| **392** | 3253 | XP_026576367.1 | 43.98 | 13 | 0 | 0 | 0 | 0 | 0 | 0 | 0 | 0 | 0 | 0 | 0 | 0 | 0 | 0 | 0 | 13 | | | | | | | | | | | | | | | | 1.7557E4 | 1 | 1 | 0 | 0 | 0 | 0 | 0 | 0 | 0 | 0 | 0 | 0 | 0 | 0 | 0 | 0 | 0 | 1 | N | 13998 | histone H2B 1/2/3/4/6-like [Pseudonaja textilis] |
| **247** | 1008 | XP_029139092.1 | 43.30 | 2 | 0 | 0 | 0 | 0 | 0 | 0 | 0 | 2 | 0 | 0 | 0 | 0 | 0 | 0 | 0 | 0 | | | | | | | | 0 | | | | | | | | | 1 | 1 | 0 | 0 | 0 | 0 | 0 | 0 | 0 | 1 | 0 | 0 | 0 | 0 | 0 | 0 | 0 | 0 | N | 127124 | collagen alpha-2(I) chain isoform X2 [Protobothrops mucrosquamatus] |
| **247** | 1009 | XP_029139091.1 | 43.30 | 2 | 0 | 0 | 0 | 0 | 0 | 0 | 0 | 2 | 0 | 0 | 0 | 0 | 0 | 0 | 0 | 0 | | | | | | | | 0 | | | | | | | | | 1 | 1 | 0 | 0 | 0 | 0 | 0 | 0 | 0 | 1 | 0 | 0 | 0 | 0 | 0 | 0 | 0 | 0 | N | 117238 | collagen alpha-2(I) chain isoform X1 [Protobothrops mucrosquamatus] |
| **353** | 2088 | XP_013923409.1 | 42.98 | 2 | 0 | 0 | 0 | 0 | 0 | 0 | 0 | 0 | 0 | 0 | 0 | 2 | 0 | 0 | 0 | 0 | | | | | | | | | | | | 0 | | | | | 1 | 1 | 0 | 0 | 0 | 0 | 0 | 0 | 0 | 0 | 0 | 0 | 0 | 1 | 0 | 0 | 0 | 0 | N | 117394 | PREDICTED: ELKS/Rab6-interacting/CAST family member 1 isoform X10 [Thamnophis sirtalis] |
| **259** | 1710 | XP_039182761.1 | 42.54 | 1 | 0 | 0 | 0 | 0 | 0 | 1 | 0 | 0 | 0 | 0 | 0 | 0 | 0 | 0 | 0 | 0 | | | | | | 0 | | | | | | | | | | | 1 | 1 | 0 | 0 | 0 | 0 | 0 | 1 | 0 | 0 | 0 | 0 | 0 | 0 | 0 | 0 | 0 | 0 | N | 351102 | nucleosome-remodeling factor subunit BPTF isoform X5 [Crotalus tigris] |
| **259** | 1711 | XP_039182758.1 | 42.54 | 1 | 0 | 0 | 0 | 0 | 0 | 1 | 0 | 0 | 0 | 0 | 0 | 0 | 0 | 0 | 0 | 0 | | | | | | 0 | | | | | | | | | | | 1 | 1 | 0 | 0 | 0 | 0 | 0 | 1 | 0 | 0 | 0 | 0 | 0 | 0 | 0 | 0 | 0 | 0 | N | 354680 | nucleosome-remodeling factor subunit BPTF isoform X3 [Crotalus tigris] |
| **259** | 1712 | XP_039182757.1 | 42.54 | 1 | 0 | 0 | 0 | 0 | 0 | 1 | 0 | 0 | 0 | 0 | 0 | 0 | 0 | 0 | 0 | 0 | | | | | | 0 | | | | | | | | | | | 1 | 1 | 0 | 0 | 0 | 0 | 0 | 1 | 0 | 0 | 0 | 0 | 0 | 0 | 0 | 0 | 0 | 0 | N | 357710 | nucleosome-remodeling factor subunit BPTF isoform X2 [Crotalus tigris] |
| **259** | 1713 | XP_039182756.1 | 42.54 | 1 | 0 | 0 | 0 | 0 | 0 | 1 | 0 | 0 | 0 | 0 | 0 | 0 | 0 | 0 | 0 | 0 | | | | | | 0 | | | | | | | | | | | 1 | 1 | 0 | 0 | 0 | 0 | 0 | 1 | 0 | 0 | 0 | 0 | 0 | 0 | 0 | 0 | 0 | 0 | N | 357839 | nucleosome-remodeling factor subunit BPTF isoform X1 [Crotalus tigris] |
| **259** | 1714 | XP_039182763.1 | 42.54 | 1 | 0 | 0 | 0 | 0 | 0 | 1 | 0 | 0 | 0 | 0 | 0 | 0 | 0 | 0 | 0 | 0 | | | | | | 0 | | | | | | | | | | | 1 | 1 | 0 | 0 | 0 | 0 | 0 | 1 | 0 | 0 | 0 | 0 | 0 | 0 | 0 | 0 | 0 | 0 | N | 342687 | nucleosome-remodeling factor subunit BPTF isoform X7 [Crotalus tigris] |
| **259** | 1715 | XP_039182762.1 | 42.54 | 1 | 0 | 0 | 0 | 0 | 0 | 1 | 0 | 0 | 0 | 0 | 0 | 0 | 0 | 0 | 0 | 0 | | | | | | 0 | | | | | | | | | | | 1 | 1 | 0 | 0 | 0 | 0 | 0 | 1 | 0 | 0 | 0 | 0 | 0 | 0 | 0 | 0 | 0 | 0 | N | 349424 | nucleosome-remodeling factor subunit BPTF isoform X6 [Crotalus tigris] |
| **259** | 1716 | XP_039182759.1 | 42.54 | 1 | 0 | 0 | 0 | 0 | 0 | 1 | 0 | 0 | 0 | 0 | 0 | 0 | 0 | 0 | 0 | 0 | | | | | | 0 | | | | | | | | | | | 1 | 1 | 0 | 0 | 0 | 0 | 0 | 1 | 0 | 0 | 0 | 0 | 0 | 0 | 0 | 0 | 0 | 0 | N | 352583 | nucleosome-remodeling factor subunit BPTF isoform X4 [Crotalus tigris] |
| **337** | 1886 | XP_034278588.1 | 42.47 | 3 | 0 | 0 | 0 | 0 | 0 | 0 | 0 | 0 | 0 | 0 | 0 | 0 | 0 | 3 | 0 | 0 | | | | | | | | | | | | | | 0 | | | 1 | 1 | 0 | 0 | 0 | 0 | 0 | 0 | 0 | 0 | 0 | 0 | 0 | 0 | 0 | 1 | 0 | 0 | N | 92899 | scaffold attachment factor B1 [Pantherophis guttatus] |
| **399** | 2436 | P82464.1 | 42.32 | 14 | 0 | 0 | 0 | 0 | 0 | 0 | 0 | 0 | 14 | 0 | 0 | 0 | 0 | 0 | 0 | 0 | | | | | | | | | 3.152E5 | | | | | | | | 1 | 1 | 0 | 0 | 0 | 0 | 0 | 0 | 0 | 0 | 1 | 0 | 0 | 0 | 0 | 0 | 0 | 0 | Y | 7624 | RecName: Full=Muscarinic toxin-like protein 3; Short=MTLP-3 |
| **357** | 1838 | XP_058025957.1 | 42.03 | 1 | 0 | 0 | 0 | 0 | 0 | 0 | 0 | 0 | 0 | 1 | 0 | 0 | 0 | 0 | 0 | 0 | | | | | | | | | | 0 | | | | | | | 1 | 1 | 0 | 0 | 0 | 0 | 0 | 0 | 0 | 0 | 0 | 1 | 0 | 0 | 0 | 0 | 0 | 0 | N | 151890 | SAM and SH3 domain-containing protein 1-like isoform X4 [Ahaetulla prasina] |
| **186** | 2816 | Q8AY45.2 | 41.37 | 11 | 0 | 0 | 0 | 0 | 11 | 0 | 0 | 0 | 0 | 0 | 0 | 0 | 0 | 0 | 0 | 0 | | | | | 3.1771E6 | | | | | | | | | | | | 1 | 1 | 0 | 0 | 0 | 0 | 1 | 0 | 0 | 0 | 0 | 0 | 0 | 0 | 0 | 0 | 0 | 0 | N | 9592 | RecName: Full=Kunitz-type serine protease inhibitor homolog beta-bungarotoxin B2a chain; Flags: Precursor |
| **186** | 2817 | AAL30066.1 | 41.37 | 10 | 0 | 0 | 0 | 0 | 10 | 0 | 0 | 0 | 0 | 0 | 0 | 0 | 0 | 0 | 0 | 0 | | | | | 3.1771E6 | | | | | | | | | | | | 1 | 1 | 0 | 0 | 0 | 0 | 1 | 0 | 0 | 0 | 0 | 0 | 0 | 0 | 0 | 0 | 0 | 0 | N | 10122 | beta bungarotoxin B2a chain, partial [Bungarus candidus] |
| **186** | 3408 | AAB32179.1 | 41.37 | 23 | 0 | 0 | 0 | 0 | 23 | 0 | 0 | 0 | 0 | 0 | 0 | 0 | 0 | 0 | 0 | 0 | | | | | 3.1771E6 | | | | | | | | | | | | 1 | 1 | 0 | 0 | 0 | 0 | 1 | 0 | 0 | 0 | 0 | 0 | 0 | 0 | 0 | 0 | 0 | 0 | N | 4616 | beta-bungarotoxin subunit SP II-B, beta-Butx subunit SP II-B=non-phospholipase A2 subunit {N-terminal} [Bungarus multicinctus=Taiwan banded kraits, venom, Peptide Partial, 39 aa] |
| **186** | 3409 | 1BUN | 41.37 | 15 | 0 | 0 | 0 | 0 | 15 | 0 | 0 | 0 | 0 | 0 | 0 | 0 | 0 | 0 | 0 | 0 | | | | | 3.1771E6 | | | | | | | | | | | | 1 | 1 | 0 | 0 | 0 | 0 | 1 | 0 | 0 | 0 | 0 | 0 | 0 | 0 | 0 | 0 | 0 | 0 | N | 7197 | Chain B, BETA2-BUNGAROTOXIN |
| **186** | 3410 | CAB62504.1 | 41.37 | 11 | 0 | 0 | 0 | 0 | 11 | 0 | 0 | 0 | 0 | 0 | 0 | 0 | 0 | 0 | 0 | 0 | | | | | 3.1771E6 | | | | | | | | | | | | 1 | 1 | 0 | 0 | 0 | 0 | 1 | 0 | 0 | 0 | 0 | 0 | 0 | 0 | 0 | 0 | 0 | 0 | N | 9120 | beta-bungarotoxin B2, partial [Bungarus multicinctus] |
| **186** | 3411 | CAJ18318.1 | 41.37 | 11 | 0 | 0 | 0 | 0 | 11 | 0 | 0 | 0 | 0 | 0 | 0 | 0 | 0 | 0 | 0 | 0 | | | | | 3.1771E6 | | | | | | | | | | | | 1 | 1 | 0 | 0 | 0 | 0 | 1 | 0 | 0 | 0 | 0 | 0 | 0 | 0 | 0 | 0 | 0 | 0 | N | 9568 | beta-bungarotoxin b2 chain precursor [Bungarus multicinctus] |
| **186** | 3412 | CAJ18319.1 | 41.37 | 11 | 0 | 0 | 0 | 0 | 11 | 0 | 0 | 0 | 0 | 0 | 0 | 0 | 0 | 0 | 0 | 0 | | | | | 3.1771E6 | | | | | | | | | | | | 1 | 1 | 0 | 0 | 0 | 0 | 1 | 0 | 0 | 0 | 0 | 0 | 0 | 0 | 0 | 0 | 0 | 0 | N | 9499 | beta-bungarotoxin b4 chain precursor [Bungarus multicinctus] |
| **186** | 3413 | Q1RPT0.1 | 41.37 | 11 | 0 | 0 | 0 | 0 | 11 | 0 | 0 | 0 | 0 | 0 | 0 | 0 | 0 | 0 | 0 | 0 | | | | | 3.1771E6 | | | | | | | | | | | | 1 | 1 | 0 | 0 | 0 | 0 | 1 | 0 | 0 | 0 | 0 | 0 | 0 | 0 | 0 | 0 | 0 | 0 | N | 9499 | RecName: Full=Kunitz-type serine protease inhibitor homolog beta-bungarotoxin B4 chain; Flags: Precursor |
| **186** | 3414 | P00989.2 | 41.37 | 11 | 0 | 0 | 0 | 0 | 11 | 0 | 0 | 0 | 0 | 0 | 0 | 0 | 0 | 0 | 0 | 0 | | | | | 3.1771E6 | | | | | | | | | | | | 1 | 1 | 0 | 0 | 0 | 0 | 1 | 0 | 0 | 0 | 0 | 0 | 0 | 0 | 0 | 0 | 0 | 0 | N | 9568 | RecName: Full=Kunitz-type serine protease inhibitor homolog beta-bungarotoxin B2 chain; AltName: Full=Beta-2-bungarotoxin; Flags: Precursor |
| **186** | 3415 | CAA72810.1 | 41.37 | 11 | 0 | 0 | 0 | 0 | 11 | 0 | 0 | 0 | 0 | 0 | 0 | 0 | 0 | 0 | 0 | 0 | | | | | 3.1771E6 | | | | | | | | | | | | 1 | 1 | 0 | 0 | 0 | 0 | 1 | 0 | 0 | 0 | 0 | 0 | 0 | 0 | 0 | 0 | 0 | 0 | N | 9568 | bungarotoxin, B2 chain [Bungarus multicinctus] |
| **186** | 3878 | ABY71037.1 | 41.37 | 11 | 0 | 0 | 0 | 0 | 11 | 0 | 0 | 0 | 0 | 0 | 0 | 0 | 0 | 0 | 0 | 0 | | | | | 3.1771E6 | | | | | | | | | | | | 1 | 1 | 0 | 0 | 0 | 0 | 1 | 0 | 0 | 0 | 0 | 0 | 0 | 0 | 0 | 0 | 0 | 0 | N | 9636 | beta bungarotoxin BF B1 [Bungarus fasciatus] |
| **186** | 3879 | B2KTG2.1 | 41.37 | 11 | 0 | 0 | 0 | 0 | 11 | 0 | 0 | 0 | 0 | 0 | 0 | 0 | 0 | 0 | 0 | 0 | | | | | 3.1771E6 | | | | | | | | | | | | 1 | 1 | 0 | 0 | 0 | 0 | 1 | 0 | 0 | 0 | 0 | 0 | 0 | 0 | 0 | 0 | 0 | 0 | N | 9636 | RecName: Full=Kunitz-type serine protease inhibitor homolog beta-bungarotoxin BF B1 chain; Flags: Precursor |
| **186** | 4266 | 0805258B | 41.37 | 15 | 0 | 0 | 0 | 0 | 15 | 0 | 0 | 0 | 0 | 0 | 0 | 0 | 0 | 0 | 0 | 0 | | | | | 3.1771E6 | | | | | | | | | | | | 1 | 1 | 0 | 0 | 0 | 0 | 1 | 0 | 0 | 0 | 0 | 0 | 0 | 0 | 0 | 0 | 0 | 0 | N | 7100 | toxin Bbeta2,bungaro |
| **367** | 2411 | XP_026523812.1 | 41.12 | 2 | 0 | 0 | 0 | 0 | 0 | 0 | 0 | 0 | 0 | 0 | 0 | 0 | 0 | 2 | 0 | 0 | | | | | | | | | | | | | | 4.8334E5 | | | 1 | 1 | 0 | 0 | 0 | 0 | 0 | 0 | 0 | 0 | 0 | 0 | 0 | 0 | 0 | 1 | 0 | 0 | Y | 89622 | protein FAM171B [Notechis scutatus] |
| **206** | 1643 | XP_026556042.1 | 41.02 | 2 | 0 | 0 | 2 | 0 | 0 | 0 | 0 | 0 | 0 | 0 | 0 | 0 | 0 | 0 | 0 | 0 | | | 1.1974E6 | | | | | | | | | | | | | | 1 | 1 | 0 | 0 | 1 | 0 | 0 | 0 | 0 | 0 | 0 | 0 | 0 | 0 | 0 | 0 | 0 | 0 | N | 160070 | rho GTPase-activating protein SYDE2 isoform X1 [Pseudonaja textilis] |

**total 568 proteins**

## AAL87004.1

back to list

| Protein Coverage | Supporting Peptides | Best Unique PSM |

**Protein Coverage:**

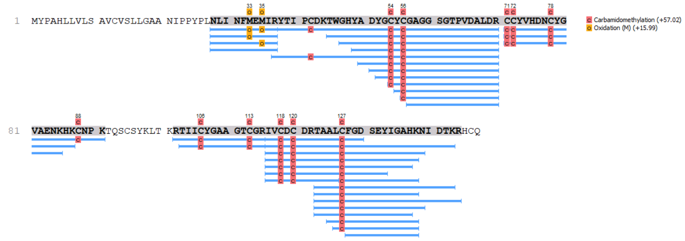


**Supporting Peptides:**

| Peptide | Uniq | -10lgP | Mass | Length | ppm | m/z | z | RT | Fraction | Scan | Source File | Area F1 | Area F2a | Area F3a | Area F4 | Area F5 | Area F6a | Area F7a | Area F7b | Area F8 | Area F9a | Area Sample 1F10a | Area F10b | Area F11a | Area F11b | Area F12a | Area F13 | #Feature | #Feature F1 | #Feature F2a | #Feature F3a | #Feature F4 | #Feature F5 | #Feature F6a | #Feature F7a | #Feature F7b | #Feature F8 | #Feature F9a | #Feature Sample 1F10a | #Feature F10b | #Feature F11a | #Feature F11b | #Feature F12a | #Feature F13 | Start | End | PTM | AScore | Found By |
|---|---|---|---|---|---|---|---|---|---|---|---|---|---|---|---|---|---|---|---|---|---|---|---|---|---|---|---|---|---|---|---|---|---|---|---|---|---|---|---|---|---|---|---|---|---|---|---|---|---|
| K.TWGHYADYGC(+57.02)YC(+57.02)GAGGSGTPVDALDR.C | Y | 123.31 | 2805.1548 | 26 | 0.1 | 936.0590 | 3 | 63.11 | 10 | F10:39407 | BuCaTN01_F9a.raw | | | | | | | | 2.5324E6 | | 9.8815E7 | | 4.0328E6 | | 1.6545E6 | 2.2952E6 | | 9 | 0 | 0 | 0 | 0 | 0 | 0 | 0 | 1 | 0 | 5 | 0 | 1 | 0 | 1 | 1 | 0 | 45 | 70 | Carbamidomethylation | C10:Carbamidomethylation:1000.00;C12:Carbamidomethylation:1000.00 | PEAKS DB |
| R.C(+57.02)C(+57.02)YVHDNC(+57.02)YGVAENK.H | Y | 118.36 | 1887.7339 | 15 | -1.0 | 944.8733 | 2 | 36.88 | 10 | F10:15373 | BuCaTN01_F9a.raw | | | | | | 8.6825E6 | 7.2754E5 | 1.7922E7 | | 2.0038E8 | | 1.582E7 | | 3.5092E5 | 5.7797E6 | | 15 | 0 | 0 | 0 | 0 | 0 | 2 | 1 | 3 | 0 | 4 | 0 | 2 | 0 | 1 | 2 | 0 | 71 | 85 | Carbamidomethylation | C1:Carbamidomethylation:1000.00;C2:Carbamidomethylation:1000.00;C8:Carbamidomethylation:1000.00 | PEAKS DB |
| R.TAALC(+57.02)FGDSEYIGAHK.N | Y | 115.33 | 1738.7985 | 16 | 0.5 | 870.4070 | 2 | 55.64 | 11 | F11:25709 | BuCaTN01_F10a.raw | | | | | | 2.2971E7 | 9.4031E5 | 3.1551E7 | 3.8456E6 | 5.0817E8 | 6.7962E7 | 9.1556E7 | 7.6662E6 | 5.867E7 | 2.7538E7 | 5.2901E6 | 119 | 0 | 0 | 0 | 0 | 0 | 2 | 1 | 11 | 2 | 12 | 26 | 53 | 3 | 4 | 2 | 3 | 123 | 138 | Carbamidomethylation | C5:Carbamidomethylation:1000.00 | PEAKS DB |
| R.IVC(+57.02)DC(+57.02)DRTAALC(+57.02)FGDSEYIGAHK.N | Y | 105.04 | 2657.1672 | 23 | -0.2 | 886.7296 | 3 | 57.28 | 11 | F11:26923 | BuCaTN01_F10a.raw | | | | | | 3.0593E7 | | 4.8915E7 | 6.1227E6 | 7.9689E8 | 8.3497E7 | 8.4757E7 | 6.009E6 | 2.1907E7 | 3.0944E7 | 3.7153E6 | 99 | 0 | 0 | 0 | 0 | 0 | 1 | 0 | 4 | 1 | 44 | 35 | 3 | 2 | 2 | 6 | 1 | 116 | 138 | Carbamidomethylation | C3:Carbamidomethylation:1000.00;C5:Carbamidomethylation:1000.00;C12:Carbamidomethylation:1000.00 | PEAKS DB |
| R.YTIPC(+57.02)DKTWGHYADYGC(+57.02)YC(+57.02)GAGGSGTPVDALDR.C | Y | 99.95 | 3682.5552 | 33 | 1.1 | 1228.5270 | 3 | 67.23 | 10 | F10:43604 | BuCaTN01_F9a.raw | | | | | | 3.4213E6 | | 9.7534E6 | | 4.2682E8 | 2.0095E6 | 1.1818E7 | | 3.7351E6 | 9.6566E6 | | 10 | 0 | 0 | 0 | 0 | 0 | 1 | 0 | 1 | 0 | 4 | 1 | 1 | 0 | 1 | 1 | 0 | 38 | 70 | Carbamidomethylation | C5:Carbamidomethylation:1000.00;C17:Carbamidomethylation:1000.00;C19:Carbamidomethylation:1000.00 | PEAKS DB |
| R.C(+57.02)C(+57.02)YVHDNC(+57.02)YGVAENKHK.C | Y | 96.11 | 2152.8877 | 17 | 0.5 | 718.6368 | 3 | 30.97 | 10 | F10:10373 | BuCaTN01_F9a.raw | | | | | | 2.7157E6 | | 9.9188E6 | | 8.1173E7 | | 7.6189E6 | | | 2.8521E6 | | 15 | 0 | 0 | 0 | 0 | 0 | 1 | 0 | 2 | 0 | 8 | 0 | 2 | 0 | 0 | 2 | 0 | 71 | 87 | Carbamidomethylation | C1:Carbamidomethylation:1000.00;C2:Carbamidomethylation:1000.00;C8:Carbamidomethylation:1000.00 | PEAKS DB |
| A.DYGC(+57.02)YC(+57.02)GAGGSGTPVDALDR.C | Y | 91.42 | 2089.8469 | 20 | -1.9 | 1045.9287 | 2 | 55.35 | 6 | F6:33988 | BuCaTN01_F6a.raw | | | | | | 3.8475E7 | 0 | 1.781E6 | | 0 | | | | | | | 5 | 0 | 0 | 0 | 0 | 0 | 3 | 0 | 2 | 0 | 0 | 0 | 0 | 0 | 0 | 0 | 0 | 51 | 70 | Carbamidomethylation | C4:Carbamidomethylation:1000.00;C6:Carbamidomethylation:1000.00 | PEAKS DB |
| R.TIIC(+57.02)YGAAGTC(+57.02)GR.I | N | 78.53 | 1398.6384 | 13 | 4.1 | 700.3258 | 2 | 41.89 | 15 | F15:19548 | BuCaTN01_F12a.raw | | 4.6764E6 | 9.2303E5 | | 5.036E5 | 4.9225E7 | 3.3654E6 | 8.9405E7 | | 9.4872E8 | 2.7124E6 | 1.1893E8 | 7.0282E5 | 5.8017E7 | 6.3576E7 | | 29 | 0 | 1 | 1 | 0 | 1 | 2 | 1 | 2 | 0 | 13 | 2 | 2 | 1 | 2 | 1 | 0 | 103 | 115 | Carbamidomethylation | C4:Carbamidomethylation:1000.00;C11:Carbamidomethylation:1000.00 | PEAKS DB |
| K.RTIIC(+57.02)YGAAGTC(+57.02)GR.I | N | 77.30 | 1554.7395 | 14 | -0.1 | 519.2537 | 3 | 39.00 | 7 | F7:15155 | BuCaTN01_F7a.raw | | | | | | 2.1318E6 | 3.0621E5 | 7.7778E6 | | 1.1073E8 | 4.1242E5 | 2.362E6 | | | | | 7 | 0 | 0 | 0 | 0 | 0 | 1 | 1 | 1 | 0 | 2 | 1 | 1 | 0 | 0 | 0 | 0 | 102 | 115 | Carbamidomethylation | C5:Carbamidomethylation:1000.00;C12:Carbamidomethylation:1000.00 | PEAKS DB |
| R.TAALC(+57.02)FGDSEYIGAHKNIDTK.R | Y | 74.86 | 2310.0950 | 21 | 0.6 | 578.5314 | 4 | 53.11 | 6 | F6:31480 | BuCaTN01_F6a.raw | | | | | | 1.6236E6 | | 1.8267E6 | 0 | 3.4849E7 | 4.5296E6 | 4.5144E6 | | 1.4851E6 | 1.4555E6 | 0 | 7 | 0 | 0 | 0 | 0 | 0 | 1 | 0 | 1 | 0 | 1 | 1 | 1 | 0 | 1 | 1 | 0 | 123 | 143 | Carbamidomethylation | C5:Carbamidomethylation:1000.00 | PEAKS DB |
| R.IVC(+57.02)DC(+57.02)DRTAALC(+57.02)FGDSEYIGAHKNIDTK.R | Y | 72.10 | 3228.4639 | 28 | 0.7 | 808.1238 | 4 | 55.94 | 10 | F10:32659 | BuCaTN01_F9a.raw | | | | | | 7.0544E6 | | 6.3419E6 | | 1.2486E8 | 1.2141E7 | | | | | 3.2776E5 | 10 | 0 | 0 | 0 | 0 | 0 | 1 | 0 | 2 | 0 | 4 | 2 | 0 | 0 | 0 | 0 | 1 | 116 | 143 | Carbamidomethylation | C3:Carbamidomethylation:1000.00;C5:Carbamidomethylation:1000.00;C12:Carbamidomethylation:1000.00 | PEAKS DB |
| Y.ADYGC(+57.02)YC(+57.02)GAGGSGTPVDALDR.C | Y | 71.00 | 2160.8840 | 21 | -1.3 | 1081.4479 | 2 | 55.10 | 10 | F10:31693 | BuCaTN01_F9a.raw | | | | | | | | | | 1.7576E5 | | | | | | | 1 | 0 | 0 | 0 | 0 | 0 | 0 | 0 | 0 | 0 | 1 | 0 | 0 | 0 | 0 | 0 | 0 | 50 | 70 | Carbamidomethylation | C5:Carbamidomethylation:1000.00;C7:Carbamidomethylation:1000.00 | PEAKS DB |
| R.IVC(+57.02)DC(+57.02)DRTAALC(+57.02)FGDSEYIGAHKN.I | Y | 70.09 | 2771.2102 | 24 | -0.8 | 693.8093 | 4 | 55.91 | 10 | F10:32604 | BuCaTN01_F9a.raw | | | | | | | | | | 2.7976E7 | | | | | | | 3 | 0 | 0 | 0 | 0 | 0 | 0 | 0 | 0 | 0 | 3 | 0 | 0 | 0 | 0 | 0 | 0 | 116 | 139 | Carbamidomethylation | C3:Carbamidomethylation:1000.00;C5:Carbamidomethylation:1000.00;C12:Carbamidomethylation:1000.00 | PEAKS DB |
| W.GHYADYGC(+57.02)YC(+57.02)GAGGSGTPVDALDR.C | Y | 69.79 | 2518.0278 | 24 | -1.3 | 840.3488 | 3 | 53.65 | 6 | F6:32035 | BuCaTN01_F6a.raw | | | | | | 1.4402E5 | | | | 1.5981E6 | | | | | | | 2 | 0 | 0 | 0 | 0 | 0 | 1 | 0 | 0 | 0 | 1 | 0 | 0 | 0 | 0 | 0 | 0 | 47 | 70 | Carbamidomethylation | C8:Carbamidomethylation:1000.00;C10:Carbamidomethylation:1000.00 | PEAKS DB |
| R.TAALC(+57.02)FGDSEYIGAHKN.I | Y | 69.10 | 1852.8413 | 17 | -0.1 | 618.6210 | 3 | 53.97 | 10 | F10:30517 | BuCaTN01_F9a.raw | | | | | | | | | | 3.4922E7 | | | | | | | 4 | 0 | 0 | 0 | 0 | 0 | 0 | 0 | 0 | 0 | 4 | 0 | 0 | 0 | 0 | 0 | 0 | 123 | 139 | Carbamidomethylation | C5:Carbamidomethylation:1000.00 | PEAKS DB |
| H.YADYGC(+57.02)YC(+57.02)GAGGSGTPVDALDR.C | Y | 68.53 | 2323.9473 | 22 | 1.1 | 1162.9822 | 2 | 58.07 | 10 | F10:34499 | BuCaTN01_F9a.raw | | | | | | | | | | 9.3947E5 | | | | | | | 1 | 0 | 0 | 0 | 0 | 0 | 0 | 0 | 0 | 0 | 1 | 0 | 0 | 0 | 0 | 0 | 0 | 49 | 70 | Carbamidomethylation | C6:Carbamidomethylation:1000.00;C8:Carbamidomethylation:1000.00 | PEAKS DB |
| R.TAALC(+57.02)FGDSEYIGAHKNIDTKR.H | Y | 66.98 | 2466.1960 | 22 | 0.6 | 617.5566 | 4 | 51.24 | 11 | F11:22179 | BuCaTN01_F10a.raw | | | | | | 0 | | 3.3818E5 | | 3.0669E7 | 4.0656E6 | 3.5786E6 | 1.4788E5 | 2.2106E5 | 2.1531E5 | | 12 | 0 | 0 | 0 | 0 | 0 | 0 | 0 | 1 | 0 | 4 | 2 | 2 | 1 | 1 | 1 | 0 | 123 | 144 | Carbamidomethylation | C5:Carbamidomethylation:1000.00 | PEAKS DB |
| C.YC(+57.02)GAGGSGTPVDALDR.C | N | 63.99 | 1594.7046 | 16 | 3.8 | 798.3602 | 2 | 45.99 | 8 | F8:22798 | BuCaTN01_F7b.raw | | | | | | 4.319E5 | | 5.8065E5 | | 9.7937E5 | | | | | | | 3 | 0 | 0 | 0 | 0 | 0 | 1 | 0 | 1 | 0 | 1 | 0 | 0 | 0 | 0 | 0 | 0 | 55 | 70 | Carbamidomethylation | C2:Carbamidomethylation:1000.00 | PEAKS DB |
| R.IVC(+57.02)DC(+57.02)DRTAALC(+57.02)FGDSEY.I | N | 61.38 | 2150.8706 | 18 | 3.7 | 1076.4434 | 2 | 62.45 | 8 | F8:37981 | BuCaTN01_F7b.raw | | | | | | | | 4.8895E6 | | | | | | | | | 1 | 0 | 0 | 0 | 0 | 0 | 0 | 0 | 1 | 0 | 0 | 0 | 0 | 0 | 0 | 0 | 0 | 116 | 133 | Carbamidomethylation | C3:Carbamidomethylation:1000.00;C5:Carbamidomethylation:1000.00;C12:Carbamidomethylation:1000.00 | PEAKS DB |
| Y.C(+57.02)GAGGSGTPVDALDR.C | N | 60.47 | 1431.6412 | 15 | 0.2 | 716.8280 | 2 | 41.58 | 10 | F10:19689 | BuCaTN01_F9a.raw | | | | | | | | 7.7921E4 | | 1.2681E7 | | | | 2.9711E5 | | | 4 | 0 | 0 | 0 | 0 | 0 | 0 | 0 | 1 | 0 | 2 | 0 | 0 | 0 | 1 | 0 | 0 | 56 | 70 | Carbamidomethylation | C1:Carbamidomethylation:1000.00 | PEAKS DB |
| A.ALC(+57.02)FGDSEYIGAHK.N | Y | 60.40 | 1566.7136 | 14 | -3.0 | 523.2436 | 3 | 53.39 | 10 | F10:30267 | BuCaTN01_F9a.raw | | | | | | | | | | 0 | | | | | | | 0 | 0 | 0 | 0 | 0 | 0 | 0 | 0 | 0 | 0 | 0 | 0 | 0 | 0 | 0 | 0 | 0 | 125 | 138 | Carbamidomethylation | C3:Carbamidomethylation:1000.00 | PEAKS DB |
| L.NLINFM(+15.99)EMIR.Y | N | 58.78 | 1295.6366 | 10 | 0.9 | 648.8262 | 2 | 75.08 | 9 | F9:48868 | BuCaTN01_F8.raw | | | | | | | | 3.2075E6 | 1.4879E8 | 6.3981E7 | | | | | | | 3 | 0 | 0 | 0 | 0 | 0 | 0 | 0 | 1 | 1 | 1 | 0 | 0 | 0 | 0 | 0 | 0 | 28 | 37 | Oxidation (M) | M6:Oxidation (M):43.88 | PEAKS DB |
| L.NLINFMEMIR.Y | N | 58.60 | 1279.6417 | 10 | 0.6 | 640.8285 | 2 | 89.02 | 11 | F11:48120 | BuCaTN01_F10a.raw | | | | | | | | 2.9837E6 | 9.8225E6 | 5.8511E8 | 1.9261E6 | 3.9106E6 | | | 4.6857E6 | | 6 | 0 | 0 | 0 | 0 | 0 | 0 | 0 | 1 | 1 | 1 | 1 | 1 | 0 | 0 | 1 | 0 | 28 | 37 | | | PEAKS DB |
| L.C(+57.02)FGDSEYIGAHK.N | Y | 57.66 | 1382.5924 | 12 | 3.3 | 461.8716 | 3 | 40.75 | 8 | F8:18546 | BuCaTN01_F7b.raw | | | | | | | | 0 | | 3.4117E6 | | | | | | | 2 | 0 | 0 | 0 | 0 | 0 | 0 | 0 | 0 | 0 | 2 | 0 | 0 | 0 | 0 | 0 | 0 | 127 | 138 | Carbamidomethylation | C1:Carbamidomethylation:1000.00 | PEAKS DB |
| R.IVC(+57.02)DC(+57.02)DRTAALC(+57.02)FGDSEYIGAH.K | Y | 57.38 | 2529.0723 | 22 | -2.5 | 844.0293 | 3 | 60.74 | 6 | F6:38979 | BuCaTN01_F6a.raw | | | | | | 0 | | | | | 2.5004E5 | | | | | | 1 | 0 | 0 | 0 | 0 | 0 | 0 | 0 | 0 | 0 | 0 | 1 | 0 | 0 | 0 | 0 | 0 | 116 | 137 | Carbamidomethylation | C3:Carbamidomethylation:1000.00;C5:Carbamidomethylation:1000.00;C12:Carbamidomethylation:1000.00 | PEAKS DB |
| D.YGC(+57.02)YC(+57.02)GAGGSGTPVDALDR.C | N | 56.96 | 1974.8199 | 19 | 0.7 | 988.4180 | 2 | 52.44 | 10 | F10:29221 | BuCaTN01_F9a.raw | | | | | | 2.2178E5 | | 2.9023E5 | | 1.5633E6 | | 0 | | | | | 3 | 0 | 0 | 0 | 0 | 0 | 1 | 0 | 1 | 0 | 1 | 0 | 0 | 0 | 0 | 0 | 0 | 52 | 70 | Carbamidomethylation | C3:Carbamidomethylation:1000.00;C5:Carbamidomethylation:1000.00 | PEAKS DB |
| R.C(+57.02)C(+57.02)YVHDNC(+57.02)YGVAENKHKC(+57.02)NPK.T | Y | 55.99 | 2652.1089 | 21 | -0.8 | 664.0339 | 4 | 30.13 | 10 | F10:9800 | BuCaTN01_F9a.raw | | | | | | | | | | 9.8196E5 | | | | | | | 1 | 0 | 0 | 0 | 0 | 0 | 0 | 0 | 0 | 0 | 1 | 0 | 0 | 0 | 0 | 0 | 0 | 71 | 91 | Carbamidomethylation | C1:Carbamidomethylation:1000.00;C2:Carbamidomethylation:1000.00;C8:Carbamidomethylation:1000.00;C18:Carbamidomethylation:1000.00 | PEAKS DB |
| L.NLINFMEM(+15.99)IR.Y | N | 53.89 | 1295.6366 | 10 | 0.4 | 648.8258 | 2 | 83.74 | 10 | F10:60895 | BuCaTN01_F9a.raw | | | | | | | 1.553E5 | 1.1201E6 | | 1.6424E8 | | 1.1443E6 | | | 1.4545E6 | | 6 | 0 | 0 | 0 | 0 | 0 | 0 | 1 | 1 | 0 | 2 | 0 | 1 | 0 | 0 | 1 | 0 | 28 | 37 | Oxidation (M) | M8:Oxidation (M):35.36 | PEAKS DB |
| R.IVC(+57.02)DC(+57.02)DRTAAL.C | N | 53.80 | 1292.5853 | 11 | 4.0 | 647.3007 | 2 | 38.90 | 8 | F8:16711 | BuCaTN01_F7b.raw | | | | | | | | 8.9609E6 | | 1.7898E7 | | 1.6803E6 | | 0 | 8.1365E5 | | 4 | 0 | 0 | 0 | 0 | 0 | 0 | 0 | 1 | 0 | 1 | 0 | 1 | 0 | 0 | 1 | 0 | 116 | 126 | Carbamidomethylation | C3:Carbamidomethylation:1000.00;C5:Carbamidomethylation:1000.00 | PEAKS DB |
| C.GAGGSGTPVDALDR.C | N | 53.19 | 1271.6106 | 14 | -0.7 | 636.8121 | 2 | 40.71 | 10 | F10:18523 | BuCaTN01_F9a.raw | | | | | | | | | | 2.1968E6 | | | | | | | 1 | 0 | 0 | 0 | 0 | 0 | 0 | 0 | 0 | 0 | 1 | 0 | 0 | 0 | 0 | 0 | 0 | 57 | 70 | | | PEAKS DB |
| I.RYTIPC(+57.02)DKTWGHYADYGC(+57.02)YC(+57.02)GAGGSGTPVDALDR.C | Y | 52.95 | 3838.6562 | 34 | -0.2 | 960.6711 | 4 | 64.11 | 6 | F6:42515 | BuCaTN01_F6a.raw | | | | | | 6.0945E6 | | | | | | | | | | | 2 | 0 | 0 | 0 | 0 | 0 | 2 | 0 | 0 | 0 | 0 | 0 | 0 | 0 | 0 | 0 | 0 | 37 | 70 | Carbamidomethylation | C6:Carbamidomethylation:1000.00;C18:Carbamidomethylation:1000.00;C20:Carbamidomethylation:1000.00 | PEAKS DB |
| C.FGDSEYIGAHK.N | Y | 51.49 | 1222.5618 | 11 | -0.4 | 408.5277 | 3 | 37.07 | 10 | F10:15542 | BuCaTN01_F9a.raw | | | | | | | | | | 2.4694E5 | | | | | | | 1 | 0 | 0 | 0 | 0 | 0 | 0 | 0 | 0 | 0 | 1 | 0 | 0 | 0 | 0 | 0 | 0 | 128 | 138 | | | PEAKS DB |
| G.C(+57.02)YC(+57.02)GAGGSGTPVDALDR.C | N | 50.80 | 1754.7352 | 17 | -1.7 | 878.3734 | 2 | 47.47 | 10 | F10:24734 | BuCaTN01_F9a.raw | | | | | | | | | | 3.0674E5 | | | | | | | 1 | 0 | 0 | 0 | 0 | 0 | 0 | 0 | 0 | 0 | 1 | 0 | 0 | 0 | 0 | 0 | 0 | 54 | 70 | Carbamidomethylation | C1:Carbamidomethylation:1000.00;C3:Carbamidomethylation:1000.00 | PEAKS DB |
| L.NLINFM(+15.99)EM(+15.99)IR.Y | N | 48.42 | 1311.6315 | 10 | 3.3 | 656.8232 | 2 | 63.35 | 8 | F8:38918 | BuCaTN01_F7b.raw | | | | | | | | 5.8966E6 | 1.1938E7 | 7.7607E7 | | | | | | | 3 | 0 | 0 | 0 | 0 | 0 | 0 | 0 | 1 | 1 | 1 | 0 | 0 | 0 | 0 | 0 | 0 | 28 | 37 | Oxidation (M) | M6:Oxidation (M):1000.00;M8:Oxidation (M):1000.00 | PEAKS DB |
| A.LC(+57.02)FGDSEYIGAHK.N | Y | 44.88 | 1495.6765 | 13 | 1.0 | 499.5666 | 3 | 51.25 | 10 | F10:28335 | BuCaTN01_F9a.raw | | | | | | | | | | 1.3935E5 | | | | | | | 1 | 0 | 0 | 0 | 0 | 0 | 0 | 0 | 0 | 0 | 1 | 0 | 0 | 0 | 0 | 0 | 0 | 126 | 138 | Carbamidomethylation | C2:Carbamidomethylation:1000.00 | PEAKS DB |
| R.TAALC(+57.02)FGDSEYIGAH.K | Y | 44.82 | 1610.7035 | 15 | 1.5 | 806.3602 | 2 | 62.33 | 10 | F10:38643 | BuCaTN01_F9a.raw | | | | | | | | | | 1.1617E6 | | | | | | | 1 | 0 | 0 | 0 | 0 | 0 | 0 | 0 | 0 | 0 | 1 | 0 | 0 | 0 | 0 | 0 | 0 | 123 | 137 | Carbamidomethylation | C5:Carbamidomethylation:1000.00 | PEAKS DB |
| R.IVC(+57.02)DC(+57.02)DRTAALC(+57.02)FGD.S | N | 44.59 | 1771.7328 | 15 | 0.8 | 886.8744 | 2 | 55.31 | 10 | F10:32257 | BuCaTN01_F9a.raw | | | | | | | | | | 1.0611E7 | | | | | | | 1 | 0 | 0 | 0 | 0 | 0 | 0 | 0 | 0 | 0 | 1 | 0 | 0 | 0 | 0 | 0 | 0 | 116 | 130 | Carbamidomethylation | C3:Carbamidomethylation:1000.00;C5:Carbamidomethylation:1000.00;C12:Carbamidomethylation:1000.00 | PEAKS DB |
| **total 37 peptides** | | | | | | | | | | | | | | | | | | | | | | | | | | | | | | | | | | | | | | | | | | | | | | | | | |

**Best Unique PSM (Scan F10:39407, m/z=936.0590, z=3, RT=63.11, ppm=0.1):**

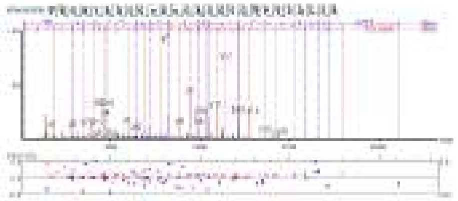

## Q8QFW3.1

back to list

| Protein Coverage | Supporting Peptides | Best Unique PSM |

**Protein Coverage:**

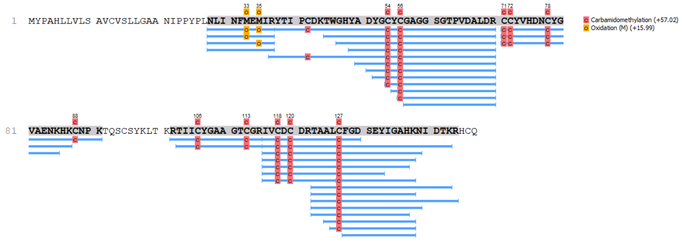


**Supporting Peptides:**

| Peptide | Uniq | -10lgP | Mass | Length | ppm | m/z | z | RT | Fraction | Scan | Source File | Area F1 | Area F2a | Area F3a | Area F4 | Area F5 | Area F6a | Area F7a | Area F7b | Area F8 | Area F9a | Area Sample 1F10a | Area F10b | Area F11a | Area F11b | Area F12a | Area F13 | #Feature | #Feature F1 | #Feature F2a | #Feature F3a | #Feature F4 | #Feature F5 | #Feature F6a | #Feature F7a | #Feature F7b | #Feature F8 | #Feature F9a | #Feature Sample 1F10a | #Feature F10b | #Feature F11a | #Feature F11b | #Feature F12a | #Feature F13 | Start | End | PTM | AScore | Found By |
|---|---|---|---|---|---|---|---|---|---|---|---|---|---|---|---|---|---|---|---|---|---|---|---|---|---|---|---|---|---|---|---|---|---|---|---|---|---|---|---|---|---|---|---|---|---|---|---|---|---|
| K.TWGHYADYGC(+57.02)YC(+57.02)GAGGSGTPVDALDR.C | Y | 123.31 | 2805.1548 | 26 | 0.1 | 936.0590 | 3 | 63.11 | 10 | F10:39407 | BuCaTN01_F9a.raw | | | | | | | | 2.5324E6 | | 9.8815E7 | | 4.0328E6 | | 1.6545E6 | 2.2952E6 | | 9 | 0 | 0 | 0 | 0 | 0 | 0 | 0 | 1 | 0 | 5 | 0 | 1 | 0 | 1 | 1 | 0 | 45 | 70 | Carbamidomethylation | C10:Carbamidomethylation:1000.00;C12:Carbamidomethylation:1000.00 | PEAKS DB |
| R.C(+57.02)C(+57.02)YVHDNC(+57.02)YGVAENK.H | Y | 118.36 | 1887.7339 | 15 | -1.0 | 944.8733 | 2 | 36.88 | 10 | F10:15373 | BuCaTN01_F9a.raw | | | | | | 8.6825E6 | 7.2754E5 | 1.7922E7 | | 2.0038E8 | | 1.582E7 | | 3.5092E5 | 5.7797E6 | | 15 | 0 | 0 | 0 | 0 | 0 | 2 | 1 | 3 | 0 | 4 | 0 | 2 | 0 | 1 | 2 | 0 | 71 | 85 | Carbamidomethylation | C1:Carbamidomethylation:1000.00;C2:Carbamidomethylation:1000.00;C8:Carbamidomethylation:1000.00 | PEAKS DB |
| R.TAALC(+57.02)FGDSEYIGAHK.N | Y | 115.33 | 1738.7985 | 16 | 0.5 | 870.4070 | 2 | 55.64 | 11 | F11:25709 | BuCaTN01_F10a.raw | | | | | | 2.2971E7 | 9.4031E5 | 3.1551E7 | 3.8456E6 | 5.0817E8 | 6.7962E7 | 9.1556E7 | 7.6662E6 | 5.867E7 | 2.7538E7 | 5.2901E6 | 119 | 0 | 0 | 0 | 0 | 0 | 2 | 1 | 11 | 2 | 12 | 26 | 53 | 3 | 4 | 2 | 3 | 123 | 138 | Carbamidomethylation | C5:Carbamidomethylation:1000.00 | PEAKS DB |
| R.IVC(+57.02)DC(+57.02)DRTAALC(+57.02)FGDSEYIGAHK.N | Y | 105.04 | 2657.1672 | 23 | -0.2 | 886.7296 | 3 | 57.28 | 11 | F11:26923 | BuCaTN01_F10a.raw | | | | | | 3.0593E7 | | 4.8915E7 | 6.1227E6 | 7.9689E8 | 8.3497E7 | 8.4757E7 | 6.009E6 | 2.1907E7 | 3.0944E7 | 3.7153E6 | 99 | 0 | 0 | 0 | 0 | 0 | 1 | 0 | 4 | 1 | 44 | 35 | 3 | 2 | 2 | 6 | 1 | 116 | 138 | Carbamidomethylation | C3:Carbamidomethylation:1000.00;C5:Carbamidomethylation:1000.00;C12:Carbamidomethylation:1000.00 | PEAKS DB |
| R.YTIPC(+57.02)DKTWGHYADYGC(+57.02)YC(+57.02)GAGGSGTPVDALDR.C | Y | 99.95 | 3682.5552 | 33 | 1.1 | 1228.5270 | 3 | 67.23 | 10 | F10:43604 | BuCaTN01_F9a.raw | | | | | | 3.4213E6 | | 9.7534E6 | | 4.2682E8 | 2.0095E6 | 1.1818E7 | | 3.7351E6 | 9.6566E6 | | 10 | 0 | 0 | 0 | 0 | 0 | 1 | 0 | 1 | 0 | 4 | 1 | 1 | 0 | 1 | 1 | 0 | 38 | 70 | Carbamidomethylation | C5:Carbamidomethylation:1000.00;C17:Carbamidomethylation:1000.00;C19:Carbamidomethylation:1000.00 | PEAKS DB |
| R.C(+57.02)C(+57.02)YVHDNC(+57.02)YGVAENKHK.C | Y | 96.11 | 2152.8877 | 17 | 0.5 | 718.6368 | 3 | 30.97 | 10 | F10:10373 | BuCaTN01_F9a.raw | | | | | | 2.7157E6 | | 9.9188E6 | | 8.1173E7 | | 7.6189E6 | | | 2.8521E6 | | 15 | 0 | 0 | 0 | 0 | 0 | 1 | 0 | 2 | 0 | 8 | 0 | 2 | 0 | 0 | 2 | 0 | 71 | 87 | Carbamidomethylation | C1:Carbamidomethylation:1000.00;C2:Carbamidomethylation:1000.00;C8:Carbamidomethylation:1000.00 | PEAKS DB |
| A.DYGC(+57.02)YC(+57.02)GAGGSGTPVDALDR.C | Y | 91.42 | 2089.8469 | 20 | -1.9 | 1045.9287 | 2 | 55.35 | 6 | F6:33988 | BuCaTN01_F6a.raw | | | | | | 3.8475E7 | 0 | 1.781E6 | | 0 | | | | | | | 5 | 0 | 0 | 0 | 0 | 0 | 3 | 0 | 2 | 0 | 0 | 0 | 0 | 0 | 0 | 0 | 0 | 51 | 70 | Carbamidomethylation | C4:Carbamidomethylation:1000.00;C6:Carbamidomethylation:1000.00 | PEAKS DB |
| R.TIIC(+57.02)YGAAGTC(+57.02)GR.I | N | 78.53 | 1398.6384 | 13 | 4.1 | 700.3258 | 2 | 41.89 | 15 | F15:19548 | BuCaTN01_F12a.raw | | 4.6764E6 | 9.2303E5 | | 5.036E5 | 4.9225E7 | 3.3654E6 | 8.9405E7 | | 9.4872E8 | 2.7124E6 | 1.1893E8 | 7.0282E5 | 5.8017E7 | 6.3576E7 | | 29 | 0 | 1 | 1 | 0 | 1 | 2 | 1 | 2 | 0 | 13 | 2 | 2 | 1 | 2 | 1 | 0 | 103 | 115 | Carbamidomethylation | C4:Carbamidomethylation:1000.00;C11:Carbamidomethylation:1000.00 | PEAKS DB |
| K.RTIIC(+57.02)YGAAGTC(+57.02)GR.I | N | 77.30 | 1554.7395 | 14 | -0.1 | 519.2537 | 3 | 39.00 | 7 | F7:15155 | BuCaTN01_F7a.raw | | | | | | 2.1318E6 | 3.0621E5 | 7.7778E6 | | 1.1073E8 | 4.1242E5 | 2.362E6 | | | | | 7 | 0 | 0 | 0 | 0 | 0 | 1 | 1 | 1 | 0 | 2 | 1 | 1 | 0 | 0 | 0 | 0 | 102 | 115 | Carbamidomethylation | C5:Carbamidomethylation:1000.00;C12:Carbamidomethylation:1000.00 | PEAKS DB |
| R.TAALC(+57.02)FGDSEYIGAHKNIDTK.R | Y | 74.86 | 2310.0950 | 21 | 0.6 | 578.5314 | 4 | 53.11 | 6 | F6:31480 | BuCaTN01_F6a.raw | | | | | | 1.6236E6 | | 1.8267E6 | 0 | 3.4849E7 | 4.5296E6 | 4.5144E6 | | 1.4851E6 | 1.4555E6 | 0 | 7 | 0 | 0 | 0 | 0 | 0 | 1 | 0 | 1 | 0 | 1 | 1 | 1 | 0 | 1 | 1 | 0 | 123 | 143 | Carbamidomethylation | C5:Carbamidomethylation:1000.00 | PEAKS DB |
| R.IVC(+57.02)DC(+57.02)DRTAALC(+57.02)FGDSEYIGAHKNIDTK.R | Y | 72.10 | 3228.4639 | 28 | 0.7 | 808.1238 | 4 | 55.94 | 10 | F10:32659 | BuCaTN01_F9a.raw | | | | | | 7.0544E6 | | 6.3419E6 | | 1.2486E8 | 1.2141E7 | | | | | 3.2776E5 | 10 | 0 | 0 | 0 | 0 | 0 | 1 | 0 | 2 | 0 | 4 | 2 | 0 | 0 | 0 | 0 | 1 | 116 | 143 | Carbamidomethylation | C3:Carbamidomethylation:1000.00;C5:Carbamidomethylation:1000.00;C12:Carbamidomethylation:1000.00 | PEAKS DB |
| Y.ADYGC(+57.02)YC(+57.02)GAGGSGTPVDALDR.C | Y | 71.00 | 2160.8840 | 21 | -1.3 | 1081.4479 | 2 | 55.10 | 10 | F10:31693 | BuCaTN01_F9a.raw | | | | | | | | | | 1.7576E5 | | | | | | | 1 | 0 | 0 | 0 | 0 | 0 | 0 | 0 | 0 | 0 | 1 | 0 | 0 | 0 | 0 | 0 | 0 | 50 | 70 | Carbamidomethylation | C5:Carbamidomethylation:1000.00;C7:Carbamidomethylation:1000.00 | PEAKS DB |
| R.IVC(+57.02)DC(+57.02)DRTAALC(+57.02)FGDSEYIGAHKN.I | Y | 70.09 | 2771.2102 | 24 | -0.8 | 693.8093 | 4 | 55.91 | 10 | F10:32604 | BuCaTN01_F9a.raw | | | | | | | | | | 2.7976E7 | | | | | | | 3 | 0 | 0 | 0 | 0 | 0 | 0 | 0 | 0 | 0 | 3 | 0 | 0 | 0 | 0 | 0 | 0 | 116 | 139 | Carbamidomethylation | C3:Carbamidomethylation:1000.00;C5:Carbamidomethylation:1000.00;C12:Carbamidomethylation:1000.00 | PEAKS DB |
| W.GHYADYGC(+57.02)YC(+57.02)GAGGSGTPVDALDR.C | Y | 69.79 | 2518.0278 | 24 | -1.3 | 840.3488 | 3 | 53.65 | 6 | F6:32035 | BuCaTN01_F6a.raw | | | | | | 1.4402E5 | | | | 1.5981E6 | | | | | | | 2 | 0 | 0 | 0 | 0 | 0 | 1 | 0 | 0 | 0 | 1 | 0 | 0 | 0 | 0 | 0 | 0 | 47 | 70 | Carbamidomethylation | C8:Carbamidomethylation:1000.00;C10:Carbamidomethylation:1000.00 | PEAKS DB |
| R.TAALC(+57.02)FGDSEYIGAHKN.I | Y | 69.10 | 1852.8413 | 17 | -0.1 | 618.6210 | 3 | 53.97 | 10 | F10:30517 | BuCaTN01_F9a.raw | | | | | | | | | | 3.4922E7 | | | | | | | 4 | 0 | 0 | 0 | 0 | 0 | 0 | 0 | 0 | 0 | 4 | 0 | 0 | 0 | 0 | 0 | 0 | 123 | 139 | Carbamidomethylation | C5:Carbamidomethylation:1000.00 | PEAKS DB |
| H.YADYGC(+57.02)YC(+57.02)GAGGSGTPVDALDR.C | Y | 68.53 | 2323.9473 | 22 | 1.1 | 1162.9822 | 2 | 58.07 | 10 | F10:34499 | BuCaTN01_F9a.raw | | | | | | | | | | 9.3947E5 | | | | | | | 1 | 0 | 0 | 0 | 0 | 0 | 0 | 0 | 0 | 0 | 1 | 0 | 0 | 0 | 0 | 0 | 0 | 49 | 70 | Carbamidomethylation | C6:Carbamidomethylation:1000.00;C8:Carbamidomethylation:1000.00 | PEAKS DB |
| R.TAALC(+57.02)FGDSEYIGAHKNIDTKR.H | Y | 66.98 | 2466.1960 | 22 | 0.6 | 617.5566 | 4 | 51.24 | 11 | F11:22179 | BuCaTN01_F10a.raw | | | | | | 0 | | 3.3818E5 | | 3.0669E7 | 4.0656E6 | 3.5786E6 | 1.4788E5 | 2.2106E5 | 2.1531E5 | | 12 | 0 | 0 | 0 | 0 | 0 | 0 | 0 | 1 | 0 | 4 | 2 | 2 | 1 | 1 | 1 | 0 | 123 | 144 | Carbamidomethylation | C5:Carbamidomethylation:1000.00 | PEAKS DB |
| C.YC(+57.02)GAGGSGTPVDALDR.C | N | 63.99 | 1594.7046 | 16 | 3.8 | 798.3602 | 2 | 45.99 | 8 | F8:22798 | BuCaTN01_F7b.raw | | | | | | 4.319E5 | | 5.8065E5 | | 9.7937E5 | | | | | | | 3 | 0 | 0 | 0 | 0 | 0 | 1 | 0 | 1 | 0 | 1 | 0 | 0 | 0 | 0 | 0 | 0 | 55 | 70 | Carbamidomethylation | C2:Carbamidomethylation:1000.00 | PEAKS DB |
| R.IVC(+57.02)DC(+57.02)DRTAALC(+57.02)FGDSEY.I | N | 61.38 | 2150.8706 | 18 | 3.7 | 1076.4434 | 2 | 62.45 | 8 | F8:37981 | BuCaTN01_F7b.raw | | | | | | | | 4.8895E6 | | | | | | | | | 1 | 0 | 0 | 0 | 0 | 0 | 0 | 0 | 1 | 0 | 0 | 0 | 0 | 0 | 0 | 0 | 0 | 116 | 133 | Carbamidomethylation | C3:Carbamidomethylation:1000.00;C5:Carbamidomethylation:1000.00;C12:Carbamidomethylation:1000.00 | PEAKS DB |
| Y.C(+57.02)GAGGSGTPVDALDR.C | N | 60.47 | 1431.6412 | 15 | 0.2 | 716.8280 | 2 | 41.58 | 10 | F10:19689 | BuCaTN01_F9a.raw | | | | | | | | 7.7921E4 | | 1.2681E7 | | | | 2.9711E5 | | | 4 | 0 | 0 | 0 | 0 | 0 | 0 | 0 | 1 | 0 | 2 | 0 | 0 | 0 | 1 | 0 | 0 | 56 | 70 | Carbamidomethylation | C1:Carbamidomethylation:1000.00 | PEAKS DB |
| A.ALC(+57.02)FGDSEYIGAHK.N | Y | 60.40 | 1566.7136 | 14 | -3.0 | 523.2436 | 3 | 53.39 | 10 | F10:30267 | BuCaTN01_F9a.raw | | | | | | | | | | 0 | | | | | | | 0 | 0 | 0 | 0 | 0 | 0 | 0 | 0 | 0 | 0 | 0 | 0 | 0 | 0 | 0 | 0 | 0 | 125 | 138 | Carbamidomethylation | C3:Carbamidomethylation:1000.00 | PEAKS DB |
| L.NLINFM(+15.99)EMIR.Y | N | 58.78 | 1295.6366 | 10 | 0.9 | 648.8262 | 2 | 75.08 | 9 | F9:48868 | BuCaTN01_F8.raw | | | | | | | | 3.2075E6 | 1.4879E8 | 6.3981E7 | | | | | | | 3 | 0 | 0 | 0 | 0 | 0 | 0 | 0 | 1 | 1 | 1 | 0 | 0 | 0 | 0 | 0 | 0 | 28 | 37 | Oxidation (M) | M6:Oxidation (M):43.88 | PEAKS DB |
| L.NLINFMEMIR.Y | N | 58.60 | 1279.6417 | 10 | 0.6 | 640.8285 | 2 | 89.02 | 11 | F11:48120 | BuCaTN01_F10a.raw | | | | | | | | 2.9837E6 | 9.8225E6 | 5.8511E8 | 1.9261E6 | 3.9106E6 | | | 4.6857E6 | | 6 | 0 | 0 | 0 | 0 | 0 | 0 | 0 | 1 | 1 | 1 | 1 | 1 | 0 | 0 | 1 | 0 | 28 | 37 | | | PEAKS DB |
| L.C(+57.02)FGDSEYIGAHK.N | Y | 57.66 | 1382.5924 | 12 | 3.3 | 461.8716 | 3 | 40.75 | 8 | F8:18546 | BuCaTN01_F7b.raw | | | | | | | | 0 | | 3.4117E6 | | | | | | | 2 | 0 | 0 | 0 | 0 | 0 | 0 | 0 | 0 | 0 | 2 | 0 | 0 | 0 | 0 | 0 | 0 | 127 | 138 | Carbamidomethylation | C1:Carbamidomethylation:1000.00 | PEAKS DB |
| R.IVC(+57.02)DC(+57.02)DRTAALC(+57.02)FGDSEYIGAH.K | Y | 57.38 | 2529.0723 | 22 | -2.5 | 844.0293 | 3 | 60.74 | 6 | F6:38979 | BuCaTN01_F6a.raw | | | | | | 0 | | | | | 2.5004E5 | | | | | | 1 | 0 | 0 | 0 | 0 | 0 | 0 | 0 | 0 | 0 | 0 | 1 | 0 | 0 | 0 | 0 | 0 | 116 | 137 | Carbamidomethylation | C3:Carbamidomethylation:1000.00;C5:Carbamidomethylation:1000.00;C12:Carbamidomethylation:1000.00 | PEAKS DB |
| D.YGC(+57.02)YC(+57.02)GAGGSGTPVDALDR.C | N | 56.96 | 1974.8199 | 19 | 0.7 | 988.4180 | 2 | 52.44 | 10 | F10:29221 | BuCaTN01_F9a.raw | | | | | | 2.2178E5 | | 2.9023E5 | | 1.5633E6 | | 0 | | | | | 3 | 0 | 0 | 0 | 0 | 0 | 1 | 0 | 1 | 0 | 1 | 0 | 0 | 0 | 0 | 0 | 0 | 52 | 70 | Carbamidomethylation | C3:Carbamidomethylation:1000.00;C5:Carbamidomethylation:1000.00 | PEAKS DB |
| R.C(+57.02)C(+57.02)YVHDNC(+57.02)YGVAENKHKC(+57.02)NPK.T | Y | 55.99 | 2652.1089 | 21 | -0.8 | 664.0339 | 4 | 30.13 | 10 | F10:9800 | BuCaTN01_F9a.raw | | | | | | | | | | 9.8196E5 | | | | | | | 1 | 0 | 0 | 0 | 0 | 0 | 0 | 0 | 0 | 0 | 1 | 0 | 0 | 0 | 0 | 0 | 0 | 71 | 91 | Carbamidomethylation | C1:Carbamidomethylation:1000.00;C2:Carbamidomethylation:1000.00;C8:Carbamidomethylation:1000.00;C18:Carbamidomethylation:1000.00 | PEAKS DB |
| L.NLINFMEM(+15.99)IR.Y | N | 53.89 | 1295.6366 | 10 | 0.4 | 648.8258 | 2 | 83.74 | 10 | F10:60895 | BuCaTN01_F9a.raw | | | | | | | 1.553E5 | 1.1201E6 | | 1.6424E8 | | 1.1443E6 | | | 1.4545E6 | | 6 | 0 | 0 | 0 | 0 | 0 | 0 | 1 | 1 | 0 | 2 | 0 | 1 | 0 | 0 | 1 | 0 | 28 | 37 | Oxidation (M) | M8:Oxidation (M):35.36 | PEAKS DB |
| R.IVC(+57.02)DC(+57.02)DRTAAL.C | N | 53.80 | 1292.5853 | 11 | 4.0 | 647.3007 | 2 | 38.90 | 8 | F8:16711 | BuCaTN01_F7b.raw | | | | | | | | 8.9609E6 | | 1.7898E7 | | 1.6803E6 | | 0 | 8.1365E5 | | 4 | 0 | 0 | 0 | 0 | 0 | 0 | 0 | 1 | 0 | 1 | 0 | 1 | 0 | 0 | 1 | 0 | 116 | 126 | Carbamidomethylation | C3:Carbamidomethylation:1000.00;C5:Carbamidomethylation:1000.00 | PEAKS DB |
| C.GAGGSGTPVDALDR.C | N | 53.19 | 1271.6106 | 14 | -0.7 | 636.8121 | 2 | 40.71 | 10 | F10:18523 | BuCaTN01_F9a.raw | | | | | | | | | | 2.1968E6 | | | | | | | 1 | 0 | 0 | 0 | 0 | 0 | 0 | 0 | 0 | 0 | 1 | 0 | 0 | 0 | 0 | 0 | 0 | 57 | 70 | | | PEAKS DB |

| | | | | | | | | | | | | | | | | | | | | | | | | | | | | | | | | | | | | | | | | | | | | | | | | | |
|---|---|---|---|---|---|---|---|---|---|---|---|---|---|---|---|---|---|---|---|---|---|---|---|---|---|---|---|---|---|---|---|---|---|---|---|---|---|---|---|---|---|---|---|---|---|---|---|---|---|
| I.RYTIPC(+57.02)DKTWGHYADYGC(+57.02)YC(+57.02)GAGGSGTPVDALDR.C | Y | 52.95 | 3838.6562 | 34 | -0.2 | 960.6711 | 4 | 64.11 | 6 | F6:42515 | BuCaTN01_F6a.raw | | | | | | 6.0945E6 | | | | | | | | | | | 2 | 0 | 0 | 0 | 0 | 0 | 2 | 0 | 0 | 0 | 0 | 0 | 0 | 0 | 0 | 0 | 0 | 37 | 70 | Carbamidomethylation | C6:Carbamidomethylation:1000.00;C18:Carbamidomethylation:1000.00;C20:Carbamidomethylation:1000.00 | PEAKS DB |
| C.FGDSEYIGAHK.N | Y | 51.49 | 1222.5618 | 11 | -0.4 | 408.5277 | 3 | 37.07 | 10 | F10:15542 | BuCaTN01_F9a.raw | | | | | | | | | | 2.4694E5 | | | | | | | 1 | 0 | 0 | 0 | 0 | 0 | 0 | 0 | 0 | 0 | 1 | 0 | 0 | 0 | 0 | 0 | 0 | 128 | 138 | | | PEAKS DB |
| G.C(+57.02)YC(+57.02)GAGGSGTPVDALDR.C | N | 50.80 | 1754.7352 | 17 | -1.7 | 878.3734 | 2 | 47.47 | 10 | F10:24734 | BuCaTN01_F9a.raw | | | | | | | | | | 3.0674E5 | | | | | | | 1 | 0 | 0 | 0 | 0 | 0 | 0 | 0 | 0 | 0 | 1 | 0 | 0 | 0 | 0 | 0 | 0 | 54 | 70 | Carbamidomethylation | C1:Carbamidomethylation:1000.00;C3:Carbamidomethylation:1000.00 | PEAKS DB |
| L.NLINFM(+15.99)EM(+15.99)IR.Y | N | 48.42 | 1311.6315 | 10 | 3.3 | 656.8232 | 2 | 63.35 | 8 | F8:38918 | BuCaTN01_F7b.raw | | | | | | | | 5.8966E6 | 1.1938E7 | 7.7607E7 | | | | | | | 3 | 0 | 0 | 0 | 0 | 0 | 0 | 0 | 1 | 1 | 1 | 0 | 0 | 0 | 0 | 0 | 0 | 28 | 37 | Oxidation (M) | M6:Oxidation (M):1000.00;M8:Oxidation (M):1000.00 | PEAKS DB |
| A.LC(+57.02)FGDSEYIGAHK.N | Y | 44.88 | 1495.6765 | 13 | 1.0 | 499.5666 | 3 | 51.25 | 10 | F10:28335 | BuCaTN01_F9a.raw | | | | | | | | | | 1.3935E5 | | | | | | | 1 | 0 | 0 | 0 | 0 | 0 | 0 | 0 | 0 | 0 | 1 | 0 | 0 | 0 | 0 | 0 | 0 | 126 | 138 | Carbamidomethylation | C2:Carbamidomethylation:1000.00 | PEAKS DB |
| R.TAALC(+57.02)FGDSEYIGAH.K | Y | 44.82 | 1610.7035 | 15 | 1.5 | 806.3602 | 2 | 62.33 | 10 | F10:38643 | BuCaTN01_F9a.raw | | | | | | | | | | 1.1617E6 | | | | | | | 1 | 0 | 0 | 0 | 0 | 0 | 0 | 0 | 0 | 0 | 1 | 0 | 0 | 0 | 0 | 0 | 0 | 123 | 137 | Carbamidomethylation | C5:Carbamidomethylation:1000.00 | PEAKS DB |
| R.IVC(+57.02)DC(+57.02)DRTAALC(+57.02)FGD.S | N | 44.59 | 1771.7328 | 15 | 0.8 | 886.8744 | 2 | 55.31 | 10 | F10:32257 | BuCaTN01_F9a.raw | | | | | | | | | | 1.0611E7 | | | | | | | 1 | 0 | 0 | 0 | 0 | 0 | 0 | 0 | 0 | 0 | 1 | 0 | 0 | 0 | 0 | 0 | 0 | 116 | 130 | Carbamidomethylation | C3:Carbamidomethylation:1000.00;C5:Carbamidomethylation:1000.00;C12:Carbamidomethylation:1000.00 | PEAKS DB |
| **total 37 peptides** | | | | | | | | | | | | | | | | | | | | | | | | | | | | | | | | | | | | | | | | | | | | | | | | | |

**Best Unique PSM (Scan F10:39407, m/z=936.0590, z=3, RT=63.11, ppm=0.1):**

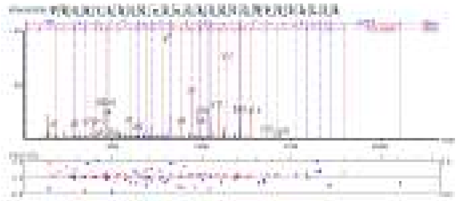

## Q8QFW4.1

back to list

| Protein Coverage | Supporting Peptides | Best Unique PSM |

**Protein Coverage:**

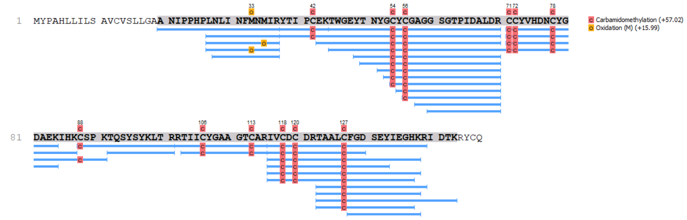


**Supporting Peptides:**

| Peptide | Uniq | -10lgP | Mass | Length | ppm | m/z | z | RT | Fraction | Scan | Source File | Area F1 | Area F2a | Area F3a | Area F4 | Area F5 | Area F6a | Area F7a | Area F7b | Area F8 | Area F9a | Area Sample 1F10a | Area F10b | Area F11a | Area F11b | Area F12a | Area F13 | #Feature | #Feature F1 | #Feature F2a | #Feature F3a | #Feature F4 | #Feature F5 | #Feature F6a | #Feature F7a | #Feature F7b | #Feature F8 | #Feature F9a | #Feature Sample 1F10a | #Feature F10b | #Feature F11a | #Feature F11b | #Feature F12a | #Feature F13 | Start | End | PTM | AScore | Found By |
|---|---|---|---|---|---|---|---|---|---|---|---|---|---|---|---|---|---|---|---|---|---|---|---|---|---|---|---|---|---|---|---|---|---|---|---|---|---|---|---|---|---|---|---|---|---|---|---|---|---|
| K.TWGEYTNYGC(+57.02)YC(+57.02)GAGGSGTPIDALDR.C | Y | 115.46 | 2840.1807 | 26 | -1.7 | 1421.0952 | 2 | 71.09 | 10 | F10:47693 | BuCaTN01_F9a.raw | | | | | | 7.4617E5 | | 3.6744E6 | | 1.6402E8 | 8.06E6 | 6.9796E6 | | 2.0354E6 | 3.8789E6 | | 12 | 0 | 0 | 0 | 0 | 0 | 1 | 0 | 2 | 0 | 3 | 2 | 2 | 0 | 1 | 1 | 0 | 45 | 70 | Carbamidomethylation | C10:Carbamidomethylation:1000.00;C12:Carbamidomethylation:1000.00 | PEAKS DB |
| R.YTIPC(+57.02)EKTWGEYTNYGC(+57.02)YC(+57.02)GAGGSGTPIDALDR.C | Y | 105.66 | 3731.5967 | 33 | 0.6 | 1244.8735 | 3 | 73.77 | 10 | F10:50317 | BuCaTN01_F9a.raw | | | | | | | | 2.7403E6 | | 2.0668E7 | 2.0757E6 | 3.8332E6 | | | 4.7628E6 | | 9 | 0 | 0 | 0 | 0 | 0 | 0 | 0 | 1 | 0 | 4 | 2 | 1 | 0 | 0 | 1 | 0 | 38 | 70 | Carbamidomethylation | C5:Carbamidomethylation:1000.00;C17:Carbamidomethylation:1000.00;C19:Carbamidomethylation:1000.00 | PEAKS DB |
| R.TAALC(+57.02)FGDSEYIEGHKR.I | Y | 102.70 | 1952.9050 | 17 | -0.5 | 977.4594 | 2 | 50.84 | 10 | F10:28262 | BuCaTN01_F9a.raw | | | | | | 1.7743E6 | 2.6506E5 | 7.3911E6 | 1.0674E6 | 2.659E8 | 2.559E7 | 4.5275E7 | 1.9414E6 | 1.6214E7 | 1.6638E7 | 1.6094E6 | 63 | 0 | 0 | 0 | 0 | 0 | 1 | 1 | 4 | 2 | 39 | 8 | 2 | 1 | 1 | 3 | 1 | 123 | 139 | Carbamidomethylation | C5:Carbamidomethylation:1000.00 | PEAKS DB |
| R.TAALC(+57.02)FGDSEYIEGHK.R | N | 101.39 | 1796.8040 | 16 | 1.6 | 599.9429 | 3 | 56.54 | 11 | F11:26491 | BuCaTN01_F10a.raw | | | | | | 4.8248E5 | | 5.9616E6 | 0 | 1.0783E7 | 1.576E7 | 2.2945E7 | 1.6573E6 | 1.8491E7 | 1.1375E7 | 1.4681E6 | 26 | 0 | 0 | 0 | 0 | 0 | 1 | 0 | 1 | 0 | 8 | 3 | 6 | 1 | 3 | 2 | 1 | 123 | 138 | Carbamidomethylation | C5:Carbamidomethylation:1000.00 | PEAKS DB |
| R.IVC(+57.02)DC(+57.02)DRTAALC(+57.02)FGDSEYIEGHK.R | Y | 100.24 | 2715.1726 | 23 | 0.1 | 906.0649 | 3 | 57.39 | 10 | F10:33965 | BuCaTN01_F9a.raw | | | | | | | | 1.7826E7 | | 2.9621E8 | 2.683E7 | 5.0181E7 | | 1.4729E7 | 1.5365E7 | 1.461E5 | 23 | 0 | 0 | 0 | 0 | 0 | 0 | 0 | 2 | 0 | 12 | 1 | 3 | 0 | 2 | 2 | 1 | 116 | 138 | Carbamidomethylation | C3:Carbamidomethylation:1000.00;C5:Carbamidomethylation:1000.00;C12:Carbamidomethylation:1000.00 | PEAKS DB |
| R.IVC(+57.02)DC(+57.02)DRTAALC(+57.02)FGDSEYIEGHKR.I | Y | 97.98 | 2871.2737 | 24 | -0.1 | 958.0984 | 3 | 53.71 | 10 | F10:30526 | BuCaTN01_F9a.raw | | | | | | 5.9362E6 | 5.5334E5 | 2.794E7 | | 5.9604E8 | 5.1326E7 | 1.1033E8 | 4.5955E6 | 1.1787E7 | 2.9659E7 | 3.6545E6 | 73 | 0 | 0 | 0 | 0 | 0 | 2 | 1 | 4 | 0 | 46 | 5 | 5 | 2 | 2 | 4 | 2 | 116 | 139 | Carbamidomethylation | C3:Carbamidomethylation:1000.00;C5:Carbamidomethylation:1000.00;C12:Carbamidomethylation:1000.00 | PEAKS DB |
| R.C(+57.02)C(+57.02)YVHDNC(+57.02)YGDAEKIHK.C | Y | 94.13 | 2167.8875 | 17 | 2.8 | 723.6368 | 3 | 34.49 | 12 | F12:13380 | BuCaTN01_F10b.raw | | | | | | 6.5879E5 | | 1.1871E7 | | 1.334E8 | | 1.625E7 | | 0 | 3.8506E6 | | 14 | 0 | 0 | 0 | 0 | 0 | 1 | 0 | 3 | 0 | 5 | 0 | 3 | 0 | 0 | 2 | 0 | 71 | 87 | Carbamidomethylation | C1:Carbamidomethylation:1000.00;C2:Carbamidomethylation:1000.00;C8:Carbamidomethylation:1000.00 | PEAKS DB |
| R.RTIIC(+57.02)YGAAGTC(+57.02)AR.I | N | 88.84 | 1568.7551 | 14 | -1.3 | 785.3839 | 2 | 38.57 | 10 | F10:16889 | BuCaTN01_F9a.raw | | | | | | | 4.0005E5 | 1.2772E7 | 3.3373E5 | 2.278E8 | 9.1937E5 | 2.7776E7 | 1.5839E5 | 1.1907E7 | 1.6814E7 | | 12 | 0 | 0 | 0 | 0 | 0 | 0 | 1 | 1 | 1 | 2 | 1 | 2 | 1 | 1 | 2 | 0 | 102 | 115 | Carbamidomethylation | C5:Carbamidomethylation:1000.00;C12:Carbamidomethylation:1000.00 | PEAKS DB |
| N.YGC(+57.02)YC(+57.02)GAGGSGTPIDALDR.C | N | 85.25 | 1988.8356 | 19 | -0.4 | 995.4246 | 2 | 57.12 | 10 | F10:33627 | BuCaTN01_F9a.raw | | | | | | | | 0 | | 5.5571E5 | | | | | | | 1 | 0 | 0 | 0 | 0 | 0 | 0 | 0 | 0 | 0 | 1 | 0 | 0 | 0 | 0 | 0 | 0 | 52 | 70 | Carbamidomethylation | C3:Carbamidomethylation:1000.00;C5:Carbamidomethylation:1000.00 | PEAKS DB |

| | | | | | | | | | | | | | | | | | | | | | | | | | | | | | | | | | | | | | | | | | | | | | | | | | |
|---|---|---|---|---|---|---|---|---|---|---|---|---|---|---|---|---|---|---|---|---|---|---|---|---|---|---|---|---|---|---|---|---|---|---|---|---|---|---|---|---|---|---|---|---|---|---|---|---|---|
| R.TIIC(+57.02)YGAAGTC(+57.02)AR.I | N | 80.45 | 1412.6541 | 13 | 0.3 | 707.3345 | 2 | 42.42 | 6 | F6:22027 | BuCaTN01_F6a.raw | | | | | | 1.0191E7 | 2.0352E6 | 6.1107E7 | 1.0219E6 | 8.86E8 | 2.6706E6 | 1.6292E8 | 7.4926E5 | 5.9873E7 | 6.133E7 | | 28 | 0 | 0 | 0 | 0 | 0 | 1 | 1 | 2 | 1 | 14 | 1 | 2 | 1 | 2 | 3 | 0 | 103 | 115 | Carbamidomethylation | C4:Carbamidomethylation:1000.00;C11:Carbamidomethylation:1000.00 | PEAKS DB |
| C.YC(+57.02)GAGGSGTPIDALDR.C | N | 79.98 | 1608.7202 | 16 | -1.8 | 805.3660 | 2 | 51.59 | 10 | F10:28809 | BuCaTN01_F9a.raw | | | | | | | | 5.1555E5 | | 1.6435E6 | | | | | | | 2 | 0 | 0 | 0 | 0 | 0 | 0 | 0 | 1 | 0 | 1 | 0 | 0 | 0 | 0 | 0 | 0 | 55 | 70 | Carbamidomethylation | C2:Carbamidomethylation:1000.00 | PEAKS DB |
| R.TAALC(+57.02)FGDSEYIEGH.K | N | 72.55 | 1668.7090 | 15 | 0.1 | 835.3618 | 2 | 62.78 | 10 | F10:39428 | BuCaTN01_F9a.raw | | | | | | | | | | 6.6743E6 | 7.7152E5 | | | 5.9943E5 | | | 3 | 0 | 0 | 0 | 0 | 0 | 0 | 0 | 0 | 0 | 1 | 1 | 0 | 0 | 1 | 0 | 0 | 123 | 137 | Carbamidomethylation | C5:Carbamidomethylation:1000.00 | PEAKS DB |
| Y.C(+57.02)GAGGSGTPIDALDR.C | N | 72.10 | 1445.6569 | 15 | 0.0 | 723.8357 | 2 | 47.77 | 10 | F10:25299 | BuCaTN01_F9a.raw | | | | | | | | | | 2.0753E7 | | | | 8.9612E5 | 5.8639E5 | | 3 | 0 | 0 | 0 | 0 | 0 | 0 | 0 | 0 | 0 | 1 | 0 | 0 | 0 | 1 | 1 | 0 | 56 | 70 | Carbamidomethylation | C1:Carbamidomethylation:1000.00 | PEAKS DB |
| R.C(+57.02)C(+57.02)YVHDNC(+57.02)YGDAEK.I | N | 71.68 | 1789.6494 | 14 | 4.2 | 597.5566 | 3 | 32.82 | 15 | F15:12456 | BuCaTN01_F12a.raw | | | | | | | | 5.5353E5 | | 7.35E7 | | 1.5588E7 | | 2.0346E6 | 4.9485E7 | | 15 | 0 | 0 | 0 | 0 | 0 | 0 | 0 | 1 | 0 | 3 | 0 | 3 | 0 | 2 | 6 | 0 | 71 | 84 | Carbamidomethylation | C1:Carbamidomethylation:1000.00;C2:Carbamidomethylation:1000.00;C8:Carbamidomethylation:1000.00 | PEAKS DB |
| Y.TNYGC(+57.02)YC(+57.02)GAGGSGTPIDALDR.C | Y | 68.28 | 2203.9263 | 21 | -1.2 | 1102.9691 | 2 | 58.37 | 10 | F10:34890 | BuCaTN01_F9a.raw | | | | | | 2.4693E5 | | 0 | | 1.9717E4 | | | | | | | 2 | 0 | 0 | 0 | 0 | 0 | 1 | 0 | 0 | 0 | 1 | 0 | 0 | 0 | 0 | 0 | 0 | 50 | 70 | Carbamidomethylation | C5:Carbamidomethylation:1000.00;C7:Carbamidomethylation:1000.00 | PEAKS DB |
| R.IVC(+57.02)DC(+57.02)DRTAALC(+57.02)FGDSEYIEGH.K | Y | 68.10 | 2587.0776 | 22 | 0.8 | 863.3672 | 3 | 62.20 | 10 | F10:38468 | BuCaTN01_F9a.raw | | | | | | | | | | 1.404E7 | 1.7108E6 | 1.8146E6 | 2.6567E4 | | | | 4 | 0 | 0 | 0 | 0 | 0 | 0 | 0 | 0 | 0 | 1 | 1 | 1 | 1 | 0 | 0 | 0 | 116 | 137 | Carbamidomethylation | C3:Carbamidomethylation:1000.00;C5:Carbamidomethylation:1000.00;C12:Carbamidomethylation:1000.00 | PEAKS DB |
| K.C(+57.02)SPKTQSYSYKLTR.R | Y | 68.10 | 1717.8457 | 14 | 0.0 | 430.4687 | 4 | 34.43 | 10 | F10:13199 | BuCaTN01_F9a.raw | | | | | | | | | | 1.5414E6 | | 3.0182E4 | | | | | 3 | 0 | 0 | 0 | 0 | 0 | 0 | 0 | 0 | 0 | 2 | 0 | 1 | 0 | 0 | 0 | 0 | 88 | 101 | Carbamidomethylation | C1:Carbamidomethylation:1000.00 | PEAKS DB |
| E.YTNYGC(+57.02)YC(+57.02)GAGGSGTPIDALDR.C | Y | 66.55 | 2366.9895 | 22 | 1.0 | 1184.5032 | 2 | 60.13 | 6 | F6:38295 | BuCaTN01_F6a.raw | | | | | | 9.1465E5 | | | | | | | | | | | 2 | 0 | 0 | 0 | 0 | 0 | 2 | 0 | 0 | 0 | 0 | 0 | 0 | 0 | 0 | 0 | 0 | 49 | 70 | Carbamidomethylation | C6:Carbamidomethylation:1000.00;C8:Carbamidomethylation:1000.00 | PEAKS DB |
| G.C(+57.02)YC(+57.02)GAGGSGTPIDALDR.C | N | 64.64 | 1768.7509 | 17 | -6.4 | 885.3770 | 2 | 52.78 | 10 | F10:29671 | BuCaTN01_F9a.raw | | | | | | | | | | 0 | | | | | | | 0 | 0 | 0 | 0 | 0 | 0 | 0 | 0 | 0 | 0 | 0 | 0 | 0 | 0 | 0 | 0 | 0 | 54 | 70 | Carbamidomethylation | C1:Carbamidomethylation:1000.00;C3:Carbamidomethylation:1000.00 | PEAKS DB |
| R.IVC(+57.02)DC(+57.02)DRTAALC(+57.02)FGDSEY.I | N | 61.38 | 2150.8706 | 18 | 3.7 | 1076.4434 | 2 | 62.45 | 8 | F8:37981 | BuCaTN01_F7b.raw | | | | | | | | 4.8895E6 | | | | | | | | | 1 | 0 | 0 | 0 | 0 | 0 | 0 | 0 | 1 | 0 | 0 | 0 | 0 | 0 | 0 | 0 | 0 | 116 | 133 | Carbamidomethylation | C3:Carbamidomethylation:1000.00;C5:Carbamidomethylation:1000.00;C12:Carbamidomethylation:1000.00 | PEAKS DB |
| L.C(+57.02)FGDSEYIEGHK.R | N | 61.30 | 1440.5979 | 12 | 2.9 | 481.2079 | 3 | 41.75 | 10 | F10:19613 | BuCaTN01_F9a.raw | | | | | | | | | | 1.7737E6 | | | | | | | 1 | 0 | 0 | 0 | 0 | 0 | 0 | 0 | 0 | 0 | 1 | 0 | 0 | 0 | 0 | 0 | 0 | 127 | 138 | Carbamidomethylation | C1:Carbamidomethylation:1000.00 | PEAKS DB |
| R.TAALC(+57.02)FGDSEYIEGHKRIDTK.R | Y | 60.38 | 2410.1587 | 21 | 0.3 | 603.5471 | 4 | 51.48 | 10 | F10:28592 | BuCaTN01_F9a.raw | | | | | | | | | | 2.0105E6 | 2.8544E5 | | | | | | 2 | 0 | 0 | 0 | 0 | 0 | 0 | 0 | 0 | 0 | 1 | 1 | 0 | 0 | 0 | 0 | 0 | 123 | 143 | Carbamidomethylation | C5:Carbamidomethylation:1000.00 | PEAKS DB |
| A.ANIPPHPLNLINFMNMIR.Y | Y | 58.77 | 2104.1074 | 18 | 1.2 | 702.3773 | 3 | 90.03 | 10 | F10:66388 | BuCaTN01_F9a.raw | | | | | | | | | | 1.7663E6 | | | | | | | 1 | 0 | 0 | 0 | 0 | 0 | 0 | 0 | 0 | 0 | 1 | 0 | 0 | 0 | 0 | 0 | 0 | 20 | 37 | | | PEAKS DB |
| R.IVC(+57.02)DC(+57.02)DRTAAL.C | N | 53.80 | 1292.5853 | 11 | 4.0 | 647.3007 | 2 | 38.90 | 8 | F8:16711 | BuCaTN01_F7b.raw | | | | | | | | 8.9609E6 | | 1.7898E7 | | 1.6803E6 | | 0 | 8.1365E5 | | 4 | 0 | 0 | 0 | 0 | 0 | 0 | 0 | 1 | 0 | 1 | 0 | 1 | 0 | 0 | 1 | 0 | 116 | 126 | Carbamidomethylation | C3:Carbamidomethylation:1000.00;C5:Carbamidomethylation:1000.00 | PEAKS DB |
| K.TQSYSYKLTR.R | Y | 52.61 | 1245.6354 | 10 | -0.3 | 416.2189 | 3 | 35.98 | 10 | F10:14842 | BuCaTN01_F9a.raw | | | | | | 1.8747E5 | 3.2566E5 | 6.7683E6 | | 1.9118E7 | 1.7917E5 | 5.9016E6 | | | | | 7 | 0 | 0 | 0 | 0 | 0 | 1 | 1 | 2 | 0 | 1 | 1 | 1 | 0 | 0 | 0 | 0 | 92 | 101 | | | PEAKS DB |
| G.AGGSGTPIDALDR.C | N | 51.35 | 1228.6047 | 13 | 0.3 | 615.3098 | 2 | 46.48 | 10 | F10:24061 | BuCaTN01_F9a.raw | | | | | | | | 2.2064E5 | | 1.0263E6 | | | | | | | 2 | 0 | 0 | 0 | 0 | 0 | 0 | 0 | 1 | 0 | 1 | 0 | 0 | 0 | 0 | 0 | 0 | 58 | 70 | | | PEAKS DB |
| Y.GC(+57.02)YC(+57.02)GAGGSGTPIDALDR.C | N | 50.11 | 1825.7723 | 18 | 0.4 | 913.8938 | 2 | 53.06 | 10 | F10:29689 | BuCaTN01_F9a.raw | | | | | | | | | | 1.6628E6 | | | | | | | 1 | 0 | 0 | 0 | 0 | 0 | 0 | 0 | 0 | 0 | 1 | 0 | 0 | 0 | 0 | 0 | 0 | 53 | 70 | Carbamidomethylation | C2:Carbamidomethylation:1000.00;C4:Carbamidomethylation:1000.00 | PEAKS DB |
| K.TWGEYTNYGC(+57.02)YC(+57.02)GAGGSGTPIDALDRC(+57.02)C(+57.02)YVHDNC(+57.02)YGDAEK.I | Y | 49.16 | 4611.8193 | 40 | -1.1 | 1153.9608 | 4 | 69.18 | 10 | F10:45570 | BuCaTN01_F9a.raw | | | | | | | | | | 0 | | | | | | | 0 | 0 | 0 | 0 | 0 | 0 | 0 | 0 | 0 | 0 | 0 | 0 | 0 | 0 | 0 | 0 | 0 | 45 | 84 | Carbamidomethylation | C10:Carbamidomethylation:1000.00;C12:Carbamidomethylation:1000.00;C27:Carbamidomethylation:1000.00;C28:Carbamidomethylation:1000.00;C34:Carbamidomethylation:1000.00 | PEAKS DB |
| G.GSGTPIDALDR.C | N | 48.75 | 1100.5461 | 11 | -0.1 | 551.2803 | 2 | 46.14 | 10 | F10:23547 | BuCaTN01_F9a.raw | | | | | | | | | | 9.7573E5 | | | | | | | 1 | 0 | 0 | 0 | 0 | 0 | 0 | 0 | 0 | 0 | 1 | 0 | 0 | 0 | 0 | 0 | 0 | 60 | 70 | | | PEAKS DB |
| P.LNLINFMNMIR.Y | Y | 47.61 | 1377.7261 | 11 | 0.5 | 460.2495 | 3 | 92.93 | 10 | F10:68257 | BuCaTN01_F9a.raw | | | | | | | | | | 8.9509E7 | | | | | | | 2 | 0 | 0 | 0 | 0 | 0 | 0 | 0 | 0 | 0 | 2 | 0 | 0 | 0 | 0 | 0 | 0 | 27 | 37 | | | PEAKS DB |
| L.NLINFMNMIR.Y | Y | 45.64 | 1264.6421 | 10 | -1.2 | 633.3276 | 2 | 85.48 | 10 | F10:62575 | BuCaTN01_F9a.raw | | | | | | | | | | 5.6426E8 | | | | | | | 1 | 0 | 0 | 0 | 0 | 0 | 0 | 0 | 0 | 0 | 1 | 0 | 0 | 0 | 0 | 0 | 0 | 28 | 37 | | | PEAKS DB |
| R.IVC(+57.02)DC(+57.02)DRTAALC(+57.02)FGD.S | N | 44.59 | 1771.7328 | 15 | 0.8 | 886.8744 | 2 | 55.31 | 10 | F10:32257 | BuCaTN01_F9a.raw | | | | | | | | | | 1.0611E7 | | | | | | | 1 | 0 | 0 | 0 | 0 | 0 | 0 | 0 | 0 | 0 | 1 | 0 | 0 | 0 | 0 | 0 | 0 | 116 | 130 | Carbamidomethylation | C3:Carbamidomethylation:1000.00;C5:Carbamidomethylation:1000.00;C12:Carbamidomethylation:1000.00 | PEAKS DB |
| C.FGDSEYIEGHK.R | N | 44.44 | 1280.5673 | 11 | 1.3 | 427.8636 | 3 | 38.21 | 10 | F10:16535 | BuCaTN01_F9a.raw | | | | | | | | | | 1.3696E5 | | | | | | | 1 | 0 | 0 | 0 | 0 | 0 | 0 | 0 | 0 | 0 | 1 | 0 | 0 | 0 | 0 | 0 | 0 | 128 | 138 | | | PEAKS DB |
| | | | | | | | | | | | | | | | | | | | | | | | | | | | | | | | | | | | | | | | | | | | | | | | C1:Carbamidomethylation:1000.00;C2:Car | |

| | | | | | | | | | | | | | | | | | | | | | | | | | | | | | | | | | | | | | | | | | | | | | | | | | |
|---|---|---|---|---|---|---|---|---|---|---|---|---|---|---|---|---|---|---|---|---|---|---|---|---|---|---|---|---|---|---|---|---|---|---|---|---|---|---|---|---|---|---|---|---|---|---|---|---|---|
| R.C(+57.02)C(+57.02)YVHDNC(+57.02)YGDAEKIHKC(+57.02)SPK.T | Y | 44.38 | 2640.0977 | 21 | 0.3 | 529.0270 | 5 | 34.51 | 10 | F10:13244 | BuCaTN01_F9a.raw | | | | | | | | | | 3.8635E6 | | | | | | | 1 | 0 | 0 | 0 | 0 | 0 | 0 | 0 | 0 | 0 | 1 | 0 | 0 | 0 | 0 | 0 | 0 | 71 | 91 | Carbamidomethylation | bamidomethylation:1000.00; C8:Carbamidomethylation:1000.00; C18:Carbamidomethylation:1000.00 | PEAKS DB |
| P.LNLINFMNM(+15.99)IR.Y | Y | 43.01 | 1393.7211 | 11 | -0.5 | 697.8675 | 2 | 86.19 | 10 | F10:63149 | BuCaTN01_F9a.raw | | | | | | | | | | 1.6676E7 | | | | | | | 1 | 0 | 0 | 0 | 0 | 0 | 0 | 0 | 0 | 0 | 1 | 0 | 0 | 0 | 0 | 0 | 0 | 27 | 37 | Oxidation (M) | M9:Oxidation (M):24.32 | PEAKS DB |
| R.YTIPC(+57.02)EK.T | N | 42.20 | 909.4266 | 7 | 2.8 | 455.7205 | 2 | 36.93 | 8 | F8:14947 | BuCaTN01_F7b.raw | | | | | | | | 7.3611E6 | | | | | | | | | 1 | 0 | 0 | 0 | 0 | 0 | 0 | 0 | 1 | 0 | 0 | 0 | 0 | 0 | 0 | 0 | 0 | 38 | 44 | Carbamidomethylation | C5:Carbamidomethylation:1000.00 | PEAKS DB |
| C.EKTWGEYTNYGC(+57.02)YC(+57.02)GAGGSGTPIDALDR.C | Y | 41.95 | 3097.3181 | 28 | -1.1 | 1033.4456 | 3 | 66.75 | 10 | F10:43147 | BuCaTN01_F9a.raw | | | | | | | | | | 3.2207E4 | | | | | | | 1 | 0 | 0 | 0 | 0 | 0 | 0 | 0 | 0 | 0 | 1 | 0 | 0 | 0 | 0 | 0 | 0 | 43 | 70 | Carbamidomethylation | C12:Carbamidomethylation:1000.00; C14:Carbamidomethylation:1000.00 | PEAKS DB |
| P.LNLINFM(+15.99)NMIR.Y | Y | 41.43 | 1393.7211 | 11 | 0.3 | 697.8680 | 2 | 80.66 | 10 | F10:57728 | BuCaTN01_F9a.raw | | | | | | | | | | 2.0689E7 | | | | | | | 1 | 0 | 0 | 0 | 0 | 0 | 0 | 0 | 0 | 0 | 1 | 0 | 0 | 0 | 0 | 0 | 0 | 27 | 37 | Oxidation (M) | M7:Oxidation (M):24.32 | PEAKS DB |
| **total 38 peptides** | | | | | | | | | | | | | | | | | | | | | | | | | | | | | | | | | | | | | | | | | | | | | | | | | |

**Best Unique PSM (Scan F10:47693, m/z=1421.0952, z=2, RT=71.09, ppm=-1.7):**

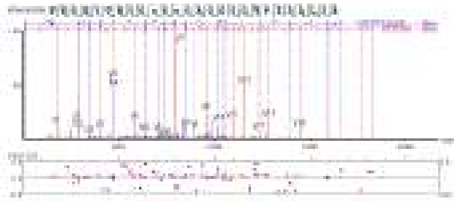

## AAL87003.1

back to list

| Protein Coverage | Supporting Peptides | Best Unique PSM |

**Protein Coverage:**

1 MYPAHLLILS AVCVSLLGAA NIPPHPLNLI NFMNMIRYTI PCEKTWGEYT NYGCYCGAGG SGTPIDALDR CCYVHDNCYG

81 DAEKIHKCSP KTQSYSYKLT RRTIICYGAA GTCARIVCDC DRTAALCFGD SEYIEGHKRI DTKRYCQ

C Carbamidomethylation (+57.02)
O Oxidation (M) (+15.99)

**Supporting Peptides:**

| Peptide | Uniq | -10lgP | Mass | Length | ppm | m/z | z | RT | Fraction | Scan | Source File | Area F1 | Area F2a | Area F3a | Area F4 | Area F5 | Area F6a | Area F7a | Area F7b | Area F8 | Area F9a | Area Sample 1F10a | Area F10b | Area F11a | Area F11b | Area F12a | Area F13 | #Feature | #Feature F1 | #Feature F2a | #Feature F3a | #Feature F4 | #Feature F5 | #Feature F6a | #Feature F7a | #Feature F7b | #Feature F8 | #Feature F9a | #Feature Sample 1F10a | #Feature F10b | #Feature F11a | #Feature F11b | #Feature F12a | #Feature F13 | Start | End | PTM | AScore | Found By |
|---|---|---|---|---|---|---|---|---|---|---|---|---|---|---|---|---|---|---|---|---|---|---|---|---|---|---|---|---|---|---|---|---|---|---|---|---|---|---|---|---|---|---|---|---|---|---|---|---|---|
| K.TWGEYTNYGC(+57.02)YC(+57.02)GAGGSGTPIDALDR.C | Y | 115.46 | 2840.1807 | 26 | -1.7 | 1421.0952 | 2 | 71.09 | 10 | F10:47693 | BuCaTN01_F9a.raw | | | | | | 7.4617E5 | | 3.6744E6 | | 1.6402E8 | 8.06E6 | 6.9796E6 | | 2.0354E6 | 3.8789E6 | | 12 | 0 | 0 | 0 | 0 | 0 | 1 | 0 | 2 | 0 | 3 | 2 | 2 | 0 | 1 | 1 | 0 | 45 | 70 | Carbamidomethylation | C10:Carbamidomethylation:1000.00; C12:Carbamidomethylation:1000.00 | PEAKS DB |
| R.YTIPC(+57.02)EKTWGEYTNYGC(+57.02)YC(+57.02)GAGGSGTPIDALDR.C | Y | 105.66 | 3731.5967 | 33 | 0.6 | 1244.8735 | 3 | 73.77 | 10 | F10:50317 | BuCaTN01_F9a.raw | | | | | | | | 2.7403E6 | | 2.0668E7 | 2.0757E6 | 3.8332E6 | | | 4.7628E6 | | 9 | 0 | 0 | 0 | 0 | 0 | 0 | 0 | 1 | 0 | 4 | 2 | 1 | 0 | 0 | 1 | 0 | 38 | 70 | Carbamidomethylation | C5:Carbamidomethylation:1000.00; C17:Carbamidomethylation:1000.00; C19:Carbamidomethylation:1000.00 | PEAKS DB |
| R.TAALC(+57.02)FGDSEYIEGHKR.I | Y | 102.70 | 1952.9050 | 17 | -0.5 | 977.4594 | 2 | 50.84 | 10 | F10:28262 | BuCaTN01_F9a.raw | | | | | | 1.7743E6 | 2.6506E5 | 7.3911E6 | 1.0674E6 | 2.659E8 | 2.559E7 | 4.5275E7 | 1.9414E6 | 1.6214E7 | 1.6638E7 | 1.6094E6 | 63 | 0 | 0 | 0 | 0 | 0 | 1 | 1 | 4 | 2 | 39 | 8 | 2 | 1 | 1 | 3 | 1 | 123 | 139 | Carbamidomethylation | C5:Carbamidomethylation:1000.00 | PEAKS DB |
| R.TAALC(+57.02)FGDSEYIEGHK.R | N | 101.39 | 1796.8040 | 16 | 1.6 | 599.9429 | 3 | 56.54 | 11 | F11:26491 | BuCaTN01_F10a.raw | | | | | | 4.8248E5 | | 5.9616E6 | 0 | 1.0783E7 | 1.576E7 | 2.2945E7 | 1.6573E6 | 1.8491E7 | 1.1375E7 | 1.4681E6 | 26 | 0 | 0 | 0 | 0 | 0 | 1 | 0 | 1 | 0 | 8 | 3 | 6 | 1 | 3 | 2 | 1 | 123 | 138 | Carbamidomethylation | C5:Carbamidomethylation:1000.00 | PEAKS DB |
| R.IVC(+57.02)DC(+57.02)DRTAALC(+57.02)FGDSEYIEGHK.R | Y | 100.24 | 2715.1726 | 23 | 0.1 | 906.0649 | 3 | 57.39 | 10 | F10:33965 | BuCaTN01_F9a.raw | | | | | | | | 1.7826E7 | | 2.9621E8 | 2.683E7 | 5.0181E7 | | 1.4729E7 | 1.5365E7 | 1.461E5 | 23 | 0 | 0 | 0 | 0 | 0 | 0 | 0 | 2 | 0 | 12 | 1 | 3 | 0 | 2 | 2 | 1 | 116 | 138 | Carbamidomethylation | C3:Carbamidomethylation:1000.00; C5:Carbamidomethylation:1000.00; C12:Carbamidomethylation:1000.00 | PEAKS DB |
| R.IVC(+57.02)DC(+57.02)DRTAALC(+57.02)FGDSEYIEGHKR.I | Y | 97.98 | 2871.2737 | 24 | -0.1 | 958.0984 | 3 | 53.71 | 10 | F10:30526 | BuCaTN01_F9a.raw | | | | | | 5.9362E6 | 5.5334E5 | 2.794E7 | | 5.9604E8 | 5.1326E7 | 1.1033E8 | 4.5955E6 | 1.1787E7 | 2.9659E7 | 3.6545E6 | 73 | 0 | 0 | 0 | 0 | 0 | 2 | 1 | 4 | 0 | 46 | 5 | 5 | 2 | 2 | 4 | 2 | 116 | 139 | Carbamidomethylation | C3:Carbamidomethylation:1000.00; C5:Carbamidomethylation:1000.00; C12:Carbamidomethylation:1000.00 | PEAKS DB |
| R.C(+57.02)C(+57.02)YVHDNC(+57.02)YGDAEKIHK.C | Y | 94.13 | 2167.8875 | 17 | 2.8 | 723.6368 | 3 | 34.49 | 12 | F12:13380 | BuCaTN01_F10b.raw | | | | | | 6.5879E5 | | 1.1871E7 | | 1.334E8 | | 1.625E7 | | 0 | 3.8506E6 | | 14 | 0 | 0 | 0 | 0 | 0 | 1 | 0 | 3 | 0 | 5 | 0 | 3 | 0 | 0 | 2 | 0 | 71 | 87 | Carbamidomethylation | C1:Carbamidomethylation:1000.00; C2:Carbamidomethylation:1000.00; C8:Carbamidomethylation:1000.00 | PEAKS DB |
| R.RTIIC(+57.02)YGAAGTC(+57.02)AR.I | N | 88.84 | 1568.7551 | 14 | -1.3 | 785.3839 | 2 | 38.57 | 10 | F10:16889 | BuCaTN01_F9a.raw | | | | | | | 4.0005E5 | 1.2772E7 | 3.3373E5 | 2.278E8 | 9.1937E5 | 2.7776E7 | 1.5839E5 | 1.1907E7 | 1.6814E7 | | 12 | 0 | 0 | 0 | 0 | 0 | 0 | 1 | 1 | 1 | 2 | 1 | 2 | 1 | 1 | 2 | 0 | 102 | 115 | Carbamidomethylation | C5:Carbamidomethylation:1000.00; C12:Carbamidomethylation:1 | PEAKS DB |

| | | | | | | | | | | | | | | | | | | | | | | | | | | | | | | | | | | | | | | | | | | | | | | | | | |
|---|---|---|---|---|---|---|---|---|---|---|---|---|---|---|---|---|---|---|---|---|---|---|---|---|---|---|---|---|---|---|---|---|---|---|---|---|---|---|---|---|---|---|---|---|---|---|---|---|---|
| | | | | | | | | | | | | | | | | | | | | | | | | | | | | | | | | | | | | | | | | | | | | | | | | 000.00 | |
| N.YGC(+57.02)YC(+57.02)GAGGSGTPIDALDR.C | N | 85.25 | 1988.8356 | 19 | -0.4 | 995.4246 | 2 | 57.12 | 10 | F10:33627 | BuCaTN01_F9a.raw | | | | | | | | 0 | | 5.5571E5 | | | | | | | 1 | 0 | 0 | 0 | 0 | 0 | 0 | 0 | 0 | 0 | 1 | 0 | 0 | 0 | 0 | 0 | 0 | 52 | 70 | Carbamidomethylation | C3:Carbamidomethylation:1000.00;C5:Carbamidomethylation:1000.00 | PEAKS DB |
| R.TIIC(+57.02)YGAAGTC(+57.02)AR.I | N | 80.45 | 1412.6541 | 13 | 0.3 | 707.3345 | 2 | 42.42 | 6 | F6:22027 | BuCaTN01_F6a.raw | | | | | | 1.0191E7 | 2.0352E6 | 6.1107E7 | 1.0219E6 | 8.86E8 | 2.6706E6 | 1.6292E8 | 7.4926E5 | 5.9873E7 | 6.133E7 | | 28 | 0 | 0 | 0 | 0 | 0 | 1 | 1 | 2 | 1 | 14 | 1 | 2 | 1 | 2 | 3 | 0 | 103 | 115 | Carbamidomethylation | C4:Carbamidomethylation:1000.00;C11:Carbamidomethylation:1000.00 | PEAKS DB |
| C.YC(+57.02)GAGGSGTPIDALDR.C | N | 79.98 | 1608.7202 | 16 | -1.8 | 805.3660 | 2 | 51.59 | 10 | F10:28809 | BuCaTN01_F9a.raw | | | | | | | | 5.1555E5 | | 1.6435E6 | | | | | | | 2 | 0 | 0 | 0 | 0 | 0 | 0 | 0 | 1 | 0 | 1 | 0 | 0 | 0 | 0 | 0 | 0 | 55 | 70 | Carbamidomethylation | C2:Carbamidomethylation:1000.00 | PEAKS DB |
| R.TAALC(+57.02)FGDSEYIEGH.K | N | 72.55 | 1668.7090 | 15 | 0.1 | 835.3618 | 2 | 62.78 | 10 | F10:39428 | BuCaTN01_F9a.raw | | | | | | | | | | 6.6743E6 | 7.7152E5 | | | 5.9943E5 | | | 3 | 0 | 0 | 0 | 0 | 0 | 0 | 0 | 0 | 0 | 1 | 1 | 0 | 0 | 1 | 0 | 0 | 123 | 137 | Carbamidomethylation | C5:Carbamidomethylation:1000.00 | PEAKS DB |
| Y.C(+57.02)GAGGSGTPIDALDR.C | N | 72.10 | 1445.6569 | 15 | 0.0 | 723.8357 | 2 | 47.77 | 10 | F10:25299 | BuCaTN01_F9a.raw | | | | | | | | | | 2.0753E7 | | | | 8.9612E5 | 5.8639E5 | | 3 | 0 | 0 | 0 | 0 | 0 | 0 | 0 | 0 | 0 | 1 | 0 | 0 | 0 | 1 | 1 | 0 | 56 | 70 | Carbamidomethylation | C1:Carbamidomethylation:1000.00 | PEAKS DB |
| R.C(+57.02)C(+57.02)YVHDNC(+57.02)YGDAEK.I | N | 71.68 | 1789.6494 | 14 | 4.2 | 597.5566 | 3 | 32.82 | 15 | F15:12456 | BuCaTN01_F12a.raw | | | | | | | | 5.5353E5 | | 7.35E7 | | 1.5588E7 | | 2.0346E6 | 4.9485E7 | | 15 | 0 | 0 | 0 | 0 | 0 | 0 | 0 | 1 | 0 | 3 | 0 | 3 | 0 | 2 | 6 | 0 | 71 | 84 | Carbamidomethylation | C1:Carbamidomethylation:1000.00;C2:Carbamidomethylation:1000.00;C8:Carbamidomethylation:1000.00 | PEAKS DB |
| Y.TNYGC(+57.02)YC(+57.02)GAGGSGTPIDALDR.C | Y | 68.28 | 2203.9263 | 21 | -1.2 | 1102.9691 | 2 | 58.37 | 10 | F10:34890 | BuCaTN01_F9a.raw | | | | | | 2.4693E5 | | 0 | | 1.9717E4 | | | | | | | 2 | 0 | 0 | 0 | 0 | 0 | 1 | 0 | 0 | 0 | 1 | 0 | 0 | 0 | 0 | 0 | 0 | 50 | 70 | Carbamidomethylation | C5:Carbamidomethylation:1000.00;C7:Carbamidomethylation:1000.00 | PEAKS DB |
| R.IVC(+57.02)DC(+57.02)DRTAALC(+57.02)FGDSEYIEGH.K | Y | 68.10 | 2587.0776 | 22 | 0.8 | 863.3672 | 3 | 62.20 | 10 | F10:38468 | BuCaTN01_F9a.raw | | | | | | | | | | 1.404E7 | 1.7108E6 | 1.8146E6 | 2.6567E4 | | | | 4 | 0 | 0 | 0 | 0 | 0 | 0 | 0 | 0 | 0 | 1 | 1 | 1 | 1 | 0 | 0 | 0 | 116 | 137 | Carbamidomethylation | C3:Carbamidomethylation:1000.00;C5:Carbamidomethylation:1000.00;C12:Carbamidomethylation:1000.00 | PEAKS DB |
| K.C(+57.02)SPKTQSYSYKLTR.R | Y | 68.10 | 1717.8457 | 14 | 0.0 | 430.4687 | 4 | 34.43 | 10 | F10:13199 | BuCaTN01_F9a.raw | | | | | | | | | | 1.5414E6 | | 3.0182E4 | | | | | 3 | 0 | 0 | 0 | 0 | 0 | 0 | 0 | 0 | 0 | 2 | 0 | 1 | 0 | 0 | 0 | 0 | 88 | 101 | Carbamidomethylation | C1:Carbamidomethylation:1000.00 | PEAKS DB |
| E.YTNYGC(+57.02)YC(+57.02)GAGGSGTPIDALDR.C | Y | 66.55 | 2366.9895 | 22 | 1.0 | 1184.5032 | 2 | 60.13 | 6 | F6:38295 | BuCaTN01_F6a.raw | | | | | | 9.1465E5 | | | | | | | | | | | 2 | 0 | 0 | 0 | 0 | 0 | 2 | 0 | 0 | 0 | 0 | 0 | 0 | 0 | 0 | 0 | 0 | 49 | 70 | Carbamidomethylation | C6:Carbamidomethylation:1000.00;C8:Carbamidomethylation:1000.00 | PEAKS DB |
| G.C(+57.02)YC(+57.02)GAGGSGTPIDALDR.C | N | 64.64 | 1768.7509 | 17 | -6.4 | 885.3770 | 2 | 52.78 | 10 | F10:29671 | BuCaTN01_F9a.raw | | | | | | | | | | 0 | | | | | | | 0 | 0 | 0 | 0 | 0 | 0 | 0 | 0 | 0 | 0 | 0 | 0 | 0 | 0 | 0 | 0 | 0 | 54 | 70 | Carbamidomethylation | C1:Carbamidomethylation:1000.00;C3:Carbamidomethylation:1000.00 | PEAKS DB |
| R.IVC(+57.02)DC(+57.02)DRTAALC(+57.02)FGDSEY.I | N | 61.38 | 2150.8706 | 18 | 3.7 | 1076.4434 | 2 | 62.45 | 8 | F8:37981 | BuCaTN01_F7b.raw | | | | | | | | 4.8895E6 | | | | | | | | | 1 | 0 | 0 | 0 | 0 | 0 | 0 | 0 | 1 | 0 | 0 | 0 | 0 | 0 | 0 | 0 | 0 | 116 | 133 | Carbamidomethylation | C3:Carbamidomethylation:1000.00;C5:Carbamidomethylation:1000.00;C12:Carbamidomethylation:1000.00 | PEAKS DB |
| L.C(+57.02)FGDSEYIEGHK.R | N | 61.30 | 1440.5979 | 12 | 2.9 | 481.2079 | 3 | 41.75 | 10 | F10:19613 | BuCaTN01_F9a.raw | | | | | | | | | | 1.7737E6 | | | | | | | 1 | 0 | 0 | 0 | 0 | 0 | 0 | 0 | 0 | 0 | 1 | 0 | 0 | 0 | 0 | 0 | 0 | 127 | 138 | Carbamidomethylation | C1:Carbamidomethylation:1000.00 | PEAKS DB |
| R.TAALC(+57.02)FGDSEYIEGHKRIDTK.R | Y | 60.38 | 2410.1587 | 21 | 0.3 | 603.5471 | 4 | 51.48 | 10 | F10:28592 | BuCaTN01_F9a.raw | | | | | | | | | | 2.0105E6 | 2.8544E5 | | | | | | 2 | 0 | 0 | 0 | 0 | 0 | 0 | 0 | 0 | 0 | 1 | 1 | 0 | 0 | 0 | 0 | 0 | 123 | 143 | Carbamidomethylation | C5:Carbamidomethylation:1000.00 | PEAKS DB |
| A.ANIPPHPLNLINFMNMIR.Y | Y | 58.77 | 2104.1074 | 18 | 1.2 | 702.3773 | 3 | 90.03 | 10 | F10:66388 | BuCaTN01_F9a.raw | | | | | | | | | | 1.7663E6 | | | | | | | 1 | 0 | 0 | 0 | 0 | 0 | 0 | 0 | 0 | 0 | 1 | 0 | 0 | 0 | 0 | 0 | 0 | 20 | 37 | | | PEAKS DB |
| R.IVC(+57.02)DC(+57.02)DRTAAL.C | N | 53.80 | 1292.5853 | 11 | 4.0 | 647.3007 | 2 | 38.90 | 8 | F8:16711 | BuCaTN01_F7b.raw | | | | | | | | 8.9609E6 | | 1.7898E7 | | 1.6803E6 | | 0 | 8.1365E5 | | 4 | 0 | 0 | 0 | 0 | 0 | 0 | 0 | 1 | 0 | 1 | 0 | 1 | 0 | 0 | 1 | 0 | 116 | 126 | Carbamidomethylation | C3:Carbamidomethylation:1000.00;C5:Carbamidomethylation:1000.00 | PEAKS DB |
| K.TQSYSYKLTR.R | Y | 52.61 | 1245.6354 | 10 | -0.3 | 416.2189 | 3 | 35.98 | 10 | F10:14842 | BuCaTN01_F9a.raw | | | | | | 1.8747E5 | 3.2566E5 | 6.7683E6 | | 1.9118E7 | 1.7917E5 | 5.9016E6 | | | | | 7 | 0 | 0 | 0 | 0 | 0 | 1 | 1 | 2 | 0 | 1 | 1 | 1 | 0 | 0 | 0 | 0 | 92 | 101 | | | PEAKS DB |
| G.AGGSGTPIDALDR.C | N | 51.35 | 1228.6047 | 13 | 0.3 | 615.3098 | 2 | 46.48 | 10 | F10:24061 | BuCaTN01_F9a.raw | | | | | | | | 2.2064E5 | | 1.0263E6 | | | | | | | 2 | 0 | 0 | 0 | 0 | 0 | 0 | 0 | 1 | 0 | 1 | 0 | 0 | 0 | 0 | 0 | 0 | 58 | 70 | | | PEAKS DB |
| Y.GC(+57.02)YC(+57.02)GAGGSGTPIDALDR.C | N | 50.11 | 1825.7723 | 18 | 0.4 | 913.8938 | 2 | 53.06 | 10 | F10:29689 | BuCaTN01_F9a.raw | | | | | | | | | | 1.6628E6 | | | | | | | 1 | 0 | 0 | 0 | 0 | 0 | 0 | 0 | 0 | 0 | 1 | 0 | 0 | 0 | 0 | 0 | 0 | 53 | 70 | Carbamidomethylation | C2:Carbamidomethylation:1000.00;C4:Carbamidomethylation:1000.00 | PEAKS DB |
| K.TWGEYTNYGC(+57.02)YC(+57.02)GAGGSGTPIDALDRC(+57.02)C(+57.02)YVHDNC(+57.02)YGDAEK.I | Y | 49.16 | 4611.8193 | 40 | -1.1 | 1153.9608 | 4 | 69.18 | 10 | F10:45570 | BuCaTN01_F9a.raw | | | | | | | | | | 0 | | | | | | | 0 | 0 | 0 | 0 | 0 | 0 | 0 | 0 | 0 | 0 | 0 | 0 | 0 | 0 | 0 | 0 | 0 | 45 | 84 | Carbamidomethylation | C10:Carbamidomethylation:1000.00;C12:Carbamidomethylation:1000.00;C27:Carbamidomethylation:1000.00;C28:Carbamidomethylation:1000.00;C34:Carbamidomethylation:1000.00 | PEAKS DB |
| G.GSGTPIDALDR.C | N | 48.75 | 1100.5461 | 11 | -0.1 | 551.2803 | 2 | 46.14 | 10 | F10:23547 | BuCaTN01_F9a.raw | | | | | | | | | | 9.7573E5 | | | | | | | 1 | 0 | 0 | 0 | 0 | 0 | 0 | 0 | 0 | 0 | 1 | 0 | 0 | 0 | 0 | 0 | 0 | 60 | 70 | | | PEAKS DB |
| P.LNLINFMNMIR.Y | Y | 47.61 | 1377.7261 | 11 | 0.5 | 460.2495 | 3 | 92.93 | 10 | F10:68257 | BuCaTN01_F9a.raw | | | | | | | | | | 8.9509E7 | | | | | | | 2 | 0 | 0 | 0 | 0 | 0 | 0 | 0 | 0 | 0 | 2 | 0 | 0 | 0 | 0 | 0 | 0 | 27 | 37 | | | PEAKS DB |
| L.NLINFMNMIR.Y | Y | 45.64 | 1264.6421 | 10 | -1.2 | 633.3276 | 2 | 85.48 | 10 | F10:62575 | BuCaTN01_F9a.raw | | | | | | | | | | 5.6426E8 | | | | | | | 1 | 0 | 0 | 0 | 0 | 0 | 0 | 0 | 0 | 0 | 1 | 0 | 0 | 0 | 0 | 0 | 0 | 28 | 37 | | | PEAKS DB |
| R.IVC(+57.02)DC(+57.02)DRTAALC(+57.02)FGD.S | N | 44.59 | 1771.7328 | 15 | 0.8 | 886.8744 | 2 | 55.31 | 10 | F10:32257 | BuCaTN01_F9a.raw | | | | | | | | | | 1.0611E7 | | | | | | | 1 | 0 | 0 | 0 | 0 | 0 | 0 | 0 | 0 | 0 | 1 | 0 | 0 | 0 | 0 | 0 | 0 | 116 | 130 | Carbamidomethylation | C3:Carbamidomethylation:1000.00;C5:Carbamidomethylation:1000.00;C12:Carbamid | PEAKS DB |

| | | | | | | | | | | | | | | | | | | | | | | | | | | | | | | | | | | | | | | | | | | | | | | | | | |
|---|---|---|---|---|---|---|---|---|---|---|---|---|---|---|---|---|---|---|---|---|---|---|---|---|---|---|---|---|---|---|---|---|---|---|---|---|---|---|---|---|---|---|---|---|---|---|---|---|---|
| | | | | | | | | | | | | | | | | | | | | | | | | | | | | | | | | | | | | | | | | | | | | | | | | omethylation:1000.00 | |
| C.FGDSEYIEGHK.R | N | 44.44 | 1280.5673 | 11 | 1.3 | 427.8636 | 3 | 38.21 | 10 | F10:16535 | BuCaTN01_F9a.raw | | | | | | | | | | 1.3696E5 | | | | | | | 1 | 0 | 0 | 0 | 0 | 0 | 0 | 0 | 0 | 0 | 1 | 0 | 0 | 0 | 0 | 0 | 0 | 128 | 138 | | | PEAKS DB |
| R.C(+57.02)C(+57.02)YVHDNC(+57.02)YGDAEKIHKC(+57.02)SPK.T | Y | 44.38 | 2640.0977 | 21 | 0.3 | 529.0270 | 5 | 34.51 | 10 | F10:13244 | BuCaTN01_F9a.raw | | | | | | | | | | 3.8635E6 | | | | | | | 1 | 0 | 0 | 0 | 0 | 0 | 0 | 0 | 0 | 0 | 1 | 0 | 0 | 0 | 0 | 0 | 0 | 71 | 91 | Carbamidomethylation | C1:Carbamidomethylation:1000.00;C2:Carbamidomethylation:1000.00;C8:Carbamidomethylation:1000.00;C18:Carbamidomethylation:1000.00 | PEAKS DB |
| P.LNLINFMNM(+15.99)IR.Y | Y | 43.01 | 1393.7211 | 11 | -0.5 | 697.8675 | 2 | 86.19 | 10 | F10:63149 | BuCaTN01_F9a.raw | | | | | | | | | | 1.6676E7 | | | | | | | 1 | 0 | 0 | 0 | 0 | 0 | 0 | 0 | 0 | 0 | 1 | 0 | 0 | 0 | 0 | 0 | 0 | 27 | 37 | Oxidation (M) | M9:Oxidation (M):24.32 | PEAKS DB |
| R.YTIPC(+57.02)EK.T | N | 42.20 | 909.4266 | 7 | 2.8 | 455.7205 | 2 | 36.93 | 8 | F8:14947 | BuCaTN01_F7b.raw | | | | | | | | 7.3611E6 | | | | | | | | | 1 | 0 | 0 | 0 | 0 | 0 | 0 | 0 | 1 | 0 | 0 | 0 | 0 | 0 | 0 | 0 | 0 | 38 | 44 | Carbamidomethylation | C5:Carbamidomethylation:1000.00 | PEAKS DB |
| C.EKTWGEYTNYGC(+57.02)YC(+57.02)GAGGSGTPIDALDR.C | Y | 41.95 | 3097.3181 | 28 | -1.1 | 1033.4456 | 3 | 66.75 | 10 | F10:43147 | BuCaTN01_F9a.raw | | | | | | | | | | 3.2207E4 | | | | | | | 1 | 0 | 0 | 0 | 0 | 0 | 0 | 0 | 0 | 0 | 1 | 0 | 0 | 0 | 0 | 0 | 0 | 43 | 70 | Carbamidomethylation | C12:Carbamidomethylation:1000.00;C14:Carbamidomethylation:1000.00 | PEAKS DB |
| P.LNLINFM(+15.99)NMIR.Y | Y | 41.43 | 1393.7211 | 11 | 0.3 | 697.8680 | 2 | 80.66 | 10 | F10:57728 | BuCaTN01_F9a.raw | | | | | | | | | | 2.0689E7 | | | | | | | 1 | 0 | 0 | 0 | 0 | 0 | 0 | 0 | 0 | 0 | 1 | 0 | 0 | 0 | 0 | 0 | 0 | 27 | 37 | Oxidation (M) | M7:Oxidation (M):24.32 | PEAKS DB |
| **total 38 peptides** | | | | | | | | | | | | | | | | | | | | | | | | | | | | | | | | | | | | | | | | | | | | | | | | | |

**Best Unique PSM (Scan F10:47693, m/z=1421.0952, z=2, RT=71.09, ppm=-1.7):**

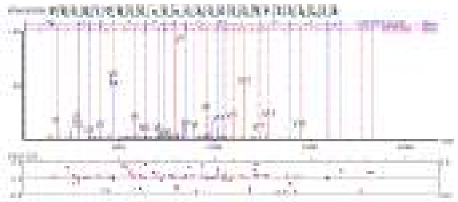

## 4QWW

back to list

| Protein Coverage | Supporting Peptides | Best Unique PSM |

**Protein Coverage:**

```
  1 GRAGELKVST QTGSVRGLSL PVLDGHVSAF LGIPFAEPPL GRMRFLRPEP VKPWQHVLDA TSYKPACYQM VDTSYPGFQG
 81 TEMWNPNRGM SEDCLYLNIW VPSPRPKDAP VLVWIYGGGF YSGAASLDVY DGRFLTYTQN VILVSLSYRV GAFGFLGLPG
161 SPEAPGNMGL LDQRLALQWI QNNIHPFGGN PRAVTVFGES AGAASVGMHL LSTQSRTLFQ RAILQSGGPN APWATVTPAE
241 SRGRAALLGK QLGCHFNNDS ELVSCLRSKN PQELIDEEWS VLPYKSIFRF PFVPVIDGDF FPDTPEAMLS SGNFKETQVL
321 LGVVKDEGSY FLIYGLPGFS KDNESLISRA DFLEGVRMSV PHANDIATDA VVLQYTDWQD QDNREKNREA LDDIVGDHNV
401 ICPVVQFAND YAKRNSKVYA YLFDHRASNL LWPPWMGVPH GYEIEFVFGL PLNDSLNYTP QEKELSRRMM RYWANFARTG
481 NPTDPADKSG AWPTYTASQP QYVQLNTQPL ATQPSLRAQI CAFWNHFLPK LLNATVDPPR AD
```

402 C

C Carbamidomethylation (+57.02)
O Oxidation (M) (+15.99)

**Supporting Peptides:**

| Peptide | Uniq | -10lgP | Mass | Length | ppm | m/z | z | RT | Fraction | Scan | Source File | Area F1 | Area F2a | Area F3a | Area F4 | Area F5 | Area F6a | Area F7a | Area F7b | Area F8 | Area F9a | Area Sample 1F10a | Area F10b | Area F11a | Area F11b | Area F12a | Area F13 | #Feature | #Feature F1 | #Feature F2a | #Feature F3a | #Feature F4 | #Feature F5 | #Feature F6a | #Feature F7a | #Feature F7b | #Feature F8 | #Feature F9a | #Feature Sample 1F10a | #Feature F10b | #Feature F11a | #Feature F11b | #Feature F12a | #Feature F13 | Start | End | PTM | AScore | Found By |
|---|---|---|---|---|---|---|---|---|---|---|---|---|---|---|---|---|---|---|---|---|---|---|---|---|---|---|---|---|---|---|---|---|---|---|---|---|---|---|---|---|---|---|---|---|---|---|---|---|---|
| R.AILQSGGPNAPWATVTPAESR.G | N | 122.40 | 2122.0806 | 21 | 0.2 | 1062.0477 | 2 | 64.33 | 16 | F16:32291 | BuCaTN01_F13.raw | | | | | | | | | 4.2489E5 | | | | | | | 2.432E7 | 5 | 0 | 0 | 0 | 0 | 0 | 0 | 0 | 0 | 2 | 0 | 0 | 0 | 0 | 0 | 0 | 3 | 222 | 242 | | | PEAKS DB |
| R.SKNPQELIDEEWSVLPYK.S | Y | 100.39 | 2174.0896 | 18 | 1.1 | 725.7046 | 3 | 78.13 | 16 | F16:42155 | BuCaTN01_F13.raw | | | | | | | | | | | | | | | | 5.585E6 | 1 | 0 | 0 | 0 | 0 | 0 | 0 | 0 | 0 | 0 | 0 | 0 | 0 | 0 | 0 | 0 | 1 | 268 | 285 | | | PEAKS DB |
| K.SGAWPTYTASQPQYVQLNTQPLATQPSLR.A | Y | 90.63 | 3202.6047 | 29 | 0.5 | 1068.5427 | 3 | 72.87 | 16 | F16:38224 | BuCaTN01_F13.raw | | | | | | | | | | | | | | | | 4.1527E6 | 1 | 0 | 0 | 0 | 0 | 0 | 0 | 0 | 0 | 0 | 0 | 0 | 0 | 0 | 0 | 0 | 1 | 489 | 517 | | | PEAKS DB |
| R.AGELKVSTQTGSVR.G | Y | 85.19 | 1431.7681 | 14 | 0.4 | 478.2635 | 3 | 32.16 | 16 | F16:11066 | BuCaTN01_F13.raw | | | | | | | | | | | | | | | | 6.4414E7 | 3 | 0 | 0 | 0 | 0 | 0 | 0 | 0 | 0 | 0 | 0 | 0 | 0 | 0 | 0 | 0 | 3 | 3 | 16 | | | PEAKS DB |
| R.TGNPTDPADKSGAWPTYTASQPQYVQLNTQPLATQPSLR.A | Y | 79.64 | 4199.0557 | 39 | 0.0 | 1050.7712 | 4 | 69.79 | 16 | F16:35951 | BuCaTN01_F13.raw | | | | | | | | | | | | | | | | 1.0938E7 | 2 | 0 | 0 | 0 | 0 | 0 | 0 | 0 | 0 | 0 | 0 | 0 | 0 | 0 | 0 | 0 | 2 | 479 | 517 | | | PEAKS DB |
| R.MMRYWANFAR.T | N | 72.98 | 1344.6219 | 10 | 0.2 | 449.2147 | 3 | 61.26 | 16 | F16:30310 | BuCaTN01_F13.raw | | | | | | | | | | | | | | | | 1.0526E7 | 2 | 0 | 0 | 0 | 0 | 0 | 0 | 0 | 0 | 0 | 0 | 0 | 0 | 0 | 0 | 0 | 2 | 469 | 478 | | | PEAKS DB |
| R.TLFQRAILQSGGPNAPWATVTPAESR.G | Y | 67.34 | 2767.4404 | 26 | -0.1 | 923.4873 | 3 | 74.20 | 16 | F16:38946 | BuCaTN01_F13.raw | | | | | | | | | | | | | | | | 8.3998E5 | 1 | 0 | 0 | 0 | 0 | 0 | 0 | 0 | 0 | 0 | 0 | 0 | 0 | 0 | 0 | 0 | 1 | 217 | 242 | | | PEAKS DB |
| S.GGPNAPWATVTPAESR.G | N | 65.09 | 1609.7848 | 16 | 0.6 | 805.9001 | 2 | 54.13 | 16 | F16:25775 | BuCaTN01_F13.raw | | | | | | | | | | | | | | | | 8.4674E5 | 1 | 0 | 0 | 0 | 0 | 0 | 0 | 0 | 0 | 0 | 0 | 0 | 0 | 0 | 0 | 0 | 1 | 227 | 242 | | | PEAKS DB |
| GRAGELKVSTQTGSVR.G | Y | 64.81 | 1644.8907 | 16 | -0.2 | 549.3041 | 3 | 30.39 | 16 | F16:10104 | BuCaTN01_F13.raw | | | | | | | | | | | | | | | | 9.435E6 | 2 | 0 | 0 | 0 | 0 | 0 | 0 | 0 | 0 | 0 | 0 | 0 | 0 | 0 | 0 | 0 | 2 | 1 | 16 | | | PEAKS DB |
| R.TGNPTDPADKSGAWPTYTA.S | Y | 64.47 | 1948.8802 | 19 | 0.9 | 975.4483 | 2 | 54.60 | 16 | F16:25922 | BuCaTN01_F13.raw | | | | | | | | | | | | | | | | 1.1925E6 | 1 | 0 | 0 | 0 | 0 | 0 | 0 | 0 | 0 | 0 | 0 | 0 | 0 | 0 | 0 | 0 | 1 | 479 | 497 | | | PEAKS DB |
| R.LALQWIQNNIHPFGGNPR.A | Y | 61.80 | 2074.0859 | 18 | 0.9 | 692.3699 | 3 | 78.32 | 16 | F16:42470 | BuCaTN01_F13.raw | | | | | | | | | | | | | | | | 3.2941E6 | 1 | 0 | 0 | 0 | 0 | 0 | 0 | 0 | 0 | 0 | 0 | 0 | 0 | 0 | 0 | 0 | 1 | 175 | 192 | | | PEAKS DB |
| R.AQIC(+57.02)AFWNHFLPK.L | N | 57.84 | 1630.8079 | 13 | -0.1 | 544.6099 | 3 | 79.80 | 16 | F16:43298 | BuCaTN01_F13.raw | | | | | | | | | | | | | | | | 1.6489E5 | 1 | 0 | 0 | 0 | 0 | 0 | 0 | 0 | 0 | 0 | 0 | 0 | 0 | 0 | 0 | 0 | 1 | 518 | 530 | Carbamidomethylation | C4:Carbamidomethylation:1000.00 | PEAKS DB |
| K.NPQELIDEEWSVLPYK.S | Y | 56.05 | 1958.9625 | 16 | -0.3 | 980.4882 | 2 | 83.04 | 16 | F16:45435 | BuCaTN01_F13.raw | | | | | | | | | | | | | | | | 3.344E5 | 1 | 0 | 0 | 0 | 0 | 0 | 0 | 0 | 0 | 0 | 0 | 0 | 0 | 0 | 0 | 0 | 1 | 270 | 285 | | | PEAKS DB |
| Q.SGGPNAPWATVTPAESR.G | N | 55.86 | 1696.8169 | 17 | -0.7 | 849.4152 | 2 | 54.07 | 16 | F16:25471 | BuCaTN01_F13.raw | | | | | | | | | | | | | | | | 1.2605E6 | 1 | 0 | 0 | 0 | 0 | 0 | 0 | 0 | 0 | 0 | 0 | 0 | 0 | 0 | 0 | 0 | 1 | 226 | 242 | | | PEAKS DB |
| R.MM(+15.99)RYWANFAR.T | N | 55.68 | 1360.6168 | 10 | 0.0 | 454.5462 | 3 | 57.31 | 16 | F16:27557 | BuCaTN01_F13.raw | | | | | | | | | | | | | | | | 2.5519E6 | 1 | 0 | 0 | 0 | 0 | 0 | 0 | 0 | 0 | 0 | 0 | 0 | 0 | 0 | 0 | 0 | 1 | 469 | 478 | Oxidation (M) | M2:Oxidation (M):0.00 | PEAKS DB |
| K.VYAYLFDHR.A | N | 50.01 | 1182.5822 | 9 | 1.2 | 395.2018 | 3 | 54.55 | 16 | F16:26050 | BuCaTN01_F13.raw | | | | | | | | | | | | | | | | 7.9089E5 | 1 | 0 | 0 | 0 | 0 | 0 | 0 | 0 | 0 | 0 | 0 | 0 | 0 | 0 | 0 | 0 | 1 | 418 | 426 | | | PEAKS DB |
| D.DIVGDHNVIC(+57.02)PVVQFANDYAK.R | N | 49.39 | 2373.1423 | 21 | 1.2 | 792.0557 | 3 | 77.07 | 16 | F16:41389 | BuCaTN01_F13.raw | | | | | | | | | | | | | | | | 1.8347E5 | 1 | 0 | 0 | 0 | 0 | 0 | 0 | 0 | 0 | 0 | 0 | 0 | 0 | 0 | 0 | 0 | 1 | 393 | 413 | Carbamidomethylation | C10:Carbamidomethylation:1000.00 | PEAKS DB |
| L.NTQPLATQPSLR.A | N | 48.75 | 1324.7098 | 12 | 0.4 | 663.3625 | 2 | 39.64 | 16 | F16:16309 | BuCaTN01_F13.raw | | | | | | | | | | | | | | | | 7.6236E5 | 1 | 0 | 0 | 0 | 0 | 0 | 0 | 0 | 0 | 0 | 0 | 0 | 0 | 0 | 0 | 0 | 1 | 506 | 517 | | | PEAKS DB |
| C.PVVQFANDYAKR.N | N | 46.60 | 1406.7306 | 12 | 0.8 | 469.9178 | 3 | 45.58 | 16 | F16:20184 | BuCaTN01_F13.raw | | | | | | | | | | | | | | | | 6.8345E4 | 1 | 0 | 0 | 0 | 0 | 0 | 0 | 0 | 0 | 0 | 0 | 0 | 0 | 0 | 0 | 0 | 1 | 403 | 414 | | | PEAKS DB |
| N.APWATVTPAESR.G | N | 46.37 | 1284.6462 | 12 | 0.3 | 643.3306 | 2 | 47.38 | 16 | F16:21485 | BuCaTN01_F13.raw | | | | | | | | | | | | | | | | 7.7721E5 | 1 | 0 | 0 | 0 | 0 | 0 | 0 | 0 | 0 | 0 | 0 | 0 | 0 | 0 | 0 | 0 | 1 | 231 | 242 | | | PEAKS DB |
| R.ADFLEGVR.M | N | 41.27 | 905.4606 | 8 | -0.1 | 453.7375 | 2 | 52.97 | 16 | F16:25185 | BuCaTN01_F13.raw | | | | | | | | | | | | | | | | 1.1983E7 | 1 | 0 | 0 | 0 | 0 | 0 | 0 | 0 | 0 | 0 | 0 | 0 | 0 | 0 | 0 | 0 | 1 | 350 | 357 | | | PEAKS DB |
| **total 21 peptides** | | | | | | | | | | | | | | | | | | | | | | | | | | | | | | | | | | | | | | | | | | | | | | | | | |

**Best Unique PSM (Scan F16:42155, m/z=725.7046, z=3, RT=78.13, ppm=1.1):**

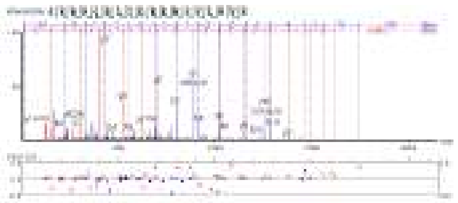

## AAC59905.1

back to list

| Protein Coverage | Supporting Peptides | Best Unique PSM |

**Protein Coverage:**

```
  1  MPSCQPGKMP APWPWWLQLL LCIPSCVAVL PGRAGELKVS TQTGSVRGLS LPVLDGHVSA FLGIPFAEPP LGRMRFLRPE
 81  PVKPWQHVLD ATSYKPACYQ MVDTSYPGFQ GTEMWNPNRG MSEDCLYLNI WVPSPRPKDA PVLVWIYGGG FYSGAASLDV
161  YDGRFLTYTQ NVILVSLSYR VGAFGFLGLP GSPEAPGNMG LLDQRLALQW IQNNIHPFGG NPRAVTVFGE SAGAASVGMH
241  LLSTQSRTLF QRAILQSGGP NAPWATVTPA ESRGRAALLG KQLGCHFNND SELVSCLRSK NPQELIDEEW SVLPYKSIFR
321  FPFVPVIDGD FFPDTPEAML SSGNFKETQV LLGVVKDEGS YFLIYGLPGF SKDNESLISR ADFLEGVRMS VPHANDIATD
                                                433
401  AVVLQYTDWQ DQDNREKNRE ALDDIVGDHN VICPVVQFAN DYAKRNSKVY AYLFDHRASN LLWPPWMGVP HGYEIEFVFG
481  LPLNDSLNYT PQEKELSRRM MRYWANFART GNPTDPADKS GAWPTYTASQ PQYVQLNTQP LATQPSLRAQ ICAFWNHFLP
561  KLLNATVDPP RADRRRRSAR A
```

C Carbamidomethylation (+57.02)
O Oxidation (M) (+15.99)

**Supporting Peptides:**

| Peptide | Uniq | -10lgP | Mass | Length | ppm | m/z | z | RT | Fraction | Scan | Source File | Area F1 | Area F2a | Area F3a | Area F4 | Area F5 | Area F6a | Area F7a | Area F7b | Area F8 | Area F9a | Area Sample 1F10a | Area F10b | Area F11a | Area F11b | Area F12a | Area F13 |
|---|---|---|---|---|---|---|---|---|---|---|---|---|---|---|---|---|---|---|---|---|---|---|---|---|---|---|---|
| R.AILQSGGPNAPWATVTPAESR.G | N | 122.40 | 2122.0806 | 21 | 0.2 | 1062.0477 | 2 | 64.33 | 16 | F16:32291 | BuCaTN01_F13.raw | | | | | | | | | 4.2489E5 | | | | | | | 2.432E7 |
| R.SKNPQELIDEEWSVLPYK.S | Y | 100.39 | 2174.0896 | 18 | 1.1 | 725.7046 | 3 | 78.13 | 16 | F16:42155 | BuCaTN01_F13.raw | | | | | | | | | | | | | | | | 5.585E6 |
| K.SGAWPTYTASQPQYVQLNTQPLATQPSLR.A | Y | 90.63 | 3202.6047 | 29 | 0.5 | 1068.5427 | 3 | 72.87 | 16 | F16:38224 | BuCaTN01_F13.raw | | | | | | | | | | | | | | | | 4.1527E6 |
| R.AGELKVSTQTGSVR.G | Y | 85.19 | 1431.7681 | 14 | 0.4 | 478.2635 | 3 | 32.16 | 16 | F16:11066 | BuCaTN01_F13.raw | | | | | | | | | | | | | | | | 6.4414E7 |
| R.TGNPTDPADKSGAWPTYTASQPQYVQLNTQPLATQPSLR.A | Y | 79.64 | 4199.0557 | 39 | 0.0 | 1050.7712 | 4 | 69.79 | 16 | F16:35951 | BuCaTN01_F13.raw | | | | | | | | | | | | | | | | 1.0938E7 |
| R.MMRYWANFAR.T | N | 72.98 | 1344.6219 | 10 | 0.2 | 449.2147 | 3 | 61.26 | 16 | F16:30310 | BuCaTN01_F13.raw | | | | | | | | | | | | | | | | 1.0526E7 |
| R.TLFQRAILQSGGPNAPWATVTPAESR.G | Y | 67.34 | 2767.4404 | 26 | -0.1 | 923.4873 | 3 | 74.20 | 16 | F16:38946 | BuCaTN01_F13.raw | | | | | | | | | | | | | | | | 8.3998E5 |
| S.GGPNAPWATVTPAESR.G | N | 65.09 | 1609.7848 | 16 | 0.6 | 805.9001 | 2 | 54.13 | 16 | F16:25775 | BuCaTN01_F13.raw | | | | | | | | | | | | | | | | 8.4674E5 |
| P.GRAGELKVSTQTGSVR.G | Y | 64.81 | 1644.8907 | 16 | -0.2 | 549.3041 | 3 | 30.39 | 16 | F16:10104 | BuCaTN01_F13.raw | | | | | | | | | | | | | | | | 9.435E6 |
| R.TGNPTDPADKSGAWPTYTA.S | Y | 64.47 | 1948.8802 | 19 | 0.9 | 975.4483 | 2 | 54.60 | 16 | F16:25922 | BuCaTN01_F13.raw | | | | | | | | | | | | | | | | 1.1925E6 |
| R.LALQWIQNNIHPFGGNPR.A | Y | 61.80 | 2074.0859 | 18 | 0.9 | 692.3699 | 3 | 78.32 | 16 | F16:42470 | BuCaTN01_F13.raw | | | | | | | | | | | | | | | | 3.2941E6 |
| R.AQIC(+57.02)AFWNHFLPK.L | N | 57.84 | 1630.8079 | 13 | -0.1 | 544.6099 | 3 | 79.80 | 16 | F16:43298 | BuCaTN01_F13.raw | | | | | | | | | | | | | | | | 1.6489E5 |
| K.NPQELIDEEWSVLPYK.S | Y | 56.05 | 1958.9625 | 16 | -0.3 | 980.4882 | 2 | 83.04 | 16 | F16:45435 | BuCaTN01_F13.raw | | | | | | | | | | | | | | | | 3.344E5 |
| Q.SGGPNAPWATVTPAESR.G | N | 55.86 | 1696.8169 | 17 | -0.7 | 849.4152 | 2 | 54.07 | 16 | F16:25471 | BuCaTN01_F13.raw | | | | | | | | | | | | | | | | 1.2605E6 |
| R.MM(+15.99)RYWANFAR.T | N | 55.68 | 1360.6168 | 10 | 0.0 | 454.5462 | 3 | 57.31 | 16 | F16:27557 | BuCaTN01_F13.raw | | | | | | | | | | | | | | | | 2.5519E6 |
| K.VYAYLFDHR.A | N | 50.01 | 1182.5822 | 9 | 1.2 | 395.2018 | 3 | 54.55 | 16 | F16:26050 | BuCaTN01_F13.raw | | | | | | | | | | | | | | | | 7.9089E5 |
| D.DIVGDHNVIC(+57.02)PVVQFANDYAK.R | N | 49.39 | 2373.1423 | 21 | 1.2 | 792.0557 | 3 | 77.07 | 16 | F16:41389 | BuCaTN01_F13.raw | | | | | | | | | | | | | | | | 1.8347E5 |
| L.NTQPLATQPSLR.A | N | 48.75 | 1324.7098 | 12 | 0.4 | 663.3625 | 2 | 39.64 | 16 | F16:16309 | BuCaTN01_F13.raw | | | | | | | | | | | | | | | | 7.6236E5 |
| C.PVVQFANDYAKR.N | N | 46.60 | 1406.7306 | 12 | 0.8 | 469.9178 | 3 | 45.58 | 16 | F16:20184 | BuCaTN01_F13.raw | | | | | | | | | | | | | | | | 6.8345E4 |
| N.APWATVTPAESR.G | N | 46.37 | 1284.6462 | 12 | 0.3 | 643.3306 | 2 | 47.38 | 16 | F16:21485 | BuCaTN01_F13.raw | | | | | | | | | | | | | | | | 7.7721E5 |
| R.ADFLEGVR.M | N | 41.27 | 905.4606 | 8 | -0.1 | 453.7375 | 2 | 52.97 | 16 | F16:25185 | BuCaTN01_F13.raw | | | | | | | | | | | | | | | | 1.1983E7 |
| total 21 peptides | | | | | | | | | | | | | | | | | | | | | | | | | | | |

| Peptide | #Feature | #Feature F1 | #Feature F2a | #Feature F3a | #Feature F4 | #Feature F5 | #Feature F6a | #Feature F7a | #Feature F7b | #Feature F8 | #Feature F9a | #Feature Sample 1F10a | #Feature F10b | #Feature F11a | #Feature F11b | #Feature F12a | #Feature F13 | Start | End | PTM | AScore | Found By |
|---|---|---|---|---|---|---|---|---|---|---|---|---|---|---|---|---|---|---|---|---|---|---|
| R.AILQSGGPNAPWATVTPAESR.G | 5 | 0 | 0 | 0 | 0 | 0 | 0 | 0 | 0 | 2 | 0 | 0 | 0 | 0 | 0 | 0 | 3 | 253 | 273 | | | PEAKS DB |
| R.SKNPQELIDEEWSVLPYK.S | 1 | 0 | 0 | 0 | 0 | 0 | 0 | 0 | 0 | 0 | 0 | 0 | 0 | 0 | 0 | 0 | 1 | 299 | 316 | | | PEAKS DB |
| K.SGAWPTYTASQPQYVQLNTQPLATQPSLR.A | 1 | 0 | 0 | 0 | 0 | 0 | 0 | 0 | 0 | 0 | 0 | 0 | 0 | 0 | 0 | 0 | 1 | 520 | 548 | | | PEAKS DB |
| R.AGELKVSTQTGSVR.G | 3 | 0 | 0 | 0 | 0 | 0 | 0 | 0 | 0 | 0 | 0 | 0 | 0 | 0 | 0 | 0 | 3 | 34 | 47 | | | PEAKS DB |
| R.TGNPTDPADKSGAWPTYTASQPQYVQLNTQPLATQPSLR.A | 2 | 0 | 0 | 0 | 0 | 0 | 0 | 0 | 0 | 0 | 0 | 0 | 0 | 0 | 0 | 0 | 2 | 510 | 548 | | | PEAKS DB |
| R.MMRYWANFAR.T | 2 | 0 | 0 | 0 | 0 | 0 | 0 | 0 | 0 | 0 | 0 | 0 | 0 | 0 | 0 | 0 | 2 | 500 | 509 | | | PEAKS DB |
| R.TLFQRAILQSGGPNAPWATVTPAESR.G | 1 | 0 | 0 | 0 | 0 | 0 | 0 | 0 | 0 | 0 | 0 | 0 | 0 | 0 | 0 | 0 | 1 | 248 | 273 | | | PEAKS DB |
| S.GGPNAPWATVTPAESR.G | 1 | 0 | 0 | 0 | 0 | 0 | 0 | 0 | 0 | 0 | 0 | 0 | 0 | 0 | 0 | 0 | 1 | 258 | 273 | | | PEAKS DB |
| P.GRAGELKVSTQTGSVR.G | 2 | 0 | 0 | 0 | 0 | 0 | 0 | 0 | 0 | 0 | 0 | 0 | 0 | 0 | 0 | 0 | 2 | 32 | 47 | | | PEAKS DB |
| R.TGNPTDPADKSGAWPTYTA.S | 1 | 0 | 0 | 0 | 0 | 0 | 0 | 0 | 0 | 0 | 0 | 0 | 0 | 0 | 0 | 0 | 1 | 510 | 528 | | | PEAKS DB |
| R.LALQWIQNNIHPFGGNPR.A | 1 | 0 | 0 | 0 | 0 | 0 | 0 | 0 | 0 | 0 | 0 | 0 | 0 | 0 | 0 | 0 | 1 | 206 | 223 | | | PEAKS DB |
| R.AQIC(+57.02)AFWNHFLPK.L | 1 | 0 | 0 | 0 | 0 | 0 | 0 | 0 | 0 | 0 | 0 | 0 | 0 | 0 | 0 | 0 | 1 | 549 | 561 | Carbamidomethylation | C4:Carbamidomethylation:1000.00 | PEAKS DB |
| K.NPQELIDEEWSVLPYK.S | 1 | 0 | 0 | 0 | 0 | 0 | 0 | 0 | 0 | 0 | 0 | 0 | 0 | 0 | 0 | 0 | 1 | 301 | 316 | | | PEAKS DB |
| Q.SGGPNAPWATVTPAESR.G | 1 | 0 | 0 | 0 | 0 | 0 | 0 | 0 | 0 | 0 | 0 | 0 | 0 | 0 | 0 | 0 | 1 | 257 | 273 | | | PEAKS DB |
| R.MM(+15.99)RYWANFAR.T | 1 | 0 | 0 | 0 | 0 | 0 | 0 | 0 | 0 | 0 | 0 | 0 | 0 | 0 | 0 | 0 | 1 | 500 | 509 | Oxidation (M) | M2:Oxidation (M):0.00 | PEAKS DB |
| K.VYAYLFDHR.A | 1 | 0 | 0 | 0 | 0 | 0 | 0 | 0 | 0 | 0 | 0 | 0 | 0 | 0 | 0 | 0 | 1 | 449 | 457 | | | PEAKS DB |
| D.DIVGDHNVIC(+57.02)PVVQFANDYAK.R | 1 | 0 | 0 | 0 | 0 | 0 | 0 | 0 | 0 | 0 | 0 | 0 | 0 | 0 | 0 | 0 | 1 | 424 | 444 | Carbamidomethylation | C10:Carbamidomethylation:1000.00 | PEAKS DB |
| L.NTQPLATQPSLR.A | 1 | 0 | 0 | 0 | 0 | 0 | 0 | 0 | 0 | 0 | 0 | 0 | 0 | 0 | 0 | 0 | 1 | 537 | 548 | | | PEAKS DB |
| C.PVVQFANDYAKR.N | 1 | 0 | 0 | 0 | 0 | 0 | 0 | 0 | 0 | 0 | 0 | 0 | 0 | 0 | 0 | 0 | 1 | 434 | 445 | | | PEAKS DB |
| N.APWATVTPAESR.G | 1 | 0 | 0 | 0 | 0 | 0 | 0 | 0 | 0 | 0 | 0 | 0 | 0 | 0 | 0 | 0 | 1 | 262 | 273 | | | PEAKS DB |
| R.ADFLEGVR.M | 1 | 0 | 0 | 0 | 0 | 0 | 0 | 0 | 0 | 0 | 0 | 0 | 0 | 0 | 0 | 0 | 1 | 381 | 388 | | | PEAKS DB |

**Best Unique PSM (Scan F16:42155, m/z=725.7046, z=3, RT=78.13, ppm=1.1):**

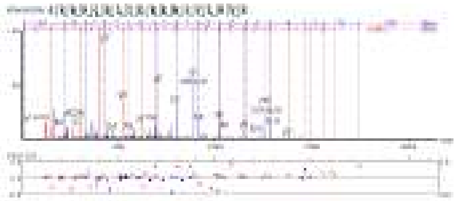

## Q92035.2

back to list

| Protein Coverage | Supporting Peptides | Best Unique PSM |

**Protein Coverage:**

```
  1  MPSCQPGKMP APWPWWLQLL LCIPSCVAVL PGRAGELKVS TQTGSVRGLS LPVLDGHVSA FLGIPFAEPP LGRMRFLRPE
 81  PVKPWQHVLD ATSYKPACYQ MVDTSYPGFQ GTEMWNPNRG MSEDCLYLNI WVPSPRPKDA PVLVWIYGGG FYSGAASLDV
161  YDGRFLTYTQ NVILVSLSYR VGAFGFLGLP GSPEAPGNMG LLDQRLALQW IQNNIHPFGG NPRAVTVFGE SAGAASVGMH
241  LLSTQSRTLF QRAILQSGGP NAPWATVTPA ESRGRAALLG KQLGCHFNND SELVSCLRSK NPQELIDEEW SVLPYKSIFR
321  FPFVPVIDGD FFPDTPEAML SSGNFKETQV LLGVVKDEGS YFLIYGLPGF SKDNESLISR ADFLEGVRMS VPHANDIATD
                                                433
401  AVVLQYTDWQ DQDNREKNRE ALDDIVGDHN VICPVVQFAN DYAKRNSKVY AYLFDHRASN LLWPPWMGVP HGYEIEFVFG
481  LPLNDSLNYT PQEKELSRRM MRYWANFART GNPTDPADKS GAWPTYTASQ PQYVQLNTQP LATQPSLRAQ ICAFWNHFLP
561  KLLNATDNIE EAERQWKLEF HLWSAYMMHW KSQFDHYNKQ DRCSEL
```

C Carbamidomethylation (+57.02)
O Oxidation (M) (+15.99)

**Supporting Peptides:**

| Peptide | Uniq | -10lgP | Mass | Length | ppm | m/z | z | RT | Fraction | Scan | Source File | Area F1 | Area F2a | Area F3a | Area F4 | Area F5 | Area F6a | Area F7a | Area F7b | Area F8 | Area F9a | Area Sample 1F10a | Area F10b | Area F11a | Area F11b | Area F12a | Area F13 |
|---|---|---|---|---|---|---|---|---|---|---|---|---|---|---|---|---|---|---|---|---|---|---|---|---|---|---|---|
| R.AILQSGGPNAPWATVTPAESR.G | N | 122.40 | 2122.0806 | 21 | 0.2 | 1062.0477 | 2 | 64.33 | 16 | F16:32291 | BuCaTN01_F13.raw | | | | | | | | | 4.2489E5 | | | | | | | 2.432E7 |
| R.SKNPQELIDEEWSVLPYK.S | Y | 100.39 | 2174.0896 | 18 | 1.1 | 725.7046 | 3 | 78.13 | 16 | F16:42155 | BuCaTN01_F13.raw | | | | | | | | | | | | | | | | 5.585E6 |

| Peptide | #Feature | #Feature F1 | #Feature F2a | #Feature F3a | #Feature F4 | #Feature F5 | #Feature F6a | #Feature F7a | #Feature F7b | #Feature F8 | #Feature F9a | #Feature Sample 1F10a | #Feature F10b | #Feature F11a | #Feature F11b | #Feature F12a | #Feature F13 | Start | End | PTM | AScore | Found By |
|---|---|---|---|---|---|---|---|---|---|---|---|---|---|---|---|---|---|---|---|---|---|---|
| R.AILQSGGPNAPWATVTPAESR.G | 5 | 0 | 0 | 0 | 0 | 0 | 0 | 0 | 0 | 2 | 0 | 0 | 0 | 0 | 0 | 0 | 3 | 253 | 273 | | | PEAKS DB |
| R.SKNPQELIDEEWSVLPYK.S | 1 | 0 | 0 | 0 | 0 | 0 | 0 | 0 | 0 | 0 | 0 | 0 | 0 | 0 | 0 | 0 | 1 | 299 | 316 | | | PEAKS DB |

| Peptide | Uniq | -10lgP | Mass | Length | ppm | m/z | z | RT | Fraction | Scan | Source File | Area F1 | Area F2a | Area F3a | Area F4 | Area F5 | Area F6a | Area F7a | Area F7b | Area F8 | Area F9a | Area Sample 1F10a | Area F10b | Area F11a | Area F11b | Area F12a | Area F13 | #Feature | #Feature F1 | #Feature F2a | #Feature F3a | #Feature F4 | #Feature F5 | #Feature F6a | #Feature F7a | #Feature F7b | #Feature F8 | #Feature F9a | #Feature Sample 1F10a | #Feature F10b | #Feature F11a | #Feature F11b | #Feature F12a | #Feature F13 | Start | End | PTM | AScore | Found By |
|---|---|---|---|---|---|---|---|---|---|---|---|---|---|---|---|---|---|---|---|---|---|---|---|---|---|---|---|---|---|---|---|---|---|---|---|---|---|---|---|---|---|---|---|---|---|---|---|---|---|
| K.SGAWPTYTASQPQYVQLNTQPLATQPSLR.A | Y | 90.63 | 3202.6047 | 29 | 0.5 | 1068.5427 | 3 | 72.87 | 16 | F16:38224 | BuCaTN01_F13.raw | | | | | | | | | | | | | | | | 4.1527E6 | 1 | 0 | 0 | 0 | 0 | 0 | 0 | 0 | 0 | 0 | 0 | 0 | 0 | 0 | 0 | 0 | 1 | 520 | 548 | | | PEAKS DB |
| R.AGELKVSTQTGSVR.G | Y | 85.19 | 1431.7681 | 14 | 0.4 | 478.2635 | 3 | 32.16 | 16 | F16:11066 | BuCaTN01_F13.raw | | | | | | | | | | | | | | | | 6.4414E7 | 3 | 0 | 0 | 0 | 0 | 0 | 0 | 0 | 0 | 0 | 0 | 0 | 0 | 0 | 0 | 0 | 3 | 34 | 47 | | | PEAKS DB |
| R.TGNPTDPADKSGAWPTYTASQPQYVQLNTQPLATQPSLR.A | Y | 79.64 | 4199.0557 | 39 | 0.0 | 1050.7712 | 4 | 69.79 | 16 | F16:35951 | BuCaTN01_F13.raw | | | | | | | | | | | | | | | | 1.0938E7 | 2 | 0 | 0 | 0 | 0 | 0 | 0 | 0 | 0 | 0 | 0 | 0 | 0 | 0 | 0 | 0 | 2 | 510 | 548 | | | PEAKS DB |
| R.MMRYWANFAR.T | N | 72.98 | 1344.6219 | 10 | 0.2 | 449.2147 | 3 | 61.26 | 16 | F16:30310 | BuCaTN01_F13.raw | | | | | | | | | | | | | | | | 1.0526E7 | 2 | 0 | 0 | 0 | 0 | 0 | 0 | 0 | 0 | 0 | 0 | 0 | 0 | 0 | 0 | 0 | 2 | 500 | 509 | | | PEAKS DB |
| R.TLFQRAILQSGGPNAPWATVTPAESR.G | Y | 67.34 | 2767.4404 | 26 | -0.1 | 923.4873 | 3 | 74.20 | 16 | F16:38946 | BuCaTN01_F13.raw | | | | | | | | | | | | | | | | 8.3998E5 | 1 | 0 | 0 | 0 | 0 | 0 | 0 | 0 | 0 | 0 | 0 | 0 | 0 | 0 | 0 | 0 | 1 | 248 | 273 | | | PEAKS DB |
| S.GGPNAPWATVTPAESR.G | N | 65.09 | 1609.7848 | 16 | 0.6 | 805.9001 | 2 | 54.13 | 16 | F16:25775 | BuCaTN01_F13.raw | | | | | | | | | | | | | | | | 8.4674E5 | 1 | 0 | 0 | 0 | 0 | 0 | 0 | 0 | 0 | 0 | 0 | 0 | 0 | 0 | 0 | 0 | 1 | 258 | 273 | | | PEAKS DB |
| P.GRAGELKVSTQTGSVR.G | Y | 64.81 | 1644.8907 | 16 | -0.2 | 549.3041 | 3 | 30.39 | 16 | F16:10104 | BuCaTN01_F13.raw | | | | | | | | | | | | | | | | 9.435E6 | 2 | 0 | 0 | 0 | 0 | 0 | 0 | 0 | 0 | 0 | 0 | 0 | 0 | 0 | 0 | 0 | 2 | 32 | 47 | | | PEAKS DB |
| R.TGNPTDPADKSGAWPTYTA.S | Y | 64.47 | 1948.8802 | 19 | 0.9 | 975.4483 | 2 | 54.60 | 16 | F16:25922 | BuCaTN01_F13.raw | | | | | | | | | | | | | | | | 1.1925E6 | 1 | 0 | 0 | 0 | 0 | 0 | 0 | 0 | 0 | 0 | 0 | 0 | 0 | 0 | 0 | 0 | 1 | 510 | 528 | | | PEAKS DB |
| R.LALQWIQNNIHPFGGNPR.A | Y | 61.80 | 2074.0859 | 18 | 0.9 | 692.3699 | 3 | 78.32 | 16 | F16:42470 | BuCaTN01_F13.raw | | | | | | | | | | | | | | | | 3.2941E6 | 1 | 0 | 0 | 0 | 0 | 0 | 0 | 0 | 0 | 0 | 0 | 0 | 0 | 0 | 0 | 0 | 1 | 206 | 223 | | | PEAKS DB |
| R.AQIC(+57.02)AFWNHFLPK.L | N | 57.84 | 1630.8079 | 13 | -0.1 | 544.6099 | 3 | 79.80 | 16 | F16:43298 | BuCaTN01_F13.raw | | | | | | | | | | | | | | | | 1.6489E5 | 1 | 0 | 0 | 0 | 0 | 0 | 0 | 0 | 0 | 0 | 0 | 0 | 0 | 0 | 0 | 0 | 1 | 549 | 561 | Carbamidomethylation | C4:Carbamidomethylation:1000.00 | PEAKS DB |
| K.NPQELIDEEWSVLPYK.S | Y | 56.05 | 1958.9625 | 16 | -0.3 | 980.4882 | 2 | 83.04 | 16 | F16:45435 | BuCaTN01_F13.raw | | | | | | | | | | | | | | | | 3.344E5 | 1 | 0 | 0 | 0 | 0 | 0 | 0 | 0 | 0 | 0 | 0 | 0 | 0 | 0 | 0 | 0 | 1 | 301 | 316 | | | PEAKS DB |
| Q.SGGPNAPWATVTPAESR.G | N | 55.86 | 1696.8169 | 17 | -0.7 | 849.4152 | 2 | 54.07 | 16 | F16:25471 | BuCaTN01_F13.raw | | | | | | | | | | | | | | | | 1.2605E6 | 1 | 0 | 0 | 0 | 0 | 0 | 0 | 0 | 0 | 0 | 0 | 0 | 0 | 0 | 0 | 0 | 1 | 257 | 273 | | | PEAKS DB |
| R.MM(+15.99)RYWANFAR.T | N | 55.68 | 1360.6168 | 10 | 0.0 | 454.5462 | 3 | 57.31 | 16 | F16:27557 | BuCaTN01_F13.raw | | | | | | | | | | | | | | | | 2.5519E6 | 1 | 0 | 0 | 0 | 0 | 0 | 0 | 0 | 0 | 0 | 0 | 0 | 0 | 0 | 0 | 0 | 1 | 500 | 509 | Oxidation (M) | M2:Oxidation (M):0.00 | PEAKS DB |
| K.VYAYLFDHR.A | N | 50.01 | 1182.5822 | 9 | 1.2 | 395.2018 | 3 | 54.55 | 16 | F16:26050 | BuCaTN01_F13.raw | | | | | | | | | | | | | | | | 7.9089E5 | 1 | 0 | 0 | 0 | 0 | 0 | 0 | 0 | 0 | 0 | 0 | 0 | 0 | 0 | 0 | 0 | 1 | 449 | 457 | | | PEAKS DB |
| D.DIVGDHNVIC(+57.02)PVVQFANDYAK.R | N | 49.39 | 2373.1423 | 21 | 1.2 | 792.0557 | 3 | 77.07 | 16 | F16:41389 | BuCaTN01_F13.raw | | | | | | | | | | | | | | | | 1.8347E5 | 1 | 0 | 0 | 0 | 0 | 0 | 0 | 0 | 0 | 0 | 0 | 0 | 0 | 0 | 0 | 0 | 1 | 424 | 444 | Carbamidomethylation | C10:Carbamidomethylation:1000.00 | PEAKS DB |
| L.NTQPLATQPSLR.A | N | 48.75 | 1324.7098 | 12 | 0.4 | 663.3625 | 2 | 39.64 | 16 | F16:16309 | BuCaTN01_F13.raw | | | | | | | | | | | | | | | | 7.6236E5 | 1 | 0 | 0 | 0 | 0 | 0 | 0 | 0 | 0 | 0 | 0 | 0 | 0 | 0 | 0 | 0 | 1 | 537 | 548 | | | PEAKS DB |
| C.PVVQFANDYAKR.N | N | 46.60 | 1406.7306 | 12 | 0.8 | 469.9178 | 3 | 45.58 | 16 | F16:20184 | BuCaTN01_F13.raw | | | | | | | | | | | | | | | | 6.8345E4 | 1 | 0 | 0 | 0 | 0 | 0 | 0 | 0 | 0 | 0 | 0 | 0 | 0 | 0 | 0 | 0 | 1 | 434 | 445 | | | PEAKS DB |
| N.APWATVTPAESR.G | N | 46.37 | 1284.6462 | 12 | 0.3 | 643.3306 | 2 | 47.38 | 16 | F16:21485 | BuCaTN01_F13.raw | | | | | | | | | | | | | | | | 7.7721E5 | 1 | 0 | 0 | 0 | 0 | 0 | 0 | 0 | 0 | 0 | 0 | 0 | 0 | 0 | 0 | 0 | 1 | 262 | 273 | | | PEAKS DB |
| R.ADFLEGVR.M | N | 41.27 | 905.4606 | 8 | -0.1 | 453.7375 | 2 | 52.97 | 16 | F16:25185 | BuCaTN01_F13.raw | | | | | | | | | | | | | | | | 1.1983E7 | 1 | 0 | 0 | 0 | 0 | 0 | 0 | 0 | 0 | 0 | 0 | 0 | 0 | 0 | 0 | 0 | 1 | 381 | 388 | | | PEAKS DB |
| total 21 peptides | | | | | | | | | | | | | | | | | | | | | | | | | | | | | | | | | | | | | | | | | | | | | | | | | |

**Best Unique PSM (Scan F16:42155, m/z=725.7046, z=3, RT=78.13, ppm=1.1):**

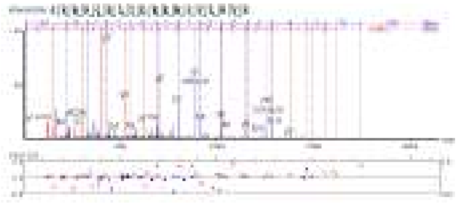

## AAZ29513.1

back to list

| **Protein Coverage** | **Supporting Peptides** | **Best Unique PSM** |

**Protein Coverage:**

1 VIAVCVSLLG ASSIPPEPLN LYQFKNMIKC TNTRHWLSFT NYGCYCGYGG SGTPVDELDR CCQVHDKCYD TAKRVHKCFP

C Carbamidomethylation (+57.02)
O Oxidation (M) (+15.99)

81 SVRTYSYDCS EGKLTCKDNN TKCKDFVCNC DRTAALCFAK APYNNKNFKI DPSRCQ

**Supporting Peptides:**

| Peptide | Uniq | -10lgP | Mass | Length | ppm | m/z | z | RT | Fraction | Scan | Source File | Area F1 | Area F2a | Area F3a | Area F4 | Area F5 | Area F6a | Area F7a | Area F7b | Area F8 | Area F9a | Area Sample 1F10a | Area F10b | Area F11a | Area F11b | Area F12a | Area F13 | #Feature | #Feature F1 | #Feature F2a | #Feature F3a | #Feature F4 | #Feature F5 | #Feature F6a | #Feature F7a | #Feature F7b | #Feature F8 | #Feature F9a | #Feature Sample 1F10a | #Feature F10b | #Feature F11a | #Feature F11b | #Feature F12a | #Feature F13 | Start | End | PTM | AScore | Found By |
|---|---|---|---|---|---|---|---|---|---|---|---|---|---|---|---|---|---|---|---|---|---|---|---|---|---|---|---|---|---|---|---|---|---|---|---|---|---|---|---|---|---|---|---|---|---|---|---|---|---|
| N.YGC(+57.02)YC(+57.02)GYGGSGTPVDELDR.C | N | 112.36 | 2124.8516 | 19 | 3.7 | 1063.4326 | 2 | 56.60 | 14 | F14:33384 | BuCaTN01_F11b.raw | | | 0 | | | 2.0097E6 | | 1.4053E7 | | 8.8531E7 | 3.4891E6 | 4.8054E8 | 6.165E7 | 1.7851E9 | 1.2628E8 | | 69 | 0 | 0 | 0 | 0 | 0 | 1 | 0 | 2 | 0 | 3 | 1 | 4 | 2 | 53 | 3 | 0 | 42 | 60 | Carbamidomethylation | C3:Carbamidomethylation:1000.00;C5:Carbamidomethylation:1000.00 | PEAKS DB |
| Y.C(+57.02)GYGGSGTPVDELDR.C | N | 88.12 | 1581.6729 | 15 | 4.4 | 791.8439 | 2 | 48.02 | 14 | F14:25306 | BuCaTN01_F11b.raw | | | | | | | | 2.166E6 | | 8.5786E6 | | 1.5853E7 | 6.495E5 | 1.7537E7 | 0 | | 6 | 0 | 0 | 0 | 0 | 0 | 0 | 0 | 1 | 0 | 1 | 0 | 2 | 1 | 1 | 0 | 0 | 46 | 60 | Carbamidomethylation | C1:Carbamidomethylation:1000.00 | PEAKS DB |
| G.C(+57.02)YC(+57.02)GYGGSGTPVDELDR.C | N | 84.40 | 1904.7668 | 17 | 2.7 | 953.3911 | 2 | 53.16 | 12 | F12:29351 | BuCaTN01_F10b.raw | | | | | | | | 4.1849E5 | | | | 3.7261E5 | | 2.1774E6 | | | 3 | 0 | 0 | 0 | 0 | 0 | 0 | 0 | 1 | 0 | 0 | 0 | 1 | 0 | 1 | 0 | 0 | 44 | 60 | Carbamidomethylation | C1:Carbamidomethylation:1000.00;C3:Carbamidomethylation:1000.00 | PEAKS DB |
| R.TYSYDC(+57.02)SEGKLTC(+57.02)K.D | N | 82.89 | 1710.7229 | 14 | 0.5 | 856.3691 | 2 | 38.29 | 7 | F7:14505 | BuCaTN01_F7a.raw | | | | | | | 1.4512E8 | 2.3155E7 | | 3.8991E5 | 4.5941E5 | | | | | | 8 | 0 | 0 | 0 | 0 | 0 | 0 | 4 | 2 | 0 | 1 | 1 | 0 | 0 | 0 | 0 | 0 | 84 | 97 | Carbamidomethylation | C6:Carbamidomethylation:1000.00;C13:Carbamidomethylation:1000.00 | PEAKS DB |
| Y.GC(+57.02)YC(+57.02)GYGGSGTPVDELDR.C | N | 82.10 | 1961.7883 | 18 | 1.4 | 981.9028 | 2 | 53.49 | 10 | F10:30232 | BuCaTN01_F9a.raw | | | | | | | | 1.7852E6 | | 3.3077E6 | | 1.5846E6 | | 3.2964E6 | 1.0428E5 | | 5 | 0 | 0 | 0 | 0 | 0 | 0 | 0 | 1 | 0 | 1 | 0 | 1 | 0 | 1 | 1 | 0 | 43 | 60 | Carbamidomethylation | C2:Carbamidomethylation:1000.00;C4:Carbamidomethylation:1000.00 | PEAKS DB |
| R.TYSYDC(+57.02)SEGKLTC(+57.02)KD.N | N | 75.87 | 1825.7499 | 15 | 2.5 | 913.8818 | 2 | 38.60 | 8 | F8:16643 | BuCaTN01_F7b.raw | | | | | | | | 7.3044E5 | | | | | | | | | 1 | 0 | 0 | 0 | 0 | 0 | 0 | 0 | 1 | 0 | 0 | 0 | 0 | 0 | 0 | 0 | 0 | 84 | 98 | Carbamidomethylation | C6:Carbamidomethylation:1000.00;C13:Carbamidomethylation:1000.00 | PEAKS DB |
| C.YC(+57.02)GYGGSGTPVDELDR.C | N | 75.62 | 1744.7362 | 16 | 4.1 | 873.3754 | 2 | 52.27 | 14 | F14:28849 | BuCaTN01_F11b.raw | | | | | | | | 7.7286E5 | | 7.3447E5 | | | | 3.0904E6 | 1.4057E5 | | 4 | 0 | 0 | 0 | 0 | 0 | 0 | 0 | 1 | 0 | 1 | 0 | 0 | 0 | 1 | 1 | 0 | 45 | 60 | Carbamidomethylation | C2:Carbamidomethylation:1000.00 | PEAKS DB |
| G.YGGSGTPVDELDR.C | N | 75.26 | 1364.6207 | 13 | 4.1 | 683.3177 | 2 | 45.29 | 14 | F14:22502 | BuCaTN01_F11b.raw | | | | | | | 6.8893E5 | | | | | | | 9.1406E6 | 6.9945E5 | | 3 | 0 | 0 | 0 | 0 | 0 | 0 | 1 | 0 | 0 | 0 | 0 | 0 | 0 | 1 | 1 | 0 | 48 | 60 | | | PEAKS DB |
| R.TAALC(+57.02)FAKAPY.N | N | 73.24 | 1211.6008 | 11 | 2.2 | 606.8076 | 2 | 55.84 | 12 | F12:32023 | BuCaTN01_F10b.raw | | | | | | | 7.3334E4 | | | 1.7166E6 | 1.3753E5 | 1.06E7 | | 8.1407E6 | 9.828E5 | | 7 | 0 | 0 | 0 | 0 | 0 | 0 | 1 | 0 | 0 | 1 | 1 | 2 | 0 | 1 | 1 | 0 | 113 | 123 | Carbamidomethylation | C5:Carbamidomethylation:1000.00 | PEAKS DB |
| T.NYGC(+57.02)YC(+57.02)GYGGSGTPVDELDR.C | N | 72.69 | 2238.8945 | 20 | 5.3 | 1120.4559 | 2 | 64.81 | 14 | F14:40195 | BuCaTN01_F11b.raw | | | | | | | | | | 2.3985E4 | | 0 | 1.1223E5 | 5.3539E5 | | | 3 | 0 | 0 | 0 | 0 | 0 | 0 | 0 | 0 | 0 | 1 | 0 | 0 | 1 | 1 | 0 | 0 | 41 | 60 | Carbamidomethylation | C4:Carbamidomethylation:1000.00;C6:Carbamidomethylation:1000.00 | PEAKS DB |
| R.TAALC(+57.02)FAKAPYN.N | N | 69.35 | 1325.6438 | 12 | -0.2 | 663.8290 | 2 | 51.91 | 6 | F6:30474 | BuCaTN01_F6a.raw | | | | | | 3.7675E6 | | 1.5632E7 | | 4.6358E6 | | 1.9346E6 | | | | | 5 | 0 | 0 | 0 | 0 | 0 | 1 | 0 | 2 | 0 | 1 | 0 | 1 | 0 | 0 | 0 | 0 | 113 | 124 | Carbamidomethylation | C5:Carbamidomethylation:1000.00 | PEAKS DB |
| C.GYGGSGTPVDELDR.C | N | 66.26 | 1421.6422 | 14 | 0.0 | 711.8284 | 2 | 46.82 | 10 | F10:24048 | BuCaTN01_F9a.raw | | | | | | | 2.9103E5 | 1.2275E6 | | 2.1867E6 | | | | 8.7883E6 | | | 5 | 0 | 0 | 0 | 0 | 0 | 0 | 1 | 1 | 0 | 1 | 0 | 0 | 0 | 2 | 0 | 0 | 47 | 60 | | | PEAKS DB |
| N.C(+57.02)DRTAALC(+57.02)FAK.A | N | 63.48 | 1311.6063 | 11 | -1.7 | 438.2076 | 3 | 37.36 | 12 | F12:15762 | BuCaTN01_F10b.raw | | | | | | | | | | | | 5.2815E4 | | 3.1918E5 | | | 2 | 0 | 0 | 0 | 0 | 0 | 0 | 0 | 0 | 0 | 0 | 0 | 1 | 0 | 1 | 0 | 0 | 110 | 120 | Carbamidomethylation | C1:Carbamidomethylation:1000.00;C8:Carbamidomethylation:1000.00 | PEAKS DB |
| R.TAALC(+57.02)FAK.A | N | 60.21 | 880.4476 | 8 | 3.4 | 441.2316 | 2 | 41.58 | 12 | F12:19288 | BuCaTN01_F10b.raw | | | | | | 9.3985E4 | 9.2429E6 | 1.2777E7 | 4.6024E5 | 3.2388E7 | 3.591E6 | 1.265E8 | 3.5731E6 | 1.1283E8 | 1.0943E8 | | 21 | 0 | 0 | 0 | 0 | 0 | 1 | 4 | 2 | 1 | 2 | 2 | 2 | 1 | 2 | 4 | 0 | 113 | 120 | Carbamidomethylation | C5:Carbamidomethylation:1000.00 | PEAKS DB |
| K.DFVC(+57.02)NC(+57.02)DRTAALC(+57.02)FAK.A | N | 59.68 | 1946.8438 | 16 | -0.4 | 974.4265 | 2 | 51.09 | 12 | F12:27773 | BuCaTN01_F10b.raw | | | | | | | | | | 1.1341E6 | | 0 | | | | | 1 | 0 | 0 | 0 | 0 | 0 | 0 | 0 | 0 | 0 | 1 | 0 | 0 | 0 | 0 | 0 | 0 | 105 | 120 | Carbamidomethylation | C4:Carbamidomethylation:1000.00;C6:Carbamidomethylation:1000.00;C13:Carbamidomethylation:1000.00 | PEAKS DB |
| G.GSGTPVDELDR.C | N | 56.36 | 1144.5360 | 11 | 3.4 | 573.2756 | 2 | 40.15 | 8 | F8:17921 | BuCaTN01_F7b.raw | | | | | | | | 1.1068E6 | | | | | | | | | 1 | 0 | 0 | 0 | 0 | 0 | 0 | 0 | 1 | 0 | 0 | 0 | 0 | 0 | 0 | 0 | 0 | 50 | 60 | | | PEAKS DB |
| R.TYSYDC(+57.02)SEGK.L | N | 55.61 | 1208.4656 | 10 | 0.1 | 605.2401 | 2 | 29.72 | 7 | F7:8746 | BuCaTN01_F7a.raw | | | | | | | 5.0914E6 | | | | | | | | | | 1 | 0 | 0 | 0 | 0 | 0 | 0 | 1 | 0 | 0 | 0 | 0 | 0 | 0 | 0 | 0 | 0 | 84 | 93 | Carbamidomethylation | C6:Carbamidomethylation:1000.00 | PEAKS DB |
| N.M(+15.99)IKC(+57.02)TNTRHWLSFTNYGC(+57.02)YC(+57.02)GYGGSGTPVDELDR.C | Y | 53.32 | 4030.7495 | 34 | 1.3 | 1008.6960 | 4 | 77.35 | 10 | F10:53957 | BuCaTN01_F9a.raw | | | | | | | | | | 0 | | | | | | | 0 | 0 | 0 | 0 | 0 | 0 | 0 | 0 | 0 | 0 | 0 | 0 | 0 | 0 | 0 | 0 | 0 | 27 | 60 | Oxidation (M); Carbamidomethylation | M1:Oxidation (M):1000.00;C4:Carbamidomethylation:1000.00;C18:Carbamidomethylation:1000.00;C20:Carbamidomethylation:1000.00 | PEAKS DB |
| R.TYSYDC(+57.02)SEGKLTC(+57.02).K | N | 52.98 | 1582.6279 | 13 | 2.9 | 792.3212 | 2 | 43.19 | 8 | F8:20253 | BuCaTN01_F7b.raw | | | | | | | | 5.7951E5 | | | | | | | | | 1 | 0 | 0 | 0 | 0 | 0 | 0 | 0 | 1 | 0 | 0 | 0 | 0 | 0 | 0 | 0 | 0 | 84 | 96 | Carbamidomethylation | C6:Carbamidomethylation:1000.00;C13:Carbamidomethylation:1000.00 | PEAKS DB |
| R.TYSYDC(+57.02)SEGKLT.C | N | 50.03 | 1422.5973 | 12 | 2.7 | 712.3058 | 2 | 41.55 | 8 | F8:18942 | BuCaTN01_F7b.raw | | | | | | | 5.0044E6 | 1.8313E6 | | | | | | | | | 2 | 0 | 0 | 0 | 0 | 0 | 0 | 1 | 1 | 0 | 0 | 0 | 0 | 0 | 0 | 0 | 0 | 84 | 95 | Carbamidomethylation | C6:Carbamidomethylation:1000.00 | PEAKS DB |
| Y.GGSGTPVDELDR.C | N | 49.09 | 1201.5575 | 12 | 0.3 | 601.7862 | 2 | 40.57 | 11 | F11:14528 | BuCaTN01_F10a.raw | | | | | | | 9.2274E5 | 2.7577E6 | | 1.1335E7 | 6.7591E5 | | 9.9086E5 | 2.6759E7 | | | 6 | 0 | 0 | 0 | 0 | 0 | 0 | 1 | 1 | 0 | 1 | 1 | 0 | 1 | 1 | 0 | 0 | 49 | 60 | | | PEAKS DB |
| R.TAALC(+57.02)FAKAP.Y | N | 47.25 | 1048.5375 | 10 | 3.6 | 525.2764 | 2 | 48.84 | 8 | F8:25881 | BuCaTN01_F7b.raw | | | | | | 3.1579E6 | | 4.0965E6 | | | | | | | | | 2 | 0 | 0 | 0 | 0 | 0 | 1 | 0 | 1 | 0 | 0 | 0 | 0 | 0 | 0 | 0 | 0 | 113 | 122 | Carbamidomethylation | C5:Carbamidomethylation:1000.00 | PEAKS DB |
| total 22 peptides | | | | | | | | | | | | | | | | | | | | | | | | | | | | | | | | | | | | | | | | | | | | | | | | |

**Best Unique PSM (Scan F10:53957, m/z=1008.6960, z=4, RT=77.35, ppm=1.3):**

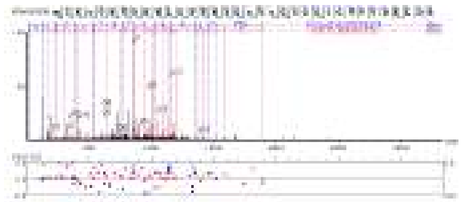

## JAI09008.1

back to list

| Protein Coverage | Supporting Peptides | Best Unique PSM |

**Protein Coverage:**

1 MNPAHLLVLA AVCVSLLGAS SIPPEPLNLY QFKNMIKCTN TRHWLSFTNY GCYCGYGGSG TPVDELDRCC QVHDKCYDTA

C Carbamidomethylation (+57.02)
O Oxidation (M) (+15.99)

81 KRVHKCFPSV RTYSYDCSEG KLTCKDNNTN CKDFVCNCDR TAALCFAKAP YNNKNFKIDP SRCQ

**Supporting Peptides:**

| Peptide | Uniq | -10lgP | Mass | Length | ppm | m/z | z | RT | Fraction | Scan | Source File |
|---|---|---|---|---|---|---|---|---|---|---|---|
| N.YGC(+57.02)YC(+57.02)GYGGSGTPVDELDR.C | N | 112.36 | 2124.8516 | 19 | 3.7 | 1063.4326 | 2 | 56.60 | 14 | F14:33384 | BuCaTN01_F11b.raw |
| Y.C(+57.02)GYGGSGTPVDELDR.C | N | 88.12 | 1581.6729 | 15 | 4.4 | 791.8439 | 2 | 48.02 | 14 | F14:25306 | BuCaTN01_F11b.raw |
| G.C(+57.02)YC(+57.02)GYGGSGTPVDELDR.C | N | 84.40 | 1904.7668 | 17 | 2.7 | 953.3911 | 2 | 53.16 | 12 | F12:29351 | BuCaTN01_F10b.raw |
| R.TYSYDC(+57.02)SEGKLTC(+57.02)K.D | N | 82.89 | 1710.7229 | 14 | 0.5 | 856.3691 | 2 | 38.29 | 7 | F7:14505 | BuCaTN01_F7a.raw |
| Y.GC(+57.02)YC(+57.02)GYGGSGTPVDELDR.C | N | 82.10 | 1961.7883 | 18 | 1.4 | 981.9028 | 2 | 53.49 | 10 | F10:30232 | BuCaTN01_F9a.raw |
| R.TYSYDC(+57.02)SEGKLTC(+57.02)KD.N | N | 75.87 | 1825.7499 | 15 | 2.5 | 913.8818 | 2 | 38.60 | 8 | F8:16643 | BuCaTN01_F7b.raw |
| C.YC(+57.02)GYGGSGTPVDELDR.C | N | 75.62 | 1744.7362 | 16 | 4.1 | 873.3754 | 2 | 52.27 | 14 | F14:28849 | BuCaTN01_F11b.raw |
| G.YGGSGTPVDELDR.C | N | 75.26 | 1364.6207 | 13 | 4.1 | 683.3177 | 2 | 45.29 | 14 | F14:22502 | BuCaTN01_F11b.raw |
| R.TAALC(+57.02)FAKAPY.N | N | 73.24 | 1211.6008 | 11 | 2.2 | 606.8076 | 2 | 55.84 | 12 | F12:32023 | BuCaTN01_F10b.raw |
| T.NYGC(+57.02)YC(+57.02)GYGGSGTPVDELDR.C | N | 72.69 | 2238.8945 | 20 | 5.3 | 1120.4559 | 2 | 64.81 | 14 | F14:40195 | BuCaTN01_F11b.raw |
| R.TAALC(+57.02)FAKAPYN.N | N | 69.35 | 1325.6438 | 12 | -0.2 | 663.8290 | 2 | 51.91 | 6 | F6:30474 | BuCaTN01_F6a.raw |
| C.GYGGSGTPVDELDR.C | N | 66.26 | 1421.6422 | 14 | 0.0 | 711.8284 | 2 | 46.82 | 10 | F10:24048 | BuCaTN01_F9a.raw |
| N.C(+57.02)DRTAALC(+57.02)FAK.A | N | 63.48 | 1311.6063 | 11 | -1.7 | 438.2076 | 3 | 37.36 | 12 | F12:15762 | BuCaTN01_F10b.raw |
| R.TAALC(+57.02)FAK.A | N | 60.21 | 880.4476 | 8 | 3.4 | 441.2316 | 2 | 41.58 | 12 | F12:19288 | BuCaTN01_F10b.raw |
| K.DFVC(+57.02)NC(+57.02)DRTAALC(+57.02)FAK.A | N | 59.68 | 1946.8438 | 16 | -0.4 | 974.4265 | 2 | 51.09 | 12 | F12:27773 | BuCaTN01_F10b.raw |
| G.GSGTPVDELDR.C | N | 56.36 | 1144.5360 | 11 | 3.4 | 573.2756 | 2 | 40.15 | 8 | F8:17921 | BuCaTN01_F7b.raw |
| R.TYSYDC(+57.02)SEGK.L | N | 55.61 | 1208.4656 | 10 | 0.1 | 605.2401 | 2 | 29.72 | 7 | F7:8746 | BuCaTN01_F7a.raw |
| N.M(+15.99)IKC(+57.02)TNTRHWLSFTNYGC(+57.02)YC(+57.02)GYGGSGTPVDELDR.C | Y | 53.32 | 4030.7495 | 34 | 1.3 | 1008.6960 | 4 | 77.35 | 10 | F10:53957 | BuCaTN01_F9a.raw |
| R.TYSYDC(+57.02)SEGKLTC(+57.02).K | N | 52.98 | 1582.6279 | 13 | 2.9 | 792.3212 | 2 | 43.19 | 8 | F8:20253 | BuCaTN01_F7b.raw |
| R.TYSYDC(+57.02)SEGKLT.C | N | 50.03 | 1422.5973 | 12 | 2.7 | 712.3058 | 2 | 41.55 | 8 | F8:18942 | BuCaTN01_F7b.raw |
| Y.GGSGTPVDELDR.C | N | 49.09 | 1201.5575 | 12 | 0.3 | 601.7862 | 2 | 40.57 | 11 | F11:14528 | BuCaTN01_F10a.raw |
| R.TAALC(+57.02)FAKAP.Y | N | 47.25 | 1048.5375 | 10 | 3.6 | 525.2764 | 2 | 48.84 | 8 | F8:25881 | BuCaTN01_F7b.raw |
| total 22 peptides | | | | | | | | | | | |

| Peptide | Area F1 | Area F2a | Area F3a | Area F4 | Area F5 | Area F6a | Area F7a | Area F7b | Area F8 | Area F9a | Area Sample 1F10a | Area F10b | Area F11a | Area F11b | Area F12a | Area F13 | #Feature |
|---|---|---|---|---|---|---|---|---|---|---|---|---|---|---|---|---|---|
| N.YGC(+57.02)YC(+57.02)GYGGSGTPVDELDR.C | | | 0 | | | 2.0097E6 | | 1.4053E7 | | 8.8531E7 | 3.4891E6 | 4.8054E8 | 6.165E7 | 1.7851E9 | 1.2628E8 | | 69 |
| Y.C(+57.02)GYGGSGTPVDELDR.C | | | | | | | | 2.166E6 | | 8.5786E6 | | 1.5853E7 | 6.495E5 | 1.7537E7 | 0 | | 6 |
| G.C(+57.02)YC(+57.02)GYGGSGTPVDELDR.C | | | | | | | | 4.1849E5 | | | | 3.7261E5 | | 2.1774E6 | | | 3 |
| R.TYSYDC(+57.02)SEGKLTC(+57.02)K.D | | | | | | | 1.4512E8 | 2.3155E7 | | 3.8991E5 | 4.5941E5 | | | | | | 8 |
| Y.GC(+57.02)YC(+57.02)GYGGSGTPVDELDR.C | | | | | | | | 1.7852E6 | | 3.3077E6 | | 1.5846E6 | | 3.2964E6 | 1.0428E5 | | 5 |
| R.TYSYDC(+57.02)SEGKLTC(+57.02)KD.N | | | | | | | | 7.3044E5 | | | | | | | | | 1 |
| C.YC(+57.02)GYGGSGTPVDELDR.C | | | | | | | | 7.7286E5 | | 7.3447E5 | | | | 3.0904E6 | 1.4057E5 | | 4 |
| G.YGGSGTPVDELDR.C | | | | | | | 6.8893E5 | | | | | | | 9.1406E6 | 6.9945E5 | | 3 |
| R.TAALC(+57.02)FAKAPY.N | | | | | | | 7.3334E4 | | | 1.7166E6 | 1.3753E5 | 1.06E7 | | 8.1407E6 | 9.828E5 | | 7 |
| T.NYGC(+57.02)YC(+57.02)GYGGSGTPVDELDR.C | | | | | | | | | | 2.3985E4 | | 0 | 1.1223E5 | 5.3539E5 | | | 3 |
| R.TAALC(+57.02)FAKAPYN.N | | | | | | 3.7675E6 | | 1.5632E7 | | 4.6358E6 | | 1.9346E6 | | | | | 5 |
| C.GYGGSGTPVDELDR.C | | | | | | | 2.9103E5 | 1.2275E6 | | 2.1867E6 | | | | 8.7883E6 | | | 5 |
| N.C(+57.02)DRTAALC(+57.02)FAK.A | | | | | | | | | | | | 5.2815E4 | | 3.1918E5 | | | 2 |
| R.TAALC(+57.02)FAK.A | | | | | | 9.3985E4 | 9.2429E6 | 1.2777E7 | 4.6024E5 | 3.2388E7 | 3.591E6 | 1.265E8 | 3.5731E6 | 1.1283E8 | 1.0943E8 | | 21 |
| K.DFVC(+57.02)NC(+57.02)DRTAALC(+57.02)FAK.A | | | | | | | | | | 1.1341E6 | | 0 | | | | | 1 |
| G.GSGTPVDELDR.C | | | | | | | | 1.1068E6 | | | | | | | | | 1 |
| R.TYSYDC(+57.02)SEGK.L | | | | | | | 5.0914E6 | | | | | | | | | | 1 |
| N.M(+15.99)IKC(+57.02)TNTRHWLSFTNYGC(+57.02)YC(+57.02)GYGGSGTPVDELDR.C | | | | | | | | | | 0 | | | | | | | 0 |
| R.TYSYDC(+57.02)SEGKLTC(+57.02).K | | | | | | | | 5.7951E5 | | | | | | | | | 1 |
| R.TYSYDC(+57.02)SEGKLT.C | | | | | | | 5.0044E6 | 1.8313E6 | | | | | | | | | 2 |
| Y.GGSGTPVDELDR.C | | | | | | | 9.2274E5 | 2.7577E6 | | 1.1335E7 | 6.7591E5 | | 9.9086E5 | 2.6759E7 | | | 6 |
| R.TAALC(+57.02)FAKAP.Y | | | | | | 3.1579E6 | | 4.0965E6 | | | | | | | | | 2 |

| Peptide | #Feature F1 | #Feature F2a | #Feature F3a | #Feature F4 | #Feature F5 | #Feature F6a | #Feature F7a | #Feature F7b | #Feature F8 | #Feature F9a | #Feature Sample 1F10a | #Feature F10b | #Feature F11a | #Feature F11b | #Feature F12a | #Feature F13 | Start | End | PTM | AScore | Found By |
|---|---|---|---|---|---|---|---|---|---|---|---|---|---|---|---|---|---|---|---|---|---|
| N.YGC(+57.02)YC(+57.02)GYGGSGTPVDELDR.C | 0 | 0 | 0 | 0 | 0 | 1 | 0 | 2 | 0 | 3 | 1 | 4 | 2 | 53 | 3 | 0 | 50 | 68 | Carbamidomethylation | C3:Carbamidomethylation:1000.00;C5:Carbamidomethylation:1000.00 | PEAKS DB |
| Y.C(+57.02)GYGGSGTPVDELDR.C | 0 | 0 | 0 | 0 | 0 | 0 | 0 | 1 | 0 | 1 | 0 | 2 | 1 | 1 | 0 | 0 | 54 | 68 | Carbamidomethylation | C1:Carbamidomethylation:1000.00 | PEAKS DB |
| G.C(+57.02)YC(+57.02)GYGGSGTPVDELDR.C | 0 | 0 | 0 | 0 | 0 | 0 | 0 | 1 | 0 | 0 | 0 | 1 | 0 | 1 | 0 | 0 | 52 | 68 | Carbamidomethylation | C1:Carbamidomethylation:1000.00;C3:Carbamidomethylation:1000.00 | PEAKS DB |
| R.TYSYDC(+57.02)SEGKLTC(+57.02)K.D | 0 | 0 | 0 | 0 | 0 | 0 | 4 | 2 | 0 | 1 | 1 | 0 | 0 | 0 | 0 | 0 | 92 | 105 | Carbamidomethylation | C6:Carbamidomethylation:1000.00;C13:Carbamidomethylation:1000.00 | PEAKS DB |
| Y.GC(+57.02)YC(+57.02)GYGGSGTPVDELDR.C | 0 | 0 | 0 | 0 | 0 | 0 | 0 | 1 | 0 | 1 | 0 | 1 | 0 | 1 | 1 | 0 | 51 | 68 | Carbamidomethylation | C2:Carbamidomethylation:1000.00;C4:Carbamidomethylation:1000.00 | PEAKS DB |
| R.TYSYDC(+57.02)SEGKLTC(+57.02)KD.N | 0 | 0 | 0 | 0 | 0 | 0 | 0 | 1 | 0 | 0 | 0 | 0 | 0 | 0 | 0 | 0 | 92 | 106 | Carbamidomethylation | C6:Carbamidomethylation:1000.00;C13:Carbamidomethylation:1000.00 | PEAKS DB |
| C.YC(+57.02)GYGGSGTPVDELDR.C | 0 | 0 | 0 | 0 | 0 | 0 | 0 | 1 | 0 | 1 | 0 | 0 | 0 | 1 | 1 | 0 | 53 | 68 | Carbamidomethylation | C2:Carbamidomethylation:1000.00 | PEAKS DB |
| G.YGGSGTPVDELDR.C | 0 | 0 | 0 | 0 | 0 | 0 | 1 | 0 | 0 | 0 | 0 | 0 | 0 | 1 | 1 | 0 | 56 | 68 | | | PEAKS DB |
| R.TAALC(+57.02)FAKAPY.N | 0 | 0 | 0 | 0 | 0 | 0 | 1 | 0 | 0 | 1 | 1 | 2 | 0 | 1 | 1 | 0 | 121 | 131 | Carbamidomethylation | C5:Carbamidomethylation:1000.00 | PEAKS DB |
| T.NYGC(+57.02)YC(+57.02)GYGGSGTPVDELDR.C | 0 | 0 | 0 | 0 | 0 | 0 | 0 | 0 | 0 | 1 | 0 | 0 | 1 | 1 | 0 | 0 | 49 | 68 | Carbamidomethylation | C4:Carbamidomethylation:1000.00;C6:Carbamidomethylation:1000.00 | PEAKS DB |
| R.TAALC(+57.02)FAKAPYN.N | 0 | 0 | 0 | 0 | 0 | 1 | 0 | 2 | 0 | 1 | 0 | 1 | 0 | 0 | 0 | 0 | 121 | 132 | Carbamidomethylation | C5:Carbamidomethylation:1000.00 | PEAKS DB |
| C.GYGGSGTPVDELDR.C | 0 | 0 | 0 | 0 | 0 | 0 | 1 | 1 | 0 | 1 | 0 | 0 | 0 | 2 | 0 | 0 | 55 | 68 | | | PEAKS DB |
| N.C(+57.02)DRTAALC(+57.02)FAK.A | 0 | 0 | 0 | 0 | 0 | 0 | 0 | 0 | 0 | 0 | 0 | 1 | 0 | 1 | 0 | 0 | 118 | 128 | Carbamidomethylation | C1:Carbamidomethylation:1000.00;C8:Carbamidomethylation:1000.00 | PEAKS DB |
| R.TAALC(+57.02)FAK.A | 0 | 0 | 0 | 0 | 0 | 1 | 4 | 2 | 1 | 2 | 2 | 2 | 1 | 2 | 4 | 0 | 121 | 128 | Carbamidomethylation | C5:Carbamidomethylation:1000.00 | PEAKS DB |
| K.DFVC(+57.02)NC(+57.02)DRTAALC(+57.02)FAK.A | 0 | 0 | 0 | 0 | 0 | 0 | 0 | 0 | 0 | 1 | 0 | 0 | 0 | 0 | 0 | 0 | 113 | 128 | Carbamidomethylation | C4:Carbamidomethylation:1000.00;C6:Carbamidomethylation:1000.00;C13:Carbamidomethylation:1000.00 | PEAKS DB |
| G.GSGTPVDELDR.C | 0 | 0 | 0 | 0 | 0 | 0 | 0 | 1 | 0 | 0 | 0 | 0 | 0 | 0 | 0 | 0 | 58 | 68 | | | PEAKS DB |
| R.TYSYDC(+57.02)SEGK.L | 0 | 0 | 0 | 0 | 0 | 0 | 1 | 0 | 0 | 0 | 0 | 0 | 0 | 0 | 0 | 0 | 92 | 101 | Carbamidomethylation | C6:Carbamidomethylation:1000.00 | PEAKS DB |
| N.M(+15.99)IKC(+57.02)TNTRHWLSFTNYGC(+57.02)YC(+57.02)GYGGSGTPVDELDR.C | 0 | 0 | 0 | 0 | 0 | 0 | 0 | 0 | 0 | 0 | 0 | 0 | 0 | 0 | 0 | 0 | 35 | 68 | Oxidation (M); Carbamidomethylation | M1:Oxidation (M):1000.00;C4:Carbamidomethylation:1000.00;C18:Carbamidomethylation:1000.00;C20:Carbamidomethylation:1000.00 | PEAKS DB |
| R.TYSYDC(+57.02)SEGKLTC(+57.02).K | 0 | 0 | 0 | 0 | 0 | 0 | 0 | 1 | 0 | 0 | 0 | 0 | 0 | 0 | 0 | 0 | 92 | 104 | Carbamidomethylation | C6:Carbamidomethylation:1000.00;C13:Carbamidomethylation:1000.00 | PEAKS DB |
| R.TYSYDC(+57.02)SEGKLT.C | 0 | 0 | 0 | 0 | 0 | 0 | 1 | 1 | 0 | 0 | 0 | 0 | 0 | 0 | 0 | 0 | 92 | 103 | Carbamidomethylation | C6:Carbamidomethylation:1000.00 | PEAKS DB |
| Y.GGSGTPVDELDR.C | 0 | 0 | 0 | 0 | 0 | 0 | 1 | 1 | 0 | 1 | 1 | 0 | 1 | 1 | 0 | 0 | 57 | 68 | | | PEAKS DB |
| R.TAALC(+57.02)FAKAP.Y | 0 | 0 | 0 | 0 | 0 | 1 | 0 | 1 | 0 | 0 | 0 | 0 | 0 | 0 | 0 | 0 | 121 | 130 | Carbamidomethylation | C5:Carbamidomethylation:1000.00 | PEAKS DB |

**Best Unique PSM (Scan F10:53957, m/z=1008.6960, z=4, RT=77.35, ppm=1.3):**

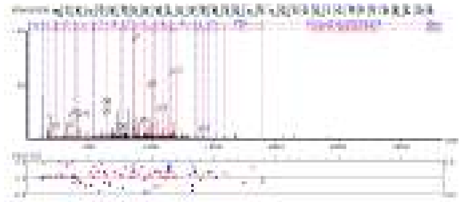

## JAS04997.1

back to list

| Protein Coverage | Supporting Peptides | Best Unique PSM |

**Protein Coverage:**

1 MNPAHLLVLA AVCVSLLGAS SIPPEPLNLY QFKNMIKCTN TRHWLSFTNY GCYCGYGGSG TPVDELDRCC QVHDKCYDTA

C Carbamidomethylation (+57.02)
O Oxidation (M) (+15.99)

81 KRVHKCFPSV RTYSYDCSEG KLTCKDNNTN CKDFVCNCDR TAALCFAKAP YNNKNFKIDP SRCQ

**Supporting Peptides:**

| Peptide | Uniq | -10lgP | Mass | Length | ppm | m/z | z | RT | Fraction | Scan | Source File |
|---|---|---|---|---|---|---|---|---|---|---|---|
| N.YGC(+57.02)YC(+57.02)GYGGSGTPVDELDR.C | N | 112.36 | 2124.8516 | 19 | 3.7 | 1063.4326 | 2 | 56.60 | 14 | F14:33384 | BuCaTN01_F11b.raw |

| Peptide | Area F1 | Area F2a | Area F3a | Area F4 | Area F5 | Area F6a | Area F7a | Area F7b | Area F8 | Area F9a | Area Sample 1F10a | Area F10b | Area F11a | Area F11b | Area F12a | Area F13 | #Feature |
|---|---|---|---|---|---|---|---|---|---|---|---|---|---|---|---|---|---|
| N.YGC(+57.02)YC(+57.02)GYGGSGTPVDELDR.C | | | 0 | | | 2.0097E6 | | 1.4053E7 | | 8.8531E7 | 3.4891E6 | 4.8054E8 | 6.165E7 | 1.7851E9 | 1.2628E8 | | 69 |

| Peptide | #Feature F1 | #Feature F2a | #Feature F3a | #Feature F4 | #Feature F5 | #Feature F6a | #Feature F7a | #Feature F7b | #Feature F8 | #Feature F9a | #Feature Sample 1F10a | #Feature F10b | #Feature F11a | #Feature F11b | #Feature F12a | #Feature F13 | Start | End | PTM | AScore | Found By |
|---|---|---|---|---|---|---|---|---|---|---|---|---|---|---|---|---|---|---|---|---|---|
| N.YGC(+57.02)YC(+57.02)GYGGSGTPVDELDR.C | 0 | 0 | 0 | 0 | 0 | 1 | 0 | 2 | 0 | 3 | 1 | 4 | 2 | 53 | 3 | 0 | 50 | 68 | Carbamidomethylation | C3:Carbamidomethylation:1000.00;C5:Carbamidomethylation:1000.00 | PEAKS DB |
| | | | | | | | | | | | | | | | | | | | | C1:Carbamidomethylatio | PEAKS |

| | | | | | | | | | | | | | | | | | | | | | | | | | | | | | | | | | | | | | | | | | | | | | | | | | |
|---|---|---|---|---|---|---|---|---|---|---|---|---|---|---|---|---|---|---|---|---|---|---|---|---|---|---|---|---|---|---|---|---|---|---|---|---|---|---|---|---|---|---|---|---|---|---|---|---|---|
| Y.C(+57.02)GYGGSGTPVDELDR.C | N | 88.12 | 1581.6729 | 15 | 4.4 | 791.8439 | 2 | 48.02 | 14 | F14:25306 | BuCaTN01_F11b.raw | | | | | | | | 2.166E6 | | 8.5786E6 | | 1.5853E7 | 6.495E5 | 1.7537E7 | 0 | | 6 | 0 | 0 | 0 | 0 | 0 | 0 | 0 | 1 | 0 | 1 | 0 | 2 | 1 | 1 | 0 | 0 | 54 | 68 | Carbamidomethylation | n:1000.00 | DB |
| G.C(+57.02)YC(+57.02)GYGGSGTPVDELDR.C | N | 84.40 | 1904.7668 | 17 | 2.7 | 953.3911 | 2 | 53.16 | 12 | F12:29351 | BuCaTN01_F10b.raw | | | | | | | | 4.1849E5 | | | | 3.7261E5 | | 2.1774E6 | | | 3 | 0 | 0 | 0 | 0 | 0 | 0 | 0 | 1 | 0 | 0 | 0 | 1 | 0 | 1 | 0 | 0 | 52 | 68 | Carbamidomethylation | C1:Carbamidomethylation:1000.00;C3:Carbamidomethylation:1000.00 | PEAKS DB |
| R.TYSYDC(+57.02)SEGKLTC(+57.02)K.D | N | 82.89 | 1710.7229 | 14 | 0.5 | 856.3691 | 2 | 38.29 | 7 | F7:14505 | BuCaTN01_F7a.raw | | | | | | | 1.4512E8 | 2.3155E7 | | 3.8991E5 | 4.5941E5 | | | | | | 8 | 0 | 0 | 0 | 0 | 0 | 0 | 4 | 2 | 0 | 1 | 1 | 0 | 0 | 0 | 0 | 0 | 92 | 105 | Carbamidomethylation | C6:Carbamidomethylation:1000.00;C13:Carbamidomethylation:1000.00 | PEAKS DB |
| Y.GC(+57.02)YC(+57.02)GYGGSGTPVDELDR.C | N | 82.10 | 1961.7883 | 18 | 1.4 | 981.9028 | 2 | 53.49 | 10 | F10:30232 | BuCaTN01_F9a.raw | | | | | | | | 1.7852E6 | | 3.3077E6 | | 1.5846E6 | | 3.2964E6 | 1.0428E5 | | 5 | 0 | 0 | 0 | 0 | 0 | 0 | 0 | 1 | 0 | 1 | 0 | 1 | 0 | 1 | 1 | 0 | 51 | 68 | Carbamidomethylation | C2:Carbamidomethylation:1000.00;C4:Carbamidomethylation:1000.00 | PEAKS DB |
| R.TYSYDC(+57.02)SEGKLTC(+57.02)KD.N | N | 75.87 | 1825.7499 | 15 | 2.5 | 913.8818 | 2 | 38.60 | 8 | F8:16643 | BuCaTN01_F7b.raw | | | | | | | | 7.3044E5 | | | | | | | | | 1 | 0 | 0 | 0 | 0 | 0 | 0 | 0 | 1 | 0 | 0 | 0 | 0 | 0 | 0 | 0 | 0 | 92 | 106 | Carbamidomethylation | C6:Carbamidomethylation:1000.00;C13:Carbamidomethylation:1000.00 | PEAKS DB |
| C.YC(+57.02)GYGGSGTPVDELDR.C | N | 75.62 | 1744.7362 | 16 | 4.1 | 873.3754 | 2 | 52.27 | 14 | F14:28849 | BuCaTN01_F11b.raw | | | | | | | | 7.7286E5 | | 7.3447E5 | | | | 3.0904E6 | 1.4057E5 | | 4 | 0 | 0 | 0 | 0 | 0 | 0 | 0 | 1 | 0 | 1 | 0 | 0 | 0 | 1 | 1 | 0 | 53 | 68 | Carbamidomethylation | C2:Carbamidomethylation:1000.00 | PEAKS DB |
| G.YGGSGTPVDELDR.C | N | 75.26 | 1364.6207 | 13 | 4.1 | 683.3177 | 2 | 45.29 | 14 | F14:22502 | BuCaTN01_F11b.raw | | | | | | | 6.8893E5 | | | | | | | 9.1406E6 | 6.9945E5 | | 3 | 0 | 0 | 0 | 0 | 0 | 0 | 1 | 0 | 0 | 0 | 0 | 0 | 0 | 1 | 1 | 0 | 56 | 68 | | | PEAKS DB |
| R.TAAIC(+57.02)FAKAPY.N | N | 73.24 | 1211.6008 | 11 | 2.2 | 606.8076 | 2 | 55.84 | 12 | F12:32023 | BuCaTN01_F10b.raw | | | | | | | 7.3334E4 | | | 1.7166E6 | 1.3753E5 | 1.06E7 | | 8.1407E6 | 9.828E5 | | 7 | 0 | 0 | 0 | 0 | 0 | 0 | 1 | 0 | 0 | 1 | 1 | 2 | 0 | 1 | 1 | 0 | 121 | 131 | Carbamidomethylation | C5:Carbamidomethylation:1000.00 | PEAKS DB |
| T.NYGC(+57.02)YC(+57.02)GYGGSGTPVDELDR.C | N | 72.69 | 2238.8945 | 20 | 5.3 | 1120.4559 | 2 | 64.81 | 14 | F14:40195 | BuCaTN01_F11b.raw | | | | | | | | | | 2.3985E4 | | 0 | 1.1223E5 | 5.3539E5 | | | 3 | 0 | 0 | 0 | 0 | 0 | 0 | 0 | 0 | 0 | 1 | 0 | 0 | 1 | 1 | 0 | 0 | 49 | 68 | Carbamidomethylation | C4:Carbamidomethylation:1000.00;C6:Carbamidomethylation:1000.00 | PEAKS DB |
| R.TAAIC(+57.02)FAKAPYN.N | N | 69.35 | 1325.6438 | 12 | -0.2 | 663.8290 | 2 | 51.91 | 6 | F6:30474 | BuCaTN01_F6a.raw | | | | | | 3.7675E6 | | 1.5632E7 | | 4.6358E6 | | 1.9346E6 | | | | | 5 | 0 | 0 | 0 | 0 | 0 | 1 | 0 | 2 | 0 | 1 | 0 | 1 | 0 | 0 | 0 | 0 | 121 | 132 | Carbamidomethylation | C5:Carbamidomethylation:1000.00 | PEAKS DB |
| C.GYGGSGTPVDELDR.C | N | 66.26 | 1421.6422 | 14 | 0.0 | 711.8284 | 2 | 46.82 | 10 | F10:24048 | BuCaTN01_F9a.raw | | | | | | | 2.9103E5 | 1.2275E6 | | 2.1867E6 | | | | 8.7883E6 | | | 5 | 0 | 0 | 0 | 0 | 0 | 0 | 1 | 1 | 0 | 1 | 0 | 0 | 0 | 2 | 0 | 0 | 55 | 68 | | | PEAKS DB |
| N.C(+57.02)DRTAAIC(+57.02)FAK.A | N | 63.48 | 1311.6063 | 11 | -1.7 | 438.2076 | 3 | 37.36 | 12 | F12:15762 | BuCaTN01_F10b.raw | | | | | | | | | | | | 5.2815E4 | | 3.1918E5 | | | 2 | 0 | 0 | 0 | 0 | 0 | 0 | 0 | 0 | 0 | 0 | 0 | 1 | 0 | 1 | 0 | 0 | 118 | 128 | Carbamidomethylation | C1:Carbamidomethylation:1000.00;C8:Carbamidomethylation:1000.00 | PEAKS DB |
| R.TAAIC(+57.02)FAK.A | N | 60.21 | 880.4476 | 8 | 3.4 | 441.2316 | 2 | 41.58 | 12 | F12:19288 | BuCaTN01_F10b.raw | | | | | | 9.3985E4 | 9.2429E6 | 1.2777E7 | 4.6024E5 | 3.2388E7 | 3.591E6 | 1.265E8 | 3.5731E6 | 1.1283E8 | 1.0943E8 | | 21 | 0 | 0 | 0 | 0 | 0 | 1 | 4 | 2 | 1 | 2 | 2 | 2 | 1 | 2 | 4 | 0 | 121 | 128 | Carbamidomethylation | C5:Carbamidomethylation:1000.00 | PEAKS DB |
| K.DFVC(+57.02)NC(+57.02)DRTAAIC(+57.02)FAK.A | N | 59.68 | 1946.8438 | 16 | -0.4 | 974.4265 | 2 | 51.09 | 12 | F12:27773 | BuCaTN01_F10b.raw | | | | | | | | | | 1.1341E6 | | 0 | | | | | 1 | 0 | 0 | 0 | 0 | 0 | 0 | 0 | 0 | 0 | 1 | 0 | 0 | 0 | 0 | 0 | 0 | 113 | 128 | Carbamidomethylation | C4:Carbamidomethylation:1000.00;C6:Carbamidomethylation:1000.00;C13:Carbamidomethylation:1000.00 | PEAKS DB |
| G.GSGTPVDELDR.C | N | 56.36 | 1144.5360 | 11 | 3.4 | 573.2756 | 2 | 40.15 | 8 | F8:17921 | BuCaTN01_F7b.raw | | | | | | | | 1.1068E6 | | | | | | | | | 1 | 0 | 0 | 0 | 0 | 0 | 0 | 0 | 1 | 0 | 0 | 0 | 0 | 0 | 0 | 0 | 0 | 58 | 68 | | | PEAKS DB |
| R.TYSYDC(+57.02)SEGK.L | N | 55.61 | 1208.4656 | 10 | 0.1 | 605.2401 | 2 | 29.72 | 7 | F7:8746 | BuCaTN01_F7a.raw | | | | | | | 5.0914E6 | | | | | | | | | | 1 | 0 | 0 | 0 | 0 | 0 | 0 | 1 | 0 | 0 | 0 | 0 | 0 | 0 | 0 | 0 | 0 | 92 | 101 | Carbamidomethylation | C6:Carbamidomethylation:1000.00 | PEAKS DB |
| N.M(+15.99)IKC(+57.02)TNTRHWLSFTNYGC(+57.02)YC(+57.02)GYGGSGTPVDELDR.C | Y | 53.32 | 4030.7495 | 34 | 1.3 | 1008.6960 | 4 | 77.35 | 10 | F10:53957 | BuCaTN01_F9a.raw | | | | | | | | | | 0 | | | | | | | 0 | 0 | 0 | 0 | 0 | 0 | 0 | 0 | 0 | 0 | 0 | 0 | 0 | 0 | 0 | 0 | 0 | 35 | 68 | Oxidation (M); Carbamidomethylation | M1:Oxidation (M):1000.00;C4:Carbamidomethylation:1000.00;C18:Carbamidomethylation:1000.00;C20:Carbamidomethylation:1000.00 | PEAKS DB |
| R.TYSYDC(+57.02)SEGKLTC(+57.02).K | N | 52.98 | 1582.6279 | 13 | 2.9 | 792.3212 | 2 | 43.19 | 8 | F8:20253 | BuCaTN01_F7b.raw | | | | | | | | 5.7951E5 | | | | | | | | | 1 | 0 | 0 | 0 | 0 | 0 | 0 | 0 | 1 | 0 | 0 | 0 | 0 | 0 | 0 | 0 | 0 | 92 | 104 | Carbamidomethylation | C6:Carbamidomethylation:1000.00;C13:Carbamidomethylation:1000.00 | PEAKS DB |
| R.TYSYDC(+57.02)SEGKLT.C | N | 50.03 | 1422.5973 | 12 | 2.7 | 712.3058 | 2 | 41.55 | 8 | F8:18942 | BuCaTN01_F7b.raw | | | | | | | 5.0044E6 | 1.8313E6 | | | | | | | | | 2 | 0 | 0 | 0 | 0 | 0 | 0 | 1 | 1 | 0 | 0 | 0 | 0 | 0 | 0 | 0 | 0 | 92 | 103 | Carbamidomethylation | C6:Carbamidomethylation:1000.00 | PEAKS DB |
| Y.GGSGTPVDELDR.C | N | 49.09 | 1201.5575 | 12 | 0.3 | 601.7862 | 2 | 40.57 | 11 | F11:14528 | BuCaTN01_F10a.raw | | | | | | | 9.2274E5 | 2.7577E6 | | 1.1335E7 | 6.7591E5 | | 9.9086E5 | 2.6759E7 | | | 6 | 0 | 0 | 0 | 0 | 0 | 0 | 1 | 1 | 0 | 1 | 1 | 0 | 1 | 1 | 0 | 0 | 57 | 68 | | | PEAKS DB |
| R.TAAIC(+57.02)FAKAP.Y | N | 47.25 | 1048.5375 | 10 | 3.6 | 525.2764 | 2 | 48.84 | 8 | F8:25881 | BuCaTN01_F7b.raw | | | | | | 3.1579E6 | | 4.0965E6 | | | | | | | | | 2 | 0 | 0 | 0 | 0 | 0 | 1 | 0 | 1 | 0 | 0 | 0 | 0 | 0 | 0 | 0 | 0 | 121 | 130 | Carbamidomethylation | C5:Carbamidomethylation:1000.00 | PEAKS DB |
| **total 22 peptides** | | | | | | | | | | | | | | | | | | | | | | | | | | | | | | | | | | | | | | | | | | | | | | | | | |

**Best Unique PSM (Scan F10:53957, m/z=1008.6960, z=4, RT=77.35, ppm=1.3):**

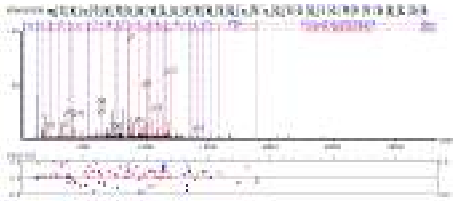

## JAB52790.1

back to list

| Protein Coverage | Supporting Peptides | Best Unique PSM |

**Protein Coverage:**

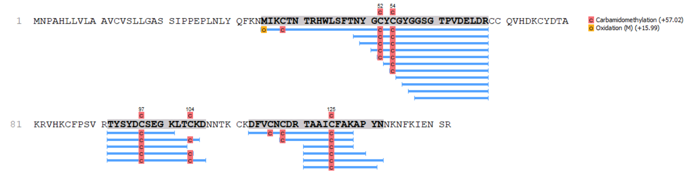


**Supporting Peptides:**

| Peptide | Uniq | -10lgP | Mass | Length | ppm | m/z | z | RT | Fraction | Scan | Source File | Area F1 | Area F2a | Area F3a | Area F4 | Area F5 | Area F6a | Area F7a | Area F7b | Area F8 | Area F9a | Area Sample 1F10a | Area F10b | Area F11a | Area F11b | Area F12a | Area F13 | #Feature | #Feature F1 | #Feature F2a | #Feature F3a | #Feature F4 | #Feature F5 | #Feature F6a | #Feature F7a | #Feature F7b | #Feature F8 | #Feature F9a | #Feature Sample 1F10a | #Feature F10b | #Feature F11a | #Feature F11b | #Feature F12a | #Feature F13 | Start | End | PTM | AScore | Found By |
|---|---|---|---|---|---|---|---|---|---|---|---|---|---|---|---|---|---|---|---|---|---|---|---|---|---|---|---|---|---|---|---|---|---|---|---|---|---|---|---|---|---|---|---|---|---|---|---|---|---|
| N.YGC(+57.02)YC(+57.02)GYGGSGTPVDELDR.C | N | 112.36 | 2124.8516 | 19 | 3.7 | 1063.4326 | 2 | 56.60 | 14 | F14:33384 | BuCaTN01_F11b.raw | | | 0 | | | 2.0097E6 | | 1.4053E7 | | 8.8531E7 | 3.4891E6 | 4.8054E8 | 6.165E7 | 1.7851E9 | 1.2628E8 | | 69 | 0 | 0 | 0 | 0 | 0 | 1 | 0 | 2 | 0 | 3 | 1 | 4 | 2 | 53 | 3 | 0 | 50 | 68 | Carbamidomethylation | C3:Carbamidomethylation:1000.00;C5:Carbamidomethylation:1000.00 | PEAKS DB |
| Y.C(+57.02)GYGGSGTPVDELDR.C | N | 88.12 | 1581.6729 | 15 | 4.4 | 791.8439 | 2 | 48.02 | 14 | F14:25306 | BuCaTN01_F11b.raw | | | | | | | | 2.166E6 | | 8.5786E6 | | 1.5853E7 | 6.495E5 | 1.7537E7 | 0 | | 6 | 0 | 0 | 0 | 0 | 0 | 0 | 0 | 1 | 0 | 1 | 0 | 2 | 1 | 1 | 0 | 0 | 54 | 68 | Carbamidomethylation | C1:Carbamidomethylation:1000.00 | PEAKS DB |
| G.C(+57.02)YC(+57.02)GYGGSGTPVDELDR.C | N | 84.40 | 1904.7668 | 17 | 2.7 | 953.3911 | 2 | 53.16 | 12 | F12:29351 | BuCaTN01_F10b.raw | | | | | | | | 4.1849E5 | | | | 3.7261E5 | | 2.1774E6 | | | 3 | 0 | 0 | 0 | 0 | 0 | 0 | 0 | 1 | 0 | 0 | 0 | 1 | 0 | 1 | 0 | 0 | 52 | 68 | Carbamidomethylation | C1:Carbamidomethylation:1000.00;C3:Carbamidomethylation:1000.00 | PEAKS DB |
| R.TYSYDC(+57.02)SEGKLTC(+57.02)K.D | N | 82.89 | 1710.7229 | 14 | 0.5 | 856.3691 | 2 | 38.29 | 7 | F7:14505 | BuCaTN01_F7a.raw | | | | | | | 1.4512E8 | 2.3155E7 | | 3.8991E5 | 4.5941E5 | | | | | | 8 | 0 | 0 | 0 | 0 | 0 | 0 | 4 | 2 | 0 | 1 | 1 | 0 | 0 | 0 | 0 | 0 | 92 | 105 | Carbamidomethylation | C6:Carbamidomethylation:1000.00;C13:Carbamidomethylation:1000.00 | PEAKS DB |
| Y.GC(+57.02)YC(+57.02)GYGGSGTPVDELDR.C | N | 82.10 | 1961.7883 | 18 | 1.4 | 981.9028 | 2 | 53.49 | 10 | F10:30232 | BuCaTN01_F9a.raw | | | | | | | | 1.7852E6 | | 3.3077E6 | | 1.5846E6 | | 3.2964E6 | 1.0428E5 | | 5 | 0 | 0 | 0 | 0 | 0 | 0 | 0 | 1 | 0 | 1 | 0 | 1 | 0 | 1 | 1 | 0 | 51 | 68 | Carbamidomethylation | C2:Carbamidomethylation:1000.00;C4:Carbamidomethylation:1000.00 | PEAKS DB |
| R.TYSYDC(+57.02)SEGKLTC(+57.02)KD.N | N | 75.87 | 1825.7499 | 15 | 2.5 | 913.8818 | 2 | 38.60 | 8 | F8:16643 | BuCaTN01_F7b.raw | | | | | | | | 7.3044E5 | | | | | | | | | 1 | 0 | 0 | 0 | 0 | 0 | 0 | 0 | 1 | 0 | 0 | 0 | 0 | 0 | 0 | 0 | 0 | 92 | 106 | Carbamidomethylation | C6:Carbamidomethylation:1000.00;C13:Carbamidomethylation:1000.00 | PEAKS DB |
| C.YC(+57.02)GYGGSGTPVDELDR.C | N | 75.62 | 1744.7362 | 16 | 4.1 | 873.3754 | 2 | 52.27 | 14 | F14:28849 | BuCaTN01_F11b.raw | | | | | | | | 7.7286E5 | | 7.3447E5 | | | | 3.0904E6 | 1.4057E5 | | 4 | 0 | 0 | 0 | 0 | 0 | 0 | 0 | 1 | 0 | 1 | 0 | 0 | 0 | 1 | 1 | 0 | 53 | 68 | Carbamidomethylation | C2:Carbamidomethylation:1000.00 | PEAKS DB |
| G.YGGSGTPVDELDR.C | N | 75.26 | 1364.6207 | 13 | 4.1 | 683.3177 | 2 | 45.29 | 14 | F14:22502 | BuCaTN01_F11b.raw | | | | | | | 6.8893E5 | | | | | | | 9.1406E6 | 6.9945E5 | | 3 | 0 | 0 | 0 | 0 | 0 | 0 | 1 | 0 | 0 | 0 | 0 | 0 | 0 | 1 | 1 | 0 | 56 | 68 | | | PEAKS DB |
| R.TAAIC(+57.02)FAKAPY.N | N | 73.24 | 1211.6008 | 11 | 2.2 | 606.8076 | 2 | 55.84 | 12 | F12:32023 | BuCaTN01_F10b.raw | | | | | | | 7.3334E4 | | | 1.7166E6 | 1.3753E5 | 1.06E7 | | 8.1407E6 | 9.828E5 | | 7 | 0 | 0 | 0 | 0 | 0 | 0 | 1 | 0 | 0 | 1 | 1 | 2 | 0 | 1 | 1 | 0 | 121 | 131 | Carbamidomethylation | C5:Carbamidomethylation:1000.00 | PEAKS DB |
| T.NYGC(+57.02)YC(+57.02)GYGGSGTPVDELDR.C | N | 72.69 | 2238.8945 | 20 | 5.3 | 1120.4559 | 2 | 64.81 | 14 | F14:40195 | BuCaTN01_F11b.raw | | | | | | | | | | 2.3985E4 | | 0 | 1.1223E5 | 5.3539E5 | | | 3 | 0 | 0 | 0 | 0 | 0 | 0 | 0 | 0 | 0 | 1 | 0 | 0 | 1 | 1 | 0 | 0 | 49 | 68 | Carbamidomethylation | C4:Carbamidomethylation:1000.00;C6:Carbamidomethylation:1000.00 | PEAKS DB |
| R.TAAIC(+57.02)FAKAPYN.N | N | 69.35 | 1325.6438 | 12 | -0.2 | 663.8290 | 2 | 51.91 | 6 | F6:30474 | BuCaTN01_F6a.raw | | | | | | 3.7675E6 | | 1.5632E7 | | 4.6358E6 | | 1.9346E6 | | | | | 5 | 0 | 0 | 0 | 0 | 0 | 1 | 0 | 2 | 0 | 1 | 0 | 1 | 0 | 0 | 0 | 0 | 121 | 132 | Carbamidomethylation | C5:Carbamidomethylation:1000.00 | PEAKS DB |
| C.GYGGSGTPVDELDR.C | N | 66.26 | 1421.6422 | 14 | 0.0 | 711.8284 | 2 | 46.82 | 10 | F10:24048 | BuCaTN01_F9a.raw | | | | | | | 2.9103E5 | 1.2275E6 | | 2.1867E6 | | | | 8.7883E6 | | | 5 | 0 | 0 | 0 | 0 | 0 | 0 | 1 | 1 | 0 | 1 | 0 | 0 | 0 | 2 | 0 | 0 | 55 | 68 | | | PEAKS DB |
| N.C(+57.02)DRTAAIC(+57.02)FAK.A | N | 63.48 | 1311.6063 | 11 | -1.7 | 438.2076 | 3 | 37.36 | 12 | F12:15762 | BuCaTN01_F10b.raw | | | | | | | | | | | | 5.2815E4 | | 3.1918E5 | | | 2 | 0 | 0 | 0 | 0 | 0 | 0 | 0 | 0 | 0 | 0 | 0 | 1 | 0 | 1 | 0 | 0 | 118 | 128 | Carbamidomethylation | C1:Carbamidomethylation:1000.00;C8:Carbamidomethylation:1000.00 | PEAKS DB |
| R.TAAIC(+57.02)FAK.A | N | 60.21 | 880.4476 | 8 | 3.4 | 441.2316 | 2 | 41.58 | 12 | F12:19288 | BuCaTN01_F10b.raw | | | | | | 9.3985E4 | 9.2429E6 | 1.2777E7 | 4.6024E5 | 3.2388E7 | 3.591E6 | 1.265E8 | 3.5731E6 | 1.1283E8 | 1.0943E8 | | 21 | 0 | 0 | 0 | 0 | 0 | 1 | 4 | 2 | 1 | 2 | 2 | 2 | 1 | 2 | 4 | 0 | 121 | 128 | Carbamidomethylation | C5:Carbamidomethylation:1000.00 | PEAKS DB |
| K.DFVC(+57.02)NC(+57.02)DRTAAIC(+57.02)FAK.A | N | 59.68 | 1946.8438 | 16 | -0.4 | 974.4265 | 2 | 51.09 | 12 | F12:27773 | BuCaTN01_F10b.raw | | | | | | | | | | 1.1341E6 | | 0 | | | | | 1 | 0 | 0 | 0 | 0 | 0 | 0 | 0 | 0 | 0 | 1 | 0 | 0 | 0 | 0 | 0 | 0 | 113 | 128 | Carbamidomethylation | C4:Carbamidomethylation:1000.00;C6:Carbamidomethylation:1000.00;C13:Carbamidomethylation:1000.00 | PEAKS DB |
| G.GSGTPVDELDR.C | N | 56.36 | 1144.5360 | 11 | 3.4 | 573.2756 | 2 | 40.15 | 8 | F8:17921 | BuCaTN01_F7b.raw | | | | | | | | 1.1068E6 | | | | | | | | | 1 | 0 | 0 | 0 | 0 | 0 | 0 | 0 | 1 | 0 | 0 | 0 | 0 | 0 | 0 | 0 | 0 | 58 | 68 | | | PEAKS DB |
| R.TYSYDC(+57.02)SEGK.L | N | 55.61 | 1208.4656 | 10 | 0.1 | 605.2401 | 2 | 29.72 | 7 | F7:8746 | BuCaTN01_F7a.raw | | | | | | | 5.0914E6 | | | | | | | | | | 1 | 0 | 0 | 0 | 0 | 0 | 0 | 1 | 0 | 0 | 0 | 0 | 0 | 0 | 0 | 0 | 0 | 92 | 101 | Carbamidomethylation | C6:Carbamidomethylation:1000.00 | PEAKS DB |
| N.M(+15.99)IKC(+57.02)TNTRHWLSFTNYGC(+57.02)YC(+57.02)GYGGSGTPVDELDR.C | Y | 53.32 | 4030.7495 | 34 | 1.3 | 1008.6960 | 4 | 77.35 | 10 | F10:53957 | BuCaTN01_F9a.raw | | | | | | | | | | 0 | | | | | | | 0 | 0 | 0 | 0 | 0 | 0 | 0 | 0 | 0 | 0 | 0 | 0 | 0 | 0 | 0 | 0 | 0 | 35 | 68 | Oxidation (M); Carbamidomethylation | M1:Oxidation (M):1000.00;C4:Carbamidomethylation:1000.00;C18:Carbamidomethylation:1000.00;C20:Carbamidomethylation:1000.00 | PEAKS DB |
| R.TYSYDC(+57.02)SEGKLTC(+57.02).K | N | 52.98 | 1582.6279 | 13 | 2.9 | 792.3212 | 2 | 43.19 | 8 | F8:20253 | BuCaTN01_F7b.raw | | | | | | | | 5.7951E5 | | | | | | | | | 1 | 0 | 0 | 0 | 0 | 0 | 0 | 0 | 1 | 0 | 0 | 0 | 0 | 0 | 0 | 0 | 0 | 92 | 104 | Carbamidomethylation | C6:Carbamidomethylation:1000.00;C13:Carbamidomethylation:1000.00 | PEAKS DB |
| R.TYSYDC(+57.02)SEGKLT.C | N | 50.03 | 1422.5973 | 12 | 2.7 | 712.3058 | 2 | 41.55 | 8 | F8:18942 | BuCaTN01_F7b.raw | | | | | | | 5.0044E6 | 1.8313E6 | | | | | | | | | 2 | 0 | 0 | 0 | 0 | 0 | 0 | 1 | 1 | 0 | 0 | 0 | 0 | 0 | 0 | 0 | 0 | 92 | 103 | Carbamidomethylation | C6:Carbamidomethylation:1000.00 | PEAKS DB |
| Y.GGSGTPVDELDR.C | N | 49.09 | 1201.5575 | 12 | 0.3 | 601.7862 | 2 | 40.57 | 11 | F11:14528 | BuCaTN01_F10a.raw | | | | | | | 9.2274E5 | 2.7577E6 | | 1.1335E7 | 6.7591E5 | | 9.9086E5 | 2.6759E7 | | | 6 | 0 | 0 | 0 | 0 | 0 | 0 | 1 | 1 | 0 | 1 | 1 | 0 | 1 | 1 | 0 | 0 | 57 | 68 | | | PEAKS DB |
| R.TAAIC(+57.02)FAKAP.Y | N | 47.25 | 1048.5375 | 10 | 3.6 | 525.2764 | 2 | 48.84 | 8 | F8:25881 | BuCaTN01_F7b.raw | | | | | | 3.1579E6 | | 4.0965E6 | | | | | | | | | 2 | 0 | 0 | 0 | 0 | 0 | 1 | 0 | 1 | 0 | 0 | 0 | 0 | 0 | 0 | 0 | 0 | 121 | 130 | Carbamidomethylation | C5:Carbamidomethylation:1000.00 | PEAKS DB |
| **total 22 peptides** | | | | | | | | | | | | | | | | | | | | | | | | | | | | | | | | | | | | | | | | | | | | | | | | | |

**Best Unique PSM (Scan F10:53957, m/z=1008.6960, z=4, RT=77.35, ppm=1.3):**

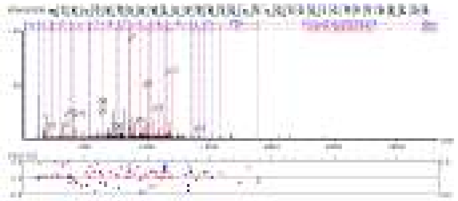

## JAB52809.1

back to list

| Protein Coverage | Supporting Peptides | Best Unique PSM |

**Protein Coverage:**

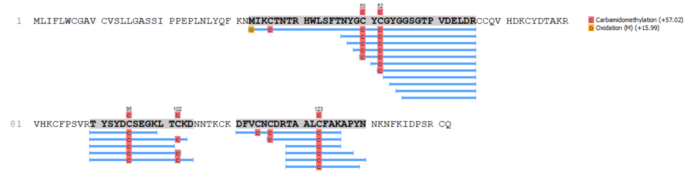


**Supporting Peptides:**

| Peptide | Uniq | -10lgP | Mass | Length | ppm | m/z | z | RT | Fraction | Scan | Source File | Area F1 | Area F2a | Area F3a | Area F4 | Area F5 | Area F6a | Area F7a | Area F7b | Area F8 | Area F9a | Area Sample 1F10a | Area F10b | Area F11a | Area F11b | Area F12a | Area F13 | #Feature | #Feature F1 | #Feature F2a | #Feature F3a | #Feature F4 | #Feature F5 | #Feature F6a | #Feature F7a | #Feature F7b | #Feature F8 | #Feature F9a | #Feature Sample 1F10a | #Feature F10b | #Feature F11a | #Feature F11b | #Feature F12a | #Feature F13 | Start | End | PTM | AScore | Found By |
|---|---|---|---|---|---|---|---|---|---|---|---|---|---|---|---|---|---|---|---|---|---|---|---|---|---|---|---|---|---|---|---|---|---|---|---|---|---|---|---|---|---|---|---|---|---|---|---|---|---|
| N.YGC(+57.02)YC(+57.02)GYGGSGTPVDELDR.C | N | 112.36 | 2124.8516 | 19 | 3.7 | 1063.4326 | 2 | 56.60 | 14 | F14:33384 | BuCaTN01_F11b.raw | | | 0 | | | 2.0097E6 | | 1.4053E7 | | 8.8531E7 | 3.4891E6 | 4.8054E8 | 6.165E7 | 1.7851E9 | 1.2628E8 | | 69 | 0 | 0 | 0 | 0 | 0 | 1 | 0 | 2 | 0 | 3 | 1 | 4 | 2 | 53 | 3 | 0 | 48 | 66 | Carbamidomethylation | C3:Carbamidomethylation:1000.00;C5:Carbamidomethylation:1000.00 | PEAKS DB |
| Y.C(+57.02)GYGGSGTPVDELDR.C | N | 88.12 | 1581.6729 | 15 | 4.4 | 791.8439 | 2 | 48.02 | 14 | F14:25306 | BuCaTN01_F11b.raw | | | | | | | | 2.166E6 | | 8.5786E6 | | 1.5853E7 | 6.495E5 | 1.7537E7 | 0 | | 6 | 0 | 0 | 0 | 0 | 0 | 0 | 0 | 1 | 0 | 1 | 0 | 2 | 1 | 1 | 0 | 0 | 52 | 66 | Carbamidomethylation | C1:Carbamidomethylation:1000.00 | PEAKS DB |
| G.C(+57.02)YC(+57.02)GYGGSGTPVDELDR.C | N | 84.40 | 1904.7668 | 17 | 2.7 | 953.3911 | 2 | 53.16 | 12 | F12:29351 | BuCaTN01_F10b.raw | | | | | | | | 4.1849E5 | | | | 3.7261E5 | | 2.1774E6 | | | 3 | 0 | 0 | 0 | 0 | 0 | 0 | 0 | 1 | 0 | 0 | 0 | 1 | 0 | 1 | 0 | 0 | 50 | 66 | Carbamidomethylation | C1:Carbamidomethylation:1000.00;C3:Carbamidomethylation:1000.00 | PEAKS DB |
| R.TYSYDC(+57.02)SEGKLTC(+57.02)K.D | N | 82.89 | 1710.7229 | 14 | 0.5 | 856.3691 | 2 | 38.29 | 7 | F7:14505 | BuCaTN01_F7a.raw | | | | | | | 1.4512E8 | 2.3155E7 | | 3.8991E5 | 4.5941E5 | | | | | | 8 | 0 | 0 | 0 | 0 | 0 | 0 | 4 | 2 | 0 | 1 | 1 | 0 | 0 | 0 | 0 | 0 | 90 | 103 | Carbamidomethylation | C6:Carbamidomethylation:1000.00;C13:Carbamidomethylation:1000.00 | PEAKS DB |
| Y.GC(+57.02)YC(+57.02)GYGGSGTPVDELDR.C | N | 82.10 | 1961.7883 | 18 | 1.4 | 981.9028 | 2 | 53.49 | 10 | F10:30232 | BuCaTN01_F9a.raw | | | | | | | | 1.7852E6 | | 3.3077E6 | | 1.5846E6 | | 3.2964E6 | 1.0428E5 | | 5 | 0 | 0 | 0 | 0 | 0 | 0 | 0 | 1 | 0 | 1 | 0 | 1 | 0 | 1 | 1 | 0 | 49 | 66 | Carbamidomethylation | C2:Carbamidomethylation:1000.00;C4:Carbamidomethylation:1000.00 | PEAKS DB |
| R.TYSYDC(+57.02)SEGKLTC(+57.02)KD.N | N | 75.87 | 1825.7499 | 15 | 2.5 | 913.8818 | 2 | 38.60 | 8 | F8:16643 | BuCaTN01_F7b.raw | | | | | | | | 7.3044E5 | | | | | | | | | 1 | 0 | 0 | 0 | 0 | 0 | 0 | 0 | 1 | 0 | 0 | 0 | 0 | 0 | 0 | 0 | 0 | 90 | 104 | Carbamidomethylation | C6:Carbamidomethylation:1000.00;C13:Carbamidomethylation:1000.00 | PEAKS DB |
| C.YC(+57.02)GYGGSGTPVDELDR.C | N | 75.62 | 1744.7362 | 16 | 4.1 | 873.3754 | 2 | 52.27 | 14 | F14:28849 | BuCaTN01_F11b.raw | | | | | | | | 7.7286E5 | | 7.3447E5 | | | | 3.0904E6 | 1.4057E5 | | 4 | 0 | 0 | 0 | 0 | 0 | 0 | 0 | 1 | 0 | 1 | 0 | 0 | 0 | 1 | 1 | 0 | 51 | 66 | Carbamidomethylation | C2:Carbamidomethylation:1000.00 | PEAKS DB |
| G.YGGSGTPVDELDR.C | N | 75.26 | 1364.6207 | 13 | 4.1 | 683.3177 | 2 | 45.29 | 14 | F14:22502 | BuCaTN01_F11b.raw | | | | | | | 6.8893E5 | | | | | | | 9.1406E6 | 6.9945E5 | | 3 | 0 | 0 | 0 | 0 | 0 | 0 | 1 | 0 | 0 | 0 | 0 | 0 | 0 | 1 | 1 | 0 | 54 | 66 | | | PEAKS DB |
| R.TAALC(+57.02)FAKAPY.N | N | 73.24 | 1211.6008 | 11 | 2.2 | 606.8076 | 2 | 55.84 | 12 | F12:32023 | BuCaTN01_F10b.raw | | | | | | | 7.3334E4 | | | 1.7166E6 | 1.3753E5 | 1.06E7 | | 8.1407E6 | 9.828E5 | | 7 | 0 | 0 | 0 | 0 | 0 | 0 | 1 | 0 | 0 | 1 | 1 | 2 | 0 | 1 | 1 | 0 | 119 | 129 | Carbamidomethylation | C5:Carbamidomethylation:1000.00 | PEAKS DB |
| T.NYGC(+57.02)YC(+57.02)GYGGSGTPVDELDR.C | N | 72.69 | 2238.8945 | 20 | 5.3 | 1120.4559 | 2 | 64.81 | 14 | F14:40195 | BuCaTN01_F11b.raw | | | | | | | | | | 2.3985E4 | | 0 | 1.1223E5 | 5.3539E5 | | | 3 | 0 | 0 | 0 | 0 | 0 | 0 | 0 | 0 | 0 | 1 | 0 | 0 | 1 | 1 | 0 | 0 | 47 | 66 | Carbamidomethylation | C4:Carbamidomethylation:1000.00;C6:Carbamidomethylation:1000.00 | PEAKS DB |
| R.TAALC(+57.02)FAKAPYN.N | N | 69.35 | 1325.6438 | 12 | -0.2 | 663.8290 | 2 | 51.91 | 6 | F6:30474 | BuCaTN01_F6a.raw | | | | | | 3.7675E6 | | 1.5632E7 | | 4.6358E6 | | 1.9346E6 | | | | | 5 | 0 | 0 | 0 | 0 | 0 | 1 | 0 | 2 | 0 | 1 | 0 | 1 | 0 | 0 | 0 | 0 | 119 | 130 | Carbamidomethylation | C5:Carbamidomethylation:1000.00 | PEAKS DB |
| C.GYGGSGTPVDELDR.C | N | 66.26 | 1421.6422 | 14 | 0.0 | 711.8284 | 2 | 46.82 | 10 | F10:24048 | BuCaTN01_F9a.raw | | | | | | | 2.9103E5 | 1.2275E6 | | 2.1867E6 | | | | 8.7883E6 | | | 5 | 0 | 0 | 0 | 0 | 0 | 0 | 1 | 1 | 0 | 1 | 0 | 0 | 0 | 2 | 0 | 0 | 53 | 66 | | | PEAKS DB |
| N.C(+57.02)DRTAALC(+57.02)FAK.A | N | 63.48 | 1311.6063 | 11 | -1.7 | 438.2076 | 3 | 37.36 | 12 | F12:15762 | BuCaTN01_F10b.raw | | | | | | | | | | | | 5.2815E4 | | 3.1918E5 | | | 2 | 0 | 0 | 0 | 0 | 0 | 0 | 0 | 0 | 0 | 0 | 0 | 1 | 0 | 1 | 0 | 0 | 116 | 126 | Carbamidomethylation | C1:Carbamidomethylation:1000.00;C8:Carbamidomethylation:1000.00 | PEAKS DB |
| R.TAALC(+57.02)FAK.A | N | 60.21 | 880.4476 | 8 | 3.4 | 441.2316 | 2 | 41.58 | 12 | F12:19288 | BuCaTN01_F10b.raw | | | | | | 9.3985E4 | 9.2429E6 | 1.2777E7 | 4.6024E5 | 3.2388E7 | 3.591E6 | 1.265E8 | 3.5731E6 | 1.1283E8 | 1.0943E8 | | 21 | 0 | 0 | 0 | 0 | 0 | 1 | 4 | 2 | 1 | 2 | 2 | 2 | 1 | 2 | 4 | 0 | 119 | 126 | Carbamidomethylation | C5:Carbamidomethylation:1000.00 | PEAKS DB |
| K.DFVC(+57.02)NC(+57.02)DRTAALC(+57.02)FAK.A | N | 59.68 | 1946.8438 | 16 | -0.4 | 974.4265 | 2 | 51.09 | 12 | F12:27773 | BuCaTN01_F10b.raw | | | | | | | | | | 1.1341E6 | | 0 | | | | | 1 | 0 | 0 | 0 | 0 | 0 | 0 | 0 | 0 | 0 | 1 | 0 | 0 | 0 | 0 | 0 | 0 | 111 | 126 | Carbamidomethylation | C4:Carbamidomethylation:1000.00;C6:Carbamidomethylation:1000.00;C13:Carbamidomethylation:1000.00 | PEAKS DB |
| G.GSGTPVDELDR.C | N | 56.36 | 1144.5360 | 11 | 3.4 | 573.2756 | 2 | 40.15 | 8 | F8:17921 | BuCaTN01_F7b.raw | | | | | | | | 1.1068E6 | | | | | | | | | 1 | 0 | 0 | 0 | 0 | 0 | 0 | 0 | 1 | 0 | 0 | 0 | 0 | 0 | 0 | 0 | 0 | 56 | 66 | | | PEAKS DB |
| R.TYSYDC(+57.02)SEGK.L | N | 55.61 | 1208.4656 | 10 | 0.1 | 605.2401 | 2 | 29.72 | 7 | F7:8746 | BuCaTN01_F7a.raw | | | | | | | 5.0914E6 | | | | | | | | | | 1 | 0 | 0 | 0 | 0 | 0 | 0 | 1 | 0 | 0 | 0 | 0 | 0 | 0 | 0 | 0 | 0 | 90 | 99 | Carbamidomethylation | C6:Carbamidomethylation:1000.00 | PEAKS DB |
| N.M(+15.99)IKC(+57.02)TNTRHWLSFTNYGC(+57.02)YC(+57.02)GYGGSGTPVDELDR.C | Y | 53.32 | 4030.7495 | 34 | 1.3 | 1008.6960 | 4 | 77.35 | 10 | F10:53957 | BuCaTN01_F9a.raw | | | | | | | | | | 0 | | | | | | | 0 | 0 | 0 | 0 | 0 | 0 | 0 | 0 | 0 | 0 | 0 | 0 | 0 | 0 | 0 | 0 | 0 | 33 | 66 | Oxidation (M); Carbamidomethylation | M1:Oxidation (M):1000.00;C4:Carbamidomethylation:1000.00;C18:Carbamidomethylation:1000.00;C20:Carbamidomethylation:1000.00 | PEAKS DB |
| R.TYSYDC(+57.02)SEGKLTC(+57.02).K | N | 52.98 | 1582.6279 | 13 | 2.9 | 792.3212 | 2 | 43.19 | 8 | F8:20253 | BuCaTN01_F7b.raw | | | | | | | | 5.7951E5 | | | | | | | | | 1 | 0 | 0 | 0 | 0 | 0 | 0 | 0 | 1 | 0 | 0 | 0 | 0 | 0 | 0 | 0 | 0 | 90 | 102 | Carbamidomethylation | C6:Carbamidomethylation:1000.00;C13:Carbamidomethylation:1000.00 | PEAKS DB |
| R.TYSYDC(+57.02)SEGKLT.C | N | 50.03 | 1422.5973 | 12 | 2.7 | 712.3058 | 2 | 41.55 | 8 | F8:18942 | BuCaTN01_F7b.raw | | | | | | | 5.0044E6 | 1.8313E6 | | | | | | | | | 2 | 0 | 0 | 0 | 0 | 0 | 0 | 1 | 1 | 0 | 0 | 0 | 0 | 0 | 0 | 0 | 0 | 90 | 101 | Carbamidomethylation | C6:Carbamidomethylation:1000.00 | PEAKS DB |
| Y.GGSGTPVDELDR.C | N | 49.09 | 1201.5575 | 12 | 0.3 | 601.7862 | 2 | 40.57 | 11 | F11:14528 | BuCaTN01_F10a.raw | | | | | | | 9.2274E5 | 2.7577E6 | | 1.1335E7 | 6.7591E5 | | 9.9086E5 | 2.6759E7 | | | 6 | 0 | 0 | 0 | 0 | 0 | 0 | 1 | 1 | 0 | 1 | 1 | 0 | 1 | 1 | 0 | 0 | 55 | 66 | | | PEAKS DB |
| R.TAALC(+57.02)FAKAP.Y | N | 47.25 | 1048.5375 | 10 | 3.6 | 525.2764 | 2 | 48.84 | 8 | F8:25881 | BuCaTN01_F7b.raw | | | | | | 3.1579E6 | | 4.0965E6 | | | | | | | | | 2 | 0 | 0 | 0 | 0 | 0 | 1 | 0 | 1 | 0 | 0 | 0 | 0 | 0 | 0 | 0 | 0 | 119 | 128 | Carbamidomethylation | C5:Carbamidomethylation:1000.00 | PEAKS DB |
| **total 22 peptides** | | | | | | | | | | | | | | | | | | | | | | | | | | | | | | | | | | | | | | | | | | | | | | | | | |

**Best Unique PSM (Scan F10:53957, m/z=1008.6960, z=4, RT=77.35, ppm=1.3):**

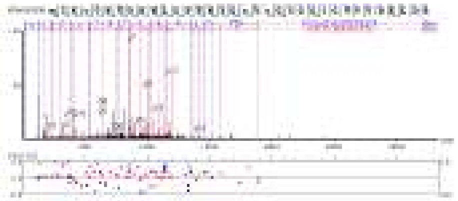

## JAS04998.1

back to list

| Protein Coverage | Supporting Peptides | Best Unique PSM |

**Protein Coverage:**

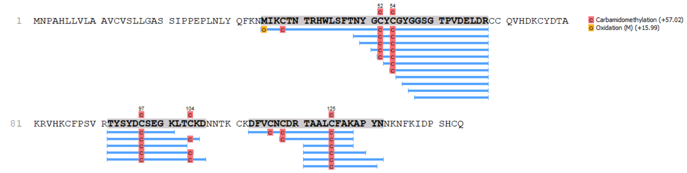


**Supporting Peptides:**

| Peptide | Uniq | -10lgP | Mass | Length | ppm | m/z | z | RT | Fraction | Scan | Source File | Area F1 | Area F2a | Area F3a | Area F4 | Area F5 | Area F6a | Area F7a | Area F7b | Area F8 | Area F9a | Area Sample 1F10a | Area F10b | Area F11a | Area F11b | Area F12a | Area F13 | #Feature | #Feature F1 | #Feature F2a | #Feature F3a | #Feature F4 | #Feature F5 | #Feature F6a | #Feature F7a | #Feature F7b | #Feature F8 | #Feature F9a | #Feature Sample 1F10a | #Feature F10b | #Feature F11a | #Feature F11b | #Feature F12a | #Feature F13 | Start | End | PTM | AScore | Found By |
|---|---|---|---|---|---|---|---|---|---|---|---|---|---|---|---|---|---|---|---|---|---|---|---|---|---|---|---|---|---|---|---|---|---|---|---|---|---|---|---|---|---|---|---|---|---|---|---|---|---|
| N.YGC(+57.02)YC(+57.02)GYGGSGTPVDELDR.C | N | 112.36 | 2124.8516 | 19 | 3.7 | 1063.4326 | 2 | 56.60 | 14 | F14:33384 | BuCaTN01_F11b.raw | | | 0 | | | 2.0097E6 | | 1.4053E7 | | 8.8531E7 | 3.4891E6 | 4.8054E8 | 6.165E7 | 1.7851E9 | 1.2628E8 | | 69 | 0 | 0 | 0 | 0 | 0 | 1 | 0 | 2 | 0 | 3 | 1 | 4 | 2 | 53 | 3 | 0 | 50 | 68 | Carbamidomethylation | C3:Carbamidomethylation:1000.00;C5:Carbamid | PEAKS DB |

| | | | | | | | | | | | | | | | | | | | | | | | | | | | | | | | | | | | | | | | | | | | | | | | | | |
|---|---|---|---|---|---|---|---|---|---|---|---|---|---|---|---|---|---|---|---|---|---|---|---|---|---|---|---|---|---|---|---|---|---|---|---|---|---|---|---|---|---|---|---|---|---|---|---|---|---|
| | | | | | | | | | | | | | | | | | | | | | | | | | | | | | | | | | | | | | | | | | | | | | | | | omethylation:1000.00 | |
| Y.C(+57.02)GYGGSGTPVDELDR.C | N | 88.12 | 1581.6729 | 15 | 4.4 | 791.8439 | 2 | 48.02 | 14 | F14:25306 | BuCaTN01_F11b.raw | | | | | | | | 2.166E6 | | 8.5786E6 | | 1.5853E7 | 6.495E5 | 1.7537E7 | 0 | | 6 | 0 | 0 | 0 | 0 | 0 | 0 | 0 | 1 | 0 | 1 | 0 | 2 | 1 | 1 | 0 | 0 | 54 | 68 | Carbamidomethylation | C1:Carbamidomethylation:1000.00 | PEAKS DB |
| G.C(+57.02)YC(+57.02)GYGGSGTPVDELDR.C | N | 84.40 | 1904.7668 | 17 | 2.7 | 953.3911 | 2 | 53.16 | 12 | F12:29351 | BuCaTN01_F10b.raw | | | | | | | | 4.1849E5 | | | | 3.7261E5 | | 2.1774E6 | | | 3 | 0 | 0 | 0 | 0 | 0 | 0 | 0 | 1 | 0 | 0 | 0 | 1 | 0 | 1 | 0 | 0 | 52 | 68 | Carbamidomethylation | C1:Carbamidomethylation:1000.00;C3:Carbamidomethylation:1000.00 | PEAKS DB |
| R.TYSYDC(+57.02)SEGKLTC(+57.02)K.D | N | 82.89 | 1710.7229 | 14 | 0.5 | 856.3691 | 2 | 38.29 | 7 | F7:14505 | BuCaTN01_F7a.raw | | | | | | | 1.4512E8 | 2.3155E7 | | 3.8991E5 | 4.5941E5 | | | | | | 8 | 0 | 0 | 0 | 0 | 0 | 0 | 4 | 2 | 0 | 1 | 1 | 0 | 0 | 0 | 0 | 0 | 92 | 105 | Carbamidomethylation | C6:Carbamidomethylation:1000.00;C13:Carbamidomethylation:1000.00 | PEAKS DB |
| Y.GC(+57.02)YC(+57.02)GYGGSGTPVDELDR.C | N | 82.10 | 1961.7883 | 18 | 1.4 | 981.9028 | 2 | 53.49 | 10 | F10:30232 | BuCaTN01_F9a.raw | | | | | | | | 1.7852E6 | | 3.3077E6 | | 1.5846E6 | | 3.2964E6 | 1.0428E5 | | 5 | 0 | 0 | 0 | 0 | 0 | 0 | 0 | 1 | 0 | 1 | 0 | 1 | 0 | 1 | 1 | 0 | 51 | 68 | Carbamidomethylation | C2:Carbamidomethylation:1000.00;C4:Carbamidomethylation:1000.00 | PEAKS DB |
| R.TYSYDC(+57.02)SEGKLTC(+57.02)KD.N | N | 75.87 | 1825.7499 | 15 | 2.5 | 913.8818 | 2 | 38.60 | 8 | F8:16643 | BuCaTN01_F7b.raw | | | | | | | | 7.3044E5 | | | | | | | | | 1 | 0 | 0 | 0 | 0 | 0 | 0 | 0 | 1 | 0 | 0 | 0 | 0 | 0 | 0 | 0 | 0 | 92 | 106 | Carbamidomethylation | C6:Carbamidomethylation:1000.00;C13:Carbamidomethylation:1000.00 | PEAKS DB |
| C.YC(+57.02)GYGGSGTPVDELDR.C | N | 75.62 | 1744.7362 | 16 | 4.1 | 873.3754 | 2 | 52.27 | 14 | F14:28849 | BuCaTN01_F11b.raw | | | | | | | | 7.7286E5 | | 7.3447E5 | | | | 3.0904E6 | 1.4057E5 | | 4 | 0 | 0 | 0 | 0 | 0 | 0 | 0 | 1 | 0 | 1 | 0 | 0 | 0 | 1 | 1 | 0 | 53 | 68 | Carbamidomethylation | C2:Carbamidomethylation:1000.00 | PEAKS DB |
| G.YGGSGTPVDELDR.C | N | 75.26 | 1364.6207 | 13 | 4.1 | 683.3177 | 2 | 45.29 | 14 | F14:22502 | BuCaTN01_F11b.raw | | | | | | | 6.8893E5 | | | | | | | 9.1406E6 | 6.9945E5 | | 3 | 0 | 0 | 0 | 0 | 0 | 0 | 1 | 0 | 0 | 0 | 0 | 0 | 0 | 1 | 1 | 0 | 56 | 68 | | | PEAKS DB |
| R.TAALC(+57.02)FAKAPY.N | N | 73.24 | 1211.6008 | 11 | 2.2 | 606.8076 | 2 | 55.84 | 12 | F12:32023 | BuCaTN01_F10b.raw | | | | | | | 7.3334E4 | | | 1.7166E6 | 1.3753E5 | 1.06E7 | | 8.1407E6 | 9.828E5 | | 7 | 0 | 0 | 0 | 0 | 0 | 0 | 1 | 0 | 0 | 1 | 1 | 2 | 0 | 1 | 1 | 0 | 121 | 131 | Carbamidomethylation | C5:Carbamidomethylation:1000.00 | PEAKS DB |
| T.NYGC(+57.02)YC(+57.02)GYGGSGTPVDELDR.C | N | 72.69 | 2238.8945 | 20 | 5.3 | 1120.4559 | 2 | 64.81 | 14 | F14:40195 | BuCaTN01_F11b.raw | | | | | | | | | | 2.3985E4 | | 0 | 1.1223E5 | 5.3539E5 | | | 3 | 0 | 0 | 0 | 0 | 0 | 0 | 0 | 0 | 0 | 1 | 0 | 0 | 1 | 1 | 0 | 0 | 49 | 68 | Carbamidomethylation | C4:Carbamidomethylation:1000.00;C6:Carbamidomethylation:1000.00 | PEAKS DB |
| R.TAALC(+57.02)FAKAPYN.N | N | 69.35 | 1325.6438 | 12 | -0.2 | 663.8290 | 2 | 51.91 | 6 | F6:30474 | BuCaTN01_F6a.raw | | | | | | 3.7675E6 | | 1.5632E7 | | 4.6358E6 | | 1.9346E6 | | | | | 5 | 0 | 0 | 0 | 0 | 0 | 1 | 0 | 2 | 0 | 1 | 0 | 1 | 0 | 0 | 0 | 0 | 121 | 132 | Carbamidomethylation | C5:Carbamidomethylation:1000.00 | PEAKS DB |
| C.GYGGSGTPVDELDR.C | N | 66.26 | 1421.6422 | 14 | 0.0 | 711.8284 | 2 | 46.82 | 10 | F10:24048 | BuCaTN01_F9a.raw | | | | | | | 2.9103E5 | 1.2275E6 | | 2.1867E6 | | | | 8.7883E6 | | | 5 | 0 | 0 | 0 | 0 | 0 | 0 | 1 | 1 | 0 | 1 | 0 | 0 | 0 | 2 | 0 | 0 | 55 | 68 | | | PEAKS DB |
| N.C(+57.02)DRTAALC(+57.02)FAK.A | N | 63.48 | 1311.6063 | 11 | -1.7 | 438.2076 | 3 | 37.36 | 12 | F12:15762 | BuCaTN01_F10b.raw | | | | | | | | | | | | 5.2815E4 | | 3.1918E5 | | | 2 | 0 | 0 | 0 | 0 | 0 | 0 | 0 | 0 | 0 | 0 | 0 | 1 | 0 | 1 | 0 | 0 | 118 | 128 | Carbamidomethylation | C1:Carbamidomethylation:1000.00;C8:Carbamidomethylation:1000.00 | PEAKS DB |
| R.TAALC(+57.02)FAK.A | N | 60.21 | 880.4476 | 8 | 3.4 | 441.2316 | 2 | 41.58 | 12 | F12:19288 | BuCaTN01_F10b.raw | | | | | | 9.3985E4 | 9.2429E6 | 1.2777E7 | 4.6024E5 | 3.2388E7 | 3.591E6 | 1.265E8 | 3.5731E6 | 1.1283E8 | 1.0943E8 | | 21 | 0 | 0 | 0 | 0 | 0 | 1 | 4 | 2 | 1 | 2 | 2 | 2 | 1 | 2 | 4 | 0 | 121 | 128 | Carbamidomethylation | C5:Carbamidomethylation:1000.00 | PEAKS DB |
| K.DFVC(+57.02)NC(+57.02)DRTAALC(+57.02)FAK.A | N | 59.68 | 1946.8438 | 16 | -0.4 | 974.4265 | 2 | 51.09 | 12 | F12:27773 | BuCaTN01_F10b.raw | | | | | | | | | | 1.1341E6 | | 0 | | | | | 1 | 0 | 0 | 0 | 0 | 0 | 0 | 0 | 0 | 0 | 1 | 0 | 0 | 0 | 0 | 0 | 0 | 113 | 128 | Carbamidomethylation | C4:Carbamidomethylation:1000.00;C6:Carbamidomethylation:1000.00;C13:Carbamidomethylation:1000.00 | PEAKS DB |
| G.GSGTPVDELDR.C | N | 56.36 | 1144.5360 | 11 | 3.4 | 573.2756 | 2 | 40.15 | 8 | F8:17921 | BuCaTN01_F7b.raw | | | | | | | | 1.1068E6 | | | | | | | | | 1 | 0 | 0 | 0 | 0 | 0 | 0 | 0 | 1 | 0 | 0 | 0 | 0 | 0 | 0 | 0 | 0 | 58 | 68 | | | PEAKS DB |
| R.TYSYDC(+57.02)SEGK.L | N | 55.61 | 1208.4656 | 10 | 0.1 | 605.2401 | 2 | 29.72 | 7 | F7:8746 | BuCaTN01_F7a.raw | | | | | | | 5.0914E6 | | | | | | | | | | 1 | 0 | 0 | 0 | 0 | 0 | 0 | 1 | 0 | 0 | 0 | 0 | 0 | 0 | 0 | 0 | 0 | 92 | 101 | Carbamidomethylation | C6:Carbamidomethylation:1000.00 | PEAKS DB |
| N.M(+15.99)IKC(+57.02)TNTRHWLSFTNYGC(+57.02)YC(+57.02)GYGGSGTPVDELDR.C | Y | 53.32 | 4030.7495 | 34 | 1.3 | 1008.6960 | 4 | 77.35 | 10 | F10:53957 | BuCaTN01_F9a.raw | | | | | | | | | | 0 | | | | | | | 0 | 0 | 0 | 0 | 0 | 0 | 0 | 0 | 0 | 0 | 0 | 0 | 0 | 0 | 0 | 0 | 0 | 35 | 68 | Oxidation (M); Carbamidomethylation | M1:Oxidation (M):1000.00;C4:Carbamidomethylation:1000.00;C18:Carbamidomethylation:1000.00;C20:Carbamidomethylation:1000.00 | PEAKS DB |
| R.TYSYDC(+57.02)SEGKLTC(+57.02).K | N | 52.98 | 1582.6279 | 13 | 2.9 | 792.3212 | 2 | 43.19 | 8 | F8:20253 | BuCaTN01_F7b.raw | | | | | | | | 5.7951E5 | | | | | | | | | 1 | 0 | 0 | 0 | 0 | 0 | 0 | 0 | 1 | 0 | 0 | 0 | 0 | 0 | 0 | 0 | 0 | 92 | 104 | Carbamidomethylation | C6:Carbamidomethylation:1000.00;C13:Carbamidomethylation:1000.00 | PEAKS DB |
| R.TYSYDC(+57.02)SEGKLT.C | N | 50.03 | 1422.5973 | 12 | 2.7 | 712.3058 | 2 | 41.55 | 8 | F8:18942 | BuCaTN01_F7b.raw | | | | | | | 5.0044E6 | 1.8313E6 | | | | | | | | | 2 | 0 | 0 | 0 | 0 | 0 | 0 | 1 | 1 | 0 | 0 | 0 | 0 | 0 | 0 | 0 | 0 | 92 | 103 | Carbamidomethylation | C6:Carbamidomethylation:1000.00 | PEAKS DB |
| Y.GGSGTPVDELDR.C | N | 49.09 | 1201.5575 | 12 | 0.3 | 601.7862 | 2 | 40.57 | 11 | F11:14528 | BuCaTN01_F10a.raw | | | | | | | 9.2274E5 | 2.7577E6 | | 1.1335E7 | 6.7591E5 | | 9.9086E5 | 2.6759E7 | | | 6 | 0 | 0 | 0 | 0 | 0 | 0 | 1 | 1 | 0 | 1 | 1 | 0 | 1 | 1 | 0 | 0 | 57 | 68 | | | PEAKS DB |
| R.TAALC(+57.02)FAKAP.Y | N | 47.25 | 1048.5375 | 10 | 3.6 | 525.2764 | 2 | 48.84 | 8 | F8:25881 | BuCaTN01_F7b.raw | | | | | | 3.1579E6 | | 4.0965E6 | | | | | | | | | 2 | 0 | 0 | 0 | 0 | 0 | 1 | 0 | 1 | 0 | 0 | 0 | 0 | 0 | 0 | 0 | 0 | 121 | 130 | Carbamidomethylation | C5:Carbamidomethylation:1000.00 | PEAKS DB |
| total 22 peptides | | | | | | | | | | | | | | | | | | | | | | | | | | | | | | | | | | | | | | | | | | | | | | | | | |

**Best Unique PSM (Scan F10:53957, m/z=1008.6960, z=4, RT=77.35, ppm=1.3):**

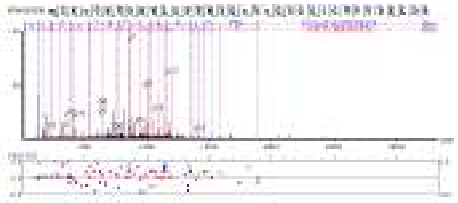

## JAB52772.1

back to list

| Protein Coverage | Supporting Peptides | Best Unique PSM |

**Protein Coverage:**

1 MILAHLLVLA AVCVSLLGAS SIPPEPLNLY QFKNMIKCTN TRHWLSFTNY GCYCGYGGSG TPVDELDRCC QVHDKCYDTA

81 KRVHKCFPSV RTYSYDCSEG KLTCKDNNTK CKDFVCNCDR TAALCFAKAP YNNKNFKIDP SRCQ

C Carbamidomethylation (+57.02)
O Oxidation (M) (+15.99)

**Supporting Peptides:**

| Peptide | Uniq | -10lgP | Mass | Length | ppm | m/z | z | RT | Fraction | Scan | Source File | Area F1 | Area F2a | Area F3a | Area F4 | Area F5 | Area F6a | Area F7a | Area F7b | Area F8 | Area F9a | Area Sample 1F10a | Area F10b | Area F11a | Area F11b | Area F12a | Area F13 | #Feature | #Feature F1 | #Feature F2a | #Feature F3a | #Feature F4 | #Feature F5 | #Feature F6a | #Feature F7a | #Feature F7b | #Feature F8 | #Feature F9a | #Feature Sample 1F10a | #Feature F10b | #Feature F11a | #Feature F11b | #Feature F12a | #Feature F13 | Start | End | PTM | AScore | Found By |
|---|---|---|---|---|---|---|---|---|---|---|---|---|---|---|---|---|---|---|---|---|---|---|---|---|---|---|---|---|---|---|---|---|---|---|---|---|---|---|---|---|---|---|---|---|---|---|---|---|---|
| N.YGC(+57.02)YC(+57.02)GYGGSGTPVDELDR.C | N | 112.36 | 2124.8516 | 19 | 3.7 | 1063.4326 | 2 | 56.60 | 14 | F14:33384 | BuCaTN01_F11b.raw | | | 0 | | | 2.0097E6 | | 1.4053E7 | | 8.8531E7 | 3.4891E6 | 4.8054E8 | 6.165E7 | 1.7851E9 | 1.2628E8 | | 69 | 0 | 0 | 0 | 0 | 0 | 1 | 0 | 2 | 0 | 3 | 1 | 4 | 2 | 53 | 3 | 0 | 50 | 68 | Carbamidomethylation | C3:Carbamidomethylation:1000.00;C5:Carbamidomethylation:1000.00 | PEAKS DB |
| Y.C(+57.02)GYGGSGTPVDELDR.C | N | 88.12 | 1581.6729 | 15 | 4.4 | 791.8439 | 2 | 48.02 | 14 | F14:25306 | BuCaTN01_F11b.raw | | | | | | | | 2.166E6 | | 8.5786E6 | | 1.5853E7 | 6.495E5 | 1.7537E7 | 0 | | 6 | 0 | 0 | 0 | 0 | 0 | 0 | 0 | 1 | 0 | 1 | 0 | 2 | 1 | 1 | 0 | 0 | 54 | 68 | Carbamidomethylation | C1:Carbamidomethylation:1000.00 | PEAKS DB |
| G.C(+57.02)YC(+57.02)GYGGSGTPVDELDR.C | N | 84.40 | 1904.7668 | 17 | 2.7 | 953.3911 | 2 | 53.16 | 12 | F12:29351 | BuCaTN01_F10b.raw | | | | | | | | 4.1849E5 | | | | 3.7261E5 | | 2.1774E6 | | | 3 | 0 | 0 | 0 | 0 | 0 | 0 | 0 | 1 | 0 | 0 | 0 | 1 | 0 | 1 | 0 | 0 | 52 | 68 | Carbamidomethylation | C1:Carbamidomethylation:1000.00;C3:Carbamidomethylation:1000.00 | PEAKS DB |
| R.TYSYDC(+57.02)SEGKLTC(+57.02)K.D | N | 82.89 | 1710.7229 | 14 | 0.5 | 856.3691 | 2 | 38.29 | 7 | F7:14505 | BuCaTN01_F7a.raw | | | | | | | 1.4512E8 | 2.3155E7 | | 3.8991E5 | 4.5941E5 | | | | | | 8 | 0 | 0 | 0 | 0 | 0 | 0 | 4 | 2 | 0 | 1 | 1 | 0 | 0 | 0 | 0 | 0 | 92 | 105 | Carbamidomethylation | C6:Carbamidomethylation:1000.00;C13:Carbamidomethylation:1000.00 | PEAKS DB |
| Y.GC(+57.02)YC(+57.02)GYGGSGTPVDELDR.C | N | 82.10 | 1961.7883 | 18 | 1.4 | 981.9028 | 2 | 53.49 | 10 | F10:30232 | BuCaTN01_F9a.raw | | | | | | | | 1.7852E6 | | 3.3077E6 | | 1.5846E6 | | 3.2964E6 | 1.0428E5 | | 5 | 0 | 0 | 0 | 0 | 0 | 0 | 0 | 1 | 0 | 1 | 0 | 1 | 0 | 1 | 1 | 0 | 51 | 68 | Carbamidomethylation | C2:Carbamidomethylation:1000.00;C4:Carbamidomethylation:1000.00 | PEAKS DB |
| R.TYSYDC(+57.02)SEGKLTC(+57.02)KD.N | N | 75.87 | 1825.7499 | 15 | 2.5 | 913.8818 | 2 | 38.60 | 8 | F8:16643 | BuCaTN01_F7b.raw | | | | | | | | 7.3044E5 | | | | | | | | | 1 | 0 | 0 | 0 | 0 | 0 | 0 | 0 | 1 | 0 | 0 | 0 | 0 | 0 | 0 | 0 | 0 | 92 | 106 | Carbamidomethylation | C6:Carbamidomethylation:1000.00;C13:Carbamidomethylation:1000.00 | PEAKS DB |
| C.YC(+57.02)GYGGSGTPVDELDR.C | N | 75.62 | 1744.7362 | 16 | 4.1 | 873.3754 | 2 | 52.27 | 14 | F14:28849 | BuCaTN01_F11b.raw | | | | | | | | 7.7286E5 | | 7.3447E5 | | | | 3.0904E6 | 1.4057E5 | | 4 | 0 | 0 | 0 | 0 | 0 | 0 | 0 | 1 | 0 | 1 | 0 | 0 | 0 | 1 | 1 | 0 | 53 | 68 | Carbamidomethylation | C2:Carbamidomethylation:1000.00 | PEAKS DB |
| G.YGGSGTPVDELDR.C | N | 75.26 | 1364.6207 | 13 | 4.1 | 683.3177 | 2 | 45.29 | 14 | F14:22502 | BuCaTN01_F11b.raw | | | | | | | 6.8893E5 | | | | | | | 9.1406E6 | 6.9945E5 | | 3 | 0 | 0 | 0 | 0 | 0 | 0 | 1 | 0 | 0 | 0 | 0 | 0 | 0 | 1 | 1 | 0 | 56 | 68 | | | PEAKS DB |
| R.TAALC(+57.02)FAKAPY.N | N | 73.24 | 1211.6008 | 11 | 2.2 | 606.8076 | 2 | 55.84 | 12 | F12:32023 | BuCaTN01_F10b.raw | | | | | | | 7.3334E4 | | | 1.7166E6 | 1.3753E5 | 1.06E7 | | 8.1407E6 | 9.828E5 | | 7 | 0 | 0 | 0 | 0 | 0 | 0 | 1 | 0 | 0 | 1 | 1 | 2 | 0 | 1 | 1 | 0 | 121 | 131 | Carbamidomethylation | C5:Carbamidomethylation:1000.00 | PEAKS DB |
| T.NYGC(+57.02)YC(+57.02)GYGGSGTPVDELDR.C | N | 72.69 | 2238.8945 | 20 | 5.3 | 1120.4559 | 2 | 64.81 | 14 | F14:40195 | BuCaTN01_F11b.raw | | | | | | | | | | 2.3985E4 | | 0 | 1.1223E5 | 5.3539E5 | | | 3 | 0 | 0 | 0 | 0 | 0 | 0 | 0 | 0 | 0 | 1 | 0 | 0 | 1 | 1 | 0 | 0 | 49 | 68 | Carbamidomethylation | C4:Carbamidomethylation:1000.00;C6:Carbamidomethylation:1000.00 | PEAKS DB |
| R.TAALC(+57.02)FAKAPYN.N | N | 69.35 | 1325.6438 | 12 | -0.2 | 663.8290 | 2 | 51.91 | 6 | F6:30474 | BuCaTN01_F6a.raw | | | | | | 3.7675E6 | | 1.5632E7 | | 4.6358E6 | | 1.9346E6 | | | | | 5 | 0 | 0 | 0 | 0 | 0 | 1 | 0 | 2 | 0 | 1 | 0 | 1 | 0 | 0 | 0 | 0 | 121 | 132 | Carbamidomethylation | C5:Carbamidomethylation:1000.00 | PEAKS DB |
| C.GYGGSGTPVDELDR.C | N | 66.26 | 1421.6422 | 14 | 0.0 | 711.8284 | 2 | 46.82 | 10 | F10:24048 | BuCaTN01_F9a.raw | | | | | | | 2.9103E5 | 1.2275E6 | | 2.1867E6 | | | | 8.7883E6 | | | 5 | 0 | 0 | 0 | 0 | 0 | 0 | 1 | 1 | 0 | 1 | 0 | 0 | 0 | 2 | 0 | 0 | 55 | 68 | | | PEAKS DB |
| N.C(+57.02)DRTAALC(+57.02)FAK.A | N | 63.48 | 1311.6063 | 11 | -1.7 | 438.2076 | 3 | 37.36 | 12 | F12:15762 | BuCaTN01_F10b.raw | | | | | | | | | | | | 5.2815E4 | | 3.1918E5 | | | 2 | 0 | 0 | 0 | 0 | 0 | 0 | 0 | 0 | 0 | 0 | 0 | 1 | 0 | 1 | 0 | 0 | 118 | 128 | Carbamidomethylation | C1:Carbamidomethylation:1000.00;C8:Carbamidomethylation:1000.00 | PEAKS DB |
| R.TAALC(+57.02)FAK.A | N | 60.21 | 880.4476 | 8 | 3.4 | 441.2316 | 2 | 41.58 | 12 | F12:19288 | BuCaTN01_F10b.raw | | | | | | 9.3985E4 | 9.2429E6 | 1.2777E7 | 4.6024E5 | 3.2388E7 | 3.591E6 | 1.265E8 | 3.5731E6 | 1.1283E8 | 1.0943E8 | | 21 | 0 | 0 | 0 | 0 | 0 | 1 | 4 | 2 | 1 | 2 | 2 | 2 | 1 | 2 | 4 | 0 | 121 | 128 | Carbamidomethylation | C5:Carbamidomethylation:1000.00 | PEAKS DB |
| K.DFVC(+57.02)NC(+57.02)DRTAALC(+57.02)FAK.A | N | 59.68 | 1946.8438 | 16 | -0.4 | 974.4265 | 2 | 51.09 | 12 | F12:27773 | BuCaTN01_F10b.raw | | | | | | | | | | 1.1341E6 | | 0 | | | | | 1 | 0 | 0 | 0 | 0 | 0 | 0 | 0 | 0 | 0 | 1 | 0 | 0 | 0 | 0 | 0 | 0 | 113 | 128 | Carbamidomethylation | C4:Carbamidomethylation:1000.00;C6:Carbamidomethylation:1000.00;C13:Carbamidomethylation:1000.00 | PEAKS DB |
| G.GSGTPVDELDR.C | N | 56.36 | 1144.5360 | 11 | 3.4 | 573.2756 | 2 | 40.15 | 8 | F8:17921 | BuCaTN01_F7b.raw | | | | | | | | 1.1068E6 | | | | | | | | | 1 | 0 | 0 | 0 | 0 | 0 | 0 | 0 | 1 | 0 | 0 | 0 | 0 | 0 | 0 | 0 | 0 | 58 | 68 | | | PEAKS DB |
| R.TYSYDC(+57.02)SEGK.L | N | 55.61 | 1208.4656 | 10 | 0.1 | 605.2401 | 2 | 29.72 | 7 | F7:8746 | BuCaTN01_F7a.raw | | | | | | | 5.0914E6 | | | | | | | | | | 1 | 0 | 0 | 0 | 0 | 0 | 0 | 1 | 0 | 0 | 0 | 0 | 0 | 0 | 0 | 0 | 0 | 92 | 101 | Carbamidomethylation | C6:Carbamidomethylation:1000.00 | PEAKS DB |
| N.M(+15.99)IKC(+57.02)TNTRHWLSFTNYGC(+57.02)YC(+57.02)GYGGSGTPVDELDR.C | Y | 53.32 | 4030.7495 | 34 | 1.3 | 1008.6960 | 4 | 77.35 | 10 | F10:53957 | BuCaTN01_F9a.raw | | | | | | | | | | 0 | | | | | | | 0 | 0 | 0 | 0 | 0 | 0 | 0 | 0 | 0 | 0 | 0 | 0 | 0 | 0 | 0 | 0 | 0 | 35 | 68 | Oxidation (M); Carbamidomethylation | M1:Oxidation (M):1000.00;C4:Carbamidomethylation:1000.00;C18:Carbamidomethylation:1000.00;C20:Carbamidomethylation:1000.00 | PEAKS DB |
| R.TYSYDC(+57.02)SEGKLTC(+57.02).K | N | 52.98 | 1582.6279 | 13 | 2.9 | 792.3212 | 2 | 43.19 | 8 | F8:20253 | BuCaTN01_F7b.raw | | | | | | | | 5.7951E5 | | | | | | | | | 1 | 0 | 0 | 0 | 0 | 0 | 0 | 0 | 1 | 0 | 0 | 0 | 0 | 0 | 0 | 0 | 0 | 92 | 104 | Carbamidomethylation | C6:Carbamidomethylation:1000.00;C13:Carbamidomethylation:1000.00 | PEAKS DB |
| R.TYSYDC(+57.02)SEGKLT.C | N | 50.03 | 1422.5973 | 12 | 2.7 | 712.3058 | 2 | 41.55 | 8 | F8:18942 | BuCaTN01_F7b.raw | | | | | | | 5.0044E6 | 1.8313E6 | | | | | | | | | 2 | 0 | 0 | 0 | 0 | 0 | 0 | 1 | 1 | 0 | 0 | 0 | 0 | 0 | 0 | 0 | 0 | 92 | 103 | Carbamidomethylation | C6:Carbamidomethylation:1000.00 | PEAKS DB |
| Y.GGSGTPVDELDR.C | N | 49.09 | 1201.5575 | 12 | 0.3 | 601.7862 | 2 | 40.57 | 11 | F11:14528 | BuCaTN01_F10a.raw | | | | | | | 9.2274E5 | 2.7577E6 | | 1.1335E7 | 6.7591E5 | | 9.9086E5 | 2.6759E7 | | | 6 | 0 | 0 | 0 | 0 | 0 | 0 | 1 | 1 | 0 | 1 | 1 | 0 | 1 | 1 | 0 | 0 | 57 | 68 | | | PEAKS DB |
| R.TAALC(+57.02)FAKAP.Y | N | 47.25 | 1048.5375 | 10 | 3.6 | 525.2764 | 2 | 48.84 | 8 | F8:25881 | BuCaTN01_F7b.raw | | | | | | 3.1579E6 | | 4.0965E6 | | | | | | | | | 2 | 0 | 0 | 0 | 0 | 0 | 1 | 0 | 1 | 0 | 0 | 0 | 0 | 0 | 0 | 0 | 0 | 121 | 130 | Carbamidomethylation | C5:Carbamidomethylation:1000.00 | PEAKS DB |

**total 22 peptides**

**Best Unique PSM (Scan F10:53957, m/z=1008.6960, z=4, RT=77.35, ppm=1.3):**

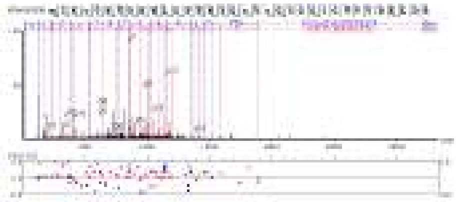

## JAS04995.1

back to list

| Protein Coverage | Supporting Peptides | Best Unique PSM |

**Protein Coverage:**

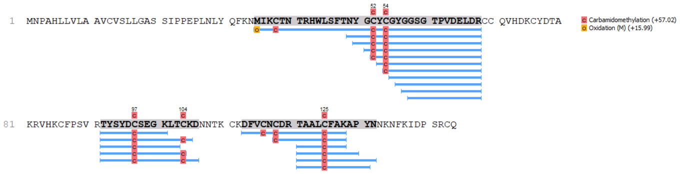


**Supporting Peptides:**

| Peptide | Uniq | -10lgP | Mass | Length | ppm | m/z | z | RT | Fraction | Scan | Source File | Area F1 | Area F2a | Area F3a | Area F4 | Area F5 | Area F6a | Area F7a | Area F7b | Area F8 | Area F9a | Area Sample 1F10a | Area F10b | Area F11a | Area F11b | Area F12a | Area F13 | #Feature | #Feature F1 | #Feature F2a | #Feature F3a | #Feature F4 | #Feature F5 | #Feature F6a | #Feature F7a | #Feature F7b | #Feature F8 | #Feature F9a | #Feature Sample 1F10a | #Feature F10b | #Feature F11a | #Feature F11b | #Feature F12a | #Feature F13 | Start | End | PTM | AScore | Found By |
|---|---|---|---|---|---|---|---|---|---|---|---|---|---|---|---|---|---|---|---|---|---|---|---|---|---|---|---|---|---|---|---|---|---|---|---|---|---|---|---|---|---|---|---|---|---|---|---|---|---|
| N.YGC(+57.02)YC(+57.02)GYGGSGTPVDELDR.C | N | 112.36 | 2124.8516 | 19 | 3.7 | 1063.4326 | 2 | 56.60 | 14 | F14:33384 | BuCaTN01_F11b.raw | | | 0 | | | 2.0097E6 | | 1.4053E7 | | 8.8531E7 | 3.4891E6 | 4.8054E8 | 6.165E7 | 1.7851E9 | 1.2628E8 | | 69 | 0 | 0 | 0 | 0 | 0 | 1 | 0 | 2 | 0 | 3 | 1 | 4 | 2 | 53 | 3 | 0 | 50 | 68 | Carbamidomethylation | C3:Carbamidomethylation:1000.00;C5:Carbamidomethylation:1000.00 | PEAKS DB |
| Y.C(+57.02)GYGGSGTPVDELDR.C | N | 88.12 | 1581.6729 | 15 | 4.4 | 791.8439 | 2 | 48.02 | 14 | F14:25306 | BuCaTN01_F11b.raw | | | | | | | | 2.166E6 | | 8.5786E6 | | 1.5853E7 | 6.495E5 | 1.7537E7 | 0 | | 6 | 0 | 0 | 0 | 0 | 0 | 0 | 0 | 1 | 0 | 1 | 0 | 2 | 1 | 1 | 0 | 0 | 54 | 68 | Carbamidomethylation | C1:Carbamidomethylation:1000.00 | PEAKS DB |
| G.C(+57.02)YC(+57.02)GYGGSGTPVDELDR.C | N | 84.40 | 1904.7668 | 17 | 2.7 | 953.3911 | 2 | 53.16 | 12 | F12:29351 | BuCaTN01_F10b.raw | | | | | | | | 4.1849E5 | | | | 3.7261E5 | | 2.1774E6 | | | 3 | 0 | 0 | 0 | 0 | 0 | 0 | 0 | 1 | 0 | 0 | 0 | 1 | 0 | 1 | 0 | 0 | 52 | 68 | Carbamidomethylation | C1:Carbamidomethylation:1000.00;C3:Carbamidomethylation:1000.00 | PEAKS DB |
| R.TYSYDC(+57.02)SEGKLTC(+57.02)K.D | N | 82.89 | 1710.7229 | 14 | 0.5 | 856.3691 | 2 | 38.29 | 7 | F7:14505 | BuCaTN01_F7a.raw | | | | | | | 1.4512E8 | 2.3155E7 | | 3.8991E5 | 4.5941E5 | | | | | | 8 | 0 | 0 | 0 | 0 | 0 | 0 | 4 | 2 | 0 | 1 | 1 | 0 | 0 | 0 | 0 | 0 | 92 | 105 | Carbamidomethylation | C6:Carbamidomethylation:1000.00;C13:Carbamidomethylation:1000.00 | PEAKS DB |
| Y.GC(+57.02)YC(+57.02)GYGGSGTPVDELDR.C | N | 82.10 | 1961.7883 | 18 | 1.4 | 981.9028 | 2 | 53.49 | 10 | F10:30232 | BuCaTN01_F9a.raw | | | | | | | | 1.7852E6 | | 3.3077E6 | | 1.5846E6 | | 3.2964E6 | 1.0428E5 | | 5 | 0 | 0 | 0 | 0 | 0 | 0 | 0 | 1 | 0 | 1 | 0 | 1 | 0 | 1 | 1 | 0 | 51 | 68 | Carbamidomethylation | C2:Carbamidomethylation:1000.00;C4:Carbamidomethylation:1000.00 | PEAKS DB |
| R.TYSYDC(+57.02)SEGKLTC(+57.02)KD.N | N | 75.87 | 1825.7499 | 15 | 2.5 | 913.8818 | 2 | 38.60 | 8 | F8:16643 | BuCaTN01_F7b.raw | | | | | | | | 7.3044E5 | | | | | | | | | 1 | 0 | 0 | 0 | 0 | 0 | 0 | 0 | 1 | 0 | 0 | 0 | 0 | 0 | 0 | 0 | 0 | 92 | 106 | Carbamidomethylation | C6:Carbamidomethylation:1000.00;C13:Carbamidomethylation:1000.00 | PEAKS DB |
| C.YC(+57.02)GYGGSGTPVDELDR.C | N | 75.62 | 1744.7362 | 16 | 4.1 | 873.3754 | 2 | 52.27 | 14 | F14:28849 | BuCaTN01_F11b.raw | | | | | | | | 7.7286E5 | | 7.3447E5 | | | | 3.0904E6 | 1.4057E5 | | 4 | 0 | 0 | 0 | 0 | 0 | 0 | 0 | 1 | 0 | 1 | 0 | 0 | 0 | 1 | 1 | 0 | 53 | 68 | Carbamidomethylation | C2:Carbamidomethylation:1000.00 | PEAKS DB |
| G.YGGSGTPVDELDR.C | N | 75.26 | 1364.6207 | 13 | 4.1 | 683.3177 | 2 | 45.29 | 14 | F14:22502 | BuCaTN01_F11b.raw | | | | | | | 6.8893E5 | | | | | | | 9.1406E6 | 6.9945E5 | | 3 | 0 | 0 | 0 | 0 | 0 | 0 | 1 | 0 | 0 | 0 | 0 | 0 | 0 | 1 | 1 | 0 | 56 | 68 | | | PEAKS DB |
| R.TAALC(+57.02)FAKAPY.N | N | 73.24 | 1211.6008 | 11 | 2.2 | 606.8076 | 2 | 55.84 | 12 | F12:32023 | BuCaTN01_F10b.raw | | | | | | | 7.3334E4 | | | 1.7166E6 | 1.3753E5 | 1.06E7 | | 8.1407E6 | 9.828E5 | | 7 | 0 | 0 | 0 | 0 | 0 | 0 | 1 | 0 | 0 | 1 | 1 | 2 | 0 | 1 | 1 | 0 | 121 | 131 | Carbamidomethylation | C5:Carbamidomethylation:1000.00 | PEAKS DB |
| T.NYGC(+57.02)YC(+57.02)GYGGSGTPVDELDR.C | N | 72.69 | 2238.8945 | 20 | 5.3 | 1120.4559 | 2 | 64.81 | 14 | F14:40195 | BuCaTN01_F11b.raw | | | | | | | | | | 2.3985E4 | | 0 | 1.1223E5 | 5.3539E5 | | | 3 | 0 | 0 | 0 | 0 | 0 | 0 | 0 | 0 | 0 | 1 | 0 | 0 | 1 | 1 | 0 | 0 | 49 | 68 | Carbamidomethylation | C4:Carbamidomethylation:1000.00;C6:Carbamidomethylation:1000.00 | PEAKS DB |
| R.TAALC(+57.02)FAKAPYN.N | N | 69.35 | 1325.6438 | 12 | -0.2 | 663.8290 | 2 | 51.91 | 6 | F6:30474 | BuCaTN01_F6a.raw | | | | | | 3.7675E6 | | 1.5632E7 | | 4.6358E6 | | 1.9346E6 | | | | | 5 | 0 | 0 | 0 | 0 | 0 | 1 | 0 | 2 | 0 | 1 | 0 | 1 | 0 | 0 | 0 | 0 | 121 | 132 | Carbamidomethylation | C5:Carbamidomethylation:1000.00 | PEAKS DB |
| C.GYGGSGTPVDELDR.C | N | 66.26 | 1421.6422 | 14 | 0.0 | 711.8284 | 2 | 46.82 | 10 | F10:24048 | BuCaTN01_F9a.raw | | | | | | | 2.9103E5 | 1.2275E6 | | 2.1867E6 | | | | 8.7883E6 | | | 5 | 0 | 0 | 0 | 0 | 0 | 0 | 1 | 1 | 0 | 1 | 0 | 0 | 0 | 2 | 0 | 0 | 55 | 68 | | | PEAKS DB |
| N.C(+57.02)DRTAALC(+57.02)FAK.A | N | 63.48 | 1311.6063 | 11 | -1.7 | 438.2076 | 3 | 37.36 | 12 | F12:15762 | BuCaTN01_F10b.raw | | | | | | | | | | | | 5.2815E4 | | 3.1918E5 | | | 2 | 0 | 0 | 0 | 0 | 0 | 0 | 0 | 0 | 0 | 0 | 0 | 1 | 0 | 1 | 0 | 0 | 118 | 128 | Carbamidomethylation | C1:Carbamidomethylation:1000.00;C8:Carbamidomethylation:1000.00 | PEAKS DB |
| R.TAALC(+57.02)FAK.A | N | 60.21 | 880.4476 | 8 | 3.4 | 441.2316 | 2 | 41.58 | 12 | F12:19288 | BuCaTN01_F10b.raw | | | | | | 9.3985E4 | 9.2429E6 | 1.2777E7 | 4.6024E5 | 3.2388E7 | 3.591E6 | 1.265E8 | 3.5731E6 | 1.1283E8 | 1.0943E8 | | 21 | 0 | 0 | 0 | 0 | 0 | 1 | 4 | 2 | 1 | 2 | 2 | 2 | 1 | 2 | 4 | 0 | 121 | 128 | Carbamidomethylation | C5:Carbamidomethylation:1000.00 | PEAKS DB |
| K.DFVC(+57.02)NC(+57.02)DRTAALC(+57.02)FAK.A | N | 59.68 | 1946.8438 | 16 | -0.4 | 974.4265 | 2 | 51.09 | 12 | F12:27773 | BuCaTN01_F10b.raw | | | | | | | | | | 1.1341E6 | | 0 | | | | | 1 | 0 | 0 | 0 | 0 | 0 | 0 | 0 | 0 | 0 | 1 | 0 | 0 | 0 | 0 | 0 | 0 | 113 | 128 | Carbamidomethylation | C4:Carbamidomethylation:1000.00;C6:Carbamidomethylation:1000.00;C13:Carbamidomethylation:1000.00 | PEAKS DB |
| G.GSGTPVDELDR.C | N | 56.36 | 1144.5360 | 11 | 3.4 | 573.2756 | 2 | 40.15 | 8 | F8:17921 | BuCaTN01_F7b.raw | | | | | | | | 1.1068E6 | | | | | | | | | 1 | 0 | 0 | 0 | 0 | 0 | 0 | 0 | 1 | 0 | 0 | 0 | 0 | 0 | 0 | 0 | 0 | 58 | 68 | | | PEAKS DB |
| R.TYSYDC(+57.02)SEGK.L | N | 55.61 | 1208.4656 | 10 | 0.1 | 605.2401 | 2 | 29.72 | 7 | F7:8746 | BuCaTN01_F7a.raw | | | | | | | 5.0914E6 | | | | | | | | | | 1 | 0 | 0 | 0 | 0 | 0 | 0 | 1 | 0 | 0 | 0 | 0 | 0 | 0 | 0 | 0 | 0 | 92 | 101 | Carbamidomethylation | C6:Carbamidomethylation:1000.00 | PEAKS DB |
| N.M(+15.99)IKC(+57.02)TNTRHWLSFTNYGC(+57.02)YC(+57.02)GYGGSGTPVDELDR.C | Y | 53.32 | 4030.7495 | 34 | 1.3 | 1008.6960 | 4 | 77.35 | 10 | F10:53957 | BuCaTN01_F9a.raw | | | | | | | | | | 0 | | | | | | | 0 | 0 | 0 | 0 | 0 | 0 | 0 | 0 | 0 | 0 | 0 | 0 | 0 | 0 | 0 | 0 | 0 | 35 | 68 | Oxidation (M); Carbamidomethylation | M1:Oxidation (M):1000.00;C4:Carbamidomethylation:1000.00;C18:Carbamidomethylation:1000.00;C20:Carbamidomethylation:1000.00 | PEAKS DB |
| R.TYSYDC(+57.02)SEGKLTC(+57.02).K | N | 52.98 | 1582.6279 | 13 | 2.9 | 792.3212 | 2 | 43.19 | 8 | F8:20253 | BuCaTN01_F7b.raw | | | | | | | | 5.7951E5 | | | | | | | | | 1 | 0 | 0 | 0 | 0 | 0 | 0 | 0 | 1 | 0 | 0 | 0 | 0 | 0 | 0 | 0 | 0 | 92 | 104 | Carbamidomethylation | C6:Carbamidomethylation:1000.00;C13:Carbamidomethylation:1000.00 | PEAKS DB |
| R.TYSYDC(+57.02)SEGKLT.C | N | 50.03 | 1422.5973 | 12 | 2.7 | 712.3058 | 2 | 41.55 | 8 | F8:18942 | BuCaTN01_F7b.raw | | | | | | | 5.0044E6 | 1.8313E6 | | | | | | | | | 2 | 0 | 0 | 0 | 0 | 0 | 0 | 1 | 1 | 0 | 0 | 0 | 0 | 0 | 0 | 0 | 0 | 92 | 103 | Carbamidomethylation | C6:Carbamidomethylation:1000.00 | PEAKS DB |
| Y.GGSGTPVDELDR.C | N | 49.09 | 1201.5575 | 12 | 0.3 | 601.7862 | 2 | 40.57 | 11 | F11:14528 | BuCaTN01_F10a.raw | | | | | | | 9.2274E5 | 2.7577E6 | | 1.1335E7 | 6.7591E5 | | 9.9086E5 | 2.6759E7 | | | 6 | 0 | 0 | 0 | 0 | 0 | 0 | 1 | 1 | 0 | 1 | 1 | 0 | 1 | 1 | 0 | 0 | 57 | 68 | | | PEAKS DB |
| R.TAALC(+57.02)FAKAP.Y | N | 47.25 | 1048.5375 | 10 | 3.6 | 525.2764 | 2 | 48.84 | 8 | F8:25881 | BuCaTN01_F7b.raw | | | | | | 3.1579E6 | | 4.0965E6 | | | | | | | | | 2 | 0 | 0 | 0 | 0 | 0 | 1 | 0 | 1 | 0 | 0 | 0 | 0 | 0 | 0 | 0 | 0 | 121 | 130 | Carbamidomethylation | C5:Carbamidomethylation:1000.00 | PEAKS DB |
| **total 22 peptides** | | | | | | | | | | | | | | | | | | | | | | | | | | | | | | | | | | | | | | | | | | | | | | | | | |

**Best Unique PSM (Scan F10:53957, m/z=1008.6960, z=4, RT=77.35, ppm=1.3):**

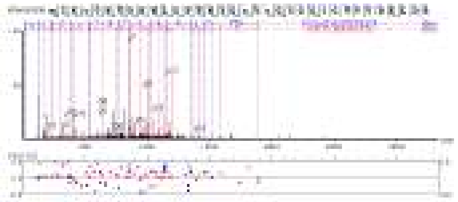

## JAS04999.1

back to list

| Protein Coverage | Supporting Peptides | Best Unique PSM |

**Protein Coverage:**

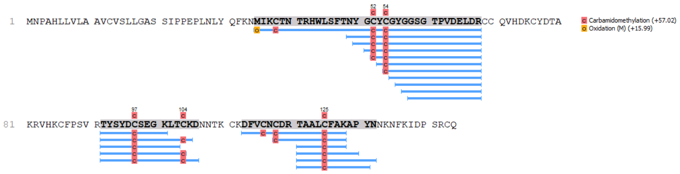


**Supporting Peptides:**

| Peptide | Uniq | -10lgP | Mass | Length | ppm | m/z | z | RT | Fraction | Scan | Source File | Area F1 | Area F2a | Area F3a | Area F4 | Area F5 | Area F6a | Area F7a | Area F7b | Area F8 | Area F9a | Area Sample 1F10a | Area F10b | Area F11a | Area F11b | Area F12a | Area F13 | #Feature | #Feature F1 | #Feature F2a | #Feature F3a | #Feature F4 | #Feature F5 | #Feature F6a | #Feature F7a | #Feature F7b | #Feature F8 | #Feature F9a | #Feature Sample 1F10a | #Feature F10b | #Feature F11a | #Feature F11b | #Feature F12a | #Feature F13 | Start | End | PTM | AScore | Found By |
|---|---|---|---|---|---|---|---|---|---|---|---|---|---|---|---|---|---|---|---|---|---|---|---|---|---|---|---|---|---|---|---|---|---|---|---|---|---|---|---|---|---|---|---|---|---|---|---|---|---|

| | | | | | | | | | | | | | | | | | | | | | | | | | | | | | | | | | | | | | | | | | | | | | | | | | |
|---|---|---|---|---|---|---|---|---|---|---|---|---|---|---|---|---|---|---|---|---|---|---|---|---|---|---|---|---|---|---|---|---|---|---|---|---|---|---|---|---|---|---|---|---|---|---|---|---|---|
| N.YGC(+57.02)YC(+57.02)GYGGSGTPVDELDR.C | N | 112.36 | 2124.8516 | 19 | 3.7 | 1063.4326 | 2 | 56.60 | 14 | F14:33384 | BuCaTN01_F11b.raw | | | 0 | | | 2.0097E6 | | 1.4053E7 | | 8.8531E7 | 3.4891E6 | 4.8054E8 | 6.165E7 | 1.7851E9 | 1.2628E8 | | 69 | 0 | 0 | 0 | 0 | 0 | 1 | 0 | 2 | 0 | 3 | 1 | 4 | 2 | 53 | 3 | 0 | 50 | 68 | Carbamidomethylation | C3:Carbamidomethylation:1000.00;C5:Carbamidomethylation:1000.00 | PEAKS DB |
| Y.C(+57.02)GYGGSGTPVDELDR.C | N | 88.12 | 1581.6729 | 15 | 4.4 | 791.8439 | 2 | 48.02 | 14 | F14:25306 | BuCaTN01_F11b.raw | | | | | | | | 2.166E6 | | 8.5786E6 | | 1.5853E7 | 6.495E5 | 1.7537E7 | 0 | | 6 | 0 | 0 | 0 | 0 | 0 | 0 | 0 | 1 | 0 | 1 | 0 | 2 | 1 | 1 | 0 | 0 | 54 | 68 | Carbamidomethylation | C1:Carbamidomethylation:1000.00 | PEAKS DB |
| G.C(+57.02)YC(+57.02)GYGGSGTPVDELDR.C | N | 84.40 | 1904.7668 | 17 | 2.7 | 953.3911 | 2 | 53.16 | 12 | F12:29351 | BuCaTN01_F10b.raw | | | | | | | | 4.1849E5 | | | | 3.7261E5 | | 2.1774E6 | | | 3 | 0 | 0 | 0 | 0 | 0 | 0 | 0 | 1 | 0 | 0 | 0 | 1 | 0 | 1 | 0 | 0 | 52 | 68 | Carbamidomethylation | C1:Carbamidomethylation:1000.00;C3:Carbamidomethylation:1000.00 | PEAKS DB |
| R.TYSYDC(+57.02)SEGKLTC(+57.02)K.D | N | 82.89 | 1710.7229 | 14 | 0.5 | 856.3691 | 2 | 38.29 | 7 | F7:14505 | BuCaTN01_F7a.raw | | | | | | | 1.4512E8 | 2.3155E7 | | 3.8991E5 | 4.5941E5 | | | | | | 8 | 0 | 0 | 0 | 0 | 0 | 0 | 4 | 2 | 0 | 1 | 1 | 0 | 0 | 0 | 0 | 0 | 92 | 105 | Carbamidomethylation | C6:Carbamidomethylation:1000.00;C13:Carbamidomethylation:1000.00 | PEAKS DB |
| Y.GC(+57.02)YC(+57.02)GYGGSGTPVDELDR.C | N | 82.10 | 1961.7883 | 18 | 1.4 | 981.9028 | 2 | 53.49 | 10 | F10:30232 | BuCaTN01_F9a.raw | | | | | | | | 1.7852E6 | | 3.3077E6 | | 1.5846E6 | | 3.2964E6 | 1.0428E5 | | 5 | 0 | 0 | 0 | 0 | 0 | 0 | 0 | 1 | 0 | 1 | 0 | 1 | 0 | 1 | 1 | 0 | 51 | 68 | Carbamidomethylation | C2:Carbamidomethylation:1000.00;C4:Carbamidomethylation:1000.00 | PEAKS DB |
| R.TYSYDC(+57.02)SEGKLTC(+57.02)KD.N | N | 75.87 | 1825.7499 | 15 | 2.5 | 913.8818 | 2 | 38.60 | 8 | F8:16643 | BuCaTN01_F7b.raw | | | | | | | | 7.3044E5 | | | | | | | | | 1 | 0 | 0 | 0 | 0 | 0 | 0 | 0 | 1 | 0 | 0 | 0 | 0 | 0 | 0 | 0 | 0 | 92 | 106 | Carbamidomethylation | C6:Carbamidomethylation:1000.00;C13:Carbamidomethylation:1000.00 | PEAKS DB |
| C.YC(+57.02)GYGGSGTPVDELDR.C | N | 75.62 | 1744.7362 | 16 | 4.1 | 873.3754 | 2 | 52.27 | 14 | F14:28849 | BuCaTN01_F11b.raw | | | | | | | | 7.7286E5 | | 7.3447E5 | | | | 3.0904E6 | 1.4057E5 | | 4 | 0 | 0 | 0 | 0 | 0 | 0 | 0 | 1 | 0 | 1 | 0 | 0 | 0 | 1 | 1 | 0 | 53 | 68 | Carbamidomethylation | C2:Carbamidomethylation:1000.00 | PEAKS DB |
| G.YGGSGTPVDELDR.C | N | 75.26 | 1364.6207 | 13 | 4.1 | 683.3177 | 2 | 45.29 | 14 | F14:22502 | BuCaTN01_F11b.raw | | | | | | | 6.8893E5 | | | | | | | 9.1406E6 | 6.9945E5 | | 3 | 0 | 0 | 0 | 0 | 0 | 0 | 1 | 0 | 0 | 0 | 0 | 0 | 0 | 1 | 1 | 0 | 56 | 68 | | | PEAKS DB |
| R.TAALC(+57.02)FAKAPY.N | N | 73.24 | 1211.6008 | 11 | 2.2 | 606.8076 | 2 | 55.84 | 12 | F12:32023 | BuCaTN01_F10b.raw | | | | | | | 7.3334E4 | | | 1.7166E6 | 1.3753E5 | 1.06E7 | | 8.1407E6 | 9.828E5 | | 7 | 0 | 0 | 0 | 0 | 0 | 0 | 1 | 0 | 0 | 1 | 1 | 2 | 0 | 1 | 1 | 0 | 121 | 131 | Carbamidomethylation | C5:Carbamidomethylation:1000.00 | PEAKS DB |
| T.NYGC(+57.02)YC(+57.02)GYGGSGTPVDELDR.C | N | 72.69 | 2238.8945 | 20 | 5.3 | 1120.4559 | 2 | 64.81 | 14 | F14:40195 | BuCaTN01_F11b.raw | | | | | | | | | | 2.3985E4 | | 0 | 1.1223E5 | 5.3539E5 | | | 3 | 0 | 0 | 0 | 0 | 0 | 0 | 0 | 0 | 0 | 1 | 0 | 0 | 1 | 1 | 0 | 0 | 49 | 68 | Carbamidomethylation | C4:Carbamidomethylation:1000.00;C6:Carbamidomethylation:1000.00 | PEAKS DB |
| R.TAALC(+57.02)FAKAPYN.N | N | 69.35 | 1325.6438 | 12 | -0.2 | 663.8290 | 2 | 51.91 | 6 | F6:30474 | BuCaTN01_F6a.raw | | | | | | 3.7675E6 | | 1.5632E7 | | 4.6358E6 | | 1.9346E6 | | | | | 5 | 0 | 0 | 0 | 0 | 0 | 1 | 0 | 2 | 0 | 1 | 0 | 1 | 0 | 0 | 0 | 0 | 121 | 132 | Carbamidomethylation | C5:Carbamidomethylation:1000.00 | PEAKS DB |
| C.GYGGSGTPVDELDR.C | N | 66.26 | 1421.6422 | 14 | 0.0 | 711.8284 | 2 | 46.82 | 10 | F10:24048 | BuCaTN01_F9a.raw | | | | | | | 2.9103E5 | 1.2275E6 | | 2.1867E6 | | | | 8.7883E6 | | | 5 | 0 | 0 | 0 | 0 | 0 | 0 | 1 | 1 | 0 | 1 | 0 | 0 | 0 | 2 | 0 | 0 | 55 | 68 | | | PEAKS DB |
| N.C(+57.02)DRTAALC(+57.02)FAK.A | N | 63.48 | 1311.6063 | 11 | -1.7 | 438.2076 | 3 | 37.36 | 12 | F12:15762 | BuCaTN01_F10b.raw | | | | | | | | | | | | 5.2815E4 | | 3.1918E5 | | | 2 | 0 | 0 | 0 | 0 | 0 | 0 | 0 | 0 | 0 | 0 | 0 | 1 | 0 | 1 | 0 | 0 | 118 | 128 | Carbamidomethylation | C1:Carbamidomethylation:1000.00;C8:Carbamidomethylation:1000.00 | PEAKS DB |
| R.TAALC(+57.02)FAK.A | N | 60.21 | 880.4476 | 8 | 3.4 | 441.2316 | 2 | 41.58 | 12 | F12:19288 | BuCaTN01_F10b.raw | | | | | | 9.3985E4 | 9.2429E6 | 1.2777E7 | 4.6024E5 | 3.2388E7 | 3.591E6 | 1.265E8 | 3.5731E6 | 1.1283E8 | 1.0943E8 | | 21 | 0 | 0 | 0 | 0 | 0 | 1 | 4 | 2 | 1 | 2 | 2 | 2 | 1 | 2 | 4 | 0 | 121 | 128 | Carbamidomethylation | C5:Carbamidomethylation:1000.00 | PEAKS DB |
| K.DFVC(+57.02)NC(+57.02)DRTAALC(+57.02)FAK.A | N | 59.68 | 1946.8438 | 16 | -0.4 | 974.4265 | 2 | 51.09 | 12 | F12:27773 | BuCaTN01_F10b.raw | | | | | | | | | | 1.1341E6 | | 0 | | | | | 1 | 0 | 0 | 0 | 0 | 0 | 0 | 0 | 0 | 0 | 1 | 0 | 0 | 0 | 0 | 0 | 0 | 113 | 128 | Carbamidomethylation | C4:Carbamidomethylation:1000.00;C6:Carbamidomethylation:1000.00;C13:Carbamidomethylation:1000.00 | PEAKS DB |
| G.GSGTPVDELDR.C | N | 56.36 | 1144.5360 | 11 | 3.4 | 573.2756 | 2 | 40.15 | 8 | F8:17921 | BuCaTN01_F7b.raw | | | | | | | | 1.1068E6 | | | | | | | | | 1 | 0 | 0 | 0 | 0 | 0 | 0 | 0 | 1 | 0 | 0 | 0 | 0 | 0 | 0 | 0 | 0 | 58 | 68 | | | PEAKS DB |
| R.TYSYDC(+57.02)SEGK.L | N | 55.61 | 1208.4656 | 10 | 0.1 | 605.2401 | 2 | 29.72 | 7 | F7:8746 | BuCaTN01_F7a.raw | | | | | | | 5.0914E6 | | | | | | | | | | 1 | 0 | 0 | 0 | 0 | 0 | 0 | 1 | 0 | 0 | 0 | 0 | 0 | 0 | 0 | 0 | 0 | 92 | 101 | Carbamidomethylation | C6:Carbamidomethylation:1000.00 | PEAKS DB |
| N.M(+15.99)IKC(+57.02)TNTRHWLSFTNYGC(+57.02)YC(+57.02)GYGGSGTPVDELDR.C | Y | 53.32 | 4030.7495 | 34 | 1.3 | 1008.6960 | 4 | 77.35 | 10 | F10:53957 | BuCaTN01_F9a.raw | | | | | | | | | | 0 | | | | | | | 0 | 0 | 0 | 0 | 0 | 0 | 0 | 0 | 0 | 0 | 0 | 0 | 0 | 0 | 0 | 0 | 0 | 35 | 68 | Oxidation (M); Carbamidomethylation | M1:Oxidation (M):1000.00;C4:Carbamidomethylation:1000.00;C18:Carbamidomethylation:1000.00;C20:Carbamidomethylation:1000.00 | PEAKS DB |
| R.TYSYDC(+57.02)SEGKLTC(+57.02).K | N | 52.98 | 1582.6279 | 13 | 2.9 | 792.3212 | 2 | 43.19 | 8 | F8:20253 | BuCaTN01_F7b.raw | | | | | | | | 5.7951E5 | | | | | | | | | 1 | 0 | 0 | 0 | 0 | 0 | 0 | 0 | 1 | 0 | 0 | 0 | 0 | 0 | 0 | 0 | 0 | 92 | 104 | Carbamidomethylation | C6:Carbamidomethylation:1000.00;C13:Carbamidomethylation:1000.00 | PEAKS DB |
| R.TYSYDC(+57.02)SEGKLT.C | N | 50.03 | 1422.5973 | 12 | 2.7 | 712.3058 | 2 | 41.55 | 8 | F8:18942 | BuCaTN01_F7b.raw | | | | | | | 5.0044E6 | 1.8313E6 | | | | | | | | | 2 | 0 | 0 | 0 | 0 | 0 | 0 | 1 | 1 | 0 | 0 | 0 | 0 | 0 | 0 | 0 | 0 | 92 | 103 | Carbamidomethylation | C6:Carbamidomethylation:1000.00 | PEAKS DB |
| Y.GGSGTPVDELDR.C | N | 49.09 | 1201.5575 | 12 | 0.3 | 601.7862 | 2 | 40.57 | 11 | F11:14528 | BuCaTN01_F10a.raw | | | | | | | 9.2274E5 | 2.7577E6 | | 1.1335E7 | 6.7591E5 | | 9.9086E5 | 2.6759E7 | | | 6 | 0 | 0 | 0 | 0 | 0 | 0 | 1 | 1 | 0 | 1 | 1 | 0 | 1 | 1 | 0 | 0 | 57 | 68 | | | PEAKS DB |
| R.TAALC(+57.02)FAKAP.Y | N | 47.25 | 1048.5375 | 10 | 3.6 | 525.2764 | 2 | 48.84 | 8 | F8:25881 | BuCaTN01_F7b.raw | | | | | | 3.1579E6 | | 4.0965E6 | | | | | | | | | 2 | 0 | 0 | 0 | 0 | 0 | 1 | 0 | 1 | 0 | 0 | 0 | 0 | 0 | 0 | 0 | 0 | 121 | 130 | Carbamidomethylation | C5:Carbamidomethylation:1000.00 | PEAKS DB |
| total 22 peptides | | | | | | | | | | | | | | | | | | | | | | | | | | | | | | | | | | | | | | | | | | | | | | | | | |

**Best Unique PSM (Scan F10:53957, m/z=1008.6960, z=4, RT=77.35, ppm=1.3):**

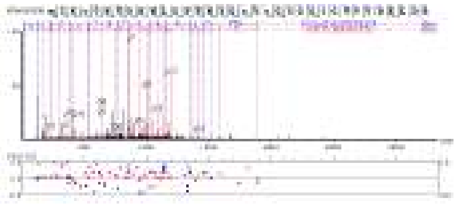

## JAB52770.1

back to list

| **Protein Coverage** | **Supporting Peptides** | **Best Unique PSM** |

**Protein Coverage:**

1 MNPAHLLVLA AVCVSLLGAS SIPPEPLNLY QFKNMIKCTN TRHWLSFTNY GCYCGYGGSG TPVDELDRCC QVHDKCYDTA

C Carbamidomethylation (+57.02)
O Oxidation (M) (+15.99)

81 KRVHKCFPSV RTYSYDCSEG KLTCKDNNTK CKDFVCNCDR TAALCFAKAP YNNKNFKIDP SHCQ

**Supporting Peptides:**

| Peptide | Uniq | -10lgP | Mass | Length | ppm | m/z | z | RT | Fraction | Scan | Source File | Area F1 | Area F2a | Area F3a | Area F4 | Area F5 | Area F6a | Area F7a | Area F7b | Area F8 | Area F9a | Area Sample 1F10a | Area F10b | Area F11a | Area F11b | Area F12a | Area F13 | #Feature | #Feature F1 | #Feature F2a | #Feature F3a | #Feature F4 | #Feature F5 | #Feature F6a | #Feature F7a | #Feature F7b | #Feature F8 | #Feature F9a | #Feature Sample 1F10a | #Feature F10b | #Feature F11a | #Feature F11b | #Feature F12a | #Feature F13 | Start | End | PTM | AScore | Found By |
|---|---|---|---|---|---|---|---|---|---|---|---|---|---|---|---|---|---|---|---|---|---|---|---|---|---|---|---|---|---|---|---|---|---|---|---|---|---|---|---|---|---|---|---|---|---|---|---|---|---|
| N.YGC(+57.02)YC(+57.02)GYGGSGTPVDELDR.C | N | 112.36 | 2124.8516 | 19 | 3.7 | 1063.4326 | 2 | 56.60 | 14 | F14:33384 | BuCaTN01_F11b.raw | | | 0 | | | 2.0097E6 | | 1.4053E7 | | 8.8531E7 | 3.4891E6 | 4.8054E8 | 6.165E7 | 1.7851E9 | 1.2628E8 | | 69 | 0 | 0 | 0 | 0 | 0 | 1 | 0 | 2 | 0 | 3 | 1 | 4 | 2 | 53 | 3 | 0 | 50 | 68 | Carbamidomethylation | C3:Carbamidomethylation:1000.00;C5:Carbamidomethylation:1000.00 | PEAKS DB |
| Y.C(+57.02)GYGGSGTPVDELDR.C | N | 88.12 | 1581.6729 | 15 | 4.4 | 791.8439 | 2 | 48.02 | 14 | F14:25306 | BuCaTN01_F11b.raw | | | | | | | | 2.166E6 | | 8.5786E6 | | 1.5853E7 | 6.495E5 | 1.7537E7 | 0 | | 6 | 0 | 0 | 0 | 0 | 0 | 0 | 0 | 1 | 0 | 1 | 0 | 2 | 1 | 1 | 0 | 0 | 54 | 68 | Carbamidomethylation | C1:Carbamidomethylation:1000.00 | PEAKS DB |
| G.C(+57.02)YC(+57.02)GYGGSGTPVDELDR.C | N | 84.40 | 1904.7668 | 17 | 2.7 | 953.3911 | 2 | 53.16 | 12 | F12:29351 | BuCaTN01_F10b.raw | | | | | | | | 4.1849E5 | | | | 3.7261E5 | | 2.1774E6 | | | 3 | 0 | 0 | 0 | 0 | 0 | 0 | 0 | 1 | 0 | 0 | 0 | 1 | 0 | 1 | 0 | 0 | 52 | 68 | Carbamidomethylation | C1:Carbamidomethylation:1000.00;C3:Carbamidomethylation:1000.00 | PEAKS DB |
| R.TYSYDC(+57.02)SEGKLTC(+57.02)K.D | N | 82.89 | 1710.7229 | 14 | 0.5 | 856.3691 | 2 | 38.29 | 7 | F7:14505 | BuCaTN01_F7a.raw | | | | | | | 1.4512E8 | 2.3155E7 | | 3.8991E5 | 4.5941E5 | | | | | | 8 | 0 | 0 | 0 | 0 | 0 | 0 | 4 | 2 | 0 | 1 | 1 | 0 | 0 | 0 | 0 | 0 | 92 | 105 | Carbamidomethylation | C6:Carbamidomethylation:1000.00;C13:Carbamidomethylation:1000.00 | PEAKS DB |
| Y.GC(+57.02)YC(+57.02)GYGGSGTPVDELDR.C | N | 82.10 | 1961.7883 | 18 | 1.4 | 981.9028 | 2 | 53.49 | 10 | F10:30232 | BuCaTN01_F9a.raw | | | | | | | | 1.7852E6 | | 3.3077E6 | | 1.5846E6 | | 3.2964E6 | 1.0428E5 | | 5 | 0 | 0 | 0 | 0 | 0 | 0 | 0 | 1 | 0 | 1 | 0 | 1 | 0 | 1 | 1 | 0 | 51 | 68 | Carbamidomethylation | C2:Carbamidomethylation:1000.00;C4:Carbamidomethylation:1000.00 | PEAKS DB |
| R.TYSYDC(+57.02)SEGKLTC(+57.02)KD.N | N | 75.87 | 1825.7499 | 15 | 2.5 | 913.8818 | 2 | 38.60 | 8 | F8:16643 | BuCaTN01_F7b.raw | | | | | | | | 7.3044E5 | | | | | | | | | 1 | 0 | 0 | 0 | 0 | 0 | 0 | 0 | 1 | 0 | 0 | 0 | 0 | 0 | 0 | 0 | 0 | 92 | 106 | Carbamidomethylation | C6:Carbamidomethylation:1000.00;C13:Carbamidomethylation:1000.00 | PEAKS DB |
| C.YC(+57.02)GYGGSGTPVDELDR.C | N | 75.62 | 1744.7362 | 16 | 4.1 | 873.3754 | 2 | 52.27 | 14 | F14:28849 | BuCaTN01_F11b.raw | | | | | | | | 7.7286E5 | | 7.3447E5 | | | | 3.0904E6 | 1.4057E5 | | 4 | 0 | 0 | 0 | 0 | 0 | 0 | 0 | 1 | 0 | 1 | 0 | 0 | 0 | 1 | 1 | 0 | 53 | 68 | Carbamidomethylation | C2:Carbamidomethylation:1000.00 | PEAKS DB |
| G.YGGSGTPVDELDR.C | N | 75.26 | 1364.6207 | 13 | 4.1 | 683.3177 | 2 | 45.29 | 14 | F14:22502 | BuCaTN01_F11b.raw | | | | | | | 6.8893E5 | | | | | | | 9.1406E6 | 6.9945E5 | | 3 | 0 | 0 | 0 | 0 | 0 | 0 | 1 | 0 | 0 | 0 | 0 | 0 | 0 | 1 | 1 | 0 | 56 | 68 | | | PEAKS DB |
| R.TAALC(+57.02)FAKAPY.N | N | 73.24 | 1211.6008 | 11 | 2.2 | 606.8076 | 2 | 55.84 | 12 | F12:32023 | BuCaTN01_F10b.raw | | | | | | | 7.3334E4 | | | 1.7166E6 | 1.3753E5 | 1.06E7 | | 8.1407E6 | 9.828E5 | | 7 | 0 | 0 | 0 | 0 | 0 | 0 | 1 | 0 | 0 | 1 | 1 | 2 | 0 | 1 | 1 | 0 | 121 | 131 | Carbamidomethylation | C5:Carbamidomethylation:1000.00 | PEAKS DB |
| T.NYGC(+57.02)YC(+57.02)GYGGSGTPVDELDR.C | N | 72.69 | 2238.8945 | 20 | 5.3 | 1120.4559 | 2 | 64.81 | 14 | F14:40195 | BuCaTN01_F11b.raw | | | | | | | | | | 2.3985E4 | | 0 | 1.1223E5 | 5.3539E5 | | | 3 | 0 | 0 | 0 | 0 | 0 | 0 | 0 | 0 | 0 | 1 | 0 | 0 | 1 | 1 | 0 | 0 | 49 | 68 | Carbamidomethylation | C4:Carbamidomethylation:1000.00;C6:Carbamidomethylation:1000.00 | PEAKS DB |
| R.TAALC(+57.02)FAKAPYN.N | N | 69.35 | 1325.6438 | 12 | -0.2 | 663.8290 | 2 | 51.91 | 6 | F6:30474 | BuCaTN01_F6a.raw | | | | | | 3.7675E6 | | 1.5632E7 | | 4.6358E6 | | 1.9346E6 | | | | | 5 | 0 | 0 | 0 | 0 | 0 | 1 | 0 | 2 | 0 | 1 | 0 | 1 | 0 | 0 | 0 | 0 | 121 | 132 | Carbamidomethylation | C5:Carbamidomethylation:1000.00 | PEAKS DB |
| C.GYGGSGTPVDELDR.C | N | 66.26 | 1421.6422 | 14 | 0.0 | 711.8284 | 2 | 46.82 | 10 | F10:24048 | BuCaTN01_F9a.raw | | | | | | | 2.9103E5 | 1.2275E6 | | 2.1867E6 | | | | 8.7883E6 | | | 5 | 0 | 0 | 0 | 0 | 0 | 0 | 1 | 1 | 0 | 1 | 0 | 0 | 0 | 2 | 0 | 0 | 55 | 68 | | | PEAKS DB |
| N.C(+57.02)DRTAALC(+57.02)FAK.A | N | 63.48 | 1311.6063 | 11 | -1.7 | 438.2076 | 3 | 37.36 | 12 | F12:15762 | BuCaTN01_F10b.raw | | | | | | | | | | | | 5.2815E4 | | 3.1918E5 | | | 2 | 0 | 0 | 0 | 0 | 0 | 0 | 0 | 0 | 0 | 0 | 0 | 1 | 0 | 1 | 0 | 0 | 118 | 128 | Carbamidomethylation | C1:Carbamidomethylation:1000.00;C8:Carbamidomethylation:1000.00 | PEAKS DB |
| R.TAALC(+57.02)FAK.A | N | 60.21 | 880.4476 | 8 | 3.4 | 441.2316 | 2 | 41.58 | 12 | F12:19288 | BuCaTN01_F10b.raw | | | | | | 9.3985E4 | 9.2429E6 | 1.2777E7 | 4.6024E5 | 3.2388E7 | 3.591E6 | 1.265E8 | 3.5731E6 | 1.1283E8 | 1.0943E8 | | 21 | 0 | 0 | 0 | 0 | 0 | 1 | 4 | 2 | 1 | 2 | 2 | 2 | 1 | 2 | 4 | 0 | 121 | 128 | Carbamidomethylation | C5:Carbamidomethylation:1000.00 | PEAKS DB |
| K.DFVC(+57.02)NC(+57.02)DRTAALC(+57.02)FAK.A | N | 59.68 | 1946.8438 | 16 | -0.4 | 974.4265 | 2 | 51.09 | 12 | F12:27773 | BuCaTN01_F10b.raw | | | | | | | | | | 1.1341E6 | | 0 | | | | | 1 | 0 | 0 | 0 | 0 | 0 | 0 | 0 | 0 | 0 | 1 | 0 | 0 | 0 | 0 | 0 | 0 | 113 | 128 | Carbamidomethylation | C4:Carbamidomethylation:1000.00;C6:Carbamidomethylation:1000.00;C13:Carbamidomethylation:1000.00 | PEAKS DB |
| G.GSGTPVDELDR.C | N | 56.36 | 1144.5360 | 11 | 3.4 | 573.2756 | 2 | 40.15 | 8 | F8:17921 | BuCaTN01_F7b.raw | | | | | | | | 1.1068E6 | | | | | | | | | 1 | 0 | 0 | 0 | 0 | 0 | 0 | 0 | 1 | 0 | 0 | 0 | 0 | 0 | 0 | 0 | 0 | 58 | 68 | | | PEAKS DB |
| R.TYSYDC(+57.02)SEGK.L | N | 55.61 | 1208.4656 | 10 | 0.1 | 605.2401 | 2 | 29.72 | 7 | F7:8746 | BuCaTN01_F7a.raw | | | | | | | 5.0914E6 | | | | | | | | | | 1 | 0 | 0 | 0 | 0 | 0 | 0 | 1 | 0 | 0 | 0 | 0 | 0 | 0 | 0 | 0 | 0 | 92 | 101 | Carbamidomethylation | C6:Carbamidomethylation:1000.00 | PEAKS DB |
| N.M(+15.99)IKC(+57.02)TNTRHWLSFTNYGC(+57.02)YC(+57.02)GYGGSGTPVDELDR.C | Y | 53.32 | 4030.7495 | 34 | 1.3 | 1008.6960 | 4 | 77.35 | 10 | F10:53957 | BuCaTN01_F9a.raw | | | | | | | | | | 0 | | | | | | | 0 | 0 | 0 | 0 | 0 | 0 | 0 | 0 | 0 | 0 | 0 | 0 | 0 | 0 | 0 | 0 | 0 | 35 | 68 | Oxidation (M); Carbamidomethylation | M1:Oxidation (M):1000.00;C4:Carbamidomethylation:1000.00;C18:Carbamidomethylation:1000.00;C20:Carbamidomethylation:1000.00 | PEAKS DB |
| R.TYSYDC(+57.02)SEGKLTC(+57.02).K | N | 52.98 | 1582.6279 | 13 | 2.9 | 792.3212 | 2 | 43.19 | 8 | F8:20253 | BuCaTN01_F7b.raw | | | | | | | | 5.7951E5 | | | | | | | | | 1 | 0 | 0 | 0 | 0 | 0 | 0 | 0 | 1 | 0 | 0 | 0 | 0 | 0 | 0 | 0 | 0 | 92 | 104 | Carbamidomethylation | C6:Carbamidomethylation:1000.00;C13:Carbamidomethylation:1000.00 | PEAKS DB |
| R.TYSYDC(+57.02)SEGKLT.C | N | 50.03 | 1422.5973 | 12 | 2.7 | 712.3058 | 2 | 41.55 | 8 | F8:18942 | BuCaTN01_F7b.raw | | | | | | | 5.0044E6 | 1.8313E6 | | | | | | | | | 2 | 0 | 0 | 0 | 0 | 0 | 0 | 1 | 1 | 0 | 0 | 0 | 0 | 0 | 0 | 0 | 0 | 92 | 103 | Carbamidomethylation | C6:Carbamidomethylation:1000.00 | PEAKS DB |
| Y.GGSGTPVDELDR.C | N | 49.09 | 1201.5575 | 12 | 0.3 | 601.7862 | 2 | 40.57 | 11 | F11:14528 | BuCaTN01_F10a.raw | | | | | | | 9.2274E5 | 2.7577E6 | | 1.1335E7 | 6.7591E5 | | 9.9086E5 | 2.6759E7 | | | 6 | 0 | 0 | 0 | 0 | 0 | 0 | 1 | 1 | 0 | 1 | 1 | 0 | 1 | 1 | 0 | 0 | 57 | 68 | | | PEAKS DB |

| | | | | | | | | | | | | | | | | | | | | | | | | | | | | | | | | | | | | | | | | | | | | | | | | | |
|---|---|---|---|---|---|---|---|---|---|---|---|---|---|---|---|---|---|---|---|---|---|---|---|---|---|---|---|---|---|---|---|---|---|---|---|---|---|---|---|---|---|---|---|---|---|---|---|---|---|
| R.TAALC(+57.02)FAKAP.Y | N | 47.25 | 1048.5375 | 10 | 3.6 | 525.2764 | 2 | 48.84 | 8 | F8:25881 | BuCaTN01_F7b.raw | | | | | | 3.1579E6 | | 4.0965E6 | | | | | | | | | 2 | 0 | 0 | 0 | 0 | 0 | 1 | 0 | 1 | 0 | 0 | 0 | 0 | 0 | 0 | 0 | 0 | 121 | 130 | Carbamidomethylation | C5:Carbamidomethylation:1000.00 | PEAKS DB |
| total 22 peptides | | | | | | | | | | | | | | | | | | | | | | | | | | | | | | | | | | | | | | | | | | | | | | | | | |

**Best Unique PSM (Scan F10:53957, m/z=1008.6960, z=4, RT=77.35, ppm=1.3):**

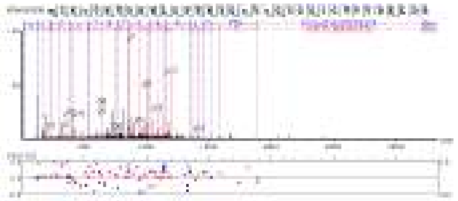

## JAB52774.1

back to list

| Protein Coverage | Supporting Peptides | Best Unique PSM |

**Protein Coverage:**

62 64

1 MNPAHLLVLA AVCVSLLGAS SIPPEPLNLY QFKNMIKCTN TRHWLSFTNY GCYCGYGGSG TPVDELDRCC QVHDKCYDTA

C Carbamidomethylation (+57.02)
O Oxidation (M) (+15.99)

97 104 125

81 KRVHKCFPSV RTYSYDCSEG KLTCKDNNTK CKDFVCNCDR TAALCFAKAP YNNKNFKIDP SRCQ

**Supporting Peptides:**

| Peptide | Uniq | -10lgP | Mass | Length | ppm | m/z | z | RT | Fraction | Scan | Source File | Area F1 | Area F2a | Area F3a | Area F4 | Area F5 | Area F6a | Area F7a | Area F7b | Area F8 | Area F9a | Area Sample 1F10a | Area F10b | Area F11a | Area F11b | Area F12a | Area F13 | #Feature | #Feature F1 | #Feature F2a | #Feature F3a | #Feature F4 | #Feature F5 | #Feature F6a | #Feature F7a | #Feature F7b | #Feature F8 | #Feature F9a | #Feature Sample 1F10a | #Feature F10b | #Feature F11a | #Feature F11b | #Feature F12a | #Feature F13 | Start | End | PTM | AScore | Found By |
|---|---|---|---|---|---|---|---|---|---|---|---|---|---|---|---|---|---|---|---|---|---|---|---|---|---|---|---|---|---|---|---|---|---|---|---|---|---|---|---|---|---|---|---|---|---|---|---|---|---|
| N.YGC(+57.02)YC(+57.02)GYGGSGTPVDELDR.C | N | 112.36 | 2124.8516 | 19 | 3.7 | 1063.4326 | 2 | 56.60 | 14 | F14:33384 | BuCaTN01_F11b.raw | | | 0 | | | 2.0097E6 | | 1.4053E7 | | 8.8531E7 | 3.4891E6 | 4.8054E8 | 6.165E7 | 1.7851E9 | 1.2628E8 | | 69 | 0 | 0 | 0 | 0 | 0 | 1 | 0 | 2 | 0 | 3 | 1 | 4 | 2 | 53 | 3 | 0 | 50 | 68 | Carbamidomethylation | C3:Carbamidomethylation:1000.00;C5:Carbamidomethylation:1000.00 | PEAKS DB |
| Y.C(+57.02)GYGGSGTPVDELDR.C | N | 88.12 | 1581.6729 | 15 | 4.4 | 791.8439 | 2 | 48.02 | 14 | F14:25306 | BuCaTN01_F11b.raw | | | | | | | | 2.166E6 | | 8.5786E6 | | 1.5853E7 | 6.495E5 | 1.7537E7 | 0 | | 6 | 0 | 0 | 0 | 0 | 0 | 0 | 0 | 1 | 0 | 1 | 0 | 2 | 1 | 1 | 0 | 0 | 54 | 68 | Carbamidomethylation | C1:Carbamidomethylation:1000.00 | PEAKS DB |
| G.C(+57.02)YC(+57.02)GYGGSGTPVDELDR.C | N | 84.40 | 1904.7668 | 17 | 2.7 | 953.3911 | 2 | 53.16 | 12 | F12:29351 | BuCaTN01_F10b.raw | | | | | | | | 4.1849E5 | | | | 3.7261E5 | | 2.1774E6 | | | 3 | 0 | 0 | 0 | 0 | 0 | 0 | 0 | 1 | 0 | 0 | 0 | 1 | 0 | 1 | 0 | 0 | 52 | 68 | Carbamidomethylation | C1:Carbamidomethylation:1000.00;C3:Carbamidomethylation:1000.00 | PEAKS DB |
| R.TYSYDC(+57.02)SEGKLTC(+57.02)K.D | N | 82.89 | 1710.7229 | 14 | 0.5 | 856.3691 | 2 | 38.29 | 7 | F7:14505 | BuCaTN01_F7a.raw | | | | | | | 1.4512E8 | 2.3155E7 | | 3.8991E5 | 4.5941E5 | | | | | | 8 | 0 | 0 | 0 | 0 | 0 | 0 | 4 | 2 | 0 | 1 | 1 | 0 | 0 | 0 | 0 | 0 | 92 | 105 | Carbamidomethylation | C6:Carbamidomethylation:1000.00;C13:Carbamidomethylation:1000.00 | PEAKS DB |
| Y.GC(+57.02)YC(+57.02)GYGGSGTPVDELDR.C | N | 82.10 | 1961.7883 | 18 | 1.4 | 981.9028 | 2 | 53.49 | 10 | F10:30232 | BuCaTN01_F9a.raw | | | | | | | | 1.7852E6 | | 3.3077E6 | | 1.5846E6 | | 3.2964E6 | 1.0428E5 | | 5 | 0 | 0 | 0 | 0 | 0 | 0 | 0 | 1 | 0 | 1 | 0 | 1 | 0 | 1 | 1 | 0 | 51 | 68 | Carbamidomethylation | C2:Carbamidomethylation:1000.00;C4:Carbamidomethylation:1000.00 | PEAKS DB |
| R.TYSYDC(+57.02)SEGKLTC(+57.02)KD.N | N | 75.87 | 1825.7499 | 15 | 2.5 | 913.8818 | 2 | 38.60 | 8 | F8:16643 | BuCaTN01_F7b.raw | | | | | | | | 7.3044E5 | | | | | | | | | 1 | 0 | 0 | 0 | 0 | 0 | 0 | 0 | 1 | 0 | 0 | 0 | 0 | 0 | 0 | 0 | 0 | 92 | 106 | Carbamidomethylation | C6:Carbamidomethylation:1000.00;C13:Carbamidomethylation:1000.00 | PEAKS DB |
| C.YC(+57.02)GYGGSGTPVDELDR.C | N | 75.62 | 1744.7362 | 16 | 4.1 | 873.3754 | 2 | 52.27 | 14 | F14:28849 | BuCaTN01_F11b.raw | | | | | | | | 7.7286E5 | | 7.3447E5 | | | | 3.0904E6 | 1.4057E5 | | 4 | 0 | 0 | 0 | 0 | 0 | 0 | 0 | 1 | 0 | 1 | 0 | 0 | 0 | 1 | 1 | 0 | 53 | 68 | Carbamidomethylation | C2:Carbamidomethylation:1000.00 | PEAKS DB |
| G.YGGSGTPVDELDR.C | N | 75.26 | 1364.6207 | 13 | 4.1 | 683.3177 | 2 | 45.29 | 14 | F14:22502 | BuCaTN01_F11b.raw | | | | | | | 6.8893E5 | | | | | | | 9.1406E6 | 6.9945E5 | | 3 | 0 | 0 | 0 | 0 | 0 | 0 | 1 | 0 | 0 | 0 | 0 | 0 | 0 | 1 | 1 | 0 | 56 | 68 | | | PEAKS DB |
| R.TAALC(+57.02)FAKAPY.N | N | 73.24 | 1211.6008 | 11 | 2.2 | 606.8076 | 2 | 55.84 | 12 | F12:32023 | BuCaTN01_F10b.raw | | | | | | | 7.3334E4 | | | 1.7166E6 | 1.3753E5 | 1.06E7 | | 8.1407E6 | 9.828E5 | | 7 | 0 | 0 | 0 | 0 | 0 | 0 | 1 | 0 | 0 | 1 | 1 | 2 | 0 | 1 | 1 | 0 | 121 | 131 | Carbamidomethylation | C5:Carbamidomethylation:1000.00 | PEAKS DB |
| T.NYGC(+57.02)YC(+57.02)GYGGSGTPVDELDR.C | N | 72.69 | 2238.8945 | 20 | 5.3 | 1120.4559 | 2 | 64.81 | 14 | F14:40195 | BuCaTN01_F11b.raw | | | | | | | | | | 2.3985E4 | | 0 | 1.1223E5 | 5.3539E5 | | | 3 | 0 | 0 | 0 | 0 | 0 | 0 | 0 | 0 | 0 | 1 | 0 | 0 | 1 | 1 | 0 | 0 | 49 | 68 | Carbamidomethylation | C4:Carbamidomethylation:1000.00;C6:Carbamidomethylation:1000.00 | PEAKS DB |
| R.TAALC(+57.02)FAKAPYN.N | N | 69.35 | 1325.6438 | 12 | -0.2 | 663.8290 | 2 | 51.91 | 6 | F6:30474 | BuCaTN01_F6a.raw | | | | | | 3.7675E6 | | 1.5632E7 | | 4.6358E6 | | 1.9346E6 | | | | | 5 | 0 | 0 | 0 | 0 | 0 | 1 | 0 | 2 | 0 | 1 | 0 | 1 | 0 | 0 | 0 | 0 | 121 | 132 | Carbamidomethylation | C5:Carbamidomethylation:1000.00 | PEAKS DB |
| C.GYGGSGTPVDELDR.C | N | 66.26 | 1421.6422 | 14 | 0.0 | 711.8284 | 2 | 46.82 | 10 | F10:24048 | BuCaTN01_F9a.raw | | | | | | | 2.9103E5 | 1.2275E6 | | 2.1867E6 | | | | 8.7883E6 | | | 5 | 0 | 0 | 0 | 0 | 0 | 0 | 1 | 1 | 0 | 1 | 0 | 0 | 0 | 2 | 0 | 0 | 55 | 68 | | | PEAKS DB |
| N.C(+57.02)DRTAALC(+57.02)FAK.A | N | 63.48 | 1311.6063 | 11 | -1.7 | 438.2076 | 3 | 37.36 | 12 | F12:15762 | BuCaTN01_F10b.raw | | | | | | | | | | | | 5.2815E4 | | 3.1918E5 | | | 2 | 0 | 0 | 0 | 0 | 0 | 0 | 0 | 0 | 0 | 0 | 0 | 1 | 0 | 1 | 0 | 0 | 118 | 128 | Carbamidomethylation | C1:Carbamidomethylation:1000.00;C8:Carbamidomethylation:1000.00 | PEAKS DB |
| R.TAALC(+57.02)FAK.A | N | 60.21 | 880.4476 | 8 | 3.4 | 441.2316 | 2 | 41.58 | 12 | F12:19288 | BuCaTN01_F10b.raw | | | | | | 9.3985E4 | 9.2429E6 | 1.2777E7 | 4.6024E5 | 3.2388E7 | 3.591E6 | 1.265E8 | 3.5731E6 | 1.1283E8 | 1.0943E8 | | 21 | 0 | 0 | 0 | 0 | 0 | 1 | 4 | 2 | 1 | 2 | 2 | 2 | 1 | 2 | 4 | 0 | 121 | 128 | Carbamidomethylation | C5:Carbamidomethylation:1000.00 | PEAKS DB |
| K.DFVC(+57.02)NC(+57.02)DRTAALC(+57.02)FAK.A | N | 59.68 | 1946.8438 | 16 | -0.4 | 974.4265 | 2 | 51.09 | 12 | F12:27773 | BuCaTN01_F10b.raw | | | | | | | | | | 1.1341E6 | | 0 | | | | | 1 | 0 | 0 | 0 | 0 | 0 | 0 | 0 | 0 | 0 | 1 | 0 | 0 | 0 | 0 | 0 | 0 | 113 | 128 | Carbamidomethylation | C4:Carbamidomethylation:1000.00;C6:Carbamidomethylation:1000.00;C13:Carbamidomethylation:1000.00 | PEAKS DB |
| G.GSGTPVDELDR.C | N | 56.36 | 1144.5360 | 11 | 3.4 | 573.2756 | 2 | 40.15 | 8 | F8:17921 | BuCaTN01_F7b.raw | | | | | | | | 1.1068E6 | | | | | | | | | 1 | 0 | 0 | 0 | 0 | 0 | 0 | 0 | 1 | 0 | 0 | 0 | 0 | 0 | 0 | 0 | 0 | 58 | 68 | | | PEAKS DB |
| R.TYSYDC(+57.02)SEGK.L | N | 55.61 | 1208.4656 | 10 | 0.1 | 605.2401 | 2 | 29.72 | 7 | F7:8746 | BuCaTN01_F7a.raw | | | | | | | 5.0914E6 | | | | | | | | | | 1 | 0 | 0 | 0 | 0 | 0 | 0 | 1 | 0 | 0 | 0 | 0 | 0 | 0 | 0 | 0 | 0 | 92 | 101 | Carbamidomethylation | C6:Carbamidomethylation:1000.00 | PEAKS DB |
| N.M(+15.99)IKC(+57.02)TNTRHWLSFTNYGC(+57.02)YC(+57.02)GYGGSGTPVDELDR.C | Y | 53.32 | 4030.7495 | 34 | 1.3 | 1008.6960 | 4 | 77.35 | 10 | F10:53957 | BuCaTN01_F9a.raw | | | | | | | | | | 0 | | | | | | | 0 | 0 | 0 | 0 | 0 | 0 | 0 | 0 | 0 | 0 | 0 | 0 | 0 | 0 | 0 | 0 | 0 | 35 | 68 | Oxidation (M); Carbamidomethylation | M1:Oxidation (M):1000.00;C4:Carbamidomethylation:1000.00;C18:Carbamidomethylation:1000.00;C20:Carbamidomethylation:1000.00 | PEAKS DB |
| R.TYSYDC(+57.02)SEGKLTC(+57.02).K | N | 52.98 | 1582.6279 | 13 | 2.9 | 792.3212 | 2 | 43.19 | 8 | F8:20253 | BuCaTN01_F7b.raw | | | | | | | | 5.7951E5 | | | | | | | | | 1 | 0 | 0 | 0 | 0 | 0 | 0 | 0 | 1 | 0 | 0 | 0 | 0 | 0 | 0 | 0 | 0 | 92 | 104 | Carbamidomethylation | C6:Carbamidomethylation:1000.00;C13:Carbamidomethylation:1000.00 | PEAKS DB |
| R.TYSYDC(+57.02)SEGKLT.C | N | 50.03 | 1422.5973 | 12 | 2.7 | 712.3058 | 2 | 41.55 | 8 | F8:18942 | BuCaTN01_F7b.raw | | | | | | | 5.0044E6 | 1.8313E6 | | | | | | | | | 2 | 0 | 0 | 0 | 0 | 0 | 0 | 1 | 1 | 0 | 0 | 0 | 0 | 0 | 0 | 0 | 0 | 92 | 103 | Carbamidomethylation | C6:Carbamidomethylation:1000.00 | PEAKS DB |
| Y.GGSGTPVDELDR.C | N | 49.09 | 1201.5575 | 12 | 0.3 | 601.7862 | 2 | 40.57 | 11 | F11:14528 | BuCaTN01_F10a.raw | | | | | | | 9.2274E5 | 2.7577E6 | | 1.1335E7 | 6.7591E5 | | 9.9086E5 | 2.6759E7 | | | 6 | 0 | 0 | 0 | 0 | 0 | 0 | 1 | 1 | 0 | 1 | 1 | 0 | 1 | 1 | 0 | 0 | 57 | 68 | | | PEAKS DB |
| R.TAALC(+57.02)FAKAP.Y | N | 47.25 | 1048.5375 | 10 | 3.6 | 525.2764 | 2 | 48.84 | 8 | F8:25881 | BuCaTN01_F7b.raw | | | | | | 3.1579E6 | | 4.0965E6 | | | | | | | | | 2 | 0 | 0 | 0 | 0 | 0 | 1 | 0 | 1 | 0 | 0 | 0 | 0 | 0 | 0 | 0 | 0 | 121 | 130 | Carbamidomethylation | C5:Carbamidomethylation:1000.00 | PEAKS DB |
| total 22 peptides | | | | | | | | | | | | | | | | | | | | | | | | | | | | | | | | | | | | | | | | | | | | | | | | | |

**Best Unique PSM (Scan F10:53957, m/z=1008.6960, z=4, RT=77.35, ppm=1.3):**

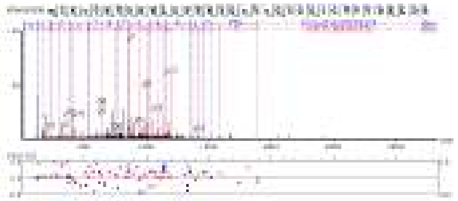

## JAI09006.1

back to list

| Protein Coverage | Supporting Peptides | Best Unique PSM |

**Protein Coverage:**

62 64

1 MNPAHLLVLA AVCVSLLGAS SIPPEPLNLY QFKNMIKCTN TRHWLSFTNY GCYCGYGGSG TPVDELDRCC QVHDKCYDTA

C Carbamidomethylation (+57.02)
O Oxidation (M) (+15.99)

97 104 125

81 KRVHKCFPSV RTYSYDCSEG KLTCKDNNTK CKDFVCNCDR TAALCFAKAP YNNKNFKIDP SRCQ

**Supporting Peptides:**

| [illegible] | [illegible] | [illegible] | [illegible] | [illegible] | [illegible] | [illegible] | [illegible] | [illegible] | [illegible] | [illegible] | [illegible] | Area | Area | Area | Area | Area | Area | Area | Area | [illegible] | Area | Area | Area | Area | Area | Area | Area | [illegible] | #Feature | #Feature | #Feature | #Feature | #Feature | #Feature | #Feature | #Feature | #Feature | #Feature | #Feature | #Feature | #Feature | #Feature | #Feature | #Feature | [illegible] | [illegible] | [illegible] | [illegible] | Found |
|---|---|---|---|---|---|---|---|---|---|---|---|---|---|---|---|---|---|---|---|---|---|---|---|---|---|---|---|---|---|---|---|---|---|---|---|---|---|---|---|---|---|---|---|---|---|---|---|---|---|

| Peptide | Uniq | -10lgP | Mass | Length | ppm | m/z | z | RT | Fraction | Scan | Source File | F1 | F2a | F3a | F4 | F5 | F6a | F7a | F7b | Area F8 | F9a | Sample 1F10a | F10b | F11a | F11b | F12a | F13 | #Feature | F1 | F2a | F3a | F4 | F5 | F6a | F7a | F7b | F8 | F9a | Sample 1F10a | F10b | F11a | F11b | F12a | F13 | Start | End | PTM | AScore | By |
|---|---|---|---|---|---|---|---|---|---|---|---|---|---|---|---|---|---|---|---|---|---|---|---|---|---|---|---|---|---|---|---|---|---|---|---|---|---|---|---|---|---|---|---|---|---|---|---|---|---|
| N.YGC(+57.02)YC(+57.02)GYGGSGTPVDELDR.C | N | 112.36 | 2124.8516 | 19 | 3.7 | 1063.4326 | 2 | 56.60 | 14 | F14:33384 | BuCaTN01_F11b.raw | | | 0 | | | 2.0097E6 | | 1.4053E7 | | 8.8531E7 | 3.4891E6 | 4.8054E8 | 6.165E7 | 1.7851E9 | 1.2628E8 | | 69 | 0 | 0 | 0 | 0 | 0 | 1 | 0 | 2 | 0 | 3 | 1 | 4 | 2 | 53 | 3 | 0 | 50 | 68 | Carbamidomethylation | C3:Carbamidomethylation:1000.00;C5:Carbamidomethylation:1000.00 | PEAKS DB |
| Y.C(+57.02)GYGGSGTPVDELDR.C | N | 88.12 | 1581.6729 | 15 | 4.4 | 791.8439 | 2 | 48.02 | 14 | F14:25306 | BuCaTN01_F11b.raw | | | | | | | | 2.166E6 | | 8.5786E6 | | 1.5853E7 | 6.495E5 | 1.7537E7 | 0 | | 6 | 0 | 0 | 0 | 0 | 0 | 0 | 0 | 1 | 0 | 1 | 0 | 2 | 1 | 1 | 0 | 0 | 54 | 68 | Carbamidomethylation | C1:Carbamidomethylation:1000.00 | PEAKS DB |
| G.C(+57.02)YC(+57.02)GYGGSGTPVDELDR.C | N | 84.40 | 1904.7668 | 17 | 2.7 | 953.3911 | 2 | 53.16 | 12 | F12:29351 | BuCaTN01_F10b.raw | | | | | | | | 4.1849E5 | | | | 3.7261E5 | | 2.1774E6 | | | 3 | 0 | 0 | 0 | 0 | 0 | 0 | 0 | 1 | 0 | 0 | 0 | 1 | 0 | 1 | 0 | 0 | 52 | 68 | Carbamidomethylation | C1:Carbamidomethylation:1000.00;C3:Carbamidomethylation:1000.00 | PEAKS DB |
| R.TYSYDC(+57.02)SEGKLTC(+57.02)K.D | N | 82.89 | 1710.7229 | 14 | 0.5 | 856.3691 | 2 | 38.29 | 7 | F7:14505 | BuCaTN01_F7a.raw | | | | | | | 1.4512E8 | 2.3155E7 | | 3.8991E5 | 4.5941E5 | | | | | | 8 | 0 | 0 | 0 | 0 | 0 | 0 | 4 | 2 | 0 | 1 | 1 | 0 | 0 | 0 | 0 | 0 | 92 | 105 | Carbamidomethylation | C6:Carbamidomethylation:1000.00;C13:Carbamidomethylation:1000.00 | PEAKS DB |
| Y.GC(+57.02)YC(+57.02)GYGGSGTPVDELDR.C | N | 82.10 | 1961.7883 | 18 | 1.4 | 981.9028 | 2 | 53.49 | 10 | F10:30232 | BuCaTN01_F9a.raw | | | | | | | | 1.7852E6 | | 3.3077E6 | | 1.5846E6 | | 3.2964E6 | 1.0428E5 | | 5 | 0 | 0 | 0 | 0 | 0 | 0 | 0 | 1 | 0 | 1 | 0 | 1 | 0 | 1 | 1 | 0 | 51 | 68 | Carbamidomethylation | C2:Carbamidomethylation:1000.00;C4:Carbamidomethylation:1000.00 | PEAKS DB |
| R.TYSYDC(+57.02)SEGKLTC(+57.02)KD.N | N | 75.87 | 1825.7499 | 15 | 2.5 | 913.8818 | 2 | 38.60 | 8 | F8:16643 | BuCaTN01_F7b.raw | | | | | | | | 7.3044E5 | | | | | | | | | 1 | 0 | 0 | 0 | 0 | 0 | 0 | 0 | 1 | 0 | 0 | 0 | 0 | 0 | 0 | 0 | 0 | 92 | 106 | Carbamidomethylation | C6:Carbamidomethylation:1000.00;C13:Carbamidomethylation:1000.00 | PEAKS DB |
| C.YC(+57.02)GYGGSGTPVDELDR.C | N | 75.62 | 1744.7362 | 16 | 4.1 | 873.3754 | 2 | 52.27 | 14 | F14:28849 | BuCaTN01_F11b.raw | | | | | | | | 7.7286E5 | | 7.3447E5 | | | | 3.0904E6 | 1.4057E5 | | 4 | 0 | 0 | 0 | 0 | 0 | 0 | 0 | 1 | 0 | 1 | 0 | 0 | 0 | 1 | 1 | 0 | 53 | 68 | Carbamidomethylation | C2:Carbamidomethylation:1000.00 | PEAKS DB |
| G.YGGSGTPVDELDR.C | N | 75.26 | 1364.6207 | 13 | 4.1 | 683.3177 | 2 | 45.29 | 14 | F14:22502 | BuCaTN01_F11b.raw | | | | | | | 6.8893E5 | | | | | | | 9.1406E6 | 6.9945E5 | | 3 | 0 | 0 | 0 | 0 | 0 | 0 | 1 | 0 | 0 | 0 | 0 | 0 | 0 | 1 | 1 | 0 | 56 | 68 | | | PEAKS DB |
| R.TAALC(+57.02)FAKAPY.N | N | 73.24 | 1211.6008 | 11 | 2.2 | 606.8076 | 2 | 55.84 | 12 | F12:32023 | BuCaTN01_F10b.raw | | | | | | | 7.3334E4 | | | 1.7166E6 | 1.3753E5 | 1.06E7 | | 8.1407E6 | 9.828E5 | | 7 | 0 | 0 | 0 | 0 | 0 | 0 | 1 | 0 | 0 | 1 | 1 | 2 | 0 | 1 | 1 | 0 | 121 | 131 | Carbamidomethylation | C5:Carbamidomethylation:1000.00 | PEAKS DB |
| T.NYGC(+57.02)YC(+57.02)GYGGSGTPVDELDR.C | N | 72.69 | 2238.8945 | 20 | 5.3 | 1120.4559 | 2 | 64.81 | 14 | F14:40195 | BuCaTN01_F11b.raw | | | | | | | | | | 2.3985E4 | | 0 | 1.1223E5 | 5.3539E5 | | | 3 | 0 | 0 | 0 | 0 | 0 | 0 | 0 | 0 | 0 | 1 | 0 | 0 | 1 | 1 | 0 | 0 | 49 | 68 | Carbamidomethylation | C4:Carbamidomethylation:1000.00;C6:Carbamidomethylation:1000.00 | PEAKS DB |
| R.TAALC(+57.02)FAKAPYN.N | N | 69.35 | 1325.6438 | 12 | -0.2 | 663.8290 | 2 | 51.91 | 6 | F6:30474 | BuCaTN01_F6a.raw | | | | | | 3.7675E6 | | 1.5632E7 | | 4.6358E6 | | 1.9346E6 | | | | | 5 | 0 | 0 | 0 | 0 | 0 | 1 | 0 | 2 | 0 | 1 | 0 | 1 | 0 | 0 | 0 | 0 | 121 | 132 | Carbamidomethylation | C5:Carbamidomethylation:1000.00 | PEAKS DB |
| C.GYGGSGTPVDELDR.C | N | 66.26 | 1421.6422 | 14 | 0.0 | 711.8284 | 2 | 46.82 | 10 | F10:24048 | BuCaTN01_F9a.raw | | | | | | | 2.9103E5 | 1.2275E6 | | 2.1867E6 | | | | 8.7883E6 | | | 5 | 0 | 0 | 0 | 0 | 0 | 0 | 1 | 1 | 0 | 1 | 0 | 0 | 0 | 2 | 0 | 0 | 55 | 68 | | | PEAKS DB |
| N.C(+57.02)DRTAALC(+57.02)FAK.A | N | 63.48 | 1311.6063 | 11 | -1.7 | 438.2076 | 3 | 37.36 | 12 | F12:15762 | BuCaTN01_F10b.raw | | | | | | | | | | | | 5.2815E4 | | 3.1918E5 | | | 2 | 0 | 0 | 0 | 0 | 0 | 0 | 0 | 0 | 0 | 0 | 0 | 1 | 0 | 1 | 0 | 0 | 118 | 128 | Carbamidomethylation | C1:Carbamidomethylation:1000.00;C8:Carbamidomethylation:1000.00 | PEAKS DB |
| R.TAALC(+57.02)FAK.A | N | 60.21 | 880.4476 | 8 | 3.4 | 441.2316 | 2 | 41.58 | 12 | F12:19288 | BuCaTN01_F10b.raw | | | | | | 9.3985E4 | 9.2429E6 | 1.2777E7 | 4.6024E5 | 3.2388E7 | 3.591E6 | 1.265E8 | 3.5731E6 | 1.1283E8 | 1.0943E8 | | 21 | 0 | 0 | 0 | 0 | 0 | 1 | 4 | 2 | 1 | 2 | 2 | 2 | 1 | 2 | 4 | 0 | 121 | 128 | Carbamidomethylation | C5:Carbamidomethylation:1000.00 | PEAKS DB |
| K.DFVC(+57.02)NC(+57.02)DRTAALC(+57.02)FAK.A | N | 59.68 | 1946.8438 | 16 | -0.4 | 974.4265 | 2 | 51.09 | 12 | F12:27773 | BuCaTN01_F10b.raw | | | | | | | | | | 1.1341E6 | | 0 | | | | | 1 | 0 | 0 | 0 | 0 | 0 | 0 | 0 | 0 | 0 | 1 | 0 | 0 | 0 | 0 | 0 | 0 | 113 | 128 | Carbamidomethylation | C4:Carbamidomethylation:1000.00;C6:Carbamidomethylation:1000.00;C13:Carbamidomethylation:1000.00 | PEAKS DB |
| G.GSGTPVDELDR.C | N | 56.36 | 1144.5360 | 11 | 3.4 | 573.2756 | 2 | 40.15 | 8 | F8:17921 | BuCaTN01_F7b.raw | | | | | | | | 1.1068E6 | | | | | | | | | 1 | 0 | 0 | 0 | 0 | 0 | 0 | 0 | 1 | 0 | 0 | 0 | 0 | 0 | 0 | 0 | 0 | 58 | 68 | | | PEAKS DB |
| R.TYSYDC(+57.02)SEGK.L | N | 55.61 | 1208.4656 | 10 | 0.1 | 605.2401 | 2 | 29.72 | 7 | F7:8746 | BuCaTN01_F7a.raw | | | | | | | 5.0914E6 | | | | | | | | | | 1 | 0 | 0 | 0 | 0 | 0 | 0 | 1 | 0 | 0 | 0 | 0 | 0 | 0 | 0 | 0 | 0 | 92 | 101 | Carbamidomethylation | C6:Carbamidomethylation:1000.00 | PEAKS DB |
| N.M(+15.99)IKC(+57.02)TNTRHWLSFTNYGC(+57.02)YC(+57.02)GYGGSGTPVDELDR.C | Y | 53.32 | 4030.7495 | 34 | 1.3 | 1008.6960 | 4 | 77.35 | 10 | F10:53957 | BuCaTN01_F9a.raw | | | | | | | | | | 0 | | | | | | | 0 | 0 | 0 | 0 | 0 | 0 | 0 | 0 | 0 | 0 | 0 | 0 | 0 | 0 | 0 | 0 | 0 | 35 | 68 | Oxidation (M); Carbamidomethylation | M1:Oxidation (M):1000.00;C4:Carbamidomethylation:1000.00;C18:Carbamidomethylation:1000.00;C20:Carbamidomethylation:1000.00 | PEAKS DB |
| R.TYSYDC(+57.02)SEGKLTC(+57.02).K | N | 52.98 | 1582.6279 | 13 | 2.9 | 792.3212 | 2 | 43.19 | 8 | F8:20253 | BuCaTN01_F7b.raw | | | | | | | | 5.7951E5 | | | | | | | | | 1 | 0 | 0 | 0 | 0 | 0 | 0 | 0 | 1 | 0 | 0 | 0 | 0 | 0 | 0 | 0 | 0 | 92 | 104 | Carbamidomethylation | C6:Carbamidomethylation:1000.00;C13:Carbamidomethylation:1000.00 | PEAKS DB |
| R.TYSYDC(+57.02)SEGKLT.C | N | 50.03 | 1422.5973 | 12 | 2.7 | 712.3058 | 2 | 41.55 | 8 | F8:18942 | BuCaTN01_F7b.raw | | | | | | | 5.0044E6 | 1.8313E6 | | | | | | | | | 2 | 0 | 0 | 0 | 0 | 0 | 0 | 1 | 1 | 0 | 0 | 0 | 0 | 0 | 0 | 0 | 0 | 92 | 103 | Carbamidomethylation | C6:Carbamidomethylation:1000.00 | PEAKS DB |
| Y.GGSGTPVDELDR.C | N | 49.09 | 1201.5575 | 12 | 0.3 | 601.7862 | 2 | 40.57 | 11 | F11:14528 | BuCaTN01_F10a.raw | | | | | | | 9.2274E5 | 2.7577E6 | | 1.1335E7 | 6.7591E5 | | 9.9086E5 | 2.6759E7 | | | 6 | 0 | 0 | 0 | 0 | 0 | 0 | 1 | 1 | 0 | 1 | 1 | 0 | 1 | 1 | 0 | 0 | 57 | 68 | | | PEAKS DB |
| R.TAALC(+57.02)FAKAP.Y | N | 47.25 | 1048.5375 | 10 | 3.6 | 525.2764 | 2 | 48.84 | 8 | F8:25881 | BuCaTN01_F7b.raw | | | | | | 3.1579E6 | | 4.0965E6 | | | | | | | | | 2 | 0 | 0 | 0 | 0 | 0 | 1 | 0 | 1 | 0 | 0 | 0 | 0 | 0 | 0 | 0 | 0 | 121 | 130 | Carbamidomethylation | C5:Carbamidomethylation:1000.00 | PEAKS DB |
| **total 22 peptides** | | | | | | | | | | | | | | | | | | | | | | | | | | | | | | | | | | | | | | | | | | | | | | | | | |

**Best Unique PSM (Scan F10:53957, m/z=1008.6960, z=4, RT=77.35, ppm=1.3):**

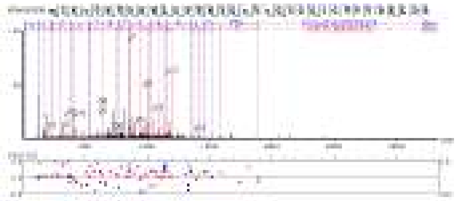

## JAI09009.1

| Protein Coverage | Supporting Peptides | Best Unique PSM |

**Protein Coverage:**

```
 1  MNPAHLLVLA AVCVSLLGAS SIPPEPLNLY QFKNMIKCTN TRHWLSFTNY GCYCGYGGSG TPVDELDRCC QVHDKCYDTA
81  KRVHKCFPSV RTYSYDCSEG KLTCKDNNTK CKDFVCNCDR TAALCFAKAP YNNKNFKIDP SHCQ
```

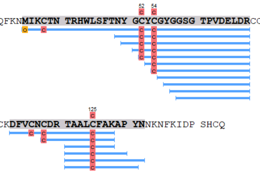


Carbamidomethylation (+57.02)
Oxidation (M) (+15.99)

**Supporting Peptides:**

| Peptide | Uniq | -10lgP | Mass | Length | ppm | m/z | z | RT | Fraction | Scan | Source File | Area F1 | Area F2a | Area F3a | Area F4 | Area F5 | Area F6a | Area F7a | Area F7b | Area F8 | Area F9a | Area Sample 1F10a | Area F10b | Area F11a | Area F11b | Area F12a | Area F13 | #Feature | #Feature F1 | #Feature F2a | #Feature F3a | #Feature F4 | #Feature F5 | #Feature F6a | #Feature F7a | #Feature F7b | #Feature F8 | #Feature F9a | #Feature Sample 1F10a | #Feature F10b | #Feature F11a | #Feature F11b | #Feature F12a | #Feature F13 | Start | End | PTM | AScore | Found By |
|---|---|---|---|---|---|---|---|---|---|---|---|---|---|---|---|---|---|---|---|---|---|---|---|---|---|---|---|---|---|---|---|---|---|---|---|---|---|---|---|---|---|---|---|---|---|---|---|---|---|
| N.YGC(+57.02)YC(+57.02)GYGGSGTPVDELDR.C | N | 112.36 | 2124.8516 | 19 | 3.7 | 1063.4326 | 2 | 56.60 | 14 | F14:33384 | BuCaTN01_F11b.raw | | | 0 | | | 2.0097E6 | | 1.4053E7 | | 8.8531E7 | 3.4891E6 | 4.8054E8 | 6.165E7 | 1.7851E9 | 1.2628E8 | | 69 | 0 | 0 | 0 | 0 | 0 | 1 | 0 | 2 | 0 | 3 | 1 | 4 | 2 | 53 | 3 | 0 | 50 | 68 | Carbamidomethylation | C3:Carbamidomethylation:1000.00;C5:Carbamidomethylation:1000.00 | PEAKS DB |
| Y.C(+57.02)GYGGSGTPVDELDR.C | N | 88.12 | 1581.6729 | 15 | 4.4 | 791.8439 | 2 | 48.02 | 14 | F14:25306 | BuCaTN01_F11b.raw | | | | | | | | 2.166E6 | | 8.5786E6 | | 1.5853E7 | 6.495E5 | 1.7537E7 | 0 | | 6 | 0 | 0 | 0 | 0 | 0 | 0 | 0 | 1 | 0 | 1 | 0 | 2 | 1 | 1 | 0 | 0 | 54 | 68 | Carbamidomethylation | C1:Carbamidomethylation:1000.00 | PEAKS DB |
| G.C(+57.02)YC(+57.02)GYGGSGTPVDELDR.C | N | 84.40 | 1904.7668 | 17 | 2.7 | 953.3911 | 2 | 53.16 | 12 | F12:29351 | BuCaTN01_F10b.raw | | | | | | | | 4.1849E5 | | | | 3.7261E5 | | 2.1774E6 | | | 3 | 0 | 0 | 0 | 0 | 0 | 0 | 0 | 1 | 0 | 0 | 0 | 1 | 0 | 1 | 0 | 0 | 52 | 68 | Carbamidomethylation | C1:Carbamidomethylation:1000.00;C3:Carbamidomethylation:1000.00 | PEAKS DB |
| R.TYSYDC(+57.02)SEGKLTC(+57.02)K.D | N | 82.89 | 1710.7229 | 14 | 0.5 | 856.3691 | 2 | 38.29 | 7 | F7:14505 | BuCaTN01_F7a.raw | | | | | | | 1.4512E8 | 2.3155E7 | | 3.8991E5 | 4.5941E5 | | | | | | 8 | 0 | 0 | 0 | 0 | 0 | 0 | 4 | 2 | 0 | 1 | 1 | 0 | 0 | 0 | 0 | 0 | 92 | 105 | Carbamidomethylation | C6:Carbamidomethylation:1000.00;C13:Carbamidomethylation:1000.00 | PEAKS DB |
| Y.GC(+57.02)YC(+57.02)GYGGSGTPVDELDR.C | N | 82.10 | 1961.7883 | 18 | 1.4 | 981.9028 | 2 | 53.49 | 10 | F10:30232 | BuCaTN01_F9a.raw | | | | | | | | 1.7852E6 | | 3.3077E6 | | 1.5846E6 | | 3.2964E6 | 1.0428E5 | | 5 | 0 | 0 | 0 | 0 | 0 | 0 | 0 | 1 | 0 | 1 | 0 | 1 | 0 | 1 | 1 | 0 | 51 | 68 | Carbamidomethylation | C2:Carbamidomethylation:1000.00;C4:Carbamidomethylation:1000.00 | PEAKS DB |
| R.TYSYDC(+57.02)SEGKLTC(+57.02)KD.N | N | 75.87 | 1825.7499 | 15 | 2.5 | 913.8818 | 2 | 38.60 | 8 | F8:16643 | BuCaTN01_F7b.raw | | | | | | | | 7.3044E5 | | | | | | | | | 1 | 0 | 0 | 0 | 0 | 0 | 0 | 0 | 1 | 0 | 0 | 0 | 0 | 0 | 0 | 0 | 0 | 92 | 106 | Carbamidomethylation | C6:Carbamidomethylation:1000.00;C13:Carbamidomethylation:1000.00 | PEAKS DB |
| C.YC(+57.02)GYGGSGTPVDELDR.C | N | 75.62 | 1744.7362 | 16 | 4.1 | 873.3754 | 2 | 52.27 | 14 | F14:28849 | BuCaTN01_F11b.raw | | | | | | | | 7.7286E5 | | 7.3447E5 | | | | 3.0904E6 | 1.4057E5 | | 4 | 0 | 0 | 0 | 0 | 0 | 0 | 0 | 1 | 0 | 1 | 0 | 0 | 0 | 1 | 1 | 0 | 53 | 68 | Carbamidomethylation | C2:Carbamidomethylation:1000.00 | PEAKS DB |
| G.YGGSGTPVDELDR.C | N | 75.26 | 1364.6207 | 13 | 4.1 | 683.3177 | 2 | 45.29 | 14 | F14:22502 | BuCaTN01_F11b.raw | | | | | | | 6.8893E5 | | | | | | | 9.1406E6 | 6.9945E5 | | 3 | 0 | 0 | 0 | 0 | 0 | 0 | 1 | 0 | 0 | 0 | 0 | 0 | 0 | 1 | 1 | 0 | 56 | 68 | | | PEAKS DB |
| R.TAALC(+57.02)FAKAPY.N | N | 73.24 | 1211.6008 | 11 | 2.2 | 606.8076 | 2 | 55.84 | 12 | F12:32023 | BuCaTN01_F10b.raw | | | | | | | 7.3334E4 | | | 1.7166E6 | 1.3753E5 | 1.06E7 | | 8.1407E6 | 9.828E5 | | 7 | 0 | 0 | 0 | 0 | 0 | 0 | 1 | 0 | 0 | 1 | 1 | 2 | 0 | 1 | 1 | 0 | 121 | 131 | Carbamidomethylation | C5:Carbamidomethylation:1000.00 | PEAKS DB |
| T.NYGC(+57.02)YC(+57.02)GYGGSGTPVDELDR.C | N | 72.69 | 2238.8945 | 20 | 5.3 | 1120.4559 | 2 | 64.81 | 14 | F14:40195 | BuCaTN01_F11b.raw | | | | | | | | | | 2.3985E4 | | 0 | 1.1223E5 | 5.3539E5 | | | 3 | 0 | 0 | 0 | 0 | 0 | 0 | 0 | 0 | 0 | 1 | 0 | 0 | 1 | 1 | 0 | 0 | 49 | 68 | Carbamidomethylation | C4:Carbamidomethylation:1000.00;C6:Carbamidomethylation:1000.00 | PEAKS DB |
| R.TAALC(+57.02)FAKAPYN.N | N | 69.35 | 1325.6438 | 12 | -0.2 | 663.8290 | 2 | 51.91 | 6 | F6:30474 | BuCaTN01_F6a.raw | | | | | | 3.7675E6 | | 1.5632E7 | | 4.6358E6 | | 1.9346E6 | | | | | 5 | 0 | 0 | 0 | 0 | 0 | 1 | 0 | 2 | 0 | 1 | 0 | 1 | 0 | 0 | 0 | 0 | 121 | 132 | Carbamidomethylation | C5:Carbamidomethylation:1000.00 | PEAKS DB |
| C.GYGGSGTPVDELDR.C | N | 66.26 | 1421.6422 | 14 | 0.0 | 711.8284 | 2 | 46.82 | 10 | F10:24048 | BuCaTN01_F9a.raw | | | | | | | 2.9103E5 | 1.2275E6 | | 2.1867E6 | | | | 8.7883E6 | | | 5 | 0 | 0 | 0 | 0 | 0 | 0 | 1 | 1 | 0 | 1 | 0 | 0 | 0 | 2 | 0 | 0 | 55 | 68 | | | PEAKS DB |
| N.C(+57.02)DRTAALC(+57.02)FAK.A | N | 63.48 | 1311.6063 | 11 | -1.7 | 438.2076 | 3 | 37.36 | 12 | F12:15762 | BuCaTN01_F10b.raw | | | | | | | | | | | | 5.2815E4 | | 3.1918E5 | | | 2 | 0 | 0 | 0 | 0 | 0 | 0 | 0 | 0 | 0 | 0 | 0 | 1 | 0 | 1 | 0 | 0 | 118 | 128 | Carbamidomethylation | C1:Carbamidomethylation:1000.00;C8:Carbamidomethylation:1000.00 | PEAKS DB |
| R.TAALC(+57.02)FAK.A | N | 60.21 | 880.4476 | 8 | 3.4 | 441.2316 | 2 | 41.58 | 12 | F12:19288 | BuCaTN01_F10b.raw | | | | | | 9.3985E4 | 9.2429E6 | 1.2777E7 | 4.6024E5 | 3.2388E7 | 3.591E6 | 1.265E8 | 3.5731E6 | 1.1283E8 | 1.0943E8 | | 21 | 0 | 0 | 0 | 0 | 0 | 1 | 4 | 2 | 1 | 2 | 2 | 2 | 1 | 2 | 4 | 0 | 121 | 128 | Carbamidomethylation | C5:Carbamidomethylation:1000.00 | PEAKS DB |
| K.DFVC(+57.02)NC(+57.02)DRTAALC(+57.02)FAK.A | N | 59.68 | 1946.8438 | 16 | -0.4 | 974.4265 | 2 | 51.09 | 12 | F12:27773 | BuCaTN01_F10b.raw | | | | | | | | | | 1.1341E6 | | 0 | | | | | 1 | 0 | 0 | 0 | 0 | 0 | 0 | 0 | 0 | 0 | 1 | 0 | 0 | 0 | 0 | 0 | 0 | 113 | 128 | Carbamidomethylation | C4:Carbamidomethylation:1000.00;C6:Carbamidomethylation:1000.00;C13:Carbamidomethylation:1000.00 | PEAKS DB |
| G.GSGTPVDELDR.C | N | 56.36 | 1144.5360 | 11 | 3.4 | 573.2756 | 2 | 40.15 | 8 | F8:17921 | BuCaTN01_F7b.raw | | | | | | | | 1.1068E6 | | | | | | | | | 1 | 0 | 0 | 0 | 0 | 0 | 0 | 0 | 1 | 0 | 0 | 0 | 0 | 0 | 0 | 0 | 0 | 58 | 68 | | | PEAKS DB |
| R.TYSYDC(+57.02)SEGK.L | N | 55.61 | 1208.4656 | 10 | 0.1 | 605.2401 | 2 | 29.72 | 7 | F7:8746 | BuCaTN01_F7a.raw | | | | | | | 5.0914E6 | | | | | | | | | | 1 | 0 | 0 | 0 | 0 | 0 | 0 | 1 | 0 | 0 | 0 | 0 | 0 | 0 | 0 | 0 | 0 | 92 | 101 | Carbamidomethylation | C6:Carbamidomethylation:1000.00 | PEAKS DB |
| N.M(+15.99)IKC(+57.02)TNTRHWLSFTNYGC(+57.02)YC(+57.02)GYGGSGTPVDELDR.C | Y | 53.32 | 4030.7495 | 34 | 1.3 | 1008.6960 | 4 | 77.35 | 10 | F10:53957 | BuCaTN01_F9a.raw | | | | | | | | | | 0 | | | | | | | 0 | 0 | 0 | 0 | 0 | 0 | 0 | 0 | 0 | 0 | 0 | 0 | 0 | 0 | 0 | 0 | 0 | 35 | 68 | Oxidation (M); Carbamidomethylation | M1:Oxidation (M):1000.00;C4:Carbamidomethylation:1000.00;C18:Carbamidomethylation:1000.00;C20:Carbamidomethylation:1000.00 | PEAKS DB |
| R.TYSYDC(+57.02)SEGKLTC(+57.02).K | N | 52.98 | 1582.6279 | 13 | 2.9 | 792.3212 | 2 | 43.19 | 8 | F8:20253 | BuCaTN01_F7b.raw | | | | | | | | 5.7951E5 | | | | | | | | | 1 | 0 | 0 | 0 | 0 | 0 | 0 | 0 | 1 | 0 | 0 | 0 | 0 | 0 | 0 | 0 | 0 | 92 | 104 | Carbamidomethylation | C6:Carbamidomethylation:1000.00;C13:Carbamidomethylation:1000.00 | PEAKS DB |
| R.TYSYDC(+57.02)SEGKLT.C | N | 50.03 | 1422.5973 | 12 | 2.7 | 712.3058 | 2 | 41.55 | 8 | F8:18942 | BuCaTN01_F7b.raw | | | | | | | 5.0044E6 | 1.8313E6 | | | | | | | | | 2 | 0 | 0 | 0 | 0 | 0 | 0 | 1 | 1 | 0 | 0 | 0 | 0 | 0 | 0 | 0 | 0 | 92 | 103 | Carbamidomethylation | C6:Carbamidomethylation:1000.00 | PEAKS DB |

| Peptide | Uniq | -10lgP | Mass | Length | ppm | m/z | z | RT | Fraction | Scan | Source File | Area F1 | Area F2a | Area F3a | Area F4 | Area F5 | Area F6a | Area F7a | Area F7b | Area F8 | Area F9a | Area Sample 1F10a | Area F10b | Area F11a | Area F11b | Area F12a | Area F13 | #Feature | #Feature F1 | #Feature F2a | #Feature F3a | #Feature F4 | #Feature F5 | #Feature F6a | #Feature F7a | #Feature F7b | #Feature F8 | #Feature F9a | #Feature Sample 1F10a | #Feature F10b | #Feature F11a | #Feature F11b | #Feature F12a | #Feature F13 | Start | End | PTM | AScore | Found By |
|---|---|---|---|---|---|---|---|---|---|---|---|---|---|---|---|---|---|---|---|---|---|---|---|---|---|---|---|---|---|---|---|---|---|---|---|---|---|---|---|---|---|---|---|---|---|---|---|---|---|
| Y.GGSGTPVDELDR.C | N | 49.09 | 1201.5575 | 12 | 0.3 | 601.7862 | 2 | 40.57 | 11 | F11:14528 | BuCaTN01_F10a.raw | | | | | | | 9.2274E5 | 2.7577E6 | | 1.1335E7 | 6.7591E5 | | 9.9086E5 | 2.6759E7 | | | 6 | 0 | 0 | 0 | 0 | 0 | 0 | 1 | 1 | 0 | 1 | 1 | 0 | 1 | 1 | 0 | 0 | 57 | 68 | | | PEAKS DB |
| R.TAALC(+57.02)FAKAP.Y | N | 47.25 | 1048.5375 | 10 | 3.6 | 525.2764 | 2 | 48.84 | 8 | F8:25881 | BuCaTN01_F7b.raw | | | | | | 3.1579E6 | | 4.0965E6 | | | | | | | | | 2 | 0 | 0 | 0 | 0 | 0 | 1 | 0 | 1 | 0 | 0 | 0 | 0 | 0 | 0 | 0 | 0 | 121 | 130 | Carbamidomethylation | C5:Carbamidomethylation:1000.00 | PEAKS DB |
| total 22 peptides | | | | | | | | | | | | | | | | | | | | | | | | | | | | | | | | | | | | | | | | | | | | | | | | | |

**Best Unique PSM (Scan F10:53957, m/z=1008.6960, z=4, RT=77.35, ppm=1.3):**

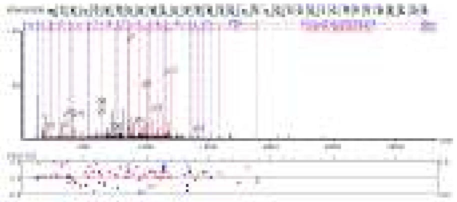

## JAB52771.1

back to list

| Protein Coverage | Supporting Peptides | Best Unique PSM |

**Protein Coverage:**

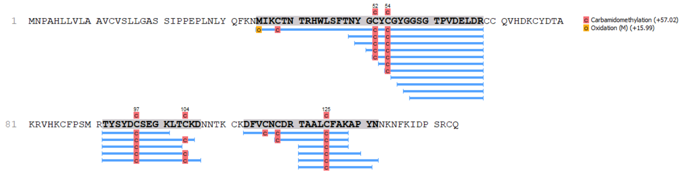


**Supporting Peptides:**

| Peptide | Uniq | -10lgP | Mass | Length | ppm | m/z | z | RT | Fraction | Scan | Source File | Area F1 | Area F2a | Area F3a | Area F4 | Area F5 | Area F6a | Area F7a | Area F7b | Area F8 | Area F9a | Area Sample 1F10a | Area F10b | Area F11a | Area F11b | Area F12a | Area F13 | #Feature | #Feature F1 | #Feature F2a | #Feature F3a | #Feature F4 | #Feature F5 | #Feature F6a | #Feature F7a | #Feature F7b | #Feature F8 | #Feature F9a | #Feature Sample 1F10a | #Feature F10b | #Feature F11a | #Feature F11b | #Feature F12a | #Feature F13 | Start | End | PTM | AScore | Found By |
|---|---|---|---|---|---|---|---|---|---|---|---|---|---|---|---|---|---|---|---|---|---|---|---|---|---|---|---|---|---|---|---|---|---|---|---|---|---|---|---|---|---|---|---|---|---|---|---|---|---|
| N.YGC(+57.02)YC(+57.02)GYGGSGTPVDELDR.C | N | 112.36 | 2124.8516 | 19 | 3.7 | 1063.4326 | 2 | 56.60 | 14 | F14:33384 | BuCaTN01_F11b.raw | | | 0 | | | 2.0097E6 | | 1.4053E7 | | 8.8531E7 | 3.4891E6 | 4.8054E8 | 6.165E7 | 1.7851E9 | 1.2628E8 | | 69 | 0 | 0 | 0 | 0 | 0 | 1 | 0 | 2 | 0 | 3 | 1 | 4 | 2 | 53 | 3 | 0 | 50 | 68 | Carbamidomethylation | C3:Carbamidomethylation:1000.00;C5:Carbamidomethylation:1000.00 | PEAKS DB |
| Y.C(+57.02)GYGGSGTPVDELDR.C | N | 88.12 | 1581.6729 | 15 | 4.4 | 791.8439 | 2 | 48.02 | 14 | F14:25306 | BuCaTN01_F11b.raw | | | | | | | | 2.166E6 | | 8.5786E6 | | 1.5853E7 | 6.495E5 | 1.7537E7 | 0 | | 6 | 0 | 0 | 0 | 0 | 0 | 0 | 0 | 1 | 0 | 1 | 0 | 2 | 1 | 1 | 0 | 0 | 54 | 68 | Carbamidomethylation | C1:Carbamidomethylation:1000.00 | PEAKS DB |
| G.C(+57.02)YC(+57.02)GYGGSGTPVDELDR.C | N | 84.40 | 1904.7668 | 17 | 2.7 | 953.3911 | 2 | 53.16 | 12 | F12:29351 | BuCaTN01_F10b.raw | | | | | | | | 4.1849E5 | | | | 3.7261E5 | | 2.1774E6 | | | 3 | 0 | 0 | 0 | 0 | 0 | 0 | 0 | 1 | 0 | 0 | 0 | 1 | 0 | 1 | 0 | 0 | 52 | 68 | Carbamidomethylation | C1:Carbamidomethylation:1000.00;C3:Carbamidomethylation:1000.00 | PEAKS DB |
| R.TYSYDC(+57.02)SEGKLTC(+57.02)K.D | N | 82.89 | 1710.7229 | 14 | 0.5 | 856.3691 | 2 | 38.29 | 7 | F7:14505 | BuCaTN01_F7a.raw | | | | | | | 1.4512E8 | 2.3155E7 | | 3.8991E5 | 4.5941E5 | | | | | | 8 | 0 | 0 | 0 | 0 | 0 | 0 | 4 | 2 | 0 | 1 | 1 | 0 | 0 | 0 | 0 | 0 | 92 | 105 | Carbamidomethylation | C6:Carbamidomethylation:1000.00;C13:Carbamidomethylation:1000.00 | PEAKS DB |
| Y.GC(+57.02)YC(+57.02)GYGGSGTPVDELDR.C | N | 82.10 | 1961.7883 | 18 | 1.4 | 981.9028 | 2 | 53.49 | 10 | F10:30232 | BuCaTN01_F9a.raw | | | | | | | | 1.7852E6 | | 3.3077E6 | | 1.5846E6 | | 3.2964E6 | 1.0428E5 | | 5 | 0 | 0 | 0 | 0 | 0 | 0 | 0 | 1 | 0 | 1 | 0 | 1 | 0 | 1 | 1 | 0 | 51 | 68 | Carbamidomethylation | C2:Carbamidomethylation:1000.00;C4:Carbamidomethylation:1000.00 | PEAKS DB |
| R.TYSYDC(+57.02)SEGKLTC(+57.02)KD.N | N | 75.87 | 1825.7499 | 15 | 2.5 | 913.8818 | 2 | 38.60 | 8 | F8:16643 | BuCaTN01_F7b.raw | | | | | | | | 7.3044E5 | | | | | | | | | 1 | 0 | 0 | 0 | 0 | 0 | 0 | 0 | 1 | 0 | 0 | 0 | 0 | 0 | 0 | 0 | 0 | 92 | 106 | Carbamidomethylation | C6:Carbamidomethylation:1000.00;C13:Carbamidomethylation:1000.00 | PEAKS DB |
| C.YC(+57.02)GYGGSGTPVDELDR.C | N | 75.62 | 1744.7362 | 16 | 4.1 | 873.3754 | 2 | 52.27 | 14 | F14:28849 | BuCaTN01_F11b.raw | | | | | | | | 7.7286E5 | | 7.3447E5 | | | | 3.0904E6 | 1.4057E5 | | 4 | 0 | 0 | 0 | 0 | 0 | 0 | 0 | 1 | 0 | 1 | 0 | 0 | 0 | 1 | 1 | 0 | 53 | 68 | Carbamidomethylation | C2:Carbamidomethylation:1000.00 | PEAKS DB |
| G.YGGSGTPVDELDR.C | N | 75.26 | 1364.6207 | 13 | 4.1 | 683.3177 | 2 | 45.29 | 14 | F14:22502 | BuCaTN01_F11b.raw | | | | | | | 6.8893E5 | | | | | | | 9.1406E6 | 6.9945E5 | | 3 | 0 | 0 | 0 | 0 | 0 | 0 | 1 | 0 | 0 | 0 | 0 | 0 | 0 | 1 | 1 | 0 | 56 | 68 | | | PEAKS DB |
| R.TAALC(+57.02)FAKAPY.N | N | 73.24 | 1211.6008 | 11 | 2.2 | 606.8076 | 2 | 55.84 | 12 | F12:32023 | BuCaTN01_F10b.raw | | | | | | | 7.3334E4 | | | 1.7166E6 | 1.3753E5 | 1.06E7 | | 8.1407E6 | 9.828E5 | | 7 | 0 | 0 | 0 | 0 | 0 | 0 | 1 | 0 | 0 | 1 | 1 | 2 | 0 | 1 | 1 | 0 | 121 | 131 | Carbamidomethylation | C5:Carbamidomethylation:1000.00 | PEAKS DB |
| T.NYGC(+57.02)YC(+57.02)GYGGSGTPVDELDR.C | N | 72.69 | 2238.8945 | 20 | 5.3 | 1120.4559 | 2 | 64.81 | 14 | F14:40195 | BuCaTN01_F11b.raw | | | | | | | | | | 2.3985E4 | | 0 | 1.1223E5 | 5.3539E5 | | | 3 | 0 | 0 | 0 | 0 | 0 | 0 | 0 | 0 | 0 | 1 | 0 | 0 | 1 | 1 | 0 | 0 | 49 | 68 | Carbamidomethylation | C4:Carbamidomethylation:1000.00;C6:Carbamidomethylation:1000.00 | PEAKS DB |
| R.TAALC(+57.02)FAKAPYN.N | N | 69.35 | 1325.6438 | 12 | -0.2 | 663.8290 | 2 | 51.91 | 6 | F6:30474 | BuCaTN01_F6a.raw | | | | | | 3.7675E6 | | 1.5632E7 | | 4.6358E6 | | 1.9346E6 | | | | | 5 | 0 | 0 | 0 | 0 | 0 | 1 | 0 | 2 | 0 | 1 | 0 | 1 | 0 | 0 | 0 | 0 | 121 | 132 | Carbamidomethylation | C5:Carbamidomethylation:1000.00 | PEAKS DB |
| C.GYGGSGTPVDELDR.C | N | 66.26 | 1421.6422 | 14 | 0.0 | 711.8284 | 2 | 46.82 | 10 | F10:24048 | BuCaTN01_F9a.raw | | | | | | | 2.9103E5 | 1.2275E6 | | 2.1867E6 | | | | 8.7883E6 | | | 5 | 0 | 0 | 0 | 0 | 0 | 0 | 1 | 1 | 0 | 1 | 0 | 0 | 0 | 2 | 0 | 0 | 55 | 68 | | | PEAKS DB |
| N.C(+57.02)DRTAALC(+57.02)FAK.A | N | 63.48 | 1311.6063 | 11 | -1.7 | 438.2076 | 3 | 37.36 | 12 | F12:15762 | BuCaTN01_F10b.raw | | | | | | | | | | | | 5.2815E4 | | 3.1918E5 | | | 2 | 0 | 0 | 0 | 0 | 0 | 0 | 0 | 0 | 0 | 0 | 0 | 1 | 0 | 1 | 0 | 0 | 118 | 128 | Carbamidomethylation | C1:Carbamidomethylation:1000.00;C8:Carbamidomethylation:1000.00 | PEAKS DB |
| R.TAALC(+57.02)FAK.A | N | 60.21 | 880.4476 | 8 | 3.4 | 441.2316 | 2 | 41.58 | 12 | F12:19288 | BuCaTN01_F10b.raw | | | | | | 9.3985E4 | 9.2429E6 | 1.2777E7 | 4.6024E5 | 3.2388E7 | 3.591E6 | 1.265E8 | 3.5731E6 | 1.1283E8 | 1.0943E8 | | 21 | 0 | 0 | 0 | 0 | 0 | 1 | 4 | 2 | 1 | 2 | 2 | 2 | 1 | 2 | 4 | 0 | 121 | 128 | Carbamidomethylation | C5:Carbamidomethylation:1000.00 | PEAKS DB |
| K.DFVC(+57.02)NC(+57.02)DRTAALC(+57.02)FAK.A | N | 59.68 | 1946.8438 | 16 | -0.4 | 974.4265 | 2 | 51.09 | 12 | F12:27773 | BuCaTN01_F10b.raw | | | | | | | | | | 1.1341E6 | | 0 | | | | | 1 | 0 | 0 | 0 | 0 | 0 | 0 | 0 | 0 | 0 | 1 | 0 | 0 | 0 | 0 | 0 | 0 | 113 | 128 | Carbamidomethylation | C4:Carbamidomethylation:1000.00;C6:Carbamidomethylation:1000.00;C13:Carbamidomethylation:1000.00 | PEAKS DB |
| G.GSGTPVDELDR.C | N | 56.36 | 1144.5360 | 11 | 3.4 | 573.2756 | 2 | 40.15 | 8 | F8:17921 | BuCaTN01_F7b.raw | | | | | | | | 1.1068E6 | | | | | | | | | 1 | 0 | 0 | 0 | 0 | 0 | 0 | 0 | 1 | 0 | 0 | 0 | 0 | 0 | 0 | 0 | 0 | 58 | 68 | | | PEAKS DB |
| R.TYSYDC(+57.02)SEGK.L | N | 55.61 | 1208.4656 | 10 | 0.1 | 605.2401 | 2 | 29.72 | 7 | F7:8746 | BuCaTN01_F7a.raw | | | | | | | 5.0914E6 | | | | | | | | | | 1 | 0 | 0 | 0 | 0 | 0 | 0 | 1 | 0 | 0 | 0 | 0 | 0 | 0 | 0 | 0 | 0 | 92 | 101 | Carbamidomethylation | C6:Carbamidomethylation:1000.00 | PEAKS DB |
| N.M(+15.99)IKC(+57.02)TNTRHWLSFTNYGC(+57.02)YC(+57.02)GYGGSGTPVDELDR.C | Y | 53.32 | 4030.7495 | 34 | 1.3 | 1008.6960 | 4 | 77.35 | 10 | F10:53957 | BuCaTN01_F9a.raw | | | | | | | | | | 0 | | | | | | | 0 | 0 | 0 | 0 | 0 | 0 | 0 | 0 | 0 | 0 | 0 | 0 | 0 | 0 | 0 | 0 | 0 | 35 | 68 | Oxidation (M); Carbamidomethylation | M1:Oxidation (M):1000.00;C4:Carbamidomethylation:1000.00;C18:Carbamidomethylation:1000.00;C20:Carbamidomethylation:1000.00 | PEAKS DB |
| R.TYSYDC(+57.02)SEGKLTC(+57.02).K | N | 52.98 | 1582.6279 | 13 | 2.9 | 792.3212 | 2 | 43.19 | 8 | F8:20253 | BuCaTN01_F7b.raw | | | | | | | | 5.7951E5 | | | | | | | | | 1 | 0 | 0 | 0 | 0 | 0 | 0 | 0 | 1 | 0 | 0 | 0 | 0 | 0 | 0 | 0 | 0 | 92 | 104 | Carbamidomethylation | C6:Carbamidomethylation:1000.00;C13:Carbamidomethylation:1000.00 | PEAKS DB |
| R.TYSYDC(+57.02)SEGKLT.C | N | 50.03 | 1422.5973 | 12 | 2.7 | 712.3058 | 2 | 41.55 | 8 | F8:18942 | BuCaTN01_F7b.raw | | | | | | | 5.0044E6 | 1.8313E6 | | | | | | | | | 2 | 0 | 0 | 0 | 0 | 0 | 0 | 1 | 1 | 0 | 0 | 0 | 0 | 0 | 0 | 0 | 0 | 92 | 103 | Carbamidomethylation | C6:Carbamidomethylation:1000.00 | PEAKS DB |
| Y.GGSGTPVDELDR.C | N | 49.09 | 1201.5575 | 12 | 0.3 | 601.7862 | 2 | 40.57 | 11 | F11:14528 | BuCaTN01_F10a.raw | | | | | | | 9.2274E5 | 2.7577E6 | | 1.1335E7 | 6.7591E5 | | 9.9086E5 | 2.6759E7 | | | 6 | 0 | 0 | 0 | 0 | 0 | 0 | 1 | 1 | 0 | 1 | 1 | 0 | 1 | 1 | 0 | 0 | 57 | 68 | | | PEAKS DB |
| R.TAALC(+57.02)FAKAP.Y | N | 47.25 | 1048.5375 | 10 | 3.6 | 525.2764 | 2 | 48.84 | 8 | F8:25881 | BuCaTN01_F7b.raw | | | | | | 3.1579E6 | | 4.0965E6 | | | | | | | | | 2 | 0 | 0 | 0 | 0 | 0 | 1 | 0 | 1 | 0 | 0 | 0 | 0 | 0 | 0 | 0 | 0 | 121 | 130 | Carbamidomethylation | C5:Carbamidomethylation:1000.00 | PEAKS DB |
| total 22 peptides | | | | | | | | | | | | | | | | | | | | | | | | | | | | | | | | | | | | | | | | | | | | | | | | | |

**Best Unique PSM (Scan F10:53957, m/z=1008.6960, z=4, RT=77.35, ppm=1.3):**

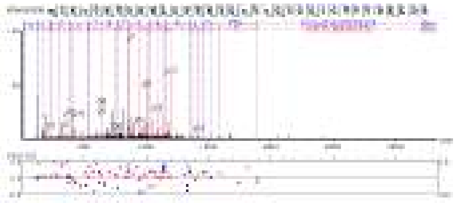

## JAB52773.1

back to list

| Protein Coverage | Supporting Peptides | Best Unique PSM |

**Protein Coverage:**

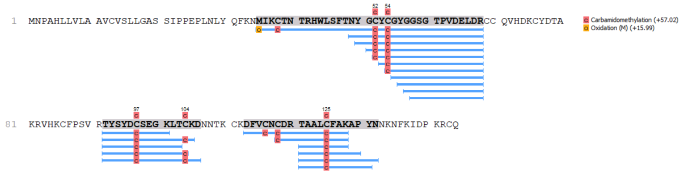


**Supporting Peptides:**

| Peptide | Uniq | -10lgP | Mass | Length | ppm | m/z | z | RT | Fraction | Scan | Source File | Area F1 | Area F2a | Area F3a | Area F4 | Area F5 | Area F6a | Area F7a | Area F7b | Area F8 | Area F9a | Area Sample 1F10a | Area F10b | Area F11a | Area F11b | Area F12a | Area F13 | #Feature | #Feature F1 | #Feature F2a | #Feature F3a | #Feature F4 | #Feature F5 | #Feature F6a | #Feature F7a | #Feature F7b | #Feature F8 | #Feature F9a | #Feature Sample 1F10a | #Feature F10b | #Feature F11a | #Feature F11b | #Feature F12a | #Feature F13 | Start | End | PTM | AScore | Found By |
|---|---|---|---|---|---|---|---|---|---|---|---|---|---|---|---|---|---|---|---|---|---|---|---|---|---|---|---|---|---|---|---|---|---|---|---|---|---|---|---|---|---|---|---|---|---|---|---|---|---|
| N.YGC(+57.02)YC(+57.02)GYGGSGTPVDELDR.C | N | 112.36 | 2124.8516 | 19 | 3.7 | 1063.4326 | 2 | 56.60 | 14 | F14:33384 | BuCaTN01_F11b.raw | | | 0 | | | 2.0097E6 | | 1.4053E7 | | 8.8531E7 | 3.4891E6 | 4.8054E8 | 6.165E7 | 1.7851E9 | 1.2628E8 | | 69 | 0 | 0 | 0 | 0 | 0 | 1 | 0 | 2 | 0 | 3 | 1 | 4 | 2 | 53 | 3 | 0 | 50 | 68 | Carbamidomethylation | C3:Carbamidomethylation:1000.00;C5:Carbamidomethylation:1000.00 | PEAKS DB |
| Y.C(+57.02)GYGGSGTPVDELDR.C | N | 88.12 | 1581.6729 | 15 | 4.4 | 791.8439 | 2 | 48.02 | 14 | F14:25306 | BuCaTN01_F11b.raw | | | | | | | | 2.166E6 | | 8.5786E6 | | 1.5853E7 | 6.495E5 | 1.7537E7 | 0 | | 6 | 0 | 0 | 0 | 0 | 0 | 0 | 0 | 1 | 0 | 1 | 0 | 2 | 1 | 1 | 0 | 0 | 54 | 68 | Carbamidomethylation | C1:Carbamidomethylation:1000.00 | PEAKS DB |
| G.C(+57.02)YC(+57.02)GYGGSGTPVDELDR.C | N | 84.40 | 1904.7668 | 17 | 2.7 | 953.3911 | 2 | 53.16 | 12 | F12:29351 | BuCaTN01_F10b.raw | | | | | | | | 4.1849E5 | | | | 3.7261E5 | | 2.1774E6 | | | 3 | 0 | 0 | 0 | 0 | 0 | 0 | 0 | 1 | 0 | 0 | 0 | 1 | 0 | 1 | 0 | 0 | 52 | 68 | Carbamidomethylation | C1:Carbamidomethylation:1000.00;C3:Carbamidomethylation:1000.00 | PEAKS DB |
| R.TYSYDC(+57.02)SEGKLTC(+57.02)K.D | N | 82.89 | 1710.7229 | 14 | 0.5 | 856.3691 | 2 | 38.29 | 7 | F7:14505 | BuCaTN01_F7a.raw | | | | | | | 1.4512E8 | 2.3155E7 | | 3.8991E5 | 4.5941E5 | | | | | | 8 | 0 | 0 | 0 | 0 | 0 | 0 | 4 | 2 | 0 | 1 | 1 | 0 | 0 | 0 | 0 | 0 | 92 | 105 | Carbamidomethylation | C6:Carbamidomethylation:1000.00;C13:Carbamidomethylation:1000.00 | PEAKS DB |
| Y.GC(+57.02)YC(+57.02)GYGGSGTPVDELDR.C | N | 82.10 | 1961.7883 | 18 | 1.4 | 981.9028 | 2 | 53.49 | 10 | F10:30232 | BuCaTN01_F9a.raw | | | | | | | | 1.7852E6 | | 3.3077E6 | | 1.5846E6 | | 3.2964E6 | 1.0428E5 | | 5 | 0 | 0 | 0 | 0 | 0 | 0 | 0 | 1 | 0 | 1 | 0 | 1 | 0 | 1 | 1 | 0 | 51 | 68 | Carbamidomethylation | C2:Carbamidomethylation:1000.00;C4:Carbamidomethylation:1000.00 | PEAKS DB |
| R.TYSYDC(+57.02)SEGKLTC(+57.02)KD.N | N | 75.87 | 1825.7499 | 15 | 2.5 | 913.8818 | 2 | 38.60 | 8 | F8:16643 | BuCaTN01_F7b.raw | | | | | | | | 7.3044E5 | | | | | | | | | 1 | 0 | 0 | 0 | 0 | 0 | 0 | 0 | 1 | 0 | 0 | 0 | 0 | 0 | 0 | 0 | 0 | 92 | 106 | Carbamidomethylation | C6:Carbamidomethylation:1000.00;C13:Carbamidomethylation:1000.00 | PEAKS DB |
| C.YC(+57.02)GYGGSGTPVDELDR.C | N | 75.62 | 1744.7362 | 16 | 4.1 | 873.3754 | 2 | 52.27 | 14 | F14:28849 | BuCaTN01_F11b.raw | | | | | | | | 7.7286E5 | | 7.3447E5 | | | | 3.0904E6 | 1.4057E5 | | 4 | 0 | 0 | 0 | 0 | 0 | 0 | 0 | 1 | 0 | 1 | 0 | 0 | 0 | 1 | 1 | 0 | 53 | 68 | Carbamidomethylation | C2:Carbamidomethylation:1000.00 | PEAKS DB |
| G.YGGSGTPVDELDR.C | N | 75.26 | 1364.6207 | 13 | 4.1 | 683.3177 | 2 | 45.29 | 14 | F14:22502 | BuCaTN01_F11b.raw | | | | | | | 6.8893E5 | | | | | | | 9.1406E6 | 6.9945E5 | | 3 | 0 | 0 | 0 | 0 | 0 | 0 | 1 | 0 | 0 | 0 | 0 | 0 | 0 | 1 | 1 | 0 | 56 | 68 | | | PEAKS DB |
| R.TAALC(+57.02)FAKAPY.N | N | 73.24 | 1211.6008 | 11 | 2.2 | 606.8076 | 2 | 55.84 | 12 | F12:32023 | BuCaTN01_F10b.raw | | | | | | | 7.3334E4 | | | 1.7166E6 | 1.3753E5 | 1.06E7 | | 8.1407E6 | 9.828E5 | | 7 | 0 | 0 | 0 | 0 | 0 | 0 | 1 | 0 | 0 | 1 | 1 | 2 | 0 | 1 | 1 | 0 | 121 | 131 | Carbamidomethylation | C5:Carbamidomethylation:1000.00 | PEAKS DB |
| T.NYGC(+57.02)YC(+57.02)GYGGSGTPVDELDR.C | N | 72.69 | 2238.8945 | 20 | 5.3 | 1120.4559 | 2 | 64.81 | 14 | F14:40195 | BuCaTN01_F11b.raw | | | | | | | | | | 2.3985E4 | | 0 | 1.1223E5 | 5.3539E5 | | | 3 | 0 | 0 | 0 | 0 | 0 | 0 | 0 | 0 | 0 | 1 | 0 | 0 | 1 | 1 | 0 | 0 | 49 | 68 | Carbamidomethylation | C4:Carbamidomethylation:1000.00;C6:Carbamidomethylation:1000.00 | PEAKS DB |
| R.TAALC(+57.02)FAKAPYN.N | N | 69.35 | 1325.6438 | 12 | -0.2 | 663.8290 | 2 | 51.91 | 6 | F6:30474 | BuCaTN01_F6a.raw | | | | | | 3.7675E6 | | 1.5632E7 | | 4.6358E6 | | 1.9346E6 | | | | | 5 | 0 | 0 | 0 | 0 | 0 | 1 | 0 | 2 | 0 | 1 | 0 | 1 | 0 | 0 | 0 | 0 | 121 | 132 | Carbamidomethylation | C5:Carbamidomethylation:1000.00 | PEAKS DB |
| C.GYGGSGTPVDELDR.C | N | 66.26 | 1421.6422 | 14 | 0.0 | 711.8284 | 2 | 46.82 | 10 | F10:24048 | BuCaTN01_F9a.raw | | | | | | | 2.9103E5 | 1.2275E6 | | 2.1867E6 | | | | 8.7883E6 | | | 5 | 0 | 0 | 0 | 0 | 0 | 0 | 1 | 1 | 0 | 1 | 0 | 0 | 0 | 2 | 0 | 0 | 55 | 68 | | | PEAKS DB |
| N.C(+57.02)DRTAALC(+57.02)FAK.A | N | 63.48 | 1311.6063 | 11 | -1.7 | 438.2076 | 3 | 37.36 | 12 | F12:15762 | BuCaTN01_F10b.raw | | | | | | | | | | | | 5.2815E4 | | 3.1918E5 | | | 2 | 0 | 0 | 0 | 0 | 0 | 0 | 0 | 0 | 0 | 0 | 0 | 1 | 0 | 1 | 0 | 0 | 118 | 128 | Carbamidomethylation | C1:Carbamidomethylation:1000.00;C8:Carbamidomethylation:1000.00 | PEAKS DB |
| R.TAALC(+57.02)FAK.A | N | 60.21 | 880.4476 | 8 | 3.4 | 441.2316 | 2 | 41.58 | 12 | F12:19288 | BuCaTN01_F10b.raw | | | | | | 9.3985E4 | 9.2429E6 | 1.2777E7 | 4.6024E5 | 3.2388E7 | 3.591E6 | 1.265E8 | 3.5731E6 | 1.1283E8 | 1.0943E8 | | 21 | 0 | 0 | 0 | 0 | 0 | 1 | 4 | 2 | 1 | 2 | 2 | 2 | 1 | 2 | 4 | 0 | 121 | 128 | Carbamidomethylation | C5:Carbamidomethylation:1000.00 | PEAKS DB |
| K.DFVC(+57.02)NC(+57.02)DRTAALC(+57.02)FAK.A | N | 59.68 | 1946.8438 | 16 | -0.4 | 974.4265 | 2 | 51.09 | 12 | F12:27773 | BuCaTN01_F10b.raw | | | | | | | | | | 1.1341E6 | | 0 | | | | | 1 | 0 | 0 | 0 | 0 | 0 | 0 | 0 | 0 | 0 | 1 | 0 | 0 | 0 | 0 | 0 | 0 | 113 | 128 | Carbamidomethylation | C4:Carbamidomethylation:1000.00;C6:Carbamidomethylation:1000.00;C13:Carbamidomethylation:1000.00 | PEAKS DB |
| G.GSGTPVDELDR.C | N | 56.36 | 1144.5360 | 11 | 3.4 | 573.2756 | 2 | 40.15 | 8 | F8:17921 | BuCaTN01_F7b.raw | | | | | | | | 1.1068E6 | | | | | | | | | 1 | 0 | 0 | 0 | 0 | 0 | 0 | 0 | 1 | 0 | 0 | 0 | 0 | 0 | 0 | 0 | 0 | 58 | 68 | | | PEAKS DB |
| R.TYSYDC(+57.02)SEGK.L | N | 55.61 | 1208.4656 | 10 | 0.1 | 605.2401 | 2 | 29.72 | 7 | F7:8746 | BuCaTN01_F7a.raw | | | | | | | 5.0914E6 | | | | | | | | | | 1 | 0 | 0 | 0 | 0 | 0 | 0 | 1 | 0 | 0 | 0 | 0 | 0 | 0 | 0 | 0 | 0 | 92 | 101 | Carbamidomethylation | C6:Carbamidomethylation:1000.00 | PEAKS DB |
| N.M(+15.99)IKC(+57.02)TNTRHWLSFTNYGC(+57.02)YC(+57.02)GYGGSGTPVDELDR.C | Y | 53.32 | 4030.7495 | 34 | 1.3 | 1008.6960 | 4 | 77.35 | 10 | F10:53957 | BuCaTN01_F9a.raw | | | | | | | | | | 0 | | | | | | | 0 | 0 | 0 | 0 | 0 | 0 | 0 | 0 | 0 | 0 | 0 | 0 | 0 | 0 | 0 | 0 | 0 | 35 | 68 | Oxidation (M); Carbamidomethylation | M1:Oxidation (M):1000.00;C4:Carbamidomethylation:1000.00;C18:Carbamidomethylation:1000.00;C20:Carbamidomethylation:1000.00 | PEAKS DB |
| R.TYSYDC(+57.02)SEGKLTC(+57.02).K | N | 52.98 | 1582.6279 | 13 | 2.9 | 792.3212 | 2 | 43.19 | 8 | F8:20253 | BuCaTN01_F7b.raw | | | | | | | | 5.7951E5 | | | | | | | | | 1 | 0 | 0 | 0 | 0 | 0 | 0 | 0 | 1 | 0 | 0 | 0 | 0 | 0 | 0 | 0 | 0 | 92 | 104 | Carbamidomethylation | C6:Carbamidomethylation:1000.00;C13:Carbamidomethylation:1000.00 | PEAKS DB |
| R.TYSYDC(+57.02)SEGKLT.C | N | 50.03 | 1422.5973 | 12 | 2.7 | 712.3058 | 2 | 41.55 | 8 | F8:18942 | BuCaTN01_F7b.raw | | | | | | | 5.0044E6 | 1.8313E6 | | | | | | | | | 2 | 0 | 0 | 0 | 0 | 0 | 0 | 1 | 1 | 0 | 0 | 0 | 0 | 0 | 0 | 0 | 0 | 92 | 103 | Carbamidomethylation | C6:Carbamidomethylation:1000.00 | PEAKS DB |
| Y.GGSGTPVDELDR.C | N | 49.09 | 1201.5575 | 12 | 0.3 | 601.7862 | 2 | 40.57 | 11 | F11:14528 | BuCaTN01_F10a.raw | | | | | | | 9.2274E5 | 2.7577E6 | | 1.1335E7 | 6.7591E5 | | 9.9086E5 | 2.6759E7 | | | 6 | 0 | 0 | 0 | 0 | 0 | 0 | 1 | 1 | 0 | 1 | 1 | 0 | 1 | 1 | 0 | 0 | 57 | 68 | | | PEAKS DB |
| R.TAALC(+57.02)FAKAP.Y | N | 47.25 | 1048.5375 | 10 | 3.6 | 525.2764 | 2 | 48.84 | 8 | F8:25881 | BuCaTN01_F7b.raw | | | | | | 3.1579E6 | | 4.0965E6 | | | | | | | | | 2 | 0 | 0 | 0 | 0 | 0 | 1 | 0 | 1 | 0 | 0 | 0 | 0 | 0 | 0 | 0 | 0 | 121 | 130 | Carbamidomethylation | C5:Carbamidomethylation:1000.00 | PEAKS DB |
| **total 22 peptides** | | | | | | | | | | | | | | | | | | | | | | | | | | | | | | | | | | | | | | | | | | | | | | | | | |

**Best Unique PSM (Scan F10:53957, m/z=1008.6960, z=4, RT=77.35, ppm=1.3):**

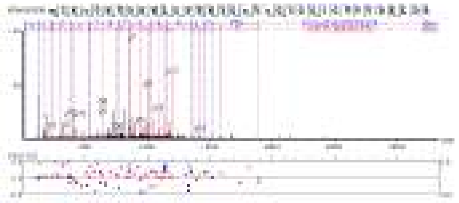

## JAI09010.1

back to list

| Protein Coverage | Supporting Peptides | Best Unique PSM |

**Protein Coverage:**

1 MNPAHLLVLA AVCVSLLGAS SIPPEPLNLY QFKN**MIKCTN TRHWLSFTNY GCYCGYGGSG TPVDELDR**CC QVHDKCYDTA

81 KRVHKCFPSV R**TYSYDCSEG KLTCKD**NNTK CK**DFVCNCDR TAALCFAKAP YN**NKNFKIDP SRCQ

C Carbamidomethylation (+57.02)
O Oxidation (M) (+15.99)

**Supporting Peptides:**


| Peptide | Uniq | -10lgP | Mass | Length | ppm | m/z | z | RT | Fraction | Scan | Source File | Area F1 | Area F2a | Area F3a | Area F4 | Area F5 | Area F6a | Area F7a | Area F7b | Area F8 | Area F9a | Area Sample 1F10a | Area F10b | Area F11a | Area F11b | Area F12a | Area F13 | #Feature | #Feature F1 | #Feature F2a | #Feature F3a | #Feature F4 | #Feature F5 | #Feature F6a | #Feature F7a | #Feature F7b | #Feature F8 | #Feature F9a | #Feature Sample 1F10a | #Feature F10b | #Feature F11a | #Feature F11b | #Feature F12a | #Feature F13 | Start | End | PTM | AScore | Found By |
|---|---|---|---|---|---|---|---|---|---|---|---|---|---|---|---|---|---|---|---|---|---|---|---|---|---|---|---|---|---|---|---|---|---|---|---|---|---|---|---|---|---|---|---|---|---|---|---|---|---|
| N.YGC(+57.02)YC(+57.02)GYGGSGTPVDELDR.C | N | 112.36 | 2124.8516 | 19 | 3.7 | 1063.4326 | 2 | 56.60 | 14 | F14:33384 | BuCaTN01_F11b.raw | | | 0 | | | 2.0097E6 | | 1.4053E7 | | 8.8531E7 | 3.4891E6 | 4.8054E8 | 6.165E7 | 1.7851E9 | 1.2628E8 | | 69 | 0 | 0 | 0 | 0 | 0 | 1 | 0 | 2 | 0 | 3 | 1 | 4 | 2 | 53 | 3 | 0 | 50 | 68 | Carbamidomethylation | C3:Carbamidomethylation:1000.00;C5:Carbamidomethylation:1000.00 | PEAKS DB |
| Y.C(+57.02)GYGGSGTPVDELDR.C | N | 88.12 | 1581.6729 | 15 | 4.4 | 791.8439 | 2 | 48.02 | 14 | F14:25306 | BuCaTN01_F11b.raw | | | | | | | | 2.166E6 | | 8.5786E6 | | 1.5853E7 | 6.495E5 | 1.7537E7 | 0 | | 6 | 0 | 0 | 0 | 0 | 0 | 0 | 0 | 1 | 0 | 1 | 0 | 2 | 1 | 1 | 0 | 0 | 54 | 68 | Carbamidomethylation | C1:Carbamidomethylation:1000.00 | PEAKS DB |
| G.C(+57.02)YC(+57.02)GYGGSGTPVDELDR.C | N | 84.40 | 1904.7668 | 17 | 2.7 | 953.3911 | 2 | 53.16 | 12 | F12:29351 | BuCaTN01_F10b.raw | | | | | | | | 4.1849E5 | | | | 3.7261E5 | | 2.1774E6 | | | 3 | 0 | 0 | 0 | 0 | 0 | 0 | 0 | 1 | 0 | 0 | 0 | 1 | 0 | 1 | 0 | 0 | 52 | 68 | Carbamidomethylation | C1:Carbamidomethylation:1000.00;C3:Carbamidomethylation:1000.00 | PEAKS DB |
| R.TYSYDC(+57.02)SEGKLTC(+57.02)K.D | N | 82.89 | 1710.7229 | 14 | 0.5 | 856.3691 | 2 | 38.29 | 7 | F7:14505 | BuCaTN01_F7a.raw | | | | | | | 1.4512E8 | 2.3155E7 | | 3.8991E5 | 4.5941E5 | | | | | | 8 | 0 | 0 | 0 | 0 | 0 | 0 | 4 | 2 | 0 | 1 | 1 | 0 | 0 | 0 | 0 | 0 | 92 | 105 | Carbamidomethylation | C6:Carbamidomethylation:1000.00;C13:Carbamidomethylation:1000.00 | PEAKS DB |
| Y.GC(+57.02)YC(+57.02)GYGGSGTPVDELDR.C | N | 82.10 | 1961.7883 | 18 | 1.4 | 981.9028 | 2 | 53.49 | 10 | F10:30232 | BuCaTN01_F9a.raw | | | | | | | | 1.7852E6 | | 3.3077E6 | | 1.5846E6 | | 3.2964E6 | 1.0428E5 | | 5 | 0 | 0 | 0 | 0 | 0 | 0 | 0 | 1 | 0 | 1 | 0 | 1 | 0 | 1 | 1 | 0 | 51 | 68 | Carbamidomethylation | C2:Carbamidomethylation:1000.00;C4:Carbamidomethylation:1000.00 | PEAKS DB |
| R.TYSYDC(+57.02)SEGKLTC(+57.02)KD.N | N | 75.87 | 1825.7499 | 15 | 2.5 | 913.8818 | 2 | 38.60 | 8 | F8:16643 | BuCaTN01_F7b.raw | | | | | | | | 7.3044E5 | | | | | | | | | 1 | 0 | 0 | 0 | 0 | 0 | 0 | 0 | 1 | 0 | 0 | 0 | 0 | 0 | 0 | 0 | 0 | 92 | 106 | Carbamidomethylation | C6:Carbamidomethylation:1000.00;C13:Carbamidomethylation:1000.00 | PEAKS DB |
| C.YC(+57.02)GYGGSGTPVDELDR.C | N | 75.62 | 1744.7362 | 16 | 4.1 | 873.3754 | 2 | 52.27 | 14 | F14:28849 | BuCaTN01_F11b.raw | | | | | | | | 7.7286E5 | | 7.3447E5 | | | | 3.0904E6 | 1.4057E5 | | 4 | 0 | 0 | 0 | 0 | 0 | 0 | 0 | 1 | 0 | 1 | 0 | 0 | 0 | 1 | 1 | 0 | 53 | 68 | Carbamidomethylation | C2:Carbamidomethylation:1000.00 | PEAKS DB |
| G.YGGSGTPVDELDR.C | N | 75.26 | 1364.6207 | 13 | 4.1 | 683.3177 | 2 | 45.29 | 14 | F14:22502 | BuCaTN01_F11b.raw | | | | | | | 6.8893E5 | | | | | | | 9.1406E6 | 6.9945E5 | | 3 | 0 | 0 | 0 | 0 | 0 | 0 | 1 | 0 | 0 | 0 | 0 | 0 | 0 | 1 | 1 | 0 | 56 | 68 | | | PEAKS DB |
| R.TAALC(+57.02)FAKAPY.N | N | 73.24 | 1211.6008 | 11 | 2.2 | 606.8076 | 2 | 55.84 | 12 | F12:32023 | BuCaTN01_F10b.raw | | | | | | | 7.3334E4 | | | 1.7166E6 | 1.3753E5 | 1.06E7 | | 8.1407E6 | 9.828E5 | | 7 | 0 | 0 | 0 | 0 | 0 | 0 | 1 | 0 | 0 | 1 | 1 | 2 | 0 | 1 | 1 | 0 | 121 | 131 | Carbamidomethylation | C5:Carbamidomethylation:1000.00 | PEAKS DB |
| T.NYGC(+57.02)YC(+57.02)GYGGSGTPVDELDR.C | N | 72.69 | 2238.8945 | 20 | 5.3 | 1120.4559 | 2 | 64.81 | 14 | F14:40195 | BuCaTN01_F11b.raw | | | | | | | | | | 2.3985E4 | | 0 | 1.1223E5 | 5.3539E5 | | | 3 | 0 | 0 | 0 | 0 | 0 | 0 | 0 | 0 | 0 | 1 | 0 | 0 | 1 | 1 | 0 | 0 | 49 | 68 | Carbamidomethylation | C4:Carbamidomethylation:1000.00;C6:Carbamidomethylation:1000.00 | PEAKS DB |
| R.TAALC(+57.02)FAKAPYN.N | N | 69.35 | 1325.6438 | 12 | -0.2 | 663.8290 | 2 | 51.91 | 6 | F6:30474 | BuCaTN01_F6a.raw | | | | | | 3.7675E6 | | 1.5632E7 | | 4.6358E6 | | 1.9346E6 | | | | | 5 | 0 | 0 | 0 | 0 | 0 | 1 | 0 | 2 | 0 | 1 | 0 | 1 | 0 | 0 | 0 | 0 | 121 | 132 | Carbamidomethylation | C5:Carbamidomethylation:1000.00 | PEAKS DB |
| C.GYGGSGTPVDELDR.C | N | 66.26 | 1421.6422 | 14 | 0.0 | 711.8284 | 2 | 46.82 | 10 | F10:24048 | BuCaTN01_F9a.raw | | | | | | | 2.9103E5 | 1.2275E6 | | 2.1867E6 | | | | 8.7883E6 | | | 5 | 0 | 0 | 0 | 0 | 0 | 0 | 1 | 1 | 0 | 1 | 0 | 0 | 0 | 2 | 0 | 0 | 55 | 68 | | | PEAKS DB |
| N.C(+57.02)DRTAALC(+57.02)FAK.A | N | 63.48 | 1311.6063 | 11 | -1.7 | 438.2076 | 3 | 37.36 | 12 | F12:15762 | BuCaTN01_F10b.raw | | | | | | | | | | | | 5.2815E4 | | 3.1918E5 | | | 2 | 0 | 0 | 0 | 0 | 0 | 0 | 0 | 0 | 0 | 0 | 0 | 1 | 0 | 1 | 0 | 0 | 118 | 128 | Carbamidomethylation | C1:Carbamidomethylation:1000.00;C8:Carbamidomethylation:1000.00 | PEAKS DB |
| R.TAALC(+57.02)FAK.A | N | 60.21 | 880.4476 | 8 | 3.4 | 441.2316 | 2 | 41.58 | 12 | F12:19288 | BuCaTN01_F10b.raw | | | | | | 9.3985E4 | 9.2429E6 | 1.2777E7 | 4.6024E5 | 3.2388E7 | 3.591E6 | 1.265E8 | 3.5731E6 | 1.1283E8 | 1.0943E8 | | 21 | 0 | 0 | 0 | 0 | 0 | 1 | 4 | 2 | 1 | 2 | 2 | 2 | 1 | 2 | 4 | 0 | 121 | 128 | Carbamidomethylation | C5:Carbamidomethylation:1000.00 | PEAKS DB |
| K.DFVC(+57.02)NC(+57.02)DRTAALC(+57.02)FAK.A | N | 59.68 | 1946.8438 | 16 | -0.4 | 974.4265 | 2 | 51.09 | 12 | F12:27773 | BuCaTN01_F10b.raw | | | | | | | | | | 1.1341E6 | | 0 | | | | | 1 | 0 | 0 | 0 | 0 | 0 | 0 | 0 | 0 | 0 | 1 | 0 | 0 | 0 | 0 | 0 | 0 | 113 | 128 | Carbamidomethylation | C4:Carbamidomethylation:1000.00;C6:Carbamidomethylation:1000.00;C13:Carbamidomethylation:1000.00 | PEAKS DB |
| G.GSGTPVDELDR.C | N | 56.36 | 1144.5360 | 11 | 3.4 | 573.2756 | 2 | 40.15 | 8 | F8:17921 | BuCaTN01_F7b.raw | | | | | | | | 1.1068E6 | | | | | | | | | 1 | 0 | 0 | 0 | 0 | 0 | 0 | 0 | 1 | 0 | 0 | 0 | 0 | 0 | 0 | 0 | 0 | 58 | 68 | | | PEAKS DB |
| R.TYSYDC(+57.02)SEGK.L | N | 55.61 | 1208.4656 | 10 | 0.1 | 605.2401 | 2 | 29.72 | 7 | F7:8746 | BuCaTN01_F7a.raw | | | | | | | 5.0914E6 | | | | | | | | | | 1 | 0 | 0 | 0 | 0 | 0 | 0 | 1 | 0 | 0 | 0 | 0 | 0 | 0 | 0 | 0 | 0 | 92 | 101 | Carbamidomethylation | C6:Carbamidomethylation:1000.00 | PEAKS DB |
| N.M(+15.99)IKC(+57.02)TNTRHWLSFTNYGC(+57.02)YC(+57.02)GYGGSGTPVDELDR.C | Y | 53.32 | 4030.7495 | 34 | 1.3 | 1008.6960 | 4 | 77.35 | 10 | F10:53957 | BuCaTN01_F9a.raw | | | | | | | | | | 0 | | | | | | | 0 | 0 | 0 | 0 | 0 | 0 | 0 | 0 | 0 | 0 | 0 | 0 | 0 | 0 | 0 | 0 | 0 | 35 | 68 | Oxidation (M); Carbamidomethylation | M1:Oxidation (M):1000.00;C4:Carbamidomethylation:1000.00;C18:Carbamidomethylation:1000.00;C20:Carbamidomethylation:1000.00 | PEAKS DB |
| R.TYSYDC(+57.02)SEGKLTC(+57.02).K | N | 52.98 | 1582.6279 | 13 | 2.9 | 792.3212 | 2 | 43.19 | 8 | F8:20253 | BuCaTN01_F7b.raw | | | | | | | | 5.7951E5 | | | | | | | | | 1 | 0 | 0 | 0 | 0 | 0 | 0 | 0 | 1 | 0 | 0 | 0 | 0 | 0 | 0 | 0 | 0 | 92 | 104 | Carbamidomethylation | C6:Carbamidomethylation:1000.00;C13:Carbamidomethylation:1000.00 | PEAKS DB |
| | | | | | | | | | | | | | | | | | | | | | | | | | | | | | | | | | | | | | | | | | | | | | | | | C6:Carbamidomethylatio | PEAKS |

| | | | | | | | | | | | | | | | | | | | | | | | | | | | | | | | | | | | | | | | | | | | | | | | | | |
|---|---|---|---|---|---|---|---|---|---|---|---|---|---|---|---|---|---|---|---|---|---|---|---|---|---|---|---|---|---|---|---|---|---|---|---|---|---|---|---|---|---|---|---|---|---|---|---|---|---|
| R.TYSYDC(+57.02)SEGKLT.C | N | 50.03 | 1422.5973 | 12 | 2.7 | 712.3058 | 2 | 41.55 | 8 | F8:18942 | BuCaTN01_F7b.raw | | | | | | | 5.0044E6 | 1.8313E6 | | | | | | | | | 2 | 0 | 0 | 0 | 0 | 0 | 0 | 1 | 1 | 0 | 0 | 0 | 0 | 0 | 0 | 0 | 0 | 92 | 103 | Carbamidomethylation | n:1000.00 | DB |
| Y.GGSGTPVDELDR.C | N | 49.09 | 1201.5575 | 12 | 0.3 | 601.7862 | 2 | 40.57 | 11 | F11:14528 | BuCaTN01_F10a.raw | | | | | | | 9.2274E5 | 2.7577E6 | | 1.1335E7 | 6.7591E5 | | 9.9086E5 | 2.6759E7 | | | 6 | 0 | 0 | 0 | 0 | 0 | 0 | 1 | 1 | 0 | 1 | 1 | 0 | 1 | 1 | 0 | 0 | 57 | 68 | | | PEAKS DB |
| R.TAALC(+57.02)FAKAP.Y | N | 47.25 | 1048.5375 | 10 | 3.6 | 525.2764 | 2 | 48.84 | 8 | F8:25881 | BuCaTN01_F7b.raw | | | | | | 3.1579E6 | | 4.0965E6 | | | | | | | | | 2 | 0 | 0 | 0 | 0 | 0 | 1 | 0 | 1 | 0 | 0 | 0 | 0 | 0 | 0 | 0 | 0 | 121 | 130 | Carbamidomethylation | C5:Carbamidomethylation:1000.00 | PEAKS DB |
| total 22 peptides | | | | | | | | | | | | | | | | | | | | | | | | | | | | | | | | | | | | | | | | | | | | | | | | | |

**Best Unique PSM (Scan F10:53957, m/z=1008.6960, z=4, RT=77.35, ppm=1.3):**

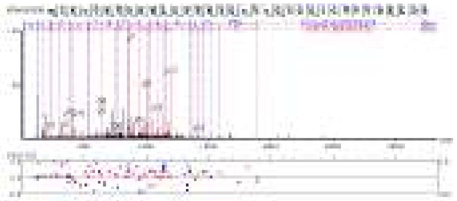

## JAB52795.1

back to list

| Protein Coverage | Supporting Peptides | Best Unique PSM |

**Protein Coverage:**

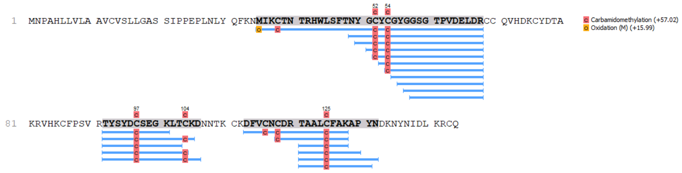


**Supporting Peptides:**

| Peptide | Uniq | -10lgP | Mass | Length | ppm | m/z | z | RT | Fraction | Scan | Source File | Area F1 | Area F2a | Area F3a | Area F4 | Area F5 | Area F6a | Area F7a | Area F7b | Area F8 | Area F9a | Area Sample 1F10a | Area F10b | Area F11a | Area F11b | Area F12a | Area F13 | #Feature | #Feature F1 | #Feature F2a | #Feature F3a | #Feature F4 | #Feature F5 | #Feature F6a | #Feature F7a | #Feature F7b | #Feature F8 | #Feature F9a | #Feature Sample 1F10a | #Feature F10b | #Feature F11a | #Feature F11b | #Feature F12a | #Feature F13 | Start | End | PTM | AScore | Found By |
|---|---|---|---|---|---|---|---|---|---|---|---|---|---|---|---|---|---|---|---|---|---|---|---|---|---|---|---|---|---|---|---|---|---|---|---|---|---|---|---|---|---|---|---|---|---|---|---|---|---|
| N.YGC(+57.02)YC(+57.02)GYGGSGTPVDELDR.C | N | 112.36 | 2124.8516 | 19 | 3.7 | 1063.4326 | 2 | 56.60 | 14 | F14:33384 | BuCaTN01_F11b.raw | | | 0 | | | 2.0097E6 | | 1.4053E7 | | 8.8531E7 | 3.4891E6 | 4.8054E8 | 6.165E7 | 1.7851E9 | 1.2628E8 | | 69 | 0 | 0 | 0 | 0 | 0 | 1 | 0 | 2 | 0 | 3 | 1 | 4 | 2 | 53 | 3 | 0 | 50 | 68 | Carbamidomethylation | C3:Carbamidomethylation:1000.00;C5:Carbamidomethylation:1000.00 | PEAKS DB |
| Y.C(+57.02)GYGGSGTPVDELDR.C | N | 88.12 | 1581.6729 | 15 | 4.4 | 791.8439 | 2 | 48.02 | 14 | F14:25306 | BuCaTN01_F11b.raw | | | | | | | | 2.166E6 | | 8.5786E6 | | 1.5853E7 | 6.495E5 | 1.7537E7 | 0 | | 6 | 0 | 0 | 0 | 0 | 0 | 0 | 0 | 1 | 0 | 1 | 0 | 2 | 1 | 1 | 0 | 0 | 54 | 68 | Carbamidomethylation | C1:Carbamidomethylation:1000.00 | PEAKS DB |
| G.C(+57.02)YC(+57.02)GYGGSGTPVDELDR.C | N | 84.40 | 1904.7668 | 17 | 2.7 | 953.3911 | 2 | 53.16 | 12 | F12:29351 | BuCaTN01_F10b.raw | | | | | | | | 4.1849E5 | | | | 3.7261E5 | | 2.1774E6 | | | 3 | 0 | 0 | 0 | 0 | 0 | 0 | 0 | 1 | 0 | 0 | 0 | 1 | 0 | 1 | 0 | 0 | 52 | 68 | Carbamidomethylation | C1:Carbamidomethylation:1000.00;C3:Carbamidomethylation:1000.00 | PEAKS DB |
| R.TYSYDC(+57.02)SEGKLTC(+57.02)K.D | N | 82.89 | 1710.7229 | 14 | 0.5 | 856.3691 | 2 | 38.29 | 7 | F7:14505 | BuCaTN01_F7a.raw | | | | | | | 1.4512E8 | 2.3155E7 | | 3.8991E5 | 4.5941E5 | | | | | | 8 | 0 | 0 | 0 | 0 | 0 | 0 | 4 | 2 | 0 | 1 | 1 | 0 | 0 | 0 | 0 | 0 | 92 | 105 | Carbamidomethylation | C6:Carbamidomethylation:1000.00;C13:Carbamidomethylation:1000.00 | PEAKS DB |
| Y.GC(+57.02)YC(+57.02)GYGGSGTPVDELDR.C | N | 82.10 | 1961.7883 | 18 | 1.4 | 981.9028 | 2 | 53.49 | 10 | F10:30232 | BuCaTN01_F9a.raw | | | | | | | | 1.7852E6 | | 3.3077E6 | | 1.5846E6 | | 3.2964E6 | 1.0428E5 | | 5 | 0 | 0 | 0 | 0 | 0 | 0 | 0 | 1 | 0 | 1 | 0 | 1 | 0 | 1 | 1 | 0 | 51 | 68 | Carbamidomethylation | C2:Carbamidomethylation:1000.00;C4:Carbamidomethylation:1000.00 | PEAKS DB |
| R.TYSYDC(+57.02)SEGKLTC(+57.02)KD.N | N | 75.87 | 1825.7499 | 15 | 2.5 | 913.8818 | 2 | 38.60 | 8 | F8:16643 | BuCaTN01_F7b.raw | | | | | | | | 7.3044E5 | | | | | | | | | 1 | 0 | 0 | 0 | 0 | 0 | 0 | 0 | 1 | 0 | 0 | 0 | 0 | 0 | 0 | 0 | 0 | 92 | 106 | Carbamidomethylation | C6:Carbamidomethylation:1000.00;C13:Carbamidomethylation:1000.00 | PEAKS DB |
| C.YC(+57.02)GYGGSGTPVDELDR.C | N | 75.62 | 1744.7362 | 16 | 4.1 | 873.3754 | 2 | 52.27 | 14 | F14:28849 | BuCaTN01_F11b.raw | | | | | | | | 7.7286E5 | | 7.3447E5 | | | | 3.0904E6 | 1.4057E5 | | 4 | 0 | 0 | 0 | 0 | 0 | 0 | 0 | 1 | 0 | 1 | 0 | 0 | 0 | 1 | 1 | 0 | 53 | 68 | Carbamidomethylation | C2:Carbamidomethylation:1000.00 | PEAKS DB |
| G.YGGSGTPVDELDR.C | N | 75.26 | 1364.6207 | 13 | 4.1 | 683.3177 | 2 | 45.29 | 14 | F14:22502 | BuCaTN01_F11b.raw | | | | | | | 6.8893E5 | | | | | | | 9.1406E6 | 6.9945E5 | | 3 | 0 | 0 | 0 | 0 | 0 | 0 | 1 | 0 | 0 | 0 | 0 | 0 | 0 | 1 | 1 | 0 | 56 | 68 | | | PEAKS DB |
| R.TAALC(+57.02)FAKAPY.N | N | 73.24 | 1211.6008 | 11 | 2.2 | 606.8076 | 2 | 55.84 | 12 | F12:32023 | BuCaTN01_F10b.raw | | | | | | | 7.3334E4 | | | 1.7166E6 | 1.3753E5 | 1.06E7 | | 8.1407E6 | 9.828E5 | | 7 | 0 | 0 | 0 | 0 | 0 | 0 | 1 | 0 | 0 | 1 | 1 | 2 | 0 | 1 | 1 | 0 | 121 | 131 | Carbamidomethylation | C5:Carbamidomethylation:1000.00 | PEAKS DB |
| T.NYGC(+57.02)YC(+57.02)GYGGSGTPVDELDR.C | N | 72.69 | 2238.8945 | 20 | 5.3 | 1120.4559 | 2 | 64.81 | 14 | F14:40195 | BuCaTN01_F11b.raw | | | | | | | | | | 2.3985E4 | | 0 | 1.1223E5 | 5.3539E5 | | | 3 | 0 | 0 | 0 | 0 | 0 | 0 | 0 | 0 | 0 | 1 | 0 | 0 | 1 | 1 | 0 | 0 | 49 | 68 | Carbamidomethylation | C4:Carbamidomethylation:1000.00;C6:Carbamidomethylation:1000.00 | PEAKS DB |
| R.TAALC(+57.02)FAKAPYN.D | N | 69.35 | 1325.6438 | 12 | -0.2 | 663.8290 | 2 | 51.91 | 6 | F6:30474 | BuCaTN01_F6a.raw | | | | | | 3.7675E6 | | 1.5632E7 | | 4.6358E6 | | 1.9346E6 | | | | | 5 | 0 | 0 | 0 | 0 | 0 | 1 | 0 | 2 | 0 | 1 | 0 | 1 | 0 | 0 | 0 | 0 | 121 | 132 | Carbamidomethylation | C5:Carbamidomethylation:1000.00 | PEAKS DB |
| C.GYGGSGTPVDELDR.C | N | 66.26 | 1421.6422 | 14 | 0.0 | 711.8284 | 2 | 46.82 | 10 | F10:24048 | BuCaTN01_F9a.raw | | | | | | | 2.9103E5 | 1.2275E6 | | 2.1867E6 | | | | 8.7883E6 | | | 5 | 0 | 0 | 0 | 0 | 0 | 0 | 1 | 1 | 0 | 1 | 0 | 0 | 0 | 2 | 0 | 0 | 55 | 68 | | | PEAKS DB |
| N.C(+57.02)DRTAALC(+57.02)FAK.A | N | 63.48 | 1311.6063 | 11 | -1.7 | 438.2076 | 3 | 37.36 | 12 | F12:15762 | BuCaTN01_F10b.raw | | | | | | | | | | | | 5.2815E4 | | 3.1918E5 | | | 2 | 0 | 0 | 0 | 0 | 0 | 0 | 0 | 0 | 0 | 0 | 0 | 1 | 0 | 1 | 0 | 0 | 118 | 128 | Carbamidomethylation | C1:Carbamidomethylation:1000.00;C8:Carbamidomethylation:1000.00 | PEAKS DB |
| R.TAALC(+57.02)FAK.A | N | 60.21 | 880.4476 | 8 | 3.4 | 441.2316 | 2 | 41.58 | 12 | F12:19288 | BuCaTN01_F10b.raw | | | | | | 9.3985E4 | 9.2429E6 | 1.2777E7 | 4.6024E5 | 3.2388E7 | 3.591E6 | 1.265E8 | 3.5731E6 | 1.1283E8 | 1.0943E8 | | 21 | 0 | 0 | 0 | 0 | 0 | 1 | 4 | 2 | 1 | 2 | 2 | 2 | 1 | 2 | 4 | 0 | 121 | 128 | Carbamidomethylation | C5:Carbamidomethylation:1000.00 | PEAKS DB |
| K.DFVC(+57.02)NC(+57.02)DRTAALC(+57.02)FAK.A | N | 59.68 | 1946.8438 | 16 | -0.4 | 974.4265 | 2 | 51.09 | 12 | F12:27773 | BuCaTN01_F10b.raw | | | | | | | | | | 1.1341E6 | | 0 | | | | | 1 | 0 | 0 | 0 | 0 | 0 | 0 | 0 | 0 | 0 | 1 | 0 | 0 | 0 | 0 | 0 | 0 | 113 | 128 | Carbamidomethylation | C4:Carbamidomethylation:1000.00;C6:Carbamidomethylation:1000.00;C13:Carbamidomethylation:1000.00 | PEAKS DB |
| G.GSGTPVDELDR.C | N | 56.36 | 1144.5360 | 11 | 3.4 | 573.2756 | 2 | 40.15 | 8 | F8:17921 | BuCaTN01_F7b.raw | | | | | | | | 1.1068E6 | | | | | | | | | 1 | 0 | 0 | 0 | 0 | 0 | 0 | 0 | 1 | 0 | 0 | 0 | 0 | 0 | 0 | 0 | 0 | 58 | 68 | | | PEAKS DB |
| R.TYSYDC(+57.02)SEGK.L | N | 55.61 | 1208.4656 | 10 | 0.1 | 605.2401 | 2 | 29.72 | 7 | F7:8746 | BuCaTN01_F7a.raw | | | | | | | 5.0914E6 | | | | | | | | | | 1 | 0 | 0 | 0 | 0 | 0 | 0 | 1 | 0 | 0 | 0 | 0 | 0 | 0 | 0 | 0 | 0 | 92 | 101 | Carbamidomethylation | C6:Carbamidomethylation:1000.00 | PEAKS DB |
| N.M(+15.99)IKC(+57.02)TNTRHWLSFTNYGC(+57.02)YC(+57.02)GYGGSGTPVDELDR.C | Y | 53.32 | 4030.7495 | 34 | 1.3 | 1008.6960 | 4 | 77.35 | 10 | F10:53957 | BuCaTN01_F9a.raw | | | | | | | | | | 0 | | | | | | | 0 | 0 | 0 | 0 | 0 | 0 | 0 | 0 | 0 | 0 | 0 | 0 | 0 | 0 | 0 | 0 | 0 | 35 | 68 | Oxidation (M); Carbamidomethylation | M1:Oxidation (M):1000.00;C4:Carbamidomethylation:1000.00;C18:Carbamidomethylation:1000.00;C20:Carbamidomethylation:1000.00 | PEAKS DB |
| R.TYSYDC(+57.02)SEGKLTC(+57.02).K | N | 52.98 | 1582.6279 | 13 | 2.9 | 792.3212 | 2 | 43.19 | 8 | F8:20253 | BuCaTN01_F7b.raw | | | | | | | | 5.7951E5 | | | | | | | | | 1 | 0 | 0 | 0 | 0 | 0 | 0 | 0 | 1 | 0 | 0 | 0 | 0 | 0 | 0 | 0 | 0 | 92 | 104 | Carbamidomethylation | C6:Carbamidomethylation:1000.00;C13:Carbamidomethylation:1000.00 | PEAKS DB |
| R.TYSYDC(+57.02)SEGKLT.C | N | 50.03 | 1422.5973 | 12 | 2.7 | 712.3058 | 2 | 41.55 | 8 | F8:18942 | BuCaTN01_F7b.raw | | | | | | | 5.0044E6 | 1.8313E6 | | | | | | | | | 2 | 0 | 0 | 0 | 0 | 0 | 0 | 1 | 1 | 0 | 0 | 0 | 0 | 0 | 0 | 0 | 0 | 92 | 103 | Carbamidomethylation | C6:Carbamidomethylation:1000.00 | PEAKS DB |
| Y.GGSGTPVDELDR.C | N | 49.09 | 1201.5575 | 12 | 0.3 | 601.7862 | 2 | 40.57 | 11 | F11:14528 | BuCaTN01_F10a.raw | | | | | | | 9.2274E5 | 2.7577E6 | | 1.1335E7 | 6.7591E5 | | 9.9086E5 | 2.6759E7 | | | 6 | 0 | 0 | 0 | 0 | 0 | 0 | 1 | 1 | 0 | 1 | 1 | 0 | 1 | 1 | 0 | 0 | 57 | 68 | | | PEAKS DB |
| R.TAALC(+57.02)FAKAP.Y | N | 47.25 | 1048.5375 | 10 | 3.6 | 525.2764 | 2 | 48.84 | 8 | F8:25881 | BuCaTN01_F7b.raw | | | | | | 3.1579E6 | | 4.0965E6 | | | | | | | | | 2 | 0 | 0 | 0 | 0 | 0 | 1 | 0 | 1 | 0 | 0 | 0 | 0 | 0 | 0 | 0 | 0 | 121 | 130 | Carbamidomethylation | C5:Carbamidomethylation:1000.00 | PEAKS DB |
| total 22 peptides | | | | | | | | | | | | | | | | | | | | | | | | | | | | | | | | | | | | | | | | | | | | | | | | | |

**Best Unique PSM (Scan F10:53957, m/z=1008.6960, z=4, RT=77.35, ppm=1.3):**

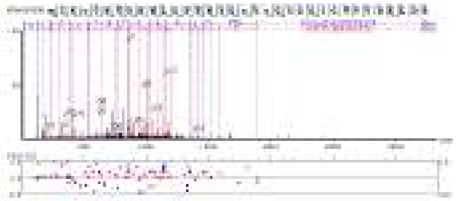

## JAI09004.1

back to list

| Protein Coverage | Supporting Peptides | Best Unique PSM |

**Protein Coverage:**

1 MNPAHLLFLA SVCVSLLGAS SIPPEPLNLY QFKNMIKCTN TRHWLSFTNY GCYCGYGGSG TPVDELDRCC QVHDKCYDTA

81 KRVHKCFPSV RTYSYDCSEG KLTCKDNNTK CKDFVCNCDR TAALCFAKAP YNDKNYNIDL KRCQ

C Carbamidomethylation (+57.02)
O Oxidation (M) (+15.99)

**Supporting Peptides:**

| Peptide | Uniq | -10lgP | Mass | Length | ppm | m/z | z | RT | Fraction | Scan | Source File | Area F1 | Area F2a | Area F3a | Area F4 | Area F5 | Area F6a | Area F7a | Area F7b | Area F8 | Area F9a | Area Sample 1F10a | Area F10b | Area F11a | Area F11b | Area F12a | Area F13 | #Feature | #Feature F1 | #Feature F2a | #Feature F3a | #Feature F4 | #Feature F5 | #Feature F6a | #Feature F7a | #Feature F7b | #Feature F8 | #Feature F9a | #Feature Sample 1F10a | #Feature F10b | #Feature F11a | #Feature F11b | #Feature F12a | #Feature F13 | Start | End | PTM | AScore | Found By |
|---|---|---|---|---|---|---|---|---|---|---|---|---|---|---|---|---|---|---|---|---|---|---|---|---|---|---|---|---|---|---|---|---|---|---|---|---|---|---|---|---|---|---|---|---|---|---|---|---|---|
| N.YGC(+57.02)YC(+57.02)GYGGSGTPVDELDR.C | N | 112.36 | 2124.8516 | 19 | 3.7 | 1063.4326 | 2 | 56.60 | 14 | F14:33384 | BuCaTN01_F11b.raw | | | 0 | | | 2.0097E6 | | 1.4053E7 | | 8.8531E7 | 3.4891E6 | 4.8054E8 | 6.165E7 | 1.7851E9 | 1.2628E8 | | 69 | 0 | 0 | 0 | 0 | 0 | 1 | 0 | 2 | 0 | 3 | 1 | 4 | 2 | 53 | 3 | 0 | 50 | 68 | Carbamidomethylation | C3:Carbamidomethylation:1000.00;C5:Carbamidomethylation:1000.00 | PEAKS DB |
| Y.C(+57.02)GYGGSGTPVDELDR.C | N | 88.12 | 1581.6729 | 15 | 4.4 | 791.8439 | 2 | 48.02 | 14 | F14:25306 | BuCaTN01_F11b.raw | | | | | | | | 2.166E6 | | 8.5786E6 | | 1.5853E7 | 6.495E5 | 1.7537E7 | 0 | | 6 | 0 | 0 | 0 | 0 | 0 | 0 | 0 | 1 | 0 | 1 | 0 | 2 | 1 | 1 | 0 | 0 | 54 | 68 | Carbamidomethylation | C1:Carbamidomethylation:1000.00 | PEAKS DB |
| G.C(+57.02)YC(+57.02)GYGGSGTPVDELDR.C | N | 84.40 | 1904.7668 | 17 | 2.7 | 953.3911 | 2 | 53.16 | 12 | F12:29351 | BuCaTN01_F10b.raw | | | | | | | | 4.1849E5 | | | | 3.7261E5 | | 2.1774E6 | | | 3 | 0 | 0 | 0 | 0 | 0 | 0 | 0 | 1 | 0 | 0 | 0 | 1 | 0 | 1 | 0 | 0 | 52 | 68 | Carbamidomethylation | C1:Carbamidomethylation:1000.00;C3:Carbamidomethylation:1000.00 | PEAKS DB |
| R.TYSYDC(+57.02)SEGKLTC(+57.02)K.D | N | 82.89 | 1710.7229 | 14 | 0.5 | 856.3691 | 2 | 38.29 | 7 | F7:14505 | BuCaTN01_F7a.raw | | | | | | | 1.4512E8 | 2.3155E7 | | 3.8991E5 | 4.5941E5 | | | | | | 8 | 0 | 0 | 0 | 0 | 0 | 0 | 4 | 2 | 0 | 1 | 1 | 0 | 0 | 0 | 0 | 0 | 92 | 105 | Carbamidomethylation | C6:Carbamidomethylation:1000.00;C13:Carbamidomethylation:1000.00 | PEAKS DB |
| Y.GC(+57.02)YC(+57.02)GYGGSGTPVDELDR.C | N | 82.10 | 1961.7883 | 18 | 1.4 | 981.9028 | 2 | 53.49 | 10 | F10:30232 | BuCaTN01_F9a.raw | | | | | | | | 1.7852E6 | | 3.3077E6 | | 1.5846E6 | | 3.2964E6 | 1.0428E5 | | 5 | 0 | 0 | 0 | 0 | 0 | 0 | 0 | 1 | 0 | 1 | 0 | 1 | 0 | 1 | 1 | 0 | 51 | 68 | Carbamidomethylation | C2:Carbamidomethylation:1000.00;C4:Carbamidomethylation:1000.00 | PEAKS DB |
| R.TYSYDC(+57.02)SEGKLTC(+57.02)KD.N | N | 75.87 | 1825.7499 | 15 | 2.5 | 913.8818 | 2 | 38.60 | 8 | F8:16643 | BuCaTN01_F7b.raw | | | | | | | | 7.3044E5 | | | | | | | | | 1 | 0 | 0 | 0 | 0 | 0 | 0 | 0 | 1 | 0 | 0 | 0 | 0 | 0 | 0 | 0 | 0 | 92 | 106 | Carbamidomethylation | C6:Carbamidomethylation:1000.00;C13:Carbamidomethylation:1000.00 | PEAKS DB |
| C.YC(+57.02)GYGGSGTPVDELDR.C | N | 75.62 | 1744.7362 | 16 | 4.1 | 873.3754 | 2 | 52.27 | 14 | F14:28849 | BuCaTN01_F11b.raw | | | | | | | | 7.7286E5 | | 7.3447E5 | | | | 3.0904E6 | 1.4057E5 | | 4 | 0 | 0 | 0 | 0 | 0 | 0 | 0 | 1 | 0 | 1 | 0 | 0 | 0 | 1 | 1 | 0 | 53 | 68 | Carbamidomethylation | C2:Carbamidomethylation:1000.00 | PEAKS DB |
| G.YGGSGTPVDELDR.C | N | 75.26 | 1364.6207 | 13 | 4.1 | 683.3177 | 2 | 45.29 | 14 | F14:22502 | BuCaTN01_F11b.raw | | | | | | | 6.8893E5 | | | | | | | 9.1406E6 | 6.9945E5 | | 3 | 0 | 0 | 0 | 0 | 0 | 0 | 1 | 0 | 0 | 0 | 0 | 0 | 0 | 1 | 1 | 0 | 56 | 68 | | | PEAKS DB |
| R.TAALC(+57.02)FAKAPY.N | N | 73.24 | 1211.6008 | 11 | 2.2 | 606.8076 | 2 | 55.84 | 12 | F12:32023 | BuCaTN01_F10b.raw | | | | | | | 7.3334E4 | | | 1.7166E6 | 1.3753E5 | 1.06E7 | | 8.1407E6 | 9.828E5 | | 7 | 0 | 0 | 0 | 0 | 0 | 0 | 1 | 0 | 0 | 1 | 1 | 2 | 0 | 1 | 1 | 0 | 121 | 131 | Carbamidomethylation | C5:Carbamidomethylation:1000.00 | PEAKS DB |
| T.NYGC(+57.02)YC(+57.02)GYGGSGTPVDELDR.C | N | 72.69 | 2238.8945 | 20 | 5.3 | 1120.4559 | 2 | 64.81 | 14 | F14:40195 | BuCaTN01_F11b.raw | | | | | | | | | | 2.3985E4 | | 0 | 1.1223E5 | 5.3539E5 | | | 3 | 0 | 0 | 0 | 0 | 0 | 0 | 0 | 0 | 0 | 1 | 0 | 0 | 1 | 1 | 0 | 0 | 49 | 68 | Carbamidomethylation | C4:Carbamidomethylation:1000.00;C6:Carbamidomethylation:1000.00 | PEAKS DB |
| R.TAALC(+57.02)FAKAPYN.D | N | 69.35 | 1325.6438 | 12 | -0.2 | 663.8290 | 2 | 51.91 | 6 | F6:30474 | BuCaTN01_F6a.raw | | | | | | 3.7675E6 | | 1.5632E7 | | 4.6358E6 | | 1.9346E6 | | | | | 5 | 0 | 0 | 0 | 0 | 0 | 1 | 0 | 2 | 0 | 1 | 0 | 1 | 0 | 0 | 0 | 0 | 121 | 132 | Carbamidomethylation | C5:Carbamidomethylation:1000.00 | PEAKS DB |
| C.GYGGSGTPVDELDR.C | N | 66.26 | 1421.6422 | 14 | 0.0 | 711.8284 | 2 | 46.82 | 10 | F10:24048 | BuCaTN01_F9a.raw | | | | | | | 2.9103E5 | 1.2275E6 | | 2.1867E6 | | | | 8.7883E6 | | | 5 | 0 | 0 | 0 | 0 | 0 | 0 | 1 | 1 | 0 | 1 | 0 | 0 | 0 | 2 | 0 | 0 | 55 | 68 | | | PEAKS DB |
| N.C(+57.02)DRTAALC(+57.02)FAK.A | N | 63.48 | 1311.6063 | 11 | -1.7 | 438.2076 | 3 | 37.36 | 12 | F12:15762 | BuCaTN01_F10b.raw | | | | | | | | | | | | 5.2815E4 | | 3.1918E5 | | | 2 | 0 | 0 | 0 | 0 | 0 | 0 | 0 | 0 | 0 | 0 | 0 | 1 | 0 | 1 | 0 | 0 | 118 | 128 | Carbamidomethylation | C1:Carbamidomethylation:1000.00;C8:Carbamidomethylation:1000.00 | PEAKS DB |
| R.TAALC(+57.02)FAK.A | N | 60.21 | 880.4476 | 8 | 3.4 | 441.2316 | 2 | 41.58 | 12 | F12:19288 | BuCaTN01_F10b.raw | | | | | | 9.3985E4 | 9.2429E6 | 1.2777E7 | 4.6024E5 | 3.2388E7 | 3.591E6 | 1.265E8 | 3.5731E6 | 1.1283E8 | 1.0943E8 | | 21 | 0 | 0 | 0 | 0 | 0 | 1 | 4 | 2 | 1 | 2 | 2 | 2 | 1 | 2 | 4 | 0 | 121 | 128 | Carbamidomethylation | C5:Carbamidomethylation:1000.00 | PEAKS DB |
| K.DFVC(+57.02)NC(+57.02)DRTAALC(+57.02)FAK.A | N | 59.68 | 1946.8438 | 16 | -0.4 | 974.4265 | 2 | 51.09 | 12 | F12:27773 | BuCaTN01_F10b.raw | | | | | | | | | | 1.1341E6 | | 0 | | | | | 1 | 0 | 0 | 0 | 0 | 0 | 0 | 0 | 0 | 0 | 1 | 0 | 0 | 0 | 0 | 0 | 0 | 113 | 128 | Carbamidomethylation | C4:Carbamidomethylation:1000.00;C6:Carbamidomethylation:1000.00;C13:Carbamidomethylation:1000.00 | PEAKS DB |
| G.GSGTPVDELDR.C | N | 56.36 | 1144.5360 | 11 | 3.4 | 573.2756 | 2 | 40.15 | 8 | F8:17921 | BuCaTN01_F7b.raw | | | | | | | | 1.1068E6 | | | | | | | | | 1 | 0 | 0 | 0 | 0 | 0 | 0 | 0 | 1 | 0 | 0 | 0 | 0 | 0 | 0 | 0 | 0 | 58 | 68 | | | PEAKS DB |
| R.TYSYDC(+57.02)SEGK.L | N | 55.61 | 1208.4656 | 10 | 0.1 | 605.2401 | 2 | 29.72 | 7 | F7:8746 | BuCaTN01_F7a.raw | | | | | | | 5.0914E6 | | | | | | | | | | 1 | 0 | 0 | 0 | 0 | 0 | 0 | 1 | 0 | 0 | 0 | 0 | 0 | 0 | 0 | 0 | 0 | 92 | 101 | Carbamidomethylation | C6:Carbamidomethylation:1000.00 | PEAKS DB |
| N.M(+15.99)IKC(+57.02)TNTRHWLSFTNYGC(+57.02)YC(+57.02)GYGGSGTPVDELDR.C | Y | 53.32 | 4030.7495 | 34 | 1.3 | 1008.6960 | 4 | 77.35 | 10 | F10:53957 | BuCaTN01_F9a.raw | | | | | | | | | | 0 | | | | | | | 0 | 0 | 0 | 0 | 0 | 0 | 0 | 0 | 0 | 0 | 0 | 0 | 0 | 0 | 0 | 0 | 0 | 35 | 68 | Oxidation (M); Carbamidomethylation | M1:Oxidation (M):1000.00;C4:Carbamidomethylation:1000.00;C18:Carbamidomethylation:1000.00;C20:Carbamidomethylation:1000.00 | PEAKS DB |
| R.TYSYDC(+57.02)SEGKLTC(+57.02).K | N | 52.98 | 1582.6279 | 13 | 2.9 | 792.3212 | 2 | 43.19 | 8 | F8:20253 | BuCaTN01_F7b.raw | | | | | | | | 5.7951E5 | | | | | | | | | 1 | 0 | 0 | 0 | 0 | 0 | 0 | 0 | 1 | 0 | 0 | 0 | 0 | 0 | 0 | 0 | 0 | 92 | 104 | Carbamidomethylation | C6:Carbamidomethylation:1000.00;C13:Carbamidomethylation:1000.00 | PEAKS DB |
| R.TYSYDC(+57.02)SEGKLT.C | N | 50.03 | 1422.5973 | 12 | 2.7 | 712.3058 | 2 | 41.55 | 8 | F8:18942 | BuCaTN01_F7b.raw | | | | | | | 5.0044E6 | 1.8313E6 | | | | | | | | | 2 | 0 | 0 | 0 | 0 | 0 | 0 | 1 | 1 | 0 | 0 | 0 | 0 | 0 | 0 | 0 | 0 | 92 | 103 | Carbamidomethylation | C6:Carbamidomethylation:1000.00 | PEAKS DB |
| Y.GGSGTPVDELDR.C | N | 49.09 | 1201.5575 | 12 | 0.3 | 601.7862 | 2 | 40.57 | 11 | F11:14528 | BuCaTN01_F10a.raw | | | | | | | 9.2274E5 | 2.7577E6 | | 1.1335E7 | 6.7591E5 | | 9.9086E5 | 2.6759E7 | | | 6 | 0 | 0 | 0 | 0 | 0 | 0 | 1 | 1 | 0 | 1 | 1 | 0 | 1 | 1 | 0 | 0 | 57 | 68 | | | PEAKS DB |
| R.TAALC(+57.02)FAKAP.Y | N | 47.25 | 1048.5375 | 10 | 3.6 | 525.2764 | 2 | 48.84 | 8 | F8:25881 | BuCaTN01_F7b.raw | | | | | | 3.1579E6 | | 4.0965E6 | | | | | | | | | 2 | 0 | 0 | 0 | 0 | 0 | 1 | 0 | 1 | 0 | 0 | 0 | 0 | 0 | 0 | 0 | 0 | 121 | 130 | Carbamidomethylation | C5:Carbamidomethylation:1000.00 | PEAKS DB |

**total 22 peptides**

**Best Unique PSM (Scan F10:53957, m/z=1008.6960, z=4, RT=77.35, ppm=1.3):**

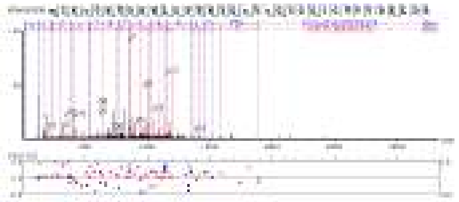

## JAS04994.1

| Protein Coverage | Supporting Peptides | Best Unique PSM |

back to list

**Protein Coverage:**

1 MNPAHLLVLA AVCVSLLGAS SIPPEPLNLY QFKNMIKCTN TRHWLSFTNY GCYCGYGGSG TPVDELDRCC QVHDKCYDTA

81 KRVHKCFPSV RTYSYDCSEG KLTCKDNNTK CKDFVCNCDR TAALCFAKAP YNDKNYNIDL KRCQ

C Carbamidomethylation (+57.02)
O Oxidation (M) (+15.99)

**Supporting Peptides:**

| Peptide | Uniq | -10lgP | Mass | Length | ppm | m/z | z | RT | Fraction | Scan | Source File | Area F1 | Area F2a | Area F3a | Area F4 | Area F5 | Area F6a | Area F7a | Area F7b | Area F8 | Area F9a | Area Sample 1F10a | Area F10b | Area F11a | Area F11b | Area F12a | Area F13 | #Feature | #Feature F1 | #Feature F2a | #Feature F3a | #Feature F4 | #Feature F5 | #Feature F6a | #Feature F7a | #Feature F7b | #Feature F8 | #Feature F9a | #Feature Sample 1F10a | #Feature F10b | #Feature F11a | #Feature F11b | #Feature F12a | #Feature F13 | Start | End | PTM | AScore | Found By |
|---|---|---|---|---|---|---|---|---|---|---|---|---|---|---|---|---|---|---|---|---|---|---|---|---|---|---|---|---|---|---|---|---|---|---|---|---|---|---|---|---|---|---|---|---|---|---|---|---|---|
| N.YGC(+57.02)YC(+57.02)GYGGSGTPVDELDR.C | N | 112.36 | 2124.8516 | 19 | 3.7 | 1063.4326 | 2 | 56.60 | 14 | F14:33384 | BuCaTN01_F11b.raw | | | 0 | | | 2.0097E6 | | 1.4053E7 | | 8.8531E7 | 3.4891E6 | 4.8054E8 | 6.165E7 | 1.7851E9 | 1.2628E8 | | 69 | 0 | 0 | 0 | 0 | 0 | 1 | 0 | 2 | 0 | 3 | 1 | 4 | 2 | 53 | 3 | 0 | 50 | 68 | Carbamidomethylation | C3:Carbamidomethylation:1000.00;C5:Carbamidomethylation:1000.00 | PEAKS DB |
| Y.C(+57.02)GYGGSGTPVDELDR.C | N | 88.12 | 1581.6729 | 15 | 4.4 | 791.8439 | 2 | 48.02 | 14 | F14:25306 | BuCaTN01_F11b.raw | | | | | | | | 2.166E6 | | 8.5786E6 | | 1.5853E7 | 6.495E5 | 1.7537E7 | 0 | | 6 | 0 | 0 | 0 | 0 | 0 | 0 | 0 | 1 | 0 | 1 | 0 | 2 | 1 | 1 | 0 | 0 | 54 | 68 | Carbamidomethylation | C1:Carbamidomethylation:1000.00 | PEAKS DB |
| G.C(+57.02)YC(+57.02)GYGGSGTPVDELDR.C | N | 84.40 | 1904.7668 | 17 | 2.7 | 953.3911 | 2 | 53.16 | 12 | F12:29351 | BuCaTN01_F10b.raw | | | | | | | | 4.1849E5 | | | | 3.7261E5 | | 2.1774E6 | | | 3 | 0 | 0 | 0 | 0 | 0 | 0 | 0 | 1 | 0 | 0 | 0 | 1 | 0 | 1 | 0 | 0 | 52 | 68 | Carbamidomethylation | C1:Carbamidomethylation:1000.00;C3:Carbamidomethylation:1000.00 | PEAKS DB |
| R.TYSYDC(+57.02)SEGKLTC(+57.02)K.D | N | 82.89 | 1710.7229 | 14 | 0.5 | 856.3691 | 2 | 38.29 | 7 | F7:14505 | BuCaTN01_F7a.raw | | | | | | | 1.4512E8 | 2.3155E7 | | 3.8991E5 | 4.5941E5 | | | | | | 8 | 0 | 0 | 0 | 0 | 0 | 0 | 4 | 2 | 0 | 1 | 1 | 0 | 0 | 0 | 0 | 0 | 92 | 105 | Carbamidomethylation | C6:Carbamidomethylation:1000.00;C13:Carbamidomethylation:1000.00 | PEAKS DB |
| Y.GC(+57.02)YC(+57.02)GYGGSGTPVDELDR.C | N | 82.10 | 1961.7883 | 18 | 1.4 | 981.9028 | 2 | 53.49 | 10 | F10:30232 | BuCaTN01_F9a.raw | | | | | | | | 1.7852E6 | | 3.3077E6 | | 1.5846E6 | | 3.2964E6 | 1.0428E5 | | 5 | 0 | 0 | 0 | 0 | 0 | 0 | 0 | 1 | 0 | 1 | 0 | 1 | 0 | 1 | 1 | 0 | 51 | 68 | Carbamidomethylation | C2:Carbamidomethylation:1000.00;C4:Carbamidomethylation:1000.00 | PEAKS DB |
| R.TYSYDC(+57.02)SEGKLTC(+57.02)KD.N | N | 75.87 | 1825.7499 | 15 | 2.5 | 913.8818 | 2 | 38.60 | 8 | F8:16643 | BuCaTN01_F7b.raw | | | | | | | | 7.3044E5 | | | | | | | | | 1 | 0 | 0 | 0 | 0 | 0 | 0 | 0 | 1 | 0 | 0 | 0 | 0 | 0 | 0 | 0 | 0 | 92 | 106 | Carbamidomethylation | C6:Carbamidomethylation:1000.00;C13:Carbamidomethylation:1000.00 | PEAKS DB |
| C.YC(+57.02)GYGGSGTPVDELDR.C | N | 75.62 | 1744.7362 | 16 | 4.1 | 873.3754 | 2 | 52.27 | 14 | F14:28849 | BuCaTN01_F11b.raw | | | | | | | | 7.7286E5 | | 7.3447E5 | | | | 3.0904E6 | 1.4057E5 | | 4 | 0 | 0 | 0 | 0 | 0 | 0 | 0 | 1 | 0 | 1 | 0 | 0 | 0 | 1 | 1 | 0 | 53 | 68 | Carbamidomethylation | C2:Carbamidomethylation:1000.00 | PEAKS DB |
| G.YGGSGTPVDELDR.C | N | 75.26 | 1364.6207 | 13 | 4.1 | 683.3177 | 2 | 45.29 | 14 | F14:22502 | BuCaTN01_F11b.raw | | | | | | | 6.8893E5 | | | | | | | 9.1406E6 | 6.9945E5 | | 3 | 0 | 0 | 0 | 0 | 0 | 0 | 1 | 0 | 0 | 0 | 0 | 0 | 0 | 1 | 1 | 0 | 56 | 68 | | | PEAKS DB |
| R.TAALC(+57.02)FAKAPY.N | N | 73.24 | 1211.6008 | 11 | 2.2 | 606.8076 | 2 | 55.84 | 12 | F12:32023 | BuCaTN01_F10b.raw | | | | | | | 7.3334E4 | | | 1.7166E6 | 1.3753E5 | 1.06E7 | | 8.1407E6 | 9.828E5 | | 7 | 0 | 0 | 0 | 0 | 0 | 0 | 1 | 0 | 0 | 1 | 1 | 2 | 0 | 1 | 1 | 0 | 121 | 131 | Carbamidomethylation | C5:Carbamidomethylation:1000.00 | PEAKS DB |
| T.NYGC(+57.02)YC(+57.02)GYGGSGTPVDELDR.C | N | 72.69 | 2238.8945 | 20 | 5.3 | 1120.4559 | 2 | 64.81 | 14 | F14:40195 | BuCaTN01_F11b.raw | | | | | | | | | | 2.3985E4 | | 0 | 1.1223E5 | 5.3539E5 | | | 3 | 0 | 0 | 0 | 0 | 0 | 0 | 0 | 0 | 0 | 1 | 0 | 0 | 1 | 1 | 0 | 0 | 49 | 68 | Carbamidomethylation | C4:Carbamidomethylation:1000.00;C6:Carbamidomethylation:1000.00 | PEAKS DB |
| R.TAALC(+57.02)FAKAPYN.D | N | 69.35 | 1325.6438 | 12 | -0.2 | 663.8290 | 2 | 51.91 | 6 | F6:30474 | BuCaTN01_F6a.raw | | | | | | 3.7675E6 | | 1.5632E7 | | 4.6358E6 | | 1.9346E6 | | | | | 5 | 0 | 0 | 0 | 0 | 0 | 1 | 0 | 2 | 0 | 1 | 0 | 1 | 0 | 0 | 0 | 0 | 121 | 132 | Carbamidomethylation | C5:Carbamidomethylation:1000.00 | PEAKS DB |
| C.GYGGSGTPVDELDR.C | N | 66.26 | 1421.6422 | 14 | 0.0 | 711.8284 | 2 | 46.82 | 10 | F10:24048 | BuCaTN01_F9a.raw | | | | | | | 2.9103E5 | 1.2275E6 | | 2.1867E6 | | | | 8.7883E6 | | | 5 | 0 | 0 | 0 | 0 | 0 | 0 | 1 | 1 | 0 | 1 | 0 | 0 | 0 | 2 | 0 | 0 | 55 | 68 | | | PEAKS DB |
| N.C(+57.02)DRTAALC(+57.02)FAK.A | N | 63.48 | 1311.6063 | 11 | -1.7 | 438.2076 | 3 | 37.36 | 12 | F12:15762 | BuCaTN01_F10b.raw | | | | | | | | | | | | 5.2815E4 | | 3.1918E5 | | | 2 | 0 | 0 | 0 | 0 | 0 | 0 | 0 | 0 | 0 | 0 | 0 | 1 | 0 | 1 | 0 | 0 | 118 | 128 | Carbamidomethylation | C1:Carbamidomethylation:1000.00;C8:Carbamid | PEAKS DB |

| | | | | | | | | | | | | | | | | | | | | | | | | | | | | | | | | | | | | | | | | | | | | | | | | | |
|---|---|---|---|---|---|---|---|---|---|---|---|---|---|---|---|---|---|---|---|---|---|---|---|---|---|---|---|---|---|---|---|---|---|---|---|---|---|---|---|---|---|---|---|---|---|---|---|---|---|
| | | | | | | | | | | | | | | | | | | | | | | | | | | | | | | | | | | | | | | | | | | | | | | | | omethylation:1000.00 | |
| R.TAALC(+57.02)FAK.A | N | 60.21 | 880.4476 | 8 | 3.4 | 441.2316 | 2 | 41.58 | 12 | F12:19288 | BuCaTN01_F10b.raw | | | | | | 9.3985E4 | 9.2429E6 | 1.2777E7 | 4.6024E5 | 3.2388E7 | 3.591E6 | 1.265E8 | 3.5731E6 | 1.1283E8 | 1.0943E8 | | 21 | 0 | 0 | 0 | 0 | 0 | 1 | 4 | 2 | 1 | 2 | 2 | 2 | 1 | 2 | 4 | 0 | 121 | 128 | Carbamidomethylation | C5:Carbamidomethylation:1000.00 | PEAKS DB |
| K.DFVC(+57.02)NC(+57.02)DRTAALC(+57.02)FAK.A | N | 59.68 | 1946.8438 | 16 | -0.4 | 974.4265 | 2 | 51.09 | 12 | F12:27773 | BuCaTN01_F10b.raw | | | | | | | | | | 1.1341E6 | | 0 | | | | | 1 | 0 | 0 | 0 | 0 | 0 | 0 | 0 | 0 | 0 | 1 | 0 | 0 | 0 | 0 | 0 | 0 | 113 | 128 | Carbamidomethylation | C4:Carbamidomethylation:1000.00;C6:Carbamidomethylation:1000.00;C13:Carbamidomethylation:1000.00 | PEAKS DB |
| G.GSGTPVDELDR.C | N | 56.36 | 1144.5360 | 11 | 3.4 | 573.2756 | 2 | 40.15 | 8 | F8:17921 | BuCaTN01_F7b.raw | | | | | | | | 1.1068E6 | | | | | | | | | 1 | 0 | 0 | 0 | 0 | 0 | 0 | 0 | 1 | 0 | 0 | 0 | 0 | 0 | 0 | 0 | 0 | 58 | 68 | | | PEAKS DB |
| R.TYSYDC(+57.02)SEGK.L | N | 55.61 | 1208.4656 | 10 | 0.1 | 605.2401 | 2 | 29.72 | 7 | F7:8746 | BuCaTN01_F7a.raw | | | | | | | 5.0914E6 | | | | | | | | | | 1 | 0 | 0 | 0 | 0 | 0 | 0 | 1 | 0 | 0 | 0 | 0 | 0 | 0 | 0 | 0 | 0 | 92 | 101 | Carbamidomethylation | C6:Carbamidomethylation:1000.00 | PEAKS DB |
| N.M(+15.99)IKC(+57.02)TNTRHWLSFTNYGC(+57.02)YC(+57.02)GYGGSGTPVDELDR.C | Y | 53.32 | 4030.7495 | 34 | 1.3 | 1008.6960 | 4 | 77.35 | 10 | F10:53957 | BuCaTN01_F9a.raw | | | | | | | | | | 0 | | | | | | | 0 | 0 | 0 | 0 | 0 | 0 | 0 | 0 | 0 | 0 | 0 | 0 | 0 | 0 | 0 | 0 | 0 | 35 | 68 | Oxidation (M); Carbamidomethylation | M1:Oxidation (M):1000.00;C4:Carbamidomethylation:1000.00;C18:Carbamidomethylation:1000.00;C20:Carbamidomethylation:1000.00 | PEAKS DB |
| R.TYSYDC(+57.02)SEGKLTC(+57.02).K | N | 52.98 | 1582.6279 | 13 | 2.9 | 792.3212 | 2 | 43.19 | 8 | F8:20253 | BuCaTN01_F7b.raw | | | | | | | | 5.7951E5 | | | | | | | | | 1 | 0 | 0 | 0 | 0 | 0 | 0 | 0 | 1 | 0 | 0 | 0 | 0 | 0 | 0 | 0 | 0 | 92 | 104 | Carbamidomethylation | C6:Carbamidomethylation:1000.00;C13:Carbamidomethylation:1000.00 | PEAKS DB |
| R.TYSYDC(+57.02)SEGKLT.C | N | 50.03 | 1422.5973 | 12 | 2.7 | 712.3058 | 2 | 41.55 | 8 | F8:18942 | BuCaTN01_F7b.raw | | | | | | | 5.0044E6 | 1.8313E6 | | | | | | | | | 2 | 0 | 0 | 0 | 0 | 0 | 0 | 1 | 1 | 0 | 0 | 0 | 0 | 0 | 0 | 0 | 0 | 92 | 103 | Carbamidomethylation | C6:Carbamidomethylation:1000.00 | PEAKS DB |
| Y.GGSGTPVDELDR.C | N | 49.09 | 1201.5575 | 12 | 0.3 | 601.7862 | 2 | 40.57 | 11 | F11:14528 | BuCaTN01_F10a.raw | | | | | | | 9.2274E5 | 2.7577E6 | | 1.1335E7 | 6.7591E5 | | 9.9086E5 | 2.6759E7 | | | 6 | 0 | 0 | 0 | 0 | 0 | 0 | 1 | 1 | 0 | 1 | 1 | 0 | 1 | 1 | 0 | 0 | 57 | 68 | | | PEAKS DB |
| R.TAALC(+57.02)FAKAP.Y | N | 47.25 | 1048.5375 | 10 | 3.6 | 525.2764 | 2 | 48.84 | 8 | F8:25881 | BuCaTN01_F7b.raw | | | | | | 3.1579E6 | | 4.0965E6 | | | | | | | | | 2 | 0 | 0 | 0 | 0 | 0 | 1 | 0 | 1 | 0 | 0 | 0 | 0 | 0 | 0 | 0 | 0 | 121 | 130 | Carbamidomethylation | C5:Carbamidomethylation:1000.00 | PEAKS DB |
| **total 22 peptides** | | | | | | | | | | | | | | | | | | | | | | | | | | | | | | | | | | | | | | | | | | | | | | | | | |

**Best Unique PSM (Scan F10:53957, m/z=1008.6960, z=4, RT=77.35, ppm=1.3):**

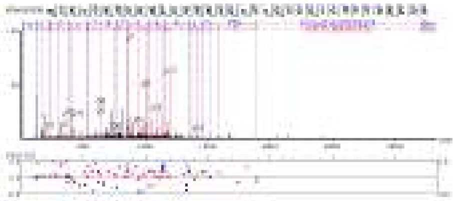

## JAI09005.1

back to list

| Protein Coverage | Supporting Peptides | Best Unique PSM |

**Protein Coverage:**

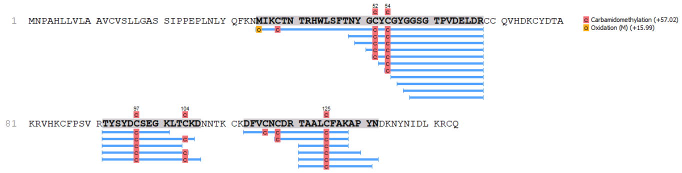


**Supporting Peptides:**

| Peptide | Uniq | -10lgP | Mass | Length | ppm | m/z | z | RT | Fraction | Scan | Source File | Area F1 | Area F2a | Area F3a | Area F4 | Area F5 | Area F6a | Area F7a | Area F7b | Area F8 | Area F9a | Area Sample 1F10a | Area F10b | Area F11a | Area F11b | Area F12a | Area F13 | #Feature | #Feature F1 | #Feature F2a | #Feature F3a | #Feature F4 | #Feature F5 | #Feature F6a | #Feature F7a | #Feature F7b | #Feature F8 | #Feature F9a | #Feature Sample 1F10a | #Feature F10b | #Feature F11a | #Feature F11b | #Feature F12a | #Feature F13 | Start | End | PTM | AScore | Found By |
|---|---|---|---|---|---|---|---|---|---|---|---|---|---|---|---|---|---|---|---|---|---|---|---|---|---|---|---|---|---|---|---|---|---|---|---|---|---|---|---|---|---|---|---|---|---|---|---|---|---|
| N.YGC(+57.02)YC(+57.02)GYGGSGTPVDELDR.C | N | 112.36 | 2124.8516 | 19 | 3.7 | 1063.4326 | 2 | 56.60 | 14 | F14:33384 | BuCaTN01_F11b.raw | | | 0 | | | 2.0097E6 | | 1.4053E7 | | 8.8531E7 | 3.4891E6 | 4.8054E8 | 6.165E7 | 1.7851E9 | 1.2628E8 | | 69 | 0 | 0 | 0 | 0 | 0 | 1 | 0 | 2 | 0 | 3 | 1 | 4 | 2 | 53 | 3 | 0 | 50 | 68 | Carbamidomethylation | C3:Carbamidomethylation:1000.00;C5:Carbamidomethylation:1000.00 | PEAKS DB |
| Y.C(+57.02)GYGGSGTPVDELDR.C | N | 88.12 | 1581.6729 | 15 | 4.4 | 791.8439 | 2 | 48.02 | 14 | F14:25306 | BuCaTN01_F11b.raw | | | | | | | | 2.166E6 | | 8.5786E6 | | 1.5853E7 | 6.495E5 | 1.7537E7 | 0 | | 6 | 0 | 0 | 0 | 0 | 0 | 0 | 0 | 1 | 0 | 1 | 0 | 2 | 1 | 1 | 0 | 0 | 54 | 68 | Carbamidomethylation | C1:Carbamidomethylation:1000.00 | PEAKS DB |
| G.C(+57.02)YC(+57.02)GYGGSGTPVDELDR.C | N | 84.40 | 1904.7668 | 17 | 2.7 | 953.3911 | 2 | 53.16 | 12 | F12:29351 | BuCaTN01_F10b.raw | | | | | | | | 4.1849E5 | | | | 3.7261E5 | | 2.1774E6 | | | 3 | 0 | 0 | 0 | 0 | 0 | 0 | 0 | 1 | 0 | 0 | 0 | 1 | 0 | 1 | 0 | 0 | 52 | 68 | Carbamidomethylation | C1:Carbamidomethylation:1000.00;C3:Carbamidomethylation:1000.00 | PEAKS DB |
| R.TYSYDC(+57.02)SEGKLTC(+57.02)K.D | N | 82.89 | 1710.7229 | 14 | 0.5 | 856.3691 | 2 | 38.29 | 7 | F7:14505 | BuCaTN01_F7a.raw | | | | | | | 1.4512E8 | 2.3155E7 | | 3.8991E5 | 4.5941E5 | | | | | | 8 | 0 | 0 | 0 | 0 | 0 | 0 | 4 | 2 | 0 | 1 | 1 | 0 | 0 | 0 | 0 | 0 | 92 | 105 | Carbamidomethylation | C6:Carbamidomethylation:1000.00;C13:Carbamidomethylation:1000.00 | PEAKS DB |
| Y.GC(+57.02)YC(+57.02)GYGGSGTPVDELDR.C | N | 82.10 | 1961.7883 | 18 | 1.4 | 981.9028 | 2 | 53.49 | 10 | F10:30232 | BuCaTN01_F9a.raw | | | | | | | | 1.7852E6 | | 3.3077E6 | | 1.5846E6 | | 3.2964E6 | 1.0428E5 | | 5 | 0 | 0 | 0 | 0 | 0 | 0 | 0 | 1 | 0 | 1 | 0 | 1 | 0 | 1 | 1 | 0 | 51 | 68 | Carbamidomethylation | C2:Carbamidomethylation:1000.00;C4:Carbamidomethylation:1000.00 | PEAKS DB |
| R.TYSYDC(+57.02)SEGKLTC(+57.02)KD.N | N | 75.87 | 1825.7499 | 15 | 2.5 | 913.8818 | 2 | 38.60 | 8 | F8:16643 | BuCaTN01_F7b.raw | | | | | | | | 7.3044E5 | | | | | | | | | 1 | 0 | 0 | 0 | 0 | 0 | 0 | 0 | 1 | 0 | 0 | 0 | 0 | 0 | 0 | 0 | 0 | 92 | 106 | Carbamidomethylation | C6:Carbamidomethylation:1000.00;C13:Carbamidomethylation:1000.00 | PEAKS DB |
| C.YC(+57.02)GYGGSGTPVDELDR.C | N | 75.62 | 1744.7362 | 16 | 4.1 | 873.3754 | 2 | 52.27 | 14 | F14:28849 | BuCaTN01_F11b.raw | | | | | | | | 7.7286E5 | | 7.3447E5 | | | | 3.0904E6 | 1.4057E5 | | 4 | 0 | 0 | 0 | 0 | 0 | 0 | 0 | 1 | 0 | 1 | 0 | 0 | 0 | 1 | 1 | 0 | 53 | 68 | Carbamidomethylation | C2:Carbamidomethylation:1000.00 | PEAKS DB |
| G.YGGSGTPVDELDR.C | N | 75.26 | 1364.6207 | 13 | 4.1 | 683.3177 | 2 | 45.29 | 14 | F14:22502 | BuCaTN01_F11b.raw | | | | | | | 6.8893E5 | | | | | | | 9.1406E6 | 6.9945E5 | | 3 | 0 | 0 | 0 | 0 | 0 | 0 | 1 | 0 | 0 | 0 | 0 | 0 | 0 | 1 | 1 | 0 | 56 | 68 | | | PEAKS DB |
| R.TAALC(+57.02)FAKAPY.N | N | 73.24 | 1211.6008 | 11 | 2.2 | 606.8076 | 2 | 55.84 | 12 | F12:32023 | BuCaTN01_F10b.raw | | | | | | | 7.3334E4 | | | 1.7166E6 | 1.3753E5 | 1.06E7 | | 8.1407E6 | 9.828E5 | | 7 | 0 | 0 | 0 | 0 | 0 | 0 | 1 | 0 | 0 | 1 | 1 | 2 | 0 | 1 | 1 | 0 | 121 | 131 | Carbamidomethylation | C5:Carbamidomethylation:1000.00 | PEAKS DB |
| T.NYGC(+57.02)YC(+57.02)GYGGSGTPVDELDR.C | N | 72.69 | 2238.8945 | 20 | 5.3 | 1120.4559 | 2 | 64.81 | 14 | F14:40195 | BuCaTN01_F11b.raw | | | | | | | | | | 2.3985E4 | | 0 | 1.1223E5 | 5.3539E5 | | | 3 | 0 | 0 | 0 | 0 | 0 | 0 | 0 | 0 | 0 | 1 | 0 | 0 | 1 | 1 | 0 | 0 | 49 | 68 | Carbamidomethylation | C4:Carbamidomethylation:1000.00;C6:Carbamidomethylation:1000.00 | PEAKS DB |
| R.TAALC(+57.02)FAKAPYN.D | N | 69.35 | 1325.6438 | 12 | -0.2 | 663.8290 | 2 | 51.91 | 6 | F6:30474 | BuCaTN01_F6a.raw | | | | | | 3.7675E6 | | 1.5632E7 | | 4.6358E6 | | 1.9346E6 | | | | | 5 | 0 | 0 | 0 | 0 | 0 | 1 | 0 | 2 | 0 | 1 | 0 | 1 | 0 | 0 | 0 | 0 | 121 | 132 | Carbamidomethylation | C5:Carbamidomethylation:1000.00 | PEAKS DB |
| C.GYGGSGTPVDELDR.C | N | 66.26 | 1421.6422 | 14 | 0.0 | 711.8284 | 2 | 46.82 | 10 | F10:24048 | BuCaTN01_F9a.raw | | | | | | | 2.9103E5 | 1.2275E6 | | 2.1867E6 | | | | 8.7883E6 | | | 5 | 0 | 0 | 0 | 0 | 0 | 0 | 1 | 1 | 0 | 1 | 0 | 0 | 0 | 2 | 0 | 0 | 55 | 68 | | | PEAKS DB |
| N.C(+57.02)DRTAALC(+57.02)FAK.A | N | 63.48 | 1311.6063 | 11 | -1.7 | 438.2076 | 3 | 37.36 | 12 | F12:15762 | BuCaTN01_F10b.raw | | | | | | | | | | | | 5.2815E4 | | 3.1918E5 | | | 2 | 0 | 0 | 0 | 0 | 0 | 0 | 0 | 0 | 0 | 0 | 0 | 1 | 0 | 1 | 0 | 0 | 118 | 128 | Carbamidomethylation | C1:Carbamidomethylation:1000.00;C8:Carbamidomethylation:1000.00 | PEAKS DB |
| R.TAALC(+57.02)FAK.A | N | 60.21 | 880.4476 | 8 | 3.4 | 441.2316 | 2 | 41.58 | 12 | F12:19288 | BuCaTN01_F10b.raw | | | | | | 9.3985E4 | 9.2429E6 | 1.2777E7 | 4.6024E5 | 3.2388E7 | 3.591E6 | 1.265E8 | 3.5731E6 | 1.1283E8 | 1.0943E8 | | 21 | 0 | 0 | 0 | 0 | 0 | 1 | 4 | 2 | 1 | 2 | 2 | 2 | 1 | 2 | 4 | 0 | 121 | 128 | Carbamidomethylation | C5:Carbamidomethylation:1000.00 | PEAKS DB |
| K.DFVC(+57.02)NC(+57.02)DRTAALC(+57.02)FAK.A | N | 59.68 | 1946.8438 | 16 | -0.4 | 974.4265 | 2 | 51.09 | 12 | F12:27773 | BuCaTN01_F10b.raw | | | | | | | | | | 1.1341E6 | | 0 | | | | | 1 | 0 | 0 | 0 | 0 | 0 | 0 | 0 | 0 | 0 | 1 | 0 | 0 | 0 | 0 | 0 | 0 | 113 | 128 | Carbamidomethylation | C4:Carbamidomethylation:1000.00;C6:Carbamidomethylation:1000.00;C13:Carbamidomethylation:1000.00 | PEAKS DB |
| G.GSGTPVDELDR.C | N | 56.36 | 1144.5360 | 11 | 3.4 | 573.2756 | 2 | 40.15 | 8 | F8:17921 | BuCaTN01_F7b.raw | | | | | | | | 1.1068E6 | | | | | | | | | 1 | 0 | 0 | 0 | 0 | 0 | 0 | 0 | 1 | 0 | 0 | 0 | 0 | 0 | 0 | 0 | 0 | 58 | 68 | | | PEAKS DB |
| R.TYSYDC(+57.02)SEGK.L | N | 55.61 | 1208.4656 | 10 | 0.1 | 605.2401 | 2 | 29.72 | 7 | F7:8746 | BuCaTN01_F7a.raw | | | | | | | 5.0914E6 | | | | | | | | | | 1 | 0 | 0 | 0 | 0 | 0 | 0 | 1 | 0 | 0 | 0 | 0 | 0 | 0 | 0 | 0 | 0 | 92 | 101 | Carbamidomethylation | C6:Carbamidomethylation:1000.00 | PEAKS DB |
| N.M(+15.99)IKC(+57.02)TNTRHWLSFTNYGC(+57.02)YC(+57.02)GYGGSGTPVDELDR.C | Y | 53.32 | 4030.7495 | 34 | 1.3 | 1008.6960 | 4 | 77.35 | 10 | F10:53957 | BuCaTN01_F9a.raw | | | | | | | | | | 0 | | | | | | | 0 | 0 | 0 | 0 | 0 | 0 | 0 | 0 | 0 | 0 | 0 | 0 | 0 | 0 | 0 | 0 | 0 | 35 | 68 | Oxidation (M); Carbamidomethylation | M1:Oxidation (M):1000.00;C4:Carbamidomethylation:1000.00;C18:Carbamidomethylation:1000.00;C20:Carbamidomethylation:1000.00 | PEAKS DB |
| R.TYSYDC(+57.02)SEGKLTC(+57.02).K | N | 52.98 | 1582.6279 | 13 | 2.9 | 792.3212 | 2 | 43.19 | 8 | F8:20253 | BuCaTN01_F7b.raw | | | | | | | | 5.7951E5 | | | | | | | | | 1 | 0 | 0 | 0 | 0 | 0 | 0 | 0 | 1 | 0 | 0 | 0 | 0 | 0 | 0 | 0 | 0 | 92 | 104 | Carbamidomethylation | C6:Carbamidomethylation:1000.00;C13:Carbamidomethylation:1000.00 | PEAKS DB |
| R.TYSYDC(+57.02)SEGKLT.C | N | 50.03 | 1422.5973 | 12 | 2.7 | 712.3058 | 2 | 41.55 | 8 | F8:18942 | BuCaTN01_F7b.raw | | | | | | | 5.0044E6 | 1.8313E6 | | | | | | | | | 2 | 0 | 0 | 0 | 0 | 0 | 0 | 1 | 1 | 0 | 0 | 0 | 0 | 0 | 0 | 0 | 0 | 92 | 103 | Carbamidomethylation | C6:Carbamidomethylation:1000.00 | PEAKS DB |
| Y.GGSGTPVDELDR.C | N | 49.09 | 1201.5575 | 12 | 0.3 | 601.7862 | 2 | 40.57 | 11 | F11:14528 | BuCaTN01_F10a.raw | | | | | | | 9.2274E5 | 2.7577E6 | | 1.1335E7 | 6.7591E5 | | 9.9086E5 | 2.6759E7 | | | 6 | 0 | 0 | 0 | 0 | 0 | 0 | 1 | 1 | 0 | 1 | 1 | 0 | 1 | 1 | 0 | 0 | 57 | 68 | | | PEAKS DB |
| R.TAALC(+57.02)FAKAP.Y | N | 47.25 | 1048.5375 | 10 | 3.6 | 525.2764 | 2 | 48.84 | 8 | F8:25881 | BuCaTN01_F7b.raw | | | | | | 3.1579E6 | | 4.0965E6 | | | | | | | | | 2 | 0 | 0 | 0 | 0 | 0 | 1 | 0 | 1 | 0 | 0 | 0 | 0 | 0 | 0 | 0 | 0 | 121 | 130 | Carbamidomethylation | C5:Carbamidomethylation:1000.00 | PEAKS DB |
| **total 22 peptides** | | | | | | | | | | | | | | | | | | | | | | | | | | | | | | | | | | | | | | | | | | | | | | | | | |

**Best Unique PSM (Scan F10:53957, m/z=1008.6960, z=4, RT=77.35, ppm=1.3):**

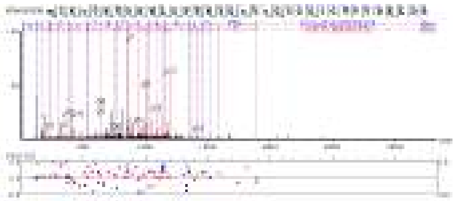

## JAS04993.1

back to list

| Protein Coverage | Supporting Peptides | Best Unique PSM |

**Protein Coverage:**

1 MNPAHLLFLA SVCVSLLGAS SIPPEPLNLY QFKNMIKCTN TRHWLSFTNY GCYCGYGGSG TPVDELDRCC QVHDKCYDTA

81 KRVHKCFPSV RTYSYDCSEG KLTCKDNNTK CKDFVCNCDR TAALCFAKAP YNDKNYNIDL KRCQ

C Carbamidomethylation (+57.02)
O Oxidation (M) (+15.99)

**Supporting Peptides:**

| Peptide | Uniq | -10lgP | Mass | Length | ppm | m/z | z | RT | Fraction | Scan | Source File | Area F1 | Area F2a | Area F3a | Area F4 | Area F5 | Area F6a | Area F7a | Area F7b | Area F8 | Area F9a | Area Sample 1F10a | Area F10b | Area F11a | Area F11b | Area F12a | Area F13 | #Feature | #Feature F1 | #Feature F2a | #Feature F3a | #Feature F4 | #Feature F5 | #Feature F6a | #Feature F7a | #Feature F7b | #Feature F8 | #Feature F9a | #Feature Sample 1F10a | #Feature F10b | #Feature F11a | #Feature F11b | #Feature F12a | #Feature F13 | Start | End | PTM | AScore | Found By |
|---|---|---|---|---|---|---|---|---|---|---|---|---|---|---|---|---|---|---|---|---|---|---|---|---|---|---|---|---|---|---|---|---|---|---|---|---|---|---|---|---|---|---|---|---|---|---|---|---|---|
| N.YGC(+57.02)YC(+57.02)GYGGSGTPVDELDR.C | N | 112.36 | 2124.8516 | 19 | 3.7 | 1063.4326 | 2 | 56.60 | 14 | F14:33384 | BuCaTN01_F11b.raw | | | 0 | | | 2.0097E6 | | 1.4053E7 | | 8.8531E7 | 3.4891E6 | 4.8054E8 | 6.165E7 | 1.7851E9 | 1.2628E8 | | 69 | 0 | 0 | 0 | 0 | 0 | 1 | 0 | 2 | 0 | 3 | 1 | 4 | 2 | 53 | 3 | 0 | 50 | 68 | Carbamidomethylation | C3:Carbamidomethylation:1000.00;C5:Carbamidomethylation:1000.00 | PEAKS DB |
| Y.C(+57.02)GYGGSGTPVDELDR.C | N | 88.12 | 1581.6729 | 15 | 4.4 | 791.8439 | 2 | 48.02 | 14 | F14:25306 | BuCaTN01_F11b.raw | | | | | | | | 2.166E6 | | 8.5786E6 | | 1.5853E7 | 6.495E5 | 1.7537E7 | 0 | | 6 | 0 | 0 | 0 | 0 | 0 | 0 | 0 | 1 | 0 | 1 | 0 | 2 | 1 | 1 | 0 | 0 | 54 | 68 | Carbamidomethylation | C1:Carbamidomethylation:1000.00 | PEAKS DB |
| G.C(+57.02)YC(+57.02)GYGGSGTPVDELDR.C | N | 84.40 | 1904.7668 | 17 | 2.7 | 953.3911 | 2 | 53.16 | 12 | F12:29351 | BuCaTN01_F10b.raw | | | | | | | | 4.1849E5 | | | | 3.7261E5 | | 2.1774E6 | | | 3 | 0 | 0 | 0 | 0 | 0 | 0 | 0 | 1 | 0 | 0 | 0 | 1 | 0 | 1 | 0 | 0 | 52 | 68 | Carbamidomethylation | C1:Carbamidomethylation:1000.00;C3:Carbamidomethylation:1000.00 | PEAKS DB |
| R.TYSYDC(+57.02)SEGKLTC(+57.02)K.D | N | 82.89 | 1710.7229 | 14 | 0.5 | 856.3691 | 2 | 38.29 | 7 | F7:14505 | BuCaTN01_F7a.raw | | | | | | | 1.4512E8 | 2.3155E7 | | 3.8991E5 | 4.5941E5 | | | | | | 8 | 0 | 0 | 0 | 0 | 0 | 0 | 4 | 2 | 0 | 1 | 1 | 0 | 0 | 0 | 0 | 0 | 92 | 105 | Carbamidomethylation | C6:Carbamidomethylation:1000.00;C13:Carbamidomethylation:1000.00 | PEAKS DB |
| Y.GC(+57.02)YC(+57.02)GYGGSGTPVDELDR.C | N | 82.10 | 1961.7883 | 18 | 1.4 | 981.9028 | 2 | 53.49 | 10 | F10:30232 | BuCaTN01_F9a.raw | | | | | | | | 1.7852E6 | | 3.3077E6 | | 1.5846E6 | | 3.2964E6 | 1.0428E5 | | 5 | 0 | 0 | 0 | 0 | 0 | 0 | 0 | 1 | 0 | 1 | 0 | 1 | 0 | 1 | 1 | 0 | 51 | 68 | Carbamidomethylation | C2:Carbamidomethylation:1000.00;C4:Carbamidomethylation:1000.00 | PEAKS DB |
| R.TYSYDC(+57.02)SEGKLTC(+57.02)KD.N | N | 75.87 | 1825.7499 | 15 | 2.5 | 913.8818 | 2 | 38.60 | 8 | F8:16643 | BuCaTN01_F7b.raw | | | | | | | | 7.3044E5 | | | | | | | | | 1 | 0 | 0 | 0 | 0 | 0 | 0 | 0 | 1 | 0 | 0 | 0 | 0 | 0 | 0 | 0 | 0 | 92 | 106 | Carbamidomethylation | C6:Carbamidomethylation:1000.00;C13:Carbamidomethylation:1000.00 | PEAKS DB |
| C.YC(+57.02)GYGGSGTPVDELDR.C | N | 75.62 | 1744.7362 | 16 | 4.1 | 873.3754 | 2 | 52.27 | 14 | F14:28849 | BuCaTN01_F11b.raw | | | | | | | | 7.7286E5 | | 7.3447E5 | | | | 3.0904E6 | 1.4057E5 | | 4 | 0 | 0 | 0 | 0 | 0 | 0 | 0 | 1 | 0 | 1 | 0 | 0 | 0 | 1 | 1 | 0 | 53 | 68 | Carbamidomethylation | C2:Carbamidomethylation:1000.00 | PEAKS DB |
| G.YGGSGTPVDELDR.C | N | 75.26 | 1364.6207 | 13 | 4.1 | 683.3177 | 2 | 45.29 | 14 | F14:22502 | BuCaTN01_F11b.raw | | | | | | | 6.8893E5 | | | | | | | 9.1406E6 | 6.9945E5 | | 3 | 0 | 0 | 0 | 0 | 0 | 0 | 1 | 0 | 0 | 0 | 0 | 0 | 0 | 1 | 1 | 0 | 56 | 68 | | | PEAKS DB |
| R.TAALC(+57.02)FAKAPY.N | N | 73.24 | 1211.6008 | 11 | 2.2 | 606.8076 | 2 | 55.84 | 12 | F12:32023 | BuCaTN01_F10b.raw | | | | | | | 7.3334E4 | | | 1.7166E6 | 1.3753E5 | 1.06E7 | | 8.1407E6 | 9.828E5 | | 7 | 0 | 0 | 0 | 0 | 0 | 0 | 1 | 0 | 0 | 1 | 1 | 2 | 0 | 1 | 1 | 0 | 121 | 131 | Carbamidomethylation | C5:Carbamidomethylation:1000.00 | PEAKS DB |
| T.NYGC(+57.02)YC(+57.02)GYGGSGTPVDELDR.C | N | 72.69 | 2238.8945 | 20 | 5.3 | 1120.4559 | 2 | 64.81 | 14 | F14:40195 | BuCaTN01_F11b.raw | | | | | | | | | | 2.3985E4 | | 0 | 1.1223E5 | 5.3539E5 | | | 3 | 0 | 0 | 0 | 0 | 0 | 0 | 0 | 0 | 0 | 1 | 0 | 0 | 1 | 1 | 0 | 0 | 49 | 68 | Carbamidomethylation | C4:Carbamidomethylation:1000.00;C6:Carbamidomethylation:1000.00 | PEAKS DB |
| R.TAALC(+57.02)FAKAPYN.D | N | 69.35 | 1325.6438 | 12 | -0.2 | 663.8290 | 2 | 51.91 | 6 | F6:30474 | BuCaTN01_F6a.raw | | | | | | 3.7675E6 | | 1.5632E7 | | 4.6358E6 | | 1.9346E6 | | | | | 5 | 0 | 0 | 0 | 0 | 0 | 1 | 0 | 2 | 0 | 1 | 0 | 1 | 0 | 0 | 0 | 0 | 121 | 132 | Carbamidomethylation | C5:Carbamidomethylation:1000.00 | PEAKS DB |
| C.GYGGSGTPVDELDR.C | N | 66.26 | 1421.6422 | 14 | 0.0 | 711.8284 | 2 | 46.82 | 10 | F10:24048 | BuCaTN01_F9a.raw | | | | | | | 2.9103E5 | 1.2275E6 | | 2.1867E6 | | | | 8.7883E6 | | | 5 | 0 | 0 | 0 | 0 | 0 | 0 | 1 | 1 | 0 | 1 | 0 | 0 | 0 | 2 | 0 | 0 | 55 | 68 | | | PEAKS DB |
| N.C(+57.02)DRTAALC(+57.02)FAK.A | N | 63.48 | 1311.6063 | 11 | -1.7 | 438.2076 | 3 | 37.36 | 12 | F12:15762 | BuCaTN01_F10b.raw | | | | | | | | | | | | 5.2815E4 | | 3.1918E5 | | | 2 | 0 | 0 | 0 | 0 | 0 | 0 | 0 | 0 | 0 | 0 | 0 | 1 | 0 | 1 | 0 | 0 | 118 | 128 | Carbamidomethylation | C1:Carbamidomethylation:1000.00;C8:Carbamidomethylation:1000.00 | PEAKS DB |
| R.TAALC(+57.02)FAK.A | N | 60.21 | 880.4476 | 8 | 3.4 | 441.2316 | 2 | 41.58 | 12 | F12:19288 | BuCaTN01_F10b.raw | | | | | | 9.3985E4 | 9.2429E6 | 1.2777E7 | 4.6024E5 | 3.2388E7 | 3.591E6 | 1.265E8 | 3.5731E6 | 1.1283E8 | 1.0943E8 | | 21 | 0 | 0 | 0 | 0 | 0 | 1 | 4 | 2 | 1 | 2 | 2 | 2 | 1 | 2 | 4 | 0 | 121 | 128 | Carbamidomethylation | C5:Carbamidomethylation:1000.00 | PEAKS DB |
| K.DFVC(+57.02)NC(+57.02)DRTAALC(+57.02)FAK.A | N | 59.68 | 1946.8438 | 16 | -0.4 | 974.4265 | 2 | 51.09 | 12 | F12:27773 | BuCaTN01_F10b.raw | | | | | | | | | | 1.1341E6 | | 0 | | | | | 1 | 0 | 0 | 0 | 0 | 0 | 0 | 0 | 0 | 0 | 1 | 0 | 0 | 0 | 0 | 0 | 0 | 113 | 128 | Carbamidomethylation | C4:Carbamidomethylation:1000.00;C6:Carbamidomethylation:1000.00;C13:Carbamidomethylation:1000.00 | PEAKS DB |
| G.GSGTPVDELDR.C | N | 56.36 | 1144.5360 | 11 | 3.4 | 573.2756 | 2 | 40.15 | 8 | F8:17921 | BuCaTN01_F7b.raw | | | | | | | | 1.1068E6 | | | | | | | | | 1 | 0 | 0 | 0 | 0 | 0 | 0 | 0 | 1 | 0 | 0 | 0 | 0 | 0 | 0 | 0 | 0 | 58 | 68 | | | PEAKS DB |
| R.TYSYDC(+57.02)SEGK.L | N | 55.61 | 1208.4656 | 10 | 0.1 | 605.2401 | 2 | 29.72 | 7 | F7:8746 | BuCaTN01_F7a.raw | | | | | | | 5.0914E6 | | | | | | | | | | 1 | 0 | 0 | 0 | 0 | 0 | 0 | 1 | 0 | 0 | 0 | 0 | 0 | 0 | 0 | 0 | 0 | 92 | 101 | Carbamidomethylation | C6:Carbamidomethylation:1000.00 | PEAKS DB |
| N.M(+15.99)IKC(+57.02)TNTRHWLSFTNYGC(+57.02)YC(+57.02)GYGGSGTPVDELDR.C | Y | 53.32 | 4030.7495 | 34 | 1.3 | 1008.6960 | 4 | 77.35 | 10 | F10:53957 | BuCaTN01_F9a.raw | | | | | | | | | | 0 | | | | | | | 0 | 0 | 0 | 0 | 0 | 0 | 0 | 0 | 0 | 0 | 0 | 0 | 0 | 0 | 0 | 0 | 0 | 35 | 68 | Oxidation (M); Carbamidomethylation | M1:Oxidation (M):1000.00;C4:Carbamidomethylation:1000.00;C18:Carbamidomethylation:1000.00;C20:Carbamidomethylation:1000.00 | PEAKS DB |
| R.TYSYDC(+57.02)SEGKLTC(+57.02).K | N | 52.98 | 1582.6279 | 13 | 2.9 | 792.3212 | 2 | 43.19 | 8 | F8:20253 | BuCaTN01_F7b.raw | | | | | | | | 5.7951E5 | | | | | | | | | 1 | 0 | 0 | 0 | 0 | 0 | 0 | 0 | 1 | 0 | 0 | 0 | 0 | 0 | 0 | 0 | 0 | 92 | 104 | Carbamidomethylation | C6:Carbamidomethylation:1000.00;C13:Carbamidomethylation:1000.00 | PEAKS DB |
| R.TYSYDC(+57.02)SEGKLT.C | N | 50.03 | 1422.5973 | 12 | 2.7 | 712.3058 | 2 | 41.55 | 8 | F8:18942 | BuCaTN01_F7b.raw | | | | | | | 5.0044E6 | 1.8313E6 | | | | | | | | | 2 | 0 | 0 | 0 | 0 | 0 | 0 | 1 | 1 | 0 | 0 | 0 | 0 | 0 | 0 | 0 | 0 | 92 | 103 | Carbamidomethylation | C6:Carbamidomethylation:1000.00 | PEAKS DB |
| Y.GGSGTPVDELDR.C | N | 49.09 | 1201.5575 | 12 | 0.3 | 601.7862 | 2 | 40.57 | 11 | F11:14528 | BuCaTN01_F10a.raw | | | | | | | 9.2274E5 | 2.7577E6 | | 1.1335E7 | 6.7591E5 | | 9.9086E5 | 2.6759E7 | | | 6 | 0 | 0 | 0 | 0 | 0 | 0 | 1 | 1 | 0 | 1 | 1 | 0 | 1 | 1 | 0 | 0 | 57 | 68 | | | PEAKS DB |
| R.TAALC(+57.02)FAKAP.Y | N | 47.25 | 1048.5375 | 10 | 3.6 | 525.2764 | 2 | 48.84 | 8 | F8:25881 | BuCaTN01_F7b.raw | | | | | | 3.1579E6 | | 4.0965E6 | | | | | | | | | 2 | 0 | 0 | 0 | 0 | 0 | 1 | 0 | 1 | 0 | 0 | 0 | 0 | 0 | 0 | 0 | 0 | 121 | 130 | Carbamidomethylation | C5:Carbamidomethylation:1000.00 | PEAKS DB |
| **total 22 peptides** | | | | | | | | | | | | | | | | | | | | | | | | | | | | | | | | | | | | | | | | | | | | | | | | | |

**Best Unique PSM (Scan F10:53957, m/z=1008.6960, z=4, RT=77.35, ppm=1.3):**

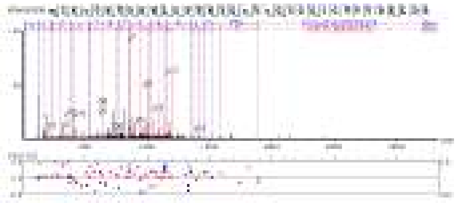

## JAB52794.1

| Protein Coverage | Supporting Peptides | Best Unique PSM |

**Protein Coverage:**

1 MLIFLWCGAV CVSLLGASSI PPEPLNLYHV SNMIKCTNTR HWLSFTNYGC YCGYGGSGTP VDELDRCCQV HDKCYDTAKR

81 VHKCFPSMRT YSYDCSEGKL TCKDNNTKCK DFVCNCDRTA ALCFAKAPYN NKNFKIDPSR CQ

C Carbamidomethylation (+57.02)
O Oxidation (M) (+15.99)

**Supporting Peptides:**

| Peptide | Uniq | -10lgP | Mass | Length | ppm | m/z | z | RT | Fraction | Scan | Source File | Area F1 | Area F2a | Area F3a | Area F4 | Area F5 | Area F6a | Area F7a | Area F7b | Area F8 | Area F9a | Area Sample 1F10a | Area F10b | Area F11a | Area F11b | Area F12a | Area F13 | #Feature | #Feature F1 | #Feature F2a | #Feature F3a | #Feature F4 | #Feature F5 | #Feature F6a | #Feature F7a | #Feature F7b | #Feature F8 | #Feature F9a | #Feature Sample 1F10a | #Feature F10b | #Feature F11a | #Feature F11b | #Feature F12a | #Feature F13 | Start | End | PTM | AScore | Found By |
|---|---|---|---|---|---|---|---|---|---|---|---|---|---|---|---|---|---|---|---|---|---|---|---|---|---|---|---|---|---|---|---|---|---|---|---|---|---|---|---|---|---|---|---|---|---|---|---|---|---|
| N.YGC(+57.02)YC(+57.02)GYGGSGTPVDELDR.C | N | 112.36 | 2124.8516 | 19 | 3.7 | 1063.4326 | 2 | 56.60 | 14 | F14:33384 | BuCaTN01_F11b.raw | | | 0 | | | 2.0097E6 | | 1.4053E7 | | 8.8531E7 | 3.4891E6 | 4.8054E8 | 6.165E7 | 1.7851E9 | 1.2628E8 | | 69 | 0 | 0 | 0 | 0 | 0 | 1 | 0 | 2 | 0 | 3 | 1 | 4 | 2 | 53 | 3 | 0 | 48 | 66 | Carbamidomethylation | C3:Carbamidomethylation:1000.00;C5:Carbamidomethylation:1000.00 | PEAKS DB |
| Y.C(+57.02)GYGGSGTPVDELDR.C | N | 88.12 | 1581.6729 | 15 | 4.4 | 791.8439 | 2 | 48.02 | 14 | F14:25306 | BuCaTN01_F11b.raw | | | | | | | | 2.166E6 | | 8.5786E6 | | 1.5853E7 | 6.495E5 | 1.7537E7 | 0 | | 6 | 0 | 0 | 0 | 0 | 0 | 0 | 0 | 1 | 0 | 1 | 0 | 2 | 1 | 1 | 0 | 0 | 52 | 66 | Carbamidomethylation | C1:Carbamidomethylation:1000.00 | PEAKS DB |
| G.C(+57.02)YC(+57.02)GYGGSGTPVDELDR.C | N | 84.40 | 1904.7668 | 17 | 2.7 | 953.3911 | 2 | 53.16 | 12 | F12:29351 | BuCaTN01_F10b.raw | | | | | | | | 4.1849E5 | | | | 3.7261E5 | | 2.1774E6 | | | 3 | 0 | 0 | 0 | 0 | 0 | 0 | 0 | 1 | 0 | 0 | 0 | 1 | 0 | 1 | 0 | 0 | 50 | 66 | Carbamidomethylation | C1:Carbamidomethylation:1000.00;C3:Carbamidomethylation:1000.00 | PEAKS DB |
| R.TYSYDC(+57.02)SEGKLTC(+57.02)K.D | N | 82.89 | 1710.7229 | 14 | 0.5 | 856.3691 | 2 | 38.29 | 7 | F7:14505 | BuCaTN01_F7a.raw | | | | | | | 1.4512E8 | 2.3155E7 | | 3.8991E5 | 4.5941E5 | | | | | | 8 | 0 | 0 | 0 | 0 | 0 | 0 | 4 | 2 | 0 | 1 | 1 | 0 | 0 | 0 | 0 | 0 | 90 | 103 | Carbamidomethylation | C6:Carbamidomethylation:1000.00;C13:Carbamidomethylation:1000.00 | PEAKS DB |
| Y.GC(+57.02)YC(+57.02)GYGGSGTPVDELDR.C | N | 82.10 | 1961.7883 | 18 | 1.4 | 981.9028 | 2 | 53.49 | 10 | F10:30232 | BuCaTN01_F9a.raw | | | | | | | | 1.7852E6 | | 3.3077E6 | | 1.5846E6 | | 3.2964E6 | 1.0428E5 | | 5 | 0 | 0 | 0 | 0 | 0 | 0 | 0 | 1 | 0 | 1 | 0 | 1 | 0 | 1 | 1 | 0 | 49 | 66 | Carbamidomethylation | C2:Carbamidomethylation:1000.00;C4:Carbamidomethylation:1000.00 | PEAKS DB |
| R.TYSYDC(+57.02)SEGKLTC(+57.02)KD.N | N | 75.87 | 1825.7499 | 15 | 2.5 | 913.8818 | 2 | 38.60 | 8 | F8:16643 | BuCaTN01_F7b.raw | | | | | | | | 7.3044E5 | | | | | | | | | 1 | 0 | 0 | 0 | 0 | 0 | 0 | 0 | 1 | 0 | 0 | 0 | 0 | 0 | 0 | 0 | 0 | 90 | 104 | Carbamidomethylation | C6:Carbamidomethylation:1000.00;C13:Carbamidomethylation:1000.00 | PEAKS DB |
| C.YC(+57.02)GYGGSGTPVDELDR.C | N | 75.62 | 1744.7362 | 16 | 4.1 | 873.3754 | 2 | 52.27 | 14 | F14:28849 | BuCaTN01_F11b.raw | | | | | | | | 7.7286E5 | | 7.3447E5 | | | | 3.0904E6 | 1.4057E5 | | 4 | 0 | 0 | 0 | 0 | 0 | 0 | 0 | 1 | 0 | 1 | 0 | 0 | 0 | 1 | 1 | 0 | 51 | 66 | Carbamidomethylation | C2:Carbamidomethylation:1000.00 | PEAKS DB |
| G.YGGSGTPVDELDR.C | N | 75.26 | 1364.6207 | 13 | 4.1 | 683.3177 | 2 | 45.29 | 14 | F14:22502 | BuCaTN01_F11b.raw | | | | | | | 6.8893E5 | | | | | | | 9.1406E6 | 6.9945E5 | | 3 | 0 | 0 | 0 | 0 | 0 | 0 | 1 | 0 | 0 | 0 | 0 | 0 | 0 | 1 | 1 | 0 | 54 | 66 | | | PEAKS DB |
| R.TAALC(+57.02)FAKAPY.N | N | 73.24 | 1211.6008 | 11 | 2.2 | 606.8076 | 2 | 55.84 | 12 | F12:32023 | BuCaTN01_F10b.raw | | | | | | | 7.3334E4 | | | 1.7166E6 | 1.3753E5 | 1.06E7 | | 8.1407E6 | 9.828E5 | | 7 | 0 | 0 | 0 | 0 | 0 | 0 | 1 | 0 | 0 | 1 | 1 | 2 | 0 | 1 | 1 | 0 | 119 | 129 | Carbamidomethylation | C5:Carbamidomethylation:1000.00 | PEAKS DB |
| T.NYGC(+57.02)YC(+57.02)GYGGSGTPVDELDR.C | N | 72.69 | 2238.8945 | 20 | 5.3 | 1120.4559 | 2 | 64.81 | 14 | F14:40195 | BuCaTN01_F11b.raw | | | | | | | | | | 2.3985E4 | | 0 | 1.1223E5 | 5.3539E5 | | | 3 | 0 | 0 | 0 | 0 | 0 | 0 | 0 | 0 | 0 | 1 | 0 | 0 | 1 | 1 | 0 | 0 | 47 | 66 | Carbamidomethylation | C4:Carbamidomethylation:1000.00;C6:Carbamidomethylation:1000.00 | PEAKS DB |
| R.TAALC(+57.02)FAKAPYN.N | N | 69.35 | 1325.6438 | 12 | -0.2 | 663.8290 | 2 | 51.91 | 6 | F6:30474 | BuCaTN01_F6a.raw | | | | | | 3.7675E6 | | 1.5632E7 | | 4.6358E6 | | 1.9346E6 | | | | | 5 | 0 | 0 | 0 | 0 | 0 | 1 | 0 | 2 | 0 | 1 | 0 | 1 | 0 | 0 | 0 | 0 | 119 | 130 | Carbamidomethylation | C5:Carbamidomethylation:1000.00 | PEAKS DB |
| C.GYGGSGTPVDELDR.C | N | 66.26 | 1421.6422 | 14 | 0.0 | 711.8284 | 2 | 46.82 | 10 | F10:24048 | BuCaTN01_F9a.raw | | | | | | | 2.9103E5 | 1.2275E6 | | 2.1867E6 | | | | 8.7883E6 | | | 5 | 0 | 0 | 0 | 0 | 0 | 0 | 1 | 1 | 0 | 1 | 0 | 0 | 0 | 2 | 0 | 0 | 53 | 66 | | | PEAKS DB |

| | | | | | | | | | | | | | | | | | | | | | | | | | | | | | | | | | | | | | | | | | | | | | | | | | |
|---|---|---|---|---|---|---|---|---|---|---|---|---|---|---|---|---|---|---|---|---|---|---|---|---|---|---|---|---|---|---|---|---|---|---|---|---|---|---|---|---|---|---|---|---|---|---|---|---|---|
| N.C(+57.02)DRTAALC(+57.02)FAK.A | N | 63.48 | 1311.6063 | 11 | -1.7 | 438.2076 | 3 | 37.36 | 12 | F12:15762 | BuCaTN01_F10b.raw | | | | | | | | | | | | 5.2815E4 | | 3.1918E5 | | | 2 | 0 | 0 | 0 | 0 | 0 | 0 | 0 | 0 | 0 | 0 | 0 | 1 | 0 | 1 | 0 | 0 | 116 | 126 | Carbamidomethylation | C1:Carbamidomethylation:1000.00;C8:Carbamidomethylation:1000.00 | PEAKS DB |
| R.TAALC(+57.02)FAK.A | N | 60.21 | 880.4476 | 8 | 3.4 | 441.2316 | 2 | 41.58 | 12 | F12:19288 | BuCaTN01_F10b.raw | | | | | | 9.3985E4 | 9.2429E6 | 1.2777E7 | 4.6024E5 | 3.2388E7 | 3.591E6 | 1.265E8 | 3.5731E6 | 1.1283E8 | 1.0943E8 | | 21 | 0 | 0 | 0 | 0 | 0 | 1 | 4 | 2 | 1 | 2 | 2 | 2 | 1 | 2 | 4 | 0 | 119 | 126 | Carbamidomethylation | C5:Carbamidomethylation:1000.00 | PEAKS DB |
| K.DFVC(+57.02)NC(+57.02)DRTAALC(+57.02)FAK.A | N | 59.68 | 1946.8438 | 16 | -0.4 | 974.4265 | 2 | 51.09 | 12 | F12:27773 | BuCaTN01_F10b.raw | | | | | | | | | | 1.1341E6 | | 0 | | | | | 1 | 0 | 0 | 0 | 0 | 0 | 0 | 0 | 0 | 0 | 1 | 0 | 0 | 0 | 0 | 0 | 0 | 111 | 126 | Carbamidomethylation | C4:Carbamidomethylation:1000.00;C6:Carbamidomethylation:1000.00;C13:Carbamidomethylation:1000.00 | PEAKS DB |
| G.GSGTPVDELDR.C | N | 56.36 | 1144.5360 | 11 | 3.4 | 573.2756 | 2 | 40.15 | 8 | F8:17921 | BuCaTN01_F7b.raw | | | | | | | | 1.1068E6 | | | | | | | | | 1 | 0 | 0 | 0 | 0 | 0 | 0 | 0 | 1 | 0 | 0 | 0 | 0 | 0 | 0 | 0 | 0 | 56 | 66 | | | PEAKS DB |
| R.TYSYDC(+57.02)SEGK.L | N | 55.61 | 1208.4656 | 10 | 0.1 | 605.2401 | 2 | 29.72 | 7 | F7:8746 | BuCaTN01_F7a.raw | | | | | | | 5.0914E6 | | | | | | | | | | 1 | 0 | 0 | 0 | 0 | 0 | 0 | 1 | 0 | 0 | 0 | 0 | 0 | 0 | 0 | 0 | 0 | 90 | 99 | Carbamidomethylation | C6:Carbamidomethylation:1000.00 | PEAKS DB |
| N.M(+15.99)IKC(+57.02)TNTRHWLSFTNYGC(+57.02)YC(+57.02)GYGGSGTPVDELDR.C | Y | 53.32 | 4030.7495 | 34 | 1.3 | 1008.6960 | 4 | 77.35 | 10 | F10:53957 | BuCaTN01_F9a.raw | | | | | | | | | | 0 | | | | | | | 0 | 0 | 0 | 0 | 0 | 0 | 0 | 0 | 0 | 0 | 0 | 0 | 0 | 0 | 0 | 0 | 0 | 33 | 66 | Oxidation (M); Carbamidomethylation | M1:Oxidation (M):1000.00;C4:Carbamidomethylation:1000.00;C18:Carbamidomethylation:1000.00;C20:Carbamidomethylation:1000.00 | PEAKS DB |
| R.TYSYDC(+57.02)SEGKLTC(+57.02).K | N | 52.98 | 1582.6279 | 13 | 2.9 | 792.3212 | 2 | 43.19 | 8 | F8:20253 | BuCaTN01_F7b.raw | | | | | | | | 5.7951E5 | | | | | | | | | 1 | 0 | 0 | 0 | 0 | 0 | 0 | 0 | 1 | 0 | 0 | 0 | 0 | 0 | 0 | 0 | 0 | 90 | 102 | Carbamidomethylation | C6:Carbamidomethylation:1000.00;C13:Carbamidomethylation:1000.00 | PEAKS DB |
| R.TYSYDC(+57.02)SEGKLT.C | N | 50.03 | 1422.5973 | 12 | 2.7 | 712.3058 | 2 | 41.55 | 8 | F8:18942 | BuCaTN01_F7b.raw | | | | | | | 5.0044E6 | 1.8313E6 | | | | | | | | | 2 | 0 | 0 | 0 | 0 | 0 | 0 | 1 | 1 | 0 | 0 | 0 | 0 | 0 | 0 | 0 | 0 | 90 | 101 | Carbamidomethylation | C6:Carbamidomethylation:1000.00 | PEAKS DB |
| Y.GGSGTPVDELDR.C | N | 49.09 | 1201.5575 | 12 | 0.3 | 601.7862 | 2 | 40.57 | 11 | F11:14528 | BuCaTN01_F10a.raw | | | | | | | 9.2274E5 | 2.7577E6 | | 1.1335E7 | 6.7591E5 | | 9.9086E5 | 2.6759E7 | | | 6 | 0 | 0 | 0 | 0 | 0 | 0 | 1 | 1 | 0 | 1 | 1 | 0 | 1 | 1 | 0 | 0 | 55 | 66 | | | PEAKS DB |
| R.TAALC(+57.02)FAKAP.Y | N | 47.25 | 1048.5375 | 10 | 3.6 | 525.2764 | 2 | 48.84 | 8 | F8:25881 | BuCaTN01_F7b.raw | | | | | | 3.1579E6 | | 4.0965E6 | | | | | | | | | 2 | 0 | 0 | 0 | 0 | 0 | 1 | 0 | 1 | 0 | 0 | 0 | 0 | 0 | 0 | 0 | 0 | 119 | 128 | Carbamidomethylation | C5:Carbamidomethylation:1000.00 | PEAKS DB |
| **total 22 peptides** | | | | | | | | | | | | | | | | | | | | | | | | | | | | | | | | | | | | | | | | | | | | | | | | | |

**Best Unique PSM (Scan F10:53957, m/z=1008.6960, z=4, RT=77.35, ppm=1.3):**

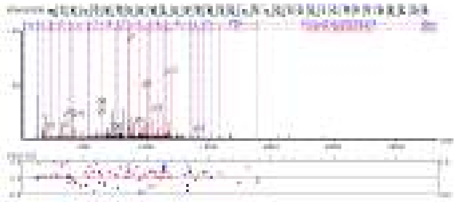

## 1G0Z

back to list

| Protein Coverage | Supporting Peptides | Best Unique PSM |

**Protein Coverage:**

1 NLKQFKNMIQ CAGTRTWTSY IGYGCYCGYG GSGTPVDELD RCCYTHDHCY NKAANIPGCN PLIKTYSYTC TKPNITCNDT

Carbamidomethylation (+57.02)

81 SDSCARFICD CDRTAAICFA SAPYNINNIM ISASTSCQ

**Supporting Peptides:**

| Peptide | Uniq | -10lgP | Mass | Length | ppm | m/z | z | RT | Fraction | Scan | Source File | Area F1 | Area F2a | Area F3a | Area F4 | Area F5 | Area F6a | Area F7a | Area F7b | Area F8 | Area F9a | Area Sample 1F10a | Area F10b | Area F11a | Area F11b | Area F12a | Area F13 | #Feature | #Feature F1 | #Feature F2a | #Feature F3a | #Feature F4 | #Feature F5 | #Feature F6a | #Feature F7a | #Feature F7b | #Feature F8 | #Feature F9a | #Feature Sample 1F10a | #Feature F10b | #Feature F11a | #Feature F11b | #Feature F12a | #Feature F13 | Start | End | PTM | AScore | Found By |
|---|---|---|---|---|---|---|---|---|---|---|---|---|---|---|---|---|---|---|---|---|---|---|---|---|---|---|---|---|---|---|---|---|---|---|---|---|---|---|---|---|---|---|---|---|---|---|---|---|---|
| G.YGC(+57.02)YC(+57.02)GYGGSGTPVDELDR.C | N | 112.36 | 2124.8516 | 19 | 3.7 | 1063.4326 | 2 | 56.60 | 14 | F14:33384 | BuCaTN01_F11b.raw | | | 0 | | | 2.0097E6 | | 1.4053E7 | | 8.8531E7 | 3.4891E6 | 4.8054E8 | 6.165E7 | 1.7851E9 | 1.2628E8 | | 69 | 0 | 0 | 0 | 0 | 0 | 1 | 0 | 2 | 0 | 3 | 1 | 4 | 2 | 53 | 3 | 0 | 23 | 41 | Carbamidomethylation | C3:Carbamidomethylation:1000.00;C5:Carbamidomethylation:1000.00 | PEAKS DB |
| I.GYGC(+57.02)YC(+57.02)GYGGSGTPVDELDR.C | Y | 100.48 | 2181.8730 | 20 | 0.3 | 1091.9441 | 2 | 57.45 | 10 | F10:33873 | BuCaTN01_F9a.raw | | | | | | | 0 | | | 2.5729E7 | 2.6663E5 | 6.1424E7 | 1.3809E7 | 2.1113E8 | 7.3334E6 | | 15 | 0 | 0 | 0 | 0 | 0 | 0 | 0 | 0 | 0 | 1 | 1 | 3 | 3 | 4 | 3 | 0 | 22 | 41 | Carbamidomethylation | C4:Carbamidomethylation:1000.00;C6:Carbamidomethylation:1000.00 | PEAKS DB |
| Y.C(+57.02)GYGGSGTPVDELDR.C | N | 88.12 | 1581.6729 | 15 | 4.4 | 791.8439 | 2 | 48.02 | 14 | F14:25306 | BuCaTN01_F11b.raw | | | | | | | | 2.166E6 | | 8.5786E6 | | 1.5853E7 | 6.495E5 | 1.7537E7 | 0 | | 6 | 0 | 0 | 0 | 0 | 0 | 0 | 0 | 1 | 0 | 1 | 0 | 2 | 1 | 1 | 0 | 0 | 27 | 41 | Carbamidomethylation | C1:Carbamidomethylation:1000.00 | PEAKS DB |
| G.C(+57.02)YC(+57.02)GYGGSGTPVDELDR.C | N | 84.40 | 1904.7668 | 17 | 2.7 | 953.3911 | 2 | 53.16 | 12 | F12:29351 | BuCaTN01_F10b.raw | | | | | | | | 4.1849E5 | | | | 3.7261E5 | | 2.1774E6 | | | 3 | 0 | 0 | 0 | 0 | 0 | 0 | 0 | 1 | 0 | 0 | 0 | 1 | 0 | 1 | 0 | 0 | 25 | 41 | Carbamidomethylation | C1:Carbamidomethylation:1000.00;C3:Carbamidomethylation:1000.00 | PEAKS DB |
| Y.GC(+57.02)YC(+57.02)GYGGSGTPVDELDR.C | N | 82.10 | 1961.7883 | 18 | 1.4 | 981.9028 | 2 | 53.49 | 10 | F10:30232 | BuCaTN01_F9a.raw | | | | | | | | 1.7852E6 | | 3.3077E6 | | 1.5846E6 | | 3.2964E6 | 1.0428E5 | | 5 | 0 | 0 | 0 | 0 | 0 | 0 | 0 | 1 | 0 | 1 | 0 | 1 | 0 | 1 | 1 | 0 | 24 | 41 | Carbamidomethylation | C2:Carbamidomethylation:1000.00;C4:Carbamidomethylation:1000.00 | PEAKS DB |
| C.YC(+57.02)GYGGSGTPVDELDR.C | N | 75.62 | 1744.7362 | 16 | 4.1 | 873.3754 | 2 | 52.27 | 14 | F14:28849 | BuCaTN01_F11b.raw | | | | | | | | 7.7286E5 | | 7.3447E5 | | | | 3.0904E6 | 1.4057E5 | | 4 | 0 | 0 | 0 | 0 | 0 | 0 | 0 | 1 | 0 | 1 | 0 | 0 | 0 | 1 | 1 | 0 | 26 | 41 | Carbamidomethylation | C2:Carbamidomethylation:1000.00 | PEAKS DB |
| G.YGGSGTPVDELDR.C | N | 75.26 | 1364.6207 | 13 | 4.1 | 683.3177 | 2 | 45.29 | 14 | F14:22502 | BuCaTN01_F11b.raw | | | | | | | 6.8893E5 | | | | | | | 9.1406E6 | 6.9945E5 | | 3 | 0 | 0 | 0 | 0 | 0 | 0 | 1 | 0 | 0 | 0 | 0 | 0 | 0 | 1 | 1 | 0 | 29 | 41 | | | PEAKS DB |
| C.GYGGSGTPVDELDR.C | N | 66.26 | 1421.6422 | 14 | 0.0 | 711.8284 | 2 | 46.82 | 10 | F10:24048 | BuCaTN01_F9a.raw | | | | | | | 2.9103E5 | 1.2275E6 | | 2.1867E6 | | | | 8.7883E6 | | | 5 | 0 | 0 | 0 | 0 | 0 | 0 | 1 | 1 | 0 | 1 | 0 | 0 | 0 | 2 | 0 | 0 | 28 | 41 | | | PEAKS DB |
| K.TYSYTC(+57.02)TKPNIT.C | N | 66.05 | 1447.6653 | 12 | 2.5 | 724.8387 | 2 | 43.32 | 14 | F14:21091 | BuCaTN01_F11b.raw | | | | | | | | 1.3481E6 | | | | 2.4842E6 | | 1.5203E7 | 9.0876E6 | | 5 | 0 | 0 | 0 | 0 | 0 | 0 | 0 | 1 | 0 | 0 | 0 | 1 | 0 | 2 | 1 | 0 | 65 | 76 | Carbamidomethylation | C6:Carbamidomethylation:1000.00 | PEAKS DB |
| R.FIC(+57.02)DC(+57.02)DRTAAIC(+57.02).F | N | 58.10 | 1500.6160 | 12 | 4.0 | 751.3152 | 2 | 47.17 | 14 | F14:24274 | BuCaTN01_F11b.raw | | | | | | | | | | | | | | 3.9576E6 | | | 1 | 0 | 0 | 0 | 0 | 0 | 0 | 0 | 0 | 0 | 0 | 0 | 0 | 0 | 1 | 0 | 0 | 87 | 98 | Carbamidomethylation | C3:Carbamidomethylation:1000.00;C5:Carbamidomethylation:1000.00;C12:Carbamidomethylation:1000.00 | PEAKS DB |
| G.GSGTPVDELDR.C | N | 56.36 | 1144.5360 | 11 | 3.4 | 573.2756 | 2 | 40.15 | 8 | F8:17921 | BuCaTN01_F7b.raw | | | | | | | | 1.1068E6 | | | | | | | | | 1 | 0 | 0 | 0 | 0 | 0 | 0 | 0 | 1 | 0 | 0 | 0 | 0 | 0 | 0 | 0 | 0 | 31 | 41 | | | PEAKS DB |
| K.TYSYTC(+57.02)TKPNITC(+57.02).N | N | 55.98 | 1607.6960 | 13 | 5.0 | 804.8560 | 2 | 43.60 | 14 | F14:21061 | BuCaTN01_F11b.raw | | | | | | | | | | | | | | 2.3535E6 | 7.2547E5 | | 3 | 0 | 0 | 0 | 0 | 0 | 0 | 0 | 0 | 0 | 0 | 0 | 0 | 0 | 2 | 1 | 0 | 65 | 77 | Carbamidomethylation | C6:Carbamidomethylation:1000.00;C13:Carbamidomethylation:1000.00 | PEAKS DB |
| N.IPGC(+57.02)NPLIK.T | Y | 52.95 | 1010.5583 | 9 | 0.9 | 506.2869 | 2 | 41.50 | 13 | F13:18979 | BuCaTN01_F11a.raw | | | | | | 3.3656E6 | 8.2953E5 | 2.7046E7 | | 6.9838E7 | 1.5423E6 | 3.4919E8 | 2.8685E7 | 1.5397E9 | 1.518E8 | | 9 | 0 | 0 | 0 | 0 | 0 | 1 | 1 | 1 | 0 | 1 | 1 | 1 | 1 | 1 | 1 | 0 | 56 | 64 | Carbamidomethylation | C4:Carbamidomethylation:1000.00 | PEAKS DB |
| R.FIC(+57.02)DC(+57.02)DRTAAIC(+57.02)F.A | N | 49.37 | 1647.6843 | 13 | 3.5 | 824.8499 | 2 | 66.79 | 8 | F8:42173 | BuCaTN01_F7b.raw | | | | | | | | 7.495E5 | | | | | | | | | 1 | 0 | 0 | 0 | 0 | 0 | 0 | 0 | 1 | 0 | 0 | 0 | 0 | 0 | 0 | 0 | 0 | 87 | 99 | Carbamidomethylation | C3:Carbamidomethylation:1000.00;C5:Carbamidomethylation:1000.00;C12:Carbamidomethylation:1000.00 | PEAKS DB |
| Y.GGSGTPVDELDR.C | N | 49.09 | 1201.5575 | 12 | 0.3 | 601.7862 | 2 | 40.57 | 11 | F11:14528 | BuCaTN01_F10a.raw | | | | | | | 9.2274E5 | 2.7577E6 | | 1.1335E7 | 6.7591E5 | | 9.9086E5 | 2.6759E7 | | | 6 | 0 | 0 | 0 | 0 | 0 | 0 | 1 | 1 | 0 | 1 | 1 | 0 | 1 | 1 | 0 | 0 | 30 | 41 | | | PEAKS DB |
| K.TYSYTC(+57.02)TKPNI.T | N | 46.29 | 1346.6177 | 11 | 5.6 | 674.3171 | 2 | 45.63 | 14 | F14:22808 | BuCaTN01_F11b.raw | | | | | | | | | | | | | | 5.6477E6 | 2.611E6 | | 2 | 0 | 0 | 0 | 0 | 0 | 0 | 0 | 0 | 0 | 0 | 0 | 0 | 0 | 1 | 1 | 0 | 65 | 75 | Carbamidomethylation | C6:Carbamidomethylation:1000.00 | PEAKS DB |
| **total 16 peptides** | | | | | | | | | | | | | | | | | | | | | | | | | | | | | | | | | | | | | | | | | | | | | | | | | |

**Best Unique PSM (Scan F10:33873, m/z=1091.9441, z=2, RT=57.45, ppm=0.3):**

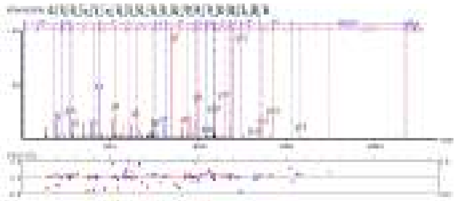

## 1U4J

back to list

| Protein Coverage | Supporting Peptides | Best Unique PSM |

**Protein Coverage:**

1 NLKQFKNMIQ CAGTRTWTSY IGYGCYCGYG GSGTPVDELD RCCYTHDHCY NKAANIPGCN PLIKTYSYTC TKPNITCNDT

Carbamidomethylation (+57.02)

81 SDSCARFICD CDRTAAICFA SAPYNINNIM ISASTSCQ

**Supporting Peptides:**

| Peptide | Uniq | -10lgP | Mass | Length | ppm | m/z | z | RT | Fraction | Scan | Source File | Area F1 | Area F2a | Area F3a | Area F4 | Area F5 | Area F6a | Area F7a | Area F7b | Area F8 | Area F9a | Area Sample 1F10a | Area F10b | Area F11a | Area F11b | Area F12a | Area F13 | #Feature | #Feature F1 | #Feature F2a | #Feature F3a | #Feature F4 | #Feature F5 | #Feature F6a | #Feature F7a | #Feature F7b | #Feature F8 | #Feature F9a | #Feature Sample 1F10a | #Feature F10b | #Feature F11a | #Feature F11b | #Feature F12a | #Feature F13 | Start | End | PTM | AScore | Found By |
|---|---|---|---|---|---|---|---|---|---|---|---|---|---|---|---|---|---|---|---|---|---|---|---|---|---|---|---|---|---|---|---|---|---|---|---|---|---|---|---|---|---|---|---|---|---|---|---|---|---|
| G.YGC(+57.02)YC(+57.02)GYGGSGTPVDELDR.C | N | 112.36 | 2124.8516 | 19 | 3.7 | 1063.4326 | 2 | 56.60 | 14 | F14:33384 | BuCaTN01_F11b.raw | | | 0 | | | 2.0097E6 | | 1.4053E7 | | 8.8531E7 | 3.4891E6 | 4.8054E8 | 6.165E7 | 1.7851E9 | 1.2628E8 | | 69 | 0 | 0 | 0 | 0 | 0 | 1 | 0 | 2 | 0 | 3 | 1 | 4 | 2 | 53 | 3 | 0 | 23 | 41 | Carbamidomethylation | C3:Carbamidomethylation:1000.00;C5:Carbamidomethylation:1000.00 | PEAKS DB |
| I.GYGC(+57.02)YC(+57.02)GYGGSGTPVDELDR.C | Y | 100.48 | 2181.8730 | 20 | 0.3 | 1091.9441 | 2 | 57.45 | 10 | F10:33873 | BuCaTN01_F9a.raw | | | | | | | 0 | | | 2.5729E7 | 2.6663E5 | 6.1424E7 | 1.3809E7 | 2.1113E8 | 7.3334E6 | | 15 | 0 | 0 | 0 | 0 | 0 | 0 | 0 | 0 | 0 | 1 | 1 | 3 | 3 | 4 | 3 | 0 | 22 | 41 | Carbamidomethylation | C4:Carbamidomethylation:1000.00;C6:Carbamidomethylation:1000.00 | PEAKS DB |

| | | | | | | | | | | | | | | | | | | | | | | | | | | | | | | | | | | | | | | | | | | | | | | | | | |
|---|---|---|---|---|---|---|---|---|---|---|---|---|---|---|---|---|---|---|---|---|---|---|---|---|---|---|---|---|---|---|---|---|---|---|---|---|---|---|---|---|---|---|---|---|---|---|---|---|---|
| Y.C(+57.02)GYGGSGTPVDELDR.C | N | 88.12 | 1581.6729 | 15 | 4.4 | 791.8439 | 2 | 48.02 | 14 | F14:25306 | BuCaTN01_F11b.raw | | | | | | | | 2.166E6 | | 8.5786E6 | | 1.5853E7 | 6.495E5 | 1.7537E7 | 0 | | 6 | 0 | 0 | 0 | 0 | 0 | 0 | 0 | 1 | 0 | 1 | 0 | 2 | 1 | 1 | 0 | 0 | 27 | 41 | Carbamidomethylation | C1:Carbamidomethylation:1000.00 | PEAKS DB |
| G.C(+57.02)YC(+57.02)GYGGSGTPVDELDR.C | N | 84.40 | 1904.7668 | 17 | 2.7 | 953.3911 | 2 | 53.16 | 12 | F12:29351 | BuCaTN01_F10b.raw | | | | | | | | 4.1849E5 | | | | 3.7261E5 | | 2.1774E6 | | | 3 | 0 | 0 | 0 | 0 | 0 | 0 | 0 | 1 | 0 | 0 | 0 | 1 | 0 | 1 | 0 | 0 | 25 | 41 | Carbamidomethylation | C1:Carbamidomethylation:1000.00;C3:Carbamidomethylation:1000.00 | PEAKS DB |
| Y.GC(+57.02)YC(+57.02)GYGGSGTPVDELDR.C | N | 82.10 | 1961.7883 | 18 | 1.4 | 981.9028 | 2 | 53.49 | 10 | F10:30232 | BuCaTN01_F9a.raw | | | | | | | | 1.7852E6 | | 3.3077E6 | | 1.5846E6 | | 3.2964E6 | 1.0428E5 | | 5 | 0 | 0 | 0 | 0 | 0 | 0 | 0 | 1 | 0 | 1 | 0 | 1 | 0 | 1 | 1 | 0 | 24 | 41 | Carbamidomethylation | C2:Carbamidomethylation:1000.00;C4:Carbamidomethylation:1000.00 | PEAKS DB |
| C.YC(+57.02)GYGGSGTPVDELDR.C | N | 75.62 | 1744.7362 | 16 | 4.1 | 873.3754 | 2 | 52.27 | 14 | F14:28849 | BuCaTN01_F11b.raw | | | | | | | | 7.7286E5 | | 7.3447E5 | | | | 3.0904E6 | 1.4057E5 | | 4 | 0 | 0 | 0 | 0 | 0 | 0 | 0 | 1 | 0 | 1 | 0 | 0 | 0 | 1 | 1 | 0 | 26 | 41 | Carbamidomethylation | C2:Carbamidomethylation:1000.00 | PEAKS DB |
| G.YGGSGTPVDELDR.C | N | 75.26 | 1364.6207 | 13 | 4.1 | 683.3177 | 2 | 45.29 | 14 | F14:22502 | BuCaTN01_F11b.raw | | | | | | | 6.8893E5 | | | | | | | 9.1406E6 | 6.9945E5 | | 3 | 0 | 0 | 0 | 0 | 0 | 0 | 1 | 0 | 0 | 0 | 0 | 0 | 0 | 1 | 1 | 0 | 29 | 41 | | | PEAKS DB |
| C.GYGGSGTPVDELDR.C | N | 66.26 | 1421.6422 | 14 | 0.0 | 711.8284 | 2 | 46.82 | 10 | F10:24048 | BuCaTN01_F9a.raw | | | | | | | 2.9103E5 | 1.2275E6 | | 2.1867E6 | | | | 8.7883E6 | | | 5 | 0 | 0 | 0 | 0 | 0 | 0 | 1 | 1 | 0 | 1 | 0 | 0 | 0 | 2 | 0 | 0 | 28 | 41 | | | PEAKS DB |
| K.TYSYTC(+57.02)TKPNIT.C | N | 66.05 | 1447.6653 | 12 | 2.5 | 724.8387 | 2 | 43.32 | 14 | F14:21091 | BuCaTN01_F11b.raw | | | | | | | | 1.3481E6 | | | | 2.4842E6 | | 1.5203E7 | 9.0876E6 | | 5 | 0 | 0 | 0 | 0 | 0 | 0 | 0 | 1 | 0 | 0 | 0 | 1 | 0 | 2 | 1 | 0 | 65 | 76 | Carbamidomethylation | C6:Carbamidomethylation:1000.00 | PEAKS DB |
| R.FIC(+57.02)DC(+57.02)DRTAAIC(+57.02).F | N | 58.10 | 1500.6160 | 12 | 4.0 | 751.3152 | 2 | 47.17 | 14 | F14:24274 | BuCaTN01_F11b.raw | | | | | | | | | | | | | | 3.9576E6 | | | 1 | 0 | 0 | 0 | 0 | 0 | 0 | 0 | 0 | 0 | 0 | 0 | 0 | 0 | 1 | 0 | 0 | 87 | 98 | Carbamidomethylation | C3:Carbamidomethylation:1000.00;C5:Carbamidomethylation:1000.00;C12:Carbamidomethylation:1000.00 | PEAKS DB |
| G.GSGTPVDELDR.C | N | 56.36 | 1144.5360 | 11 | 3.4 | 573.2756 | 2 | 40.15 | 8 | F8:17921 | BuCaTN01_F7b.raw | | | | | | | | 1.1068E6 | | | | | | | | | 1 | 0 | 0 | 0 | 0 | 0 | 0 | 0 | 1 | 0 | 0 | 0 | 0 | 0 | 0 | 0 | 0 | 31 | 41 | | | PEAKS DB |
| K.TYSYTC(+57.02)TKPNITC(+57.02).N | N | 55.98 | 1607.6960 | 13 | 5.0 | 804.8560 | 2 | 43.60 | 14 | F14:21061 | BuCaTN01_F11b.raw | | | | | | | | | | | | | | 2.3535E6 | 7.2547E5 | | 3 | 0 | 0 | 0 | 0 | 0 | 0 | 0 | 0 | 0 | 0 | 0 | 0 | 0 | 2 | 1 | 0 | 65 | 77 | Carbamidomethylation | C6:Carbamidomethylation:1000.00;C13:Carbamidomethylation:1000.00 | PEAKS DB |
| N.IPGC(+57.02)NPLIK.T | Y | 52.95 | 1010.5583 | 9 | 0.9 | 506.2869 | 2 | 41.50 | 13 | F13:18979 | BuCaTN01_F11a.raw | | | | | | 3.3656E6 | 8.2953E5 | 2.7046E7 | | 6.9838E7 | 1.5423E6 | 3.4919E8 | 2.8685E7 | 1.5397E9 | 1.518E8 | | 9 | 0 | 0 | 0 | 0 | 0 | 1 | 1 | 1 | 0 | 1 | 1 | 1 | 1 | 1 | 1 | 0 | 56 | 64 | Carbamidomethylation | C4:Carbamidomethylation:1000.00 | PEAKS DB |
| R.FIC(+57.02)DC(+57.02)DRTAAIC(+57.02)F.A | N | 49.37 | 1647.6843 | 13 | 3.5 | 824.8499 | 2 | 66.79 | 8 | F8:42173 | BuCaTN01_F7b.raw | | | | | | | | 7.495E5 | | | | | | | | | 1 | 0 | 0 | 0 | 0 | 0 | 0 | 0 | 1 | 0 | 0 | 0 | 0 | 0 | 0 | 0 | 0 | 87 | 99 | Carbamidomethylation | C3:Carbamidomethylation:1000.00;C5:Carbamidomethylation:1000.00;C12:Carbamidomethylation:1000.00 | PEAKS DB |
| Y.GGSGTPVDELDR.C | N | 49.09 | 1201.5575 | 12 | 0.3 | 601.7862 | 2 | 40.57 | 11 | F11:14528 | BuCaTN01_F10a.raw | | | | | | | 9.2274E5 | 2.7577E6 | | 1.1335E7 | 6.7591E5 | | 9.9086E5 | 2.6759E7 | | | 6 | 0 | 0 | 0 | 0 | 0 | 0 | 1 | 1 | 0 | 1 | 1 | 0 | 1 | 1 | 0 | 0 | 30 | 41 | | | PEAKS DB |
| K.TYSYTC(+57.02)TKPNI.T | N | 46.29 | 1346.6177 | 11 | 5.6 | 674.3171 | 2 | 45.63 | 14 | F14:22808 | BuCaTN01_F11b.raw | | | | | | | | | | | | | | 5.6477E6 | 2.611E6 | | 2 | 0 | 0 | 0 | 0 | 0 | 0 | 0 | 0 | 0 | 0 | 0 | 0 | 0 | 1 | 1 | 0 | 65 | 75 | Carbamidomethylation | C6:Carbamidomethylation:1000.00 | PEAKS DB |

**total 16 peptides**

**Best Unique PSM (Scan F10:33873, m/z=1091.9441, z=2, RT=57.45, ppm=0.3):**

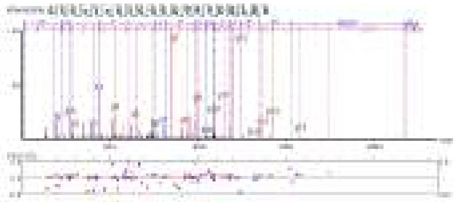

## Q6SLM1.1

back to list

| **Protein Coverage** | **Supporting Peptides** | **Best Unique PSM** |

**Protein Coverage:**

1 LVAVCVSLLG AANIPPQPLN LKQFKNMIQC AGTRTWTSYI **GYGCYCGYGG SGTPVDELDR** CCYTHDHCYN KAAN**IPGCNP**

Carbamidomethylation (+57.02)

81 **LIKTYSYTCT KPNITC**NDTS DSCAR**FICDC DRTAAICF**AS APYNINNIMI SASTSCQ

**Supporting Peptides:**

| Peptide | Uniq | -10lgP | Mass | Length | ppm | m/z | z | RT | Fraction | Scan | Source File | Area F1 | Area F2a | Area F3a | Area F4 | Area F5 | Area F6a | Area F7a | Area F7b | Area F8 | Area F9a | Area Sample 1F10a | Area F10b | Area F11a | Area F11b | Area F12a | Area F13 | #Feature | #Feature F1 | #Feature F2a | #Feature F3a | #Feature F4 | #Feature F5 | #Feature F6a | #Feature F7a | #Feature F7b | #Feature F8 | #Feature F9a | #Feature Sample 1F10a | #Feature F10b | #Feature F11a | #Feature F11b | #Feature F12a | #Feature F13 | Start | End | PTM | AScore | Found By |
|---|---|---|---|---|---|---|---|---|---|---|---|---|---|---|---|---|---|---|---|---|---|---|---|---|---|---|---|---|---|---|---|---|---|---|---|---|---|---|---|---|---|---|---|---|---|---|---|---|---|
| G.YGC(+57.02)YC(+57.02)GYGGSGTPVDELDR.C | N | 112.36 | 2124.8516 | 19 | 3.7 | 1063.4326 | 2 | 56.60 | 14 | F14:33384 | BuCaTN01_F11b.raw | | | 0 | | | 2.0097E6 | | 1.4053E7 | | 8.8531E7 | 3.4891E6 | 4.8054E8 | 6.165E7 | 1.7851E9 | 1.2628E8 | | 69 | 0 | 0 | 0 | 0 | 0 | 1 | 0 | 2 | 0 | 3 | 1 | 4 | 2 | 53 | 3 | 0 | 42 | 60 | Carbamidomethylation | C3:Carbamidomethylation:1000.00;C5:Carbamidomethylation:1000.00 | PEAKS DB |
| I.GYGC(+57.02)YC(+57.02)GYGGSGTPVDELDR.C | Y | 100.48 | 2181.8730 | 20 | 0.3 | 1091.9441 | 2 | 57.45 | 10 | F10:33873 | BuCaTN01_F9a.raw | | | | | | | 0 | | | 2.5729E7 | 2.6663E5 | 6.1424E7 | 1.3809E7 | 2.1113E8 | 7.3334E6 | | 15 | 0 | 0 | 0 | 0 | 0 | 0 | 0 | 0 | 0 | 1 | 1 | 3 | 3 | 4 | 3 | 0 | 41 | 60 | Carbamidomethylation | C4:Carbamidomethylation:1000.00;C6:Carbamidomethylation:1000.00 | PEAKS DB |
| Y.C(+57.02)GYGGSGTPVDELDR.C | N | 88.12 | 1581.6729 | 15 | 4.4 | 791.8439 | 2 | 48.02 | 14 | F14:25306 | BuCaTN01_F11b.raw | | | | | | | | 2.166E6 | | 8.5786E6 | | 1.5853E7 | 6.495E5 | 1.7537E7 | 0 | | 6 | 0 | 0 | 0 | 0 | 0 | 0 | 0 | 1 | 0 | 1 | 0 | 2 | 1 | 1 | 0 | 0 | 46 | 60 | Carbamidomethylation | C1:Carbamidomethylation:1000.00 | PEAKS DB |
| G.C(+57.02)YC(+57.02)GYGGSGTPVDELDR.C | N | 84.40 | 1904.7668 | 17 | 2.7 | 953.3911 | 2 | 53.16 | 12 | F12:29351 | BuCaTN01_F10b.raw | | | | | | | | 4.1849E5 | | | | 3.7261E5 | | 2.1774E6 | | | 3 | 0 | 0 | 0 | 0 | 0 | 0 | 0 | 1 | 0 | 0 | 0 | 1 | 0 | 1 | 0 | 0 | 44 | 60 | Carbamidomethylation | C1:Carbamidomethylation:1000.00;C3:Carbamidomethylation:1000.00 | PEAKS DB |
| Y.GC(+57.02)YC(+57.02)GYGGSGTPVDELDR.C | N | 82.10 | 1961.7883 | 18 | 1.4 | 981.9028 | 2 | 53.49 | 10 | F10:30232 | BuCaTN01_F9a.raw | | | | | | | | 1.7852E6 | | 3.3077E6 | | 1.5846E6 | | 3.2964E6 | 1.0428E5 | | 5 | 0 | 0 | 0 | 0 | 0 | 0 | 0 | 1 | 0 | 1 | 0 | 1 | 0 | 1 | 1 | 0 | 43 | 60 | Carbamidomethylation | C2:Carbamidomethylation:1000.00;C4:Carbamidomethylation:1000.00 | PEAKS DB |
| C.YC(+57.02)GYGGSGTPVDELDR.C | N | 75.62 | 1744.7362 | 16 | 4.1 | 873.3754 | 2 | 52.27 | 14 | F14:28849 | BuCaTN01_F11b.raw | | | | | | | | 7.7286E5 | | 7.3447E5 | | | | 3.0904E6 | 1.4057E5 | | 4 | 0 | 0 | 0 | 0 | 0 | 0 | 0 | 1 | 0 | 1 | 0 | 0 | 0 | 1 | 1 | 0 | 45 | 60 | Carbamidomethylation | C2:Carbamidomethylation:1000.00 | PEAKS DB |
| G.YGGSGTPVDELDR.C | N | 75.26 | 1364.6207 | 13 | 4.1 | 683.3177 | 2 | 45.29 | 14 | F14:22502 | BuCaTN01_F11b.raw | | | | | | | 6.8893E5 | | | | | | | 9.1406E6 | 6.9945E5 | | 3 | 0 | 0 | 0 | 0 | 0 | 0 | 1 | 0 | 0 | 0 | 0 | 0 | 0 | 1 | 1 | 0 | 48 | 60 | | | PEAKS DB |
| C.GYGGSGTPVDELDR.C | N | 66.26 | 1421.6422 | 14 | 0.0 | 711.8284 | 2 | 46.82 | 10 | F10:24048 | BuCaTN01_F9a.raw | | | | | | | 2.9103E5 | 1.2275E6 | | 2.1867E6 | | | | 8.7883E6 | | | 5 | 0 | 0 | 0 | 0 | 0 | 0 | 1 | 1 | 0 | 1 | 0 | 0 | 0 | 2 | 0 | 0 | 47 | 60 | | | PEAKS DB |
| K.TYSYTC(+57.02)TKPNIT.C | N | 66.05 | 1447.6653 | 12 | 2.5 | 724.8387 | 2 | 43.32 | 14 | F14:21091 | BuCaTN01_F11b.raw | | | | | | | | 1.3481E6 | | | | 2.4842E6 | | 1.5203E7 | 9.0876E6 | | 5 | 0 | 0 | 0 | 0 | 0 | 0 | 0 | 1 | 0 | 0 | 0 | 1 | 0 | 2 | 1 | 0 | 84 | 95 | Carbamidomethylation | C6:Carbamidomethylation:1000.00 | PEAKS DB |
| R.FIC(+57.02)DC(+57.02)DRTAAIC(+57.02).F | N | 58.10 | 1500.6160 | 12 | 4.0 | 751.3152 | 2 | 47.17 | 14 | F14:24274 | BuCaTN01_F11b.raw | | | | | | | | | | | | | | 3.9576E6 | | | 1 | 0 | 0 | 0 | 0 | 0 | 0 | 0 | 0 | 0 | 0 | 0 | 0 | 0 | 1 | 0 | 0 | 106 | 117 | Carbamidomethylation | C3:Carbamidomethylation:1000.00;C5:Carbamidomethylation:1000.00;C12:Carbamidomethylation:1000.00 | PEAKS DB |
| G.GSGTPVDELDR.C | N | 56.36 | 1144.5360 | 11 | 3.4 | 573.2756 | 2 | 40.15 | 8 | F8:17921 | BuCaTN01_F7b.raw | | | | | | | | 1.1068E6 | | | | | | | | | 1 | 0 | 0 | 0 | 0 | 0 | 0 | 0 | 1 | 0 | 0 | 0 | 0 | 0 | 0 | 0 | 0 | 50 | 60 | | | PEAKS DB |
| K.TYSYTC(+57.02)TKPNITC(+57.02).N | N | 55.98 | 1607.6960 | 13 | 5.0 | 804.8560 | 2 | 43.60 | 14 | F14:21061 | BuCaTN01_F11b.raw | | | | | | | | | | | | | | 2.3535E6 | 7.2547E5 | | 3 | 0 | 0 | 0 | 0 | 0 | 0 | 0 | 0 | 0 | 0 | 0 | 0 | 0 | 2 | 1 | 0 | 84 | 96 | Carbamidomethylation | C6:Carbamidomethylation:1000.00;C13:Carbamidomethylation:1000.00 | PEAKS DB |
| N.IPGC(+57.02)NPLIK.T | Y | 52.95 | 1010.5583 | 9 | 0.9 | 506.2869 | 2 | 41.50 | 13 | F13:18979 | BuCaTN01_F11a.raw | | | | | | 3.3656E6 | 8.2953E5 | 2.7046E7 | | 6.9838E7 | 1.5423E6 | 3.4919E8 | 2.8685E7 | 1.5397E9 | 1.518E8 | | 9 | 0 | 0 | 0 | 0 | 0 | 1 | 1 | 1 | 0 | 1 | 1 | 1 | 1 | 1 | 1 | 0 | 75 | 83 | Carbamidomethylation | C4:Carbamidomethylation:1000.00 | PEAKS DB |
| R.FIC(+57.02)DC(+57.02)DRTAAIC(+57.02)F.A | N | 49.37 | 1647.6843 | 13 | 3.5 | 824.8499 | 2 | 66.79 | 8 | F8:42173 | BuCaTN01_F7b.raw | | | | | | | | 7.495E5 | | | | | | | | | 1 | 0 | 0 | 0 | 0 | 0 | 0 | 0 | 1 | 0 | 0 | 0 | 0 | 0 | 0 | 0 | 0 | 106 | 118 | Carbamidomethylation | C3:Carbamidomethylation:1000.00;C5:Carbamidomethylation:1000.00;C12:Carbamidomethylation:1000.00 | PEAKS DB |
| Y.GGSGTPVDELDR.C | N | 49.09 | 1201.5575 | 12 | 0.3 | 601.7862 | 2 | 40.57 | 11 | F11:14528 | BuCaTN01_F10a.raw | | | | | | | 9.2274E5 | 2.7577E6 | | 1.1335E7 | 6.7591E5 | | 9.9086E5 | 2.6759E7 | | | 6 | 0 | 0 | 0 | 0 | 0 | 0 | 1 | 1 | 0 | 1 | 1 | 0 | 1 | 1 | 0 | 0 | 49 | 60 | | | PEAKS DB |
| K.TYSYTC(+57.02)TKPNI.T | N | 46.29 | 1346.6177 | 11 | 5.6 | 674.3171 | 2 | 45.63 | 14 | F14:22808 | BuCaTN01_F11b.raw | | | | | | | | | | | | | | 5.6477E6 | 2.611E6 | | 2 | 0 | 0 | 0 | 0 | 0 | 0 | 0 | 0 | 0 | 0 | 0 | 0 | 0 | 1 | 1 | 0 | 84 | 94 | Carbamidomethylation | C6:Carbamidomethylation:1000.00 | PEAKS DB |

**total 16 peptides**

**Best Unique PSM (Scan F10:33873, m/z=1091.9441, z=2, RT=57.45, ppm=0.3):**

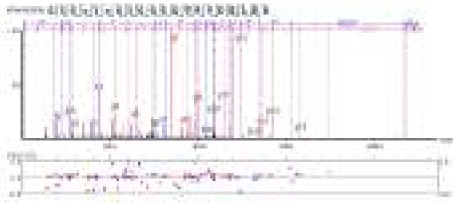

## AAR19228.1

back to list

| **Protein Coverage** | **Supporting Peptides** | **Best Unique PSM** |

**Protein Coverage:**

1 LVAVCVSLLG AANIPPQPLN LKQFKNMIQC AGTRTWTSYI **GYGCYCGYGG SGTPVDELDR** CCYTHDHCYN KAAN**IPGCNP**

Carbamidomethylation (+57.02)

81 **LIKTYSYTCT KPNITC**NDTS DSCAR**FICDC DRTAAICF**AS APYNINNIMI SASTSCQ

**Supporting Peptides:**

| Peptide | Uniq | -10lgP | Mass | Length | ppm | m/z | z | RT | Fraction | Scan | Source File | Area F1 | Area F2a | Area F3a | Area F4 | Area F5 | Area F6a | Area F7a | Area F7b | Area F8 | Area F9a | Area Sample 1F10a | Area F10b | Area F11a | Area F11b | Area F12a | Area F13 | #Feature | #Feature F1 | #Feature F2a | #Feature F3a | #Feature F4 | #Feature F5 | #Feature F6a | #Feature F7a | #Feature F7b | #Feature F8 | #Feature F9a | #Feature Sample 1F10a | #Feature F10b | #Feature F11a | #Feature F11b | #Feature F12a | #Feature F13 | Start | End | PTM | AScore | Found By |
|---|---|---|---|---|---|---|---|---|---|---|---|---|---|---|---|---|---|---|---|---|---|---|---|---|---|---|---|---|---|---|---|---|---|---|---|---|---|---|---|---|---|---|---|---|---|---|---|---|---|

| | | | | | | | | | | | | | | | | | | | | | | | | | | | | | | | | | | | | | | | | | | | | | | | | | |
|---|---|---|---|---|---|---|---|---|---|---|---|---|---|---|---|---|---|---|---|---|---|---|---|---|---|---|---|---|---|---|---|---|---|---|---|---|---|---|---|---|---|---|---|---|---|---|---|---|---|
| G.YGC(+57.02)YC(+57.02)GYGGSGTPVDELDR.C | N | 112.36 | 2124.8516 | 19 | 3.7 | 1063.4326 | 2 | 56.60 | 14 | F14:33384 | BuCaTN01_F11b.raw | | | 0 | | | 2.0097E6 | | 1.4053E7 | | 8.8531E7 | 3.4891E6 | 4.8054E8 | 6.165E7 | 1.7851E9 | 1.2628E8 | | 69 | 0 | 0 | 0 | 0 | 0 | 1 | 0 | 2 | 0 | 3 | 1 | 4 | 2 | 53 | 3 | 0 | 42 | 60 | Carbamidomethylation | C3:Carbamidomethylation:1000.00;C5:Carbamidomethylation:1000.00 | PEAKS DB |
| I.GYGC(+57.02)YC(+57.02)GYGGSGTPVDELDR.C | Y | 100.48 | 2181.8730 | 20 | 0.3 | 1091.9441 | 2 | 57.45 | 10 | F10:33873 | BuCaTN01_F9a.raw | | | | | | | 0 | | | 2.5729E7 | 2.6663E5 | 6.1424E7 | 1.3809E7 | 2.1113E8 | 7.3334E6 | | 15 | 0 | 0 | 0 | 0 | 0 | 0 | 0 | 0 | 0 | 1 | 1 | 3 | 3 | 4 | 3 | 0 | 41 | 60 | Carbamidomethylation | C4:Carbamidomethylation:1000.00;C6:Carbamidomethylation:1000.00 | PEAKS DB |
| Y.C(+57.02)GYGGSGTPVDELDR.C | N | 88.12 | 1581.6729 | 15 | 4.4 | 791.8439 | 2 | 48.02 | 14 | F14:25306 | BuCaTN01_F11b.raw | | | | | | | | 2.166E6 | | 8.5786E6 | | 1.5853E7 | 6.495E5 | 1.7537E7 | 0 | | 6 | 0 | 0 | 0 | 0 | 0 | 0 | 0 | 1 | 0 | 1 | 0 | 2 | 1 | 1 | 0 | 0 | 46 | 60 | Carbamidomethylation | C1:Carbamidomethylation:1000.00 | PEAKS DB |
| G.C(+57.02)YC(+57.02)GYGGSGTPVDELDR.C | N | 84.40 | 1904.7668 | 17 | 2.7 | 953.3911 | 2 | 53.16 | 12 | F12:29351 | BuCaTN01_F10b.raw | | | | | | | | 4.1849E5 | | | | 3.7261E5 | | 2.1774E6 | | | 3 | 0 | 0 | 0 | 0 | 0 | 0 | 0 | 1 | 0 | 0 | 0 | 1 | 0 | 1 | 0 | 0 | 44 | 60 | Carbamidomethylation | C1:Carbamidomethylation:1000.00;C3:Carbamidomethylation:1000.00 | PEAKS DB |
| Y.GC(+57.02)YC(+57.02)GYGGSGTPVDELDR.C | N | 82.10 | 1961.7883 | 18 | 1.4 | 981.9028 | 2 | 53.49 | 10 | F10:30232 | BuCaTN01_F9a.raw | | | | | | | | 1.7852E6 | | 3.3077E6 | | 1.5846E6 | | 3.2964E6 | 1.0428E5 | | 5 | 0 | 0 | 0 | 0 | 0 | 0 | 0 | 1 | 0 | 1 | 0 | 1 | 0 | 1 | 1 | 0 | 43 | 60 | Carbamidomethylation | C2:Carbamidomethylation:1000.00;C4:Carbamidomethylation:1000.00 | PEAKS DB |
| C.YC(+57.02)GYGGSGTPVDELDR.C | N | 75.62 | 1744.7362 | 16 | 4.1 | 873.3754 | 2 | 52.27 | 14 | F14:28849 | BuCaTN01_F11b.raw | | | | | | | | 7.7286E5 | | 7.3447E5 | | | | 3.0904E6 | 1.4057E5 | | 4 | 0 | 0 | 0 | 0 | 0 | 0 | 0 | 1 | 0 | 1 | 0 | 0 | 0 | 1 | 1 | 0 | 45 | 60 | Carbamidomethylation | C2:Carbamidomethylation:1000.00 | PEAKS DB |
| G.YGGSGTPVDELDR.C | N | 75.26 | 1364.6207 | 13 | 4.1 | 683.3177 | 2 | 45.29 | 14 | F14:22502 | BuCaTN01_F11b.raw | | | | | | | 6.8893E5 | | | | | | | 9.1406E6 | 6.9945E5 | | 3 | 0 | 0 | 0 | 0 | 0 | 0 | 1 | 0 | 0 | 0 | 0 | 0 | 0 | 1 | 1 | 0 | 48 | 60 | | | PEAKS DB |
| C.GYGGSGTPVDELDR.C | N | 66.26 | 1421.6422 | 14 | 0.0 | 711.8284 | 2 | 46.82 | 10 | F10:24048 | BuCaTN01_F9a.raw | | | | | | | 2.9103E5 | 1.2275E6 | | 2.1867E6 | | | | 8.7883E6 | | | 5 | 0 | 0 | 0 | 0 | 0 | 0 | 1 | 1 | 0 | 1 | 0 | 0 | 0 | 2 | 0 | 0 | 47 | 60 | | | PEAKS DB |
| K.TYSYTC(+57.02)TKPNIT.C | N | 66.05 | 1447.6653 | 12 | 2.5 | 724.8387 | 2 | 43.32 | 14 | F14:21091 | BuCaTN01_F11b.raw | | | | | | | | 1.3481E6 | | | | 2.4842E6 | | 1.5203E7 | 9.0876E6 | | 5 | 0 | 0 | 0 | 0 | 0 | 0 | 0 | 1 | 0 | 0 | 0 | 1 | 0 | 2 | 1 | 0 | 84 | 95 | Carbamidomethylation | C6:Carbamidomethylation:1000.00 | PEAKS DB |
| R.FIC(+57.02)DC(+57.02)DRTAAIC(+57.02).F | N | 58.10 | 1500.6160 | 12 | 4.0 | 751.3152 | 2 | 47.17 | 14 | F14:24274 | BuCaTN01_F11b.raw | | | | | | | | | | | | | | 3.9576E6 | | | 1 | 0 | 0 | 0 | 0 | 0 | 0 | 0 | 0 | 0 | 0 | 0 | 0 | 0 | 1 | 0 | 0 | 106 | 117 | Carbamidomethylation | C3:Carbamidomethylation:1000.00;C5:Carbamidomethylation:1000.00;C12:Carbamidomethylation:1000.00 | PEAKS DB |
| G.GSGTPVDELDR.C | N | 56.36 | 1144.5360 | 11 | 3.4 | 573.2756 | 2 | 40.15 | 8 | F8:17921 | BuCaTN01_F7b.raw | | | | | | | | 1.1068E6 | | | | | | | | | 1 | 0 | 0 | 0 | 0 | 0 | 0 | 0 | 1 | 0 | 0 | 0 | 0 | 0 | 0 | 0 | 0 | 50 | 60 | | | PEAKS DB |
| K.TYSYTC(+57.02)TKPNITC(+57.02).N | N | 55.98 | 1607.6960 | 13 | 5.0 | 804.8560 | 2 | 43.60 | 14 | F14:21061 | BuCaTN01_F11b.raw | | | | | | | | | | | | | | 2.3535E6 | 7.2547E5 | | 3 | 0 | 0 | 0 | 0 | 0 | 0 | 0 | 0 | 0 | 0 | 0 | 0 | 0 | 2 | 1 | 0 | 84 | 96 | Carbamidomethylation | C6:Carbamidomethylation:1000.00;C13:Carbamidomethylation:1000.00 | PEAKS DB |
| N.IPGC(+57.02)NPLIK.T | Y | 52.95 | 1010.5583 | 9 | 0.9 | 506.2869 | 2 | 41.50 | 13 | F13:18979 | BuCaTN01_F11a.raw | | | | | | 3.3656E6 | 8.2953E5 | 2.7046E7 | | 6.9838E7 | 1.5423E6 | 3.4919E8 | 2.8685E7 | 1.5397E9 | 1.518E8 | | 9 | 0 | 0 | 0 | 0 | 0 | 1 | 1 | 1 | 0 | 1 | 1 | 1 | 1 | 1 | 1 | 0 | 75 | 83 | Carbamidomethylation | C4:Carbamidomethylation:1000.00 | PEAKS DB |
| R.FIC(+57.02)DC(+57.02)DRTAAIC(+57.02)F.A | N | 49.37 | 1647.6843 | 13 | 3.5 | 824.8499 | 2 | 66.79 | 8 | F8:42173 | BuCaTN01_F7b.raw | | | | | | | | 7.495E5 | | | | | | | | | 1 | 0 | 0 | 0 | 0 | 0 | 0 | 0 | 1 | 0 | 0 | 0 | 0 | 0 | 0 | 0 | 0 | 106 | 118 | Carbamidomethylation | C3:Carbamidomethylation:1000.00;C5:Carbamidomethylation:1000.00;C12:Carbamidomethylation:1000.00 | PEAKS DB |
| Y.GGSGTPVDELDR.C | N | 49.09 | 1201.5575 | 12 | 0.3 | 601.7862 | 2 | 40.57 | 11 | F11:14528 | BuCaTN01_F10a.raw | | | | | | | 9.2274E5 | 2.7577E6 | | 1.1335E7 | 6.7591E5 | | 9.9086E5 | 2.6759E7 | | | 6 | 0 | 0 | 0 | 0 | 0 | 0 | 1 | 1 | 0 | 1 | 1 | 0 | 1 | 1 | 0 | 0 | 49 | 60 | | | PEAKS DB |
| K.TYSYTC(+57.02)TKPNI.T | N | 46.29 | 1346.6177 | 11 | 5.6 | 674.3171 | 2 | 45.63 | 14 | F14:22808 | BuCaTN01_F11b.raw | | | | | | | | | | | | | | 5.6477E6 | 2.611E6 | | 2 | 0 | 0 | 0 | 0 | 0 | 0 | 0 | 0 | 0 | 0 | 0 | 0 | 0 | 1 | 1 | 0 | 84 | 94 | Carbamidomethylation | C6:Carbamidomethylation:1000.00 | PEAKS DB |
| **total 16 peptides** | | | | | | | | | | | | | | | | | | | | | | | | | | | | | | | | | | | | | | | | | | | | | | | | | |

**Best Unique PSM (Scan F10:33873, m/z=1091.9441, z=2, RT=57.45, ppm=0.3):**

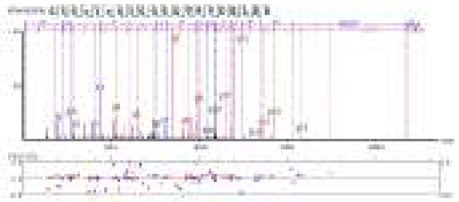

## AAB25729.1

back to list

| **Protein Coverage** | **Supporting Peptides** | **Best Unique PSM** |

**Protein Coverage:**

C Carbamidomethylation (+57.02)

```
  1  MSMLCYTLII AFLIGIWAAP KSEDNVPLGS PAKSDFSDTN CAQTHEGLKT SRNTDQHHPT PKKSEDQELG SAANIIVDPK
 81  LFQKRRFQSP RVLFSTQPPP LSRDEQSVKF LDTEDTLNRN IWANNENHPV HNQGEHSVCD SISVWVTNKT KATDIKGNTV
                        182                        204                         232 234
161  TVMVDVNLNN EVYKQYFFET KCRNPNPVPS GCRGIDSRHW NSYCTTTDTF VKALTMEGNR ASWRFIRIDT ACVCVISRKT
241  ENF
```

**Supporting Peptides:**

| Peptide | Uniq | -10lgP | Mass | Length | ppm | m/z | z | RT | Fraction | Scan | Source File | Area F1 | Area F2a | Area F3a | Area F4 | Area F5 | Area F6a | Area F7a | Area F7b | Area F8 | Area F9a | Area Sample 1F10a | Area F10b | Area F11a | Area F11b | Area F12a | Area F13 | #Feature | #Feature F1 | #Feature F2a | #Feature F3a | #Feature F4 | #Feature F5 | #Feature F6a | #Feature F7a | #Feature F7b | #Feature F8 | #Feature F9a | #Feature Sample 1F10a | #Feature F10b | #Feature F11a | #Feature F11b | #Feature F12a | #Feature F13 | Start | End | PTM | AScore | Found By |
|---|---|---|---|---|---|---|---|---|---|---|---|---|---|---|---|---|---|---|---|---|---|---|---|---|---|---|---|---|---|---|---|---|---|---|---|---|---|---|---|---|---|---|---|---|---|---|---|---|---|
| R.HWNSYC(+57.02)TTTDTFVK.A | Y | 118.89 | 1758.7672 | 14 | 2.8 | 880.3908 | 2 | 49.91 | 8 | F8:26632 | BuCaTN01_F7b.raw | | | | | | | 2.7032E7 | 1.4368E7 | 5.9762E5 | 3.3814E5 | | | | | | | 6 | 0 | 0 | 0 | 0 | 0 | 0 | 2 | 2 | 1 | 1 | 0 | 0 | 0 | 0 | 0 | 0 | 199 | 212 | Carbamidomethylation | C6:Carbamidomethylation:1000.00 | PEAKS DB |
| R.IDTAC(+57.02)VC(+57.02)VISR.K | N | 81.21 | 1292.6217 | 11 | 0.1 | 647.3182 | 2 | 47.01 | 7 | F7:21996 | BuCaTN01_F7a.raw | | | | | | 2.9158E5 | 3.0809E7 | 1.2168E7 | 2.4316E5 | 2.6689E5 | | | | | | | 6 | 0 | 0 | 0 | 0 | 0 | 1 | 2 | 1 | 1 | 1 | 0 | 0 | 0 | 0 | 0 | 0 | 228 | 238 | Carbamidomethylation | C5:Carbamidomethylation:1000.00;C7:Carbamidomethylation:1000.00 | PEAKS DB |
| R.FIRIDTAC(+57.02)VC(+57.02)VISR.K | N | 79.64 | 1708.8752 | 14 | 1.0 | 570.6329 | 3 | 63.02 | 7 | F7:35237 | BuCaTN01_F7a.raw | | | | | | | 3.425E7 | 1.536E7 | 1.7143E6 | 6.4042E4 | | | | 4.6395E4 | | | 26 | 0 | 0 | 0 | 0 | 0 | 0 | 19 | 4 | 1 | 1 | 0 | 0 | 0 | 1 | 0 | 0 | 225 | 238 | Carbamidomethylation | C8:Carbamidomethylation:1000.00;C10:Carbamidomethylation:1000.00 | PEAKS DB |
| R.FIRIDTAC(+57.02)VC(+57.02)VISRK.T | N | 66.71 | 1836.9702 | 15 | 0.6 | 613.3311 | 3 | 56.65 | 7 | F7:29482 | BuCaTN01_F7a.raw | | | | | | | 2.0277E6 | 3.8493E5 | | | | | | | | | 3 | 0 | 0 | 0 | 0 | 0 | 0 | 2 | 1 | 0 | 0 | 0 | 0 | 0 | 0 | 0 | 0 | 225 | 239 | Carbamidomethylation | C8:Carbamidomethylation:1000.00;C10:Carbamidomethylation:1000.00 | PEAKS DB |
| R.HWNSYC(+57.02)TTTDTFVKALTMEGNR.A | Y | 63.84 | 2631.1846 | 22 | -0.9 | 878.0680 | 3 | 74.60 | 7 | F7:45433 | BuCaTN01_F7a.raw | | | | | | | 5.6818E4 | | | | | | | | | | 1 | 0 | 0 | 0 | 0 | 0 | 0 | 1 | 0 | 0 | 0 | 0 | 0 | 0 | 0 | 0 | 0 | 199 | 220 | Carbamidomethylation | C6:Carbamidomethylation:1000.00 | PEAKS DB |
| R.NPNPVPSGC(+57.02)R.G | N | 63.70 | 1096.5083 | 10 | -0.4 | 549.2612 | 2 | 26.38 | 7 | F7:7077 | BuCaTN01_F7a.raw | | | | | | | 8.9262E5 | | | | | | | | | | 1 | 0 | 0 | 0 | 0 | 0 | 0 | 1 | 0 | 0 | 0 | 0 | 0 | 0 | 0 | 0 | 0 | 184 | 193 | Carbamidomethylation | C9:Carbamidomethylation:1000.00 | PEAKS DB |
| K.C(+57.02)RNPNPVPSGC(+57.02)R.G | N | 62.43 | 1412.6401 | 12 | 0.4 | 471.8875 | 3 | 24.90 | 7 | F7:6664 | BuCaTN01_F7a.raw | | | | | | | 5.7693E6 | 2.8804E5 | | | | | | | | | 3 | 0 | 0 | 0 | 0 | 0 | 0 | 2 | 1 | 0 | 0 | 0 | 0 | 0 | 0 | 0 | 0 | 182 | 193 | Carbamidomethylation | C1:Carbamidomethylation:1000.00;C11:Carbamidomethylation:1000.00 | PEAKS DB |
| K.ATDIKGNTVTVMVDVNLNNEVYK.Q | Y | 57.75 | 2536.2842 | 23 | -0.7 | 846.4348 | 3 | 68.45 | 7 | F7:39485 | BuCaTN01_F7a.raw | | | | | | | 1.5338E5 | | | | | | | | | | 2 | 0 | 0 | 0 | 0 | 0 | 0 | 2 | 0 | 0 | 0 | 0 | 0 | 0 | 0 | 0 | 0 | 152 | 174 | | | PEAKS DB |
| E.VYKQYFFETK.C | N | 54.63 | 1351.6812 | 10 | 2.9 | 451.5676 | 3 | 50.63 | 8 | F8:27078 | BuCaTN01_F7b.raw | | | | | | | | 5.2856E5 | | | | | | | | | 1 | 0 | 0 | 0 | 0 | 0 | 0 | 0 | 1 | 0 | 0 | 0 | 0 | 0 | 0 | 0 | 0 | 172 | 181 | | | PEAKS DB |
| K.QYFFETK.C | N | 54.60 | 961.4545 | 7 | 0.2 | 481.7346 | 2 | 53.27 | 7 | F7:26821 | BuCaTN01_F7a.raw | | | | | | | 2.4891E7 | 7.2632E6 | | | | | | | | | 2 | 0 | 0 | 0 | 0 | 0 | 0 | 1 | 1 | 0 | 0 | 0 | 0 | 0 | 0 | 0 | 0 | 175 | 181 | | | PEAKS DB |
| R.GIDSRHWNSYC(+57.02)TTTDTFVK.A | Y | 53.49 | 2287.0327 | 19 | 0.1 | 572.7655 | 4 | 54.55 | 7 | F7:27579 | BuCaTN01_F7a.raw | | | | | | | 5.0443E6 | 1.0265E6 | | | | | | | | | 2 | 0 | 0 | 0 | 0 | 0 | 0 | 1 | 1 | 0 | 0 | 0 | 0 | 0 | 0 | 0 | 0 | 194 | 212 | Carbamidomethylation | C11:Carbamidomethylation:1000.00 | PEAKS DB |
| R.IDTAC(+57.02)VC(+57.02)VISRKTENF | Y | 52.36 | 1911.9182 | 16 | 0.0 | 638.3134 | 3 | 56.13 | 7 | F7:29024 | BuCaTN01_F7a.raw | | | | | | | 1.5151E6 | | | | | | | | | | 1 | 0 | 0 | 0 | 0 | 0 | 0 | 1 | 0 | 0 | 0 | 0 | 0 | 0 | 0 | 0 | 0 | 228 | 243 | Carbamidomethylation | C5:Carbamidomethylation:1000.00;C7:Carbamidomethylation:1000.00 | PEAKS DB |
| N.SYC(+57.02)TTTDTFVK.A | Y | 52.05 | 1321.5861 | 11 | 0.7 | 661.8008 | 2 | 43.31 | 7 | F7:18733 | BuCaTN01_F7a.raw | | | | | | | 7.6053E5 | | | | | | | | | | 1 | 0 | 0 | 0 | 0 | 0 | 0 | 1 | 0 | 0 | 0 | 0 | 0 | 0 | 0 | 0 | 0 | 202 | 212 | Carbamidomethylation | C3:Carbamidomethylation:1000.00 | PEAKS DB |
| R.FIRIDTAC(+57.02)VC(+57.02).V | N | 49.72 | 1253.5896 | 10 | 3.0 | 627.8021 | 2 | 54.32 | 8 | F8:30301 | BuCaTN01_F7b.raw | | | | | | | | 3.8159E5 | | | | | | | | | 1 | 0 | 0 | 0 | 0 | 0 | 0 | 0 | 1 | 0 | 0 | 0 | 0 | 0 | 0 | 0 | 0 | 225 | 234 | Carbamidomethylation | C8:Carbamidomethylation:1000.00;C10:Carbamidomethylation:1000.00 | PEAKS DB |
| R.IDTAC(+57.02)VC(+57.02)VISRK.T | N | 47.61 | 1420.7167 | 12 | 0.1 | 474.5795 | 3 | 41.27 | 7 | F7:17257 | BuCaTN01_F7a.raw | | | | | | | 1.7324E6 | | | | | | | | | | 1 | 0 | 0 | 0 | 0 | 0 | 0 | 1 | 0 | 0 | 0 | 0 | 0 | 0 | 0 | 0 | 0 | 228 | 239 | Carbamidomethylation | C5:Carbamidomethylation:1000.00;C7:Carbamidomethylation:1000.00 | PEAKS DB |
| W.NSYC(+57.02)TTTDTFVK.A | Y | 42.93 | 1435.6289 | 12 | -0.1 | 718.8217 | 2 | 43.85 | 7 | F7:19088 | BuCaTN01_F7a.raw | | | | | | | 1.2687E6 | | | | | | | | | | 1 | 0 | 0 | 0 | 0 | 0 | 0 | 1 | 0 | 0 | 0 | 0 | 0 | 0 | 0 | 0 | 0 | 201 | 212 | Carbamidomethylation | C4:Carbamidomethylation:1000.00 | PEAKS DB |
| L.NNEVYKQYFFETK.C | N | 42.27 | 1708.8097 | 13 | 3.3 | 855.4124 | 2 | 57.90 | 8 | F8:33658 | BuCaTN01_F7b.raw | | | | | | | | 3.8306E4 | | | | | | | | | 1 | 0 | 0 | 0 | 0 | 0 | 0 | 0 | 1 | 0 | 0 | 0 | 0 | 0 | 0 | 0 | 0 | 169 | 181 | | | PEAKS DB |
| K.ATDIKGNTVTVMVDVNLNNEVYKQYFFETK.C | Y | 42.12 | 3479.7283 | 30 | 0.5 | 870.9398 | 4 | 82.05 | 7 | F7:51994 | BuCaTN01_F7a.raw | | | | | | | 1.3243E5 | | | | | | | | | | 1 | 0 | 0 | 0 | 0 | 0 | 0 | 1 | 0 | 0 | 0 | 0 | 0 | 0 | 0 | 0 | 0 | 152 | 181 | | | PEAKS DB |
| K.QYFFETKC(+57.02)R.N | N | 41.76 | 1277.5863 | 9 | -0.7 | 426.8691 | 3 | 46.69 | 7 | F7:21495 | BuCaTN01_F7a.raw | | | | | | | 3.7535E5 | | | | | | | | | | 1 | 0 | 0 | 0 | 0 | 0 | 0 | 1 | 0 | 0 | 0 | 0 | 0 | 0 | 0 | 0 | 0 | 175 | 183 | Carbamidomethylation | C8:Carbamidomethylation:1000.00 | PEAKS DB |
| **total 19 peptides** | | | | | | | | | | | | | | | | | | | | | | | | | | | | | | | | | | | | | | | | | | | | | | | | | |

**Best Unique PSM (Scan F8:26632, m/z=880.3908, z=2, RT=49.91, ppm=2.8):**

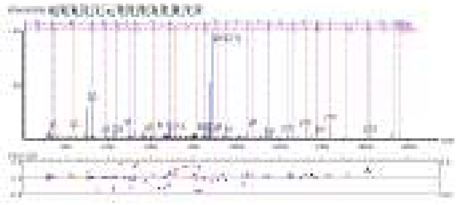

## P34128.1

back to list

| **Protein Coverage** | **Supporting Peptides** | **Best Unique PSM** |

**Protein Coverage:**

1 MSMLCYTLII AFLIGIWAAP KSEDNVPLGS PAKSDFSDTN CAQTHEGLKT SRNTDQHHPT PKKSEDQELG SAANIIVDPK
81 LFQKRRFQSP RVLFSTQPPP LSRDEQSVKF LDTEDTLNRN IWANNENHPV HNQGEHSVCD SISVWVTNKT KATDIKGNTV
161 TVMVDVNLNN EVYKQYFFET KCRNPNPVPS GCRGIDSRHW NSYCTTTDTF VKALTMEGNR ASWRFIRIDT ACVCVISRKT
241 ENF

Carbamidomethylation (+57.02)

**Supporting Peptides:**

| Peptide | Uniq | -10lgP | Mass | Length | ppm | m/z | z | RT | Fraction | Scan | Source File | Area F1 | Area F2a | Area F3a | Area F4 | Area F5 | Area F6a | Area F7a | Area F7b | Area F8 | Area F9a | Area Sample 1F10a | Area F10b | Area F11a | Area F11b | Area F12a | Area F13 | #Feature | #Feature F1 | #Feature F2a | #Feature F3a | #Feature F4 | #Feature F5 | #Feature F6a | #Feature F7a | #Feature F7b | #Feature F8 | #Feature F9a | #Feature Sample 1F10a | #Feature F10b | #Feature F11a | #Feature F11b | #Feature F12a | #Feature F13 | Start | End | PTM | AScore | Found By |
|---|---|---|---|---|---|---|---|---|---|---|---|---|---|---|---|---|---|---|---|---|---|---|---|---|---|---|---|---|---|---|---|---|---|---|---|---|---|---|---|---|---|---|---|---|---|---|---|---|---|
| R.HWNSYC(+57.02)TTTDTFVK.A | Y | 118.89 | 1758.7672 | 14 | 2.8 | 880.3908 | 2 | 49.91 | 8 | F8:26632 | BuCaTN01_F7b.raw | | | | | | | 2.7032E7 | 1.4368E7 | 5.9762E5 | 3.3814E5 | | | | | | | 6 | 0 | 0 | 0 | 0 | 0 | 0 | 2 | 2 | 1 | 1 | 0 | 0 | 0 | 0 | 0 | 0 | 199 | 212 | Carbamidomethylation | C6:Carbamidomethylation:1000.00 | PEAKS DB |
| R.IDTAC(+57.02)VC(+57.02)VISR.K | N | 81.21 | 1292.6217 | 11 | 0.1 | 647.3182 | 2 | 47.01 | 7 | F7:21996 | BuCaTN01_F7a.raw | | | | | | 2.9158E5 | 3.0809E7 | 1.2168E7 | 2.4316E5 | 2.6689E5 | | | | | | | 6 | 0 | 0 | 0 | 0 | 0 | 1 | 2 | 1 | 1 | 1 | 0 | 0 | 0 | 0 | 0 | 0 | 228 | 238 | Carbamidomethylation | C5:Carbamidomethylation:1000.00;C7:Carbamidomethylation:1000.00 | PEAKS DB |
| R.FIRIDTAC(+57.02)VC(+57.02)VISR.K | N | 79.64 | 1708.8752 | 14 | 1.0 | 570.6329 | 3 | 63.02 | 7 | F7:35237 | BuCaTN01_F7a.raw | | | | | | | 3.425E7 | 1.536E7 | 1.7143E6 | 6.4042E4 | | | | 4.6395E4 | | | 26 | 0 | 0 | 0 | 0 | 0 | 0 | 19 | 4 | 1 | 1 | 0 | 0 | 0 | 1 | 0 | 0 | 225 | 238 | Carbamidomethylation | C8:Carbamidomethylation:1000.00;C10:Carbamidomethylation:1000.00 | PEAKS DB |
| R.FIRIDTAC(+57.02)VC(+57.02)VISRK.T | N | 66.71 | 1836.9702 | 15 | 0.6 | 613.3311 | 3 | 56.65 | 7 | F7:29482 | BuCaTN01_F7a.raw | | | | | | | 2.0277E6 | 3.8493E5 | | | | | | | | | 3 | 0 | 0 | 0 | 0 | 0 | 0 | 2 | 1 | 0 | 0 | 0 | 0 | 0 | 0 | 0 | 0 | 225 | 239 | Carbamidomethylation | C8:Carbamidomethylation:1000.00;C10:Carbamidomethylation:1000.00 | PEAKS DB |
| R.HWNSYC(+57.02)TTTDTFVKALTMEGNR.A | Y | 63.84 | 2631.1846 | 22 | -0.9 | 878.0680 | 3 | 74.60 | 7 | F7:45433 | BuCaTN01_F7a.raw | | | | | | | 5.6818E4 | | | | | | | | | | 1 | 0 | 0 | 0 | 0 | 0 | 0 | 1 | 0 | 0 | 0 | 0 | 0 | 0 | 0 | 0 | 0 | 199 | 220 | Carbamidomethylation | C6:Carbamidomethylation:1000.00 | PEAKS DB |
| R.NPNPVPSGC(+57.02)R.G | N | 63.70 | 1096.5083 | 10 | -0.4 | 549.2612 | 2 | 26.38 | 7 | F7:7077 | BuCaTN01_F7a.raw | | | | | | | 8.9262E5 | | | | | | | | | | 1 | 0 | 0 | 0 | 0 | 0 | 0 | 1 | 0 | 0 | 0 | 0 | 0 | 0 | 0 | 0 | 0 | 184 | 193 | Carbamidomethylation | C9:Carbamidomethylation:1000.00 | PEAKS DB |
| K.C(+57.02)RNPNPVPSGC(+57.02)R.G | N | 62.43 | 1412.6401 | 12 | 0.4 | 471.8875 | 3 | 24.90 | 7 | F7:6664 | BuCaTN01_F7a.raw | | | | | | | 5.7693E6 | 2.8804E5 | | | | | | | | | 3 | 0 | 0 | 0 | 0 | 0 | 0 | 2 | 1 | 0 | 0 | 0 | 0 | 0 | 0 | 0 | 0 | 182 | 193 | Carbamidomethylation | C1:Carbamidomethylation:1000.00;C11:Carbamidomethylation:1000.00 | PEAKS DB |
| K.ATDIKGNTVTVMVDVNLNNEVYK.Q | Y | 57.75 | 2536.2842 | 23 | -0.7 | 846.4348 | 3 | 68.45 | 7 | F7:39485 | BuCaTN01_F7a.raw | | | | | | | 1.5338E5 | | | | | | | | | | 2 | 0 | 0 | 0 | 0 | 0 | 0 | 2 | 0 | 0 | 0 | 0 | 0 | 0 | 0 | 0 | 0 | 152 | 174 | | | PEAKS DB |
| E.VYKQYFFETK.C | N | 54.63 | 1351.6812 | 10 | 2.9 | 451.5676 | 3 | 50.63 | 8 | F8:27078 | BuCaTN01_F7b.raw | | | | | | | | 5.2856E5 | | | | | | | | | 1 | 0 | 0 | 0 | 0 | 0 | 0 | 0 | 1 | 0 | 0 | 0 | 0 | 0 | 0 | 0 | 0 | 172 | 181 | | | PEAKS DB |
| K.QYFFETK.C | N | 54.60 | 961.4545 | 7 | 0.2 | 481.7346 | 2 | 53.27 | 7 | F7:26821 | BuCaTN01_F7a.raw | | | | | | | 2.4891E7 | 7.2632E6 | | | | | | | | | 2 | 0 | 0 | 0 | 0 | 0 | 0 | 1 | 1 | 0 | 0 | 0 | 0 | 0 | 0 | 0 | 0 | 175 | 181 | | | PEAKS DB |
| R.GIDSRHWNSYC(+57.02)TTTDTFVK.A | Y | 53.49 | 2287.0327 | 19 | 0.1 | 572.7655 | 4 | 54.55 | 7 | F7:27579 | BuCaTN01_F7a.raw | | | | | | | 5.0443E6 | 1.0265E6 | | | | | | | | | 2 | 0 | 0 | 0 | 0 | 0 | 0 | 1 | 1 | 0 | 0 | 0 | 0 | 0 | 0 | 0 | 0 | 194 | 212 | Carbamidomethylation | C11:Carbamidomethylation:1000.00 | PEAKS DB |
| R.IDTAC(+57.02)VC(+57.02)VISRKTENF | Y | 52.36 | 1911.9182 | 16 | 0.0 | 638.3134 | 3 | 56.13 | 7 | F7:29024 | BuCaTN01_F7a.raw | | | | | | | 1.5151E6 | | | | | | | | | | 1 | 0 | 0 | 0 | 0 | 0 | 0 | 1 | 0 | 0 | 0 | 0 | 0 | 0 | 0 | 0 | 0 | 228 | 243 | Carbamidomethylation | C5:Carbamidomethylation:1000.00;C7:Carbamidomethylation:1000.00 | PEAKS DB |
| N.SYC(+57.02)TTTDTFVK.A | Y | 52.05 | 1321.5861 | 11 | 0.7 | 661.8008 | 2 | 43.31 | 7 | F7:18733 | BuCaTN01_F7a.raw | | | | | | | 7.6053E5 | | | | | | | | | | 1 | 0 | 0 | 0 | 0 | 0 | 0 | 1 | 0 | 0 | 0 | 0 | 0 | 0 | 0 | 0 | 0 | 202 | 212 | Carbamidomethylation | C3:Carbamidomethylation:1000.00 | PEAKS DB |
| R.FIRIDTAC(+57.02)VC(+57.02).V | N | 49.72 | 1253.5896 | 10 | 3.0 | 627.8021 | 2 | 54.32 | 8 | F8:30301 | BuCaTN01_F7b.raw | | | | | | | | 3.8159E5 | | | | | | | | | 1 | 0 | 0 | 0 | 0 | 0 | 0 | 0 | 1 | 0 | 0 | 0 | 0 | 0 | 0 | 0 | 0 | 225 | 234 | Carbamidomethylation | C8:Carbamidomethylation:1000.00;C10:Carbamidomethylation:1000.00 | PEAKS DB |
| R.IDTAC(+57.02)VC(+57.02)VISRK.T | N | 47.61 | 1420.7167 | 12 | 0.1 | 474.5795 | 3 | 41.27 | 7 | F7:17257 | BuCaTN01_F7a.raw | | | | | | | 1.7324E6 | | | | | | | | | | 1 | 0 | 0 | 0 | 0 | 0 | 0 | 1 | 0 | 0 | 0 | 0 | 0 | 0 | 0 | 0 | 0 | 228 | 239 | Carbamidomethylation | C5:Carbamidomethylation:1000.00;C7:Carbamidomethylation:1000.00 | PEAKS DB |
| W.NSYC(+57.02)TTTDTFVK.A | Y | 42.93 | 1435.6289 | 12 | -0.1 | 718.8217 | 2 | 43.85 | 7 | F7:19088 | BuCaTN01_F7a.raw | | | | | | | 1.2687E6 | | | | | | | | | | 1 | 0 | 0 | 0 | 0 | 0 | 0 | 1 | 0 | 0 | 0 | 0 | 0 | 0 | 0 | 0 | 0 | 201 | 212 | Carbamidomethylation | C4:Carbamidomethylation:1000.00 | PEAKS DB |
| L.NNEVYKQYFFETK.C | N | 42.27 | 1708.8097 | 13 | 3.3 | 855.4124 | 2 | 57.90 | 8 | F8:33658 | BuCaTN01_F7b.raw | | | | | | | | 3.8306E4 | | | | | | | | | 1 | 0 | 0 | 0 | 0 | 0 | 0 | 0 | 1 | 0 | 0 | 0 | 0 | 0 | 0 | 0 | 0 | 169 | 181 | | | PEAKS DB |
| K.ATDIKGNTVTVMVDVNLNNEVYKQYFFETK.C | Y | 42.12 | 3479.7283 | 30 | 0.5 | 870.9398 | 4 | 82.05 | 7 | F7:51994 | BuCaTN01_F7a.raw | | | | | | | 1.3243E5 | | | | | | | | | | 1 | 0 | 0 | 0 | 0 | 0 | 0 | 1 | 0 | 0 | 0 | 0 | 0 | 0 | 0 | 0 | 0 | 152 | 181 | | | PEAKS DB |
| K.QYFFETKC(+57.02)R.N | N | 41.76 | 1277.5863 | 9 | -0.7 | 426.8691 | 3 | 46.69 | 7 | F7:21495 | BuCaTN01_F7a.raw | | | | | | | 3.7535E5 | | | | | | | | | | 1 | 0 | 0 | 0 | 0 | 0 | 0 | 1 | 0 | 0 | 0 | 0 | 0 | 0 | 0 | 0 | 0 | 175 | 183 | Carbamidomethylation | C8:Carbamidomethylation:1000.00 | PEAKS DB |
| total 19 peptides | | | | | | | | | | | | | | | | | | | | | | | | | | | | | | | | | | | | | | | | | | | | | | | | | |

**Best Unique PSM (Scan F8:26632, m/z=880.3908, z=2, RT=49.91, ppm=2.8):**

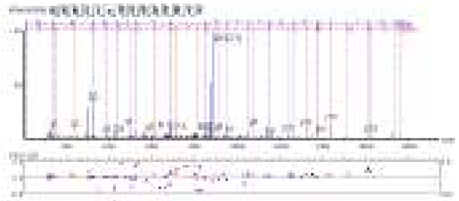

## ACE73577.1

| Protein Coverage | Supporting Peptides | Best Unique PSM |

**Protein Coverage:**

1 MIAFVVLLSL AAVLQQSSGT VDFASESSNK RENQKQIVDK HNALRRSVRP TARNMLQMEW NSNAAQNAKR WADRCTFAHS
81 PPHLRTVGKF SCGENLFMSS QPYAWSKVIQ SWYDENKKFV YGVGANPPGS VIGHYTQIVW YKSHLLGCAV TRCSSSKYIY
161 VCQYCPAGNI IGSTATPYKS GPPCGDCPSA CVNGLCTNPC KYNDDYTNCK SLAKQDRCQT EWIKSKCGAS CFCRTEII

Carbamidomethylation (+57.02)
Oxidation (M) (+15.99)

**Supporting Peptides:**

| Peptide | Uniq | -10lgP | Mass | Length | ppm | m/z | z | RT | Fraction | Scan | Source File | Area F1 | Area F2a | Area F3a | Area F4 | Area F5 | Area F6a | Area F7a | Area F7b | Area F8 | Area F9a | Area Sample 1F10a | Area F10b | Area F11a | Area F11b | Area F12a | Area F13 | #Feature | #Feature F1 | #Feature F2a | #Feature F3a | #Feature F4 | #Feature F5 | #Feature F6a | #Feature F7a | #Feature F7b | #Feature F8 | #Feature F9a | #Feature Sample 1F10a | #Feature F10b | #Feature F11a | #Feature F11b | #Feature F12a | #Feature F13 | Start | End | PTM | AScore | Found By |
|---|---|---|---|---|---|---|---|---|---|---|---|---|---|---|---|---|---|---|---|---|---|---|---|---|---|---|---|---|---|---|---|---|---|---|---|---|---|---|---|---|---|---|---|---|---|---|---|---|---|
| K.SGPPC(+57.02)GDC(+57.02)PSAC(+57.02)VNGLC(+57.02)TNPC(+57.02)K.Y | N | 112.66 | 2406.9482 | 22 | 0.0 | 803.3234 | 3 | 46.85 | 11 | F11:19049 | BuCaTN01_F10a.raw | | | | | | | | | | | 8.2354E6 | | 4.6901E7 | 1.7605E6 | | | 6 | 0 | 0 | 0 | 0 | 0 | 0 | 0 | 0 | 0 | 0 | 2 | 0 | 3 | 1 | 0 | 0 | 180 | 201 | Carbamidomethylation | C5:Carbamidomethylation:1000.00;C8:Carbamidomethylation:1000.00;C12:Carbamidomethylation:1000.00;C17:Carbamidomethylation:1000.00;C21:Carbamidomethylation:1000.00 | PEAKS DB |
| R.NMLQM(+15.99)EWNSNAAQNAK.R | N | 92.39 | 1864.8196 | 16 | 1.2 | 933.4182 | 2 | 54.45 | 13 | F13:29949 | BuCaTN01_F11a.raw | | | | | | | | | | | 2.4645E5 | | 1.8788E6 | | | 6.546E5 | 7 | 0 | 0 | 0 | 0 | 0 | 0 | 0 | 0 | 0 | 0 | 2 | 0 | 2 | 0 | 0 | 3 | 54 | 69 | Oxidation (M) | M5:Oxidation (M):77.24 | PEAKS DB |
| R.NM(+15.99)LQM(+15.99)EWNSNAAQNAK.R | N | 91.55 | 1880.8146 | 16 | -0.5 | 941.4141 | 2 | 45.31 | 13 | F13:21815 | BuCaTN01_F11a.raw | | | | | | | | | | | 8.0152E5 | | 4.7799E6 | | | | 7 | 0 | 0 | 0 | 0 | 0 | 0 | 0 | 0 | 0 | 0 | 5 | 0 | 2 | 0 | 0 | 0 | 54 | 69 | Oxidation (M) | M2:Oxidation (M):1000.00;M5:Oxidation (M):1000.00 | PEAKS DB |
| R.NM(+15.99)LQM(+15.99)EWNSNAAQNAKR.W | N | 85.70 | 2036.9156 | 17 | -0.2 | 679.9790 | 3 | 42.31 | 13 | F13:19106 | BuCaTN01_F11a.raw | | | | | | | | | | | 1.5703E6 | | 9.863E6 | | | | 2 | 0 | 0 | 0 | 0 | 0 | 0 | 0 | 0 | 0 | 0 | 1 | 0 | 1 | 0 | 0 | 0 | 54 | 70 | Oxidation (M) | M2:Oxidation (M):1000.00;M5:Oxidation (M):1000.00 | PEAKS DB |
| R.NMLQM(+15.99)EWNSNAAQNAKR.W | N | 84.44 | 2020.9208 | 17 | 0.4 | 674.6478 | 3 | 51.10 | 13 | F13:26282 | BuCaTN01_F11a.raw | | | | | | | | | | | 4.5326E5 | | 2.6157E6 | | | 7.526E5 | 3 | 0 | 0 | 0 | 0 | 0 | 0 | 0 | 0 | 0 | 0 | 1 | 0 | 1 | 0 | 0 | 1 | 54 | 70 | Oxidation (M) | M5:Oxidation (M):26.52 | PEAKS DB |
| K.SGPPC(+57.02)GDC(+57.02)PSAC(+57.02)VNGLC(+57.02)TNPC(+57.02)KYNDDYTNC(+57.02)K.S | Y | 82.55 | 3580.3879 | 31 | -3.4 | 1194.4658 | 3 | 51.22 | 11 | F11:23182 | BuCaTN01_F10a.raw | | | | | | | | | | | 3.7851E7 | | 3.0898E7 | | | | 5 | 0 | 0 | 0 | 0 | 0 | 0 | 0 | 0 | 0 | 0 | 3 | 0 | 2 | 0 | 0 | 0 | 180 | 210 | Carbamidomethylation | C5:Carbamidomethylation:1000.00;C8:Carbamidomethylation:1000.00;C12:Carbamidomethylation:1000.00;C17:Carbamidomethylation:1000.00;C21:Carbamidomethylation:1000.00;C30:Carbamidomethylation:1000.00 | PEAKS DB |
| R.NMLQMEWNSNAAQNAK.R | N | 78.48 | 1848.8247 | 16 | -0.1 | 925.4196 | 2 | 61.65 | 16 | F16:30581 | BuCaTN01_F13.raw | | | | | | | | | | | 8.2478E5 | | 1.6062E6 | | | 3.3797E6 | 4 | 0 | 0 | 0 | 0 | 0 | 0 | 0 | 0 | 0 | 0 | 2 | 0 | 1 | 0 | 0 | 1 | 54 | 69 | | | PEAKS DB |
| K.SGPPC(+57.02)GDC(+57.02)PSAC(+57.02)VNGL.C | N | 70.35 | 1646.6487 | 16 | 0.5 | 824.3320 | 2 | 51.37 | 13 | F13:26935 | BuCaTN01_F11a.raw | | | | | | | | | | | 1.5119E6 | | 5.7825E6 | | | | 2 | 0 | 0 | 0 | 0 | 0 | 0 | 0 | 0 | 0 | 0 | 1 | 0 | 1 | 0 | 0 | 0 | 180 | 195 | Carbamidomethylation | C5:Carbamidomethylation:1000.00;C8:Carbamidomethylation:1000.00;C12:Carbamidomethylation:1000.00 | PEAKS DB |
| R.NMLQMEWNSNAAQNAKR.W | N | 69.63 | 2004.9258 | 17 | 0.4 | 669.3162 | 3 | 56.51 | 13 | F13:31303 | BuCaTN01_F11a.raw | | | | | | | | | | | 6.3305E5 | | 6.8801E6 | | | 4.9021E6 | 4 | 0 | 0 | 0 | 0 | 0 | 0 | 0 | 0 | 0 | 0 | 1 | 0 | 2 | 0 | 0 | 1 | 54 | 70 | | | PEAKS DB |
| K.SGPPC(+57.02)GDC(+57.02)PSAC(+57.02)VN.G | N | 59.14 | 1476.5432 | 14 | 0.4 | 739.2792 | 2 | 34.53 | 13 | F13:12981 | BuCaTN01_F11a.raw | | | | | | | | | | | 8.238E5 | | 3.037E6 | | | | 2 | 0 | 0 | 0 | 0 | 0 | 0 | 0 | 0 | 0 | 0 | 1 | 0 | 1 | 0 | 0 | 0 | 180 | 193 | Carbamidomethylation | C5:Carbamidomethylation:1000.00;C8:Carbamidomethylation:1000.00;C12:Carbamidomethylation:1000.00 | PEAKS DB |
| P.GSVIGHYTQIVWYK.S | N | 59.00 | 1649.8566 | 14 | 2.7 | 825.9344 | 2 | 67.12 | 14 | F14:42294 | BuCaTN01_F11b.raw | | | | | | | | | | | | | | 4.2892E5 | | | 2 | 0 | 0 | 0 | 0 | 0 | 0 | 0 | 0 | 0 | 0 | 0 | 0 | 0 | 2 | 0 | 0 | 129 | 142 | | | PEAKS DB |
| K.YIYVC(+57.02)QYC(+57.02)PAGN.I | N | 58.24 | 1506.6272 | 12 | 0.2 | 754.3210 | 2 | 59.53 | 13 | F13:34348 | BuCaTN01_F11a.raw | | | | | | | | | | | | | 8.7629E6 | | | | 1 | 0 | 0 | 0 | 0 | 0 | 0 | 0 | 0 | 0 | 0 | 0 | 0 | 1 | 0 | 0 | 0 | 158 | 169 | Carbamidomethylation | C5:Carbamidomethylation:1000.00;C8:Carbamidomethylation:1000.00 | PEAKS DB |
| Y.KSGPPC(+57.02)GDC(+57.02)PSAC(+57.02)VNGLC(+57.02)TNPC(+57.02)K.Y | N | 57.65 | 2535.0432 | 23 | -2.0 | 846.0200 | 3 | 42.60 | 13 | F13:19440 | BuCaTN01_F11a.raw | | | | | | | | | | | | | 6.1313E5 | | | | 1 | 0 | 0 | 0 | 0 | 0 | 0 | 0 | 0 | 0 | 0 | 0 | 0 | 1 | 0 | 0 | 0 | 179 | 201 | Carbamidomethylation | C6:Carbamidomethylation:1000.00;C9:Carbamidomethylation:1000.00;C13:Carbamidomethylation:1000.00;C18:Carbamidomethylation:1000.00;C22:Carbamidomethylation:1000.00 | PEAKS DB |
| K.SGPPC(+57.02)GDC(+57.02)PSAC(+57.02)VNGLC(+57.02)TNPC(+57.02)KYNDDYTNC(+57.02)KSLAK.Q | Y | 54.24 | 3979.6362 | 35 | -0.7 | 995.9156 | 4 | 53.01 | 13 | F13:28294 | BuCaTN01_F11a.raw | | | | | | | | | | | 2.264E6 | | 1.6339E7 | | | | 2 | 0 | 0 | 0 | 0 | 0 | 0 | 0 | 0 | 0 | 0 | 1 | 0 | 1 | 0 | 0 | 0 | 180 | 214 | Carbamidomethylation | C5:Carbamidomethylation:1000.00;C8:Carbamidomethylation:1000.00;C12:Carbamidomethylation:1000.00;C17:Carbamidomethylation:1000.00;C21:Carbamidomethylation:1000.00;C30:Carbamidomethylation:1000.00 | PEAKS DB |
| R.NM(+15.99)LQMEWNSNAAQNAKR.W | N | 54.15 | 2020.9208 | 17 | 0.1 | 674.6476 | 3 | 47.52 | 13 | F13:23429 | BuCaTN01_F11a.raw | | | | | | | | | | | | | 1.6196E6 | | | | 1 | 0 | 0 | 0 | 0 | 0 | 0 | 0 | 0 | 0 | 0 | 0 | 0 | 1 | 0 | 0 | 0 | 54 | 70 | Oxidation (M) | M2:Oxidation (M):47.09 | PEAKS DB |

| | | | | | | | | | | | | | | | | | | | | | | | | | | | | | | | | | | | | | | | | | | | | | | | | | |
|---|---|---|---|---|---|---|---|---|---|---|---|---|---|---|---|---|---|---|---|---|---|---|---|---|---|---|---|---|---|---|---|---|---|---|---|---|---|---|---|---|---|---|---|---|---|---|---|---|---|
| K.QIVDKHNALR.R | N | 53.10 | 1192.6676 | 10 | -0.4 | 398.5630 | 3 | 23.28 | 13 | F13:6364 | BuCaTN01_F11a.raw | | | | | | | | | | | 1.3327E6 | | 2.9907E6 | | | | 2 | 0 | 0 | 0 | 0 | 0 | 0 | 0 | 0 | 0 | 0 | 1 | 0 | 1 | 0 | 0 | 0 | 36 | 45 | | | PEAKS DB |
| K.YNDDYTNC(+57.02)K.S | Y | 52.62 | 1191.4502 | 9 | 1.2 | 596.7331 | 2 | 28.31 | 13 | F13:8853 | BuCaTN01_F11a.raw | | | | | | | | | | | 1.3452E5 | | 5.2674E5 | | | | 2 | 0 | 0 | 0 | 0 | 0 | 0 | 0 | 0 | 0 | 0 | 1 | 0 | 1 | 0 | 0 | 0 | 202 | 210 | Carbamidomethylation | C8:Carbamidomethylation:1000.00 | PEAKS DB |
| R.NM(+15.99)LQMEWNSNAAQNAK.R | N | 52.57 | 1864.8196 | 16 | 1.7 | 622.6149 | 3 | 52.23 | 13 | F13:27379 | BuCaTN01_F11a.raw | | | | | | | | | | | | | 2.2136E5 | | | | 2 | 0 | 0 | 0 | 0 | 0 | 0 | 0 | 0 | 0 | 0 | 0 | 0 | 2 | 0 | 0 | 0 | 54 | 69 | Oxidation (M) | M2:Oxidation (M):56.34 | PEAKS DB |
| K.YIYVC(+57.02)QYC(+57.02)PAGNIIG.S | N | 51.03 | 1789.8168 | 15 | 6.5 | 895.9215 | 2 | 60.28 | 13 | F13:34997 | BuCaTN01_F11a.raw | | | | | | | | | | | | | 7.8778E6 | 0 | | | 1 | 0 | 0 | 0 | 0 | 0 | 0 | 0 | 0 | 0 | 0 | 0 | 0 | 1 | 0 | 0 | 0 | 158 | 172 | Carbamidomethylation | C5:Carbamidomethylation:1000.00;C8:Carbamidomethylation:1000.00 | PEAKS DB |
| G.DC(+57.02)PSAC(+57.02)VNGLC(+57.02)TNPC(+57.02)K.Y | N | 43.28 | 1851.7372 | 16 | -0.3 | 926.8756 | 2 | 43.52 | 13 | F13:19924 | BuCaTN01_F11a.raw | | | | | | | | | | | | | 1.1644E5 | | | | 1 | 0 | 0 | 0 | 0 | 0 | 0 | 0 | 0 | 0 | 0 | 0 | 0 | 1 | 0 | 0 | 0 | 186 | 201 | Carbamidomethylation | C2:Carbamidomethylation:1000.00;C6:Carbamidomethylation:1000.00;C11:Carbamidomethylation:1000.00;C15:Carbamidomethylation:1000.00 | PEAKS DB |
| **total 20 peptides** | | | | | | | | | | | | | | | | | | | | | | | | | | | | | | | | | | | | | | | | | | | | | | | | | |

**Best Unique PSM (Scan F11:23182, m/z=1194.4658, z=3, RT=51.22, ppm=-3.4):**

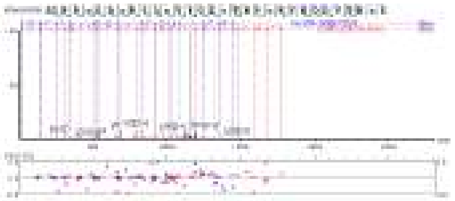

## ACE73578.1

back to list

| Protein Coverage | Supporting Peptides | Best Unique PSM |

**Protein Coverage:**

```
  1 MIAFVVLLSL AAVLQQSSGT VDFASESSNK RENQKQIVDK HNALRRSVRP TARNMLQMEW NSNAAQNAKR WADRCTFAHS
 81 PPHLRTVGIF SCGENLFMSS QPYAWSKVIQ SWYDENKKFV YGVGANPPGS VIGHYTQIVW YKSHLLGCAV TRCSSSKYIY
161 VCQYCPAGNI IGSTATPYKS GPPCGDCPSA CVNGLCTNPC KYNDDYTNCK SLAKQDRCQT EWIKSKCGAS CFCRTEII
```

55 58 162 165 184 187 191 196 200 209

C Carbamidomethylation (+57.02)
O Oxidation (M) (+15.99)

**Supporting Peptides:**

| Peptide | Uniq | -10lgP | Mass | Length | ppm | m/z | z | RT | Fraction | Scan | Source File | Area F1 | Area F2a | Area F3a | Area F4 | Area F5 | Area F6a | Area F7a | Area F7b | Area F8 | Area F9a | Area Sample 1F10a | Area F10b | Area F11a | Area F11b | Area F12a | Area F13 | #Feature | #Feature F1 | #Feature F2a | #Feature F3a | #Feature F4 | #Feature F5 | #Feature F6a | #Feature F7a | #Feature F7b | #Feature F8 | #Feature F9a | #Feature Sample 1F10a | #Feature F10b | #Feature F11a | #Feature F11b | #Feature F12a | #Feature F13 | Start | End | PTM | AScore | Found By |
|---|---|---|---|---|---|---|---|---|---|---|---|---|---|---|---|---|---|---|---|---|---|---|---|---|---|---|---|---|---|---|---|---|---|---|---|---|---|---|---|---|---|---|---|---|---|---|---|---|---|
| K.SGPPC(+57.02)GDC(+57.02)PSAC(+57.02)VNGLC(+57.02)TNPC(+57.02)K.Y | N | 112.66 | 2406.9482 | 22 | 0.0 | 803.3234 | 3 | 46.85 | 11 | F11:19049 | BuCaTN01_F10a.raw | | | | | | | | | | | 8.2354E6 | | 4.6901E7 | 1.7605E6 | | | 6 | 0 | 0 | 0 | 0 | 0 | 0 | 0 | 0 | 0 | 0 | 2 | 0 | 3 | 1 | 0 | 0 | 180 | 201 | Carbamidomethylation | C5:Carbamidomethylation:1000.00;C8:Carbamidomethylation:1000.00;C12:Carbamidomethylation:1000.00;C17:Carbamidomethylation:1000.00;C21:Carbamidomethylation:1000.00 | PEAKS DB |
| R.NMLQM(+15.99)EWNSNAAQNAK.R | N | 92.39 | 1864.8196 | 16 | 1.2 | 933.4182 | 2 | 54.45 | 13 | F13:29949 | BuCaTN01_F11a.raw | | | | | | | | | | | 2.4645E5 | | 1.8788E6 | | | 6.546E5 | 7 | 0 | 0 | 0 | 0 | 0 | 0 | 0 | 0 | 0 | 0 | 2 | 0 | 2 | 0 | 0 | 3 | 54 | 69 | Oxidation (M) | M5:Oxidation (M):77.24 | PEAKS DB |
| R.NM(+15.99)LQM(+15.99)EWNSNAAQNAK.R | N | 91.55 | 1880.8146 | 16 | -0.5 | 941.4141 | 2 | 45.31 | 13 | F13:21815 | BuCaTN01_F11a.raw | | | | | | | | | | | 8.0152E5 | | 4.7799E6 | | | | 7 | 0 | 0 | 0 | 0 | 0 | 0 | 0 | 0 | 0 | 0 | 5 | 0 | 2 | 0 | 0 | 0 | 54 | 69 | Oxidation (M) | M2:Oxidation (M):1000.00;M5:Oxidation (M):1000.00 | PEAKS DB |
| R.NM(+15.99)LQM(+15.99)EWNSNAAQNAKR.W | N | 85.70 | 2036.9156 | 17 | -0.2 | 679.9790 | 3 | 42.31 | 13 | F13:19106 | BuCaTN01_F11a.raw | | | | | | | | | | | 1.5703E6 | | 9.863E6 | | | | 2 | 0 | 0 | 0 | 0 | 0 | 0 | 0 | 0 | 0 | 0 | 1 | 0 | 1 | 0 | 0 | 0 | 54 | 70 | Oxidation (M) | M2:Oxidation (M):1000.00;M5:Oxidation (M):1000.00 | PEAKS DB |
| R.NMLQM(+15.99)EWNSNAAQNAKR.W | N | 84.44 | 2020.9208 | 17 | 0.4 | 674.6478 | 3 | 51.10 | 13 | F13:26282 | BuCaTN01_F11a.raw | | | | | | | | | | | 4.5326E5 | | 2.6157E6 | | | 7.526E5 | 3 | 0 | 0 | 0 | 0 | 0 | 0 | 0 | 0 | 0 | 0 | 1 | 0 | 1 | 0 | 0 | 1 | 54 | 70 | Oxidation (M) | M5:Oxidation (M):26.52 | PEAKS DB |
| K.SGPPC(+57.02)GDC(+57.02)PSAC(+57.02)VNGLC(+57.02)TNPC(+57.02)KYNDDYTNC(+57.02)K.S | Y | 82.55 | 3580.3879 | 31 | -3.4 | 1194.4658 | 3 | 51.22 | 11 | F11:23182 | BuCaTN01_F10a.raw | | | | | | | | | | | 3.7851E7 | | 3.0898E7 | | | | 5 | 0 | 0 | 0 | 0 | 0 | 0 | 0 | 0 | 0 | 0 | 3 | 0 | 2 | 0 | 0 | 0 | 180 | 210 | Carbamidomethylation | C5:Carbamidomethylation:1000.00;C8:Carbamidomethylation:1000.00;C12:Carbamidomethylation:1000.00;C17:Carbamidomethylation:1000.00;C21:Carbamidomethylation:1000.00;C30:Carbamidomethylation:1000.00 | PEAKS DB |
| R.NMLQMEWNSNAAQNAK.R | N | 78.48 | 1848.8247 | 16 | -0.1 | 925.4196 | 2 | 61.65 | 16 | F16:30581 | BuCaTN01_F13.raw | | | | | | | | | | | 8.2478E5 | | 1.6062E6 | | | 3.3797E6 | 4 | 0 | 0 | 0 | 0 | 0 | 0 | 0 | 0 | 0 | 0 | 2 | 0 | 1 | 0 | 0 | 1 | 54 | 69 | | | PEAKS DB |
| K.SGPPC(+57.02)GDC(+57.02)PSAC(+57.02)VNGL.C | N | 70.35 | 1646.6487 | 16 | 0.5 | 824.3320 | 2 | 51.37 | 13 | F13:26935 | BuCaTN01_F11a.raw | | | | | | | | | | | 1.5119E6 | | 5.7825E6 | | | | 2 | 0 | 0 | 0 | 0 | 0 | 0 | 0 | 0 | 0 | 0 | 1 | 0 | 1 | 0 | 0 | 0 | 180 | 195 | Carbamidomethylation | C5:Carbamidomethylation:1000.00;C8:Carbamidomethylation:1000.00;C12:Carbamidomethylation:1000.00 | PEAKS DB |
| R.NMLQMEWNSNAAQNAKR.W | N | 69.63 | 2004.9258 | 17 | 0.4 | 669.3162 | 3 | 56.51 | 13 | F13:31303 | BuCaTN01_F11a.raw | | | | | | | | | | | 6.3305E5 | | 6.8801E6 | | | 4.9021E6 | 4 | 0 | 0 | 0 | 0 | 0 | 0 | 0 | 0 | 0 | 0 | 1 | 0 | 2 | 0 | 0 | 1 | 54 | 70 | | | PEAKS DB |
| K.SGPPC(+57.02)GDC(+57.02)PSAC(+57.02)VN.G | N | 59.14 | 1476.5432 | 14 | 0.4 | 739.2792 | 2 | 34.53 | 13 | F13:12981 | BuCaTN01_F11a.raw | | | | | | | | | | | 8.238E5 | | 3.037E6 | | | | 2 | 0 | 0 | 0 | 0 | 0 | 0 | 0 | 0 | 0 | 0 | 1 | 0 | 1 | 0 | 0 | 0 | 180 | 193 | Carbamidomethylation | C5:Carbamidomethylation:1000.00;C8:Carbamidomethylation:1000.00;C12:Carbamidomethylation:1000.00 | PEAKS DB |
| P.GSVIGHYTQIVWYK.S | N | 59.00 | 1649.8566 | 14 | 2.7 | 825.9344 | 2 | 67.12 | 14 | F14:42294 | BuCaTN01_F11b.raw | | | | | | | | | | | | | | 4.2892E5 | | | 2 | 0 | 0 | 0 | 0 | 0 | 0 | 0 | 0 | 0 | 0 | 0 | 0 | 0 | 2 | 0 | 0 | 129 | 142 | | | PEAKS DB |
| K.YIYVC(+57.02)QYC(+57.02)PAGN.I | N | 58.24 | 1506.6272 | 12 | 0.2 | 754.3210 | 2 | 59.53 | 13 | F13:34348 | BuCaTN01_F11a.raw | | | | | | | | | | | | | 8.7629E6 | | | | 1 | 0 | 0 | 0 | 0 | 0 | 0 | 0 | 0 | 0 | 0 | 0 | 0 | 1 | 0 | 0 | 0 | 158 | 169 | Carbamidomethylation | C5:Carbamidomethylation:1000.00;C8:Carbamidomethylation:1000.00 | PEAKS DB |
| Y.KSGPPC(+57.02)GDC(+57.02)PSAC(+57.02)VNGLC(+57.02)TNPC(+57.02)K.Y | N | 57.65 | 2535.0432 | 23 | -2.0 | 846.0200 | 3 | 42.60 | 13 | F13:19440 | BuCaTN01_F11a.raw | | | | | | | | | | | | | 6.1313E5 | | | | 1 | 0 | 0 | 0 | 0 | 0 | 0 | 0 | 0 | 0 | 0 | 0 | 0 | 1 | 0 | 0 | 0 | 179 | 201 | Carbamidomethylation | C6:Carbamidomethylation:1000.00;C9:Carbamidomethylation:1000.00;C13:Carbamidomethylation:1000.00;C18:Carbamidomethylation:1000.00;C22:Carbamidomethylation:1000.00 | PEAKS DB |
| K.SGPPC(+57.02)GDC(+57.02)PSAC(+57.02)VNGLC(+57.02)TNPC(+57.02)KYNDDYTNC(+57.02)KSLAK.Q | Y | 54.24 | 3979.6362 | 35 | -0.7 | 995.9156 | 4 | 53.01 | 13 | F13:28294 | BuCaTN01_F11a.raw | | | | | | | | | | | 2.264E6 | | 1.6339E7 | | | | 2 | 0 | 0 | 0 | 0 | 0 | 0 | 0 | 0 | 0 | 0 | 1 | 0 | 1 | 0 | 0 | 0 | 180 | 214 | Carbamidomethylation | C5:Carbamidomethylation:1000.00;C8:Carbamidomethylation:1000.00;C12:Carbamidomethylation:1000.00;C17:Carbamidomethylation:1000.00;C21:Carbamidomethylation:1000.00;C30:Carbamidomethylation:1000.00 | PEAKS DB |
| R.NM(+15.99)LQMEWNSNAAQNAKR.W | N | 54.15 | 2020.9208 | 17 | 0.1 | 674.6476 | 3 | 47.52 | 13 | F13:23429 | BuCaTN01_F11a.raw | | | | | | | | | | | | | 1.6196E6 | | | | 1 | 0 | 0 | 0 | 0 | 0 | 0 | 0 | 0 | 0 | 0 | 0 | 0 | 1 | 0 | 0 | 0 | 54 | 70 | Oxidation (M) | M2:Oxidation (M):47.09 | PEAKS DB |
| K.QIVDKHNALR.R | N | 53.10 | 1192.6676 | 10 | -0.4 | 398.5630 | 3 | 23.28 | 13 | F13:6364 | BuCaTN01_F11a.raw | | | | | | | | | | | 1.3327E6 | | 2.9907E6 | | | | 2 | 0 | 0 | 0 | 0 | 0 | 0 | 0 | 0 | 0 | 0 | 1 | 0 | 1 | 0 | 0 | 0 | 36 | 45 | | | PEAKS DB |
| K.YNDDYTNC(+57.02)K.S | Y | 52.62 | 1191.4502 | 9 | 1.2 | 596.7331 | 2 | 28.31 | 13 | F13:8853 | BuCaTN01_F11a.raw | | | | | | | | | | | 1.3452E5 | | 5.2674E5 | | | | 2 | 0 | 0 | 0 | 0 | 0 | 0 | 0 | 0 | 0 | 0 | 1 | 0 | 1 | 0 | 0 | 0 | 202 | 210 | Carbamidomethylation | C8:Carbamidomethylation:1000.00 | PEAKS DB |
| R.NM(+15.99)LQMEWNSNAAQNAK.R | N | 52.57 | 1864.8196 | 16 | 1.7 | 622.6149 | 3 | 52.23 | 13 | F13:27379 | BuCaTN01_F11a.raw | | | | | | | | | | | | | 2.2136E5 | | | | 2 | 0 | 0 | 0 | 0 | 0 | 0 | 0 | 0 | 0 | 0 | 0 | 0 | 2 | 0 | 0 | 0 | 54 | 69 | Oxidation (M) | M2:Oxidation (M):56.34 | PEAKS DB |
| K.YIYVC(+57.02)QYC(+57.02)PAGNIIG.S | N | 51.03 | 1789.8168 | 15 | 6.5 | 895.9215 | 2 | 60.28 | 13 | F13:34997 | BuCaTN01_F11a.raw | | | | | | | | | | | | | 7.8778E6 | 0 | | | 1 | 0 | 0 | 0 | 0 | 0 | 0 | 0 | 0 | 0 | 0 | 0 | 0 | 1 | 0 | 0 | 0 | 158 | 172 | Carbamidomethylation | C5:Carbamidomethylation:1000.00;C8:Carbamidomethylation:1000.00 | PEAKS DB |
| G.DC(+57.02)PSAC(+57.02)VNGLC(+57.02)TNPC(+57.02)K.Y | N | 43.28 | 1851.7372 | 16 | -0.3 | 926.8756 | 2 | 43.52 | 13 | F13:19924 | BuCaTN01_F11a.raw | | | | | | | | | | | | | 1.1644E5 | | | | 1 | 0 | 0 | 0 | 0 | 0 | 0 | 0 | 0 | 0 | 0 | 0 | 0 | 1 | 0 | 0 | 0 | 186 | 201 | Carbamidomethylation | C2:Carbamidomethylation:1000.00;C6:Carbamidomethylation:1000.00;C11:Carbamidomethylation:1000.00;C15:Carbamidomethylation:1000.00 | PEAKS DB |
| **total 20 peptides** | | | | | | | | | | | | | | | | | | | | | | | | | | | | | | | | | | | | | | | | | | | | | | | | | |

**Best Unique PSM (Scan F11:23182, m/z=1194.4658, z=3, RT=51.22, ppm=-3.4):**

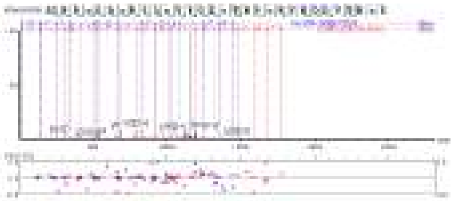

## AAL30054.1

back to list

| Protein Coverage | Supporting Peptides | Best Unique PSM |

**Protein Coverage:**

1 MKTLLLTLVV VTIVCLDLGY TRTCLISPSS TPQTCPQGQD ICFLKAQCDK FCSIRGPVIE QGCVATCPQF RSNYRSLLCC

C Carbamidomethylation (+57.02)

81 TTDNCNH

**Supporting Peptides:**

| Peptide | Uniq | -10lgP | Mass | Length | ppm | m/z | z | RT | Fraction | Scan | Source File | Area F1 | Area F2a | Area F3a | Area F4 | Area F5 | Area F6a | Area F7a | Area F7b | Area F8 | Area F9a | Area Sample 1F10a | Area F10b | Area F11a | Area F11b | Area F12a | Area F13 | #Feature | #Feature F1 | #Feature F2a | #Feature F3a | #Feature F4 | #Feature F5 | #Feature F6a | #Feature F7a | #Feature F7b | #Feature F8 | #Feature F9a | #Feature Sample 1F10a | #Feature F10b | #Feature F11a | #Feature F11b | #Feature F12a | #Feature F13 | Start | End | PTM | AScore | Found By |
|---|---|---|---|---|---|---|---|---|---|---|---|---|---|---|---|---|---|---|---|---|---|---|---|---|---|---|---|---|---|---|---|---|---|---|---|---|---|---|---|---|---|---|---|---|---|---|---|---|---|
| T.RTC(+57.02)LISPSSTPQTC(+57.02)PQGQDIC(+57.02)FLK.A | Y | 101.81 | 2793.3247 | 24 | 0.2 | 932.1138 | 3 | 60.45 | 3 | F3:35015 | BuCaTN01_F3a.raw | | | 2.1521E7 | | | 1.0482E9 | 1.4449E6 | 3.6542E7 | | 3.8552E6 | | 7.4117E6 | | 4.7284E6 | | | 28 | 0 | 0 | 1 | 0 | 0 | 21 | 1 | 2 | 0 | 1 | 0 | 1 | 0 | 1 | 0 | 0 | 22 | 45 | Carbamidomethylation | C3:Carbamidomethylation:1000.00;C14:Carbamidomethylation:1000.00;C21:Carbamidomethylation:1000.00 | PEAKS DB |
| R.TC(+57.02)LISPSSTPQTC(+57.02)PQGQDIC(+57.02)FLK.A | Y | 100.66 | 2637.2236 | 23 | -1.8 | 880.0802 | 3 | 66.27 | 6 | F6:44950 | BuCaTN01_F6a.raw | | | 5.3882E6 | | | 2.1537E8 | | 5.4292E6 | | 3.7823E4 | | | | 2.3049E6 | | | 8 | 0 | 0 | 2 | 0 | 0 | 2 | 0 | 2 | 0 | 1 | 0 | 0 | 0 | 1 | 0 | 0 | 23 | 45 | Carbamidomethylation | C2:Carbamidomethylation:1000.00;C13:Carbamidomethylation:1000.00;C20:Carbamidomethylation:1000.00 | PEAKS DB |
| R.GPVIEQGC(+57.02)VATC(+57.02)PQFR.S | N | 96.70 | 1817.8552 | 16 | 1.3 | 909.9360 | 2 | 51.36 | 6 | F6:30037 | BuCaTN01_F6a.raw | | | | | | 4.938E5 | | | | | | | | 0 | | | 1 | 0 | 0 | 0 | 0 | 0 | 1 | 0 | 0 | 0 | 0 | 0 | 0 | 0 | 0 | 0 | 0 | 56 | 71 | Carbamidomethylation | C8:Carbamidomethylation:1000.00;C12:Carbamidomethylation:1000.00 | PEAKS DB |
| R.SLLC(+57.02)C(+57.02)TTDNC(+57.02)NH | N | 79.81 | 1493.5698 | 12 | -0.3 | 747.7920 | 2 | 37.43 | 6 | F6:17551 | BuCaTN01_F6a.raw | | | 4.1658E6 | | | 7.1212E8 | 3.0441E6 | 4.2545E7 | | 1.9164E6 | | 4.7006E6 | | 5.2769E6 | 7.5251E5 | | 12 | 0 | 0 | 1 | 0 | 0 | 4 | 1 | 2 | 0 | 1 | 0 | 1 | 0 | 1 | 1 | 0 | 76 | 87 | Carbamidomethylation | C4:Carbamidomethylation:1000.00;C5:Carbamidomethylation:1000.00;C10:Carbamidomethylation:1000.00 | PEAKS DB |
| P.VIEQGC(+57.02)VATC(+57.02)PQFR.S | N | 70.62 | 1663.7810 | 14 | 3.0 | 832.8978 | 2 | 46.31 | 8 | F8:22974 | BuCaTN01_F7b.raw | | | | | | | | 7.7386E6 | | | | | | | | | 1 | 0 | 0 | 0 | 0 | 0 | 0 | 0 | 1 | 0 | 0 | 0 | 0 | 0 | 0 | 0 | 0 | 58 | 71 | Carbamidomethylation | C6:Carbamidomethylation:1000.00;C10:Carbamidomethylation:1000.00 | PEAKS DB |
| R.SNYRSLLC(+57.02)C(+57.02)TTDNC(+57.02)NH | N | 62.84 | 2013.8092 | 16 | -1.7 | 672.2759 | 3 | 46.75 | 6 | F6:25700 | BuCaTN01_F6a.raw | | | | | | 1.3691E6 | | 9.5627E5 | | | | | | | | | 2 | 0 | 0 | 0 | 0 | 0 | 1 | 0 | 1 | 0 | 0 | 0 | 0 | 0 | 0 | 0 | 0 | 72 | 87 | Carbamidomethylation | C8:Carbamidomethylation:1000.00;C9:Carbamidomethylation:1000.00;C14:Carbamidomethylation:1000.00 | PEAKS DB |
| T.RTC(+57.02)LISPSSTPQTC(+57.02)PQGQDIC(+57.02)FLKAQC(+57.02)DK.F | Y | 61.53 | 3395.5730 | 29 | -0.1 | 849.9005 | 4 | 57.35 | 6 | F6:35870 | BuCaTN01_F6a.raw | | | | | | 8.3012E6 | | | | | | | | | | | 1 | 0 | 0 | 0 | 0 | 0 | 1 | 0 | 0 | 0 | 0 | 0 | 0 | 0 | 0 | 0 | 0 | 22 | 50 | Carbamidomethylation | C3:Carbamidomethylation:1000.00;C14:Carbamidomethylation:1000.00;C21:Carbamidomethylation:1000.00;C27:Carbamidomethylation:1000.00 | PEAKS DB |
| S.STPQTC(+57.02)PQGQDIC(+57.02)FLK.A | N | 58.36 | 1878.8604 | 16 | -0.9 | 940.4366 | 2 | 55.34 | 6 | F6:33965 | BuCaTN01_F6a.raw | | | | | | 1.4915E7 | | | | | | | | | | | 1 | 0 | 0 | 0 | 0 | 0 | 1 | 0 | 0 | 0 | 0 | 0 | 0 | 0 | 0 | 0 | 0 | 30 | 45 | Carbamidomethylation | C6:Carbamidomethylation:1000.00;C13:Carbamidomethylation:1000.00 | PEAKS DB |
| R.TC(+57.02)LISPSSTPQTC(+57.02)PQGQDIC(+57.02)FLKAQC(+57.02)DK.F | Y | 53.44 | 3239.4719 | 28 | 0.9 | 1080.8323 | 3 | 61.37 | 6 | F6:39331 | BuCaTN01_F6a.raw | | | | | | 3.7826E6 | | | | | | | | | | | 1 | 0 | 0 | 0 | 0 | 0 | 1 | 0 | 0 | 0 | 0 | 0 | 0 | 0 | 0 | 0 | 0 | 23 | 50 | Carbamidomethylation | C2:Carbamidomethylation:1000.00;C13:Carbamidomethylation:1000.00;C20:Carbamidomethylation:1000.00;C26:Carbamidomethylation:1000.00 | PEAKS DB |
| C.LISPSSTPQTC(+57.02)PQGQDIC(+57.02)FLK.A | Y | 51.11 | 2376.1453 | 21 | -0.3 | 793.0555 | 3 | 62.66 | 6 | F6:41180 | BuCaTN01_F6a.raw | | | | | | 6.8584E6 | | | | | | | | | | | 1 | 0 | 0 | 0 | 0 | 0 | 1 | 0 | 0 | 0 | 0 | 0 | 0 | 0 | 0 | 0 | 0 | 25 | 45 | Carbamidomethylation | C11:Carbamidomethylation:1000.00;C18:Carbamidomethylation:1000.00 | PEAKS DB |
| R.GPVIEQGC(+57.02)VAT.C | N | 50.22 | 1129.5437 | 11 | 0.5 | 565.7794 | 2 | 41.92 | 6 | F6:21363 | BuCaTN01_F6a.raw | | | | | | 6.58E5 | | | | | | | | | | | 1 | 0 | 0 | 0 | 0 | 0 | 1 | 0 | 0 | 0 | 0 | 0 | 0 | 0 | 0 | 0 | 0 | 56 | 66 | Carbamidomethylation | C8:Carbamidomethylation:1000.00 | PEAKS DB |
| Q.TC(+57.02)PQGQDIC(+57.02)FLK.A | N | 48.70 | 1465.6693 | 12 | 0.0 | 733.8419 | 2 | 53.66 | 6 | F6:32244 | BuCaTN01_F6a.raw | | | | | | 8.6923E6 | | | | | | | | | | | 1 | 0 | 0 | 0 | 0 | 0 | 1 | 0 | 0 | 0 | 0 | 0 | 0 | 0 | 0 | 0 | 0 | 34 | 45 | Carbamidomethylation | C2:Carbamidomethylation:1000.00;C9:Carbamidomethylation:1000.00 | PEAKS DB |
| S.TPQTC(+57.02)PQGQDIC(+57.02)FLK.A | N | 42.63 | 1791.8284 | 15 | -0.9 | 896.9207 | 2 | 55.31 | 6 | F6:33757 | BuCaTN01_F6a.raw | | | | | | 1.7896E4 | | | | | | | | | | | 1 | 0 | 0 | 0 | 0 | 0 | 1 | 0 | 0 | 0 | 0 | 0 | 0 | 0 | 0 | 0 | 0 | 31 | 45 | Carbamidomethylation | C5:Carbamidomethylation:1000.00;C12:Carbamidomethylation:1000.00 | PEAKS DB |
| R.GPVIEQGC(+57.02)VA.T | N | 42.28 | 1028.4961 | 10 | 0.2 | 515.2554 | 2 | 41.42 | 6 | F6:20784 | BuCaTN01_F6a.raw | | | | | | 2.0497E6 | | | | | | | | | | | 1 | 0 | 0 | 0 | 0 | 0 | 1 | 0 | 0 | 0 | 0 | 0 | 0 | 0 | 0 | 0 | 0 | 56 | 65 | Carbamidomethylation | C8:Carbamidomethylation:1000.00 | PEAKS DB |
| V.IEQGC(+57.02)VATC(+57.02)PQFR.S | N | 41.92 | 1564.7126 | 13 | 1.4 | 783.3624 | 2 | 43.03 | 8 | F8:20158 | BuCaTN01_F7b.raw | | | | | | | | 1.5548E5 | | | | | | | | | 1 | 0 | 0 | 0 | 0 | 0 | 0 | 0 | 1 | 0 | 0 | 0 | 0 | 0 | 0 | 0 | 0 | 59 | 71 | Carbamidomethylation | C5:Carbamidomethylation:1000.00;C9:Carbamidomethylation:1000.00 | PEAKS DB |
| **total 15 peptides** | | | | | | | | | | | | | | | | | | | | | | | | | | | | | | | | | | | | | | | | | | | | | | | | | |

**Best Unique PSM (Scan F3:35015, m/z=932.1138, z=3, RT=60.45, ppm=0.2):**

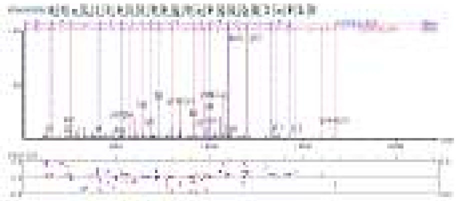

## Q8AY56.1

back to list

| Protein Coverage | Supporting Peptides | Best Unique PSM |

**Protein Coverage:**


1 MKTLLLTLVV VTIVCLDLGY TRTCLISPSS TPQTCPQGQD ICFLKAQCDK FCSIRGPVIE QGCVATCPQF RSNYRSLLCC

C Carbamidomethylation (+57.02)

81 TTDNCNH

**Supporting Peptides:**

| Peptide | Uniq | -10lgP | Mass | Length | ppm | m/z | z | RT | Fraction | Scan | Source File | Area F1 | Area F2a | Area F3a | Area F4 | Area F5 | Area F6a | Area F7a | Area F7b | Area F8 | Area F9a | Area Sample 1F10a | Area F10b | Area F11a | Area F11b | Area F12a | Area F13 | #Feature | #Feature F1 | #Feature F2a | #Feature F3a | #Feature F4 | #Feature F5 | #Feature F6a | #Feature F7a | #Feature F7b | #Feature F8 | #Feature F9a | #Feature Sample 1F10a | #Feature F10b | #Feature F11a | #Feature F11b | #Feature F12a | #Feature F13 | Start | End | PTM | AScore | Found By |
|---|---|---|---|---|---|---|---|---|---|---|---|---|---|---|---|---|---|---|---|---|---|---|---|---|---|---|---|---|---|---|---|---|---|---|---|---|---|---|---|---|---|---|---|---|---|---|---|---|---|
| T.RTC(+57.02)LISPSSTPQTC(+57.02)PQGQDIC(+57.02)FLK.A | Y | 101.81 | 2793.3247 | 24 | 0.2 | 932.1138 | 3 | 60.45 | 3 | F3:35015 | BuCaTN01_F3a.raw | | | 2.1521E7 | | | 1.0482E9 | 1.4449E6 | 3.6542E7 | | 3.8552E6 | | 7.4117E6 | | 4.7284E6 | | | 28 | 0 | 0 | 1 | 0 | 0 | 21 | 1 | 2 | 0 | 1 | 0 | 1 | 0 | 1 | 0 | 0 | 22 | 45 | Carbamidomethylation | C3:Carbamidomethylation:1000.00;C14:Carbamidomethylation:1000.00;C21:Carbamidomethylation:1000.00 | PEAKS DB |
| R.TC(+57.02)LISPSSTPQTC(+57.02)PQGQDIC(+57.02)FLK.A | Y | 100.66 | 2637.2236 | 23 | -1.8 | 880.0802 | 3 | 66.27 | 6 | F6:44950 | BuCaTN01_F6a.raw | | | 5.3882E6 | | | 2.1537E8 | | 5.4292E6 | | 3.7823E4 | | | | 2.3049E6 | | | 8 | 0 | 0 | 2 | 0 | 0 | 2 | 0 | 2 | 0 | 1 | 0 | 0 | 0 | 1 | 0 | 0 | 23 | 45 | Carbamidomethylation | C2:Carbamidomethylation:1000.00;C13:Carbamidomethylation:1000.00;C20:Carbamidomethylation:1000.00 | PEAKS DB |
| R.GPVIEQGC(+57.02)VATC(+57.02)PQFR.S | N | 96.70 | 1817.8552 | 16 | 1.3 | 909.9360 | 2 | 51.36 | 6 | F6:30037 | BuCaTN01_F6a.raw | | | | | | 4.938E5 | | | | | | | | 0 | | | 1 | 0 | 0 | 0 | 0 | 0 | 1 | 0 | 0 | 0 | 0 | 0 | 0 | 0 | 0 | 0 | 0 | 56 | 71 | Carbamidomethylation | C8:Carbamidomethylation:1000.00;C12:Carbamidomethylation:1000.00 | PEAKS DB |
| R.SLLC(+57.02)C(+57.02)TTDNC(+57.02)NH | N | 79.81 | 1493.5698 | 12 | -0.3 | 747.7920 | 2 | 37.43 | 6 | F6:17551 | BuCaTN01_F6a.raw | | | 4.1658E6 | | | 7.1212E8 | 3.0441E6 | 4.2545E7 | | 1.9164E6 | | 4.7006E6 | | 5.2769E6 | 7.5251E5 | | 12 | 0 | 0 | 1 | 0 | 0 | 4 | 1 | 2 | 0 | 1 | 0 | 1 | 0 | 1 | 1 | 0 | 76 | 87 | Carbamidomethylation | C4:Carbamidomethylation:1000.00;C5:Carbamidomethylation:1000.00;C10:Carbamidomethylation:1000.00 | PEAKS DB |
| P.VIEQGC(+57.02)VATC(+57.02)PQFR.S | N | 70.62 | 1663.7810 | 14 | 3.0 | 832.8978 | 2 | 46.31 | 8 | F8:22974 | BuCaTN01_F7b.raw | | | | | | | | 7.7386E6 | | | | | | | | | 1 | 0 | 0 | 0 | 0 | 0 | 0 | 0 | 1 | 0 | 0 | 0 | 0 | 0 | 0 | 0 | 0 | 58 | 71 | Carbamidomethylation | C6:Carbamidomethylation:1000.00;C10:Carbamidomethylation:1000.00 | PEAKS DB |
| R.SNYRSLLC(+57.02)C(+57.02)TTDNC(+57.02)NH | N | 62.84 | 2013.8092 | 16 | -1.7 | 672.2759 | 3 | 46.75 | 6 | F6:25700 | BuCaTN01_F6a.raw | | | | | | 1.3691E6 | | 9.5627E5 | | | | | | | | | 2 | 0 | 0 | 0 | 0 | 0 | 1 | 0 | 1 | 0 | 0 | 0 | 0 | 0 | 0 | 0 | 0 | 72 | 87 | Carbamidomethylation | C8:Carbamidomethylation:1000.00;C9:Carbamidomethylation:1000.00;C14:Carbamidomethylation:1000.00 | PEAKS DB |
| T.RTC(+57.02)LISPSSTPQTC(+57.02)PQGQDIC(+57.02)FLKAQC(+57.02)DK.F | Y | 61.53 | 3395.5730 | 29 | -0.1 | 849.9005 | 4 | 57.35 | 6 | F6:35870 | BuCaTN01_F6a.raw | | | | | | 8.3012E6 | | | | | | | | | | | 1 | 0 | 0 | 0 | 0 | 0 | 1 | 0 | 0 | 0 | 0 | 0 | 0 | 0 | 0 | 0 | 0 | 22 | 50 | Carbamidomethylation | C3:Carbamidomethylation:1000.00;C14:Carbamidomethylation:1000.00;C21:Carbamidomethylation:1000.00;C27:Carbamidomethylation:1000.00 | PEAKS DB |
| S.STPQTC(+57.02)PQGQDIC(+57.02)FLK.A | N | 58.36 | 1878.8604 | 16 | -0.9 | 940.4366 | 2 | 55.34 | 6 | F6:33965 | BuCaTN01_F6a.raw | | | | | | 1.4915E7 | | | | | | | | | | | 1 | 0 | 0 | 0 | 0 | 0 | 1 | 0 | 0 | 0 | 0 | 0 | 0 | 0 | 0 | 0 | 0 | 30 | 45 | Carbamidomethylation | C6:Carbamidomethylation:1000.00;C13:Carbamidomethylation:1000.00 | PEAKS DB |
| R.TC(+57.02)LISPSSTPQTC(+57.02)PQGQDIC(+57.02)FLKAQC(+57.02)DK.F | Y | 53.44 | 3239.4719 | 28 | 0.9 | 1080.8323 | 3 | 61.37 | 6 | F6:39331 | BuCaTN01_F6a.raw | | | | | | 3.7826E6 | | | | | | | | | | | 1 | 0 | 0 | 0 | 0 | 0 | 1 | 0 | 0 | 0 | 0 | 0 | 0 | 0 | 0 | 0 | 0 | 23 | 50 | Carbamidomethylation | C2:Carbamidomethylation:1000.00;C13:Carbamidomethylation:1000.00;C20:Carbamidomethylation:1000.00;C26:Carbamidomethylation:1000.00 | PEAKS DB |
| C.LISPSSTPQTC(+57.02)PQGQDIC(+57.02)FLK.A | Y | 51.11 | 2376.1453 | 21 | -0.3 | 793.0555 | 3 | 62.66 | 6 | F6:41180 | BuCaTN01_F6a.raw | | | | | | 6.8584E6 | | | | | | | | | | | 1 | 0 | 0 | 0 | 0 | 0 | 1 | 0 | 0 | 0 | 0 | 0 | 0 | 0 | 0 | 0 | 0 | 25 | 45 | Carbamidomethylation | C11:Carbamidomethylation:1000.00;C18:Carbamidomethylation:1000.00 | PEAKS DB |
| R.GPVIEQGC(+57.02)VAT.C | N | 50.22 | 1129.5437 | 11 | 0.5 | 565.7794 | 2 | 41.92 | 6 | F6:21363 | BuCaTN01_F6a.raw | | | | | | 6.58E5 | | | | | | | | | | | 1 | 0 | 0 | 0 | 0 | 0 | 1 | 0 | 0 | 0 | 0 | 0 | 0 | 0 | 0 | 0 | 0 | 56 | 66 | Carbamidomethylation | C8:Carbamidomethylation:1000.00 | PEAKS DB |
| Q.TC(+57.02)PQGQDIC(+57.02)FLK.A | N | 48.70 | 1465.6693 | 12 | 0.0 | 733.8419 | 2 | 53.66 | 6 | F6:32244 | BuCaTN01_F6a.raw | | | | | | 8.6923E6 | | | | | | | | | | | 1 | 0 | 0 | 0 | 0 | 0 | 1 | 0 | 0 | 0 | 0 | 0 | 0 | 0 | 0 | 0 | 0 | 34 | 45 | Carbamidomethylation | C2:Carbamidomethylation:1000.00;C9:Carbamidomethylation:1000.00 | PEAKS DB |
| S.TPQTC(+57.02)PQGQDIC(+57.02)FLK.A | N | 42.63 | 1791.8284 | 15 | -0.9 | 896.9207 | 2 | 55.31 | 6 | F6:33757 | BuCaTN01_F6a.raw | | | | | | 1.7896E4 | | | | | | | | | | | 1 | 0 | 0 | 0 | 0 | 0 | 1 | 0 | 0 | 0 | 0 | 0 | 0 | 0 | 0 | 0 | 0 | 31 | 45 | Carbamidomethylation | C5:Carbamidomethylation:1000.00;C12:Carbamidomethylation:1000.00 | PEAKS DB |
| R.GPVIEQGC(+57.02)VA.T | N | 42.28 | 1028.4961 | 10 | 0.2 | 515.2554 | 2 | 41.42 | 6 | F6:20784 | BuCaTN01_F6a.raw | | | | | | 2.0497E6 | | | | | | | | | | | 1 | 0 | 0 | 0 | 0 | 0 | 1 | 0 | 0 | 0 | 0 | 0 | 0 | 0 | 0 | 0 | 0 | 56 | 65 | Carbamidomethylation | C8:Carbamidomethylation:1000.00 | PEAKS DB |
| V.IEQGC(+57.02)VATC(+57.02)PQFR.S | N | 41.92 | 1564.7126 | 13 | 1.4 | 783.3624 | 2 | 43.03 | 8 | F8:20158 | BuCaTN01_F7b.raw | | | | | | | | 1.5548E5 | | | | | | | | | 1 | 0 | 0 | 0 | 0 | 0 | 0 | 0 | 1 | 0 | 0 | 0 | 0 | 0 | 0 | 0 | 0 | 59 | 71 | Carbamidomethylation | C5:Carbamidomethylation:1000.00;C9:Carbamidomethylation:1000.00 | PEAKS DB |
| **total 15 peptides** | | | | | | | | | | | | | | | | | | | | | | | | | | | | | | | | | | | | | | | | | | | | | | | | | |

**Best Unique PSM (Scan F3:35015, m/z=932.1138, z=3, RT=60.45, ppm=0.2):**

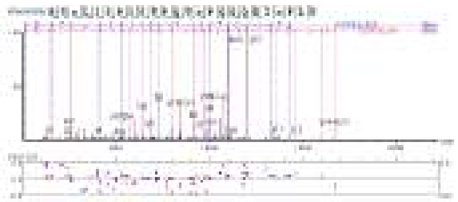

## AAS20530.1

back to list

| Protein Coverage | Supporting Peptides | Best Unique PSM |

**Protein Coverage:**

```
1   LVAVCVSLLG AANIPPQPLN LYQLMNMIQC ANTRTWPSYT NYGCYCGKGG SGTPVDDLDR CCYTHDHCYN DAKNIDGCNP
81  VTKTYSYTCT EPTITCNDSK DKCARFVCDC DRTAAICFAK APYNTSNVMI RSTNSCQ
```

C Carbamidomethylation (+57.02)

108 110 117

**Supporting Peptides:**

| Peptide | Uniq | -10lgP | Mass | Length | ppm | m/z | z | RT | Fraction | Scan | Source File | Area F1 | Area F2a | Area F3a | Area F4 | Area F5 | Area F6a | Area F7a | Area F7b | Area F8 | Area F9a | Area Sample 1F10a | Area F10b | Area F11a | Area F11b | Area F12a | Area F13 | #Feature | #Feature F1 | #Feature F2a | #Feature F3a | #Feature F4 | #Feature F5 | #Feature F6a | #Feature F7a | #Feature F7b | #Feature F8 | #Feature F9a | #Feature Sample 1F10a | #Feature F10b | #Feature F11a | #Feature F11b | #Feature F12a | #Feature F13 | Start | End | PTM | AScore | Found By |
|---|---|---|---|---|---|---|---|---|---|---|---|---|---|---|---|---|---|---|---|---|---|---|---|---|---|---|---|---|---|---|---|---|---|---|---|---|---|---|---|---|---|---|---|---|---|---|---|---|---|
| R.FVC(+57.02)DC(+57.02)DRTAAIC(+57.02)FAK.A | Y | 114.63 | 1832.8008 | 15 | 4.5 | 917.4072 | 2 | 49.59 | 15 | F15:26245 | BuCaTN01_F12a.raw | | | | | | 2.5208E7 | 1.6707E8 | 4.9947E8 | | 2.6145E9 | 5.2226E7 | 6.4722E9 | 1.7192E7 | 1.7308E9 | 3.9989E9 | | 42 | 0 | 0 | 0 | 0 | 0 | 2 | 4 | 3 | 0 | 6 | 3 | 9 | 2 | 4 | 9 | 0 | 106 | 120 | Carbamidomethylation | C3:Carbamidomethylation:1000.00;C5:Carbamidomethylation:1000.00;C12:Carbamidomethylation:1000.00 | PEAKS DB |
| A.RFVC(+57.02)DC(+57.02)DRTAAIC(+57.02)FAK.A | Y | 78.26 | 1988.9019 | 16 | 1.8 | 498.2325 | 4 | 47.48 | 12 | F12:24632 | BuCaTN01_F10b.raw | | | | | | | | 1.808E6 | | | 9.0711E5 | 2.0703E8 | | 7.0193E6 | | | 8 | 0 | 0 | 0 | 0 | 0 | 0 | 0 | 1 | 0 | 0 | 1 | 4 | 0 | 2 | 0 | 0 | 105 | 120 | Carbamidomethylation | C4:Carbamidomethylation:1000.00;C6:Carbamidomethylation:1000.00;C13:Carbamidomethylation:1000.00 | PEAKS DB |
| R.TAAIC(+57.02)FAKAPY.N | N | 73.24 | 1211.6008 | 11 | 2.2 | 606.8076 | 2 | 55.84 | 12 | F12:32023 | BuCaTN01_F10b.raw | | | | | | | 7.3334E4 | | | 1.7166E6 | 1.3753E5 | 1.06E7 | | 8.1407E6 | 9.828E5 | | 7 | 0 | 0 | 0 | 0 | 0 | 0 | 1 | 0 | 0 | 1 | 1 | 2 | 0 | 1 | 1 | 0 | 113 | 123 | Carbamidomethylation | C5:Carbamidomethylation:1000.00 | PEAKS DB |
| R.FVC(+57.02)DC(+57.02)DRTAAIC(+57.02)FAKAPYN.T | Y | 71.28 | 2277.9968 | 19 | 0.1 | 760.3397 | 3 | 58.06 | 7 | F7:30880 | BuCaTN01_F7a.raw | | | | | | 1.4903E7 | 2.0995E6 | 8.9111E7 | | 1.7533E7 | | | | | 3.5967E6 | | 7 | 0 | 0 | 0 | 0 | 0 | 2 | 1 | 2 | 0 | 1 | 0 | 0 | 0 | 0 | 1 | 0 | 106 | 124 | Carbamidomethylation | C3:Carbamidomethylation:1000.00;C5:Carbamidomethylation:1000.00;C12:Carbamidomethylation:1000.00 | PEAKS DB |
| R.TAAIC(+57.02)FAKAPYN.T | N | 69.35 | 1325.6438 | 12 | -0.2 | 663.8290 | 2 | 51.91 | 6 | F6:30474 | BuCaTN01_F6a.raw | | | | | | 3.7675E6 | | 1.5632E7 | | 4.6358E6 | | 1.9346E6 | | | | | 5 | 0 | 0 | 0 | 0 | 0 | 1 | 0 | 2 | 0 | 1 | 0 | 1 | 0 | 0 | 0 | 0 | 113 | 124 | Carbamidomethylation | C5:Carbamidomethylation:1000.00 | PEAKS DB |
| K.GGSGTPVDDLDR.C | N | 69.16 | 1187.5417 | 12 | -1.2 | 594.7775 | 2 | 38.65 | 10 | F10:17034 | BuCaTN01_F9a.raw | | | | | | | | | | 5.2316E6 | | | | | | | 1 | 0 | 0 | 0 | 0 | 0 | 0 | 0 | 0 | 0 | 1 | 0 | 0 | 0 | 0 | 0 | 0 | 49 | 60 | | | PEAKS DB |
| D.C(+57.02)DRTAAIC(+57.02)FAK.A | N | 63.48 | 1311.6063 | 11 | -1.7 | 438.2076 | 3 | 37.36 | 12 | F12:15762 | BuCaTN01_F10b.raw | | | | | | | | | | | | 5.2815E4 | | 3.1918E5 | | | 2 | 0 | 0 | 0 | 0 | 0 | 0 | 0 | 0 | 0 | 0 | 0 | 1 | 0 | 1 | 0 | 0 | 110 | 120 | Carbamidomethylation | C1:Carbamidomethylation:1000.00;C8:Carbamidomethylation:1000.00 | PEAKS DB |
| R.FVC(+57.02)DC(+57.02)DRTAAIC(+57.02).F | N | 62.55 | 1486.6003 | 12 | 2.9 | 744.3079 | 2 | 41.59 | 12 | F12:19336 | BuCaTN01_F10b.raw | | | | | | | 3.2591E5 | 5.6967E6 | | 1.9877E6 | | 2.0528E7 | | 2.7882E6 | 1.1445E7 | | 7 | 0 | 0 | 0 | 0 | 0 | 0 | 1 | 1 | 0 | 1 | 0 | 2 | 0 | 1 | 1 | 0 | 106 | 117 | Carbamidomethylation | C3:Carbamidomethylation:1000.00;C5:Carbamidomethylation:1000.00;C12:Carbamidomethylation:1000.00 | PEAKS DB |
| C.DC(+57.02)DRTAAIC(+57.02)FAK.A | Y | 60.32 | 1426.6333 | 12 | 2.6 | 476.5518 | 3 | 40.33 | 12 | F12:18176 | BuCaTN01_F10b.raw | | | | | | | | | | | | 0 | | 4.6798E5 | | | 1 | 0 | 0 | 0 | 0 | 0 | 0 | 0 | 0 | 0 | 0 | 0 | 0 | 0 | 1 | 0 | 0 | 109 | 120 | Carbamidomethylation | C2:Carbamidomethylation:1000.00;C9:Carbamidomethylation:1000.00 | PEAKS DB |
| R.TAAIC(+57.02)FAK.A | N | 60.21 | 880.4476 | 8 | 3.4 | 441.2316 | 2 | 41.58 | 12 | F12:19288 | BuCaTN01_F10b.raw | | | | | | 9.3985E4 | 9.2429E6 | 1.2777E7 | 4.6024E5 | 3.2388E7 | 3.591E6 | 1.265E8 | 3.5731E6 | 1.1283E8 | 1.0943E8 | | 21 | 0 | 0 | 0 | 0 | 0 | 1 | 4 | 2 | 1 | 2 | 2 | 2 | 1 | 2 | 4 | 0 | 113 | 120 | Carbamidomethylation | C5:Carbamidomethylation:1000.00 | PEAKS DB |
| R.FVC(+57.02)DC(+57.02)DRTAAI.C | N | 53.25 | 1326.5696 | 11 | -0.5 | 664.2917 | 2 | 44.27 | 7 | F7:19638 | BuCaTN01_F7a.raw | | | | | | | 4.353E5 | 1.0134E6 | | | | | | | 6.8105E6 | | 3 | 0 | 0 | 0 | 0 | 0 | 0 | 1 | 1 | 0 | 0 | 0 | 0 | 0 | 0 | 1 | 0 | 106 | 116 | Carbamidomethylation | C3:Carbamidomethylation:1000.00;C5:Carbamidomethylation:1000.00 | PEAKS DB |
| S.GTPVDDLDR.C | N | 51.39 | 986.4669 | 9 | 0.6 | 494.2410 | 2 | 37.62 | 7 | F7:14221 | BuCaTN01_F7a.raw | | | | | | | 4.0904E6 | | | | | | | | | | 1 | 0 | 0 | 0 | 0 | 0 | 0 | 1 | 0 | 0 | 0 | 0 | 0 | 0 | 0 | 0 | 0 | 52 | 60 | | | PEAKS DB |
| G.TPVDDLDR.C | N | 48.70 | 929.4454 | 8 | -0.4 | 465.7298 | 2 | 34.76 | 7 | F7:11899 | BuCaTN01_F7a.raw | | | | | | | 6.3757E5 | | | | | | | | | | 1 | 0 | 0 | 0 | 0 | 0 | 0 | 1 | 0 | 0 | 0 | 0 | 0 | 0 | 0 | 0 | 0 | 53 | 60 | | | PEAKS DB |
| R.TAAIC(+57.02)FAKAP.Y | N | 47.25 | 1048.5375 | 10 | 3.6 | 525.2764 | 2 | 48.84 | 8 | F8:25881 | BuCaTN01_F7b.raw | | | | | | 3.1579E6 | | 4.0965E6 | | | | | | | | | 2 | 0 | 0 | 0 | 0 | 0 | 1 | 0 | 1 | 0 | 0 | 0 | 0 | 0 | 0 | 0 | 0 | 113 | 122 | Carbamidomethylation | C5:Carbamidomethylation:1000.00 | PEAKS DB |
| R.FVC(+57.02)DC(+57.02)DRTAAIC(+57.02)F.A | N | 47.20 | 1633.6687 | 13 | 5.0 | 817.8416 | 2 | 60.81 | 15 | F15:36004 | BuCaTN01_F12a.raw | | | | | | | | | | | | | | | 1.0006E7 | | 1 | 0 | 0 | 0 | 0 | 0 | 0 | 0 | 0 | 0 | 0 | 0 | 0 | 0 | 0 | 1 | 0 | 106 | 118 | Carbamidomethylation | C3:Carbamidomethylation:1000.00;C5:Carbamidomethylation:1000.00;C12:Carbamidomethylation:1000.00 | PEAKS DB |
| R.FVC(+57.02)DC(+57.02)DRTAAIC(+57.02)FAKAPY.N | Y | 46.45 | 2163.9539 | 18 | 1.6 | 722.3247 | 3 | 61.77 | 12 | F12:37977 | BuCaTN01_F10b.raw | | | | | | | | | | | | 3.9497E6 | | | 1.3752E7 | | 2 | 0 | 0 | 0 | 0 | 0 | 0 | 0 | 0 | 0 | 0 | 0 | 1 | 0 | 0 | 1 | 0 | 106 | 123 | Carbamidomethylation | C3:Carbamidomethylation:1000.00;C5:Carbamidomethylation:1000.00;C12:Carbamidomethylation:1000.00 | PEAKS DB |
| R.FVC(+57.02)DC(+57.02)DRTAAIC(+57.02)FAKA.P | Y | 45.80 | 1903.8379 | 16 | 1.7 | 952.9250 | 2 | 55.04 | 8 | F8:30870 | BuCaTN01_F7b.raw | | | | | | | | 2.9142E5 | | | | | | | | | 1 | 0 | 0 | 0 | 0 | 0 | 0 | 0 | 1 | 0 | 0 | 0 | 0 | 0 | 0 | 0 | 0 | 106 | 121 | Carbamidomethylation | C3:Carbamidomethylation:1000.00;C5:Carbamidomethylation:1000.00;C12:Carbamidomethylation:1000.00 | PEAKS DB |
| total 17 peptides | | | | | | | | | | | | | | | | | | | | | | | | | | | | | | | | | | | | | | | | | | | | | | | | | |

**Best Unique PSM (Scan F15:26245, m/z=917.4072, z=2, RT=49.59, ppm=4.5):**

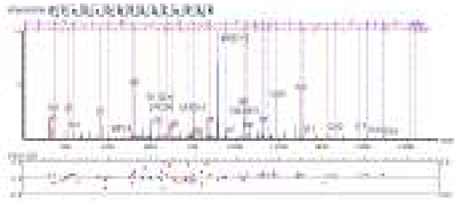

## Q6SLM2.1

back to list

| Protein Coverage | Supporting Peptides | Best Unique PSM |

**Protein Coverage:**

```
1   LVAVCVSLLG AANIPPQPLN LYQLMNMIQC ANTRTWPSYT NYGCYCGKGG SGTPVDDLDR CCYTHDHCYN DAKNIDGCNP
81  VTKTYSYTCT EPTITCNDSK DKCARFVCDC DRTAAICFAK APYNTSNVMI RSTNSCQ
```

C Carbamidomethylation (+57.02)

108 110 117

**Supporting Peptides:**

| Peptide | Uniq | -10lgP | Mass | Length | ppm | m/z | z | RT | Fraction | Scan | Source File | Area F1 | Area F2a | Area F3a | Area F4 | Area F5 | Area F6a | Area F7a | Area F7b | Area F8 | Area F9a | Area Sample 1F10a | Area F10b | Area F11a | Area F11b | Area F12a | Area F13 | #Feature | #Feature F1 | #Feature F2a | #Feature F3a | #Feature F4 | #Feature F5 | #Feature F6a | #Feature F7a | #Feature F7b | #Feature F8 | #Feature F9a | #Feature Sample 1F10a | #Feature F10b | #Feature F11a | #Feature F11b | #Feature F12a | #Feature F13 | Start | End | PTM | AScore | Found By |
|---|---|---|---|---|---|---|---|---|---|---|---|---|---|---|---|---|---|---|---|---|---|---|---|---|---|---|---|---|---|---|---|---|---|---|---|---|---|---|---|---|---|---|---|---|---|---|---|---|---|
| R.FVC(+57.02)DC(+57.02)DRTAAIC(+57.02)FAK.A | Y | 114.63 | 1832.8008 | 15 | 4.5 | 917.4072 | 2 | 49.59 | 15 | F15:26245 | BuCaTN01_F12a.raw | | | | | | 2.5208E7 | 1.6707E8 | 4.9947E8 | | 2.6145E9 | 5.2226E7 | 6.4722E9 | 1.7192E7 | 1.7308E9 | 3.9989E9 | | 42 | 0 | 0 | 0 | 0 | 0 | 2 | 4 | 3 | 0 | 6 | 3 | 9 | 2 | 4 | 9 | 0 | 106 | 120 | Carbamidomethylation | C3:Carbamidomethylation:1000.00;C5:Carbamidomethylation:1000.00;C12:Carbamidomethylation:1000.00 | PEAKS DB |
| A.RFVC(+57.02)DC(+57.02)DRTAAIC(+57.02)FAK.A | Y | 78.26 | 1988.9019 | 16 | 1.8 | 498.2325 | 4 | 47.48 | 12 | F12:24632 | BuCaTN01_F10b.raw | | | | | | | | 1.808E6 | | | 9.0711E5 | 2.0703E8 | | 7.0193E6 | | | 8 | 0 | 0 | 0 | 0 | 0 | 0 | 0 | 1 | 0 | 0 | 1 | 4 | 0 | 2 | 0 | 0 | 105 | 120 | Carbamidomethylation | C4:Carbamidomethylation:1000.00;C6:Carbamidomethylation:1000.00;C13:Carbamidomethylation:1000.00 | PEAKS DB |
| R.TAAIC(+57.02)FAKAPY.N | N | 73.24 | 1211.6008 | 11 | 2.2 | 606.8076 | 2 | 55.84 | 12 | F12:32023 | BuCaTN01_F10b.raw | | | | | | | 7.3334E4 | | | 1.7166E6 | 1.3753E5 | 1.06E7 | | 8.1407E6 | 9.828E5 | | 7 | 0 | 0 | 0 | 0 | 0 | 0 | 1 | 0 | 0 | 1 | 1 | 2 | 0 | 1 | 1 | 0 | 113 | 123 | Carbamidomethylation | C5:Carbamidomethylation:1000.00 | PEAKS DB |
| R.FVC(+57.02)DC(+57.02)DRTAAIC(+57.02)FAKAPYN.T | Y | 71.28 | 2277.9968 | 19 | 0.1 | 760.3397 | 3 | 58.06 | 7 | F7:30880 | BuCaTN01_F7a.raw | | | | | | 1.4903E7 | 2.0995E6 | 8.9111E7 | | 1.7533E7 | | | | | 3.5967E6 | | 7 | 0 | 0 | 0 | 0 | 0 | 2 | 1 | 2 | 0 | 1 | 0 | 0 | 0 | 0 | 1 | 0 | 106 | 124 | Carbamidomethylation | C3:Carbamidomethylation:1000.00;C5:Carbamidomethylation:1000.00;C12:Carbamidomethylation:1000.00 | PEAKS DB |
| R.TAAIC(+57.02)FAKAPYN.T | N | 69.35 | 1325.6438 | 12 | -0.2 | 663.8290 | 2 | 51.91 | 6 | F6:30474 | BuCaTN01_F6a.raw | | | | | | 3.7675E6 | | 1.5632E7 | | 4.6358E6 | | 1.9346E6 | | | | | 5 | 0 | 0 | 0 | 0 | 0 | 1 | 0 | 2 | 0 | 1 | 0 | 1 | 0 | 0 | 0 | 0 | 113 | 124 | Carbamidomethylation | C5:Carbamidomethylation:1000.00 | PEAKS DB |
| K.GGSGTPVDDLDR.C | N | 69.16 | 1187.5417 | 12 | -1.2 | 594.7775 | 2 | 38.65 | 10 | F10:17034 | BuCaTN01_F9a.raw | | | | | | | | | | 5.2316E6 | | | | | | | 1 | 0 | 0 | 0 | 0 | 0 | 0 | 0 | 0 | 0 | 1 | 0 | 0 | 0 | 0 | 0 | 0 | 49 | 60 | | | PEAKS DB |
| D.C(+57.02)DRTAAIC(+57.02)FAK.A | N | 63.48 | 1311.6063 | 11 | -1.7 | 438.2076 | 3 | 37.36 | 12 | F12:15762 | BuCaTN01_F10b.raw | | | | | | | | | | | | 5.2815E4 | | 3.1918E5 | | | 2 | 0 | 0 | 0 | 0 | 0 | 0 | 0 | 0 | 0 | 0 | 0 | 1 | 0 | 1 | 0 | 0 | 110 | 120 | Carbamidomethylation | C1:Carbamidomethylation:1000.00;C8:Carbamidomethylation:1000.00 | PEAKS DB |
| R.FVC(+57.02)DC(+57.02)DRTAAIC(+57.02).F | N | 62.55 | 1486.6003 | 12 | 2.9 | 744.3079 | 2 | 41.59 | 12 | F12:19336 | BuCaTN01_F10b.raw | | | | | | | 3.2591E5 | 5.6967E6 | | 1.9877E6 | | 2.0528E7 | | 2.7882E6 | 1.1445E7 | | 7 | 0 | 0 | 0 | 0 | 0 | 0 | 1 | 1 | 0 | 1 | 0 | 2 | 0 | 1 | 1 | 0 | 106 | 117 | Carbamidomethylation | C3:Carbamidomethylation:1000.00;C5:Carbamidomethylation:1000.00;C12:Carbamidomethylation:1000.00 | PEAKS DB |
| C.DC(+57.02)DRTAAIC(+57.02)FAK.A | Y | 60.32 | 1426.6333 | 12 | 2.6 | 476.5518 | 3 | 40.33 | 12 | F12:18176 | BuCaTN01_F10b.raw | | | | | | | | | | | | 0 | | 4.6798E5 | | | 1 | 0 | 0 | 0 | 0 | 0 | 0 | 0 | 0 | 0 | 0 | 0 | 0 | 0 | 1 | 0 | 0 | 109 | 120 | Carbamidomethylation | C2:Carbamidomethylation:1000.00;C9:Carbamidomethylation:1000.00 | PEAKS DB |
| R.TAAIC(+57.02)FAK.A | N | 60.21 | 880.4476 | 8 | 3.4 | 441.2316 | 2 | 41.58 | 12 | F12:19288 | BuCaTN01_F10b.raw | | | | | | 9.3985E4 | 9.2429E6 | 1.2777E7 | 4.6024E5 | 3.2388E7 | 3.591E6 | 1.265E8 | 3.5731E6 | 1.1283E8 | 1.0943E8 | | 21 | 0 | 0 | 0 | 0 | 0 | 1 | 4 | 2 | 1 | 2 | 2 | 2 | 1 | 2 | 4 | 0 | 113 | 120 | Carbamidomethylation | C5:Carbamidomethylation:1000.00 | PEAKS |

| | | | | | | | | | | | | | | | | | | | | | | | | | | | | | | | | | | | | | | | | | | | | | | | | | |
|---|---|---|---|---|---|---|---|---|---|---|---|---|---|---|---|---|---|---|---|---|---|---|---|---|---|---|---|---|---|---|---|---|---|---|---|---|---|---|---|---|---|---|---|---|---|---|---|---|---|
| | | | | | | | | | | | | | | | | | | | | | | | | | | | | | | | | | | | | | | | | | | | | | | | | | DB |
| R.FVC(+57.02)DC(+57.02)DRTAAI.C | N | 53.25 | 1326.5696 | 11 | -0.5 | 664.2917 | 2 | 44.27 | 7 | F7:19638 | BuCaTN01_F7a.raw | | | | | | | 4.353E5 | 1.0134E6 | | | | | | | 6.8105E6 | | 3 | 0 | 0 | 0 | 0 | 0 | 0 | 1 | 1 | 0 | 0 | 0 | 0 | 0 | 0 | 1 | 0 | 106 | 116 | Carbamidomethylation | C3:Carbamidomethylation:1000.00;C5:Carbamidomethylation:1000.00 | PEAKS DB |
| S.GTPVDDLDR.C | N | 51.39 | 986.4669 | 9 | 0.6 | 494.2410 | 2 | 37.62 | 7 | F7:14221 | BuCaTN01_F7a.raw | | | | | | | 4.0904E6 | | | | | | | | | | 1 | 0 | 0 | 0 | 0 | 0 | 0 | 1 | 0 | 0 | 0 | 0 | 0 | 0 | 0 | 0 | 0 | 52 | 60 | | | PEAKS DB |
| G.TPVDDLDR.C | N | 48.70 | 929.4454 | 8 | -0.4 | 465.7298 | 2 | 34.76 | 7 | F7:11899 | BuCaTN01_F7a.raw | | | | | | | 6.3757E5 | | | | | | | | | | 1 | 0 | 0 | 0 | 0 | 0 | 0 | 1 | 0 | 0 | 0 | 0 | 0 | 0 | 0 | 0 | 0 | 53 | 60 | | | PEAKS DB |
| R.TAAIC(+57.02)FAKAP.Y | N | 47.25 | 1048.5375 | 10 | 3.6 | 525.2764 | 2 | 48.84 | 8 | F8:25881 | BuCaTN01_F7b.raw | | | | | | 3.1579E6 | | 4.0965E6 | | | | | | | | | 2 | 0 | 0 | 0 | 0 | 0 | 1 | 0 | 1 | 0 | 0 | 0 | 0 | 0 | 0 | 0 | 0 | 113 | 122 | Carbamidomethylation | C5:Carbamidomethylation:1000.00 | PEAKS DB |
| R.FVC(+57.02)DC(+57.02)DRTAAIC(+57.02)F.A | N | 47.20 | 1633.6687 | 13 | 5.0 | 817.8416 | 2 | 60.81 | 15 | F15:36004 | BuCaTN01_F12a.raw | | | | | | | | | | | | | | | 1.0006E7 | | 1 | 0 | 0 | 0 | 0 | 0 | 0 | 0 | 0 | 0 | 0 | 0 | 0 | 0 | 0 | 1 | 0 | 106 | 118 | Carbamidomethylation | C3:Carbamidomethylation:1000.00;C5:Carbamidomethylation:1000.00;C12:Carbamidomethylation:1000.00 | PEAKS DB |
| R.FVC(+57.02)DC(+57.02)DRTAAIC(+57.02)FAKAPY.N | Y | 46.45 | 2163.9539 | 18 | 1.6 | 722.3247 | 3 | 61.77 | 12 | F12:37977 | BuCaTN01_F10b.raw | | | | | | | | | | | | 3.9497E6 | | | 1.3752E7 | | 2 | 0 | 0 | 0 | 0 | 0 | 0 | 0 | 0 | 0 | 0 | 0 | 1 | 0 | 0 | 1 | 0 | 106 | 123 | Carbamidomethylation | C3:Carbamidomethylation:1000.00;C5:Carbamidomethylation:1000.00;C12:Carbamidomethylation:1000.00 | PEAKS DB |
| R.FVC(+57.02)DC(+57.02)DRTAAIC(+57.02)FAKA.P | Y | 45.80 | 1903.8379 | 16 | 1.7 | 952.9250 | 2 | 55.04 | 8 | F8:30870 | BuCaTN01_F7b.raw | | | | | | | | 2.9142E5 | | | | | | | | | 1 | 0 | 0 | 0 | 0 | 0 | 0 | 0 | 1 | 0 | 0 | 0 | 0 | 0 | 0 | 0 | 0 | 106 | 121 | Carbamidomethylation | C3:Carbamidomethylation:1000.00;C5:Carbamidomethylation:1000.00;C12:Carbamidomethylation:1000.00 | PEAKS DB |
| **total 17 peptides** | | | | | | | | | | | | | | | | | | | | | | | | | | | | | | | | | | | | | | | | | | | | | | | | | |

**Best Unique PSM (Scan F15:26245, m/z=917.4072, z=2, RT=49.59, ppm=4.5):**

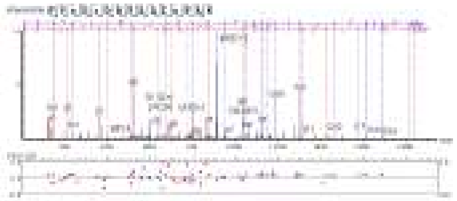

## 1TC8

back to list

| Protein Coverage | Supporting Peptides | Best Unique PSM |

**Protein Coverage:**

1 NLYQLMNMIQ CANTRTWPSY TNYGCYCGKG GSGTPVDDLD RCCYTHDHCY NDAKNIDGCN PVTKTYSYTC TEPTITCNDS  C Carbamidomethylation (+57.02)

81 KDKCARFVCD CDRTAAICFA KAPYNTSNVM IRSTNSCQ

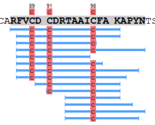

**Supporting Peptides:**

| Peptide | Uniq | -10lgP | Mass | Length | ppm | m/z | z | RT | Fraction | Scan | Source File | Area F1 | Area F2a | Area F3a | Area F4 | Area F5 | Area F6a | Area F7a | Area F7b | Area F8 | Area F9a | Area Sample 1F10a | Area F10b | Area F11a | Area F11b | Area F12a | Area F13 | #Feature | #Feature F1 | #Feature F2a | #Feature F3a | #Feature F4 | #Feature F5 | #Feature F6a | #Feature F7a | #Feature F7b | #Feature F8 | #Feature F9a | #Feature Sample 1F10a | #Feature F10b | #Feature F11a | #Feature F11b | #Feature F12a | #Feature F13 | Start | End | PTM | AScore | Found By |
|---|---|---|---|---|---|---|---|---|---|---|---|---|---|---|---|---|---|---|---|---|---|---|---|---|---|---|---|---|---|---|---|---|---|---|---|---|---|---|---|---|---|---|---|---|---|---|---|---|---|
| R.FVC(+57.02)DC(+57.02)DRTAAIC(+57.02)FAK.A | Y | 114.63 | 1832.8008 | 15 | 4.5 | 917.4072 | 2 | 49.59 | 15 | F15:26245 | BuCaTN01_F12a.raw | | | | | | 2.5208E7 | 1.6707E8 | 4.9947E8 | | 2.6145E9 | 5.2226E7 | 6.4722E9 | 1.7192E7 | 1.7308E9 | 3.9989E9 | | 42 | 0 | 0 | 0 | 0 | 0 | 2 | 4 | 3 | 0 | 6 | 3 | 9 | 2 | 4 | 9 | 0 | 87 | 101 | Carbamidomethylation | C3:Carbamidomethylation:1000.00;C5:Carbamidomethylation:1000.00;C12:Carbamidomethylation:1000.00 | PEAKS DB |
| A.RFVC(+57.02)DC(+57.02)DRTAAIC(+57.02)FAK.A | Y | 78.26 | 1988.9019 | 16 | 1.8 | 498.2325 | 4 | 47.48 | 12 | F12:24632 | BuCaTN01_F10b.raw | | | | | | | | 1.808E6 | | | 9.0711E5 | 2.0703E8 | | 7.0193E6 | | | 8 | 0 | 0 | 0 | 0 | 0 | 0 | 0 | 1 | 0 | 0 | 1 | 4 | 0 | 2 | 0 | 0 | 86 | 101 | Carbamidomethylation | C4:Carbamidomethylation:1000.00;C6:Carbamidomethylation:1000.00;C13:Carbamidomethylation:1000.00 | PEAKS DB |
| R.TAAIC(+57.02)FAKAPY.N | N | 73.24 | 1211.6008 | 11 | 2.2 | 606.8076 | 2 | 55.84 | 12 | F12:32023 | BuCaTN01_F10b.raw | | | | | | | 7.3334E4 | | | 1.7166E6 | 1.3753E5 | 1.06E7 | | 8.1407E6 | 9.828E5 | | 7 | 0 | 0 | 0 | 0 | 0 | 0 | 1 | 0 | 0 | 1 | 1 | 2 | 0 | 1 | 1 | 0 | 94 | 104 | Carbamidomethylation | C5:Carbamidomethylation:1000.00 | PEAKS DB |
| R.FVC(+57.02)DC(+57.02)DRTAAIC(+57.02)FAKAPYN.T | Y | 71.28 | 2277.9968 | 19 | 0.1 | 760.3397 | 3 | 58.06 | 7 | F7:30880 | BuCaTN01_F7a.raw | | | | | | 1.4903E7 | 2.0995E6 | 8.9111E7 | | 1.7533E7 | | | | | 3.5967E6 | | 7 | 0 | 0 | 0 | 0 | 0 | 2 | 1 | 2 | 0 | 1 | 0 | 0 | 0 | 0 | 1 | 0 | 87 | 105 | Carbamidomethylation | C3:Carbamidomethylation:1000.00;C5:Carbamidomethylation:1000.00;C12:Carbamidomethylation:1000.00 | PEAKS DB |
| R.TAAIC(+57.02)FAKAPYN.T | N | 69.35 | 1325.6438 | 12 | -0.2 | 663.8290 | 2 | 51.91 | 6 | F6:30474 | BuCaTN01_F6a.raw | | | | | | 3.7675E6 | | 1.5632E7 | | 4.6358E6 | | 1.9346E6 | | | | | 5 | 0 | 0 | 0 | 0 | 0 | 1 | 0 | 2 | 0 | 1 | 0 | 1 | 0 | 0 | 0 | 0 | 94 | 105 | Carbamidomethylation | C5:Carbamidomethylation:1000.00 | PEAKS DB |
| K.GGSGTPVDDLDR.C | N | 69.16 | 1187.5417 | 12 | -1.2 | 594.7775 | 2 | 38.65 | 10 | F10:17034 | BuCaTN01_F9a.raw | | | | | | | | | | 5.2316E6 | | | | | | | 1 | 0 | 0 | 0 | 0 | 0 | 0 | 0 | 0 | 0 | 1 | 0 | 0 | 0 | 0 | 0 | 0 | 30 | 41 | | | PEAKS DB |
| D.C(+57.02)DRTAAIC(+57.02)FAK.A | N | 63.48 | 1311.6063 | 11 | -1.7 | 438.2076 | 3 | 37.36 | 12 | F12:15762 | BuCaTN01_F10b.raw | | | | | | | | | | | | 5.2815E4 | | 3.1918E5 | | | 2 | 0 | 0 | 0 | 0 | 0 | 0 | 0 | 0 | 0 | 0 | 0 | 1 | 0 | 1 | 0 | 0 | 91 | 101 | Carbamidomethylation | C1:Carbamidomethylation:1000.00;C8:Carbamidomethylation:1000.00 | PEAKS DB |
| R.FVC(+57.02)DC(+57.02)DRTAAIC(+57.02).F | N | 62.55 | 1486.6003 | 12 | 2.9 | 744.3079 | 2 | 41.59 | 12 | F12:19336 | BuCaTN01_F10b.raw | | | | | | | 3.2591E5 | 5.6967E6 | | 1.9877E6 | | 2.0528E7 | | 2.7882E6 | 1.1445E7 | | 7 | 0 | 0 | 0 | 0 | 0 | 0 | 1 | 1 | 0 | 1 | 0 | 2 | 0 | 1 | 1 | 0 | 87 | 98 | Carbamidomethylation | C3:Carbamidomethylation:1000.00;C5:Carbamidomethylation:1000.00;C12:Carbamidomethylation:1000.00 | PEAKS DB |
| C.DC(+57.02)DRTAAIC(+57.02)FAK.A | Y | 60.32 | 1426.6333 | 12 | 2.6 | 476.5518 | 3 | 40.33 | 12 | F12:18176 | BuCaTN01_F10b.raw | | | | | | | | | | | | 0 | | 4.6798E5 | | | 1 | 0 | 0 | 0 | 0 | 0 | 0 | 0 | 0 | 0 | 0 | 0 | 0 | 0 | 1 | 0 | 0 | 90 | 101 | Carbamidomethylation | C2:Carbamidomethylation:1000.00;C9:Carbamidomethylation:1000.00 | PEAKS DB |
| R.TAAIC(+57.02)FAK.A | N | 60.21 | 880.4476 | 8 | 3.4 | 441.2316 | 2 | 41.58 | 12 | F12:19288 | BuCaTN01_F10b.raw | | | | | | 9.3985E4 | 9.2429E6 | 1.2777E7 | 4.6024E5 | 3.2388E7 | 3.591E6 | 1.265E8 | 3.5731E6 | 1.1283E8 | 1.0943E8 | | 21 | 0 | 0 | 0 | 0 | 0 | 1 | 4 | 2 | 1 | 2 | 2 | 2 | 1 | 2 | 4 | 0 | 94 | 101 | Carbamidomethylation | C5:Carbamidomethylation:1000.00 | PEAKS DB |
| R.FVC(+57.02)DC(+57.02)DRTAAI.C | N | 53.25 | 1326.5696 | 11 | -0.5 | 664.2917 | 2 | 44.27 | 7 | F7:19638 | BuCaTN01_F7a.raw | | | | | | | 4.353E5 | 1.0134E6 | | | | | | | 6.8105E6 | | 3 | 0 | 0 | 0 | 0 | 0 | 0 | 1 | 1 | 0 | 0 | 0 | 0 | 0 | 0 | 1 | 0 | 87 | 97 | Carbamidomethylation | C3:Carbamidomethylation:1000.00;C5:Carbamidomethylation:1000.00 | PEAKS DB |
| S.GTPVDDLDR.C | N | 51.39 | 986.4669 | 9 | 0.6 | 494.2410 | 2 | 37.62 | 7 | F7:14221 | BuCaTN01_F7a.raw | | | | | | | 4.0904E6 | | | | | | | | | | 1 | 0 | 0 | 0 | 0 | 0 | 0 | 1 | 0 | 0 | 0 | 0 | 0 | 0 | 0 | 0 | 0 | 33 | 41 | | | PEAKS DB |
| G.TPVDDLDR.C | N | 48.70 | 929.4454 | 8 | -0.4 | 465.7298 | 2 | 34.76 | 7 | F7:11899 | BuCaTN01_F7a.raw | | | | | | | 6.3757E5 | | | | | | | | | | 1 | 0 | 0 | 0 | 0 | 0 | 0 | 1 | 0 | 0 | 0 | 0 | 0 | 0 | 0 | 0 | 0 | 34 | 41 | | | PEAKS DB |
| R.TAAIC(+57.02)FAKAP.Y | N | 47.25 | 1048.5375 | 10 | 3.6 | 525.2764 | 2 | 48.84 | 8 | F8:25881 | BuCaTN01_F7b.raw | | | | | | 3.1579E6 | | 4.0965E6 | | | | | | | | | 2 | 0 | 0 | 0 | 0 | 0 | 1 | 0 | 1 | 0 | 0 | 0 | 0 | 0 | 0 | 0 | 0 | 94 | 103 | Carbamidomethylation | C5:Carbamidomethylation:1000.00 | PEAKS DB |
| R.FVC(+57.02)DC(+57.02)DRTAAIC(+57.02)F.A | N | 47.20 | 1633.6687 | 13 | 5.0 | 817.8416 | 2 | 60.81 | 15 | F15:36004 | BuCaTN01_F12a.raw | | | | | | | | | | | | | | | 1.0006E7 | | 1 | 0 | 0 | 0 | 0 | 0 | 0 | 0 | 0 | 0 | 0 | 0 | 0 | 0 | 0 | 1 | 0 | 87 | 99 | Carbamidomethylation | C3:Carbamidomethylation:1000.00;C5:Carbamidomethylation:1000.00;C12:Carbamidomethylation:1000.00 | PEAKS DB |
| R.FVC(+57.02)DC(+57.02)DRTAAIC(+57.02)FAKAPY.N | Y | 46.45 | 2163.9539 | 18 | 1.6 | 722.3247 | 3 | 61.77 | 12 | F12:37977 | BuCaTN01_F10b.raw | | | | | | | | | | | | 3.9497E6 | | | 1.3752E7 | | 2 | 0 | 0 | 0 | 0 | 0 | 0 | 0 | 0 | 0 | 0 | 0 | 1 | 0 | 0 | 1 | 0 | 87 | 104 | Carbamidomethylation | C3:Carbamidomethylation:1000.00;C5:Carbamidomethylation:1000.00;C12:Carbamidomethylation:1000.00 | PEAKS DB |
| R.FVC(+57.02)DC(+57.02)DRTAAIC(+57.02)FAKA.P | Y | 45.80 | 1903.8379 | 16 | 1.7 | 952.9250 | 2 | 55.04 | 8 | F8:30870 | BuCaTN01_F7b.raw | | | | | | | | 2.9142E5 | | | | | | | | | 1 | 0 | 0 | 0 | 0 | 0 | 0 | 0 | 1 | 0 | 0 | 0 | 0 | 0 | 0 | 0 | 0 | 87 | 102 | Carbamidomethylation | C3:Carbamidomethylation:1000.00;C5:Carbamidomethylation:1000.00;C12:Carbamidomethylation:1000.00 | PEAKS DB |
| **total 17 peptides** | | | | | | | | | | | | | | | | | | | | | | | | | | | | | | | | | | | | | | | | | | | | | | | | | |

**Best Unique PSM (Scan F15:26245, m/z=917.4072, z=2, RT=49.59, ppm=4.5):**

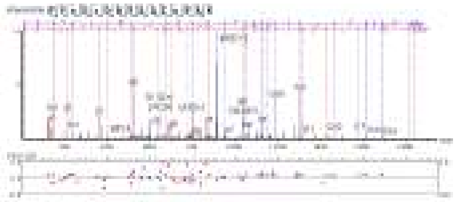

## 1PO8

back to list

| Protein Coverage | Supporting Peptides | Best Unique PSM |

**Protein Coverage:**

1 NLYQLMNMIQ CANTRTWPSY TNYGCYCGKG GSGTPVDDLD RCCYTHDHCY NDAKNIDGCN PVTKTYSYTC TEPTITCNDS  C Carbamidomethylation (+57.02)

81 KDKCARFVCD CDRTAAICFA KAPYNTSNVM IRSTNSCQ

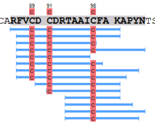

**Supporting Peptides:**

| Peptide | Uniq | -10lgP | Mass | Length | ppm | m/z | z | RT | Fraction | Scan | Source File | Area F1 | Area F2a | Area F3a | Area F4 | Area F5 | Area F6a | Area F7a | Area F7b | Area F8 | Area F9a | Area Sample 1F10a | Area F10b | Area F11a | Area F11b | Area F12a | Area F13 | #Feature | #Feature F1 | #Feature F2a | #Feature F3a | #Feature F4 | #Feature F5 | #Feature F6a | #Feature F7a | #Feature F7b | #Feature F8 | #Feature F9a | #Feature Sample 1F10a | #Feature F10b | #Feature F11a | #Feature F11b | #Feature F12a | #Feature F13 | Start | End | PTM | AScore | Found By |
|---|---|---|---|---|---|---|---|---|---|---|---|---|---|---|---|---|---|---|---|---|---|---|---|---|---|---|---|---|---|---|---|---|---|---|---|---|---|---|---|---|---|---|---|---|---|---|---|---|---|
| R.FVC(+57.02)DC(+57.02)DRTAAIC(+57.02)FAK.A | Y | 114.63 | 1832.8008 | 15 | 4.5 | 917.4072 | 2 | 49.59 | 15 | F15:26245 | BuCaTN01_F12a.raw | | | | | | 2.5208E7 | 1.6707E8 | 4.9947E8 | | 2.6145E9 | 5.2226E7 | 6.4722E9 | 1.7192E7 | 1.7308E9 | 3.9989E9 | | 42 | 0 | 0 | 0 | 0 | 0 | 2 | 4 | 3 | 0 | 6 | 3 | 9 | 2 | 4 | 9 | 0 | 87 | 101 | Carbamidomethylation | C3:Carbamidomethylation:1000.00;C5:Carbamidomethylation:1000.00;C12:Carbamidomethylation:1000.00 | PEAKS |

| | | | | | | | | | | | | | | | | | | | | | | | | | | | | | | | | | | | | | | | | | | | | | | | | | |
|---|---|---|---|---|---|---|---|---|---|---|---|---|---|---|---|---|---|---|---|---|---|---|---|---|---|---|---|---|---|---|---|---|---|---|---|---|---|---|---|---|---|---|---|---|---|---|---|---|---|
| | | | | | | | | | | | | | | | | | | | | | | | | | | | | | | | | | | | | | | | | | | | | | | | | hylation:1000.00 | DB |
| A.RFVC(+57.02)DC(+57.02)DRTAAIC(+57.02)FAK.A | Y | 78.26 | 1988.9019 | 16 | 1.8 | 498.2325 | 4 | 47.48 | 12 | F12:24632 | BuCaTN01_F10b.raw | | | | | | | | 1.808E6 | | | 9.0711E5 | 2.0703E8 | | 7.0193E6 | | | 8 | 0 | 0 | 0 | 0 | 0 | 0 | 0 | 1 | 0 | 0 | 1 | 4 | 0 | 2 | 0 | 0 | 86 | 101 | Carbamidomethylation | C4:Carbamidomethylation:1000.00;C6:Carbamidomethylation:1000.00;C13:Carbamidomethylation:1000.00 | PEAKS DB |
| R.TAAIC(+57.02)FAKAPY.N | N | 73.24 | 1211.6008 | 11 | 2.2 | 606.8076 | 2 | 55.84 | 12 | F12:32023 | BuCaTN01_F10b.raw | | | | | | | 7.3334E4 | | | 1.7166E6 | 1.3753E5 | 1.06E7 | | 8.1407E6 | 9.828E5 | | 7 | 0 | 0 | 0 | 0 | 0 | 0 | 1 | 0 | 0 | 1 | 1 | 2 | 0 | 1 | 1 | 0 | 94 | 104 | Carbamidomethylation | C5:Carbamidomethylation:1000.00 | PEAKS DB |
| R.FVC(+57.02)DC(+57.02)DRTAAIC(+57.02)FAKAPYN.T | Y | 71.28 | 2277.9968 | 19 | 0.1 | 760.3397 | 3 | 58.06 | 7 | F7:30880 | BuCaTN01_F7a.raw | | | | | | 1.4903E7 | 2.0995E6 | 8.9111E7 | | 1.7533E7 | | | | | 3.5967E6 | | 7 | 0 | 0 | 0 | 0 | 0 | 2 | 1 | 2 | 0 | 1 | 0 | 0 | 0 | 0 | 1 | 0 | 87 | 105 | Carbamidomethylation | C3:Carbamidomethylation:1000.00;C5:Carbamidomethylation:1000.00;C12:Carbamidomethylation:1000.00 | PEAKS DB |
| R.TAAIC(+57.02)FAKAPYN.T | N | 69.35 | 1325.6438 | 12 | -0.2 | 663.8290 | 2 | 51.91 | 6 | F6:30474 | BuCaTN01_F6a.raw | | | | | | 3.7675E6 | | 1.5632E7 | | 4.6358E6 | | 1.9346E6 | | | | | 5 | 0 | 0 | 0 | 0 | 0 | 1 | 0 | 2 | 0 | 1 | 0 | 1 | 0 | 0 | 0 | 0 | 94 | 105 | Carbamidomethylation | C5:Carbamidomethylation:1000.00 | PEAKS DB |
| K.GGSGTPVDDLDR.C | N | 69.16 | 1187.5417 | 12 | -1.2 | 594.7775 | 2 | 38.65 | 10 | F10:17034 | BuCaTN01_F9a.raw | | | | | | | | | | 5.2316E6 | | | | | | | 1 | 0 | 0 | 0 | 0 | 0 | 0 | 0 | 0 | 0 | 1 | 0 | 0 | 0 | 0 | 0 | 0 | 30 | 41 | | | PEAKS DB |
| D.C(+57.02)DRTAAIC(+57.02)FAK.A | N | 63.48 | 1311.6063 | 11 | -1.7 | 438.2076 | 3 | 37.36 | 12 | F12:15762 | BuCaTN01_F10b.raw | | | | | | | | | | | | 5.2815E4 | | 3.1918E5 | | | 2 | 0 | 0 | 0 | 0 | 0 | 0 | 0 | 0 | 0 | 0 | 0 | 1 | 0 | 1 | 0 | 0 | 91 | 101 | Carbamidomethylation | C1:Carbamidomethylation:1000.00;C8:Carbamidomethylation:1000.00 | PEAKS DB |
| R.FVC(+57.02)DC(+57.02)DRTAAIC(+57.02).F | N | 62.55 | 1486.6003 | 12 | 2.9 | 744.3079 | 2 | 41.59 | 12 | F12:19336 | BuCaTN01_F10b.raw | | | | | | | 3.2591E5 | 5.6967E6 | | 1.9877E6 | | 2.0528E7 | | 2.7882E6 | 1.1445E7 | | 7 | 0 | 0 | 0 | 0 | 0 | 0 | 1 | 1 | 0 | 1 | 0 | 2 | 0 | 1 | 1 | 0 | 87 | 98 | Carbamidomethylation | C3:Carbamidomethylation:1000.00;C5:Carbamidomethylation:1000.00;C12:Carbamidomethylation:1000.00 | PEAKS DB |
| C.DC(+57.02)DRTAAIC(+57.02)FAK.A | Y | 60.32 | 1426.6333 | 12 | 2.6 | 476.5518 | 3 | 40.33 | 12 | F12:18176 | BuCaTN01_F10b.raw | | | | | | | | | | | | 0 | | 4.6798E5 | | | 1 | 0 | 0 | 0 | 0 | 0 | 0 | 0 | 0 | 0 | 0 | 0 | 0 | 0 | 1 | 0 | 0 | 90 | 101 | Carbamidomethylation | C2:Carbamidomethylation:1000.00;C9:Carbamidomethylation:1000.00 | PEAKS DB |
| R.TAAIC(+57.02)FAK.A | N | 60.21 | 880.4476 | 8 | 3.4 | 441.2316 | 2 | 41.58 | 12 | F12:19288 | BuCaTN01_F10b.raw | | | | | | 9.3985E4 | 9.2429E6 | 1.2777E7 | 4.6024E5 | 3.2388E7 | 3.591E6 | 1.265E8 | 3.5731E6 | 1.1283E8 | 1.0943E8 | | 21 | 0 | 0 | 0 | 0 | 0 | 1 | 4 | 2 | 1 | 2 | 2 | 2 | 1 | 2 | 4 | 0 | 94 | 101 | Carbamidomethylation | C5:Carbamidomethylation:1000.00 | PEAKS DB |
| R.FVC(+57.02)DC(+57.02)DRTAAI.C | N | 53.25 | 1326.5696 | 11 | -0.5 | 664.2917 | 2 | 44.27 | 7 | F7:19638 | BuCaTN01_F7a.raw | | | | | | | 4.353E5 | 1.0134E6 | | | | | | | 6.8105E6 | | 3 | 0 | 0 | 0 | 0 | 0 | 0 | 1 | 1 | 0 | 0 | 0 | 0 | 0 | 0 | 1 | 0 | 87 | 97 | Carbamidomethylation | C3:Carbamidomethylation:1000.00;C5:Carbamidomethylation:1000.00 | PEAKS DB |
| S.GTPVDDLDR.C | N | 51.39 | 986.4669 | 9 | 0.6 | 494.2410 | 2 | 37.62 | 7 | F7:14221 | BuCaTN01_F7a.raw | | | | | | | 4.0904E6 | | | | | | | | | | 1 | 0 | 0 | 0 | 0 | 0 | 0 | 1 | 0 | 0 | 0 | 0 | 0 | 0 | 0 | 0 | 0 | 33 | 41 | | | PEAKS DB |
| G.TPVDDLDR.C | N | 48.70 | 929.4454 | 8 | -0.4 | 465.7298 | 2 | 34.76 | 7 | F7:11899 | BuCaTN01_F7a.raw | | | | | | | 6.3757E5 | | | | | | | | | | 1 | 0 | 0 | 0 | 0 | 0 | 0 | 1 | 0 | 0 | 0 | 0 | 0 | 0 | 0 | 0 | 0 | 34 | 41 | | | PEAKS DB |
| R.TAAIC(+57.02)FAKAP.Y | N | 47.25 | 1048.5375 | 10 | 3.6 | 525.2764 | 2 | 48.84 | 8 | F8:25881 | BuCaTN01_F7b.raw | | | | | | 3.1579E6 | | 4.0965E6 | | | | | | | | | 2 | 0 | 0 | 0 | 0 | 0 | 1 | 0 | 1 | 0 | 0 | 0 | 0 | 0 | 0 | 0 | 0 | 94 | 103 | Carbamidomethylation | C5:Carbamidomethylation:1000.00 | PEAKS DB |
| R.FVC(+57.02)DC(+57.02)DRTAAIC(+57.02)F.A | N | 47.20 | 1633.6687 | 13 | 5.0 | 817.8416 | 2 | 60.81 | 15 | F15:36004 | BuCaTN01_F12a.raw | | | | | | | | | | | | | | | 1.0006E7 | | 1 | 0 | 0 | 0 | 0 | 0 | 0 | 0 | 0 | 0 | 0 | 0 | 0 | 0 | 0 | 1 | 0 | 87 | 99 | Carbamidomethylation | C3:Carbamidomethylation:1000.00;C5:Carbamidomethylation:1000.00;C12:Carbamidomethylation:1000.00 | PEAKS DB |
| R.FVC(+57.02)DC(+57.02)DRTAAIC(+57.02)FAKAPY.N | Y | 46.45 | 2163.9539 | 18 | 1.6 | 722.3247 | 3 | 61.77 | 12 | F12:37977 | BuCaTN01_F10b.raw | | | | | | | | | | | | 3.9497E6 | | | 1.3752E7 | | 2 | 0 | 0 | 0 | 0 | 0 | 0 | 0 | 0 | 0 | 0 | 0 | 1 | 0 | 0 | 1 | 0 | 87 | 104 | Carbamidomethylation | C3:Carbamidomethylation:1000.00;C5:Carbamidomethylation:1000.00;C12:Carbamidomethylation:1000.00 | PEAKS DB |
| R.FVC(+57.02)DC(+57.02)DRTAAIC(+57.02)FAKA.P | Y | 45.80 | 1903.8379 | 16 | 1.7 | 952.9250 | 2 | 55.04 | 8 | F8:30870 | BuCaTN01_F7b.raw | | | | | | | | 2.9142E5 | | | | | | | | | 1 | 0 | 0 | 0 | 0 | 0 | 0 | 0 | 1 | 0 | 0 | 0 | 0 | 0 | 0 | 0 | 0 | 87 | 102 | Carbamidomethylation | C3:Carbamidomethylation:1000.00;C5:Carbamidomethylation:1000.00;C12:Carbamidomethylation:1000.00 | PEAKS DB |
| **total 17 peptides** | | | | | | | | | | | | | | | | | | | | | | | | | | | | | | | | | | | | | | | | | | | | | | | | | |

**Best Unique PSM (Scan F15:26245, m/z=917.4072, z=2, RT=49.59, ppm=4.5):**

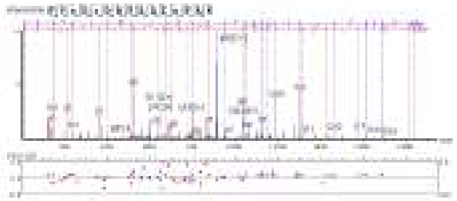

## ABU63166.1

[back to list]

| **Protein Coverage** | **Supporting Peptides** | **Best Unique PSM** |

**Protein Coverage:**

1 MNPAHLLVLS AVCVSLLGAA NIPPQPLNLF QFENMIECAG TRSWTHYVSY GCYCGYGGSG TPVDELDRCC YVHDHCYGEA

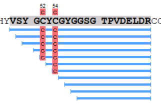

C Carbamidomethylation (+57.02)

81 EKIFKCNPKT KTYSYTCNKP DLTRTDAKGT CERFVCDCDR AAAICFAAAP YNNNNFMMKP KTHC

**Supporting Peptides:**

| Peptide | Uniq | -10lgP | Mass | Length | ppm | m/z | z | RT | Fraction | Scan | Source File | Area F1 | Area F2a | Area F3a | Area F4 | Area F5 | Area F6a | Area F7a | Area F7b | Area F8 | Area F9a | Area Sample 1F10a | Area F10b | Area F11a | Area F11b | Area F12a | Area F13 | #Feature | #Feature F1 | #Feature F2a | #Feature F3a | #Feature F4 | #Feature F5 | #Feature F6a | #Feature F7a | #Feature F7b | #Feature F8 | #Feature F9a | #Feature Sample 1F10a | #Feature F10b | #Feature F11a | #Feature F11b | #Feature F12a | #Feature F13 | Start | End | PTM | AScore | Found By |
|---|---|---|---|---|---|---|---|---|---|---|---|---|---|---|---|---|---|---|---|---|---|---|---|---|---|---|---|---|---|---|---|---|---|---|---|---|---|---|---|---|---|---|---|---|---|---|---|---|---|
| S.YGC(+57.02)YC(+57.02)GYGGSGTPVDELDR.C | N | 112.36 | 2124.8516 | 19 | 3.7 | 1063.4326 | 2 | 56.60 | 14 | F14:33384 | BuCaTN01_F11b.raw | | | 0 | | | 2.0097E6 | | 1.4053E7 | | 8.8531E7 | 3.4891E6 | 4.8054E8 | 6.165E7 | 1.7851E9 | 1.2628E8 | | 69 | 0 | 0 | 0 | 0 | 0 | 1 | 0 | 2 | 0 | 3 | 1 | 4 | 2 | 53 | 3 | 0 | 50 | 68 | Carbamidomethylation | C3:Carbamidomethylation:1000.00;C5:Carbamidomethylation:1000.00 | PEAKS DB |
| Y.C(+57.02)GYGGSGTPVDELDR.C | N | 88.12 | 1581.6729 | 15 | 4.4 | 791.8439 | 2 | 48.02 | 14 | F14:25306 | BuCaTN01_F11b.raw | | | | | | | | 2.166E6 | | 8.5786E6 | | 1.5853E7 | 6.495E5 | 1.7537E7 | 0 | | 6 | 0 | 0 | 0 | 0 | 0 | 0 | 0 | 1 | 0 | 1 | 0 | 2 | 1 | 1 | 0 | 0 | 54 | 68 | Carbamidomethylation | C1:Carbamidomethylation:1000.00 | PEAKS DB |
| G.C(+57.02)YC(+57.02)GYGGSGTPVDELDR.C | N | 84.40 | 1904.7668 | 17 | 2.7 | 953.3911 | 2 | 53.16 | 12 | F12:29351 | BuCaTN01_F10b.raw | | | | | | | | 4.1849E5 | | | | 3.7261E5 | | 2.1774E6 | | | 3 | 0 | 0 | 0 | 0 | 0 | 0 | 0 | 1 | 0 | 0 | 0 | 1 | 0 | 1 | 0 | 0 | 52 | 68 | Carbamidomethylation | C1:Carbamidomethylation:1000.00;C3:Carbamidomethylation:1000.00 | PEAKS DB |
| Y.GC(+57.02)YC(+57.02)GYGGSGTPVDELDR.C | N | 82.10 | 1961.7883 | 18 | 1.4 | 981.9028 | 2 | 53.49 | 10 | F10:30232 | BuCaTN01_F9a.raw | | | | | | | | 1.7852E6 | | 3.3077E6 | | 1.5846E6 | | 3.2964E6 | 1.0428E5 | | 5 | 0 | 0 | 0 | 0 | 0 | 0 | 0 | 1 | 0 | 1 | 0 | 1 | 0 | 1 | 1 | 0 | 51 | 68 | Carbamidomethylation | C2:Carbamidomethylation:1000.00;C4:Carbamidomethylation:1000.00 | PEAKS DB |
| C.YC(+57.02)GYGGSGTPVDELDR.C | N | 75.62 | 1744.7362 | 16 | 4.1 | 873.3754 | 2 | 52.27 | 14 | F14:28849 | BuCaTN01_F11b.raw | | | | | | | | 7.7286E5 | | 7.3447E5 | | | | 3.0904E6 | 1.4057E5 | | 4 | 0 | 0 | 0 | 0 | 0 | 0 | 0 | 1 | 0 | 1 | 0 | 0 | 0 | 1 | 1 | 0 | 53 | 68 | Carbamidomethylation | C2:Carbamidomethylation:1000.00 | PEAKS DB |
| G.YGGSGTPVDELDR.C | N | 75.26 | 1364.6207 | 13 | 4.1 | 683.3177 | 2 | 45.29 | 14 | F14:22502 | BuCaTN01_F11b.raw | | | | | | | 6.8893E5 | | | | | | | 9.1406E6 | 6.9945E5 | | 3 | 0 | 0 | 0 | 0 | 0 | 0 | 1 | 0 | 0 | 0 | 0 | 0 | 0 | 1 | 1 | 0 | 56 | 68 | | | PEAKS DB |
| C.GYGGSGTPVDELDR.C | N | 66.26 | 1421.6422 | 14 | 0.0 | 711.8284 | 2 | 46.82 | 10 | F10:24048 | BuCaTN01_F9a.raw | | | | | | | 2.9103E5 | 1.2275E6 | | 2.1867E6 | | | | 8.7883E6 | | | 5 | 0 | 0 | 0 | 0 | 0 | 0 | 1 | 1 | 0 | 1 | 0 | 0 | 0 | 2 | 0 | 0 | 55 | 68 | | | PEAKS DB |
| Y.VSYGC(+57.02)YC(+57.02)GYGGSGTPVDELDR.C | Y | 60.66 | 2310.9521 | 21 | 0.0 | 1156.4834 | 2 | 60.24 | 10 | F10:36465 | BuCaTN01_F9a.raw | | | | | | | | 1.1687E6 | | 5.7042E6 | | | | | | | 2 | 0 | 0 | 0 | 0 | 0 | 0 | 0 | 1 | 0 | 1 | 0 | 0 | 0 | 0 | 0 | 0 | 48 | 68 | Carbamidomethylation | C5:Carbamidomethylation:1000.00;C7:Carbamidomethylation:1000.00 | PEAKS DB |
| V.SYGC(+57.02)YC(+57.02)GYGGSGTPVDELDR.C | Y | 56.72 | 2211.8838 | 20 | -6.4 | 1106.9376 | 2 | 57.52 | 14 | F14:33849 | BuCaTN01_F11b.raw | | | | | | | | | | | | | | 8.9925E5 | | | 1 | 0 | 0 | 0 | 0 | 0 | 0 | 0 | 0 | 0 | 0 | 0 | 0 | 0 | 1 | 0 | 0 | 49 | 68 | Carbamidomethylation | C4:Carbamidomethylation:1000.00;C6:Carbamidomethylation:1000.00 | PEAKS DB |
| G.GSGTPVDELDR.C | N | 56.36 | 1144.5360 | 11 | 3.4 | 573.2756 | 2 | 40.15 | 8 | F8:17921 | BuCaTN01_F7b.raw | | | | | | | | 1.1068E6 | | | | | | | | | 1 | 0 | 0 | 0 | 0 | 0 | 0 | 0 | 1 | 0 | 0 | 0 | 0 | 0 | 0 | 0 | 0 | 58 | 68 | | | PEAKS DB |
| Y.GGSGTPVDELDR.C | N | 49.09 | 1201.5575 | 12 | 0.3 | 601.7862 | 2 | 40.57 | 11 | F11:14528 | BuCaTN01_F10a.raw | | | | | | | 9.2274E5 | 2.7577E6 | | 1.1335E7 | 6.7591E5 | | 9.9086E5 | 2.6759E7 | | | 6 | 0 | 0 | 0 | 0 | 0 | 0 | 1 | 1 | 0 | 1 | 1 | 0 | 1 | 1 | 0 | 0 | 57 | 68 | | | PEAKS DB |
| **total 11 peptides** | | | | | | | | | | | | | | | | | | | | | | | | | | | | | | | | | | | | | | | | | | | | | | | | | |

**Best Unique PSM (Scan F10:36465, m/z=1156.4834, z=2, RT=60.24, ppm=0.0):**

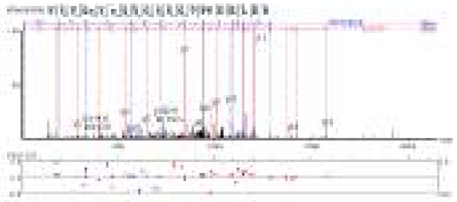

## ABU63164.1

[back to list]

| **Protein Coverage** | **Supporting Peptides** | **Best Unique PSM** |

**Protein Coverage:**

1 MNPAHLLVLS AVCVSLLGAA NIPPQPLNLF QFKNMIECAG TRSWTHYVSY GCYCGYGGSG TPVDELDRCC YVHDHCYGEA

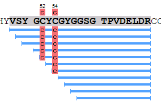

C Carbamidomethylation (+57.02)

81 EKIFKCNPKT KTYSYTCNKP DLTCTDAKGT CERFVCDCDR AAAICFAAAP YNNNNFMMKP KTHC

**Supporting Peptides:**

| Peptide | Uniq | -10lgP | Mass | Length | ppm | m/z | z | RT | Fraction | Scan | Source File | Area F1 | Area F2a | Area F3a | Area F4 | Area F5 | Area F6a | Area F7a | Area F7b | Area F8 | Area F9a | Area Sample 1F10a | Area F10b | Area F11a | Area F11b | Area F12a | Area F13 | #Feature | #Feature F1 | #Feature F2a | #Feature F3a | #Feature F4 | #Feature F5 | #Feature F6a | #Feature F7a | #Feature F7b | #Feature F8 | #Feature F9a | #Feature Sample 1F10a | #Feature F10b | #Feature F11a | #Feature F11b | #Feature F12a | #Feature F13 | Start | End | PTM | AScore | Found By |
|---|---|---|---|---|---|---|---|---|---|---|---|---|---|---|---|---|---|---|---|---|---|---|---|---|---|---|---|---|---|---|---|---|---|---|---|---|---|---|---|---|---|---|---|---|---|---|---|---|---|
| S.YGC(+57.02)YC(+57.02)GYGGSGTPVDELDR.C | N | 112.36 | 2124.8516 | 19 | 3.7 | 1063.4326 | 2 | 56.60 | 14 | F14:33384 | BuCaTN01_F11b.raw | | | 0 | | | 2.0097E6 | | 1.4053E7 | | 8.8531E7 | 3.4891E6 | 4.8054E8 | 6.165E7 | 1.7851E9 | 1.2628E8 | | 69 | 0 | 0 | 0 | 0 | 0 | 1 | 0 | 2 | 0 | 3 | 1 | 4 | 2 | 53 | 3 | 0 | 50 | 68 | Carbamidomethylation | C3:Carbamidomethylation:1000.00;C5:Carbamidomethylation:1000.00 | PEAKS DB |
| Y.C(+57.02)GYGGSGTPVDELDR.C | N | 88.12 | 1581.6729 | 15 | 4.4 | 791.8439 | 2 | 48.02 | 14 | F14:25306 | BuCaTN01_F11b.raw | | | | | | | | 2.166E6 | | 8.5786E6 | | 1.5853E7 | 6.495E5 | 1.7537E7 | 0 | | 6 | 0 | 0 | 0 | 0 | 0 | 0 | 0 | 1 | 0 | 1 | 0 | 2 | 1 | 1 | 0 | 0 | 54 | 68 | Carbamidomethylation | C1:Carbamidomethylation:1000.00 | PEAKS DB |
| G.C(+57.02)YC(+57.02)GYGGSGTPVDELDR.C | N | 84.40 | 1904.7668 | 17 | 2.7 | 953.3911 | 2 | 53.16 | 12 | F12:29351 | BuCaTN01_F10b.raw | | | | | | | | 4.1849E5 | | | | 3.7261E5 | | 2.1774E6 | | | 3 | 0 | 0 | 0 | 0 | 0 | 0 | 0 | 1 | 0 | 0 | 0 | 1 | 0 | 1 | 0 | 0 | 52 | 68 | Carbamidomethylation | C1:Carbamidomethylation:1000.00;C3:Carbamidomethylation:1000.00 | PEAKS DB |
| Y.GC(+57.02)YC(+57.02)GYGGSGTPVDELDR.C | N | 82.10 | 1961.7883 | 18 | 1.4 | 981.9028 | 2 | 53.49 | 10 | F10:30232 | BuCaTN01_F9a.raw | | | | | | | | 1.7852E6 | | 3.3077E6 | | 1.5846E6 | | 3.2964E6 | 1.0428E5 | | 5 | 0 | 0 | 0 | 0 | 0 | 0 | 0 | 1 | 0 | 1 | 0 | 1 | 0 | 1 | 1 | 0 | 51 | 68 | Carbamidomethylation | C2:Carbamidomethylation:1000.00;C4:Carbamidomethylation:1000.00 | PEAKS DB |
| | | | | | | | | | | | | | | | | | | | | | | | | | | | | | | | | | | | | | | | | | | | | | | | | | PEAKS |

| | | | | | | | | | | | | | | | | | | | | | | | | | | | | | | | | | | | | | | | | | | | | | | | | | |
|---|---|---|---|---|---|---|---|---|---|---|---|---|---|---|---|---|---|---|---|---|---|---|---|---|---|---|---|---|---|---|---|---|---|---|---|---|---|---|---|---|---|---|---|---|---|---|---|---|---|
| C.YC(+57.02)GYGGSGTPVDELDR.C | N | 75.62 | 1744.7362 | 16 | 4.1 | 873.3754 | 2 | 52.27 | 14 | F14:28849 | BuCaTN01_F11b.raw | | | | | | | | 7.7286E5 | | 7.3447E5 | | | | 3.0904E6 | 1.4057E5 | | 4 | 0 | 0 | 0 | 0 | 0 | 0 | 0 | 1 | 0 | 1 | 0 | 0 | 0 | 1 | 1 | 0 | 53 | 68 | Carbamidomethylation | C2:Carbamidomethylation:1000.00 | DB |
| G.YGGSGTPVDELDR.C | N | 75.26 | 1364.6207 | 13 | 4.1 | 683.3177 | 2 | 45.29 | 14 | F14:22502 | BuCaTN01_F11b.raw | | | | | | | 6.8893E5 | | | | | | | 9.1406E6 | 6.9945E5 | | 3 | 0 | 0 | 0 | 0 | 0 | 0 | 1 | 0 | 0 | 0 | 0 | 0 | 0 | 1 | 1 | 0 | 56 | 68 | | | PEAKS DB |
| C.GYGGSGTPVDELDR.C | N | 66.26 | 1421.6422 | 14 | 0.0 | 711.8284 | 2 | 46.82 | 10 | F10:24048 | BuCaTN01_F9a.raw | | | | | | | 2.9103E5 | 1.2275E6 | | 2.1867E6 | | | | 8.7883E6 | | | 5 | 0 | 0 | 0 | 0 | 0 | 0 | 1 | 1 | 0 | 1 | 0 | 0 | 0 | 2 | 0 | 0 | 55 | 68 | | | PEAKS DB |
| Y.VSYGC(+57.02)YC(+57.02)GYGGSGTPVDELDR.C | Y | 60.66 | 2310.9521 | 21 | 0.0 | 1156.4834 | 2 | 60.24 | 10 | F10:36465 | BuCaTN01_F9a.raw | | | | | | | | 1.1687E6 | | 5.7042E6 | | | | | | | 2 | 0 | 0 | 0 | 0 | 0 | 0 | 0 | 1 | 0 | 1 | 0 | 0 | 0 | 0 | 0 | 0 | 48 | 68 | Carbamidomethylation | C5:Carbamidomethylation:1000.00;C7:Carbamidomethylation:1000.00 | PEAKS DB |
| V.SYGC(+57.02)YC(+57.02)GYGGSGTPVDELDR.C | Y | 56.72 | 2211.8838 | 20 | -6.4 | 1106.9376 | 2 | 57.52 | 14 | F14:33849 | BuCaTN01_F11b.raw | | | | | | | | | | | | | | 8.9925E5 | | | 1 | 0 | 0 | 0 | 0 | 0 | 0 | 0 | 0 | 0 | 0 | 0 | 0 | 0 | 1 | 0 | 0 | 49 | 68 | Carbamidomethylation | C4:Carbamidomethylation:1000.00;C6:Carbamidomethylation:1000.00 | PEAKS DB |
| G.GSGTPVDELDR.C | N | 56.36 | 1144.5360 | 11 | 3.4 | 573.2756 | 2 | 40.15 | 8 | F8:17921 | BuCaTN01_F7b.raw | | | | | | | | 1.1068E6 | | | | | | | | | 1 | 0 | 0 | 0 | 0 | 0 | 0 | 0 | 1 | 0 | 0 | 0 | 0 | 0 | 0 | 0 | 0 | 58 | 68 | | | PEAKS DB |
| Y.GGSGTPVDELDR.C | N | 49.09 | 1201.5575 | 12 | 0.3 | 601.7862 | 2 | 40.57 | 11 | F11:14528 | BuCaTN01_F10a.raw | | | | | | | 9.2274E5 | 2.7577E6 | | 1.1335E7 | 6.7591E5 | | 9.9086E5 | 2.6759E7 | | | 6 | 0 | 0 | 0 | 0 | 0 | 0 | 1 | 1 | 0 | 1 | 1 | 0 | 1 | 1 | 0 | 0 | 57 | 68 | | | PEAKS DB |
| **total 11 peptides** | | | | | | | | | | | | | | | | | | | | | | | | | | | | | | | | | | | | | | | | | | | | | | | | | |

**Best Unique PSM (Scan F10:36465, m/z=1156.4834, z=2, RT=60.24, ppm=0.0):**

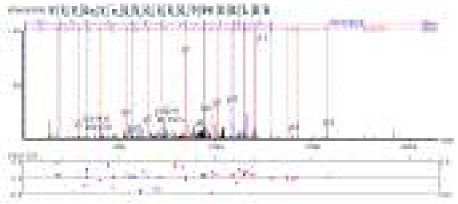

## C0HLB8.1

back to list

| Protein Coverage | Supporting Peptides | Best Unique PSM |

**Protein Coverage:**

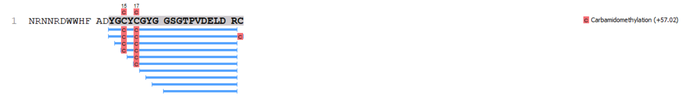


**Supporting Peptides:**

| Peptide | Uniq | -10lgP | Mass | Length | ppm | m/z | z | RT | Fraction | Scan | Source File | Area F1 | Area F2a | Area F3a | Area F4 | Area F5 | Area F6a | Area F7a | Area F7b | Area F8 | Area F9a | Area Sample 1F10a | Area F10b | Area F11a | Area F11b | Area F12a | Area F13 | #Feature | #Feature F1 | #Feature F2a | #Feature F3a | #Feature F4 | #Feature F5 | #Feature F6a | #Feature F7a | #Feature F7b | #Feature F8 | #Feature F9a | #Feature Sample 1F10a | #Feature F10b | #Feature F11a | #Feature F11b | #Feature F12a | #Feature F13 | Start | End | PTM | AScore | Found By |
|---|---|---|---|---|---|---|---|---|---|---|---|---|---|---|---|---|---|---|---|---|---|---|---|---|---|---|---|---|---|---|---|---|---|---|---|---|---|---|---|---|---|---|---|---|---|---|---|---|---|
| D.YGC(+57.02)YC(+57.02)GYGGSGTPVDELDR.C | N | 112.36 | 2124.8516 | 19 | 3.7 | 1063.4326 | 2 | 56.60 | 14 | F14:33384 | BuCaTN01_F11b.raw | | | 0 | | | 2.0097E6 | | 1.4053E7 | | 8.8531E7 | 3.4891E6 | 4.8054E8 | 6.165E7 | 1.7851E9 | 1.2628E8 | | 69 | 0 | 0 | 0 | 0 | 0 | 1 | 0 | 2 | 0 | 3 | 1 | 4 | 2 | 53 | 3 | 0 | 13 | 31 | Carbamidomethylation | C3:Carbamidomethylation:1000.00;C5:Carbamidomethylation:1000.00 | PEAKS DB |
| Y.C(+57.02)GYGGSGTPVDELDR.C | N | 88.12 | 1581.6729 | 15 | 4.4 | 791.8439 | 2 | 48.02 | 14 | F14:25306 | BuCaTN01_F11b.raw | | | | | | | | 2.166E6 | | 8.5786E6 | | 1.5853E7 | 6.495E5 | 1.7537E7 | 0 | | 6 | 0 | 0 | 0 | 0 | 0 | 0 | 0 | 1 | 0 | 1 | 0 | 2 | 1 | 1 | 0 | 0 | 17 | 31 | Carbamidomethylation | C1:Carbamidomethylation:1000.00 | PEAKS DB |
| G.C(+57.02)YC(+57.02)GYGGSGTPVDELDR.C | N | 84.40 | 1904.7668 | 17 | 2.7 | 953.3911 | 2 | 53.16 | 12 | F12:29351 | BuCaTN01_F10b.raw | | | | | | | | 4.1849E5 | | | | 3.7261E5 | | 2.1774E6 | | | 3 | 0 | 0 | 0 | 0 | 0 | 0 | 0 | 1 | 0 | 0 | 0 | 1 | 0 | 1 | 0 | 0 | 15 | 31 | Carbamidomethylation | C1:Carbamidomethylation:1000.00;C3:Carbamidomethylation:1000.00 | PEAKS DB |
| Y.GC(+57.02)YC(+57.02)GYGGSGTPVDELDR.C | N | 82.10 | 1961.7883 | 18 | 1.4 | 981.9028 | 2 | 53.49 | 10 | F10:30232 | BuCaTN01_F9a.raw | | | | | | | | 1.7852E6 | | 3.3077E6 | | 1.5846E6 | | 3.2964E6 | 1.0428E5 | | 5 | 0 | 0 | 0 | 0 | 0 | 0 | 0 | 1 | 0 | 1 | 0 | 1 | 0 | 1 | 1 | 0 | 14 | 31 | Carbamidomethylation | C2:Carbamidomethylation:1000.00;C4:Carbamidomethylation:1000.00 | PEAKS DB |
| C.YC(+57.02)GYGGSGTPVDELDR.C | N | 75.62 | 1744.7362 | 16 | 4.1 | 873.3754 | 2 | 52.27 | 14 | F14:28849 | BuCaTN01_F11b.raw | | | | | | | | 7.7286E5 | | 7.3447E5 | | | | 3.0904E6 | 1.4057E5 | | 4 | 0 | 0 | 0 | 0 | 0 | 0 | 0 | 1 | 0 | 1 | 0 | 0 | 0 | 1 | 1 | 0 | 16 | 31 | Carbamidomethylation | C2:Carbamidomethylation:1000.00 | PEAKS DB |
| G.YGGSGTPVDELDR.C | N | 75.26 | 1364.6207 | 13 | 4.1 | 683.3177 | 2 | 45.29 | 14 | F14:22502 | BuCaTN01_F11b.raw | | | | | | | 6.8893E5 | | | | | | | 9.1406E6 | 6.9945E5 | | 3 | 0 | 0 | 0 | 0 | 0 | 0 | 1 | 0 | 0 | 0 | 0 | 0 | 0 | 1 | 1 | 0 | 19 | 31 | | | PEAKS DB |
| C.GYGGSGTPVDELDR.C | N | 66.26 | 1421.6422 | 14 | 0.0 | 711.8284 | 2 | 46.82 | 10 | F10:24048 | BuCaTN01_F9a.raw | | | | | | | 2.9103E5 | 1.2275E6 | | 2.1867E6 | | | | 8.7883E6 | | | 5 | 0 | 0 | 0 | 0 | 0 | 0 | 1 | 1 | 0 | 1 | 0 | 0 | 0 | 2 | 0 | 0 | 18 | 31 | | | PEAKS DB |
| D.YGC(+57.02)YC(+57.02)GYGGSGTPVDELDRC(+57.02) | Y | 62.53 | 2284.8823 | 20 | 4.0 | 1143.4504 | 2 | 58.45 | 12 | F12:34536 | BuCaTN01_F10b.raw | | | | | | | | | | | | 1.5544E5 | | 1.0963E6 | | | 2 | 0 | 0 | 0 | 0 | 0 | 0 | 0 | 0 | 0 | 0 | 0 | 1 | 0 | 1 | 0 | 0 | 13 | 32 | Carbamidomethylation | C3:Carbamidomethylation:1000.00;C5:Carbamidomethylation:1000.00;C20:Carbamidomethylation:1000.00 | PEAKS DB |
| G.GSGTPVDELDR.C | N | 56.36 | 1144.5360 | 11 | 3.4 | 573.2756 | 2 | 40.15 | 8 | F8:17921 | BuCaTN01_F7b.raw | | | | | | | | 1.1068E6 | | | | | | | | | 1 | 0 | 0 | 0 | 0 | 0 | 0 | 0 | 1 | 0 | 0 | 0 | 0 | 0 | 0 | 0 | 0 | 21 | 31 | | | PEAKS DB |
| Y.GGSGTPVDELDR.C | N | 49.09 | 1201.5575 | 12 | 0.3 | 601.7862 | 2 | 40.57 | 11 | F11:14528 | BuCaTN01_F10a.raw | | | | | | | 9.2274E5 | 2.7577E6 | | 1.1335E7 | 6.7591E5 | | 9.9086E5 | 2.6759E7 | | | 6 | 0 | 0 | 0 | 0 | 0 | 0 | 1 | 1 | 0 | 1 | 1 | 0 | 1 | 1 | 0 | 0 | 20 | 31 | | | PEAKS DB |
| **total 10 peptides** | | | | | | | | | | | | | | | | | | | | | | | | | | | | | | | | | | | | | | | | | | | | | | | | | |

**Best Unique PSM (Scan F12:34536, m/z=1143.4504, z=2, RT=58.45, ppm=4.0):**

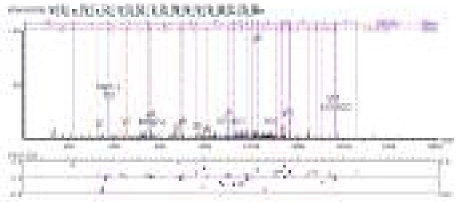

## P00606.2

back to list

| Protein Coverage | Supporting Peptides | Best Unique PSM |

**Protein Coverage:**

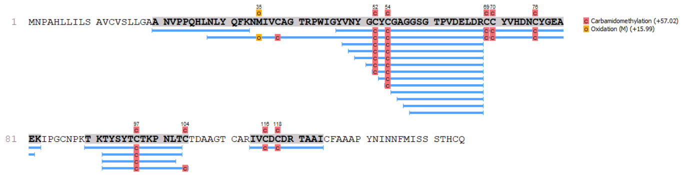


**Supporting Peptides:**

| Peptide | Uniq | -10lgP | Mass | Length | ppm | m/z | z | RT | Fraction | Scan | Source File | Area F1 | Area F2a | Area F3a | Area F4 | Area F5 | Area F6a | Area F7a | Area F7b | Area F8 | Area F9a | Area Sample 1F10a | Area F10b | Area F11a | Area F11b | Area F12a | Area F13 | #Feature | #Feature F1 | #Feature F2a | #Feature F3a | #Feature F4 | #Feature F5 | #Feature F6a | #Feature F7a | #Feature F7b | #Feature F8 | #Feature F9a | #Feature Sample 1F10a | #Feature F10b | #Feature F11a | #Feature F11b | #Feature F12a | #Feature F13 | Start | End | PTM | AScore | Found By |
|---|---|---|---|---|---|---|---|---|---|---|---|---|---|---|---|---|---|---|---|---|---|---|---|---|---|---|---|---|---|---|---|---|---|---|---|---|---|---|---|---|---|---|---|---|---|---|---|---|---|
| Y.VNYGC(+57.02)YC(+57.02)GAGGSGTPVDELDR.C | Y | 79.29 | 2245.9368 | 21 | 1.6 | 749.6523 | 3 | 55.84 | 12 | F12:31893 | BuCaTN01_F10b.raw | | | | | | | 8.8358E3 | 2.8116E5 | | 7.1274E5 | 2.079E5 | 7.0896E6 | | 2.5499E6 | | | 8 | 0 | 0 | 0 | 0 | 0 | 0 | 1 | 1 | 0 | 2 | 1 | 2 | 0 | 1 | 0 | 0 | 48 | 68 | Carbamidomethylation | C5:Carbamidomethylation:1000.00;C7:Carbamidomethylation:1000.00 | PEAKS DB |
| R.C(+57.02)C(+57.02)YVHDNC(+57.02)YGEAEK.I | N | 78.69 | 1803.6650 | 14 | 1.5 | 602.2285 | 3 | 33.04 | 12 | F12:12015 | BuCaTN01_F10b.raw | | | | | | | | | | | | 2.8186E7 | | 1.4756E7 | | | 4 | 0 | 0 | 0 | 0 | 0 | 0 | 0 | 0 | 0 | 0 | 0 | 2 | 0 | 2 | 0 | 0 | 69 | 82 | Carbamidomethylation | C1:Carbamidomethylation:1000.00;C2:Carbamidomethylation:1000.00;C8:Carbamidomethylation:1000.00 | PEAKS DB |
| C.YC(+57.02)GAGGSGTPVDELDR.C | N | 76.90 | 1652.7100 | 16 | 3.1 | 827.3629 | 2 | 45.67 | 12 | F12:22664 | BuCaTN01_F10b.raw | | | | | | | | | | 1.6847E5 | | 2.8019E6 | | 7.7562E5 | | | 3 | 0 | 0 | 0 | 0 | 0 | 0 | 0 | 0 | 0 | 1 | 0 | 1 | 0 | 1 | 0 | 0 | 53 | 68 | Carbamidomethylation | C2:Carbamidomethylation:1000.00 | PEAKS DB |
| Y.GC(+57.02)YC(+57.02)GAGGSGTPVDELDR.C | N | 69.43 | 1869.7621 | 18 | 1.9 | 935.8880 | 2 | 47.48 | 12 | F12:24483 | BuCaTN01_F10b.raw | | | | | | | | | | | | 2.3111E6 | | | | | 1 | 0 | 0 | 0 | 0 | 0 | 0 | 0 | 0 | 0 | 0 | 0 | 1 | 0 | 0 | 0 | 0 | 51 | 68 | Carbamidomethylation | C2:Carbamidomethylation:1000.00;C4:Carbamidomethylation:1000.00 | PEAKS DB |
| Y.C(+57.02)GAGGSGTPVDELDR.C | N | 67.25 | 1489.6467 | 15 | 4.1 | 745.8307 | 2 | 41.63 | 14 | F14:19582 | BuCaTN01_F11b.raw | | | | | | | | | | | 5.1011E5 | 3.3028E7 | | 9.0909E6 | | | 3 | 0 | 0 | 0 | 0 | 0 | 0 | 0 | 0 | 0 | 0 | 1 | 1 | 0 | 1 | 0 | 0 | 54 | 68 | Carbamidomethylation | C1:Carbamidomethylation:1000.00 | PEAKS DB |
| K.TYSYTC(+57.02)TKPNLT.C | N | 66.05 | 1447.6653 | 12 | 2.5 | 724.8387 | 2 | 43.32 | 14 | F14:21091 | BuCaTN01_F11b.raw | | | | | | | | 1.3481E6 | | | | 2.4842E6 | | 1.5203E7 | 9.0876E6 | | 5 | 0 | 0 | 0 | 0 | 0 | 0 | 0 | 1 | 0 | 0 | 0 | 1 | 0 | 2 | 1 | 0 | 92 | 103 | Carbamidomethylation | C6:Carbamidomethylation:1000.00 | PEAKS DB |
| N.YGC(+57.02)YC(+57.02)GAGGSGTPVDELDR.C | N | 65.18 | 2032.8254 | 19 | 7.1 | 1017.4242 | 2 | 52.27 | 8 | F8:28495 | BuCaTN01_F7b.raw | | | | | | | | 5.527E4 | | 2.3803E5 | | 1.1215E7 | | 1.5685E6 | | | 5 | 0 | 0 | 0 | 0 | 0 | 0 | 0 | 1 | 0 | 1 | 0 | 2 | 0 | 1 | 0 | 0 | 50 | 68 | Carbamidomethylation | C3:Carbamidomethylation:1000.00;C5:Carbamidomethylation:1000.00 | PEAKS DB |
| G.AGGSGTPVDELDR.C | N | 61.00 | 1272.5946 | 13 | 2.3 | 637.3046 | 2 | 39.79 | 12 | F12:17765 | BuCaTN01_F10b.raw | | | | | | | | | | | | 6.0848E6 | | | | | 1 | 0 | 0 | 0 | 0 | 0 | 0 | 0 | 0 | 0 | 0 | 0 | 1 | 0 | 0 | 0 | 0 | 56 | 68 | | | PEAKS DB |
| K.TKTYSYTC(+57.02)TKPNLT.C | Y | 57.97 | 1676.8080 | 14 | 1.8 | 839.4109 | 2 | 38.12 | 12 | F12:16363 | BuCaTN01_F10b.raw | | | | | | | | | | | | 0 | | | | | 0 | 0 | 0 | 0 | 0 | 0 | 0 | 0 | 0 | 0 | 0 | 0 | 0 | 0 | 0 | 0 | 0 | 90 | 103 | Carbamidomethylation | C8:Carbamidomethylation:1000.00 | PEAKS DB |
| G.YVNYGC(+57.02)YC(+57.02)GAGGSGTPVDELDR.C | Y | 57.61 | 2409.0000 | 22 | 2.8 | 1205.5079 | 2 | 58.75 | 12 | F12:34796 | BuCaTN01_F10b.raw | | | | | | | | | | | | 5.0304E5 | | | | | 1 | 0 | 0 | 0 | 0 | 0 | 0 | 0 | 0 | 0 | 0 | 0 | 1 | 0 | 0 | 0 | 0 | 47 | 68 | Carbamidomethylation | C6:Carbamidomethylation:1000.00;C8:Carbamidomethylation:1000.00 | PEAKS DB |
| G.C(+57.02)YC(+57.02)GAGGSGTPVDELDR.C | N | 56.73 | 1812.7407 | 17 | 1.1 | 907.3765 | 2 | 47.13 | 12 | F12:24185 | BuCaTN01_F10b.raw | | | | | | | | | | | | 1.2993E6 | | 1.447E5 | | | 2 | 0 | 0 | 0 | 0 | 0 | 0 | 0 | 0 | 0 | 0 | 0 | 1 | 0 | 1 | 0 | 0 | 52 | 68 | Carbamidomethylation | C1:Carbamidomethylation:1000.00;C3:Carbamidomethylation:1000.00 | PEAKS DB |
| G.GSGTPVDELDR.C | N | 56.36 | 1144.5360 | 11 | 3.4 | 573.2756 | 2 | 40.15 | 8 | F8:17921 | BuCaTN01_F7b.raw | | | | | | | | 1.1068E6 | | | | | | | | | 1 | 0 | 0 | 0 | 0 | 0 | 0 | 0 | 1 | 0 | 0 | 0 | 0 | 0 | 0 | 0 | 0 | 58 | 68 | | | PEAKS DB |

| | | | | | | | | | | | | | | | | | | | | | | | | | | | | | | | | | | | | | | | | | | | | | | | | | |
|---|---|---|---|---|---|---|---|---|---|---|---|---|---|---|---|---|---|---|---|---|---|---|---|---|---|---|---|---|---|---|---|---|---|---|---|---|---|---|---|---|---|---|---|---|---|---|---|---|---|
| K.TYSYTC(+57.02)TKPNLTC(+57.02).T | N | 55.98 | 1607.6960 | 13 | 5.0 | 804.8560 | 2 | 43.60 | 14 | F14:21061 | BuCaTN01_F11b.raw | | | | | | | | | | | | | | 2.3535E6 | 7.2547E5 | | 3 | 0 | 0 | 0 | 0 | 0 | 0 | 0 | 0 | 0 | 0 | 0 | 0 | 0 | 2 | 1 | 0 | 92 | 104 | Carbamidomethylation | C6:Carbamidomethylation:1000.00;C13:Carbamidomethylation:1000.00 | PEAKS DB |
| C.GAGGSGTPVDELDR.C | N | 54.07 | 1329.6160 | 14 | 3.0 | 665.8145 | 2 | 40.54 | 14 | F14:18567 | BuCaTN01_F11b.raw | | | | | | | | | | | | 3.4593E6 | | 1.1632E6 | | | 2 | 0 | 0 | 0 | 0 | 0 | 0 | 0 | 0 | 0 | 0 | 0 | 1 | 0 | 1 | 0 | 0 | 55 | 68 | | | PEAKS DB |
| R.IVC(+57.02)DC(+57.02)DRTAAI.C | N | 53.80 | 1292.5853 | 11 | 4.0 | 647.3007 | 2 | 38.90 | 8 | F8:16711 | BuCaTN01_F7b.raw | | | | | | | | 8.9609E6 | | 1.7898E7 | | 1.6803E6 | | 0 | 8.1365E5 | | 4 | 0 | 0 | 0 | 0 | 0 | 0 | 0 | 1 | 0 | 1 | 0 | 1 | 0 | 0 | 1 | 0 | 114 | 124 | Carbamidomethylation | C3:Carbamidomethylation:1000.00;C5:Carbamidomethylation:1000.00 | PEAKS DB |
| L.NLYQFKNM(+15.99)IVC(+57.02)AGTRPWIGYVNYGC(+57.02)YC(+57.02)GAGGSGTPVDELDR.C | Y | 50.74 | 4674.1187 | 41 | -1.3 | 1169.5327 | 4 | 89.73 | 12 | F12:65102 | BuCaTN01_F10b.raw | | | | | | | | | | | | 1.0473E5 | | | | | 1 | 0 | 0 | 0 | 0 | 0 | 0 | 0 | 0 | 0 | 0 | 0 | 1 | 0 | 0 | 0 | 0 | 28 | 68 | Oxidation (M); Carbamidomethylation | M8:Oxidation (M):1000.00;C11:Carbamidomethylation:1000.00;C25:Carbamidomethylation:1000.00;C27:Carbamidomethylation:1000.00 | PEAKS DB |
| A.GGSGTPVDELDR.C | N | 49.09 | 1201.5575 | 12 | 0.3 | 601.7862 | 2 | 40.57 | 11 | F11:14528 | BuCaTN01_F10a.raw | | | | | | | 9.2274E5 | 2.7577E6 | | 1.1335E7 | 6.7591E5 | | 9.9086E5 | 2.6759E7 | | | 6 | 0 | 0 | 0 | 0 | 0 | 0 | 1 | 1 | 0 | 1 | 1 | 0 | 1 | 1 | 0 | 0 | 57 | 68 | | | PEAKS DB |
| K.TYSYTC(+57.02)TKPNL.T | N | 46.29 | 1346.6177 | 11 | 5.6 | 674.3171 | 2 | 45.63 | 14 | F14:22808 | BuCaTN01_F11b.raw | | | | | | | | | | | | | | 5.6477E6 | 2.611E6 | | 2 | 0 | 0 | 0 | 0 | 0 | 0 | 0 | 0 | 0 | 0 | 0 | 0 | 0 | 1 | 1 | 0 | 92 | 102 | Carbamidomethylation | C6:Carbamidomethylation:1000.00 | PEAKS DB |
| R.C(+57.02)C(+57.02)YVHDNC(+57.02)YGEAE.K | N | 45.95 | 1675.5701 | 13 | 1.2 | 838.7914 | 2 | 37.10 | 12 | F12:15600 | BuCaTN01_F10b.raw | | | | | | | | | | | | 0 | | | | | 0 | 0 | 0 | 0 | 0 | 0 | 0 | 0 | 0 | 0 | 0 | 0 | 0 | 0 | 0 | 0 | 0 | 69 | 81 | Carbamidomethylation | C1:Carbamidomethylation:1000.00;C2:Carbamidomethylation:1000.00;C8:Carbamidomethylation:1000.00 | PEAKS DB |
| V.NYGC(+57.02)YC(+57.02)GAGGSGTPVDELDR.C | N | 42.85 | 2146.8684 | 20 | 1.5 | 1074.4387 | 2 | 63.24 | 14 | F14:38697 | BuCaTN01_F11b.raw | | | | | | | | | | | | | | 0 | | | 0 | 0 | 0 | 0 | 0 | 0 | 0 | 0 | 0 | 0 | 0 | 0 | 0 | 0 | 0 | 0 | 0 | 49 | 68 | Carbamidomethylation | C4:Carbamidomethylation:1000.00;C6:Carbamidomethylation:1000.00 | PEAKS DB |
| A.ANVPPQHLNLYQFK.N | Y | 41.00 | 1667.8783 | 14 | 4.2 | 556.9668 | 3 | 55.58 | 14 | F14:32520 | BuCaTN01_F11b.raw | | | | | | | | | | | | | | 1.0577E7 | | | 1 | 0 | 0 | 0 | 0 | 0 | 0 | 0 | 0 | 0 | 0 | 0 | 0 | 0 | 1 | 0 | 0 | 20 | 33 | | | PEAKS DB |
| total 21 peptides | | | | | | | | | | | | | | | | | | | | | | | | | | | | | | | | | | | | | | | | | | | | | | | | | |

**Best Unique PSM (Scan F12:31893, m/z=749.6523, z=3, RT=55.84, ppm=1.6):**

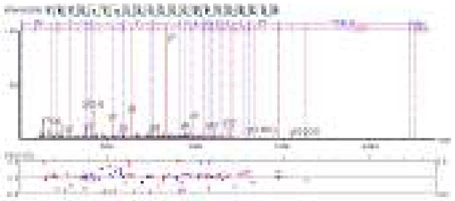

## CAA37482.1

back to list

| Protein Coverage | Supporting Peptides | Best Unique PSM |

**Protein Coverage:**

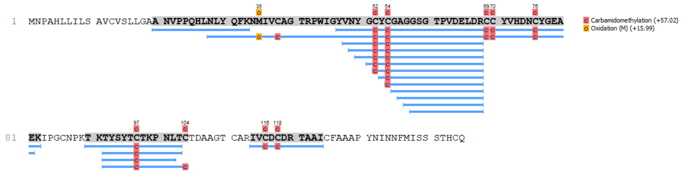


**Supporting Peptides:**

| Peptide | Uniq | -10lgP | Mass | Length | ppm | m/z | z | RT | Fraction | Scan | Source File | Area F1 | Area F2a | Area F3a | Area F4 | Area F5 | Area F6a | Area F7a | Area F7b | Area F8 | Area F9a | Area Sample 1F10a | Area F10b | Area F11a | Area F11b | Area F12a | Area F13 | #Feature | #Feature F1 | #Feature F2a | #Feature F3a | #Feature F4 | #Feature F5 | #Feature F6a | #Feature F7a | #Feature F7b | #Feature F8 | #Feature F9a | #Feature Sample 1F10a | #Feature F10b | #Feature F11a | #Feature F11b | #Feature F12a | #Feature F13 | Start | End | PTM | AScore | Found By |
|---|---|---|---|---|---|---|---|---|---|---|---|---|---|---|---|---|---|---|---|---|---|---|---|---|---|---|---|---|---|---|---|---|---|---|---|---|---|---|---|---|---|---|---|---|---|---|---|---|---|
| Y.VNYGC(+57.02)YC(+57.02)GAGGSGTPVDELDR.C | Y | 79.29 | 2245.9368 | 21 | 1.6 | 749.6523 | 3 | 55.84 | 12 | F12:31893 | BuCaTN01_F10b.raw | | | | | | | 8.8358E3 | 2.8116E5 | | 7.1274E5 | 2.079E5 | 7.0896E6 | | 2.5499E6 | | | 8 | 0 | 0 | 0 | 0 | 0 | 0 | 1 | 1 | 0 | 2 | 1 | 2 | 0 | 1 | 0 | 0 | 48 | 68 | Carbamidomethylation | C5:Carbamidomethylation:1000.00;C7:Carbamidomethylation:1000.00 | PEAKS DB |
| R.C(+57.02)C(+57.02)YVHDNC(+57.02)YGEAEK.I | N | 78.69 | 1803.6650 | 14 | 1.5 | 602.2285 | 3 | 33.04 | 12 | F12:12015 | BuCaTN01_F10b.raw | | | | | | | | | | | | 2.8186E7 | | 1.4756E7 | | | 4 | 0 | 0 | 0 | 0 | 0 | 0 | 0 | 0 | 0 | 0 | 0 | 2 | 0 | 2 | 0 | 0 | 69 | 82 | Carbamidomethylation | C1:Carbamidomethylation:1000.00;C2:Carbamidomethylation:1000.00;C8:Carbamidomethylation:1000.00 | PEAKS DB |
| C.YC(+57.02)GAGGSGTPVDELDR.C | N | 76.90 | 1652.7100 | 16 | 3.1 | 827.3629 | 2 | 45.67 | 12 | F12:22664 | BuCaTN01_F10b.raw | | | | | | | | | | 1.6847E5 | | 2.8019E6 | | 7.7562E5 | | | 3 | 0 | 0 | 0 | 0 | 0 | 0 | 0 | 0 | 0 | 1 | 0 | 1 | 0 | 1 | 0 | 0 | 53 | 68 | Carbamidomethylation | C2:Carbamidomethylation:1000.00 | PEAKS DB |
| Y.GC(+57.02)YC(+57.02)GAGGSGTPVDELDR.C | N | 69.43 | 1869.7621 | 18 | 1.9 | 935.8880 | 2 | 47.48 | 12 | F12:24483 | BuCaTN01_F10b.raw | | | | | | | | | | | | 2.3111E6 | | | | | 1 | 0 | 0 | 0 | 0 | 0 | 0 | 0 | 0 | 0 | 0 | 0 | 1 | 0 | 0 | 0 | 0 | 51 | 68 | Carbamidomethylation | C2:Carbamidomethylation:1000.00;C4:Carbamidomethylation:1000.00 | PEAKS DB |
| Y.C(+57.02)GAGGSGTPVDELDR.C | N | 67.25 | 1489.6467 | 15 | 4.1 | 745.8307 | 2 | 41.63 | 14 | F14:19582 | BuCaTN01_F11b.raw | | | | | | | | | | | 5.1011E5 | 3.3028E7 | | 9.0909E6 | | | 3 | 0 | 0 | 0 | 0 | 0 | 0 | 0 | 0 | 0 | 0 | 1 | 1 | 0 | 1 | 0 | 0 | 54 | 68 | Carbamidomethylation | C1:Carbamidomethylation:1000.00 | PEAKS DB |
| K.TYSYTC(+57.02)TKPNLT.C | N | 66.05 | 1447.6653 | 12 | 2.5 | 724.8387 | 2 | 43.32 | 14 | F14:21091 | BuCaTN01_F11b.raw | | | | | | | | 1.3481E6 | | | | 2.4842E6 | | 1.5203E7 | 9.0876E6 | | 5 | 0 | 0 | 0 | 0 | 0 | 0 | 0 | 1 | 0 | 0 | 0 | 1 | 0 | 2 | 1 | 0 | 92 | 103 | Carbamidomethylation | C6:Carbamidomethylation:1000.00 | PEAKS DB |
| N.YGC(+57.02)YC(+57.02)GAGGSGTPVDELDR.C | N | 65.18 | 2032.8254 | 19 | 7.1 | 1017.4242 | 2 | 52.27 | 8 | F8:28495 | BuCaTN01_F7b.raw | | | | | | | | 5.527E4 | | 2.3803E5 | | 1.1215E7 | | 1.5685E6 | | | 5 | 0 | 0 | 0 | 0 | 0 | 0 | 0 | 1 | 0 | 1 | 0 | 2 | 0 | 1 | 0 | 0 | 50 | 68 | Carbamidomethylation | C3:Carbamidomethylation:1000.00;C5:Carbamidomethylation:1000.00 | PEAKS DB |
| G.AGGSGTPVDELDR.C | N | 61.00 | 1272.5946 | 13 | 2.3 | 637.3046 | 2 | 39.79 | 12 | F12:17765 | BuCaTN01_F10b.raw | | | | | | | | | | | | 6.0848E6 | | | | | 1 | 0 | 0 | 0 | 0 | 0 | 0 | 0 | 0 | 0 | 0 | 0 | 1 | 0 | 0 | 0 | 0 | 56 | 68 | | | PEAKS DB |
| K.TKTYSYTC(+57.02)TKPNLT.C | Y | 57.97 | 1676.8080 | 14 | 1.8 | 839.4109 | 2 | 38.12 | 12 | F12:16363 | BuCaTN01_F10b.raw | | | | | | | | | | | | 0 | | | | | 0 | 0 | 0 | 0 | 0 | 0 | 0 | 0 | 0 | 0 | 0 | 0 | 0 | 0 | 0 | 0 | 0 | 90 | 103 | Carbamidomethylation | C8:Carbamidomethylation:1000.00 | PEAKS DB |
| G.YVNYGC(+57.02)YC(+57.02)GAGGSGTPVDELDR.C | Y | 57.61 | 2409.0000 | 22 | 2.8 | 1205.5079 | 2 | 58.75 | 12 | F12:34796 | BuCaTN01_F10b.raw | | | | | | | | | | | | 5.0304E5 | | | | | 1 | 0 | 0 | 0 | 0 | 0 | 0 | 0 | 0 | 0 | 0 | 0 | 1 | 0 | 0 | 0 | 0 | 47 | 68 | Carbamidomethylation | C6:Carbamidomethylation:1000.00;C8:Carbamidomethylation:1000.00 | PEAKS DB |
| G.C(+57.02)YC(+57.02)GAGGSGTPVDELDR.C | N | 56.73 | 1812.7407 | 17 | 1.1 | 907.3765 | 2 | 47.13 | 12 | F12:24185 | BuCaTN01_F10b.raw | | | | | | | | | | | | 1.2993E6 | | 1.447E5 | | | 2 | 0 | 0 | 0 | 0 | 0 | 0 | 0 | 0 | 0 | 0 | 0 | 1 | 0 | 1 | 0 | 0 | 52 | 68 | Carbamidomethylation | C1:Carbamidomethylation:1000.00;C3:Carbamidomethylation:1000.00 | PEAKS DB |
| G.GSGTPVDELDR.C | N | 56.36 | 1144.5360 | 11 | 3.4 | 573.2756 | 2 | 40.15 | 8 | F8:17921 | BuCaTN01_F7b.raw | | | | | | | | 1.1068E6 | | | | | | | | | 1 | 0 | 0 | 0 | 0 | 0 | 0 | 0 | 1 | 0 | 0 | 0 | 0 | 0 | 0 | 0 | 0 | 58 | 68 | | | PEAKS DB |
| K.TYSYTC(+57.02)TKPNLTC(+57.02).T | N | 55.98 | 1607.6960 | 13 | 5.0 | 804.8560 | 2 | 43.60 | 14 | F14:21061 | BuCaTN01_F11b.raw | | | | | | | | | | | | | | 2.3535E6 | 7.2547E5 | | 3 | 0 | 0 | 0 | 0 | 0 | 0 | 0 | 0 | 0 | 0 | 0 | 0 | 0 | 2 | 1 | 0 | 92 | 104 | Carbamidomethylation | C6:Carbamidomethylation:1000.00;C13:Carbamidomethylation:1000.00 | PEAKS DB |
| C.GAGGSGTPVDELDR.C | N | 54.07 | 1329.6160 | 14 | 3.0 | 665.8145 | 2 | 40.54 | 14 | F14:18567 | BuCaTN01_F11b.raw | | | | | | | | | | | | 3.4593E6 | | 1.1632E6 | | | 2 | 0 | 0 | 0 | 0 | 0 | 0 | 0 | 0 | 0 | 0 | 0 | 1 | 0 | 1 | 0 | 0 | 55 | 68 | | | PEAKS DB |
| R.IVC(+57.02)DC(+57.02)DRTAAI.C | N | 53.80 | 1292.5853 | 11 | 4.0 | 647.3007 | 2 | 38.90 | 8 | F8:16711 | BuCaTN01_F7b.raw | | | | | | | | 8.9609E6 | | 1.7898E7 | | 1.6803E6 | | 0 | 8.1365E5 | | 4 | 0 | 0 | 0 | 0 | 0 | 0 | 0 | 1 | 0 | 1 | 0 | 1 | 0 | 0 | 1 | 0 | 114 | 124 | Carbamidomethylation | C3:Carbamidomethylation:1000.00;C5:Carbamidomethylation:1000.00 | PEAKS DB |
| L.NLYQFKNM(+15.99)IVC(+57.02)AGTRPWIGYVNYGC(+57.02)YC(+57.02)GAGGSGTPVDELDR.C | Y | 50.74 | 4674.1187 | 41 | -1.3 | 1169.5327 | 4 | 89.73 | 12 | F12:65102 | BuCaTN01_F10b.raw | | | | | | | | | | | | 1.0473E5 | | | | | 1 | 0 | 0 | 0 | 0 | 0 | 0 | 0 | 0 | 0 | 0 | 0 | 1 | 0 | 0 | 0 | 0 | 28 | 68 | Oxidation (M); Carbamidomethylation | M8:Oxidation (M):1000.00;C11:Carbamidomethylation:1000.00;C25:Carbamidomethylation:1000.00;C27:Carbamidomethylation:1000.00 | PEAKS DB |
| A.GGSGTPVDELDR.C | N | 49.09 | 1201.5575 | 12 | 0.3 | 601.7862 | 2 | 40.57 | 11 | F11:14528 | BuCaTN01_F10a.raw | | | | | | | 9.2274E5 | 2.7577E6 | | 1.1335E7 | 6.7591E5 | | 9.9086E5 | 2.6759E7 | | | 6 | 0 | 0 | 0 | 0 | 0 | 0 | 1 | 1 | 0 | 1 | 1 | 0 | 1 | 1 | 0 | 0 | 57 | 68 | | | PEAKS DB |
| K.TYSYTC(+57.02)TKPNL.T | N | 46.29 | 1346.6177 | 11 | 5.6 | 674.3171 | 2 | 45.63 | 14 | F14:22808 | BuCaTN01_F11b.raw | | | | | | | | | | | | | | 5.6477E6 | 2.611E6 | | 2 | 0 | 0 | 0 | 0 | 0 | 0 | 0 | 0 | 0 | 0 | 0 | 0 | 0 | 1 | 1 | 0 | 92 | 102 | Carbamidomethylation | C6:Carbamidomethylation:1000.00 | PEAKS DB |
| R.C(+57.02)C(+57.02)YVHDNC(+57.02)YGEAE.K | N | 45.95 | 1675.5701 | 13 | 1.2 | 838.7914 | 2 | 37.10 | 12 | F12:15600 | BuCaTN01_F10b.raw | | | | | | | | | | | | 0 | | | | | 0 | 0 | 0 | 0 | 0 | 0 | 0 | 0 | 0 | 0 | 0 | 0 | 0 | 0 | 0 | 0 | 0 | 69 | 81 | Carbamidomethylation | C1:Carbamidomethylation:1000.00;C2:Carbamidomethylation:1000.00;C8:Carbamidomethylation:1000.00 | PEAKS DB |
| V.NYGC(+57.02)YC(+57.02)GAGGSGTPVDELDR.C | N | 42.85 | 2146.8684 | 20 | 1.5 | 1074.4387 | 2 | 63.24 | 14 | F14:38697 | BuCaTN01_F11b.raw | | | | | | | | | | | | | | 0 | | | 0 | 0 | 0 | 0 | 0 | 0 | 0 | 0 | 0 | 0 | 0 | 0 | 0 | 0 | 0 | 0 | 0 | 49 | 68 | Carbamidomethylation | C4:Carbamidomethylation:1000.00;C6:Carbamidomethylation:1000.00 | PEAKS DB |
| A.ANVPPQHLNLYQFK.N | Y | 41.00 | 1667.8783 | 14 | 4.2 | 556.9668 | 3 | 55.58 | 14 | F14:32520 | BuCaTN01_F11b.raw | | | | | | | | | | | | | | 1.0577E7 | | | 1 | 0 | 0 | 0 | 0 | 0 | 0 | 0 | 0 | 0 | 0 | 0 | 0 | 0 | 1 | 0 | 0 | 20 | 33 | | | PEAKS DB |
| total 21 peptides | | | | | | | | | | | | | | | | | | | | | | | | | | | | | | | | | | | | | | | | | | | | | | | | | |

**Best Unique PSM (Scan F12:31893, m/z=749.6523, z=3, RT=55.84, ppm=1.6):**

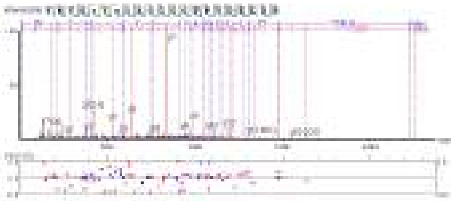

## Q3SB03.1

back to list

| **Protein Coverage** | **Supporting Peptides** | **Best Unique PSM** |

**Protein Coverage:**

1 MIAFIVLLSL AAVLQQSSGT VDFASESSNK KDYQKEIVDK HNALRRSVKP TARNMLRMKW NSRAAQNAKR WADRCTFAHS

81 PPHTRTVGKL RCGENIFMSS QPFAWSGVVQ AWYDEVKKFV YGIGAKPPGS VIGHYTQVVW YKSHLLGCAS AKCSSTKYLY

161 VCQYCPAGNI RGSIATPYKS GPPCADCPSA CVNGLCTNPC KHEDDFSNCK ALAKNSKCQT AWIKSKCPAT CFCHNKII

C Carbamidomethylation (+57.02)

**Supporting Peptides:**

| Peptide | Uniq | -10lgP | Mass | Length | ppm | m/z | z | RT | Fraction | Scan | Source File | Area F1 | Area F2a | Area F3a | Area F4 | Area F5 | Area F6a | Area F7a | Area F7b | Area F8 | Area F9a | Area Sample 1F10a | Area F10b | Area F11a | Area F11b | Area F12a | Area F13 | #Feature | #Feature F1 | #Feature F2a | #Feature F3a | #Feature F4 | #Feature F5 | #Feature F6a | #Feature F7a | #Feature F7b | #Feature F8 | #Feature F9a | #Feature Sample 1F10a | #Feature F10b | #Feature F11a | #Feature F11b | #Feature F12a | #Feature F13 | Start | End | PTM | AScore | Found By |
|---|---|---|---|---|---|---|---|---|---|---|---|---|---|---|---|---|---|---|---|---|---|---|---|---|---|---|---|---|---|---|---|---|---|---|---|---|---|---|---|---|---|---|---|---|---|---|---|---|---|
| K.SGPPC(+57.02)ADC(+57.02)PSAC(+57.02)VNGLC(+57.02)TNPC(+57.02)K.H | Y | 98.41 | 2420.9641 | 22 | -0.1 | 807.9952 | 3 | 47.34 | 11 | F11:19473 | BuCaTN01_F10a.raw | | | | | | | | | | | 3.0597E6 | | 2.3529E7 | 3.8278E5 | | | 5 | 0 | 0 | 0 | 0 | 0 | 0 | 0 | 0 | 0 | 0 | 1 | 0 | 3 | 1 | 0 | 0 | 180 | 201 | Carbamidomethylation | C5:Carbamidomethylation:1000.00;C8:Carbamidomethylation:1000.00;C12:Carbamidomethylation:1000.00;C17:Carbamidomethylation:1000.00;C21:Carbamidomethylation:1000.00 | PEAKS DB |
| K.YLYVC(+57.02)QYC(+57.02)PAGNIR.G | N | 95.63 | 1775.8124 | 14 | 5.4 | 888.9147 | 2 | 59.09 | 14 | F14:35374 | BuCaTN01_F11b.raw | | | | | | | 7.3804E5 | 2.1772E5 | | | 6.518E7 | 1.8222E7 | 5.8095E8 | 1.531E8 | 5.0325E6 | | 41 | 0 | 0 | 0 | 0 | 0 | 0 | 1 | 1 | 0 | 0 | 2 | 3 | 31 | 2 | 1 | 0 | 158 | 171 | Carbamidomethylation | C5:Carbamidomethylation:1000.00;C8:Carbamidomethylation:1000.00 | PEAKS DB |
| K.YLYVC(+57.02)QYC(+57.02)PAGNIRG.S | N | 85.28 | 1832.8337 | 15 | 0.2 | 917.4243 | 2 | 59.91 | 13 | F13:34345 | BuCaTN01_F11a.raw | | | | | | | | | | | 1.8785E6 | | 4.3156E7 | | | | 3 | 0 | 0 | 0 | 0 | 0 | 0 | 0 | 0 | 0 | 0 | 1 | 0 | 2 | 0 | 0 | 0 | 158 | 172 | Carbamidomethylation | C5:Carbamidomethylation:1000.00;C8:Carbamidomethylation:1000.00 | PEAKS DB |
| R.GSIATPYKSGPPC(+57.02)ADC(+57.02)PSAC(+57.02)VNGLC(+57.02)TNPC(+57.02)K.H | Y | 62.79 | 3238.3975 | 30 | 0.7 | 1080.4739 | 3 | 52.03 | 13 | F13:26984 | BuCaTN01_F11a.raw | | | | | | | | | | | | | 2.7434E6 | | | | 1 | 0 | 0 | 0 | 0 | 0 | 0 | 0 | 0 | 0 | 0 | 0 | 0 | 1 | 0 | 0 | 0 | 172 | 201 | Carbamidomethylation | C13:Carbamidomethylation:1000.00;C16:Carbamidomethylation:1000.00;C20:Carbamidomethylation:1000.00;C25:Carbamidomethylation:1000.00;C29:Carbamidomethylation:1000.00 | PEAKS DB |
| K.YLYVC(+57.02)QYC(+57.02)PAGN.I | N | 58.24 | 1506.6272 | 12 | 0.2 | 754.3210 | 2 | 59.53 | 13 | F13:34348 | BuCaTN01_F11a.raw | | | | | | | | | | | | | 8.7629E6 | | | | 1 | 0 | 0 | 0 | 0 | 0 | 0 | 0 | 0 | 0 | 0 | 0 | 0 | 1 | 0 | 0 | 0 | 158 | 169 | Carbamidomethylation | C5:Carbamidomethylation:1000.00;C8:Carbamidomethylation:1000.00 | PEAKS DB |
| K.C(+57.02)SSTKYLYVC(+57.02)QYC(+57.02)PAGNIR.G | N | 57.40 | 2339.0496 | 19 | -1.1 | 780.6896 | 3 | 56.06 | 13 | F13:30956 | BuCaTN01_F11a.raw | | | | | | | | | | | 3.0152E5 | | 8.8383E5 | | | | 3 | 0 | 0 | 0 | 0 | 0 | 0 | 0 | 0 | 0 | 0 | 1 | 0 | 2 | 0 | 0 | 0 | 153 | 171 | Carbamidomethylation | C1:Carbamidomethylation:1000.00;C10:Carbamidomethylation:1000.00;C13:Carbamidomethylation:1000.00 | PEAKS DB |
| R.GSIATPYK.S | N | 53.31 | 835.4440 | 8 | 0.0 | 418.7293 | 2 | 31.21 | 11 | F11:9319 | BuCaTN01_F10a.raw | | | | | | | | | | | 5.4744E5 | | 2.1054E6 | 2.2918E5 | | | 3 | 0 | 0 | 0 | 0 | 0 | 0 | 0 | 0 | 0 | 0 | 1 | 0 | 1 | 1 | 0 | 0 | 172 | 179 | | | PEAKS DB |
| K.SGPPC(+57.02)ADC(+57.02)PSAC(+57.02)VNGL.C | Y | 49.43 | 1660.6643 | 16 | 1.8 | 831.3409 | 2 | 52.23 | 13 | F13:27838 | BuCaTN01_F11a.raw | | | | | | | | | | | | | 1.7893E6 | | | | 1 | 0 | 0 | 0 | 0 | 0 | 0 | 0 | 0 | 0 | 0 | 0 | 0 | 1 | 0 | 0 | 0 | 180 | 195 | Carbamidomethylation | C5:Carbamidomethylation:1000.00;C8:Carbamidomethylation:1000.00;C12:Carbamidomethylation:1000.00 | PEAKS DB |
| Y.LYVC(+57.02)QYC(+57.02)PAGNIR.G | N | 48.17 | 1612.7490 | 13 | -0.1 | 807.3817 | 2 | 53.30 | 11 | F11:23819 | BuCaTN01_F10a.raw | | | | | | | | | | | 1.0604E6 | | 6.0911E5 | | | | 3 | 0 | 0 | 0 | 0 | 0 | 0 | 0 | 0 | 0 | 0 | 2 | 0 | 1 | 0 | 0 | 0 | 159 | 171 | Carbamidomethylation | C4:Carbamidomethylation:1000.00;C7:Carbamidomethylation:1000.00 | PEAKS DB |
| Y.VC(+57.02)QYC(+57.02)PAGNIR.G | N | 45.45 | 1336.6016 | 11 | 0.5 | 669.3084 | 2 | 37.77 | 13 | F13:15521 | BuCaTN01_F11a.raw | | | | | | | | | | | | | 7.7069E5 | | | | 1 | 0 | 0 | 0 | 0 | 0 | 0 | 0 | 0 | 0 | 0 | 0 | 0 | 1 | 0 | 0 | 0 | 161 | 171 | Carbamidomethylation | C2:Carbamidomethylation:1000.00;C5:Carbamidomethylation:1000.00 | PEAKS DB |
| R.GSIATPYKSGPPC(+57.02)AD.C | N | 43.36 | 1519.6976 | 15 | 0.5 | 760.8564 | 2 | 42.06 | 13 | F13:18896 | BuCaTN01_F11a.raw | | | | | | | | | | | | | 7.221E5 | | | | 1 | 0 | 0 | 0 | 0 | 0 | 0 | 0 | 0 | 0 | 0 | 0 | 0 | 1 | 0 | 0 | 0 | 172 | 186 | Carbamidomethylation | C13:Carbamidomethylation:1000.00 | PEAKS DB |
| A.DC(+57.02)PSAC(+57.02)VNGLC(+57.02)TNPC(+57.02)K.H | N | 43.28 | 1851.7372 | 16 | -0.3 | 926.8756 | 2 | 43.52 | 13 | F13:19924 | BuCaTN01_F11a.raw | | | | | | | | | | | | | 1.1644E5 | | | | 1 | 0 | 0 | 0 | 0 | 0 | 0 | 0 | 0 | 0 | 0 | 0 | 0 | 1 | 0 | 0 | 0 | 186 | 201 | Carbamidomethylation | C2:Carbamidomethylation:1000.00;C6:Carbamidomethylation:1000.00;C11:Carbamidomethylation:1000.00;C15:Carbamidomethylation:1000.00 | PEAKS DB |
| **total 12 peptides** | | | | | | | | | | | | | | | | | | | | | | | | | | | | | | | | | | | | | | | | | | | | | | | | | |

**Best Unique PSM (Scan F11:19473, m/z=807.9952, z=3, RT=47.34, ppm=-0.1):**

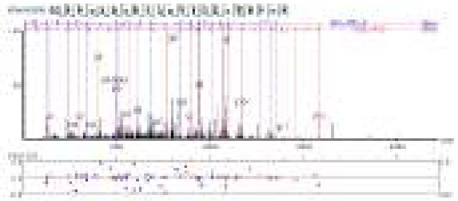

## AAZ38984.1

back to list

| **Protein Coverage** | **Supporting Peptides** | **Best Unique PSM** |

**Protein Coverage:**

1 MIAFIVLLSL AAVLQQSSGT VDFASESSNK KDYQKEIVDK HNALRRSVKP TARNMLRMKW NSRAAQNAKR WADRCTFAHS

81 PPHTRTVGKL RCGENIFMSS QPFAWSGVVQ AWYDEVKKFV YGIGAKPPGS VIGHYTQVVW YKSHLLGCAS AKCSSTKYLY

161 VCQYCPAGNI RGSIATPYKS GPPCADCPSA CVNGLCTNPC KHEDDFSNCK ALAKNSKCQT AWIKSKCPAT CFCHNKII

C Carbamidomethylation (+57.02)

**Supporting Peptides:**

| Peptide | Uniq | -10lgP | Mass | Length | ppm | m/z | z | RT | Fraction | Scan | Source File | Area F1 | Area F2a | Area F3a | Area F4 | Area F5 | Area F6a | Area F7a | Area F7b | Area F8 | Area F9a | Area Sample 1F10a | Area F10b | Area F11a | Area F11b | Area F12a | Area F13 | #Feature | #Feature F1 | #Feature F2a | #Feature F3a | #Feature F4 | #Feature F5 | #Feature F6a | #Feature F7a | #Feature F7b | #Feature F8 | #Feature F9a | #Feature Sample 1F10a | #Feature F10b | #Feature F11a | #Feature F11b | #Feature F12a | #Feature F13 | Start | End | PTM | AScore | Found By |
|---|---|---|---|---|---|---|---|---|---|---|---|---|---|---|---|---|---|---|---|---|---|---|---|---|---|---|---|---|---|---|---|---|---|---|---|---|---|---|---|---|---|---|---|---|---|---|---|---|---|
| K.SGPPC(+57.02)ADC(+57.02)PSAC(+57.02)VNGLC(+57.02)TNPC(+57.02)K.H | Y | 98.41 | 2420.9641 | 22 | -0.1 | 807.9952 | 3 | 47.34 | 11 | F11:19473 | BuCaTN01_F10a.raw | | | | | | | | | | | 3.0597E6 | | 2.3529E7 | 3.8278E5 | | | 5 | 0 | 0 | 0 | 0 | 0 | 0 | 0 | 0 | 0 | 0 | 1 | 0 | 3 | 1 | 0 | 0 | 180 | 201 | Carbamidomethylation | C5:Carbamidomethylation:1000.00;C8:Carbamidomethylation:1000.00;C12:Carbamidomethylation:1000.00;C17:Carbamidomethylation:1000.00;C21:Carbamidomethylation:1000.00 | PEAKS DB |
| K.YLYVC(+57.02)QYC(+57.02)PAGNIR.G | N | 95.63 | 1775.8124 | 14 | 5.4 | 888.9147 | 2 | 59.09 | 14 | F14:35374 | BuCaTN01_F11b.raw | | | | | | | 7.3804E5 | 2.1772E5 | | | 6.518E7 | 1.8222E7 | 5.8095E8 | 1.531E8 | 5.0325E6 | | 41 | 0 | 0 | 0 | 0 | 0 | 0 | 1 | 1 | 0 | 0 | 2 | 3 | 31 | 2 | 1 | 0 | 158 | 171 | Carbamidomethylation | C5:Carbamidomethylation:1000.00;C8:Carbamidomethylation:1000.00 | PEAKS DB |
| K.YLYVC(+57.02)QYC(+57.02)PAGNIRG.S | N | 85.28 | 1832.8337 | 15 | 0.2 | 917.4243 | 2 | 59.91 | 13 | F13:34345 | BuCaTN01_F11a.raw | | | | | | | | | | | 1.8785E6 | | 4.3156E7 | | | | 3 | 0 | 0 | 0 | 0 | 0 | 0 | 0 | 0 | 0 | 0 | 1 | 0 | 2 | 0 | 0 | 0 | 158 | 172 | Carbamidomethylation | C5:Carbamidomethylation:1000.00;C8:Carbamidomethylation:1000.00 | PEAKS DB |
| R.GSIATPYKSGPPC(+57.02)ADC(+57.02)PSAC(+57.02)VNGLC(+57.02)TNPC(+57.02)K.H | Y | 62.79 | 3238.3975 | 30 | 0.7 | 1080.4739 | 3 | 52.03 | 13 | F13:26984 | BuCaTN01_F11a.raw | | | | | | | | | | | | | 2.7434E6 | | | | 1 | 0 | 0 | 0 | 0 | 0 | 0 | 0 | 0 | 0 | 0 | 0 | 0 | 1 | 0 | 0 | 0 | 172 | 201 | Carbamidomethylation | C13:Carbamidomethylation:1000.00;C16:Carbamidomethylation:1000.00;C20:Carbamidomethylation:1000.00;C25:Carbamidomethylation:1000.00;C29:Carbamidomethylation:1000.00 | PEAKS DB |
| K.YLYVC(+57.02)QYC(+57.02)PAGN.I | N | 58.24 | 1506.6272 | 12 | 0.2 | 754.3210 | 2 | 59.53 | 13 | F13:34348 | BuCaTN01_F11a.raw | | | | | | | | | | | | | 8.7629E6 | | | | 1 | 0 | 0 | 0 | 0 | 0 | 0 | 0 | 0 | 0 | 0 | 0 | 0 | 1 | 0 | 0 | 0 | 158 | 169 | Carbamidomethylation | C5:Carbamidomethylation:1000.00;C8:Carbamidomethylation:1000.00 | PEAKS DB |
| K.C(+57.02)SSTKYLYVC(+57.02)QYC(+57.02)PAGNIR.G | N | 57.40 | 2339.0496 | 19 | -1.1 | 780.6896 | 3 | 56.06 | 13 | F13:30956 | BuCaTN01_F11a.raw | | | | | | | | | | | 3.0152E5 | | 8.8383E5 | | | | 3 | 0 | 0 | 0 | 0 | 0 | 0 | 0 | 0 | 0 | 0 | 1 | 0 | 2 | 0 | 0 | 0 | 153 | 171 | Carbamidomethylation | C1:Carbamidomethylation:1000.00;C10:Carbamidomethylation:1000.00;C13:Carbamidomethylation:1000.00 | PEAKS DB |
| R.GSIATPYK.S | N | 53.31 | 835.4440 | 8 | 0.0 | 418.7293 | 2 | 31.21 | 11 | F11:9319 | BuCaTN01_F10a.raw | | | | | | | | | | | 5.4744E5 | | 2.1054E6 | 2.2918E5 | | | 3 | 0 | 0 | 0 | 0 | 0 | 0 | 0 | 0 | 0 | 0 | 1 | 0 | 1 | 1 | 0 | 0 | 172 | 179 | | | PEAKS DB |
| K.SGPPC(+57.02)ADC(+57.02)PSAC(+57.02)VNGL.C | Y | 49.43 | 1660.6643 | 16 | 1.8 | 831.3409 | 2 | 52.23 | 13 | F13:27838 | BuCaTN01_F11a.raw | | | | | | | | | | | | | 1.7893E6 | | | | 1 | 0 | 0 | 0 | 0 | 0 | 0 | 0 | 0 | 0 | 0 | 0 | 0 | 1 | 0 | 0 | 0 | 180 | 195 | Carbamidomethylation | C5:Carbamidomethylation:1000.00;C8:Carbamidomethylation:1000.00;C12:Carbamidomethylation:1000.00 | PEAKS DB |
| Y.LYVC(+57.02)QYC(+57.02)PAGNIR.G | N | 48.17 | 1612.7490 | 13 | -0.1 | 807.3817 | 2 | 53.30 | 11 | F11:23819 | BuCaTN01_F10a.raw | | | | | | | | | | | 1.0604E6 | | 6.0911E5 | | | | 3 | 0 | 0 | 0 | 0 | 0 | 0 | 0 | 0 | 0 | 0 | 2 | 0 | 1 | 0 | 0 | 0 | 159 | 171 | Carbamidomethylation | C4:Carbamidomethylation:1000.00;C7:Carbamidomethylation:1000.00 | PEAKS DB |
| Y.VC(+57.02)QYC(+57.02)PAGNIR.G | N | 45.45 | 1336.6016 | 11 | 0.5 | 669.3084 | 2 | 37.77 | 13 | F13:15521 | BuCaTN01_F11a.raw | | | | | | | | | | | | | 7.7069E5 | | | | 1 | 0 | 0 | 0 | 0 | 0 | 0 | 0 | 0 | 0 | 0 | 0 | 0 | 1 | 0 | 0 | 0 | 161 | 171 | Carbamidomethylation | C2:Carbamidomethylation:1000.00;C5:Carbamidomethylation:1000.00 | PEAKS DB |
| R.GSIATPYKSGPPC(+57.02)AD.C | N | 43.36 | 1519.6976 | 15 | 0.5 | 760.8564 | 2 | 42.06 | 13 | F13:18896 | BuCaTN01_F11a.raw | | | | | | | | | | | | | 7.221E5 | | | | 1 | 0 | 0 | 0 | 0 | 0 | 0 | 0 | 0 | 0 | 0 | 0 | 0 | 1 | 0 | 0 | 0 | 172 | 186 | Carbamidomethylation | C13:Carbamidomethylation:1000.00 | PEAKS DB |
| A.DC(+57.02)PSAC(+57.02)VNGLC(+57.02)TNPC(+57.02)K.H | N | 43.28 | 1851.7372 | 16 | -0.3 | 926.8756 | 2 | 43.52 | 13 | F13:19924 | BuCaTN01_F11a.raw | | | | | | | | | | | | | 1.1644E5 | | | | 1 | 0 | 0 | 0 | 0 | 0 | 0 | 0 | 0 | 0 | 0 | 0 | 0 | 1 | 0 | 0 | 0 | 186 | 201 | Carbamidomethylation | C2:Carbamidomethylation:1000.00;C6:Carbamidomethylation:1000.00;C11:Carbamidomethylation:1000.00;C15:Carbamidomethylation:1000.00 | PEAKS DB |
| **total 12 peptides** | | | | | | | | | | | | | | | | | | | | | | | | | | | | | | | | | | | | | | | | | | | | | | | | | |

**Best Unique PSM (Scan F11:19473, m/z=807.9952, z=3, RT=47.34, ppm=-0.1):**

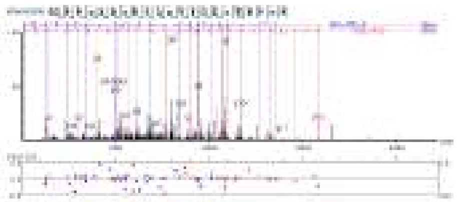

## CAM11302.1

back to list

| Protein Coverage | Supporting Peptides | Best Unique PSM |

**Protein Coverage:**

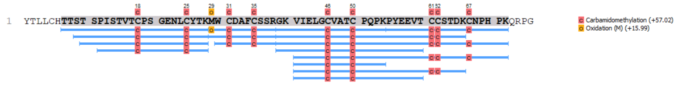


**Supporting Peptides:**

| Peptide | Uniq | -10lgP | Mass | Length | ppm | m/z | z | RT | Fraction | Scan | Source File | Area F1 | Area F2a | Area F3a | Area F4 | Area F5 | Area F6a | Area F7a | Area F7b | Area F8 | Area F9a | Area Sample 1F10a | Area F10b | Area F11a | Area F11b | Area F12a | Area F13 | #Feature | #Feature F1 | #Feature F2a | #Feature F3a | #Feature F4 | #Feature F5 | #Feature F6a | #Feature F7a | #Feature F7b | #Feature F8 | #Feature F9a | #Feature Sample 1F10a | #Feature F10b | #Feature F11a | #Feature F11b | #Feature F12a | #Feature F13 | Start | End | PTM | AScore | Found By |
|---|---|---|---|---|---|---|---|---|---|---|---|---|---|---|---|---|---|---|---|---|---|---|---|---|---|---|---|---|---|---|---|---|---|---|---|---|---|---|---|---|---|---|---|---|---|---|---|---|---|
| T.STSPISTVTC(+57.02)PSGENLC(+57.02)YTK.M | Y | 86.24 | 2200.9980 | 20 | -0.4 | 1101.5059 | 2 | 54.17 | 6 | F6:32677 | BuCaTN01_F6a.raw | | | | | | 1.6668E7 | | | | | | | | | | | 2 | 0 | 0 | 0 | 0 | 0 | 2 | 0 | 0 | 0 | 0 | 0 | 0 | 0 | 0 | 0 | 0 | 9 | 28 | Carbamidomethylation | C10:Carbamidomethylation:1000.00;C17:Carbamidomethylation:1000.00 | PEAKS DB |
| R.GKVIELGC(+57.02)VATC(+57.02)PQPKPYEEVTC(+57.02)C(+57.02)STDK.C | Y | 84.43 | 3225.4814 | 28 | -0.5 | 1076.1672 | 3 | 54.32 | 10 | F10:30972 | BuCaTN01_F9a.raw | | | | | | | | 0 | | 2.507E7 | | 1.0519E6 | | | | | 3 | 0 | 0 | 0 | 0 | 0 | 0 | 0 | 0 | 0 | 2 | 0 | 1 | 0 | 0 | 0 | 0 | 39 | 66 | Carbamidomethylation | C8:Carbamidomethylation:1000.00;C12:Carbamidomethylation:1000.00;C23:Carbamidomethylation:1000.00;C24:Carbamidomethylation:1000.00 | PEAKS DB |
| K.VIELGC(+57.02)VATC(+57.02)PQPKPYEEVTC(+57.02)C(+57.02)STDK.C | Y | 78.78 | 3040.3650 | 26 | 0.9 | 1014.4631 | 3 | 58.23 | 10 | F10:34928 | BuCaTN01_F9a.raw | | | | | | | | | | 5.7183E7 | | 4.1361E6 | | | | | 4 | 0 | 0 | 0 | 0 | 0 | 0 | 0 | 0 | 0 | 2 | 0 | 2 | 0 | 0 | 0 | 0 | 41 | 66 | Carbamidomethylation | C6:Carbamidomethylation:1000.00;C10:Carbamidomethylation:1000.00;C21:Carbamidomethylation:1000.00;C22:Carbamidomethylation:1000.00 | PEAKS DB |
| K.VIELGC(+57.02)VATC(+57.02)PQPK.P | Y | 75.48 | 1570.7847 | 14 | 3.2 | 786.3998 | 2 | 51.11 | 8 | F8:27668 | BuCaTN01_F7b.raw | | | | | | 1.7303E5 | 1.8122E5 | 8.9185E6 | | 1.2697E8 | | 5.4066E6 | | 3.7932E6 | 1.9368E6 | | 10 | 0 | 0 | 0 | 0 | 0 | 1 | 1 | 1 | 0 | 2 | 0 | 2 | 0 | 1 | 2 | 0 | 41 | 54 | Carbamidomethylation | C6:Carbamidomethylation:1000.00;C10:Carbamidomethylation:1000.00 | PEAKS DB |
| R.GKVIELGC(+57.02)VATC(+57.02)PQPK.P | Y | 64.32 | 1755.9011 | 16 | 0.3 | 878.9581 | 2 | 45.61 | 10 | F10:22895 | BuCaTN01_F9a.raw | | | | | | | | | | 2.88E7 | | | | | | | 2 | 0 | 0 | 0 | 0 | 0 | 0 | 0 | 0 | 0 | 2 | 0 | 0 | 0 | 0 | 0 | 0 | 39 | 54 | Carbamidomethylation | C8:Carbamidomethylation:1000.00;C12:Carbamidomethylation:1000.00 | PEAKS DB |
| S.TSPISTVTC(+57.02)PSGENLC(+57.02)YTK.M | Y | 63.33 | 2113.9661 | 19 | -1.2 | 1057.9890 | 2 | 53.66 | 6 | F6:32206 | BuCaTN01_F6a.raw | | | | | | 1.545E6 | | | | | | | | | | | 1 | 0 | 0 | 0 | 0 | 0 | 1 | 0 | 0 | 0 | 0 | 0 | 0 | 0 | 0 | 0 | 0 | 10 | 28 | Carbamidomethylation | C9:Carbamidomethylation:1000.00;C16:Carbamidomethylation:1000.00 | PEAKS DB |
| K.MWC(+57.02)DAFC(+57.02)SSR.G | Y | 57.87 | 1318.4893 | 10 | 0.0 | 660.2519 | 2 | 56.42 | 6 | F6:34475 | BuCaTN01_F6a.raw | | | | | | 3.0586E7 | | | | | | | | | | | 1 | 0 | 0 | 0 | 0 | 0 | 1 | 0 | 0 | 0 | 0 | 0 | 0 | 0 | 0 | 0 | 0 | 29 | 38 | Carbamidomethylation | C3:Carbamidomethylation:1000.00;C7:Carbamidomethylation:1000.00 | PEAKS DB |
| K.VIELGC(+57.02)VATC(+57.02)PQPKPYEEVTC(+57.02)C(+57.02)STDKC(+57.02)NPHPK.Q | Y | 52.88 | 3773.6980 | 32 | 2.9 | 755.7468 | 5 | 53.01 | 8 | F8:29141 | BuCaTN01_F7b.raw | | | | | | | | 1.4768E6 | | | | | | | | | 1 | 0 | 0 | 0 | 0 | 0 | 0 | 0 | 1 | 0 | 0 | 0 | 0 | 0 | 0 | 0 | 0 | 41 | 72 | Carbamidomethylation | C6:Carbamidomethylation:1000.00;C10:Carbamidomethylation:1000.00;C21:Carbamidomethylation:1000.00;C22:Carbamidomethylation:1000.00;C27:Carbamidomethylation:1000.00 | PEAKS DB |
| K.M(+15.99)WC(+57.02)DAFC(+57.02)SSR.G | Y | 50.69 | 1334.4843 | 10 | 0.8 | 668.2499 | 2 | 51.87 | 6 | F6:30789 | BuCaTN01_F6a.raw | | | | | | 1.5628E7 | | | | | | | | | | | 1 | 0 | 0 | 0 | 0 | 0 | 1 | 0 | 0 | 0 | 0 | 0 | 0 | 0 | 0 | 0 | 0 | 29 | 38 | Oxidation (M); Carbamidomethylation | M1:Oxidation (M):1000.00;C3:Carbamidomethylation:1000.00;C7:Carbamidomethylation:1000.00 | PEAKS DB |
| R.GKVIELGC(+57.02)VATC(+57.02)PQPKPYEEVT.C | Y | 48.69 | 2474.2185 | 22 | 2.9 | 825.7491 | 3 | 56.58 | 10 | F10:33227 | BuCaTN01_F9a.raw | | | | | | | | | | 0 | | | | | | | 0 | 0 | 0 | 0 | 0 | 0 | 0 | 0 | 0 | 0 | 0 | 0 | 0 | 0 | 0 | 0 | 0 | 39 | 60 | Carbamidomethylation | C8:Carbamidomethylation:1000.00;C12:Carbamidomethylation:1000.00 | PEAKS DB |
| K.PYEEVTC(+57.02)C(+57.02)STDKC(+57.02)NPHPK.Q | N | 46.34 | 2220.9238 | 18 | 0.7 | 556.2386 | 4 | 29.53 | 6 | F6:10966 | BuCaTN01_F6a.raw | | | | | | 1.5168E6 | | | | | | | | | | | 1 | 0 | 0 | 0 | 0 | 0 | 1 | 0 | 0 | 0 | 0 | 0 | 0 | 0 | 0 | 0 | 0 | 55 | 72 | Carbamidomethylation | C7:Carbamidomethylation:1000.00;C8:Carbamidomethylation:1000.00;C13:Carbamidomethylation:1000.00 | PEAKS DB |
| R.GKVIELGC(+57.02)VATC(+57.02)PQPKPYEEVTC(+57.02)C(+57.02)STDKC(+57.02)NPHPK.Q | Y | 45.00 | 3958.8145 | 34 | 0.0 | 792.7701 | 5 | 49.86 | 10 | F10:27061 | BuCaTN01_F9a.raw | | | | | | | | | | 2.4613E6 | | | | | | | 1 | 0 | 0 | 0 | 0 | 0 | 0 | 0 | 0 | 0 | 1 | 0 | 0 | 0 | 0 | 0 | 0 | 39 | 72 | Carbamidomethylation | C8:Carbamidomethylation:1000.00;C12:Carbamidomethylation:1000.00;C23:Carbamidomethylation:1000.00;C24:Carbamidomethylation:1000.00;C29:Carbamidomethylation:1000.00 | PEAKS DB |
| M.WC(+57.02)DAFC(+57.02)SSR.G | Y | 44.41 | 1187.4489 | 9 | -0.8 | 594.7312 | 2 | 46.96 | 6 | F6:25735 | BuCaTN01_F6a.raw | | | | | | 1.5955E8 | | | | | | | | | | | 1 | 0 | 0 | 0 | 0 | 0 | 1 | 0 | 0 | 0 | 0 | 0 | 0 | 0 | 0 | 0 | 0 | 30 | 38 | Carbamidomethylation | C2:Carbamidomethylation:1000.00;C6:Carbamidomethylation:1000.00 | PEAKS DB |
| K.VIELGC(+57.02)VATC(+57.02)PQPKPYEEVT.C | Y | 43.75 | 2289.1021 | 20 | 4.0 | 1145.5629 | 2 | 61.95 | 10 | F10:38507 | BuCaTN01_F9a.raw | | | | | | | | | | 5.9744E5 | | | | | | | 1 | 0 | 0 | 0 | 0 | 0 | 0 | 0 | 0 | 0 | 1 | 0 | 0 | 0 | 0 | 0 | 0 | 41 | 60 | Carbamidomethylation | C6:Carbamidomethylation:1000.00;C10:Carbamidomethylation:1000.00 | PEAKS DB |
| S.PISTVTC(+57.02)PSGENLC(+57.02)YTK.M | Y | 41.83 | 1925.8862 | 17 | 0.9 | 963.9484 | 2 | 49.07 | 8 | F8:25888 | BuCaTN01_F7b.raw | | | | | | | | 0 | | | | | | | | | 0 | 0 | 0 | 0 | 0 | 0 | 0 | 0 | 0 | 0 | 0 | 0 | 0 | 0 | 0 | 0 | 0 | 12 | 28 | Carbamidomethylation | C7:Carbamidomethylation:1000.00;C14:Carbamidomethylation:1000.00 | PEAKS DB |
| H.TTSTSPISTVTC(+57.02)PSGENLC(+57.02)YTK.M | Y | 41.81 | 2403.0935 | 22 | -0.6 | 1202.5533 | 2 | 54.92 | 6 | F6:33376 | BuCaTN01_F6a.raw | | | | | | 0 | | | | | | | | | | | 0 | 0 | 0 | 0 | 0 | 0 | 0 | 0 | 0 | 0 | 0 | 0 | 0 | 0 | 0 | 0 | 0 | 7 | 28 | Carbamidomethylation | C12:Carbamidomethylation:1000.00;C19:Carbamidomethylation:1000.00 | PEAKS DB |

**total 16 peptides**

**Best Unique PSM (Scan F6:32677, m/z=1101.5059, z=2, RT=54.17, ppm=-0.4):**

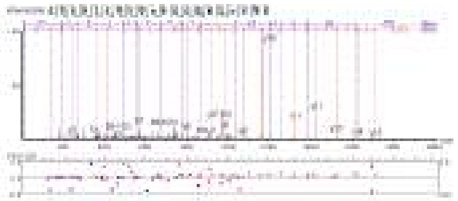

## D2N116.1

back to list

| Protein Coverage | Supporting Peptides | Best Unique PSM |

**Protein Coverage:**

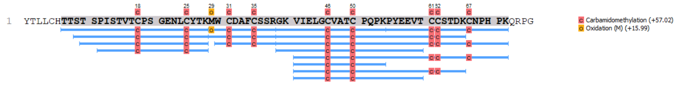


**Supporting Peptides:**

| Peptide | Uniq | -10lgP | Mass | Length | ppm | m/z | z | RT | Fraction | Scan | Source File | Area F1 | Area F2a | Area F3a | Area F4 | Area F5 | Area F6a | Area F7a | Area F7b | Area F8 | Area F9a | Area Sample 1F10a | Area F10b | Area F11a | Area F11b | Area F12a | Area F13 | #Feature | #Feature F1 | #Feature F2a | #Feature F3a | #Feature F4 | #Feature F5 | #Feature F6a | #Feature F7a | #Feature F7b | #Feature F8 | #Feature F9a | #Feature Sample 1F10a | #Feature F10b | #Feature F11a | #Feature F11b | #Feature F12a | #Feature F13 | Start | End | PTM | AScore | Found By |
|---|---|---|---|---|---|---|---|---|---|---|---|---|---|---|---|---|---|---|---|---|---|---|---|---|---|---|---|---|---|---|---|---|---|---|---|---|---|---|---|---|---|---|---|---|---|---|---|---|---|
| T.STSPISTVTC(+57.02)PSGENLC(+57.02)YTK.M | Y | 86.24 | 2200.9980 | 20 | -0.4 | 1101.5059 | 2 | 54.17 | 6 | F6:32677 | BuCaTN01_F6a.raw | | | | | | 1.6668E7 | | | | | | | | | | | 2 | 0 | 0 | 0 | 0 | 0 | 2 | 0 | 0 | 0 | 0 | 0 | 0 | 0 | 0 | 0 | 0 | 9 | 28 | Carbamidomethylation | C10:Carbamidomethylation:1000.00;C17:Carbamidomethylation:1000.00 | PEAKS DB |
| R.GKVIELGC(+57.02)VATC(+57.02)PQPKPYEEVTC(+57.02)C(+57.02)STDK.C | Y | 84.43 | 3225.4814 | 28 | -0.5 | 1076.1672 | 3 | 54.32 | 10 | F10:30972 | BuCaTN01_F9a.raw | | | | | | | | 0 | | 2.507E7 | | 1.0519E6 | | | | | 3 | 0 | 0 | 0 | 0 | 0 | 0 | 0 | 0 | 0 | 2 | 0 | 1 | 0 | 0 | 0 | 0 | 39 | 66 | Carbamidomethylation | C8:Carbamidomethylation:1000.00;C12:Carbamidomethylation:1000.00;C23:Carbamidomethylation:1000.00;C24:Carbamidomethylation:1000.00 | PEAKS DB |
| K.VIELGC(+57.02)VATC(+57.02)PQPKPYEEVTC(+57.02)C(+57.02)STDK.C | Y | 78.78 | 3040.3650 | 26 | 0.9 | 1014.4631 | 3 | 58.23 | 10 | F10:34928 | BuCaTN01_F9a.raw | | | | | | | | | | 5.7183E7 | | 4.1361E6 | | | | | 4 | 0 | 0 | 0 | 0 | 0 | 0 | 0 | 0 | 0 | 2 | 0 | 2 | 0 | 0 | 0 | 0 | 41 | 66 | Carbamidomethylation | C6:Carbamidomethylation:1000.00;C10:Carbamidomethylation:1000.00;C21:Carbamidomethylation:1000.00;C22:Carbamidomethylation:1000.00 | PEAKS DB |
| K.VIELGC(+57.02)VATC(+57.02)PQPK.P | Y | 75.48 | 1570.7847 | 14 | 3.2 | 786.3998 | 2 | 51.11 | 8 | F8:27668 | BuCaTN01_F7b.raw | | | | | | 1.7303E5 | 1.8122E5 | 8.9185E6 | | 1.2697E8 | | 5.4066E6 | | 3.7932E6 | 1.9368E6 | | 10 | 0 | 0 | 0 | 0 | 0 | 1 | 1 | 1 | 0 | 2 | 0 | 2 | 0 | 1 | 2 | 0 | 41 | 54 | Carbamidomethylation | C6:Carbamidomethylation:1000.00;C10:Carbamidomethylation:1000.00 | PEAKS DB |
| R.GKVIELGC(+57.02)VATC(+57.02)PQPK.P | Y | 64.32 | 1755.9011 | 16 | 0.3 | 878.9581 | 2 | 45.61 | 10 | F10:22895 | BuCaTN01_F9a.raw | | | | | | | | | | 2.88E7 | | | | | | | 2 | 0 | 0 | 0 | 0 | 0 | 0 | 0 | 0 | 0 | 2 | 0 | 0 | 0 | 0 | 0 | 0 | 39 | 54 | Carbamidomethylation | C8:Carbamidomethylation:1000.00;C12:Carbamidomethylation:1000.00 | PEAKS DB |
| S.TSPISTVTC(+57.02)PSGENLC(+57.02)YTK.M | Y | 63.33 | 2113.9661 | 19 | -1.2 | 1057.9890 | 2 | 53.66 | 6 | F6:32206 | BuCaTN01_F6a.raw | | | | | | 1.545E6 | | | | | | | | | | | 1 | 0 | 0 | 0 | 0 | 0 | 1 | 0 | 0 | 0 | 0 | 0 | 0 | 0 | 0 | 0 | 0 | 10 | 28 | Carbamidomethylation | C9:Carbamidomethylation:1000.00;C16:Carbamidomethylation:1000.00 | PEAKS DB |

| | | | | | | | | | | | | | | | | | | | | | | | | | | | | | | | | | | | | | | | | | | | | | | | | | |
|---|---|---|---|---|---|---|---|---|---|---|---|---|---|---|---|---|---|---|---|---|---|---|---|---|---|---|---|---|---|---|---|---|---|---|---|---|---|---|---|---|---|---|---|---|---|---|---|---|---|
| K.MWC(+57.02)DAFC(+57.02)SSR.G | Y | 57.87 | 1318.4893 | 10 | 0.0 | 660.2519 | 2 | 56.42 | 6 | F6:34475 | BuCaTN01_F6a.raw | | | | | | 3.0586E7 | | | | | | | | | | | 1 | 0 | 0 | 0 | 0 | 0 | 1 | 0 | 0 | 0 | 0 | 0 | 0 | 0 | 0 | 0 | 0 | 29 | 38 | Carbamidomethylation | C3:Carbamidomethylation:1000.00;C7:Carbamidomethylation:1000.00 | PEAKS DB |
| K.VIELGC(+57.02)VATC(+57.02)PQPKPYEEVTC(+57.02)C(+57.02)STDKC(+57.02)NPHPK.Q | Y | 52.88 | 3773.6980 | 32 | 2.9 | 755.7468 | 5 | 53.01 | 8 | F8:29141 | BuCaTN01_F7b.raw | | | | | | | | 1.4768E6 | | | | | | | | | 1 | 0 | 0 | 0 | 0 | 0 | 0 | 0 | 1 | 0 | 0 | 0 | 0 | 0 | 0 | 0 | 0 | 41 | 72 | Carbamidomethylation | C6:Carbamidomethylation:1000.00;C10:Carbamidomethylation:1000.00;C21:Carbamidomethylation:1000.00;C22:Carbamidomethylation:1000.00;C27:Carbamidomethylation:1000.00 | PEAKS DB |
| K.M(+15.99)WC(+57.02)DAFC(+57.02)SSR.G | Y | 50.69 | 1334.4843 | 10 | 0.8 | 668.2499 | 2 | 51.87 | 6 | F6:30789 | BuCaTN01_F6a.raw | | | | | | 1.5628E7 | | | | | | | | | | | 1 | 0 | 0 | 0 | 0 | 0 | 1 | 0 | 0 | 0 | 0 | 0 | 0 | 0 | 0 | 0 | 0 | 29 | 38 | Oxidation (M); Carbamidomethylation | M1:Oxidation (M):1000.00;C3:Carbamidomethylation:1000.00;C7:Carbamidomethylation:1000.00 | PEAKS DB |
| R.GKVIELGC(+57.02)VATC(+57.02)PQPKPYEEVT.C | Y | 48.69 | 2474.2185 | 22 | 2.9 | 825.7491 | 3 | 56.58 | 10 | F10:33227 | BuCaTN01_F9a.raw | | | | | | | | | | 0 | | | | | | | 0 | 0 | 0 | 0 | 0 | 0 | 0 | 0 | 0 | 0 | 0 | 0 | 0 | 0 | 0 | 0 | 0 | 39 | 60 | Carbamidomethylation | C8:Carbamidomethylation:1000.00;C12:Carbamidomethylation:1000.00 | PEAKS DB |
| K.PYEEVTC(+57.02)C(+57.02)STDKC(+57.02)NPHPK.Q | N | 46.34 | 2220.9238 | 18 | 0.7 | 556.2386 | 4 | 29.53 | 6 | F6:10966 | BuCaTN01_F6a.raw | | | | | | 1.5168E6 | | | | | | | | | | | 1 | 0 | 0 | 0 | 0 | 0 | 1 | 0 | 0 | 0 | 0 | 0 | 0 | 0 | 0 | 0 | 0 | 55 | 72 | Carbamidomethylation | C7:Carbamidomethylation:1000.00;C8:Carbamidomethylation:1000.00;C13:Carbamidomethylation:1000.00 | PEAKS DB |
| R.GKVIELGC(+57.02)VATC(+57.02)PQPKPYEEVTC(+57.02)C(+57.02)STDKC(+57.02)NPHPK.Q | Y | 45.00 | 3958.8145 | 34 | 0.0 | 792.7701 | 5 | 49.86 | 10 | F10:27061 | BuCaTN01_F9a.raw | | | | | | | | | | 2.4613E6 | | | | | | | 1 | 0 | 0 | 0 | 0 | 0 | 0 | 0 | 0 | 0 | 1 | 0 | 0 | 0 | 0 | 0 | 0 | 39 | 72 | Carbamidomethylation | C8:Carbamidomethylation:1000.00;C12:Carbamidomethylation:1000.00;C23:Carbamidomethylation:1000.00;C24:Carbamidomethylation:1000.00;C29:Carbamidomethylation:1000.00 | PEAKS DB |
| M.WC(+57.02)DAFC(+57.02)SSR.G | Y | 44.41 | 1187.4489 | 9 | -0.8 | 594.7312 | 2 | 46.96 | 6 | F6:25735 | BuCaTN01_F6a.raw | | | | | | 1.5955E8 | | | | | | | | | | | 1 | 0 | 0 | 0 | 0 | 0 | 1 | 0 | 0 | 0 | 0 | 0 | 0 | 0 | 0 | 0 | 0 | 30 | 38 | Carbamidomethylation | C2:Carbamidomethylation:1000.00;C6:Carbamidomethylation:1000.00 | PEAKS DB |
| K.VIELGC(+57.02)VATC(+57.02)PQPKPYEEVT.C | Y | 43.75 | 2289.1021 | 20 | 4.0 | 1145.5629 | 2 | 61.95 | 10 | F10:38507 | BuCaTN01_F9a.raw | | | | | | | | | | 5.9744E5 | | | | | | | 1 | 0 | 0 | 0 | 0 | 0 | 0 | 0 | 0 | 0 | 1 | 0 | 0 | 0 | 0 | 0 | 0 | 41 | 60 | Carbamidomethylation | C6:Carbamidomethylation:1000.00;C10:Carbamidomethylation:1000.00 | PEAKS DB |
| S.PISTVTC(+57.02)PSGENLC(+57.02)YTK.M | Y | 41.83 | 1925.8862 | 17 | 0.9 | 963.9484 | 2 | 49.07 | 8 | F8:25888 | BuCaTN01_F7b.raw | | | | | | | | 0 | | | | | | | | | 0 | 0 | 0 | 0 | 0 | 0 | 0 | 0 | 0 | 0 | 0 | 0 | 0 | 0 | 0 | 0 | 0 | 12 | 28 | Carbamidomethylation | C7:Carbamidomethylation:1000.00;C14:Carbamidomethylation:1000.00 | PEAKS DB |
| H.TTSTSPISTVTC(+57.02)PSGENLC(+57.02)YTK.M | Y | 41.81 | 2403.0935 | 22 | -0.6 | 1202.5533 | 2 | 54.92 | 6 | F6:33376 | BuCaTN01_F6a.raw | | | | | | 0 | | | | | | | | | | | 0 | 0 | 0 | 0 | 0 | 0 | 0 | 0 | 0 | 0 | 0 | 0 | 0 | 0 | 0 | 0 | 0 | 7 | 28 | Carbamidomethylation | C12:Carbamidomethylation:1000.00;C19:Carbamidomethylation:1000.00 | PEAKS DB |

**total 16 peptides**

**Best Unique PSM (Scan F6:32677, m/z=1101.5059, z=2, RT=54.17, ppm=-0.4):**

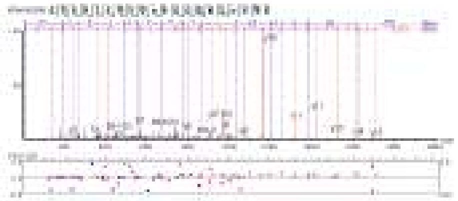

## ABN72539.1

back to list

| Protein Coverage | Supporting Peptides | Best Unique PSM |

**Protein Coverage:**

```
  1 MNVFSIFSLV FLAAFGSCAD DRRSPLEECF READYEEFLE IARNGLKKTS NPKHVVVVGA GMAGLSAAYV LEKAGHRVTL
 81 LEASDRVGGR ANTYRDEKEG WYVNMGPMRL PERHRIVRTY IAKFGLKLNE FFQENENAWY FIRNIRKRVW EVKKDPGVFK
161 YPVKPSEEGK SASQLYRESL KKIIEELKRT NCSYILDKYD SYSTKEYLIK EGNLSRGAVD MIGDLLNEDS SYYLSFIESL
241 KNDDLFSYEK RFDEISGGFD QLPKSMHQDI AEMVHLNAQV TKIQHDAEKV RVAYQTPAKT LSYVTADYVI VCATSRAVRR
321 ISFEPPLPSK KAHALRSIHY KSATKIFLTC TQKFWEADGI HGGKSTTDLP SRFIYYPNHN FTSGVGVIVA YVLADDSDFF
401 QALDIKTSAD IVINDLSLIH QLPKNEIQAL CYPSLIKKWS LDKYTMGALT SFTPYQFQDY SETVAAPVGR IYFAGEYTAR
481 VHGWLDSTIK SGLTAARDVN RASQKPSRIH LISDNQL
```

**Supporting Peptides:**

| Peptide | Uniq | -10lgP | Mass | Length | ppm | m/z | z | RT | Fraction | Scan | Source File | Area F1 | Area F2a | Area F3a | Area F4 | Area F5 | Area F6a | Area F7a | Area F7b | Area F8 | Area F9a | Area Sample 1F10a | Area F10b | Area F11a | Area F11b | Area F12a | Area F13 | #Feature | #Feature F1 | #Feature F2a | #Feature F3a | #Feature F4 | #Feature F5 | #Feature F6a | #Feature F7a | #Feature F7b | #Feature F8 | #Feature F9a | #Feature Sample 1F10a | #Feature F10b | #Feature F11a | #Feature F11b | #Feature F12a | #Feature F13 | Start | End | PTM | AScore | Found By |
|---|---|---|---|---|---|---|---|---|---|---|---|---|---|---|---|---|---|---|---|---|---|---|---|---|---|---|---|---|---|---|---|---|---|---|---|---|---|---|---|---|---|---|---|---|---|---|---|---|---|
| K.HVVVVGAGMAGLSAAYVLEKAGHR.V | Y | 93.24 | 2391.2844 | 24 | 0.0 | 598.8284 | 4 | 71.65 | 16 | F16:37157 | BuCaTN01_F13.raw | | | | | | | | | | | | | | | | 4.1275E5 | 1 | 0 | 0 | 0 | 0 | 0 | 0 | 0 | 0 | 0 | 0 | 0 | 0 | 0 | 0 | 0 | 1 | 54 | 77 | | | PEAKS DB |
| R.VHGWLDSTIKSGLTAAR.D | N | 85.08 | 1810.9690 | 17 | -0.1 | 453.7495 | 4 | 63.71 | 16 | F16:31762 | BuCaTN01_F13.raw | | | | | | | | | | | | | | | | 9.4969E5 | 2 | 0 | 0 | 0 | 0 | 0 | 0 | 0 | 0 | 0 | 0 | 0 | 0 | 0 | 0 | 0 | 2 | 481 | 497 | | | PEAKS DB |
| K.RFDEISGGFDQLPK.S | Y | 71.50 | 1607.7943 | 14 | 0.2 | 536.9388 | 3 | 60.82 | 16 | F16:29757 | BuCaTN01_F13.raw | | | | | | | | | | | | | | | | 3.254E6 | 1 | 0 | 0 | 0 | 0 | 0 | 0 | 0 | 0 | 0 | 0 | 0 | 0 | 0 | 0 | 0 | 1 | 251 | 264 | | | PEAKS DB |
| K.KDPGVFKYPVKPSEEGK.S | N | 68.67 | 1904.0043 | 17 | 0.4 | 477.0085 | 4 | 39.18 | 16 | F16:15857 | BuCaTN01_F13.raw | | | | | | | | | | | | | | | | 5.3545E5 | 1 | 0 | 0 | 0 | 0 | 0 | 0 | 0 | 0 | 0 | 0 | 0 | 0 | 0 | 0 | 0 | 1 | 154 | 170 | | | PEAKS DB |
| K.HVVVVGAGMAGLSAAYVLEK.A | Y | 64.42 | 1970.0659 | 20 | 1.0 | 657.6966 | 3 | 72.52 | 16 | F16:37875 | BuCaTN01_F13.raw | | | | | | | | | | | | | | | | 1.6667E5 | 2 | 0 | 0 | 0 | 0 | 0 | 0 | 0 | 0 | 0 | 0 | 0 | 0 | 0 | 0 | 0 | 2 | 54 | 73 | | | PEAKS DB |
| R.FDEISGGFDQLPK.S | Y | 56.72 | 1451.6932 | 13 | -0.9 | 726.8532 | 2 | 63.80 | 16 | F16:31925 | BuCaTN01_F13.raw | | | | | | | | | | | | | | | | 2.8552E6 | 1 | 0 | 0 | 0 | 0 | 0 | 0 | 0 | 0 | 0 | 0 | 0 | 0 | 0 | 0 | 0 | 1 | 252 | 264 | | | PEAKS DB |
| R.IYFAGEYTAR.V | N | 54.01 | 1189.5768 | 10 | -0.1 | 595.7956 | 2 | 51.76 | 16 | F16:24412 | BuCaTN01_F13.raw | | | | | | | | | | | | | | | | 5.5615E6 | 1 | 0 | 0 | 0 | 0 | 0 | 0 | 0 | 0 | 0 | 0 | 0 | 0 | 0 | 0 | 0 | 1 | 471 | 480 | | | PEAKS DB |
| K.HVVVVGAGMAG.L | N | 42.23 | 995.5222 | 11 | 0.2 | 498.7685 | 2 | 40.63 | 16 | F16:16823 | BuCaTN01_F13.raw | | | | | | | | | | | | | | | | 3.257E4 | 1 | 0 | 0 | 0 | 0 | 0 | 0 | 0 | 0 | 0 | 0 | 0 | 0 | 0 | 0 | 0 | 1 | 54 | 64 | | | PEAKS DB |
| Y.QFQDYSETVAAPVGR.I | Y | 42.19 | 1666.7950 | 15 | -2.7 | 834.4025 | 2 | 51.10 | 16 | F16:23805 | BuCaTN01_F13.raw | | | | | | | | | | | | | | | | 2.0267E4 | 1 | 0 | 0 | 0 | 0 | 0 | 0 | 0 | 0 | 0 | 0 | 0 | 0 | 0 | 0 | 0 | 1 | 456 | 470 | | | PEAKS DB |
| R.VHGWLDSTIK.S | N | 42.06 | 1154.6084 | 10 | 1.0 | 385.8771 | 3 | 51.32 | 16 | F16:23843 | BuCaTN01_F13.raw | | | | | | | | | | | | | | | | 5.3693E5 | 1 | 0 | 0 | 0 | 0 | 0 | 0 | 0 | 0 | 0 | 0 | 0 | 0 | 0 | 0 | 0 | 1 | 481 | 490 | | | PEAKS DB |
| K.TSADIVINDLSLIHQLPK.N | N | 41.52 | 1976.0942 | 18 | 1.6 | 659.7064 | 3 | 81.28 | 16 | F16:44375 | BuCaTN01_F13.raw | | | | | | | | | | | | | | | | 0 | 0 | 0 | 0 | 0 | 0 | 0 | 0 | 0 | 0 | 0 | 0 | 0 | 0 | 0 | 0 | 0 | 0 | 407 | 424 | | | PEAKS DB |

**total 11 peptides**

**Best Unique PSM (Scan F16:37157, m/z=598.8284, z=4, RT=71.65, ppm=0.0):**

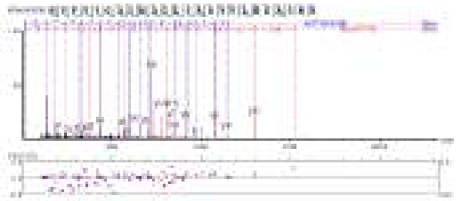

## A8QL51.1

back to list

| Protein Coverage | Supporting Peptides | Best Unique PSM |

**Protein Coverage:**

```
  1 MNVFSIFSLV FLAAFGSCAD DRRSPLEECF READYEEFLE IARNGLKKTS NPKHVVVVGA GMAGLSAAYV LEKAGHRVTL
 81 LEASDRVGGR ANTYRDEKEG WYVNMGPMRL PERHRIVRTY IAKFGLKLNE FFQENENAWY FIRNIRKRVW EVKKDPGVFK
161 YPVKPSEEGK SASQLYRESL KKIIEELKRT NCSYILDKYD SYSTKEYLIK EGNLSRGAVD MIGDLLNEDS SYYLSFIESL
241 KNDDLFSYEK RFDEISGGFD QLPKSMHQDI AEMVHLNAQV TKIQHDAEKV RVAYQTPAKT LSYVTADYVI VCATSRAVRR
321 ISFEPPLPSK KAHALRSIHY KSATKIFLTC TQKFWEADGI HGGKSTTDLP SRFIYYPNHN FTSGVGVIVA YVLADDSDFF
401 QALDIKTSAD IVINDLSLIH QLPKNEIQAL CYPSLIKKWS LDKYTMGALT SFTPYQFQDY SETVAAPVGR IYFAGEYTAR
481 VHGWLDSTIK SGLTAARDVN RASQKPSRIH LISDNQL
```

**Supporting Peptides:**

proteins

| Peptide | Uniq | -10lgP | Mass | Length | ppm | m/z | z | RT | Fraction | Scan | Source File | Area F1 | Area F2a | Area F3a | Area F4 | Area F5 | Area F6a | Area F7a | Area F7b | Area F8 | Area F9a | Area Sample 1F10a | Area F10b | Area F11a | Area F11b | Area F12a | Area F13 | #Feature | #Feature F1 | #Feature F2a | #Feature F3a | #Feature F4 | #Feature F5 | #Feature F6a | #Feature F7a | #Feature F7b | #Feature F8 | #Feature F9a | #Feature Sample 1F10a | #Feature F10b | #Feature F11a | #Feature F11b | #Feature F12a | #Feature F13 | Start | End | PTM | AScore | Found By |
|---|---|---|---|---|---|---|---|---|---|---|---|---|---|---|---|---|---|---|---|---|---|---|---|---|---|---|---|---|---|---|---|---|---|---|---|---|---|---|---|---|---|---|---|---|---|---|---|---|---|
| K.HVVVVGAGMAGLSAAYVLEKAGHR.V | Y | 93.24 | 2391.2844 | 24 | 0.0 | 598.8284 | 4 | 71.65 | 16 | F16:37157 | BuCaTN01_F13.raw | | | | | | | | | | | | | | | | 4.1275E5 | 1 | 0 | 0 | 0 | 0 | 0 | 0 | 0 | 0 | 0 | 0 | 0 | 0 | 0 | 0 | 0 | 1 | 54 | 77 | | | PEAKS DB |
| R.VHGWLDSTIKSGLTAAR.D | N | 85.08 | 1810.9690 | 17 | -0.1 | 453.7495 | 4 | 63.71 | 16 | F16:31762 | BuCaTN01_F13.raw | | | | | | | | | | | | | | | | 9.4969E5 | 2 | 0 | 0 | 0 | 0 | 0 | 0 | 0 | 0 | 0 | 0 | 0 | 0 | 0 | 0 | 0 | 2 | 481 | 497 | | | PEAKS DB |
| K.RFDEISGGFDQLPK.S | Y | 71.50 | 1607.7943 | 14 | 0.2 | 536.9388 | 3 | 60.82 | 16 | F16:29757 | BuCaTN01_F13.raw | | | | | | | | | | | | | | | | 3.254E6 | 1 | 0 | 0 | 0 | 0 | 0 | 0 | 0 | 0 | 0 | 0 | 0 | 0 | 0 | 0 | 0 | 1 | 251 | 264 | | | PEAKS DB |
| K.KDPGVFKYPVKPSEEGK.S | N | 68.67 | 1904.0043 | 17 | 0.4 | 477.0085 | 4 | 39.18 | 16 | F16:15857 | BuCaTN01_F13.raw | | | | | | | | | | | | | | | | 5.3545E5 | 1 | 0 | 0 | 0 | 0 | 0 | 0 | 0 | 0 | 0 | 0 | 0 | 0 | 0 | 0 | 0 | 1 | 154 | 170 | | | PEAKS DB |
| K.HVVVVGAGMAGLSAAYVLEK.A | Y | 64.42 | 1970.0659 | 20 | 1.0 | 657.6966 | 3 | 72.52 | 16 | F16:37875 | BuCaTN01_F13.raw | | | | | | | | | | | | | | | | 1.6667E5 | 2 | 0 | 0 | 0 | 0 | 0 | 0 | 0 | 0 | 0 | 0 | 0 | 0 | 0 | 0 | 0 | 2 | 54 | 73 | | | PEAKS DB |
| R.FDEISGGFDQLPK.S | Y | 56.72 | 1451.6932 | 13 | -0.9 | 726.8532 | 2 | 63.80 | 16 | F16:31925 | BuCaTN01_F13.raw | | | | | | | | | | | | | | | | 2.8552E6 | 1 | 0 | 0 | 0 | 0 | 0 | 0 | 0 | 0 | 0 | 0 | 0 | 0 | 0 | 0 | 0 | 1 | 252 | 264 | | | PEAKS DB |
| R.IYFAGEYTAR.V | N | 54.01 | 1189.5768 | 10 | -0.1 | 595.7956 | 2 | 51.76 | 16 | F16:24412 | BuCaTN01_F13.raw | | | | | | | | | | | | | | | | 5.5615E6 | 1 | 0 | 0 | 0 | 0 | 0 | 0 | 0 | 0 | 0 | 0 | 0 | 0 | 0 | 0 | 0 | 1 | 471 | 480 | | | PEAKS DB |
| K.HVVVVGAGMAG.L | N | 42.23 | 995.5222 | 11 | 0.2 | 498.7685 | 2 | 40.63 | 16 | F16:16823 | BuCaTN01_F13.raw | | | | | | | | | | | | | | | | 3.257E4 | 1 | 0 | 0 | 0 | 0 | 0 | 0 | 0 | 0 | 0 | 0 | 0 | 0 | 0 | 0 | 0 | 1 | 54 | 64 | | | PEAKS DB |
| Y.QFQDYSETVAAPVGR.I | Y | 42.19 | 1666.7950 | 15 | -2.7 | 834.4025 | 2 | 51.10 | 16 | F16:23805 | BuCaTN01_F13.raw | | | | | | | | | | | | | | | | 2.0267E4 | 1 | 0 | 0 | 0 | 0 | 0 | 0 | 0 | 0 | 0 | 0 | 0 | 0 | 0 | 0 | 0 | 1 | 456 | 470 | | | PEAKS DB |
| R.VHGWLDSTIK.S | N | 42.06 | 1154.6084 | 10 | 1.0 | 385.8771 | 3 | 51.32 | 16 | F16:23843 | BuCaTN01_F13.raw | | | | | | | | | | | | | | | | 5.3693E5 | 1 | 0 | 0 | 0 | 0 | 0 | 0 | 0 | 0 | 0 | 0 | 0 | 0 | 0 | 0 | 0 | 1 | 481 | 490 | | | PEAKS DB |
| K.TSADIVINDLSLIHQLPK.N | N | 41.52 | 1976.0942 | 18 | 1.6 | 659.7064 | 3 | 81.28 | 16 | F16:44375 | BuCaTN01_F13.raw | | | | | | | | | | | | | | | | 0 | 0 | 0 | 0 | 0 | 0 | 0 | 0 | 0 | 0 | 0 | 0 | 0 | 0 | 0 | 0 | 0 | 0 | 407 | 424 | | | PEAKS DB |
| total 11 peptides | | | | | | | | | | | | | | | | | | | | | | | | | | | | | | | | | | | | | | | | | | | | | | | | | | | |

**Best Unique PSM (Scan F16:37157, m/z=598.8284, z=4, RT=71.65, ppm=0.0):**

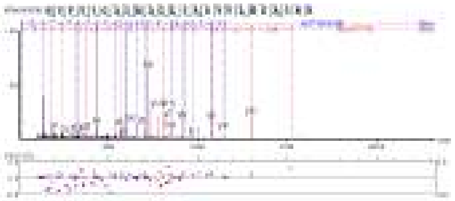

## ABN72537.1

[back to list]

| **Protein Coverage** | **Supporting Peptides** | **Best Unique PSM** |

**Protein Coverage:**

```
  1 MIQALLVTIC LAVFPYQGSS IILESGNVND YEVVYPQKVP LLPKGGVQNP QPKTKYEDTV QYEFEVNGEP VVLHLERNKG
 81 LFSEDYTEAH YAPDGREITT RPPVQDHCYY HGYIQNEADS SAAISACDGL KGHFKHQGET YFIEPLKLSD SEAHAIYKDE
161 NVEEENETPK ICGLTETTWE SDEPIRNASL LIYTPEQNRY LKVKKYIEFY VAVDNRMYRH YKRNKPIIKR RVYELVNILN
241 TILRRLNFHI ALIGLEIWSK RDKINVQSDV KATLKSFGKW REKKLLPRKR NDNAQLLTRI DFNGNTVGLA ALGSLCSVKY
321 SVAVIQDYSK RTSMVASTMA HEMGHNLGIN HDRASCTSCG SNKCIMATKR TKPASRFSSC SVREHQRYLL RDRPQCILNK
401 PLITDIVAPA ICGNYFVEVG EECDCGSPRD CRSACCNAAT CKLKHEAQCD SGECCGKCKF KKVGAKCRAA KDDCDLPERC
481 TGRSAECPTD IFRRNGLPCQ NKQGYCYNGK CPTLTNQCIA LMGPNVKVSR DSCFTLNQRG KGCGYCRMQN GAKIPCAAKD
561 IKCGKLFCKK RNSGVCNCLI LPDDPNYGMV ETGTKCGDGM VCSDRKCVKL QTVY
```

C Carbamidomethylation (+57.02)
O Oxidation (M) (+15.99)

423 487 511 518 522 533 607

**Supporting Peptides:**

| Peptide | Uniq | -10lgP | Mass | Length | ppm | m/z | z | RT | Fraction | Scan | Source File | Area F1 | Area F2a | Area F3a | Area F4 | Area F5 | Area F6a | Area F7a | Area F7b | Area F8 | Area F9a | Area Sample 1F10a | Area F10b | Area F11a | Area F11b | Area F12a | Area F13 | #Feature | #Feature F1 | #Feature F2a | #Feature F3a | #Feature F4 | #Feature F5 | #Feature F6a | #Feature F7a | #Feature F7b | #Feature F8 | #Feature F9a | #Feature Sample 1F10a | #Feature F10b | #Feature F11a | #Feature F11b | #Feature F12a | #Feature F13 | Start | End | PTM | AScore | Found By |
|---|---|---|---|---|---|---|---|---|---|---|---|---|---|---|---|---|---|---|---|---|---|---|---|---|---|---|---|---|---|---|---|---|---|---|---|---|---|---|---|---|---|---|---|---|---|---|---|---|---|
| N.YFVEVGEEC(+57.02)DC(+57.02)GSPR.D | N | 78.13 | 1802.7240 | 15 | -1.2 | 902.3682 | 2 | 51.19 | 7 | F7:25194 | BuCaTN01_F7a.raw | | | | | | | 6.8002E5 | | | | | | | | | | 1 | 0 | 0 | 0 | 0 | 0 | 0 | 1 | 0 | 0 | 0 | 0 | 0 | 0 | 0 | 0 | 0 | 415 | 429 | Carbamidomethylation | C9:Carbamidomethylation:1000.00;C11:Carbamidomethylation:1000.00 | PEAKS DB |
| R.SAEC(+57.02)PTDIFR.R | N | 77.69 | 1194.5339 | 10 | 0.3 | 598.2744 | 2 | 52.54 | 6 | F6:31248 | BuCaTN01_F6a.raw | | | | | | 5.7631E5 | 0 | | | | | | | | | 6.1447E5 | 2 | 0 | 0 | 0 | 0 | 0 | 1 | 0 | 0 | 0 | 0 | 0 | 0 | 0 | 0 | 0 | 1 | 484 | 493 | Carbamidomethylation | C4:Carbamidomethylation:1000.00 | PEAKS DB |
| K.C(+57.02)PTLTNQC(+57.02)IALMGPNVK.V | Y | 75.87 | 1915.9318 | 17 | -0.1 | 958.9730 | 2 | 61.86 | 7 | F7:34256 | BuCaTN01_F7a.raw | | | | | | | 9.4698E5 | | | | | | | | | | 1 | 0 | 0 | 0 | 0 | 0 | 0 | 1 | 0 | 0 | 0 | 0 | 0 | 0 | 0 | 0 | 0 | 511 | 527 | Carbamidomethylation | C1:Carbamidomethylation:1000.00;C8:Carbamidomethylation:1000.00 | PEAKS DB |
| K.YSVAVIQDYSKR.T | N | 65.96 | 1427.7408 | 12 | -0.4 | 714.8774 | 2 | 50.38 | 16 | F16:23330 | BuCaTN01_F13.raw | | | | | | 3.8617E4 | | 2.1157E5 | 7.2103E5 | | | | | | | 1.3262E5 | 5 | 0 | 0 | 0 | 0 | 0 | 1 | 0 | 1 | 2 | 0 | 0 | 0 | 0 | 0 | 0 | 1 | 320 | 331 | | | PEAKS DB |
| R.SAEC(+57.02)PTDIFRR.N | N | 63.53 | 1350.6350 | 11 | -0.3 | 676.3246 | 2 | 47.99 | 7 | F7:22811 | BuCaTN01_F7a.raw | | | | | | | 5.2593E6 | | | | | | | | | | 1 | 0 | 0 | 0 | 0 | 0 | 0 | 1 | 0 | 0 | 0 | 0 | 0 | 0 | 0 | 0 | 0 | 484 | 494 | Carbamidomethylation | C4:Carbamidomethylation:1000.00 | PEAKS DB |
| R.DSC(+57.02)FTLNQR.G | N | 61.56 | 1139.5029 | 9 | 0.7 | 570.7592 | 2 | 42.59 | 7 | F7:18306 | BuCaTN01_F7a.raw | | | | | | 2.5225E6 | 1.4188E7 | | | | | | | | | 1.2128E6 | 4 | 0 | 0 | 0 | 0 | 0 | 1 | 2 | 0 | 0 | 0 | 0 | 0 | 0 | 0 | 0 | 1 | 531 | 539 | Carbamidomethylation | C3:Carbamidomethylation:1000.00 | PEAKS DB |
| K.YSVAVIQDYSK.R | N | 59.91 | 1271.6398 | 11 | -1.4 | 636.8263 | 2 | 54.22 | 16 | F16:25601 | BuCaTN01_F13.raw | | | | | | | | | | | | | | | | 1.3887E5 | 1 | 0 | 0 | 0 | 0 | 0 | 0 | 0 | 0 | 0 | 0 | 0 | 0 | 0 | 0 | 0 | 1 | 320 | 330 | | | PEAKS DB |
| N.GNTVGLAALGSLC(+57.02)SVK.Y | Y | 58.77 | 1545.8185 | 16 | 4.6 | 773.9169 | 2 | 68.86 | 14 | F14:43881 | BuCaTN01_F11b.raw | | | | | | | | | | | | | | 1.7694E5 | | | 1 | 0 | 0 | 0 | 0 | 0 | 0 | 0 | 0 | 0 | 0 | 0 | 0 | 0 | 1 | 0 | 0 | 304 | 319 | Carbamidomethylation | C13:Carbamidomethylation:1000.00 | PEAKS DB |
| R.NDNAQLLTR.I | N | 56.90 | 1043.5360 | 9 | -1.2 | 522.7747 | 2 | 34.37 | 16 | F16:12308 | BuCaTN01_F13.raw | | | | | | | | | | | | | | | | 4.5342E5 | 1 | 0 | 0 | 0 | 0 | 0 | 0 | 0 | 0 | 0 | 0 | 0 | 0 | 0 | 0 | 0 | 1 | 291 | 299 | | | PEAKS DB |
| K.C(+57.02)PTLTNQC(+57.02)IALM(+15.99)GPNVK.V | Y | 55.66 | 1931.9268 | 17 | 0.5 | 644.9832 | 3 | 55.08 | 7 | F7:28076 | BuCaTN01_F7a.raw | | | | | | | 4.7884E5 | | | | | | | | | | 2 | 0 | 0 | 0 | 0 | 0 | 0 | 2 | 0 | 0 | 0 | 0 | 0 | 0 | 0 | 0 | 0 | 511 | 527 | Carbamidomethylation; Oxidation (M) | C1:Carbamidomethylation:1000.00;C8:Carbamidomethylation:1000.00;M12:Oxidation (M):1000.00 | PEAKS DB |
| S.VKYSVAVIQDYSKR.T | N | 51.68 | 1654.9042 | 14 | -0.1 | 552.6420 | 3 | 49.70 | 9 | F9:28243 | BuCaTN01_F8.raw | | | | | | | | | 1.4456E5 | | | | | | | | 1 | 0 | 0 | 0 | 0 | 0 | 0 | 0 | 0 | 1 | 0 | 0 | 0 | 0 | 0 | 0 | 0 | 318 | 331 | | | PEAKS DB |
| D.PNYGMVETGTK.C | Y | 51.49 | 1195.5543 | 11 | 4.2 | 598.7869 | 2 | 37.39 | 16 | F16:14710 | BuCaTN01_F13.raw | | | | | | | | | | | | | | | | 2.0955E5 | 2 | 0 | 0 | 0 | 0 | 0 | 0 | 0 | 0 | 0 | 0 | 0 | 0 | 0 | 0 | 0 | 2 | 585 | 595 | | | PEAKS DB |
| S.VKYSVAVIQDYSK.R | N | 44.22 | 1498.8031 | 13 | 0.5 | 500.6086 | 3 | 52.57 | 9 | F9:30635 | BuCaTN01_F8.raw | | | | | | | | | 5.0127E4 | | | | | | | | 1 | 0 | 0 | 0 | 0 | 0 | 0 | 0 | 0 | 1 | 0 | 0 | 0 | 0 | 0 | 0 | 0 | 318 | 330 | | | PEAKS DB |
| G.VC(+57.02)NC(+57.02)LILPDDPNYGMVETGTK.C | Y | 43.93 | 2395.0857 | 21 | -1.7 | 799.3678 | 3 | 67.90 | 7 | F7:38826 | BuCaTN01_F7a.raw | | | | | | | 0 | | | | | | | | | | 0 | 0 | 0 | 0 | 0 | 0 | 0 | 0 | 0 | 0 | 0 | 0 | 0 | 0 | 0 | 0 | 0 | 575 | 595 | Carbamidomethylation | C2:Carbamidomethylation:1000.00;C4:Carbamidomethylation:1000.00 | PEAKS DB |
| R.KC(+57.02)VKLQTVY | Y | 41.46 | 1137.6216 | 9 | 1.4 | 569.8188 | 2 | 37.52 | 7 | F7:14028 | BuCaTN01_F7a.raw | | | | | | | 1.5697E5 | | | | | | | | | | 1 | 0 | 0 | 0 | 0 | 0 | 0 | 1 | 0 | 0 | 0 | 0 | 0 | 0 | 0 | 0 | 0 | 606 | 614 | Carbamidomethylation | C2:Carbamidomethylation:1000.00 | PEAKS DB |
| total 15 peptides | | | | | | | | | | | | | | | | | | | | | | | | | | | | | | | | | | | | | | | | | | | | | | | | | | | |

**Best Unique PSM (Scan F7:34256, m/z=958.9730, z=2, RT=61.86, ppm=-0.1):**

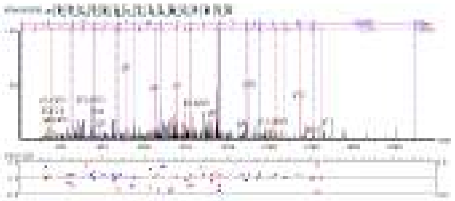

## A8QL49.1

[back to list]

| **Protein Coverage** | **Supporting Peptides** | **Best Unique PSM** |

**Protein Coverage:**

1 MIQALLVTIC LAVFPYQGSS IILESGNVND YEVVYPQKVP LLPKGGVQNP QPKTKYEDTV QYEFEVNGEP VVLHLERNKG
81 LFSEDYTEAH YAPDGREITT RPPVQDHCYY HGYIQNEADS SAAISACDGL KGHFKHQGET YFIEPLKLSD SEAHAIYKDE
161 NVEEENETPK ICGLTETTWE SDEPIRNASL LIYTPEQNRY LKVKKYIEFY VAVDNRMYRH YKRNKPIIKR RVYELVNILN
241 TILRRLNFHI ALIGLEIWSK RDKINVQSDV KATLKSFGKW REKKLLPRKR **NDNAQLLTR**I DFN**GNTVGLA ALGSLCSVKY**
321 **SVAVIQDYSK R**TSMVASTMA HEMGHNLGIN HDRASCTSCG SNKCIMATKR TKPASRFSSC SVREHQRYLL RDRPQCILNK
401 PLITDIVAPA ICGN**YFVEVG EECDCGSPR**D CRSACCNAAT CKLKHEAQCD SGECCGKCKF KKVGAKCRAA KDDCDLPERC
481 TGR**SAECPTD IFRR**NGLPCQ NKQGYCYNGK **CPTLTNQCIA LMGPNVK**VSR **DSCFTLNQR**G KGCGYCRMQN GAKIPCAAKD
561 IKCGKLFCKK RNSG**VCNCLI LPDDPNYGMV ETGTK**CGDGM VCSDR**KCVKL QTVY**

C Carbamidomethylation (+57.02)
O Oxidation (M) (+15.99)

423 487 511 518 522 533 607

**Supporting Peptides:**

| Peptide | Uniq | -10lgP | Mass | Length | ppm | m/z | z | RT | Fraction | Scan | Source File | Area F1 | Area F2a | Area F3a | Area F4 | Area F5 | Area F6a | Area F7a | Area F7b | Area F8 | Area F9a | Area Sample 1F10a | Area F10b | Area F11a | Area F11b | Area F12a | Area F13 | #Feature | #Feature F1 | #Feature F2a | #Feature F3a | #Feature F4 | #Feature F5 | #Feature F6a | #Feature F7a | #Feature F7b | #Feature F8 | #Feature F9a | #Feature Sample 1F10a | #Feature F10b | #Feature F11a | #Feature F11b | #Feature F12a | #Feature F13 | Start | End | PTM | AScore | Found By |
|---|---|---|---|---|---|---|---|---|---|---|---|---|---|---|---|---|---|---|---|---|---|---|---|---|---|---|---|---|---|---|---|---|---|---|---|---|---|---|---|---|---|---|---|---|---|---|---|---|---|
| N.YFVEVGEEC(+57.02)DC(+57.02)GSPR.D | N | 78.13 | 1802.7240 | 15 | -1.2 | 902.3682 | 2 | 51.19 | 7 | F7:25194 | BuCaTN01_F7a.raw | | | | | | | 6.8002E5 | | | | | | | | | | 1 | 0 | 0 | 0 | 0 | 0 | 0 | 1 | 0 | 0 | 0 | 0 | 0 | 0 | 0 | 0 | 0 | 415 | 429 | Carbamidomethylation | C9:Carbamidomethylation:1000.00;C11:Carbamidomethylation:1000.00 | PEAKS DB |
| R.SAEC(+57.02)PTDIFR.R | N | 77.69 | 1194.5339 | 10 | 0.3 | 598.2744 | 2 | 52.54 | 6 | F6:31248 | BuCaTN01_F6a.raw | | | | | | 5.7631E5 | 0 | | | | | | | | | 6.1447E5 | 2 | 0 | 0 | 0 | 0 | 0 | 1 | 0 | 0 | 0 | 0 | 0 | 0 | 0 | 0 | 0 | 1 | 484 | 493 | Carbamidomethylation | C4:Carbamidomethylation:1000.00 | PEAKS DB |
| K.C(+57.02)PTLTNQC(+57.02)IALMGPNVK.V | Y | 75.87 | 1915.9318 | 17 | -0.1 | 958.9730 | 2 | 61.86 | 7 | F7:34256 | BuCaTN01_F7a.raw | | | | | | | 9.4698E5 | | | | | | | | | | 1 | 0 | 0 | 0 | 0 | 0 | 0 | 1 | 0 | 0 | 0 | 0 | 0 | 0 | 0 | 0 | 0 | 511 | 527 | Carbamidomethylation | C1:Carbamidomethylation:1000.00;C8:Carbamidomethylation:1000.00 | PEAKS DB |
| K.YSVAVIQDYSKR.T | N | 65.96 | 1427.7408 | 12 | -0.4 | 714.8774 | 2 | 50.38 | 16 | F16:23330 | BuCaTN01_F13.raw | | | | | | 3.8617E4 | | 2.1157E5 | 7.2103E5 | | | | | | | 1.3262E5 | 5 | 0 | 0 | 0 | 0 | 0 | 1 | 0 | 1 | 2 | 0 | 0 | 0 | 0 | 0 | 0 | 1 | 320 | 331 | | | PEAKS DB |
| R.SAEC(+57.02)PTDIFRR.N | N | 63.53 | 1350.6350 | 11 | -0.3 | 676.3246 | 2 | 47.99 | 7 | F7:22811 | BuCaTN01_F7a.raw | | | | | | | 5.2593E6 | | | | | | | | | | 1 | 0 | 0 | 0 | 0 | 0 | 0 | 1 | 0 | 0 | 0 | 0 | 0 | 0 | 0 | 0 | 0 | 484 | 494 | Carbamidomethylation | C4:Carbamidomethylation:1000.00 | PEAKS DB |
| R.DSC(+57.02)FTLNQR.G | N | 61.56 | 1139.5029 | 9 | 0.7 | 570.7592 | 2 | 42.59 | 7 | F7:18306 | BuCaTN01_F7a.raw | | | | | | 2.5225E6 | 1.4188E7 | | | | | | | | | 1.2128E6 | 4 | 0 | 0 | 0 | 0 | 0 | 1 | 2 | 0 | 0 | 0 | 0 | 0 | 0 | 0 | 0 | 1 | 531 | 539 | Carbamidomethylation | C3:Carbamidomethylation:1000.00 | PEAKS DB |
| K.YSVAVIQDYSK.R | N | 59.91 | 1271.6398 | 11 | -1.4 | 636.8263 | 2 | 54.22 | 16 | F16:25601 | BuCaTN01_F13.raw | | | | | | | | | | | | | | | | 1.3887E5 | 1 | 0 | 0 | 0 | 0 | 0 | 0 | 0 | 0 | 0 | 0 | 0 | 0 | 0 | 0 | 0 | 1 | 320 | 330 | | | PEAKS DB |
| N.GNTVGLAALGSLC(+57.02)SVK.Y | Y | 58.77 | 1545.8185 | 16 | 4.6 | 773.9169 | 2 | 68.86 | 14 | F14:43881 | BuCaTN01_F11b.raw | | | | | | | | | | | | | | 1.7694E5 | | | 1 | 0 | 0 | 0 | 0 | 0 | 0 | 0 | 0 | 0 | 0 | 0 | 0 | 0 | 1 | 0 | 0 | 304 | 319 | Carbamidomethylation | C13:Carbamidomethylation:1000.00 | PEAKS DB |
| R.NDNAQLLTR.I | N | 56.90 | 1043.5360 | 9 | -1.2 | 522.7747 | 2 | 34.37 | 16 | F16:12308 | BuCaTN01_F13.raw | | | | | | | | | | | | | | | | 4.5342E5 | 1 | 0 | 0 | 0 | 0 | 0 | 0 | 0 | 0 | 0 | 0 | 0 | 0 | 0 | 0 | 0 | 1 | 291 | 299 | | | PEAKS DB |
| K.C(+57.02)PTLTNQC(+57.02)IALM(+15.99)GPNVK.V | Y | 55.66 | 1931.9268 | 17 | 0.5 | 644.9832 | 3 | 55.08 | 7 | F7:28076 | BuCaTN01_F7a.raw | | | | | | | 4.7884E5 | | | | | | | | | | 2 | 0 | 0 | 0 | 0 | 0 | 0 | 2 | 0 | 0 | 0 | 0 | 0 | 0 | 0 | 0 | 0 | 511 | 527 | Carbamidomethylation; Oxidation (M) | C1:Carbamidomethylation:1000.00;C8:Carbamidomethylation:1000.00;M12:Oxidation (M):1000.00 | PEAKS DB |
| S.VKYSVAVIQDYSKR.T | N | 51.68 | 1654.9042 | 14 | -0.1 | 552.6420 | 3 | 49.70 | 9 | F9:28243 | BuCaTN01_F8.raw | | | | | | | | | 1.4456E5 | | | | | | | | 1 | 0 | 0 | 0 | 0 | 0 | 0 | 0 | 0 | 1 | 0 | 0 | 0 | 0 | 0 | 0 | 0 | 318 | 331 | | | PEAKS DB |
| D.PNYGMVETGTK.C | Y | 51.49 | 1195.5543 | 11 | 4.2 | 598.7869 | 2 | 37.39 | 16 | F16:14710 | BuCaTN01_F13.raw | | | | | | | | | | | | | | | | 2.0955E5 | 2 | 0 | 0 | 0 | 0 | 0 | 0 | 0 | 0 | 0 | 0 | 0 | 0 | 0 | 0 | 0 | 2 | 585 | 595 | | | PEAKS DB |
| S.VKYSVAVIQDYSK.R | N | 44.22 | 1498.8031 | 13 | 0.5 | 500.6086 | 3 | 52.57 | 9 | F9:30635 | BuCaTN01_F8.raw | | | | | | | | | 5.0127E4 | | | | | | | | 1 | 0 | 0 | 0 | 0 | 0 | 0 | 0 | 0 | 1 | 0 | 0 | 0 | 0 | 0 | 0 | 0 | 318 | 330 | | | PEAKS DB |
| G.VC(+57.02)NC(+57.02)LILPDDPNYGMVETGTK.C | Y | 43.93 | 2395.0857 | 21 | -1.7 | 799.3678 | 3 | 67.90 | 7 | F7:38826 | BuCaTN01_F7a.raw | | | | | | | 0 | | | | | | | | | | 0 | 0 | 0 | 0 | 0 | 0 | 0 | 0 | 0 | 0 | 0 | 0 | 0 | 0 | 0 | 0 | 0 | 575 | 595 | Carbamidomethylation | C2:Carbamidomethylation:1000.00;C4:Carbamidomethylation:1000.00 | PEAKS DB |
| R.KC(+57.02)VKLQTVY | Y | 41.46 | 1137.6216 | 9 | 1.4 | 569.8188 | 2 | 37.52 | 7 | F7:14028 | BuCaTN01_F7a.raw | | | | | | | 1.5697E5 | | | | | | | | | | 1 | 0 | 0 | 0 | 0 | 0 | 0 | 1 | 0 | 0 | 0 | 0 | 0 | 0 | 0 | 0 | 0 | 606 | 614 | Carbamidomethylation | C2:Carbamidomethylation:1000.00 | PEAKS DB |
| total 15 peptides | | | | | | | | | | | | | | | | | | | | | | | | | | | | | | | | | | | | | | | | | | | | | | | | | |

**Best Unique PSM (Scan F7:34256, m/z=958.9730, z=2, RT=61.86, ppm=-0.1):**

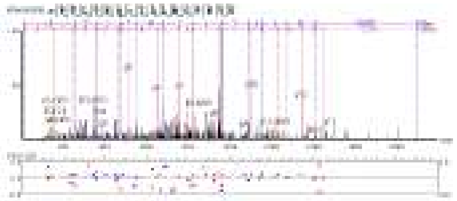

## 7ULR

back to list

| Protein Coverage | Supporting Peptides | Best Unique PSM |

**Protein Coverage:**

1 M**RTCLISPSS TPQTCPNGQD ICFLKA**QCDK FCSIR**GPVIE QGCVATCPQF RSNYRSLLCC TTDNCNH**

15 43 47 59 60 65

C Carbamidomethylation (+57.02)

**Supporting Peptides:**

| Peptide | Uniq | -10lgP | Mass | Length | ppm | m/z | z | RT | Fraction | Scan | Source File | Area F1 | Area F2a | Area F3a | Area F4 | Area F5 | Area F6a | Area F7a | Area F7b | Area F8 | Area F9a | Area Sample 1F10a | Area F10b | Area F11a | Area F11b | Area F12a | Area F13 | #Feature | #Feature F1 | #Feature F2a | #Feature F3a | #Feature F4 | #Feature F5 | #Feature F6a | #Feature F7a | #Feature F7b | #Feature F8 | #Feature F9a | #Feature Sample 1F10a | #Feature F10b | #Feature F11a | #Feature F11b | #Feature F12a | #Feature F13 | Start | End | PTM | AScore | Found By |
|---|---|---|---|---|---|---|---|---|---|---|---|---|---|---|---|---|---|---|---|---|---|---|---|---|---|---|---|---|---|---|---|---|---|---|---|---|---|---|---|---|---|---|---|---|---|---|---|---|---|
| R.GPVIEQGC(+57.02)VATC(+57.02)PQFR.S | N | 96.70 | 1817.8552 | 16 | 1.3 | 909.9360 | 2 | 51.36 | 6 | F6:30037 | BuCaTN01_F6a.raw | | | | | | 4.938E5 | | | | | | | | 0 | | | 1 | 0 | 0 | 0 | 0 | 0 | 1 | 0 | 0 | 0 | 0 | 0 | 0 | 0 | 0 | 0 | 0 | 36 | 51 | Carbamidomethylation | C8:Carbamidomethylation:1000.00;C12:Carbamidomethylation:1000.00 | PEAKS DB |
| R.SLLC(+57.02)C(+57.02)TTDNC(+57.02)NH | N | 79.81 | 1493.5698 | 12 | -0.3 | 747.7920 | 2 | 37.43 | 6 | F6:17551 | BuCaTN01_F6a.raw | | | 4.1658E6 | | | 7.1212E8 | 3.0441E6 | 4.2545E7 | | 1.9164E6 | | 4.7006E6 | | 5.2769E6 | 7.5251E5 | | 12 | 0 | 0 | 1 | 0 | 0 | 4 | 1 | 2 | 0 | 1 | 0 | 1 | 0 | 1 | 1 | 0 | 56 | 67 | Carbamidomethylation | C4:Carbamidomethylation:1000.00;C5:Carbamidomethylation:1000.00;C10:Carbamidomethylation:1000.00 | PEAKS DB |
| P.VIEQGC(+57.02)VATC(+57.02)PQFR.S | N | 70.62 | 1663.7810 | 14 | 3.0 | 832.8978 | 2 | 46.31 | 8 | F8:22974 | BuCaTN01_F7b.raw | | | | | | | | 7.7386E6 | | | | | | | | | 1 | 0 | 0 | 0 | 0 | 0 | 0 | 0 | 1 | 0 | 0 | 0 | 0 | 0 | 0 | 0 | 0 | 38 | 51 | Carbamidomethylation | C6:Carbamidomethylation:1000.00;C10:Carbamidomethylation:1000.00 | PEAKS DB |
| R.SNYRSLLC(+57.02)C(+57.02)TTDNC(+57.02)NH | N | 62.84 | 2013.8092 | 16 | -1.7 | 672.2759 | 3 | 46.75 | 6 | F6:25700 | BuCaTN01_F6a.raw | | | | | | 1.3691E6 | | 9.5627E5 | | | | | | | | | 2 | 0 | 0 | 0 | 0 | 0 | 1 | 0 | 1 | 0 | 0 | 0 | 0 | 0 | 0 | 0 | 0 | 52 | 67 | Carbamidomethylation | C8:Carbamidomethylation:1000.00;C9:Carbamidomethylation:1000.00;C14:Carbamidomethylation:1000.00 | PEAKS DB |
| M.RTC(+57.02)LISPSSTPQTC(+57.02)PNGQDIC(+57.02)FLKA.Q | Y | 53.10 | 2850.3462 | 25 | -0.5 | 951.1222 | 3 | 61.02 | 6 | F6:38720 | BuCaTN01_F6a.raw | | | | | | 8.6023E7 | | | | | | | | | | | 1 | 0 | 0 | 0 | 0 | 0 | 1 | 0 | 0 | 0 | 0 | 0 | 0 | 0 | 0 | 0 | 0 | 2 | 26 | Carbamidomethylation | C3:Carbamidomethylation:1000.00;C14:Carbamidomethylation:1000.00;C21:Carbamidomethylation:1000.00 | PEAKS DB |
| R.GPVIEQGC(+57.02)VAT.C | N | 50.22 | 1129.5437 | 11 | 0.5 | 565.7794 | 2 | 41.92 | 6 | F6:21363 | BuCaTN01_F6a.raw | | | | | | 6.58E5 | | | | | | | | | | | 1 | 0 | 0 | 0 | 0 | 0 | 1 | 0 | 0 | 0 | 0 | 0 | 0 | 0 | 0 | 0 | 0 | 36 | 46 | Carbamidomethylation | C8:Carbamidomethylation:1000.00 | PEAKS DB |
| M.RTC(+57.02)LISPSSTPQT.C | Y | 46.36 | 1446.7136 | 13 | 0.5 | 724.3644 | 2 | 40.18 | 6 | F6:19763 | BuCaTN01_F6a.raw | | | | | | 1.458E6 | | | | | | | | | | | 1 | 0 | 0 | 0 | 0 | 0 | 1 | 0 | 0 | 0 | 0 | 0 | 0 | 0 | 0 | 0 | 0 | 2 | 14 | Carbamidomethylation | C3:Carbamidomethylation:1000.00 | PEAKS DB |
| R.GPVIEQGC(+57.02)VA.T | N | 42.28 | 1028.4961 | 10 | 0.2 | 515.2554 | 2 | 41.42 | 6 | F6:20784 | BuCaTN01_F6a.raw | | | | | | 2.0497E6 | | | | | | | | | | | 1 | 0 | 0 | 0 | 0 | 0 | 1 | 0 | 0 | 0 | 0 | 0 | 0 | 0 | 0 | 0 | 0 | 36 | 45 | Carbamidomethylation | C8:Carbamidomethylation:1000.00 | PEAKS DB |
| V.IEQGC(+57.02)VATC(+57.02)PQFR.S | N | 41.92 | 1564.7126 | 13 | 1.4 | 783.3624 | 2 | 43.03 | 8 | F8:20158 | BuCaTN01_F7b.raw | | | | | | | | 1.5548E5 | | | | | | | | | 1 | 0 | 0 | 0 | 0 | 0 | 0 | 0 | 1 | 0 | 0 | 0 | 0 | 0 | 0 | 0 | 0 | 39 | 51 | Carbamidomethylation | C5:Carbamidomethylation:1000.00;C9:Carbamidomethylation:1000.00 | PEAKS DB |
| total 9 peptides | | | | | | | | | | | | | | | | | | | | | | | | | | | | | | | | | | | | | | | | | | | | | | | | | |

**Best Unique PSM (Scan F6:38720, m/z=951.1222, z=3, RT=61.02, ppm=-0.5):**

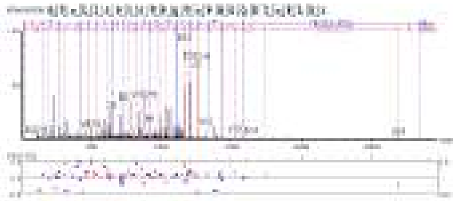

## 1KBA

back to list

| Protein Coverage | Supporting Peptides | Best Unique PSM |

**Protein Coverage:**

1 **RTCLISPSST PQTCPNGQDI CFLKA**QCDKF CSIR**GPVIEQ GCVATCPQFR SNYRSLLCCT TDNCNH**

14 42 46 58 59 64

C Carbamidomethylation (+57.02)

**Supporting Peptides:**

| | Area | #Feature |
|---|---|---|

| Peptide | Uniq | -10lgP | Mass | Length | ppm | m/z | z | RT | Fraction | Scan | Source File | Area F1 | Area F2a | Area F3a | Area F4 | Area F5 | Area F6a | Area F7a | Area F7b | Area F8 | Area F9a | Sample 1F10a | Area F10b | Area F11a | Area F11b | Area F12a | Area F13 | #Feature | #Feature F1 | #Feature F2a | #Feature F3a | #Feature F4 | #Feature F5 | #Feature F6a | #Feature F7a | #Feature F7b | #Feature F8 | #Feature F9a | Sample 1F10a | #Feature F10b | #Feature F11a | #Feature F11b | #Feature F12a | #Feature F13 | Start | End | PTM | AScore | Found By |
|---|---|---|---|---|---|---|---|---|---|---|---|---|---|---|---|---|---|---|---|---|---|---|---|---|---|---|---|---|---|---|---|---|---|---|---|---|---|---|---|---|---|---|---|---|---|---|---|---|---|
| R.GPVIEQGC(+57.02)VATC(+57.02)PQFR.S | N | 96.70 | 1817.8552 | 16 | 1.3 | 909.9360 | 2 | 51.36 | 6 | F6:30037 | BuCaTN01_F6a.raw |  |  |  |  |  | 4.938E5 |  |  |  |  |  |  |  | 0 |  |  | 1 | 0 | 0 | 0 | 0 | 0 | 1 | 0 | 0 | 0 | 0 | 0 | 0 | 0 | 0 | 0 | 0 | 35 | 50 | Carbamidomethylation | C8:Carbamidomethylation:1000.00;C12:Carbamidomethylation:1000.00 | PEAKS DB |
| R.SLLC(+57.02)C(+57.02)TTDNC(+57.02)NH | N | 79.81 | 1493.5698 | 12 | -0.3 | 747.7920 | 2 | 37.43 | 6 | F6:17551 | BuCaTN01_F6a.raw |  |  | 4.1658E6 |  |  | 7.1212E8 | 3.0441E6 | 4.2545E7 |  | 1.9164E6 |  | 4.7006E6 |  | 5.2769E6 | 7.5251E5 |  | 12 | 0 | 0 | 1 | 0 | 0 | 4 | 1 | 2 | 0 | 1 | 0 | 1 | 0 | 1 | 1 | 0 | 55 | 66 | Carbamidomethylation | C4:Carbamidomethylation:1000.00;C5:Carbamidomethylation:1000.00;C10:Carbamidomethylation:1000.00 | PEAKS DB |
| P.VIEQGC(+57.02)VATC(+57.02)PQFR.S | N | 70.62 | 1663.7810 | 14 | 3.0 | 832.8978 | 2 | 46.31 | 8 | F8:22974 | BuCaTN01_F7b.raw |  |  |  |  |  |  |  | 7.7386E6 |  |  |  |  |  |  |  |  | 1 | 0 | 0 | 0 | 0 | 0 | 0 | 0 | 1 | 0 | 0 | 0 | 0 | 0 | 0 | 0 | 0 | 37 | 50 | Carbamidomethylation | C6:Carbamidomethylation:1000.00;C10:Carbamidomethylation:1000.00 | PEAKS DB |
| R.SNYRSLLC(+57.02)C(+57.02)TTDNC(+57.02)NH | N | 62.84 | 2013.8092 | 16 | -1.7 | 672.2759 | 3 | 46.75 | 6 | F6:25700 | BuCaTN01_F6a.raw |  |  |  |  |  | 1.3691E6 |  | 9.5627E5 |  |  |  |  |  |  |  |  | 2 | 0 | 0 | 0 | 0 | 0 | 1 | 0 | 1 | 0 | 0 | 0 | 0 | 0 | 0 | 0 | 0 | 51 | 66 | Carbamidomethylation | C8:Carbamidomethylation:1000.00;C9:Carbamidomethylation:1000.00;C14:Carbamidomethylation:1000.00 | PEAKS DB |
| RTC(+57.02)LISPSSTPQTC(+57.02)PNGQDIC(+57.02)FLKA.Q | Y | 53.10 | 2850.3462 | 25 | -0.5 | 951.1222 | 3 | 61.02 | 6 | F6:38720 | BuCaTN01_F6a.raw |  |  |  |  |  | 8.6023E7 |  |  |  |  |  |  |  |  |  |  | 1 | 0 | 0 | 0 | 0 | 0 | 1 | 0 | 0 | 0 | 0 | 0 | 0 | 0 | 0 | 0 | 0 | 1 | 25 | Carbamidomethylation | C3:Carbamidomethylation:1000.00;C14:Carbamidomethylation:1000.00;C21:Carbamidomethylation:1000.00 | PEAKS DB |
| R.GPVIEQGC(+57.02)VAT.C | N | 50.22 | 1129.5437 | 11 | 0.5 | 565.7794 | 2 | 41.92 | 6 | F6:21363 | BuCaTN01_F6a.raw |  |  |  |  |  | 6.58E5 |  |  |  |  |  |  |  |  |  |  | 1 | 0 | 0 | 0 | 0 | 0 | 1 | 0 | 0 | 0 | 0 | 0 | 0 | 0 | 0 | 0 | 0 | 35 | 45 | Carbamidomethylation | C8:Carbamidomethylation:1000.00 | PEAKS DB |
| RTC(+57.02)LISPSSTPQT.C | Y | 46.36 | 1446.7136 | 13 | 0.5 | 724.3644 | 2 | 40.18 | 6 | F6:19763 | BuCaTN01_F6a.raw |  |  |  |  |  | 1.458E6 |  |  |  |  |  |  |  |  |  |  | 1 | 0 | 0 | 0 | 0 | 0 | 1 | 0 | 0 | 0 | 0 | 0 | 0 | 0 | 0 | 0 | 0 | 1 | 13 | Carbamidomethylation | C3:Carbamidomethylation:1000.00 | PEAKS DB |
| R.GPVIEQGC(+57.02)VA.T | N | 42.28 | 1028.4961 | 10 | 0.2 | 515.2554 | 2 | 41.42 | 6 | F6:20784 | BuCaTN01_F6a.raw |  |  |  |  |  | 2.0497E6 |  |  |  |  |  |  |  |  |  |  | 1 | 0 | 0 | 0 | 0 | 0 | 1 | 0 | 0 | 0 | 0 | 0 | 0 | 0 | 0 | 0 | 0 | 35 | 44 | Carbamidomethylation | C8:Carbamidomethylation:1000.00 | PEAKS DB |
| V.IEQGC(+57.02)VATC(+57.02)PQFR.S | N | 41.92 | 1564.7126 | 13 | 1.4 | 783.3624 | 2 | 43.03 | 8 | F8:20158 | BuCaTN01_F7b.raw |  |  |  |  |  |  |  | 1.5548E5 |  |  |  |  |  |  |  |  | 1 | 0 | 0 | 0 | 0 | 0 | 0 | 0 | 1 | 0 | 0 | 0 | 0 | 0 | 0 | 0 | 0 | 38 | 50 | Carbamidomethylation | C5:Carbamidomethylation:1000.00;C9:Carbamidomethylation:1000.00 | PEAKS DB |
| total 9 peptides |  |  |  |  |  |  |  |  |  |  |  |  |  |  |  |  |  |  |  |  |  |  |  |  |  |  |  |  |  |  |  |  |  |  |  |  |  |  |  |  |  |  |  |  |  |  |  |  |  |

**Best Unique PSM (Scan F6:38720, m/z=951.1222, z=3, RT=61.02, ppm=-0.5):**

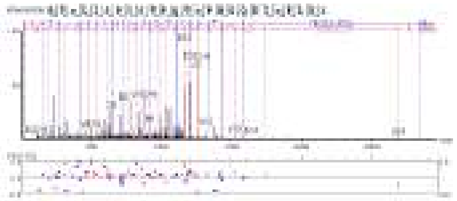

## 2NBT

back to list

| Protein Coverage | Supporting Peptides | Best Unique PSM |

**Protein Coverage:**

1 RTCLISPSST PQTCPNGQDI CFLKAQCDKF CSIRGPVIEQ GCVATCPQFR SNYRSLLCCT TDNCNH

C Carbamidomethylation (+57.02)

**Supporting Peptides:**

| Peptide | Uniq | -10lgP | Mass | Length | ppm | m/z | z | RT | Fraction | Scan | Source File | Area F1 | Area F2a | Area F3a | Area F4 | Area F5 | Area F6a | Area F7a | Area F7b | Area F8 | Area F9a | Area Sample 1F10a | Area F10b | Area F11a | Area F11b | Area F12a | Area F13 | #Feature | #Feature F1 | #Feature F2a | #Feature F3a | #Feature F4 | #Feature F5 | #Feature F6a | #Feature F7a | #Feature F7b | #Feature F8 | #Feature F9a | #Feature Sample 1F10a | #Feature F10b | #Feature F11a | #Feature F11b | #Feature F12a | #Feature F13 | Start | End | PTM | AScore | Found By |
|---|---|---|---|---|---|---|---|---|---|---|---|---|---|---|---|---|---|---|---|---|---|---|---|---|---|---|---|---|---|---|---|---|---|---|---|---|---|---|---|---|---|---|---|---|---|---|---|---|---|
| R.GPVIEQGC(+57.02)VATC(+57.02)PQFR.S | N | 96.70 | 1817.8552 | 16 | 1.3 | 909.9360 | 2 | 51.36 | 6 | F6:30037 | BuCaTN01_F6a.raw |  |  |  |  |  | 4.938E5 |  |  |  |  |  |  |  | 0 |  |  | 1 | 0 | 0 | 0 | 0 | 0 | 1 | 0 | 0 | 0 | 0 | 0 | 0 | 0 | 0 | 0 | 0 | 35 | 50 | Carbamidomethylation | C8:Carbamidomethylation:1000.00;C12:Carbamidomethylation:1000.00 | PEAKS DB |
| R.SLLC(+57.02)C(+57.02)TTDNC(+57.02)NH | N | 79.81 | 1493.5698 | 12 | -0.3 | 747.7920 | 2 | 37.43 | 6 | F6:17551 | BuCaTN01_F6a.raw |  |  | 4.1658E6 |  |  | 7.1212E8 | 3.0441E6 | 4.2545E7 |  | 1.9164E6 |  | 4.7006E6 |  | 5.2769E6 | 7.5251E5 |  | 12 | 0 | 0 | 1 | 0 | 0 | 4 | 1 | 2 | 0 | 1 | 0 | 1 | 0 | 1 | 1 | 0 | 55 | 66 | Carbamidomethylation | C4:Carbamidomethylation:1000.00;C5:Carbamidomethylation:1000.00;C10:Carbamidomethylation:1000.00 | PEAKS DB |
| P.VIEQGC(+57.02)VATC(+57.02)PQFR.S | N | 70.62 | 1663.7810 | 14 | 3.0 | 832.8978 | 2 | 46.31 | 8 | F8:22974 | BuCaTN01_F7b.raw |  |  |  |  |  |  |  | 7.7386E6 |  |  |  |  |  |  |  |  | 1 | 0 | 0 | 0 | 0 | 0 | 0 | 0 | 1 | 0 | 0 | 0 | 0 | 0 | 0 | 0 | 0 | 37 | 50 | Carbamidomethylation | C6:Carbamidomethylation:1000.00;C10:Carbamidomethylation:1000.00 | PEAKS DB |
| R.SNYRSLLC(+57.02)C(+57.02)TTDNC(+57.02)NH | N | 62.84 | 2013.8092 | 16 | -1.7 | 672.2759 | 3 | 46.75 | 6 | F6:25700 | BuCaTN01_F6a.raw |  |  |  |  |  | 1.3691E6 |  | 9.5627E5 |  |  |  |  |  |  |  |  | 2 | 0 | 0 | 0 | 0 | 0 | 1 | 0 | 1 | 0 | 0 | 0 | 0 | 0 | 0 | 0 | 0 | 51 | 66 | Carbamidomethylation | C8:Carbamidomethylation:1000.00;C9:Carbamidomethylation:1000.00;C14:Carbamidomethylation:1000.00 | PEAKS DB |
| RTC(+57.02)LISPSSTPQTC(+57.02)PNGQDIC(+57.02)FLKA.Q | Y | 53.10 | 2850.3462 | 25 | -0.5 | 951.1222 | 3 | 61.02 | 6 | F6:38720 | BuCaTN01_F6a.raw |  |  |  |  |  | 8.6023E7 |  |  |  |  |  |  |  |  |  |  | 1 | 0 | 0 | 0 | 0 | 0 | 1 | 0 | 0 | 0 | 0 | 0 | 0 | 0 | 0 | 0 | 0 | 1 | 25 | Carbamidomethylation | C3:Carbamidomethylation:1000.00;C14:Carbamidomethylation:1000.00;C21:Carbamidomethylation:1000.00 | PEAKS DB |
| R.GPVIEQGC(+57.02)VAT.C | N | 50.22 | 1129.5437 | 11 | 0.5 | 565.7794 | 2 | 41.92 | 6 | F6:21363 | BuCaTN01_F6a.raw |  |  |  |  |  | 6.58E5 |  |  |  |  |  |  |  |  |  |  | 1 | 0 | 0 | 0 | 0 | 0 | 1 | 0 | 0 | 0 | 0 | 0 | 0 | 0 | 0 | 0 | 0 | 35 | 45 | Carbamidomethylation | C8:Carbamidomethylation:1000.00 | PEAKS DB |
| RTC(+57.02)LISPSSTPQT.C | Y | 46.36 | 1446.7136 | 13 | 0.5 | 724.3644 | 2 | 40.18 | 6 | F6:19763 | BuCaTN01_F6a.raw |  |  |  |  |  | 1.458E6 |  |  |  |  |  |  |  |  |  |  | 1 | 0 | 0 | 0 | 0 | 0 | 1 | 0 | 0 | 0 | 0 | 0 | 0 | 0 | 0 | 0 | 0 | 1 | 13 | Carbamidomethylation | C3:Carbamidomethylation:1000.00 | PEAKS DB |
| R.GPVIEQGC(+57.02)VA.T | N | 42.28 | 1028.4961 | 10 | 0.2 | 515.2554 | 2 | 41.42 | 6 | F6:20784 | BuCaTN01_F6a.raw |  |  |  |  |  | 2.0497E6 |  |  |  |  |  |  |  |  |  |  | 1 | 0 | 0 | 0 | 0 | 0 | 1 | 0 | 0 | 0 | 0 | 0 | 0 | 0 | 0 | 0 | 0 | 35 | 44 | Carbamidomethylation | C8:Carbamidomethylation:1000.00 | PEAKS DB |
| V.IEQGC(+57.02)VATC(+57.02)PQFR.S | N | 41.92 | 1564.7126 | 13 | 1.4 | 783.3624 | 2 | 43.03 | 8 | F8:20158 | BuCaTN01_F7b.raw |  |  |  |  |  |  |  | 1.5548E5 |  |  |  |  |  |  |  |  | 1 | 0 | 0 | 0 | 0 | 0 | 0 | 0 | 1 | 0 | 0 | 0 | 0 | 0 | 0 | 0 | 0 | 38 | 50 | Carbamidomethylation | C5:Carbamidomethylation:1000.00;C9:Carbamidomethylation:1000.00 | PEAKS DB |
| total 9 peptides |  |  |  |  |  |  |  |  |  |  |  |  |  |  |  |  |  |  |  |  |  |  |  |  |  |  |  |  |  |  |  |  |  |  |  |  |  |  |  |  |  |  |  |  |  |  |  |  |  |

**Best Unique PSM (Scan F6:38720, m/z=951.1222, z=3, RT=61.02, ppm=-0.5):**

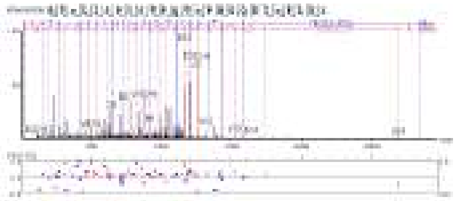

## ACF70483.1

back to list

| Protein Coverage | Supporting Peptides | Best Unique PSM |

**Protein Coverage:**

1 MNVFFMFSLL FLATLGSCAD DKNPLEECFR EDDYEEFLEI AKNGLKKTSN PKHIVIVGAG MSGLSAAYVL AGAGHKVTVL

O Oxidation (M) (+15.99)

81 EASERPGGRV RTHRNVKEGW YANLGPMRVP EKHRIIREYI RKFGLKLNEF VQETENGWYF IKNIRKRVGE VKKDPGLLKY

161 PVKPSEAGKS AGQLYQESLG KAVEELKRTN CSYILNKYDT YSTKEYLIKE GNLSPGAVDM IGDLLNEDSG YYVSFIESLK

241 HDDIFAYEKR FDEIVGGMDQ LPTSMYRAIE ESVHFKARVI KIQQNAEKVT VTYQTTQKNL LLETADYVIV CTTSRAARRI

321 TFKPPLPPKK AHALRSVHYR SGTKIFLTCT KKFWEDDGIQ GGKSTTDLPS RFIYYPNHNF TTGVGVIIAY GIGDDANFFQ

401 ALNLNECADI VFNDLSSIHQ LPKKDLQTFC YPSIIQKWSL DKYAMGAITT FTPYQFQHFS EALTAPVGRI FFAGEYTANA

481 HGWIDSTIKS GLTAARDVNR ASEL

**Supporting Peptides:**

| Peptide | Uniq | -10lgP | Mass | Length | ppm | m/z | z | RT | Fraction | Scan | Source File | Area F1 | Area F2a | Area F3a | Area F4 | Area F5 | Area F6a | Area F7a | Area F7b | Area F8 | Area F9a | Area Sample 1F10a | Area F10b | Area F11a | Area F11b | Area F12a | Area F13 | #Feature | #Feature F1 | #Feature F2a | #Feature F3a | #Feature F4 | #Feature F5 | #Feature F6a | #Feature F7a | #Feature F7b | #Feature F8 | #Feature F9a | #Feature Sample 1F10a | #Feature F10b | #Feature F11a | #Feature F11b | #Feature F12a | #Feature F13 | Start | End | PTM | AScore | Found By |
|---|---|---|---|---|---|---|---|---|---|---|---|---|---|---|---|---|---|---|---|---|---|---|---|---|---|---|---|---|---|---|---|---|---|---|---|---|---|---|---|---|---|---|---|---|---|---|---|---|---|
| K.HIVIVGAGM(+15.99)SGLSAAYVLAGAGHK.V | Y | 105.63 | 2294.2205 | 24 | -0.2 | 574.5623 | 4 | 67.31 | 16 | F16:34457 | BuCaTN01_F13.raw |  |  |  |  |  |  |  |  |  |  |  |  |  |  |  | 3.8376E6 | 2 | 0 | 0 | 0 | 0 | 0 | 0 | 0 | 0 | 0 | 0 | 0 | 0 | 0 | 0 | 0 | 2 | 53 | 76 | Oxidation (M) | M9:Oxidation (M):1000.00 | PEAKS DB |
| K.HIVIVGAGMSGLSAAYVLAGAGHK.V | Y | 78.91 | 2278.2256 | 24 | -0.2 | 570.5635 | 4 | 74.97 | 16 | F16:39447 | BuCaTN01_F13.raw |  |  |  |  |  |  |  |  |  |  |  |  |  |  |  | 4.5812E5 | 2 | 0 | 0 | 0 | 0 | 0 | 0 | 0 | 0 | 0 | 0 | 0 | 0 | 0 | 0 | 0 | 2 | 53 | 76 |  |  | PEAKS DB |
| V.GAGM(+15.99)SGLSAAYVLAGAGHK.V | N | 71.56 | 1732.8566 | 19 | -0.1 | 578.6261 | 3 | 56.99 | 16 | F16:27415 | BuCaTN01_F13.raw |  |  |  |  |  |  |  |  |  |  |  |  |  |  |  | 1.9804E5 | 1 | 0 | 0 | 0 | 0 | 0 | 0 | 0 | 0 | 0 | 0 | 0 | 0 | 0 | 0 | 0 | 1 | 58 | 76 | Oxidation (M) | M4:Oxidation (M):1000.00 | PEAKS DB |
| D.PGLLKYPVKPSEAGK.S | N | 67.14 | 1582.9082 | 15 | -0.5 | 528.6431 | 3 | 41.67 | 16 | F16:17748 | BuCaTN01_F13.raw |  |  |  |  |  |  |  |  |  |  |  |  |  |  |  | 4.8066E6 | 2 | 0 | 0 | 0 | 0 | 0 | 0 | 0 | 0 | 0 | 0 | 0 | 0 | 0 | 0 | 0 | 2 | 155 | 169 |  |  | PEAKS DB |
| K.KDPGLLKYPVKPSEAGK.S | N | 50.04 | 1826.0302 | 17 | -0.4 | 457.5146 | 4 | 39.92 | 16 | F16:16202 | BuCaTN01_F13.raw |  |  |  |  |  |  |  |  |  |  |  |  |  |  |  | 1.9288E5 | 1 | 0 | 0 | 0 | 0 | 0 | 0 | 0 | 0 | 0 | 0 | 0 | 0 | 0 | 0 | 0 | 1 | 153 | 169 |  |  | PEAKS DB |
| K.VTVLEASERPGGR.V | N | 47.48 | 1369.7313 | 13 | 0.0 | 457.5844 | 3 | 35.95 | 16 | F16:13465 | BuCaTN01_F13.raw |  |  |  |  |  |  |  |  |  |  |  |  |  |  |  | 1.3097E7 | 1 | 0 | 0 | 0 | 0 | 0 | 0 | 0 | 0 | 0 | 0 | 0 | 0 | 0 | 0 | 0 | 1 | 77 | 89 |  |  | PEAKS DB |
| K.VTVLEASERPG.G | Y | 45.59 | 1156.6088 | 11 | 0.3 | 579.3118 | 2 | 39.81 | 16 | F16:16182 | BuCaTN01_F13.raw |  |  |  |  |  |  |  |  |  |  |  |  |  |  |  | 7.1027E5 | 1 | 0 | 0 | 0 | 0 | 0 | 0 | 0 | 0 | 0 | 0 | 0 | 0 | 0 | 0 | 0 | 1 | 77 | 87 |  |  | PEAKS DB |
| R.ITFKPPLPPK.K | Y | 45.34 | 1136.6957 | 10 | 0.5 | 379.9060 | 3 | 49.85 | 16 | F16:23165 | BuCaTN01_F13.raw |  |  |  |  |  |  |  |  |  |  |  |  |  |  |  | 6.1028E4 | 1 | 0 | 0 | 0 | 0 | 0 | 0 | 0 | 0 | 0 | 0 | 0 | 0 | 0 | 0 | 0 | 1 | 320 | 329 |  |  | PEAKS DB |
| K.TSNPKHIVIVGAGM(+15.99)SGLSAAYVLAGAGHK.V | Y | 44.82 | 2821.4907 | 29 | 4.3 | 565.3079 | 5 | 64.36 | 16 | F16:32066 | BuCaTN01_F13.raw |  |  |  |  |  |  |  |  |  |  |  |  |  |  |  | 2.3574E5 | 1 | 0 | 0 | 0 | 0 | 0 | 0 | 0 | 0 | 0 | 0 | 0 | 0 | 0 | 0 | 0 | 1 | 48 | 76 | Oxidation (M) | M14:Oxidation (M):1000.00 | PEAKS DB |
| K.HIVIVGAGM(+15.99)SGL.S | Y | 44.47 | 1168.6274 | 12 | 0.2 | 585.3211 | 2 | 54.26 | 16 | F16:25682 | BuCaTN01_F13.raw |  |  |  |  |  |  |  |  |  |  |  |  |  |  |  | 2.87E4 | 1 | 0 | 0 | 0 | 0 | 0 | 0 | 0 | 0 | 0 | 0 | 0 | 0 | 0 | 0 | 0 | 1 | 53 | 64 | Oxidation (M) | M9:Oxidation (M):1000.00 | PEAKS DB |
| R.NVKEGWYANLGPMR.V | Y | 43.06 | 1633.8035 | 14 | -0.7 | 545.6080 | 3 | 59.55 | 16 | F16:29105 | BuCaTN01_F13.raw |  |  |  |  |  |  |  |  |  |  |  |  |  |  |  | 0 | 0 | 0 | 0 | 0 | 0 | 0 | 0 | 0 | 0 | 0 | 0 | 0 | 0 | 0 | 0 | 0 | 0 | 95 | 108 |  |  | PEAKS DB |
| total 11 peptides |  |  |  |  |  |  |  |  |  |  |  |  |  |  |  |  |  |  |  |  |  |  |  |  |  |  |  |  |  |  |  |  |  |  |  |  |  |  |  |  |  |  |  |  |  |  |  |  |  |

**Best Unique PSM (Scan F16:34457, m/z=574.5623, z=4, RT=67.31, ppm=-0.2):**

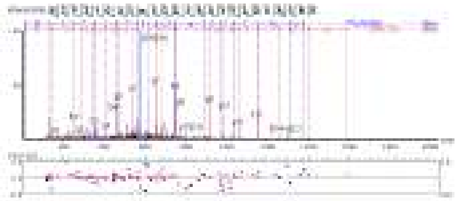

## G8XQX1.1

back to list

| Protein Coverage | Supporting Peptides | Best Unique PSM |

**Protein Coverage:**

```
  1 MNVFFMFSLL FLATLGSCAD DKNPLEECFR EDDYEEFLEI AKNGLKKTSN PKHIVIVGAG MSGLSAAYVL AGAGHKVTVL
 81 EASERPGGRV RTHRNVKEGW YANLGPMRVP EKHRIIREYI RKFGLKLNEF VQETENGWYF IKNIRKRVGE VKKDPGLLKY
161 PVKPSEAGKS AGQLYQESLG KAVEELKRTN CSYILNKYDT YSTKEYLIKE GNLSPGAVDM IGDLLNEDSG YYVSFIESLK
241 HDDIFAYEKR FDEIVGGMDQ LPTSMYRAIE ESVHFKARVI KIQQNAEKVT VTYQTTQKNL LLETADYVIV CTTSRAARRI
321 TFKPPLPPKK AHALRSVHYR SGTKIFLTCT KKFWEDDGIQ GGKSTTDLPS RFIYYPNHNF TTGVGVIIAY GIGDDANFFQ
401 ALNLNECADI VFNDLSSIHQ LPKKDLQTFC YPSIIQKWSL DKYAMGAITT FTPYQFQHFS EALTAPVGRI FFAGEYTANA
481 HGWIDSTIKS GLTAARDVNR ASEL
```

61 Oxidation (M) (+15.99)

**Supporting Peptides:**

| Peptide | Uniq | -10lgP | Mass | Length | ppm | m/z | z | RT | Fraction | Scan | Source File | Area F1 | Area F2a | Area F3a | Area F4 | Area F5 | Area F6a | Area F7a | Area F7b | Area F8 | Area F9a | Area Sample 1F10a | Area F10b | Area F11a | Area F11b | Area F12a | Area F13 | #Feature | #Feature F1 | #Feature F2a | #Feature F3a | #Feature F4 | #Feature F5 | #Feature F6a | #Feature F7a | #Feature F7b | #Feature F8 | #Feature F9a | #Feature Sample 1F10a | #Feature F10b | #Feature F11a | #Feature F11b | #Feature F12a | #Feature F13 | Start | End | PTM | AScore | Found By |
|---|---|---|---|---|---|---|---|---|---|---|---|---|---|---|---|---|---|---|---|---|---|---|---|---|---|---|---|---|---|---|---|---|---|---|---|---|---|---|---|---|---|---|---|---|---|---|---|---|---|
| K.HIVIVGAGM(+15.99)SGLSAAYVLAGAGHK.V | Y | 105.63 | 2294.2205 | 24 | -0.2 | 574.5623 | 4 | 67.31 | 16 | F16:34457 | BuCaTN01_F13.raw | | | | | | | | | | | | | | | | 3.8376E6 | 2 | 0 | 0 | 0 | 0 | 0 | 0 | 0 | 0 | 0 | 0 | 0 | 0 | 0 | 0 | 0 | 2 | 53 | 76 | Oxidation (M) | M9:Oxidation (M):1000.00 | PEAKS DB |
| K.HIVIVGAGMSGLSAAYVLAGAGHK.V | Y | 78.91 | 2278.2256 | 24 | -0.2 | 570.5635 | 4 | 74.97 | 16 | F16:39447 | BuCaTN01_F13.raw | | | | | | | | | | | | | | | | 4.5812E5 | 2 | 0 | 0 | 0 | 0 | 0 | 0 | 0 | 0 | 0 | 0 | 0 | 0 | 0 | 0 | 0 | 2 | 53 | 76 | | | PEAKS DB |
| V.GAGM(+15.99)SGLSAAYVLAGAGHK.V | N | 71.56 | 1732.8566 | 19 | -0.1 | 578.6261 | 3 | 56.99 | 16 | F16:27415 | BuCaTN01_F13.raw | | | | | | | | | | | | | | | | 1.9804E5 | 1 | 0 | 0 | 0 | 0 | 0 | 0 | 0 | 0 | 0 | 0 | 0 | 0 | 0 | 0 | 0 | 1 | 58 | 76 | Oxidation (M) | M4:Oxidation (M):1000.00 | PEAKS DB |
| D.PGLLKYPVKPSEAGK.S | N | 67.14 | 1582.9082 | 15 | -0.5 | 528.6431 | 3 | 41.67 | 16 | F16:17748 | BuCaTN01_F13.raw | | | | | | | | | | | | | | | | 4.8066E6 | 2 | 0 | 0 | 0 | 0 | 0 | 0 | 0 | 0 | 0 | 0 | 0 | 0 | 0 | 0 | 0 | 2 | 155 | 169 | | | PEAKS DB |
| K.KDPGLLKYPVKPSEAGK.S | N | 50.04 | 1826.0302 | 17 | -0.4 | 457.5146 | 4 | 39.92 | 16 | F16:16202 | BuCaTN01_F13.raw | | | | | | | | | | | | | | | | 1.9288E5 | 1 | 0 | 0 | 0 | 0 | 0 | 0 | 0 | 0 | 0 | 0 | 0 | 0 | 0 | 0 | 0 | 1 | 153 | 169 | | | PEAKS DB |
| K.VTVLEASERPGGR.V | N | 47.48 | 1369.7313 | 13 | 0.0 | 457.5844 | 3 | 35.95 | 16 | F16:13465 | BuCaTN01_F13.raw | | | | | | | | | | | | | | | | 1.3097E7 | 1 | 0 | 0 | 0 | 0 | 0 | 0 | 0 | 0 | 0 | 0 | 0 | 0 | 0 | 0 | 0 | 1 | 77 | 89 | | | PEAKS DB |
| K.VTVLEASERPG.G | Y | 45.59 | 1156.6088 | 11 | 0.3 | 579.3118 | 2 | 39.81 | 16 | F16:16182 | BuCaTN01_F13.raw | | | | | | | | | | | | | | | | 7.1027E5 | 1 | 0 | 0 | 0 | 0 | 0 | 0 | 0 | 0 | 0 | 0 | 0 | 0 | 0 | 0 | 0 | 1 | 77 | 87 | | | PEAKS DB |
| R.ITFKPPLPPK.K | Y | 45.34 | 1136.6957 | 10 | 0.5 | 379.9060 | 3 | 49.85 | 16 | F16:23165 | BuCaTN01_F13.raw | | | | | | | | | | | | | | | | 6.1028E4 | 1 | 0 | 0 | 0 | 0 | 0 | 0 | 0 | 0 | 0 | 0 | 0 | 0 | 0 | 0 | 0 | 1 | 320 | 329 | | | PEAKS DB |
| K.TSNPKHIVIVGAGM(+15.99)SGLSAAYVLAGAGHK.V | Y | 44.82 | 2821.4907 | 29 | 4.3 | 565.3079 | 5 | 64.36 | 16 | F16:32066 | BuCaTN01_F13.raw | | | | | | | | | | | | | | | | 2.3574E5 | 1 | 0 | 0 | 0 | 0 | 0 | 0 | 0 | 0 | 0 | 0 | 0 | 0 | 0 | 0 | 0 | 1 | 48 | 76 | Oxidation (M) | M14:Oxidation (M):1000.00 | PEAKS DB |
| K.HIVIVGAGM(+15.99)SGL.S | Y | 44.47 | 1168.6274 | 12 | 0.2 | 585.3211 | 2 | 54.26 | 16 | F16:25682 | BuCaTN01_F13.raw | | | | | | | | | | | | | | | | 2.87E4 | 1 | 0 | 0 | 0 | 0 | 0 | 0 | 0 | 0 | 0 | 0 | 0 | 0 | 0 | 0 | 0 | 1 | 53 | 64 | Oxidation (M) | M9:Oxidation (M):1000.00 | PEAKS DB |
| R.NVKEGWYANLGPMR.V | Y | 43.06 | 1633.8035 | 14 | -0.7 | 545.6080 | 3 | 59.55 | 16 | F16:29105 | BuCaTN01_F13.raw | | | | | | | | | | | | | | | | 0 | 0 | 0 | 0 | 0 | 0 | 0 | 0 | 0 | 0 | 0 | 0 | 0 | 0 | 0 | 0 | 0 | 0 | 95 | 108 | | | PEAKS DB |
| total 11 peptides | | | | | | | | | | | | | | | | | | | | | | | | | | | | | | | | | | | | | | | | | | | | | | | | | | |

**Best Unique PSM (Scan F16:34457, m/z=574.5623, z=4, RT=67.31, ppm=-0.2):**

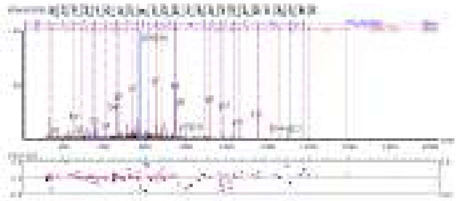

## 1F94

back to list

| Protein Coverage | Supporting Peptides | Best Unique PSM |

**Protein Coverage:**

```
  1 MECYRCGVSG CHLKITCSAE ETFCYKWLNK ISNERWLGCA KTCTEIDTWN VYNKCCTTNL CNT
```

17 24 55 56 61

C Carbamidomethylation (+57.02)

**Supporting Peptides:**

| Peptide | Uniq | -10lgP | Mass | Length | ppm | m/z | z | RT | Fraction | Scan | Source File | Area F1 | Area F2a | Area F3a | Area F4 | Area F5 | Area F6a | Area F7a | Area F7b | Area F8 | Area F9a | Area Sample 1F10a | Area F10b | Area F11a | Area F11b | Area F12a | Area F13 | #Feature | #Feature F1 | #Feature F2a | #Feature F3a | #Feature F4 | #Feature F5 | #Feature F6a | #Feature F7a | #Feature F7b | #Feature F8 | #Feature F9a | #Feature Sample 1F10a | #Feature F10b | #Feature F11a | #Feature F11b | #Feature F12a | #Feature F13 | Start | End | PTM | AScore | Found By |
|---|---|---|---|---|---|---|---|---|---|---|---|---|---|---|---|---|---|---|---|---|---|---|---|---|---|---|---|---|---|---|---|---|---|---|---|---|---|---|---|---|---|---|---|---|---|---|---|---|---|
| K.ITC(+57.02)SAEETFC(+57.02)YK.W | Y | 90.16 | 1507.6323 | 12 | 2.8 | 754.8234 | 2 | 47.60 | 8 | F8:24678 | BuCaTN01_F7b.raw | | | | | | 5.6529E8 | 1.1028E6 | 2.8689E7 | 1.2868E6 | 2.0316E6 | | 0 | | | | | 23 | 0 | 0 | 0 | 0 | 0 | 19 | 1 | 1 | 1 | 1 | 0 | 0 | 0 | 0 | 0 | 0 | 15 | 26 | Carbamidomethylation | C3:Carbamidomethylation:1000.00;C10:Carbamidomethylation:1000.00 | PEAKS DB |
| R.C(+57.02)GVSGC(+57.02)HLKITC(+57.02)SAEETFC(+57.02)YK.W | Y | 88.69 | 2506.0750 | 21 | -2.0 | 836.3639 | 3 | 53.55 | 6 | F6:32082 | BuCaTN01_F6a.raw | | | | | | 1.1029E7 | | | | | | | | | | | 3 | 0 | 0 | 0 | 0 | 0 | 3 | 0 | 0 | 0 | 0 | 0 | 0 | 0 | 0 | 0 | 0 | 6 | 26 | Carbamidomethylation | C1:Carbamidomethylation:1000.00;C6:Carbamidomethylation:1000.00;C12:Carbamidomethylation:1000.00;C19:Carbamidomethylation:1000.00 | PEAKS DB |
| K.ITC(+57.02)SAEETFC(+57.02)YKWLNK.I | Y | 72.94 | 2048.9336 | 16 | 0.8 | 683.9857 | 3 | 63.58 | 6 | F6:41890 | BuCaTN01_F6a.raw | | | | | | 4.7109E7 | | | | | | | | | | | 3 | 0 | 0 | 0 | 0 | 0 | 3 | 0 | 0 | 0 | 0 | 0 | 0 | 0 | 0 | 0 | 0 | 15 | 30 | Carbamidomethylation | C3:Carbamidomethylation:1000.00;C10:Carbamidomethylation:1000.00 | PEAKS DB |
| K.ITC(+57.02)SAEETFC(+57.02)YKWLNKISNER.W | Y | 70.93 | 2648.2363 | 21 | 1.8 | 883.7543 | 3 | 70.00 | 6 | F6:48784 | BuCaTN01_F6a.raw | | | | | | 2.6461E8 | | | | | | | | | | | 4 | 0 | 0 | 0 | 0 | 0 | 4 | 0 | 0 | 0 | 0 | 0 | 0 | 0 | 0 | 0 | 0 | 15 | 35 | Carbamidomethylation | C3:Carbamidomethylation:1000.00;C10:Carbamidomethylation:1000.00 | PEAKS DB |
| C.SAEETFC(+57.02)YKWLNK.I | Y | 59.35 | 1674.7711 | 13 | -1.1 | 838.3919 | 2 | 59.22 | 6 | F6:37505 | BuCaTN01_F6a.raw | | | | | | 1.3168E6 | | | | | | | | | | | 1 | 0 | 0 | 0 | 0 | 0 | 1 | 0 | 0 | 0 | 0 | 0 | 0 | 0 | 0 | 0 | 0 | 18 | 30 | Carbamidomethylation | C7:Carbamidomethylation:1000.00 | PEAKS DB |
| L.KITC(+57.02)SAEETFC(+57.02)YK.W | Y | 52.26 | 1635.7273 | 13 | 0.1 | 546.2498 | 3 | 42.98 | 6 | F6:22384 | BuCaTN01_F6a.raw | | | | | | 2.7893E5 | | | | | | | | | | | 1 | 0 | 0 | 0 | 0 | 0 | 1 | 0 | 0 | 0 | 0 | 0 | 0 | 0 | 0 | 0 | 0 | 14 | 26 | Carbamidomethylation | C4:Carbamidomethylation:1000.00;C11:Carbamidomethylation:1000.00 | PEAKS DB |
| I.DTWNVYNKC(+57.02)C(+57.02)TTNLC(+57.02)NT | Y | 43.96 | 2162.8821 | 17 | -1.1 | 1082.4471 | 2 | 54.91 | 6 | F6:33458 | BuCaTN01_F6a.raw | | | | | | 3.2186E6 | | | | | | | | | | | 1 | 0 | 0 | 0 | 0 | 0 | 1 | 0 | 0 | 0 | 0 | 0 | 0 | 0 | 0 | 0 | 0 | 47 | 63 | Carbamidomethylation | C9:Carbamidomethylation:1000.00;C10:Carbamidomethylation:1000.00;C15:Carbamidomethylation:1000.00 | PEAKS DB |
| K.WLNKISNER.W | Y | 41.68 | 1158.6145 | 9 | 0.1 | 580.3146 | 2 | 36.65 | 6 | F6:16419 | BuCaTN01_F6a.raw | | | | | | 5.9942E6 | | | | | | | | | | | 1 | 0 | 0 | 0 | 0 | 0 | 1 | 0 | 0 | 0 | 0 | 0 | 0 | 0 | 0 | 0 | 0 | 27 | 35 | | | PEAKS DB |
| total 8 peptides | | | | | | | | | | | | | | | | | | | | | | | | | | | | | | | | | | | | | | | | | | | | | | | | | |

**Best Unique PSM (Scan F8:24678, m/z=754.8234, z=2, RT=47.60, ppm=2.8):**

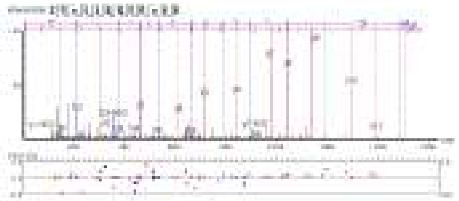

## 1IJC

back to list

| **Protein Coverage** | **Supporting Peptides** | **Best Unique PSM** |

**Protein Coverage:**

1 MECYRCGVSG CHLKITCSAE ETFCYKWLNK ISNERWLGCA KTCTEIDTWN VYNKCCTTNL CNT

Carbamidomethylation (+57.02)

**Supporting Peptides:**

| Peptide | Uniq | -10lgP | Mass | Length | ppm | m/z | z | RT | Fraction | Scan | Source File | Area F1 | Area F2a | Area F3a | Area F4 | Area F5 | Area F6a | Area F7a | Area F7b | Area F8 | Area F9a | Area Sample 1F10a | Area F10b | Area F11a | Area F11b | Area F12a | Area F13 | #Feature | #Feature F1 | #Feature F2a | #Feature F3a | #Feature F4 | #Feature F5 | #Feature F6a | #Feature F7a | #Feature F7b | #Feature F8 | #Feature F9a | #Feature Sample 1F10a | #Feature F10b | #Feature F11a | #Feature F11b | #Feature F12a | #Feature F13 | Start | End | PTM | AScore | Found By |
|---|---|---|---|---|---|---|---|---|---|---|---|---|---|---|---|---|---|---|---|---|---|---|---|---|---|---|---|---|---|---|---|---|---|---|---|---|---|---|---|---|---|---|---|---|---|---|---|---|---|
| K.ITC(+57.02)SAEETFC(+57.02)YK.W | Y | 90.16 | 1507.6323 | 12 | 2.8 | 754.8234 | 2 | 47.60 | 8 | F8:24678 | BuCaTN01_F7b.raw | | | | | | 5.6529E8 | 1.1028E6 | 2.8689E7 | 1.2868E6 | 2.0316E6 | | 0 | | | | | 23 | 0 | 0 | 0 | 0 | 0 | 19 | 1 | 1 | 1 | 1 | 0 | 0 | 0 | 0 | 0 | 0 | 15 | 26 | Carbamidomethylation | C3:Carbamidomethylation:1000.00;C10:Carbamidomethylation:1000.00 | PEAKS DB |
| R.C(+57.02)GVSGC(+57.02)HLKITC(+57.02)SAEETFC(+57.02)YK.W | Y | 88.69 | 2506.0750 | 21 | -2.0 | 836.3639 | 3 | 53.55 | 6 | F6:32082 | BuCaTN01_F6a.raw | | | | | | 1.1029E7 | | | | | | | | | | | 3 | 0 | 0 | 0 | 0 | 0 | 3 | 0 | 0 | 0 | 0 | 0 | 0 | 0 | 0 | 0 | 0 | 6 | 26 | Carbamidomethylation | C1:Carbamidomethylation:1000.00;C6:Carbamidomethylation:1000.00;C12:Carbamidomethylation:1000.00;C19:Carbamidomethylation:1000.00 | PEAKS DB |
| K.ITC(+57.02)SAEETFC(+57.02)YKWLNK.I | Y | 72.94 | 2048.9336 | 16 | 0.8 | 683.9857 | 3 | 63.58 | 6 | F6:41890 | BuCaTN01_F6a.raw | | | | | | 4.7109E7 | | | | | | | | | | | 3 | 0 | 0 | 0 | 0 | 0 | 3 | 0 | 0 | 0 | 0 | 0 | 0 | 0 | 0 | 0 | 0 | 15 | 30 | Carbamidomethylation | C3:Carbamidomethylation:1000.00;C10:Carbamidomethylation:1000.00 | PEAKS DB |
| K.ITC(+57.02)SAEETFC(+57.02)YKWLNKISNER.W | Y | 70.93 | 2648.2363 | 21 | 1.8 | 883.7543 | 3 | 70.00 | 6 | F6:48784 | BuCaTN01_F6a.raw | | | | | | 2.6461E8 | | | | | | | | | | | 4 | 0 | 0 | 0 | 0 | 0 | 4 | 0 | 0 | 0 | 0 | 0 | 0 | 0 | 0 | 0 | 0 | 15 | 35 | Carbamidomethylation | C3:Carbamidomethylation:1000.00;C10:Carbamidomethylation:1000.00 | PEAKS DB |
| C.SAEETFC(+57.02)YKWLNK.I | Y | 59.35 | 1674.7711 | 13 | -1.1 | 838.3919 | 2 | 59.22 | 6 | F6:37505 | BuCaTN01_F6a.raw | | | | | | 1.3168E6 | | | | | | | | | | | 1 | 0 | 0 | 0 | 0 | 0 | 1 | 0 | 0 | 0 | 0 | 0 | 0 | 0 | 0 | 0 | 0 | 18 | 30 | Carbamidomethylation | C7:Carbamidomethylation:1000.00 | PEAKS DB |
| L.KITC(+57.02)SAEETFC(+57.02)YK.W | Y | 52.26 | 1635.7273 | 13 | 0.1 | 546.2498 | 3 | 42.98 | 6 | F6:22384 | BuCaTN01_F6a.raw | | | | | | 2.7893E5 | | | | | | | | | | | 1 | 0 | 0 | 0 | 0 | 0 | 1 | 0 | 0 | 0 | 0 | 0 | 0 | 0 | 0 | 0 | 0 | 14 | 26 | Carbamidomethylation | C4:Carbamidomethylation:1000.00;C11:Carbamidomethylation:1000.00 | PEAKS DB |
| I.DTWNVYNKC(+57.02)C(+57.02)TTNLC(+57.02)NT | Y | 43.96 | 2162.8821 | 17 | -1.1 | 1082.4471 | 2 | 54.91 | 6 | F6:33458 | BuCaTN01_F6a.raw | | | | | | 3.2186E6 | | | | | | | | | | | 1 | 0 | 0 | 0 | 0 | 0 | 1 | 0 | 0 | 0 | 0 | 0 | 0 | 0 | 0 | 0 | 0 | 47 | 63 | Carbamidomethylation | C9:Carbamidomethylation:1000.00;C10:Carbamidomethylation:1000.00;C15:Carbamidomethylation:1000.00 | PEAKS DB |
| K.WLNKISNER.W | Y | 41.68 | 1158.6145 | 9 | 0.1 | 580.3146 | 2 | 36.65 | 6 | F6:16419 | BuCaTN01_F6a.raw | | | | | | 5.9942E6 | | | | | | | | | | | 1 | 0 | 0 | 0 | 0 | 0 | 1 | 0 | 0 | 0 | 0 | 0 | 0 | 0 | 0 | 0 | 0 | 27 | 35 | | | PEAKS DB |
| **total 8 peptides** | | | | | | | | | | | | | | | | | | | | | | | | | | | | | | | | | | | | | | | | | | | | | | | | | |

**Best Unique PSM (Scan F8:24678, m/z=754.8234, z=2, RT=47.60, ppm=2.8):**

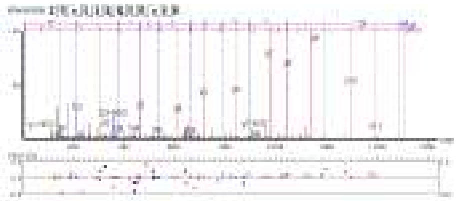

## P81782.1

back to list

| **Protein Coverage** | **Supporting Peptides** | **Best Unique PSM** |

**Protein Coverage:**

1 MECYRCGVSG CHLKITCSAE ETFCYKWLNK ISNERWLGCA KTCTEIDTWN VYNKCCTTNL CNT

Carbamidomethylation (+57.02)

**Supporting Peptides:**

| Peptide | Uniq | -10lgP | Mass | Length | ppm | m/z | z | RT | Fraction | Scan | Source File | Area F1 | Area F2a | Area F3a | Area F4 | Area F5 | Area F6a | Area F7a | Area F7b | Area F8 | Area F9a | Area Sample 1F10a | Area F10b | Area F11a | Area F11b | Area F12a | Area F13 | #Feature | #Feature F1 | #Feature F2a | #Feature F3a | #Feature F4 | #Feature F5 | #Feature F6a | #Feature F7a | #Feature F7b | #Feature F8 | #Feature F9a | #Feature Sample 1F10a | #Feature F10b | #Feature F11a | #Feature F11b | #Feature F12a | #Feature F13 | Start | End | PTM | AScore | Found By |
|---|---|---|---|---|---|---|---|---|---|---|---|---|---|---|---|---|---|---|---|---|---|---|---|---|---|---|---|---|---|---|---|---|---|---|---|---|---|---|---|---|---|---|---|---|---|---|---|---|---|
| K.ITC(+57.02)SAEETFC(+57.02)YK.W | Y | 90.16 | 1507.6323 | 12 | 2.8 | 754.8234 | 2 | 47.60 | 8 | F8:24678 | BuCaTN01_F7b.raw | | | | | | 5.6529E8 | 1.1028E6 | 2.8689E7 | 1.2868E6 | 2.0316E6 | | 0 | | | | | 23 | 0 | 0 | 0 | 0 | 0 | 19 | 1 | 1 | 1 | 1 | 0 | 0 | 0 | 0 | 0 | 0 | 15 | 26 | Carbamidomethylation | C3:Carbamidomethylation:1000.00;C10:Carbamidomethylation:1000.00 | PEAKS DB |
| R.C(+57.02)GVSGC(+57.02)HLKITC(+57.02)SAEETFC(+57.02)YK.W | Y | 88.69 | 2506.0750 | 21 | -2.0 | 836.3639 | 3 | 53.55 | 6 | F6:32082 | BuCaTN01_F6a.raw | | | | | | 1.1029E7 | | | | | | | | | | | 3 | 0 | 0 | 0 | 0 | 0 | 3 | 0 | 0 | 0 | 0 | 0 | 0 | 0 | 0 | 0 | 0 | 6 | 26 | Carbamidomethylation | C1:Carbamidomethylation:1000.00;C6:Carbamidomethylation:1000.00;C12:Carbamidomethylation:1000.00;C19:Carbamidomethylation:1000.00 | PEAKS DB |
| K.ITC(+57.02)SAEETFC(+57.02)YKWLNK.I | Y | 72.94 | 2048.9336 | 16 | 0.8 | 683.9857 | 3 | 63.58 | 6 | F6:41890 | BuCaTN01_F6a.raw | | | | | | 4.7109E7 | | | | | | | | | | | 3 | 0 | 0 | 0 | 0 | 0 | 3 | 0 | 0 | 0 | 0 | 0 | 0 | 0 | 0 | 0 | 0 | 15 | 30 | Carbamidomethylation | C3:Carbamidomethylation:1000.00;C10:Carbamidomethylation:1000.00 | PEAKS DB |
| K.ITC(+57.02)SAEETFC(+57.02)YKWLNKISNER.W | Y | 70.93 | 2648.2363 | 21 | 1.8 | 883.7543 | 3 | 70.00 | 6 | F6:48784 | BuCaTN01_F6a.raw | | | | | | 2.6461E8 | | | | | | | | | | | 4 | 0 | 0 | 0 | 0 | 0 | 4 | 0 | 0 | 0 | 0 | 0 | 0 | 0 | 0 | 0 | 0 | 15 | 35 | Carbamidomethylation | C3:Carbamidomethylation:1000.00;C10:Carbamidomethylation:1000.00 | PEAKS DB |
| C.SAEETFC(+57.02)YKWLNK.I | Y | 59.35 | 1674.7711 | 13 | -1.1 | 838.3919 | 2 | 59.22 | 6 | F6:37505 | BuCaTN01_F6a.raw | | | | | | 1.3168E6 | | | | | | | | | | | 1 | 0 | 0 | 0 | 0 | 0 | 1 | 0 | 0 | 0 | 0 | 0 | 0 | 0 | 0 | 0 | 0 | 18 | 30 | Carbamidomethylation | C7:Carbamidomethylation:1000.00 | PEAKS DB |
| L.KITC(+57.02)SAEETFC(+57.02)YK.W | Y | 52.26 | 1635.7273 | 13 | 0.1 | 546.2498 | 3 | 42.98 | 6 | F6:22384 | BuCaTN01_F6a.raw | | | | | | 2.7893E5 | | | | | | | | | | | 1 | 0 | 0 | 0 | 0 | 0 | 1 | 0 | 0 | 0 | 0 | 0 | 0 | 0 | 0 | 0 | 0 | 14 | 26 | Carbamidomethylation | C4:Carbamidomethylation:1000.00;C11:Carbamidomethylation:1000.00 | PEAKS DB |
| I.DTWNVYNKC(+57.02)C(+57.02)TTNLC(+57.02)NT | Y | 43.96 | 2162.8821 | 17 | -1.1 | 1082.4471 | 2 | 54.91 | 6 | F6:33458 | BuCaTN01_F6a.raw | | | | | | 3.2186E6 | | | | | | | | | | | 1 | 0 | 0 | 0 | 0 | 0 | 1 | 0 | 0 | 0 | 0 | 0 | 0 | 0 | 0 | 0 | 0 | 47 | 63 | Carbamidomethylation | C9:Carbamidomethylation:1000.00;C10:Carbamidomethylation:1000.00;C15:Carbamidomethylation:1000.00 | PEAKS DB |
| K.WLNKISNER.W | Y | 41.68 | 1158.6145 | 9 | 0.1 | 580.3146 | 2 | 36.65 | 6 | F6:16419 | BuCaTN01_F6a.raw | | | | | | 5.9942E6 | | | | | | | | | | | 1 | 0 | 0 | 0 | 0 | 0 | 1 | 0 | 0 | 0 | 0 | 0 | 0 | 0 | 0 | 0 | 0 | 27 | 35 | | | PEAKS DB |
| **total 8 peptides** | | | | | | | | | | | | | | | | | | | | | | | | | | | | | | | | | | | | | | | | | | | | | | | | | |

**Best Unique PSM (Scan F8:24678, m/z=754.8234, z=2, RT=47.60, ppm=2.8):**

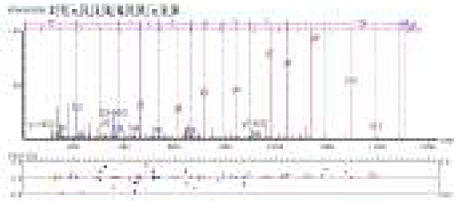

## AAL55556.1

back to list

| **Protein Coverage** | **Supporting Peptides** | **Best Unique PSM** |

**Protein Coverage:**

1 LVQFSYVITC ANHNRRSSLD YADYGCYCGA GGSGTPVDEL DRCCKIHDDC YGEAEKQGCY PKMLIYDYDC GSNGPYCKNV

Carbamidomethylation (+57.02)

81 TKKCNRKVCD CDVAAAKCFA RNAYNNANYN IDTKKRCK

**Supporting Peptides:**

| Peptide | Uniq | -10lgP | Mass | Length | ppm | m/z | z | RT | Fraction | Scan | Source File | Area F1 | Area F2a | Area F3a | Area F4 | Area F5 | Area F6a | Area F7a | Area F7b | Area F8 | Area F9a | Area Sample 1F10a | Area F10b | Area F11a | Area F11b | Area F12a | Area F13 | #Feature | #Feature F1 | #Feature F2a | #Feature F3a | #Feature F4 | #Feature F5 | #Feature F6a | #Feature F7a | #Feature F7b | #Feature F8 | #Feature F9a | #Feature Sample 1F10a | #Feature F10b | #Feature F11a | #Feature F11b | #Feature F12a | #Feature F13 | Start | End | PTM | AScore | Found By |
|---|---|---|---|---|---|---|---|---|---|---|---|---|---|---|---|---|---|---|---|---|---|---|---|---|---|---|---|---|---|---|---|---|---|---|---|---|---|---|---|---|---|---|---|---|---|---|---|---|---|
| C.YC(+57.02)GAGGSGTPVDELDR.C | N | 76.90 | 1652.7100 | 16 | 3.1 | 827.3629 | 2 | 45.67 | 12 | F12:22664 | BuCaTN01_F10b.raw | | | | | | | | | | 1.6847E5 | | 2.8019E6 | | 7.7562E5 | | | 3 | 0 | 0 | 0 | 0 | 0 | 0 | 0 | 0 | 0 | 1 | 0 | 1 | 0 | 1 | 0 | 0 | 27 | 42 | Carbamidomethylation | C2:Carbamidomethylation:1000.00 | PEAKS DB |
| Y.GC(+57.02)YC(+57.02)GAGGSGTPVDELDR.C | N | 69.43 | 1869.7621 | 18 | 1.9 | 935.8880 | 2 | 47.48 | 12 | F12:24483 | BuCaTN01_F10b.raw | | | | | | | | | | | | 2.3111E6 | | | | | 1 | 0 | 0 | 0 | 0 | 0 | 0 | 0 | 0 | 0 | 0 | 0 | 1 | 0 | 0 | 0 | 0 | 25 | 42 | Carbamidomethylation | C2:Carbamidomethylation:1000.00;C4:Carbamidomethylation:1000.00 | PEAKS DB |
| Y.C(+57.02)GAGGSGTPVDELDR.C | N | 67.25 | 1489.6467 | 15 | 4.1 | 745.8307 | 2 | 41.63 | 14 | F14:19582 | BuCaTN01_F11b.raw | | | | | | | | | | | 5.1011E5 | 3.3028E7 | | 9.0909E6 | | | 3 | 0 | 0 | 0 | 0 | 0 | 0 | 0 | 0 | 0 | 0 | 1 | 1 | 0 | 1 | 0 | 0 | 28 | 42 | Carbamidomethylation | C1:Carbamidomethylation:1000.00 | PEAKS DB |
| D.YGC(+57.02)YC(+57.02)GAGGSGTPVDELDR.C | N | 65.18 | 2032.8254 | 19 | 7.1 | 1017.4242 | 2 | 52.27 | 8 | F8:28495 | BuCaTN01_F7b.raw | | | | | | | | 5.527E4 | | 2.3803E5 | | 1.1215E7 | | 1.5685E6 | | | 5 | 0 | 0 | 0 | 0 | 0 | 0 | 0 | 1 | 0 | 1 | 0 | 2 | 0 | 1 | 0 | 0 | 24 | 42 | Carbamidomethylation | C3:Carbamidomethylation:1000.00;C5:Carbamidomethylation:1000.00 | PEAKS DB |
| G.AGGSGTPVDELDR.C | N | 61.00 | 1272.5946 | 13 | 2.3 | 637.3046 | 2 | 39.79 | 12 | F12:17765 | BuCaTN01_F10b.raw | | | | | | | | | | | | 6.0848E6 | | | | | 1 | 0 | 0 | 0 | 0 | 0 | 0 | 0 | 0 | 0 | 0 | 0 | 1 | 0 | 0 | 0 | 0 | 30 | 42 | | | PEAKS DB |
| G.C(+57.02)YC(+57.02)GAGGSGTPVDELDR.C | N | 56.73 | 1812.7407 | 17 | 1.1 | 907.3765 | 2 | 47.13 | 12 | F12:24185 | BuCaTN01_F10b.raw | | | | | | | | | | | | 1.2993E6 | | 1.447E5 | | | 2 | 0 | 0 | 0 | 0 | 0 | 0 | 0 | 0 | 0 | 0 | 0 | 1 | 0 | 1 | 0 | 0 | 26 | 42 | Carbamidomethylation | C1:Carbamidomethylation:1000.00;C3:Carbamidomethylation:1000.00 | PEAKS DB |
| G.GSGTPVDELDR.C | N | 56.36 | 1144.5360 | 11 | 3.4 | 573.2756 | 2 | 40.15 | 8 | F8:17921 | BuCaTN01_F7b.raw | | | | | | | | 1.1068E6 | | | | | | | | | 1 | 0 | 0 | 0 | 0 | 0 | 0 | 0 | 1 | 0 | 0 | 0 | 0 | 0 | 0 | 0 | 0 | 32 | 42 | | | PEAKS DB |
| R.RSSLDYADYGC(+57.02)YC(+57.02)GAGGSGTPVDELDR.C | Y | 54.51 | 2940.2290 | 27 | 9.3 | 981.0878 | 3 | 76.69 | 15 | F15:49606 | BuCaTN01_F12a.raw | | | | | | | | | | 7.812E6 | | | | | 1.299E5 | | 2 | 0 | 0 | 0 | 0 | 0 | 0 | 0 | 0 | 0 | 1 | 0 | 0 | 0 | 0 | 1 | 0 | 16 | 42 | Carbamidomethylation | C11:Carbamidomethylation:1000.00;C13:Carbamidomethylation:1000.00 | PEAKS DB |
| C.GAGGSGTPVDELDR.C | N | 54.07 | 1329.6160 | 14 | 3.0 | 665.8145 | 2 | 40.54 | 14 | F14:18567 | BuCaTN01_F11b.raw | | | | | | | | | | | | 3.4593E6 | | 1.1632E6 | | | 2 | 0 | 0 | 0 | 0 | 0 | 0 | 0 | 0 | 0 | 0 | 0 | 1 | 0 | 1 | 0 | 0 | 29 | 42 | | | PEAKS DB |
| A.GGSGTPVDELDR.C | N | 49.09 | 1201.5575 | 12 | 0.3 | 601.7862 | 2 | 40.57 | 11 | F11:14528 | BuCaTN01_F10a.raw | | | | | | | 9.2274E5 | 2.7577E6 | | 1.1335E7 | 6.7591E5 | | 9.9086E5 | 2.6759E7 | | | 6 | 0 | 0 | 0 | 0 | 0 | 0 | 1 | 1 | 0 | 1 | 1 | 0 | 1 | 1 | 0 | 0 | 31 | 42 | | | PEAKS DB |
| **total 10 peptides** | | | | | | | | | | | | | | | | | | | | | | | | | | | | | | | | | | | | | | | | | | | | | | | | | |

**Best Unique PSM (Scan F15:49606, m/z=981.0878, z=3, RT=76.69, ppm=9.3):**

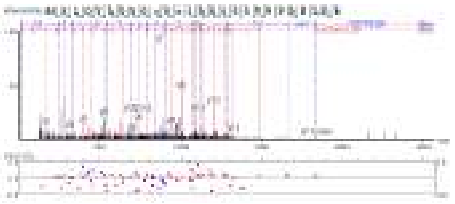

## ABN54807.1

back to list

| Protein Coverage | Supporting Peptides | Best Unique PSM |

**Protein Coverage:**

1 MYPAHLLVLL AVCVSLLGAA SIPPLPLNLV QFSYVITCAN HNRRSSLDYA DYGCYCGAGG SGTPVDELDR CCKIHDDCYG

Carbamidomethylation (+57.02)

81 EAEKQGCYPK MLIYDYDCGS NGPYCKNVTK KCNRKVCDCD VAAAKCFARN AYNNANYNID TKKRCK

**Supporting Peptides:**

| Peptide | Uniq | -10lgP | Mass | Length | ppm | m/z | z | RT | Fraction | Scan | Source File | Area F1 | Area F2a | Area F3a | Area F4 | Area F5 | Area F6a | Area F7a | Area F7b | Area F8 | Area F9a | Area Sample 1F10a | Area F10b | Area F11a | Area F11b | Area F12a | Area F13 | #Feature | #Feature F1 | #Feature F2a | #Feature F3a | #Feature F4 | #Feature F5 | #Feature F6a | #Feature F7a | #Feature F7b | #Feature F8 | #Feature F9a | #Feature Sample 1F10a | #Feature F10b | #Feature F11a | #Feature F11b | #Feature F12a | #Feature F13 | Start | End | PTM | AScore | Found By |
|---|---|---|---|---|---|---|---|---|---|---|---|---|---|---|---|---|---|---|---|---|---|---|---|---|---|---|---|---|---|---|---|---|---|---|---|---|---|---|---|---|---|---|---|---|---|---|---|---|---|
| C.YC(+57.02)GAGGSGTPVDELDR.C | N | 76.90 | 1652.7100 | 16 | 3.1 | 827.3629 | 2 | 45.67 | 12 | F12:22664 | BuCaTN01_F10b.raw | | | | | | | | | | 1.6847E5 | | 2.8019E6 | | 7.7562E5 | | | 3 | 0 | 0 | 0 | 0 | 0 | 0 | 0 | 0 | 0 | 1 | 0 | 1 | 0 | 1 | 0 | 0 | 55 | 70 | Carbamidomethylation | C2:Carbamidomethylation:1000.00 | PEAKS DB |
| Y.GC(+57.02)YC(+57.02)GAGGSGTPVDELDR.C | N | 69.43 | 1869.7621 | 18 | 1.9 | 935.8880 | 2 | 47.48 | 12 | F12:24483 | BuCaTN01_F10b.raw | | | | | | | | | | | | 2.3111E6 | | | | | 1 | 0 | 0 | 0 | 0 | 0 | 0 | 0 | 0 | 0 | 0 | 0 | 1 | 0 | 0 | 0 | 0 | 53 | 70 | Carbamidomethylation | C2:Carbamidomethylation:1000.00;C4:Carbamidomethylation:1000.00 | PEAKS DB |
| Y.C(+57.02)GAGGSGTPVDELDR.C | N | 67.25 | 1489.6467 | 15 | 4.1 | 745.8307 | 2 | 41.63 | 14 | F14:19582 | BuCaTN01_F11b.raw | | | | | | | | | | | 5.1011E5 | 3.3028E7 | | 9.0909E6 | | | 3 | 0 | 0 | 0 | 0 | 0 | 0 | 0 | 0 | 0 | 0 | 1 | 1 | 0 | 1 | 0 | 0 | 56 | 70 | Carbamidomethylation | C1:Carbamidomethylation:1000.00 | PEAKS DB |
| D.YGC(+57.02)YC(+57.02)GAGGSGTPVDELDR.C | N | 65.18 | 2032.8254 | 19 | 7.1 | 1017.4242 | 2 | 52.27 | 8 | F8:28495 | BuCaTN01_F7b.raw | | | | | | | | 5.527E4 | | 2.3803E5 | | 1.1215E7 | | 1.5685E6 | | | 5 | 0 | 0 | 0 | 0 | 0 | 0 | 0 | 1 | 0 | 1 | 0 | 2 | 0 | 1 | 0 | 0 | 52 | 70 | Carbamidomethylation | C3:Carbamidomethylation:1000.00;C5:Carbamidomethylation:1000.00 | PEAKS DB |
| G.AGGSGTPVDELDR.C | N | 61.00 | 1272.5946 | 13 | 2.3 | 637.3046 | 2 | 39.79 | 12 | F12:17765 | BuCaTN01_F10b.raw | | | | | | | | | | | | 6.0848E6 | | | | | 1 | 0 | 0 | 0 | 0 | 0 | 0 | 0 | 0 | 0 | 0 | 0 | 1 | 0 | 0 | 0 | 0 | 58 | 70 | | | PEAKS DB |
| G.C(+57.02)YC(+57.02)GAGGSGTPVDELDR.C | N | 56.73 | 1812.7407 | 17 | 1.1 | 907.3765 | 2 | 47.13 | 12 | F12:24185 | BuCaTN01_F10b.raw | | | | | | | | | | | | 1.2993E6 | | 1.447E5 | | | 2 | 0 | 0 | 0 | 0 | 0 | 0 | 0 | 0 | 0 | 0 | 0 | 1 | 0 | 1 | 0 | 0 | 54 | 70 | Carbamidomethylation | C1:Carbamidomethylation:1000.00;C3:Carbamidomethylation:1000.00 | PEAKS DB |
| G.GSGTPVDELDR.C | N | 56.36 | 1144.5360 | 11 | 3.4 | 573.2756 | 2 | 40.15 | 8 | F8:17921 | BuCaTN01_F7b.raw | | | | | | | | 1.1068E6 | | | | | | | | | 1 | 0 | 0 | 0 | 0 | 0 | 0 | 0 | 1 | 0 | 0 | 0 | 0 | 0 | 0 | 0 | 0 | 60 | 70 | | | PEAKS DB |
| R.RSSLDYADYGC(+57.02)YC(+57.02)GAGGSGTPVDELDR.C | Y | 54.51 | 2940.2290 | 27 | 9.3 | 981.0878 | 3 | 76.69 | 15 | F15:49606 | BuCaTN01_F12a.raw | | | | | | | | | | 7.812E6 | | | | | 1.299E5 | | 2 | 0 | 0 | 0 | 0 | 0 | 0 | 0 | 0 | 0 | 1 | 0 | 0 | 0 | 0 | 1 | 0 | 44 | 70 | Carbamidomethylation | C11:Carbamidomethylation:1000.00;C13:Carbamidomethylation:1000.00 | PEAKS DB |
| C.GAGGSGTPVDELDR.C | N | 54.07 | 1329.6160 | 14 | 3.0 | 665.8145 | 2 | 40.54 | 14 | F14:18567 | BuCaTN01_F11b.raw | | | | | | | | | | | | 3.4593E6 | | 1.1632E6 | | | 2 | 0 | 0 | 0 | 0 | 0 | 0 | 0 | 0 | 0 | 0 | 0 | 1 | 0 | 1 | 0 | 0 | 57 | 70 | | | PEAKS DB |
| A.GGSGTPVDELDR.C | N | 49.09 | 1201.5575 | 12 | 0.3 | 601.7862 | 2 | 40.57 | 11 | F11:14528 | BuCaTN01_F10a.raw | | | | | | | 9.2274E5 | 2.7577E6 | | 1.1335E7 | 6.7591E5 | | 9.9086E5 | 2.6759E7 | | | 6 | 0 | 0 | 0 | 0 | 0 | 0 | 1 | 1 | 0 | 1 | 1 | 0 | 1 | 1 | 0 | 0 | 59 | 70 | | | PEAKS DB |
| total 10 peptides | | | | | | | | | | | | | | | | | | | | | | | | | | | | | | | | | | | | | | | | | | | | | | | | | |

**Best Unique PSM (Scan F15:49606, m/z=981.0878, z=3, RT=76.69, ppm=9.3):**

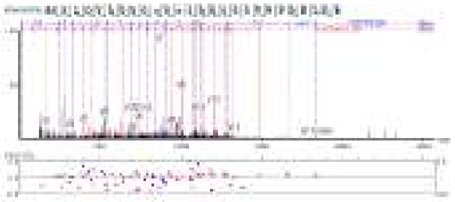

## Q8UW30.2

back to list

| Protein Coverage | Supporting Peptides | Best Unique PSM |

**Protein Coverage:**

1 MYPAHLLVLL AVCVSLLGAA SIPPLPLNLV QFSYVITCAN HNRRSSLDYA DYGCYCGAGG SGTPVDELDR CCKIHDDCYG

Carbamidomethylation (+57.02)

81 EAEKQGCYPK MLIYDYDCGS NGPYCKNVTK KCNRKVCDCD VAAAKCFARN AYNNANYNID TKKRCK

**Supporting Peptides:**


| Peptide | Uniq | -10lgP | Mass | Length | ppm | m/z | z | RT | Fraction | Scan | Source File | Area F1 | Area F2a | Area F3a | Area F4 | Area F5 | Area F6a | Area F7a | Area F7b | Area F8 | Area F9a | Area Sample 1F10a | Area F10b | Area F11a | Area F11b | Area F12a | Area F13 | #Feature | #Feature F1 | #Feature F2a | #Feature F3a | #Feature F4 | #Feature F5 | #Feature F6a | #Feature F7a | #Feature F7b | #Feature F8 | #Feature F9a | #Feature Sample 1F10a | #Feature F10b | #Feature F11a | #Feature F11b | #Feature F12a | #Feature F13 | Start | End | PTM | AScore | Found By |
|---|---|---|---|---|---|---|---|---|---|---|---|---|---|---|---|---|---|---|---|---|---|---|---|---|---|---|---|---|---|---|---|---|---|---|---|---|---|---|---|---|---|---|---|---|---|---|---|---|---|
| C.YC(+57.02)GAGGSGTPVDELDR.C | N | 76.90 | 1652.7100 | 16 | 3.1 | 827.3629 | 2 | 45.67 | 12 | F12:22664 | BuCaTN01_F10b.raw | | | | | | | | | | 1.6847E5 | | 2.8019E6 | | 7.7562E5 | | | 3 | 0 | 0 | 0 | 0 | 0 | 0 | 0 | 0 | 0 | 1 | 0 | 1 | 0 | 1 | 0 | 0 | 55 | 70 | Carbamidomethylation | C2:Carbamidomethylation:1000.00 | PEAKS DB |
| Y.GC(+57.02)YC(+57.02)GAGGSGTPVDELDR.C | N | 69.43 | 1869.7621 | 18 | 1.9 | 935.8880 | 2 | 47.48 | 12 | F12:24483 | BuCaTN01_F10b.raw | | | | | | | | | | | | 2.3111E6 | | | | | 1 | 0 | 0 | 0 | 0 | 0 | 0 | 0 | 0 | 0 | 0 | 0 | 1 | 0 | 0 | 0 | 0 | 53 | 70 | Carbamidomethylation | C2:Carbamidomethylation:1000.00;C4:Carbamidomethylation:1000.00 | PEAKS DB |
| Y.C(+57.02)GAGGSGTPVDELDR.C | N | 67.25 | 1489.6467 | 15 | 4.1 | 745.8307 | 2 | 41.63 | 14 | F14:19582 | BuCaTN01_F11b.raw | | | | | | | | | | | 5.1011E5 | 3.3028E7 | | 9.0909E6 | | | 3 | 0 | 0 | 0 | 0 | 0 | 0 | 0 | 0 | 0 | 0 | 1 | 1 | 0 | 1 | 0 | 0 | 56 | 70 | Carbamidomethylation | C1:Carbamidomethylation:1000.00 | PEAKS DB |
| D.YGC(+57.02)YC(+57.02)GAGGSGTPVDELDR.C | N | 65.18 | 2032.8254 | 19 | 7.1 | 1017.4242 | 2 | 52.27 | 8 | F8:28495 | BuCaTN01_F7b.raw | | | | | | | | 5.527E4 | | 2.3803E5 | | 1.1215E7 | | 1.5685E6 | | | 5 | 0 | 0 | 0 | 0 | 0 | 0 | 0 | 1 | 0 | 1 | 0 | 2 | 0 | 1 | 0 | 0 | 52 | 70 | Carbamidomethylation | C3:Carbamidomethylation:1000.00;C5:Carbamidomethylation:1000.00 | PEAKS DB |
| G.AGGSGTPVDELDR.C | N | 61.00 | 1272.5946 | 13 | 2.3 | 637.3046 | 2 | 39.79 | 12 | F12:17765 | BuCaTN01_F10b.raw | | | | | | | | | | | | 6.0848E6 | | | | | 1 | 0 | 0 | 0 | 0 | 0 | 0 | 0 | 0 | 0 | 0 | 0 | 1 | 0 | 0 | 0 | 0 | 58 | 70 | | | PEAKS DB |
| G.C(+57.02)YC(+57.02)GAGGSGTPVDELDR.C | N | 56.73 | 1812.7407 | 17 | 1.1 | 907.3765 | 2 | 47.13 | 12 | F12:24185 | BuCaTN01_F10b.raw | | | | | | | | | | | | 1.2993E6 | | 1.447E5 | | | 2 | 0 | 0 | 0 | 0 | 0 | 0 | 0 | 0 | 0 | 0 | 0 | 1 | 0 | 1 | 0 | 0 | 54 | 70 | Carbamidomethylation | C1:Carbamidomethylation:1000.00;C3:Carbamidomethylation:1000.00 | PEAKS DB |
| G.GSGTPVDELDR.C | N | 56.36 | 1144.5360 | 11 | 3.4 | 573.2756 | 2 | 40.15 | 8 | F8:17921 | BuCaTN01_F7b.raw | | | | | | | | 1.1068E6 | | | | | | | | | 1 | 0 | 0 | 0 | 0 | 0 | 0 | 0 | 1 | 0 | 0 | 0 | 0 | 0 | 0 | 0 | 0 | 60 | 70 | | | PEAKS DB |
| R.RSSLDYADYGC(+57.02)YC(+57.02)GAGGSGTPVDELDR.C | Y | 54.51 | 2940.2290 | 27 | 9.3 | 981.0878 | 3 | 76.69 | 15 | F15:49606 | BuCaTN01_F12a.raw | | | | | | | | | | 7.812E6 | | | | | 1.299E5 | | 2 | 0 | 0 | 0 | 0 | 0 | 0 | 0 | 0 | 0 | 1 | 0 | 0 | 0 | 0 | 1 | 0 | 44 | 70 | Carbamidomethylation | C11:Carbamidomethylation:1000.00;C13:Carbamidomethylation:1000.00 | PEAKS DB |
| C.GAGGSGTPVDELDR.C | N | 54.07 | 1329.6160 | 14 | 3.0 | 665.8145 | 2 | 40.54 | 14 | F14:18567 | BuCaTN01_F11b.raw | | | | | | | | | | | | 3.4593E6 | | 1.1632E6 | | | 2 | 0 | 0 | 0 | 0 | 0 | 0 | 0 | 0 | 0 | 0 | 0 | 1 | 0 | 1 | 0 | 0 | 57 | 70 | | | PEAKS DB |
| A.GGSGTPVDELDR.C | N | 49.09 | 1201.5575 | 12 | 0.3 | 601.7862 | 2 | 40.57 | 11 | F11:14528 | BuCaTN01_F10a.raw | | | | | | | 9.2274E5 | 2.7577E6 | | 1.1335E7 | 6.7591E5 | | 9.9086E5 | 2.6759E7 | | | 6 | 0 | 0 | 0 | 0 | 0 | 0 | 1 | 1 | 0 | 1 | 1 | 0 | 1 | 1 | 0 | 0 | 59 | 70 | | | PEAKS DB |
| total 10 peptides | | | | | | | | | | | | | | | | | | | | | | | | | | | | | | | | | | | | | | | | | | | | | | | | | |


**Best Unique PSM (Scan F15:49606, m/z=981.0878, z=3, RT=76.69, ppm=9.3):**

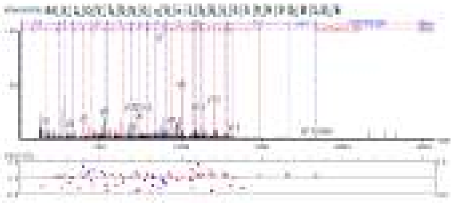

## P00610.1

back to list

| Protein Coverage | Supporting Peptides | Best Unique PSM |

**Protein Coverage:**

proteins

1 NLVQFSYVIT CANHNR**RSSL DYADYGCYCG AGGSGTPVDE LDR**CCKIHDD CYGEAEKQGC YPKMLMYDYY CGSNGPYCRN

27 29

C Carbamidomethylation (+57.02)

81 VKKKCNRKVC DCDVAAAECF ARNAYNNANY NIDTKKRCK

**Supporting Peptides:**

| Peptide | Uniq | -10lgP | Mass | Length | ppm | m/z | z | RT | Fraction | Scan | Source File | Area F1 | Area F2a | Area F3a | Area F4 | Area F5 | Area F6a | Area F7a | Area F7b | Area F8 | Area F9a | Area Sample 1F10a | Area F10b | Area F11a | Area F11b | Area F12a | Area F13 | #Feature | #Feature F1 | #Feature F2a | #Feature F3a | #Feature F4 | #Feature F5 | #Feature F6a | #Feature F7a | #Feature F7b | #Feature F8 | #Feature F9a | #Feature Sample 1F10a | #Feature F10b | #Feature F11a | #Feature F11b | #Feature F12a | #Feature F13 | Start | End | PTM | AScore | Found By |
|---|---|---|---|---|---|---|---|---|---|---|---|---|---|---|---|---|---|---|---|---|---|---|---|---|---|---|---|---|---|---|---|---|---|---|---|---|---|---|---|---|---|---|---|---|---|---|---|---|---|
| C.YC(+57.02)GAGGSGTPVDELDR.C | N | 76.90 | 1652.7100 | 16 | 3.1 | 827.3629 | 2 | 45.67 | 12 | F12:22664 | BuCaTN01_F10b.raw | | | | | | | | | | 1.6847E5 | | 2.8019E6 | | 7.7562E5 | | | 3 | 0 | 0 | 0 | 0 | 0 | 0 | 0 | 0 | 0 | 1 | 0 | 1 | 0 | 1 | 0 | 0 | 28 | 43 | Carbamidomethylation | C2:Carbamidomethylation:1000.00 | PEAKS DB |
| Y.GC(+57.02)YC(+57.02)GAGGSGTPVDELDR.C | N | 69.43 | 1869.7621 | 18 | 1.9 | 935.8880 | 2 | 47.48 | 12 | F12:24483 | BuCaTN01_F10b.raw | | | | | | | | | | | | 2.3111E6 | | | | | 1 | 0 | 0 | 0 | 0 | 0 | 0 | 0 | 0 | 0 | 0 | 0 | 1 | 0 | 0 | 0 | 0 | 26 | 43 | Carbamidomethylation | C2:Carbamidomethylation:1000.00;C4:Carbamidomethylation:1000.00 | PEAKS DB |
| Y.C(+57.02)GAGGSGTPVDELDR.C | N | 67.25 | 1489.6467 | 15 | 4.1 | 745.8307 | 2 | 41.63 | 14 | F14:19582 | BuCaTN01_F11b.raw | | | | | | | | | | | 5.1011E5 | 3.3028E7 | | 9.0909E6 | | | 3 | 0 | 0 | 0 | 0 | 0 | 0 | 0 | 0 | 0 | 0 | 1 | 1 | 0 | 1 | 0 | 0 | 29 | 43 | Carbamidomethylation | C1:Carbamidomethylation:1000.00 | PEAKS DB |
| D.YGC(+57.02)YC(+57.02)GAGGSGTPVDELDR.C | N | 65.18 | 2032.8254 | 19 | 7.1 | 1017.4242 | 2 | 52.27 | 8 | F8:28495 | BuCaTN01_F7b.raw | | | | | | | | 5.527E4 | | 2.3803E5 | | 1.1215E7 | | 1.5685E6 | | | 5 | 0 | 0 | 0 | 0 | 0 | 0 | 0 | 1 | 0 | 1 | 0 | 2 | 0 | 1 | 0 | 0 | 25 | 43 | Carbamidomethylation | C3:Carbamidomethylation:1000.00;C5:Carbamidomethylation:1000.00 | PEAKS DB |
| G.AGGSGTPVDELDR.C | N | 61.00 | 1272.5946 | 13 | 2.3 | 637.3046 | 2 | 39.79 | 12 | F12:17765 | BuCaTN01_F10b.raw | | | | | | | | | | | | 6.0848E6 | | | | | 1 | 0 | 0 | 0 | 0 | 0 | 0 | 0 | 0 | 0 | 0 | 0 | 1 | 0 | 0 | 0 | 0 | 31 | 43 | | | PEAKS DB |
| G.C(+57.02)YC(+57.02)GAGGSGTPVDELDR.C | N | 56.73 | 1812.7407 | 17 | 1.1 | 907.3765 | 2 | 47.13 | 12 | F12:24185 | BuCaTN01_F10b.raw | | | | | | | | | | | | 1.2993E6 | | 1.447E5 | | | 2 | 0 | 0 | 0 | 0 | 0 | 0 | 0 | 0 | 0 | 0 | 0 | 1 | 0 | 1 | 0 | 0 | 27 | 43 | Carbamidomethylation | C1:Carbamidomethylation:1000.00;C3:Carbamidomethylation:1000.00 | PEAKS DB |
| G.GSGTPVDELDR.C | N | 56.36 | 1144.5360 | 11 | 3.4 | 573.2756 | 2 | 40.15 | 8 | F8:17921 | BuCaTN01_F7b.raw | | | | | | | | 1.1068E6 | | | | | | | | | 1 | 0 | 0 | 0 | 0 | 0 | 0 | 0 | 1 | 0 | 0 | 0 | 0 | 0 | 0 | 0 | 0 | 33 | 43 | | | PEAKS DB |
| R.RSSLDYADYGC(+57.02)YC(+57.02)GAGGSGTPVDELDR.C | Y | 54.51 | 2940.2290 | 27 | 9.3 | 981.0878 | 3 | 76.69 | 15 | F15:49606 | BuCaTN01_F12a.raw | | | | | | | | | | 7.812E6 | | | | | 1.299E5 | | 2 | 0 | 0 | 0 | 0 | 0 | 0 | 0 | 0 | 0 | 1 | 0 | 0 | 0 | 0 | 1 | 0 | 17 | 43 | Carbamidomethylation | C11:Carbamidomethylation:1000.00;C13:Carbamidomethylation:1000.00 | PEAKS DB |
| C.GAGGSGTPVDELDR.C | N | 54.07 | 1329.6160 | 14 | 3.0 | 665.8145 | 2 | 40.54 | 14 | F14:18567 | BuCaTN01_F11b.raw | | | | | | | | | | | | 3.4593E6 | | 1.1632E6 | | | 2 | 0 | 0 | 0 | 0 | 0 | 0 | 0 | 0 | 0 | 0 | 0 | 1 | 0 | 1 | 0 | 0 | 30 | 43 | | | PEAKS DB |
| A.GGSGTPVDELDR.C | N | 49.09 | 1201.5575 | 12 | 0.3 | 601.7862 | 2 | 40.57 | 11 | F11:14528 | BuCaTN01_F10a.raw | | | | | | | 9.2274E5 | 2.7577E6 | | 1.1335E7 | 6.7591E5 | | 9.9086E5 | 2.6759E7 | | | 6 | 0 | 0 | 0 | 0 | 0 | 0 | 1 | 1 | 0 | 1 | 1 | 0 | 1 | 1 | 0 | 0 | 32 | 43 | | | PEAKS DB |
| total 10 peptides | | | | | | | | | | | | | | | | | | | | | | | | | | | | | | | | | | | | | | | | | | | | | | | | | |

**Best Unique PSM (Scan F15:49606, m/z=981.0878, z=3, RT=76.69, ppm=9.3):**

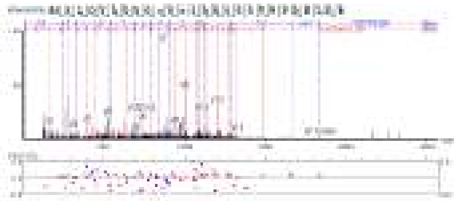

## AAB20783.1

back to list

| Protein Coverage | Supporting Peptides | Best Unique PSM |

**Protein Coverage:**

1 **NLYQFGNMIQ CANHGRRPTL AYADYGCYCG AGGSGTPVDE LDR**CCKAHDD CYGEAGKKGC YPTLTLYSWQ CIEKTPTCNS

27 29

C Carbamidomethylation (+57.02)

81 KTGCERSVCD CDATAAKCFA KAPYNKKNYN IDTEKRCQ

**Supporting Peptides:**

| Peptide | Uniq | -10lgP | Mass | Length | ppm | m/z | z | RT | Fraction | Scan | Source File | Area F1 | Area F2a | Area F3a | Area F4 | Area F5 | Area F6a | Area F7a | Area F7b | Area F8 | Area F9a | Area Sample 1F10a | Area F10b | Area F11a | Area F11b | Area F12a | Area F13 | #Feature | #Feature F1 | #Feature F2a | #Feature F3a | #Feature F4 | #Feature F5 | #Feature F6a | #Feature F7a | #Feature F7b | #Feature F8 | #Feature F9a | #Feature Sample 1F10a | #Feature F10b | #Feature F11a | #Feature F11b | #Feature F12a | #Feature F13 | Start | End | PTM | AScore | Found By |
|---|---|---|---|---|---|---|---|---|---|---|---|---|---|---|---|---|---|---|---|---|---|---|---|---|---|---|---|---|---|---|---|---|---|---|---|---|---|---|---|---|---|---|---|---|---|---|---|---|---|
| C.YC(+57.02)GAGGSGTPVDELDR.C | N | 76.90 | 1652.7100 | 16 | 3.1 | 827.3629 | 2 | 45.67 | 12 | F12:22664 | BuCaTN01_F10b.raw | | | | | | | | | | 1.6847E5 | | 2.8019E6 | | 7.7562E5 | | | 3 | 0 | 0 | 0 | 0 | 0 | 0 | 0 | 0 | 0 | 1 | 0 | 1 | 0 | 1 | 0 | 0 | 28 | 43 | Carbamidomethylation | C2:Carbamidomethylation:1000.00 | PEAKS DB |
| Y.GC(+57.02)YC(+57.02)GAGGSGTPVDELDR.C | N | 69.43 | 1869.7621 | 18 | 1.9 | 935.8880 | 2 | 47.48 | 12 | F12:24483 | BuCaTN01_F10b.raw | | | | | | | | | | | | 2.3111E6 | | | | | 1 | 0 | 0 | 0 | 0 | 0 | 0 | 0 | 0 | 0 | 0 | 0 | 1 | 0 | 0 | 0 | 0 | 26 | 43 | Carbamidomethylation | C2:Carbamidomethylation:1000.00;C4:Carbamidomethylation:1000.00 | PEAKS DB |
| Y.C(+57.02)GAGGSGTPVDELDR.C | N | 67.25 | 1489.6467 | 15 | 4.1 | 745.8307 | 2 | 41.63 | 14 | F14:19582 | BuCaTN01_F11b.raw | | | | | | | | | | | 5.1011E5 | 3.3028E7 | | 9.0909E6 | | | 3 | 0 | 0 | 0 | 0 | 0 | 0 | 0 | 0 | 0 | 0 | 1 | 1 | 0 | 1 | 0 | 0 | 29 | 43 | Carbamidomethylation | C1:Carbamidomethylation:1000.00 | PEAKS DB |
| D.YGC(+57.02)YC(+57.02)GAGGSGTPVDELDR.C | N | 65.18 | 2032.8254 | 19 | 7.1 | 1017.4242 | 2 | 52.27 | 8 | F8:28495 | BuCaTN01_F7b.raw | | | | | | | | 5.527E4 | | 2.3803E5 | | 1.1215E7 | | 1.5685E6 | | | 5 | 0 | 0 | 0 | 0 | 0 | 0 | 0 | 1 | 0 | 1 | 0 | 2 | 0 | 1 | 0 | 0 | 25 | 43 | Carbamidomethylation | C3:Carbamidomethylation:1000.00;C5:Carbamidomethylation:1000.00 | PEAKS DB |
| G.AGGSGTPVDELDR.C | N | 61.00 | 1272.5946 | 13 | 2.3 | 637.3046 | 2 | 39.79 | 12 | F12:17765 | BuCaTN01_F10b.raw | | | | | | | | | | | | 6.0848E6 | | | | | 1 | 0 | 0 | 0 | 0 | 0 | 0 | 0 | 0 | 0 | 0 | 0 | 1 | 0 | 0 | 0 | 0 | 31 | 43 | | | PEAKS DB |
| G.C(+57.02)YC(+57.02)GAGGSGTPVDELDR.C | N | 56.73 | 1812.7407 | 17 | 1.1 | 907.3765 | 2 | 47.13 | 12 | F12:24185 | BuCaTN01_F10b.raw | | | | | | | | | | | | 1.2993E6 | | 1.447E5 | | | 2 | 0 | 0 | 0 | 0 | 0 | 0 | 0 | 0 | 0 | 0 | 0 | 1 | 0 | 1 | 0 | 0 | 27 | 43 | Carbamidomethylation | C1:Carbamidomethylation:1000.00;C3:Carbamidomethylation:1000.00 | PEAKS DB |
| G.GSGTPVDELDR.C | N | 56.36 | 1144.5360 | 11 | 3.4 | 573.2756 | 2 | 40.15 | 8 | F8:17921 | BuCaTN01_F7b.raw | | | | | | | | 1.1068E6 | | | | | | | | | 1 | 0 | 0 | 0 | 0 | 0 | 0 | 0 | 1 | 0 | 0 | 0 | 0 | 0 | 0 | 0 | 0 | 33 | 43 | | | PEAKS DB |
| C.GAGGSGTPVDELDR.C | N | 54.07 | 1329.6160 | 14 | 3.0 | 665.8145 | 2 | 40.54 | 14 | F14:18567 | BuCaTN01_F11b.raw | | | | | | | | | | | | 3.4593E6 | | 1.1632E6 | | | 2 | 0 | 0 | 0 | 0 | 0 | 0 | 0 | 0 | 0 | 0 | 0 | 1 | 0 | 1 | 0 | 0 | 30 | 43 | | | PEAKS DB |
| NLYQFGNMIQC(+57.02)ANHGRRPTLAYADYGC(+57.02)YC(+57.02)GAGGSGTPVDELDR.C | Y | 51.08 | 4824.1323 | 43 | 8.0 | 965.8414 | 5 | 78.59 | 11 | F11:42220 | BuCaTN01_F10a.raw | | | | | | | | | | | 4.0825E5 | | | | | | 1 | 0 | 0 | 0 | 0 | 0 | 0 | 0 | 0 | 0 | 0 | 1 | 0 | 0 | 0 | 0 | 0 | 1 | 43 | Carbamidomethylation | C11:Carbamidomethylation:1000.00;C27:Carbamidomethylation:1000.00;C29:Carbamidomethylation:1000.00 | PEAKS DB |
| A.GGSGTPVDELDR.C | N | 49.09 | 1201.5575 | 12 | 0.3 | 601.7862 | 2 | 40.57 | 11 | F11:14528 | BuCaTN01_F10a.raw | | | | | | | 9.2274E5 | 2.7577E6 | | 1.1335E7 | 6.7591E5 | | 9.9086E5 | 2.6759E7 | | | 6 | 0 | 0 | 0 | 0 | 0 | 0 | 1 | 1 | 0 | 1 | 1 | 0 | 1 | 1 | 0 | 0 | 32 | 43 | | | PEAKS DB |
| total 10 peptides | | | | | | | | | | | | | | | | | | | | | | | | | | | | | | | | | | | | | | | | | | | | | | | | | |

**Best Unique PSM (Scan F11:42220, m/z=965.8414, z=5, RT=78.59, ppm=8.0):**

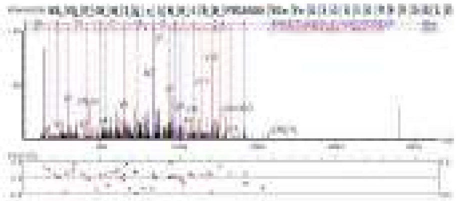

## P08873.1

back to list

| Protein Coverage | Supporting Peptides | Best Unique PSM |

**Protein Coverage:**

1 MYPAHLLVLL TVCVSLLEAS SIPARPL**NLY QFGNMIQCAN HGRRPTLAYA DYGCYCGAGG SGTPVDELDR** CCKAHDDCYG

54 56

C Carbamidomethylation (+57.02)

81 EAGKKGCYPT LTLYSWQCIE KTPTCNSKTG CERSVCDCDA TAAKCFAKAP YNKKNYNIDT EKRCQ

**Supporting Peptides:**

| Peptide | Uniq | -10lgP | Mass | Length | ppm | m/z | z | RT | Fraction | Scan | Source File | Area F1 | Area F2a | Area F3a | Area F4 | Area F5 | Area F6a | Area F7a | Area F7b | Area F8 | Area F9a | Area Sample 1F10a | Area F10b | Area F11a | Area F11b | Area F12a | Area F13 | #Feature | #Feature F1 | #Feature F2a | #Feature F3a | #Feature F4 | #Feature F5 | #Feature F6a | #Feature F7a | #Feature F7b | #Feature F8 | #Feature F9a | #Feature Sample 1F10a | #Feature F10b | #Feature F11a | #Feature F11b | #Feature F12a | #Feature F13 | Start | End | PTM | AScore | Found By |
|---|---|---|---|---|---|---|---|---|---|---|---|---|---|---|---|---|---|---|---|---|---|---|---|---|---|---|---|---|---|---|---|---|---|---|---|---|---|---|---|---|---|---|---|---|---|---|---|---|---|
| C.YC(+57.02)GAGGSGTPVDELDR.C | N | 76.90 | 1652.7100 | 16 | 3.1 | 827.3629 | 2 | 45.67 | 12 | F12:22664 | BuCaTN01_F10b.raw | | | | | | | | | | 1.6847E5 | | 2.8019E6 | | 7.7562E5 | | | 3 | 0 | 0 | 0 | 0 | 0 | 0 | 0 | 0 | 0 | 1 | 0 | 1 | 0 | 1 | 0 | 0 | 55 | 70 | Carbamidomethylation | C2:Carbamidomethylation:1000.00 | PEAKS DB |
| Y.GC(+57.02)YC(+57.02)GAGGSGTPVDELDR.C | N | 69.43 | 1869.7621 | 18 | 1.9 | 935.8880 | 2 | 47.48 | 12 | F12:24483 | BuCaTN01_F10b.raw | | | | | | | | | | | | 2.3111E6 | | | | | 1 | 0 | 0 | 0 | 0 | 0 | 0 | 0 | 0 | 0 | 0 | 0 | 1 | 0 | 0 | 0 | 0 | 53 | 70 | Carbamidomethylation | C2:Carbamidomethylation:1000.00;C4:Carbamidomethylation:1000.00 | PEAKS DB |
| Y.C(+57.02)GAGGSGTPVDELDR.C | N | 67.25 | 1489.6467 | 15 | 4.1 | 745.8307 | 2 | 41.63 | 14 | F14:19582 | BuCaTN01_F11b.raw | | | | | | | | | | | 5.1011E5 | 3.3028E7 | | 9.0909E6 | | | 3 | 0 | 0 | 0 | 0 | 0 | 0 | 0 | 0 | 0 | 0 | 1 | 1 | 0 | 1 | 0 | 0 | 56 | 70 | Carbamidomethylation | C1:Carbamidomethylation:1000.00 | PEAKS DB |
| D.YGC(+57.02)YC(+57.02)GAGGSGTPVDELDR.C | N | 65.18 | 2032.8254 | 19 | 7.1 | 1017.4242 | 2 | 52.27 | 8 | F8:28495 | BuCaTN01_F7b.raw | | | | | | | | 5.527E4 | | 2.3803E5 | | 1.1215E7 | | 1.5685E6 | | | 5 | 0 | 0 | 0 | 0 | 0 | 0 | 0 | 1 | 0 | 1 | 0 | 2 | 0 | 1 | 0 | 0 | 52 | 70 | Carbamidomethylation | C3:Carbamidomethylation:1000.00;C5:Carbamidomethyl | PEAKS DB |

| | | | | | | | | | | | | | | | | | | | | | | | | | | | | | | | | | | | | | | | | | | | | | | | | | |
|---|---|---|---|---|---|---|---|---|---|---|---|---|---|---|---|---|---|---|---|---|---|---|---|---|---|---|---|---|---|---|---|---|---|---|---|---|---|---|---|---|---|---|---|---|---|---|---|---|---|
| | | | | | | | | | | | | | | | | | | | | | | | | | | | | | | | | | | | | | | | | | | | | | | | | ation:1000.00 | |
| G.AGGSGTPVDELDR.C | N | 61.00 | 1272.5946 | 13 | 2.3 | 637.3046 | 2 | 39.79 | 12 | F12:17765 | BuCaTN01_F10b.raw | | | | | | | | | | | | 6.0848E6 | | | | | 1 | 0 | 0 | 0 | 0 | 0 | 0 | 0 | 0 | 0 | 0 | 0 | 1 | 0 | 0 | 0 | 0 | 58 | 70 | | | PEAKS DB |
| G.C(+57.02)YC(+57.02)GAGGSGTPVDELDR.C | N | 56.73 | 1812.7407 | 17 | 1.1 | 907.3765 | 2 | 47.13 | 12 | F12:24185 | BuCaTN01_F10b.raw | | | | | | | | | | | | 1.2993E6 | | 1.447E5 | | | 2 | 0 | 0 | 0 | 0 | 0 | 0 | 0 | 0 | 0 | 0 | 0 | 1 | 0 | 1 | 0 | 0 | 54 | 70 | Carbamidomethylation | C1:Carbamidomethylation:1000.00;C3:Carbamidomethylation:1000.00 | PEAKS DB |
| G.GSGTPVDELDR.C | N | 56.36 | 1144.5360 | 11 | 3.4 | 573.2756 | 2 | 40.15 | 8 | F8:17921 | BuCaTN01_F7b.raw | | | | | | | | 1.1068E6 | | | | | | | | | 1 | 0 | 0 | 0 | 0 | 0 | 0 | 0 | 1 | 0 | 0 | 0 | 0 | 0 | 0 | 0 | 0 | 60 | 70 | | | PEAKS DB |
| C.GAGGSGTPVDELDR.C | N | 54.07 | 1329.6160 | 14 | 3.0 | 665.8145 | 2 | 40.54 | 14 | F14:18567 | BuCaTN01_F11b.raw | | | | | | | | | | | | 3.4593E6 | | 1.1632E6 | | | 2 | 0 | 0 | 0 | 0 | 0 | 0 | 0 | 0 | 0 | 0 | 0 | 1 | 0 | 1 | 0 | 0 | 57 | 70 | | | PEAKS DB |
| L.NLYQFGNMIQC(+57.02)ANHGRRPTLAYADYGC(+57.02)YC(+57.02)GAGGSGTPVDELDR.C | Y | 51.08 | 4824.1323 | 43 | 8.0 | 965.8414 | 5 | 78.59 | 11 | F11:42220 | BuCaTN01_F10a.raw | | | | | | | | | | | 4.0825E5 | | | | | | 1 | 0 | 0 | 0 | 0 | 0 | 0 | 0 | 0 | 0 | 0 | 1 | 0 | 0 | 0 | 0 | 0 | 28 | 70 | Carbamidomethylation | C11:Carbamidomethylation:1000.00;C27:Carbamidomethylation:1000.00;C29:Carbamidomethylation:1000.00 | PEAKS DB |
| A.GGSGTPVDELDR.C | N | 49.09 | 1201.5575 | 12 | 0.3 | 601.7862 | 2 | 40.57 | 11 | F11:14528 | BuCaTN01_F10a.raw | | | | | | | 9.2274E5 | 2.7577E6 | | 1.1335E7 | 6.7591E5 | | 9.9086E5 | 2.6759E7 | | | 6 | 0 | 0 | 0 | 0 | 0 | 0 | 1 | 1 | 0 | 1 | 1 | 0 | 1 | 1 | 0 | 0 | 59 | 70 | | | PEAKS DB |
| total 10 peptides | | | | | | | | | | | | | | | | | | | | | | | | | | | | | | | | | | | | | | | | | | | | | | | | | |

**Best Unique PSM (Scan F11:42220, m/z=965.8414, z=5, RT=78.59, ppm=8.0):**

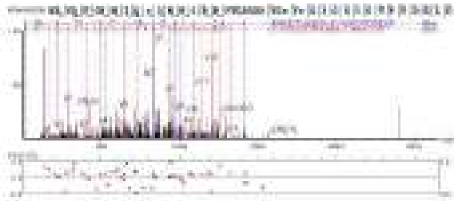

## CAA31125.1

back to list

| Protein Coverage | Supporting Peptides | Best Unique PSM |

**Protein Coverage:**

1 MYPAHLLVLL TVCVSLLEAS SIPARPLNLY QFGNMIQCAN HGRRPTLAYA DYGCYCGAGG SGTPVDELDR CCKAHDDCYG

C Carbamidomethylation (+57.02)

81 EAGKKGCYPT LTLYSWQCIE KTPTCNSKTG CERSVCDCDA TAAKCFAKAP YNKKNYNIDT EKRCQ

**Supporting Peptides:**

| Peptide | Uniq | -10lgP | Mass | Length | ppm | m/z | z | RT | Fraction | Scan | Source File | Area F1 | Area F2a | Area F3a | Area F4 | Area F5 | Area F6a | Area F7a | Area F7b | Area F8 | Area F9a | Area Sample 1F10a | Area F10b | Area F11a | Area F11b | Area F12a | Area F13 | #Feature | #Feature F1 | #Feature F2a | #Feature F3a | #Feature F4 | #Feature F5 | #Feature F6a | #Feature F7a | #Feature F7b | #Feature F8 | #Feature F9a | #Feature Sample 1F10a | #Feature F10b | #Feature F11a | #Feature F11b | #Feature F12a | #Feature F13 | Start | End | PTM | AScore | Found By |
|---|---|---|---|---|---|---|---|---|---|---|---|---|---|---|---|---|---|---|---|---|---|---|---|---|---|---|---|---|---|---|---|---|---|---|---|---|---|---|---|---|---|---|---|---|---|---|---|---|---|
| C.YC(+57.02)GAGGSGTPVDELDR.C | N | 76.90 | 1652.7100 | 16 | 3.1 | 827.3629 | 2 | 45.67 | 12 | F12:22664 | BuCaTN01_F10b.raw | | | | | | | | | | 1.6847E5 | | 2.8019E6 | | 7.7562E5 | | | 3 | 0 | 0 | 0 | 0 | 0 | 0 | 0 | 0 | 0 | 1 | 0 | 1 | 0 | 1 | 0 | 0 | 55 | 70 | Carbamidomethylation | C2:Carbamidomethylation:1000.00 | PEAKS DB |
| Y.GC(+57.02)YC(+57.02)GAGGSGTPVDELDR.C | N | 69.43 | 1869.7621 | 18 | 1.9 | 935.8880 | 2 | 47.48 | 12 | F12:24483 | BuCaTN01_F10b.raw | | | | | | | | | | | | 2.3111E6 | | | | | 1 | 0 | 0 | 0 | 0 | 0 | 0 | 0 | 0 | 0 | 0 | 0 | 1 | 0 | 0 | 0 | 0 | 53 | 70 | Carbamidomethylation | C2:Carbamidomethylation:1000.00;C4:Carbamidomethylation:1000.00 | PEAKS DB |
| Y.C(+57.02)GAGGSGTPVDELDR.C | N | 67.25 | 1489.6467 | 15 | 4.1 | 745.8307 | 2 | 41.63 | 14 | F14:19582 | BuCaTN01_F11b.raw | | | | | | | | | | | 5.1011E5 | 3.3028E7 | | 9.0909E6 | | | 3 | 0 | 0 | 0 | 0 | 0 | 0 | 0 | 0 | 0 | 0 | 1 | 1 | 0 | 1 | 0 | 0 | 56 | 70 | Carbamidomethylation | C1:Carbamidomethylation:1000.00 | PEAKS DB |
| D.YGC(+57.02)YC(+57.02)GAGGSGTPVDELDR.C | N | 65.18 | 2032.8254 | 19 | 7.1 | 1017.4242 | 2 | 52.27 | 8 | F8:28495 | BuCaTN01_F7b.raw | | | | | | | | 5.527E4 | | 2.3803E5 | | 1.1215E7 | | 1.5685E6 | | | 5 | 0 | 0 | 0 | 0 | 0 | 0 | 0 | 1 | 0 | 1 | 0 | 2 | 0 | 1 | 0 | 0 | 52 | 70 | Carbamidomethylation | C3:Carbamidomethylation:1000.00;C5:Carbamidomethylation:1000.00 | PEAKS DB |
| G.AGGSGTPVDELDR.C | N | 61.00 | 1272.5946 | 13 | 2.3 | 637.3046 | 2 | 39.79 | 12 | F12:17765 | BuCaTN01_F10b.raw | | | | | | | | | | | | 6.0848E6 | | | | | 1 | 0 | 0 | 0 | 0 | 0 | 0 | 0 | 0 | 0 | 0 | 0 | 1 | 0 | 0 | 0 | 0 | 58 | 70 | | | PEAKS DB |
| G.C(+57.02)YC(+57.02)GAGGSGTPVDELDR.C | N | 56.73 | 1812.7407 | 17 | 1.1 | 907.3765 | 2 | 47.13 | 12 | F12:24185 | BuCaTN01_F10b.raw | | | | | | | | | | | | 1.2993E6 | | 1.447E5 | | | 2 | 0 | 0 | 0 | 0 | 0 | 0 | 0 | 0 | 0 | 0 | 0 | 1 | 0 | 1 | 0 | 0 | 54 | 70 | Carbamidomethylation | C1:Carbamidomethylation:1000.00;C3:Carbamidomethylation:1000.00 | PEAKS DB |
| G.GSGTPVDELDR.C | N | 56.36 | 1144.5360 | 11 | 3.4 | 573.2756 | 2 | 40.15 | 8 | F8:17921 | BuCaTN01_F7b.raw | | | | | | | | 1.1068E6 | | | | | | | | | 1 | 0 | 0 | 0 | 0 | 0 | 0 | 0 | 1 | 0 | 0 | 0 | 0 | 0 | 0 | 0 | 0 | 60 | 70 | | | PEAKS DB |
| C.GAGGSGTPVDELDR.C | N | 54.07 | 1329.6160 | 14 | 3.0 | 665.8145 | 2 | 40.54 | 14 | F14:18567 | BuCaTN01_F11b.raw | | | | | | | | | | | | 3.4593E6 | | 1.1632E6 | | | 2 | 0 | 0 | 0 | 0 | 0 | 0 | 0 | 0 | 0 | 0 | 0 | 1 | 0 | 1 | 0 | 0 | 57 | 70 | | | PEAKS DB |
| L.NLYQFGNMIQC(+57.02)ANHGRRPTLAYADYGC(+57.02)YC(+57.02)GAGGSGTPVDELDR.C | Y | 51.08 | 4824.1323 | 43 | 8.0 | 965.8414 | 5 | 78.59 | 11 | F11:42220 | BuCaTN01_F10a.raw | | | | | | | | | | | 4.0825E5 | | | | | | 1 | 0 | 0 | 0 | 0 | 0 | 0 | 0 | 0 | 0 | 0 | 1 | 0 | 0 | 0 | 0 | 0 | 28 | 70 | Carbamidomethylation | C11:Carbamidomethylation:1000.00;C27:Carbamidomethylation:1000.00;C29:Carbamidomethylation:1000.00 | PEAKS DB |
| A.GGSGTPVDELDR.C | N | 49.09 | 1201.5575 | 12 | 0.3 | 601.7862 | 2 | 40.57 | 11 | F11:14528 | BuCaTN01_F10a.raw | | | | | | | 9.2274E5 | 2.7577E6 | | 1.1335E7 | 6.7591E5 | | 9.9086E5 | 2.6759E7 | | | 6 | 0 | 0 | 0 | 0 | 0 | 0 | 1 | 1 | 0 | 1 | 1 | 0 | 1 | 1 | 0 | 0 | 59 | 70 | | | PEAKS DB |
| total 10 peptides | | | | | | | | | | | | | | | | | | | | | | | | | | | | | | | | | | | | | | | | | | | | | | | | | |

**Best Unique PSM (Scan F11:42220, m/z=965.8414, z=5, RT=78.59, ppm=8.0):**

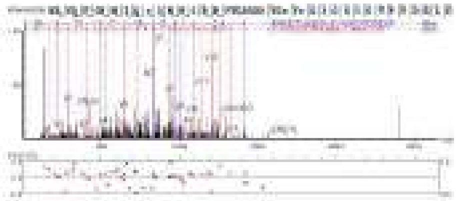

## BAP39981.1

back to list

| Protein Coverage | Supporting Peptides | Best Unique PSM |

**Protein Coverage:**

1 NQYRGGAVLQ ATSTDCQLPV VQQFDPDWLQ VDSAFHPSEN LALLENIKVH SVMSMLCYTL IIAFLIGIWA APKSEDNVPL

C Carbamidomethylation (+57.02)

81 GSPATSDLSD TSCAKTHEAL KTSRNTDQHY PAPKKAEDQE FGSAANIIVD PKLFQKRRFQ SPRVLFSTQP PPLSRDEQSV

231

161 DNANSLNRNI RSKREDHPVH NRGEYSVCDS VSVWVANKTT ATDIRGNLVT VMVDVNLNNN VYKQYFFETK CRNPNPVPTG

281 283

241 CRGIDSRHWN SYCTTTHTFV RALTMEGNQA SWRFIRIDTA CVCVISRKNE NFG

**Supporting Peptides:**

| Peptide | Uniq | -10lgP | Mass | Length | ppm | m/z | z | RT | Fraction | Scan | Source File | Area F1 | Area F2a | Area F3a | Area F4 | Area F5 | Area F6a | Area F7a | Area F7b | Area F8 | Area F9a | Area Sample 1F10a | Area F10b | Area F11a | Area F11b | Area F12a | Area F13 | #Feature | #Feature F1 | #Feature F2a | #Feature F3a | #Feature F4 | #Feature F5 | #Feature F6a | #Feature F7a | #Feature F7b | #Feature F8 | #Feature F9a | #Feature Sample 1F10a | #Feature F10b | #Feature F11a | #Feature F11b | #Feature F12a | #Feature F13 | Start | End | PTM | AScore | Found By |
|---|---|---|---|---|---|---|---|---|---|---|---|---|---|---|---|---|---|---|---|---|---|---|---|---|---|---|---|---|---|---|---|---|---|---|---|---|---|---|---|---|---|---|---|---|---|---|---|---|---|
| R.IDTAC(+57.02)VC(+57.02)VISR.K | N | 81.21 | 1292.6217 | 11 | 0.1 | 647.3182 | 2 | 47.01 | 7 | F7:21996 | BuCaTN01_F7a.raw | | | | | | 2.9158E5 | 3.0809E7 | 1.2168E7 | 2.4316E5 | 2.6689E5 | | | | | | | 6 | 0 | 0 | 0 | 0 | 0 | 1 | 2 | 1 | 1 | 1 | 0 | 0 | 0 | 0 | 0 | 0 | 277 | 287 | Carbamidomethylation | C5:Carbamidomethylation:1000.00;C7:Carbamidomethylation:1000.00 | PEAKS DB |
| R.FIRIDTAC(+57.02)VC(+57.02)VISR.K | N | 79.64 | 1708.8752 | 14 | 1.0 | 570.6329 | 3 | 63.02 | 7 | F7:35237 | BuCaTN01_F7a.raw | | | | | | | 3.425E7 | 1.536E7 | 1.7143E6 | 6.4042E4 | | | | 4.6395E4 | | | 26 | 0 | 0 | 0 | 0 | 0 | 0 | 19 | 4 | 1 | 1 | 0 | 0 | 0 | 1 | 0 | 0 | 274 | 287 | Carbamidomethylation | C8:Carbamidomethylation:1000.00;C10:Carbamidomethylation:1000.00 | PEAKS DB |
| R.FIRIDTAC(+57.02)VC(+57.02)VISRK.N | N | 66.71 | 1836.9702 | 15 | 0.6 | 613.3311 | 3 | 56.65 | 7 | F7:29482 | BuCaTN01_F7a.raw | | | | | | | 2.0277E6 | 3.8493E5 | | | | | | | | | 3 | 0 | 0 | 0 | 0 | 0 | 0 | 2 | 1 | 0 | 0 | 0 | 0 | 0 | 0 | 0 | 0 | 274 | 288 | Carbamidomethylation | C8:Carbamidomethylation:1000.00;C10:Carbamidomethylation:1000.00 | PEAKS DB |
| N.VYKQYFFETK.C | N | 54.63 | 1351.6812 | 10 | 2.9 | 451.5676 | 3 | 50.63 | 8 | F8:27078 | BuCaTN01_F7b.raw | | | | | | | | 5.2856E5 | | | | | | | | | 1 | 0 | 0 | 0 | 0 | 0 | 0 | 0 | 1 | 0 | 0 | 0 | 0 | 0 | 0 | 0 | 0 | 221 | 230 | | | PEAKS DB |
| K.QYFFETK.C | N | 54.60 | 961.4545 | 7 | 0.2 | 481.7346 | 2 | 53.27 | 7 | F7:26821 | BuCaTN01_F7a.raw | | | | | | | 2.4891E7 | 7.2632E6 | | | | | | | | | 2 | 0 | 0 | 0 | 0 | 0 | 0 | 1 | 1 | 0 | 0 | 0 | 0 | 0 | 0 | 0 | 0 | 224 | 230 | | | PEAKS DB |
| K.C(+57.02)RNPNPVPTGC(+57.02)R.G | Y | 51.04 | 1426.6558 | 12 | 0.2 | 476.5593 | 3 | 25.43 | 7 | F7:6915 | BuCaTN01_F7a.raw | | | | | | | 1.494E6 | | | | | | | | | | 1 | 0 | 0 | 0 | 0 | 0 | 0 | 1 | 0 | 0 | 0 | 0 | 0 | 0 | 0 | 0 | 0 | 231 | 242 | Carbamidomethylation | C1:Carbamidomethylation:1000.00;C11:Carbamidomethylation:1000.00 | PEAKS DB |
| R.NPNPVPTGC(+57.02)R.G | Y | 50.81 | 1110.5240 | 10 | 0.0 | 556.2693 | 2 | 27.71 | 7 | F7:7627 | BuCaTN01_F7a.raw | | | | | | | 2.2345E5 | | | | | | | | | | 1 | 0 | 0 | 0 | 0 | 0 | 0 | 1 | 0 | 0 | 0 | 0 | 0 | 0 | 0 | 0 | 0 | 233 | 242 | Carbamidomethylation | C9:Carbamidomethylation:1000.00 | PEAKS DB |
| R.FIRIDTAC(+57.02)VC(+57.02).V | N | 49.72 | 1253.5896 | 10 | 3.0 | 627.8021 | 2 | 54.32 | 8 | F8:30301 | BuCaTN01_F7b.raw | | | | | | | | 3.8159E5 | | | | | | | | | 1 | 0 | 0 | 0 | 0 | 0 | 0 | 0 | 1 | 0 | 0 | 0 | 0 | 0 | 0 | 0 | 0 | 274 | 283 | Carbamidomethylation | C8:Carbamidomethylation:1000.00;C10:Carbamidomethylation:1000.00 | PEAKS DB |
| R.IDTAC(+57.02)VC(+57.02)VISRK.N | N | 47.61 | 1420.7167 | 12 | 0.1 | 474.5795 | 3 | 41.27 | 7 | F7:17257 | BuCaTN01_F7a.raw | | | | | | | 1.7324E6 | | | | | | | | | | 1 | 0 | 0 | 0 | 0 | 0 | 0 | 1 | 0 | 0 | 0 | 0 | 0 | 0 | 0 | 0 | 0 | 277 | 288 | Carbamidomethylation | C5:Carbamidomethylation:1000.00;C7:Carbamidomethylation:1000.00 | PEAKS DB |
| K.QYFFETKC(+57.02)R.N | N | 41.76 | 1277.5863 | 9 | -0.7 | 426.8691 | 3 | 46.69 | 7 | F7:21495 | BuCaTN01_F7a.raw | | | | | | | 3.7535E5 | | | | | | | | | | 1 | 0 | 0 | 0 | 0 | 0 | 0 | 1 | 0 | 0 | 0 | 0 | 0 | 0 | 0 | 0 | 0 | 224 | 232 | Carbamidomethylation | C8:Carbamidomethylation:1000.00 | PEAKS DB |
| total 10 peptides | | | | | | | | | | | | | | | | | | | | | | | | | | | | | | | | | | | | | | | | | | | | | | | | | |

**Best Unique PSM (Scan F7:6915, m/z=476.5593, z=3, RT=25.43, ppm=0.2):**

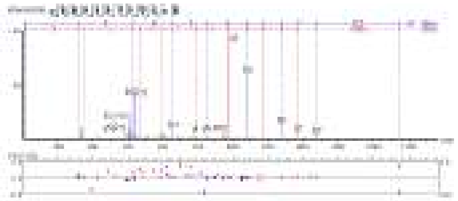

## BAN82015.1

back to list

| Protein Coverage | Supporting Peptides | Best Unique PSM |

**Protein Coverage:**

```
  1 MSMLCYTLII AFLIGIWAAP KSEDNVPLGS PATSDLSDTS CAKTHEALKT SRNTDQHYPA PKKAEDQEFG SAANIIVDPK
 81 LFQKRRFQSP RVLFSTQPPP LSRDEQSVDN ANSLNRNIRS KREDHPVHNR GEYSVCDSVS VWVANKTTAT DIRGNLVTVM
161 VDVNLNNNVY KQYFFETKCR NPNPVPTGCR GIDSRHWNSY CTTTHTFVRA LTMEGNQASW RFIRIDTACV CVISRKNENF
241 G
```

C Carbamidomethylation (+57.02) — modified positions: 179, 229, 231

**Supporting Peptides:**

| Peptide | Uniq | -10lgP | Mass | Length | ppm | m/z | z | RT | Fraction | Scan | Source File | Area F1 | Area F2a | Area F3a | Area F4 | Area F5 | Area F6a | Area F7a | Area F7b | Area F8 | Area F9a | Area Sample 1F10a | Area F10b | Area F11a | Area F11b | Area F12a | Area F13 | #Feature | #Feature F1 | #Feature F2a | #Feature F3a | #Feature F4 | #Feature F5 | #Feature F6a | #Feature F7a | #Feature F7b | #Feature F8 | #Feature F9a | #Feature Sample 1F10a | #Feature F10b | #Feature F11a | #Feature F11b | #Feature F12a | #Feature F13 | Start | End | PTM | AScore | Found By |
|---|---|---|---|---|---|---|---|---|---|---|---|---|---|---|---|---|---|---|---|---|---|---|---|---|---|---|---|---|---|---|---|---|---|---|---|---|---|---|---|---|---|---|---|---|---|---|---|---|---|
| R.IDTAC(+57.02)VC(+57.02)VISR.K | N | 81.21 | 1292.6217 | 11 | 0.1 | 647.3182 | 2 | 47.01 | 7 | F7:21996 | BuCaTN01_F7a.raw | | | | | | 2.9158E5 | 3.0809E7 | 1.2168E7 | 2.4316E5 | 2.6689E5 | | | | | | | 6 | 0 | 0 | 0 | 0 | 0 | 1 | 2 | 1 | 1 | 1 | 0 | 0 | 0 | 0 | 0 | 0 | 225 | 235 | Carbamidomethylation | C5:Carbamidomethylation:1000.00;C7:Carbamidomethylation:1000.00 | PEAKS DB |
| R.FIRIDTAC(+57.02)VC(+57.02)VISR.K | N | 79.64 | 1708.8752 | 14 | 1.0 | 570.6329 | 3 | 63.02 | 7 | F7:35237 | BuCaTN01_F7a.raw | | | | | | | 3.425E7 | 1.536E7 | 1.7143E6 | 6.4042E4 | | | | 4.6395E4 | | | 26 | 0 | 0 | 0 | 0 | 0 | 0 | 19 | 4 | 1 | 1 | 0 | 0 | 0 | 1 | 0 | 0 | 222 | 235 | Carbamidomethylation | C8:Carbamidomethylation:1000.00;C10:Carbamidomethylation:1000.00 | PEAKS DB |
| R.FIRIDTAC(+57.02)VC(+57.02)VISRK.N | N | 66.71 | 1836.9702 | 15 | 0.6 | 613.3311 | 3 | 56.65 | 7 | F7:29482 | BuCaTN01_F7a.raw | | | | | | | 2.0277E6 | 3.8493E5 | | | | | | | | | 3 | 0 | 0 | 0 | 0 | 0 | 0 | 2 | 1 | 0 | 0 | 0 | 0 | 0 | 0 | 0 | 0 | 222 | 236 | Carbamidomethylation | C8:Carbamidomethylation:1000.00;C10:Carbamidomethylation:1000.00 | PEAKS DB |
| N.VYKQYFFETK.C | N | 54.63 | 1351.6812 | 10 | 2.9 | 451.5676 | 3 | 50.63 | 8 | F8:27078 | BuCaTN01_F7b.raw | | | | | | | | 5.2856E5 | | | | | | | | | 1 | 0 | 0 | 0 | 0 | 0 | 0 | 0 | 1 | 0 | 0 | 0 | 0 | 0 | 0 | 0 | 0 | 169 | 178 | | | PEAKS DB |
| K.QYFFETK.C | N | 54.60 | 961.4545 | 7 | 0.2 | 481.7346 | 2 | 53.27 | 7 | F7:26821 | BuCaTN01_F7a.raw | | | | | | | 2.4891E7 | 7.2632E6 | | | | | | | | | 2 | 0 | 0 | 0 | 0 | 0 | 0 | 1 | 1 | 0 | 0 | 0 | 0 | 0 | 0 | 0 | 0 | 172 | 178 | | | PEAKS DB |
| K.C(+57.02)RNPNPVPTGC(+57.02)R.G | Y | 51.04 | 1426.6558 | 12 | 0.2 | 476.5593 | 3 | 25.43 | 7 | F7:6915 | BuCaTN01_F7a.raw | | | | | | | 1.494E6 | | | | | | | | | | 1 | 0 | 0 | 0 | 0 | 0 | 0 | 1 | 0 | 0 | 0 | 0 | 0 | 0 | 0 | 0 | 0 | 179 | 190 | Carbamidomethylation | C1:Carbamidomethylation:1000.00;C11:Carbamidomethylation:1000.00 | PEAKS DB |
| R.NPNPVPTGC(+57.02)R.G | Y | 50.81 | 1110.5240 | 10 | 0.0 | 556.2693 | 2 | 27.71 | 7 | F7:7627 | BuCaTN01_F7a.raw | | | | | | | 2.2345E5 | | | | | | | | | | 1 | 0 | 0 | 0 | 0 | 0 | 0 | 1 | 0 | 0 | 0 | 0 | 0 | 0 | 0 | 0 | 0 | 181 | 190 | Carbamidomethylation | C9:Carbamidomethylation:1000.00 | PEAKS DB |
| R.FIRIDTAC(+57.02)VC(+57.02).V | N | 49.72 | 1253.5896 | 10 | 3.0 | 627.8021 | 2 | 54.32 | 8 | F8:30301 | BuCaTN01_F7b.raw | | | | | | | | 3.8159E5 | | | | | | | | | 1 | 0 | 0 | 0 | 0 | 0 | 0 | 0 | 1 | 0 | 0 | 0 | 0 | 0 | 0 | 0 | 0 | 222 | 231 | Carbamidomethylation | C8:Carbamidomethylation:1000.00;C10:Carbamidomethylation:1000.00 | PEAKS DB |
| R.IDTAC(+57.02)VC(+57.02)VISRK.N | N | 47.61 | 1420.7167 | 12 | 0.1 | 474.5795 | 3 | 41.27 | 7 | F7:17257 | BuCaTN01_F7a.raw | | | | | | | 1.7324E6 | | | | | | | | | | 1 | 0 | 0 | 0 | 0 | 0 | 0 | 1 | 0 | 0 | 0 | 0 | 0 | 0 | 0 | 0 | 0 | 225 | 236 | Carbamidomethylation | C5:Carbamidomethylation:1000.00;C7:Carbamidomethylation:1000.00 | PEAKS DB |
| K.QYFFETKC(+57.02)R.N | N | 41.76 | 1277.5863 | 9 | -0.7 | 426.8691 | 3 | 46.69 | 7 | F7:21495 | BuCaTN01_F7a.raw | | | | | | | 3.7535E5 | | | | | | | | | | 1 | 0 | 0 | 0 | 0 | 0 | 0 | 1 | 0 | 0 | 0 | 0 | 0 | 0 | 0 | 0 | 0 | 172 | 180 | Carbamidomethylation | C8:Carbamidomethylation:1000.00 | PEAKS DB |
| total 10 peptides | | | | | | | | | | | | | | | | | | | | | | | | | | | | | | | | | | | | | | | | | | | | | | | | | |

**Best Unique PSM (Scan F7:6915, m/z=476.5593, z=3, RT=25.43, ppm=0.2):**

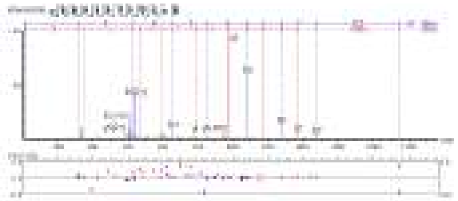

## AAG12169.1

back to list

| Protein Coverage | Supporting Peptides | Best Unique PSM |

**Protein Coverage:**

```
  1 MSMLCYTLII TLLIGIWAAP KSEDNVPLGS PATSDLSVTS CTKTHEALKT SRNTDQHYPA PKKEEDQEFG SAANIIVDPK
 81 LFQKRRFQSP RVLFSTQPPP LSRDEQSVDD ANSLNRNIRA KREDHPVHNR GEYSVCDSVN VWVANKTTAT DIRGNVVTVM
161 VDVNINNNVY KQYFFETKCR NPNPVPTGCR GIDARHWNSY CTTTNTFVKA LTMEGNQASW RFIRIDTACV CVISRKNENF
241 G
```

C Carbamidomethylation (+57.02) — modified positions: 179, 229, 231

**Supporting Peptides:**

| Peptide | Uniq | -10lgP | Mass | Length | ppm | m/z | z | RT | Fraction | Scan | Source File | Area F1 | Area F2a | Area F3a | Area F4 | Area F5 | Area F6a | Area F7a | Area F7b | Area F8 | Area F9a | Area Sample 1F10a | Area F10b | Area F11a | Area F11b | Area F12a | Area F13 | #Feature | #Feature F1 | #Feature F2a | #Feature F3a | #Feature F4 | #Feature F5 | #Feature F6a | #Feature F7a | #Feature F7b | #Feature F8 | #Feature F9a | #Feature Sample 1F10a | #Feature F10b | #Feature F11a | #Feature F11b | #Feature F12a | #Feature F13 | Start | End | PTM | AScore | Found By |
|---|---|---|---|---|---|---|---|---|---|---|---|---|---|---|---|---|---|---|---|---|---|---|---|---|---|---|---|---|---|---|---|---|---|---|---|---|---|---|---|---|---|---|---|---|---|---|---|---|---|
| R.IDTAC(+57.02)VC(+57.02)VISR.K | N | 81.21 | 1292.6217 | 11 | 0.1 | 647.3182 | 2 | 47.01 | 7 | F7:21996 | BuCaTN01_F7a.raw | | | | | | 2.9158E5 | 3.0809E7 | 1.2168E7 | 2.4316E5 | 2.6689E5 | | | | | | | 6 | 0 | 0 | 0 | 0 | 0 | 1 | 2 | 1 | 1 | 1 | 0 | 0 | 0 | 0 | 0 | 0 | 225 | 235 | Carbamidomethylation | C5:Carbamidomethylation:1000.00;C7:Carbamidomethylation:1000.00 | PEAKS DB |
| R.FIRIDTAC(+57.02)VC(+57.02)VISR.K | N | 79.64 | 1708.8752 | 14 | 1.0 | 570.6329 | 3 | 63.02 | 7 | F7:35237 | BuCaTN01_F7a.raw | | | | | | | 3.425E7 | 1.536E7 | 1.7143E6 | 6.4042E4 | | | | 4.6395E4 | | | 26 | 0 | 0 | 0 | 0 | 0 | 0 | 19 | 4 | 1 | 1 | 0 | 0 | 0 | 1 | 0 | 0 | 222 | 235 | Carbamidomethylation | C8:Carbamidomethylation:1000.00;C10:Carbamidomethylation:1000.00 | PEAKS DB |
| R.FIRIDTAC(+57.02)VC(+57.02)VISRK.N | N | 66.71 | 1836.9702 | 15 | 0.6 | 613.3311 | 3 | 56.65 | 7 | F7:29482 | BuCaTN01_F7a.raw | | | | | | | 2.0277E6 | 3.8493E5 | | | | | | | | | 3 | 0 | 0 | 0 | 0 | 0 | 0 | 2 | 1 | 0 | 0 | 0 | 0 | 0 | 0 | 0 | 0 | 222 | 236 | Carbamidomethylation | C8:Carbamidomethylation:1000.00;C10:Carbamidomethylation:1000.00 | PEAKS DB |
| N.VYKQYFFETK.C | N | 54.63 | 1351.6812 | 10 | 2.9 | 451.5676 | 3 | 50.63 | 8 | F8:27078 | BuCaTN01_F7b.raw | | | | | | | | 5.2856E5 | | | | | | | | | 1 | 0 | 0 | 0 | 0 | 0 | 0 | 0 | 1 | 0 | 0 | 0 | 0 | 0 | 0 | 0 | 0 | 169 | 178 | | | PEAKS DB |
| K.QYFFETK.C | N | 54.60 | 961.4545 | 7 | 0.2 | 481.7346 | 2 | 53.27 | 7 | F7:26821 | BuCaTN01_F7a.raw | | | | | | | 2.4891E7 | 7.2632E6 | | | | | | | | | 2 | 0 | 0 | 0 | 0 | 0 | 0 | 1 | 1 | 0 | 0 | 0 | 0 | 0 | 0 | 0 | 0 | 172 | 178 | | | PEAKS DB |
| K.C(+57.02)RNPNPVPTGC(+57.02)R.G | Y | 51.04 | 1426.6558 | 12 | 0.2 | 476.5593 | 3 | 25.43 | 7 | F7:6915 | BuCaTN01_F7a.raw | | | | | | | 1.494E6 | | | | | | | | | | 1 | 0 | 0 | 0 | 0 | 0 | 0 | 1 | 0 | 0 | 0 | 0 | 0 | 0 | 0 | 0 | 0 | 179 | 190 | Carbamidomethylation | C1:Carbamidomethylation:1000.00;C11:Carbamidomethylation:1000.00 | PEAKS DB |
| R.NPNPVPTGC(+57.02)R.G | Y | 50.81 | 1110.5240 | 10 | 0.0 | 556.2693 | 2 | 27.71 | 7 | F7:7627 | BuCaTN01_F7a.raw | | | | | | | 2.2345E5 | | | | | | | | | | 1 | 0 | 0 | 0 | 0 | 0 | 0 | 1 | 0 | 0 | 0 | 0 | 0 | 0 | 0 | 0 | 0 | 181 | 190 | Carbamidomethylation | C9:Carbamidomethylation:1000.00 | PEAKS DB |
| R.FIRIDTAC(+57.02)VC(+57.02).V | N | 49.72 | 1253.5896 | 10 | 3.0 | 627.8021 | 2 | 54.32 | 8 | F8:30301 | BuCaTN01_F7b.raw | | | | | | | | 3.8159E5 | | | | | | | | | 1 | 0 | 0 | 0 | 0 | 0 | 0 | 0 | 1 | 0 | 0 | 0 | 0 | 0 | 0 | 0 | 0 | 222 | 231 | Carbamidomethylation | C8:Carbamidomethylation:1000.00;C10:Carbamidomethylation:1000.00 | PEAKS DB |
| R.IDTAC(+57.02)VC(+57.02)VISRK.N | N | 47.61 | 1420.7167 | 12 | 0.1 | 474.5795 | 3 | 41.27 | 7 | F7:17257 | BuCaTN01_F7a.raw | | | | | | | 1.7324E6 | | | | | | | | | | 1 | 0 | 0 | 0 | 0 | 0 | 0 | 1 | 0 | 0 | 0 | 0 | 0 | 0 | 0 | 0 | 0 | 225 | 236 | Carbamidomethylation | C5:Carbamidomethylation:1000.00;C7:Carbamidomethylation:1000.00 | PEAKS DB |
| K.QYFFETKC(+57.02)R.N | N | 41.76 | 1277.5863 | 9 | -0.7 | 426.8691 | 3 | 46.69 | 7 | F7:21495 | BuCaTN01_F7a.raw | | | | | | | 3.7535E5 | | | | | | | | | | 1 | 0 | 0 | 0 | 0 | 0 | 0 | 1 | 0 | 0 | 0 | 0 | 0 | 0 | 0 | 0 | 0 | 172 | 180 | Carbamidomethylation | C8:Carbamidomethylation:1000.00 | PEAKS DB |
| total 10 peptides | | | | | | | | | | | | | | | | | | | | | | | | | | | | | | | | | | | | | | | | | | | | | | | | | |

**Best Unique PSM (Scan F7:6915, m/z=476.5593, z=3, RT=25.43, ppm=0.2):**

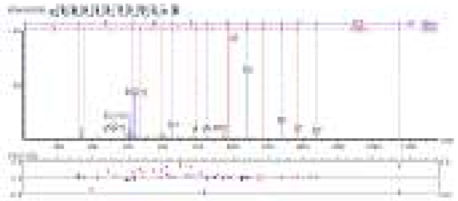

## 1MR6

back to list

| Protein Coverage | Supporting Peptides | Best Unique PSM |

**Protein Coverage:**

1 MQCKTCSFYT CPNSETCPDG KNICVKRSWT AVRGDGPKRE IRRECAATCP PSKLGLTVFC CTTDNCNH

Carbamidomethylation (+57.02)
Oxidation (M) (+15.99)

**Supporting Peptides:**

| Peptide | Uniq | -10lgP | Mass | Length | ppm | m/z | z | RT | Fraction | Scan | Source File | Area F1 | Area F2a | Area F3a | Area F4 | Area F5 | Area F6a | Area F7a | Area F7b | Area F8 | Area F9a | Area Sample 1F10a | Area F10b | Area F11a | Area F11b | Area F12a | Area F13 | #Feature | #Feature F1 | #Feature F2a | #Feature F3a | #Feature F4 | #Feature F5 | #Feature F6a | #Feature F7a | #Feature F7b | #Feature F8 | #Feature F9a | #Feature Sample 1F10a | #Feature F10b | #Feature F11a | #Feature F11b | #Feature F12a | #Feature F13 | Start | End | PTM | AScore | Found By |
|---|---|---|---|---|---|---|---|---|---|---|---|---|---|---|---|---|---|---|---|---|---|---|---|---|---|---|---|---|---|---|---|---|---|---|---|---|---|---|---|---|---|---|---|---|---|---|---|---|---|
| S.FYTC(+57.02)PNSETC(+57.02)PDGKNIC(+57.02)VK.R | Y | 103.15 | 2288.9863 | 19 | 1.6 | 1145.5000 | 2 | 42.38 | 3 | F3:19627 | BuCaTN01_F3a.raw | | 1.8803E8 | 6.3454E9 | 1.2201E6 | | 2.4142E8 | 0 | 6.7345E7 | | 1.4377E7 | | 1.7088E7 | | | | | 12 | 0 | 3 | 3 | 1 | 0 | 2 | 0 | 1 | 0 | 1 | 0 | 1 | 0 | 0 | 0 | 0 | 8 | 26 | Carbamidomethylation | C4:Carbamidomethylation:1000.00; C10:Carbamidomethylation:1000.00; C17:Carbamidomethylation:1000.00 | PEAKS DB |
| S.FYTC(+57.02)PNSETC(+57.02)PDGK.N | Y | 82.69 | 1674.6654 | 14 | -0.4 | 838.3397 | 2 | 36.79 | 10 | F10:15352 | BuCaTN01_F9a.raw | | 7.415E8 | 1.4821E10 | 2.6498E6 | | 5.4853E8 | 3.3952E6 | 1.2621E8 | | 2.8544E7 | | 3.1496E7 | | 1.6806E7 | 1.1184E7 | | 31 | 0 | 2 | 19 | 1 | 0 | 2 | 1 | 1 | 0 | 1 | 0 | 1 | 0 | 1 | 2 | 0 | 8 | 21 | Carbamidomethylation | C4:Carbamidomethylation:1000.00; C10:Carbamidomethylation:1000.00 | PEAKS DB |
| F.YTC(+57.02)PNSETC(+57.02)PDGKNIC(+57.02)VK.R | Y | 54.84 | 2141.9180 | 18 | 3.9 | 1071.9683 | 2 | 36.35 | 3 | F3:13810 | BuCaTN01_F3a.raw | | | 0 | | | | | | | | | | | | | | 0 | 0 | 0 | 0 | 0 | 0 | 0 | 0 | 0 | 0 | 0 | 0 | 0 | 0 | 0 | 0 | 0 | 9 | 26 | Carbamidomethylation | C3:Carbamidomethylation:1000.00; C9:Carbamidomethylation:1000.00; C16:Carbamidomethylation:1000.00 | PEAKS DB |
| M(+15.99)QC(+57.02)KTC(+57.02)SFYTC(+57.02)PNSETC(+57.02)PDGKNIC(+57.02)VK.R | Y | 49.78 | 3200.3164 | 26 | 5.6 | 1067.7832 | 3 | 50.04 | 3 | F3:26140 | BuCaTN01_F3a.raw | | | 3.2461E6 | | | | | | | | | | | | | | 1 | 0 | 0 | 1 | 0 | 0 | 0 | 0 | 0 | 0 | 0 | 0 | 0 | 0 | 0 | 0 | 0 | 1 | 26 | Oxidation (M); Carbamidomethylation | M1:Oxidation (M): 1000.00;C3:Carbamidomethylation:1000.00;C6:Carbamidomethylation:1000.00;C11:Carbamidomethylation:1000.00;C17:Carbamidomethylation:1000.00;C24:Carbamidomethylation:1000.00 | PEAKS DB |
| C.PNSETC(+57.02)PDGKNIC(+57.02)VK.R | Y | 43.85 | 1717.7764 | 15 | 3.5 | 573.6002 | 3 | 29.98 | 3 | F3:9299 | BuCaTN01_F3a.raw | | | 7.0058E5 | | | | | | | | | | | | | | 1 | 0 | 0 | 1 | 0 | 0 | 0 | 0 | 0 | 0 | 0 | 0 | 0 | 0 | 0 | 0 | 0 | 12 | 26 | Carbamidomethylation | C6:Carbamidomethylation:1000.00; C13:Carbamidomethylation:1000.00 | PEAKS DB |
| S.FYTC(+57.02)PNSETC(+57.02)PDGKNIC(+57.02)VKR.S | Y | 43.09 | 2445.0874 | 20 | 1.6 | 612.2789 | 4 | 39.69 | 3 | F3:17166 | BuCaTN01_F3a.raw | | | 4.7889E6 | | | | | | | | | | | | | | 1 | 0 | 0 | 1 | 0 | 0 | 0 | 0 | 0 | 0 | 0 | 0 | 0 | 0 | 0 | 0 | 0 | 8 | 27 | Carbamidomethylation | C4:Carbamidomethylation:1000.00; C10:Carbamidomethylation:1000.00; C17:Carbamidomethylation:1000.00 | PEAKS DB |
| **total 6 peptides** | | | | | | | | | | | | | | | | | | | | | | | | | | | | | | | | | | | | | | | | | | | | | | | | | |

**Best Unique PSM (Scan F3:19627, m/z=1145.5000, z=2, RT=42.38, ppm=1.6):**

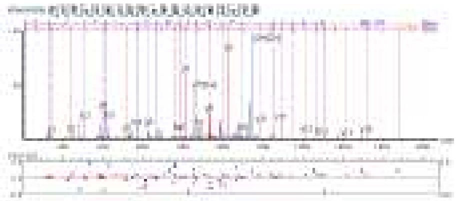

## CAA06885.1

back to list

| Protein Coverage | Supporting Peptides | Best Unique PSM |

**Protein Coverage:**

1 MKTLLLTLVV VTIVCLDLGY TMQCKTCSFY TCPNSETCPD GKNICVKRSW TAVRGDGPKR EIRRECAATC PPSKLGLTVF

Carbamidomethylation (+57.02)
Oxidation (M) (+15.99)

81 CCTTDNCNH

**Supporting Peptides:**

| Peptide | Uniq | -10lgP | Mass | Length | ppm | m/z | z | RT | Fraction | Scan | Source File | Area F1 | Area F2a | Area F3a | Area F4 | Area F5 | Area F6a | Area F7a | Area F7b | Area F8 | Area F9a | Area Sample 1F10a | Area F10b | Area F11a | Area F11b | Area F12a | Area F13 | #Feature | #Feature F1 | #Feature F2a | #Feature F3a | #Feature F4 | #Feature F5 | #Feature F6a | #Feature F7a | #Feature F7b | #Feature F8 | #Feature F9a | #Feature Sample 1F10a | #Feature F10b | #Feature F11a | #Feature F11b | #Feature F12a | #Feature F13 | Start | End | PTM | AScore | Found By |
|---|---|---|---|---|---|---|---|---|---|---|---|---|---|---|---|---|---|---|---|---|---|---|---|---|---|---|---|---|---|---|---|---|---|---|---|---|---|---|---|---|---|---|---|---|---|---|---|---|---|
| S.FYTC(+57.02)PNSETC(+57.02)PDGKNIC(+57.02)VK.R | Y | 103.15 | 2288.9863 | 19 | 1.6 | 1145.5000 | 2 | 42.38 | 3 | F3:19627 | BuCaTN01_F3a.raw | | 1.8803E8 | 6.3454E9 | 1.2201E6 | | 2.4142E8 | 0 | 6.7345E7 | | 1.4377E7 | | 1.7088E7 | | | | | 12 | 0 | 3 | 3 | 1 | 0 | 2 | 0 | 1 | 0 | 1 | 0 | 1 | 0 | 0 | 0 | 0 | 29 | 47 | Carbamidomethylation | C4:Carbamidomethylation:1000.00;C10:Carbamidomethylation:1000.00;C17:Carbamidomethylation:1000.00 | PEAKS DB |
| S.FYTC(+57.02)PNSETC(+57.02)PDGK.N | Y | 82.69 | 1674.6654 | 14 | -0.4 | 838.3397 | 2 | 36.79 | 10 | F10:15352 | BuCaTN01_F9a.raw | | 7.415E8 | 1.4821E10 | 2.6498E6 | | 5.4853E8 | 3.3952E6 | 1.2621E8 | | 2.8544E7 | | 3.1496E7 | | 1.6806E7 | 1.1184E7 | | 31 | 0 | 2 | 19 | 1 | 0 | 2 | 1 | 1 | 0 | 1 | 0 | 1 | 0 | 1 | 2 | 0 | 29 | 42 | Carbamidomethylation | C4:Carbamidomethylation:1000.00;C10:Carbamidomethylation:1000.00 | PEAKS DB |
| F.YTC(+57.02)PNSETC(+57.02)PDGKNIC(+57.02)VK.R | Y | 54.84 | 2141.9180 | 18 | 3.9 | 1071.9683 | 2 | 36.35 | 3 | F3:13810 | BuCaTN01_F3a.raw | | | 0 | | | | | | | | | | | | | | 0 | 0 | 0 | 0 | 0 | 0 | 0 | 0 | 0 | 0 | 0 | 0 | 0 | 0 | 0 | 0 | 0 | 30 | 47 | Carbamidomethylation | C3:Carbamidomethylation:1000.00;C9:Carbamidomethylation:1000.00;C16:Carbamidomethylation:1000.00 | PEAKS DB |
| T.M(+15.99)QC(+57.02)KTC(+57.02)SFYTC(+57.02)PNSETC(+57.02)PDGKNIC(+57.02)VK.R | Y | 49.78 | 3200.3164 | 26 | 5.6 | 1067.7832 | 3 | 50.04 | 3 | F3:26140 | BuCaTN01_F3a.raw | | | 3.2461E6 | | | | | | | | | | | | | | 1 | 0 | 0 | 1 | 0 | 0 | 0 | 0 | 0 | 0 | 0 | 0 | 0 | 0 | 0 | 0 | 0 | 22 | 47 | Oxidation (M); Carbamidomethylation | M1:Oxidation (M):1000.00;C3:Carbamidomethylation:1000.00;C6:Carbamidomethylation:1000.00;C11:Carbamidomethylation:1000.00;C17:Carbamidomethylation:1000.00;C24:Carbamidomethylation:1000.00 | PEAKS DB |
| C.PNSETC(+57.02)PDGKNIC(+57.02)VK.R | Y | 43.85 | 1717.7764 | 15 | 3.5 | 573.6002 | 3 | 29.98 | 3 | F3:9299 | BuCaTN01_F3a.raw | | | 7.0058E5 | | | | | | | | | | | | | | 1 | 0 | 0 | 1 | 0 | 0 | 0 | 0 | 0 | 0 | 0 | 0 | 0 | 0 | 0 | 0 | 0 | 33 | 47 | Carbamidomethylation | C6:Carbamidomethylation:1000.00;C13:Carbamidomethylation:1000.00 | PEAKS DB |
| S.FYTC(+57.02)PNSETC(+57.02)PDGKNIC(+57.02)VKR.S | Y | 43.09 | 2445.0874 | 20 | 1.6 | 612.2789 | 4 | 39.69 | 3 | F3:17166 | BuCaTN01_F3a.raw | | | 4.7889E6 | | | | | | | | | | | | | | 1 | 0 | 0 | 1 | 0 | 0 | 0 | 0 | 0 | 0 | 0 | 0 | 0 | 0 | 0 | 0 | 0 | 29 | 48 | Carbamidomethylation | C4:Carbamidomethylation:1000.00;C10:Carbamidomethylation:1000.00;C17:Carbamidomethylation:1000.00 | PEAKS DB |
| **total 6 peptides** | | | | | | | | | | | | | | | | | | | | | | | | | | | | | | | | | | | | | | | | | | | | | | | | | |

**Best Unique PSM (Scan F3:19627, m/z=1145.5000, z=2, RT=42.38, ppm=1.6):**

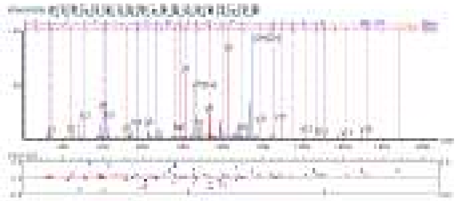

## Q9YGJ0.1

back to list

| Protein Coverage | Supporting Peptides | Best Unique PSM |

**Protein Coverage:**

1 MKTLLLTLVV VTIVCLDLGY TMQCKTCSFY TCPNSETCPD GKNICVKRSW TAVRGDGPKR EIRRECAATC PPSKLGLTVF

C Carbamidomethylation (+57.02)
O Oxidation (M) (+15.99)

81 CCTTDNCNH

**Supporting Peptides:**

| Peptide | Uniq | -10lgP | Mass | Length | ppm | m/z | z | RT | Fraction | Scan | Source File | Area F1 | Area F2a | Area F3a | Area F4 | Area F5 | Area F6a | Area F7a | Area F7b | Area F8 | Area F9a | Area Sample 1F10a | Area F10b | Area F11a | Area F11b | Area F12a | Area F13 | #Feature | #Feature F1 | #Feature F2a | #Feature F3a | #Feature F4 | #Feature F5 | #Feature F6a | #Feature F7a | #Feature F7b | #Feature F8 | #Feature F9a | #Feature Sample 1F10a | #Feature F10b | #Feature F11a | #Feature F11b | #Feature F12a | #Feature F13 | Start | End | PTM | AScore | Found By |
|---|---|---|---|---|---|---|---|---|---|---|---|---|---|---|---|---|---|---|---|---|---|---|---|---|---|---|---|---|---|---|---|---|---|---|---|---|---|---|---|---|---|---|---|---|---|---|---|---|---|
| S.FYTC(+57.02)PNSETC(+57.02)PDGKNIC(+57.02)VK.R | Y | 103.15 | 2288.9863 | 19 | 1.6 | 1145.5000 | 2 | 42.38 | 3 | F3:19627 | BuCaTN01_F3a.raw | | 1.8803E8 | 6.3454E9 | 1.2201E6 | | 2.4142E8 | 0 | 6.7345E7 | | 1.4377E7 | | 1.7088E7 | | | | | 12 | 0 | 3 | 3 | 1 | 0 | 2 | 0 | 1 | 0 | 1 | 0 | 1 | 0 | 0 | 0 | 0 | 29 | 47 | Carbamidomethylation | C4:Carbamidomethylation:1000.00;C10:Carbamidomethylation:1000.00;C17:Carbamidomethylation:1000.00 | PEAKS DB |
| S.FYTC(+57.02)PNSETC(+57.02)PDGK.N | Y | 82.69 | 1674.6654 | 14 | -0.4 | 838.3397 | 2 | 36.79 | 10 | F10:15352 | BuCaTN01_F9a.raw | | 7.415E8 | 1.4821E10 | 2.6498E6 | | 5.4853E8 | 3.3952E6 | 1.2621E8 | | 2.8544E7 | | 3.1496E7 | | 1.6806E7 | 1.1184E7 | | 31 | 0 | 2 | 19 | 1 | 0 | 2 | 1 | 1 | 0 | 1 | 0 | 1 | 0 | 1 | 2 | 0 | 29 | 42 | Carbamidomethylation | C4:Carbamidomethylation:1000.00;C10:Carbamidomethylation:1000.00 | PEAKS DB |
| F.YTC(+57.02)PNSETC(+57.02)PDGKNIC(+57.02)VK.R | Y | 54.84 | 2141.9180 | 18 | 3.9 | 1071.9683 | 2 | 36.35 | 3 | F3:13810 | BuCaTN01_F3a.raw | | | 0 | | | | | | | | | | | | | | 0 | 0 | 0 | 0 | 0 | 0 | 0 | 0 | 0 | 0 | 0 | 0 | 0 | 0 | 0 | 0 | 0 | 30 | 47 | Carbamidomethylation | C3:Carbamidomethylation:1000.00;C9:Carbamidomethylation:1000.00;C16:Carbamidomethylation:1000.00 | PEAKS DB |
| T.M(+15.99)QC(+57.02)KTC(+57.02)SFYTC(+57.02)PNSETC(+57.02)PDGKNIC(+57.02)VK.R | Y | 49.78 | 3200.3164 | 26 | 5.6 | 1067.7832 | 3 | 50.04 | 3 | F3:26140 | BuCaTN01_F3a.raw | | | 3.2461E6 | | | | | | | | | | | | | | 1 | 0 | 0 | 1 | 0 | 0 | 0 | 0 | 0 | 0 | 0 | 0 | 0 | 0 | 0 | 0 | 0 | 22 | 47 | Oxidation (M); Carbamidomethylation | M1:Oxidation (M):1000.00;C3:Carbamidomethylation:1000.00;C6:Carbamidomethylation:1000.00;C11:Carbamidomethylation:1000.00;C17:Carbamidomethylation:1000.00;C24:Carbamidomethylation:1000.00 | PEAKS DB |
| C.PNSETC(+57.02)PDGKNIC(+57.02)VK.R | Y | 43.85 | 1717.7764 | 15 | 3.5 | 573.6002 | 3 | 29.98 | 3 | F3:9299 | BuCaTN01_F3a.raw | | | 7.0058E5 | | | | | | | | | | | | | | 1 | 0 | 0 | 1 | 0 | 0 | 0 | 0 | 0 | 0 | 0 | 0 | 0 | 0 | 0 | 0 | 0 | 33 | 47 | Carbamidomethylation | C6:Carbamidomethylation:1000.00;C13:Carbamidomethylation:1000.00 | PEAKS DB |
| S.FYTC(+57.02)PNSETC(+57.02)PDGKNIC(+57.02)VKR.S | Y | 43.09 | 2445.0874 | 20 | 1.6 | 612.2789 | 4 | 39.69 | 3 | F3:17166 | BuCaTN01_F3a.raw | | | 4.7889E6 | | | | | | | | | | | | | | 1 | 0 | 0 | 1 | 0 | 0 | 0 | 0 | 0 | 0 | 0 | 0 | 0 | 0 | 0 | 0 | 0 | 29 | 48 | Carbamidomethylation | C4:Carbamidomethylation:1000.00;C10:Carbamidomethylation:1000.00;C17:Carbamidomethylation:1000.00 | PEAKS DB |
| total 6 peptides | | | | | | | | | | | | | | | | | | | | | | | | | | | | | | | | | | | | | | | | | | | | | | | | | |

**Best Unique PSM (Scan F3:19627, m/z=1145.5000, z=2, RT=42.38, ppm=1.6):**

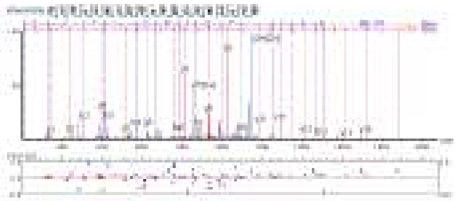

## CAD01082.1

back to list

| **Protein Coverage** | **Supporting Peptides** | **Best Unique PSM** |

**Protein Coverage:**

1 MKTLLLTLVV VTIVCLDLGY TMQCKTCSFY TCPNSETCPD GKNICVKRSW TAVRGDGPKR EIRRECAATC PPSKLGLTVF

C Carbamidomethylation (+57.02)
O Oxidation (M) (+15.99)

81 CCTTDNCNH

**Supporting Peptides:**

| Peptide | Uniq | -10lgP | Mass | Length | ppm | m/z | z | RT | Fraction | Scan | Source File | Area F1 | Area F2a | Area F3a | Area F4 | Area F5 | Area F6a | Area F7a | Area F7b | Area F8 | Area F9a | Area Sample 1F10a | Area F10b | Area F11a | Area F11b | Area F12a | Area F13 | #Feature | #Feature F1 | #Feature F2a | #Feature F3a | #Feature F4 | #Feature F5 | #Feature F6a | #Feature F7a | #Feature F7b | #Feature F8 | #Feature F9a | #Feature Sample 1F10a | #Feature F10b | #Feature F11a | #Feature F11b | #Feature F12a | #Feature F13 | Start | End | PTM | AScore | Found By |
|---|---|---|---|---|---|---|---|---|---|---|---|---|---|---|---|---|---|---|---|---|---|---|---|---|---|---|---|---|---|---|---|---|---|---|---|---|---|---|---|---|---|---|---|---|---|---|---|---|---|
| S.FYTC(+57.02)PNSETC(+57.02)PDGKNIC(+57.02)VK.R | Y | 103.15 | 2288.9863 | 19 | 1.6 | 1145.5000 | 2 | 42.38 | 3 | F3:19627 | BuCaTN01_F3a.raw | | 1.8803E8 | 6.3454E9 | 1.2201E6 | | 2.4142E8 | 0 | 6.7345E7 | | 1.4377E7 | | 1.7088E7 | | | | | 12 | 0 | 3 | 3 | 1 | 0 | 2 | 0 | 1 | 0 | 1 | 0 | 1 | 0 | 0 | 0 | 0 | 29 | 47 | Carbamidomethylation | C4:Carbamidomethylation:1000.00;C10:Carbamidomethylation:1000.00;C17:Carbamidomethylation:1000.00 | PEAKS DB |
| S.FYTC(+57.02)PNSETC(+57.02)PDGK.N | Y | 82.69 | 1674.6654 | 14 | -0.4 | 838.3397 | 2 | 36.79 | 10 | F10:15352 | BuCaTN01_F9a.raw | | 7.415E8 | 1.4821E10 | 2.6498E6 | | 5.4853E8 | 3.3952E6 | 1.2621E8 | | 2.8544E7 | | 3.1496E7 | | 1.6806E7 | 1.1184E7 | | 31 | 0 | 2 | 19 | 1 | 0 | 2 | 1 | 1 | 0 | 1 | 0 | 1 | 0 | 1 | 2 | 0 | 29 | 42 | Carbamidomethylation | C4:Carbamidomethylation:1000.00;C10:Carbamidomethylation:1000.00 | PEAKS DB |
| F.YTC(+57.02)PNSETC(+57.02)PDGKNIC(+57.02)VK.R | Y | 54.84 | 2141.9180 | 18 | 3.9 | 1071.9683 | 2 | 36.35 | 3 | F3:13810 | BuCaTN01_F3a.raw | | | 0 | | | | | | | | | | | | | | 0 | 0 | 0 | 0 | 0 | 0 | 0 | 0 | 0 | 0 | 0 | 0 | 0 | 0 | 0 | 0 | 0 | 30 | 47 | Carbamidomethylation | C3:Carbamidomethylation:1000.00;C9:Carbamidomethylation:1000.00;C16:Carbamidomethylation:1000.00 | PEAKS DB |
| T.M(+15.99)QC(+57.02)KTC(+57.02)SFYTC(+57.02)PNSETC(+57.02)PDGKNIC(+57.02)VK.R | Y | 49.78 | 3200.3164 | 26 | 5.6 | 1067.7832 | 3 | 50.04 | 3 | F3:26140 | BuCaTN01_F3a.raw | | | 3.2461E6 | | | | | | | | | | | | | | 1 | 0 | 0 | 1 | 0 | 0 | 0 | 0 | 0 | 0 | 0 | 0 | 0 | 0 | 0 | 0 | 0 | 22 | 47 | Oxidation (M); Carbamidomethylation | M1:Oxidation (M):1000.00;C3:Carbamidomethylation:1000.00;C6:Carbamidomethylation:1000.00;C11:Carbamidomethylation:1000.00;C17:Carbamidomethylation:1000.00;C24:Carbamidomethylation:1000.00 | PEAKS DB |
| C.PNSETC(+57.02)PDGKNIC(+57.02)VK.R | Y | 43.85 | 1717.7764 | 15 | 3.5 | 573.6002 | 3 | 29.98 | 3 | F3:9299 | BuCaTN01_F3a.raw | | | 7.0058E5 | | | | | | | | | | | | | | 1 | 0 | 0 | 1 | 0 | 0 | 0 | 0 | 0 | 0 | 0 | 0 | 0 | 0 | 0 | 0 | 0 | 33 | 47 | Carbamidomethylation | C6:Carbamidomethylation:1000.00;C13:Carbamidomethylation:1000.00 | PEAKS DB |
| S.FYTC(+57.02)PNSETC(+57.02)PDGKNIC(+57.02)VKR.S | Y | 43.09 | 2445.0874 | 20 | 1.6 | 612.2789 | 4 | 39.69 | 3 | F3:17166 | BuCaTN01_F3a.raw | | | 4.7889E6 | | | | | | | | | | | | | | 1 | 0 | 0 | 1 | 0 | 0 | 0 | 0 | 0 | 0 | 0 | 0 | 0 | 0 | 0 | 0 | 0 | 29 | 48 | Carbamidomethylation | C4:Carbamidomethylation:1000.00;C10:Carbamidomethylation:1000.00;C17:Carbamidomethylation:1000.00 | PEAKS DB |
| total 6 peptides | | | | | | | | | | | | | | | | | | | | | | | | | | | | | | | | | | | | | | | | | | | | | | | | | |

**Best Unique PSM (Scan F3:19627, m/z=1145.5000, z=2, RT=42.38, ppm=1.6):**

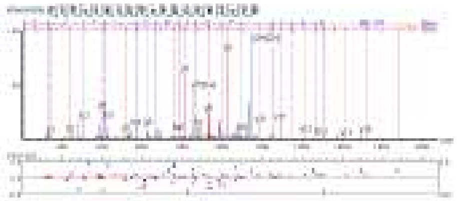

## O12963.1

back to list

| **Protein Coverage** | **Supporting Peptides** | **Best Unique PSM** |

**Protein Coverage:**

1 MKTLLLTLVV VTIVCLDLGY TMQCKTCSFY TCPNSETCPD GKNICVKRSW TAVAGDGLKR EIRRECAATC PPSKLGLTVF

Carbamidomethylation (+57.02)
Oxidation (M) (+15.99)

81 CCTTDNCNH

**Supporting Peptides:**

| Peptide | Uniq | -10lgP | Mass | Length | ppm | m/z | z | RT | Fraction | Scan | Source File | Area F1 | Area F2a | Area F3a | Area F4 | Area F5 | Area F6a | Area F7a | Area F7b | Area F8 | Area F9a | Area Sample 1F10a | Area F10b | Area F11a | Area F11b | Area F12a | Area F13 | #Feature | #Feature F1 | #Feature F2a | #Feature F3a | #Feature F4 | #Feature F5 | #Feature F6a | #Feature F7a | #Feature F7b | #Feature F8 | #Feature F9a | #Feature Sample 1F10a | #Feature F10b | #Feature F11a | #Feature F11b | #Feature F12a | #Feature F13 | Start | End | PTM | AScore | Found By |
|---|---|---|---|---|---|---|---|---|---|---|---|---|---|---|---|---|---|---|---|---|---|---|---|---|---|---|---|---|---|---|---|---|---|---|---|---|---|---|---|---|---|---|---|---|---|---|---|---|---|
| S.FYTC(+57.02)PNSETC(+57.02)PDGKNIC(+57.02)VK.R | Y | 103.15 | 2288.9863 | 19 | 1.6 | 1145.5000 | 2 | 42.38 | 3 | F3:19627 | BuCaTN01_F3a.raw | | 1.8803E8 | 6.3454E9 | 1.2201E6 | | 2.4142E8 | 0 | 6.7345E7 | | 1.4377E7 | | 1.7088E7 | | | | | 12 | 0 | 3 | 3 | 1 | 0 | 2 | 0 | 1 | 0 | 1 | 0 | 1 | 0 | 0 | 0 | 0 | 29 | 47 | Carbamidomethylation | C4:Carbamidomethylation:1000.00;C10:Carbamidomethylation:1000.00;C17:Carbamidomethylation:1000.00 | PEAKS DB |
| S.FYTC(+57.02)PNSETC(+57.02)PDGK.N | Y | 82.69 | 1674.6654 | 14 | -0.4 | 838.3397 | 2 | 36.79 | 10 | F10:15352 | BuCaTN01_F9a.raw | | 7.415E8 | 1.4821E10 | 2.6498E6 | | 5.4853E8 | 3.3952E6 | 1.2621E8 | | 2.8544E7 | | 3.1496E7 | | 1.6806E7 | 1.1184E7 | | 31 | 0 | 2 | 19 | 1 | 0 | 2 | 1 | 1 | 0 | 1 | 0 | 1 | 0 | 1 | 2 | 0 | 29 | 42 | Carbamidomethylation | C4:Carbamidomethylation:1000.00;C10:Carbamidomethylation:1000.00 | PEAKS DB |
| F.YTC(+57.02)PNSETC(+57.02)PDGKNIC(+57.02)VK.R | Y | 54.84 | 2141.9180 | 18 | 3.9 | 1071.9683 | 2 | 36.35 | 3 | F3:13810 | BuCaTN01_F3a.raw | | | 0 | | | | | | | | | | | | | | 0 | 0 | 0 | 0 | 0 | 0 | 0 | 0 | 0 | 0 | 0 | 0 | 0 | 0 | 0 | 0 | 0 | 30 | 47 | Carbamidomethylation | C3:Carbamidomethylation:1000.00;C9:Carbamidomethylation:1000.00;C16:Carbamidomethylation:1000.00 | PEAKS DB |
| T.M(+15.99)QC(+57.02)KTC(+57.02)SFYTC(+57.02)PNSETC(+57.02)PDGKNIC(+57.02)VK.R | Y | 49.78 | 3200.3164 | 26 | 5.6 | 1067.7832 | 3 | 50.04 | 3 | F3:26140 | BuCaTN01_F3a.raw | | | 3.2461E6 | | | | | | | | | | | | | | 1 | 0 | 0 | 1 | 0 | 0 | 0 | 0 | 0 | 0 | 0 | 0 | 0 | 0 | 0 | 0 | 0 | 22 | 47 | Oxidation (M); Carbamidomethylation | M1:Oxidation (M):1000.00;C3:Carbamidomethylation:1000.00;C6:Carbamidomethylation:1000.00;C11:Carbamidomethylation:1000.00;C17:Carbamidomethylation:1000.00;C24:Carbamidomethylation:1000.00 | PEAKS DB |
| C.PNSETC(+57.02)PDGKNIC(+57.02)VK.R | Y | 43.85 | 1717.7764 | 15 | 3.5 | 573.6002 | 3 | 29.98 | 3 | F3:9299 | BuCaTN01_F3a.raw | | | 7.0058E5 | | | | | | | | | | | | | | 1 | 0 | 0 | 1 | 0 | 0 | 0 | 0 | 0 | 0 | 0 | 0 | 0 | 0 | 0 | 0 | 0 | 33 | 47 | Carbamidomethylation | C6:Carbamidomethylation:1000.00;C13:Carbamidomethylation:1000.00 | PEAKS DB |
| S.FYTC(+57.02)PNSETC(+57.02)PDGKNIC(+57.02)VKR.S | Y | 43.09 | 2445.0874 | 20 | 1.6 | 612.2789 | 4 | 39.69 | 3 | F3:17166 | BuCaTN01_F3a.raw | | | 4.7889E6 | | | | | | | | | | | | | | 1 | 0 | 0 | 1 | 0 | 0 | 0 | 0 | 0 | 0 | 0 | 0 | 0 | 0 | 0 | 0 | 0 | 29 | 48 | Carbamidomethylation | C4:Carbamidomethylation:1000.00;C10:Carbamidomethylation:1000.00;C17:Carbamidomethylation:1000.00 | PEAKS DB |
| total 6 peptides | | | | | | | | | | | | | | | | | | | | | | | | | | | | | | | | | | | | | | | | | | | | | | | | | |

**Best Unique PSM (Scan F3:19627, m/z=1145.5000, z=2, RT=42.38, ppm=1.6):**

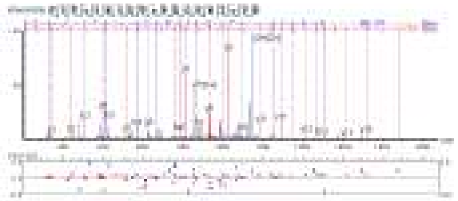

## AAD41806.1

back to list

| Protein Coverage | Supporting Peptides | Best Unique PSM |

**Protein Coverage:**

1 METLLLTLVV VTIVCLDLGY TMQCKTCSFY TCPNSETCPD GKNICVKRSW TAVRGDGPKR EIRRECAATC PPSKLGLTVF

Carbamidomethylation (+57.02)
Oxidation (M) (+15.99)

81 CCTTDNCNH

**Supporting Peptides:**

| Peptide | Uniq | -10lgP | Mass | Length | ppm | m/z | z | RT | Fraction | Scan | Source File | Area F1 | Area F2a | Area F3a | Area F4 | Area F5 | Area F6a | Area F7a | Area F7b | Area F8 | Area F9a | Area Sample 1F10a | Area F10b | Area F11a | Area F11b | Area F12a | Area F13 | #Feature | #Feature F1 | #Feature F2a | #Feature F3a | #Feature F4 | #Feature F5 | #Feature F6a | #Feature F7a | #Feature F7b | #Feature F8 | #Feature F9a | #Feature Sample 1F10a | #Feature F10b | #Feature F11a | #Feature F11b | #Feature F12a | #Feature F13 | Start | End | PTM | AScore | Found By |
|---|---|---|---|---|---|---|---|---|---|---|---|---|---|---|---|---|---|---|---|---|---|---|---|---|---|---|---|---|---|---|---|---|---|---|---|---|---|---|---|---|---|---|---|---|---|---|---|---|---|
| S.FYTC(+57.02)PNSETC(+57.02)PDGKNIC(+57.02)VK.R | Y | 103.15 | 2288.9863 | 19 | 1.6 | 1145.5000 | 2 | 42.38 | 3 | F3:19627 | BuCaTN01_F3a.raw | | 1.8803E8 | 6.3454E9 | 1.2201E6 | | 2.4142E8 | 0 | 6.7345E7 | | 1.4377E7 | | 1.7088E7 | | | | | 12 | 0 | 3 | 3 | 1 | 0 | 2 | 0 | 1 | 0 | 1 | 0 | 1 | 0 | 0 | 0 | 0 | 29 | 47 | Carbamidomethylation | C4:Carbamidomethylation:1000.00;C10:Carbamidomethylation:1000.00;C17:Carbamidomethylation:1000.00 | PEAKS DB |
| S.FYTC(+57.02)PNSETC(+57.02)PDGK.N | Y | 82.69 | 1674.6654 | 14 | -0.4 | 838.3397 | 2 | 36.79 | 10 | F10:15352 | BuCaTN01_F9a.raw | | 7.415E8 | 1.4821E10 | 2.6498E6 | | 5.4853E8 | 3.3952E6 | 1.2621E8 | | 2.8544E7 | | 3.1496E7 | | 1.6806E7 | 1.1184E7 | | 31 | 0 | 2 | 19 | 1 | 0 | 2 | 1 | 1 | 0 | 1 | 0 | 1 | 0 | 1 | 2 | 0 | 29 | 42 | Carbamidomethylation | C4:Carbamidomethylation:1000.00;C10:Carbamidomethylation:1000.00 | PEAKS DB |
| F.YTC(+57.02)PNSETC(+57.02)PDGKNIC(+57.02)VK.R | Y | 54.84 | 2141.9180 | 18 | 3.9 | 1071.9683 | 2 | 36.35 | 3 | F3:13810 | BuCaTN01_F3a.raw | | | 0 | | | | | | | | | | | | | | 0 | 0 | 0 | 0 | 0 | 0 | 0 | 0 | 0 | 0 | 0 | 0 | 0 | 0 | 0 | 0 | 0 | 30 | 47 | Carbamidomethylation | C3:Carbamidomethylation:1000.00;C9:Carbamidomethylation:1000.00;C16:Carbamidomethylation:1000.00 | PEAKS DB |
| T.M(+15.99)QC(+57.02)KTC(+57.02)SFYTC(+57.02)PNSETC(+57.02)PDGKNIC(+57.02)VK.R | Y | 49.78 | 3200.3164 | 26 | 5.6 | 1067.7832 | 3 | 50.04 | 3 | F3:26140 | BuCaTN01_F3a.raw | | | 3.2461E6 | | | | | | | | | | | | | | 1 | 0 | 0 | 1 | 0 | 0 | 0 | 0 | 0 | 0 | 0 | 0 | 0 | 0 | 0 | 0 | 0 | 22 | 47 | Oxidation (M); Carbamidomethylation | M1:Oxidation (M):1000.00;C3:Carbamidomethylation:1000.00;C6:Carbamidomethylation:1000.00;C11:Carbamidomethylation:1000.00;C17:Carbamidomethylation:1000.00;C24:Carbamidomethylation:1000.00 | PEAKS DB |
| C.PNSETC(+57.02)PDGKNIC(+57.02)VK.R | Y | 43.85 | 1717.7764 | 15 | 3.5 | 573.6002 | 3 | 29.98 | 3 | F3:9299 | BuCaTN01_F3a.raw | | | 7.0058E5 | | | | | | | | | | | | | | 1 | 0 | 0 | 1 | 0 | 0 | 0 | 0 | 0 | 0 | 0 | 0 | 0 | 0 | 0 | 0 | 0 | 33 | 47 | Carbamidomethylation | C6:Carbamidomethylation:1000.00;C13:Carbamidomethylation:1000.00 | PEAKS DB |
| S.FYTC(+57.02)PNSETC(+57.02)PDGKNIC(+57.02)VKR.S | Y | 43.09 | 2445.0874 | 20 | 1.6 | 612.2789 | 4 | 39.69 | 3 | F3:17166 | BuCaTN01_F3a.raw | | | 4.7889E6 | | | | | | | | | | | | | | 1 | 0 | 0 | 1 | 0 | 0 | 0 | 0 | 0 | 0 | 0 | 0 | 0 | 0 | 0 | 0 | 0 | 29 | 48 | Carbamidomethylation | C4:Carbamidomethylation:1000.00;C10:Carbamidomethylation:1000.00;C17:Carbamidomethylation:1000.00 | PEAKS DB |
| total 6 peptides | | | | | | | | | | | | | | | | | | | | | | | | | | | | | | | | | | | | | | | | | | | | | | | | | |

**Best Unique PSM (Scan F3:19627, m/z=1145.5000, z=2, RT=42.38, ppm=1.6):**

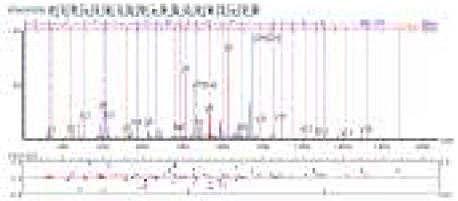

## CAA72941.1

back to list

| Protein Coverage | Supporting Peptides | Best Unique PSM |

**Protein Coverage:**

1 MKTLLLTLVV VTIVCLDLGY TMQCKTCSFY TCPNSETCPD GKNICVKRSW TAVAGDGLKR EIRRECAATC PPSKLGLTVF

Carbamidomethylation (+57.02)
Oxidation (M) (+15.99)

81 CCTTDNCNH

**Supporting Peptides:**

| Peptide | Uniq | -10lgP | Mass | Length | ppm | m/z | z | RT | Fraction | Scan | Source File | Area F1 | Area F2a | Area F3a | Area F4 | Area F5 | Area F6a | Area F7a | Area F7b | Area F8 | Area F9a | Area Sample 1F10a | Area F10b | Area F11a | Area F11b | Area F12a | Area F13 | #Feature | #Feature F1 | #Feature F2a | #Feature F3a | #Feature F4 | #Feature F5 | #Feature F6a | #Feature F7a | #Feature F7b | #Feature F8 | #Feature F9a | #Feature Sample 1F10a | #Feature F10b | #Feature F11a | #Feature F11b | #Feature F12a | #Feature F13 | Start | End | PTM | AScore | Found By |
|---|---|---|---|---|---|---|---|---|---|---|---|---|---|---|---|---|---|---|---|---|---|---|---|---|---|---|---|---|---|---|---|---|---|---|---|---|---|---|---|---|---|---|---|---|---|---|---|---|---|
| S.FYTC(+57.02)PNSETC(+57.02)PDGKNIC(+57.02)VK.R | Y | 103.15 | 2288.9863 | 19 | 1.6 | 1145.5000 | 2 | 42.38 | 3 | F3:19627 | BuCaTN01_F3a.raw | | 1.8803E8 | 6.3454E9 | 1.2201E6 | | 2.4142E8 | 0 | 6.7345E7 | | 1.4377E7 | | 1.7088E7 | | | | | 12 | 0 | 3 | 3 | 1 | 0 | 2 | 0 | 1 | 0 | 1 | 0 | 1 | 0 | 0 | 0 | 0 | 29 | 47 | Carbamidomethylation | C4:Carbamidomethylation:1000.00;C10:Carbamidomethylation:1000.00;C17:Carbamidomethylation:1000.00 | PEAKS DB |
| S.FYTC(+57.02)PNSETC(+57.02)PDGK.N | Y | 82.69 | 1674.6654 | 14 | -0.4 | 838.3397 | 2 | 36.79 | 10 | F10:15352 | BuCaTN01_F9a.raw | | 7.415E8 | 1.4821E10 | 2.6498E6 | | 5.4853E8 | 3.3952E6 | 1.2621E8 | | 2.8544E7 | | 3.1496E7 | | 1.6806E7 | 1.1184E7 | | 31 | 0 | 2 | 19 | 1 | 0 | 2 | 1 | 1 | 0 | 1 | 0 | 1 | 0 | 1 | 2 | 0 | 29 | 42 | Carbamidomethylation | C4:Carbamidomethylation:1000.00;C10:Carbamidomethylation:1000.00 | PEAKS DB |
| F.YTC(+57.02)PNSETC(+57.02)PDGKNIC(+57.02)VK.R | Y | 54.84 | 2141.9180 | 18 | 3.9 | 1071.9683 | 2 | 36.35 | 3 | F3:13810 | BuCaTN01_F3a.raw | | | 0 | | | | | | | | | | | | | | 0 | 0 | 0 | 0 | 0 | 0 | 0 | 0 | 0 | 0 | 0 | 0 | 0 | 0 | 0 | 0 | 0 | 30 | 47 | Carbamidomethylation | C3:Carbamidomethylation:1000.00;C9:Carbamidomethylation:1000.00;C16:Carbamidomethylation:1000.00 | PEAKS DB |
| T.M(+15.99)QC(+57.02)KTC(+57.02)SFYTC(+57.02)PNSETC(+57.02)PDGKNIC(+57.02)VK.R | Y | 49.78 | 3200.3164 | 26 | 5.6 | 1067.7832 | 3 | 50.04 | 3 | F3:26140 | BuCaTN01_F3a.raw | | | 3.2461E6 | | | | | | | | | | | | | | 1 | 0 | 0 | 1 | 0 | 0 | 0 | 0 | 0 | 0 | 0 | 0 | 0 | 0 | 0 | 0 | 0 | 22 | 47 | Oxidation (M); Carbamidomethylation | M1:Oxidation (M):1000.00;C3:Carbamidomethylation:1000.00;C6:Carbamidomethylation:1000.00;C11:Carbamidomethylation:1000.00;C17:Carbamidomethylation:1000.00;C24:Carbamidomethylation:1000.00 | PEAKS DB |
| C.PNSETC(+57.02)PDGKNIC(+57.02)VK.R | Y | 43.85 | 1717.7764 | 15 | 3.5 | 573.6002 | 3 | 29.98 | 3 | F3:9299 | BuCaTN01_F3a.raw | | | 7.0058E5 | | | | | | | | | | | | | | 1 | 0 | 0 | 1 | 0 | 0 | 0 | 0 | 0 | 0 | 0 | 0 | 0 | 0 | 0 | 0 | 0 | 33 | 47 | Carbamidomethylation | C6:Carbamidomethylation:1000.00;C13:Carbamidomethylation:1000.00 | PEAKS DB |
| S.FYTC(+57.02)PNSETC(+57.02)PDGKNIC(+57.02)VKR.S | Y | 43.09 | 2445.0874 | 20 | 1.6 | 612.2789 | 4 | 39.69 | 3 | F3:17166 | BuCaTN01_F3a.raw | | | 4.7889E6 | | | | | | | | | | | | | | 1 | 0 | 0 | 1 | 0 | 0 | 0 | 0 | 0 | 0 | 0 | 0 | 0 | 0 | 0 | 0 | 0 | 29 | 48 | Carbamidomethylation | C4:Carbamidomethylation:1000.00;C10:Carbamidomethylation:1000.00;C17:Carbamidomethylation:1000.00 | PEAKS DB |
| total 6 peptides | | | | | | | | | | | | | | | | | | | | | | | | | | | | | | | | | | | | | | | | | | | | | | | | | |

**Best Unique PSM (Scan F3:19627, m/z=1145.5000, z=2, RT=42.38, ppm=1.6):**

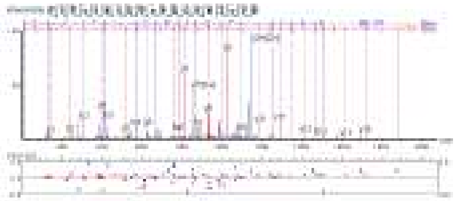

## CAA11214.1

back to list

| Protein Coverage | Supporting Peptides | Best Unique PSM |

**Protein Coverage:**

1 MKTLLLSLVV VIIVCLDLGY TMQCKTCSFY TCPNSETCPD GKNICVKRSW TAVRGDGPKR EIRRECAATC PPSKLGLTVF

Carbamidomethylation (+57.02)
Oxidation (M) (+15.99)

81 CCTTDNCYH

**Supporting Peptides:**

| Peptide | Uniq | -10lgP | Mass | Length | ppm | m/z | z | RT | Fraction | Scan | Source File | Area F1 | Area F2a | Area F3a | Area F4 | Area F5 | Area F6a | Area F7a | Area F7b | Area F8 | Area F9a | Area Sample 1F10a | Area F10b | Area F11a | Area F11b | Area F12a | Area F13 | #Feature | #Feature F1 | #Feature F2a | #Feature F3a | #Feature F4 | #Feature F5 | #Feature F6a | #Feature F7a | #Feature F7b | #Feature F8 | #Feature F9a | #Feature Sample 1F10a | #Feature F10b | #Feature F11a | #Feature F11b | #Feature F12a | #Feature F13 | Start | End | PTM | AScore | Found By |
|---|---|---|---|---|---|---|---|---|---|---|---|---|---|---|---|---|---|---|---|---|---|---|---|---|---|---|---|---|---|---|---|---|---|---|---|---|---|---|---|---|---|---|---|---|---|---|---|---|---|
| S.FYTC(+57.02)PNSETC(+57.02)PDGKNIC(+57.02)VK.R | Y | 103.15 | 2288.9863 | 19 | 1.6 | 1145.5000 | 2 | 42.38 | 3 | F3:19627 | BuCaTN01_F3a.raw | | 1.8803E8 | 6.3454E9 | 1.2201E6 | | 2.4142E8 | 0 | 6.7345E7 | | 1.4377E7 | | 1.7088E7 | | | | | 12 | 0 | 3 | 3 | 1 | 0 | 2 | 0 | 1 | 0 | 1 | 0 | 1 | 0 | 0 | 0 | 0 | 29 | 47 | Carbamidomethylation | C4:Carbamidomethylation:1000.00;C10:Carbamidomethylation:1000.00;C17:Carbamidomethylation:1000.00 | PEAKS DB |
| S.FYTC(+57.02)PNSETC(+57.02)PDGK.N | Y | 82.69 | 1674.6654 | 14 | -0.4 | 838.3397 | 2 | 36.79 | 10 | F10:15352 | BuCaTN01_F9a.raw | | 7.415E8 | 1.4821E10 | 2.6498E6 | | 5.4853E8 | 3.3952E6 | 1.2621E8 | | 2.8544E7 | | 3.1496E7 | | 1.6806E7 | 1.1184E7 | | 31 | 0 | 2 | 19 | 1 | 0 | 2 | 1 | 1 | 0 | 1 | 0 | 1 | 0 | 1 | 2 | 0 | 29 | 42 | Carbamidomethylation | C4:Carbamidomethylation:1000.00;C10:Carbamidomethylation:1000.00 | PEAKS DB |
| F.YTC(+57.02)PNSETC(+57.02)PDGKNIC(+57.02)VK.R | Y | 54.84 | 2141.9180 | 18 | 3.9 | 1071.9683 | 2 | 36.35 | 3 | F3:13810 | BuCaTN01_F3a.raw | | | 0 | | | | | | | | | | | | | | 0 | 0 | 0 | 0 | 0 | 0 | 0 | 0 | 0 | 0 | 0 | 0 | 0 | 0 | 0 | 0 | 0 | 30 | 47 | Carbamidomethylation | C3:Carbamidomethylation:1000.00;C9:Carbamidomethylation:1000.00;C16:Carbamidomethylation:1000.00 | PEAKS DB |
| T.M(+15.99)QC(+57.02)KTC(+57.02)SFYTC(+57.02)PNSETC(+57.02)PDGKNIC(+57.02)VK.R | Y | 49.78 | 3200.3164 | 26 | 5.6 | 1067.7832 | 3 | 50.04 | 3 | F3:26140 | BuCaTN01_F3a.raw | | | 3.2461E6 | | | | | | | | | | | | | | 1 | 0 | 0 | 1 | 0 | 0 | 0 | 0 | 0 | 0 | 0 | 0 | 0 | 0 | 0 | 0 | 0 | 22 | 47 | Oxidation (M); Carbamidomethylation | M1:Oxidation (M):1000.00;C3:Carbamidomethylation:1000.00;C6:Carbamidomethylation:1000.00;C11:Carbamidomethylation:1000.00;C17:Carbamidomethylation:1000.00;C24:Carbamidomethylation:1000.00 | PEAKS DB |
| C.PNSETC(+57.02)PDGKNIC(+57.02)VK.R | Y | 43.85 | 1717.7764 | 15 | 3.5 | 573.6002 | 3 | 29.98 | 3 | F3:9299 | BuCaTN01_F3a.raw | | | 7.0058E5 | | | | | | | | | | | | | | 1 | 0 | 0 | 1 | 0 | 0 | 0 | 0 | 0 | 0 | 0 | 0 | 0 | 0 | 0 | 0 | 0 | 33 | 47 | Carbamidomethylation | C6:Carbamidomethylation:1000.00;C13:Carbamidomethylation:1000.00 | PEAKS DB |
| S.FYTC(+57.02)PNSETC(+57.02)PDGKNIC(+57.02)VKR.S | Y | 43.09 | 2445.0874 | 20 | 1.6 | 612.2789 | 4 | 39.69 | 3 | F3:17166 | BuCaTN01_F3a.raw | | | 4.7889E6 | | | | | | | | | | | | | | 1 | 0 | 0 | 1 | 0 | 0 | 0 | 0 | 0 | 0 | 0 | 0 | 0 | 0 | 0 | 0 | 0 | 29 | 48 | Carbamidomethylation | C4:Carbamidomethylation:1000.00;C10:Carbamidomethylation:1000.00;C17:Carbamidomethylation:1000.00 | PEAKS DB |
| total 6 peptides | | | | | | | | | | | | | | | | | | | | | | | | | | | | | | | | | | | | | | | | | | | | | | | | | |

**Best Unique PSM (Scan F3:19627, m/z=1145.5000, z=2, RT=42.38, ppm=1.6):**

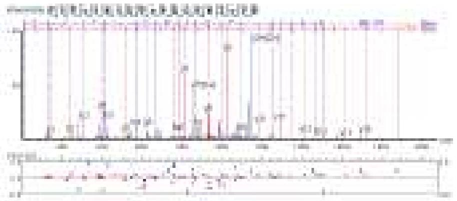

## Q9YGH9.1

back to list

| Protein Coverage | Supporting Peptides | Best Unique PSM |

**Protein Coverage:**

1 MKTLLLSLVV VIIVCLDLGY TMQCKTCSFY TCPNSETCPD GKNICVKRSW TAVRGDGPKR EIRRECAATC PPSKLGLTVF

81 CCTTDNCYH

Carbamidomethylation (+57.02)
Oxidation (M) (+15.99)

**Supporting Peptides:**

| Peptide | Uniq | -10lgP | Mass | Length | ppm | m/z | z | RT | Fraction | Scan | Source File | Area F1 | Area F2a | Area F3a | Area F4 | Area F5 | Area F6a | Area F7a | Area F7b | Area F8 | Area F9a | Area Sample 1F10a | Area F10b | Area F11a | Area F11b | Area F12a | Area F13 | #Feature | #Feature F1 | #Feature F2a | #Feature F3a | #Feature F4 | #Feature F5 | #Feature F6a | #Feature F7a | #Feature F7b | #Feature F8 | #Feature F9a | #Feature Sample 1F10a | #Feature F10b | #Feature F11a | #Feature F11b | #Feature F12a | #Feature F13 | Start | End | PTM | AScore | Found By |
|---|---|---|---|---|---|---|---|---|---|---|---|---|---|---|---|---|---|---|---|---|---|---|---|---|---|---|---|---|---|---|---|---|---|---|---|---|---|---|---|---|---|---|---|---|---|---|---|---|---|
| S.FYTC(+57.02)PNSETC(+57.02)PDGKNIC(+57.02)VK.R | Y | 103.15 | 2288.9863 | 19 | 1.6 | 1145.5000 | 2 | 42.38 | 3 | F3:19627 | BuCaTN01_F3a.raw | | 1.8803E8 | 6.3454E9 | 1.2201E6 | | 2.4142E8 | 0 | 6.7345E7 | | 1.4377E7 | | 1.7088E7 | | | | | 12 | 0 | 3 | 3 | 1 | 0 | 2 | 0 | 1 | 0 | 1 | 0 | 1 | 0 | 0 | 0 | 0 | 29 | 47 | Carbamidomethylation | C4:Carbamidomethylation:1000.00;C10:Carbamidomethylation:1000.00;C17:Carbamidomethylation:1000.00 | PEAKS DB |
| S.FYTC(+57.02)PNSETC(+57.02)PDGK.N | Y | 82.69 | 1674.6654 | 14 | -0.4 | 838.3397 | 2 | 36.79 | 10 | F10:15352 | BuCaTN01_F9a.raw | | 7.415E8 | 1.4821E10 | 2.6498E6 | | 5.4853E8 | 3.3952E6 | 1.2621E8 | | 2.8544E7 | | 3.1496E7 | | 1.6806E7 | 1.1184E7 | | 31 | 0 | 2 | 19 | 1 | 0 | 2 | 1 | 1 | 0 | 1 | 0 | 1 | 0 | 1 | 2 | 0 | 29 | 42 | Carbamidomethylation | C4:Carbamidomethylation:1000.00;C10:Carbamidomethylation:1000.00 | PEAKS DB |
| F.YTC(+57.02)PNSETC(+57.02)PDGKNIC(+57.02)VK.R | Y | 54.84 | 2141.9180 | 18 | 3.9 | 1071.9683 | 2 | 36.35 | 3 | F3:13810 | BuCaTN01_F3a.raw | | | 0 | | | | | | | | | | | | | | 0 | 0 | 0 | 0 | 0 | 0 | 0 | 0 | 0 | 0 | 0 | 0 | 0 | 0 | 0 | 0 | 0 | 30 | 47 | Carbamidomethylation | C3:Carbamidomethylation:1000.00;C9:Carbamidomethylation:1000.00;C16:Carbamidomethylation:1000.00 | PEAKS DB |
| T.M(+15.99)QC(+57.02)KTC(+57.02)SFYTC(+57.02)PNSETC(+57.02)PDGKNIC(+57.02)VK.R | Y | 49.78 | 3200.3164 | 26 | 5.6 | 1067.7832 | 3 | 50.04 | 3 | F3:26140 | BuCaTN01_F3a.raw | | | 3.2461E6 | | | | | | | | | | | | | | 1 | 0 | 0 | 1 | 0 | 0 | 0 | 0 | 0 | 0 | 0 | 0 | 0 | 0 | 0 | 0 | 0 | 22 | 47 | Oxidation (M); Carbamidomethylation | M1:Oxidation (M):1000.00;C3:Carbamidomethylation:1000.00;C6:Carbamidomethylation:1000.00;C11:Carbamidomethylation:1000.00;C17:Carbamidomethylation:1000.00;C24:Carbamidomethylation:1000.00 | PEAKS DB |
| C.PNSETC(+57.02)PDGKNIC(+57.02)VK.R | Y | 43.85 | 1717.7764 | 15 | 3.5 | 573.6002 | 3 | 29.98 | 3 | F3:9299 | BuCaTN01_F3a.raw | | | 7.0058E5 | | | | | | | | | | | | | | 1 | 0 | 0 | 1 | 0 | 0 | 0 | 0 | 0 | 0 | 0 | 0 | 0 | 0 | 0 | 0 | 0 | 33 | 47 | Carbamidomethylation | C6:Carbamidomethylation:1000.00;C13:Carbamidomethylation:1000.00 | PEAKS DB |
| S.FYTC(+57.02)PNSETC(+57.02)PDGKNIC(+57.02)VKR.S | Y | 43.09 | 2445.0874 | 20 | 1.6 | 612.2789 | 4 | 39.69 | 3 | F3:17166 | BuCaTN01_F3a.raw | | | 4.7889E6 | | | | | | | | | | | | | | 1 | 0 | 0 | 1 | 0 | 0 | 0 | 0 | 0 | 0 | 0 | 0 | 0 | 0 | 0 | 0 | 0 | 29 | 48 | Carbamidomethylation | C4:Carbamidomethylation:1000.00;C10:Carbamidomethylation:1000.00;C17:Carbamidomethylation:1000.00 | PEAKS DB |
| total 6 peptides | | | | | | | | | | | | | | | | | | | | | | | | | | | | | | | | | | | | | | | | | | | | | | | | | |

**Best Unique PSM (Scan F3:19627, m/z=1145.5000, z=2, RT=42.38, ppm=1.6):**

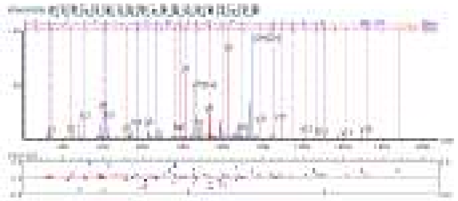

## JAS05143.1

| Protein Coverage | Supporting Peptides | Best Unique PSM |

**Protein Coverage:**

1 MPTSRRRSGA HGCPRSAPSA MWRLVGAVCF CAALSVAAAG SFKLTILHTN DVHARVEQTS RDSGKCTRED CYGGVARRAT

81 KIREIRASHR NVLLLDAGDQ YQGTVWFNYF KGREVVHFMN SLRYDAMALG NHEFDNGLNG LLDPLLKNVK FPILSANIRP

161 KGPIASNISG YILPYKIINV GSEKVGIIGY TTKETPVLSN PGPYLEFRDE VEELQKQADK LTTLGVNKII ALGHSGFKED

241 CRIAQKVKGV DVVVGGHTNT FLYTGSPPSN EVPAGNYPFM QLSDDGRQVP VVQAYAFGKY LGYLNVIFDD KGKVIKASGN

321 PILLNKSIQE DPAVKAEVSR MKVQLQKYSK QEIGKTIVYL NGTTHACRFH ECNLGNLICD AVVYNNLRHP DDNEWNHVSM

401 CIVNGGGIRS PIDEKANNGI ITLEELTAVL PFGGTFDLLQ IKGSALRQAF EHSVHRHGQG TGELLQVSGI KVLYDLSQKP

481 GKRVVSLNVL CTECRVPTYV PLQMEKTYKV LLPSFLAAGG DGYYMLKGNS SNHSSGDLDI SIVSDYIKRM GKVFPAMEGR

561 VMFSAGTLFQ AHHS

Carbamidomethylation (+57.02)
Oxidation (M) (+15.99)

**Supporting Peptides:**

| Peptide | Uniq | -10lgP | Mass | Length | ppm | m/z | z | RT | Fraction | Scan | Source File | Area F1 | Area F2a | Area F3a | Area F4 | Area F5 | Area F6a | Area F7a | Area F7b | Area F8 | Area F9a | Area Sample 1F10a | Area F10b | Area F11a | Area F11b | Area F12a | Area F13 | #Feature | #Feature F1 | #Feature F2a | #Feature F3a | #Feature F4 | #Feature F5 | #Feature F6a | #Feature F7a | #Feature F7b | #Feature F8 | #Feature F9a | #Feature Sample 1F10a | #Feature F10b | #Feature F11a | #Feature F11b | #Feature F12a | #Feature F13 | Start | End | PTM | AScore | Found By |
|---|---|---|---|---|---|---|---|---|---|---|---|---|---|---|---|---|---|---|---|---|---|---|---|---|---|---|---|---|---|---|---|---|---|---|---|---|---|---|---|---|---|---|---|---|---|---|---|---|---|
| K.ASGNPILLNK.S | N | 71.96 | 1025.5869 | 10 | -0.1 | 513.8007 | 2 | 40.65 | 16 | F16:17022 | BuCaTN01_F13.raw | | | | | | | | | | | | | | | | 2.2075E6 | 1 | 0 | 0 | 0 | 0 | 0 | 0 | 0 | 0 | 0 | 0 | 0 | 0 | 0 | 0 | 0 | 1 | 317 | 326 | | | PEAKS DB |
| A.GSFKLTILHTNDVHAR.V | N | 68.40 | 1807.9692 | 16 | -1.1 | 452.9991 | 4 | 50.67 | 16 | F16:23710 | BuCaTN01_F13.raw | | | | | | | | | | | | | | | | 1.0157E6 | 1 | 0 | 0 | 0 | 0 | 0 | 0 | 0 | 0 | 0 | 0 | 0 | 0 | 0 | 0 | 0 | 1 | 40 | 55 | | | PEAKS DB |
| K.SIQEDPAVKAEVSR.M | Y | 61.28 | 1527.7892 | 14 | 0.9 | 510.2708 | 3 | 36.48 | 16 | F16:13815 | BuCaTN01_F13.raw | | | | | | | | | | | | | | | | 1.2835E6 | 1 | 0 | 0 | 0 | 0 | 0 | 0 | 0 | 0 | 0 | 0 | 0 | 0 | 0 | 0 | 0 | 1 | 327 | 340 | | | PEAKS DB |
| N.ISGYILPYKIINVGSEK.V | N | 59.75 | 1893.0610 | 17 | -0.2 | 632.0275 | 3 | 71.29 | 16 | F16:37082 | BuCaTN01_F13.raw | | | | | | | | | | | | | | | | 4.9796E5 | 1 | 0 | 0 | 0 | 0 | 0 | 0 | 0 | 0 | 0 | 0 | 0 | 0 | 0 | 0 | 0 | 1 | 168 | 184 | | | PEAKS DB |
| R.VVSLNVLC(+57.02)TEC(+57.02)R.V | N | 59.19 | 1448.7115 | 12 | 2.9 | 725.3630 | 2 | 59.59 | 8 | F8:35185 | BuCaTN01_F7b.raw | | | | | | | | 4.7661E5 | | | | | | | | | 1 | 0 | 0 | 0 | 0 | 0 | 0 | 0 | 1 | 0 | 0 | 0 | 0 | 0 | 0 | 0 | 0 | 484 | 495 | Carbamidomethylation | C8:Carbamidomethylation:1000.00;C11:Carbamidomethylation:1000.00 | PEAKS DB |
| K.NVKFPILSANIRPK.G | N | 59.13 | 1595.9510 | 14 | 1.2 | 532.9916 | 3 | 58.57 | 16 | F16:28436 | BuCaTN01_F13.raw | | | | | | | | | | | | | | | | 3.5508E5 | 1 | 0 | 0 | 0 | 0 | 0 | 0 | 0 | 0 | 0 | 0 | 0 | 0 | 0 | 0 | 0 | 1 | 148 | 161 | | | PEAKS DB |
| R.QVPVVQAYAFGK.Y | N | 57.72 | 1305.7081 | 12 | 0.0 | 653.8613 | 2 | 58.36 | 16 | F16:28377 | BuCaTN01_F13.raw | | | | | | | | | | | | | | | | 2.6708E5 | 1 | 0 | 0 | 0 | 0 | 0 | 0 | 0 | 0 | 0 | 0 | 0 | 0 | 0 | 0 | 0 | 1 | 288 | 299 | | | PEAKS DB |
| K.FPILSANIRPK.G | N | 55.81 | 1254.7448 | 11 | 0.4 | 419.2557 | 3 | 55.55 | 16 | F16:26305 | BuCaTN01_F13.raw | | | | | | | | | | | | | | | | 4.8037E5 | 1 | 0 | 0 | 0 | 0 | 0 | 0 | 0 | 0 | 0 | 0 | 0 | 0 | 0 | 0 | 0 | 1 | 151 | 161 | | | PEAKS DB |
| K.LTILHTNDVHAR.V | N | 52.16 | 1388.7524 | 12 | -1.7 | 463.9240 | 3 | 37.10 | 16 | F16:14164 | BuCaTN01_F13.raw | | | | | | | | | | | | | | | | 0 | 0 | 0 | 0 | 0 | 0 | 0 | 0 | 0 | 0 | 0 | 0 | 0 | 0 | 0 | 0 | 0 | 0 | 44 | 55 | | | PEAKS DB |
| N.ISGYILPYK.I | N | 50.21 | 1052.5906 | 9 | 0.3 | 527.3027 | 2 | 57.19 | 16 | F16:27479 | BuCaTN01_F13.raw | | | | | | | | | | | | | | | | 5.5906E5 | 1 | 0 | 0 | 0 | 0 | 0 | 0 | 0 | 0 | 0 | 0 | 0 | 0 | 0 | 0 | 0 | 1 | 168 | 176 | | | PEAKS DB |
| K.VFPAMEGR.V | N | 41.93 | 905.4429 | 8 | -0.1 | 453.7287 | 2 | 42.75 | 16 | F16:18519 | BuCaTN01_F13.raw | | | | | | | | | | | | | | | | 4.3727E5 | 1 | 0 | 0 | 0 | 0 | 0 | 0 | 0 | 0 | 0 | 0 | 0 | 0 | 0 | 0 | 0 | 1 | 553 | 560 | | | PEAKS DB |
| R.MGKVFPAM(+15.99)EGR.V | N | 41.68 | 1237.5947 | 11 | 0.8 | 413.5392 | 3 | 33.99 | 16 | F16:12305 | BuCaTN01_F13.raw | | | | | | | | | | | | | | | | 9.9954E4 | 1 | 0 | 0 | 0 | 0 | 0 | 0 | 0 | 0 | 0 | 0 | 0 | 0 | 0 | 0 | 0 | 1 | 550 | 560 | Oxidation (M) | M8:Oxidation (M):79.97 | PEAKS DB |
| total 12 peptides | | | | | | | | | | | | | | | | | | | | | | | | | | | | | | | | | | | | | | | | | | | | | | | | | |

**Best Unique PSM (Scan F16:13815, m/z=510.2708, z=3, RT=36.48, ppm=0.9):**

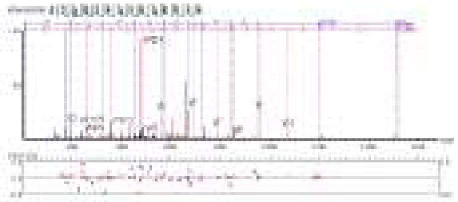

## JAI09047.1

| Protein Coverage | Supporting Peptides | Best Unique PSM |

**Protein Coverage:**

1 MPTSRRRSGA HGCPRSAPSA MWRLVGAVCF CAALSVAAAG SFKLTILHTN DVHARVEQTS RDSGKCTRED CYGGVARRAT

81 KIREIRASHR NVLLLDAGDQ YQGTVWFNYF KGREVVHFMN SLRYDAMALG NHEFDNGLNG LLDPLLKNVK FPILSANIRP

161 KGPIASNISG YILPYKIINV GSEKVGIIGY TTKETPVLSN PGPYLEFRDE VEELQKQADK LTTLGVNKII ALGHSGFKED

241 CRIAQKVKGV DVVVGGHTNT FLYTGSPPSN EVPAGNYPFM QLSDDGRQVP VVQAYAFGKY LGYLNVIFDD KGKVIKASGN

321 PILLNKSIQE DPAVKAEVSR MKVQLQKYSK QEIGKTIVYL NGTTHACRFH ECNLGNLICD AVVYNNLRHP DDNEWNHVSM

401 CIVNGGGIRS PIDEKANNGI ITLEELTAVL PFGGTFDLLQ IKGSALRQAF EHSVHRHGQG TGELLQVSGI KVLYDLSQKP

481 GKRVVSLNVL CTECRVPAYV PLQMEKTYKV LLPSFLAAGG DGYYMLKGDS SNHSSGDLDI SIVSDYIKRM GKVFPAMEGR

561 VMFSAGTLFQ AHHS

C Carbamidomethylation (+57.02)
O Oxidation (M) (+15.99)

**Supporting Peptides:**

| Peptide | Uniq | -10lgP | Mass | Length | ppm | m/z | z | RT | Fraction | Scan | Source File | Area F1 | Area F2a | Area F3a | Area F4 | Area F5 | Area F6a | Area F7a | Area F7b | Area F8 | Area F9a | Area Sample 1F10a | Area F10b | Area F11a | Area F11b | Area F12a | Area F13 | #Feature | #Feature F1 | #Feature F2a | #Feature F3a | #Feature F4 | #Feature F5 | #Feature F6a | #Feature F7a | #Feature F7b | #Feature F8 | #Feature F9a | #Feature Sample 1F10a | #Feature F10b | #Feature F11a | #Feature F11b | #Feature F12a | #Feature F13 | Start | End | PTM | AScore | Found By |
|---|---|---|---|---|---|---|---|---|---|---|---|---|---|---|---|---|---|---|---|---|---|---|---|---|---|---|---|---|---|---|---|---|---|---|---|---|---|---|---|---|---|---|---|---|---|---|---|---|---|
| K.ASGNPILLNK.S | N | 71.96 | 1025.5869 | 10 | -0.1 | 513.8007 | 2 | 40.65 | 16 | F16:17022 | BuCaTN01_F13.raw | | | | | | | | | | | | | | | | 2.2075E6 | 1 | 0 | 0 | 0 | 0 | 0 | 0 | 0 | 0 | 0 | 0 | 0 | 0 | 0 | 0 | 0 | 1 | 317 | 326 | | | PEAKS DB |
| A.GSFKLTILHTNDVHAR.V | N | 68.40 | 1807.9692 | 16 | -1.1 | 452.9991 | 4 | 50.67 | 16 | F16:23710 | BuCaTN01_F13.raw | | | | | | | | | | | | | | | | 1.0157E6 | 1 | 0 | 0 | 0 | 0 | 0 | 0 | 0 | 0 | 0 | 0 | 0 | 0 | 0 | 0 | 0 | 1 | 40 | 55 | | | PEAKS DB |
| K.SIQEDPAVKAEVSR.M | Y | 61.28 | 1527.7892 | 14 | 0.9 | 510.2708 | 3 | 36.48 | 16 | F16:13815 | BuCaTN01_F13.raw | | | | | | | | | | | | | | | | 1.2835E6 | 1 | 0 | 0 | 0 | 0 | 0 | 0 | 0 | 0 | 0 | 0 | 0 | 0 | 0 | 0 | 0 | 1 | 327 | 340 | | | PEAKS DB |
| N.ISGYILPYKIINVGSEK.V | N | 59.75 | 1893.0610 | 17 | -0.2 | 632.0275 | 3 | 71.29 | 16 | F16:37082 | BuCaTN01_F13.raw | | | | | | | | | | | | | | | | 4.9796E5 | 1 | 0 | 0 | 0 | 0 | 0 | 0 | 0 | 0 | 0 | 0 | 0 | 0 | 0 | 0 | 0 | 1 | 168 | 184 | | | PEAKS DB |
| R.VVSLNVLC(+57.02)TEC(+57.02)R.V | N | 59.19 | 1448.7115 | 12 | 2.9 | 725.3630 | 2 | 59.59 | 8 | F8:35185 | BuCaTN01_F7b.raw | | | | | | | | 4.7661E5 | | | | | | | | | 1 | 0 | 0 | 0 | 0 | 0 | 0 | 0 | 1 | 0 | 0 | 0 | 0 | 0 | 0 | 0 | 0 | 484 | 495 | Carbamidomethylation | C8:Carbamidomethylation:1000.00;C11:Carbamidomethylation:1000.00 | PEAKS DB |
| K.NVKFPILSANIRPK.G | N | 59.13 | 1595.9510 | 14 | 1.2 | 532.9916 | 3 | 58.57 | 16 | F16:28436 | BuCaTN01_F13.raw | | | | | | | | | | | | | | | | 3.5508E5 | 1 | 0 | 0 | 0 | 0 | 0 | 0 | 0 | 0 | 0 | 0 | 0 | 0 | 0 | 0 | 0 | 1 | 148 | 161 | | | PEAKS DB |
| R.QVPVVQAYAFGK.Y | N | 57.72 | 1305.7081 | 12 | 0.0 | 653.8613 | 2 | 58.36 | 16 | F16:28377 | BuCaTN01_F13.raw | | | | | | | | | | | | | | | | 2.6708E5 | 1 | 0 | 0 | 0 | 0 | 0 | 0 | 0 | 0 | 0 | 0 | 0 | 0 | 0 | 0 | 0 | 1 | 288 | 299 | | | PEAKS DB |
| K.FPILSANIRPK.G | N | 55.81 | 1254.7448 | 11 | 0.4 | 419.2557 | 3 | 55.55 | 16 | F16:26305 | BuCaTN01_F13.raw | | | | | | | | | | | | | | | | 4.8037E5 | 1 | 0 | 0 | 0 | 0 | 0 | 0 | 0 | 0 | 0 | 0 | 0 | 0 | 0 | 0 | 0 | 1 | 151 | 161 | | | PEAKS DB |
| K.LTILHTNDVHAR.V | N | 52.16 | 1388.7524 | 12 | -1.7 | 463.9240 | 3 | 37.10 | 16 | F16:14164 | BuCaTN01_F13.raw | | | | | | | | | | | | | | | | 0 | 0 | 0 | 0 | 0 | 0 | 0 | 0 | 0 | 0 | 0 | 0 | 0 | 0 | 0 | 0 | 0 | 0 | 44 | 55 | | | PEAKS DB |
| N.ISGYILPYK.I | N | 50.21 | 1052.5906 | 9 | 0.3 | 527.3027 | 2 | 57.19 | 16 | F16:27479 | BuCaTN01_F13.raw | | | | | | | | | | | | | | | | 5.5906E5 | 1 | 0 | 0 | 0 | 0 | 0 | 0 | 0 | 0 | 0 | 0 | 0 | 0 | 0 | 0 | 0 | 1 | 168 | 176 | | | PEAKS DB |
| K.VFPAMEGR.V | N | 41.93 | 905.4429 | 8 | -0.1 | 453.7287 | 2 | 42.75 | 16 | F16:18519 | BuCaTN01_F13.raw | | | | | | | | | | | | | | | | 4.3727E5 | 1 | 0 | 0 | 0 | 0 | 0 | 0 | 0 | 0 | 0 | 0 | 0 | 0 | 0 | 0 | 0 | 1 | 553 | 560 | | | PEAKS DB |
| R.MGKVFPAM(+15.99)EGR.V | N | 41.68 | 1237.5947 | 11 | 0.8 | 413.5392 | 3 | 33.99 | 16 | F16:12305 | BuCaTN01_F13.raw | | | | | | | | | | | | | | | | 9.9954E4 | 1 | 0 | 0 | 0 | 0 | 0 | 0 | 0 | 0 | 0 | 0 | 0 | 0 | 0 | 0 | 0 | 1 | 550 | 560 | Oxidation (M) | M8:Oxidation (M):79.97 | PEAKS DB |
| **total 12 peptides** | | | | | | | | | | | | | | | | | | | | | | | | | | | | | | | | | | | | | | | | | | | | | | | | | |

**Best Unique PSM (Scan F16:13815, m/z=510.2708, z=3, RT=36.48, ppm=0.9):**

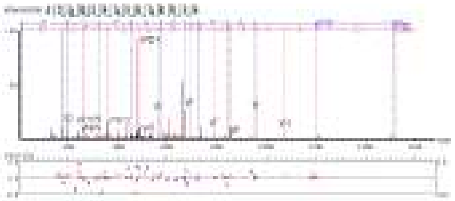

## JAB52815.1

back to list

| Protein Coverage | Supporting Peptides | Best Unique PSM |

**Protein Coverage:**

1 MPTSRRRSGA HGCPRSAPSA MWRLVGAVCF CAALSVAAAG SFKLTILHTN DVHARVEQTS RDSGKCTRED CYGGVARRAT

81 KIREIRASHR NVLLLDAGDQ YQGTVWFNYF KGREVVHFMN SLRYDAMALG NHEFDNGLNG LLDPLLKNVK FPILSANIRP

161 KGPIASNISG YILPYKIINV GSEKVGIIGY TTKETPVLSN PGPYLEFRDE VEELQKQADK LTTLGVNKII ALGHSGFKED

241 CRIAQKVKGV DVVVGGHTNT FLYTGSPPSN EVPAGNYPFM QLSDDGRQVP VVQAYAFGKY LGYLNVIFDD KGKVIKASGN

321 PILLNKSIQE DPAVKAEVSR MKVQLQKYSK QEIGKTIVYL NGTTHACRFH ECNLGNLICD AVVYNNLRHP DDNEWNHVSM

401 CIVNGGGIRS PIDEKANNGI ITLEELTAVL PFGGTFDLLQ IKGSALRQAF EHSVHRHGQG TGELLQVSGI KVLYDLSQKP

481 GKRVVSLNVL CTECRVPAYV PLQMEKTYKV LLPSFLAAGG DGYYMLKGDS SNHSSGDLDI SIVSDYIKRM GKVFPAMEGR

561 VMFSAGTLFQ AHHS

C Carbamidomethylation (+57.02)
O Oxidation (M) (+15.99)

**Supporting Peptides:**

| Peptide | Uniq | -10lgP | Mass | Length | ppm | m/z | z | RT | Fraction | Scan | Source File | Area F1 | Area F2a | Area F3a | Area F4 | Area F5 | Area F6a | Area F7a | Area F7b | Area F8 | Area F9a | Area Sample 1F10a | Area F10b | Area F11a | Area F11b | Area F12a | Area F13 | #Feature | #Feature F1 | #Feature F2a | #Feature F3a | #Feature F4 | #Feature F5 | #Feature F6a | #Feature F7a | #Feature F7b | #Feature F8 | #Feature F9a | #Feature Sample 1F10a | #Feature F10b | #Feature F11a | #Feature F11b | #Feature F12a | #Feature F13 | Start | End | PTM | AScore | Found By |
|---|---|---|---|---|---|---|---|---|---|---|---|---|---|---|---|---|---|---|---|---|---|---|---|---|---|---|---|---|---|---|---|---|---|---|---|---|---|---|---|---|---|---|---|---|---|---|---|---|---|
| K.ASGNPILLNK.S | N | 71.96 | 1025.5869 | 10 | -0.1 | 513.8007 | 2 | 40.65 | 16 | F16:17022 | BuCaTN01_F13.raw | | | | | | | | | | | | | | | | 2.2075E6 | 1 | 0 | 0 | 0 | 0 | 0 | 0 | 0 | 0 | 0 | 0 | 0 | 0 | 0 | 0 | 0 | 1 | 317 | 326 | | | PEAKS DB |
| A.GSFKLTILHTNDVHAR.V | N | 68.40 | 1807.9692 | 16 | -1.1 | 452.9991 | 4 | 50.67 | 16 | F16:23710 | BuCaTN01_F13.raw | | | | | | | | | | | | | | | | 1.0157E6 | 1 | 0 | 0 | 0 | 0 | 0 | 0 | 0 | 0 | 0 | 0 | 0 | 0 | 0 | 0 | 0 | 1 | 40 | 55 | | | PEAKS DB |
| K.SIQEDPAVKAEVSR.M | Y | 61.28 | 1527.7892 | 14 | 0.9 | 510.2708 | 3 | 36.48 | 16 | F16:13815 | BuCaTN01_F13.raw | | | | | | | | | | | | | | | | 1.2835E6 | 1 | 0 | 0 | 0 | 0 | 0 | 0 | 0 | 0 | 0 | 0 | 0 | 0 | 0 | 0 | 0 | 1 | 327 | 340 | | | PEAKS DB |
| N.ISGYILPYKIINVGSEK.V | N | 59.75 | 1893.0610 | 17 | -0.2 | 632.0275 | 3 | 71.29 | 16 | F16:37082 | BuCaTN01_F13.raw | | | | | | | | | | | | | | | | 4.9796E5 | 1 | 0 | 0 | 0 | 0 | 0 | 0 | 0 | 0 | 0 | 0 | 0 | 0 | 0 | 0 | 0 | 1 | 168 | 184 | | | PEAKS DB |
| R.VVSLNVLC(+57.02)TEC(+57.02)R.V | N | 59.19 | 1448.7115 | 12 | 2.9 | 725.3630 | 2 | 59.59 | 8 | F8:35185 | BuCaTN01_F7b.raw | | | | | | | | 4.7661E5 | | | | | | | | | 1 | 0 | 0 | 0 | 0 | 0 | 0 | 0 | 1 | 0 | 0 | 0 | 0 | 0 | 0 | 0 | 0 | 484 | 495 | Carbamidomethylation | C8:Carbamidomethylation:1000.00;C11:Carbamidomethylation:1000.00 | PEAKS DB |
| K.NVKFPILSANIRPK.G | N | 59.13 | 1595.9510 | 14 | 1.2 | 532.9916 | 3 | 58.57 | 16 | F16:28436 | BuCaTN01_F13.raw | | | | | | | | | | | | | | | | 3.5508E5 | 1 | 0 | 0 | 0 | 0 | 0 | 0 | 0 | 0 | 0 | 0 | 0 | 0 | 0 | 0 | 0 | 1 | 148 | 161 | | | PEAKS DB |
| R.QVPVVQAYAFGK.Y | N | 57.72 | 1305.7081 | 12 | 0.0 | 653.8613 | 2 | 58.36 | 16 | F16:28377 | BuCaTN01_F13.raw | | | | | | | | | | | | | | | | 2.6708E5 | 1 | 0 | 0 | 0 | 0 | 0 | 0 | 0 | 0 | 0 | 0 | 0 | 0 | 0 | 0 | 0 | 1 | 288 | 299 | | | PEAKS DB |
| K.FPILSANIRPK.G | N | 55.81 | 1254.7448 | 11 | 0.4 | 419.2557 | 3 | 55.55 | 16 | F16:26305 | BuCaTN01_F13.raw | | | | | | | | | | | | | | | | 4.8037E5 | 1 | 0 | 0 | 0 | 0 | 0 | 0 | 0 | 0 | 0 | 0 | 0 | 0 | 0 | 0 | 0 | 1 | 151 | 161 | | | PEAKS DB |
| K.LTILHTNDVHAR.V | N | 52.16 | 1388.7524 | 12 | -1.7 | 463.9240 | 3 | 37.10 | 16 | F16:14164 | BuCaTN01_F13.raw | | | | | | | | | | | | | | | | 0 | 0 | 0 | 0 | 0 | 0 | 0 | 0 | 0 | 0 | 0 | 0 | 0 | 0 | 0 | 0 | 0 | 0 | 44 | 55 | | | PEAKS DB |
| N.ISGYILPYK.I | N | 50.21 | 1052.5906 | 9 | 0.3 | 527.3027 | 2 | 57.19 | 16 | F16:27479 | BuCaTN01_F13.raw | | | | | | | | | | | | | | | | 5.5906E5 | 1 | 0 | 0 | 0 | 0 | 0 | 0 | 0 | 0 | 0 | 0 | 0 | 0 | 0 | 0 | 0 | 1 | 168 | 176 | | | PEAKS DB |
| K.VFPAMEGR.V | N | 41.93 | 905.4429 | 8 | -0.1 | 453.7287 | 2 | 42.75 | 16 | F16:18519 | BuCaTN01_F13.raw | | | | | | | | | | | | | | | | 4.3727E5 | 1 | 0 | 0 | 0 | 0 | 0 | 0 | 0 | 0 | 0 | 0 | 0 | 0 | 0 | 0 | 0 | 1 | 553 | 560 | | | PEAKS DB |
| R.MGKVFPAM(+15.99)EGR.V | N | 41.68 | 1237.5947 | 11 | 0.8 | 413.5392 | 3 | 33.99 | 16 | F16:12305 | BuCaTN01_F13.raw | | | | | | | | | | | | | | | | 9.9954E4 | 1 | 0 | 0 | 0 | 0 | 0 | 0 | 0 | 0 | 0 | 0 | 0 | 0 | 0 | 0 | 0 | 1 | 550 | 560 | Oxidation (M) | M8:Oxidation (M):79.97 | PEAKS DB |
| **total 12 peptides** | | | | | | | | | | | | | | | | | | | | | | | | | | | | | | | | | | | | | | | | | | | | | | | | | |

**Best Unique PSM (Scan F16:13815, m/z=510.2708, z=3, RT=36.48, ppm=0.9):**

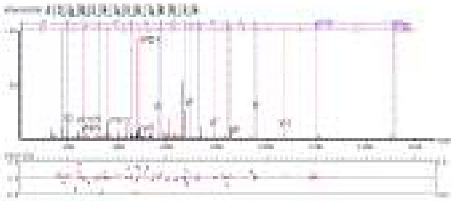

## JAI09048.1

back to list

| Protein Coverage | Supporting Peptides | Best Unique PSM |

**Protein Coverage:**

1 MPTSRRRSGA HGCPRSAPSA MWRLVGAVCF CAALSVAAA**G SFKLTILHTN DVHAR**VEQTS RDSGKCTRED CYGGVARRAT

81 KIREIRASHR NVLLLDAGDQ YQGTVWFNYF KGREVVHFMN SLRYDAMALG NHEFDNGLNG LLDPLLK**NVK FPILSANIRP**

161 **K**GPIASN**ISG YILPYKIINV GSEK**VGIIGY TTKETPVLSN PGPYLEFRDE VEELQKQADK LTTLGVNKII ALGHSGFKED

241 CRIAQKVKGV DVVVGGHTNT FLYTGSPPSN EVPAGNYPFM QLSDDGR**QVP VVQAYAFGK**Y LGYLNVIFDD KGKVIK**ASGN**

321 **PILLNKSIQE DPAVKAEVSR** MKVQLQKYSK QEIGKTIVYL NGTTHACRFH ECNLGNLICD AVVYNNLRHP DDNEWNHVSM

401 CIVNGGGIRS PIDEKANNGI ITLEELTAVL PFGGTFDLLQ IKGSALRQAF EHSVHRHGQG TGELLQVSGI KVLYDLSQKP

481 GKR**VVSLNVL CTECR**VPIYV PLQMEKTYKV LLPSFLAAGG DGYYMLKGDS SNHSSGDLDI SIVSDYIKR**M GKVFPAMEGR**

561 VMFSAGTLFQ AHHS

C Carbamidomethylation (+57.02)
O Oxidation (M) (+15.99)

491 C; 557 O

**Supporting Peptides:**

| Peptide | Uniq | -10lgP | Mass | Length | ppm | m/z | z | RT | Fraction | Scan | Source File | Area F1 | Area F2a | Area F3a | Area F4 | Area F5 | Area F6a | Area F7a | Area F7b | Area F8 | Area F9a | Area Sample 1F10a | Area F10b | Area F11a | Area F11b | Area F12a | Area F13 | #Feature | #Feature F1 | #Feature F2a | #Feature F3a | #Feature F4 | #Feature F5 | #Feature F6a | #Feature F7a | #Feature F7b | #Feature F8 | #Feature F9a | #Feature Sample 1F10a | #Feature F10b | #Feature F11a | #Feature F11b | #Feature F12a | #Feature F13 | Start | End | PTM | AScore | Found By |
|---|---|---|---|---|---|---|---|---|---|---|---|---|---|---|---|---|---|---|---|---|---|---|---|---|---|---|---|---|---|---|---|---|---|---|---|---|---|---|---|---|---|---|---|---|---|---|---|---|---|
| K.ASGNPILLNK.S | N | 71.96 | 1025.5869 | 10 | -0.1 | 513.8007 | 2 | 40.65 | 16 | F16:17022 | BuCaTN01_F13.raw | | | | | | | | | | | | | | | | 2.2075E6 | 1 | 0 | 0 | 0 | 0 | 0 | 0 | 0 | 0 | 0 | 0 | 0 | 0 | 0 | 0 | 0 | 1 | 317 | 326 | | | PEAKS DB |
| A.GSFKLTILHTNDVHAR.V | N | 68.40 | 1807.9692 | 16 | -1.1 | 452.9991 | 4 | 50.67 | 16 | F16:23710 | BuCaTN01_F13.raw | | | | | | | | | | | | | | | | 1.0157E6 | 1 | 0 | 0 | 0 | 0 | 0 | 0 | 0 | 0 | 0 | 0 | 0 | 0 | 0 | 0 | 0 | 1 | 40 | 55 | | | PEAKS DB |
| K.SIQEDPAVKAEVSR.M | Y | 61.28 | 1527.7892 | 14 | 0.9 | 510.2708 | 3 | 36.48 | 16 | F16:13815 | BuCaTN01_F13.raw | | | | | | | | | | | | | | | | 1.2835E6 | 1 | 0 | 0 | 0 | 0 | 0 | 0 | 0 | 0 | 0 | 0 | 0 | 0 | 0 | 0 | 0 | 1 | 327 | 340 | | | PEAKS DB |
| N.ISGYILPYKIINVGSEK.V | N | 59.75 | 1893.0610 | 17 | -0.2 | 632.0275 | 3 | 71.29 | 16 | F16:37082 | BuCaTN01_F13.raw | | | | | | | | | | | | | | | | 4.9796E5 | 1 | 0 | 0 | 0 | 0 | 0 | 0 | 0 | 0 | 0 | 0 | 0 | 0 | 0 | 0 | 0 | 1 | 168 | 184 | | | PEAKS DB |
| R.VVSLNVLC(+57.02)TEC(+57.02)R.V | N | 59.19 | 1448.7115 | 12 | 2.9 | 725.3630 | 2 | 59.59 | 8 | F8:35185 | BuCaTN01_F7b.raw | | | | | | | | 4.7661E5 | | | | | | | | | 1 | 0 | 0 | 0 | 0 | 0 | 0 | 0 | 1 | 0 | 0 | 0 | 0 | 0 | 0 | 0 | 0 | 484 | 495 | Carbamidomethylation | C8:Carbamidomethylation:1000.00;C11:Carbamidomethylation:1000.00 | PEAKS DB |
| K.NVKFPILSANIRPK.G | N | 59.13 | 1595.9510 | 14 | 1.2 | 532.9916 | 3 | 58.57 | 16 | F16:28436 | BuCaTN01_F13.raw | | | | | | | | | | | | | | | | 3.5508E5 | 1 | 0 | 0 | 0 | 0 | 0 | 0 | 0 | 0 | 0 | 0 | 0 | 0 | 0 | 0 | 0 | 1 | 148 | 161 | | | PEAKS DB |
| R.QVPVVQAYAFGK.Y | N | 57.72 | 1305.7081 | 12 | 0.0 | 653.8613 | 2 | 58.36 | 16 | F16:28377 | BuCaTN01_F13.raw | | | | | | | | | | | | | | | | 2.6708E5 | 1 | 0 | 0 | 0 | 0 | 0 | 0 | 0 | 0 | 0 | 0 | 0 | 0 | 0 | 0 | 0 | 1 | 288 | 299 | | | PEAKS DB |
| K.FPILSANIRPK.G | N | 55.81 | 1254.7448 | 11 | 0.4 | 419.2557 | 3 | 55.55 | 16 | F16:26305 | BuCaTN01_F13.raw | | | | | | | | | | | | | | | | 4.8037E5 | 1 | 0 | 0 | 0 | 0 | 0 | 0 | 0 | 0 | 0 | 0 | 0 | 0 | 0 | 0 | 0 | 1 | 151 | 161 | | | PEAKS DB |
| K.LTILHTNDVHAR.V | N | 52.16 | 1388.7524 | 12 | -1.7 | 463.9240 | 3 | 37.10 | 16 | F16:14164 | BuCaTN01_F13.raw | | | | | | | | | | | | | | | | 0 | 0 | 0 | 0 | 0 | 0 | 0 | 0 | 0 | 0 | 0 | 0 | 0 | 0 | 0 | 0 | 0 | 0 | 44 | 55 | | | PEAKS DB |
| N.ISGYILPYK.I | N | 50.21 | 1052.5906 | 9 | 0.3 | 527.3027 | 2 | 57.19 | 16 | F16:27479 | BuCaTN01_F13.raw | | | | | | | | | | | | | | | | 5.5906E5 | 1 | 0 | 0 | 0 | 0 | 0 | 0 | 0 | 0 | 0 | 0 | 0 | 0 | 0 | 0 | 0 | 1 | 168 | 176 | | | PEAKS DB |
| K.VFPAMEGR.V | N | 41.93 | 905.4429 | 8 | -0.1 | 453.7287 | 2 | 42.75 | 16 | F16:18519 | BuCaTN01_F13.raw | | | | | | | | | | | | | | | | 4.3727E5 | 1 | 0 | 0 | 0 | 0 | 0 | 0 | 0 | 0 | 0 | 0 | 0 | 0 | 0 | 0 | 0 | 1 | 553 | 560 | | | PEAKS DB |
| R.MGKVFPAM(+15.99)EGR.V | N | 41.68 | 1237.5947 | 11 | 0.8 | 413.5392 | 3 | 33.99 | 16 | F16:12305 | BuCaTN01_F13.raw | | | | | | | | | | | | | | | | 9.9954E4 | 1 | 0 | 0 | 0 | 0 | 0 | 0 | 0 | 0 | 0 | 0 | 0 | 0 | 0 | 0 | 0 | 1 | 550 | 560 | Oxidation (M) | M8:Oxidation (M):79.97 | PEAKS DB |

**total 12 peptides**

**Best Unique PSM (Scan F16:13815, m/z=510.2708, z=3, RT=36.48, ppm=0.9):**

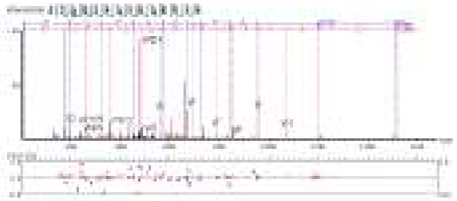

## JAS05038.1

back to list

| Protein Coverage | Supporting Peptides | Best Unique PSM |

**Protein Coverage:**

1 MPTSRRRSGA HGCPRSAPSA MWRLVGAVCF CAALSVAAA**G SFKLTILHTN DVHAR**VEQTS RDSGKCTRED CYGGVARRAT

81 KIREIRASHR NVLLLDAGDQ YQGTVWFNYF KGREVVHFMN SLRYDAMALG NHEFDNGLNG LLDPLLK**NVK FPILSANIRP**

161 **K**GPIASN**ISG YILPYKIINV GSEK**VGIIGY TTKETPVLSN PGPYLEFRDE VEELQKQADK LTTLGVNKII ALGHSGFKED

241 CRIAQKVKGV DVVVGGHTNT FLYTGSPPSN EVPAGNYPFM QLSDDGR**QVP VVQAYAFGK**Y LGYLNVIFDD KGKVIK**ASGN**

321 **PILLNKSIQE DPAVKAEVSR** MKVQLQKYSK QEIGKTIVYL NGTTHACRFH ECNLGNLICD AVVYNNLRHP DDNEWNHVSM

401 CIVNGGGIRS PIDEKANNGI ITLEELTAVL PFGGTFDLLQ IKGSALRQAF EHSVHRHGQG TGELLQVSGI KVLYDLSQKP

481 GKR**VVSLNVL CTECR**VPIYV PLQMEKTYKV LLPSFLAAGG DGYYMLKGDS SNHSSGDLDI SIVSDYIKR**M GKVFPAMEGR**

561 VMFSAGTLFQ AHHS

C Carbamidomethylation (+57.02)
O Oxidation (M) (+15.99)

491 C; 557 O

**Supporting Peptides:**

| Peptide | Uniq | -10lgP | Mass | Length | ppm | m/z | z | RT | Fraction | Scan | Source File | Area F1 | Area F2a | Area F3a | Area F4 | Area F5 | Area F6a | Area F7a | Area F7b | Area F8 | Area F9a | Area Sample 1F10a | Area F10b | Area F11a | Area F11b | Area F12a | Area F13 | #Feature | #Feature F1 | #Feature F2a | #Feature F3a | #Feature F4 | #Feature F5 | #Feature F6a | #Feature F7a | #Feature F7b | #Feature F8 | #Feature F9a | #Feature Sample 1F10a | #Feature F10b | #Feature F11a | #Feature F11b | #Feature F12a | #Feature F13 | Start | End | PTM | AScore | Found By |
|---|---|---|---|---|---|---|---|---|---|---|---|---|---|---|---|---|---|---|---|---|---|---|---|---|---|---|---|---|---|---|---|---|---|---|---|---|---|---|---|---|---|---|---|---|---|---|---|---|---|
| K.ASGNPILLNK.S | N | 71.96 | 1025.5869 | 10 | -0.1 | 513.8007 | 2 | 40.65 | 16 | F16:17022 | BuCaTN01_F13.raw | | | | | | | | | | | | | | | | 2.2075E6 | 1 | 0 | 0 | 0 | 0 | 0 | 0 | 0 | 0 | 0 | 0 | 0 | 0 | 0 | 0 | 0 | 1 | 317 | 326 | | | PEAKS DB |
| A.GSFKLTILHTNDVHAR.V | N | 68.40 | 1807.9692 | 16 | -1.1 | 452.9991 | 4 | 50.67 | 16 | F16:23710 | BuCaTN01_F13.raw | | | | | | | | | | | | | | | | 1.0157E6 | 1 | 0 | 0 | 0 | 0 | 0 | 0 | 0 | 0 | 0 | 0 | 0 | 0 | 0 | 0 | 0 | 1 | 40 | 55 | | | PEAKS DB |
| K.SIQEDPAVKAEVSR.M | Y | 61.28 | 1527.7892 | 14 | 0.9 | 510.2708 | 3 | 36.48 | 16 | F16:13815 | BuCaTN01_F13.raw | | | | | | | | | | | | | | | | 1.2835E6 | 1 | 0 | 0 | 0 | 0 | 0 | 0 | 0 | 0 | 0 | 0 | 0 | 0 | 0 | 0 | 0 | 1 | 327 | 340 | | | PEAKS DB |
| N.ISGYILPYKIINVGSEK.V | N | 59.75 | 1893.0610 | 17 | -0.2 | 632.0275 | 3 | 71.29 | 16 | F16:37082 | BuCaTN01_F13.raw | | | | | | | | | | | | | | | | 4.9796E5 | 1 | 0 | 0 | 0 | 0 | 0 | 0 | 0 | 0 | 0 | 0 | 0 | 0 | 0 | 0 | 0 | 1 | 168 | 184 | | | PEAKS DB |
| R.VVSLNVLC(+57.02)TEC(+57.02)R.V | N | 59.19 | 1448.7115 | 12 | 2.9 | 725.3630 | 2 | 59.59 | 8 | F8:35185 | BuCaTN01_F7b.raw | | | | | | | | 4.7661E5 | | | | | | | | | 1 | 0 | 0 | 0 | 0 | 0 | 0 | 0 | 1 | 0 | 0 | 0 | 0 | 0 | 0 | 0 | 0 | 484 | 495 | Carbamidomethylation | C8:Carbamidomethylation:1000.00;C11:Carbamidomethylation:1000.00 | PEAKS DB |
| K.NVKFPILSANIRPK.G | N | 59.13 | 1595.9510 | 14 | 1.2 | 532.9916 | 3 | 58.57 | 16 | F16:28436 | BuCaTN01_F13.raw | | | | | | | | | | | | | | | | 3.5508E5 | 1 | 0 | 0 | 0 | 0 | 0 | 0 | 0 | 0 | 0 | 0 | 0 | 0 | 0 | 0 | 0 | 1 | 148 | 161 | | | PEAKS DB |
| R.QVPVVQAYAFGK.Y | N | 57.72 | 1305.7081 | 12 | 0.0 | 653.8613 | 2 | 58.36 | 16 | F16:28377 | BuCaTN01_F13.raw | | | | | | | | | | | | | | | | 2.6708E5 | 1 | 0 | 0 | 0 | 0 | 0 | 0 | 0 | 0 | 0 | 0 | 0 | 0 | 0 | 0 | 0 | 1 | 288 | 299 | | | PEAKS DB |
| K.FPILSANIRPK.G | N | 55.81 | 1254.7448 | 11 | 0.4 | 419.2557 | 3 | 55.55 | 16 | F16:26305 | BuCaTN01_F13.raw | | | | | | | | | | | | | | | | 4.8037E5 | 1 | 0 | 0 | 0 | 0 | 0 | 0 | 0 | 0 | 0 | 0 | 0 | 0 | 0 | 0 | 0 | 1 | 151 | 161 | | | PEAKS DB |
| K.LTILHTNDVHAR.V | N | 52.16 | 1388.7524 | 12 | -1.7 | 463.9240 | 3 | 37.10 | 16 | F16:14164 | BuCaTN01_F13.raw | | | | | | | | | | | | | | | | 0 | 0 | 0 | 0 | 0 | 0 | 0 | 0 | 0 | 0 | 0 | 0 | 0 | 0 | 0 | 0 | 0 | 0 | 44 | 55 | | | PEAKS DB |
| N.ISGYILPYK.I | N | 50.21 | 1052.5906 | 9 | 0.3 | 527.3027 | 2 | 57.19 | 16 | F16:27479 | BuCaTN01_F13.raw | | | | | | | | | | | | | | | | 5.5906E5 | 1 | 0 | 0 | 0 | 0 | 0 | 0 | 0 | 0 | 0 | 0 | 0 | 0 | 0 | 0 | 0 | 1 | 168 | 176 | | | PEAKS DB |
| K.VFPAMEGR.V | N | 41.93 | 905.4429 | 8 | -0.1 | 453.7287 | 2 | 42.75 | 16 | F16:18519 | BuCaTN01_F13.raw | | | | | | | | | | | | | | | | 4.3727E5 | 1 | 0 | 0 | 0 | 0 | 0 | 0 | 0 | 0 | 0 | 0 | 0 | 0 | 0 | 0 | 0 | 1 | 553 | 560 | | | PEAKS DB |
| R.MGKVFPAM(+15.99)EGR.V | N | 41.68 | 1237.5947 | 11 | 0.8 | 413.5392 | 3 | 33.99 | 16 | F16:12305 | BuCaTN01_F13.raw | | | | | | | | | | | | | | | | 9.9954E4 | 1 | 0 | 0 | 0 | 0 | 0 | 0 | 0 | 0 | 0 | 0 | 0 | 0 | 0 | 0 | 0 | 1 | 550 | 560 | Oxidation (M) | M8:Oxidation (M):79.97 | PEAKS DB |

**total 12 peptides**

**Best Unique PSM (Scan F16:13815, m/z=510.2708, z=3, RT=36.48, ppm=0.9):**

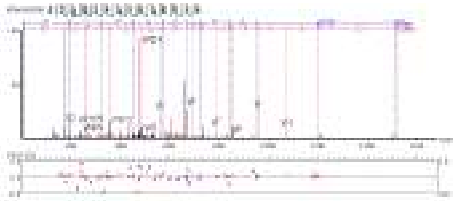

## JAS05036.1

back to list

| Protein Coverage | Supporting Peptides | Best Unique PSM |

**Protein Coverage:**

1 MPTSRRRSGA HGCPRSAPSA MWRLVGAVCF CAALSVAAA**G SFKLTILHTN DVHAR**VEQTS RDSGKCTRED CYGGVARRAT

C Carbamidomethylation (+57.02)
O Oxidation (M) (+15.99)

81 KIREIRASHR NVLLLDAGDQ YQGTVWFNYF KGREVVHFMN SLRYDAMALG NHEFDNGLNG LLDPLLK**NVK FPILSANIRP**

161 **K**GPIASN**ISG YILPYKIINV GSEK**VGIIGY TTKETPVLSN PGPYLEFRDE VEELQKQADK LTTLGVNKII ALGHSGFKED

241 CRIAQKVKGV DVVVGGHTNT FLYTGSPPSN EVPAGNYPFM QLSDDGR**QVP VVQAYAFGK**Y LGYLNVIFDD KGKVIK**ASGN**

321 **PILLNKSIQE DPAVKAEVSR** MKVQLQKYSK QEIGKTIVYL NGTTHACRFH ECNLGNLICD AVVYNNLRHP DDNEWNHVSM

401 CIVNGGGIRS PIDEKANNGI ITLEELTAVL PFGGTFDLLQ IKGSALRQAF EHSVHRHGQG TGELLQVSGI KVLYDLSQKP

491 557
481 GKR**VVSLNVL CTECR**VPAYV PLQMEKTYKV LLPSFLAAGG DGYYMLKGDS SNHSSGDLDI SIVSDYIKR**M GKVFPAMEGR**

561 VMFSAGTLFQ AHHS

**Supporting Peptides:**

| Peptide | Uniq | -10lgP | Mass | Length | ppm | m/z | z | RT | Fraction | Scan | Source File | Area F1 | Area F2a | Area F3a | Area F4 | Area F5 | Area F6a | Area F7a | Area F7b | Area F8 | Area F9a | Area Sample 1F10a | Area F10b | Area F11a | Area F11b | Area F12a | Area F13 | #Feature | #Feature F1 | #Feature F2a | #Feature F3a | #Feature F4 | #Feature F5 | #Feature F6a | #Feature F7a | #Feature F7b | #Feature F8 | #Feature F9a | #Feature Sample 1F10a | #Feature F10b | #Feature F11a | #Feature F11b | #Feature F12a | #Feature F13 | Start | End | PTM | AScore | Found By |
|---|---|---|---|---|---|---|---|---|---|---|---|---|---|---|---|---|---|---|---|---|---|---|---|---|---|---|---|---|---|---|---|---|---|---|---|---|---|---|---|---|---|---|---|---|---|---|---|---|---|
| K.ASGNPILLNK.S | N | 71.96 | 1025.5869 | 10 | -0.1 | 513.8007 | 2 | 40.65 | 16 | F16:17022 | BuCaTN01_F13.raw | | | | | | | | | | | | | | | | 2.2075E6 | 1 | 0 | 0 | 0 | 0 | 0 | 0 | 0 | 0 | 0 | 0 | 0 | 0 | 0 | 0 | 0 | 1 | 317 | 326 | | | PEAKS DB |
| A.GSFKLTILHTNDVHAR.V | N | 68.40 | 1807.9692 | 16 | -1.1 | 452.9991 | 4 | 50.67 | 16 | F16:23710 | BuCaTN01_F13.raw | | | | | | | | | | | | | | | | 1.0157E6 | 1 | 0 | 0 | 0 | 0 | 0 | 0 | 0 | 0 | 0 | 0 | 0 | 0 | 0 | 0 | 0 | 1 | 40 | 55 | | | PEAKS DB |
| K.SIQEDPAVKAEVSR.M | Y | 61.28 | 1527.7892 | 14 | 0.9 | 510.2708 | 3 | 36.48 | 16 | F16:13815 | BuCaTN01_F13.raw | | | | | | | | | | | | | | | | 1.2835E6 | 1 | 0 | 0 | 0 | 0 | 0 | 0 | 0 | 0 | 0 | 0 | 0 | 0 | 0 | 0 | 0 | 1 | 327 | 340 | | | PEAKS DB |
| N.ISGYILPYKIINVGSEK.V | N | 59.75 | 1893.0610 | 17 | -0.2 | 632.0275 | 3 | 71.29 | 16 | F16:37082 | BuCaTN01_F13.raw | | | | | | | | | | | | | | | | 4.9796E5 | 1 | 0 | 0 | 0 | 0 | 0 | 0 | 0 | 0 | 0 | 0 | 0 | 0 | 0 | 0 | 0 | 1 | 168 | 184 | | | PEAKS DB |
| R.VVSLNVLC(+57.02)TEC(+57.02)R.V | N | 59.19 | 1448.7115 | 12 | 2.9 | 725.3630 | 2 | 59.59 | 8 | F8:35185 | BuCaTN01_F7b.raw | | | | | | | | 4.7661E5 | | | | | | | | | 1 | 0 | 0 | 0 | 0 | 0 | 0 | 0 | 1 | 0 | 0 | 0 | 0 | 0 | 0 | 0 | 0 | 484 | 495 | Carbamidomethylation | C8:Carbamidomethylation:1000.00;C11:Carbamidomethylation:1000.00 | PEAKS DB |
| K.NVKFPILSANIRPK.G | N | 59.13 | 1595.9510 | 14 | 1.2 | 532.9916 | 3 | 58.57 | 16 | F16:28436 | BuCaTN01_F13.raw | | | | | | | | | | | | | | | | 3.5508E5 | 1 | 0 | 0 | 0 | 0 | 0 | 0 | 0 | 0 | 0 | 0 | 0 | 0 | 0 | 0 | 0 | 1 | 148 | 161 | | | PEAKS DB |
| R.QVPVVQAYAFGK.Y | N | 57.72 | 1305.7081 | 12 | 0.0 | 653.8613 | 2 | 58.36 | 16 | F16:28377 | BuCaTN01_F13.raw | | | | | | | | | | | | | | | | 2.6708E5 | 1 | 0 | 0 | 0 | 0 | 0 | 0 | 0 | 0 | 0 | 0 | 0 | 0 | 0 | 0 | 0 | 1 | 288 | 299 | | | PEAKS DB |
| K.FPILSANIRPK.G | N | 55.81 | 1254.7448 | 11 | 0.4 | 419.2557 | 3 | 55.55 | 16 | F16:26305 | BuCaTN01_F13.raw | | | | | | | | | | | | | | | | 4.8037E5 | 1 | 0 | 0 | 0 | 0 | 0 | 0 | 0 | 0 | 0 | 0 | 0 | 0 | 0 | 0 | 0 | 1 | 151 | 161 | | | PEAKS DB |
| K.LTILHTNDVHAR.V | N | 52.16 | 1388.7524 | 12 | -1.7 | 463.9240 | 3 | 37.10 | 16 | F16:14164 | BuCaTN01_F13.raw | | | | | | | | | | | | | | | | 0 | 0 | 0 | 0 | 0 | 0 | 0 | 0 | 0 | 0 | 0 | 0 | 0 | 0 | 0 | 0 | 0 | 0 | 44 | 55 | | | PEAKS DB |
| N.ISGYILPYK.I | N | 50.21 | 1052.5906 | 9 | 0.3 | 527.3027 | 2 | 57.19 | 16 | F16:27479 | BuCaTN01_F13.raw | | | | | | | | | | | | | | | | 5.5906E5 | 1 | 0 | 0 | 0 | 0 | 0 | 0 | 0 | 0 | 0 | 0 | 0 | 0 | 0 | 0 | 0 | 1 | 168 | 176 | | | PEAKS DB |
| K.VFPAMEGR.V | N | 41.93 | 905.4429 | 8 | -0.1 | 453.7287 | 2 | 42.75 | 16 | F16:18519 | BuCaTN01_F13.raw | | | | | | | | | | | | | | | | 4.3727E5 | 1 | 0 | 0 | 0 | 0 | 0 | 0 | 0 | 0 | 0 | 0 | 0 | 0 | 0 | 0 | 0 | 1 | 553 | 560 | | | PEAKS DB |
| R.MGKVFPAM(+15.99)EGR.V | N | 41.68 | 1237.5947 | 11 | 0.8 | 413.5392 | 3 | 33.99 | 16 | F16:12305 | BuCaTN01_F13.raw | | | | | | | | | | | | | | | | 9.9954E4 | 1 | 0 | 0 | 0 | 0 | 0 | 0 | 0 | 0 | 0 | 0 | 0 | 0 | 0 | 0 | 0 | 1 | 550 | 560 | Oxidation (M) | M8:Oxidation (M):79.97 | PEAKS DB |
| **total 12 peptides** | | | | | | | | | | | | | | | | | | | | | | | | | | | | | | | | | | | | | | | | | | | | | | | | | |

**Best Unique PSM (Scan F16:13815, m/z=510.2708, z=3, RT=36.48, ppm=0.9):**

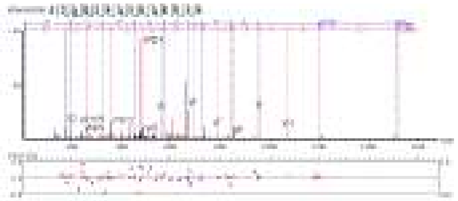

## JAS05037.1

back to list

| Protein Coverage | Supporting Peptides | Best Unique PSM |

**Protein Coverage:**

1 MPTSRRRSGA HGCPRSAPSA MWRLVGAVCF CAALSVAAA**G SFKLTILHTN DVHAR**VEQTS RDSGKCTRED CYGGVARRAT

C Carbamidomethylation (+57.02)
O Oxidation (M) (+15.99)

81 KIREIRASHR NVLLLDAGDQ YQGTVWFNYF KGREVVHFMN SLRYDAMALG NHEFDNGLNG LLDPLLK**NVK FPILSANIRP**

161 **K**GPIASN**ISG YILPYKIINV GSEK**VGIIGY TTKETPVLSN PGPYLEFRDE VEELQKQADK LTTLGVNKII ALGHSGFKED

241 CRIAQKVKGV DVVVGGHTNT FLYTGSPPSN EVPAGNYPFM QLSDDGR**QVP VVQAYAFGK**Y LGYLNVIFDD KGKVIK**ASGN**

321 **PILLNKSIQE DPAVKAEVSR** MKVQLQKYSK QEIGKTIVYL NGTTHACRFH ECNLGNLICD AVVYNNLRHP DDNEWNHVSM

401 CIVNGGGIRS PIDEKANNGI ITLEELTAVL PFGGTFDLLQ IKGSALRQAF EHSVHRHGQG TGELLQVSGI KVLYDLSQKP

491 557
481 GKR**VVSLNVL CTECR**VPIYV PLQMEKTYKV LLPSFLAAGG DGYYMLKGDS SNHSSGDLDI SIVSDYIKR**M GKVFPAMEGR**

561 VMFSAGTLFQ AHHS

**Supporting Peptides:**

| Peptide | Uniq | -10lgP | Mass | Length | ppm | m/z | z | RT | Fraction | Scan | Source File | Area F1 | Area F2a | Area F3a | Area F4 | Area F5 | Area F6a | Area F7a | Area F7b | Area F8 | Area F9a | Area Sample 1F10a | Area F10b | Area F11a | Area F11b | Area F12a | Area F13 | #Feature | #Feature F1 | #Feature F2a | #Feature F3a | #Feature F4 | #Feature F5 | #Feature F6a | #Feature F7a | #Feature F7b | #Feature F8 | #Feature F9a | #Feature Sample 1F10a | #Feature F10b | #Feature F11a | #Feature F11b | #Feature F12a | #Feature F13 | Start | End | PTM | AScore | Found By |
|---|---|---|---|---|---|---|---|---|---|---|---|---|---|---|---|---|---|---|---|---|---|---|---|---|---|---|---|---|---|---|---|---|---|---|---|---|---|---|---|---|---|---|---|---|---|---|---|---|---|
| K.ASGNPILLNK.S | N | 71.96 | 1025.5869 | 10 | -0.1 | 513.8007 | 2 | 40.65 | 16 | F16:17022 | BuCaTN01_F13.raw | | | | | | | | | | | | | | | | 2.2075E6 | 1 | 0 | 0 | 0 | 0 | 0 | 0 | 0 | 0 | 0 | 0 | 0 | 0 | 0 | 0 | 0 | 1 | 317 | 326 | | | PEAKS DB |
| A.GSFKLTILHTNDVHAR.V | N | 68.40 | 1807.9692 | 16 | -1.1 | 452.9991 | 4 | 50.67 | 16 | F16:23710 | BuCaTN01_F13.raw | | | | | | | | | | | | | | | | 1.0157E6 | 1 | 0 | 0 | 0 | 0 | 0 | 0 | 0 | 0 | 0 | 0 | 0 | 0 | 0 | 0 | 0 | 1 | 40 | 55 | | | PEAKS DB |
| K.SIQEDPAVKAEVSR.M | Y | 61.28 | 1527.7892 | 14 | 0.9 | 510.2708 | 3 | 36.48 | 16 | F16:13815 | BuCaTN01_F13.raw | | | | | | | | | | | | | | | | 1.2835E6 | 1 | 0 | 0 | 0 | 0 | 0 | 0 | 0 | 0 | 0 | 0 | 0 | 0 | 0 | 0 | 0 | 1 | 327 | 340 | | | PEAKS DB |
| N.ISGYILPYKIINVGSEK.V | N | 59.75 | 1893.0610 | 17 | -0.2 | 632.0275 | 3 | 71.29 | 16 | F16:37082 | BuCaTN01_F13.raw | | | | | | | | | | | | | | | | 4.9796E5 | 1 | 0 | 0 | 0 | 0 | 0 | 0 | 0 | 0 | 0 | 0 | 0 | 0 | 0 | 0 | 0 | 1 | 168 | 184 | | | PEAKS DB |
| R.VVSLNVLC(+57.02)TEC(+57.02)R.V | N | 59.19 | 1448.7115 | 12 | 2.9 | 725.3630 | 2 | 59.59 | 8 | F8:35185 | BuCaTN01_F7b.raw | | | | | | | | 4.7661E5 | | | | | | | | | 1 | 0 | 0 | 0 | 0 | 0 | 0 | 0 | 1 | 0 | 0 | 0 | 0 | 0 | 0 | 0 | 0 | 484 | 495 | Carbamidomethylation | C8:Carbamidomethylation:1000.00;C11:Carbamidomethylation:1000.00 | PEAKS DB |
| K.NVKFPILSANIRPK.G | N | 59.13 | 1595.9510 | 14 | 1.2 | 532.9916 | 3 | 58.57 | 16 | F16:28436 | BuCaTN01_F13.raw | | | | | | | | | | | | | | | | 3.5508E5 | 1 | 0 | 0 | 0 | 0 | 0 | 0 | 0 | 0 | 0 | 0 | 0 | 0 | 0 | 0 | 0 | 1 | 148 | 161 | | | PEAKS DB |
| R.QVPVVQAYAFGK.Y | N | 57.72 | 1305.7081 | 12 | 0.0 | 653.8613 | 2 | 58.36 | 16 | F16:28377 | BuCaTN01_F13.raw | | | | | | | | | | | | | | | | 2.6708E5 | 1 | 0 | 0 | 0 | 0 | 0 | 0 | 0 | 0 | 0 | 0 | 0 | 0 | 0 | 0 | 0 | 1 | 288 | 299 | | | PEAKS DB |
| K.FPILSANIRPK.G | N | 55.81 | 1254.7448 | 11 | 0.4 | 419.2557 | 3 | 55.55 | 16 | F16:26305 | BuCaTN01_F13.raw | | | | | | | | | | | | | | | | 4.8037E5 | 1 | 0 | 0 | 0 | 0 | 0 | 0 | 0 | 0 | 0 | 0 | 0 | 0 | 0 | 0 | 0 | 1 | 151 | 161 | | | PEAKS DB |
| K.LTILHTNDVHAR.V | N | 52.16 | 1388.7524 | 12 | -1.7 | 463.9240 | 3 | 37.10 | 16 | F16:14164 | BuCaTN01_F13.raw | | | | | | | | | | | | | | | | 0 | 0 | 0 | 0 | 0 | 0 | 0 | 0 | 0 | 0 | 0 | 0 | 0 | 0 | 0 | 0 | 0 | 0 | 44 | 55 | | | PEAKS DB |
| N.ISGYILPYK.I | N | 50.21 | 1052.5906 | 9 | 0.3 | 527.3027 | 2 | 57.19 | 16 | F16:27479 | BuCaTN01_F13.raw | | | | | | | | | | | | | | | | 5.5906E5 | 1 | 0 | 0 | 0 | 0 | 0 | 0 | 0 | 0 | 0 | 0 | 0 | 0 | 0 | 0 | 0 | 1 | 168 | 176 | | | PEAKS DB |
| K.VFPAMEGR.V | N | 41.93 | 905.4429 | 8 | -0.1 | 453.7287 | 2 | 42.75 | 16 | F16:18519 | BuCaTN01_F13.raw | | | | | | | | | | | | | | | | 4.3727E5 | 1 | 0 | 0 | 0 | 0 | 0 | 0 | 0 | 0 | 0 | 0 | 0 | 0 | 0 | 0 | 0 | 1 | 553 | 560 | | | PEAKS DB |
| R.MGKVFPAM(+15.99)EGR.V | N | 41.68 | 1237.5947 | 11 | 0.8 | 413.5392 | 3 | 33.99 | 16 | F16:12305 | BuCaTN01_F13.raw | | | | | | | | | | | | | | | | 9.9954E4 | 1 | 0 | 0 | 0 | 0 | 0 | 0 | 0 | 0 | 0 | 0 | 0 | 0 | 0 | 0 | 0 | 1 | 550 | 560 | Oxidation (M) | M8:Oxidation (M):79.97 | PEAKS DB |
| **total 12 peptides** | | | | | | | | | | | | | | | | | | | | | | | | | | | | | | | | | | | | | | | | | | | | | | | | | |

**Best Unique PSM (Scan F16:13815, m/z=510.2708, z=3, RT=36.48, ppm=0.9):**

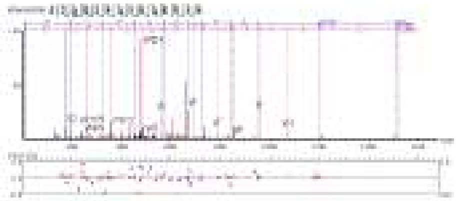

## JAI09049.1

back to list

| Protein Coverage | Supporting Peptides | Best Unique PSM |

**Protein Coverage:**

1 MPTSRRRSGA HGCPRSAPSA MWRLVGAVCF CAALSVAAA**G SFKLTILHTN DVHAR**VEQTS RDSGKCTRED CYGGVARRAT

Carbamidomethylation (+57.02)
Oxidation (M) (+15.99)

81 KIREIRASHR NVLLLDAGDQ YQGTVWFNYF KGREVVHFMN SLRYDAMALG NHEFDNGLNG LLDPLLK**NVK FPILSANIRP**

161 **K**GPIASN**ISG YILPYKIINV GSEK**VGIIGY TTKETPVLSN PGPYLEFRDE VEELQKQADK LTTLGVNKII ALGHSGFKED

241 CRIAQKVKGV DVVVGGHTNT FLYTGSPPSN EVPAGNYPFM QLSDDGR**QVP VVQAYAFGK**Y LGYLNVIFDD KGKVIK**ASGN**

321 **PILLNKSIQE DPAVKAEVSR** MKVQLQKYSK QEIGKTIVYL NGTTHACRFH ECNLGNLICD AVVYNNLRHP DDNEWNHVSM

401 CIVNGGGIRS PIDEKANNGI ITLEELTAVL PFGGTFDLLQ IKGSALRQAF EHSVHRHGQG TGELLQVSGI KVLYDLSQKP

491 557

481 GKR**VVSLNVL CTECR**VPIYV PLQMEKTYKV LLPSFLAAGG DGYYMLKGDS SNHSSGDLDI SIVSDYIKR**M GKVFPAMEGR**

561 VMFSAGTLFQ AHHS

**Supporting Peptides:**

| Peptide | Uniq | -10lgP | Mass | Length | ppm | m/z | z | RT | Fraction | Scan | Source File | Area F1 | Area F2a | Area F3a | Area F4 | Area F5 | Area F6a | Area F7a | Area F7b | Area F8 | Area F9a | Area Sample 1F10a | Area F10b | Area F11a | Area F11b | Area F12a | Area F13 | #Feature | #Feature F1 | #Feature F2a | #Feature F3a | #Feature F4 | #Feature F5 | #Feature F6a | #Feature F7a | #Feature F7b | #Feature F8 | #Feature F9a | #Feature Sample 1F10a | #Feature F10b | #Feature F11a | #Feature F11b | #Feature F12a | #Feature F13 | Start | End | PTM | AScore | Found By |
|---|---|---|---|---|---|---|---|---|---|---|---|---|---|---|---|---|---|---|---|---|---|---|---|---|---|---|---|---|---|---|---|---|---|---|---|---|---|---|---|---|---|---|---|---|---|---|---|---|---|
| K.ASGNPILLNK.S | N | 71.96 | 1025.5869 | 10 | -0.1 | 513.8007 | 2 | 40.65 | 16 | F16:17022 | BuCaTN01_F13.raw | | | | | | | | | | | | | | | | 2.2075E6 | 1 | 0 | 0 | 0 | 0 | 0 | 0 | 0 | 0 | 0 | 0 | 0 | 0 | 0 | 0 | 0 | 1 | 317 | 326 | | | PEAKS DB |
| A.GSFKLTILHTNDVHAR.V | N | 68.40 | 1807.9692 | 16 | -1.1 | 452.9991 | 4 | 50.67 | 16 | F16:23710 | BuCaTN01_F13.raw | | | | | | | | | | | | | | | | 1.0157E6 | 1 | 0 | 0 | 0 | 0 | 0 | 0 | 0 | 0 | 0 | 0 | 0 | 0 | 0 | 0 | 0 | 1 | 40 | 55 | | | PEAKS DB |
| K.SIQEDPAVKAEVSR.M | Y | 61.28 | 1527.7892 | 14 | 0.9 | 510.2708 | 3 | 36.48 | 16 | F16:13815 | BuCaTN01_F13.raw | | | | | | | | | | | | | | | | 1.2835E6 | 1 | 0 | 0 | 0 | 0 | 0 | 0 | 0 | 0 | 0 | 0 | 0 | 0 | 0 | 0 | 0 | 1 | 327 | 340 | | | PEAKS DB |
| N.ISGYILPYKIINVGSEK.V | N | 59.75 | 1893.0610 | 17 | -0.2 | 632.0275 | 3 | 71.29 | 16 | F16:37082 | BuCaTN01_F13.raw | | | | | | | | | | | | | | | | 4.9796E5 | 1 | 0 | 0 | 0 | 0 | 0 | 0 | 0 | 0 | 0 | 0 | 0 | 0 | 0 | 0 | 0 | 1 | 168 | 184 | | | PEAKS DB |
| R.VVSLNVLC(+57.02)TEC(+57.02)R.V | N | 59.19 | 1448.7115 | 12 | 2.9 | 725.3630 | 2 | 59.59 | 8 | F8:35185 | BuCaTN01_F7b.raw | | | | | | | | 4.7661E5 | | | | | | | | | 1 | 0 | 0 | 0 | 0 | 0 | 0 | 0 | 1 | 0 | 0 | 0 | 0 | 0 | 0 | 0 | 0 | 484 | 495 | Carbamidomethylation | C8:Carbamidomethylation:1000.00;C11:Carbamidomethylation:1000.00 | PEAKS DB |
| K.NVKFPILSANIRPK.G | N | 59.13 | 1595.9510 | 14 | 1.2 | 532.9916 | 3 | 58.57 | 16 | F16:28436 | BuCaTN01_F13.raw | | | | | | | | | | | | | | | | 3.5508E5 | 1 | 0 | 0 | 0 | 0 | 0 | 0 | 0 | 0 | 0 | 0 | 0 | 0 | 0 | 0 | 0 | 1 | 148 | 161 | | | PEAKS DB |
| R.QVPVVQAYAFGK.Y | N | 57.72 | 1305.7081 | 12 | 0.0 | 653.8613 | 2 | 58.36 | 16 | F16:28377 | BuCaTN01_F13.raw | | | | | | | | | | | | | | | | 2.6708E5 | 1 | 0 | 0 | 0 | 0 | 0 | 0 | 0 | 0 | 0 | 0 | 0 | 0 | 0 | 0 | 0 | 1 | 288 | 299 | | | PEAKS DB |
| K.FPILSANIRPK.G | N | 55.81 | 1254.7448 | 11 | 0.4 | 419.2557 | 3 | 55.55 | 16 | F16:26305 | BuCaTN01_F13.raw | | | | | | | | | | | | | | | | 4.8037E5 | 1 | 0 | 0 | 0 | 0 | 0 | 0 | 0 | 0 | 0 | 0 | 0 | 0 | 0 | 0 | 0 | 1 | 151 | 161 | | | PEAKS DB |
| K.LTILHTNDVHAR.V | N | 52.16 | 1388.7524 | 12 | -1.7 | 463.9240 | 3 | 37.10 | 16 | F16:14164 | BuCaTN01_F13.raw | | | | | | | | | | | | | | | | 0 | 0 | 0 | 0 | 0 | 0 | 0 | 0 | 0 | 0 | 0 | 0 | 0 | 0 | 0 | 0 | 0 | 0 | 44 | 55 | | | PEAKS DB |
| N.ISGYILPYK.I | N | 50.21 | 1052.5906 | 9 | 0.3 | 527.3027 | 2 | 57.19 | 16 | F16:27479 | BuCaTN01_F13.raw | | | | | | | | | | | | | | | | 5.5906E5 | 1 | 0 | 0 | 0 | 0 | 0 | 0 | 0 | 0 | 0 | 0 | 0 | 0 | 0 | 0 | 0 | 1 | 168 | 176 | | | PEAKS DB |
| K.VFPAMEGR.V | N | 41.93 | 905.4429 | 8 | -0.1 | 453.7287 | 2 | 42.75 | 16 | F16:18519 | BuCaTN01_F13.raw | | | | | | | | | | | | | | | | 4.3727E5 | 1 | 0 | 0 | 0 | 0 | 0 | 0 | 0 | 0 | 0 | 0 | 0 | 0 | 0 | 0 | 0 | 1 | 553 | 560 | | | PEAKS DB |
| R.MGKVFPAM(+15.99)EGR.V | N | 41.68 | 1237.5947 | 11 | 0.8 | 413.5392 | 3 | 33.99 | 16 | F16:12305 | BuCaTN01_F13.raw | | | | | | | | | | | | | | | | 9.9954E4 | 1 | 0 | 0 | 0 | 0 | 0 | 0 | 0 | 0 | 0 | 0 | 0 | 0 | 0 | 0 | 0 | 1 | 550 | 560 | Oxidation (M) | M8:Oxidation (M):79.97 | PEAKS DB |
| total 12 peptides | | | | | | | | | | | | | | | | | | | | | | | | | | | | | | | | | | | | | | | | | | | | | | | | | |

**Best Unique PSM (Scan F16:13815, m/z=510.2708, z=3, RT=36.48, ppm=0.9):**

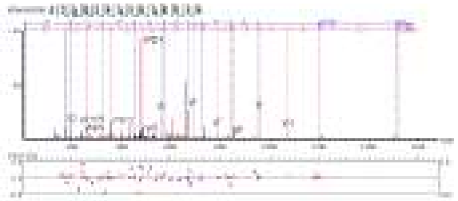

## CAA06887.1

back to list

| Protein Coverage | Supporting Peptides | Best Unique PSM |

**Protein Coverage:**

38 63 67 78 79

1 MKTLLLTLVV VTIICLDLGY TRKCLIK**YSQ ANESSKTCPS GQLLCLKKWE IGNPSGK**EVK **RGCVATCPKP KKNEIIQCCA**

Carbamidomethylation (+57.02)

81 **KDK**CNK

**Supporting Peptides:**

| Peptide | Uniq | -10lgP | Mass | Length | ppm | m/z | z | RT | Fraction | Scan | Source File | Area F1 | Area F2a | Area F3a | Area F4 | Area F5 | Area F6a | Area F7a | Area F7b | Area F8 | Area F9a | Area Sample 1F10a | Area F10b | Area F11a | Area F11b | Area F12a | Area F13 | #Feature | #Feature F1 | #Feature F2a | #Feature F3a | #Feature F4 | #Feature F5 | #Feature F6a | #Feature F7a | #Feature F7b | #Feature F8 | #Feature F9a | #Feature Sample 1F10a | #Feature F10b | #Feature F11a | #Feature F11b | #Feature F12a | #Feature F13 | Start | End | PTM | AScore | Found By |
|---|---|---|---|---|---|---|---|---|---|---|---|---|---|---|---|---|---|---|---|---|---|---|---|---|---|---|---|---|---|---|---|---|---|---|---|---|---|---|---|---|---|---|---|---|---|---|---|---|---|
| K.YSQANESSKTC(+57.02)PSGQLL.C | Y | 87.01 | 1868.8574 | 17 | 0.2 | 935.4362 | 2 | 44.07 | 2 | F2:20441 | BuCaTN01_F2a.raw | | 3.1931E6 | | 2.2557E5 | | | | | | | | | | | | | 2 | 0 | 1 | 0 | 1 | 0 | 0 | 0 | 0 | 0 | 0 | 0 | 0 | 0 | 0 | 0 | 0 | 28 | 44 | Carbamidomethylation | C11:Carbamidomethylation:1000.00 | PEAKS DB |
| K.KWEIGNPSGK.E | Y | 71.37 | 1114.5770 | 10 | 3.1 | 558.2964 | 2 | 79.97 | 3 | F3:58424 | BuCaTN01_F3a.raw | | 3.1577E8 | 1.9955E8 | 4.7388E6 | 2.7601E6 | 4.3415E6 | | 1.1039E7 | | 1.778E6 | | 1.1533E7 | | 4.477E6 | 2.6445E5 | | 51 | 0 | 19 | 10 | 2 | 2 | 2 | 0 | 5 | 0 | 2 | 0 | 5 | 0 | 3 | 1 | 0 | 48 | 57 | | | PEAKS DB |
| K.KNEIIQC(+57.02)C(+57.02)AK.D | Y | 58.97 | 1262.6111 | 10 | 0.7 | 632.3132 | 2 | 25.62 | 2 | F2:7701 | BuCaTN01_F2a.raw | | 1.5779E7 | | | | | | | | | | | | | | | 2 | 0 | 2 | 0 | 0 | 0 | 0 | 0 | 0 | 0 | 0 | 0 | 0 | 0 | 0 | 0 | 0 | 72 | 81 | Carbamidomethylation | C7:Carbamidomethylation:1000.00;C8:Carbamidomethylation:1000.00 | PEAKS DB |
| K.KNEIIQC(+57.02)C(+57.02)AKDK.C | Y | 58.02 | 1505.7330 | 12 | 0.2 | 502.9184 | 3 | 23.49 | 6 | F6:7056 | BuCaTN01_F6a.raw | | 4.8764E6 | 4.3875E5 | | | 1.6994E5 | | | | | | | | | | | 3 | 0 | 1 | 1 | 0 | 0 | 1 | 0 | 0 | 0 | 0 | 0 | 0 | 0 | 0 | 0 | 0 | 72 | 83 | Carbamidomethylation | C7:Carbamidomethylation:1000.00;C8:Carbamidomethylation:1000.00 | PEAKS DB |
| K.WEIGNPSGK.E | Y | 56.58 | 986.4821 | 9 | -1.0 | 494.2478 | 2 | 44.83 | 6 | F6:24053 | BuCaTN01_F6a.raw | | 4.7306E8 | 4.0587E7 | 6.5954E6 | 3.8246E6 | 1.6019E7 | 4.752E4 | 8.5129E6 | | 4.6662E6 | | 2.5934E6 | | 2.3146E6 | | | 14 | 0 | 5 | 1 | 1 | 1 | 1 | 1 | 1 | 0 | 1 | 0 | 1 | 0 | 1 | 0 | 0 | 49 | 57 | | | PEAKS DB |
| R.GC(+57.02)VATC(+57.02)PKPKKNEIIQC(+57.02)C(+57.02)AK.D | Y | 55.95 | 2361.1426 | 20 | 0.7 | 591.2933 | 4 | 30.17 | 2 | F2:10208 | BuCaTN01_F2a.raw | | 4.4703E6 | 4.5874E5 | | | | | 4.4615E5 | | | | | | | | | 4 | 0 | 2 | 1 | 0 | 0 | 0 | 0 | 1 | 0 | 0 | 0 | 0 | 0 | 0 | 0 | 0 | 62 | 81 | Carbamidomethylation | C2:Carbamidomethylation:1000.00;C6:Carbamidomethylation:1000.00;C17:Carbamidomethylation:1000.00;C18:Carbamidomethylation:1000.00 | PEAKS DB |
| K.TC(+57.02)PSGQLLC(+57.02)LKKWEIGNPSGK.E | Y | 46.52 | 2372.1980 | 21 | -0.9 | 594.0562 | 4 | 66.49 | 2 | F2:37798 | BuCaTN01_F2a.raw | | 1.2123E6 | | | | | | | | | | | | | | | 1 | 0 | 1 | 0 | 0 | 0 | 0 | 0 | 0 | 0 | 0 | 0 | 0 | 0 | 0 | 0 | 0 | 37 | 57 | Carbamidomethylation | C2:Carbamidomethylation:1000.00;C9:Carbamidomethylation:1000.00 | PEAKS DB |
| K.RGC(+57.02)VATC(+57.02)PKPK.K | Y | 45.87 | 1272.6431 | 11 | 1.4 | 425.2213 | 3 | 17.91 | 3 | F3:4361 | BuCaTN01_F3a.raw | | 3.0699E6 | 2.5951E5 | | | | | | | | | | | | | | 2 | 0 | 1 | 1 | 0 | 0 | 0 | 0 | 0 | 0 | 0 | 0 | 0 | 0 | 0 | 0 | 0 | 61 | 71 | Carbamidomethylation | C3:Carbamidomethylation:1000.00;C7:Carbamidomethylation:1000.00 | PEAKS DB |
| R.GC(+57.02)VATC(+57.02)PKPK.K | Y | 44.97 | 1116.5420 | 10 | -0.8 | 559.2778 | 2 | 18.98 | 2 | F2:4696 | BuCaTN01_F2a.raw | | 7.8636E5 | | | | | | | | | | | | | | | 1 | 0 | 1 | 0 | 0 | 0 | 0 | 0 | 0 | 0 | 0 | 0 | 0 | 0 | 0 | 0 | 0 | 62 | 71 | Carbamidomethylation | C2:Carbamidomethylation:1000.00;C6:Carbamidomethylation:1000.00 | PEAKS DB |
| K.TC(+57.02)PSGQLLC(+57.02)LKK.W | Y | 44.46 | 1403.7264 | 12 | -1.7 | 468.9153 | 3 | 49.32 | 2 | F2:23951 | BuCaTN01_F2a.raw | | 1.4116E6 | | | | | | | | | | | | | | | 1 | 0 | 1 | 0 | 0 | 0 | 0 | 0 | 0 | 0 | 0 | 0 | 0 | 0 | 0 | 0 | 0 | 37 | 48 | Carbamidomethylation | C2:Carbamidomethylation:1000.00;C9:Carbamidomethylation:1000.00 | PEAKS DB |
| total 10 peptides | | | | | | | | | | | | | | | | | | | | | | | | | | | | | | | | | | | | | | | | | | | | | | | | | |

**Best Unique PSM (Scan F2:20441, m/z=935.4362, z=2, RT=44.07, ppm=0.2):**

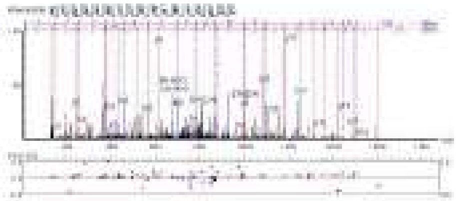

## Q9YGI8.1

back to list

| Protein Coverage | Supporting Peptides | Best Unique PSM |

**Protein Coverage:**

38 63 67 78 79

1 MKTLLLTLVV VTIICLDLGY TRKCLIK**YSQ ANESSKTCPS GQLLCLKKWE IGNPSGK**EVK **RGCVATCPKP KKNEIIQCCA**

Carbamidomethylation (+57.02)

81 **KDK**CNK

**Supporting Peptides:**

| Peptide | Uniq | -10lgP | Mass | Length | ppm | m/z | z | RT | Fraction | Scan | Source File | Area F1 | Area F2a | Area F3a | Area F4 | Area F5 | Area F6a | Area F7a | Area F7b | Area F8 | Area F9a | Area Sample 1F10a | Area F10b | Area F11a | Area F11b | Area F12a | Area F13 | #Feature | #Feature F1 | #Feature F2a | #Feature F3a | #Feature F4 | #Feature F5 | #Feature F6a | #Feature F7a | #Feature F7b | #Feature F8 | #Feature F9a | #Feature Sample 1F10a | #Feature F10b | #Feature F11a | #Feature F11b | #Feature F12a | #Feature F13 | Start | End | PTM | AScore | Found By |
|---|---|---|---|---|---|---|---|---|---|---|---|---|---|---|---|---|---|---|---|---|---|---|---|---|---|---|---|---|---|---|---|---|---|---|---|---|---|---|---|---|---|---|---|---|---|---|---|---|---|
| K.YSQANESSKTC(+57.02)PSGQLL.C | Y | 87.01 | 1868.8574 | 17 | 0.2 | 935.4362 | 2 | 44.07 | 2 | F2:20441 | BuCaTN01_F2a.raw | | 3.1931E6 | | 2.2557E5 | | | | | | | | | | | | | 2 | 0 | 1 | 0 | 1 | 0 | 0 | 0 | 0 | 0 | 0 | 0 | 0 | 0 | 0 | 0 | 0 | 28 | 44 | Carbamidomethylation | C11:Carbamidomethylation:1000.00 | PEAKS DB |
| | | | | | | | | | | | | | | | | | | | | | | | | | | | | | | | | | | | | | | | | | | | | | | | | | PEAKS |

| | | | | | | | | | | | | | | | | | | | | | | | | | | | | | | | | | | | | | | | | | | | | | | | | | |
|---|---|---|---|---|---|---|---|---|---|---|---|---|---|---|---|---|---|---|---|---|---|---|---|---|---|---|---|---|---|---|---|---|---|---|---|---|---|---|---|---|---|---|---|---|---|---|---|---|---|
| K.KWEIGNPSGK.E | Y | 71.37 | 1114.5770 | 10 | 3.1 | 558.2964 | 2 | 79.97 | 3 | F3:58424 | BuCaTN01_F3a.raw | | 3.1577E8 | 1.9955E8 | 4.7388E6 | 2.7601E6 | 4.3415E6 | | 1.1039E7 | | 1.778E6 | | 1.1533E7 | | 4.477E6 | 2.6445E5 | | 51 | 0 | 19 | 10 | 2 | 2 | 2 | 0 | 5 | 0 | 2 | 0 | 5 | 0 | 3 | 1 | 0 | 48 | 57 | | | DB |
| K.KNEIIQC(+57.02)C(+57.02)AK.D | Y | 58.97 | 1262.6111 | 10 | 0.7 | 632.3132 | 2 | 25.62 | 2 | F2:7701 | BuCaTN01_F2a.raw | | 1.5779E7 | | | | | | | | | | | | | | | 2 | 0 | 2 | 0 | 0 | 0 | 0 | 0 | 0 | 0 | 0 | 0 | 0 | 0 | 0 | 0 | 0 | 72 | 81 | Carbamidomethylation | C7:Carbamidomethylation:1000.00;C8:Carbamidomethylation:1000.00 | PEAKS DB |
| K.KNEIIQC(+57.02)C(+57.02)AKDK.C | Y | 58.02 | 1505.7330 | 12 | 0.2 | 502.9184 | 3 | 23.49 | 6 | F6:7056 | BuCaTN01_F6a.raw | | 4.8764E6 | 4.3875E5 | | | 1.6994E5 | | | | | | | | | | | 3 | 0 | 1 | 1 | 0 | 0 | 1 | 0 | 0 | 0 | 0 | 0 | 0 | 0 | 0 | 0 | 0 | 72 | 83 | Carbamidomethylation | C7:Carbamidomethylation:1000.00;C8:Carbamidomethylation:1000.00 | PEAKS DB |
| K.WEIGNPSGK.E | Y | 56.58 | 986.4821 | 9 | -1.0 | 494.2478 | 2 | 44.83 | 6 | F6:24053 | BuCaTN01_F6a.raw | | 4.7306E8 | 4.0587E7 | 6.5954E6 | 3.8246E6 | 1.6019E7 | 4.752E4 | 8.5129E6 | | 4.6662E6 | | 2.5934E6 | | 2.3146E6 | | | 14 | 0 | 5 | 1 | 1 | 1 | 1 | 1 | 1 | 0 | 1 | 0 | 1 | 0 | 1 | 0 | 0 | 49 | 57 | | | PEAKS DB |
| R.GC(+57.02)VATC(+57.02)PKPKKNEIIQC(+57.02)C(+57.02)AK.D | Y | 55.95 | 2361.1426 | 20 | 0.7 | 591.2933 | 4 | 30.17 | 2 | F2:10208 | BuCaTN01_F2a.raw | | 4.4703E6 | 4.5874E5 | | | | | 4.4615E5 | | | | | | | | | 4 | 0 | 2 | 1 | 0 | 0 | 0 | 0 | 1 | 0 | 0 | 0 | 0 | 0 | 0 | 0 | 0 | 62 | 81 | Carbamidomethylation | C2:Carbamidomethylation:1000.00;C6:Carbamidomethylation:1000.00;C17:Carbamidomethylation:1000.00;C18:Carbamidomethylation:1000.00 | PEAKS DB |
| K.TC(+57.02)PSGQLLC(+57.02)LKKWEIGNPSGK.E | Y | 46.52 | 2372.1980 | 21 | -0.9 | 594.0562 | 4 | 66.49 | 2 | F2:37798 | BuCaTN01_F2a.raw | | 1.2123E6 | | | | | | | | | | | | | | | 1 | 0 | 1 | 0 | 0 | 0 | 0 | 0 | 0 | 0 | 0 | 0 | 0 | 0 | 0 | 0 | 0 | 37 | 57 | Carbamidomethylation | C2:Carbamidomethylation:1000.00;C9:Carbamidomethylation:1000.00 | PEAKS DB |
| K.RGC(+57.02)VATC(+57.02)PKPK.K | Y | 45.87 | 1272.6431 | 11 | 1.4 | 425.2213 | 3 | 17.91 | 3 | F3:4361 | BuCaTN01_F3a.raw | | 3.0699E6 | 2.5951E5 | | | | | | | | | | | | | | 2 | 0 | 1 | 1 | 0 | 0 | 0 | 0 | 0 | 0 | 0 | 0 | 0 | 0 | 0 | 0 | 0 | 61 | 71 | Carbamidomethylation | C3:Carbamidomethylation:1000.00;C7:Carbamidomethylation:1000.00 | PEAKS DB |
| R.GC(+57.02)VATC(+57.02)PKPK.K | Y | 44.97 | 1116.5420 | 10 | -0.8 | 559.2778 | 2 | 18.98 | 2 | F2:4696 | BuCaTN01_F2a.raw | | 7.8636E5 | | | | | | | | | | | | | | | 1 | 0 | 1 | 0 | 0 | 0 | 0 | 0 | 0 | 0 | 0 | 0 | 0 | 0 | 0 | 0 | 0 | 62 | 71 | Carbamidomethylation | C2:Carbamidomethylation:1000.00;C6:Carbamidomethylation:1000.00 | PEAKS DB |
| K.TC(+57.02)PSGQLLC(+57.02)LKK.W | Y | 44.46 | 1403.7264 | 12 | -1.7 | 468.9153 | 3 | 49.32 | 2 | F2:23951 | BuCaTN01_F2a.raw | | 1.4116E6 | | | | | | | | | | | | | | | 1 | 0 | 1 | 0 | 0 | 0 | 0 | 0 | 0 | 0 | 0 | 0 | 0 | 0 | 0 | 0 | 0 | 37 | 48 | Carbamidomethylation | C2:Carbamidomethylation:1000.00;C9:Carbamidomethylation:1000.00 | PEAKS DB |
| total 10 peptides | | | | | | | | | | | | | | | | | | | | | | | | | | | | | | | | | | | | | | | | | | | | | | | | | |

**Best Unique PSM (Scan F2:20441, m/z=935.4362, z=2, RT=44.07, ppm=0.2):**

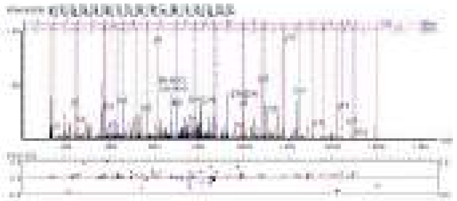

## Q9W729.1

back to list

| Protein Coverage | Supporting Peptides | Best Unique PSM |

**Protein Coverage:**

42 63 67

1 MKTLLLSLVV VTIVCLDLGY TRTCHISTSS TPQTCPKGQD ICFRKTQCDK FCSIRGAVIE QGCVATCPEF RSNYRSLLCC

C Carbamidomethylation (+57.02)

81 RTDNCNP

**Supporting Peptides:**

| Peptide | Uniq | -10lgP | Mass | Length | ppm | m/z | z | RT | Fraction | Scan | Source File | Area F1 | Area F2a | Area F3a | Area F4 | Area F5 | Area F6a | Area F7a | Area F7b | Area F8 | Area F9a | Area Sample 1F10a | Area F10b | Area F11a | Area F11b | Area F12a | Area F13 | #Feature | #Feature F1 | #Feature F2a | #Feature F3a | #Feature F4 | #Feature F5 | #Feature F6a | #Feature F7a | #Feature F7b | #Feature F8 | #Feature F9a | #Feature Sample 1F10a | #Feature F10b | #Feature F11a | #Feature F11b | #Feature F12a | #Feature F13 | Start | End | PTM | AScore | Found By |
|---|---|---|---|---|---|---|---|---|---|---|---|---|---|---|---|---|---|---|---|---|---|---|---|---|---|---|---|---|---|---|---|---|---|---|---|---|---|---|---|---|---|---|---|---|---|---|---|---|---|
| R.GAVIEQGC(+57.02)VATC(+57.02)PEFR.S | Y | 101.41 | 1792.8236 | 16 | 1.3 | 897.4203 | 2 | 51.79 | 6 | F6:30393 | BuCaTN01_F6a.raw | | | 2.4705E5 | | | 7.1182E6 | 9.3683E6 | 4.4703E8 | 5.9609E6 | 6.6479E6 | 1.7237E4 | 3.1182E6 | | 3.5463E6 | | | 20 | 0 | 0 | 2 | 0 | 0 | 2 | 3 | 3 | 3 | 2 | 1 | 2 | 0 | 2 | 0 | 0 | 56 | 71 | Carbamidomethylation | C8:Carbamidomethylation:1000.00;C12:Carbamidomethylation:1000.00 | PEAKS DB |
| A.VIEQGC(+57.02)VATC(+57.02)PEFR.S | Y | 73.41 | 1664.7650 | 14 | 3.3 | 833.3901 | 2 | 48.13 | 8 | F8:24812 | BuCaTN01_F7b.raw | | | | | | 1.9648E6 | | 5.0327E5 | | | | | | | | | 2 | 0 | 0 | 0 | 0 | 0 | 1 | 0 | 1 | 0 | 0 | 0 | 0 | 0 | 0 | 0 | 0 | 58 | 71 | Carbamidomethylation | C6:Carbamidomethylation:1000.00;C10:Carbamidomethylation:1000.00 | PEAKS DB |
| T.RTC(+57.02)HISTSSTPQTC(+57.02)PKGQDIC(+57.02)FR.K | Y | 58.96 | 2736.2529 | 23 | -9.8 | 913.0800 | 3 | 41.93 | 8 | F8:19468 | BuCaTN01_F7b.raw | | | | | | | | 1.0942E8 | | | | | | | | | 1 | 0 | 0 | 0 | 0 | 0 | 0 | 0 | 1 | 0 | 0 | 0 | 0 | 0 | 0 | 0 | 0 | 22 | 44 | Carbamidomethylation | C3:Carbamidomethylation:1000.00;C14:Carbamidomethylation:1000.00;C21:Carbamidomethylation:1000.00 | PEAKS DB |
| R.GAVIEQGC(+57.02)VATC(+57.02)PEFRSNYR.S | Y | 52.37 | 2313.0630 | 20 | 5.1 | 772.0299 | 3 | 50.84 | 8 | F8:27348 | BuCaTN01_F7b.raw | | | | | | | | 9.9421E7 | 0 | | | | | | | | 1 | 0 | 0 | 0 | 0 | 0 | 0 | 0 | 1 | 0 | 0 | 0 | 0 | 0 | 0 | 0 | 0 | 56 | 75 | Carbamidomethylation | C8:Carbamidomethylation:1000.00;C12:Carbamidomethylation:1000.00 | PEAKS DB |
| R.GAVIEQGC(+57.02)VAT.C | Y | 49.46 | 1103.5281 | 11 | 3.0 | 552.7714 | 2 | 40.25 | 8 | F8:18076 | BuCaTN01_F7b.raw | | | | | | | | 1.9556E6 | | | | | | | | | 1 | 0 | 0 | 0 | 0 | 0 | 0 | 0 | 1 | 0 | 0 | 0 | 0 | 0 | 0 | 0 | 0 | 56 | 66 | Carbamidomethylation | C8:Carbamidomethylation:1000.00 | PEAKS DB |
| Q.GC(+57.02)VATC(+57.02)PEFR.S | Y | 45.84 | 1195.5114 | 10 | 3.2 | 598.7631 | 2 | 39.55 | 8 | F8:17413 | BuCaTN01_F7b.raw | | | | | | | | 3.6105E5 | | | | | | | | | 1 | 0 | 0 | 0 | 0 | 0 | 0 | 0 | 1 | 0 | 0 | 0 | 0 | 0 | 0 | 0 | 0 | 62 | 71 | Carbamidomethylation | C2:Carbamidomethylation:1000.00;C6:Carbamidomethylation:1000.00 | PEAKS DB |
| total 6 peptides | | | | | | | | | | | | | | | | | | | | | | | | | | | | | | | | | | | | | | | | | | | | | | | | | |

**Best Unique PSM (Scan F6:30393, m/z=897.4203, z=2, RT=51.79, ppm=1.3):**

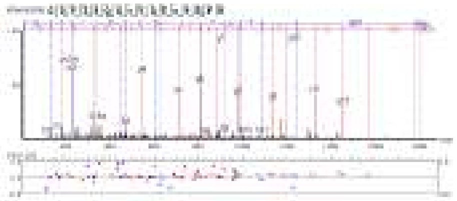

## CAB46659.1

back to list

| Protein Coverage | Supporting Peptides | Best Unique PSM |

**Protein Coverage:**

42 63 67

1 MKTLLLSLVV VTIVCLDLGY TRTCHISTSS TPQTCPKGQD ICFRKTQCDK FCSIRGAVIE QGCVATCPEF RSNYRSLLCC

C Carbamidomethylation (+57.02)

81 RTDNCNP

**Supporting Peptides:**

| Peptide | Uniq | -10lgP | Mass | Length | ppm | m/z | z | RT | Fraction | Scan | Source File | Area F1 | Area F2a | Area F3a | Area F4 | Area F5 | Area F6a | Area F7a | Area F7b | Area F8 | Area F9a | Area Sample 1F10a | Area F10b | Area F11a | Area F11b | Area F12a | Area F13 | #Feature | #Feature F1 | #Feature F2a | #Feature F3a | #Feature F4 | #Feature F5 | #Feature F6a | #Feature F7a | #Feature F7b | #Feature F8 | #Feature F9a | #Feature Sample 1F10a | #Feature F10b | #Feature F11a | #Feature F11b | #Feature F12a | #Feature F13 | Start | End | PTM | AScore | Found By |
|---|---|---|---|---|---|---|---|---|---|---|---|---|---|---|---|---|---|---|---|---|---|---|---|---|---|---|---|---|---|---|---|---|---|---|---|---|---|---|---|---|---|---|---|---|---|---|---|---|---|
| R.GAVIEQGC(+57.02)VATC(+57.02)PEFR.S | Y | 101.41 | 1792.8236 | 16 | 1.3 | 897.4203 | 2 | 51.79 | 6 | F6:30393 | BuCaTN01_F6a.raw | | | 2.4705E5 | | | 7.1182E6 | 9.3683E6 | 4.4703E8 | 5.9609E6 | 6.6479E6 | 1.7237E4 | 3.1182E6 | | 3.5463E6 | | | 20 | 0 | 0 | 2 | 0 | 0 | 2 | 3 | 3 | 3 | 2 | 1 | 2 | 0 | 2 | 0 | 0 | 56 | 71 | Carbamidomethylation | C8:Carbamidomethylation:1000.00;C12:Carbamidomethylation:1000.00 | PEAKS DB |
| A.VIEQGC(+57.02)VATC(+57.02)PEFR.S | Y | 73.41 | 1664.7650 | 14 | 3.3 | 833.3901 | 2 | 48.13 | 8 | F8:24812 | BuCaTN01_F7b.raw | | | | | | 1.9648E6 | | 5.0327E5 | | | | | | | | | 2 | 0 | 0 | 0 | 0 | 0 | 1 | 0 | 1 | 0 | 0 | 0 | 0 | 0 | 0 | 0 | 0 | 58 | 71 | Carbamidomethylation | C6:Carbamidomethylation:1000.00;C10:Carbamidomethylation:1000.00 | PEAKS DB |
| T.RTC(+57.02)HISTSSTPQTC(+57.02)PKGQDIC(+57.02)FR.K | Y | 58.96 | 2736.2529 | 23 | -9.8 | 913.0800 | 3 | 41.93 | 8 | F8:19468 | BuCaTN01_F7b.raw | | | | | | | | 1.0942E8 | | | | | | | | | 1 | 0 | 0 | 0 | 0 | 0 | 0 | 0 | 1 | 0 | 0 | 0 | 0 | 0 | 0 | 0 | 0 | 22 | 44 | Carbamidomethylation | C3:Carbamidomethylation:1000.00;C14:Carbamidomethylation:1000.00;C21:Carbamidomethylation:1000.00 | PEAKS DB |
| R.GAVIEQGC(+57.02)VATC(+57.02)PEFRSNYR.S | Y | 52.37 | 2313.0630 | 20 | 5.1 | 772.0299 | 3 | 50.84 | 8 | F8:27348 | BuCaTN01_F7b.raw | | | | | | | | 9.9421E7 | 0 | | | | | | | | 1 | 0 | 0 | 0 | 0 | 0 | 0 | 0 | 1 | 0 | 0 | 0 | 0 | 0 | 0 | 0 | 0 | 56 | 75 | Carbamidomethylation | C8:Carbamidomethylation:1000.00;C12:Carbamidomethylation:1000.00 | PEAKS DB |
| R.GAVIEQGC(+57.02)VAT.C | Y | 49.46 | 1103.5281 | 11 | 3.0 | 552.7714 | 2 | 40.25 | 8 | F8:18076 | BuCaTN01_F7b.raw | | | | | | | | 1.9556E6 | | | | | | | | | 1 | 0 | 0 | 0 | 0 | 0 | 0 | 0 | 1 | 0 | 0 | 0 | 0 | 0 | 0 | 0 | 0 | 56 | 66 | Carbamidomethylation | C8:Carbamidomethylation:1000.00 | PEAKS DB |
| Q.GC(+57.02)VATC(+57.02)PEFR.S | Y | 45.84 | 1195.5114 | 10 | 3.2 | 598.7631 | 2 | 39.55 | 8 | F8:17413 | BuCaTN01_F7b.raw | | | | | | | | 3.6105E5 | | | | | | | | | 1 | 0 | 0 | 0 | 0 | 0 | 0 | 0 | 1 | 0 | 0 | 0 | 0 | 0 | 0 | 0 | 0 | 62 | 71 | Carbamidomethylation | C2:Carbamidomethylation:1000.00;C6:Carbamidomethylation:1000.00 | PEAKS DB |
| total 6 peptides | | | | | | | | | | | | | | | | | | | | | | | | | | | | | | | | | | | | | | | | | | | | | | | | | |

**Best Unique PSM (Scan F6:30393, m/z=897.4203, z=2, RT=51.79, ppm=1.3):**

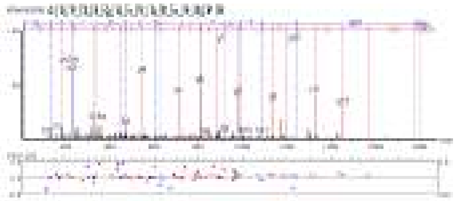

## 1POB

back to list

| Protein Coverage | Supporting Peptides | Best Unique PSM |

**Protein Coverage:**

1 NLYQFKNMIQ CTVPSRSWWD FADYGCYCGR GGSGTPVDDL DRCCQVHDNC YNEAEKISGC WPYFKTYSYE CSQGTLTCKG  Carbamidomethylation (+57.02)

81 GNNACAAAVC DCDRLAAICF AGAPYNDNDY NINLKARC

**Supporting Peptides:**

| Peptide | Uniq | -10lgP | Mass | Length | ppm | m/z | z | RT | Fraction | Scan | Source File | Area F1 | Area F2a | Area F3a | Area F4 | Area F5 | Area F6a | Area F7a | Area F7b | Area F8 | Area F9a | Area Sample 1F10a | Area F10b | Area F11a | Area F11b | Area F12a | Area F13 | #Feature | #Feature F1 | #Feature F2a | #Feature F3a | #Feature F4 | #Feature F5 | #Feature F6a | #Feature F7a | #Feature F7b | #Feature F8 | #Feature F9a | #Feature Sample 1F10a | #Feature F10b | #Feature F11a | #Feature F11b | #Feature F12a | #Feature F13 | Start | End | PTM | AScore | Found By |
|---|---|---|---|---|---|---|---|---|---|---|---|---|---|---|---|---|---|---|---|---|---|---|---|---|---|---|---|---|---|---|---|---|---|---|---|---|---|---|---|---|---|---|---|---|---|---|---|---|---|
| K.TYSYEC(+57.02)SQGTLTC(+57.02)K.G | N | 86.67 | 1696.7073 | 14 | 0.1 | 849.3610 | 2 | 41.09 | 10 | F10:18921 | BuCaTN01_F9a.raw | | | | | | | | | | 3.0796E6 | | | | | | | 1 | 0 | 0 | 0 | 0 | 0 | 0 | 0 | 0 | 0 | 1 | 0 | 0 | 0 | 0 | 0 | 0 | 66 | 79 | Carbamidomethylation | C6:Carbamidomethylation:1000.00;C13:Carbamidomethylation:1000.00 | PEAKS DB |
| R.GGSGTPVDDLDR.C | N | 69.16 | 1187.5417 | 12 | -1.2 | 594.7775 | 2 | 38.65 | 10 | F10:17034 | BuCaTN01_F9a.raw | | | | | | | | | | 5.2316E6 | | | | | | | 1 | 0 | 0 | 0 | 0 | 0 | 0 | 0 | 0 | 0 | 1 | 0 | 0 | 0 | 0 | 0 | 0 | 31 | 42 | | | PEAKS DB |
| R.LAAIC(+57.02)FAGAPYNDNDYNINLK.A | Y | 55.21 | 2356.1157 | 21 | 0.0 | 1179.0652 | 2 | 73.58 | 10 | F10:50390 | BuCaTN01_F9a.raw | | | | | | | | | | 5.8457E6 | | | | | | | 1 | 0 | 0 | 0 | 0 | 0 | 0 | 0 | 0 | 0 | 1 | 0 | 0 | 0 | 0 | 0 | 0 | 95 | 115 | Carbamidomethylation | C5:Carbamidomethylation:1000.00 | PEAKS DB |
| K.ISGC(+57.02)WPYFK.T | N | 53.17 | 1156.5375 | 9 | 1.1 | 579.2767 | 2 | 66.21 | 10 | F10:42322 | BuCaTN01_F9a.raw | | | | | | | | | | 3.4937E6 | | | | | | | 1 | 0 | 0 | 0 | 0 | 0 | 0 | 0 | 0 | 0 | 1 | 0 | 0 | 0 | 0 | 0 | 0 | 57 | 65 | Carbamidomethylation | C4:Carbamidomethylation:1000.00 | PEAKS DB |
| S.GTPVDDLDR.C | N | 51.39 | 986.4669 | 9 | 0.6 | 494.2410 | 2 | 37.62 | 7 | F7:14221 | BuCaTN01_F7a.raw | | | | | | | 4.0904E6 | | | | | | | | | | 1 | 0 | 0 | 0 | 0 | 0 | 0 | 1 | 0 | 0 | 0 | 0 | 0 | 0 | 0 | 0 | 0 | 34 | 42 | | | PEAKS DB |
| NLYQFKNMIQC(+57.02)TVPS.R | N | 50.68 | 1841.8804 | 15 | 0.8 | 921.9482 | 2 | 72.92 | 9 | F9:46618 | BuCaTN01_F8.raw | | | | | | | | | 1.0192E6 | | | | | | | | 1 | 0 | 0 | 0 | 0 | 0 | 0 | 0 | 0 | 1 | 0 | 0 | 0 | 0 | 0 | 0 | 0 | 1 | 15 | Carbamidomethylation | C11:Carbamidomethylation:1000.00 | PEAKS DB |
| G.TPVDDLDR.C | N | 48.70 | 929.4454 | 8 | -0.4 | 465.7298 | 2 | 34.76 | 7 | F7:11899 | BuCaTN01_F7a.raw | | | | | | | 6.3757E5 | | | | | | | | | | 1 | 0 | 0 | 0 | 0 | 0 | 0 | 1 | 0 | 0 | 0 | 0 | 0 | 0 | 0 | 0 | 0 | 35 | 42 | | | PEAKS DB |
| R.SWWDFADYGC(+57.02)YC(+57.02)GR.G | N | 46.98 | 1841.6926 | 14 | -0.7 | 921.8530 | 2 | 83.50 | 10 | F10:60456 | BuCaTN01_F9a.raw | | | | | | | | | | 0 | | | | | | | 0 | 0 | 0 | 0 | 0 | 0 | 0 | 0 | 0 | 0 | 0 | 0 | 0 | 0 | 0 | 0 | 0 | 17 | 30 | Carbamidomethylation | C10:Carbamidomethylation:1000.00;C12:Carbamidomethylation:1000.00 | PEAKS DB |
| total 8 peptides | | | | | | | | | | | | | | | | | | | | | | | | | | | | | | | | | | | | | | | | | | | | | | | | | |

**Best Unique PSM (Scan F10:50390, m/z=1179.0652, z=2, RT=73.58, ppm=0.0):**

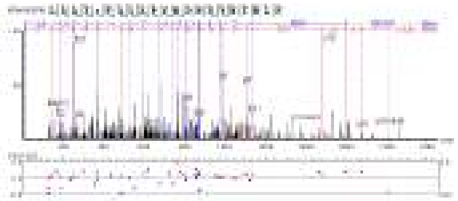

## 1POA

back to list

| Protein Coverage | Supporting Peptides | Best Unique PSM |

**Protein Coverage:**

1 NLYQFKNMIQ CTVPSRSWWD FADYGCYCGR GGSGTPVDDL DRCCQVHDNC YNEAEKISGC WPYFKTYSYE CSQGTLTCKG  Carbamidomethylation (+57.02)

81 GNNACAAAVC DCDRLAAICF AGAPYNDNDY NINLKARC

**Supporting Peptides:**

| Peptide | Uniq | -10lgP | Mass | Length | ppm | m/z | z | RT | Fraction | Scan | Source File | Area F1 | Area F2a | Area F3a | Area F4 | Area F5 | Area F6a | Area F7a | Area F7b | Area F8 | Area F9a | Area Sample 1F10a | Area F10b | Area F11a | Area F11b | Area F12a | Area F13 | #Feature | #Feature F1 | #Feature F2a | #Feature F3a | #Feature F4 | #Feature F5 | #Feature F6a | #Feature F7a | #Feature F7b | #Feature F8 | #Feature F9a | #Feature Sample 1F10a | #Feature F10b | #Feature F11a | #Feature F11b | #Feature F12a | #Feature F13 | Start | End | PTM | AScore | Found By |
|---|---|---|---|---|---|---|---|---|---|---|---|---|---|---|---|---|---|---|---|---|---|---|---|---|---|---|---|---|---|---|---|---|---|---|---|---|---|---|---|---|---|---|---|---|---|---|---|---|---|
| K.TYSYEC(+57.02)SQGTLTC(+57.02)K.G | N | 86.67 | 1696.7073 | 14 | 0.1 | 849.3610 | 2 | 41.09 | 10 | F10:18921 | BuCaTN01_F9a.raw | | | | | | | | | | 3.0796E6 | | | | | | | 1 | 0 | 0 | 0 | 0 | 0 | 0 | 0 | 0 | 0 | 1 | 0 | 0 | 0 | 0 | 0 | 0 | 66 | 79 | Carbamidomethylation | C6:Carbamidomethylation:1000.00;C13:Carbamidomethylation:1000.00 | PEAKS DB |
| R.GGSGTPVDDLDR.C | N | 69.16 | 1187.5417 | 12 | -1.2 | 594.7775 | 2 | 38.65 | 10 | F10:17034 | BuCaTN01_F9a.raw | | | | | | | | | | 5.2316E6 | | | | | | | 1 | 0 | 0 | 0 | 0 | 0 | 0 | 0 | 0 | 0 | 1 | 0 | 0 | 0 | 0 | 0 | 0 | 31 | 42 | | | PEAKS DB |
| R.LAAIC(+57.02)FAGAPYNDNDYNINLK.A | Y | 55.21 | 2356.1157 | 21 | 0.0 | 1179.0652 | 2 | 73.58 | 10 | F10:50390 | BuCaTN01_F9a.raw | | | | | | | | | | 5.8457E6 | | | | | | | 1 | 0 | 0 | 0 | 0 | 0 | 0 | 0 | 0 | 0 | 1 | 0 | 0 | 0 | 0 | 0 | 0 | 95 | 115 | Carbamidomethylation | C5:Carbamidomethylation:1000.00 | PEAKS DB |
| K.ISGC(+57.02)WPYFK.T | N | 53.17 | 1156.5375 | 9 | 1.1 | 579.2767 | 2 | 66.21 | 10 | F10:42322 | BuCaTN01_F9a.raw | | | | | | | | | | 3.4937E6 | | | | | | | 1 | 0 | 0 | 0 | 0 | 0 | 0 | 0 | 0 | 0 | 1 | 0 | 0 | 0 | 0 | 0 | 0 | 57 | 65 | Carbamidomethylation | C4:Carbamidomethylation:1000.00 | PEAKS DB |
| S.GTPVDDLDR.C | N | 51.39 | 986.4669 | 9 | 0.6 | 494.2410 | 2 | 37.62 | 7 | F7:14221 | BuCaTN01_F7a.raw | | | | | | | 4.0904E6 | | | | | | | | | | 1 | 0 | 0 | 0 | 0 | 0 | 0 | 1 | 0 | 0 | 0 | 0 | 0 | 0 | 0 | 0 | 0 | 34 | 42 | | | PEAKS DB |
| NLYQFKNMIQC(+57.02)TVPS.R | N | 50.68 | 1841.8804 | 15 | 0.8 | 921.9482 | 2 | 72.92 | 9 | F9:46618 | BuCaTN01_F8.raw | | | | | | | | | 1.0192E6 | | | | | | | | 1 | 0 | 0 | 0 | 0 | 0 | 0 | 0 | 0 | 1 | 0 | 0 | 0 | 0 | 0 | 0 | 0 | 1 | 15 | Carbamidomethylation | C11:Carbamidomethylation:1000.00 | PEAKS DB |
| G.TPVDDLDR.C | N | 48.70 | 929.4454 | 8 | -0.4 | 465.7298 | 2 | 34.76 | 7 | F7:11899 | BuCaTN01_F7a.raw | | | | | | | 6.3757E5 | | | | | | | | | | 1 | 0 | 0 | 0 | 0 | 0 | 0 | 1 | 0 | 0 | 0 | 0 | 0 | 0 | 0 | 0 | 0 | 35 | 42 | | | PEAKS DB |
| R.SWWDFADYGC(+57.02)YC(+57.02)GR.G | N | 46.98 | 1841.6926 | 14 | -0.7 | 921.8530 | 2 | 83.50 | 10 | F10:60456 | BuCaTN01_F9a.raw | | | | | | | | | | 0 | | | | | | | 0 | 0 | 0 | 0 | 0 | 0 | 0 | 0 | 0 | 0 | 0 | 0 | 0 | 0 | 0 | 0 | 0 | 17 | 30 | Carbamidomethylation | C10:Carbamidomethylation:1000.00;C12:Carbamidomethylation:1000.00 | PEAKS DB |
| total 8 peptides | | | | | | | | | | | | | | | | | | | | | | | | | | | | | | | | | | | | | | | | | | | | | | | | | |

**Best Unique PSM (Scan F10:50390, m/z=1179.0652, z=2, RT=73.58, ppm=0.0):**

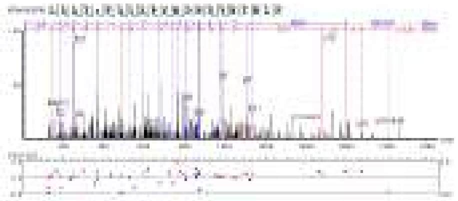

## CAA72434.1

back to list

| Protein Coverage | Supporting Peptides | Best Unique PSM |

**Protein Coverage:**

1 MKTLLLTLVV VTIVCLDLGY TRTCLISPSS TPQTCPNGQD ICFRKAQCDN FCHSRGPVIE QGCVATCPQF RSNYRSLLCC  Carbamidomethylation (+57.02)

81 RTDNCNH

**Supporting Peptides:**

| Peptide | Uniq | -10lgP | Mass | Length | ppm | m/z | z | RT | Fraction | Scan | Source File | Area F1 | Area F2a | Area F3a | Area F4 | Area F5 | Area F6a | Area F7a | Area F7b | Area F8 | Area F9a | Area Sample 1F10a | Area F10b | Area F11a | Area F11b | Area F12a | Area F13 | #Feature | #Feature F1 | #Feature F2a | #Feature F3a | #Feature F4 | #Feature F5 | #Feature F6a | #Feature F7a | #Feature F7b | #Feature F8 | #Feature F9a | #Feature Sample 1F10a | #Feature F10b | #Feature F11a | #Feature F11b | #Feature F12a | #Feature F13 | Start | End | PTM | AScore | Found By |
|---|---|---|---|---|---|---|---|---|---|---|---|---|---|---|---|---|---|---|---|---|---|---|---|---|---|---|---|---|---|---|---|---|---|---|---|---|---|---|---|---|---|---|---|---|---|---|---|---|---|
| R.GPVIEQGC(+57.02)VATC(+57.02)PQFR.S | N | 96.70 | 1817.8552 | 16 | 1.3 | 909.9360 | 2 | 51.36 | 6 | F6:30037 | BuCaTN01_F6a.raw | | | | | | 4.938E5 | | | | | | | | 0 | | | 1 | 0 | 0 | 0 | 0 | 0 | 1 | 0 | 0 | 0 | 0 | 0 | 0 | 0 | 0 | 0 | 0 | 56 | 71 | Carbamidomethylation | C8:Carbamidomethylation:1000.00;C12:Carbamidomethylation:1000.00 | PEAKS DB |
| P.VIEQGC(+57.02)VATC(+57.02)PQFR.S | N | 70.62 | 1663.7810 | 14 | 3.0 | 832.8978 | 2 | 46.31 | 8 | F8:22974 | BuCaTN01_F7b.raw | | | | | | | | 7.7386E6 | | | | | | | | | 1 | 0 | 0 | 0 | 0 | 0 | 0 | 0 | 1 | 0 | 0 | 0 | 0 | 0 | 0 | 0 | 0 | 58 | 71 | Carbamidomethylation | C6:Carbamidomethylation:1000.00;C10:Carbamidomethylation:1000.00 | PEAKS DB |
| R.GPVIEQGC(+57.02)VAT.C | N | 50.22 | 1129.5437 | 11 | 0.5 | 565.7794 | 2 | 41.92 | 6 | F6:21363 | BuCaTN01_F6a.raw | | | | | | 6.58E5 | | | | | | | | | | | 1 | 0 | 0 | 0 | 0 | 0 | 1 | 0 | 0 | 0 | 0 | 0 | 0 | 0 | 0 | 0 | 0 | 56 | 66 | Carbamidomethylation | C8:Carbamidomethylation:1000.00 | PEAKS DB |
| S.STPQTC(+57.02)PNGQDIC(+57.02)FR.K | Y | 48.47 | 1779.7668 | 15 | 3.1 | 890.8908 | 2 | 45.52 | 8 | F8:22181 | BuCaTN01_F7b.raw | | | | | | | | 8.9982E5 | | | | | | | | | 1 | 0 | 0 | 0 | 0 | 0 | 0 | 0 | 1 | 0 | 0 | 0 | 0 | 0 | 0 | 0 | 0 | 30 | 44 | Carbamidomethylation | C6:Carbamidomethylation:1000.00;C13:Carbamidomethylation:1000.00 | PEAKS DB |
| R.GPVIEQGC(+57.02)VA.T | N | 42.28 | 1028.4961 | 10 | 0.2 | 515.2554 | 2 | 41.42 | 6 | F6:20784 | BuCaTN01_F6a.raw | | | | | | 2.0497E6 | | | | | | | | | | | 1 | 0 | 0 | 0 | 0 | 0 | 1 | 0 | 0 | 0 | 0 | 0 | 0 | 0 | 0 | 0 | 0 | 56 | 65 | Carbamidomethylation | C8:Carbamidomethylation:1000.00 | PEAKS DB |
| V.IEQGC(+57.02)VATC(+57.02)PQFR.S | N | 41.92 | 1564.7126 | 13 | 1.4 | 783.3624 | 2 | 43.03 | 8 | F8:20158 | BuCaTN01_F7b.raw | | | | | | | | 1.5548E5 | | | | | | | | | 1 | 0 | 0 | 0 | 0 | 0 | 0 | 0 | 1 | 0 | 0 | 0 | 0 | 0 | 0 | 0 | 0 | 59 | 71 | Carbamidomethylation | C5:Carbamidomethylation:1000.00;C9:Carbamidomethylation:1000.00 | PEAKS DB |
| total 6 peptides | | | | | | | | | | | | | | | | | | | | | | | | | | | | | | | | | | | | | | | | | | | | | | | | | |

**Best Unique PSM (Scan F8:22181, m/z=890.8908, z=2, RT=45.52, ppm=3.1):**

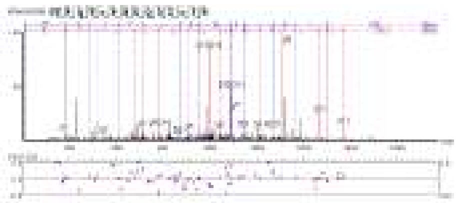

## CAA35775.1

back to list

| Protein Coverage | Supporting Peptides | Best Unique PSM |

**Protein Coverage:**

1 MKTLLLSLVV VTIVCLDLGY TRTCLISPSS TPQTCPNGQD ICFRKAQCDN FCHSRGPVIE QGCVATCPQF RSNYRSLLCC

81 RTDNCNH

C Carbamidomethylation (+57.02)

**Supporting Peptides:**

| Peptide | Uniq | -10lgP | Mass | Length | ppm | m/z | z | RT | Fraction | Scan | Source File | Area F1 | Area F2a | Area F3a | Area F4 | Area F5 | Area F6a | Area F7a | Area F7b | Area F8 | Area F9a | Area Sample 1F10a | Area F10b | Area F11a | Area F11b | Area F12a | Area F13 | #Feature | #Feature F1 | #Feature F2a | #Feature F3a | #Feature F4 | #Feature F5 | #Feature F6a | #Feature F7a | #Feature F7b | #Feature F8 | #Feature F9a | #Feature Sample 1F10a | #Feature F10b | #Feature F11a | #Feature F11b | #Feature F12a | #Feature F13 | Start | End | PTM | AScore | Found By |
|---|---|---|---|---|---|---|---|---|---|---|---|---|---|---|---|---|---|---|---|---|---|---|---|---|---|---|---|---|---|---|---|---|---|---|---|---|---|---|---|---|---|---|---|---|---|---|---|---|---|
| R.GPVIEQGC(+57.02)VATC(+57.02)PQFR.S | N | 96.70 | 1817.8552 | 16 | 1.3 | 909.9360 | 2 | 51.36 | 6 | F6:30037 | BuCaTN01_F6a.raw | | | | | | 4.938E5 | | | | | | | | 0 | | | 1 | 0 | 0 | 0 | 0 | 0 | 1 | 0 | 0 | 0 | 0 | 0 | 0 | 0 | 0 | 0 | 0 | 56 | 71 | Carbamidomethylation | C8:Carbamidomethylation:1000.00;C12:Carbamidomethylation:1000.00 | PEAKS DB |
| P.VIEQGC(+57.02)VATC(+57.02)PQFR.S | N | 70.62 | 1663.7810 | 14 | 3.0 | 832.8978 | 2 | 46.31 | 8 | F8:22974 | BuCaTN01_F7b.raw | | | | | | | | 7.7386E6 | | | | | | | | | 1 | 0 | 0 | 0 | 0 | 0 | 0 | 0 | 1 | 0 | 0 | 0 | 0 | 0 | 0 | 0 | 0 | 58 | 71 | Carbamidomethylation | C6:Carbamidomethylation:1000.00;C10:Carbamidomethylation:1000.00 | PEAKS DB |
| R.GPVIEQGC(+57.02)VAT.C | N | 50.22 | 1129.5437 | 11 | 0.5 | 565.7794 | 2 | 41.92 | 6 | F6:21363 | BuCaTN01_F6a.raw | | | | | | 6.58E5 | | | | | | | | | | | 1 | 0 | 0 | 0 | 0 | 0 | 1 | 0 | 0 | 0 | 0 | 0 | 0 | 0 | 0 | 0 | 0 | 56 | 66 | Carbamidomethylation | C8:Carbamidomethylation:1000.00 | PEAKS DB |
| S.STPQTC(+57.02)PNGQDIC(+57.02)FR.K | Y | 48.47 | 1779.7668 | 15 | 3.1 | 890.8908 | 2 | 45.52 | 8 | F8:22181 | BuCaTN01_F7b.raw | | | | | | | | 8.9982E5 | | | | | | | | | 1 | 0 | 0 | 0 | 0 | 0 | 0 | 0 | 1 | 0 | 0 | 0 | 0 | 0 | 0 | 0 | 0 | 30 | 44 | Carbamidomethylation | C6:Carbamidomethylation:1000.00;C13:Carbamidomethylation:1000.00 | PEAKS DB |
| R.GPVIEQGC(+57.02)VA.T | N | 42.28 | 1028.4961 | 10 | 0.2 | 515.2554 | 2 | 41.42 | 6 | F6:20784 | BuCaTN01_F6a.raw | | | | | | 2.0497E6 | | | | | | | | | | | 1 | 0 | 0 | 0 | 0 | 0 | 1 | 0 | 0 | 0 | 0 | 0 | 0 | 0 | 0 | 0 | 0 | 56 | 65 | Carbamidomethylation | C8:Carbamidomethylation:1000.00 | PEAKS DB |
| V.IEQGC(+57.02)VATC(+57.02)PQFR.S | N | 41.92 | 1564.7126 | 13 | 1.4 | 783.3624 | 2 | 43.03 | 8 | F8:20158 | BuCaTN01_F7b.raw | | | | | | | | 1.5548E5 | | | | | | | | | 1 | 0 | 0 | 0 | 0 | 0 | 0 | 0 | 1 | 0 | 0 | 0 | 0 | 0 | 0 | 0 | 0 | 59 | 71 | Carbamidomethylation | C5:Carbamidomethylation:1000.00;C9:Carbamidomethylation:1000.00 | PEAKS DB |
| total 6 peptides | | | | | | | | | | | | | | | | | | | | | | | | | | | | | | | | | | | | | | | | | | | | | | | | | |

**Best Unique PSM (Scan F8:22181, m/z=890.8908, z=2, RT=45.52, ppm=3.1):**

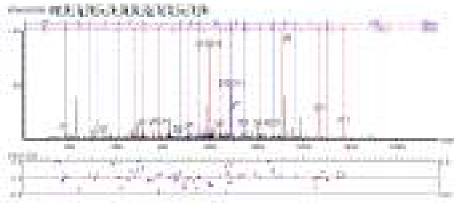

## P15817.1

back to list

| Protein Coverage | Supporting Peptides | Best Unique PSM |

**Protein Coverage:**

1 MKTLLLSLVV VTIVCLDLGY TRTCLISPSS TPQTCPNGQD ICFRKAQCDN FCHSRGPVIE QGCVATCPQF RSNYRSLLCC

81 RTDNCNH

C Carbamidomethylation (+57.02)

**Supporting Peptides:**

| Peptide | Uniq | -10lgP | Mass | Length | ppm | m/z | z | RT | Fraction | Scan | Source File | Area F1 | Area F2a | Area F3a | Area F4 | Area F5 | Area F6a | Area F7a | Area F7b | Area F8 | Area F9a | Area Sample 1F10a | Area F10b | Area F11a | Area F11b | Area F12a | Area F13 | #Feature | #Feature F1 | #Feature F2a | #Feature F3a | #Feature F4 | #Feature F5 | #Feature F6a | #Feature F7a | #Feature F7b | #Feature F8 | #Feature F9a | #Feature Sample 1F10a | #Feature F10b | #Feature F11a | #Feature F11b | #Feature F12a | #Feature F13 | Start | End | PTM | AScore | Found By |
|---|---|---|---|---|---|---|---|---|---|---|---|---|---|---|---|---|---|---|---|---|---|---|---|---|---|---|---|---|---|---|---|---|---|---|---|---|---|---|---|---|---|---|---|---|---|---|---|---|---|
| R.GPVIEQGC(+57.02)VATC(+57.02)PQFR.S | N | 96.70 | 1817.8552 | 16 | 1.3 | 909.9360 | 2 | 51.36 | 6 | F6:30037 | BuCaTN01_F6a.raw | | | | | | 4.938E5 | | | | | | | | 0 | | | 1 | 0 | 0 | 0 | 0 | 0 | 1 | 0 | 0 | 0 | 0 | 0 | 0 | 0 | 0 | 0 | 0 | 56 | 71 | Carbamidomethylation | C8:Carbamidomethylation:1000.00;C12:Carbamidomethylation:1000.00 | PEAKS DB |
| P.VIEQGC(+57.02)VATC(+57.02)PQFR.S | N | 70.62 | 1663.7810 | 14 | 3.0 | 832.8978 | 2 | 46.31 | 8 | F8:22974 | BuCaTN01_F7b.raw | | | | | | | | 7.7386E6 | | | | | | | | | 1 | 0 | 0 | 0 | 0 | 0 | 0 | 0 | 1 | 0 | 0 | 0 | 0 | 0 | 0 | 0 | 0 | 58 | 71 | Carbamidomethylation | C6:Carbamidomethylation:1000.00;C10:Carbamidomethylation:1000.00 | PEAKS DB |
| R.GPVIEQGC(+57.02)VAT.C | N | 50.22 | 1129.5437 | 11 | 0.5 | 565.7794 | 2 | 41.92 | 6 | F6:21363 | BuCaTN01_F6a.raw | | | | | | 6.58E5 | | | | | | | | | | | 1 | 0 | 0 | 0 | 0 | 0 | 1 | 0 | 0 | 0 | 0 | 0 | 0 | 0 | 0 | 0 | 0 | 56 | 66 | Carbamidomethylation | C8:Carbamidomethylation:1000.00 | PEAKS DB |
| S.STPQTC(+57.02)PNGQDIC(+57.02)FR.K | Y | 48.47 | 1779.7668 | 15 | 3.1 | 890.8908 | 2 | 45.52 | 8 | F8:22181 | BuCaTN01_F7b.raw | | | | | | | | 8.9982E5 | | | | | | | | | 1 | 0 | 0 | 0 | 0 | 0 | 0 | 0 | 1 | 0 | 0 | 0 | 0 | 0 | 0 | 0 | 0 | 30 | 44 | Carbamidomethylation | C6:Carbamidomethylation:1000.00;C13:Carbamidomethylation:1000.00 | PEAKS DB |
| R.GPVIEQGC(+57.02)VA.T | N | 42.28 | 1028.4961 | 10 | 0.2 | 515.2554 | 2 | 41.42 | 6 | F6:20784 | BuCaTN01_F6a.raw | | | | | | 2.0497E6 | | | | | | | | | | | 1 | 0 | 0 | 0 | 0 | 0 | 1 | 0 | 0 | 0 | 0 | 0 | 0 | 0 | 0 | 0 | 0 | 56 | 65 | Carbamidomethylation | C8:Carbamidomethylation:1000.00 | PEAKS DB |
| V.IEQGC(+57.02)VATC(+57.02)PQFR.S | N | 41.92 | 1564.7126 | 13 | 1.4 | 783.3624 | 2 | 43.03 | 8 | F8:20158 | BuCaTN01_F7b.raw | | | | | | | | 1.5548E5 | | | | | | | | | 1 | 0 | 0 | 0 | 0 | 0 | 0 | 0 | 1 | 0 | 0 | 0 | 0 | 0 | 0 | 0 | 0 | 59 | 71 | Carbamidomethylation | C5:Carbamidomethylation:1000.00;C9:Carbamidomethylation:1000.00 | PEAKS DB |
| total 6 peptides | | | | | | | | | | | | | | | | | | | | | | | | | | | | | | | | | | | | | | | | | | | | | | | | | |

**Best Unique PSM (Scan F8:22181, m/z=890.8908, z=2, RT=45.52, ppm=3.1):**

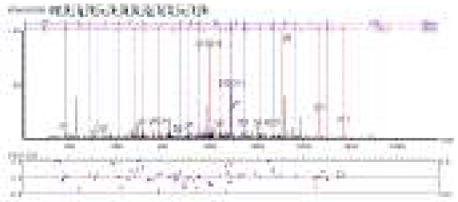

## CAD24463.1

back to list

| Protein Coverage | Supporting Peptides | Best Unique PSM |

**Protein Coverage:**

1 MNPAHLLVLS AVCVSLLGAA NIPPHPLNLI NFMEMIRYTI PCEKTWGEYA DYGCYCGAGG SGRPIDALDR CCYVHDNCYG

81 DAEKKHKCNP KTQSYSYKLT KRTIICYDAA GTCARIVCDC DRTAALCFGN SEYIGAHKNI DTARYC

C Carbamidomethylation (+57.02)
O Oxidation (M) (+15.99)

**Supporting Peptides:**

| Peptide | Uniq | -10lgP | Mass | Length | ppm | m/z | z | RT | Fraction | Scan | Source File | Area F1 | Area F2a | Area F3a | Area F4 | Area F5 | Area F6a | Area F7a | Area F7b | Area F8 | Area F9a | Area Sample 1F10a | Area F10b | Area F11a | Area F11b | Area F12a | Area F13 | #Feature | #Feature F1 | #Feature F2a | #Feature F3a | #Feature F4 | #Feature F5 | #Feature F6a | #Feature F7a | #Feature F7b | #Feature F8 | #Feature F9a | #Feature Sample 1F10a | #Feature F10b | #Feature F11a | #Feature F11b | #Feature F12a | #Feature F13 | Start | End | PTM | AScore | Found By |
|---|---|---|---|---|---|---|---|---|---|---|---|---|---|---|---|---|---|---|---|---|---|---|---|---|---|---|---|---|---|---|---|---|---|---|---|---|---|---|---|---|---|---|---|---|---|---|---|---|---|
| R.C(+57.02)C(+57.02)YVHDNC(+57.02)YGDAEK.K | N | 71.68 | 1789.6494 | 14 | 4.2 | 597.5566 | 3 | 32.82 | 15 | F15:12456 | BuCaTN01_F12a.raw | | | | | | | | 5.5353E5 | | 7.35E7 | | 1.5588E7 | | 2.0346E6 | 4.9485E7 | | 15 | 0 | 0 | 0 | 0 | 0 | 0 | 0 | 1 | 0 | 3 | 0 | 3 | 0 | 2 | 6 | 0 | 71 | 84 | Carbamidomethylation | C1:Carbamidomethylation:1000.00;C2:Carbamidomethylation:1000.00;C8:Carbamidomethylation:1000.00 | PEAKS DB |
| R.IVC(+57.02)DC(+57.02)DRTAALC(+57.02)FGNSEYIGAHK.N | Y | 65.31 | 2656.1831 | 23 | -5.2 | 886.3970 | 3 | 58.81 | 10 | F10:35303 | BuCaTN01_F9a.raw | | | | | | | | | | 9.8379E5 | | | | | | | 1 | 0 | 0 | 0 | 0 | 0 | 0 | 0 | 0 | 0 | 1 | 0 | 0 | 0 | 0 | 0 | 0 | 116 | 138 | Carbamidomethylation | C3:Carbamidomethylation:1000.00;C5:Carbamidomethylation:1000.00;C12:Carbamidomethylation:1000.00 | PEAKS DB |
| L.NLINFM(+15.99)EMIR.Y | N | 58.78 | 1295.6366 | 10 | 0.9 | 648.8262 | 2 | 75.08 | 9 | F9:48868 | BuCaTN01_F8.raw | | | | | | | | 3.2075E6 | 1.4879E8 | 6.3981E7 | | | | | | | 3 | 0 | 0 | 0 | 0 | 0 | 0 | 0 | 1 | 1 | 1 | 0 | 0 | 0 | 0 | 0 | 0 | 28 | 37 | Oxidation (M) | M6:Oxidation (M):43.88 | PEAKS DB |
| L.NLINFMEMIR.Y | N | 58.60 | 1279.6417 | 10 | 0.6 | 640.8285 | 2 | 89.02 | 11 | F11:48120 | BuCaTN01_F10a.raw | | | | | | | | 2.9837E6 | 9.8225E6 | 5.8511E8 | 1.9261E6 | 3.9106E6 | | | 4.6857E6 | | 6 | 0 | 0 | 0 | 0 | 0 | 0 | 0 | 1 | 1 | 1 | 1 | 1 | 0 | 0 | 1 | 0 | 28 | 37 | | | PEAKS DB |

| Peptide | Uniq | -10lgP | Mass | Length | ppm | m/z | z | RT | Fraction | Scan | Source File | Area F1 | Area F2a | Area F3a | Area F4 | Area F5 | Area F6a | Area F7a | Area F7b | Area F8 | Area F9a | Area Sample 1F10a | Area F10b | Area F11a | Area F11b | Area F12a | Area F13 | #Feature | #Feature F1 | #Feature F2a | #Feature F3a | #Feature F4 | #Feature F5 | #Feature F6a | #Feature F7a | #Feature F7b | #Feature F8 | #Feature F9a | #Feature Sample 1F10a | #Feature F10b | #Feature F11a | #Feature F11b | #Feature F12a | #Feature F13 | Start | End | PTM | AScore | Found By |
|---|---|---|---|---|---|---|---|---|---|---|---|---|---|---|---|---|---|---|---|---|---|---|---|---|---|---|---|---|---|---|---|---|---|---|---|---|---|---|---|---|---|---|---|---|---|---|---|---|---|
| L.NLINFMEM(+15.99)IR.Y | N | 53.89 | 1295.6366 | 10 | 0.4 | 648.8258 | 2 | 83.74 | 10 | F10:60895 | BuCaTN01_F9a.raw | | | | | | | 1.553E5 | 1.1201E6 | | 1.6424E8 | | 1.1443E6 | | | 1.4545E6 | | 6 | 0 | 0 | 0 | 0 | 0 | 0 | 1 | 1 | 0 | 2 | 0 | 1 | 0 | 0 | 1 | 0 | 28 | 37 | Oxidation (M) | M8:Oxidation (M):35.36 | PEAKS DB |
| R.IVC(+57.02)DC(+57.02)DRTAAL.C | N | 53.80 | 1292.5853 | 11 | 4.0 | 647.3007 | 2 | 38.90 | 8 | F8:16711 | BuCaTN01_F7b.raw | | | | | | | | 8.9609E6 | | 1.7898E7 | | 1.6803E6 | | 0 | 8.1365E5 | | 4 | 0 | 0 | 0 | 0 | 0 | 0 | 0 | 1 | 0 | 1 | 0 | 1 | 0 | 0 | 1 | 0 | 116 | 126 | Carbamidomethylation | C3:Carbamidomethylation:1000.00;C5:Carbamidomethylation:1000.00 | PEAKS DB |
| R.C(+57.02)C(+57.02)YVHDNC(+57.02)YGDAEKK.H | N | 52.61 | 1917.7444 | 15 | 2.4 | 640.2555 | 3 | 30.04 | 12 | F12:9754 | BuCaTN01_F10b.raw | | | | | | | | | | | | 4.9696E6 | | | 6.7487E6 | | 5 | 0 | 0 | 0 | 0 | 0 | 0 | 0 | 0 | 0 | 0 | 0 | 3 | 0 | 0 | 2 | 0 | 71 | 85 | Carbamidomethylation | C1:Carbamidomethylation:1000.00;C2:Carbamidomethylation:1000.00;C8:Carbamidomethylation:1000.00 | PEAKS DB |
| K.TQSYSYKLTK.R | N | 50.80 | 1217.6292 | 10 | 0.4 | 609.8221 | 2 | 34.34 | 10 | F10:13162 | BuCaTN01_F9a.raw | | | | | | | | 1.9531E6 | | 8.0324E6 | | 8.5846E5 | | | 4.8061E5 | | 6 | 0 | 0 | 0 | 0 | 0 | 0 | 0 | 2 | 0 | 2 | 0 | 1 | 0 | 0 | 1 | 0 | 92 | 101 | | | PEAKS DB |
| K.C(+57.02)NPKTQSYSYKLTK.R | N | 49.91 | 1716.8505 | 14 | -0.2 | 573.2906 | 3 | 32.90 | 10 | F10:11935 | BuCaTN01_F9a.raw | | | | | | | | | | 2.5785E5 | | | | | | | 1 | 0 | 0 | 0 | 0 | 0 | 0 | 0 | 0 | 0 | 1 | 0 | 0 | 0 | 0 | 0 | 0 | 88 | 101 | Carbamidomethylation | C1:Carbamidomethylation:1000.00 | PEAKS DB |
| L.NLINFM(+15.99)EM(+15.99)IR.Y | N | 48.42 | 1311.6315 | 10 | 3.3 | 656.8232 | 2 | 63.35 | 8 | F8:38918 | BuCaTN01_F7b.raw | | | | | | | | 5.8966E6 | 1.1938E7 | 7.7607E7 | | | | | | | 3 | 0 | 0 | 0 | 0 | 0 | 0 | 0 | 1 | 1 | 1 | 0 | 0 | 0 | 0 | 0 | 0 | 28 | 37 | Oxidation (M) | M6:Oxidation (M):1000.00;M8:Oxidation (M):1000.00 | PEAKS DB |
| R.IVC(+57.02)DC(+57.02)DRTAALC(+57.02)FGN.S | N | 46.45 | 1770.7488 | 15 | -4.9 | 886.3773 | 2 | 53.43 | 10 | F10:30303 | BuCaTN01_F9a.raw | | | | | | | | | | 0 | | | | | | | 0 | 0 | 0 | 0 | 0 | 0 | 0 | 0 | 0 | 0 | 0 | 0 | 0 | 0 | 0 | 0 | 0 | 116 | 130 | Carbamidomethylation | C3:Carbamidomethylation:1000.00;C5:Carbamidomethylation:1000.00;C12:Carbamidomethylation:1000.00 | PEAKS DB |
| R.TAALC(+57.02)FGNSEYIGAHK.N | Y | 45.29 | 1737.8145 | 16 | -2.9 | 580.2771 | 3 | 66.10 | 10 | F10:42504 | BuCaTN01_F9a.raw | | | | | | | | | | 0 | | 0 | | | 0 | | 0 | 0 | 0 | 0 | 0 | 0 | 0 | 0 | 0 | 0 | 0 | 0 | 0 | 0 | 0 | 0 | 0 | 123 | 138 | Carbamidomethylation | C5:Carbamidomethylation:1000.00 | PEAKS DB |
| R.YTIPC(+57.02)EK.T | N | 42.20 | 909.4266 | 7 | 2.8 | 455.7205 | 2 | 36.93 | 8 | F8:14947 | BuCaTN01_F7b.raw | | | | | | | | 7.3611E6 | | | | | | | | | 1 | 0 | 0 | 0 | 0 | 0 | 0 | 0 | 1 | 0 | 0 | 0 | 0 | 0 | 0 | 0 | 0 | 38 | 44 | Carbamidomethylation | C5:Carbamidomethylation:1000.00 | PEAKS DB |
| total 13 peptides | | | | | | | | | | | | | | | | | | | | | | | | | | | | | | | | | | | | | | | | | | | | | | | | | |

**Best Unique PSM (Scan F10:35303, m/z=886.3970, z=3, RT=58.81, ppm=-5.2):**

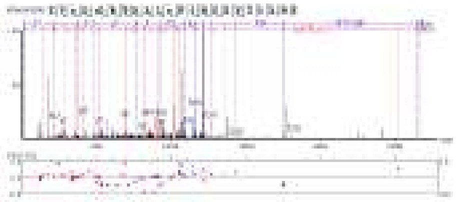

## 0412250A

back to list

| Protein Coverage | Supporting Peptides | Best Unique PSM |

**Protein Coverage:**

1 NLINFMEMIR YTIPCEKTWG EYADYGCYCG AGGSGRPIDA LDRCCYVHDN CYGDAEKKHK CNPKTSQYSY KLTKRTIICY

81 GAAGGTCRIV CDCDRTAALC FGQSDYIEGH KNIDTARFCQ

C Carbamidomethylation (+57.02)
O Oxidation (M) (+15.99)

**Supporting Peptides:**

| Peptide | Uniq | -10lgP | Mass | Length | ppm | m/z | z | RT | Fraction | Scan | Source File | Area F1 | Area F2a | Area F3a | Area F4 | Area F5 | Area F6a | Area F7a | Area F7b | Area F8 | Area F9a | Area Sample 1F10a | Area F10b | Area F11a | Area F11b | Area F12a | Area F13 | #Feature | #Feature F1 | #Feature F2a | #Feature F3a | #Feature F4 | #Feature F5 | #Feature F6a | #Feature F7a | #Feature F7b | #Feature F8 | #Feature F9a | #Feature Sample 1F10a | #Feature F10b | #Feature F11a | #Feature F11b | #Feature F12a | #Feature F13 | Start | End | PTM | AScore | Found By |
|---|---|---|---|---|---|---|---|---|---|---|---|---|---|---|---|---|---|---|---|---|---|---|---|---|---|---|---|---|---|---|---|---|---|---|---|---|---|---|---|---|---|---|---|---|---|---|---|---|---|
| R.C(+57.02)C(+57.02)YVHDNC(+57.02)YGDAEK.K | N | 71.68 | 1789.6494 | 14 | 4.2 | 597.5566 | 3 | 32.82 | 15 | F15:12456 | BuCaTN01_F12a.raw | | | | | | | | 5.5353E5 | | 7.35E7 | | 1.5588E7 | | 2.0346E6 | 4.9485E7 | | 15 | 0 | 0 | 0 | 0 | 0 | 0 | 0 | 1 | 0 | 3 | 0 | 3 | 0 | 2 | 6 | 0 | 44 | 57 | Carbamidomethylation | C1:Carbamidomethylation:1000.00;C2:Carbamidomethylation:1000.00;C8:Carbamidomethylation:1000.00 | PEAKS DB |
| R.IVC(+57.02)DC(+57.02)DRTAALC(+57.02)FGQSDYIEGHK.N | N | 69.28 | 2714.1887 | 23 | -0.4 | 905.7365 | 3 | 56.13 | 10 | F10:33105 | BuCaTN01_F9a.raw | | | | | | | | | | 2.7393E8 | | 6.5608E6 | | | | | 5 | 0 | 0 | 0 | 0 | 0 | 0 | 0 | 0 | 0 | 4 | 0 | 1 | 0 | 0 | 0 | 0 | 89 | 111 | Carbamidomethylation | C3:Carbamidomethylation:1000.00;C5:Carbamidomethylation:1000.00;C12:Carbamidomethylation:1000.00 | PEAKS DB |
| R.TIIC(+57.02)YGAAGGTC(+57.02)R.I | Y | 67.19 | 1398.6384 | 13 | 4.0 | 700.3264 | 2 | 42.08 | 14 | F14:20439 | BuCaTN01_F11b.raw | | 4.6764E6 | 9.2303E5 | | | | | | 1.3558E6 | 2.1784E5 | | 0 | | 5.708E7 | | | 8 | 0 | 1 | 1 | 0 | 0 | 0 | 0 | 0 | 1 | 4 | 0 | 0 | 0 | 1 | 0 | 0 | 76 | 88 | Carbamidomethylation | C4:Carbamidomethylation:1000.00;C12:Carbamidomethylation:1000.00 | PEAKS DB |
| R.TAALC(+57.02)FGQSDYIEGHK.N | N | 61.41 | 1795.8199 | 16 | 0.4 | 599.6141 | 3 | 58.30 | 11 | F11:27836 | BuCaTN01_F10a.raw | | | | | | | | | | 7.7087E7 | 1.0888E7 | | | | | | 6 | 0 | 0 | 0 | 0 | 0 | 0 | 0 | 0 | 0 | 3 | 3 | 0 | 0 | 0 | 0 | 0 | 96 | 111 | Carbamidomethylation | C5:Carbamidomethylation:1000.00 | PEAKS DB |
| NLINFM(+15.99)EMIR.Y | N | 58.78 | 1295.6366 | 10 | 0.9 | 648.8262 | 2 | 75.08 | 9 | F9:48868 | BuCaTN01_F8.raw | | | | | | | | 3.2075E6 | 1.4879E8 | 6.3981E7 | | | | | | | 3 | 0 | 0 | 0 | 0 | 0 | 0 | 0 | 1 | 1 | 1 | 0 | 0 | 0 | 0 | 0 | 0 | 1 | 10 | Oxidation (M) | M6:Oxidation (M):43.88 | PEAKS DB |
| NLINFMEMIR.Y | N | 58.60 | 1279.6417 | 10 | 0.6 | 640.8285 | 2 | 89.02 | 11 | F11:48120 | BuCaTN01_F10a.raw | | | | | | | | 2.9837E6 | 9.8225E6 | 5.8511E8 | 1.9261E6 | 3.9106E6 | | | 4.6857E6 | | 6 | 0 | 0 | 0 | 0 | 0 | 0 | 0 | 1 | 1 | 1 | 1 | 1 | 0 | 0 | 1 | 0 | 1 | 10 | | | PEAKS DB |
| NLINFMEM(+15.99)IR.Y | N | 53.89 | 1295.6366 | 10 | 0.4 | 648.8258 | 2 | 83.74 | 10 | F10:60895 | BuCaTN01_F9a.raw | | | | | | | 1.553E5 | 1.1201E6 | | 1.6424E8 | | 1.1443E6 | | | 1.4545E6 | | 6 | 0 | 0 | 0 | 0 | 0 | 0 | 1 | 1 | 0 | 2 | 0 | 1 | 0 | 0 | 1 | 0 | 1 | 10 | Oxidation (M) | M8:Oxidation (M):35.36 | PEAKS DB |
| R.IVC(+57.02)DC(+57.02)DRTAAL.C | N | 53.80 | 1292.5853 | 11 | 4.0 | 647.3007 | 2 | 38.90 | 8 | F8:16711 | BuCaTN01_F7b.raw | | | | | | | | 8.9609E6 | | 1.7898E7 | | 1.6803E6 | | 0 | 8.1365E5 | | 4 | 0 | 0 | 0 | 0 | 0 | 0 | 0 | 1 | 0 | 1 | 0 | 1 | 0 | 0 | 1 | 0 | 89 | 99 | Carbamidomethylation | C3:Carbamidomethylation:1000.00;C5:Carbamidomethylation:1000.00 | PEAKS DB |
| R.C(+57.02)C(+57.02)YVHDNC(+57.02)YGDAEKK.H | N | 52.61 | 1917.7444 | 15 | 2.4 | 640.2555 | 3 | 30.04 | 12 | F12:9754 | BuCaTN01_F10b.raw | | | | | | | | | | | | 4.9696E6 | | | 6.7487E6 | | 5 | 0 | 0 | 0 | 0 | 0 | 0 | 0 | 0 | 0 | 0 | 0 | 3 | 0 | 0 | 2 | 0 | 44 | 58 | Carbamidomethylation | C1:Carbamidomethylation:1000.00;C2:Carbamidomethylation:1000.00;C8:Carbamidomethylation:1000.00 | PEAKS DB |
| NLINFM(+15.99)EM(+15.99)IR.Y | N | 48.42 | 1311.6315 | 10 | 3.3 | 656.8232 | 2 | 63.35 | 8 | F8:38918 | BuCaTN01_F7b.raw | | | | | | | | 5.8966E6 | 1.1938E7 | 7.7607E7 | | | | | | | 3 | 0 | 0 | 0 | 0 | 0 | 0 | 0 | 1 | 1 | 1 | 0 | 0 | 0 | 0 | 0 | 0 | 1 | 10 | Oxidation (M) | M6:Oxidation (M):1000.00;M8:Oxidation (M):1000.00 | PEAKS DB |
| R.YTIPC(+57.02)EK.T | N | 42.20 | 909.4266 | 7 | 2.8 | 455.7205 | 2 | 36.93 | 8 | F8:14947 | BuCaTN01_F7b.raw | | | | | | | | 7.3611E6 | | | | | | | | | 1 | 0 | 0 | 0 | 0 | 0 | 0 | 0 | 1 | 0 | 0 | 0 | 0 | 0 | 0 | 0 | 0 | 11 | 17 | Carbamidomethylation | C5:Carbamidomethylation:1000.00 | PEAKS DB |
| total 11 peptides | | | | | | | | | | | | | | | | | | | | | | | | | | | | | | | | | | | | | | | | | | | | | | | | | |

**Best Unique PSM (Scan F14:20439, m/z=700.3264, z=2, RT=42.08, ppm=4.0):**

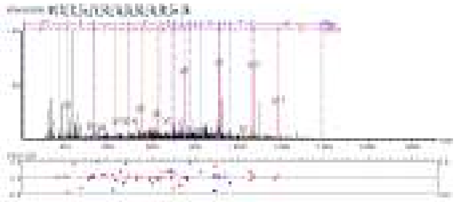

## Q75S48.1

back to list

| Protein Coverage | Supporting Peptides | Best Unique PSM |

**Protein Coverage:**

1 MNPAYFLVLA AVCVSLLGAA NIPPQPLSFY RYTEMIQCTI RGSLTYLDYM DYGCYCGTGS RGTPVDELDR CCQTHDNCYA

81 EAEEHPKCSS LVKSPYMNLY SYTCSGGTIT CNADNDECGA FICNCDRTAA LCFAKAPYNE ENKEIDISKR CQ

C Carbamidomethylation (+57.02)

**Supporting Peptides:**

| Peptide | Uniq | -10lgP | Mass | Length | ppm | m/z | z | RT | Fraction | Scan | Source File | Area F1 | Area F2a | Area F3a | Area F4 | Area F5 | Area F6a | Area F7a | Area F7b | Area F8 | Area F9a | Area Sample 1F10a | Area F10b | Area F11a | Area F11b | Area F12a | Area F13 | #Feature | #Feature F1 | #Feature F2a | #Feature F3a | #Feature F4 | #Feature F5 | #Feature F6a | #Feature F7a | #Feature F7b | #Feature F8 | #Feature F9a | #Feature Sample 1F10a | #Feature F10b | #Feature F11a | #Feature F11b | #Feature F12a | #Feature F13 | Start | End | PTM | AScore | Found By |
|---|---|---|---|---|---|---|---|---|---|---|---|---|---|---|---|---|---|---|---|---|---|---|---|---|---|---|---|---|---|---|---|---|---|---|---|---|---|---|---|---|---|---|---|---|---|---|---|---|---|
| R.TAALC(+57.02)FAKAPY.N | N | 73.24 | 1211.6008 | 11 | 2.2 | 606.8076 | 2 | 55.84 | 12 | F12:32023 | BuCaTN01_F10b.raw | | | | | | | 7.3334E4 | | | 1.7166E6 | 1.3753E5 | 1.06E7 | | 8.1407E6 | 9.828E5 | | 7 | 0 | 0 | 0 | 0 | 0 | 0 | 1 | 0 | 0 | 1 | 1 | 2 | 0 | 1 | 1 | 0 | 128 | 138 | Carbamidomethylation | C5:Carbamidomethylation:1000.00 | PEAKS DB |
| R.TAALC(+57.02)FAKAPYN.E | N | 69.35 | 1325.6438 | 12 | -0.2 | 663.8290 | 2 | 51.91 | 6 | F6:30474 | BuCaTN01_F6a.raw | | | | | | 3.7675E6 | | 1.5632E7 | | 4.6358E6 | | 1.9346E6 | | | | | 5 | 0 | 0 | 0 | 0 | 0 | 1 | 0 | 2 | 0 | 1 | 0 | 1 | 0 | 0 | 0 | 0 | 128 | 139 | Carbamidomethylation | C5:Carbamidomethylation:1000.00 | PEAKS DB |
| N.C(+57.02)DRTAALC(+57.02)FAK.A | N | 63.48 | 1311.6063 | 11 | -1.7 | 438.2076 | 3 | 37.36 | 12 | F12:15762 | BuCaTN01_F10b.raw | | | | | | | | | | | | 5.2815E4 | | 3.1918E5 | | | 2 | 0 | 0 | 0 | 0 | 0 | 0 | 0 | 0 | 0 | 0 | 0 | 1 | 0 | 1 | 0 | 0 | 125 | 135 | Carbamidomethylation | C1:Carbamidomethylation:1000.00;C8:Carbamidomethylation:1000.00 | PEAKS DB |
| R.TAALC(+57.02)FAK.A | N | 60.21 | 880.4476 | 8 | 3.4 | 441.2316 | 2 | 41.58 | 12 | F12:19288 | BuCaTN01_F10b.raw | | | | | | 9.3985E4 | 9.2429E6 | 1.2777E7 | 4.6024E5 | 3.2388E7 | 3.591E6 | 1.265E8 | 3.5731E6 | 1.1283E8 | 1.0943E8 | | 21 | 0 | 0 | 0 | 0 | 0 | 1 | 4 | 2 | 1 | 2 | 2 | 2 | 1 | 2 | 4 | 0 | 128 | 135 | Carbamidomethylation | C5:Carbamidomethylation:1000.00 | PEAKS DB |
| R.GSLTYLDYMDYGC(+57.02)YC(+57.02)GTGSR.G | Y | 60.09 | 2337.9341 | 20 | 5.4 | 1169.9747 | 2 | 74.07 | 15 | F15:47420 | BuCaTN01_F12a.raw | | | | | | | | | | 0 | | | | | 2.0525E6 | | 1 | 0 | 0 | 0 | 0 | 0 | 0 | 0 | 0 | 0 | 0 | 0 | 0 | 0 | 0 | 1 | 0 | 42 | 61 | Carbamidomethylation | C13:Carbamidomethylation:1000.00;C15:Carbamidomethylation:1000.00 | PEAKS DB |
| R.GTPVDELDRC(+57.02)C(+57.02)QTHDNC(+57.02)YAEAEEHPK.C | Y | 57.32 | 3130.2815 | 26 | 1.1 | 783.5762 | 4 | 45.17 | 8 | F8:22039 | BuCaTN01_F7b.raw | | | | | | | | 2.2127E6 | | | | | | | 3.9229E7 | | 4 | 0 | 0 | 0 | 0 | 0 | 0 | 0 | 1 | 0 | 0 | 0 | 0 | 0 | 0 | 3 | 0 | 62 | 87 | Carbamidomethylation | C10:Carbamidomethylation:1000.00;C11:Carbamidomethylation:1000.00;C17:Carbamidomethylation:1000.00 | PEAKS DB |
| R.TAALC(+57.02)FAKAP.Y | N | 47.25 | 1048.5375 | 10 | 3.6 | 525.2764 | 2 | 48.84 | 8 | F8:25881 | BuCaTN01_F7b.raw | | | | | | 3.1579E6 | | 4.0965E6 | | | | | | | | | 2 | 0 | 0 | 0 | 0 | 0 | 1 | 0 | 1 | 0 | 0 | 0 | 0 | 0 | 0 | 0 | 0 | 128 | 137 | Carbamidomethylation | C5:Carbamidomethylation:1000.00 | PEAKS DB |
| total 7 peptides | | | | | | | | | | | | | | | | | | | | | | | | | | | | | | | | | | | | | | | | | | | | | | | | | |

**Best Unique PSM (Scan F15:47420, m/z=1169.9747, z=2, RT=74.07, ppm=5.4):**

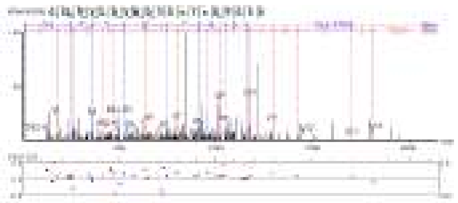

## BAD06270.1

back to list

| Protein Coverage | Supporting Peptides | Best Unique PSM |

**Protein Coverage:**

```
1  MNPAYFLVLA AVCVSLLGAA NIPPQPLSFY RYTEMIQCTI RGSLTYLDYM DYGCYCGTGS RGTPVDELDR CCQTHDNCYA
81 EAEEHPKCSS LVKSPYMNLY SYTCSGGTIT CNADNDECGA FICNCDRTAA LCFAKAPYNE ENKEIDISKR CQ
```

C Carbamidomethylation (+57.02)

**Supporting Peptides:**

| Peptide | Uniq | -10lgP | Mass | Length | ppm | m/z | z | RT | Fraction | Scan | Source File | Area F1 | Area F2a | Area F3a | Area F4 | Area F5 | Area F6a | Area F7a | Area F7b | Area F8 | Area F9a | Area Sample 1F10a | Area F10b | Area F11a | Area F11b | Area F12a | Area F13 | #Feature | #Feature F1 | #Feature F2a | #Feature F3a | #Feature F4 | #Feature F5 | #Feature F6a | #Feature F7a | #Feature F7b | #Feature F8 | #Feature F9a | #Feature Sample 1F10a | #Feature F10b | #Feature F11a | #Feature F11b | #Feature F12a | #Feature F13 | Start | End | PTM | AScore | Found By |
|---|---|---|---|---|---|---|---|---|---|---|---|---|---|---|---|---|---|---|---|---|---|---|---|---|---|---|---|---|---|---|---|---|---|---|---|---|---|---|---|---|---|---|---|---|---|---|---|---|---|
| R.TAALC(+57.02)FAKAPY.N | N | 73.24 | 1211.6008 | 11 | 2.2 | 606.8076 | 2 | 55.84 | 12 | F12:32023 | BuCaTN01_F10b.raw | | | | | | | 7.3334E4 | | | 1.7166E6 | 1.3753E5 | 1.06E7 | | 8.1407E6 | 9.828E5 | | 7 | 0 | 0 | 0 | 0 | 0 | 0 | 1 | 0 | 0 | 1 | 1 | 2 | 0 | 1 | 1 | 0 | 128 | 138 | Carbamidomethylation | C5:Carbamidomethylation:1000.00 | PEAKS DB |
| R.TAALC(+57.02)FAKAPYN.E | N | 69.35 | 1325.6438 | 12 | -0.2 | 663.8290 | 2 | 51.91 | 6 | F6:30474 | BuCaTN01_F6a.raw | | | | | | 3.7675E6 | | 1.5632E7 | | 4.6358E6 | | 1.9346E6 | | | | | 5 | 0 | 0 | 0 | 0 | 0 | 1 | 0 | 2 | 0 | 1 | 0 | 1 | 0 | 0 | 0 | 0 | 128 | 139 | Carbamidomethylation | C5:Carbamidomethylation:1000.00 | PEAKS DB |
| N.C(+57.02)DRTAALC(+57.02)FAK.A | N | 63.48 | 1311.6063 | 11 | -1.7 | 438.2076 | 3 | 37.36 | 12 | F12:15762 | BuCaTN01_F10b.raw | | | | | | | | | | | | 5.2815E4 | | 3.1918E5 | | | 2 | 0 | 0 | 0 | 0 | 0 | 0 | 0 | 0 | 0 | 0 | 0 | 1 | 0 | 1 | 0 | 0 | 125 | 135 | Carbamidomethylation | C1:Carbamidomethylation:1000.00;C8:Carbamidomethylation:1000.00 | PEAKS DB |
| R.TAALC(+57.02)FAK.A | N | 60.21 | 880.4476 | 8 | 3.4 | 441.2316 | 2 | 41.58 | 12 | F12:19288 | BuCaTN01_F10b.raw | | | | | | 9.3985E4 | 9.2429E6 | 1.2777E7 | 4.6024E5 | 3.2388E7 | 3.591E6 | 1.265E8 | 3.5731E6 | 1.1283E8 | 1.0943E8 | | 21 | 0 | 0 | 0 | 0 | 0 | 1 | 4 | 2 | 1 | 2 | 2 | 2 | 1 | 2 | 4 | 0 | 128 | 135 | Carbamidomethylation | C5:Carbamidomethylation:1000.00 | PEAKS DB |
| R.GSLTYLDYMDYGC(+57.02)YC(+57.02)GTGSR.G | Y | 60.09 | 2337.9341 | 20 | 5.4 | 1169.9747 | 2 | 74.07 | 15 | F15:47420 | BuCaTN01_F12a.raw | | | | | | | | | | 0 | | | | | 2.0525E6 | | 1 | 0 | 0 | 0 | 0 | 0 | 0 | 0 | 0 | 0 | 0 | 0 | 0 | 0 | 0 | 1 | 0 | 42 | 61 | Carbamidomethylation | C13:Carbamidomethylation:1000.00; C15:Carbamidomethylation:1000.00 | PEAKS DB |
| R.GTPVDELDRC(+57.02)C(+57.02)QTHDNC(+57.02)YAEAEEHPK.C | Y | 57.32 | 3130.2815 | 26 | 1.1 | 783.5762 | 4 | 45.17 | 8 | F8:22039 | BuCaTN01_F7b.raw | | | | | | | | 2.2127E6 | | | | | | | 3.9229E7 | | 4 | 0 | 0 | 0 | 0 | 0 | 0 | 0 | 1 | 0 | 0 | 0 | 0 | 0 | 0 | 3 | 0 | 62 | 87 | Carbamidomethylation | C10:Carbamidomethylation:1000.00; C11:Carbamidomethylation:1000.00; C17:Carbamidomethylation:1000.00 | PEAKS DB |
| R.TAALC(+57.02)FAKAP.Y | N | 47.25 | 1048.5375 | 10 | 3.6 | 525.2764 | 2 | 48.84 | 8 | F8:25881 | BuCaTN01_F7b.raw | | | | | | 3.1579E6 | | 4.0965E6 | | | | | | | | | 2 | 0 | 0 | 0 | 0 | 0 | 1 | 0 | 1 | 0 | 0 | 0 | 0 | 0 | 0 | 0 | 0 | 128 | 137 | Carbamidomethylation | C5:Carbamidomethylation:1000.00 | PEAKS DB |
| total 7 peptides | | | | | | | | | | | | | | | | | | | | | | | | | | | | | | | | | | | | | | | | | | | | | | | | | |

**Best Unique PSM (Scan F15:47420, m/z=1169.9747, z=2, RT=74.07, ppm=5.4):**

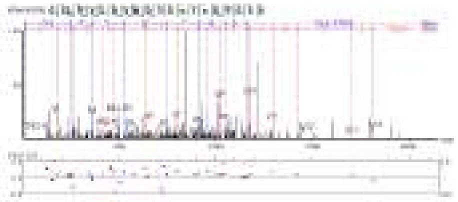

## P81993.1

back to list

| Protein Coverage | Supporting Peptides | Best Unique PSM |

**Protein Coverage:**

```
1  ESSNKRENQK QIVDKHNALR RSVRPTARNM LQMEWNSNAA QNAKRFADRC TFAHSPPHLR TVGIFSC
```

C Carbamidomethylation (+57.02)
O Oxidation (M) (+15.99)

**Supporting Peptides:**

| Peptide | Uniq | -10lgP | Mass | Length | ppm | m/z | z | RT | Fraction | Scan | Source File | Area F1 | Area F2a | Area F3a | Area F4 | Area F5 | Area F6a | Area F7a | Area F7b | Area F8 | Area F9a | Area Sample 1F10a | Area F10b | Area F11a | Area F11b | Area F12a | Area F13 | #Feature | #Feature F1 | #Feature F2a | #Feature F3a | #Feature F4 | #Feature F5 | #Feature F6a | #Feature F7a | #Feature F7b | #Feature F8 | #Feature F9a | #Feature Sample 1F10a | #Feature F10b | #Feature F11a | #Feature F11b | #Feature F12a | #Feature F13 | Start | End | PTM | AScore | Found By |
|---|---|---|---|---|---|---|---|---|---|---|---|---|---|---|---|---|---|---|---|---|---|---|---|---|---|---|---|---|---|---|---|---|---|---|---|---|---|---|---|---|---|---|---|---|---|---|---|---|---|
| R.NMLQM(+15.99)EWNSNAAQNAK.R | N | 92.39 | 1864.8196 | 16 | 1.2 | 933.4182 | 2 | 54.45 | 13 | F13:29949 | BuCaTN01_F11a.raw | | | | | | | | | | | 2.4645E5 | | 1.8788E6 | | | 6.546E5 | 7 | 0 | 0 | 0 | 0 | 0 | 0 | 0 | 0 | 0 | 0 | 2 | 0 | 2 | 0 | 0 | 3 | 29 | 44 | Oxidation (M) | M5:Oxidation (M):77.24 | PEAKS DB |
| R.NM(+15.99)LQM(+15.99)EWNSNAAQNAK.R | N | 91.55 | 1880.8146 | 16 | -0.5 | 941.4141 | 2 | 45.31 | 13 | F13:21815 | BuCaTN01_F11a.raw | | | | | | | | | | | 8.0152E5 | | 4.7799E6 | | | | 7 | 0 | 0 | 0 | 0 | 0 | 0 | 0 | 0 | 0 | 0 | 5 | 0 | 2 | 0 | 0 | 0 | 29 | 44 | Oxidation (M) | M2:Oxidation (M):1000.00;M5:Oxidation (M):1000.00 | PEAKS DB |
| R.NM(+15.99)LQM(+15.99)EWNSNAAQNAKR.F | N | 85.70 | 2036.9156 | 17 | -0.2 | 679.9790 | 3 | 42.31 | 13 | F13:19106 | BuCaTN01_F11a.raw | | | | | | | | | | | 1.5703E6 | | 9.863E6 | | | | 2 | 0 | 0 | 0 | 0 | 0 | 0 | 0 | 0 | 0 | 0 | 1 | 0 | 1 | 0 | 0 | 0 | 29 | 45 | Oxidation (M) | M2:Oxidation (M):1000.00;M5:Oxidation (M):1000.00 | PEAKS DB |
| R.NMLQM(+15.99)EWNSNAAQNAKR.F | N | 84.44 | 2020.9208 | 17 | 0.4 | 674.6478 | 3 | 51.10 | 13 | F13:26282 | BuCaTN01_F11a.raw | | | | | | | | | | | 4.5326E5 | | 2.6157E6 | | | 7.526E5 | 3 | 0 | 0 | 0 | 0 | 0 | 0 | 0 | 0 | 0 | 0 | 1 | 0 | 1 | 0 | 0 | 1 | 29 | 45 | Oxidation (M) | M5:Oxidation (M):26.52 | PEAKS DB |
| R.NMLQMEWNSNAAQNAK.R | N | 78.48 | 1848.8247 | 16 | -0.1 | 925.4196 | 2 | 61.65 | 16 | F16:30581 | BuCaTN01_F13.raw | | | | | | | | | | | 8.2478E5 | | 1.6062E6 | | | 3.3797E6 | 4 | 0 | 0 | 0 | 0 | 0 | 0 | 0 | 0 | 0 | 0 | 2 | 0 | 1 | 0 | 0 | 1 | 29 | 44 | | | PEAKS DB |
| R.NMLQMEWNSNAAQNAKR.F | N | 69.63 | 2004.9258 | 17 | 0.4 | 669.3162 | 3 | 56.51 | 13 | F13:31303 | BuCaTN01_F11a.raw | | | | | | | | | | | 6.3305E5 | | 6.8801E6 | | | 4.9021E6 | 4 | 0 | 0 | 0 | 0 | 0 | 0 | 0 | 0 | 0 | 0 | 1 | 0 | 2 | 0 | 0 | 1 | 29 | 45 | | | PEAKS DB |
| K.RFADRC(+57.02)TFAHSPPHLR.T | Y | 68.18 | 1966.9696 | 16 | -9.5 | 492.7450 | 4 | 80.99 | 13 | F13:52654 | BuCaTN01_F11a.raw | | | | | | | | | | | | 0 | 4.7351E7 | 8.3018E6 | | | 3 | 0 | 0 | 0 | 0 | 0 | 0 | 0 | 0 | 0 | 0 | 0 | 0 | 2 | 1 | 0 | 0 | 45 | 60 | Carbamidomethylation | C6:Carbamidomethylation:1000.00 | PEAKS DB |
| R.NM(+15.99)LQMEWNSNAAQNAKR.F | N | 54.15 | 2020.9208 | 17 | 0.1 | 674.6476 | 3 | 47.52 | 13 | F13:23429 | BuCaTN01_F11a.raw | | | | | | | | | | | | | 1.6196E6 | | | | 1 | 0 | 0 | 0 | 0 | 0 | 0 | 0 | 0 | 0 | 0 | 0 | 0 | 1 | 0 | 0 | 0 | 29 | 45 | Oxidation (M) | M2:Oxidation (M):47.09 | PEAKS DB |
| K.QIVDKHNALR.R | N | 53.10 | 1192.6676 | 10 | -0.4 | 398.5630 | 3 | 23.28 | 13 | F13:6364 | BuCaTN01_F11a.raw | | | | | | | | | | | 1.3327E6 | | 2.9907E6 | | | | 2 | 0 | 0 | 0 | 0 | 0 | 0 | 0 | 0 | 0 | 0 | 1 | 0 | 1 | 0 | 0 | 0 | 11 | 20 | | | PEAKS DB |
| R.NM(+15.99)LQMEWNSNAAQNAK.R | N | 52.57 | 1864.8196 | 16 | 1.7 | 622.6149 | 3 | 52.23 | 13 | F13:27379 | BuCaTN01_F11a.raw | | | | | | | | | | | | | 2.2136E5 | | | | 2 | 0 | 0 | 0 | 0 | 0 | 0 | 0 | 0 | 0 | 0 | 0 | 0 | 2 | 0 | 0 | 0 | 29 | 44 | Oxidation (M) | M2:Oxidation (M):56.34 | PEAKS DB |
| total 10 peptides | | | | | | | | | | | | | | | | | | | | | | | | | | | | | | | | | | | | | | | | | | | | | | | | | |

**Best Unique PSM (Scan F13:52654, m/z=492.7450, z=4, RT=80.99, ppm=-9.5):**

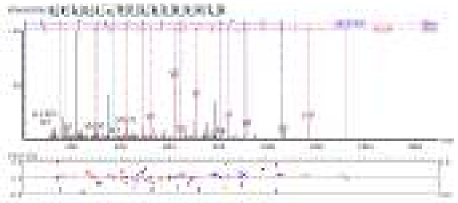

## P25668.1

back to list

| Protein Coverage | Supporting Peptides | Best Unique PSM |

**Protein Coverage:**

```
1  IRCFITPDIT SKDCPNGHVC YTKTWCDGFC SIRGKRVDLG CAATCPTVRT GVDIQCCSTD DCDPFPTRKR P
```

C Carbamidomethylation (+57.02)

**Supporting Peptides:**

| Peptide | Uniq | -10lgP | Mass | Length | ppm | m/z | z | RT | Fraction | Scan | Source File | Area F1 | Area F2a | Area F3a | Area F4 | Area F5 | Area F6a | Area F7a | Area F7b | Area F8 | Area F9a | Area Sample 1F10a | Area F10b | Area F11a | Area F11b | Area F12a | Area F13 | #Feature | #Feature F1 | #Feature F2a | #Feature F3a | #Feature F4 | #Feature F5 | #Feature F6a | #Feature F7a | #Feature F7b | #Feature F8 | #Feature F9a | #Feature Sample 1F10a | #Feature F10b | #Feature F11a | #Feature F11b | #Feature F12a | #Feature F13 | Start | End | PTM | AScore | Found By |
|---|---|---|---|---|---|---|---|---|---|---|---|---|---|---|---|---|---|---|---|---|---|---|---|---|---|---|---|---|---|---|---|---|---|---|---|---|---|---|---|---|---|---|---|---|---|---|---|---|---|
| K.RVDLGC(+57.02)AATC(+57.02)PTVR.T | Y | 76.14 | 1574.7657 | 14 | -0.7 | 525.9288 | 3 | 39.07 | 6 | F6:18725 | BuCaTN01_F6a.raw | | | | | 1.3891E6 | 1.1148E7 | | 1.9376E6 | | | | | | | | | 4 | 0 | 0 | 0 | 0 | 1 | 2 | 0 | 1 | 0 | 0 | 0 | 0 | 0 | 0 | 0 | 0 | 36 | 49 | Carbamidomethylation | C6:Carbamidomethylation:1000.00;C10:Carbamidomethylation:1000.00 | PEAKS DB |
| | | | | | | | | | | | | | | | | | | | | | | | | | | | | | | | | | | | | | | | | | | | | | | | C5:Carbamidomethylation:1000.00;C9:Carba | PEAKS |

| | | | | | | | | | | | | | | | | | | | | | | | | | | | | | | | | | | | | | | | | | | | | | | | | | | |
|---|---|---|---|---|---|---|---|---|---|---|---|---|---|---|---|---|---|---|---|---|---|---|---|---|---|---|---|---|---|---|---|---|---|---|---|---|---|---|---|---|---|---|---|---|---|---|---|---|---|---|
| R.VDLGC(+57.02)AATC(+57.02)PTVR.T | Y | 72.60 | 1418.6646 | 13 | 0.2 | 710.3397 | 2 | 42.98 | 6 | F6:22646 | BuCaTN01_F6a.raw | | | | | 6.5182E5 | 1.1319E7 | 5.9731E4 | | | | | | | | | | 4 | 0 | 0 | 0 | 0 | 1 | 2 | 1 | 0 | 0 | 0 | 0 | 0 | 0 | 0 | 0 | 0 | 37 | 49 | Carbamidomethylation | midomethylation:1000.00 | DB |
| R.GKRVDLGC(+57.02)AATC(+57.02)PTVR.T | Y | 72.05 | 1759.8821 | 16 | 0.7 | 440.9781 | 4 | 34.58 | 6 | F6:14950 | BuCaTN01_F6a.raw | | | | | 0 | 0 | | | | | | | | | | | 0 | 0 | 0 | 0 | 0 | 0 | 0 | 0 | 0 | 0 | 0 | 0 | 0 | 0 | 0 | 0 | 0 | 34 | 49 | Carbamidomethylation | C8:Carbamidomethylation:1000.00;C12:Carbamidomethylation:1000.00 | PEAKS DB |
| R.C(+57.02)FITPDITSK.D | N | 59.45 | 1180.5798 | 10 | -0.7 | 591.2968 | 2 | 52.58 | 6 | F6:31076 | BuCaTN01_F6a.raw | | | | | | 1.0443E7 | | | | | | | | | | | 1 | 0 | 0 | 0 | 0 | 0 | 1 | 0 | 0 | 0 | 0 | 0 | 0 | 0 | 0 | 0 | 0 | 3 | 12 | Carbamidomethylation | C1:Carbamidomethylation:1000.00 | PEAKS DB |
| IRC(+57.02)FITPDITSK.D | N | 53.97 | 1449.7650 | 12 | 0.2 | 484.2624 | 3 | 51.57 | 6 | F6:29993 | BuCaTN01_F6a.raw | | | | | | 2.8124E6 | 0 | 7.1601E5 | | | | | | | | | 2 | 0 | 0 | 0 | 0 | 0 | 1 | 0 | 1 | 0 | 0 | 0 | 0 | 0 | 0 | 0 | 0 | 1 | 12 | Carbamidomethylation | C3:Carbamidomethylation:1000.00 | PEAKS DB |
| **total 5 peptides** | | | | | | | | | | | | | | | | | | | | | | | | | | | | | | | | | | | | | | | | | | | | | | | | | | |

**Best Unique PSM (Scan F6:18725, m/z=525.9288, z=3, RT=39.07, ppm=-0.7):**

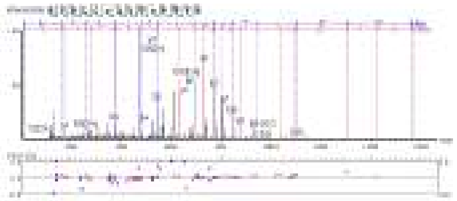

## Q3HXY1.1

back to list

| Protein Coverage | Supporting Peptides | Best Unique PSM |

**Protein Coverage:**

```
  1  MSMLCYTLII AFLIGIWAAP QSEDNVPLGS PATSDLSDTS CAQTHEGLKT SRNTDQRHPA PKKVDDQEPG SVANIIVDPK
 81  LFQKRQFQSS RVLFSTQPPP LSRDEQSVEF LNNEDALNRN IQAKRQNHPV HDLGEHSVCD SISEWVTKTT ATDIKGNTVT
                                181                                                     231 233
161  VEVDVNLNNE VYKQYFFETK CRNPNPVPSG CRGIDSRLWN SYCTTTQTFV KALTMEGNQA SWRFIRIDTA CVCVITKKTD

241  NL
```

C Carbamidomethylation (+57.02)

**Supporting Peptides:**

| Peptide | Uniq | -10lgP | Mass | Length | ppm | m/z | z | RT | Fraction | Scan | Source File | Area F1 | Area F2a | Area F3a | Area F4 | Area F5 | Area F6a | Area F7a | Area F7b | Area F8 | Area F9a | Area Sample 1F10a | Area F10b | Area F11a | Area F11b | Area F12a | Area F13 | #Feature | #Feature F1 | #Feature F2a | #Feature F3a | #Feature F4 | #Feature F5 | #Feature F6a | #Feature F7a | #Feature F7b | #Feature F8 | #Feature F9a | #Feature Sample 1F10a | #Feature F10b | #Feature F11a | #Feature F11b | #Feature F12a | #Feature F13 | Start | End | PTM | AScore | Found By |
|---|---|---|---|---|---|---|---|---|---|---|---|---|---|---|---|---|---|---|---|---|---|---|---|---|---|---|---|---|---|---|---|---|---|---|---|---|---|---|---|---|---|---|---|---|---|---|---|---|---|---|
| R.FIRIDTAC(+57.02)VC(+57.02)VITK.K | Y | 66.96 | 1694.8848 | 14 | 3.7 | 565.9693 | 3 | 61.17 | 8 | F8:36635 | BuCaTN01_F7b.raw | | | | | | | | 2.4636E5 | | | | | | | | | 1 | 0 | 0 | 0 | 0 | 0 | 0 | 0 | 1 | 0 | 0 | 0 | 0 | 0 | 0 | 0 | 0 | 224 | 237 | Carbamidomethylation | C8:Carbamidomethylation:1000.00;C10:Carbamidomethylation:1000.00 | PEAKS DB |
| R.NPNPVPSGC(+57.02)R.G | N | 63.70 | 1096.5083 | 10 | -0.4 | 549.2612 | 2 | 26.38 | 7 | F7:7077 | BuCaTN01_F7a.raw | | | | | | | 8.9262E5 | | | | | | | | | | 1 | 0 | 0 | 0 | 0 | 0 | 0 | 1 | 0 | 0 | 0 | 0 | 0 | 0 | 0 | 0 | 0 | 183 | 192 | Carbamidomethylation | C9:Carbamidomethylation:1000.00 | PEAKS DB |
| K.C(+57.02)RNPNPVPSGC(+57.02)R.G | N | 62.43 | 1412.6401 | 12 | 0.4 | 471.8875 | 3 | 24.90 | 7 | F7:6664 | BuCaTN01_F7a.raw | | | | | | | 5.7693E6 | 2.8804E5 | | | | | | | | | 3 | 0 | 0 | 0 | 0 | 0 | 0 | 2 | 1 | 0 | 0 | 0 | 0 | 0 | 0 | 0 | 0 | 181 | 192 | Carbamidomethylation | C1:Carbamidomethylation:1000.00;C11:Carbamidomethylation:1000.00 | PEAKS DB |
| E.VYKQYFFETK.C | N | 54.63 | 1351.6812 | 10 | 2.9 | 451.5676 | 3 | 50.63 | 8 | F8:27078 | BuCaTN01_F7b.raw | | | | | | | | 5.2856E5 | | | | | | | | | 1 | 0 | 0 | 0 | 0 | 0 | 0 | 0 | 1 | 0 | 0 | 0 | 0 | 0 | 0 | 0 | 0 | 171 | 180 | | | PEAKS DB |
| K.QYFFETK.C | N | 54.60 | 961.4545 | 7 | 0.2 | 481.7346 | 2 | 53.27 | 7 | F7:26821 | BuCaTN01_F7a.raw | | | | | | | 2.4891E7 | 7.2632E6 | | | | | | | | | 2 | 0 | 0 | 0 | 0 | 0 | 0 | 1 | 1 | 0 | 0 | 0 | 0 | 0 | 0 | 0 | 0 | 174 | 180 | | | PEAKS DB |
| R.FIRIDTAC(+57.02)VC(+57.02)VITKK.T | Y | 52.21 | 1822.9797 | 15 | 1.3 | 456.7515 | 4 | 56.15 | 8 | F8:31913 | BuCaTN01_F7b.raw | | | | | | | | 2.2079E5 | | | | | | | | | 1 | 0 | 0 | 0 | 0 | 0 | 0 | 0 | 1 | 0 | 0 | 0 | 0 | 0 | 0 | 0 | 0 | 224 | 238 | Carbamidomethylation | C8:Carbamidomethylation:1000.00;C10:Carbamidomethylation:1000.00 | PEAKS DB |
| R.FIRIDTAC(+57.02)VC(+57.02).V | N | 49.72 | 1253.5896 | 10 | 3.0 | 627.8021 | 2 | 54.32 | 8 | F8:30301 | BuCaTN01_F7b.raw | | | | | | | | 3.8159E5 | | | | | | | | | 1 | 0 | 0 | 0 | 0 | 0 | 0 | 0 | 1 | 0 | 0 | 0 | 0 | 0 | 0 | 0 | 0 | 224 | 233 | Carbamidomethylation | C8:Carbamidomethylation:1000.00;C10:Carbamidomethylation:1000.00 | PEAKS DB |
| L.NNEVYKQYFFETK.C | N | 42.27 | 1708.8097 | 13 | 3.3 | 855.4124 | 2 | 57.90 | 8 | F8:33658 | BuCaTN01_F7b.raw | | | | | | | | 3.8306E4 | | | | | | | | | 1 | 0 | 0 | 0 | 0 | 0 | 0 | 0 | 1 | 0 | 0 | 0 | 0 | 0 | 0 | 0 | 0 | 168 | 180 | | | PEAKS DB |
| K.QYFFETKC(+57.02)R.N | N | 41.76 | 1277.5863 | 9 | -0.7 | 426.8691 | 3 | 46.69 | 7 | F7:21495 | BuCaTN01_F7a.raw | | | | | | | 3.7535E5 | | | | | | | | | | 1 | 0 | 0 | 0 | 0 | 0 | 0 | 1 | 0 | 0 | 0 | 0 | 0 | 0 | 0 | 0 | 0 | 174 | 182 | Carbamidomethylation | C8:Carbamidomethylation:1000.00 | PEAKS DB |
| **total 9 peptides** | | | | | | | | | | | | | | | | | | | | | | | | | | | | | | | | | | | | | | | | | | | | | | | | | | |

**Best Unique PSM (Scan F8:36635, m/z=565.9693, z=3, RT=61.17, ppm=3.7):**

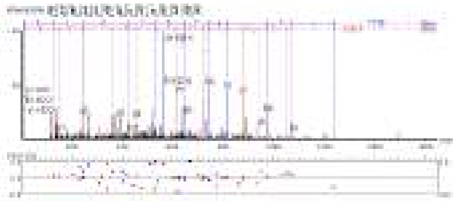

## ABA60126.1

back to list

| Protein Coverage | Supporting Peptides | Best Unique PSM |

**Protein Coverage:**


```
  1  MSMLCYTLII AFLIGIWAAP QSEDNVPLGS PATSDLSDTS CAQTHEGLKT SRNTDQRHPA PKKVDDQEPG SVANIIVDPK
 81  LFQKRQFQSS RVLFSTQPPP LSRDEQSVEF LNNEDALNRN IQAKRQNHPV HDLGEHSVCD SISEWVTKTT ATDIKGNTVT
                                181                                                     231 233
161  VEVDVNLNNE VYKQYFFETK CRNPNPVPSG CRGIDSRLWN SYCTTTQTFV KALTMEGNQA SWRFIRIDTA CVCVITKKTD

241  NL
```

C Carbamidomethylation (+57.02)


**Supporting Peptides:**


| Peptide | Uniq | -10lgP | Mass | Length | ppm | m/z | z | RT | Fraction | Scan | Source File | Area F1 | Area F2a | Area F3a | Area F4 | Area F5 | Area F6a | Area F7a | Area F7b | Area F8 | Area F9a | Area Sample 1F10a | Area F10b | Area F11a | Area F11b | Area F12a | Area F13 | #Feature | #Feature F1 | #Feature F2a | #Feature F3a | #Feature F4 | #Feature F5 | #Feature F6a | #Feature F7a | #Feature F7b | #Feature F8 | #Feature F9a | #Feature Sample 1F10a | #Feature F10b | #Feature F11a | #Feature F11b | #Feature F12a | #Feature F13 | Start | End | PTM | AScore | Found By |
|---|---|---|---|---|---|---|---|---|---|---|---|---|---|---|---|---|---|---|---|---|---|---|---|---|---|---|---|---|---|---|---|---|---|---|---|---|---|---|---|---|---|---|---|---|---|---|---|---|---|---|
| R.FIRIDTAC(+57.02)VC(+57.02)VITK.K | Y | 66.96 | 1694.8848 | 14 | 3.7 | 565.9693 | 3 | 61.17 | 8 | F8:36635 | BuCaTN01_F7b.raw | | | | | | | | 2.4636E5 | | | | | | | | | 1 | 0 | 0 | 0 | 0 | 0 | 0 | 0 | 1 | 0 | 0 | 0 | 0 | 0 | 0 | 0 | 0 | 224 | 237 | Carbamidomethylation | C8:Carbamidomethylation:1000.00;C10:Carbamidomethylation:1000.00 | PEAKS DB |
| R.NPNPVPSGC(+57.02)R.G | N | 63.70 | 1096.5083 | 10 | -0.4 | 549.2612 | 2 | 26.38 | 7 | F7:7077 | BuCaTN01_F7a.raw | | | | | | | 8.9262E5 | | | | | | | | | | 1 | 0 | 0 | 0 | 0 | 0 | 0 | 1 | 0 | 0 | 0 | 0 | 0 | 0 | 0 | 0 | 0 | 183 | 192 | Carbamidomethylation | C9:Carbamidomethylation:1000.00 | PEAKS DB |
| K.C(+57.02)RNPNPVPSGC(+57.02)R.G | N | 62.43 | 1412.6401 | 12 | 0.4 | 471.8875 | 3 | 24.90 | 7 | F7:6664 | BuCaTN01_F7a.raw | | | | | | | 5.7693E6 | 2.8804E5 | | | | | | | | | 3 | 0 | 0 | 0 | 0 | 0 | 0 | 2 | 1 | 0 | 0 | 0 | 0 | 0 | 0 | 0 | 0 | 181 | 192 | Carbamidomethylation | C1:Carbamidomethylation:1000.00;C11:Carbamidomethylation:1000.00 | PEAKS DB |
| E.VYKQYFFETK.C | N | 54.63 | 1351.6812 | 10 | 2.9 | 451.5676 | 3 | 50.63 | 8 | F8:27078 | BuCaTN01_F7b.raw | | | | | | | | 5.2856E5 | | | | | | | | | 1 | 0 | 0 | 0 | 0 | 0 | 0 | 0 | 1 | 0 | 0 | 0 | 0 | 0 | 0 | 0 | 0 | 171 | 180 | | | PEAKS DB |
| K.QYFFETK.C | N | 54.60 | 961.4545 | 7 | 0.2 | 481.7346 | 2 | 53.27 | 7 | F7:26821 | BuCaTN01_F7a.raw | | | | | | | 2.4891E7 | 7.2632E6 | | | | | | | | | 2 | 0 | 0 | 0 | 0 | 0 | 0 | 1 | 1 | 0 | 0 | 0 | 0 | 0 | 0 | 0 | 0 | 174 | 180 | | | PEAKS DB |
| R.FIRIDTAC(+57.02)VC(+57.02)VITKK.T | Y | 52.21 | 1822.9797 | 15 | 1.3 | 456.7515 | 4 | 56.15 | 8 | F8:31913 | BuCaTN01_F7b.raw | | | | | | | | 2.2079E5 | | | | | | | | | 1 | 0 | 0 | 0 | 0 | 0 | 0 | 0 | 1 | 0 | 0 | 0 | 0 | 0 | 0 | 0 | 0 | 224 | 238 | Carbamidomethylation | C8:Carbamidomethylation:1000.00;C10:Carbamidomethylation:1000.00 | PEAKS DB |
| R.FIRIDTAC(+57.02)VC(+57.02).V | N | 49.72 | 1253.5896 | 10 | 3.0 | 627.8021 | 2 | 54.32 | 8 | F8:30301 | BuCaTN01_F7b.raw | | | | | | | | 3.8159E5 | | | | | | | | | 1 | 0 | 0 | 0 | 0 | 0 | 0 | 0 | 1 | 0 | 0 | 0 | 0 | 0 | 0 | 0 | 0 | 224 | 233 | Carbamidomethylation | C8:Carbamidomethylation:1000.00;C10:Carbamidomethylation:1000.00 | PEAKS DB |
| L.NNEVYKQYFFETK.C | N | 42.27 | 1708.8097 | 13 | 3.3 | 855.4124 | 2 | 57.90 | 8 | F8:33658 | BuCaTN01_F7b.raw | | | | | | | | 3.8306E4 | | | | | | | | | 1 | 0 | 0 | 0 | 0 | 0 | 0 | 0 | 1 | 0 | 0 | 0 | 0 | 0 | 0 | 0 | 0 | 168 | 180 | | | PEAKS DB |
| K.QYFFETKC(+57.02)R.N | N | 41.76 | 1277.5863 | 9 | -0.7 | 426.8691 | 3 | 46.69 | 7 | F7:21495 | BuCaTN01_F7a.raw | | | | | | | 3.7535E5 | | | | | | | | | | 1 | 0 | 0 | 0 | 0 | 0 | 0 | 1 | 0 | 0 | 0 | 0 | 0 | 0 | 0 | 0 | 0 | 174 | 182 | Carbamidomethylation | C8:Carbamidomethylation:1000.00 | PEAKS DB |
| **total 9 peptides** | | | | | | | | | | | | | | | | | | | | | | | | | | | | | | | | | | | | | | | | | | | | | | | | | | |


**Best Unique PSM (Scan F8:36635, m/z=565.9693, z=3, RT=61.17, ppm=3.7):**

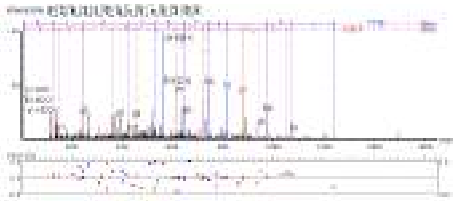

## Q3HXY3.1

back to list

| Protein Coverage | Supporting Peptides | Best Unique PSM |

**Protein Coverage:**

```
  1 MSMLCYTLII AFLIGIWAAP QSEDNVPLGS PATSDLSDTS CAQTHEGLKT SRNTDQRHPA PKKVDDQELG SVANIIVDPK
 81 LFQKRQFQSS RVLFSTQPPP LSRDEQSVEF LDNEDALNRN IQAKRQNHPV HDLGEHSVCD SISEWVTKTT ATDIKGNTVT
                           181                                                  231 233
161 VEVDVNLNNE VYKQYFFETK CRNPNPVPSG CRGIDSRLWN SYCTTTQTFV KALTMEGNQA SWRFIRIDTA CVCVITKKTD
241 NL
```

C Carbamidomethylation (+57.02)

**Supporting Peptides:**

| Peptide | Uniq | -10lgP | Mass | Length | ppm | m/z | z | RT | Fraction | Scan | Source File | Area F1 | Area F2a | Area F3a | Area F4 | Area F5 | Area F6a | Area F7a | Area F7b | Area F8 | Area F9a | Area Sample 1F10a | Area F10b | Area F11a | Area F11b | Area F12a | Area F13 | #Feature | #Feature F1 | #Feature F2a | #Feature F3a | #Feature F4 | #Feature F5 | #Feature F6a | #Feature F7a | #Feature F7b | #Feature F8 | #Feature F9a | #Feature Sample 1F10a | #Feature F10b | #Feature F11a | #Feature F11b | #Feature F12a | #Feature F13 | Start | End | PTM | AScore | Found By |
|---|---|---|---|---|---|---|---|---|---|---|---|---|---|---|---|---|---|---|---|---|---|---|---|---|---|---|---|---|---|---|---|---|---|---|---|---|---|---|---|---|---|---|---|---|---|---|---|---|---|
| R.FIRIDTAC(+57.02)VC(+57.02)VITK.K | Y | 66.96 | 1694.8848 | 14 | 3.7 | 565.9693 | 3 | 61.17 | 8 | F8:36635 | BuCaTN01_F7b.raw | | | | | | | | 2.4636E5 | | | | | | | | | 1 | 0 | 0 | 0 | 0 | 0 | 0 | 0 | 1 | 0 | 0 | 0 | 0 | 0 | 0 | 0 | 0 | 224 | 237 | Carbamidomethylation | C8:Carbamidomethylation:1000.00;C10:Carbamidomethylation:1000.00 | PEAKS DB |
| R.NPNPVPSGC(+57.02)R.G | N | 63.70 | 1096.5083 | 10 | -0.4 | 549.2612 | 2 | 26.38 | 7 | F7:7077 | BuCaTN01_F7a.raw | | | | | | | 8.9262E5 | | | | | | | | | | 1 | 0 | 0 | 0 | 0 | 0 | 0 | 1 | 0 | 0 | 0 | 0 | 0 | 0 | 0 | 0 | 0 | 183 | 192 | Carbamidomethylation | C9:Carbamidomethylation:1000.00 | PEAKS DB |
| K.C(+57.02)RNPNPVPSGC(+57.02)R.G | N | 62.43 | 1412.6401 | 12 | 0.4 | 471.8875 | 3 | 24.90 | 7 | F7:6664 | BuCaTN01_F7a.raw | | | | | | | 5.7693E6 | 2.8804E5 | | | | | | | | | 3 | 0 | 0 | 0 | 0 | 0 | 0 | 2 | 1 | 0 | 0 | 0 | 0 | 0 | 0 | 0 | 0 | 181 | 192 | Carbamidomethylation | C1:Carbamidomethylation:1000.00;C11:Carbamidomethylation:1000.00 | PEAKS DB |
| E.VYKQYFFETK.C | N | 54.63 | 1351.6812 | 10 | 2.9 | 451.5676 | 3 | 50.63 | 8 | F8:27078 | BuCaTN01_F7b.raw | | | | | | | | 5.2856E5 | | | | | | | | | 1 | 0 | 0 | 0 | 0 | 0 | 0 | 0 | 1 | 0 | 0 | 0 | 0 | 0 | 0 | 0 | 0 | 171 | 180 | | | PEAKS DB |
| K.QYFFETK.C | N | 54.60 | 961.4545 | 7 | 0.2 | 481.7346 | 2 | 53.27 | 7 | F7:26821 | BuCaTN01_F7a.raw | | | | | | | 2.4891E7 | 7.2632E6 | | | | | | | | | 2 | 0 | 0 | 0 | 0 | 0 | 0 | 1 | 1 | 0 | 0 | 0 | 0 | 0 | 0 | 0 | 0 | 174 | 180 | | | PEAKS DB |
| R.FIRIDTAC(+57.02)VC(+57.02)VITKK.T | Y | 52.21 | 1822.9797 | 15 | 1.3 | 456.7515 | 4 | 56.15 | 8 | F8:31913 | BuCaTN01_F7b.raw | | | | | | | | 2.2079E5 | | | | | | | | | 1 | 0 | 0 | 0 | 0 | 0 | 0 | 0 | 1 | 0 | 0 | 0 | 0 | 0 | 0 | 0 | 0 | 224 | 238 | Carbamidomethylation | C8:Carbamidomethylation:1000.00;C10:Carbamidomethylation:1000.00 | PEAKS DB |
| R.FIRIDTAC(+57.02)VC(+57.02).V | N | 49.72 | 1253.5896 | 10 | 3.0 | 627.8021 | 2 | 54.32 | 8 | F8:30301 | BuCaTN01_F7b.raw | | | | | | | | 3.8159E5 | | | | | | | | | 1 | 0 | 0 | 0 | 0 | 0 | 0 | 0 | 1 | 0 | 0 | 0 | 0 | 0 | 0 | 0 | 0 | 224 | 233 | Carbamidomethylation | C8:Carbamidomethylation:1000.00;C10:Carbamidomethylation:1000.00 | PEAKS DB |
| L.NNEVYKQYFFETK.C | N | 42.27 | 1708.8097 | 13 | 3.3 | 855.4124 | 2 | 57.90 | 8 | F8:33658 | BuCaTN01_F7b.raw | | | | | | | | 3.8306E4 | | | | | | | | | 1 | 0 | 0 | 0 | 0 | 0 | 0 | 0 | 1 | 0 | 0 | 0 | 0 | 0 | 0 | 0 | 0 | 168 | 180 | | | PEAKS DB |
| K.QYFFETKC(+57.02)R.N | N | 41.76 | 1277.5863 | 9 | -0.7 | 426.8691 | 3 | 46.69 | 7 | F7:21495 | BuCaTN01_F7a.raw | | | | | | | 3.7535E5 | | | | | | | | | | 1 | 0 | 0 | 0 | 0 | 0 | 0 | 1 | 0 | 0 | 0 | 0 | 0 | 0 | 0 | 0 | 0 | 174 | 182 | Carbamidomethylation | C8:Carbamidomethylation:1000.00 | PEAKS DB |
| total 9 peptides | | | | | | | | | | | | | | | | | | | | | | | | | | | | | | | | | | | | | | | | | | | | | | | | | |

**Best Unique PSM (Scan F8:36635, m/z=565.9693, z=3, RT=61.17, ppm=3.7):**

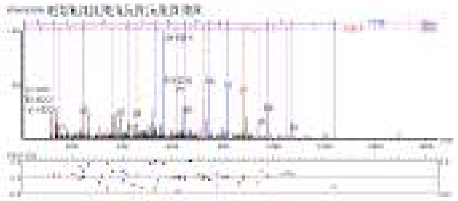

## ABA60124.1

back to list

| Protein Coverage | Supporting Peptides | Best Unique PSM |

**Protein Coverage:**

```
  1 MSMLCYTLII AFLIGIWAAP QSEDNVPLGS PATSDLSDTS CAQTHEGLKT SRNTDQRHPA PKKVDDQELG SVANIIVDPK
 81 LFQKRQFQSS RVLFSTQPPP LSRDEQSVEF LDNEDALNRN IQAKRQNHPV HDLGEHSVCD SISEWVTKTT ATDIKGNTVT
                           181                                                  231 233
161 VEVDVNLNNE VYKQYFFETK CRNPNPVPSG CRGIDSRLWN SYCTTTQTFV KALTMEGNQA SWRFIRIDTA CVCVITKKTD
241 NL
```

C Carbamidomethylation (+57.02)

**Supporting Peptides:**

| Peptide | Uniq | -10lgP | Mass | Length | ppm | m/z | z | RT | Fraction | Scan | Source File | Area F1 | Area F2a | Area F3a | Area F4 | Area F5 | Area F6a | Area F7a | Area F7b | Area F8 | Area F9a | Area Sample 1F10a | Area F10b | Area F11a | Area F11b | Area F12a | Area F13 | #Feature | #Feature F1 | #Feature F2a | #Feature F3a | #Feature F4 | #Feature F5 | #Feature F6a | #Feature F7a | #Feature F7b | #Feature F8 | #Feature F9a | #Feature Sample 1F10a | #Feature F10b | #Feature F11a | #Feature F11b | #Feature F12a | #Feature F13 | Start | End | PTM | AScore | Found By |
|---|---|---|---|---|---|---|---|---|---|---|---|---|---|---|---|---|---|---|---|---|---|---|---|---|---|---|---|---|---|---|---|---|---|---|---|---|---|---|---|---|---|---|---|---|---|---|---|---|---|
| R.FIRIDTAC(+57.02)VC(+57.02)VITK.K | Y | 66.96 | 1694.8848 | 14 | 3.7 | 565.9693 | 3 | 61.17 | 8 | F8:36635 | BuCaTN01_F7b.raw | | | | | | | | 2.4636E5 | | | | | | | | | 1 | 0 | 0 | 0 | 0 | 0 | 0 | 0 | 1 | 0 | 0 | 0 | 0 | 0 | 0 | 0 | 0 | 224 | 237 | Carbamidomethylation | C8:Carbamidomethylation:1000.00;C10:Carbamidomethylation:1000.00 | PEAKS DB |
| R.NPNPVPSGC(+57.02)R.G | N | 63.70 | 1096.5083 | 10 | -0.4 | 549.2612 | 2 | 26.38 | 7 | F7:7077 | BuCaTN01_F7a.raw | | | | | | | 8.9262E5 | | | | | | | | | | 1 | 0 | 0 | 0 | 0 | 0 | 0 | 1 | 0 | 0 | 0 | 0 | 0 | 0 | 0 | 0 | 0 | 183 | 192 | Carbamidomethylation | C9:Carbamidomethylation:1000.00 | PEAKS DB |
| K.C(+57.02)RNPNPVPSGC(+57.02)R.G | N | 62.43 | 1412.6401 | 12 | 0.4 | 471.8875 | 3 | 24.90 | 7 | F7:6664 | BuCaTN01_F7a.raw | | | | | | | 5.7693E6 | 2.8804E5 | | | | | | | | | 3 | 0 | 0 | 0 | 0 | 0 | 0 | 2 | 1 | 0 | 0 | 0 | 0 | 0 | 0 | 0 | 0 | 181 | 192 | Carbamidomethylation | C1:Carbamidomethylation:1000.00;C11:Carbamidomethylation:1000.00 | PEAKS DB |
| E.VYKQYFFETK.C | N | 54.63 | 1351.6812 | 10 | 2.9 | 451.5676 | 3 | 50.63 | 8 | F8:27078 | BuCaTN01_F7b.raw | | | | | | | | 5.2856E5 | | | | | | | | | 1 | 0 | 0 | 0 | 0 | 0 | 0 | 0 | 1 | 0 | 0 | 0 | 0 | 0 | 0 | 0 | 0 | 171 | 180 | | | PEAKS DB |
| K.QYFFETK.C | N | 54.60 | 961.4545 | 7 | 0.2 | 481.7346 | 2 | 53.27 | 7 | F7:26821 | BuCaTN01_F7a.raw | | | | | | | 2.4891E7 | 7.2632E6 | | | | | | | | | 2 | 0 | 0 | 0 | 0 | 0 | 0 | 1 | 1 | 0 | 0 | 0 | 0 | 0 | 0 | 0 | 0 | 174 | 180 | | | PEAKS DB |
| R.FIRIDTAC(+57.02)VC(+57.02)VITKK.T | Y | 52.21 | 1822.9797 | 15 | 1.3 | 456.7515 | 4 | 56.15 | 8 | F8:31913 | BuCaTN01_F7b.raw | | | | | | | | 2.2079E5 | | | | | | | | | 1 | 0 | 0 | 0 | 0 | 0 | 0 | 0 | 1 | 0 | 0 | 0 | 0 | 0 | 0 | 0 | 0 | 224 | 238 | Carbamidomethylation | C8:Carbamidomethylation:1000.00;C10:Carbamidomethylation:1000.00 | PEAKS DB |
| R.FIRIDTAC(+57.02)VC(+57.02).V | N | 49.72 | 1253.5896 | 10 | 3.0 | 627.8021 | 2 | 54.32 | 8 | F8:30301 | BuCaTN01_F7b.raw | | | | | | | | 3.8159E5 | | | | | | | | | 1 | 0 | 0 | 0 | 0 | 0 | 0 | 0 | 1 | 0 | 0 | 0 | 0 | 0 | 0 | 0 | 0 | 224 | 233 | Carbamidomethylation | C8:Carbamidomethylation:1000.00;C10:Carbamidomethylation:1000.00 | PEAKS DB |
| L.NNEVYKQYFFETK.C | N | 42.27 | 1708.8097 | 13 | 3.3 | 855.4124 | 2 | 57.90 | 8 | F8:33658 | BuCaTN01_F7b.raw | | | | | | | | 3.8306E4 | | | | | | | | | 1 | 0 | 0 | 0 | 0 | 0 | 0 | 0 | 1 | 0 | 0 | 0 | 0 | 0 | 0 | 0 | 0 | 168 | 180 | | | PEAKS DB |
| K.QYFFETKC(+57.02)R.N | N | 41.76 | 1277.5863 | 9 | -0.7 | 426.8691 | 3 | 46.69 | 7 | F7:21495 | BuCaTN01_F7a.raw | | | | | | | 3.7535E5 | | | | | | | | | | 1 | 0 | 0 | 0 | 0 | 0 | 0 | 1 | 0 | 0 | 0 | 0 | 0 | 0 | 0 | 0 | 0 | 174 | 182 | Carbamidomethylation | C8:Carbamidomethylation:1000.00 | PEAKS DB |
| total 9 peptides | | | | | | | | | | | | | | | | | | | | | | | | | | | | | | | | | | | | | | | | | | | | | | | | | |

**Best Unique PSM (Scan F8:36635, m/z=565.9693, z=3, RT=61.17, ppm=3.7):**

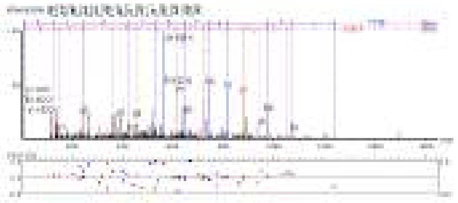

## AAO84769.1

back to list

| Protein Coverage | Supporting Peptides | Best Unique PSM |

**Protein Coverage:**

```
                                                                                 78
  1 AVCVSLLGAA NIPPQPLTFL QFNEMIECTI PGSFPLLDNM DCGCYCGTGG RGTPVDMLDR CCKEHDDCYA QIKENLKCSS
                                 113 115     122
 81 LLNVPYVKQY SFTCSEGDLT CSDAAGTCAR IVCDCDRTAA LCFAEAPYKR RNFKIDYKTR CQ
```

C Carbamidomethylation (+57.02)

**Supporting Peptides:**

| Peptide | Uniq | -10lgP | Mass | Length | ppm | m/z | z | RT | Fraction | Scan | Source File | Area F1 | Area F2a | Area F3a | Area F4 | Area F5 | Area F6a | Area F7a | Area F7b | Area F8 | Area F9a | Area Sample 1F10a | Area F10b | Area F11a | Area F11b | Area F12a | Area F13 | #Feature | #Feature F1 | #Feature F2a | #Feature F3a | #Feature F4 | #Feature F5 | #Feature F6a | #Feature F7a | #Feature F7b | #Feature F8 | #Feature F9a | #Feature Sample 1F10a | #Feature F10b | #Feature F11a | #Feature F11b | #Feature F12a | #Feature F13 | Start | End | PTM | AScore | Found By |
|---|---|---|---|---|---|---|---|---|---|---|---|---|---|---|---|---|---|---|---|---|---|---|---|---|---|---|---|---|---|---|---|---|---|---|---|---|---|---|---|---|---|---|---|---|---|---|---|---|---|
| K.C(+57.02)SSLLNVPYVK.Q | N | 83.60 | 1278.6642 | 11 | 4.4 | 640.3396 | 2 | 61.00 | 14 | F14:36687 | BuCaTN01_F11b.raw | | | | | | | | 3.8205E5 | | 3.3131E6 | 0 | 2.8562E6 | 2.7996E5 | 7.3534E7 | 4.4745E6 | | 6 | 0 | 0 | 0 | 0 | 0 | 0 | 0 | 1 | 0 | 1 | 0 | 1 | 1 | 1 | 1 | 0 | 78 | 88 | Carbamidomethylation | C1:Carbamidomethylation:1000.00 | PEAKS DB |
| R.TAALC(+57.02)FAEAPYK.R | Y | 79.51 | 1340.6434 | 12 | 1.5 | 671.3285 | 2 | 56.61 | 12 | F12:32686 | BuCaTN01_F10b.raw | | | | | | | | 4.5975E5 | | 4.0569E6 | | 3.3872E6 | 3.1907E5 | 1.0278E8 | 5.3528E6 | | 6 | 0 | 0 | 0 | 0 | 0 | 0 | 0 | 1 | 0 | 1 | 0 | 1 | 1 | 1 | 1 | 0 | 118 | 129 | Carbamidomethylation | C5:Carbamidomethylation:1000.00 | PEAKS DB |
| R.TAALC(+57.02)FAEAPYKR.R | Y | 71.55 | 1496.7445 | 13 | 2.9 | 499.9215 | 3 | 50.04 | 14 | F14:27067 | BuCaTN01_F11b.raw | | | | | | | | | | 1.0359E6 | | 0 | | 4.1271E7 | 0 | | 3 | 0 | 0 | 0 | 0 | 0 | 0 | 0 | 0 | 0 | 1 | 0 | 0 | 0 | 2 | 0 | 0 | 118 | 130 | Carbamidomethylation | C5:Carbamidomethylation:1000.00 | PEAKS DB |
| R.IVC(+57.02)DC(+57.02)DRTAAL.C | N | 53.80 | 1292.5853 | 11 | 4.0 | 647.3007 | 2 | 38.90 | 8 | F8:16711 | BuCaTN01_F7b.raw | | | | | | | | 8.9609E6 | | 1.7898E7 | | 1.6803E6 | | 0 | 8.1365E5 | | 4 | 0 | 0 | 0 | 0 | 0 | 0 | 0 | 1 | 0 | 1 | 0 | 1 | 0 | 0 | 1 | 0 | 111 | 121 | Carbamidomethylation | C3:Carbamidomethylation:1000.00;C5:Carbamidomethylation:1000.00 | PEAKS DB |
| C.SSLLNVPYVK.Q | N | 53.49 | 1118.6335 | 10 | 3.1 | 560.3235 | 2 | 60.43 | 14 | F14:36274 | BuCaTN01_F11b.raw | | | | | | | | | | | | | | 5.9096E5 | | | 1 | 0 | 0 | 0 | 0 | 0 | 0 | 0 | 0 | 0 | 0 | 0 | 0 | 0 | 1 | 0 | 0 | 79 | 88 | | | PEAKS DB |
| total 5 peptides | | | | | | | | | | | | | | | | | | | | | | | | | | | | | | | | | | | | | | | | | | | | | | | | | |

**Best Unique PSM (Scan F12:32686, m/z=671.3285, z=2, RT=56.61, ppm=1.5):**

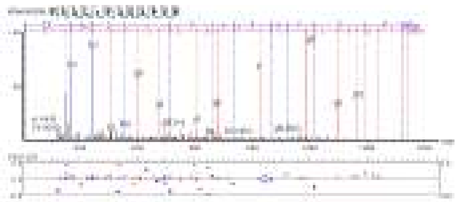

## Q802I1.1

back to list

| Protein Coverage | Supporting Peptides | Best Unique PSM |

**Protein Coverage:**

1 AVCVSLLGAA NIPPQPLTFL QFNEMIECTI PGSFPLLDNM DCGCYCGTGG RGTPVDMLDR CCKEHDDCYA QIKENLK**CSS**

81 **LLNVPYVK**QY SFTCSEGDLT CSDAAGTCAR **IVCDCDRTAA LCFAEAPYKR** RNFKIDYKTR CQ

C Carbamidomethylation (+57.02)

**Supporting Peptides:**

| Peptide | Uniq | -10lgP | Mass | Length | ppm | m/z | z | RT | Fraction | Scan | Source File | Area F1 | Area F2a | Area F3a | Area F4 | Area F5 | Area F6a | Area F7a | Area F7b | Area F8 | Area F9a | Area Sample 1F10a | Area F10b | Area F11a | Area F11b | Area F12a | Area F13 | #Feature | #Feature F1 | #Feature F2a | #Feature F3a | #Feature F4 | #Feature F5 | #Feature F6a | #Feature F7a | #Feature F7b | #Feature F8 | #Feature F9a | #Feature Sample 1F10a | #Feature F10b | #Feature F11a | #Feature F11b | #Feature F12a | #Feature F13 | Start | End | PTM | AScore | Found By |
|---|---|---|---|---|---|---|---|---|---|---|---|---|---|---|---|---|---|---|---|---|---|---|---|---|---|---|---|---|---|---|---|---|---|---|---|---|---|---|---|---|---|---|---|---|---|---|---|---|---|
| K.C(+57.02)SSLLNVPYVK.Q | N | 83.60 | 1278.6642 | 11 | 4.4 | 640.3396 | 2 | 61.00 | 14 | F14:36687 | BuCaTN01_F11b.raw | | | | | | | | 3.8205E5 | | 3.3131E6 | 0 | 2.8562E6 | 2.7996E5 | 7.3534E7 | 4.4745E6 | | 6 | 0 | 0 | 0 | 0 | 0 | 0 | 0 | 1 | 0 | 1 | 0 | 1 | 1 | 1 | 1 | 0 | 78 | 88 | Carbamidomethylation | C1:Carbamidomethylation:1000.00 | PEAKS DB |
| R.TAALC(+57.02)FAEAPYK.R | Y | 79.51 | 1340.6434 | 12 | 1.5 | 671.3285 | 2 | 56.61 | 12 | F12:32686 | BuCaTN01_F10b.raw | | | | | | | | 4.5975E5 | | 4.0569E6 | | 3.3872E6 | 3.1907E5 | 1.0278E8 | 5.3528E6 | | 6 | 0 | 0 | 0 | 0 | 0 | 0 | 0 | 1 | 0 | 1 | 0 | 1 | 1 | 1 | 1 | 0 | 118 | 129 | Carbamidomethylation | C5:Carbamidomethylation:1000.00 | PEAKS DB |
| R.TAALC(+57.02)FAEAPYKR.R | Y | 71.55 | 1496.7445 | 13 | 2.9 | 499.9215 | 3 | 50.04 | 14 | F14:27067 | BuCaTN01_F11b.raw | | | | | | | | | | 1.0359E6 | | 0 | | 4.1271E7 | 0 | | 3 | 0 | 0 | 0 | 0 | 0 | 0 | 0 | 0 | 0 | 1 | 0 | 0 | 0 | 2 | 0 | 0 | 118 | 130 | Carbamidomethylation | C5:Carbamidomethylation:1000.00 | PEAKS DB |
| R.IVC(+57.02)DC(+57.02)DRTAAL.C | N | 53.80 | 1292.5853 | 11 | 4.0 | 647.3007 | 2 | 38.90 | 8 | F8:16711 | BuCaTN01_F7b.raw | | | | | | | | 8.9609E6 | | 1.7898E7 | | 1.6803E6 | | 0 | 8.1365E5 | | 4 | 0 | 0 | 0 | 0 | 0 | 0 | 0 | 1 | 0 | 1 | 0 | 1 | 0 | 0 | 1 | 0 | 111 | 121 | Carbamidomethylation | C3:Carbamidomethylation:1000.00;C5:Carbamidomethylation:1000.00 | PEAKS DB |
| C.SSLLNVPYVK.Q | N | 53.49 | 1118.6335 | 10 | 3.1 | 560.3235 | 2 | 60.43 | 14 | F14:36274 | BuCaTN01_F11b.raw | | | | | | | | | | | | | | 5.9096E5 | | | 1 | 0 | 0 | 0 | 0 | 0 | 0 | 0 | 0 | 0 | 0 | 0 | 0 | 0 | 1 | 0 | 0 | 79 | 88 | | | PEAKS DB |
| total 5 peptides | | | | | | | | | | | | | | | | | | | | | | | | | | | | | | | | | | | | | | | | | | | | | | | | | |

**Best Unique PSM (Scan F12:32686, m/z=671.3285, z=2, RT=56.61, ppm=1.5):**

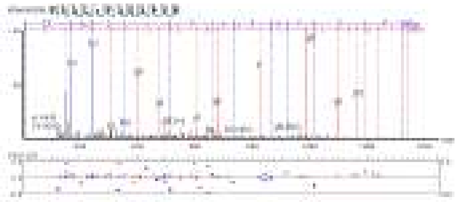

## XP_026582322.1

back to list

| Protein Coverage | Supporting Peptides | Best Unique PSM |

**Protein Coverage:**

1 MRTGAGNQKS NCLLVSSTWG ACVHWEDDLV LELMPPRLPV SGTHALGNHE FDNGLNGLLD PLLK**NVKFPI LSANIRPK**GP

C Carbamidomethylation (+57.02)
O Oxidation (M) (+15.99)

81 IASN**ISGYIL PYKIINVGSE K**VGIIGYTTK ETPFLSNPGP YLEFRDEVEE LQKHADKLTT LGVNKIIALG HSGFKEDCRI

161 AQKVKGVDVV VGGHTNTFLY TGSPPSNEVA AGNYPFMQLS DDGREVPVVQ AYAFGKYLGY LNVTFDDKGK VIK**ASGNPIL**

241 **LNK**NIQEDPA VKAEVTRMKD QLQNYSSQVI GRTIVYLNGT THACRFHECN LGNLICDAVV YNNLRHPDDS EWNHVSMCIV

321 NGGGIRSPID EQANNGIITL EELTAVLPFG GTFDLLQIKG STLRQAFEHS VHR**HGQGSGE LLQVSGIK**VV YDLSQKPGER

401 **VVSLNVLCTE CR**VPAYVPLE MEKTYKVLLP SFLAAGGDGY YMLKGDSSNH SSGNLDISIV SDYIKR**MGKV FPAMEGR**MMF

481 SAGTLFQVHL FLTWGLCISL LYFIL

**Supporting Peptides:**

| Peptide | Uniq | -10lgP | Mass | Length | ppm | m/z | z | RT | Fraction | Scan | Source File | Area F1 | Area F2a | Area F3a | Area F4 | Area F5 | Area F6a | Area F7a | Area F7b | Area F8 | Area F9a | Area Sample 1F10a | Area F10b | Area F11a | Area F11b | Area F12a | Area F13 | #Feature | #Feature F1 | #Feature F2a | #Feature F3a | #Feature F4 | #Feature F5 | #Feature F6a | #Feature F7a | #Feature F7b | #Feature F8 | #Feature F9a | #Feature Sample 1F10a | #Feature F10b | #Feature F11a | #Feature F11b | #Feature F12a | #Feature F13 | Start | End | PTM | AScore | Found By |
|---|---|---|---|---|---|---|---|---|---|---|---|---|---|---|---|---|---|---|---|---|---|---|---|---|---|---|---|---|---|---|---|---|---|---|---|---|---|---|---|---|---|---|---|---|---|---|---|---|---|
| K.ASGNPILLNK.N | N | 71.96 | 1025.5869 | 10 | -0.1 | 513.8007 | 2 | 40.65 | 16 | F16:17022 | BuCaTN01_F13.raw | | | | | | | | | | | | | | | | 2.2075E6 | 1 | 0 | 0 | 0 | 0 | 0 | 0 | 0 | 0 | 0 | 0 | 0 | 0 | 0 | 0 | 0 | 1 | 234 | 243 | | | PEAKS DB |
| N.ISGYILPYKIINVGSEK.V | N | 59.75 | 1893.0610 | 17 | -0.2 | 632.0275 | 3 | 71.29 | 16 | F16:37082 | BuCaTN01_F13.raw | | | | | | | | | | | | | | | | 4.9796E5 | 1 | 0 | 0 | 0 | 0 | 0 | 0 | 0 | 0 | 0 | 0 | 0 | 0 | 0 | 0 | 0 | 1 | 85 | 101 | | | PEAKS DB |
| R.VVSLNVLC(+57.02)TEC(+57.02)R.V | N | 59.19 | 1448.7115 | 12 | 2.9 | 725.3630 | 2 | 59.59 | 8 | F8:35185 | BuCaTN01_F7b.raw | | | | | | | | 4.7661E5 | | | | | | | | | 1 | 0 | 0 | 0 | 0 | 0 | 0 | 0 | 1 | 0 | 0 | 0 | 0 | 0 | 0 | 0 | 0 | 401 | 412 | Carbamidomethylation | C8:Carbamidomethylation:1000.00;C11:Carbamidomethylation:1000.00 | PEAKS DB |
| K.NVKFPILSANIRPK.G | N | 59.13 | 1595.9510 | 14 | 1.2 | 532.9916 | 3 | 58.57 | 16 | F16:28436 | BuCaTN01_F13.raw | | | | | | | | | | | | | | | | 3.5508E5 | 1 | 0 | 0 | 0 | 0 | 0 | 0 | 0 | 0 | 0 | 0 | 0 | 0 | 0 | 0 | 0 | 1 | 65 | 78 | | | PEAKS DB |
| K.FPILSANIRPK.G | N | 55.81 | 1254.7448 | 11 | 0.4 | 419.2557 | 3 | 55.55 | 16 | F16:26305 | BuCaTN01_F13.raw | | | | | | | | | | | | | | | | 4.8037E5 | 1 | 0 | 0 | 0 | 0 | 0 | 0 | 0 | 0 | 0 | 0 | 0 | 0 | 0 | 0 | 0 | 1 | 68 | 78 | | | PEAKS DB |
| N.ISGYILPYK.I | N | 50.21 | 1052.5906 | 9 | 0.3 | 527.3027 | 2 | 57.19 | 16 | F16:27479 | BuCaTN01_F13.raw | | | | | | | | | | | | | | | | 5.5906E5 | 1 | 0 | 0 | 0 | 0 | 0 | 0 | 0 | 0 | 0 | 0 | 0 | 0 | 0 | 0 | 0 | 1 | 85 | 93 | | | PEAKS DB |
| R.HGQGSGELLQVSGIK.V | Y | 47.08 | 1508.7947 | 15 | 0.5 | 503.9391 | 3 | 49.38 | 16 | F16:22692 | BuCaTN01_F13.raw | | | | | | | | | | | | | | | | 0 | 0 | 0 | 0 | 0 | 0 | 0 | 0 | 0 | 0 | 0 | 0 | 0 | 0 | 0 | 0 | 0 | 0 | 374 | 388 | | | PEAKS DB |
| K.VFPAMEGR.M | N | 41.93 | 905.4429 | 8 | -0.1 | 453.7287 | 2 | 42.75 | 16 | F16:18519 | BuCaTN01_F13.raw | | | | | | | | | | | | | | | | 4.3727E5 | 1 | 0 | 0 | 0 | 0 | 0 | 0 | 0 | 0 | 0 | 0 | 0 | 0 | 0 | 0 | 0 | 1 | 470 | 477 | | | PEAKS DB |
| R.MGKVFPAM(+15.99)EGR.M | N | 41.68 | 1237.5947 | 11 | 0.8 | 413.5392 | 3 | 33.99 | 16 | F16:12305 | BuCaTN01_F13.raw | | | | | | | | | | | | | | | | 9.9954E4 | 1 | 0 | 0 | 0 | 0 | 0 | 0 | 0 | 0 | 0 | 0 | 0 | 0 | 0 | 0 | 0 | 1 | 467 | 477 | Oxidation (M) | M8:Oxidation (M):79.97 | PEAKS DB |
| total 9 peptides | | | | | | | | | | | | | | | | | | | | | | | | | | | | | | | | | | | | | | | | | | | | | | | | | |

**Best Unique PSM (Scan F16:22692, m/z=503.9391, z=3, RT=49.38, ppm=0.5):**

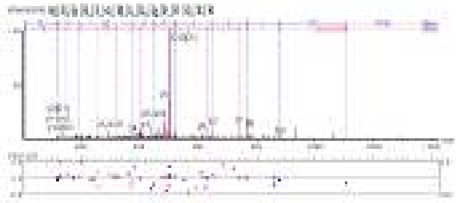

## Q4JHE1.1

back to list

| Protein Coverage | Supporting Peptides | Best Unique PSM |

**Protein Coverage:**

1 MNVFFMFSLL FLAALGSCAD DRRRPLEECF READYEEFLE IAKNGLQRTS NPKRVVVVGA GMAGLSAAYV LAGAGHQ**VTL**

81 **LEASER**VGGR VNTYRNEKDG WYVNLGPMRL PERHRIIREY IRKFGLELNE FIQENDNAWY FIKNIRKRVS EVK**KDPGVFK**

161 **YPVKPSEEGK** SASQLYRESL QKVIEELKRT NCSYILNKYD TYSTKEYLIK EGNLSPGAVD MIGDLLNEDS SYYLSFIESL

241 KSDDIFSYEK RFDEIVGGFD QLPRSMYQAI AEKVHLNAQV IKIQQNAEDV RVTYQTPAKT LSYVIADYVI VCSTSRAARR

321 IHFEPPLPPK KAHALRSIHY RSSTK**IFLTC SQKFWEADGI HGGK**STTDLP SRFIYYPNHS FTSGIGVIVA YTLADDTDFF

401 QALDIE**TSAD IVINDLSLIH QLPK**EQIQAL CYPSKIQKWS LDEYAMGAIT SFTPYQFQDF FEIVAAPVGR IYFAGEYTAS

481 **VHGWLDSTIK SGLTAAR**DVN LASQKPSRIQ LSNDNEL

C Carbamidomethylation (+57.02)

**Supporting Peptides:**

| Peptide | Uniq | -10lgP | Mass | Length | ppm | m/z | z | RT | Fraction | Scan | Source File | Area F1 | Area F2a | Area F3a | Area F4 | Area F5 | Area F6a | Area F7a | Area F7b | Area F8 | Area F9a | Area Sample 1F10a | Area F10b | Area F11a | Area F11b | Area F12a | Area F13 | #Feature | #Feature F1 | #Feature F2a | #Feature F3a | #Feature F4 | #Feature F5 | #Feature F6a | #Feature F7a | #Feature F7b | #Feature F8 | #Feature F9a | #Feature Sample 1F10a | #Feature F10b | #Feature F11a | #Feature F11b | #Feature F12a | #Feature F13 | Start | End | PTM | AScore | Found By |
|---|---|---|---|---|---|---|---|---|---|---|---|---|---|---|---|---|---|---|---|---|---|---|---|---|---|---|---|---|---|---|---|---|---|---|---|---|---|---|---|---|---|---|---|---|---|---|---|---|---|
| S.VHGWLDSTIKSGLTAAR.D | N | 85.08 | 1810.9690 | 17 | -0.1 | 453.7495 | 4 | 63.71 | 16 | F16:31762 | BuCaTN01_F13.raw | | | | | | | | | | | | | | | | 9.4969E5 | 2 | 0 | 0 | 0 | 0 | 0 | 0 | 0 | 0 | 0 | 0 | 0 | 0 | 0 | 0 | 0 | 2 | 481 | 497 | | | PEAKS DB |
| K.KDPGVFKYPVKPSEEGK.S | N | 68.67 | 1904.0043 | 17 | 0.4 | 477.0085 | 4 | 39.18 | 16 | F16:15857 | BuCaTN01_F13.raw | | | | | | | | | | | | | | | | 5.3545E5 | 1 | 0 | 0 | 0 | 0 | 0 | 0 | 0 | 0 | 0 | 0 | 0 | 0 | 0 | 0 | 0 | 1 | 154 | 170 | | | PEAKS DB |
| K.IFLTC(+57.02)SQKFWEADGIHGGK.S | Y | 45.26 | 2193.0676 | 19 | 3.5 | 549.2733 | 4 | 65.32 | 15 | F15:39201 | BuCaTN01_F12a.raw | | | | | | | | | | | | | | | 1.2745E5 | | 1 | 0 | 0 | 0 | 0 | 0 | 0 | 0 | 0 | 0 | 0 | 0 | 0 | 0 | 0 | 1 | 0 | 346 | 364 | Carbamidomethylation | C5:Carbamidomethylation:1000.00 | PEAKS DB |
| S.VHGWLDSTIK.S | N | 42.06 | 1154.6084 | 10 | 1.0 | 385.8771 | 3 | 51.32 | 16 | F16:23843 | BuCaTN01_F13.raw | | | | | | | | | | | | | | | | 5.3693E5 | 1 | 0 | 0 | 0 | 0 | 0 | 0 | 0 | 0 | 0 | 0 | 0 | 0 | 0 | 0 | 0 | 1 | 481 | 490 | | | PEAKS DB |
| Q.VTLLEASER.V | N | 41.88 | 1016.5502 | 9 | -0.5 | 509.2821 | 2 | 44.09 | 16 | F16:19432 | BuCaTN01_F13.raw | | | | | | | | | | | | | | | | 1.9042E6 | 1 | 0 | 0 | 0 | 0 | 0 | 0 | 0 | 0 | 0 | 0 | 0 | 0 | 0 | 0 | 0 | 1 | 78 | 86 | | | PEAKS DB |
| E.TSADIVINDLSLIHQLPK.E | N | 41.52 | 1976.0942 | 18 | 1.6 | 659.7064 | 3 | 81.28 | 16 | F16:44375 | BuCaTN01_F13.raw | | | | | | | | | | | | | | | | 0 | 0 | 0 | 0 | 0 | 0 | 0 | 0 | 0 | 0 | 0 | 0 | 0 | 0 | 0 | 0 | 0 | 0 | 407 | 424 | | | PEAKS DB |
| **total 6 peptides** | | | | | | | | | | | | | | | | | | | | | | | | | | | | | | | | | | | | | | | | | | | | | | | | | |

**Best Unique PSM (Scan F15:39201, m/z=549.2733, z=4, RT=65.32, ppm=3.5):**

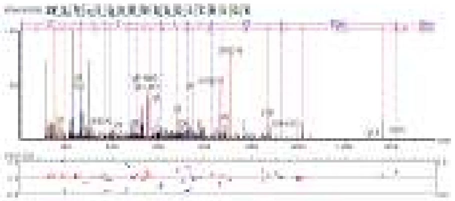

## AAY89682.1

back to list

| Protein Coverage | Supporting Peptides | Best Unique PSM |

**Protein Coverage:**

1 MNVFFMFSLL FLAALGSCAD DRRRPLEECF READYEEFLE IAKNGLQRTS NPKRVVVVGA GMAGLSAAYV LAGAGHQ**VTL**

81 **LEASER**VGGR VNTYRNEKDG WYVNLGPMRL PERHRIIREY IRKFGLELNE FIQENDNAWY FIKNIRKRVS EVK**KDPGVFK**

161 **YPVKPSEEGK** SASQLYRESL QKVIEELKRT NCSYILNKYD TYSTKEYLIK EGNLSPGAVD MIGDLLNEDS SYYLSFIESL

241 KSDDIFSYEK RFDEIVGGFD QLPRSMYQAI AEKVHLNAQV IKIQQNAEDV RVTYQTPAKT LSYVIADYVI VCSTSRAARR

321 IHFEPPLPPK KAHALRSIHY RSSTK**IFLTC SQKFWEADGI HGGK**STTDLP SRFIYYPNHS FTSGIGVIVA YTLADDTDFF

401 QALDIE**TSAD IVINDLSLIH QLPK**EQIQAL CYPSKIQKWS LDEYAMGAIT SFTPYQFQDF FEIVAAPVGR IYFAGEYTAS

481 **VHGWLDSTIK SGLTAAR**DVN LASQKPSRIQ LSNDNEL

C Carbamidomethylation (+57.02)

**Supporting Peptides:**

| Peptide | Uniq | -10lgP | Mass | Length | ppm | m/z | z | RT | Fraction | Scan | Source File | Area F1 | Area F2a | Area F3a | Area F4 | Area F5 | Area F6a | Area F7a | Area F7b | Area F8 | Area F9a | Area Sample 1F10a | Area F10b | Area F11a | Area F11b | Area F12a | Area F13 | #Feature | #Feature F1 | #Feature F2a | #Feature F3a | #Feature F4 | #Feature F5 | #Feature F6a | #Feature F7a | #Feature F7b | #Feature F8 | #Feature F9a | #Feature Sample 1F10a | #Feature F10b | #Feature F11a | #Feature F11b | #Feature F12a | #Feature F13 | Start | End | PTM | AScore | Found By |
|---|---|---|---|---|---|---|---|---|---|---|---|---|---|---|---|---|---|---|---|---|---|---|---|---|---|---|---|---|---|---|---|---|---|---|---|---|---|---|---|---|---|---|---|---|---|---|---|---|---|
| S.VHGWLDSTIKSGLTAAR.D | N | 85.08 | 1810.9690 | 17 | -0.1 | 453.7495 | 4 | 63.71 | 16 | F16:31762 | BuCaTN01_F13.raw | | | | | | | | | | | | | | | | 9.4969E5 | 2 | 0 | 0 | 0 | 0 | 0 | 0 | 0 | 0 | 0 | 0 | 0 | 0 | 0 | 0 | 0 | 2 | 481 | 497 | | | PEAKS DB |
| K.KDPGVFKYPVKPSEEGK.S | N | 68.67 | 1904.0043 | 17 | 0.4 | 477.0085 | 4 | 39.18 | 16 | F16:15857 | BuCaTN01_F13.raw | | | | | | | | | | | | | | | | 5.3545E5 | 1 | 0 | 0 | 0 | 0 | 0 | 0 | 0 | 0 | 0 | 0 | 0 | 0 | 0 | 0 | 0 | 1 | 154 | 170 | | | PEAKS DB |
| K.IFLTC(+57.02)SQKFWEADGIHGGK.S | Y | 45.26 | 2193.0676 | 19 | 3.5 | 549.2733 | 4 | 65.32 | 15 | F15:39201 | BuCaTN01_F12a.raw | | | | | | | | | | | | | | | 1.2745E5 | | 1 | 0 | 0 | 0 | 0 | 0 | 0 | 0 | 0 | 0 | 0 | 0 | 0 | 0 | 0 | 1 | 0 | 346 | 364 | Carbamidomethylation | C5:Carbamidomethylation:1000.00 | PEAKS DB |
| S.VHGWLDSTIK.S | N | 42.06 | 1154.6084 | 10 | 1.0 | 385.8771 | 3 | 51.32 | 16 | F16:23843 | BuCaTN01_F13.raw | | | | | | | | | | | | | | | | 5.3693E5 | 1 | 0 | 0 | 0 | 0 | 0 | 0 | 0 | 0 | 0 | 0 | 0 | 0 | 0 | 0 | 0 | 1 | 481 | 490 | | | PEAKS DB |
| Q.VTLLEASER.V | N | 41.88 | 1016.5502 | 9 | -0.5 | 509.2821 | 2 | 44.09 | 16 | F16:19432 | BuCaTN01_F13.raw | | | | | | | | | | | | | | | | 1.9042E6 | 1 | 0 | 0 | 0 | 0 | 0 | 0 | 0 | 0 | 0 | 0 | 0 | 0 | 0 | 0 | 0 | 1 | 78 | 86 | | | PEAKS DB |
| E.TSADIVINDLSLIHQLPK.E | N | 41.52 | 1976.0942 | 18 | 1.6 | 659.7064 | 3 | 81.28 | 16 | F16:44375 | BuCaTN01_F13.raw | | | | | | | | | | | | | | | | 0 | 0 | 0 | 0 | 0 | 0 | 0 | 0 | 0 | 0 | 0 | 0 | 0 | 0 | 0 | 0 | 0 | 0 | 407 | 424 | | | PEAKS DB |
| **total 6 peptides** | | | | | | | | | | | | | | | | | | | | | | | | | | | | | | | | | | | | | | | | | | | | | | | | | |

**Best Unique PSM (Scan F15:39201, m/z=549.2733, z=4, RT=65.32, ppm=3.5):**

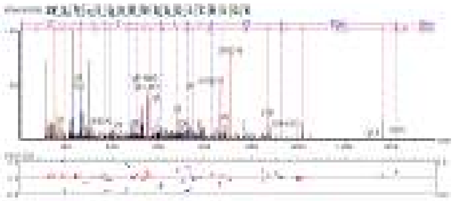

## Q9PVK7.1

back to list

| Protein Coverage | Supporting Peptides | Best Unique PSM |

**Protein Coverage:**

1 MIQLSWSSII LESGNVNDYE VVYPQKVPAL LKGGVQNPQP ETKYEDTMQY EFQVNGEPVV LHLERNKGLF SEDYTETHYA

81 PDGREITTSP PVQDHCYYHG YFQNEADSSA VISACDGLKG HFKLQGEIYF IEPLKISDSE AHAIYKDENV EEEDETPKIC

161 GVTDTTWESD EPIKKTSLLT NTPEQDRYLQ AEKYIEFYMV VDNIMYRHYK RNQLVIKRKV YEMINTMNMI YRRLNFHIAL

241 IGLEIWSNIN EINVQSDVKA TLDLFGEWRE KKLLPRKRND NAQLLTGIDF NGTPVGLAYI GSICNPK**TSA AVVQDYSKST**

321 **R**MVAITMAHE MGHNLGMNHD KGFCTCGFNK CVMSTR**RTKP AYQFSSCSVR** EHQRYLLRDR PQCILNKPLS TDIVSPPICG

401 NYFVEVGEEC DCGSPADCQS ACCNATTCKL QHEAQCDSEE CCEKCKFKGA GAECR**AAKDD CDLPELCTGQ SAECPTDVFQ**

481 **R**NGLPCQNNG YCYNGKCPIM TNQCIALRGP GVKVSR**DSCF TLNQR**TRGCG LCRMEYGRKI PCAAKDVKCG RLFCKKRNSM

561 ICNCSISPR**D PSYGMVEPGT K**CGDGMVCSN RQCVDVKTAY

C Carbamidomethylation (+57.02)
O Oxidation (M) (+15.99)

**Supporting Peptides:**

| Peptide | Uniq | -10lgP | Mass | Length | ppm | m/z | z | RT | Fraction | Scan | Source File | Area F1 | Area F2a | Area F3a | Area F4 | Area F5 | Area F6a | Area F7a | Area F7b | Area F8 | Area F9a | Area Sample 1F10a | Area F10b | Area F11a | Area F11b | Area F12a | Area F13 | #Feature | #Feature F1 | #Feature F2a | #Feature F3a | #Feature F4 | #Feature F5 | #Feature F6a | #Feature F7a | #Feature F7b | #Feature F8 | #Feature F9a | #Feature Sample 1F10a | #Feature F10b | #Feature F11a | #Feature F11b | #Feature F12a | #Feature F13 | Start | End | PTM | AScore | Found By |
|---|---|---|---|---|---|---|---|---|---|---|---|---|---|---|---|---|---|---|---|---|---|---|---|---|---|---|---|---|---|---|---|---|---|---|---|---|---|---|---|---|---|---|---|---|---|---|---|---|---|
| R.DSC(+57.02)FTLNQR.T | N | 61.56 | 1139.5029 | 9 | 0.7 | 570.7592 | 2 | 42.59 | 7 | F7:18306 | BuCaTN01_F7a.raw | | | | | | 2.5225E6 | 1.4188E7 | | | | | | | | | 1.2128E6 | 4 | 0 | 0 | 0 | 0 | 0 | 1 | 2 | 0 | 0 | 0 | 0 | 0 | 0 | 0 | 0 | 1 | 517 | 525 | Carbamidomethylation | C3:Carbamidomethylation:1000.00 | PEAKS DB |
| R.TKPAYQFSSC(+57.02)SVR.E | N | 60.87 | 1529.7296 | 13 | 1.5 | 510.9179 | 3 | 39.55 | 7 | F7:15581 | BuCaTN01_F7a.raw | | | | | | | 1.0745E5 | 0 | | | | | | | | 4.5504E4 | 2 | 0 | 0 | 0 | 0 | 0 | 0 | 1 | 0 | 0 | 0 | 0 | 0 | 0 | 0 | 0 | 1 | 358 | 370 | Carbamidomethylation | C10:Carbamidomethylation:1000.00 | PEAKS DB |
| K.TSAAVVQDYSKSTR.M | Y | 59.58 | 1511.7579 | 14 | 0.7 | 504.9269 | 3 | 33.48 | 16 | F16:11634 | BuCaTN01_F13.raw | | | | | | | | | 5.9107E4 | | | | | | | 3.6793E5 | 2 | 0 | 0 | 0 | 0 | 0 | 0 | 0 | 0 | 1 | 0 | 0 | 0 | 0 | 0 | 0 | 1 | 308 | 321 | | | PEAKS DB |
| | | | | | | | | | | | | | | | | | | | | | | | | | | | | | | | | | | | | | | | | | | | | | | | | | PEAKS |

| | | | | | | | | | | | | | | | | | | | | | | | | | | | | | | | | | | | | | | | | | | | | | | | | |
|---|---|---|---|---|---|---|---|---|---|---|---|---|---|---|---|---|---|---|---|---|---|---|---|---|---|---|---|---|---|---|---|---|---|---|---|---|---|---|---|---|---|---|---|---|---|---|---|---|---|
| R.DPSYGMVEPGTK.C | Y | 57.71 | 1279.5754 | 12 | 0.5 | 640.7953 | 2 | 42.58 | 16 | F16:18420 | BuCaTN01_F13.raw | | | | | | | 6.6604E4 | | | | | | | | | 5.0391E5 | 2 | 0 | 0 | 0 | 0 | 0 | 0 | 1 | 0 | 0 | 0 | 0 | 0 | 0 | 0 | 0 | 1 | 570 | 581 | | | DB |
| K.TSAAVVQDYSK.S | Y | 55.94 | 1167.5771 | 11 | 0.0 | 584.7958 | 2 | 33.62 | 16 | F16:11683 | BuCaTN01_F13.raw | | | | | | | | | | | | | | | | 4.9289E5 | 1 | 0 | 0 | 0 | 0 | 0 | 0 | 0 | 0 | 0 | 0 | 0 | 0 | 0 | 0 | 0 | 1 | 308 | 318 | | | PEAKS DB |
| R.AAKDDC(+57.02)DLPELC(+57.02)TGQSAEC(+57.02)PTDVFQR.N | N | 50.36 | 2982.2793 | 26 | 0.2 | 995.1006 | 3 | 63.64 | 7 | F7:35647 | BuCaTN01_F7a.raw | | | | | | | 2.9943E4 | | | | | | | | | | 1 | 0 | 0 | 0 | 0 | 0 | 0 | 1 | 0 | 0 | 0 | 0 | 0 | 0 | 0 | 0 | 0 | 456 | 481 | Carbamidomethylation | C6:Carbamidomethylation:1000.00;C12:Carbamidomethylation:1000.00;C19:Carbamidomethylation:1000.00 | PEAKS DB |
| R.DPSYGM(+15.99)VEPGTK.C | Y | 50.17 | 1295.5703 | 12 | 0.3 | 648.7926 | 2 | 34.12 | 16 | F16:12336 | BuCaTN01_F13.raw | | | | | | | | | | | | | | | | 2.7203E5 | 1 | 0 | 0 | 0 | 0 | 0 | 0 | 0 | 0 | 0 | 0 | 0 | 0 | 0 | 0 | 0 | 1 | 570 | 581 | Oxidation (M) | M6:Oxidation (M):1000.00 | PEAKS DB |
| R.RTKPAYQFSSC(+57.02)SVR.E | N | 43.46 | 1685.8307 | 14 | 2.5 | 562.9506 | 3 | 35.91 | 8 | F8:14347 | BuCaTN01_F7b.raw | | | | | | | | 0 | | | | | | | | | 0 | 0 | 0 | 0 | 0 | 0 | 0 | 0 | 0 | 0 | 0 | 0 | 0 | 0 | 0 | 0 | 0 | 357 | 370 | Carbamidomethylation | C11:Carbamidomethylation:1000.00 | PEAKS DB |
| total 8 peptides | | | | | | | | | | | | | | | | | | | | | | | | | | | | | | | | | | | | | | | | | | | | | | | | | |

**Best Unique PSM (Scan F16:11634, m/z=504.9269, z=3, RT=33.48, ppm=0.7):**

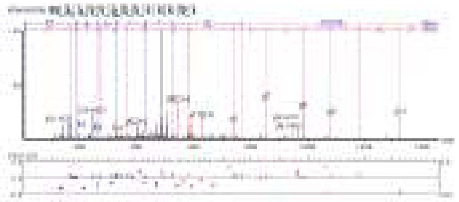

## AAF00693.1

back to list

| Protein Coverage | Supporting Peptides | Best Unique PSM |

**Protein Coverage:**

```
  1 MIQLSWSSII LESGNVNDYE VVYPQKVPAL LKGGVQNPQP ETKYEDTMQY EFQVNGEPVV LHLERNKGLF SEDYTETHYA
 81 PDGREITTSP PVQDHCYYHG YFQNEADSSA VISACDGLKG HFKLQGEIYF IEPLKISDSE AHAIYKDENV EEEDETPKIC
161 GVTDTTWESD EPIKKTSLLT NTPEQDRYLQ AEKYIEFYMV VDNIMYRHYK RNQLVIKRKV YEMINTMNMI YRRLNFHIAL
241 IGLEIWSNIN EINVQSDVKA TLDLFGEWRE KKLLPRKRND NAQLLTGIDF NGTPVGLAYI GSICNPKTSA AVVQDYSKST
321 RMVAITMAHE MGHNLGMNHD KGFCTCGFNK CVMSTRRTKP AYQFSSCSVR EHQRYLLRDR PQCILNKPLS TDIVSPPICG
401 NYFVEVGEEC DCGSPADCQS ACCNATTCKL QHEAQCDSEE CCEKCKFKGA GAECRAAKDD CDLPELCTGQ SAECPTDVFQ
481 RNGLPCQNNG YCYNGKCPIM TNQCIALRGP GVKVSRDSCF TLNQRTRGCG LCRMEYGRKI PCAAKDVKCG RLFCKKRNSM
561 ICNCSISPRD PSYGMVEPGT KCGDGMVCSN RQCVDVKTAY
```

C Carbamidomethylation (+57.02)
O Oxidation (M) (+15.99)

367, 474, 519, 575

**Supporting Peptides:**

| Peptide | Uniq | -10lgP | Mass | Length | ppm | m/z | z | RT | Fraction | Scan | Source File | Area F1 | Area F2a | Area F3a | Area F4 | Area F5 | Area F6a | Area F7a | Area F7b | Area F8 | Area F9a | Area Sample 1F10a | Area F10b | Area F11a | Area F11b | Area F12a | Area F13 | #Feature | #Feature F1 | #Feature F2a | #Feature F3a | #Feature F4 | #Feature F5 | #Feature F6a | #Feature F7a | #Feature F7b | #Feature F8 | #Feature F9a | #Feature Sample 1F10a | #Feature F10b | #Feature F11a | #Feature F11b | #Feature F12a | #Feature F13 | Start | End | PTM | AScore | Found By |
|---|---|---|---|---|---|---|---|---|---|---|---|---|---|---|---|---|---|---|---|---|---|---|---|---|---|---|---|---|---|---|---|---|---|---|---|---|---|---|---|---|---|---|---|---|---|---|---|---|---|
| R.DSC(+57.02)FTLNQR.T | N | 61.56 | 1139.5029 | 9 | 0.7 | 570.7592 | 2 | 42.59 | 7 | F7:18306 | BuCaTN01_F7a.raw | | | | | | 2.5225E6 | 1.4188E7 | | | | | | | | | 1.2128E6 | 4 | 0 | 0 | 0 | 0 | 0 | 1 | 2 | 0 | 0 | 0 | 0 | 0 | 0 | 0 | 0 | 1 | 517 | 525 | Carbamidomethylation | C3:Carbamidomethylation:1000.00 | PEAKS DB |
| R.TKPAYQFSSC(+57.02)SVR.E | N | 60.87 | 1529.7296 | 13 | 1.5 | 510.9179 | 3 | 39.55 | 7 | F7:15581 | BuCaTN01_F7a.raw | | | | | | | 1.0745E5 | 0 | | | | | | | | 4.5504E4 | 2 | 0 | 0 | 0 | 0 | 0 | 0 | 1 | 0 | 0 | 0 | 0 | 0 | 0 | 0 | 0 | 1 | 358 | 370 | Carbamidomethylation | C10:Carbamidomethylation:1000.00 | PEAKS DB |
| K.TSAAVVQDYSKSTR.M | Y | 59.58 | 1511.7579 | 14 | 0.7 | 504.9269 | 3 | 33.48 | 16 | F16:11634 | BuCaTN01_F13.raw | | | | | | | | | 5.9107E4 | | | | | | | 3.6793E5 | 2 | 0 | 0 | 0 | 0 | 0 | 0 | 0 | 0 | 1 | 0 | 0 | 0 | 0 | 0 | 0 | 1 | 308 | 321 | | | PEAKS DB |
| R.DPSYGMVEPGTK.C | Y | 57.71 | 1279.5754 | 12 | 0.5 | 640.7953 | 2 | 42.58 | 16 | F16:18420 | BuCaTN01_F13.raw | | | | | | | 6.6604E4 | | | | | | | | | 5.0391E5 | 2 | 0 | 0 | 0 | 0 | 0 | 0 | 1 | 0 | 0 | 0 | 0 | 0 | 0 | 0 | 0 | 1 | 570 | 581 | | | PEAKS DB |
| K.TSAAVVQDYSK.S | Y | 55.94 | 1167.5771 | 11 | 0.0 | 584.7958 | 2 | 33.62 | 16 | F16:11683 | BuCaTN01_F13.raw | | | | | | | | | | | | | | | | 4.9289E5 | 1 | 0 | 0 | 0 | 0 | 0 | 0 | 0 | 0 | 0 | 0 | 0 | 0 | 0 | 0 | 0 | 1 | 308 | 318 | | | PEAKS DB |
| R.AAKDDC(+57.02)DLPELC(+57.02)TGQSAEC(+57.02)PTDVFQR.N | N | 50.36 | 2982.2793 | 26 | 0.2 | 995.1006 | 3 | 63.64 | 7 | F7:35647 | BuCaTN01_F7a.raw | | | | | | | 2.9943E4 | | | | | | | | | | 1 | 0 | 0 | 0 | 0 | 0 | 0 | 1 | 0 | 0 | 0 | 0 | 0 | 0 | 0 | 0 | 0 | 456 | 481 | Carbamidomethylation | C6:Carbamidomethylation:1000.00;C12:Carbamidomethylation:1000.00;C19:Carbamidomethylation:1000.00 | PEAKS DB |
| R.DPSYGM(+15.99)VEPGTK.C | Y | 50.17 | 1295.5703 | 12 | 0.3 | 648.7926 | 2 | 34.12 | 16 | F16:12336 | BuCaTN01_F13.raw | | | | | | | | | | | | | | | | 2.7203E5 | 1 | 0 | 0 | 0 | 0 | 0 | 0 | 0 | 0 | 0 | 0 | 0 | 0 | 0 | 0 | 0 | 1 | 570 | 581 | Oxidation (M) | M6:Oxidation (M):1000.00 | PEAKS DB |
| R.RTKPAYQFSSC(+57.02)SVR.E | N | 43.46 | 1685.8307 | 14 | 2.5 | 562.9506 | 3 | 35.91 | 8 | F8:14347 | BuCaTN01_F7b.raw | | | | | | | | 0 | | | | | | | | | 0 | 0 | 0 | 0 | 0 | 0 | 0 | 0 | 0 | 0 | 0 | 0 | 0 | 0 | 0 | 0 | 0 | 357 | 370 | Carbamidomethylation | C11:Carbamidomethylation:1000.00 | PEAKS DB |
| total 8 peptides | | | | | | | | | | | | | | | | | | | | | | | | | | | | | | | | | | | | | | | | | | | | | | | | | |

**Best Unique PSM (Scan F16:11634, m/z=504.9269, z=3, RT=33.48, ppm=0.7):**

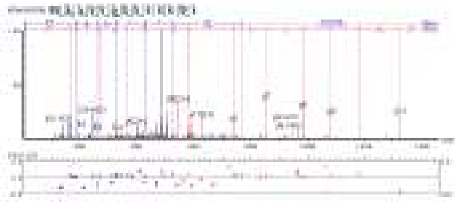

## 1DPY

back to list

| Protein Coverage | Supporting Peptides | Best Unique PSM |

**Protein Coverage:**

```
 1 NLIQFKNMIQ CAGTRIWTAY VAYGCYCGKG GSGTPVDELD RCCYTHDHCY NEAEKIPGCN PNIKTYSYTC TQPNLTCTDS
81 ADTCAQFLCE CDRTAAICFA SAPYNSNNIM LSSSTSCQ
```

C Carbamidomethylation (+57.02)
O Oxidation (M) (+15.99)

11

**Supporting Peptides:**

| Peptide | Uniq | -10lgP | Mass | Length | ppm | m/z | z | RT | Fraction | Scan | Source File | Area F1 | Area F2a | Area F3a | Area F4 | Area F5 | Area F6a | Area F7a | Area F7b | Area F8 | Area F9a | Area Sample 1F10a | Area F10b | Area F11a | Area F11b | Area F12a | Area F13 | #Feature | #Feature F1 | #Feature F2a | #Feature F3a | #Feature F4 | #Feature F5 | #Feature F6a | #Feature F7a | #Feature F7b | #Feature F8 | #Feature F9a | #Feature Sample 1F10a | #Feature F10b | #Feature F11a | #Feature F11b | #Feature F12a | #Feature F13 | Start | End | PTM | AScore | Found By |
|---|---|---|---|---|---|---|---|---|---|---|---|---|---|---|---|---|---|---|---|---|---|---|---|---|---|---|---|---|---|---|---|---|---|---|---|---|---|---|---|---|---|---|---|---|---|---|---|---|---|
| R.IWTAYVAYGC(+57.02)YC(+57.02)GKGGSGTPVDELDR.C | Y | 84.92 | 2894.3003 | 26 | -7.6 | 965.7628 | 3 | 76.75 | 14 | F14:51694 | BuCaTN01_F11b.raw | | | | | | | | | | | | 1.1216E7 | | 2.2097E7 | 1.2222E6 | | 3 | 0 | 0 | 0 | 0 | 0 | 0 | 0 | 0 | 0 | 0 | 0 | 1 | 0 | 1 | 1 | 0 | 16 | 41 | Carbamidomethylation | C10:Carbamidomethylation:1000.00;C12:Carbamidomethylation:1000.00 | PEAKS DB |
| G.GSGTPVDELDR.C | N | 56.36 | 1144.5360 | 11 | 3.4 | 573.2756 | 2 | 40.15 | 8 | F8:17921 | BuCaTN01_F7b.raw | | | | | | | | 1.1068E6 | | | | | | | | | 1 | 0 | 0 | 0 | 0 | 0 | 0 | 0 | 1 | 0 | 0 | 0 | 0 | 0 | 0 | 0 | 0 | 31 | 41 | | | PEAKS DB |
| K.GGSGTPVDELDR.C | N | 49.09 | 1201.5575 | 12 | 0.3 | 601.7862 | 2 | 40.57 | 11 | F11:14528 | BuCaTN01_F10a.raw | | | | | | | 9.2274E5 | 2.7577E6 | | 1.1335E7 | 6.7591E5 | | 9.9086E5 | 2.6759E7 | | | 6 | 0 | 0 | 0 | 0 | 0 | 0 | 1 | 1 | 0 | 1 | 1 | 0 | 1 | 1 | 0 | 0 | 30 | 41 | | | PEAKS DB |
| NLIQFKNM(+15.99)IQC(+57.02)AGTRIWTAY.V | Y | 41.70 | 2443.2141 | 20 | 1.4 | 815.4131 | 3 | 84.36 | 9 | F9:58682 | BuCaTN01_F8.raw | | | | | | | | | 5.6665E7 | | | | | | | | 1 | 0 | 0 | 0 | 0 | 0 | 0 | 0 | 0 | 1 | 0 | 0 | 0 | 0 | 0 | 0 | 0 | 1 | 20 | Oxidation (M); Carbamidomethylation | M8:Oxidation (M):1000.00;C11:Carbamidomethylation:1000.00 | PEAKS DB |
| total 4 peptides | | | | | | | | | | | | | | | | | | | | | | | | | | | | | | | | | | | | | | | | | | | | | | | | | |

**Best Unique PSM (Scan F14:51694, m/z=965.7628, z=3, RT=76.75, ppm=-7.6):**

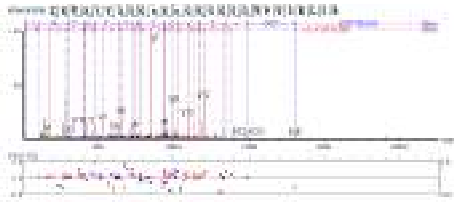

## 1ZM6

back to list

| Protein Coverage | Supporting Peptides | Best Unique PSM |

**Protein Coverage:**

1 NLYQFKNMIQ CTVPSRSWAD FADYGCYCGK GGSGTPVDDL DRCCQTHDNC YNEAENISGC RPYFKTYSYE CTQGTLTCKG

81 DNNACAASVC DCDRLAAICF AGAPYNDDNY NIDLKARCN

Carbamidomethylation (+57.02)

**Supporting Peptides:**

| Peptide | Uniq | -10lgP | Mass | Length | ppm | m/z | z | RT | Fraction | Scan | Source File | Area F1 | Area F2a | Area F3a | Area F4 | Area F5 | Area F6a | Area F7a | Area F7b | Area F8 | Area F9a | Area Sample 1F10a | Area F10b | Area F11a | Area F11b | Area F12a | Area F13 | #Feature | #Feature F1 | #Feature F2a | #Feature F3a | #Feature F4 | #Feature F5 | #Feature F6a | #Feature F7a | #Feature F7b | #Feature F8 | #Feature F9a | #Feature Sample 1F10a | #Feature F10b | #Feature F11a | #Feature F11b | #Feature F12a | #Feature F13 | Start | End | PTM | AScore | Found By |
|---|---|---|---|---|---|---|---|---|---|---|---|---|---|---|---|---|---|---|---|---|---|---|---|---|---|---|---|---|---|---|---|---|---|---|---|---|---|---|---|---|---|---|---|---|---|---|---|---|---|
| K.GGSGTPVDDLDR.C | N | 69.16 | 1187.5417 | 12 | -1.2 | 594.7775 | 2 | 38.65 | 10 | F10:17034 | BuCaTN01_F9a.raw | | | | | | | | | | 5.2316E6 | | | | | | | 1 | 0 | 0 | 0 | 0 | 0 | 0 | 0 | 0 | 0 | 1 | 0 | 0 | 0 | 0 | 0 | 0 | 31 | 42 | | | PEAKS DB |
| S.GTPVDDLDR.C | N | 51.39 | 986.4669 | 9 | 0.6 | 494.2410 | 2 | 37.62 | 7 | F7:14221 | BuCaTN01_F7a.raw | | | | | | | 4.0904E6 | | | | | | | | | | 1 | 0 | 0 | 0 | 0 | 0 | 0 | 1 | 0 | 0 | 0 | 0 | 0 | 0 | 0 | 0 | 0 | 34 | 42 | | | PEAKS DB |
| NLYQFKNMIQC(+57.02)TVPS.R | N | 50.68 | 1841.8804 | 15 | 0.8 | 921.9482 | 2 | 72.92 | 9 | F9:46618 | BuCaTN01_F8.raw | | | | | | | | | 1.0192E6 | | | | | | | | 1 | 0 | 0 | 0 | 0 | 0 | 0 | 0 | 0 | 1 | 0 | 0 | 0 | 0 | 0 | 0 | 0 | 1 | 15 | Carbamidomethylation | C11:Carbamidomethylation:1000.00 | PEAKS DB |
| K.TYSYEC(+57.02)TQGTLTC(+57.02)K.G | Y | 50.11 | 1710.7229 | 14 | 1.6 | 856.3701 | 2 | 41.67 | 10 | F10:19604 | BuCaTN01_F9a.raw | | | | | | | | | | 9.234E4 | | | | | | | 1 | 0 | 0 | 0 | 0 | 0 | 0 | 0 | 0 | 0 | 1 | 0 | 0 | 0 | 0 | 0 | 0 | 66 | 79 | Carbamidomethylation | C6:Carbamidomethylation:1000.00;C13:Carbamidomethylation:1000.00 | PEAKS DB |
| G.TPVDDLDR.C | N | 48.70 | 929.4454 | 8 | -0.4 | 465.7298 | 2 | 34.76 | 7 | F7:11899 | BuCaTN01_F7a.raw | | | | | | | 6.3757E5 | | | | | | | | | | 1 | 0 | 0 | 0 | 0 | 0 | 0 | 1 | 0 | 0 | 0 | 0 | 0 | 0 | 0 | 0 | 0 | 35 | 42 | | | PEAKS DB |
| total 5 peptides | | | | | | | | | | | | | | | | | | | | | | | | | | | | | | | | | | | | | | | | | | | | | | | | | |

**Best Unique PSM (Scan F10:19604, m/z=856.3701, z=2, RT=41.67, ppm=1.6):**

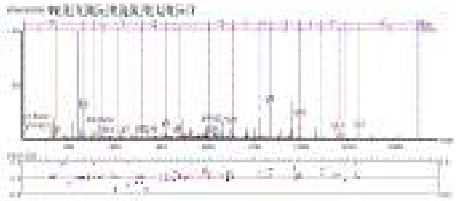

## Q9DF52.1

back to list

| Protein Coverage | Supporting Peptides | Best Unique PSM |

**Protein Coverage:**

1 MYPAHLLVLV AVCVSLLGAA NIPPQPLNLI QFKNMIQCAG TRPWTAYVNY GCYCGKGGSG TPVDELDRCC YTHDNCYNEA

81 EKIPGCNPNI KTYSYTCTEP NLTCTDTADT CARFLCDCDR TAAICFASAP YNSNNVMISS STNCQ

Carbamidomethylation (+57.02)
Oxidation (M) (+15.99)

**Supporting Peptides:**

| Peptide | Uniq | -10lgP | Mass | Length | ppm | m/z | z | RT | Fraction | Scan | Source File | Area F1 | Area F2a | Area F3a | Area F4 | Area F5 | Area F6a | Area F7a | Area F7b | Area F8 | Area F9a | Area Sample 1F10a | Area F10b | Area F11a | Area F11b | Area F12a | Area F13 | #Feature | #Feature F1 | #Feature F2a | #Feature F3a | #Feature F4 | #Feature F5 | #Feature F6a | #Feature F7a | #Feature F7b | #Feature F8 | #Feature F9a | #Feature Sample 1F10a | #Feature F10b | #Feature F11a | #Feature F11b | #Feature F12a | #Feature F13 | Start | End | PTM | AScore | Found By |
|---|---|---|---|---|---|---|---|---|---|---|---|---|---|---|---|---|---|---|---|---|---|---|---|---|---|---|---|---|---|---|---|---|---|---|---|---|---|---|---|---|---|---|---|---|---|---|---|---|---|
| R.FLC(+57.02)DC(+57.02)DRTAAIC(+57.02).F | N | 58.10 | 1500.6160 | 12 | 4.0 | 751.3152 | 2 | 47.17 | 14 | F14:24274 | BuCaTN01_F11b.raw | | | | | | | | | | | | | | 3.9576E6 | | | 1 | 0 | 0 | 0 | 0 | 0 | 0 | 0 | 0 | 0 | 0 | 0 | 0 | 0 | 1 | 0 | 0 | 114 | 125 | Carbamidomethylation | C3:Carbamidomethylation:1000.00;C5:Carbamidomethylation:1000.00;C12:Carbamidomethylation:1000.00 | PEAKS DB |
| G.GSGTPVDELDR.C | N | 56.36 | 1144.5360 | 11 | 3.4 | 573.2756 | 2 | 40.15 | 8 | F8:17921 | BuCaTN01_F7b.raw | | | | | | | | 1.1068E6 | | | | | | | | | 1 | 0 | 0 | 0 | 0 | 0 | 0 | 0 | 1 | 0 | 0 | 0 | 0 | 0 | 0 | 0 | 0 | 58 | 68 | | | PEAKS DB |
| R.FLC(+57.02)DC(+57.02)DRTAAIC(+57.02)F.A | N | 49.37 | 1647.6843 | 13 | 3.5 | 824.8499 | 2 | 66.79 | 8 | F8:42173 | BuCaTN01_F7b.raw | | | | | | | | 7.495E5 | | | | | | | | | 1 | 0 | 0 | 0 | 0 | 0 | 0 | 0 | 1 | 0 | 0 | 0 | 0 | 0 | 0 | 0 | 0 | 114 | 126 | Carbamidomethylation | C3:Carbamidomethylation:1000.00;C5:Carbamidomethylation:1000.00;C12:Carbamidomethylation:1000.00 | PEAKS DB |
| K.GGSGTPVDELDR.C | N | 49.09 | 1201.5575 | 12 | 0.3 | 601.7862 | 2 | 40.57 | 11 | F11:14528 | BuCaTN01_F10a.raw | | | | | | | 9.2274E5 | 2.7577E6 | | 1.1335E7 | 6.7591E5 | | 9.9086E5 | 2.6759E7 | | | 6 | 0 | 0 | 0 | 0 | 0 | 0 | 1 | 1 | 0 | 1 | 1 | 0 | 1 | 1 | 0 | 0 | 57 | 68 | | | PEAKS DB |
| K.NM(+15.99)IQC(+57.02)AGTRPWTAYVNYGC(+57.02)YC(+57.02)GKGGSGTPVDELDR.C | Y | 45.08 | 3968.7339 | 35 | 2.8 | 993.1912 | 4 | 71.41 | 12 | F12:47267 | BuCaTN01_F10b.raw | | | | | | | | | | | 1.3115E6 | 2.2032E7 | | | | | 2 | 0 | 0 | 0 | 0 | 0 | 0 | 0 | 0 | 0 | 0 | 1 | 1 | 0 | 0 | 0 | 0 | 34 | 68 | Oxidation (M); Carbamidomethylation | M2:Oxidation (M):1000.00;C5:Carbamidomethylation:1000.00;C19:Carbamidomethylation:1000.00;C21:Carbamidomethylation:1000.00 | PEAKS DB |
| K.NMIQC(+57.02)AGTRP.W | Y | 41.67 | 1146.5273 | 10 | 1.1 | 574.2716 | 2 | 36.79 | 9 | F9:17073 | BuCaTN01_F8.raw | | | | | | | | | 2.992E5 | | | | | | | | 1 | 0 | 0 | 0 | 0 | 0 | 0 | 0 | 0 | 1 | 0 | 0 | 0 | 0 | 0 | 0 | 0 | 34 | 43 | Carbamidomethylation | C5:Carbamidomethylation:1000.00 | PEAKS DB |
| total 6 peptides | | | | | | | | | | | | | | | | | | | | | | | | | | | | | | | | | | | | | | | | | | | | | | | | | |

**Best Unique PSM (Scan F12:47267, m/z=993.1912, z=4, RT=71.41, ppm=2.8):**

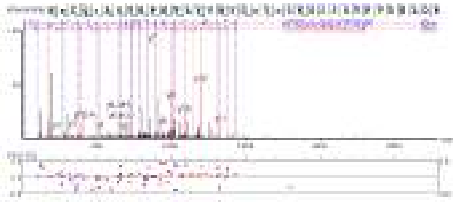

## AAG13412.1

back to list

| Protein Coverage | Supporting Peptides | Best Unique PSM |

**Protein Coverage:**

1 MYPAHLLVLV AVCVSLLGAA NIPPQPLNLI QFKNMIQCAG TRPWTAYVNY GCYCGKGGSG TPVDELDRCC YTHDNCYNEA

81 EKIPGCNPNI KTYSYTCTEP NLTCTDTADT CARFLCDCDR TAAICFASAP YNSNNVMISS STNCQ

Carbamidomethylation (+57.02)
Oxidation (M) (+15.99)

**Supporting Peptides:**

| Peptide | Uniq | -10lgP | Mass | Length | ppm | m/z | z | RT | Fraction | Scan | Source File | Area F1 | Area F2a | Area F3a | Area F4 | Area F5 | Area F6a | Area F7a | Area F7b | Area F8 | Area F9a | Area Sample 1F10a | Area F10b | Area F11a | Area F11b | Area F12a | Area F13 | #Feature | #Feature F1 | #Feature F2a | #Feature F3a | #Feature F4 | #Feature F5 | #Feature F6a | #Feature F7a | #Feature F7b | #Feature F8 | #Feature F9a | #Feature Sample 1F10a | #Feature F10b | #Feature F11a | #Feature F11b | #Feature F12a | #Feature F13 | Start | End | PTM | AScore | Found By |
|---|---|---|---|---|---|---|---|---|---|---|---|---|---|---|---|---|---|---|---|---|---|---|---|---|---|---|---|---|---|---|---|---|---|---|---|---|---|---|---|---|---|---|---|---|---|---|---|---|---|
| R.FLC(+57.02)DC(+57.02)DRTAAIC(+57.02).F | N | 58.10 | 1500.6160 | 12 | 4.0 | 751.3152 | 2 | 47.17 | 14 | F14:24274 | BuCaTN01_F11b.raw | | | | | | | | | | | | | | 3.9576E6 | | | 1 | 0 | 0 | 0 | 0 | 0 | 0 | 0 | 0 | 0 | 0 | 0 | 0 | 0 | 1 | 0 | 0 | 114 | 125 | Carbamidomethylation | C3:Carbamidomethylation:1000.00;C5:Carbamidomethylation:1000.00;C12:Carbamidomethylation:1000.00 | PEAKS DB |
| G.GSGTPVDELDR.C | N | 56.36 | 1144.5360 | 11 | 3.4 | 573.2756 | 2 | 40.15 | 8 | F8:17921 | BuCaTN01_F7b.raw | | | | | | | | 1.1068E6 | | | | | | | | | 1 | 0 | 0 | 0 | 0 | 0 | 0 | 0 | 1 | 0 | 0 | 0 | 0 | 0 | 0 | 0 | 0 | 58 | 68 | | | PEAKS DB |
| R.FLC(+57.02)DC(+57.02)DRTAAIC(+57.02)F.A | N | 49.37 | 1647.6843 | 13 | 3.5 | 824.8499 | 2 | 66.79 | 8 | F8:42173 | BuCaTN01_F7b.raw | | | | | | | | 7.495E5 | | | | | | | | | 1 | 0 | 0 | 0 | 0 | 0 | 0 | 0 | 1 | 0 | 0 | 0 | 0 | 0 | 0 | 0 | 0 | 114 | 126 | Carbamidomethylation | C3:Carbamidomethylation:1000.00;C5:Carbamidomethylation:1000.00;C12:Carbamidomethylation:1000.00 | PEAKS DB |
| K.GGSGTPVDELDR.C | N | 49.09 | 1201.5575 | 12 | 0.3 | 601.7862 | 2 | 40.57 | 11 | F11:14528 | BuCaTN01_F10a.raw | | | | | | | 9.2274E5 | 2.7577E6 | | 1.1335E7 | 6.7591E5 | | 9.9086E5 | 2.6759E7 | | | 6 | 0 | 0 | 0 | 0 | 0 | 0 | 1 | 1 | 0 | 1 | 1 | 0 | 1 | 1 | 0 | 0 | 57 | 68 | | | PEAKS DB |
| K.NM(+15.99)IQC(+57.02)AGTRPWTAYVNYGC(+57.02)YC(+57.02)GKGGSGTPVDELDR.C | Y | 45.08 | 3968.7339 | 35 | 2.8 | 993.1912 | 4 | 71.41 | 12 | F12:47267 | BuCaTN01_F10b.raw | | | | | | | | | | | 1.3115E6 | 2.2032E7 | | | | | 2 | 0 | 0 | 0 | 0 | 0 | 0 | 0 | 0 | 0 | 0 | 1 | 1 | 0 | 0 | 0 | 0 | 34 | 68 | Oxidation (M); Carbamidomethylation | M2:Oxidation (M):1000.00;C5:Carbamidomethylation:1000.00;C19:Carbamidomethylation:1000.00;C21:Carbamidomethylation:1000.00 | PEAKS DB |
| K.NMIQC(+57.02)AGTRP.W | Y | 41.67 | 1146.5273 | 10 | 1.1 | 574.2716 | 2 | 36.79 | 9 | F9:17073 | BuCaTN01_F8.raw | | | | | | | | | 2.992E5 | | | | | | | | 1 | 0 | 0 | 0 | 0 | 0 | 0 | 0 | 0 | 1 | 0 | 0 | 0 | 0 | 0 | 0 | 0 | 34 | 43 | Carbamidomethylation | C5:Carbamidomethylation:1000.00 | PEAKS DB |
| total 6 peptides | | | | | | | | | | | | | | | | | | | | | | | | | | | | | | | | | | | | | | | | | | | | | | | | | |

**Best Unique PSM (Scan F12:47267, m/z=993.1912, z=4, RT=71.41, ppm=2.8):**

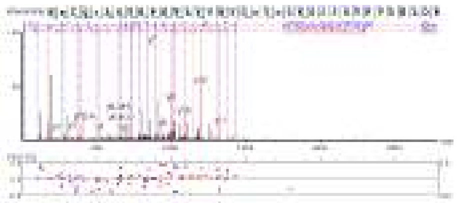

## AAZ39880.1

back to list

| Protein Coverage | Supporting Peptides | Best Unique PSM |

**Protein Coverage:**

```
  1 MMQVLLVTIC LAVFPYHGSS IILESGNVND YEVVYPQKVT AMPKEAVKQP EQKYEDAMQY KFEVNGEPVL LHLEKNKDLF
 81 SEDYSETHYS PDGREITTKP LVQDHCYYHG HIQNDAHSSA SISACNGLKG HFKLRGEMYL IEPLKLSDSE AHAVYKYENV
161 EKEDEALKMC GVTQTNWESD EPIKKASLLV ATSERNRYFN PYSYVELIIT VDHSMVTKYK NDLTAIRTWV FELVNTINEI
241 FKYLYIRVPL VGLEIWKNRD LINVTSAANV TLDLFGEWRK SYLLPRKIHD NSQLLTAIDL NGLTIGMAYV STMCQSKYSV
                                                                               394
321 GVVQDHSKIN LRVAVTMAHE IGHNLGLTHD GVYCTCGGYS CIMSAVLGDQ PSKYFSNCSY NQYRRFLTEH NPECIINPPL
401 RTDIVSPPAC GNELLERGEE CDCGSPENCR DPCCDAASCK LHSWVECESG KCCNQCRFKR AGTECRPARD ECDKAEQCTG
481 RSANCPVDEF HENGRPCLHN FGYCYNGKCP IMYHQCHALF GQNVTGVQDS CFQYNRLGVY YAYCRKENGR KIPCAPKDEK
561 CGRLYCSYKS PGNQIPCLPY YIPSDENKGM VDHGTKCGDG KVCSNGQCVD LNIAY
```

C Carbamidomethylation (+57.02)

**Supporting Peptides:**

| Peptide | Uniq | -10lgP | Mass | Length | ppm | m/z | z | RT | Fraction | Scan | Source File | Area F1 | Area F2a | Area F3a | Area F4 | Area F5 | Area F6a | Area F7a | Area F7b | Area F8 | Area F9a | Area Sample 1F10a | Area F10b | Area F11a | Area F11b | Area F12a | Area F13 | #Feature | #Feature F1 | #Feature F2a | #Feature F3a | #Feature F4 | #Feature F5 | #Feature F6a | #Feature F7a | #Feature F7b | #Feature F8 | #Feature F9a | #Feature Sample 1F10a | #Feature F10b | #Feature F11a | #Feature F11b | #Feature F12a | #Feature F13 | Start | End | PTM | AScore | Found By |
|---|---|---|---|---|---|---|---|---|---|---|---|---|---|---|---|---|---|---|---|---|---|---|---|---|---|---|---|---|---|---|---|---|---|---|---|---|---|---|---|---|---|---|---|---|---|---|---|---|---|
| R.FLTEHNPEC(+57.02)IINPPLR.T | Y | 74.47 | 1948.9829 | 16 | 0.5 | 650.6686 | 3 | 60.70 | 16 | F16:29736 | BuCaTN01_F13.raw | | | | | | | | | | | | | | | | 4.4846E5 | 1 | 0 | 0 | 0 | 0 | 0 | 0 | 0 | 0 | 0 | 0 | 0 | 0 | 0 | 0 | 0 | 1 | 386 | 401 | Carbamidomethylation | C9:Carbamidomethylation:1000.00 | PEAKS DB |
| R.LGVYYAYC(+57.02)R.K | Y | 55.43 | 1163.5433 | 9 | 0.1 | 582.7790 | 2 | 51.02 | 13 | F13:26096 | BuCaTN01_F11a.raw | | | | | | | | | | | | | 3.2243E5 | | | | 1 | 0 | 0 | 0 | 0 | 0 | 0 | 0 | 0 | 0 | 0 | 0 | 0 | 1 | 0 | 0 | 0 | 537 | 545 | Carbamidomethylation | C8:Carbamidomethylation:1000.00 | PEAKS DB |
| R.VAVTMAHEIGHNLGLTHD.G | Y | 41.87 | 1913.9418 | 18 | 0.5 | 638.9882 | 3 | 60.10 | 16 | F16:29323 | BuCaTN01_F13.raw | | | | | | | | | | | | | | | | 8.0387E5 | 1 | 0 | 0 | 0 | 0 | 0 | 0 | 0 | 0 | 0 | 0 | 0 | 0 | 0 | 0 | 0 | 1 | 333 | 350 | | | PEAKS DB |
| total 3 peptides | | | | | | | | | | | | | | | | | | | | | | | | | | | | | | | | | | | | | | | | | | | | | | | | | |

**Best Unique PSM (Scan F16:29736, m/z=650.6686, z=3, RT=60.70, ppm=0.5):**

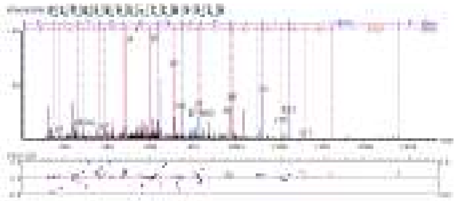

## B8K1W0.1

back to list

| Protein Coverage | Supporting Peptides | Best Unique PSM |

**Protein Coverage:**

```
  1 MMQVLLVTIC LAVFPYHGSS IILESGNVND YEVVYPQKVT AMPKEAVKQP EQKYEDAMQY KFEVNGEPVL LHLEKNKDLF
 81 SEDYSETHYS PDGREITTKP LVQDHCYYHG HIQNDAHSSA SISACNGLKG HFKLRGEMYL IEPLKLSDSE AHAVYKYENV
161 EKEDEALKMC GVTQTNWESD EPIKKASLLV ATSERNRYFN PYSYVELIIT VDHSMVTKYK NDLTAIRTWV FELVNTINEI
241 FKYLYIRVPL VGLEIWKNRD LINVTSAANV TLDLFGEWRK SYLLPRKIHD NSQLLTAIDL NGLTIGMAYV STMCQSKYSV
                                                                               394
321 GVVQDHSKIN LRVAVTMAHE IGHNLGLTHD GVYCTCGGYS CIMSAVLGDQ PSKYFSNCSY NQYRRFLTEH NPECIINPPL
401 RTDIVSPPAC GNELLERGEE CDCGSPENCR DPCCDAASCK LHSWVECESG KCCNQCRFKR AGTECRPARD ECDKAEQCTG
481 RSANCPVDEF HENGRPCLHN FGYCYNGKCP IMYHQCHALF GQNVTGVQDS CFQYNRLGVY YAYCRKENGR KIPCAPKDEK
561 CGRLYCSYKS PGNQIPCLPY YIPSDENKGM VDHGTKCGDG KVCSNGQCVD LNIAY
```

C Carbamidomethylation (+57.02)

**Supporting Peptides:**

| Peptide | Uniq | -10lgP | Mass | Length | ppm | m/z | z | RT | Fraction | Scan | Source File | Area F1 | Area F2a | Area F3a | Area F4 | Area F5 | Area F6a | Area F7a | Area F7b | Area F8 | Area F9a | Area Sample 1F10a | Area F10b | Area F11a | Area F11b | Area F12a | Area F13 | #Feature | #Feature F1 | #Feature F2a | #Feature F3a | #Feature F4 | #Feature F5 | #Feature F6a | #Feature F7a | #Feature F7b | #Feature F8 | #Feature F9a | #Feature Sample 1F10a | #Feature F10b | #Feature F11a | #Feature F11b | #Feature F12a | #Feature F13 | Start | End | PTM | AScore | Found By |
|---|---|---|---|---|---|---|---|---|---|---|---|---|---|---|---|---|---|---|---|---|---|---|---|---|---|---|---|---|---|---|---|---|---|---|---|---|---|---|---|---|---|---|---|---|---|---|---|---|---|
| R.FLTEHNPEC(+57.02)IINPPLR.T | Y | 74.47 | 1948.9829 | 16 | 0.5 | 650.6686 | 3 | 60.70 | 16 | F16:29736 | BuCaTN01_F13.raw | | | | | | | | | | | | | | | | 4.4846E5 | 1 | 0 | 0 | 0 | 0 | 0 | 0 | 0 | 0 | 0 | 0 | 0 | 0 | 0 | 0 | 0 | 1 | 386 | 401 | Carbamidomethylation | C9:Carbamidomethylation:1000.00 | PEAKS DB |
| R.LGVYYAYC(+57.02)R.K | Y | 55.43 | 1163.5433 | 9 | 0.1 | 582.7790 | 2 | 51.02 | 13 | F13:26096 | BuCaTN01_F11a.raw | | | | | | | | | | | | | 3.2243E5 | | | | 1 | 0 | 0 | 0 | 0 | 0 | 0 | 0 | 0 | 0 | 0 | 0 | 0 | 1 | 0 | 0 | 0 | 537 | 545 | Carbamidomethylation | C8:Carbamidomethylation:1000.00 | PEAKS DB |
| R.VAVTMAHEIGHNLGLTHD.G | Y | 41.87 | 1913.9418 | 18 | 0.5 | 638.9882 | 3 | 60.10 | 16 | F16:29323 | BuCaTN01_F13.raw | | | | | | | | | | | | | | | | 8.0387E5 | 1 | 0 | 0 | 0 | 0 | 0 | 0 | 0 | 0 | 0 | 0 | 0 | 0 | 0 | 0 | 0 | 1 | 333 | 350 | | | PEAKS DB |
| total 3 peptides | | | | | | | | | | | | | | | | | | | | | | | | | | | | | | | | | | | | | | | | | | | | | | | | | |

**Best Unique PSM (Scan F16:29736, m/z=650.6686, z=3, RT=60.70, ppm=0.5):**

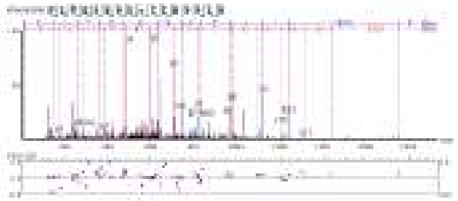

## XP_058034421.1

back to list

| Protein Coverage | Supporting Peptides | Best Unique PSM |

**Protein Coverage:**

```
  1 MSMLCYTLII AFLIGIWAAP KSEDNVPLGS PATSDLSDTS CAKTHEGLKA SRNTDQRHPA PKKAEDQEIG SAANIIVDPK
 81 LFQKRRFQSP RVLFSTQPPP LSRDEQSVDN ADSLNRNIRS KREAHPVHNR GEYSVCDSIS VWVANKTTAT DIKGKPVTVM
                        179                                                      229  231
161 VDVNLNNNVY KQYFFETKCR NPKPVPSGCR GIDSRHWNSY CTTTHTFVKA LTMEDNRASW RFIRIDTACV CIISRKTENF
```

C Carbamidomethylation (+57.02)

**Supporting Peptides:**

| Peptide | Uniq | -10lgP | Mass | Length | ppm | m/z | z | RT | Fraction | Scan | Source File | Area F1 | Area F2a | Area F3a | Area F4 | Area F5 | Area F6a | Area F7a | Area F7b | Area F8 | Area F9a | Area Sample 1F10a | Area F10b | Area F11a | Area F11b | Area F12a | Area F13 | #Feature | #Feature F1 | #Feature F2a | #Feature F3a | #Feature F4 | #Feature F5 | #Feature F6a | #Feature F7a | #Feature F7b | #Feature F8 | #Feature F9a | #Feature Sample 1F10a | #Feature F10b | #Feature F11a | #Feature F11b | #Feature F12a | #Feature F13 | Start | End | PTM | AScore | Found By |
|---|---|---|---|---|---|---|---|---|---|---|---|---|---|---|---|---|---|---|---|---|---|---|---|---|---|---|---|---|---|---|---|---|---|---|---|---|---|---|---|---|---|---|---|---|---|---|---|---|---|
| R.IDTAC(+57.02)VC(+57.02)IISR.K | Y | 59.00 | 1306.6373 | 11 | -0.3 | 654.3257 | 2 | 47.77 | 7 | F7:22494 | BuCaTN01_F7a.raw | | | | | | | 2.2386E6 | | | | | | | | | | 1 | 0 | 0 | 0 | 0 | 0 | 0 | 1 | 0 | 0 | 0 | 0 | 0 | 0 | 0 | 0 | 0 | 225 | 235 | Carbamidomethylation | C5:Carbamidomethylation:1000.00;C7:Carbamidomethylation:1000.00 | PEAKS DB |
| N.VYKQYFFETK.C | N | 54.63 | 1351.6812 | 10 | 2.9 | 451.5676 | 3 | 50.63 | 8 | F8:27078 | BuCaTN01_F7b.raw | | | | | | | | 5.2856E5 | | | | | | | | | 1 | 0 | 0 | 0 | 0 | 0 | 0 | 0 | 1 | 0 | 0 | 0 | 0 | 0 | 0 | 0 | 0 | 169 | 178 | | | PEAKS DB |
| K.QYFFETK.C | N | 54.60 | 961.4545 | 7 | 0.2 | 481.7346 | 2 | 53.27 | 7 | F7:26821 | BuCaTN01_F7a.raw | | | | | | | 2.4891E7 | 7.2632E6 | | | | | | | | | 2 | 0 | 0 | 0 | 0 | 0 | 0 | 1 | 1 | 0 | 0 | 0 | 0 | 0 | 0 | 0 | 0 | 172 | 178 | | | PEAKS DB |
| R.FIRIDTAC(+57.02)VC(+57.02).I | N | 49.72 | 1253.5896 | 10 | 3.0 | 627.8021 | 2 | 54.32 | 8 | F8:30301 | BuCaTN01_F7b.raw | | | | | | | | 3.8159E5 | | | | | | | | | 1 | 0 | 0 | 0 | 0 | 0 | 0 | 0 | 1 | 0 | 0 | 0 | 0 | 0 | 0 | 0 | 0 | 222 | 231 | Carbamidomethylation | C8:Carbamidomethylation:1000.00;C10:Carbamidomethylation:1000.00 | PEAKS DB |
| | | | | | | | | | | | | | | | | | | | | | | | | | | | | | | | | | | | | | | | | | | | | | | | C8:Carbamidomethylation:1000.00;C10:Carbam | PEAKS |

| | | | | | | | | | | | | | | | | | | | | | | | | | | | | | | | | | | | | | | | | | | | | | | | | | |
|---|---|---|---|---|---|---|---|---|---|---|---|---|---|---|---|---|---|---|---|---|---|---|---|---|---|---|---|---|---|---|---|---|---|---|---|---|---|---|---|---|---|---|---|---|---|---|---|---|---|
| R.FIRIDTAC(+57.02)VC(+57.02)IISR.K | Y | 42.69 | 1722.8909 | 14 | 0.7 | 575.3046 | 3 | 61.95 | 7 | F7:34298 | BuCaTN01_F7a.raw | | | | | | | 1.9199E6 | | | | | | | | | | 1 | 0 | 0 | 0 | 0 | 0 | 0 | 1 | 0 | 0 | 0 | 0 | 0 | 0 | 0 | 0 | 0 | 222 | 235 | Carbamidomethylation | idomethylation:1000.00 | DB |
| K.QYFFETKC(+57.02)R.N | N | 41.76 | 1277.5863 | 9 | -0.7 | 426.8691 | 3 | 46.69 | 7 | F7:21495 | BuCaTN01_F7a.raw | | | | | | | 3.7535E5 | | | | | | | | | | 1 | 0 | 0 | 0 | 0 | 0 | 0 | 1 | 0 | 0 | 0 | 0 | 0 | 0 | 0 | 0 | 0 | 172 | 180 | Carbamidomethylation | C8:Carbamidomethylation:1000.00 | PEAKS DB |

**total 6 peptides**

**Best Unique PSM (Scan F7:22494, m/z=654.3257, z=2, RT=47.77, ppm=-0.3):**

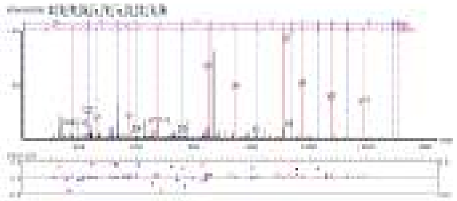

## XP_058034419.1

back to list

| Protein Coverage | Supporting Peptides | Best Unique PSM |

**Protein Coverage:**

```
  1  MSMLCYTLII AFLIGIWAAP KSEDNVPLGS PATSDLSDTS CAKTHEGLKA SRNTDQRHPA PKKAEDQEIG SAANIIVDPK
 81  LFQKRRFQSP RVLFSTQPPP LSRDEQSVDN ADSLNRNIRS KREAHPVHNR GEYSVCDSIS VWVANKTTAT DIKGKPVTVM
                    179                                                              229 231
161  VDVNLNNNVY KQYFFETKCR NPKPVPSGCR GIDSRHWNSY CTTTHTFVKA LTMEDNRASW RFIRIDTACV CIISRKTENF
```

C Carbamidomethylation (+57.02)

**Supporting Peptides:**

| Peptide | Uniq | -10lgP | Mass | Length | ppm | m/z | z | RT | Fraction | Scan | Source File | Area F1 | Area F2a | Area F3a | Area F4 | Area F5 | Area F6a | Area F7a | Area F7b | Area F8 | Area F9a | Area Sample 1F10a | Area F10b | Area F11a | Area F11b | Area F12a | Area F13 | #Feature | #Feature F1 | #Feature F2a | #Feature F3a | #Feature F4 | #Feature F5 | #Feature F6a | #Feature F7a | #Feature F7b | #Feature F8 | #Feature F9a | #Feature Sample 1F10a | #Feature F10b | #Feature F11a | #Feature F11b | #Feature F12a | #Feature F13 | Start | End | PTM | AScore | Found By |
|---|---|---|---|---|---|---|---|---|---|---|---|---|---|---|---|---|---|---|---|---|---|---|---|---|---|---|---|---|---|---|---|---|---|---|---|---|---|---|---|---|---|---|---|---|---|---|---|---|---|
| R.IDTAC(+57.02)VC(+57.02)IISR.K | Y | 59.00 | 1306.6373 | 11 | -0.3 | 654.3257 | 2 | 47.77 | 7 | F7:22494 | BuCaTN01_F7a.raw | | | | | | | 2.2386E6 | | | | | | | | | | 1 | 0 | 0 | 0 | 0 | 0 | 0 | 1 | 0 | 0 | 0 | 0 | 0 | 0 | 0 | 0 | 0 | 225 | 235 | Carbamidomethylation | C5:Carbamidomethylation:1000.00;C7:Carbamidomethylation:1000.00 | PEAKS DB |
| N.VYKQYFFETK.C | N | 54.63 | 1351.6812 | 10 | 2.9 | 451.5676 | 3 | 50.63 | 8 | F8:27078 | BuCaTN01_F7b.raw | | | | | | | | 5.2856E5 | | | | | | | | | 1 | 0 | 0 | 0 | 0 | 0 | 0 | 0 | 1 | 0 | 0 | 0 | 0 | 0 | 0 | 0 | 0 | 169 | 178 | | | PEAKS DB |
| K.QYFFETK.C | N | 54.60 | 961.4545 | 7 | 0.2 | 481.7346 | 2 | 53.27 | 7 | F7:26821 | BuCaTN01_F7a.raw | | | | | | | 2.4891E7 | 7.2632E6 | | | | | | | | | 2 | 0 | 0 | 0 | 0 | 0 | 0 | 1 | 1 | 0 | 0 | 0 | 0 | 0 | 0 | 0 | 0 | 172 | 178 | | | PEAKS DB |
| R.FIRIDTAC(+57.02)VC(+57.02).I | N | 49.72 | 1253.5896 | 10 | 3.0 | 627.8021 | 2 | 54.32 | 8 | F8:30301 | BuCaTN01_F7b.raw | | | | | | | | 3.8159E5 | | | | | | | | | 1 | 0 | 0 | 0 | 0 | 0 | 0 | 0 | 1 | 0 | 0 | 0 | 0 | 0 | 0 | 0 | 0 | 222 | 231 | Carbamidomethylation | C8:Carbamidomethylation:1000.00;C10:Carbamidomethylation:1000.00 | PEAKS DB |
| R.FIRIDTAC(+57.02)VC(+57.02)IISR.K | Y | 42.69 | 1722.8909 | 14 | 0.7 | 575.3046 | 3 | 61.95 | 7 | F7:34298 | BuCaTN01_F7a.raw | | | | | | | 1.9199E6 | | | | | | | | | | 1 | 0 | 0 | 0 | 0 | 0 | 0 | 1 | 0 | 0 | 0 | 0 | 0 | 0 | 0 | 0 | 0 | 222 | 235 | Carbamidomethylation | C8:Carbamidomethylation:1000.00;C10:Carbamidomethylation:1000.00 | PEAKS DB |
| K.QYFFETKC(+57.02)R.N | N | 41.76 | 1277.5863 | 9 | -0.7 | 426.8691 | 3 | 46.69 | 7 | F7:21495 | BuCaTN01_F7a.raw | | | | | | | 3.7535E5 | | | | | | | | | | 1 | 0 | 0 | 0 | 0 | 0 | 0 | 1 | 0 | 0 | 0 | 0 | 0 | 0 | 0 | 0 | 0 | 172 | 180 | Carbamidomethylation | C8:Carbamidomethylation:1000.00 | PEAKS DB |

**total 6 peptides**

**Best Unique PSM (Scan F7:22494, m/z=654.3257, z=2, RT=47.77, ppm=-0.3):**

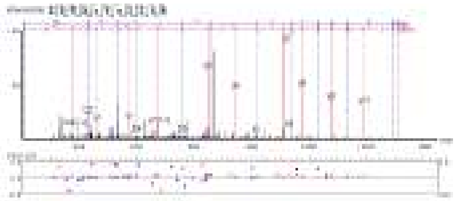

## XP_058034418.1

back to list

| Protein Coverage | Supporting Peptides | Best Unique PSM |

**Protein Coverage:**

```
  1  MSMLCYTLII AFLIGIWAAP KSEDNVPLGS PATSDLSDTS CAKTHEGLKA SRNTDQRHPA PKKAEDQEIG SAANIIVDPK
 81  LFQKRRFQSP RVLFSTQPPP LSRDEQSVDN ADSLNRNIRS KREAHPVHNR GEYSVCDSIS VWVANKTTAT DIKGKPVTVM
                    179                                                              229 231
161  VDVNLNNNVY KQYFFETKCR NPKPVPSGCR GIDSRHWNSY CTTTHTFVKA LTMEDNRASW RFIRIDTACV CIISRKTENF
```

C Carbamidomethylation (+57.02)

**Supporting Peptides:**

| Peptide | Uniq | -10lgP | Mass | Length | ppm | m/z | z | RT | Fraction | Scan | Source File | Area F1 | Area F2a | Area F3a | Area F4 | Area F5 | Area F6a | Area F7a | Area F7b | Area F8 | Area F9a | Area Sample 1F10a | Area F10b | Area F11a | Area F11b | Area F12a | Area F13 | #Feature | #Feature F1 | #Feature F2a | #Feature F3a | #Feature F4 | #Feature F5 | #Feature F6a | #Feature F7a | #Feature F7b | #Feature F8 | #Feature F9a | #Feature Sample 1F10a | #Feature F10b | #Feature F11a | #Feature F11b | #Feature F12a | #Feature F13 | Start | End | PTM | AScore | Found By |
|---|---|---|---|---|---|---|---|---|---|---|---|---|---|---|---|---|---|---|---|---|---|---|---|---|---|---|---|---|---|---|---|---|---|---|---|---|---|---|---|---|---|---|---|---|---|---|---|---|---|
| R.IDTAC(+57.02)VC(+57.02)IISR.K | Y | 59.00 | 1306.6373 | 11 | -0.3 | 654.3257 | 2 | 47.77 | 7 | F7:22494 | BuCaTN01_F7a.raw | | | | | | | 2.2386E6 | | | | | | | | | | 1 | 0 | 0 | 0 | 0 | 0 | 0 | 1 | 0 | 0 | 0 | 0 | 0 | 0 | 0 | 0 | 0 | 225 | 235 | Carbamidomethylation | C5:Carbamidomethylation:1000.00;C7:Carbamidomethylation:1000.00 | PEAKS DB |
| N.VYKQYFFETK.C | N | 54.63 | 1351.6812 | 10 | 2.9 | 451.5676 | 3 | 50.63 | 8 | F8:27078 | BuCaTN01_F7b.raw | | | | | | | | 5.2856E5 | | | | | | | | | 1 | 0 | 0 | 0 | 0 | 0 | 0 | 0 | 1 | 0 | 0 | 0 | 0 | 0 | 0 | 0 | 0 | 169 | 178 | | | PEAKS DB |
| K.QYFFETK.C | N | 54.60 | 961.4545 | 7 | 0.2 | 481.7346 | 2 | 53.27 | 7 | F7:26821 | BuCaTN01_F7a.raw | | | | | | | 2.4891E7 | 7.2632E6 | | | | | | | | | 2 | 0 | 0 | 0 | 0 | 0 | 0 | 1 | 1 | 0 | 0 | 0 | 0 | 0 | 0 | 0 | 0 | 172 | 178 | | | PEAKS DB |
| R.FIRIDTAC(+57.02)VC(+57.02).I | N | 49.72 | 1253.5896 | 10 | 3.0 | 627.8021 | 2 | 54.32 | 8 | F8:30301 | BuCaTN01_F7b.raw | | | | | | | | 3.8159E5 | | | | | | | | | 1 | 0 | 0 | 0 | 0 | 0 | 0 | 0 | 1 | 0 | 0 | 0 | 0 | 0 | 0 | 0 | 0 | 222 | 231 | Carbamidomethylation | C8:Carbamidomethylation:1000.00;C10:Carbamidomethylation:1000.00 | PEAKS DB |
| R.FIRIDTAC(+57.02)VC(+57.02)IISR.K | Y | 42.69 | 1722.8909 | 14 | 0.7 | 575.3046 | 3 | 61.95 | 7 | F7:34298 | BuCaTN01_F7a.raw | | | | | | | 1.9199E6 | | | | | | | | | | 1 | 0 | 0 | 0 | 0 | 0 | 0 | 1 | 0 | 0 | 0 | 0 | 0 | 0 | 0 | 0 | 0 | 222 | 235 | Carbamidomethylation | C8:Carbamidomethylation:1000.00;C10:Carbamidomethylation:1000.00 | PEAKS DB |
| K.QYFFETKC(+57.02)R.N | N | 41.76 | 1277.5863 | 9 | -0.7 | 426.8691 | 3 | 46.69 | 7 | F7:21495 | BuCaTN01_F7a.raw | | | | | | | 3.7535E5 | | | | | | | | | | 1 | 0 | 0 | 0 | 0 | 0 | 0 | 1 | 0 | 0 | 0 | 0 | 0 | 0 | 0 | 0 | 0 | 172 | 180 | Carbamidomethylation | C8:Carbamidomethylation:1000.00 | PEAKS DB |

**total 6 peptides**

**Best Unique PSM (Scan F7:22494, m/z=654.3257, z=2, RT=47.77, ppm=-0.3):**

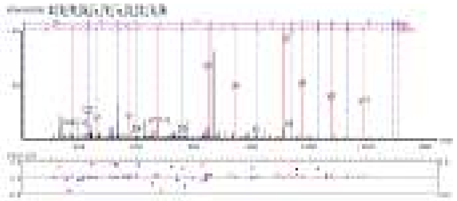

## XP_058034420.1

back to list

| Protein Coverage | Supporting Peptides | Best Unique PSM |

**Protein Coverage:**

```
  1  MSMLCYTLII AFLIGIWAAP KSEDNVPLGS PATSDLSDTS CAKTHEGLKA SRNTDQRHPA PKKAEDQEIG SAANIIVDPK
 81  LFQKRRFQSP RVLFSTQPPP LSRDEQSVDN ADSLNRNIRS KREAHPVHNR GEYSVCDSIS VWVANKTTAT DIKGKPVTVM
                    179                                                              229 231
161  VDVNLNNNVY KQYFFETKCR NPKPVPSGCR GIDSRHWNSY CTTTHTFVKA LTMEDNRASW RFIRIDTACV CIISRKTENF
```

C Carbamidomethylation (+57.02)

**Supporting Peptides:**

| Peptide | Uniq | -10lgP | Mass | Length | ppm | m/z | z | RT | Fraction | Scan | Source File | Area F1 | Area F2a | Area F3a | Area F4 | Area F5 | Area F6a | Area F7a | Area F7b | Area F8 | Area F9a | Area Sample 1F10a | Area F10b | Area F11a | Area F11b | Area F12a | Area F13 | #Feature | #Feature F1 | #Feature F2a | #Feature F3a | #Feature F4 | #Feature F5 | #Feature F6a | #Feature F7a | #Feature F7b | #Feature F8 | #Feature F9a | #Feature Sample 1F10a | #Feature F10b | #Feature F11a | #Feature F11b | #Feature F12a | #Feature F13 | Start | End | PTM | AScore | Found By |
|---|---|---|---|---|---|---|---|---|---|---|---|---|---|---|---|---|---|---|---|---|---|---|---|---|---|---|---|---|---|---|---|---|---|---|---|---|---|---|---|---|---|---|---|---|---|---|---|---|---|
| R.IDTAC(+57.02)VC(+57.02)IISR.K | Y | 59.00 | 1306.6373 | 11 | -0.3 | 654.3257 | 2 | 47.77 | 7 | F7:22494 | BuCaTN01_F7a.raw | | | | | | | 2.2386E6 | | | | | | | | | | 1 | 0 | 0 | 0 | 0 | 0 | 0 | 1 | 0 | 0 | 0 | 0 | 0 | 0 | 0 | 0 | 0 | 225 | 235 | Carbamidomethylation | C5:Carbamidomethylation:1000.00;C7:Carbamidomethylation:1000.00 | PEAKS DB |
| N.VYKQYFFETK.C | N | 54.63 | 1351.6812 | 10 | 2.9 | 451.5676 | 3 | 50.63 | 8 | F8:27078 | BuCaTN01_F7b.raw | | | | | | | | 5.2856E5 | | | | | | | | | 1 | 0 | 0 | 0 | 0 | 0 | 0 | 0 | 1 | 0 | 0 | 0 | 0 | 0 | 0 | 0 | 0 | 169 | 178 | | | PEAKS DB |
| K.QYFFETK.C | N | 54.60 | 961.4545 | 7 | 0.2 | 481.7346 | 2 | 53.27 | 7 | F7:26821 | BuCaTN01_F7a.raw | | | | | | | 2.4891E7 | 7.2632E6 | | | | | | | | | 2 | 0 | 0 | 0 | 0 | 0 | 0 | 1 | 1 | 0 | 0 | 0 | 0 | 0 | 0 | 0 | 0 | 172 | 178 | | | PEAKS DB |

| | | | | | | | | | | | | | | | | | | | | | | | | | | | | | | | | | | | | | | | | | | | | | | | | | |
|---|---|---|---|---|---|---|---|---|---|---|---|---|---|---|---|---|---|---|---|---|---|---|---|---|---|---|---|---|---|---|---|---|---|---|---|---|---|---|---|---|---|---|---|---|---|---|---|---|---|
| R.FIRIDTAC(+57.02)VC(+57.02).I | N | 49.72 | 1253.5896 | 10 | 3.0 | 627.8021 | 2 | 54.32 | 8 | F8:30301 | BuCaTN01_F7b.raw | | | | | | | | 3.8159E5 | | | | | | | | | 1 | 0 | 0 | 0 | 0 | 0 | 0 | 0 | 1 | 0 | 0 | 0 | 0 | 0 | 0 | 0 | 0 | 222 | 231 | Carbamidomethylation | C8:Carbamidomethylation:1000.00;C10:Carbamidomethylation:1000.00 | PEAKS DB |
| R.FIRIDTAC(+57.02)VC(+57.02)IISR.K | Y | 42.69 | 1722.8909 | 14 | 0.7 | 575.3046 | 3 | 61.95 | 7 | F7:34298 | BuCaTN01_F7a.raw | | | | | | | 1.9199E6 | | | | | | | | | | 1 | 0 | 0 | 0 | 0 | 0 | 0 | 1 | 0 | 0 | 0 | 0 | 0 | 0 | 0 | 0 | 0 | 222 | 235 | Carbamidomethylation | C8:Carbamidomethylation:1000.00;C10:Carbamidomethylation:1000.00 | PEAKS DB |
| K.QYFFETKC(+57.02)R.N | N | 41.76 | 1277.5863 | 9 | -0.7 | 426.8691 | 3 | 46.69 | 7 | F7:21495 | BuCaTN01_F7a.raw | | | | | | | 3.7535E5 | | | | | | | | | | 1 | 0 | 0 | 0 | 0 | 0 | 0 | 1 | 0 | 0 | 0 | 0 | 0 | 0 | 0 | 0 | 0 | 172 | 180 | Carbamidomethylation | C8:Carbamidomethylation:1000.00 | PEAKS DB |
| total 6 peptides | | | | | | | | | | | | | | | | | | | | | | | | | | | | | | | | | | | | | | | | | | | | | | | | | |

**Best Unique PSM (Scan F7:22494, m/z=654.3257, z=2, RT=47.77, ppm=-0.3):**

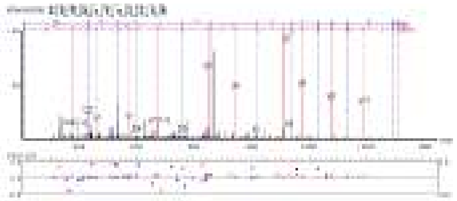

## XP_058034417.1

| Protein Coverage | Supporting Peptides | Best Unique PSM |

**Protein Coverage:**

```
  1 MKVHSVMSML CYTLIIAFLI GIWAAPKSED NVPLGSPATS DLSDTSCAKT HEGLKASRNT DQRHPAPKKA EDQEIGSAAN
 81 IIVDPKLFQK RRFQSPRVLF STQPPPLSRD EQSVDNADSL NRNIRSKREA HPVHNRGEYS VCDSISVWVA NKTTATDIKG
                                     185                                                         235 237
161 KPVTVMVDVN LNNNVYKQYF FETKCRNPKP VPSGCRGIDS RHWNSYCTTT HTFVKALTME DNRASWRFIR IDTACVCIIS
241 RKTENF
```

C Carbamidomethylation (+57.02)

**Supporting Peptides:**

| Peptide | Uniq | -10lgP | Mass | Length | ppm | m/z | z | RT | Fraction | Scan | Source File | Area F1 | Area F2a | Area F3a | Area F4 | Area F5 | Area F6a | Area F7a | Area F7b | Area F8 | Area F9a | Area Sample 1F10a | Area F10b | Area F11a | Area F11b | Area F12a | Area F13 | #Feature | #Feature F1 | #Feature F2a | #Feature F3a | #Feature F4 | #Feature F5 | #Feature F6a | #Feature F7a | #Feature F7b | #Feature F8 | #Feature F9a | #Feature Sample 1F10a | #Feature F10b | #Feature F11a | #Feature F11b | #Feature F12a | #Feature F13 | Start | End | PTM | AScore | Found By |
|---|---|---|---|---|---|---|---|---|---|---|---|---|---|---|---|---|---|---|---|---|---|---|---|---|---|---|---|---|---|---|---|---|---|---|---|---|---|---|---|---|---|---|---|---|---|---|---|---|---|
| R.IDTAC(+57.02)VC(+57.02)IISR.K | Y | 59.00 | 1306.6373 | 11 | -0.3 | 654.3257 | 2 | 47.77 | 7 | F7:22494 | BuCaTN01_F7a.raw | | | | | | | 2.2386E6 | | | | | | | | | | 1 | 0 | 0 | 0 | 0 | 0 | 0 | 1 | 0 | 0 | 0 | 0 | 0 | 0 | 0 | 0 | 0 | 231 | 241 | Carbamidomethylation | C5:Carbamidomethylation:1000.00;C7:Carbamidomethylation:1000.00 | PEAKS DB |
| N.VYKQYFFETK.C | N | 54.63 | 1351.6812 | 10 | 2.9 | 451.5676 | 3 | 50.63 | 8 | F8:27078 | BuCaTN01_F7b.raw | | | | | | | | 5.2856E5 | | | | | | | | | 1 | 0 | 0 | 0 | 0 | 0 | 0 | 0 | 1 | 0 | 0 | 0 | 0 | 0 | 0 | 0 | 0 | 175 | 184 | | | PEAKS DB |
| K.QYFFETK.C | N | 54.60 | 961.4545 | 7 | 0.2 | 481.7346 | 2 | 53.27 | 7 | F7:26821 | BuCaTN01_F7a.raw | | | | | | | 2.4891E7 | 7.2632E6 | | | | | | | | | 2 | 0 | 0 | 0 | 0 | 0 | 0 | 1 | 1 | 0 | 0 | 0 | 0 | 0 | 0 | 0 | 0 | 178 | 184 | | | PEAKS DB |
| R.FIRIDTAC(+57.02)VC(+57.02).I | N | 49.72 | 1253.5896 | 10 | 3.0 | 627.8021 | 2 | 54.32 | 8 | F8:30301 | BuCaTN01_F7b.raw | | | | | | | | 3.8159E5 | | | | | | | | | 1 | 0 | 0 | 0 | 0 | 0 | 0 | 0 | 1 | 0 | 0 | 0 | 0 | 0 | 0 | 0 | 0 | 228 | 237 | Carbamidomethylation | C8:Carbamidomethylation:1000.00;C10:Carbamidomethylation:1000.00 | PEAKS DB |
| R.FIRIDTAC(+57.02)VC(+57.02)IISR.K | Y | 42.69 | 1722.8909 | 14 | 0.7 | 575.3046 | 3 | 61.95 | 7 | F7:34298 | BuCaTN01_F7a.raw | | | | | | | 1.9199E6 | | | | | | | | | | 1 | 0 | 0 | 0 | 0 | 0 | 0 | 1 | 0 | 0 | 0 | 0 | 0 | 0 | 0 | 0 | 0 | 228 | 241 | Carbamidomethylation | C8:Carbamidomethylation:1000.00;C10:Carbamidomethylation:1000.00 | PEAKS DB |
| K.QYFFETKC(+57.02)R.N | N | 41.76 | 1277.5863 | 9 | -0.7 | 426.8691 | 3 | 46.69 | 7 | F7:21495 | BuCaTN01_F7a.raw | | | | | | | 3.7535E5 | | | | | | | | | | 1 | 0 | 0 | 0 | 0 | 0 | 0 | 1 | 0 | 0 | 0 | 0 | 0 | 0 | 0 | 0 | 0 | 178 | 186 | Carbamidomethylation | C8:Carbamidomethylation:1000.00 | PEAKS DB |
| total 6 peptides | | | | | | | | | | | | | | | | | | | | | | | | | | | | | | | | | | | | | | | | | | | | | | | | | |

**Best Unique PSM (Scan F7:22494, m/z=654.3257, z=2, RT=47.77, ppm=-0.3):**

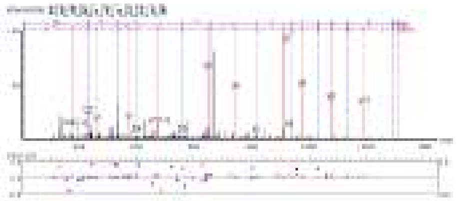

## AVX27607.1

| Protein Coverage | Supporting Peptides | Best Unique PSM |

**Protein Coverage:**

```
  1 MNVLFIFSLL FLAALESCAD DRRSPLEKCF QEADYEDFLE IARNGLKETS NPKHVVVVGA GMAGLSAAYV LAGAGHKVTL
 81 LEASERVGGR VITYHNDREG WYVNMGPMRL PERHRIVREY IRKFGLKLNE FFQENENAWY YINNIRKRVW EVKKDPSLLK
161 YPVKPSEEGK SASQLYQESL RKVIEELKRT NCSYILNKYD SYSTKEYLIK EGNLSRGAVD MIGDLLNEDS SYHLSFMESL
241 KSDALFSYEK RFDEIVGGFD QLPISMYQAI AEMVHLNARV IKIQYDAEKV RVTYQTPAKT FVTADYVIVC STSRAARRIY
321 FEPPLPPKKA HALRSIHYRS ATKIFLTCSK KFWEADGIHG GKSTTDLPSR FIHYPNHNFT SGIGVIMAYV LADDSDFFQA
401 LDTKTCADIV INDLSLIHDL PKREIQALCY PSIKKWNLDK YTMGSITSFT PYQFQDYFES AAAPVGRIHF AGEYTGRFHG
481 WIDSTIMTGL RAARDVNRAS QKPSKIR
```

o Oxidation (M) (+15.99)

**Supporting Peptides:**

| Peptide | Uniq | -10lgP | Mass | Length | ppm | m/z | z | RT | Fraction | Scan | Source File | Area F1 | Area F2a | Area F3a | Area F4 | Area F5 | Area F6a | Area F7a | Area F7b | Area F8 | Area F9a | Area Sample 1F10a | Area F10b | Area F11a | Area F11b | Area F12a | Area F13 | #Feature | #Feature F1 | #Feature F2a | #Feature F3a | #Feature F4 | #Feature F5 | #Feature F6a | #Feature F7a | #Feature F7b | #Feature F8 | #Feature F9a | #Feature Sample 1F10a | #Feature F10b | #Feature F11a | #Feature F11b | #Feature F12a | #Feature F13 | Start | End | PTM | AScore | Found By |
|---|---|---|---|---|---|---|---|---|---|---|---|---|---|---|---|---|---|---|---|---|---|---|---|---|---|---|---|---|---|---|---|---|---|---|---|---|---|---|---|---|---|---|---|---|---|---|---|---|---|
| K.HVVVVGAGM(+15.99)AGLSAAYVLAGAGHK.V | N | 66.75 | 2250.1943 | 24 | 0.1 | 563.5559 | 4 | 65.96 | 16 | F16:33316 | BuCaTN01_F13.raw | | | | | | | | | | | | | | | | 0 | 0 | 0 | 0 | 0 | 0 | 0 | 0 | 0 | 0 | 0 | 0 | 0 | 0 | 0 | 0 | 0 | 0 | 54 | 77 | Oxidation (M) | M9:Oxidation (M):1000.00 | PEAKS DB |
| R.IHFAGEYTGR.F | Y | 49.04 | 1149.5566 | 10 | 0.9 | 384.1932 | 3 | 40.04 | 16 | F16:16395 | BuCaTN01_F13.raw | | | | | | | | | | | | | | | | 9.9121E4 | 1 | 0 | 0 | 0 | 0 | 0 | 0 | 0 | 0 | 0 | 0 | 0 | 0 | 0 | 0 | 0 | 1 | 468 | 477 | | | PEAKS DB |
| K.SASQLYQESLR.K | Y | 48.08 | 1280.6360 | 11 | -1.5 | 641.3243 | 2 | 45.12 | 16 | F16:19933 | BuCaTN01_F13.raw | | | | | | | | | | | | | | | | 1.4485E4 | 1 | 0 | 0 | 0 | 0 | 0 | 0 | 0 | 0 | 0 | 0 | 0 | 0 | 0 | 0 | 0 | 1 | 171 | 181 | | | PEAKS DB |
| K.HVVVVGAGMAG.L | N | 42.23 | 995.5222 | 11 | 0.2 | 498.7685 | 2 | 40.63 | 16 | F16:16823 | BuCaTN01_F13.raw | | | | | | | | | | | | | | | | 3.257E4 | 1 | 0 | 0 | 0 | 0 | 0 | 0 | 0 | 0 | 0 | 0 | 0 | 0 | 0 | 0 | 0 | 1 | 54 | 64 | | | PEAKS DB |
| K.VTLLEASER.V | N | 41.88 | 1016.5502 | 9 | -0.5 | 509.2821 | 2 | 44.09 | 16 | F16:19432 | BuCaTN01_F13.raw | | | | | | | | | | | | | | | | 1.9042E6 | 1 | 0 | 0 | 0 | 0 | 0 | 0 | 0 | 0 | 0 | 0 | 0 | 0 | 0 | 0 | 0 | 1 | 78 | 86 | | | PEAKS DB |
| D.PSLLKYPVKPSEEGK.S | Y | 41.70 | 1670.9242 | 15 | 0.2 | 418.7384 | 4 | 42.14 | 16 | F16:18094 | BuCaTN01_F13.raw | | | | | | | | | | | | | | | | 4.9879E5 | 1 | 0 | 0 | 0 | 0 | 0 | 0 | 0 | 0 | 0 | 0 | 0 | 0 | 0 | 0 | 0 | 1 | 156 | 170 | | | PEAKS DB |
| total 6 peptides | | | | | | | | | | | | | | | | | | | | | | | | | | | | | | | | | | | | | | | | | | | | | | | | | |

**Best Unique PSM (Scan F16:16395, m/z=384.1932, z=3, RT=40.04, ppm=0.9):**

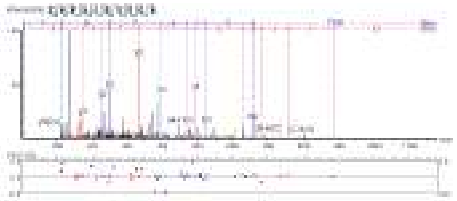

## 5Z2G

| Protein Coverage | Supporting Peptides | Best Unique PSM |

**Protein Coverage:**

1 MNVLFIFSLL FLAALESCAD DRRSPLEKCF QEADYEDFLE IARNGLKETS NPK**HVVVVGA GMAGLSAAYV LAGAGHKVTL** Oxidation (M) (+15.99)

81 **LEASER**VGGR VITYHNDREG WYVNMGPMRL PERHRIVREY IRKFGLKLNE FFQENENAWY YINNIRKRVW EVKKD**PSLLK**

161 **YPVKPSEEGK SASQLYQESL R**KVIEELKRT NCSYILNKYD SYSTKEYLIK EGNLSRGAVD MIGDLLNEDS SYHLSFMESL

241 KSDALFSYEK RFDEIVGGFD QLPISMYQAI AEMVHLNARV IKIQYDAEKV RVTYQTPAKT FVTADYVICV STSRAARRIY
321 FEPPLPPKKA HALRSIHYRS ATKIFLTCSK KFWEADGIHG GKSTTDLPSR FIHYPNHNFT SGIGVIMAYV LADDSDFFQA
401 LDTKTCADIV INDLSLIHDL PKREIQALCY PSIKKWNLDK YTMGSITSFT PYQFQDYFES AAAPVGR**IHF AGEYTGR**FHG

481 WIDSTIMTGL RAARDVNRAS QKPSKIR

**Supporting Peptides:**

| Peptide | Uniq | -10lgP | Mass | Length | ppm | m/z | z | RT | Fraction | Scan | Source File | Area F1 | Area F2a | Area F3a | Area F4 | Area F5 | Area F6a | Area F7a | Area F7b | Area F8 | Area F9a | Area Sample 1F10a | Area F10b | Area F11a | Area F11b | Area F12a | Area F13 | #Feature | #Feature F1 | #Feature F2a | #Feature F3a | #Feature F4 | #Feature F5 | #Feature F6a | #Feature F7a | #Feature F7b | #Feature F8 | #Feature F9a | #Feature Sample 1F10a | #Feature F10b | #Feature F11a | #Feature F11b | #Feature F12a | #Feature F13 | Start | End | PTM | AScore | Found By |
|---|---|---|---|---|---|---|---|---|---|---|---|---|---|---|---|---|---|---|---|---|---|---|---|---|---|---|---|---|---|---|---|---|---|---|---|---|---|---|---|---|---|---|---|---|---|---|---|---|---|
| K.HVVVVGAGM(+15.99)AGLSAAYVLAGAGHK.V | N | 66.75 | 2250.1943 | 24 | 0.1 | 563.5559 | 4 | 65.96 | 16 | F16:33316 | BuCaTN01_F13.raw | | | | | | | | | | | | | | | | 0 | 0 | 0 | 0 | 0 | 0 | 0 | 0 | 0 | 0 | 0 | 0 | 0 | 0 | 0 | 0 | 0 | 0 | 54 | 77 | Oxidation (M) | M9:Oxidation (M):1000.00 | PEAKS DB |
| R.IHFAGEYTGR.F | Y | 49.04 | 1149.5566 | 10 | 0.9 | 384.1932 | 3 | 40.04 | 16 | F16:16395 | BuCaTN01_F13.raw | | | | | | | | | | | | | | | | 9.9121E4 | 1 | 0 | 0 | 0 | 0 | 0 | 0 | 0 | 0 | 0 | 0 | 0 | 0 | 0 | 0 | 0 | 1 | 468 | 477 | | | PEAKS DB |
| K.SASQLYQESLR.K | Y | 48.08 | 1280.6360 | 11 | -1.5 | 641.3243 | 2 | 45.12 | 16 | F16:19933 | BuCaTN01_F13.raw | | | | | | | | | | | | | | | | 1.4485E4 | 1 | 0 | 0 | 0 | 0 | 0 | 0 | 0 | 0 | 0 | 0 | 0 | 0 | 0 | 0 | 0 | 1 | 171 | 181 | | | PEAKS DB |
| K.HVVVVGAGMAG.L | N | 42.23 | 995.5222 | 11 | 0.2 | 498.7685 | 2 | 40.63 | 16 | F16:16823 | BuCaTN01_F13.raw | | | | | | | | | | | | | | | | 3.257E4 | 1 | 0 | 0 | 0 | 0 | 0 | 0 | 0 | 0 | 0 | 0 | 0 | 0 | 0 | 0 | 0 | 1 | 54 | 64 | | | PEAKS DB |
| K.VTLLEASER.V | N | 41.88 | 1016.5502 | 9 | -0.5 | 509.2821 | 2 | 44.09 | 16 | F16:19432 | BuCaTN01_F13.raw | | | | | | | | | | | | | | | | 1.9042E6 | 1 | 0 | 0 | 0 | 0 | 0 | 0 | 0 | 0 | 0 | 0 | 0 | 0 | 0 | 0 | 0 | 1 | 78 | 86 | | | PEAKS DB |
| D.PSLLKYPVKPSEEGK.S | Y | 41.70 | 1670.9242 | 15 | 0.2 | 418.7384 | 4 | 42.14 | 16 | F16:18094 | BuCaTN01_F13.raw | | | | | | | | | | | | | | | | 4.9879E5 | 1 | 0 | 0 | 0 | 0 | 0 | 0 | 0 | 0 | 0 | 0 | 0 | 0 | 0 | 0 | 0 | 1 | 156 | 170 | | | PEAKS DB |
| total 6 peptides | | | | | | | | | | | | | | | | | | | | | | | | | | | | | | | | | | | | | | | | | | | | | | | | | |

**Best Unique PSM (Scan F16:16395, m/z=384.1932, z=3, RT=40.04, ppm=0.9):**

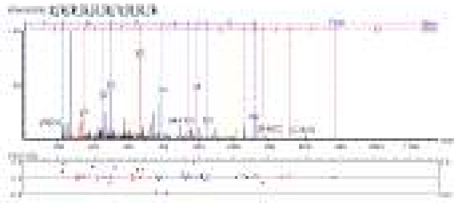

## A8QL58.2

back to list

| Protein Coverage | Supporting Peptides | Best Unique PSM |

**Protein Coverage:**

1 MNVLFIFSLL FLAALESCAD DRRSPLEKCF QEADYEDFLE IARNGLKETS NPK**HVVVVGA GMAGLSAAYV LAGAGHKVTL** Oxidation (M) (+15.99)

81 **LEASER**VGGR VITYHNDREG WYVNMGPMRL PERHRIVREY IRKFGLKLNE FFQENENAWY YINNIRKRVW EVKKD**PSLLK**

161 **YPVKPSEEGK SASQLYQESL R**KVIEELKRT NCSYILNKYD SYSTKEYLIK EGNLSRGAVD MIGDLLNEDS SYHLSFMESL

241 KSDALFSYEK RFDEIVGGFD QLPISMYQAI AEMVHLNARV IKIQYDAEKV RVTYQTPAKT FVTADYVICV STSRAARRIY
321 FEPPLPPKKA HALRSIHYRS ATKIFLTCSK KFWEADGIHG GKSTTDLPSR FIHYPNHNFT SGIGVIMAYV LADDSDFFQA
401 LDTKTCADIV INDLSLIHDL PKREIQALCY PSIKKWNLDK YTMGSITSFT PYQFQDYFES AAAPVGR**IHF AGEYTGR**FHG

481 WIDSTIMTGL RAARDVNRAS QKPSKIR

**Supporting Peptides:**

| Peptide | Uniq | -10lgP | Mass | Length | ppm | m/z | z | RT | Fraction | Scan | Source File | Area F1 | Area F2a | Area F3a | Area F4 | Area F5 | Area F6a | Area F7a | Area F7b | Area F8 | Area F9a | Area Sample 1F10a | Area F10b | Area F11a | Area F11b | Area F12a | Area F13 | #Feature | #Feature F1 | #Feature F2a | #Feature F3a | #Feature F4 | #Feature F5 | #Feature F6a | #Feature F7a | #Feature F7b | #Feature F8 | #Feature F9a | #Feature Sample 1F10a | #Feature F10b | #Feature F11a | #Feature F11b | #Feature F12a | #Feature F13 | Start | End | PTM | AScore | Found By |
|---|---|---|---|---|---|---|---|---|---|---|---|---|---|---|---|---|---|---|---|---|---|---|---|---|---|---|---|---|---|---|---|---|---|---|---|---|---|---|---|---|---|---|---|---|---|---|---|---|---|
| K.HVVVVGAGM(+15.99)AGLSAAYVLAGAGHK.V | N | 66.75 | 2250.1943 | 24 | 0.1 | 563.5559 | 4 | 65.96 | 16 | F16:33316 | BuCaTN01_F13.raw | | | | | | | | | | | | | | | | 0 | 0 | 0 | 0 | 0 | 0 | 0 | 0 | 0 | 0 | 0 | 0 | 0 | 0 | 0 | 0 | 0 | 0 | 54 | 77 | Oxidation (M) | M9:Oxidation (M):1000.00 | PEAKS DB |
| R.IHFAGEYTGR.F | Y | 49.04 | 1149.5566 | 10 | 0.9 | 384.1932 | 3 | 40.04 | 16 | F16:16395 | BuCaTN01_F13.raw | | | | | | | | | | | | | | | | 9.9121E4 | 1 | 0 | 0 | 0 | 0 | 0 | 0 | 0 | 0 | 0 | 0 | 0 | 0 | 0 | 0 | 0 | 1 | 468 | 477 | | | PEAKS DB |
| K.SASQLYQESLR.K | Y | 48.08 | 1280.6360 | 11 | -1.5 | 641.3243 | 2 | 45.12 | 16 | F16:19933 | BuCaTN01_F13.raw | | | | | | | | | | | | | | | | 1.4485E4 | 1 | 0 | 0 | 0 | 0 | 0 | 0 | 0 | 0 | 0 | 0 | 0 | 0 | 0 | 0 | 0 | 1 | 171 | 181 | | | PEAKS DB |
| K.HVVVVGAGMAG.L | N | 42.23 | 995.5222 | 11 | 0.2 | 498.7685 | 2 | 40.63 | 16 | F16:16823 | BuCaTN01_F13.raw | | | | | | | | | | | | | | | | 3.257E4 | 1 | 0 | 0 | 0 | 0 | 0 | 0 | 0 | 0 | 0 | 0 | 0 | 0 | 0 | 0 | 0 | 1 | 54 | 64 | | | PEAKS DB |
| K.VTLLEASER.V | N | 41.88 | 1016.5502 | 9 | -0.5 | 509.2821 | 2 | 44.09 | 16 | F16:19432 | BuCaTN01_F13.raw | | | | | | | | | | | | | | | | 1.9042E6 | 1 | 0 | 0 | 0 | 0 | 0 | 0 | 0 | 0 | 0 | 0 | 0 | 0 | 0 | 0 | 0 | 1 | 78 | 86 | | | PEAKS DB |
| D.PSLLKYPVKPSEEGK.S | Y | 41.70 | 1670.9242 | 15 | 0.2 | 418.7384 | 4 | 42.14 | 16 | F16:18094 | BuCaTN01_F13.raw | | | | | | | | | | | | | | | | 4.9879E5 | 1 | 0 | 0 | 0 | 0 | 0 | 0 | 0 | 0 | 0 | 0 | 0 | 0 | 0 | 0 | 0 | 1 | 156 | 170 | | | PEAKS DB |
| total 6 peptides | | | | | | | | | | | | | | | | | | | | | | | | | | | | | | | | | | | | | | | | | | | | | | | | | |

**Best Unique PSM (Scan F16:16395, m/z=384.1932, z=3, RT=40.04, ppm=0.9):**

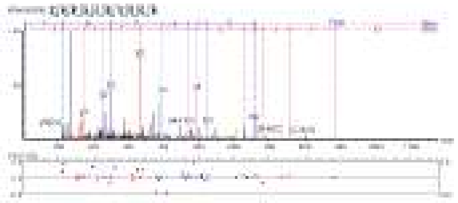

## XP_026579406.1

back to list

| Protein Coverage | Supporting Peptides | Best Unique PSM |

**Protein Coverage:**

1 MGSGRSPLLL LLLLLLFWGD FALWGSLHSV SG**LFEGSCSI FPNDSQR**CGE PKNRFFYNIT SKSCEPFVYH GCPGNRNRYH Carbamidomethylation (+57.02)

81 TIEECQRYCG NIEKPGFCPP SPRGASGPCW TRCLHDGSCP EMEKCCSYGC ALYCMKPVTD LCQLPSEPGP CDLKVKRWFY

161 DPKIQRCKK**F TYGGCAGNAN NFKK**HLECRL RCRKRGIH

**Supporting Peptides:**

| Peptide | Uniq | -10lgP | Mass | Length | ppm | m/z | z | RT | Fraction | Scan | Source File | Area F1 | Area F2a | Area F3a | Area F4 | Area F5 | Area F6a | Area F7a | Area F7b | Area F8 | Area F9a | Area Sample 1F10a | Area F10b | Area F11a | Area F11b | Area F12a | Area F13 | #Feature | #Feature F1 | #Feature F2a | #Feature F3a | #Feature F4 | #Feature F5 | #Feature F6a | #Feature F7a | #Feature F7b | #Feature F8 | #Feature F9a | #Feature Sample 1F10a | #Feature F10b | #Feature F11a | #Feature F11b | #Feature F12a | #Feature F13 | Start | End | PTM | AScore | Found By |
|---|---|---|---|---|---|---|---|---|---|---|---|---|---|---|---|---|---|---|---|---|---|---|---|---|---|---|---|---|---|---|---|---|---|---|---|---|---|---|---|---|---|---|---|---|---|---|---|---|---|
| K.FTYGGC(+57.02)AGNANNFK.K | Y | 93.70 | 1519.6514 | 14 | 4.0 | 760.8345 | 2 | 42.50 | 3 | F3:19119 | BuCaTN01_F3a.raw | | 9.87E6 | 3.7722E8 | | | 2.0917E7 | 1.7985E5 | 3.983E6 | | | | 0 | | 6.0577E5 | | | 9 | 0 | 1 | 3 | 0 | 0 | 1 | 1 | 1 | 0 | 0 | 0 | 0 | 0 | 2 | 0 | 0 | 170 | 183 | Carbamidomethylation | C6:Carbamidomethylation:1000.00 | PEAKS DB |
| G.LFEGSC(+57.02)SIFPNDSQR.C | Y | 49.11 | 1755.7886 | 15 | 0.6 | 878.9021 | 2 | 63.05 | 7 | F7:35242 | BuCaTN01_F7a.raw | | | | | | | 2.6284E5 | | | | | | | | | | 1 | 0 | 0 | 0 | 0 | 0 | 0 | 1 | 0 | 0 | 0 | 0 | 0 | 0 | 0 | 0 | 0 | 33 | 47 | Carbamidomethylation | C6:Carbamidomethylation:1000.00 | PEAKS DB |
| K.FTYGGC(+57.02)AGNANNFKK.H | Y | 41.93 | 1647.7463 | 15 | 1.9 | 550.2560 | 3 | 36.56 | 3 | F3:13974 | BuCaTN01_F3a.raw | | | 1.7131E5 | | | | | | | | | | | | | | 1 | 0 | 0 | 1 | 0 | 0 | 0 | 0 | 0 | 0 | 0 | 0 | 0 | 0 | 0 | 0 | 0 | 170 | 184 | Carbamidomethylation | C6:Carbamidomethylation:1000.00 | PEAKS DB |
| total 3 peptides | | | | | | | | | | | | | | | | | | | | | | | | | | | | | | | | | | | | | | | | | | | | | | | | | |

**Best Unique PSM (Scan F3:19119, m/z=760.8345, z=2, RT=42.50, ppm=4.0):**

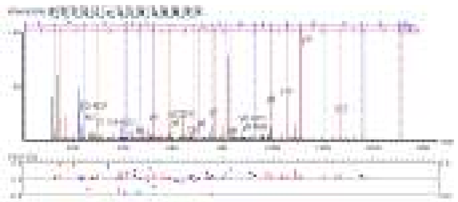

## XP_026579408.1

back to list

| Protein Coverage | Supporting Peptides | Best Unique PSM |

**Protein Coverage:**

```
  1 MGSGRSPLLL LLLLLLFWGD FALWGSLHSV SGLFEGSCSI FPNDSQRCGE PKNRFFYNIT SKSCEPFVYH GCPGNRNRYH
 81 TIEECQRYCG NIEKPGFCPP SPRGASGPCW TRCLHDGSCP EMEKCCSYGC ALYCMKPVTD LCQLPSEPGP CDLKVKRWFY
161 DPKIQRCKKF TYGGCAGNAN NFKKHLECRL RCRKRGTP
```

C Carbamidomethylation (+57.02)

**Supporting Peptides:**

| Peptide | Uniq | -10lgP | Mass | Length | ppm | m/z | z | RT | Fraction | Scan | Source File | Area F1 | Area F2a | Area F3a | Area F4 | Area F5 | Area F6a | Area F7a | Area F7b | Area F8 | Area F9a | Area Sample 1F10a | Area F10b | Area F11a | Area F11b | Area F12a | Area F13 | #Feature | #Feature F1 | #Feature F2a | #Feature F3a | #Feature F4 | #Feature F5 | #Feature F6a | #Feature F7a | #Feature F7b | #Feature F8 | #Feature F9a | #Feature Sample 1F10a | #Feature F10b | #Feature F11a | #Feature F11b | #Feature F12a | #Feature F13 | Start | End | PTM | AScore | Found By |
|---|---|---|---|---|---|---|---|---|---|---|---|---|---|---|---|---|---|---|---|---|---|---|---|---|---|---|---|---|---|---|---|---|---|---|---|---|---|---|---|---|---|---|---|---|---|---|---|---|---|
| K.FTYGGC(+57.02)AGNANNFK.K | Y | 93.70 | 1519.6514 | 14 | 4.0 | 760.8345 | 2 | 42.50 | 3 | F3:19119 | BuCaTN01_F3a.raw | | 9.87E6 | 3.7722E8 | | | 2.0917E7 | 1.7985E5 | 3.983E6 | | | | 0 | | 6.0577E5 | | | 9 | 0 | 1 | 3 | 0 | 0 | 1 | 1 | 1 | 0 | 0 | 0 | 0 | 0 | 2 | 0 | 0 | 170 | 183 | Carbamidomethylation | C6:Carbamidomethylation:1000.00 | PEAKS DB |
| G.LFEGSC(+57.02)SIFPNDSQR.C | Y | 49.11 | 1755.7886 | 15 | 0.6 | 878.9021 | 2 | 63.05 | 7 | F7:35242 | BuCaTN01_F7a.raw | | | | | | | 2.6284E5 | | | | | | | | | | 1 | 0 | 0 | 0 | 0 | 0 | 0 | 1 | 0 | 0 | 0 | 0 | 0 | 0 | 0 | 0 | 0 | 33 | 47 | Carbamidomethylation | C6:Carbamidomethylation:1000.00 | PEAKS DB |
| K.FTYGGC(+57.02)AGNANNFKK.H | Y | 41.93 | 1647.7463 | 15 | 1.9 | 550.2560 | 3 | 36.56 | 3 | F3:13974 | BuCaTN01_F3a.raw | | | 1.7131E5 | | | | | | | | | | | | | | 1 | 0 | 0 | 1 | 0 | 0 | 0 | 0 | 0 | 0 | 0 | 0 | 0 | 0 | 0 | 0 | 0 | 170 | 184 | Carbamidomethylation | C6:Carbamidomethylation:1000.00 | PEAKS DB |
| total 3 peptides | | | | | | | | | | | | | | | | | | | | | | | | | | | | | | | | | | | | | | | | | | | | | | | | | |

**Best Unique PSM (Scan F3:19119, m/z=760.8345, z=2, RT=42.50, ppm=4.0):**

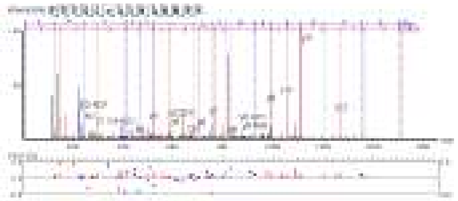

## XP_026579405.1

back to list

| Protein Coverage | Supporting Peptides | Best Unique PSM |

**Protein Coverage:**

```
  1 MGSGRSPLLL LLLLLLFWGD FALWGSLHSV SGLFEGSCSI FPNDSQRCGE PKNRFFYNIT SKSCEPFVYH GCPGNRNRYH
 81 TIEECQRYCG NIEKPGFCPP SPRGASGPCW TRCLHDGSCP EMEKCCSYGC ALYCMKPVTD LCQLPSEPGP CDLKVKRWFY
161 DPKIQRCKKF TYGGCAGNAN NFKKHLECRL RCRKRGPP
```

C Carbamidomethylation (+57.02)

**Supporting Peptides:**

| Peptide | Uniq | -10lgP | Mass | Length | ppm | m/z | z | RT | Fraction | Scan | Source File | Area F1 | Area F2a | Area F3a | Area F4 | Area F5 | Area F6a | Area F7a | Area F7b | Area F8 | Area F9a | Area Sample 1F10a | Area F10b | Area F11a | Area F11b | Area F12a | Area F13 | #Feature | #Feature F1 | #Feature F2a | #Feature F3a | #Feature F4 | #Feature F5 | #Feature F6a | #Feature F7a | #Feature F7b | #Feature F8 | #Feature F9a | #Feature Sample 1F10a | #Feature F10b | #Feature F11a | #Feature F11b | #Feature F12a | #Feature F13 | Start | End | PTM | AScore | Found By |
|---|---|---|---|---|---|---|---|---|---|---|---|---|---|---|---|---|---|---|---|---|---|---|---|---|---|---|---|---|---|---|---|---|---|---|---|---|---|---|---|---|---|---|---|---|---|---|---|---|---|
| K.FTYGGC(+57.02)AGNANNFK.K | Y | 93.70 | 1519.6514 | 14 | 4.0 | 760.8345 | 2 | 42.50 | 3 | F3:19119 | BuCaTN01_F3a.raw | | 9.87E6 | 3.7722E8 | | | 2.0917E7 | 1.7985E5 | 3.983E6 | | | | 0 | | 6.0577E5 | | | 9 | 0 | 1 | 3 | 0 | 0 | 1 | 1 | 1 | 0 | 0 | 0 | 0 | 0 | 2 | 0 | 0 | 170 | 183 | Carbamidomethylation | C6:Carbamidomethylation:1000.00 | PEAKS DB |
| G.LFEGSC(+57.02)SIFPNDSQR.C | Y | 49.11 | 1755.7886 | 15 | 0.6 | 878.9021 | 2 | 63.05 | 7 | F7:35242 | BuCaTN01_F7a.raw | | | | | | | 2.6284E5 | | | | | | | | | | 1 | 0 | 0 | 0 | 0 | 0 | 0 | 1 | 0 | 0 | 0 | 0 | 0 | 0 | 0 | 0 | 0 | 33 | 47 | Carbamidomethylation | C6:Carbamidomethylation:1000.00 | PEAKS DB |
| K.FTYGGC(+57.02)AGNANNFKK.H | Y | 41.93 | 1647.7463 | 15 | 1.9 | 550.2560 | 3 | 36.56 | 3 | F3:13974 | BuCaTN01_F3a.raw | | | 1.7131E5 | | | | | | | | | | | | | | 1 | 0 | 0 | 1 | 0 | 0 | 0 | 0 | 0 | 0 | 0 | 0 | 0 | 0 | 0 | 0 | 0 | 170 | 184 | Carbamidomethylation | C6:Carbamidomethylation:1000.00 | PEAKS DB |
| total 3 peptides | | | | | | | | | | | | | | | | | | | | | | | | | | | | | | | | | | | | | | | | | | | | | | | | | |

**Best Unique PSM (Scan F3:19119, m/z=760.8345, z=2, RT=42.50, ppm=4.0):**

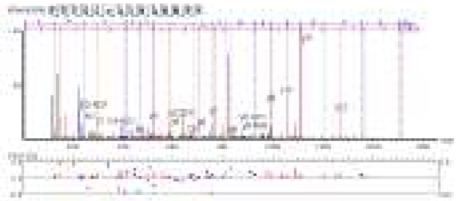

## XP_026579404.1

back to list

| Protein Coverage | Supporting Peptides | Best Unique PSM |

**Protein Coverage:**

```
  1 MGSGRSPLLL LLLLLLFWGD FALWGSLHSV SGLFEGSCSI FPNDSQRCGE PKNRFFYNIT SKSCEPFVYH GCPGNRNRYH
 81 TIEECQRYCG NIEKPGFCPP SPRGASGPCW TRCLHDGSCP EMEKCCSYGC ALYCMKPVTD LCQLPSEPGP CDLKVKRWFY
161 DPKIQRCKKF TYGGCAGNAN NFKKHLECRL RCRKRGTP
```

C Carbamidomethylation (+57.02)

**Supporting Peptides:**

| Peptide | Uniq | -10lgP | Mass | Length | ppm | m/z | z | RT | Fraction | Scan | Source File | Area F1 | Area F2a | Area F3a | Area F4 | Area F5 | Area F6a | Area F7a | Area F7b | Area F8 | Area F9a | Area Sample 1F10a | Area F10b | Area F11a | Area F11b | Area F12a | Area F13 | #Feature | #Feature F1 | #Feature F2a | #Feature F3a | #Feature F4 | #Feature F5 | #Feature F6a | #Feature F7a | #Feature F7b | #Feature F8 | #Feature F9a | #Feature Sample 1F10a | #Feature F10b | #Feature F11a | #Feature F11b | #Feature F12a | #Feature F13 | Start | End | PTM | AScore | Found By |
|---|---|---|---|---|---|---|---|---|---|---|---|---|---|---|---|---|---|---|---|---|---|---|---|---|---|---|---|---|---|---|---|---|---|---|---|---|---|---|---|---|---|---|---|---|---|---|---|---|---|
| K.FTYGGC(+57.02)AGNANNFK.K | Y | 93.70 | 1519.6514 | 14 | 4.0 | 760.8345 | 2 | 42.50 | 3 | F3:19119 | BuCaTN01_F3a.raw | | 9.87E6 | 3.7722E8 | | | 2.0917E7 | 1.7985E5 | 3.983E6 | | | | 0 | | 6.0577E5 | | | 9 | 0 | 1 | 3 | 0 | 0 | 1 | 1 | 1 | 0 | 0 | 0 | 0 | 0 | 2 | 0 | 0 | 170 | 183 | Carbamidomethylation | C6:Carbamidomethylation:1000.00 | PEAKS DB |
| G.LFEGSC(+57.02)SIFPNDSQR.C | Y | 49.11 | 1755.7886 | 15 | 0.6 | 878.9021 | 2 | 63.05 | 7 | F7:35242 | BuCaTN01_F7a.raw | | | | | | | 2.6284E5 | | | | | | | | | | 1 | 0 | 0 | 0 | 0 | 0 | 0 | 1 | 0 | 0 | 0 | 0 | 0 | 0 | 0 | 0 | 0 | 33 | 47 | Carbamidomethylation | C6:Carbamidomethylation:1000.00 | PEAKS DB |
| K.FTYGGC(+57.02)AGNANNFKK.H | Y | 41.93 | 1647.7463 | 15 | 1.9 | 550.2560 | 3 | 36.56 | 3 | F3:13974 | BuCaTN01_F3a.raw | | | 1.7131E5 | | | | | | | | | | | | | | 1 | 0 | 0 | 1 | 0 | 0 | 0 | 0 | 0 | 0 | 0 | 0 | 0 | 0 | 0 | 0 | 0 | 170 | 184 | Carbamidomethylation | C6:Carbamidomethylation:1000.00 | PEAKS DB |
| total 3 peptides | | | | | | | | | | | | | | | | | | | | | | | | | | | | | | | | | | | | | | | | | | | | | | | | | |

**Best Unique PSM (Scan F3:19119, m/z=760.8345, z=2, RT=42.50, ppm=4.0):**

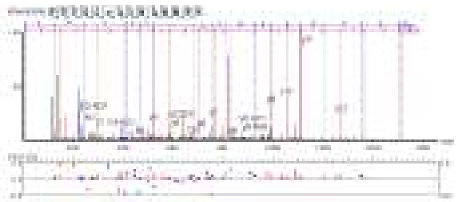

## XP_026579409.1

back to list

| Protein Coverage | Supporting Peptides | Best Unique PSM |

**Protein Coverage:**

```
  1 MGSGRSPLLL LLLLLLFWGD FALWGSLHSV SGLFEGSCSI FPNDSQRCGE PKNRFFYNIT SKSCEPFVYH GCPGNRNRYH
 81 TIEECQRYCG NIEKPGFCPP SPRGASGPCW TRCLHDGSCP EMEKCCSYGC ALYCMKPVTD LCQLPSEPGP CDLKVKRWFY
161 DPKIQRCKKF TYGGCAGNAN NFKKHLECRL RCRKRGPP
```

C Carbamidomethylation (+57.02)

**Supporting Peptides:**

| Peptide | Uniq | -10lgP | Mass | Length | ppm | m/z | z | RT | Fraction | Scan | Source File | Area F1 | Area F2a | Area F3a | Area F4 | Area F5 | Area F6a | Area F7a | Area F7b | Area F8 | Area F9a | Area Sample 1F10a | Area F10b | Area F11a | Area F11b | Area F12a | Area F13 | #Feature | #Feature F1 | #Feature F2a | #Feature F3a | #Feature F4 | #Feature F5 | #Feature F6a | #Feature F7a | #Feature F7b | #Feature F8 | #Feature F9a | #Feature Sample 1F10a | #Feature F10b | #Feature F11a | #Feature F11b | #Feature F12a | #Feature F13 | Start | End | PTM | AScore | Found By |
|---|---|---|---|---|---|---|---|---|---|---|---|---|---|---|---|---|---|---|---|---|---|---|---|---|---|---|---|---|---|---|---|---|---|---|---|---|---|---|---|---|---|---|---|---|---|---|---|---|---|
| K.FTYGGC(+57.02)AGNANNFK.K | Y | 93.70 | 1519.6514 | 14 | 4.0 | 760.8345 | 2 | 42.50 | 3 | F3:19119 | BuCaTN01_F3a.raw | | 9.87E6 | 3.7722E8 | | | 2.0917E7 | 1.7985E5 | 3.983E6 | | | | 0 | | 6.0577E5 | | | 9 | 0 | 1 | 3 | 0 | 0 | 1 | 1 | 1 | 0 | 0 | 0 | 0 | 0 | 2 | 0 | 0 | 170 | 183 | Carbamidomethylation | C6:Carbamidomethylation:1000.00 | PEAKS DB |
| G.LFEGSC(+57.02)SIFPNDSQR.C | Y | 49.11 | 1755.7886 | 15 | 0.6 | 878.9021 | 2 | 63.05 | 7 | F7:35242 | BuCaTN01_F7a.raw | | | | | | | 2.6284E5 | | | | | | | | | | 1 | 0 | 0 | 0 | 0 | 0 | 0 | 1 | 0 | 0 | 0 | 0 | 0 | 0 | 0 | 0 | 0 | 33 | 47 | Carbamidomethylation | C6:Carbamidomethylation:1000.00 | PEAKS DB |
| K.FTYGGC(+57.02)AGNANNFKK.H | Y | 41.93 | 1647.7463 | 15 | 1.9 | 550.2560 | 3 | 36.56 | 3 | F3:13974 | BuCaTN01_F3a.raw | | | 1.7131E5 | | | | | | | | | | | | | | 1 | 0 | 0 | 1 | 0 | 0 | 0 | 0 | 0 | 0 | 0 | 0 | 0 | 0 | 0 | 0 | 0 | 170 | 184 | Carbamidomethylation | C6:Carbamidomethylation:1000.00 | PEAKS DB |
| total 3 peptides | | | | | | | | | | | | | | | | | | | | | | | | | | | | | | | | | | | | | | | | | | | | | | | | | |

**Best Unique PSM (Scan F3:19119, m/z=760.8345, z=2, RT=42.50, ppm=4.0):**

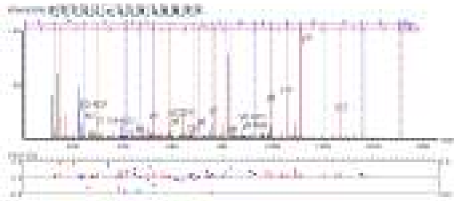

## P00600.1

back to list

| Protein Coverage | Supporting Peptides | Best Unique PSM |

**Protein Coverage:**

```
  1 NLYQFKNMIQ CTVPNRSWWH FANYGCYCGR GGSGTPVDDL DRCCQIHDNC YGEAEKISGC WPYIKTYTYE SCQGTLTSCG
 81 ANNKCAASVC DCDRVAANCF ARATYNDKNY NIDFNARCQ
```

C Carbamidomethylation (+57.02)

**Supporting Peptides:**

| Peptide | Uniq | -10lgP | Mass | Length | ppm | m/z | z | RT | Fraction | Scan | Source File | Area F1 | Area F2a | Area F3a | Area F4 | Area F5 | Area F6a | Area F7a | Area F7b | Area F8 | Area F9a | Area Sample 1F10a | Area F10b | Area F11a | Area F11b | Area F12a | Area F13 | #Feature | #Feature F1 | #Feature F2a | #Feature F3a | #Feature F4 | #Feature F5 | #Feature F6a | #Feature F7a | #Feature F7b | #Feature F8 | #Feature F9a | #Feature Sample 1F10a | #Feature F10b | #Feature F11a | #Feature F11b | #Feature F12a | #Feature F13 | Start | End | PTM | AScore | Found By |
|---|---|---|---|---|---|---|---|---|---|---|---|---|---|---|---|---|---|---|---|---|---|---|---|---|---|---|---|---|---|---|---|---|---|---|---|---|---|---|---|---|---|---|---|---|---|---|---|---|---|
| R.GGSGTPVDDLDR.C | N | 69.16 | 1187.5417 | 12 | -1.2 | 594.7775 | 2 | 38.65 | 10 | F10:17034 | BuCaTN01_F9a.raw | | | | | | | | | | 5.2316E6 | | | | | | | 1 | 0 | 0 | 0 | 0 | 0 | 0 | 0 | 0 | 0 | 1 | 0 | 0 | 0 | 0 | 0 | 0 | 31 | 42 | | | PEAKS DB |
| S.GTPVDDLDR.C | N | 51.39 | 986.4669 | 9 | 0.6 | 494.2410 | 2 | 37.62 | 7 | F7:14221 | BuCaTN01_F7a.raw | | | | | | | 4.0904E6 | | | | | | | | | | 1 | 0 | 0 | 0 | 0 | 0 | 0 | 1 | 0 | 0 | 0 | 0 | 0 | 0 | 0 | 0 | 0 | 34 | 42 | | | PEAKS DB |
| G.TPVDDLDR.C | N | 48.70 | 929.4454 | 8 | -0.4 | 465.7298 | 2 | 34.76 | 7 | F7:11899 | BuCaTN01_F7a.raw | | | | | | | 6.3757E5 | | | | | | | | | | 1 | 0 | 0 | 0 | 0 | 0 | 0 | 1 | 0 | 0 | 0 | 0 | 0 | 0 | 0 | 0 | 0 | 35 | 42 | | | PEAKS DB |
| K.ISGC(+57.02)WPYIK.T | Y | 48.44 | 1122.5532 | 9 | 0.2 | 562.2840 | 2 | 62.17 | 10 | F10:38769 | BuCaTN01_F9a.raw | | | | | | | | | | 1.9309E6 | | | | | | | 1 | 0 | 0 | 0 | 0 | 0 | 0 | 0 | 0 | 0 | 1 | 0 | 0 | 0 | 0 | 0 | 0 | 57 | 65 | Carbamidomethylation | C4:Carbamidomethylation:1000.00 | PEAKS DB |
| total 4 peptides | | | | | | | | | | | | | | | | | | | | | | | | | | | | | | | | | | | | | | | | | | | | | | | | | |

**Best Unique PSM (Scan F10:38769, m/z=562.2840, z=2, RT=62.17, ppm=0.2):**

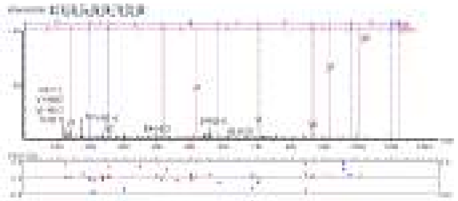

## ETE67131.1

back to list

| Protein Coverage | Supporting Peptides | Best Unique PSM |

**Protein Coverage:**

1 LSEQIKRRIL ALLFIGAQLY SELGKPCLKK SLPPASYPLI TMSRQSCGIS VSGGNKGFSS SSACFSGGQR MSFNSVSTSR
81 GCSVRSGGGG AAFGSRSLYN LNQGGNKRIS YGGVGSGFGA GFGGGCGQGG GSFGFGGGYG AGFGAGGGFG GAFQACPPGG
161 IKEVTINQHL LQPLNLEIDP EIQKVRVEER EQIK**TLNNKF ASFIDKVR**FL EQQNKVLETK WKLLQEQGHG TGGQKNLDHF
241 FEAYINNLRR QLDGLLNERR GLDSELKNFQ DLVEDFKNKY EEEINKRTAA ENDFVLLKKE LNQMQQTVSD TSVVLQMDNN
321 RNLDLNSIIA EVKAQYEDIA QKSRAEAESW YQGKYEELQV SAGRHGDNLR DTKAEIAELN RTIQRMRAEI ENVKKQASIA
401 EAEERGELAL KDARDKLSHL EEALQKSKEE MARLLKEYQE LMNVK**LALDI EIATYR**KLLE GEESRMSGEC QSAVSVSVIG
481 SSMTSSGGGI GGSYGGGSCF GGASGGFGGF SSGSGRGVCS IGGGGVGSVG GGMSFQAICQ GSKGSRRGYS SCSAFGGGTG
561 GGIRHSYSSC RGYGGGMRGQ YGSRSLHNYC GVTRISYGSG YGGGICGGYG GGYGGIGGGY GGIGGGYGGM VGGLGGRGLG
641 GLGSGGIGSV QVDPSLLRPV RVDIDPQIQQ VKNQEKEQIK VLNNQFASFI DKVRFLEQQN KVLQTKWQIL QQQSQGIGPS
721 MNLDAEFQQF LNSLRNQIEN IRSQKAQLSI ELQSMQHYVE DYKNRYEEEI NRRTTAENDF VVLKKDVDCS YLSKSNLEVR
801 VGALAEQINF LRRIFDVEIS QYQTGGQDTS VVVSMDNNIH LNLEGIIDEV RSQYEEIARS SRAEAEAWYH SQFEALQHTA
881 GRHGDNLRNT KQEIQELTRN IQRLRSEIEH VKKQCVKLQS DIAEVEERGE LALRDAKQKL HDLECALAKD KEELAGLLKE
961 YQDLLNIKIA LDIEIAMYRK LLEGEEDRIS GSGSQVHVSV RGGSIGSGVV GGIGGGSCGF GNGGYGGVIS GGGAGGSVCG
1041 GGGGFSHGSG STMSRQFSSR SGGGIRTSRG YTSSTTVIPS GTKASFSSVS LSRGVKGSAG LGGGFGSRSL YNLGGGKRTS
1121 LGISHSSIGG GFGSGLGYGG GGGGGGFGSG YGYGVGGFGL GVGVGVGGYD GGLRMGPMVP PGGIQKVTVN PNLLSPLHLE
1201 IDPDIQKVRQ QEREQIKTLN NKFASFIDKV RFLEQQNKVL ETKWNLLQQQ GSSAPKFNID LLFETYICNL RKNVDQLISD
1281 RAKLDGELRN VQDLVEDYRR KYEDEINQRT AAENDFVGIK KDVDVSFMQK VDLQAKTDRL IDELNFLRTL FEAELIQIRG
1361 QISDTNVVLQ MDNNRDLDLK SIIAEVKAQY EDIANRSRAE AEAWYQGKFQ ELQITAVGHG DDLKDMKNQI AELTRHIQRL
1441 KAEIDNMRKV NEGLQKAIAD AEQRGELALK DARVKLSDLQ LALQNSKDEL ARLLRDYQEL LNTKLSLDIE IATYRTLLEG
1521 EECRISGELT APVSMSVISS SSTMSGGGRV SSGGFGLGVG GGGGGSGAGG SLGLTMGGVG AGGFGISTGG SSYSTGSYGG
1601 GIGVGGGGGS SSMKYVSTTT SSGFRVGNSS TSVIGGVGSF GGGFGSRSLY NVGGSKRISM GLASGGGGGA HFGRSLGGGL
1681 GFSSYSTGYG GYGGGVALGG GIGLGSHVGG FGGGGGYALS GSMPCPPPGR IHNVIVNKNL LTPLHVEVDP EIQRVRVQER
1761 EQIKTLNNKF ASFIDKVRFL EQQNKVLETK WDLLYQQGSP ALRDNLEPIY EAYIETLRRQ LDSLCNDKNH LDIELKNTQE
1841 LVEDFK**NKYE DEINKR**TACE NDFVVLKKDV DGSYMHKIDL GSKSDSLTQY IQFLRALFDA ELAQLHSQVK ETNVILQMDN
1921 NR**DLDLDSII AEVK**AQYEDI ANRSRAEAEA WYQTKFQELQ STAFKHGDDL KSTKDEIADM SRFVQRIRSE IEILKKSITS
2001 LQATLSEAEQ RGELALKDAR TKLLDLQTAL TTAKDELARL LRDYQELMNV K**LALDVEIAT YR**TLLEGEEC RMSGEFETPV
2081 NISVISSSST ISGAGAGGYG VGGGSGYGAG GGAGYTIGGG AGGLSLGGGG GGGGHSHISV GGGKMSRFSS GGSLSGSGSL
2161 TGGGYAYGGG SSSMKNNLQS SKDELARLLR DYQNLLNLKL PLDIEIAMYK SLLEGEENRS PGHVSELQYE YAVIKKNTES
2241 DDTLSIERAA I

**Supporting Peptides:**

| Peptide | Uniq | -10lgP | Mass | Length | ppm | m/z | z | RT | Fraction | Scan | Source File | Area F1 | Area F2a | Area F3a | Area F4 | Area F5 | Area F6a | Area F7a | Area F7b | Area F8 | Area F9a | Area Sample 1F10a | Area F10b | Area F11a | Area F11b | Area F12a | Area F13 | #Feature | #Feature F1 | #Feature F2a | #Feature F3a | #Feature F4 | #Feature F5 | #Feature F6a | #Feature F7a | #Feature F7b | #Feature F8 | #Feature F9a | #Feature Sample 1F10a | #Feature F10b | #Feature F11a | #Feature F11b | #Feature F12a | #Feature F13 | Start | End | PTM | AScore | Found By |
|---|---|---|---|---|---|---|---|---|---|---|---|---|---|---|---|---|---|---|---|---|---|---|---|---|---|---|---|---|---|---|---|---|---|---|---|---|---|---|---|---|---|---|---|---|---|---|---|---|---|
| K.FASFIDKVR.F | N | 52.96 | 1081.5920 | 9 | 4.5 | 361.5378 | 3 | 50.93 | 15 | F15:26707 | BuCaTN01_F12a.raw | 7.0769E4 | | | | | 5.8464E4 | | 2.0416E5 | | 6.8682E5 | | 0 | 1.8562E5 | | 1.0105E5 | | 6 | 1 | 0 | 0 | 0 | 0 | 1 | 0 | 1 | 0 | 1 | 0 | 0 | 1 | 0 | 1 | 0 | 200 | 208 | | | PEAKS DB |
| K.TLNNKFASFIDKVR.F | N | 52.29 | 1651.9045 | 14 | 0.6 | 551.6425 | 3 | 64.93 | 7 | F7:36739 | BuCaTN01_F7a.raw | | 1.0212E5 | | | | | 5.3175E4 | | | | | 0 | | | | | 2 | 0 | 1 | 0 | 0 | 0 | 0 | 1 | 0 | 0 | 0 | 0 | 0 | 0 | 0 | 0 | 0 | 195 | 208 | | | PEAKS DB |
| K.LALDIEIATYR.K | N | 48.98 | 1276.7026 | 11 | 3.4 | 639.3589 | 2 | 72.56 | 8 | F8:48459 | BuCaTN01_F7b.raw | | 5.015E5 | | | | | | 8.9534E5 | | | 3.3691E5 | | 6.8742E5 | | | | 4 | 0 | 1 | 0 | 0 | 0 | 0 | 0 | 1 | 0 | 0 | 1 | 0 | 1 | 0 | 0 | 0 | 446 | 456 | | | PEAKS DB |
| K.LALDVEIATYR.T | N | 46.88 | 1262.6870 | 11 | 1.9 | 632.3502 | 2 | 67.49 | 8 | F8:43237 | BuCaTN01_F7b.raw | | | | | | | | 1.3824E5 | | | | | 0 | | | | 1 | 0 | 0 | 0 | 0 | 0 | 0 | 0 | 1 | 0 | 0 | 0 | 0 | 0 | 0 | 0 | 0 | 2052 | 2062 | | | PEAKS DB |
| R.DLDLDSIIAEVK.A | Y | 45.60 | 1329.7028 | 12 | 0.4 | 665.8589 | 2 | 91.52 | 13 | F13:60556 | BuCaTN01_F11a.raw | | | | | | | | | | | | | 0 | | | | 0 | 0 | 0 | 0 | 0 | 0 | 0 | 0 | 0 | 0 | 0 | 0 | 0 | 0 | 0 | 0 | 0 | 1923 | 1934 | | | PEAKS DB |
| K.NKYEDEINKR.T | N | 45.46 | 1307.6470 | 10 | -0.2 | 436.8895 | 3 | 29.95 | 16 | F16:9612 | BuCaTN01_F13.raw | | | | | | | | | | | | | 1.5043E5 | | | 1.0452E5 | 2 | 0 | 0 | 0 | 0 | 0 | 0 | 0 | 0 | 0 | 0 | 0 | 0 | 1 | 0 | 0 | 1 | 1847 | 1856 | | | PEAKS DB |
| total 6 peptides | | | | | | | | | | | | | | | | | | | | | | | | | | | | | | | | | | | | | | | | | | | | | | | | | |

**Best Unique PSM (Scan F13:60556, m/z=665.8589, z=2, RT=91.52, ppm=0.4):**

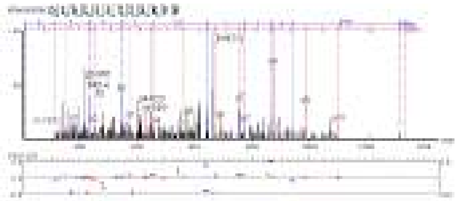

## Q8JFX7.1

back to list

| Protein Coverage | Supporting Peptides | Best Unique PSM |

**Protein Coverage:**

1 MKTLLLTLVV VTIICLDLGY TEMCNMCVRP YPFMSSCCPE GQDR**CYKSYW VNENGKQK**KY HGKYPVILER GCVTACTGPG

C Carbamidomethylation (+57.02)

81 SGSIYNLYTC CPTNRCGSSS TSG

**Supporting Peptides:**

| Peptide | Uniq | -10lgP | Mass | Length | ppm | m/z | z | RT | Fraction | Scan | Source File | Area F1 | Area F2a | Area F3a | Area F4 | Area F5 | Area F6a | Area F7a | Area F7b | Area F8 | Area F9a | Area Sample 1F10a | Area F10b | Area F11a | Area F11b | Area F12a | Area F13 | #Feature | #Feature F1 | #Feature F2a | #Feature F3a | #Feature F4 | #Feature F5 | #Feature F6a | #Feature F7a | #Feature F7b | #Feature F8 | #Feature F9a | #Feature Sample 1F10a | #Feature F10b | #Feature F11a | #Feature F11b | #Feature F12a | #Feature F13 | Start | End | PTM | AScore | Found By |
|---|---|---|---|---|---|---|---|---|---|---|---|---|---|---|---|---|---|---|---|---|---|---|---|---|---|---|---|---|---|---|---|---|---|---|---|---|---|---|---|---|---|---|---|---|---|---|---|---|---|
| K.SYWVNENGK.Q | N | 67.93 | 1095.4985 | 9 | 0.3 | 548.7567 | 2 | 39.85 | 5 | F5:22799 | BuCaTN01_F5.raw | | | | | 2.6101E7 | 1.023E6 | | | | | | | | | | | 2 | 0 | 0 | 0 | 0 | 1 | 1 | 0 | 0 | 0 | 0 | 0 | 0 | 0 | 0 | 0 | 0 | 48 | 56 | | | PEAKS DB |
| R.C(+57.02)YKSYWVNENGK.Q | N | 67.82 | 1546.6875 | 12 | 0.1 | 774.3511 | 2 | 40.88 | 4 | F4:19419 | BuCaTN01_F4.raw | | | | 6.9125E5 | 6.7159E5 | 1.369E6 | | | | | | | | | | | 3 | 0 | 0 | 0 | 1 | 1 | 1 | 0 | 0 | 0 | 0 | 0 | 0 | 0 | 0 | 0 | 0 | 45 | 56 | Carbamidomethylation | C1:Carbamidomethylation:1000.00 | PEAKS DB |
| K.SYWVNENGKQK.K | Y | 46.96 | 1351.6521 | 11 | 0.6 | 451.5582 | 3 | 32.73 | 5 | F5:15837 | BuCaTN01_F5.raw | | | | | 2.945E6 | | | | | | | | | | | | 1 | 0 | 0 | 0 | 0 | 1 | 0 | 0 | 0 | 0 | 0 | 0 | 0 | 0 | 0 | 0 | 0 | 48 | 58 | | | PEAKS DB |
| R.C(+57.02)YKSYWVNENGKQK.K | Y | 43.98 | 1802.8409 | 14 | 4.9 | 902.4322 | 2 | 35.46 | 4 | F4:15387 | BuCaTN01_F4.raw | | | | 0 | | | | | | | | | | | | | 0 | 0 | 0 | 0 | 0 | 0 | 0 | 0 | 0 | 0 | 0 | 0 | 0 | 0 | 0 | 0 | 0 | 45 | 58 | Carbamidomethylation | C1:Carbamidomethylation:1000.00 | PEAKS DB |
| total 4 peptides | | | | | | | | | | | | | | | | | | | | | | | | | | | | | | | | | | | | | | | | | | | | | | | | | |

**Best Unique PSM (Scan F5:15837, m/z=451.5582, z=3, RT=32.73, ppm=0.6):**

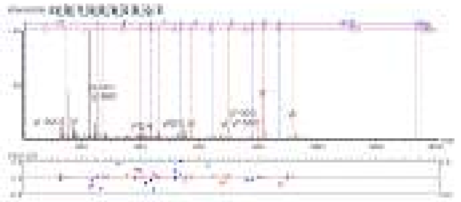

## CAD18848.1

back to list

| Protein Coverage | Supporting Peptides | Best Unique PSM |

**Protein Coverage:**

1 MKTLLLTLVV VTIICLDLGY TEMCNMCVRP YPFMSSCCPE GQDR**CYKSYW VNENGKQK**KY HGKYPVILER GCVTACTGPG

C Carbamidomethylation (+57.02)

81 SGSIYNLYTC CPTNRCGSSS TSG

**Supporting Peptides:**

| Peptide | Uniq | -10lgP | Mass | Length | ppm | m/z | z | RT | Fraction | Scan | Source File | Area F1 | Area F2a | Area F3a | Area F4 | Area F5 | Area F6a | Area F7a | Area F7b | Area F8 | Area F9a | Area Sample 1F10a | Area F10b | Area F11a | Area F11b | Area F12a | Area F13 | #Feature | #Feature F1 | #Feature F2a | #Feature F3a | #Feature F4 | #Feature F5 | #Feature F6a | #Feature F7a | #Feature F7b | #Feature F8 | #Feature F9a | #Feature Sample 1F10a | #Feature F10b | #Feature F11a | #Feature F11b | #Feature F12a | #Feature F13 | Start | End | PTM | AScore | Found By |
|---|---|---|---|---|---|---|---|---|---|---|---|---|---|---|---|---|---|---|---|---|---|---|---|---|---|---|---|---|---|---|---|---|---|---|---|---|---|---|---|---|---|---|---|---|---|---|---|---|---|
| K.SYWVNENGK.Q | N | 67.93 | 1095.4985 | 9 | 0.3 | 548.7567 | 2 | 39.85 | 5 | F5:22799 | BuCaTN01_F5.raw | | | | | 2.6101E7 | 1.023E6 | | | | | | | | | | | 2 | 0 | 0 | 0 | 0 | 1 | 1 | 0 | 0 | 0 | 0 | 0 | 0 | 0 | 0 | 0 | 0 | 48 | 56 | | | PEAKS DB |
| R.C(+57.02)YKSYWVNENGK.Q | N | 67.82 | 1546.6875 | 12 | 0.1 | 774.3511 | 2 | 40.88 | 4 | F4:19419 | BuCaTN01_F4.raw | | | | 6.9125E5 | 6.7159E5 | 1.369E6 | | | | | | | | | | | 3 | 0 | 0 | 0 | 1 | 1 | 1 | 0 | 0 | 0 | 0 | 0 | 0 | 0 | 0 | 0 | 0 | 45 | 56 | Carbamidomethylation | C1:Carbamidomethylation:1000.00 | PEAKS DB |
| K.SYWVNENGKQK.K | Y | 46.96 | 1351.6521 | 11 | 0.6 | 451.5582 | 3 | 32.73 | 5 | F5:15837 | BuCaTN01_F5.raw | | | | | 2.945E6 | | | | | | | | | | | | 1 | 0 | 0 | 0 | 0 | 1 | 0 | 0 | 0 | 0 | 0 | 0 | 0 | 0 | 0 | 0 | 0 | 48 | 58 | | | PEAKS |

| | | | | | | | | | | | | | | | | | | | | | | | | | | | | | | | | | | | | | | | | | | | | | | | | | |
|---|---|---|---|---|---|---|---|---|---|---|---|---|---|---|---|---|---|---|---|---|---|---|---|---|---|---|---|---|---|---|---|---|---|---|---|---|---|---|---|---|---|---|---|---|---|---|---|---|---|
| | | | | | | | | | | | | | | | | | | | | | | | | | | | | | | | | | | | | | | | | | | | | | | | | | DB |
| R.C(+57.02)YKSYWVNENGKQK.K | Y | 43.98 | 1802.8409 | 14 | 4.9 | 902.4322 | 2 | 35.46 | 4 | F4:15387 | BuCaTN01_F4.raw | | | | 0 | | | | | | | | | | | | | 0 | 0 | 0 | 0 | 0 | 0 | 0 | 0 | 0 | 0 | 0 | 0 | 0 | 0 | 0 | 0 | 0 | 45 | 58 | Carbamidomethylation | C1:Carbamidomethylation:1000.00 | PEAKS DB |
| total 4 peptides | | | | | | | | | | | | | | | | | | | | | | | | | | | | | | | | | | | | | | | | | | | | | | | | | |

**Best Unique PSM (Scan F5:15837, m/z=451.5582, z=3, RT=32.73, ppm=0.6):**

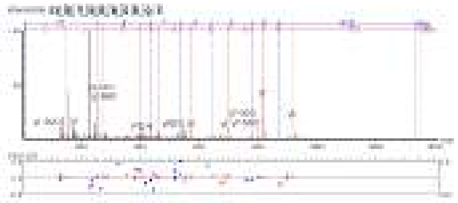

## P59069.1

back to list

| Protein Coverage | Supporting Peptides | Best Unique PSM |

**Protein Coverage:**

1 NLIQLSNMIK CAIPGSR**PLL HYTDYGCYCG KGGSGTPVDE LDR**CCK

C Carbamidomethylation (+57.02)

**Supporting Peptides:**

| Peptide | Uniq | -10lgP | Mass | Length | ppm | m/z | z | RT | Fraction | Scan | Source File | Area F1 | Area F2a | Area F3a | Area F4 | Area F5 | Area F6a | Area F7a | Area F7b | Area F8 | Area F9a | Area Sample 1F10a | Area F10b | Area F11a | Area F11b | Area F12a | Area F13 | #Feature | #Feature F1 | #Feature F2a | #Feature F3a | #Feature F4 | #Feature F5 | #Feature F6a | #Feature F7a | #Feature F7b | #Feature F8 | #Feature F9a | #Feature Sample 1F10a | #Feature F10b | #Feature F11a | #Feature F11b | #Feature F12a | #Feature F13 | Start | End | PTM | AScore | Found By |
|---|---|---|---|---|---|---|---|---|---|---|---|---|---|---|---|---|---|---|---|---|---|---|---|---|---|---|---|---|---|---|---|---|---|---|---|---|---|---|---|---|---|---|---|---|---|---|---|---|---|
| G.GSGTPVDELDR.C | N | 56.36 | 1144.5360 | 11 | 3.4 | 573.2756 | 2 | 40.15 | 8 | F8:17921 | BuCaTN01_F7b.raw | | | | | | | | 1.1068E6 | | | | | | | | | 1 | 0 | 0 | 0 | 0 | 0 | 0 | 0 | 1 | 0 | 0 | 0 | 0 | 0 | 0 | 0 | 0 | 33 | 43 | | | PEAKS DB |
| R.PLLHYTDYGC(+57.02)YC(+57.02)GKGGSGTPVDELDR.C | Y | 50.91 | 2929.3010 | 26 | -6.7 | 977.4322 | 3 | 69.34 | 12 | F12:45144 | BuCaTN01_F10b.raw | | | | | | | | | | | | 0 | | | | | 0 | 0 | 0 | 0 | 0 | 0 | 0 | 0 | 0 | 0 | 0 | 0 | 0 | 0 | 0 | 0 | 0 | 18 | 43 | Carbamidomethylation | C10:Carbamidomethylation:1000.00;C12: Carbamidomethylation:1000.00 | PEAKS DB |
| K.GGSGTPVDELDR.C | N | 49.09 | 1201.5575 | 12 | 0.3 | 601.7862 | 2 | 40.57 | 11 | F11:14528 | BuCaTN01_F10a.raw | | | | | | | 9.2274E5 | 2.7577E6 | | 1.1335E7 | 6.7591E5 | | 9.9086E5 | 2.6759E7 | | | 6 | 0 | 0 | 0 | 0 | 0 | 0 | 1 | 1 | 0 | 1 | 1 | 0 | 1 | 1 | 0 | 0 | 32 | 43 | | | PEAKS DB |
| total 3 peptides | | | | | | | | | | | | | | | | | | | | | | | | | | | | | | | | | | | | | | | | | | | | | | | | | |

**Best Unique PSM (Scan F12:45144, m/z=977.4322, z=3, RT=69.34, ppm=-6.7):**

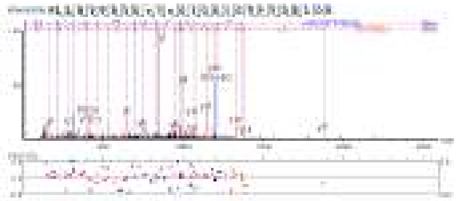

## CAB53358.1

back to list

| Protein Coverage | Supporting Peptides | Best Unique PSM |

**Protein Coverage:**

1 MKTLLLTLVV VTIICLDLGY TEMCNMCVRP YPFMSSCCPE GQDR**CYKSYW VNENGKQE**AY HGKYPVILER GCVTACTGPG

81 SGSIYNLYTC CPTNRCGSSS TSG

C Carbamidomethylation (+57.02)

**Supporting Peptides:**

| Peptide | Uniq | -10lgP | Mass | Length | ppm | m/z | z | RT | Fraction | Scan | Source File | Area F1 | Area F2a | Area F3a | Area F4 | Area F5 | Area F6a | Area F7a | Area F7b | Area F8 | Area F9a | Area Sample 1F10a | Area F10b | Area F11a | Area F11b | Area F12a | Area F13 | #Feature | #Feature F1 | #Feature F2a | #Feature F3a | #Feature F4 | #Feature F5 | #Feature F6a | #Feature F7a | #Feature F7b | #Feature F8 | #Feature F9a | #Feature Sample 1F10a | #Feature F10b | #Feature F11a | #Feature F11b | #Feature F12a | #Feature F13 | Start | End | PTM | AScore | Found By |
|---|---|---|---|---|---|---|---|---|---|---|---|---|---|---|---|---|---|---|---|---|---|---|---|---|---|---|---|---|---|---|---|---|---|---|---|---|---|---|---|---|---|---|---|---|---|---|---|---|---|
| K.SYWVNENGK.Q | N | 67.93 | 1095.4985 | 9 | 0.3 | 548.7567 | 2 | 39.85 | 5 | F5:22799 | BuCaTN01_F5.raw | | | | | 2.6101E7 | 1.023E6 | | | | | | | | | | | 2 | 0 | 0 | 0 | 0 | 1 | 1 | 0 | 0 | 0 | 0 | 0 | 0 | 0 | 0 | 0 | 0 | 48 | 56 | | | PEAKS DB |
| R.C(+57.02)YKSYWVNENGK.Q | N | 67.82 | 1546.6875 | 12 | 0.1 | 774.3511 | 2 | 40.88 | 4 | F4:19419 | BuCaTN01_F4.raw | | | | 6.9125E5 | 6.7159E5 | 1.369E6 | | | | | | | | | | | 3 | 0 | 0 | 0 | 1 | 1 | 1 | 0 | 0 | 0 | 0 | 0 | 0 | 0 | 0 | 0 | 0 | 45 | 56 | Carbamidomethylation | C1:Carbamidomethylation:1000.00 | PEAKS DB |
| R.C(+57.02)YKSYWVNENGKQE.A | Y | 55.89 | 1803.7886 | 14 | 0.3 | 902.9018 | 2 | 44.12 | 6 | F6:23208 | BuCaTN01_F6a.raw | | | | | | 3.8075E6 | | | | | | | | | | | 1 | 0 | 0 | 0 | 0 | 0 | 1 | 0 | 0 | 0 | 0 | 0 | 0 | 0 | 0 | 0 | 0 | 45 | 58 | Carbamidomethylation | C1:Carbamidomethylation:1000.00 | PEAKS DB |
| total 3 peptides | | | | | | | | | | | | | | | | | | | | | | | | | | | | | | | | | | | | | | | | | | | | | | | | | |

**Best Unique PSM (Scan F6:23208, m/z=902.9018, z=2, RT=44.12, ppm=0.3):**

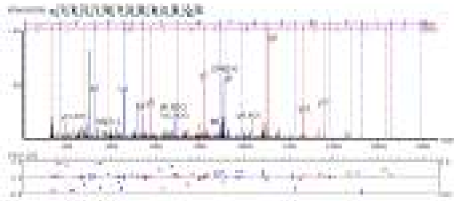

## Q9PW19.1

back to list

| Protein Coverage | Supporting Peptides | Best Unique PSM |

**Protein Coverage:**

1 MKTLLLTLVV VTIICLDLGY TEMCNMCVRP YPFMSSCCPE GQDR**CYKSYW VNENGKQE**AY HGKYPVILER GCVTACTGPG

81 SGSIYNLYTC CPTNRCGSSS TSG

C Carbamidomethylation (+57.02)

**Supporting Peptides:**

| Peptide | Uniq | -10lgP | Mass | Length | ppm | m/z | z | RT | Fraction | Scan | Source File | Area F1 | Area F2a | Area F3a | Area F4 | Area F5 | Area F6a | Area F7a | Area F7b | Area F8 | Area F9a | Area Sample 1F10a | Area F10b | Area F11a | Area F11b | Area F12a | Area F13 | #Feature | #Feature F1 | #Feature F2a | #Feature F3a | #Feature F4 | #Feature F5 | #Feature F6a | #Feature F7a | #Feature F7b | #Feature F8 | #Feature F9a | #Feature Sample 1F10a | #Feature F10b | #Feature F11a | #Feature F11b | #Feature F12a | #Feature F13 | Start | End | PTM | AScore | Found By |
|---|---|---|---|---|---|---|---|---|---|---|---|---|---|---|---|---|---|---|---|---|---|---|---|---|---|---|---|---|---|---|---|---|---|---|---|---|---|---|---|---|---|---|---|---|---|---|---|---|---|
| K.SYWVNENGK.Q | N | 67.93 | 1095.4985 | 9 | 0.3 | 548.7567 | 2 | 39.85 | 5 | F5:22799 | BuCaTN01_F5.raw | | | | | 2.6101E7 | 1.023E6 | | | | | | | | | | | 2 | 0 | 0 | 0 | 0 | 1 | 1 | 0 | 0 | 0 | 0 | 0 | 0 | 0 | 0 | 0 | 0 | 48 | 56 | | | PEAKS DB |
| R.C(+57.02)YKSYWVNENGK.Q | N | 67.82 | 1546.6875 | 12 | 0.1 | 774.3511 | 2 | 40.88 | 4 | F4:19419 | BuCaTN01_F4.raw | | | | 6.9125E5 | 6.7159E5 | 1.369E6 | | | | | | | | | | | 3 | 0 | 0 | 0 | 1 | 1 | 1 | 0 | 0 | 0 | 0 | 0 | 0 | 0 | 0 | 0 | 0 | 45 | 56 | Carbamidomethylation | C1:Carbamidomethylation:1000.00 | PEAKS DB |
| R.C(+57.02)YKSYWVNENGKQE.A | Y | 55.89 | 1803.7886 | 14 | 0.3 | 902.9018 | 2 | 44.12 | 6 | F6:23208 | BuCaTN01_F6a.raw | | | | | | 3.8075E6 | | | | | | | | | | | 1 | 0 | 0 | 0 | 0 | 0 | 1 | 0 | 0 | 0 | 0 | 0 | 0 | 0 | 0 | 0 | 0 | 45 | 58 | Carbamidomethylation | C1:Carbamidomethylation:1000.00 | PEAKS DB |
| total 3 peptides | | | | | | | | | | | | | | | | | | | | | | | | | | | | | | | | | | | | | | | | | | | | | | | | | |

**Best Unique PSM (Scan F6:23208, m/z=902.9018, z=2, RT=44.12, ppm=0.3):**

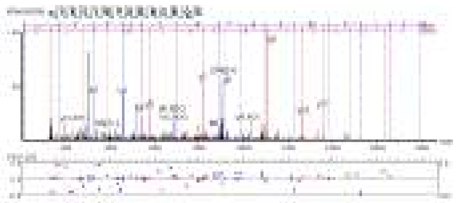

## XP_025030548.1

back to list

| Protein Coverage | Supporting Peptides | Best Unique PSM |

**Protein Coverage:**

```
  1 MSRVSYRASS GGANRGFTSG SAIVGGGGGG GGRTSFSSYS VTRVGGARGG GGGGYGLGGF GSRSLHNMGG SKRIAYSTIG
 81 GGLRSCYGGG GYGFGGGAGF GYGMGAGGAG YGMGGGPGGG FMVGGPGFVR AGPGYPVCPP GGIQEVTINQ HLLQPLKLEI
161 DPEIQRVRSQ EREQIKTLNN KFASFIDKVR FLEQQNKVLE TKWELLQQQG TTVRQHNIEP LFEGYINNLR RQLDSLLNER
241 GRLDLELRNM QDMVEDFKNK YEDEINKRTG AENEFVVLKK DVDAAYMNKI ELGGKSDSLT DEMEFLKALF EAELAQMQQQ
321 VSDTSVILSM DNNRNLDLNS IIAEVKAQYE DIANRSRTEA ESWYQSKYEE LQVTAGRHGD DLRSTKTEIS ELNRIVQRLR
401 GEIDALKKQC ANLQGTIAEA EDRGELALKD AKQKLSELEE ALQRAKQELA RQLKEYQELM NVKLSLDIEI ATYRKLLEGE
481 EIRLAEGVGN VSYSVVSSTT TAGYGGGSGI ALGGGFSGGS AGRLGGGFGV GVSGGGGGGG GGYVISSGSN YGGSGFGSMI
561 SSGGGSSYGS GSGFGSMISS GGGSSYGSGG GRLSSGGGGS SSVKIVSKTS TSRKTAY
```

**Supporting Peptides:**

| Peptide | Uniq | -10lgP | Mass | Length | ppm | m/z | z | RT | Fraction | Scan | Source File | Area F1 | Area F2a | Area F3a | Area F4 | Area F5 | Area F6a | Area F7a | Area F7b | Area F8 | Area F9a | Area Sample 1F10a | Area F10b | Area F11a | Area F11b | Area F12a | Area F13 |
|---|---|---|---|---|---|---|---|---|---|---|---|---|---|---|---|---|---|---|---|---|---|---|---|---|---|---|---|
| K.FASFIDKVR.F | N | 52.96 | 1081.5920 | 9 | 4.5 | 361.5378 | 3 | 50.93 | 15 | F15:26707 | BuCaTN01_F12a.raw | 7.0769E4 | | | | | 5.8464E4 | | 2.0416E5 | | 6.8682E5 | | 0 | 1.8562E5 | | 1.0105E5 | |
| K.TLNNKFASFIDKVR.F | N | 52.29 | 1651.9045 | 14 | 0.6 | 551.6425 | 3 | 64.93 | 7 | F7:36739 | BuCaTN01_F7a.raw | | 1.0212E5 | | | | | 5.3175E4 | | | | | 0 | | | | |
| K.NKYEDEINKR.T | N | 45.46 | 1307.6470 | 10 | -0.2 | 436.8895 | 3 | 29.95 | 16 | F16:9612 | BuCaTN01_F13.raw | | | | | | | | | | | | | 1.5043E5 | | | 1.0452E5 |
| R.GFTSGSAIVGGGGGGGGR.T | Y | 44.76 | 1449.6960 | 18 | -8.8 | 725.8459 | 2 | 53.83 | 14 | F14:30523 | BuCaTN01_F11b.raw | | | | | | | | | | | | | | 0 | | |
| K.YEELQVTAGR.H | N | 41.44 | 1164.5775 | 10 | -0.9 | 583.2955 | 2 | 41.24 | 2 | F2:18103 | BuCaTN01_F2a.raw | | 2.0495E4 | | | | | | | | | | | | | | |

| Peptide | #Feature | #Feature F1 | #Feature F2a | #Feature F3a | #Feature F4 | #Feature F5 | #Feature F6a | #Feature F7a | #Feature F7b | #Feature F8 | #Feature F9a | #Feature Sample 1F10a | #Feature F10b | #Feature F11a | #Feature F11b | #Feature F12a | #Feature F13 | Start | End | PTM | AScore | Found By |
|---|---|---|---|---|---|---|---|---|---|---|---|---|---|---|---|---|---|---|---|---|---|---|
| K.FASFIDKVR.F | 6 | 1 | 0 | 0 | 0 | 0 | 1 | 0 | 1 | 0 | 1 | 0 | 0 | 1 | 0 | 1 | 0 | 182 | 190 | | | PEAKS DB |
| K.TLNNKFASFIDKVR.F | 2 | 0 | 1 | 0 | 0 | 0 | 0 | 1 | 0 | 0 | 0 | 0 | 0 | 0 | 0 | 0 | 0 | 177 | 190 | | | PEAKS DB |
| K.NKYEDEINKR.T | 2 | 0 | 0 | 0 | 0 | 0 | 0 | 0 | 0 | 0 | 0 | 0 | 0 | 1 | 0 | 0 | 1 | 259 | 268 | | | PEAKS DB |
| R.GFTSGSAIVGGGGGGGGR.T | 0 | 0 | 0 | 0 | 0 | 0 | 0 | 0 | 0 | 0 | 0 | 0 | 0 | 0 | 0 | 0 | 0 | 16 | 33 | | | PEAKS DB |
| K.YEELQVTAGR.H | 1 | 0 | 1 | 0 | 0 | 0 | 0 | 0 | 0 | 0 | 0 | 0 | 0 | 0 | 0 | 0 | 0 | 368 | 377 | | | PEAKS DB |

total 5 peptides

**Best Unique PSM (Scan F14:30523, m/z=725.8459, z=2, RT=53.83, ppm=-8.8):**

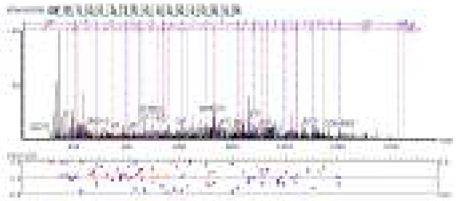

## BAC77656.1

back to list

| Protein Coverage | Supporting Peptides | Best Unique PSM |

**Protein Coverage:**

71 72 78

```
  1 MNPAHLLVLS AVCVSLLGAA IVPPQPLNLI QFSSLIQCAN GGSRATWHYA DYGCYCGKGG GGTPVDELDR CCQTHDNCYG
 81 EAEKLTKCSP YYKTYKYDCS EGKLTCNDAP GSCERSVCDC DRVAAICFAG APYNDKNFMI NTETNCQ
```

C Carbamidomethylation (+57.02)

**Supporting Peptides:**

| Peptide | Uniq | -10lgP | Mass | Length | ppm | m/z | z | RT | Fraction | Scan | Source File | Area F1 | Area F2a | Area F3a | Area F4 | Area F5 | Area F6a | Area F7a | Area F7b | Area F8 | Area F9a | Area Sample 1F10a | Area F10b | Area F11a | Area F11b | Area F12a | Area F13 |
|---|---|---|---|---|---|---|---|---|---|---|---|---|---|---|---|---|---|---|---|---|---|---|---|---|---|---|---|
| K.GGGGTPVDELDR.C | N | 61.54 | 1171.5469 | 12 | 1.0 | 586.7813 | 2 | 40.17 | 7 | F7:16143 | BuCaTN01_F7a.raw | | | | | | | 2.5577E6 | | 2.8377E5 | | | | | | | |
| R.C(+57.02)C(+57.02)QTHDNC(+57.02)YGEAEKL.T | N | 51.93 | 1883.7236 | 15 | 0.5 | 628.9155 | 3 | 37.42 | 7 | F7:13894 | BuCaTN01_F7a.raw | | | | | | | 1.1837E6 | | | | | | | | | |
| K.GGGGTPVDELDRC(+57.02)C(+57.02)QTHDNC(+57.02)YGEAEK.L | Y | 47.57 | 2924.1760 | 26 | 0.0 | 732.0513 | 4 | 43.77 | 7 | F7:19182 | BuCaTN01_F7a.raw | | | | | | | 7.1389E6 | | | | | | | | | |

| Peptide | #Feature | #Feature F1 | #Feature F2a | #Feature F3a | #Feature F4 | #Feature F5 | #Feature F6a | #Feature F7a | #Feature F7b | #Feature F8 | #Feature F9a | #Feature Sample 1F10a | #Feature F10b | #Feature F11a | #Feature F11b | #Feature F12a | #Feature F13 | Start | End | PTM | AScore | Found By |
|---|---|---|---|---|---|---|---|---|---|---|---|---|---|---|---|---|---|---|---|---|---|---|
| K.GGGGTPVDELDR.C | 4 | 0 | 0 | 0 | 0 | 0 | 0 | 3 | 0 | 1 | 0 | 0 | 0 | 0 | 0 | 0 | 0 | 59 | 70 | | | PEAKS DB |
| R.C(+57.02)C(+57.02)QTHDNC(+57.02)YGEAEKL.T | 1 | 0 | 0 | 0 | 0 | 0 | 0 | 1 | 0 | 0 | 0 | 0 | 0 | 0 | 0 | 0 | 0 | 71 | 85 | Carbamidomethylation | C1:Carbamidomethylation:1000.00;C2:Carbamidomethylation:1000.00;C8:Carbamidomethylation:1000.00 | PEAKS DB |
| K.GGGGTPVDELDRC(+57.02)C(+57.02)QTHDNC(+57.02)YGEAEK.L | 1 | 0 | 0 | 0 | 0 | 0 | 0 | 1 | 0 | 0 | 0 | 0 | 0 | 0 | 0 | 0 | 0 | 59 | 84 | Carbamidomethylation | C13:Carbamidomethylation:1000.00;C14:Carbamidomethylation:1000.00;C20:Carbamidomethylation:1000.00 | PEAKS DB |

total 3 peptides

**Best Unique PSM (Scan F7:19182, m/z=732.0513, z=4, RT=43.77, ppm=0.0):**

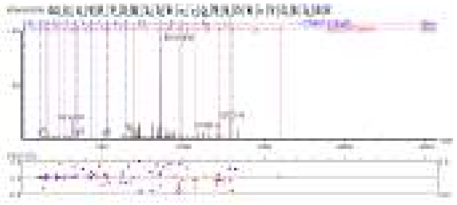

## Q7T2Q4.1

back to list

| Protein Coverage | Supporting Peptides | Best Unique PSM |

**Protein Coverage:**

71 72 78

```
  1 MNPAHLLVLS AVCVSLLGAA IVPPQPLNLI QFSSLIQCAN GGSRATWHYA DYGCYCGKGG GGTPVDELDR CCQTHDNCYG
 81 EAEKLTKCSP YYKTYKYDCS EGKLTCNDAP GSCERSVCDC DRVAAICFAG APYNDKNFMI NTETNCQ
```

C Carbamidomethylation (+57.02)

**Supporting Peptides:**

| Peptide | Uniq | -10lgP | Mass | Length | ppm | m/z | z | RT | Fraction | Scan | Source File | Area F1 | Area F2a | Area F3a | Area F4 | Area F5 | Area F6a | Area F7a | Area F7b | Area F8 | Area F9a | Area Sample 1F10a | Area F10b | Area F11a | Area F11b | Area F12a | Area F13 |
|---|---|---|---|---|---|---|---|---|---|---|---|---|---|---|---|---|---|---|---|---|---|---|---|---|---|---|---|
| K.GGGGTPVDELDR.C | N | 61.54 | 1171.5469 | 12 | 1.0 | 586.7813 | 2 | 40.17 | 7 | F7:16143 | BuCaTN01_F7a.raw | | | | | | | 2.5577E6 | | 2.8377E5 | | | | | | | |
| R.C(+57.02)C(+57.02)QTHDNC(+57.02)YGEAEKL.T | N | 51.93 | 1883.7236 | 15 | 0.5 | 628.9155 | 3 | 37.42 | 7 | F7:13894 | BuCaTN01_F7a.raw | | | | | | | 1.1837E6 | | | | | | | | | |
| K.GGGGTPVDELDRC(+57.02)C(+57.02)QTHDNC(+57.02)YGEAEK.L | Y | 47.57 | 2924.1760 | 26 | 0.0 | 732.0513 | 4 | 43.77 | 7 | F7:19182 | BuCaTN01_F7a.raw | | | | | | | 7.1389E6 | | | | | | | | | |

| Peptide | #Feature | #Feature F1 | #Feature F2a | #Feature F3a | #Feature F4 | #Feature F5 | #Feature F6a | #Feature F7a | #Feature F7b | #Feature F8 | #Feature F9a | #Feature Sample 1F10a | #Feature F10b | #Feature F11a | #Feature F11b | #Feature F12a | #Feature F13 | Start | End | PTM | AScore | Found By |
|---|---|---|---|---|---|---|---|---|---|---|---|---|---|---|---|---|---|---|---|---|---|---|
| K.GGGGTPVDELDR.C | 4 | 0 | 0 | 0 | 0 | 0 | 0 | 3 | 0 | 1 | 0 | 0 | 0 | 0 | 0 | 0 | 0 | 59 | 70 | | | PEAKS DB |
| R.C(+57.02)C(+57.02)QTHDNC(+57.02)YGEAEKL.T | 1 | 0 | 0 | 0 | 0 | 0 | 0 | 1 | 0 | 0 | 0 | 0 | 0 | 0 | 0 | 0 | 0 | 71 | 85 | Carbamidomethylation | C1:Carbamidomethylation:1000.00;C2:Carbamidomethylation:1000.00;C8:Carbamidomethylation:1000.00 | PEAKS DB |
| K.GGGGTPVDELDRC(+57.02)C(+57.02)QTHDNC(+57.02)YGEAEK.L | 1 | 0 | 0 | 0 | 0 | 0 | 0 | 1 | 0 | 0 | 0 | 0 | 0 | 0 | 0 | 0 | 0 | 59 | 84 | Carbamidomethylation | C13:Carbamidomethylation:1000.00;C14:Carbamidomethylation:1000.00;C20:Carbamidomethylation:1000.00 | PEAKS DB |

total 3 peptides

**Best Unique PSM (Scan F7:19182, m/z=732.0513, z=4, RT=43.77, ppm=0.0):**

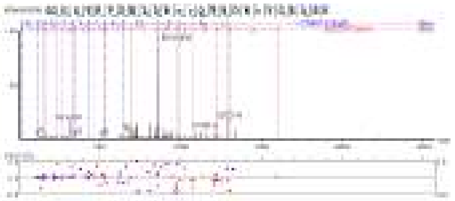

## ADF50035.1

back to list

| Protein Coverage | Supporting Peptides | Best Unique PSM |

**Protein Coverage:**

```
                                                                                   71 72      78
 1 MNPAHLLVLS AVCVSLLGAA IVPPQPLNLI QFSSLIQCAN GGSRATWHYA DYGCYCGKGG GGTPVDELDR CCQTHDNCYG    Carbamidomethylation (+57.02)
81 EAEKLTKCSP YYKTYKYDCS EGKLTCNDAP GSCERSVCDC DRVAAICFAG APYNDKNFMI NTEANCQ
```

**Supporting Peptides:**

| Peptide | Uniq | -10lgP | Mass | Length | ppm | m/z | z | RT | Fraction | Scan | Source File | Area F1 | Area F2a | Area F3a | Area F4 | Area F5 | Area F6a | Area F7a | Area F7b | Area F8 | Area F9a | Area Sample 1F10a | Area F10b | Area F11a | Area F11b | Area F12a | Area F13 | #Feature | #Feature F1 | #Feature F2a | #Feature F3a | #Feature F4 | #Feature F5 | #Feature F6a | #Feature F7a | #Feature F7b | #Feature F8 | #Feature F9a | #Feature Sample 1F10a | #Feature F10b | #Feature F11a | #Feature F11b | #Feature F12a | #Feature F13 | Start | End | PTM | AScore | Found By |
|---|---|---|---|---|---|---|---|---|---|---|---|---|---|---|---|---|---|---|---|---|---|---|---|---|---|---|---|---|---|---|---|---|---|---|---|---|---|---|---|---|---|---|---|---|---|---|---|---|---|
| K.GGGGTPVDELDR.C | N | 61.54 | 1171.5469 | 12 | 1.0 | 586.7813 | 2 | 40.17 | 7 | F7:16143 | BuCaTN01_F7a.raw | | | | | | | 2.5577E6 | | 2.8377E5 | | | | | | | | 4 | 0 | 0 | 0 | 0 | 0 | 0 | 3 | 0 | 1 | 0 | 0 | 0 | 0 | 0 | 0 | 0 | 59 | 70 | | | PEAKS DB |
| R.C(+57.02)C(+57.02)QTHDNC(+57.02)YGEAEKL.T | N | 51.93 | 1883.7236 | 15 | 0.5 | 628.9155 | 3 | 37.42 | 7 | F7:13894 | BuCaTN01_F7a.raw | | | | | | | 1.1837E6 | | | | | | | | | | 1 | 0 | 0 | 0 | 0 | 0 | 0 | 1 | 0 | 0 | 0 | 0 | 0 | 0 | 0 | 0 | 0 | 71 | 85 | Carbamidomethylation | C1:Carbamidomethylation:1000.00;C2:Carbamidomethylation:1000.00;C8:Carbamidomethylation:1000.00 | PEAKS DB |
| K.GGGGTPVDELDRC(+57.02)C(+57.02)QTHDNC(+57.02)YGEAEK.L | Y | 47.57 | 2924.1760 | 26 | 0.0 | 732.0513 | 4 | 43.77 | 7 | F7:19182 | BuCaTN01_F7a.raw | | | | | | | 7.1389E6 | | | | | | | | | | 1 | 0 | 0 | 0 | 0 | 0 | 0 | 1 | 0 | 0 | 0 | 0 | 0 | 0 | 0 | 0 | 0 | 59 | 84 | Carbamidomethylation | C13:Carbamidomethylation:1000.00;C14:Carbamidomethylation:1000.00;C20:Carbamidomethylation:1000.00 | PEAKS DB |
| total 3 peptides | | | | | | | | | | | | | | | | | | | | | | | | | | | | | | | | | | | | | | | | | | | | | | | | | |

**Best Unique PSM (Scan F7:19182, m/z=732.0513, z=4, RT=43.77, ppm=0.0):**

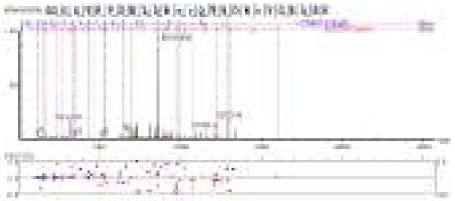

## P01388.1

back to list

| Protein Coverage | Supporting Peptides | Best Unique PSM |

**Protein Coverage:**

```
                                           41  45
 1 IRCFITPDVT SQICADGHVC YTKTWCDNFC ASRGKRVDLG CAATCPTVKP GVNIKCCSTD NCNPFPTRNR P    Carbamidomethylation (+57.02)
```

**Supporting Peptides:**

| Peptide | Uniq | -10lgP | Mass | Length | ppm | m/z | z | RT | Fraction | Scan | Source File | Area F1 | Area F2a | Area F3a | Area F4 | Area F5 | Area F6a | Area F7a | Area F7b | Area F8 | Area F9a | Area Sample 1F10a | Area F10b | Area F11a | Area F11b | Area F12a | Area F13 | #Feature | #Feature F1 | #Feature F2a | #Feature F3a | #Feature F4 | #Feature F5 | #Feature F6a | #Feature F7a | #Feature F7b | #Feature F8 | #Feature F9a | #Feature Sample 1F10a | #Feature F10b | #Feature F11a | #Feature F11b | #Feature F12a | #Feature F13 | Start | End | PTM | AScore | Found By |
|---|---|---|---|---|---|---|---|---|---|---|---|---|---|---|---|---|---|---|---|---|---|---|---|---|---|---|---|---|---|---|---|---|---|---|---|---|---|---|---|---|---|---|---|---|---|---|---|---|---|
| R.VDLGC(+57.02)AATC(+57.02)PTVK.P | N | 96.12 | 1390.6584 | 13 | 0.0 | 696.3365 | 2 | 41.75 | 6 | F6:21108 | BuCaTN01_F6a.raw | | | | | | 3.9617E6 | | | | | | | | | | | 1 | 0 | 0 | 0 | 0 | 0 | 1 | 0 | 0 | 0 | 0 | 0 | 0 | 0 | 0 | 0 | 0 | 37 | 49 | Carbamidomethylation | C5:Carbamidomethylation:1000.00;C9:Carbamidomethylation:1000.00 | PEAKS DB |
| V.DLGC(+57.02)AATC(+57.02)PTVKPGVNIK.C | Y | 46.43 | 1899.9546 | 18 | -0.4 | 634.3239 | 3 | 57.43 | 3 | F3:32474 | BuCaTN01_F3a.raw | | | 2.007E6 | | | | | | | | | | | | | | 2 | 0 | 0 | 2 | 0 | 0 | 0 | 0 | 0 | 0 | 0 | 0 | 0 | 0 | 0 | 0 | 0 | 38 | 55 | Carbamidomethylation | C4:Carbamidomethylation:1000.00;C8:Carbamidomethylation:1000.00 | PEAKS DB |
| total 2 peptides | | | | | | | | | | | | | | | | | | | | | | | | | | | | | | | | | | | | | | | | | | | | | | | | | |

**Best Unique PSM (Scan F3:32474, m/z=634.3239, z=3, RT=57.43, ppm=-0.4):**

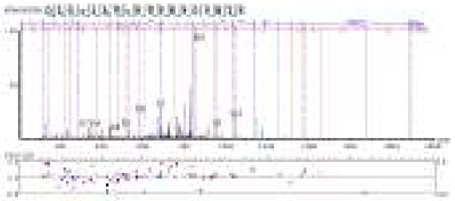

## KAG8131486.1

back to list

| Protein Coverage | Supporting Peptides | Best Unique PSM |

**Protein Coverage:**

```
                                    29
 1 MSSGHLLLLL GLLTLWVELT PVSGRPGFCE LPAAKGRCNA YITAFYYNPD SRRCKKFTYG GCGGNANRFR TIDECNRTCV    Carbamidomethylation (+57.02)
81 G
```

**Supporting Peptides:**

| Peptide | Uniq | -10lgP | Mass | Length | ppm | m/z | z | RT | Fraction | Scan | Source File | Area F1 | Area F2a | Area F3a | Area F4 | Area F5 | Area F6a | Area F7a | Area F7b | Area F8 | Area F9a | Area Sample 1F10a | Area F10b | Area F11a | Area F11b | Area F12a | Area F13 | #Feature | #Feature F1 | #Feature F2a | #Feature F3a | #Feature F4 | #Feature F5 | #Feature F6a | #Feature F7a | #Feature F7b | #Feature F8 | #Feature F9a | #Feature Sample 1F10a | #Feature F10b | #Feature F11a | #Feature F11b | #Feature F12a | #Feature F13 | Start | End | PTM | AScore | Found By |
|---|---|---|---|---|---|---|---|---|---|---|---|---|---|---|---|---|---|---|---|---|---|---|---|---|---|---|---|---|---|---|---|---|---|---|---|---|---|---|---|---|---|---|---|---|---|---|---|---|---|
| R.C(+57.02)NAYITAFYYNPDSR.R | Y | 72.57 | 1853.8042 | 15 | -0.6 | 927.9088 | 2 | 68.44 | 2 | F2:39964 | BuCaTN01_F2a.raw | | 5.1067E6 | 4.9528E5 | | | | | | | | | | | | | | 3 | 0 | 2 | 1 | 0 | 0 | 0 | 0 | 0 | 0 | 0 | 0 | 0 | 0 | 0 | 0 | 0 | 38 | 52 | Carbamidomethylation | C1:Carbamidomethylation:1000.00 | PEAKS DB |
| K.GRC(+57.02)NAYITAFYYNPDSR.R | Y | 59.90 | 2066.9268 | 17 | 1.1 | 689.9836 | 3 | 62.53 | 2 | F2:34713 | BuCaTN01_F2a.raw | | 1.3916E6 | | | | | | | | | | | | | | | 1 | 0 | 1 | 0 | 0 | 0 | 0 | 0 | 0 | 0 | 0 | 0 | 0 | 0 | 0 | 0 | 0 | 36 | 52 | Carbamidomethylation | C3:Carbamidomethylation:1000.00 | PEAKS DB |
| G.FC(+57.02)ELPAAK.G | Y | 48.05 | 934.4582 | 8 | 0.2 | 468.2365 | 2 | 37.50 | 2 | F2:15298 | BuCaTN01_F2a.raw | | 2.0672E6 | | | | | | | | | | | | | | | 1 | 0 | 1 | 0 | 0 | 0 | 0 | 0 | 0 | 0 | 0 | 0 | 0 | 0 | 0 | 0 | 0 | 28 | 35 | Carbamidomethylation | C2:Carbamidomethylation:1000.00 | PEAKS DB |
| total 3 peptides | | | | | | | | | | | | | | | | | | | | | | | | | | | | | | | | | | | | | | | | | | | | | | | | | |

**Best Unique PSM (Scan F2:39964, m/z=927.9088, z=2, RT=68.44, ppm=-0.6):**

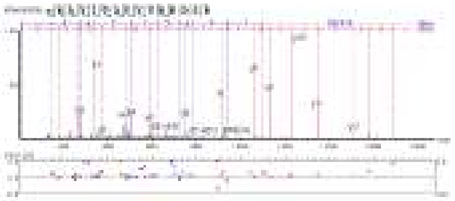

## BAC77655.1

back to list

| Protein Coverage | Supporting Peptides | Best Unique PSM |

**Protein Coverage:**

```
 1  MNPAHLLVLA AVCVSLLGAA IVPPQPLNLY QFNNMIQCAN HGNRPTWHYA HYGCYCGKGG GGTAVDELDR CCQTHDNCYG   Carbamidomethylation (+57.02)
81  EAEKLPKCSP YYKTYKYDCS EGKLTCKDAP GSCERSVCDC DRVAANCFAG APYNNDNFFI SFKENCQ
```

**Supporting Peptides:**

| Peptide | Uniq | -10lgP | Mass | Length | ppm | m/z | z | RT | Fraction | Scan | Source File | Area F1 | Area F2a | Area F3a | Area F4 | Area F5 | Area F6a | Area F7a | Area F7b | Area F8 | Area F9a | Area Sample 1F10a | Area F10b | Area F11a | Area F11b | Area F12a | Area F13 | #Feature | #Feature F1 | #Feature F2a | #Feature F3a | #Feature F4 | #Feature F5 | #Feature F6a | #Feature F7a | #Feature F7b | #Feature F8 | #Feature F9a | #Feature Sample 1F10a | #Feature F10b | #Feature F11a | #Feature F11b | #Feature F12a | #Feature F13 | Start | End | PTM | AScore | Found By |
|---|---|---|---|---|---|---|---|---|---|---|---|---|---|---|---|---|---|---|---|---|---|---|---|---|---|---|---|---|---|---|---|---|---|---|---|---|---|---|---|---|---|---|---|---|---|---|---|---|---|
| R.PTWHYAHYGC(+57.02)YC(+57.02)GK.G | Y | 69.30 | 1798.7344 | 14 | 1.1 | 450.6913 | 4 | 43.61 | 10 | F10:21171 | BuCaTN01_F9a.raw | | | | | | | 3.6093E5 | 1.8578E6 | | 2.5918E6 | | | | | | | 4 | 0 | 0 | 0 | 0 | 0 | 0 | 1 | 1 | 0 | 2 | 0 | 0 | 0 | 0 | 0 | 0 | 45 | 58 | Carbamidomethylation | C10:Carbamidomethylation:1000.00;C12:Carbamidomethylation:1000.00 | PEAKS DB |
| R.C(+57.02)C(+57.02)QTHDNC(+57.02)YGEAEKL.P | N | 51.93 | 1883.7236 | 15 | 0.5 | 628.9155 | 3 | 37.42 | 7 | F7:13894 | BuCaTN01_F7a.raw | | | | | | | 1.1837E6 | | | | | | | | | | 1 | 0 | 0 | 0 | 0 | 0 | 0 | 1 | 0 | 0 | 0 | 0 | 0 | 0 | 0 | 0 | 0 | 71 | 85 | Carbamidomethylation | C1:Carbamidomethylation:1000.00;C2:Carbamidomethylation:1000.00;C8:Carbamidomethylation:1000.00 | PEAKS DB |
| total 2 peptides | | | | | | | | | | | | | | | | | | | | | | | | | | | | | | | | | | | | | | | | | | | | | | | | | |

**Best Unique PSM (Scan F10:21171, m/z=450.6913, z=4, RT=43.61, ppm=1.1):**

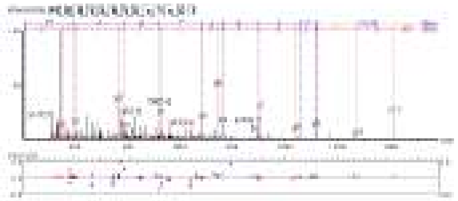

## Q7T2Q5.1

back to list

| Protein Coverage | Supporting Peptides | Best Unique PSM |

**Protein Coverage:**

```
 1  MNPAHLLVLA AVCVSLLGAA IVPPQPLNLY QFNNMIQCAN HGNRPTWHYA HYGCYCGKGG GGTAVDELDR CCQTHDNCYG   Carbamidomethylation (+57.02)
81  EAEKLPKCSP YYKTYKYDCS EGKLTCKDAP GSCERSVCDC DRVAANCFAG APYNNDNFFI SFKENCQ
```

**Supporting Peptides:**

| Peptide | Uniq | -10lgP | Mass | Length | ppm | m/z | z | RT | Fraction | Scan | Source File | Area F1 | Area F2a | Area F3a | Area F4 | Area F5 | Area F6a | Area F7a | Area F7b | Area F8 | Area F9a | Area Sample 1F10a | Area F10b | Area F11a | Area F11b | Area F12a | Area F13 | #Feature | #Feature F1 | #Feature F2a | #Feature F3a | #Feature F4 | #Feature F5 | #Feature F6a | #Feature F7a | #Feature F7b | #Feature F8 | #Feature F9a | #Feature Sample 1F10a | #Feature F10b | #Feature F11a | #Feature F11b | #Feature F12a | #Feature F13 | Start | End | PTM | AScore | Found By |
|---|---|---|---|---|---|---|---|---|---|---|---|---|---|---|---|---|---|---|---|---|---|---|---|---|---|---|---|---|---|---|---|---|---|---|---|---|---|---|---|---|---|---|---|---|---|---|---|---|---|
| R.PTWHYAHYGC(+57.02)YC(+57.02)GK.G | Y | 69.30 | 1798.7344 | 14 | 1.1 | 450.6913 | 4 | 43.61 | 10 | F10:21171 | BuCaTN01_F9a.raw | | | | | | | 3.6093E5 | 1.8578E6 | | 2.5918E6 | | | | | | | 4 | 0 | 0 | 0 | 0 | 0 | 0 | 1 | 1 | 0 | 2 | 0 | 0 | 0 | 0 | 0 | 0 | 45 | 58 | Carbamidomethylation | C10:Carbamidomethylation:1000.00;C12:Carbamidomethylation:1000.00 | PEAKS DB |
| R.C(+57.02)C(+57.02)QTHDNC(+57.02)YGEAEKL.P | N | 51.93 | 1883.7236 | 15 | 0.5 | 628.9155 | 3 | 37.42 | 7 | F7:13894 | BuCaTN01_F7a.raw | | | | | | | 1.1837E6 | | | | | | | | | | 1 | 0 | 0 | 0 | 0 | 0 | 0 | 1 | 0 | 0 | 0 | 0 | 0 | 0 | 0 | 0 | 0 | 71 | 85 | Carbamidomethylation | C1:Carbamidomethylation:1000.00;C2:Carbamidomethylation:1000.00;C8:Carbamidomethylation:1000.00 | PEAKS DB |
| total 2 peptides | | | | | | | | | | | | | | | | | | | | | | | | | | | | | | | | | | | | | | | | | | | | | | | | | |

**Best Unique PSM (Scan F10:21171, m/z=450.6913, z=4, RT=43.61, ppm=1.1):**

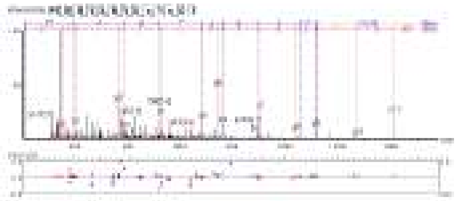

## Q8AY42.2

back to list

| Protein Coverage | Supporting Peptides | Best Unique PSM |

**Protein Coverage:**

```
 1  MSSGGLLLLV GLLTLCAELT PVSSKDRPKF CNVPPEPGRC NANVRAFYYN PRLRKCIEFT YGGCGGNANN FKSGGECKRA   Carbamidomethylation (+57.02)
81  CGE
```

**Supporting Peptides:**

| Peptide | Uniq | -10lgP | Mass | Length | ppm | m/z | z | RT | Fraction | Scan | Source File | Area F1 | Area F2a | Area F3a | Area F4 | Area F5 | Area F6a | Area F7a | Area F7b | Area F8 | Area F9a | Area Sample 1F10a | Area F10b | Area F11a | Area F11b | Area F12a | Area F13 | #Feature | #Feature F1 | #Feature F2a | #Feature F3a | #Feature F4 | #Feature F5 | #Feature F6a | #Feature F7a | #Feature F7b | #Feature F8 | #Feature F9a | #Feature Sample 1F10a | #Feature F10b | #Feature F11a | #Feature F11b | #Feature F12a | #Feature F13 | Start | End | PTM | AScore | Found By |
|---|---|---|---|---|---|---|---|---|---|---|---|---|---|---|---|---|---|---|---|---|---|---|---|---|---|---|---|---|---|---|---|---|---|---|---|---|---|---|---|---|---|---|---|---|---|---|---|---|---|
| E.FTYGGC(+57.02)GGNANNFK.S | Y | 86.88 | 1505.6357 | 14 | -1.1 | 753.8243 | 2 | 42.80 | 6 | F6:22061 | BuCaTN01_F6a.raw | | 9.4887E7 | 2.5801E9 | 1.0399E9 | 1.5114E7 | 1.4385E8 | 2.0429E6 | 3.6801E7 | | 8.7639E6 | | 9.9524E6 | | 6.8982E6 | 1.398E6 | | 30 | 0 | 1 | 15 | 3 | 1 | 2 | 1 | 3 | 0 | 1 | 0 | 1 | 0 | 1 | 1 | 0 | 59 | 72 | Carbamidomethylation | C6:Carbamidomethylation:1000.00 | PEAKS DB |
| total 1 peptides | | | | | | | | | | | | | | | | | | | | | | | | | | | | | | | | | | | | | | | | | | | | | | | | | |

**Best Unique PSM (Scan F6:22061, m/z=753.8243, z=2, RT=42.80, ppm=-1.1):**

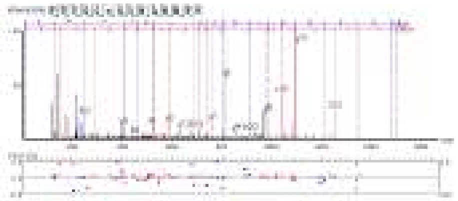

## AAL30069.1

back to list

| Protein Coverage | Supporting Peptides | Best Unique PSM |

**Protein Coverage:**

```
 1  PGGFIMSSGG LLLLVGLLTL CAELTPVSSK DRPKFCNVPP EPGRCNANVR AFYYNPRLRK CIEFTYGGCG GNANNFKSGG   Carbamidomethylation (+57.02)
81  ECKRACGE
```

**Supporting Peptides:**

| Peptide | Uniq | -10lgP | Mass | Length | ppm | m/z | z | RT | Fraction | Scan | Source File | Area F1 | Area F2a | Area F3a | Area F4 | Area F5 | Area F6a | Area F7a | Area F7b | Area F8 | Area F9a | Area Sample 1F10a | Area F10b | Area F11a | Area F11b | Area F12a | Area F13 | #Feature | #Feature F1 | #Feature F2a | #Feature F3a | #Feature F4 | #Feature F5 | #Feature F6a | #Feature F7a | #Feature F7b | #Feature F8 | #Feature F9a | #Feature Sample 1F10a | #Feature F10b | #Feature F11a | #Feature F11b | #Feature F12a | #Feature F13 | Start | End | PTM | AScore | Found By |
|---|---|---|---|---|---|---|---|---|---|---|---|---|---|---|---|---|---|---|---|---|---|---|---|---|---|---|---|---|---|---|---|---|---|---|---|---|---|---|---|---|---|---|---|---|---|---|---|---|---|
| E.FTYGGC(+57.02)GGNANNFK.S | Y | 86.88 | 1505.6357 | 14 | -1.1 | 753.8243 | 2 | 42.80 | 6 | F6:22061 | BuCaTN01_F6a.raw | | 9.4887E7 | 2.5801E9 | 1.0399E9 | 1.5114E7 | 1.4385E8 | 2.0429E6 | 3.6801E7 | | 8.7639E6 | | 9.9524E6 | | 6.8982E6 | 1.398E6 | | 30 | 0 | 1 | 15 | 3 | 1 | 2 | 1 | 3 | 0 | 1 | 0 | 1 | 0 | 1 | 1 | 0 | 64 | 77 | Carbamidomethylation | C6:Carbamidomethylation:1000.00 | PEAKS DB |
| total 1 peptides | | | | | | | | | | | | | | | | | | | | | | | | | | | | | | | | | | | | | | | | | | | | | | | | | |

**Best Unique PSM (Scan F6:22061, m/z=753.8243, z=2, RT=42.80, ppm=-1.1):**

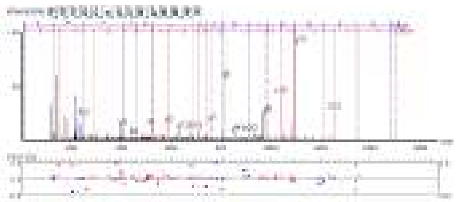

## Q8AY43.2

back to list

| Protein Coverage | Supporting Peptides | Best Unique PSM |

**Protein Coverage:**

64

1 MSSGGLLLLL GLLTLCAELT PVSSKDRPKF CNVPPEPGRC NANVRAFYYN PRLRKCIE**FT YGGCGGNANN FK**SRGECKRT    Carbamidomethylation (+57.02)

81 CAE

**Supporting Peptides:**

| Peptide | Uniq | -10lgP | Mass | Length | ppm | m/z | z | RT | Fraction | Scan | Source File | Area F1 | Area F2a | Area F3a | Area F4 | Area F5 | Area F6a | Area F7a | Area F7b | Area F8 | Area F9a | Area Sample 1F10a | Area F10b | Area F11a | Area F11b | Area F12a | Area F13 | #Feature | #Feature F1 | #Feature F2a | #Feature F3a | #Feature F4 | #Feature F5 | #Feature F6a | #Feature F7a | #Feature F7b | #Feature F8 | #Feature F9a | #Feature Sample 1F10a | #Feature F10b | #Feature F11a | #Feature F11b | #Feature F12a | #Feature F13 | Start | End | PTM | AScore | Found By |
|---|---|---|---|---|---|---|---|---|---|---|---|---|---|---|---|---|---|---|---|---|---|---|---|---|---|---|---|---|---|---|---|---|---|---|---|---|---|---|---|---|---|---|---|---|---|---|---|---|---|
| E.FTYGGC(+57.02)GGNANNFK.S | Y | 86.88 | 1505.6357 | 14 | -1.1 | 753.8243 | 2 | 42.80 | 6 | F6:22061 | BuCaTN01_F6a.raw | | 9.4887E7 | 2.5801E9 | 1.0399E9 | 1.5114E7 | 1.4385E8 | 2.0429E6 | 3.6801E7 | | 8.7639E6 | | 9.9524E6 | | 6.8982E6 | 1.398E6 | | 30 | 0 | 1 | 15 | 3 | 1 | 2 | 1 | 3 | 0 | 1 | 0 | 1 | 0 | 1 | 1 | 0 | 59 | 72 | Carbamidomethylation | C6:Carbamidomethylation:1000.00 | PEAKS DB |
| total 1 peptides | | | | | | | | | | | | | | | | | | | | | | | | | | | | | | | | | | | | | | | | | | | | | | | | | |

**Best Unique PSM (Scan F6:22061, m/z=753.8243, z=2, RT=42.80, ppm=-1.1):**

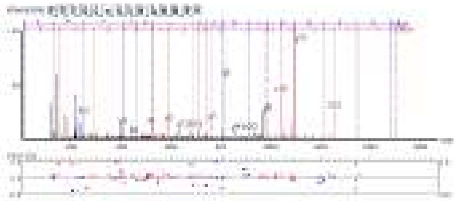

## AAL30068.1

back to list

| Protein Coverage | Supporting Peptides | Best Unique PSM |

**Protein Coverage:**

69

1 PDGFIMSSGG LLLLLGLLTL CAELTPVSSK DRPKFCNVPP EPGRCNANVR AFYYNPRLRK CIE**FTYGGCG GNANNFK**SRG    Carbamidomethylation (+57.02)

81 ECKRTCAE

**Supporting Peptides:**

| Peptide | Uniq | -10lgP | Mass | Length | ppm | m/z | z | RT | Fraction | Scan | Source File | Area F1 | Area F2a | Area F3a | Area F4 | Area F5 | Area F6a | Area F7a | Area F7b | Area F8 | Area F9a | Area Sample 1F10a | Area F10b | Area F11a | Area F11b | Area F12a | Area F13 | #Feature | #Feature F1 | #Feature F2a | #Feature F3a | #Feature F4 | #Feature F5 | #Feature F6a | #Feature F7a | #Feature F7b | #Feature F8 | #Feature F9a | #Feature Sample 1F10a | #Feature F10b | #Feature F11a | #Feature F11b | #Feature F12a | #Feature F13 | Start | End | PTM | AScore | Found By |
|---|---|---|---|---|---|---|---|---|---|---|---|---|---|---|---|---|---|---|---|---|---|---|---|---|---|---|---|---|---|---|---|---|---|---|---|---|---|---|---|---|---|---|---|---|---|---|---|---|---|
| E.FTYGGC(+57.02)GGNANNFK.S | Y | 86.88 | 1505.6357 | 14 | -1.1 | 753.8243 | 2 | 42.80 | 6 | F6:22061 | BuCaTN01_F6a.raw | | 9.4887E7 | 2.5801E9 | 1.0399E9 | 1.5114E7 | 1.4385E8 | 2.0429E6 | 3.6801E7 | | 8.7639E6 | | 9.9524E6 | | 6.8982E6 | 1.398E6 | | 30 | 0 | 1 | 15 | 3 | 1 | 2 | 1 | 3 | 0 | 1 | 0 | 1 | 0 | 1 | 1 | 0 | 64 | 77 | Carbamidomethylation | C6:Carbamidomethylation:1000.00 | PEAKS DB |
| total 1 peptides | | | | | | | | | | | | | | | | | | | | | | | | | | | | | | | | | | | | | | | | | | | | | | | | | |

**Best Unique PSM (Scan F6:22061, m/z=753.8243, z=2, RT=42.80, ppm=-1.1):**

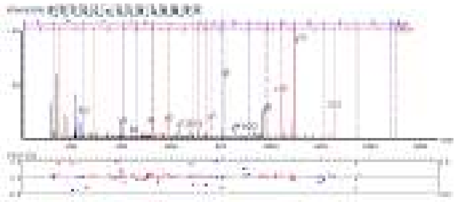

## P0DI84.1

back to list

| Protein Coverage | Supporting Peptides | Best Unique PSM |

**Protein Coverage:**

41

1 DRNPLEECFR ETDYEEFLEI ARNGLKKTSN PK**HVVVVGAG MSGLSAAYVL AGAGHK**VTVL EASERAGGRV RTHRNSKEGW    Oxidation (M) (+15.99)

81 YANLGPMRIP EKHRIVREYI RKFGLNLNEF SQENDNAWYF IKNIRKRVGE VNKDPGLLKY PVKPSEEGKS AGQLYEESLG
161 SAVKDLKRTN CSYILNKYDT YSTKEYLIKE GNLSPGAVDM IGDLLNEDSG YYVSFIESLK HDDIFAYEKR FDEIVGGMDQ
241 LPTSMYRAIE EKVKFNARVI KIQQNANQVT VTYQTPEKDT SSNTADYVIV CTTSRAARRI QFEPPLPPKK QHALRSVHYR
321 SGTKIFLTCS SKFWEDDGIH GGKSTTDLPS RFIYYPNHNF STGVGVIIAY GIGDDANFFQ ALKFKDCADI VFNDLSLIHQ
401 LPKEEIQSFC YPSMIQKWSL DKYAMGAITT FTPYQFQRFS EALTAPQGRI FFAGEYTAEA HGWIDSTIKS GLTAARDVNR
481 ASEQ

**Supporting Peptides:**

| Peptide | Uniq | -10lgP | Mass | Length | ppm | m/z | z | RT | Fraction | Scan | Source File | Area F1 | Area F2a | Area F3a | Area F4 | Area F5 | Area F6a | Area F7a | Area F7b | Area F8 | Area F9a | Area Sample 1F10a | Area F10b | Area F11a | Area F11b | Area F12a | Area F13 | #Feature | #Feature F1 | #Feature F2a | #Feature F3a | #Feature F4 | #Feature F5 | #Feature F6a | #Feature F7a | #Feature F7b | #Feature F8 | #Feature F9a | #Feature Sample 1F10a | #Feature F10b | #Feature F11a | #Feature F11b | #Feature F12a | #Feature F13 | Start | End | PTM | AScore | Found By |
|---|---|---|---|---|---|---|---|---|---|---|---|---|---|---|---|---|---|---|---|---|---|---|---|---|---|---|---|---|---|---|---|---|---|---|---|---|---|---|---|---|---|---|---|---|---|---|---|---|---|
| V.GAGM(+15.99)SGLSAAYVLAGAGHK.V | N | 71.56 | 1732.8566 | 19 | -0.1 | 578.6261 | 3 | 56.99 | 16 | F16:27415 | BuCaTN01_F13.raw | | | | | | | | | | | | | | | | 1.9804E5 | 1 | 0 | 0 | 0 | 0 | 0 | 0 | 0 | 0 | 0 | 0 | 0 | 0 | 0 | 0 | 0 | 1 | 38 | 56 | Oxidation (M) | M4:Oxidation (M):1000.00 | PEAKS DB |
| K.HVVVVGAGMSGLSAAYVLAGAGHK.V | Y | 43.57 | 2250.1943 | 24 | -0.3 | 563.5557 | 4 | 65.81 | 16 | F16:33239 | BuCaTN01_F13.raw | | | | | | | | | | | | | | | | 2.0715E4 | 1 | 0 | 0 | 0 | 0 | 0 | 0 | 0 | 0 | 0 | 0 | 0 | 0 | 0 | 0 | 0 | 1 | 33 | 56 | | | PEAKS DB |
| total 2 peptides | | | | | | | | | | | | | | | | | | | | | | | | | | | | | | | | | | | | | | | | | | | | | | | | | |

**Best Unique PSM (Scan F16:33239, m/z=563.5557, z=4, RT=65.81, ppm=-0.3):**

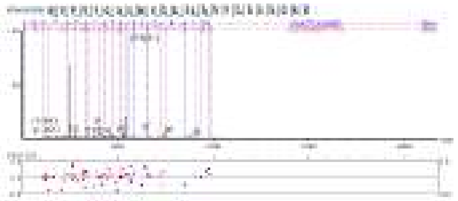

## 3KVE

back to list

| Protein Coverage | Supporting Peptides | Best Unique PSM |

**Protein Coverage:**

43

1 ANDRNPLEEC FRETDYEEFL EIARNGLKKT SNPK**HVVVVG AGMSGLSAAY VLAGAGHK**VT VLEASERAGG RVRTHRNSKE   Oxidation (M) (+15.99)

81 GWYANLGPMR IPEKHRIVRE YIRKFGLNLN EFSQENDNAW YFIKNIRKRV GEVNKDPGLL KYPVKPSEEG KSAGQLYEES
161 LGSAVKDLKR TNCSYILNKY DTYSTKEYLI KEGNLSPGAV DMIGDLLNED SGYYVSFIES LKHDDIFAYE KRFDEIVGGM
241 DQLPTSMYRA IEEKVKFNAR VIKIQQNANQ VTVTYQTPEK DTSSNTADYV IVCTTSRAAR RIQFEPPLPP KKQHALRSVH
321 YRSGTKIFLT CSSKFWEDDG IHGGKSTTDL PSRFIYYPNH NFSTGVGVII AYGIGDDANF FQALKFKDCA DIVFNDLSLI
401 HQLPKEEIQS FCYPSMIQKW SLDKYAMGAI TTFTPYQFQR FSEALTAPQG RIFFAGEYTA EAHGWIDSTI KSGLTAARDV
481 NRASEQ

**Supporting Peptides:**

| Peptide | Uniq | -10lgP | Mass | Length | ppm | m/z | z | RT | Fraction | Scan | Source File | Area F1 | Area F2a | Area F3a | Area F4 | Area F5 | Area F6a | Area F7a | Area F7b | Area F8 | Area F9a | Area Sample 1F10a | Area F10b | Area F11a | Area F11b | Area F12a | Area F13 | #Feature | #Feature F1 | #Feature F2a | #Feature F3a | #Feature F4 | #Feature F5 | #Feature F6a | #Feature F7a | #Feature F7b | #Feature F8 | #Feature F9a | #Feature Sample 1F10a | #Feature F10b | #Feature F11a | #Feature F11b | #Feature F12a | #Feature F13 | Start | End | PTM | AScore | Found By |
|---|---|---|---|---|---|---|---|---|---|---|---|---|---|---|---|---|---|---|---|---|---|---|---|---|---|---|---|---|---|---|---|---|---|---|---|---|---|---|---|---|---|---|---|---|---|---|---|---|---|
| V.GAGM(+15.99)SGLSAAYVLAGAGHK.V | N | 71.56 | 1732.8566 | 19 | -0.1 | 578.6261 | 3 | 56.99 | 16 | F16:27415 | BuCaTN01_F13.raw | | | | | | | | | | | | | | | | 1.9804E5 | 1 | 0 | 0 | 0 | 0 | 0 | 0 | 0 | 0 | 0 | 0 | 0 | 0 | 0 | 0 | 0 | 1 | 40 | 58 | Oxidation (M) | M4:Oxidation (M):1000.00 | PEAKS DB |
| K.HVVVVGAGMSGLSAAYVLAGAGHK.V | Y | 43.57 | 2250.1943 | 24 | -0.3 | 563.5557 | 4 | 65.81 | 16 | F16:33239 | BuCaTN01_F13.raw | | | | | | | | | | | | | | | | 2.0715E4 | 1 | 0 | 0 | 0 | 0 | 0 | 0 | 0 | 0 | 0 | 0 | 0 | 0 | 0 | 0 | 0 | 1 | 35 | 58 | | | PEAKS DB |
| total 2 peptides | | | | | | | | | | | | | | | | | | | | | | | | | | | | | | | | | | | | | | | | | | | | | | | | | |

**Best Unique PSM (Scan F16:33239, m/z=563.5557, z=4, RT=65.81, ppm=-0.3):**

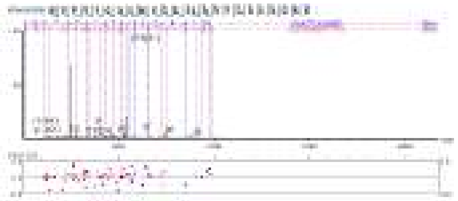

## CAB42053.1

back to list

| Protein Coverage | Supporting Peptides | Best Unique PSM |

**Protein Coverage:**

35 63 7475 80

1 MKTLLLTLVV VTIVCLDLGY TLKCNK**LVPL FYKTCPAGK**N LCYKMFMVAT PKVPVKR**GCI DVCPKNSLLV KYVCCNTDRC**   Carbamidomethylation (+57.02)

81 **N**

**Supporting Peptides:**

| Peptide | Uniq | -10lgP | Mass | Length | ppm | m/z | z | RT | Fraction | Scan | Source File | Area F1 | Area F2a | Area F3a | Area F4 | Area F5 | Area F6a | Area F7a | Area F7b | Area F8 | Area F9a | Area Sample 1F10a | Area F10b | Area F11a | Area F11b | Area F12a | Area F13 | #Feature | #Feature F1 | #Feature F2a | #Feature F3a | #Feature F4 | #Feature F5 | #Feature F6a | #Feature F7a | #Feature F7b | #Feature F8 | #Feature F9a | #Feature Sample 1F10a | #Feature F10b | #Feature F11a | #Feature F11b | #Feature F12a | #Feature F13 | Start | End | PTM | AScore | Found By |
|---|---|---|---|---|---|---|---|---|---|---|---|---|---|---|---|---|---|---|---|---|---|---|---|---|---|---|---|---|---|---|---|---|---|---|---|---|---|---|---|---|---|---|---|---|---|---|---|---|---|
| K.NSLLVKYVC(+57.02)C(+57.02)NTDRC(+57.02)N | N | 74.78 | 2014.9023 | 16 | 5.9 | 672.6434 | 3 | 52.92 | 8 | F8:29061 | BuCaTN01_F7b.raw | | | | | | | | 2.1966E5 | | | | | | | | | 1 | 0 | 0 | 0 | 0 | 0 | 0 | 0 | 1 | 0 | 0 | 0 | 0 | 0 | 0 | 0 | 0 | 66 | 81 | Carbamidomethylation | C9:Carbamidomethylation:1000.00;C10:Carbamidomethylation:1000.00;C15:Carbamidomethylation:1000.00 | PEAKS DB |
| R.GC(+57.02)IDVC(+57.02)PKNSLLVK.Y | N | 50.76 | 1601.8269 | 14 | 2.2 | 801.9202 | 2 | 49.87 | 8 | F8:26668 | BuCaTN01_F7b.raw | | | | | | | | 0 | | | | | | | | | 0 | 0 | 0 | 0 | 0 | 0 | 0 | 0 | 0 | 0 | 0 | 0 | 0 | 0 | 0 | 0 | 0 | 58 | 71 | Carbamidomethylation | C2:Carbamidomethylation:1000.00;C6:Carbamidomethylation:1000.00 | PEAKS DB |
| K.LVPLFYKTC(+57.02)PAGK.N | Y | 43.41 | 1492.8112 | 13 | 2.9 | 498.6110 | 3 | 54.37 | 8 | F8:30331 | BuCaTN01_F7b.raw | | | | | | | | 3.3915E5 | | | | | | | | | 1 | 0 | 0 | 0 | 0 | 0 | 0 | 0 | 1 | 0 | 0 | 0 | 0 | 0 | 0 | 0 | 0 | 27 | 39 | Carbamidomethylation | C9:Carbamidomethylation:1000.00 | PEAKS DB |
| total 3 peptides | | | | | | | | | | | | | | | | | | | | | | | | | | | | | | | | | | | | | | | | | | | | | | | | | |

**Best Unique PSM (Scan F8:30331, m/z=498.6110, z=3, RT=54.37, ppm=2.9):**

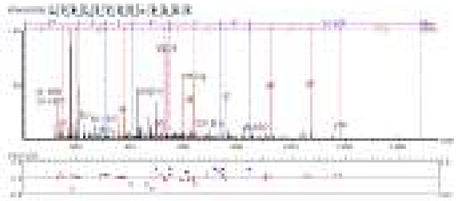

## AAB18383.1

back to list

| Protein Coverage | Supporting Peptides | Best Unique PSM |

**Protein Coverage:**

35 63 7475 80

1 MKTLLLTLVV VTIVCLDLGY TLKCNK**LVPL FYKTCPAGK**N LCYKMFMVAT PKVPVKR**GCI DVCPKNSLLV KYVCCNTDRC**   Carbamidomethylation (+57.02)

81 **N**

**Supporting Peptides:**

| Peptide | Uniq | -10lgP | Mass | Length | ppm | m/z | z | RT | Fraction | Scan | Source File | Area F1 | Area F2a | Area F3a | Area F4 | Area F5 | Area F6a | Area F7a | Area F7b | Area F8 | Area F9a | Area Sample 1F10a | Area F10b | Area F11a | Area F11b | Area F12a | Area F13 | #Feature | #Feature F1 | #Feature F2a | #Feature F3a | #Feature F4 | #Feature F5 | #Feature F6a | #Feature F7a | #Feature F7b | #Feature F8 | #Feature F9a | #Feature Sample 1F10a | #Feature F10b | #Feature F11a | #Feature F11b | #Feature F12a | #Feature F13 | Start | End | PTM | AScore | Found By |
|---|---|---|---|---|---|---|---|---|---|---|---|---|---|---|---|---|---|---|---|---|---|---|---|---|---|---|---|---|---|---|---|---|---|---|---|---|---|---|---|---|---|---|---|---|---|---|---|---|---|
| K.NSLLVKYVC(+57.02)C(+57.02)NTDRC(+57.02)N | N | 74.78 | 2014.9023 | 16 | 5.9 | 672.6434 | 3 | 52.92 | 8 | F8:29061 | BuCaTN01_F7b.raw | | | | | | | | 2.1966E5 | | | | | | | | | 1 | 0 | 0 | 0 | 0 | 0 | 0 | 0 | 1 | 0 | 0 | 0 | 0 | 0 | 0 | 0 | 0 | 66 | 81 | Carbamidomethylation | C9:Carbamidomethylation:1000.00;C10:Carbamidomethylation:1000.00;C15:Carbamidomethylation:1000.00 | PEAKS DB |
| R.GC(+57.02)IDVC(+57.02)PKNSLLVK.Y | N | 50.76 | 1601.8269 | 14 | 2.2 | 801.9202 | 2 | 49.87 | 8 | F8:26668 | BuCaTN01_F7b.raw | | | | | | | | 0 | | | | | | | | | 0 | 0 | 0 | 0 | 0 | 0 | 0 | 0 | 0 | 0 | 0 | 0 | 0 | 0 | 0 | 0 | 0 | 58 | 71 | Carbamidomethylation | C2:Carbamidomethylation:1000.00;C6:Carbamidomethylation:1000.00 | PEAKS DB |
| K.LVPLFYKTC(+57.02)PAGK.N | Y | 43.41 | 1492.8112 | 13 | 2.9 | 498.6110 | 3 | 54.37 | 8 | F8:30331 | BuCaTN01_F7b.raw | | | | | | | | 3.3915E5 | | | | | | | | | 1 | 0 | 0 | 0 | 0 | 0 | 0 | 0 | 1 | 0 | 0 | 0 | 0 | 0 | 0 | 0 | 0 | 27 | 39 | Carbamidomethylation | C9:Carbamidomethylation:1000.00 | PEAKS DB |
| total 3 peptides | | | | | | | | | | | | | | | | | | | | | | | | | | | | | | | | | | | | | | | | | | | | | | | | | |

**Best Unique PSM (Scan F8:30331, m/z=498.6110, z=3, RT=54.37, ppm=2.9):**

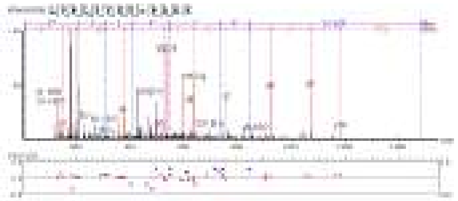

## JAA74725.1

back to list

| Protein Coverage | Supporting Peptides | Best Unique PSM |

**Protein Coverage:**

78

1 MYPVHLLVLL AVCVSLLGAV SIPPRPLNLF QFKDMIECAN HGS**RGALDYA DYGCYCGKGG SGTPVDELDR CCQTHDDCYG**   Carbamidomethylation (+57.02)

81 **EAEK**LPACNY MLSGPYYNTF KYDCVEHQLT CKDDKDECRA FICNCDRTAA ICFAGAPYNN TNYNIDTKTH CQ

**Supporting Peptides:**

| Peptide | Uniq | -10lgP | Mass | Length | ppm | m/z | z | RT | Fraction | Scan | Source File | Area F1 | Area F2a | Area F3a | Area F4 | Area F5 | Area F6a | Area F7a | Area F7b | Area F8 | Area F9a | Area Sample 1F10a | Area F10b | Area F11a | Area F11b | Area F12a | Area F13 | #Feature | #Feature F1 | #Feature F2a | #Feature F3a | #Feature F4 | #Feature F5 | #Feature F6a | #Feature F7a | #Feature F7b | #Feature F8 | #Feature F9a | #Feature Sample 1F10a | #Feature F10b | #Feature F11a | #Feature F11b | #Feature F12a | #Feature F13 | Start | End | PTM | AScore | Found By |
|---|---|---|---|---|---|---|---|---|---|---|---|---|---|---|---|---|---|---|---|---|---|---|---|---|---|---|---|---|---|---|---|---|---|---|---|---|---|---|---|---|---|---|---|---|---|---|---|---|---|
| G.GSGTPVDELDR.C | N | 56.36 | 1144.5360 | 11 | 3.4 | 573.2756 | 2 | 40.15 | 8 | F8:17921 | BuCaTN01_F7b.raw | | | | | | | | 1.1068E6 | | | | | | | | | 1 | 0 | 0 | 0 | 0 | 0 | 0 | 0 | 1 | 0 | 0 | 0 | 0 | 0 | 0 | 0 | 0 | 60 | 70 | | | PEAKS DB |

| | | | | | | | | | | | | | | | | | | | | | | | | | | | | | | | | | | | | | | | | | | | | | | | | | |
|---|---|---|---|---|---|---|---|---|---|---|---|---|---|---|---|---|---|---|---|---|---|---|---|---|---|---|---|---|---|---|---|---|---|---|---|---|---|---|---|---|---|---|---|---|---|---|---|---|---|
| K.GGSGTPVDELDR.C | N | 49.09 | 1201.5575 | 12 | 0.3 | 601.7862 | 2 | 40.57 | 11 | F11:14528 | BuCaTN01_F10a.raw | | | | | | | 9.2274E5 | 2.7577E6 | | 1.1335E7 | 6.7591E5 | | 9.9086E5 | 2.6759E7 | | | 6 | 0 | 0 | 0 | 0 | 0 | 0 | 1 | 1 | 0 | 1 | 1 | 0 | 1 | 1 | 0 | 0 | 59 | 70 | | | PEAKS DB |
| S.RGALDYADYGC(+57.02)YC(+57.02)GKGGSGTPVDELDR.C | Y | 44.99 | 2951.2812 | 27 | 2.9 | 984.7706 | 3 | 76.45 | 13 | F13:48024 | BuCaTN01_F11a.raw | | | | | | | | | | | | 2.4671E4 | 1.0132E5 | | | | 2 | 0 | 0 | 0 | 0 | 0 | 0 | 0 | 0 | 0 | 0 | 0 | 1 | 1 | 0 | 0 | 0 | 44 | 70 | Carbamidomethylation | C11:Carbamidomethylation:1000.00;C13:Carbamidomethylation:1000.00 | PEAKS DB |
| R.C(+57.02)C(+57.02)QTHDDC(+57.02)YGEAEK.L | N | 44.85 | 1771.6237 | 14 | 0.2 | 591.5486 | 3 | 24.28 | 7 | F7:6301 | BuCaTN01_F7a.raw | | | | | | | 1.7946E5 | | | | | | | | | | 1 | 0 | 0 | 0 | 0 | 0 | 0 | 1 | 0 | 0 | 0 | 0 | 0 | 0 | 0 | 0 | 0 | 71 | 84 | Carbamidomethylation | C1:Carbamidomethylation:1000.00;C2:Carbamidomethylation:1000.00;C8:Carbamidomethylation:1000.00 | PEAKS DB |
| total 4 peptides | | | | | | | | | | | | | | | | | | | | | | | | | | | | | | | | | | | | | | | | | | | | | | | | | |

**Best Unique PSM (Scan F13:48024, m/z=984.7706, z=3, RT=76.45, ppm=2.9):**

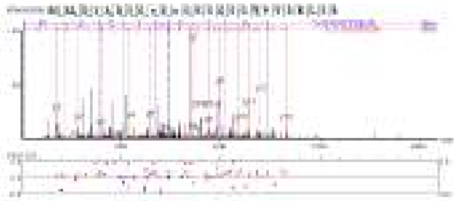

## AED89576.1

back to list

| Protein Coverage | Supporting Peptides | Best Unique PSM |

**Protein Coverage:**

1 MNPAHLLVLA AVCISLLGAS SIAPQPLNLY QFGNMINCTM PGGSPLLDYA DYGCYCGS**GG GGTPVDDLDR** CCQAHDNCYG

81 EAETVHKCNP FWTFYSYECS EGQLTCRDND TNCKEFVCNC DLEAANCFAK APYIEENYNI NLNRCT

**Supporting Peptides:**

| Peptide | Uniq | -10lgP | Mass | Length | ppm | m/z | z | RT | Fraction | Scan | Source File | Area F1 | Area F2a | Area F3a | Area F4 | Area F5 | Area F6a | Area F7a | Area F7b | Area F8 | Area F9a | Area Sample 1F10a | Area F10b | Area F11a | Area F11b | Area F12a | Area F13 | #Feature | #Feature F1 | #Feature F2a | #Feature F3a | #Feature F4 | #Feature F5 | #Feature F6a | #Feature F7a | #Feature F7b | #Feature F8 | #Feature F9a | #Feature Sample 1F10a | #Feature F10b | #Feature F11a | #Feature F11b | #Feature F12a | #Feature F13 | Start | End | PTM | AScore | Found By |
|---|---|---|---|---|---|---|---|---|---|---|---|---|---|---|---|---|---|---|---|---|---|---|---|---|---|---|---|---|---|---|---|---|---|---|---|---|---|---|---|---|---|---|---|---|---|---|---|---|---|
| S.GGGGTPVDDLDR.C | Y | 63.05 | 1157.5312 | 12 | 0.3 | 579.7731 | 2 | 40.47 | 7 | F7:16369 | BuCaTN01_F7a.raw | | | | | | 5.1797E5 | 6.2655E8 | 9.9245E7 | 2.0593E6 | 4.4931E7 | 3.4374E6 | | | | | | 10 | 0 | 0 | 0 | 0 | 0 | 1 | 5 | 1 | 1 | 1 | 1 | 0 | 0 | 0 | 0 | 0 | 59 | 70 | | | PEAKS DB |
| G.GTPVDDLDR.C | N | 51.39 | 986.4669 | 9 | 0.6 | 494.2410 | 2 | 37.62 | 7 | F7:14221 | BuCaTN01_F7a.raw | | | | | | | 4.0904E6 | | | | | | | | | | 1 | 0 | 0 | 0 | 0 | 0 | 0 | 1 | 0 | 0 | 0 | 0 | 0 | 0 | 0 | 0 | 0 | 62 | 70 | | | PEAKS DB |
| G.TPVDDLDR.C | N | 48.70 | 929.4454 | 8 | -0.4 | 465.7298 | 2 | 34.76 | 7 | F7:11899 | BuCaTN01_F7a.raw | | | | | | | 6.3757E5 | | | | | | | | | | 1 | 0 | 0 | 0 | 0 | 0 | 0 | 1 | 0 | 0 | 0 | 0 | 0 | 0 | 0 | 0 | 0 | 63 | 70 | | | PEAKS DB |
| total 3 peptides | | | | | | | | | | | | | | | | | | | | | | | | | | | | | | | | | | | | | | | | | | | | | | | | | |

**Best Unique PSM (Scan F7:16369, m/z=579.7731, z=2, RT=40.47, ppm=0.3):**

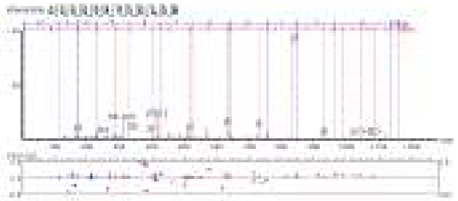

## XP_058026748.1

back to list

| Protein Coverage | Supporting Peptides | Best Unique PSM |

**Protein Coverage:**

1 MSQMTSFSSR SATAPSNFSQ VRISSVSSSR GIRGSGSFRK PGGFGSASLF NLGSGSRRLA PSYPLSPSLG YGGGIQEVTI

81 NTNLLSPFNF EIDPAIQKVR KEEKEQIKVL NNKFATFIDK VRFLEQQNKI LETKWSLLQQ QKTTSRSNIT PIFETYINNL

161 RRQLESLHND KGRLEGELKS MQDLVEDFKN KYEDEINRRT SAENEFVVLK KDVDASYMNK VELESKVDGL TDEINFLRTL

241 FDAELAEMQT QISDTSVVLQ IDNNR**SLDLD SIIAEVKAQY EEIANK**SRME AESWYQSKFE VLQATAGKHG DDLRHTKNEI

321 AEINRTILRM QAEIDNVKNQ RAKLEASVAE AEERGTLAVK DAKAKLEELE LALNKAKQEM AHQLREYHEL MNVK**LALDIE**

401 **IATYR**KLLEG EESRLSGDGA GSVSMTLVSS SGGGTSSSGL GYGGGFTLGS GMGSGALGFT SGGGSRKSTY SVTSTSRRSV

481 RN

**Supporting Peptides:**

| Peptide | Uniq | -10lgP | Mass | Length | ppm | m/z | z | RT | Fraction | Scan | Source File | Area F1 | Area F2a | Area F3a | Area F4 | Area F5 | Area F6a | Area F7a | Area F7b | Area F8 | Area F9a | Area Sample 1F10a | Area F10b | Area F11a | Area F11b | Area F12a | Area F13 | #Feature | #Feature F1 | #Feature F2a | #Feature F3a | #Feature F4 | #Feature F5 | #Feature F6a | #Feature F7a | #Feature F7b | #Feature F8 | #Feature F9a | #Feature Sample 1F10a | #Feature F10b | #Feature F11a | #Feature F11b | #Feature F12a | #Feature F13 | Start | End | PTM | AScore | Found By |
|---|---|---|---|---|---|---|---|---|---|---|---|---|---|---|---|---|---|---|---|---|---|---|---|---|---|---|---|---|---|---|---|---|---|---|---|---|---|---|---|---|---|---|---|---|---|---|---|---|---|
| R.SLDLDSIIAEVK.A | N | 73.59 | 1301.7078 | 12 | 0.6 | 651.8616 | 2 | 82.95 | 1 | F1:39411 | BuCaTN01_F1.raw | 3.873E6 | 1.0735E7 | 3.3784E6 | | 5.5592E5 | 9.862E5 | 5.7757E5 | 9.5711E6 | 1.8421E6 | 8.7299E6 | 1.198E6 | 3.4491E6 | 2.3574E6 | 4.4275E6 | 7.5446E5 | 3.385E6 | 17 | 2 | 2 | 1 | 0 | 1 | 1 | 1 | 1 | 1 | 1 | 1 | 1 | 1 | 1 | 1 | 1 | 266 | 277 | | | PEAKS DB |
| K.LALDIEIATYR.K | N | 48.98 | 1276.7026 | 11 | 3.4 | 639.3589 | 2 | 72.56 | 8 | F8:48459 | BuCaTN01_F7b.raw | | 5.015E5 | | | | | | 8.9534E5 | | | 3.3691E5 | | 6.8742E5 | | | | 4 | 0 | 1 | 0 | 0 | 0 | 0 | 0 | 1 | 0 | 0 | 1 | 0 | 1 | 0 | 0 | 0 | 395 | 405 | | | PEAKS DB |
| R.SLDLDSIIAEVKAQYEEIANK.S | Y | 46.48 | 2348.2109 | 21 | 0.2 | 783.7444 | 3 | 98.25 | 2 | F2:60548 | BuCaTN01_F2a.raw | | 1.0416E6 | 2.6124E6 | | | | | | | | | | | | | | 2 | 0 | 1 | 1 | 0 | 0 | 0 | 0 | 0 | 0 | 0 | 0 | 0 | 0 | 0 | 0 | 0 | 266 | 286 | | | PEAKS DB |
| total 3 peptides | | | | | | | | | | | | | | | | | | | | | | | | | | | | | | | | | | | | | | | | | | | | | | | | | |

**Best Unique PSM (Scan F2:60548, m/z=783.7444, z=3, RT=98.25, ppm=0.2):**

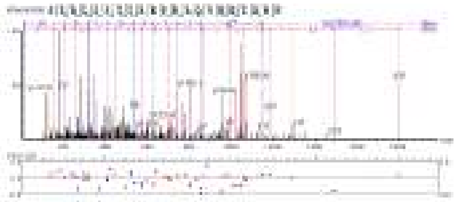

## ADF50022.1

back to list

| Protein Coverage | Supporting Peptides | Best Unique PSM |

**Protein Coverage:**

1 MKSLLLTLVV VTIVCLDLG**Y TLTCLICPEK YCQK**VHTCRD GEKICFKRFY EGKRFGKKFP RGCAATCPEA KPHEIVECCS

C Carbamidomethylation (+57.02)

81 TDKCNK

**Supporting Peptides:**

| Peptide | Uniq | -10lgP | Mass | Length | ppm | m/z | z | RT | Fraction | Scan | Source File | Area F1 | Area F2a | Area F3a | Area F4 | Area F5 | Area F6a | Area F7a | Area F7b | Area F8 | Area F9a | Area Sample 1F10a | Area F10b | Area F11a | Area F11b | Area F12a | Area F13 | #Feature | #Feature F1 | #Feature F2a | #Feature F3a | #Feature F4 | #Feature F5 | #Feature F6a | #Feature F7a | #Feature F7b | #Feature F8 | #Feature F9a | #Feature Sample 1F10a | #Feature F10b | #Feature F11a | #Feature F11b | #Feature F12a | #Feature F13 | Start | End | PTM | AScore | Found By |
|---|---|---|---|---|---|---|---|---|---|---|---|---|---|---|---|---|---|---|---|---|---|---|---|---|---|---|---|---|---|---|---|---|---|---|---|---|---|---|---|---|---|---|---|---|---|---|---|---|---|
| T.LTC(+57.02)LIC(+57.02)PEKYC(+57.02)QK.V | Y | 70.27 | 1711.8096 | 13 | -1.4 | 856.9109 | 2 | 49.62 | 6 | F6:28451 | BuCaTN01_F6a.raw | | | | | 2.0983E6 | 3.0728E7 | | 1.9351E5 | | | | | | | | | 4 | 0 | 0 | 0 | 0 | 1 | 2 | 0 | 1 | 0 | 0 | 0 | 0 | 0 | 0 | 0 | 0 | 22 | 34 | Carbamidomethylation | C3:Carbamidomethylation:1000.00;C6:Carbamidomethylation:1000.00;C11:Carbamidomethylation:1000.00 | PEAKS DB |
| T.LTC(+57.02)LIC(+57.02)PEK.Y | Y | 58.09 | 1132.5620 | 9 | 1.1 | 567.2889 | 2 | 45.40 | 6 | F6:24566 | BuCaTN01_F6a.raw | | | 1.5108E6 | | 3.9943E7 | 5.2042E6 | 0 | 9.1618E5 | | | | | | | | | 4 | 0 | 0 | 1 | 0 | 1 | 1 | 0 | 1 | 0 | 0 | 0 | 0 | 0 | 0 | 0 | 0 | 22 | 30 | Carbamidomethylation | C3:Carbamidomethylation:1000.00;C6:Carbamidomethylation:1000.00 | PEAKS DB |
| G.YTLTC(+57.02)LIC(+57.02)PEKYC(+57.02)QK.V | Y | 42.49 | 1975.9205 | 15 | 0.2 | 659.6476 | 3 | 64.39 | 6 | F6:42719 | BuCaTN01_F6a.raw | | | | | | 2.6811E6 | | | | | | | | | | | 1 | 0 | 0 | 0 | 0 | 0 | 1 | 0 | 0 | 0 | 0 | 0 | 0 | 0 | 0 | 0 | 0 | 20 | 34 | Carbamidomethylation | C5:Carbamidomethylation:1000.00;C8:Carbamidomethylation:1000.00;C13:Carbamidomethylation:1000.00 | PEAKS DB |
| total 3 peptides | | | | | | | | | | | | | | | | | | | | | | | | | | | | | | | | | | | | | | | | | | | | | | | | | |

**Best Unique PSM (Scan F6:28451, m/z=856.9109, z=2, RT=49.62, ppm=-1.4):**

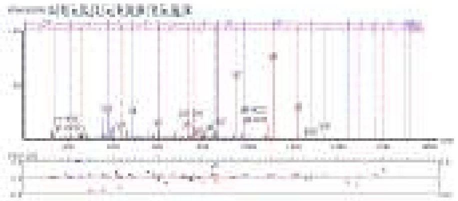

## Q6IZ95.1

back to list

| Protein Coverage | Supporting Peptides | Best Unique PSM |

**Protein Coverage:**

1 MKTLLLTLVV VTIVCLDLGY TLTCLICPEK YCQKVHTCRD GENLCVKRFY EGKRFGKKYP RGCAATCPEA KPHEIVECCS

81 TDKCNK

C Carbamidomethylation (+57.02)

**Supporting Peptides:**

| Peptide | Uniq | -10lgP | Mass | Length | ppm | m/z | z | RT | Fraction | Scan | Source File | Area F1 | Area F2a | Area F3a | Area F4 | Area F5 | Area F6a | Area F7a | Area F7b | Area F8 | Area F9a | Area Sample 1F10a | Area F10b | Area F11a | Area F11b | Area F12a | Area F13 | #Feature | #Feature F1 | #Feature F2a | #Feature F3a | #Feature F4 | #Feature F5 | #Feature F6a | #Feature F7a | #Feature F7b | #Feature F8 | #Feature F9a | #Feature Sample 1F10a | #Feature F10b | #Feature F11a | #Feature F11b | #Feature F12a | #Feature F13 | Start | End | PTM | AScore | Found By |
|---|---|---|---|---|---|---|---|---|---|---|---|---|---|---|---|---|---|---|---|---|---|---|---|---|---|---|---|---|---|---|---|---|---|---|---|---|---|---|---|---|---|---|---|---|---|---|---|---|---|
| T.LTC(+57.02)LIC(+57.02)PEKYC(+57.02)QK.V | Y | 70.27 | 1711.8096 | 13 | -1.4 | 856.9109 | 2 | 49.62 | 6 | F6:28451 | BuCaTN01_F6a.raw | | | | | 2.0983E6 | 3.0728E7 | | 1.9351E5 | | | | | | | | | 4 | 0 | 0 | 0 | 0 | 1 | 2 | 0 | 1 | 0 | 0 | 0 | 0 | 0 | 0 | 0 | 0 | 22 | 34 | Carbamidomethylation | C3:Carbamidomethylation:1000.00;C6:Carbamidomethylation:1000.00;C11:Carbamidomethylation:1000.00 | PEAKS DB |
| T.LTC(+57.02)LIC(+57.02)PEK.Y | Y | 58.09 | 1132.5620 | 9 | 1.1 | 567.2889 | 2 | 45.40 | 6 | F6:24566 | BuCaTN01_F6a.raw | | | 1.5108E6 | | 3.9943E7 | 5.2042E6 | 0 | 9.1618E5 | | | | | | | | | 4 | 0 | 0 | 1 | 0 | 1 | 1 | 0 | 1 | 0 | 0 | 0 | 0 | 0 | 0 | 0 | 0 | 22 | 30 | Carbamidomethylation | C3:Carbamidomethylation:1000.00;C6:Carbamidomethylation:1000.00 | PEAKS DB |
| G.YTLTC(+57.02)LIC(+57.02)PEKYC(+57.02)QK.V | Y | 42.49 | 1975.9205 | 15 | 0.2 | 659.6476 | 3 | 64.39 | 6 | F6:42719 | BuCaTN01_F6a.raw | | | | | | 2.6811E6 | | | | | | | | | | | 1 | 0 | 0 | 0 | 0 | 0 | 1 | 0 | 0 | 0 | 0 | 0 | 0 | 0 | 0 | 0 | 0 | 20 | 34 | Carbamidomethylation | C5:Carbamidomethylation:1000.00;C8:Carbamidomethylation:1000.00;C13:Carbamidomethylation:1000.00 | PEAKS DB |
| total 3 peptides | | | | | | | | | | | | | | | | | | | | | | | | | | | | | | | | | | | | | | | | | | | | | | | | | |

**Best Unique PSM (Scan F6:28451, m/z=856.9109, z=2, RT=49.62, ppm=-1.4):**

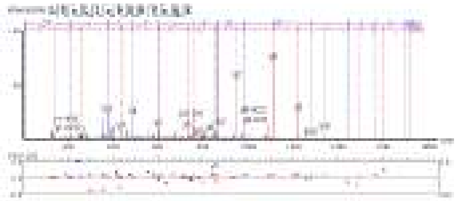

## ADF50021.1

back to list

| Protein Coverage | Supporting Peptides | Best Unique PSM |

**Protein Coverage:**

1 MKTLLLNLVG VTIVCLDLGY TLTCLICPEK YCQKVHTCRD GEKICFKRFY EGKRFGKKFP RGCAATCPEA KPHEIVECCS

81 TDKCNK

C Carbamidomethylation (+57.02)

**Supporting Peptides:**

| Peptide | Uniq | -10lgP | Mass | Length | ppm | m/z | z | RT | Fraction | Scan | Source File | Area F1 | Area F2a | Area F3a | Area F4 | Area F5 | Area F6a | Area F7a | Area F7b | Area F8 | Area F9a | Area Sample 1F10a | Area F10b | Area F11a | Area F11b | Area F12a | Area F13 | #Feature | #Feature F1 | #Feature F2a | #Feature F3a | #Feature F4 | #Feature F5 | #Feature F6a | #Feature F7a | #Feature F7b | #Feature F8 | #Feature F9a | #Feature Sample 1F10a | #Feature F10b | #Feature F11a | #Feature F11b | #Feature F12a | #Feature F13 | Start | End | PTM | AScore | Found By |
|---|---|---|---|---|---|---|---|---|---|---|---|---|---|---|---|---|---|---|---|---|---|---|---|---|---|---|---|---|---|---|---|---|---|---|---|---|---|---|---|---|---|---|---|---|---|---|---|---|---|
| T.LTC(+57.02)LIC(+57.02)PEKYC(+57.02)QK.V | Y | 70.27 | 1711.8096 | 13 | -1.4 | 856.9109 | 2 | 49.62 | 6 | F6:28451 | BuCaTN01_F6a.raw | | | | | 2.0983E6 | 3.0728E7 | | 1.9351E5 | | | | | | | | | 4 | 0 | 0 | 0 | 0 | 1 | 2 | 0 | 1 | 0 | 0 | 0 | 0 | 0 | 0 | 0 | 0 | 22 | 34 | Carbamidomethylation | C3:Carbamidomethylation:1000.00;C6:Carbamidomethylation:1000.00;C11:Carbamidomethylation:1000.00 | PEAKS DB |
| T.LTC(+57.02)LIC(+57.02)PEK.Y | Y | 58.09 | 1132.5620 | 9 | 1.1 | 567.2889 | 2 | 45.40 | 6 | F6:24566 | BuCaTN01_F6a.raw | | | 1.5108E6 | | 3.9943E7 | 5.2042E6 | 0 | 9.1618E5 | | | | | | | | | 4 | 0 | 0 | 1 | 0 | 1 | 1 | 0 | 1 | 0 | 0 | 0 | 0 | 0 | 0 | 0 | 0 | 22 | 30 | Carbamidomethylation | C3:Carbamidomethylation:1000.00;C6:Carbamidomethylation:1000.00 | PEAKS DB |
| G.YTLTC(+57.02)LIC(+57.02)PEKYC(+57.02)QK.V | Y | 42.49 | 1975.9205 | 15 | 0.2 | 659.6476 | 3 | 64.39 | 6 | F6:42719 | BuCaTN01_F6a.raw | | | | | | 2.6811E6 | | | | | | | | | | | 1 | 0 | 0 | 0 | 0 | 0 | 1 | 0 | 0 | 0 | 0 | 0 | 0 | 0 | 0 | 0 | 0 | 20 | 34 | Carbamidomethylation | C5:Carbamidomethylation:1000.00;C8:Carbamidomethylation:1000.00;C13:Carbamidomethylation:1000.00 | PEAKS DB |
| total 3 peptides | | | | | | | | | | | | | | | | | | | | | | | | | | | | | | | | | | | | | | | | | | | | | | | | | |

**Best Unique PSM (Scan F6:28451, m/z=856.9109, z=2, RT=49.62, ppm=-1.4):**

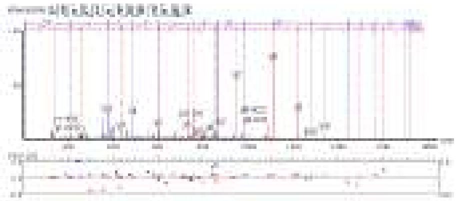

## AAT38875.1

back to list

| Protein Coverage | Supporting Peptides | Best Unique PSM |

**Protein Coverage:**

1 MKTLLLTLVV VTIVCLDLGY TLTCLICPEK YCQKVHTCRD GENLCVKRFY EGKRFGKKYP RGCAATCPEA KPHEIVECCS

81 TDKCNK

C Carbamidomethylation (+57.02)

**Supporting Peptides:**

| Peptide | Uniq | -10lgP | Mass | Length | ppm | m/z | z | RT | Fraction | Scan | Source File | Area F1 | Area F2a | Area F3a | Area F4 | Area F5 | Area F6a | Area F7a | Area F7b | Area F8 | Area F9a | Area Sample 1F10a | Area F10b | Area F11a | Area F11b | Area F12a | Area F13 | #Feature | #Feature F1 | #Feature F2a | #Feature F3a | #Feature F4 | #Feature F5 | #Feature F6a | #Feature F7a | #Feature F7b | #Feature F8 | #Feature F9a | #Feature Sample 1F10a | #Feature F10b | #Feature F11a | #Feature F11b | #Feature F12a | #Feature F13 | Start | End | PTM | AScore | Found By |
|---|---|---|---|---|---|---|---|---|---|---|---|---|---|---|---|---|---|---|---|---|---|---|---|---|---|---|---|---|---|---|---|---|---|---|---|---|---|---|---|---|---|---|---|---|---|---|---|---|---|
| T.LTC(+57.02)LIC(+57.02)PEKYC(+57.02)QK.V | Y | 70.27 | 1711.8096 | 13 | -1.4 | 856.9109 | 2 | 49.62 | 6 | F6:28451 | BuCaTN01_F6a.raw | | | | | 2.0983E6 | 3.0728E7 | | 1.9351E5 | | | | | | | | | 4 | 0 | 0 | 0 | 0 | 1 | 2 | 0 | 1 | 0 | 0 | 0 | 0 | 0 | 0 | 0 | 0 | 22 | 34 | Carbamidomethylation | C3:Carbamidomethylation:1000.00;C6:Carbamidomethylation:1000.00;C11:Carbamidomethylation:1000.00 | PEAKS DB |
| T.LTC(+57.02)LIC(+57.02)PEK.Y | Y | 58.09 | 1132.5620 | 9 | 1.1 | 567.2889 | 2 | 45.40 | 6 | F6:24566 | BuCaTN01_F6a.raw | | | 1.5108E6 | | 3.9943E7 | 5.2042E6 | 0 | 9.1618E5 | | | | | | | | | 4 | 0 | 0 | 1 | 0 | 1 | 1 | 0 | 1 | 0 | 0 | 0 | 0 | 0 | 0 | 0 | 0 | 22 | 30 | Carbamidomethylation | C3:Carbamidomethylation:1000.00;C6:Carbamidomethylation:1000.00 | PEAKS DB |
| G.YTLTC(+57.02)LIC(+57.02)PEKYC(+57.02)QK.V | Y | 42.49 | 1975.9205 | 15 | 0.2 | 659.6476 | 3 | 64.39 | 6 | F6:42719 | BuCaTN01_F6a.raw | | | | | | 2.6811E6 | | | | | | | | | | | 1 | 0 | 0 | 0 | 0 | 0 | 1 | 0 | 0 | 0 | 0 | 0 | 0 | 0 | 0 | 0 | 0 | 20 | 34 | Carbamidomethylation | C5:Carbamidomethylation:1000.00;C8:Carbamidomethylation:1000.00;C13:Carbamidomethylation:1000.00 | PEAKS DB |
| total 3 peptides | | | | | | | | | | | | | | | | | | | | | | | | | | | | | | | | | | | | | | | | | | | | | | | | | |

**Best Unique PSM (Scan F6:28451, m/z=856.9109, z=2, RT=49.62, ppm=-1.4):**

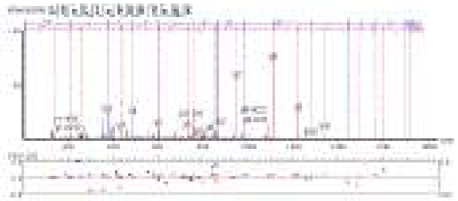

## A2CKF7.1

back to list

| Protein Coverage | Supporting Peptides | Best Unique PSM |

**Protein Coverage:**

1 MKTLLLTLVV VTIVCLDLGY TLTCLICPEK YCQKVHTCRD GENLCVKRFY EGKRFGKKYP RGCAATCPEA KPHEIVECCS

81 TDKCNK

C Carbamidomethylation (+57.02)

**Supporting Peptides:**

| Peptide | Uniq | -10lgP | Mass | Length | ppm | m/z | z | RT | Fraction | Scan | Source File | Area F1 | Area F2a | Area F3a | Area F4 | Area F5 | Area F6a | Area F7a | Area F7b | Area F8 | Area F9a | Area Sample 1F10a | Area F10b | Area F11a | Area F11b | Area F12a | Area F13 | #Feature | #Feature F1 | #Feature F2a | #Feature F3a | #Feature F4 | #Feature F5 | #Feature F6a | #Feature F7a | #Feature F7b | #Feature F8 | #Feature F9a | #Feature Sample 1F10a | #Feature F10b | #Feature F11a | #Feature F11b | #Feature F12a | #Feature F13 | Start | End | PTM | AScore | Found By |
|---|---|---|---|---|---|---|---|---|---|---|---|---|---|---|---|---|---|---|---|---|---|---|---|---|---|---|---|---|---|---|---|---|---|---|---|---|---|---|---|---|---|---|---|---|---|---|---|---|---|
| T.LTC(+57.02)LIC(+57.02)PEKYC(+57.02)QK.V | Y | 70.27 | 1711.8096 | 13 | -1.4 | 856.9109 | 2 | 49.62 | 6 | F6:28451 | BuCaTN01_F6a.raw | | | | | 2.0983E6 | 3.0728E7 | | 1.9351E5 | | | | | | | | | 4 | 0 | 0 | 0 | 0 | 1 | 2 | 0 | 1 | 0 | 0 | 0 | 0 | 0 | 0 | 0 | 0 | 22 | 34 | Carbamidomethylation | C3:Carbamidomethylation:1000.00;C6:Carbamidomethylation:1000.00;C11:Carbamidomethylation:1000.00 | PEAKS DB |
| T.LTC(+57.02)LIC(+57.02)PEK.Y | Y | 58.09 | 1132.5620 | 9 | 1.1 | 567.2889 | 2 | 45.40 | 6 | F6:24566 | BuCaTN01_F6a.raw | | | 1.5108E6 | | 3.9943E7 | 5.2042E6 | 0 | 9.1618E5 | | | | | | | | | 4 | 0 | 0 | 1 | 0 | 1 | 1 | 0 | 1 | 0 | 0 | 0 | 0 | 0 | 0 | 0 | 0 | 22 | 30 | Carbamidomethylation | C3:Carbamidomethylation:1000.00;C6:Carbamidomethylation:1000.00 | PEAKS DB |
| G.YTLTC(+57.02)LIC(+57.02)PEKYC(+57.02)QK.V | Y | 42.49 | 1975.9205 | 15 | 0.2 | 659.6476 | 3 | 64.39 | 6 | F6:42719 | BuCaTN01_F6a.raw | | | | | | 2.6811E6 | | | | | | | | | | | 1 | 0 | 0 | 0 | 0 | 0 | 1 | 0 | 0 | 0 | 0 | 0 | 0 | 0 | 0 | 0 | 0 | 20 | 34 | Carbamidomethylation | C5:Carbamidomethylation:1000.00;C8:Carbamidomethylation:1000.00;C13:Carbamidomethylation:1000.00 | PEAKS DB |
| total 3 peptides | | | | | | | | | | | | | | | | | | | | | | | | | | | | | | | | | | | | | | | | | | | | | | | | | |

**Best Unique PSM (Scan F6:28451, m/z=856.9109, z=2, RT=49.62, ppm=-1.4):**

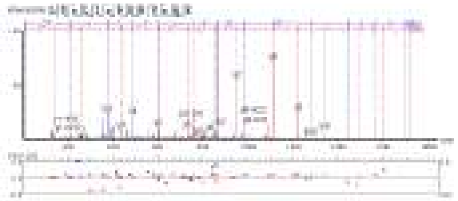

## ABI33871.1

back to list

| Protein Coverage | Supporting Peptides | Best Unique PSM |

**Protein Coverage:**

1 MKTLLLTLVV VTIVCLDLGY TLTCLICPEK YCQKVHTCRD GENLCVKRFY EGKRFGKKYP RGCAATCPEA KPHEIVECCS

81 TDKCNK

C Carbamidomethylation (+57.02)

**Supporting Peptides:**

| Peptide | Uniq | -10lgP | Mass | Length | ppm | m/z | z | RT | Fraction | Scan | Source File | Area F1 | Area F2a | Area F3a | Area F4 | Area F5 | Area F6a | Area F7a | Area F7b | Area F8 | Area F9a | Area Sample 1F10a | Area F10b | Area F11a | Area F11b | Area F12a | Area F13 | #Feature | #Feature F1 | #Feature F2a | #Feature F3a | #Feature F4 | #Feature F5 | #Feature F6a | #Feature F7a | #Feature F7b | #Feature F8 | #Feature F9a | #Feature Sample 1F10a | #Feature F10b | #Feature F11a | #Feature F11b | #Feature F12a | #Feature F13 | Start | End | PTM | AScore | Found By |
|---|---|---|---|---|---|---|---|---|---|---|---|---|---|---|---|---|---|---|---|---|---|---|---|---|---|---|---|---|---|---|---|---|---|---|---|---|---|---|---|---|---|---|---|---|---|---|---|---|---|
| T.LTC(+57.02)LIC(+57.02)PEKYC(+57.02)QK.V | Y | 70.27 | 1711.8096 | 13 | -1.4 | 856.9109 | 2 | 49.62 | 6 | F6:28451 | BuCaTN01_F6a.raw | | | | | 2.0983E6 | 3.0728E7 | | 1.9351E5 | | | | | | | | | 4 | 0 | 0 | 0 | 0 | 1 | 2 | 0 | 1 | 0 | 0 | 0 | 0 | 0 | 0 | 0 | 0 | 22 | 34 | Carbamidomethylation | C3:Carbamidomethylation:1000.00;C6:Carbamidomethylation:1000.00;C11:Carbamidomethylation:1000.00 | PEAKS DB |
| T.LTC(+57.02)LIC(+57.02)PEK.Y | Y | 58.09 | 1132.5620 | 9 | 1.1 | 567.2889 | 2 | 45.40 | 6 | F6:24566 | BuCaTN01_F6a.raw | | | 1.5108E6 | | 3.9943E7 | 5.2042E6 | 0 | 9.1618E5 | | | | | | | | | 4 | 0 | 0 | 1 | 0 | 1 | 1 | 0 | 1 | 0 | 0 | 0 | 0 | 0 | 0 | 0 | 0 | 22 | 30 | Carbamidomethylation | C3:Carbamidomethylation:1000.00;C6:Carbamidomethylation:1000.00 | PEAKS DB |
| G.YTLTC(+57.02)LIC(+57.02)PEKYC(+57.02)QK.V | Y | 42.49 | 1975.9205 | 15 | 0.2 | 659.6476 | 3 | 64.39 | 6 | F6:42719 | BuCaTN01_F6a.raw | | | | | | 2.6811E6 | | | | | | | | | | | 1 | 0 | 0 | 0 | 0 | 0 | 1 | 0 | 0 | 0 | 0 | 0 | 0 | 0 | 0 | 0 | 0 | 20 | 34 | Carbamidomethylation | C5:Carbamidomethylation:1000.00;C8:Carbamidomethylation:1000.00;C13:Carbamidomethylation:1000.00 | PEAKS DB |
| total 3 peptides | | | | | | | | | | | | | | | | | | | | | | | | | | | | | | | | | | | | | | | | | | | | | | | | | |

**Best Unique PSM (Scan F6:28451, m/z=856.9109, z=2, RT=49.62, ppm=-1.4):**

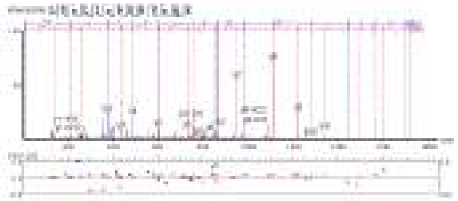

## ADF50020.1

back to list

| Protein Coverage | Supporting Peptides | Best Unique PSM |

**Protein Coverage:**

1 MKSLLLTLVV VTIVCLDLGY TLTCLICPEK YCQKVHTCQD AEKICFKRFY EGKQLGKKFP RGCAATCPEA KPHEIVECCS

81 TDKCNK

C Carbamidomethylation (+57.02)

**Supporting Peptides:**

| Peptide | Uniq | -10lgP | Mass | Length | ppm | m/z | z | RT | Fraction | Scan | Source File | Area F1 | Area F2a | Area F3a | Area F4 | Area F5 | Area F6a | Area F7a | Area F7b | Area F8 | Area F9a | Area Sample 1F10a | Area F10b | Area F11a | Area F11b | Area F12a | Area F13 | #Feature | #Feature F1 | #Feature F2a | #Feature F3a | #Feature F4 | #Feature F5 | #Feature F6a | #Feature F7a | #Feature F7b | #Feature F8 | #Feature F9a | #Feature Sample 1F10a | #Feature F10b | #Feature F11a | #Feature F11b | #Feature F12a | #Feature F13 | Start | End | PTM | AScore | Found By |
|---|---|---|---|---|---|---|---|---|---|---|---|---|---|---|---|---|---|---|---|---|---|---|---|---|---|---|---|---|---|---|---|---|---|---|---|---|---|---|---|---|---|---|---|---|---|---|---|---|---|
| T.LTC(+57.02)LIC(+57.02)PEKYC(+57.02)QK.V | Y | 70.27 | 1711.8096 | 13 | -1.4 | 856.9109 | 2 | 49.62 | 6 | F6:28451 | BuCaTN01_F6a.raw | | | | | 2.0983E6 | 3.0728E7 | | 1.9351E5 | | | | | | | | | 4 | 0 | 0 | 0 | 0 | 1 | 2 | 0 | 1 | 0 | 0 | 0 | 0 | 0 | 0 | 0 | 0 | 22 | 34 | Carbamidomethylation | C3:Carbamidomethylation:1000.00;C6:Carbamidomethylation:1000.00;C11:Carbamidomethylation:1000.00 | PEAKS DB |
| T.LTC(+57.02)LIC(+57.02)PEK.Y | Y | 58.09 | 1132.5620 | 9 | 1.1 | 567.2889 | 2 | 45.40 | 6 | F6:24566 | BuCaTN01_F6a.raw | | | 1.5108E6 | | 3.9943E7 | 5.2042E6 | 0 | 9.1618E5 | | | | | | | | | 4 | 0 | 0 | 1 | 0 | 1 | 1 | 0 | 1 | 0 | 0 | 0 | 0 | 0 | 0 | 0 | 0 | 22 | 30 | Carbamidomethylation | C3:Carbamidomethylation:1000.00;C6:Carbamidomethylation:1000.00 | PEAKS DB |
| G.YTLTC(+57.02)LIC(+57.02)PEKYC(+57.02)QK.V | Y | 42.49 | 1975.9205 | 15 | 0.2 | 659.6476 | 3 | 64.39 | 6 | F6:42719 | BuCaTN01_F6a.raw | | | | | | 2.6811E6 | | | | | | | | | | | 1 | 0 | 0 | 0 | 0 | 0 | 1 | 0 | 0 | 0 | 0 | 0 | 0 | 0 | 0 | 0 | 0 | 20 | 34 | Carbamidomethylation | C5:Carbamidomethylation:1000.00;C8:Carbamidomethylation:1000.00;C13:Carbamidomethylation:1000.00 | PEAKS DB |
| total 3 peptides | | | | | | | | | | | | | | | | | | | | | | | | | | | | | | | | | | | | | | | | | | | | | | | | | |

**Best Unique PSM (Scan F6:28451, m/z=856.9109, z=2, RT=49.62, ppm=-1.4):**

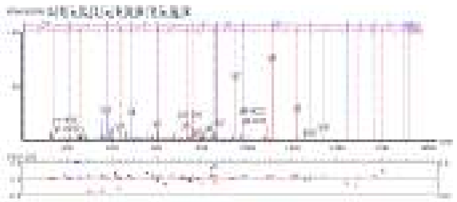

## P20255.1

back to list

| Protein Coverage | Supporting Peptides | Best Unique PSM |

**Protein Coverage:**

1 **NLIQFGNMIQ CANKGSRPSL NYADYGCYCG WGGSGTPVDE LDR**CCQVHDN CYEQAGKKGC FPKLTLYSWK CTGNVPTCNS

81 KTGCKSFVCA CDAAAAKCFA KAPYKKENYN IDTKKRCK

C Carbamidomethylation (+57.02)

**Supporting Peptides:**

| Peptide | Uniq | -10lgP | Mass | Length | ppm | m/z | z | RT | Fraction | Scan | Source File | Area F1 | Area F2a | Area F3a | Area F4 | Area F5 | Area F6a | Area F7a | Area F7b | Area F8 | Area F9a | Area Sample 1F10a | Area F10b | Area F11a | Area F11b | Area F12a | Area F13 | #Feature | #Feature F1 | #Feature F2a | #Feature F3a | #Feature F4 | #Feature F5 | #Feature F6a | #Feature F7a | #Feature F7b | #Feature F8 | #Feature F9a | #Feature Sample 1F10a | #Feature F10b | #Feature F11a | #Feature F11b | #Feature F12a | #Feature F13 | Start | End | PTM | AScore | Found By |
|---|---|---|---|---|---|---|---|---|---|---|---|---|---|---|---|---|---|---|---|---|---|---|---|---|---|---|---|---|---|---|---|---|---|---|---|---|---|---|---|---|---|---|---|---|---|---|---|---|---|
| G.GSGTPVDELDR.C | N | 56.36 | 1144.5360 | 11 | 3.4 | 573.2756 | 2 | 40.15 | 8 | F8:17921 | BuCaTN01_F7b.raw | | | | | | | | 1.1068E6 | | | | | | | | | 1 | 0 | 0 | 0 | 0 | 0 | 0 | 0 | 1 | 0 | 0 | 0 | 0 | 0 | 0 | 0 | 0 | 33 | 43 | | | PEAKS DB |
| W.GGSGTPVDELDR.C | N | 49.09 | 1201.5575 | 12 | 0.3 | 601.7862 | 2 | 40.57 | 11 | F11:14528 | BuCaTN01_F10a.raw | | | | | | | 9.2274E5 | 2.7577E6 | | 1.1335E7 | 6.7591E5 | | 9.9086E5 | 2.6759E7 | | | 6 | 0 | 0 | 0 | 0 | 0 | 0 | 1 | 1 | 0 | 1 | 1 | 0 | 1 | 1 | 0 | 0 | 32 | 43 | | | PEAKS DB |
| NLIQFGNMIQC(+57.02)ANKGSRPSLNYADYGC(+57.02)YC(+57.02)GWGGSGTPVDELDR.C | Y | 43.47 | 4840.1528 | 43 | 1.8 | 1211.0449 | 4 | 78.19 | 12 | F12:54070 | BuCaTN01_F10b.raw | | | | | | | | | | | | 1.126E5 | | | | | 1 | 0 | 0 | 0 | 0 | 0 | 0 | 0 | 0 | 0 | 0 | 0 | 1 | 0 | 0 | 0 | 0 | 1 | 43 | Carbamidomethylation | C11:Carbamidomethylation:1000.00;C27:Carbamidomethylation:1000.00;C29:Carbamidomethylation:1000.00 | PEAKS DB |
| total 3 peptides | | | | | | | | | | | | | | | | | | | | | | | | | | | | | | | | | | | | | | | | | | | | | | | | | |

**Best Unique PSM (Scan F12:54070, m/z=1211.0449, z=4, RT=78.19, ppm=1.8):**

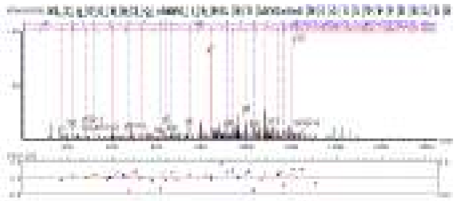

## ADF50023.1

back to list

| Protein Coverage | Supporting Peptides | Best Unique PSM |

**Protein Coverage:**

1 LISPSSTSQT CPKGQDICFT KAFCDRWCSS R**GPVIEQGCA ATCPEFTS**RY K**SLLCCTTDN CNH**

39 55 56 61

C Carbamidomethylation (+57.02)

**Supporting Peptides:**

| Peptide | Uniq | -10lgP | Mass | Length | ppm | m/z | z | RT | Fraction | Scan | Source File | Area F1 | Area F2a | Area F3a | Area F4 | Area F5 | Area F6a | Area F7a | Area F7b | Area F8 | Area F9a | Area Sample 1F10a | Area F10b | Area F11a | Area F11b | Area F12a | Area F13 | #Feature | #Feature F1 | #Feature F2a | #Feature F3a | #Feature F4 | #Feature F5 | #Feature F6a | #Feature F7a | #Feature F7b | #Feature F8 | #Feature F9a | #Feature Sample 1F10a | #Feature F10b | #Feature F11a | #Feature F11b | #Feature F12a | #Feature F13 | Start | End | PTM | AScore | Found By |
|---|---|---|---|---|---|---|---|---|---|---|---|---|---|---|---|---|---|---|---|---|---|---|---|---|---|---|---|---|---|---|---|---|---|---|---|---|---|---|---|---|---|---|---|---|---|---|---|---|---|
| K.SLLC(+57.02)C(+57.02)TTDNC(+57.02)NH | N | 79.81 | 1493.5698 | 12 | -0.3 | 747.7920 | 2 | 37.43 | 6 | F6:17551 | BuCaTN01_F6a.raw | | | 4.1658E6 | | | 7.1212E8 | 3.0441E6 | 4.2545E7 | | 1.9164E6 | | 4.7006E6 | | 5.2769E6 | 7.5251E5 | | 12 | 0 | 0 | 1 | 0 | 0 | 4 | 1 | 2 | 0 | 1 | 0 | 1 | 0 | 1 | 1 | 0 | 52 | 63 | Carbamidomethylation | C4:Carbamidomethylation:1000.00;C5:Carbamidomethylation:1000.00;C10:Carbamidomethylation:1000.00 | PEAKS DB |
| R.GPVIEQGC(+57.02)AATC(+57.02)PEFTS.R | Y | 62.63 | 1822.7866 | 17 | 5.3 | 912.4055 | 2 | 48.71 | 6 | F6:27673 | BuCaTN01_F6a.raw | | | | | | 1.2004E6 | | | | | | | | | | | 1 | 0 | 0 | 0 | 0 | 0 | 1 | 0 | 0 | 0 | 0 | 0 | 0 | 0 | 0 | 0 | 0 | 32 | 48 | Carbamidomethylation | C8:Carbamidomethylation:1000.00;C12:Carbamidomethylation:1000.00 | PEAKS DB |
| total 2 peptides | | | | | | | | | | | | | | | | | | | | | | | | | | | | | | | | | | | | | | | | | | | | | | | | | |

**Best Unique PSM (Scan F6:27673, m/z=912.4055, z=2, RT=48.71, ppm=5.3):**

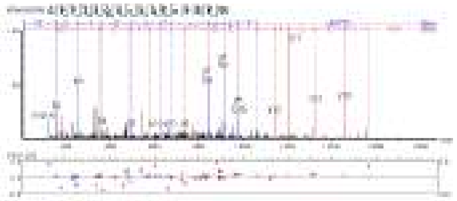

## P15815.1

back to list

| Protein Coverage | Supporting Peptides | Best Unique PSM |

**Protein Coverage:**

1 RTCLISPSST SQTCPKGQDI CFTKAFCDRW CSSR**GPVIEQ GCAATCPEFT S**RYK**SLLCCT TDNCNH**

42 58 59 64

C Carbamidomethylation (+57.02)

**Supporting Peptides:**

| Peptide | Uniq | -10lgP | Mass | Length | ppm | m/z | z | RT | Fraction | Scan | Source File | Area F1 | Area F2a | Area F3a | Area F4 | Area F5 | Area F6a | Area F7a | Area F7b | Area F8 | Area F9a | Area Sample 1F10a | Area F10b | Area F11a | Area F11b | Area F12a | Area F13 | #Feature | #Feature F1 | #Feature F2a | #Feature F3a | #Feature F4 | #Feature F5 | #Feature F6a | #Feature F7a | #Feature F7b | #Feature F8 | #Feature F9a | #Feature Sample 1F10a | #Feature F10b | #Feature F11a | #Feature F11b | #Feature F12a | #Feature F13 | Start | End | PTM | AScore | Found By |
|---|---|---|---|---|---|---|---|---|---|---|---|---|---|---|---|---|---|---|---|---|---|---|---|---|---|---|---|---|---|---|---|---|---|---|---|---|---|---|---|---|---|---|---|---|---|---|---|---|---|
| K.SLLC(+57.02)C(+57.02)TTDNC(+57.02)NH | N | 79.81 | 1493.5698 | 12 | -0.3 | 747.7920 | 2 | 37.43 | 6 | F6:17551 | BuCaTN01_F6a.raw | | | 4.1658E6 | | | 7.1212E8 | 3.0441E6 | 4.2545E7 | | 1.9164E6 | | 4.7006E6 | | 5.2769E6 | 7.5251E5 | | 12 | 0 | 0 | 1 | 0 | 0 | 4 | 1 | 2 | 0 | 1 | 0 | 1 | 0 | 1 | 1 | 0 | 55 | 66 | Carbamidomethylation | C4:Carbamidomethylation:1000.00;C5:Carbamidomethylation:1000.00;C10:Carbamidomethylation:1000.00 | PEAKS DB |
| R.GPVIEQGC(+57.02)AATC(+57.02)PEFTS.R | Y | 62.63 | 1822.7866 | 17 | 5.3 | 912.4055 | 2 | 48.71 | 6 | F6:27673 | BuCaTN01_F6a.raw | | | | | | 1.2004E6 | | | | | | | | | | | 1 | 0 | 0 | 0 | 0 | 0 | 1 | 0 | 0 | 0 | 0 | 0 | 0 | 0 | 0 | 0 | 0 | 35 | 51 | Carbamidomethylation | C8:Carbamidomethylation:1000.00;C12:Carbamidomethylation:1000.00 | PEAKS DB |
| total 2 peptides | | | | | | | | | | | | | | | | | | | | | | | | | | | | | | | | | | | | | | | | | | | | | | | | | |

**Best Unique PSM (Scan F6:27673, m/z=912.4055, z=2, RT=48.71, ppm=5.3):**

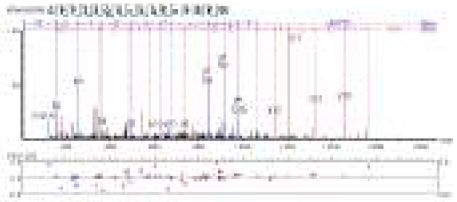

## P01383.1

back to list

| Protein Coverage | Supporting Peptides | Best Unique PSM |

**Protein Coverage:**

1 KRCYRTPDLK SQTCPPGEDL CYTKKWCADW CTSRGKVIEL GCVATCPKVK PYEQITCCST DNCNPHPKMK P

Carbamidomethylation (+57.02)

**Supporting Peptides:**

| Peptide | Uniq | -10lgP | Mass | Length | ppm | m/z | z | RT | Fraction | Scan | Source File | Area F1 | Area F2a | Area F3a | Area F4 | Area F5 | Area F6a | Area F7a | Area F7b | Area F8 | Area F9a | Area Sample 1F10a | Area F10b | Area F11a | Area F11b | Area F12a | Area F13 | #Feature | #Feature F1 | #Feature F2a | #Feature F3a | #Feature F4 | #Feature F5 | #Feature F6a | #Feature F7a | #Feature F7b | #Feature F8 | #Feature F9a | #Feature Sample 1F10a | #Feature F10b | #Feature F11a | #Feature F11b | #Feature F12a | #Feature F13 | Start | End | PTM | AScore | Found By |
|---|---|---|---|---|---|---|---|---|---|---|---|---|---|---|---|---|---|---|---|---|---|---|---|---|---|---|---|---|---|---|---|---|---|---|---|---|---|---|---|---|---|---|---|---|---|---|---|---|---|
| R.GKVIELGC(+57.02)VATC(+57.02)PKVKPYEQITC(+57.02)C(+57.02)STDNC(+57.02)NPHPK.M | Y | 59.45 | 3959.8459 | 34 | -8.0 | 792.9701 | 5 | 49.99 | 10 | F10:27338 | BuCaTN01_F9a.raw | | | | | | | | | | 1.5789E5 | | | | | | | 1 | 0 | 0 | 0 | 0 | 0 | 0 | 0 | 0 | 0 | 1 | 0 | 0 | 0 | 0 | 0 | 0 | 35 | 68 | Carbamidomethylation | C8:Carbamidomethylation:1000.00;C12:Carbamidomethylation:1000.00;C23:Carbamidomethylation:1000.00;C24:Carbamidomethylation:1000.00;C29:Carbamidomethylation:1000.00 | PEAKS DB |
| K.KWC(+57.02)ADWC(+57.02)TSR.G | Y | 47.31 | 1368.5703 | 10 | -1.7 | 457.1966 | 3 | 47.04 | 6 | F6:25907 | BuCaTN01_F6a.raw | | | | | | 3.6091E6 | | | | | | | | | | | 1 | 0 | 0 | 0 | 0 | 0 | 1 | 0 | 0 | 0 | 0 | 0 | 0 | 0 | 0 | 0 | 0 | 25 | 34 | Carbamidomethylation | C3:Carbamidomethylation:1000.00;C7:Carbamidomethylation:1000.00 | PEAKS DB |
| R.GKVIELGC(+57.02)VATC(+57.02)PKVKPYEQITC(+57.02)C(+57.02)STDN.C | Y | 45.28 | 3226.5129 | 28 | -8.8 | 1076.5021 | 3 | 54.26 | 10 | F10:31007 | BuCaTN01_F9a.raw | | | | | | | | | | 0 | | | | | | | 0 | 0 | 0 | 0 | 0 | 0 | 0 | 0 | 0 | 0 | 0 | 0 | 0 | 0 | 0 | 0 | 0 | 35 | 62 | Carbamidomethylation | C8:Carbamidomethylation:1000.00;C12:Carbamidomethylation:1000.00;C23:Carbamidomethylation:1000.00;C24:Carbamidomethylation:1000.00 | PEAKS DB |
| K.VIELGC(+57.02)VATC(+57.02)PKVKPYEQITC(+57.02)C(+57.02)STDNC(+57.02)NPHPK.M | Y | 41.15 | 3774.7295 | 32 | -5.2 | 755.9470 | 5 | 52.94 | 8 | F8:29107 | BuCaTN01_F7b.raw | | | | | | | | 0 | | | | | | | | | 0 | 0 | 0 | 0 | 0 | 0 | 0 | 0 | 0 | 0 | 0 | 0 | 0 | 0 | 0 | 0 | 0 | 37 | 68 | Carbamidomethylation | C6:Carbamidomethylation:1000.00;C10:Carbamidomethylation:1000.00;C21:Carbamidomethylation:1000.00;C22:Carbamidomethylation:1000.00;C27:Carbamidomethylation:1000.00 | PEAKS DB |
| total 4 peptides | | | | | | | | | | | | | | | | | | | | | | | | | | | | | | | | | | | | | | | | | | | | | | | | | |

**Best Unique PSM (Scan F10:27338, m/z=792.9701, z=5, RT=49.99, ppm=-8.0):**

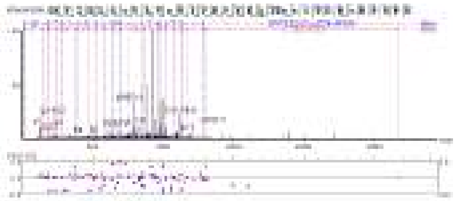

## P00605.1

back to list

| Protein Coverage | Supporting Peptides | Best Unique PSM |

**Protein Coverage:**

1 NLYQFKNMIH CTVPSRPWWH FADYGCYCGR GGKGTPVDDL DRCCQVHDNC YEKAGKMGCW PYLTLYKYKC SQGKLTCSGG

81 NSKCGAAVCN CDLVAANCFA GARYIDANYN INFKKRCQ

Carbamidomethylation (+57.02)
Oxidation (M) (+15.99)

**Supporting Peptides:**

| Peptide | Uniq | -10lgP | Mass | Length | ppm | m/z | z | RT | Fraction | Scan | Source File | Area F1 | Area F2a | Area F3a | Area F4 | Area F5 | Area F6a | Area F7a | Area F7b | Area F8 | Area F9a | Area Sample 1F10a | Area F10b | Area F11a | Area F11b | Area F12a | Area F13 | #Feature | #Feature F1 | #Feature F2a | #Feature F3a | #Feature F4 | #Feature F5 | #Feature F6a | #Feature F7a | #Feature F7b | #Feature F8 | #Feature F9a | #Feature Sample 1F10a | #Feature F10b | #Feature F11a | #Feature F11b | #Feature F12a | #Feature F13 | Start | End | PTM | AScore | Found By |
|---|---|---|---|---|---|---|---|---|---|---|---|---|---|---|---|---|---|---|---|---|---|---|---|---|---|---|---|---|---|---|---|---|---|---|---|---|---|---|---|---|---|---|---|---|---|---|---|---|---|
| K.M(+15.99)GC(+57.02)WPYLTLYK.Y | Y | 65.21 | 1446.6676 | 11 | -0.3 | 724.3409 | 2 | 74.24 | 10 | F10:50779 | BuCaTN01_F9a.raw | | | | | | | | | | 0 | | | | | | | 0 | 0 | 0 | 0 | 0 | 0 | 0 | 0 | 0 | 0 | 0 | 0 | 0 | 0 | 0 | 0 | 0 | 57 | 67 | Oxidation (M); Carbamidomethylation | M1:Oxidation (M):1000.00;C3:Carbamidomethylation:1000.00 | PEAKS DB |
| K.GTPVDDLDR.C | N | 51.39 | 986.4669 | 9 | 0.6 | 494.2410 | 2 | 37.62 | 7 | F7:14221 | BuCaTN01_F7a.raw | | | | | | | 4.0904E6 | | | | | | | | | | 1 | 0 | 0 | 0 | 0 | 0 | 0 | 1 | 0 | 0 | 0 | 0 | 0 | 0 | 0 | 0 | 0 | 34 | 42 | | | PEAKS DB |
| G.TPVDDLDR.C | N | 48.70 | 929.4454 | 8 | -0.4 | 465.7298 | 2 | 34.76 | 7 | F7:11899 | BuCaTN01_F7a.raw | | | | | | | 6.3757E5 | | | | | | | | | | 1 | 0 | 0 | 0 | 0 | 0 | 0 | 1 | 0 | 0 | 0 | 0 | 0 | 0 | 0 | 0 | 0 | 35 | 42 | | | PEAKS DB |
| total 3 peptides | | | | | | | | | | | | | | | | | | | | | | | | | | | | | | | | | | | | | | | | | | | | | | | | | |

**Best Unique PSM (Scan F10:50779, m/z=724.3409, z=2, RT=74.24, ppm=-0.3):**

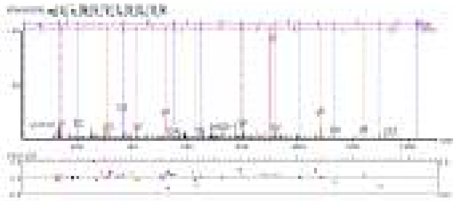

## PSNJ3B

back to list

| Protein Coverage | Supporting Peptides | Best Unique PSM |

**Protein Coverage:**

1 NLYQFKNMIH CTVPSRPWWH FADYGCYCGR GGKGTPVDDL DRCCQVHDNC YEKAGKMGCW PYLTLYKYKC SQGKLTCSGG

81 NSKCGAAVCN CDLVAANCFA GARYIDANYN INFKKRCQ

Carbamidomethylation (+57.02)
Oxidation (M) (+15.99)

**Supporting Peptides:**

| Peptide | Uniq | -10lgP | Mass | Length | ppm | m/z | z | RT | Fraction | Scan | Source File | Area F1 | Area F2a | Area F3a | Area F4 | Area F5 | Area F6a | Area F7a | Area F7b | Area F8 | Area F9a | Area Sample 1F10a | Area F10b | Area F11a | Area F11b | Area F12a | Area F13 | #Feature | #Feature F1 | #Feature F2a | #Feature F3a | #Feature F4 | #Feature F5 | #Feature F6a | #Feature F7a | #Feature F7b | #Feature F8 | #Feature F9a | #Feature Sample 1F10a | #Feature F10b | #Feature F11a | #Feature F11b | #Feature F12a | #Feature F13 | Start | End | PTM | AScore | Found By |
|---|---|---|---|---|---|---|---|---|---|---|---|---|---|---|---|---|---|---|---|---|---|---|---|---|---|---|---|---|---|---|---|---|---|---|---|---|---|---|---|---|---|---|---|---|---|---|---|---|---|
| K.M(+15.99)GC(+57.02)WPYLTLYK.Y | Y | 65.21 | 1446.6676 | 11 | -0.3 | 724.3409 | 2 | 74.24 | 10 | F10:50779 | BuCaTN01_F9a.raw | | | | | | | | | | 0 | | | | | | | 0 | 0 | 0 | 0 | 0 | 0 | 0 | 0 | 0 | 0 | 0 | 0 | 0 | 0 | 0 | 0 | 0 | 57 | 67 | Oxidation (M); Carbamidomethylation | M1:Oxidation (M):1000.00;C3:Carbamidomethylation:1000.00 | PEAKS DB |
| K.GTPVDDLDR.C | N | 51.39 | 986.4669 | 9 | 0.6 | 494.2410 | 2 | 37.62 | 7 | F7:14221 | BuCaTN01_F7a.raw | | | | | | | 4.0904E6 | | | | | | | | | | 1 | 0 | 0 | 0 | 0 | 0 | 0 | 1 | 0 | 0 | 0 | 0 | 0 | 0 | 0 | 0 | 0 | 34 | 42 | | | PEAKS DB |
| G.TPVDDLDR.C | N | 48.70 | 929.4454 | 8 | -0.4 | 465.7298 | 2 | 34.76 | 7 | F7:11899 | BuCaTN01_F7a.raw | | | | | | | 6.3757E5 | | | | | | | | | | 1 | 0 | 0 | 0 | 0 | 0 | 0 | 1 | 0 | 0 | 0 | 0 | 0 | 0 | 0 | 0 | 0 | 35 | 42 | | | PEAKS DB |
| total 3 peptides | | | | | | | | | | | | | | | | | | | | | | | | | | | | | | | | | | | | | | | | | | | | | | | | | |

**Best Unique PSM (Scan F10:50779, m/z=724.3409, z=2, RT=74.24, ppm=-0.3):**

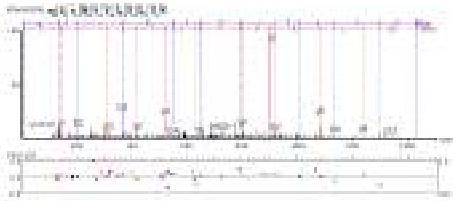

## UNW14928.1

back to list

| Protein Coverage | Supporting Peptides | Best Unique PSM |

**Protein Coverage:**

1 HPAHLLVPLA VCVSLLGAAR IPPLPLSLLQ FRILIKCANH NSRDVLDYAN YGCYCGKGGS GTPVDELDRC CQAHDYCYDD

81 AEKLPACNYR FSGPYWNPYS YKCNEGEVTC TDDNDECKAF ICNCDRTAAI CFAGALTTTK TS

Carbamidomethylation (+57.02)

**Supporting Peptides:**

| Peptide | Uniq | -10lgP | Mass | Length | ppm | m/z | z | RT | Fraction | Scan | Source File | Area F1 | Area F2a | Area F3a | Area F4 | Area F5 | Area F6a | Area F7a | Area F7b | Area F8 | Area F9a | Area Sample 1F10a | Area F10b | Area F11a | Area F11b | Area F12a | Area F13 | #Feature | #Feature F1 | #Feature F2a | #Feature F3a | #Feature F4 | #Feature F5 | #Feature F6a | #Feature F7a | #Feature F7b | #Feature F8 | #Feature F9a | #Feature Sample 1F10a | #Feature F10b | #Feature F11a | #Feature F11b | #Feature F12a | #Feature F13 | Start | End | PTM | AScore | Found By |
|---|---|---|---|---|---|---|---|---|---|---|---|---|---|---|---|---|---|---|---|---|---|---|---|---|---|---|---|---|---|---|---|---|---|---|---|---|---|---|---|---|---|---|---|---|---|---|---|---|---|
| G.GSGTPVDELDR.C | N | 56.36 | 1144.5360 | 11 | 3.4 | 573.2756 | 2 | 40.15 | 8 | F8:17921 | BuCaTN01_F7b.raw | | | | | | | | 1.1068E6 | | | | | | | | | 1 | 0 | 0 | 0 | 0 | 0 | 0 | 0 | 1 | 0 | 0 | 0 | 0 | 0 | 0 | 0 | 0 | 59 | 69 | | | PEAKS DB |
| R.DVLDYANYGC(+57.02)YC(+57.02)GKGGSGTPVDELDR.C | Y | 50.95 | 2880.2329 | 26 | 8.2 | 961.0906 | 3 | 76.66 | 12 | F12:52584 | BuCaTN01_F10b.raw | | | | | | | | | | 4.3389E7 | | 1.3553E8 | | | | | 3 | 0 | 0 | 0 | 0 | 0 | 0 | 0 | 0 | 0 | 1 | 0 | 2 | 0 | 0 | 0 | 0 | 44 | 69 | Carbamidomethylation | C10:Carbamidomethylation:1000.00;C12:Carbamidomethylation:1000.00 | PEAKS DB |
| K.GGSGTPVDELDR.C | N | 49.09 | 1201.5575 | 12 | 0.3 | 601.7862 | 2 | 40.57 | 11 | F11:14528 | BuCaTN01_F10a.raw | | | | | | | 9.2274E5 | 2.7577E6 | | 1.1335E7 | 6.7591E5 | | 9.9086E5 | 2.6759E7 | | | 6 | 0 | 0 | 0 | 0 | 0 | 0 | 1 | 1 | 0 | 1 | 1 | 0 | 1 | 1 | 0 | 0 | 58 | 69 | | | PEAKS DB |
| total 3 peptides | | | | | | | | | | | | | | | | | | | | | | | | | | | | | | | | | | | | | | | | | | | | | | | | | |

**Best Unique PSM (Scan F12:52584, m/z=961.0906, z=3, RT=76.66, ppm=8.2):**

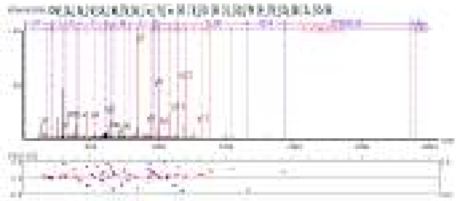

## QSI84030.1

[back to list]

| **Protein Coverage** | **Supporting Peptides** | **Best Unique PSM** |

**Protein Coverage:**

```
 1  MTPAHLLVLA AVCVSFLAAS AIPPQPLSHC QFKKMIKCTV SKSLSWRSFV DYGCYCGKGG SGTPVDELDR CCQTHDNCYT
81  EAEKQPACNS FCEGPYYKIY SYTCSEGTLT CTDKKDECAA FVCNCDRVAA NCFAKAPYNK ENVNIDTKKR CK
```

C Carbamidomethylation (+57.02)

**Supporting Peptides:**

| Peptide | Uniq | -10lgP | Mass | Length | ppm | m/z | z | RT | Fraction | Scan | Source File | Area F1 | Area F2a | Area F3a | Area F4 | Area F5 | Area F6a | Area F7a | Area F7b | Area F8 | Area F9a | Area Sample 1F10a | Area F10b | Area F11a | Area F11b | Area F12a | Area F13 | #Feature | #Feature F1 | #Feature F2a | #Feature F3a | #Feature F4 | #Feature F5 | #Feature F6a | #Feature F7a | #Feature F7b | #Feature F8 | #Feature F9a | #Feature Sample 1F10a | #Feature F10b | #Feature F11a | #Feature F11b | #Feature F12a | #Feature F13 | Start | End | PTM | AScore | Found By |
|---|---|---|---|---|---|---|---|---|---|---|---|---|---|---|---|---|---|---|---|---|---|---|---|---|---|---|---|---|---|---|---|---|---|---|---|---|---|---|---|---|---|---|---|---|---|---|---|---|---|
| G.GSGTPVDELDR.C | N | 56.36 | 1144.5360 | 11 | 3.4 | 573.2756 | 2 | 40.15 | 8 | F8:17921 | BuCaTN01_F7b.raw | | | | | | | | 1.1068E6 | | | | | | | | | 1 | 0 | 0 | 0 | 0 | 0 | 0 | 0 | 1 | 0 | 0 | 0 | 0 | 0 | 0 | 0 | 0 | 60 | 70 | | | PEAKS DB |
| S.WRSFVDYGC(+57.02)YC(+57.02)GKGGSGTPVDELDR.C | Y | 50.71 | 2880.2595 | 25 | 1.0 | 961.0908 | 3 | 76.53 | 14 | F14:51287 | BuCaTN01_F11b.raw | | | | | | | | | | | | | | 4.582E7 | | | 1 | 0 | 0 | 0 | 0 | 0 | 0 | 0 | 0 | 0 | 0 | 0 | 0 | 0 | 1 | 0 | 0 | 46 | 70 | Carbamidomethylation | C9:Carbamidomethylation:1000.00;C11:Carbamidomethylation:1000.00 | PEAKS DB |
| K.GGSGTPVDELDR.C | N | 49.09 | 1201.5575 | 12 | 0.3 | 601.7862 | 2 | 40.57 | 11 | F11:14528 | BuCaTN01_F10a.raw | | | | | | | 9.2274E5 | 2.7577E6 | | 1.1335E7 | 6.7591E5 | | 9.9086E5 | 2.6759E7 | | | 6 | 0 | 0 | 0 | 0 | 0 | 0 | 1 | 1 | 0 | 1 | 1 | 0 | 1 | 1 | 0 | 0 | 59 | 70 | | | PEAKS DB |
| total 3 peptides | | | | | | | | | | | | | | | | | | | | | | | | | | | | | | | | | | | | | | | | | | | | | | | | | |

**Best Unique PSM (Scan F14:51287, m/z=961.0908, z=3, RT=76.53, ppm=1.0):**

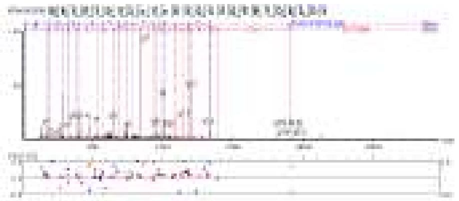

## QSI84025.1

[back to list]

| **Protein Coverage** | **Supporting Peptides** | **Best Unique PSM** |

**Protein Coverage:**

```
 1  MTPAHLLVLA AVCVSLLAAS AIPPQPLNLY QFGNMIQCTI PKRLSWRSFV DYGCYCGKGG SGTPVDELDR CCQTHDNCYT
81  EAEKQPACNS FWEGPYYKIY SYTCSEGTLT CTDKKDECAA FVCNCDRVAA NCFAKAPYNK ENVNIDTKKR CK
```

C Carbamidomethylation (+57.02)

**Supporting Peptides:**

| Peptide | Uniq | -10lgP | Mass | Length | ppm | m/z | z | RT | Fraction | Scan | Source File | Area F1 | Area F2a | Area F3a | Area F4 | Area F5 | Area F6a | Area F7a | Area F7b | Area F8 | Area F9a | Area Sample 1F10a | Area F10b | Area F11a | Area F11b | Area F12a | Area F13 | #Feature | #Feature F1 | #Feature F2a | #Feature F3a | #Feature F4 | #Feature F5 | #Feature F6a | #Feature F7a | #Feature F7b | #Feature F8 | #Feature F9a | #Feature Sample 1F10a | #Feature F10b | #Feature F11a | #Feature F11b | #Feature F12a | #Feature F13 | Start | End | PTM | AScore | Found By |
|---|---|---|---|---|---|---|---|---|---|---|---|---|---|---|---|---|---|---|---|---|---|---|---|---|---|---|---|---|---|---|---|---|---|---|---|---|---|---|---|---|---|---|---|---|---|---|---|---|---|
| G.GSGTPVDELDR.C | N | 56.36 | 1144.5360 | 11 | 3.4 | 573.2756 | 2 | 40.15 | 8 | F8:17921 | BuCaTN01_F7b.raw | | | | | | | | 1.1068E6 | | | | | | | | | 1 | 0 | 0 | 0 | 0 | 0 | 0 | 0 | 1 | 0 | 0 | 0 | 0 | 0 | 0 | 0 | 0 | 60 | 70 | | | PEAKS DB |
| S.WRSFVDYGC(+57.02)YC(+57.02)GKGGSGTPVDELDR.C | Y | 50.71 | 2880.2595 | 25 | 1.0 | 961.0908 | 3 | 76.53 | 14 | F14:51287 | BuCaTN01_F11b.raw | | | | | | | | | | | | | | 4.582E7 | | | 1 | 0 | 0 | 0 | 0 | 0 | 0 | 0 | 0 | 0 | 0 | 0 | 0 | 0 | 1 | 0 | 0 | 46 | 70 | Carbamidomethylation | C9:Carbamidomethylation:1000.00;C11:Carbamidomethylation:1000.00 | PEAKS DB |
| K.GGSGTPVDELDR.C | N | 49.09 | 1201.5575 | 12 | 0.3 | 601.7862 | 2 | 40.57 | 11 | F11:14528 | BuCaTN01_F10a.raw | | | | | | | 9.2274E5 | 2.7577E6 | | 1.1335E7 | 6.7591E5 | | 9.9086E5 | 2.6759E7 | | | 6 | 0 | 0 | 0 | 0 | 0 | 0 | 1 | 1 | 0 | 1 | 1 | 0 | 1 | 1 | 0 | 0 | 59 | 70 | | | PEAKS DB |
| total 3 peptides | | | | | | | | | | | | | | | | | | | | | | | | | | | | | | | | | | | | | | | | | | | | | | | | | |

**Best Unique PSM (Scan F14:51287, m/z=961.0908, z=3, RT=76.53, ppm=1.0):**

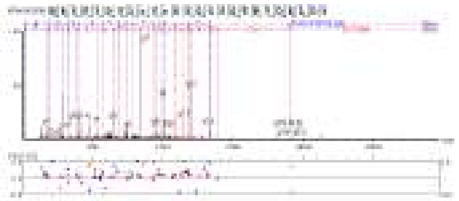

## QSI84033.1

[back to list]

| **Protein Coverage** | **Supporting Peptides** | **Best Unique PSM** |

**Protein Coverage:**

```
 1  MTPAHLLVLA AVCVSFLAAS AIPPQPLSHC QFKKMIKCTV SKSLSWRSFV DYGCYCGKGG SGTPVDELDR CCQTHDNCYT
81  AAGKHPDCNH FWERPFTKRY SYTCSEGTLT CTDKKDKCAA FVCNCDRVAA NCFAKAPAVP TFSHSRCLTN TQP
```

C Carbamidomethylation (+57.02)

**Supporting Peptides:**

| Peptide | Uniq | -10lgP | Mass | Length | ppm | m/z | z | RT | Fraction | Scan | Source File | Area F1 | Area F2a | Area F3a | Area F4 | Area F5 | Area F6a | Area F7a | Area F7b | Area F8 | Area F9a | Area Sample 1F10a | Area F10b | Area F11a | Area F11b | Area F12a | Area F13 | #Feature | #Feature F1 | #Feature F2a | #Feature F3a | #Feature F4 | #Feature F5 | #Feature F6a | #Feature F7a | #Feature F7b | #Feature F8 | #Feature F9a | #Feature Sample 1F10a | #Feature F10b | #Feature F11a | #Feature F11b | #Feature F12a | #Feature F13 | Start | End | PTM | AScore | Found By |
|---|---|---|---|---|---|---|---|---|---|---|---|---|---|---|---|---|---|---|---|---|---|---|---|---|---|---|---|---|---|---|---|---|---|---|---|---|---|---|---|---|---|---|---|---|---|---|---|---|---|
| G.GSGTPVDELDR.C | N | 56.36 | 1144.5360 | 11 | 3.4 | 573.2756 | 2 | 40.15 | 8 | F8:17921 | BuCaTN01_F7b.raw | | | | | | | | 1.1068E6 | | | | | | | | | 1 | 0 | 0 | 0 | 0 | 0 | 0 | 0 | 1 | 0 | 0 | 0 | 0 | 0 | 0 | 0 | 0 | 60 | 70 | | | PEAKS DB |
| S.WRSFVDYGC(+57.02)YC(+57.02)GKGGSGTPVDELDR.C | Y | 50.71 | 2880.2595 | 25 | 1.0 | 961.0908 | 3 | 76.53 | 14 | F14:51287 | BuCaTN01_F11b.raw | | | | | | | | | | | | | | 4.582E7 | | | 1 | 0 | 0 | 0 | 0 | 0 | 0 | 0 | 0 | 0 | 0 | 0 | 0 | 0 | 1 | 0 | 0 | 46 | 70 | Carbamidomethylation | C9:Carbamidomethylation:1000.00;C11:Carbamidomethylation:1000.00 | PEAKS DB |
| K.GGSGTPVDELDR.C | N | 49.09 | 1201.5575 | 12 | 0.3 | 601.7862 | 2 | 40.57 | 11 | F11:14528 | BuCaTN01_F10a.raw | | | | | | | 9.2274E5 | 2.7577E6 | | 1.1335E7 | 6.7591E5 | | 9.9086E5 | 2.6759E7 | | | 6 | 0 | 0 | 0 | 0 | 0 | 0 | 1 | 1 | 0 | 1 | 1 | 0 | 1 | 1 | 0 | 0 | 59 | 70 | | | PEAKS DB |
| total 3 peptides | | | | | | | | | | | | | | | | | | | | | | | | | | | | | | | | | | | | | | | | | | | | | | | | | |

**Best Unique PSM (Scan F14:51287, m/z=961.0908, z=3, RT=76.53, ppm=1.0):**

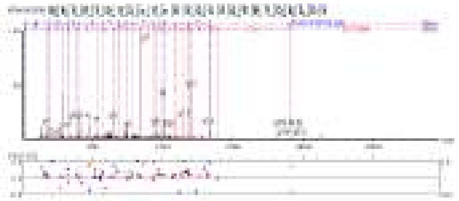

## QSI84029.1 back to list

| Protein Coverage | Supporting Peptides | Best Unique PSM |

**Protein Coverage:**

1 MTPAHLLVLA AVCVSFLAAS AIPPQPLSHC QFKKMIKCTV SKSLS**WRSFV DYGCYCGKGG SGTPVDELDR** CCQTHDNCYT

Carbamidomethylation (+57.02)

81 AAGKHPDCNH FWERPFTKRY SYTCSEGTLT CTDKKDKCAA FVCNCDRVAA NCFAKAPYNK ENVNIDTKKR CK

**Supporting Peptides:**

| Peptide | Uniq | -10lgP | Mass | Length | ppm | m/z | z | RT | Fraction | Scan | Source File | Area F1 | Area F2a | Area F3a | Area F4 | Area F5 | Area F6a | Area F7a | Area F7b | Area F8 | Area F9a | Area Sample 1F10a | Area F10b | Area F11a | Area F11b | Area F12a | Area F13 | #Feature | #Feature F1 | #Feature F2a | #Feature F3a | #Feature F4 | #Feature F5 | #Feature F6a | #Feature F7a | #Feature F7b | #Feature F8 | #Feature F9a | #Feature Sample 1F10a | #Feature F10b | #Feature F11a | #Feature F11b | #Feature F12a | #Feature F13 | Start | End | PTM | AScore | Found By |
|---|---|---|---|---|---|---|---|---|---|---|---|---|---|---|---|---|---|---|---|---|---|---|---|---|---|---|---|---|---|---|---|---|---|---|---|---|---|---|---|---|---|---|---|---|---|---|---|---|---|
| G.GSGTPVDELDR.C | N | 56.36 | 1144.5360 | 11 | 3.4 | 573.2756 | 2 | 40.15 | 8 | F8:17921 | BuCaTN01_F7b.raw | | | | | | | | 1.1068E6 | | | | | | | | | 1 | 0 | 0 | 0 | 0 | 0 | 0 | 0 | 1 | 0 | 0 | 0 | 0 | 0 | 0 | 0 | 0 | 60 | 70 | | | PEAKS DB |
| S.WRSFVDYGC(+57.02)YC(+57.02)GKGGSGTPVDELDR.C | Y | 50.71 | 2880.2595 | 25 | 1.0 | 961.0908 | 3 | 76.53 | 14 | F14:51287 | BuCaTN01_F11b.raw | | | | | | | | | | | | | | 4.582E7 | | | 1 | 0 | 0 | 0 | 0 | 0 | 0 | 0 | 0 | 0 | 0 | 0 | 0 | 0 | 1 | 0 | 0 | 46 | 70 | Carbamidomethylation | C9:Carbamidomethylation:1000.00;C11: Carbamidomethylation:1000.00 | PEAKS DB |
| K.GGSGTPVDELDR.C | N | 49.09 | 1201.5575 | 12 | 0.3 | 601.7862 | 2 | 40.57 | 11 | F11:14528 | BuCaTN01_F10a.raw | | | | | | | 9.2274E5 | 2.7577E6 | | 1.1335E7 | 6.7591E5 | | 9.9086E5 | 2.6759E7 | | | 6 | 0 | 0 | 0 | 0 | 0 | 0 | 1 | 1 | 0 | 1 | 1 | 0 | 1 | 1 | 0 | 0 | 59 | 70 | | | PEAKS DB |
| total 3 peptides | | | | | | | | | | | | | | | | | | | | | | | | | | | | | | | | | | | | | | | | | | | | | | | | | |

**Best Unique PSM (Scan F14:51287, m/z=961.0908, z=3, RT=76.53, ppm=1.0):**

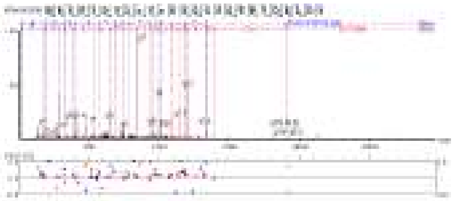

## QSI84028.1 back to list

| Protein Coverage | Supporting Peptides | Best Unique PSM |

**Protein Coverage:**

1 MTPAHLLVLA AVCVSFLAAS AIPPQPLSHC QFKKMIKCTV SKSLS**WRSFV DYGCYCGKGG SGTPVDELDR** CCQTHDNCYT

Carbamidomethylation (+57.02)

81 EAGKHPDCKY FWEHAYTKTY SYTCSEGTLT CTDKKDKCAA FVCNCDRVAA NCFAKAPYNQ ENVSIDTKKR CK

**Supporting Peptides:**

| Peptide | Uniq | -10lgP | Mass | Length | ppm | m/z | z | RT | Fraction | Scan | Source File | Area F1 | Area F2a | Area F3a | Area F4 | Area F5 | Area F6a | Area F7a | Area F7b | Area F8 | Area F9a | Area Sample 1F10a | Area F10b | Area F11a | Area F11b | Area F12a | Area F13 | #Feature | #Feature F1 | #Feature F2a | #Feature F3a | #Feature F4 | #Feature F5 | #Feature F6a | #Feature F7a | #Feature F7b | #Feature F8 | #Feature F9a | #Feature Sample 1F10a | #Feature F10b | #Feature F11a | #Feature F11b | #Feature F12a | #Feature F13 | Start | End | PTM | AScore | Found By |
|---|---|---|---|---|---|---|---|---|---|---|---|---|---|---|---|---|---|---|---|---|---|---|---|---|---|---|---|---|---|---|---|---|---|---|---|---|---|---|---|---|---|---|---|---|---|---|---|---|---|
| G.GSGTPVDELDR.C | N | 56.36 | 1144.5360 | 11 | 3.4 | 573.2756 | 2 | 40.15 | 8 | F8:17921 | BuCaTN01_F7b.raw | | | | | | | | 1.1068E6 | | | | | | | | | 1 | 0 | 0 | 0 | 0 | 0 | 0 | 0 | 1 | 0 | 0 | 0 | 0 | 0 | 0 | 0 | 0 | 60 | 70 | | | PEAKS DB |
| S.WRSFVDYGC(+57.02)YC(+57.02)GKGGSGTPVDELDR.C | Y | 50.71 | 2880.2595 | 25 | 1.0 | 961.0908 | 3 | 76.53 | 14 | F14:51287 | BuCaTN01_F11b.raw | | | | | | | | | | | | | | 4.582E7 | | | 1 | 0 | 0 | 0 | 0 | 0 | 0 | 0 | 0 | 0 | 0 | 0 | 0 | 0 | 1 | 0 | 0 | 46 | 70 | Carbamidomethylation | C9:Carbamidomethylation:1000.00;C11: Carbamidomethylation:1000.00 | PEAKS DB |
| K.GGSGTPVDELDR.C | N | 49.09 | 1201.5575 | 12 | 0.3 | 601.7862 | 2 | 40.57 | 11 | F11:14528 | BuCaTN01_F10a.raw | | | | | | | 9.2274E5 | 2.7577E6 | | 1.1335E7 | 6.7591E5 | | 9.9086E5 | 2.6759E7 | | | 6 | 0 | 0 | 0 | 0 | 0 | 0 | 1 | 1 | 0 | 1 | 1 | 0 | 1 | 1 | 0 | 0 | 59 | 70 | | | PEAKS DB |
| total 3 peptides | | | | | | | | | | | | | | | | | | | | | | | | | | | | | | | | | | | | | | | | | | | | | | | | | |

**Best Unique PSM (Scan F14:51287, m/z=961.0908, z=3, RT=76.53, ppm=1.0):**

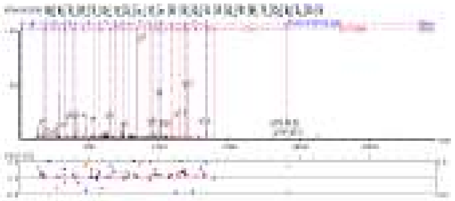

## QSI84035.1 back to list

| Protein Coverage | Supporting Peptides | Best Unique PSM |

**Protein Coverage:**

1 MTPAHLLVLA AVCVSFLAAS AIPPQPLSHC QFKKMIKCTV SKSLS**WRSFV DYGCYCGKGG SGTPVDELDR** CCQTHDNCYT

Carbamidomethylation (+57.02)

81 AAGKHPDCNH FWERPFTKRY SYTCSEGTLT CTDKKDKCAA FVCNCDSTAA NCRVSAPPVP TFSHSRCLTN TQP

**Supporting Peptides:**

| Peptide | Uniq | -10lgP | Mass | Length | ppm | m/z | z | RT | Fraction | Scan | Source File | Area F1 | Area F2a | Area F3a | Area F4 | Area F5 | Area F6a | Area F7a | Area F7b | Area F8 | Area F9a | Area Sample 1F10a | Area F10b | Area F11a | Area F11b | Area F12a | Area F13 | #Feature | #Feature F1 | #Feature F2a | #Feature F3a | #Feature F4 | #Feature F5 | #Feature F6a | #Feature F7a | #Feature F7b | #Feature F8 | #Feature F9a | #Feature Sample 1F10a | #Feature F10b | #Feature F11a | #Feature F11b | #Feature F12a | #Feature F13 | Start | End | PTM | AScore | Found By |
|---|---|---|---|---|---|---|---|---|---|---|---|---|---|---|---|---|---|---|---|---|---|---|---|---|---|---|---|---|---|---|---|---|---|---|---|---|---|---|---|---|---|---|---|---|---|---|---|---|---|
| G.GSGTPVDELDR.C | N | 56.36 | 1144.5360 | 11 | 3.4 | 573.2756 | 2 | 40.15 | 8 | F8:17921 | BuCaTN01_F7b.raw | | | | | | | | 1.1068E6 | | | | | | | | | 1 | 0 | 0 | 0 | 0 | 0 | 0 | 0 | 1 | 0 | 0 | 0 | 0 | 0 | 0 | 0 | 0 | 60 | 70 | | | PEAKS DB |
| S.WRSFVDYGC(+57.02)YC(+57.02)GKGGSGTPVDELDR.C | Y | 50.71 | 2880.2595 | 25 | 1.0 | 961.0908 | 3 | 76.53 | 14 | F14:51287 | BuCaTN01_F11b.raw | | | | | | | | | | | | | | 4.582E7 | | | 1 | 0 | 0 | 0 | 0 | 0 | 0 | 0 | 0 | 0 | 0 | 0 | 0 | 0 | 1 | 0 | 0 | 46 | 70 | Carbamidomethylation | C9:Carbamidomethylation:1000.00;C11: Carbamidomethylation:1000.00 | PEAKS DB |
| K.GGSGTPVDELDR.C | N | 49.09 | 1201.5575 | 12 | 0.3 | 601.7862 | 2 | 40.57 | 11 | F11:14528 | BuCaTN01_F10a.raw | | | | | | | 9.2274E5 | 2.7577E6 | | 1.1335E7 | 6.7591E5 | | 9.9086E5 | 2.6759E7 | | | 6 | 0 | 0 | 0 | 0 | 0 | 0 | 1 | 1 | 0 | 1 | 1 | 0 | 1 | 1 | 0 | 0 | 59 | 70 | | | PEAKS DB |
| total 3 peptides | | | | | | | | | | | | | | | | | | | | | | | | | | | | | | | | | | | | | | | | | | | | | | | | | |

**Best Unique PSM (Scan F14:51287, m/z=961.0908, z=3, RT=76.53, ppm=1.0):**

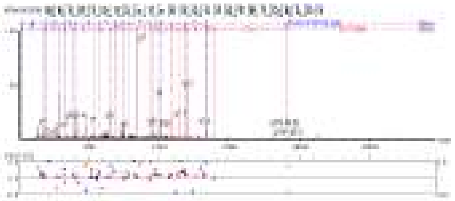

## QSI84034.1 back to list

| Protein Coverage | Supporting Peptides | Best Unique PSM |

**Protein Coverage:**

1 MTPAHLLVLA AVCVSFLAAS AIPPQPLSHC QFKKMIKCTV SKSLS**WRSFV DYGCYCGKGG SGTPVDELDR** CCQTHDNCYT

Carbamidomethylation (+57.02)

81 AAGKHPDCNH FWERPFTKRY SYTCSEGTLT CTDKKDKCAA FVCNCDRVAA PCLARAPAVP TFSHSRCLTN TQP

**Supporting Peptides:**

| Peptide | Uniq | -10lgP | Mass | Length | ppm | m/z | z | RT | Fraction | Scan | Source File | Area F1 | Area F2a | Area F3a | Area F4 | Area F5 | Area F6a | Area F7a | Area F7b | Area F8 | Area F9a | Area Sample 1F10a | Area F10b | Area F11a | Area F11b | Area F12a | Area F13 | #Feature | #Feature F1 | #Feature F2a | #Feature F3a | #Feature F4 | #Feature F5 | #Feature F6a | #Feature F7a | #Feature F7b | #Feature F8 | #Feature F9a | #Feature Sample 1F10a | #Feature F10b | #Feature F11a | #Feature F11b | #Feature F12a | #Feature F13 | Start | End | PTM | AScore | Found By |
|---|---|---|---|---|---|---|---|---|---|---|---|---|---|---|---|---|---|---|---|---|---|---|---|---|---|---|---|---|---|---|---|---|---|---|---|---|---|---|---|---|---|---|---|---|---|---|---|---|---|
| G.GSGTPVDELDR.C | N | 56.36 | 1144.5360 | 11 | 3.4 | 573.2756 | 2 | 40.15 | 8 | F8:17921 | BuCaTN01_F7b.raw | | | | | | | | 1.1068E6 | | | | | | | | | 1 | 0 | 0 | 0 | 0 | 0 | 0 | 0 | 1 | 0 | 0 | 0 | 0 | 0 | 0 | 0 | 0 | 60 | 70 | | | PEAKS DB |
| S.WRSFVDYGC(+57.02)YC(+57.02)GKGGSGTPVDELDR.C | Y | 50.71 | 2880.2595 | 25 | 1.0 | 961.0908 | 3 | 76.53 | 14 | F14:51287 | BuCaTN01_F11b.raw | | | | | | | | | | | | | | 4.582E7 | | | 1 | 0 | 0 | 0 | 0 | 0 | 0 | 0 | 0 | 0 | 0 | 0 | 0 | 0 | 1 | 0 | 0 | 46 | 70 | Carbamidomethylation | C9:Carbamidomethylation:1000.00;C11: Carbamidomethylation:1000.00 | PEAKS DB |
| K.GGSGTPVDELDR.C | N | 49.09 | 1201.5575 | 12 | 0.3 | 601.7862 | 2 | 40.57 | 11 | F11:14528 | BuCaTN01_F10a.raw | | | | | | | 9.2274E5 | 2.7577E6 | | 1.1335E7 | 6.7591E5 | | 9.9086E5 | 2.6759E7 | | | 6 | 0 | 0 | 0 | 0 | 0 | 0 | 1 | 1 | 0 | 1 | 1 | 0 | 1 | 1 | 0 | 0 | 59 | 70 | | | PEAKS DB |
| total 3 peptides | | | | | | | | | | | | | | | | | | | | | | | | | | | | | | | | | | | | | | | | | | | | | | | | | |

**Best Unique PSM (Scan F14:51287, m/z=961.0908, z=3, RT=76.53, ppm=1.0):**

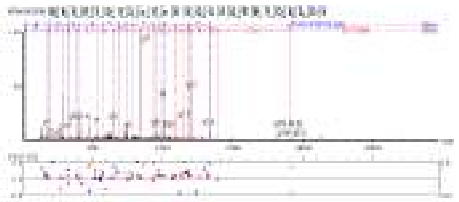

## LAB37309.1

back to list

| Protein Coverage | Supporting Peptides | Best Unique PSM |

**Protein Coverage:**

```
 1 LTCSFTMDKM NPAHLLVLAA VCVSLLGASS VPPRPLNLIN FQRMIECTTG RSALDFTNYG CNCGRGGSGT PVDELDRCCK
81 VHDDCYAAAE KYHGCSPKMT LYTSTCSSRT GNVTCKGNST
```

C Carbamidomethylation (+57.02)

**Supporting Peptides:**

| Peptide | Uniq | -10lgP | Mass | Length | ppm | m/z | z | RT | Fraction | Scan | Source File | Area F1 | Area F2a | Area F3a | Area F4 | Area F5 | Area F6a | Area F7a | Area F7b | Area F8 | Area F9a | Area Sample 1F10a | Area F10b | Area F11a | Area F11b | Area F12a | Area F13 | #Feature | #Feature F1 | #Feature F2a | #Feature F3a | #Feature F4 | #Feature F5 | #Feature F6a | #Feature F7a | #Feature F7b | #Feature F8 | #Feature F9a | #Feature Sample 1F10a | #Feature F10b | #Feature F11a | #Feature F11b | #Feature F12a | #Feature F13 | Start | End | PTM | AScore | Found By |
|---|---|---|---|---|---|---|---|---|---|---|---|---|---|---|---|---|---|---|---|---|---|---|---|---|---|---|---|---|---|---|---|---|---|---|---|---|---|---|---|---|---|---|---|---|---|---|---|---|---|
| G.GSGTPVDELDR.C | N | 56.36 | 1144.5360 | 11 | 3.4 | 573.2756 | 2 | 40.15 | 8 | F8:17921 | BuCaTN01_F7b.raw | | | | | | | | 1.1068E6 | | | | | | | | | 1 | 0 | 0 | 0 | 0 | 0 | 0 | 0 | 1 | 0 | 0 | 0 | 0 | 0 | 0 | 0 | 0 | 67 | 77 | | | PEAKS DB |
| N.YGC(+57.02)NC(+57.02)GRGGSGTPVDELDR.C | Y | 49.73 | 2068.8691 | 19 | -9.9 | 1035.4274 | 2 | 56.61 | 14 | F14:32976 | BuCaTN01_F11b.raw | | | | | | | | | | | | | | 0 | | | 0 | 0 | 0 | 0 | 0 | 0 | 0 | 0 | 0 | 0 | 0 | 0 | 0 | 0 | 0 | 0 | 0 | 59 | 77 | Carbamidomethylation | C3:Carbamidomethylation:1000.00;C5:Carbamidomethylation:1000.00 | PEAKS DB |
| R.GGSGTPVDELDR.C | N | 49.09 | 1201.5575 | 12 | 0.3 | 601.7862 | 2 | 40.57 | 11 | F11:14528 | BuCaTN01_F10a.raw | | | | | | | 9.2274E5 | 2.7577E6 | | 1.1335E7 | 6.7591E5 | | 9.9086E5 | 2.6759E7 | | | 6 | 0 | 0 | 0 | 0 | 0 | 0 | 1 | 1 | 0 | 1 | 1 | 0 | 1 | 1 | 0 | 0 | 66 | 77 | | | PEAKS DB |
| total 3 peptides | | | | | | | | | | | | | | | | | | | | | | | | | | | | | | | | | | | | | | | | | | | | | | | | | |

**Best Unique PSM (Scan F14:32976, m/z=1035.4274, z=2, RT=56.61, ppm=-9.9):**

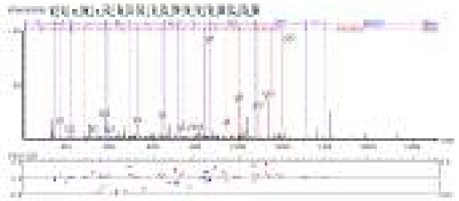

## XP_034272439.1

back to list

| Protein Coverage | Supporting Peptides | Best Unique PSM |

**Protein Coverage:**

```
  1 MNTYSFRQSS SNIGGGGFGG SSSFRTPSIH GGSGGYGISV SSARLASSGF GGSLGGGYGG GYGGGSFNFG SGCFGGAGGA
 81 GGNINASDGL LSGNEKVTMQ NLNDRLANYL DKVRALEEAN TELEVKIREW YQKQGPTATR DYSPYYKTIE DLRDKILVAT
161 IENNKMILQI DNARLAADDF KTKFETEQAL RMSVEADING LRRVLDELTL ARTDLELQIE SLKEELAYLK KNHEEEMSAL
241 SGQLGGQVSV EVDSAPGIDL TKILADMRDQ YELLADKNRR DAEAWFTSKT EELTKEVAIN TEQLQTSKTE ITDLRRTLQG
321 LEIELQSQLS MKSALEGTLG DTEARYGAQL CQIQSLISNL EAQLVDVRSD MERQNNDYKM LMDIKSRLEQ EIETYRQLLD
401 GQDFNFSGNA SGMYGKTKQL LATSLEDYPL RYAQIFNTQI SMAGIIEWTQ LELDNLDQKT RKLMTMHCAL HPKSDIDQLY
481 LPRSQDGKGL LQVKQTVEEE KHSLNDYIQK EKEPLLKETK PSNSLASELS FTMSHYYQSS SSSYGGGGGG GSGRVASVRH
561 GPSSIHGGAG GRVSASSARF VSSGSGYGGG YGYGSGLGGG CAIGYGSVGG LGYGNYFGSS GGYGGGVGGG YGCGVGGGYG
641 GGVGGGYGGG LDFVVGGGGG GDGGLLTGNE KITMQNLNDR LANYLDKVRA LEEANTELEI KIRDWHQKQA PSSPAKDYSH
721 YYKIISDLRD KIHDATIDNS RTILEIDNAR LAADDFRLKY ENELHLRQGV ESDINGLRRV LDELTLFKSD MEMQIEEKKE
801 DLAYLKKNHE ELMKEFSTQL TGKVSVEMDA APGVDLTKIL ADMREQYEQM AERNRKDAEA WFFTKTEELN REVATHTEQL
881 QTSKTEISEL RRTIQSLEIE LQSQLSMKAG LETSLAETEG RYCAQLAQIQ NLITNVEEQL AELRNDMERQ NHEYRMLMDI
961 KTRLEQEIAT YRNLLEGQDS KLPGWSPKDA TYGGSSTINY SSGGSGSGGG GAISGSGYSS GGSSVTTSKV RSGNF
```

**Supporting Peptides:**

| Peptide | Uniq | -10lgP | Mass | Length | ppm | m/z | z | RT | Fraction | Scan | Source File | Area F1 | Area F2a | Area F3a | Area F4 | Area F5 | Area F6a | Area F7a | Area F7b | Area F8 | Area F9a | Area Sample 1F10a | Area F10b | Area F11a | Area F11b | Area F12a | Area F13 | #Feature | #Feature F1 | #Feature F2a | #Feature F3a | #Feature F4 | #Feature F5 | #Feature F6a | #Feature F7a | #Feature F7b | #Feature F8 | #Feature F9a | #Feature Sample 1F10a | #Feature F10b | #Feature F11a | #Feature F11b | #Feature F12a | #Feature F13 | Start | End | PTM | AScore | Found By |
|---|---|---|---|---|---|---|---|---|---|---|---|---|---|---|---|---|---|---|---|---|---|---|---|---|---|---|---|---|---|---|---|---|---|---|---|---|---|---|---|---|---|---|---|---|---|---|---|---|---|
| K.TRLEQEIATYR.N | Y | 55.56 | 1378.7205 | 11 | 2.6 | 460.5806 | 3 | 49.88 | 8 | F8:26641 | BuCaTN01_F7b.raw | | | | | | | | 1.0047E5 | | | | | | | | | 1 | 0 | 0 | 0 | 0 | 0 | 0 | 0 | 1 | 0 | 0 | 0 | 0 | 0 | 0 | 0 | 0 | 962 | 972 | | | PEAKS DB |
| R.VLDELTLAR.T | Y | 50.48 | 1028.5865 | 9 | 2.0 | 515.3000 | 2 | 55.27 | 8 | F8:31057 | BuCaTN01_F7b.raw | | | | | | | 4.9848E4 | 4.0418E5 | | | | | | | | | 2 | 0 | 0 | 0 | 0 | 0 | 0 | 1 | 1 | 0 | 0 | 0 | 0 | 0 | 0 | 0 | 0 | 204 | 212 | | | PEAKS DB |
| K.VTMQNLNDR.L | N | 44.39 | 1089.5237 | 9 | -0.1 | 545.7690 | 2 | 34.52 | 1 | F1:13259 | BuCaTN01_F1.raw | 8.1561E5 | | | | | | | | | | | | | | | | 1 | 1 | 0 | 0 | 0 | 0 | 0 | 0 | 0 | 0 | 0 | 0 | 0 | 0 | 0 | 0 | 0 | 97 | 105 | | | PEAKS DB |
| R.LAADDFR.L | N | 42.34 | 806.3922 | 7 | 0.4 | 404.2036 | 2 | 38.46 | 7 | F7:14678 | BuCaTN01_F7a.raw | | | | | | | 7.0174E4 | | | | | | | | | | 1 | 0 | 0 | 0 | 0 | 0 | 0 | 1 | 0 | 0 | 0 | 0 | 0 | 0 | 0 | 0 | 0 | 751 | 757 | | | PEAKS DB |
| total 4 peptides | | | | | | | | | | | | | | | | | | | | | | | | | | | | | | | | | | | | | | | | | | | | | | | | | |

**Best Unique PSM (Scan F8:26641, m/z=460.5806, z=3, RT=49.88, ppm=2.6):**

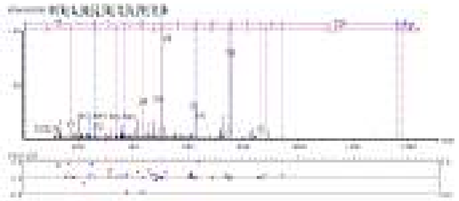

## XP_039209904.1

back to list

| Protein Coverage | Supporting Peptides | Best Unique PSM |

**Protein Coverage:**

1 MNTYSFRQSS SNIGGGGFGG SSSFRTPSIH GGSGGYGISV SSARLASSGF GGGLGSGYGG GSFSYGSGCF GGAGGAGGAG
81 GVFGGNLNVS DSLLSGNEK**V TMQNLNDR**LA NYLDKVRALE EANTELEVKI REWYQKQGPT ASRDYSPFYK TIEDLRDKIL
161 EATIENNKMV LQIDNARLAA DDFKTKFETE QALRMSVEAD INGLRR**VLDE LTLAR**ADLEM QIENLKEELA YLKKNHEEEM
241 STLSGHLGGQ VSVEIDSAPG IDLTKILADM RDQYELLAEK NRRDAEAWFM SKTEELNKEV AVNTEQLQTS KTEITDLRRT
321 LQSLEIELQS QLSMKTALEG TLADTEARYG NRLCQIQGLI SNIEAQLVEV RSDMERQNND YKMLMDIKTR LEQEIETYRQ
401 LLDGQDFNFS GNASGMYDKA VDETKPSNSL ATELSFTMST YYYQSSSSSH GGGGGGGSGR VASVRLGPSS IHGGAGGRVS
481 ASSARFVSSG SGYGSNYGYG SGLGGGCGVG YVSGGGLGYG SCFGGGVGGG YGGGVGGGYG GGVGGGYGGG LDFVVSGGGG
561 DGGLLTGNEK ITMQNLNDRL ANYLDKVRAL EEANTELEIR IRDWHQKQAP TSPAKDYSNY YKIISDLRDK IADSTIDNSR
641 VILEIDNAR**L AADDFR**LKYE NELHLRQGVE SDINGLRRVL DELTLFKSDM EMQIEEKKED LAYLKKNHEE LMKEYSTQLT
721 GQVNVEMDAA PGVDLTKILA DMREQYEQMA EKNRRDAEAW FFTKTEELNR EVATHTEQLQ TSKTEISELR RTIQGLEIEL
801 QSHLSMKAGL ETNLADTEGR YCAQLAQIQN VISSVEEQLA DLRNDMERQN QEYRMLMDIK **TRLEQEIATY R**NLLEGQDSK
881 LPGWSPKDAS YGGGSTTITY SSGGSGSGGG AISGGGYSSG GGSSITTSKV RSGNF

**Supporting Peptides:**

| Peptide | Uniq | -10lgP | Mass | Length | ppm | m/z | z | RT | Fraction | Scan | Source File | Area F1 | Area F2a | Area F3a | Area F4 | Area F5 | Area F6a | Area F7a | Area F7b | Area F8 | Area F9a | Area Sample 1F10a | Area F10b | Area F11a | Area F11b | Area F12a | Area F13 | #Feature | #Feature F1 | #Feature F2a | #Feature F3a | #Feature F4 | #Feature F5 | #Feature F6a | #Feature F7a | #Feature F7b | #Feature F8 | #Feature F9a | #Feature Sample 1F10a | #Feature F10b | #Feature F11a | #Feature F11b | #Feature F12a | #Feature F13 | Start | End | PTM | AScore | Found By |
|---|---|---|---|---|---|---|---|---|---|---|---|---|---|---|---|---|---|---|---|---|---|---|---|---|---|---|---|---|---|---|---|---|---|---|---|---|---|---|---|---|---|---|---|---|---|---|---|---|---|
| K.TRLEQEIATYR.N | Y | 55.56 | 1378.7205 | 11 | 2.6 | 460.5806 | 3 | 49.88 | 8 | F8:26641 | BuCaTN01_F7b.raw | | | | | | | | 1.0047E5 | | | | | | | | | 1 | 0 | 0 | 0 | 0 | 0 | 0 | 0 | 1 | 0 | 0 | 0 | 0 | 0 | 0 | 0 | 0 | 861 | 871 | | | PEAKS DB |
| R.VLDELTLAR.A | Y | 50.48 | 1028.5865 | 9 | 2.0 | 515.3000 | 2 | 55.27 | 8 | F8:31057 | BuCaTN01_F7b.raw | | | | | | | 4.9848E4 | 4.0418E5 | | | | | | | | | 2 | 0 | 0 | 0 | 0 | 0 | 0 | 1 | 1 | 0 | 0 | 0 | 0 | 0 | 0 | 0 | 0 | 207 | 215 | | | PEAKS DB |
| K.VTMQNLNDR.L | N | 44.39 | 1089.5237 | 9 | -0.1 | 545.7690 | 2 | 34.52 | 1 | F1:13259 | BuCaTN01_F1.raw | 8.1561E5 | | | | | | | | | | | | | | | | 1 | 1 | 0 | 0 | 0 | 0 | 0 | 0 | 0 | 0 | 0 | 0 | 0 | 0 | 0 | 0 | 0 | 100 | 108 | | | PEAKS DB |
| R.LAADDFR.L | N | 42.34 | 806.3922 | 7 | 0.4 | 404.2036 | 2 | 38.46 | 7 | F7:14678 | BuCaTN01_F7a.raw | | | | | | | 7.0174E4 | | | | | | | | | | 1 | 0 | 0 | 0 | 0 | 0 | 0 | 1 | 0 | 0 | 0 | 0 | 0 | 0 | 0 | 0 | 0 | 650 | 656 | | | PEAKS DB |
| **total 4 peptides** | | | | | | | | | | | | | | | | | | | | | | | | | | | | | | | | | | | | | | | | | | | | | | | | | |

**Best Unique PSM (Scan F8:26641, m/z=460.5806, z=3, RT=49.88, ppm=2.6):**

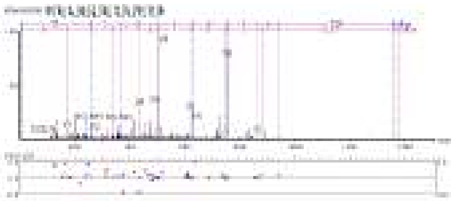

## UNW14927.1

back to list

| Protein Coverage | Supporting Peptides | Best Unique PSM |

**Protein Coverage:**

1 HPAHLLVLLG VCVSLLGATR IPPLPLSLVQ FRILIKCANH SSR**NVLDYAD YGCYCGKGGS GTPVDELDR**C CQAHDYCYDD

Carbamidomethylation (+57.02)

81 AEKLPACNYR FSGPYWNPYS YKCNEGEVTC TDDNDECKAF ICNCDRTAAI CFAGAPLQRR KLHDHTKKK

**Supporting Peptides:**

| Peptide | Uniq | -10lgP | Mass | Length | ppm | m/z | z | RT | Fraction | Scan | Source File | Area F1 | Area F2a | Area F3a | Area F4 | Area F5 | Area F6a | Area F7a | Area F7b | Area F8 | Area F9a | Area Sample 1F10a | Area F10b | Area F11a | Area F11b | Area F12a | Area F13 | #Feature | #Feature F1 | #Feature F2a | #Feature F3a | #Feature F4 | #Feature F5 | #Feature F6a | #Feature F7a | #Feature F7b | #Feature F8 | #Feature F9a | #Feature Sample 1F10a | #Feature F10b | #Feature F11a | #Feature F11b | #Feature F12a | #Feature F13 | Start | End | PTM | AScore | Found By |
|---|---|---|---|---|---|---|---|---|---|---|---|---|---|---|---|---|---|---|---|---|---|---|---|---|---|---|---|---|---|---|---|---|---|---|---|---|---|---|---|---|---|---|---|---|---|---|---|---|---|
| G.GSGTPVDELDR.C | N | 56.36 | 1144.5360 | 11 | 3.4 | 573.2756 | 2 | 40.15 | 8 | F8:17921 | BuCaTN01_F7b.raw | | | | | | | | 1.1068E6 | | | | | | | | | 1 | 0 | 0 | 0 | 0 | 0 | 0 | 0 | 1 | 0 | 0 | 0 | 0 | 0 | 0 | 0 | 0 | 59 | 69 | | | PEAKS DB |
| K.GGSGTPVDELDR.C | N | 49.09 | 1201.5575 | 12 | 0.3 | 601.7862 | 2 | 40.57 | 11 | F11:14528 | BuCaTN01_F10a.raw | | | | | | | 9.2274E5 | 2.7577E6 | | 1.1335E7 | 6.7591E5 | | 9.9086E5 | 2.6759E7 | | | 6 | 0 | 0 | 0 | 0 | 0 | 0 | 1 | 1 | 0 | 1 | 1 | 0 | 1 | 1 | 0 | 0 | 58 | 69 | | | PEAKS DB |
| R.NVLDYADYGC(+57.02)YC(+57.02)GKGGSGTPVDELDR.C | Y | 47.70 | 2880.2329 | 26 | 6.5 | 961.0912 | 3 | 76.75 | 11 | F11:40958 | BuCaTN01_F10a.raw | | | | | | | | | | | 1.0327E7 | 0 | | | | | 1 | 0 | 0 | 0 | 0 | 0 | 0 | 0 | 0 | 0 | 0 | 1 | 0 | 0 | 0 | 0 | 0 | 44 | 69 | Carbamidomethylation | C10:Carbamidomethylation:1000.00;C12: Carbamidomethylation:1000.00 | PEAKS DB |
| **total 3 peptides** | | | | | | | | | | | | | | | | | | | | | | | | | | | | | | | | | | | | | | | | | | | | | | | | | |

**Best Unique PSM (Scan F11:40958, m/z=961.0912, z=3, RT=76.75, ppm=6.5):**

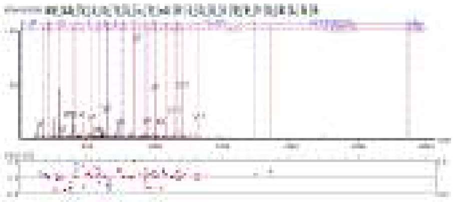

## UNW14922.1

back to list

| Protein Coverage | Supporting Peptides | Best Unique PSM |

**Protein Coverage:**

1 HPAHLLVLLG VCVSLLGAAR IPPLPLSLVQ FRILIKCANH NSR**NVLDYAD YGCYCGKGGS GTPVDELDR**C CQAHDYCYDD

Carbamidomethylation (+57.02)

81 AEKLPACNYR FSGPYWNPYS YKCNEGEVTC TDDNDECKAF ICNCDRTAAI CFAGAPYNDE NFMITTKKKN ICQ

**Supporting Peptides:**

| Peptide | Uniq | -10lgP | Mass | Length | ppm | m/z | z | RT | Fraction | Scan | Source File | Area F1 | Area F2a | Area F3a | Area F4 | Area F5 | Area F6a | Area F7a | Area F7b | Area F8 | Area F9a | Area Sample 1F10a | Area F10b | Area F11a | Area F11b | Area F12a | Area F13 | #Feature | #Feature F1 | #Feature F2a | #Feature F3a | #Feature F4 | #Feature F5 | #Feature F6a | #Feature F7a | #Feature F7b | #Feature F8 | #Feature F9a | #Feature Sample 1F10a | #Feature F10b | #Feature F11a | #Feature F11b | #Feature F12a | #Feature F13 | Start | End | PTM | AScore | Found By |
|---|---|---|---|---|---|---|---|---|---|---|---|---|---|---|---|---|---|---|---|---|---|---|---|---|---|---|---|---|---|---|---|---|---|---|---|---|---|---|---|---|---|---|---|---|---|---|---|---|---|
| G.GSGTPVDELDR.C | N | 56.36 | 1144.5360 | 11 | 3.4 | 573.2756 | 2 | 40.15 | 8 | F8:17921 | BuCaTN01_F7b.raw | | | | | | | | 1.1068E6 | | | | | | | | | 1 | 0 | 0 | 0 | 0 | 0 | 0 | 0 | 1 | 0 | 0 | 0 | 0 | 0 | 0 | 0 | 0 | 59 | 69 | | | PEAKS DB |
| K.GGSGTPVDELDR.C | N | 49.09 | 1201.5575 | 12 | 0.3 | 601.7862 | 2 | 40.57 | 11 | F11:14528 | BuCaTN01_F10a.raw | | | | | | | 9.2274E5 | 2.7577E6 | | 1.1335E7 | 6.7591E5 | | 9.9086E5 | 2.6759E7 | | | 6 | 0 | 0 | 0 | 0 | 0 | 0 | 1 | 1 | 0 | 1 | 1 | 0 | 1 | 1 | 0 | 0 | 58 | 69 | | | PEAKS DB |
| R.NVLDYADYGC(+57.02)YC(+57.02)GKGGSGTPVDELDR.C | Y | 47.70 | 2880.2329 | 26 | 6.5 | 961.0912 | 3 | 76.75 | 11 | F11:40958 | BuCaTN01_F10a.raw | | | | | | | | | | | 1.0327E7 | 0 | | | | | 1 | 0 | 0 | 0 | 0 | 0 | 0 | 0 | 0 | 0 | 0 | 1 | 0 | 0 | 0 | 0 | 0 | 44 | 69 | Carbamidomethylation | C10:Carbamidomethylation:1000.00;C12: Carbamidomethylation:1000.00 | PEAKS DB |
| **total 3 peptides** | | | | | | | | | | | | | | | | | | | | | | | | | | | | | | | | | | | | | | | | | | | | | | | | | | |

**Best Unique PSM (Scan F11:40958, m/z=961.0912, z=3, RT=76.75, ppm=6.5):**

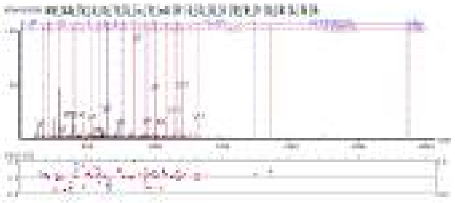

## AAD40976.1

back to list

| Protein Coverage | Supporting Peptides | Best Unique PSM |

**Protein Coverage:**

1 MHPAHLLVPL GVCVSLLGAA RIPPLPLSLV EFRILIKCAN HNSR**NVLDYA DYGCYCGKGG SGTPVDELDR** CCQAHDYCYD

Carbamidomethylation (+57.02)

81 DAEKLPACNY RFSGPYWNPY SYKCNEGEVT CTDDNDECKA FICNCDRTAA ICFAGAPYND ENFMITIKKK NICQ

**Supporting Peptides:**

| Peptide | Uniq | -10lgP | Mass | Length | ppm | m/z | z | RT | Fraction | Scan | Source File | Area F1 | Area F2a | Area F3a | Area F4 | Area F5 | Area F6a | Area F7a | Area F7b | Area F8 | Area F9a | Area Sample 1F10a | Area F10b | Area F11a | Area F11b | Area F12a | Area F13 | #Feature | #Feature F1 | #Feature F2a | #Feature F3a | #Feature F4 | #Feature F5 | #Feature F6a | #Feature F7a | #Feature F7b | #Feature F8 | #Feature F9a | #Feature Sample 1F10a | #Feature F10b | #Feature F11a | #Feature F11b | #Feature F12a | #Feature F13 | Start | End | PTM | AScore | Found By |
|---|---|---|---|---|---|---|---|---|---|---|---|---|---|---|---|---|---|---|---|---|---|---|---|---|---|---|---|---|---|---|---|---|---|---|---|---|---|---|---|---|---|---|---|---|---|---|---|---|---|
| G.GSGTPVDELDR.C | N | 56.36 | 1144.5360 | 11 | 3.4 | 573.2756 | 2 | 40.15 | 8 | F8:17921 | BuCaTN01_F7b.raw | | | | | | | | 1.1068E6 | | | | | | | | | 1 | 0 | 0 | 0 | 0 | 0 | 0 | 0 | 1 | 0 | 0 | 0 | 0 | 0 | 0 | 0 | 0 | 60 | 70 | | | PEAKS DB |
| K.GGSGTPVDELDR.C | N | 49.09 | 1201.5575 | 12 | 0.3 | 601.7862 | 2 | 40.57 | 11 | F11:14528 | BuCaTN01_F10a.raw | | | | | | | 9.2274E5 | 2.7577E6 | | 1.1335E7 | 6.7591E5 | | 9.9086E5 | 2.6759E7 | | | 6 | 0 | 0 | 0 | 0 | 0 | 0 | 1 | 1 | 0 | 1 | 1 | 0 | 1 | 1 | 0 | 0 | 59 | 70 | | | PEAKS DB |
| R.NVLDYADYGC(+57.02)YC(+57.02)GKGGSGTPVDELDR.C | Y | 47.70 | 2880.2329 | 26 | 6.5 | 961.0912 | 3 | 76.75 | 11 | F11:40958 | BuCaTN01_F10a.raw | | | | | | | | | | | 1.0327E7 | 0 | | | | | 1 | 0 | 0 | 0 | 0 | 0 | 0 | 0 | 0 | 0 | 0 | 1 | 0 | 0 | 0 | 0 | 0 | 45 | 70 | Carbamidomethylation | C10:Carbamidomethylation:1000.00;C12: Carbamidomethylation:1000.00 | PEAKS DB |
| total 3 peptides | | | | | | | | | | | | | | | | | | | | | | | | | | | | | | | | | | | | | | | | | | | | | | | | | |

**Best Unique PSM (Scan F11:40958, m/z=961.0912, z=3, RT=76.75, ppm=6.5):**

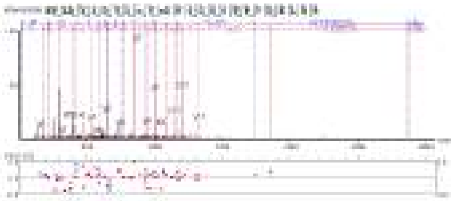

## AAK15776.1

back to list

| Protein Coverage | Supporting Peptides | Best Unique PSM |

**Protein Coverage:**

1 MHPAHLLVPL GVCVSLLGAA RIPPLPLSLV EFRILIKCAN HNSR**NVLDYA DYGCYCGKGG SGTPVDELDR** CCQAHDYCYD

81 DAEKLPACNY RFSGPYWNPY SYKCNEGEVT CTDDNDECKA FICNCDRTAA ICFAGAPYND ENFMITIKKK NICQ

C Carbamidomethylation (+57.02)

**Supporting Peptides:**

| Peptide | Uniq | -10lgP | Mass | Length | ppm | m/z | z | RT | Fraction | Scan | Source File | Area F1 | Area F2a | Area F3a | Area F4 | Area F5 | Area F6a | Area F7a | Area F7b | Area F8 | Area F9a | Area Sample 1F10a | Area F10b | Area F11a | Area F11b | Area F12a | Area F13 | #Feature | #Feature F1 | #Feature F2a | #Feature F3a | #Feature F4 | #Feature F5 | #Feature F6a | #Feature F7a | #Feature F7b | #Feature F8 | #Feature F9a | #Feature Sample 1F10a | #Feature F10b | #Feature F11a | #Feature F11b | #Feature F12a | #Feature F13 | Start | End | PTM | AScore | Found By |
|---|---|---|---|---|---|---|---|---|---|---|---|---|---|---|---|---|---|---|---|---|---|---|---|---|---|---|---|---|---|---|---|---|---|---|---|---|---|---|---|---|---|---|---|---|---|---|---|---|---|
| G.GSGTPVDELDR.C | N | 56.36 | 1144.5360 | 11 | 3.4 | 573.2756 | 2 | 40.15 | 8 | F8:17921 | BuCaTN01_F7b.raw | | | | | | | | 1.1068E6 | | | | | | | | | 1 | 0 | 0 | 0 | 0 | 0 | 0 | 0 | 1 | 0 | 0 | 0 | 0 | 0 | 0 | 0 | 0 | 60 | 70 | | | PEAKS DB |
| K.GGSGTPVDELDR.C | N | 49.09 | 1201.5575 | 12 | 0.3 | 601.7862 | 2 | 40.57 | 11 | F11:14528 | BuCaTN01_F10a.raw | | | | | | | 9.2274E5 | 2.7577E6 | | 1.1335E7 | 6.7591E5 | | 9.9086E5 | 2.6759E7 | | | 6 | 0 | 0 | 0 | 0 | 0 | 0 | 1 | 1 | 0 | 1 | 1 | 0 | 1 | 1 | 0 | 0 | 59 | 70 | | | PEAKS DB |
| R.NVLDYADYGC(+57.02)YC(+57.02)GKGGSGTPVDELDR.C | Y | 47.70 | 2880.2329 | 26 | 6.5 | 961.0912 | 3 | 76.75 | 11 | F11:40958 | BuCaTN01_F10a.raw | | | | | | | | | | | 1.0327E7 | 0 | | | | | 1 | 0 | 0 | 0 | 0 | 0 | 0 | 0 | 0 | 0 | 0 | 1 | 0 | 0 | 0 | 0 | 0 | 45 | 70 | Carbamidomethylation | C10:Carbamidomethylation:1000.00;C12: Carbamidomethylation:1000.00 | PEAKS DB |
| total 3 peptides | | | | | | | | | | | | | | | | | | | | | | | | | | | | | | | | | | | | | | | | | | | | | | | | | |

**Best Unique PSM (Scan F11:40958, m/z=961.0912, z=3, RT=76.75, ppm=6.5):**

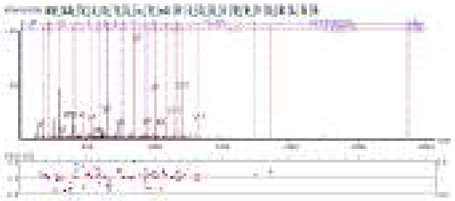

## Q9W7J3.1

back to list

| Protein Coverage | Supporting Peptides | Best Unique PSM |

**Protein Coverage:**

1 MHPAHLLVPL GVCVSLLGAA RIPPLPLSLV EFRILIKCAN HNSR**NVLDYA DYGCYCGKGG SGTPVDELDR** CCQAHDYCYD

81 DAEKLPACNY RFSGPYWNPY SYKCNEGEVT CTDDNDECKA FICNCDRTAA ICFAGAPYND ENFMITIKKK NICQ

C Carbamidomethylation (+57.02)

**Supporting Peptides:**

| Peptide | Uniq | -10lgP | Mass | Length | ppm | m/z | z | RT | Fraction | Scan | Source File | Area F1 | Area F2a | Area F3a | Area F4 | Area F5 | Area F6a | Area F7a | Area F7b | Area F8 | Area F9a | Area Sample 1F10a | Area F10b | Area F11a | Area F11b | Area F12a | Area F13 | #Feature | #Feature F1 | #Feature F2a | #Feature F3a | #Feature F4 | #Feature F5 | #Feature F6a | #Feature F7a | #Feature F7b | #Feature F8 | #Feature F9a | #Feature Sample 1F10a | #Feature F10b | #Feature F11a | #Feature F11b | #Feature F12a | #Feature F13 | Start | End | PTM | AScore | Found By |
|---|---|---|---|---|---|---|---|---|---|---|---|---|---|---|---|---|---|---|---|---|---|---|---|---|---|---|---|---|---|---|---|---|---|---|---|---|---|---|---|---|---|---|---|---|---|---|---|---|---|
| G.GSGTPVDELDR.C | N | 56.36 | 1144.5360 | 11 | 3.4 | 573.2756 | 2 | 40.15 | 8 | F8:17921 | BuCaTN01_F7b.raw | | | | | | | | 1.1068E6 | | | | | | | | | 1 | 0 | 0 | 0 | 0 | 0 | 0 | 0 | 1 | 0 | 0 | 0 | 0 | 0 | 0 | 0 | 0 | 60 | 70 | | | PEAKS DB |
| K.GGSGTPVDELDR.C | N | 49.09 | 1201.5575 | 12 | 0.3 | 601.7862 | 2 | 40.57 | 11 | F11:14528 | BuCaTN01_F10a.raw | | | | | | | 9.2274E5 | 2.7577E6 | | 1.1335E7 | 6.7591E5 | | 9.9086E5 | 2.6759E7 | | | 6 | 0 | 0 | 0 | 0 | 0 | 0 | 1 | 1 | 0 | 1 | 1 | 0 | 1 | 1 | 0 | 0 | 59 | 70 | | | PEAKS DB |
| R.NVLDYADYGC(+57.02)YC(+57.02)GKGGSGTPVDELDR.C | Y | 47.70 | 2880.2329 | 26 | 6.5 | 961.0912 | 3 | 76.75 | 11 | F11:40958 | BuCaTN01_F10a.raw | | | | | | | | | | | 1.0327E7 | 0 | | | | | 1 | 0 | 0 | 0 | 0 | 0 | 0 | 0 | 0 | 0 | 0 | 1 | 0 | 0 | 0 | 0 | 0 | 45 | 70 | Carbamidomethylation | C10:Carbamidomethylation:1000.00;C12: Carbamidomethylation:1000.00 | PEAKS DB |
| total 3 peptides | | | | | | | | | | | | | | | | | | | | | | | | | | | | | | | | | | | | | | | | | | | | | | | | | |

**Best Unique PSM (Scan F11:40958, m/z=961.0912, z=3, RT=76.75, ppm=6.5):**

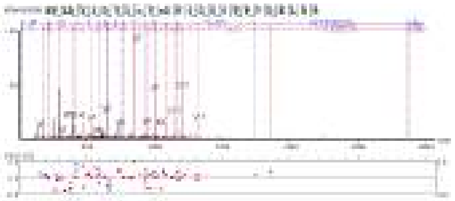

## JAA75026.1

back to list

| Protein Coverage | Supporting Peptides | Best Unique PSM |

**Protein Coverage:**

1 MYPAQLLVLL GVCVSLLGAA SIPPQPLNLY QFKNMIQCAN HGRRPTWHYM DYGCY**CGPGG SGTPVDELDR** CCKIHDDCYA

81 KAETSECNPK LQIYIWQCGS DGPQCGNSET GCKSSVCACD AAAAKCFAEA HYNDANWDIK TDTPCQ

C Carbamidomethylation (+57.02)

**Supporting Peptides:**

| Peptide | Uniq | -10lgP | Mass | Length | ppm | m/z | z | RT | Fraction | Scan | Source File | Area F1 | Area F2a | Area F3a | Area F4 | Area F5 | Area F6a | Area F7a | Area F7b | Area F8 | Area F9a | Area Sample 1F10a | Area F10b | Area F11a | Area F11b | Area F12a | Area F13 | #Feature | #Feature F1 | #Feature F2a | #Feature F3a | #Feature F4 | #Feature F5 | #Feature F6a | #Feature F7a | #Feature F7b | #Feature F8 | #Feature F9a | #Feature Sample 1F10a | #Feature F10b | #Feature F11a | #Feature F11b | #Feature F12a | #Feature F13 | Start | End | PTM | AScore | Found By |
|---|---|---|---|---|---|---|---|---|---|---|---|---|---|---|---|---|---|---|---|---|---|---|---|---|---|---|---|---|---|---|---|---|---|---|---|---|---|---|---|---|---|---|---|---|---|---|---|---|---|
| G.GSGTPVDELDR.C | N | 56.36 | 1144.5360 | 11 | 3.4 | 573.2756 | 2 | 40.15 | 8 | F8:17921 | BuCaTN01_F7b.raw | | | | | | | | 1.1068E6 | | | | | | | | | 1 | 0 | 0 | 0 | 0 | 0 | 0 | 0 | 1 | 0 | 0 | 0 | 0 | 0 | 0 | 0 | 0 | 60 | 70 | | | PEAKS DB |
| P.GGSGTPVDELDR.C | N | 49.09 | 1201.5575 | 12 | 0.3 | 601.7862 | 2 | 40.57 | 11 | F11:14528 | BuCaTN01_F10a.raw | | | | | | | 9.2274E5 | 2.7577E6 | | 1.1335E7 | 6.7591E5 | | 9.9086E5 | 2.6759E7 | | | 6 | 0 | 0 | 0 | 0 | 0 | 0 | 1 | 1 | 0 | 1 | 1 | 0 | 1 | 1 | 0 | 0 | 59 | 70 | | | PEAKS DB |
| Y.C(+57.02)GPGGSGTPVDELDR.C | Y | 44.56 | 1515.6624 | 15 | 2.8 | 758.8375 | 2 | 50.80 | 14 | F14:27539 | BuCaTN01_F11b.raw | | | | | | | | | | | | | | 1.0361E5 | | | 1 | 0 | 0 | 0 | 0 | 0 | 0 | 0 | 0 | 0 | 0 | 0 | 0 | 0 | 1 | 0 | 0 | 56 | 70 | Carbamidomethylation | C1:Carbamidomethylati on:1000.00 | PEAKS DB |
| total 3 peptides | | | | | | | | | | | | | | | | | | | | | | | | | | | | | | | | | | | | | | | | | | | | | | | | | |

**Best Unique PSM (Scan F14:27539, m/z=758.8375, z=2, RT=50.80, ppm=2.8):**

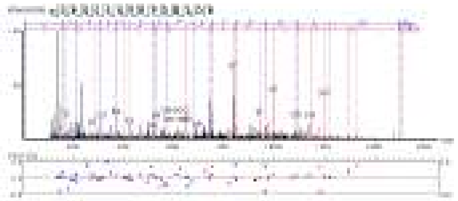

## CAA68449.1

back to list

| Protein Coverage | Supporting Peptides | Best Unique PSM |

**Protein Coverage:**

1 MYPAHLLLLL AVCVSLLGAS AIPPLPLNLA QFALVIKCAD KGKRPRWHYM DYGCY**CGPGG SGTPVDELDR** CCKTHDQCYA

C Carbamidomethylation (+57.02)

81 QAEKKGCYPK LTMYSYYCGG DGPYCNSKTE CQRFVCDCDV RAADCFARYP YNNKNYNINT SKRCK

**Supporting Peptides:**

| Peptide | Uniq | -10lgP | Mass | Length | ppm | m/z | z | RT | Fraction | Scan | Source File | Area F1 | Area F2a | Area F3a | Area F4 | Area F5 | Area F6a | Area F7a | Area F7b | Area F8 | Area F9a | Area Sample 1F10a | Area F10b | Area F11a | Area F11b | Area F12a | Area F13 | #Feature | #Feature F1 | #Feature F2a | #Feature F3a | #Feature F4 | #Feature F5 | #Feature F6a | #Feature F7a | #Feature F7b | #Feature F8 | #Feature F9a | #Feature Sample 1F10a | #Feature F10b | #Feature F11a | #Feature F11b | #Feature F12a | #Feature F13 | Start | End | PTM | AScore | Found By |
|---|---|---|---|---|---|---|---|---|---|---|---|---|---|---|---|---|---|---|---|---|---|---|---|---|---|---|---|---|---|---|---|---|---|---|---|---|---|---|---|---|---|---|---|---|---|---|---|---|---|
| G.GSGTPVDELDR.C | N | 56.36 | 1144.5360 | 11 | 3.4 | 573.2756 | 2 | 40.15 | 8 | F8:17921 | BuCaTN01_F7b.raw | | | | | | | | 1.1068E6 | | | | | | | | | 1 | 0 | 0 | 0 | 0 | 0 | 0 | 0 | 1 | 0 | 0 | 0 | 0 | 0 | 0 | 0 | 0 | 60 | 70 | | | PEAKS DB |
| P.GGSGTPVDELDR.C | N | 49.09 | 1201.5575 | 12 | 0.3 | 601.7862 | 2 | 40.57 | 11 | F11:14528 | BuCaTN01_F10a.raw | | | | | | | 9.2274E5 | 2.7577E6 | | 1.1335E7 | 6.7591E5 | | 9.9086E5 | 2.6759E7 | | | 6 | 0 | 0 | 0 | 0 | 0 | 0 | 1 | 1 | 0 | 1 | 1 | 0 | 1 | 1 | 0 | 0 | 59 | 70 | | | PEAKS DB |
| Y.C(+57.02)GPGGSGTPVDELDR.C | Y | 44.56 | 1515.6624 | 15 | 2.8 | 758.8375 | 2 | 50.80 | 14 | F14:27539 | BuCaTN01_F11b.raw | | | | | | | | | | | | | | 1.0361E5 | | | 1 | 0 | 0 | 0 | 0 | 0 | 0 | 0 | 0 | 0 | 0 | 0 | 0 | 0 | 1 | 0 | 0 | 56 | 70 | Carbamidomethylation | C1:Carbamidomethylation:1000.00 | PEAKS DB |
| total 3 peptides | | | | | | | | | | | | | | | | | | | | | | | | | | | | | | | | | | | | | | | | | | | | | | | | | |

**Best Unique PSM (Scan F14:27539, m/z=758.8375, z=2, RT=50.80, ppm=2.8):**

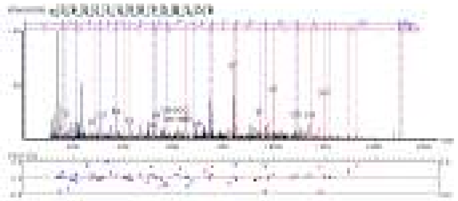

## P19000.1

back to list

| Protein Coverage | Supporting Peptides | Best Unique PSM |

**Protein Coverage:**

1 MYPAHLLLLL AVCVSLLGAS AIPPLPLNLA QFALVIKCAD KGKRPRWHYM DYGCY**CGPGG SGTPVDELDR** CCKTHDQCYA

C Carbamidomethylation (+57.02)

81 QAEKKGCYPK LTMYSYYCGG DGPYCNSKTE CQRFVCDCDV RAADCFARYP YNNKNYNINT SKRCK

**Supporting Peptides:**

| Peptide | Uniq | -10lgP | Mass | Length | ppm | m/z | z | RT | Fraction | Scan | Source File | Area F1 | Area F2a | Area F3a | Area F4 | Area F5 | Area F6a | Area F7a | Area F7b | Area F8 | Area F9a | Area Sample 1F10a | Area F10b | Area F11a | Area F11b | Area F12a | Area F13 | #Feature | #Feature F1 | #Feature F2a | #Feature F3a | #Feature F4 | #Feature F5 | #Feature F6a | #Feature F7a | #Feature F7b | #Feature F8 | #Feature F9a | #Feature Sample 1F10a | #Feature F10b | #Feature F11a | #Feature F11b | #Feature F12a | #Feature F13 | Start | End | PTM | AScore | Found By |
|---|---|---|---|---|---|---|---|---|---|---|---|---|---|---|---|---|---|---|---|---|---|---|---|---|---|---|---|---|---|---|---|---|---|---|---|---|---|---|---|---|---|---|---|---|---|---|---|---|---|
| G.GSGTPVDELDR.C | N | 56.36 | 1144.5360 | 11 | 3.4 | 573.2756 | 2 | 40.15 | 8 | F8:17921 | BuCaTN01_F7b.raw | | | | | | | | 1.1068E6 | | | | | | | | | 1 | 0 | 0 | 0 | 0 | 0 | 0 | 0 | 1 | 0 | 0 | 0 | 0 | 0 | 0 | 0 | 0 | 60 | 70 | | | PEAKS DB |
| P.GGSGTPVDELDR.C | N | 49.09 | 1201.5575 | 12 | 0.3 | 601.7862 | 2 | 40.57 | 11 | F11:14528 | BuCaTN01_F10a.raw | | | | | | | 9.2274E5 | 2.7577E6 | | 1.1335E7 | 6.7591E5 | | 9.9086E5 | 2.6759E7 | | | 6 | 0 | 0 | 0 | 0 | 0 | 0 | 1 | 1 | 0 | 1 | 1 | 0 | 1 | 1 | 0 | 0 | 59 | 70 | | | PEAKS DB |
| Y.C(+57.02)GPGGSGTPVDELDR.C | Y | 44.56 | 1515.6624 | 15 | 2.8 | 758.8375 | 2 | 50.80 | 14 | F14:27539 | BuCaTN01_F11b.raw | | | | | | | | | | | | | | 1.0361E5 | | | 1 | 0 | 0 | 0 | 0 | 0 | 0 | 0 | 0 | 0 | 0 | 0 | 0 | 0 | 1 | 0 | 0 | 56 | 70 | Carbamidomethylation | C1:Carbamidomethylation:1000.00 | PEAKS DB |
| total 3 peptides | | | | | | | | | | | | | | | | | | | | | | | | | | | | | | | | | | | | | | | | | | | | | | | | | |

**Best Unique PSM (Scan F14:27539, m/z=758.8375, z=2, RT=50.80, ppm=2.8):**

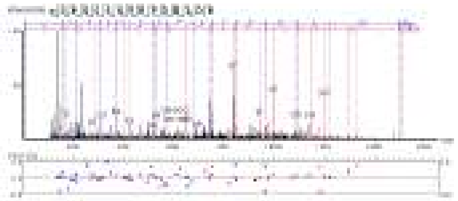

## Q8UUI4.1

back to list

| Protein Coverage | Supporting Peptides | Best Unique PSM |

**Protein Coverage:**

1 MYPAHLLLLL AVCVSLLGAS AIPPLPLNLA QFALVIKCAD KGKRPRWHYM DYGCY**CGPGG SGTPVDELDR** CCKTHDECYA

C Carbamidomethylation (+57.02)

81 QAEKKGCYPK LTMYSYYCGG DGPYCNSKTE CQRFVCDCDV RAADCFARYP YNNKNYNINT SKRCE

**Supporting Peptides:**

| Peptide | Uniq | -10lgP | Mass | Length | ppm | m/z | z | RT | Fraction | Scan | Source File | Area F1 | Area F2a | Area F3a | Area F4 | Area F5 | Area F6a | Area F7a | Area F7b | Area F8 | Area F9a | Area Sample 1F10a | Area F10b | Area F11a | Area F11b | Area F12a | Area F13 | #Feature | #Feature F1 | #Feature F2a | #Feature F3a | #Feature F4 | #Feature F5 | #Feature F6a | #Feature F7a | #Feature F7b | #Feature F8 | #Feature F9a | #Feature Sample 1F10a | #Feature F10b | #Feature F11a | #Feature F11b | #Feature F12a | #Feature F13 | Start | End | PTM | AScore | Found By |
|---|---|---|---|---|---|---|---|---|---|---|---|---|---|---|---|---|---|---|---|---|---|---|---|---|---|---|---|---|---|---|---|---|---|---|---|---|---|---|---|---|---|---|---|---|---|---|---|---|---|
| G.GSGTPVDELDR.C | N | 56.36 | 1144.5360 | 11 | 3.4 | 573.2756 | 2 | 40.15 | 8 | F8:17921 | BuCaTN01_F7b.raw | | | | | | | | 1.1068E6 | | | | | | | | | 1 | 0 | 0 | 0 | 0 | 0 | 0 | 0 | 1 | 0 | 0 | 0 | 0 | 0 | 0 | 0 | 0 | 60 | 70 | | | PEAKS DB |
| P.GGSGTPVDELDR.C | N | 49.09 | 1201.5575 | 12 | 0.3 | 601.7862 | 2 | 40.57 | 11 | F11:14528 | BuCaTN01_F10a.raw | | | | | | | 9.2274E5 | 2.7577E6 | | 1.1335E7 | 6.7591E5 | | 9.9086E5 | 2.6759E7 | | | 6 | 0 | 0 | 0 | 0 | 0 | 0 | 1 | 1 | 0 | 1 | 1 | 0 | 1 | 1 | 0 | 0 | 59 | 70 | | | PEAKS DB |
| Y.C(+57.02)GPGGSGTPVDELDR.C | Y | 44.56 | 1515.6624 | 15 | 2.8 | 758.8375 | 2 | 50.80 | 14 | F14:27539 | BuCaTN01_F11b.raw | | | | | | | | | | | | | | 1.0361E5 | | | 1 | 0 | 0 | 0 | 0 | 0 | 0 | 0 | 0 | 0 | 0 | 0 | 0 | 0 | 1 | 0 | 0 | 56 | 70 | Carbamidomethylation | C1:Carbamidomethylation:1000.00 | PEAKS DB |
| total 3 peptides | | | | | | | | | | | | | | | | | | | | | | | | | | | | | | | | | | | | | | | | | | | | | | | | | |

**Best Unique PSM (Scan F14:27539, m/z=758.8375, z=2, RT=50.80, ppm=2.8):**

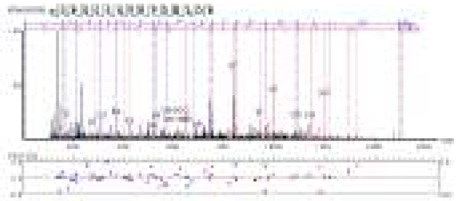

## BAB72248.1

back to list

| Protein Coverage | Supporting Peptides | Best Unique PSM |

**Protein Coverage:**

```
 1  MYPAHLLLLL AVCVSLLGAS AIPPLPLNLA QFALVIKCAD KGKRPRWHYM DYGCYCGPGG SGTPVDELDR CCKTHDECYA
81  QAEKKGCYPK LTMYSYYCGG DGPYCNSKTE CQRFVCDCDV RAADCFARYP YNNKNYNINT SKRCE
```

C Carbamidomethylation (+57.02)

**Supporting Peptides:**

| Peptide | Uniq | -10lgP | Mass | Length | ppm | m/z | z | RT | Fraction | Scan | Source File | Area F1 | Area F2a | Area F3a | Area F4 | Area F5 | Area F6a | Area F7a | Area F7b | Area F8 | Area F9a | Area Sample 1F10a | Area F10b | Area F11a | Area F11b | Area F12a | Area F13 | #Feature | #Feature F1 | #Feature F2a | #Feature F3a | #Feature F4 | #Feature F5 | #Feature F6a | #Feature F7a | #Feature F7b | #Feature F8 | #Feature F9a | #Feature Sample 1F10a | #Feature F10b | #Feature F11a | #Feature F11b | #Feature F12a | #Feature F13 | Start | End | PTM | AScore | Found By |
|---|---|---|---|---|---|---|---|---|---|---|---|---|---|---|---|---|---|---|---|---|---|---|---|---|---|---|---|---|---|---|---|---|---|---|---|---|---|---|---|---|---|---|---|---|---|---|---|---|---|---|
| G.GSGTPVDELDR.C | N | 56.36 | 1144.5360 | 11 | 3.4 | 573.2756 | 2 | 40.15 | 8 | F8:17921 | BuCaTN01_F7b.raw | | | | | | | | 1.1068E6 | | | | | | | | | 1 | 0 | 0 | 0 | 0 | 0 | 0 | 0 | 1 | 0 | 0 | 0 | 0 | 0 | 0 | 0 | 0 | 60 | 70 | | | PEAKS DB |
| P.GGSGTPVDELDR.C | N | 49.09 | 1201.5575 | 12 | 0.3 | 601.7862 | 2 | 40.57 | 11 | F11:14528 | BuCaTN01_F10a.raw | | | | | | | 9.2274E5 | 2.7577E6 | | 1.1335E7 | 6.7591E5 | | 9.9086E5 | 2.6759E7 | | | 6 | 0 | 0 | 0 | 0 | 0 | 0 | 1 | 1 | 0 | 1 | 1 | 0 | 1 | 1 | 0 | 0 | 59 | 70 | | | PEAKS DB |
| Y.C(+57.02)GPGGSGTPVDELDR.C | Y | 44.56 | 1515.6624 | 15 | 2.8 | 758.8375 | 2 | 50.80 | 14 | F14:27539 | BuCaTN01_F11b.raw | | | | | | | | | | | | | | 1.0361E5 | | | 1 | 0 | 0 | 0 | 0 | 0 | 0 | 0 | 0 | 0 | 0 | 0 | 0 | 0 | 1 | 0 | 0 | 56 | 70 | Carbamidomethylation | C1:Carbamidomethylation:1000.00 | PEAKS DB |
| total 3 peptides | | | | | | | | | | | | | | | | | | | | | | | | | | | | | | | | | | | | | | | | | | | | | | | | | | |

**Best Unique PSM (Scan F14:27539, m/z=758.8375, z=2, RT=50.80, ppm=2.8):**

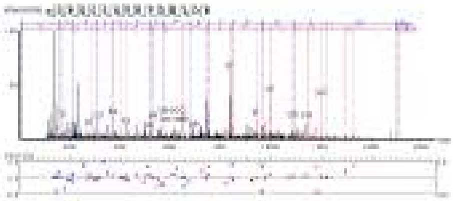

## Q7LZQ6.1

back to list

| Protein Coverage | Supporting Peptides | Best Unique PSM |

**Protein Coverage:**

```
 1  NLYQFKNMIE CAGTRTWIAY VKYGAYTYAY T
```

C Carbamidomethylation (+57.02)

**Supporting Peptides:**

| Peptide | Uniq | -10lgP | Mass | Length | ppm | m/z | z | RT | Fraction | Scan | Source File | Area F1 | Area F2a | Area F3a | Area F4 | Area F5 | Area F6a | Area F7a | Area F7b | Area F8 | Area F9a | Area Sample 1F10a | Area F10b | Area F11a | Area F11b | Area F12a | Area F13 | #Feature | #Feature F1 | #Feature F2a | #Feature F3a | #Feature F4 | #Feature F5 | #Feature F6a | #Feature F7a | #Feature F7b | #Feature F8 | #Feature F9a | #Feature Sample 1F10a | #Feature F10b | #Feature F11a | #Feature F11b | #Feature F12a | #Feature F13 | Start | End | PTM | AScore | Found By |
|---|---|---|---|---|---|---|---|---|---|---|---|---|---|---|---|---|---|---|---|---|---|---|---|---|---|---|---|---|---|---|---|---|---|---|---|---|---|---|---|---|---|---|---|---|---|---|---|---|---|---|
| NLYQFKNMIEC(+57.02)AGTR.T | Y | 62.30 | 1843.8708 | 15 | 0.7 | 615.6313 | 3 | 65.95 | 9 | F9:40585 | BuCaTN01_F8.raw | | | | | | | | | 3.0437E6 | | | 0 | | | | | 3 | 0 | 0 | 0 | 0 | 0 | 0 | 0 | 0 | 3 | 0 | 0 | 0 | 0 | 0 | 0 | 0 | 1 | 15 | Carbamidomethylation | C11:Carbamidomethylation:1000.00 | PEAKS DB |
| NLYQFKNMIEC(+57.02)AG.T | Y | 56.93 | 1586.7222 | 13 | 0.2 | 794.3685 | 2 | 70.77 | 9 | F9:44278 | BuCaTN01_F8.raw | | | | | | | | | 2.9376E6 | | | | | | | | 2 | 0 | 0 | 0 | 0 | 0 | 0 | 0 | 0 | 2 | 0 | 0 | 0 | 0 | 0 | 0 | 0 | 1 | 13 | Carbamidomethylation | C11:Carbamidomethylation:1000.00 | PEAKS DB |
| total 2 peptides | | | | | | | | | | | | | | | | | | | | | | | | | | | | | | | | | | | | | | | | | | | | | | | | | |

**Best Unique PSM (Scan F9:40585, m/z=615.6313, z=3, RT=65.95, ppm=0.7):**

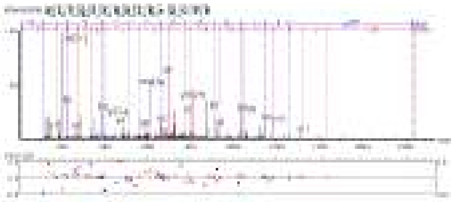

## P81383.3

back to list

| Protein Coverage | Supporting Peptides | Best Unique PSM |

**Protein Coverage:**

```
  1  MNDFLLLLLV LFLGVPRSEN HVINLEECFQ EPEYENWLAT ASHGLTKTLN PKKIVIVGAG ISGLTAAKLF REAGHEVVIL
 81  EASDRVGGRI KTHREDGWYV DVGPMRVPQT HRIVREYIKK FNISLNPFRQ TDENAWYLIK HVRQKMSANN PENFGYQLNP
161  NERGKSASQL FDETLDKVTD DCTLQKEKYD SFSTKEYLIK EGKLSTGAVE MIGDFLNEEA GFHNSFLISV MDHFLFLNNS
241  FDEITGGFDQ LPERFFKDMD SIVHLNSTVE KIVHINNKVT VFYEGLSTNM RLVADYVLIT ATARATRLIK FVPPLSIPKT
321  RALRSLIYAS ATKIILVCTD KFWEKDGIHG GRSITDLPSR VIYYPNHDFT NGIGVLLASY TWYSDSEFYT TLSDEKCVDV
401  VMDDLVEIHN VSKDYLKSVC GKHVVQKWAL DQYSMGAFST YTPYQITHYS QMLAQNEGRI YFAGEYTAHP HGWIETSMKS
481  AIREAINIHN A
```

**Supporting Peptides:**

| Peptide | Uniq | -10lgP | Mass | Length | ppm | m/z | z | RT | Fraction | Scan | Source File | Area F1 | Area F2a | Area F3a | Area F4 | Area F5 | Area F6a | Area F7a | Area F7b | Area F8 | Area F9a | Area Sample 1F10a | Area F10b | Area F11a | Area F11b | Area F12a | Area F13 | #Feature | #Feature F1 | #Feature F2a | #Feature F3a | #Feature F4 | #Feature F5 | #Feature F6a | #Feature F7a | #Feature F7b | #Feature F8 | #Feature F9a | #Feature Sample 1F10a | #Feature F10b | #Feature F11a | #Feature F11b | #Feature F12a | #Feature F13 | Start | End | PTM | AScore | Found By |
|---|---|---|---|---|---|---|---|---|---|---|---|---|---|---|---|---|---|---|---|---|---|---|---|---|---|---|---|---|---|---|---|---|---|---|---|---|---|---|---|---|---|---|---|---|---|---|---|---|---|---|
| K.KIVIVGAGISGLTAAK.L | Y | 54.62 | 1496.9290 | 16 | 1.1 | 499.9841 | 3 | 58.94 | 16 | F16:28577 | BuCaTN01_F13.raw | | | | | | | | | | | | | | | | 8.0907E5 | 1 | 0 | 0 | 0 | 0 | 0 | 0 | 0 | 0 | 0 | 0 | 0 | 0 | 0 | 0 | 0 | 1 | 53 | 68 | | | PEAKS DB |
| R.EAGHEVVILEASDR.V | Y | 47.75 | 1523.7579 | 14 | 0.0 | 508.9266 | 3 | 45.03 | 16 | F16:19881 | BuCaTN01_F13.raw | | | | | | | | | | | | | | | | 6.2067E3 | 1 | 0 | 0 | 0 | 0 | 0 | 0 | 0 | 0 | 0 | 0 | 0 | 0 | 0 | 0 | 0 | 1 | 72 | 85 | | | PEAKS DB |
| K.IVIVGAGISGLTAAK.L | Y | 43.78 | 1368.8340 | 15 | 0.6 | 685.4247 | 2 | 65.00 | 16 | F16:32574 | BuCaTN01_F13.raw | | | | | | | | | | | | | | | | 2.9522E5 | 1 | 0 | 0 | 0 | 0 | 0 | 0 | 0 | 0 | 0 | 0 | 0 | 0 | 0 | 0 | 0 | 1 | 54 | 68 | | | PEAKS DB |
| total 3 peptides | | | | | | | | | | | | | | | | | | | | | | | | | | | | | | | | | | | | | | | | | | | | | | | | | | |

**Best Unique PSM (Scan F16:28577, m/z=499.9841, z=3, RT=58.94, ppm=1.1):**

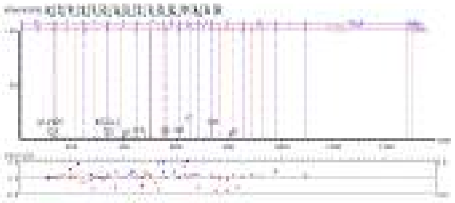

## ABN72538.1

back to list

| Protein Coverage | Supporting Peptides | Best Unique PSM |

**Protein Coverage:**

```
  1  MNDFLLLLLV LFLGVPRSEN HVINLEECFQ EPEYENWLAT ASHGLTKTLN PKKIVIVGAG ISGLTAAKLF REAGHEVVIL
 81  EASDRVGGRI KTHREDGWYV DVGPMRVPQT HRIVREYIKK FNISLNPFRQ TDENAWYLIK HVRQKMSANN PENFGYQLNP
161  NERGKSASQL FDETLDKVTD DCTLQKEKYD SFSTKEYLIK EGKLSTGAVE MIGDFLNEEA GFHNSFLISV MDHFLFLNNS
241  FDEITGGFDQ LPERFFKDMD SIVHLNSTVE KIVHINNKVT VFYEGLSTNM RLVADYVLIT ATARATRLIK FVPPLSIPKT
321  RALRSLIYAS ATKIILVCTD KFWEKDGIHG GRSITDLPSR VIYYPNHDFT NGIGVLLASY TWYSDSEFYT TLSDEKCVDV
401  VMDDLVEIHN VSKDYLKSVC GKHVVQKWAL DQYSMGAFST YTPYQITHYS QMLAQNEGRI YFAGEYTAHP HGWIETSMKS
481  AIREAINIHN A
```

**Supporting Peptides:**

| Peptide | Uniq | -10lgP | Mass | Length | ppm | m/z | z | RT | Fraction | Scan | Source File | Area | Area | Area | Area | Area | Area | Area | Area | Area | Area | Area Sample | Area | Area | Area | Area | Area | #Feature | #Feature | #Feature | #Feature | #Feature | #Feature | #Feature | #Feature | #Feature | #Feature | #Feature | #Feature Sample | #Feature | #Feature | #Feature | #Feature | #Feature | Start | End | PTM | AScore | Found |
|---|---|---|---|---|---|---|---|---|---|---|---|---|---|---|---|---|---|---|---|---|---|---|---|---|---|---|---|---|---|---|---|---|---|---|---|---|---|---|---|---|---|---|---|---|---|---|---|---|---|---|

| | | | | | | | | | | | | F1 | F2a | F3a | F4 | F5 | F6a | F7a | F7b | F8 | F9a | 1F10a | F10b | F11a | F11b | F12a | F13 | | F1 | F2a | F3a | F4 | F5 | F6a | F7a | F7b | F8 | F9a | 1F10a | F10b | F11a | F11b | F12a | F13 | | | | | By |
|---|---|---|---|---|---|---|---|---|---|---|---|---|---|---|---|---|---|---|---|---|---|---|---|---|---|---|---|---|---|---|---|---|---|---|---|---|---|---|---|---|---|---|---|---|---|---|---|---|---|
| K.KIVIVGAGISGLTAAK.L | Y | 54.62 | 1496.9290 | 16 | 1.1 | 499.9841 | 3 | 58.94 | 16 | F16:28577 | BuCaTN01_F13.raw | | | | | | | | | | | | | | | | 8.0907E5 | 1 | 0 | 0 | 0 | 0 | 0 | 0 | 0 | 0 | 0 | 0 | 0 | 0 | 0 | 0 | 0 | 1 | 53 | 68 | | | PEAKS DB |
| R.EAGHEVVILEASDR.V | Y | 47.75 | 1523.7579 | 14 | 0.0 | 508.9266 | 3 | 45.03 | 16 | F16:19881 | BuCaTN01_F13.raw | | | | | | | | | | | | | | | | 6.2067E3 | 1 | 0 | 0 | 0 | 0 | 0 | 0 | 0 | 0 | 0 | 0 | 0 | 0 | 0 | 0 | 0 | 1 | 72 | 85 | | | PEAKS DB |
| K.IVIVGAGISGLTAAK.L | Y | 43.78 | 1368.8340 | 15 | 0.6 | 685.4247 | 2 | 65.00 | 16 | F16:32574 | BuCaTN01_F13.raw | | | | | | | | | | | | | | | | 2.9522E5 | 1 | 0 | 0 | 0 | 0 | 0 | 0 | 0 | 0 | 0 | 0 | 0 | 0 | 0 | 0 | 0 | 1 | 54 | 68 | | | PEAKS DB |
| total 3 peptides | | | | | | | | | | | | | | | | | | | | | | | | | | | | | | | | | | | | | | | | | | | | | | | | | |

**Best Unique PSM (Scan F16:28577, m/z=499.9841, z=3, RT=58.94, ppm=1.1):**

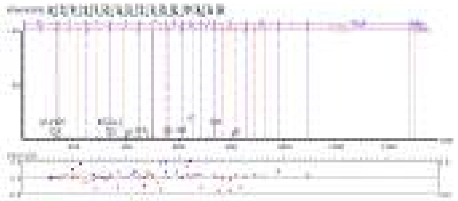

## ETE56914.1

back to list

| Protein Coverage | Supporting Peptides | Best Unique PSM |

**Protein Coverage:**

```
  1 LFLVLLLLVL FLGVPRSENH VINLEECFQE PEYENWLATA SHGLTKTLNP KKIVIVGAGI SGLTAAKLFR EAGHEVVILE
 81 ASDRVGGRIK THREDGWYVD VGPMRVPKTH RIVREYIKKF NISLNPFRQT DENAWYLIKH VRQKMSANNP ENFGYQLNPN
161 ERGKSASQLF DETLDKVVLR SRRDVQADKS NALEYLIKEG KLSTGAVEMI GDFLNEEAGF HNSFLISVMD HFLFLNNSFD
241 EITGGFDQLP ESFFKDMDSI VHLNSTVEKI VHINNKVTVF YEGLSTNMRL VADYVLITAT ARATRLIKFV PPLSIPKTRA
321 LRSLIYASAT KIILVCTDKF WEKDGIHGGR SITDLPSRVI YYPNHDFTNG IGVLLASYTW YSDSEFYTTL SDEKCVDVVM
401 DDLVEIHKVS KDYLKSVCGK HVVQKWALDQ YSMGAFSTYT PYQITHYSQM LAQNEGRIYF AGEYTAHPHG WIETSMKSAI
481 REAINIHNA
```

**Supporting Peptides:**

| Peptide | Uniq | -10lgP | Mass | Length | ppm | m/z | z | RT | Fraction | Scan | Source File | Area F1 | Area F2a | Area F3a | Area F4 | Area F5 | Area F6a | Area F7a | Area F7b | Area F8 | Area F9a | Area Sample 1F10a | Area F10b | Area F11a | Area F11b | Area F12a | Area F13 | #Feature | #Feature F1 | #Feature F2a | #Feature F3a | #Feature F4 | #Feature F5 | #Feature F6a | #Feature F7a | #Feature F7b | #Feature F8 | #Feature F9a | #Feature Sample 1F10a | #Feature F10b | #Feature F11a | #Feature F11b | #Feature F12a | #Feature F13 | Start | End | PTM | AScore | Found By |
|---|---|---|---|---|---|---|---|---|---|---|---|---|---|---|---|---|---|---|---|---|---|---|---|---|---|---|---|---|---|---|---|---|---|---|---|---|---|---|---|---|---|---|---|---|---|---|---|---|---|
| K.KIVIVGAGISGLTAAK.L | Y | 54.62 | 1496.9290 | 16 | 1.1 | 499.9841 | 3 | 58.94 | 16 | F16:28577 | BuCaTN01_F13.raw | | | | | | | | | | | | | | | | 8.0907E5 | 1 | 0 | 0 | 0 | 0 | 0 | 0 | 0 | 0 | 0 | 0 | 0 | 0 | 0 | 0 | 0 | 1 | 52 | 67 | | | PEAKS DB |
| R.EAGHEVVILEASDR.V | Y | 47.75 | 1523.7579 | 14 | 0.0 | 508.9266 | 3 | 45.03 | 16 | F16:19881 | BuCaTN01_F13.raw | | | | | | | | | | | | | | | | 6.2067E3 | 1 | 0 | 0 | 0 | 0 | 0 | 0 | 0 | 0 | 0 | 0 | 0 | 0 | 0 | 0 | 0 | 1 | 71 | 84 | | | PEAKS DB |
| K.IVIVGAGISGLTAAK.L | Y | 43.78 | 1368.8340 | 15 | 0.6 | 685.4247 | 2 | 65.00 | 16 | F16:32574 | BuCaTN01_F13.raw | | | | | | | | | | | | | | | | 2.9522E5 | 1 | 0 | 0 | 0 | 0 | 0 | 0 | 0 | 0 | 0 | 0 | 0 | 0 | 0 | 0 | 0 | 1 | 53 | 67 | | | PEAKS DB |
| total 3 peptides | | | | | | | | | | | | | | | | | | | | | | | | | | | | | | | | | | | | | | | | | | | | | | | | | |

**Best Unique PSM (Scan F16:28577, m/z=499.9841, z=3, RT=58.94, ppm=1.1):**

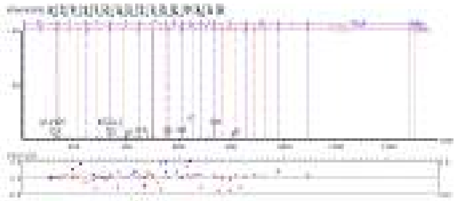

## XP_013922516.1

back to list

| Protein Coverage | Supporting Peptides | Best Unique PSM |

**Protein Coverage:**

```
  1 MNFLLTWIHW GLAALLYFHN AKVLQAAPAQ GDGDRQQGEV ISFMKVFERS ACRSIETMVD IFQEYPDEVE YIFKPSCVPL
 81 MRCAGCCNDE ALECVPTEIY NVTMEIMKLK HFQSQHIHPM SFQQHSKCEC RQKKEVTVRQ ENHCEPCSER RKHLYKQDPL
161 TCKCSCKFTD SRCKSKQLEL NERTCRCEKP RR
```

C Carbamidomethylation (+57.02)

162 C

**Supporting Peptides:**

| Peptide | Uniq | -10lgP | Mass | Length | ppm | m/z | z | RT | Fraction | Scan | Source File | Area F1 | Area F2a | Area F3a | Area F4 | Area F5 | Area F6a | Area F7a | Area F7b | Area F8 | Area F9a | Area Sample 1F10a | Area F10b | Area F11a | Area F11b | Area F12a | Area F13 | #Feature | #Feature F1 | #Feature F2a | #Feature F3a | #Feature F4 | #Feature F5 | #Feature F6a | #Feature F7a | #Feature F7b | #Feature F8 | #Feature F9a | #Feature Sample 1F10a | #Feature F10b | #Feature F11a | #Feature F11b | #Feature F12a | #Feature F13 | Start | End | PTM | AScore | Found By |
|---|---|---|---|---|---|---|---|---|---|---|---|---|---|---|---|---|---|---|---|---|---|---|---|---|---|---|---|---|---|---|---|---|---|---|---|---|---|---|---|---|---|---|---|---|---|---|---|---|---|
| K.HLYKQDPLTC(+57.02)K.C | Y | 79.51 | 1401.7074 | 11 | -0.9 | 468.2427 | 3 | 32.91 | 11 | F11:10027 | BuCaTN01_F10a.raw | | | | | | | 3.2403E5 | | | | 4.3374E5 | | | | | | 2 | 0 | 0 | 0 | 0 | 0 | 0 | 1 | 0 | 0 | 0 | 1 | 0 | 0 | 0 | 0 | 0 | 153 | 163 | Carbamidomethylation | C10:Carbamidomethylation:1000.00 | PEAKS DB |
| K.HFQSQHIHPMSFQQHSK.C | Y | 44.08 | 2102.9856 | 17 | -0.1 | 421.6044 | 5 | 37.10 | 10 | F10:15639 | BuCaTN01_F9a.raw | | | | | | | | | | 2.5195E4 | | | | | | | 1 | 0 | 0 | 0 | 0 | 0 | 0 | 0 | 0 | 0 | 1 | 0 | 0 | 0 | 0 | 0 | 0 | 111 | 127 | | | PEAKS DB |
| total 2 peptides | | | | | | | | | | | | | | | | | | | | | | | | | | | | | | | | | | | | | | | | | | | | | | | | | |

**Best Unique PSM (Scan F11:10027, m/z=468.2427, z=3, RT=32.91, ppm=-0.9):**

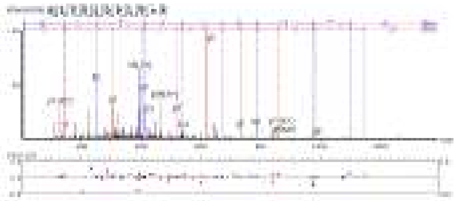

## JAB52756.1

back to list

| Protein Coverage | Supporting Peptides | Best Unique PSM |

**Protein Coverage:**

```
  1 MNFLLTWIHC GLAALLYFHN AKVLQAAPAQ EDGEKQPDEV ITFMTVYEHS VCRPIETMVD IFQEYPDEVE YIFKPSCVLL
 81 MKCGGCCNDE ALECVPTEVY NVSMEIMKLK HFQSQHIHPM SFQQHSKCQC RPKKAMRIKQ ENHCEPCSER RKHLYKQDPL
161 TCKCSCKFTD SRCKSKQLEL NERTCRYLHL LQHVKESSLC LETLALSQEI N
```

C Carbamidomethylation (+57.02)

162 C

**Supporting Peptides:**

| Peptide | Uniq | -10lgP | Mass | Length | ppm | m/z | z | RT | Fraction | Scan | Source File | Area F1 | Area F2a | Area F3a | Area F4 | Area F5 | Area F6a | Area F7a | Area F7b | Area F8 | Area F9a | Area Sample 1F10a | Area F10b | Area F11a | Area F11b | Area F12a | Area F13 | #Feature | #Feature F1 | #Feature F2a | #Feature F3a | #Feature F4 | #Feature F5 | #Feature F6a | #Feature F7a | #Feature F7b | #Feature F8 | #Feature F9a | #Feature Sample 1F10a | #Feature F10b | #Feature F11a | #Feature F11b | #Feature F12a | #Feature F13 | Start | End | PTM | AScore | Found By |
|---|---|---|---|---|---|---|---|---|---|---|---|---|---|---|---|---|---|---|---|---|---|---|---|---|---|---|---|---|---|---|---|---|---|---|---|---|---|---|---|---|---|---|---|---|---|---|---|---|---|
| K.HLYKQDPLTC(+57.02)K.C | Y | 79.51 | 1401.7074 | 11 | -0.9 | 468.2427 | 3 | 32.91 | 11 | F11:10027 | BuCaTN01_F10a.raw | | | | | | | 3.2403E5 | | | | 4.3374E5 | | | | | | 2 | 0 | 0 | 0 | 0 | 0 | 0 | 1 | 0 | 0 | 0 | 1 | 0 | 0 | 0 | 0 | 0 | 153 | 163 | Carbamidomethylation | C10:Carbamidomethylation:1000.00 | PEAKS DB |
| K.HFQSQHIHPMSFQQHSK.C | Y | 44.08 | 2102.9856 | 17 | -0.1 | 421.6044 | 5 | 37.10 | 10 | F10:15639 | BuCaTN01_F9a.raw | | | | | | | | | | 2.5195E4 | | | | | | | 1 | 0 | 0 | 0 | 0 | 0 | 0 | 0 | 0 | 0 | 1 | 0 | 0 | 0 | 0 | 0 | 0 | 111 | 127 | | | PEAKS DB |
| total 2 peptides | | | | | | | | | | | | | | | | | | | | | | | | | | | | | | | | | | | | | | | | | | | | | | | | | |

**Best Unique PSM (Scan F11:10027, m/z=468.2427, z=3, RT=32.91, ppm=-0.9):**

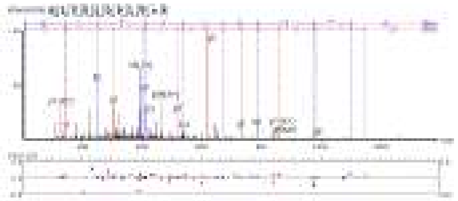

## AXL96619.1

back to list

| Protein Coverage | Supporting Peptides | Best Unique PSM |

**Protein Coverage:**

```
  1  MNFLLTWIHW GLAALLYFHN AKVLQAAPAQ GDGERQQGEV ISFMKVFERS ACRSIETMVD IFQEYPDEVE YIFKPSCVPL
 81  MRCAGCCNDE ALECVPTEVY NVTMEIMKLK HFQSQHIHPM SFQQHSKCEC RQKKEVRIRQ ENHCEPCSER RKHLYKQDPL
161  TCKCSCKFTD SRCKSKQLEL NERTCRCEKP RR
```

C Carbamidomethylation (+57.02)

**Supporting Peptides:**

| Peptide | Uniq | -10lgP | Mass | Length | ppm | m/z | z | RT | Fraction | Scan | Source File | Area F1 | Area F2a | Area F3a | Area F4 | Area F5 | Area F6a | Area F7a | Area F7b | Area F8 | Area F9a | Area Sample 1F10a | Area F10b | Area F11a | Area F11b | Area F12a | Area F13 | #Feature | #Feature F1 | #Feature F2a | #Feature F3a | #Feature F4 | #Feature F5 | #Feature F6a | #Feature F7a | #Feature F7b | #Feature F8 | #Feature F9a | #Feature Sample 1F10a | #Feature F10b | #Feature F11a | #Feature F11b | #Feature F12a | #Feature F13 | Start | End | PTM | AScore | Found By |
|---|---|---|---|---|---|---|---|---|---|---|---|---|---|---|---|---|---|---|---|---|---|---|---|---|---|---|---|---|---|---|---|---|---|---|---|---|---|---|---|---|---|---|---|---|---|---|---|---|---|
| K.HLYKQDPLTC(+57.02)K.C | Y | 79.51 | 1401.7074 | 11 | -0.9 | 468.2427 | 3 | 32.91 | 11 | F11:10027 | BuCaTN01_F10a.raw | | | | | | | 3.2403E5 | | | | 4.3374E5 | | | | | | 2 | 0 | 0 | 0 | 0 | 0 | 0 | 1 | 0 | 0 | 0 | 1 | 0 | 0 | 0 | 0 | 0 | 153 | 163 | Carbamidomethylation | C10:Carbamidomethylation:1000.00 | PEAKS DB |
| K.HFQSQHIHPMSFQQHSK.C | Y | 44.08 | 2102.9856 | 17 | -0.1 | 421.6044 | 5 | 37.10 | 10 | F10:15639 | BuCaTN01_F9a.raw | | | | | | | | | | 2.5195E4 | | | | | | | 1 | 0 | 0 | 0 | 0 | 0 | 0 | 0 | 0 | 0 | 1 | 0 | 0 | 0 | 0 | 0 | 0 | 111 | 127 | | | PEAKS DB |
| total 2 peptides | | | | | | | | | | | | | | | | | | | | | | | | | | | | | | | | | | | | | | | | | | | | | | | | | |

**Best Unique PSM (Scan F11:10027, m/z=468.2427, z=3, RT=32.91, ppm=-0.9):**

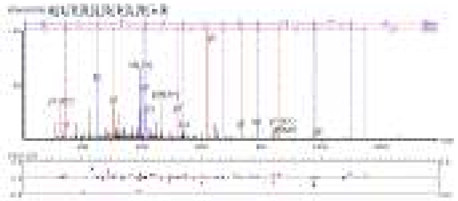

## XP_058018264.1

back to list

| Protein Coverage | Supporting Peptides | Best Unique PSM |

**Protein Coverage:**

```
  1  MNFLLTWIHW GLAALLYFHN AKVLQAAPAQ GDGERQQGEV ISFMKVFERS ACRSIETMVD IFQEYPDEVE YIFKPSCVPL
 81  MRCAGCCNDE ALECVPTEVY NVTMEIMKLK HFQSQHIHPM SFQQHSKCEC RQKKEVRIRQ ENHCEPCSER RKHLYKQDPL
161  TCKCSCKFTD SRCKSKQLEL NERTCRCEKP RR
```

C Carbamidomethylation (+57.02)

**Supporting Peptides:**

| Peptide | Uniq | -10lgP | Mass | Length | ppm | m/z | z | RT | Fraction | Scan | Source File | Area F1 | Area F2a | Area F3a | Area F4 | Area F5 | Area F6a | Area F7a | Area F7b | Area F8 | Area F9a | Area Sample 1F10a | Area F10b | Area F11a | Area F11b | Area F12a | Area F13 | #Feature | #Feature F1 | #Feature F2a | #Feature F3a | #Feature F4 | #Feature F5 | #Feature F6a | #Feature F7a | #Feature F7b | #Feature F8 | #Feature F9a | #Feature Sample 1F10a | #Feature F10b | #Feature F11a | #Feature F11b | #Feature F12a | #Feature F13 | Start | End | PTM | AScore | Found By |
|---|---|---|---|---|---|---|---|---|---|---|---|---|---|---|---|---|---|---|---|---|---|---|---|---|---|---|---|---|---|---|---|---|---|---|---|---|---|---|---|---|---|---|---|---|---|---|---|---|---|
| K.HLYKQDPLTC(+57.02)K.C | Y | 79.51 | 1401.7074 | 11 | -0.9 | 468.2427 | 3 | 32.91 | 11 | F11:10027 | BuCaTN01_F10a.raw | | | | | | | 3.2403E5 | | | | 4.3374E5 | | | | | | 2 | 0 | 0 | 0 | 0 | 0 | 0 | 1 | 0 | 0 | 0 | 1 | 0 | 0 | 0 | 0 | 0 | 153 | 163 | Carbamidomethylation | C10:Carbamidomethylation:1000.00 | PEAKS DB |
| K.HFQSQHIHPMSFQQHSK.C | Y | 44.08 | 2102.9856 | 17 | -0.1 | 421.6044 | 5 | 37.10 | 10 | F10:15639 | BuCaTN01_F9a.raw | | | | | | | | | | 2.5195E4 | | | | | | | 1 | 0 | 0 | 0 | 0 | 0 | 0 | 0 | 0 | 0 | 1 | 0 | 0 | 0 | 0 | 0 | 0 | 111 | 127 | | | PEAKS DB |
| total 2 peptides | | | | | | | | | | | | | | | | | | | | | | | | | | | | | | | | | | | | | | | | | | | | | | | | | |

**Best Unique PSM (Scan F11:10027, m/z=468.2427, z=3, RT=32.91, ppm=-0.9):**

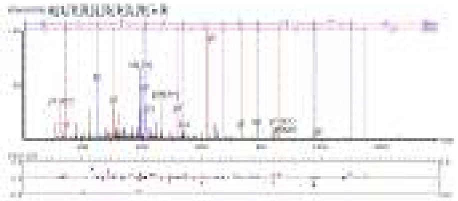

## XP_058018253.1

back to list

| Protein Coverage | Supporting Peptides | Best Unique PSM |

**Protein Coverage:**

```
  1  MLRLKTCVRS ARGSRGPKGS PHTRRRRAGP QRRQVLQAAP AQGDGERQQG EVISFMKVFE RSACRSIETM VDIFQEYPDE
 81  VEYIFKPSCV PLMRCAGCCN DEALECVPTE VYNVTMEIMK LKHFQSQHIH PMSFQQHSKC ECRQKKEVRI RQENHCEPCS
161  ERRKHLYKQD PLTCKCSCKF TDSRCKSKQL ELNERTCRCE KPRR
```

C Carbamidomethylation (+57.02)

**Supporting Peptides:**

| Peptide | Uniq | -10lgP | Mass | Length | ppm | m/z | z | RT | Fraction | Scan | Source File | Area F1 | Area F2a | Area F3a | Area F4 | Area F5 | Area F6a | Area F7a | Area F7b | Area F8 | Area F9a | Area Sample 1F10a | Area F10b | Area F11a | Area F11b | Area F12a | Area F13 | #Feature | #Feature F1 | #Feature F2a | #Feature F3a | #Feature F4 | #Feature F5 | #Feature F6a | #Feature F7a | #Feature F7b | #Feature F8 | #Feature F9a | #Feature Sample 1F10a | #Feature F10b | #Feature F11a | #Feature F11b | #Feature F12a | #Feature F13 | Start | End | PTM | AScore | Found By |
|---|---|---|---|---|---|---|---|---|---|---|---|---|---|---|---|---|---|---|---|---|---|---|---|---|---|---|---|---|---|---|---|---|---|---|---|---|---|---|---|---|---|---|---|---|---|---|---|---|---|
| K.HLYKQDPLTC(+57.02)K.C | Y | 79.51 | 1401.7074 | 11 | -0.9 | 468.2427 | 3 | 32.91 | 11 | F11:10027 | BuCaTN01_F10a.raw | | | | | | | 3.2403E5 | | | | 4.3374E5 | | | | | | 2 | 0 | 0 | 0 | 0 | 0 | 0 | 1 | 0 | 0 | 0 | 1 | 0 | 0 | 0 | 0 | 0 | 165 | 175 | Carbamidomethylation | C10:Carbamidomethylation:1000.00 | PEAKS DB |
| K.HFQSQHIHPMSFQQHSK.C | Y | 44.08 | 2102.9856 | 17 | -0.1 | 421.6044 | 5 | 37.10 | 10 | F10:15639 | BuCaTN01_F9a.raw | | | | | | | | | | 2.5195E4 | | | | | | | 1 | 0 | 0 | 0 | 0 | 0 | 0 | 0 | 0 | 0 | 1 | 0 | 0 | 0 | 0 | 0 | 0 | 123 | 139 | | | PEAKS DB |
| total 2 peptides | | | | | | | | | | | | | | | | | | | | | | | | | | | | | | | | | | | | | | | | | | | | | | | | | |

**Best Unique PSM (Scan F11:10027, m/z=468.2427, z=3, RT=32.91, ppm=-0.9):**

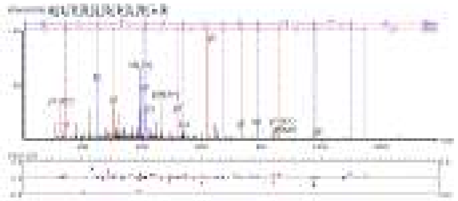

## XP_058018245.1

back to list

| Protein Coverage | Supporting Peptides | Best Unique PSM |

**Protein Coverage:**

1 MNFLLTWIHW GLAALLYFHN AKVLQAAPAQ GDGERQQGEV ISFMKVFERS ACRSIETMVD IFQEYPDEVE YIFKPSCVPL

81 MRCAGCCNDE ALECVPTEVY NVTMEIMKLK **HFQSQHIHPM SFQQHSK**CEC RQKKEVRIRQ EKKSKRGKGK GQKRKRKRGR

161 YKPQNFHCEP CSERRK**HLYK QDPLTCK**CSC KFTDSRCKSK QLELNERTCR CEKPRR

C Carbamidomethylation (+57.02)

**Supporting Peptides:**

| Peptide | Uniq | -10lgP | Mass | Length | ppm | m/z | z | RT | Fraction | Scan | Source File | Area F1 | Area F2a | Area F3a | Area F4 | Area F5 | Area F6a | Area F7a | Area F7b | Area F8 | Area F9a | Area Sample 1F10a | Area F10b | Area F11a | Area F11b | Area F12a | Area F13 | #Feature | #Feature F1 | #Feature F2a | #Feature F3a | #Feature F4 | #Feature F5 | #Feature F6a | #Feature F7a | #Feature F7b | #Feature F8 | #Feature F9a | #Feature Sample 1F10a | #Feature F10b | #Feature F11a | #Feature F11b | #Feature F12a | #Feature F13 | Start | End | PTM | AScore | Found By |
|---|---|---|---|---|---|---|---|---|---|---|---|---|---|---|---|---|---|---|---|---|---|---|---|---|---|---|---|---|---|---|---|---|---|---|---|---|---|---|---|---|---|---|---|---|---|---|---|---|---|
| K.HLYKQDPLTC(+57.02)K.C | Y | 79.51 | 1401.7074 | 11 | -0.9 | 468.2427 | 3 | 32.91 | 11 | F11:10027 | BuCaTN01_F10a.raw | | | | | | | 3.2403E5 | | | | 4.3374E5 | | | | | | 2 | 0 | 0 | 0 | 0 | 0 | 0 | 1 | 0 | 0 | 0 | 1 | 0 | 0 | 0 | 0 | 0 | 177 | 187 | Carbamidomethylation | C10:Carbamidomethylation:1000.00 | PEAKS DB |
| K.HFQSQHIHPMSFQQHSK.C | Y | 44.08 | 2102.9856 | 17 | -0.1 | 421.6044 | 5 | 37.10 | 10 | F10:15639 | BuCaTN01_F9a.raw | | | | | | | | | | 2.5195E4 | | | | | | | 1 | 0 | 0 | 0 | 0 | 0 | 0 | 0 | 0 | 0 | 1 | 0 | 0 | 0 | 0 | 0 | 0 | 111 | 127 | | | PEAKS DB |
| total 2 peptides | | | | | | | | | | | | | | | | | | | | | | | | | | | | | | | | | | | | | | | | | | | | | | | | | |

**Best Unique PSM (Scan F11:10027, m/z=468.2427, z=3, RT=32.91, ppm=-0.9):**

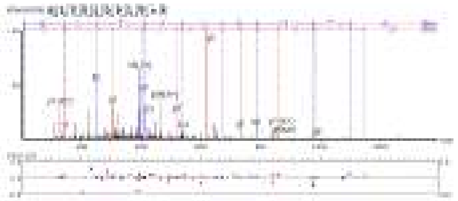

## XP_058018236.1

back to list

| Protein Coverage | Supporting Peptides | Best Unique PSM |

**Protein Coverage:**

1 MLRLKTCVRS ARGSRGPKGS PHTRRRRAGP QRRQVLQAAP AQGDGERQQG EVISFMKVFE RSACRSIETM VDIFQEYPDE

81 VEYIFKPSCV PLMRCAGCCN DEALECVPTE VYNVTMEIMK LK**HFQSQHIH PMSFQQHSK**C ECRQKKEVRI RQEKKSKRGK

161 GKGQKRKRKR GRHCEPCSER RK**HLYKQDPL TCK**CSCKFTD SRCKSKQLEL NERTCRCEKP RR

C Carbamidomethylation (+57.02)

**Supporting Peptides:**

| Peptide | Uniq | -10lgP | Mass | Length | ppm | m/z | z | RT | Fraction | Scan | Source File | Area F1 | Area F2a | Area F3a | Area F4 | Area F5 | Area F6a | Area F7a | Area F7b | Area F8 | Area F9a | Area Sample 1F10a | Area F10b | Area F11a | Area F11b | Area F12a | Area F13 | #Feature | #Feature F1 | #Feature F2a | #Feature F3a | #Feature F4 | #Feature F5 | #Feature F6a | #Feature F7a | #Feature F7b | #Feature F8 | #Feature F9a | #Feature Sample 1F10a | #Feature F10b | #Feature F11a | #Feature F11b | #Feature F12a | #Feature F13 | Start | End | PTM | AScore | Found By |
|---|---|---|---|---|---|---|---|---|---|---|---|---|---|---|---|---|---|---|---|---|---|---|---|---|---|---|---|---|---|---|---|---|---|---|---|---|---|---|---|---|---|---|---|---|---|---|---|---|---|
| K.HLYKQDPLTC(+57.02)K.C | Y | 79.51 | 1401.7074 | 11 | -0.9 | 468.2427 | 3 | 32.91 | 11 | F11:10027 | BuCaTN01_F10a.raw | | | | | | | 3.2403E5 | | | | 4.3374E5 | | | | | | 2 | 0 | 0 | 0 | 0 | 0 | 0 | 1 | 0 | 0 | 0 | 1 | 0 | 0 | 0 | 0 | 0 | 183 | 193 | Carbamidomethylation | C10:Carbamidomethylation:1000.00 | PEAKS DB |
| K.HFQSQHIHPMSFQQHSK.C | Y | 44.08 | 2102.9856 | 17 | -0.1 | 421.6044 | 5 | 37.10 | 10 | F10:15639 | BuCaTN01_F9a.raw | | | | | | | | | | 2.5195E4 | | | | | | | 1 | 0 | 0 | 0 | 0 | 0 | 0 | 0 | 0 | 0 | 1 | 0 | 0 | 0 | 0 | 0 | 0 | 123 | 139 | | | PEAKS DB |
| total 2 peptides | | | | | | | | | | | | | | | | | | | | | | | | | | | | | | | | | | | | | | | | | | | | | | | | | |

**Best Unique PSM (Scan F11:10027, m/z=468.2427, z=3, RT=32.91, ppm=-0.9):**

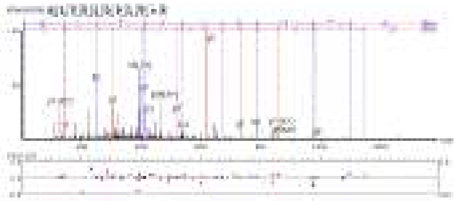

## XP_058018228.1

back to list

| Protein Coverage | Supporting Peptides | Best Unique PSM |

**Protein Coverage:**

1 MLRLKTCVRS ARGSRGPKGS PHTRRRRAGP QRRQVLQAAP AQGDGERQQG EVISFMKVFE RSACRSIETM VDIFQEYPDE

81 VEYIFKPSCV PLMRCAGCCN DEALECVPTE VYNVTMEIMK LK**HFQSQHIH PMSFQQHSK**C ECRQKKEVRI RQEKKSKRGK

161 GKGQKRKRKR GRYKPQNFHC EPCSERRK**HL YKQDPLTCK**C SCKFTDSRCK SKQLELNERT CRCEKPRR

C Carbamidomethylation (+57.02)

**Supporting Peptides:**

| Peptide | Uniq | -10lgP | Mass | Length | ppm | m/z | z | RT | Fraction | Scan | Source File | Area F1 | Area F2a | Area F3a | Area F4 | Area F5 | Area F6a | Area F7a | Area F7b | Area F8 | Area F9a | Area Sample 1F10a | Area F10b | Area F11a | Area F11b | Area F12a | Area F13 | #Feature | #Feature F1 | #Feature F2a | #Feature F3a | #Feature F4 | #Feature F5 | #Feature F6a | #Feature F7a | #Feature F7b | #Feature F8 | #Feature F9a | #Feature Sample 1F10a | #Feature F10b | #Feature F11a | #Feature F11b | #Feature F12a | #Feature F13 | Start | End | PTM | AScore | Found By |
|---|---|---|---|---|---|---|---|---|---|---|---|---|---|---|---|---|---|---|---|---|---|---|---|---|---|---|---|---|---|---|---|---|---|---|---|---|---|---|---|---|---|---|---|---|---|---|---|---|---|
| K.HLYKQDPLTC(+57.02)K.C | Y | 79.51 | 1401.7074 | 11 | -0.9 | 468.2427 | 3 | 32.91 | 11 | F11:10027 | BuCaTN01_F10a.raw | | | | | | | 3.2403E5 | | | | 4.3374E5 | | | | | | 2 | 0 | 0 | 0 | 0 | 0 | 0 | 1 | 0 | 0 | 0 | 1 | 0 | 0 | 0 | 0 | 0 | 189 | 199 | Carbamidomethylation | C10:Carbamidomethylation:1000.00 | PEAKS DB |
| K.HFQSQHIHPMSFQQHSK.C | Y | 44.08 | 2102.9856 | 17 | -0.1 | 421.6044 | 5 | 37.10 | 10 | F10:15639 | BuCaTN01_F9a.raw | | | | | | | | | | 2.5195E4 | | | | | | | 1 | 0 | 0 | 0 | 0 | 0 | 0 | 0 | 0 | 0 | 1 | 0 | 0 | 0 | 0 | 0 | 0 | 123 | 139 | | | PEAKS DB |
| total 2 peptides | | | | | | | | | | | | | | | | | | | | | | | | | | | | | | | | | | | | | | | | | | | | | | | | | |

**Best Unique PSM (Scan F11:10027, m/z=468.2427, z=3, RT=32.91, ppm=-0.9):**

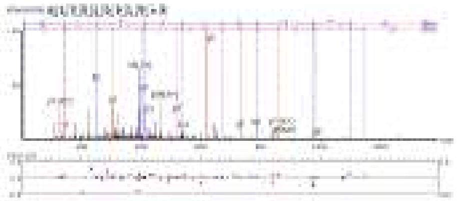

## AWX67617.1

back to list

| Protein Coverage | Supporting Peptides | Best Unique PSM |

**Protein Coverage:**

1 MNFLLTWIHW GLAALLYFHN AKVLQAAPAQ GDGDRQQGEV ISFMKVFERS ACRSIETMVD IFLEYPDEVE YIFKPSCVPL

81 MRCAGCCNDE ALECVPTEVY NVTMEIMKLK **HFQSQHIHPM SFQQHSK**CEC RQKKEVRITQ ENHCEPCSER RK**HLYKQDPL**

161 **TCK**CSCKFTD SRCKSKQLEL NERTCRCEKP RR

C Carbamidomethylation (+57.02)

**Supporting Peptides:**

| Peptide | Uniq | -10lgP | Mass | Length | ppm | m/z | z | RT | Fraction | Scan | Source File | Area F1 | Area F2a | Area F3a | Area F4 | Area F5 | Area F6a | Area F7a | Area F7b | Area F8 | Area F9a | Area Sample 1F10a | Area F10b | Area F11a | Area F11b | Area F12a | Area F13 | #Feature | #Feature F1 | #Feature F2a | #Feature F3a | #Feature F4 | #Feature F5 | #Feature F6a | #Feature F7a | #Feature F7b | #Feature F8 | #Feature F9a | #Feature Sample 1F10a | #Feature F10b | #Feature F11a | #Feature F11b | #Feature F12a | #Feature F13 | Start | End | PTM | AScore | Found By |
|---|---|---|---|---|---|---|---|---|---|---|---|---|---|---|---|---|---|---|---|---|---|---|---|---|---|---|---|---|---|---|---|---|---|---|---|---|---|---|---|---|---|---|---|---|---|---|---|---|---|
| K.HLYKQDPLTC(+57.02)K.C | Y | 79.51 | 1401.7074 | 11 | -0.9 | 468.2427 | 3 | 32.91 | 11 | F11:10027 | BuCaTN01_F10a.raw | | | | | | | 3.2403E5 | | | | 4.3374E5 | | | | | | 2 | 0 | 0 | 0 | 0 | 0 | 0 | 1 | 0 | 0 | 0 | 1 | 0 | 0 | 0 | 0 | 0 | 153 | 163 | Carbamidomethylation | C10:Carbamidomethylation:1000.00 | PEAKS DB |
| K.HFQSQHIHPMSFQQHSK.C | Y | 44.08 | 2102.9856 | 17 | -0.1 | 421.6044 | 5 | 37.10 | 10 | F10:15639 | BuCaTN01_F9a.raw | | | | | | | | | | 2.5195E4 | | | | | | | 1 | 0 | 0 | 0 | 0 | 0 | 0 | 0 | 0 | 0 | 1 | 0 | 0 | 0 | 0 | 0 | 0 | 111 | 127 | | | PEAKS DB |
| total 2 peptides | | | | | | | | | | | | | | | | | | | | | | | | | | | | | | | | | | | | | | | | | | | | | | | | | |

**Best Unique PSM (Scan F11:10027, m/z=468.2427, z=3, RT=32.91, ppm=-0.9):**

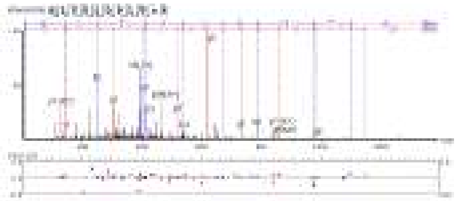

## JAS04556.1

back to list

| Protein Coverage | Supporting Peptides | Best Unique PSM |

**Protein Coverage:**

```
  1 MNFLLTWIHW GLAALLYFHN AKVLQAAPAQ GDGDRQQGEV ISFMKVFERS ACRSIETMVD IFLEYPDEVE YIFKPSCVPL
 81 MRCAGCCNDE ALECVPTEVY NVTMEIMKLK HFQSQHIHPM SFQQHSKCEC RQKKEVRITQ ENHCEPCSER RKHLYKQDPL
161 TCKCSCKFTD SRCKSKQLEL NERTCRCEKP RR
```

C Carbamidomethylation (+57.02); 162 C

**Supporting Peptides:**

| Peptide | Uniq | -10lgP | Mass | Length | ppm | m/z | z | RT | Fraction | Scan | Source File | Area F1 | Area F2a | Area F3a | Area F4 | Area F5 | Area F6a | Area F7a | Area F7b | Area F8 | Area F9a | Area Sample 1F10a | Area F10b | Area F11a | Area F11b | Area F12a | Area F13 | #Feature | #Feature F1 | #Feature F2a | #Feature F3a | #Feature F4 | #Feature F5 | #Feature F6a | #Feature F7a | #Feature F7b | #Feature F8 | #Feature F9a | #Feature Sample 1F10a | #Feature F10b | #Feature F11a | #Feature F11b | #Feature F12a | #Feature F13 | Start | End | PTM | AScore | Found By |
|---|---|---|---|---|---|---|---|---|---|---|---|---|---|---|---|---|---|---|---|---|---|---|---|---|---|---|---|---|---|---|---|---|---|---|---|---|---|---|---|---|---|---|---|---|---|---|---|---|---|
| K.HLYKQDPLTC(+57.02)K.C | Y | 79.51 | 1401.7074 | 11 | -0.9 | 468.2427 | 3 | 32.91 | 11 | F11:10027 | BuCaTN01_F10a.raw | | | | | | | 3.2403E5 | | | | 4.3374E5 | | | | | | 2 | 0 | 0 | 0 | 0 | 0 | 0 | 1 | 0 | 0 | 0 | 1 | 0 | 0 | 0 | 0 | 0 | 153 | 163 | Carbamidomethylation | C10:Carbamidomethylation:1000.00 | PEAKS DB |
| K.HFQSQHIHPMSFQQHSK.C | Y | 44.08 | 2102.9856 | 17 | -0.1 | 421.6044 | 5 | 37.10 | 10 | F10:15639 | BuCaTN01_F9a.raw | | | | | | | | | | 2.5195E4 | | | | | | | 1 | 0 | 0 | 0 | 0 | 0 | 0 | 0 | 0 | 0 | 1 | 0 | 0 | 0 | 0 | 0 | 0 | 111 | 127 | | | PEAKS DB |
| total 2 peptides | | | | | | | | | | | | | | | | | | | | | | | | | | | | | | | | | | | | | | | | | | | | | | | | | |

**Best Unique PSM (Scan F11:10027, m/z=468.2427, z=3, RT=32.91, ppm=-0.9):**

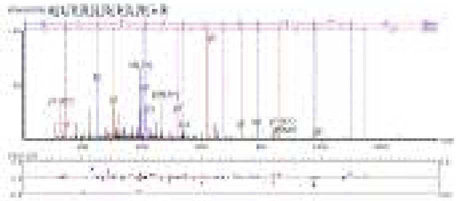

## XP_032072241.1

back to list

| Protein Coverage | Supporting Peptides | Best Unique PSM |

**Protein Coverage:**

```
  1 MNFLLTWIHW GLAALLYFHN AKVLQAAPAQ GDGDRQQGEV ISFMKVFERS ACRSIETMVD IFQEYPDEVE YIFKPSCVPL
 81 MRCAGCCNDE ALECVPTEIY NVTMEIMKLK HFQSQHIHPM SFQQHSKCEC RQKKEVRVRQ ENHCEPCSER RKHLYKQDPL
161 TCKCSCKFTD SRCKSKQLEL NERTCRCEKP RR
```

C Carbamidomethylation (+57.02); 162 C

**Supporting Peptides:**

| Peptide | Uniq | -10lgP | Mass | Length | ppm | m/z | z | RT | Fraction | Scan | Source File | Area F1 | Area F2a | Area F3a | Area F4 | Area F5 | Area F6a | Area F7a | Area F7b | Area F8 | Area F9a | Area Sample 1F10a | Area F10b | Area F11a | Area F11b | Area F12a | Area F13 | #Feature | #Feature F1 | #Feature F2a | #Feature F3a | #Feature F4 | #Feature F5 | #Feature F6a | #Feature F7a | #Feature F7b | #Feature F8 | #Feature F9a | #Feature Sample 1F10a | #Feature F10b | #Feature F11a | #Feature F11b | #Feature F12a | #Feature F13 | Start | End | PTM | AScore | Found By |
|---|---|---|---|---|---|---|---|---|---|---|---|---|---|---|---|---|---|---|---|---|---|---|---|---|---|---|---|---|---|---|---|---|---|---|---|---|---|---|---|---|---|---|---|---|---|---|---|---|---|
| K.HLYKQDPLTC(+57.02)K.C | Y | 79.51 | 1401.7074 | 11 | -0.9 | 468.2427 | 3 | 32.91 | 11 | F11:10027 | BuCaTN01_F10a.raw | | | | | | | 3.2403E5 | | | | 4.3374E5 | | | | | | 2 | 0 | 0 | 0 | 0 | 0 | 0 | 1 | 0 | 0 | 0 | 1 | 0 | 0 | 0 | 0 | 0 | 153 | 163 | Carbamidomethylation | C10:Carbamidomethylation:1000.00 | PEAKS DB |
| K.HFQSQHIHPMSFQQHSK.C | Y | 44.08 | 2102.9856 | 17 | -0.1 | 421.6044 | 5 | 37.10 | 10 | F10:15639 | BuCaTN01_F9a.raw | | | | | | | | | | 2.5195E4 | | | | | | | 1 | 0 | 0 | 0 | 0 | 0 | 0 | 0 | 0 | 0 | 1 | 0 | 0 | 0 | 0 | 0 | 0 | 111 | 127 | | | PEAKS DB |
| total 2 peptides | | | | | | | | | | | | | | | | | | | | | | | | | | | | | | | | | | | | | | | | | | | | | | | | | |

**Best Unique PSM (Scan F11:10027, m/z=468.2427, z=3, RT=32.91, ppm=-0.9):**

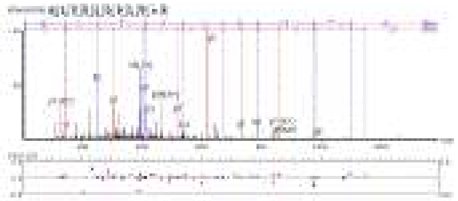

## JAG68919.1

back to list

| Protein Coverage | Supporting Peptides | Best Unique PSM |

**Protein Coverage:**

```
  1 MNFLLTWIHW GLAALLYFHN AKVLQAAPAQ GDGDRQQGEV ISFMKVFERS ACRSIETMVD IFLEYPDEVE YIFKPSCVPL
 81 MRCAGCCNDE ALECVPTEVY NVTMEIMKLK HFQSQHIHPM SFQQHSKCEC RQKKEVRITQ ENHCEPCSER RKHLYKQDPL
161 TCKCSCKFTD SRCKSKQLEL NERTCRCEKP RR
```

C Carbamidomethylation (+57.02); 162 C

**Supporting Peptides:**

| Peptide | Uniq | -10lgP | Mass | Length | ppm | m/z | z | RT | Fraction | Scan | Source File | Area F1 | Area F2a | Area F3a | Area F4 | Area F5 | Area F6a | Area F7a | Area F7b | Area F8 | Area F9a | Area Sample 1F10a | Area F10b | Area F11a | Area F11b | Area F12a | Area F13 | #Feature | #Feature F1 | #Feature F2a | #Feature F3a | #Feature F4 | #Feature F5 | #Feature F6a | #Feature F7a | #Feature F7b | #Feature F8 | #Feature F9a | #Feature Sample 1F10a | #Feature F10b | #Feature F11a | #Feature F11b | #Feature F12a | #Feature F13 | Start | End | PTM | AScore | Found By |
|---|---|---|---|---|---|---|---|---|---|---|---|---|---|---|---|---|---|---|---|---|---|---|---|---|---|---|---|---|---|---|---|---|---|---|---|---|---|---|---|---|---|---|---|---|---|---|---|---|---|
| K.HLYKQDPLTC(+57.02)K.C | Y | 79.51 | 1401.7074 | 11 | -0.9 | 468.2427 | 3 | 32.91 | 11 | F11:10027 | BuCaTN01_F10a.raw | | | | | | | 3.2403E5 | | | | 4.3374E5 | | | | | | 2 | 0 | 0 | 0 | 0 | 0 | 0 | 1 | 0 | 0 | 0 | 1 | 0 | 0 | 0 | 0 | 0 | 153 | 163 | Carbamidomethylation | C10:Carbamidomethylation:1000.00 | PEAKS DB |
| K.HFQSQHIHPMSFQQHSK.C | Y | 44.08 | 2102.9856 | 17 | -0.1 | 421.6044 | 5 | 37.10 | 10 | F10:15639 | BuCaTN01_F9a.raw | | | | | | | | | | 2.5195E4 | | | | | | | 1 | 0 | 0 | 0 | 0 | 0 | 0 | 0 | 0 | 0 | 1 | 0 | 0 | 0 | 0 | 0 | 0 | 111 | 127 | | | PEAKS DB |
| total 2 peptides | | | | | | | | | | | | | | | | | | | | | | | | | | | | | | | | | | | | | | | | | | | | | | | | | |

**Best Unique PSM (Scan F11:10027, m/z=468.2427, z=3, RT=32.91, ppm=-0.9):**

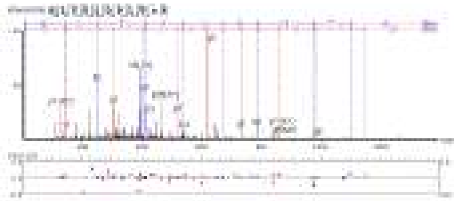

## XP_013922515.1

back to list

| Protein Coverage | Supporting Peptides | Best Unique PSM |

**Protein Coverage:**

```
  1 MNFLLTWIHW GLAALLYFHN AKVLQAAPAQ GDGDRQQGEV ISFMKVFERS ACRSIETMVD IFQEYPDEVE YIFKPSCVPL
 81 MRCAGCCNDE ALECVPTEIY NVTMEIMKLK HFQSQHIHPM SFQQHSKCEC RQKKEVTVRQ EKKSKRGKGK GQKRKRRRGR
161 HCEPCSERRK HLYKQDPLTC KCSCKFTDSR CKSKQLELNE RTCRCEKPRR
```

C Carbamidomethylation (+57.02)

**Supporting Peptides:**

| Peptide | Uniq | -10lgP | Mass | Length | ppm | m/z | z | RT | Fraction | Scan | Source File | Area F1 | Area F2a | Area F3a | Area F4 | Area F5 | Area F6a | Area F7a | Area F7b | Area F8 | Area F9a | Area Sample 1F10a | Area F10b | Area F11a | Area F11b | Area F12a | Area F13 | #Feature | #Feature F1 | #Feature F2a | #Feature F3a | #Feature F4 | #Feature F5 | #Feature F6a | #Feature F7a | #Feature F7b | #Feature F8 | #Feature F9a | #Feature Sample 1F10a | #Feature F10b | #Feature F11a | #Feature F11b | #Feature F12a | #Feature F13 | Start | End | PTM | AScore | Found By |
|---|---|---|---|---|---|---|---|---|---|---|---|---|---|---|---|---|---|---|---|---|---|---|---|---|---|---|---|---|---|---|---|---|---|---|---|---|---|---|---|---|---|---|---|---|---|---|---|---|---|
| K.HLYKQDPLTC(+57.02)K.C | Y | 79.51 | 1401.7074 | 11 | -0.9 | 468.2427 | 3 | 32.91 | 11 | F11:10027 | BuCaTN01_F10a.raw | | | | | | | 3.2403E5 | | | | 4.3374E5 | | | | | | 2 | 0 | 0 | 0 | 0 | 0 | 0 | 1 | 0 | 0 | 0 | 1 | 0 | 0 | 0 | 0 | 0 | 171 | 181 | Carbamidomethylation | C10:Carbamidomethylation:1000.00 | PEAKS DB |
| K.HFQSQHIHPMSFQQHSK.C | Y | 44.08 | 2102.9856 | 17 | -0.1 | 421.6044 | 5 | 37.10 | 10 | F10:15639 | BuCaTN01_F9a.raw | | | | | | | | | | 2.5195E4 | | | | | | | 1 | 0 | 0 | 0 | 0 | 0 | 0 | 0 | 0 | 0 | 1 | 0 | 0 | 0 | 0 | 0 | 0 | 111 | 127 | | | PEAKS DB |
| total 2 peptides | | | | | | | | | | | | | | | | | | | | | | | | | | | | | | | | | | | | | | | | | | | | | | | | | |

**Best Unique PSM (Scan F11:10027, m/z=468.2427, z=3, RT=32.91, ppm=-0.9):**

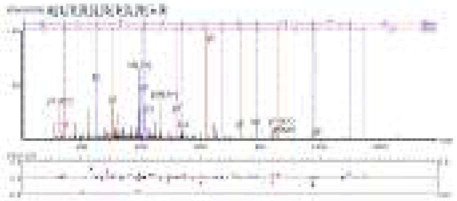

## XP_032072240.1

back to list

| Protein Coverage | Supporting Peptides | Best Unique PSM |

**Protein Coverage:**

```
  1 MNFLLTWIHW GLAALLYFHN AKVLQAAPAQ GDGDRQQGEV ISFMKVFERS ACRSIETMVD IFQEYPDEVE YIFKPSCVPL
 81 MRCAGCCNDE ALECVPTEIY NVTMEIMKLK HFQSQHIHPM SFQQHSKCEC RQKKEVRVRQ EKKSKRGKGK GQKRKRRRGR
161 YKPQNFHCEP CSERRKHLYK QDPLTCKCSC KFTDSRCKSK QLELNERTCR CEKPRR
```

C Carbamidomethylation (+57.02)

**Supporting Peptides:**

| Peptide | Uniq | -10lgP | Mass | Length | ppm | m/z | z | RT | Fraction | Scan | Source File | Area F1 | Area F2a | Area F3a | Area F4 | Area F5 | Area F6a | Area F7a | Area F7b | Area F8 | Area F9a | Area Sample 1F10a | Area F10b | Area F11a | Area F11b | Area F12a | Area F13 | #Feature | #Feature F1 | #Feature F2a | #Feature F3a | #Feature F4 | #Feature F5 | #Feature F6a | #Feature F7a | #Feature F7b | #Feature F8 | #Feature F9a | #Feature Sample 1F10a | #Feature F10b | #Feature F11a | #Feature F11b | #Feature F12a | #Feature F13 | Start | End | PTM | AScore | Found By |
|---|---|---|---|---|---|---|---|---|---|---|---|---|---|---|---|---|---|---|---|---|---|---|---|---|---|---|---|---|---|---|---|---|---|---|---|---|---|---|---|---|---|---|---|---|---|---|---|---|---|
| K.HLYKQDPLTC(+57.02)K.C | Y | 79.51 | 1401.7074 | 11 | -0.9 | 468.2427 | 3 | 32.91 | 11 | F11:10027 | BuCaTN01_F10a.raw | | | | | | | 3.2403E5 | | | | 4.3374E5 | | | | | | 2 | 0 | 0 | 0 | 0 | 0 | 0 | 1 | 0 | 0 | 0 | 1 | 0 | 0 | 0 | 0 | 0 | 177 | 187 | Carbamidomethylation | C10:Carbamidomethylation:1000.00 | PEAKS DB |
| K.HFQSQHIHPMSFQQHSK.C | Y | 44.08 | 2102.9856 | 17 | -0.1 | 421.6044 | 5 | 37.10 | 10 | F10:15639 | BuCaTN01_F9a.raw | | | | | | | | | | 2.5195E4 | | | | | | | 1 | 0 | 0 | 0 | 0 | 0 | 0 | 0 | 0 | 0 | 1 | 0 | 0 | 0 | 0 | 0 | 0 | 111 | 127 | | | PEAKS DB |
| total 2 peptides | | | | | | | | | | | | | | | | | | | | | | | | | | | | | | | | | | | | | | | | | | | | | | | | | |

**Best Unique PSM (Scan F11:10027, m/z=468.2427, z=3, RT=32.91, ppm=-0.9):**

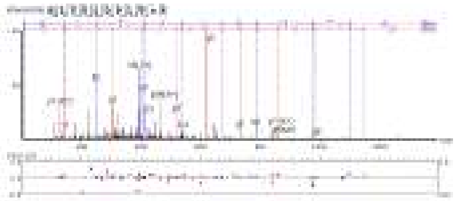

## XP_013922514.1

back to list

| Protein Coverage | Supporting Peptides | Best Unique PSM |

**Protein Coverage:**

```
  1 MNFLLTWIHW GLAALLYFHN AKVLQAAPAQ GDGDRQQGEV ISFMKVFERS ACRSIETMVD IFQEYPDEVE YIFKPSCVPL
 81 MRCAGCCNDE ALECVPTEIY NVTMEIMKLK HFQSQHIHPM SFQQHSKCEC RQKKEVTVRQ EKKSKRGKGK GQKRKRRRGR
161 YKPQNFHCEP CSERRKHLYK QDPLTCKCSC KFTDSRCKSK QLELNERTCR CEKPRR
```

C Carbamidomethylation (+57.02)

**Supporting Peptides:**

| Peptide | Uniq | -10lgP | Mass | Length | ppm | m/z | z | RT | Fraction | Scan | Source File | Area F1 | Area F2a | Area F3a | Area F4 | Area F5 | Area F6a | Area F7a | Area F7b | Area F8 | Area F9a | Area Sample 1F10a | Area F10b | Area F11a | Area F11b | Area F12a | Area F13 | #Feature | #Feature F1 | #Feature F2a | #Feature F3a | #Feature F4 | #Feature F5 | #Feature F6a | #Feature F7a | #Feature F7b | #Feature F8 | #Feature F9a | #Feature Sample 1F10a | #Feature F10b | #Feature F11a | #Feature F11b | #Feature F12a | #Feature F13 | Start | End | PTM | AScore | Found By |
|---|---|---|---|---|---|---|---|---|---|---|---|---|---|---|---|---|---|---|---|---|---|---|---|---|---|---|---|---|---|---|---|---|---|---|---|---|---|---|---|---|---|---|---|---|---|---|---|---|---|
| K.HLYKQDPLTC(+57.02)K.C | Y | 79.51 | 1401.7074 | 11 | -0.9 | 468.2427 | 3 | 32.91 | 11 | F11:10027 | BuCaTN01_F10a.raw | | | | | | | 3.2403E5 | | | | 4.3374E5 | | | | | | 2 | 0 | 0 | 0 | 0 | 0 | 0 | 1 | 0 | 0 | 0 | 1 | 0 | 0 | 0 | 0 | 0 | 177 | 187 | Carbamidomethylation | C10:Carbamidomethylation:1000.00 | PEAKS DB |
| K.HFQSQHIHPMSFQQHSK.C | Y | 44.08 | 2102.9856 | 17 | -0.1 | 421.6044 | 5 | 37.10 | 10 | F10:15639 | BuCaTN01_F9a.raw | | | | | | | | | | 2.5195E4 | | | | | | | 1 | 0 | 0 | 0 | 0 | 0 | 0 | 0 | 0 | 0 | 1 | 0 | 0 | 0 | 0 | 0 | 0 | 111 | 127 | | | PEAKS DB |
| total 2 peptides | | | | | | | | | | | | | | | | | | | | | | | | | | | | | | | | | | | | | | | | | | | | | | | | | |

**Best Unique PSM (Scan F11:10027, m/z=468.2427, z=3, RT=32.91, ppm=-0.9):**

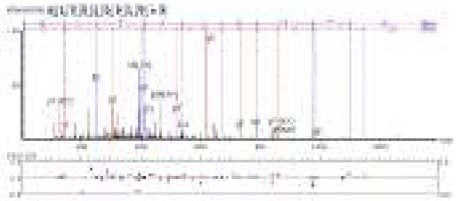

## AWX67680.1

back to list

| Protein Coverage | Supporting Peptides | Best Unique PSM |

**Protein Coverage:**

```
  1 MNFLLTWIHW GLAALLYFHN AKVLQAAPAQ GDGDRQQGEV ISFMKVFERS ACRSIETMVD IFLEYPDEVE YIFKPSCVPL
 81 MRCAGCCNDE ALECVPTEVY NVTMEIMKLK HFQSQHIHPM SFQQHSKCEC RQKKEVRITQ EKKSKRGKGK GQKRKRKRGR
161 YKPQNFHCEP CSERRKHLYK QDPLTCKCSC KFTDSRCKSK QLELNERTCR CEKPRR
```

C Carbamidomethylation (+57.02)

**Supporting Peptides:**

| Peptide | Uniq | -10lgP | Mass | Length | ppm | m/z | z | RT | Fraction | Scan | Source File | Area F1 | Area F2a | Area F3a | Area F4 | Area F5 | Area F6a | Area F7a | Area F7b | Area F8 | Area F9a | Area Sample 1F10a | Area F10b | Area F11a | Area F11b | Area F12a | Area F13 | #Feature | #Feature F1 | #Feature F2a | #Feature F3a | #Feature F4 | #Feature F5 | #Feature F6a | #Feature F7a | #Feature F7b | #Feature F8 | #Feature F9a | #Feature Sample 1F10a | #Feature F10b | #Feature F11a | #Feature F11b | #Feature F12a | #Feature F13 | Start | End | PTM | AScore | Found By |
|---|---|---|---|---|---|---|---|---|---|---|---|---|---|---|---|---|---|---|---|---|---|---|---|---|---|---|---|---|---|---|---|---|---|---|---|---|---|---|---|---|---|---|---|---|---|---|---|---|---|
| K.HLYKQDPLTC(+57.02)K.C | Y | 79.51 | 1401.7074 | 11 | -0.9 | 468.2427 | 3 | 32.91 | 11 | F11:10027 | BuCaTN01_F10a.raw | | | | | | | 3.2403E5 | | | | 4.3374E5 | | | | | | 2 | 0 | 0 | 0 | 0 | 0 | 0 | 1 | 0 | 0 | 0 | 1 | 0 | 0 | 0 | 0 | 0 | 177 | 187 | Carbamidomethylation | C10:Carbamidomethylation:1000.00 | PEAKS DB |
| K.HFQSQHIHPMSFQQHSK.C | Y | 44.08 | 2102.9856 | 17 | -0.1 | 421.6044 | 5 | 37.10 | 10 | F10:15639 | BuCaTN01_F9a.raw | | | | | | | | | | 2.5195E4 | | | | | | | 1 | 0 | 0 | 0 | 0 | 0 | 0 | 0 | 0 | 0 | 1 | 0 | 0 | 0 | 0 | 0 | 0 | 111 | 127 | | | PEAKS DB |
| total 2 peptides | | | | | | | | | | | | | | | | | | | | | | | | | | | | | | | | | | | | | | | | | | | | | | | | | |

**Best Unique PSM (Scan F11:10027, m/z=468.2427, z=3, RT=32.91, ppm=-0.9):**

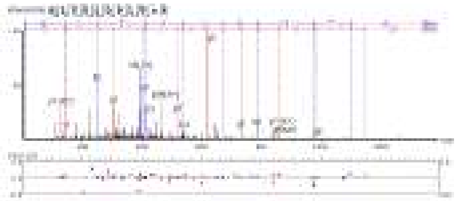

## JAC94979.1

back to list

| Protein Coverage | Supporting Peptides | Best Unique PSM |

**Protein Coverage:**

```
  1  MNFLLTWIHW GLAALLYFHN AKVLQAAPAQ GDGDRQQGEV ISFMKVFERS ACRSIETMVD IFQEYPDEVE YIFKPSCVPL
 81  MRCAGCCNDE ALECVPTEVY NVTMEIMKLK HFQSQHIHPM SFQQHSKCEC RQKKEVRIRQ EKKSKRGKGK GQKRKRKRGR
                                                   186
161  YKPQNFHCEP CSERRKHLYK QDPLTCKCSC KFTDSRCKSK QLELNERTCR CEKPRR
```

C Carbamidomethylation (+57.02)

**Supporting Peptides:**

| Peptide | Uniq | -10lgP | Mass | Length | ppm | m/z | z | RT | Fraction | Scan | Source File | Area F1 | Area F2a | Area F3a | Area F4 | Area F5 | Area F6a | Area F7a | Area F7b | Area F8 | Area F9a | Area Sample 1F10a | Area F10b | Area F11a | Area F11b | Area F12a | Area F13 | #Feature | #Feature F1 | #Feature F2a | #Feature F3a | #Feature F4 | #Feature F5 | #Feature F6a | #Feature F7a | #Feature F7b | #Feature F8 | #Feature F9a | #Feature Sample 1F10a | #Feature F10b | #Feature F11a | #Feature F11b | #Feature F12a | #Feature F13 | Start | End | PTM | AScore | Found By |
|---|---|---|---|---|---|---|---|---|---|---|---|---|---|---|---|---|---|---|---|---|---|---|---|---|---|---|---|---|---|---|---|---|---|---|---|---|---|---|---|---|---|---|---|---|---|---|---|---|---|
| K.HLYKQDPLTC(+57.02)K.C | Y | 79.51 | 1401.7074 | 11 | -0.9 | 468.2427 | 3 | 32.91 | 11 | F11:10027 | BuCaTN01_F10a.raw | | | | | | | 3.2403E5 | | | | 4.3374E5 | | | | | | 2 | 0 | 0 | 0 | 0 | 0 | 0 | 1 | 0 | 0 | 0 | 1 | 0 | 0 | 0 | 0 | 0 | 177 | 187 | Carbamidomethylation | C10:Carbamidomethylation:1000.00 | PEAKS DB |
| K.HFQSQHIHPMSFQQHSK.C | Y | 44.08 | 2102.9856 | 17 | -0.1 | 421.6044 | 5 | 37.10 | 10 | F10:15639 | BuCaTN01_F9a.raw | | | | | | | | | | 2.5195E4 | | | | | | | 1 | 0 | 0 | 0 | 0 | 0 | 0 | 0 | 0 | 0 | 1 | 0 | 0 | 0 | 0 | 0 | 0 | 111 | 127 | | | PEAKS DB |
| total 2 peptides | | | | | | | | | | | | | | | | | | | | | | | | | | | | | | | | | | | | | | | | | | | | | | | | | |

**Best Unique PSM (Scan F11:10027, m/z=468.2427, z=3, RT=32.91, ppm=-0.9):**

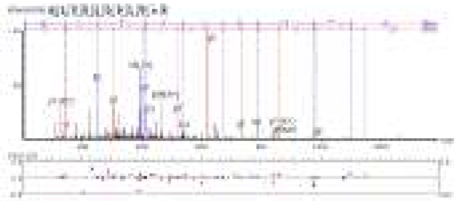

## XP_034283975.1

back to list

| Protein Coverage | Supporting Peptides | Best Unique PSM |

**Protein Coverage:**

```
  1  MNFLLTWIHW GLAALLYFHN AKVLQAAPAQ GDGDRQQSEV ISFMKVFERS ACRSIETMVD IFQEYPDEVE YIFKPSCVPL
 81  MRCAGCCNDE ALECVPTEVY NVTMEIMKLK HFQSQHIHPM SFQQHSKCEC RQKKEVRIRQ ENHCEPCSER RKHLYKQDPL
     162
161  TCKCSCKFTD SRCKSKQLEL NERTCRCEKP RR
```

C Carbamidomethylation (+57.02)

**Supporting Peptides:**

| Peptide | Uniq | -10lgP | Mass | Length | ppm | m/z | z | RT | Fraction | Scan | Source File | Area F1 | Area F2a | Area F3a | Area F4 | Area F5 | Area F6a | Area F7a | Area F7b | Area F8 | Area F9a | Area Sample 1F10a | Area F10b | Area F11a | Area F11b | Area F12a | Area F13 | #Feature | #Feature F1 | #Feature F2a | #Feature F3a | #Feature F4 | #Feature F5 | #Feature F6a | #Feature F7a | #Feature F7b | #Feature F8 | #Feature F9a | #Feature Sample 1F10a | #Feature F10b | #Feature F11a | #Feature F11b | #Feature F12a | #Feature F13 | Start | End | PTM | AScore | Found By |
|---|---|---|---|---|---|---|---|---|---|---|---|---|---|---|---|---|---|---|---|---|---|---|---|---|---|---|---|---|---|---|---|---|---|---|---|---|---|---|---|---|---|---|---|---|---|---|---|---|---|
| K.HLYKQDPLTC(+57.02)K.C | Y | 79.51 | 1401.7074 | 11 | -0.9 | 468.2427 | 3 | 32.91 | 11 | F11:10027 | BuCaTN01_F10a.raw | | | | | | | 3.2403E5 | | | | 4.3374E5 | | | | | | 2 | 0 | 0 | 0 | 0 | 0 | 0 | 1 | 0 | 0 | 0 | 1 | 0 | 0 | 0 | 0 | 0 | 153 | 163 | Carbamidomethylation | C10:Carbamidomethylation:1000.00 | PEAKS DB |
| K.HFQSQHIHPMSFQQHSK.C | Y | 44.08 | 2102.9856 | 17 | -0.1 | 421.6044 | 5 | 37.10 | 10 | F10:15639 | BuCaTN01_F9a.raw | | | | | | | | | | 2.5195E4 | | | | | | | 1 | 0 | 0 | 0 | 0 | 0 | 0 | 0 | 0 | 0 | 1 | 0 | 0 | 0 | 0 | 0 | 0 | 111 | 127 | | | PEAKS DB |
| total 2 peptides | | | | | | | | | | | | | | | | | | | | | | | | | | | | | | | | | | | | | | | | | | | | | | | | | |

**Best Unique PSM (Scan F11:10027, m/z=468.2427, z=3, RT=32.91, ppm=-0.9):**

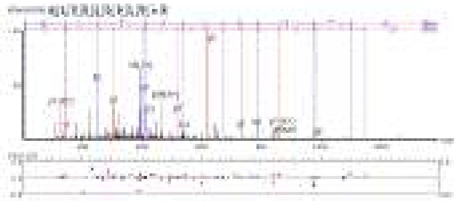

## JAC95039.1

back to list

| Protein Coverage | Supporting Peptides | Best Unique PSM |

**Protein Coverage:**

```
  1  MNFLLTWIHW GLAALLYFHN AKVLQAAPAQ GDGDRQQSEV ISFMKVFERS ACRSIETMVD IFQEYPDEVE YIFKPSCVPL
 81  MRCAGCCNDE ALECVPTEVY NVTMEIMKLK HFQSQHIHPM SFQQHSKCEC RQKKEVRIRQ ENHCEPCSER RKHLYKQDPL
     162
161  TCKCSCKFTD SRCKSKQLEL NERTCRCEKP RR
```

C Carbamidomethylation (+57.02)

**Supporting Peptides:**

| Peptide | Uniq | -10lgP | Mass | Length | ppm | m/z | z | RT | Fraction | Scan | Source File | Area F1 | Area F2a | Area F3a | Area F4 | Area F5 | Area F6a | Area F7a | Area F7b | Area F8 | Area F9a | Area Sample 1F10a | Area F10b | Area F11a | Area F11b | Area F12a | Area F13 | #Feature | #Feature F1 | #Feature F2a | #Feature F3a | #Feature F4 | #Feature F5 | #Feature F6a | #Feature F7a | #Feature F7b | #Feature F8 | #Feature F9a | #Feature Sample 1F10a | #Feature F10b | #Feature F11a | #Feature F11b | #Feature F12a | #Feature F13 | Start | End | PTM | AScore | Found By |
|---|---|---|---|---|---|---|---|---|---|---|---|---|---|---|---|---|---|---|---|---|---|---|---|---|---|---|---|---|---|---|---|---|---|---|---|---|---|---|---|---|---|---|---|---|---|---|---|---|---|
| K.HLYKQDPLTC(+57.02)K.C | Y | 79.51 | 1401.7074 | 11 | -0.9 | 468.2427 | 3 | 32.91 | 11 | F11:10027 | BuCaTN01_F10a.raw | | | | | | | 3.2403E5 | | | | 4.3374E5 | | | | | | 2 | 0 | 0 | 0 | 0 | 0 | 0 | 1 | 0 | 0 | 0 | 1 | 0 | 0 | 0 | 0 | 0 | 153 | 163 | Carbamidomethylation | C10:Carbamidomethylation:1000.00 | PEAKS DB |
| K.HFQSQHIHPMSFQQHSK.C | Y | 44.08 | 2102.9856 | 17 | -0.1 | 421.6044 | 5 | 37.10 | 10 | F10:15639 | BuCaTN01_F9a.raw | | | | | | | | | | 2.5195E4 | | | | | | | 1 | 0 | 0 | 0 | 0 | 0 | 0 | 0 | 0 | 0 | 1 | 0 | 0 | 0 | 0 | 0 | 0 | 111 | 127 | | | PEAKS DB |
| total 2 peptides | | | | | | | | | | | | | | | | | | | | | | | | | | | | | | | | | | | | | | | | | | | | | | | | | |

**Best Unique PSM (Scan F11:10027, m/z=468.2427, z=3, RT=32.91, ppm=-0.9):**

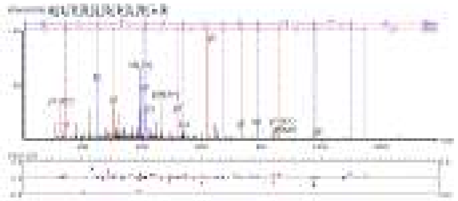

## XP_034283974.1

back to list

| Protein Coverage | Supporting Peptides | Best Unique PSM |

**Protein Coverage:**

```
  1  MNFLLTWIHW GLAALLYFHN AKVLQAAPAQ GDGDRQQSEV ISFMKVFERS ACRSIETMVD IFQEYPDEVE YIFKPSCVPL
 81  MRCAGCCNDE ALECVPTEVY NVTMEIMKLK HFQSQHIHPM SFQQHSKCEC RQKKEVRIRQ EKKSKRGKGK GQKRKRKRGR
161  HCEPCSERRK HLYKQDPLTC KCSCKFTDSR CKSKQLELNE RTCRCEKPRR
```

C Carbamidomethylation (+57.02); 180

**Supporting Peptides:**

| Peptide | Uniq | -10lgP | Mass | Length | ppm | m/z | z | RT | Fraction | Scan | Source File | Area F1 | Area F2a | Area F3a | Area F4 | Area F5 | Area F6a | Area F7a | Area F7b | Area F8 | Area F9a | Area Sample 1F10a | Area F10b | Area F11a | Area F11b | Area F12a | Area F13 | #Feature | #Feature F1 | #Feature F2a | #Feature F3a | #Feature F4 | #Feature F5 | #Feature F6a | #Feature F7a | #Feature F7b | #Feature F8 | #Feature F9a | #Feature Sample 1F10a | #Feature F10b | #Feature F11a | #Feature F11b | #Feature F12a | #Feature F13 | Start | End | PTM | AScore | Found By |
|---|---|---|---|---|---|---|---|---|---|---|---|---|---|---|---|---|---|---|---|---|---|---|---|---|---|---|---|---|---|---|---|---|---|---|---|---|---|---|---|---|---|---|---|---|---|---|---|---|---|
| K.HLYKQDPLTC(+57.02)K.C | Y | 79.51 | 1401.7074 | 11 | -0.9 | 468.2427 | 3 | 32.91 | 11 | F11:10027 | BuCaTN01_F10a.raw | | | | | | | 3.2403E5 | | | | 4.3374E5 | | | | | | 2 | 0 | 0 | 0 | 0 | 0 | 0 | 1 | 0 | 0 | 0 | 1 | 0 | 0 | 0 | 0 | 0 | 171 | 181 | Carbamidomethylation | C10:Carbamidomethylation:1000.00 | PEAKS DB |
| K.HFQSQHIHPMSFQQHSK.C | Y | 44.08 | 2102.9856 | 17 | -0.1 | 421.6044 | 5 | 37.10 | 10 | F10:15639 | BuCaTN01_F9a.raw | | | | | | | | | | 2.5195E4 | | | | | | | 1 | 0 | 0 | 0 | 0 | 0 | 0 | 0 | 0 | 0 | 1 | 0 | 0 | 0 | 0 | 0 | 0 | 111 | 127 | | | PEAKS DB |
| total 2 peptides | | | | | | | | | | | | | | | | | | | | | | | | | | | | | | | | | | | | | | | | | | | | | | | | | |

**Best Unique PSM (Scan F11:10027, m/z=468.2427, z=3, RT=32.91, ppm=-0.9):**

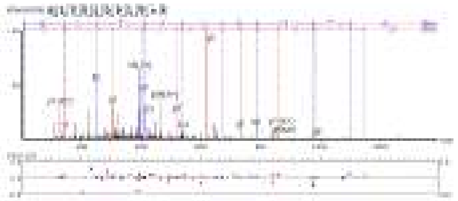

## XP_034283973.1

back to list

| Protein Coverage | Supporting Peptides | Best Unique PSM |

**Protein Coverage:**

```
  1  MNFLLTWIHW GLAALLYFHN AKVLQAAPAQ GDGDRQQSEV ISFMKVFERS ACRSIETMVD IFQEYPDEVE YIFKPSCVPL
 81  MRCAGCCNDE ALECVPTEVY NVTMEIMKLK HFQSQHIHPM SFQQHSKCEC RQKKEVRIRQ EKKSKRGKGK GQKRKRKRGR
161  YKPQNFHCEP CSERRKHLYK QDPLTCKCSC KFTDSRCKSK QLELNERTCR CEKPRR
```

C Carbamidomethylation (+57.02); 186

**Supporting Peptides:**

| Peptide | Uniq | -10lgP | Mass | Length | ppm | m/z | z | RT | Fraction | Scan | Source File | Area F1 | Area F2a | Area F3a | Area F4 | Area F5 | Area F6a | Area F7a | Area F7b | Area F8 | Area F9a | Area Sample 1F10a | Area F10b | Area F11a | Area F11b | Area F12a | Area F13 | #Feature | #Feature F1 | #Feature F2a | #Feature F3a | #Feature F4 | #Feature F5 | #Feature F6a | #Feature F7a | #Feature F7b | #Feature F8 | #Feature F9a | #Feature Sample 1F10a | #Feature F10b | #Feature F11a | #Feature F11b | #Feature F12a | #Feature F13 | Start | End | PTM | AScore | Found By |
|---|---|---|---|---|---|---|---|---|---|---|---|---|---|---|---|---|---|---|---|---|---|---|---|---|---|---|---|---|---|---|---|---|---|---|---|---|---|---|---|---|---|---|---|---|---|---|---|---|---|
| K.HLYKQDPLTC(+57.02)K.C | Y | 79.51 | 1401.7074 | 11 | -0.9 | 468.2427 | 3 | 32.91 | 11 | F11:10027 | BuCaTN01_F10a.raw | | | | | | | 3.2403E5 | | | | 4.3374E5 | | | | | | 2 | 0 | 0 | 0 | 0 | 0 | 0 | 1 | 0 | 0 | 0 | 1 | 0 | 0 | 0 | 0 | 0 | 177 | 187 | Carbamidomethylation | C10:Carbamidomethylation:1000.00 | PEAKS DB |
| K.HFQSQHIHPMSFQQHSK.C | Y | 44.08 | 2102.9856 | 17 | -0.1 | 421.6044 | 5 | 37.10 | 10 | F10:15639 | BuCaTN01_F9a.raw | | | | | | | | | | 2.5195E4 | | | | | | | 1 | 0 | 0 | 0 | 0 | 0 | 0 | 0 | 0 | 0 | 1 | 0 | 0 | 0 | 0 | 0 | 0 | 111 | 127 | | | PEAKS DB |
| total 2 peptides | | | | | | | | | | | | | | | | | | | | | | | | | | | | | | | | | | | | | | | | | | | | | | | | | |

**Best Unique PSM (Scan F11:10027, m/z=468.2427, z=3, RT=32.91, ppm=-0.9):**

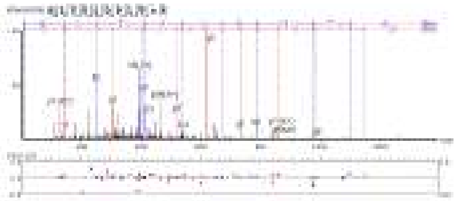

## JAC96418.1

back to list

| Protein Coverage | Supporting Peptides | Best Unique PSM |

**Protein Coverage:**

```
  1  MNFLLTWIHW GLAALLYFHN AKVLQAAPAQ GDGDRPQGEV ISFMKVFELS ACRSIETMVD IFQEYPDEVE YIFKPSCVSL
 81  MRCAGCCNDE ALECVPTEVY NISMEIMKLK HFQSQHIHPM SFQQHSKCEC RQKKEVRIRQ ENHCEPCSER RKHLYKQDPL
161  TCKCSCKFTD SRCKSKQLEL NERTCRCEKP RR
```

C Carbamidomethylation (+57.02); 162

**Supporting Peptides:**

| Peptide | Uniq | -10lgP | Mass | Length | ppm | m/z | z | RT | Fraction | Scan | Source File | Area F1 | Area F2a | Area F3a | Area F4 | Area F5 | Area F6a | Area F7a | Area F7b | Area F8 | Area F9a | Area Sample 1F10a | Area F10b | Area F11a | Area F11b | Area F12a | Area F13 | #Feature | #Feature F1 | #Feature F2a | #Feature F3a | #Feature F4 | #Feature F5 | #Feature F6a | #Feature F7a | #Feature F7b | #Feature F8 | #Feature F9a | #Feature Sample 1F10a | #Feature F10b | #Feature F11a | #Feature F11b | #Feature F12a | #Feature F13 | Start | End | PTM | AScore | Found By |
|---|---|---|---|---|---|---|---|---|---|---|---|---|---|---|---|---|---|---|---|---|---|---|---|---|---|---|---|---|---|---|---|---|---|---|---|---|---|---|---|---|---|---|---|---|---|---|---|---|---|
| K.HLYKQDPLTC(+57.02)K.C | Y | 79.51 | 1401.7074 | 11 | -0.9 | 468.2427 | 3 | 32.91 | 11 | F11:10027 | BuCaTN01_F10a.raw | | | | | | | 3.2403E5 | | | | 4.3374E5 | | | | | | 2 | 0 | 0 | 0 | 0 | 0 | 0 | 1 | 0 | 0 | 0 | 1 | 0 | 0 | 0 | 0 | 0 | 153 | 163 | Carbamidomethylation | C10:Carbamidomethylation:1000.00 | PEAKS DB |
| K.HFQSQHIHPMSFQQHSK.C | Y | 44.08 | 2102.9856 | 17 | -0.1 | 421.6044 | 5 | 37.10 | 10 | F10:15639 | BuCaTN01_F9a.raw | | | | | | | | | | 2.5195E4 | | | | | | | 1 | 0 | 0 | 0 | 0 | 0 | 0 | 0 | 0 | 0 | 1 | 0 | 0 | 0 | 0 | 0 | 0 | 111 | 127 | | | PEAKS DB |
| total 2 peptides | | | | | | | | | | | | | | | | | | | | | | | | | | | | | | | | | | | | | | | | | | | | | | | | | |

**Best Unique PSM (Scan F11:10027, m/z=468.2427, z=3, RT=32.91, ppm=-0.9):**

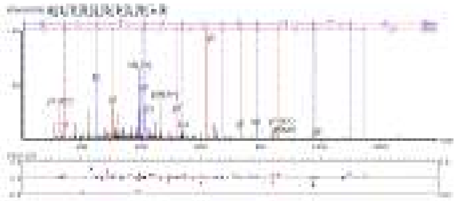

## JAB52755.1

back to list

| Protein Coverage | Supporting Peptides | Best Unique PSM |

**Protein Coverage:**

```
  1  MNFLLTWIHC GLAALLYFHN AKVLQAAPAQ EDGEKQPDEV ITFMTVYEHS VCRPIETMVD IFQEYPDEVE YIFKPSCVLL
 81  MKCGGCCNDE ALECVPTEVY NVSMEIMKLK HFQSQHIHPM SFQQHSKCQC RPKKAMRIKQ ENHCEPCSER RKHLYKQDPL
161  TCKCSCKFTD SRCKSKQLEL NERTCRCEKP RR
```

C Carbamidomethylation (+57.02); 162

**Supporting Peptides:**

| Peptide | Uniq | -10lgP | Mass | Length | ppm | m/z | z | RT | Fraction | Scan | Source File | Area F1 | Area F2a | Area F3a | Area F4 | Area F5 | Area F6a | Area F7a | Area F7b | Area F8 | Area F9a | Area Sample 1F10a | Area F10b | Area F11a | Area F11b | Area F12a | Area F13 | #Feature | #Feature F1 | #Feature F2a | #Feature F3a | #Feature F4 | #Feature F5 | #Feature F6a | #Feature F7a | #Feature F7b | #Feature F8 | #Feature F9a | #Feature Sample 1F10a | #Feature F10b | #Feature F11a | #Feature F11b | #Feature F12a | #Feature F13 | Start | End | PTM | AScore | Found By |
|---|---|---|---|---|---|---|---|---|---|---|---|---|---|---|---|---|---|---|---|---|---|---|---|---|---|---|---|---|---|---|---|---|---|---|---|---|---|---|---|---|---|---|---|---|---|---|---|---|---|
| K.HLYKQDPLTC(+57.02)K.C | Y | 79.51 | 1401.7074 | 11 | -0.9 | 468.2427 | 3 | 32.91 | 11 | F11:10027 | BuCaTN01_F10a.raw | | | | | | | 3.2403E5 | | | | 4.3374E5 | | | | | | 2 | 0 | 0 | 0 | 0 | 0 | 0 | 1 | 0 | 0 | 0 | 1 | 0 | 0 | 0 | 0 | 0 | 153 | 163 | Carbamidomethylation | C10:Carbamidomethylation:1000.00 | PEAKS DB |
| K.HFQSQHIHPMSFQQHSK.C | Y | 44.08 | 2102.9856 | 17 | -0.1 | 421.6044 | 5 | 37.10 | 10 | F10:15639 | BuCaTN01_F9a.raw | | | | | | | | | | 2.5195E4 | | | | | | | 1 | 0 | 0 | 0 | 0 | 0 | 0 | 0 | 0 | 0 | 1 | 0 | 0 | 0 | 0 | 0 | 0 | 111 | 127 | | | PEAKS DB |
| total 2 peptides | | | | | | | | | | | | | | | | | | | | | | | | | | | | | | | | | | | | | | | | | | | | | | | | | |

**Best Unique PSM (Scan F11:10027, m/z=468.2427, z=3, RT=32.91, ppm=-0.9):**

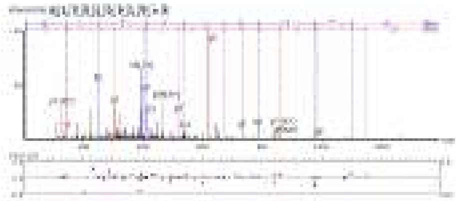

## JAI08989.1

back to list

| Protein Coverage | Supporting Peptides | Best Unique PSM |

**Protein Coverage:**

```
  1  MNFLLTWIHC GLAALLYFHN AKVLQAAPAQ EDGEKQPDEV ITFMTVYEHS VCRPIETMVD IFQEYPDEVE YIFKPSCVLL
 81  MKCGGCCNDE ALECVPTEVY NVSMEIMKLK HFQSQHIHPM SFQQHSKCQC RPKKAMRIKQ ENHCEPCSER RKHLYKQDPL
161  TCKCSCKFTD SRCKSKQLEL NERTCRCEKP RR
```

C Carbamidomethylation (+57.02)

162

**Supporting Peptides:**

| Peptide | Uniq | -10lgP | Mass | Length | ppm | m/z | z | RT | Fraction | Scan | Source File | Area F1 | Area F2a | Area F3a | Area F4 | Area F5 | Area F6a | Area F7a | Area F7b | Area F8 | Area F9a | Area Sample 1F10a | Area F10b | Area F11a | Area F11b | Area F12a | Area F13 | #Feature | #Feature F1 | #Feature F2a | #Feature F3a | #Feature F4 | #Feature F5 | #Feature F6a | #Feature F7a | #Feature F7b | #Feature F8 | #Feature F9a | #Feature Sample 1F10a | #Feature F10b | #Feature F11a | #Feature F11b | #Feature F12a | #Feature F13 | Start | End | PTM | AScore | Found By |
|---|---|---|---|---|---|---|---|---|---|---|---|---|---|---|---|---|---|---|---|---|---|---|---|---|---|---|---|---|---|---|---|---|---|---|---|---|---|---|---|---|---|---|---|---|---|---|---|---|---|
| K.HLYKQDPLTC(+57.02)K.C | Y | 79.51 | 1401.7074 | 11 | -0.9 | 468.2427 | 3 | 32.91 | 11 | F11:10027 | BuCaTN01_F10a.raw | | | | | | | 3.2403E5 | | | | 4.3374E5 | | | | | | 2 | 0 | 0 | 0 | 0 | 0 | 0 | 1 | 0 | 0 | 0 | 1 | 0 | 0 | 0 | 0 | 0 | 153 | 163 | Carbamidomethylation | C10:Carbamidomethylation:1000.00 | PEAKS DB |
| K.HFQSQHIHPMSFQQHSK.C | Y | 44.08 | 2102.9856 | 17 | -0.1 | 421.6044 | 5 | 37.10 | 10 | F10:15639 | BuCaTN01_F9a.raw | | | | | | | | | | 2.5195E4 | | | | | | | 1 | 0 | 0 | 0 | 0 | 0 | 0 | 0 | 0 | 0 | 1 | 0 | 0 | 0 | 0 | 0 | 0 | 111 | 127 | | | PEAKS DB |
| total 2 peptides | | | | | | | | | | | | | | | | | | | | | | | | | | | | | | | | | | | | | | | | | | | | | | | | | |

**Best Unique PSM (Scan F11:10027, m/z=468.2427, z=3, RT=32.91, ppm=-0.9):**

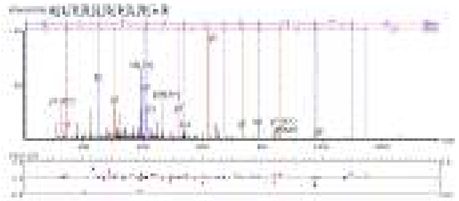

## ETE70050.1

back to list

| Protein Coverage | Supporting Peptides | Best Unique PSM |

**Protein Coverage:**

```
  1  MFATVPSCRW RRNPKTACHS LLAHKDYVLY AMLEKWADTC LTRSCGNLPP HQRAACLKRL ETQQRLCLEL IVAACWPMKA
 81  LVLQAAPAQE DGEKQPDEVI TFMTVFERSA CRPIETMVDI FQEYPDEVEY IFKPSCVLLM KCGGCCNDEA LECVPIEVYN
161  VSMEIMKLKH FQSQHIHPMS FQQHSKCQCS HCEPCSERRK HLYKQDPLTC KCSCKFTDSR CKSKQLELNE RTCRFVS
```

C Carbamidomethylation (+57.02)

210

**Supporting Peptides:**

| Peptide | Uniq | -10lgP | Mass | Length | ppm | m/z | z | RT | Fraction | Scan | Source File | Area F1 | Area F2a | Area F3a | Area F4 | Area F5 | Area F6a | Area F7a | Area F7b | Area F8 | Area F9a | Area Sample 1F10a | Area F10b | Area F11a | Area F11b | Area F12a | Area F13 | #Feature | #Feature F1 | #Feature F2a | #Feature F3a | #Feature F4 | #Feature F5 | #Feature F6a | #Feature F7a | #Feature F7b | #Feature F8 | #Feature F9a | #Feature Sample 1F10a | #Feature F10b | #Feature F11a | #Feature F11b | #Feature F12a | #Feature F13 | Start | End | PTM | AScore | Found By |
|---|---|---|---|---|---|---|---|---|---|---|---|---|---|---|---|---|---|---|---|---|---|---|---|---|---|---|---|---|---|---|---|---|---|---|---|---|---|---|---|---|---|---|---|---|---|---|---|---|---|
| K.HLYKQDPLTC(+57.02)K.C | Y | 79.51 | 1401.7074 | 11 | -0.9 | 468.2427 | 3 | 32.91 | 11 | F11:10027 | BuCaTN01_F10a.raw | | | | | | | 3.2403E5 | | | | 4.3374E5 | | | | | | 2 | 0 | 0 | 0 | 0 | 0 | 0 | 1 | 0 | 0 | 0 | 1 | 0 | 0 | 0 | 0 | 0 | 201 | 211 | Carbamidomethylation | C10:Carbamidomethylation:1000.00 | PEAKS DB |
| K.HFQSQHIHPMSFQQHSK.C | Y | 44.08 | 2102.9856 | 17 | -0.1 | 421.6044 | 5 | 37.10 | 10 | F10:15639 | BuCaTN01_F9a.raw | | | | | | | | | | 2.5195E4 | | | | | | | 1 | 0 | 0 | 0 | 0 | 0 | 0 | 0 | 0 | 0 | 1 | 0 | 0 | 0 | 0 | 0 | 0 | 170 | 186 | | | PEAKS DB |
| total 2 peptides | | | | | | | | | | | | | | | | | | | | | | | | | | | | | | | | | | | | | | | | | | | | | | | | | |

**Best Unique PSM (Scan F11:10027, m/z=468.2427, z=3, RT=32.91, ppm=-0.9):**

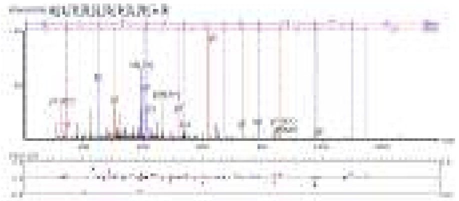

## XP_015681610.1

back to list

| Protein Coverage | Supporting Peptides | Best Unique PSM |

**Protein Coverage:**

```
  1  MKYAIVCLLL VGSVFAAEDD DKREEVGTVV GIDLGTTYSC VGVFKNGRVE IIANDQGNRI TPSYVAFTPE GERLIGDAAK
 81  NQLTSNPENT VFDAKRLIGR TWNDPSVQQD IKYLPFKVIE KKTKPYIQVD IGGGQTKTFA PEEISAMVLT KMKETAEAYL
161  GKKVTHAVVT VPAYFNDAQR QATKDAGTIA GLNVMRIINE PTAAAIAYGL DKREGEKNIL VFDLGGGTFD VSLLTIDNGV
241  FEVVATNGDT HLGGEDFDQR VMEHFIKLYK KKTGKDVRKD NRAVQKLRRE VEKAKRALSA QHQARIEIES FFEGEDFSET
321  LTRAKFEELN MDLFRSTMKP VQKVLEDADL KKPDIDEIVL VGGSTRIPKI QQLVKEFFNG KEPSRGINPD EAVAYGAAVQ
401  AGVLSGDQDT GDLVLLDVCP LTLGIETVGG VMTKLIPRNT VVPTKKSQIF STASDNQPTV TIKVYEGERP LTKDNHLLGT
481  FDLTGIPPAP RGVPQIEVTF EIDVNGILRV TAEDKGTGNK NKITITNDQN RLTPEEIERM VNDAEKFAEE DKRLKERIDA
561  RNELESYAYS LKNQIGDKEK LGGKLSSEEK ETIEKAVEEK IEWLESHQEA DIEDFKAQKK ELEEVVQPIV GKLYGGAGPP
641  PGGEEETAEK DEL
```

**Supporting Peptides:**

| Peptide | Uniq | -10lgP | Mass | Length | ppm | m/z | z | RT | Fraction | Scan | Source File | Area F1 | Area F2a | Area F3a | Area F4 | Area F5 | Area F6a | Area F7a | Area F7b | Area F8 | Area F9a | Area Sample 1F10a | Area F10b | Area F11a | Area F11b | Area F12a | Area F13 | #Feature | #Feature F1 | #Feature F2a | #Feature F3a | #Feature F4 | #Feature F5 | #Feature F6a | #Feature F7a | #Feature F7b | #Feature F8 | #Feature F9a | #Feature Sample 1F10a | #Feature F10b | #Feature F11a | #Feature F11b | #Feature F12a | #Feature F13 | Start | End | PTM | AScore | Found By |
|---|---|---|---|---|---|---|---|---|---|---|---|---|---|---|---|---|---|---|---|---|---|---|---|---|---|---|---|---|---|---|---|---|---|---|---|---|---|---|---|---|---|---|---|---|---|---|---|---|---|
| R.IINEPTAAAIAYGLDKR.E | Y | 45.90 | 1814.9890 | 17 | 1.3 | 606.0044 | 3 | 64.58 | 16 | F16:32262 | BuCaTN01_F13.raw | | | | | | | | | | | | | | | | 2.2698E5 | 1 | 0 | 0 | 0 | 0 | 0 | 0 | 0 | 0 | 0 | 0 | 0 | 0 | 0 | 0 | 0 | 1 | 197 | 213 | | | PEAKS DB |
| K.DAGTIAGLNVMR.I | Y | 45.60 | 1216.6234 | 12 | 1.5 | 609.3199 | 2 | 59.49 | 16 | F16:28937 | BuCaTN01_F13.raw | | | | | | | | | | | | | | | | 1.4052E5 | 1 | 0 | 0 | 0 | 0 | 0 | 0 | 0 | 0 | 0 | 0 | 0 | 0 | 0 | 0 | 0 | 1 | 185 | 196 | | | PEAKS DB |
| R.TWNDPSVQQDIK.Y | Y | 43.32 | 1429.6837 | 12 | 0.5 | 715.8495 | 2 | 47.71 | 16 | F16:21538 | BuCaTN01_F13.raw | | | | | | | | | | | | | | | | 7.1401E4 | 1 | 0 | 0 | 0 | 0 | 0 | 0 | 0 | 0 | 0 | 0 | 0 | 0 | 0 | 0 | 0 | 1 | 101 | 112 | | | PEAKS DB |
| total 3 peptides | | | | | | | | | | | | | | | | | | | | | | | | | | | | | | | | | | | | | | | | | | | | | | | | | |

**Best Unique PSM (Scan F16:32262, m/z=606.0044, z=3, RT=64.58, ppm=1.3):**

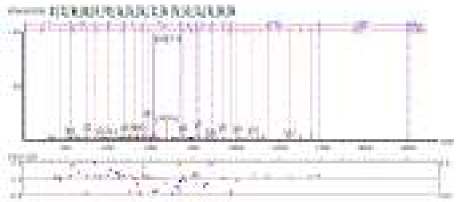

## XP_026568326.1

back to list

| Protein Coverage | Supporting Peptides | Best Unique PSM |

**Protein Coverage:**

```
  1 MKYYAVLCLL LVGSALATDD DDKKEEVGTV VGIDLGTTYS CVGVFKNGRV EIIANDQGNR ITPSYVAFTP EGERLIGDAA
 81 KNQLTSNPEN TVFDAKRLIG RTWNDPSVQQ DIKYLPFKVI EKKAKPYIQV DIGGGQTKTF APEEISAMVL TKMKETAEAY
161 LGKKVTHAVV TVPAYFNDAQ RQATKDAGTI AGLNVMRIIN EPTAAAIAYG LDKREGEKNI LVFDLGGGTF DVSLLTIDNG
241 VFEVVATNGD THLGGEDFDQ RVMEHFIKLY KKKTGKDVRK DNRAVQKLRR EVEKAKRALS SQHQARIEIE SFFEGEDFSE
321 TLTRAKFEEL NMDLFRSTMK PVQKVLEDAD LKKSDIDEIV LVGGSTRIPK IQQLVKEFFN GKEPSRGINP DEAVAYGAAV
401 QAGVLSGDQD TGDLVLLDVC PLTLGIETVG GVMTKLIPRN TVVPTKKSQI FSTASDNQPT VTIKVYEGER PLTKDNHLLG
481 TFDLTGIPPA PRGVPQIEVT FEIDVNGILR VTAEDKGTGN KNKITITNDQ NRLTPEEIER MVNDAEKFAE EDKKLKERID
561 ARNELESYAY SLKNQIGDKE KLGGKLSPED KETIEKAVEE KIEWLESHQD GDIEEFKAQK KELEEVVQPI VGKLYGGAGP
641 PPGGDEEAAG EKDEL
```

**Supporting Peptides:**

| Peptide | Uniq | -10lgP | Mass | Length | ppm | m/z | z | RT | Fraction | Scan | Source File | Area F1 | Area F2a | Area F3a | Area F4 | Area F5 | Area F6a | Area F7a | Area F7b | Area F8 | Area F9a | Area Sample 1F10a | Area F10b | Area F11a | Area F11b | Area F12a | Area F13 | #Feature | #Feature F1 | #Feature F2a | #Feature F3a | #Feature F4 | #Feature F5 | #Feature F6a | #Feature F7a | #Feature F7b | #Feature F8 | #Feature F9a | #Feature Sample 1F10a | #Feature F10b | #Feature F11a | #Feature F11b | #Feature F12a | #Feature F13 | Start | End | PTM | AScore | Found By |
|---|---|---|---|---|---|---|---|---|---|---|---|---|---|---|---|---|---|---|---|---|---|---|---|---|---|---|---|---|---|---|---|---|---|---|---|---|---|---|---|---|---|---|---|---|---|---|---|---|---|
| R.IINEPTAAAIAYGLDKR.E | Y | 45.90 | 1814.9890 | 17 | 1.3 | 606.0044 | 3 | 64.58 | 16 | F16:32262 | BuCaTN01_F13.raw | | | | | | | | | | | | | | | | 2.2698E5 | 1 | 0 | 0 | 0 | 0 | 0 | 0 | 0 | 0 | 0 | 0 | 0 | 0 | 0 | 0 | 0 | 1 | 198 | 214 | | | PEAKS DB |
| K.DAGTIAGLNVMR.I | Y | 45.60 | 1216.6234 | 12 | 1.5 | 609.3199 | 2 | 59.49 | 16 | F16:28937 | BuCaTN01_F13.raw | | | | | | | | | | | | | | | | 1.4052E5 | 1 | 0 | 0 | 0 | 0 | 0 | 0 | 0 | 0 | 0 | 0 | 0 | 0 | 0 | 0 | 0 | 1 | 186 | 197 | | | PEAKS DB |
| R.TWNDPSVQQDIK.Y | Y | 43.32 | 1429.6837 | 12 | 0.5 | 715.8495 | 2 | 47.71 | 16 | F16:21538 | BuCaTN01_F13.raw | | | | | | | | | | | | | | | | 7.1401E4 | 1 | 0 | 0 | 0 | 0 | 0 | 0 | 0 | 0 | 0 | 0 | 0 | 0 | 0 | 0 | 0 | 1 | 102 | 113 | | | PEAKS DB |
| total 3 peptides | | | | | | | | | | | | | | | | | | | | | | | | | | | | | | | | | | | | | | | | | | | | | | | | | |

**Best Unique PSM (Scan F16:32262, m/z=606.0044, z=3, RT=64.58, ppm=1.3):**

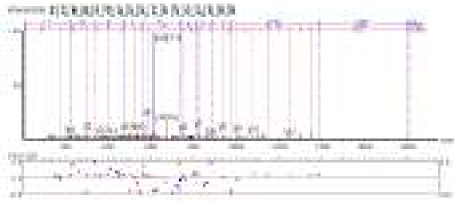

## XP_058015571.1

back to list

| Protein Coverage | Supporting Peptides | Best Unique PSM |

**Protein Coverage:**

```
  1 MKYYAVVCLL LTGSVFAADD DDKKEDVGTV VGIDLGTTYS CVGVFKNGRV EIIANDQGNR ITPSYVAFTP EGERLIGDAA
 81 KNQLTSNPEN TVFDAKRLIG RTWNDPSVQQ DIKYLPFKVI EKKAKPYIQV DIGGGQIKTF APEEISAMVL TKMKETAEAY
161 LGKKVTHAVV TVPAYFNDAQ RQATKDAGTI AGLNVMRIIN EPTAAAIAYG LDKREGEKNI LVFDLGGGTF DVSLLTIDNG
241 VFEVVATNGD THLGGEDFDQ RVMEHFIKLY KKKTGKDVRK DNRAVQKLRR EVEKAKRALS SQHQARIEIE SFFEGEDFSE
321 TLTRAKFEEL NMDLFRSTMK PVQKVLEDAD LKKSDIDEIV LVGGSTRIPK IQQLVKEFFN GKEPSRGINP DEAVAYGAAV
401 QAGVLSGDQD TGDLVLLDVC PLTLGIETVG GVMTKLIPRN TVVPTKKSQI FSTASDNQPT VTIKVYEGER PLTKDNHLLG
481 TFDLTGIPPA PRGVPQIEVT FEIDVNGILR VTAEDKGTGN KNKITITNDQ NRLTPEEIER MVNDAEKFAE EDKKLKERID
561 ARNELESYAY SLKNQIGDKE KLGGKLSPED KETIEKAVEE KIEWLESHQD GDIEEFKAQK KELEEVVQPI VGKLYGGAGP
641 PPGGEEAGEK DEL
```

**Supporting Peptides:**

| Peptide | Uniq | -10lgP | Mass | Length | ppm | m/z | z | RT | Fraction | Scan | Source File | Area F1 | Area F2a | Area F3a | Area F4 | Area F5 | Area F6a | Area F7a | Area F7b | Area F8 | Area F9a | Area Sample 1F10a | Area F10b | Area F11a | Area F11b | Area F12a | Area F13 | #Feature | #Feature F1 | #Feature F2a | #Feature F3a | #Feature F4 | #Feature F5 | #Feature F6a | #Feature F7a | #Feature F7b | #Feature F8 | #Feature F9a | #Feature Sample 1F10a | #Feature F10b | #Feature F11a | #Feature F11b | #Feature F12a | #Feature F13 | Start | End | PTM | AScore | Found By |
|---|---|---|---|---|---|---|---|---|---|---|---|---|---|---|---|---|---|---|---|---|---|---|---|---|---|---|---|---|---|---|---|---|---|---|---|---|---|---|---|---|---|---|---|---|---|---|---|---|---|
| R.IINEPTAAAIAYGLDKR.E | Y | 45.90 | 1814.9890 | 17 | 1.3 | 606.0044 | 3 | 64.58 | 16 | F16:32262 | BuCaTN01_F13.raw | | | | | | | | | | | | | | | | 2.2698E5 | 1 | 0 | 0 | 0 | 0 | 0 | 0 | 0 | 0 | 0 | 0 | 0 | 0 | 0 | 0 | 0 | 1 | 198 | 214 | | | PEAKS DB |
| K.DAGTIAGLNVMR.I | Y | 45.60 | 1216.6234 | 12 | 1.5 | 609.3199 | 2 | 59.49 | 16 | F16:28937 | BuCaTN01_F13.raw | | | | | | | | | | | | | | | | 1.4052E5 | 1 | 0 | 0 | 0 | 0 | 0 | 0 | 0 | 0 | 0 | 0 | 0 | 0 | 0 | 0 | 0 | 1 | 186 | 197 | | | PEAKS DB |
| R.TWNDPSVQQDIK.Y | Y | 43.32 | 1429.6837 | 12 | 0.5 | 715.8495 | 2 | 47.71 | 16 | F16:21538 | BuCaTN01_F13.raw | | | | | | | | | | | | | | | | 7.1401E4 | 1 | 0 | 0 | 0 | 0 | 0 | 0 | 0 | 0 | 0 | 0 | 0 | 0 | 0 | 0 | 0 | 1 | 102 | 113 | | | PEAKS DB |
| total 3 peptides | | | | | | | | | | | | | | | | | | | | | | | | | | | | | | | | | | | | | | | | | | | | | | | | | |

**Best Unique PSM (Scan F16:32262, m/z=606.0044, z=3, RT=64.58, ppm=1.3):**

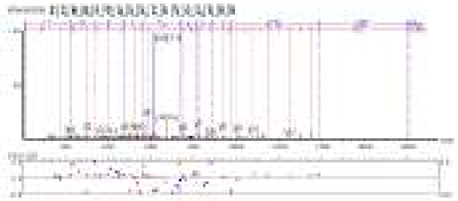

## JAC95528.1

back to list

| Protein Coverage | Supporting Peptides | Best Unique PSM |

**Protein Coverage:**

```
  1 MKYYAVVCLL LVGSVFAAED DDKKEDVGTV VGIDLGTTYS CVGVFKNGRV EIIANDQGNR ITPSYVAFTP EGERLIGDAA
 81 KNQLTSNPEN TVFDAKRLIG RTWNDPSVQQ DIKYLPFKVI EKKTKPYIQV DIGGGQTKTF APEEISAMVL TKMKETAEAY
161 LGKKVTHAVV TVPAYFNDAQ RQATKDAGTI AGLNVMRIIN EPTAAAIAYG LDKREGEKNI LVFDLGGGTF DVSLLTIDNG
241 VFEVVATNGD THLGGEDFDQ RVMEHFIKLY KKKTGKDVRK DNRAVQKLRR EVEKAKRALS SQHQARIEIE SFFESEDFSE
321 TLTRAKFEEL NMDLFRSTMK PVQKVLEDAD LKKSDIDEIV LVGGSTRIPK IQQLVKEFFN GKEPSRGINP DEAVAYGAAV
401 QAGVLSGDQD TGDLVLLDVC PLTLGIETVG GVMTKLIPRN TVVPTKKSQI FSTASDNQPT VTIKVYEGER PLTKDNHLLG
481 TFDLTGIPPA PRGVPQIEVT FEIDVNGILR VTAEDKGTGN KNKITITNDQ NRLTPEEIER MVNDAEKFAE EDKKLKERID
561 ARNELESYAY SLKNQIGDKE KLGGKLSSED KETIEKAVEE KIEWLESHQD GDIEEFKAQK KELEEVVQPI VGKLYGGAGP
641 PPSGEEAEAG EKDEL
```

**Supporting Peptides:**

| Peptide | Uniq | -10lgP | Mass | Length | ppm | m/z | z | RT | Fraction | Scan | Source File | Area F1 | Area F2a | Area F3a | Area F4 | Area F5 | Area F6a | Area F7a | Area F7b | Area F8 | Area F9a | Area Sample 1F10a | Area F10b | Area F11a | Area F11b | Area F12a | Area F13 | #Feature | #Feature F1 | #Feature F2a | #Feature F3a | #Feature F4 | #Feature F5 | #Feature F6a | #Feature F7a | #Feature F7b | #Feature F8 | #Feature F9a | #Feature Sample 1F10a | #Feature F10b | #Feature F11a | #Feature F11b | #Feature F12a | #Feature F13 | Start | End | PTM | AScore | Found By |
|---|---|---|---|---|---|---|---|---|---|---|---|---|---|---|---|---|---|---|---|---|---|---|---|---|---|---|---|---|---|---|---|---|---|---|---|---|---|---|---|---|---|---|---|---|---|---|---|---|---|
| R.IINEPTAAAIAYGLDKR.E | Y | 45.90 | 1814.9890 | 17 | 1.3 | 606.0044 | 3 | 64.58 | 16 | F16:32262 | BuCaTN01_F13.raw | | | | | | | | | | | | | | | | 2.2698E5 | 1 | 0 | 0 | 0 | 0 | 0 | 0 | 0 | 0 | 0 | 0 | 0 | 0 | 0 | 0 | 0 | 1 | 198 | 214 | | | PEAKS DB |

| | | | | | | | | | | | | | | | | | | | | | | | | | | | | | | | | | | | | | | | | | | | | | | | | | |
|---|---|---|---|---|---|---|---|---|---|---|---|---|---|---|---|---|---|---|---|---|---|---|---|---|---|---|---|---|---|---|---|---|---|---|---|---|---|---|---|---|---|---|---|---|---|---|---|---|---|
| K.DAGTIAGLNVMR.I | Y | 45.60 | 1216.6234 | 12 | 1.5 | 609.3199 | 2 | 59.49 | 16 | F16:28937 | BuCaTN01_F13.raw | | | | | | | | | | | | | | | | 1.4052E5 | 1 | 0 | 0 | 0 | 0 | 0 | 0 | 0 | 0 | 0 | 0 | 0 | 0 | 0 | 0 | 0 | 1 | 186 | 197 | | | PEAKS DB |
| R.TWNDPSVQQDIK.Y | Y | 43.32 | 1429.6837 | 12 | 0.5 | 715.8495 | 2 | 47.71 | 16 | F16:21538 | BuCaTN01_F13.raw | | | | | | | | | | | | | | | | 7.1401E4 | 1 | 0 | 0 | 0 | 0 | 0 | 0 | 0 | 0 | 0 | 0 | 0 | 0 | 0 | 0 | 0 | 1 | 102 | 113 | | | PEAKS DB |
| total 3 peptides | | | | | | | | | | | | | | | | | | | | | | | | | | | | | | | | | | | | | | | | | | | | | | | | | |

**Best Unique PSM (Scan F16:32262, m/z=606.0044, z=3, RT=64.58, ppm=1.3):**

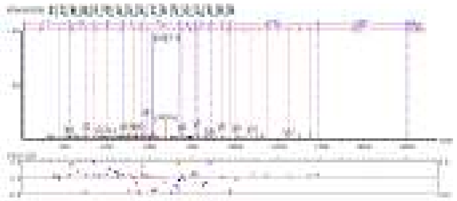

## XP_025022112.1

back to list

| Protein Coverage | Supporting Peptides | Best Unique PSM |

**Protein Coverage:**

```
  1 MKYALVCLLL VSSVFADEDE KKEDVGTVVG IDLGTTYSCV GVFKNGRVEI IANDQGNRIT PSYVAFTPEG ERLIGDAAKN
 81 QLTSNPENTV FDAKRLIGRT WNDPSVQQDI KYLPFKVIEK KAKPHIQVDI GGGQTKTFAP EEISAMVLTK MKETAEAYLG
161 KKVTHAVVTV PAYFNDAQRQ ATKDAGTIAG LNVMRIINEP TAAAIAYGLD KREGEKNILV FDLGGGTFDV SLLTIDNGVF
241 EVVATNGDTH LGGEDFDQRV MEHFIKLYKK KTGKDVRKDN RAVQKLRREV EKAKRALSSQ HQARIEIESF FEGEDFSETL
321 TRAKFEELNM DLFRSTMKPV QKVLEDADLK KSDIDEIVLV GGSTRIPKIQ QLVKEFFNGK EPSRGINPDE AVAYGAAVQA
401 GVLSGDQDTG DLVLLDVCPL TLGIETVGGV MTKLIPRNTV VPTKKSQIFS TASDNQPTVT IKVYEGERPL TKDNHLLGTF
481 DLTGIPPAPR GVPQIEVTFE IDVNGILRVT AEDKGTGNKN KITITNDQNR LTPEEIERMV NDAEKFAEED KKLKERIDAR
561 NELESYAYSL KNQIGDKEKL GGKLSSEDKD TIEKAVEEKI EWLESHQDAD IEDFKAQKKE LEEVVQPIVG KLYGGAGPPP
641 GEEEAGEKDE L
```

**Supporting Peptides:**

| Peptide | Uniq | -10lgP | Mass | Length | ppm | m/z | z | RT | Fraction | Scan | Source File | Area F1 | Area F2a | Area F3a | Area F4 | Area F5 | Area F6a | Area F7a | Area F7b | Area F8 | Area F9a | Area Sample 1F10a | Area F10b | Area F11a | Area F11b | Area F12a | Area F13 | #Feature | #Feature F1 | #Feature F2a | #Feature F3a | #Feature F4 | #Feature F5 | #Feature F6a | #Feature F7a | #Feature F7b | #Feature F8 | #Feature F9a | #Feature Sample 1F10a | #Feature F10b | #Feature F11a | #Feature F11b | #Feature F12a | #Feature F13 | Start | End | PTM | AScore | Found By |
|---|---|---|---|---|---|---|---|---|---|---|---|---|---|---|---|---|---|---|---|---|---|---|---|---|---|---|---|---|---|---|---|---|---|---|---|---|---|---|---|---|---|---|---|---|---|---|---|---|---|
| R.IINEPTAAAIAYGLDKR.E | Y | 45.90 | 1814.9890 | 17 | 1.3 | 606.0044 | 3 | 64.58 | 16 | F16:32262 | BuCaTN01_F13.raw | | | | | | | | | | | | | | | | 2.2698E5 | 1 | 0 | 0 | 0 | 0 | 0 | 0 | 0 | 0 | 0 | 0 | 0 | 0 | 0 | 0 | 0 | 1 | 196 | 212 | | | PEAKS DB |
| K.DAGTIAGLNVMR.I | Y | 45.60 | 1216.6234 | 12 | 1.5 | 609.3199 | 2 | 59.49 | 16 | F16:28937 | BuCaTN01_F13.raw | | | | | | | | | | | | | | | | 1.4052E5 | 1 | 0 | 0 | 0 | 0 | 0 | 0 | 0 | 0 | 0 | 0 | 0 | 0 | 0 | 0 | 0 | 1 | 184 | 195 | | | PEAKS DB |
| R.TWNDPSVQQDIK.Y | Y | 43.32 | 1429.6837 | 12 | 0.5 | 715.8495 | 2 | 47.71 | 16 | F16:21538 | BuCaTN01_F13.raw | | | | | | | | | | | | | | | | 7.1401E4 | 1 | 0 | 0 | 0 | 0 | 0 | 0 | 0 | 0 | 0 | 0 | 0 | 0 | 0 | 0 | 0 | 1 | 100 | 111 | | | PEAKS DB |
| total 3 peptides | | | | | | | | | | | | | | | | | | | | | | | | | | | | | | | | | | | | | | | | | | | | | | | | | |

**Best Unique PSM (Scan F16:32262, m/z=606.0044, z=3, RT=64.58, ppm=1.3):**

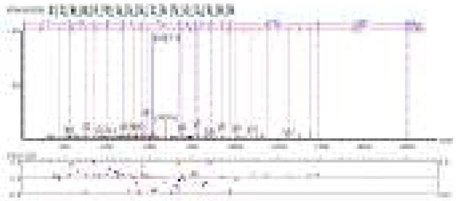

## JAB54174.1

back to list

| Protein Coverage | Supporting Peptides | Best Unique PSM |

**Protein Coverage:**

```
  1 MKYYYAVLCL LLVGSALAAD DDKKEEVGTV VGIDLGTTYS CVGVFKNGRV EIIANDQGNR ITPSYVAFTP EGERLIGDAA
 81 KNQLTSNPEN TVFDAKRLIG RTWNDPSVQQ DIKYLPFKVI EKKTKPYIQV DIGGGQTKTF APEEISAMVL TKMKETAEAY
161 LGKKVTHAVV TVPAYFNDAQ RQATKDAGTI AGLNVMRIIN EPTAAAIAYG LDKREGEKNI LVFDLGGGTF DVSLLTIDNG
241 VFEVVATNGD THLGGEDFDQ RVMEHFIKLY KKKTGKDVRK DNRAVQKLRR EVEKAKRALS SQHQARIEIE SFFEGEDFSE
321 TLTRAKFEEL NMDLFRSTMK PVQKVLEDAD LKKSDIDEIV LVGGSTRIPK IQQLVKEFFN GKEPSRGINP DEAVAYGAAV
401 QAGVLSGDQD TGDLVLLDVC PLTLGIETVG GVMTKLIPRN TVVPTKKSQI FSTASDNQPT VTIKVYEGER PLTKDNHLLG
481 TFDLTGIPPA PRGVPQIEVT FEIDVNGILR VTAEDKGTGN KNKITITNDQ NRLTPEEIER MVNDAEKFAD EDKKLKERID
561 ARNELESYAY SLKNQIGDKE KLGGKLSPED KETIEKAVEE KIEWLESHQD GDIEEFKAQK KELEEVVQPI VGKLYGGAGP
641 PPGGEEAAGE KDEL
```

**Supporting Peptides:**

| Peptide | Uniq | -10lgP | Mass | Length | ppm | m/z | z | RT | Fraction | Scan | Source File | Area F1 | Area F2a | Area F3a | Area F4 | Area F5 | Area F6a | Area F7a | Area F7b | Area F8 | Area F9a | Area Sample 1F10a | Area F10b | Area F11a | Area F11b | Area F12a | Area F13 | #Feature | #Feature F1 | #Feature F2a | #Feature F3a | #Feature F4 | #Feature F5 | #Feature F6a | #Feature F7a | #Feature F7b | #Feature F8 | #Feature F9a | #Feature Sample 1F10a | #Feature F10b | #Feature F11a | #Feature F11b | #Feature F12a | #Feature F13 | Start | End | PTM | AScore | Found By |
|---|---|---|---|---|---|---|---|---|---|---|---|---|---|---|---|---|---|---|---|---|---|---|---|---|---|---|---|---|---|---|---|---|---|---|---|---|---|---|---|---|---|---|---|---|---|---|---|---|---|
| R.IINEPTAAAIAYGLDKR.E | Y | 45.90 | 1814.9890 | 17 | 1.3 | 606.0044 | 3 | 64.58 | 16 | F16:32262 | BuCaTN01_F13.raw | | | | | | | | | | | | | | | | 2.2698E5 | 1 | 0 | 0 | 0 | 0 | 0 | 0 | 0 | 0 | 0 | 0 | 0 | 0 | 0 | 0 | 0 | 1 | 198 | 214 | | | PEAKS DB |
| K.DAGTIAGLNVMR.I | Y | 45.60 | 1216.6234 | 12 | 1.5 | 609.3199 | 2 | 59.49 | 16 | F16:28937 | BuCaTN01_F13.raw | | | | | | | | | | | | | | | | 1.4052E5 | 1 | 0 | 0 | 0 | 0 | 0 | 0 | 0 | 0 | 0 | 0 | 0 | 0 | 0 | 0 | 0 | 1 | 186 | 197 | | | PEAKS DB |
| R.TWNDPSVQQDIK.Y | Y | 43.32 | 1429.6837 | 12 | 0.5 | 715.8495 | 2 | 47.71 | 16 | F16:21538 | BuCaTN01_F13.raw | | | | | | | | | | | | | | | | 7.1401E4 | 1 | 0 | 0 | 0 | 0 | 0 | 0 | 0 | 0 | 0 | 0 | 0 | 0 | 0 | 0 | 0 | 1 | 102 | 113 | | | PEAKS DB |
| total 3 peptides | | | | | | | | | | | | | | | | | | | | | | | | | | | | | | | | | | | | | | | | | | | | | | | | | |

**Best Unique PSM (Scan F16:32262, m/z=606.0044, z=3, RT=64.58, ppm=1.3):**

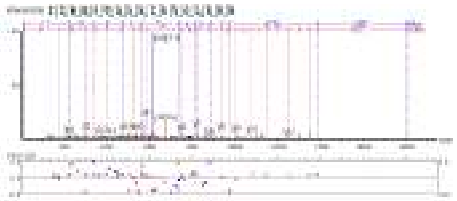

## JAG67806.1

back to list

| Protein Coverage | Supporting Peptides | Best Unique PSM |

**Protein Coverage:**

1 MKYYAVICLL LVGSVFADDD DKKEDVGTVV GIDLGTTYSC VGVFKNGRVE IIANDQGNRI TPSYVAFTPE GERLIGDAAK
81 NQLTSNPENT VFDAKRLIGR **TWNDPSVQQD IK**YLPFKVIE KKSKPYIQVD IGGGQTKTFA PEEISAMVLT KMKETAEAYL
161 GKKVTHAVVT VPAYFNDAQR QATK**DAGTIA GLNVMRIINE PTAAAIAYGL DKR**EGEKNIL VFDLGGTFD VSLLTIDNGV
241 FEVVATNGDT HLGGEDFDQR VMEHFIKLYK KKTGKDVRKD NRAVQKLRRE VEKAKRALSS QHQARIEIES FFEGEDFSET
321 LTRAKFEELN MDLFRSTMKP VQKVLEDADL KKSDIDEIVL VGGSTRIPKI QQLVKEFFNG KEPSRGINPD EAVAYGAAVQ
401 AGVLSGDQDT GDLVLLDVCP LTLGIETVGG VMTKLIPRNT VVPTKKSQIF STASDNQPTV TIKVYEGERP LTKDNHLLGT
481 FDLTGIPPAP RGVPQIEVTF EIDVNGILRV TAEDKGTGNK NKITITNDQN RLTPEEIERM VTDAEKFAEE DKKLKERIDA
561 RNELESYAYS LKNQIGDKEK LGGKLSSEDK ETIEKAVEEK IEWLESHQDG DIEEFKAQKK ELEEVVQPIV GKLYGGAGPP
641 PGGEEAGEKD EL

**Supporting Peptides:**

| Peptide | Uniq | -10lgP | Mass | Length | ppm | m/z | z | RT | Fraction | Scan | Source File | Area F1 | Area F2a | Area F3a | Area F4 | Area F5 | Area F6a | Area F7a | Area F7b | Area F8 | Area F9a | Area Sample 1F10a | Area F10b | Area F11a | Area F11b | Area F12a | Area F13 | #Feature | #Feature F1 | #Feature F2a | #Feature F3a | #Feature F4 | #Feature F5 | #Feature F6a | #Feature F7a | #Feature F7b | #Feature F8 | #Feature F9a | #Feature Sample 1F10a | #Feature F10b | #Feature F11a | #Feature F11b | #Feature F12a | #Feature F13 | Start | End | PTM | AScore | Found By |
|---|---|---|---|---|---|---|---|---|---|---|---|---|---|---|---|---|---|---|---|---|---|---|---|---|---|---|---|---|---|---|---|---|---|---|---|---|---|---|---|---|---|---|---|---|---|---|---|---|---|
| R.IINEPTAAAIAYGLDKR.E | Y | 45.90 | 1814.9890 | 17 | 1.3 | 606.0044 | 3 | 64.58 | 16 | F16:32262 | BuCaTN01_F13.raw | | | | | | | | | | | | | | | | 2.2698E5 | 1 | 0 | 0 | 0 | 0 | 0 | 0 | 0 | 0 | 0 | 0 | 0 | 0 | 0 | 0 | 0 | 1 | 197 | 213 | | | PEAKS DB |
| K.DAGTIAGLNVMR.I | Y | 45.60 | 1216.6234 | 12 | 1.5 | 609.3199 | 2 | 59.49 | 16 | F16:28937 | BuCaTN01_F13.raw | | | | | | | | | | | | | | | | 1.4052E5 | 1 | 0 | 0 | 0 | 0 | 0 | 0 | 0 | 0 | 0 | 0 | 0 | 0 | 0 | 0 | 0 | 1 | 185 | 196 | | | PEAKS DB |
| R.TWNDPSVQQDIK.Y | Y | 43.32 | 1429.6837 | 12 | 0.5 | 715.8495 | 2 | 47.71 | 16 | F16:21538 | BuCaTN01_F13.raw | | | | | | | | | | | | | | | | 7.1401E4 | 1 | 0 | 0 | 0 | 0 | 0 | 0 | 0 | 0 | 0 | 0 | 0 | 0 | 0 | 0 | 0 | 1 | 101 | 112 | | | PEAKS DB |
| **total 3 peptides** | | | | | | | | | | | | | | | | | | | | | | | | | | | | | | | | | | | | | | | | | | | | | | | | | |

**Best Unique PSM (Scan F16:32262, m/z=606.0044, z=3, RT=64.58, ppm=1.3):**

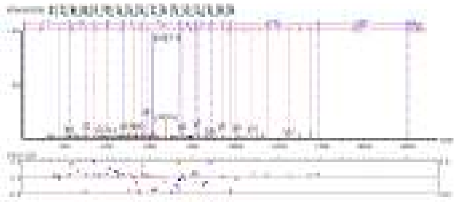

## XP_026542482.1

back to list

| Protein Coverage | Supporting Peptides | Best Unique PSM |

**Protein Coverage:**

1 MKYYAILCLL LVGSALATDD EDRKEEVGTV VGIDLGTTYS CVGVFKNGRV EIIANDQGNR ITPSYVAFTP EGERLIGDAA
81 KNQLTSNPEN TVFDAKRLIG R**TWNDPSVQQ DIK**YLPFKVI EKKTKPYIQV DIGGGQTKTF APEEISAMVL TKMKETAEAY
161 LGKKVTHAVV TVPAYFNDAQ RQATK**DAGTI AGLNVMRIIN EPTAAAIAYG LDKR**EGEKNI LVFDLGGGTF DVSLLTIDNG
241 VFEVVATNGD THLGGEDFDQ RVMEHFIKLY KKKTGKDVRK DNRAVQKLRR EVEKAKRALS SQHQARIEIE SFFEGEDFSE
321 TLTRAKFEEL NMDLFRSTMK PVQKVLEDAD LKKSDIDEIV LVGGSTRIPK IQQLVKEFFN GKEPSRGINP DEAVAYGAAV
401 QAGVLSGDQD TGDLVLLDVC PLTLGIETVG GVMTKLIPRN TVVPTKKSQI FSTASDNQPT VTIKVYEGER PLTKDNHLLG
481 TFDLTGIPPA PRGVPQIEVT FEIDVNGILR VTAEDKGTGN KNKITITNDQ NRLTPEEIER MVNDAEKFAE EDKKLKERID
561 ARNELESYAY SLKNQIGDKE KLGGKLSPED KETIEKAVEE KIEWLESHQD GNIEEFKAQK KELEEVVQPI VGKLYGGAGP
641 PPGGEEEAAG EKDEL

**Supporting Peptides:**

| Peptide | Uniq | -10lgP | Mass | Length | ppm | m/z | z | RT | Fraction | Scan | Source File | Area F1 | Area F2a | Area F3a | Area F4 | Area F5 | Area F6a | Area F7a | Area F7b | Area F8 | Area F9a | Area Sample 1F10a | Area F10b | Area F11a | Area F11b | Area F12a | Area F13 | #Feature | #Feature F1 | #Feature F2a | #Feature F3a | #Feature F4 | #Feature F5 | #Feature F6a | #Feature F7a | #Feature F7b | #Feature F8 | #Feature F9a | #Feature Sample 1F10a | #Feature F10b | #Feature F11a | #Feature F11b | #Feature F12a | #Feature F13 | Start | End | PTM | AScore | Found By |
|---|---|---|---|---|---|---|---|---|---|---|---|---|---|---|---|---|---|---|---|---|---|---|---|---|---|---|---|---|---|---|---|---|---|---|---|---|---|---|---|---|---|---|---|---|---|---|---|---|---|
| R.IINEPTAAAIAYGLDKR.E | Y | 45.90 | 1814.9890 | 17 | 1.3 | 606.0044 | 3 | 64.58 | 16 | F16:32262 | BuCaTN01_F13.raw | | | | | | | | | | | | | | | | 2.2698E5 | 1 | 0 | 0 | 0 | 0 | 0 | 0 | 0 | 0 | 0 | 0 | 0 | 0 | 0 | 0 | 0 | 1 | 198 | 214 | | | PEAKS DB |
| K.DAGTIAGLNVMR.I | Y | 45.60 | 1216.6234 | 12 | 1.5 | 609.3199 | 2 | 59.49 | 16 | F16:28937 | BuCaTN01_F13.raw | | | | | | | | | | | | | | | | 1.4052E5 | 1 | 0 | 0 | 0 | 0 | 0 | 0 | 0 | 0 | 0 | 0 | 0 | 0 | 0 | 0 | 0 | 1 | 186 | 197 | | | PEAKS DB |
| R.TWNDPSVQQDIK.Y | Y | 43.32 | 1429.6837 | 12 | 0.5 | 715.8495 | 2 | 47.71 | 16 | F16:21538 | BuCaTN01_F13.raw | | | | | | | | | | | | | | | | 7.1401E4 | 1 | 0 | 0 | 0 | 0 | 0 | 0 | 0 | 0 | 0 | 0 | 0 | 0 | 0 | 0 | 0 | 1 | 102 | 113 | | | PEAKS DB |
| **total 3 peptides** | | | | | | | | | | | | | | | | | | | | | | | | | | | | | | | | | | | | | | | | | | | | | | | | |

**Best Unique PSM (Scan F16:32262, m/z=606.0044, z=3, RT=64.58, ppm=1.3):**

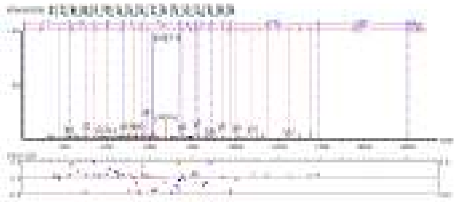

## XP_034262421.1

back to list

| Protein Coverage | Supporting Peptides | Best Unique PSM |

**Protein Coverage:**

1 TILLSSVGVF KNGRVEIIAN DQGNRITPSY VAFTPEGERL IGDAAKNQLT SNPENTVFDA KRLIGR**TWND PSVQQDIK**YL
81 PFKVIEKKTK PYIQVDIGGG QTKTFAPEEI SAMVLTKMKE TAEAYLGKKV THAVVTVPAY FNDAQRQATK **DAGTIAGLNV**
161 **MRIINEPTAA AIAYGLDKR**E GEKNILVFDL GGGTFDVSLL TIDNGVFEVV ATNGDTHLGG EDFDQRVMEH FIKLYKKKTG
241 KDVRKDNRAV QKLRREVEKA KRALSSQHQA RIEIESFFEG EDFSETLTRA KFEELNMDLF RSTMKPVQKV LEDADLKKSD
321 IDEIVLVGGS TRIPKIQQLV KEFFNGKEPS RGINPDEAVA YGAAVQAGVL SGDQDTGDLV LLDVCPLTLG IETVGGVMTK
401 LIPRNTVVPT KKSQIFSTAS DNQPTVTIKV YEGERPLTKD NHLLGTFDLT GIPPAPRGVP QIEVTFEIDV NGILRVTAED
481 KGTGNKNKIT ITNDQNRLTP EEIERMVTDA EKFAEEDKKL KERIDARNEL ESYAYSLKNQ IGDKEKLGGK LSSEDKETIE
561 KAVEEKIEWL ESHQDGDIEE FKAQKKELEE VVQPIVGKLY GGAGPPPSGE EAGEKDEL

**Supporting Peptides:**

| Peptide | Uniq | -10lgP | Mass | Length | ppm | m/z | z | RT | Fraction | Scan | Source File | Area F1 | Area F2a | Area F3a | Area F4 | Area F5 | Area F6a | Area F7a | Area F7b | Area F8 | Area F9a | Area Sample 1F10a | Area F10b | Area F11a | Area F11b | Area F12a | Area F13 | #Feature | #Feature F1 | #Feature F2a | #Feature F3a | #Feature F4 | #Feature F5 | #Feature F6a | #Feature F7a | #Feature F7b | #Feature F8 | #Feature F9a | #Feature Sample 1F10a | #Feature F10b | #Feature F11a | #Feature F11b | #Feature F12a | #Feature F13 | Start | End | PTM | AScore | Found By |
|---|---|---|---|---|---|---|---|---|---|---|---|---|---|---|---|---|---|---|---|---|---|---|---|---|---|---|---|---|---|---|---|---|---|---|---|---|---|---|---|---|---|---|---|---|---|---|---|---|---|
| R.IINEPTAAAIAYGLDKR.E | Y | 45.90 | 1814.9890 | 17 | 1.3 | 606.0044 | 3 | 64.58 | 16 | F16:32262 | BuCaTN01_F13.raw | | | | | | | | | | | | | | | | 2.2698E5 | 1 | 0 | 0 | 0 | 0 | 0 | 0 | 0 | 0 | 0 | 0 | 0 | 0 | 0 | 0 | 0 | 1 | 163 | 179 | | | PEAKS DB |
| K.DAGTIAGLNVMR.I | Y | 45.60 | 1216.6234 | 12 | 1.5 | 609.3199 | 2 | 59.49 | 16 | F16:28937 | BuCaTN01_F13.raw | | | | | | | | | | | | | | | | 1.4052E5 | 1 | 0 | 0 | 0 | 0 | 0 | 0 | 0 | 0 | 0 | 0 | 0 | 0 | 0 | 0 | 0 | 1 | 151 | 162 | | | PEAKS DB |
| R.TWNDPSVQQDIK.Y | Y | 43.32 | 1429.6837 | 12 | 0.5 | 715.8495 | 2 | 47.71 | 16 | F16:21538 | BuCaTN01_F13.raw | | | | | | | | | | | | | | | | 7.1401E4 | 1 | 0 | 0 | 0 | 0 | 0 | 0 | 0 | 0 | 0 | 0 | 0 | 0 | 0 | 0 | 0 | 1 | 67 | 78 | | | PEAKS DB |
| **total 3 peptides** | | | | | | | | | | | | | | | | | | | | | | | | | | | | | | | | | | | | | | | | | | | | | | | | | |

**Best Unique PSM (Scan F16:32262, m/z=606.0044, z=3, RT=64.58, ppm=1.3):**

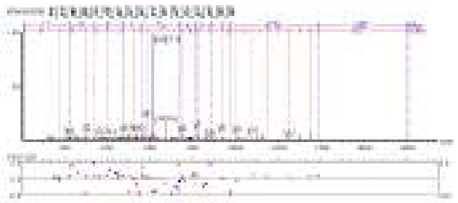

## XP_032088936.1

back to list

| Protein Coverage | Supporting Peptides | Best Unique PSM |

**Protein Coverage:**

1 MKYYAVFCLL LAGSVFADDD DKKEEVGTVV GIDLGTTYSC VGVFKNGRVE IIANDQGNRI TPSYVAFTPE GERLIGDAAK
81 NQLTSNPENT VFDAKRLIGR **TWNDPSVQQD IK**YLPFKVLE KKSKPYIQVD IGGGQMKTFA PEEISAMVLT KMKETAEAYL
161 GKKVTHAVVT VPAYFNDAQR QATK**DAGTIA GLNVMRIINE PTAAAIAYGL DKR**EGEKNIL VFDLGGGTFD VSLLTIDNGV
241 FEVVATNGDT HLGGEDFDQR VMEHFIKLYK KKTGKDVRKD VRAVQKLRRE VEKAKRALSS QHQARIEIES FFESEDFSET
321 LTRAKFEELN MDLFRSTMKP VQKVLEDADL KKSDIDEIVL VGGSTRIPKI QQLVKEFFNG KEPSRGINPD EAVAYGAAVQ
401 AGVLSGDQDT GDLVLLDVCP LTLGIETVGG VMTKLIPRNT VVPTKKSQIF STASDNQPTV TIKVYEGERP LTKDNHLLGT
481 FDLTGIPPAP RGVPQIEVTF EIDVNGILRV TAEDKGTGNK NKITITNDQN RLTPEEIERM VTDAEKFAEE DKKLKERIDA
561 RNELESYAYS LKNQIGDKEK LGGKLSSEDK ETIEKAVEEK IEWLESHQDG DIEEFKAQKK ELEEVVQPIV GKLYGGAGPP
641 PGGEEAGEKD EL

**Supporting Peptides:**

| Peptide | Uniq | -10lgP | Mass | Length | ppm | m/z | z | RT | Fraction | Scan | Source File | Area F1 | Area F2a | Area F3a | Area F4 | Area F5 | Area F6a | Area F7a | Area F7b | Area F8 | Area F9a | Area Sample 1F10a | Area F10b | Area F11a | Area F11b | Area F12a | Area F13 | #Feature | #Feature F1 | #Feature F2a | #Feature F3a | #Feature F4 | #Feature F5 | #Feature F6a | #Feature F7a | #Feature F7b | #Feature F8 | #Feature F9a | #Feature Sample 1F10a | #Feature F10b | #Feature F11a | #Feature F11b | #Feature F12a | #Feature F13 | Start | End | PTM | AScore | Found By |
|---|---|---|---|---|---|---|---|---|---|---|---|---|---|---|---|---|---|---|---|---|---|---|---|---|---|---|---|---|---|---|---|---|---|---|---|---|---|---|---|---|---|---|---|---|---|---|---|---|---|
| R.IINEPTAAAIAYGLDKR.E | Y | 45.90 | 1814.9890 | 17 | 1.3 | 606.0044 | 3 | 64.58 | 16 | F16:32262 | BuCaTN01_F13.raw | | | | | | | | | | | | | | | | 2.2698E5 | 1 | 0 | 0 | 0 | 0 | 0 | 0 | 0 | 0 | 0 | 0 | 0 | 0 | 0 | 0 | 0 | 1 | 197 | 213 | | | PEAKS DB |
| K.DAGTIAGLNVMR.I | Y | 45.60 | 1216.6234 | 12 | 1.5 | 609.3199 | 2 | 59.49 | 16 | F16:28937 | BuCaTN01_F13.raw | | | | | | | | | | | | | | | | 1.4052E5 | 1 | 0 | 0 | 0 | 0 | 0 | 0 | 0 | 0 | 0 | 0 | 0 | 0 | 0 | 0 | 0 | 1 | 185 | 196 | | | PEAKS DB |
| R.TWNDPSVQQDIK.Y | Y | 43.32 | 1429.6837 | 12 | 0.5 | 715.8495 | 2 | 47.71 | 16 | F16:21538 | BuCaTN01_F13.raw | | | | | | | | | | | | | | | | 7.1401E4 | 1 | 0 | 0 | 0 | 0 | 0 | 0 | 0 | 0 | 0 | 0 | 0 | 0 | 0 | 0 | 0 | 1 | 101 | 112 | | | PEAKS DB |
| total 3 peptides | | | | | | | | | | | | | | | | | | | | | | | | | | | | | | | | | | | | | | | | | | | | | | | | | |

**Best Unique PSM (Scan F16:32262, m/z=606.0044, z=3, RT=64.58, ppm=1.3):**

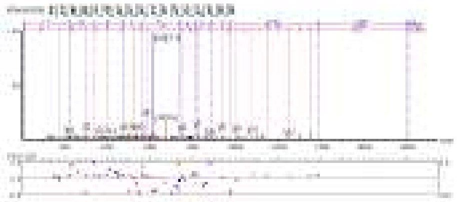

## XP_013910585.1

back to list

| Protein Coverage | Supporting Peptides | Best Unique PSM |

**Protein Coverage:**

1 MKYYAVFCLL LAGSVFADDD DKKEEVGTVV GIDLGTTYSC VGVFKNGRVE IIANDQGNRI TPSYVAFTPE GERLIGDAAK
81 NQLTSNPENT VFDAKRLIGR **TWNDPSVQQD IK**YLPFKVLE KKSKPYIQVD IGGGQMKTFA PEEISAMVLT KMKETAEAYL
161 GKKVTHAVVT VPAYFNDAQR QATK**DAGTIA GLNVMRIINE PTAAAIAYGL DKR**EGEKNIL VFDLGGGTFD VSLLTIDNGV
241 FEVVATNGDT HLGGEDFDQR VMEHFIKLYK KKTGKDVRKD VRAVQKLRRE VEKAKRALSS QHQARIEIES FFESEDFSET
321 LTRAKFEELN MDLFRSTMKP VQKVLEDADL KKSDIDEIVL VGGSTRIPKI QQLVKEFFNG KEPSRGINPD EAVAYGAAVQ
401 AGVLSGDQDT GDLVLLDVCP LTLGIETVGG VMTKLIPRNT VVPTKKSQIF STASDNQPTV TIKVYEGERP LTKDNHLLGT
481 FDLTGIPPAP RGVPQIEVTF EIDVNGILRV TAEDKGTGNK NKITITNDQN RLTPEEIERM VTDAEKFAEE DKKLKERIDA
561 RNELESYAYS LKNQIGDKEK LGGKLSSEDK ETIEKAVEEK IEWLESHQDG DIEEFKAQKK ELEEVVQPIV GKLYGGAGPP
641 PGGEEAGEKD EL

**Supporting Peptides:**

| Peptide | Uniq | -10lgP | Mass | Length | ppm | m/z | z | RT | Fraction | Scan | Source File | Area F1 | Area F2a | Area F3a | Area F4 | Area F5 | Area F6a | Area F7a | Area F7b | Area F8 | Area F9a | Area Sample 1F10a | Area F10b | Area F11a | Area F11b | Area F12a | Area F13 | #Feature | #Feature F1 | #Feature F2a | #Feature F3a | #Feature F4 | #Feature F5 | #Feature F6a | #Feature F7a | #Feature F7b | #Feature F8 | #Feature F9a | #Feature Sample 1F10a | #Feature F10b | #Feature F11a | #Feature F11b | #Feature F12a | #Feature F13 | Start | End | PTM | AScore | Found By |
|---|---|---|---|---|---|---|---|---|---|---|---|---|---|---|---|---|---|---|---|---|---|---|---|---|---|---|---|---|---|---|---|---|---|---|---|---|---|---|---|---|---|---|---|---|---|---|---|---|---|
| R.IINEPTAAAIAYGLDKR.E | Y | 45.90 | 1814.9890 | 17 | 1.3 | 606.0044 | 3 | 64.58 | 16 | F16:32262 | BuCaTN01_F13.raw | | | | | | | | | | | | | | | | 2.2698E5 | 1 | 0 | 0 | 0 | 0 | 0 | 0 | 0 | 0 | 0 | 0 | 0 | 0 | 0 | 0 | 0 | 1 | 197 | 213 | | | PEAKS DB |
| K.DAGTIAGLNVMR.I | Y | 45.60 | 1216.6234 | 12 | 1.5 | 609.3199 | 2 | 59.49 | 16 | F16:28937 | BuCaTN01_F13.raw | | | | | | | | | | | | | | | | 1.4052E5 | 1 | 0 | 0 | 0 | 0 | 0 | 0 | 0 | 0 | 0 | 0 | 0 | 0 | 0 | 0 | 0 | 1 | 185 | 196 | | | PEAKS DB |
| R.TWNDPSVQQDIK.Y | Y | 43.32 | 1429.6837 | 12 | 0.5 | 715.8495 | 2 | 47.71 | 16 | F16:21538 | BuCaTN01_F13.raw | | | | | | | | | | | | | | | | 7.1401E4 | 1 | 0 | 0 | 0 | 0 | 0 | 0 | 0 | 0 | 0 | 0 | 0 | 0 | 0 | 0 | 0 | 1 | 101 | 112 | | | PEAKS DB |
| total 3 peptides | | | | | | | | | | | | | | | | | | | | | | | | | | | | | | | | | | | | | | | | | | | | | | | | | |

**Best Unique PSM (Scan F16:32262, m/z=606.0044, z=3, RT=64.58, ppm=1.3):**

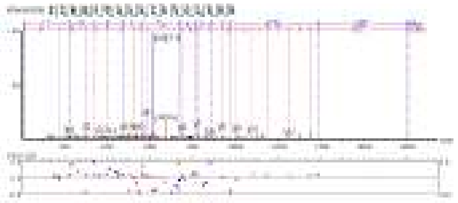

## JAV50419.1

back to list

| Protein Coverage | Supporting Peptides | Best Unique PSM |

**Protein Coverage:**

1 MKYAIVCLLL VGSVFAAEDD DKKEDVGTVV GIDLGTTYSC VGVFKNGRVE IIANDQGNRI TPSYVAFTPE GERLIGDAAK
81 NQLTSNPENT VFDAKRLIGR **TWNDPSVQQD IK**YLPFKVME KKTKPYIQVD IGGGQTKTFA PEEISAMVLT KMKETAEAYL
161 GKKVTHAVVT VPAYFNDAQR QATK**DAGTIA GLNVMRIINE PTAAAIAYGL DKR**EGEKNIL VFDLGGGTFD VSLLTIDNGV
241 FEVVATNGDT HLGGEDFDQR VMEHFIKLYK KKTGKDVRKD NRAVQKLRRE VEKAKRALSS QHQARIEIES FFEGEDFSET
321 LTRAKFEELN MDLFRSTMKP VQKVLEDADL KKPDIDEIVL VGGSTRIPKI QQLVKEFFNG KEPSRGINPD EAVAYGAAVQ
401 AGVLSGDQDT GDLVLLDVCP LTLGIETVGG VMTKLIPRNT VVPTKKSQIF STASDNQPTV TIKVYEGERP LTKDNHLLGT
481 FDLTGIPPAP RGVPQIEVTF EIDVNGILRV TAEDKGTGNK NKITITNDQN RLTPEEIERM VNDAEKFAEE DKKLKERIDA
561 RNELESYAYS LKNQIGDKEK LGGKLSSEEK ETIEKAVEEK IEWLESHQEA DIEDFKAQKK ELEEVVQPIV GKLYGGAGPP
641 HRRGRRDRGE G

**Supporting Peptides:**

| Peptide | Uniq | -10lgP | Mass | Length | ppm | m/z | z | RT | Fraction | Scan | Source File | Area F1 | Area F2a | Area F3a | Area F4 | Area F5 | Area F6a | Area F7a | Area F7b | Area F8 | Area F9a | Area Sample 1F10a | Area F10b | Area F11a | Area F11b | Area F12a | Area F13 | #Feature | #Feature F1 | #Feature F2a | #Feature F3a | #Feature F4 | #Feature F5 | #Feature F6a | #Feature F7a | #Feature F7b | #Feature F8 | #Feature F9a | #Feature Sample 1F10a | #Feature F10b | #Feature F11a | #Feature F11b | #Feature F12a | #Feature F13 | Start | End | PTM | AScore | Found By |
|---|---|---|---|---|---|---|---|---|---|---|---|---|---|---|---|---|---|---|---|---|---|---|---|---|---|---|---|---|---|---|---|---|---|---|---|---|---|---|---|---|---|---|---|---|---|---|---|---|---|
| R.IINEPTAAAIAYGLDKR.E | Y | 45.90 | 1814.9890 | 17 | 1.3 | 606.0044 | 3 | 64.58 | 16 | F16:32262 | BuCaTN01_F13.raw | | | | | | | | | | | | | | | | 2.2698E5 | 1 | 0 | 0 | 0 | 0 | 0 | 0 | 0 | 0 | 0 | 0 | 0 | 0 | 0 | 0 | 0 | 1 | 197 | 213 | | | PEAKS DB |

| | | | | | | | | | | | | | | | | | | | | | | | | | | | | | | | | | | | | | | | | | | | | | | | | | |
|---|---|---|---|---|---|---|---|---|---|---|---|---|---|---|---|---|---|---|---|---|---|---|---|---|---|---|---|---|---|---|---|---|---|---|---|---|---|---|---|---|---|---|---|---|---|---|---|---|---|
| K.DAGTIAGLNVMR.I | Y | 45.60 | 1216.6234 | 12 | 1.5 | 609.3199 | 2 | 59.49 | 16 | F16:28937 | BuCaTN01_F13.raw | | | | | | | | | | | | | | | | 1.4052E5 | 1 | 0 | 0 | 0 | 0 | 0 | 0 | 0 | 0 | 0 | 0 | 0 | 0 | 0 | 0 | 0 | 1 | 185 | 196 | | | PEAKS DB |
| R.TWNDPSVQQDIK.Y | Y | 43.32 | 1429.6837 | 12 | 0.5 | 715.8495 | 2 | 47.71 | 16 | F16:21538 | BuCaTN01_F13.raw | | | | | | | | | | | | | | | | 7.1401E4 | 1 | 0 | 0 | 0 | 0 | 0 | 0 | 0 | 0 | 0 | 0 | 0 | 0 | 0 | 0 | 0 | 1 | 101 | 112 | | | PEAKS DB |
| total 3 peptides | | | | | | | | | | | | | | | | | | | | | | | | | | | | | | | | | | | | | | | | | | | | | | | | | |

**Best Unique PSM (Scan F16:32262, m/z=606.0044, z=3, RT=64.58, ppm=1.3):**

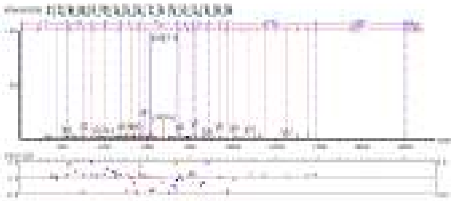

## XP_039178976.1

back to list

| Protein Coverage | Supporting Peptides | Best Unique PSM |

**Protein Coverage:**

```
  1 MKYAIVCLLL VGSVFAAEDD DKKEDVGTVV GIDLGTTYSC VGVFKNGRVE IIANDQGNRI TPSYVAFTPE GERLIGDAAK
 81 NQLTSNPENT VFDAKRLIGR TWNDPSVQQD IKYLPFKVME KKTKPYIQVD IGGGQTKTFA PEEISAMVLT KMKETAEAYL
161 GKKVTHAVVT VPAYFNDAQR QATKDAGTIA GLNVMRIINE PTAAAIAYGL DKREGEKNIL VFDLGGGTFD VSLLTIDNGV
241 FEVVATNGDT HLGGEDFDQR VMEHFIKLYK KKTGKDVRKD NRAVQKLRRE VEKAKRALSS QHQARIEIES FFEGEDFSET
321 LTRAKFEELN MDLFRSTMKP VQKVLEDADL KKPDIDEIVL VGGSTRIPKI QQLVKEFFNG KEPSRGINPD EAVAYGAAVQ
401 AGVLSGDQDT GDLVLLDVCP LTLGIETVGG VMTKLIPRNT VVPTKKSQIF STASDNQPTV TIKVYEGERP LTKDNHLLGT
481 FDLTGIPPAP RGVPQIEVTF EIDVNGILRV TAEDKGTGNK NKITITNDQN RLTPEEIERM VNDAEKFAEE DKKLKERIDA
561 RNELESYAYS LKNQIGDKEK LGGKLSSEEK ETIEKAVEEK IEWLESHQEA DIEDFKAQKK ELEEVVQPIV GKLYGGAGPP
641 PGGEEETGEK DEL
```

**Supporting Peptides:**

| Peptide | Uniq | -10lgP | Mass | Length | ppm | m/z | z | RT | Fraction | Scan | Source File | Area F1 | Area F2a | Area F3a | Area F4 | Area F5 | Area F6a | Area F7a | Area F7b | Area F8 | Area F9a | Area Sample 1F10a | Area F10b | Area F11a | Area F11b | Area F12a | Area F13 | #Feature | #Feature F1 | #Feature F2a | #Feature F3a | #Feature F4 | #Feature F5 | #Feature F6a | #Feature F7a | #Feature F7b | #Feature F8 | #Feature F9a | #Feature Sample 1F10a | #Feature F10b | #Feature F11a | #Feature F11b | #Feature F12a | #Feature F13 | Start | End | PTM | AScore | Found By |
|---|---|---|---|---|---|---|---|---|---|---|---|---|---|---|---|---|---|---|---|---|---|---|---|---|---|---|---|---|---|---|---|---|---|---|---|---|---|---|---|---|---|---|---|---|---|---|---|---|---|
| R.IINEPTAAAIAYGLDKR.E | Y | 45.90 | 1814.9890 | 17 | 1.3 | 606.0044 | 3 | 64.58 | 16 | F16:32262 | BuCaTN01_F13.raw | | | | | | | | | | | | | | | | 2.2698E5 | 1 | 0 | 0 | 0 | 0 | 0 | 0 | 0 | 0 | 0 | 0 | 0 | 0 | 0 | 0 | 0 | 1 | 197 | 213 | | | PEAKS DB |
| K.DAGTIAGLNVMR.I | Y | 45.60 | 1216.6234 | 12 | 1.5 | 609.3199 | 2 | 59.49 | 16 | F16:28937 | BuCaTN01_F13.raw | | | | | | | | | | | | | | | | 1.4052E5 | 1 | 0 | 0 | 0 | 0 | 0 | 0 | 0 | 0 | 0 | 0 | 0 | 0 | 0 | 0 | 0 | 1 | 185 | 196 | | | PEAKS DB |
| R.TWNDPSVQQDIK.Y | Y | 43.32 | 1429.6837 | 12 | 0.5 | 715.8495 | 2 | 47.71 | 16 | F16:21538 | BuCaTN01_F13.raw | | | | | | | | | | | | | | | | 7.1401E4 | 1 | 0 | 0 | 0 | 0 | 0 | 0 | 0 | 0 | 0 | 0 | 0 | 0 | 0 | 0 | 0 | 1 | 101 | 112 | | | PEAKS DB |
| total 3 peptides | | | | | | | | | | | | | | | | | | | | | | | | | | | | | | | | | | | | | | | | | | | | | | | | | |

**Best Unique PSM (Scan F16:32262, m/z=606.0044, z=3, RT=64.58, ppm=1.3):**

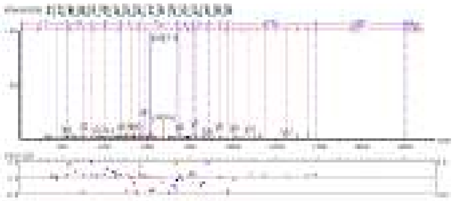

## JAA96909.1

back to list

| Protein Coverage | Supporting Peptides | Best Unique PSM |

**Protein Coverage:**

```
  1 MKYAIVCLLL VGSVFAAEDD DKKEDVGTVV GIDLGTTYSC VGVFKNGRVE IIANDQGNRI TPSYVAFTPE GERLIGDAAK
 81 NQLTSNPENT VFDAKRLIGR TWNDPSVQQD IKYLPFKVME KKTKPYIQVD IGGGQTKTFA PEEISAMVLT KMKETAEAYL
161 GKKVTHAVVT VPAYFNDAQR QATKDAGTIA GLNVMRIINE PTAAAIAYGL DKREGEKNIL VFDLGGGTFD VSLLTIDNGV
241 FEVVATNGDT HLGGEDFDQR VMEHFIKLYK KKTGKDVRKD NRAVQKLRRE VEKAKRALSS QHQARIEIES FFEGEDFSET
321 LTRAKFEELN MDLFRSTMKP VQKVLEDADL KKPDIDEIVL VGGSTRIPKI QQLVKEFFNG KEPSRGINPD EAVAYGAAVQ
401 AGVLSGDQDT GDLVLLDVCP LTLGIETVGG VMTKLIPRNT VVPTKKSQIF STASDNQPTV TIKVYEGERP LTKDNHLLGT
481 FDLTGIPPAP RGVPQIEVTF EIDVNGILRV TAEDKGTGNK NKITITNDQN RLTPEEIERM VNDAEKFAEE DKKLKERIDA
561 RNELESYAYS LKNQIGDKEK LGGKLSSEEK ETIEKAVEEK IEWLESHQEA DIEDFKAQKK ELEEVVQPIV GKLYGGAGPP
641 PGGEEETGEK DEL
```

**Supporting Peptides:**

| Peptide | Uniq | -10lgP | Mass | Length | ppm | m/z | z | RT | Fraction | Scan | Source File | Area F1 | Area F2a | Area F3a | Area F4 | Area F5 | Area F6a | Area F7a | Area F7b | Area F8 | Area F9a | Area Sample 1F10a | Area F10b | Area F11a | Area F11b | Area F12a | Area F13 | #Feature | #Feature F1 | #Feature F2a | #Feature F3a | #Feature F4 | #Feature F5 | #Feature F6a | #Feature F7a | #Feature F7b | #Feature F8 | #Feature F9a | #Feature Sample 1F10a | #Feature F10b | #Feature F11a | #Feature F11b | #Feature F12a | #Feature F13 | Start | End | PTM | AScore | Found By |
|---|---|---|---|---|---|---|---|---|---|---|---|---|---|---|---|---|---|---|---|---|---|---|---|---|---|---|---|---|---|---|---|---|---|---|---|---|---|---|---|---|---|---|---|---|---|---|---|---|---|
| R.IINEPTAAAIAYGLDKR.E | Y | 45.90 | 1814.9890 | 17 | 1.3 | 606.0044 | 3 | 64.58 | 16 | F16:32262 | BuCaTN01_F13.raw | | | | | | | | | | | | | | | | 2.2698E5 | 1 | 0 | 0 | 0 | 0 | 0 | 0 | 0 | 0 | 0 | 0 | 0 | 0 | 0 | 0 | 0 | 1 | 197 | 213 | | | PEAKS DB |
| K.DAGTIAGLNVMR.I | Y | 45.60 | 1216.6234 | 12 | 1.5 | 609.3199 | 2 | 59.49 | 16 | F16:28937 | BuCaTN01_F13.raw | | | | | | | | | | | | | | | | 1.4052E5 | 1 | 0 | 0 | 0 | 0 | 0 | 0 | 0 | 0 | 0 | 0 | 0 | 0 | 0 | 0 | 0 | 1 | 185 | 196 | | | PEAKS DB |
| R.TWNDPSVQQDIK.Y | Y | 43.32 | 1429.6837 | 12 | 0.5 | 715.8495 | 2 | 47.71 | 16 | F16:21538 | BuCaTN01_F13.raw | | | | | | | | | | | | | | | | 7.1401E4 | 1 | 0 | 0 | 0 | 0 | 0 | 0 | 0 | 0 | 0 | 0 | 0 | 0 | 0 | 0 | 0 | 1 | 101 | 112 | | | PEAKS DB |
| total 3 peptides | | | | | | | | | | | | | | | | | | | | | | | | | | | | | | | | | | | | | | | | | | | | | | | | | |

**Best Unique PSM (Scan F16:32262, m/z=606.0044, z=3, RT=64.58, ppm=1.3):**

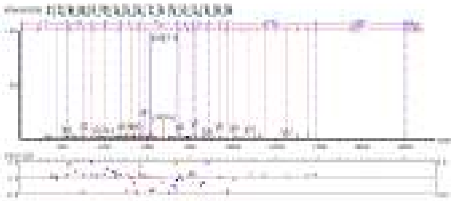

## JAG44540.1

back to list

| Protein Coverage | Supporting Peptides | Best Unique PSM |

**Protein Coverage:**

1 MKYAIVCLLL VGSVFAAEDD DKKEDVGTVV GIDLGTTYSC VGVFKNGRVE IIANDQGNRI TPSYVAFTPE GERLIGDAAK
81 NQLTSNPENT VFDAKRLIGR TWNDPSVQQD IKYLPFKVME KKTKPYIQVD IGGGQTKTFA PEEISAMVLT KMKETAEAYL
161 GKKVTHAVVT VPAYFNDAQR QATKDAGTIA GLNVMRIINE PTAAAIAYGL DKREGEKNIL VFDLGGGTFD VSLLTIDNGV
241 FEVVATNGDT HLGGEDFDQR VMEHFIKLYK KKTGKDVRKD NRAVQKLRRE VEKAKRALSS QHQARIEIES FFEGEDFSET
321 LTRAKFEELN MDLFRSTMKP VQKVLEDADL KKPDIDEIVL VGGSTRIPKI QQLVKEFFNG KEPSRGINPD EAVAYGAAVQ
401 AGVLSGDQDT GDLVLLDVCP LTLGIETVGG VMTKLIPRNT VVPTKKSQIF STASDNQPTV TIKVYEGERP LTKDNHLLGT
481 FDLTGIPPAP RGVPQIEVTF EIDVNGILRV TAEDKGTGNK NKITITNDQN RLTPEEIERM VNDAEKFAEE DKKLKERIDA
561 RNELESYAYS LKNQIGDKEK LGGKLSSEEK ETIEKAVEEK IEWLESHQEA DIEDFKAQKK ELEEVVQPIV GKLYGGAGPP
641 PGGEEETGEK DEL

**Supporting Peptides:**

| Peptide | Uniq | -10lgP | Mass | Length | ppm | m/z | z | RT | Fraction | Scan | Source File | Area F1 | Area F2a | Area F3a | Area F4 | Area F5 | Area F6a | Area F7a | Area F7b | Area F8 | Area F9a | Area Sample 1F10a | Area F10b | Area F11a | Area F11b | Area F12a | Area F13 | #Feature | #Feature F1 | #Feature F2a | #Feature F3a | #Feature F4 | #Feature F5 | #Feature F6a | #Feature F7a | #Feature F7b | #Feature F8 | #Feature F9a | #Feature Sample 1F10a | #Feature F10b | #Feature F11a | #Feature F11b | #Feature F12a | #Feature F13 | Start | End | PTM | AScore | Found By |
|---|---|---|---|---|---|---|---|---|---|---|---|---|---|---|---|---|---|---|---|---|---|---|---|---|---|---|---|---|---|---|---|---|---|---|---|---|---|---|---|---|---|---|---|---|---|---|---|---|---|
| R.IINEPTAAAIAYGLDKR.E | Y | 45.90 | 1814.9890 | 17 | 1.3 | 606.0044 | 3 | 64.58 | 16 | F16:32262 | BuCaTN01_F13.raw | | | | | | | | | | | | | | | | 2.2698E5 | 1 | 0 | 0 | 0 | 0 | 0 | 0 | 0 | 0 | 0 | 0 | 0 | 0 | 0 | 0 | 0 | 1 | 197 | 213 | | | PEAKS DB |
| K.DAGTIAGLNVMR.I | Y | 45.60 | 1216.6234 | 12 | 1.5 | 609.3199 | 2 | 59.49 | 16 | F16:28937 | BuCaTN01_F13.raw | | | | | | | | | | | | | | | | 1.4052E5 | 1 | 0 | 0 | 0 | 0 | 0 | 0 | 0 | 0 | 0 | 0 | 0 | 0 | 0 | 0 | 0 | 1 | 185 | 196 | | | PEAKS DB |
| R.TWNDPSVQQDIK.Y | Y | 43.32 | 1429.6837 | 12 | 0.5 | 715.8495 | 2 | 47.71 | 16 | F16:21538 | BuCaTN01_F13.raw | | | | | | | | | | | | | | | | 7.1401E4 | 1 | 0 | 0 | 0 | 0 | 0 | 0 | 0 | 0 | 0 | 0 | 0 | 0 | 0 | 0 | 0 | 1 | 101 | 112 | | | PEAKS DB |
| total 3 peptides | | | | | | | | | | | | | | | | | | | | | | | | | | | | | | | | | | | | | | | | | | | | | | | | | | |

**Best Unique PSM (Scan F16:32262, m/z=606.0044, z=3, RT=64.58, ppm=1.3):**

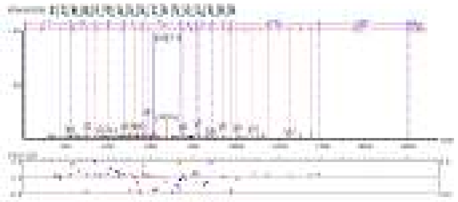

## JAI13107.1

back to list

| Protein Coverage | Supporting Peptides | Best Unique PSM |

**Protein Coverage:**

1 MKYAIVCLLL VGSVFAAEDD DKKEDVGTVV GIDLGTTYSC VGVFKNGRVE IIANDQGNRI TPSYVAFTPE GERLIGDAAK
81 NQLTSNPENT VFDAKRLIGR TWNDPSVQQD IKYLPFKVME KKTKPYIQVD IGGGQTKTFA PEEISAMVLT KMKETAEAYL
161 GKKVTHAVVT VPAYFNDAQR QATKDAGTIA GLNVMRIINE PTAAAIAYGL DKREGEKNIL VFDLGGGTFD VSLLTIDNGV
241 FEVVATNGDT HLGGEDFDQR VMEHFIKLYK KKTGKDVRKD NRAVQKLRRE VEKAKRALSS QHQARIEIES FFEGEDFSET
321 LTRAKFEELN MDLFRSTMKP VQKVLEDADL KKPDIDEIVL VGGSTRIPKI QQLVKEFFNG KEPSRGINPD EAVAYGAAVQ
401 AGVLSGDQDT GDLVLLDVCP LTLGIETVGG VMTKLIPRNT VVPTKKSQIF STASDNQPTV TIKVYEGERP LTKDNHLLGT
481 FDLTGIPPAP RGVPQIEVTF EIDVNGILRV TAEDKGTGNK NKITITNDQN RLTPEEIERM VNDAEKFAEE DKKLKERIDA
561 RNELESYAYS LKNQIGDKEK LGGKLSSEEK ETIEKAVEEK IEWLESHQEA DIEDFKAQKK ELEEVVQPIV GKLYGGAGPP
641 PGGEEETGEK DEL

**Supporting Peptides:**

| Peptide | Uniq | -10lgP | Mass | Length | ppm | m/z | z | RT | Fraction | Scan | Source File | Area F1 | Area F2a | Area F3a | Area F4 | Area F5 | Area F6a | Area F7a | Area F7b | Area F8 | Area F9a | Area Sample 1F10a | Area F10b | Area F11a | Area F11b | Area F12a | Area F13 | #Feature | #Feature F1 | #Feature F2a | #Feature F3a | #Feature F4 | #Feature F5 | #Feature F6a | #Feature F7a | #Feature F7b | #Feature F8 | #Feature F9a | #Feature Sample 1F10a | #Feature F10b | #Feature F11a | #Feature F11b | #Feature F12a | #Feature F13 | Start | End | PTM | AScore | Found By |
|---|---|---|---|---|---|---|---|---|---|---|---|---|---|---|---|---|---|---|---|---|---|---|---|---|---|---|---|---|---|---|---|---|---|---|---|---|---|---|---|---|---|---|---|---|---|---|---|---|---|
| R.IINEPTAAAIAYGLDKR.E | Y | 45.90 | 1814.9890 | 17 | 1.3 | 606.0044 | 3 | 64.58 | 16 | F16:32262 | BuCaTN01_F13.raw | | | | | | | | | | | | | | | | 2.2698E5 | 1 | 0 | 0 | 0 | 0 | 0 | 0 | 0 | 0 | 0 | 0 | 0 | 0 | 0 | 0 | 0 | 1 | 197 | 213 | | | PEAKS DB |
| K.DAGTIAGLNVMR.I | Y | 45.60 | 1216.6234 | 12 | 1.5 | 609.3199 | 2 | 59.49 | 16 | F16:28937 | BuCaTN01_F13.raw | | | | | | | | | | | | | | | | 1.4052E5 | 1 | 0 | 0 | 0 | 0 | 0 | 0 | 0 | 0 | 0 | 0 | 0 | 0 | 0 | 0 | 0 | 1 | 185 | 196 | | | PEAKS DB |
| R.TWNDPSVQQDIK.Y | Y | 43.32 | 1429.6837 | 12 | 0.5 | 715.8495 | 2 | 47.71 | 16 | F16:21538 | BuCaTN01_F13.raw | | | | | | | | | | | | | | | | 7.1401E4 | 1 | 0 | 0 | 0 | 0 | 0 | 0 | 0 | 0 | 0 | 0 | 0 | 0 | 0 | 0 | 0 | 1 | 101 | 112 | | | PEAKS DB |
| total 3 peptides | | | | | | | | | | | | | | | | | | | | | | | | | | | | | | | | | | | | | | | | | | | | | | | | | | |

**Best Unique PSM (Scan F16:32262, m/z=606.0044, z=3, RT=64.58, ppm=1.3):**

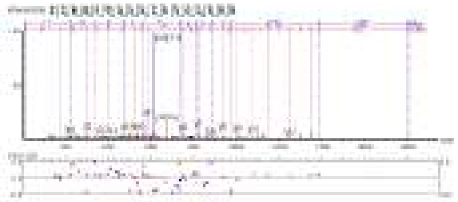

## JAG46692.1

back to list

| Protein Coverage | Supporting Peptides | Best Unique PSM |

**Protein Coverage:**

1 MKYAIVCLLL VGSVFAAEDD DKKEDVGTVV GIDLGTTYSC VGVFKNGRVE IIANDQGNRI TPSYVAFTPE GERLIGDAAK
81 NQLTSNPENT VFDAKRLIGR TWNDPSVQQD IKYLPFKVME KKTKPYIQVD IGGGQTKTFA PEEISAMVLT KMKETAEAYL
161 GKKVTHAVVT VPAYFNDAQR QATKDAGTIA GLNVMRIINE PTAAAIAYGL DKREGEKNIL VFDLGGGTFD VSLLTIDNGV
241 FEVVATNGDT HLGGEDFDQR VMEHFIKLYK KKTGKDVRKD NRAVQKLRRE VEKAKRALSS QHQARIEIES FFEGEDFSET
321 LTRAKFEELN MDLFRSTMKP VQKVLEDADL KKPDIDEIVL VGGSTRIPKI QQLVKEFFNG KEPSRGINPD EAVAYGAAVQ
401 AGVLSGDQDT GDLVLLDVCP LTLGIETVGG VMTKLIPRNT VVPTKKSQIF STASDNQPTV TIKVYEGERP LTKDNHLLGT
481 FDLTGIPPAP RGVPQIEVTF EIDVNGILRV TAEDKGTGNK NKITITNDQN RLTPEEIERM VNDAEKFAEE DKKLKERIDA
561 RNELESYAYS LKNQIGDKEK LGGKLSSEEK ETIEKAVEEK IEWLESHQEA DIEDFKAQKK ELEEVVQPIV GKLYGGAGPP
641 PGGEEETGEK DEL

**Supporting Peptides:**

| Peptide | Uniq | -10lgP | Mass | Length | ppm | m/z | z | RT | Fraction | Scan | Source File | Area F1 | Area F2a | Area F3a | Area F4 | Area F5 | Area F6a | Area F7a | Area F7b | Area F8 | Area F9a | Area Sample 1F10a | Area F10b | Area F11a | Area F11b | Area F12a | Area F13 | #Feature | #Feature F1 | #Feature F2a | #Feature F3a | #Feature F4 | #Feature F5 | #Feature F6a | #Feature F7a | #Feature F7b | #Feature F8 | #Feature F9a | #Feature Sample 1F10a | #Feature F10b | #Feature F11a | #Feature F11b | #Feature F12a | #Feature F13 | Start | End | PTM | AScore | Found By |
|---|---|---|---|---|---|---|---|---|---|---|---|---|---|---|---|---|---|---|---|---|---|---|---|---|---|---|---|---|---|---|---|---|---|---|---|---|---|---|---|---|---|---|---|---|---|---|---|---|---|
| R.IINEPTAAAIAYGLDKR.E | Y | 45.90 | 1814.9890 | 17 | 1.3 | 606.0044 | 3 | 64.58 | 16 | F16:32262 | BuCaTN01_F13.raw | | | | | | | | | | | | | | | | 2.2698E5 | 1 | 0 | 0 | 0 | 0 | 0 | 0 | 0 | 0 | 0 | 0 | 0 | 0 | 0 | 0 | 0 | 1 | 197 | 213 | | | PEAKS DB |
| K.DAGTIAGLNVMR.I | Y | 45.60 | 1216.6234 | 12 | 1.5 | 609.3199 | 2 | 59.49 | 16 | F16:28937 | BuCaTN01_F13.raw | | | | | | | | | | | | | | | | 1.4052E5 | 1 | 0 | 0 | 0 | 0 | 0 | 0 | 0 | 0 | 0 | 0 | 0 | 0 | 0 | 0 | 0 | 1 | 185 | 196 | | | PEAKS DB |
| R.TWNDPSVQQDIK.Y | Y | 43.32 | 1429.6837 | 12 | 0.5 | 715.8495 | 2 | 47.71 | 16 | F16:21538 | BuCaTN01_F13.raw | | | | | | | | | | | | | | | | 7.1401E4 | 1 | 0 | 0 | 0 | 0 | 0 | 0 | 0 | 0 | 0 | 0 | 0 | 0 | 0 | 0 | 0 | 1 | 101 | 112 | | | PEAKS DB |
| total 3 peptides | | | | | | | | | | | | | | | | | | | | | | | | | | | | | | | | | | | | | | | | | | | | | | | | | | |

**Best Unique PSM (Scan F16:32262, m/z=606.0044, z=3, RT=64.58, ppm=1.3):**

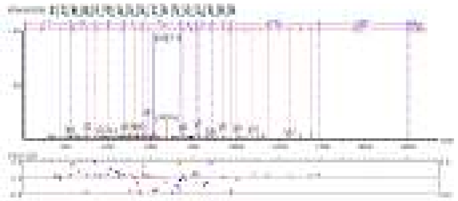

## AFJ50327.1

back to list

| Protein Coverage | Supporting Peptides | Best Unique PSM |

**Protein Coverage:**

```
  1 MKYAIVCLLL VGSVFAAEDD DKKEDVGTVV GIDLGTTYSC VGVFKNGRVE IIANDQGNRI TPSYVAFTPE GERLIGDAAK
 81 NQLTSNPENT VFDAKRLIGR TWNDPSVQQD IKYLPFKVME KKTKPYIQVD IGGGQTKTFA PEEISAMVLT KMKETAEAYL
161 GKKVTHAVVT VPAYFNDAQR QATKDAGTIA GLNVMRIINE PTAAAIAYGL DKREGEKNIL VFDLGGGTFD VSLLTIDNGV
241 FEVVATNGDT HLGGEDFDQR VMEHFIKLYK KKTGKDVRKD NRAVQKLRRE VEKAKRALSS QHQARIEIES FFEGEDFSET
321 LTRAKFEELN MDLFRSTMKP VQKVLEDADL KKPDIDEIVL VGGSTRIPKI QQLVKEFFNG KEPSRGINPD EAVAYGAAVQ
401 AGVLSGDQDT GDLVLLDVCP LTLGIETVGG VMTKLIPRNT VVPTKKSQIF STASDNQPTV TIKVYEGERP LTKDNHLLGT
481 FDLTGIPPAP RGVPQIEVTF EIDVNGILRV TAEDKGTGNK NKITITNDQN RLTPEEIERM VNDAEKFAEE DKKLKERIDA
561 RNELESYAYS LKNQIGDKEK LGGKLSSEEK ETIEKAVEEK IEWLESHQEA DIEDFKAQKK ELEEVVQPIV GKLYGGAGPP
641 PGGEEETGEK DEL
```

**Supporting Peptides:**

| Peptide | Uniq | -10lgP | Mass | Length | ppm | m/z | z | RT | Fraction | Scan | Source File | Area F1 | Area F2a | Area F3a | Area F4 | Area F5 | Area F6a | Area F7a | Area F7b | Area F8 | Area F9a | Area Sample 1F10a | Area F10b | Area F11a | Area F11b | Area F12a | Area F13 | #Feature | #Feature F1 | #Feature F2a | #Feature F3a | #Feature F4 | #Feature F5 | #Feature F6a | #Feature F7a | #Feature F7b | #Feature F8 | #Feature F9a | #Feature Sample 1F10a | #Feature F10b | #Feature F11a | #Feature F11b | #Feature F12a | #Feature F13 | Start | End | PTM | AScore | Found By |
|---|---|---|---|---|---|---|---|---|---|---|---|---|---|---|---|---|---|---|---|---|---|---|---|---|---|---|---|---|---|---|---|---|---|---|---|---|---|---|---|---|---|---|---|---|---|---|---|---|---|
| R.IINEPTAAAIAYGLDKR.E | Y | 45.90 | 1814.9890 | 17 | 1.3 | 606.0044 | 3 | 64.58 | 16 | F16:32262 | BuCaTN01_F13.raw | | | | | | | | | | | | | | | | 2.2698E5 | 1 | 0 | 0 | 0 | 0 | 0 | 0 | 0 | 0 | 0 | 0 | 0 | 0 | 0 | 0 | 0 | 1 | 197 | 213 | | | PEAKS DB |
| K.DAGTIAGLNVMR.I | Y | 45.60 | 1216.6234 | 12 | 1.5 | 609.3199 | 2 | 59.49 | 16 | F16:28937 | BuCaTN01_F13.raw | | | | | | | | | | | | | | | | 1.4052E5 | 1 | 0 | 0 | 0 | 0 | 0 | 0 | 0 | 0 | 0 | 0 | 0 | 0 | 0 | 0 | 0 | 1 | 185 | 196 | | | PEAKS DB |
| R.TWNDPSVQQDIK.Y | Y | 43.32 | 1429.6837 | 12 | 0.5 | 715.8495 | 2 | 47.71 | 16 | F16:21538 | BuCaTN01_F13.raw | | | | | | | | | | | | | | | | 7.1401E4 | 1 | 0 | 0 | 0 | 0 | 0 | 0 | 0 | 0 | 0 | 0 | 0 | 0 | 0 | 0 | 0 | 1 | 101 | 112 | | | PEAKS DB |
| total 3 peptides | | | | | | | | | | | | | | | | | | | | | | | | | | | | | | | | | | | | | | | | | | | | | | | | | |

**Best Unique PSM (Scan F16:32262, m/z=606.0044, z=3, RT=64.58, ppm=1.3):**

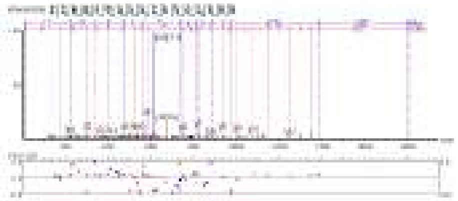

## AFJ50239.1

back to list

| Protein Coverage | Supporting Peptides | Best Unique PSM |

**Protein Coverage:**

```
  1 MKYAIVCLLL VGSVFAAEDD DKKEDVGTVV GIDLGTTYSC VGVFKNGRVE IIANDQGNRI TPSYVAFTPE GERLIGDAAK
 81 NQLTSNPENT VFDAKRLIGR TWNDPSVQQD IKYLPFKVME KKTKPYIQVD IGGGQTKTFA PEEISAMVLT KMKETAEAYL
161 GKKVTHAVVT VPAYFNDAQR QATKDAGTIA GLNVMRIINE PTAAAIAYGL DKREGEKNIL VFDLGGGTFD VSLLTIDNGV
241 FEVVATNGDT HLGGEDFDQR VMEHFIKLYK KKTGKDVRKD NRAVQKLRRE VEKAKRALSS QHQARIEIES FFEGEDFSET
321 LTRAKFEELN MDLFRSTMKP VQKVLEDADL KKPDIDEIVL VGGSTRIPKI QQLVKEFFNG KEPSRGINPD EAVAYGAAVQ
401 AGVLSGDQDT GDLVLLDVCP LTLGIETVGG VMTKLIPRNT VVPTKKSQIF STASDNQPTV TIKVYEGERP LTKDNHLLGT
481 FDLTGIPPAP RGVPQIEVTF EIDVTGIPPA PRGVPQIEVT FEIDVNGILR VTAEDKGTGN KNKITITNDQ NRLTPEEIER
561 MVNDAEKFAE EDKKLKERID ARNELESYAY SLKNQIGDKE KLGGKLSSEE KETIEKAVEE KETIEKAVEE KIEWLESHQE
641 ADIEDFKAQK KELEEVVQPI VGKLYGGAGP PPGGEEETGE KDEL
```

**Supporting Peptides:**

| Peptide | Uniq | -10lgP | Mass | Length | ppm | m/z | z | RT | Fraction | Scan | Source File | Area F1 | Area F2a | Area F3a | Area F4 | Area F5 | Area F6a | Area F7a | Area F7b | Area F8 | Area F9a | Area Sample 1F10a | Area F10b | Area F11a | Area F11b | Area F12a | Area F13 | #Feature | #Feature F1 | #Feature F2a | #Feature F3a | #Feature F4 | #Feature F5 | #Feature F6a | #Feature F7a | #Feature F7b | #Feature F8 | #Feature F9a | #Feature Sample 1F10a | #Feature F10b | #Feature F11a | #Feature F11b | #Feature F12a | #Feature F13 | Start | End | PTM | AScore | Found By |
|---|---|---|---|---|---|---|---|---|---|---|---|---|---|---|---|---|---|---|---|---|---|---|---|---|---|---|---|---|---|---|---|---|---|---|---|---|---|---|---|---|---|---|---|---|---|---|---|---|---|
| R.IINEPTAAAIAYGLDKR.E | Y | 45.90 | 1814.9890 | 17 | 1.3 | 606.0044 | 3 | 64.58 | 16 | F16:32262 | BuCaTN01_F13.raw | | | | | | | | | | | | | | | | 2.2698E5 | 1 | 0 | 0 | 0 | 0 | 0 | 0 | 0 | 0 | 0 | 0 | 0 | 0 | 0 | 0 | 0 | 1 | 197 | 213 | | | PEAKS DB |
| K.DAGTIAGLNVMR.I | Y | 45.60 | 1216.6234 | 12 | 1.5 | 609.3199 | 2 | 59.49 | 16 | F16:28937 | BuCaTN01_F13.raw | | | | | | | | | | | | | | | | 1.4052E5 | 1 | 0 | 0 | 0 | 0 | 0 | 0 | 0 | 0 | 0 | 0 | 0 | 0 | 0 | 0 | 0 | 1 | 185 | 196 | | | PEAKS DB |
| R.TWNDPSVQQDIK.Y | Y | 43.32 | 1429.6837 | 12 | 0.5 | 715.8495 | 2 | 47.71 | 16 | F16:21538 | BuCaTN01_F13.raw | | | | | | | | | | | | | | | | 7.1401E4 | 1 | 0 | 0 | 0 | 0 | 0 | 0 | 0 | 0 | 0 | 0 | 0 | 0 | 0 | 0 | 0 | 1 | 101 | 112 | | | PEAKS DB |
| total 3 peptides | | | | | | | | | | | | | | | | | | | | | | | | | | | | | | | | | | | | | | | | | | | | | | | | | |

**Best Unique PSM (Scan F16:32262, m/z=606.0044, z=3, RT=64.58, ppm=1.3):**

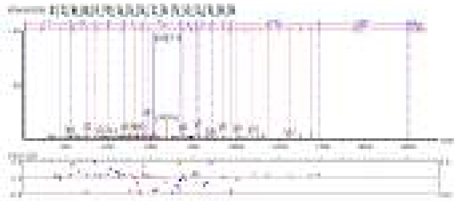

## ETE59912.1

back to list

| Protein Coverage | Supporting Peptides | Best Unique PSM |

**Protein Coverage:**

```
  1 MKYYAVLCLL LVGSVLAADD DDRKEEVGTV VGIDLGTTYS CVGVFKNGRV EIIANDQGNR ITPSYVAFTP EGERLIGDAA
 81 KNQLTSNPEN TVFDAKRLIG RTWNDPSVQQ DIKYLPFKVI EKKAKPYIQV DIGGGQTKTF APEEISAMVL TKMKETAEAY
161 LGKKVTHAVV TVPAYFNDAQ RQATKDAGTI AGLNVMRIIN EPTAAAIAYG LDKREGEKNI LVFDLGGGTF DVSLLTIDNG
241 VFEVVATNGD THLGGEDFDQ RVMEHFIKLY KKKTGKDVRK DNRAVQKLRR EVEKAKRALS SQHQARIEIE SFFEGEDFSE
321 TLTRAKFEEL NMDLFRSTMK PVQKVLEDAD LKKSDIDEIV LVGGSTRIPK IQQLVKEFFN GKEPSRGINP DEAVAYRSIT
401 PDEAVAYGAA VQAGVLSGDQ DTGERPLTKD NHLLGTFDLT GIPPAPRGVP QIEVTFEIDV NGILRVTAED KGTGNKNKIT
481 ITNDQNRLTP EEIERMVNDA EKFAEEDKKL KERIDARNEL ESYAYSLKNQ IGDKEKLGGK LSSEDKETIE KAVEEKIEWL
561 ESHQDGDIEE FKAQKKELEE VVQPIVGKLY GGAGPPPGGE EEAGEKDEL
```

**Supporting Peptides:**

| Peptide | Uniq | -10lgP | Mass | Length | ppm | m/z | z | RT | Fraction | Scan | Source File | Area F1 | Area F2a | Area F3a | Area F4 | Area F5 | Area F6a | Area F7a | Area F7b | Area F8 | Area F9a | Area Sample 1F10a | Area F10b | Area F11a | Area F11b | Area F12a | Area F13 | #Feature | #Feature F1 | #Feature F2a | #Feature F3a | #Feature F4 | #Feature F5 | #Feature F6a | #Feature F7a | #Feature F7b | #Feature F8 | #Feature F9a | #Feature Sample 1F10a | #Feature F10b | #Feature F11a | #Feature F11b | #Feature F12a | #Feature F13 | Start | End | PTM | AScore | Found By |
|---|---|---|---|---|---|---|---|---|---|---|---|---|---|---|---|---|---|---|---|---|---|---|---|---|---|---|---|---|---|---|---|---|---|---|---|---|---|---|---|---|---|---|---|---|---|---|---|---|---|
| R.IINEPTAAAIAYGLDKR.E | Y | 45.90 | 1814.9890 | 17 | 1.3 | 606.0044 | 3 | 64.58 | 16 | F16:32262 | BuCaTN01_F13.raw | | | | | | | | | | | | | | | | 2.2698E5 | 1 | 0 | 0 | 0 | 0 | 0 | 0 | 0 | 0 | 0 | 0 | 0 | 0 | 0 | 0 | 0 | 1 | 198 | 214 | | | PEAKS DB |
| K.DAGTIAGLNVMR.I | Y | 45.60 | 1216.6234 | 12 | 1.5 | 609.3199 | 2 | 59.49 | 16 | F16:28937 | BuCaTN01_F13.raw | | | | | | | | | | | | | | | | 1.4052E5 | 1 | 0 | 0 | 0 | 0 | 0 | 0 | 0 | 0 | 0 | 0 | 0 | 0 | 0 | 0 | 0 | 1 | 186 | 197 | | | PEAKS |

| | | | | | | | | | | | | | | | | | | | | | | | | | | | | | | | | | | | | | | | | | | | | | | | | | |
|---|---|---|---|---|---|---|---|---|---|---|---|---|---|---|---|---|---|---|---|---|---|---|---|---|---|---|---|---|---|---|---|---|---|---|---|---|---|---|---|---|---|---|---|---|---|---|---|---|---|
| | | | | | | | | | | | | | | | | | | | | | | | | | | | | | | | | | | | | | | | | | | | | | | | | | DB |
| R.TWNDPSVQQDIK.Y | Y | 43.32 | 1429.6837 | 12 | 0.5 | 715.8495 | 2 | 47.71 | 16 | F16:21538 | BuCaTN01_F13.raw | | | | | | | | | | | | | | | | 7.1401E4 | 1 | 0 | 0 | 0 | 0 | 0 | 0 | 0 | 0 | 0 | 0 | 0 | 0 | 0 | 0 | 0 | 1 | 102 | 113 | | | PEAKS DB |

**total 3 peptides**

**Best Unique PSM (Scan F16:32262, m/z=606.0044, z=3, RT=64.58, ppm=1.3):**

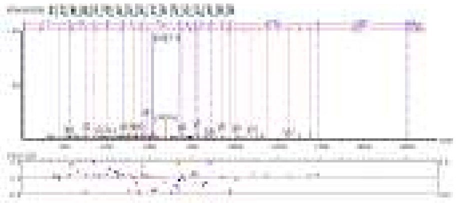

## QSI83986.1

back to list

| Protein Coverage | Supporting Peptides | Best Unique PSM |

**Protein Coverage:**

Carbamidomethylation (+57.02)

1 MIAFIVLLSL AAVLQQSSGI VDFPSVSTNK RENQKQIVDK HNDLRRSVRP TASNMLQMEW NSKAAQNAKR WSDKCSFAHS
81 PPRLRTVGKI RCGENIFMSS QPYEWSGAIQ AWYDEYKNFI YGVGANPPGS VIGHYTQVVW YRSYLLGCAA TKCSSSK**YIY**
161 **VCQYCPAGN**I V**GSIATPYKS GPPCGDCPSA CDNKLCTNPC K**HHNEYSNCQ SLAKQPGCQT EWVKSKCPAS CFCRTEIK

(162 C, 165 C, 184 C, 187 C, 191 C, 196 C, 200 C)

**Supporting Peptides:**

| Peptide | Uniq | -10lgP | Mass | Length | ppm | m/z | z | RT | Fraction | Scan | Source File | Area F1 | Area F2a | Area F3a | Area F4 | Area F5 | Area F6a | Area F7a | Area F7b | Area F8 | Area F9a | Area Sample 1F10a | Area F10b | Area F11a | Area F11b | Area F12a | Area F13 | #Feature | #Feature F1 | #Feature F2a | #Feature F3a | #Feature F4 | #Feature F5 | #Feature F6a | #Feature F7a | #Feature F7b | #Feature F8 | #Feature F9a | #Feature Sample 1F10a | #Feature F10b | #Feature F11a | #Feature F11b | #Feature F12a | #Feature F13 | Start | End | PTM | AScore | Found By |
|---|---|---|---|---|---|---|---|---|---|---|---|---|---|---|---|---|---|---|---|---|---|---|---|---|---|---|---|---|---|---|---|---|---|---|---|---|---|---|---|---|---|---|---|---|---|---|---|---|---|
| K.YIYVC(+57.02)QYC(+57.02)PAGN.I | N | 58.24 | 1506.6272 | 12 | 0.2 | 754.3210 | 2 | 59.53 | 13 | F13:34348 | BuCaTN01_F11a.raw | | | | | | | | | | | | | 8.7629E6 | | | | 1 | 0 | 0 | 0 | 0 | 0 | 0 | 0 | 0 | 0 | 0 | 0 | 0 | 1 | 0 | 0 | 0 | 158 | 169 | Carbamidomethylation | C5:Carbamidomethylation:1000.00;C8:Carbamidomethylation:1000.00 | PEAKS DB |
| V.GSIATPYK.S | N | 53.31 | 835.4440 | 8 | 0.0 | 418.7293 | 2 | 31.21 | 11 | F11:9319 | BuCaTN01_F10a.raw | | | | | | | | | | | 5.4744E5 | | 2.1054E6 | 2.2918E5 | | | 3 | 0 | 0 | 0 | 0 | 0 | 0 | 0 | 0 | 0 | 0 | 1 | 0 | 1 | 1 | 0 | 0 | 172 | 179 | | | PEAKS DB |
| K.SGPPC(+57.02)GDC(+57.02)PSAC(+57.02)DNKLC(+57.02)TNPC(+57.02)K.H | Y | 47.20 | 2493.9805 | 22 | 1.3 | 832.3352 | 3 | 46.34 | 13 | F13:22273 | BuCaTN01_F11a.raw | | | | | | | | | | | | | 2.3815E4 | | | | 1 | 0 | 0 | 0 | 0 | 0 | 0 | 0 | 0 | 0 | 0 | 0 | 0 | 1 | 0 | 0 | 0 | 180 | 201 | Carbamidomethylation | C5:Carbamidomethylation:1000.00;C8:Carbamidomethylation:1000.00;C12:Carbamidomethylation:1000.00;C17:Carbamidomethylation:1000.00;C21:Carbamidomethylation:1000.00 | PEAKS DB |

**total 3 peptides**

**Best Unique PSM (Scan F13:22273, m/z=832.3352, z=3, RT=46.34, ppm=1.3):**

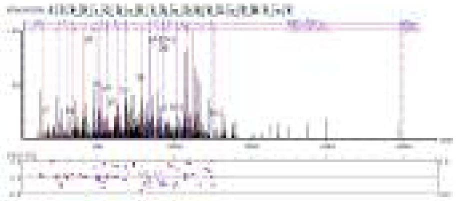

## XP_026539628.1

back to list

| Protein Coverage | Supporting Peptides | Best Unique PSM |

**Protein Coverage:**

1 MSFQTICQGS KGSRRGYSSC SAFGGGTGGG IRRSYSSCRG YGGGMRGQYG SRSLHNYGGV TRISYGSGYG GGVCGGYGGG
81 YGGIGGGYGG IGGSCGGMGG GYGGIGG**GYG GMGGGYGGMG GGYGGMGGGL GGR**GFGGLGS GGIGSVQVDP NLLRPVRVDI
161 DPQIQQVKNQ EKEQIKVLNN Q**FASFIDKVR** FLEQQNKVLQ TKWQILQQQS QGIGPTMNLD AQFQQFLNSL RNQIENIRSQ
241 KGQLATELQS MQHYVEDFKN KYEEEINRRT TAENDFVVLK KDVDCSYLSK SNLEVRVGAL AEQINFLRKI FDVEISQYQT
321 GGQDTSVVVS MDNNTHLNLE GIIDEVRSQY EEIARSSRAE AESWYHSQFE ALQHTAGRHG DNLRNTRQEI QELTRNIQRL
401 RSEIEHVKKQ CAKLQSDIAE VEERGELALR DAKQKLHDLE CALAKDKEEL ARLLKEYQEL LNVKLALDIE IAMYRKLLEG
481 EESRLCGSGS QVHVSVRRGS IGGGIGGGIG GGIGGGLGGG ICGGIGSGVV GSIGGGSCGY GSGGYGGIIS GGGAGGSVCG
561 GGGGFSHGSG SGSSTVIRRT TTSSSCTKSS GRY

**Supporting Peptides:**

| Peptide | Uniq | -10lgP | Mass | Length | ppm | m/z | z | RT | Fraction | Scan | Source File | Area F1 | Area F2a | Area F3a | Area F4 | Area F5 | Area F6a | Area F7a | Area F7b | Area F8 | Area F9a | Area Sample 1F10a | Area F10b | Area F11a | Area F11b | Area F12a | Area F13 | #Feature | #Feature F1 | #Feature F2a | #Feature F3a | #Feature F4 | #Feature F5 | #Feature F6a | #Feature F7a | #Feature F7b | #Feature F8 | #Feature F9a | #Feature Sample 1F10a | #Feature F10b | #Feature F11a | #Feature F11b | #Feature F12a | #Feature F13 | Start | End | PTM | AScore | Found By |
|---|---|---|---|---|---|---|---|---|---|---|---|---|---|---|---|---|---|---|---|---|---|---|---|---|---|---|---|---|---|---|---|---|---|---|---|---|---|---|---|---|---|---|---|---|---|---|---|---|---|
| Q.FASFIDKVR.F | N | 52.96 | 1081.5920 | 9 | 4.5 | 361.5378 | 3 | 50.93 | 15 | F15:26707 | BuCaTN01_F12a.raw | 7.0769E4 | | | | | 5.8464E4 | | 2.0416E5 | | 6.8682E5 | | 0 | 1.8562E5 | | 1.0105E5 | | 6 | 1 | 0 | 0 | 0 | 0 | 1 | 0 | 1 | 0 | 1 | 0 | 0 | 1 | 0 | 1 | 0 | 182 | 190 | | | PEAKS DB |
| G.GYGGMGGGYGGMGGGYGGMGGGLGGR.G | Y | 47.11 | 2195.8936 | 26 | 3.7 | 1098.9526 | 2 | 58.56 | 15 | F15:33865 | BuCaTN01_F12a.raw | | | | | | | | | | 1.3253E8 | | | | 1.1967E8 | 2.7284E8 | | 4 | 0 | 0 | 0 | 0 | 0 | 0 | 0 | 0 | 0 | 1 | 0 | 0 | 0 | 1 | 2 | 0 | 108 | 133 | | | PEAKS DB |

**total 2 peptides**

**Best Unique PSM (Scan F15:33865, m/z=1098.9526, z=2, RT=58.56, ppm=3.7):**

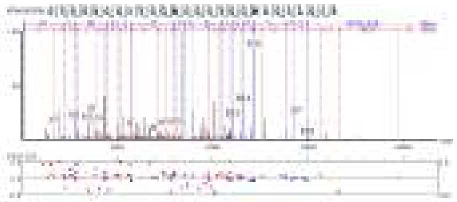

## XP_026531043.1

back to list

| Protein Coverage | Supporting Peptides | Best Unique PSM |

**Protein Coverage:**

1 MPKPVNVRVI TMDAELEFAI QPNTTGKQLF DQVVKTIGLR EVWYFGLQYM DNKGFPTWLK LEKKVSAQEI KKENPLQFKF
81 RSKFFPEDVS VELIQDITQK LFFLQVKEGI LSDEIYCPPE TAVLLGSYAV QSKFGDYNKE LHKAGYLSSE RLVPQRVMDQ
161 HKLSREQWEE RIQVWHAEHG GMLKENAMLE YLKIAQDLEM YGINYFEIKN KKGTDLWLGV DALGLNIYEK EDKLTPKIGF
241 PWSEIRNISF NDKKFVIKPI DKK**SPDFIFY APR**LRINKRI LQLCMGNHEL YMRRRKPDTI EVQQMKAQAR EEKHQKELER
321 EMLENEKKKR ETIEKEKEQM MKEKEELMMR LQEYEVKTQK AERELSDQIE KARQLEEERT RAQEEAERLE NERLMALQAK
401 EELEKQAVDQ MK**SQEQLATE LAEYTAR**IAL LEEARKRKES EVEEWQHRAK EAQDDLVKTK EELHLVMTAP PPPPPPVYEP
481 VNYHVHDNLQ DEGTESSGYS AEFSSEGILN DRNEEKRITE AEKNERVQRQ LKTLTDELAQ ARDEDMKTQN DLIHTENMRQ
561 GRDKYKTLRQ IRQGNTKQRI DEFEAM

**Supporting Peptides:**

| Peptide | Uniq | -10lgP | Mass | Length | ppm | m/z | z | RT | Fraction | Scan | Source File | Area F1 | Area F2a | Area F3a | Area F4 | Area F5 | Area F6a | Area F7a | Area F7b | Area F8 | Area F9a | Area Sample 1F10a | Area F10b | Area F11a | Area F11b | Area F12a | Area F13 | #Feature | #Feature F1 | #Feature F2a | #Feature F3a | #Feature F4 | #Feature F5 | #Feature F6a | #Feature F7a | #Feature F7b | #Feature F8 | #Feature F9a | #Feature Sample 1F10a | #Feature F10b | #Feature F11a | #Feature F11b | #Feature F12a | #Feature F13 | Start | End | PTM | AScore | Found By |
|---|---|---|---|---|---|---|---|---|---|---|---|---|---|---|---|---|---|---|---|---|---|---|---|---|---|---|---|---|---|---|---|---|---|---|---|---|---|---|---|---|---|---|---|---|---|---|---|---|---|
| K.SQEQLATELAEYTAR.I | Y | 54.53 | 1708.8268 | 15 | 0.7 | 570.6166 | 3 | 68.43 | 16 | F16:34844 | BuCaTN01_F13.raw | | | | | | | | | | | | | | | | 3.8068E5 | 2 | 0 | 0 | 0 | 0 | 0 | 0 | 0 | 0 | 0 | 0 | 0 | 0 | 0 | 0 | 0 | 2 | 413 | 427 | | | PEAKS DB |
| K.SPDFIFYAPR.L | Y | 46.25 | 1211.5974 | 10 | -0.3 | 606.8058 | 2 | 69.02 | 16 | F16:35486 | BuCaTN01_F13.raw | | | | | | | | | | | | | | | | 6.0937E5 | 1 | 0 | 0 | 0 | 0 | 0 | 0 | 0 | 0 | 0 | 0 | 0 | 0 | 0 | 0 | 0 | 1 | 264 | 273 | | | PEAKS DB |

**total 2 peptides**

**Best Unique PSM (Scan F16:34844, m/z=570.6166, z=3, RT=68.43, ppm=0.7):**

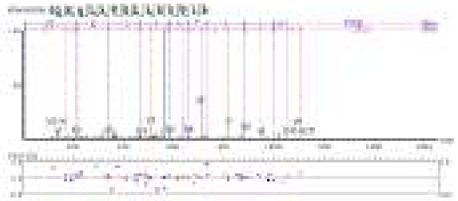

## XP_026531042.1

back to list

| Protein Coverage | Supporting Peptides | Best Unique PSM |

**Protein Coverage:**

```
  1 MPKPVNVRVI TMDAELEFAI QPNTTGKQLF DQVVKTIGLR EVWYFGLQYM DNKGFPTWLK LEKKVSAQEI KKENPLQFKF
 81 RSKFFPEDVS VELIQDITQK LFFLQVKEGI LSDEIYCPPE TAVLLGSYAV QSKFGDYNKE LHKAGYLSSE RLVPQRVMDQ
161 HKLSREQWEE RIQVWHAEHG GMLKENAMLE YLKIAQDLEM YGINYFEIKN KKGTDLWLGV DALGLNIYEK EDKLTPKIGF
241 PWSEIRNISF NDKKFVIKPI DKKSPDFIFY APRLRINKRI LQLCMGNHEL YMRRRKPDTI EVQQMKAQAR EEKHQKELER

321 EMLENEKKKR ETIEKEKEQM MKEKEELMMR LQEYEVKTQK AERELSDQIE KARQLEEERT RAQEEAERLE NERLMALQAK
401 EELEKQAVDQ MKSQEQLATE LAEYTARIAL LEEARKRKES EVEEWQHRAK EAQDDLVKTK EELHLVMTAP PPPPPPVYEP

481 VNYHVHDNLQ DEGTESSGYS AEFSSEGILN DRNEEKRITE AEKNERVQRQ LKTLTDELAQ ARDEDMKTQN DLIHTENMRQ
561 GRDKYKTLRQ IRQGNTKQRI DEFEAM
```

**Supporting Peptides:**

| Peptide | Uniq | -10lgP | Mass | Length | ppm | m/z | z | RT | Fraction | Scan | Source File | Area F1 | Area F2a | Area F3a | Area F4 | Area F5 | Area F6a | Area F7a | Area F7b | Area F8 | Area F9a | Area Sample 1F10a | Area F10b | Area F11a | Area F11b | Area F12a | Area F13 | #Feature | #Feature F1 | #Feature F2a | #Feature F3a | #Feature F4 | #Feature F5 | #Feature F6a | #Feature F7a | #Feature F7b | #Feature F8 | #Feature F9a | #Feature Sample 1F10a | #Feature F10b | #Feature F11a | #Feature F11b | #Feature F12a | #Feature F13 | Start | End | PTM | AScore | Found By |
|---|---|---|---|---|---|---|---|---|---|---|---|---|---|---|---|---|---|---|---|---|---|---|---|---|---|---|---|---|---|---|---|---|---|---|---|---|---|---|---|---|---|---|---|---|---|---|---|---|---|
| K.SQEQLATELAEYTAR.I | Y | 54.53 | 1708.8268 | 15 | 0.7 | 570.6166 | 3 | 68.43 | 16 | F16:34844 | BuCaTN01_F13.raw | | | | | | | | | | | | | | | | 3.8068E5 | 2 | 0 | 0 | 0 | 0 | 0 | 0 | 0 | 0 | 0 | 0 | 0 | 0 | 0 | 0 | 0 | 2 | 413 | 427 | | | PEAKS DB |
| K.SPDFIFYAPR.L | Y | 46.25 | 1211.5974 | 10 | -0.3 | 606.8058 | 2 | 69.02 | 16 | F16:35486 | BuCaTN01_F13.raw | | | | | | | | | | | | | | | | 6.0937E5 | 1 | 0 | 0 | 0 | 0 | 0 | 0 | 0 | 0 | 0 | 0 | 0 | 0 | 0 | 0 | 0 | 1 | 264 | 273 | | | PEAKS DB |
| total 2 peptides | | | | | | | | | | | | | | | | | | | | | | | | | | | | | | | | | | | | | | | | | | | | | | | | | |

**Best Unique PSM (Scan F16:34844, m/z=570.6166, z=3, RT=68.43, ppm=0.7):**

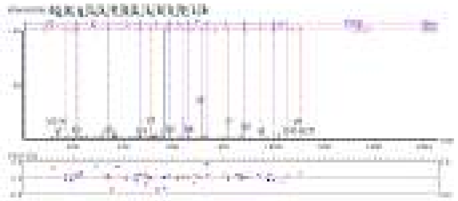

## XP_026560859.1

back to list

| Protein Coverage | Supporting Peptides | Best Unique PSM |

**Protein Coverage:**

```
  1 MPKPVNVRVI TMDAELEFAI QPNTTGKQLF DQVVKTIGLR EVWYFGLQYM DNKGFPTWLK LEKKVSAQEI RKENPLQFKF
 81 RAKFFPEDVS VELIQDITQK LFFLQVKEGI LSDEIYCPPE TAVLLGSYAV QSKFGDYNKD LHKAGYLSSE RLVPQRVMDQ
161 HKLSREQWEE RIQVWHAEHG GMLKENAMLE YLKIAQDLEM YGINYFEIKN KKGTDLWLGV DALGLNIYEK EDKLTPKIGF
241 PWSEIRNISF NDKKFVIKPI DKKSPDFIFY APRLRINKRI LQLCMGNHEL YMRRRKPDTI EVQQMKAQAR EEKHQKELER

321 EMLENEKKKR ETIEKEKEQM MKEKEELMMR LQEYEVKTQR AERELSDQIE KARQLEEERT RAQEEAERLE NERLMALQAK
401 EELEKQAVDQ MKSQEQLATE LAEYTARIAL LEEARKRKES EVEEWQHRAK EAQDDLVKTK EELHLVMTAP PPPPPPVYEP

481 VNYHVHDNLQ DEGTESSGYS AEFSSEGILN DRNEEKRITE AEKNERVQRQ LKTLTDELAQ ARDEDMKTQN DLIHTENMRQ
561 GRDKYKTLRQ IRQGNTKQRI DEFEAM
```

**Supporting Peptides:**

| Peptide | Uniq | -10lgP | Mass | Length | ppm | m/z | z | RT | Fraction | Scan | Source File | Area F1 | Area F2a | Area F3a | Area F4 | Area F5 | Area F6a | Area F7a | Area F7b | Area F8 | Area F9a | Area Sample 1F10a | Area F10b | Area F11a | Area F11b | Area F12a | Area F13 | #Feature | #Feature F1 | #Feature F2a | #Feature F3a | #Feature F4 | #Feature F5 | #Feature F6a | #Feature F7a | #Feature F7b | #Feature F8 | #Feature F9a | #Feature Sample 1F10a | #Feature F10b | #Feature F11a | #Feature F11b | #Feature F12a | #Feature F13 | Start | End | PTM | AScore | Found By |
|---|---|---|---|---|---|---|---|---|---|---|---|---|---|---|---|---|---|---|---|---|---|---|---|---|---|---|---|---|---|---|---|---|---|---|---|---|---|---|---|---|---|---|---|---|---|---|---|---|---|
| K.SQEQLATELAEYTAR.I | Y | 54.53 | 1708.8268 | 15 | 0.7 | 570.6166 | 3 | 68.43 | 16 | F16:34844 | BuCaTN01_F13.raw | | | | | | | | | | | | | | | | 3.8068E5 | 2 | 0 | 0 | 0 | 0 | 0 | 0 | 0 | 0 | 0 | 0 | 0 | 0 | 0 | 0 | 0 | 2 | 413 | 427 | | | PEAKS DB |
| K.SPDFIFYAPR.L | Y | 46.25 | 1211.5974 | 10 | -0.3 | 606.8058 | 2 | 69.02 | 16 | F16:35486 | BuCaTN01_F13.raw | | | | | | | | | | | | | | | | 6.0937E5 | 1 | 0 | 0 | 0 | 0 | 0 | 0 | 0 | 0 | 0 | 0 | 0 | 0 | 0 | 0 | 0 | 1 | 264 | 273 | | | PEAKS DB |
| total 2 peptides | | | | | | | | | | | | | | | | | | | | | | | | | | | | | | | | | | | | | | | | | | | | | | | | | |

**Best Unique PSM (Scan F16:34844, m/z=570.6166, z=3, RT=68.43, ppm=0.7):**

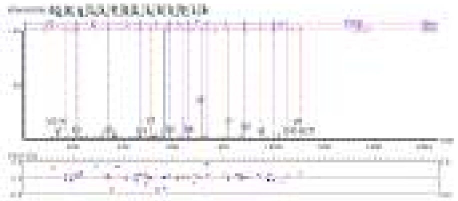

## XP_026560860.1

back to list

| Protein Coverage | Supporting Peptides | Best Unique PSM |

**Protein Coverage:**

```
  1 MPKPVNVRVI TMDAELEFAI QPNTTGKQLF DQVMKTIGLR EVWYFGLQYM DNKGFPTWLK LEKKVSAQEI RKENPLQFKF
 81 RAKFFPEDVS VELIQDITQK LFFLQVKEGI LSDEIYCPPE TAVLLGSYAV QSKFGDYNKD LHKAGYLSSE RLVPQRVMDQ
161 HKLSREQWEE RIQVWHAEHG GMLKENAMLE YLKIAQDLEM YGINYFEIKN KKGTDLWLGV DALGLNIYEK EDKLTPKIGF
241 PWSEIRNISF NDKKFVIKPI DKKSPDFIFY APRLRINKRI LQLCMGNHEL YMRRRKPDTI EVQQMKAQAR EEKHQKELER

321 EMLENEKKKR ETIEKEKEQM MKEKEELMMR LQEYEVKTQR AERELSDQIE KARQLEEERT RAQEEAERLE NERLMALQAK
401 EELEKQAVDQ MKSQEQLATE LAEYTARIAL LEEARKRKES EVEEWQHRAK EAQDDLVKTK EELHLVMTAP PPPPPPVYEP

481 VNYHVHDNLQ DEGTESSGYS AEFSSEGILN DRNEEKRITE AEKNERVQRQ LKTLTDELAQ ARDEDMKTQN DLIHTENMRQ
561 GRDKYKTLRQ IRQGNTKQRI DEFEAM
```

**Supporting Peptides:**

| Peptide | Uniq | -10lgP | Mass | Length | ppm | m/z | z | RT | Fraction | Scan | Source File | Area F1 | Area F2a | Area F3a | Area F4 | Area F5 | Area F6a | Area F7a | Area F7b | Area F8 | Area F9a | Area Sample 1F10a | Area F10b | Area F11a | Area F11b | Area F12a | Area F13 | #Feature | #Feature F1 | #Feature F2a | #Feature F3a | #Feature F4 | #Feature F5 | #Feature F6a | #Feature F7a | #Feature F7b | #Feature F8 | #Feature F9a | #Feature Sample 1F10a | #Feature F10b | #Feature F11a | #Feature F11b | #Feature F12a | #Feature F13 | Start | End | PTM | AScore | Found By |
|---|---|---|---|---|---|---|---|---|---|---|---|---|---|---|---|---|---|---|---|---|---|---|---|---|---|---|---|---|---|---|---|---|---|---|---|---|---|---|---|---|---|---|---|---|---|---|---|---|---|
| K.SQEQLATELAEYTAR.I | Y | 54.53 | 1708.8268 | 15 | 0.7 | 570.6166 | 3 | 68.43 | 16 | F16:34844 | BuCaTN01_F13.raw | | | | | | | | | | | | | | | | 3.8068E5 | 2 | 0 | 0 | 0 | 0 | 0 | 0 | 0 | 0 | 0 | 0 | 0 | 0 | 0 | 0 | 0 | 2 | 413 | 427 | | | PEAKS DB |
| K.SPDFIFYAPR.L | Y | 46.25 | 1211.5974 | 10 | -0.3 | 606.8058 | 2 | 69.02 | 16 | F16:35486 | BuCaTN01_F13.raw | | | | | | | | | | | | | | | | 6.0937E5 | 1 | 0 | 0 | 0 | 0 | 0 | 0 | 0 | 0 | 0 | 0 | 0 | 0 | 0 | 0 | 0 | 1 | 264 | 273 | | | PEAKS DB |
| total 2 peptides | | | | | | | | | | | | | | | | | | | | | | | | | | | | | | | | | | | | | | | | | | | | | | | | | |

**Best Unique PSM (Scan F16:34844, m/z=570.6166, z=3, RT=68.43, ppm=0.7):**

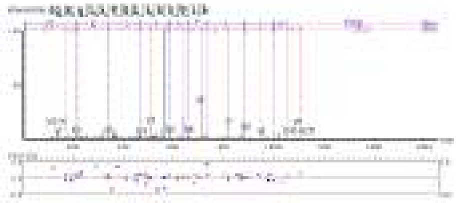

## XP_026560858.1

back to list

| Protein Coverage | Supporting Peptides | Best Unique PSM |

**Protein Coverage:**

```
  1 MPKPVNVRVI TMDAELEFAI QPNTTGKQLF DQVVKTIGLR EVWYFGLQYM DNKGFPTWLK LEKKVSAQEI RKENPLQFKF
 81 RAKFFPEDVS VELIQDITQK LFFLQVKEGI LSDEIYCPPE TAVLLGSYAV QSKFGDYNKD LHKAGYLSSE RLVPQRVMDQ
161 HKLSREQWEE RIQVWHAEHG GMLKENAMLE YLKIAQDLEM YGINYFEIKN KKGTDLWLGV DALGLNIYEK EDKLTPKIGF
241 PWSEIRNISF NDKKFVIKPI DKKSPDFIFY APRLRINKRI LQLCMGNHEL YMRRRKPDTI EVQQMKAQAR EEKHQKELER
321 EMLENEKKKR ETIEKEKEQM MKEKEELMMR LQEYEVKTQR AERELSDQIE KARQLEEERT RAQEEAERLE NERLMALQAK
401 EELEKQAVDQ MKSQEQLATE LAEYTARIAL LEEARKRKES EVEEWQHRAK EAQDDLVKTK EELHLVMTAP PPPPPPVYEP
481 VNYHVHDNLQ DEGTESSGYS AEFSSEGILN DRNEEKRITE AEKNERVQRQ LKTLTDELAQ ARDEDMKTQN DLIHTENMRQ
561 GRDKYKTLRQ IRQGNTKQRI DEFEAM
```

**Supporting Peptides:**

| Peptide | Uniq | -10lgP | Mass | Length | ppm | m/z | z | RT | Fraction | Scan | Source File | Area F1 | Area F2a | Area F3a | Area F4 | Area F5 | Area F6a | Area F7a | Area F7b | Area F8 | Area F9a | Area Sample 1F10a | Area F10b | Area F11a | Area F11b | Area F12a | Area F13 | #Feature | #Feature F1 | #Feature F2a | #Feature F3a | #Feature F4 | #Feature F5 | #Feature F6a | #Feature F7a | #Feature F7b | #Feature F8 | #Feature F9a | #Feature Sample 1F10a | #Feature F10b | #Feature F11a | #Feature F11b | #Feature F12a | #Feature F13 | Start | End | PTM | AScore | Found By |
|---|---|---|---|---|---|---|---|---|---|---|---|---|---|---|---|---|---|---|---|---|---|---|---|---|---|---|---|---|---|---|---|---|---|---|---|---|---|---|---|---|---|---|---|---|---|---|---|---|---|
| K.SQEQLATELAEYTAR.I | Y | 54.53 | 1708.8268 | 15 | 0.7 | 570.6166 | 3 | 68.43 | 16 | F16:34844 | BuCaTN01_F13.raw | | | | | | | | | | | | | | | | 3.8068E5 | 2 | 0 | 0 | 0 | 0 | 0 | 0 | 0 | 0 | 0 | 0 | 0 | 0 | 0 | 0 | 0 | 2 | 413 | 427 | | | PEAKS DB |
| K.SPDFIFYAPR.L | Y | 46.25 | 1211.5974 | 10 | -0.3 | 606.8058 | 2 | 69.02 | 16 | F16:35486 | BuCaTN01_F13.raw | | | | | | | | | | | | | | | | 6.0937E5 | 1 | 0 | 0 | 0 | 0 | 0 | 0 | 0 | 0 | 0 | 0 | 0 | 0 | 0 | 0 | 0 | 1 | 264 | 273 | | | PEAKS DB |
| total 2 peptides | | | | | | | | | | | | | | | | | | | | | | | | | | | | | | | | | | | | | | | | | | | | | | | | | |

**Best Unique PSM (Scan F16:34844, m/z=570.6166, z=3, RT=68.43, ppm=0.7):**

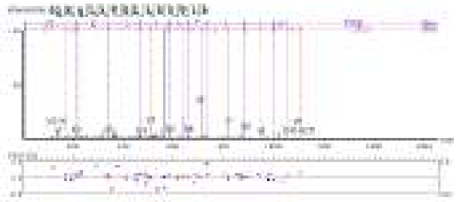

## JAC95627.1

back to list

| Protein Coverage | Supporting Peptides | Best Unique PSM |

**Protein Coverage:**

```
  1 MPKPVNVRVT TMDAELEFAI QPNTTGKQLF DQVVKTVGLR EVWYFGLQYT DNKGFPTWLK LEKKVSAQEI RKENPLQFKF
 81 RAKFFPEDVS VELIQDITQK LFFLQVKEGI LSDEIYCPPE TAVLLGSYAV QSKFGDYNKE LHKAGYLSSE RLVPQRVMDQ
161 HKLSREQWEE RIQVWHAEHG GMLKENAMLE YLKIAQDLEM YGINYFEIKN KKGTDLWLGV DALGLNIYEK EDKLTPKIGF
241 PWSEIRNISF NDKKFVIKPI DKKSPDFIFY APRLRINKRI LQLCMGNHEL YMRRRKPDTI EVQQMKAQAR EEKHQKELER
321 EMLENEKKKR ETIEKEKEQM LREKEELMLR LEEYQVKTQK AEKELSDQIE KARQLEEERT RAQLEAERLE NERLMALKAK
401 EELERQAVDQ MKSQEQLATE LAEYTARISL LEEARKRKES EVEEWQHRAR EAQDDLVKTK EELHLVMTAP PPPPPPVYEP
481 MNYHVHDNLQ DEGPESTGYS AEFSSEGILN DRNEEKRITE AEKNERVQRQ LKTLTDELAQ ARDEEMKTPN DLIHTENMRQ
561 GRDKYKTLRQ IRQGNTKQRI DEFEAM
```

**Supporting Peptides:**

| Peptide | Uniq | -10lgP | Mass | Length | ppm | m/z | z | RT | Fraction | Scan | Source File | Area F1 | Area F2a | Area F3a | Area F4 | Area F5 | Area F6a | Area F7a | Area F7b | Area F8 | Area F9a | Area Sample 1F10a | Area F10b | Area F11a | Area F11b | Area F12a | Area F13 | #Feature | #Feature F1 | #Feature F2a | #Feature F3a | #Feature F4 | #Feature F5 | #Feature F6a | #Feature F7a | #Feature F7b | #Feature F8 | #Feature F9a | #Feature Sample 1F10a | #Feature F10b | #Feature F11a | #Feature F11b | #Feature F12a | #Feature F13 | Start | End | PTM | AScore | Found By |
|---|---|---|---|---|---|---|---|---|---|---|---|---|---|---|---|---|---|---|---|---|---|---|---|---|---|---|---|---|---|---|---|---|---|---|---|---|---|---|---|---|---|---|---|---|---|---|---|---|---|
| K.SQEQLATELAEYTAR.I | Y | 54.53 | 1708.8268 | 15 | 0.7 | 570.6166 | 3 | 68.43 | 16 | F16:34844 | BuCaTN01_F13.raw | | | | | | | | | | | | | | | | 3.8068E5 | 2 | 0 | 0 | 0 | 0 | 0 | 0 | 0 | 0 | 0 | 0 | 0 | 0 | 0 | 0 | 0 | 2 | 413 | 427 | | | PEAKS DB |
| K.SPDFIFYAPR.L | Y | 46.25 | 1211.5974 | 10 | -0.3 | 606.8058 | 2 | 69.02 | 16 | F16:35486 | BuCaTN01_F13.raw | | | | | | | | | | | | | | | | 6.0937E5 | 1 | 0 | 0 | 0 | 0 | 0 | 0 | 0 | 0 | 0 | 0 | 0 | 0 | 0 | 0 | 0 | 1 | 264 | 273 | | | PEAKS DB |
| total 2 peptides | | | | | | | | | | | | | | | | | | | | | | | | | | | | | | | | | | | | | | | | | | | | | | | | | |

**Best Unique PSM (Scan F16:34844, m/z=570.6166, z=3, RT=68.43, ppm=0.7):**

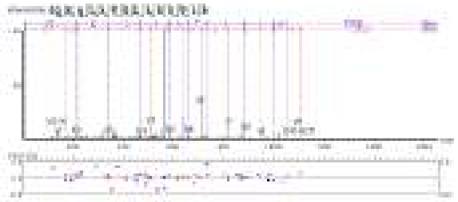

## UNW14903.1

back to list

| Protein Coverage | Supporting Peptides | Best Unique PSM |

**Protein Coverage:**

```
                                                               62   66
 1 KTLLLTLVVV TIVCLDLGYT RTCFITPDVK SKPCPPGQEV CYTKTWCDGF CSIRGKRVDL GCAATCPREK TGIDVICCSE
81 DDCNPFPVRP RGRLSSIMDH P
```

C Carbamidomethylation (+57.02)

**Supporting Peptides:**

| Peptide | Uniq | -10lgP | Mass | Length | ppm | m/z | z | RT | Fraction | Scan | Source File | Area F1 | Area F2a | Area F3a | Area F4 | Area F5 | Area F6a | Area F7a | Area F7b | Area F8 | Area F9a | Area Sample 1F10a | Area F10b | Area F11a | Area F11b | Area F12a | Area F13 | #Feature | #Feature F1 | #Feature F2a | #Feature F3a | #Feature F4 | #Feature F5 | #Feature F6a | #Feature F7a | #Feature F7b | #Feature F8 | #Feature F9a | #Feature Sample 1F10a | #Feature F10b | #Feature F11a | #Feature F11b | #Feature F12a | #Feature F13 | Start | End | PTM | AScore | Found By |
|---|---|---|---|---|---|---|---|---|---|---|---|---|---|---|---|---|---|---|---|---|---|---|---|---|---|---|---|---|---|---|---|---|---|---|---|---|---|---|---|---|---|---|---|---|---|---|---|---|---|
| K.RVDLGC(+57.02)AATC(+57.02)PREK.T | Y | 54.78 | 1631.7871 | 14 | 6.7 | 544.9399 | 3 | 40.05 | 6 | F6:19637 | BuCaTN01_F6a.raw | | | | | | 3.7657E4 | | | | | | | | | | | 1 | 0 | 0 | 0 | 0 | 0 | 1 | 0 | 0 | 0 | 0 | 0 | 0 | 0 | 0 | 0 | 0 | 57 | 70 | Carbamidomethylation | C6:Carbamidomethylation:1000.00;C10:Carbamidomethylation:1000.00 | PEAKS DB |
| total 1 peptides | | | | | | | | | | | | | | | | | | | | | | | | | | | | | | | | | | | | | | | | | | | | | | | | | |

**Best Unique PSM (Scan F6:19637, m/z=544.9399, z=3, RT=40.05, ppm=6.7):**

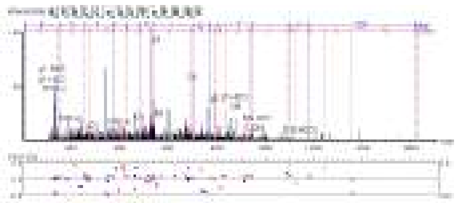

## Q7ZT13.1

back to list

| Protein Coverage | Supporting Peptides | Best Unique PSM |

**Protein Coverage:**

1 MKTLLLTLVV LTIACLDLGY TKTCFNDDLT NPKTTELCRH SMYFCFKNSW IAGGVER**IER GCSLTCPDIK** YNGKYIYCCT

81 RDNCNA

C Carbamidomethylation (+57.02)

**Supporting Peptides:**

| Peptide | Uniq | -10lgP | Mass | Length | ppm | m/z | z | RT | Fraction | Scan | Source File | Area F1 | Area F2a | Area F3a | Area F4 | Area F5 | Area F6a | Area F7a | Area F7b | Area F8 | Area F9a | Area Sample 1F10a | Area F10b | Area F11a | Area F11b | Area F12a | Area F13 | #Feature | #Feature F1 | #Feature F2a | #Feature F3a | #Feature F4 | #Feature F5 | #Feature F6a | #Feature F7a | #Feature F7b | #Feature F8 | #Feature F9a | #Feature Sample 1F10a | #Feature F10b | #Feature F11a | #Feature F11b | #Feature F12a | #Feature F13 | Start | End | PTM | AScore | Found By |
|---|---|---|---|---|---|---|---|---|---|---|---|---|---|---|---|---|---|---|---|---|---|---|---|---|---|---|---|---|---|---|---|---|---|---|---|---|---|---|---|---|---|---|---|---|---|---|---|---|---|
| R.GC(+57.02)SLTC(+57.02)PDIK.Y | Y | 52.39 | 1149.5159 | 10 | 3.7 | 575.7656 | 2 | 40.22 | 8 | F8:18058 | BuCaTN01_F7b.raw | | | | | | | | 2.6353E6 | | | | | | | | | 1 | 0 | 0 | 0 | 0 | 0 | 0 | 0 | 1 | 0 | 0 | 0 | 0 | 0 | 0 | 0 | 0 | 61 | 70 | Carbamidomethylation | C2:Carbamidomethylation:1000.00;C6:Carbamidomethylation:1000.00 | PEAKS DB |
| R.IERGC(+57.02)SLTC(+57.02)PDIK.Y | Y | 43.69 | 1547.7435 | 13 | 3.7 | 516.9222 | 3 | 39.13 | 8 | F8:16955 | BuCaTN01_F7b.raw | | | | | | | | 5.7834E5 | | | | | | | | | 1 | 0 | 0 | 0 | 0 | 0 | 0 | 0 | 1 | 0 | 0 | 0 | 0 | 0 | 0 | 0 | 0 | 58 | 70 | Carbamidomethylation | C5:Carbamidomethylation:1000.00;C9:Carbamidomethylation:1000.00 | PEAKS DB |
| **total 2 peptides** | | | | | | | | | | | | | | | | | | | | | | | | | | | | | | | | | | | | | | | | | | | | | | | | | |

**Best Unique PSM (Scan F8:18058, m/z=575.7656, z=2, RT=40.22, ppm=3.7):**

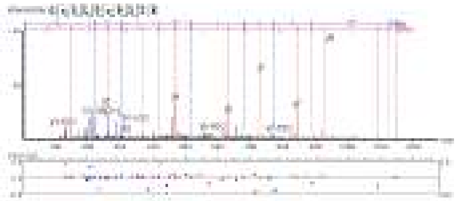

## AAL30058.1

back to list

| Protein Coverage | Supporting Peptides | Best Unique PSM |

**Protein Coverage:**

1 MKTLLLTLVV LTIACLDLGY TKTCFNDNLA NPKTTELCRH SMYFCFKNSW IAGGVER**IER GCSLTCPDIK** YNGKYIYCCT

81 RDNCNA

C Carbamidomethylation (+57.02)

**Supporting Peptides:**

| Peptide | Uniq | -10lgP | Mass | Length | ppm | m/z | z | RT | Fraction | Scan | Source File | Area F1 | Area F2a | Area F3a | Area F4 | Area F5 | Area F6a | Area F7a | Area F7b | Area F8 | Area F9a | Area Sample 1F10a | Area F10b | Area F11a | Area F11b | Area F12a | Area F13 | #Feature | #Feature F1 | #Feature F2a | #Feature F3a | #Feature F4 | #Feature F5 | #Feature F6a | #Feature F7a | #Feature F7b | #Feature F8 | #Feature F9a | #Feature Sample 1F10a | #Feature F10b | #Feature F11a | #Feature F11b | #Feature F12a | #Feature F13 | Start | End | PTM | AScore | Found By |
|---|---|---|---|---|---|---|---|---|---|---|---|---|---|---|---|---|---|---|---|---|---|---|---|---|---|---|---|---|---|---|---|---|---|---|---|---|---|---|---|---|---|---|---|---|---|---|---|---|---|
| R.GC(+57.02)SLTC(+57.02)PDIK.Y | Y | 52.39 | 1149.5159 | 10 | 3.7 | 575.7656 | 2 | 40.22 | 8 | F8:18058 | BuCaTN01_F7b.raw | | | | | | | | 2.6353E6 | | | | | | | | | 1 | 0 | 0 | 0 | 0 | 0 | 0 | 0 | 1 | 0 | 0 | 0 | 0 | 0 | 0 | 0 | 0 | 61 | 70 | Carbamidomethylation | C2:Carbamidomethylation:1000.00;C6:Carbamidomethylation:1000.00 | PEAKS DB |
| R.IERGC(+57.02)SLTC(+57.02)PDIK.Y | Y | 43.69 | 1547.7435 | 13 | 3.7 | 516.9222 | 3 | 39.13 | 8 | F8:16955 | BuCaTN01_F7b.raw | | | | | | | | 5.7834E5 | | | | | | | | | 1 | 0 | 0 | 0 | 0 | 0 | 0 | 0 | 1 | 0 | 0 | 0 | 0 | 0 | 0 | 0 | 0 | 58 | 70 | Carbamidomethylation | C5:Carbamidomethylation:1000.00;C9:Carbamidomethylation:1000.00 | PEAKS DB |
| **total 2 peptides** | | | | | | | | | | | | | | | | | | | | | | | | | | | | | | | | | | | | | | | | | | | | | | | | | |

**Best Unique PSM (Scan F8:18058, m/z=575.7656, z=2, RT=40.22, ppm=3.7):**

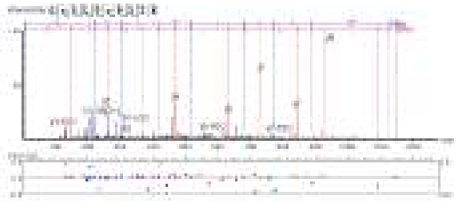

## AAO47840.1

back to list

| Protein Coverage | Supporting Peptides | Best Unique PSM |

**Protein Coverage:**

1 MKTLLLTLVV LTIACLDLGY TKTCFNDDLT NPKTTELCRH SMYFCFKNSW IAGGVER**IER GCSLTCPDIK** YNGKYIYCCT

81 RDNCNA

C Carbamidomethylation (+57.02)

**Supporting Peptides:**

| Peptide | Uniq | -10lgP | Mass | Length | ppm | m/z | z | RT | Fraction | Scan | Source File | Area F1 | Area F2a | Area F3a | Area F4 | Area F5 | Area F6a | Area F7a | Area F7b | Area F8 | Area F9a | Area Sample 1F10a | Area F10b | Area F11a | Area F11b | Area F12a | Area F13 | #Feature | #Feature F1 | #Feature F2a | #Feature F3a | #Feature F4 | #Feature F5 | #Feature F6a | #Feature F7a | #Feature F7b | #Feature F8 | #Feature F9a | #Feature Sample 1F10a | #Feature F10b | #Feature F11a | #Feature F11b | #Feature F12a | #Feature F13 | Start | End | PTM | AScore | Found By |
|---|---|---|---|---|---|---|---|---|---|---|---|---|---|---|---|---|---|---|---|---|---|---|---|---|---|---|---|---|---|---|---|---|---|---|---|---|---|---|---|---|---|---|---|---|---|---|---|---|---|
| R.GC(+57.02)SLTC(+57.02)PDIK.Y | Y | 52.39 | 1149.5159 | 10 | 3.7 | 575.7656 | 2 | 40.22 | 8 | F8:18058 | BuCaTN01_F7b.raw | | | | | | | | 2.6353E6 | | | | | | | | | 1 | 0 | 0 | 0 | 0 | 0 | 0 | 0 | 1 | 0 | 0 | 0 | 0 | 0 | 0 | 0 | 0 | 61 | 70 | Carbamidomethylation | C2:Carbamidomethylation:1000.00;C6:Carbamidomethylation:1000.00 | PEAKS DB |
| R.IERGC(+57.02)SLTC(+57.02)PDIK.Y | Y | 43.69 | 1547.7435 | 13 | 3.7 | 516.9222 | 3 | 39.13 | 8 | F8:16955 | BuCaTN01_F7b.raw | | | | | | | | 5.7834E5 | | | | | | | | | 1 | 0 | 0 | 0 | 0 | 0 | 0 | 0 | 1 | 0 | 0 | 0 | 0 | 0 | 0 | 0 | 0 | 58 | 70 | Carbamidomethylation | C5:Carbamidomethylation:1000.00;C9:Carbamidomethylation:1000.00 | PEAKS DB |
| **total 2 peptides** | | | | | | | | | | | | | | | | | | | | | | | | | | | | | | | | | | | | | | | | | | | | | | | | | |

**Best Unique PSM (Scan F8:18058, m/z=575.7656, z=2, RT=40.22, ppm=3.7):**

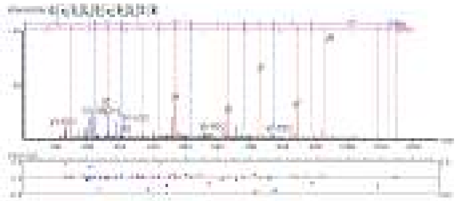

## AAO47842.1

back to list

| Protein Coverage | Supporting Peptides | Best Unique PSM |

**Protein Coverage:**

1 MKTLLLTLVV LTIACLDLGY TKTCFNDDLT NPKTTELCRH SMYFCFKNSW IAGGVERIER GCSLTCPDIK YNGKYIYCCT Carbamidomethylation (+57.02)

81 RDNCNA

**Supporting Peptides:**

| Peptide | Uniq | -10lgP | Mass | Length | ppm | m/z | z | RT | Fraction | Scan | Source File | Area F1 | Area F2a | Area F3a | Area F4 | Area F5 | Area F6a | Area F7a | Area F7b | Area F8 | Area F9a | Area Sample 1F10a | Area F10b | Area F11a | Area F11b | Area F12a | Area F13 | #Feature | #Feature F1 | #Feature F2a | #Feature F3a | #Feature F4 | #Feature F5 | #Feature F6a | #Feature F7a | #Feature F7b | #Feature F8 | #Feature F9a | #Feature Sample 1F10a | #Feature F10b | #Feature F11a | #Feature F11b | #Feature F12a | #Feature F13 | Start | End | PTM | AScore | Found By |
|---|---|---|---|---|---|---|---|---|---|---|---|---|---|---|---|---|---|---|---|---|---|---|---|---|---|---|---|---|---|---|---|---|---|---|---|---|---|---|---|---|---|---|---|---|---|---|---|---|---|
| R.GC(+57.02)SLTC(+57.02)PDIK.Y | Y | 52.39 | 1149.5159 | 10 | 3.7 | 575.7656 | 2 | 40.22 | 8 | F8:18058 | BuCaTN01_F7b.raw | | | | | | | | 2.6353E6 | | | | | | | | | 1 | 0 | 0 | 0 | 0 | 0 | 0 | 0 | 1 | 0 | 0 | 0 | 0 | 0 | 0 | 0 | 0 | 61 | 70 | Carbamidomethylation | C2:Carbamidomethylation:1000.00;C6:Carbamidomethylation:1000.00 | PEAKS DB |
| R.IERGC(+57.02)SLTC(+57.02)PDIK.Y | Y | 43.69 | 1547.7435 | 13 | 3.7 | 516.9222 | 3 | 39.13 | 8 | F8:16955 | BuCaTN01_F7b.raw | | | | | | | | 5.7834E5 | | | | | | | | | 1 | 0 | 0 | 0 | 0 | 0 | 0 | 0 | 1 | 0 | 0 | 0 | 0 | 0 | 0 | 0 | 0 | 58 | 70 | Carbamidomethylation | C5:Carbamidomethylation:1000.00;C9:Carbamidomethylation:1000.00 | PEAKS DB |
| total 2 peptides | | | | | | | | | | | | | | | | | | | | | | | | | | | | | | | | | | | | | | | | | | | | | | | | | |

**Best Unique PSM (Scan F8:18058, m/z=575.7656, z=2, RT=40.22, ppm=3.7):**

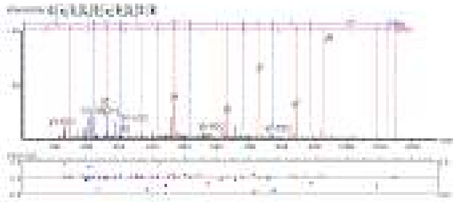

## Q8AY53.1 back to list

| Protein Coverage | Supporting Peptides | Best Unique PSM |

**Protein Coverage:**

1 MKTLLLTLVV LTIACLDLGY TKTCFNDDLA NPKTTELCRH SMYFCFKNSW IAGGVERIER GCSLTCPDIK YNGKYIYCCT Carbamidomethylation (+57.02)

81 RDNCNA

**Supporting Peptides:**

| Peptide | Uniq | -10lgP | Mass | Length | ppm | m/z | z | RT | Fraction | Scan | Source File | Area F1 | Area F2a | Area F3a | Area F4 | Area F5 | Area F6a | Area F7a | Area F7b | Area F8 | Area F9a | Area Sample 1F10a | Area F10b | Area F11a | Area F11b | Area F12a | Area F13 | #Feature | #Feature F1 | #Feature F2a | #Feature F3a | #Feature F4 | #Feature F5 | #Feature F6a | #Feature F7a | #Feature F7b | #Feature F8 | #Feature F9a | #Feature Sample 1F10a | #Feature F10b | #Feature F11a | #Feature F11b | #Feature F12a | #Feature F13 | Start | End | PTM | AScore | Found By |
|---|---|---|---|---|---|---|---|---|---|---|---|---|---|---|---|---|---|---|---|---|---|---|---|---|---|---|---|---|---|---|---|---|---|---|---|---|---|---|---|---|---|---|---|---|---|---|---|---|---|
| R.GC(+57.02)SLTC(+57.02)PDIK.Y | Y | 52.39 | 1149.5159 | 10 | 3.7 | 575.7656 | 2 | 40.22 | 8 | F8:18058 | BuCaTN01_F7b.raw | | | | | | | | 2.6353E6 | | | | | | | | | 1 | 0 | 0 | 0 | 0 | 0 | 0 | 0 | 1 | 0 | 0 | 0 | 0 | 0 | 0 | 0 | 0 | 61 | 70 | Carbamidomethylation | C2:Carbamidomethylation:1000.00;C6:Carbamidomethylation:1000.00 | PEAKS DB |
| R.IERGC(+57.02)SLTC(+57.02)PDIK.Y | Y | 43.69 | 1547.7435 | 13 | 3.7 | 516.9222 | 3 | 39.13 | 8 | F8:16955 | BuCaTN01_F7b.raw | | | | | | | | 5.7834E5 | | | | | | | | | 1 | 0 | 0 | 0 | 0 | 0 | 0 | 0 | 1 | 0 | 0 | 0 | 0 | 0 | 0 | 0 | 0 | 58 | 70 | Carbamidomethylation | C5:Carbamidomethylation:1000.00;C9:Carbamidomethylation:1000.00 | PEAKS DB |
| total 2 peptides | | | | | | | | | | | | | | | | | | | | | | | | | | | | | | | | | | | | | | | | | | | | | | | | | |

**Best Unique PSM (Scan F8:18058, m/z=575.7656, z=2, RT=40.22, ppm=3.7):**

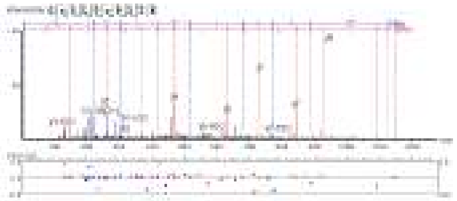

## AAO47839.1 back to list

| Protein Coverage | Supporting Peptides | Best Unique PSM |

**Protein Coverage:**

1 MKTLLLTLVV LTIACLDLGY TKTCFNDDLT NPKTTELCRH SMYFCFKNSW IAGGVERIER GCSLTCPDIK YNGKYIYCCT Carbamidomethylation (+57.02)

81 RDNCNA

**Supporting Peptides:**

| Peptide | Uniq | -10lgP | Mass | Length | ppm | m/z | z | RT | Fraction | Scan | Source File | Area F1 | Area F2a | Area F3a | Area F4 | Area F5 | Area F6a | Area F7a | Area F7b | Area F8 | Area F9a | Area Sample 1F10a | Area F10b | Area F11a | Area F11b | Area F12a | Area F13 | #Feature | #Feature F1 | #Feature F2a | #Feature F3a | #Feature F4 | #Feature F5 | #Feature F6a | #Feature F7a | #Feature F7b | #Feature F8 | #Feature F9a | #Feature Sample 1F10a | #Feature F10b | #Feature F11a | #Feature F11b | #Feature F12a | #Feature F13 | Start | End | PTM | AScore | Found By |
|---|---|---|---|---|---|---|---|---|---|---|---|---|---|---|---|---|---|---|---|---|---|---|---|---|---|---|---|---|---|---|---|---|---|---|---|---|---|---|---|---|---|---|---|---|---|---|---|---|---|
| R.GC(+57.02)SLTC(+57.02)PDIK.Y | Y | 52.39 | 1149.5159 | 10 | 3.7 | 575.7656 | 2 | 40.22 | 8 | F8:18058 | BuCaTN01_F7b.raw | | | | | | | | 2.6353E6 | | | | | | | | | 1 | 0 | 0 | 0 | 0 | 0 | 0 | 0 | 1 | 0 | 0 | 0 | 0 | 0 | 0 | 0 | 0 | 61 | 70 | Carbamidomethylation | C2:Carbamidomethylation:1000.00;C6:Carbamidomethylation:1000.00 | PEAKS DB |
| R.IERGC(+57.02)SLTC(+57.02)PDIK.Y | Y | 43.69 | 1547.7435 | 13 | 3.7 | 516.9222 | 3 | 39.13 | 8 | F8:16955 | BuCaTN01_F7b.raw | | | | | | | | 5.7834E5 | | | | | | | | | 1 | 0 | 0 | 0 | 0 | 0 | 0 | 0 | 1 | 0 | 0 | 0 | 0 | 0 | 0 | 0 | 0 | 58 | 70 | Carbamidomethylation | C5:Carbamidomethylation:1000.00;C9:Carbamidomethylation:1000.00 | PEAKS DB |
| total 2 peptides | | | | | | | | | | | | | | | | | | | | | | | | | | | | | | | | | | | | | | | | | | | | | | | | | |

**Best Unique PSM (Scan F8:18058, m/z=575.7656, z=2, RT=40.22, ppm=3.7):**

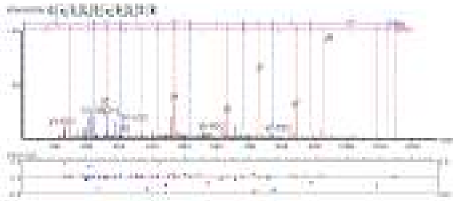

## CAA11213.1 back to list

| Protein Coverage | Supporting Peptides | Best Unique PSM |

**Protein Coverage:**

1 MKTLLLSLVV LTIACLDLGY TKTCFNDDLT NPKTTELCRH SMYFCFKNSW IAGGVERIER GCSLTCPDIK YNGKYIYCCT Carbamidomethylation (+57.02)

81 RDNCNA

**Supporting Peptides:**

| Peptide | Uniq | -10lgP | Mass | Length | ppm | m/z | z | RT | Fraction | Scan | Source File | Area F1 | Area F2a | Area F3a | Area F4 | Area F5 | Area F6a | Area F7a | Area F7b | Area F8 | Area F9a | Area Sample 1F10a | Area F10b | Area F11a | Area F11b | Area F12a | Area F13 | #Feature | #Feature F1 | #Feature F2a | #Feature F3a | #Feature F4 | #Feature F5 | #Feature F6a | #Feature F7a | #Feature F7b | #Feature F8 | #Feature F9a | #Feature Sample 1F10a | #Feature F10b | #Feature F11a | #Feature F11b | #Feature F12a | #Feature F13 | Start | End | PTM | AScore | Found By |
|---|---|---|---|---|---|---|---|---|---|---|---|---|---|---|---|---|---|---|---|---|---|---|---|---|---|---|---|---|---|---|---|---|---|---|---|---|---|---|---|---|---|---|---|---|---|---|---|---|---|
| R.GC(+57.02)SLTC(+57.02)PDIK.Y | Y | 52.39 | 1149.5159 | 10 | 3.7 | 575.7656 | 2 | 40.22 | 8 | F8:18058 | BuCaTN01_F7b.raw | | | | | | | | 2.6353E6 | | | | | | | | | 1 | 0 | 0 | 0 | 0 | 0 | 0 | 0 | 1 | 0 | 0 | 0 | 0 | 0 | 0 | 0 | 0 | 61 | 70 | Carbamidomethylation | C2:Carbamidomethylation:1000.00;C6:Carbamidomethylation:1000.00 | PEAKS DB |
| R.IERGC(+57.02)SLTC(+57.02)PDIK.Y | Y | 43.69 | 1547.7435 | 13 | 3.7 | 516.9222 | 3 | 39.13 | 8 | F8:16955 | BuCaTN01_F7b.raw | | | | | | | | 5.7834E5 | | | | | | | | | 1 | 0 | 0 | 0 | 0 | 0 | 0 | 0 | 1 | 0 | 0 | 0 | 0 | 0 | 0 | 0 | 0 | 58 | 70 | Carbamidomethylation | C5:Carbamidomethylation:1000.00;C9:Carbamidomethylation:1000.00 | PEAKS DB |
| total 2 peptides | | | | | | | | | | | | | | | | | | | | | | | | | | | | | | | | | | | | | | | | | | | | | | | | | |

**Best Unique PSM (Scan F8:18058, m/z=575.7656, z=2, RT=40.22, ppm=3.7):**

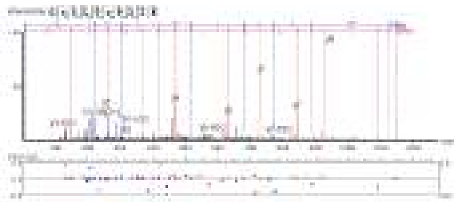

## Q8AY52.1

back to list

| Protein Coverage | Supporting Peptides | Best Unique PSM |

**Protein Coverage:**

1 MKTLLLTLVV LTIACLDLGY TKTCFNDNLA NPKTTELCRH SMYFCFKNSW IAGGVERIER GCSLTCPDIK YNGKYIYCCT

81 RDNCNA

66 C Carbamidomethylation (+57.02)

**Supporting Peptides:**

| Peptide | Uniq | -10lgP | Mass | Length | ppm | m/z | z | RT | Fraction | Scan | Source File | Area F1 | Area F2a | Area F3a | Area F4 | Area F5 | Area F6a | Area F7a | Area F7b | Area F8 | Area F9a | Area Sample 1F10a | Area F10b | Area F11a | Area F11b | Area F12a | Area F13 | #Feature | #Feature F1 | #Feature F2a | #Feature F3a | #Feature F4 | #Feature F5 | #Feature F6a | #Feature F7a | #Feature F7b | #Feature F8 | #Feature F9a | #Feature Sample 1F10a | #Feature F10b | #Feature F11a | #Feature F11b | #Feature F12a | #Feature F13 | Start | End | PTM | AScore | Found By |
|---|---|---|---|---|---|---|---|---|---|---|---|---|---|---|---|---|---|---|---|---|---|---|---|---|---|---|---|---|---|---|---|---|---|---|---|---|---|---|---|---|---|---|---|---|---|---|---|---|---|
| R.GC(+57.02)SLTC(+57.02)PDIK.Y | Y | 52.39 | 1149.5159 | 10 | 3.7 | 575.7656 | 2 | 40.22 | 8 | F8:18058 | BuCaTN01_F7b.raw | | | | | | | | 2.6353E6 | | | | | | | | | 1 | 0 | 0 | 0 | 0 | 0 | 0 | 0 | 1 | 0 | 0 | 0 | 0 | 0 | 0 | 0 | 0 | 61 | 70 | Carbamidomethylation | C2:Carbamidomethylation:1000.00;C6:Carbamidomethylation:1000.00 | PEAKS DB |
| R.IERGC(+57.02)SLTC(+57.02)PDIK.Y | Y | 43.69 | 1547.7435 | 13 | 3.7 | 516.9222 | 3 | 39.13 | 8 | F8:16955 | BuCaTN01_F7b.raw | | | | | | | | 5.7834E5 | | | | | | | | | 1 | 0 | 0 | 0 | 0 | 0 | 0 | 0 | 1 | 0 | 0 | 0 | 0 | 0 | 0 | 0 | 0 | 58 | 70 | Carbamidomethylation | C5:Carbamidomethylation:1000.00;C9:Carbamidomethylation:1000.00 | PEAKS DB |
| total 2 peptides | | | | | | | | | | | | | | | | | | | | | | | | | | | | | | | | | | | | | | | | | | | | | | | | | |

**Best Unique PSM (Scan F8:18058, m/z=575.7656, z=2, RT=40.22, ppm=3.7):**

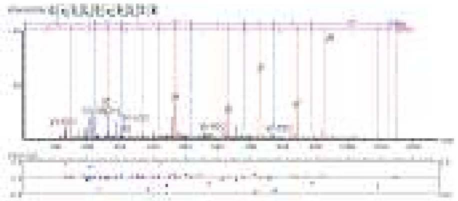

## Q9YGI0.1

back to list

| Protein Coverage | Supporting Peptides | Best Unique PSM |

**Protein Coverage:**

1 MKTLLLSLVV LTIACLDLGY TKTCFNDDLT NPKTTELCRH SMYFCFKNSW IAGGVERIER GCSLTCPDIK YNGKYIYCCT

81 RDNCNA

66 C Carbamidomethylation (+57.02)

**Supporting Peptides:**

| Peptide | Uniq | -10lgP | Mass | Length | ppm | m/z | z | RT | Fraction | Scan | Source File | Area F1 | Area F2a | Area F3a | Area F4 | Area F5 | Area F6a | Area F7a | Area F7b | Area F8 | Area F9a | Area Sample 1F10a | Area F10b | Area F11a | Area F11b | Area F12a | Area F13 | #Feature | #Feature F1 | #Feature F2a | #Feature F3a | #Feature F4 | #Feature F5 | #Feature F6a | #Feature F7a | #Feature F7b | #Feature F8 | #Feature F9a | #Feature Sample 1F10a | #Feature F10b | #Feature F11a | #Feature F11b | #Feature F12a | #Feature F13 | Start | End | PTM | AScore | Found By |
|---|---|---|---|---|---|---|---|---|---|---|---|---|---|---|---|---|---|---|---|---|---|---|---|---|---|---|---|---|---|---|---|---|---|---|---|---|---|---|---|---|---|---|---|---|---|---|---|---|---|
| R.GC(+57.02)SLTC(+57.02)PDIK.Y | Y | 52.39 | 1149.5159 | 10 | 3.7 | 575.7656 | 2 | 40.22 | 8 | F8:18058 | BuCaTN01_F7b.raw | | | | | | | | 2.6353E6 | | | | | | | | | 1 | 0 | 0 | 0 | 0 | 0 | 0 | 0 | 1 | 0 | 0 | 0 | 0 | 0 | 0 | 0 | 0 | 61 | 70 | Carbamidomethylation | C2:Carbamidomethylation:1000.00;C6:Carbamidomethylation:1000.00 | PEAKS DB |
| R.IERGC(+57.02)SLTC(+57.02)PDIK.Y | Y | 43.69 | 1547.7435 | 13 | 3.7 | 516.9222 | 3 | 39.13 | 8 | F8:16955 | BuCaTN01_F7b.raw | | | | | | | | 5.7834E5 | | | | | | | | | 1 | 0 | 0 | 0 | 0 | 0 | 0 | 0 | 1 | 0 | 0 | 0 | 0 | 0 | 0 | 0 | 0 | 58 | 70 | Carbamidomethylation | C5:Carbamidomethylation:1000.00;C9:Carbamidomethylation:1000.00 | PEAKS DB |
| total 2 peptides | | | | | | | | | | | | | | | | | | | | | | | | | | | | | | | | | | | | | | | | | | | | | | | | | |

**Best Unique PSM (Scan F8:18058, m/z=575.7656, z=2, RT=40.22, ppm=3.7):**

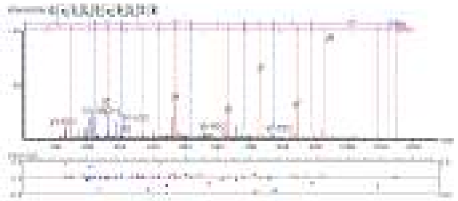

## AAL30057.1

back to list

| Protein Coverage | Supporting Peptides | Best Unique PSM |

**Protein Coverage:**

1 MKTLLLTLVV LTIACLDLGY TKTCFNDDLA NPKTTELCRH SMYFCFKNSW IAGGVERIER GCSLTCPDIK YNGKYIYCCT

81 RDNCNA

66 C Carbamidomethylation (+57.02)

**Supporting Peptides:**

| Peptide | Uniq | -10lgP | Mass | Length | ppm | m/z | z | RT | Fraction | Scan | Source File | Area F1 | Area F2a | Area F3a | Area F4 | Area F5 | Area F6a | Area F7a | Area F7b | Area F8 | Area F9a | Area Sample 1F10a | Area F10b | Area F11a | Area F11b | Area F12a | Area F13 | #Feature | #Feature F1 | #Feature F2a | #Feature F3a | #Feature F4 | #Feature F5 | #Feature F6a | #Feature F7a | #Feature F7b | #Feature F8 | #Feature F9a | #Feature Sample 1F10a | #Feature F10b | #Feature F11a | #Feature F11b | #Feature F12a | #Feature F13 | Start | End | PTM | AScore | Found By |
|---|---|---|---|---|---|---|---|---|---|---|---|---|---|---|---|---|---|---|---|---|---|---|---|---|---|---|---|---|---|---|---|---|---|---|---|---|---|---|---|---|---|---|---|---|---|---|---|---|---|
| R.GC(+57.02)SLTC(+57.02)PDIK.Y | Y | 52.39 | 1149.5159 | 10 | 3.7 | 575.7656 | 2 | 40.22 | 8 | F8:18058 | BuCaTN01_F7b.raw | | | | | | | | 2.6353E6 | | | | | | | | | 1 | 0 | 0 | 0 | 0 | 0 | 0 | 0 | 1 | 0 | 0 | 0 | 0 | 0 | 0 | 0 | 0 | 61 | 70 | Carbamidomethylation | C2:Carbamidomethylation:1000.00;C6:Carbamidomethylation:1000.00 | PEAKS DB |
| R.IERGC(+57.02)SLTC(+57.02)PDIK.Y | Y | 43.69 | 1547.7435 | 13 | 3.7 | 516.9222 | 3 | 39.13 | 8 | F8:16955 | BuCaTN01_F7b.raw | | | | | | | | 5.7834E5 | | | | | | | | | 1 | 0 | 0 | 0 | 0 | 0 | 0 | 0 | 1 | 0 | 0 | 0 | 0 | 0 | 0 | 0 | 0 | 58 | 70 | Carbamidomethylation | C5:Carbamidomethylation:1000.00;C9:Carbamidomethylation:1000.00 | PEAKS DB |
| total 2 peptides | | | | | | | | | | | | | | | | | | | | | | | | | | | | | | | | | | | | | | | | | | | | | | | | | |

**Best Unique PSM (Scan F8:18058, m/z=575.7656, z=2, RT=40.22, ppm=3.7):**

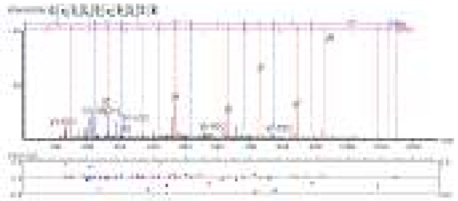

## AAO47843.1

back to list

| Protein Coverage | Supporting Peptides | Best Unique PSM |

**Protein Coverage:**

1 MKTLLLTLVV LTIACLDLGY TKTCFNDDLT NPKTTELCRH SMYFCFKNSW IAGGVER**IER GCSLTCPDIK** YNGKYIYCCT    Carbamidomethylation (+57.02)

81 RDNCNA

**Supporting Peptides:**

| Peptide | Uniq | -10lgP | Mass | Length | ppm | m/z | z | RT | Fraction | Scan | Source File | Area F1 | Area F2a | Area F3a | Area F4 | Area F5 | Area F6a | Area F7a | Area F7b | Area F8 | Area F9a | Area Sample 1F10a | Area F10b | Area F11a | Area F11b | Area F12a | Area F13 | #Feature | #Feature F1 | #Feature F2a | #Feature F3a | #Feature F4 | #Feature F5 | #Feature F6a | #Feature F7a | #Feature F7b | #Feature F8 | #Feature F9a | #Feature Sample 1F10a | #Feature F10b | #Feature F11a | #Feature F11b | #Feature F12a | #Feature F13 | Start | End | PTM | AScore | Found By |
|---|---|---|---|---|---|---|---|---|---|---|---|---|---|---|---|---|---|---|---|---|---|---|---|---|---|---|---|---|---|---|---|---|---|---|---|---|---|---|---|---|---|---|---|---|---|---|---|---|---|
| R.GC(+57.02)SLTC(+57.02)PDIK.Y | Y | 52.39 | 1149.5159 | 10 | 3.7 | 575.7656 | 2 | 40.22 | 8 | F8:18058 | BuCaTN01_F7b.raw | | | | | | | | 2.6353E6 | | | | | | | | | 1 | 0 | 0 | 0 | 0 | 0 | 0 | 0 | 1 | 0 | 0 | 0 | 0 | 0 | 0 | 0 | 0 | 61 | 70 | Carbamidomethylation | C2:Carbamidomethylation:1000.00;C6:Carbamidomethylation:1000.00 | PEAKS DB |
| R.IERGC(+57.02)SLTC(+57.02)PDIK.Y | Y | 43.69 | 1547.7435 | 13 | 3.7 | 516.9222 | 3 | 39.13 | 8 | F8:16955 | BuCaTN01_F7b.raw | | | | | | | | 5.7834E5 | | | | | | | | | 1 | 0 | 0 | 0 | 0 | 0 | 0 | 0 | 1 | 0 | 0 | 0 | 0 | 0 | 0 | 0 | 0 | 58 | 70 | Carbamidomethylation | C5:Carbamidomethylation:1000.00;C9:Carbamidomethylation:1000.00 | PEAKS DB |
| total 2 peptides | | | | | | | | | | | | | | | | | | | | | | | | | | | | | | | | | | | | | | | | | | | | | | | | | |

**Best Unique PSM (Scan F8:18058, m/z=575.7656, z=2, RT=40.22, ppm=3.7):**

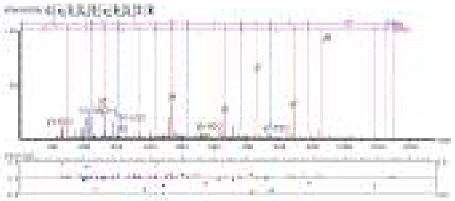

## E5L0E4.1

back to list

| Protein Coverage | Supporting Peptides | Best Unique PSM |

**Protein Coverage:**

1 MVLIKVLANL LVLQLSYAQK SSELVVGGDE CNINEHRSLV FLYNNSFGCS GTLINQQWVL SAVHCDMENV RIYLGVHNLT
81 LRNNAEIRLP EERFFCLSNK NYTKWDKDIM LIKLDRPVK**T STYIAPLSLP SSPPR**VGSVC RIMGWGAITS PNETFPGVTH

161 CANINILPYS VCR**AAYKGLP AQSR**TLCGGI LEGGIGSCMG DSGGPLICNG EMHGIVAWGD DTCAQPHKPV HYTKVYDYTD

241 WIQSIIAGNT AATCPP

**Supporting Peptides:**

| Peptide | Uniq | -10lgP | Mass | Length | ppm | m/z | z | RT | Fraction | Scan | Source File | Area F1 | Area F2a | Area F3a | Area F4 | Area F5 | Area F6a | Area F7a | Area F7b | Area F8 | Area F9a | Area Sample 1F10a | Area F10b | Area F11a | Area F11b | Area F12a | Area F13 | #Feature | #Feature F1 | #Feature F2a | #Feature F3a | #Feature F4 | #Feature F5 | #Feature F6a | #Feature F7a | #Feature F7b | #Feature F8 | #Feature F9a | #Feature Sample 1F10a | #Feature F10b | #Feature F11a | #Feature F11b | #Feature F12a | #Feature F13 | Start | End | PTM | AScore | Found By |
|---|---|---|---|---|---|---|---|---|---|---|---|---|---|---|---|---|---|---|---|---|---|---|---|---|---|---|---|---|---|---|---|---|---|---|---|---|---|---|---|---|---|---|---|---|---|---|---|---|---|
| K.TSTYIAPLSLPSSPPR.V | Y | 70.94 | 1685.8988 | 16 | 0.7 | 843.9573 | 2 | 63.72 | 16 | F16:31707 | BuCaTN01_F13.raw | | | | | | | | | | | 0 | | | | | 9.2723E5 | 2 | 0 | 0 | 0 | 0 | 0 | 0 | 0 | 0 | 0 | 0 | 0 | 0 | 0 | 0 | 0 | 2 | 120 | 135 | | | PEAKS DB |
| R.AAYKGLPAQSR.T | Y | 47.16 | 1160.6301 | 11 | 2.4 | 387.8849 | 3 | 28.26 | 16 | F16:8862 | BuCaTN01_F13.raw | | | | | | | | | | | | | | | | 2.1424E4 | 1 | 0 | 0 | 0 | 0 | 0 | 0 | 0 | 0 | 0 | 0 | 0 | 0 | 0 | 0 | 0 | 1 | 174 | 184 | | | PEAKS DB |
| total 2 peptides | | | | | | | | | | | | | | | | | | | | | | | | | | | | | | | | | | | | | | | | | | | | | | | | | |

**Best Unique PSM (Scan F16:31707, m/z=843.9573, z=2, RT=63.72, ppm=0.7):**

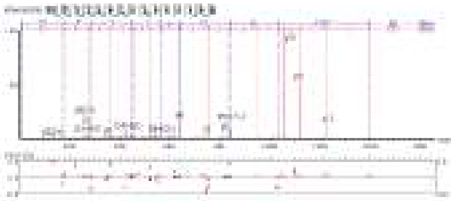

## ADP88560.1

back to list

| Protein Coverage | Supporting Peptides | Best Unique PSM |

**Protein Coverage:**

1 MVLIKVLANL LVLQLSYAQK SSELVVGGDE CNINEHRSLV FLYNNSFGCS GTLINQQWVL SAVHCDMENV RIYLGVHNLT
81 LRNNAEIRLP EERFFCLSNK NYTKWDKDIM LIKLDRPVK**T STYIAPLSLP SSPPR**VGSVC RIMGWGAITS PNETFPGVTH

161 CANINILPYS VCR**AAYKGLP AQSR**TLCGGI LEGGIGSCMG DSGGPLICNG EMHGIVAWGD DTCAQPHKPV HYTKVYDYTD

241 WIQSIIAGNT AATCPP

**Supporting Peptides:**

| Peptide | Uniq | -10lgP | Mass | Length | ppm | m/z | z | RT | Fraction | Scan | Source File | Area F1 | Area F2a | Area F3a | Area F4 | Area F5 | Area F6a | Area F7a | Area F7b | Area F8 | Area F9a | Area Sample 1F10a | Area F10b | Area F11a | Area F11b | Area F12a | Area F13 | #Feature | #Feature F1 | #Feature F2a | #Feature F3a | #Feature F4 | #Feature F5 | #Feature F6a | #Feature F7a | #Feature F7b | #Feature F8 | #Feature F9a | #Feature Sample 1F10a | #Feature F10b | #Feature F11a | #Feature F11b | #Feature F12a | #Feature F13 | Start | End | PTM | AScore | Found By |
|---|---|---|---|---|---|---|---|---|---|---|---|---|---|---|---|---|---|---|---|---|---|---|---|---|---|---|---|---|---|---|---|---|---|---|---|---|---|---|---|---|---|---|---|---|---|---|---|---|---|
| K.TSTYIAPLSLPSSPPR.V | Y | 70.94 | 1685.8988 | 16 | 0.7 | 843.9573 | 2 | 63.72 | 16 | F16:31707 | BuCaTN01_F13.raw | | | | | | | | | | | 0 | | | | | 9.2723E5 | 2 | 0 | 0 | 0 | 0 | 0 | 0 | 0 | 0 | 0 | 0 | 0 | 0 | 0 | 0 | 0 | 2 | 120 | 135 | | | PEAKS DB |
| R.AAYKGLPAQSR.T | Y | 47.16 | 1160.6301 | 11 | 2.4 | 387.8849 | 3 | 28.26 | 16 | F16:8862 | BuCaTN01_F13.raw | | | | | | | | | | | | | | | | 2.1424E4 | 1 | 0 | 0 | 0 | 0 | 0 | 0 | 0 | 0 | 0 | 0 | 0 | 0 | 0 | 0 | 0 | 1 | 174 | 184 | | | PEAKS DB |
| total 2 peptides | | | | | | | | | | | | | | | | | | | | | | | | | | | | | | | | | | | | | | | | | | | | | | | | | |

**Best Unique PSM (Scan F16:31707, m/z=843.9573, z=2, RT=63.72, ppm=0.7):**

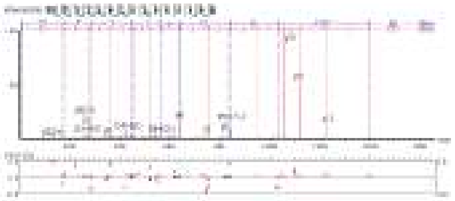

## CAJ77819.1

back to list

| Protein Coverage | Supporting Peptides | Best Unique PSM |

**Protein Coverage:**

1 YTLLCYKTPS PINAETCPPG ENLCYTKMWC DAWCSSR**GKV IELGCAAT**CP SKK**PYEEVTC CSTDKCNPHP K**QRPG    Carbamidomethylation (+57.02)

**Supporting Peptides:**

| Peptide | Uniq | -10lgP | Mass | Length | ppm | m/z | z | RT | Fraction | Scan | Source File | Area F1 | Area F2a | Area F3a | Area F4 | Area F5 | Area F6a | Area F7a | Area F7b | Area F8 | Area F9a | Area Sample 1F10a | Area F10b | Area F11a | Area F11b | Area F12a | Area F13 | #Feature | #Feature F1 | #Feature F2a | #Feature F3a | #Feature F4 | #Feature F5 | #Feature F6a | #Feature F7a | #Feature F7b | #Feature F8 | #Feature F9a | #Feature Sample 1F10a | #Feature F10b | #Feature F11a | #Feature F11b | #Feature F12a | #Feature F13 | Start | End | PTM | AScore | Found By |
|---|---|---|---|---|---|---|---|---|---|---|---|---|---|---|---|---|---|---|---|---|---|---|---|---|---|---|---|---|---|---|---|---|---|---|---|---|---|---|---|---|---|---|---|---|---|---|---|---|---|
| R.GKVIELGC(+57.02)AAT.C | Y | 47.40 | 1117.5801 | 11 | 1.6 | 559.7982 | 2 | 41.67 | 6 | F6:21010 | BuCaTN01_F6a.raw | | | | | | 1.8135E6 | | | | 3.8172E5 | | | | | | | 2 | 0 | 0 | 0 | 0 | 0 | 1 | 0 | 0 | 0 | 1 | 0 | 0 | 0 | 0 | 0 | 0 | 38 | 48 | Carbamidomethylation | C8:Carbamidomethylation:1000.00 | PEAKS DB |
| K.PYEEVTC(+57.02)C(+57.02)STDKC(+57.02)NPHPK.Q | N | 46.34 | 2220.9238 | 18 | 0.7 | 556.2386 | 4 | 29.53 | 6 | F6:10966 | BuCaTN01_F6a.raw | | | | | | 1.5168E6 | | | | | | | | | | | 1 | 0 | 0 | 0 | 0 | 0 | 1 | 0 | 0 | 0 | 0 | 0 | 0 | 0 | 0 | 0 | 0 | 54 | 71 | Carbamidomethylation | C7:Carbamidomethylation:1000.00;C8:Carbamidomethylation:1000.00;C13:Carbamidomethylation:1000.00 | PEAKS DB |
| total 2 peptides | | | | | | | | | | | | | | | | | | | | | | | | | | | | | | | | | | | | | | | | | | | | | | | | | |

**Best Unique PSM (Scan F6:21010, m/z=559.7982, z=2, RT=41.67, ppm=1.6):**

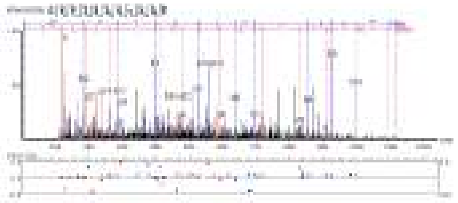

## A1IVR8.1

back to list

| Protein Coverage | Supporting Peptides | Best Unique PSM |

**Protein Coverage:**

```
                                        45                               66
  1 YTLLCYKTPS PINAETCPPG ENLCYTKMWC DAWCSSRGKV IELGCAATCP SKKPYEEVTC CSTDKCNPHP KQRPG
```

Carbamidomethylation (+57.02)

**Supporting Peptides:**

| Peptide | Uniq | -10lgP | Mass | Length | ppm | m/z | z | RT | Fraction | Scan | Source File | Area F1 | Area F2a | Area F3a | Area F4 | Area F5 | Area F6a | Area F7a | Area F7b | Area F8 | Area F9a | Area Sample 1F10a | Area F10b | Area F11a | Area F11b | Area F12a | Area F13 | #Feature | #Feature F1 | #Feature F2a | #Feature F3a | #Feature F4 | #Feature F5 | #Feature F6a | #Feature F7a | #Feature F7b | #Feature F8 | #Feature F9a | #Feature Sample 1F10a | #Feature F10b | #Feature F11a | #Feature F11b | #Feature F12a | #Feature F13 | Start | End | PTM | AScore | Found By |
|---|---|---|---|---|---|---|---|---|---|---|---|---|---|---|---|---|---|---|---|---|---|---|---|---|---|---|---|---|---|---|---|---|---|---|---|---|---|---|---|---|---|---|---|---|---|---|---|---|---|
| R.GKVIELGC(+57.02)AAT.C | Y | 47.40 | 1117.5801 | 11 | 1.6 | 559.7982 | 2 | 41.67 | 6 | F6:21010 | BuCaTN01_F6a.raw | | | | | | 1.8135E6 | | | | 3.8172E5 | | | | | | | 2 | 0 | 0 | 0 | 0 | 0 | 1 | 0 | 0 | 0 | 1 | 0 | 0 | 0 | 0 | 0 | 0 | 38 | 48 | Carbamidomethylation | C8:Carbamidomethylation:1000.00 | PEAKS DB |
| K.PYEEVTC(+57.02)C(+57.02)STDKC(+57.02)NPHPK.Q | N | 46.34 | 2220.9238 | 18 | 0.7 | 556.2386 | 4 | 29.53 | 6 | F6:10966 | BuCaTN01_F6a.raw | | | | | | 1.5168E6 | | | | | | | | | | | 1 | 0 | 0 | 0 | 0 | 0 | 1 | 0 | 0 | 0 | 0 | 0 | 0 | 0 | 0 | 0 | 0 | 54 | 71 | Carbamidomethylation | C7:Carbamidomethylation:1000.00;C8:Carbamidomethylation:1000.00;C13:Carbamidomethylation:1000.00 | PEAKS DB |
| total 2 peptides | | | | | | | | | | | | | | | | | | | | | | | | | | | | | | | | | | | | | | | | | | | | | | | | | |

**Best Unique PSM (Scan F6:21010, m/z=559.7982, z=2, RT=41.67, ppm=1.6):**

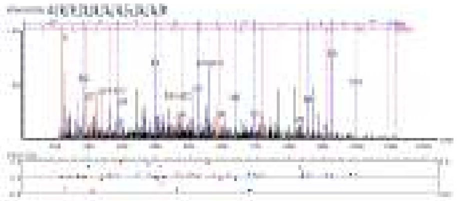

## XP_026541740.1

back to list

| Protein Coverage | Supporting Peptides | Best Unique PSM |

**Protein Coverage:**

```
  1 MTAMSPLWLC LCYLAAWPAW TRSESQTPPP ANGDDQPPPA QFPADISQHA VLPDAISREF LLQKIQENLE DGQQLPILVH
 81 GEPQAVPLQP RGSGRHCKEV GCTRKPPFYK LEEFPPGRPS LSNLGALCSA GRTKPSYGPW NLPQTSFSHL SRQGEALNQL
161 ERQFTRCCQL AEDQKLPCAS NEWEKCLARY CKQEFSTKTL PFHCCRAGGR KDRLNCFARQ APFPKYEGEG EWLDLANLTT
241 PILDSICSQA TSQDEKPRPA LVQNITESCC KLPESERTLC AQGVKSQFIT AFCSSQRRSW KDPQKCCAQT EEAARNDCFD

321 RSYLGHVSLL QRQQEEEEEE AEEVVEEIVL PTQAAP
```

Carbamidomethylation (+57.02)

**Supporting Peptides:**

| Peptide | Uniq | -10lgP | Mass | Length | ppm | m/z | z | RT | Fraction | Scan | Source File | Area F1 | Area F2a | Area F3a | Area F4 | Area F5 | Area F6a | Area F7a | Area F7b | Area F8 | Area F9a | Area Sample 1F10a | Area F10b | Area F11a | Area F11b | Area F12a | Area F13 | #Feature | #Feature F1 | #Feature F2a | #Feature F3a | #Feature F4 | #Feature F5 | #Feature F6a | #Feature F7a | #Feature F7b | #Feature F8 | #Feature F9a | #Feature Sample 1F10a | #Feature F10b | #Feature F11a | #Feature F11b | #Feature F12a | #Feature F13 | Start | End | PTM | AScore | Found By |
|---|---|---|---|---|---|---|---|---|---|---|---|---|---|---|---|---|---|---|---|---|---|---|---|---|---|---|---|---|---|---|---|---|---|---|---|---|---|---|---|---|---|---|---|---|---|---|---|---|---|
| R.SYLGHVSLLQR.Q | Y | 54.13 | 1271.6986 | 11 | -0.3 | 424.9067 | 3 | 54.95 | 7 | F7:28010 | BuCaTN01_F7a.raw | | | | | | | 6.9503E4 | | | | | | | | | | 1 | 0 | 0 | 0 | 0 | 0 | 0 | 1 | 0 | 0 | 0 | 0 | 0 | 0 | 0 | 0 | 0 | 322 | 332 | | | PEAKS DB |
| K.SQFITAFC(+57.02)SSQR.R | Y | 46.12 | 1430.6613 | 12 | -2.6 | 716.3361 | 2 | 57.59 | 7 | F7:30373 | BuCaTN01_F7a.raw | | | | | | | 2.6295E5 | | | | | | | | | | 1 | 0 | 0 | 0 | 0 | 0 | 0 | 1 | 0 | 0 | 0 | 0 | 0 | 0 | 0 | 0 | 0 | 286 | 297 | Carbamidomethylation | C8:Carbamidomethylation:1000.00 | PEAKS DB |
| total 2 peptides | | | | | | | | | | | | | | | | | | | | | | | | | | | | | | | | | | | | | | | | | | | | | | | | | |

**Best Unique PSM (Scan F7:28010, m/z=424.9067, z=3, RT=54.95, ppm=-0.3):**

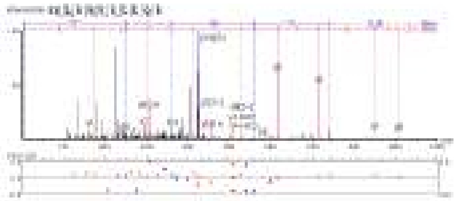

## XP_026541739.1

back to list

| Protein Coverage | Supporting Peptides | Best Unique PSM |

**Protein Coverage:**

```
  1 MTAMSPLWLC LCYLAAWPAW TRSESQTPPP ANGDDQPPPA QFPADISQHA VLPDAISREF LLQKIQENLE DGQQLPILVH
 81 GEPQAVPLQP RGSGRHCKEV GCTRKPPFYK LEEFPPGRPS LSNLGALCSA GRTKPSYGPW NLPQTSFSHL SRQGEALNQL
161 ERQFTRCCQL AEDQKLPCAS NEWEKCLARY CKQEFSTKTL PFHCCRAGGR KDRLNCFARQ APFPKYEGEG EWLDLANLTT
241 PILDSICSQA TSQDEQKPRP ALVQNITESC CKLPESERTL CAQGVKSQFI TAFCSSQRRS WKDPQKCCAQ TEEAARNDCF

321 DRSYLGHVSL LQRQQEEEEE EAEEVVEEIV LPTQAAP
```

Carbamidomethylation (+57.02)

**Supporting Peptides:**

| Peptide | Uniq | -10lgP | Mass | Length | ppm | m/z | z | RT | Fraction | Scan | Source File | Area F1 | Area F2a | Area F3a | Area F4 | Area F5 | Area F6a | Area F7a | Area F7b | Area F8 | Area F9a | Area Sample 1F10a | Area F10b | Area F11a | Area F11b | Area F12a | Area F13 | #Feature | #Feature F1 | #Feature F2a | #Feature F3a | #Feature F4 | #Feature F5 | #Feature F6a | #Feature F7a | #Feature F7b | #Feature F8 | #Feature F9a | #Feature Sample 1F10a | #Feature F10b | #Feature F11a | #Feature F11b | #Feature F12a | #Feature F13 | Start | End | PTM | AScore | Found By |
|---|---|---|---|---|---|---|---|---|---|---|---|---|---|---|---|---|---|---|---|---|---|---|---|---|---|---|---|---|---|---|---|---|---|---|---|---|---|---|---|---|---|---|---|---|---|---|---|---|---|
| R.SYLGHVSLLQR.Q | Y | 54.13 | 1271.6986 | 11 | -0.3 | 424.9067 | 3 | 54.95 | 7 | F7:28010 | BuCaTN01_F7a.raw | | | | | | | 6.9503E4 | | | | | | | | | | 1 | 0 | 0 | 0 | 0 | 0 | 0 | 1 | 0 | 0 | 0 | 0 | 0 | 0 | 0 | 0 | 0 | 323 | 333 | | | PEAKS DB |
| K.SQFITAFC(+57.02)SSQR.R | Y | 46.12 | 1430.6613 | 12 | -2.6 | 716.3361 | 2 | 57.59 | 7 | F7:30373 | BuCaTN01_F7a.raw | | | | | | | 2.6295E5 | | | | | | | | | | 1 | 0 | 0 | 0 | 0 | 0 | 0 | 1 | 0 | 0 | 0 | 0 | 0 | 0 | 0 | 0 | 0 | 287 | 298 | Carbamidomethylation | C8:Carbamidomethylation:1000.00 | PEAKS DB |
| total 2 peptides | | | | | | | | | | | | | | | | | | | | | | | | | | | | | | | | | | | | | | | | | | | | | | | | | |

**Best Unique PSM (Scan F7:28010, m/z=424.9067, z=3, RT=54.95, ppm=-0.3):**

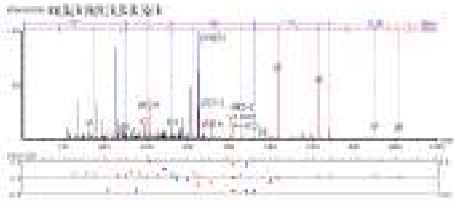

## KAG8142835.1

back to list

| [Protein Coverage](#) | [Supporting Peptides](#) | [Best Unique PSM](#) |

**Protein Coverage:**

```
  1 MFQHIFELKV SEKLHIMCHL WIKCLATWIL LKRFNGVHVM QARAPMYPNE PFLVFWNAPT TQCQLRYKVD LDLKTFHIVP
 81 NAKESLSGSA VTIFYPTQLG VYPHIDDHGH FVHGIIPQNE SITKHLNKTK SDINHMIPLK TFHGLGVIDW ENWRPQWDRN
161 WGSKNVYRTR SIQFAKQLHP ELSEAAIKRL AKQEYEKAGK HFMRDTLLLA ENMRPGGFWG YYLYPDCYNY NYKKNPEQYT
241 GKCPKLEILR NDQLLWLWRD STALFPSIYL EIILKSSANA LKFVHHRLEE SMRIASMARK DYALPVFVYA RPFYAYTFEP
321 LTK
```

**Supporting Peptides:**

| Peptide | Uniq | -10lgP | Mass | Length | ppm | m/z | z | RT | Fraction | Scan | Source File | Area F1 | Area F2a | Area F3a | Area F4 | Area F5 | Area F6a | Area F7a | Area F7b | Area F8 | Area F9a | Area Sample 1F10a | Area F10b | Area F11a | Area F11b | Area F12a | Area F13 | #Feature | #Feature F1 | #Feature F2a | #Feature F3a | #Feature F4 | #Feature F5 | #Feature F6a | #Feature F7a | #Feature F7b | #Feature F8 | #Feature F9a | #Feature Sample 1F10a | #Feature F10b | #Feature F11a | #Feature F11b | #Feature F12a | #Feature F13 | Start | End | PTM | AScore | Found By |
|---|---|---|---|---|---|---|---|---|---|---|---|---|---|---|---|---|---|---|---|---|---|---|---|---|---|---|---|---|---|---|---|---|---|---|---|---|---|---|---|---|---|---|---|---|---|---|---|---|---|
| K.QLHPELSEAAIK.R | Y | 69.94 | 1334.7194 | 12 | 0.7 | 445.9140 | 3 | 43.75 | 16 | F16:18956 | BuCaTN01_F13.raw | | | | | | | | | | | | | | | | 3.5323E6 | 1 | 0 | 0 | 0 | 0 | 0 | 0 | 0 | 0 | 0 | 0 | 0 | 0 | 0 | 0 | 0 | 1 | 177 | 188 | | | PEAKS DB |
| total 1 peptides | | | | | | | | | | | | | | | | | | | | | | | | | | | | | | | | | | | | | | | | | | | | | | | | | |

**Best Unique PSM (Scan F16:18956, m/z=445.9140, z=3, RT=43.75, ppm=0.7):**

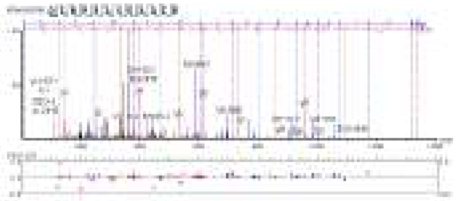

## KAG8142834.1

[back to list](#)

| [Protein Coverage](#) | [Supporting Peptides](#) | [Best Unique PSM](#) |

**Protein Coverage:**

```
  1 LKELKVSEKL HIMCHLWIKC LATWILLKRF NGVHVMQARA PMYPNEPFLV FWNAPTTQCQ LRYKVDLDLK TFHIVPNAKE
 81 SLSGSAVTIF YPTQLGVYPH IDDHGHFVHG IIPQNESITK HLNKTKSDIN HMIPLKTFHG LGVIDWENWR PQWDRNWGSK
161 NVYRTRSIQF AKQLHPELSE AAIKRLAKQE YEKAGKHFMR DTLLLAENMR PGGFWGYYLY PDCYNYNYKK NPEQYTGKCP
241 KLEILRNDQL LWLWRDSTAL FPSIYLEIIL KSSANALKFV HHRLEESMRI ASMARKDYAL PVFVYARPFY AYTFEPLTKE
321 DLVSTVGETA AMGAAGIVFW GSMQYASTIE SCQRVKDYMN GPFGRYIFNV TSAAKICSHF LCKKKGRCVR KHSDSNAFLH
401 LFPESFRIMV HANATHKKAF VKGKLELENL RYLRKNFMCQ CYQGWKGLYC EEHYKKKGNK I
```

**Supporting Peptides:**

| Peptide | Uniq | -10lgP | Mass | Length | ppm | m/z | z | RT | Fraction | Scan | Source File | Area F1 | Area F2a | Area F3a | Area F4 | Area F5 | Area F6a | Area F7a | Area F7b | Area F8 | Area F9a | Area Sample 1F10a | Area F10b | Area F11a | Area F11b | Area F12a | Area F13 | #Feature | #Feature F1 | #Feature F2a | #Feature F3a | #Feature F4 | #Feature F5 | #Feature F6a | #Feature F7a | #Feature F7b | #Feature F8 | #Feature F9a | #Feature Sample 1F10a | #Feature F10b | #Feature F11a | #Feature F11b | #Feature F12a | #Feature F13 | Start | End | PTM | AScore | Found By |
|---|---|---|---|---|---|---|---|---|---|---|---|---|---|---|---|---|---|---|---|---|---|---|---|---|---|---|---|---|---|---|---|---|---|---|---|---|---|---|---|---|---|---|---|---|---|---|---|---|---|
| K.QLHPELSEAAIK.R | Y | 69.94 | 1334.7194 | 12 | 0.7 | 445.9140 | 3 | 43.75 | 16 | F16:18956 | BuCaTN01_F13.raw | | | | | | | | | | | | | | | | 3.5323E6 | 1 | 0 | 0 | 0 | 0 | 0 | 0 | 0 | 0 | 0 | 0 | 0 | 0 | 0 | 0 | 0 | 1 | 173 | 184 | | | PEAKS DB |
| total 1 peptides | | | | | | | | | | | | | | | | | | | | | | | | | | | | | | | | | | | | | | | | | | | | | | | | | |

**Best Unique PSM (Scan F16:18956, m/z=445.9140, z=3, RT=43.75, ppm=0.7):**

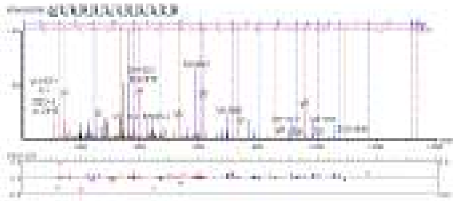

## XP_026522979.1

[back to list](#)

| [Protein Coverage](#) | [Supporting Peptides](#) | [Best Unique PSM](#) |

**Protein Coverage:**

```
  1 MHLLGLFSLG CYLAAAAVVL LVAREPAIAA AYESGQGYYE EEPDLGEAKN YANKELEDQL RSVSSVDELM TVLYPEYWKM
 81 FKCQLRKGGW PHREQSNFDG RTGDSNPIKF AAAHYNTEIL KSIDNEWRKT QCMPREVTVD VGKESGATTN IFFKPPCVSV
161 YRCGGCCNSE GLQCMNISTS YVSKTLFEIT VPLSHGPKPV TISFANHTSC RCMSKLDVYR QVHSIIRRSL PVLQAQCQIT
241 NKTCPKSHIW NNHLCRCLAQ HDFSFSSYPP DTDTAEGFHD ICGSNKELDE ETCQCVCKGG VTPSSCGIHK ELDRTSCQCT
321 CKNKLLPSSC GLNKEFDEEK CQCVCKRTCP KHQPLNPTKC ICECVESPNK CFLKGERFHH QTCSCYRRPC TVRKKRCDSG
401 FYYSEEVCRC VPTYWKLPLI N
```

C Carbamidomethylation (+57.02)

**Supporting Peptides:**

| Peptide | Uniq | -10lgP | Mass | Length | ppm | m/z | z | RT | Fraction | Scan | Source File | Area F1 | Area F2a | Area F3a | Area F4 | Area F5 | Area F6a | Area F7a | Area F7b | Area F8 | Area F9a | Area Sample 1F10a | Area F10b | Area F11a | Area F11b | Area F12a | Area F13 | #Feature | #Feature F1 | #Feature F2a | #Feature F3a | #Feature F4 | #Feature F5 | #Feature F6a | #Feature F7a | #Feature F7b | #Feature F8 | #Feature F9a | #Feature Sample 1F10a | #Feature F10b | #Feature F11a | #Feature F11b | #Feature F12a | #Feature F13 | Start | End | PTM | AScore | Found By |
|---|---|---|---|---|---|---|---|---|---|---|---|---|---|---|---|---|---|---|---|---|---|---|---|---|---|---|---|---|---|---|---|---|---|---|---|---|---|---|---|---|---|---|---|---|---|---|---|---|---|
| R.C(+57.02)DSGFYYSEEVC(+57.02)R.C | Y | 64.83 | 1670.6340 | 13 | 0.9 | 836.3251 | 2 | 52.61 | 7 | F7:26144 | BuCaTN01_F7a.raw | | | | | | | 3.5029E5 | | | | | | | | | | 1 | 0 | 0 | 0 | 0 | 0 | 0 | 1 | 0 | 0 | 0 | 0 | 0 | 0 | 0 | 0 | 0 | 397 | 409 | Carbamidomethylation | C1:Carbamidomethylation:1000.00;C12:Carbamidomethylation:1000.00 | PEAKS DB |
| R.EVTVDVGKESGATTNIFFKPPC(+57.02)VSVYR.C | Y | 47.16 | 2999.5061 | 27 | -1.2 | 750.8829 | 4 | 70.61 | 7 | F7:41039 | BuCaTN01_F7a.raw | | | | | | | 1.5942E5 | | | | | | | | | | 1 | 0 | 0 | 0 | 0 | 0 | 0 | 1 | 0 | 0 | 0 | 0 | 0 | 0 | 0 | 0 | 0 | 136 | 162 | Carbamidomethylation | C22:Carbamidomethylation:1000.00 | PEAKS DB |
| total 2 peptides | | | | | | | | | | | | | | | | | | | | | | | | | | | | | | | | | | | | | | | | | | | | | | | | | |

**Best Unique PSM (Scan F7:26144, m/z=836.3251, z=2, RT=52.61, ppm=0.9):**

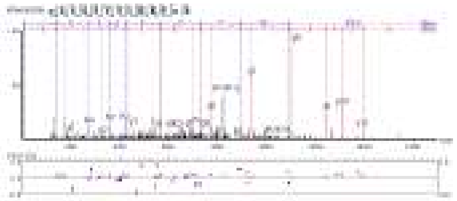

## LAB17921.1

[back to list](#)

| [Protein Coverage](#) | [Supporting Peptides](#) | [Best Unique PSM](#) |

**Protein Coverage:**

```
  1 AKGRAGGGFR GPFLCSIPGS PLLKGLPREC QERRGALQRQ PHAVFTASAV ALAAFCTMHL LGLFSLGCYL AAAAVVLLVA
 81 REPAIAAAYE SGQGYYEEEP DLGEAKNYAN KELEEQLRSV SSVDELMTVL YPEYWKMFKC QLRKGGWPHR EQSNFDGRTG
161 DSNPIKFAAA HYNTEILKSI DNEWRKTQCM PREVTVDVGK ESGATTNIFF KPPCVSVYRC GGCCNSEGLQ CMNISTSYVS
241 KTLFEITVPL SHGPKPVTIS FANHTSCRCM SKLDVYRQVH SIIRRSLPVI QAQCQITNKT CPKSHIWNNH LCRCLAQQDF
321 SFSSYPPDTD TAEGYHDICG SNKELDEETC QCVCKGGVTP SSCGIHKELD RTSCQCTCKN KLLPSSCGLN KEFDEEKCQC
401 VCKRTCPKHQ PLNPTKCICE CVESPNKCFL KGERFHHQTC SCYRRPCTVR KKRCDSGFYY SEEVCRCVPT YWKLPLIN
```

C Carbamidomethylation (+57.02)

**Supporting Peptides:**

| Peptide | Uniq | -10lgP | Mass | Length | ppm | m/z | z | RT | Fraction | Scan | Source File | Area F1 | Area F2a | Area F3a | Area F4 | Area F5 | Area F6a | Area F7a | Area F7b | Area F8 | Area F9a | Area Sample 1F10a | Area F10b | Area F11a | Area F11b | Area F12a | Area F13 | #Feature | #Feature F1 | #Feature F2a | #Feature F3a | #Feature F4 | #Feature F5 | #Feature F6a | #Feature F7a | #Feature F7b | #Feature F8 | #Feature F9a | #Feature Sample 1F10a | #Feature F10b | #Feature F11a | #Feature F11b | #Feature F12a | #Feature F13 | Start | End | PTM | AScore | Found By |
|---|---|---|---|---|---|---|---|---|---|---|---|---|---|---|---|---|---|---|---|---|---|---|---|---|---|---|---|---|---|---|---|---|---|---|---|---|---|---|---|---|---|---|---|---|---|---|---|---|---|

| Peptide | Uniq | -10lgP | Mass | Length | ppm | m/z | z | RT | Fraction | Scan | Source File | Area F1 | Area F2a | Area F3a | Area F4 | Area F5 | Area F6a | Area F7a | Area F7b | Area F8 | Area F9a | Area Sample 1F10a | Area F10b | Area F11a | Area F11b | Area F12a | Area F13 | #Feature | #Feature F1 | #Feature F2a | #Feature F3a | #Feature F4 | #Feature F5 | #Feature F6a | #Feature F7a | #Feature F7b | #Feature F8 | #Feature F9a | #Feature Sample 1F10a | #Feature F10b | #Feature F11a | #Feature F11b | #Feature F12a | #Feature F13 | Start | End | PTM | AScore | Found By |
|---|---|---|---|---|---|---|---|---|---|---|---|---|---|---|---|---|---|---|---|---|---|---|---|---|---|---|---|---|---|---|---|---|---|---|---|---|---|---|---|---|---|---|---|---|---|---|---|---|---|
| R.C(+57.02)DSGFYYSEEVC(+57.02)R.C | Y | 64.83 | 1670.6340 | 13 | 0.9 | 836.3251 | 2 | 52.61 | 7 | F7:26144 | BuCaTN01_F7a.raw | | | | | | | 3.5029E5 | | | | | | | | | | 1 | 0 | 0 | 0 | 0 | 0 | 0 | 1 | 0 | 0 | 0 | 0 | 0 | 0 | 0 | 0 | 0 | 454 | 466 | Carbamidomethylation | C1:Carbamidomethylation:1000.00;C12:Carbamidomethylation:1000.00 | PEAKS DB |
| R.EVTVDVGKESGATTNIFFKPPC(+57.02)VSVYR.C | Y | 47.16 | 2999.5061 | 27 | -1.2 | 750.8829 | 4 | 70.61 | 7 | F7:41039 | BuCaTN01_F7a.raw | | | | | | | 1.5942E5 | | | | | | | | | | 1 | 0 | 0 | 0 | 0 | 0 | 0 | 1 | 0 | 0 | 0 | 0 | 0 | 0 | 0 | 0 | 0 | 193 | 219 | Carbamidomethylation | C22:Carbamidomethylation:1000.00 | PEAKS DB |
| **total 2 peptides** | | | | | | | | | | | | | | | | | | | | | | | | | | | | | | | | | | | | | | | | | | | | | | | | | |

**Best Unique PSM (Scan F7:26144, m/z=836.3251, z=2, RT=52.61, ppm=0.9):**

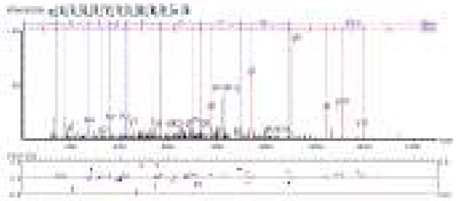

## LAA17506.1

back to list

| Protein Coverage | Supporting Peptides | Best Unique PSM |

**Protein Coverage:**

```
  1 YMGEFRGPFL RSIPGSPLLK GLPRECQERR GALQRQPHAV FTASAVALAA FCTMHLLGLF SLGCYLAAAA VVLLVAREPA
 81 IAAAYESGQG YYEEEPDLGE AKNYANKELE EQLRSVSSVD ELMTVLYPEY WKMFKCQLRK GGWPHREQSN FDGRTGDSNP
161 IKFAAAHYNT EFLKSIDNEW RKTQCMPREV TVDVGKESGA TTNIFFKPPC VSVYRCGGCC NSEGLQCMNI STSYVSKTLF
241 EITVPLSHGP KPVTISFANH TSCRCMSKLD VYRQVHSIIR RSLPVIQAQC QITNKTCPKS HIWNNHLCRC LAQQDFSFSS
321 YPPDTDTAEG YHDICGSNKE LDEETCQCVC KGGVTPSSCG IHKELDRTSC QCTCKNKLLP SSCGLNKEFD EEKCQCVCKR
401 TCPKHQPLNP TKCICECVES PNKCFLKGER FHHQTCSCYR RPCTVRKKRC DSGFYYSEEV CRCVPTYWKL PLIN
```

C Carbamidomethylation (+57.02)

**Supporting Peptides:**

| Peptide | Uniq | -10lgP | Mass | Length | ppm | m/z | z | RT | Fraction | Scan | Source File | Area F1 | Area F2a | Area F3a | Area F4 | Area F5 | Area F6a | Area F7a | Area F7b | Area F8 | Area F9a | Area Sample 1F10a | Area F10b | Area F11a | Area F11b | Area F12a | Area F13 | #Feature | #Feature F1 | #Feature F2a | #Feature F3a | #Feature F4 | #Feature F5 | #Feature F6a | #Feature F7a | #Feature F7b | #Feature F8 | #Feature F9a | #Feature Sample 1F10a | #Feature F10b | #Feature F11a | #Feature F11b | #Feature F12a | #Feature F13 | Start | End | PTM | AScore | Found By |
|---|---|---|---|---|---|---|---|---|---|---|---|---|---|---|---|---|---|---|---|---|---|---|---|---|---|---|---|---|---|---|---|---|---|---|---|---|---|---|---|---|---|---|---|---|---|---|---|---|---|
| R.C(+57.02)DSGFYYSEEVC(+57.02)R.C | Y | 64.83 | 1670.6340 | 13 | 0.9 | 836.3251 | 2 | 52.61 | 7 | F7:26144 | BuCaTN01_F7a.raw | | | | | | | 3.5029E5 | | | | | | | | | | 1 | 0 | 0 | 0 | 0 | 0 | 0 | 1 | 0 | 0 | 0 | 0 | 0 | 0 | 0 | 0 | 0 | 450 | 462 | Carbamidomethylation | C1:Carbamidomethylation:1000.00;C12:Carbamidomethylation:1000.00 | PEAKS DB |
| R.EVTVDVGKESGATTNIFFKPPC(+57.02)VSVYR.C | Y | 47.16 | 2999.5061 | 27 | -1.2 | 750.8829 | 4 | 70.61 | 7 | F7:41039 | BuCaTN01_F7a.raw | | | | | | | 1.5942E5 | | | | | | | | | | 1 | 0 | 0 | 0 | 0 | 0 | 0 | 1 | 0 | 0 | 0 | 0 | 0 | 0 | 0 | 0 | 0 | 189 | 215 | Carbamidomethylation | C22:Carbamidomethylation:1000.00 | PEAKS DB |
| **total 2 peptides** | | | | | | | | | | | | | | | | | | | | | | | | | | | | | | | | | | | | | | | | | | | | | | | | | |

**Best Unique PSM (Scan F7:26144, m/z=836.3251, z=2, RT=52.61, ppm=0.9):**

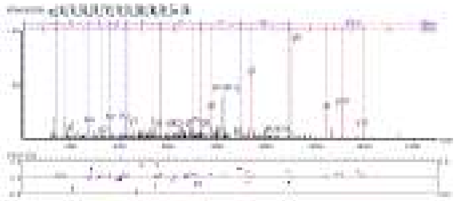

## ETE60014.1

back to list

| Protein Coverage | Supporting Peptides | Best Unique PSM |

**Protein Coverage:**

```
  1 MHLLGLFSLG CYLAAAAVVL LVAREPAIAA AYESGQGYYE EEPDLGEAKN YANKELEEQL RSVSSVDELM TVLYPEYWKM
 81 FKCQLRKGGW PHREQSNFDG RTADSNPIKF AAAHYNTEIL KSIDNEWRKT QCMPREVTVD VGKESGATTN IFFKPPCVSV
161 YRCGGCCNSE GLQCMNISTS YVSKTLFEIT VPLSHGPKPV TISFANHTSC RCMSKLDVYR QVHSIIRRSL PVIQAQCQIT
241 NKTCPKSHIW NNHLCRCLAQ HEFGFSSYPP DTDTAEGYHD ICGSNKELDE ETCQCVCKGG VTPSSCGIHK ELDRTSCQCT
321 CKNKLLPSSC GLNKEFDEEK CQCVCKRTCP KHQPLNPTKC ICECVESPNK CFLKGERFHH QTCSCYRRPC TVRKKRCDSG
401 FYYSEEVCRC VPTYWKLPLI N
```

C Carbamidomethylation (+57.02)

**Supporting Peptides:**

| Peptide | Uniq | -10lgP | Mass | Length | ppm | m/z | z | RT | Fraction | Scan | Source File | Area F1 | Area F2a | Area F3a | Area F4 | Area F5 | Area F6a | Area F7a | Area F7b | Area F8 | Area F9a | Area Sample 1F10a | Area F10b | Area F11a | Area F11b | Area F12a | Area F13 | #Feature | #Feature F1 | #Feature F2a | #Feature F3a | #Feature F4 | #Feature F5 | #Feature F6a | #Feature F7a | #Feature F7b | #Feature F8 | #Feature F9a | #Feature Sample 1F10a | #Feature F10b | #Feature F11a | #Feature F11b | #Feature F12a | #Feature F13 | Start | End | PTM | AScore | Found By |
|---|---|---|---|---|---|---|---|---|---|---|---|---|---|---|---|---|---|---|---|---|---|---|---|---|---|---|---|---|---|---|---|---|---|---|---|---|---|---|---|---|---|---|---|---|---|---|---|---|---|
| R.C(+57.02)DSGFYYSEEVC(+57.02)R.C | Y | 64.83 | 1670.6340 | 13 | 0.9 | 836.3251 | 2 | 52.61 | 7 | F7:26144 | BuCaTN01_F7a.raw | | | | | | | 3.5029E5 | | | | | | | | | | 1 | 0 | 0 | 0 | 0 | 0 | 0 | 1 | 0 | 0 | 0 | 0 | 0 | 0 | 0 | 0 | 0 | 397 | 409 | Carbamidomethylation | C1:Carbamidomethylation:1000.00;C12:Carbamidomethylation:1000.00 | PEAKS DB |
| R.EVTVDVGKESGATTNIFFKPPC(+57.02)VSVYR.C | Y | 47.16 | 2999.5061 | 27 | -1.2 | 750.8829 | 4 | 70.61 | 7 | F7:41039 | BuCaTN01_F7a.raw | | | | | | | 1.5942E5 | | | | | | | | | | 1 | 0 | 0 | 0 | 0 | 0 | 0 | 1 | 0 | 0 | 0 | 0 | 0 | 0 | 0 | 0 | 0 | 136 | 162 | Carbamidomethylation | C22:Carbamidomethylation:1000.00 | PEAKS DB |
| **total 2 peptides** | | | | | | | | | | | | | | | | | | | | | | | | | | | | | | | | | | | | | | | | | | | | | | | | | |

**Best Unique PSM (Scan F7:26144, m/z=836.3251, z=2, RT=52.61, ppm=0.9):**

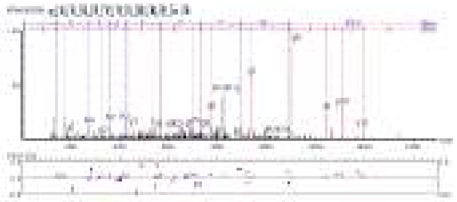

## XP_026555459.1

back to list

| Protein Coverage | Supporting Peptides | Best Unique PSM |

**Protein Coverage:**

```
  1 MHLLGLFSLG CYLAAAAVVL LVAREPAIAA AYESGQGYYE EEPDLGEAKN YANKELEEQL RSVSSVDELM TVLYPEYWKM
 81 FKCQLRKGGW PHREQSSFDG RTRDSNPIKF AAAHYNTEIL KSIDNEWRKT QCMPREVTVD VGKESGATTN IFFKPPCVSV
161 YRCGGCCNSE GLQCMNISTS YVSKTLFEIT VPLSHGPKPV TISFANHTSC RCMSKLDVYR QVHSIIRRSL PVIQAQCQIT
241 NKTCPKSHIW NNHLCRCLAQ HDFSFSSYPP DTDTAEGFHD ICGSNKELDE ETCQCVCKGG VTPSSCGIHK ELDRTSCQCT
321 CKNKLLPSSC GLNKEFDEEK CQCVCKRTCP KHQPLNPTKC ICECVESPNK CFLKGERFHH QTCSCYRRPC TVRKKRCDSG
401 FYYSEEVCRC VPTYWKLPLI N
```

C Carbamidomethylation (+57.02)

**Supporting Peptides:**

| Peptide | Uniq | -10lgP | Mass | Length | ppm | m/z | z | RT | Fraction | Scan | Source File | Area F1 | Area F2a | Area F3a | Area F4 | Area F5 | Area F6a | Area F7a | Area F7b | Area F8 | Area F9a | Area Sample 1F10a | Area F10b | Area F11a | Area F11b | Area F12a | Area F13 | #Feature | #Feature F1 | #Feature F2a | #Feature F3a | #Feature F4 | #Feature F5 | #Feature F6a | #Feature F7a | #Feature F7b | #Feature F8 | #Feature F9a | #Feature Sample 1F10a | #Feature F10b | #Feature F11a | #Feature F11b | #Feature F12a | #Feature F13 | Start | End | PTM | AScore | Found By |
|---|---|---|---|---|---|---|---|---|---|---|---|---|---|---|---|---|---|---|---|---|---|---|---|---|---|---|---|---|---|---|---|---|---|---|---|---|---|---|---|---|---|---|---|---|---|---|---|---|---|
| R.C(+57.02)DSGFYYSEEVC(+57.02)R.C | Y | 64.83 | 1670.6340 | 13 | 0.9 | 836.3251 | 2 | 52.61 | 7 | F7:26144 | BuCaTN01_F7a.raw | | | | | | | 3.5029E5 | | | | | | | | | | 1 | 0 | 0 | 0 | 0 | 0 | 0 | 1 | 0 | 0 | 0 | 0 | 0 | 0 | 0 | 0 | 0 | 397 | 409 | Carbamidomethylation | C1:Carbamidomethylation:1000.00;C12:Carbamidomethylation:1000.00 | PEAKS DB |
| R.EVTVDVGKESGATTNIFFKPPC(+57.02)VSVYR.C | Y | 47.16 | 2999.5061 | 27 | -1.2 | 750.8829 | 4 | 70.61 | 7 | F7:41039 | BuCaTN01_F7a.raw | | | | | | | 1.5942E5 | | | | | | | | | | 1 | 0 | 0 | 0 | 0 | 0 | 0 | 1 | 0 | 0 | 0 | 0 | 0 | 0 | 0 | 0 | 0 | 136 | 162 | Carbamidomethylation | C22:Carbamidomethylation:1000.00 | PEAKS DB |
| **total 2 peptides** | | | | | | | | | | | | | | | | | | | | | | | | | | | | | | | | | | | | | | | | | | | | | | | | | |

**Best Unique PSM (Scan F7:26144, m/z=836.3251, z=2, RT=52.61, ppm=0.9):**

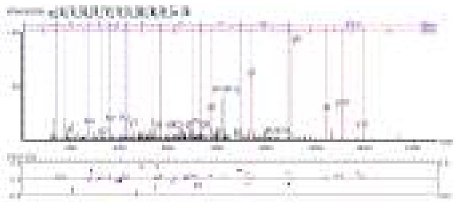

## LAB17920.1

back to list

| Protein Coverage | Supporting Peptides | Best Unique PSM |

**Protein Coverage:**

```
  1 MPREVTVDVG KESGATTNIF FKPPCVSVYR CGGCCNSEGL QCMNISTSYV SKTLFEITVP LSHGPKPVTI SFANHTSCRC
 81 MSKLDVYRQV HSIIRRSLPV IQAQCQITNK TCPKSHIWNN HLCRCLAQQD FSFSSYPPDT DTAEGYHDIC GSNKELDEET
161 CQCVCKGGVT PSSCGIHKEL DRTSCQCTCK NKLLPSSCGL NKEFDEEKCQ CVCKRTCPKH QPLNPTKCIC ECVESPNKCF
241 LKGERFHHQT CSCYRRPCTV RKKRCDSGFY YSEEVCRCVP TYWKLPLIN
```

C Carbamidomethylation (+57.02)

**Supporting Peptides:**

| Peptide | Uniq | -10lgP | Mass | Length | ppm | m/z | z | RT | Fraction | Scan | Source File | Area F1 | Area F2a | Area F3a | Area F4 | Area F5 | Area F6a | Area F7a | Area F7b | Area F8 | Area F9a | Area Sample 1F10a | Area F10b | Area F11a | Area F11b | Area F12a | Area F13 | #Feature | #Feature F1 | #Feature F2a | #Feature F3a | #Feature F4 | #Feature F5 | #Feature F6a | #Feature F7a | #Feature F7b | #Feature F8 | #Feature F9a | #Feature Sample 1F10a | #Feature F10b | #Feature F11a | #Feature F11b | #Feature F12a | #Feature F13 | Start | End | PTM | AScore | Found By |
|---|---|---|---|---|---|---|---|---|---|---|---|---|---|---|---|---|---|---|---|---|---|---|---|---|---|---|---|---|---|---|---|---|---|---|---|---|---|---|---|---|---|---|---|---|---|---|---|---|---|
| R.C(+57.02)DSGFYYSEEVC(+57.02)R.C | Y | 64.83 | 1670.6340 | 13 | 0.9 | 836.3251 | 2 | 52.61 | 7 | F7:26144 | BuCaTN01_F7a.raw | | | | | | | 3.5029E5 | | | | | | | | | | 1 | 0 | 0 | 0 | 0 | 0 | 0 | 1 | 0 | 0 | 0 | 0 | 0 | 0 | 0 | 0 | 0 | 265 | 277 | Carbamidomethylation | C1:Carbamidomethylation:1000.00;C12:Carbamidomethylation:1000.00 | PEAKS DB |
| R.EVTVDVGKESGATTNIFFKPPC(+57.02)VSVYR.C | Y | 47.16 | 2999.5061 | 27 | -1.2 | 750.8829 | 4 | 70.61 | 7 | F7:41039 | BuCaTN01_F7a.raw | | | | | | | 1.5942E5 | | | | | | | | | | 1 | 0 | 0 | 0 | 0 | 0 | 0 | 1 | 0 | 0 | 0 | 0 | 0 | 0 | 0 | 0 | 0 | 4 | 30 | Carbamidomethylation | C22:Carbamidomethylation:1000.00 | PEAKS DB |
| total 2 peptides | | | | | | | | | | | | | | | | | | | | | | | | | | | | | | | | | | | | | | | | | | | | | | | | | |

**Best Unique PSM (Scan F7:26144, m/z=836.3251, z=2, RT=52.61, ppm=0.9):**

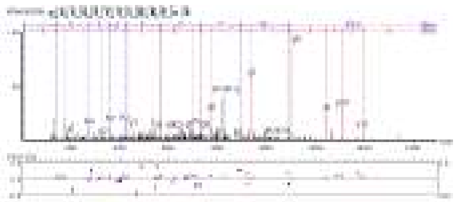

## KAG8131487.1

back to list

| Protein Coverage | Supporting Peptides | Best Unique PSM |

**Protein Coverage:**

```
  1 MSSGRLLLLL GLLTLWAELT PVSGRPRFCE LAPSAGSCFG FVSSYYYNRY SNTCHSFTYS GCGGNANRMT NEETHPEWIQ
 81 LDPKTLLLPW TTLETLLPNP TLGSFLFLCN KALVPAALLV FGVSFGPFVG FPAPL
```

C Carbamidomethylation (+57.02)

**Supporting Peptides:**

| Peptide | Uniq | -10lgP | Mass | Length | ppm | m/z | z | RT | Fraction | Scan | Source File | Area F1 | Area F2a | Area F3a | Area F4 | Area F5 | Area F6a | Area F7a | Area F7b | Area F8 | Area F9a | Area Sample 1F10a | Area F10b | Area F11a | Area F11b | Area F12a | Area F13 | #Feature | #Feature F1 | #Feature F2a | #Feature F3a | #Feature F4 | #Feature F5 | #Feature F6a | #Feature F7a | #Feature F7b | #Feature F8 | #Feature F9a | #Feature Sample 1F10a | #Feature F10b | #Feature F11a | #Feature F11b | #Feature F12a | #Feature F13 | Start | End | PTM | AScore | Found By |
|---|---|---|---|---|---|---|---|---|---|---|---|---|---|---|---|---|---|---|---|---|---|---|---|---|---|---|---|---|---|---|---|---|---|---|---|---|---|---|---|---|---|---|---|---|---|---|---|---|---|
| R.YSNTC(+57.02)HSFTYSGC(+57.02)GGNANR.M | Y | 66.33 | 2151.8486 | 19 | 0.9 | 718.2908 | 3 | 36.24 | 6 | F6:16305 | BuCaTN01_F6a.raw | | | | | | 0 | | | | | | | | | | | 0 | 0 | 0 | 0 | 0 | 0 | 0 | 0 | 0 | 0 | 0 | 0 | 0 | 0 | 0 | 0 | 0 | 50 | 68 | Carbamidomethylation | C5:Carbamidomethylation:1000.00;C13:Carbamidomethylation:1000.00 | PEAKS DB |
| total 1 peptides | | | | | | | | | | | | | | | | | | | | | | | | | | | | | | | | | | | | | | | | | | | | | | | | | |

**Best Unique PSM (Scan F6:16305, m/z=718.2908, z=3, RT=36.24, ppm=0.9):**

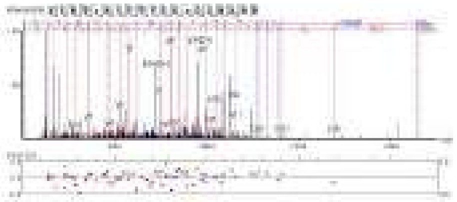

## ETE70041.1

back to list

| Protein Coverage | Supporting Peptides | Best Unique PSM |

**Protein Coverage:**

```
  1 MSSQKPEDGI QFNKYEKNIE SLRGTIITLM EIHGDTCSSA SPPLRPTSVT ELRDFFLVNN VTYLALIFDK DDSFLSREVA
 81 LDMSQFENIA VRRVLQSNEE LVKQFNITTF PSGLLVTNNG SCRSIPGLED WRSQYTNFLR NLPGVFRRDV FIPTVSPTAQ
161 STTAFTIWKV ADRRKIYMAD LESAVLYSLR IEAAVFPNLE GERLSALKDF VSVLVQHFAG RLVVKNFLYN LDLWLRPKTI
241 VLQSEWEEAL KNKKEFPNAT LPETPHWVGC QGSKPQFRGF FCGLWTLFHH LTVQASCQKE SASSGIPEVL PTMRGYIRNF
321 FGCRMCAEHF EGMAAESMHE VKNRDEAVLW LWSRHNRVNA RLAGTATDDP MFPKIQWPPK SLCSSCHYTS TDSSLQDGKK
401 FMWDENRVLM FLKWHFSEKN IYMDSPLEHQ TRNGRNVAQE NYEAEWKLEG EGGGEENDQG NQKEPDEDKK TEQLETGKVE
481 KKKFDLGKPV SPELHKPSIV KMSTKAKEMV EDIVDLDTFS EQHFKSKALK PARQSSIRER RTKRDTRLFL MENEGHHSLD
561 YIRESLRHKN LGAKQFSGIQ VEKENTLRKN QWIYILGMGF SRLDVSLCVL LYLLSSVCLL GMYIFFRMRM SYRKARLGYS
641 LT
```

**Supporting Peptides:**

| Peptide | Uniq | -10lgP | Mass | Length | ppm | m/z | z | RT | Fraction | Scan | Source File | Area F1 | Area F2a | Area F3a | Area F4 | Area F5 | Area F6a | Area F7a | Area F7b | Area F8 | Area F9a | Area Sample 1F10a | Area F10b | Area F11a | Area F11b | Area F12a | Area F13 | #Feature | #Feature F1 | #Feature F2a | #Feature F3a | #Feature F4 | #Feature F5 | #Feature F6a | #Feature F7a | #Feature F7b | #Feature F8 | #Feature F9a | #Feature Sample 1F10a | #Feature F10b | #Feature F11a | #Feature F11b | #Feature F12a | #Feature F13 | Start | End | PTM | AScore | Found By |
|---|---|---|---|---|---|---|---|---|---|---|---|---|---|---|---|---|---|---|---|---|---|---|---|---|---|---|---|---|---|---|---|---|---|---|---|---|---|---|---|---|---|---|---|---|---|---|---|---|---|
| R.RVLQSNEELVK.Q | Y | 54.80 | 1313.7302 | 11 | 0.2 | 438.9174 | 3 | 34.77 | 16 | F16:12614 | BuCaTN01_F13.raw | | | | | | | | | | | | | | | | 2.3744E4 | 1 | 0 | 0 | 0 | 0 | 0 | 0 | 0 | 0 | 0 | 0 | 0 | 0 | 0 | 0 | 0 | 1 | 93 | 103 | | | PEAKS DB |
| total 1 peptides | | | | | | | | | | | | | | | | | | | | | | | | | | | | | | | | | | | | | | | | | | | | | | | | | |

**Best Unique PSM (Scan F16:12614, m/z=438.9174, z=3, RT=34.77, ppm=0.2):**

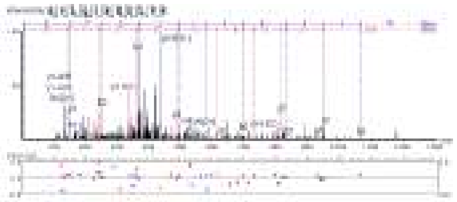

## XP_026559083.1
back to list

| Protein Coverage | Supporting Peptides | Best Unique PSM |

**Protein Coverage:**

```
  1 MPRWLSGAGS RHLMPPARLL RAVAFLLLVQ GPGAHGLGLY SPDDPLVELD TDTLERRIFN SSGVWVVEFY ASWCGHCKAF
 81 APLWKGLAND LKDWRPAVML GAIDCADFSN KNICYKFGIK SYPTVKFFKV SSQKPEDGVQ FNKYEKTIES LRGTIITLME
161 IHGDTWPSAS PPLRPTSVTE LRDFFLVNNV TYLALIFEKD DSFLSREVAL DMSQFENIAV RRVLQSNEEL VKQFNITTFP
241 SGLLVTNNGS CRSIPGLEDW RSQYTNFLRN LPGVFRRDVF IPTVSPTAQS TTAFTIWKVA DKRKIYMADI ESAVLYSLRI
321 EAAVFPSLEG ERLSALKDFV SVLVQHFAGR LVVKNFLYNL DLWLRPKTIV LQSEWEEALK NKKEFPNATL PERPHWVGCQ
401 GSKPHFRGFP CGLWTLFHHL TVQASCQKES ASSGIPEVLP TMRGYIRNFF GCRMCAEHFE GMAAESMHEV KNRDEAVLWL
481 WSRHNRVNAR LAGTATGDPM FPKIQWPPKS LCSSCHYTPT DSSLQDGNKF MWDENRVLMF LKWHFSEKNI YMDSPLEHQT
561 RSGRNVAQQN YEAEWKLEEG GRREENDQGN QKEPDEDKKT EQLETGKVEK KKFDLGKPGS PELHKPSIVK MSTKAKEMVE
641 DIVDLDTFSE QHFKSKALKL ARQSSIRERR TKRDTRLFLM ENEGRHSLDY IRESLRHKNL GAKRFSGIQV EKENTLRKNR
721 WIYILGMGFS RLDVSLCVLL YLLSSVCLLG MYTFFRMRMS YRKARLGYSL T
```

**Supporting Peptides:**

| Peptide | Uniq | -10lgP | Mass | Length | ppm | m/z | z | RT | Fraction | Scan | Source File | Area F1 | Area F2a | Area F3a | Area F4 | Area F5 | Area F6a | Area F7a | Area F7b | Area F8 | Area F9a | Area Sample 1F10a | Area F10b | Area F11a | Area F11b | Area F12a | Area F13 | #Feature | #Feature F1 | #Feature F2a | #Feature F3a | #Feature F4 | #Feature F5 | #Feature F6a | #Feature F7a | #Feature F7b | #Feature F8 | #Feature F9a | #Feature Sample 1F10a | #Feature F10b | #Feature F11a | #Feature F11b | #Feature F12a | #Feature F13 | Start | End | PTM | AScore | Found By |
|---|---|---|---|---|---|---|---|---|---|---|---|---|---|---|---|---|---|---|---|---|---|---|---|---|---|---|---|---|---|---|---|---|---|---|---|---|---|---|---|---|---|---|---|---|---|---|---|---|---|
| R.RVLQSNEELVK.Q | Y | 54.80 | 1313.7302 | 11 | 0.2 | 438.9174 | 3 | 34.77 | 16 | F16:12614 | BuCaTN01_F13.raw | | | | | | | | | | | | | | | | 2.3744E4 | 1 | 0 | 0 | 0 | 0 | 0 | 0 | 0 | 0 | 0 | 0 | 0 | 0 | 0 | 0 | 0 | 1 | 222 | 232 | | | PEAKS DB |
| total 1 peptides | | | | | | | | | | | | | | | | | | | | | | | | | | | | | | | | | | | | | | | | | | | | | | | | | |

**Best Unique PSM (Scan F16:12614, m/z=438.9174, z=3, RT=34.77, ppm=0.2):**

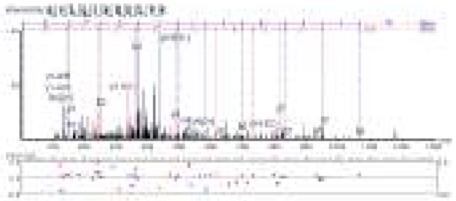

## JAG68843.1
back to list

| Protein Coverage | Supporting Peptides | Best Unique PSM |

**Protein Coverage:**

```
  1 MEEEIAALVI DNGSGMCKAG FAGDDAPRAV FPSIVGRPRH QGVMVGMGQK DSYVGDEAQS KRGILTLKYP IEHGIVTNWD
 81 DMEKIWHHTF YNELRVAPEE HPVLLTEAPL NPKANREKMT QIMFETFNTP AMYVAIQAVL SLYASGRTTG IVMDSGDGVT
161 HTVPIYEGYA LPHAILRLDL AGRDLTDYLM KILTERGYSF TTTAEREIVR DIKEKLCYVA LDFEQEMATA ASSSSLEKSY
241 ELPDGQVITI GNERFRCPEA LFQPSFLGME SCGIHETTFN SIMKCDVDIR KDLYANTVLS GGTTMYPGIA DRMQKEITAL
321 APSTMKIKII APPERKYSVW IGGSILASLS TFQQMWISKQ EYDESGPSIV HRKCF
```

**Supporting Peptides:**

| Peptide | Uniq | -10lgP | Mass | Length | ppm | m/z | z | RT | Fraction | Scan | Source File | Area F1 | Area F2a | Area F3a | Area F4 | Area F5 | Area F6a | Area F7a | Area F7b | Area F8 | Area F9a | Area Sample 1F10a | Area F10b | Area F11a | Area F11b | Area F12a | Area F13 | #Feature | #Feature F1 | #Feature F2a | #Feature F3a | #Feature F4 | #Feature F5 | #Feature F6a | #Feature F7a | #Feature F7b | #Feature F8 | #Feature F9a | #Feature Sample 1F10a | #Feature F10b | #Feature F11a | #Feature F11b | #Feature F12a | #Feature F13 | Start | End | PTM | AScore | Found By |
|---|---|---|---|---|---|---|---|---|---|---|---|---|---|---|---|---|---|---|---|---|---|---|---|---|---|---|---|---|---|---|---|---|---|---|---|---|---|---|---|---|---|---|---|---|---|---|---|---|---|
| R.KYSVWIGGSILASLSTFQQMWISK.Q | Y | 57.10 | 2729.4250 | 24 | 0.3 | 910.8159 | 3 | 102.40 | 1 | F1:49862 | BuCaTN01_F1.raw | 1.0126E5 | | | | | | | | | | | | | | | | 1 | 1 | 0 | 0 | 0 | 0 | 0 | 0 | 0 | 0 | 0 | 0 | 0 | 0 | 0 | 0 | 0 | 336 | 359 | | | PEAKS DB |
| K.DLYANTVLSGGTTMYPGIADR.M | Y | 50.22 | 2214.0627 | 21 | 0.8 | 1108.0396 | 2 | 73.08 | 1 | F1:33231 | BuCaTN01_F1.raw | 4.7954E4 | | | | | | | | | | | | | | | | 1 | 1 | 0 | 0 | 0 | 0 | 0 | 0 | 0 | 0 | 0 | 0 | 0 | 0 | 0 | 0 | 0 | 292 | 312 | | | PEAKS DB |
| total 2 peptides | | | | | | | | | | | | | | | | | | | | | | | | | | | | | | | | | | | | | | | | | | | | | | | | | |

**Best Unique PSM (Scan F1:49862, m/z=910.8159, z=3, RT=102.40, ppm=0.3):**

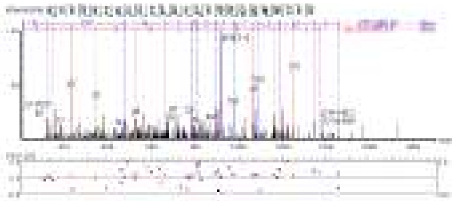

## XP_013921350.1
back to list

| Protein Coverage | Supporting Peptides | Best Unique PSM |

**Protein Coverage:**

```
  1 MEEEIAALVI DNGSGMCKAG FAGDDAPRAV FPSIVGRPRH QGVMVGMGQK DSYVGDEAQS KRGILTLKYP IEHGIVTNWD
 81 DMEKIWHHTF YNELRVAPEE HPVLLTEAPL NPKANREKMT QIMFETFNTP AMYVAIQAVL SLYASGRTTG IVMDSGDGVT
161 HTVPIYEGYA LPHAILRLDL AGRDLTDYLM KILTERGYSF TTTAEREIVR DIKEKLCYVA LDFEQEMATA ASSSSLEKSY
241 ELPDGQVITI GNERFRCPEA LFQPSFLGME SCGIHETTFN SIMKCDVDIR KDLYANTVLS GGTTMYPGIA DRMQKEITAL
321 APSTMKIKII APPERKYSVW IGGSILASLS TFQQMWISKQ EYDESGPSIV HRKCF
```

**Supporting Peptides:**

| Peptide | Uniq | -10lgP | Mass | Length | ppm | m/z | z | RT | Fraction | Scan | Source File | Area F1 | Area F2a | Area F3a | Area F4 | Area F5 | Area F6a | Area F7a | Area F7b | Area F8 | Area F9a | Area Sample 1F10a | Area F10b | Area F11a | Area F11b | Area F12a | Area F13 | #Feature | #Feature F1 | #Feature F2a | #Feature F3a | #Feature F4 | #Feature F5 | #Feature F6a | #Feature F7a | #Feature F7b | #Feature F8 | #Feature F9a | #Feature Sample 1F10a | #Feature F10b | #Feature F11a | #Feature F11b | #Feature F12a | #Feature F13 | Start | End | PTM | AScore | Found By |
|---|---|---|---|---|---|---|---|---|---|---|---|---|---|---|---|---|---|---|---|---|---|---|---|---|---|---|---|---|---|---|---|---|---|---|---|---|---|---|---|---|---|---|---|---|---|---|---|---|---|
| R.KYSVWIGGSILASLSTFQQMWISK.Q | Y | 57.10 | 2729.4250 | 24 | 0.3 | 910.8159 | 3 | 102.40 | 1 | F1:49862 | BuCaTN01_F1.raw | 1.0126E5 | | | | | | | | | | | | | | | | 1 | 1 | 0 | 0 | 0 | 0 | 0 | 0 | 0 | 0 | 0 | 0 | 0 | 0 | 0 | 0 | 0 | 336 | 359 | | | PEAKS DB |
| K.DLYANTVLSGGTTMYPGIADR.M | Y | 50.22 | 2214.0627 | 21 | 0.8 | 1108.0396 | 2 | 73.08 | 1 | F1:33231 | BuCaTN01_F1.raw | 4.7954E4 | | | | | | | | | | | | | | | | 1 | 1 | 0 | 0 | 0 | 0 | 0 | 0 | 0 | 0 | 0 | 0 | 0 | 0 | 0 | 0 | 0 | 292 | 312 | | | PEAKS DB |
| total 2 peptides | | | | | | | | | | | | | | | | | | | | | | | | | | | | | | | | | | | | | | | | | | | | | | | | | |

**Best Unique PSM (Scan F1:49862, m/z=910.8159, z=3, RT=102.40, ppm=0.3):**

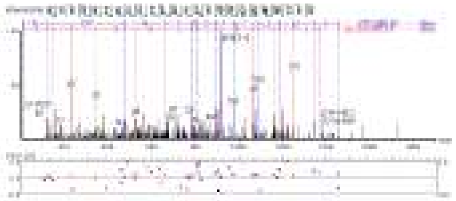

## JAV51372.1
back to list

| Protein Coverage | Supporting Peptides | Best Unique PSM |

**Protein Coverage:**

1 MEEEIAALVI DNGSGMCKAG FAGDDAPRAV FPSIVGRPRH QGVMVGMGQK DSYVGDEAQS KRGILTLKYP IEHGIVTNWD
81 DMEKIWHHTF YNELRVAPEE HPVLLTEAPL NPKANREKMT QIMFETFNTP AMYVAIQAVL SLYASGRTTG IVMDSGDGVT
161 HTVPIYEGYA LPHAILRLDL AGRDLTDYLM KILTERGYSF TTTAEREIVR DIKEKLCYVA LDFEQEMATA ASSSSLEKSY
241 ELPDGQVITI GNERFRCPEA LFQPSFLGME SCGIHETTFN SIMKCDVDIR K**DLYANTVLS GGTTMYPGIA DR**MQKEITAL

321 APSTMKIKII APPER**KYSVW IGGSILASLS TFQQMWISK**Q EYDESGPSIV HRKCF

**Supporting Peptides:**

| Peptide | Uniq | -10lgP | Mass | Length | ppm | m/z | z | RT | Fraction | Scan | Source File | Area F1 | Area F2a | Area F3a | Area F4 | Area F5 | Area F6a | Area F7a | Area F7b | Area F8 | Area F9a | Area Sample 1F10a | Area F10b | Area F11a | Area F11b | Area F12a | Area F13 | #Feature | #Feature F1 | #Feature F2a | #Feature F3a | #Feature F4 | #Feature F5 | #Feature F6a | #Feature F7a | #Feature F7b | #Feature F8 | #Feature F9a | #Feature Sample 1F10a | #Feature F10b | #Feature F11a | #Feature F11b | #Feature F12a | #Feature F13 | Start | End | PTM | AScore | Found By |
|---|---|---|---|---|---|---|---|---|---|---|---|---|---|---|---|---|---|---|---|---|---|---|---|---|---|---|---|---|---|---|---|---|---|---|---|---|---|---|---|---|---|---|---|---|---|---|---|---|---|
| R.KYSVWIGGSILASLSTFQQMWISK.Q | Y | 57.10 | 2729.4250 | 24 | 0.3 | 910.8159 | 3 | 102.40 | 1 | F1:49862 | BuCaTN01_F1.raw | 1.0126E5 | | | | | | | | | | | | | | | | 1 | 1 | 0 | 0 | 0 | 0 | 0 | 0 | 0 | 0 | 0 | 0 | 0 | 0 | 0 | 0 | 0 | 336 | 359 | | | PEAKS DB |
| K.DLYANTVLSGGTTMYPGIADR.M | Y | 50.22 | 2214.0627 | 21 | 0.8 | 1108.0396 | 2 | 73.08 | 1 | F1:33231 | BuCaTN01_F1.raw | 4.7954E4 | | | | | | | | | | | | | | | | 1 | 1 | 0 | 0 | 0 | 0 | 0 | 0 | 0 | 0 | 0 | 0 | 0 | 0 | 0 | 0 | 0 | 292 | 312 | | | PEAKS DB |
| total 2 peptides | | | | | | | | | | | | | | | | | | | | | | | | | | | | | | | | | | | | | | | | | | | | | | | | | |

**Best Unique PSM (Scan F1:49862, m/z=910.8159, z=3, RT=102.40, ppm=0.3):**

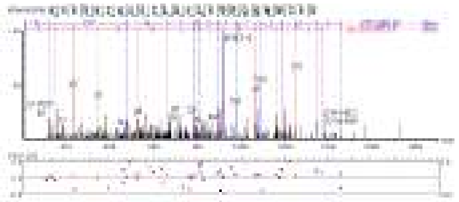

## JAB54395.1

back to list

| Protein Coverage | Supporting Peptides | Best Unique PSM |

**Protein Coverage:**

1 MEEEIAALVI DNGSGMCKAG FAGDDAPRAV FPSIVGRPRH QGVMVGMGQK DSYVGDEAQS KRGILTLKYP IEHGIVTNWD
81 DMEKIWHHTF YNELRVAPEE HPVLLTEAPL NPKANREKMT QIMFETFNTP AMYVAIQAVL SLYASGRTTG IVMDSGDGVT
161 HTVPIYEGYA LPHAILRLDL AGRDLTDYLM KILTERGYSF TTTAEREIVR DIKEKLCYVA LDFEQEMATA ASSSSLEKSY
241 ELPDGQVITI GNERFRCPEA LFQPSFLGME SCGIHETTFN SIMKCDVDIR K**DLYANTVLS GGTTMYPGIA DR**MQKEITAL

321 APSTMKIKII APPER**KYSVW IGGSILASLS TFQQMWISK**Q EYDESGPSIV HRKCF

**Supporting Peptides:**

| Peptide | Uniq | -10lgP | Mass | Length | ppm | m/z | z | RT | Fraction | Scan | Source File | Area F1 | Area F2a | Area F3a | Area F4 | Area F5 | Area F6a | Area F7a | Area F7b | Area F8 | Area F9a | Area Sample 1F10a | Area F10b | Area F11a | Area F11b | Area F12a | Area F13 | #Feature | #Feature F1 | #Feature F2a | #Feature F3a | #Feature F4 | #Feature F5 | #Feature F6a | #Feature F7a | #Feature F7b | #Feature F8 | #Feature F9a | #Feature Sample 1F10a | #Feature F10b | #Feature F11a | #Feature F11b | #Feature F12a | #Feature F13 | Start | End | PTM | AScore | Found By |
|---|---|---|---|---|---|---|---|---|---|---|---|---|---|---|---|---|---|---|---|---|---|---|---|---|---|---|---|---|---|---|---|---|---|---|---|---|---|---|---|---|---|---|---|---|---|---|---|---|---|
| R.KYSVWIGGSILASLSTFQQMWISK.Q | Y | 57.10 | 2729.4250 | 24 | 0.3 | 910.8159 | 3 | 102.40 | 1 | F1:49862 | BuCaTN01_F1.raw | 1.0126E5 | | | | | | | | | | | | | | | | 1 | 1 | 0 | 0 | 0 | 0 | 0 | 0 | 0 | 0 | 0 | 0 | 0 | 0 | 0 | 0 | 0 | 336 | 359 | | | PEAKS DB |
| K.DLYANTVLSGGTTMYPGIADR.M | Y | 50.22 | 2214.0627 | 21 | 0.8 | 1108.0396 | 2 | 73.08 | 1 | F1:33231 | BuCaTN01_F1.raw | 4.7954E4 | | | | | | | | | | | | | | | | 1 | 1 | 0 | 0 | 0 | 0 | 0 | 0 | 0 | 0 | 0 | 0 | 0 | 0 | 0 | 0 | 0 | 292 | 312 | | | PEAKS DB |
| total 2 peptides | | | | | | | | | | | | | | | | | | | | | | | | | | | | | | | | | | | | | | | | | | | | | | | | | |

**Best Unique PSM (Scan F1:49862, m/z=910.8159, z=3, RT=102.40, ppm=0.3):**

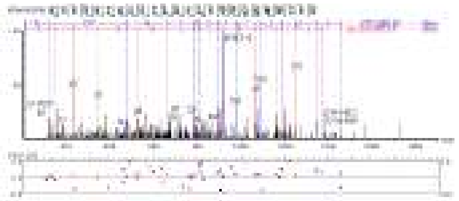

## JAC96363.1

back to list

| Protein Coverage | Supporting Peptides | Best Unique PSM |

**Protein Coverage:**

1 MEEEIAALVI DNGSGMCKAG FAGDDAPRAV FPSIVGRPRH QGVMVGMGQK DSYVGDEAQS KRGILTLKYP IEHGIVTNWD
81 DMEKIWHHTF YNELRVAPEE HPVLLTEAPL NPKANREKMT QIMFETFNTP AMYVAIQAVL SLYASGRTTG IVMDSGDGVT
161 HTVPIYEGYA LPHAILRLDL AGRDLTDYLM KILTERGYSF TTTAEREIVR DIKEKLCYVA LDFEQEMATA ASSSSLEKSY
241 ELPDGQVITI GNERFRCPEA LFQPSFLGME SCGIHETTFN SIMKCDVDIR K**DLYANTVLS GGTTMYPGIA DR**MQKEITAL

321 APSTMKIKII APPER**KYSVW IGGSILASLS TFQQMWISK**Q EYDESGPSIV HRKCF

**Supporting Peptides:**

| Peptide | Uniq | -10lgP | Mass | Length | ppm | m/z | z | RT | Fraction | Scan | Source File | Area F1 | Area F2a | Area F3a | Area F4 | Area F5 | Area F6a | Area F7a | Area F7b | Area F8 | Area F9a | Area Sample 1F10a | Area F10b | Area F11a | Area F11b | Area F12a | Area F13 | #Feature | #Feature F1 | #Feature F2a | #Feature F3a | #Feature F4 | #Feature F5 | #Feature F6a | #Feature F7a | #Feature F7b | #Feature F8 | #Feature F9a | #Feature Sample 1F10a | #Feature F10b | #Feature F11a | #Feature F11b | #Feature F12a | #Feature F13 | Start | End | PTM | AScore | Found By |
|---|---|---|---|---|---|---|---|---|---|---|---|---|---|---|---|---|---|---|---|---|---|---|---|---|---|---|---|---|---|---|---|---|---|---|---|---|---|---|---|---|---|---|---|---|---|---|---|---|---|
| R.KYSVWIGGSILASLSTFQQMWISK.Q | Y | 57.10 | 2729.4250 | 24 | 0.3 | 910.8159 | 3 | 102.40 | 1 | F1:49862 | BuCaTN01_F1.raw | 1.0126E5 | | | | | | | | | | | | | | | | 1 | 1 | 0 | 0 | 0 | 0 | 0 | 0 | 0 | 0 | 0 | 0 | 0 | 0 | 0 | 0 | 0 | 336 | 359 | | | PEAKS DB |
| K.DLYANTVLSGGTTMYPGIADR.M | Y | 50.22 | 2214.0627 | 21 | 0.8 | 1108.0396 | 2 | 73.08 | 1 | F1:33231 | BuCaTN01_F1.raw | 4.7954E4 | | | | | | | | | | | | | | | | 1 | 1 | 0 | 0 | 0 | 0 | 0 | 0 | 0 | 0 | 0 | 0 | 0 | 0 | 0 | 0 | 0 | 292 | 312 | | | PEAKS DB |
| total 2 peptides | | | | | | | | | | | | | | | | | | | | | | | | | | | | | | | | | | | | | | | | | | | | | | | | | |

**Best Unique PSM (Scan F1:49862, m/z=910.8159, z=3, RT=102.40, ppm=0.3):**

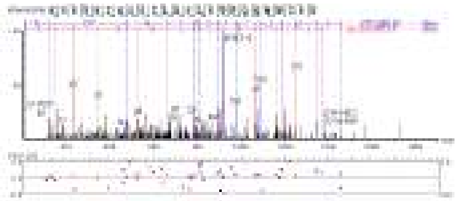

## JAA97900.1

back to list

| Protein Coverage | Supporting Peptides | Best Unique PSM |

**Protein Coverage:**

1 MEEEIAALVI DNGSGMCKAG FAGDDAPRAV FPSIVGRPRH QGVMVGMGQK DSYVGDEAQS KRGILTLKYP IEHGIVTNWD
81 DMEKIWHHTF YNELRVAPEE HPVLLTEAPL NPKANREKMT QIMFETFNTP AMYVAIQAVL SLYASGRTTG IVMDSGDGVT
161 HTVPIYEGYA LPHAILRLDL AGRDLTDYLM KILTERGYSF TTTAEREIVR DIKEKLCYVA LDFEQEMATA ASSSSLEKSY
241 ELPDGQVITI GNERFRCPEA LFQPSFLGME SCGIHETTFN SIMKCDVDIR K**DLYANTVLS GGTTMYPGIA DR**MQKEITAL

321 APSTMKIKII APPER**KYSVW IGGSILASLS TFQQMWISK**Q EYDESGPSIV HRKCF

**Supporting Peptides:**

| Peptide | Uniq | -10lgP | Mass | Length | ppm | m/z | z | RT | Fraction | Scan | Source File | Area F1 | Area F2a | Area F3a | Area F4 | Area F5 | Area F6a | Area F7a | Area F7b | Area F8 | Area F9a | Area Sample 1F10a | Area F10b | Area F11a | Area F11b | Area F12a | Area F13 | #Feature | #Feature F1 | #Feature F2a | #Feature F3a | #Feature F4 | #Feature F5 | #Feature F6a | #Feature F7a | #Feature F7b | #Feature F8 | #Feature F9a | #Feature Sample 1F10a | #Feature F10b | #Feature F11a | #Feature F11b | #Feature F12a | #Feature F13 | Start | End | PTM | AScore | Found By |
|---|---|---|---|---|---|---|---|---|---|---|---|---|---|---|---|---|---|---|---|---|---|---|---|---|---|---|---|---|---|---|---|---|---|---|---|---|---|---|---|---|---|---|---|---|---|---|---|---|---|
| R.KYSVWIGGSILASLSTFQQMWISK.Q | Y | 57.10 | 2729.4250 | 24 | 0.3 | 910.8159 | 3 | 102.40 | 1 | F1:49862 | BuCaTN01_F1.raw | 1.0126E5 | | | | | | | | | | | | | | | | 1 | 1 | 0 | 0 | 0 | 0 | 0 | 0 | 0 | 0 | 0 | 0 | 0 | 0 | 0 | 0 | 0 | 336 | 359 | | | PEAKS DB |
| K.DLYANTVLSGGTTMYPGIADR.M | Y | 50.22 | 2214.0627 | 21 | 0.8 | 1108.0396 | 2 | 73.08 | 1 | F1:33231 | BuCaTN01_F1.raw | 4.7954E4 | | | | | | | | | | | | | | | | 1 | 1 | 0 | 0 | 0 | 0 | 0 | 0 | 0 | 0 | 0 | 0 | 0 | 0 | 0 | 0 | 0 | 292 | 312 | | | PEAKS DB |

| |
|---|
| total 2 peptides |

Best Unique PSM (Scan F1:49862, m/z=910.8159, z=3, RT=102.40, ppm=0.3):

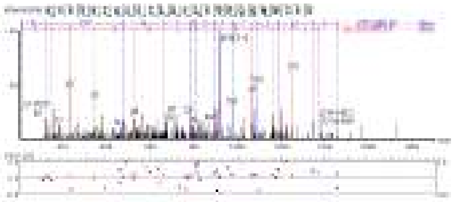

## LAA21099.1

back to list

| Protein Coverage | Supporting Peptides | Best Unique PSM |

Protein Coverage:

```
  1 MEEEIAALVI DNGSGMCKAG FAGDDAPRAV FPSIVGRPRH QGVMVGMGQK DSYVGDEAQS KRGILTLKYP IEHGIVTNWD
 81 DMEKIWHHTF YNELRVAPEE HPVLLTEAPL NPKANREKMT QIMFETFNTP AMYVAIQAVL SLYASGRTTG IVMDSGDGVT
161 HTVPIYEGYA LPHAILRLDL AGRDLTDYLM KILTERGYSF TTTAEREIVR DIKEKLCYVA LDFEQEMATA ASSSSLEKSY
241 ELPDGQVITI GNERFRCPEA LFQPSFLGME SCGIHETTFN SIMKCDVDIR KDLYANTVLS GGTTMYPGIA DRMQKEITAL

321 APSTMKIKII APPERKYSVW IGGSILASLS TFQQMWISKQ EYDESGPSIV HRKCF
```

Supporting Peptides:

| Peptide | Uniq | -10lgP | Mass | Length | ppm | m/z | z | RT | Fraction | Scan | Source File | Area F1 | Area F2a | Area F3a | Area F4 | Area F5 | Area F6a | Area F7a | Area F7b | Area F8 | Area F9a | Area Sample 1F10a | Area F10b | Area F11a | Area F11b | Area F12a | Area F13 | #Feature | #Feature F1 | #Feature F2a | #Feature F3a | #Feature F4 | #Feature F5 | #Feature F6a | #Feature F7a | #Feature F7b | #Feature F8 | #Feature F9a | #Feature Sample 1F10a | #Feature F10b | #Feature F11a | #Feature F11b | #Feature F12a | #Feature F13 | Start | End | PTM | AScore | Found By |
|---|---|---|---|---|---|---|---|---|---|---|---|---|---|---|---|---|---|---|---|---|---|---|---|---|---|---|---|---|---|---|---|---|---|---|---|---|---|---|---|---|---|---|---|---|---|---|---|---|---|
| R.KYSVWIGGSILASLSTFQQMWISK.Q | Y | 57.10 | 2729.4250 | 24 | 0.3 | 910.8159 | 3 | 102.40 | 1 | F1:49862 | BuCaTN01_F1.raw | 1.0126E5 | | | | | | | | | | | | | | | | 1 | 1 | 0 | 0 | 0 | 0 | 0 | 0 | 0 | 0 | 0 | 0 | 0 | 0 | 0 | 0 | 0 | 336 | 359 | | | PEAKS DB |
| K.DLYANTVLSGGTTMYPGIADR.M | Y | 50.22 | 2214.0627 | 21 | 0.8 | 1108.0396 | 2 | 73.08 | 1 | F1:33231 | BuCaTN01_F1.raw | 4.7954E4 | | | | | | | | | | | | | | | | 1 | 1 | 0 | 0 | 0 | 0 | 0 | 0 | 0 | 0 | 0 | 0 | 0 | 0 | 0 | 0 | 0 | 292 | 312 | | | PEAKS DB |
| total 2 peptides | | | | | | | | | | | | | | | | | | | | | | | | | | | | | | | | | | | | | | | | | | | | | | | | | |

Best Unique PSM (Scan F1:49862, m/z=910.8159, z=3, RT=102.40, ppm=0.3):

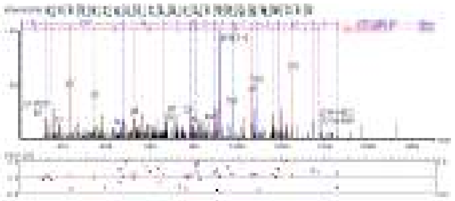

## XP_026531551.1

back to list

| Protein Coverage | Supporting Peptides | Best Unique PSM |

Protein Coverage:

```
  1 MEEEIAALVI DNGSGMCKAG FAGDDAPRAV FPSIVGRPRH QGVMVGMGQK DSYVGDEAQS KRGILTLKYP IEHGIVTNWD
 81 DMEKIWHHTF YNELRVAPEE HPVLLTEAPL NPKANREKMT QIMFETFNTP AMYVAIQAVL SLYASGRTTG IVMDSGDGVT
161 HTVPIYEGYA LPHAILRLDL AGRDLTDYLM KILTERGYSF TTTAEREIVR DIKEKLCYVA LDFEQEMATA ASSSSLEKSY
241 ELPDGQVITI GNERFRCPEA LFQPSFLGME SCGIHETTFN SIMKCDVDIR KDLYANTVLS GGTTMYPGIA DRMQKEITAL

321 APSTMKIKII APPERKYSVW IGGSILASLS TFQQMWISKQ EYDESGPSIV HRKCF
```

Supporting Peptides:

| Peptide | Uniq | -10lgP | Mass | Length | ppm | m/z | z | RT | Fraction | Scan | Source File | Area F1 | Area F2a | Area F3a | Area F4 | Area F5 | Area F6a | Area F7a | Area F7b | Area F8 | Area F9a | Area Sample 1F10a | Area F10b | Area F11a | Area F11b | Area F12a | Area F13 | #Feature | #Feature F1 | #Feature F2a | #Feature F3a | #Feature F4 | #Feature F5 | #Feature F6a | #Feature F7a | #Feature F7b | #Feature F8 | #Feature F9a | #Feature Sample 1F10a | #Feature F10b | #Feature F11a | #Feature F11b | #Feature F12a | #Feature F13 | Start | End | PTM | AScore | Found By |
|---|---|---|---|---|---|---|---|---|---|---|---|---|---|---|---|---|---|---|---|---|---|---|---|---|---|---|---|---|---|---|---|---|---|---|---|---|---|---|---|---|---|---|---|---|---|---|---|---|---|
| R.KYSVWIGGSILASLSTFQQMWISK.Q | Y | 57.10 | 2729.4250 | 24 | 0.3 | 910.8159 | 3 | 102.40 | 1 | F1:49862 | BuCaTN01_F1.raw | 1.0126E5 | | | | | | | | | | | | | | | | 1 | 1 | 0 | 0 | 0 | 0 | 0 | 0 | 0 | 0 | 0 | 0 | 0 | 0 | 0 | 0 | 0 | 336 | 359 | | | PEAKS DB |
| K.DLYANTVLSGGTTMYPGIADR.M | Y | 50.22 | 2214.0627 | 21 | 0.8 | 1108.0396 | 2 | 73.08 | 1 | F1:33231 | BuCaTN01_F1.raw | 4.7954E4 | | | | | | | | | | | | | | | | 1 | 1 | 0 | 0 | 0 | 0 | 0 | 0 | 0 | 0 | 0 | 0 | 0 | 0 | 0 | 0 | 0 | 292 | 312 | | | PEAKS DB |
| total 2 peptides | | | | | | | | | | | | | | | | | | | | | | | | | | | | | | | | | | | | | | | | | | | | | | | | | |

Best Unique PSM (Scan F1:49862, m/z=910.8159, z=3, RT=102.40, ppm=0.3):

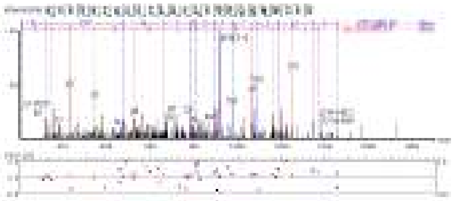

## LAA21098.1

back to list

| Protein Coverage | Supporting Peptides | Best Unique PSM |

Protein Coverage:

```
  1 MEEEIAALVI DNGSGMCKAG FAGDDAPRAV FPSIVGRPRH QGVMVGMGQK DSYVGDEAQS KRGILTLKYP IEHGIVTNWD
 81 DMEKIWHHTF YNELRVAPEE HPVLLTEAPL NPKANREKMT QIMFETFNTP AMYVAIQAVL SLYASGRTTG IVMDSGDGVT
161 HTVPIYEGYA LPHAILRLDL AGRDLTDYLM KILTERGYSF TTTAEREIVR DIKEKLCYVA LDFEQEMATA ASSSSLEKSY
241 ELPDGQVITI GNERFRCPEA LFQPSFLGME SCGIHETTFN SIMKCDVDIR KDLYANTVLS GGTTMYPGIA DRMQKEITAL

321 APSTMKIKII APPERKYSVW IGGSILASLS TFQQMWISKQ EYDESGPSIV HRKCF
```

Supporting Peptides:

| Peptide | Uniq | -10lgP | Mass | Length | ppm | m/z | z | RT | Fraction | Scan | Source File | Area F1 | Area F2a | Area F3a | Area F4 | Area F5 | Area F6a | Area F7a | Area F7b | Area F8 | Area F9a | Area Sample 1F10a | Area F10b | Area F11a | Area F11b | Area F12a | Area F13 | #Feature | #Feature F1 | #Feature F2a | #Feature F3a | #Feature F4 | #Feature F5 | #Feature F6a | #Feature F7a | #Feature F7b | #Feature F8 | #Feature F9a | #Feature Sample 1F10a | #Feature F10b | #Feature F11a | #Feature F11b | #Feature F12a | #Feature F13 | Start | End | PTM | AScore | Found By |
|---|---|---|---|---|---|---|---|---|---|---|---|---|---|---|---|---|---|---|---|---|---|---|---|---|---|---|---|---|---|---|---|---|---|---|---|---|---|---|---|---|---|---|---|---|---|---|---|---|---|
| R.KYSVWIGGSILASLSTFQQMWISK.Q | Y | 57.10 | 2729.4250 | 24 | 0.3 | 910.8159 | 3 | 102.40 | 1 | F1:49862 | BuCaTN01_F1.raw | 1.0126E5 | | | | | | | | | | | | | | | | 1 | 1 | 0 | 0 | 0 | 0 | 0 | 0 | 0 | 0 | 0 | 0 | 0 | 0 | 0 | 0 | 0 | 336 | 359 | | | PEAKS DB |
| K.DLYANTVLSGGTTMYPGIADR.M | Y | 50.22 | 2214.0627 | 21 | 0.8 | 1108.0396 | 2 | 73.08 | 1 | F1:33231 | BuCaTN01_F1.raw | 4.7954E4 | | | | | | | | | | | | | | | | 1 | 1 | 0 | 0 | 0 | 0 | 0 | 0 | 0 | 0 | 0 | 0 | 0 | 0 | 0 | 0 | 0 | 292 | 312 | | | PEAKS DB |
| total 2 peptides | | | | | | | | | | | | | | | | | | | | | | | | | | | | | | | | | | | | | | | | | | | | | | | | | |

Best Unique PSM (Scan F1:49862, m/z=910.8159, z=3, RT=102.40, ppm=0.3):

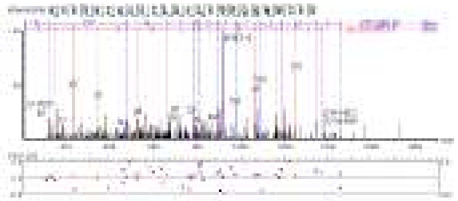

## AFJ49303.1

back to list

| Protein Coverage | Supporting Peptides | Best Unique PSM |

**Protein Coverage:**

```
  1 MEEEIAALVI DNGSGMCKAG FAGDDAPRAV FPSIVGRPRH QGVMVGMGQK DSYVGDEAQS KRGILTLKYP IEHGIVTNWD
 81 DMEKIWHHTF YNELRVAPEE HPVLLTEAPL NPKANREKMT QIMFETFNTP AMYVAIQAVL SLYASGRTTG IVMDSGDGVT
161 HTVPIYEGYA LPHAILRLDL AGRDLTDYLM KILTERGYSF TTTAERENVR DIKEKLCYVA LDFEQEMATA ASSSSLEKSY
241 ELPDGQVITI GNERFRCPEA LFQPSFLGME SCGIHETTFN SIMKCDVDIR KDLYANTVLS GGTTMYPGIA DRMQKEITAL
321 APSTMKIKII APPERKYSVW IGGSILASLS TFQQMWISKQ EYDESGPSIV HRKCF
```

**Supporting Peptides:**

| Peptide | Uniq | -10lgP | Mass | Length | ppm | m/z | z | RT | Fraction | Scan | Source File | Area F1 | Area F2a | Area F3a | Area F4 | Area F5 | Area F6a | Area F7a | Area F7b | Area F8 | Area F9a | Area Sample 1F10a | Area F10b | Area F11a | Area F11b | Area F12a | Area F13 | #Feature | #Feature F1 | #Feature F2a | #Feature F3a | #Feature F4 | #Feature F5 | #Feature F6a | #Feature F7a | #Feature F7b | #Feature F8 | #Feature F9a | #Feature Sample 1F10a | #Feature F10b | #Feature F11a | #Feature F11b | #Feature F12a | #Feature F13 | Start | End | PTM | AScore | Found By |
|---|---|---|---|---|---|---|---|---|---|---|---|---|---|---|---|---|---|---|---|---|---|---|---|---|---|---|---|---|---|---|---|---|---|---|---|---|---|---|---|---|---|---|---|---|---|---|---|---|---|
| R.KYSVWIGGSILASLSTFQQMWISK.Q | Y | 57.10 | 2729.4250 | 24 | 0.3 | 910.8159 | 3 | 102.40 | 1 | F1:49862 | BuCaTN01_F1.raw | 1.0126E5 | | | | | | | | | | | | | | | | 1 | 1 | 0 | 0 | 0 | 0 | 0 | 0 | 0 | 0 | 0 | 0 | 0 | 0 | 0 | 0 | 0 | 336 | 359 | | | PEAKS DB |
| K.DLYANTVLSGGTTMYPGIADR.M | Y | 50.22 | 2214.0627 | 21 | 0.8 | 1108.0396 | 2 | 73.08 | 1 | F1:33231 | BuCaTN01_F1.raw | 4.7954E4 | | | | | | | | | | | | | | | | 1 | 1 | 0 | 0 | 0 | 0 | 0 | 0 | 0 | 0 | 0 | 0 | 0 | 0 | 0 | 0 | 0 | 292 | 312 | | | PEAKS DB |
| total 2 peptides | | | | | | | | | | | | | | | | | | | | | | | | | | | | | | | | | | | | | | | | | | | | | | | | | |

**Best Unique PSM (Scan F1:49862, m/z=910.8159, z=3, RT=102.40, ppm=0.3):**

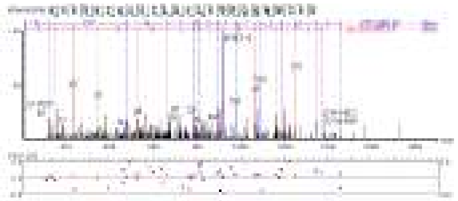

## JAI10341.1

back to list

| Protein Coverage | Supporting Peptides | Best Unique PSM |

**Protein Coverage:**

```
  1 MEEEIAALVI DNGSGMCKAG FAGDDAPRAV FPSIVGRPRH QGVMVGMGQK DSYVGDEAQS KRGILTLKYP IEHGIVTNWD
 81 DMEKIWHHTF YNELRVAPEE HPVLLTEAPL NPKANREKMT QIMFETFNTP AMYVAIQAVL SLYASGRTTG IVMDSGDGVT
161 HTVPIYEGYA LPHAILRLDL AGRDLTDYLM KILTERGYSF TTTAERENVR DIKEKLCYVA LDFEQEMATA ASSSSLEKSY
241 ELPDGQVITI GNERFRCPEA LFQPSFLGME SCGIHETTFN SIMKCDVDIR KDLYANTVLS GGTTMYPGIA DRMQKEITAL
321 APSTMKIKII APPERKYSVW IGGSILASLS TFQQMWISKQ EYDESGPSIV HRKCF
```

**Supporting Peptides:**

| Peptide | Uniq | -10lgP | Mass | Length | ppm | m/z | z | RT | Fraction | Scan | Source File | Area F1 | Area F2a | Area F3a | Area F4 | Area F5 | Area F6a | Area F7a | Area F7b | Area F8 | Area F9a | Area Sample 1F10a | Area F10b | Area F11a | Area F11b | Area F12a | Area F13 | #Feature | #Feature F1 | #Feature F2a | #Feature F3a | #Feature F4 | #Feature F5 | #Feature F6a | #Feature F7a | #Feature F7b | #Feature F8 | #Feature F9a | #Feature Sample 1F10a | #Feature F10b | #Feature F11a | #Feature F11b | #Feature F12a | #Feature F13 | Start | End | PTM | AScore | Found By |
|---|---|---|---|---|---|---|---|---|---|---|---|---|---|---|---|---|---|---|---|---|---|---|---|---|---|---|---|---|---|---|---|---|---|---|---|---|---|---|---|---|---|---|---|---|---|---|---|---|---|
| R.KYSVWIGGSILASLSTFQQMWISK.Q | Y | 57.10 | 2729.4250 | 24 | 0.3 | 910.8159 | 3 | 102.40 | 1 | F1:49862 | BuCaTN01_F1.raw | 1.0126E5 | | | | | | | | | | | | | | | | 1 | 1 | 0 | 0 | 0 | 0 | 0 | 0 | 0 | 0 | 0 | 0 | 0 | 0 | 0 | 0 | 0 | 336 | 359 | | | PEAKS DB |
| K.DLYANTVLSGGTTMYPGIADR.M | Y | 50.22 | 2214.0627 | 21 | 0.8 | 1108.0396 | 2 | 73.08 | 1 | F1:33231 | BuCaTN01_F1.raw | 4.7954E4 | | | | | | | | | | | | | | | | 1 | 1 | 0 | 0 | 0 | 0 | 0 | 0 | 0 | 0 | 0 | 0 | 0 | 0 | 0 | 0 | 0 | 292 | 312 | | | PEAKS DB |
| total 2 peptides | | | | | | | | | | | | | | | | | | | | | | | | | | | | | | | | | | | | | | | | | | | | | | | | | |

**Best Unique PSM (Scan F1:49862, m/z=910.8159, z=3, RT=102.40, ppm=0.3):**

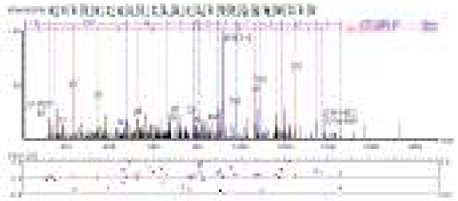

## JAI14495.1

back to list

| Protein Coverage | Supporting Peptides | Best Unique PSM |

**Protein Coverage:**

```
  1 MEEEIAALVI DNGSGMCKAG FAGDDAPRAV FPSIVGRPRH QGVMVGMGQK DSYVGDEAQS KRGILTLKYP IEHGIVTNWD
 81 DMEKIWHHTF YNELRVAPEE HPVLLTEAPL NPKANREKMT QIMFETFNTP AMYVAIQAVL SLYASGRTTG IVMDSGDGVT
161 HTVPIYEGYA LPHAILRLDL AGRDLTDYLM KILTERGYSF TTTAERENVR DIKEKLCYVA LDFEQEMATA ASSSSLEKSY
241 ELPDGQVITI GNERFRCPEA LFQPSFLGME SCGIHETTFN SIMKCDVDIR KDLYANTVLS GGTTMYPGIA DRMQKEITAL
321 APSTMKIKII APPERKYSVW IGGSILASLS TFQQMWISKQ EYDESGPSIV HRKCF
```

**Supporting Peptides:**

| Peptide | Uniq | -10lgP | Mass | Length | ppm | m/z | z | RT | Fraction | Scan | Source File | Area F1 | Area F2a | Area F3a | Area F4 | Area F5 | Area F6a | Area F7a | Area F7b | Area F8 | Area F9a | Area Sample 1F10a | Area F10b | Area F11a | Area F11b | Area F12a | Area F13 | #Feature | #Feature F1 | #Feature F2a | #Feature F3a | #Feature F4 | #Feature F5 | #Feature F6a | #Feature F7a | #Feature F7b | #Feature F8 | #Feature F9a | #Feature Sample 1F10a | #Feature F10b | #Feature F11a | #Feature F11b | #Feature F12a | #Feature F13 | Start | End | PTM | AScore | Found By |
|---|---|---|---|---|---|---|---|---|---|---|---|---|---|---|---|---|---|---|---|---|---|---|---|---|---|---|---|---|---|---|---|---|---|---|---|---|---|---|---|---|---|---|---|---|---|---|---|---|---|
| R.KYSVWIGGSILASLSTFQQMWISK.Q | Y | 57.10 | 2729.4250 | 24 | 0.3 | 910.8159 | 3 | 102.40 | 1 | F1:49862 | BuCaTN01_F1.raw | 1.0126E5 | | | | | | | | | | | | | | | | 1 | 1 | 0 | 0 | 0 | 0 | 0 | 0 | 0 | 0 | 0 | 0 | 0 | 0 | 0 | 0 | 0 | 336 | 359 | | | PEAKS DB |
| K.DLYANTVLSGGTTMYPGIADR.M | Y | 50.22 | 2214.0627 | 21 | 0.8 | 1108.0396 | 2 | 73.08 | 1 | F1:33231 | BuCaTN01_F1.raw | 4.7954E4 | | | | | | | | | | | | | | | | 1 | 1 | 0 | 0 | 0 | 0 | 0 | 0 | 0 | 0 | 0 | 0 | 0 | 0 | 0 | 0 | 0 | 292 | 312 | | | PEAKS DB |
| total 2 peptides | | | | | | | | | | | | | | | | | | | | | | | | | | | | | | | | | | | | | | | | | | | | | | | | | |

**Best Unique PSM (Scan F1:49862, m/z=910.8159, z=3, RT=102.40, ppm=0.3):**

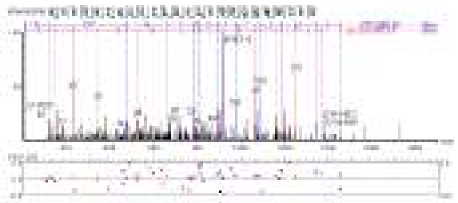

## XP_007419915.2

back to list

| Protein Coverage | Supporting Peptides | Best Unique PSM |

**Protein Coverage:**

```
  1 MEEEIAALVI DNGSGMCKAG FAGDDAPRAV FPSIVGRPRH QGVMVGMGQK DSYVGDEAQS KRGILTLKYP IEHGIVTNWD
 81 DMEKIWHHTF YNELRVAPEE HPVLLTEAPL NPKANREKMT QIMFETFNTP AMYVAIQAVL SLYASGRTTG IVMDSGDGVT
161 HTVPIYEGYA LPHAILRLDL AGRDLTDYLM KILTERGYSF TTTAEREIVR DIKEKLCYVA LDFEQEMATA ASSSSLEKSY
241 ELPDGQVITI GNERFRCPEA LFQPSFLGME SCGIHETTFN SIMKCDVDIR KDLYANTVLS GGTTMYPGIA DRMQKEITAL
321 APSTMKIKII APPERKYSVW IGGSILASLS TFQQMWISKQ EYDESGPSIV HRKCF
```

**Supporting Peptides:**

| Peptide | Uniq | -10lgP | Mass | Length | ppm | m/z | z | RT | Fraction | Scan | Source File | Area F1 | Area F2a | Area F3a | Area F4 | Area F5 | Area F6a | Area F7a | Area F7b | Area F8 | Area F9a | Area Sample 1F10a | Area F10b | Area F11a | Area F11b | Area F12a | Area F13 | #Feature | #Feature F1 | #Feature F2a | #Feature F3a | #Feature F4 | #Feature F5 | #Feature F6a | #Feature F7a | #Feature F7b | #Feature F8 | #Feature F9a | #Feature Sample 1F10a | #Feature F10b | #Feature F11a | #Feature F11b | #Feature F12a | #Feature F13 | Start | End | PTM | AScore | Found By |
|---|---|---|---|---|---|---|---|---|---|---|---|---|---|---|---|---|---|---|---|---|---|---|---|---|---|---|---|---|---|---|---|---|---|---|---|---|---|---|---|---|---|---|---|---|---|---|---|---|---|
| R.KYSVWIGGSILASLSTFQQMWISK.Q | Y | 57.10 | 2729.4250 | 24 | 0.3 | 910.8159 | 3 | 102.40 | 1 | F1:49862 | BuCaTN01_F1.raw | 1.0126E5 | | | | | | | | | | | | | | | | 1 | 1 | 0 | 0 | 0 | 0 | 0 | 0 | 0 | 0 | 0 | 0 | 0 | 0 | 0 | 0 | 0 | 336 | 359 | | | PEAKS DB |
| K.DLYANTVLSGGTTMYPGIADR.M | Y | 50.22 | 2214.0627 | 21 | 0.8 | 1108.0396 | 2 | 73.08 | 1 | F1:33231 | BuCaTN01_F1.raw | 4.7954E4 | | | | | | | | | | | | | | | | 1 | 1 | 0 | 0 | 0 | 0 | 0 | 0 | 0 | 0 | 0 | 0 | 0 | 0 | 0 | 0 | 0 | 292 | 312 | | | PEAKS DB |
| total 2 peptides | | | | | | | | | | | | | | | | | | | | | | | | | | | | | | | | | | | | | | | | | | | | | | | | | |

**Best Unique PSM (Scan F1:49862, m/z=910.8159, z=3, RT=102.40, ppm=0.3):**

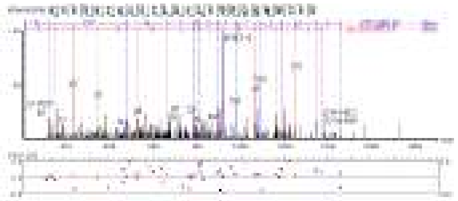

## JAG47579.1

back to list

| Protein Coverage | Supporting Peptides | Best Unique PSM |

**Protein Coverage:**

```
  1 MEEEIAALVI DNGSGMCKAG FAGDDAPRAV FPSIVGRPRH QGVMVGMGQK DSYVGDEAQS KRGILTLKYP IEHGIVTNWD
 81 DMEKIWHHTF YNELRVAPEE HPVLLTEAPL NPKANREKMT QIMFETFNTP AMYVAIQAVL SLYASGRTTG IVMDSGDGVT
161 HTVPIYEGYA LPHAILRLDL AGRDLTDYLM KILTERGYSF TTTAEREIVR DIKEKLCYVA LDFEQEMATA ASSSSLEKSY
241 ELPDGQVITI GNERFRCPEA LFQPSFLGME SCGIHETTFN SIMKCDVDIR KDLYANTVLS GGTTMYPGIA DRMQKEITAL
321 APSTMKIKII APPERKYSVW IGGSILASLS TFQQMWISKQ EYDESGPSIV HRKCF
```

**Supporting Peptides:**

| Peptide | Uniq | -10lgP | Mass | Length | ppm | m/z | z | RT | Fraction | Scan | Source File | Area F1 | Area F2a | Area F3a | Area F4 | Area F5 | Area F6a | Area F7a | Area F7b | Area F8 | Area F9a | Area Sample 1F10a | Area F10b | Area F11a | Area F11b | Area F12a | Area F13 | #Feature | #Feature F1 | #Feature F2a | #Feature F3a | #Feature F4 | #Feature F5 | #Feature F6a | #Feature F7a | #Feature F7b | #Feature F8 | #Feature F9a | #Feature Sample 1F10a | #Feature F10b | #Feature F11a | #Feature F11b | #Feature F12a | #Feature F13 | Start | End | PTM | AScore | Found By |
|---|---|---|---|---|---|---|---|---|---|---|---|---|---|---|---|---|---|---|---|---|---|---|---|---|---|---|---|---|---|---|---|---|---|---|---|---|---|---|---|---|---|---|---|---|---|---|---|---|---|
| R.KYSVWIGGSILASLSTFQQMWISK.Q | Y | 57.10 | 2729.4250 | 24 | 0.3 | 910.8159 | 3 | 102.40 | 1 | F1:49862 | BuCaTN01_F1.raw | 1.0126E5 | | | | | | | | | | | | | | | | 1 | 1 | 0 | 0 | 0 | 0 | 0 | 0 | 0 | 0 | 0 | 0 | 0 | 0 | 0 | 0 | 0 | 336 | 359 | | | PEAKS DB |
| K.DLYANTVLSGGTTMYPGIADR.M | Y | 50.22 | 2214.0627 | 21 | 0.8 | 1108.0396 | 2 | 73.08 | 1 | F1:33231 | BuCaTN01_F1.raw | 4.7954E4 | | | | | | | | | | | | | | | | 1 | 1 | 0 | 0 | 0 | 0 | 0 | 0 | 0 | 0 | 0 | 0 | 0 | 0 | 0 | 0 | 0 | 292 | 312 | | | PEAKS DB |
| total 2 peptides | | | | | | | | | | | | | | | | | | | | | | | | | | | | | | | | | | | | | | | | | | | | | | | | | |

**Best Unique PSM (Scan F1:49862, m/z=910.8159, z=3, RT=102.40, ppm=0.3):**

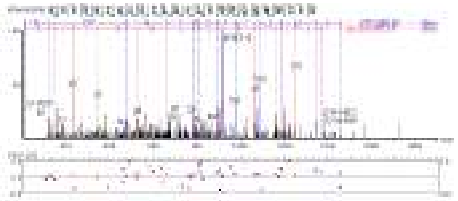

## JAG45996.1

back to list

| Protein Coverage | Supporting Peptides | Best Unique PSM |

**Protein Coverage:**

```
  1 MEEEIAALVI DNGSGMCKAG FAGDDAPRAV FPSIVGRPRH QGVMVGMGQK DSYVGDEAQS KRGILTLKYP IEHGIVTNWD
 81 DMEKIWHHTF YNELRVAPEE HPVLLTEAPL NPKANREKMT QIMFETFNTP AMYVAIQAVL SLYASGRTTG IVMDSGDGVT
161 HTVPIYEGYA LPHAILRLDL AGRDLTDYLM KILTERGYSF TTTAEREIVR DIKEKLCYVA LDFEQEMATA ASSSSLEKSY
241 ELPDGQVITI GNERFRCPEA LFQPSFLGME SCGIHETTFN SIMKCDVDIR KDLYANTVLS GGTTMYPGIA DRMQKEITAL
321 APSTMKIKII APPERKYSVW IGGSILASLS TFQQMWISKQ EYDESGPSIV HRKCF
```

**Supporting Peptides:**

| Peptide | Uniq | -10lgP | Mass | Length | ppm | m/z | z | RT | Fraction | Scan | Source File | Area F1 | Area F2a | Area F3a | Area F4 | Area F5 | Area F6a | Area F7a | Area F7b | Area F8 | Area F9a | Area Sample 1F10a | Area F10b | Area F11a | Area F11b | Area F12a | Area F13 | #Feature | #Feature F1 | #Feature F2a | #Feature F3a | #Feature F4 | #Feature F5 | #Feature F6a | #Feature F7a | #Feature F7b | #Feature F8 | #Feature F9a | #Feature Sample 1F10a | #Feature F10b | #Feature F11a | #Feature F11b | #Feature F12a | #Feature F13 | Start | End | PTM | AScore | Found By |
|---|---|---|---|---|---|---|---|---|---|---|---|---|---|---|---|---|---|---|---|---|---|---|---|---|---|---|---|---|---|---|---|---|---|---|---|---|---|---|---|---|---|---|---|---|---|---|---|---|---|
| R.KYSVWIGGSILASLSTFQQMWISK.Q | Y | 57.10 | 2729.4250 | 24 | 0.3 | 910.8159 | 3 | 102.40 | 1 | F1:49862 | BuCaTN01_F1.raw | 1.0126E5 | | | | | | | | | | | | | | | | 1 | 1 | 0 | 0 | 0 | 0 | 0 | 0 | 0 | 0 | 0 | 0 | 0 | 0 | 0 | 0 | 0 | 336 | 359 | | | PEAKS DB |
| K.DLYANTVLSGGTTMYPGIADR.M | Y | 50.22 | 2214.0627 | 21 | 0.8 | 1108.0396 | 2 | 73.08 | 1 | F1:33231 | BuCaTN01_F1.raw | 4.7954E4 | | | | | | | | | | | | | | | | 1 | 1 | 0 | 0 | 0 | 0 | 0 | 0 | 0 | 0 | 0 | 0 | 0 | 0 | 0 | 0 | 0 | 292 | 312 | | | PEAKS DB |
| total 2 peptides | | | | | | | | | | | | | | | | | | | | | | | | | | | | | | | | | | | | | | | | | | | | | | | | | |

**Best Unique PSM (Scan F1:49862, m/z=910.8159, z=3, RT=102.40, ppm=0.3):**

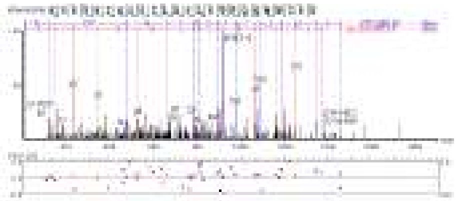

## ETE74025.1

back to list

| Protein Coverage | Supporting Peptides | Best Unique PSM |

**Protein Coverage:**

```
  1 MEEEIAALVI DNGSGMCKAG FAGDDAPRAV FPSIVGRPRH QGVMVGMGQK DSYVGDEAQS KRGILTLKYP IEHGIVTNWD
 81 DMEKIWHHTF YNELRVAPEE HPVLLTEAPL NPKANREKMT QIMFETFNTP AMYVAIQAVL SLYASGRTTG IVMDSGDGVT
161 HTVPIYEGYA LPHAILRLDL AGRDLTDYLM KILTERGYSF TTTAEREIVR DIKEKLCYVA LDFEQEMATA ASSSSLEKSY
241 ELPDGQVITI GNERFRCPEA LFQPSFLGME SCGIHETTFN SIMKCDVDIR KDLYANTVLS GGTTMYPGIA DRMQKEITAL

321 APSTMKIKII APPERKYSVW IGGSILASLS TFQQMWISKQ EYDESGPSIV HRKCF
```

**Supporting Peptides:**

| Peptide | Uniq | -10lgP | Mass | Length | ppm | m/z | z | RT | Fraction | Scan | Source File | Area F1 | Area F2a | Area F3a | Area F4 | Area F5 | Area F6a | Area F7a | Area F7b | Area F8 | Area F9a | Area Sample 1F10a | Area F10b | Area F11a | Area F11b | Area F12a | Area F13 | #Feature | #Feature F1 | #Feature F2a | #Feature F3a | #Feature F4 | #Feature F5 | #Feature F6a | #Feature F7a | #Feature F7b | #Feature F8 | #Feature F9a | #Feature Sample 1F10a | #Feature F10b | #Feature F11a | #Feature F11b | #Feature F12a | #Feature F13 | Start | End | PTM | AScore | Found By |
|---|---|---|---|---|---|---|---|---|---|---|---|---|---|---|---|---|---|---|---|---|---|---|---|---|---|---|---|---|---|---|---|---|---|---|---|---|---|---|---|---|---|---|---|---|---|---|---|---|---|
| R.KYSVWIGGSILASLSTFQQMWISK.Q | Y | 57.10 | 2729.4250 | 24 | 0.3 | 910.8159 | 3 | 102.40 | 1 | F1:49862 | BuCaTN01_F1.raw | 1.0126E5 | | | | | | | | | | | | | | | | 1 | 1 | 0 | 0 | 0 | 0 | 0 | 0 | 0 | 0 | 0 | 0 | 0 | 0 | 0 | 0 | 0 | 336 | 359 | | | PEAKS DB |
| K.DLYANTVLSGGTTMYPGIADR.M | Y | 50.22 | 2214.0627 | 21 | 0.8 | 1108.0396 | 2 | 73.08 | 1 | F1:33231 | BuCaTN01_F1.raw | 4.7954E4 | | | | | | | | | | | | | | | | 1 | 1 | 0 | 0 | 0 | 0 | 0 | 0 | 0 | 0 | 0 | 0 | 0 | 0 | 0 | 0 | 0 | 292 | 312 | | | PEAKS DB |
| total 2 peptides | | | | | | | | | | | | | | | | | | | | | | | | | | | | | | | | | | | | | | | | | | | | | | | | | |

**Best Unique PSM (Scan F1:49862, m/z=910.8159, z=3, RT=102.40, ppm=0.3):**

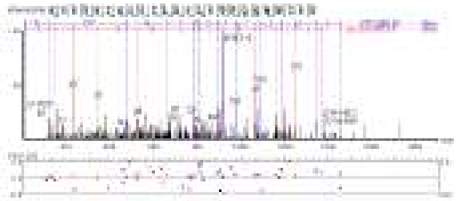

## LAA58652.1

back to list

| Protein Coverage | Supporting Peptides | Best Unique PSM |

**Protein Coverage:**

```
  1 MDDDIAALVV DNGSGMCKAG FAGDDAPRAV FPSIVGRPRH QGVMVGMGQK DSYVGDEAQS KRGILTLKYP IEHGIVTNWD
 81 DMEKIWHHTF YNELRVAPEE HPVLLTEAPL NPKANREKMT QIMFETFNTP AMYVAIQAVL SLYASGRTTG IVMDSGDGVT
161 HTVPIYEGYA LPHAILRLDL AGRDLTDYLM KILTERGYSF TTTAEREIVR DIKEKLCYVA LDFEQEMATA ASSSSLEKSY
241 ELPDGQVITI GNERFRCPEA LFQPSFLGME SCGIHETTFN SIMKCDVDIR KDLYANTVLS GGTTMYPGIA DRMQKEITAL

321 APSTMKIKII APPERKYSVW IGGSILASLS TFQQMWISKQ EYDESGPSIV HRKCF
```

**Supporting Peptides:**

| Peptide | Uniq | -10lgP | Mass | Length | ppm | m/z | z | RT | Fraction | Scan | Source File | Area F1 | Area F2a | Area F3a | Area F4 | Area F5 | Area F6a | Area F7a | Area F7b | Area F8 | Area F9a | Area Sample 1F10a | Area F10b | Area F11a | Area F11b | Area F12a | Area F13 | #Feature | #Feature F1 | #Feature F2a | #Feature F3a | #Feature F4 | #Feature F5 | #Feature F6a | #Feature F7a | #Feature F7b | #Feature F8 | #Feature F9a | #Feature Sample 1F10a | #Feature F10b | #Feature F11a | #Feature F11b | #Feature F12a | #Feature F13 | Start | End | PTM | AScore | Found By |
|---|---|---|---|---|---|---|---|---|---|---|---|---|---|---|---|---|---|---|---|---|---|---|---|---|---|---|---|---|---|---|---|---|---|---|---|---|---|---|---|---|---|---|---|---|---|---|---|---|---|
| R.KYSVWIGGSILASLSTFQQMWISK.Q | Y | 57.10 | 2729.4250 | 24 | 0.3 | 910.8159 | 3 | 102.40 | 1 | F1:49862 | BuCaTN01_F1.raw | 1.0126E5 | | | | | | | | | | | | | | | | 1 | 1 | 0 | 0 | 0 | 0 | 0 | 0 | 0 | 0 | 0 | 0 | 0 | 0 | 0 | 0 | 0 | 336 | 359 | | | PEAKS DB |
| K.DLYANTVLSGGTTMYPGIADR.M | Y | 50.22 | 2214.0627 | 21 | 0.8 | 1108.0396 | 2 | 73.08 | 1 | F1:33231 | BuCaTN01_F1.raw | 4.7954E4 | | | | | | | | | | | | | | | | 1 | 1 | 0 | 0 | 0 | 0 | 0 | 0 | 0 | 0 | 0 | 0 | 0 | 0 | 0 | 0 | 0 | 292 | 312 | | | PEAKS DB |
| total 2 peptides | | | | | | | | | | | | | | | | | | | | | | | | | | | | | | | | | | | | | | | | | | | | | | | | | |

**Best Unique PSM (Scan F1:49862, m/z=910.8159, z=3, RT=102.40, ppm=0.3):**

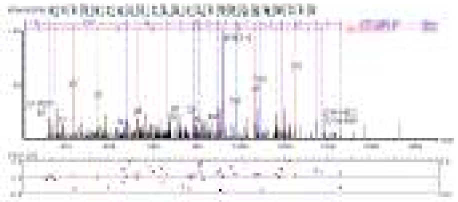

## JAA97901.1

back to list

| Protein Coverage | Supporting Peptides | Best Unique PSM |

**Protein Coverage:**

```
  1 MDDDIAALVV DNGSGMCKAG FAGDDAPRAV FPSIVGRPRH QGVMVGMGQK DSYVGDEAQS KRGILTLKYP IEHGIVTNWD
 81 DMEKIWHHTF YNELRVAPEE HPVLLTEAPL NPKANREKMT QIMFETFNTP AMYVAIQAVL SLYASGRTTG IVMDSGDGVT
161 HTVPIYEGYA LPHAILRLDL AGRDLTDYLM KILTERGYSF TTTAEREIVR DIKEKLCYVA LDFEQEMATA ASSSSLEKSY
241 ELPDGQVITI GNERFRCPEA LFQPSFLGME SCGIHETTFN SIMKCDVDIR KDLYANTVLS GGTTMYPGIA DRMQKEITAL

321 APSTMKIKII APPERKYSVW IGGSILASLS TFQQMWISKQ EYDESGPSIV HRKCF
```

**Supporting Peptides:**

| Peptide | Uniq | -10lgP | Mass | Length | ppm | m/z | z | RT | Fraction | Scan | Source File | Area F1 | Area F2a | Area F3a | Area F4 | Area F5 | Area F6a | Area F7a | Area F7b | Area F8 | Area F9a | Area Sample 1F10a | Area F10b | Area F11a | Area F11b | Area F12a | Area F13 | #Feature | #Feature F1 | #Feature F2a | #Feature F3a | #Feature F4 | #Feature F5 | #Feature F6a | #Feature F7a | #Feature F7b | #Feature F8 | #Feature F9a | #Feature Sample 1F10a | #Feature F10b | #Feature F11a | #Feature F11b | #Feature F12a | #Feature F13 | Start | End | PTM | AScore | Found By |
|---|---|---|---|---|---|---|---|---|---|---|---|---|---|---|---|---|---|---|---|---|---|---|---|---|---|---|---|---|---|---|---|---|---|---|---|---|---|---|---|---|---|---|---|---|---|---|---|---|---|
| R.KYSVWIGGSILASLSTFQQMWISK.Q | Y | 57.10 | 2729.4250 | 24 | 0.3 | 910.8159 | 3 | 102.40 | 1 | F1:49862 | BuCaTN01_F1.raw | 1.0126E5 | | | | | | | | | | | | | | | | 1 | 1 | 0 | 0 | 0 | 0 | 0 | 0 | 0 | 0 | 0 | 0 | 0 | 0 | 0 | 0 | 0 | 336 | 359 | | | PEAKS DB |
| K.DLYANTVLSGGTTMYPGIADR.M | Y | 50.22 | 2214.0627 | 21 | 0.8 | 1108.0396 | 2 | 73.08 | 1 | F1:33231 | BuCaTN01_F1.raw | 4.7954E4 | | | | | | | | | | | | | | | | 1 | 1 | 0 | 0 | 0 | 0 | 0 | 0 | 0 | 0 | 0 | 0 | 0 | 0 | 0 | 0 | 0 | 292 | 312 | | | PEAKS DB |
| total 2 peptides | | | | | | | | | | | | | | | | | | | | | | | | | | | | | | | | | | | | | | | | | | | | | | | | | |

**Best Unique PSM (Scan F1:49862, m/z=910.8159, z=3, RT=102.40, ppm=0.3):**

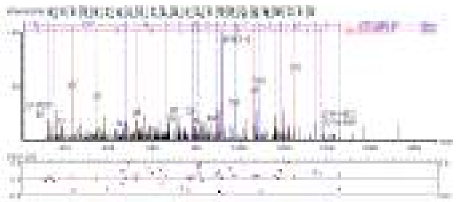

## JAG47580.1

back to list

| Protein Coverage | Supporting Peptides | Best Unique PSM |

**Protein Coverage:**

```
  1 MDDDIAALVV DNGSGMCKAG FAGDDAPRAV FPSIVGRPRH QGVMVGMGQK DSYVGDEAQS KRGILTLKYP IEHGIVTNWD
 81 DMEKIWHHTF YNELRVAPEE HPVLLTEAPL NPKANREKMT QIMFETFNTP AMYVAIQAVL SLYASGRTTG IVMDSGDGVT
161 HTVPIYEGYA LPHAILRLDL AGRDLTDYLM KILTERGYSF TTTAEREIVR DIKEKLCYVA LDFEQEMATA ASSSSLEKSY
241 ELPDGQVITI GNERFRCPEA LFQPSFLGME SCGIHETTFN SIMKCDVDIR KDLYANTVLS GGTTMYPGIA DRMQKEITAL

321 APSTMKIKII APPERKYSVW IGGSILASLS TFQQMWISKQ EYDESGPSIV HRKCF
```

**Supporting Peptides:**

| Peptide | Uniq | -10lgP | Mass | Length | ppm | m/z | z | RT | Fraction | Scan | Source File | Area F1 | Area F2a | Area F3a | Area F4 | Area F5 | Area F6a | Area F7a | Area F7b | Area F8 | Area F9a | Area Sample 1F10a | Area F10b | Area F11a | Area F11b | Area F12a | Area F13 | #Feature | #Feature F1 | #Feature F2a | #Feature F3a | #Feature F4 | #Feature F5 | #Feature F6a | #Feature F7a | #Feature F7b | #Feature F8 | #Feature F9a | #Feature Sample 1F10a | #Feature F10b | #Feature F11a | #Feature F11b | #Feature F12a | #Feature F13 | Start | End | PTM | AScore | Found By |
|---|---|---|---|---|---|---|---|---|---|---|---|---|---|---|---|---|---|---|---|---|---|---|---|---|---|---|---|---|---|---|---|---|---|---|---|---|---|---|---|---|---|---|---|---|---|---|---|---|---|
| R.KYSVWIGGSILASLSTFQQMWISK.Q | Y | 57.10 | 2729.4250 | 24 | 0.3 | 910.8159 | 3 | 102.40 | 1 | F1:49862 | BuCaTN01_F1.raw | 1.0126E5 | | | | | | | | | | | | | | | | 1 | 1 | 0 | 0 | 0 | 0 | 0 | 0 | 0 | 0 | 0 | 0 | 0 | 0 | 0 | 0 | 0 | 336 | 359 | | | PEAKS DB |
| K.DLYANTVLSGGTTMYPGIADR.M | Y | 50.22 | 2214.0627 | 21 | 0.8 | 1108.0396 | 2 | 73.08 | 1 | F1:33231 | BuCaTN01_F1.raw | 4.7954E4 | | | | | | | | | | | | | | | | 1 | 1 | 0 | 0 | 0 | 0 | 0 | 0 | 0 | 0 | 0 | 0 | 0 | 0 | 0 | 0 | 0 | 292 | 312 | | | PEAKS DB |
| total 2 peptides | | | | | | | | | | | | | | | | | | | | | | | | | | | | | | | | | | | | | | | | | | | | | | | | | |

**Best Unique PSM (Scan F1:49862, m/z=910.8159, z=3, RT=102.40, ppm=0.3):**

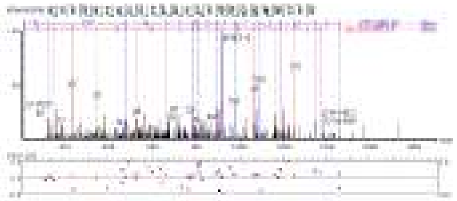

## JAI14496.1

back to list

| Protein Coverage | Supporting Peptides | Best Unique PSM |

**Protein Coverage:**

```
  1 MDDDIAALVV DNGSGMCKAG FAGDDAPRAV FPSIVGRPRH QGVMVGMGQK DSYVGDEAQS KRGILTLKYP IEHGIVTNWD
 81 DMEKIWHHTF YNELRVAPEE HPVLLTEAPL NPKANREKMT QIMFETFNTP AMYVAIQAVL SLYASGRTTG IVMDSGDGVT
161 HTVPIYEGYA LPHAILRLDL AGRDLTDYLM KILTERGYSF TTTAEREIVR DIKEKLCYVA LDFEQEMATA ASSSSLEKSY
241 ELPDGQVITI GNERFRCPEA LFQPSFLGME SCGIHETTFN SIMKCDVDIR KDLYANTVLS GGTTMYPGIA DRMQKEITAL

321 APSTMKIKII APPERKYSVW IGGSILASLS TFQQMWISKQ EYDESGPSIV HRKCF
```

**Supporting Peptides:**

| Peptide | Uniq | -10lgP | Mass | Length | ppm | m/z | z | RT | Fraction | Scan | Source File | Area F1 | Area F2a | Area F3a | Area F4 | Area F5 | Area F6a | Area F7a | Area F7b | Area F8 | Area F9a | Area Sample 1F10a | Area F10b | Area F11a | Area F11b | Area F12a | Area F13 | #Feature | #Feature F1 | #Feature F2a | #Feature F3a | #Feature F4 | #Feature F5 | #Feature F6a | #Feature F7a | #Feature F7b | #Feature F8 | #Feature F9a | #Feature Sample 1F10a | #Feature F10b | #Feature F11a | #Feature F11b | #Feature F12a | #Feature F13 | Start | End | PTM | AScore | Found By |
|---|---|---|---|---|---|---|---|---|---|---|---|---|---|---|---|---|---|---|---|---|---|---|---|---|---|---|---|---|---|---|---|---|---|---|---|---|---|---|---|---|---|---|---|---|---|---|---|---|---|
| R.KYSVWIGGSILASLSTFQQMWISK.Q | Y | 57.10 | 2729.4250 | 24 | 0.3 | 910.8159 | 3 | 102.40 | 1 | F1:49862 | BuCaTN01_F1.raw | 1.0126E5 | | | | | | | | | | | | | | | | 1 | 1 | 0 | 0 | 0 | 0 | 0 | 0 | 0 | 0 | 0 | 0 | 0 | 0 | 0 | 0 | 0 | 336 | 359 | | | PEAKS DB |
| K.DLYANTVLSGGTTMYPGIADR.M | Y | 50.22 | 2214.0627 | 21 | 0.8 | 1108.0396 | 2 | 73.08 | 1 | F1:33231 | BuCaTN01_F1.raw | 4.7954E4 | | | | | | | | | | | | | | | | 1 | 1 | 0 | 0 | 0 | 0 | 0 | 0 | 0 | 0 | 0 | 0 | 0 | 0 | 0 | 0 | 0 | 292 | 312 | | | PEAKS DB |
| total 2 peptides | | | | | | | | | | | | | | | | | | | | | | | | | | | | | | | | | | | | | | | | | | | | | | | | | |

**Best Unique PSM (Scan F1:49862, m/z=910.8159, z=3, RT=102.40, ppm=0.3):**

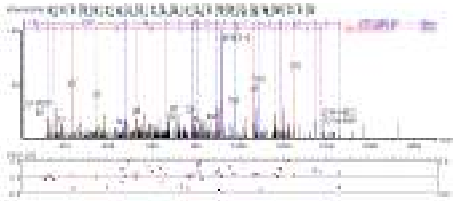

## XP_007436312.1

back to list

| Protein Coverage | Supporting Peptides | Best Unique PSM |

**Protein Coverage:**

```
  1 MDDDIAALVV DNGSGMCKAG FAGDDAPRAV FPSIVGRPRH QGVMVGMGQK DSYVGDEAQS KRGILTLKYP IEHGIVTNWD
 81 DMEKIWHHTF YNELRVAPEE HPVLLTEAPL NPKANREKMT QIMFETFNTP AMYVAIQAVL SLYASGRTTG IVMDSGDGVT
161 HTVPIYEGYA LPHAILRLDL AGRDLTDYLM KILTERGYSF TTTAEREIVR DIKEKLCYVA LDFEQEMATA ASSSSLEKSY
241 ELPDGQVITI GNERFRCPEA LFQPSFLGME SCGIHETTFN SIMKCDVDIR KDLYANTVLS GGTTMYPGIA DRMQKEITAL

321 APSTMKIKII APPERKYSVW IGGSILASLS TFQQMWISKQ EYDESGPSIV HRKCF
```

**Supporting Peptides:**

| Peptide | Uniq | -10lgP | Mass | Length | ppm | m/z | z | RT | Fraction | Scan | Source File | Area F1 | Area F2a | Area F3a | Area F4 | Area F5 | Area F6a | Area F7a | Area F7b | Area F8 | Area F9a | Area Sample 1F10a | Area F10b | Area F11a | Area F11b | Area F12a | Area F13 | #Feature | #Feature F1 | #Feature F2a | #Feature F3a | #Feature F4 | #Feature F5 | #Feature F6a | #Feature F7a | #Feature F7b | #Feature F8 | #Feature F9a | #Feature Sample 1F10a | #Feature F10b | #Feature F11a | #Feature F11b | #Feature F12a | #Feature F13 | Start | End | PTM | AScore | Found By |
|---|---|---|---|---|---|---|---|---|---|---|---|---|---|---|---|---|---|---|---|---|---|---|---|---|---|---|---|---|---|---|---|---|---|---|---|---|---|---|---|---|---|---|---|---|---|---|---|---|---|
| R.KYSVWIGGSILASLSTFQQMWISK.Q | Y | 57.10 | 2729.4250 | 24 | 0.3 | 910.8159 | 3 | 102.40 | 1 | F1:49862 | BuCaTN01_F1.raw | 1.0126E5 | | | | | | | | | | | | | | | | 1 | 1 | 0 | 0 | 0 | 0 | 0 | 0 | 0 | 0 | 0 | 0 | 0 | 0 | 0 | 0 | 0 | 336 | 359 | | | PEAKS DB |
| K.DLYANTVLSGGTTMYPGIADR.M | Y | 50.22 | 2214.0627 | 21 | 0.8 | 1108.0396 | 2 | 73.08 | 1 | F1:33231 | BuCaTN01_F1.raw | 4.7954E4 | | | | | | | | | | | | | | | | 1 | 1 | 0 | 0 | 0 | 0 | 0 | 0 | 0 | 0 | 0 | 0 | 0 | 0 | 0 | 0 | 0 | 292 | 312 | | | PEAKS DB |
| total 2 peptides | | | | | | | | | | | | | | | | | | | | | | | | | | | | | | | | | | | | | | | | | | | | | | | | | |

**Best Unique PSM (Scan F1:49862, m/z=910.8159, z=3, RT=102.40, ppm=0.3):**

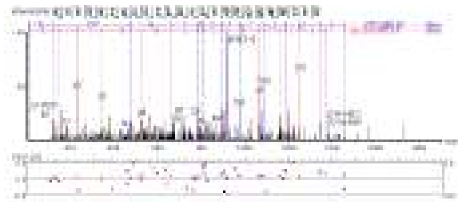

## JAC96364.1

back to list

| Protein Coverage | Supporting Peptides | Best Unique PSM |

**Protein Coverage:**

```
  1 MDDDIAALVV DNGSGMCKAG FAGDDAPRAV FPSIVGRPRH QGVMVGMGQK DSYVGDEAQS KRGILTLKYP IEHGIVTNWD
 81 DMEKIWHHTF YNELRVAPEE HPVLLTEAPL NPKANREKMT QIMFETFNTP AMYVAIQAVL SLYASGRTTG IVMDSGDGVT
161 HTVPIYEGYA LPHAILRLDL AGRDLTDYLM KILTERGYSF TTTAEREIVR DIKEKLCYVA LDFEQEMATA ASSSSLEKSY
241 ELPDGQVITI GNERFRCPEA LFQPSFLGME SCGIHETTFN SIMKCDVDIR KDLYANTVLS GGTTMYPGIA DRMQKEITAL
321 APSTMKIKII APPERKYSVW IGGSILASLS TFQQMWISKQ EYDESGPSIV HRKCF
```

**Supporting Peptides:**

| Peptide | Uniq | -10lgP | Mass | Length | ppm | m/z | z | RT | Fraction | Scan | Source File | Area F1 | Area F2a | Area F3a | Area F4 | Area F5 | Area F6a | Area F7a | Area F7b | Area F8 | Area F9a | Area Sample 1F10a | Area F10b | Area F11a | Area F11b | Area F12a | Area F13 | #Feature | #Feature F1 | #Feature F2a | #Feature F3a | #Feature F4 | #Feature F5 | #Feature F6a | #Feature F7a | #Feature F7b | #Feature F8 | #Feature F9a | #Feature Sample 1F10a | #Feature F10b | #Feature F11a | #Feature F11b | #Feature F12a | #Feature F13 | Start | End | PTM | AScore | Found By |
|---|---|---|---|---|---|---|---|---|---|---|---|---|---|---|---|---|---|---|---|---|---|---|---|---|---|---|---|---|---|---|---|---|---|---|---|---|---|---|---|---|---|---|---|---|---|---|---|---|---|
| R.KYSVWIGGSILASLSTFQQMWISK.Q | Y | 57.10 | 2729.4250 | 24 | 0.3 | 910.8159 | 3 | 102.40 | 1 | F1:49862 | BuCaTN01_F1.raw | 1.0126E5 | | | | | | | | | | | | | | | | 1 | 1 | 0 | 0 | 0 | 0 | 0 | 0 | 0 | 0 | 0 | 0 | 0 | 0 | 0 | 0 | 0 | 336 | 359 | | | PEAKS DB |
| K.DLYANTVLSGGTTMYPGIADR.M | Y | 50.22 | 2214.0627 | 21 | 0.8 | 1108.0396 | 2 | 73.08 | 1 | F1:33231 | BuCaTN01_F1.raw | 4.7954E4 | | | | | | | | | | | | | | | | 1 | 1 | 0 | 0 | 0 | 0 | 0 | 0 | 0 | 0 | 0 | 0 | 0 | 0 | 0 | 0 | 0 | 292 | 312 | | | PEAKS DB |
| total 2 peptides | | | | | | | | | | | | | | | | | | | | | | | | | | | | | | | | | | | | | | | | | | | | | | | | | |

**Best Unique PSM (Scan F1:49862, m/z=910.8159, z=3, RT=102.40, ppm=0.3):**

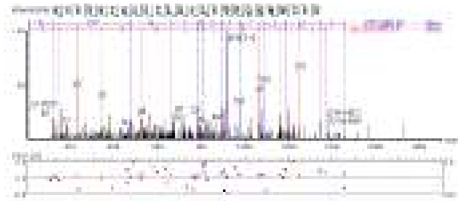

## BAQ22177.1

back to list

| Protein Coverage | Supporting Peptides | Best Unique PSM |

**Protein Coverage:**

```
  1 MDDDIAALVV DNGSGMCKAG FAGDDAPRAV FPSIVGRPRH QGVMVGMGQK DSYVGDEAQS KRGILTLKYP IEHGIVTNWD
 81 DMEKIWHHTF YNELRVAPEE HPVLLTEAPL NPKANREKMT QIMFETFNTP AMYVAIQAVL SLYASGRTTG IVMDSGDGVT
161 HTVPIYEGYA LPHAILRLDL AGRDLTDYLM KILTERGYSF TTTAEREIVR DIKEKLCYVA LDFEQEMATA ASSSSLEKSY
241 ELPDGQVITI GNERFRCPEA LFQPSFLGME SCGIHETTFN SIMKCDVDIR KDLYANTVLS GGTTMYPGIA DRMQKEITAL
321 APSTMKIKII APPERKYSVW IGGSILASLS TFQQMWISKQ EYDESGPSIV HRKCF
```

**Supporting Peptides:**

| Peptide | Uniq | -10lgP | Mass | Length | ppm | m/z | z | RT | Fraction | Scan | Source File | Area F1 | Area F2a | Area F3a | Area F4 | Area F5 | Area F6a | Area F7a | Area F7b | Area F8 | Area F9a | Area Sample 1F10a | Area F10b | Area F11a | Area F11b | Area F12a | Area F13 | #Feature | #Feature F1 | #Feature F2a | #Feature F3a | #Feature F4 | #Feature F5 | #Feature F6a | #Feature F7a | #Feature F7b | #Feature F8 | #Feature F9a | #Feature Sample 1F10a | #Feature F10b | #Feature F11a | #Feature F11b | #Feature F12a | #Feature F13 | Start | End | PTM | AScore | Found By |
|---|---|---|---|---|---|---|---|---|---|---|---|---|---|---|---|---|---|---|---|---|---|---|---|---|---|---|---|---|---|---|---|---|---|---|---|---|---|---|---|---|---|---|---|---|---|---|---|---|---|
| R.KYSVWIGGSILASLSTFQQMWISK.Q | Y | 57.10 | 2729.4250 | 24 | 0.3 | 910.8159 | 3 | 102.40 | 1 | F1:49862 | BuCaTN01_F1.raw | 1.0126E5 | | | | | | | | | | | | | | | | 1 | 1 | 0 | 0 | 0 | 0 | 0 | 0 | 0 | 0 | 0 | 0 | 0 | 0 | 0 | 0 | 0 | 336 | 359 | | | PEAKS DB |
| K.DLYANTVLSGGTTMYPGIADR.M | Y | 50.22 | 2214.0627 | 21 | 0.8 | 1108.0396 | 2 | 73.08 | 1 | F1:33231 | BuCaTN01_F1.raw | 4.7954E4 | | | | | | | | | | | | | | | | 1 | 1 | 0 | 0 | 0 | 0 | 0 | 0 | 0 | 0 | 0 | 0 | 0 | 0 | 0 | 0 | 0 | 292 | 312 | | | PEAKS DB |
| total 2 peptides | | | | | | | | | | | | | | | | | | | | | | | | | | | | | | | | | | | | | | | | | | | | | | | | | |

**Best Unique PSM (Scan F1:49862, m/z=910.8159, z=3, RT=102.40, ppm=0.3):**

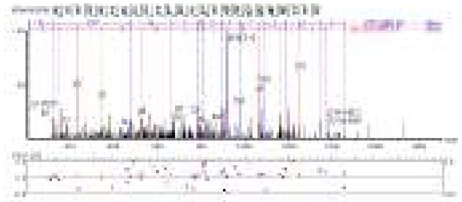

## JAG45999.1

back to list

| Protein Coverage | Supporting Peptides | Best Unique PSM |

**Protein Coverage:**

```
  1 MDDDIAALVV DNGSGMCKAG FAGDDAPRAV FPSIVGRPRH QGVMVGMGQK DSYVGDEAQS KRGILTLKYP IEHGIVTNWD
 81 DMEKIWHHTF YNELRVAPEE HPVLLTEAPL NPKANREKMT QIMFETFNTP AMYVAIQAVL SLYASGRTTG IVMDSGDGVT
161 HTVPIYEGYA LPHAILRLDL AGRDLTDYLM KILTERGYSF TTTAEREIVR DIKEKLCYVA LDFEQEMATA ASSSSLEKSY
241 ELPDGQVITI GNERFRCPEA LFQPSFLGME SCGIHETTFN SIMKCDVDIR KDLYANTVLS GGTTMYPGIA DRMQKEITAL
321 APSTMKIKII APPERKYSVW IGGSILASLS TFQQMWISKQ EYDESGPSIV HRKCF
```

**Supporting Peptides:**

| Peptide | Uniq | -10lgP | Mass | Length | ppm | m/z | z | RT | Fraction | Scan | Source File | Area F1 | Area F2a | Area F3a | Area F4 | Area F5 | Area F6a | Area F7a | Area F7b | Area F8 | Area F9a | Area Sample 1F10a | Area F10b | Area F11a | Area F11b | Area F12a | Area F13 | #Feature | #Feature F1 | #Feature F2a | #Feature F3a | #Feature F4 | #Feature F5 | #Feature F6a | #Feature F7a | #Feature F7b | #Feature F8 | #Feature F9a | #Feature Sample 1F10a | #Feature F10b | #Feature F11a | #Feature F11b | #Feature F12a | #Feature F13 | Start | End | PTM | AScore | Found By |
|---|---|---|---|---|---|---|---|---|---|---|---|---|---|---|---|---|---|---|---|---|---|---|---|---|---|---|---|---|---|---|---|---|---|---|---|---|---|---|---|---|---|---|---|---|---|---|---|---|---|
| R.KYSVWIGGSILASLSTFQQMWISK.Q | Y | 57.10 | 2729.4250 | 24 | 0.3 | 910.8159 | 3 | 102.40 | 1 | F1:49862 | BuCaTN01_F1.raw | 1.0126E5 | | | | | | | | | | | | | | | | 1 | 1 | 0 | 0 | 0 | 0 | 0 | 0 | 0 | 0 | 0 | 0 | 0 | 0 | 0 | 0 | 0 | 336 | 359 | | | PEAKS DB |
| K.DLYANTVLSGGTTMYPGIADR.M | Y | 50.22 | 2214.0627 | 21 | 0.8 | 1108.0396 | 2 | 73.08 | 1 | F1:33231 | BuCaTN01_F1.raw | 4.7954E4 | | | | | | | | | | | | | | | | 1 | 1 | 0 | 0 | 0 | 0 | 0 | 0 | 0 | 0 | 0 | 0 | 0 | 0 | 0 | 0 | 0 | 292 | 312 | | | PEAKS DB |
| total 2 peptides | | | | | | | | | | | | | | | | | | | | | | | | | | | | | | | | | | | | | | | | | | | | | | | | | |

**Best Unique PSM (Scan F1:49862, m/z=910.8159, z=3, RT=102.40, ppm=0.3):**

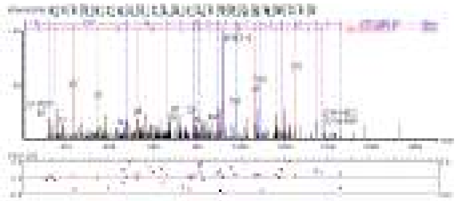

## JAI10342.1

back to list

| Protein Coverage | Supporting Peptides | Best Unique PSM |

**Protein Coverage:**

```
  1 MDDDIAALVV DNGSGMCKAG FAGDDAPRAV FPSIVGRPRH QGVMVGMGQK DSYVGDEAQS KRGILTLKYP IEHGIVTNWD
 81 DMEKIWHHTF YNELRVAPEE HPVLLTEAPL NPKANREKMT QIMFETFNTP AMYVAIQAVL SLYASGRTTG IVMDSGDGVT
161 HTVPIYEGYA LPHAILRLDL AGRDLTDYLM KILTERGYSF TTTAEREIVR DIKEKLCYVA LDFEQEMATA ASSSSLEKSY
241 ELPDGQVITI GNERFRCPEA LFQPSFLGME SCGIHETTFN SIMKCDVDIR KDLYANTVLS GGTTMYPGIA DRMQKEITAL
321 APSTMKIKII APPERKYSVW IGGSILASLS TFQQMWISKQ EYDESGPSIV HRKCF
```

**Supporting Peptides:**

| Peptide | Uniq | -10lgP | Mass | Length | ppm | m/z | z | RT | Fraction | Scan | Source File | Area F1 | Area F2a | Area F3a | Area F4 | Area F5 | Area F6a | Area F7a | Area F7b | Area F8 | Area F9a | Area Sample 1F10a | Area F10b | Area F11a | Area F11b | Area F12a | Area F13 | #Feature | #Feature F1 | #Feature F2a | #Feature F3a | #Feature F4 | #Feature F5 | #Feature F6a | #Feature F7a | #Feature F7b | #Feature F8 | #Feature F9a | #Feature Sample 1F10a | #Feature F10b | #Feature F11a | #Feature F11b | #Feature F12a | #Feature F13 | Start | End | PTM | AScore | Found By |
|---|---|---|---|---|---|---|---|---|---|---|---|---|---|---|---|---|---|---|---|---|---|---|---|---|---|---|---|---|---|---|---|---|---|---|---|---|---|---|---|---|---|---|---|---|---|---|---|---|---|
| R.KYSVWIGGSILASLSTFQQMWISK.Q | Y | 57.10 | 2729.4250 | 24 | 0.3 | 910.8159 | 3 | 102.40 | 1 | F1:49862 | BuCaTN01_F1.raw | 1.0126E5 | | | | | | | | | | | | | | | | 1 | 1 | 0 | 0 | 0 | 0 | 0 | 0 | 0 | 0 | 0 | 0 | 0 | 0 | 0 | 0 | 0 | 336 | 359 | | | PEAKS DB |
| K.DLYANTVLSGGTTMYPGIADR.M | Y | 50.22 | 2214.0627 | 21 | 0.8 | 1108.0396 | 2 | 73.08 | 1 | F1:33231 | BuCaTN01_F1.raw | 4.7954E4 | | | | | | | | | | | | | | | | 1 | 1 | 0 | 0 | 0 | 0 | 0 | 0 | 0 | 0 | 0 | 0 | 0 | 0 | 0 | 0 | 0 | 292 | 312 | | | PEAKS DB |
| total 2 peptides | | | | | | | | | | | | | | | | | | | | | | | | | | | | | | | | | | | | | | | | | | | | | | | | | |

**Best Unique PSM (Scan F1:49862, m/z=910.8159, z=3, RT=102.40, ppm=0.3):**

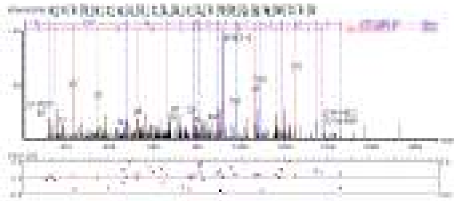

## JAB54771.1

back to list

| Protein Coverage | Supporting Peptides | Best Unique PSM |

**Protein Coverage:**

```
  1 MDDDIAALVV DNGSGMCKAG FAGDDAPRAV FPSIVGRPRH QGVMVGMGQK DSYVGDEAQS KRGILTLKYP IEHGIVTNWD
 81 DMEKIWHHTF YNELRVAPEE HPVLLTEAPL NPKANREKMT QIMFETFNTP AMYVAIQAVL SLYASGRTTG IVMDSGDGVT
161 HTVPIYEGYA LPHAILRLDL AGRDLTDYLM KILTERGYSF TTTAEREIVR DIKEKLCYVA LDFEQEMATA ASSSSLEKSY
241 ELPDGQVITI GNERFRCPEA LFQPSFLGME SCGIHETTFN SIMKCDVDIR KDLYANTVLS GGTTMYPGIA DRMQKEITAL
321 APSTMKIKII APPERKYSVW IGGSILASLS TFQQMWISKQ EYDESGPSIV HRKCF
```

**Supporting Peptides:**

| Peptide | Uniq | -10lgP | Mass | Length | ppm | m/z | z | RT | Fraction | Scan | Source File | Area F1 | Area F2a | Area F3a | Area F4 | Area F5 | Area F6a | Area F7a | Area F7b | Area F8 | Area F9a | Area Sample 1F10a | Area F10b | Area F11a | Area F11b | Area F12a | Area F13 | #Feature | #Feature F1 | #Feature F2a | #Feature F3a | #Feature F4 | #Feature F5 | #Feature F6a | #Feature F7a | #Feature F7b | #Feature F8 | #Feature F9a | #Feature Sample 1F10a | #Feature F10b | #Feature F11a | #Feature F11b | #Feature F12a | #Feature F13 | Start | End | PTM | AScore | Found By |
|---|---|---|---|---|---|---|---|---|---|---|---|---|---|---|---|---|---|---|---|---|---|---|---|---|---|---|---|---|---|---|---|---|---|---|---|---|---|---|---|---|---|---|---|---|---|---|---|---|---|
| R.KYSVWIGGSILASLSTFQQMWISK.Q | Y | 57.10 | 2729.4250 | 24 | 0.3 | 910.8159 | 3 | 102.40 | 1 | F1:49862 | BuCaTN01_F1.raw | 1.0126E5 | | | | | | | | | | | | | | | | 1 | 1 | 0 | 0 | 0 | 0 | 0 | 0 | 0 | 0 | 0 | 0 | 0 | 0 | 0 | 0 | 0 | 336 | 359 | | | PEAKS DB |
| K.DLYANTVLSGGTTMYPGIADR.M | Y | 50.22 | 2214.0627 | 21 | 0.8 | 1108.0396 | 2 | 73.08 | 1 | F1:33231 | BuCaTN01_F1.raw | 4.7954E4 | | | | | | | | | | | | | | | | 1 | 1 | 0 | 0 | 0 | 0 | 0 | 0 | 0 | 0 | 0 | 0 | 0 | 0 | 0 | 0 | 0 | 292 | 312 | | | PEAKS DB |
| total 2 peptides | | | | | | | | | | | | | | | | | | | | | | | | | | | | | | | | | | | | | | | | | | | | | | | | | |

**Best Unique PSM (Scan F1:49862, m/z=910.8159, z=3, RT=102.40, ppm=0.3):**

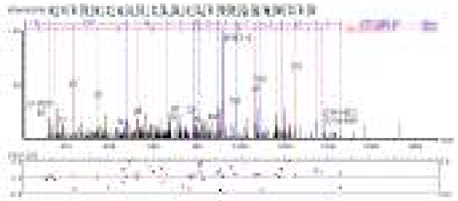

## JAG68844.1

back to list

| Protein Coverage | Supporting Peptides | Best Unique PSM |

**Protein Coverage:**

```
  1 MDDDIAALVV DNGSGMCKAG FAGDDAPRAV FPSIVGRPRH QGVMVGMGQK DSYVGDEAQS KRGILTLKYP IEHGIVTNWD
 81 DMEKIWHHTF YNELRVAPEE HPVLLTEAPL NPKANREKMT QIMFETFNTP AMYVAIQAVL SLYASGRTTG IVMDSGDGVT
161 HTVPIYEGYA LPHAILRLDL AGRDLTDYLM KILTERGYSF TTTAEREIVR DIKEKLCYVA LDFEQEMATA ASSSSLEKSY
241 ELPDGQVITI GNERFRCPEA LFQPSFLGME SCGIHETTFN SIMKCDVDIR KDLYANTVLS GGTTMYPGIA DRMQKEITAL
321 APSTMKIKII APPERKYSVW IGGSILASLS TFQQMWISKQ EYDESGPSIV HRKCF
```

**Supporting Peptides:**

| Peptide | Uniq | -10lgP | Mass | Length | ppm | m/z | z | RT | Fraction | Scan | Source File | Area F1 | Area F2a | Area F3a | Area F4 | Area F5 | Area F6a | Area F7a | Area F7b | Area F8 | Area F9a | Area Sample 1F10a | Area F10b | Area F11a | Area F11b | Area F12a | Area F13 | #Feature | #Feature F1 | #Feature F2a | #Feature F3a | #Feature F4 | #Feature F5 | #Feature F6a | #Feature F7a | #Feature F7b | #Feature F8 | #Feature F9a | #Feature Sample 1F10a | #Feature F10b | #Feature F11a | #Feature F11b | #Feature F12a | #Feature F13 | Start | End | PTM | AScore | Found By |
|---|---|---|---|---|---|---|---|---|---|---|---|---|---|---|---|---|---|---|---|---|---|---|---|---|---|---|---|---|---|---|---|---|---|---|---|---|---|---|---|---|---|---|---|---|---|---|---|---|---|
| R.KYSVWIGGSILASLSTFQQMWISK.Q | Y | 57.10 | 2729.4250 | 24 | 0.3 | 910.8159 | 3 | 102.40 | 1 | F1:49862 | BuCaTN01_F1.raw | 1.0126E5 | | | | | | | | | | | | | | | | 1 | 1 | 0 | 0 | 0 | 0 | 0 | 0 | 0 | 0 | 0 | 0 | 0 | 0 | 0 | 0 | 0 | 336 | 359 | | | PEAKS DB |
| K.DLYANTVLSGGTTMYPGIADR.M | Y | 50.22 | 2214.0627 | 21 | 0.8 | 1108.0396 | 2 | 73.08 | 1 | F1:33231 | BuCaTN01_F1.raw | 4.7954E4 | | | | | | | | | | | | | | | | 1 | 1 | 0 | 0 | 0 | 0 | 0 | 0 | 0 | 0 | 0 | 0 | 0 | 0 | 0 | 0 | 0 | 292 | 312 | | | PEAKS DB |
| total 2 peptides | | | | | | | | | | | | | | | | | | | | | | | | | | | | | | | | | | | | | | | | | | | | | | | | | |

**Best Unique PSM (Scan F1:49862, m/z=910.8159, z=3, RT=102.40, ppm=0.3):**

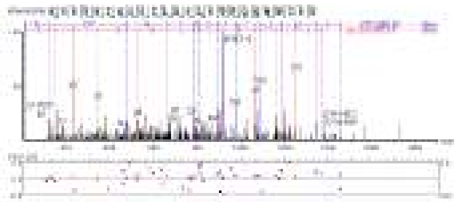

## JAV51374.1

back to list

| Protein Coverage | Supporting Peptides | Best Unique PSM |

**Protein Coverage:**

```
  1 MDDDIAALVV DNGSGMCKAG FAGDDAPRAV FPSIVGRPRH QGVMVGMGQK DSYVGDEAQS KRGILTLKYP IEHGIVTNWD
 81 DMEKIWHHTF YNELRVAPEE HPVLLTEAPL NPKANREKMT QIMFETFNTP AMYVAIQAVL SLYASGRTTG IVMDSGDGVT
161 HTVPIYEGYA LPHAILRLDL AGRDLTDYLM KILTERGYSF TTTAEREIVR DIKEKLCYVA LDFEQEMATA ASSSSLEKSY
241 ELPDGQVITI GNERFRCPEA LFQPSFLGME SCGIHETTFN SIMKCDVDIR KDLYANTVLS GGTTMYPGIA DRMQKEITAL
321 APSTMKIKII APPERKYSVW IGGSILASLS TFQQMWISKQ EYDESGPSIV HRKCF
```

**Supporting Peptides:**

| Peptide | Uniq | -10lgP | Mass | Length | ppm | m/z | z | RT | Fraction | Scan | Source File | Area F1 | Area F2a | Area F3a | Area F4 | Area F5 | Area F6a | Area F7a | Area F7b | Area F8 | Area F9a | Area Sample 1F10a | Area F10b | Area F11a | Area F11b | Area F12a | Area F13 | #Feature | #Feature F1 | #Feature F2a | #Feature F3a | #Feature F4 | #Feature F5 | #Feature F6a | #Feature F7a | #Feature F7b | #Feature F8 | #Feature F9a | #Feature Sample 1F10a | #Feature F10b | #Feature F11a | #Feature F11b | #Feature F12a | #Feature F13 | Start | End | PTM | AScore | Found By |
|---|---|---|---|---|---|---|---|---|---|---|---|---|---|---|---|---|---|---|---|---|---|---|---|---|---|---|---|---|---|---|---|---|---|---|---|---|---|---|---|---|---|---|---|---|---|---|---|---|---|
| R.KYSVWIGGSILASLSTFQQMWISK.Q | Y | 57.10 | 2729.4250 | 24 | 0.3 | 910.8159 | 3 | 102.40 | 1 | F1:49862 | BuCaTN01_F1.raw | 1.0126E5 | | | | | | | | | | | | | | | | 1 | 1 | 0 | 0 | 0 | 0 | 0 | 0 | 0 | 0 | 0 | 0 | 0 | 0 | 0 | 0 | 0 | 336 | 359 | | | PEAKS DB |
| K.DLYANTVLSGGTTMYPGIADR.M | Y | 50.22 | 2214.0627 | 21 | 0.8 | 1108.0396 | 2 | 73.08 | 1 | F1:33231 | BuCaTN01_F1.raw | 4.7954E4 | | | | | | | | | | | | | | | | 1 | 1 | 0 | 0 | 0 | 0 | 0 | 0 | 0 | 0 | 0 | 0 | 0 | 0 | 0 | 0 | 0 | 292 | 312 | | | PEAKS DB |
| total 2 peptides | | | | | | | | | | | | | | | | | | | | | | | | | | | | | | | | | | | | | | | | | | | | | | | | | |

**Best Unique PSM (Scan F1:49862, m/z=910.8159, z=3, RT=102.40, ppm=0.3):**

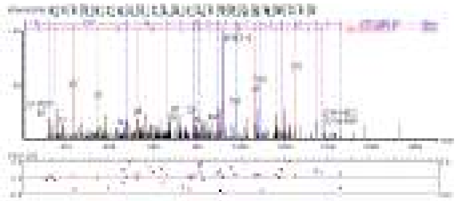

## AFJ49302.1

back to list

| Protein Coverage | Supporting Peptides | Best Unique PSM |

**Protein Coverage:**

```
  1 MDDDIAALVV DNGSGMCKAG FAGDDAPRAV FPSIVGRPRH QGVMVGMGQK DSYVGDEAQS KRGILTLKYP IEHGIVTNWD
 81 DMEKIWHHTF YNELRVAPEE HPVLLTEAPL NPKANREKMT QIMFETFNTP AMYVAIQAVL SLYASGRTTG IVMDSGDGVT
161 HTVPIYEGYA LPHAILRLDL AGRDLTDYLM KILTERGYSF TTTAEREIVR DIKEKLCYVA LDFEQEMATA ASSSSLEKSY
241 ELPDGQVITI GNERFRCPEA LFQPSFLGME SCGIHETTFN SIMKCDVDIR KDLYANTVLS GGTTMYPGIA DRMQKEITAL
321 APSTMKIKII APPERKYSVW IGGSILASLS TFQQMWISKQ EYDESGPSIV HRKCF
```

**Supporting Peptides:**

| Peptide | Uniq | -10lgP | Mass | Length | ppm | m/z | z | RT | Fraction | Scan | Source File | Area F1 | Area F2a | Area F3a | Area F4 | Area F5 | Area F6a | Area F7a | Area F7b | Area F8 | Area F9a | Area Sample 1F10a | Area F10b | Area F11a | Area F11b | Area F12a | Area F13 | #Feature | #Feature F1 | #Feature F2a | #Feature F3a | #Feature F4 | #Feature F5 | #Feature F6a | #Feature F7a | #Feature F7b | #Feature F8 | #Feature F9a | #Feature Sample 1F10a | #Feature F10b | #Feature F11a | #Feature F11b | #Feature F12a | #Feature F13 | Start | End | PTM | AScore | Found By |
|---|---|---|---|---|---|---|---|---|---|---|---|---|---|---|---|---|---|---|---|---|---|---|---|---|---|---|---|---|---|---|---|---|---|---|---|---|---|---|---|---|---|---|---|---|---|---|---|---|---|
| R.KYSVWIGGSILASLSTFQQMWISK.Q | Y | 57.10 | 2729.4250 | 24 | 0.3 | 910.8159 | 3 | 102.40 | 1 | F1:49862 | BuCaTN01_F1.raw | 1.0126E5 | | | | | | | | | | | | | | | | 1 | 1 | 0 | 0 | 0 | 0 | 0 | 0 | 0 | 0 | 0 | 0 | 0 | 0 | 0 | 0 | 0 | 336 | 359 | | | PEAKS DB |
| K.DLYANTVLSGGTTMYPGIADR.M | Y | 50.22 | 2214.0627 | 21 | 0.8 | 1108.0396 | 2 | 73.08 | 1 | F1:33231 | BuCaTN01_F1.raw | 4.7954E4 | | | | | | | | | | | | | | | | 1 | 1 | 0 | 0 | 0 | 0 | 0 | 0 | 0 | 0 | 0 | 0 | 0 | 0 | 0 | 0 | 0 | 292 | 312 | | | PEAKS DB |
| total 2 peptides | | | | | | | | | | | | | | | | | | | | | | | | | | | | | | | | | | | | | | | | | | | | | | | | | |

**Best Unique PSM (Scan F1:49862, m/z=910.8159, z=3, RT=102.40, ppm=0.3):**

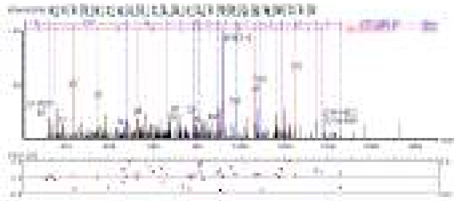

## ETE57948.1

back to list

| Protein Coverage | Supporting Peptides | Best Unique PSM |

**Protein Coverage:**

```
  1 MCKAGFAGDD APRAVFPSIV GRPRHQGVMV GMGQKDSYVG DEAQSKRGIL TLKYPIEHGI VTNWDDMEKI WHHTFYNELR
 81 VAPEEHPVLL TEAPLNPKAN REKMTQIMFE TFNTPAMYVA IQAVLSLYAS GRTTGIVMDS GDGVTHTVPI YEGYALPHAI
161 LRLDLAGRDL TDYLMKILTE RGYSFTTTAE REIVRDIKEK LCYVALDFEQ EMATAASSSS LEKSYELPDG QVITIGNERF
241 RCPEALFQPS FLGMESCGIH ETTFNSIMKC DVDIRKDLYA NTVLSGGTTM YPGIADRMQK EITALAPSTM KIKIIAPPER
321 KYSVWIGGSI LASLSTFQQM WISKQEYDES GPSIVHRKCF
```

**Supporting Peptides:**

| Peptide | Uniq | -10lgP | Mass | Length | ppm | m/z | z | RT | Fraction | Scan | Source File | Area F1 | Area F2a | Area F3a | Area F4 | Area F5 | Area F6a | Area F7a | Area F7b | Area F8 | Area F9a | Area Sample 1F10a | Area F10b | Area F11a | Area F11b | Area F12a | Area F13 | #Feature | #Feature F1 | #Feature F2a | #Feature F3a | #Feature F4 | #Feature F5 | #Feature F6a | #Feature F7a | #Feature F7b | #Feature F8 | #Feature F9a | #Feature Sample 1F10a | #Feature F10b | #Feature F11a | #Feature F11b | #Feature F12a | #Feature F13 | Start | End | PTM | AScore | Found By |
|---|---|---|---|---|---|---|---|---|---|---|---|---|---|---|---|---|---|---|---|---|---|---|---|---|---|---|---|---|---|---|---|---|---|---|---|---|---|---|---|---|---|---|---|---|---|---|---|---|---|
| R.KYSVWIGGSILASLSTFQQMWISK.Q | Y | 57.10 | 2729.4250 | 24 | 0.3 | 910.8159 | 3 | 102.40 | 1 | F1:49862 | BuCaTN01_F1.raw | 1.0126E5 | | | | | | | | | | | | | | | | 1 | 1 | 0 | 0 | 0 | 0 | 0 | 0 | 0 | 0 | 0 | 0 | 0 | 0 | 0 | 0 | 0 | 321 | 344 | | | PEAKS DB |
| K.DLYANTVLSGGTTMYPGIADR.M | Y | 50.22 | 2214.0627 | 21 | 0.8 | 1108.0396 | 2 | 73.08 | 1 | F1:33231 | BuCaTN01_F1.raw | 4.7954E4 | | | | | | | | | | | | | | | | 1 | 1 | 0 | 0 | 0 | 0 | 0 | 0 | 0 | 0 | 0 | 0 | 0 | 0 | 0 | 0 | 0 | 277 | 297 | | | PEAKS DB |
| total 2 peptides | | | | | | | | | | | | | | | | | | | | | | | | | | | | | | | | | | | | | | | | | | | | | | | | | |

**Best Unique PSM (Scan F1:49862, m/z=910.8159, z=3, RT=102.40, ppm=0.3):**

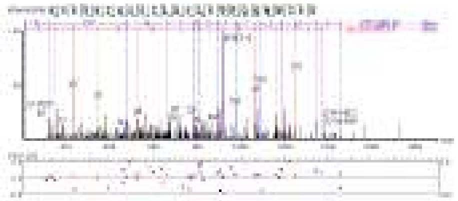

## ABW24180.1

back to list

| Protein Coverage | Supporting Peptides | Best Unique PSM |

**Protein Coverage:**

```
 1  MYPAHLLVLL AVCVSLLGAA SIPPQPLNLV QFSYLIQCAN HGSRATWHYT DYGCYCGSGG TGTPVDELDR CCQTHDNCYA
81  EAEKKGCYPK MSAYDYYCGE NGPYCRNIKK ECQRFVCDCD VEAAKCFARA PYNDANWNID TKKRCQ
```

C Carbamidomethylation (+57.02)

**Supporting Peptides:**

| Peptide | Uniq | -10lgP | Mass | Length | ppm | m/z | z | RT | Fraction | Scan | Source File | Area F1 | Area F2a | Area F3a | Area F4 | Area F5 | Area F6a | Area F7a | Area F7b | Area F8 | Area F9a | Area Sample 1F10a | Area F10b | Area F11a | Area F11b | Area F12a | Area F13 | #Feature | #Feature F1 | #Feature F2a | #Feature F3a | #Feature F4 | #Feature F5 | #Feature F6a | #Feature F7a | #Feature F7b | #Feature F8 | #Feature F9a | #Feature Sample 1F10a | #Feature F10b | #Feature F11a | #Feature F11b | #Feature F12a | #Feature F13 | Start | End | PTM | AScore | Found By |
|---|---|---|---|---|---|---|---|---|---|---|---|---|---|---|---|---|---|---|---|---|---|---|---|---|---|---|---|---|---|---|---|---|---|---|---|---|---|---|---|---|---|---|---|---|---|---|---|---|---|
| D.YGC(+57.02)YC(+57.02)GSGGTGTPVDELDR.C | Y | 46.48 | 2062.8359 | 19 | -3.4 | 1032.4165 | 2 | 62.52 | 15 | F15:37112 | BuCaTN01_F12a.raw | | | | | | | | | | | | | 0 | | 0 | | 0 | 0 | 0 | 0 | 0 | 0 | 0 | 0 | 0 | 0 | 0 | 0 | 0 | 0 | 0 | 0 | 0 | 52 | 70 | Carbamidomethylation | C3:Carbamidomethylation:1000.00;C5:Carbamidomethylation:1000.00 | PEAKS DB |
| S.GGTGTPVDELDR.C | Y | 43.22 | 1215.5731 | 12 | 4.5 | 608.7935 | 2 | 43.43 | 15 | F15:20196 | BuCaTN01_F12a.raw | | | | | | | | | | | | 6.303E6 | | | 4.7967E7 | | 2 | 0 | 0 | 0 | 0 | 0 | 0 | 0 | 0 | 0 | 0 | 0 | 1 | 0 | 0 | 1 | 0 | 59 | 70 | | | PEAKS DB |
| total 2 peptides | | | | | | | | | | | | | | | | | | | | | | | | | | | | | | | | | | | | | | | | | | | | | | | | | |

**Best Unique PSM (Scan F15:37112, m/z=1032.4165, z=2, RT=62.52, ppm=-3.4):**

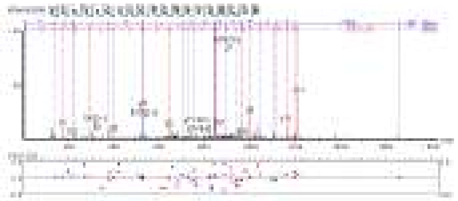

## QBF53421.1

back to list

| Protein Coverage | Supporting Peptides | Best Unique PSM |

**Protein Coverage:**

```
  1  MIRFGNPSSS DKRRQRCWSW YWGGLLLLWA VAETRADIHY ATVYWLEAEK SFQIKDVLDK NGDAYGYYND TIQSTGWGIL
 81  EIKAGYGNQR ISNEILMYAA GFLEGYLTAS HMSDHFANLF PVMIKNVIIE QKVKDFIQKQ DEWTRQQIKN NKDDPFWRNA
161  GYVIAQLDGL YMGNVEWAKR QKRTPLTDFE ISFLNALGDL LDIIPALNSQ LRKSDFRSMP DVSRIYQWDM GHCSALIKVL
241  PGYENIYFAH SSWFTYAATL RIYKHWDFKI TDPQTKTGRA SFSSYPGLLV SLDDFYILGS GLIMLQTTNS VYNLFLLKQV
321  VPESLFAWER VRIANMMADS GKTWAQTFEK ENSGTYNNQY MILDTKKIKL QRSLEDGTLY IVEQIPKLVK YSDQTKVLRN
401  GYWPSYNIPF HKVIYNMSGY TEYVQRYGLE FSYEMAPRAK IFRRDQGKVT DMESMKHIMR YNNYKEDPYT KHNPCNTICC
481  RQDLSHKSPV PAGCYDSKVA DISMAAKFTA YAINGPPVEK DLPVFSWVHF NQTKHQGLPE SYNFDFVTMK PVL
```

**Supporting Peptides:**

| Peptide | Uniq | -10lgP | Mass | Length | ppm | m/z | z | RT | Fraction | Scan | Source File | Area F1 | Area F2a | Area F3a | Area F4 | Area F5 | Area F6a | Area F7a | Area F7b | Area F8 | Area F9a | Area Sample 1F10a | Area F10b | Area F11a | Area F11b | Area F12a | Area F13 | #Feature | #Feature F1 | #Feature F2a | #Feature F3a | #Feature F4 | #Feature F5 | #Feature F6a | #Feature F7a | #Feature F7b | #Feature F8 | #Feature F9a | #Feature Sample 1F10a | #Feature F10b | #Feature F11a | #Feature F11b | #Feature F12a | #Feature F13 | Start | End | PTM | AScore | Found By |
|---|---|---|---|---|---|---|---|---|---|---|---|---|---|---|---|---|---|---|---|---|---|---|---|---|---|---|---|---|---|---|---|---|---|---|---|---|---|---|---|---|---|---|---|---|---|---|---|---|---|
| K.FTAYAINGPPVEK.D | Y | 57.42 | 1405.7241 | 13 | 0.6 | 703.8698 | 2 | 52.52 | 16 | F16:24918 | BuCaTN01_F13.raw | | | | | | | | | | | | | | | | 2.1472E5 | 1 | 0 | 0 | 0 | 0 | 0 | 0 | 0 | 0 | 0 | 0 | 0 | 0 | 0 | 0 | 0 | 1 | 508 | 520 | | | PEAKS DB |
| total 1 peptides | | | | | | | | | | | | | | | | | | | | | | | | | | | | | | | | | | | | | | | | | | | | | | | | | |

**Best Unique PSM (Scan F16:24918, m/z=703.8698, z=2, RT=52.52, ppm=0.6):**

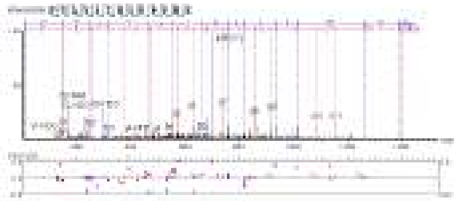

## XP_026550416.1

back to list

| Protein Coverage | Supporting Peptides | Best Unique PSM |

**Protein Coverage:**

```
  1  MVRFGSAASS DNRRRRCWSW YWGGLLLLWA VAETRADLHY ATVYWLEAEK SFQVKDLLDK NGDAYGYYND TVQSTGWGIL
 81  EIKAGYGSQL VSNEILMYAA GFLEGYLTAL RMSDHVANLY HQLIKNVITE KKVKDFMQKQ DEWTRQQIKN NKDDPFWRNA
161  GYIIAQLDGL YMGNLEWAKR QKRTPLTKFE ISFLNAIGDM LDLIPALSPE SRNDDFLSMS EVSKMYEWDM GHCSALIKVL
241  PGYENIYFAH SSWFTYAATL RIYKHWDFRI IDPQTKTGHA SFSSYPGLLA SLDDFYILGS GLIMLQTTNS VFNISLLQQV
321  VPESLFAWER VRIANMMADS GKTWAQTFKK QNSGTYNNQY MILDTKKIKL RRSIEDGTLY VIEQVPNLVE YSDQTTILRK
401  GYWPSYNIPF HKVIYNMSGY REYVQKYGLD FSYEMAPRAK IFRRDQGKVI DIESMKRIMR YNNYKKDPYT KHNPCNTICC
481  RQDLYYMTPV PAGCYDSKVA DINMAAKFTA YAINGPPVEK GLPIFSWVHF NETTHQGLPE SYNFDFVTMK PVL
```

**Supporting Peptides:**

| Peptide | Uniq | -10lgP | Mass | Length | ppm | m/z | z | RT | Fraction | Scan | Source File | Area F1 | Area F2a | Area F3a | Area F4 | Area F5 | Area F6a | Area F7a | Area F7b | Area F8 | Area F9a | Area Sample 1F10a | Area F10b | Area F11a | Area F11b | Area F12a | Area F13 | #Feature | #Feature F1 | #Feature F2a | #Feature F3a | #Feature F4 | #Feature F5 | #Feature F6a | #Feature F7a | #Feature F7b | #Feature F8 | #Feature F9a | #Feature Sample 1F10a | #Feature F10b | #Feature F11a | #Feature F11b | #Feature F12a | #Feature F13 | Start | End | PTM | AScore | Found By |
|---|---|---|---|---|---|---|---|---|---|---|---|---|---|---|---|---|---|---|---|---|---|---|---|---|---|---|---|---|---|---|---|---|---|---|---|---|---|---|---|---|---|---|---|---|---|---|---|---|---|
| K.FTAYAINGPPVEK.G | Y | 57.42 | 1405.7241 | 13 | 0.6 | 703.8698 | 2 | 52.52 | 16 | F16:24918 | BuCaTN01_F13.raw | | | | | | | | | | | | | | | | 2.1472E5 | 1 | 0 | 0 | 0 | 0 | 0 | 0 | 0 | 0 | 0 | 0 | 0 | 0 | 0 | 0 | 0 | 1 | 508 | 520 | | | PEAKS DB |
| total 1 peptides | | | | | | | | | | | | | | | | | | | | | | | | | | | | | | | | | | | | | | | | | | | | | | | | | |

**Best Unique PSM (Scan F16:24918, m/z=703.8698, z=2, RT=52.52, ppm=0.6):**

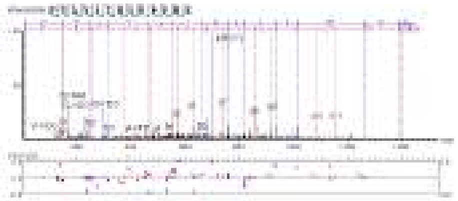

## ACR78473.1

back to list

| Protein Coverage | Supporting Peptides | Best Unique PSM |

**Protein Coverage:**

```
  1 MVRFGSAASS DNRRRRCWSW YWGGLLLLWA VAETRADLHY ATVYWLEAEK SFQVKDLLDK NGDAYGYYND TVQSTGWGIL
 81 EIKAGYGSQL VSNEILMYAA GFLEGYLTAS RMSDHVANLY HQMIKNVITE QKVKDFMQKQ DEWTRQQIKN NKDDPFWRNA
161 GYIIAQLDGL YMGNLEWAKR QKRTPLTKFE ISFLNALGDL LDLIPALSPE SRNNGFLSMS EISKMYEWDM GHCSALIKVL
241 PGYENIYFAH SSWFTYAATL RIYKHLDFRI IDPQTKTGRA SFSSYPGLLA SLDDFYILGS GLIMLQTTNS VFNISLLQQV
321 VPESLFAWER VRIANMMADS GKTWAQTFKK QNSGTYNNQY MILDTKKIKL RRSIEDGTLY IIEQVPNLVE YSDQTTILRK
401 GYWPSYNIPF HKVIYNMSGY REYVQKYGLD FSYEMAPRAK IFRRDQGKVI DIESMKRIMR YNNYKKDPYT KHNPCNTICC
481 RQDLYYMTPV PAGCYDSKVA DINMAAKFTA YAINGPPVEK GLPIFSWVHF NETTHQGLPE SYNFDFVTMK PVL
```

**Supporting Peptides:**

| Peptide | Uniq | -10lgP | Mass | Length | ppm | m/z | z | RT | Fraction | Scan | Source File | Area F1 | Area F2a | Area F3a | Area F4 | Area F5 | Area F6a | Area F7a | Area F7b | Area F8 | Area F9a | Area Sample 1F10a | Area F10b | Area F11a | Area F11b | Area F12a | Area F13 | #Feature | #Feature F1 | #Feature F2a | #Feature F3a | #Feature F4 | #Feature F5 | #Feature F6a | #Feature F7a | #Feature F7b | #Feature F8 | #Feature F9a | #Feature Sample 1F10a | #Feature F10b | #Feature F11a | #Feature F11b | #Feature F12a | #Feature F13 | Start | End | PTM | AScore | Found By |
|---|---|---|---|---|---|---|---|---|---|---|---|---|---|---|---|---|---|---|---|---|---|---|---|---|---|---|---|---|---|---|---|---|---|---|---|---|---|---|---|---|---|---|---|---|---|---|---|---|---|
| K.FTAYAINGPPVEK.G | Y | 57.42 | 1405.7241 | 13 | 0.6 | 703.8698 | 2 | 52.52 | 16 | F16:24918 | BuCaTN01_F13.raw | | | | | | | | | | | | | | | | 2.1472E5 | 1 | 0 | 0 | 0 | 0 | 0 | 0 | 0 | 0 | 0 | 0 | 0 | 0 | 0 | 0 | 0 | 1 | 508 | 520 | | | PEAKS DB |
| total 1 peptides | | | | | | | | | | | | | | | | | | | | | | | | | | | | | | | | | | | | | | | | | | | | | | | | | |

**Best Unique PSM (Scan F16:24918, m/z=703.8698, z=2, RT=52.52, ppm=0.6):**

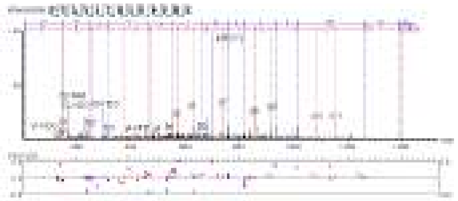

## F8J2D3.1

back to list

| Protein Coverage | Supporting Peptides | Best Unique PSM |

**Protein Coverage:**

```
  1 MVRFGSAASS DNRRRRCWSW YWGGLLLLWA VAETRADLHY ATVYWLEAEK SFQVKDLLDK NGDAYGYYND TVQSTGWGIL
 81 EIKAGYGSQL VSNEILMYAA GFLEGYLTAS RMSDHVANLY HQMIKNVITE QKVKDFMQKQ DEWTRQQIKN NKDDPFWRNA
161 GYIIAQLDGL YMGNLEWAKR QKRTPLTKFE ISFLNALGDL LDLIPALSPE SRNNGFLSMS EISKMYEWDM GHCSALIKVL
241 PGYENIYFAH SSWFTYAATL RIYKHLDFRI IDPQTKTGRA SFSSYPGLLA SLDDFYILGS GLIMLQTTNS VFNISLLQQV
321 VPESLFAWER VRIANMMADS GKTWAQTFKK QNSGTYNNQY MILDTKKIKL RRSIEDGTLY IIEQVPNLVE YSDQTTILRK
401 GYWPSYNIPF HKVIYNMSGY REYVQKYGLD FSYEMAPRAK IFRRDQGKVI DIESMKRIMR YNNYKKDPYT KHNPCNTICC
481 RQDLYYMTPV PAGCYDSKVA DINMAAKFTA YAINGPPVEK GLPIFSWVHF NETTHQGLPE SYNFDFVTMK PVL
```

**Supporting Peptides:**

| Peptide | Uniq | -10lgP | Mass | Length | ppm | m/z | z | RT | Fraction | Scan | Source File | Area F1 | Area F2a | Area F3a | Area F4 | Area F5 | Area F6a | Area F7a | Area F7b | Area F8 | Area F9a | Area Sample 1F10a | Area F10b | Area F11a | Area F11b | Area F12a | Area F13 | #Feature | #Feature F1 | #Feature F2a | #Feature F3a | #Feature F4 | #Feature F5 | #Feature F6a | #Feature F7a | #Feature F7b | #Feature F8 | #Feature F9a | #Feature Sample 1F10a | #Feature F10b | #Feature F11a | #Feature F11b | #Feature F12a | #Feature F13 | Start | End | PTM | AScore | Found By |
|---|---|---|---|---|---|---|---|---|---|---|---|---|---|---|---|---|---|---|---|---|---|---|---|---|---|---|---|---|---|---|---|---|---|---|---|---|---|---|---|---|---|---|---|---|---|---|---|---|---|
| K.FTAYAINGPPVEK.G | Y | 57.42 | 1405.7241 | 13 | 0.6 | 703.8698 | 2 | 52.52 | 16 | F16:24918 | BuCaTN01_F13.raw | | | | | | | | | | | | | | | | 2.1472E5 | 1 | 0 | 0 | 0 | 0 | 0 | 0 | 0 | 0 | 0 | 0 | 0 | 0 | 0 | 0 | 0 | 1 | 508 | 520 | | | PEAKS DB |
| total 1 peptides | | | | | | | | | | | | | | | | | | | | | | | | | | | | | | | | | | | | | | | | | | | | | | | | | |

**Best Unique PSM (Scan F16:24918, m/z=703.8698, z=2, RT=52.52, ppm=0.6):**

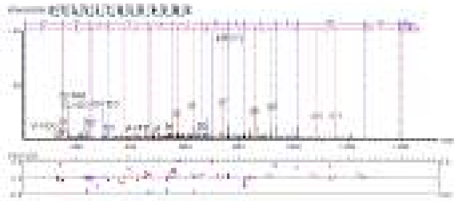

## AWX67672.1

back to list

| Protein Coverage | Supporting Peptides | Best Unique PSM |

**Protein Coverage:**

```
  1 MVPFSSASSS DNRRQRCWSW YWGGLLLLWA VAETRADLHY ATVYWLEAEK TFQVRDVLDK NGDAYGYYND TVQSTGWGIL
 81 EIKAGYGNQP VSNEIFMYSA GFLEGYLTAS RMSDHVANLY PQLIKNVIIE QKIKEFMQKQ DEWTRQQIKN NKDDPFWRNA
161 GYVIAQLDGL YMGNMEWAKR QKRTPLTVFE IIFLNAIGDL LDLIPALTSD SRNNDFLSMP EVSRMYQWDM GHCSALIKVL
241 PGYENIYFGH SSWFTYAATL RIYKHWDFRI TDPQTKTGRA SFSSYPGFLV SLDDFYILGS GLIMLQTTNS VFNLSLLKQV
321 VPESLFAWQR VRIANMMADS GKTWAQTFEK QNSGTYNNQY MILDTKKIKL RRSIDDGALY IIEQVPNLVE YSDQTNILRT
401 GYWPSYNIPF HNIIYNMSGY GEYVQKYGLD FSYELAPRAK IFRRDQGKVI DIESMKYIMR YNNYKKDPYT RHNPCNTICC
481 REDLKYKTPV PAGCYDSKVA DINMAAEFTA YAINGPPVEK GLPIFSWVHF NKTKHQGLPE SYNFDFVTMK PVL
```

**Supporting Peptides:**

| Peptide | Uniq | -10lgP | Mass | Length | ppm | m/z | z | RT | Fraction | Scan | Source File | Area F1 | Area F2a | Area F3a | Area F4 | Area F5 | Area F6a | Area F7a | Area F7b | Area F8 | Area F9a | Area Sample 1F10a | Area F10b | Area F11a | Area F11b | Area F12a | Area F13 | #Feature | #Feature F1 | #Feature F2a | #Feature F3a | #Feature F4 | #Feature F5 | #Feature F6a | #Feature F7a | #Feature F7b | #Feature F8 | #Feature F9a | #Feature Sample 1F10a | #Feature F10b | #Feature F11a | #Feature F11b | #Feature F12a | #Feature F13 | Start | End | PTM | AScore | Found By |
|---|---|---|---|---|---|---|---|---|---|---|---|---|---|---|---|---|---|---|---|---|---|---|---|---|---|---|---|---|---|---|---|---|---|---|---|---|---|---|---|---|---|---|---|---|---|---|---|---|---|
| E.FTAYAINGPPVEK.G | Y | 57.42 | 1405.7241 | 13 | 0.6 | 703.8698 | 2 | 52.52 | 16 | F16:24918 | BuCaTN01_F13.raw | | | | | | | | | | | | | | | | 2.1472E5 | 1 | 0 | 0 | 0 | 0 | 0 | 0 | 0 | 0 | 0 | 0 | 0 | 0 | 0 | 0 | 0 | 1 | 508 | 520 | | | PEAKS DB |
| total 1 peptides | | | | | | | | | | | | | | | | | | | | | | | | | | | | | | | | | | | | | | | | | | | | | | | | | |

**Best Unique PSM (Scan F16:24918, m/z=703.8698, z=2, RT=52.52, ppm=0.6):**

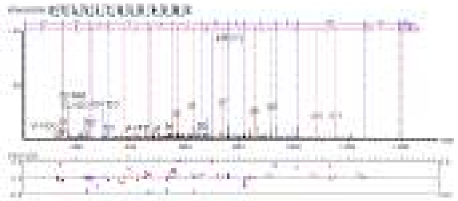

## JAG67025.1

back to list

| Protein Coverage | Supporting Peptides | Best Unique PSM |

**Protein Coverage:**

1 MVPFSSASSS DNRRQRCWSW YWGGLLLLWA VAETRADLHY ATVYWLEAEK TFQVRDVLDK NGDAYGYYND TVQSSGWGIL
81 EIKAGYGNQP VSNEIFMYSA GFLEGYLTAS RMSDHVANLY PQLIKNVIIE QKIKEFTQKQ DEWTRQQIKN NKDDPFWRNA
161 GYVIAQLDGL YMGNVEWAKR QKRTPLTVFE IIFLNAIGDL LDLIPALTSD SRNNDFLSMP EVSRMYQWDM GHCSALIKVL
241 PGYENIYFGH SSWFTYAATL RIYKHWDFRI TDPQTKTGRA SFSSYPGFLV SLDDFYILGS GLIMLQTTNS VFNLSLLKQV
321 VPESLFAWQR VRIANMMADS GKTWAQTFEK QNSGTYNNQY MILDTKKIKL RRSIDDGALY IIEQVPNLVE YSDQTNILRT
401 GYWPSYNIPF HNIIYNMSGY GEYVQKYGLD FSYELAPRAK IFRRDQGKVI DIESMKYIMR YNNYKKDPYT RHNPCNTICC
481 REDLKYKTPV PAGCYDSKVA DINMAAE**FTA YAINGPPVEK** GLPIFSWVHF NKTKHQGLPE SYNFDFVTMK PVL

**Supporting Peptides:**

| Peptide | Uniq | -10lgP | Mass | Length | ppm | m/z | z | RT | Fraction | Scan | Source File | Area F1 | Area F2a | Area F3a | Area F4 | Area F5 | Area F6a | Area F7a | Area F7b | Area F8 | Area F9a | Area Sample 1F10a | Area F10b | Area F11a | Area F11b | Area F12a | Area F13 | #Feature | #Feature F1 | #Feature F2a | #Feature F3a | #Feature F4 | #Feature F5 | #Feature F6a | #Feature F7a | #Feature F7b | #Feature F8 | #Feature F9a | #Feature Sample 1F10a | #Feature F10b | #Feature F11a | #Feature F11b | #Feature F12a | #Feature F13 | Start | End | PTM | AScore | Found By |
|---|---|---|---|---|---|---|---|---|---|---|---|---|---|---|---|---|---|---|---|---|---|---|---|---|---|---|---|---|---|---|---|---|---|---|---|---|---|---|---|---|---|---|---|---|---|---|---|---|---|
| E.FTAYAINGPPVEK.G | Y | 57.42 | 1405.7241 | 13 | 0.6 | 703.8698 | 2 | 52.52 | 16 | F16:24918 | BuCaTN01_F13.raw | | | | | | | | | | | | | | | | 2.1472E5 | 1 | 0 | 0 | 0 | 0 | 0 | 0 | 0 | 0 | 0 | 0 | 0 | 0 | 0 | 0 | 0 | 1 | 508 | 520 | | | PEAKS DB |
| total 1 peptides | | | | | | | | | | | | | | | | | | | | | | | | | | | | | | | | | | | | | | | | | | | | | | | | | |

**Best Unique PSM (Scan F16:24918, m/z=703.8698, z=2, RT=52.52, ppm=0.6):**

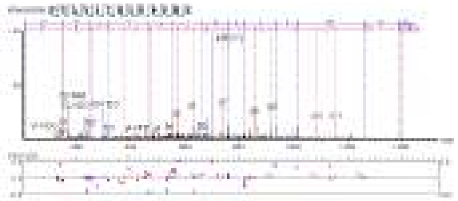

## BAN82155.1

back to list

| Protein Coverage | Supporting Peptides | Best Unique PSM |

**Protein Coverage:**

1 MIRFGNPSSS VKRRQRCRSW YWGGLLLLWA VAETRADIHY ATVYWLEAEK SFQIKAVLDK NGDAYGYYND TIQSTGWGIL
81 EIKAGYGNQP ISNEILMYAA GFLEGYLTAS HMSDHFANLF PLMIKNVIIE QKVKDFIQKQ DEWTRQQIKN NKDDPFWRNA
161 GYVIVQLDGL YMGNVEWAKQ QKRTPLTNFE ISFLNVIGDL LDLIPALYSE LRKSDFRSMP DVSRIYQWDM GHCSALIKVL
241 PGYENIYFAH SSWFTYAATL RIYKHLDFKI TDPQTKTGRA SFSSYPGFLS SLDDFYILGS GLIMLQTTNS VFNLSLLKKV
321 VPESLFAWER VRIANMMADS GKTWAETFEK ENSGTYNNQY MILDTKKIKL QRSLEDGTLY IIEQIPKLVK YSDQTKVLRN
401 GYWPSYNIPF DKVIYNMSGY REYVQRHGLE FSYEMAPRAK IFRRDQGKVT DMESMKSIMR YNNYKEDPYA KRNPCNTICC
481 RQDLDRRTPV PAGCYDSKVA DISMASK**FTA YAINGPPVEK** GLPVFSWVHF NKTKHQGLPE SYNFDFVTMK PVL

**Supporting Peptides:**

| Peptide | Uniq | -10lgP | Mass | Length | ppm | m/z | z | RT | Fraction | Scan | Source File | Area F1 | Area F2a | Area F3a | Area F4 | Area F5 | Area F6a | Area F7a | Area F7b | Area F8 | Area F9a | Area Sample 1F10a | Area F10b | Area F11a | Area F11b | Area F12a | Area F13 | #Feature | #Feature F1 | #Feature F2a | #Feature F3a | #Feature F4 | #Feature F5 | #Feature F6a | #Feature F7a | #Feature F7b | #Feature F8 | #Feature F9a | #Feature Sample 1F10a | #Feature F10b | #Feature F11a | #Feature F11b | #Feature F12a | #Feature F13 | Start | End | PTM | AScore | Found By |
|---|---|---|---|---|---|---|---|---|---|---|---|---|---|---|---|---|---|---|---|---|---|---|---|---|---|---|---|---|---|---|---|---|---|---|---|---|---|---|---|---|---|---|---|---|---|---|---|---|---|
| K.FTAYAINGPPVEK.G | Y | 57.42 | 1405.7241 | 13 | 0.6 | 703.8698 | 2 | 52.52 | 16 | F16:24918 | BuCaTN01_F13.raw | | | | | | | | | | | | | | | | 2.1472E5 | 1 | 0 | 0 | 0 | 0 | 0 | 0 | 0 | 0 | 0 | 0 | 0 | 0 | 0 | 0 | 0 | 1 | 508 | 520 | | | PEAKS DB |
| total 1 peptides | | | | | | | | | | | | | | | | | | | | | | | | | | | | | | | | | | | | | | | | | | | | | | | | | |

**Best Unique PSM (Scan F16:24918, m/z=703.8698, z=2, RT=52.52, ppm=0.6):**

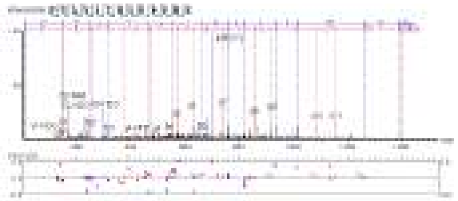

## BAP39922.1

back to list

| Protein Coverage | Supporting Peptides | Best Unique PSM |

**Protein Coverage:**

1 MIRFGTPSSS DKRPQRCRRW YWGGLLLFWA VAETRADIHY ATVYWLEAEK SFQIKDVLDK NGDAYGYYND TIQSTGWGIL
81 EIKAGYGNQP ISNEILMYAA GFLEGYLTAS HMSDHFANLY PQMIKNVIIE QKVKDFMQKQ DEWTRQQIKN NKDDPFWRNA
161 GYVIAQLDGL YMGNVEWAKR QKRTPLTDFE ISFLNAIGDL LDLIPALHSE LRKSYFRSMP DVSRIYQWDM GHCSALIKVL
241 PGYENIYFAH SSWFTYAATL RIYKHLDFKI TDPQTKTGRA SFSSYPGFLV STDDFYILGS GLIMLQTTNS VLDLSLLKKV
321 VPESLFAWER VRIANMMADS GKTWAETFEK QNSGTYNNQY MILDTKKIKL QRSLEDGTLY IIEQVPKLVK YSDQTNILRN
401 GYWPSYNIPF DKEIYNMSGY GEYVQKHGLE FSYEMAPRAK IFRRDQGKVT DMESMKSIMR YNNYKEDPYA KHNPCNTICC
481 RQDLDRRTPV PAGCYDSKVA DISMAAK**FTA YAINGPPVEK** GLPVFNWVHF NKTKHQGLPE SYNFDFVTMK PVL

**Supporting Peptides:**

| Peptide | Uniq | -10lgP | Mass | Length | ppm | m/z | z | RT | Fraction | Scan | Source File | Area F1 | Area F2a | Area F3a | Area F4 | Area F5 | Area F6a | Area F7a | Area F7b | Area F8 | Area F9a | Area Sample 1F10a | Area F10b | Area F11a | Area F11b | Area F12a | Area F13 | #Feature | #Feature F1 | #Feature F2a | #Feature F3a | #Feature F4 | #Feature F5 | #Feature F6a | #Feature F7a | #Feature F7b | #Feature F8 | #Feature F9a | #Feature Sample 1F10a | #Feature F10b | #Feature F11a | #Feature F11b | #Feature F12a | #Feature F13 | Start | End | PTM | AScore | Found By |
|---|---|---|---|---|---|---|---|---|---|---|---|---|---|---|---|---|---|---|---|---|---|---|---|---|---|---|---|---|---|---|---|---|---|---|---|---|---|---|---|---|---|---|---|---|---|---|---|---|---|
| K.FTAYAINGPPVEK.G | Y | 57.42 | 1405.7241 | 13 | 0.6 | 703.8698 | 2 | 52.52 | 16 | F16:24918 | BuCaTN01_F13.raw | | | | | | | | | | | | | | | | 2.1472E5 | 1 | 0 | 0 | 0 | 0 | 0 | 0 | 0 | 0 | 0 | 0 | 0 | 0 | 0 | 0 | 0 | 1 | 508 | 520 | | | PEAKS DB |
| total 1 peptides | | | | | | | | | | | | | | | | | | | | | | | | | | | | | | | | | | | | | | | | | | | | | | | | | |

**Best Unique PSM (Scan F16:24918, m/z=703.8698, z=2, RT=52.52, ppm=0.6):**

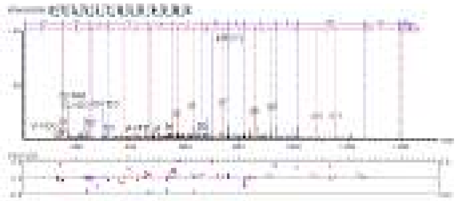

## BAN82026.1

back to list

| Protein Coverage | Supporting Peptides | Best Unique PSM |

**Protein Coverage:**

1 MIRFGTPSSS DKRRQRCRSW YWGGLLLFWA VAETRADIHY ATVYWLEAEK SFQIKDVLDR NGDAYGYYND TIQSTGWGIL
81 EIKAGYGNQP ISNEILMYAA GFLEGYLTAS HMSDHFANLY PQMIKNVIIE QKVKDFMQKQ DEWTRQQIKN NKDDPFWRNA
161 GYVIAQLDGL YMGNVEWAKR QKRTPLTDFE ISFLNAIGDL LDLIPALHSE LRKSYFRSMP DVSRIYQWDM GHCSALIKVL
241 PGYENIYFAH SSWFTYAATL RIYKHLDFKI TDPQTKTGRA SFSSYPGFLV SLDDFYILGN GLIMLQTTNS VFNLSLLKKV
321 VPESLFAWER VRIANMMADS GKTWAETFEK QNSGTYNNQY MILDTKKIKL QRSLEDGTLY IIEQVPKLVK YSDQTKVLRN
401 GYWPSYNIPF DKEIYNMSGY GEYVQRHGLE FSYEMAPRAK IFRRDQGKVT DMESMKSIMR YNNYKEDPYA KHNPCNTICC
481 REDLNRRTPV PAGCYDSKVA DISMAAK**FTA YAINGPPVEK** GLPVFNWVHF NKTKHQGLPE SYNFDFVTMK PVL

**Supporting Peptides:**

| Peptide | Uniq | -10lgP | Mass | Length | ppm | m/z | z | RT | Fraction | Scan | Source File | Area F1 | Area F2a | Area F3a | Area F4 | Area F5 | Area F6a | Area F7a | Area F7b | Area F8 | Area F9a | Area Sample 1F10a | Area F10b | Area F11a | Area F11b | Area F12a | Area F13 | #Feature | #Feature F1 | #Feature F2a | #Feature F3a | #Feature F4 | #Feature F5 | #Feature F6a | #Feature F7a | #Feature F7b | #Feature F8 | #Feature F9a | #Feature Sample 1F10a | #Feature F10b | #Feature F11a | #Feature F11b | #Feature F12a | #Feature F13 | Start | End | PTM | AScore | Found By |
|---|---|---|---|---|---|---|---|---|---|---|---|---|---|---|---|---|---|---|---|---|---|---|---|---|---|---|---|---|---|---|---|---|---|---|---|---|---|---|---|---|---|---|---|---|---|---|---|---|---|

| Peptide | Uniq | -10lgP | Mass | Length | ppm | m/z | z | RT | Fraction | Scan | Source File | Area F1 | Area F2a | Area F3a | Area F4 | Area F5 | Area F6a | Area F7a | Area F7b | Area F8 | Area F9a | Area Sample 1F10a | Area F10b | Area F11a | Area F11b | Area F12a | Area F13 | #Feature | #Feature F1 | #Feature F2a | #Feature F3a | #Feature F4 | #Feature F5 | #Feature F6a | #Feature F7a | #Feature F7b | #Feature F8 | #Feature F9a | #Feature Sample 1F10a | #Feature F10b | #Feature F11a | #Feature F11b | #Feature F12a | #Feature F13 | Start | End | PTM | AScore | Found By |
|---|---|---|---|---|---|---|---|---|---|---|---|---|---|---|---|---|---|---|---|---|---|---|---|---|---|---|---|---|---|---|---|---|---|---|---|---|---|---|---|---|---|---|---|---|---|---|---|---|---|
| K.FTAYAINGPPVEK.G | Y | 57.42 | 1405.7241 | 13 | 0.6 | 703.8698 | 2 | 52.52 | 16 | F16:24918 | BuCaTN01_F13.raw | | | | | | | | | | | | | | | | 2.1472E5 | 1 | 0 | 0 | 0 | 0 | 0 | 0 | 0 | 0 | 0 | 0 | 0 | 0 | 0 | 0 | 0 | 1 | 508 | 520 | | | PEAKS DB |
| total 1 peptides | | | | | | | | | | | | | | | | | | | | | | | | | | | | | | | | | | | | | | | | | | | | | | | | | |

**Best Unique PSM (Scan F16:24918, m/z=703.8698, z=2, RT=52.52, ppm=0.6):**

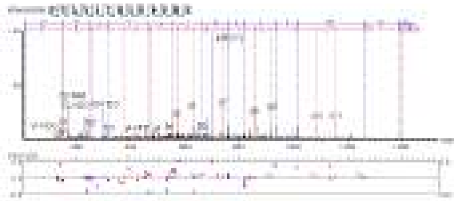

## BAP39973.1

back to list

| Protein Coverage | Supporting Peptides | Best Unique PSM |

**Protein Coverage:**

```
  1 MIRFGTPSSS DKRRQRCRSW YWGGLLLFWA VAETRADIHY ATVYWLEAEK SFQIKDVLDR NGDAYGYYND TIQSTGWGIL
 81 EIKAGYGNQP ISNEILMYAA GFLEGYLTAS HMSDHFANLY PQMIKNVIIE QKVKDFMQKQ DEWTRQQIKN NKDDPFWRNA
161 GYVIAQLDGL YMGNVEWAKR QKRTPLTDFE ISFLNAIGDL LDLIPALHSE LRKSYFRSMP DVSRIYQWDM GHCSALIKVL
241 PGYENIYFAH SSWFTYAATL RIYKHLDFKI TDPQTKTGRA SFSSYPGFLV SLDDFYILGN GLIMLQTTNS VFNLSLLKKV
321 VPESLFAWER VRIANMMADS GKTWAETFEK QNSGTYNNQY MILDTKKIKL QRSLEDGTLY IIEQVPKLVK YSDQTKVLRN
401 GYWPSYNIPF DKEIYNMSGY GEYVQRHGLE FSYEMAPRAK IFRRDQGKVT DMESMKSIMR YNNYKEDPYA KHNPCNTICC
481 REDLNRRTPV PAGCYDSKVA DISMAAKFTA YAINGPPVEK GLPVFNWVHF NKTKHQGLPE SYNFDFVTMK PVL
```

**Supporting Peptides:**

| Peptide | Uniq | -10lgP | Mass | Length | ppm | m/z | z | RT | Fraction | Scan | Source File | Area F1 | Area F2a | Area F3a | Area F4 | Area F5 | Area F6a | Area F7a | Area F7b | Area F8 | Area F9a | Area Sample 1F10a | Area F10b | Area F11a | Area F11b | Area F12a | Area F13 | #Feature | #Feature F1 | #Feature F2a | #Feature F3a | #Feature F4 | #Feature F5 | #Feature F6a | #Feature F7a | #Feature F7b | #Feature F8 | #Feature F9a | #Feature Sample 1F10a | #Feature F10b | #Feature F11a | #Feature F11b | #Feature F12a | #Feature F13 | Start | End | PTM | AScore | Found By |
|---|---|---|---|---|---|---|---|---|---|---|---|---|---|---|---|---|---|---|---|---|---|---|---|---|---|---|---|---|---|---|---|---|---|---|---|---|---|---|---|---|---|---|---|---|---|---|---|---|---|
| K.FTAYAINGPPVEK.G | Y | 57.42 | 1405.7241 | 13 | 0.6 | 703.8698 | 2 | 52.52 | 16 | F16:24918 | BuCaTN01_F13.raw | | | | | | | | | | | | | | | | 2.1472E5 | 1 | 0 | 0 | 0 | 0 | 0 | 0 | 0 | 0 | 0 | 0 | 0 | 0 | 0 | 0 | 0 | 1 | 508 | 520 | | | PEAKS DB |
| total 1 peptides | | | | | | | | | | | | | | | | | | | | | | | | | | | | | | | | | | | | | | | | | | | | | | | | | |

**Best Unique PSM (Scan F16:24918, m/z=703.8698, z=2, RT=52.52, ppm=0.6):**

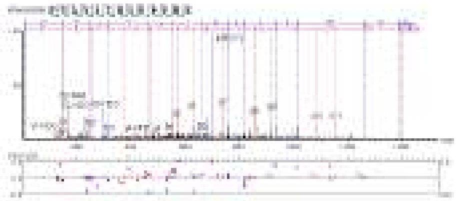

## JAI12031.1

back to list

| Protein Coverage | Supporting Peptides | Best Unique PSM |

**Protein Coverage:**

```
  1 MIRFGNPSSS DKRRQRCRSW YWGGLLLLWA VAETRADIHY ATVYWLEAEK SFQIKDVLDK NGDAYGYYND AIQSTGWGIL
 81 EIKAGYGNQP ISNEILMYAA GFLEGYLTAS HMSDHFANLF PLMIKNVIIE QKVKDFIQKQ DEWTRQQIKN NKDDPFWRNA
161 GYVIAQLDGL YMGNVEWAKR QKRTPLTDFE ISFLNAIGDL LDLIPALHSE LRKSDFRSMP DVSRIYQWDM GHCSALIKVL
241 PGYENIYFAH SSWFTYAATL RIYKHLDFRI TDPQTKTGRA SFSSYPGFLV SLDDFYILGS GLIMLQTTNS VFNLSLLKKV
321 VPESLFAWER VRIANMMADS GKTWAETFEK QNSGTYNNQY MILDTKKIKL QRSLEDGTLY IIEQVPKLVK YSDQTKVLRN
401 GYWPSYNIPF DKEIYNMSGY GEYVQRHGLE FSYEMAPRAK IFRRDQGKVT DMESMKFIMR YNNYKEDPYA KHNPCNTICC
481 RQDLDRRTPV PAGCYDSKVA DISMAAKFTA YAINGPPVEK GLPVFSWVHF NKTKHQGLPE SYNFDFVTMK PVL
```

**Supporting Peptides:**

| Peptide | Uniq | -10lgP | Mass | Length | ppm | m/z | z | RT | Fraction | Scan | Source File | Area F1 | Area F2a | Area F3a | Area F4 | Area F5 | Area F6a | Area F7a | Area F7b | Area F8 | Area F9a | Area Sample 1F10a | Area F10b | Area F11a | Area F11b | Area F12a | Area F13 | #Feature | #Feature F1 | #Feature F2a | #Feature F3a | #Feature F4 | #Feature F5 | #Feature F6a | #Feature F7a | #Feature F7b | #Feature F8 | #Feature F9a | #Feature Sample 1F10a | #Feature F10b | #Feature F11a | #Feature F11b | #Feature F12a | #Feature F13 | Start | End | PTM | AScore | Found By |
|---|---|---|---|---|---|---|---|---|---|---|---|---|---|---|---|---|---|---|---|---|---|---|---|---|---|---|---|---|---|---|---|---|---|---|---|---|---|---|---|---|---|---|---|---|---|---|---|---|---|
| K.FTAYAINGPPVEK.G | Y | 57.42 | 1405.7241 | 13 | 0.6 | 703.8698 | 2 | 52.52 | 16 | F16:24918 | BuCaTN01_F13.raw | | | | | | | | | | | | | | | | 2.1472E5 | 1 | 0 | 0 | 0 | 0 | 0 | 0 | 0 | 0 | 0 | 0 | 0 | 0 | 0 | 0 | 0 | 1 | 508 | 520 | | | PEAKS DB |
| total 1 peptides | | | | | | | | | | | | | | | | | | | | | | | | | | | | | | | | | | | | | | | | | | | | | | | | | |

**Best Unique PSM (Scan F16:24918, m/z=703.8698, z=2, RT=52.52, ppm=0.6):**

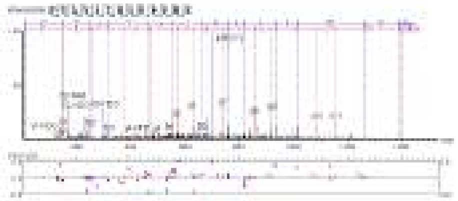

## F8S101.1

back to list

| Protein Coverage | Supporting Peptides | Best Unique PSM |

**Protein Coverage:**

```
  1 MIRFGNPSSS DKRRQRCRSW YWGGLLLLWA VAETRADIHY ATVYWLEAEK SFQIKDVLDK NGDAYGYYND AIQSTGWGIL
 81 EIKAGYGNQP ISNEILMYAA GFLEGYLTAS HMSDHFANLF PLMIKNVIIE QKVKDFIQKQ DEWTRQQIKN NKDDPFWRNA
161 GYVIAQLDGL YMGNVEWAKR QKRTPLTDFE ISFLNAIGDL LDLIPALHSE LRKSDFRSMP DVSRIYQWDM GHCSALIKVL
241 PGYENIYFAH SSWFTYAATL RIYKHLDFRI TDPQTKTGRA SFSSYPGLFG SLDDFYILGS GLIMLQTTNS VFNLSLLKKV
321 VPESLFAWER VRIANMMADS GKTWAETFEK QNSGTYNNQY MILDTKKIKL QRSLEDGTLY IIEQVPKLVK YSDQTKVLRN
401 GYWPSYNIPF DKEIYNMSGY GEYVQRHGLE FSYEMAPRAK IFRRDQGKVT DMESMKFIMR YNNYKEDPYA KHNPCNTICC
481 RQDLDRRTPV PAGCYDSKVA DISMAAKFTA YAINGPPVEK GLPVFSWVHF NKTKHQGLPE SYNFDFVTMK PVL
```

**Supporting Peptides:**

| Peptide | Uniq | -10lgP | Mass | Length | ppm | m/z | z | RT | Fraction | Scan | Source File | Area F1 | Area F2a | Area F3a | Area F4 | Area F5 | Area F6a | Area F7a | Area F7b | Area F8 | Area F9a | Area Sample 1F10a | Area F10b | Area F11a | Area F11b | Area F12a | Area F13 | #Feature | #Feature F1 | #Feature F2a | #Feature F3a | #Feature F4 | #Feature F5 | #Feature F6a | #Feature F7a | #Feature F7b | #Feature F8 | #Feature F9a | #Feature Sample 1F10a | #Feature F10b | #Feature F11a | #Feature F11b | #Feature F12a | #Feature F13 | Start | End | PTM | AScore | Found By |
|---|---|---|---|---|---|---|---|---|---|---|---|---|---|---|---|---|---|---|---|---|---|---|---|---|---|---|---|---|---|---|---|---|---|---|---|---|---|---|---|---|---|---|---|---|---|---|---|---|---|
| K.FTAYAINGPPVEK.G | Y | 57.42 | 1405.7241 | 13 | 0.6 | 703.8698 | 2 | 52.52 | 16 | F16:24918 | BuCaTN01_F13.raw | | | | | | | | | | | | | | | | 2.1472E5 | 1 | 0 | 0 | 0 | 0 | 0 | 0 | 0 | 0 | 0 | 0 | 0 | 0 | 0 | 0 | 0 | 1 | 508 | 520 | | | PEAKS DB |
| total 1 peptides | | | | | | | | | | | | | | | | | | | | | | | | | | | | | | | | | | | | | | | | | | | | | | | | | |

**Best Unique PSM (Scan F16:24918, m/z=703.8698, z=2, RT=52.52, ppm=0.6):**

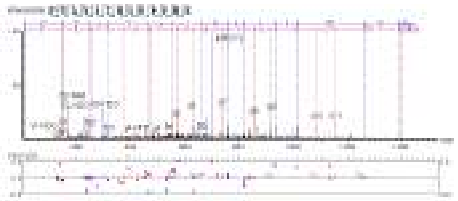

## AFJ50957.1

back to list

| Protein Coverage | Supporting Peptides | Best Unique PSM |

**Protein Coverage:**

```
  1 MIRFGNPSSS DKRRQRCRSW YWGGLLLLWA VAETRADIHY ATVYWLEAEK SFQIKDVLDK NGDAYGYYND AIQSTGWGIL
 81 EIKAGYGNQP ISNEILMYAA GFLEGYLTAS HMSDHFANLF PLMIKNVIIE QKVKDFIQKQ DEWTRQQIKN NKDDPFWRNA
161 GYVIAQLDGL YMGNVEWAKR QKRTPLTDFE ISFLNAIGDL LDLIPALHSE LRKSDFRSMP DVSRIYQWDM GHCSALIKVL
241 PGYENIYFAH SSWFTYAATL RIYKHLDFRI TDPQTKTGRA SFSSYPGFLV SLDDFYILGS GLIMLQTTNS VFNLSLLKKV
321 VPESLFAWER VRIANMMADS GKTWAETFEK QNSGTYNNQY MILDTKKIKL QRSLEDGTLY IIEQVPKLVK YSDQTKVLRN
401 GYWPSYNIPF DKEIYNMSGY GEYVQRHGLE FSYEMAPRAK IFRRDQGKVT DMESMKFIMR YNNYKEDPYA KHNPCNTICC
481 RQDLDRRTPV PAGCYDSKVA DISMAAKFTA YAINGPPVEK GLPVFSWVHF NKTKHQGLPE SYNFDFVTMK PVL
```

**Supporting Peptides:**

| Peptide | Uniq | -10lgP | Mass | Length | ppm | m/z | z | RT | Fraction | Scan | Source File | Area F1 | Area F2a | Area F3a | Area F4 | Area F5 | Area F6a | Area F7a | Area F7b | Area F8 | Area F9a | Area Sample 1F10a | Area F10b | Area F11a | Area F11b | Area F12a | Area F13 | #Feature | #Feature F1 | #Feature F2a | #Feature F3a | #Feature F4 | #Feature F5 | #Feature F6a | #Feature F7a | #Feature F7b | #Feature F8 | #Feature F9a | #Feature Sample 1F10a | #Feature F10b | #Feature F11a | #Feature F11b | #Feature F12a | #Feature F13 | Start | End | PTM | AScore | Found By |
|---|---|---|---|---|---|---|---|---|---|---|---|---|---|---|---|---|---|---|---|---|---|---|---|---|---|---|---|---|---|---|---|---|---|---|---|---|---|---|---|---|---|---|---|---|---|---|---|---|---|
| K.FTAYAINGPPVEK.G | Y | 57.42 | 1405.7241 | 13 | 0.6 | 703.8698 | 2 | 52.52 | 16 | F16:24918 | BuCaTN01_F13.raw | | | | | | | | | | | | | | | | 2.1472E5 | 1 | 0 | 0 | 0 | 0 | 0 | 0 | 0 | 0 | 0 | 0 | 0 | 0 | 0 | 0 | 0 | 1 | 508 | 520 | | | PEAKS DB |
| total 1 peptides | | | | | | | | | | | | | | | | | | | | | | | | | | | | | | | | | | | | | | | | | | | | | | | | | |

**Best Unique PSM (Scan F16:24918, m/z=703.8698, z=2, RT=52.52, ppm=0.6):**

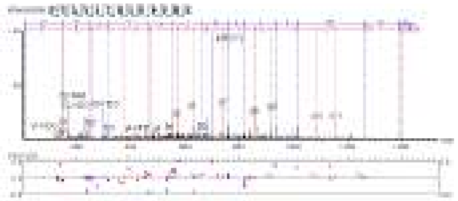

## AEJ31983.1

back to list

| Protein Coverage | Supporting Peptides | Best Unique PSM |

**Protein Coverage:**

```
  1 MIRFGNPSSS DKRRQRCRSW YWGGLLLLWA VAETRADIHY ATVYWLEAEK SFQIKDVLDK NGDAYGYYND AIQSTGWGIL
 81 EIKAGYGNQP ISNEILMYAA GFLEGYLTAS HMSDHFANLF PLMIKNVIIE QKVKDFIQKQ DEWTRQQIKN NKDDPFWRNA
161 GYVIAQLDGL YMGNVEWAKR QKRTPLTDFE ISFLNAIGDL LDLIPALHSE LRKSDFRSMP DVSRIYQWDM GHCSALIKVL
241 PGYENIYFAH SSWFTYAATL RIYKHLDFRI TDPQTKTGRA SFSSYPGLFG SLDDFYILGS GLIMLQTTNS VFNLSLLKKV
321 VPESLFAWER VRIANMMADS GKTWAETFEK QNSGTYNNQY MILDTKKIKL QRSLEDGTLY IIEQVPKLVK YSDQTKVLRN
401 GYWPSYNIPF DKEIYNMSGY GEYVQRHGLE FSYEMAPRAK IFRRDQGKVT DMESMKFIMR YNNYKEDPYA KHNPCNTICC
481 RQDLDRRTPV PAGCYDSKVA DISMAAKFTA YAINGPPVEK GLPVFSWVHF NKTKHQGLPE SYNFDFVTMK PVL
```

**Supporting Peptides:**

| Peptide | Uniq | -10lgP | Mass | Length | ppm | m/z | z | RT | Fraction | Scan | Source File | Area F1 | Area F2a | Area F3a | Area F4 | Area F5 | Area F6a | Area F7a | Area F7b | Area F8 | Area F9a | Area Sample 1F10a | Area F10b | Area F11a | Area F11b | Area F12a | Area F13 | #Feature | #Feature F1 | #Feature F2a | #Feature F3a | #Feature F4 | #Feature F5 | #Feature F6a | #Feature F7a | #Feature F7b | #Feature F8 | #Feature F9a | #Feature Sample 1F10a | #Feature F10b | #Feature F11a | #Feature F11b | #Feature F12a | #Feature F13 | Start | End | PTM | AScore | Found By |
|---|---|---|---|---|---|---|---|---|---|---|---|---|---|---|---|---|---|---|---|---|---|---|---|---|---|---|---|---|---|---|---|---|---|---|---|---|---|---|---|---|---|---|---|---|---|---|---|---|---|
| K.FTAYAINGPPVEK.G | Y | 57.42 | 1405.7241 | 13 | 0.6 | 703.8698 | 2 | 52.52 | 16 | F16:24918 | BuCaTN01_F13.raw | | | | | | | | | | | | | | | | 2.1472E5 | 1 | 0 | 0 | 0 | 0 | 0 | 0 | 0 | 0 | 0 | 0 | 0 | 0 | 0 | 0 | 0 | 1 | 508 | 520 | | | PEAKS DB |
| total 1 peptides | | | | | | | | | | | | | | | | | | | | | | | | | | | | | | | | | | | | | | | | | | | | | | | | |

**Best Unique PSM (Scan F16:24918, m/z=703.8698, z=2, RT=52.52, ppm=0.6):**

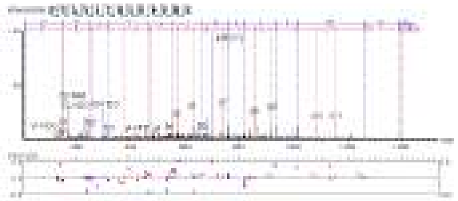

## JAA96126.1

back to list

| Protein Coverage | Supporting Peptides | Best Unique PSM |

**Protein Coverage:**

```
  1 MIRFGNPSSS DKRRQRCRSW YWGGLLLLWA VAETRADIHY ATVYWLEAEK SFQIKDVLDK NGDAYGYYND TIQSTGWGIL
 81 EIKAGYGNQP ISNEILMYAA GFLEGYLTAS HMSDHFANLF PLMIKNVIIE QKVKDFIQKQ DEWTRQQIKN NKDDPFWRNA
161 GYVIAQLDGL YMGNVEWAKQ QKRTPLTYFE ISFLNAIGDL LDLILALHSE LRKSDFRSMP DVSRMYQWDM GHCSALIKVL
241 PGYENIYFAH SSWFTYAATL RIYKHLDFKI TDPQTKTGRA SFSSYPGFLV SLDDFYILGS GLIMLQTTNS VFNLSLLKKI
321 VPESLFAWER VRIANMMADS GKTWAETFEK QNSGTYNNQY MILDTKKIKL QRSLEDGTLY IIEQVPKLVK YSDQTKVLRN
401 GYWPSYNIPF DKEIYNMSGY GEYVQRHGLE FSYEMAPRAK IFRRDQGKVT DMESMKFIMR YNNYKEDPYA KRNPCNTICC
481 RQDLDRRTPV PAGCYDSKVA DISMAAKFTA YAINGPPVEK GLPVFSWVHF NKTKHQGLPE SYNFDFVTMK PVL
```

**Supporting Peptides:**

| Peptide | Uniq | -10lgP | Mass | Length | ppm | m/z | z | RT | Fraction | Scan | Source File | Area F1 | Area F2a | Area F3a | Area F4 | Area F5 | Area F6a | Area F7a | Area F7b | Area F8 | Area F9a | Area Sample 1F10a | Area F10b | Area F11a | Area F11b | Area F12a | Area F13 | #Feature | #Feature F1 | #Feature F2a | #Feature F3a | #Feature F4 | #Feature F5 | #Feature F6a | #Feature F7a | #Feature F7b | #Feature F8 | #Feature F9a | #Feature Sample 1F10a | #Feature F10b | #Feature F11a | #Feature F11b | #Feature F12a | #Feature F13 | Start | End | PTM | AScore | Found By |
|---|---|---|---|---|---|---|---|---|---|---|---|---|---|---|---|---|---|---|---|---|---|---|---|---|---|---|---|---|---|---|---|---|---|---|---|---|---|---|---|---|---|---|---|---|---|---|---|---|---|
| K.FTAYAINGPPVEK.G | Y | 57.42 | 1405.7241 | 13 | 0.6 | 703.8698 | 2 | 52.52 | 16 | F16:24918 | BuCaTN01_F13.raw | | | | | | | | | | | | | | | | 2.1472E5 | 1 | 0 | 0 | 0 | 0 | 0 | 0 | 0 | 0 | 0 | 0 | 0 | 0 | 0 | 0 | 0 | 1 | 508 | 520 | | | PEAKS DB |
| total 1 peptides | | | | | | | | | | | | | | | | | | | | | | | | | | | | | | | | | | | | | | | | | | | | | | | | |

**Best Unique PSM (Scan F16:24918, m/z=703.8698, z=2, RT=52.52, ppm=0.6):**

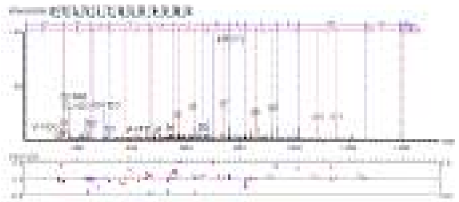

## JAC94989.1

back to list

| Protein Coverage | Supporting Peptides | Best Unique PSM |

**Protein Coverage:**

```
  1 MVSFGSASSS DNHRQRCWSW SWGGLLLLWA VAETRADLHY ATVYWLEAEK TFQVKDVLDK NGDAYGYYND TVQSTGWGIL
 81 EIKAGYGNQP VSNEILMYAA GFLEGYLTAS RMSDHVANLY PQLIKNVIIE QKIKDFMQKQ DEWTRQQIKN NKDDPFWRNA
161 GYVIAQLDGL YMGNVEWAKR QKRTPLTDFE ISFLNAIGDL LDLIPALTSD SRNNDFLSMP EVSRMYQWNM GHCSALIKVL
241 PGYENIYFAH SSWFTYAATL RIYKHWDFRI TDPQTKTGRA SFSSYPGFLI SLDDFYILGS GLIMLQTTNS VFNLSLLKQV
321 VPESLLAWER VRIANMMADS GKTWAQIFEK QNSGTYNNQY MILDTKKIKL QRSIDDGTLY IIEQVPNLVE YSDQTNTLRK
401 GYWPSYNIPF HNIIYNMSGY GEYVQKYGLD FSYEMAPRAK IFRRDQGKVT DMESMKYIMR YNNYKKDPYT RHNPCNTICC
481 REDLNHKNPV PSGCYDSKVA DINMAAKFTA YAINGPPVEK GLPIFSWVNF NKTKHQGLPE SYNFDFVTMK PVL
```

**Supporting Peptides:**

| Peptide | Uniq | -10lgP | Mass | Length | ppm | m/z | z | RT | Fraction | Scan | Source File | Area F1 | Area F2a | Area F3a | Area F4 | Area F5 | Area F6a | Area F7a | Area F7b | Area F8 | Area F9a | Area Sample 1F10a | Area F10b | Area F11a | Area F11b | Area F12a | Area F13 | #Feature | #Feature F1 | #Feature F2a | #Feature F3a | #Feature F4 | #Feature F5 | #Feature F6a | #Feature F7a | #Feature F7b | #Feature F8 | #Feature F9a | #Feature Sample 1F10a | #Feature F10b | #Feature F11a | #Feature F11b | #Feature F12a | #Feature F13 | Start | End | PTM | AScore | Found By |
|---|---|---|---|---|---|---|---|---|---|---|---|---|---|---|---|---|---|---|---|---|---|---|---|---|---|---|---|---|---|---|---|---|---|---|---|---|---|---|---|---|---|---|---|---|---|---|---|---|---|
| K.FTAYAINGPPVEK.G | Y | 57.42 | 1405.7241 | 13 | 0.6 | 703.8698 | 2 | 52.52 | 16 | F16:24918 | BuCaTN01_F13.raw | | | | | | | | | | | | | | | | 2.1472E5 | 1 | 0 | 0 | 0 | 0 | 0 | 0 | 0 | 0 | 0 | 0 | 0 | 0 | 0 | 0 | 0 | 1 | 508 | 520 | | | PEAKS DB |
| total 1 peptides | | | | | | | | | | | | | | | | | | | | | | | | | | | | | | | | | | | | | | | | | | | | | | | | | |

**Best Unique PSM (Scan F16:24918, m/z=703.8698, z=2, RT=52.52, ppm=0.6):**

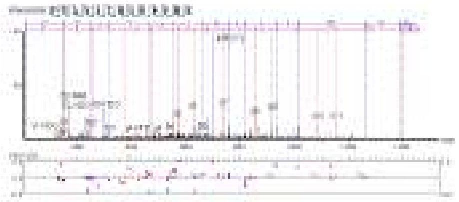

## SMD28241.1

back to list

| Protein Coverage | Supporting Peptides | Best Unique PSM |

**Protein Coverage:**

```
  1 MIRFGNPSSS DKRRQRCRSW YWGGLLLLWA VAETRADIHY ATVYWLEAEK SFQIKDVLDK NGDAYGYYND TIQSTGWGIL
 81 EIKAGYGNQP VSNEILMYAA GFLEGYLTAS HMSDHFANLF PLMIKNVIIE QKVKDFIQKQ DEWTRQQIKN NKDDPFWRNA
161 GYVIAQLDGL YMGNVEWAKR QKRTPLTDFE ISFLNAIGDL LDLIPALHSE LRKSDFRSMP DVSRIYQWDM GHCSALIKVL
241 PGYENIYFAH SSWFTYAATL RIYKHLDFKI TDPQTKTGRA SFSSYPVVPE SLFAWERVRI ANMMADSGKT WAETFEKQNS
321 GTYNNQYMIL DTKKIKLQRS LEDGTLYIIE QVPKLVKYSD QTKVLRNGYW PSYNIPFDKE IYNMSGYGEY VQRHGLEFSY
401 EMAPRAKILR RDQGKVTDME SMKFIMRYNN YKEDPYAKRN PCNTICCRQD LDRRTPVPAG CYDSKVADIS MAAKFTAYAI

481 NGPPVEKGLP VFSWVHFNKT KHQGLPESYN FDFVTMKPVL
```

**Supporting Peptides:**

| Peptide | Uniq | -10lgP | Mass | Length | ppm | m/z | z | RT | Fraction | Scan | Source File | Area F1 | Area F2a | Area F3a | Area F4 | Area F5 | Area F6a | Area F7a | Area F7b | Area F8 | Area F9a | Area Sample 1F10a | Area F10b | Area F11a | Area F11b | Area F12a | Area F13 | #Feature | #Feature F1 | #Feature F2a | #Feature F3a | #Feature F4 | #Feature F5 | #Feature F6a | #Feature F7a | #Feature F7b | #Feature F8 | #Feature F9a | #Feature Sample 1F10a | #Feature F10b | #Feature F11a | #Feature F11b | #Feature F12a | #Feature F13 | Start | End | PTM | AScore | Found By |
|---|---|---|---|---|---|---|---|---|---|---|---|---|---|---|---|---|---|---|---|---|---|---|---|---|---|---|---|---|---|---|---|---|---|---|---|---|---|---|---|---|---|---|---|---|---|---|---|---|---|
| K.FTAYAINGPPVEK.G | Y | 57.42 | 1405.7241 | 13 | 0.6 | 703.8698 | 2 | 52.52 | 16 | F16:24918 | BuCaTN01_F13.raw | | | | | | | | | | | | | | | | 2.1472E5 | 1 | 0 | 0 | 0 | 0 | 0 | 0 | 0 | 0 | 0 | 0 | 0 | 0 | 0 | 0 | 0 | 1 | 475 | 487 | | | PEAKS DB |
| total 1 peptides | | | | | | | | | | | | | | | | | | | | | | | | | | | | | | | | | | | | | | | | | | | | | | | | | |

**Best Unique PSM (Scan F16:24918, m/z=703.8698, z=2, RT=52.52, ppm=0.6):**

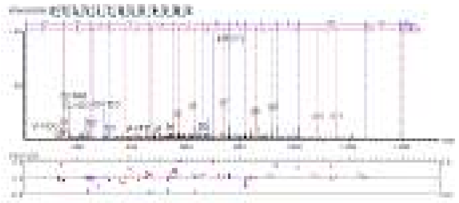

## SMD28240.1

back to list

| Protein Coverage | Supporting Peptides | Best Unique PSM |

**Protein Coverage:**

```
  1 MIRFGNPSSS DKRRQRCRSW YWGGLLLLWA VAETRADIHY ATVYWLEAEK SFQIKDVLDK NGDAYGYYND TIQSTGWGIL
 81 EIKAGYGNQP VSNEILMYAA GFLEGYLTAS HMSDHFANLF PLMIKNVIIE QKVKDFIQKQ DEWTRQQIKN NKDDPFWRNA
161 GYVIAQLDGL YMGNVEWAKR QKRTPLTDFE ISFLNAIGDL LDLIPALHSE LRKSDFRSMP DVSRIYQWDM GHCSALIKVL
241 PGYENIYFAH SSWFTYAATL RIYKHLDFKI TDPQTKTGRA SFSSYPGFLV SLDDFYILDS GLIILQTTNS VFNLSLLKKV
321 VPESLFAWER VRIANMMADS GKTWAETFEK QNSGTYNNQY MILDTKKIKL QRSLEDGTLY IIEQVPKLVK YSDQTKVLRN
401 GYWPSYNIPF DKEIYNMSGY GEYVQRHGLE FSYEMAPRAK ILRRDQGKVT DMESMKFIMR YNNYKEDPYA KRNPCNTICC
481 RQDLDRRTPV PAGCYDSKVA DISMAAKFTA YAINGPPVEK GLPVFSWVHF NKTKHQGLPE SYNFDFVTMK PVL
```

**Supporting Peptides:**

| Peptide | Uniq | -10lgP | Mass | Length | ppm | m/z | z | RT | Fraction | Scan | Source File | Area F1 | Area F2a | Area F3a | Area F4 | Area F5 | Area F6a | Area F7a | Area F7b | Area F8 | Area F9a | Area Sample 1F10a | Area F10b | Area F11a | Area F11b | Area F12a | Area F13 | #Feature | #Feature F1 | #Feature F2a | #Feature F3a | #Feature F4 | #Feature F5 | #Feature F6a | #Feature F7a | #Feature F7b | #Feature F8 | #Feature F9a | #Feature Sample 1F10a | #Feature F10b | #Feature F11a | #Feature F11b | #Feature F12a | #Feature F13 | Start | End | PTM | AScore | Found By |
|---|---|---|---|---|---|---|---|---|---|---|---|---|---|---|---|---|---|---|---|---|---|---|---|---|---|---|---|---|---|---|---|---|---|---|---|---|---|---|---|---|---|---|---|---|---|---|---|---|---|
| K.FTAYAINGPPVEK.G | Y | 57.42 | 1405.7241 | 13 | 0.6 | 703.8698 | 2 | 52.52 | 16 | F16:24918 | BuCaTN01_F13.raw | | | | | | | | | | | | | | | | 2.1472E5 | 1 | 0 | 0 | 0 | 0 | 0 | 0 | 0 | 0 | 0 | 0 | 0 | 0 | 0 | 0 | 0 | 1 | 508 | 520 | | | PEAKS DB |
| total 1 peptides | | | | | | | | | | | | | | | | | | | | | | | | | | | | | | | | | | | | | | | | | | | | | | | | | |

**Best Unique PSM (Scan F16:24918, m/z=703.8698, z=2, RT=52.52, ppm=0.6):**

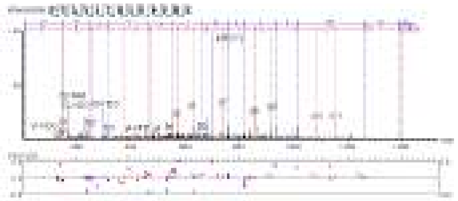

## JAV49636.1

back to list

| Protein Coverage | Supporting Peptides | Best Unique PSM |

**Protein Coverage:**

```
  1 MIRFGNPSSS DKRLQRCRSW YWGGLLLLWA VAETRADIHY ATVYWLEAEK SFQIKDVLDK NGDAYGYYND TIQSTGWGIL
 81 EIKAGYGNQP ISNEILMYAA GFLEGYLTAS HMNDHFANLF PLMIKNVIIE QKVKDFIQKQ DEWTRQQIKN NKDDPFWRNA
161 GYVIAQLDGL YMGNVEWAKR QKRTPLTDFE ISFLNAIGDL LDLIPALRSE LRKSDFRSMP DVSRIYQWDM GHCSALIKVL
241 PGYENIYFAH SSWFTYAATL RIYKHLDFKI TDPQTKTGRA SFSSYPGFLI SLDDFYILGS GLIMLQTTNS VFNLSLLKKV
321 VPESLFAWER VRIANMMADS GKTWAETFEK QNSGTYNNQY MILDTKKIKL QRSLEDGTLY IIEQVPKLVK YSDQTKVLRN
401 GYWPSYNIPF DKEIYNMSGY GEYVQRHGLE FSYEMAPRAK IFRRDQGKVT DMESMKFIMR YNNYKEDPYA KRNPCNTICC
481 RQDLDRRTPV PAGCYDSKVA DISMAAKFTA YAINGPPVEK GLPVFSWVHF NKTKHQGLPE SYNFDFVTMK PVL
```

**Supporting Peptides:**

| Peptide | Uniq | -10lgP | Mass | Length | ppm | m/z | z | RT | Fraction | Scan | Source File | Area F1 | Area F2a | Area F3a | Area F4 | Area F5 | Area F6a | Area F7a | Area F7b | Area F8 | Area F9a | Area Sample 1F10a | Area F10b | Area F11a | Area F11b | Area F12a | Area F13 | #Feature | #Feature F1 | #Feature F2a | #Feature F3a | #Feature F4 | #Feature F5 | #Feature F6a | #Feature F7a | #Feature F7b | #Feature F8 | #Feature F9a | #Feature Sample 1F10a | #Feature F10b | #Feature F11a | #Feature F11b | #Feature F12a | #Feature F13 | Start | End | PTM | AScore | Found By |
|---|---|---|---|---|---|---|---|---|---|---|---|---|---|---|---|---|---|---|---|---|---|---|---|---|---|---|---|---|---|---|---|---|---|---|---|---|---|---|---|---|---|---|---|---|---|---|---|---|---|
| K.FTAYAINGPPVEK.G | Y | 57.42 | 1405.7241 | 13 | 0.6 | 703.8698 | 2 | 52.52 | 16 | F16:24918 | BuCaTN01_F13.raw | | | | | | | | | | | | | | | | 2.1472E5 | 1 | 0 | 0 | 0 | 0 | 0 | 0 | 0 | 0 | 0 | 0 | 0 | 0 | 0 | 0 | 0 | 1 | 508 | 520 | | | PEAKS DB |
| total 1 peptides | | | | | | | | | | | | | | | | | | | | | | | | | | | | | | | | | | | | | | | | | | | | | | | | | |

**Best Unique PSM (Scan F16:24918, m/z=703.8698, z=2, RT=52.52, ppm=0.6):**

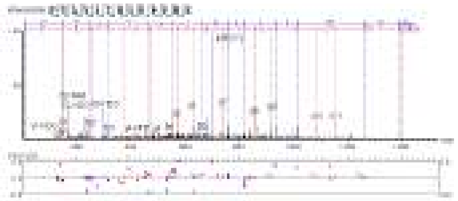

## XP_039191573.1

back to list

| Protein Coverage | Supporting Peptides | Best Unique PSM |

**Protein Coverage:**

```
  1 MIRFGNPSSS DKRRQRCRSW YWGGLLLLWA VAETRADIHY ATVYWLEAEK SFQIKDVLDK NGDAYGYYND AIQSTGWGIL
 81 EIKAGYGNQP ISNEILMYAA GFLEGYLTAS HMSDHFANLF PLMIKNVIIE QKVKDFIQKQ DEWTRQQIKN NKDDPFWRNA
161 GYVIAQLDGL YMGNVEWAKR QKRTPLTDFE ISFLNAIGDL LDLIPALHSE LRKSDFRSMP DVSRIYQWDM GHCSALIKVL
241 PGYENIYFAH SSWFTYASTL RIYKHLDFKI TDPQTKTGRA SFSSYPGFLV SLDDFYILGS GLIMLQTTNS VFNLSLLKKV
321 VPESLFAWER VRIANMMADS GKTWAETFEK QNSGTYNNQY MILDTKKIKL QRSLEDGTLY IIEQVPKLVK YSDQTKVLRN
401 GYWPSYNIPF DKEIYNMSGY GEYVQRHGLE FSYEMAPRAK IFRRDQGKVT DMESMKFIMR YNNYKEDPYA KRNPCNTICC
481 RQDLDRRTPV PAGCYDSKVA DISMAAKFTA YAINGPPVEK GLPVFSWVHF NKTKHQGLPE SYNFDFVTMK PVL
```

**Supporting Peptides:**

| Peptide | Uniq | -10lgP | Mass | Length | ppm | m/z | z | RT | Fraction | Scan | Source File | Area F1 | Area F2a | Area F3a | Area F4 | Area F5 | Area F6a | Area F7a | Area F7b | Area F8 | Area F9a | Area Sample 1F10a | Area F10b | Area F11a | Area F11b | Area F12a | Area F13 | #Feature | #Feature F1 | #Feature F2a | #Feature F3a | #Feature F4 | #Feature F5 | #Feature F6a | #Feature F7a | #Feature F7b | #Feature F8 | #Feature F9a | #Feature Sample 1F10a | #Feature F10b | #Feature F11a | #Feature F11b | #Feature F12a | #Feature F13 | Start | End | PTM | AScore | Found By |
|---|---|---|---|---|---|---|---|---|---|---|---|---|---|---|---|---|---|---|---|---|---|---|---|---|---|---|---|---|---|---|---|---|---|---|---|---|---|---|---|---|---|---|---|---|---|---|---|---|---|
| K.FTAYAINGPPVEK.G | Y | 57.42 | 1405.7241 | 13 | 0.6 | 703.8698 | 2 | 52.52 | 16 | F16:24918 | BuCaTN01_F13.raw | | | | | | | | | | | | | | | | 2.1472E5 | 1 | 0 | 0 | 0 | 0 | 0 | 0 | 0 | 0 | 0 | 0 | 0 | 0 | 0 | 0 | 0 | 1 | 508 | 520 | | | PEAKS DB |
| total 1 peptides | | | | | | | | | | | | | | | | | | | | | | | | | | | | | | | | | | | | | | | | | | | | | | | | | |

**Best Unique PSM (Scan F16:24918, m/z=703.8698, z=2, RT=52.52, ppm=0.6):**

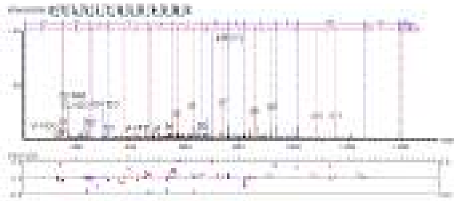

## JAV48419.1

back to list

| Protein Coverage | Supporting Peptides | Best Unique PSM |

**Protein Coverage:**

```
  1 MIRFGNPSSS DKRRQRCRSW YWGGLLLLWA VAETRADIHY ATVYWLEAEK SFQIKDVLDK NGDAYGYYND AIQSTGWGIL
 81 EIKAGYGNQP ISNEILMYAA GFLEGYLTAS HMSDHFANLF PLMIKNVIIE QKVKDFIQKQ DEWTRQQIKN NKDDPFWRNA
161 GYVIAQLDGL YMGNVEWAKR QKRTPLTDFE ISFLNAIGDL LDLIPALHSE LRKSDFRSMP DVSRIYQWDM GHCSALIKVL
241 PGYENIYFAH SSWFTYAATL RIYKHLDFRI TDPQTKTGRA SFSSYPGFLV SLDDFYILGS GLIMLQTTNS VFNLSLLKKV
321 VPESLFAWER VRIANMMADS GKTWAETFEK QNSGTYNNQY MILDTKKIKL QRSLEDGTLY IIEQVPKLVK YSDQTKVLRN
401 GYWPSYNIPF DKEIYNMSGY GEYVQRHGLE FSYEMAPRAK IFRRDQGKVT DMESMKFIMR YNNYKEDPYA KRNPCNTICC
481 RQDLDRRTPV PAGCYDSKVA DISMAAKFTA YAINGPPVEK GLPVFSWVHF NKTKHQGLPE SYNFDFVTMK PVL
```

**Supporting Peptides:**

| Peptide | Uniq | -10lgP | Mass | Length | ppm | m/z | z | RT | Fraction | Scan | Source File | Area F1 | Area F2a | Area F3a | Area F4 | Area F5 | Area F6a | Area F7a | Area F7b | Area F8 | Area F9a | Area Sample 1F10a | Area F10b | Area F11a | Area F11b | Area F12a | Area F13 | #Feature | #Feature F1 | #Feature F2a | #Feature F3a | #Feature F4 | #Feature F5 | #Feature F6a | #Feature F7a | #Feature F7b | #Feature F8 | #Feature F9a | #Feature Sample 1F10a | #Feature F10b | #Feature F11a | #Feature F11b | #Feature F12a | #Feature F13 | Start | End | PTM | AScore | Found By |
|---|---|---|---|---|---|---|---|---|---|---|---|---|---|---|---|---|---|---|---|---|---|---|---|---|---|---|---|---|---|---|---|---|---|---|---|---|---|---|---|---|---|---|---|---|---|---|---|---|---|
| K.FTAYAINGPPVEK.G | Y | 57.42 | 1405.7241 | 13 | 0.6 | 703.8698 | 2 | 52.52 | 16 | F16:24918 | BuCaTN01_F13.raw | | | | | | | | | | | | | | | | 2.1472E5 | 1 | 0 | 0 | 0 | 0 | 0 | 0 | 0 | 0 | 0 | 0 | 0 | 0 | 0 | 0 | 0 | 1 | 508 | 520 | | | PEAKS DB |
| total 1 peptides | | | | | | | | | | | | | | | | | | | | | | | | | | | | | | | | | | | | | | | | | | | | | | | | | |

**Best Unique PSM (Scan F16:24918, m/z=703.8698, z=2, RT=52.52, ppm=0.6):**

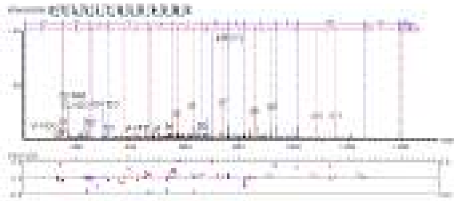

## ETE59578.1

back to list

| Protein Coverage | Supporting Peptides | Best Unique PSM |

**Protein Coverage:**

```
  1 DLHYATVYWL EAEKSFQVKD LLDKNGDAYG YYNDTVQSTG WGILEIKAGY GNQLVSNEIL MYAAGFLEGY LTASRMRDHV
 81 ANLYHQLIKN VTIEQKVKDF MQKQDEWTRQ QIKNNKDDPF WRHAGYIIAQ LDGLYMGNLE WAKRQKRTPL TEFEVSFLNA
161 IGDLLDLISA LSPESRNNDS NMYQWDMGHC SALIKVLPGY ENIYFAHSSW FTYAATLRIY KHWDFRITDP QTKTGRASFS
241 SYPGFLISLD DFYILGSGLI MLQTTNSVFN LSLLKQVVPE SLFAWERVRI ANMMADSGKT WAQTFEKQNS GTYNNQYMIL
321 DTKKIKLRRS IEDGSLYIIE QVPNLVEYSD QTTILRKGYW PSYNIPFHKV IYNMSGYREY VQKYGLDFSY ELAPRAKIFR
401 RDQGKVTDME SMKHIMRYNN YKNDPYAKHN PCNTICCRQD LNYKTPVADI NMAAKFTAYA INGPPVEKGL PIFSWVHFNK

481 TTHQGLPESY NFDFVTMKPV L
```

**Supporting Peptides:**

| Peptide | Uniq | -10lgP | Mass | Length | ppm | m/z | z | RT | Fraction | Scan | Source File | Area F1 | Area F2a | Area F3a | Area F4 | Area F5 | Area F6a | Area F7a | Area F7b | Area F8 | Area F9a | Area Sample 1F10a | Area F10b | Area F11a | Area F11b | Area F12a | Area F13 | #Feature | #Feature F1 | #Feature F2a | #Feature F3a | #Feature F4 | #Feature F5 | #Feature F6a | #Feature F7a | #Feature F7b | #Feature F8 | #Feature F9a | #Feature Sample 1F10a | #Feature F10b | #Feature F11a | #Feature F11b | #Feature F12a | #Feature F13 | Start | End | PTM | AScore | Found By |
|---|---|---|---|---|---|---|---|---|---|---|---|---|---|---|---|---|---|---|---|---|---|---|---|---|---|---|---|---|---|---|---|---|---|---|---|---|---|---|---|---|---|---|---|---|---|---|---|---|---|
| K.FTAYAINGPPVEK.G | Y | 57.42 | 1405.7241 | 13 | 0.6 | 703.8698 | 2 | 52.52 | 16 | F16:24918 | BuCaTN01_F13.raw | | | | | | | | | | | | | | | | 2.1472E5 | 1 | 0 | 0 | 0 | 0 | 0 | 0 | 0 | 0 | 0 | 0 | 0 | 0 | 0 | 0 | 0 | 1 | 456 | 468 | | | PEAKS DB |
| total 1 peptides | | | | | | | | | | | | | | | | | | | | | | | | | | | | | | | | | | | | | | | | | | | | | | | | | |

**Best Unique PSM (Scan F16:24918, m/z=703.8698, z=2, RT=52.52, ppm=0.6):**

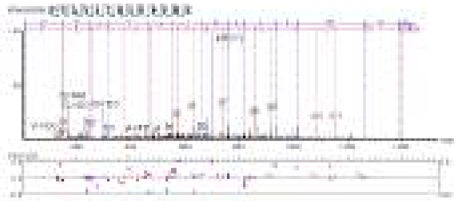

## XP_015687635.1

back to list

| Protein Coverage | Supporting Peptides | Best Unique PSM |

**Protein Coverage:**

```
  1 MIRFGTPSSS DKRRQRCRSW YWGGLLLFWA VAETRADIHY ATVYWLEAEK SFQIKDVLDK NGDAYGYYND TIQSTGWGIL
 81 EIKAGYGNQP ISNEILMYAA GFLEGYLTAS HMSDHFANLY PQMIKNVIIE QKVKDFMQKQ DEWTRQQIKN NKDDPFWRNA
161 GYVIAQLDGL YMGNVEWAKR QKRTPLTDFE ISFLNALGDL LDLIPALRKS YFRSMPDVSR IYQWDMGHCS ALIKVLPGYE
241 NIYFAHSSWF TYAATLRIYK HLDFKITDPQ TKTGRASFSS YPGFLVSLDD FYILGSGLIM LQTTNSVFNL SLLKKVVPES
321 LFAWERVRIA NMMADSGKTW AETFEKQNSG TYNNQYMILD TKKIKLQRSL EDGTLYIIEQ VPKLVKYSDQ TKVLRNGYWP
401 SYNIPFDKEI YNMSGYGEYV RRHGLEFSYE MAPRAKIFRR DQGKVTDIES MKSIMRYNNY KEDPYTKHDP CNTICCREDL
481 DHRTPVPAGC YDSKVADISM AAKFTAYAIN GPPVEKGLPV FSWVHFNKTK HQGLPESYNF DFVTMKPVL
```

**Supporting Peptides:**

| Peptide | Uniq | -10lgP | Mass | Length | ppm | m/z | z | RT | Fraction | Scan | Source File | Area F1 | Area F2a | Area F3a | Area F4 | Area F5 | Area F6a | Area F7a | Area F7b | Area F8 | Area F9a | Area Sample 1F10a | Area F10b | Area F11a | Area F11b | Area F12a | Area F13 | #Feature | #Feature F1 | #Feature F2a | #Feature F3a | #Feature F4 | #Feature F5 | #Feature F6a | #Feature F7a | #Feature F7b | #Feature F8 | #Feature F9a | #Feature Sample 1F10a | #Feature F10b | #Feature F11a | #Feature F11b | #Feature F12a | #Feature F13 | Start | End | PTM | AScore | Found By |
|---|---|---|---|---|---|---|---|---|---|---|---|---|---|---|---|---|---|---|---|---|---|---|---|---|---|---|---|---|---|---|---|---|---|---|---|---|---|---|---|---|---|---|---|---|---|---|---|---|---|
| K.FTAYAINGPPVEK.G | Y | 57.42 | 1405.7241 | 13 | 0.6 | 703.8698 | 2 | 52.52 | 16 | F16:24918 | BuCaTN01_F13.raw | | | | | | | | | | | | | | | | 2.1472E5 | 1 | 0 | 0 | 0 | 0 | 0 | 0 | 0 | 0 | 0 | 0 | 0 | 0 | 0 | 0 | 0 | 1 | 504 | 516 | | | PEAKS DB |
| total 1 peptides | | | | | | | | | | | | | | | | | | | | | | | | | | | | | | | | | | | | | | | | | | | | | | | | | |

**Best Unique PSM (Scan F16:24918, m/z=703.8698, z=2, RT=52.52, ppm=0.6):**

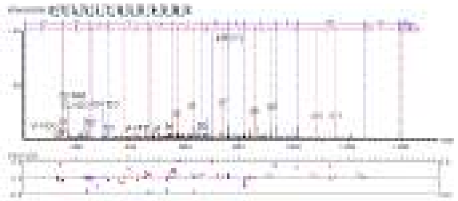

## JAI09658.1

back to list

| Protein Coverage | Supporting Peptides | Best Unique PSM |

**Protein Coverage:**

```
  1 MVRFGSAASS DNRRQRCWSW YWRGLLLLWA VAETRADLHY ATVYWLEAEK SFQVKDLLDK NGDAYGYYND SVQSTGWGIL
 81 EIKAGYGNQL VSNEILMYAA GFLEGYLTAS RMSDHVANMY HQLIKDVIVE QKVKDFMQKQ DEWTRQQIKN NKDDPFWRNA
161 GYIIAQLDGL YMGNLEWAKR QKRTPLTEFE ISFLNAIGDL LDLIPALSSK SRNDDSPSMS GASNMYEWDM GHCSALIKVL
241 PGYENIYFGH SSWFTYAATL RIYKHWDFRI IDPQTKTGRA SFSSYPGLLV SLDDFYILGS GLIMLQTTNT VFNLSLLKQV
321 VPESLFAWER VRIANMMADS GKTWAQTFEK QNSGTYNNQY MILDTKKIIL RKSIEDGALY IIEQVPNLVE YSDQTTILRK
401 GYWPSYNIPF HKVIYNMSGY KKFGLDFSYE MAPRAKIFRR DQGKVTDMKS MKYIMRYNNY KKDPYTKHNP CNTICCRQDL
481 NYKAPVPDGC YDSKVSDINM AAKFTAYAIN GPPVEKGLPI FSWIHFNKTT HQGLPESYNF DFVTMKPVL
```

**Supporting Peptides:**

| Peptide | Uniq | -10lgP | Mass | Length | ppm | m/z | z | RT | Fraction | Scan | Source File | Area F1 | Area F2a | Area F3a | Area F4 | Area F5 | Area F6a | Area F7a | Area F7b | Area F8 | Area F9a | Area Sample 1F10a | Area F10b | Area F11a | Area F11b | Area F12a | Area F13 | #Feature | #Feature F1 | #Feature F2a | #Feature F3a | #Feature F4 | #Feature F5 | #Feature F6a | #Feature F7a | #Feature F7b | #Feature F8 | #Feature F9a | #Feature Sample 1F10a | #Feature F10b | #Feature F11a | #Feature F11b | #Feature F12a | #Feature F13 | Start | End | PTM | AScore | Found By |
|---|---|---|---|---|---|---|---|---|---|---|---|---|---|---|---|---|---|---|---|---|---|---|---|---|---|---|---|---|---|---|---|---|---|---|---|---|---|---|---|---|---|---|---|---|---|---|---|---|---|
| K.FTAYAINGPPVEK.G | Y | 57.42 | 1405.7241 | 13 | 0.6 | 703.8698 | 2 | 52.52 | 16 | F16:24918 | BuCaTN01_F13.raw | | | | | | | | | | | | | | | | 2.1472E5 | 1 | 0 | 0 | 0 | 0 | 0 | 0 | 0 | 0 | 0 | 0 | 0 | 0 | 0 | 0 | 0 | 1 | 504 | 516 | | | PEAKS DB |
| total 1 peptides | | | | | | | | | | | | | | | | | | | | | | | | | | | | | | | | | | | | | | | | | | | | | | | | | |

**Best Unique PSM (Scan F16:24918, m/z=703.8698, z=2, RT=52.52, ppm=0.6):**

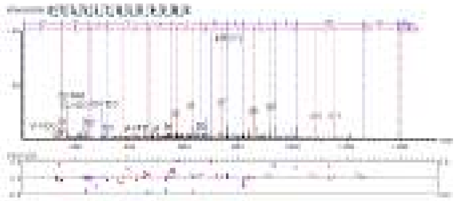

## JAB52759.1

back to list

| Protein Coverage | Supporting Peptides | Best Unique PSM |

**Protein Coverage:**

```
  1 MVRFGSAASS DNRRQRCWSW YWRGLLLLWA VAETRADLHY ATVYWLEAEK SFQVKDLLDK NGDAYGYYND SVQSTGWGIL
 81 EIKAGYGNQL VSNEILMYAA GFLEGYLTAS RMSDHVANMY HQLIKDVIVE QKVKDFMQKQ DEWTRQQIKN NKDDPFWRNA
161 GYIIAQLDGL YMGNLEWAKR QKRTPLTEFE ISFLNAIGDL LDLIPALSSK SRNDDSPSMS GASNMYEWDM GHCSALIKVL
241 PGYENIYFGH SSWFTYAATL RIYKHWDFRI IDPQTKTGRA SFSSYPGLLV SLDDFYILGS GLIMLQTTNT VFNLSLLKQV
321 VPESLFAWER VRIANMMADS GKTWAQTFEK QNSGTYNNQY MILDTKKIIL RKSIEDGALY IIEQVPNLVE YSDQTTILRK
401 GYWPSYNIPF HKVIYNMSGY KKFGLDFSYE MAPRAKIFRR DQGKVTDMKS MKYIMRYNNY KKDPYTKHNP CNTICCRQDL
481 NYKAPVPDGC YDSKVSDINM AAKFTAYAIN GPPVEKGLPI FSWIHFNKTT HQGLPESYNF DFVTMKPVL
```

**Supporting Peptides:**

| Peptide | Uniq | -10lgP | Mass | Length | ppm | m/z | z | RT | Fraction | Scan | Source File | Area F1 | Area F2a | Area F3a | Area F4 | Area F5 | Area F6a | Area F7a | Area F7b | Area F8 | Area F9a | Area Sample 1F10a | Area F10b | Area F11a | Area F11b | Area F12a | Area F13 | #Feature | #Feature F1 | #Feature F2a | #Feature F3a | #Feature F4 | #Feature F5 | #Feature F6a | #Feature F7a | #Feature F7b | #Feature F8 | #Feature F9a | #Feature Sample 1F10a | #Feature F10b | #Feature F11a | #Feature F11b | #Feature F12a | #Feature F13 | Start | End | PTM | AScore | Found By |
|---|---|---|---|---|---|---|---|---|---|---|---|---|---|---|---|---|---|---|---|---|---|---|---|---|---|---|---|---|---|---|---|---|---|---|---|---|---|---|---|---|---|---|---|---|---|---|---|---|---|
| K.FTAYAINGPPVEK.G | Y | 57.42 | 1405.7241 | 13 | 0.6 | 703.8698 | 2 | 52.52 | 16 | F16:24918 | BuCaTN01_F13.raw | | | | | | | | | | | | | | | | 2.1472E5 | 1 | 0 | 0 | 0 | 0 | 0 | 0 | 0 | 0 | 0 | 0 | 0 | 0 | 0 | 0 | 0 | 1 | 504 | 516 | | | PEAKS DB |
| total 1 peptides | | | | | | | | | | | | | | | | | | | | | | | | | | | | | | | | | | | | | | | | | | | | | | | | | |

**Best Unique PSM (Scan F16:24918, m/z=703.8698, z=2, RT=52.52, ppm=0.6):**

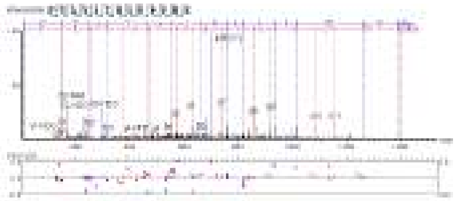

## XP_039177250.1

back to list

| Protein Coverage | Supporting Peptides | Best Unique PSM |

**Protein Coverage:**

```
  1 MQIFVKTLTG KTITLEVEPS DTIENVKAKI QDKEGIPPDQ QRLIFAGKQL EDGRTLSDYN IQKESTLHLV LRLRGGMQIF
 81 VKTLTGKTIT LEVEPSDTIE NVKAKIQDKE GIPPDQQRLI FAGKQLEDGR TLSDYNIQKE STLHLVLRLR GGMQIFVKTL
161 TGKTITLEVE PSDTIENVKA KIQDKEGIPP DQQRLIFAGK QLEDGRTLSD YNIQKESTLH LVLRLRGGMQ IFVKTLTGKT
241 ITLEVEPSDT IENVKAKIQD KEGIPPDQQR LIFAGKQLED GRTLSDYTIQ KESTLHLVLR LRGGMQIFVK TLTGKTITLE
321 VEPSDTIENV KAKIQDKEGI PPDQQRLIFA GKQLEDGRTL SDYNIQKEST LHLVLRLRGG MQIFVKTLTG KTITLEVEPS
401 DTIENVKAKI QDKEGIPPDQ QRLIFAGKQL EDGRTLSDYN IQKESTLHLV LRLRGGI
```

**Supporting Peptides:**

| Peptide | Uniq | -10lgP | Mass | Length | ppm | m/z | z | RT | Fraction | Scan | Source File | Area F1 | Area F2a | Area F3a | Area F4 | Area F5 | Area F6a | Area F7a | Area F7b | Area F8 | Area F9a | Area Sample 1F10a | Area F10b | Area F11a | Area F11b | Area F12a | Area F13 | #Feature | #Feature F1 | #Feature F2a | #Feature F3a | #Feature F4 | #Feature F5 | #Feature F6a | #Feature F7a | #Feature F7b | #Feature F8 | #Feature F9a | #Feature Sample 1F10a | #Feature F10b | #Feature F11a | #Feature F11b | #Feature F12a | #Feature F13 | Start | End | PTM | AScore | Found By |
|---|---|---|---|---|---|---|---|---|---|---|---|---|---|---|---|---|---|---|---|---|---|---|---|---|---|---|---|---|---|---|---|---|---|---|---|---|---|---|---|---|---|---|---|---|---|---|---|---|---|
| K.TITLEVEPSDTIENVK.A | Y | 52.24 | 1786.9200 | 16 | 2.9 | 894.4678 | 2 | 61.96 | 12 | F12:38000 | BuCaTN01_F10b.raw | | | | | | | | | | | | 1.8169E5 | | | | | 1 | 0 | 0 | 0 | 0 | 0 | 0 | 0 | 0 | 0 | 0 | 0 | 1 | 0 | 0 | 0 | 0 | 12 | 27 | | | PEAKS DB |
| R.TLSDYNIQKESTLHLVLR.L | Y | 50.38 | 2129.1479 | 18 | 1.9 | 533.2941 | 4 | 62.60 | 12 | F12:38710 | BuCaTN01_F10b.raw | | | | | | | | | | | | 1.0541E5 | | | | | 1 | 0 | 0 | 0 | 0 | 0 | 0 | 0 | 0 | 0 | 0 | 0 | 1 | 0 | 0 | 0 | 0 | 55 | 72 | | | PEAKS DB |
| total 2 peptides | | | | | | | | | | | | | | | | | | | | | | | | | | | | | | | | | | | | | | | | | | | | | | | | | |

**Best Unique PSM (Scan F12:38000, m/z=894.4678, z=2, RT=61.96, ppm=2.9):**

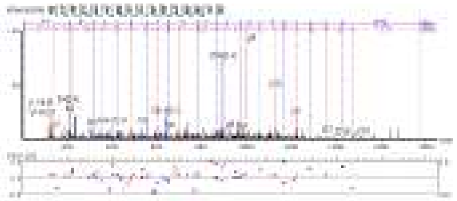

## JAG66642.1

back to list

| Protein Coverage | Supporting Peptides | Best Unique PSM |

**Protein Coverage:**

```
  1 MQIFVKTLTG KTITLEVEPS DTIENVKAKI QDKEGIPPDQ QRLIFAGKQL EDGRTLSDYN IQKESTLHLV LRLRGGAKKR
 81 KKKSYTTPKK NKHKRKKVKL AVLKYYKVDE NGKISRLRRE CPSEECGAGV FMASHFDRHY CGKCCLTYCF NKPEDK
```

**Supporting Peptides:**

| Peptide | Uniq | -10lgP | Mass | Length | ppm | m/z | z | RT | Fraction | Scan | Source File | Area F1 | Area F2a | Area F3a | Area F4 | Area F5 | Area F6a | Area F7a | Area F7b | Area F8 | Area F9a | Area Sample 1F10a | Area F10b | Area F11a | Area F11b | Area F12a | Area F13 | #Feature | #Feature F1 | #Feature F2a | #Feature F3a | #Feature F4 | #Feature F5 | #Feature F6a | #Feature F7a | #Feature F7b | #Feature F8 | #Feature F9a | #Feature Sample 1F10a | #Feature F10b | #Feature F11a | #Feature F11b | #Feature F12a | #Feature F13 | Start | End | PTM | AScore | Found By |
|---|---|---|---|---|---|---|---|---|---|---|---|---|---|---|---|---|---|---|---|---|---|---|---|---|---|---|---|---|---|---|---|---|---|---|---|---|---|---|---|---|---|---|---|---|---|---|---|---|---|
| K.TITLEVEPSDTIENVK.A | Y | 52.24 | 1786.9200 | 16 | 2.9 | 894.4678 | 2 | 61.96 | 12 | F12:38000 | BuCaTN01_F10b.raw | | | | | | | | | | | | 1.8169E5 | | | | | 1 | 0 | 0 | 0 | 0 | 0 | 0 | 0 | 0 | 0 | 0 | 0 | 1 | 0 | 0 | 0 | 0 | 12 | 27 | | | PEAKS DB |
| R.TLSDYNIQKESTLHLVLR.L | Y | 50.38 | 2129.1479 | 18 | 1.9 | 533.2941 | 4 | 62.60 | 12 | F12:38710 | BuCaTN01_F10b.raw | | | | | | | | | | | | 1.0541E5 | | | | | 1 | 0 | 0 | 0 | 0 | 0 | 0 | 0 | 0 | 0 | 0 | 0 | 1 | 0 | 0 | 0 | 0 | 55 | 72 | | | PEAKS DB |
| total 2 peptides | | | | | | | | | | | | | | | | | | | | | | | | | | | | | | | | | | | | | | | | | | | | | | | | | |

**Best Unique PSM (Scan F12:38000, m/z=894.4678, z=2, RT=61.96, ppm=2.9):**

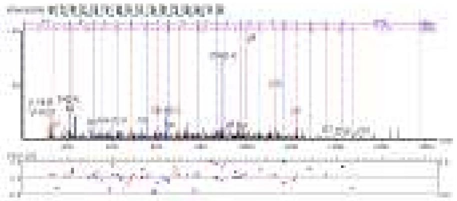

## JAB53407.1

back to list

| Protein Coverage | Supporting Peptides | Best Unique PSM |

**Protein Coverage:**

1 MQIFVKTLTG K**TITLEVEPS DTIENVK**AKI QDKEGIPPDQ QRLIFAGKQL EDGR**TLSDYN IQKESTLHLV LR**LRGGAKKR

81 KKKSYTTPKK NKHKRKKVKL AVLKYYKVDE NGKISRLRRE CPSEECGAGV FMASHFDRHY CGKCCLTYCF NKPEDK

**Supporting Peptides:**

| Peptide | Uniq | -10lgP | Mass | Length | ppm | m/z | z | RT | Fraction | Scan | Source File | Area F1 | Area F2a | Area F3a | Area F4 | Area F5 | Area F6a | Area F7a | Area F7b | Area F8 | Area F9a | Area Sample 1F10a | Area F10b | Area F11a | Area F11b | Area F12a | Area F13 | #Feature | #Feature F1 | #Feature F2a | #Feature F3a | #Feature F4 | #Feature F5 | #Feature F6a | #Feature F7a | #Feature F7b | #Feature F8 | #Feature F9a | #Feature Sample 1F10a | #Feature F10b | #Feature F11a | #Feature F11b | #Feature F12a | #Feature F13 | Start | End | PTM | AScore | Found By |
|---|---|---|---|---|---|---|---|---|---|---|---|---|---|---|---|---|---|---|---|---|---|---|---|---|---|---|---|---|---|---|---|---|---|---|---|---|---|---|---|---|---|---|---|---|---|---|---|---|---|
| K.TITLEVEPSDTIENVK.A | Y | 52.24 | 1786.9200 | 16 | 2.9 | 894.4678 | 2 | 61.96 | 12 | F12:38000 | BuCaTN01_F10b.raw | | | | | | | | | | | | 1.8169E5 | | | | | 1 | 0 | 0 | 0 | 0 | 0 | 0 | 0 | 0 | 0 | 0 | 0 | 1 | 0 | 0 | 0 | 0 | 12 | 27 | | | PEAKS DB |
| R.TLSDYNIQKESTLHLVLR.L | Y | 50.38 | 2129.1479 | 18 | 1.9 | 533.2941 | 4 | 62.60 | 12 | F12:38710 | BuCaTN01_F10b.raw | | | | | | | | | | | | 1.0541E5 | | | | | 1 | 0 | 0 | 0 | 0 | 0 | 0 | 0 | 0 | 0 | 0 | 0 | 1 | 0 | 0 | 0 | 0 | 55 | 72 | | | PEAKS DB |
| total 2 peptides | | | | | | | | | | | | | | | | | | | | | | | | | | | | | | | | | | | | | | | | | | | | | | | | | |

**Best Unique PSM (Scan F12:38000, m/z=894.4678, z=2, RT=61.96, ppm=2.9):**

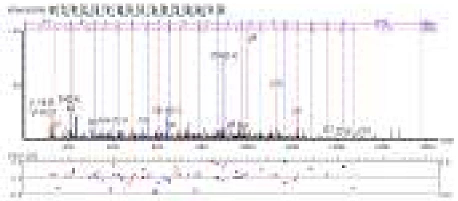

## JAI11580.1

back to list

| Protein Coverage | Supporting Peptides | Best Unique PSM |

**Protein Coverage:**

1 MQIFVKTLTG K**TITLEVEPS DTIENVK**AKI QDKEGIPPDQ QRLIFAGKQL EDGR**TLSDYN IQKESTLHLV LR**LRGGAKKR

81 KKKSYTTPKK NKHKRKKVKL AVLKYYKVDE NGKISRLRRE CPSEECGAGV FMASHFDRHY CGKCCLTYCF NKPEDK

**Supporting Peptides:**

| Peptide | Uniq | -10lgP | Mass | Length | ppm | m/z | z | RT | Fraction | Scan | Source File | Area F1 | Area F2a | Area F3a | Area F4 | Area F5 | Area F6a | Area F7a | Area F7b | Area F8 | Area F9a | Area Sample 1F10a | Area F10b | Area F11a | Area F11b | Area F12a | Area F13 | #Feature | #Feature F1 | #Feature F2a | #Feature F3a | #Feature F4 | #Feature F5 | #Feature F6a | #Feature F7a | #Feature F7b | #Feature F8 | #Feature F9a | #Feature Sample 1F10a | #Feature F10b | #Feature F11a | #Feature F11b | #Feature F12a | #Feature F13 | Start | End | PTM | AScore | Found By |
|---|---|---|---|---|---|---|---|---|---|---|---|---|---|---|---|---|---|---|---|---|---|---|---|---|---|---|---|---|---|---|---|---|---|---|---|---|---|---|---|---|---|---|---|---|---|---|---|---|---|
| K.TITLEVEPSDTIENVK.A | Y | 52.24 | 1786.9200 | 16 | 2.9 | 894.4678 | 2 | 61.96 | 12 | F12:38000 | BuCaTN01_F10b.raw | | | | | | | | | | | | 1.8169E5 | | | | | 1 | 0 | 0 | 0 | 0 | 0 | 0 | 0 | 0 | 0 | 0 | 0 | 1 | 0 | 0 | 0 | 0 | 12 | 27 | | | PEAKS DB |
| R.TLSDYNIQKESTLHLVLR.L | Y | 50.38 | 2129.1479 | 18 | 1.9 | 533.2941 | 4 | 62.60 | 12 | F12:38710 | BuCaTN01_F10b.raw | | | | | | | | | | | | 1.0541E5 | | | | | 1 | 0 | 0 | 0 | 0 | 0 | 0 | 0 | 0 | 0 | 0 | 0 | 1 | 0 | 0 | 0 | 0 | 55 | 72 | | | PEAKS DB |
| total 2 peptides | | | | | | | | | | | | | | | | | | | | | | | | | | | | | | | | | | | | | | | | | | | | | | | | | |

**Best Unique PSM (Scan F12:38000, m/z=894.4678, z=2, RT=61.96, ppm=2.9):**

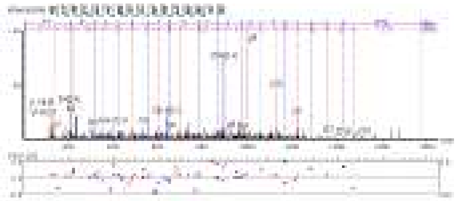

## LAA53283.1

back to list

| Protein Coverage | Supporting Peptides | Best Unique PSM |

**Protein Coverage:**

1 MQIFVKTLTG K**TITLEVEPS DTIENVK**AKI QDKEGIPPDQ QRLIFAGKQL EDGR**TLSDYN IQKESTLHLV LR**LRGGAKKR

81 KKKSYTTPKK NKHKRKKVKL AVLKYYKVDE NGKISRLRRE CPSEECGAGV FMASHFDRHY CGKCCLTYCF NKPEDK

**Supporting Peptides:**

| Peptide | Uniq | -10lgP | Mass | Length | ppm | m/z | z | RT | Fraction | Scan | Source File | Area F1 | Area F2a | Area F3a | Area F4 | Area F5 | Area F6a | Area F7a | Area F7b | Area F8 | Area F9a | Area Sample 1F10a | Area F10b | Area F11a | Area F11b | Area F12a | Area F13 | #Feature | #Feature F1 | #Feature F2a | #Feature F3a | #Feature F4 | #Feature F5 | #Feature F6a | #Feature F7a | #Feature F7b | #Feature F8 | #Feature F9a | #Feature Sample 1F10a | #Feature F10b | #Feature F11a | #Feature F11b | #Feature F12a | #Feature F13 | Start | End | PTM | AScore | Found By |
|---|---|---|---|---|---|---|---|---|---|---|---|---|---|---|---|---|---|---|---|---|---|---|---|---|---|---|---|---|---|---|---|---|---|---|---|---|---|---|---|---|---|---|---|---|---|---|---|---|---|
| K.TITLEVEPSDTIENVK.A | Y | 52.24 | 1786.9200 | 16 | 2.9 | 894.4678 | 2 | 61.96 | 12 | F12:38000 | BuCaTN01_F10b.raw | | | | | | | | | | | | 1.8169E5 | | | | | 1 | 0 | 0 | 0 | 0 | 0 | 0 | 0 | 0 | 0 | 0 | 0 | 1 | 0 | 0 | 0 | 0 | 12 | 27 | | | PEAKS DB |
| R.TLSDYNIQKESTLHLVLR.L | Y | 50.38 | 2129.1479 | 18 | 1.9 | 533.2941 | 4 | 62.60 | 12 | F12:38710 | BuCaTN01_F10b.raw | | | | | | | | | | | | 1.0541E5 | | | | | 1 | 0 | 0 | 0 | 0 | 0 | 0 | 0 | 0 | 0 | 0 | 0 | 1 | 0 | 0 | 0 | 0 | 55 | 72 | | | PEAKS DB |
| total 2 peptides | | | | | | | | | | | | | | | | | | | | | | | | | | | | | | | | | | | | | | | | | | | | | | | | | |

**Best Unique PSM (Scan F12:38000, m/z=894.4678, z=2, RT=61.96, ppm=2.9):**

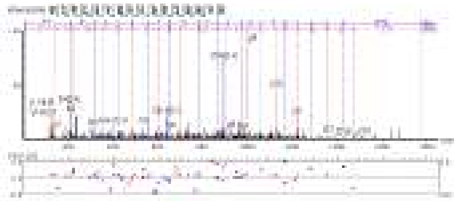

## AFJ51304.1

back to list

| Protein Coverage | Supporting Peptides | Best Unique PSM |

**Protein Coverage:**

1 MQIFVKTLTG K**TITLEVEPS DTIENVK**AKI QDKEGIPPDQ QRLIFAGKQL EDGR**TLSDYN IQKESTLHLV LR**LRGGAKKR

81 KKKSYTTPKK NKHKRKKVKL AVLKYYKVDE NGKISRLRRE CPSEECGAGV FMASHFDRHY CGKCCLTYCF NKPEDK

**Supporting Peptides:**

| Peptide | Uniq | -10lgP | Mass | Length | ppm | m/z | z | RT | Fraction | Scan | Source File | Area F1 | Area F2a | Area F3a | Area F4 | Area F5 | Area F6a | Area F7a | Area F7b | Area F8 | Area F9a | Area Sample 1F10a | Area F10b | Area F11a | Area F11b | Area F12a | Area F13 | #Feature | #Feature F1 | #Feature F2a | #Feature F3a | #Feature F4 | #Feature F5 | #Feature F6a | #Feature F7a | #Feature F7b | #Feature F8 | #Feature F9a | #Feature Sample 1F10a | #Feature F10b | #Feature F11a | #Feature F11b | #Feature F12a | #Feature F13 | Start | End | PTM | AScore | Found By |
|---|---|---|---|---|---|---|---|---|---|---|---|---|---|---|---|---|---|---|---|---|---|---|---|---|---|---|---|---|---|---|---|---|---|---|---|---|---|---|---|---|---|---|---|---|---|---|---|---|---|
| K.TITLEVEPSDTIENVK.A | Y | 52.24 | 1786.9200 | 16 | 2.9 | 894.4678 | 2 | 61.96 | 12 | F12:38000 | BuCaTN01_F10b.raw | | | | | | | | | | | | 1.8169E5 | | | | | 1 | 0 | 0 | 0 | 0 | 0 | 0 | 0 | 0 | 0 | 0 | 0 | 1 | 0 | 0 | 0 | 0 | 12 | 27 | | | PEAKS DB |
| R.TLSDYNIQKESTLHLVLR.L | Y | 50.38 | 2129.1479 | 18 | 1.9 | 533.2941 | 4 | 62.60 | 12 | F12:38710 | BuCaTN01_F10b.raw | | | | | | | | | | | | 1.0541E5 | | | | | 1 | 0 | 0 | 0 | 0 | 0 | 0 | 0 | 0 | 0 | 0 | 0 | 1 | 0 | 0 | 0 | 0 | 55 | 72 | | | PEAKS DB |
| total 2 peptides | | | | | | | | | | | | | | | | | | | | | | | | | | | | | | | | | | | | | | | | | | | | | | | | | |

**Best Unique PSM (Scan F12:38000, m/z=894.4678, z=2, RT=61.96, ppm=2.9):**

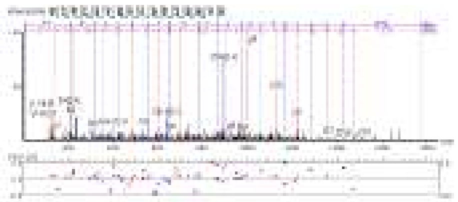

## JAA95751.1

back to list

| Protein Coverage | Supporting Peptides | Best Unique PSM |

**Protein Coverage:**

1 MQIFVKTLTG K**TITLEVEPS DTIENVK**AKI QDKEGIPPDQ QRLIFAGKQL EDGR**TLSDYN IQKESTLHLV LR**LRGGAKKR

81 KKKSYTTPKK NKHKRKKVKL AVLKYYKVDE NGKISRLRRE CPSEECGAGV FMASHFDRHY CGKCCLTYCF NKPEDK

**Supporting Peptides:**

| Peptide | Uniq | -10lgP | Mass | Length | ppm | m/z | z | RT | Fraction | Scan | Source File | Area F1 | Area F2a | Area F3a | Area F4 | Area F5 | Area F6a | Area F7a | Area F7b | Area F8 | Area F9a | Area Sample 1F10a | Area F10b | Area F11a | Area F11b | Area F12a | Area F13 | #Feature | #Feature F1 | #Feature F2a | #Feature F3a | #Feature F4 | #Feature F5 | #Feature F6a | #Feature F7a | #Feature F7b | #Feature F8 | #Feature F9a | #Feature Sample 1F10a | #Feature F10b | #Feature F11a | #Feature F11b | #Feature F12a | #Feature F13 | Start | End | PTM | AScore | Found By |
|---|---|---|---|---|---|---|---|---|---|---|---|---|---|---|---|---|---|---|---|---|---|---|---|---|---|---|---|---|---|---|---|---|---|---|---|---|---|---|---|---|---|---|---|---|---|---|---|---|---|
| K.TITLEVEPSDTIENVK.A | Y | 52.24 | 1786.9200 | 16 | 2.9 | 894.4678 | 2 | 61.96 | 12 | F12:38000 | BuCaTN01_F10b.raw | | | | | | | | | | | | 1.8169E5 | | | | | 1 | 0 | 0 | 0 | 0 | 0 | 0 | 0 | 0 | 0 | 0 | 0 | 1 | 0 | 0 | 0 | 0 | 12 | 27 | | | PEAKS DB |
| R.TLSDYNIQKESTLHLVLR.L | Y | 50.38 | 2129.1479 | 18 | 1.9 | 533.2941 | 4 | 62.60 | 12 | F12:38710 | BuCaTN01_F10b.raw | | | | | | | | | | | | 1.0541E5 | | | | | 1 | 0 | 0 | 0 | 0 | 0 | 0 | 0 | 0 | 0 | 0 | 0 | 1 | 0 | 0 | 0 | 0 | 55 | 72 | | | PEAKS DB |
| total 2 peptides | | | | | | | | | | | | | | | | | | | | | | | | | | | | | | | | | | | | | | | | | | | | | | | | | |

**Best Unique PSM (Scan F12:38000, m/z=894.4678, z=2, RT=61.96, ppm=2.9):**

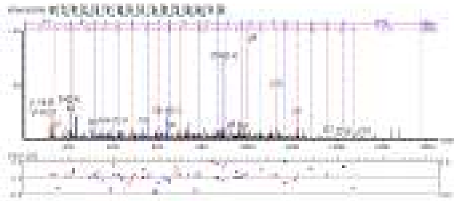

## JAI09470.1

back to list

| Protein Coverage | Supporting Peptides | Best Unique PSM |

**Protein Coverage:**

1 MQIFVKTLTG K**TITLEVEPS DTIENVK**AKI QDKEGIPPDQ QRLIFAGKQL EDGR**TLSDYN IQKESTLHLV LR**LRGGAKKR

81 KKKSYTTPKK NKHKRKKVKL AVLKYYKVDE NGKISRLRRE CPSEECGAGV FMASHFDRHY CGKCCLTYCF NKPEDK

**Supporting Peptides:**

| Peptide | Uniq | -10lgP | Mass | Length | ppm | m/z | z | RT | Fraction | Scan | Source File | Area F1 | Area F2a | Area F3a | Area F4 | Area F5 | Area F6a | Area F7a | Area F7b | Area F8 | Area F9a | Area Sample 1F10a | Area F10b | Area F11a | Area F11b | Area F12a | Area F13 | #Feature | #Feature F1 | #Feature F2a | #Feature F3a | #Feature F4 | #Feature F5 | #Feature F6a | #Feature F7a | #Feature F7b | #Feature F8 | #Feature F9a | #Feature Sample 1F10a | #Feature F10b | #Feature F11a | #Feature F11b | #Feature F12a | #Feature F13 | Start | End | PTM | AScore | Found By |
|---|---|---|---|---|---|---|---|---|---|---|---|---|---|---|---|---|---|---|---|---|---|---|---|---|---|---|---|---|---|---|---|---|---|---|---|---|---|---|---|---|---|---|---|---|---|---|---|---|---|
| K.TITLEVEPSDTIENVK.A | Y | 52.24 | 1786.9200 | 16 | 2.9 | 894.4678 | 2 | 61.96 | 12 | F12:38000 | BuCaTN01_F10b.raw | | | | | | | | | | | | 1.8169E5 | | | | | 1 | 0 | 0 | 0 | 0 | 0 | 0 | 0 | 0 | 0 | 0 | 0 | 1 | 0 | 0 | 0 | 0 | 12 | 27 | | | PEAKS DB |
| R.TLSDYNIQKESTLHLVLR.L | Y | 50.38 | 2129.1479 | 18 | 1.9 | 533.2941 | 4 | 62.60 | 12 | F12:38710 | BuCaTN01_F10b.raw | | | | | | | | | | | | 1.0541E5 | | | | | 1 | 0 | 0 | 0 | 0 | 0 | 0 | 0 | 0 | 0 | 0 | 0 | 1 | 0 | 0 | 0 | 0 | 55 | 72 | | | PEAKS DB |
| total 2 peptides | | | | | | | | | | | | | | | | | | | | | | | | | | | | | | | | | | | | | | | | | | | | | | | | | |

**Best Unique PSM (Scan F12:38000, m/z=894.4678, z=2, RT=61.96, ppm=2.9):**

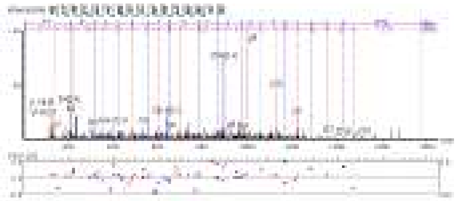

## JAI10738.1

back to list

| Protein Coverage | Supporting Peptides | Best Unique PSM |

**Protein Coverage:**

1 MQIFVKTLTG K**TITLEVEPS DTIENVK**AKI QDKEGIPPDQ QRLIFAGKQL EDGR**TLSDYN IQKESTLHLV LR**LRGGMQIF

81 VKTLTGKTIT LEVGCRSL

**Supporting Peptides:**

| Peptide | Uniq | -10lgP | Mass | Length | ppm | m/z | z | RT | Fraction | Scan | Source File | Area F1 | Area F2a | Area F3a | Area F4 | Area F5 | Area F6a | Area F7a | Area F7b | Area F8 | Area F9a | Area Sample 1F10a | Area F10b | Area F11a | Area F11b | Area F12a | Area F13 | #Feature | #Feature F1 | #Feature F2a | #Feature F3a | #Feature F4 | #Feature F5 | #Feature F6a | #Feature F7a | #Feature F7b | #Feature F8 | #Feature F9a | #Feature Sample 1F10a | #Feature F10b | #Feature F11a | #Feature F11b | #Feature F12a | #Feature F13 | Start | End | PTM | AScore | Found By |
|---|---|---|---|---|---|---|---|---|---|---|---|---|---|---|---|---|---|---|---|---|---|---|---|---|---|---|---|---|---|---|---|---|---|---|---|---|---|---|---|---|---|---|---|---|---|---|---|---|---|
| K.TITLEVEPSDTIENVK.A | Y | 52.24 | 1786.9200 | 16 | 2.9 | 894.4678 | 2 | 61.96 | 12 | F12:38000 | BuCaTN01_F10b.raw | | | | | | | | | | | | 1.8169E5 | | | | | 1 | 0 | 0 | 0 | 0 | 0 | 0 | 0 | 0 | 0 | 0 | 0 | 1 | 0 | 0 | 0 | 0 | 12 | 27 | | | PEAKS DB |
| R.TLSDYNIQKESTLHLVLR.L | Y | 50.38 | 2129.1479 | 18 | 1.9 | 533.2941 | 4 | 62.60 | 12 | F12:38710 | BuCaTN01_F10b.raw | | | | | | | | | | | | 1.0541E5 | | | | | 1 | 0 | 0 | 0 | 0 | 0 | 0 | 0 | 0 | 0 | 0 | 0 | 1 | 0 | 0 | 0 | 0 | 55 | 72 | | | PEAKS DB |
| total 2 peptides | | | | | | | | | | | | | | | | | | | | | | | | | | | | | | | | | | | | | | | | | | | | | | | | | |

**Best Unique PSM (Scan F12:38000, m/z=894.4678, z=2, RT=61.96, ppm=2.9):**

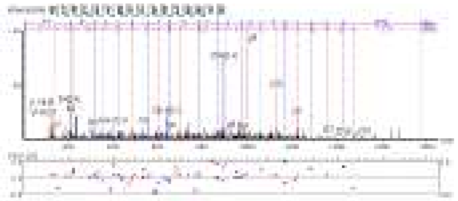

## LAA24946.1

back to list

| Protein Coverage | Supporting Peptides | Best Unique PSM |

**Protein Coverage:**

1 IFAGKQLEDG R**TLSDYNIQK ESTLHLVLR**L RGGMQIFVKT LTGK**TITLEV EPSDTIENVK** AKIQDKEGIP PDQQRLIFAG

81 KQLEDGRTLS DYNIQKESTL HLVLRLRG

**Supporting Peptides:**

| Peptide | Uniq | -10lgP | Mass | Length | ppm | m/z | z | RT | Fraction | Scan | Source File | Area F1 | Area F2a | Area F3a | Area F4 | Area F5 | Area F6a | Area F7a | Area F7b | Area F8 | Area F9a | Area Sample 1F10a | Area F10b | Area F11a | Area F11b | Area F12a | Area F13 | #Feature | #Feature F1 | #Feature F2a | #Feature F3a | #Feature F4 | #Feature F5 | #Feature F6a | #Feature F7a | #Feature F7b | #Feature F8 | #Feature F9a | #Feature Sample 1F10a | #Feature F10b | #Feature F11a | #Feature F11b | #Feature F12a | #Feature F13 | Start | End | PTM | AScore | Found By |
|---|---|---|---|---|---|---|---|---|---|---|---|---|---|---|---|---|---|---|---|---|---|---|---|---|---|---|---|---|---|---|---|---|---|---|---|---|---|---|---|---|---|---|---|---|---|---|---|---|---|

| | | | | | | | | | | | | | | | | | | | | | | | | | | | | | | | | | | | | | | | | | | | | | | | | | |
|---|---|---|---|---|---|---|---|---|---|---|---|---|---|---|---|---|---|---|---|---|---|---|---|---|---|---|---|---|---|---|---|---|---|---|---|---|---|---|---|---|---|---|---|---|---|---|---|---|---|
| K.TITLEVEPSDTIENVK.A | Y | 52.24 | 1786.9200 | 16 | 2.9 | 894.4678 | 2 | 61.96 | 12 | F12:38000 | BuCaTN01_F10b.raw | | | | | | | | | | | | 1.8169E5 | | | | | 1 | 0 | 0 | 0 | 0 | 0 | 0 | 0 | 0 | 0 | 0 | 0 | 1 | 0 | 0 | 0 | 0 | 45 | 60 | | | PEAKS DB |
| R.TLSDYNIQKESTLHLVLR.L | Y | 50.38 | 2129.1479 | 18 | 1.9 | 533.2941 | 4 | 62.60 | 12 | F12:38710 | BuCaTN01_F10b.raw | | | | | | | | | | | | 1.0541E5 | | | | | 1 | 0 | 0 | 0 | 0 | 0 | 0 | 0 | 0 | 0 | 0 | 0 | 1 | 0 | 0 | 0 | 0 | 12 | 29 | | | PEAKS DB |
| **total 2 peptides** | | | | | | | | | | | | | | | | | | | | | | | | | | | | | | | | | | | | | | | | | | | | | | | | | |

**Best Unique PSM (Scan F12:38000, m/z=894.4678, z=2, RT=61.96, ppm=2.9):**

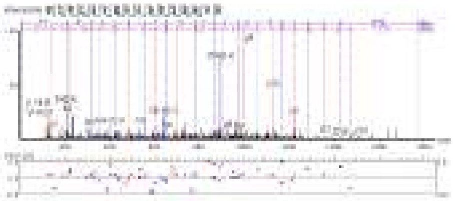

## XP_013929631.1

back to list

| Protein Coverage | Supporting Peptides | Best Unique PSM |

**Protein Coverage:**

1 MQIFVKTLTG K**TITLEVEPS DTIENVK**AKI QDKEGIPPDQ QRLIFAGKQL EDGR**TLSDYN IQKESTLHLV LR**LCGGIIEP

81 SLRQLAQKYN CNKMICRKCY ARLHPRAVNC RKKKCGHTNN LRPKKKVK

**Supporting Peptides:**

| Peptide | Uniq | -10lgP | Mass | Length | ppm | m/z | z | RT | Fraction | Scan | Source File | Area F1 | Area F2a | Area F3a | Area F4 | Area F5 | Area F6a | Area F7a | Area F7b | Area F8 | Area F9a | Area Sample 1F10a | Area F10b | Area F11a | Area F11b | Area F12a | Area F13 | #Feature | #Feature F1 | #Feature F2a | #Feature F3a | #Feature F4 | #Feature F5 | #Feature F6a | #Feature F7a | #Feature F7b | #Feature F8 | #Feature F9a | #Feature Sample 1F10a | #Feature F10b | #Feature F11a | #Feature F11b | #Feature F12a | #Feature F13 | Start | End | PTM | AScore | Found By |
|---|---|---|---|---|---|---|---|---|---|---|---|---|---|---|---|---|---|---|---|---|---|---|---|---|---|---|---|---|---|---|---|---|---|---|---|---|---|---|---|---|---|---|---|---|---|---|---|---|---|
| K.TITLEVEPSDTIENVK.A | Y | 52.24 | 1786.9200 | 16 | 2.9 | 894.4678 | 2 | 61.96 | 12 | F12:38000 | BuCaTN01_F10b.raw | | | | | | | | | | | | 1.8169E5 | | | | | 1 | 0 | 0 | 0 | 0 | 0 | 0 | 0 | 0 | 0 | 0 | 0 | 1 | 0 | 0 | 0 | 0 | 12 | 27 | | | PEAKS DB |
| R.TLSDYNIQKESTLHLVLR.L | Y | 50.38 | 2129.1479 | 18 | 1.9 | 533.2941 | 4 | 62.60 | 12 | F12:38710 | BuCaTN01_F10b.raw | | | | | | | | | | | | 1.0541E5 | | | | | 1 | 0 | 0 | 0 | 0 | 0 | 0 | 0 | 0 | 0 | 0 | 0 | 1 | 0 | 0 | 0 | 0 | 55 | 72 | | | PEAKS DB |
| **total 2 peptides** | | | | | | | | | | | | | | | | | | | | | | | | | | | | | | | | | | | | | | | | | | | | | | | | | |

**Best Unique PSM (Scan F12:38000, m/z=894.4678, z=2, RT=61.96, ppm=2.9):**

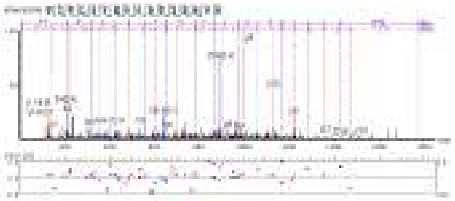

## JAI09497.1

back to list

| Protein Coverage | Supporting Peptides | Best Unique PSM |

**Protein Coverage:**

1 MQIFVKTLTG K**TITLEVEPS DTIENVK**AKI QDKEGIPPDQ QRLIFAGKQL EDGR**TLSDYN IQKESTLHLV LR**LRGGIIEP

81 SLRQLAQKYN CDKMICRKCY ARLHPRAVNC RKKKCGHTNN LRPKKKVK

**Supporting Peptides:**

| Peptide | Uniq | -10lgP | Mass | Length | ppm | m/z | z | RT | Fraction | Scan | Source File | Area F1 | Area F2a | Area F3a | Area F4 | Area F5 | Area F6a | Area F7a | Area F7b | Area F8 | Area F9a | Area Sample 1F10a | Area F10b | Area F11a | Area F11b | Area F12a | Area F13 | #Feature | #Feature F1 | #Feature F2a | #Feature F3a | #Feature F4 | #Feature F5 | #Feature F6a | #Feature F7a | #Feature F7b | #Feature F8 | #Feature F9a | #Feature Sample 1F10a | #Feature F10b | #Feature F11a | #Feature F11b | #Feature F12a | #Feature F13 | Start | End | PTM | AScore | Found By |
|---|---|---|---|---|---|---|---|---|---|---|---|---|---|---|---|---|---|---|---|---|---|---|---|---|---|---|---|---|---|---|---|---|---|---|---|---|---|---|---|---|---|---|---|---|---|---|---|---|---|
| K.TITLEVEPSDTIENVK.A | Y | 52.24 | 1786.9200 | 16 | 2.9 | 894.4678 | 2 | 61.96 | 12 | F12:38000 | BuCaTN01_F10b.raw | | | | | | | | | | | | 1.8169E5 | | | | | 1 | 0 | 0 | 0 | 0 | 0 | 0 | 0 | 0 | 0 | 0 | 0 | 1 | 0 | 0 | 0 | 0 | 12 | 27 | | | PEAKS DB |
| R.TLSDYNIQKESTLHLVLR.L | Y | 50.38 | 2129.1479 | 18 | 1.9 | 533.2941 | 4 | 62.60 | 12 | F12:38710 | BuCaTN01_F10b.raw | | | | | | | | | | | | 1.0541E5 | | | | | 1 | 0 | 0 | 0 | 0 | 0 | 0 | 0 | 0 | 0 | 0 | 0 | 1 | 0 | 0 | 0 | 0 | 55 | 72 | | | PEAKS DB |
| **total 2 peptides** | | | | | | | | | | | | | | | | | | | | | | | | | | | | | | | | | | | | | | | | | | | | | | | | | |

**Best Unique PSM (Scan F12:38000, m/z=894.4678, z=2, RT=61.96, ppm=2.9):**

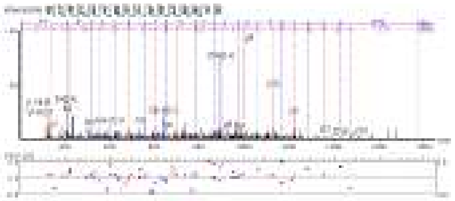

## JAB53439.1

back to list

| Protein Coverage | Supporting Peptides | Best Unique PSM |

**Protein Coverage:**

1 MQIFVKTLTG K**TITLEVEPS DTIENVK**AKI QDKEGIPPDQ QRLIFAGKQL EDGR**TLSDYN IQKESTLHLV LR**LRGGIIEP

81 SLRQLAQKYN CDKMICRKCY ARLHPRAVNC RKKKCGHTNN LRPKKKVK

**Supporting Peptides:**

| Peptide | Uniq | -10lgP | Mass | Length | ppm | m/z | z | RT | Fraction | Scan | Source File | Area F1 | Area F2a | Area F3a | Area F4 | Area F5 | Area F6a | Area F7a | Area F7b | Area F8 | Area F9a | Area Sample 1F10a | Area F10b | Area F11a | Area F11b | Area F12a | Area F13 | #Feature | #Feature F1 | #Feature F2a | #Feature F3a | #Feature F4 | #Feature F5 | #Feature F6a | #Feature F7a | #Feature F7b | #Feature F8 | #Feature F9a | #Feature Sample 1F10a | #Feature F10b | #Feature F11a | #Feature F11b | #Feature F12a | #Feature F13 | Start | End | PTM | AScore | Found By |
|---|---|---|---|---|---|---|---|---|---|---|---|---|---|---|---|---|---|---|---|---|---|---|---|---|---|---|---|---|---|---|---|---|---|---|---|---|---|---|---|---|---|---|---|---|---|---|---|---|---|
| K.TITLEVEPSDTIENVK.A | Y | 52.24 | 1786.9200 | 16 | 2.9 | 894.4678 | 2 | 61.96 | 12 | F12:38000 | BuCaTN01_F10b.raw | | | | | | | | | | | | 1.8169E5 | | | | | 1 | 0 | 0 | 0 | 0 | 0 | 0 | 0 | 0 | 0 | 0 | 0 | 1 | 0 | 0 | 0 | 0 | 12 | 27 | | | PEAKS DB |
| R.TLSDYNIQKESTLHLVLR.L | Y | 50.38 | 2129.1479 | 18 | 1.9 | 533.2941 | 4 | 62.60 | 12 | F12:38710 | BuCaTN01_F10b.raw | | | | | | | | | | | | 1.0541E5 | | | | | 1 | 0 | 0 | 0 | 0 | 0 | 0 | 0 | 0 | 0 | 0 | 0 | 1 | 0 | 0 | 0 | 0 | 55 | 72 | | | PEAKS DB |
| **total 2 peptides** | | | | | | | | | | | | | | | | | | | | | | | | | | | | | | | | | | | | | | | | | | | | | | | | | |

**Best Unique PSM (Scan F12:38000, m/z=894.4678, z=2, RT=61.96, ppm=2.9):**

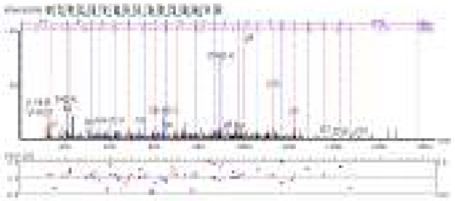

## JAI11613.1

back to list

| Protein Coverage | Supporting Peptides | Best Unique PSM |

**Protein Coverage:**

1 MQIFVKTLTG KTITLEVEPS DTIENVKAKI QDKEGIPPDQ QRLIFAGKQL EDGRTLSDYN IQKESTLHLV LRLRGGIIEP

81 SLRQLAQKYN CDKMICRKCY ARLHPRAVNC RKKKCGHTNN LRPKKKVK

**Supporting Peptides:**

| Peptide | Uniq | -10lgP | Mass | Length | ppm | m/z | z | RT | Fraction | Scan | Source File | Area F1 | Area F2a | Area F3a | Area F4 | Area F5 | Area F6a | Area F7a | Area F7b | Area F8 | Area F9a | Area Sample 1F10a | Area F10b | Area F11a | Area F11b | Area F12a | Area F13 | #Feature | #Feature F1 | #Feature F2a | #Feature F3a | #Feature F4 | #Feature F5 | #Feature F6a | #Feature F7a | #Feature F7b | #Feature F8 | #Feature F9a | #Feature Sample 1F10a | #Feature F10b | #Feature F11a | #Feature F11b | #Feature F12a | #Feature F13 | Start | End | PTM | AScore | Found By |
|---|---|---|---|---|---|---|---|---|---|---|---|---|---|---|---|---|---|---|---|---|---|---|---|---|---|---|---|---|---|---|---|---|---|---|---|---|---|---|---|---|---|---|---|---|---|---|---|---|---|
| K.TITLEVEPSDTIENVK.A | Y | 52.24 | 1786.9200 | 16 | 2.9 | 894.4678 | 2 | 61.96 | 12 | F12:38000 | BuCaTN01_F10b.raw | | | | | | | | | | | | 1.8169E5 | | | | | 1 | 0 | 0 | 0 | 0 | 0 | 0 | 0 | 0 | 0 | 0 | 0 | 1 | 0 | 0 | 0 | 0 | 12 | 27 | | | PEAKS DB |
| R.TLSDYNIQKESTLHLVLR.L | Y | 50.38 | 2129.1479 | 18 | 1.9 | 533.2941 | 4 | 62.60 | 12 | F12:38710 | BuCaTN01_F10b.raw | | | | | | | | | | | | 1.0541E5 | | | | | 1 | 0 | 0 | 0 | 0 | 0 | 0 | 0 | 0 | 0 | 0 | 0 | 1 | 0 | 0 | 0 | 0 | 55 | 72 | | | PEAKS DB |
| total 2 peptides | | | | | | | | | | | | | | | | | | | | | | | | | | | | | | | | | | | | | | | | | | | | | | | | | |

**Best Unique PSM (Scan F12:38000, m/z=894.4678, z=2, RT=61.96, ppm=2.9):**

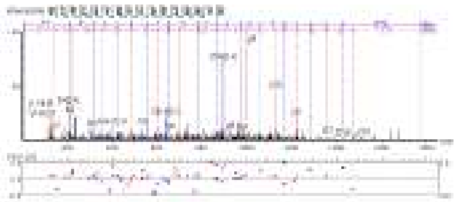

## XP_013929630.1 back to list

| Protein Coverage | Supporting Peptides | Best Unique PSM |

**Protein Coverage:**

1 MQIFVKTLTG KTITLEVEPS DTIENVKAKI QDKEGIPPDQ QRLIFAGKQL EDGRTLSDYN IQKESTLHLV LRLCGGIIEP

81 SLRQLAQKYN CNKMICRKCY ARLHPRAVNC RKKKCGHTNN LRPKKKVK

**Supporting Peptides:**

| Peptide | Uniq | -10lgP | Mass | Length | ppm | m/z | z | RT | Fraction | Scan | Source File | Area F1 | Area F2a | Area F3a | Area F4 | Area F5 | Area F6a | Area F7a | Area F7b | Area F8 | Area F9a | Area Sample 1F10a | Area F10b | Area F11a | Area F11b | Area F12a | Area F13 | #Feature | #Feature F1 | #Feature F2a | #Feature F3a | #Feature F4 | #Feature F5 | #Feature F6a | #Feature F7a | #Feature F7b | #Feature F8 | #Feature F9a | #Feature Sample 1F10a | #Feature F10b | #Feature F11a | #Feature F11b | #Feature F12a | #Feature F13 | Start | End | PTM | AScore | Found By |
|---|---|---|---|---|---|---|---|---|---|---|---|---|---|---|---|---|---|---|---|---|---|---|---|---|---|---|---|---|---|---|---|---|---|---|---|---|---|---|---|---|---|---|---|---|---|---|---|---|---|
| K.TITLEVEPSDTIENVK.A | Y | 52.24 | 1786.9200 | 16 | 2.9 | 894.4678 | 2 | 61.96 | 12 | F12:38000 | BuCaTN01_F10b.raw | | | | | | | | | | | | 1.8169E5 | | | | | 1 | 0 | 0 | 0 | 0 | 0 | 0 | 0 | 0 | 0 | 0 | 0 | 1 | 0 | 0 | 0 | 0 | 12 | 27 | | | PEAKS DB |
| R.TLSDYNIQKESTLHLVLR.L | Y | 50.38 | 2129.1479 | 18 | 1.9 | 533.2941 | 4 | 62.60 | 12 | F12:38710 | BuCaTN01_F10b.raw | | | | | | | | | | | | 1.0541E5 | | | | | 1 | 0 | 0 | 0 | 0 | 0 | 0 | 0 | 0 | 0 | 0 | 0 | 1 | 0 | 0 | 0 | 0 | 55 | 72 | | | PEAKS DB |
| total 2 peptides | | | | | | | | | | | | | | | | | | | | | | | | | | | | | | | | | | | | | | | | | | | | | | | | | |

**Best Unique PSM (Scan F12:38000, m/z=894.4678, z=2, RT=61.96, ppm=2.9):**

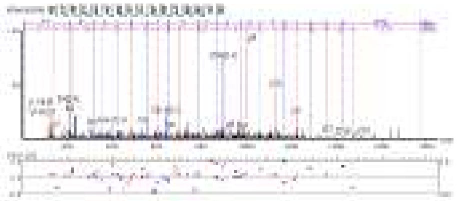

## AFJ51273.1 back to list

| Protein Coverage | Supporting Peptides | Best Unique PSM |

**Protein Coverage:**

1 MQIFVKTLTG KTITLEVEPS DTIENVKAKI QDKEGIPPDQ QRLIFAGKQL EDGRTLSDYN IQKESTLHLV LRLRGGIIEP

81 SLRQLAQKYN CDKMICRKCY ARLHPRAVNC RKKKCGHTNN LRPKKKVK

**Supporting Peptides:**

| Peptide | Uniq | -10lgP | Mass | Length | ppm | m/z | z | RT | Fraction | Scan | Source File | Area F1 | Area F2a | Area F3a | Area F4 | Area F5 | Area F6a | Area F7a | Area F7b | Area F8 | Area F9a | Area Sample 1F10a | Area F10b | Area F11a | Area F11b | Area F12a | Area F13 | #Feature | #Feature F1 | #Feature F2a | #Feature F3a | #Feature F4 | #Feature F5 | #Feature F6a | #Feature F7a | #Feature F7b | #Feature F8 | #Feature F9a | #Feature Sample 1F10a | #Feature F10b | #Feature F11a | #Feature F11b | #Feature F12a | #Feature F13 | Start | End | PTM | AScore | Found By |
|---|---|---|---|---|---|---|---|---|---|---|---|---|---|---|---|---|---|---|---|---|---|---|---|---|---|---|---|---|---|---|---|---|---|---|---|---|---|---|---|---|---|---|---|---|---|---|---|---|---|
| K.TITLEVEPSDTIENVK.A | Y | 52.24 | 1786.9200 | 16 | 2.9 | 894.4678 | 2 | 61.96 | 12 | F12:38000 | BuCaTN01_F10b.raw | | | | | | | | | | | | 1.8169E5 | | | | | 1 | 0 | 0 | 0 | 0 | 0 | 0 | 0 | 0 | 0 | 0 | 0 | 1 | 0 | 0 | 0 | 0 | 12 | 27 | | | PEAKS DB |
| R.TLSDYNIQKESTLHLVLR.L | Y | 50.38 | 2129.1479 | 18 | 1.9 | 533.2941 | 4 | 62.60 | 12 | F12:38710 | BuCaTN01_F10b.raw | | | | | | | | | | | | 1.0541E5 | | | | | 1 | 0 | 0 | 0 | 0 | 0 | 0 | 0 | 0 | 0 | 0 | 0 | 1 | 0 | 0 | 0 | 0 | 55 | 72 | | | PEAKS DB |
| total 2 peptides | | | | | | | | | | | | | | | | | | | | | | | | | | | | | | | | | | | | | | | | | | | | | | | | | |

**Best Unique PSM (Scan F12:38000, m/z=894.4678, z=2, RT=61.96, ppm=2.9):**

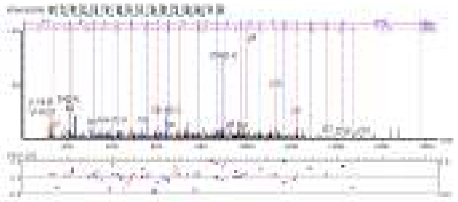

## JAG66673.1 back to list

| Protein Coverage | Supporting Peptides | Best Unique PSM |

**Protein Coverage:**

1 MQIFVKTLTG KTITLEVEPS DTIENVKAKI QDKEGIPPDQ QRLIFAGKQL EDGRTLSDYN IQKESTLHLV LRLRGGIIEP

81 SLRQLAQKYN CDKMICRKCY ARLHPRAVNC RKKKCGHTNN LRPKKKVK

**Supporting Peptides:**

| Peptide | Uniq | -10lgP | Mass | Length | ppm | m/z | z | RT | Fraction | Scan | Source File | Area F1 | Area F2a | Area F3a | Area F4 | Area F5 | Area F6a | Area F7a | Area F7b | Area F8 | Area F9a | Area Sample 1F10a | Area F10b | Area F11a | Area F11b | Area F12a | Area F13 | #Feature | #Feature F1 | #Feature F2a | #Feature F3a | #Feature F4 | #Feature F5 | #Feature F6a | #Feature F7a | #Feature F7b | #Feature F8 | #Feature F9a | #Feature Sample 1F10a | #Feature F10b | #Feature F11a | #Feature F11b | #Feature F12a | #Feature F13 | Start | End | PTM | AScore | Found By |
|---|---|---|---|---|---|---|---|---|---|---|---|---|---|---|---|---|---|---|---|---|---|---|---|---|---|---|---|---|---|---|---|---|---|---|---|---|---|---|---|---|---|---|---|---|---|---|---|---|---|
| K.TITLEVEPSDTIENVK.A | Y | 52.24 | 1786.9200 | 16 | 2.9 | 894.4678 | 2 | 61.96 | 12 | F12:38000 | BuCaTN01_F10b.raw | | | | | | | | | | | | 1.8169E5 | | | | | 1 | 0 | 0 | 0 | 0 | 0 | 0 | 0 | 0 | 0 | 0 | 0 | 1 | 0 | 0 | 0 | 0 | 12 | 27 | | | PEAKS DB |
| R.TLSDYNIQKESTLHLVLR.L | Y | 50.38 | 2129.1479 | 18 | 1.9 | 533.2941 | 4 | 62.60 | 12 | F12:38710 | BuCaTN01_F10b.raw | | | | | | | | | | | | 1.0541E5 | | | | | 1 | 0 | 0 | 0 | 0 | 0 | 0 | 0 | 0 | 0 | 0 | 0 | 1 | 0 | 0 | 0 | 0 | 55 | 72 | | | PEAKS DB |
| total 2 peptides | | | | | | | | | | | | | | | | | | | | | | | | | | | | | | | | | | | | | | | | | | | | | | | | | |

**Best Unique PSM (Scan F12:38000, m/z=894.4678, z=2, RT=61.96, ppm=2.9):**

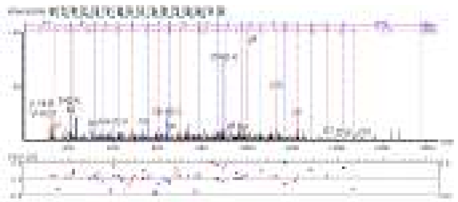

## JAA95781.1

back to list

| Protein Coverage | Supporting Peptides | Best Unique PSM |

**Protein Coverage:**

```
  1  MQIFVKTLTG KTITLEVEPS DTIENVKAKI QDKEGIPPDQ QRLIFAGKQL EDGRTLSDYN IQKESTLHLV LRLRGGIIEP
 81  SLRQLAQKYN CDKMICRKCY ARLHPRAVNC RKKKCGHTNN LRPKKKVK
```

**Supporting Peptides:**

| Peptide | Uniq | -10lgP | Mass | Length | ppm | m/z | z | RT | Fraction | Scan | Source File | Area F1 | Area F2a | Area F3a | Area F4 | Area F5 | Area F6a | Area F7a | Area F7b | Area F8 | Area F9a | Area Sample 1F10a | Area F10b | Area F11a | Area F11b | Area F12a | Area F13 | #Feature | #Feature F1 | #Feature F2a | #Feature F3a | #Feature F4 | #Feature F5 | #Feature F6a | #Feature F7a | #Feature F7b | #Feature F8 | #Feature F9a | #Feature Sample 1F10a | #Feature F10b | #Feature F11a | #Feature F11b | #Feature F12a | #Feature F13 | Start | End | PTM | AScore | Found By |
|---|---|---|---|---|---|---|---|---|---|---|---|---|---|---|---|---|---|---|---|---|---|---|---|---|---|---|---|---|---|---|---|---|---|---|---|---|---|---|---|---|---|---|---|---|---|---|---|---|---|
| K.TITLEVEPSDTIENVK.A | Y | 52.24 | 1786.9200 | 16 | 2.9 | 894.4678 | 2 | 61.96 | 12 | F12:38000 | BuCaTN01_F10b.raw | | | | | | | | | | | | 1.8169E5 | | | | | 1 | 0 | 0 | 0 | 0 | 0 | 0 | 0 | 0 | 0 | 0 | 0 | 1 | 0 | 0 | 0 | 0 | 12 | 27 | | | PEAKS DB |
| R.TLSDYNIQKESTLHLVLR.L | Y | 50.38 | 2129.1479 | 18 | 1.9 | 533.2941 | 4 | 62.60 | 12 | F12:38710 | BuCaTN01_F10b.raw | | | | | | | | | | | | 1.0541E5 | | | | | 1 | 0 | 0 | 0 | 0 | 0 | 0 | 0 | 0 | 0 | 0 | 0 | 1 | 0 | 0 | 0 | 0 | 55 | 72 | | | PEAKS DB |
| total 2 peptides | | | | | | | | | | | | | | | | | | | | | | | | | | | | | | | | | | | | | | | | | | | | | | | | | |

**Best Unique PSM (Scan F12:38000, m/z=894.4678, z=2, RT=61.96, ppm=2.9):**

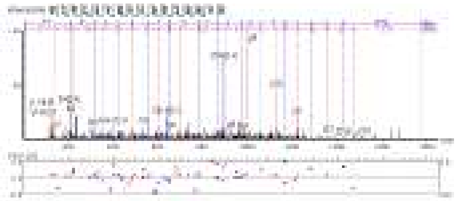

## JAB53440.1

back to list

| Protein Coverage | Supporting Peptides | Best Unique PSM |

**Protein Coverage:**

```
  1  MQTFVKTLTG KTITLEVEPS DTIENVKAKI QDKEGIPPDQ QRLIFAGKQL EDGRTLSDYN IQKESTLHLV LRLRGGIIEP
 81  SLRQLAQKYN CDKMICRKCY ARLHPRAVNC RKKKCGHTNN LRPKKKVK
```

**Supporting Peptides:**

| Peptide | Uniq | -10lgP | Mass | Length | ppm | m/z | z | RT | Fraction | Scan | Source File | Area F1 | Area F2a | Area F3a | Area F4 | Area F5 | Area F6a | Area F7a | Area F7b | Area F8 | Area F9a | Area Sample 1F10a | Area F10b | Area F11a | Area F11b | Area F12a | Area F13 | #Feature | #Feature F1 | #Feature F2a | #Feature F3a | #Feature F4 | #Feature F5 | #Feature F6a | #Feature F7a | #Feature F7b | #Feature F8 | #Feature F9a | #Feature Sample 1F10a | #Feature F10b | #Feature F11a | #Feature F11b | #Feature F12a | #Feature F13 | Start | End | PTM | AScore | Found By |
|---|---|---|---|---|---|---|---|---|---|---|---|---|---|---|---|---|---|---|---|---|---|---|---|---|---|---|---|---|---|---|---|---|---|---|---|---|---|---|---|---|---|---|---|---|---|---|---|---|---|
| K.TITLEVEPSDTIENVK.A | Y | 52.24 | 1786.9200 | 16 | 2.9 | 894.4678 | 2 | 61.96 | 12 | F12:38000 | BuCaTN01_F10b.raw | | | | | | | | | | | | 1.8169E5 | | | | | 1 | 0 | 0 | 0 | 0 | 0 | 0 | 0 | 0 | 0 | 0 | 0 | 1 | 0 | 0 | 0 | 0 | 12 | 27 | | | PEAKS DB |
| R.TLSDYNIQKESTLHLVLR.L | Y | 50.38 | 2129.1479 | 18 | 1.9 | 533.2941 | 4 | 62.60 | 12 | F12:38710 | BuCaTN01_F10b.raw | | | | | | | | | | | | 1.0541E5 | | | | | 1 | 0 | 0 | 0 | 0 | 0 | 0 | 0 | 0 | 0 | 0 | 0 | 1 | 0 | 0 | 0 | 0 | 55 | 72 | | | PEAKS DB |
| total 2 peptides | | | | | | | | | | | | | | | | | | | | | | | | | | | | | | | | | | | | | | | | | | | | | | | | | |

**Best Unique PSM (Scan F12:38000, m/z=894.4678, z=2, RT=61.96, ppm=2.9):**

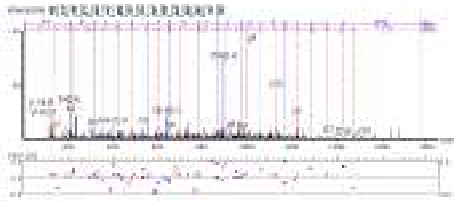

## AAG17445.1

back to list

| Protein Coverage | Supporting Peptides | Best Unique PSM |

**Protein Coverage:**

```
  1  MQIFVKTLTG KTITLEVEPS DTIENVKAKI QDKEGIPPDQ QRLIFAGKQL EDGRTLSDYN IQKESTLHLV LRLRGGIIEP
 81  SLRQLAQKYN CDKMICRKCY ARLHPRAVNC RKKKCGHTNN LRPKKKVK
```

**Supporting Peptides:**

| Peptide | Uniq | -10lgP | Mass | Length | ppm | m/z | z | RT | Fraction | Scan | Source File | Area F1 | Area F2a | Area F3a | Area F4 | Area F5 | Area F6a | Area F7a | Area F7b | Area F8 | Area F9a | Area Sample 1F10a | Area F10b | Area F11a | Area F11b | Area F12a | Area F13 | #Feature | #Feature F1 | #Feature F2a | #Feature F3a | #Feature F4 | #Feature F5 | #Feature F6a | #Feature F7a | #Feature F7b | #Feature F8 | #Feature F9a | #Feature Sample 1F10a | #Feature F10b | #Feature F11a | #Feature F11b | #Feature F12a | #Feature F13 | Start | End | PTM | AScore | Found By |
|---|---|---|---|---|---|---|---|---|---|---|---|---|---|---|---|---|---|---|---|---|---|---|---|---|---|---|---|---|---|---|---|---|---|---|---|---|---|---|---|---|---|---|---|---|---|---|---|---|---|
| K.TITLEVEPSDTIENVK.A | Y | 52.24 | 1786.9200 | 16 | 2.9 | 894.4678 | 2 | 61.96 | 12 | F12:38000 | BuCaTN01_F10b.raw | | | | | | | | | | | | 1.8169E5 | | | | | 1 | 0 | 0 | 0 | 0 | 0 | 0 | 0 | 0 | 0 | 0 | 0 | 1 | 0 | 0 | 0 | 0 | 12 | 27 | | | PEAKS DB |
| R.TLSDYNIQKESTLHLVLR.L | Y | 50.38 | 2129.1479 | 18 | 1.9 | 533.2941 | 4 | 62.60 | 12 | F12:38710 | BuCaTN01_F10b.raw | | | | | | | | | | | | 1.0541E5 | | | | | 1 | 0 | 0 | 0 | 0 | 0 | 0 | 0 | 0 | 0 | 0 | 0 | 1 | 0 | 0 | 0 | 0 | 55 | 72 | | | PEAKS DB |
| total 2 peptides | | | | | | | | | | | | | | | | | | | | | | | | | | | | | | | | | | | | | | | | | | | | | | | | | |

**Best Unique PSM (Scan F12:38000, m/z=894.4678, z=2, RT=61.96, ppm=2.9):**

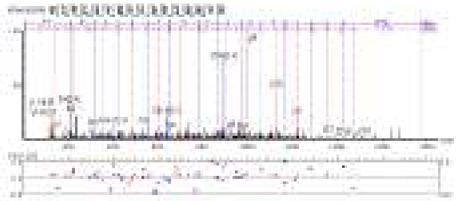

## XP_013928637.1

back to list

| Protein Coverage | Supporting Peptides | Best Unique PSM |

**Protein Coverage:**

```
  1  MQIFVKTLTG KTITLEVEPS DTIENVKAKI QDKEGIPPDQ QRLIFAGKQL EDGRTLSDYN IQKESTLHLV LRLRGGMQIF
 81  VKTLTGKTIT LEVEPSDTIE NVKAKIQDKE GIPPDQQRLI FAGKQLEDGR TLSDYNIQKE STLHLVLRLR GGFQIFVKTL
161  TGKTITLEVE PSDTIENVKA KIQDKEGIPP DQQRLIFAGK QLEDGRTLSD YNIQKESTLH LVLRLRGGF
```

**Supporting Peptides:**

| Peptide | Uniq | -10lgP | Mass | Length | ppm | m/z | z | RT | Fraction | Scan | Source File | Area F1 | Area F2a | Area F3a | Area F4 | Area F5 | Area F6a | Area F7a | Area F7b | Area F8 | Area F9a | Area Sample 1F10a | Area F10b | Area F11a | Area F11b | Area F12a | Area F13 | #Feature | #Feature F1 | #Feature F2a | #Feature F3a | #Feature F4 | #Feature F5 | #Feature F6a | #Feature F7a | #Feature F7b | #Feature F8 | #Feature F9a | #Feature Sample 1F10a | #Feature F10b | #Feature F11a | #Feature F11b | #Feature F12a | #Feature F13 | Start | End | PTM | AScore | Found By |
|---|---|---|---|---|---|---|---|---|---|---|---|---|---|---|---|---|---|---|---|---|---|---|---|---|---|---|---|---|---|---|---|---|---|---|---|---|---|---|---|---|---|---|---|---|---|---|---|---|---|
| K.TITLEVEPSDTIENVK.A | Y | 52.24 | 1786.9200 | 16 | 2.9 | 894.4678 | 2 | 61.96 | 12 | F12:38000 | BuCaTN01_F10b.raw | | | | | | | | | | | | 1.8169E5 | | | | | 1 | 0 | 0 | 0 | 0 | 0 | 0 | 0 | 0 | 0 | 0 | 0 | 1 | 0 | 0 | 0 | 0 | 12 | 27 | | | PEAKS DB |
| R.TLSDYNIQKESTLHLVLR.L | Y | 50.38 | 2129.1479 | 18 | 1.9 | 533.2941 | 4 | 62.60 | 12 | F12:38710 | BuCaTN01_F10b.raw | | | | | | | | | | | | 1.0541E5 | | | | | 1 | 0 | 0 | 0 | 0 | 0 | 0 | 0 | 0 | 0 | 0 | 0 | 1 | 0 | 0 | 0 | 0 | 55 | 72 | | | PEAKS DB |
| total 2 peptides | | | | | | | | | | | | | | | | | | | | | | | | | | | | | | | | | | | | | | | | | | | | | | | | | |

**Best Unique PSM (Scan F12:38000, m/z=894.4678, z=2, RT=61.96, ppm=2.9):**

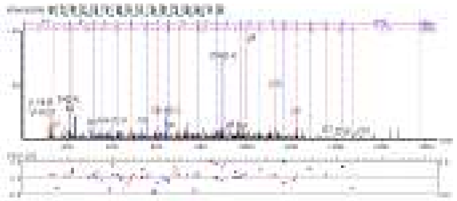

## AFJ51941.1

back to list

| Protein Coverage | Supporting Peptides | Best Unique PSM |

**Protein Coverage:**

```
  1 MQIFVKTLTG KTITLEVEPS DTIENVKAKI QDKEGIPPDQ QRLIFAGKQL EDGRTLSDYN IQKESTLHLV LRLRGGMQIF
 81 VKTLTGKTIT LEVEPSDTIE NVKAKIQDKE GIPPDQQRLI FAGKQLEDGR TLSDYNIQKE STLHLVLRLR GGMQIFVKTL
161 TGKTITLEVE PSDTIENVKA KIQDKEGIPP DQQRLIFAGK QLEDGRTLSD YNIQKESTLH LVLRLRGGF
```

**Supporting Peptides:**

| Peptide | Uniq | -10lgP | Mass | Length | ppm | m/z | z | RT | Fraction | Scan | Source File | Area F1 | Area F2a | Area F3a | Area F4 | Area F5 | Area F6a | Area F7a | Area F7b | Area F8 | Area F9a | Area Sample 1F10a | Area F10b | Area F11a | Area F11b | Area F12a | Area F13 | #Feature | #Feature F1 | #Feature F2a | #Feature F3a | #Feature F4 | #Feature F5 | #Feature F6a | #Feature F7a | #Feature F7b | #Feature F8 | #Feature F9a | #Feature Sample 1F10a | #Feature F10b | #Feature F11a | #Feature F11b | #Feature F12a | #Feature F13 | Start | End | PTM | AScore | Found By |
|---|---|---|---|---|---|---|---|---|---|---|---|---|---|---|---|---|---|---|---|---|---|---|---|---|---|---|---|---|---|---|---|---|---|---|---|---|---|---|---|---|---|---|---|---|---|---|---|---|---|
| K.TITLEVEPSDTIENVK.A | Y | 52.24 | 1786.9200 | 16 | 2.9 | 894.4678 | 2 | 61.96 | 12 | F12:38000 | BuCaTN01_F10b.raw | | | | | | | | | | | | 1.8169E5 | | | | | 1 | 0 | 0 | 0 | 0 | 0 | 0 | 0 | 0 | 0 | 0 | 0 | 1 | 0 | 0 | 0 | 0 | 12 | 27 | | | PEAKS DB |
| R.TLSDYNIQKESTLHLVLR.L | Y | 50.38 | 2129.1479 | 18 | 1.9 | 533.2941 | 4 | 62.60 | 12 | F12:38710 | BuCaTN01_F10b.raw | | | | | | | | | | | | 1.0541E5 | | | | | 1 | 0 | 0 | 0 | 0 | 0 | 0 | 0 | 0 | 0 | 0 | 0 | 1 | 0 | 0 | 0 | 0 | 55 | 72 | | | PEAKS DB |
| total 2 peptides | | | | | | | | | | | | | | | | | | | | | | | | | | | | | | | | | | | | | | | | | | | | | | | | | |

**Best Unique PSM (Scan F12:38000, m/z=894.4678, z=2, RT=61.96, ppm=2.9):**

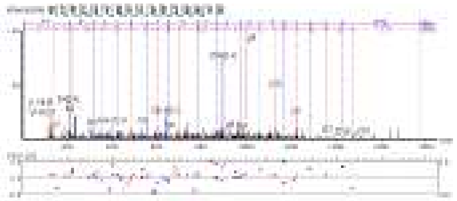

## JAB53023.1

back to list

| Protein Coverage | Supporting Peptides | Best Unique PSM |

**Protein Coverage:**

```
  1 MQIFVKTLTG KTITLEVEPS DTIENVKAKI QDKEGIPPDQ QRLIFAGKQL EDGRTLSDYN IQKESTLHLV LRLRGGMQIF
 81 VKTLTGKTIT LEVEPSDTIE NVKAKIQDKE GIPPDQQRLI FAGKQLEDGR TLSDYNIQKE STLHLVLRLR GGMQIFVKTL
161 TGKTITLEVE PSDTIENVKA KIQDKEGIPP DQQRLIFAGK QLEDGRTLSD YNIQKESTLH LVLRLRGGMQ IFVKTLTGKT
241 ITLEVEPSDT IENVKAKIQD KEGIPPDQQR LIFAGKQLED GRTLSDYNIQ KESTLHLVLR LRGGMQIFVK TLTGKTITLE
321 VEPSDTIENV KAKIQDKEGI PPDQQRLIFA GKQLEDGRTL SDYNIQKEST LHLVLRLRGG I
```

**Supporting Peptides:**

| Peptide | Uniq | -10lgP | Mass | Length | ppm | m/z | z | RT | Fraction | Scan | Source File | Area F1 | Area F2a | Area F3a | Area F4 | Area F5 | Area F6a | Area F7a | Area F7b | Area F8 | Area F9a | Area Sample 1F10a | Area F10b | Area F11a | Area F11b | Area F12a | Area F13 | #Feature | #Feature F1 | #Feature F2a | #Feature F3a | #Feature F4 | #Feature F5 | #Feature F6a | #Feature F7a | #Feature F7b | #Feature F8 | #Feature F9a | #Feature Sample 1F10a | #Feature F10b | #Feature F11a | #Feature F11b | #Feature F12a | #Feature F13 | Start | End | PTM | AScore | Found By |
|---|---|---|---|---|---|---|---|---|---|---|---|---|---|---|---|---|---|---|---|---|---|---|---|---|---|---|---|---|---|---|---|---|---|---|---|---|---|---|---|---|---|---|---|---|---|---|---|---|---|
| K.TITLEVEPSDTIENVK.A | Y | 52.24 | 1786.9200 | 16 | 2.9 | 894.4678 | 2 | 61.96 | 12 | F12:38000 | BuCaTN01_F10b.raw | | | | | | | | | | | | 1.8169E5 | | | | | 1 | 0 | 0 | 0 | 0 | 0 | 0 | 0 | 0 | 0 | 0 | 0 | 1 | 0 | 0 | 0 | 0 | 12 | 27 | | | PEAKS DB |
| R.TLSDYNIQKESTLHLVLR.L | Y | 50.38 | 2129.1479 | 18 | 1.9 | 533.2941 | 4 | 62.60 | 12 | F12:38710 | BuCaTN01_F10b.raw | | | | | | | | | | | | 1.0541E5 | | | | | 1 | 0 | 0 | 0 | 0 | 0 | 0 | 0 | 0 | 0 | 0 | 0 | 1 | 0 | 0 | 0 | 0 | 55 | 72 | | | PEAKS DB |
| total 2 peptides | | | | | | | | | | | | | | | | | | | | | | | | | | | | | | | | | | | | | | | | | | | | | | | | | |

**Best Unique PSM (Scan F12:38000, m/z=894.4678, z=2, RT=61.96, ppm=2.9):**

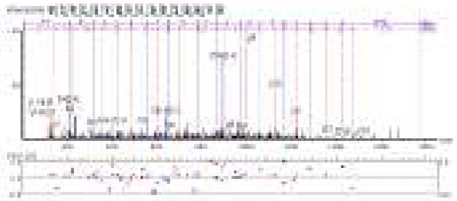

## XP_015684186.1

back to list

| Protein Coverage | Supporting Peptides | Best Unique PSM |

**Protein Coverage:**

```
  1 MQIFVKTLTG KTITLEVEPS DTIENVKAKI QDKEGIPPDQ QRLIFAGKQL EDGRTLSDYN IQKESTLHLV LRLRGGMQIF
 81 VKTLTGKTIT LEVEPSDTIE NVKAKIQDKE GIPPDQQRLI FAGKQLEDGR TLSDYNIQKE STLHLVLRLR GGMQIFVKTL
161 TGKTITLEVE PSDTIENVKA KIQDKEGIPP DQQRLIFAGK QLEDGRTLSD YNIQKESTLH LVLRLRGGMQ IFVKTLTGKT
241 ITLEVEPSDT IENVKAKIQD KEGIPPDQQR LIFAGKQLED GRTLSDYNIQ KESTLHLVLR LRGGMQIFVK TLTGKTITLE
321 VEPSDTIENV KAKIQDKEGI PPDQQRLIFA GKQLEDGRTL SDYNIQKEST LHLVLRLRGG MQIFVKTLTG KTITLEVEPS
401 DTIENVKAKI QDKEGIPPDQ QRLIFAGKQL EDGRTLSDYN IQKESTLHLV LRLRGGMQIF VKTLTGKTIT LEVEPSDTIE
481 NVKAKIQDKE GIPPDQQRLI FAGKQLEDGR TLSDYNIQKE STLHLVLRLR GGMQIFVKTL TGKTITLEVE PSDTIENVKA
561 KIQDKEGIPP DQQRLIFAGK QLEDGRTLSD YNIQKESTLH LVLRLRGGI
```

**Supporting Peptides:**

| Peptide | Uniq | -10lgP | Mass | Length | ppm | m/z | z | RT | Fraction | Scan | Source File | Area F1 | Area F2a | Area F3a | Area F4 | Area F5 | Area F6a | Area F7a | Area F7b | Area F8 | Area F9a | Area Sample 1F10a | Area F10b | Area F11a | Area F11b | Area F12a | Area F13 | #Feature | #Feature F1 | #Feature F2a | #Feature F3a | #Feature F4 | #Feature F5 | #Feature F6a | #Feature F7a | #Feature F7b | #Feature F8 | #Feature F9a | #Feature Sample 1F10a | #Feature F10b | #Feature F11a | #Feature F11b | #Feature F12a | #Feature F13 | Start | End | PTM | AScore | Found By |
|---|---|---|---|---|---|---|---|---|---|---|---|---|---|---|---|---|---|---|---|---|---|---|---|---|---|---|---|---|---|---|---|---|---|---|---|---|---|---|---|---|---|---|---|---|---|---|---|---|---|
| K.TITLEVEPSDTIENVK.A | Y | 52.24 | 1786.9200 | 16 | 2.9 | 894.4678 | 2 | 61.96 | 12 | F12:38000 | BuCaTN01_F10b.raw | | | | | | | | | | | | 1.8169E5 | | | | | 1 | 0 | 0 | 0 | 0 | 0 | 0 | 0 | 0 | 0 | 0 | 0 | 1 | 0 | 0 | 0 | 0 | 12 | 27 | | | PEAKS DB |
| R.TLSDYNIQKESTLHLVLR.L | Y | 50.38 | 2129.1479 | 18 | 1.9 | 533.2941 | 4 | 62.60 | 12 | F12:38710 | BuCaTN01_F10b.raw | | | | | | | | | | | | 1.0541E5 | | | | | 1 | 0 | 0 | 0 | 0 | 0 | 0 | 0 | 0 | 0 | 0 | 0 | 1 | 0 | 0 | 0 | 0 | 55 | 72 | | | PEAKS DB |
| total 2 peptides | | | | | | | | | | | | | | | | | | | | | | | | | | | | | | | | | | | | | | | | | | | | | | | | | |

**Best Unique PSM (Scan F12:38000, m/z=894.4678, z=2, RT=61.96, ppm=2.9):**

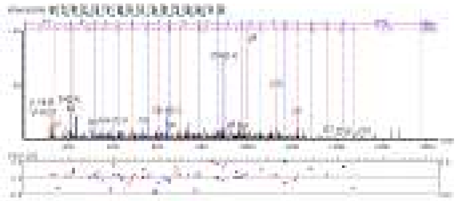

## XP_015666146.1

back to list

| Protein Coverage | Supporting Peptides | Best Unique PSM |

**Protein Coverage:**

```
  1 MQIFVKTLTG KTITLEVEPS DTIENVKAKI QDKEGIPPDQ QRLIFAGKQL EDGRTLSDYN IQKESTLHLV LRLRGGMQIF
 81 VKTLTGKTIT LEVEPSDTIE NVKAKIQDKE GIPPDQQRLI FAGKQLEDGR TLSDYNIQKE STLHLVLRLR GGMQIFVKTL
161 TGKTITLEVE PSDTIENVKA KIQDKEGIPP DQQRLIFAGK QLEDGRTLSD YNIQKESTLH LVLRLRGGMQ IFVKTLTGKT
241 ITLEVEPSDT IENVKAKIQD KEGIPPDQQR LIFAGKQLED GRTLSDYNIQ KESTLHLVLR LRGGMQIFVK TLTGKTITLE
321 VEPSDTIENV KAKIQDKEGI PPDQQRLIFA GKQLEDGRTL SDYNIQKEST LHLVLRLRGG MQIFVKTLTG KTITLEVEPS
401 DTIENVKAKI QDKEGIPPDQ QRLIFAGKQL EDGRTLSDYN IQKESTLHLV LRLRGGMQIF VKTLTGKTIT LEVEPSDTIE
481 NVKAKIQDKE GIPPDQQRLI FAGKQLEDGR TLSDYNIQKE STLHLVLRLR GGMQIFVKTL TGKTITLEVE PSDTIENVKA
561 KIQDKEGIPP DQQRLIFAGK QLEDGRTLSD YNIQKESTLH LVLRLRGGMQ IFVKTLTGKT ITLEVEPSDT IENVKAKIQD
641 KEGIPPDQQR LIFAGKQLED GRTLSDYNIQ KESTLHLVLR LRGGMQIFVK TLTGKTITLE VEPSDTIENV KAKIQDKEGI
721 PPDQQRLIFA GKQLEDGRTL SDYNIQKEST LHLVLRLRGG F
```

**Supporting Peptides:**

| Peptide | Uniq | -10lgP | Mass | Length | ppm | m/z | z | RT | Fraction | Scan | Source File | Area F1 | Area F2a | Area F3a | Area F4 | Area F5 | Area F6a | Area F7a | Area F7b | Area F8 | Area F9a | Area Sample 1F10a | Area F10b | Area F11a | Area F11b | Area F12a | Area F13 | #Feature | #Feature F1 | #Feature F2a | #Feature F3a | #Feature F4 | #Feature F5 | #Feature F6a | #Feature F7a | #Feature F7b | #Feature F8 | #Feature F9a | #Feature Sample 1F10a | #Feature F10b | #Feature F11a | #Feature F11b | #Feature F12a | #Feature F13 | Start | End | PTM | AScore | Found By |
|---|---|---|---|---|---|---|---|---|---|---|---|---|---|---|---|---|---|---|---|---|---|---|---|---|---|---|---|---|---|---|---|---|---|---|---|---|---|---|---|---|---|---|---|---|---|---|---|---|---|
| K.TITLEVEPSDTIENVK.A | Y | 52.24 | 1786.9200 | 16 | 2.9 | 894.4678 | 2 | 61.96 | 12 | F12:38000 | BuCaTN01_F10b.raw | | | | | | | | | | | | 1.8169E5 | | | | | 1 | 0 | 0 | 0 | 0 | 0 | 0 | 0 | 0 | 0 | 0 | 0 | 1 | 0 | 0 | 0 | 0 | 12 | 27 | | | PEAKS DB |
| R.TLSDYNIQKESTLHLVLR.L | Y | 50.38 | 2129.1479 | 18 | 1.9 | 533.2941 | 4 | 62.60 | 12 | F12:38710 | BuCaTN01_F10b.raw | | | | | | | | | | | | 1.0541E5 | | | | | 1 | 0 | 0 | 0 | 0 | 0 | 0 | 0 | 0 | 0 | 0 | 0 | 1 | 0 | 0 | 0 | 0 | 55 | 72 | | | PEAKS DB |
| total 2 peptides | | | | | | | | | | | | | | | | | | | | | | | | | | | | | | | | | | | | | | | | | | | | | | | | | |

**Best Unique PSM (Scan F12:38000, m/z=894.4678, z=2, RT=61.96, ppm=2.9):**

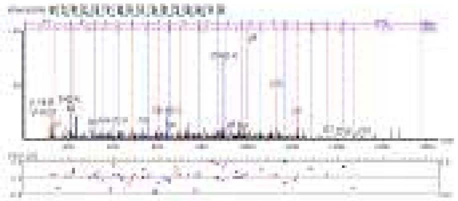

## TIKFBU

back to list

| Protein Coverage | Supporting Peptides | Best Unique PSM |

**Protein Coverage:**

52 54 56

```
  1 RQRHRDCDKP PDKGNCGPVR AFYYDTRLKT CKAFQYRGCD GDHGNFKTET LCRCECLVYP
```

C Carbamidomethylation (+57.02)

**Supporting Peptides:**

| Peptide | Uniq | -10lgP | Mass | Length | ppm | m/z | z | RT | Fraction | Scan | Source File | Area F1 | Area F2a | Area F3a | Area F4 | Area F5 | Area F6a | Area F7a | Area F7b | Area F8 | Area F9a | Area Sample 1F10a | Area F10b | Area F11a | Area F11b | Area F12a | Area F13 | #Feature | #Feature F1 | #Feature F2a | #Feature F3a | #Feature F4 | #Feature F5 | #Feature F6a | #Feature F7a | #Feature F7b | #Feature F8 | #Feature F9a | #Feature Sample 1F10a | #Feature F10b | #Feature F11a | #Feature F11b | #Feature F12a | #Feature F13 | Start | End | PTM | AScore | Found By |
|---|---|---|---|---|---|---|---|---|---|---|---|---|---|---|---|---|---|---|---|---|---|---|---|---|---|---|---|---|---|---|---|---|---|---|---|---|---|---|---|---|---|---|---|---|---|---|---|---|---|
| K.TETLC(+57.02)RC(+57.02)EC(+57.02)LVY.P | N | 52.97 | 1602.6841 | 12 | 3.4 | 802.3497 | 2 | 54.06 | 8 | F8:30032 | BuCaTN01_F7b.raw | | | | | | | | 6.3121E6 | | | | | | | | | 1 | 0 | 0 | 0 | 0 | 0 | 0 | 0 | 1 | 0 | 0 | 0 | 0 | 0 | 0 | 0 | 0 | 48 | 59 | Carbamidomethylation | C5:Carbamidomethylation:1000.00;C7:Carbamidomethylation:1000.00;C9:Carbamidomethylation:1000.00 | PEAKS DB |
| D.GDHGNFKTETLC(+57.02)R.C | Y | 48.70 | 1533.6995 | 13 | 2.1 | 512.2404 | 3 | 32.87 | 12 | F12:11910 | BuCaTN01_F10b.raw | | | | | | 5.284E6 | | 0 | | 6.3042E5 | | 2.1794E6 | | | | | 4 | 0 | 0 | 0 | 0 | 0 | 2 | 0 | 0 | 0 | 1 | 0 | 1 | 0 | 0 | 0 | 0 | 41 | 53 | Carbamidomethylation | C12:Carbamidomethylation:1000.00 | PEAKS DB |
| total 2 peptides | | | | | | | | | | | | | | | | | | | | | | | | | | | | | | | | | | | | | | | | | | | | | | | | | |

**Best Unique PSM (Scan F12:11910, m/z=512.2404, z=3, RT=32.87, ppm=2.1):**

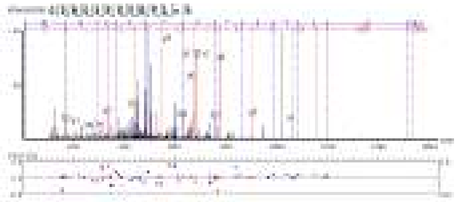

## 0402253B

back to list

| Protein Coverage | Supporting Peptides | Best Unique PSM |

**Protein Coverage:**

52 54 56

```
  1 RQRHRDCDKP PDKGNCGPVR AFYYDTRLKT CKAFQYRGCD GDHGNFKTET LCRCECLVYP
```

C Carbamidomethylation (+57.02)

**Supporting Peptides:**

| Peptide | Uniq | -10lgP | Mass | Length | ppm | m/z | z | RT | Fraction | Scan | Source File | Area F1 | Area F2a | Area F3a | Area F4 | Area F5 | Area F6a | Area F7a | Area F7b | Area F8 | Area F9a | Area Sample 1F10a | Area F10b | Area F11a | Area F11b | Area F12a | Area F13 | #Feature | #Feature F1 | #Feature F2a | #Feature F3a | #Feature F4 | #Feature F5 | #Feature F6a | #Feature F7a | #Feature F7b | #Feature F8 | #Feature F9a | #Feature Sample 1F10a | #Feature F10b | #Feature F11a | #Feature F11b | #Feature F12a | #Feature F13 | Start | End | PTM | AScore | Found By |
|---|---|---|---|---|---|---|---|---|---|---|---|---|---|---|---|---|---|---|---|---|---|---|---|---|---|---|---|---|---|---|---|---|---|---|---|---|---|---|---|---|---|---|---|---|---|---|---|---|---|
| K.TETLC(+57.02)RC(+57.02)EC(+57.02)LVY.P | N | 52.97 | 1602.6841 | 12 | 3.4 | 802.3497 | 2 | 54.06 | 8 | F8:30032 | BuCaTN01_F7b.raw | | | | | | | | 6.3121E6 | | | | | | | | | 1 | 0 | 0 | 0 | 0 | 0 | 0 | 0 | 1 | 0 | 0 | 0 | 0 | 0 | 0 | 0 | 0 | 48 | 59 | Carbamidomethylation | C5:Carbamidomethylation:1000.00;C7:Carbamidomethylation:1000.00;C9:Carbamidomethylation:1000.00 | PEAKS DB |
| D.GDHGNFKTETLC(+57.02)R.C | Y | 48.70 | 1533.6995 | 13 | 2.1 | 512.2404 | 3 | 32.87 | 12 | F12:11910 | BuCaTN01_F10b.raw | | | | | | 5.284E6 | | 0 | | 6.3042E5 | | 2.1794E6 | | | | | 4 | 0 | 0 | 0 | 0 | 0 | 2 | 0 | 0 | 0 | 1 | 0 | 1 | 0 | 0 | 0 | 0 | 41 | 53 | Carbamidomethylation | C12:Carbamidomethylation:1000.00 | PEAKS DB |
| total 2 peptides | | | | | | | | | | | | | | | | | | | | | | | | | | | | | | | | | | | | | | | | | | | | | | | | | |

**Best Unique PSM (Scan F12:11910, m/z=512.2404, z=3, RT=32.87, ppm=2.1):**

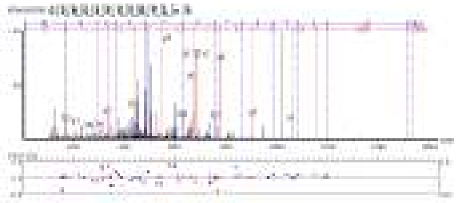

## B4ESA4.1

back to list

| Protein Coverage | Supporting Peptides | Best Unique PSM |

**Protein Coverage:**

64

1 MSSGGLLLLL GLLTLWAELT PVSSRKRHQF CNVPPEPGRC NANVRAFYYN PRLR**KCIEFS YGGCGGNANN FK**SRGECKRT  C Carbamidomethylation (+57.02)

81 CAE

**Supporting Peptides:**

| Peptide | Uniq | -10lgP | Mass | Length | ppm | m/z | z | RT | Fraction | Scan | Source File | Area F1 | Area F2a | Area F3a | Area F4 | Area F5 | Area F6a | Area F7a | Area F7b | Area F8 | Area F9a | Area Sample 1F10a | Area F10b | Area F11a | Area F11b | Area F12a | Area F13 | #Feature | #Feature F1 | #Feature F2a | #Feature F3a | #Feature F4 | #Feature F5 | #Feature F6a | #Feature F7a | #Feature F7b | #Feature F8 | #Feature F9a | #Feature Sample 1F10a | #Feature F10b | #Feature F11a | #Feature F11b | #Feature F12a | #Feature F13 | Start | End | PTM | AScore | Found By |
|---|---|---|---|---|---|---|---|---|---|---|---|---|---|---|---|---|---|---|---|---|---|---|---|---|---|---|---|---|---|---|---|---|---|---|---|---|---|---|---|---|---|---|---|---|---|---|---|---|---|
| R.KC(+57.02)IEFSYGGC(+57.02)GGNANNFK.S | Y | 58.51 | 2021.8723 | 18 | 1.5 | 674.9644 | 3 | 52.31 | 3 | F3:28185 | BuCaTN01_F3a.raw | | | 4.0964E5 | | | | | | | | | | | | | | 1 | 0 | 0 | 1 | 0 | 0 | 0 | 0 | 0 | 0 | 0 | 0 | 0 | 0 | 0 | 0 | 0 | 55 | 72 | Carbamidomethylation | C2:Carbamidomethylation:1000.00;C10:Carbamidomethylation:1000.00 | PEAKS DB |
| total 1 peptides | | | | | | | | | | | | | | | | | | | | | | | | | | | | | | | | | | | | | | | | | | | | | | | | | |

**Best Unique PSM (Scan F3:28185, m/z=674.9644, z=3, RT=52.31, ppm=1.5):**

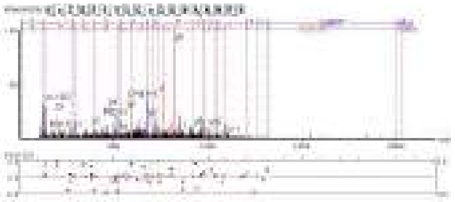

## CAP74383.1

back to list

| Protein Coverage | Supporting Peptides | Best Unique PSM |

**Protein Coverage:**

64

1 MSSGGLLLLL GLLTLWAELT PVSSRKRHQF CNVPPEPGRC NANVRAFYYN PRLR**KCIEFS YGGCGGNANN FK**SRGECKRT  C Carbamidomethylation (+57.02)

81 CAE

**Supporting Peptides:**

| Peptide | Uniq | -10lgP | Mass | Length | ppm | m/z | z | RT | Fraction | Scan | Source File | Area F1 | Area F2a | Area F3a | Area F4 | Area F5 | Area F6a | Area F7a | Area F7b | Area F8 | Area F9a | Area Sample 1F10a | Area F10b | Area F11a | Area F11b | Area F12a | Area F13 | #Feature | #Feature F1 | #Feature F2a | #Feature F3a | #Feature F4 | #Feature F5 | #Feature F6a | #Feature F7a | #Feature F7b | #Feature F8 | #Feature F9a | #Feature Sample 1F10a | #Feature F10b | #Feature F11a | #Feature F11b | #Feature F12a | #Feature F13 | Start | End | PTM | AScore | Found By |
|---|---|---|---|---|---|---|---|---|---|---|---|---|---|---|---|---|---|---|---|---|---|---|---|---|---|---|---|---|---|---|---|---|---|---|---|---|---|---|---|---|---|---|---|---|---|---|---|---|---|
| R.KC(+57.02)IEFSYGGC(+57.02)GGNANNFK.S | Y | 58.51 | 2021.8723 | 18 | 1.5 | 674.9644 | 3 | 52.31 | 3 | F3:28185 | BuCaTN01_F3a.raw | | | 4.0964E5 | | | | | | | | | | | | | | 1 | 0 | 0 | 1 | 0 | 0 | 0 | 0 | 0 | 0 | 0 | 0 | 0 | 0 | 0 | 0 | 0 | 55 | 72 | Carbamidomethylation | C2:Carbamidomethylation:1000.00;C10:Carbamidomethylation:1000.00 | PEAKS DB |
| total 1 peptides | | | | | | | | | | | | | | | | | | | | | | | | | | | | | | | | | | | | | | | | | | | | | | | | | |

**Best Unique PSM (Scan F3:28185, m/z=674.9644, z=3, RT=52.31, ppm=1.5):**

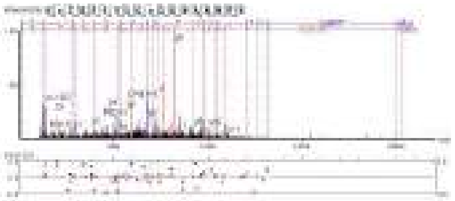

## Q75S49.1

back to list

| Protein Coverage | Supporting Peptides | Best Unique PSM |

**Protein Coverage:**

76 78 80

1 SSGGLLLLLG LLTLCAELIP VSSRQRHRDC DKPPDKGNCG SVRRAFYYDT RLKTCKAFPY RGCN**GNGNHF KTETLCRCEC**  C Carbamidomethylation (+57.02)

81 **LVY**P

**Supporting Peptides:**

| Peptide | Uniq | -10lgP | Mass | Length | ppm | m/z | z | RT | Fraction | Scan | Source File | Area F1 | Area F2a | Area F3a | Area F4 | Area F5 | Area F6a | Area F7a | Area F7b | Area F8 | Area F9a | Area Sample 1F10a | Area F10b | Area F11a | Area F11b | Area F12a | Area F13 | #Feature | #Feature F1 | #Feature F2a | #Feature F3a | #Feature F4 | #Feature F5 | #Feature F6a | #Feature F7a | #Feature F7b | #Feature F8 | #Feature F9a | #Feature Sample 1F10a | #Feature F10b | #Feature F11a | #Feature F11b | #Feature F12a | #Feature F13 | Start | End | PTM | AScore | Found By |
|---|---|---|---|---|---|---|---|---|---|---|---|---|---|---|---|---|---|---|---|---|---|---|---|---|---|---|---|---|---|---|---|---|---|---|---|---|---|---|---|---|---|---|---|---|---|---|---|---|---|
| K.TETLC(+57.02)RC(+57.02)EC(+57.02)LVY.P | N | 52.97 | 1602.6841 | 12 | 3.4 | 802.3497 | 2 | 54.06 | 8 | F8:30032 | BuCaTN01_F7b.raw | | | | | | | | 6.3121E6 | | | | | | | | | 1 | 0 | 0 | 0 | 0 | 0 | 0 | 0 | 1 | 0 | 0 | 0 | 0 | 0 | 0 | 0 | 0 | 72 | 83 | Carbamidomethylation | C5:Carbamidomethylation:1000.00;C7:Carbamidomethylation:1000.00;C9:Carbamidomethylation:1000.00 | PEAKS DB |
| N.GNGNHFKTETLC(+57.02)R.C | Y | 47.23 | 1532.7155 | 13 | 2.9 | 511.9124 | 3 | 29.79 | 8 | F8:9649 | BuCaTN01_F7b.raw | | | | | | | | 3.5322E6 | | | | | | | | | 1 | 0 | 0 | 0 | 0 | 0 | 0 | 0 | 1 | 0 | 0 | 0 | 0 | 0 | 0 | 0 | 0 | 65 | 77 | Carbamidomethylation | C12:Carbamidomethylation:1000.00 | PEAKS DB |
| total 2 peptides | | | | | | | | | | | | | | | | | | | | | | | | | | | | | | | | | | | | | | | | | | | | | | | | | |

**Best Unique PSM (Scan F8:9649, m/z=511.9124, z=3, RT=29.79, ppm=2.9):**

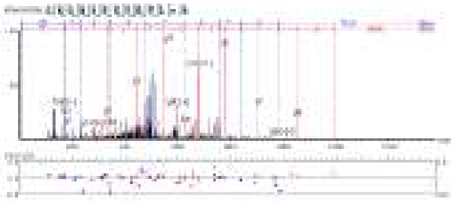

## BAD06269.1

back to list

| Protein Coverage | Supporting Peptides | Best Unique PSM |

**Protein Coverage:**

76 78 80

1 SSGGLLLLLG LLTLCAELIP VSSRQRHRDC DKPPDKGNCG SVRRAFYYDT RLKTCKAFPY RGCN**GNGNHF KTETLCRCEC**  C Carbamidomethylation (+57.02)

81 **LVY**P

**Supporting Peptides:**

| Peptide | Uniq | -10lgP | Mass | Length | ppm | m/z | z | RT | Fraction | Scan | Source File | Area F1 | Area F2a | Area F3a | Area F4 | Area F5 | Area F6a | Area F7a | Area F7b | Area F8 | Area F9a | Area Sample 1F10a | Area F10b | Area F11a | Area F11b | Area F12a | Area F13 | #Feature | #Feature F1 | #Feature F2a | #Feature F3a | #Feature F4 | #Feature F5 | #Feature F6a | #Feature F7a | #Feature F7b | #Feature F8 | #Feature F9a | #Feature Sample 1F10a | #Feature F10b | #Feature F11a | #Feature F11b | #Feature F12a | #Feature F13 | Start | End | PTM | AScore | Found By |
|---|---|---|---|---|---|---|---|---|---|---|---|---|---|---|---|---|---|---|---|---|---|---|---|---|---|---|---|---|---|---|---|---|---|---|---|---|---|---|---|---|---|---|---|---|---|---|---|---|---|
| K.TETLC(+57.02)RC(+57.02)EC(+57.02)LVY.P | N | 52.97 | 1602.6841 | 12 | 3.4 | 802.3497 | 2 | 54.06 | 8 | F8:30032 | BuCaTN01_F7b.raw | | | | | | | | 6.3121E6 | | | | | | | | | 1 | 0 | 0 | 0 | 0 | 0 | 0 | 0 | 1 | 0 | 0 | 0 | 0 | 0 | 0 | 0 | 0 | 72 | 83 | Carbamidomethylation | C5:Carbamidomethylation:1000.00;C7:Carbamidomethylation:1000.00;C9:Carbamidomethylation:1000.00 | PEAKS DB |
| N.GNGNHFKTETLC(+57.02)R.C | Y | 47.23 | 1532.7155 | 13 | 2.9 | 511.9124 | 3 | 29.79 | 8 | F8:9649 | BuCaTN01_F7b.raw | | | | | | | | 3.5322E6 | | | | | | | | | 1 | 0 | 0 | 0 | 0 | 0 | 0 | 0 | 1 | 0 | 0 | 0 | 0 | 0 | 0 | 0 | 0 | 65 | 77 | Carbamidomethylation | C12:Carbamidomethylation:1000.00 | PEAKS DB |
| total 2 peptides | | | | | | | | | | | | | | | | | | | | | | | | | | | | | | | | | | | | | | | | | | | | | | | | | |

**Best Unique PSM (Scan F8:9649, m/z=511.9124, z=3, RT=29.79, ppm=2.9):**

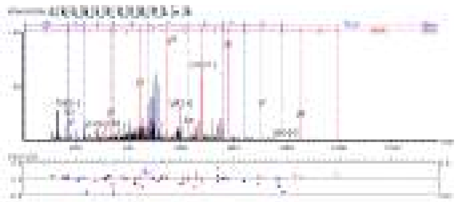

## Q8AY46.2

back to list

| Protein Coverage | Supporting Peptides | Best Unique PSM |

**Protein Coverage:**

1 MSSGGLLLLL GLLTLCAELT PVSSRQRHRD CDKPPDKGNC GSVRRAFYYD TRLKTCKAFP YRGCNGNGNH FKTETLCRCE

81 CLVYP

C Carbamidomethylation (+57.02)

**Supporting Peptides:**

| Peptide | Uniq | -10lgP | Mass | Length | ppm | m/z | z | RT | Fraction | Scan | Source File | Area F1 | Area F2a | Area F3a | Area F4 | Area F5 | Area F6a | Area F7a | Area F7b | Area F8 | Area F9a | Area Sample 1F10a | Area F10b | Area F11a | Area F11b | Area F12a | Area F13 | #Feature | #Feature F1 | #Feature F2a | #Feature F3a | #Feature F4 | #Feature F5 | #Feature F6a | #Feature F7a | #Feature F7b | #Feature F8 | #Feature F9a | #Feature Sample 1F10a | #Feature F10b | #Feature F11a | #Feature F11b | #Feature F12a | #Feature F13 | Start | End | PTM | AScore | Found By |
|---|---|---|---|---|---|---|---|---|---|---|---|---|---|---|---|---|---|---|---|---|---|---|---|---|---|---|---|---|---|---|---|---|---|---|---|---|---|---|---|---|---|---|---|---|---|---|---|---|---|
| K.TETLC(+57.02)RC(+57.02)EC(+57.02)LVY.P | N | 52.97 | 1602.6841 | 12 | 3.4 | 802.3497 | 2 | 54.06 | 8 | F8:30032 | BuCaTN01_F7b.raw | | | | | | | | 6.3121E6 | | | | | | | | | 1 | 0 | 0 | 0 | 0 | 0 | 0 | 0 | 1 | 0 | 0 | 0 | 0 | 0 | 0 | 0 | 0 | 73 | 84 | Carbamidomethylation | C5:Carbamidomethylation:1000.00;C7:Carbamidomethylation:1000.00;C9:Carbamidomethylation:1000.00 | PEAKS DB |
| N.GNGNHFKTETLC(+57.02)R.C | Y | 47.23 | 1532.7155 | 13 | 2.9 | 511.9124 | 3 | 29.79 | 8 | F8:9649 | BuCaTN01_F7b.raw | | | | | | | | 3.5322E6 | | | | | | | | | 1 | 0 | 0 | 0 | 0 | 0 | 0 | 0 | 1 | 0 | 0 | 0 | 0 | 0 | 0 | 0 | 0 | 66 | 78 | Carbamidomethylation | C12:Carbamidomethylation:1000.00 | PEAKS DB |
| total 2 peptides | | | | | | | | | | | | | | | | | | | | | | | | | | | | | | | | | | | | | | | | | | | | | | | | | |

**Best Unique PSM (Scan F8:9649, m/z=511.9124, z=3, RT=29.79, ppm=2.9):**

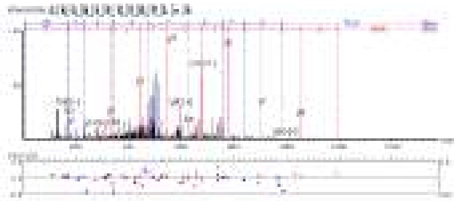

## AAL30065.1

back to list

| Protein Coverage | Supporting Peptides | Best Unique PSM |

**Protein Coverage:**

1 PDGFIMSSGG LLLLLGLLTL CAELTPVSSR QRHRDCDKPP DKGNCGSVRR AFYYDTRLKT CKAFPYRGCN GNGNHFKTET

81 LCRCECLVYP

C Carbamidomethylation (+57.02)

**Supporting Peptides:**

| Peptide | Uniq | -10lgP | Mass | Length | ppm | m/z | z | RT | Fraction | Scan | Source File | Area F1 | Area F2a | Area F3a | Area F4 | Area F5 | Area F6a | Area F7a | Area F7b | Area F8 | Area F9a | Area Sample 1F10a | Area F10b | Area F11a | Area F11b | Area F12a | Area F13 | #Feature | #Feature F1 | #Feature F2a | #Feature F3a | #Feature F4 | #Feature F5 | #Feature F6a | #Feature F7a | #Feature F7b | #Feature F8 | #Feature F9a | #Feature Sample 1F10a | #Feature F10b | #Feature F11a | #Feature F11b | #Feature F12a | #Feature F13 | Start | End | PTM | AScore | Found By |
|---|---|---|---|---|---|---|---|---|---|---|---|---|---|---|---|---|---|---|---|---|---|---|---|---|---|---|---|---|---|---|---|---|---|---|---|---|---|---|---|---|---|---|---|---|---|---|---|---|---|
| K.TETLC(+57.02)RC(+57.02)EC(+57.02)LVY.P | N | 52.97 | 1602.6841 | 12 | 3.4 | 802.3497 | 2 | 54.06 | 8 | F8:30032 | BuCaTN01_F7b.raw | | | | | | | | 6.3121E6 | | | | | | | | | 1 | 0 | 0 | 0 | 0 | 0 | 0 | 0 | 1 | 0 | 0 | 0 | 0 | 0 | 0 | 0 | 0 | 78 | 89 | Carbamidomethylation | C5:Carbamidomethylation:1000.00;C7:Carbamidomethylation:1000.00;C9:Carbamidomethylation:1000.00 | PEAKS DB |
| N.GNGNHFKTETLC(+57.02)R.C | Y | 47.23 | 1532.7155 | 13 | 2.9 | 511.9124 | 3 | 29.79 | 8 | F8:9649 | BuCaTN01_F7b.raw | | | | | | | | 3.5322E6 | | | | | | | | | 1 | 0 | 0 | 0 | 0 | 0 | 0 | 0 | 1 | 0 | 0 | 0 | 0 | 0 | 0 | 0 | 0 | 71 | 83 | Carbamidomethylation | C12:Carbamidomethylation:1000.00 | PEAKS DB |
| total 2 peptides | | | | | | | | | | | | | | | | | | | | | | | | | | | | | | | | | | | | | | | | | | | | | | | | | |

**Best Unique PSM (Scan F8:9649, m/z=511.9124, z=3, RT=29.79, ppm=2.9):**

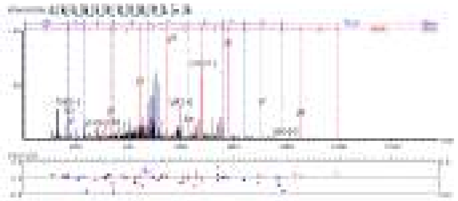

## CAB62503.1

back to list

| Protein Coverage | Supporting Peptides | Best Unique PSM |

**Protein Coverage:**

1 MSSGGLLLLL GLLTLSAELI PVSSRQRHRD CDKPPDKGNC GPVRRAFYYD TRLKTCKAFQ YRGCNGNGNH FKTETLCRCE

81 CLVYP

C Carbamidomethylation (+57.02)

**Supporting Peptides:**

| Peptide | Uniq | -10lgP | Mass | Length | ppm | m/z | z | RT | Fraction | Scan | Source File | Area F1 | Area F2a | Area F3a | Area F4 | Area F5 | Area F6a | Area F7a | Area F7b | Area F8 | Area F9a | Area Sample 1F10a | Area F10b | Area F11a | Area F11b | Area F12a | Area F13 | #Feature | #Feature F1 | #Feature F2a | #Feature F3a | #Feature F4 | #Feature F5 | #Feature F6a | #Feature F7a | #Feature F7b | #Feature F8 | #Feature F9a | #Feature Sample 1F10a | #Feature F10b | #Feature F11a | #Feature F11b | #Feature F12a | #Feature F13 | Start | End | PTM | AScore | Found By |
|---|---|---|---|---|---|---|---|---|---|---|---|---|---|---|---|---|---|---|---|---|---|---|---|---|---|---|---|---|---|---|---|---|---|---|---|---|---|---|---|---|---|---|---|---|---|---|---|---|---|
| K.TETLC(+57.02)RC(+57.02)EC(+57.02)LVY.P | N | 52.97 | 1602.6841 | 12 | 3.4 | 802.3497 | 2 | 54.06 | 8 | F8:30032 | BuCaTN01_F7b.raw | | | | | | | | 6.3121E6 | | | | | | | | | 1 | 0 | 0 | 0 | 0 | 0 | 0 | 0 | 1 | 0 | 0 | 0 | 0 | 0 | 0 | 0 | 0 | 73 | 84 | Carbamidomethylation | C5:Carbamidomethylation:1000.00;C7:Carbamidomethylation:1000.00;C9:Carbamidomethylation:1000.00 | PEAKS DB |
| N.GNGNHFKTETLC(+57.02)R.C | Y | 47.23 | 1532.7155 | 13 | 2.9 | 511.9124 | 3 | 29.79 | 8 | F8:9649 | BuCaTN01_F7b.raw | | | | | | | | 3.5322E6 | | | | | | | | | 1 | 0 | 0 | 0 | 0 | 0 | 0 | 0 | 1 | 0 | 0 | 0 | 0 | 0 | 0 | 0 | 0 | 66 | 78 | Carbamidomethylation | C12:Carbamidomethylation:1000.00 | PEAKS DB |
| total 2 peptides | | | | | | | | | | | | | | | | | | | | | | | | | | | | | | | | | | | | | | | | | | | | | | | | | |

**Best Unique PSM (Scan F8:9649, m/z=511.9124, z=3, RT=29.79, ppm=2.9):**

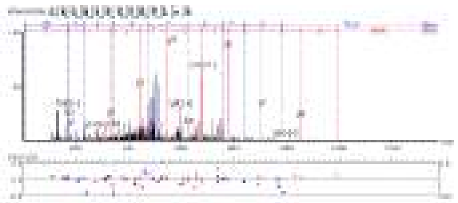

proteins

## CAA72809.1

back to list

| Protein Coverage | Supporting Peptides | Best Unique PSM |

**Protein Coverage:**

```
 1 MSSGGLLLLL GLLTLCAELI PVSSRQRHRD CDKPPDKGNC GPVRRAFYYD TRLKTCKAFQ YRGCNGNGNH FKTETLCRCE
81 CLVYP
```

C Carbamidomethylation (+57.02)

**Supporting Peptides:**

| Peptide | Uniq | -10lgP | Mass | Length | ppm | m/z | z | RT | Fraction | Scan | Source File | Area F1 | Area F2a | Area F3a | Area F4 | Area F5 | Area F6a | Area F7a | Area F7b | Area F8 | Area F9a | Area Sample 1F10a | Area F10b | Area F11a | Area F11b | Area F12a | Area F13 | #Feature | #Feature F1 | #Feature F2a | #Feature F3a | #Feature F4 | #Feature F5 | #Feature F6a | #Feature F7a | #Feature F7b | #Feature F8 | #Feature F9a | #Feature Sample 1F10a | #Feature F10b | #Feature F11a | #Feature F11b | #Feature F12a | #Feature F13 | Start | End | PTM | AScore | Found By |
|---|---|---|---|---|---|---|---|---|---|---|---|---|---|---|---|---|---|---|---|---|---|---|---|---|---|---|---|---|---|---|---|---|---|---|---|---|---|---|---|---|---|---|---|---|---|---|---|---|---|
| K.TETLC(+57.02)RC(+57.02)EC(+57.02)LVY.P | N | 52.97 | 1602.6841 | 12 | 3.4 | 802.3497 | 2 | 54.06 | 8 | F8:30032 | BuCaTN01_F7b.raw | | | | | | | | 6.3121E6 | | | | | | | | | 1 | 0 | 0 | 0 | 0 | 0 | 0 | 0 | 1 | 0 | 0 | 0 | 0 | 0 | 0 | 0 | 0 | 73 | 84 | Carbamidomethylation | C5:Carbamidomethylation:1000.00;C7:Carbamidomethylation:1000.00;C9:Carbamidomethylation:1000.00 | PEAKS DB |
| N.GNGNHFKTETLC(+57.02)R.C | Y | 47.23 | 1532.7155 | 13 | 2.9 | 511.9124 | 3 | 29.79 | 8 | F8:9649 | BuCaTN01_F7b.raw | | | | | | | | 3.5322E6 | | | | | | | | | 1 | 0 | 0 | 0 | 0 | 0 | 0 | 0 | 1 | 0 | 0 | 0 | 0 | 0 | 0 | 0 | 0 | 66 | 78 | Carbamidomethylation | C12:Carbamidomethylation:1000.00 | PEAKS DB |
| total 2 peptides | | | | | | | | | | | | | | | | | | | | | | | | | | | | | | | | | | | | | | | | | | | | | | | | | |

**Best Unique PSM (Scan F8:9649, m/z=511.9124, z=3, RT=29.79, ppm=2.9):**

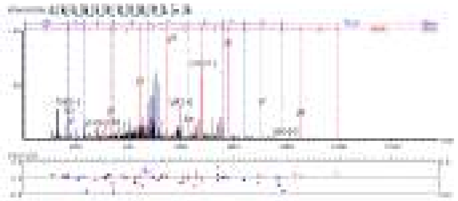

## P00987.2

back to list

| Protein Coverage | Supporting Peptides | Best Unique PSM |

**Protein Coverage:**

```
 1 MSSGGLLLLL GLLTLCAELI PVSSRQRHRD CDKPPDKGNC GPVRRAFYYD TRLKTCKAFQ YRGCNGNGNH FKTETLCRCE
81 CLVYP
```

C Carbamidomethylation (+57.02)

**Supporting Peptides:**

| Peptide | Uniq | -10lgP | Mass | Length | ppm | m/z | z | RT | Fraction | Scan | Source File | Area F1 | Area F2a | Area F3a | Area F4 | Area F5 | Area F6a | Area F7a | Area F7b | Area F8 | Area F9a | Area Sample 1F10a | Area F10b | Area F11a | Area F11b | Area F12a | Area F13 | #Feature | #Feature F1 | #Feature F2a | #Feature F3a | #Feature F4 | #Feature F5 | #Feature F6a | #Feature F7a | #Feature F7b | #Feature F8 | #Feature F9a | #Feature Sample 1F10a | #Feature F10b | #Feature F11a | #Feature F11b | #Feature F12a | #Feature F13 | Start | End | PTM | AScore | Found By |
|---|---|---|---|---|---|---|---|---|---|---|---|---|---|---|---|---|---|---|---|---|---|---|---|---|---|---|---|---|---|---|---|---|---|---|---|---|---|---|---|---|---|---|---|---|---|---|---|---|---|
| K.TETLC(+57.02)RC(+57.02)EC(+57.02)LVY.P | N | 52.97 | 1602.6841 | 12 | 3.4 | 802.3497 | 2 | 54.06 | 8 | F8:30032 | BuCaTN01_F7b.raw | | | | | | | | 6.3121E6 | | | | | | | | | 1 | 0 | 0 | 0 | 0 | 0 | 0 | 0 | 1 | 0 | 0 | 0 | 0 | 0 | 0 | 0 | 0 | 73 | 84 | Carbamidomethylation | C5:Carbamidomethylation:1000.00;C7:Carbamidomethylation:1000.00;C9:Carbamidomethylation:1000.00 | PEAKS DB |
| N.GNGNHFKTETLC(+57.02)R.C | Y | 47.23 | 1532.7155 | 13 | 2.9 | 511.9124 | 3 | 29.79 | 8 | F8:9649 | BuCaTN01_F7b.raw | | | | | | | | 3.5322E6 | | | | | | | | | 1 | 0 | 0 | 0 | 0 | 0 | 0 | 0 | 1 | 0 | 0 | 0 | 0 | 0 | 0 | 0 | 0 | 66 | 78 | Carbamidomethylation | C12:Carbamidomethylation:1000.00 | PEAKS DB |
| total 2 peptides | | | | | | | | | | | | | | | | | | | | | | | | | | | | | | | | | | | | | | | | | | | | | | | | | |

**Best Unique PSM (Scan F8:9649, m/z=511.9124, z=3, RT=29.79, ppm=2.9):**

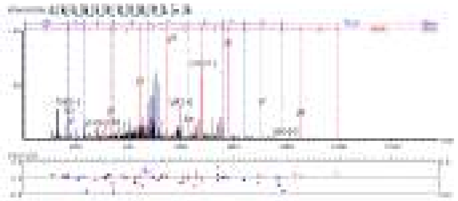

## W8E7D1.1

back to list

| Protein Coverage | Supporting Peptides | Best Unique PSM |

**Protein Coverage:**

```
  1 MIQQKVLFIS LVAVALGLGL GLGLKESVEP QVSCRYRCNE TFSKMASGCS CDDKCTERQA CCQDYEDTCV LPTQSWSCSK
 81 LRCSEKRMAN VLCSCSEDCL EKKDCCTDYK SICKGETSWL KDQCASSSAA QCPSGFEQSP LILFSMDGFR AGYLETWDSL
161 MPNINKLKTC GTHAKYMRAV YPTKTFVNHY TIVTGLYPES HGIIDNNIYD VTLNLNFSLS APTMTNPAWW GGQPIWHTVT
241 YQGLKAATYF WPGSEVKING SYPTIYKVYN KSIPFEARVT EVLKWLDLPK AERPDFVTLY IEEPDTTGHK FGPVSGEIIM
321 ALQMADRTLG MLMEGLKQRN LHNCVNLILL ADHGMEQISC NRLEYMTDYF DKVDFFMYEG PAPRIRSKNV PKDFYTFDSE
401 GIVRNLTCQK PKQYFKAYLA KDLPKRLHYV NNIRIDKVNL MVDQQWMAVR NKNYNRCNGG THGYDNEFKS MQAIFLAHGP
481 GFKGKNEVTS FENIEVYNLM CDLLKLKPAP NNGTHGSLNH LLKNPFYNPS PAKEQTSPLS CPFGPVPSPD VSGCKCSSIT
561 DLGKVNERLN LNNQAKTESE AHNLPYGRPQ VLQNHSKYCL LHQAKYISAY SQDVLMPLWS SYTINKSPPT SVPPSASDCL
641 RLDVRIPAAQ SQTCSNYQPD LTITPGFLYP PNFGSSNFEQ YDALITSNLV PMFKGFTRLW NYFHGTLLPK YARERNGLNV
721 ISGPIFDYNY DGHFDSYDTI KEYVNDTKIP IPTHFFVVLT SCENQINTPL NCPGSLKVLS FILPHRPDNS ESCADTSPDN
801 LWVEERIQTH TARVRDVELL TGLNFYSGLK QPLPETLQLK TFLPIFVNPV N
```

**Supporting Peptides:**

| Peptide | Uniq | -10lgP | Mass | Length | ppm | m/z | z | RT | Fraction | Scan | Source File | Area F1 | Area F2a | Area F3a | Area F4 | Area F5 | Area F6a | Area F7a | Area F7b | Area F8 | Area F9a | Area Sample 1F10a | Area F10b | Area F11a | Area F11b | Area F12a | Area F13 | #Feature | #Feature F1 | #Feature F2a | #Feature F3a | #Feature F4 | #Feature F5 | #Feature F6a | #Feature F7a | #Feature F7b | #Feature F8 | #Feature F9a | #Feature Sample 1F10a | #Feature F10b | #Feature F11a | #Feature F11b | #Feature F12a | #Feature F13 | Start | End | PTM | AScore | Found By |
|---|---|---|---|---|---|---|---|---|---|---|---|---|---|---|---|---|---|---|---|---|---|---|---|---|---|---|---|---|---|---|---|---|---|---|---|---|---|---|---|---|---|---|---|---|---|---|---|---|---|
| K.RLHYVNNIR.I | Y | 56.01 | 1183.6573 | 9 | -0.5 | 395.5595 | 3 | 32.30 | 16 | F16:10971 | BuCaTN01_F13.raw | | | | | | | | | | | | | | | | 3.0155E5 | 1 | 0 | 0 | 0 | 0 | 0 | 0 | 0 | 0 | 0 | 0 | 0 | 0 | 0 | 0 | 0 | 1 | 426 | 434 | | | PEAKS DB |
| total 1 peptides | | | | | | | | | | | | | | | | | | | | | | | | | | | | | | | | | | | | | | | | | | | | | | | | | |

**Best Unique PSM (Scan F16:10971, m/z=395.5595, z=3, RT=32.30, ppm=-0.5):**

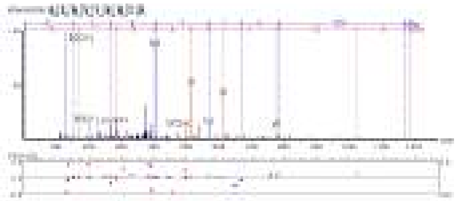

## AHJ80885.1

back to list

| Protein Coverage | Supporting Peptides | Best Unique PSM |

**Protein Coverage:**

```
  1 MIQQKVLFIS LVAVALGLGL GLGLKESVEP QVSCRYRCNE TFSKMASGCS CDDKCTERQA CCQDYEDTCV LPTQSWSCSK
 81 LRCSEKRMAN VLCSCSEDCL EKKDCCTDYK SICKGETSWL KDQCASSSAA QCPSGFEQSP LILFSMDGFR AGYLETWDSL
161 MPNINKLKTC GTHAKYMRAV YPTKTFVNHY TIVTGLYPES HGIIDNNIYD VTLNLNFSLS APTMTNPAWW GGQPIWHTVT
241 YQGLKAATYF WPGSEVKING SYPTIYKVYN KSIPFEARVT EVLKWLDLPK AERPDFVTLY IEEPDTTGHK FGPVSGEIIM
321 ALQMADRTLG MLMEGLKQRN LHNCVNLILL ADHGMEQISC NRLEYMTDYF DKVDFFMYEG PAPRIRSKNV PKDFYTFDSE
401 GIVRNLTCQK PKQYFKAYLA KDLPKRLHYV NNIRIDKVNL MVDQQWMAVR NKNYNRCNGG THGYDNEFKS MQAIFLAHGP
481 GFKGKNEVTS FENIEVYNLM CDLLKLKPAP NNGTHGSLNH LLKNPFYNPS PAKEQTSPLS CPFGPVPSPD VSGCKCSSIT
561 DLGKVNERLN LNNQAKTESE AHNLPYGRPQ VLQNHSKYCL LHQAKYISAY SQDVLMPLWS SYTINKSPPT SVPPSASDCL
641 RLDVRIPAAQ SQTCSNYQPD LTITPGFLYP PNFGSSNFEQ YDALITSNLV PMFKGFTRLW NYFHGTLLPK YARERNGLNV
721 ISGPIFDYNY DGHFDSYDTI KEYVNDTKIP IPTHFFVVLT SCENQINTPL NCPGSLKVLS FILPHRPDNS ESCADTSPDN
801 LWVEERIQTH TARVRDVELL TGLNFYSGLK QPLPETLQLK TFLPIFVNPV N
```

**Supporting Peptides:**

| Peptide | Uniq | -10lgP | Mass | Length | ppm | m/z | z | RT | Fraction | Scan | Source File | Area F1 | Area F2a | Area F3a | Area F4 | Area F5 | Area F6a | Area F7a | Area F7b | Area F8 | Area F9a | Area Sample 1F10a | Area F10b | Area F11a | Area F11b | Area F12a | Area F13 | #Feature | #Feature F1 | #Feature F2a | #Feature F3a | #Feature F4 | #Feature F5 | #Feature F6a | #Feature F7a | #Feature F7b | #Feature F8 | #Feature F9a | #Feature Sample 1F10a | #Feature F10b | #Feature F11a | #Feature F11b | #Feature F12a | #Feature F13 | Start | End | PTM | AScore | Found By |
|---|---|---|---|---|---|---|---|---|---|---|---|---|---|---|---|---|---|---|---|---|---|---|---|---|---|---|---|---|---|---|---|---|---|---|---|---|---|---|---|---|---|---|---|---|---|---|---|---|---|
| K.RLHYVNNIR.I | Y | 56.01 | 1183.6573 | 9 | -0.5 | 395.5595 | 3 | 32.30 | 16 | F16:10971 | BuCaTN01_F13.raw | | | | | | | | | | | | | | | | 3.0155E5 | 1 | 0 | 0 | 0 | 0 | 0 | 0 | 0 | 0 | 0 | 0 | 0 | 0 | 0 | 0 | 0 | 1 | 426 | 434 | | | PEAKS DB |
| total 1 peptides | | | | | | | | | | | | | | | | | | | | | | | | | | | | | | | | | | | | | | | | | | | | | | | | | |

**Best Unique PSM (Scan F16:10971, m/z=395.5595, z=3, RT=32.30, ppm=-0.5):**

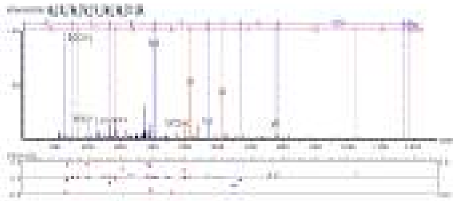

## JAS05169.1

back to list

| Protein Coverage | Supporting Peptides | Best Unique PSM |

**Protein Coverage:**

```
  1 MCHLWIKCLA TWILLKRFNG VHVMQTRAPM YPNEPFLVFW NAPTTQCQLR YKVALDLKTF HIVTNANESL SGSAVTIFYP
 81 TQLGIYPHID DHGNFVNGII PQNESITKHL NKTKSNINHM IPLKTFHGLG VIDWENWRPQ WDRNWGSKNV YRNRSIQFAK
161 ELHPDLSEHA IKRLAKEEFE KAGKSFMRDT LLLAENMRPD GYWGYYLYPD CYNYNYKKKP EQYTGKCPDI EMSRNDQLIW
241 LWRDSTALFP SIYLETILKS SANALKFVHH RLKESMRIAS MARKDYALPV FVYARPFYAY TFEPLTEEDW VTTIGETAAM
321 GAAGIVFWGS MQYASTIESC QKVKDYINGP FGHYIINVTS AAKICSHFLC KGKGRCVRKH SDSNAFLHLF PESFRIMVQA
401 NATHKKAIVK GKLELENLKY LRKNFICQCY QGWKGLYCEE HHNKEGN
```

**Supporting Peptides:**

| Peptide | Uniq | -10lgP | Mass | Length | ppm | m/z | z | RT | Fraction | Scan | Source File | Area F1 | Area F2a | Area F3a | Area F4 | Area F5 | Area F6a | Area F7a | Area F7b | Area F8 | Area F9a | Area Sample 1F10a | Area F10b | Area F11a | Area F11b | Area F12a | Area F13 | #Feature | #Feature F1 | #Feature F2a | #Feature F3a | #Feature F4 | #Feature F5 | #Feature F6a | #Feature F7a | #Feature F7b | #Feature F8 | #Feature F9a | #Feature Sample 1F10a | #Feature F10b | #Feature F11a | #Feature F11b | #Feature F12a | #Feature F13 | Start | End | PTM | AScore | Found By |
|---|---|---|---|---|---|---|---|---|---|---|---|---|---|---|---|---|---|---|---|---|---|---|---|---|---|---|---|---|---|---|---|---|---|---|---|---|---|---|---|---|---|---|---|---|---|---|---|---|---|
| K.LELENLKYLR.K | Y | 42.76 | 1289.7343 | 10 | -0.1 | 430.9187 | 3 | 61.68 | 16 | F16:30620 | BuCaTN01_F13.raw | | | | | | | | | | | | | | | | 3.8116E6 | 1 | 0 | 0 | 0 | 0 | 0 | 0 | 0 | 0 | 0 | 0 | 0 | 0 | 0 | 0 | 0 | 1 | 413 | 422 | | | PEAKS DB |
| total 1 peptides | | | | | | | | | | | | | | | | | | | | | | | | | | | | | | | | | | | | | | | | | | | | | | | | | |

**Best Unique PSM (Scan F16:30620, m/z=430.9187, z=3, RT=61.68, ppm=-0.1):**

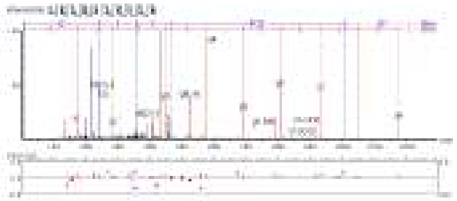

## JAB52840.1

back to list

| Protein Coverage | Supporting Peptides | Best Unique PSM |

**Protein Coverage:**

```
  1 MCHLWIKCLA TWILLKRFNG VHVMQTRTPM YPNEPFLVFW NAPTTQCQLR YKVALDLKTF HIVTNANESL SGSAVTIFYP
 81 TQLGIYPHID DHGNFVNGII PQNESITKHL NKTKSNINHM IPLKTFHGLG VIDWENWRPQ WDRNWGSKNV YRNRSIQFAK
161 ELHPDLSEHA IKRLAKEEFE KAGKSFMRDT LLLAENMRPD GYWGYYLYPD CYNYNYKKKP EQYTGKCPDI EMSRNDQLIW
241 LWRDSTALFP SIYLETILKS SANALKFVHH RLKESMRISS MARKDYALPV FVYARPFYAY TFEPLTEEDW VTTIGETAAM
321 GAAGIVFWGS MQYASTIESC QKVKDYIKGP FGHYIINVTS AAKICSHFLC KGKGRCVRKH SDSNAFLHLF PESFRIMVQA
401 NATHKKAIVK GKLELENLKY LRKNFICQCY QGWKGLYCEE HHNKEGN
```

**Supporting Peptides:**

| Peptide | Uniq | -10lgP | Mass | Length | ppm | m/z | z | RT | Fraction | Scan | Source File | Area F1 | Area F2a | Area F3a | Area F4 | Area F5 | Area F6a | Area F7a | Area F7b | Area F8 | Area F9a | Area Sample 1F10a | Area F10b | Area F11a | Area F11b | Area F12a | Area F13 | #Feature | #Feature F1 | #Feature F2a | #Feature F3a | #Feature F4 | #Feature F5 | #Feature F6a | #Feature F7a | #Feature F7b | #Feature F8 | #Feature F9a | #Feature Sample 1F10a | #Feature F10b | #Feature F11a | #Feature F11b | #Feature F12a | #Feature F13 | Start | End | PTM | AScore | Found By |
|---|---|---|---|---|---|---|---|---|---|---|---|---|---|---|---|---|---|---|---|---|---|---|---|---|---|---|---|---|---|---|---|---|---|---|---|---|---|---|---|---|---|---|---|---|---|---|---|---|---|
| K.LELENLKYLR.K | Y | 42.76 | 1289.7343 | 10 | -0.1 | 430.9187 | 3 | 61.68 | 16 | F16:30620 | BuCaTN01_F13.raw | | | | | | | | | | | | | | | | 3.8116E6 | 1 | 0 | 0 | 0 | 0 | 0 | 0 | 0 | 0 | 0 | 0 | 0 | 0 | 0 | 0 | 0 | 1 | 413 | 422 | | | PEAKS DB |
| total 1 peptides | | | | | | | | | | | | | | | | | | | | | | | | | | | | | | | | | | | | | | | | | | | | | | | | | |

**Best Unique PSM (Scan F16:30620, m/z=430.9187, z=3, RT=61.68, ppm=-0.1):**

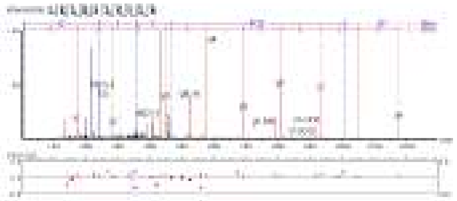

## JAS05059.1

back to list

| Protein Coverage | Supporting Peptides | Best Unique PSM |

**Protein Coverage:**

```
  1 MCHLWIKCLA TWILLKRFNG VHVMQTRTPM YPNEPFLVFW NAPTTQCQLR YKVALDLKTF HIVTNANESL SGSAVTIFYP
 81 TQLGIYPHID DHGNFVNGII PQNESITKHL NKTKSNINHM IPLKTFHGLG VIDWENWRPQ WDRNWGSKNV YRNRSIQFAK
161 ELHPDLSEHA IKRLAKEEFE KAGKSFMRDT LLLAENMRPD GYWGYYLYPD CYNYNYKKKP EQYTGKCPDI EMSRNDQLIW
241 LWRDSTALFP SIYLETILKS SANALKFVHH RLKESMRISS MARKDYALPV FVYARPFYAY TFEPLTEEDW VTTIGETAAM
321 GAAGIVFWGS MQYASTIESC QKVKDYIKGP FGHYIINVTS AAKICSHFLC KGKGRCVRKH SDSNAFLHLF PESFRIMVQA
401 NATHKKAIVK GKLELENLKY LRKNFICQCY QGWKGLYCEE HHNKEGN
```

**Supporting Peptides:**

| Peptide | Uniq | -10lgP | Mass | Length | ppm | m/z | z | RT | Fraction | Scan | Source File | Area F1 | Area F2a | Area F3a | Area F4 | Area F5 | Area F6a | Area F7a | Area F7b | Area F8 | Area F9a | Area Sample 1F10a | Area F10b | Area F11a | Area F11b | Area F12a | Area F13 | #Feature | #Feature F1 | #Feature F2a | #Feature F3a | #Feature F4 | #Feature F5 | #Feature F6a | #Feature F7a | #Feature F7b | #Feature F8 | #Feature F9a | #Feature Sample 1F10a | #Feature F10b | #Feature F11a | #Feature F11b | #Feature F12a | #Feature F13 | Start | End | PTM | AScore | Found By |
|---|---|---|---|---|---|---|---|---|---|---|---|---|---|---|---|---|---|---|---|---|---|---|---|---|---|---|---|---|---|---|---|---|---|---|---|---|---|---|---|---|---|---|---|---|---|---|---|---|---|
| K.LELENLKYLR.K | Y | 42.76 | 1289.7343 | 10 | -0.1 | 430.9187 | 3 | 61.68 | 16 | F16:30620 | BuCaTN01_F13.raw | | | | | | | | | | | | | | | | 3.8116E6 | 1 | 0 | 0 | 0 | 0 | 0 | 0 | 0 | 0 | 0 | 0 | 0 | 0 | 0 | 0 | 0 | 1 | 413 | 422 | | | PEAKS DB |
| total 1 peptides | | | | | | | | | | | | | | | | | | | | | | | | | | | | | | | | | | | | | | | | | | | | | | | | | |

**Best Unique PSM (Scan F16:30620, m/z=430.9187, z=3, RT=61.68, ppm=-0.1):**

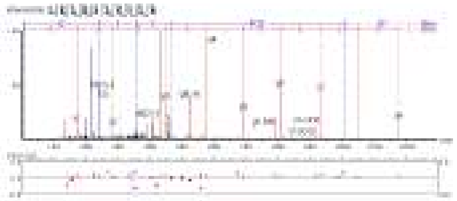

## JAI09070.1

back to list

| Protein Coverage | Supporting Peptides | Best Unique PSM |

**Protein Coverage:**

```
  1 MCHLWIKCLA TWILLKRFNG VHVMQTRTPM YPNEPFLVFW NAPTTQCQLR YKVALDLKTF HIVTNANESL SGSAVTIFYP
 81 TQLGIYPHID DHGNFVNGII PQNESITKHL NKTKSNINHM IPLKTFHGLG VIDWENWRPQ WDRNWGSKNV YRNRSIQFAK
161 ELHPDLSEHA IKRLAKEEFE KAGKSFMRDT LLLAENMRPD GYWGYYLYPD CYNYNYKKKP EQYTGKCPDI EMSRNDQLIW
241 LWRDSTALFP SIYLETILKS SANALKFVHH RLKESMRISS MARKDYALPV FVYARPFYAY TFEPLTEEDW VTTIGETAAM
321 GAAGIVFWGS MQYASTIESC QKVKDYIKGP FGHYIINVTS AAKICSHFLC KGKGRCVRKH SDSNAFLHLF PESFRIMVQA
401 NATHKKAIVK GKLELENLKY LRKNFICQCY QGWKGLYCEE HHNKEGN
```

**Supporting Peptides:**

| Peptide | Uniq | -10lgP | Mass | Length | ppm | m/z | z | RT | Fraction | Scan | Source File | Area F1 | Area F2a | Area F3a | Area F4 | Area F5 | Area F6a | Area F7a | Area F7b | Area F8 | Area F9a | Area Sample 1F10a | Area F10b | Area F11a | Area F11b | Area F12a | Area F13 | #Feature | #Feature F1 | #Feature F2a | #Feature F3a | #Feature F4 | #Feature F5 | #Feature F6a | #Feature F7a | #Feature F7b | #Feature F8 | #Feature F9a | #Feature Sample 1F10a | #Feature F10b | #Feature F11a | #Feature F11b | #Feature F12a | #Feature F13 | Start | End | PTM | AScore | Found By |
|---|---|---|---|---|---|---|---|---|---|---|---|---|---|---|---|---|---|---|---|---|---|---|---|---|---|---|---|---|---|---|---|---|---|---|---|---|---|---|---|---|---|---|---|---|---|---|---|---|---|
| K.LELENLKYLR.K | Y | 42.76 | 1289.7343 | 10 | -0.1 | 430.9187 | 3 | 61.68 | 16 | F16:30620 | BuCaTN01_F13.raw | | | | | | | | | | | | | | | | 3.8116E6 | 1 | 0 | 0 | 0 | 0 | 0 | 0 | 0 | 0 | 0 | 0 | 0 | 0 | 0 | 0 | 0 | 1 | 413 | 422 | | | PEAKS DB |
| total 1 peptides | | | | | | | | | | | | | | | | | | | | | | | | | | | | | | | | | | | | | | | | | | | | | | | | | |

**Best Unique PSM (Scan F16:30620, m/z=430.9187, z=3, RT=61.68, ppm=-0.1):**

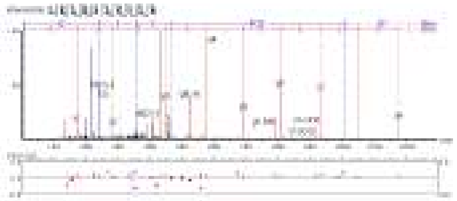

## LAA86767.1

back to list

| Protein Coverage | Supporting Peptides | Best Unique PSM |

**Protein Coverage:**

```
  1 MGAAGIVFWG SMQYASTIES CQKVKDYING PFGHYIINVT SAAKICSHFL CKGKGRCVRK HSDSNAFLHL FPESFRIMVH
 81 ANATHKKAIV KGKLELENLK YLRNNFVCQC YQGWKGLDCE EHYNKEGN
```

**Supporting Peptides:**

| Peptide | Uniq | -10lgP | Mass | Length | ppm | m/z | z | RT | Fraction | Scan | Source File | Area F1 | Area F2a | Area F3a | Area F4 | Area F5 | Area F6a | Area F7a | Area F7b | Area F8 | Area F9a | Area Sample 1F10a | Area F10b | Area F11a | Area F11b | Area F12a | Area F13 | #Feature | #Feature F1 | #Feature F2a | #Feature F3a | #Feature F4 | #Feature F5 | #Feature F6a | #Feature F7a | #Feature F7b | #Feature F8 | #Feature F9a | #Feature Sample 1F10a | #Feature F10b | #Feature F11a | #Feature F11b | #Feature F12a | #Feature F13 | Start | End | PTM | AScore | Found By |
|---|---|---|---|---|---|---|---|---|---|---|---|---|---|---|---|---|---|---|---|---|---|---|---|---|---|---|---|---|---|---|---|---|---|---|---|---|---|---|---|---|---|---|---|---|---|---|---|---|---|
| K.LELENLKYLR.N | Y | 42.76 | 1289.7343 | 10 | -0.1 | 430.9187 | 3 | 61.68 | 16 | F16:30620 | BuCaTN01_F13.raw | | | | | | | | | | | | | | | | 3.8116E6 | 1 | 0 | 0 | 0 | 0 | 0 | 0 | 0 | 0 | 0 | 0 | 0 | 0 | 0 | 0 | 0 | 1 | 94 | 103 | | | PEAKS DB |
| total 1 peptides | | | | | | | | | | | | | | | | | | | | | | | | | | | | | | | | | | | | | | | | | | | | | | | | | |

**Best Unique PSM (Scan F16:30620, m/z=430.9187, z=3, RT=61.68, ppm=-0.1):**

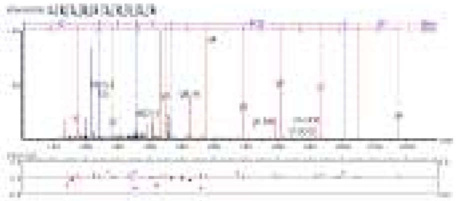

## LAA86741.1

back to list

| Protein Coverage | Supporting Peptides | Best Unique PSM |

**Protein Coverage:**

```
  1 MGAAGIVFWG SMQYASTIES CQKVKDYING PFGHYIINVT SAAKICSHFL CKGKGRCVRK HSDSNAFLHL FPESFRIMVH
 81 ANATHKKAIV KGKLELENLK YLRNNFVCQC YQGWKGLDCE EHYNKEGN
```

**Supporting Peptides:**

| Peptide | Uniq | -10lgP | Mass | Length | ppm | m/z | z | RT | Fraction | Scan | Source File | Area F1 | Area F2a | Area F3a | Area F4 | Area F5 | Area F6a | Area F7a | Area F7b | Area F8 | Area F9a | Area Sample 1F10a | Area F10b | Area F11a | Area F11b | Area F12a | Area F13 | #Feature | #Feature F1 | #Feature F2a | #Feature F3a | #Feature F4 | #Feature F5 | #Feature F6a | #Feature F7a | #Feature F7b | #Feature F8 | #Feature F9a | #Feature Sample 1F10a | #Feature F10b | #Feature F11a | #Feature F11b | #Feature F12a | #Feature F13 | Start | End | PTM | AScore | Found By |
|---|---|---|---|---|---|---|---|---|---|---|---|---|---|---|---|---|---|---|---|---|---|---|---|---|---|---|---|---|---|---|---|---|---|---|---|---|---|---|---|---|---|---|---|---|---|---|---|---|---|
| K.LELENLKYLR.N | Y | 42.76 | 1289.7343 | 10 | -0.1 | 430.9187 | 3 | 61.68 | 16 | F16:30620 | BuCaTN01_F13.raw | | | | | | | | | | | | | | | | 3.8116E6 | 1 | 0 | 0 | 0 | 0 | 0 | 0 | 0 | 0 | 0 | 0 | 0 | 0 | 0 | 0 | 0 | 1 | 94 | 103 | | | PEAKS DB |
| total 1 peptides | | | | | | | | | | | | | | | | | | | | | | | | | | | | | | | | | | | | | | | | | | | | | | | | | |

**Best Unique PSM (Scan F16:30620, m/z=430.9187, z=3, RT=61.68, ppm=-0.1):**

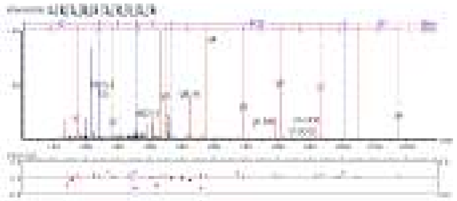

## LAA86748.1

back to list

| Protein Coverage | Supporting Peptides | Best Unique PSM |

**Protein Coverage:**

1 MGAAGIVFWG SMQYASTIES CQKVKDYING PFGHYIINVT SAAKICSHFL CKGKGRCVRK HSDSNAFLHL FPESFRIMVH
81 ANATHKKAIV KGK**LELENLK YLR**NNFVCQC YQGWKGLDCE EHYNKEGN

**Supporting Peptides:**

| Peptide | Uniq | -10lgP | Mass | Length | ppm | m/z | z | RT | Fraction | Scan | Source File | Area F1 | Area F2a | Area F3a | Area F4 | Area F5 | Area F6a | Area F7a | Area F7b | Area F8 | Area F9a | Area Sample 1F10a | Area F10b | Area F11a | Area F11b | Area F12a | Area F13 | #Feature | #Feature F1 | #Feature F2a | #Feature F3a | #Feature F4 | #Feature F5 | #Feature F6a | #Feature F7a | #Feature F7b | #Feature F8 | #Feature F9a | #Feature Sample 1F10a | #Feature F10b | #Feature F11a | #Feature F11b | #Feature F12a | #Feature F13 | Start | End | PTM | AScore | Found By |
|---|---|---|---|---|---|---|---|---|---|---|---|---|---|---|---|---|---|---|---|---|---|---|---|---|---|---|---|---|---|---|---|---|---|---|---|---|---|---|---|---|---|---|---|---|---|---|---|---|---|
| K.LELENLKYLR.N | Y | 42.76 | 1289.7343 | 10 | -0.1 | 430.9187 | 3 | 61.68 | 16 | F16:30620 | BuCaTN01_F13.raw | | | | | | | | | | | | | | | | 3.8116E6 | 1 | 0 | 0 | 0 | 0 | 0 | 0 | 0 | 0 | 0 | 0 | 0 | 0 | 0 | 0 | 0 | 1 | 94 | 103 | | | PEAKS DB |
| total 1 peptides | | | | | | | | | | | | | | | | | | | | | | | | | | | | | | | | | | | | | | | | | | | | | | | | | |

**Best Unique PSM (Scan F16:30620, m/z=430.9187, z=3, RT=61.68, ppm=-0.1):**

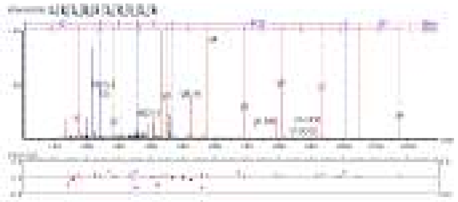

## LAA86751.1

back to list

| Protein Coverage | Supporting Peptides | Best Unique PSM |

**Protein Coverage:**

1 MGAAGIVFWG SMQYASTIES CQKVKDYING PFGHYIINVT SAAKICSHFL CKGKGRCVRK HSDSNAFLHL FPESFRIMVH
81 ANATHKKAIV KGK**LELENLK YLR**NNFVCQC YQGWKGLDCE EHYNKEGN

**Supporting Peptides:**

| Peptide | Uniq | -10lgP | Mass | Length | ppm | m/z | z | RT | Fraction | Scan | Source File | Area F1 | Area F2a | Area F3a | Area F4 | Area F5 | Area F6a | Area F7a | Area F7b | Area F8 | Area F9a | Area Sample 1F10a | Area F10b | Area F11a | Area F11b | Area F12a | Area F13 | #Feature | #Feature F1 | #Feature F2a | #Feature F3a | #Feature F4 | #Feature F5 | #Feature F6a | #Feature F7a | #Feature F7b | #Feature F8 | #Feature F9a | #Feature Sample 1F10a | #Feature F10b | #Feature F11a | #Feature F11b | #Feature F12a | #Feature F13 | Start | End | PTM | AScore | Found By |
|---|---|---|---|---|---|---|---|---|---|---|---|---|---|---|---|---|---|---|---|---|---|---|---|---|---|---|---|---|---|---|---|---|---|---|---|---|---|---|---|---|---|---|---|---|---|---|---|---|---|
| K.LELENLKYLR.N | Y | 42.76 | 1289.7343 | 10 | -0.1 | 430.9187 | 3 | 61.68 | 16 | F16:30620 | BuCaTN01_F13.raw | | | | | | | | | | | | | | | | 3.8116E6 | 1 | 0 | 0 | 0 | 0 | 0 | 0 | 0 | 0 | 0 | 0 | 0 | 0 | 0 | 0 | 0 | 1 | 94 | 103 | | | PEAKS DB |
| total 1 peptides | | | | | | | | | | | | | | | | | | | | | | | | | | | | | | | | | | | | | | | | | | | | | | | | | |

**Best Unique PSM (Scan F16:30620, m/z=430.9187, z=3, RT=61.68, ppm=-0.1):**

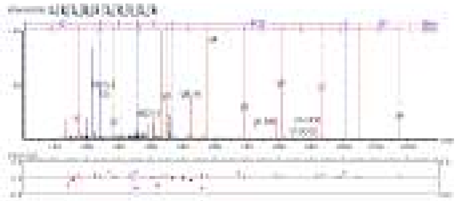

## LAA86759.1

back to list

| Protein Coverage | Supporting Peptides | Best Unique PSM |

**Protein Coverage:**

1 MGAAGIVFWG SMQYASTIES CQKVKDYING PFGHYIINVT SAAKICSHFL CKGKGRCVRK HSDSNAFLHL FPESFRIMVH
81 ANATHKKAIV KGK**LELENLK YLR**NNFVCQC YQGWKGLDCE EHYNKEGN

**Supporting Peptides:**

| Peptide | Uniq | -10lgP | Mass | Length | ppm | m/z | z | RT | Fraction | Scan | Source File | Area F1 | Area F2a | Area F3a | Area F4 | Area F5 | Area F6a | Area F7a | Area F7b | Area F8 | Area F9a | Area Sample 1F10a | Area F10b | Area F11a | Area F11b | Area F12a | Area F13 | #Feature | #Feature F1 | #Feature F2a | #Feature F3a | #Feature F4 | #Feature F5 | #Feature F6a | #Feature F7a | #Feature F7b | #Feature F8 | #Feature F9a | #Feature Sample 1F10a | #Feature F10b | #Feature F11a | #Feature F11b | #Feature F12a | #Feature F13 | Start | End | PTM | AScore | Found By |
|---|---|---|---|---|---|---|---|---|---|---|---|---|---|---|---|---|---|---|---|---|---|---|---|---|---|---|---|---|---|---|---|---|---|---|---|---|---|---|---|---|---|---|---|---|---|---|---|---|---|
| K.LELENLKYLR.N | Y | 42.76 | 1289.7343 | 10 | -0.1 | 430.9187 | 3 | 61.68 | 16 | F16:30620 | BuCaTN01_F13.raw | | | | | | | | | | | | | | | | 3.8116E6 | 1 | 0 | 0 | 0 | 0 | 0 | 0 | 0 | 0 | 0 | 0 | 0 | 0 | 0 | 0 | 0 | 1 | 94 | 103 | | | PEAKS DB |
| total 1 peptides | | | | | | | | | | | | | | | | | | | | | | | | | | | | | | | | | | | | | | | | | | | | | | | | | |

**Best Unique PSM (Scan F16:30620, m/z=430.9187, z=3, RT=61.68, ppm=-0.1):**

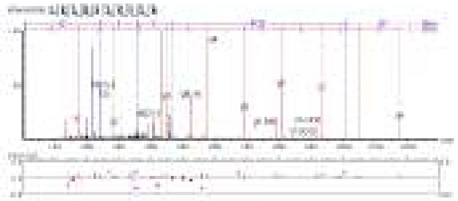

## LAA86757.1

back to list

| Protein Coverage | Supporting Peptides | Best Unique PSM |

**Protein Coverage:**

1 MGAAGIVFWG SMQYASTIES CQKVKDYING PFGHYIINVT SAAKICSHFL CKGKGRCVRK HSDSNAFLHL FPESFRIMVH
81 ANATHKKAIV KGK**LELENLK YLR**NNFVCQC YQGWKGLDCE EHYNKEGN

**Supporting Peptides:**

| Peptide | Uniq | -10lgP | Mass | Length | ppm | m/z | z | RT | Fraction | Scan | Source File | Area F1 | Area F2a | Area F3a | Area F4 | Area F5 | Area F6a | Area F7a | Area F7b | Area F8 | Area F9a | Area Sample 1F10a | Area F10b | Area F11a | Area F11b | Area F12a | Area F13 | #Feature | #Feature F1 | #Feature F2a | #Feature F3a | #Feature F4 | #Feature F5 | #Feature F6a | #Feature F7a | #Feature F7b | #Feature F8 | #Feature F9a | #Feature Sample 1F10a | #Feature F10b | #Feature F11a | #Feature F11b | #Feature F12a | #Feature F13 | Start | End | PTM | AScore | Found By |
|---|---|---|---|---|---|---|---|---|---|---|---|---|---|---|---|---|---|---|---|---|---|---|---|---|---|---|---|---|---|---|---|---|---|---|---|---|---|---|---|---|---|---|---|---|---|---|---|---|---|
| K.LELENLKYLR.N | Y | 42.76 | 1289.7343 | 10 | -0.1 | 430.9187 | 3 | 61.68 | 16 | F16:30620 | BuCaTN01_F13.raw | | | | | | | | | | | | | | | | 3.8116E6 | 1 | 0 | 0 | 0 | 0 | 0 | 0 | 0 | 0 | 0 | 0 | 0 | 0 | 0 | 0 | 0 | 1 | 94 | 103 | | | PEAKS DB |
| total 1 peptides | | | | | | | | | | | | | | | | | | | | | | | | | | | | | | | | | | | | | | | | | | | | | | | | | |

**Best Unique PSM (Scan F16:30620, m/z=430.9187, z=3, RT=61.68, ppm=-0.1):**

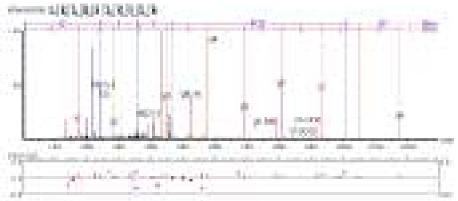

## LAA86750.1

back to list

| Protein Coverage | Supporting Peptides | Best Unique PSM |

**Protein Coverage:**

1 MGAAGIVFWG SMQYASTIES CQKVKDYING PFGHYIINVT SAAKICSHFL CKGKGRCVRK HSDSNAFLHL FPESFRIMVH
81 ANATHKKAIV KGKLELENLK YLRNNFVCQC YQGWKGLDCE EHYNKEGN

**Supporting Peptides:**

| Peptide | Uniq | -10lgP | Mass | Length | ppm | m/z | z | RT | Fraction | Scan | Source File | Area F1 | Area F2a | Area F3a | Area F4 | Area F5 | Area F6a | Area F7a | Area F7b | Area F8 | Area F9a | Area Sample 1F10a | Area F10b | Area F11a | Area F11b | Area F12a | Area F13 | #Feature | #Feature F1 | #Feature F2a | #Feature F3a | #Feature F4 | #Feature F5 | #Feature F6a | #Feature F7a | #Feature F7b | #Feature F8 | #Feature F9a | #Feature Sample 1F10a | #Feature F10b | #Feature F11a | #Feature F11b | #Feature F12a | #Feature F13 | Start | End | PTM | AScore | Found By |
|---|---|---|---|---|---|---|---|---|---|---|---|---|---|---|---|---|---|---|---|---|---|---|---|---|---|---|---|---|---|---|---|---|---|---|---|---|---|---|---|---|---|---|---|---|---|---|---|---|---|
| K.LELENLKYLR.N | Y | 42.76 | 1289.7343 | 10 | -0.1 | 430.9187 | 3 | 61.68 | 16 | F16:30620 | BuCaTN01_F13.raw | | | | | | | | | | | | | | | | 3.8116E6 | 1 | 0 | 0 | 0 | 0 | 0 | 0 | 0 | 0 | 0 | 0 | 0 | 0 | 0 | 0 | 0 | 1 | 94 | 103 | | | PEAKS DB |
| total 1 peptides | | | | | | | | | | | | | | | | | | | | | | | | | | | | | | | | | | | | | | | | | | | | | | | | | |

**Best Unique PSM (Scan F16:30620, m/z=430.9187, z=3, RT=61.68, ppm=-0.1):**

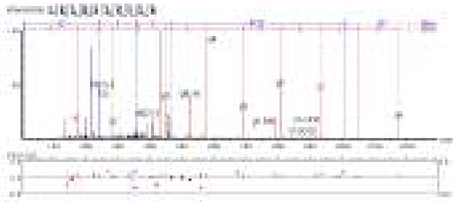

## LAA86749.1

back to list

| Protein Coverage | Supporting Peptides | Best Unique PSM |

**Protein Coverage:**

1 MGAAGIVFWG SMQYASTIES CQKVKDYING PFGHYIINVT SAAKICSHFL CKGKGRCVRK HSDSNAFLHL FPESFRIMVH
81 ANATHKKAIV KGKLELENLK YLRNNFVCQC YQGWKGLDCE EHYNKEGN

**Supporting Peptides:**

| Peptide | Uniq | -10lgP | Mass | Length | ppm | m/z | z | RT | Fraction | Scan | Source File | Area F1 | Area F2a | Area F3a | Area F4 | Area F5 | Area F6a | Area F7a | Area F7b | Area F8 | Area F9a | Area Sample 1F10a | Area F10b | Area F11a | Area F11b | Area F12a | Area F13 | #Feature | #Feature F1 | #Feature F2a | #Feature F3a | #Feature F4 | #Feature F5 | #Feature F6a | #Feature F7a | #Feature F7b | #Feature F8 | #Feature F9a | #Feature Sample 1F10a | #Feature F10b | #Feature F11a | #Feature F11b | #Feature F12a | #Feature F13 | Start | End | PTM | AScore | Found By |
|---|---|---|---|---|---|---|---|---|---|---|---|---|---|---|---|---|---|---|---|---|---|---|---|---|---|---|---|---|---|---|---|---|---|---|---|---|---|---|---|---|---|---|---|---|---|---|---|---|---|
| K.LELENLKYLR.N | Y | 42.76 | 1289.7343 | 10 | -0.1 | 430.9187 | 3 | 61.68 | 16 | F16:30620 | BuCaTN01_F13.raw | | | | | | | | | | | | | | | | 3.8116E6 | 1 | 0 | 0 | 0 | 0 | 0 | 0 | 0 | 0 | 0 | 0 | 0 | 0 | 0 | 0 | 0 | 1 | 94 | 103 | | | PEAKS DB |
| total 1 peptides | | | | | | | | | | | | | | | | | | | | | | | | | | | | | | | | | | | | | | | | | | | | | | | | | |

**Best Unique PSM (Scan F16:30620, m/z=430.9187, z=3, RT=61.68, ppm=-0.1):**

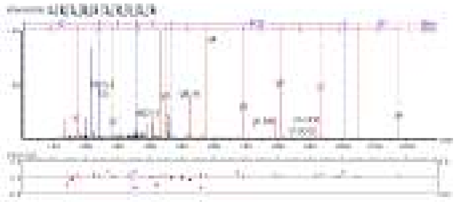

## LAA86742.1

back to list

| Protein Coverage | Supporting Peptides | Best Unique PSM |

**Protein Coverage:**

1 MGAAGIVFWG SMQYASTIES CQKVKDYING PFGHYIINVT SAAKICSHFL CKGKGRCVRK HSDSNAFLHL FPESFRIMVH
81 ANATHKKAIV KGKLELENLK YLRNNFVCQC YQGWKGLDCE EHYNKEGN

**Supporting Peptides:**

| Peptide | Uniq | -10lgP | Mass | Length | ppm | m/z | z | RT | Fraction | Scan | Source File | Area F1 | Area F2a | Area F3a | Area F4 | Area F5 | Area F6a | Area F7a | Area F7b | Area F8 | Area F9a | Area Sample 1F10a | Area F10b | Area F11a | Area F11b | Area F12a | Area F13 | #Feature | #Feature F1 | #Feature F2a | #Feature F3a | #Feature F4 | #Feature F5 | #Feature F6a | #Feature F7a | #Feature F7b | #Feature F8 | #Feature F9a | #Feature Sample 1F10a | #Feature F10b | #Feature F11a | #Feature F11b | #Feature F12a | #Feature F13 | Start | End | PTM | AScore | Found By |
|---|---|---|---|---|---|---|---|---|---|---|---|---|---|---|---|---|---|---|---|---|---|---|---|---|---|---|---|---|---|---|---|---|---|---|---|---|---|---|---|---|---|---|---|---|---|---|---|---|---|
| K.LELENLKYLR.N | Y | 42.76 | 1289.7343 | 10 | -0.1 | 430.9187 | 3 | 61.68 | 16 | F16:30620 | BuCaTN01_F13.raw | | | | | | | | | | | | | | | | 3.8116E6 | 1 | 0 | 0 | 0 | 0 | 0 | 0 | 0 | 0 | 0 | 0 | 0 | 0 | 0 | 0 | 0 | 1 | 94 | 103 | | | PEAKS DB |
| total 1 peptides | | | | | | | | | | | | | | | | | | | | | | | | | | | | | | | | | | | | | | | | | | | | | | | | | |

**Best Unique PSM (Scan F16:30620, m/z=430.9187, z=3, RT=61.68, ppm=-0.1):**

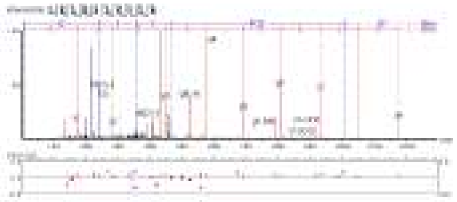

## LAA86765.1

back to list

| Protein Coverage | Supporting Peptides | Best Unique PSM |

**Protein Coverage:**

1 MGAAGIVFWG SMQYASTIES CQKVKDYING PFGHYIINVT SAAKICSHFL CKGKGRCVRK HSDSNAFLHL FPESFRIMVH
81 ANATHKKAIV KGKLELENLK YLRNNFVCQC YQGWKGLDCE EHYNKEGN

**Supporting Peptides:**

| Peptide | Uniq | -10lgP | Mass | Length | ppm | m/z | z | RT | Fraction | Scan | Source File | Area F1 | Area F2a | Area F3a | Area F4 | Area F5 | Area F6a | Area F7a | Area F7b | Area F8 | Area F9a | Area Sample 1F10a | Area F10b | Area F11a | Area F11b | Area F12a | Area F13 | #Feature | #Feature F1 | #Feature F2a | #Feature F3a | #Feature F4 | #Feature F5 | #Feature F6a | #Feature F7a | #Feature F7b | #Feature F8 | #Feature F9a | #Feature Sample 1F10a | #Feature F10b | #Feature F11a | #Feature F11b | #Feature F12a | #Feature F13 | Start | End | PTM | AScore | Found By |
|---|---|---|---|---|---|---|---|---|---|---|---|---|---|---|---|---|---|---|---|---|---|---|---|---|---|---|---|---|---|---|---|---|---|---|---|---|---|---|---|---|---|---|---|---|---|---|---|---|---|
| K.LELENLKYLR.N | Y | 42.76 | 1289.7343 | 10 | -0.1 | 430.9187 | 3 | 61.68 | 16 | F16:30620 | BuCaTN01_F13.raw | | | | | | | | | | | | | | | | 3.8116E6 | 1 | 0 | 0 | 0 | 0 | 0 | 0 | 0 | 0 | 0 | 0 | 0 | 0 | 0 | 0 | 0 | 1 | 94 | 103 | | | PEAKS DB |
| total 1 peptides | | | | | | | | | | | | | | | | | | | | | | | | | | | | | | | | | | | | | | | | | | | | | | | | | |

**Best Unique PSM (Scan F16:30620, m/z=430.9187, z=3, RT=61.68, ppm=-0.1):**

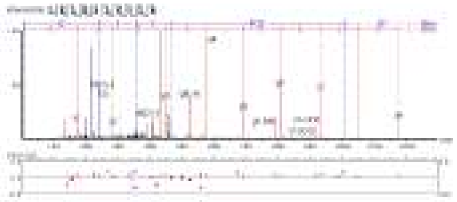

## LAA86747.1

back to list

| Protein Coverage | Supporting Peptides | Best Unique PSM |

**Protein Coverage:**

1 MGAAGIVFWG SMQYASTIES CQKVKDYING PFGHYIINVT SAAKICSHFL CKGKGRCVRK HSDSNAFLHL FPESFRIMVH
81 ANATHKKAIV KGKLELENLK YLRNNFVCQC YQGWKGLDCE EHYNKEGN

**Supporting Peptides:**

| Peptide | Uniq | -10lgP | Mass | Length | ppm | m/z | z | RT | Fraction | Scan | Source File | Area F1 | Area F2a | Area F3a | Area F4 | Area F5 | Area F6a | Area F7a | Area F7b | Area F8 | Area F9a | Area Sample 1F10a | Area F10b | Area F11a | Area F11b | Area F12a | Area F13 | #Feature | #Feature F1 | #Feature F2a | #Feature F3a | #Feature F4 | #Feature F5 | #Feature F6a | #Feature F7a | #Feature F7b | #Feature F8 | #Feature F9a | #Feature Sample 1F10a | #Feature F10b | #Feature F11a | #Feature F11b | #Feature F12a | #Feature F13 | Start | End | PTM | AScore | Found By |
|---|---|---|---|---|---|---|---|---|---|---|---|---|---|---|---|---|---|---|---|---|---|---|---|---|---|---|---|---|---|---|---|---|---|---|---|---|---|---|---|---|---|---|---|---|---|---|---|---|---|
| K.LELENLKYLR.N | Y | 42.76 | 1289.7343 | 10 | -0.1 | 430.9187 | 3 | 61.68 | 16 | F16:30620 | BuCaTN01_F13.raw | | | | | | | | | | | | | | | | 3.8116E6 | 1 | 0 | 0 | 0 | 0 | 0 | 0 | 0 | 0 | 0 | 0 | 0 | 0 | 0 | 0 | 0 | 1 | 94 | 103 | | | PEAKS DB |
| total 1 peptides | | | | | | | | | | | | | | | | | | | | | | | | | | | | | | | | | | | | | | | | | | | | | | | | | |

**Best Unique PSM (Scan F16:30620, m/z=430.9187, z=3, RT=61.68, ppm=-0.1):**

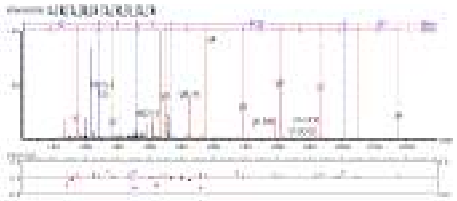

## LAA86770.1

back to list

| **Protein Coverage** | **Supporting Peptides** | **Best Unique PSM** |

**Protein Coverage:**

1 MGAAGIVFWG SMQYASTIES CQKVKDYING PFGHYIINVT SAAKICSHFL CKGKGRCVRK HSDSNAFLHL FPESFRIMVH
81 ANATHKKAIV KGKLELENLK YLRNNFVCQC YQGWKGLDCE EHYNKEGN

**Supporting Peptides:**

| Peptide | Uniq | -10lgP | Mass | Length | ppm | m/z | z | RT | Fraction | Scan | Source File | Area F1 | Area F2a | Area F3a | Area F4 | Area F5 | Area F6a | Area F7a | Area F7b | Area F8 | Area F9a | Area Sample 1F10a | Area F10b | Area F11a | Area F11b | Area F12a | Area F13 | #Feature | #Feature F1 | #Feature F2a | #Feature F3a | #Feature F4 | #Feature F5 | #Feature F6a | #Feature F7a | #Feature F7b | #Feature F8 | #Feature F9a | #Feature Sample 1F10a | #Feature F10b | #Feature F11a | #Feature F11b | #Feature F12a | #Feature F13 | Start | End | PTM | AScore | Found By |
|---|---|---|---|---|---|---|---|---|---|---|---|---|---|---|---|---|---|---|---|---|---|---|---|---|---|---|---|---|---|---|---|---|---|---|---|---|---|---|---|---|---|---|---|---|---|---|---|---|---|
| K.LELENLKYLR.N | Y | 42.76 | 1289.7343 | 10 | -0.1 | 430.9187 | 3 | 61.68 | 16 | F16:30620 | BuCaTN01_F13.raw | | | | | | | | | | | | | | | | 3.8116E6 | 1 | 0 | 0 | 0 | 0 | 0 | 0 | 0 | 0 | 0 | 0 | 0 | 0 | 0 | 0 | 0 | 1 | 94 | 103 | | | PEAKS DB |
| total 1 peptides | | | | | | | | | | | | | | | | | | | | | | | | | | | | | | | | | | | | | | | | | | | | | | | | | |

**Best Unique PSM (Scan F16:30620, m/z=430.9187, z=3, RT=61.68, ppm=-0.1):**

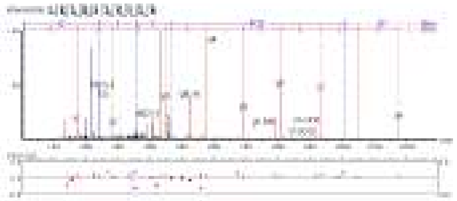

## LAA86752.1

back to list

| **Protein Coverage** | **Supporting Peptides** | **Best Unique PSM** |

**Protein Coverage:**

1 MGAAGIVFWG SMQYASTIES CQKVKDYING PFGHYIINVT SAAKICSHFL CKGKGRCVRK HSDSNAFLHL FPESFRIMVH
81 ANATHKKAIV KGKLELENLK YLRNNFVCQC YQGWKGLDCE EHYNKEGN

**Supporting Peptides:**

| Peptide | Uniq | -10lgP | Mass | Length | ppm | m/z | z | RT | Fraction | Scan | Source File | Area F1 | Area F2a | Area F3a | Area F4 | Area F5 | Area F6a | Area F7a | Area F7b | Area F8 | Area F9a | Area Sample 1F10a | Area F10b | Area F11a | Area F11b | Area F12a | Area F13 | #Feature | #Feature F1 | #Feature F2a | #Feature F3a | #Feature F4 | #Feature F5 | #Feature F6a | #Feature F7a | #Feature F7b | #Feature F8 | #Feature F9a | #Feature Sample 1F10a | #Feature F10b | #Feature F11a | #Feature F11b | #Feature F12a | #Feature F13 | Start | End | PTM | AScore | Found By |
|---|---|---|---|---|---|---|---|---|---|---|---|---|---|---|---|---|---|---|---|---|---|---|---|---|---|---|---|---|---|---|---|---|---|---|---|---|---|---|---|---|---|---|---|---|---|---|---|---|---|
| K.LELENLKYLR.N | Y | 42.76 | 1289.7343 | 10 | -0.1 | 430.9187 | 3 | 61.68 | 16 | F16:30620 | BuCaTN01_F13.raw | | | | | | | | | | | | | | | | 3.8116E6 | 1 | 0 | 0 | 0 | 0 | 0 | 0 | 0 | 0 | 0 | 0 | 0 | 0 | 0 | 0 | 0 | 1 | 94 | 103 | | | PEAKS DB |
| total 1 peptides | | | | | | | | | | | | | | | | | | | | | | | | | | | | | | | | | | | | | | | | | | | | | | | | | |

**Best Unique PSM (Scan F16:30620, m/z=430.9187, z=3, RT=61.68, ppm=-0.1):**

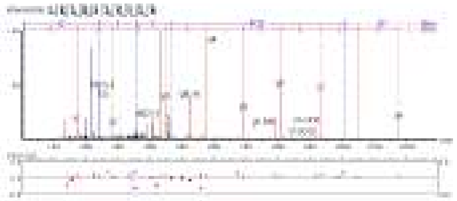

## LAA86761.1

back to list

| **Protein Coverage** | **Supporting Peptides** | **Best Unique PSM** |

**Protein Coverage:**

1 MGAAGIVFWG SMQYASTIES CQKVKDYING PFGHYIINVT SAAKICSHFL CKGKGRCVRK HSDSNAFLHL FPESFRIMVH
81 ANATHKKAIV KGKLELENLK YLRNNFVCQC YQGWKGLDCE EHYNKEGN

**Supporting Peptides:**

| Peptide | Uniq | -10lgP | Mass | Length | ppm | m/z | z | RT | Fraction | Scan | Source File | Area F1 | Area F2a | Area F3a | Area F4 | Area F5 | Area F6a | Area F7a | Area F7b | Area F8 | Area F9a | Area Sample 1F10a | Area F10b | Area F11a | Area F11b | Area F12a | Area F13 | #Feature | #Feature F1 | #Feature F2a | #Feature F3a | #Feature F4 | #Feature F5 | #Feature F6a | #Feature F7a | #Feature F7b | #Feature F8 | #Feature F9a | #Feature Sample 1F10a | #Feature F10b | #Feature F11a | #Feature F11b | #Feature F12a | #Feature F13 | Start | End | PTM | AScore | Found By |
|---|---|---|---|---|---|---|---|---|---|---|---|---|---|---|---|---|---|---|---|---|---|---|---|---|---|---|---|---|---|---|---|---|---|---|---|---|---|---|---|---|---|---|---|---|---|---|---|---|---|
| K.LELENLKYLR.N | Y | 42.76 | 1289.7343 | 10 | -0.1 | 430.9187 | 3 | 61.68 | 16 | F16:30620 | BuCaTN01_F13.raw | | | | | | | | | | | | | | | | 3.8116E6 | 1 | 0 | 0 | 0 | 0 | 0 | 0 | 0 | 0 | 0 | 0 | 0 | 0 | 0 | 0 | 0 | 1 | 94 | 103 | | | PEAKS DB |
| total 1 peptides | | | | | | | | | | | | | | | | | | | | | | | | | | | | | | | | | | | | | | | | | | | | | | | | | |

**Best Unique PSM (Scan F16:30620, m/z=430.9187, z=3, RT=61.68, ppm=-0.1):**

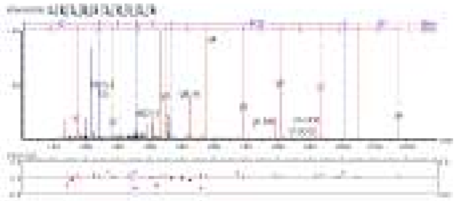

## LAA86756.1

back to list

| **Protein Coverage** | **Supporting Peptides** | **Best Unique PSM** |

**Protein Coverage:**

1 MGAAGIVFWG SMQYASTIES CQKVKDYING PFGHYIINVT SAAKICSHFL CKGKGRCVRK HSDSNAFLHL FPESFRIMVH
81 ANATHKKAIV KGK**LELENLK YLR**NNFVCQC YQGWKGLDCE EHYNKEGN

**Supporting Peptides:**

| Peptide | Uniq | -10lgP | Mass | Length | ppm | m/z | z | RT | Fraction | Scan | Source File | Area F1 | Area F2a | Area F3a | Area F4 | Area F5 | Area F6a | Area F7a | Area F7b | Area F8 | Area F9a | Area Sample 1F10a | Area F10b | Area F11a | Area F11b | Area F12a | Area F13 | #Feature | #Feature F1 | #Feature F2a | #Feature F3a | #Feature F4 | #Feature F5 | #Feature F6a | #Feature F7a | #Feature F7b | #Feature F8 | #Feature F9a | #Feature Sample 1F10a | #Feature F10b | #Feature F11a | #Feature F11b | #Feature F12a | #Feature F13 | Start | End | PTM | AScore | Found By |
|---|---|---|---|---|---|---|---|---|---|---|---|---|---|---|---|---|---|---|---|---|---|---|---|---|---|---|---|---|---|---|---|---|---|---|---|---|---|---|---|---|---|---|---|---|---|---|---|---|---|
| K.LELENLKYLR.N | Y | 42.76 | 1289.7343 | 10 | -0.1 | 430.9187 | 3 | 61.68 | 16 | F16:30620 | BuCaTN01_F13.raw | | | | | | | | | | | | | | | | 3.8116E6 | 1 | 0 | 0 | 0 | 0 | 0 | 0 | 0 | 0 | 0 | 0 | 0 | 0 | 0 | 0 | 0 | 1 | 94 | 103 | | | PEAKS DB |
| total 1 peptides | | | | | | | | | | | | | | | | | | | | | | | | | | | | | | | | | | | | | | | | | | | | | | | | | |

**Best Unique PSM (Scan F16:30620, m/z=430.9187, z=3, RT=61.68, ppm=-0.1):**

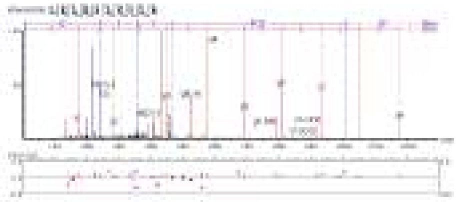

## LAA86746.1

back to list

| Protein Coverage | Supporting Peptides | Best Unique PSM |

**Protein Coverage:**

1 MGAAGIVFWG SMQYASTIES CQKVKDYING PFGHYIINVT SAAKICSHFL CKGKGRCVRK HSDSNAFLHL FPESFRIMVH
81 ANATHKKAIV KGK**LELENLK YLR**NNFVCQC YQGWKGLDCE EHYNKEGN

**Supporting Peptides:**

| Peptide | Uniq | -10lgP | Mass | Length | ppm | m/z | z | RT | Fraction | Scan | Source File | Area F1 | Area F2a | Area F3a | Area F4 | Area F5 | Area F6a | Area F7a | Area F7b | Area F8 | Area F9a | Area Sample 1F10a | Area F10b | Area F11a | Area F11b | Area F12a | Area F13 | #Feature | #Feature F1 | #Feature F2a | #Feature F3a | #Feature F4 | #Feature F5 | #Feature F6a | #Feature F7a | #Feature F7b | #Feature F8 | #Feature F9a | #Feature Sample 1F10a | #Feature F10b | #Feature F11a | #Feature F11b | #Feature F12a | #Feature F13 | Start | End | PTM | AScore | Found By |
|---|---|---|---|---|---|---|---|---|---|---|---|---|---|---|---|---|---|---|---|---|---|---|---|---|---|---|---|---|---|---|---|---|---|---|---|---|---|---|---|---|---|---|---|---|---|---|---|---|---|
| K.LELENLKYLR.N | Y | 42.76 | 1289.7343 | 10 | -0.1 | 430.9187 | 3 | 61.68 | 16 | F16:30620 | BuCaTN01_F13.raw | | | | | | | | | | | | | | | | 3.8116E6 | 1 | 0 | 0 | 0 | 0 | 0 | 0 | 0 | 0 | 0 | 0 | 0 | 0 | 0 | 0 | 0 | 1 | 94 | 103 | | | PEAKS DB |
| total 1 peptides | | | | | | | | | | | | | | | | | | | | | | | | | | | | | | | | | | | | | | | | | | | | | | | | | |

**Best Unique PSM (Scan F16:30620, m/z=430.9187, z=3, RT=61.68, ppm=-0.1):**

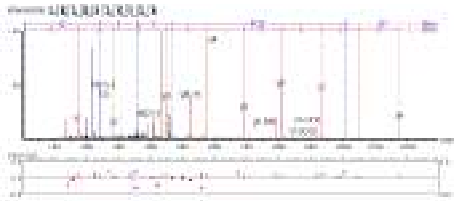

## LAA86771.1

back to list

| Protein Coverage | Supporting Peptides | Best Unique PSM |

**Protein Coverage:**

1 MGAAGIVFWG SMQYASTIES CQKVKDYING PFGHYIINVT SAAKICSHFL CKGKGRCVRK HSDSNAFLHL FPESFRIMVH
81 ANATHKKAIV KGK**LELENLK YLR**NNFVCQC YQGWKGLDCE EHYNKEGN

**Supporting Peptides:**

| Peptide | Uniq | -10lgP | Mass | Length | ppm | m/z | z | RT | Fraction | Scan | Source File | Area F1 | Area F2a | Area F3a | Area F4 | Area F5 | Area F6a | Area F7a | Area F7b | Area F8 | Area F9a | Area Sample 1F10a | Area F10b | Area F11a | Area F11b | Area F12a | Area F13 | #Feature | #Feature F1 | #Feature F2a | #Feature F3a | #Feature F4 | #Feature F5 | #Feature F6a | #Feature F7a | #Feature F7b | #Feature F8 | #Feature F9a | #Feature Sample 1F10a | #Feature F10b | #Feature F11a | #Feature F11b | #Feature F12a | #Feature F13 | Start | End | PTM | AScore | Found By |
|---|---|---|---|---|---|---|---|---|---|---|---|---|---|---|---|---|---|---|---|---|---|---|---|---|---|---|---|---|---|---|---|---|---|---|---|---|---|---|---|---|---|---|---|---|---|---|---|---|---|
| K.LELENLKYLR.N | Y | 42.76 | 1289.7343 | 10 | -0.1 | 430.9187 | 3 | 61.68 | 16 | F16:30620 | BuCaTN01_F13.raw | | | | | | | | | | | | | | | | 3.8116E6 | 1 | 0 | 0 | 0 | 0 | 0 | 0 | 0 | 0 | 0 | 0 | 0 | 0 | 0 | 0 | 0 | 1 | 94 | 103 | | | PEAKS DB |
| total 1 peptides | | | | | | | | | | | | | | | | | | | | | | | | | | | | | | | | | | | | | | | | | | | | | | | | | |

**Best Unique PSM (Scan F16:30620, m/z=430.9187, z=3, RT=61.68, ppm=-0.1):**

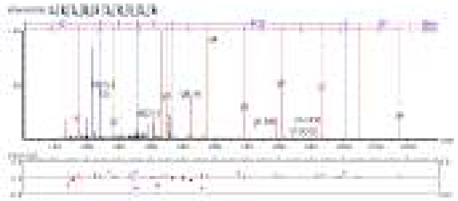

## LAA86763.1

back to list

| Protein Coverage | Supporting Peptides | Best Unique PSM |

**Protein Coverage:**

1 MGAAGIVFWG SMQYASTIES CQKVKDYING PFGHYIINVT SAAKICSHFL CKGKGRCVRK HSDSNAFLHL FPESFRIMVH
81 ANATHKKAIV KGK**LELENLK YLR**NNFVCQC YQGWKGLDCE EHYNKEGN

**Supporting Peptides:**

| Peptide | Uniq | -10lgP | Mass | Length | ppm | m/z | z | RT | Fraction | Scan | Source File | Area F1 | Area F2a | Area F3a | Area F4 | Area F5 | Area F6a | Area F7a | Area F7b | Area F8 | Area F9a | Area Sample 1F10a | Area F10b | Area F11a | Area F11b | Area F12a | Area F13 | #Feature | #Feature F1 | #Feature F2a | #Feature F3a | #Feature F4 | #Feature F5 | #Feature F6a | #Feature F7a | #Feature F7b | #Feature F8 | #Feature F9a | #Feature Sample 1F10a | #Feature F10b | #Feature F11a | #Feature F11b | #Feature F12a | #Feature F13 | Start | End | PTM | AScore | Found By |
|---|---|---|---|---|---|---|---|---|---|---|---|---|---|---|---|---|---|---|---|---|---|---|---|---|---|---|---|---|---|---|---|---|---|---|---|---|---|---|---|---|---|---|---|---|---|---|---|---|---|
| K.LELENLKYLR.N | Y | 42.76 | 1289.7343 | 10 | -0.1 | 430.9187 | 3 | 61.68 | 16 | F16:30620 | BuCaTN01_F13.raw | | | | | | | | | | | | | | | | 3.8116E6 | 1 | 0 | 0 | 0 | 0 | 0 | 0 | 0 | 0 | 0 | 0 | 0 | 0 | 0 | 0 | 0 | 1 | 94 | 103 | | | PEAKS DB |
| total 1 peptides | | | | | | | | | | | | | | | | | | | | | | | | | | | | | | | | | | | | | | | | | | | | | | | | | |

**Best Unique PSM (Scan F16:30620, m/z=430.9187, z=3, RT=61.68, ppm=-0.1):**

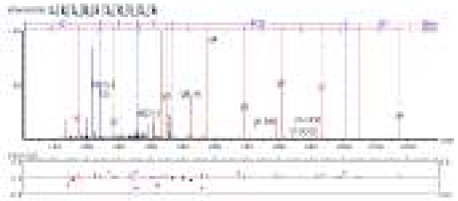

## LAA86754.1

back to list

| Protein Coverage | Supporting Peptides | Best Unique PSM |

**Protein Coverage:**

1 MGAAGIVFWG SMQYASTIES CQKVKDYING PFGHYIINVT SAAKICSHFL CKGKGRCVRK HSDSNAFLHL FPESFRIMVH
81 ANATHKKAIV KGK**LELENLK YLR**NNFVCQC YQGWKGLDCE EHYNKEGN

**Supporting Peptides:**

| Peptide | Uniq | -10lgP | Mass | Length | ppm | m/z | z | RT | Fraction | Scan | Source File | Area F1 | Area F2a | Area F3a | Area F4 | Area F5 | Area F6a | Area F7a | Area F7b | Area F8 | Area F9a | Area Sample 1F10a | Area F10b | Area F11a | Area F11b | Area F12a | Area F13 | #Feature | #Feature F1 | #Feature F2a | #Feature F3a | #Feature F4 | #Feature F5 | #Feature F6a | #Feature F7a | #Feature F7b | #Feature F8 | #Feature F9a | #Feature Sample 1F10a | #Feature F10b | #Feature F11a | #Feature F11b | #Feature F12a | #Feature F13 | Start | End | PTM | AScore | Found By |
|---|---|---|---|---|---|---|---|---|---|---|---|---|---|---|---|---|---|---|---|---|---|---|---|---|---|---|---|---|---|---|---|---|---|---|---|---|---|---|---|---|---|---|---|---|---|---|---|---|---|
| K.LELENLKYLR.N | Y | 42.76 | 1289.7343 | 10 | -0.1 | 430.9187 | 3 | 61.68 | 16 | F16:30620 | BuCaTN01_F13.raw | | | | | | | | | | | | | | | | 3.8116E6 | 1 | 0 | 0 | 0 | 0 | 0 | 0 | 0 | 0 | 0 | 0 | 0 | 0 | 0 | 0 | 0 | 1 | 94 | 103 | | | PEAKS DB |
| total 1 peptides | | | | | | | | | | | | | | | | | | | | | | | | | | | | | | | | | | | | | | | | | | | | | | | | | |

**Best Unique PSM (Scan F16:30620, m/z=430.9187, z=3, RT=61.68, ppm=-0.1):**

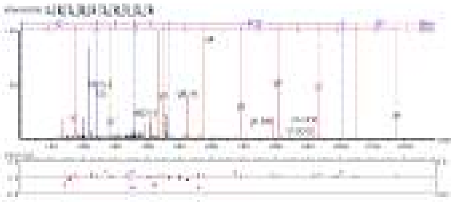

## XP_015678372.1 back to list

| Protein Coverage | Supporting Peptides | Best Unique PSM |

**Protein Coverage:**

1 MIAFVALLCF AAVLQQSSGT VDFASESANK RETQKEILDK HNALRRSVRP TARNMLQMEW NFNAAQNAKR WADRCSFDHS

C Carbamidomethylation (+57.02)

81 PRHLRTVGKL KCGENLFMSS HPFPWTRVIQ SWYDENKNFK HGVGANPPNA VIGHYTQIVW YRSYLLGCAA ARCHSSSYNY

186 193 198 202

161 YYVCHYCPAG NIIGKIATPY K**SGPPCEDCP SACVNGLCTN PCK**HIDRYTN CDSLVQQIGC QNNNMKSDCP ASCFCHNEIK

**Supporting Peptides:**

| Peptide | Uniq | -10lgP | Mass | Length | ppm | m/z | z | RT | Fraction | Scan | Source File | Area F1 | Area F2a | Area F3a | Area F4 | Area F5 | Area F6a | Area F7a | Area F7b | Area F8 | Area F9a | Area Sample 1F10a | Area F10b | Area F11a | Area F11b | Area F12a | Area F13 | #Feature | #Feature F1 | #Feature F2a | #Feature F3a | #Feature F4 | #Feature F5 | #Feature F6a | #Feature F7a | #Feature F7b | #Feature F8 | #Feature F9a | #Feature Sample 1F10a | #Feature F10b | #Feature F11a | #Feature F11b | #Feature F12a | #Feature F13 | Start | End | PTM | AScore | Found By |
|---|---|---|---|---|---|---|---|---|---|---|---|---|---|---|---|---|---|---|---|---|---|---|---|---|---|---|---|---|---|---|---|---|---|---|---|---|---|---|---|---|---|---|---|---|---|---|---|---|---|
| K.SGPPC(+57.02)EDC(+57.02)PSAC(+57.02)VNGLC(+57.02)TNPC(+57.02)K.H | Y | 50.54 | 2478.9695 | 22 | 0.2 | 827.3306 | 3 | 39.81 | 13 | F13:17534 | BuCaTN01_F11a.raw | | | | | | | | | | | | | 2.5656E6 | | | | 1 | 0 | 0 | 0 | 0 | 0 | 0 | 0 | 0 | 0 | 0 | 0 | 0 | 1 | 0 | 0 | 0 | 182 | 203 | Carbamidomethylation | C5:Carbamidomethylation:1000.00;C8:Carbamidomethylation:1000.00;C12:Carbamidomethylation:1000.00;C17:Carbamidomethylation:1000.00;C21:Carbamidomethylation:1000.00 | PEAKS DB |
| E.DC(+57.02)PSAC(+57.02)VNGLC(+57.02)TNPC(+57.02)K.H | N | 43.28 | 1851.7372 | 16 | -0.3 | 926.8756 | 2 | 43.52 | 13 | F13:19924 | BuCaTN01_F11a.raw | | | | | | | | | | | | | 1.1644E5 | | | | 1 | 0 | 0 | 0 | 0 | 0 | 0 | 0 | 0 | 0 | 0 | 0 | 0 | 1 | 0 | 0 | 0 | 188 | 203 | Carbamidomethylation | C2:Carbamidomethylation:1000.00;C6:Carbamidomethylation:1000.00;C11:Carbamidomethylation:1000.00;C15:Carbamidomethylation:1000.00 | PEAKS DB |
| total 2 peptides | | | | | | | | | | | | | | | | | | | | | | | | | | | | | | | | | | | | | | | | | | | | | | | | | |

**Best Unique PSM (Scan F13:17534, m/z=827.3306, z=3, RT=39.81, ppm=0.2):**

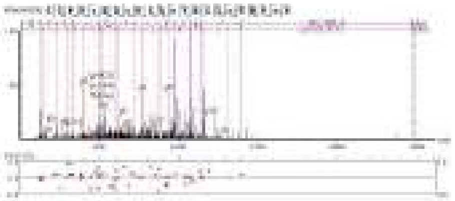

## P82463.1 back to list

| Protein Coverage | Supporting Peptides | Best Unique PSM |

**Protein Coverage:**

17 24

1 LTCVKEK**SIF GVTTEDCPDG QNLCFKR**WHM IVPGRYKKTR GCAATCPIAE NRDVIECCST DKCNL

C Carbamidomethylation (+57.02)

**Supporting Peptides:**

| Peptide | Uniq | -10lgP | Mass | Length | ppm | m/z | z | RT | Fraction | Scan | Source File | Area F1 | Area F2a | Area F3a | Area F4 | Area F5 | Area F6a | Area F7a | Area F7b | Area F8 | Area F9a | Area Sample 1F10a | Area F10b | Area F11a | Area F11b | Area F12a | Area F13 | #Feature | #Feature F1 | #Feature F2a | #Feature F3a | #Feature F4 | #Feature F5 | #Feature F6a | #Feature F7a | #Feature F7b | #Feature F8 | #Feature F9a | #Feature Sample 1F10a | #Feature F10b | #Feature F11a | #Feature F11b | #Feature F12a | #Feature F13 | Start | End | PTM | AScore | Found By |
|---|---|---|---|---|---|---|---|---|---|---|---|---|---|---|---|---|---|---|---|---|---|---|---|---|---|---|---|---|---|---|---|---|---|---|---|---|---|---|---|---|---|---|---|---|---|---|---|---|---|
| K.SIFGVTTEDC(+57.02)PDGQNLC(+57.02)FKR.W | Y | 47.98 | 2343.0623 | 20 | -0.6 | 782.0276 | 3 | 62.88 | 6 | F6:41068 | BuCaTN01_F6a.raw | | | | | | 1.8722E6 | | | | | | | | | | | 1 | 0 | 0 | 0 | 0 | 0 | 1 | 0 | 0 | 0 | 0 | 0 | 0 | 0 | 0 | 0 | 0 | 8 | 27 | Carbamidomethylation | C10:Carbamidomethylation:1000.00;C17:Carbamidomethylation:1000.00 | PEAKS DB |
| K.SIFGVTTEDC(+57.02)PDGQNLC(+57.02)FK.R | Y | 44.50 | 2186.9612 | 19 | 5.6 | 1094.4940 | 2 | 69.31 | 6 | F6:48014 | BuCaTN01_F6a.raw | | | | | | 3.2015E4 | | | | | | | | | | | 1 | 0 | 0 | 0 | 0 | 0 | 1 | 0 | 0 | 0 | 0 | 0 | 0 | 0 | 0 | 0 | 0 | 8 | 26 | Carbamidomethylation | C10:Carbamidomethylation:1000.00;C17:Carbamidomethylation:1000.00 | PEAKS DB |
| total 2 peptides | | | | | | | | | | | | | | | | | | | | | | | | | | | | | | | | | | | | | | | | | | | | | | | | | |

**Best Unique PSM (Scan F6:41068, m/z=782.0276, z=3, RT=62.88, ppm=-0.6):**

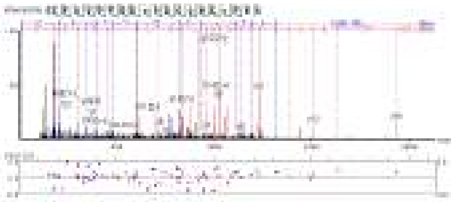

## AUF41653.1 back to list

| Protein Coverage | Supporting Peptides | Best Unique PSM |

**Protein Coverage:**

1 MMQVLLVTIS LAVFPYQGSS IILESGNVND YEVVYPQKVT ALPKGAVQQP EQKYEDTMQY EFEVNGEPVV LHLEKNKILF
81 SEDYSETHYS PDGREITTNP PVEDHCYYHG RIQNDAHSSA SISACNGLKG HFKLRGEMYF IEPLKLSNNE AHAVYKYENI
161 EKEDETPKMC GVTQTNWESD EPIKKASQLV ATSAQFNKAF IELIIIVDHS MAKKCNSTAT NTKIYEIVNS ANEIFNPLNI
241 HVTLIGVEFW CDRDLINVTS SADETLDSFG EWRASDLMTR K**SHDNALLFT DMR**FDLNTLG ITFLAGMCQA YRSVGIVQVQ

321 GNRNFKTAVI MAHELSHNLG MYHDGKNCIC NDSSCVMSPV LSDQPSKLFS NCSIHDYQRY LTRYKPKCIL YPPLRKDIVS
401 PPVCGNEIWE EGEECDCGSP ADCQNPCCDA ATCKLKPGAE CGNGLCCYQC KIKTAGTVCR RARNECDVPE HCTGQSAECP
481 RDQLQQNGQP CQNNRGYCYN GDCPIMRNQC ISLFGSRANV AKDSCFQENL KGSYYGYCRK ENGRKIPCAP QDVKCGRLFC
561 LNNSPGNKNP CNMHYSCMDQ HKGMVDPGTK CEDGKVCNNK RQCVDVNTAY QSTTGFSQI

**Supporting Peptides:**

| Peptide | Uniq | -10lgP | Mass | Length | ppm | m/z | z | RT | Fraction | Scan | Source File | Area F1 | Area F2a | Area F3a | Area F4 | Area F5 | Area F6a | Area F7a | Area F7b | Area F8 | Area F9a | Area Sample 1F10a | Area F10b | Area F11a | Area F11b | Area F12a | Area F13 | #Feature | #Feature F1 | #Feature F2a | #Feature F3a | #Feature F4 | #Feature F5 | #Feature F6a | #Feature F7a | #Feature F7b | #Feature F8 | #Feature F9a | #Feature Sample 1F10a | #Feature F10b | #Feature F11a | #Feature F11b | #Feature F12a | #Feature F13 | Start | End | PTM | AScore | Found By |
|---|---|---|---|---|---|---|---|---|---|---|---|---|---|---|---|---|---|---|---|---|---|---|---|---|---|---|---|---|---|---|---|---|---|---|---|---|---|---|---|---|---|---|---|---|---|---|---|---|---|
| K.SHDNALLFTDMR.F | Y | 49.84 | 1418.6613 | 12 | -0.5 | 473.8941 | 3 | 58.17 | 16 | F16:28198 | BuCaTN01_F13.raw | | | | | | | | | | | | | | | | 1.7363E4 | 1 | 0 | 0 | 0 | 0 | 0 | 0 | 0 | 0 | 0 | 0 | 0 | 0 | 0 | 0 | 0 | 1 | 282 | 293 | | | PEAKS DB |
| total 1 peptides | | | | | | | | | | | | | | | | | | | | | | | | | | | | | | | | | | | | | | | | | | | | | | | | | |

**Best Unique PSM (Scan F16:28198, m/z=473.8941, z=3, RT=58.17, ppm=-0.5):**

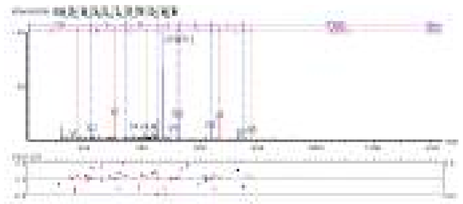

## AUF41652.1

back to list

| Protein Coverage | Supporting Peptides | Best Unique PSM |

**Protein Coverage:**

```
  1 MMQVLLVTIS LAVFPYQGSS IILESGNVND YEVVYPQKVT ALPKGAVQQP EQKYEDTMQY EFEVNGEPVV LHLEKNKILF
 81 SEDYSETHYS PDGREITTNP PVEDHCYYHG RIQNDAHSSA SISACNGLKG HFKLRGEMYF IEPLKLSNNE AHAVYKYENI
161 EKEDETPKMC GVTQTNWESD EPIKKASQLV ATSAQFNKAF IELIIIVDHS MAKKCNSTAT NTKIYEIVNS ANEIFNPLNI
241 HVTLIGVEFW CDRDLINVTS SADETLDSFG EWRASDLMTR KSHDNALLFT DMRFDLNTLG ITFLAGMCQA YRSVGIVQVQ

321 GNRNFKTAVI MAHELSHNLG MYHDGKNCIC NDSSCVMSPV LSDQPSKLFS NCSIHDYQRY LTRYKPKCIL YPPLRKDIVS
401 PPVCGNEIWE EGEECDCGSP ADCQNPCCDA ATCKLKPGAE CGNGLCCYQC KIKTAGTVCR RARNECDVPE HCTGQSAECP
481 RDQLQQNGQP CQNNRGYCYN GDCPIMRNQC ISLFGSRANV AKDSCFQENL KGSYYGYCRK ENGRKIPCAP QDVKCGRLFC
561 LNNSPGNKNP CNMHYSCMDQ HKGMVDPGTK CEDGKVCNNK RQCVDVNTAY QSTTGFSQI
```

**Supporting Peptides:**

| Peptide | Uniq | -10lgP | Mass | Length | ppm | m/z | z | RT | Fraction | Scan | Source File | Area F1 | Area F2a | Area F3a | Area F4 | Area F5 | Area F6a | Area F7a | Area F7b | Area F8 | Area F9a | Area Sample 1F10a | Area F10b | Area F11a | Area F11b | Area F12a | Area F13 | #Feature | #Feature F1 | #Feature F2a | #Feature F3a | #Feature F4 | #Feature F5 | #Feature F6a | #Feature F7a | #Feature F7b | #Feature F8 | #Feature F9a | #Feature Sample 1F10a | #Feature F10b | #Feature F11a | #Feature F11b | #Feature F12a | #Feature F13 | Start | End | PTM | AScore | Found By |
|---|---|---|---|---|---|---|---|---|---|---|---|---|---|---|---|---|---|---|---|---|---|---|---|---|---|---|---|---|---|---|---|---|---|---|---|---|---|---|---|---|---|---|---|---|---|---|---|---|---|
| K.SHDNALLFTDMR.F | Y | 49.84 | 1418.6613 | 12 | -0.5 | 473.8941 | 3 | 58.17 | 16 | F16:28198 | BuCaTN01_F13.raw | | | | | | | | | | | | | | | | 1.7363E4 | 1 | 0 | 0 | 0 | 0 | 0 | 0 | 0 | 0 | 0 | 0 | 0 | 0 | 0 | 0 | 0 | 1 | 282 | 293 | | | PEAKS DB |
| total 1 peptides | | | | | | | | | | | | | | | | | | | | | | | | | | | | | | | | | | | | | | | | | | | | | | | | | |

**Best Unique PSM (Scan F16:28198, m/z=473.8941, z=3, RT=58.17, ppm=-0.5):**

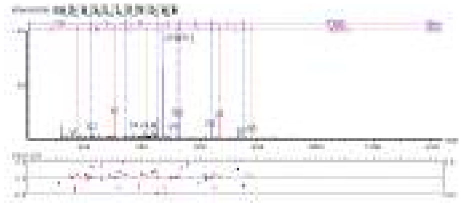

## ADJ67475.1

back to list

| Protein Coverage | Supporting Peptides | Best Unique PSM |

**Protein Coverage:**

```
  1 MMQVLLVTIS LAVFPYQGSS IILESGNVND YEVVYPQKVT AMPKGAVKQP EQKYEDTMQY EFEVNGEPVV LHLEKNKILF
 81 SEDYSETHYY PDGREITTNP PVEDHCYYHG HIQNDGHSSA SISACNGLKG HFKLRGEMYF IEPLKLSNNE AHAVYKYENI
161 EKEDETPKMC GVTQTNWESD KPIKKASQLV STSAQFNKAF IELIIIVDHS MAKKCNSTAT NTKIYEIVNS ANEIFNPLNI
241 HVTLIGVEFW CDRDLINVTS SADETLDSFG EWRASDLMTR KSHDNALLFT DMRFDLNTLG ITFLAGMCQA YRSVGIVQVQ

321 GNRNFKTAVI MAHELSHNLG MYHDGKNCIC NDSSCVMSPV LSDQPSKLFS NCSIHDYQRY LTRYKPKCIL YPPLRKDIVS
401 PPVCGNEIWE EGEECDCGSP ADCQNPCCDA ATCKLKPGAE CGNGLCCYQC KIKTAGTVCR RARNECDVPE HCTGQSAECP
481 RDQLQQNGQP CQNNRGYCYN GDCPIMRNQC ISLFGSRATV AKDSCFQENL KGSYYGYCRK ENGRKIPCAP QDVKCGRLFC
561 LNNSPRNKNP CNMHYSCMDQ HKGMVDPGTK CEDGKVCNNK RQCVDVNTAY QSTTGFSQI
```

**Supporting Peptides:**

| Peptide | Uniq | -10lgP | Mass | Length | ppm | m/z | z | RT | Fraction | Scan | Source File | Area F1 | Area F2a | Area F3a | Area F4 | Area F5 | Area F6a | Area F7a | Area F7b | Area F8 | Area F9a | Area Sample 1F10a | Area F10b | Area F11a | Area F11b | Area F12a | Area F13 | #Feature | #Feature F1 | #Feature F2a | #Feature F3a | #Feature F4 | #Feature F5 | #Feature F6a | #Feature F7a | #Feature F7b | #Feature F8 | #Feature F9a | #Feature Sample 1F10a | #Feature F10b | #Feature F11a | #Feature F11b | #Feature F12a | #Feature F13 | Start | End | PTM | AScore | Found By |
|---|---|---|---|---|---|---|---|---|---|---|---|---|---|---|---|---|---|---|---|---|---|---|---|---|---|---|---|---|---|---|---|---|---|---|---|---|---|---|---|---|---|---|---|---|---|---|---|---|---|
| K.SHDNALLFTDMR.F | Y | 49.84 | 1418.6613 | 12 | -0.5 | 473.8941 | 3 | 58.17 | 16 | F16:28198 | BuCaTN01_F13.raw | | | | | | | | | | | | | | | | 1.7363E4 | 1 | 0 | 0 | 0 | 0 | 0 | 0 | 0 | 0 | 0 | 0 | 0 | 0 | 0 | 0 | 0 | 1 | 282 | 293 | | | PEAKS DB |
| total 1 peptides | | | | | | | | | | | | | | | | | | | | | | | | | | | | | | | | | | | | | | | | | | | | | | | | | |

**Best Unique PSM (Scan F16:28198, m/z=473.8941, z=3, RT=58.17, ppm=-0.5):**

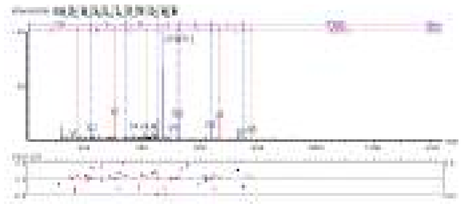

## AAZ39881.1

back to list

| Protein Coverage | Supporting Peptides | Best Unique PSM |

**Protein Coverage:**

```
  1 MMQVLLVTIS LAVFPYQGSS IILESGNVND YEVVYPQKVT ALPKGAVQQP EQKYEDTMQY EFEVNGEPVV LHLEKNKILF
 81 SEDYSETHYY PDGREITTNP PVEDHCYYHG RIQNDAHSSA SISACNGLKG HFKLRGEMYF IEPLKLSNSE AHAVYKYENI
161 EKEDEIPKMC GVTQTNWESD KPIKKASQLV STSAQFNKIF IELVIIVDHS MAKKCNSTAT NTKIYEIVNS ANEIFNPLNI
241 HVTLIGVEFW CDRDLINVTS SADETLNSFG EWRASDLMTR KSHDNALLFT DMRFDLNTLG ITFLAGMCQA YRSVEIVQEQ

321 GNRNFKTAVI MAHELSHNLG MYHDGKNCIC NDSSCVMSPV LSDQPSKLFS NCSIHDYQRY LTRYKPKCIF NPPLRKDIVS
401 PPVCGNEIWE EGEECDCGSP ANCQNPCCDA ATCKLKPGAE CGNGLCCYQC KIKTAGTVCR RARDECDVPE HCTGQSAECP
481 RDQLQQNGKP CQNNRGYCYN GDCPIMRNQC ISLFGSRANV AKDSCFQENL KGSYYGYCRK ENGRKIPCAP QDVKCGRLFC
561 LNNSPRNKNP CNMHYSCMDQ HKGMVDPGTK CEDGKVCNNK RQCVDVNTAY QSTTGFSQI
```

**Supporting Peptides:**

| Peptide | Uniq | -10lgP | Mass | Length | ppm | m/z | z | RT | Fraction | Scan | Source File | Area F1 | Area F2a | Area F3a | Area F4 | Area F5 | Area F6a | Area F7a | Area F7b | Area F8 | Area F9a | Area Sample 1F10a | Area F10b | Area F11a | Area F11b | Area F12a | Area F13 | #Feature | #Feature F1 | #Feature F2a | #Feature F3a | #Feature F4 | #Feature F5 | #Feature F6a | #Feature F7a | #Feature F7b | #Feature F8 | #Feature F9a | #Feature Sample 1F10a | #Feature F10b | #Feature F11a | #Feature F11b | #Feature F12a | #Feature F13 | Start | End | PTM | AScore | Found By |
|---|---|---|---|---|---|---|---|---|---|---|---|---|---|---|---|---|---|---|---|---|---|---|---|---|---|---|---|---|---|---|---|---|---|---|---|---|---|---|---|---|---|---|---|---|---|---|---|---|---|
| K.SHDNALLFTDMR.F | Y | 49.84 | 1418.6613 | 12 | -0.5 | 473.8941 | 3 | 58.17 | 16 | F16:28198 | BuCaTN01_F13.raw | | | | | | | | | | | | | | | | 1.7363E4 | 1 | 0 | 0 | 0 | 0 | 0 | 0 | 0 | 0 | 0 | 0 | 0 | 0 | 0 | 0 | 0 | 1 | 282 | 293 | | | PEAKS DB |
| total 1 peptides | | | | | | | | | | | | | | | | | | | | | | | | | | | | | | | | | | | | | | | | | | | | | | | | | |

**Best Unique PSM (Scan F16:28198, m/z=473.8941, z=3, RT=58.17, ppm=-0.5):**

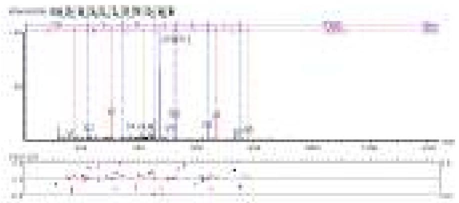

## Q7LZ61.2

back to list

| Protein Coverage | Supporting Peptides | Best Unique PSM |

**Protein Coverage:**

```
  1 MMQVLLVTIS LAVFPYQGSS IILESGNVND YEVVYPQKVT ALPKGAVQQP EQKYEDTMQY EFEVNGEPVV LHLEKNKILF
 81 SEDYSETHYY PDGREITTNP PVEDHCYYHG RIQNDAHSSA SISACNGLKG HFKLRGEMYF IEPLKLSNSE AHAVYKYENI
161 EKEDEIPKMC GVTQTNWESD KPIKKASQLV STSAQFNKIF IELVIIVDHS MAKKCNSTAT NTKIYEIVNS ANEIFNPLNI
241 HVTLIGVEFW CDRDLINVTS SADETLNSFG EWRASDLMTR KSHDNALLFT DMRFDLNTLG ITFLAGMCQA YRSVEIVQEQ
321 GNRNFKTAVI MAHELSHNLG MYHDGKNCIC NDSSCVMSPV LSDQPSKLFS NCSIHDYQRY LTRYKPKCIF NPPLRKDIVS
401 PPVCGNEIWE EGEECDCGSP ANCQNPCCDA ATCKLKPGAE CGNGLCCYQC KIKTAGTVCR RARDECDVPE HCTGQSAECP
481 RDQLQQNGKP CQNNRGYCYN GDCPIMRNQC ISLFGSRANV AKDSCFQENL KGSYYGYCRK ENGRKIPCAP QDVKCGRLFC
561 LNNSPRNKNP CNMHYSCMDQ HKGMVDPGTK CEDGKVCNNK RQCVDVNTAY QSTTGFSQI
```

**Supporting Peptides:**

| Peptide | Uniq | -10lgP | Mass | Length | ppm | m/z | z | RT | Fraction | Scan | Source File | Area F1 | Area F2a | Area F3a | Area F4 | Area F5 | Area F6a | Area F7a | Area F7b | Area F8 | Area F9a | Area Sample 1F10a | Area F10b | Area F11a | Area F11b | Area F12a | Area F13 | #Feature | #Feature F1 | #Feature F2a | #Feature F3a | #Feature F4 | #Feature F5 | #Feature F6a | #Feature F7a | #Feature F7b | #Feature F8 | #Feature F9a | #Feature Sample 1F10a | #Feature F10b | #Feature F11a | #Feature F11b | #Feature F12a | #Feature F13 | Start | End | PTM | AScore | Found By |
|---|---|---|---|---|---|---|---|---|---|---|---|---|---|---|---|---|---|---|---|---|---|---|---|---|---|---|---|---|---|---|---|---|---|---|---|---|---|---|---|---|---|---|---|---|---|---|---|---|---|---|
| K.SHDNALLFTDMR.F | Y | 49.84 | 1418.6613 | 12 | -0.5 | 473.8941 | 3 | 58.17 | 16 | F16:28198 | BuCaTN01_F13.raw | | | | | | | | | | | | | | | | 1.7363E4 | 1 | 0 | 0 | 0 | 0 | 0 | 0 | 0 | 0 | 0 | 0 | 0 | 0 | 0 | 0 | 0 | 1 | 282 | 293 | | | PEAKS DB |
| total 1 peptides | | | | | | | | | | | | | | | | | | | | | | | | | | | | | | | | | | | | | | | | | | | | | | | | | | | |

**Best Unique PSM (Scan F16:28198, m/z=473.8941, z=3, RT=58.17, ppm=-0.5):**

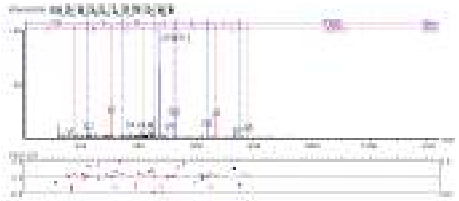

## AAB22477.1

back to list

| Protein Coverage | Supporting Peptides | Best Unique PSM |

**Protein Coverage:**

```
  1 LVSTSAQFNK IFIELVIIVD HSMAKKCNST ATNTKIYEIV NSANEIFNPL NIHVTLIGVE FWCDRDLINV TSSADETLNS
 81 FGEWRASDLM TRKSHDNALL FTDMRFDLNT LGITFLAGMC QAYRSVEIVQ EQGNRNFKTA VIMAHELSHN LGMYHDGKNC
161 ICNDSSCVMS PVLSDQPSKL FSNCSIHDYQ RYLTRYKPKC IFNPPLRKDI VSPPVCGNEI WEEGEECDCG SPANCQNPCC
241 DAATCKLKPG AECGNGLCCY QCKIKTAGTV CRTRRARDEC DVPEHCTGQS AECPRDQLQQ NGKPCQNNRG YCYNGDCPIM
321 RNQCISLFGS RANVAKDSCF QENLKGSYYG YCRKENGRKI PCAPQDVKCG RLFCLNNSPR NKNPCNMHYS CMDQHKGMVD
401 PGTKCEDGKV CNNKRQCVDV NTAYQSTTG
```

**Supporting Peptides:**

| Peptide | Uniq | -10lgP | Mass | Length | ppm | m/z | z | RT | Fraction | Scan | Source File | Area F1 | Area F2a | Area F3a | Area F4 | Area F5 | Area F6a | Area F7a | Area F7b | Area F8 | Area F9a | Area Sample 1F10a | Area F10b | Area F11a | Area F11b | Area F12a | Area F13 | #Feature | #Feature F1 | #Feature F2a | #Feature F3a | #Feature F4 | #Feature F5 | #Feature F6a | #Feature F7a | #Feature F7b | #Feature F8 | #Feature F9a | #Feature Sample 1F10a | #Feature F10b | #Feature F11a | #Feature F11b | #Feature F12a | #Feature F13 | Start | End | PTM | AScore | Found By |
|---|---|---|---|---|---|---|---|---|---|---|---|---|---|---|---|---|---|---|---|---|---|---|---|---|---|---|---|---|---|---|---|---|---|---|---|---|---|---|---|---|---|---|---|---|---|---|---|---|---|---|
| K.SHDNALLFTDMR.F | Y | 49.84 | 1418.6613 | 12 | -0.5 | 473.8941 | 3 | 58.17 | 16 | F16:28198 | BuCaTN01_F13.raw | | | | | | | | | | | | | | | | 1.7363E4 | 1 | 0 | 0 | 0 | 0 | 0 | 0 | 0 | 0 | 0 | 0 | 0 | 0 | 0 | 0 | 0 | 1 | 94 | 105 | | | PEAKS DB |
| total 1 peptides | | | | | | | | | | | | | | | | | | | | | | | | | | | | | | | | | | | | | | | | | | | | | | | | | | | |

**Best Unique PSM (Scan F16:28198, m/z=473.8941, z=3, RT=58.17, ppm=-0.5):**

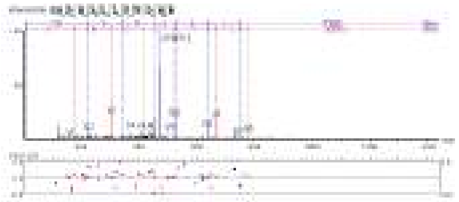

## AAM51550.1

back to list

| Protein Coverage | Supporting Peptides | Best Unique PSM |

**Protein Coverage:**

```
  1 MIQALLVAIC LAVFPYQGSS IILESGNVND YEVVYPQKVP ALSKGGVQNP QPETKYEDTM QYEFHVNGEP VVLHLERNKG
 81 LFSEDYTETH YAPDGREITT SSPVQDHCYY HGYIQNEADS SAVISACDGL KGHFKHQGET YFIEPLELSD SEAHAIYKDE
161 NVEEEEEIPK ICGVTQTTWE SDEPIEKSSQ LTNTPEQDRY LQAKKYIEFY VVVDNVMYRK YTGKLHVITR RVYEMVNALN
241 TMYRRLNFHI ALIGLEIWSN GNEINVQSDV QATLDLFGEW RENKLLPRKR NDNAQLLTST EFNGTTTGLG YIGSLCSPKK
321 SVAVVQDHSK STSMVAITMA HQMGHNLGMN DDRASCTCGS NKCIMSTKYY ESLSEFSSCS VQEHREYLLR DRPQCILNKP
401 SRKAIVTPPV CGNYFVERGE ECDCGSPEDC QNTCCDAATC KLQHEAQCDS GECCEKCKFK GAGAECRAAK NDCDFPELCT
481 GRSAKCPKDS FQRNGHPCQN NQGYCYNGTC PTLTNQCATL WGPGAKMSPG LCFMLNWNAR SCGLCRKENG RKILCAAKDV
561 KCGRLFCKKK NSMICHCPPP SKDPNYGMVA PGTKCGVKKV CRNRQCVKV
```

o Oxidation (M) (+15.99); 588 o

**Supporting Peptides:**

| Peptide | Uniq | -10lgP | Mass | Length | ppm | m/z | z | RT | Fraction | Scan | Source File | Area F1 | Area F2a | Area F3a | Area F4 | Area F5 | Area F6a | Area F7a | Area F7b | Area F8 | Area F9a | Area Sample 1F10a | Area F10b | Area F11a | Area F11b | Area F12a | Area F13 | #Feature | #Feature F1 | #Feature F2a | #Feature F3a | #Feature F4 | #Feature F5 | #Feature F6a | #Feature F7a | #Feature F7b | #Feature F8 | #Feature F9a | #Feature Sample 1F10a | #Feature F10b | #Feature F11a | #Feature F11b | #Feature F12a | #Feature F13 | Start | End | PTM | AScore | Found By |
|---|---|---|---|---|---|---|---|---|---|---|---|---|---|---|---|---|---|---|---|---|---|---|---|---|---|---|---|---|---|---|---|---|---|---|---|---|---|---|---|---|---|---|---|---|---|---|---|---|---|---|
| D.PNYGMVAPGTK.C | Y | 69.34 | 1133.5540 | 11 | 0.7 | 567.7847 | 2 | 38.92 | 16 | F16:15774 | BuCaTN01_F13.raw | | | | | | | | | | | | | | | | 4.636E6 | 1 | 0 | 0 | 0 | 0 | 0 | 0 | 0 | 0 | 0 | 0 | 0 | 0 | 0 | 0 | 0 | 1 | 584 | 594 | | | PEAKS DB |
| D.PNYGM(+15.99)VAPGTK.C | Y | 52.68 | 1149.5488 | 11 | 0.9 | 575.7822 | 2 | 30.43 | 16 | F16:10053 | BuCaTN01_F13.raw | | | | | | | | | | | | | | | | 4.4034E5 | 1 | 0 | 0 | 0 | 0 | 0 | 0 | 0 | 0 | 0 | 0 | 0 | 0 | 0 | 0 | 0 | 1 | 584 | 594 | Oxidation (M) | M5:Oxidation (M):100 0.00 | PEAKS DB |
| total 2 peptides | | | | | | | | | | | | | | | | | | | | | | | | | | | | | | | | | | | | | | | | | | | | | | | | | | | |

**Best Unique PSM (Scan F16:15774, m/z=567.7847, z=2, RT=38.92, ppm=0.7):**

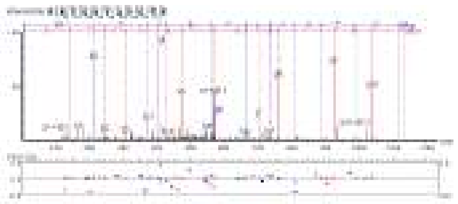

## Q10749.3

back to list

| Protein Coverage | Supporting Peptides | Best Unique PSM |

**Protein Coverage:**

```
  1 MIQALLVAIC LAVFPYQGSS IILESGNVND YEVVYPQKVP ALSKGGVQNP QPETKYEDTM QYEFHVNGEP VVLHLERNKG
 81 LFSEDYTETH YAPDGREITT SSPVQDHCYY HGYIQNEADS SAVISACDGL KGHFKHQGET YFIEPLELSD SEAHAIYKDE
161 NVEEEEEIPK ICGVTQTTWE SDEPIEKSSQ LTNTPEQDRY LQAKKYIEFY VVVDNVMYRK YTGKLHVITR RVYEMVNALN
241 TMYRRLNFHI ALIGLEIWSN GNEINVQSDV QATLDLFGEW RENKLLPRKR NDNAQLLTST EFNGTTTGLG YIGSLCSPKK
321 SVAVVQDHSK STSMVAITMA HQMGHNLGMN DDRASCTCGS NKCIMSTKYY ESLSEFSSCS VQEHREYLLR DRPQCILNKP
401 SRKAIVTPPV CGNYFVERGE ECDCGSPEDC QNTCCDAATC KLQHEAQCDS GECCEKCKFK GAGAECRAAK NDCDFPELCT
481 GRSAKCPKDS FQRNGHPCQN NQGYCYNGTC PTLTNQCATL WGPGAKMSPG LCFMLNWNAR SCGLCRKENG RKILCAAKDV
561 KCGRLFCKKK NSMICHCPPP SKDPNYGMVA PGTKCGVKKV CRNRQCVKV
```

O Oxidation (M) (+15.99)

588

**Supporting Peptides:**

| Peptide | Uniq | -10lgP | Mass | Length | ppm | m/z | z | RT | Fraction | Scan | Source File | Area F1 | Area F2a | Area F3a | Area F4 | Area F5 | Area F6a | Area F7a | Area F7b | Area F8 | Area F9a | Area Sample 1F10a | Area F10b | Area F11a | Area F11b | Area F12a | Area F13 | #Feature | #Feature F1 | #Feature F2a | #Feature F3a | #Feature F4 | #Feature F5 | #Feature F6a | #Feature F7a | #Feature F7b | #Feature F8 | #Feature F9a | #Feature Sample 1F10a | #Feature F10b | #Feature F11a | #Feature F11b | #Feature F12a | #Feature F13 | Start | End | PTM | AScore | Found By |
|---|---|---|---|---|---|---|---|---|---|---|---|---|---|---|---|---|---|---|---|---|---|---|---|---|---|---|---|---|---|---|---|---|---|---|---|---|---|---|---|---|---|---|---|---|---|---|---|---|---|
| D.PNYGMVAPGTK.C | Y | 69.34 | 1133.5540 | 11 | 0.7 | 567.7847 | 2 | 38.92 | 16 | F16:15774 | BuCaTN01_F13.raw | | | | | | | | | | | | | | | | 4.636E6 | 1 | 0 | 0 | 0 | 0 | 0 | 0 | 0 | 0 | 0 | 0 | 0 | 0 | 0 | 0 | 0 | 1 | 584 | 594 | | | PEAKS DB |
| D.PNYGM(+15.99)VAPGTK.C | Y | 52.68 | 1149.5488 | 11 | 0.9 | 575.7822 | 2 | 30.43 | 16 | F16:10053 | BuCaTN01_F13.raw | | | | | | | | | | | | | | | | 4.4034E5 | 1 | 0 | 0 | 0 | 0 | 0 | 0 | 0 | 0 | 0 | 0 | 0 | 0 | 0 | 0 | 0 | 1 | 584 | 594 | Oxidation (M) | M5:Oxidation (M):1000.00 | PEAKS DB |
| total 2 peptides | | | | | | | | | | | | | | | | | | | | | | | | | | | | | | | | | | | | | | | | | | | | | | | | | |

**Best Unique PSM (Scan F16:15774, m/z=567.7847, z=2, RT=38.92, ppm=0.7):**

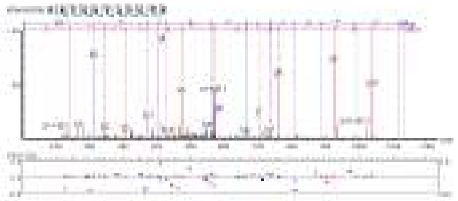

## 1CDT

back to list

| Protein Coverage | Supporting Peptides | Best Unique PSM |

**Protein Coverage:**

3 14

```
  1 LKCNKLIPIA YKTCPEGKNL CYKMMLASKK MVPVKRGCIN VCPKNSALVK YVCCSTDRCN
```

C Carbamidomethylation (+57.02)

**Supporting Peptides:**

| Peptide | Uniq | -10lgP | Mass | Length | ppm | m/z | z | RT | Fraction | Scan | Source File | Area F1 | Area F2a | Area F3a | Area F4 | Area F5 | Area F6a | Area F7a | Area F7b | Area F8 | Area F9a | Area Sample 1F10a | Area F10b | Area F11a | Area F11b | Area F12a | Area F13 | #Feature | #Feature F1 | #Feature F2a | #Feature F3a | #Feature F4 | #Feature F5 | #Feature F6a | #Feature F7a | #Feature F7b | #Feature F8 | #Feature F9a | #Feature Sample 1F10a | #Feature F10b | #Feature F11a | #Feature F11b | #Feature F12a | #Feature F13 | Start | End | PTM | AScore | Found By |
|---|---|---|---|---|---|---|---|---|---|---|---|---|---|---|---|---|---|---|---|---|---|---|---|---|---|---|---|---|---|---|---|---|---|---|---|---|---|---|---|---|---|---|---|---|---|---|---|---|---|
| LKC(+57.02)NKLIPIAYK.T | N | 45.23 | 1459.8584 | 12 | 1.4 | 365.9713 | 4 | 47.63 | 8 | F8:24407 | BuCaTN01_F7b.raw | | | | | | | | 1.2604E5 | | | | | | | | | 1 | 0 | 0 | 0 | 0 | 0 | 0 | 0 | 1 | 0 | 0 | 0 | 0 | 0 | 0 | 0 | 0 | 1 | 12 | Carbamidomethylation | C3:Carbamidomethylation:1000.00 | PEAKS DB |
| K.LIPIAYKTC(+57.02)PEGK.N | Y | 42.68 | 1488.8010 | 13 | 2.7 | 497.2742 | 3 | 44.28 | 8 | F8:21159 | BuCaTN01_F7b.raw | | | | | | | | 2.9139E5 | | | | | | | | | 1 | 0 | 0 | 0 | 0 | 0 | 0 | 0 | 1 | 0 | 0 | 0 | 0 | 0 | 0 | 0 | 0 | 6 | 18 | Carbamidomethylation | C9:Carbamidomethylation:1000.00 | PEAKS DB |
| total 2 peptides | | | | | | | | | | | | | | | | | | | | | | | | | | | | | | | | | | | | | | | | | | | | | | | | | |

**Best Unique PSM (Scan F8:21159, m/z=497.2742, z=3, RT=44.28, ppm=2.7):**

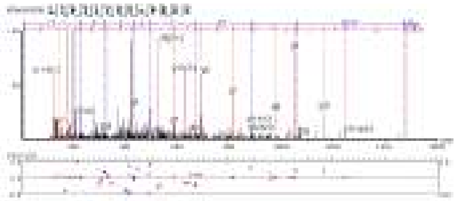

## P01452.1

back to list

| Protein Coverage | Supporting Peptides | Best Unique PSM |

**Protein Coverage:**

3 14

```
  1 LKCNKLIPIA YKTCPEGKNL CYKMMLASKK MVPVKRGCIN VCPKNSALVK YVCCSTDRCN
```

C Carbamidomethylation (+57.02)

**Supporting Peptides:**

| Peptide | Uniq | -10lgP | Mass | Length | ppm | m/z | z | RT | Fraction | Scan | Source File | Area F1 | Area F2a | Area F3a | Area F4 | Area F5 | Area F6a | Area F7a | Area F7b | Area F8 | Area F9a | Area Sample 1F10a | Area F10b | Area F11a | Area F11b | Area F12a | Area F13 | #Feature | #Feature F1 | #Feature F2a | #Feature F3a | #Feature F4 | #Feature F5 | #Feature F6a | #Feature F7a | #Feature F7b | #Feature F8 | #Feature F9a | #Feature Sample 1F10a | #Feature F10b | #Feature F11a | #Feature F11b | #Feature F12a | #Feature F13 | Start | End | PTM | AScore | Found By |
|---|---|---|---|---|---|---|---|---|---|---|---|---|---|---|---|---|---|---|---|---|---|---|---|---|---|---|---|---|---|---|---|---|---|---|---|---|---|---|---|---|---|---|---|---|---|---|---|---|---|
| LKC(+57.02)NKLIPIAYK.T | N | 45.23 | 1459.8584 | 12 | 1.4 | 365.9713 | 4 | 47.63 | 8 | F8:24407 | BuCaTN01_F7b.raw | | | | | | | | 1.2604E5 | | | | | | | | | 1 | 0 | 0 | 0 | 0 | 0 | 0 | 0 | 1 | 0 | 0 | 0 | 0 | 0 | 0 | 0 | 0 | 1 | 12 | Carbamidomethylation | C3:Carbamidomethylation:1000.00 | PEAKS DB |
| K.LIPIAYKTC(+57.02)PEGK.N | Y | 42.68 | 1488.8010 | 13 | 2.7 | 497.2742 | 3 | 44.28 | 8 | F8:21159 | BuCaTN01_F7b.raw | | | | | | | | 2.9139E5 | | | | | | | | | 1 | 0 | 0 | 0 | 0 | 0 | 0 | 0 | 1 | 0 | 0 | 0 | 0 | 0 | 0 | 0 | 0 | 6 | 18 | Carbamidomethylation | C9:Carbamidomethylation:1000.00 | PEAKS DB |
| total 2 peptides | | | | | | | | | | | | | | | | | | | | | | | | | | | | | | | | | | | | | | | | | | | | | | | | | |

**Best Unique PSM (Scan F8:21159, m/z=497.2742, z=3, RT=44.28, ppm=2.7):**

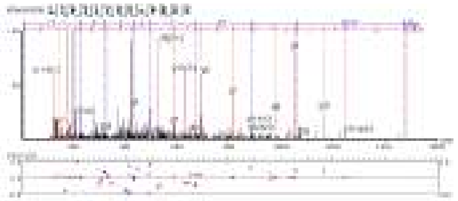

## XP_026579252.1

back to list

| Protein Coverage | Supporting Peptides | Best Unique PSM |

**Protein Coverage:**

```
 1 MSSGGLLLLL GLLTLWAELT PVSGQDRPKY CDLPANPGPC RAKITRFYYN SDSKRCEKFI YGGCHGNANN FETKEKCHYT
81 CVEKPGVCPK AHPGTITICP VRCQSDWQCD EKRKCCTYAC ITDCMDAI
```

C Carbamidomethylation (+57.02)

99

**Supporting Peptides:**

| Peptide | Uniq | -10lgP | Mass | Length | ppm | m/z | z | RT | Fraction | Scan | Source File | Area F1 | Area F2a | Area F3a | Area F4 | Area F5 | Area F6a | Area F7a | Area F7b | Area F8 | Area F9a | Area Sample 1F10a | Area F10b | Area F11a | Area F11b | Area F12a | Area F13 | #Feature | #Feature F1 | #Feature F2a | #Feature F3a | #Feature F4 | #Feature F5 | #Feature F6a | #Feature F7a | #Feature F7b | #Feature F8 | #Feature F9a | #Feature Sample 1F10a | #Feature F10b | #Feature F11a | #Feature F11b | #Feature F12a | #Feature F13 | Start | End | PTM | AScore | Found By |
|---|---|---|---|---|---|---|---|---|---|---|---|---|---|---|---|---|---|---|---|---|---|---|---|---|---|---|---|---|---|---|---|---|---|---|---|---|---|---|---|---|---|---|---|---|---|---|---|---|---|
| K.AHPGTITIC(+57.02)PVR.C | Y | 66.07 | 1320.6973 | 12 | 2.4 | 441.2395 | 3 | 40.88 | 8 | F8:18553 | BuCaTN01_F7b.raw | | | | | | 3.1615E6 | 5.8354E4 | 1.8042E6 | | | | | | | | | 3 | 0 | 0 | 0 | 0 | 0 | 1 | 1 | 1 | 0 | 0 | 0 | 0 | 0 | 0 | 0 | 0 | 91 | 102 | Carbamidomethylation | C9:Carbamidomethylation:1000.00 | PEAKS DB |
| total 1 peptides | | | | | | | | | | | | | | | | | | | | | | | | | | | | | | | | | | | | | | | | | | | | | | | | | |

**Best Unique PSM (Scan F8:18553, m/z=441.2395, z=3, RT=40.88, ppm=2.4):**

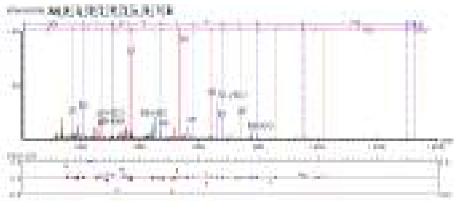

## XP_034285002.1

back to list

| Protein Coverage | Supporting Peptides | Best Unique PSM |

**Protein Coverage:**

```
   1 MSINGSHPRL NTLGRMVRSE SGPDMRYDMN SHMMMGGGGG GGGGGGSSHA HKMYYVQKCY VGDINSEGYT QNGTATVSRR
  81 QNTIQDLLQA CSDCLMRAEL IVQPELKYGD GIQISRNREL EDCFGQANEH MDILDGLIRE MRQMGQPCDL YQKRLLQLQE
 161 QMRALYKAIS VPRVRRASSK GGGYSSQSAS GWDEYTRRVT SECLNWMRQQ KTEMELVKWG FDAAAIEQQI AEHRRFHNSI
 241 GDYRWHLDKV KTDLREKAAV HQLEEEYEYL LRFSFERMDQ LRQLQNIIQA TSREIMWIND CEEEELLYDW SDRNTDIARK
 321 QEVFSKRMSQ LEVKEKELNK LKQECDQLVL SQHPASDKIE AYMDTLQTQW SWILQITKCI DVHLKENAAY FQFFEEAHST
 401 ENYLKNLQDA IRKRFICDKN MSLQVLLEQI KELEAEREKI LEYKRQVQNL VNKSKKIVQL KPRNPEYKSN KPIILRALCD
 481 YKQDLKTMRK GDECILKDNS ERSKWHVTGP GGLDMLVPSV SLIIPPPNPL AVDLATKIEQ YYDAIMALWN QLYINMKSLV
 561 SWHYCMIDVE KIRAMTIAKL KTMRREDYQR IIADLEIHYQ EFIRNSQGSE MFGDDDKRNM QTQITDAQKH YQTLVMTLPR
 641 QTQTGPPIVT EVITIEKEKQ EAWLLMELQK LRRQIENCEF RMVQRTPFQL DQGASHNLSV RISELESIQI ESQSMAEALN
 721 RQKDYLPNFR GSEKYHYLQS EISALFQKLE NINGVSACYL DSLNALRSLL QAILQTDDVI RVFEIRLTEE DTLSLDPDKV
 801 EGYRVCLKKM KADLTMKKSL LGTLEAELQK ALQVHSQSSQ TYPHYDLDLG KFADRVSQLR DRWQRLEKQL DDRSWDLEKQ
 881 VKQLRIYREL YQALNKWICD ARRRQDTIEA MKLGDVSTVM RYLQEQKNLH SEITSKRDRV EDVVKNAEVC SLAIKDYELQ
 961 AAAYSSGLET LLNIPVKRSM VQSPSGLILQ EAGEIQSRYI ELLTRSGDYY KFLSEMLKSL EDIKMKSTRI ELLEEELRLA
1041 KDANSDSNNK HKFLEQNLQK YQVECSQLKA KFISLEEMKR QVEMDGSTAK QNLDKCYAQI KDLNERITRL TYEIEDEKRK
1121 RKLLEDRYDQ QKSDFDQLQK TRHNEKETLG WQKIESEKII KEKEYEIERL RVLLQDEGTR KRECENELAK TSHRIQESKS
1201 QCNHIMQERE TLLVKIKTLE QDKARLLRLE EDLNRTKGML ESESRLKARL ENEKQQILND LNQWKSQYSR KEETIRKTEC
1281 ERDKSEREKS SLLIEIERLQ AEIKRIEERY RCRLEETSRK SQAEMETERS RWQKEIDKLK KRPYGAHRST QTEEDLMIDP
1361 SKLLFNGLRK KITAVQLYEC QLIDKCTLDK LLRGQKSVEE VAAELEPYLK GAGAIAGASL NTKEKYSLVE AKRKQLLTPE
1441 NTILLLEAQA ATGGVIDPHR NELLSVDSAV ARDLVDFDDR EQIYMAEKAI TGFKDPFSGK TMSVAEAIKK NLVDRETGMR
1521 LLEAQLASGG IVDPINSIFL PKDIALSRGL IDRDLYRALN NPSKPLIDPL SKNAISYMKL RERCRIEPHT GLLLLPVQKR
1601 SMSFQGIRKL ITVSELVDSG IIRESTANEL ETGAITVEEV TDRIKDFLQG SSCIAGIYNE ATGEKLGVYQ AMKIGLVRPG
1681 TALELLEAQA ATGFIVDPVN NLRLPVEEGY KRGLVGIEFK EKLLSAERAV TGYRDPETGN IISLFQAMNK ELIEKGHGVR
1761 LLEAQIATGG IIDPKESHRL PVHTAYQRGY FNEELNDILS DPSDDTKGFF DPNTEENLTY LQLKGRCKID ELTGLCLLPL
1841 REKKKVVQTS QKNTLRKRRV VIVDPDTSKE MSVQEAYNKG LIDYSTYMEL SDQECEWEEI TTTGSDGSTR VVLVDRKTGH
1921 QYDIEDAIEK GLVDRKFFEQ YRSGSLSLTQ FADMIASRNG GDEVFKHESI IRSPTVLSVK SSSILLKSSS GSFSDSPEEA
2001 TPIAAIFDTE NLEKISIPEA VQRGIVDTIT AQRLLEAQSC TGGIICPTTG QRLSLQDAVA QGIIDHEMAT RLKPAQKAFL
2081 GFEGIKGRKR MSAAEAVKEK WLPYEAGQRF LEFQFITGGL VDPEVHGRIS TEEAIRKGLI DGRTSQRLQD IGNYAKILTC
2161 PKTKLKISYR EAMNRSMVED QTGLRLLEAS SVSSKGISSP YNVSSAPGSR SGSRSGSRSG SRSGSRRGSF DAGTSSSYSY
2241 SYTTVSSGSL GR
```

**Supporting Peptides:**

| Peptide | Uniq | -10lgP | Mass | Length | ppm | m/z | z | RT | Fraction | Scan | Source File | Area F1 | Area F2a | Area F3a | Area F4 | Area F5 | Area F6a | Area F7a | Area F7b | Area F8 | Area F9a | Area Sample 1F10a | Area F10b | Area F11a | Area F11b | Area F12a | Area F13 | #Feature | #Feature F1 | #Feature F2a | #Feature F3a | #Feature F4 | #Feature F5 | #Feature F6a | #Feature F7a | #Feature F7b | #Feature F8 | #Feature F9a | #Feature Sample 1F10a | #Feature F10b | #Feature F11a | #Feature F11b | #Feature F12a | #Feature F13 | Start | End | PTM | AScore | Found By |
|---|---|---|---|---|---|---|---|---|---|---|---|---|---|---|---|---|---|---|---|---|---|---|---|---|---|---|---|---|---|---|---|---|---|---|---|---|---|---|---|---|---|---|---|---|---|---|---|---|---|
| R.LTYEIEDEKR.K | Y | 47.12 | 1294.6405 | 10 | 8.7 | 432.5566 | 3 | 38.20 | 8 | F8:16270 | BuCaTN01_F7b.raw | | | | | | | | 0 | 9.2313E4 | | | | | | | | 1 | 0 | 0 | 0 | 0 | 0 | 0 | 0 | 0 | 1 | 0 | 0 | 0 | 0 | 0 | 0 | 0 | 1110 | 1119 | | | PEAKS DB |
| total 1 peptides | | | | | | | | | | | | | | | | | | | | | | | | | | | | | | | | | | | | | | | | | | | | | | | | | |

**Best Unique PSM (Scan F8:16270, m/z=432.5566, z=3, RT=38.20, ppm=8.7):**

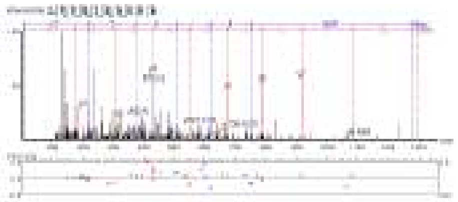

## XP_034285001.1

back to list

| Protein Coverage | Supporting Peptides | Best Unique PSM |

**Protein Coverage:**

1 MSINGSHPRL NTLGRMVRSE SGPDMRYDMN SHMMMGGGGG GGGGGGSSHA HKMYYVQKCY VGDINSEGYT QNGTATVSRR
81 QNTIQDLLQA CSDCLMRAEL IVQPELKYGD GIQISRNREL EDCFGQANEH MDILDGLIRE MRQMGQPCDL YQKRLLQLQE
161 QMRALYKAIS VPRVRRASSK GGGYSSQSAS GWDEYTRRVT SECLNWMRQQ KTEMELVKWG FDAAAIEQQI AEHRRFHNSI
241 GDYRWHLDKV KTDLREKAAV HQLEEEYEYL LRFSFERMDQ LRQLQNIIQA TSREIMWIND CEEEELLYDW SDRNTDIARK
321 QEVFSKRMSQ LEVKEKELNK LKQECDQLVL SQHPASDKIE AYMDTLQTQW SWILQITKCI DVHLKENAAY FQFFEEAHST
401 ENYLKNLQDA IRKRFICDKN MSLQVLLEQI KELEAEREKI LEYKRQVQNL VNKSKKIVQL KPRNPEYKSN KPIILRALCD
481 YKQDLKTMRK GDECILKDNS ERSKWHVTGP GGLDMLVPSV SLIIPPPNPL AVDLATKIEQ YYDAIMALWN QLYINMKSLV
561 SWHYCMIDVE KIRAMTIAKL KTMRREDYQR IIADLEIHYQ EFIRNSQGSE MFGDDDKRNM QTQITDAQKH YQTLVMTLPR
641 QTQTGPPIVT EVITIEKEKQ EAWLLMELQK LRRQIENCEF RMVQRTPFQL DQGASHNLSV RISELESIQI ESQSMAEALN
721 RQKDYLPNFR GSEKYHYLQS EISALFQKLE NINGVSACYL DSLNALRSLL QAILQTDDVI RVFEIRLTEE DTLSLDPDKV
801 EGYRVCLKKM KADLTMKKSL LGTLEAELQK ALQVHSQSSQ TYPHYDLDLG KFADRVSQLR DRWQRLEKQL DDRSWDLEKQ
881 VKQLRIYREL YQALNKWICD ARRRQDTIEA MKLGDVSTVM RYLQEQKNLH SEITSKRDRV EDVVKNAEVC SLAIKDYELQ
961 AAAYSSGLET LLNIPVKRSM VQSPSGLILQ EAGEIQSRYI ELLTRSGDYY KFLSEMLKSL EDIKMKSTRI ELLEEELRLA
1041 KDANSDSNNK HKFLEQNLQK YQVECSQLKA KFISLEEMKR QVEMDGSTAK QNLDKCYAQI KDLNERITR**L TYEIEDEKR**K
1121 RKLLEDRYDQ QKSDFDQLQK TRHNEKETLG WQKIESEKII KEKEYEIERL RVLLQDEGTR KRECENELAK VRNQFNEEMS
1201 NIKNKYETEI NIKKTTIHQI AAQKEDDAKN LRTQIERLQR ENRDLKDEIV RLNEAILDTT EQRRRAEENV LQQKACGSEV
1281 IQQKQQIELE LKQIIQLRND DNTRYKQALE DATSTIHEKV KELERLKIQL QEEAKSRWEL ENELAKTSHR IQESKSQCNH
1361 IMQERETLLV KIKTLEQDKA RLLRLEEDLN RTKGMLESES RLKARLENEK QQILNDLNQW KSQYSRKEET IRKTECERDK
1441 SEREKSSLLI EIERLQAEIK RIEERYRCRL EETSRKSQAE METERSRWQK EIDKLKKRPY GAHRSTQTEE DLMIDPSKLL
1521 FNGLRKKITA VQLYECQLID KCTLDKLLRG QKSVEEVAAE LEPYLKGAGA IAGASLNTKE KYSLVEAKRK QLLTPENTIL
1601 LLEAQAATGG VIDPHRNELL SVDSAVARDL VDFDDREQIY MAEKAITGFK DPFSGKTMSV AEAIKKNLVD RETGMRLLEA
1681 QLASGGIVDP INSIFLPKDI ALSRGLIDRD LYRALNNPSK PLIDPLSKNA ISYMKLRERC RIEPHTGLLL LPVQKRSMSF
1761 QGIRKLITVS ELVDSGIIRE STANELETGA ITVEEVTDRI KDFLQGSSCI AGIYNEATGE KLGVYQAMKI GLVRPGTALE
1841 LLEAQAATGF IVDPVNNLRL PVEEGYKRGL VGIEFKEKLL SAERAVTGYR DPETGNIISL FQAMNKELIE KGHGVRLLEA
1921 QIATGGIIDP KESHRLPVHT AYQRGYFNEE LNDILSDPSD DTKGFFDPNT EENLTYLQLK GRCKIDELTG LCLLPLREKK
2001 KVVQTSQKNT LRKRRVVIVD PDTSKEMSVQ EAYNKGLIDY STYMELSDQE CEWEEITTTG SDGSTRVVLV DRKTGHQYDI
2081 EDAIEKGLVD RKFFEQYRSG SLSLTQFADM IASRNGGDEV FKHESIIRSP TVLSVKSSSI LLKSSSGSFS DSPEEATPIA
2161 AIFDTENLEK ISIPEAVQRG IVDTITAQRL LEAQSCTGGI ICPTTGQRLS LQDAVAQGII DHEMATRLKP AQKAFLGFEG
2241 IKGRKRMSAA EAVKEKWLPY EAGQRFLEFQ FITGGLVDPE VHGRISTEEA IRKGLIDGRT SQRLQDIGNY AKILTCPKTK
2321 LKISYREAMN RSMVEDQTGL RLLEASSVSS KGISSPYNVS SAPGSRSGSR SGSRSGSRSG SRRGSFDAGT SSSYSYSYTT
2401 VSSGSLGR

**Supporting Peptides:**

| Peptide | Uniq | -10lgP | Mass | Length | ppm | m/z | z | RT | Fraction | Scan | Source File | Area F1 | Area F2a | Area F3a | Area F4 | Area F5 | Area F6a | Area F7a | Area F7b | Area F8 | Area F9a | Area Sample 1F10a | Area F10b | Area F11a | Area F11b | Area F12a | Area F13 | #Feature | #Feature F1 | #Feature F2a | #Feature F3a | #Feature F4 | #Feature F5 | #Feature F6a | #Feature F7a | #Feature F7b | #Feature F8 | #Feature F9a | #Feature Sample 1F10a | #Feature F10b | #Feature F11a | #Feature F11b | #Feature F12a | #Feature F13 | Start | End | PTM | AScore | Found By |
|---|---|---|---|---|---|---|---|---|---|---|---|---|---|---|---|---|---|---|---|---|---|---|---|---|---|---|---|---|---|---|---|---|---|---|---|---|---|---|---|---|---|---|---|---|---|---|---|---|---|
| R.LTYEIEDEKR.K | Y | 47.12 | 1294.6405 | 10 | 8.7 | 432.5566 | 3 | 38.20 | 8 | F8:16270 | BuCaTN01_F7b.raw | | | | | | | | 0 | 9.2313E4 | | | | | | | | 1 | 0 | 0 | 0 | 0 | 0 | 0 | 0 | 0 | 1 | 0 | 0 | 0 | 0 | 0 | 0 | 0 | 1110 | 1119 | | | PEAKS DB |
| total 1 peptides | | | | | | | | | | | | | | | | | | | | | | | | | | | | | | | | | | | | | | | | | | | | | | | | | |

**Best Unique PSM (Scan F8:16270, m/z=432.5566, z=3, RT=38.20, ppm=8.7):**

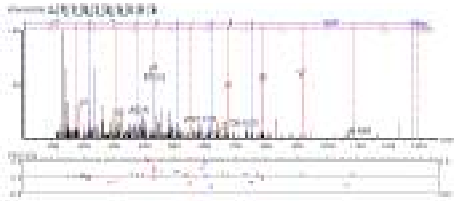

## XP_034285000.1

back to list

| Protein Coverage | Supporting Peptides | Best Unique PSM |

**Protein Coverage:**

1 MSINGSHPRL NTLGRMVRSE SGPDMRYDMN SHMMMGGGGG GGGGGGSSHA HKMYYVQKCY VGDINSEGYT QNGTATVSRR
81 QNTIQDLLQA CSDCLMRAEL IVQPELKYGD GIQISRNREL EDCFGQANEH MDILDGLIRE MRQMGQPCDL YQKRLLQLQE
161 QMRALYKAIS VPRVRRASSK GGGYSSQSAS GWDEYTRRVT SECLNWMRQQ KTEMELVKWG FDAAAIEQQI AEHRRFHNSI
241 GDYRWHLDKV KTDLREKAAV HQLEEEYEYL LRFSFERMDQ LRQLQNIIQA TSREIMWIND CEEEELLYDW SDRNTDIARK
321 QEVFSKRMSQ LEVKEKELNK LKQECDQLVL SQHPASDKIE AYMDTLQTQW SWILQITKCI DVHLKENAAY FQFFEEAHST
401 ENYLKNLQDA IRKRFICDKN MSLQVLLEQI KELEAEREKI LEYKRQVQNL VNKSKKIVQL KPRNPEYKSN KPIILRALCD
481 YKQDLKTMRK GDECILKDNS ERSKWHVTGP GGLDMLVPSV SLIIPPPNPL AVDLATKIEQ YYDAIMALWN QLYINMKSLV
561 SWHYCMIDVE KIRAMTIAKL KTMRREDYQR IIADLEIHYQ EFIRNSQGSE MFGDDDKRNM QTQITDAQKH YQTLVMTLPR
641 QTQTGPPIVT EVITIEKEKQ EAWLLMELQK LRRQIENCEF RMVQRTPFQL DQGASHNLSV RISELESIQI ESQSMAEALN
721 RQKDYLPNFR GSEKYHYLQS EISALFQKLE NINGVSACYL DSLNALRSLL QAILQTDDVI RVFEIRLTEE DTLSLDPDKV
801 EGYRVCLKKM KADLTMKKSL LGTLEAELQK ALQVHSQSSQ TYPHYDLDLG KFADRVSQLR DRWQRLEKQL DDRSWDLEKQ
881 VKQLRIYREL YQALNKWICD ARRRQDTIEA MKLGDVSTVM RYLQEQKNLH SEITSKRDRV EDVVKNAEVC SLAIKDYELQ
961 AAAYSSGLET LLNIPVKRSM VQSPSGLILQ EAGEIQSRYI ELLTRSGDYY KFLSEMLKSL EDIKMKSTRI ELLEEELRLA
1041 KDANSDSNNK HKFLEQNLQK YQVECSQLKA KFISLEEMKR QVEMDGSTAK QNLDKCYAQI KDLNERITR**L TYEIEDEKR**K
1121 RKLLEDRYDQ QKSDFDQLQK TRHNEKETLG WQKIESEKII KEKEYEIERL RVLLQDEGTR KRECENELAK VRNQFNEEMS
1201 NIKNKYETEI NIKKTTIHQI AAQKEDDAKN LRTQIERLQR ENRDLKDEIV RLNEAILDTT EQRRRAEENV LQQKACGSEV
1281 IQQKQQIELE LKQIIQLRND DNTRYKQALE DATSTIHEKV KELERLKIQL QEEAKSRWEL ENELAKVRNS YDEEIISLKN
1361 KYETEINITK TTINQVTVKK EEEASNYRAQ LDNAVRENRS LCEEIRRLKN TLSQTTENLR KVEENAHQQK AIGSEISQKK
1441 QHLEKELKQT VQKFTEEIAH YKQSLDEAGR TLKEKNKEIE KNRKLLDAEI CQRKSLEEEN IRLQRIQYDL EKANINATET
1521 ISRLKVQEQD LGRLKSEYER LAQDKKGKDQ ESTRLQSTIK EMQHQKHMLE EELNRQSRIA ADETSKRKNA EEELEAMRRT
1601 LREQAVKITN LTQQIEEISI VKKRSEDDLR HQREVLDSQM RDKQRYMEEI RKYTSEVEAL RRQMVQEQEN LKQAHIRNEH
1681 LQKAIEDKSK SLNECKIEIE RLQCLTENLT KEHLMLEEEL RNLRLEYDDL RRGRSEVDEE KNATILDLKN QLQTSNKHAL
1761 ELQGVINGLQ KERENLRQEI EKFQKQALET SHRIQESKSQ CNHIMQERET LLVKIKTLEQ DKARLLRLEE DLNRTKGMLE
1841 SESRLKARLE NEKQQILNDL NQWKSQYSRK EETIRKTECE RDKSEREKSS LLIEIERLQA EIKRIEERYR CRLEETSRKS
1921 QAEMETERSR WQKEIDKLKK RPYGAHRSTQ TEEDLMIDPS KLLFNGLRKK ITAVQLYECQ LIDKCTLDKL LRGQKSVEEV
2001 AAELEPYLKG AGAIAGASLN TKEKYSLVEA KRKQLLTPEN TILLLEAQAA TGGVIDPHRN ELLSVDSAVA RDLVDFDDRE
2081 QIYMAEKAIT GFKDPFSGKT MSVAEAIKKN LVDRETGMRL LEAQLASGGI VDPINSIFLP KDIALSRGLI DRDLYRALNN
2161 PSKPLIDPLS KNAISYMKLR ERCRIEPHTG LLLLPVQKRS MSFQGIRKLI TVSELVDSGI IRESTANELE TGAITVEEVT
2241 DRIKDFLQGS SCIAGIYNEA TGEKLGVYQA MKIGLVRPGT ALELLEAQAA TGFIVDPVNN LRLPVEEGYK RGLVGIEFKE
2321 KLLSAERAVT GYRDPETGNI ISLFQAMNKE LIEKGHGVRL LEAQIATGGI IDPKESHRLP VHTAYQRGYF NEELNDILSD
2401 PSDDTKGFFD PNTEENLTYL QLKGRCKIDE LTGLCLLPLR EKKKVVQTSQ KNTLRKRRVV IVDPDTSKEM SVQEAYNKGL
2481 IDYSTYMELS DQECEWEEIT TTGSDGSTRV VLVDRKTGHQ YDIEDAIEKG LVDRKFFEQY RSGSLSLTQF ADMIASRNGG
2561 DEVFKHESII RSPTVLSVKS SSILLKSSSG SFSDSPEEAT PIAAIFDTEN LEKISIPEAV QRGIVDTITA QRLLEAQSCT
2641 GGIICPTTGQ RLSLQDAVAQ GIIDHEMATR LKPAQKAFLG FEGIKGRKRM SAAEAVKEKW LPYEAGQRFL EFQFITGGLV
2721 DPEVHGRIST EEAIRKGLID GRTSQRLQDI GNYAKILTCP KTKLKISYRE AMNRSMVEDQ TGLRLLEASS VSSKGISSPY
2801 NVSSAPGSRS GSRSGSRSGS RSGSRRGSFD AGTSSSYSYS YTTVSSGSLG R

**Supporting Peptides:**

| Peptide | Uniq | -10lgP | Mass | Length | ppm | m/z | z | RT | Fraction | Scan | Source File | Area F1 | Area F2a | Area F3a | Area F4 | Area F5 | Area F6a | Area F7a | Area F7b | Area F8 | Area F9a | Area Sample 1F10a | Area F10b | Area F11a | Area F11b | Area F12a | Area F13 | #Feature | #Feature F1 | #Feature F2a | #Feature F3a | #Feature F4 | #Feature F5 | #Feature F6a | #Feature F7a | #Feature F7b | #Feature F8 | #Feature F9a | #Feature Sample 1F10a | #Feature F10b | #Feature F11a | #Feature F11b | #Feature F12a | #Feature F13 | Start | End | PTM | AScore | Found By |
|---|---|---|---|---|---|---|---|---|---|---|---|---|---|---|---|---|---|---|---|---|---|---|---|---|---|---|---|---|---|---|---|---|---|---|---|---|---|---|---|---|---|---|---|---|---|---|---|---|---|
| R.LTYEIEDEKR.K | Y | 47.12 | 1294.6405 | 10 | 8.7 | 432.5566 | 3 | 38.20 | 8 | F8:16270 | BuCaTN01_F7b.raw | | | | | | | | 0 | 9.2313E4 | | | | | | | | 1 | 0 | 0 | 0 | 0 | 0 | 0 | 0 | 0 | 1 | 0 | 0 | 0 | 0 | 0 | 0 | 0 | 1110 | 1119 | | | PEAKS DB |
| total 1 peptides | | | | | | | | | | | | | | | | | | | | | | | | | | | | | | | | | | | | | | | | | | | | | | | | | |

**Best Unique PSM (Scan F8:16270, m/z=432.5566, z=3, RT=38.20, ppm=8.7):**

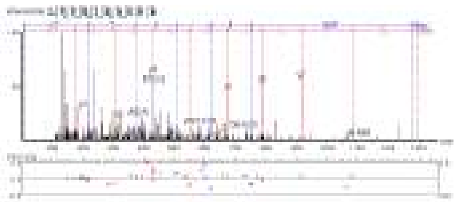

## JAC95787.1

back to list

| Protein Coverage | Supporting Peptides | Best Unique PSM |

**Protein Coverage:**

```
   1 MSINGSHPRL NTLGRMVRAE SGPDMRYDMN SHMMMGGGGG GGGSGSHTHK MYYVQKSYVG DINSDGYTQN GTATMSRRQN
  81 TIQDLLQACS DCLMRAELIV QPELKYGDGI QISRNRELED CFGQANEHMD ILDGLIREMR QMGQPCDLYQ KRLLQLQEQM
 161 RALYKAISVP RVRRASSKGG GYSSQSASGW DEYTRRVTSE CLNWMRQQKT EMELVKWGFD AAAIEQQIAE HRRCHNSIGD
 241 YRWHLDKVKT DLREKAAVHQ LEEEYEYLLR FSFERMDQQR QLQNIIQATS REIMWINDCE EEELLYDWSD RNTDIARKQE
 321 AFSKRMSQLE VKEKELNKLK QECDQLVLSQ HPASDKIEAY MDTLQTQWSW ILQITKCIDV HLKENAAYFQ FFEEAQSTEN
 401 YLKNLQDAIR KRFICDKNMS LQVLLEQIKE LETEREKILE YKRQVQNLVN KSKKIVQLKP RNPDYKSNKA IMLKALCDYK
 481 QDLKTMRKGD ECILKDNSER SKWHVTGPGG LDMLVPSVSL IIPPPNPLAV DLATKIEQYY DAIMALWNQL YINMKSLVSW
 561 HYCMIDIEKI RAMTIAKLKT MRKEDYQRII ADLEIHYQEF IRNSQGSEMF GDDDKRTMQT QITDAQKHYQ TLIIQIPNHG
 641 HPPPPPGPGI ITIETEKQEA WMLMELQKLR RQIENCEFRM LQRTPFQLDQ GASHNLSVRI SELESIAIES QSMADALNRQ
 721 KDYLPNFRGS EKYHYLQSEI SALFQKLENI NGVSACYLDS LNALRSLLQA ILQTDDVIRV FEIRLTEEDT LSLDPDKVEA
 801 YRVCLKKMKA DLSMKKSLLG TLEAELQKAL QVHSQSSQTY PHYDLDLGKF ADRVSQLRDR WQRLEKQLDD RSWDLEKQVK
 881 QLRIYRELYQ ALNKWICDAR RRQDTIEAMK LGDVSTVMRY LQEQKNLHTE IKSKRDKVEE VIKNAEVCSL AIKDYELQAA
 961 AYSSGLETLL NIPVKRSMVQ SPSGLILQEA GDIQSRYIEL LTKSGDYYKF LSEMLKSLED IKMKSTRIEL LEEELRLSKD
1041 ANSDSNNKHK FLEQNLHKYQ VECSQLKAKF ISLEEMKRQV EMDGSTAKQN LDKCYAQIKD LNDRITRLTY EIEDEKRKRK
1121 LLEDRYDQQK SDFDQLQKTR HNEKETLGWQ KIESEKIIKE KEYEIERLRV LLQDEGTRKR ECENELAKTS HRIQESKSQC
1201 NHIMQERETL LVKIKTMEQD KARMLRLEED LNRTKGMLES ESRLKVRLEN EKQQILNDLN QWKSQYSRKE ETTRLAECER
1281 DKSEREKSAL SIEIDRLQAE IKRIEERYRC RLEETSRKSQ ADMETERLRW QREYDKLKKR ACGTPRGTQT EEDLMIDPSK
1361 LLFSGLRKKI TAVQLYECQL IDKCTLDKLL KGQKSVEEVA AELEPYLRGA GAIAGVYVNL KQKYSLVEAK RKQLLTPENT
1441 ILLLEAQAAT GGVIDPHRNE LLSVDSAVAR DLIDFDDREQ IYMAEKAITG FKDPFSGKTM CVAEAMKKNL VDRETGMRLL
1521 EAQLATGGIV DPINSIFLPK DIALSRGLID RDLYRALNNP SKPLIEPLKN KAISYVMLRE KCRIEPHTGL LLLSVQKRTT
1601 SFQGIRKLIS LSELVNSGII RESTANELET GVITVEEVTD RIKDFLQGSS CIAGIYNEAT GEKLGVYQAM KIGLVRPGTA
1681 LELLEAQAAT GFIVDPVNNL RLPVEEAYKR GLVGIEFKEK LLSAEKAVTG YKDPETGNII SLFQAMNKEL IEKGHGVRLL
1761 EAQIATGGII DPKESHRLPV HTAYQRGYFN EELNDILSDP NDDTKGFFDP NTEENLTYLQ LKGRCKIDEL TGLCLLPLRE
1841 KKKVVQTSQK NTLRKRRVVI VDPDTNKEMS VQEAYNKGLI DYSTYMELSD QECEWEEITT TGSDGSTRVV LVDRKTGHQY
1921 DIEDAIDKGL VDRKFFDQYR SGSLSLTQFA DMIASRNGGD EVFKHESVIR SPTVLSVKSS SILLKSSSGS FSDSPEEATP
2001 IAAIFDTENL EKISIPEAVQ RGIVDTITGQ RLLEAQACTG GIICPTTGQR LSLQDAVAQG IIDHEMATRL KPAQKAFLGF
2081 EGIKGRKRMS AAEAVKEKWL PYEAGQRFLE FQFITGGLVD PEVHGRISTE EAIRKGLIDG RTSQRLQDIG NYAKILTCPK
2161 TKLKISYRDA MNRSMVEDQT GLRLLEASSV SSKGISSPYN VSSAPGSRSG SRSGSRSGSR SGSRRGSFDA GTSSSYTYSY
2241 TAVSSNSNVG R
```

**Supporting Peptides:**

| Peptide | Uniq | -10lgP | Mass | Length | ppm | m/z | z | RT | Fraction | Scan | Source File | Area F1 | Area F2a | Area F3a | Area F4 | Area F5 | Area F6a | Area F7a | Area F7b | Area F8 | Area F9a | Area Sample 1F10a | Area F10b | Area F11a | Area F11b | Area F12a | Area F13 | #Feature | #Feature F1 | #Feature F2a | #Feature F3a | #Feature F4 | #Feature F5 | #Feature F6a | #Feature F7a | #Feature F7b | #Feature F8 | #Feature F9a | #Feature Sample 1F10a | #Feature F10b | #Feature F11a | #Feature F11b | #Feature F12a | #Feature F13 | Start | End | PTM | AScore | Found By |
|---|---|---|---|---|---|---|---|---|---|---|---|---|---|---|---|---|---|---|---|---|---|---|---|---|---|---|---|---|---|---|---|---|---|---|---|---|---|---|---|---|---|---|---|---|---|---|---|---|---|
| R.LTYEIEDEKR.K | Y | 47.12 | 1294.6405 | 10 | 8.7 | 432.5566 | 3 | 38.20 | 8 | F8:16270 | BuCaTN01_F7b.raw | | | | | | | | 0 | 9.2313E4 | | | | | | | | 1 | 0 | 0 | 0 | 0 | 0 | 0 | 0 | 0 | 1 | 0 | 0 | 0 | 0 | 0 | 0 | 0 | 1108 | 1117 | | | PEAKS DB |
| total 1 peptides | | | | | | | | | | | | | | | | | | | | | | | | | | | | | | | | | | | | | | | | | | | | | | | | | |

**Best Unique PSM (Scan F8:16270, m/z=432.5566, z=3, RT=38.20, ppm=8.7):**

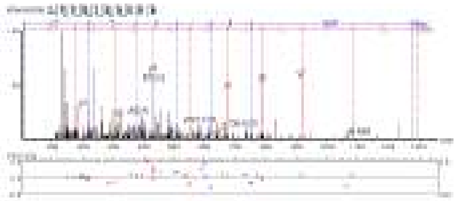

## XP_007422203.1

back to list

| Protein Coverage | Supporting Peptides | Best Unique PSM |

**Protein Coverage:**

```
   1 MSMNGGSHPR LNTLGRMIRA ESGPDMRYEM NSHMMGGGGG GGSSHTHKMY YVQKSYVGDI NSEGYTQNGT ATVSRRQNTI
  81 QDLLQNCSDC LMRAELIVQP ELKYGDGIQI SRNRELEDCF AQANEHMDIL DGLIREMRQM GQPCELYQKR LLQLQEQMRA
 161 LYKAISVPRV RRASSKGGGY SSQSASGWDE YTRRVTSECL NWMRQQKTDM ELVKWGFDAA AVEQQIAEHR RFHNSIGDYR
 241 WHLDKVKTDL REKAAVHQLE EEYEYLLRFS FERMDQLRQL QNIIQATSRE IMWINDCEEE ELLYDWSDRN TDIARKQEAF
 321 SKRMSQLEVK EKELNKLKQE CDQLVLSQHP ASDKIEAYMD TLQTQWSWIL QITKCIDVHL KENAAYFQFF EEAQSTENYL
 401 KNLQDAIRKK FICDKNMSLQ VLLEQIKELE AEREKILEYR RQVQNLVNKS KKIVQLKPRN PDYKTNKPII LRALCDYKQD
 481 LKTMRKGDEC ILKDNSERSK WHVTGPGGVD MLVPSVSLII PPPNPLAVDL ATKIEQYYEA IMALWNQLYI NMKSLVSWHY
 561 CIIDIEKIKA MTIAKLKTMR REDYQRIIAD LEIHYQEFIR NSQGSEMFGD DDKRKIQTQF TDAQKHYQTL IIQLPGSHPT
 641 PIIPVTVEQS LEKGREQEKQ EAWLLMELQK LRRQIENCEL RMVQRTTLQL DQGASHNLSV RISELEGIQN ESQSMAEALN
 721 RQKDYLPNFR GSEKYLYLQS EINALFQKLE NINGVSDGYL DSLKALRSLL QAILQTDDVI RVFEIRLTEE DTLSLDPDKV
 801 EAYRACLKKM KADLSMKKSL LGTLEAELQK ALQIHSQSSQ IYPLYDLDLG KFADRVSQLT DRWQRVEKQL DDRSWDLEKQ
 881 VKQLRTYRDL YQALNKWICD AKRRQDTIEA MKLGDVSTVM RYLQEQKNLH SEITGKRDRV EEVIKNAEVC SLAIKDYELQ
 961 AAAYSSGLET LLNIPVKRSM VQSPSGLILQ EAAEIQSRYI ELLTRSGDYY KFLSEMLKSL EDIKMKSTRI ELLEEELRLA
1041 KDANSDSSNK HKFLEQNLQK YQVECSQLKA KFISLEEMKR QVEMDGSTAK QNLDKCYAQI KDLNDRITRL TYEIEDEKRK
1121 RKLLEDRYDQ QKTDFDQLQK TRQNEKESLG WQKIESEKIV KEKEYEIERL RVLLQDEGTR KREYENELAK TSHRIQESKN
1201 QCNHIMQERE ALLMKIKNLE QDKARLLRLE EELNRTKGTL DSESRLKVRL ENEKQQILND LNQWKSQYSR KEETIRKIES
1281 EREKSEREKS SLLVEIERLQ AEIKRIEERY RCRLEETSRK SQSEMEAERL RLQREIEKLK QRPYVAHRGT QTEEDFMIDP
1361 SKLLFNGLRK KITAVQLYEC QLIDKSTLDK LLRGQKSVEE VAAELEPYLR GAGAIAGASL NPKDKYSLVE AKRKQLLTPE
1441 NTVLLLEAQA ATGGVIDPHR NELLSVDSAI ARDLIDFDDR EQIYTAEKAI TGFKDPFSGK TVPIAEAIKK NFVDRETGIR
1521 LLEAQLAAGG IVDPINSVFL PKDIALSRGV IDRDLYRALN NPSKPLIDPV TKKAISYMKL REKCRIEPHT GLLLLPVQKR
1601 SMSFQGIRKP IPVSELVESG IIRESTANDL ETGVITVEEV TDRIKDFLQG SSCIAGIYNE ATREKLGVYQ AMKIGLVRPG
1681 TALELLEAQA ATGFIVDPVS NSRLTVEEAY KRGLVGIEFK EKLLSAEKAV TGYKDPETGN IISLFQAMNK ELIEKGHGIR
1761 LLEAQIATGG IIDPKESHRL PVRTAYQRGY FNEELNDILS DPSDDTKGFF DPNTEENLTY LQLKERCKID EGTGLCLLPL
1841 RERKKVVQTS QKNTLRKRRV VIVDPDTGKE MSVQEAYNKG LIDYDTYMEL SDQECEWEEI TTTGSDGSTR VVLVDRKTGH
1921 QYDIQDAIDK GLVDRKFFDQ YRSGSLSLTQ FADMIASRNG GDEVFKHESI VRSPTVVSVK SSSILLKSSS GSFSDSPEET
2001 TPIAAIFDTE NLEKISISEA VQRGIVDTIT AQRLLEAQAC TGGIICPTTG QRVSLQDAVA QGIIDHEMAT RLKPAQKAFI
2081 GFEGIKGRKR MSAAEAVKEK WLPYEAGQRF LEFQFITGGL VDPEVHGRIS TEEAIRKGLI DGRTSQRLQD VGNYAKILTC
2161 PKTKLKISYR EAMNRSMVED QTGLRLLEAS SVSSKGISSP YNVSSAPGSR SGSRSGSRSG SRSGSRSGSR RGSFDAGTSS
2241 SYSYSYTTVS SSTLGR
```

**Supporting Peptides:**

| Peptide | Uniq | -10lgP | Mass | Length | ppm | m/z | z | RT | Fraction | Scan | Source File | Area | Area | Area | Area | Area | Area | Area | Area | Area F8 | Area | Area Sample | Area | Area | Area | Area | Area | #Feature | #Feature | #Feature | #Feature | #Feature | #Feature | #Feature | #Feature | #Feature | #Feature | #Feature | #Feature Sample | #Feature | #Feature | #Feature | #Feature | #Feature | Start | End | PTM | AScore | Found |
|---|---|---|---|---|---|---|---|---|---|---|---|---|---|---|---|---|---|---|---|---|---|---|---|---|---|---|---|---|---|---|---|---|---|---|---|---|---|---|---|---|---|---|---|---|---|---|---|---|---|

| | | | | | | | | | | | | F1 | F2a | F3a | F4 | F5 | F6a | F7a | F7b | | F9a | 1F10a | F10b | F11a | F11b | F12a | F13 | | F1 | F2a | F3a | F4 | F5 | F6a | F7a | F7b | F8 | F9a | 1F10a | F10b | F11a | F11b | F12a | F13 | | | | | By |
|---|---|---|---|---|---|---|---|---|---|---|---|---|---|---|---|---|---|---|---|---|---|---|---|---|---|---|---|---|---|---|---|---|---|---|---|---|---|---|---|---|---|---|---|---|---|---|---|---|---|
| R.LTYEIEDEKR.K | Y | 47.12 | 1294.6405 | 10 | 8.7 | 432.5566 | 3 | 38.20 | 8 | F8:16270 | BuCaTN01_F7b.raw | | | | | | | | 0 | 9.2313E4 | | | | | | | | 1 | 0 | 0 | 0 | 0 | 0 | 0 | 0 | 0 | 1 | 0 | 0 | 0 | 0 | 0 | 0 | 0 | 1110 | 1119 | | | PEAKS DB |
| total 1 peptides | | | | | | | | | | | | | | | | | | | | | | | | | | | | | | | | | | | | | | | | | | | | | | | | | |

**Best Unique PSM (Scan F8:16270, m/z=432.5566, z=3, RT=38.20, ppm=8.7):**

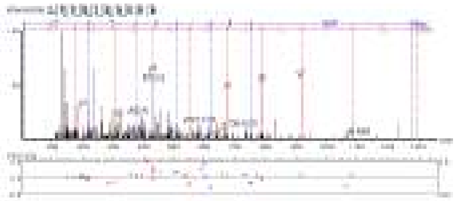

## XP_007422202.1

back to list

| Protein Coverage | Supporting Peptides | Best Unique PSM |

**Protein Coverage:**

```
   1 MSMNGGSHPR LNTLGRMIRA ESGPDMRYEM NSHMMGGGGG GGSSHTHKMY YVQKSYVGDI NSEGYTQNGT ATVSRRQNTI
  81 QDLLQNCSDC LMRAELIVQP ELKYGDGIQI SRNRELEDCF AQANEHMDIL DGLIREMRQM GQPCELYQKR LLQLQEQMRA
 161 LYKAISVPRV RRASSKGGGY SSQSASGWDE YTRRVTSECL NWMRQQKTDM ELVKWGFDAA AVEQQIAEHR RFHNSIGDYR
 241 WHLDKVKTDL REKAAVHQLE EEYEYLLRFS FERMDQLRQL QNIIQATSRE IMWINDCEEE ELLYDWSDRN TDIARKQEAF
 321 SKRMSQLEVK EKELNKLKQE CDQLVLSQHP ASDKIEAYMD TLQTQWSWIL QITKCIDVHL KENAAYFQFF EEAQSTENYL
 401 KNLQDAIRKK FICDKNMSLQ VLLEQIKELE AEREKILEYR RQVQNLVNKS KKIVQLKPRN PDYKTNKPII LRALCDYKQD
 481 LKTMRKGDEC ILKDNSERSK WHVTGPGGVD MLVPSVSLII PPPNPLAVDL ATKIEQYYEA IMALWNQLYI NMKSLVSWHY
 561 CIIDIEKIKA MTIAKLKTMR REDYQRIIAD LEIHYQEFIR NSQGSEMFGD DDKRKIQTQF TDAQKHYQTL IIQLPGSHPT
 641 PIIPVTVEQS LEKGREQEKQ EAWLLMELQK LRRQIENCEL RMVQRTTLQL DQGASHNLSV RISELEGIQN ESQSMAEALN
 721 RQKDYLPNFR GSEKYLYLQS EINALFQKLE NINGVSDGYL DSLKALRSLL QAILQTDDVI RVFEIRLTEE DTLSLDPDKV
 801 EAYRACLKKM KADLSMKKSL LGTLEAELQK ALQIHSQSSQ IYPLYDLDLG KFADRVSQLT DRWQRVEKQL DDRSWDLEKQ
 881 VKQLRTYRDL YQALNKWICD AKRRQDTIEA MKLGDVSTVM RYLQEQKNLH SEITGKRDRV EEVIKNAEVC SLAIKDYELQ
 961 AAAYSSGLET LLNIPVKRSM VQSPSGLILQ EAAEIQSRYI ELLTRSGDYY KFLSEMLKSL EDIKMKSTRI ELLEEELRLA
1041 KDANSDSSNK HKFLEQNLQK YQVECSQLKA KFISLEEMKR QVEMDGSTAK QNLDKCYAQI KDLNDRITRL TYEIEDEKRK
1121 RKLLEDRYDQ QKTDFDQLQK TRQNEKESLG WQKIESEKIV KEKEYEIERL RVLLQDEGTR KREYENELAK VRNQFNEEMS
1201 NIKNKYETEI NIKKTTIHQI AAQKEDDAKN LRAQIDRLQR ENRDLKDEIV RLNDAILDTT EQRRRAEENV LQQKACGSEV
1281 MQQKQQIELE LKQIIQLRNE DNTRYKQALE DAASTIQEKA KEIERLKIQL QEEAKGRWEL ENELAKTSHR IQESKNQCNH
1361 IMQEREALLM KIKNLEQDKA RLLRLEEELN RTKGTLDSES RLKVRLENEK QQILNDLNQW KSQYSRKEET IRKIESEREK
1441 SEREKSSLLV EIERLQAEIK RIEERYRCRL EETSRKSQSE MEAERLRLQR EIEKLKQRPY VAHRGTQTEE DFMIDPSKLL
1521 FNGLRKKITA VQLYECQLID KSTLDKLLRG QKSVEEVAAE LEPYLRGAGA IAGASLNPKD KYSLVEAKRK QLLTPENTVL
1601 LLEAQAATGG VIDPHRNELL SVDSAIARDL IDFDDREQIY TAEKAITGFK DPFSGKTVPI AEAIKKNFVD RETGIRLLEA
1681 QLAAGGIVDP INSVFLPKDI ALSRGVIDRD LYRALNNPSK PLIDPVTKKA ISYMKLREKC RIEPHTGLLL LPVQKRSMSF
1761 QGIRKPIPVS ELVESGIIRE STANDLETGV ITVEEVTDRI KDFLQGSSCI AGIYNEATRE KLGVYQAMKI GLVRPGTALE
1841 LLEAQAATGF IVDPVSNSRL TVEEAYKRGL VGIEFKEKLL SAEKAVTGYK DPETGNIISL FQAMNKELIE KGHGIRLLEA
1921 QIATGGIIDP KESHRLPVRT AYQRGYFNEE LNDILSDPSD DTKGFFDPNT EENLTYLQLK ERCKIDEGTG LCLLPLRERK
2001 KVVQTSQKNT LRKRRVVIVD PDTGKEMSVQ EAYNKGLIDY DTYMELSDQE CEWEEITTTG SDGSTRVVLV DRKTGHQYDI
2081 QDAIDKGLVD RKFFDQYRSG SLSLTQFADM IASRNGGDEV FKHESIVRSP TVVSVKSSSI LLKSSSGSFS DSPEETTPIA
2161 AIFDTENLEK ISISEAVQRG IVDTITAQRL LEAQACTGGI ICPTTGQRVS LQDAVAQGII DHEMATRLKP AQKAFIGFEG
2241 IKGRKRMSAA EAVKEKWLPY EAGQRFLEFQ FITGGLVDPE VHGRISTEEA IRKGLIDGRT SQRLQDVGNY AKILTCPKTK
2321 LKISYREAMN RSMVEDQTGL RLLEASSVSS KGISSPYNVS SAPGSRSGSR SGSRSGSRSG SRSGSRRGSF DAGTSSSYSY
2401 SYTTVSSSTL GR
```

**Supporting Peptides:**

| Peptide | Uniq | -10lgP | Mass | Length | ppm | m/z | z | RT | Fraction | Scan | Source File | Area F1 | Area F2a | Area F3a | Area F4 | Area F5 | Area F6a | Area F7a | Area F7b | Area F8 | Area F9a | Area Sample 1F10a | Area F10b | Area F11a | Area F11b | Area F12a | Area F13 | #Feature | #Feature F1 | #Feature F2a | #Feature F3a | #Feature F4 | #Feature F5 | #Feature F6a | #Feature F7a | #Feature F7b | #Feature F8 | #Feature F9a | #Feature Sample 1F10a | #Feature F10b | #Feature F11a | #Feature F11b | #Feature F12a | #Feature F13 | Start | End | PTM | AScore | Found By |
|---|---|---|---|---|---|---|---|---|---|---|---|---|---|---|---|---|---|---|---|---|---|---|---|---|---|---|---|---|---|---|---|---|---|---|---|---|---|---|---|---|---|---|---|---|---|---|---|---|---|
| R.LTYEIEDEKR.K | Y | 47.12 | 1294.6405 | 10 | 8.7 | 432.5566 | 3 | 38.20 | 8 | F8:16270 | BuCaTN01_F7b.raw | | | | | | | | 0 | 9.2313E4 | | | | | | | | 1 | 0 | 0 | 0 | 0 | 0 | 0 | 0 | 0 | 1 | 0 | 0 | 0 | 0 | 0 | 0 | 0 | 1110 | 1119 | | | PEAKS DB |
| total 1 peptides | | | | | | | | | | | | | | | | | | | | | | | | | | | | | | | | | | | | | | | | | | | | | | | | | |

**Best Unique PSM (Scan F8:16270, m/z=432.5566, z=3, RT=38.20, ppm=8.7):**

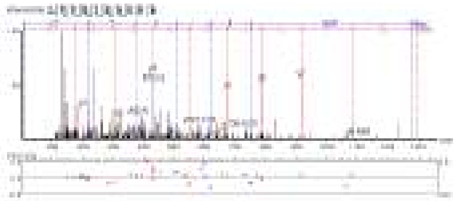

## XP_026570833.1

back to list

| Protein Coverage | Supporting Peptides | Best Unique PSM |

**Protein Coverage:**

1 MSINGSHPRL NTLGRMVRAE SGPDMRYDMN SHMMMGGGGG GSSSHTHKMY YVQKSYAGDI NSEGYTCLSL FESPDRTMEE
81 LKYGDGIQIS RNRELEDCFG QANEHMDILD GLIREMRQMG QPCDLYQKRL LQLQEQMRAL YKAISVPRVR RASSKGGGYS
161 SQSASGWDEY TRRVTTECLN WMRQQKTEME LIKWGFDASA IEQQIAEHRR FHNSIGDYRW HLDKVKTDLR EKAAVHQLEE
241 EYEYLLRFSF ERMDQLRQLQ NIIQATSREI MWINDCEEEE LLYDWSDRNT DIARKQEAFS KRMSQLEVKE KELNKLKQEC
321 DQLVLSQHPA SDKIEAYMDT LQTQWSWILQ ITKCIDVHLK ENAAYFQFFE EAQSTENYLK NLQDAIRKKF ICDKNMSLQV
401 LLEQIKELEA EREKILEYKR QVQNLVNKSK KIVQLKPRNP DYKSSKTIIL RALCDYKQDL KTMRKGDECI LKDNSERSKW
481 HVTGPGGLDM LVPSVSLIIP PPNPLAVDLA TKIEQYYDAI MALWNQLYIN MKSLVSWHYC MIDVEKIRAM TIAKLKTMRK
561 EDYQRIIADL EIHYQEFIRN SQGSEMFGDD DKRKMQTQIT DAQKHYQTLV IQLPRQTHTA APPIVTEVIT IEREKQEAWL
641 LMELQKLRRQ IENCELRMVQ RTPFQLDQGA THNLSVRISD LESIQIESQS MAEALNRQKD YLPNFRGSEK YHYLQSEISA
721 LFQKLENING VSACYLDSLN ALRSLLQAIL QTDDVIRVFE IRLTEEDTLS LDPDKVEAYR VCLKKMKADL SMKKSLLGTL
801 EAELQKALQV HSQSSQTYPH YDLDLGKFAD RVCQLTDRWQ RLEKQLDDRS WDLEKQVKQL RTYRDLYQAL NKWICDARRR
881 QDTIEAMKLG DVSTVMRYLQ EQKNLHSEIT SKRDRVEEVI KNAEVCSLAI KDYELQAAAY SSGLETLLNI PVKRSMVQSP
961 SGLILQEAGD IQSRYIELLT RSGDYYKFLS EMLKNLEDIK MKSTRIELLE EELRLAKDAN SDSNNKHKFL EQNMQKYQVE
1041 CSQLKAKFIS LEEMKRQVEM DGCTAKQNLD KCYAQIKDLN ERITR**LTYEI EDEKR**KRKLL EDRYDQQKSE FEQLQKTRQN
1121 EKESLGWQKI ESEKIIKEKE YEIERLRVLL QDEGTRKREC ENELAKVRNQ FNEEMSNIKN KYETEINIKK TTIHQIAAQK
1201 EDDAKNLRTQ IDRLQRENRD LKDEIIRLNE TILDTTEQRR RAEENVLQQK ACGSEVMQQK QQIELELKQI IQLRNDDNTR
1281 YKQALEDATL TIHEKVKELE RLKIQLQEEA KSRWELENEL AKVRNSYDEE IISLKNKYET EINITKTTIN QVTVKKEEEA
1361 SNYRAQLDNA VRENRSLCEE IRRLKNSLSQ TTENLRKVEE NAHQQKAIGS EISQKKQHLE KELKQTVQKF TEEIAHYKQS
1441 LDEAGRTLKE KNKEIEKNRK LLDAEISQRK SVEEENIRLQ RVQYDLEKAN INATETISRL KVQEQELGRL KSEYERLAQD
1521 KKGKDQESTR LQCTIKELQH QKHMLEEELN RQSRIASDET SKRKNAEEEL EAMRRTLRDQ AVKITNLTQQ IEEVSIVKKR
1601 NEDDLRHQRE VLDNHMRDKQ RYMEEIRKYT SEIEALRRQM VQEQENLKQA HIRNEHLQKA IEDKSKSLNE CKIEIERLQC
1681 LTENLTKEHL MLEEELRNLR LEYDDLRRGR SEVDEEKNAT ILDLRNQLQT SNKHSLELQG VINGLQKERE NLRQEIEKFQ
1761 KQSLEATHMI QESKSQCNHI KQERETLLVK IKTLEQDKAR LLRLEEDLNR TKGMLESESR LKVRLENEKQ QILNDLNQWK
1841 SQYSRKEETI RKTECERDKS EREKSAMLIE IERLQAEIKR IEERYRCRLE ETSRKSQAEM DAERLNWRKE IEKLKQRPYG
1921 ANRSTQTDED LMIDPSKLLF SGLRKKITAV QLYECQLIDK CTLDKLLRGQ KSVEEVAAEL EPYLRGAGAI AGASLNTKEK
2001 YSLVEAKRKQ LLTPENTILL LEAQAATGGV IDPHRNESLS VDSAIARDLI DFDDREQIYM AEKAITGFKD PFSGKTMSVA
2081 EAMKKNLVDR ETGMRLLEAQ LASGGIVDPI NSVFLPKDIA LSRGLIDRDL YRALNNPSKP LIDPLSKNAI SYMKLRERCR
2161 IESHTGLLLL PVQKRSMSFQ GIRKLITVSE LVDSGIIRES TANELETGVI SVEEVTDRIK DFLQGSSCIA GIYNEATGEK
2241 LGIYQAMKIG LVRPGTALEL LEAQAATGFI VDPVNNLRLP VEEAYKRGLV GIEFKEKLLS AERAVTGYKD PETGNIISLF
2321 QAMNKELIEK GHGVRLLEAQ IATGGIIDPK ESHRLPVHTA YQRGYFNEEL NDILSDPSDD TKGFFDPNTE ENLTYLQLKG
2401 RCKIDELTGL CLLPLREKKK VVQTSQKNTL RKRRVVIVDP DTNKEMSVQE AYNKGLIDYS TYMELSDQEC EWEEITTTGS
2481 DGSTRVVLVD RKTGHQYDIE DAIDKGLVDR KFFDQYRSGS LSLTQFADMI ASRNGGDEVF KHESVIRSPT VLSVKSSSIL
2561 LKSSSGSFSD SPEEATPIAA IFDTENLEKI SIPEAVQRGI VDTITAQRLL EAQACTGGII CPTTGQRLSL QDAVAQGIID
2641 HEMATRLKPS QKAFLGFEGI KGRKRMSAAE AVKEKWLPYE AGQRFLEFQF ITGGLVDPEV HGRISIEEAI RKGLIDGRTS
2721 QRLQDIGSYA KILTCPKTKL KISYREAMNR SMVEDQTGLR LLEASSVSSK GISSPYNVSS APGSRSGSRS GSRSGSRSGS
2801 RRGSFDAGTN SSYSYSYTTV SSSSLGR

**Supporting Peptides:**

| Peptide | Uniq | -10lgP | Mass | Length | ppm | m/z | z | RT | Fraction | Scan | Source File | Area F1 | Area F2a | Area F3a | Area F4 | Area F5 | Area F6a | Area F7a | Area F7b | Area F8 | Area F9a | Area Sample 1F10a | Area F10b | Area F11a | Area F11b | Area F12a | Area F13 | #Feature | #Feature F1 | #Feature F2a | #Feature F3a | #Feature F4 | #Feature F5 | #Feature F6a | #Feature F7a | #Feature F7b | #Feature F8 | #Feature F9a | #Feature Sample 1F10a | #Feature F10b | #Feature F11a | #Feature F11b | #Feature F12a | #Feature F13 | Start | End | PTM | AScore | Found By |
|---|---|---|---|---|---|---|---|---|---|---|---|---|---|---|---|---|---|---|---|---|---|---|---|---|---|---|---|---|---|---|---|---|---|---|---|---|---|---|---|---|---|---|---|---|---|---|---|---|---|
| R.LTYEIEDEKR.K | Y | 47.12 | 1294.6405 | 10 | 8.7 | 432.5566 | 3 | 38.20 | 8 | F8:16270 | BuCaTN01_F7b.raw | | | | | | | | 0 | 9.2313E4 | | | | | | | | 1 | 0 | 0 | 0 | 0 | 0 | 0 | 0 | 0 | 1 | 0 | 0 | 0 | 0 | 0 | 0 | 0 | 1086 | 1095 | | | PEAKS DB |
| total 1 peptides | | | | | | | | | | | | | | | | | | | | | | | | | | | | | | | | | | | | | | | | | | | | | | | | | |

**Best Unique PSM (Scan F8:16270, m/z=432.5566, z=3, RT=38.20, ppm=8.7):**

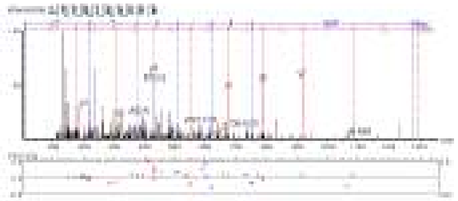

## XP_007422201.1

back to list

| Protein Coverage | Supporting Peptides | Best Unique PSM |

**Protein Coverage:**

1 MSMNGGSHPR LNTLGRMIRA ESGPDMRYEM NSHMMGGGGG GGSSHTHKMY YVQKSYVGDI NSEGYTQNGT ATVSRRQNTI
81 QDLLQNCSDC LMRAELIVQP ELKYGDGIQI SRNRELEDCF AQANEHMDIL DGLIREMRQM GQPCELYQKR LLQLQEQMRA
161 LYKAISVPRV RRASSKGGGY SSQSASGWDE YTRRVTSECL NWMRQQKTDM ELVKWGFDAA AVEQQIAEHR RFHNSIGDYR
241 WHLDKVKTDL REKAAVHQLE EEYEYLLRFS FERMDQLRQL QNIIQATSRE IMWINDCEEE ELLYDWSDRN TDIARKQEAF
321 SKRMSQLEVK EKELNKLKQE CDQLVLSQHP ASDKIEAYMD TLQTQWSWIL QITKCIDVHL KENAAYFQFF EEAQSTENYL
401 KNLQDAIRKK FICDKNMSLQ VLLEQIKELE AEREKILEYR RQVQNLVNKS KKIVQLKPRN PDYKTNKPII LRALCDYKQD
481 LKTMRKGDEC ILKDNSERSK WHVTGPGGVD MLVPSVSLII PPPNPLAVDL ATKIEQYYEA IMALWNQLYI NMKSLVSWHY
561 CIIDIEKIKA MTIAKLKTMR REDYQRIIAD LEIHYQEFIR NSQGSEMFGD DDKRKIQTQF TDAQKHYQTL IIQLPGSHPT
641 PIIPVTVEQS LEKGREQEKQ EAWLLMELQK LRRQIENCEL RMVQRTTLQL DQGASHNLSV RISELEGIQN ESQSMAEALN
721 RQKDYLPNFR GSEKYLYLQS EINALFQKLE NINGVSDGYL DSLKALRSLL QAILQTDDVI RVFEIRLTEE DTLSLDPDKV
801 EAYRACLKKM KADLSMKKSL LGTLEAELQK ALQIHSQSSQ IYPLYDLDLG KFADRVSQLT DRWQRVEKQL DDRSWDLEKQ
881 VKQLRTYRDL YQALNKWICD AKRRQDTIEA MKLGDVSTVM RYLQEQKNLH SEITGKRDRV EEVIKNAEVC SLAIKDYELQ
961 AAAYSSGLET LLNIPVKRSM VQSPSGLILQ EAAEIQSRYI ELLTRSGDYY KFLSEMLKSL EDIKMKSTRI ELLEEELRLA
1041 KDANSDSSNK HKFLEQNLQK YQVECSQLKA KFISLEEMKR QVEMDGSTAK QNLDKCYAQI KDLNDRITR**L TYEIEDEKR**K
1121 RKLLEDRYDQ QKTDFDQLQK TRQNEKESLG WQKIESEKIV KEKEYEIERL RVLLQDEGTR KREYENELAK VRNQFNEEMS
1201 NIKNKYETEI NIKKTTIHQI AAQKEDDAKN LRAQIDRLQR ENRDLKDEIV RLNDAILDTT EQRRRAEENV LQQKACGSEV
1281 MQQKQQIELE LKQIIQLRNE DNTRYKQALE DAASTIQEKA KEIERLKIQL QEEAKGRWEL ENELAKVRNS YDEEIISLKN
1361 KYETEINITK TTINQVTVKK EEEASNYRAQ LDNAVRENRS LCEEIRRLKN TLSQTTENLR QVEESAHQQK AIGSEISQKK
1441 QQLEKELKQT VQTFTEEITH YKQSLDDAGR TVKEKNKEIE KIRKLLDAEI SQRKSMEEEN IRLQRIQYDL EKANINATET
1521 ISRLKVQEQE LGRLKSEYER LAQDKKGKDQ ESTRLQSTIK EMQHKKHMLE EELNRQSRIV ADETSKRKNV EEELEAMRRT
1601 LRDQAVKITN LTQQIEEISI VKKRSEDDLR HQRDVLDSHM RDKQRYMEEI RKYTSEVETL RRQMVQEQEN LKQAHIRNEH
1681 LQKAIEDKSK SLNECKIEIE RLQSLTENLT KEHLMLEEEL RNLRLEYDDL RRGRSEVDEE KNATILELKN QLQTSNKHTL
1761 ELQGVINGLQ KERENLRQEI EKFQKQALET SHRIQESKNQ CNHIMQEREA LLMKIKNLEQ DKARLLRLEE ELNRTKGTLD
1841 SESRLKVRLE NEKQQILNDL NQWKSQYSRK EETIRKIESE REKSEREKSS LLVEIERLQA EIKRIEERYR CRLEETSRKS
1921 QSEMEAERLR LQREIEKLKQ RPYVAHRGTQ TEEDFMIDPS KLLFNGLRKK ITAVQLYECQ LIDKSTLDKL LRGQKSVEEV
2001 AAELEPYLRG AGAIAGASLN PKDKYSLVEA KRKQLLTPEN TVLLLEAQAA TGGVIDPHRN ELLSVDSAIA RDLIDFDDRE
2081 QIYTAEKAIT GFKDPFSGKT VPIAEAIKKN FVDRETGIRL LEAQLAAGGI VDPINSVFLP KDIALSRGVI DRDLYRALNN
2161 PSKPLIDPVT KKAISYMKLR EKCRIEPHTG LLLLPVQKRS MSFQGIRKPI PVSELVESGI IRESTANDLE TGVITVEEVT
2241 DRIKDFLQGS SCIAGIYNEA TREKLGVYQA MKIGLVRPGT ALELLEAQAA TGFIVDPVSN SRLTVEEAYK RGLVGIEFKE
2321 KLLSAEKAVT GYKDPETGNI ISLFQAMNKE LIEKGHGIRL LEAQIATGGI IDPKESHRLP VRTAYQRGYF NEELNDILSD
2401 PSDDTKGFFD PNTEENLTYL QLKERCKIDE GTGLCLLPLR ERKKVVQTSQ KNTLRKRRVV IVDPDTGKEM SVQEAYNKGL
2481 IDYDTYMELS DQECEWEEIT TTGSDGSTRV VLVDRKTGHQ YDIQDAIDKG LVDRKFFDQY RSGSLSLTQF ADMIASRNGG
2561 DEVFKHESIV RSPTVVSVKS SSILLKSSSG SFSDSPEETT PIAAIFDTEN LEKISISEAV QRGIVDTITA QRLLEAQACT
2641 GGIICPTTGQ RVSLQDAVAQ GIIDHEMATR LKPAQKAFIG FEGIKGRKRM SAAEAVKEKW LPYEAGQRFL EFQFITGGLV
2721 DPEVHGRIST EEAIRKGLID GRTSQRLQDV GNYAKILTCP KTKLKISYRE AMNRSMVEDQ TGLRLLEASS VSSKGISSPY
2801 NVSSAPGSRS GSRSGSRSGS RSGSRSGSRR GSFDAGTSSS YSYSYTTVSS STLGR

**Supporting Peptides:**

| Peptide | Uniq | -10lgP | Mass | Length | ppm | m/z | z | RT | Fraction | Scan | Source File | Area F1 | Area F2a | Area F3a | Area F4 | Area F5 | Area F6a | Area F7a | Area F7b | Area F8 | Area F9a | Area Sample 1F10a | Area F10b | Area F11a | Area F11b | Area F12a | Area F13 | #Feature | #Feature F1 | #Feature F2a | #Feature F3a | #Feature F4 | #Feature F5 | #Feature F6a | #Feature F7a | #Feature F7b | #Feature F8 | #Feature F9a | #Feature Sample 1F10a | #Feature F10b | #Feature F11a | #Feature F11b | #Feature F12a | #Feature F13 | Start | End | PTM | AScore | Found By |
|---|---|---|---|---|---|---|---|---|---|---|---|---|---|---|---|---|---|---|---|---|---|---|---|---|---|---|---|---|---|---|---|---|---|---|---|---|---|---|---|---|---|---|---|---|---|---|---|---|---|
| R.LTYEIEDEKR.K | Y | 47.12 | 1294.6405 | 10 | 8.7 | 432.5566 | 3 | 38.20 | 8 | F8:16270 | BuCaTN01_F7b.raw | | | | | | | | 0 | 9.2313E4 | | | | | | | | 1 | 0 | 0 | 0 | 0 | 0 | 0 | 0 | 0 | 1 | 0 | 0 | 0 | 0 | 0 | 0 | 0 | 1110 | 1119 | | | PEAKS DB |
| total 1 peptides | | | | | | | | | | | | | | | | | | | | | | | | | | | | | | | | | | | | | | | | | | | | | | | | | |

**Best Unique PSM (Scan F8:16270, m/z=432.5566, z=3, RT=38.20, ppm=8.7):**

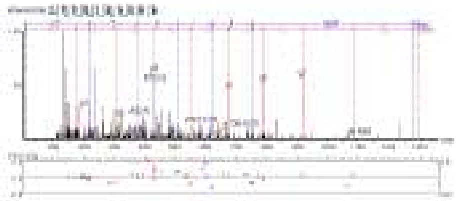

## ETE64922.1

back to list

| Protein Coverage | Supporting Peptides | Best Unique PSM |

**Protein Coverage:**

```
   1 MKLGDVSTVM RYLQEQKNLH SEITSKRDRV EEVIKNAEVC SLAIKDYELQ AAAYSSGLET LLNIPVKRSM VQSPSGLILQ
  81 EAGDIQSRYI ELLTKSGDYY KFLSEMLKNL EDIKMKSTRI ELLEEELRLA KDANSDSNNK HKFLEQNMQK YQVECSQLKA
 161 KFISLEEMKR QVEMDGSTAK QNLDKCYAQI KDLNERITRL TYEIEDEKRK RKLLEDRYDQ QKSDYEQLQK ARQNEKEKLG
 241 WQKIESEKII KEKEYEIERL RVLLQDEGTR KRESENELAK VRNQFNEEMS NIKNKYETEI NIKKTTILQI AAQKEDDAKN
 321 LRTQIDRLQR ENRDLKDEII RLNETILDTT EQRRRAEENV IQQKACSSEV IQQKQQIELE LKQIIQLRND DNTRYKQALE
 401 DATLTIHEKV KELERLKIQL QEEAKSRWEL ENELAKVRNS YDEEIISLKN KYETEINITK TTINQVTVKK EEEASNYRAQ
 481 LDNAVRENRS LCEEIRRLKN SLSQTTENLR KVEENAHQQK AIGSEISQKK QHLEKELKQT VQKFTEEIAH YKQSLDEAGR
 561 TLKEKNKEIE KNRKLLDAEI SQRKSVEEEN IRLQRIQYDL EKANINATET ISRLKVQEQE LGRLKSEYER LAQDKKGKDQ
 641 ESTRLQSTIK ELQHQKHMLE EELNRQSRIA SDEISKRKNA EEELEAMRRT LRDQAVKITN LTQQIEEVSI VKKRSEDDLR
 721 HQREVLDNHM RDKQRYMEEI RKYTSEVEAL RRQMVQEQEN LKQAHIRNEH LQKAIEDKSK SLNECKIEIE RLQCLTENLT
 801 KEHLMLEEEL RNLRLEYDDL RRGRSEVDEE KNATILDLRN QLQTSNKHAL ELQGVINGLQ KERENLRQEI EKFQKQSLEA
 881 THMIQESKSQ CNHIKQERET LLVKIKTLEQ DRARLLRLEE DLNRTKGMLE SESRLKVRLE NEKQQILNDL NQWKSQYSRK
 961 EETIRITECE RDKSEREKSA LLIEIERLQA EIKRIEERYQ CRLEETSRKS QAEMDAERSN WLREIEKLKQ RPYGANRSTQ
1041 TEEDLMIDPS KLLFSGLRKK ITAVQLYECQ LIDKCTLDKL LRGQKSVEEV AAELEPYLKG AGAIAGASLN TKEKYSLVEA
1121 KRKQLLTPEN TILLLEAQAA TGGVIDPHRI ESLSVDSAVA RDLIDFDDRE QIYMAEKAIT GFKDPFSGKT MSVAEAMKKN
1201 LVDRETGMRL LEAQLASGGI VDPINSIFLP KDIALSRGLI DRDLYRALNN PSKPLIDPLS KNAISYMKLR ERCRIESHTG
1281 LLLLPVQKRS MSFQGIRKLI TVSELVDSGI IRESTANELE TGVISVEEVT DRIKDFLQGS SCIAGIYNEA TGEKLGVYQA
1361 MKIGLVRPGT ALELLEAQAA TGFIVDPVNN LRLPVEEGYK RGLVGIEFKE KLLSAERAVT GYKDPETGNI ISLFQAMNKE
1441 LIEKGHGVRL LEAQIATGGI IDPKESHRLP VHTAYQRGYF NEELNDILSD PSDDTKGFFD PNTEENLTYL QLKGRCKIDE
1521 LTGLCLLPLR EKKKVVQTSQ KNTLRKRRVV IVDPDTNKEM SVQEAYNKGL IDYSTYMELS DQECEWEEIT TTGSDGSTRV
1601 VLVDRKTGHQ YDIEDAIDKG LVDRKFFDQY RSGSLSLTQF ADMIASRNGG DEVFKHESVI RSPTVLSVKS SSILLKSSSG
1681 SFSDSPEEAT PIAAIFDTEN LEKISIPEAV QRGIVDTITA QRLLEAQACT GGIICPTTGQ RLSLQDAVAQ GIIDHEMATR
1761 LKPSQKAFLG FEGIKGRKRM SAAEAVKEKW LPYEAGQRFL EFQFITGGLV DPEVHGRIST EEAIRKGLID GRTSQRLQDI
1841 GNYAKILTCP KTKLKISYRE AMNRSMVEDQ TGLRLLEASS VSSKGISSPY NVSSAPGSRS GSRSGSRSGS RSGSRRGSFD
1921 AGTSSSYSYS YTTVSSSSLG R
```

**Supporting Peptides:**

| Peptide | Uniq | -10lgP | Mass | Length | ppm | m/z | z | RT | Fraction | Scan | Source File | Area F1 | Area F2a | Area F3a | Area F4 | Area F5 | Area F6a | Area F7a | Area F7b | Area F8 | Area F9a | Area Sample 1F10a | Area F10b | Area F11a | Area F11b | Area F12a | Area F13 | #Feature | #Feature F1 | #Feature F2a | #Feature F3a | #Feature F4 | #Feature F5 | #Feature F6a | #Feature F7a | #Feature F7b | #Feature F8 | #Feature F9a | #Feature Sample 1F10a | #Feature F10b | #Feature F11a | #Feature F11b | #Feature F12a | #Feature F13 | Start | End | PTM | AScore | Found By |
|---|---|---|---|---|---|---|---|---|---|---|---|---|---|---|---|---|---|---|---|---|---|---|---|---|---|---|---|---|---|---|---|---|---|---|---|---|---|---|---|---|---|---|---|---|---|---|---|---|---|
| R.LTYEIEDEKR.K | Y | 47.12 | 1294.6405 | 10 | 8.7 | 432.5566 | 3 | 38.20 | 8 | F8:16270 | BuCaTN01_F7b.raw | | | | | | | | 0 | 9.2313E4 | | | | | | | | 1 | 0 | 0 | 0 | 0 | 0 | 0 | 0 | 0 | 1 | 0 | 0 | 0 | 0 | 0 | 0 | 0 | 200 | 209 | | | PEAKS DB |
| total 1 peptides | | | | | | | | | | | | | | | | | | | | | | | | | | | | | | | | | | | | | | | | | | | | | | | | | |

**Best Unique PSM (Scan F8:16270, m/z=432.5566, z=3, RT=38.20, ppm=8.7):**

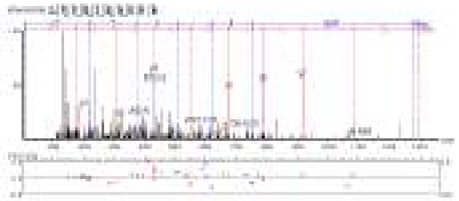

## XP_026532454.1

back to list

| Protein Coverage | Supporting Peptides | Best Unique PSM |

**Protein Coverage:**

```
   1 MRAELIVQPE LKYGDGIQIS RNRELEDCFG QANEHMDILD GLIREMRQMG QPCDLYQKRL LQLQEQMRAL YKAISVPRVR
  81 RASSKGGGYS SQSASGWDEY TRRVTTECLN WMRQQKTEME LIKWGFDAAA IEQQIAEHRR FHNSIGDYRW HLDKVKTDLR
 161 EKAAVHQLEE EYEYLLRFSF ERMDQLRQLQ NIIQATCREI MWINDCEEEE LLYDWSDRNT DIARKQEAFS KRMSQLEVKE
 241 KELNKLKQEC DQLVLSQHPA SDKIEAYMDT LQTQWSWILQ ITKCIDVHLK ENAAYFQFFE EAQSTENYLK NLQDAIRKKF
 321 ICDKNMSLQV LLEQIKELEA EREKILEYKR QVQNLVNKSK KIVQLKPRNP EYKSSKTIIL RALCDYKQDL KTMRKGDECI
 401 LKDNSERSKW HVTGPGGLDM LVPSVSLIIP PPNPLAVDLA TKIEQYYDAI MALWNQLYIN MKSLVSWHYC MIDVEKIRAM
 481 TIAKLKTMRK EDYQRIIADL EIHYQEFIRN SQGSEMFGDD DKRKMQTQIT DAQKHYQTLV IQLPRQTHTA PPIVTEVITI
 561 EREKQEAWLL MELQKLRRQI ENCELRMVQR TPFQLDQGAT HNLSVRISEL ESIQIESQSM AEALNRQKDY LPNFRGSEKY
 641 HYLQSEISAL FQKLENINGV SACYLDSLNA LRSLLQAILQ TDDMIRVFEI RLTEEDTLSL DPDKVEAYRV CLKKMKADLS
 721 MKKSLLGTLE AELQKALQVH SQSSQTYPHY DLDLGKFADR VCQLTDRWQR LEKQLDDRSW DLEKQVKQLR TYRDLYQALN
 801 KWICDARRRQ DTIEAMKLGD VSTVMRYLQE QKNLHSEITS KRDRVEEVIK NAEVCSLAIK DYELQAAAYS SGLETLLNIP
 881 VKRSMVQSPS GLILQEAGDI QSRYIELLTR SGDYYKFLSE MLKNLEDIKM KSTRIELLEE ELRLAKDANS DSNNKHKFLE
 961 QNMQKYQVEC SQLKAKFISL EEMKRQVEMD GSTAKQNLDK CYAQIKDLNE RITRLTYEIE DEKRKRKLLE DRYDQQKSEF
1041 EQLQKTRQNE KESLGWQKIE SEKIVKEKEY EIERLRVLLQ DEGTRKRECE NELAKTTHMI QESKSQCNHI KQERETLLVK
1121 IKTLEQDKAR LLRLEEDLNR TKGMLESESR LKVRLENEKQ QILNDLNQWK SQYSRKEETI RKTECERDKS EREKSAMLVE
1201 IERLQAEIKR IEERYRCRLE ETSRKSQAEM DAERLNWRKE IEKLKQRPYE ANRSTQTDED LMIDPSKLLF SGLRKKITAV
1281 QLYECQLIDK CTLDKLLRGQ KSVEEVAAEL EPYLRGAGAI AGASLNTKEK YSLVEAKRKQ LLTPENTILL LEAQAATGGV
1361 IDPHRNESLS VDSAIARDLI DFDDREQIYM AEKAITGFKD PFSGKTMSVA EAMKKNLVDR ETGMRLLEAQ LASGGIVDPI
1441 NSVFLPKDIA LSRGLIDRDL YRALNNPSKP LIDPLSKNAI SYMKLRERCR IEPHTGLLLL PVQKRSMSFQ GIRKLITVSE
1521 LVDSGIIRES TANELETGVI SVEEVTDRIK DFLQGSSCIA GIYNEATGEK LGVYQAMKIG LVRPGTALEL LEAQAATGFI
1601 VDPVNNLRLP VEEAYKRGLV GIEFKEKLLS AERAVTGYKD PETGNIISLF QAMNKELIEK GHGVRLLEAQ IATGGIIDPK
1681 ESHRLPVHTA YQRGYFNEEL NDILSDPSDD TKGFFDPNTE ENLTYLQLKG RCKIDELTGL CLLPLREKKK VVQTSQKNTL
1761 RKRRVVIVDP DTNKEMSVQE AYNKGLIDYS TYMELSDQEC EWEEITTTGS DGSTRVVLVD RKTGHQYDIE DAIDKGLVDR
1841 KFFDQYRSGS LSLTQFADMI ASRNGGDEVF KHESVIRSPT VLSVKSSSIL LKSSSGSFSD SPEEATPIAA IFDTENLEKI
1921 SIPEAVQRGI VDTITAQRLL EAQACTGGII CPTTGQRLSL QDAVAQGIID HEMATRLKPS QKAFLGFEGI KGRKRMSAAE
2001 AVKEKWLPYE AGQRFLEFQF ITGGLVDPEV HGRISTEEAI RKGLIDGRTS QRLQDIGSYA KILTCPKTKL KISYREAMNR
2081 SMVEDQTGLR LLEASSVSSK GISSPYNVSS APGSRSGSRS GSRSGSRSGS RRGSFDAGTN SSYSYSYTTV SSSSLGR
```

**Supporting Peptides:**

| Peptide | Uniq | -10lgP | Mass | Length | ppm | m/z | z | RT | Fraction | Scan | Source File | Area F1 | Area F2a | Area F3a | Area F4 | Area F5 | Area F6a | Area F7a | Area F7b | Area F8 | Area F9a | Area Sample 1F10a | Area F10b | Area F11a | Area F11b | Area F12a | Area F13 | #Feature | #Feature F1 | #Feature F2a | #Feature F3a | #Feature F4 | #Feature F5 | #Feature F6a | #Feature F7a | #Feature F7b | #Feature F8 | #Feature F9a | #Feature Sample 1F10a | #Feature F10b | #Feature F11a | #Feature F11b | #Feature F12a | #Feature F13 | Start | End | PTM | AScore | Found By |
|---|---|---|---|---|---|---|---|---|---|---|---|---|---|---|---|---|---|---|---|---|---|---|---|---|---|---|---|---|---|---|---|---|---|---|---|---|---|---|---|---|---|---|---|---|---|---|---|---|---|
| R.LTYEIEDEKR.K | Y | 47.12 | 1294.6405 | 10 | 8.7 | 432.5566 | 3 | 38.20 | 8 | F8:16270 | BuCaTN01_F7b.raw | | | | | | | | 0 | 9.2313E4 | | | | | | | | 1 | 0 | 0 | 0 | 0 | 0 | 0 | 0 | 0 | 1 | 0 | 0 | 0 | 0 | 0 | 0 | 0 | 1015 | 1024 | | | PEAKS DB |
| total 1 peptides | | | | | | | | | | | | | | | | | | | | | | | | | | | | | | | | | | | | | | | | | | | | | | | | |

**Best Unique PSM (Scan F8:16270, m/z=432.5566, z=3, RT=38.20, ppm=8.7):**

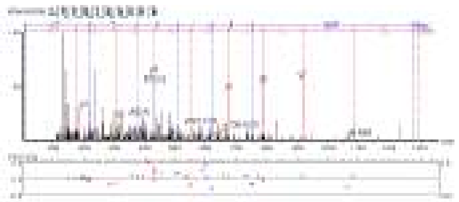

## XP_026532453.1

back to list

| Protein Coverage | Supporting Peptides | Best Unique PSM |

**Protein Coverage:**

```
   1 MRAELIVQPE LKYGDGIQIS RNRELEDCFG QANEHMDILD GLIREMRQMG QPCDLYQKRL LQLQEQMRAL YKAISVPRVR
  81 RASSKGGGYS SQSASGWDEY TRRVTTECLN WMRQQKTEME LIKWGFDAAA IEQQIAEHRR FHNSIGDYRW HLDKVKTDLR
 161 EKAAVHQLEE EYEYLLRFSF ERMDQLRQLQ NIIQATCREI MWINDCEEEE LLYDWSDRNT DIARKQEAFS KRMSQLEVKE
 241 KELNKLKQEC DQLVLSQHPA SDKIEAYMDT LQTQWSWILQ ITKCIDVHLK ENAAYFQFFE EAQSTENYLK NLQDAIRKKF
 321 ICDKNMSLQV LLEQIKELEA EREKILEYKR QVQNLVNKSK KIVQLKPRNP EYKSSKTIIL RALCDYKQDL KTMRKGDECI
 401 LKDNSERSKW HVTGPGGLDM LVPSVSLIIP PPNPLAVDLA TKIEQYYDAI MALWNQLYIN MKSLVSWHYC MIDVEKIRAM
 481 TIAKLKTMRK EDYQRIIADL EIHYQEFIRN SQGSEMFGDD DKRKMQTQIT DAQKHYQTLV IQLPRQTHTA PPIVTEVITI
 561 EREKQEAWLL MELQKLRRQI ENCELRMVQR TPFQLDQGAT HNLSVRISEL ESIQIESQSM AEALNRQKDY LPNFRGSEKY
 641 HYLQSEISAL FQKLENINGV SACYLDSLNA LRSLLQAILQ TDDMIRVFEI RLTEEDTLSL DPDKVEAYRV CLKKMKADLS
 721 MKKSLLGTLE AELQKALQVH SQSSQTYPHY DLDLGKFADR VCQLTDRWQR LEKQLDDRSW DLEKQVKQLR TYRDLYQALN
 801 KWICDARRRQ DTIEAMKLGD VSTVMRYLQE QKNLHSEITS KRDRVEEVIK NAEVCSLAIK DYELQAAAYS SGLETLLNIP
 881 VKRSMVQSPS GLILQEAGDI QSRYIELLTR SGDYYKFLSE MLKNLEDIKM KSTRIELLEE ELRLAKDANS DSNNKHKFLE
 961 QNMQKYQVEC SQLKAKFISL EEMKRQVEMD GSTAKQNLDK CYAQIKDLNE RITRLTYEIE DEKRKRKLLE DRYDQQKSEF

1041 EQLQKTRQNE KESLGWQKIE SEKIVKEKEY EIERLRVLLQ DEGTRKRECE NELAKVRNQF NEEMSNIKNK YETEINIKKT
1121 TIHQIAAQKE DDAKNLRTQI DRLQRENRDL KDEIIRLNES ILDTTEQRRR AEENVLQQKA CGSEVMQQKQ QIELELKQII
1201 QLRNDDNTRY KQALEDATLT IHEKVKELER LKIQLQEEAK SRWELENELA KTTHMIQESK SQCNHIKQER ETLLVKIKTL
1281 EQDKARLLRL EEDLNRTKGM LESESRLKVR LENEKQQILN DLNQWKSQYS RKEETIRKTE CERDKSEREK SAMLVEIERL
1361 QAEIKRIEER YRCRLEETSR KSQAEMDAER LNWRKEIEKL KQRPYEANRS TQTDEDLMID PSKLLFSGLR KKITAVQLYE
1441 CQLIDKCTLD KLLRGQKSVE EVAAELEPYL RGAGAIAGAS LNTKEKYSLV EAKRKQLLTP ENTILLLEAQ AATGGVIDPH
1521 RNESLSVDSA IARDLIDFDD REQIYMAEKA ITGFKDPFSG KTMSVAEAMK KNLVDRETGM RLLEAQLASG GIVDPINSVF
1601 LPKDIALSRG LIDRDLYRAL NNPSKPLIDP LSKNAISYMK LRERCRIEPH TGLLLLPVQK RSMSFQGIRK LITVSELVDS
1681 GIIRESTANE LETGVISVEE VTDRIKDFLQ GSSCIAGIYN EATGEKLGVY QAMKIGLVRP GTALELLEAQ AATGFIVDPV
1761 NNLRLPVEEA YKRGLVGIEF KEKLLSAERA VTGYKDPETG NIISLFQAMN KELIEKGHGV RLLEAQIATG GIIDPKESHR
1841 LPVHTAYQRG YFNEELNDIL SDPSDDTKGF FDPNTEENLT YLQLKGRCKI DELTGLCLLP LREKKKVVQT SQKNTLRKRR
1921 VVIVDPDTNK EMSVQEAYNK GLIDYSTYME LSDQECEWEE ITTTGSDGST RVVLVDRKTG HQYDIEDAID KGLVDRKFFD
2001 QYRSGSLSLT QFADMIASRN GGDEVFKHES VIRSPTVLSV KSSSILLKSS SGSFSDSPEE ATPIAAIFDT ENLEKISIPE
2081 AVQRGIVDTI TAQRLLEAQA CTGGIICPTT GQRLSLQDAV AQGIIDHEMA TRLKPSQKAF LGFEGIKGRK RMSAAEAVKE
2161 KWLPYEAGQR FLEFQFITGG LVDPEVHGRI STEEAIRKGL IDGRTSQRLQ DIGSYAKILT CPKTKLKISY REAMNRSMVE
2241 DQTGLRLLEA SSVSSKGISS PYNVSSAPGS RSGSRSGSRS GSRSGSRRGS FDAGTNSSYS YSYTTVSSSS LGR
```

**Supporting Peptides:**

| Peptide | Uniq | -10lgP | Mass | Length | ppm | m/z | z | RT | Fraction | Scan | Source File | Area F1 | Area F2a | Area F3a | Area F4 | Area F5 | Area F6a | Area F7a | Area F7b | Area F8 | Area F9a | Area Sample 1F10a | Area F10b | Area F11a | Area F11b | Area F12a | Area F13 | #Feature | #Feature F1 | #Feature F2a | #Feature F3a | #Feature F4 | #Feature F5 | #Feature F6a | #Feature F7a | #Feature F7b | #Feature F8 | #Feature F9a | #Feature Sample 1F10a | #Feature F10b | #Feature F11a | #Feature F11b | #Feature F12a | #Feature F13 | Start | End | PTM | AScore | Found By |
|---|---|---|---|---|---|---|---|---|---|---|---|---|---|---|---|---|---|---|---|---|---|---|---|---|---|---|---|---|---|---|---|---|---|---|---|---|---|---|---|---|---|---|---|---|---|---|---|---|---|
| R.LTYEIEDEKR.K | Y | 47.12 | 1294.6405 | 10 | 8.7 | 432.5566 | 3 | 38.20 | 8 | F8:16270 | BuCaTN01_F7b.raw | | | | | | | | 0 | 9.2313E4 | | | | | | | | 1 | 0 | 0 | 0 | 0 | 0 | 0 | 0 | 0 | 1 | 0 | 0 | 0 | 0 | 0 | 0 | 0 | 1015 | 1024 | | | PEAKS DB |
| total 1 peptides | | | | | | | | | | | | | | | | | | | | | | | | | | | | | | | | | | | | | | | | | | | | | | | | | |

**Best Unique PSM (Scan F8:16270, m/z=432.5566, z=3, RT=38.20, ppm=8.7):**

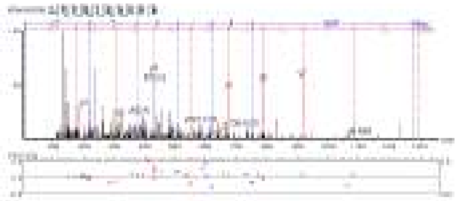

## XP_026532452.1

back to list

| Protein Coverage | Supporting Peptides | Best Unique PSM |

**Protein Coverage:**

1 MRAELIVQPE LKYGDGIQIS RNRELEDCFG QANEHMDILD GLIREMRQMG QPCDLYQKRL LQLQEQMRAL YKAISVPRVR
81 RASSKGGGYS SQSASGWDEY TRRVTTECLN WMRQQKTEME LIKWGFDAAA IEQQIAEHRR FHNSIGDYRW HLDKVKTDLR
161 EKAAVHQLEE EYEYLLRFSF ERMDQLRQLQ NIIQATCREI MWINDCEEEE LLYDWSDRNT DIARKQEAFS KRMSQLEVKE
241 KELNKLKQEC DQLVLSQHPA SDKIEAYMDT LQTQWSWILQ ITKCIDVHLK ENAAYFQFFE EAQSTENYLK NLQDAIRKKF
321 ICDKNMSLQV LLEQIKELEA EREKILEYKR QVQNLVNKSK KIVQLKPRNP EYKSSKTIIL RALCDYKQDL KTMRKGDECI
401 LKDNSERSKW HVTGPGGLDM LVPSVSLIIP PPNPLAVDLA TKIEQYYDAI MALWNQLYIN MKSLVSWHYC MIDVEKIRAM
481 TIAKLKTMRK EDYQRIIADL EIHYQEFIRN SQGSEMFGDD DKRKMQTQIT DAQKHYQTLV IQLPRQTHTA PPIVTEVITI
561 EREKQEAWLL MELQKLRRQI ENCELRMVQR TPFQLDQGAT HNLSVRISEL ESIQIESQSM AEALNRQKDY LPNFRGSEKY
641 HYLQSEISAL FQKLENINGV SACYLDSLNA LRSLLQAILQ TDDMIRVFEI RLTEEDTLSL DPDKVEAYRV CLKKMKADLS
721 MKKSLLGTLE AELQKALQVH SQSSQTYPHY DLDLGKFADR VCQLTDRWQR LEKQLDDRSW DLEKQVKQLR TYRDLYQALN
801 KWICDARRRQ DTIEAMKLGD VSTVMRYLQE QKNLHSEITS KRDRVEEVIK NAEVCSLAIK DYELQAAAYS SGLETLLNIP
881 VKRSMVQSPS GLILQEAGDI QSRYIELLTR SGDYYKFLSE MLKNLEDIKM KSTRIELLEE ELRLAKDANS DSNNKHKFLE
961 QNMQKYQVEC SQLKAKFISL EEMKRQVEMD GSTAKQNLDK CYAQIKDLNE RITR**LTYEIE DEKR**KRKLLE DRYDQQKSEF

1041 EQLQKTRQNE KESLGWQKIE SEKIVKEKEY EIERLRVLLQ DEGTRKRECE NELAKVRNQF NEEMSNIKNK YETEINIKKT
1121 TIHQIAAQKE DDAKNLRTQI DRLQRENRDL KDEIIRLNES ILDTTEQRRR AEENVLQQKA CGSEVMQQKQ QIELELKQII
1201 QLRNDDNTRY KQALEDATLT IHEKVKELER LKIQLQEEAK SRWELENELA KVRNSYDEEI ISLKNKYETE INITKTTINQ
1281 VTVKKEEEAS NYRAQLDNAV RENRSLCEEI RRLKNSLSQT TENLRKVEEN AHQQKAIGSE ISQKKQHLEK ELKQTVQKFT
1361 EEIAHYKQSL DEAGRTLKEK NKEIEKNRKL LDAEVSQRKS VEEENIRLQR VQYDLEKANI NATETISRLK VQEQELGRLK
1441 SEYERLAQDK KGKDQESTRL QCTIKELQHQ KHMLEEELNR QSRIASDEAS KRKNAEEELE AMRRTLRDQA VKITNLTQQI
1521 EEVSIVKKRS EDDLRHQREV LDNHMRDKQR YMEEIRKYTS EVEALRRQLV QEQENLKQAH IRNEHLQKAI EDKSKSLNEC
1601 KIEIERLQCL TENLTKEHLM LEEELRNLRL EYDDLRRGRS EVDEEKNATI LDLRNQLQTS NKHSLELQGV INGLQKEREN
1681 LRQEIEKFQK QSLETTHMIQ ESKSQCNHIK QERETLLVKI KTLEQDKARL LRLEEDLNRT KGMLESESRL KVRLENEKQQ
1761 ILNDLNQWKS QYSRKEETIR KTECERDKSE REKSAMLVEI ERLQAEIKRI EERYRCRLEE TSRKSQAEMD AERLNWRKEI
1841 EKLKQRPYEA NRSTQTDEDL MIDPSKLLFS GLRKKITAVQ LYECQLIDKC TLDKLLRGQK SVEEVAAELE PYLRGAGAIA
1921 GASLNTKEKY SLVEAKRKQL LTPENTILLL EAQAATGGVI DPHRNESLSV DSAIARDLID FDDREQIYMA EKAITGFKDP
2001 FSGKTMSVAE AMKKNLVDRE TGMRLLEAQL ASGGIVDPIN SVFLPKDIAL SRGLIDRDLY RALNNPSKPL IDPLSKNAIS
2081 YMKLRERCRI EPHTGLLLLP VQKRSMSFQG IRKLITVSEL VDSGIIREST ANELETGVIS VEEVTDRIKD FLQGSSCIAG
2161 IYNEATGEKL GVYQAMKIGL VRPGTALELL EAQAATGFIV DPVNNLRLPV EEAYKRGLVG IEFKEKLLSA ERAVTGYKDP
2241 ETGNIISLFQ AMNKELIEKG HGVRLLEAQI ATGGIIDPKE SHRLPVHTAY QRGYFNEELN DILSDPSDDT KGFFDPNTEE
2321 NLTYLQLKGR CKIDELTGLC LLPLREKKKV VQTSQKNTLR KRRVVIVDPD TNKEMSVQEA YNKGLIDYST YMELSDQECE
2401 WEEITTTGSD GSTRVVLVDR KTGHQYDIED AIDKGLVDRK FFDQYRSGSL SLTQFADMIA SRNGGDEVFK HESVIRSPTV
2481 LSVKSSSILL KSSSGSFSDS PEEATPIAAI FDTENLEKIS IPEAVQRGIV DTITAQRLLE AQACTGGIIC PTTGQRLSLQ
2561 DAVAQGIIDH EMATRLKPSQ KAFLGFEGIK GRKRMSAAEA VKEKWLPYEA GQRFLEFQFI TGGLVDPEVH GRISTEEAIR
2641 KGLIDGRTSQ RLQDIGSYAK ILTCPKTKLK ISYREAMNRS MVEDQTGLRL LEASSVSSKG ISSPYNVSSA PGSRSGSRSG
2721 SRSGSRSGSR RGSFDAGTNS SYSYSYTTVS SSSLGR

**Supporting Peptides:**

| Peptide | Uniq | -10lgP | Mass | Length | ppm | m/z | z | RT | Fraction | Scan | Source File | Area F1 | Area F2a | Area F3a | Area F4 | Area F5 | Area F6a | Area F7a | Area F7b | Area F8 | Area F9a | Area Sample 1F10a | Area F10b | Area F11a | Area F11b | Area F12a | Area F13 | #Feature | #Feature F1 | #Feature F2a | #Feature F3a | #Feature F4 | #Feature F5 | #Feature F6a | #Feature F7a | #Feature F7b | #Feature F8 | #Feature F9a | #Feature Sample 1F10a | #Feature F10b | #Feature F11a | #Feature F11b | #Feature F12a | #Feature F13 | Start | End | PTM | AScore | Found By |
|---|---|---|---|---|---|---|---|---|---|---|---|---|---|---|---|---|---|---|---|---|---|---|---|---|---|---|---|---|---|---|---|---|---|---|---|---|---|---|---|---|---|---|---|---|---|---|---|---|---|
| R.LTYEIEDEKR.K | Y | 47.12 | 1294.6405 | 10 | 8.7 | 432.5566 | 3 | 38.20 | 8 | F8:16270 | BuCaTN01_F7b.raw | | | | | | | | 0 | 9.2313E4 | | | | | | | | 1 | 0 | 0 | 0 | 0 | 0 | 0 | 0 | 0 | 1 | 0 | 0 | 0 | 0 | 0 | 0 | 0 | 1015 | 1024 | | | PEAKS DB |
| total 1 peptides | | | | | | | | | | | | | | | | | | | | | | | | | | | | | | | | | | | | | | | | | | | | | | | | | |

**Best Unique PSM (Scan F8:16270, m/z=432.5566, z=3, RT=38.20, ppm=8.7):**

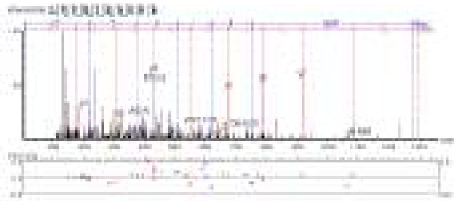

## LAA69945.1

back to list

| Protein Coverage | Supporting Peptides | Best Unique PSM |

**Protein Coverage:**

1 MSINGSHPRL NTLGRMVRAE SGPDMRYDMN SHMMMGGGGG GGGSSHTHKM YYVQKSYVGD INSEGYTQNG TATVSRRQNT
81 IQDLLQACSD CLMRAELIVQ PELKYGEGIQ ISRNRELEDC FGQANEHMDI LDGLIREMRQ MGQPCDLYQK RLLQLQEQMR
161 ALYKAISVPR VRRASSKGGY SSQSASGWDE YTRRVTTECL NWMRQQKAEM ELIKWGFDAA AIEQQITEHR RFHNSIGDYR
241 WHLDKVKTDL REKAAVHQLE EEYEYLLRFS FERMDQLRQL QGIIQATSRE IMWINDCEEE ELLYDWSDRN TDIARKQEAF
321 SKRMSQLEVK EKELNKLKQE CDQLVLSQHP ASDKIEAYMD TLQTQWSWIL QITKCIDVHL KENAAYFQFF EEAQSTENYL
401 KNLQDAIRKR FICDKNMSLH VLLEQIKELE AEREKILEYK RQVQNLVNKS KKIVQLKPRN PDYKSNKPII LRALCDYKQD
481 LKTMRKGDEC ILKDNTERSK WHVTGPGGLD MLVPSVSLII PPPNPLAVDL ATKIEQYYDA IMALWNQLYI NMKSLVSWHY
561 CMIDVEKIRA MTIAKLKTMR KEDYQRIIAD LEIHYQEFIR NSQGSEMFGD DDKRKMQTQI TDAQKHYQKL VIQILPQTHT
641 APPIVTEVIT IEREKQETWL LMELQKLRRQ IENCELRMVQ RTPFQLDQGA THNLSVRISE LESIQIESQS MADALNRQKD
721 YLPNFRGSEK YHYLQSEISA LFQKLENING VSACYLDSLN ALRSLLQAIL QTDDVIRVFE IRLTEEDTLS LDPDKVEAYR
801 VCLKKMKADL SMKKSLLGTL EAELQKALQV HSQSSQTYPH YDLDLGKFAD RVCQLTDRWQ RLEKQLDDRS WDLEKQVKQL
881 RTYRDLYQAL NKWICDARRR QDTIEAMKLG DVSTVMRYLQ EQKNLHSEIT SKRDRVEEVI KNAEVCSLAI KDYELQAAAY
961 SSGLETLLNI PVKRSMVQSP SGLILQEAGD IHSRYIELLT RSGDYYKFLS EMLKSLEDIK MKSTRIELLE EELRLAKDAN
1041 SDSNNKHKFL EQNMQKYQIE CSQLKAKFIS LEEIKRQVEM DGSTAKQNLD KCYAQIKDLN ERITR**LTYEI EDEKR**KRKLL

1121 EDRYDQQKSD FDQLQKTRQN EKESLGWQKI ESEKIIKEKE YEIERLRVLL QDEGTRKREC DNELAKVRNQ FNEEMTNIKN
1201 KYETEINIKK TTIHQIAAQK EDDAKNLRTQ IDRLQRENRD LKDEIIRLNE TILDATEQRR RAEENVLQQK ACGSEVIQQK
1281 QQIELELKQI IQLRNDDNTR YKQALEDATL TIHEKVKELE RLKIQLQEEA KSRWELENEL AKVRNSYDEE IISLKNKYET
1361 EINITKTTIN QVTVKKEEEA SNYRAQLDNA VRENRSLCEE IRRLKNSLSQ TTENLRKVEE NAHQQKAIGS EISQKKQHLE
1441 KELKQTIHKF TEEIAHYKQS LDEAGRTLKE KNKEIEKNRK LLDAEISQRK SVEEENIRLQ RIQYDLEKAN INATETISRL
1521 KVQEQELGRL KSEYERLAQD KKGKDQESTR LQCTIKELQH QKHMLEEELS RQSRIASDET SKRKNAEEEL EAMRRTLRDQ
1601 AVKITNLTQQ IEEVSIVKKR SEDDLRHQRE VLDNHMRDKQ RYMEEIRKYT SEIEALRRQM VQEQENLKQA HIRNEHLQKA
1681 IEDKSKSLNE CKIEIERLQC LTENLTKEHL MLEEELRNLR LEYDDLRRGR SEVDEEKNAT IIDLRNQLQT SNKHALELQG
1761 VLNGLQKERE NLRQEIEKFQ KQSLEATHRI QESKSQCNQI IQERETLLVK IKTLEQDKAR LLRLEEDLNR TKGMLESESR
1841 LKVRLENEKQ QILNDLNQWK SQCSRKEETI RKTECERDKS KMEKSALLIE IERLQAEIKR IEERYRCRLE ETSRKSQAEM
1921 EAERSNWLRE MEKLKQRPYE ANRGTQTEED LMIDPSKLLF SGLRKKITAV QLYECQLIDK CTLDKLLRGQ KSVEEIAAEL
2001 EPYLRGAGAI AGASLNTKEK YSLVEAKRKQ LLTPENTVLL LEAQAATGGV IDPHRNESLS VDSAIARDLI DFDDREQIYM
2081 AEKAITGFKD PFSGKTMSVA EAMKKNLVDR ETGMRLLEAQ LASGGIVDPI NSIFLPKDIA LSRGLIDRDL YRALNNPSKP
2161 LIDPLSKNAI SYMKLRERCR IESHTGLLLL PVQKRSMSFQ GIRKLITVSE LVDSGIIRES TANELETGVI SVEEVTDRIK
2241 DFLQGSSCIA GIYNEATGEK FGVYQAMKIG LVRPGTALEL LEAQAATGFI VDPVNNLRLP VEEAYKRGLV GIEFKEKLLS
2321 AERAVTGYRD PETGNIISLF QAMNKELIEK GHGVRLLEAQ IATGGIIDPK ESHRLPVHTA YQRGYFNEEL NDILSDPSDD
2401 TKGFFDPNTE ENLTYLQLKG RCKIDELTGL CLLPLREKKK VVQTSQKNTL RKRRVVIVDP DTNKEMSVQE AYNKGLIDYS
2481 TYMELSDQEC EWEEITTTGS DGSTRVVLVD RKTGHQYDIE DAIDKGLVDR KFFDQYRSGS LSLTQFADMI ASRNGGDEVF
2561 KHESVIRSPT VLSVKSSSIL LKSSSGSFSD SPEEATPIAA IFDTENLEKI SIPEAVQRGI VDTITAQRLL EAQACTGGII
2641 CPTTGQRLSL QDAVAQGIID HEMATRLKPS QKAFLGFEGI KGRKRMSAAE AVKEKWLPYE AGQRFLEFQF ITGGLVDPEV
2721 HGRISTEEAI RKGLIDGRTS QRLQDIGNYA KILTCPKTKL KISYREAMNR SMVEDQTGLR LLEASSVSSK GISSPYNVSS
2801 APGSRSGSRS GSRSGSRSGS RRGSFDAGTN SSYSYSYTTV SSSSLGR

**Supporting Peptides:**

| Peptide | Uniq | -10lgP | Mass | Length | ppm | m/z | z | RT | Fraction | Scan | Source File | Area F1 | Area F2a | Area F3a | Area F4 | Area F5 | Area F6a | Area F7a | Area F7b | Area F8 | Area F9a | Area Sample 1F10a | Area F10b | Area F11a | Area F11b | Area F12a | Area F13 | #Feature | #Feature F1 | #Feature F2a | #Feature F3a | #Feature F4 | #Feature F5 | #Feature F6a | #Feature F7a | #Feature F7b | #Feature F8 | #Feature F9a | #Feature Sample 1F10a | #Feature F10b | #Feature F11a | #Feature F11b | #Feature F12a | #Feature F13 | Start | End | PTM | AScore | Found By |
|---|---|---|---|---|---|---|---|---|---|---|---|---|---|---|---|---|---|---|---|---|---|---|---|---|---|---|---|---|---|---|---|---|---|---|---|---|---|---|---|---|---|---|---|---|---|---|---|---|---|
| R.LTYEIEDEKR.K | Y | 47.12 | 1294.6405 | 10 | 8.7 | 432.5566 | 3 | 38.20 | 8 | F8:16270 | BuCaTN01_F7b.raw | | | | | | | | 0 | 9.2313E4 | | | | | | | | 1 | 0 | 0 | 0 | 0 | 0 | 0 | 0 | 0 | 1 | 0 | 0 | 0 | 0 | 0 | 0 | 0 | 1106 | 1115 | | | PEAKS DB |
| total 1 peptides | | | | | | | | | | | | | | | | | | | | | | | | | | | | | | | | | | | | | | | | | | | | | | | | | |

**Best Unique PSM (Scan F8:16270, m/z=432.5566, z=3, RT=38.20, ppm=8.7):**

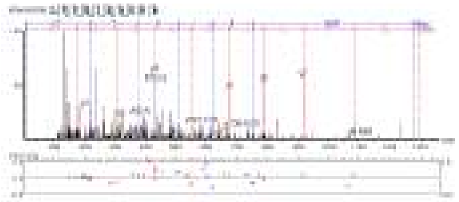

## XP_058034978.1

back to list

| Protein Coverage | Supporting Peptides | Best Unique PSM |

**Protein Coverage:**

```
   1 MSINGSHPRL NTLGRMVRAE SGPDMRYDMN SHMMMGGGGG GGGSSHTHKM YYVQKSYVGD INSEGYTQNG TATVSRRQNT
  81 IQDLLQACSD CLMRAELIVQ PELKYGDGIQ ISRNRELEDC FGQANEHMDI LDGLIREMRQ MGQPCDLYQK RLLQLQEQMR
 161 ALYKAISVPR VRRASSKGGG YSSQSASGWD EYTRRVTSEC LNWMRQQKTE MELVKWGFDA AAIEQQIAEH RRFHNSIGDY
 241 RWHLDKVKTD LREKAAVHQL EEEYEYLLRF SFERMDQLRQ LQNIIQATSR EIMWINDCEE EELLYDWSDR NTDIARKQEA
 321 FSKRMSQLEV KEKELNKLKQ ECDQLVLSQH PASDKIEAYM DTLQTQWSWI LQITKCIDVH LKENAAYFQF FEEAQSTENY
 401 LKNLQDAIRK KFICDKNMSL QVLLEQIKEL EAEREKILEY KRQVQNLVNK SKKIVQLKPR NPDYKSNKPI ILRALCDYKQ
 481 DLKTMRKGDE CILKDNSERS KWHVTGPGGL DMLVPSVSLI IPPPNPLAVD LATKIEQYYD AIMALWNQLY INMKSLVSWH
 561 YCMIDIEKIR AMTIAKLKTM RREDYQRIIT DLEIHYQEFI RNSQGSEMFG DDDKRNMQTQ ITDAQKHYQT LVMTLPRQQP
 641 TGPPIVTGVI TIEKEKQEAW LLMELQKLRR QIENCEFRMV QRTPFQLDQG ASHNLSVRIS ELESIQTESQ SMAEALNRQQ
 721 DYLPNFRGSE KYHYLQSEIS ALFQKLENIN GVSACYLDSL NALRSLLQAI LQTDDVIRVF EIRLTEEDTL SLDPDKVEAY
 801 RVCLKKMKAD LSMKKSLLGT LEAELQKALQ VHSQSSQTYP HYDLDLGKFA DRVSQLRDRW QRLEKQLDDR SWDLEKQVKQ
 881 LRVYRDLYQA FNKWICDARR RQDTIEAMKL GDVGTVMRYL QEQKNLHSEI TSKRDRVEEV VKNAEVCSLA IKDYELQAAA
 961 YSSGLETLLN IPVKRSMVQS PSGLILQEAG EIQSRYIELL TRSGDYYKFL SEMLKSLEDI KMKSTRIELL EEELRLAKDA
1041 NSDSNNKHKF LEQNLQKYQV ECSQLKAKFI SLEETKRQVE MDGSTAKQNL DKCYAQIKDL NERITRLTYE IEDEKRKRKL
1121 LEDRYDQLKS DFEQLQKARQ NEKETLGWQK IESEKIIKEK EYEIERLRVL LQDEGTRKRE CENELAKTSH RIQESKSQCN
1201 HIMQERETLL VKIKTLEQDK ARLLRLEEDL NRTKGMLESE SRLKARLENE KQQILNDLNQ WKSQFSRKEE IIRITECERD
1281 KSEREKSSLL IEIERLQAEI KRIEERYRCR LEETSRKSQA EMERERERLE KEIEKLKKRP YGAHRGTQTE EHLMIDPSKL
1361 LFNGLRKKIT AVQLYECQLI DKCTLDKLLR GQKSVEEVAA ELEPYLRGAG AIAGASLNTK EKYSLVEAKR KQLLTPENTI
1441 LLLEAQAATG GVIDPHRNEL LTVDNAVARD LVDFDDREQI YMAEKAITGF KDPFSGKTMS VAEAIKKNLV DRETGMRLLE
1521 AQLASGGIVD PINSIFLPKD IALSRGLIDR DLYRALNNPS KPLIDPLSKN AISYMKLRER CRIEPHTGLL LLPVQKRSMS
1601 FQGIRKLITV SELVDSGIIR ESTANELETG AITVEEVTDR IKDFLQGSSC IAGIYNEATG EKLGVYQAMK IGLVRPGTAL
1681 ELLEAQAATG FIVDPVNNLR LPVEEAYKRG LVGIEFKEKL LSAERAVTGY RDPETGNIIS LFQAMNKELI EKGHGVRLLE
1761 AQIATGGIID PKESHRLPVH TAYQRGYFNE ELNGILSDPS DDTKGFFDPN TEENLTYLQL KGRCKIDELT GLCLLPLREK
1841 KKVVQTSQKN TLRKRRVVIV DPDTNKEMSV QEAYNKGLID YSTYMELSDQ ECEWEEITTT GSDGSTRVVL VDRKTGHQYD
1921 IEDAIDKGLV DRKFFDQYRS GSLSLTQFAD MIANRNGGDE VFRHESVIRS PTILSVKSSS ILLKSSSGSF SDSPEEATPI
2001 AAIFDTENLE KISIPEAVQR GIVDTITAQR LLEAQACTGG IICPTTGQRL TLQDAVAQGI IDHEMATRLK PAQKAFLGFE
2081 GIKGRKRMSA AEAVKEKWLP YEAGQRFLEF QFITGGLVDP EVHGRISTEE AIRKGLIDGR TSQRLQDIGN YAKILTCPKT
2161 KLKISYREAM NRSMVEDQTG LRLLEASSVS SKGISSPYNV SSAPGSRSGS RSGSRSGSRS GSRRGSFDAG TSSSYSYSYT
2241 AVSNSSSFGR
```

**Supporting Peptides:**

| Peptide | Uniq | -10lgP | Mass | Length | ppm | m/z | z | RT | Fraction | Scan | Source File | Area F1 | Area F2a | Area F3a | Area F4 | Area F5 | Area F6a | Area F7a | Area F7b | Area F8 | Area F9a | Area Sample 1F10a | Area F10b | Area F11a | Area F11b | Area F12a | Area F13 | #Feature | #Feature F1 | #Feature F2a | #Feature F3a | #Feature F4 | #Feature F5 | #Feature F6a | #Feature F7a | #Feature F7b | #Feature F8 | #Feature F9a | #Feature Sample 1F10a | #Feature F10b | #Feature F11a | #Feature F11b | #Feature F12a | #Feature F13 | Start | End | PTM | AScore | Found By |
|---|---|---|---|---|---|---|---|---|---|---|---|---|---|---|---|---|---|---|---|---|---|---|---|---|---|---|---|---|---|---|---|---|---|---|---|---|---|---|---|---|---|---|---|---|---|---|---|---|---|
| R.LTYEIEDEKR.K | Y | 47.12 | 1294.6405 | 10 | 8.7 | 432.5566 | 3 | 38.20 | 8 | F8:16270 | BuCaTN01_F7b.raw | | | | | | | | 0 | 9.2313E4 | | | | | | | | 1 | 0 | 0 | 0 | 0 | 0 | 0 | 0 | 0 | 1 | 0 | 0 | 0 | 0 | 0 | 0 | 0 | 1107 | 1116 | | | PEAKS DB |
| total 1 peptides | | | | | | | | | | | | | | | | | | | | | | | | | | | | | | | | | | | | | | | | | | | | | | | | | |

**Best Unique PSM (Scan F8:16270, m/z=432.5566, z=3, RT=38.20, ppm=8.7):**

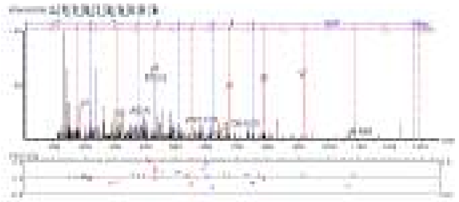

## XP_058034977.1

back to list

| Protein Coverage | Supporting Peptides | Best Unique PSM |

**Protein Coverage:**

```
   1 MSINGSHPRL NTLGRMVRAE SGPDMRYDMN SHMMMGGGGG GGGSSHTHKM YYVQKSYVGD INSEGYTQNG TATVSRRQNT
  81 IQDLLQACSD CLMRAELIVQ PELKYGDGIQ ISRNRELEDC FGQANEHMDI LDGLIREMRQ MGQPCDLYQK RLLQLQEQMR
 161 ALYKAISVPR VRRASSKGGG YSSQSASGWD EYTRRVTSEC LNWMRQQKTE MELVKWGFDA AAIEQQIAEH RRFHNSIGDY
 241 RWHLDKVKTD LREKAAVHQL EEEYEYLLRF SFERMDQLRQ LQNIIQATSR EIMWINDCEE EELLYDWSDR NTDIARKQEA
 321 FSKRMSQLEV KEKELNKLKQ ECDQLVLSQH PASDKIEAYM DTLQTQWSWI LQITKCIDVH LKENAAYFQF FEEAQSTENY
 401 LKNLQDAIRK KFICDKNMSL QVLLEQIKEL EAEREKILEY KRQVQNLVNK SKKIVQLKPR NPDYKSNKPI ILRALCDYKQ
 481 DLKTMRKGDE CILKDNSERS KWHVTGPGGL DMLVPSVSLI IPPPNPLAVD LATKIEQYYD AIMALWNQLY INMKSLVSWH
 561 YCMIDIEKIR AMTIAKLKTM RREDYQRIIT DLEIHYQEFI RNSQGSEMFG DDDKRNMQTQ ITDAQKHYQT LVMTLPRQQP
 641 TGPPIVTGVI TIEKEKQEAW LLMELQKLRR QIENCEFRMV QRTPFQLDQG ASHNLSVRIS ELESIQTESQ SMAEALNRQQ
 721 DYLPNFRGSE KYHYLQSEIS ALFQKLENIN GVSACYLDSL NALRSLLQAI LQTDDVIRVF EIRLTEEDTL SLDPDKVEAY
 801 RVCLKKMKAD LSMKKSLLGT LEAELQKALQ VHSQSSQTYP HYDLDLGKFA DRVSQLRDRW QRLEKQLDDR SWDLEKQVKQ
 881 LRVYRDLYQA FNKWICDARR RQDTIEAMKL GDVGTVMRYL QEQKNLHSEI TSKRDRVEEV VKNAEVCSLA IKDYELQAAA
 961 YSSGLETLLN IPVKRSMVQS PSGLILQEAG EIQSRYIELL TRSGDYYKFL SEMLKSLEDI KMKSTRIELL EEELRLAKDA
1041 NSDSNNKHKF LEQNLQKYQV ECSQLKAKFI SLEETKRQVE MDGSTAKQNL DKCYAQIKDL NERITRLTYE IEDEKRKRKL
1121 LEDRYDQLKS DFEQLQKARQ NEKETLGWQK IESEKIIKEK EYEIERLRVL LQDEGTRKRE CENELAKVRN QFNEEMSNIK
1201 NKYETEINIK KTTIHQIAAQ KEDDAKNLRT QIDRLQRENR DLKDEIVRLN ETILDTTEQR RREEENVLQQ KACVSEVIQQ
1281 KQQIELELKQ IIQLRNDDNT RYKQALEDAT STIHEKVKEL ERLKIQLQEE AKSRWELENE LAKTSHRIQE SKSQCNHIMQ
1361 ERETLLVKIK TLEQDKARLL RLEEDLNRTK GMLESESRLK ARLENEKQQI LNDLNQWKSQ FSRKEEIIRI TECERDKSER
1441 EKSSLLIEIE RLQAEIKRIE ERYRCRLEET SRKSQAEMER ERERLEKEIE KLKKRPYGAH RGTQTEEHLM IDPSKLLFNG
1521 LRKKITAVQL YECQLIDKCT LDKLLRGQKS VEEVAAELEP YLRGAGAIAG ASLNTKEKYS LVEAKRKQLL TPENTILLLE
1601 AQAATGGVID PHRNELLTVD NAVARDLVDF DDREQIYMAE KAITGFKDPF SGKTMSVAEA IKKNLVDRET GMRLLEAQLA
1681 SGGIVDPINS IFLPKDIALS RGLIDRDLYR ALNNPSKPLI DPLSKNAISY MKLRERCRIE PHTGLLLLPV QKRSMSFQGI
1761 RKLITVSELV DSGIIRESTA NELETGAITV EEVTDRIKDF LQGSSCIAGI YNEATGEKLG VYQAMKIGLV RPGTALELLE
1841 AQAATGFIVD PVNNLRLPVE EAYKRGLVGI EFKEKLLSAE RAVTGYRDPE TGNIISLFQA MNKELIEKGH GVRLLEAQIA
1921 TGGIIDPKES HRLPVHTAYQ RGYFNEELNG ILSDPSDDTK GFFDPNTEEN LTYLQLKGRC KIDELTGLCL LPLREKKKVV
2001 QTSQKNTLRK RRVVIVDPDT NKEMSVQEAY NKGLIDYSTY MELSDQECEW EEITTTGSDG STRVVLVDRK TGHQYDIEDA
2081 IDKGLVDRKF FDQYRSGSLS LTQFADMIAN RNGGDEVFRH ESVIRSPTIL SVKSSSILLK SSSGSFSDSP EEATPIAAIF
2161 DTENLEKISI PEAVQRGIVD TITAQRLLEA QACTGGIICP TTGQRLTLQD AVAQGIIDHE MATRLKPAQK AFLGFEGIKG
2241 RKRMSAAEAV KEKWLPYEAG QRFLEFQFIT GGLVDPEVHG RISTEEAIRK GLIDGRTSQR LQDIGNYAKI LTCPKTKLKI
2321 SYREAMNRSM VEDQTGLRLL EASSVSSKGI SSPYNVSSAP GSRSGSRSGS RSGSRSGSRR GSFDAGTSSS YSYSYTAVSN
2401 SSSFGR
```

**Supporting Peptides:**

| Peptide | Uniq | -10lgP | Mass | Length | ppm | m/z | z | RT | Fraction | Scan | Source File | Area F1 | Area F2a | Area F3a | Area F4 | Area F5 | Area F6a | Area F7a | Area F7b | Area F8 | Area F9a | Area Sample 1F10a | Area F10b | Area F11a | Area F11b | Area F12a | Area F13 | #Feature | #Feature F1 | #Feature F2a | #Feature F3a | #Feature F4 | #Feature F5 | #Feature F6a | #Feature F7a | #Feature F7b | #Feature F8 | #Feature F9a | #Feature Sample 1F10a | #Feature F10b | #Feature F11a | #Feature F11b | #Feature F12a | #Feature F13 | Start | End | PTM | AScore | Found By |
|---|---|---|---|---|---|---|---|---|---|---|---|---|---|---|---|---|---|---|---|---|---|---|---|---|---|---|---|---|---|---|---|---|---|---|---|---|---|---|---|---|---|---|---|---|---|---|---|---|---|
| R.LTYEIEDEKR.K | Y | 47.12 | 1294.6405 | 10 | 8.7 | 432.5566 | 3 | 38.20 | 8 | F8:16270 | BuCaTN01_F7b.raw | | | | | | | | 0 | 9.2313E4 | | | | | | | | 1 | 0 | 0 | 0 | 0 | 0 | 0 | 0 | 0 | 1 | 0 | 0 | 0 | 0 | 0 | 0 | 0 | 1107 | 1116 | | | PEAKS DB |
| total 1 peptides | | | | | | | | | | | | | | | | | | | | | | | | | | | | | | | | | | | | | | | | | | | | | | | | | |

**Best Unique PSM (Scan F8:16270, m/z=432.5566, z=3, RT=38.20, ppm=8.7):**

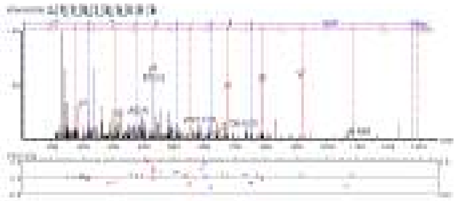

## XP_058034976.1

back to list

| Protein Coverage | Supporting Peptides | Best Unique PSM |

**Protein Coverage:**

```
   1 MSINGSHPRL NTLGRMVRAE SGPDMRYDMN SHMMMGGGGG GGGSSHTHKM YYVQKSYVGD INSEGYTQNG TATVSRRQNT
  81 IQDLLQACSD CLMRAELIVQ PELKYGDGIQ ISRNRELEDC FGQANEHMDI LDGLIREMRQ MGQPCDLYQK RLLQLQEQMR
 161 ALYKAISVPR VRRASSKGGG YSSQSASGWD EYTRRVTSEC LNWMRQQKTE MELVKWGFDA AAIEQQIAEH RRFHNSIGDY
 241 RWHLDKVKTD LREKAAVHQL EEEYEYLLRF SFERMDQLRQ LQNIIQATSR EIMWINDCEE EELLYDWSDR NTDIARKQEA
 321 FSKRMSQLEV KEKELNKLKQ ECDQLVLSQH PASDKIEAYM DTLQTQWSWI LQITKCIDVH LKENAAYFQF FEEAQSTENY
 401 LKNLQDAIRK KFICDKNMSL QVLLEQIKEL EAEREKILEY KRQVQNLVNK SKKIVQLKPR NPDYKSNKPI ILRALCDYKQ
 481 DLKTMRKGDE CILKDNSERS KWHVTGPGGL DMLVPSVSLI IPPPNPLAVD LATKIEQYYD AIMALWNQLY INMKSLVSWH
 561 YCMIDIEKIR AMTIAKLKTM RREDYQRIIT DLEIHYQEFI RNSQGSEMFG DDDKRNMQTQ ITDAQKHYQT LVMTLPRQQP
 641 TGPPIVTGVI TIEKEKQEAW LLMELQKLRR QIENCEFRMV QRTPFQLDQG ASHNLSVRIS ELESIQTESQ SMAEALNRQQ
 721 DYLPNFRGSE KYHYLQSEIS ALFQKLENIN GVSACYLDSL NALRSLLQAI LQTDDVIRVF EIRLTEEDTL SLDPDKVEAY
 801 RVCLKKMKAD LSMKKSLLGT LEAELQKALQ VHSQSSQTYP HYDLDLGKFA DRVSQLRDRW QRLEKQLDDR SWDLEKQVKQ
 881 LRVYRDLYQA FNKWICDARR RQDTIEAMKL GDVGTVMRYL QEQKNLHSEI TSKRDRVEEV VKNAEVCSLA IKDYELQAAA
 961 YSSGLETLLN IPVKRSMVQS PSGLILQEAG EIQSRYIELL TRSGDYYKFL SEMLKSLEDI KMKSTRIELL EEELRLAKDA
1041 NSDSNNKHKF LEQNLQKYQV ECSQLKAKFI SLEETKRQVE MDGSTAKQNL DKCYAQIKDL NERITRLTYE IEDEKRKRKL
1121 LEDRYDQLKS DFEQLQKARQ NEKETLGWQK IESEKIIKEK EYEIERLRVL LQDEGTRKRE CENELAKVRN QFNEEMSNIK
1201 NKYETEINIK KTTIHQIAAQ KEDDAKNLRT QIDRLQRENR DLKDEIVRLN ETILDTTEQR RREEENVLQQ KACVSEVIQQ
1281 KQQIELELKQ IIQLRNDDNT RYKQALEDAT STIHEKVKEL ERLKIQLQEE AKSRWELENE LAKVRNSYDE EIISLKNKYE
1361 TEINITKTTI NQVTVKKEEE ASNYRAQLDN AVRENRSLCE EIRRLKNTLS QTTDNLRKVE ENAHQQKAIG SEISQKKQHL
1441 EKELKQTVQK FTEEIAHYKQ SLDEAGRTLK EKNKEIEKNR KLLDAEICQR KSMEEENIRL QRIQYDLEKA NINATETISR
1521 LKVQEQELGR LKSEYERLAQ DKKGKDQEST RLQSTIKEMQ HQKHMLEEEL NRQSRIAAEE TSKRKNAEEE LEAMRRTLRE
1601 QAVKITNLTQ QIEEISIVKK RSEDDLRHQR EVLDNQMRDK QRYMEEIRKY TSEIEALRRQ LVQEQENLKQ AHIRNEHLQK
1681 AIEDKSKSLN ECKIEIERLQ CLTENLTKEH LMLEEELRNV RLEYDDLRRG RSEVNEEKNA TILDLKNQLQ TSNKHALELQ
1761 GVINGLQKER ENLRQEIEKF QKQAVETSHR IQESKSQCNH IMQERETLLV KIKTLEQDKA RLLRLEEDLN RTKGMLESES
1841 RLKARLENEK QQILNDLNQW KSQFSRKEEI IRITECERDK SEREKSSLLI EIERLQAEIK RIEERYRCRL EETSRKSQAE
1921 MERERERLEK EIEKLKKRPY GAHRGTQTEE HLMIDPSKLL FNGLRKKITA VQLYECQLID KCTLDKLLRG QKSVEEVAAE
2001 LEPYLRGAGA IAGASLNTKE KYSLVEAKRK QLLTPENTIL LLEAQAATGG VIDPHRNELL TVDNAVARDL VDFDDREQIY
2081 MAEKAITGFK DPFSGKTMSV AEAIKKNLVD RETGMRLLEA QLASGGIVDP INSIFLPKDI ALSRGLIDRD LYRALNNPSK
2161 PLIDPLSKNA ISYMKLRERC RIEPHTGLLL LPVQKRSMSF QGIRKLITVS ELVDSGIIRE STANELETGA ITVEEVTDRI
2241 KDFLQGSSCI AGIYNEATGE KLGVYQAMKI GLVRPGTALE LLEAQAATGF IVDPVNNLRL PVEEAYKRGL VGIEFKEKLL
2321 SAERAVTGYR DPETGNIISL FQAMNKELIE KGHGVRLLEA QIATGGIIDP KESHRLPVHT AYQRGYFNEE LNGILSDPSD
2401 DTKGFFDPNT EENLTYLQLK GRCKIDELTG LCLLPLREKK KVVQTSQKNT LRKRRVVIVD PDTNKEMSVQ EAYNKGLIDY
2481 STYMELSDQE CEWEEITTTG SDGSTRVVLV DRKTGHQYDI EDAIDKGLVD RKFFDQYRSG SLSLTQFADM IANRNGGDEV
2561 FRHESVIRSP TILSVKSSSI LLKSSSGSFS DSPEEATPIA AIFDTENLEK ISIPEAVQRG IVDTITAQRL LEAQACTGGI
2641 ICPTTGQRLT LQDAVAQGII DHEMATRLKP AQKAFLGFEG IKGRKRMSAA EAVKEKWLPY EAGQRFLEFQ FITGGLVDPE
2721 VHGRISTEEA IRKGLIDGRT SQRLQDIGNY AKILTCPKTK LKISYREAMN RSMVEDQTGL RLLEASSVSS KGISSPYNVS
2801 SAPGSRSGSR SGSRSGSRSG SRRGSFDAGT SSSYSYSYTA VSNSSSFGR
```

**Supporting Peptides:**

| Peptide | Uniq | -10lgP | Mass | Length | ppm | m/z | z | RT | Fraction | Scan | Source File | Area F1 | Area F2a | Area F3a | Area F4 | Area F5 | Area F6a | Area F7a | Area F7b | Area F8 | Area F9a | Area Sample 1F10a | Area F10b | Area F11a | Area F11b | Area F12a | Area F13 | #Feature | #Feature F1 | #Feature F2a | #Feature F3a | #Feature F4 | #Feature F5 | #Feature F6a | #Feature F7a | #Feature F7b | #Feature F8 | #Feature F9a | #Feature Sample 1F10a | #Feature F10b | #Feature F11a | #Feature F11b | #Feature F12a | #Feature F13 | Start | End | PTM | AScore | Found By |
|---|---|---|---|---|---|---|---|---|---|---|---|---|---|---|---|---|---|---|---|---|---|---|---|---|---|---|---|---|---|---|---|---|---|---|---|---|---|---|---|---|---|---|---|---|---|---|---|---|---|
| R.LTYEIEDEKR.K | Y | 47.12 | 1294.6405 | 10 | 8.7 | 432.5566 | 3 | 38.20 | 8 | F8:16270 | BuCaTN01_F7b.raw | | | | | | | | 0 | 9.2313E4 | | | | | | | | 1 | 0 | 0 | 0 | 0 | 0 | 0 | 0 | 0 | 1 | 0 | 0 | 0 | 0 | 0 | 0 | 0 | 1107 | 1116 | | | PEAKS DB |
| total 1 peptides | | | | | | | | | | | | | | | | | | | | | | | | | | | | | | | | | | | | | | | | | | | | | | | | | |

**Best Unique PSM (Scan F8:16270, m/z=432.5566, z=3, RT=38.20, ppm=8.7):**

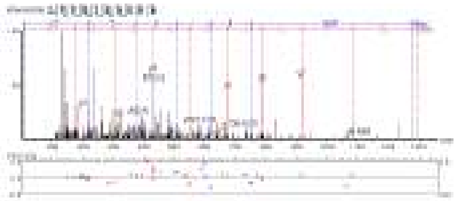

## XP_032079163.1

back to list

| Protein Coverage | Supporting Peptides | Best Unique PSM |

**Protein Coverage:**

```
   1 MSINGSHPRI NTLGRMVRAE SGPDMRYDVN SHMMMGGGGV GVGGSNHSHK QMYYVQKSYV GDINSEGYTQ NGNATMSRRQ
  81 NTIQDLMQSC SDCLMRAELI VQPELKYGEG IQISRNRELE ECFGQANEHM DILDGLIREM RQMGQPCDLY QKRLLQLQEQ
 161 MRALYKAISV PRVRRASSKG GGYSSQSASG WDEYTRRVTS ECLNWMRQQK TEMELVKWGF DATAIEQQIA EHRRFHNSIG
 241 DYRWHLDKVK TDLREKAAVH QLEEEYEYLL RFSFERMDQL RQLQNIIQAT SREIMWINDC EEEELLYDWS DRNTEISRKQ
 321 ETFSKRMSQL EVKEKELNKL KQECDQLVLS QHPASDKIEA YMDTLQTQWS WILQITKCID VHLKENAAYF QFFEEAQSTE
 401 NYLKNLQDVI RKRFICDKNM SLQVLLEQIK ELEAERDKIL EYKRQVQNLV NKSKKIVQLK PRNPEYKSSK PIILRALCDY
 481 KQDLKTMRKG DECILKDNSE RSKWHVTGPG GLDMLVPSVS LIIPPLNPFA VDLATKIEQY YDAIMALWNQ LYINMKSLVS
 561 WHYCMIDVEK IRAMTIAKLK TMRREDYQRI IADLEIHYQE FIRNSQGSEM FCDDDKRKMQ TQITDAQKHY QTLVIQIREP
 641 TKTPPVVIVP EAPEREKQEA WLLMELQKLR RQIENCEFRM GQRIPLQLDQ GASHNLSVRI SELESIQNES QSMAEVLNRQ
 721 NDYLPNFRGS EKYHYLQSEI SALFQKLENI NGVSACYLDS LNALRSLLQA ILQTDDMIRV FEIRLTEEET LSLDPEKVEA
 801 YRLCLKKMKA DFGMKKSLLG TLEAELQKAL QVHSQSSQSY PHYDLDLGKF ADRVSQLRDR WQRLEKQLDD RSWDLEKQVK
 881 QLRVYRELYQ ALNKWICDAR RRQDTIEGMK LGDVSTVMRY LQEQKNLHSE ITSKRDRVEE VVKNAEVCSL AIKDYELQAA
 961 AYSSGLETLL NIPVKRSMVQ SPSGLILQEA GEIQSRYIEL LTKSGDYYKF LNEILKNLED IKMKSTRIEL LEEELRLSKD
1041 ANSDSNNKHK FLEQNLQKYQ MECSQLKAKF ISLEEMKRQV EMDGCSAKQN LDKCYAQIKD LNERITRLTY EIEDEKRKRK
1121 LLEDRYDQQK SDFEQLQKTR HNEKETLGWQ KIESEKIVKE KEYEIERLRV LLQDEGTRKR ECENELAKTS HRIQESKSQC
1201 NHIMQERETL LVKIKSLEQD KARLLRLEED LNRTKGMLES ESRLKVRLEN EKQQILNDLN QWKSQYSRKE ETIRKTECER
1281 DKSEREKSAL LIEVERLQAE IKRIEERYRC RLEETSRKSQ AEMDTERLKL QKEIEKLKKR PYGAHRATQT EEDLMIDPSK
1361 LLFSGLRKKI TAVQLHECQL IDKCTLDKLL RGQKSVEEVA ADLEPYLRGA GAIAGASLNT KEKYSLVEAK RKQLLTPENT
1441 ILLLEAQAAT GGVIDPHRNE SLSVDSAVAR DLVDFDDREQ IYMAEKAITG FKDPFSGKTM SVAEAMKKNL VDREIGMRLL
1521 EAQLASGGIV DPVNSIFLPK DIALSRGLID RDLYRALNNP SKPLIDPLSK NAISYMKLRE RCRIEPHTGL LLLPVQKRSM
1601 SFQGIRKLIT VSELVDSGII RESTANELET GVITVEEVTD RIKDFLQGSS CIAGIYNEAT GEKLGVYQAM KIGLVRPGTA
1681 LELLEAQAAT GFIVDPVNNL RLPVEEAYKR GLVGIEFKEK LLSAERAVTG YKDPETGNII SLFQAMNKEL IEKGHGVRLL
1761 EAQIATGGII DPKESHRLPV HTAYQRGYFN QELNDILSDP SDDTKGFFDP NTEENLTYLQ LKGRCKIDEL TGLCLLPLRE
1841 KKKVVQTSQK NTLRKRRVVI VDPDTNKEMS VQEAYNKGLI DYSTYMELSD QECEWEEITT TASDGSTRVV LVDRKTGHQY
1921 DIEDSIDKGL VDRKFFDQYR SGSLSLTQFA DMIASRNGGD EVFKHESVIR SPTVLSVKSS SIVLKSSSGS FSDSPEEATP
2001 IAAIFDTENL EKISIPEAVQ RGIVDTITAQ RLLEAQACTG GIICPITGQR LSLQDAVAQG IIDHEMATRL KPAQKAFLGF
2081 EGIKGRKRMS AAEAVKEKWL PYEAGQRFLE FQFITGGLVD PDVHGRISTE EAIRKGLIDG RTSQRLQDIG NYGKILTCPK
2161 TKLKISYREA MNRSMVEDLT GLRLLEASSV SSKGISSPYN VSSAPGSRSG SRSGSRSGSR SGSRRGSFDA GTSSSYSYSY
2241 TSVSNSGSIG R
```

**Supporting Peptides:**

| Peptide | Uniq | -10lgP | Mass | Length | ppm | m/z | z | RT | Fraction | Scan | Source File | Area F1 | Area F2a | Area F3a | Area F4 | Area F5 | Area F6a | Area F7a | Area F7b | Area F8 | Area F9a | Area Sample 1F10a | Area F10b | Area F11a | Area F11b | Area F12a | Area F13 | #Feature | #Feature F1 | #Feature F2a | #Feature F3a | #Feature F4 | #Feature F5 | #Feature F6a | #Feature F7a | #Feature F7b | #Feature F8 | #Feature F9a | #Feature Sample 1F10a | #Feature F10b | #Feature F11a | #Feature F11b | #Feature F12a | #Feature F13 | Start | End | PTM | AScore | Found By |
|---|---|---|---|---|---|---|---|---|---|---|---|---|---|---|---|---|---|---|---|---|---|---|---|---|---|---|---|---|---|---|---|---|---|---|---|---|---|---|---|---|---|---|---|---|---|---|---|---|---|
| R.LTYEIEDEKR.K | Y | 47.12 | 1294.6405 | 10 | 8.7 | 432.5566 | 3 | 38.20 | 8 | F8:16270 | BuCaTN01_F7b.raw | | | | | | | | 0 | 9.2313E4 | | | | | | | | 1 | 0 | 0 | 0 | 0 | 0 | 0 | 0 | 0 | 1 | 0 | 0 | 0 | 0 | 0 | 0 | 0 | 1108 | 1117 | | | PEAKS DB |
| total 1 peptides | | | | | | | | | | | | | | | | | | | | | | | | | | | | | | | | | | | | | | | | | | | | | | | | | |

**Best Unique PSM (Scan F8:16270, m/z=432.5566, z=3, RT=38.20, ppm=8.7):**

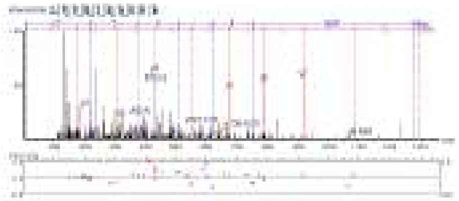

## XP_032079162.1

[back to list]

| **Protein Coverage** | **Supporting Peptides** | **Best Unique PSM** |

**Protein Coverage:**

```
   1 MSINGSHPRI NTLGRMVRAE SGPDMRYDVN SHMMMGGGGV GVGGSNHSHK QMYYVQKSYV GDINSEGYTQ NGNATMSRRQ
  81 NTIQDLMQSC SDCLMRAELI VQPELKYGEG IQISRNRELE ECFGQANEHM DILDGLIREM RQMGQPCDLY QKRLLQLQEQ
 161 MRALYKAISV PRVRRASSKG GGYSSQSASG WDEYTRRVTS ECLNWMRQQK TEMELVKWGF DATAIEQQIA EHRRFHNSIG
 241 DYRWHLDKVK TDLREKAAVH QLEEEYEYLL RFSFERMDQL RQLQNIIQAT SREIMWINDC EEEELLYDWS DRNTEISRKQ
 321 ETFSKRMSQL EVKEKELNKL KQECDQLVLS QHPASDKIEA YMDTLQTQWS WILQITKCID VHLKENAAYF QFFEEAQSTE
 401 NYLKNLQDVI RKRFICDKNM SLQVLLEQIK ELEAERDKIL EYKRQVQNLV NKSKKIVQLK PRNPEYKSSK PIILRALCDY
 481 KQDLKTMRKG DECILKDNSE RSKWHVTGPG GLDMLVPSVS LIIPPLNPFA VDLATKIEQY YDAIMALWNQ LYINMKSLVS
 561 WHYCMIDVEK IRAMTIAKLK TMRREDYQRI IADLEIHYQE FIRNSQGSEM FCDDDKRKMQ TQITDAQKHY QTLVIQIREP
 641 TKTPPVVIVP EAPEREKQEA WLLMELQKLR RQIENCEFRM GQRIPLQLDQ GASHNLSVRI SELESIQNES QSMAEVLNRQ
 721 NDYLPNFRGS EKYHYLQSEI SALFQKLENI NGVSACYLDS LNALRSLLQA ILQTDDMIRV FEIRLTEEET LSLDPEKVEA
 801 YRLCLKKMKA DFGMKKSLLG TLEAELQKAL QVHSQSSQSY PHYDLDLGKF ADRVSQLRDR WQRLEKQLDD RSWDLEKQVK
 881 QLRVYRELYQ ALNKWICDAR RRQDTIEGMK LGDVSTVMRY LQEQKNLHSE ITSKRDRVEE VVKNAEVCSL AIKDYELQAA
 961 AYSSGLETLL NIPVKRSMVQ SPSGLILQEA GEIQSRYIEL LTKSGDYYKF LNEILKNLED IKMKSTRIEL LEEELRLSKD
1041 ANSDSNNKHK FLEQNLQKYQ MECSQLKAKF ISLEEMKRQV EMDGCSAKQN LDKCYAQIKD LNERITRLTY EIEDEKRKRK
1121 LLEDRYDQQK SDFEQLQKTR HNEKETLGWQ KIESEKIVKE KEYEIERLRV LLQDEGTRKR ECENELAKVR NQFNEEMSNV
1201 KNKYETEINI KKTTIHQIAA QKEDDAKNLR TQIDRLQREN RDLKDEIVRL NETILDTTEQ RRRAEENVLQ QKACGSEVIQ
1281 QKQQIELELK QIIQLRNDDN ARYKQALEDA SSTIHEKVKE LERLKIQLQE EAKSRWELEN ELAKTSHRIQ ESKSQCNHIM
1361 QERETLLVKI KSLEQDKARL LRLEEDLNRT KGMLESESRL KVRLENEKQQ ILNDLNQWKS QYSRKEETIR KTECERDKSE
1441 REKSALLIEV ERLQAEIKRI EERYRCRLEE TSRKSQAEMD TERLKLQKEI EKLKKRPYGA HRATQTEEDL MIDPSKLLFS
1521 GLRKKITAVQ LHECQLIDKC TLDKLLRGQK SVEEVAADLE PYLRGAGAIA GASLNTKEKY SLVEAKRKQL LTPENTILLL
1601 EAQAATGGVI DPHRNESLSV DSAVARDLVD FDDREQIYMA EKAITGFKDP FSGKTMSVAE AMKKNLVDRE IGMRLLEAQL
1681 ASGGIVDPVN SIFLPKDIAL SRGLIDRDLY RALNNPSKPL IDPLSKNAIS YMKLRERCRI EPHTGLLLLP VQKRSMSFQG
1761 IRKLITVSEL VDSGIIREST ANELETGVIT VEEVTDRIKD FLQGSSCIAG IYNEATGEKL GVYQAMKIGL VRPGTALELL
1841 EAQAATGFIV DPVNNLRLPV EEAYKRGLVG IEFKEKLLSA ERAVTGYKDP ETGNIISLFQ AMNKELIEKG HGVRLLEAQI
1921 ATGGIIDPKE SHRLPVHTAY QRGYFNQELN DILSDPSDDT KGFFDPNTEE NLTYLQLKGR CKIDELTGLC LLPLREKKKV
2001 VQTSQKNTLR KRRVVIVDPD TNKEMSVQEA YNKGLIDYST YMELSDQECE WEEITTTASD GSTRVVLVDR KTGHQYDIED
2081 SIDKGLVDRK FFDQYRSGSL SLTQFADMIA SRNGGDEVFK HESVIRSPTV LSVKSSSIVL KSSSGSFSDS PEEATPIAAI
2161 FDTENLEKIS IPEAVQRGIV DTITAQRLLE AQACTGGIIC PITGQRLSLQ DAVAQGIIDH EMATRLKPAQ KAFLGFEGIK
2241 GRKRMSAAEA VKEKWLPYEA GQRFLEFQFI TGGLVDPDVH GRISTEEAIR KGLIDGRTSQ RLQDIGNYGK ILTCPKTKLK
2321 ISYREAMNRS MVEDLTGLRL LEASSVSSKG ISSPYNVSSA PGSRSGSRSG SRSGSRSGSR RGSFDAGTSS SYSYSYTSVS
2401 NSGSIGR
```

**Supporting Peptides:**

| Peptide | Uniq | -10lgP | Mass | Length | ppm | m/z | z | RT | Fraction | Scan | Source File | Area F1 | Area F2a | Area F3a | Area F4 | Area F5 | Area F6a | Area F7a | Area F7b | Area F8 | Area F9a | Area Sample 1F10a | Area F10b | Area F11a | Area F11b | Area F12a | Area F13 | #Feature | #Feature F1 | #Feature F2a | #Feature F3a | #Feature F4 | #Feature F5 | #Feature F6a | #Feature F7a | #Feature F7b | #Feature F8 | #Feature F9a | #Feature Sample 1F10a | #Feature F10b | #Feature F11a | #Feature F11b | #Feature F12a | #Feature F13 | Start | End | PTM | AScore | Found By |
|---|---|---|---|---|---|---|---|---|---|---|---|---|---|---|---|---|---|---|---|---|---|---|---|---|---|---|---|---|---|---|---|---|---|---|---|---|---|---|---|---|---|---|---|---|---|---|---|---|---|
| R.LTYEIEDEKR.K | Y | 47.12 | 1294.6405 | 10 | 8.7 | 432.5566 | 3 | 38.20 | 8 | F8:16270 | BuCaTN01_F7b.raw | | | | | | | | 0 | 9.2313E4 | | | | | | | | 1 | 0 | 0 | 0 | 0 | 0 | 0 | 0 | 0 | 1 | 0 | 0 | 0 | 0 | 0 | 0 | 0 | 1108 | 1117 | | | PEAKS DB |
| total 1 peptides | | | | | | | | | | | | | | | | | | | | | | | | | | | | | | | | | | | | | | | | | | | | | | | | | |

**Best Unique PSM (Scan F8:16270, m/z=432.5566, z=3, RT=38.20, ppm=8.7):**

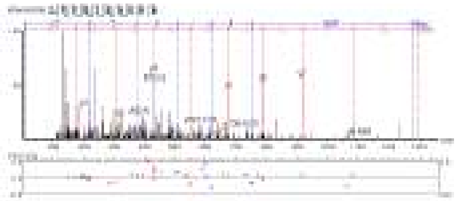

## XP_013912202.1

back to list

| Protein Coverage | Supporting Peptides | Best Unique PSM |

**Protein Coverage:**

```
   1 MRQMGQPCDH YQKKLLQLQE QMRALYKAIS VPRVRRASSK GGGYSSQSAS GWDEYTRRVT SECLNWMRQQ KTEMELVKWG
  81 FDATAIEQQI AEHRRFHNSI GDYRWHLDKV KTDLREKAAV HQLEEEYEYL LRFSFERMDQ LRQLQNIIQA TSREIMWIND
 161 CEEEELLYDW SDRNTEISRK QETFSKRMSQ LEVKEKELNK LKQECDQLVL SQHPASDKIE AYMDTLQTQW SWILQITKCI
 241 DVHLKENAAY FQFFEEAQST ENYLKNLQDV IRKRFICDKN MSLQVLLEQI KELEAERDKI LEYKRQVQNL VNKSKKIVQL
 321 KPRNPEYKSS KPIILRALCD YKQDLKTMRK GDECILKDNS ERSKWHVTGP GGLDMLVPSV SLIIPPLNPL AVDLATKIEQ
 401 YYDAIMALWN QLYINMKSLV SWHYCMIDVE KIRAMTIAKL KTMRREDYQR IIADLEIHYQ EFIRNSQGSE MFCDDDKRKM
 481 QTQITDAQKH YQTLVIQIRE PTKTPPVVIV PEAPEREKQE AWLLMELQKL RRQIENCEFR MGQRIPLQLD QGASHNLSVR
 561 ISELESIQNE SQSMAEVLNR QNDYLPNFRG SEKYHYLQSE ISALFQKLEN INGVSACYLD SLNALRSLLQ AILQTDDMIR
 641 VFEIRLTEEE TLSLDPEKVE AYRLCLKKMK ADLGMKKSLL GTLEAELQKA LQVHSQSSQS YPHYDLDLGK FADRVSQLRD
 721 RWQRLEKQLD DRSWDLEKQV KQLRVYRELY QALNKWICDA RRRQDTIEGM KLGDVSTVMR YLQEQKNLHS EITSKRDRVE
 801 EVVKNAEVCS LAIKDYELQA AAYSSGLETL LNIPVKRSMV QSPSGLILQE AGEIQSRYIE LLTKSGDYYK FLNEILKNLE
 881 DIKMKSTRIE LLEEELRLSK DANSDSNNKH KFLEQNLQKY QMECSQLKAK FISLEEMKRQ VEMDGCSAKQ NLDKCYAQIK
 961 DLNERITRLT YEIEDEKRKR KLLEDRYDQQ KSDFEQLQKT RHNEKETLGW QKIESEKIVK EKEYEIERLR VLLQDEGTRK
1041 RECENELAKV RNQFNEEMSN IKNKYETEIN IKKTTIHQIA AQKEDDAKNL RTQIDRLQRE NRDLKDEIVR LNESILDTTE
1121 QRRRAEENVL QQKACGSEVI QQKQQIELEL KQIIQLRNDD NARYKQALED ASSTIHEKVK ELERLKIQLQ EEAKSRWELE
1201 NELAKVRNSY DEEMISLKNK YETEINITKT TINQVTVKKE EEASNYRAQL DNSLRENRSL CEEIRRLKNS LSQTTESLRK
1281 VEENAHQQKA IGSEISQKKQ HLEKELKQTV QKFTEEIAHY KQSLDEAGRT LKEKNKEIEK NRKLLDAEIC QRKSLEEENI
1361 RLQRIQYDLE KANINATETI SRLKAQEQEL GRLKSEYERL AQDKKGKDQE STRLQSAMKE MQHQKHVLEE ELNRQSRIAA
1441 EETSKRKNAE EELEAMRRTL REQACKITHL TQQIEEISIV KKRSDDDLRH QREVLDSQVR DKQRYMEEIR KYTSEVEALR
1521 RQLVQEQENL KQAHIRNEHL QKAIEDKSKS LNECKIEIER LQCLTENLTK EHLMLEEELR NLRLEYDDLR RGRSEVDEEK
1601 NATILDLKNQ LQTTNKHVLE LQGVINGLHK ERENLRQEIE KFQKQALETS HRIQESKSQC NHIMQERETL LVKIKSLEQD
1681 KARLLRLEED LNRTKGMLES ESRLKVRLEN EKQQILNDLN QWKSQYSRKE ETIRKTECER DKSEREKSAL LIEIERLQAE
1761 IKRIEERYRC RLEETSRKSQ AEMDTERLKL QKEIEKLKKR PYGAHRATQT EEDLMIDPSK LLFSGLRKKI TAVQLHECQL
1841 IDKCTLDKLL RGQKSVEEVA ADLEPYLRGA GAIAGASLNT KEKYSLVEAK RKQLLTPENT ILLLEAQAAT GGVIDPHRNE
1921 SLSVDSAVAR DLVDFDDREQ IYMAEKAITG FKDPFSGKTM SVAEAMKKNL VDREIGMRLL EAQLASGGIV DPVNSIFLPK
2001 DIALSRGLID RDLYRALNNP SKPLIDPLSK NAISYMKLRE RCRIEPHTGL LLLPVQKRSM SFQGIRKLIT VSELVDSGII
2081 RESTANELET GVITVEEVTD RIKDFLQGSS CIAGIYNEAT GEKLGVYQAM KIGLVRPGTA LELLEAQAAT GFIVDPVNNL
2161 RLPVEEGYKR GLVGIEFKEK LLSAERAVTG YKDPETGNII SLFQAMNKEL IEKGHGVRLL EAQIATGGII DPKESHRLPV
2241 HTAYQRGYFN QELNDILSDP SDDTKGFFDP NTEENLTYLQ LKGRCKIDEL TGLCLLPLRE KKKVVQTSQK NTLRKRRVVI
2321 VDPDTNKEMS VQEAYNKGLI DYSTYMELSD QECEWEEITT TASDGSTRVV LVDRKTGHQY DIEDSIDKGL VDRKFFDQYR
2401 SGSLSLTQFA DMIASRNGGD EVFKHESVIR SPTVLSVKSS SIVLKSSSGS FSDSPEEATP IAAIFDTENL EKISIPEAVQ
2481 RGIVDTITAQ RLLEAQACTG GIICPITGQR LSLQDAVAQG IIDHEMATRL KPAQKAFLGF EGIKGRKRMS AAEAVKEKWL
2561 PYEAGQRFLE FQFITGGLVD PDVHGRISTE EAIRKGLIDG RTSQRLQDIG NYGKILTCPK TKLKISYREA MNRSMVEDLT
2641 GLRLLEASSV SSKGISSPYN VSSAPGSRSG SRSGSRSGSR SGSRRGSFDA GTSSSYSYSY TAVSNSGSIG R
```

**Supporting Peptides:**

| Peptide | Uniq | -10lgP | Mass | Length | ppm | m/z | z | RT | Fraction | Scan | Source File | Area F1 | Area F2a | Area F3a | Area F4 | Area F5 | Area F6a | Area F7a | Area F7b | Area F8 | Area F9a | Area Sample 1F10a | Area F10b | Area F11a | Area F11b | Area F12a | Area F13 | #Feature | #Feature F1 | #Feature F2a | #Feature F3a | #Feature F4 | #Feature F5 | #Feature F6a | #Feature F7a | #Feature F7b | #Feature F8 | #Feature F9a | #Feature Sample 1F10a | #Feature F10b | #Feature F11a | #Feature F11b | #Feature F12a | #Feature F13 | Start | End | PTM | AScore | Found By |
|---|---|---|---|---|---|---|---|---|---|---|---|---|---|---|---|---|---|---|---|---|---|---|---|---|---|---|---|---|---|---|---|---|---|---|---|---|---|---|---|---|---|---|---|---|---|---|---|---|---|
| R.LTYEIEDEKR.K | Y | 47.12 | 1294.6405 | 10 | 8.7 | 432.5566 | 3 | 38.20 | 8 | F8:16270 | BuCaTN01_F7b.raw | | | | | | | | 0 | 9.2313E4 | | | | | | | | 1 | 0 | 0 | 0 | 0 | 0 | 0 | 0 | 0 | 1 | 0 | 0 | 0 | 0 | 0 | 0 | 0 | 969 | 978 | | | PEAKS DB |
| total 1 peptides | | | | | | | | | | | | | | | | | | | | | | | | | | | | | | | | | | | | | | | | | | | | | | | | | |

**Best Unique PSM (Scan F8:16270, m/z=432.5566, z=3, RT=38.20, ppm=8.7):**

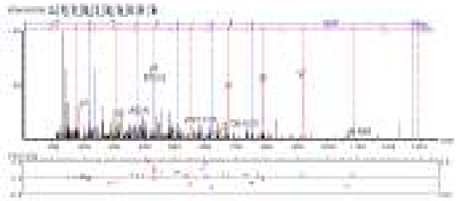

## XP_032079161.1

back to list

| Protein Coverage | Supporting Peptides | Best Unique PSM |

**Protein Coverage:**

1 MSINGSHPRI NTLGRMVRAE SGPDMRYDVN SHMMMGGGGV GVGGSNHSHK QMYYVQKSYV GDINSEGYTQ NGNATMSRRQ
81 NTIQDLMQSC SDCLMRAELI VQPELKYGEG IQISRNRELE ECFGQANEHM DILDGLIREM RQMGQPCDLY QKRLLQLQEQ
161 MRALYKAISV PRVRRASSKG GGYSSQSASG WDEYTRRVTS ECLNWMRQQK TEMELVKWGF DATAIEQQIA EHRRFHNSIG
241 DYRWHLDKVK TDLREKAAVH QLEEEYEYLL RFSFERMDQL RQLQNIIQAT SREIMWINDC EEEELLYDWS DRNTEISRKQ
321 ETFSKRMSQL EVKEKELNKL KQECDQLVLS QHPASDKIEA YMDTLQTQWS WILQITKCID VHLKENAAYF QFFEEAQSTE
401 NYLKNLQDVI RKRFICDKNM SLQVLLEQIK ELEAERDKIL EYKRQVQNLV NKSKKIVQLK PRNPEYKSSK PIILRALCDY
481 KQDLKTMRKG DECILKDNSE RSKWHVTGPG GLDMLVPSVS LIIPPLNPFA VDLATKIEQY YDAIMALWNQ LYINMKSLVS
561 WHYCMIDVEK IRAMTIAKLK TMRREDYQRI IADLEIHYQE FIRNSQGSEM FCDDDKRKMQ TQITDAQKHY QTLVIQIREP
641 TKTPPVVIVP EAPEREKQEA WLLMELQKLR RQIENCEFRM GQRIPLQLDQ GASHNLSVRI SELESIQNES QSMAEVLNRQ
721 NDYLPNFRGS EKYHYLQSEI SALFQKLENI NGVSACYLDS LNALRSLLQA ILQTDDMIRV FEIRLTEEET LSLDPEKVEA
801 YRLCLKKMKA DFGMKKSLLG TLEAELQKAL QVHSQSSQSY PHYDLDLGKF ADRVSQLRDR WQRLEKQLDD RSWDLEKQVK
881 QLRVYRELYQ ALNKWICDAR RRQDTIEGMK LGDVSTVMRY LQEQKNLHSE ITSKRDRVEE VVKNAEVCSL AIKDYELQAA
961 AYSSGLETLL NIPVKRSMVQ SPSGLILQEA GEIQSRYIEL LTKSGDYYKF LNEILKNLED IKMKSTRIEL LEEELRLSKD
1041 ANSDSNNKHK FLEQNLQKYQ MECSQLKAKF ISLEEMKRQV EMDGCSAKQN LDKCYAQIKD LNERITR**LTY EIEDEKR**KRK

1121 LLEDRYDQQK SDFEQLQKTR HNEKETLGWQ KIESEKIVKE KEYEIERLRV LLQDEGTRKR ECENELAKVR NQFNEEMSNV
1201 KNKYETEINI KKTTIHQIAA QKEDDAKNLR TQIDRLQREN RDLKDEIVRL NETILDTTEQ RRRAEENVLQ QKACGSEVIQ
1281 QKQQIELELK QIIQLRNDDN ARYKQALEDA SSTIHEKVKE LERLKIQLQE EAKSRWELEN ELAKVRNSYD EEMISLKNKY
1361 ETEINITKTT INQVTVKKEE EASNYRAQLD NSLRENRSLC EEIRRLKNSL SQTTESLRKV EENAHQQKAI GSEISQKKQH
1441 LEKELKQTVQ KFTEEIAHYK QSLDEAGRTL KEKNKEIEKN RKLLDAEICQ RKSLEEENIR LQRIQYDLEK ANINATETIS
1521 RLKAQEQELG RLKSEYERLA QDKKGKDQES TRLQSTMKEM QHQKHVLEEE LNRQSRIAAE ETSKRKNAEE ELEAMRRTLR
1601 EQACKITHLT QQIEEISIVK KRSDDDLRHQ REVLDSQVRD KQRYMEEIRK YTSEVEALRR QLVQEQENLK QAHIRNEHLQ
1681 KAIEDKSKSL NECKIEIERL QCLTENLTKE HLMLEEELRN LRLEYDDLRR GRSEVDEEKN ATILDLKNQL QTTNKHVLEL
1761 QGVINGLHKE RENLRQEIEK FQKQALETSH RIQESKSQCN HIMQERETLL VKIKSLEQDK ARLLRLEEDL NRTKGMLESE
1841 SRLKVRLENE KQQILNDLNQ WKSQYSRKEE TIRKTECERD KSEREKSALL IEVERLQAEI KRIEERYRCR LEETSRKSQA
1921 EMDTERLKLQ KEIEKLKKRP YGAHRATQTE EDLMIDPSKL LFSGLRKKIT AVQLHECQLI DKCTLDKLLR GQKSVEEVAA
2001 DLEPYLRGAG AIAGASLNTK EKYSLVEAKR KQLLTPENTI LLLEAQAATG GVIDPHRNES LSVDSAVARD LVDFDDREQI
2081 YMAEKAITGF KDPFSGKTMS VAEAMKKNLV DREIGMRLLE AQLASGGIVD PVNSIFLPKD IALSRGLIDR DLYRALNNPS
2161 KPLIDPLSKN AISYMKLRER CRIEPHTGLL LLPVQKRSMS FQGIRKLITV SELVDSGIIR ESTANELETG VITVEEVTDR
2241 IKDFLQGSSC IAGIYNEATG EKLGVYQAMK IGLVRPGTAL ELLEAQAATG FIVDPVNNLR LPVEEAYKRG LVGIEFKEKL
2321 LSAERAVTGY KDPETGNIIS LFQAMNKELI EKGHGVRLLE AQIATGGIID PKESHRLPVH TAYQRGYFNQ ELNDILSDPS
2401 DDTKGFFDPN TEENLTYLQL KGRCKIDELT GLCLLPLREK KKVVQTSQKN TLRKRRVVIV DPDTNKEMSV QEAYNKGLID
2481 YSTYMELSDQ ECEWEEITTT ASDGSTRVVL VDRKTGHQYD IEDSIDKGLV DRKFFDQYRS GSLSLTQFAD MIASRNGGDE
2561 VFKHESVIRS PTVLSVKSSS IVLKSSSGSF SDSPEEATPI AAIFDTENLE KISIPEAVQR GIVDTITAQR LLEAQACTGG
2641 IICPITGQRL SLQDAVAQGI IDHEMATRLK PAQKAFLGFE GIKGRKRMSA AEAVKEKWLP YEAGQRFLEF QFITGGLVDP
2721 DVHGRISTEE AIRKGLIDGR TSQRLQDIGN YGKILTCPKT KLKISYREAM NRSMVEDLTG LRLLEASSVS SKGISSPYNV
2801 SSAPGSRSGS RSGSRSGSRS GSRRGSFDAG TSSSYSYSYT SVSNSGSIGR

**Supporting Peptides:**

| Peptide | Uniq | -10lgP | Mass | Length | ppm | m/z | z | RT | Fraction | Scan | Source File | Area F1 | Area F2a | Area F3a | Area F4 | Area F5 | Area F6a | Area F7a | Area F7b | Area F8 | Area F9a | Area Sample 1F10a | Area F10b | Area F11a | Area F11b | Area F12a | Area F13 | #Feature | #Feature F1 | #Feature F2a | #Feature F3a | #Feature F4 | #Feature F5 | #Feature F6a | #Feature F7a | #Feature F7b | #Feature F8 | #Feature F9a | #Feature Sample 1F10a | #Feature F10b | #Feature F11a | #Feature F11b | #Feature F12a | #Feature F13 | Start | End | PTM | AScore | Found By |
|---|---|---|---|---|---|---|---|---|---|---|---|---|---|---|---|---|---|---|---|---|---|---|---|---|---|---|---|---|---|---|---|---|---|---|---|---|---|---|---|---|---|---|---|---|---|---|---|---|---|
| R.LTYEIEDEKR.K | Y | 47.12 | 1294.6405 | 10 | 8.7 | 432.5566 | 3 | 38.20 | 8 | F8:16270 | BuCaTN01_F7b.raw | | | | | | | | 0 | 9.2313E4 | | | | | | | | 1 | 0 | 0 | 0 | 0 | 0 | 0 | 0 | 0 | 1 | 0 | 0 | 0 | 0 | 0 | 0 | 0 | 1108 | 1117 | | | PEAKS DB |
| total 1 peptides | | | | | | | | | | | | | | | | | | | | | | | | | | | | | | | | | | | | | | | | | | | | | | | | | |

**Best Unique PSM (Scan F8:16270, m/z=432.5566, z=3, RT=38.20, ppm=8.7):**

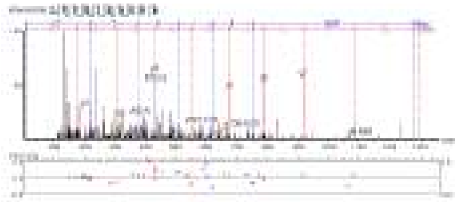

## XP_015666325.1

back to list

| Protein Coverage | Supporting Peptides | Best Unique PSM |

**Protein Coverage:**

1 MSINGSHPRL NTLGRMVRAE SGPDMRYDMN SHMMMGGGGG GGGGSSHTHK MYYVQKSYVG DINSEGYTQN GTATVSRRQN
81 TIQDLLQACS DCLMRAELIV QPELKYGDGI QISRNRELEE CFAQANEHME ILEGLIREMR QMGQPCELYQ KRLLQLQEQM
161 RALYKAISVP RVRRASSKGG GYSSQSASGW DEYTRRVTSE CLNWMRQQKT EMELIKWGFD AAAIDQQIAE HRRFHNSIGD
241 YRWHLDKVKT DLREKAAVHQ LEEEYEYLLR FSFERMDQLR QLQNIIQATS REIMWINDCE EEELLYDWSD RNTDIARKQE
321 VFSKRMSQLE VKEKELNKLK QECDQLVLSQ HPASDKIEAY MDTLQTQWSW ILQITKCIDV HLKENAAYFQ FFEEAQSTEN
401 YLKNLQDVIR KRFICDKNMS LQVLLEQIKE LETEREKILE YKRQVQNLVN KSKKIVQLKP RNPDYKTSKP IILRALCDYK
481 QDLKTMRKGD ECILKDNSER SKWHVTGPGG LDMLVPSVSL IIPPPNSLAV DLAAKIEQYY DAIMALWNQL YINMKSLVSW
561 HYCMIDIEKI KVMTIAKLKT MRREDYQRII SDLEIHYQEF IRNSQGSEMF GDDDKRTMQT QITDAQKHYQ TLVIQLPGSQ
641 PPPTIQRPIV TEVINIEKQE AWLLMELQKL RRQIENCELR TMQRTPFQLD QGATHNLCVR ISELESIQNE SQSMAEILNR
721 QNDYLPNFRG SEKYHYLQSE ISALFQKLEN INGISACYLD SLNALRSLLQ AILQTDDMIR VFEIRLTEED TLSLDPDKVE
801 AYRACLKKMK ADLSMKKSLL GTLEAELQKA LQVHSQSSQT YPHYELDLGK FADRVSQLTD RWQRLEKQLD DRSWDLEKQV
881 KQLRIYRDLY QALNKWICDA RRRQDTIEAM KLGDVSTVMR YLQEQKNLHG EITSKRDRVE EVIKNAEVCS LAIKDYELQA
961 AAYSSGLETL LNIPVKRSMV QSPSGLILQE AGEIQSRYIE LLTRSGDYYR FLSEMLKSLE DIKMKSTRIE LLEEELRLAK
1041 DANSDSNNKH KFLEQNLQKY QVECSQLKAK FISLEEMKRQ VEMDGSTSKQ NLDKCYAQIK DLNDRITR**LT YEIEDEKR**KR

1121 KLLEDRYDQQ KADFDQLQKT RQNEKETLGW QKIESEKIVK EKEYELERLR VLLQDEGTRK RECENELAKV RNQFNEEMSN
1201 IKNKYETEIN IKKTTIHQIA VQKEDDAKNL RTQIDRLQRE NRDLKDEIVR LNEAILDTTE QRRRAEENIL QQKACGSEVM
1281 QQKQQIELEL KQIIQLRNDD NTRYKQALED ATSTIHEKVK EIERVKIQLK EEAKSRWELE NELAKVRNSY DEEIISLKNK
1361 YETEINITKT TINQVTVKKE EEASNYRAQL DNAVRENRSL CEELRRLKNT LSQTTENLRK VEESAHQQKS IGSEISQKKQ
1441 QLEKELKQTV QKFTEEIAHY KQSLDEAGRT IKEKNKEIEK NRKLLDAEIS QRKSMEEENI RLQRIQYDLE KANINATETI
1521 SRLKVQEQEL GRLKSEYERL AQDKKGKDQE STRLQSTIKE LQHQKHILEE ELNRQSRMVA DETSKRKNAE EELEAMRRTL
1601 RDQAVKITNL TQQIEEISIV KKRGEDDLRH QREVLDNHMR DKQRYMEEIR KYTSEVESLR RQMVQEQENL KQAHIRNEHL
1681 QKAIEDKSKS LNECKIEIER LQCLTENLTK EHLMLEEELR NLRLEYDDLR RGRSEVDEEK NATILDLKNQ LQTSNKHALE
1761 LQGVINGLQK ERENLRQEIE KFQKQSLETS HRIQESKNQC NHIMQERETL LIKVKTLEQD KARLLRLEED LNRTKAMLES
1841 ESRLKVRLEN EKQQILNDLN QWKGQYSRKE ETIRKTECER DKSEREKSAL LIEIERLQAE IKRVEERYRC RLEETSRKSQ
1921 AEMETERLKS QREIEKLKQR PKGAPKGTQT EEDLMIDPSK LLFNGLRKKI TAVQLYECQL IDKCTLDKLL RGQKSVEEVA
2001 AELEPYLRGA GAIAGASLNT KAKFSLVEAK RKQLLTPENT VLLLEAQAAT GGVIDPHRNE LLSVDSAVAR DLIEFDDREH
2081 IYMAEKAITG FKDPFSGKIM PVAEAMKKNL VDREIGLRLL EAQLASGGIV DPINSIFLPK DIALSRGLID RDLYRALNNP
2161 SKPLIDPLSK TAISYMKLRE KCRIEPHTGL LLLPVQKRST SFQGIRKLIS VSELVESGII RESTANELES GVITVEEVTD
2241 RIKDFLQGSS CIAGIYNEAT GEKLGVYQAM KIGLVRPGTA LELLEAQAAT GFIVDPVNNL RLPVEEGYKR GLVGIEFKEK
2321 LLSAERAVTG YRDPETGNII SLFQAMNKEL IEKGHGIRLL EAQIATGGII DPKESHRLPV HIAYQRGYFN EELNDILSDP
2401 NDDTKGFFDP NTEENLTYLQ LKGRCKIDEL TGLCLLPLRE KKKVVQTSQK NTLRKRRVVI VDPDTNKEMS VQEAYNKGLI
2481 DYSTYLELSD QECEWEEITT TGSDGSTRVV LVDRKTGHQY DIEDALDKGL VDRKFFDQYR SGSLSLTQFA DMIASRNGGD
2561 EVFRHESVIR SPTVVSVKSS SILLKSSSGS FSDSPEETTP IAAIFDTENL EKISISEAVQ RGIVDTITAQ RLLEAQTCTG
2641 GIICPTTGQR LSLQDAVAQG IIDHEMATRL KPAQKAFLGF EGIKGRKRMS AAEAVKEKWL PYEAGQRFLE FQFITGGLID
2721 PEVHGRISTE EAIRKGLIDG RTSQRLQDIS NYAKILTCPK TKLKISYREA MNRSMVEDQT GLRLLEASSV SSKGISSPYN
2801 VSSAPGSRSG SRSGSRSGSR SGSRRGSFDA GTSSSYSYSY TAVSSSSLGR

**Supporting Peptides:**

| Peptide | Uniq | -10lgP | Mass | Length | ppm | m/z | z | RT | Fraction | Scan | Source File | Area F1 | Area F2a | Area F3a | Area F4 | Area F5 | Area F6a | Area F7a | Area F7b | Area F8 | Area F9a | Area Sample 1F10a | Area F10b | Area F11a | Area F11b | Area F12a | Area F13 | #Feature | #Feature F1 | #Feature F2a | #Feature F3a | #Feature F4 | #Feature F5 | #Feature F6a | #Feature F7a | #Feature F7b | #Feature F8 | #Feature F9a | #Feature Sample 1F10a | #Feature F10b | #Feature F11a | #Feature F11b | #Feature F12a | #Feature F13 | Start | End | PTM | AScore | Found By |
|---|---|---|---|---|---|---|---|---|---|---|---|---|---|---|---|---|---|---|---|---|---|---|---|---|---|---|---|---|---|---|---|---|---|---|---|---|---|---|---|---|---|---|---|---|---|---|---|---|---|
| R.LTYEIEDEKR.K | Y | 47.12 | 1294.6405 | 10 | 8.7 | 432.5566 | 3 | 38.20 | 8 | F8:16270 | BuCaTN01_F7b.raw | | | | | | | | 0 | 9.2313E4 | | | | | | | | 1 | 0 | 0 | 0 | 0 | 0 | 0 | 0 | 0 | 1 | 0 | 0 | 0 | 0 | 0 | 0 | 0 | 1109 | 1118 | | | PEAKS DB |
| total 1 peptides | | | | | | | | | | | | | | | | | | | | | | | | | | | | | | | | | | | | | | | | | | | | | | | | | |

**Best Unique PSM (Scan F8:16270, m/z=432.5566, z=3, RT=38.20, ppm=8.7):**

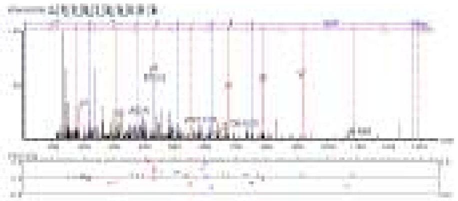

## XP_039206270.1

back to list

| Protein Coverage | Supporting Peptides | Best Unique PSM |

**Protein Coverage:**

```
   1 MSMNGSHPRL NTLGRMVRAE SGPDMRYDMN SHMMMGGGGG GGGGAGSSSH THKMYYVQKS YVGDINSEGY TQNGTATVSR
  81 RQNTIQDLLQ VCSDCLMRAE LIVQPELKYG DGIQISRNRE LEECFAQANE HMDILEGLIR EMRQMGQPCE LYQKRLLQLQ
 161 EQMRALYKAI SVPRVRRASS KGGGYSSQSA SGWDEYTRRV TSECLNWMRQ QKTEMELIKW GFDAAAIEQQ IAEHRRFHNS
 241 IGDYRWHLDK VKTDLREKAA VHQLEEEYEY LLRFSFERMD QLRQLQNIIQ ATCREIMWIN DCEEEELLYD WSDRNTDIAR
 321 KQEAFSKRMS QLEVKEKELN KLKQECDQLV LSQHPASDKI EAYMDTLQTQ WSWILQITKC IDVHLKENAA YFQFFEEAQS
 401 TENYLKNLQD VIRKRFICDK NMSLQVLLEQ IKELETEREK ILEYKRQVQN LVNKSKKIVQ LKPRNPDYKT SKPIILRALC
 481 DYKQDLKTMR KGDECILKDN SERSKWHVTG PGGLDMLVPS VSLIIPPPNP LAVDLATKIE QYYDAIMALW NQLYINMKSL
 561 VSWHYCMIDI EKIKVMTIAK LKTMRREDYQ RIISDLEIHY QEFIRNSQGS EMFGDDDKRT MQTQITDAQK HYQTLVIQLP
 641 GSQPPPTIQR PIVTEVINIE KQEAWLLMEL QKLRRQIENC ELRMMQRTPF QLDQGATHNL CVRISELESI QNESQSMAEI
 721 LNRQNDYLPN FRGSEKYHYL QSEISALFQK LENINGISAC YLDSLNALRS LLQAILQTDD MIRVFEIRLT EEDTLSLDPD
 801 KVEAYRACLK KMKADLSMKK SLLGTLEAEL QKALQVHSQS SQTYPHYELD LGKFADRVSQ LTDRWQRLEK QLDDRSWDLE
 881 KQVKQLRIYR DLYQALNKWI CDARHRQDTI EAIKLGDVST VMRYLQEQKN LHSEITSKRD RVEEVIKNAE ACSLAIKDYE
 961 LQAAAYSSGL ETLLNIPVKR SMVQSPSGLI LQEAGEIQSR YIELLTRSGD YYRFLSEMLK SLEDIKMKST RIELLEEELR
1041 LAKDANSDSN NKHKFLEQNL QKYQVECSQL KAKFISLEEM KRQVEMDSST SKQNLDKCYA QIKDLNDRIT RLTYEIEDEK
1121 RKRKLLEDRY DQQKADFDQL QKTRQNEKET LGWQKIESEK IIKEKEYELE RLRVLLQDEG TRKRECENEL AKTSHRIQES
1201 KNQCNHIMQE RETLLIKVKT LEQDKARLLR LEEDLNRTKG MLESESRLKV RLENEKQQIL NDLNQWKSQY SRKEETIRKI
1281 ESERDKSERE KSALLIETER LQAEIKRVEE RYRCRLEETS RKSQAEMETE RLRSQREIEK LKQRPKGAPK GTQTEEDLMI
1361 DPSKLLFNGL RKKITAVQLY ECQLIDKCTL DKLLRGQKSV EEVAAELEPY LKGAGAIAGA SLNTKAKFSL VEAKRKQLLT
1441 PENTVLLLEA QAATGGVIDP HRNELLSVDS AVARDLIEFD DREQIYMAEK AITGFKDPFS GKIMPVAEAM KKNLVDREIG
1521 LRLLEAQLAS GGIVDPINSI FLPKDIALSR GLIDRDLYRA LNNPSKPLID PLSKTAISYM KLREKCRIEP HTGLLLLPVQ
1601 KRSTSFQGIR KLISVSELVD SGIIRESTAN ELETGAITVE EVTDRIKDFL QGSSCIAGIY NEATGEKLGV YQAMKIGLVR
1681 PGTALELLEA QAATGFIVDP VNNLRLPVEE GYKRGLVGIE FKEKLLSAER AVTGYRDPET GNIISLFQAM NKELIEKGHG
1761 IRLLEAQIAT GGIIDPKESH RLPVHTAYQR GYFNEELNDI LSDPNDDTKG FFDPNTEENL TYLQLKGRCK IDELTGLCLL
1841 PLREKKKVVQ TSQKNTLRKR RVVIVDPDTN KEMSVQEAYN KGLIDYSTYM ELSDQECEWE EITTTGSDGS TRVVLVDRKT
1921 GHQYDIEDAL DKGLVDRKFF DQYRSGSLSL TQFADMIASR NGGDEVFRHE SVIRSPTVVS VKSSSILLKS SSGSFSDSPE
2001 ETTPIAAIFD TENLEKISIS EAVQRGIVDT ITAQRLLEAQ TCTGGIICPT TGQRLSLQDA VAQGIIDHEM ATRLKPAQKA
2081 FLGFEGIKGR KRMSAAEAVK EKWLPYEAGQ RFLEFQFITG GLIDPEVHGR ISTEEAIRKG LIDGRTSQRL QDISNYAKIL
2161 TCPKTKLKIS YREAMNRSMV EDQTGLRLLE ASSVSSKGIS SPYNVSSAPG SRSGSRSGSR SGSRSGSRRG SFDAGTTSSY
2241 SYSYTAVSSN SSLGR
```

**Supporting Peptides:**

| Peptide | Uniq | -10lgP | Mass | Length | ppm | m/z | z | RT | Fraction | Scan | Source File | Area F1 | Area F2a | Area F3a | Area F4 | Area F5 | Area F6a | Area F7a | Area F7b | Area F8 | Area F9a | Area Sample 1F10a | Area F10b | Area F11a | Area F11b | Area F12a | Area F13 | #Feature | #Feature F1 | #Feature F2a | #Feature F3a | #Feature F4 | #Feature F5 | #Feature F6a | #Feature F7a | #Feature F7b | #Feature F8 | #Feature F9a | #Feature Sample 1F10a | #Feature F10b | #Feature F11a | #Feature F11b | #Feature F12a | #Feature F13 | Start | End | PTM | AScore | Found By |
|---|---|---|---|---|---|---|---|---|---|---|---|---|---|---|---|---|---|---|---|---|---|---|---|---|---|---|---|---|---|---|---|---|---|---|---|---|---|---|---|---|---|---|---|---|---|---|---|---|---|
| R.LTYEIEDEKR.K | Y | 47.12 | 1294.6405 | 10 | 8.7 | 432.5566 | 3 | 38.20 | 8 | F8:16270 | BuCaTN01_F7b.raw | | | | | | | | 0 | 9.2313E4 | | | | | | | | 1 | 0 | 0 | 0 | 0 | 0 | 0 | 0 | 0 | 1 | 0 | 0 | 0 | 0 | 0 | 0 | 0 | 1112 | 1121 | | | PEAKS DB |
| total 1 peptides | | | | | | | | | | | | | | | | | | | | | | | | | | | | | | | | | | | | | | | | | | | | | | | | | |

**Best Unique PSM (Scan F8:16270, m/z=432.5566, z=3, RT=38.20, ppm=8.7):**

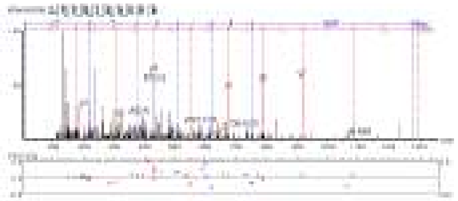

## XP_039206261.1

back to list

| Protein Coverage | Supporting Peptides | Best Unique PSM |

**Protein Coverage:**

1 MSMNGSHPRL NTLGRMVRAE SGPDMRYDMN SHMMMGGGGG GGGGAGSSSH THKMYYVQKS YVGDINSEGY TQNGTATVSR
81 RQNTIQDLLQ VCSDCLMRAE LIVQPELKYG DGIQISRNRE LEECFAQANE HMDILEGLIR EMRQMGQPCE LYQKRLLQLQ
161 EQMRALYKAI SVPRVRRASS KGGGYSSQSA SGWDEYTRRV TSECLNWMRQ QKTEMELIKW GFDAAAIEQQ IAEHRRFHNS
241 IGDYRWHLDK VKTDLREKAA VHQLEEEYEY LLRFSFERMD QLRQLQNIIQ ATCREIMWIN DCEEEELLYD WSDRNTDIAR
321 KQEAFSKRMS QLEVKEKELN KLKQECDQLV LSQHPASDKI EAYMDTLQTQ WSWILQITKC IDVHLKENAA YFQFFEEAQS
401 TENYLKNLQD VIRKRFICDK NMSLQVLLEQ IKELETEREK ILEYKRQVQN LVNKSKKIVQ LKPRNPDYKT SKPIILRALC
481 DYKQDLKTMR KGDECILKDN SERSKWHVTG PGGLDMLVPS VSLIIPPPNP LAVDLATKIE QYYDAIMALW NQLYINMKSL
561 VSWHYCMIDI EKIKVMTIAK LKTMRREDYQ RIISDLEIHY QEFIRNSQGS EMFGDDDKRT MQTQITDAQK HYQTLVIQLP
641 GSQPPPTIQR PIVTEVINIE KQEAWLLMEL QKLRRQIENC ELRMMQRTPF QLDQGATHNL CVRISELESI QNESQSMAEI
721 LNRQNDYLPN FRGSEKYHYL QSEISALFQK LENINGISAC YLDSLNALRS LLQAILQTDD MIRVFEIRLT EEDTLSLDPD
801 KVEAYRACLK KMKADLSMKK SLLGTLEAEL QKALQVHSQS SQTYPHYELD LGKFADRVSQ LTDRWQRLEK QLDDRSWDLE
881 KQVKQLRIYR DLYQALNKWI CDARHRQDTI EAIKLGDVST VMRYLQEQKN LHSEITSKRD RVEEVIKNAE ACSLAIKDYE
961 LQAAAYSSGL ETLLNIPVKR SMVQSPSGLI LQEAGEIQSR YIELLTRSGD YYRFLSEMLK SLEDIKMKST RIELLEEELR
1041 LAKDANSDSN NKHKFLEQNL QKYQVECSQL KAKFISLEEM KRQVEMDSST SKQNLDKCYA QIKDLNDRIT R**LTYEIEDEK**
1121 **R**KRKLLEDRY DQQKADFDQL QKTRQNEKET LGWQKIESEK IIKEKEYELE RLRVLLQDEG TRKRECENEL AKVRNQFNEE
1201 MSNIKNKYET EINIKKTTIH QIAAQKEDDA KNLRTQIDRL QRENRDLKDE IVRLNEAILD TTEQRRRAEE NILQQKACGS
1281 EVMQQKQQIE LELKQIIQLR NDDNSRYTQA LEDATSTIHE KVKEIERLKI QLKEEAKSQW ELENELAKTS HRIQESKNQC
1361 NHIMQERETL LIKVKTLEQD KARLLRLEED LNRTKGMLES ESRLKVRLEN EKQQILNDLN QWKSQYSRKE ETIRKIESER
1441 DKSEREKSAL LIETERLQAE IKRVEERYRC RLEETSRKSQ AEMETERLRS QREIEKLKQR PKGAPKGTQT EEDLMIDPSK
1521 LLFNGLRKKI TAVQLYECQL IDKCTLDKLL RGQKSVEEVA AELEPYLKGA GAIAGASLNT KAKFSLVEAK RKQLLTPENT
1601 VLLLEAQAAT GGVIDPHRNE LLSVDSAVAR DLIEFDDREQ IYMAEKAITG FKDPFSGKIM PVAEAMKKNL VDREIGLRLL
1681 EAQLASGGIV DPINSIFLPK DIALSRGLID RDLYRALNNP SKPLIDPLSK TAISYMKLRE KCRIEPHTGL LLLPVQKRST
1761 SFQGIRKLIS VSELVDSGII RESTANELET GAITVEEVTD RIKDFLQGSS CIAGIYNEAT GEKLGVYQAM KIGLVRPGTA
1841 LELLEAQAAT GFIVDPVNNL RLPVEEGYKR GLVGIEFKEK LLSAERAVTG YRDPETGNII SLFQAMNKEL IEKGHGIRLL
1921 EAQIATGGII DPKESHRLPV HTAYQRGYFN EELNDILSDP NDDTKGFFDP NTEENLTYLQ LKGRCKIDEL TGLCLLPLRE
2001 KKKVVQTSQK NTLRKRRVVI VDPDTNKEMS VQEAYNKGLI DYSTYMELSD QECEWEEITT TGSDGSTRVV LVDRKTGHQY
2081 DIEDALDKGL VDRKFFDQYR SGSLSLTQFA DMIASRNGGD EVFRHESVIR SPTVVSVKSS SILLKSSSGS FSDSPEETTP
2161 IAAIFDTENL EKISISEAVQ RGIVDTITAQ RLLEAQTCTG GIICPTTGQR LSLQDAVAQG IIDHEMATRL KPAQKAFLGF
2241 EGIKGRKRMS AAEAVKEKWL PYEAGQRFLE FQFITGGLID PEVHGRISTE EAIRKGLIDG RTSQRLQDIS NYAKILTCPK
2321 TKLKISYREA MNRSMVEDQT GLRLLEASSV SSKGISSPYN VSSAPGSRSG SRSGSRSGSR SGSRRGSFDA GTTSSYSYSY
2401 TAVSSNSSLG R

**Supporting Peptides:**

| Peptide | Uniq | -10lgP | Mass | Length | ppm | m/z | z | RT | Fraction | Scan | Source File | Area F1 | Area F2a | Area F3a | Area F4 | Area F5 | Area F6a | Area F7a | Area F7b | Area F8 | Area F9a | Area Sample 1F10a | Area F10b | Area F11a | Area F11b | Area F12a | Area F13 | #Feature | #Feature F1 | #Feature F2a | #Feature F3a | #Feature F4 | #Feature F5 | #Feature F6a | #Feature F7a | #Feature F7b | #Feature F8 | #Feature F9a | #Feature Sample 1F10a | #Feature F10b | #Feature F11a | #Feature F11b | #Feature F12a | #Feature F13 | Start | End | PTM | AScore | Found By |
|---|---|---|---|---|---|---|---|---|---|---|---|---|---|---|---|---|---|---|---|---|---|---|---|---|---|---|---|---|---|---|---|---|---|---|---|---|---|---|---|---|---|---|---|---|---|---|---|---|---|
| R.LTYEIEDEKR.K | Y | 47.12 | 1294.6405 | 10 | 8.7 | 432.5566 | 3 | 38.20 | 8 | F8:16270 | BuCaTN01_F7b.raw | | | | | | | | 0 | 9.2313E4 | | | | | | | | 1 | 0 | 0 | 0 | 0 | 0 | 0 | 0 | 0 | 1 | 0 | 0 | 0 | 0 | 0 | 0 | 0 | 1112 | 1121 | | | PEAKS DB |
| **total 1 peptides** | | | | | | | | | | | | | | | | | | | | | | | | | | | | | | | | | | | | | | | | | | | | | | | | | |

**Best Unique PSM (Scan F8:16270, m/z=432.5566, z=3, RT=38.20, ppm=8.7):**

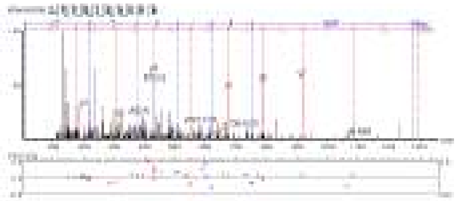

## XP_039206254.1

| Protein Coverage | Supporting Peptides | Best Unique PSM |

back to list

**Protein Coverage:**

1 MRAELIVQPE LKYGDGIQIS RNRELEECFA QANEHMDILE GLIREMRQMG QPCELYQKRL LQLQEQMRAL YKAISVPRVR
81 RASSKGGGYS SQSASGWDEY TRRVTSECLN WMRQQKTEME LIKWGFDAAA IEQQIAEHRR FHNSIGDYRW HLDKVKTDLR
161 EKAAVHQLEE EYEYLLRFSF ERMDQLRQLQ NIIQATCREI MWINDCEEEE LLYDWSDRNT DIARKQEAFS KRMSQLEVKE
241 KELNKLKQEC DQLVLSQHPA SDKIEAYMDT LQTQWSWILQ ITKCIDVHLK ENAAYFQFFE EAQSTENYLK NLQDVIRKRF
321 ICDKNMSLQV LLEQIKELET EREKILEYKR QVQNLVNKSK KIVQLKPRNP DYKTSKPIIL RALCDYKQDL KTMRKGDECI
401 LKDNSERSKW HVTGPGGLDM LVPSVSLIIP PPNPLAVDLA TKIEQYYDAI MALWNQLYIN MKSLVSWHYC MIDIEKIKVM
481 TIAKLKTMRR EDYQRIISDL EIHYQEFIRN SQGSEMFGDD DKRTMQTQIT DAQKHYQTLV IQLPGSQPPP TIQRPIVTEV
561 INIEKQEAWL LMELQKLRRQ IENCELRMMQ RTPFQLDQGA THNLCVRISE LESIQNESQS MAEILNRQND YLPNFRGSEK
641 YHYLQSEISA LFQKLENING ISACYLDSLN ALRSLLQAIL QTDDMIRVFE IRLTEEDTLS LDPDKVEAYR ACLKKMKADL
721 SMKKSLLGTL EAELQKALQV HSQSSQTYPH YELDLGKFAD RVSQLTDRWQ RLEKQLDDRS WDLEKQVKQL RIYRDLYQAL
801 NKWICDARHR QDTIEAIKLG DVSTVMRYLQ EQKNLHSEIT SKRDRVEEVI KNAEACSLAI KDYELQAAAY SSGLETLLNI
881 PVKRSMVQSP SGLILQEAGE IQSRYIELLT RSGDYYRFLS EMLKSLEDIK MKSTRIELLE EELRLAKDAN SDSNNKHKFL
961 EQNLQKYQVE CSQLKAKFIS LEEMKRQVEM DSSTSKQNLD KCYAQIKDLN DRITR**LTYEI EDEKR**KRKLL EDRYDQQKAD
1041 FDQLQKTRQN EKETLGWQKI ESEKIIKEKE YELERLRVLL QDEGTRKREC ENELAKVRNQ FNEEMSNIKN KYETEINIKK
1121 TTIHQIAAQK EDDAKNLRTQ IDRLQRENRD LKDEIVRLNE AILDTTEQRR RAEENILQQK ACGSEVMQQK QQIELELKQI
1201 IQLRNDDNSR YTQALEDATS TIHEKVKEIE RLKIQLKEEA KSQWELENEL AKVRNSYDEE IISLKNKYET EINITKTTIN
1281 QVTVKKEEEA SNYRAQLDNA VRENRSLCEE LRRLKNTLSQ TTENLRKVEE SAHQQKSIGS EISQKKQQLE KELKQTVQKF
1361 TEEIAHYKQS LDEAGRTIKE KNKEIEKNRK LLDAEISQRK SMEEENIRLQ RIQYDLEKAN INATETISRL KVQEQELGRL
1441 KSEYERLAQD KKGKDQESTR LQSTVKELQH QKHILEEELN RQSRIVADET SKRKNAEEEL EAMRRTLRDQ AIKITNLTQQ
1521 IEEISIVKKR SEDDLRHQRE VLDNHMRDKQ RYMEEIRKYT SEVESLRRQM VQEQENLKQA HIRNEHLQKA IEDKSKSLNE
1601 CKIEIERLQC LTENLTKEHL MLEEELRNLR LEYDDLRRGR SEVDEEKNAT IVDLKNQLQT SNKHALELQG VINGLQKERE
1681 NLRQEIEKFQ KQSLETSHRI QESKNQCNHI MQERETLLIK VKTLEQDKAR LLRLEEDLNR TKGMLESESR LKVRLENEKQ
1761 QILNDLNQWK SQYSRKEETI RKIESERDKS EREKSALLIE TERLQAEIKR VEERYRCRLE ETSRKSQAEM ETERLRSQRE
1841 IEKLKQRPKG APKGTQTEED LMIDPSKLLF NGLRKKITAV QLYECQLIDK CTLDKLLRGQ KSVEEVAAEL EPYLKGAGAI
1921 AGASLNTKAK FSLVEAKRKQ LLTPENTVLL LEAQAATGGV IDPHRNELLS VDSAVARDLI EFDDREQIYM AEKAITGFKD
2001 PFSGKIMPVA EAMKKNLVDR EIGLRLLEAQ LASGGIVDPI NSIFLPKDIA LSRGLIDRDL YRALNNPSKP LIDPLSKTAI
2081 SYMKLREKCR IEPHTGLLLL PVQKRSTSFQ GIRKLISVSE LVDSGIIRES TANELETGAI TVEEVTDRIK DFLQGSSCIA
2161 GIYNEATGEK LGVYQAMKIG LVRPGTALEL LEAQAATGFI VDPVNNLRLP VEEGYKRGLV GIEFKEKLLS AERAVTGYRD
2241 PETGNIISLF QAMNKELIEK GHGIRLLEAQ IATGGIIDPK ESHRLPVHTA YQRGYFNEEL NDILSDPNDD TKGFFDPNTE
2321 ENLTYLQLKG RCKIDELTGL CLLPLREKKK VVQTSQKNTL RKRRVVIVDP DTNKEMSVQE AYNKGLIDYS TYMELSDQEC
2401 EWEEITTTGS DGSTRVVLVD RKTGHQYDIE DALDKGLVDR KFFDQYRSGS LSLTQFADMI ASRNGGDEVF RHESVIRSPT
2481 VVSVKSSSIL LKSSSGSFSD SPEETTPIAA IFDTENLEKI SISEAVQRGI VDTITAQRLL EAQTCTGGII CPTTGQRLSL
2561 QDAVAQGIID HEMATRLKPA QKAFLGFEGI KGRKRMSAAE AVKEKWLPYE AGQRFLEFQF ITGGLIDPEV HGRISTEEAI
2641 RKGLIDGRTS QRLQDISNYA KILTCPKTKL KISYREAMNR SMVEDQTGLR LLEASSVSSK GISSPYNVSS APGSRSGSRS
2721 GSRSGSRSGS RRGSFDAGTT SSYSYSYTAV SSNSSLGR

**Supporting Peptides:**

| Peptide | Uniq | -10lgP | Mass | Length | ppm | m/z | z | RT | Fraction | Scan | Source File | Area F1 | Area F2a | Area F3a | Area F4 | Area F5 | Area F6a | Area F7a | Area F7b | Area F8 | Area F9a | Area Sample 1F10a | Area F10b | Area F11a | Area F11b | Area F12a | Area F13 | #Feature | #Feature F1 | #Feature F2a | #Feature F3a | #Feature F4 | #Feature F5 | #Feature F6a | #Feature F7a | #Feature F7b | #Feature F8 | #Feature F9a | #Feature Sample 1F10a | #Feature F10b | #Feature F11a | #Feature F11b | #Feature F12a | #Feature F13 | Start | End | PTM | AScore | Found By |
|---|---|---|---|---|---|---|---|---|---|---|---|---|---|---|---|---|---|---|---|---|---|---|---|---|---|---|---|---|---|---|---|---|---|---|---|---|---|---|---|---|---|---|---|---|---|---|---|---|---|
| R.LTYEIEDEKR.K | Y | 47.12 | 1294.6405 | 10 | 8.7 | 432.5566 | 3 | 38.20 | 8 | F8:16270 | BuCaTN01_F7b.raw | | | | | | | | 0 | 9.2313E4 | | | | | | | | 1 | 0 | 0 | 0 | 0 | 0 | 0 | 0 | 0 | 1 | 0 | 0 | 0 | 0 | 0 | 0 | 0 | 1016 | 1025 | | | PEAKS DB |
| **total 1 peptides** | | | | | | | | | | | | | | | | | | | | | | | | | | | | | | | | | | | | | | | | | | | | | | | | | |

**Best Unique PSM (Scan F8:16270, m/z=432.5566, z=3, RT=38.20, ppm=8.7):**

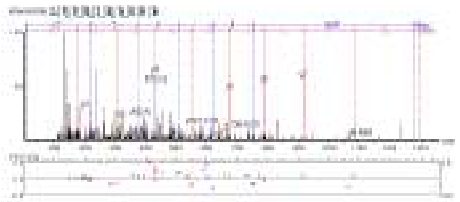

## XP_039206245.1

back to list

| Protein Coverage | Supporting Peptides | Best Unique PSM |

**Protein Coverage:**

```
   1 MSMNGSHPRL NTLGRMVRAE SGPDMRYDMN SHMMMGGGGG GGGGAGSSSH THKMYYVQKS YVGDINSEGY TQNGTATVSR
  81 RQNTIQDLLQ VCSDCLMRAE LIVQPELKYG DGIQISRNRE LEECFAQANE HMDILEGLIR EMRQMGQPCE LYQKRLLQLQ
 161 EQMRALYKAI SVPRVRRASS KGGGYSSQSA SGWDEYTRRV TSECLNWMRQ QKTEMELIKW GFDAAAIEQQ IAEHRRFHNS
 241 IGDYRWHLDK VKTDLREKAA VHQLEEEYEY LLRFSFERMD QLRQLQNIIQ ATCREIMWIN DCEEEELLYD WSDRNTDIAR
 321 KQEAFSKRMS QLEVKEKELN KLKQECDQLV LSQHPASDKI EAYMDTLQTQ WSWILQITKC IDVHLKENAA YFQFFEEAQS
 401 TENYLKNLQD VIRKRFICDK NMSLQVLLEQ IKELETEREK ILEYKRQVQN LVNKSKKIVQ LKPRNPDYKT SKPIILRALC
 481 DYKQDLKTMR KGDECILKDN SERSKWHVTG PGGLDMLVPS VSLIIPPPNP LAVDLATKIE QYYDAIMALW NQLYINMKSL
 561 VSWHYCMIDI EKIKVMTIAK LKTMRREDYQ RIISDLEIHY QEFIRNSQGS EMFGDDDKRT MQTQITDAQK HYQTLVIQLP
 641 GSQPPPTIQR PIVTEVINIE KQEAWLLMEL QKLRRQIENC ELRMMQRTPF QLDQGATHNL CVRISELESI QNESQSMAEI
 721 LNRQNDYLPN FRGSEKYHYL QSEISALFQK LENINGISAC YLDSLNALRS LLQAILQTDD MIRVFEIRLT EEDTLSLDPD
 801 KVEAYRACLK KMKADLSMKK SLLGTLEAEL QKALQVHSQS SQTYPHYELD LGKFADRVSQ LTDRWQRLEK QLDDRSWDLE
 881 KQVKQLRIYR DLYQALNKWI CDARHRQDTI EAIKLGDVST VMRYLQEQKN LHSEITSKRD RVEEVIKNAE ACSLAIKDYE
 961 LQAAAYSSGL ETLLNIPVKR SMVQSPSGLI LQEAGEIQSR YIELLTRSGD YYRFLSEMLK SLEDIKMKST RIELLEEELR
1041 LAKDANSDSN NKHKFLEQNL QKYQVECSQL KAKFISLEEM KRQVEMDSST SKQNLDKCYA QIKDLNDRIT RLTYEIEDEK
1121 RKRKLLEDRY DQQKADFDQL QKTRQNEKET LGWQKIESEK IIKEKEYELE RLRVLLQDEG TRKRECENEL AKVRNQFNEE
1201 MSNIKNKYET EINIKKTTIH QIAAQKEDDA KNLRTQIDRL QRENRDLKDE IVRLNEAILD TTEQRRRAEE NILQQKACGS
1281 EVMQQKQQIE LELKQIIQLR NDDNSRYTQA LEDATSTIHE KVKEIERLKI QLKEEAKSQW ELENELAKVR NSYDEEIISL
1361 KNKYETEINI TKTTINQVTV KKEEEASNYR AQLDNAVREN RSLCEELRRL KNTLSQTTEN LRKVEESAHQ QKSIGSEISQ
1441 KKQQLEKELK QTVQKFTEEI AHYKQSLDEA GRTIKEKNKE IEKNRKLLDA EISQRKSMEE ENIRLQRIQY DLEKANINAT
1521 ETISRLKVQE QELGRLKSEY ERLAQDKKGK DQESTRLQST VKELQHQKHI LEEELNRQSR IVADETSKRK NAEEELEAMR
1601 RTLRDQAIKI TNLTQQIEEI SIVKKRSEDD LRHQREVLDN HMRDKQRYME EIRKYTSEVE SLRRQMVQEQ ENLKQAHIRN
1681 EHLQKAIEDK SKSLNECKIE IERLQCLTEN LTKEHLMLEE ELRNLRLEYD DLRRGRSEVD EEKNATIVDL KNQLQTSNKH
1761 ALELQGVING LQKERENLRQ EIEKFQKQSL ETSHRIQESK NQCNHIMQER ETLLIKVKTL EQDKARLLRL EEDLNRTKGM
1841 LESESRLKVR LENEKQQILN DLNQWKSQYS RKEETIRKIE SERDKSEREK SALLIETERL QAEIKRVEER YRCRLEETSR
1921 KSQAEMETER LRSQREIEKL KQRPKGAPKG TQTEEDLMID PSKLLFNGLR KKITAVQLYE CQLIDKCTLD KLLRGQKSVE
2001 EVAAELEPYL KGAGAIAGAS LNTKAKFSLV EAKRKQLLTP ENTVLLLEAQ AATGGVIDPH RNELLSVDSA VARDLIEFDD
2081 REQIYMAEKA ITGFKDPFSG KIMPVAEAMK KNLVDREIGL RLLEAQLASG GIVDPINSIF LPKDIALSRG LIDRDLYRAL
2161 NNPSKPLIDP LSKTAISYMK LREKCRIEPH TGLLLLPVQK RSTSFQGIRK LISVSELVDS GIIRESTANE LETGAITVEE
2241 VTDRIKDFLQ GSSCIAGIYN EATGEKLGVY QAMKIGLVRP GTALELLEAQ AATGFIVDPV NNLRLPVEEG YKRGLVGIEF
2321 KEKLLSAERA VTGYRDPETG NIISLFQAMN KELIEKGHGI RLLEAQIATG GIIDPKESHR LPVHTAYQRG YFNEELNDIL
2401 SDPNDDTKGF FDPNTEENLT YLQLKGRCKI DELTGLCLLP LREKKKVVQT SQKNTLRKRR VVIVDPDTNK EMSVQEAYNK
2481 GLIDYSTYME LSDQECEWEE ITTTGSDGST RVVLVDRKTG HQYDIEDALD KGLVDRKFFD QYRSGSLSLT QFADMIASRN
2561 GGDEVFRHES VIRSPTVVSV KSSSILLKSS SGSFSDSPEE TTPIAAIFDT ENLEKISISE AVQRGIVDTI TAQRLLEAQT
2641 CTGGIICPTT GQRLSLQDAV AQGIIDHEMA TRLKPAQKAF LGFEGIKGRK RMSAAEAVKE KWLPYEAGQR FLEFQFITGG
2721 LIDPEVHGRI STEEAIRKGL IDGRTSQRLQ DISNYAKILT CPKTKLKISY REAMNRSMVE DQTGLRLLEA SSVSSKGISS
2801 PYNVSSAPGS RSGSRSGSRS GSRSGSRRGS FDAGTTSSYS YSYTAVSSNS SLGR
```

**Supporting Peptides:**

| Peptide | Uniq | -10lgP | Mass | Length | ppm | m/z | z | RT | Fraction | Scan | Source File | Area F1 | Area F2a | Area F3a | Area F4 | Area F5 | Area F6a | Area F7a | Area F7b | Area F8 | Area F9a | Area Sample 1F10a | Area F10b | Area F11a | Area F11b | Area F12a | Area F13 | #Feature | #Feature F1 | #Feature F2a | #Feature F3a | #Feature F4 | #Feature F5 | #Feature F6a | #Feature F7a | #Feature F7b | #Feature F8 | #Feature F9a | #Feature Sample 1F10a | #Feature F10b | #Feature F11a | #Feature F11b | #Feature F12a | #Feature F13 | Start | End | PTM | AScore | Found By |
|---|---|---|---|---|---|---|---|---|---|---|---|---|---|---|---|---|---|---|---|---|---|---|---|---|---|---|---|---|---|---|---|---|---|---|---|---|---|---|---|---|---|---|---|---|---|---|---|---|---|
| R.LTYEIEDEKR.K | Y | 47.12 | 1294.6405 | 10 | 8.7 | 432.5566 | 3 | 38.20 | 8 | F8:16270 | BuCaTN01_F7b.raw | | | | | | | | 0 | 9.2313E4 | | | | | | | | 1 | 0 | 0 | 0 | 0 | 0 | 0 | 0 | 0 | 1 | 0 | 0 | 0 | 0 | 0 | 0 | 0 | 1112 | 1121 | | | PEAKS DB |
| total 1 peptides | | | | | | | | | | | | | | | | | | | | | | | | | | | | | | | | | | | | | | | | | | | | | | | | | |

**Best Unique PSM (Scan F8:16270, m/z=432.5566, z=3, RT=38.20, ppm=8.7):**

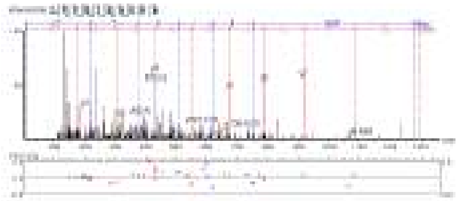

## JAI13616.1

back to list

| Protein Coverage | Supporting Peptides | Best Unique PSM |

**Protein Coverage:**

1 MYYVQKSYVG DINSEGYTQN GTATVSRRQN TIQDLLQVCS DCLMRAELIV QPELKYGDGI QISRNRELEE CFAQANEHMD
81 ILEGLIREMR QMGQPCELYQ KRLLQLQEQM RALYKAISVP RVRRASSKGG GYSSQSASGW DEYTRRVTSE CLNWMRQQKT
161 EMELIKWGFD AAAIEQQIAE HRRFHNSIGD YRWHLDKVKT DLREKAAVHQ LEEEYEYLLR FSFERMDQLR QLQNIIQATC
241 REIMWINDCE EEELLYDWSD RNTDITRKQE AFSKRMSQLE VKEKELNKLK QECDQLVLSQ HPASDKIEAY MDTLQTQWSW
321 ILQITKCIDV HLKENAAYFQ FFEEAQSTEN YLKNLQDVIR KRFICDKNMS LQVLLEQIKE LETEREKILE YKRQVQNLVN
401 KSKKIVQLKP RNPDYKTSKP IMLRALCDYK QDLKTMRKGD ECILKDNSER SKWHVTGPGG LDMLVPSVSL IIPPPNPLAV
481 DLATKIEQYY DAIMALWNQL YINMKSLVSW HYCMIDIEKI KVMTIAKLKT MRREDYQRII SDLEIHYQEF IRNSQGSEMF
561 GDDDKRTMQT QITDAQKHYQ TLVIQLPGSQ PPPTIQRPIV TEVINIEKQE AWLLMELQKL RRQIENCELR MMQRTPFQLD
641 QGATHNLCVR ISELESIQNE SQSMAEILNR QNDYLPNFRG SEKYHYLQSE ISALFQKLEN INGISACYLD SLNALRSLLQ
721 AILQTDDMIR VFEIRLTEED TLSLDPDKVE AYRACLKKMK ADLSMKKSLL GTLEAELQKA LQVHSQSSQT YPHYELDLGK
801 FADRVSQLTD RWQRLEKQLD DRSWDLEKQV KQLRIYRDLY QALNKWICDA RRRQDTIEAI KLGDVSTVMR YLQEQKNLHS
881 EITSKRDRVE EVIKNAEACS LAIKDYELQA AAYSSGLETL LNIPVKRSMV QSPSGLILQE AGEIQSRYIE LLTRSGDYYR
961 FLSEMLKSLE DIKMKSTRIE LLEEELRLAK DANSDSNNKH KFLEQNLQKY QVECSQLKAK FISLEEMKRQ VEMDSSTSKQ
1041 NLDKCYAQIK DLNDRITR**LT YEIEDEKR**KR KLLEDRYDQQ KADFDQLQKT RQNEKETLGW QKIESEKIIK EKEYELERLR
1121 VLLQDEGTRK RECENELAKV RNQFNEEMSN IKNKYETEIN IKKTTIHQIA AQKEDDAKNL RTQIDRLQRE NRDLKDEIVR
1201 LNEAILDTTE QRRRAEENIL QQKACGSEVM QQKQQIELEL KQIIQLRNDD NSRYKQALED ATSTIHEKVK EIERLKIQLK
1281 EEAKSQWELE NELAKVRNSY DEEIISLKNK YETEINITKT TINQVTVKKE EEASNYRAQL DNAVRENRSL CEELRRLKNT
1361 LSQTTENLRK VEESAHQQKS IGSEISQKKQ QLEKELKQTV QKFTEEIAHY KQSLDEAGRT IKEKNKEIEK NRKLLDAEIS
1441 QRKSMEEENI RLQRIQYDLE KANINATETI SRLKVQEQEL GRLKSEYERL AQDKKGKDQE STRLQSTVKE LQHQKHILEE
1521 ELNRQSRIVA DETSKRKNAE EELEAMRRTL RDQAIKITNL TQQIEEISIV KKRSEDDLRH QREVLDNHMR DKQRYMEEIR
1601 KYTSEVESLR RQMVQEQENL KQAHIRNEHL QKAIEDKSKS LNECKIEIER LQCLTENLTK EHLMLEEELR NLRLEYDDLR
1681 RGRSEVDEEK NATIVDLKNQ LQTSNKHALE LQGVINGLQK ERENLRQEIE KFQKQSLETS HRIQESKNQC NHIMQERETL
1761 LIKVKTLEQD KARLLRLEED LNRTKGMLES ESRLKVRLEN EKQQILNDLN QWKSQYSRKE ETIRKIESER DKSEREKSAL
1841 LIETERLQAE IKRVEERYRC RLEETSRKSQ AEMETERLRS QREIEKLKQR PKGAPKGTQT EEDLMIDPSK LLFNGLRKKI
1921 TAVQLYECQL IDKCTLDKLL RGQKSVEEVA AELEPYLRGA GAIAGASLNT KAKFSLVEAK RKQLLTPENT VLLLEAQAAT
2001 GGVIDPHRNE LLSVDSAVAR DLIEFDDREQ IYMAEKAITG FKDPFSGKIM PVAEAMKKNL VDREIGLRLL EAQLASGGIV
2081 DPINSIFLPK DIALSRGLID RDLYRALNNP SKPLIDPLSK TAISYMKLRE KCRIEPHTGL LLLPVQKRST SFQGIRKLIS
2161 VSELVDSGII RESTANELET GAITVEEVTD RIKDFLQGSS CIAGIYNEAT GEKLGVYQAM KIGLVRPGTA LELLEAQAAT
2241 GFIVDPVNNL RLPVEEGYKR GLVGIEFKEK LLSAERAVTG YRDPETGNII SLFQAMNKEL IEKGHGIRLL EAQIATGGII
2321 DPKESHRLPV HTAYQRGYFN EELNDILSDP NDDTKGFFDP NTEENLTYLQ LKGRCKIDEL TGLCLLPLRE KKKVVQTSQK
2401 NTLRKRRVVI VDPDTNKEMS VQEAYNKGLI DYSTYMELSD QECEWEEITT TGSDGSTRVV LVDRKTGHQY DIEDALDKGL
2481 VDRKFFDQYR SGSLSLTQFA DMIASKNGGD EVFRHESVIR SPTVVSVKSS SILLKSSSGS FSDSPEETTP IAAIFDTENL
2561 EKISISEAVQ RGIVDTITAQ RLLEAQTCTG GIICPTTGQR LSLQDAVAQG IIDHEMATRL KPAQKAFLGF EGIKGRKRMS
2641 AAEAVKEKWL PYEAGQRFLE FQFITGGLID PEVHGRISTE EAIRKGLIDG RTSQRLQDIS NYAKILTCPK TKLKISYREA
2721 MNRSMVEDQT GLRLLEASSV SSKGISSPYN VSSAPGSRSG SRSGSRSGSR SGSRRGSFDA GTTSSYSYSY TAVSSNSSLG
2801 R

**Supporting Peptides:**

| Peptide | Uniq | -10lgP | Mass | Length | ppm | m/z | z | RT | Fraction | Scan | Source File | Area F1 | Area F2a | Area F3a | Area F4 | Area F5 | Area F6a | Area F7a | Area F7b | Area F8 | Area F9a | Area Sample 1F10a | Area F10b | Area F11a | Area F11b | Area F12a | Area F13 | #Feature | #Feature F1 | #Feature F2a | #Feature F3a | #Feature F4 | #Feature F5 | #Feature F6a | #Feature F7a | #Feature F7b | #Feature F8 | #Feature F9a | #Feature Sample 1F10a | #Feature F10b | #Feature F11a | #Feature F11b | #Feature F12a | #Feature F13 | Start | End | PTM | AScore | Found By |
|---|---|---|---|---|---|---|---|---|---|---|---|---|---|---|---|---|---|---|---|---|---|---|---|---|---|---|---|---|---|---|---|---|---|---|---|---|---|---|---|---|---|---|---|---|---|---|---|---|---|
| R.LTYEIEDEKR.K | Y | 47.12 | 1294.6405 | 10 | 8.7 | 432.5566 | 3 | 38.20 | 8 | F8:16270 | BuCaTN01_F7b.raw | | | | | | | | 0 | 9.2313E4 | | | | | | | | 1 | 0 | 0 | 0 | 0 | 0 | 0 | 0 | 0 | 1 | 0 | 0 | 0 | 0 | 0 | 0 | 0 | 1059 | 1068 | | | PEAKS DB |
| total 1 peptides | | | | | | | | | | | | | | | | | | | | | | | | | | | | | | | | | | | | | | | | | | | | | | | | | |

**Best Unique PSM (Scan F8:16270, m/z=432.5566, z=3, RT=38.20, ppm=8.7):**

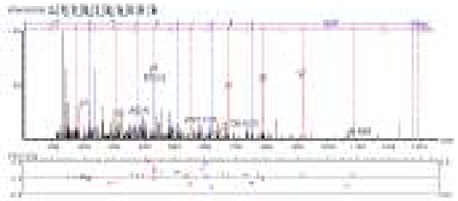

## XP_034282052.1

| Protein Coverage | Supporting Peptides | Best Unique PSM |

**Protein Coverage:**

1 MNLAHFLVPA AVCVSLLAAS AIPPLPRNLI QFGNMIKCTI PGSRPLLDYA D**YGCYCGYGG SGTPVDQLDR** CCQTHDECYS

C Carbamidomethylation (+57.02)

81 QVKKHPACQS LLDSPYTKIY SYTCSEGNVT CEDDNDECAA FICDCDRSAA TCFAGAPYNE EYKKLDTSIH CK

**Supporting Peptides:**

| Peptide | Uniq | -10lgP | Mass | Length | ppm | m/z | z | RT | Fraction | Scan | Source File | Area F1 | Area F2a | Area F3a | Area F4 | Area F5 | Area F6a | Area F7a | Area F7b | Area F8 | Area F9a | Area Sample 1F10a | Area F10b | Area F11a | Area F11b | Area F12a | Area F13 | #Feature | #Feature F1 | #Feature F2a | #Feature F3a | #Feature F4 | #Feature F5 | #Feature F6a | #Feature F7a | #Feature F7b | #Feature F8 | #Feature F9a | #Feature Sample 1F10a | #Feature F10b | #Feature F11a | #Feature F11b | #Feature F12a | #Feature F13 | Start | End | PTM | AScore | Found By |
|---|---|---|---|---|---|---|---|---|---|---|---|---|---|---|---|---|---|---|---|---|---|---|---|---|---|---|---|---|---|---|---|---|---|---|---|---|---|---|---|---|---|---|---|---|---|---|---|---|---|
| C.GYGGSGTPVDQLDR.C | Y | 42.21 | 1420.6582 | 14 | -3.4 | 711.3340 | 2 | 47.12 | 9 | F9:26037 | BuCaTN01_F8.raw | | | | | | | | | 2.7841E5 | | | | | | | | 1 | 0 | 0 | 0 | 0 | 0 | 0 | 0 | 0 | 1 | 0 | 0 | 0 | 0 | 0 | 0 | 0 | 57 | 70 | | | PEAKS DB |
| D.YGC(+57.02)YC(+57.02)GYGGSGTPVDQLDR.C | Y | 41.23 | 2123.8677 | 19 | -7.3 | 1062.9291 | 2 | 57.85 | 14 | F14:34217 | BuCaTN01_F11b.raw | | | | | | | | | | | | | | 0 | | | 0 | 0 | 0 | 0 | 0 | 0 | 0 | 0 | 0 | 0 | 0 | 0 | 0 | 0 | 0 | 0 | 0 | 52 | 70 | Carbamidomethylation | C3:Carbamidomethylation:1000.00;C5:Carbamidomethylation:1000.00 | PEAKS DB |
| total 2 peptides | | | | | | | | | | | | | | | | | | | | | | | | | | | | | | | | | | | | | | | | | | | | | | | | | |

**Best Unique PSM (Scan F9:26037, m/z=711.3340, z=2, RT=47.12, ppm=-3.4):**

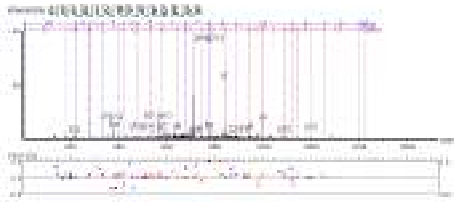

## C1IC46.1

| Protein Coverage | Supporting Peptides | Best Unique PSM |

**Protein Coverage:**

1 AVCVSLLGAS SIRPLPLHLG QFNNMIKCTI PGSTPWWDFS D**YGCYCGYGG SGTPVDQLDR** CCQTHDNCYT EAQKFSGCSP

C Carbamidomethylation (+57.02)

81 YTRKYSYECS EGTLTCKSDN DECAAFVCNC DRSAAICFAR APYNSNNVDI DLEAHCQ

**Supporting Peptides:**

| Peptide | Uniq | -10lgP | Mass | Length | ppm | m/z | z | RT | Fraction | Scan | Source File | Area F1 | Area F2a | Area F3a | Area F4 | Area F5 | Area F6a | Area F7a | Area F7b | Area F8 | Area F9a | Area Sample 1F10a | Area F10b | Area F11a | Area F11b | Area F12a | Area F13 | #Feature | #Feature F1 | #Feature F2a | #Feature F3a | #Feature F4 | #Feature F5 | #Feature F6a | #Feature F7a | #Feature F7b | #Feature F8 | #Feature F9a | #Feature Sample 1F10a | #Feature F10b | #Feature F11a | #Feature F11b | #Feature F12a | #Feature F13 | Start | End | PTM | AScore | Found By |
|---|---|---|---|---|---|---|---|---|---|---|---|---|---|---|---|---|---|---|---|---|---|---|---|---|---|---|---|---|---|---|---|---|---|---|---|---|---|---|---|---|---|---|---|---|---|---|---|---|---|
| C.GYGGSGTPVDQLDR.C | Y | 42.21 | 1420.6582 | 14 | -3.4 | 711.3340 | 2 | 47.12 | 9 | F9:26037 | BuCaTN01_F8.raw | | | | | | | | | 2.7841E5 | | | | | | | | 1 | 0 | 0 | 0 | 0 | 0 | 0 | 0 | 0 | 1 | 0 | 0 | 0 | 0 | 0 | 0 | 0 | 47 | 60 | | | PEAKS DB |
| D.YGC(+57.02)YC(+57.02)GYGGSGTPVDQLDR.C | Y | 41.23 | 2123.8677 | 19 | -7.3 | 1062.9291 | 2 | 57.85 | 14 | F14:34217 | BuCaTN01_F11b.raw | | | | | | | | | | | | | | 0 | | | 0 | 0 | 0 | 0 | 0 | 0 | 0 | 0 | 0 | 0 | 0 | 0 | 0 | 0 | 0 | 0 | 0 | 42 | 60 | Carbamidomethylation | C3:Carbamidomethylation:1000.00;C5:Carbamidomethylation:1000.00 | PEAKS DB |
| total 2 peptides | | | | | | | | | | | | | | | | | | | | | | | | | | | | | | | | | | | | | | | | | | | | | | | | | |

**Best Unique PSM (Scan F9:26037, m/z=711.3340, z=2, RT=47.12, ppm=-3.4):**

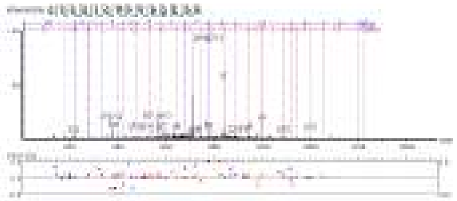

## ABX82863.1

back to list

| Protein Coverage | Supporting Peptides | Best Unique PSM |

**Protein Coverage:**

1 AVCVSLLGAS SIRPLPLHLG QFNNMIKCTI PGSTPWWDFS D**YGCYCGYGG SGTPVDQLDR** CCQTHDNCYT EAQKFSGCSP    C Carbamidomethylation (+57.02)

81 YTRKYSYECS EGTLTCKSDN DECAAFVCNC DRSAAICFAR APYNSNNVDI DLEAHCQ

**Supporting Peptides:**

| Peptide | Uniq | -10lgP | Mass | Length | ppm | m/z | z | RT | Fraction | Scan | Source File | Area F1 | Area F2a | Area F3a | Area F4 | Area F5 | Area F6a | Area F7a | Area F7b | Area F8 | Area F9a | Area Sample 1F10a | Area F10b | Area F11a | Area F11b | Area F12a | Area F13 | #Feature | #Feature F1 | #Feature F2a | #Feature F3a | #Feature F4 | #Feature F5 | #Feature F6a | #Feature F7a | #Feature F7b | #Feature F8 | #Feature F9a | #Feature Sample 1F10a | #Feature F10b | #Feature F11a | #Feature F11b | #Feature F12a | #Feature F13 | Start | End | PTM | AScore | Found By |
|---|---|---|---|---|---|---|---|---|---|---|---|---|---|---|---|---|---|---|---|---|---|---|---|---|---|---|---|---|---|---|---|---|---|---|---|---|---|---|---|---|---|---|---|---|---|---|---|---|---|
| C.GYGGSGTPVDQLDR.C | Y | 42.21 | 1420.6582 | 14 | -3.4 | 711.3340 | 2 | 47.12 | 9 | F9:26037 | BuCaTN01_F8.raw | | | | | | | | | 2.7841E5 | | | | | | | | 1 | 0 | 0 | 0 | 0 | 0 | 0 | 0 | 0 | 1 | 0 | 0 | 0 | 0 | 0 | 0 | 0 | 47 | 60 | | | PEAKS DB |
| D.YGC(+57.02)YC(+57.02)GYGGSGTPVDQLDR.C | Y | 41.23 | 2123.8677 | 19 | -7.3 | 1062.9291 | 2 | 57.85 | 14 | F14:34217 | BuCaTN01_F11b.raw | | | | | | | | | | | | | | 0 | | | 0 | 0 | 0 | 0 | 0 | 0 | 0 | 0 | 0 | 0 | 0 | 0 | 0 | 0 | 0 | 0 | 0 | 42 | 60 | Carbamidomethylation | C3:Carbamidomethylation:1000.00;C5:Carbamidomethylation:1000.00 | PEAKS DB |
| total 2 peptides | | | | | | | | | | | | | | | | | | | | | | | | | | | | | | | | | | | | | | | | | | | | | | | | | |

**Best Unique PSM (Scan F9:26037, m/z=711.3340, z=2, RT=47.12, ppm=-3.4):**

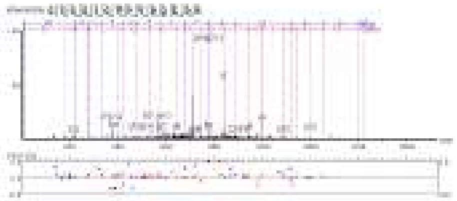

## ABX82862.1

back to list

| Protein Coverage | Supporting Peptides | Best Unique PSM |

**Protein Coverage:**

1 AVCVSLLGAS SIRPLPLHLG QFNNMIKCTI PGSTPWWDFS D**YGCYCGYGG SGTPVDQLDR** CCQTHDNCYT EAQKFSGCSP    C Carbamidomethylation (+57.02)

81 YRRKYSYECS EGTLTCKSDN DECAAFVCNC DRLAAICFAG APYNSNNVDI DLEARCQ

**Supporting Peptides:**

| Peptide | Uniq | -10lgP | Mass | Length | ppm | m/z | z | RT | Fraction | Scan | Source File | Area F1 | Area F2a | Area F3a | Area F4 | Area F5 | Area F6a | Area F7a | Area F7b | Area F8 | Area F9a | Area Sample 1F10a | Area F10b | Area F11a | Area F11b | Area F12a | Area F13 | #Feature | #Feature F1 | #Feature F2a | #Feature F3a | #Feature F4 | #Feature F5 | #Feature F6a | #Feature F7a | #Feature F7b | #Feature F8 | #Feature F9a | #Feature Sample 1F10a | #Feature F10b | #Feature F11a | #Feature F11b | #Feature F12a | #Feature F13 | Start | End | PTM | AScore | Found By |
|---|---|---|---|---|---|---|---|---|---|---|---|---|---|---|---|---|---|---|---|---|---|---|---|---|---|---|---|---|---|---|---|---|---|---|---|---|---|---|---|---|---|---|---|---|---|---|---|---|---|
| C.GYGGSGTPVDQLDR.C | Y | 42.21 | 1420.6582 | 14 | -3.4 | 711.3340 | 2 | 47.12 | 9 | F9:26037 | BuCaTN01_F8.raw | | | | | | | | | 2.7841E5 | | | | | | | | 1 | 0 | 0 | 0 | 0 | 0 | 0 | 0 | 0 | 1 | 0 | 0 | 0 | 0 | 0 | 0 | 0 | 47 | 60 | | | PEAKS DB |
| D.YGC(+57.02)YC(+57.02)GYGGSGTPVDQLDR.C | Y | 41.23 | 2123.8677 | 19 | -7.3 | 1062.9291 | 2 | 57.85 | 14 | F14:34217 | BuCaTN01_F11b.raw | | | | | | | | | | | | | | 0 | | | 0 | 0 | 0 | 0 | 0 | 0 | 0 | 0 | 0 | 0 | 0 | 0 | 0 | 0 | 0 | 0 | 0 | 42 | 60 | Carbamidomethylation | C3:Carbamidomethylation:1000.00;C5:Carbamidomethylation:1000.00 | PEAKS DB |
| total 2 peptides | | | | | | | | | | | | | | | | | | | | | | | | | | | | | | | | | | | | | | | | | | | | | | | | | |

**Best Unique PSM (Scan F9:26037, m/z=711.3340, z=2, RT=47.12, ppm=-3.4):**

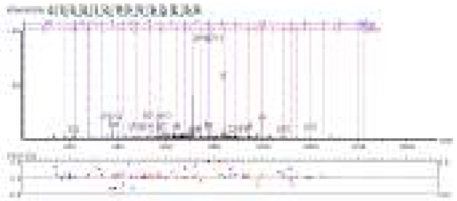

## C1IC45.1

back to list

| Protein Coverage | Supporting Peptides | Best Unique PSM |

**Protein Coverage:**

1 AVCVSLLGAS SIRPLPLHLG QFNNMIKCTI PGSTPWWDFS D**YGCYCGYGG SGTPVDQLDR** CCQTHDNCYT EAQKFSGCSP    C Carbamidomethylation (+57.02)

81 YRRKYSYECS EGTLTCKSDN DECAAFVCNC DRLAAICFAG APYNSNNVDI DLEARCQ

**Supporting Peptides:**

| Peptide | Uniq | -10lgP | Mass | Length | ppm | m/z | z | RT | Fraction | Scan | Source File | Area F1 | Area F2a | Area F3a | Area F4 | Area F5 | Area F6a | Area F7a | Area F7b | Area F8 | Area F9a | Area Sample 1F10a | Area F10b | Area F11a | Area F11b | Area F12a | Area F13 | #Feature | #Feature F1 | #Feature F2a | #Feature F3a | #Feature F4 | #Feature F5 | #Feature F6a | #Feature F7a | #Feature F7b | #Feature F8 | #Feature F9a | #Feature Sample 1F10a | #Feature F10b | #Feature F11a | #Feature F11b | #Feature F12a | #Feature F13 | Start | End | PTM | AScore | Found By |
|---|---|---|---|---|---|---|---|---|---|---|---|---|---|---|---|---|---|---|---|---|---|---|---|---|---|---|---|---|---|---|---|---|---|---|---|---|---|---|---|---|---|---|---|---|---|---|---|---|---|
| C.GYGGSGTPVDQLDR.C | Y | 42.21 | 1420.6582 | 14 | -3.4 | 711.3340 | 2 | 47.12 | 9 | F9:26037 | BuCaTN01_F8.raw | | | | | | | | | 2.7841E5 | | | | | | | | 1 | 0 | 0 | 0 | 0 | 0 | 0 | 0 | 0 | 1 | 0 | 0 | 0 | 0 | 0 | 0 | 0 | 47 | 60 | | | PEAKS DB |
| D.YGC(+57.02)YC(+57.02)GYGGSGTPVDQLDR.C | Y | 41.23 | 2123.8677 | 19 | -7.3 | 1062.9291 | 2 | 57.85 | 14 | F14:34217 | BuCaTN01_F11b.raw | | | | | | | | | | | | | | 0 | | | 0 | 0 | 0 | 0 | 0 | 0 | 0 | 0 | 0 | 0 | 0 | 0 | 0 | 0 | 0 | 0 | 0 | 42 | 60 | Carbamidomethylation | C3:Carbamidomethylation:1000.00;C5:Carbamidomethylation:1000.00 | PEAKS DB |
| total 2 peptides | | | | | | | | | | | | | | | | | | | | | | | | | | | | | | | | | | | | | | | | | | | | | | | | | | |

**Best Unique PSM (Scan F9:26037, m/z=711.3340, z=2, RT=47.12, ppm=-3.4):**

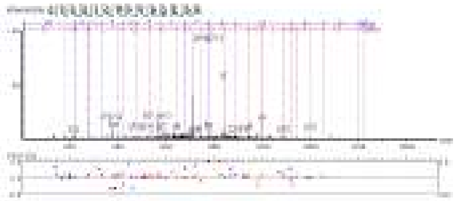

## XP_015671648.1

back to list

| Protein Coverage | Supporting Peptides | Best Unique PSM |

**Protein Coverage:**

1 MEELTAFVSK SFDQK**SKESS GGGGGGGGGG GGGGGGHKK**E SITYREVLES GLARTRELGG SDATGLQELA EGGPHCPVHL
81 FKEPGESDKD KLKDFNHANR TSEGIYECKD KREEVKSEDE DGQTKLKQRR SRTNFTLEQL NELERLFDET HYPDAFMREE
161 LSQRLGLSEA RVQVWFQNRR AKCRKQENQM HKGVILGTAS HLDACRVAPY VNMGALRMPF QQVQAQLQLE GVTHAHPHLH
241 PHLAAHAPYL MFPPPPFGLP IASLADSASA AAVVAAAAKS NSKNSSIADL RLKARKHAEA LGL

**Supporting Peptides:**

| Peptide | Uniq | -10lgP | Mass | Length | ppm | m/z | z | RT | Fraction | Scan | Source File | Area F1 | Area F2a | Area F3a | Area F4 | Area F5 | Area F6a | Area F7a | Area F7b | Area F8 | Area F9a | Area Sample 1F10a | Area F10b | Area F11a | Area F11b | Area F12a | Area F13 | #Feature | #Feature F1 | #Feature F2a | #Feature F3a | #Feature F4 | #Feature F5 | #Feature F6a | #Feature F7a | #Feature F7b | #Feature F8 | #Feature F9a | #Feature Sample 1F10a | #Feature F10b | #Feature F11a | #Feature F11b | #Feature F12a | #Feature F13 | Start | End | PTM | AScore | Found By |
|---|---|---|---|---|---|---|---|---|---|---|---|---|---|---|---|---|---|---|---|---|---|---|---|---|---|---|---|---|---|---|---|---|---|---|---|---|---|---|---|---|---|---|---|---|---|---|---|---|---|
| K.SKESSGGGGGGGGGGGGGGGGHKK.E | Y | 45.42 | 1841.8364 | 24 | -6.0 | 921.9178 | 2 | 75.83 | 12 | F12:51559 | BuCaTN01_F10b.raw | | | | | | | | | | | | 8.2966E4 | | | | | 1 | 0 | 0 | 0 | 0 | 0 | 0 | 0 | 0 | 0 | 0 | 0 | 1 | 0 | 0 | 0 | 0 | 16 | 39 | | | PEAKS DB |
| total 1 peptides | | | | | | | | | | | | | | | | | | | | | | | | | | | | | | | | | | | | | | | | | | | | | | | | | |

**Best Unique PSM (Scan F12:51559, m/z=921.9178, z=2, RT=75.83, ppm=-6.0):**

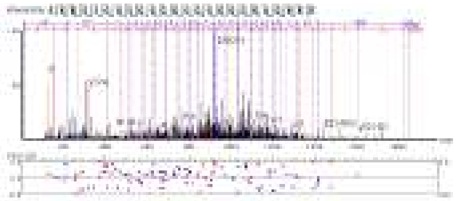

## XP_029140011.1

back to list

| Protein Coverage | Supporting Peptides | Best Unique PSM |

**Protein Coverage:**

1 MEELTAFVSK SFDQK**SKESS GGGGGGGGGG GGGGGGHKK**E SITYREVLES GLARTRELGG SDATGLQELA EGGPHCPVHL
81 FKEPGESDKD KLKDFNHANR TSEGIYECKD KREEVKSEDE DGQTKLKQRR SRTNFTLEQL NELERLFDET HYPDAFMREE
161 LSQRLGLSEA RVQVWFQNRR AKCRKQENQM HKGVILGTAS HLDACRVAPY VNMGALRMPF QQVVQAQLQL EGVTHAHPHL
241 HPHLAAHAPY LMFPPPPFGL PIASLADSAS AAAVVAAAAK SNSKNSSIAD LRLKARKHAE ALGL

**Supporting Peptides:**

| Peptide | Uniq | -10lgP | Mass | Length | ppm | m/z | z | RT | Fraction | Scan | Source File | Area F1 | Area F2a | Area F3a | Area F4 | Area F5 | Area F6a | Area F7a | Area F7b | Area F8 | Area F9a | Area Sample 1F10a | Area F10b | Area F11a | Area F11b | Area F12a | Area F13 | #Feature | #Feature F1 | #Feature F2a | #Feature F3a | #Feature F4 | #Feature F5 | #Feature F6a | #Feature F7a | #Feature F7b | #Feature F8 | #Feature F9a | #Feature Sample 1F10a | #Feature F10b | #Feature F11a | #Feature F11b | #Feature F12a | #Feature F13 | Start | End | PTM | AScore | Found By |
|---|---|---|---|---|---|---|---|---|---|---|---|---|---|---|---|---|---|---|---|---|---|---|---|---|---|---|---|---|---|---|---|---|---|---|---|---|---|---|---|---|---|---|---|---|---|---|---|---|---|
| K.SKESSGGGGGGGGGGGGGGGGHKK.E | Y | 45.42 | 1841.8364 | 24 | -6.0 | 921.9178 | 2 | 75.83 | 12 | F12:51559 | BuCaTN01_F10b.raw | | | | | | | | | | | | 8.2966E4 | | | | | 1 | 0 | 0 | 0 | 0 | 0 | 0 | 0 | 0 | 0 | 0 | 0 | 1 | 0 | 0 | 0 | 0 | 16 | 39 | | | PEAKS DB |
| total 1 peptides | | | | | | | | | | | | | | | | | | | | | | | | | | | | | | | | | | | | | | | | | | | | | | | | | |

**Best Unique PSM (Scan F12:51559, m/z=921.9178, z=2, RT=75.83, ppm=-6.0):**

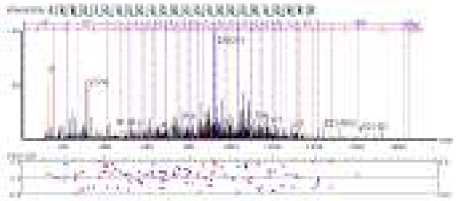

## P00602.1

back to list

| Protein Coverage | Supporting Peptides | Best Unique PSM |

**Protein Coverage:**

1 NLYQFKNMIH CTVPSRPWWH FADYGCYCGR GGKGTAVDDL DRCCQVHDNC YGEAEKLGCW PYLTLYKYEC SQGKLTCSGG
81 NNKCEAAVCN CDLVAANCFA GAP**YIDANYN VNLK**ERCQ

**Supporting Peptides:**

| Peptide | Uniq | -10lgP | Mass | Length | ppm | m/z | z | RT | Fraction | Scan | Source File | Area F1 | Area F2a | Area F3a | Area F4 | Area F5 | Area F6a | Area F7a | Area F7b | Area F8 | Area F9a | Area Sample 1F10a | Area F10b | Area F11a | Area F11b | Area F12a | Area F13 | #Feature | #Feature F1 | #Feature F2a | #Feature F3a | #Feature F4 | #Feature F5 | #Feature F6a | #Feature F7a | #Feature F7b | #Feature F8 | #Feature F9a | #Feature Sample 1F10a | #Feature F10b | #Feature F11a | #Feature F11b | #Feature F12a | #Feature F13 | Start | End | PTM | AScore | Found By |
|---|---|---|---|---|---|---|---|---|---|---|---|---|---|---|---|---|---|---|---|---|---|---|---|---|---|---|---|---|---|---|---|---|---|---|---|---|---|---|---|---|---|---|---|---|---|---|---|---|---|
| P.YIDANYNVNLK.E | Y | 42.85 | 1325.6615 | 11 | -1.3 | 663.8372 | 2 | 50.28 | 10 | F10:27561 | BuCaTN01_F9a.raw | | | | | | | | | | 7.878E5 | | | | | | | 1 | 0 | 0 | 0 | 0 | 0 | 0 | 0 | 0 | 0 | 1 | 0 | 0 | 0 | 0 | 0 | 0 | 104 | 114 | | | PEAKS DB |
| total 1 peptides | | | | | | | | | | | | | | | | | | | | | | | | | | | | | | | | | | | | | | | | | | | | | | | | | |

**Best Unique PSM (Scan F10:27561, m/z=663.8372, z=2, RT=50.28, ppm=-1.3):**

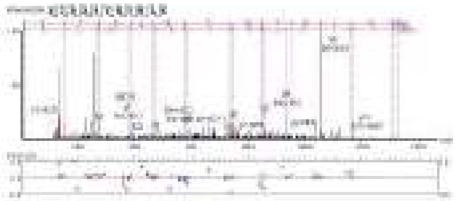

## UNJ97059.1

back to list

| Protein Coverage | Supporting Peptides | Best Unique PSM |

**Protein Coverage:**

1 MSSGHLLLLL GLLTLWAELT PVSGQGRPEF CHLPHEVGSC KALVPRFYYN STSKKCQE**FE YGGCGGNANN FK**TRDECHYT   C Carbamidomethylation (+57.02)
81 CVEKPGVCPQ GPQQHPCLEK CQDDWKCPGQ QKCCRYGCMT ECKDILYETH ALPTRRPTRI GTTVRP

**Supporting Peptides:**

| Peptide | Uniq | -10lgP | Mass | Length | ppm | m/z | z | RT | Fraction | Scan | Source File | Area F1 | Area F2a | Area F3a | Area F4 | Area F5 | Area F6a | Area F7a | Area F7b | Area F8 | Area F9a | Area Sample 1F10a | Area F10b | Area F11a | Area F11b | Area F12a | Area F13 | #Feature | #Feature F1 | #Feature F2a | #Feature F3a | #Feature F4 | #Feature F5 | #Feature F6a | #Feature F7a | #Feature F7b | #Feature F8 | #Feature F9a | #Feature Sample 1F10a | #Feature F10b | #Feature F11a | #Feature F11b | #Feature F12a | #Feature F13 | Start | End | PTM | AScore | Found By |
|---|---|---|---|---|---|---|---|---|---|---|---|---|---|---|---|---|---|---|---|---|---|---|---|---|---|---|---|---|---|---|---|---|---|---|---|---|---|---|---|---|---|---|---|---|---|---|---|---|---|
| E.FEYGGC(+57.02)GGNANNFK.T | Y | 43.67 | 1533.6306 | 14 | -0.3 | 767.8224 | 2 | 57.89 | 4 | F4:36507 | BuCaTN01_F4.raw | | | | 1.2175E7 | | | | | | | | | | | | | 1 | 0 | 0 | 0 | 1 | 0 | 0 | 0 | 0 | 0 | 0 | 0 | 0 | 0 | 0 | 0 | 0 | 59 | 72 | Carbamidomethylation | C6:Carbamidomethylation:1000.00 | PEAKS DB |
| total 1 peptides | | | | | | | | | | | | | | | | | | | | | | | | | | | | | | | | | | | | | | | | | | | | | | | | | |

**Best Unique PSM (Scan F4:36507, m/z=767.8224, z=2, RT=57.89, ppm=-0.3):**

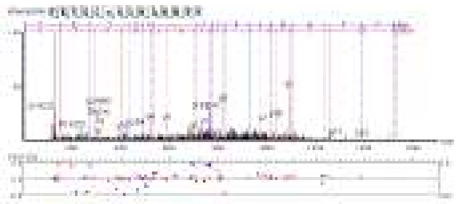

## UNJ97095.1

back to list

| Protein Coverage | Supporting Peptides | Best Unique PSM |

**Protein Coverage:**

1 MPSGGLLLLL GLLTLWAELT PVSGQGRPEF CHLPPEVGSC KALVPRFYYN STSKKCQG**FE YGGCGGNANN FK**TRDECQYT

81 CVEKPGVCPQ GPQQHPCLEK CQDDWKCPGQ QKCCRYGCMT ECKDPIFVK

64 C: Carbamidomethylation (+57.02)

**Supporting Peptides:**

| Peptide | Uniq | -10lgP | Mass | Length | ppm | m/z | z | RT | Fraction | Scan | Source File | Area F1 | Area F2a | Area F3a | Area F4 | Area F5 | Area F6a | Area F7a | Area F7b | Area F8 | Area F9a | Area Sample 1F10a | Area F10b | Area F11a | Area F11b | Area F12a | Area F13 | #Feature | #Feature F1 | #Feature F2a | #Feature F3a | #Feature F4 | #Feature F5 | #Feature F6a | #Feature F7a | #Feature F7b | #Feature F8 | #Feature F9a | #Feature Sample 1F10a | #Feature F10b | #Feature F11a | #Feature F11b | #Feature F12a | #Feature F13 | Start | End | PTM | AScore | Found By |
|---|---|---|---|---|---|---|---|---|---|---|---|---|---|---|---|---|---|---|---|---|---|---|---|---|---|---|---|---|---|---|---|---|---|---|---|---|---|---|---|---|---|---|---|---|---|---|---|---|---|
| G.FEYGGC(+57.02)GGNANNFK.T | Y | 43.67 | 1533.6306 | 14 | -0.3 | 767.8224 | 2 | 57.89 | 4 | F4:36507 | BuCaTN01_F4.raw | | | | 1.2175E7 | | | | | | | | | | | | | 1 | 0 | 0 | 0 | 1 | 0 | 0 | 0 | 0 | 0 | 0 | 0 | 0 | 0 | 0 | 0 | 0 | 59 | 72 | Carbamidomethylation | C6:Carbamidomethylation:1000.00 | PEAKS DB |
| **total 1 peptides** | | | | | | | | | | | | | | | | | | | | | | | | | | | | | | | | | | | | | | | | | | | | | | | | | |

**Best Unique PSM (Scan F4:36507, m/z=767.8224, z=2, RT=57.89, ppm=-0.3):**

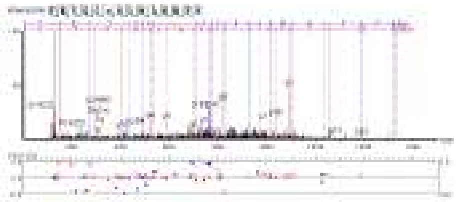

## UNJ97090.1

back to list

| Protein Coverage | Supporting Peptides | Best Unique PSM |

**Protein Coverage:**

1 MPSGGLLLLL GFLTLWAELT PVSGQGRPKF CHLPPEVGSC KALVPRFYYN STSKKCQG**FE YGGCGGNANN FK**TRDECHYT

81 CVEKPGVCPQ GPQQRPCLEK CQDDWKCPGQ QKCCRYGCTT ECKDPIFVK

64 C: Carbamidomethylation (+57.02)

**Supporting Peptides:**

| Peptide | Uniq | -10lgP | Mass | Length | ppm | m/z | z | RT | Fraction | Scan | Source File | Area F1 | Area F2a | Area F3a | Area F4 | Area F5 | Area F6a | Area F7a | Area F7b | Area F8 | Area F9a | Area Sample 1F10a | Area F10b | Area F11a | Area F11b | Area F12a | Area F13 | #Feature | #Feature F1 | #Feature F2a | #Feature F3a | #Feature F4 | #Feature F5 | #Feature F6a | #Feature F7a | #Feature F7b | #Feature F8 | #Feature F9a | #Feature Sample 1F10a | #Feature F10b | #Feature F11a | #Feature F11b | #Feature F12a | #Feature F13 | Start | End | PTM | AScore | Found By |
|---|---|---|---|---|---|---|---|---|---|---|---|---|---|---|---|---|---|---|---|---|---|---|---|---|---|---|---|---|---|---|---|---|---|---|---|---|---|---|---|---|---|---|---|---|---|---|---|---|---|
| G.FEYGGC(+57.02)GGNANNFK.T | Y | 43.67 | 1533.6306 | 14 | -0.3 | 767.8224 | 2 | 57.89 | 4 | F4:36507 | BuCaTN01_F4.raw | | | | 1.2175E7 | | | | | | | | | | | | | 1 | 0 | 0 | 0 | 1 | 0 | 0 | 0 | 0 | 0 | 0 | 0 | 0 | 0 | 0 | 0 | 0 | 59 | 72 | Carbamidomethylation | C6:Carbamidomethylation:1000.00 | PEAKS DB |
| **total 1 peptides** | | | | | | | | | | | | | | | | | | | | | | | | | | | | | | | | | | | | | | | | | | | | | | | | | |

**Best Unique PSM (Scan F4:36507, m/z=767.8224, z=2, RT=57.89, ppm=-0.3):**

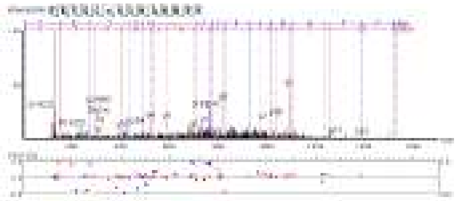

## UNJ97082.1

back to list

| Protein Coverage | Supporting Peptides | Best Unique PSM |

**Protein Coverage:**

1 MPSGGHLLLL GLLTLWAELT PVSGQGRPEF CHLPPEVGSC KALVPRFYYN STSKKCQG**FE YGGCGGNANN FK**TRDECHYT

81 CVEKPGVCPQ GPQQRPCLEK CQDDWKCPGQ QKCCRYGCMT ECKDILYETH ALPTRRPSRI GTTVRT

64 C: Carbamidomethylation (+57.02)

**Supporting Peptides:**

| Peptide | Uniq | -10lgP | Mass | Length | ppm | m/z | z | RT | Fraction | Scan | Source File | Area F1 | Area F2a | Area F3a | Area F4 | Area F5 | Area F6a | Area F7a | Area F7b | Area F8 | Area F9a | Area Sample 1F10a | Area F10b | Area F11a | Area F11b | Area F12a | Area F13 | #Feature | #Feature F1 | #Feature F2a | #Feature F3a | #Feature F4 | #Feature F5 | #Feature F6a | #Feature F7a | #Feature F7b | #Feature F8 | #Feature F9a | #Feature Sample 1F10a | #Feature F10b | #Feature F11a | #Feature F11b | #Feature F12a | #Feature F13 | Start | End | PTM | AScore | Found By |
|---|---|---|---|---|---|---|---|---|---|---|---|---|---|---|---|---|---|---|---|---|---|---|---|---|---|---|---|---|---|---|---|---|---|---|---|---|---|---|---|---|---|---|---|---|---|---|---|---|---|
| G.FEYGGC(+57.02)GGNANNFK.T | Y | 43.67 | 1533.6306 | 14 | -0.3 | 767.8224 | 2 | 57.89 | 4 | F4:36507 | BuCaTN01_F4.raw | | | | 1.2175E7 | | | | | | | | | | | | | 1 | 0 | 0 | 0 | 1 | 0 | 0 | 0 | 0 | 0 | 0 | 0 | 0 | 0 | 0 | 0 | 0 | 59 | 72 | Carbamidomethylation | C6:Carbamidomethylation:1000.00 | PEAKS DB |
| **total 1 peptides** | | | | | | | | | | | | | | | | | | | | | | | | | | | | | | | | | | | | | | | | | | | | | | | | | |

**Best Unique PSM (Scan F4:36507, m/z=767.8224, z=2, RT=57.89, ppm=-0.3):**

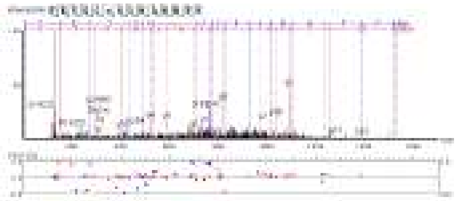

## UNJ97094.1

back to list

| Protein Coverage | Supporting Peptides | Best Unique PSM |

**Protein Coverage:**

1 MPSGGLLLLL GLLTLWAELT PVSGQGRPEF CHLPPEVGSC KALVPRFYYN STSKKCQG**FE YGGCGGNANN FK**TRDECQYT

81 CVEKPGVCPQ GPQQHPCLEK CQDDWKCPGQ QKCCRYGCMT ECKDVLYETH ALPTRRPSRI GTTVRT

64 C: Carbamidomethylation (+57.02)

**Supporting Peptides:**

| Peptide | Uniq | -10lgP | Mass | Length | ppm | m/z | z | RT | Fraction | Scan | Source File | Area F1 | Area F2a | Area F3a | Area F4 | Area F5 | Area F6a | Area F7a | Area F7b | Area F8 | Area F9a | Area Sample 1F10a | Area F10b | Area F11a | Area F11b | Area F12a | Area F13 | #Feature | #Feature F1 | #Feature F2a | #Feature F3a | #Feature F4 | #Feature F5 | #Feature F6a | #Feature F7a | #Feature F7b | #Feature F8 | #Feature F9a | #Feature Sample 1F10a | #Feature F10b | #Feature F11a | #Feature F11b | #Feature F12a | #Feature F13 | Start | End | PTM | AScore | Found By |
|---|---|---|---|---|---|---|---|---|---|---|---|---|---|---|---|---|---|---|---|---|---|---|---|---|---|---|---|---|---|---|---|---|---|---|---|---|---|---|---|---|---|---|---|---|---|---|---|---|---|

| Peptide | Uniq | -10lgP | Mass | Length | ppm | m/z | z | RT | Fraction | Scan | Source File | Area F1 | Area F2a | Area F3a | Area F4 | Area F5 | Area F6a | Area F7a | Area F7b | Area F8 | Area F9a | Area Sample 1F10a | Area F10b | Area F11a | Area F11b | Area F12a | Area F13 | #Feature | #Feature F1 | #Feature F2a | #Feature F3a | #Feature F4 | #Feature F5 | #Feature F6a | #Feature F7a | #Feature F7b | #Feature F8 | #Feature F9a | #Feature Sample 1F10a | #Feature F10b | #Feature F11a | #Feature F11b | #Feature F12a | #Feature F13 | Start | End | PTM | AScore | Found By |
|---|---|---|---|---|---|---|---|---|---|---|---|---|---|---|---|---|---|---|---|---|---|---|---|---|---|---|---|---|---|---|---|---|---|---|---|---|---|---|---|---|---|---|---|---|---|---|---|---|---|
| G.FEYGGC(+57.02)GGNANNFK.T | Y | 43.67 | 1533.6306 | 14 | -0.3 | 767.8224 | 2 | 57.89 | 4 | F4:36507 | BuCaTN01_F4.raw | | | | 1.2175E7 | | | | | | | | | | | | | 1 | 0 | 0 | 0 | 1 | 0 | 0 | 0 | 0 | 0 | 0 | 0 | 0 | 0 | 0 | 0 | 0 | 59 | 72 | Carbamidomethylation | C6:Carbamidomethylation:1000.00 | PEAKS DB |
| total 1 peptides | | | | | | | | | | | | | | | | | | | | | | | | | | | | | | | | | | | | | | | | | | | | | | | | | |

**Best Unique PSM (Scan F4:36507, m/z=767.8224, z=2, RT=57.89, ppm=-0.3):**

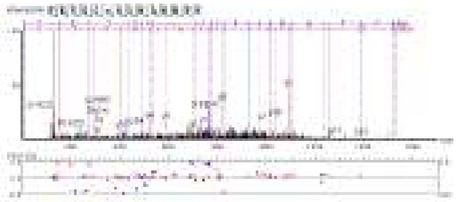

## P62377.1

back to list

| Protein Coverage | Supporting Peptides | Best Unique PSM |

**Protein Coverage:**

1 LKCHNTQLPF IYKTCPEGKN LCFKATLK**KF PLKFPVK**RGC ADNCPKNSAL LKYVCCSTDK CN

**Supporting Peptides:**

| Peptide | Uniq | -10lgP | Mass | Length | ppm | m/z | z | RT | Fraction | Scan | Source File | Area F1 | Area F2a | Area F3a | Area F4 | Area F5 | Area F6a | Area F7a | Area F7b | Area F8 | Area F9a | Area Sample 1F10a | Area F10b | Area F11a | Area F11b | Area F12a | Area F13 | #Feature | #Feature F1 | #Feature F2a | #Feature F3a | #Feature F4 | #Feature F5 | #Feature F6a | #Feature F7a | #Feature F7b | #Feature F8 | #Feature F9a | #Feature Sample 1F10a | #Feature F10b | #Feature F11a | #Feature F11b | #Feature F12a | #Feature F13 | Start | End | PTM | AScore | Found By |
|---|---|---|---|---|---|---|---|---|---|---|---|---|---|---|---|---|---|---|---|---|---|---|---|---|---|---|---|---|---|---|---|---|---|---|---|---|---|---|---|---|---|---|---|---|---|---|---|---|---|
| K.KFPLKFPVK.R | Y | 43.49 | 1102.6902 | 9 | -0.3 | 368.5706 | 3 | 50.55 | 10 | F10:27776 | BuCaTN01_F9a.raw | | | | | | | | | | 0 | | | | | | | 0 | 0 | 0 | 0 | 0 | 0 | 0 | 0 | 0 | 0 | 0 | 0 | 0 | 0 | 0 | 0 | 0 | 29 | 37 | | | PEAKS DB |
| total 1 peptides | | | | | | | | | | | | | | | | | | | | | | | | | | | | | | | | | | | | | | | | | | | | | | | | | |

**Best Unique PSM (Scan F10:27776, m/z=368.5706, z=3, RT=50.55, ppm=-0.3):**

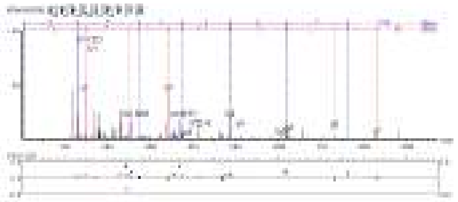

## 1CVO

back to list

| Protein Coverage | Supporting Peptides | Best Unique PSM |

**Protein Coverage:**

1 LKCHNTQLPF IYKTCPEGKN LCFKATLK**KF PLKFPVK**RGC ADNCPKNSAL LKYVCCSTDK CN

**Supporting Peptides:**

| Peptide | Uniq | -10lgP | Mass | Length | ppm | m/z | z | RT | Fraction | Scan | Source File | Area F1 | Area F2a | Area F3a | Area F4 | Area F5 | Area F6a | Area F7a | Area F7b | Area F8 | Area F9a | Area Sample 1F10a | Area F10b | Area F11a | Area F11b | Area F12a | Area F13 | #Feature | #Feature F1 | #Feature F2a | #Feature F3a | #Feature F4 | #Feature F5 | #Feature F6a | #Feature F7a | #Feature F7b | #Feature F8 | #Feature F9a | #Feature Sample 1F10a | #Feature F10b | #Feature F11a | #Feature F11b | #Feature F12a | #Feature F13 | Start | End | PTM | AScore | Found By |
|---|---|---|---|---|---|---|---|---|---|---|---|---|---|---|---|---|---|---|---|---|---|---|---|---|---|---|---|---|---|---|---|---|---|---|---|---|---|---|---|---|---|---|---|---|---|---|---|---|---|
| K.KFPLKFPVK.R | Y | 43.49 | 1102.6902 | 9 | -0.3 | 368.5706 | 3 | 50.55 | 10 | F10:27776 | BuCaTN01_F9a.raw | | | | | | | | | | 0 | | | | | | | 0 | 0 | 0 | 0 | 0 | 0 | 0 | 0 | 0 | 0 | 0 | 0 | 0 | 0 | 0 | 0 | 0 | 29 | 37 | | | PEAKS DB |
| total 1 peptides | | | | | | | | | | | | | | | | | | | | | | | | | | | | | | | | | | | | | | | | | | | | | | | | | |

**Best Unique PSM (Scan F10:27776, m/z=368.5706, z=3, RT=50.55, ppm=-0.3):**

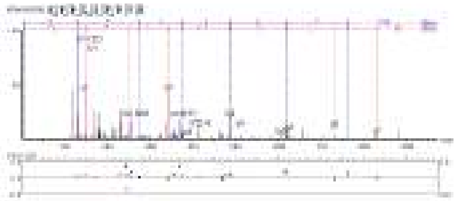

## 1KXI

back to list

| Protein Coverage | Supporting Peptides | Best Unique PSM |

**Protein Coverage:**

1 LKCHNTQLPF IYKTCPEGKN LCFKATLK**KF PLKFPVK**RGC ADNCPKNSAL LKYVCCSTDK CN

**Supporting Peptides:**

| Peptide | Uniq | -10lgP | Mass | Length | ppm | m/z | z | RT | Fraction | Scan | Source File | Area F1 | Area F2a | Area F3a | Area F4 | Area F5 | Area F6a | Area F7a | Area F7b | Area F8 | Area F9a | Area Sample 1F10a | Area F10b | Area F11a | Area F11b | Area F12a | Area F13 | #Feature | #Feature F1 | #Feature F2a | #Feature F3a | #Feature F4 | #Feature F5 | #Feature F6a | #Feature F7a | #Feature F7b | #Feature F8 | #Feature F9a | #Feature Sample 1F10a | #Feature F10b | #Feature F11a | #Feature F11b | #Feature F12a | #Feature F13 | Start | End | PTM | AScore | Found By |
|---|---|---|---|---|---|---|---|---|---|---|---|---|---|---|---|---|---|---|---|---|---|---|---|---|---|---|---|---|---|---|---|---|---|---|---|---|---|---|---|---|---|---|---|---|---|---|---|---|---|
| K.KFPLKFPVK.R | Y | 43.49 | 1102.6902 | 9 | -0.3 | 368.5706 | 3 | 50.55 | 10 | F10:27776 | BuCaTN01_F9a.raw | | | | | | | | | | 0 | | | | | | | 0 | 0 | 0 | 0 | 0 | 0 | 0 | 0 | 0 | 0 | 0 | 0 | 0 | 0 | 0 | 0 | 0 | 29 | 37 | | | PEAKS DB |
| total 1 peptides | | | | | | | | | | | | | | | | | | | | | | | | | | | | | | | | | | | | | | | | | | | | | | | | | |

**Best Unique PSM (Scan F10:27776, m/z=368.5706, z=3, RT=50.55, ppm=-0.3):**

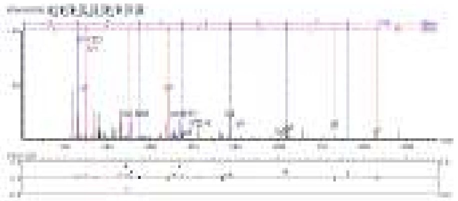

## Q9W716.1

back to list

| Protein Coverage | Supporting Peptides | Best Unique PSM |

**Protein Coverage:**

1 MKTLLLTLVV VTIVCLDLGY TLKCHNTQLP FIYKTCPEGK NLCFKATLR**K FPLKFPVK**RG CADNCPKNSA LLKYVCCSTD

81 KCN

**Supporting Peptides:**

| Peptide | Uniq | -10lgP | Mass | Length | ppm | m/z | z | RT | Fraction | Scan | Source File | Area F1 | Area F2a | Area F3a | Area F4 | Area F5 | Area F6a | Area F7a | Area F7b | Area F8 | Area F9a | Area Sample 1F10a | Area F10b | Area F11a | Area F11b | Area F12a | Area F13 | #Feature | #Feature F1 | #Feature F2a | #Feature F3a | #Feature F4 | #Feature F5 | #Feature F6a | #Feature F7a | #Feature F7b | #Feature F8 | #Feature F9a | #Feature Sample 1F10a | #Feature F10b | #Feature F11a | #Feature F11b | #Feature F12a | #Feature F13 | Start | End | PTM | AScore | Found By |
|---|---|---|---|---|---|---|---|---|---|---|---|---|---|---|---|---|---|---|---|---|---|---|---|---|---|---|---|---|---|---|---|---|---|---|---|---|---|---|---|---|---|---|---|---|---|---|---|---|---|
| R.KFPLKFPVK.R | Y | 43.49 | 1102.6902 | 9 | -0.3 | 368.5706 | 3 | 50.55 | 10 | F10:27776 | BuCaTN01_F9a.raw | | | | | | | | | | 0 | | | | | | | 0 | 0 | 0 | 0 | 0 | 0 | 0 | 0 | 0 | 0 | 0 | 0 | 0 | 0 | 0 | 0 | 0 | 50 | 58 | | | PEAKS DB |
| total 1 peptides | | | | | | | | | | | | | | | | | | | | | | | | | | | | | | | | | | | | | | | | | | | | | | | | | |

**Best Unique PSM (Scan F10:27776, m/z=368.5706, z=3, RT=50.55, ppm=-0.3):**

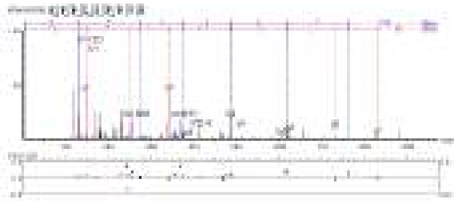

## AAB01540.1

back to list

| Protein Coverage | Supporting Peptides | Best Unique PSM |

**Protein Coverage:**

1 MKTLLLTLVV VTIVCLDLGY TLKCHNTQLP FIYKTCPEGK NLCFKATLKK FPLKFPVKRG CADNCPKNSA LLKYVCCSTD

81 KCN

**Supporting Peptides:**

| Peptide | Uniq | -10lgP | Mass | Length | ppm | m/z | z | RT | Fraction | Scan | Source File | Area F1 | Area F2a | Area F3a | Area F4 | Area F5 | Area F6a | Area F7a | Area F7b | Area F8 | Area F9a | Area Sample 1F10a | Area F10b | Area F11a | Area F11b | Area F12a | Area F13 | #Feature | #Feature F1 | #Feature F2a | #Feature F3a | #Feature F4 | #Feature F5 | #Feature F6a | #Feature F7a | #Feature F7b | #Feature F8 | #Feature F9a | #Feature Sample 1F10a | #Feature F10b | #Feature F11a | #Feature F11b | #Feature F12a | #Feature F13 | Start | End | PTM | AScore | Found By |
|---|---|---|---|---|---|---|---|---|---|---|---|---|---|---|---|---|---|---|---|---|---|---|---|---|---|---|---|---|---|---|---|---|---|---|---|---|---|---|---|---|---|---|---|---|---|---|---|---|---|
| K.KFPLKFPVK.R | Y | 43.49 | 1102.6902 | 9 | -0.3 | 368.5706 | 3 | 50.55 | 10 | F10:27776 | BuCaTN01_F9a.raw | | | | | | | | | | 0 | | | | | | | 0 | 0 | 0 | 0 | 0 | 0 | 0 | 0 | 0 | 0 | 0 | 0 | 0 | 0 | 0 | 0 | 0 | 50 | 58 | | | PEAKS DB |
| total 1 peptides | | | | | | | | | | | | | | | | | | | | | | | | | | | | | | | | | | | | | | | | | | | | | | | | | |

**Best Unique PSM (Scan F10:27776, m/z=368.5706, z=3, RT=50.55, ppm=-0.3):**

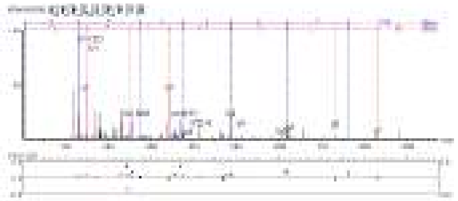

## CAB50697.1

back to list

| Protein Coverage | Supporting Peptides | Best Unique PSM |

**Protein Coverage:**

1 MKTLLLTLVV VTIVCLDLGY TLKCHNTQLP FIYKTCPEGK NLCFKATLRK FPLKFPVKRG CADNCPKNSA LLKYVCCSTD

81 KCN

**Supporting Peptides:**

| Peptide | Uniq | -10lgP | Mass | Length | ppm | m/z | z | RT | Fraction | Scan | Source File | Area F1 | Area F2a | Area F3a | Area F4 | Area F5 | Area F6a | Area F7a | Area F7b | Area F8 | Area F9a | Area Sample 1F10a | Area F10b | Area F11a | Area F11b | Area F12a | Area F13 | #Feature | #Feature F1 | #Feature F2a | #Feature F3a | #Feature F4 | #Feature F5 | #Feature F6a | #Feature F7a | #Feature F7b | #Feature F8 | #Feature F9a | #Feature Sample 1F10a | #Feature F10b | #Feature F11a | #Feature F11b | #Feature F12a | #Feature F13 | Start | End | PTM | AScore | Found By |
|---|---|---|---|---|---|---|---|---|---|---|---|---|---|---|---|---|---|---|---|---|---|---|---|---|---|---|---|---|---|---|---|---|---|---|---|---|---|---|---|---|---|---|---|---|---|---|---|---|---|
| R.KFPLKFPVK.R | Y | 43.49 | 1102.6902 | 9 | -0.3 | 368.5706 | 3 | 50.55 | 10 | F10:27776 | BuCaTN01_F9a.raw | | | | | | | | | | 0 | | | | | | | 0 | 0 | 0 | 0 | 0 | 0 | 0 | 0 | 0 | 0 | 0 | 0 | 0 | 0 | 0 | 0 | 0 | 50 | 58 | | | PEAKS DB |
| total 1 peptides | | | | | | | | | | | | | | | | | | | | | | | | | | | | | | | | | | | | | | | | | | | | | | | | | |

**Best Unique PSM (Scan F10:27776, m/z=368.5706, z=3, RT=50.55, ppm=-0.3):**

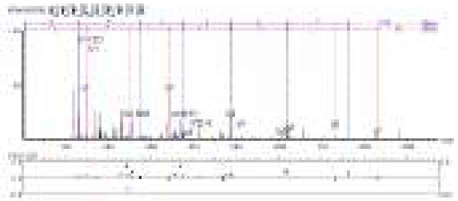

## AAB36928.1

back to list

| Protein Coverage | Supporting Peptides | Best Unique PSM |

**Protein Coverage:**

1 MKTLLLTLVV VTIVCLDLGY TLKCHNTQLP FIYKTCPEGK NLCFKATLKK FPLKFPVKRG CADNCPKNSA LLKYVCCSTD

81 KCN

**Supporting Peptides:**

| Peptide | Uniq | -10lgP | Mass | Length | ppm | m/z | z | RT | Fraction | Scan | Source File | Area F1 | Area F2a | Area F3a | Area F4 | Area F5 | Area F6a | Area F7a | Area F7b | Area F8 | Area F9a | Area Sample 1F10a | Area F10b | Area F11a | Area F11b | Area F12a | Area F13 | #Feature | #Feature F1 | #Feature F2a | #Feature F3a | #Feature F4 | #Feature F5 | #Feature F6a | #Feature F7a | #Feature F7b | #Feature F8 | #Feature F9a | #Feature Sample 1F10a | #Feature F10b | #Feature F11a | #Feature F11b | #Feature F12a | #Feature F13 | Start | End | PTM | AScore | Found By |
|---|---|---|---|---|---|---|---|---|---|---|---|---|---|---|---|---|---|---|---|---|---|---|---|---|---|---|---|---|---|---|---|---|---|---|---|---|---|---|---|---|---|---|---|---|---|---|---|---|---|
| K.KFPLKFPVK.R | Y | 43.49 | 1102.6902 | 9 | -0.3 | 368.5706 | 3 | 50.55 | 10 | F10:27776 | BuCaTN01_F9a.raw | | | | | | | | | | 0 | | | | | | | 0 | 0 | 0 | 0 | 0 | 0 | 0 | 0 | 0 | 0 | 0 | 0 | 0 | 0 | 0 | 0 | 0 | 50 | 58 | | | PEAKS DB |
| total 1 peptides | | | | | | | | | | | | | | | | | | | | | | | | | | | | | | | | | | | | | | | | | | | | | | | | | |

**Best Unique PSM (Scan F10:27776, m/z=368.5706, z=3, RT=50.55, ppm=-0.3):**

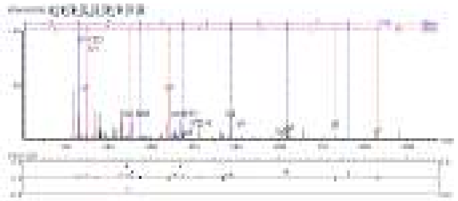

## Q91996.1

back to list

| Protein Coverage | Supporting Peptides | Best Unique PSM |

**Protein Coverage:**

1 MKTLLLTLVV VTIVCLDLGY TLKCHNTQLP FIYKTCPEGK NLCFKATLKK FPLKFPVKRG CADNCPKNSA LLKYVCCSTD

81 KCN

**Supporting Peptides:**

| Peptide | Uniq | -10lgP | Mass | Length | ppm | m/z | z | RT | Fraction | Scan | Source File | Area F1 | Area F2a | Area F3a | Area F4 | Area F5 | Area F6a | Area F7a | Area F7b | Area F8 | Area F9a | Area Sample 1F10a | Area F10b | Area F11a | Area F11b | Area F12a | Area F13 | #Feature | #Feature F1 | #Feature F2a | #Feature F3a | #Feature F4 | #Feature F5 | #Feature F6a | #Feature F7a | #Feature F7b | #Feature F8 | #Feature F9a | #Feature Sample 1F10a | #Feature F10b | #Feature F11a | #Feature F11b | #Feature F12a | #Feature F13 | Start | End | PTM | AScore | Found By |
|---|---|---|---|---|---|---|---|---|---|---|---|---|---|---|---|---|---|---|---|---|---|---|---|---|---|---|---|---|---|---|---|---|---|---|---|---|---|---|---|---|---|---|---|---|---|---|---|---|---|
| K.KFPLKFPVK.R | Y | 43.49 | 1102.6902 | 9 | -0.3 | 368.5706 | 3 | 50.55 | 10 | F10:27776 | BuCaTN01_F9a.raw | | | | | | | | | | 0 | | | | | | | 0 | 0 | 0 | 0 | 0 | 0 | 0 | 0 | 0 | 0 | 0 | 0 | 0 | 0 | 0 | 0 | 0 | 50 | 58 | | | PEAKS DB |
| total 1 peptides | | | | | | | | | | | | | | | | | | | | | | | | | | | | | | | | | | | | | | | | | | | | | | | | | |

**Best Unique PSM (Scan F10:27776, m/z=368.5706, z=3, RT=50.55, ppm=-0.3):**

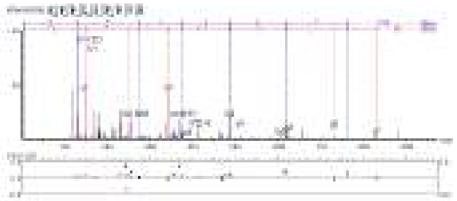

## AAB36929.1

back to list

| Protein Coverage | Supporting Peptides | Best Unique PSM |

**Protein Coverage:**

1 MKTLLLTLVV VTIVCLDLGY TLKCHNTQLP FIYKTCPEGK NLCFKATLKK FPLKFPVKRG CADNCPKNSA LLKYVCCSTE

81 KCN

**Supporting Peptides:**

| Peptide | Uniq | -10lgP | Mass | Length | ppm | m/z | z | RT | Fraction | Scan | Source File | Area F1 | Area F2a | Area F3a | Area F4 | Area F5 | Area F6a | Area F7a | Area F7b | Area F8 | Area F9a | Area Sample 1F10a | Area F10b | Area F11a | Area F11b | Area F12a | Area F13 | #Feature | #Feature F1 | #Feature F2a | #Feature F3a | #Feature F4 | #Feature F5 | #Feature F6a | #Feature F7a | #Feature F7b | #Feature F8 | #Feature F9a | #Feature Sample 1F10a | #Feature F10b | #Feature F11a | #Feature F11b | #Feature F12a | #Feature F13 | Start | End | PTM | AScore | Found By |
|---|---|---|---|---|---|---|---|---|---|---|---|---|---|---|---|---|---|---|---|---|---|---|---|---|---|---|---|---|---|---|---|---|---|---|---|---|---|---|---|---|---|---|---|---|---|---|---|---|---|
| K.KFPLKFPVK.R | Y | 43.49 | 1102.6902 | 9 | -0.3 | 368.5706 | 3 | 50.55 | 10 | F10:27776 | BuCaTN01_F9a.raw | | | | | | | | | | 0 | | | | | | | 0 | 0 | 0 | 0 | 0 | 0 | 0 | 0 | 0 | 0 | 0 | 0 | 0 | 0 | 0 | 0 | 0 | 50 | 58 | | | PEAKS DB |
| total 1 peptides | | | | | | | | | | | | | | | | | | | | | | | | | | | | | | | | | | | | | | | | | | | | | | | | | |

**Best Unique PSM (Scan F10:27776, m/z=368.5706, z=3, RT=50.55, ppm=-0.3):**

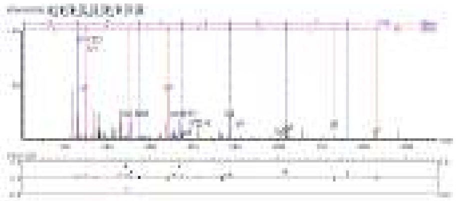

## AAB03219.1

back to list

| Protein Coverage | Supporting Peptides | Best Unique PSM |

**Protein Coverage:**

1 MKTLLLTLVV VTIVCLDLGY TLKCHNTQLP FIYKTCPEGK NLCFKATLKK FPLKFPVKRG CADNCPKNSA LLKYVCCSTD

81 KCN

**Supporting Peptides:**

| Peptide | Uniq | -10lgP | Mass | Length | ppm | m/z | z | RT | Fraction | Scan | Source File | Area F1 | Area F2a | Area F3a | Area F4 | Area F5 | Area F6a | Area F7a | Area F7b | Area F8 | Area F9a | Area Sample 1F10a | Area F10b | Area F11a | Area F11b | Area F12a | Area F13 | #Feature | #Feature F1 | #Feature F2a | #Feature F3a | #Feature F4 | #Feature F5 | #Feature F6a | #Feature F7a | #Feature F7b | #Feature F8 | #Feature F9a | #Feature Sample 1F10a | #Feature F10b | #Feature F11a | #Feature F11b | #Feature F12a | #Feature F13 | Start | End | PTM | AScore | Found By |
|---|---|---|---|---|---|---|---|---|---|---|---|---|---|---|---|---|---|---|---|---|---|---|---|---|---|---|---|---|---|---|---|---|---|---|---|---|---|---|---|---|---|---|---|---|---|---|---|---|---|
| K.KFPLKFPVK.R | Y | 43.49 | 1102.6902 | 9 | -0.3 | 368.5706 | 3 | 50.55 | 10 | F10:27776 | BuCaTN01_F9a.raw | | | | | | | | | | 0 | | | | | | | 0 | 0 | 0 | 0 | 0 | 0 | 0 | 0 | 0 | 0 | 0 | 0 | 0 | 0 | 0 | 0 | 0 | 50 | 58 | | | PEAKS DB |
| total 1 peptides | | | | | | | | | | | | | | | | | | | | | | | | | | | | | | | | | | | | | | | | | | | | | | | | | |

**Best Unique PSM (Scan F10:27776, m/z=368.5706, z=3, RT=50.55, ppm=-0.3):**

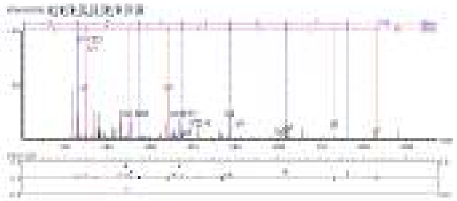

## CAA90964.1

back to list

| Protein Coverage | Supporting Peptides | Best Unique PSM |

**Protein Coverage:**

1 MKTLLLTMVV VTIVCLDLGY TLKCHNTQLP FIYKTCPEGK NLCFKATLKK FPLKFPVKRG CADNCPKNSA LLKYVCCSTD

81 KCN

**Supporting Peptides:**

| Peptide | Uniq | -10lgP | Mass | Length | ppm | m/z | z | RT | Fraction | Scan | Source File | Area F1 | Area F2a | Area F3a | Area F4 | Area F5 | Area F6a | Area F7a | Area F7b | Area F8 | Area F9a | Area Sample 1F10a | Area F10b | Area F11a | Area F11b | Area F12a | Area F13 | #Feature | #Feature F1 | #Feature F2a | #Feature F3a | #Feature F4 | #Feature F5 | #Feature F6a | #Feature F7a | #Feature F7b | #Feature F8 | #Feature F9a | #Feature Sample 1F10a | #Feature F10b | #Feature F11a | #Feature F11b | #Feature F12a | #Feature F13 | Start | End | PTM | AScore | Found By |
|---|---|---|---|---|---|---|---|---|---|---|---|---|---|---|---|---|---|---|---|---|---|---|---|---|---|---|---|---|---|---|---|---|---|---|---|---|---|---|---|---|---|---|---|---|---|---|---|---|---|
| K.KFPLKFPVK.R | Y | 43.49 | 1102.6902 | 9 | -0.3 | 368.5706 | 3 | 50.55 | 10 | F10:27776 | BuCaTN01_F9a.raw | | | | | | | | | | 0 | | | | | | | 0 | 0 | 0 | 0 | 0 | 0 | 0 | 0 | 0 | 0 | 0 | 0 | 0 | 0 | 0 | 0 | 0 | 50 | 58 | | | PEAKS DB |
| total 1 peptides | | | | | | | | | | | | | | | | | | | | | | | | | | | | | | | | | | | | | | | | | | | | | | | | | |

**Best Unique PSM (Scan F10:27776, m/z=368.5706, z=3, RT=50.55, ppm=-0.3):**

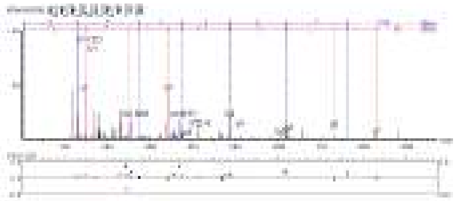

## CAA63979.1

back to list

| Protein Coverage | Supporting Peptides | Best Unique PSM |

**Protein Coverage:**

1 MKTLLLTMVV VTIVCLDLGY TLKCHNTQLP FIYKTCPEGK NLCFKATLKK FPLKFPVKRG CADNCPKNSA LLKYVCCSSD

81 KCN

**Supporting Peptides:**

| Peptide | Uniq | -10lgP | Mass | Length | ppm | m/z | z | RT | Fraction | Scan | Source File | Area F1 | Area F2a | Area F3a | Area F4 | Area F5 | Area F6a | Area F7a | Area F7b | Area F8 | Area F9a | Area Sample 1F10a | Area F10b | Area F11a | Area F11b | Area F12a | Area F13 | #Feature | #Feature F1 | #Feature F2a | #Feature F3a | #Feature F4 | #Feature F5 | #Feature F6a | #Feature F7a | #Feature F7b | #Feature F8 | #Feature F9a | #Feature Sample 1F10a | #Feature F10b | #Feature F11a | #Feature F11b | #Feature F12a | #Feature F13 | Start | End | PTM | AScore | Found By |
|---|---|---|---|---|---|---|---|---|---|---|---|---|---|---|---|---|---|---|---|---|---|---|---|---|---|---|---|---|---|---|---|---|---|---|---|---|---|---|---|---|---|---|---|---|---|---|---|---|---|
| K.KFPLKFPVK.R | Y | 43.49 | 1102.6902 | 9 | -0.3 | 368.5706 | 3 | 50.55 | 10 | F10:27776 | BuCaTN01_F9a.raw | | | | | | | | | | 0 | | | | | | | 0 | 0 | 0 | 0 | 0 | 0 | 0 | 0 | 0 | 0 | 0 | 0 | 0 | 0 | 0 | 0 | 0 | 50 | 58 | | | PEAKS DB |
| total 1 peptides | | | | | | | | | | | | | | | | | | | | | | | | | | | | | | | | | | | | | | | | | | | | | | | | | |

**Best Unique PSM (Scan F10:27776, m/z=368.5706, z=3, RT=50.55, ppm=-0.3):**

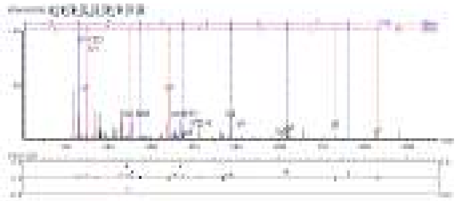

## AAB03220.1

back to list

| Protein Coverage | Supporting Peptides | Best Unique PSM |

**Protein Coverage:**

1 MKTLLLTLVV VTIVCLDLGY TLKCHNTQLP FIYKTCPEGK NLCFKATLK**K FPLKFPVK**RG CADNCPKNSA LLKYVCCSTE

81 KCN

**Supporting Peptides:**

| Peptide | Uniq | -10lgP | Mass | Length | ppm | m/z | z | RT | Fraction | Scan | Source File | Area F1 | Area F2a | Area F3a | Area F4 | Area F5 | Area F6a | Area F7a | Area F7b | Area F8 | Area F9a | Area Sample 1F10a | Area F10b | Area F11a | Area F11b | Area F12a | Area F13 | #Feature | #Feature F1 | #Feature F2a | #Feature F3a | #Feature F4 | #Feature F5 | #Feature F6a | #Feature F7a | #Feature F7b | #Feature F8 | #Feature F9a | #Feature Sample 1F10a | #Feature F10b | #Feature F11a | #Feature F11b | #Feature F12a | #Feature F13 | Start | End | PTM | AScore | Found By |
|---|---|---|---|---|---|---|---|---|---|---|---|---|---|---|---|---|---|---|---|---|---|---|---|---|---|---|---|---|---|---|---|---|---|---|---|---|---|---|---|---|---|---|---|---|---|---|---|---|---|
| K.KFPLKFPVK.R | Y | 43.49 | 1102.6902 | 9 | -0.3 | 368.5706 | 3 | 50.55 | 10 | F10:27776 | BuCaTN01_F9a.raw | | | | | | | | | | 0 | | | | | | | 0 | 0 | 0 | 0 | 0 | 0 | 0 | 0 | 0 | 0 | 0 | 0 | 0 | 0 | 0 | 0 | 0 | 50 | 58 | | | PEAKS DB |
| total 1 peptides | | | | | | | | | | | | | | | | | | | | | | | | | | | | | | | | | | | | | | | | | | | | | | | | | |

**Best Unique PSM (Scan F10:27776, m/z=368.5706, z=3, RT=50.55, ppm=-0.3):**

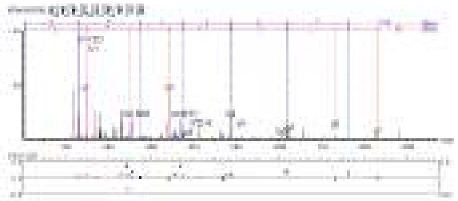

## AAB18387.1

back to list

| Protein Coverage | Supporting Peptides | Best Unique PSM |

**Protein Coverage:**

1 MKTLLLTLVV VTIVCLDLGY TLKCHNTQLP FIYKTCPEGK NLCFKATLK**K FPLKFPVK**RG CADNCPKNSA LLKYVCCSTD

81 KCN

**Supporting Peptides:**

| Peptide | Uniq | -10lgP | Mass | Length | ppm | m/z | z | RT | Fraction | Scan | Source File | Area F1 | Area F2a | Area F3a | Area F4 | Area F5 | Area F6a | Area F7a | Area F7b | Area F8 | Area F9a | Area Sample 1F10a | Area F10b | Area F11a | Area F11b | Area F12a | Area F13 | #Feature | #Feature F1 | #Feature F2a | #Feature F3a | #Feature F4 | #Feature F5 | #Feature F6a | #Feature F7a | #Feature F7b | #Feature F8 | #Feature F9a | #Feature Sample 1F10a | #Feature F10b | #Feature F11a | #Feature F11b | #Feature F12a | #Feature F13 | Start | End | PTM | AScore | Found By |
|---|---|---|---|---|---|---|---|---|---|---|---|---|---|---|---|---|---|---|---|---|---|---|---|---|---|---|---|---|---|---|---|---|---|---|---|---|---|---|---|---|---|---|---|---|---|---|---|---|---|
| K.KFPLKFPVK.R | Y | 43.49 | 1102.6902 | 9 | -0.3 | 368.5706 | 3 | 50.55 | 10 | F10:27776 | BuCaTN01_F9a.raw | | | | | | | | | | 0 | | | | | | | 0 | 0 | 0 | 0 | 0 | 0 | 0 | 0 | 0 | 0 | 0 | 0 | 0 | 0 | 0 | 0 | 0 | 50 | 58 | | | PEAKS DB |
| total 1 peptides | | | | | | | | | | | | | | | | | | | | | | | | | | | | | | | | | | | | | | | | | | | | | | | | | |

**Best Unique PSM (Scan F10:27776, m/z=368.5706, z=3, RT=50.55, ppm=-0.3):**

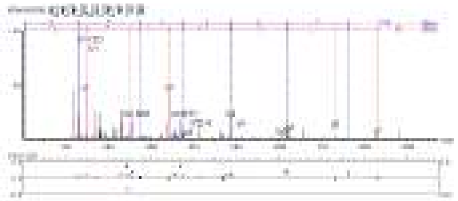

## Q91137.1

back to list

| Protein Coverage | Supporting Peptides | Best Unique PSM |

**Protein Coverage:**

1 MKTLLLTMVV VTIVCLDLGY TLKCHNTQLP FIYKTCPEGK NLCFKATLK**K FPLKFPVK**RG CADNCPKNSA LLKYVCCSSD

81 KCN

**Supporting Peptides:**

| Peptide | Uniq | -10lgP | Mass | Length | ppm | m/z | z | RT | Fraction | Scan | Source File | Area F1 | Area F2a | Area F3a | Area F4 | Area F5 | Area F6a | Area F7a | Area F7b | Area F8 | Area F9a | Area Sample 1F10a | Area F10b | Area F11a | Area F11b | Area F12a | Area F13 | #Feature | #Feature F1 | #Feature F2a | #Feature F3a | #Feature F4 | #Feature F5 | #Feature F6a | #Feature F7a | #Feature F7b | #Feature F8 | #Feature F9a | #Feature Sample 1F10a | #Feature F10b | #Feature F11a | #Feature F11b | #Feature F12a | #Feature F13 | Start | End | PTM | AScore | Found By |
|---|---|---|---|---|---|---|---|---|---|---|---|---|---|---|---|---|---|---|---|---|---|---|---|---|---|---|---|---|---|---|---|---|---|---|---|---|---|---|---|---|---|---|---|---|---|---|---|---|---|
| K.KFPLKFPVK.R | Y | 43.49 | 1102.6902 | 9 | -0.3 | 368.5706 | 3 | 50.55 | 10 | F10:27776 | BuCaTN01_F9a.raw | | | | | | | | | | 0 | | | | | | | 0 | 0 | 0 | 0 | 0 | 0 | 0 | 0 | 0 | 0 | 0 | 0 | 0 | 0 | 0 | 0 | 0 | 50 | 58 | | | PEAKS DB |
| total 1 peptides | | | | | | | | | | | | | | | | | | | | | | | | | | | | | | | | | | | | | | | | | | | | | | | | | |

**Best Unique PSM (Scan F10:27776, m/z=368.5706, z=3, RT=50.55, ppm=-0.3):**

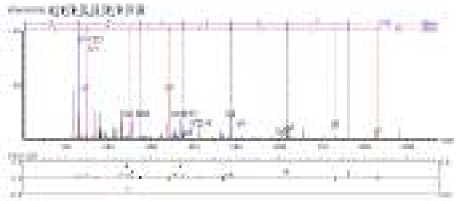

## P62375.1

back to list

| Protein Coverage | Supporting Peptides | Best Unique PSM |

**Protein Coverage:**

1 MKTLLLTMVV VTIVCLDLGY TLKCHNTQLP FIYKTCPEGK NLCFKATLK**K FPLKFPVK**RG CADNCPKNSA LLKYVCCSTD

81 KCN

**Supporting Peptides:**

| Peptide | Uniq | -10lgP | Mass | Length | ppm | m/z | z | RT | Fraction | Scan | Source File | Area F1 | Area F2a | Area F3a | Area F4 | Area F5 | Area F6a | Area F7a | Area F7b | Area F8 | Area F9a | Area Sample 1F10a | Area F10b | Area F11a | Area F11b | Area F12a | Area F13 | #Feature | #Feature F1 | #Feature F2a | #Feature F3a | #Feature F4 | #Feature F5 | #Feature F6a | #Feature F7a | #Feature F7b | #Feature F8 | #Feature F9a | #Feature Sample 1F10a | #Feature F10b | #Feature F11a | #Feature F11b | #Feature F12a | #Feature F13 | Start | End | PTM | AScore | Found By |
|---|---|---|---|---|---|---|---|---|---|---|---|---|---|---|---|---|---|---|---|---|---|---|---|---|---|---|---|---|---|---|---|---|---|---|---|---|---|---|---|---|---|---|---|---|---|---|---|---|---|
| K.KFPLKFPVK.R | Y | 43.49 | 1102.6902 | 9 | -0.3 | 368.5706 | 3 | 50.55 | 10 | F10:27776 | BuCaTN01_F9a.raw | | | | | | | | | | 0 | | | | | | | 0 | 0 | 0 | 0 | 0 | 0 | 0 | 0 | 0 | 0 | 0 | 0 | 0 | 0 | 0 | 0 | 0 | 50 | 58 | | | PEAKS DB |
| total 1 peptides | | | | | | | | | | | | | | | | | | | | | | | | | | | | | | | | | | | | | | | | | | | | | | | | | |

**Best Unique PSM (Scan F10:27776, m/z=368.5706, z=3, RT=50.55, ppm=-0.3):**

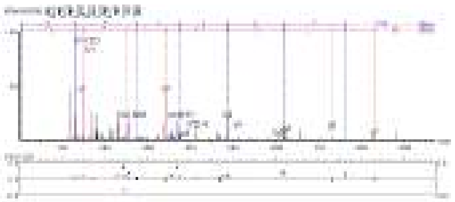

## Q91126.1
back to list

| Protein Coverage | Supporting Peptides | Best Unique PSM |

**Protein Coverage:**

1 MKTLLLTLVV VTIVCLDLGY TLKCHNTQLP FIYKTCPEGK NLCFKATLK**K FPLKFPVK**RG CADNCPKNSA LLKYVCCSTE

81 KCN

**Supporting Peptides:**

| Peptide | Uniq | -10lgP | Mass | Length | ppm | m/z | z | RT | Fraction | Scan | Source File | Area F1 | Area F2a | Area F3a | Area F4 | Area F5 | Area F6a | Area F7a | Area F7b | Area F8 | Area F9a | Area Sample 1F10a | Area F10b | Area F11a | Area F11b | Area F12a | Area F13 | #Feature | #Feature F1 | #Feature F2a | #Feature F3a | #Feature F4 | #Feature F5 | #Feature F6a | #Feature F7a | #Feature F7b | #Feature F8 | #Feature F9a | #Feature Sample 1F10a | #Feature F10b | #Feature F11a | #Feature F11b | #Feature F12a | #Feature F13 | Start | End | PTM | AScore | Found By |
|---|---|---|---|---|---|---|---|---|---|---|---|---|---|---|---|---|---|---|---|---|---|---|---|---|---|---|---|---|---|---|---|---|---|---|---|---|---|---|---|---|---|---|---|---|---|---|---|---|---|
| K.KFPLKFPVK.R | Y | 43.49 | 1102.6902 | 9 | -0.3 | 368.5706 | 3 | 50.55 | 10 | F10:27776 | BuCaTN01_F9a.raw | | | | | | | | | | 0 | | | | | | | 0 | 0 | 0 | 0 | 0 | 0 | 0 | 0 | 0 | 0 | 0 | 0 | 0 | 0 | 0 | 0 | 0 | 50 | 58 | | | PEAKS DB |
| total 1 peptides | | | | | | | | | | | | | | | | | | | | | | | | | | | | | | | | | | | | | | | | | | | | | | | | | |

**Best Unique PSM (Scan F10:27776, m/z=368.5706, z=3, RT=50.55, ppm=-0.3):**

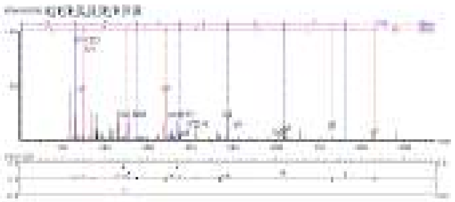

## XP_026579206.1
back to list

| Protein Coverage | Supporting Peptides | Best Unique PSM |

**Protein Coverage:**

1 MLLCFILLSL SWSQTPTSGE VVDSFQDHCP QFFLRETPPV IGVPLSPVRI CQRYQNLYRF ATLYDKHNRI PVYSAYVYNP
81 GKAKRPGKWM VEPQLISSTF QSEMETERDF LNKKGPKAAM QDSQAVLEDY KNLTDCNRGH LNPNGHQPDY SAK**SSTFTLT**

161 **NIVPQFIK**LN GGAWNNYEQT TMQQMTRGCH QTFAVVGAVP GDTYISGGRV NRPSHLWSAA CCVIDNNHLR SWAILARNDQ

241 NVVQRFTLGQ LEEKLAQLYN VNHVSLFHSD CPRQ

**Supporting Peptides:**

| Peptide | Uniq | -10lgP | Mass | Length | ppm | m/z | z | RT | Fraction | Scan | Source File | Area F1 | Area F2a | Area F3a | Area F4 | Area F5 | Area F6a | Area F7a | Area F7b | Area F8 | Area F9a | Area Sample 1F10a | Area F10b | Area F11a | Area F11b | Area F12a | Area F13 | #Feature | #Feature F1 | #Feature F2a | #Feature F3a | #Feature F4 | #Feature F5 | #Feature F6a | #Feature F7a | #Feature F7b | #Feature F8 | #Feature F9a | #Feature Sample 1F10a | #Feature F10b | #Feature F11a | #Feature F11b | #Feature F12a | #Feature F13 | Start | End | PTM | AScore | Found By |
|---|---|---|---|---|---|---|---|---|---|---|---|---|---|---|---|---|---|---|---|---|---|---|---|---|---|---|---|---|---|---|---|---|---|---|---|---|---|---|---|---|---|---|---|---|---|---|---|---|---|
| K.SSTFTLTNIVPQFIK.L | Y | 60.38 | 1694.9243 | 15 | 0.9 | 848.4702 | 2 | 85.97 | 1 | F1:41441 | BuCaTN01_F1.raw | 2.6276E5 | | | | | | | | | | | | | | | | 1 | 1 | 0 | 0 | 0 | 0 | 0 | 0 | 0 | 0 | 0 | 0 | 0 | 0 | 0 | 0 | 0 | 154 | 168 | | | PEAKS DB |
| total 1 peptides | | | | | | | | | | | | | | | | | | | | | | | | | | | | | | | | | | | | | | | | | | | | | | | | | |

**Best Unique PSM (Scan F1:41441, m/z=848.4702, z=2, RT=85.97, ppm=0.9):**

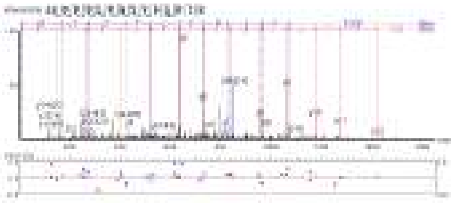

## XP_026579207.1
back to list

| Protein Coverage | Supporting Peptides | Best Unique PSM |

**Protein Coverage:**

1 MLLCFILLSL SWSQTPTSGE VVDSFQDHCP QFFLRETPPV IGVPLSPVRI CQRYQNLYRF ATLYDKHNRI PVYSAYVYNP
81 GKAKRPGKWM VEPQLISSTF QSEMETERDF LNKKGPKAAM QDSQAVLEDY KNLTDCNRGH LNPNGHQPDY SAK**SSTFTLT**

161 **NIVPQFIK**LN GGAWNNYEQT TMQQMTRGCH QTFAVVGAVP GDTYISGGRV NRPSHLWSAA CCVIDNNHLR SWAILARNDQ

241 NVVQRFTLGQ LEEKLAQLYN VNHVSLFHSD CPRQ

**Supporting Peptides:**

| Peptide | Uniq | -10lgP | Mass | Length | ppm | m/z | z | RT | Fraction | Scan | Source File | Area F1 | Area F2a | Area F3a | Area F4 | Area F5 | Area F6a | Area F7a | Area F7b | Area F8 | Area F9a | Area Sample 1F10a | Area F10b | Area F11a | Area F11b | Area F12a | Area F13 | #Feature | #Feature F1 | #Feature F2a | #Feature F3a | #Feature F4 | #Feature F5 | #Feature F6a | #Feature F7a | #Feature F7b | #Feature F8 | #Feature F9a | #Feature Sample 1F10a | #Feature F10b | #Feature F11a | #Feature F11b | #Feature F12a | #Feature F13 | Start | End | PTM | AScore | Found By |
|---|---|---|---|---|---|---|---|---|---|---|---|---|---|---|---|---|---|---|---|---|---|---|---|---|---|---|---|---|---|---|---|---|---|---|---|---|---|---|---|---|---|---|---|---|---|---|---|---|---|
| K.SSTFTLTNIVPQFIK.L | Y | 60.38 | 1694.9243 | 15 | 0.9 | 848.4702 | 2 | 85.97 | 1 | F1:41441 | BuCaTN01_F1.raw | 2.6276E5 | | | | | | | | | | | | | | | | 1 | 1 | 0 | 0 | 0 | 0 | 0 | 0 | 0 | 0 | 0 | 0 | 0 | 0 | 0 | 0 | 0 | 154 | 168 | | | PEAKS DB |
| total 1 peptides | | | | | | | | | | | | | | | | | | | | | | | | | | | | | | | | | | | | | | | | | | | | | | | | | |

**Best Unique PSM (Scan F1:41441, m/z=848.4702, z=2, RT=85.97, ppm=0.9):**

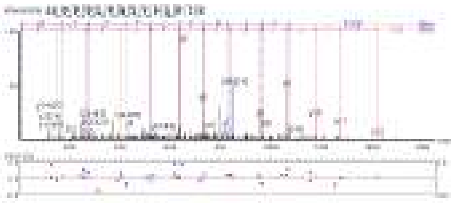

## JAB54304.1
back to list

| Protein Coverage | Supporting Peptides | Best Unique PSM |

**Protein Coverage:**

1 MLLFFILLFL SWSQMPTSGE VVDSFQDHCP QFFLRETPPV IGVSRYHVRI CQRYQNLYRF ATLYDQDNHI PVYSAYVYNP
81 GKAKRPRKWM IEPQLINSMF QSDMETEGDF LNKKGPREAL HDSQAVLEDY KNLTDCNRGH LNPNGHQPDY SAK**SSTFTLT**
161 **NIVPQFIK**LN GGAWNNYEQT TMQQMTRGCQ QTFAVVGAVP GDTNIARGRV NRPSHLWSAA CCLIDNNHLR SWAVLARNDQ
241 NVVQKFTLGH LEEKLAQLYN VNRVSLFHSD CPRQ

**Supporting Peptides:**

| Peptide | Uniq | -10lgP | Mass | Length | ppm | m/z | z | RT | Fraction | Scan | Source File | Area F1 | Area F2a | Area F3a | Area F4 | Area F5 | Area F6a | Area F7a | Area F7b | Area F8 | Area F9a | Area Sample 1F10a | Area F10b | Area F11a | Area F11b | Area F12a | Area F13 | #Feature | #Feature F1 | #Feature F2a | #Feature F3a | #Feature F4 | #Feature F5 | #Feature F6a | #Feature F7a | #Feature F7b | #Feature F8 | #Feature F9a | #Feature Sample 1F10a | #Feature F10b | #Feature F11a | #Feature F11b | #Feature F12a | #Feature F13 | Start | End | PTM | AScore | Found By |
|---|---|---|---|---|---|---|---|---|---|---|---|---|---|---|---|---|---|---|---|---|---|---|---|---|---|---|---|---|---|---|---|---|---|---|---|---|---|---|---|---|---|---|---|---|---|---|---|---|---|
| K.SSTFTLTNIVPQFIK.L | Y | 60.38 | 1694.9243 | 15 | 0.9 | 848.4702 | 2 | 85.97 | 1 | F1:41441 | BuCaTN01_F1.raw | 2.6276E5 | | | | | | | | | | | | | | | | 1 | 1 | 0 | 0 | 0 | 0 | 0 | 0 | 0 | 0 | 0 | 0 | 0 | 0 | 0 | 0 | 0 | 154 | 168 | | | PEAKS DB |
| total 1 peptides | | | | | | | | | | | | | | | | | | | | | | | | | | | | | | | | | | | | | | | | | | | | | | | | | |

**Best Unique PSM (Scan F1:41441, m/z=848.4702, z=2, RT=85.97, ppm=0.9):**

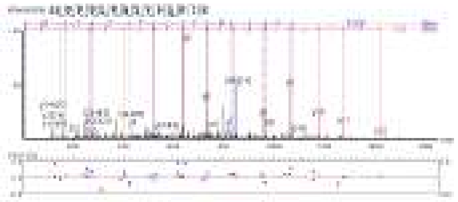

## ETE56723.1

back to list

| Protein Coverage | Supporting Peptides | Best Unique PSM |

**Protein Coverage:**

1 METEWDFLNK KGSPKALQDS QAVLEDYKNL ADCNRGHLNP NGHQPDYSAK **SSTFTLTNIV PQFIK**LNGGA WNNYEQTTMQ
81 QMTRGCQQTF AVVGAVPGDT YIARGRVNRP SHLWSAACCL IDNNHLRSWA ILARNXLEEK LAQLYNVNHV SLFHSDCPRQ

**Supporting Peptides:**

| Peptide | Uniq | -10lgP | Mass | Length | ppm | m/z | z | RT | Fraction | Scan | Source File | Area F1 | Area F2a | Area F3a | Area F4 | Area F5 | Area F6a | Area F7a | Area F7b | Area F8 | Area F9a | Area Sample 1F10a | Area F10b | Area F11a | Area F11b | Area F12a | Area F13 | #Feature | #Feature F1 | #Feature F2a | #Feature F3a | #Feature F4 | #Feature F5 | #Feature F6a | #Feature F7a | #Feature F7b | #Feature F8 | #Feature F9a | #Feature Sample 1F10a | #Feature F10b | #Feature F11a | #Feature F11b | #Feature F12a | #Feature F13 | Start | End | PTM | AScore | Found By |
|---|---|---|---|---|---|---|---|---|---|---|---|---|---|---|---|---|---|---|---|---|---|---|---|---|---|---|---|---|---|---|---|---|---|---|---|---|---|---|---|---|---|---|---|---|---|---|---|---|---|
| K.SSTFTLTNIVPQFIK.L | Y | 60.38 | 1694.9243 | 15 | 0.9 | 848.4702 | 2 | 85.97 | 1 | F1:41441 | BuCaTN01_F1.raw | 2.6276E5 | | | | | | | | | | | | | | | | 1 | 1 | 0 | 0 | 0 | 0 | 0 | 0 | 0 | 0 | 0 | 0 | 0 | 0 | 0 | 0 | 0 | 51 | 65 | | | PEAKS DB |
| total 1 peptides | | | | | | | | | | | | | | | | | | | | | | | | | | | | | | | | | | | | | | | | | | | | | | | | | |

**Best Unique PSM (Scan F1:41441, m/z=848.4702, z=2, RT=85.97, ppm=0.9):**

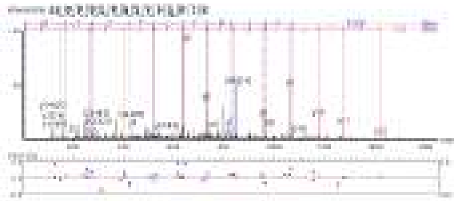

## XP_058047681.1

back to list

| Protein Coverage | Supporting Peptides | Best Unique PSM |

**Protein Coverage:**

1 MRLQRQLLPK KDLTLQTALE EAQHQKCQIN PLLISGNSIT LPTRPQGQTR ILSRLSSTCS IPWAQALFLP SWIWLRLTSS
81 SRWTDDASAQ VQTIVTHWGA FKCRCLQFGI SIAPGLFQNL MAERLLHGVP GIVPYFDDVL VSTGCRSELI ERVRAVLTRF
161 RQSGLKLKRS KCVIGVPQDN LFKPLQDWNT VPILQCAEDP QITSNRHPTP SQSFLVMLLL LIALSLSWSQ MPTSGEVVNS
241 FQDHCPQFFL RETPPLIRLP PSPVRICQRY QNLYRFATLY DKDNRIPVYS AYVYNPGKAK RPKKWMVEPQ LINSTFQNEM
321 ETEGDFLNKK GPQEALRNSQ AILQDYKNLT DCNRGHLNPN GHQPNYSAK**S STFTLTNIVP QFIK**LNGGAW NNYEQTTMLQ
401 MTEGCQETFA LVGAVPGDTY IAGGRVNRPS HLWSAACCVI DNNHLRSWGI LARNDQNVVK KFTLGQLEAQ LAHLYNVNRV
481 SLFHRDCPRQ

**Supporting Peptides:**

| Peptide | Uniq | -10lgP | Mass | Length | ppm | m/z | z | RT | Fraction | Scan | Source File | Area F1 | Area F2a | Area F3a | Area F4 | Area F5 | Area F6a | Area F7a | Area F7b | Area F8 | Area F9a | Area Sample 1F10a | Area F10b | Area F11a | Area F11b | Area F12a | Area F13 | #Feature | #Feature F1 | #Feature F2a | #Feature F3a | #Feature F4 | #Feature F5 | #Feature F6a | #Feature F7a | #Feature F7b | #Feature F8 | #Feature F9a | #Feature Sample 1F10a | #Feature F10b | #Feature F11a | #Feature F11b | #Feature F12a | #Feature F13 | Start | End | PTM | AScore | Found By |
|---|---|---|---|---|---|---|---|---|---|---|---|---|---|---|---|---|---|---|---|---|---|---|---|---|---|---|---|---|---|---|---|---|---|---|---|---|---|---|---|---|---|---|---|---|---|---|---|---|---|
| K.SSTFTLTNIVPQFIK.L | Y | 60.38 | 1694.9243 | 15 | 0.9 | 848.4702 | 2 | 85.97 | 1 | F1:41441 | BuCaTN01_F1.raw | 2.6276E5 | | | | | | | | | | | | | | | | 1 | 1 | 0 | 0 | 0 | 0 | 0 | 0 | 0 | 0 | 0 | 0 | 0 | 0 | 0 | 0 | 0 | 370 | 384 | | | PEAKS DB |
| total 1 peptides | | | | | | | | | | | | | | | | | | | | | | | | | | | | | | | | | | | | | | | | | | | | | | | | | |

**Best Unique PSM (Scan F1:41441, m/z=848.4702, z=2, RT=85.97, ppm=0.9):**

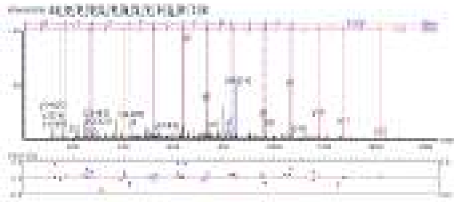

## ETE59939.1

back to list

| Protein Coverage | Supporting Peptides | Best Unique PSM |

**Protein Coverage:**

1 TPTSSHDAAD EFFLRETPPV IGVSPSPVRI CQCYQNLYRF ATLYDKQNRI PVYSVYVYNP GKATRPGKWM VEPQLINSTF
81 QSDMETERDF LNKKGPWSSL QDSQSVMKDY KNLKDYNRGH LIPNAHQPDY SAK**SSTFTLT NIVPQFIK**LN GGAWKNYEQA
161 TMQQMTRGCQ QTFAVQTAML LLWILSLAAS FLLPATGEVV PSFNPCKTFF LDGKPPNLKP MNPARICQLY KNKYRFATMY
241 DRDRRIPLFS AYQYKTGRGT RSEEWMIEPQ LALPGDRLRK NMELDVTSTI QRTLLKKSQA VDQDYKEGVQ YDRGHLAPDS
321 HQSDQDSKAA TFTLTNIVPQ FHALNNGEWR FYEEHINTAG CKQTYVIVGA VPGNAFLPGD RVNVPSHIWA AGCCVSNQNN
401 QKKSWAVIAE NKQNTVVRLT LGKLQEQLAK LYGKRNIDLF SNACNTPAGP

**Supporting Peptides:**

| Peptide | Uniq | -10lgP | Mass | Length | ppm | m/z | z | RT | Fraction | Scan | Source File | Area F1 | Area F2a | Area F3a | Area F4 | Area F5 | Area F6a | Area F7a | Area F7b | Area F8 | Area F9a | Area Sample 1F10a | Area F10b | Area F11a | Area F11b | Area F12a | Area F13 | #Feature | #Feature F1 | #Feature F2a | #Feature F3a | #Feature F4 | #Feature F5 | #Feature F6a | #Feature F7a | #Feature F7b | #Feature F8 | #Feature F9a | #Feature Sample 1F10a | #Feature F10b | #Feature F11a | #Feature F11b | #Feature F12a | #Feature F13 | Start | End | PTM | AScore | Found By |
|---|---|---|---|---|---|---|---|---|---|---|---|---|---|---|---|---|---|---|---|---|---|---|---|---|---|---|---|---|---|---|---|---|---|---|---|---|---|---|---|---|---|---|---|---|---|---|---|---|---|
| K.SSTFTLTNIVPQFIK.L | Y | 60.38 | 1694.9243 | 15 | 0.9 | 848.4702 | 2 | 85.97 | 1 | F1:41441 | BuCaTN01_F1.raw | 2.6276E5 | | | | | | | | | | | | | | | | 1 | 1 | 0 | 0 | 0 | 0 | 0 | 0 | 0 | 0 | 0 | 0 | 0 | 0 | 0 | 0 | 0 | 134 | 148 | | | PEAKS DB |
| total 1 peptides | | | | | | | | | | | | | | | | | | | | | | | | | | | | | | | | | | | | | | | | | | | | | | | | | |

**Best Unique PSM (Scan F1:41441, m/z=848.4702, z=2, RT=85.97, ppm=0.9):**

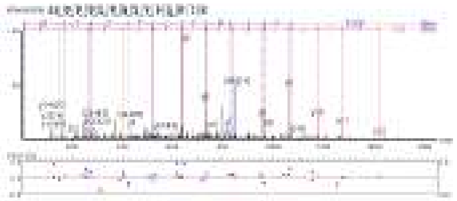

## XP_039225468.1

back to list

| Protein Coverage | Supporting Peptides | Best Unique PSM |

**Protein Coverage:**

```
  1 MLLLIFLLSA SWSNLPTNGE VVDSFEETCP QFFLRETPPV IRHPPRSARI CQRYQNLYRF ATLYDKDNRV PVYSAYIYKP
 81 GTAKRPKKWM IEPQLINSSY QPEMETEGEF LNQKGPKEAL KDSQAILQDY KNLTSYNRGH LNPNGHQPDY AAKSSTFTLT
161 NIVPQFIKLN GGAWNNYEQT TMIQMTRGCQ ETFAVVGAVP GNTYISGGRV NRPSHLWSAA CCVIDNNHLR SWAILARNDQ
241 NVVEKITLGQ LENRLARLYN VNHVSLFHGD CPRQ
```

**Supporting Peptides:**

| Peptide | Uniq | -10lgP | Mass | Length | ppm | m/z | z | RT | Fraction | Scan | Source File | Area F1 | Area F2a | Area F3a | Area F4 | Area F5 | Area F6a | Area F7a | Area F7b | Area F8 | Area F9a | Area Sample 1F10a | Area F10b | Area F11a | Area F11b | Area F12a | Area F13 | #Feature | #Feature F1 | #Feature F2a | #Feature F3a | #Feature F4 | #Feature F5 | #Feature F6a | #Feature F7a | #Feature F7b | #Feature F8 | #Feature F9a | #Feature Sample 1F10a | #Feature F10b | #Feature F11a | #Feature F11b | #Feature F12a | #Feature F13 | Start | End | PTM | AScore | Found By |
|---|---|---|---|---|---|---|---|---|---|---|---|---|---|---|---|---|---|---|---|---|---|---|---|---|---|---|---|---|---|---|---|---|---|---|---|---|---|---|---|---|---|---|---|---|---|---|---|---|---|
| K.SSTFTLTNIVPQFIK.L | Y | 60.38 | 1694.9243 | 15 | 0.9 | 848.4702 | 2 | 85.97 | 1 | F1:41441 | BuCaTN01_F1.raw | 2.6276E5 | | | | | | | | | | | | | | | | 1 | 1 | 0 | 0 | 0 | 0 | 0 | 0 | 0 | 0 | 0 | 0 | 0 | 0 | 0 | 0 | 0 | 154 | 168 | | | PEAKS DB |
| total 1 peptides | | | | | | | | | | | | | | | | | | | | | | | | | | | | | | | | | | | | | | | | | | | | | | | | | |

**Best Unique PSM (Scan F1:41441, m/z=848.4702, z=2, RT=85.97, ppm=0.9):**

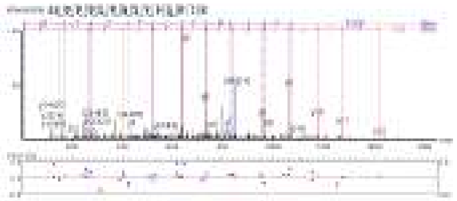

## XP_039225467.1

back to list

| Protein Coverage | Supporting Peptides | Best Unique PSM |

**Protein Coverage:**

```
  1 MLLLIFLLSA SWSNLPTNGE VVDSFEETCP QFFLRETPPV IRHPPRSARI CQRYQNLYRF ATLYDKDNRV PVYSAYIYKP
 81 GTAKRPKKWM IEPQLINSSY QPEMETEGEF LNQKGPKEAL KDSQAILQDY KNLTSYNRGH LNPNGHQPDY AAKSSTFTLT
161 NIVPQFIKLN GGAWNNYEQT TMIQMTRGCQ ETFAVVGAVP GNTYISGGRV NRPSHLWSAA CCVIDNNHLR SWAILARNDQ
241 NVVEKITLGQ LENRLARLYN VNHVSLFHGD CPRQ
```

**Supporting Peptides:**

| Peptide | Uniq | -10lgP | Mass | Length | ppm | m/z | z | RT | Fraction | Scan | Source File | Area F1 | Area F2a | Area F3a | Area F4 | Area F5 | Area F6a | Area F7a | Area F7b | Area F8 | Area F9a | Area Sample 1F10a | Area F10b | Area F11a | Area F11b | Area F12a | Area F13 | #Feature | #Feature F1 | #Feature F2a | #Feature F3a | #Feature F4 | #Feature F5 | #Feature F6a | #Feature F7a | #Feature F7b | #Feature F8 | #Feature F9a | #Feature Sample 1F10a | #Feature F10b | #Feature F11a | #Feature F11b | #Feature F12a | #Feature F13 | Start | End | PTM | AScore | Found By |
|---|---|---|---|---|---|---|---|---|---|---|---|---|---|---|---|---|---|---|---|---|---|---|---|---|---|---|---|---|---|---|---|---|---|---|---|---|---|---|---|---|---|---|---|---|---|---|---|---|---|
| K.SSTFTLTNIVPQFIK.L | Y | 60.38 | 1694.9243 | 15 | 0.9 | 848.4702 | 2 | 85.97 | 1 | F1:41441 | BuCaTN01_F1.raw | 2.6276E5 | | | | | | | | | | | | | | | | 1 | 1 | 0 | 0 | 0 | 0 | 0 | 0 | 0 | 0 | 0 | 0 | 0 | 0 | 0 | 0 | 0 | 154 | 168 | | | PEAKS DB |
| total 1 peptides | | | | | | | | | | | | | | | | | | | | | | | | | | | | | | | | | | | | | | | | | | | | | | | | | |

**Best Unique PSM (Scan F1:41441, m/z=848.4702, z=2, RT=85.97, ppm=0.9):**

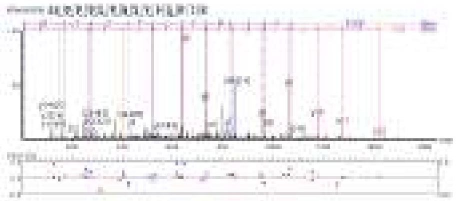

## XP_032076462.1

back to list

| Protein Coverage | Supporting Peptides | Best Unique PSM |

**Protein Coverage:**

```
  1 MRLLFILLSV SWSQMPTSGE VVDSFQDTCP QFFLRETPPL IRIPPSPVRI CQRYQNLYRF ATLYDKDNRI PVYSAYVYNP
 81 GSAKRPKKWM VEPQLINSSF QPEMETEWDF LNKKGPKEAL HVSQAVLEDY KNLSDYNRGH LNPNGHQPDY SAKSSTFTLT
161 NIVPQFIKLN GGAWNNYEQT TMVQMTKGCQ ETFAVVGAVP GDTYVAGGRV NKPSHMWSAA CCMIDNNHLR SWAILARNDQ
241 NVVEKLTLGQ LEAHLAQLYN INQVSLFHSD CPRQ
```

**Supporting Peptides:**

| Peptide | Uniq | -10lgP | Mass | Length | ppm | m/z | z | RT | Fraction | Scan | Source File | Area F1 | Area F2a | Area F3a | Area F4 | Area F5 | Area F6a | Area F7a | Area F7b | Area F8 | Area F9a | Area Sample 1F10a | Area F10b | Area F11a | Area F11b | Area F12a | Area F13 | #Feature | #Feature F1 | #Feature F2a | #Feature F3a | #Feature F4 | #Feature F5 | #Feature F6a | #Feature F7a | #Feature F7b | #Feature F8 | #Feature F9a | #Feature Sample 1F10a | #Feature F10b | #Feature F11a | #Feature F11b | #Feature F12a | #Feature F13 | Start | End | PTM | AScore | Found By |
|---|---|---|---|---|---|---|---|---|---|---|---|---|---|---|---|---|---|---|---|---|---|---|---|---|---|---|---|---|---|---|---|---|---|---|---|---|---|---|---|---|---|---|---|---|---|---|---|---|---|
| K.SSTFTLTNIVPQFIK.L | Y | 60.38 | 1694.9243 | 15 | 0.9 | 848.4702 | 2 | 85.97 | 1 | F1:41441 | BuCaTN01_F1.raw | 2.6276E5 | | | | | | | | | | | | | | | | 1 | 1 | 0 | 0 | 0 | 0 | 0 | 0 | 0 | 0 | 0 | 0 | 0 | 0 | 0 | 0 | 0 | 154 | 168 | | | PEAKS DB |
| total 1 peptides | | | | | | | | | | | | | | | | | | | | | | | | | | | | | | | | | | | | | | | | | | | | | | | | | |

**Best Unique PSM (Scan F1:41441, m/z=848.4702, z=2, RT=85.97, ppm=0.9):**

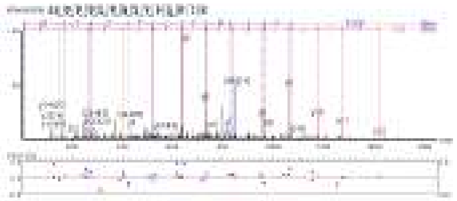

## XP_032076461.1

back to list

| Protein Coverage | Supporting Peptides | Best Unique PSM |

**Protein Coverage:**

1 MYPEFGFLLT FSKILDWKRH PTPSHTFHTM RLLFILLSVS WSQMPTSGEV VDSFQDTCPQ FFLRETPPLI RIPPSPVRIC
81 QRYQNLYRFA TLYDKDNRIP VYSAYVYNPG SAKRPKKWMV EPQLINSSFQ PEMETEWDFL NKKGPKEALH VSQAVLEDYK
161 NLSDYNRGHL NPNGHQPDYS AK**SSTFTLTN IVPQFIK**LNG GAWNNYEQTT MVQMTKGCQE TFAVVGAVPG DTYVAGGRVN
241 KPSHMWSAAC CMIDNNHLRS WAILARNDQN VVEKLTLGQL EAHLAQLYNI NQVSLFHSDC PRQ

**Supporting Peptides:**

| Peptide | Uniq | -10lgP | Mass | Length | ppm | m/z | z | RT | Fraction | Scan | Source File | Area F1 | Area F2a | Area F3a | Area F4 | Area F5 | Area F6a | Area F7a | Area F7b | Area F8 | Area F9a | Area Sample 1F10a | Area F10b | Area F11a | Area F11b | Area F12a | Area F13 | #Feature | #Feature F1 | #Feature F2a | #Feature F3a | #Feature F4 | #Feature F5 | #Feature F6a | #Feature F7a | #Feature F7b | #Feature F8 | #Feature F9a | #Feature Sample 1F10a | #Feature F10b | #Feature F11a | #Feature F11b | #Feature F12a | #Feature F13 | Start | End | PTM | AScore | Found By |
|---|---|---|---|---|---|---|---|---|---|---|---|---|---|---|---|---|---|---|---|---|---|---|---|---|---|---|---|---|---|---|---|---|---|---|---|---|---|---|---|---|---|---|---|---|---|---|---|---|---|
| K.SSTFTLTNIVPQFIK.L | Y | 60.38 | 1694.9243 | 15 | 0.9 | 848.4702 | 2 | 85.97 | 1 | F1:41441 | BuCaTN01_F1.raw | 2.6276E5 | | | | | | | | | | | | | | | | 1 | 1 | 0 | 0 | 0 | 0 | 0 | 0 | 0 | 0 | 0 | 0 | 0 | 0 | 0 | 0 | 0 | 183 | 197 | | | PEAKS DB |
| **total 1 peptides** | | | | | | | | | | | | | | | | | | | | | | | | | | | | | | | | | | | | | | | | | | | | | | | | | |

**Best Unique PSM (Scan F1:41441, m/z=848.4702, z=2, RT=85.97, ppm=0.9):**

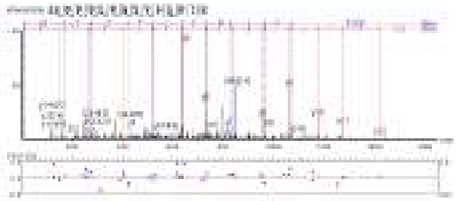

## LAB21297.1

back to list

| Protein Coverage | Supporting Peptides | Best Unique PSM |

**Protein Coverage:**

1 GASSIAPKPL **NLYQFKNMIQ CTNTR**SWWNF IDYGCYCGYG GSGKPVDELD RCCQTHFNCY SQAMDNPACT PILDSPYIKT
81 YSYTCSEGSL TCNGDNDVCG ALVCNCDLSA ANCFAGAPFI EENYNMDPER CQ

C Carbamidomethylation (+57.02)

**Supporting Peptides:**

| Peptide | Uniq | -10lgP | Mass | Length | ppm | m/z | z | RT | Fraction | Scan | Source File | Area F1 | Area F2a | Area F3a | Area F4 | Area F5 | Area F6a | Area F7a | Area F7b | Area F8 | Area F9a | Area Sample 1F10a | Area F10b | Area F11a | Area F11b | Area F12a | Area F13 | #Feature | #Feature F1 | #Feature F2a | #Feature F3a | #Feature F4 | #Feature F5 | #Feature F6a | #Feature F7a | #Feature F7b | #Feature F8 | #Feature F9a | #Feature Sample 1F10a | #Feature F10b | #Feature F11a | #Feature F11b | #Feature F12a | #Feature F13 | Start | End | PTM | AScore | Found By |
|---|---|---|---|---|---|---|---|---|---|---|---|---|---|---|---|---|---|---|---|---|---|---|---|---|---|---|---|---|---|---|---|---|---|---|---|---|---|---|---|---|---|---|---|---|---|---|---|---|---|
| L.NLYQFKNMIQC(+57.02)TNTR.S | Y | 60.18 | 1929.9189 | 15 | 0.2 | 644.3137 | 3 | 64.12 | 9 | F9:38990 | BuCaTN01_F8.raw | | | | | | | | | 1.7685E6 | | | | | | | | 1 | 0 | 0 | 0 | 0 | 0 | 0 | 0 | 0 | 1 | 0 | 0 | 0 | 0 | 0 | 0 | 0 | 11 | 25 | Carbamidomethylation | C11:Carbamidomethylation:1000.00 | PEAKS DB |
| **total 1 peptides** | | | | | | | | | | | | | | | | | | | | | | | | | | | | | | | | | | | | | | | | | | | | | | | | | |

**Best Unique PSM (Scan F9:38990, m/z=644.3137, z=3, RT=64.12, ppm=0.2):**

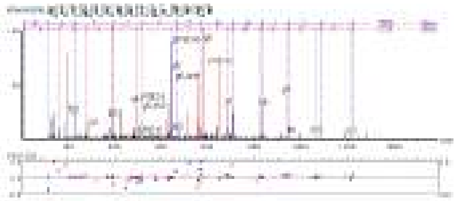

## XP_026545503.1

back to list

| Protein Coverage | Supporting Peptides | Best Unique PSM |

**Protein Coverage:**

1 MSSRYTVGPT ETERCIESLL AVFQRYSGKE GDTKTLSKKE FK**TFMDTELA SFTK**NQKDPG VVDRMMKKLD INSDGQLDFA
81 EFLNLIGGLA VAVHEMASAP AGGAPRRP

**Supporting Peptides:**

| Peptide | Uniq | -10lgP | Mass | Length | ppm | m/z | z | RT | Fraction | Scan | Source File | Area F1 | Area F2a | Area F3a | Area F4 | Area F5 | Area F6a | Area F7a | Area F7b | Area F8 | Area F9a | Area Sample 1F10a | Area F10b | Area F11a | Area F11b | Area F12a | Area F13 | #Feature | #Feature F1 | #Feature F2a | #Feature F3a | #Feature F4 | #Feature F5 | #Feature F6a | #Feature F7a | #Feature F7b | #Feature F8 | #Feature F9a | #Feature Sample 1F10a | #Feature F10b | #Feature F11a | #Feature F11b | #Feature F12a | #Feature F13 | Start | End | PTM | AScore | Found By |
|---|---|---|---|---|---|---|---|---|---|---|---|---|---|---|---|---|---|---|---|---|---|---|---|---|---|---|---|---|---|---|---|---|---|---|---|---|---|---|---|---|---|---|---|---|---|---|---|---|---|
| K.TFMDTELASFTK.N | Y | 58.63 | 1389.6486 | 12 | 1.4 | 695.8325 | 2 | 72.95 | 16 | F16:38090 | BuCaTN01_F13.raw | | | | | | | | | | | | | | | | 1.0855E5 | 1 | 0 | 0 | 0 | 0 | 0 | 0 | 0 | 0 | 0 | 0 | 0 | 0 | 0 | 0 | 0 | 1 | 43 | 54 | | | PEAKS DB |
| **total 1 peptides** | | | | | | | | | | | | | | | | | | | | | | | | | | | | | | | | | | | | | | | | | | | | | | | | | |

**Best Unique PSM (Scan F16:38090, m/z=695.8325, z=2, RT=72.95, ppm=1.4):**

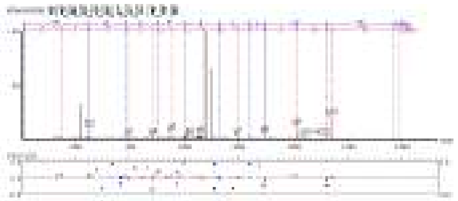

## XP_034291543.1

back to list

| Protein Coverage | Supporting Peptides | Best Unique PSM |

**Protein Coverage:**

1 MSSKYPVGPT ETERCIESLL AVFQSYAGRD GDGNTLSRKE FKTFMNIELG SFTKNQKDPG VVDRMMKALD RDNNEKLDFS
81 EFLNLIGSLA LTCHAQVASP PTTGPHPKFL NRRGRDRRGS TGEGPNPPFT TPVSNQLTSN SQVSSLPSQL PTKAMSSRYP
161 VGPTETERCI ESLLAVFQRY AGKDGKSSTL SKREFK**TFMD TELASFTK**SQ KDPGVVDRMM KKLDMNNDGQ LDFPEFLNLI
241 GGLATACHEH MASAPEPHKR L

**Supporting Peptides:**

| Peptide | Uniq | -10lgP | Mass | Length | ppm | m/z | z | RT | Fraction | Scan | Source File | Area F1 | Area F2a | Area F3a | Area F4 | Area F5 | Area F6a | Area F7a | Area F7b | Area F8 | Area F9a | Area Sample 1F10a | Area F10b | Area F11a | Area F11b | Area F12a | Area F13 | #Feature | #Feature F1 | #Feature F2a | #Feature F3a | #Feature F4 | #Feature F5 | #Feature F6a | #Feature F7a | #Feature F7b | #Feature F8 | #Feature F9a | #Feature Sample 1F10a | #Feature F10b | #Feature F11a | #Feature F11b | #Feature F12a | #Feature F13 | Start | End | PTM | AScore | Found By |
|---|---|---|---|---|---|---|---|---|---|---|---|---|---|---|---|---|---|---|---|---|---|---|---|---|---|---|---|---|---|---|---|---|---|---|---|---|---|---|---|---|---|---|---|---|---|---|---|---|---|
| K.TFMDTELASFTK.S | Y | 58.63 | 1389.6486 | 12 | 1.4 | 695.8325 | 2 | 72.95 | 16 | F16:38090 | BuCaTN01_F13.raw | | | | | | | | | | | | | | | | 1.0855E5 | 1 | 0 | 0 | 0 | 0 | 0 | 0 | 0 | 0 | 0 | 0 | 0 | 0 | 0 | 0 | 0 | 1 | 197 | 208 | | | PEAKS DB |
| **total 1 peptides** | | | | | | | | | | | | | | | | | | | | | | | | | | | | | | | | | | | | | | | | | | | | | | | | | |

**Best Unique PSM (Scan F16:38090, m/z=695.8325, z=2, RT=72.95, ppm=1.4):**

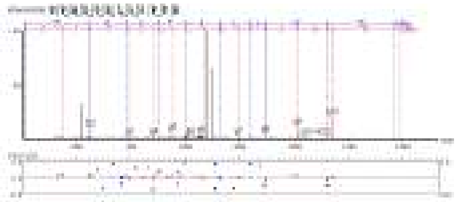

## XP_026580631.1

back to list

| Protein Coverage | Supporting Peptides | Best Unique PSM |

**Protein Coverage:**

1 MSSRYTVGPT ETERCIESLL AVFQRYAGKE GDSKTLSKKE FR**TFMDTELA SFTK**NQKDPG VVDRMMKKLD INSDGQLDFA

81 EFLNLIGGLA VAVHEMASAP VGGAPRRL

**Supporting Peptides:**

| Peptide | Uniq | -10lgP | Mass | Length | ppm | m/z | z | RT | Fraction | Scan | Source File | Area F1 | Area F2a | Area F3a | Area F4 | Area F5 | Area F6a | Area F7a | Area F7b | Area F8 | Area F9a | Area Sample 1F10a | Area F10b | Area F11a | Area F11b | Area F12a | Area F13 | #Feature | #Feature F1 | #Feature F2a | #Feature F3a | #Feature F4 | #Feature F5 | #Feature F6a | #Feature F7a | #Feature F7b | #Feature F8 | #Feature F9a | #Feature Sample 1F10a | #Feature F10b | #Feature F11a | #Feature F11b | #Feature F12a | #Feature F13 | Start | End | PTM | AScore | Found By |
|---|---|---|---|---|---|---|---|---|---|---|---|---|---|---|---|---|---|---|---|---|---|---|---|---|---|---|---|---|---|---|---|---|---|---|---|---|---|---|---|---|---|---|---|---|---|---|---|---|---|
| R.TFMDTELASFTK.N | Y | 58.63 | 1389.6486 | 12 | 1.4 | 695.8325 | 2 | 72.95 | 16 | F16:38090 | BuCaTN01_F13.raw | | | | | | | | | | | | | | | | 1.0855E5 | 1 | 0 | 0 | 0 | 0 | 0 | 0 | 0 | 0 | 0 | 0 | 0 | 0 | 0 | 0 | 0 | 1 | 43 | 54 | | | PEAKS DB |
| total 1 peptides | | | | | | | | | | | | | | | | | | | | | | | | | | | | | | | | | | | | | | | | | | | | | | | | | |

**Best Unique PSM (Scan F16:38090, m/z=695.8325, z=2, RT=72.95, ppm=1.4):**

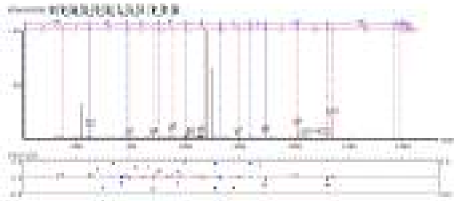

## XP_026580634.1

back to list

| Protein Coverage | Supporting Peptides | Best Unique PSM |

**Protein Coverage:**

1 MSSRYTVGPT ETERCIESLL AVFQRYAGKE GDSKTLSKKE FR**TFMDTELA SFTK**NQKDPG VVDRMMKKLD INSDGQLDFA

81 EFLNLIGGLA VAVHEMASAP VGGAPRRL

**Supporting Peptides:**

| Peptide | Uniq | -10lgP | Mass | Length | ppm | m/z | z | RT | Fraction | Scan | Source File | Area F1 | Area F2a | Area F3a | Area F4 | Area F5 | Area F6a | Area F7a | Area F7b | Area F8 | Area F9a | Area Sample 1F10a | Area F10b | Area F11a | Area F11b | Area F12a | Area F13 | #Feature | #Feature F1 | #Feature F2a | #Feature F3a | #Feature F4 | #Feature F5 | #Feature F6a | #Feature F7a | #Feature F7b | #Feature F8 | #Feature F9a | #Feature Sample 1F10a | #Feature F10b | #Feature F11a | #Feature F11b | #Feature F12a | #Feature F13 | Start | End | PTM | AScore | Found By |
|---|---|---|---|---|---|---|---|---|---|---|---|---|---|---|---|---|---|---|---|---|---|---|---|---|---|---|---|---|---|---|---|---|---|---|---|---|---|---|---|---|---|---|---|---|---|---|---|---|---|
| R.TFMDTELASFTK.N | Y | 58.63 | 1389.6486 | 12 | 1.4 | 695.8325 | 2 | 72.95 | 16 | F16:38090 | BuCaTN01_F13.raw | | | | | | | | | | | | | | | | 1.0855E5 | 1 | 0 | 0 | 0 | 0 | 0 | 0 | 0 | 0 | 0 | 0 | 0 | 0 | 0 | 0 | 0 | 1 | 43 | 54 | | | PEAKS DB |
| total 1 peptides | | | | | | | | | | | | | | | | | | | | | | | | | | | | | | | | | | | | | | | | | | | | | | | | | |

**Best Unique PSM (Scan F16:38090, m/z=695.8325, z=2, RT=72.95, ppm=1.4):**

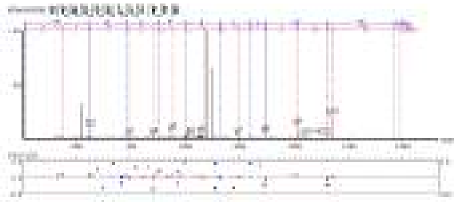

## XP_026580630.1

back to list

| Protein Coverage | Supporting Peptides | Best Unique PSM |

**Protein Coverage:**

1 MSSRYTVGPT ETERCIESLL AVFQRYAGKE GDSKTLSKKE FR**TFMDTELA SFTK**NQKDPG VVDRMMKKLD INSDGQLDFA

81 EFLNLIGGLA VAVHEMASAP VGGAPRRL

**Supporting Peptides:**

| Peptide | Uniq | -10lgP | Mass | Length | ppm | m/z | z | RT | Fraction | Scan | Source File | Area F1 | Area F2a | Area F3a | Area F4 | Area F5 | Area F6a | Area F7a | Area F7b | Area F8 | Area F9a | Area Sample 1F10a | Area F10b | Area F11a | Area F11b | Area F12a | Area F13 | #Feature | #Feature F1 | #Feature F2a | #Feature F3a | #Feature F4 | #Feature F5 | #Feature F6a | #Feature F7a | #Feature F7b | #Feature F8 | #Feature F9a | #Feature Sample 1F10a | #Feature F10b | #Feature F11a | #Feature F11b | #Feature F12a | #Feature F13 | Start | End | PTM | AScore | Found By |
|---|---|---|---|---|---|---|---|---|---|---|---|---|---|---|---|---|---|---|---|---|---|---|---|---|---|---|---|---|---|---|---|---|---|---|---|---|---|---|---|---|---|---|---|---|---|---|---|---|---|
| R.TFMDTELASFTK.N | Y | 58.63 | 1389.6486 | 12 | 1.4 | 695.8325 | 2 | 72.95 | 16 | F16:38090 | BuCaTN01_F13.raw | | | | | | | | | | | | | | | | 1.0855E5 | 1 | 0 | 0 | 0 | 0 | 0 | 0 | 0 | 0 | 0 | 0 | 0 | 0 | 0 | 0 | 0 | 1 | 43 | 54 | | | PEAKS DB |
| total 1 peptides | | | | | | | | | | | | | | | | | | | | | | | | | | | | | | | | | | | | | | | | | | | | | | | | | |

**Best Unique PSM (Scan F16:38090, m/z=695.8325, z=2, RT=72.95, ppm=1.4):**

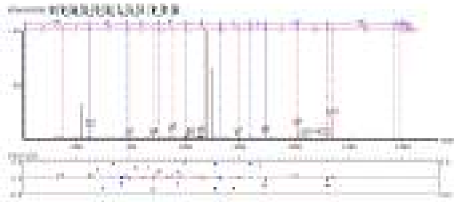

## XP_026580635.1

back to list

| Protein Coverage | Supporting Peptides | Best Unique PSM |

**Protein Coverage:**

1 MSSRYTVGPT ETERCIESLL AVFQRYAGKE GDSKTLSKKE FR**TFMDTELA SFTK**NQKDPG VVDRMMKKLD INSDGQLDFA

81 EFLNLIGGLA VAVHEMASAP VGGAPRRL

**Supporting Peptides:**

| Peptide | Uniq | -10lgP | Mass | Length | ppm | m/z | z | RT | Fraction | Scan | Source File | Area F1 | Area F2a | Area F3a | Area F4 | Area F5 | Area F6a | Area F7a | Area F7b | Area F8 | Area F9a | Area Sample 1F10a | Area F10b | Area F11a | Area F11b | Area F12a | Area F13 | #Feature | #Feature F1 | #Feature F2a | #Feature F3a | #Feature F4 | #Feature F5 | #Feature F6a | #Feature F7a | #Feature F7b | #Feature F8 | #Feature F9a | #Feature Sample 1F10a | #Feature F10b | #Feature F11a | #Feature F11b | #Feature F12a | #Feature F13 | Start | End | PTM | AScore | Found By |
|---|---|---|---|---|---|---|---|---|---|---|---|---|---|---|---|---|---|---|---|---|---|---|---|---|---|---|---|---|---|---|---|---|---|---|---|---|---|---|---|---|---|---|---|---|---|---|---|---|---|
| R.TFMDTELASFTK.N | Y | 58.63 | 1389.6486 | 12 | 1.4 | 695.8325 | 2 | 72.95 | 16 | F16:38090 | BuCaTN01_F13.raw | | | | | | | | | | | | | | | | 1.0855E5 | 1 | 0 | 0 | 0 | 0 | 0 | 0 | 0 | 0 | 0 | 0 | 0 | 0 | 0 | 0 | 0 | 1 | 43 | 54 | | | PEAKS DB |
| total 1 peptides | | | | | | | | | | | | | | | | | | | | | | | | | | | | | | | | | | | | | | | | | | | | | | | | | |

**Best Unique PSM (Scan F16:38090, m/z=695.8325, z=2, RT=72.95, ppm=1.4):**

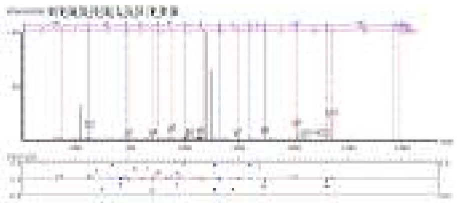

## XP_015668861.1

back to list

| Protein Coverage | Supporting Peptides | Best Unique PSM |

**Protein Coverage:**

1 MSSRYTAGPT ETERCIESLL AVFQRYAGRD SNAKTLSKKE FR**TFMDTELA SFTK**NQKDPA VIDRMMKKLD MNCDGQVDFS

81 EFLNLIGGLA QACHEHVASA PGGEAPKRP

**Supporting Peptides:**

| Peptide | Uniq | -10lgP | Mass | Length | ppm | m/z | z | RT | Fraction | Scan | Source File | Area F1 | Area F2a | Area F3a | Area F4 | Area F5 | Area F6a | Area F7a | Area F7b | Area F8 | Area F9a | Area Sample 1F10a | Area F10b | Area F11a | Area F11b | Area F12a | Area F13 | #Feature | #Feature F1 | #Feature F2a | #Feature F3a | #Feature F4 | #Feature F5 | #Feature F6a | #Feature F7a | #Feature F7b | #Feature F8 | #Feature F9a | #Feature Sample 1F10a | #Feature F10b | #Feature F11a | #Feature F11b | #Feature F12a | #Feature F13 | Start | End | PTM | AScore | Found By |
|---|---|---|---|---|---|---|---|---|---|---|---|---|---|---|---|---|---|---|---|---|---|---|---|---|---|---|---|---|---|---|---|---|---|---|---|---|---|---|---|---|---|---|---|---|---|---|---|---|---|
| R.TFMDTELASFTK.N | Y | 58.63 | 1389.6486 | 12 | 1.4 | 695.8325 | 2 | 72.95 | 16 | F16:38090 | BuCaTN01_F13.raw | | | | | | | | | | | | | | | | 1.0855E5 | 1 | 0 | 0 | 0 | 0 | 0 | 0 | 0 | 0 | 0 | 0 | 0 | 0 | 0 | 0 | 0 | 1 | 43 | 54 | | | PEAKS DB |
| total 1 peptides | | | | | | | | | | | | | | | | | | | | | | | | | | | | | | | | | | | | | | | | | | | | | | | | | |

**Best Unique PSM (Scan F16:38090, m/z=695.8325, z=2, RT=72.95, ppm=1.4):**

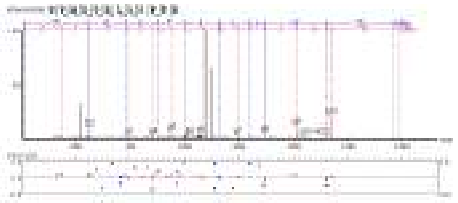

## BAK64382.1

back to list

| Protein Coverage | Supporting Peptides | Best Unique PSM |

**Protein Coverage:**

1 MKVFFSLIIF SLMLAACQGA CFSGPFEVEI KDGKPVSKTC LDIYDGRK**HL IGSTWNTAHC FR**CKCSKNGL HCCHRYGGIG  C Carbamidomethylation (+57.02)

81 VVQGCKTVIN PMTCELEYYR LDDPFRRCVA

**Supporting Peptides:**

| Peptide | Uniq | -10lgP | Mass | Length | ppm | m/z | z | RT | Fraction | Scan | Source File | Area F1 | Area F2a | Area F3a | Area F4 | Area F5 | Area F6a | Area F7a | Area F7b | Area F8 | Area F9a | Area Sample 1F10a | Area F10b | Area F11a | Area F11b | Area F12a | Area F13 | #Feature | #Feature F1 | #Feature F2a | #Feature F3a | #Feature F4 | #Feature F5 | #Feature F6a | #Feature F7a | #Feature F7b | #Feature F8 | #Feature F9a | #Feature Sample 1F10a | #Feature F10b | #Feature F11a | #Feature F11b | #Feature F12a | #Feature F13 | Start | End | PTM | AScore | Found By |
|---|---|---|---|---|---|---|---|---|---|---|---|---|---|---|---|---|---|---|---|---|---|---|---|---|---|---|---|---|---|---|---|---|---|---|---|---|---|---|---|---|---|---|---|---|---|---|---|---|---|
| K.HLIGSTWNTAHC(+57.02)FR.C | Y | 58.41 | 1698.8049 | 14 | 5.1 | 567.2768 | 3 | 49.85 | 8 | F8:26644 | BuCaTN01_F7b.raw | | | | | | 7.3485E3 | | 0 | | | | | | | | | 1 | 0 | 0 | 0 | 0 | 0 | 1 | 0 | 0 | 0 | 0 | 0 | 0 | 0 | 0 | 0 | 0 | 49 | 62 | Carbamidomethylation | C12:Carbamidomethylation:1000.00 | PEAKS DB |
| total 1 peptides | | | | | | | | | | | | | | | | | | | | | | | | | | | | | | | | | | | | | | | | | | | | | | | | | |

**Best Unique PSM (Scan F8:26644, m/z=567.2768, z=3, RT=49.85, ppm=5.1):**

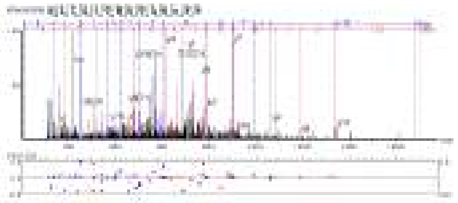

## BAJ14707.1

back to list

| Protein Coverage | Supporting Peptides | Best Unique PSM |

**Protein Coverage:**

1 MRVFFSLIIF SFMLATCQGA CGRSPHVPEF MDGKAVAPKT CIDEYDGSK**H LLGSTWNTAH CFR**CECNNYG MSCCHRYGGL  C Carbamidomethylation (+57.02)

81 AGREGCKTVL NQVTCEYEFY RLDDPSKRCG A

**Supporting Peptides:**

| Peptide | Uniq | -10lgP | Mass | Length | ppm | m/z | z | RT | Fraction | Scan | Source File | Area F1 | Area F2a | Area F3a | Area F4 | Area F5 | Area F6a | Area F7a | Area F7b | Area F8 | Area F9a | Area Sample 1F10a | Area F10b | Area F11a | Area F11b | Area F12a | Area F13 | #Feature | #Feature F1 | #Feature F2a | #Feature F3a | #Feature F4 | #Feature F5 | #Feature F6a | #Feature F7a | #Feature F7b | #Feature F8 | #Feature F9a | #Feature Sample 1F10a | #Feature F10b | #Feature F11a | #Feature F11b | #Feature F12a | #Feature F13 | Start | End | PTM | AScore | Found By |
|---|---|---|---|---|---|---|---|---|---|---|---|---|---|---|---|---|---|---|---|---|---|---|---|---|---|---|---|---|---|---|---|---|---|---|---|---|---|---|---|---|---|---|---|---|---|---|---|---|---|
| K.HLLGSTWNTAHC(+57.02)FR.C | Y | 58.41 | 1698.8049 | 14 | 5.1 | 567.2768 | 3 | 49.85 | 8 | F8:26644 | BuCaTN01_F7b.raw | | | | | | 7.3485E3 | | 0 | | | | | | | | | 1 | 0 | 0 | 0 | 0 | 0 | 1 | 0 | 0 | 0 | 0 | 0 | 0 | 0 | 0 | 0 | 0 | 50 | 63 | Carbamidomethylation | C12:Carbamidomethylation:1000.00 | PEAKS DB |
| total 1 peptides | | | | | | | | | | | | | | | | | | | | | | | | | | | | | | | | | | | | | | | | | | | | | | | | | |

**Best Unique PSM (Scan F8:26644, m/z=567.2768, z=3, RT=49.85, ppm=5.1):**

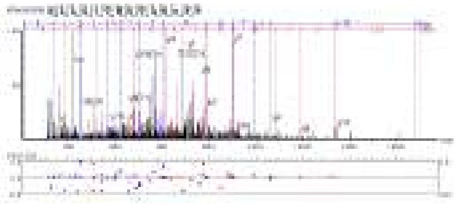

## BBV14780.1

back to list

| Protein Coverage | Supporting Peptides | Best Unique PSM |

**Protein Coverage:**

1 MKVFFSLIIF SLMLATSQGA CFTWGFEAEI KDGKPVEPNF CLDTYDGRK**H LIGSTWNTAH CFR**CNCGTDG LHCCHRYGGI  C Carbamidomethylation (+57.02)

81 AEVQGCTTVV NPVTCKHEHY RIDDPTQRCR V

**Supporting Peptides:**

| Peptide | Uniq | -10lgP | Mass | Length | ppm | m/z | z | RT | Fraction | Scan | Source File | Area F1 | Area F2a | Area F3a | Area F4 | Area F5 | Area F6a | Area F7a | Area F7b | Area F8 | Area F9a | Area Sample 1F10a | Area F10b | Area F11a | Area F11b | Area F12a | Area F13 | #Feature | #Feature F1 | #Feature F2a | #Feature F3a | #Feature F4 | #Feature F5 | #Feature F6a | #Feature F7a | #Feature F7b | #Feature F8 | #Feature F9a | #Feature Sample 1F10a | #Feature F10b | #Feature F11a | #Feature F11b | #Feature F12a | #Feature F13 | Start | End | PTM | AScore | Found By |
|---|---|---|---|---|---|---|---|---|---|---|---|---|---|---|---|---|---|---|---|---|---|---|---|---|---|---|---|---|---|---|---|---|---|---|---|---|---|---|---|---|---|---|---|---|---|---|---|---|---|
| K.HLIGSTWNTAHC(+57.02)FR.C | Y | 58.41 | 1698.8049 | 14 | 5.1 | 567.2768 | 3 | 49.85 | 8 | F8:26644 | BuCaTN01_F7b.raw | | | | | | 7.3485E3 | | 0 | | | | | | | | | 1 | 0 | 0 | 0 | 0 | 0 | 1 | 0 | 0 | 0 | 0 | 0 | 0 | 0 | 0 | 0 | 0 | 50 | 63 | Carbamidomethylation | C12:Carbamidomethylation:1000.00 | PEAKS DB |

total 1 peptides

**Best Unique PSM (Scan F8:26644, m/z=567.2768, z=3, RT=49.85, ppm=5.1):**

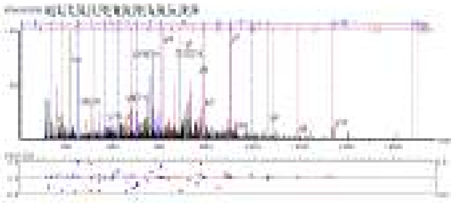

## XP_026571031.1

back to list

| Protein Coverage | Supporting Peptides | Best Unique PSM |

**Protein Coverage:**

```
 1 MKVFFSLIIF SLMLAACQGA CFSAPFEVET EDGKQVVPNT CLDRHDGRKH LIGSTWNTAH CFRCECGTDG LHCCHRYGGI
81 GVVEGCTTVV NPVTCKHEHY RIDDPSQPCG V
```

61 C — Carbamidomethylation (+57.02)

**Supporting Peptides:**

| Peptide | Uniq | -10lgP | Mass | Length | ppm | m/z | z | RT | Fraction | Scan | Source File | Area F1 | Area F2a | Area F3a | Area F4 | Area F5 | Area F6a | Area F7a | Area F7b | Area F8 | Area F9a | Area Sample 1F10a | Area F10b | Area F11a | Area F11b | Area F12a | Area F13 | #Feature | #Feature F1 | #Feature F2a | #Feature F3a | #Feature F4 | #Feature F5 | #Feature F6a | #Feature F7a | #Feature F7b | #Feature F8 | #Feature F9a | #Feature Sample 1F10a | #Feature F10b | #Feature F11a | #Feature F11b | #Feature F12a | #Feature F13 | Start | End | PTM | AScore | Found By |
|---|---|---|---|---|---|---|---|---|---|---|---|---|---|---|---|---|---|---|---|---|---|---|---|---|---|---|---|---|---|---|---|---|---|---|---|---|---|---|---|---|---|---|---|---|---|---|---|---|---|
| K.HLIGSTWNTAHC(+57.02)FR.C | Y | 58.41 | 1698.8049 | 14 | 5.1 | 567.2768 | 3 | 49.85 | 8 | F8:26644 | BuCaTN01_F7b.raw | | | | | | 7.3485E3 | | 0 | | | | | | | | | 1 | 0 | 0 | 0 | 0 | 0 | 1 | 0 | 0 | 0 | 0 | 0 | 0 | 0 | 0 | 0 | 0 | 50 | 63 | Carbamidomethylation | C12:Carbamidomethylation:1000.00 | PEAKS DB |

total 1 peptides

**Best Unique PSM (Scan F8:26644, m/z=567.2768, z=3, RT=49.85, ppm=5.1):**

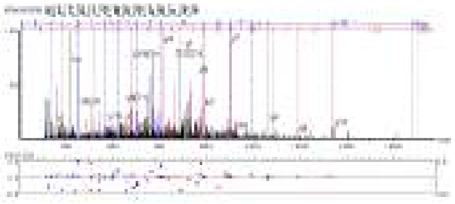

## BBV14781.1

back to list

| Protein Coverage | Supporting Peptides | Best Unique PSM |

**Protein Coverage:**

```
 1 MKVFFSLIIF SLMLATCQGA CYRGLFEAEI KDGKPVLPNF CLDTHDGRKH LIGSTWNTAH CFRCNCGTDG LHCCHRYGGI
81 ADVQGCTTVV NPVTCKHEHY RIDDPSQRCD V
```

61 C — Carbamidomethylation (+57.02)

**Supporting Peptides:**

| Peptide | Uniq | -10lgP | Mass | Length | ppm | m/z | z | RT | Fraction | Scan | Source File | Area F1 | Area F2a | Area F3a | Area F4 | Area F5 | Area F6a | Area F7a | Area F7b | Area F8 | Area F9a | Area Sample 1F10a | Area F10b | Area F11a | Area F11b | Area F12a | Area F13 | #Feature | #Feature F1 | #Feature F2a | #Feature F3a | #Feature F4 | #Feature F5 | #Feature F6a | #Feature F7a | #Feature F7b | #Feature F8 | #Feature F9a | #Feature Sample 1F10a | #Feature F10b | #Feature F11a | #Feature F11b | #Feature F12a | #Feature F13 | Start | End | PTM | AScore | Found By |
|---|---|---|---|---|---|---|---|---|---|---|---|---|---|---|---|---|---|---|---|---|---|---|---|---|---|---|---|---|---|---|---|---|---|---|---|---|---|---|---|---|---|---|---|---|---|---|---|---|---|
| K.HLIGSTWNTAHC(+57.02)FR.C | Y | 58.41 | 1698.8049 | 14 | 5.1 | 567.2768 | 3 | 49.85 | 8 | F8:26644 | BuCaTN01_F7b.raw | | | | | | 7.3485E3 | | 0 | | | | | | | | | 1 | 0 | 0 | 0 | 0 | 0 | 1 | 0 | 0 | 0 | 0 | 0 | 0 | 0 | 0 | 0 | 0 | 50 | 63 | Carbamidomethylation | C12:Carbamidomethylation:1000.00 | PEAKS DB |

total 1 peptides

**Best Unique PSM (Scan F8:26644, m/z=567.2768, z=3, RT=49.85, ppm=5.1):**

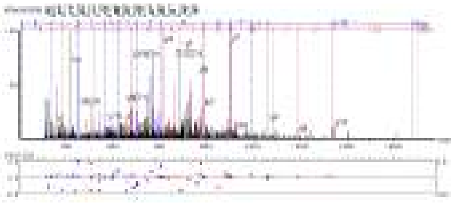

## XP_058050813.1

back to list

| Protein Coverage | Supporting Peptides | Best Unique PSM |

**Protein Coverage:**

```
 1 MKAFFCLIIF SLMLAACQGA CFGSNFKAEF KDGKEVRPNT CVDIYDKSKH LIGSTWNTAH CFRCECGRNG LSCCHRYGGI
81 GVVQGCKRVI NPVTCEQEYY RLDDPSQRCD VPSLHKDGPE
```

61 C — Carbamidomethylation (+57.02)

**Supporting Peptides:**

| Peptide | Uniq | -10lgP | Mass | Length | ppm | m/z | z | RT | Fraction | Scan | Source File | Area F1 | Area F2a | Area F3a | Area F4 | Area F5 | Area F6a | Area F7a | Area F7b | Area F8 | Area F9a | Area Sample 1F10a | Area F10b | Area F11a | Area F11b | Area F12a | Area F13 | #Feature | #Feature F1 | #Feature F2a | #Feature F3a | #Feature F4 | #Feature F5 | #Feature F6a | #Feature F7a | #Feature F7b | #Feature F8 | #Feature F9a | #Feature Sample 1F10a | #Feature F10b | #Feature F11a | #Feature F11b | #Feature F12a | #Feature F13 | Start | End | PTM | AScore | Found By |
|---|---|---|---|---|---|---|---|---|---|---|---|---|---|---|---|---|---|---|---|---|---|---|---|---|---|---|---|---|---|---|---|---|---|---|---|---|---|---|---|---|---|---|---|---|---|---|---|---|---|
| K.HLIGSTWNTAHC(+57.02)FR.C | Y | 58.41 | 1698.8049 | 14 | 5.1 | 567.2768 | 3 | 49.85 | 8 | F8:26644 | BuCaTN01_F7b.raw | | | | | | 7.3485E3 | | 0 | | | | | | | | | 1 | 0 | 0 | 0 | 0 | 0 | 1 | 0 | 0 | 0 | 0 | 0 | 0 | 0 | 0 | 0 | 0 | 50 | 63 | Carbamidomethylation | C12:Carbamidomethylation:1000.00 | PEAKS DB |

total 1 peptides

**Best Unique PSM (Scan F8:26644, m/z=567.2768, z=3, RT=49.85, ppm=5.1):**

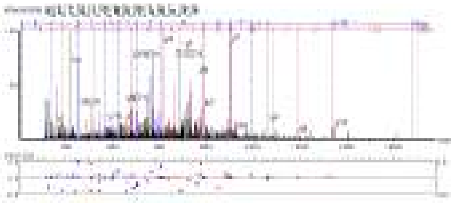

## KAG8139853.1

back to list

| Protein Coverage | Supporting Peptides | Best Unique PSM |

**Protein Coverage:**

1 MWKGSIVLSV DCSVSLCPDA SDCMILFMAR MWVHFIVFLV LSTTASSLQC PDGQKCEGSS ICCKLPEGDK YTCCSQPQFT
81 GASLRMLPPE SRNEVSGVVC PDASTCPVAH SCQLSLNSTF ACCPWEEAIS CVDGCHCCPL GTHCSTDGRV CIENEDSTSA
161 VIVRAVQCPD SEWECPDGST CCLMADESWG CCPMPEVRNL DMGKIGDREN GYVPPASCCS DKIHCCPHDT TCDLVEARCL
241 ETFGEQPLSK KFPAKKRTPV LNLTQKNICP GNHSSCPDAA TCCLLPTGQY GCCALQNAVC CSDHLHCCPQ DTHCDLKHSI
321 CTMTPKGSWP ITHLNAGLQT AHDIQCDSEF SCPDGYTCCR TTSGSWGCCP MEEAVCCSDQ IHCCPKGYQC VLSEGSCQKD
401 GQSISLVLKI DTSSTTASRI DSSSTASSIG RMVQCDPYTA CHDGQTCCRL VTGAWGCCLL TQATCCQDQI HCCPSGHKCD
481 SSSGTCLKDG YRVPWVEK**TT AIVGNLPSSR** DVKCDDQFSC ADGQTCCMRS GAWACCPLFQ AVCCEDQAHC CPHGYICDVK
561 HSLCIKSGGL SQGISMTPIL ARQETAIKCD NFSSCKDKET CCKMASGNWG HCPFPKAVCC EDHQHCCPFG YTCNVTAQTC
641 EKQHWPGLAD AAVDLLTSSQ NAVSSQDVLC GEQHYCHTGQ TCCKARSSGW ACCPYEKGTC CYDRRHCCPS GYYCSLLKHR
721 CYRRKPLRWD TGNVSPHSID AHVLL

**Supporting Peptides:**

| Peptide | Uniq | -10lgP | Mass | Length | ppm | m/z | z | RT | Fraction | Scan | Source File | Area F1 | Area F2a | Area F3a | Area F4 | Area F5 | Area F6a | Area F7a | Area F7b | Area F8 | Area F9a | Area Sample 1F10a | Area F10b | Area F11a | Area F11b | Area F12a | Area F13 | #Feature | #Feature F1 | #Feature F2a | #Feature F3a | #Feature F4 | #Feature F5 | #Feature F6a | #Feature F7a | #Feature F7b | #Feature F8 | #Feature F9a | #Feature Sample 1F10a | #Feature F10b | #Feature F11a | #Feature F11b | #Feature F12a | #Feature F13 | Start | End | PTM | AScore | Found By |
|---|---|---|---|---|---|---|---|---|---|---|---|---|---|---|---|---|---|---|---|---|---|---|---|---|---|---|---|---|---|---|---|---|---|---|---|---|---|---|---|---|---|---|---|---|---|---|---|---|---|
| K.TTAIVGNLPSSR.D | Y | 53.13 | 1214.6619 | 12 | 0.1 | 608.3383 | 2 | 43.88 | 7 | F7:19196 | BuCaTN01_F7a.raw | | | | | | | 3.9332E5 | | | | | | | | | | 1 | 0 | 0 | 0 | 0 | 0 | 0 | 1 | 0 | 0 | 0 | 0 | 0 | 0 | 0 | 0 | 0 | 499 | 510 | | | PEAKS DB |
| total 1 peptides | | | | | | | | | | | | | | | | | | | | | | | | | | | | | | | | | | | | | | | | | | | | | | | | | |

**Best Unique PSM (Scan F7:19196, m/z=608.3383, z=2, RT=43.88, ppm=0.1):**

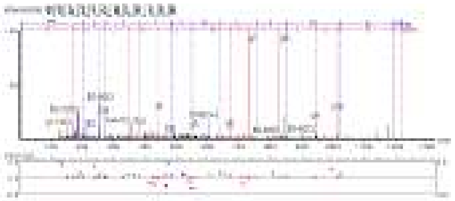

## XP_026573999.1

back to list

| Protein Coverage | Supporting Peptides | Best Unique PSM |

**Protein Coverage:**

1 MARMWVHFIV FLVLSTTASS LKCPDGQKCG GSSICCKLPE GDKYTCCGQP QFTGASLRML PPESRNEVSG VVCPDASTCP
81 VTHSCQLSLN STFACCPWEE AISCVDGHHC CPLGTHCSTD GRVCIENEDL TSAVIVRAVQ CPDSEWECPD GSTCCLMADE
161 SWGCCPMPEA SCCADKIHCC PHGTTCDLVE ARCLETFGEQ PLSKKFPAKK RTPVLNLTQK KICPGNHSSC PDAATCCLLP
241 TGQYGCCPLQ NAVCCSDHLH CCPQGTQCDL KHSICTMTPK ESWPITHLSA GFHTAHDVQC DSEFACPDGN TCCKTTSGSW
321 GCCPLEEAVC CPDKIHCCPK GYQCDLSEGS CLKDGQSIPL VLKMDASSTT ASEIDSSSTA SSIGRMVQCD PYTACQDGQT
401 CCRLATGAWG CCPLTQATCC QDQIHCCPSG YNCDSSSGTC LKDGDRMPWV EK**TTAIVGNL PSSR**DVKCDD QYSCPDGQTC
481 CLRSGAWACC PLFQAICCED HQHCCPFGYT CNVAAQSCEK RHRPRLADAA GDLLTSLQNA ASNQDVLCGE QHYCHVGQTC
561 CKAKNSSWAC CPYEKGTCCH DRRHCCPSGF YCSVLGYHCY RKPSRWDTGS ASPHSIDAHV LL

**Supporting Peptides:**

| Peptide | Uniq | -10lgP | Mass | Length | ppm | m/z | z | RT | Fraction | Scan | Source File | Area F1 | Area F2a | Area F3a | Area F4 | Area F5 | Area F6a | Area F7a | Area F7b | Area F8 | Area F9a | Area Sample 1F10a | Area F10b | Area F11a | Area F11b | Area F12a | Area F13 | #Feature | #Feature F1 | #Feature F2a | #Feature F3a | #Feature F4 | #Feature F5 | #Feature F6a | #Feature F7a | #Feature F7b | #Feature F8 | #Feature F9a | #Feature Sample 1F10a | #Feature F10b | #Feature F11a | #Feature F11b | #Feature F12a | #Feature F13 | Start | End | PTM | AScore | Found By |
|---|---|---|---|---|---|---|---|---|---|---|---|---|---|---|---|---|---|---|---|---|---|---|---|---|---|---|---|---|---|---|---|---|---|---|---|---|---|---|---|---|---|---|---|---|---|---|---|---|---|
| K.TTAIVGNLPSSR.D | Y | 53.13 | 1214.6619 | 12 | 0.1 | 608.3383 | 2 | 43.88 | 7 | F7:19196 | BuCaTN01_F7a.raw | | | | | | | 3.9332E5 | | | | | | | | | | 1 | 0 | 0 | 0 | 0 | 0 | 0 | 1 | 0 | 0 | 0 | 0 | 0 | 0 | 0 | 0 | 0 | 453 | 464 | | | PEAKS DB |
| total 1 peptides | | | | | | | | | | | | | | | | | | | | | | | | | | | | | | | | | | | | | | | | | | | | | | | | | |

**Best Unique PSM (Scan F7:19196, m/z=608.3383, z=2, RT=43.88, ppm=0.1):**

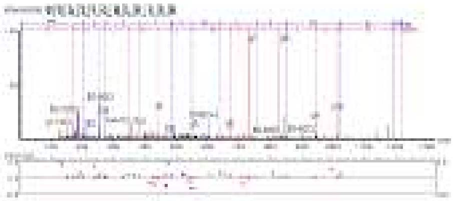

## XP_026573998.1

back to list

| Protein Coverage | Supporting Peptides | Best Unique PSM |

**Protein Coverage:**

1 MARMWVHFIV FLVLSTTASS LKCPDGQKCG GSSICCKLPE GDKYTCCGQP QFTGASLRML PPESRNEVSG VVCPDASTCP
81 VTHSCQLSLN STFACCPWEE AISCVDGHHC CPLGTHCSTD GRVCIENEGI WRQNLTSAVI VRAVQCPDSE WECPDGSTCC
161 LMADESWGCC PMPEASCCAD KIHCCPHGTT CDLVEARCLE TFGEQPLSKK FPAKKRTPVL NLTQKKICPG NHSSCPDAAT
241 CCLLPTGQYG CCPLQNAVCC SDHLHCCPQG TQCDLKHSIC TMTPKESWPI THLSAGFHTA HDVQCDSEFA CPDGNTCCKT
321 TSGSWGCCPL EEAVCCPDKI HCCPKGYQCD LSEGSCLKDG QSIPLVLKMD ASSTTASEID SSSTASSIGR MVQCDPYTAC
401 QDGQTCCRLA TGAWGCCPLT QATCCQDQIH CCPSGYNCDS SSGTCLKDGD RMPWVEK**TTA IVGNLPSSR**D VKCDDQYSCP
481 DGQTCCLRSG AWACCPLFQA ICCEDHQHCC PFGYTCNVAA QSCEKRHRPR LADAAGDLLT SLQNAASNQD VLCGEQHYCH
561 VGQTCCKAKN SSWACCPYEK GTCCHDRRHC CPSGFYCSVL GYHCYRKPSR WDTGSASPHS IDAHVLL

**Supporting Peptides:**

| Peptide | Uniq | -10lgP | Mass | Length | ppm | m/z | z | RT | Fraction | Scan | Source File | Area F1 | Area F2a | Area F3a | Area F4 | Area F5 | Area F6a | Area F7a | Area F7b | Area F8 | Area F9a | Area Sample 1F10a | Area F10b | Area F11a | Area F11b | Area F12a | Area F13 | #Feature | #Feature F1 | #Feature F2a | #Feature F3a | #Feature F4 | #Feature F5 | #Feature F6a | #Feature F7a | #Feature F7b | #Feature F8 | #Feature F9a | #Feature Sample 1F10a | #Feature F10b | #Feature F11a | #Feature F11b | #Feature F12a | #Feature F13 | Start | End | PTM | AScore | Found By |
|---|---|---|---|---|---|---|---|---|---|---|---|---|---|---|---|---|---|---|---|---|---|---|---|---|---|---|---|---|---|---|---|---|---|---|---|---|---|---|---|---|---|---|---|---|---|---|---|---|---|
| K.TTAIVGNLPSSR.D | Y | 53.13 | 1214.6619 | 12 | 0.1 | 608.3383 | 2 | 43.88 | 7 | F7:19196 | BuCaTN01_F7a.raw | | | | | | | 3.9332E5 | | | | | | | | | | 1 | 0 | 0 | 0 | 0 | 0 | 0 | 1 | 0 | 0 | 0 | 0 | 0 | 0 | 0 | 0 | 0 | 458 | 469 | | | PEAKS DB |
| total 1 peptides | | | | | | | | | | | | | | | | | | | | | | | | | | | | | | | | | | | | | | | | | | | | | | | | | |

**Best Unique PSM (Scan F7:19196, m/z=608.3383, z=2, RT=43.88, ppm=0.1):**

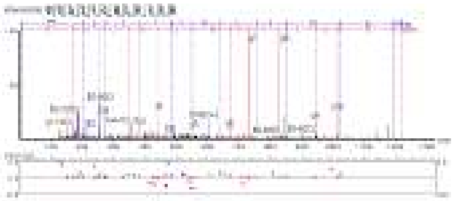

## XP_026539436.1

back to list

| Protein Coverage | Supporting Peptides | Best Unique PSM |

**Protein Coverage:**

1 MARMWVHFIV FLVLSTTASS LKCPDGQKCE GSSICCKLPE GDKYTCCGQP QFTGASLRML PPESRNEVSG VVCPDASTCP
81 VTHSCQLSLN STFACCPWEE ATSCVDGHHC CPLGTHCSTD GRVCIENEGI WRQNSTSAVI VRAVQCPDSE WECPDGSTCC
161 LMADESWGCC PMPEASCCAD KIHCCPHGTT CDLVEARCLE TFGEQPLSKK FPAKKRTPVL NLTQKKICPG NHSSCPDAAT
241 CCLLPTGQYG CCPLQNAVCC SDHLHCCPQG TQCDLKQSKC TMTPKESWPI THLSAGFHTA HDIQCDSEFT CPDGNTCCKT
321 TSGSWGCCPM EEAVCCPDKI HCCPKGYQCD LSEGSCLKDG QSIPLVLKKD TSSTTASEID SSSTASSIGR MVQCDPYTAC
401 HDGQTCCRLA TGAWGCCPLT QATCCQDQIH CCPSGYNCDS SSGTCLKDGD RMPWVEK**TTA IVGNLPSSR**D VKCDDQYSCP
481 DGQTCCLRSG AWACCPLFQA TCCEDHQHCC PFGYTCNVAA QSCEKQHRPG LADAAGDLLT SLQNAVSNQD VLCGEQHYCH
561 VGQTCCKAKS GSWACCPYEK GTCCHDRRHC CPSGFYCSRF GYHCYRKSSR WDTGSVSPHS IDAHVLL

**Supporting Peptides:**

| Peptide | Uniq | -10lgP | Mass | Length | ppm | m/z | z | RT | Fraction | Scan | Source File | Area F1 | Area F2a | Area F3a | Area F4 | Area F5 | Area F6a | Area F7a | Area F7b | Area F8 | Area F9a | Area Sample 1F10a | Area F10b | Area F11a | Area F11b | Area F12a | Area F13 | #Feature | #Feature F1 | #Feature F2a | #Feature F3a | #Feature F4 | #Feature F5 | #Feature F6a | #Feature F7a | #Feature F7b | #Feature F8 | #Feature F9a | #Feature Sample 1F10a | #Feature F10b | #Feature F11a | #Feature F11b | #Feature F12a | #Feature F13 | Start | End | PTM | AScore | Found By |
|---|---|---|---|---|---|---|---|---|---|---|---|---|---|---|---|---|---|---|---|---|---|---|---|---|---|---|---|---|---|---|---|---|---|---|---|---|---|---|---|---|---|---|---|---|---|---|---|---|---|
| K.TTAIVGNLPSSR.D | Y | 53.13 | 1214.6619 | 12 | 0.1 | 608.3383 | 2 | 43.88 | 7 | F7:19196 | BuCaTN01_F7a.raw | | | | | | | 3.9332E5 | | | | | | | | | | 1 | 0 | 0 | 0 | 0 | 0 | 0 | 1 | 0 | 0 | 0 | 0 | 0 | 0 | 0 | 0 | 0 | 458 | 469 | | | PEAKS DB |
| total 1 peptides | | | | | | | | | | | | | | | | | | | | | | | | | | | | | | | | | | | | | | | | | | | | | | | | | |

**Best Unique PSM (Scan F7:19196, m/z=608.3383, z=2, RT=43.88, ppm=0.1):**

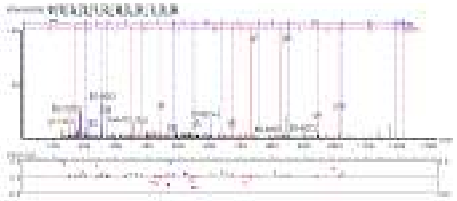

## XP_026539435.1

back to list

| Protein Coverage | Supporting Peptides | Best Unique PSM |

**Protein Coverage:**

1 MARMWVHFIV FLVLSTTASS LKCPDGQKCE GSSICCKLPE GDKYTCCGQP QFTGASLRML PPESRNEVSG VVCPDASTCP
81 VTHSCQLSLN STFACCPWEE ATSCVDGHHC CPLGTHCSTD GRVCIENEGI WRQNSTSAVI VRAVQCPDSE WECPDGSTCC
161 LMADESWGCC PMPEASCCAD KIHCCPHGTT CDLVEARCLE TFGEQPLSKK FPAKKRTPVL NLTQKKICPG NHSSCPDAAT
241 CCLLPTGQYG CCPLQNAVCC SDHLHCCPQG TQCDLKQSKC TMTPKESWPI THLSAGFHTA HDIQCDSEFT CPDGNTCCKT
321 TSGSWGCCPM EEAVCCPDKI HCCPKGYQCD LSEGSCLKDG QSIPLVLKKD TSSTTASEID SSSTASSIGR MVQCDPYTAC
401 HDGQTCCRLA TGAWGCCPLT QATCCQDQIH CCPSGYNCDS SSGTCLKDGD RMPWVEK**TTA IVGNLPSSR**D VKCDDQYSCP
481 DGQTCCLRSG AWACCPLFQA TCCEDHQHCC PFGYTCNVAA QSCEKQHRPG LADAAGDLLT SLQNAVSNQD VLCGEQHYCH
561 VGQTCCKAKS GSWACCPYEK GTCCHDRRHC CPSGFYCSRF GYHCYRKSSR WDTGSVSPHS IDAHVLL

**Supporting Peptides:**

| Peptide | Uniq | -10lgP | Mass | Length | ppm | m/z | z | RT | Fraction | Scan | Source File | Area F1 | Area F2a | Area F3a | Area F4 | Area F5 | Area F6a | Area F7a | Area F7b | Area F8 | Area F9a | Area Sample 1F10a | Area F10b | Area F11a | Area F11b | Area F12a | Area F13 | #Feature | #Feature F1 | #Feature F2a | #Feature F3a | #Feature F4 | #Feature F5 | #Feature F6a | #Feature F7a | #Feature F7b | #Feature F8 | #Feature F9a | #Feature Sample 1F10a | #Feature F10b | #Feature F11a | #Feature F11b | #Feature F12a | #Feature F13 | Start | End | PTM | AScore | Found By |
|---|---|---|---|---|---|---|---|---|---|---|---|---|---|---|---|---|---|---|---|---|---|---|---|---|---|---|---|---|---|---|---|---|---|---|---|---|---|---|---|---|---|---|---|---|---|---|---|---|---|
| K.TTAIVGNLPSSR.D | Y | 53.13 | 1214.6619 | 12 | 0.1 | 608.3383 | 2 | 43.88 | 7 | F7:19196 | BuCaTN01_F7a.raw | | | | | | | 3.9332E5 | | | | | | | | | | 1 | 0 | 0 | 0 | 0 | 0 | 0 | 1 | 0 | 0 | 0 | 0 | 0 | 0 | 0 | 0 | 0 | 458 | 469 | | | PEAKS DB |
| total 1 peptides | | | | | | | | | | | | | | | | | | | | | | | | | | | | | | | | | | | | | | | | | | | | | | | | | |

**Best Unique PSM (Scan F7:19196, m/z=608.3383, z=2, RT=43.88, ppm=0.1):**

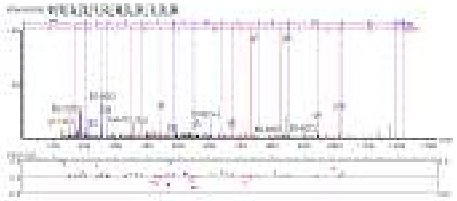

## XP_026539437.1

back to list

| Protein Coverage | Supporting Peptides | Best Unique PSM |

**Protein Coverage:**

1 MARMWVHFIV FLVLSTTASS LKCPDGQKCE GSSICCKLPE GDKYTCCGQP QFTGASLRML PPESRNEVSG VVCPDASTCP
81 VTHSCQLSLN STFACCPWEE ATSCVDGHHC CPLGTHCSTD GRVCIENEDS TSAVIVRAVQ CPDSEWECPD GSTCCLMADE
161 SWGCCPMPEA SCCADKIHCC PHGTTCDLVE ARCLETFGEQ PLSKKFPAKK RTPVLNLTQK KICPGNHSSC PDAATCCLLP
241 TGQYGCCPLQ NAVCCSDHLH CCPQGTQCDL KQSKCTMTPK ESWPITHLSA GFHTAHDIQC DSEFTCPDGN TCCKTTSGSW
321 GCCPMEEAVC CPDKIHCCPK GYQCDLSEGS CLKDGQSIPL VLKKDTSSTT ASEIDSSSTA SSIGRMVQCD PYTACHDGQT
401 CCRLATGAWG CCPLTQATCC QDQIHCCPSG YNCDSSSGTC LKDGDRMPWV EK**TTAIVGNL PSSR**DVKCDD QYSCPDGQTC
481 CLRSGAWACC PLFQATCCED HQHCCPFGYT CNVAAQSCEK QHRPGLADAA GDLLTSLQNA VSNQDVLCGE QHYCHVGQTC
561 CKAKSGSWAC CPYEKGTCCH DRRHCCPSGF YCSRFGYHCY RKSSRWDTGS VSPHSIDAHV LL

**Supporting Peptides:**

| Peptide | Uniq | -10lgP | Mass | Length | ppm | m/z | z | RT | Fraction | Scan | Source File | Area F1 | Area F2a | Area F3a | Area F4 | Area F5 | Area F6a | Area F7a | Area F7b | Area F8 | Area F9a | Area Sample 1F10a | Area F10b | Area F11a | Area F11b | Area F12a | Area F13 | #Feature | #Feature F1 | #Feature F2a | #Feature F3a | #Feature F4 | #Feature F5 | #Feature F6a | #Feature F7a | #Feature F7b | #Feature F8 | #Feature F9a | #Feature Sample 1F10a | #Feature F10b | #Feature F11a | #Feature F11b | #Feature F12a | #Feature F13 | Start | End | PTM | AScore | Found By |
|---|---|---|---|---|---|---|---|---|---|---|---|---|---|---|---|---|---|---|---|---|---|---|---|---|---|---|---|---|---|---|---|---|---|---|---|---|---|---|---|---|---|---|---|---|---|---|---|---|---|
| K.TTAIVGNLPSSR.D | Y | 53.13 | 1214.6619 | 12 | 0.1 | 608.3383 | 2 | 43.88 | 7 | F7:19196 | BuCaTN01_F7a.raw | | | | | | | 3.9332E5 | | | | | | | | | | 1 | 0 | 0 | 0 | 0 | 0 | 0 | 1 | 0 | 0 | 0 | 0 | 0 | 0 | 0 | 0 | 0 | 453 | 464 | | | PEAKS DB |
| total 1 peptides | | | | | | | | | | | | | | | | | | | | | | | | | | | | | | | | | | | | | | | | | | | | | | | | | |

**Best Unique PSM (Scan F7:19196, m/z=608.3383, z=2, RT=43.88, ppm=0.1):**

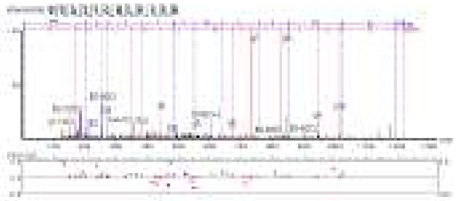

## ETE73811.1

back to list

| Protein Coverage | Supporting Peptides | Best Unique PSM |

**Protein Coverage:**

1 MARMWVHFIV FLVLSTTTSS LRCPDGQKCE GSSICCKLPE GDKYTCCGQP QFTGVSLRML PPESRNEVSG VVCPDASTCP
81 VTHSCQLSLN STFACCPWEE AISCVDGHHC CPLGTHCSTD GRVCIENEDS TFAVIVRAVQ CPDSEWECPD GSTCCLMADE
161 SWGCCPMPEA SCCADKIHCC PHGTTCDLVE ARCLETFGEQ PLSKKFPAKK RTPVLNLTQK NICPGNRSSC PDASTCCLLP
241 TGQYGCCPLQ NAVCCSDHLH CCPQGTQCDL KHSICTMTPK GSRPITHLNA GLQIAHDIQC DSEFACPDGN TCCKTTSGSW
321 GCCPLEEAVC CPDQIHCCPK GYQCKLPEGS CLKDGQSIPL VLKIDTSSTT ASEIDSSSTA SSIGRMVQCD PYTACHDGQT
401 CCRLVTGAWG CCLLTQATCC QDQIHCCPSG YNCDSSSDTC LKDGDRVPWV EK**TTAIVGNL PSSR**DVKCDD QFSCADGQTC
481 CMRSGAWACC PLFQAVCCKD QAHCCPHGYT CDVKHSFCTK SGGPSQGISV TPILARQETA IKCDNFSSCK DKETCCKMAS
561 GNWGCCPFPK VSRLQKAWWS AAQTTYIAAP VALFLIQPQA AVKNQNTLCL GHPNCMPRYC KTMAFSTMAV PAARKGRPAA
641 RAFLAVCCED HQHCCPFGYT CNVAAQSCEK QHWPGLADAA GDLLTSSQNA VSSQDVLCGE QHYCHVGQTC CKARRGGWAC
721 CPYEKNFLTC IDKTNKASEV REPAVMTGAT AVLLATIVPC VDITAIERSL SVGTLAMSPH ILLVPMFCSE VPLWVDLFEA
801 LN

**Supporting Peptides:**

| Peptide | Uniq | -10lgP | Mass | Length | ppm | m/z | z | RT | Fraction | Scan | Source File | Area F1 | Area F2a | Area F3a | Area F4 | Area F5 | Area F6a | Area F7a | Area F7b | Area F8 | Area F9a | Area Sample 1F10a | Area F10b | Area F11a | Area F11b | Area F12a | Area F13 | #Feature | #Feature F1 | #Feature F2a | #Feature F3a | #Feature F4 | #Feature F5 | #Feature F6a | #Feature F7a | #Feature F7b | #Feature F8 | #Feature F9a | #Feature Sample 1F10a | #Feature F10b | #Feature F11a | #Feature F11b | #Feature F12a | #Feature F13 | Start | End | PTM | AScore | Found By |
|---|---|---|---|---|---|---|---|---|---|---|---|---|---|---|---|---|---|---|---|---|---|---|---|---|---|---|---|---|---|---|---|---|---|---|---|---|---|---|---|---|---|---|---|---|---|---|---|---|---|
| K.TTAIVGNLPSSR.D | Y | 53.13 | 1214.6619 | 12 | 0.1 | 608.3383 | 2 | 43.88 | 7 | F7:19196 | BuCaTN01_F7a.raw | | | | | | | 3.9332E5 | | | | | | | | | | 1 | 0 | 0 | 0 | 0 | 0 | 0 | 1 | 0 | 0 | 0 | 0 | 0 | 0 | 0 | 0 | 0 | 453 | 464 | | | PEAKS DB |
| total 1 peptides | | | | | | | | | | | | | | | | | | | | | | | | | | | | | | | | | | | | | | | | | | | | | | | | | |

**Best Unique PSM (Scan F7:19196, m/z=608.3383, z=2, RT=43.88, ppm=0.1):**

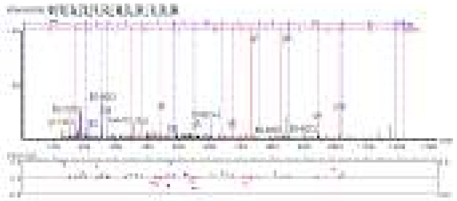

## LAA86163.1

back to list

| Protein Coverage | Supporting Peptides | Best Unique PSM |

**Protein Coverage:**

1 MARMWVHFVV FLVLSTTASS LQCPDGQKCE GSSICCKLPE GDKYTCCGQP QFTGASLRML PPESRNEVSG VVCPDASTCP
81 VTHSCQLSLN FTFACCPWEE AISCVDGHHC CPLGTHCSTD GRVCIENEDS TSAVIVRAVQ CPDSEWECPD GSTCCLMTDE
161 SWGCCPMPEA SCCADKIHCC PHGTTCDLVE ARCLETFGEQ PLSKKFPAKK RTPVLNLTQK NICPGNHSSC PDADTCCLLP
241 TGQYGCCPLQ NAVCCSDHLH CCPQGTQCDL KHSICTITPK GSWPITHLIA GLQTAHDIQC DSEFTCQDGN TCCKTTSGSW
321 GCCPLEEAVC CPDKIHCCPK GYQCNLSEGS CLKDGQSISL VLKIDTSSTT ASEIDSSSTA PSIGRMVQCD PYTACHDGQT
401 CCRLVTGAWG CCLLTQATCC QDQIHCCPSG YNCDSSSGTC LKDRDRIPWV EK**TTAIVGNL PSSR**DVKCDD QFSCADGQTC
481 CMRSGAWACC PLFQAVCCED HQHCCPFGYT CNVAAQSCEK QHWPGLANAA GDLLTSSQNA VSSQDVLCGE QHYCHVGQTC
561 CKAKSGGWAC CPYEKGTCCY DRRHCCPSGF RCSLFGYHCY RKKPLRWDTG NVYPHSIDAH VLL

**Supporting Peptides:**

| Peptide | Uniq | -10lgP | Mass | Length | ppm | m/z | z | RT | Fraction | Scan | Source File | Area F1 | Area F2a | Area F3a | Area F4 | Area F5 | Area F6a | Area F7a | Area F7b | Area F8 | Area F9a | Area Sample 1F10a | Area F10b | Area F11a | Area F11b | Area F12a | Area F13 | #Feature | #Feature F1 | #Feature F2a | #Feature F3a | #Feature F4 | #Feature F5 | #Feature F6a | #Feature F7a | #Feature F7b | #Feature F8 | #Feature F9a | #Feature Sample 1F10a | #Feature F10b | #Feature F11a | #Feature F11b | #Feature F12a | #Feature F13 | Start | End | PTM | AScore | Found By |
|---|---|---|---|---|---|---|---|---|---|---|---|---|---|---|---|---|---|---|---|---|---|---|---|---|---|---|---|---|---|---|---|---|---|---|---|---|---|---|---|---|---|---|---|---|---|---|---|---|---|
| K.TTAIVGNLPSSR.D | Y | 53.13 | 1214.6619 | 12 | 0.1 | 608.3383 | 2 | 43.88 | 7 | F7:19196 | BuCaTN01_F7a.raw | | | | | | | 3.9332E5 | | | | | | | | | | 1 | 0 | 0 | 0 | 0 | 0 | 0 | 1 | 0 | 0 | 0 | 0 | 0 | 0 | 0 | 0 | 0 | 453 | 464 | | | PEAKS DB |
| total 1 peptides | | | | | | | | | | | | | | | | | | | | | | | | | | | | | | | | | | | | | | | | | | | | | | | | | |

**Best Unique PSM (Scan F7:19196, m/z=608.3383, z=2, RT=43.88, ppm=0.1):**

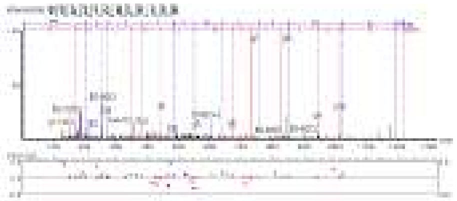

## XP_026574000.1

back to list

| Protein Coverage | Supporting Peptides | Best Unique PSM |

**Protein Coverage:**

1 MARMWVHFIV FLVLSTTASS LKCPDGQKCG GSSICCKLPE GDKYTCCGQP QFTGASLRML PPESRNEVSG VVCPDASTCP
81 VTHSCQLSLN STFACCPWEE AISCVDGHHC CPLGTHCSTD GRVCIENEGI WRQNLTSAVI VRAVQCPDSE WECPDGSTCC
161 LMADESWGCC PMPEASCCAD KIHCCPHGTT CDLVEARCLE TFGEQPLSKK FPAKKRTPVL NLTQKKICPG NHSSCPDAAT
241 CCLLPTGQYG CCPLQNAVCC SDHLHCCPQG TQCDLKHSIC TMTPKESWPI THLSAGFHTA HDVQCDSEFA CPDGNTCCKT
321 TSGSWGCCPL EEATCCQDQI HCCPSGYNCD SSSGTCLKDG DRMPWVEK**TT AIVGNLPSSR** DVKCDDQYSC PDGQTCCLRS
401 GAWACCPLFQ AICCEDHQHC CPFGYTCNVA AQSCEKRHRP RLADAAGDLL TSLQNAASNQ DVLCGEQHYC HVGQTCCKAK
481 NSSWACCPYE KGTCCHDRRH CCPSGFYCSV LGYHCYRKPS RWDTGSASPH SIDAHVLL

**Supporting Peptides:**

| Peptide | Uniq | -10lgP | Mass | Length | ppm | m/z | z | RT | Fraction | Scan | Source File | Area F1 | Area F2a | Area F3a | Area F4 | Area F5 | Area F6a | Area F7a | Area F7b | Area F8 | Area F9a | Area Sample 1F10a | Area F10b | Area F11a | Area F11b | Area F12a | Area F13 | #Feature | #Feature F1 | #Feature F2a | #Feature F3a | #Feature F4 | #Feature F5 | #Feature F6a | #Feature F7a | #Feature F7b | #Feature F8 | #Feature F9a | #Feature Sample 1F10a | #Feature F10b | #Feature F11a | #Feature F11b | #Feature F12a | #Feature F13 | Start | End | PTM | AScore | Found By |
|---|---|---|---|---|---|---|---|---|---|---|---|---|---|---|---|---|---|---|---|---|---|---|---|---|---|---|---|---|---|---|---|---|---|---|---|---|---|---|---|---|---|---|---|---|---|---|---|---|---|
| K.TTAIVGNLPSSR.D | Y | 53.13 | 1214.6619 | 12 | 0.1 | 608.3383 | 2 | 43.88 | 7 | F7:19196 | BuCaTN01_F7a.raw | | | | | | | 3.9332E5 | | | | | | | | | | 1 | 0 | 0 | 0 | 0 | 0 | 0 | 1 | 0 | 0 | 0 | 0 | 0 | 0 | 0 | 0 | 0 | 369 | 380 | | | PEAKS DB |
| total 1 peptides | | | | | | | | | | | | | | | | | | | | | | | | | | | | | | | | | | | | | | | | | | | | | | | | | |

**Best Unique PSM (Scan F7:19196, m/z=608.3383, z=2, RT=43.88, ppm=0.1):**

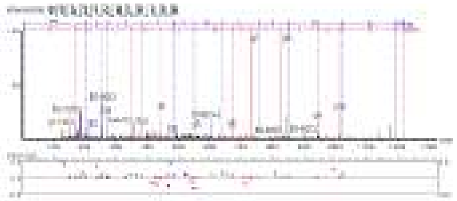

## XP_026539438.1

back to list

| Protein Coverage | Supporting Peptides | Best Unique PSM |

**Protein Coverage:**

1 MARMWVHFIV FLVLSTTASS LKCPDGQKCE GSSICCKLPE GDKYTCCGQP QFTGASLRML PPESRNEVSG VVCPDASTCP
81 VTHSCQLSLN STFACCPWEE ATSCVDGHHC CPLGTHCSTD GRVCIENEGI WRQNSTSAVI VRAVQCPDSE WECPDGSTCC
161 LMADESWGCC PMPEASCCAD KIHCCPHGTT CDLVEARCLE TFGEQPLSKK FPAKKRTPVL NLTQKKICPG NHSSCPDAAT
241 CCLLPTGQYG CCPLQNAVCC SDHLHCCPQG TQCDLKQSKC TMTPKESWPI THLSAGFHTA HDIQCDSEFT CPDGNTCCKT
321 TSGSWGCCPM EEATCCQDQI HCCPSGYNCD SSSGTCLKDG DRMPWVEK**TT AIVGNLPSSR** DVKCDDQYSC PDGQTCCLRS

401 GAWACCPLFQ ATCCEDHQHC CPFGYTCNVA AQSCEKQHRP GLADAAGDLL TSLQNAVSNQ DVLCGEQHYC HVGQTCCKAK
481 SGSWACCPYE KGTCCHDRRH CCPSGFYCSR FGYHCYRKSS RWDTGSVSPH SIDAHVLL

**Supporting Peptides:**

| Peptide | Uniq | -10lgP | Mass | Length | ppm | m/z | z | RT | Fraction | Scan | Source File | Area F1 | Area F2a | Area F3a | Area F4 | Area F5 | Area F6a | Area F7a | Area F7b | Area F8 | Area F9a | Area Sample 1F10a | Area F10b | Area F11a | Area F11b | Area F12a | Area F13 | #Feature | #Feature F1 | #Feature F2a | #Feature F3a | #Feature F4 | #Feature F5 | #Feature F6a | #Feature F7a | #Feature F7b | #Feature F8 | #Feature F9a | #Feature Sample 1F10a | #Feature F10b | #Feature F11a | #Feature F11b | #Feature F12a | #Feature F13 | Start | End | PTM | AScore | Found By |
|---|---|---|---|---|---|---|---|---|---|---|---|---|---|---|---|---|---|---|---|---|---|---|---|---|---|---|---|---|---|---|---|---|---|---|---|---|---|---|---|---|---|---|---|---|---|---|---|---|---|
| K.TTAIVGNLPSSR.D | Y | 53.13 | 1214.6619 | 12 | 0.1 | 608.3383 | 2 | 43.88 | 7 | F7:19196 | BuCaTN01_F7a.raw | | | | | | | 3.9332E5 | | | | | | | | | | 1 | 0 | 0 | 0 | 0 | 0 | 0 | 1 | 0 | 0 | 0 | 0 | 0 | 0 | 0 | 0 | 0 | 369 | 380 | | | PEAKS DB |
| total 1 peptides | | | | | | | | | | | | | | | | | | | | | | | | | | | | | | | | | | | | | | | | | | | | | | | | | |

**Best Unique PSM (Scan F7:19196, m/z=608.3383, z=2, RT=43.88, ppm=0.1):**

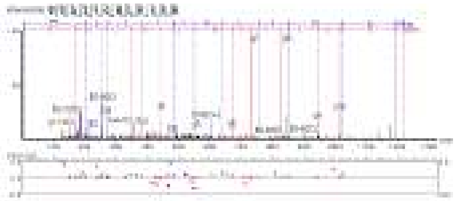

## LAA38501.1

back to list

| Protein Coverage | Supporting Peptides | Best Unique PSM |

**Protein Coverage:**

1 MARMWVHFVV FLVLSTTASS LQCPDGQKCE GSSICCKLPE GDKYTCCGQP QFTGASLRML PPESRNEVSG VACPDASTCP
81 VTHSCQLSLN FTFACCPWEE AISCVDGHHC CPLGTHCSTD GRVCIENEGI WRQDSTSAVI VRAVQCPDSE WECPDGSTCC
161 LMTDESWGCC PMPEASCCAD KIHCCPHGTT CDLVAARCLE TFGEQPLSKK FPAKKRTPVL NLTQKNICPG NHSSCPDTDT
241 CCLLPTGQYG CCPLQNAVCC RDHIHCCPQG TQCDLKHSIC TMTPKGSWPI THLSAGLQTA HDIQCDSEFT CLDGNTCCKT
321 TSGSWGCCPL EEATCCQDQI HCCPSGYNCD SSSGTCRKDG DRIPWVEK**TT AIVGNLPSSR** DVKCDDQFSC ADGQTCCMRS

401 GAWACCPLFQ AVCCEDHQHC CPFGYTCNVA AQSCEKQHWP GLADATGDLL TSSQNAVSSQ DVLCGEQHYC HVGQTCCKAK
481 SGGWACCPYE KGTCCYDRRH CCPSGFHCSL FGYHCYRKKP LRWDTGNVYS HSIDAHVLL

**Supporting Peptides:**

| Peptide | Uniq | -10lgP | Mass | Length | ppm | m/z | z | RT | Fraction | Scan | Source File | Area F1 | Area F2a | Area F3a | Area F4 | Area F5 | Area F6a | Area F7a | Area F7b | Area F8 | Area F9a | Area Sample 1F10a | Area F10b | Area F11a | Area F11b | Area F12a | Area F13 | #Feature | #Feature F1 | #Feature F2a | #Feature F3a | #Feature F4 | #Feature F5 | #Feature F6a | #Feature F7a | #Feature F7b | #Feature F8 | #Feature F9a | #Feature Sample 1F10a | #Feature F10b | #Feature F11a | #Feature F11b | #Feature F12a | #Feature F13 | Start | End | PTM | AScore | Found By |
|---|---|---|---|---|---|---|---|---|---|---|---|---|---|---|---|---|---|---|---|---|---|---|---|---|---|---|---|---|---|---|---|---|---|---|---|---|---|---|---|---|---|---|---|---|---|---|---|---|---|
| K.TTAIVGNLPSSR.D | Y | 53.13 | 1214.6619 | 12 | 0.1 | 608.3383 | 2 | 43.88 | 7 | F7:19196 | BuCaTN01_F7a.raw | | | | | | | 3.9332E5 | | | | | | | | | | 1 | 0 | 0 | 0 | 0 | 0 | 0 | 1 | 0 | 0 | 0 | 0 | 0 | 0 | 0 | 0 | 0 | 369 | 380 | | | PEAKS DB |
| total 1 peptides | | | | | | | | | | | | | | | | | | | | | | | | | | | | | | | | | | | | | | | | | | | | | | | | | |

**Best Unique PSM (Scan F7:19196, m/z=608.3383, z=2, RT=43.88, ppm=0.1):**

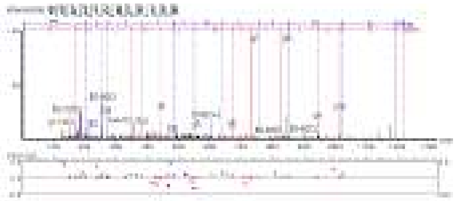

## LAB57775.1

back to list

| Protein Coverage | Supporting Peptides | Best Unique PSM |

**Protein Coverage:**

1 CNLSEGSCLK DGQSISLVLK IDTSSTIASE IDSSSTASSI GRMVQCDPYT ACHDGQTCCR LVTGAWGCCL LTQATCCQDQ
81 IHCCPSGYNC DSSSGTCLKD RDRIPWVEK**T TAIVGNLPSS R**DVKCDDQFS CADGQTCCMR SGAWACCPLF QAVCCEDHQH

161 CCPFGYTCNV AAQSCEKQHW PGLANAAMDL LTSSQNAVSS QDVLCGEQHY CHVGQTCCKA KSGGWACCPY EKGTCCYDRR
241 HCCPSGFHCS VFGYHCYRKK PLRWDTGSVY PHSIDAHVLL

**Supporting Peptides:**

| Peptide | Uniq | -10lgP | Mass | Length | ppm | m/z | z | RT | Fraction | Scan | Source File | Area F1 | Area F2a | Area F3a | Area F4 | Area F5 | Area F6a | Area F7a | Area F7b | Area F8 | Area F9a | Area Sample 1F10a | Area F10b | Area F11a | Area F11b | Area F12a | Area F13 | #Feature | #Feature F1 | #Feature F2a | #Feature F3a | #Feature F4 | #Feature F5 | #Feature F6a | #Feature F7a | #Feature F7b | #Feature F8 | #Feature F9a | #Feature Sample 1F10a | #Feature F10b | #Feature F11a | #Feature F11b | #Feature F12a | #Feature F13 | Start | End | PTM | AScore | Found By |
|---|---|---|---|---|---|---|---|---|---|---|---|---|---|---|---|---|---|---|---|---|---|---|---|---|---|---|---|---|---|---|---|---|---|---|---|---|---|---|---|---|---|---|---|---|---|---|---|---|---|
| K.TTAIVGNLPSSR.D | Y | 53.13 | 1214.6619 | 12 | 0.1 | 608.3383 | 2 | 43.88 | 7 | F7:19196 | BuCaTN01_F7a.raw | | | | | | | 3.9332E5 | | | | | | | | | | 1 | 0 | 0 | 0 | 0 | 0 | 0 | 1 | 0 | 0 | 0 | 0 | 0 | 0 | 0 | 0 | 0 | 110 | 121 | | | PEAKS DB |
| total 1 peptides | | | | | | | | | | | | | | | | | | | | | | | | | | | | | | | | | | | | | | | | | | | | | | | | | |

**Best Unique PSM (Scan F7:19196, m/z=608.3383, z=2, RT=43.88, ppm=0.1):**

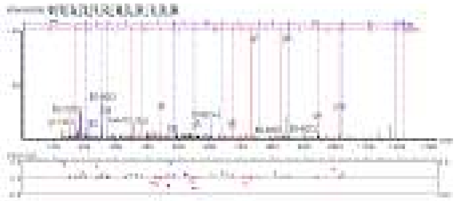

## LAA20302.1

back to list

| Protein Coverage | Supporting Peptides | Best Unique PSM |

**Protein Coverage:**

1 PDADTCCLLP TGQYGCCPLQ NAVCCSDHLH CCPQGTQCDL KHSICTITPK GSWPITHLIA GLQTAHDIQC DSEFTCQDGN
81 TCCKTTSGSW GCCPLEEAVC CPDKIHCCPK GYQCNLSEGS CLKDGQSISL VLKIDTSSTT ASEIDSSSTA SSIGRMVQCD
161 PYTACHDGQT CCRLVTGAWG CCLLTQATCC QDQIHCCPSG YNCDSSSGTC LKDRDRIPWV EK**TTAIVGNL PSSR**DVKCDD

241 QFSCADGQTC CMRSGAWACC PLFQAVCCED HQHCCPFGYT CNVAAQSCEK QHWPGLANAA GDLLTSSQNA VSSQDVLCGE
321 QHYCHVGQTC CKAKSGGWAC CPYEKGTCCY DRRHCCPSGF HCSLFGYHCY RKKPLRW

**Supporting Peptides:**

| Peptide | Uniq | -10lgP | Mass | Length | ppm | m/z | z | RT | Fraction | Scan | Source File | Area F1 | Area F2a | Area F3a | Area F4 | Area F5 | Area F6a | Area F7a | Area F7b | Area F8 | Area F9a | Area Sample 1F10a | Area F10b | Area F11a | Area F11b | Area F12a | Area F13 | #Feature | #Feature F1 | #Feature F2a | #Feature F3a | #Feature F4 | #Feature F5 | #Feature F6a | #Feature F7a | #Feature F7b | #Feature F8 | #Feature F9a | #Feature Sample 1F10a | #Feature F10b | #Feature F11a | #Feature F11b | #Feature F12a | #Feature F13 | Start | End | PTM | AScore | Found By |
|---|---|---|---|---|---|---|---|---|---|---|---|---|---|---|---|---|---|---|---|---|---|---|---|---|---|---|---|---|---|---|---|---|---|---|---|---|---|---|---|---|---|---|---|---|---|---|---|---|---|
| K.TTAIVGNLPSSR.D | Y | 53.13 | 1214.6619 | 12 | 0.1 | 608.3383 | 2 | 43.88 | 7 | F7:19196 | BuCaTN01_F7a.raw | | | | | | | 3.9332E5 | | | | | | | | | | 1 | 0 | 0 | 0 | 0 | 0 | 0 | 1 | 0 | 0 | 0 | 0 | 0 | 0 | 0 | 0 | 0 | 223 | 234 | | | PEAKS DB |
| total 1 peptides | | | | | | | | | | | | | | | | | | | | | | | | | | | | | | | | | | | | | | | | | | | | | | | | | |

**Best Unique PSM (Scan F7:19196, m/z=608.3383, z=2, RT=43.88, ppm=0.1):**

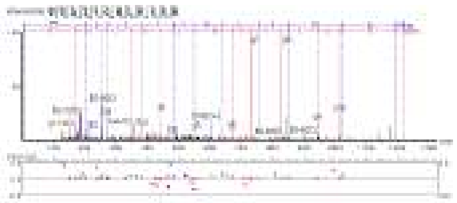

## LAA38500.1

back to list

| Protein Coverage | Supporting Peptides | Best Unique PSM |

**Protein Coverage:**

```
  1 MVQCGPYTAC HDGQTCCRLV TGVWGCCLFT QATCCQDQIH CCPSGYNCDS SSGTCRKDGD RIPWVEKTTA IVGNLPSSRD
 81 VKCDDQFSCA DGQTCCMRSG AWACCPLFQA VCCEDHQHCC PFGYTCNVAA QSCEKQHWPG LADATGDLLT SSQNAVSSQD
161 VLCGEQHYCH VGQTCCKAKS GGWACCPYEK GTCCYDRRHC CPSGFHCSLF GYHCYRKKPL RWDTGNVYSH SIDAHVLL
```

**Supporting Peptides:**

| Peptide | Uniq | -10lgP | Mass | Length | ppm | m/z | z | RT | Fraction | Scan | Source File | Area F1 | Area F2a | Area F3a | Area F4 | Area F5 | Area F6a | Area F7a | Area F7b | Area F8 | Area F9a | Area Sample 1F10a | Area F10b | Area F11a | Area F11b | Area F12a | Area F13 | #Feature | #Feature F1 | #Feature F2a | #Feature F3a | #Feature F4 | #Feature F5 | #Feature F6a | #Feature F7a | #Feature F7b | #Feature F8 | #Feature F9a | #Feature Sample 1F10a | #Feature F10b | #Feature F11a | #Feature F11b | #Feature F12a | #Feature F13 | Start | End | PTM | AScore | Found By |
|---|---|---|---|---|---|---|---|---|---|---|---|---|---|---|---|---|---|---|---|---|---|---|---|---|---|---|---|---|---|---|---|---|---|---|---|---|---|---|---|---|---|---|---|---|---|---|---|---|---|
| K.TTAIVGNLPSSR.D | Y | 53.13 | 1214.6619 | 12 | 0.1 | 608.3383 | 2 | 43.88 | 7 | F7:19196 | BuCaTN01_F7a.raw | | | | | | | 3.9332E5 | | | | | | | | | | 1 | 0 | 0 | 0 | 0 | 0 | 0 | 1 | 0 | 0 | 0 | 0 | 0 | 0 | 0 | 0 | 0 | 68 | 79 | | | PEAKS DB |
| total 1 peptides | | | | | | | | | | | | | | | | | | | | | | | | | | | | | | | | | | | | | | | | | | | | | | | | | |

**Best Unique PSM (Scan F7:19196, m/z=608.3383, z=2, RT=43.88, ppm=0.1):**

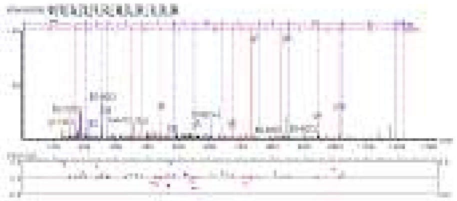

## LAB57770.1

back to list

| Protein Coverage | Supporting Peptides | Best Unique PSM |

**Protein Coverage:**

```
  1 ARMWVHFVVF LVLSTTASSL QCPDGQKCEG SSICCKLPEG DKYTCCGQPQ FTGASLRMLP PESRNEVSGV VCPDASTCPV
 81 THSCQLSLNF TFACCPWEEA ISCVDGHHCC PLGTHCSTDG RVCIENEGIW RQDSTSAVIL RAVQCPDSEW ECPDGSTFTV
161 VHRAPQCDLK HSICTITPKG SWPITHLIAG LQTAHDIQCD SEFTCLDGNT CCKTTSGSWG CCPLEEATCC QDQIHCCPSG
241 YNCDSSSGTC LKDRDRIPWV EKTTAIVGNL PSSRDVKCDD QFSCADGQTC CMRSGAWACC PLFQAVCCED HQHCCPFGYT
321 CNVAAQSCEK QHWPGLANAA MDLLTSSQNA VSSQDVLCGE QHYCHVGQTC CKAKSGGWAC CPYEKGTCCY DRRHCCPSGF
401 HCSVFGYHCY RKKPLRWDTG SVYPHSIDAH VLL
```

**Supporting Peptides:**

| Peptide | Uniq | -10lgP | Mass | Length | ppm | m/z | z | RT | Fraction | Scan | Source File | Area F1 | Area F2a | Area F3a | Area F4 | Area F5 | Area F6a | Area F7a | Area F7b | Area F8 | Area F9a | Area Sample 1F10a | Area F10b | Area F11a | Area F11b | Area F12a | Area F13 | #Feature | #Feature F1 | #Feature F2a | #Feature F3a | #Feature F4 | #Feature F5 | #Feature F6a | #Feature F7a | #Feature F7b | #Feature F8 | #Feature F9a | #Feature Sample 1F10a | #Feature F10b | #Feature F11a | #Feature F11b | #Feature F12a | #Feature F13 | Start | End | PTM | AScore | Found By |
|---|---|---|---|---|---|---|---|---|---|---|---|---|---|---|---|---|---|---|---|---|---|---|---|---|---|---|---|---|---|---|---|---|---|---|---|---|---|---|---|---|---|---|---|---|---|---|---|---|---|
| K.TTAIVGNLPSSR.D | Y | 53.13 | 1214.6619 | 12 | 0.1 | 608.3383 | 2 | 43.88 | 7 | F7:19196 | BuCaTN01_F7a.raw | | | | | | | 3.9332E5 | | | | | | | | | | 1 | 0 | 0 | 0 | 0 | 0 | 0 | 1 | 0 | 0 | 0 | 0 | 0 | 0 | 0 | 0 | 0 | 263 | 274 | | | PEAKS DB |
| total 1 peptides | | | | | | | | | | | | | | | | | | | | | | | | | | | | | | | | | | | | | | | | | | | | | | | | | |

**Best Unique PSM (Scan F7:19196, m/z=608.3383, z=2, RT=43.88, ppm=0.1):**

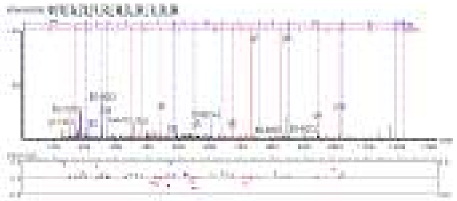

## C0HKZ8.2

back to list

| Protein Coverage | Supporting Peptides | Best Unique PSM |

**Protein Coverage:**

```
  1 MECYKCGVSG CHLKITCSAE EKFCYKWRDK ISNERWLGCA KTCTEENTWR VYNSCCTTNL CNP
```

C Carbamidomethylation (+57.02)
O Oxidation (M) (+15.99)

**Supporting Peptides:**

| Peptide | Uniq | -10lgP | Mass | Length | ppm | m/z | z | RT | Fraction | Scan | Source File | Area F1 | Area F2a | Area F3a | Area F4 | Area F5 | Area F6a | Area F7a | Area F7b | Area F8 | Area F9a | Area Sample 1F10a | Area F10b | Area F11a | Area F11b | Area F12a | Area F13 | #Feature | #Feature F1 | #Feature F2a | #Feature F3a | #Feature F4 | #Feature F5 | #Feature F6a | #Feature F7a | #Feature F7b | #Feature F8 | #Feature F9a | #Feature Sample 1F10a | #Feature F10b | #Feature F11a | #Feature F11b | #Feature F12a | #Feature F13 | Start | End | PTM | AScore | Found By |
|---|---|---|---|---|---|---|---|---|---|---|---|---|---|---|---|---|---|---|---|---|---|---|---|---|---|---|---|---|---|---|---|---|---|---|---|---|---|---|---|---|---|---|---|---|---|---|---|---|---|
| MEC(+57.02)YKC(+57.02)GVSGC(+57.02)HLK.I | Y | 49.41 | 1727.7252 | 14 | -0.2 | 576.9156 | 3 | 32.27 | 6 | F6:13066 | BuCaTN01_F6a.raw | | | | | | 2.5296E5 | | | | | | | | | | | 1 | 0 | 0 | 0 | 0 | 0 | 1 | 0 | 0 | 0 | 0 | 0 | 0 | 0 | 0 | 0 | 0 | 1 | 14 | Carbamidomethylation | C3:Carbamidomethylation:1000.00;C6:Carbamidomethylation:1000.00;C11:Carbamidomethylation:1000.00 | PEAKS DB |
| M(+15.99)EC(+57.02)YKC(+57.02)GVSGC(+57.02)HLK.I | Y | 46.61 | 1743.7201 | 14 | 3.7 | 582.2477 | 3 | 29.58 | 8 | F8:9356 | BuCaTN01_F7b.raw | | | | | | | | 1.4348E5 | | | | | | | | | 1 | 0 | 0 | 0 | 0 | 0 | 0 | 0 | 1 | 0 | 0 | 0 | 0 | 0 | 0 | 0 | 0 | 1 | 14 | Oxidation (M); Carbamidomethylation | M1:Oxidation (M):1000.00;C3:Carbamidomethylation:1000.00;C6:Carbamidomethylation:1000.00;C11:Carbamidomethylation:1000.00 | PEAKS DB |
| total 2 peptides | | | | | | | | | | | | | | | | | | | | | | | | | | | | | | | | | | | | | | | | | | | | | | | | | |

**Best Unique PSM (Scan F6:13066, m/z=576.9156, z=3, RT=32.27, ppm=-0.2):**

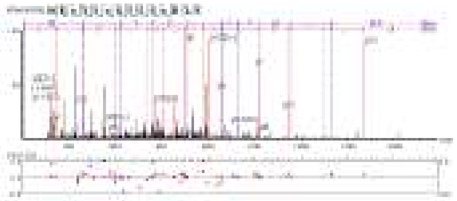

## XP_039188891.1

back to list

| Protein Coverage | Supporting Peptides | Best Unique PSM |

**Protein Coverage:**

1 MAPPPRSLAR PRLVGRAGAF EVCCRPELLD VRLGERKGFF FFFLPLRLSL GPVRSSLPLP PPPLPPLPRR ALPGFSRCGA
81 RLSPPFPPSP ALPYPTPALP GSLPQMSGGS PAGSNARRSP SGSSSSGGGG GGGGGEGSAA AWSAQPSGAV CPALSHLSPE
161 DPLRQANRLP IRILKMLSAH SGHLLHPEYL QPLSSTPVSP IELDAKKSPL ALLAQTCSQI GKPDPPPASK LNSGAAAGLS
241 GPEKESSGRA SGAGAGGSLG KQLGDAPAED KSSFKPYSKG GGGGGGGDPR KDGSLPPSQA GSGAEKNGFR VPSAACPPFP
321 PLSAASAAAS SSSTASSSSP GGSRGNSPQQ ADCRGGPDDK KESEIAAKGS P**DGAPGGGGG GGGGGSR**SGA GSVEPGAQSE
401 ASSGRKSEPP ALAGHVAPVS PYKPGHSVFP LPPSGIGYHG SIVGAYAGYP SQFVPGLDPT KASLVSGGQL PGALGLPGKA
481 SSSSPLTGAS PPSFLQGLCR DPYCLSYHPA SHLGGGNCSS CVHDPGSLKS AYPLVYPSHP LHGALSSSAT PALAGHSLYT
561 YGFMLQNDAL PHICNWVSAG GPCDKRFATS EELLAHLRTH TTLPGAEKLL AGYPTSGLGS GASCHLHLPP AGPGSPGSLP
641 GSLSLRSPQT LGLNRYHPYG KSHLPTPGSL PVASLPAAGP YYSPYALYGQ RLTSASALGY Q

**Supporting Peptides:**

| Peptide | Uniq | -10lgP | Mass | Length | ppm | m/z | z | RT | Fraction | Scan | Source File | Area F1 | Area F2a | Area F3a | Area F4 | Area F5 | Area F6a | Area F7a | Area F7b | Area F8 | Area F9a | Area Sample 1F10a | Area F10b | Area F11a | Area F11b | Area F12a | Area F13 | #Feature | #Feature F1 | #Feature F2a | #Feature F3a | #Feature F4 | #Feature F5 | #Feature F6a | #Feature F7a | #Feature F7b | #Feature F8 | #Feature F9a | #Feature Sample 1F10a | #Feature F10b | #Feature F11a | #Feature F11b | #Feature F12a | #Feature F13 | Start | End | PTM | AScore | Found By |
|---|---|---|---|---|---|---|---|---|---|---|---|---|---|---|---|---|---|---|---|---|---|---|---|---|---|---|---|---|---|---|---|---|---|---|---|---|---|---|---|---|---|---|---|---|---|---|---|---|---|
| P.DGAPGGGGGGGGGGSR.S | Y | 48.76 | 1171.4966 | 16 | -3.6 | 586.7523 | 2 | 47.78 | 3 | F3:23629 | BuCaTN01_F3a.raw | | | 7.2678E4 | | | | | | | | | | | | | | 1 | 0 | 0 | 1 | 0 | 0 | 0 | 0 | 0 | 0 | 0 | 0 | 0 | 0 | 0 | 0 | 0 | 372 | 387 | | | PEAKS DB |
| total 1 peptides | | | | | | | | | | | | | | | | | | | | | | | | | | | | | | | | | | | | | | | | | | | | | | | | | |

**Best Unique PSM (Scan F3:23629, m/z=586.7523, z=2, RT=47.78, ppm=-3.6):**

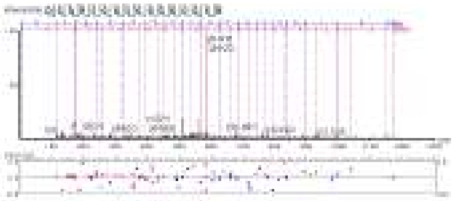

## KAG8122958.1

back to list

| Protein Coverage | Supporting Peptides | Best Unique PSM |

**Protein Coverage:**

1 MYERRILSAG IVVVFSSPGS ETSGEYVEGV SDQDVLLIHS CRQWTTVTAH SLEEGHYVIG PKIDIPLQYP GKFKLLDQDR
81 DVREPVQYFN SVEEVARIFP DRVVSGEFSE DSEVYNFTLH AGDELTLMGQ AEILCAKAAK EKSRLNTLLR **KLGKAGVPGT**
161 **GGGNGGSSNS GSGNTG**GGSG KQLKGKMPCL ICMNHRTNES LSLPFQCKGR FSTRSPLELQ MQEGEHTIRS IIEKVRLPVN
241 VIVPSRPPRN PYDLHAIREG HCYKLVSIVS KTVVLCCILR KDEVAPFHFL LLTDMPCFLL PEGMLRVGGD PQLEKLLRET
321 AALCQECFDP NEYSKAVREA KPDFSEECAS PRHVRLCLQG YSRDELAQSF QRLSLCLYAA SESSCQDPAQ GRRAQRLLLP
401 SHQEPSSLIS DTSDSEREYM TPDWVEPTFR TQELPYEELW TNQNPENYSE SSSTVPGLEN ASKGQRDLIS FGGSPCHRGL
481 LQEDLNMDAP PPVPPKSEAV REECRLLVAP PVPPRGAHTS ATSSPPMPVR FPKLQAAVSP SANLSYYSSG LHDISRPRSG
561 SCSPSPDSYS LYCYPCTWSD CKVGESTSRQ LGSPQAQLPS QPPPAQMSSW SDPWPHNDPG SSVATGRSTP LLGSIDANAI
641 KSYHSCPRLK PPPPQKRFAP FGALNPFSNP AYSSSPASSG SSDWLESLDW QKTTASSIDT FDLFEGSSSP EGHCSPAPPP
721 RSSKPFDTAE NIVIRTSVAP LSSTIVHGSE GGITHLYLAQ GVIEAPPARL NGDGSPGSLL VTYQRCHLRR YLSPCASSAS
801 LRMWSASLPG NALMAVSLYN SLKRSYLRIF ILPNFKSKK

**Supporting Peptides:**

| Peptide | Uniq | -10lgP | Mass | Length | ppm | m/z | z | RT | Fraction | Scan | Source File | Area F1 | Area F2a | Area F3a | Area F4 | Area F5 | Area F6a | Area F7a | Area F7b | Area F8 | Area F9a | Area Sample 1F10a | Area F10b | Area F11a | Area F11b | Area F12a | Area F13 | #Feature | #Feature F1 | #Feature F2a | #Feature F3a | #Feature F4 | #Feature F5 | #Feature F6a | #Feature F7a | #Feature F7b | #Feature F8 | #Feature F9a | #Feature Sample 1F10a | #Feature F10b | #Feature F11a | #Feature F11b | #Feature F12a | #Feature F13 | Start | End | PTM | AScore | Found By |
|---|---|---|---|---|---|---|---|---|---|---|---|---|---|---|---|---|---|---|---|---|---|---|---|---|---|---|---|---|---|---|---|---|---|---|---|---|---|---|---|---|---|---|---|---|---|---|---|---|---|
| R.KLGKAGVPGTGGGNGGSSNSGSGNTG.G | Y | 47.27 | 2174.0312 | 26 | 7.5 | 725.6898 | 3 | 66.86 | 1 | F1:29743 | BuCaTN01_F1.raw | 4.5257E5 | | | | | | | | | | | | | | | | 1 | 1 | 0 | 0 | 0 | 0 | 0 | 0 | 0 | 0 | 0 | 0 | 0 | 0 | 0 | 0 | 0 | 151 | 176 | | | PEAKS DB |
| total 1 peptides | | | | | | | | | | | | | | | | | | | | | | | | | | | | | | | | | | | | | | | | | | | | | | | | | |

**Best Unique PSM (Scan F1:29743, m/z=725.6898, z=3, RT=66.86, ppm=7.5):**

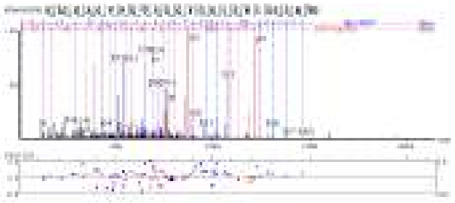

## KAG8137413.1

back to list

| Protein Coverage | Supporting Peptides | Best Unique PSM |

**Protein Coverage:**

1 MEELTAFVSK SFDQKSK**ESS GGGGGGGGGG GGGGGGGGGH KK**ESITYREV LESGLARTRE LGGSDATSLQ ELAEGGPHCP
81 VHLFKEPGES DKDKLKDFNH SSRTSEGVYE CKDKREEVKS EDEDGQTKLK QRRSRTNFTL EQLNELERLF DETHYPDAFM
161 REELSQRLGL SEARVQVWFQ NRRAKCRKQE NQMHKGRCDL GDGQPFRCLP SGSLREYGSP KDAFQQVAYL NFHLQTDLAQ
241 IVVKLVQAQL QLEGVTHAHP HLHPHLAAHA PYLMFPPPPF GLPIASLADS ASAAAVVAAA AKSNSKNSSI ADLRLKARKH
321 AEALGL

**Supporting Peptides:**

| Peptide | Uniq | -10lgP | Mass | Length | ppm | m/z | z | RT | Fraction | Scan | Source File | Area F1 | Area F2a | Area F3a | Area F4 | Area F5 | Area F6a | Area F7a | Area F7b | Area F8 | Area F9a | Area Sample 1F10a | Area F10b | Area F11a | Area F11b | Area F12a | Area F13 | #Feature | #Feature F1 | #Feature F2a | #Feature F3a | #Feature F4 | #Feature F5 | #Feature F6a | #Feature F7a | #Feature F7b | #Feature F8 | #Feature F9a | #Feature Sample 1F10a | #Feature F10b | #Feature F11a | #Feature F11b | #Feature F12a | #Feature F13 | Start | End | PTM | AScore | Found By |
|---|---|---|---|---|---|---|---|---|---|---|---|---|---|---|---|---|---|---|---|---|---|---|---|---|---|---|---|---|---|---|---|---|---|---|---|---|---|---|---|---|---|---|---|---|---|---|---|---|---|
| K.ESSGGGGGGGGGGGGGGGGGGHKK.E | Y | 46.68 | 1797.7738 | 25 | -4.6 | 600.2600 | 3 | 49.64 | 14 | F14:26682 | BuCaTN01_F11b.raw | | | | | | | | | | | | | | 0 | | | 0 | 0 | 0 | 0 | 0 | 0 | 0 | 0 | 0 | 0 | 0 | 0 | 0 | 0 | 0 | 0 | 0 | 18 | 42 | | | PEAKS DB |
| total 1 peptides | | | | | | | | | | | | | | | | | | | | | | | | | | | | | | | | | | | | | | | | | | | | | | | | | |

**Best Unique PSM (Scan F14:26682, m/z=600.2600, z=3, RT=49.64, ppm=-4.6):**

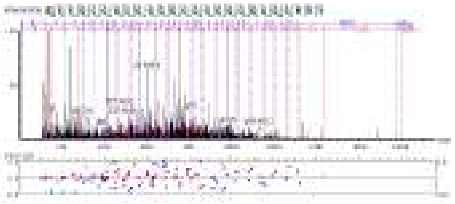

## KAG8138472.1

back to list

| Protein Coverage | Supporting Peptides | Best Unique PSM |

**Protein Coverage:**

1 MLLYFILLSL SWSPMPTSGE VVDSFQDHCP QFFLR**ETPPV IGVPPSPVR**I CQRYQNVYRF ATLYDKHNRI PVYSAYVYNP
81 GKAKRPRTWM VEPQLVNSMF QSEMETERDF LNKKGPKQAL QDSQALLEDY KNWKIATGAI

**Supporting Peptides:**

| Peptide | Uniq | -10lgP | Mass | Length | ppm | m/z | z | RT | Fraction | Scan | Source File | Area F1 | Area F2a | Area F3a | Area F4 | Area F5 | Area F6a | Area F7a | Area F7b | Area F8 | Area F9a | Area Sample 1F10a | Area F10b | Area F11a | Area F11b | Area F12a | Area F13 | #Feature | #Feature F1 | #Feature F2a | #Feature F3a | #Feature F4 | #Feature F5 | #Feature F6a | #Feature F7a | #Feature F7b | #Feature F8 | #Feature F9a | #Feature Sample 1F10a | #Feature F10b | #Feature F11a | #Feature F11b | #Feature F12a | #Feature F13 | Start | End | PTM | AScore | Found By |
|---|---|---|---|---|---|---|---|---|---|---|---|---|---|---|---|---|---|---|---|---|---|---|---|---|---|---|---|---|---|---|---|---|---|---|---|---|---|---|---|---|---|---|---|---|---|---|---|---|---|
| | | | | | | | | | | | | | | | | | | | | | | | | | | | | | | | | | | | | | | | | | | | | | | | | | PEAKS |

| Peptide | Uniq | -10lgP | Mass | Length | ppm | m/z | z | RT | Fraction | Scan | Source File | Area F1 | Area F2a | Area F3a | Area F4 | Area F5 | Area F6a | Area F7a | Area F7b | Area F8 | Area F9a | Area Sample 1F10a | Area F10b | Area F11a | Area F11b | Area F12a | Area F13 | #Feature | #Feature F1 | #Feature F2a | #Feature F3a | #Feature F4 | #Feature F5 | #Feature F6a | #Feature F7a | #Feature F7b | #Feature F8 | #Feature F9a | #Feature Sample 1F10a | #Feature F10b | #Feature F11a | #Feature F11b | #Feature F12a | #Feature F13 | Start | End | PTM | AScore | Found By |
|---|---|---|---|---|---|---|---|---|---|---|---|---|---|---|---|---|---|---|---|---|---|---|---|---|---|---|---|---|---|---|---|---|---|---|---|---|---|---|---|---|---|---|---|---|---|---|---|---|---|
| R.ETPPVIGVPPSPVR.I | Y | 46.62 | 1443.8085 | 14 | -0.1 | 722.9114 | 2 | 54.64 | 1 | F1:23224 | BuCaTN01_F1.raw | 3.8789E5 | | | | | | | | | | | | | | | | 1 | 1 | 0 | 0 | 0 | 0 | 0 | 0 | 0 | 0 | 0 | 0 | 0 | 0 | 0 | 0 | 0 | 36 | 49 | | | DB |
| **total 1 peptides** | | | | | | | | | | | | | | | | | | | | | | | | | | | | | | | | | | | | | | | | | | | | | | | | | |

**Best Unique PSM (Scan F1:23224, m/z=722.9114, z=2, RT=54.64, ppm=-0.1):**

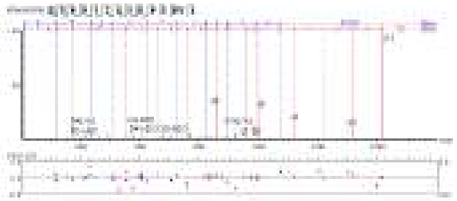

## XP_026569519.1

back to list

| Protein Coverage | Supporting Peptides | Best Unique PSM |

**Protein Coverage:**

1 MPEPAKSAPA PKKGSKKAVT KTQKKGDKKR RKSRKESYSI YVYKVLKQVH PDTGISSKAM SIMNSFVNDI FERIAGEASR
81 LAHYNKRSTI TSREIQTAVR LLLPGELAKH AVSEGTKAVT KYTSSK

**Supporting Peptides:**

| Peptide | Uniq | -10lgP | Mass | Length | ppm | m/z | z | RT | Fraction | Scan | Source File | Area F1 | Area F2a | Area F3a | Area F4 | Area F5 | Area F6a | Area F7a | Area F7b | Area F8 | Area F9a | Area Sample 1F10a | Area F10b | Area F11a | Area F11b | Area F12a | Area F13 | #Feature | #Feature F1 | #Feature F2a | #Feature F3a | #Feature F4 | #Feature F5 | #Feature F6a | #Feature F7a | #Feature F7b | #Feature F8 | #Feature F9a | #Feature Sample 1F10a | #Feature F10b | #Feature F11a | #Feature F11b | #Feature F12a | #Feature F13 | Start | End | PTM | AScore | Found By |
|---|---|---|---|---|---|---|---|---|---|---|---|---|---|---|---|---|---|---|---|---|---|---|---|---|---|---|---|---|---|---|---|---|---|---|---|---|---|---|---|---|---|---|---|---|---|---|---|---|---|
| K.HAVSEGTKAVTKYTSSK | Y | 43.98 | 1792.9319 | 17 | -0.3 | 449.2401 | 4 | 30.74 | 16 | F16:10152 | BuCaTN01_F13.raw | | | | | | | | | | | | | | | | 1.7557E4 | 1 | 0 | 0 | 0 | 0 | 0 | 0 | 0 | 0 | 0 | 0 | 0 | 0 | 0 | 0 | 0 | 1 | 110 | 126 | | | PEAKS DB |
| **total 1 peptides** | | | | | | | | | | | | | | | | | | | | | | | | | | | | | | | | | | | | | | | | | | | | | | | | | |

**Best Unique PSM (Scan F16:10152, m/z=449.2401, z=4, RT=30.74, ppm=-0.3):**

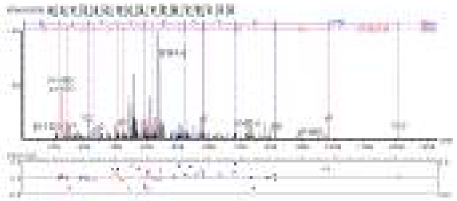

## XP_026576366.1

back to list

| Protein Coverage | Supporting Peptides | Best Unique PSM |

**Protein Coverage:**

1 MPEPAKSAPA PKKGSKKAVT KTQKKGDKKR RKSRKESYSI YVYKVLKQVH PDTGISSKAM SIMNSFVNDI FERIAGEASR
81 LAHYNKRSTI TSREIQTAVR LLLPGELAKH AVSEGTKAVT KYTSSK

**Supporting Peptides:**

| Peptide | Uniq | -10lgP | Mass | Length | ppm | m/z | z | RT | Fraction | Scan | Source File | Area F1 | Area F2a | Area F3a | Area F4 | Area F5 | Area F6a | Area F7a | Area F7b | Area F8 | Area F9a | Area Sample 1F10a | Area F10b | Area F11a | Area F11b | Area F12a | Area F13 | #Feature | #Feature F1 | #Feature F2a | #Feature F3a | #Feature F4 | #Feature F5 | #Feature F6a | #Feature F7a | #Feature F7b | #Feature F8 | #Feature F9a | #Feature Sample 1F10a | #Feature F10b | #Feature F11a | #Feature F11b | #Feature F12a | #Feature F13 | Start | End | PTM | AScore | Found By |
|---|---|---|---|---|---|---|---|---|---|---|---|---|---|---|---|---|---|---|---|---|---|---|---|---|---|---|---|---|---|---|---|---|---|---|---|---|---|---|---|---|---|---|---|---|---|---|---|---|---|
| K.HAVSEGTKAVTKYTSSK | Y | 43.98 | 1792.9319 | 17 | -0.3 | 449.2401 | 4 | 30.74 | 16 | F16:10152 | BuCaTN01_F13.raw | | | | | | | | | | | | | | | | 1.7557E4 | 1 | 0 | 0 | 0 | 0 | 0 | 0 | 0 | 0 | 0 | 0 | 0 | 0 | 0 | 0 | 0 | 1 | 110 | 126 | | | PEAKS DB |
| **total 1 peptides** | | | | | | | | | | | | | | | | | | | | | | | | | | | | | | | | | | | | | | | | | | | | | | | | | |

**Best Unique PSM (Scan F16:10152, m/z=449.2401, z=4, RT=30.74, ppm=-0.3):**

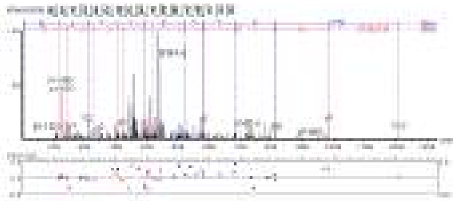

## XP_026569640.1

back to list

| Protein Coverage | Supporting Peptides | Best Unique PSM |

**Protein Coverage:**

1 MPEPAKSAPA PKKGSKKAVT KTQKKGDKKR RKSRKESYSI YVYKVLKQVH PDTGISSKAM SIMNSFVNDI FERIAGEASR
81 LAHYNKRSTI TSREIQTAVR LLLPGELAKH AVSEGTKAVT KYTSSK

**Supporting Peptides:**

| Peptide | Uniq | -10lgP | Mass | Length | ppm | m/z | z | RT | Fraction | Scan | Source File | Area F1 | Area F2a | Area F3a | Area F4 | Area F5 | Area F6a | Area F7a | Area F7b | Area F8 | Area F9a | Area Sample 1F10a | Area F10b | Area F11a | Area F11b | Area F12a | Area F13 | #Feature | #Feature F1 | #Feature F2a | #Feature F3a | #Feature F4 | #Feature F5 | #Feature F6a | #Feature F7a | #Feature F7b | #Feature F8 | #Feature F9a | #Feature Sample 1F10a | #Feature F10b | #Feature F11a | #Feature F11b | #Feature F12a | #Feature F13 | Start | End | PTM | AScore | Found By |
|---|---|---|---|---|---|---|---|---|---|---|---|---|---|---|---|---|---|---|---|---|---|---|---|---|---|---|---|---|---|---|---|---|---|---|---|---|---|---|---|---|---|---|---|---|---|---|---|---|---|
| K.HAVSEGTKAVTKYTSSK | Y | 43.98 | 1792.9319 | 17 | -0.3 | 449.2401 | 4 | 30.74 | 16 | F16:10152 | BuCaTN01_F13.raw | | | | | | | | | | | | | | | | 1.7557E4 | 1 | 0 | 0 | 0 | 0 | 0 | 0 | 0 | 0 | 0 | 0 | 0 | 0 | 0 | 0 | 0 | 1 | 110 | 126 | | | PEAKS DB |
| **total 1 peptides** | | | | | | | | | | | | | | | | | | | | | | | | | | | | | | | | | | | | | | | | | | | | | | | | | |

**Best Unique PSM (Scan F16:10152, m/z=449.2401, z=4, RT=30.74, ppm=-0.3):**

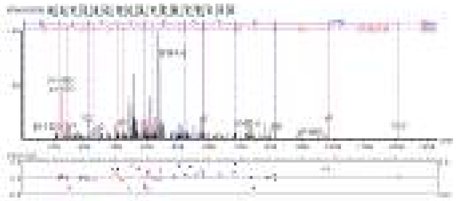

## XP_015678747.1

back to list

| Protein Coverage | Supporting Peptides | Best Unique PSM |

**Protein Coverage:**

1 MPEPAKSAPA PKKGSKKAVT KTQKKGDKKR RKTRKESYSI YVYKVLKQVH PDTGISSKAM SIMNSFVNDI FERIAGEASR
81 LAHYNKRSTI TSREIQTAVR LLLPGELAKH AVSEGTKAVT KYTSSK

**Supporting Peptides:**

| Peptide | Uniq | -10lgP | Mass | Length | ppm | m/z | z | RT | Fraction | Scan | Source File | Area F1 | Area F2a | Area F3a | Area F4 | Area F5 | Area F6a | Area F7a | Area F7b | Area F8 | Area F9a | Area Sample 1F10a | Area F10b | Area F11a | Area F11b | Area F12a | Area F13 | #Feature | #Feature F1 | #Feature F2a | #Feature F3a | #Feature F4 | #Feature F5 | #Feature F6a | #Feature F7a | #Feature F7b | #Feature F8 | #Feature F9a | #Feature Sample 1F10a | #Feature F10b | #Feature F11a | #Feature F11b | #Feature F12a | #Feature F13 | Start | End | PTM | AScore | Found By |
|---|---|---|---|---|---|---|---|---|---|---|---|---|---|---|---|---|---|---|---|---|---|---|---|---|---|---|---|---|---|---|---|---|---|---|---|---|---|---|---|---|---|---|---|---|---|---|---|---|---|
| | | | | | | | | | | | | | | | | | | | | | | | | | | | | | | | | | | | | | | | | | | | | | | | | | PEAKS |

| | | | | | | | | | | | | | | | | | | | | | | | | | | | | | | | | | | | | | | | | | | | | | | | | | | |
|---|---|---|---|---|---|---|---|---|---|---|---|---|---|---|---|---|---|---|---|---|---|---|---|---|---|---|---|---|---|---|---|---|---|---|---|---|---|---|---|---|---|---|---|---|---|---|---|---|---|---|
| K.HAVSEGTKAVTKYTSSK | Y | 43.98 | 1792.9319 | 17 | -0.3 | 449.2401 | 4 | 30.74 | 16 | F16:10152 | BuCaTN01_F13.raw | | | | | | | | | | | | | | | | 1.7557E4 | 1 | 0 | 0 | 0 | 0 | 0 | 0 | 0 | 0 | 0 | 0 | 0 | 0 | 0 | 0 | 0 | 1 | 110 | 126 | | | DB |
| total 1 peptides | | | | | | | | | | | | | | | | | | | | | | | | | | | | | | | | | | | | | | | | | | | | | | | | | | |

**Best Unique PSM (Scan F16:10152, m/z=449.2401, z=4, RT=30.74, ppm=-0.3):**

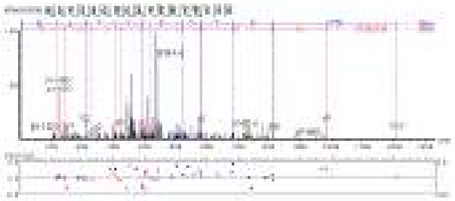

## XP_026529644.1

back to list

| Protein Coverage | Supporting Peptides | Best Unique PSM |

**Protein Coverage:**

1 MPEPAKSAPA PKKGSKKAVT KTQKKGDKKR RKSRKESYSI YVYKVLKQVH PDTGISSKAM SIMNSFVNDI FERIAGEASR
81 LAHYNKRSTI TSREIQTAVR LLLPGELAK**H AVSEGTKAVT KYTSSK**

**Supporting Peptides:**

| Peptide | Uniq | -10lgP | Mass | Length | ppm | m/z | z | RT | Fraction | Scan | Source File | Area F1 | Area F2a | Area F3a | Area F4 | Area F5 | Area F6a | Area F7a | Area F7b | Area F8 | Area F9a | Area Sample 1F10a | Area F10b | Area F11a | Area F11b | Area F12a | Area F13 | #Feature | #Feature F1 | #Feature F2a | #Feature F3a | #Feature F4 | #Feature F5 | #Feature F6a | #Feature F7a | #Feature F7b | #Feature F8 | #Feature F9a | #Feature Sample 1F10a | #Feature F10b | #Feature F11a | #Feature F11b | #Feature F12a | #Feature F13 | Start | End | PTM | AScore | Found By |
|---|---|---|---|---|---|---|---|---|---|---|---|---|---|---|---|---|---|---|---|---|---|---|---|---|---|---|---|---|---|---|---|---|---|---|---|---|---|---|---|---|---|---|---|---|---|---|---|---|---|---|
| K.HAVSEGTKAVTKYTSSK | Y | 43.98 | 1792.9319 | 17 | -0.3 | 449.2401 | 4 | 30.74 | 16 | F16:10152 | BuCaTN01_F13.raw | | | | | | | | | | | | | | | | 1.7557E4 | 1 | 0 | 0 | 0 | 0 | 0 | 0 | 0 | 0 | 0 | 0 | 0 | 0 | 0 | 0 | 0 | 1 | 110 | 126 | | | PEAKS DB |
| total 1 peptides | | | | | | | | | | | | | | | | | | | | | | | | | | | | | | | | | | | | | | | | | | | | | | | | | | |

**Best Unique PSM (Scan F16:10152, m/z=449.2401, z=4, RT=30.74, ppm=-0.3):**

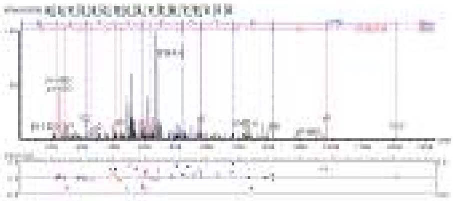

## XP_026539722.1

back to list

| Protein Coverage | Supporting Peptides | Best Unique PSM |

**Protein Coverage:**

1 MPEPAKSAPA PKKGSKKAVT KTQKKGDKKR RKSRKESYSI YVYKVLKQVH PDTGISSKAM SIMNSFVNDI FERIAGEASR
81 LAHYNKRSTI TSREIQTAVR LLLPGELAK**H AVSEGTKAVT KYTSSK**

**Supporting Peptides:**

| Peptide | Uniq | -10lgP | Mass | Length | ppm | m/z | z | RT | Fraction | Scan | Source File | Area F1 | Area F2a | Area F3a | Area F4 | Area F5 | Area F6a | Area F7a | Area F7b | Area F8 | Area F9a | Area Sample 1F10a | Area F10b | Area F11a | Area F11b | Area F12a | Area F13 | #Feature | #Feature F1 | #Feature F2a | #Feature F3a | #Feature F4 | #Feature F5 | #Feature F6a | #Feature F7a | #Feature F7b | #Feature F8 | #Feature F9a | #Feature Sample 1F10a | #Feature F10b | #Feature F11a | #Feature F11b | #Feature F12a | #Feature F13 | Start | End | PTM | AScore | Found By |
|---|---|---|---|---|---|---|---|---|---|---|---|---|---|---|---|---|---|---|---|---|---|---|---|---|---|---|---|---|---|---|---|---|---|---|---|---|---|---|---|---|---|---|---|---|---|---|---|---|---|---|
| K.HAVSEGTKAVTKYTSSK | Y | 43.98 | 1792.9319 | 17 | -0.3 | 449.2401 | 4 | 30.74 | 16 | F16:10152 | BuCaTN01_F13.raw | | | | | | | | | | | | | | | | 1.7557E4 | 1 | 0 | 0 | 0 | 0 | 0 | 0 | 0 | 0 | 0 | 0 | 0 | 0 | 0 | 0 | 0 | 1 | 110 | 126 | | | PEAKS DB |
| total 1 peptides | | | | | | | | | | | | | | | | | | | | | | | | | | | | | | | | | | | | | | | | | | | | | | | | | | |

**Best Unique PSM (Scan F16:10152, m/z=449.2401, z=4, RT=30.74, ppm=-0.3):**

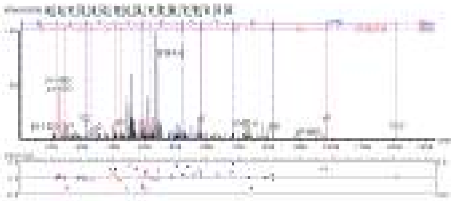

## LAA79020.1

back to list

| Protein Coverage | Supporting Peptides | Best Unique PSM |

**Protein Coverage:**

1 MPEPAKSAPA PKKGSKKAVT KTQKKGDKKR RKSRKESYSI YVYKVLKQVH PDTGISSKAM SIMNSFVNDI FERIAGEASR
81 LAHYNKRSTI TSREIQTAVR LLLPGELAK**H AVSEGTKAVT KYTSSK**

**Supporting Peptides:**

| Peptide | Uniq | -10lgP | Mass | Length | ppm | m/z | z | RT | Fraction | Scan | Source File | Area F1 | Area F2a | Area F3a | Area F4 | Area F5 | Area F6a | Area F7a | Area F7b | Area F8 | Area F9a | Area Sample 1F10a | Area F10b | Area F11a | Area F11b | Area F12a | Area F13 | #Feature | #Feature F1 | #Feature F2a | #Feature F3a | #Feature F4 | #Feature F5 | #Feature F6a | #Feature F7a | #Feature F7b | #Feature F8 | #Feature F9a | #Feature Sample 1F10a | #Feature F10b | #Feature F11a | #Feature F11b | #Feature F12a | #Feature F13 | Start | End | PTM | AScore | Found By |
|---|---|---|---|---|---|---|---|---|---|---|---|---|---|---|---|---|---|---|---|---|---|---|---|---|---|---|---|---|---|---|---|---|---|---|---|---|---|---|---|---|---|---|---|---|---|---|---|---|---|---|
| K.HAVSEGTKAVTKYTSSK | Y | 43.98 | 1792.9319 | 17 | -0.3 | 449.2401 | 4 | 30.74 | 16 | F16:10152 | BuCaTN01_F13.raw | | | | | | | | | | | | | | | | 1.7557E4 | 1 | 0 | 0 | 0 | 0 | 0 | 0 | 0 | 0 | 0 | 0 | 0 | 0 | 0 | 0 | 0 | 1 | 110 | 126 | | | PEAKS DB |
| total 1 peptides | | | | | | | | | | | | | | | | | | | | | | | | | | | | | | | | | | | | | | | | | | | | | | | | | | |

**Best Unique PSM (Scan F16:10152, m/z=449.2401, z=4, RT=30.74, ppm=-0.3):**

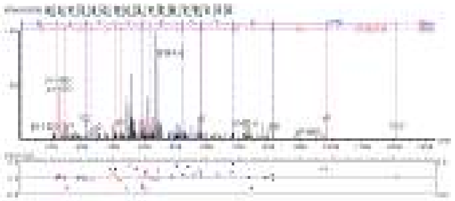

## XP_015678744.1

back to list

| Protein Coverage | Supporting Peptides | Best Unique PSM |

**Protein Coverage:**

1 MPEPAKSAPA PKKGSKKAVT KTQKKGDKKR RKSRKESYSI YVYKVLKQVH PDTGISSKAM SIMNSFVNDI FERIAGEASR
81 LAHYNKRSTI TSREIQTAVR LLLPGELAK**H AVSEGTKAVT KYTSSK**

**Supporting Peptides:**

| Peptide | Uniq | -10lgP | Mass | Length | ppm | m/z | z | RT | Fraction | Scan | Source File | Area F1 | Area F2a | Area F3a | Area F4 | Area F5 | Area F6a | Area F7a | Area F7b | Area F8 | Area F9a | Area Sample 1F10a | Area F10b | Area F11a | Area F11b | Area F12a | Area F13 | #Feature | #Feature F1 | #Feature F2a | #Feature F3a | #Feature F4 | #Feature F5 | #Feature F6a | #Feature F7a | #Feature F7b | #Feature F8 | #Feature F9a | #Feature Sample 1F10a | #Feature F10b | #Feature F11a | #Feature F11b | #Feature F12a | #Feature F13 | Start | End | PTM | AScore | Found By |
|---|---|---|---|---|---|---|---|---|---|---|---|---|---|---|---|---|---|---|---|---|---|---|---|---|---|---|---|---|---|---|---|---|---|---|---|---|---|---|---|---|---|---|---|---|---|---|---|---|---|---|
| | | | | | | | | | | | | | | | | | | | | | | | | | | | | | | | | | | | | | | | | | | | | | | | | | | PEAKS |

| | | | | | | | | | | | | | | | | | | | | | | | | | | | | | | | | | | | | | | | | | | | | | | | | | |
|---|---|---|---|---|---|---|---|---|---|---|---|---|---|---|---|---|---|---|---|---|---|---|---|---|---|---|---|---|---|---|---|---|---|---|---|---|---|---|---|---|---|---|---|---|---|---|---|---|---|
| K.HAVSEGTKAVTKYTSSK | Y | 43.98 | 1792.9319 | 17 | -0.3 | 449.2401 | 4 | 30.74 | 16 | F16:10152 | BuCaTN01_F13.raw | | | | | | | | | | | | | | | | 1.7557E4 | 1 | 0 | 0 | 0 | 0 | 0 | 0 | 0 | 0 | 0 | 0 | 0 | 0 | 0 | 0 | 0 | 1 | 110 | 126 | | | DB |
| total 1 peptides | | | | | | | | | | | | | | | | | | | | | | | | | | | | | | | | | | | | | | | | | | | | | | | | | |

**Best Unique PSM (Scan F16:10152, m/z=449.2401, z=4, RT=30.74, ppm=-0.3):**

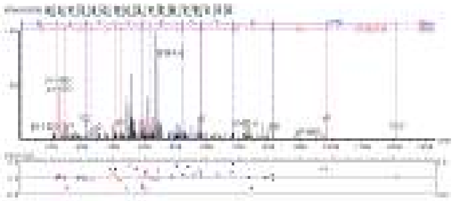

## XP_026529646.1

back to list

| Protein Coverage | Supporting Peptides | Best Unique PSM |

**Protein Coverage:**

1 MPEPAKSAPA PKKGSKKAVT KTQKKGDKKR RKSRKESYSI YVYKVLKQVH PDTGISSKAM SIMNSFVNDI FERIAGEASR
81 LAHYNKRSTI TSREIQTAVR LLLPGELAKH AVSEGTKAVT KYTSSK

**Supporting Peptides:**

| Peptide | Uniq | -10lgP | Mass | Length | ppm | m/z | z | RT | Fraction | Scan | Source File | Area F1 | Area F2a | Area F3a | Area F4 | Area F5 | Area F6a | Area F7a | Area F7b | Area F8 | Area F9a | Area Sample 1F10a | Area F10b | Area F11a | Area F11b | Area F12a | Area F13 | #Feature | #Feature F1 | #Feature F2a | #Feature F3a | #Feature F4 | #Feature F5 | #Feature F6a | #Feature F7a | #Feature F7b | #Feature F8 | #Feature F9a | #Feature Sample 1F10a | #Feature F10b | #Feature F11a | #Feature F11b | #Feature F12a | #Feature F13 | Start | End | PTM | AScore | Found By |
|---|---|---|---|---|---|---|---|---|---|---|---|---|---|---|---|---|---|---|---|---|---|---|---|---|---|---|---|---|---|---|---|---|---|---|---|---|---|---|---|---|---|---|---|---|---|---|---|---|---|
| K.HAVSEGTKAVTKYTSSK | Y | 43.98 | 1792.9319 | 17 | -0.3 | 449.2401 | 4 | 30.74 | 16 | F16:10152 | BuCaTN01_F13.raw | | | | | | | | | | | | | | | | 1.7557E4 | 1 | 0 | 0 | 0 | 0 | 0 | 0 | 0 | 0 | 0 | 0 | 0 | 0 | 0 | 0 | 0 | 1 | 110 | 126 | | | PEAKS DB |
| total 1 peptides | | | | | | | | | | | | | | | | | | | | | | | | | | | | | | | | | | | | | | | | | | | | | | | | | |

**Best Unique PSM (Scan F16:10152, m/z=449.2401, z=4, RT=30.74, ppm=-0.3):**

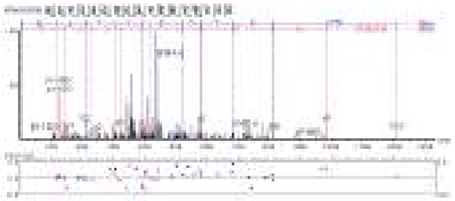

## XP_025022125.1

back to list

| Protein Coverage | Supporting Peptides | Best Unique PSM |

**Protein Coverage:**

1 MPEPAKSAPA PKKGSKKAVT KTQKKGDKKR RKSRKESYSI YVYKVLKQVH PDTGISSKAM SIMNSFVNDI FERIAGEASR
81 LAHYNKRSTI TSREIQTAVR LLLPGELAKH AVSEGTKAVT KYTSSK

**Supporting Peptides:**

| Peptide | Uniq | -10lgP | Mass | Length | ppm | m/z | z | RT | Fraction | Scan | Source File | Area F1 | Area F2a | Area F3a | Area F4 | Area F5 | Area F6a | Area F7a | Area F7b | Area F8 | Area F9a | Area Sample 1F10a | Area F10b | Area F11a | Area F11b | Area F12a | Area F13 | #Feature | #Feature F1 | #Feature F2a | #Feature F3a | #Feature F4 | #Feature F5 | #Feature F6a | #Feature F7a | #Feature F7b | #Feature F8 | #Feature F9a | #Feature Sample 1F10a | #Feature F10b | #Feature F11a | #Feature F11b | #Feature F12a | #Feature F13 | Start | End | PTM | AScore | Found By |
|---|---|---|---|---|---|---|---|---|---|---|---|---|---|---|---|---|---|---|---|---|---|---|---|---|---|---|---|---|---|---|---|---|---|---|---|---|---|---|---|---|---|---|---|---|---|---|---|---|---|
| K.HAVSEGTKAVTKYTSSK | Y | 43.98 | 1792.9319 | 17 | -0.3 | 449.2401 | 4 | 30.74 | 16 | F16:10152 | BuCaTN01_F13.raw | | | | | | | | | | | | | | | | 1.7557E4 | 1 | 0 | 0 | 0 | 0 | 0 | 0 | 0 | 0 | 0 | 0 | 0 | 0 | 0 | 0 | 0 | 1 | 110 | 126 | | | PEAKS DB |
| total 1 peptides | | | | | | | | | | | | | | | | | | | | | | | | | | | | | | | | | | | | | | | | | | | | | | | | | |

**Best Unique PSM (Scan F16:10152, m/z=449.2401, z=4, RT=30.74, ppm=-0.3):**

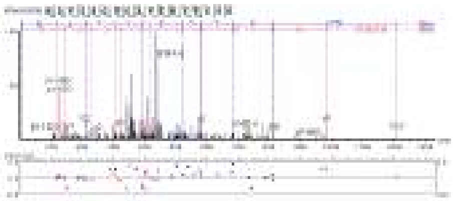

## XP_007429384.1

back to list

| Protein Coverage | Supporting Peptides | Best Unique PSM |

**Protein Coverage:**

1 MPEPAKSAPA PKKGSKKAVT KTQKKGDKKR KKSRKESYSI YVYKVLKQVH PDTAISSKAK SIMTSFFNAI FERIAGEASR
81 LAHRNRMITT HSREIQNFFR LLLPGELAKH AVSEGTKAVT KYTSSK

**Supporting Peptides:**

| Peptide | Uniq | -10lgP | Mass | Length | ppm | m/z | z | RT | Fraction | Scan | Source File | Area F1 | Area F2a | Area F3a | Area F4 | Area F5 | Area F6a | Area F7a | Area F7b | Area F8 | Area F9a | Area Sample 1F10a | Area F10b | Area F11a | Area F11b | Area F12a | Area F13 | #Feature | #Feature F1 | #Feature F2a | #Feature F3a | #Feature F4 | #Feature F5 | #Feature F6a | #Feature F7a | #Feature F7b | #Feature F8 | #Feature F9a | #Feature Sample 1F10a | #Feature F10b | #Feature F11a | #Feature F11b | #Feature F12a | #Feature F13 | Start | End | PTM | AScore | Found By |
|---|---|---|---|---|---|---|---|---|---|---|---|---|---|---|---|---|---|---|---|---|---|---|---|---|---|---|---|---|---|---|---|---|---|---|---|---|---|---|---|---|---|---|---|---|---|---|---|---|---|
| K.HAVSEGTKAVTKYTSSK | Y | 43.98 | 1792.9319 | 17 | -0.3 | 449.2401 | 4 | 30.74 | 16 | F16:10152 | BuCaTN01_F13.raw | | | | | | | | | | | | | | | | 1.7557E4 | 1 | 0 | 0 | 0 | 0 | 0 | 0 | 0 | 0 | 0 | 0 | 0 | 0 | 0 | 0 | 0 | 1 | 110 | 126 | | | PEAKS DB |
| total 1 peptides | | | | | | | | | | | | | | | | | | | | | | | | | | | | | | | | | | | | | | | | | | | | | | | | | |

**Best Unique PSM (Scan F16:10152, m/z=449.2401, z=4, RT=30.74, ppm=-0.3):**

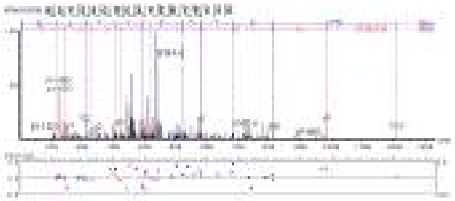

## XP_015672062.1

back to list

| Protein Coverage | Supporting Peptides | Best Unique PSM |

**Protein Coverage:**

1 MPEPAKSAPV PKKGSKKAIT KTQKKGDKKR RKTRKESYSI YVYKVLKQVH PDTGISSKAM SIMNSFVNDI FERIAAEASR
81 LAHYNKRSTI TSREVQTAVR LLLPGELAKH AVSEGTKAVT KYTSSK

**Supporting Peptides:**

| Peptide | Uniq | -10lgP | Mass | Length | ppm | m/z | z | RT | Fraction | Scan | Source File | Area F1 | Area F2a | Area F3a | Area F4 | Area F5 | Area F6a | Area F7a | Area F7b | Area F8 | Area F9a | Area Sample 1F10a | Area F10b | Area F11a | Area F11b | Area F12a | Area F13 | #Feature | #Feature F1 | #Feature F2a | #Feature F3a | #Feature F4 | #Feature F5 | #Feature F6a | #Feature F7a | #Feature F7b | #Feature F8 | #Feature F9a | #Feature Sample 1F10a | #Feature F10b | #Feature F11a | #Feature F11b | #Feature F12a | #Feature F13 | Start | End | PTM | AScore | Found By |
|---|---|---|---|---|---|---|---|---|---|---|---|---|---|---|---|---|---|---|---|---|---|---|---|---|---|---|---|---|---|---|---|---|---|---|---|---|---|---|---|---|---|---|---|---|---|---|---|---|---|
| | | | | | | | | | | | | | | | | | | | | | | | | | | | | | | | | | | | | | | | | | | | | | | | | | PEAKS |

| | | | | | | | | | | | | | | | | | | | | | | | | | | | | | | | | | | | | | | | | | | | | | | | | | |
|---|---|---|---|---|---|---|---|---|---|---|---|---|---|---|---|---|---|---|---|---|---|---|---|---|---|---|---|---|---|---|---|---|---|---|---|---|---|---|---|---|---|---|---|---|---|---|---|---|---|
| K.HAVSEGTKAVTKYTSSK | Y | 43.98 | 1792.9319 | 17 | -0.3 | 449.2401 | 4 | 30.74 | 16 | F16:10152 | BuCaTN01_F13.raw | | | | | | | | | | | | | | | | 1.7557E4 | 1 | 0 | 0 | 0 | 0 | 0 | 0 | 0 | 0 | 0 | 0 | 0 | 0 | 0 | 0 | 0 | 1 | 110 | 126 | | | DB |
| total 1 peptides | | | | | | | | | | | | | | | | | | | | | | | | | | | | | | | | | | | | | | | | | | | | | | | | | |

**Best Unique PSM (Scan F16:10152, m/z=449.2401, z=4, RT=30.74, ppm=-0.3):**

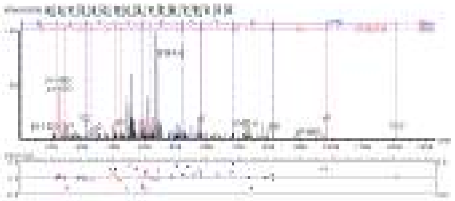

## XP_026545426.1

back to list

| Protein Coverage | Supporting Peptides | Best Unique PSM |

**Protein Coverage:**

1 MPEPAKSAPV PKKGSKKAIT KTQKKGDKKR RKTRKESYSI YVYKVLKQVH PDTGISSKAM SIMNSFVNDI FERIAAEASR
81 LAHYNKRSTI TSREIQTAVR LLLPGELAKH AVSEGTKAVT KYTSSK

**Supporting Peptides:**

| Peptide | Uniq | -10lgP | Mass | Length | ppm | m/z | z | RT | Fraction | Scan | Source File | Area F1 | Area F2a | Area F3a | Area F4 | Area F5 | Area F6a | Area F7a | Area F7b | Area F8 | Area F9a | Area Sample 1F10a | Area F10b | Area F11a | Area F11b | Area F12a | Area F13 | #Feature | #Feature F1 | #Feature F2a | #Feature F3a | #Feature F4 | #Feature F5 | #Feature F6a | #Feature F7a | #Feature F7b | #Feature F8 | #Feature F9a | #Feature Sample 1F10a | #Feature F10b | #Feature F11a | #Feature F11b | #Feature F12a | #Feature F13 | Start | End | PTM | AScore | Found By |
|---|---|---|---|---|---|---|---|---|---|---|---|---|---|---|---|---|---|---|---|---|---|---|---|---|---|---|---|---|---|---|---|---|---|---|---|---|---|---|---|---|---|---|---|---|---|---|---|---|---|
| K.HAVSEGTKAVTKYTSSK | Y | 43.98 | 1792.9319 | 17 | -0.3 | 449.2401 | 4 | 30.74 | 16 | F16:10152 | BuCaTN01_F13.raw | | | | | | | | | | | | | | | | 1.7557E4 | 1 | 0 | 0 | 0 | 0 | 0 | 0 | 0 | 0 | 0 | 0 | 0 | 0 | 0 | 0 | 0 | 1 | 110 | 126 | | | PEAKS DB |
| total 1 peptides | | | | | | | | | | | | | | | | | | | | | | | | | | | | | | | | | | | | | | | | | | | | | | | | | |

**Best Unique PSM (Scan F16:10152, m/z=449.2401, z=4, RT=30.74, ppm=-0.3):**

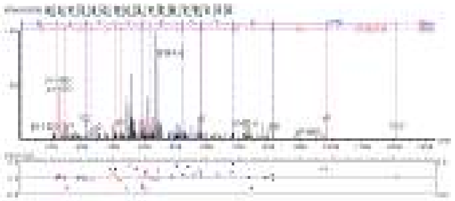

## XP_026547833.1

back to list

| Protein Coverage | Supporting Peptides | Best Unique PSM |

**Protein Coverage:**

1 MPEPAKSAPV PKKGSKKAIT KTQKKGDKKR KKSRKESYSI YVYKVLKQVH PDTGISSKAM SIMNSFVNDI FERIAAEASR
81 LAHYNKRSTI TSREIQTAVR LLLPGELAKH AVSEGTKAVT KYTSSK

**Supporting Peptides:**

| Peptide | Uniq | -10lgP | Mass | Length | ppm | m/z | z | RT | Fraction | Scan | Source File | Area F1 | Area F2a | Area F3a | Area F4 | Area F5 | Area F6a | Area F7a | Area F7b | Area F8 | Area F9a | Area Sample 1F10a | Area F10b | Area F11a | Area F11b | Area F12a | Area F13 | #Feature | #Feature F1 | #Feature F2a | #Feature F3a | #Feature F4 | #Feature F5 | #Feature F6a | #Feature F7a | #Feature F7b | #Feature F8 | #Feature F9a | #Feature Sample 1F10a | #Feature F10b | #Feature F11a | #Feature F11b | #Feature F12a | #Feature F13 | Start | End | PTM | AScore | Found By |
|---|---|---|---|---|---|---|---|---|---|---|---|---|---|---|---|---|---|---|---|---|---|---|---|---|---|---|---|---|---|---|---|---|---|---|---|---|---|---|---|---|---|---|---|---|---|---|---|---|---|
| K.HAVSEGTKAVTKYTSSK | Y | 43.98 | 1792.9319 | 17 | -0.3 | 449.2401 | 4 | 30.74 | 16 | F16:10152 | BuCaTN01_F13.raw | | | | | | | | | | | | | | | | 1.7557E4 | 1 | 0 | 0 | 0 | 0 | 0 | 0 | 0 | 0 | 0 | 0 | 0 | 0 | 0 | 0 | 0 | 1 | 110 | 126 | | | PEAKS DB |
| total 1 peptides | | | | | | | | | | | | | | | | | | | | | | | | | | | | | | | | | | | | | | | | | | | | | | | | | |

**Best Unique PSM (Scan F16:10152, m/z=449.2401, z=4, RT=30.74, ppm=-0.3):**

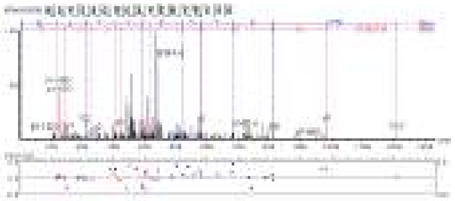

## XP_015672174.1

back to list

| Protein Coverage | Supporting Peptides | Best Unique PSM |

**Protein Coverage:**

1 MPEPAKSAPA AKKGSKKAIT KTQKKGDKKR RKTRKESYSI YVYKVLKQVH PDTGISSKAM SIMNSFVNDI FERIAAEASR
81 LAHYNKRSTI TSREIQTAVR LLLPGELAKH AVSEGTKAVT KYTSSK

**Supporting Peptides:**

| Peptide | Uniq | -10lgP | Mass | Length | ppm | m/z | z | RT | Fraction | Scan | Source File | Area F1 | Area F2a | Area F3a | Area F4 | Area F5 | Area F6a | Area F7a | Area F7b | Area F8 | Area F9a | Area Sample 1F10a | Area F10b | Area F11a | Area F11b | Area F12a | Area F13 | #Feature | #Feature F1 | #Feature F2a | #Feature F3a | #Feature F4 | #Feature F5 | #Feature F6a | #Feature F7a | #Feature F7b | #Feature F8 | #Feature F9a | #Feature Sample 1F10a | #Feature F10b | #Feature F11a | #Feature F11b | #Feature F12a | #Feature F13 | Start | End | PTM | AScore | Found By |
|---|---|---|---|---|---|---|---|---|---|---|---|---|---|---|---|---|---|---|---|---|---|---|---|---|---|---|---|---|---|---|---|---|---|---|---|---|---|---|---|---|---|---|---|---|---|---|---|---|---|
| K.HAVSEGTKAVTKYTSSK | Y | 43.98 | 1792.9319 | 17 | -0.3 | 449.2401 | 4 | 30.74 | 16 | F16:10152 | BuCaTN01_F13.raw | | | | | | | | | | | | | | | | 1.7557E4 | 1 | 0 | 0 | 0 | 0 | 0 | 0 | 0 | 0 | 0 | 0 | 0 | 0 | 0 | 0 | 0 | 1 | 110 | 126 | | | PEAKS DB |
| total 1 peptides | | | | | | | | | | | | | | | | | | | | | | | | | | | | | | | | | | | | | | | | | | | | | | | | | |

**Best Unique PSM (Scan F16:10152, m/z=449.2401, z=4, RT=30.74, ppm=-0.3):**

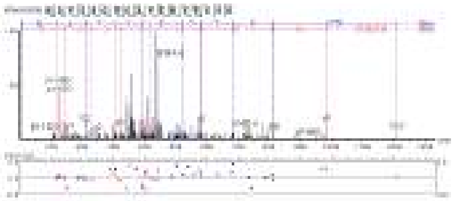

## XP_026576334.1

back to list

| Protein Coverage | Supporting Peptides | Best Unique PSM |

**Protein Coverage:**

1 MPEPAKSAPV PKKGSKKAIT KTQKKGDKKR KKSRKESYSI YVYKVLKQVH PDTGISSKAM SIMNSFVNDI FERIAAEASR
81 LAHYNKRSTI TSREIQTAVR LLLPGELAKH AVSEGTKAVT KYTSSK

**Supporting Peptides:**

| Peptide | Uniq | -10lgP | Mass | Length | ppm | m/z | z | RT | Fraction | Scan | Source File | Area F1 | Area F2a | Area F3a | Area F4 | Area F5 | Area F6a | Area F7a | Area F7b | Area F8 | Area F9a | Area Sample 1F10a | Area F10b | Area F11a | Area F11b | Area F12a | Area F13 | #Feature | #Feature F1 | #Feature F2a | #Feature F3a | #Feature F4 | #Feature F5 | #Feature F6a | #Feature F7a | #Feature F7b | #Feature F8 | #Feature F9a | #Feature Sample 1F10a | #Feature F10b | #Feature F11a | #Feature F11b | #Feature F12a | #Feature F13 | Start | End | PTM | AScore | Found By |
|---|---|---|---|---|---|---|---|---|---|---|---|---|---|---|---|---|---|---|---|---|---|---|---|---|---|---|---|---|---|---|---|---|---|---|---|---|---|---|---|---|---|---|---|---|---|---|---|---|---|
| | | | | | | | | | | | | | | | | | | | | | | | | | | | | | | | | | | | | | | | | | | | | | | | | | PEAKS |

| | | | | | | | | | | | | | | | | | | | | | | | | | | | | | | | | | | | | | | | | | | | | | | | | | |
|---|---|---|---|---|---|---|---|---|---|---|---|---|---|---|---|---|---|---|---|---|---|---|---|---|---|---|---|---|---|---|---|---|---|---|---|---|---|---|---|---|---|---|---|---|---|---|---|---|---|
| K.HAVSEGTKAVTKYTSSK | Y | 43.98 | 1792.9319 | 17 | -0.3 | 449.2401 | 4 | 30.74 | 16 | F16:10152 | BuCaTN01_F13.raw | | | | | | | | | | | | | | | | 1.7557E4 | 1 | 0 | 0 | 0 | 0 | 0 | 0 | 0 | 0 | 0 | 0 | 0 | 0 | 0 | 0 | 0 | 1 | 110 | 126 | | | DB |
| total 1 peptides | | | | | | | | | | | | | | | | | | | | | | | | | | | | | | | | | | | | | | | | | | | | | | | | | |

**Best Unique PSM (Scan F16:10152, m/z=449.2401, z=4, RT=30.74, ppm=-0.3):**

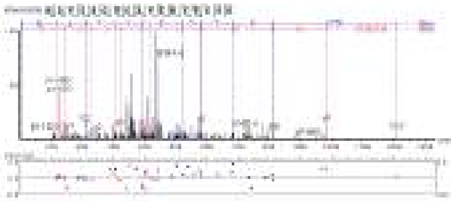

## ETE56555.1

back to list

| Protein Coverage | Supporting Peptides | Best Unique PSM |

**Protein Coverage:**

```
 1 MPEPAKSAPV PKKGSKKAIT KTQKKGDKKR KKSRKESYSI YVYKVLKQVH PDTGISSKAM SIMNSFVNDI FERIAAEASR
81 LAHYNKRSTI TSREIQTAVR LLLPGELAKH AVSEGTKAVT KYTSSK
```

**Supporting Peptides:**

| Peptide | Uniq | -10lgP | Mass | Length | ppm | m/z | z | RT | Fraction | Scan | Source File | Area F1 | Area F2a | Area F3a | Area F4 | Area F5 | Area F6a | Area F7a | Area F7b | Area F8 | Area F9a | Area Sample 1F10a | Area F10b | Area F11a | Area F11b | Area F12a | Area F13 | #Feature | #Feature F1 | #Feature F2a | #Feature F3a | #Feature F4 | #Feature F5 | #Feature F6a | #Feature F7a | #Feature F7b | #Feature F8 | #Feature F9a | #Feature Sample 1F10a | #Feature F10b | #Feature F11a | #Feature F11b | #Feature F12a | #Feature F13 | Start | End | PTM | AScore | Found By |
|---|---|---|---|---|---|---|---|---|---|---|---|---|---|---|---|---|---|---|---|---|---|---|---|---|---|---|---|---|---|---|---|---|---|---|---|---|---|---|---|---|---|---|---|---|---|---|---|---|---|
| K.HAVSEGTKAVTKYTSSK | Y | 43.98 | 1792.9319 | 17 | -0.3 | 449.2401 | 4 | 30.74 | 16 | F16:10152 | BuCaTN01_F13.raw | | | | | | | | | | | | | | | | 1.7557E4 | 1 | 0 | 0 | 0 | 0 | 0 | 0 | 0 | 0 | 0 | 0 | 0 | 0 | 0 | 0 | 0 | 1 | 110 | 126 | | | PEAKS DB |
| total 1 peptides | | | | | | | | | | | | | | | | | | | | | | | | | | | | | | | | | | | | | | | | | | | | | | | | | |

**Best Unique PSM (Scan F16:10152, m/z=449.2401, z=4, RT=30.74, ppm=-0.3):**

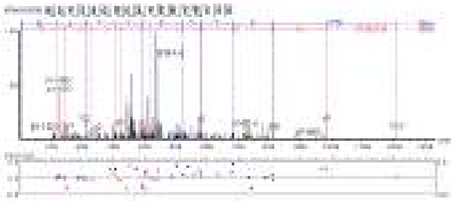

## XP_013925621.1

back to list

| Protein Coverage | Supporting Peptides | Best Unique PSM |

**Protein Coverage:**

```
 1 MPEPAKSAPV PKKGSKKAIT KTQKKGDKKR RKSRKESYSI YVYKVLKQVH PDTGISSKAM SIMNSFVNDI FERIAAEASR
81 LAHYNKRSTI TSREIQTAVR LLLPGELAKH AVSEGTKAVT KYTSSK
```

**Supporting Peptides:**

| Peptide | Uniq | -10lgP | Mass | Length | ppm | m/z | z | RT | Fraction | Scan | Source File | Area F1 | Area F2a | Area F3a | Area F4 | Area F5 | Area F6a | Area F7a | Area F7b | Area F8 | Area F9a | Area Sample 1F10a | Area F10b | Area F11a | Area F11b | Area F12a | Area F13 | #Feature | #Feature F1 | #Feature F2a | #Feature F3a | #Feature F4 | #Feature F5 | #Feature F6a | #Feature F7a | #Feature F7b | #Feature F8 | #Feature F9a | #Feature Sample 1F10a | #Feature F10b | #Feature F11a | #Feature F11b | #Feature F12a | #Feature F13 | Start | End | PTM | AScore | Found By |
|---|---|---|---|---|---|---|---|---|---|---|---|---|---|---|---|---|---|---|---|---|---|---|---|---|---|---|---|---|---|---|---|---|---|---|---|---|---|---|---|---|---|---|---|---|---|---|---|---|---|
| K.HAVSEGTKAVTKYTSSK | Y | 43.98 | 1792.9319 | 17 | -0.3 | 449.2401 | 4 | 30.74 | 16 | F16:10152 | BuCaTN01_F13.raw | | | | | | | | | | | | | | | | 1.7557E4 | 1 | 0 | 0 | 0 | 0 | 0 | 0 | 0 | 0 | 0 | 0 | 0 | 0 | 0 | 0 | 0 | 1 | 110 | 126 | | | PEAKS DB |
| total 1 peptides | | | | | | | | | | | | | | | | | | | | | | | | | | | | | | | | | | | | | | | | | | | | | | | | | |

**Best Unique PSM (Scan F16:10152, m/z=449.2401, z=4, RT=30.74, ppm=-0.3):**

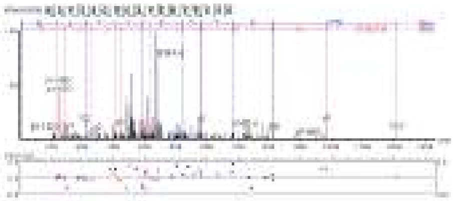

## XP_007429385.1

back to list

| Protein Coverage | Supporting Peptides | Best Unique PSM |

**Protein Coverage:**

```
 1 MPEPAKSAPV PKKGSKKAIT KTQKKGDKKR KKSRKESYSI YVYKVLKQVH PDTGISSKAM SIMNSFVNDI FERIAAEASR
81 LAHYNKRSTI TSREVQTAVR LLLPGELAKH AVSEGTKAVT KYTSSK
```

**Supporting Peptides:**

| Peptide | Uniq | -10lgP | Mass | Length | ppm | m/z | z | RT | Fraction | Scan | Source File | Area F1 | Area F2a | Area F3a | Area F4 | Area F5 | Area F6a | Area F7a | Area F7b | Area F8 | Area F9a | Area Sample 1F10a | Area F10b | Area F11a | Area F11b | Area F12a | Area F13 | #Feature | #Feature F1 | #Feature F2a | #Feature F3a | #Feature F4 | #Feature F5 | #Feature F6a | #Feature F7a | #Feature F7b | #Feature F8 | #Feature F9a | #Feature Sample 1F10a | #Feature F10b | #Feature F11a | #Feature F11b | #Feature F12a | #Feature F13 | Start | End | PTM | AScore | Found By |
|---|---|---|---|---|---|---|---|---|---|---|---|---|---|---|---|---|---|---|---|---|---|---|---|---|---|---|---|---|---|---|---|---|---|---|---|---|---|---|---|---|---|---|---|---|---|---|---|---|---|
| K.HAVSEGTKAVTKYTSSK | Y | 43.98 | 1792.9319 | 17 | -0.3 | 449.2401 | 4 | 30.74 | 16 | F16:10152 | BuCaTN01_F13.raw | | | | | | | | | | | | | | | | 1.7557E4 | 1 | 0 | 0 | 0 | 0 | 0 | 0 | 0 | 0 | 0 | 0 | 0 | 0 | 0 | 0 | 0 | 1 | 110 | 126 | | | PEAKS DB |
| total 1 peptides | | | | | | | | | | | | | | | | | | | | | | | | | | | | | | | | | | | | | | | | | | | | | | | | | |

**Best Unique PSM (Scan F16:10152, m/z=449.2401, z=4, RT=30.74, ppm=-0.3):**

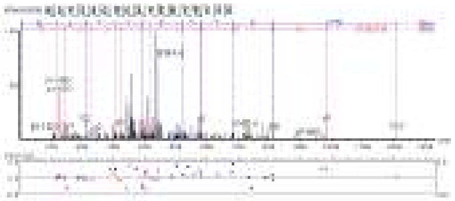

## XP_026580816.1

back to list

| Protein Coverage | Supporting Peptides | Best Unique PSM |

**Protein Coverage:**

```
 1 MPEPAKSAPV PKKGSKKAIT KTQKKGDKKR RKTRKESYSI YVYKVLKQVH PDTGISSKAM SIMNSFVNDI FERIAAEASR
81 LAHYNKRSTI TSREIQTAVR LLLPGELAKH AVSEGTKAVT KYTSSK
```

**Supporting Peptides:**

| Peptide | Uniq | -10lgP | Mass | Length | ppm | m/z | z | RT | Fraction | Scan | Source File | Area F1 | Area F2a | Area F3a | Area F4 | Area F5 | Area F6a | Area F7a | Area F7b | Area F8 | Area F9a | Area Sample 1F10a | Area F10b | Area F11a | Area F11b | Area F12a | Area F13 | #Feature | #Feature F1 | #Feature F2a | #Feature F3a | #Feature F4 | #Feature F5 | #Feature F6a | #Feature F7a | #Feature F7b | #Feature F8 | #Feature F9a | #Feature Sample 1F10a | #Feature F10b | #Feature F11a | #Feature F11b | #Feature F12a | #Feature F13 | Start | End | PTM | AScore | Found By |
|---|---|---|---|---|---|---|---|---|---|---|---|---|---|---|---|---|---|---|---|---|---|---|---|---|---|---|---|---|---|---|---|---|---|---|---|---|---|---|---|---|---|---|---|---|---|---|---|---|---|
| | | | | | | | | | | | | | | | | | | | | | | | | | | | | | | | | | | | | | | | | | | | | | | | | | PEAKS |

| | | | | | | | | | | | | | | | | | | | | | | | | | | | | | | | | | | | | | | | | | | | | | | | | | |
|---|---|---|---|---|---|---|---|---|---|---|---|---|---|---|---|---|---|---|---|---|---|---|---|---|---|---|---|---|---|---|---|---|---|---|---|---|---|---|---|---|---|---|---|---|---|---|---|---|---|
| K.HAVSEGTKAVTKYTSSK | Y | 43.98 | 1792.9319 | 17 | -0.3 | 449.2401 | 4 | 30.74 | 16 | F16:10152 | BuCaTN01_F13.raw | | | | | | | | | | | | | | | | 1.7557E4 | 1 | 0 | 0 | 0 | 0 | 0 | 0 | 0 | 0 | 0 | 0 | 0 | 0 | 0 | 0 | 0 | 1 | 110 | 126 | | | DB |
| total 1 peptides | | | | | | | | | | | | | | | | | | | | | | | | | | | | | | | | | | | | | | | | | | | | | | | | | |

**Best Unique PSM (Scan F16:10152, m/z=449.2401, z=4, RT=30.74, ppm=-0.3):**

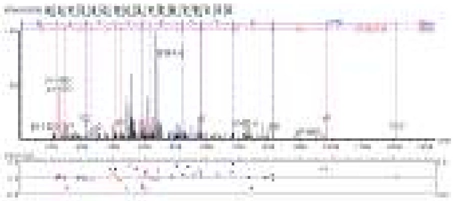

## XP_026548170.1

back to list

| **Protein Coverage** | **Supporting Peptides** | **Best Unique PSM** |

**Protein Coverage:**

1 MPEPAKSAPV PKKGSKKAIT KTQKKGDKKR KKSRKESYSI YVYKVLKQVH PDTGISSKAM SIMNSFVNDI FERIAAEASR
81 LAHYNKRSTI TSREVQTAVR LLLPGELAKH AVSEGTKAVT KYTSSK

**Supporting Peptides:**

| Peptide | Uniq | -10lgP | Mass | Length | ppm | m/z | z | RT | Fraction | Scan | Source File | Area F1 | Area F2a | Area F3a | Area F4 | Area F5 | Area F6a | Area F7a | Area F7b | Area F8 | Area F9a | Area Sample 1F10a | Area F10b | Area F11a | Area F11b | Area F12a | Area F13 | #Feature | #Feature F1 | #Feature F2a | #Feature F3a | #Feature F4 | #Feature F5 | #Feature F6a | #Feature F7a | #Feature F7b | #Feature F8 | #Feature F9a | #Feature Sample 1F10a | #Feature F10b | #Feature F11a | #Feature F11b | #Feature F12a | #Feature F13 | Start | End | PTM | AScore | Found By |
|---|---|---|---|---|---|---|---|---|---|---|---|---|---|---|---|---|---|---|---|---|---|---|---|---|---|---|---|---|---|---|---|---|---|---|---|---|---|---|---|---|---|---|---|---|---|---|---|---|---|
| K.HAVSEGTKAVTKYTSSK | Y | 43.98 | 1792.9319 | 17 | -0.3 | 449.2401 | 4 | 30.74 | 16 | F16:10152 | BuCaTN01_F13.raw | | | | | | | | | | | | | | | | 1.7557E4 | 1 | 0 | 0 | 0 | 0 | 0 | 0 | 0 | 0 | 0 | 0 | 0 | 0 | 0 | 0 | 0 | 1 | 110 | 126 | | | PEAKS DB |
| total 1 peptides | | | | | | | | | | | | | | | | | | | | | | | | | | | | | | | | | | | | | | | | | | | | | | | | | |

**Best Unique PSM (Scan F16:10152, m/z=449.2401, z=4, RT=30.74, ppm=-0.3):**

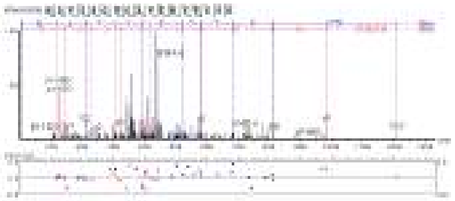

## XP_026576364.1

back to list

| **Protein Coverage** | **Supporting Peptides** | **Best Unique PSM** |

**Protein Coverage:**

1 MPEPAKSAPA PKKGSKKAIT KTQKKGDKKR KKSRKESYSI YVYKVLKQVH PDTGISSKAM SIMNSFVNDI FERIAAEASR
81 LAHYNKRSTI TSREIQTAVR LLLPGELAKH AVSEGTKAVT KYTSSK

**Supporting Peptides:**

| Peptide | Uniq | -10lgP | Mass | Length | ppm | m/z | z | RT | Fraction | Scan | Source File | Area F1 | Area F2a | Area F3a | Area F4 | Area F5 | Area F6a | Area F7a | Area F7b | Area F8 | Area F9a | Area Sample 1F10a | Area F10b | Area F11a | Area F11b | Area F12a | Area F13 | #Feature | #Feature F1 | #Feature F2a | #Feature F3a | #Feature F4 | #Feature F5 | #Feature F6a | #Feature F7a | #Feature F7b | #Feature F8 | #Feature F9a | #Feature Sample 1F10a | #Feature F10b | #Feature F11a | #Feature F11b | #Feature F12a | #Feature F13 | Start | End | PTM | AScore | Found By |
|---|---|---|---|---|---|---|---|---|---|---|---|---|---|---|---|---|---|---|---|---|---|---|---|---|---|---|---|---|---|---|---|---|---|---|---|---|---|---|---|---|---|---|---|---|---|---|---|---|---|
| K.HAVSEGTKAVTKYTSSK | Y | 43.98 | 1792.9319 | 17 | -0.3 | 449.2401 | 4 | 30.74 | 16 | F16:10152 | BuCaTN01_F13.raw | | | | | | | | | | | | | | | | 1.7557E4 | 1 | 0 | 0 | 0 | 0 | 0 | 0 | 0 | 0 | 0 | 0 | 0 | 0 | 0 | 0 | 0 | 1 | 110 | 126 | | | PEAKS DB |
| total 1 peptides | | | | | | | | | | | | | | | | | | | | | | | | | | | | | | | | | | | | | | | | | | | | | | | | | |

**Best Unique PSM (Scan F16:10152, m/z=449.2401, z=4, RT=30.74, ppm=-0.3):**

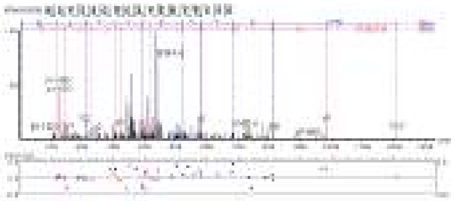

## XP_026545427.1

back to list

| **Protein Coverage** | **Supporting Peptides** | **Best Unique PSM** |

**Protein Coverage:**

1 MPEPAKSAPV PKKGSKKAIT KTQKKGDKKR RKTRKESYSI YVYKVLKQVH PDTGISSKAM SIMNSFVNDI FERIAAEASR
81 LAHYNKRSTI TSREIQTAVR LLLPGELAKH AVSEGTKAVT KYTSSK

**Supporting Peptides:**

| Peptide | Uniq | -10lgP | Mass | Length | ppm | m/z | z | RT | Fraction | Scan | Source File | Area F1 | Area F2a | Area F3a | Area F4 | Area F5 | Area F6a | Area F7a | Area F7b | Area F8 | Area F9a | Area Sample 1F10a | Area F10b | Area F11a | Area F11b | Area F12a | Area F13 | #Feature | #Feature F1 | #Feature F2a | #Feature F3a | #Feature F4 | #Feature F5 | #Feature F6a | #Feature F7a | #Feature F7b | #Feature F8 | #Feature F9a | #Feature Sample 1F10a | #Feature F10b | #Feature F11a | #Feature F11b | #Feature F12a | #Feature F13 | Start | End | PTM | AScore | Found By |
|---|---|---|---|---|---|---|---|---|---|---|---|---|---|---|---|---|---|---|---|---|---|---|---|---|---|---|---|---|---|---|---|---|---|---|---|---|---|---|---|---|---|---|---|---|---|---|---|---|---|
| K.HAVSEGTKAVTKYTSSK | Y | 43.98 | 1792.9319 | 17 | -0.3 | 449.2401 | 4 | 30.74 | 16 | F16:10152 | BuCaTN01_F13.raw | | | | | | | | | | | | | | | | 1.7557E4 | 1 | 0 | 0 | 0 | 0 | 0 | 0 | 0 | 0 | 0 | 0 | 0 | 0 | 0 | 0 | 0 | 1 | 110 | 126 | | | PEAKS DB |
| total 1 peptides | | | | | | | | | | | | | | | | | | | | | | | | | | | | | | | | | | | | | | | | | | | | | | | | | |

**Best Unique PSM (Scan F16:10152, m/z=449.2401, z=4, RT=30.74, ppm=-0.3):**

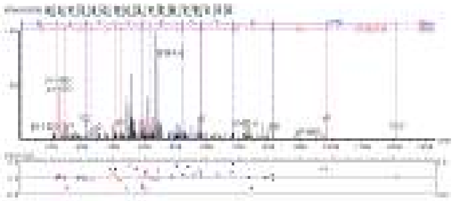

## XP_026576365.1

back to list

| **Protein Coverage** | **Supporting Peptides** | **Best Unique PSM** |

**Protein Coverage:**

1 MPEPAKSAPV PKKGSKKAIT KTQKKGDKKR KKSRKESYSI YVYKVLKQVH PDTGISSKAM SIMNSFVNDI FERIAAEASR
81 LAHYNKRSTI TSREVQTAVR LLLPGELAKH AVSEGTKAVT KYTSSK

**Supporting Peptides:**

| Peptide | Uniq | -10lgP | Mass | Length | ppm | m/z | z | RT | Fraction | Scan | Source File | Area F1 | Area F2a | Area F3a | Area F4 | Area F5 | Area F6a | Area F7a | Area F7b | Area F8 | Area F9a | Area Sample 1F10a | Area F10b | Area F11a | Area F11b | Area F12a | Area F13 | #Feature | #Feature F1 | #Feature F2a | #Feature F3a | #Feature F4 | #Feature F5 | #Feature F6a | #Feature F7a | #Feature F7b | #Feature F8 | #Feature F9a | #Feature Sample 1F10a | #Feature F10b | #Feature F11a | #Feature F11b | #Feature F12a | #Feature F13 | Start | End | PTM | AScore | Found By |
|---|---|---|---|---|---|---|---|---|---|---|---|---|---|---|---|---|---|---|---|---|---|---|---|---|---|---|---|---|---|---|---|---|---|---|---|---|---|---|---|---|---|---|---|---|---|---|---|---|---|
| | | | | | | | | | | | | | | | | | | | | | | | | | | | | | | | | | | | | | | | | | | | | | | | | | PEAKS |

| | | | | | | | | | | | | | | | | | | | | | | | | | | | | | | | | | | | | | | | | | | | | | | | | | |
|---|---|---|---|---|---|---|---|---|---|---|---|---|---|---|---|---|---|---|---|---|---|---|---|---|---|---|---|---|---|---|---|---|---|---|---|---|---|---|---|---|---|---|---|---|---|---|---|---|---|
| K.HAVSEGTKAVTKYTSSK | Y | 43.98 | 1792.9319 | 17 | -0.3 | 449.2401 | 4 | 30.74 | 16 | F16:10152 | BuCaTN01_F13.raw | | | | | | | | | | | | | | | | 1.7557E4 | 1 | 0 | 0 | 0 | 0 | 0 | 0 | 0 | 0 | 0 | 0 | 0 | 0 | 0 | 0 | 0 | 1 | 110 | 126 | | | DB |
| total 1 peptides | | | | | | | | | | | | | | | | | | | | | | | | | | | | | | | | | | | | | | | | | | | | | | | | | |

**Best Unique PSM (Scan F16:10152, m/z=449.2401, z=4, RT=30.74, ppm=-0.3):**

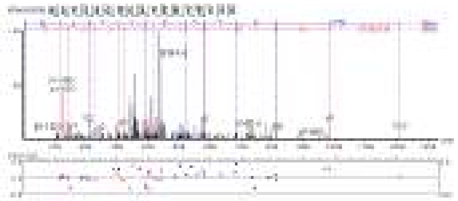

## XP_013930808.1

back to list

| Protein Coverage | Supporting Peptides | Best Unique PSM |

**Protein Coverage:**

1 MPEPAKSAPV PKKGSKKAIT KTQKKGDKKR KKSRKESYSI YVYKVLKQVH PDTGISSKAM SIMTPSVNDI FERIAAEASR
81 LAHYNKRSTI TSREVQTAVR LLLPGELAKH AVSEGTKAVT KYTSSK

**Supporting Peptides:**

| Peptide | Uniq | -10lgP | Mass | Length | ppm | m/z | z | RT | Fraction | Scan | Source File | Area F1 | Area F2a | Area F3a | Area F4 | Area F5 | Area F6a | Area F7a | Area F7b | Area F8 | Area F9a | Area Sample 1F10a | Area F10b | Area F11a | Area F11b | Area F12a | Area F13 | #Feature | #Feature F1 | #Feature F2a | #Feature F3a | #Feature F4 | #Feature F5 | #Feature F6a | #Feature F7a | #Feature F7b | #Feature F8 | #Feature F9a | #Feature Sample 1F10a | #Feature F10b | #Feature F11a | #Feature F11b | #Feature F12a | #Feature F13 | Start | End | PTM | AScore | Found By |
|---|---|---|---|---|---|---|---|---|---|---|---|---|---|---|---|---|---|---|---|---|---|---|---|---|---|---|---|---|---|---|---|---|---|---|---|---|---|---|---|---|---|---|---|---|---|---|---|---|---|
| K.HAVSEGTKAVTKYTSSK | Y | 43.98 | 1792.9319 | 17 | -0.3 | 449.2401 | 4 | 30.74 | 16 | F16:10152 | BuCaTN01_F13.raw | | | | | | | | | | | | | | | | 1.7557E4 | 1 | 0 | 0 | 0 | 0 | 0 | 0 | 0 | 0 | 0 | 0 | 0 | 0 | 0 | 0 | 0 | 1 | 110 | 126 | | | PEAKS DB |
| total 1 peptides | | | | | | | | | | | | | | | | | | | | | | | | | | | | | | | | | | | | | | | | | | | | | | | | | |

**Best Unique PSM (Scan F16:10152, m/z=449.2401, z=4, RT=30.74, ppm=-0.3):**

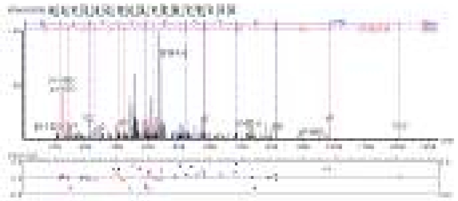

## XP_026539701.1

back to list

| Protein Coverage | Supporting Peptides | Best Unique PSM |

**Protein Coverage:**

1 MPEPAKSAPV PKKGSKKAIT KTQKKGDKKR KKSRKESYSI YVYKVLKQVH PDTGISSKAM SIMNSFVNDI FERIAAEASR
81 LAHYNKRSTI TSREIQTAVR LLLPGELAKH AVSEGTKAVT KYTSSK

**Supporting Peptides:**

| Peptide | Uniq | -10lgP | Mass | Length | ppm | m/z | z | RT | Fraction | Scan | Source File | Area F1 | Area F2a | Area F3a | Area F4 | Area F5 | Area F6a | Area F7a | Area F7b | Area F8 | Area F9a | Area Sample 1F10a | Area F10b | Area F11a | Area F11b | Area F12a | Area F13 | #Feature | #Feature F1 | #Feature F2a | #Feature F3a | #Feature F4 | #Feature F5 | #Feature F6a | #Feature F7a | #Feature F7b | #Feature F8 | #Feature F9a | #Feature Sample 1F10a | #Feature F10b | #Feature F11a | #Feature F11b | #Feature F12a | #Feature F13 | Start | End | PTM | AScore | Found By |
|---|---|---|---|---|---|---|---|---|---|---|---|---|---|---|---|---|---|---|---|---|---|---|---|---|---|---|---|---|---|---|---|---|---|---|---|---|---|---|---|---|---|---|---|---|---|---|---|---|---|
| K.HAVSEGTKAVTKYTSSK | Y | 43.98 | 1792.9319 | 17 | -0.3 | 449.2401 | 4 | 30.74 | 16 | F16:10152 | BuCaTN01_F13.raw | | | | | | | | | | | | | | | | 1.7557E4 | 1 | 0 | 0 | 0 | 0 | 0 | 0 | 0 | 0 | 0 | 0 | 0 | 0 | 0 | 0 | 0 | 1 | 110 | 126 | | | PEAKS DB |
| total 1 peptides | | | | | | | | | | | | | | | | | | | | | | | | | | | | | | | | | | | | | | | | | | | | | | | | | |

**Best Unique PSM (Scan F16:10152, m/z=449.2401, z=4, RT=30.74, ppm=-0.3):**

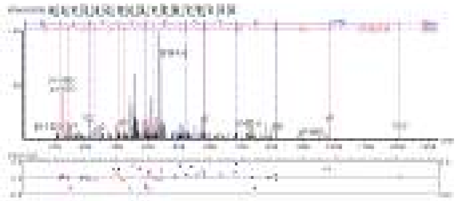

## XP_015675791.1

back to list

| Protein Coverage | Supporting Peptides | Best Unique PSM |

**Protein Coverage:**

1 MDRNHPLLTK AVSIMNSFVN DIFERIAAEA SRLAHYNKRS TITSREIQTA VRLLLPGELA KHAVSEGTKA VTKYTSSK

**Supporting Peptides:**

| Peptide | Uniq | -10lgP | Mass | Length | ppm | m/z | z | RT | Fraction | Scan | Source File | Area F1 | Area F2a | Area F3a | Area F4 | Area F5 | Area F6a | Area F7a | Area F7b | Area F8 | Area F9a | Area Sample 1F10a | Area F10b | Area F11a | Area F11b | Area F12a | Area F13 | #Feature | #Feature F1 | #Feature F2a | #Feature F3a | #Feature F4 | #Feature F5 | #Feature F6a | #Feature F7a | #Feature F7b | #Feature F8 | #Feature F9a | #Feature Sample 1F10a | #Feature F10b | #Feature F11a | #Feature F11b | #Feature F12a | #Feature F13 | Start | End | PTM | AScore | Found By |
|---|---|---|---|---|---|---|---|---|---|---|---|---|---|---|---|---|---|---|---|---|---|---|---|---|---|---|---|---|---|---|---|---|---|---|---|---|---|---|---|---|---|---|---|---|---|---|---|---|---|
| K.HAVSEGTKAVTKYTSSK | Y | 43.98 | 1792.9319 | 17 | -0.3 | 449.2401 | 4 | 30.74 | 16 | F16:10152 | BuCaTN01_F13.raw | | | | | | | | | | | | | | | | 1.7557E4 | 1 | 0 | 0 | 0 | 0 | 0 | 0 | 0 | 0 | 0 | 0 | 0 | 0 | 0 | 0 | 0 | 1 | 62 | 78 | | | PEAKS DB |
| total 1 peptides | | | | | | | | | | | | | | | | | | | | | | | | | | | | | | | | | | | | | | | | | | | | | | | | | |

**Best Unique PSM (Scan F16:10152, m/z=449.2401, z=4, RT=30.74, ppm=-0.3):**

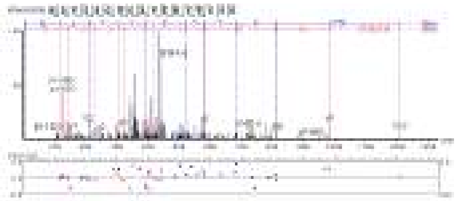

## XP_026576367.1

back to list

| Protein Coverage | Supporting Peptides | Best Unique PSM |

**Protein Coverage:**

1 MAEPAKSTPV PKKGSKKSIT KTPKKGDKKR KKSRKESYSI YIYKVLKQVH PDTGISSKAM SIMNSFVNDI FERIAAEASR
81 LAHYTKRSTI TSREIQTAVR LLLPGELAKH AVSEGTKAVT KYTSSK

**Supporting Peptides:**

| Peptide | Uniq | -10lgP | Mass | Length | ppm | m/z | z | RT | Fraction | Scan | Source File | Area F1 | Area F2a | Area F3a | Area F4 | Area F5 | Area F6a | Area F7a | Area F7b | Area F8 | Area F9a | Area Sample 1F10a | Area F10b | Area F11a | Area F11b | Area F12a | Area F13 | #Feature | #Feature F1 | #Feature F2a | #Feature F3a | #Feature F4 | #Feature F5 | #Feature F6a | #Feature F7a | #Feature F7b | #Feature F8 | #Feature F9a | #Feature Sample 1F10a | #Feature F10b | #Feature F11a | #Feature F11b | #Feature F12a | #Feature F13 | Start | End | PTM | AScore | Found By |
|---|---|---|---|---|---|---|---|---|---|---|---|---|---|---|---|---|---|---|---|---|---|---|---|---|---|---|---|---|---|---|---|---|---|---|---|---|---|---|---|---|---|---|---|---|---|---|---|---|---|
| K.HAVSEGTKAVTKYTSSK | Y | 43.98 | 1792.9319 | 17 | -0.3 | 449.2401 | 4 | 30.74 | 16 | F16:10152 | BuCaTN01_F13.raw | | | | | | | | | | | | | | | | 1.7557E4 | 1 | 0 | 0 | 0 | 0 | 0 | 0 | 0 | 0 | 0 | 0 | 0 | 0 | 0 | 0 | 0 | 1 | 110 | 126 | | | PEAKS DB |

| total 1 peptides |
|---|

**Best Unique PSM (Scan F16:10152, m/z=449.2401, z=4, RT=30.74, ppm=-0.3):**

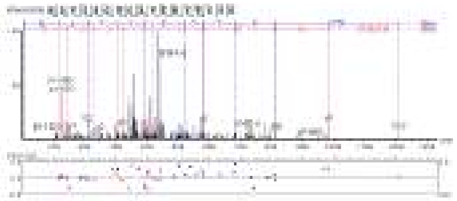

## XP_029139092.1

back to list

| Protein Coverage | Supporting Peptides | Best Unique PSM |

**Protein Coverage:**

```
   1 MLSFVDTRIL LLLAVTSCLA TSQYVNEVKR GPTGPPGRDG NDGPQGPPGP PGPPGLGENF AAQYDMSKSA DMGHGPMGLM
  81 GPRGPPGTNG PPGSPGFQGP SGEPGEPGQA GPQGSRGPMG PPGKSGEDGH PGKSGRSGER GVPGPQGTRG FPGTSGLPGF
 161 KGIRGHNGLD GRKGEPGATG NKGEPGAAGE NGTPGQAGAR GLPGERGRVG APGSVGARGS DGSVGPVGSA GPIGAAGPSG
 241 FPGAPGAKGE IGSAGNVGPS GPSGPRGDIG LPGAAGPVGP AGNPGANGLS GAKGAAGLPG VAGAPGLPGP RGIPGPTGAS
 321 GAAGARGLAG EPGPPGSKGE TGNKGEPGIA GPAGPAGSSG EEGKKGPNGE AGSSGLPGSA GLRGVPGSRG LPGSDGRAGG
 401 LGMAGSRGAT GPAGAKGPSG DTGRPGEPGL MGARGLPGQS GSPGPAGKEG PSGSAGPDGR SGPTGPAGPR GEPGNIGFPG
 481 PKGPNGEPGK PGDRGSVGLS GSQGPPGSDG NNGAQGPPGL AGNAGSKGET GPAGAPGFQG LPGPSGPVGE AGKPGERGAH
 561 GDFGLPGPAG ARGERGAPGE SGALGPAGPL GSRGPSGPAG ADGNKGEPGA VGALGSAGPS GPSGVHGERG AAGGPGGKGE
 641 KGPSGFRGDA GATGRDGARG APGAVGAPGP AGASGERGEG GSAGPAGPAG PRGSSGERGE GGPAGPPGFA GPAGAAGQTG
 721 AKGERGQRGP KGDVGPHGSI GAVGSAGPAG PVGQAGPSGA RGDGGPPGPN GFPGNAGRSG PPGPAGITGP SGPPGPAGKE
 801 GIRGPRGDTG PMGRSGETGI SGPPGFSGEK GPSGEAGAAG APGSSGPQGI LGAPGILGLP GSRGERGLPG LSGALGDHGP
 881 VGISGPSGAR GPSGPIGSSG VNGAPGESGR DGNPGNDGPP GRDGAPGYKG ERGAPGNTGP AGAVGAPGPH GSVGPVGKSG
 961 NRGERGPAGV TGPSGPAGSR GPAGSQGVRG EKGVGGDKGP RGLPGFKGHN GLQGLPGIAG HHGEQGSAGI TGPAGPRGPS
1041 GPSGPAGKDG RNGLPGPVGP AGLRGSHGSQ GPSGPPGSPG LPGPAGPNGG GYEIGYDMEY YRADQPSLRP KDYEVDATLK
1121 ILNNQIETLL TPEGSKKNPA RTCRDLKLSH PEWNNGFYWI DPNQGCTMDA IRAYCDFSTG ESCISAKPGN FPAKNWYTGR
1201 KPDEKNLVWF GETINGGTQF EYNEEGVSTK DMATQLAFLR LLANHASQNI TYHCKNSIAY MNEETGNLKK AVVLQGSNDV
1281 EFRAEGNGRF TFTVIEDGCS RKTNEWGKTV IEYRTNKPSR LPFLDIAPLD IGGADQEFRL DVGPACFK
```

**Supporting Peptides:**

| Peptide | Uniq | -10lgP | Mass | Length | ppm | m/z | z | RT | Fraction | Scan | Source File | Area F1 | Area F2a | Area F3a | Area F4 | Area F5 | Area F6a | Area F7a | Area F7b | Area F8 | Area F9a | Area Sample 1F10a | Area F10b | Area F11a | Area F11b | Area F12a | Area F13 | #Feature | #Feature F1 | #Feature F2a | #Feature F3a | #Feature F4 | #Feature F5 | #Feature F6a | #Feature F7a | #Feature F7b | #Feature F8 | #Feature F9a | #Feature Sample 1F10a | #Feature F10b | #Feature F11a | #Feature F11b | #Feature F12a | #Feature F13 | Start | End | PTM | AScore | Found By |
|---|---|---|---|---|---|---|---|---|---|---|---|---|---|---|---|---|---|---|---|---|---|---|---|---|---|---|---|---|---|---|---|---|---|---|---|---|---|---|---|---|---|---|---|---|---|---|---|---|---|
| R.DGARGAPGAVGAPGPAGASGER.G | Y | 43.30 | 1876.9139 | 22 | -8.3 | 626.6382 | 3 | 44.93 | 8 | F8:21924 | BuCaTN01_F7b.raw | | | | | | | | 0 | | | | | | | | | 0 | 0 | 0 | 0 | 0 | 0 | 0 | 0 | 0 | 0 | 0 | 0 | 0 | 0 | 0 | 0 | 0 | 656 | 677 | | | PEAKS DB |
| total 1 peptides | | | | | | | | | | | | | | | | | | | | | | | | | | | | | | | | | | | | | | | | | | | | | | | | | |

**Best Unique PSM (Scan F8:21924, m/z=626.6382, z=3, RT=44.93, ppm=-8.3):**

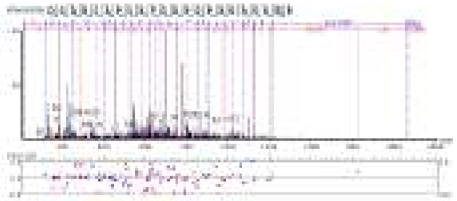

## XP_029139091.1

back to list

| Protein Coverage | Supporting Peptides | Best Unique PSM |

**Protein Coverage:**

```
   1 MSKSADMGHG PMGLMGPRGP PGTNGPPGSP GFQGPSGEPG EPGQAGPQGS RGPMGPPGKS GEDGHPGKSG RSGERGVPGP
  81 QGTRGFPGTS GLPGFKGIRG HNGLDGRKGE PGATGNKGEP GAAGENGTPG QAGARGLPGE RGRVGAPGSV GARGSDGSVG
 161 PVGSAGPIGA AGPSGFPGAP GAKGEIGSAG NVGPSGPSGP RGDIGLPGAA GPVGPAGNPG ANGLSGAKGA AGLPGVAGAP
 241 GLPGPRGIPG PTGASGAAGA RGLAGEPGPP GSKGETGNKG EPGIAGPAGP AGSSGEEGKK GPNGEAGSSG LPGSAGLRGV
 321 PGSRGLPGSD GRAGGLGMAG SRGATGPAGA KGPSGDTGRP GEPGLMGARG LPGQSGSPGP AGKEGPSGSA GPDGRSGPTG
 401 PAGPRGEPGN IGFPGPKGPN GEPGKPGDRG SVGLSGSQGP PGSDGNNGAQ GPPGLAGNAG SKGETGPAGA PGFQGLPGPS
 481 GPVGEAGKPG ERGAHGDFGL PGPAGARGER GAPGESGALG PAGPLGSRGP SGPAGADGNK GEPGAVGALG SAGPSGPSGV
 561 HGERGAAGGP GGKGEKGPSG FRGDAGATGR DGARGAPGAV GAPGPAGASG ERGEGGSAGP AGPAGPRGSS GERGEGGPAG
 641 PPGFAGPAGP VGQAGPSGAR GDGGPPGPNG FPGNAGRSGP PGPAGITGPS GPPGPAGKEG IRGPRGDTGP MGRSGETGIS
 721 GPPGFSGEKG PSGEAGAAGA PGSSGPQGIL GAPGILGLPG SRGERGLPGL SGALGDHGPV GISGPSGARG PSGPIGSSGV
 801 NGAPGESGRD GNPGNDGPPG RDGAPGYKGE RGAPGNTGPA GAVGAPGPHG SVGPVGKSGN RGERGPAGVT GPSGPAGSRG
 881 PAGSQGVRGE KGVGGDKGPR GLPGFKGHNG LQGLPGIAGH HGEQGSAGIT GPAGPRGPSG PSGPAGKDGR NGLPGPVGPA
 961 GLRGSHGSQG PSGPPGSPGL PGPAGPNGGG YEIGYDMEYY RADQPSLRPK DYEVDATLKI LNNQIETLLT PEGSKKNPAR
1041 TCRDLKLSHP EWNNGFYWID PNQGCTMDAI RAYCDFSTGE SCISAKPGNF PAKNWYTGRK PDEKNLVWFG ETINGGTQFE
1121 YNEEGVSTKD MATQLAFLRL LANHASQNIT YHCKNSIAYM NEETGNLKKA VVLQGSNDVE FRAEGNGRFT FTVIEDGCSR
1201 KTNEWGKTVI EYRTNKPSRL PFLDIAPLDI GGADQEFRLD VGPACFK
```

**Supporting Peptides:**

| Peptide | Uniq | -10lgP | Mass | Length | ppm | m/z | z | RT | Fraction | Scan | Source File | Area F1 | Area F2a | Area F3a | Area F4 | Area F5 | Area F6a | Area F7a | Area F7b | Area F8 | Area F9a | Area Sample 1F10a | Area F10b | Area F11a | Area F11b | Area F12a | Area F13 | #Feature | #Feature F1 | #Feature F2a | #Feature F3a | #Feature F4 | #Feature F5 | #Feature F6a | #Feature F7a | #Feature F7b | #Feature F8 | #Feature F9a | #Feature Sample 1F10a | #Feature F10b | #Feature F11a | #Feature F11b | #Feature F12a | #Feature F13 | Start | End | PTM | AScore | Found By |
|---|---|---|---|---|---|---|---|---|---|---|---|---|---|---|---|---|---|---|---|---|---|---|---|---|---|---|---|---|---|---|---|---|---|---|---|---|---|---|---|---|---|---|---|---|---|---|---|---|---|
| R.DGARGAPGAVGAPGPAGASGER.G | Y | 43.30 | 1876.9139 | 22 | -8.3 | 626.6382 | 3 | 44.93 | 8 | F8:21924 | BuCaTN01_F7b.raw | | | | | | | | 0 | | | | | | | | | 0 | 0 | 0 | 0 | 0 | 0 | 0 | 0 | 0 | 0 | 0 | 0 | 0 | 0 | 0 | 0 | 0 | 591 | 612 | | | PEAKS DB |
| total 1 peptides | | | | | | | | | | | | | | | | | | | | | | | | | | | | | | | | | | | | | | | | | | | | | | | | | |

**Best Unique PSM (Scan F8:21924, m/z=626.6382, z=3, RT=44.93, ppm=-8.3):**

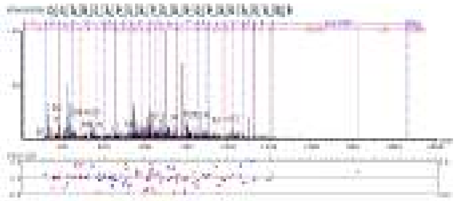

## XP_013923409.1

back to list

| Protein Coverage | Supporting Peptides | Best Unique PSM |

**Protein Coverage:**

1 MYGSARSVGK IEPSSQSPGR SPRLPRSPRL GHR**RTNSTGG SSGGSAGGGS GK**TLSMENIQ SLNAAYATSG PMYLSEHENV
81 GADTPKSTMT LGRSGGRLPY GVRMTAMGSS PNIASSGVAS DTIAFGEHHL PPVSMASTVP HSLRQARDNT IMDLQTQLKE
161 VLRENDILRK DVEVKESKLS SSMNSIKTFW SPELKKERAL RKDEASKITI WKEQYRVVQE ENQHMQMTIQ ALQDELRIQR
241 DLNQLFQQDT SSRSSEPFLT ELTEENFQRL HTEHERQAKE LFLLRKTLEE MELRIETQKQ TLNARDESIK KLLEMLQSKG
321 LSAKATEEDH ERTRRLAEAE MHVHHLETLL EQKEKESSLL REEMHRRFEN APDSAKTKAL QTVIEMKDSK ISSMERGLRD
401 LEEEIQMLKS NGALSTEERE EEIKQMEVYR SHSKFMKNKV EQLKEELNTK EVQGEELKKK VADFQTEIGQ VKQELSRKDT
481 ELLALQTKLE TLTNQFSDSK QHIDVLKESL TAKEQRAAIL QTEVDALRLR LEEKEAMLNK KTKQIQEMAE EKGTQAGEIH
561 DLKDMLDVKE RKVNILQKKI ENLQEQLRDK EKQMSSLKER VKSLQADTTN TDTALTTLEE ALAEKERTIE RLKEQRDRDE
641 REKQEEIDSH KKDIKDLKEK VSILQADLTE KETTLLDLKE HASSLASAGL KKDSRLKTLD IALEQKKEEC LKFESQLKKA
721 YEATIEARAN PEMNDRFQQF EKEVAQYREE STKAQAEVDR LLEILKEMEN EKNDKDKKIA ELESLTSRQI KDQNKKVANL
801 KHKEQVEKKK SAQMLEEARR REDNLNDSSQ QLQDSLHKKD DRIEELEEAL RESVQITAER EMVLAQEESA RTQAEKQVEE
881 LMMAMEKVKQ ELESMKAKLS STQQSLAEKE THLTNLRAER RKHLEEVLEM KQEALLAAIS EKDANIALLE LSSSKKKTQE
961 EVAGLKREKD RLVQQLKQQT QNRMKLMADN YEDDNLKSSH FNQTNHKPSP DQDEEEGIWA

**Supporting Peptides:**

| Peptide | Uniq | -10lgP | Mass | Length | ppm | m/z | z | RT | Fraction | Scan | Source File | Area F1 | Area F2a | Area F3a | Area F4 | Area F5 | Area F6a | Area F7a | Area F7b | Area F8 | Area F9a | Area Sample 1F10a | Area F10b | Area F11a | Area F11b | Area F12a | Area F13 | #Feature | #Feature F1 | #Feature F2a | #Feature F3a | #Feature F4 | #Feature F5 | #Feature F6a | #Feature F7a | #Feature F7b | #Feature F8 | #Feature F9a | #Feature Sample 1F10a | #Feature F10b | #Feature F11a | #Feature F11b | #Feature F12a | #Feature F13 | Start | End | PTM | AScore | Found By |
|---|---|---|---|---|---|---|---|---|---|---|---|---|---|---|---|---|---|---|---|---|---|---|---|---|---|---|---|---|---|---|---|---|---|---|---|---|---|---|---|---|---|---|---|---|---|---|---|---|---|
| R.RTNSTGGSSGGSAGGGSGK.T | Y | 42.98 | 1580.7139 | 19 | -6.1 | 791.3576 | 2 | 55.78 | 12 | F12:31919 | BuCaTN01_F10b.raw | | | | | | | | | | | | 0 | | | | | 0 | 0 | 0 | 0 | 0 | 0 | 0 | 0 | 0 | 0 | 0 | 0 | 0 | 0 | 0 | 0 | 0 | 34 | 52 | | | PEAKS DB |
| total 1 peptides | | | | | | | | | | | | | | | | | | | | | | | | | | | | | | | | | | | | | | | | | | | | | | | | | |

**Best Unique PSM (Scan F12:31919, m/z=791.3576, z=2, RT=55.78, ppm=-6.1):**

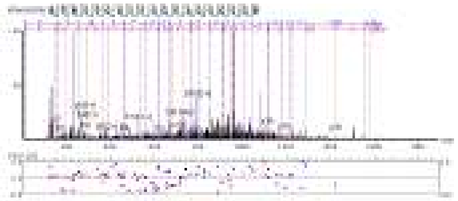

## XP_039182761.1

back to list

| Protein Coverage | Supporting Peptides | Best Unique PSM |

**Protein Coverage:**

1 MRGRRGRPPK HQQPAAAPAR ASTPAPPAPP GPIGGLRSRL RGSSRGRWAA SGQAAGAAGP RSRGSGAGQA STAAASSSSS
81 RGGGRRKGGG GSSSTPGGGG SSGRGRGR**AG AAAAAAGGGG GGGSRSSSSS QGRAAASSR**R SGGGKVVYDD HESDEEEEEE
161 SVGSDEDEED GEAEEKPDSQ EEEAVMEDDD DDDDSDYPEE MEDEDDASYC TESSFRSHST YSSTPGRRRQ RIHRPRSPIL
241 EEKDIPFLDL PKSSGDLLVP NEHIMNVIAI YEVLRNFGTV LRLSPFRFED FCAALVSQEQ CTLMAEMHIV LLKAVLREED
321 TSNTTFGPAD LKDSVNSTLY FIDGMTWPEV LRVYCESDKE YHHVLPFQEA EDYPYGPVKN KIQVLQFLVD QFLTTNIARE
401 ELMSEGVIQY DDHCRVCHKL GDLLCCETCS AVYHLECVKP PLEEVPEDEW QCEVCVAHKV PGVTDCIAEI QKNKPYIRHE
481 PIGYDRNRRK YWFLNRRIVI EEDSECEKHK RIWYYSTKVQ LAELTECLDK DYWEADICKT LEEMREEIHR HMDITEDLTN
561 KARGSNKSFL TAANEEILET IRGEKGELLE DRSLVADEEK TTVENKECTD TENGKEELSN QQMEVNKEIV AEESSEKGRK
641 EESADACNEN DSVLSLPGSS IPNVSAEAAD ASEGKTTVGC DSETPDDNKA DKNMASEDLK AISDETSKTI PNLNIDSSAE
721 THLSDQEHSS SKEPNSMQSE SVKNSDEVEI TEGDSQNSED PGDKSNGERS ESPNSGIGTP GSTRMVTRLR NPDSKLSQLK
801 IQQVAAAAHE ANKLFKEGKE VLVVNSQGEI SRMNTKKEIV MKGNISNYFK LGQEGKYRVY HNQYATNSFA LNKHQHREDH
881 DKRRHLSHKF CLSPAGEFKW NGSVHGSKVL TISTLRLTII QLENNIPSSF LHPNWASHRS NWIKAVQMCS KPREFALALA
961 ILECAIKPVV MLPIWRESLG HTRLRRMTAI EREEKEKVKK KERKQEEEET MQQATWVKYT FPIKHQVWKQ KGEEYRVTGY
1041 GGWIWISKTH VHRFVPRLPG NTNISYRKLL LAKNNTAAIT EQQYELDKRK NPAKVKVEHC SLKSRIKNFH EPHKKDQKET
1121 EHSETTKKSQ TKEENELNEE KAEIDGLFEK PDHAKEDEKG ICENDKDIKE EPMEIDEVKM DTSPKCEETP ISADLINVSE
1201 GFHLRTCYKR KVKTSKLDGL LERRVKQFTL EEKQRLERIK LKAASNSSDC KSLSPQRNTD ALPLVKTETD EKRAHSLGTK
1281 QSEDPVKDCL SPNNIQFNKN SESVQSLSCL NRLLDEESDL ISDQCSDTQC LIAANAHGSA SEPADTEKNK MLNKEDDNST
1361 MEHSQAIDNK ESTKCSVLED NEKSSEEITG PNCEDNIQPE GIEKNCEKDT ASLIKEENET LKTENQGRDS VEACNLEDHH
1441 AANKINVSLP KSSEDASEPK IALNPKPDLE GLKSLRTELI SEKAFEAQIP DIIGENIIDE ITHCKIAETN GEKLEQAKCD
1521 PPTSISKCID QIKSIADKNN NKNGEHDVQM DRGKSAPYQM NGKDADFKVL PNEECLKREA LAEAESNLDS KVNNINKNNL
1601 LGKTFPSNES VKPFMNGDIA VEDVDDRRNS DNEFSIGSSV DPNCDSRGGV LPQGDTSLSA QDSGRKQCSS ERSSPVDDAS
1681 ITAIHSCNEN SISSETECME IESSCVKKAA LSPVISCEES SLSNDFADQN GLQTHRIKNI NGENAVKAVV TEVTTTSTIS
1761 TESKTVRKMS ELSAVKEDTE RVMSSTENCS ISTITTTTTT TVTKLSTPTT NSSTDVISVK EHSKTVVTTT VTDSLTTPSG
1841 TLLTSMTVSK EYSTRNKVKL MKFTRPKKTR SGTALPSYRK FVTKSSKKSI FVLPSDDLKK LARRGGIREV PCFNYTAKPA
1921 LDIWPYPSPR PTFGITWRYR LQTVKSLAGV SLMLRLLWAC LKWDDMAAKP PPGGGTTRTE TSEMEITTTE IIKRRDVGPY
2001 GIRSEYCIRK IICPIGVPEA PKETPTPQRK GLRSSALRPK RPETPKQTGP VIIETWVAEE ELELWEIKAF SERVEKEKAQ
2081 AVEQQAKVSE QKKAEEFKAQ METQLKQQRL AAQQKRLEQQ KQVPIVCGAA TATTTSSTVT TVSTPQKVVV GQLPRSMPSG
2161 AKVVLATKMG SPATVTFQQN KNFHQTFATW VKQGQPSTAT STAATSATTI SSSGQTFQIA SSPVTMAGKV ITKLPLPANS
2241 KIVAVNVPAT QGGVVQVQQK VLGIIPSTTG ANQQTFTSFQ PRTATVTIRP NNTGTVCTTS ASQVMPGTPL RPGMTVIRAP
2321 LQQSSLGKAI IRTPVVVQQG VLPTSQTQQV VTQIIRGQPV STAVSSPTTV STSQKVATAP SQHMQPQAAP PHPPRPQQGQ
2401 VKLTMAQLTQ LTQGQGGSQG LTVVIQGQGQ TTGQLQLIPQ GVTVIPGPGQ QLMQAAMPNG TIQRFLFTPL STAATAASTT
2481 STTVSTSTSA AGEQKQTSLA QTQTQPTPAL PPAEPKPAPT SFSAPPPSSS AQTVMPAQTQ TVQAPVPVEP HPFPEAQVPP
2561 SLPSPTVTSE AQPPRTQSTV TPTVAQPPPQ SLAQGLPFPP VQNQAQPVMH LQTPPKGQTI QPTQVQTAAP QQVQIQPHPS
2641 VQIQTQQSQV PTSVPPLPPA STVNQVTVLP PRPQLQIQPP QTKVITMPQL QQQVQVFSQL PSHMVAQIQA QQSGLPQQIK
2721 LQLPIQIQQA SAVQTHQIQN VVTVQASVQE QLQRVQQLRE QQQKKKQQHI EIKREHSLQA SNQSDIIQKQ VVMKHNAVIE
2801 HLKQKKTLTP AEREENQRMI VCNQVMKYIL DKIDKEEKQA AKKRKREESV EQKRSKQNAS KLSALLFKHK EQLKAEILKK
2881 RALLDKDLQI EVQEELKKDL TKIKREKEKA QAAAAAAAAA AALSPPPPPP PLPPPPPPPP PPPPPPLPPP ASSSSIPSTA
2961 SGTSYISSHK RKREEEKESA SSKSKKKKMI STTSKETKKD TKLYCICKTP YDESKFYIGC DRCQNWYHGR CVGILQSEAD
3041 LIDEYVCPQC QSTEDAMTVL SPLTDKDYEG LKRVLRSLQA HKMAWPFLEP VDPNDAPDYY GVIKEPMDLS TMEERILKRY
3121 YKKVTEFVAD MTKIFDNCRY YNPSDSPFYQ CAEVLESFFV QKLKGFKASR SHNNKLQSTA S

**Supporting Peptides:**

| Peptide | Uniq | -10lgP | Mass | Length | ppm | m/z | z | RT | Fraction | Scan | Source File | Area F1 | Area F2a | Area F3a | Area F4 | Area F5 | Area F6a | Area F7a | Area F7b | Area F8 | Area F9a | Area Sample 1F10a | Area F10b | Area F11a | Area F11b | Area F12a | Area F13 | #Feature | #Feature F1 | #Feature F2a | #Feature F3a | #Feature F4 | #Feature F5 | #Feature F6a | #Feature F7a | #Feature F7b | #Feature F8 | #Feature F9a | #Feature Sample 1F10a | #Feature F10b | #Feature F11a | #Feature F11b | #Feature F12a | #Feature F13 | Start | End | PTM | AScore | Found By |
|---|---|---|---|---|---|---|---|---|---|---|---|---|---|---|---|---|---|---|---|---|---|---|---|---|---|---|---|---|---|---|---|---|---|---|---|---|---|---|---|---|---|---|---|---|---|---|---|---|---|
| R.AGAAAAAAGGGGGGGSRSSSSSQGRAAASSR.R | Y | 42.54 | 2534.1929 | 31 | -4.4 | 845.7345 | 3 | 68.02 | 6 | F6:46654 | BuCaTN01_F6a.raw | | | | | | 0 | | | | | | | | | | | 0 | 0 | 0 | 0 | 0 | 0 | 0 | 0 | 0 | 0 | 0 | 0 | 0 | 0 | 0 | 0 | 0 | 109 | 139 | | | PEAKS DB |
| total 1 peptides | | | | | | | | | | | | | | | | | | | | | | | | | | | | | | | | | | | | | | | | | | | | | | | | | |

**Best Unique PSM (Scan F6:46654, m/z=845.7345, z=3, RT=68.02, ppm=-4.4):**

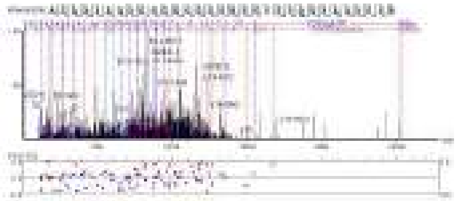

## XP_039182758.1

back to list

| Protein Coverage | Supporting Peptides | Best Unique PSM |

**Protein Coverage:**

1 MRGRRGRPPK HQQPAAAPAR ASTPAPPAPP GPIGGLRSRL RGSSRGRWAA SGQAAGAAGP RSRGSGAGQA STAAASSSSS
81 RGGGRRKGGG GSSSTPGGGG SSGRGRGR**AG AAAAAAGGGG GGGSRSSSSS QGRAAASSR**R SGGGKVVYDD HESDEEEEEE
161 SVGSDEDEED GEAEEKPDSQ EEEAVMEDDD DDDDSDYPEE MEDEDDASYC TESSFRSHST YSSTPGRRRQ RIHRPRSPIL
241 EEKDIPFLDL PKSSGDLLVP NEHIMNVIAI YEVLRNFGTV LRLSPFRFED FCAALVSQEQ CTLMAEMHIV LLKAVLREED
321 TSNTTFGPAD LKDSVNSTLY FIDGMTWPEV LRVYCESDKE YHHVLPFQEA EDYPYGPVKN KIQVLQFLVD QFLTTNIARE
401 ELMSEGVIQY DDHCRVCHKL GDLLCCETCS AVYHLECVKP PLEEVPEDEW QCEVCVAHKV PGVTDCIAEI QKNKPYIRHE
481 PIGYDRNRRK YWFLNRRIVI EEDSECEKHK RIWYYSTKVQ LAELTECLDK DYWEADICKT LEEMREEIHR HMDITEDLTN
561 KARGSNKSFL TAANEEILET IRGEKGELLE DRSLVADEEK TTVENKECTD TENGKEELSN QQMEVNKEIV AEESSEKGRK
641 EESADACNEN DSVLSLPGSS IPNVSAEAAD ASEGKTTVGC DSETPDDNKA DKNMASEDLK AISDETSKTI PNLNIDSSAE
721 THLSDQEHSS SKEPNSMQSE SVKNSDEVEI TEGDSQNSED PGDKSNGERS ESPNSGIGTP GSTRMVTRLR NPDSKLSQLK
801 IQQVAAAAHE ANKLFKEGKE VLVVNSQGEI SRMNTKKEIV MKGNISNYFK LGQEGKYRVY HNQYATNSFA LNKHQHREDH
881 DKRRHLSHKF CLSPAGEFKW NGSVHGSKVL TISTLRLTII QLENNIPSSF LHPNWASHRS NWIKAVQMCS KPREFALALA
961 ILECAIKPVV MLPIWRESLG HTRLRRMTAI EREEKEKVKK KERKQEEEET MQQATWVKYT FPIKHQVWKQ KGEEYRVTGY
1041 GGWIWISKTH VHRFVPRLPG NTNISYRKLL LAKNNTAAIT EQQYELDKRK NPAKVKVEHC SLKSRIKNFH EPHKKDQKET
1121 EHSETTKKSQ TKEENELNEE KAEIDGLFEK PDHAKEDEKG ICENDKDIKE EPMEIDEVKM DTSPKCEETP ISADLINVSE
1201 GFHLRTCYKR KVKTSKLDGL LERRVKQFTL EEKQRLERIK LKAASNSSDC KSLSPQRNTD ALPLVKTETD EKRAHSLGTK
1281 QSEDPVKDCL SPNNIQFNKN SESVQSLSCL NRLLDEESDL ISDQCSDTQC LIAANAHGSA SEPADTEKNK MLNKEDDNST
1361 MEHSQAIDNK ESTKCSVLED NEKSSEEITG PNCEDNIQPE GIEKNCEKDT ASLIKEENET LKTENQGRDS VEACNLEDHH
1441 AANKINVSLP KSSEDASEPK IALNPKPDLE GLKSLRTELI SEKAFEAQIP DIIGENIIDE ITHCKIAETN GEKLEQAKCD
1521 PPTSISKCID QIKSIADKNN NKNGEHDVQM DRGKSAPYQM NGKDADFKVL PNEECLKREA LAEAESNLDS KVNNINKNNL
1601 LGKTFPSNES VKPFMNGDIA VEDVDDRRNS DNEFSIGSSV DPNCDSRGGV LPQGDTSLSA QDSGRKQCSS ERSSPVDDAS
1681 ITAIHSCNEN SISSETECME IESSCVKKAA LSPVISCEES SLSNDFADQN GLQTHRIKNI NGENAVKAVV TEVTTTSTIS
1761 TESKTVRKMS ELSAVKEDTE RVMSSTENCS ISTITTTTTT TVTKLSTPTT NSSTDVISVK EHSKTVVTTT VTDSLTTPSG
1841 TLLTSMTVSK EYSTRNKVKL MKFTRPKKTR SGTALPSYRK FVTKSSKKSI FVLPSDDLKK LARRGGIREV PCFNYTAKPA
1921 LDIWPYPSPR PTFGITWRYR LQTVKSLAGV SLMLRLLWAC LKWDDMAAKP PPGGGTTRTE TSEMEITTTE IIKRRDVGPY
2001 GIRSEYCIRK IICPIGVPEA PKETPTPQRK GLRSSALRPK RPETPKQTGP VIIETWVAEE ELELWEIKAF SERVEKEKAQ
2081 AVEQQAKKRL EQQKQVPIVC GAATATTTSS TVTTVSTPQK VVVGQLPRSM PSGAKVVLAT KMGSPATVTF QQNKNFHQTF
2161 ATWVKQGQPS TATSTAATSA TTISSSGQTF QIASSPVTMA GKVITKLPLP ANSKIVAVNV PATQGGVVQV QQKVLGIIPS
2241 TTGANQQTFT SFQPRTATVT IRPNNTGTVC TTSASQVMPG TPLRPGMTVI RAPLQQSSLG KAIIRTPVVV QQGVLPTSQT
2321 QQVVTQIIRG QPVSTAVSSP TTVSTSQKVA TAPSQHMQPQ AAPPHPPRPQ QGQVKLTMAQ LTQLTQGQGG SQGLTVVIQG
2401 QGQTTGQLQL IPQGVTVIPG PGQQLMQAAM PNGTIQRFLF TPLSTAATAA STTSTTVSTS TSAAGEQKQT SLAQTQTQPT
2481 PALPPAEPKP APTSFSAPPP SSSAQTVMPA QTQTVQAPVP VEPHPFPEAQ VPPSLPSPTV TSEAQPPRTQ STVTPTVAQP
2561 PPQSLAQGLP FPPVQNQAQP VMHLQTPPKG QTIQPTQVQT AAPQQVQIQP HPSVQIQTQQ SQVPTSVPPL PPASTVNQVT
2641 VLPPRPQLQI QPPQTKVITM PQLQQQVQVF SQLPSHMVAQ IQAQQSGLPQ QIKLQLPIQI QQASAVQTHQ IQNVVTVQAS
2721 VQEQLQRVQQ LREQQQKKKQ QHIEIKREHS LQASNQSDII QKQVVMKHNA VIEHLKQKKT LTPAEREENQ RMIVCNQVMK
2801 YILDKIDKEE KQAAKKRKRE ESVEQKRSKQ NASKLSALLF KHKEQLKAEI LKKRALLDKD LQIEVQEELK KDLTKIKREK
2881 EKAQAAAAAA AAAAALSPPP PPPPLPPPPP PPPPPPPPPL PPPASSSSIP STASGTSYIS SHKRKREEEK ESASSKSKKK
2961 KMISTTSKET KKDTKLYCIC KTPYDESKFY IGCDLCTNWY HGECVGITEK EAKKMDVYIC NECKRAQEGS SEELYCICRT
3041 PYDESQFYIG CDRCQNWYHG RCVGILQSEA DLIDEYVCPQ CQSTEDAMTV LSPLTDKDYE GLKRVLRSLQ AHKMAWPFLE
3121 PVDPNDAPDY YGVIKEPMDL STMEERILKR YYKKVTEFVA DMTKIFDNCR YYNPSDSPFY QCAEVLESFF VQKLKGFKAS
3201 RSHNNKLQST AS

**Supporting Peptides:**

| Peptide | Uniq | -10lgP | Mass | Length | ppm | m/z | z | RT | Fraction | Scan | Source File | Area F1 | Area F2a | Area F3a | Area F4 | Area F5 | Area F6a | Area F7a | Area F7b | Area F8 | Area F9a | Area Sample 1F10a | Area F10b | Area F11a | Area F11b | Area F12a | Area F13 | #Feature | #Feature F1 | #Feature F2a | #Feature F3a | #Feature F4 | #Feature F5 | #Feature F6a | #Feature F7a | #Feature F7b | #Feature F8 | #Feature F9a | #Feature Sample 1F10a | #Feature F10b | #Feature F11a | #Feature F11b | #Feature F12a | #Feature F13 | Start | End | PTM | AScore | Found By |
|---|---|---|---|---|---|---|---|---|---|---|---|---|---|---|---|---|---|---|---|---|---|---|---|---|---|---|---|---|---|---|---|---|---|---|---|---|---|---|---|---|---|---|---|---|---|---|---|---|---|
| R.AGAAAAAAGGGGGGGSRSSSSSQGRAAASSR.R | Y | 42.54 | 2534.1929 | 31 | -4.4 | 845.7345 | 3 | 68.02 | 6 | F6:46654 | BuCaTN01_F6a.raw | | | | | | 0 | | | | | | | | | | | 0 | 0 | 0 | 0 | 0 | 0 | 0 | 0 | 0 | 0 | 0 | 0 | 0 | 0 | 0 | 0 | 0 | 109 | 139 | | | PEAKS DB |
| **total 1 peptides** | | | | | | | | | | | | | | | | | | | | | | | | | | | | | | | | | | | | | | | | | | | | | | | | | |

**Best Unique PSM (Scan F6:46654, m/z=845.7345, z=3, RT=68.02, ppm=-4.4):**

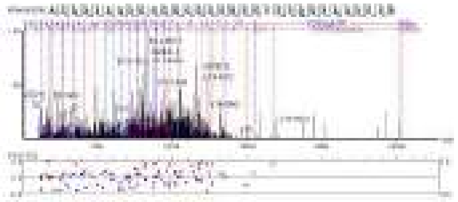

## XP_039182757.1

back to list

| Protein Coverage | Supporting Peptides | Best Unique PSM |

**Protein Coverage:**

```
   1 MRGRRGRPPK HQQPAAAPAR ASTPAPPAPP GPIGGLRSRL RGSSRGRWAA SGQAAGAAGP RSRGSGAGQA STAAASSSSS
  81 RGGGRRKGGG GSSSTPGGGG SSGRGRGRAG AAAAAAGGGG GGGSRSSSSS QGRAAASSRR SGGGKVVYDD HESDEEEEEE
 161 SVGSDEDEED GEAEEKPDSQ EEEAVMEDDD DDDDSDYPEE MEDEDDASYC TESSFRSHST YSSTPGRRRQ RIHRPRSPIL
 241 EEKDIPFLDL PKSSGDLLVP NEHIMNVIAI YEVLRNFGTV LRLSPFRFED FCAALVSQEQ CTLMAEMHIV LLKAVLREED
 321 TSNTTFGPAD LKDSVNSTLY FIDGMTWPEV LRVYCESDKE YHHVLPFQEA EDYPYGPVKN KIQVLQFLVD QFLTTNIARE
 401 ELMSEGVIQY DDHCRVCHKL GDLLCCETCS AVYHLECVKP PLEEVPEDEW QCEVCVAHKV PGVTDCIAEI QKNKPYIRHE
 481 PIGYDRNRRK YWFLNRRIVI EEDSECEKHK RIWYYSTKVQ LAELTECLDK DYWEADICKT LEEMREEIHR HMDITEDLTN
 561 KARGSNKSFL TAANEILETI RGEKGELLED RSLVADEEKT TVENKECTDT ENGKEELSNQ QMEVNKEIVA EESSEKGRKE
 641 ESADACNEND SVLSLPGSSI PNVSAEAADA SEGKTTVGCD SETPDDNKAD KNMASEDLKA ISDETSKTIP NLNIDSSAET
 721 HLSDQEHSSS KEPNSMQSES VKNSDEVEIT EGDSQNSEDP GDKSNGERSE SPNSGIGTPG STRMVTRLRN PDSKLSQLKI
 801 QQVAAAAHEA NKLFKEGKEV LVVNSQGEIS RMNTKKEIVM KGNISNYFKL GQEGKYRVYH NQYATNSFAL NKHQHREDHD
 881 KRRHLSHKFC LSPAGEFKWN GSVHGSKVLT ISTLRLTIIQ LENNIPSSFL HPNWASHRSN WIKAVQMCSK PREFALALAI
 961 LECAIKPVVM LPIWRESLGH TRLRRMTAIE REEKEKVKKK ERKQEEEETM QQATWVKYTF PIKHQVWKQK GEEYRVTGYG
1041 GWIWISKTHV HRFVPRLPGN TNISYRKLLL AKNNTAAITE QQYELDKRKN PAKVKVEHCS LKSRIKNFHE PHKKDQKETE
1121 HSETTKKSQT KEENELNEEK AEIDGLFEKP DHAKEDEKGI CENDKDIKEE PMEIDEVKMD TSPKCEETPI SADLINVSEG
1201 FHLRTCYKRK VKTSKLDGLL ERRVKQFTLE EKQRLERIKL KAASNSSDCK SLSPQRNTDA LPLVKTETDE KRAHSLGTKQ
1281 SEDPVKDCLS PNNIQFNKNS ESVQSLSCLN RLLDEESDLI SDQCSDTQCL IAANAHGSAS EPADTEKNKM LNKEDDNSTM
1361 EHSQAIDNKE STKCSVLEDN EKSSEEITGP NCEDNIQPEG IEKNCEKDTA SLIKEENETL KTENQGRDSV EACNLEDHHA
1441 ANKINVSLPK SSEDASEPKI ALNPKPDLEG LKSLRTELIS EKAFEAQIPD IIGENIIDEI THCKIAETNG EKLEQAKCDP
1521 PTSISKCIDQ IKSIADKNNN KNGEHDVQMD RGKSAPYQMN GKDADFKVLP NEECLKREAL AEAESNLDSK VNNINKNNLL
1601 GKTFPSNESV KPFMNGDIAV EDVDDRRNSD NEFSIGSSVD PNCDSRGGVL PQGDTSLSAQ DSGRKQCSSE RSSPVDDASI
1681 TAIHSCNENS ISSETECMEI ESSCVKKAAL SPVISCEESS LSNDFADQNG LQTHRIKNIN GENAVKAVVT EVTTTSTIST
1761 ESKTVRKMSE LSAVKEDTER VMSSTENCSI STITTTTTTT VTKLSTPTTN SSTDVISVKE HSKTVVTTTV TDSLTTPSGT
1841 LLTSMTVSKE YSTRNKVKLM KFTRPKKTRS GTALPSYRKF VTKSSKKSIF VLPSDDLKKL ARRGGIREVP CFNYTAKPAL
1921 DIWPYPSPRP TFGITWRYRL QTVKSLAGVS LMLRLLWACL KWDDMAAKPP PGGGTTRTET SEMEITTTEI IKRRDVGPYG
2001 IRSEYCIRKI ICPIGVPEAP KETPTPQRKG LRSSALRPKR PETPKQTGPV IIETWVAEEE LELWEIKAFS ERVEKEKAQA
2081 VEQQAKVSEQ KKAEEFKAQM ETQLKQQRLA AQQKRLEQQK QVPIVCGAAT ATTTSSTVTT VSTPQKVVVG QLPRSMPSGA
2161 KVVLATKMGS PATVTFQQNK NFHQTFATWV KQGQPSTATS TAATSATTIS SSGQTFQIAS SPVTMAGKVI TKLPLPANSK
2241 IVAVNVPATQ GGVVQVQQKV LGIIPSTTGA NQQTFTSFQP RTATVTIRPN NTGTVCTTSA SQVMPGTPLR PGMTVIRAPL
2321 QQSSLGKAII RTPVVVQQGV LPTSQTQQVV TQIIRGQPVS TAVSSPTTVS TSQKVATAPS QHMQPQAAPP HPPRPQQGQV
2401 KLTMAQLTQL TQGQGGSQGL TVVIQGQGQT TGQLQLIPQG VTVIPGPGQQ LMQAAMPNGT IQRFLFTPLS TAATAASTTS
2481 TTVSTSTSAA GEQKQTSLAQ TQTQPTPALP PAEPKPAPTS FSAPPPSSSA QTVMPAQTQT VQAPVPVEPH PFPEAQVPPS
2561 LPSPTVTSEA QPPRTQSTVT PTVAQPPPQS LAQGLPFPPV QNQAQPVMHL QTPPKGQTIQ PTQVQTAAPQ QVQIQPHPSV
2641 QIQTQQSQVP TSVPPLPPAS TVNQVTVLPP RPQLQIQPPQ TKVITMPQLQ QQVQVFSQLP SHMVAQIQAQ QSGLPQQIKL
2721 QLPIQIQQAS AVQTHQIQNV VTVQASVQEQ LQRVQQLREQ QQKKKQQHIE IKREHSLQAS NQSDIIQKQV VMKHNAVIEH
2801 LKQKKTLTPA EREENQRMIV CNQVMKYILD KIDKEEKQAA KKRKREESVE QKRSKQNASK LSALLFKHKE QLKAEILKKR
2881 ALLDKDLQIE VQEELKKDLT KIKREKEKAQ AAAAAAAAAA ALSPPPPPPP LPPPPPPPPP PPPPPLPPPA SSSSIPSTAS
2961 GTSYISSHKR KREEEKESAS SKSKKKKMIS TTSKETKKDT KLYCICKTPY DESKFYIGCD LCTNWYHGEC VGITEKEAKK
3041 MDVYICNECK RAQEGSSEEL YCICRTPYDE SQFYIGCDRC QNWYHGRCVG ILQSEADLID EYVCPQCQST EDAMTVLSPL
3121 TDKDYEGLKR VLRSLQAHKM AWPFLEPVDP NDAPDYYGVI KEPMDLSTME ERILKRYYKK VTEFVADMTK IFDNCRYYNP
3201 SDSPFYQCAE VLESFFVQKL KGFKASRSHN NKLQSTAS
```

**Supporting Peptides:**

| Peptide | Uniq | -10lgP | Mass | Length | ppm | m/z | z | RT | Fraction | Scan | Source File | Area F1 | Area F2a | Area F3a | Area F4 | Area F5 | Area F6a | Area F7a | Area F7b | Area F8 | Area F9a | Area Sample 1F10a | Area F10b | Area F11a | Area F11b | Area F12a | Area F13 |
|---|---|---|---|---|---|---|---|---|---|---|---|---|---|---|---|---|---|---|---|---|---|---|---|---|---|---|---|
| R.AGAAAAAAGGGGGGGSRSSSSSQGRAAASSR.R | Y | 42.54 | 2534.1929 | 31 | -4.4 | 845.7345 | 3 | 68.02 | 6 | F6:46654 | BuCaTN01_F6a.raw | | | | | | 0 | | | | | | | | | | |
| total 1 peptides | | | | | | | | | | | | | | | | | | | | | | | | | | | |

| Peptide | #Feature | #Feature F1 | #Feature F2a | #Feature F3a | #Feature F4 | #Feature F5 | #Feature F6a | #Feature F7a | #Feature F7b | #Feature F8 | #Feature F9a | #Feature Sample 1F10a | #Feature F10b | #Feature F11a | #Feature F11b | #Feature F12a | #Feature F13 | Start | End | PTM | AScore | Found By |
|---|---|---|---|---|---|---|---|---|---|---|---|---|---|---|---|---|---|---|---|---|---|---|
| R.AGAAAAAAGGGGGGGSRSSSSSQGRAAASSR.R | 0 | 0 | 0 | 0 | 0 | 0 | 0 | 0 | 0 | 0 | 0 | 0 | 0 | 0 | 0 | 0 | 0 | 109 | 139 | | | PEAKS DB |
| total 1 peptides | | | | | | | | | | | | | | | | | | | | | | |

**Best Unique PSM (Scan F6:46654, m/z=845.7345, z=3, RT=68.02, ppm=-4.4):**

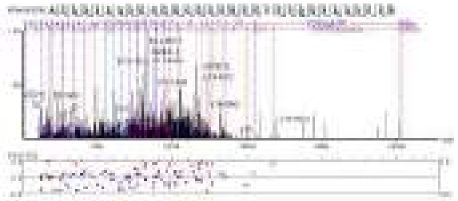

## XP_039182756.1

back to list

| Protein Coverage | Supporting Peptides | Best Unique PSM |

**Protein Coverage:**

```
   1 MRGRRGRPPK HQQPAAAPAR ASTPAPPAPP GPIGGLRSRL RGSSRGRWAA SGQAAGAAGP RSRGSGAGQA STAAASSSSS
  81 RGGGRRKGGG GSSSTPGGGG SSGRGRGRAG AAAAAAGGGG GGGSRSSSSS QGRAAASSRR SGGGKVVYDD HESDEEEEEE
 161 SVGSDEDEED GEAEEKPDSQ EEEAVMEDDD DDDDSDYPEE MEDEDDASYC TESSFRSHST YSSTPGRRRQ RIHRPRSPIL
 241 EEKDIPFLDL PKSSGDLLVP NEHIMNVIAI YEVLRNFGTV LRLSPFRFED FCAALVSQEQ CTLMAEMHIV LLKAVLREED
 321 TSNTTFGPAD LKDSVNSTLY FIDGMTWPEV LRVYCESDKE YHHVLPFQEA EDYPYGPVKN KIQVLQFLVD QFLTTNIARE
 401 ELMSEGVIQY DDHCRVCHKL GDLLCCETCS AVYHLECVKP PLEEVPEDEW QCEVCVAHKV PGVTDCIAEI QKNKPYIRHE
 481 PIGYDRNRRK YWFLNRRIVI EEDSECEKHK RIWYYSTKVQ LAELTECLDK DYWEADICKT LEEMREEIHR HMDITEDLTN
 561 KARGSNKSFL TAANEEILET IRGEKGELLE DRSLVADEEK TTVENKECTD TENGKEELSN QQMEVNKEIV AEESSEKGRK
 641 EESADACNEN DSVLSLPGSS IPNVSAEAAD ASEGKTTVGC DSETPDDNKA DKNMASEDLK AISDETSKTI PNLNIDSSAE
 721 THLSDQEHSS SKEPNSMQSE SVKNSDEVEI TEGDSQNSED PGDKSNGERS ESPNSGIGTP GSTRMVTRLR NPDSKLSQLK
 801 IQQVAAAAHE ANKLFKEGKE VLVVNSQGEI SRMNTKKEIV MKGNISNYFK LGQEGKYRVY HNQYATNSFA LNKHQHREDH
 881 DKRRHLSHKF CLSPAGEFKW NGSVHGSKVL TISTLRLTII QLENNIPSSF LHPNWASHRS NWIKAVQMCS KPREFALALA
 961 ILECAIKPVV MLPIWRESLG HTRLRRMTAI EREEKEKVKK KERKQEEEET MQQATWVKYT FPIKHQVWKQ KGEEYRVTGY
1041 GGWIWISKTH VHRFVPRLPG NTNISYRKLL LAKNNTAAIT EQQYELDKRK NPAKVKVEHC SLKSRIKNFH EPHKKDQKET
1121 EHSETTKKSQ TKEENELNEE KAEIDGLFEK PDHAKEDEKG ICENDKDIKE EPMEIDEVKM DTSPKCEETP ISADLINVSE
1201 GFHLRTCYKR KVKTSKLDGL LERRVKQFTL EEKQRLERIK LKAASNSSDC KSLSPQRNTD ALPLVKTETD EKRAHSLGTK
1281 QSEDPVKDCL SPNNIQFNKN SESVQSLSCL NRLLDEESDL ISDQCSDTQC LIAANAHGSA SEPADTEKNK MLNKEDDNST
1361 MEHSQAIDNK ESTKCSVLED NEKSSEEITG PNCEDNIQPE GIEKNCEKDT ASLIKEENET LKTENQGRDS VEACNLEDHH
1441 AANKINVSLP KSSEDASEPK IALNPKPDLE GLKSLRTELI SEKAFEAQIP DIIGENIIDE ITHCKIAETN GEKLEQAKCD
1521 PPTSISKCID QIKSIADKNN NKNGEHDVQM DRGKSAPYQM NGKDADFKVL PNEECLKREA LAEAESNLDS KVNNINKNNL
1601 LGKTFPSNES VKPFMNGDIA VEDVDDRRNS DNEFSIGSSV DPNCDSRGGV LPQGDTSLSA QDSGRKQCSS ERSSPVDDAS
1681 ITAIHSCNEN SISSETECME IESSCVKKAA LSPVISCEES SLSNDFADQN GLQTHRIKNI NGENAVKAVV TEVTTTSTIS
1761 TESKTVRKMS ELSAVKEDTE RVMSSTENCS ISTITTTTTT TVTKLSTPTT NSSTDVISVK EHSKTVVTTT VTDSLTTPSG
1841 TLLTSMTVSK EYSTRNKVKL MKFTRPKKTR SGTALPSYRK FVTKSSKKSI FVLPSDDLKK LARRGGIREV PCFNYTAKPA
1921 LDIWPYPSPR PTFGITWRYR LQTVKSLAGV SLMLRLLWAC LKWDDMAAKP PPGGGTTRTE TSEMEITTTE IIKRRDVGPY
2001 GIRSEYCIRK IICPIGVPEA PKETPTPQRK GLRSSALRPK RPETPKQTGP VIIETWVAEE ELELWEIKAF SERVEKEKAQ
2081 AVEQQAKVSE QKKAEEFKAQ METQLKQQRL AAQQKRLEQQ KQVPIVCGAA TATTTSSTVT TVSTPQKVVV GQLPRSMPSG
2161 AKVVLATKMG SPATVTFQQN KNFHQTFATW VKQGQPSTAT STAATSATTI SSSGQTFQIA SSPVTMAGKV ITKLPLPANS
2241 KIVAVNVPAT QGGVVQVQQK VLGIIPSTTG ANQQTFTSFQ PRTATVTIRP NNTGTVCTTS ASQVMPGTPL RPGMTVIRAP
2321 LQQSSLGKAI IRTPVVVQQG VLPTSQTQQV VTQIIRGQPV STAVSSPTTV STSQKVATAP SQHMQPQAAP PHPPRPQQGQ
2401 VKLTMAQLTQ LTQGQGGSQG LTVVIQGQGQ TTGQLQLIPQ GVTVIPGPGQ QLMQAAMPNG TIQRFLFTPL STAATAASTT
2481 STTVSTSTSA AGEQKQTSLA QTQTQPTPAL PPAEPKPAPT SFSAPPPSSS AQTVMPAQTQ TVQAPVPVEP HPFPEAQVPP
2561 SLPSPTVTSE AQPPRTQSTV TPTVAQPPPQ SLAQGLPFPP VQNQAQPVMH LQTPPKGQTI QPTQVQTAAP QQVQIQPHPS
2641 VQIQTQQSQV PTSVPPLPPA STVNQVTVLP PRPQLQIQPP QTKVITMPQL QQQVQVFSQL PSHMVAQIQA QQSGLPQQIK
2721 LQLPIQIQQA SAVQTHQIQN VVTVQASVQE QLQRVQQLRE QQQKKKQQHI EIKREHSLQA SNQSDIIQKQ VVMKHNAVIE
2801 HLKQKKTLTP AEREENQRMI VCNQVMKYIL DKIDKEEKQA AKKRKREESV EQKRSKQNAS KLSALLFKHK EQLKAEILKK
2881 RALLDKDLQI EVQEELKKDL TKIKREKEKA QAAAAAAAAA AALSPPPPPP PLPPPPPPPP PPPPPPLPPP ASSSSIPSTA
2961 SGTSYISSHK RKREEEKESA SSKSKKKKMI STTSKETKKD TKLYCICKTP YDESKFYIGC DLCTNWYHGE CVGITEKEAK
3041 KMDVYICNEC KRAQEGSSEE LYCICRTPYD ESQFYIGCDR CQNWYHGRCV GILQSEADLI DEYVCPQCQS TEDAMTVLSP
3121 LTDKDYEGLK RVLRSLQAHK MAWPFLEPVD PNDAPDYYGV IKEPMDLSTM EERILKRYYK KVTEFVADMT KIFDNCRYYN
3201 PSDSPFYQCA EVLESFFVQK LKGFKASRSH NNKLQSTAS
```

**Supporting Peptides:**

| Peptide | Uniq | -10lgP | Mass | Length | ppm | m/z | z | RT | Fraction | Scan | Source File | Area F1 | Area F2a | Area F3a | Area F4 | Area F5 | Area F6a | Area F7a | Area F7b | Area F8 | Area F9a | Area Sample 1F10a | Area F10b | Area F11a | Area F11b | Area F12a | Area F13 |
|---|---|---|---|---|---|---|---|---|---|---|---|---|---|---|---|---|---|---|---|---|---|---|---|---|---|---|---|
| R.AGAAAAAAGGGGGGGSRSSSSSQGRAAASSR.R | Y | 42.54 | 2534.1929 | 31 | -4.4 | 845.7345 | 3 | 68.02 | 6 | F6:46654 | BuCaTN01_F6a.raw | | | | | | 0 | | | | | | | | | | |
| total 1 peptides | | | | | | | | | | | | | | | | | | | | | | | | | | | |

| Peptide | #Feature | #Feature F1 | #Feature F2a | #Feature F3a | #Feature F4 | #Feature F5 | #Feature F6a | #Feature F7a | #Feature F7b | #Feature F8 | #Feature F9a | #Feature Sample 1F10a | #Feature F10b | #Feature F11a | #Feature F11b | #Feature F12a | #Feature F13 | Start | End | PTM | AScore | Found By |
|---|---|---|---|---|---|---|---|---|---|---|---|---|---|---|---|---|---|---|---|---|---|---|
| R.AGAAAAAAGGGGGGGSRSSSSSQGRAAASSR.R | 0 | 0 | 0 | 0 | 0 | 0 | 0 | 0 | 0 | 0 | 0 | 0 | 0 | 0 | 0 | 0 | 0 | 109 | 139 | | | PEAKS DB |

**Best Unique PSM (Scan F6:46654, m/z=845.7345, z=3, RT=68.02, ppm=-4.4):**

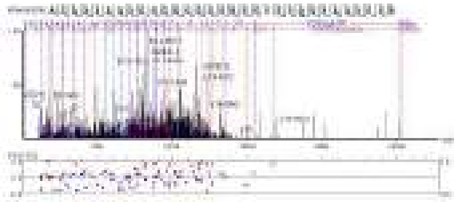

## XP_039182763.1

back to list

| Protein Coverage | Supporting Peptides | Best Unique PSM |

**Protein Coverage:**

```
   1 MRGRRGRPPK HQQPAAAPAR ASTPAPPAPP GPIGGLRSRL RGSSRGRWAA SGQAAGAAGP RSRGSGAGQA STAAASSSSS
  81 RGGGRRKGGG GSSSTPGGGG SSGRGRGRAG AAAAAAGGGG GGGSRSSSSS QGRAAASSRR SGGGKVVYDD HESDEEEEEE
 161 SVGSDEDEED GEAEEKPDSQ EEEAVMEDDD DDDDSDYPEE MEDEDDASYC TESSFRSHST YSSTPGRRRQ RIHRPRSPIL
 241 EEKDIPFLDL PKSSGDLLVP NEHIMNVIAI YEVLRNFGTV LRLSPFRFED FCAALVSQEQ CTLMAEMHIV LLKAVLREED
 321 TSNTTFGPAD LKDSVNSTLY FIDGMTWPEV LRVYCESDKE YHHVLPFQEA EDYPYGPVKN KIQVLQFLVD QFLTTNIARE
 401 ELMSEGVIQY DDHCRVCHKL GDLLCCETCS AVYHLECVKP PLEEVPEDEW QCEVCVAHKV PGVTDCIAEI QKNKPYIRHE
 481 PIGYDRNRRK YWFLNRRIVI EEDSECEKHK RIWYYSTKVQ LAELTECLDK DYWEADICKT LEEMREEIHR HMDITEDLTN
 561 KARGSNKSFL TAANEEILET IRGEKGELLE DRSLVADEEK TTVENKECTD TENGKEELSN QQMEVNKEIV AEESSEKGRK
 641 EESADACNEN DSVLSLPGSS IPNVSAEAAD ASEGKTTVGC DSETPDDNKA DKNMASEDLK AISDETSKTI PNLNIDSSAE
 721 THLSDQEHSS SKEPNSMQSE SVKNSDEVEI TEGDSQNSED PGDKSNGERS ESPNSGIGTP GSTRMVTRLR NPDSKLSQLK
 801 IQQVAAAAHE ANKLFKEGKE VLVVNSQGEI SRMNTKKEIV MKGNISNYFK LGQEGKYRVY HNQYATNSFA LNKHQHREDH
 881 DKRRHLSHKF CLSPAGEFKW NGSVHGSKVL TISTLRLTII QLENNIPSSF LHPNWASHRS NWIKAVQMCS KPREFALALA
 961 ILECAIKPVV MLPIWRESLG HTRLRRMTAI EREEKEKVKK KERKQEEEET MQQATWVKYT FPIKHQVWKQ KGEEYRVTGY
1041 GGWIWISKTH VHRFVPRLPG NTNISYRKLL LAKNNTAAIT EQQYELDKRK NPAKVKVEHC SLKSRIKNFH EPHKKDQKET
1121 EHSETTKKSQ TKEENELNEE KAEIDGLFEK PDHAKEDEKG ICENDKDIKE EPMEIDEVKM DTSPKCEETP ISADLINVSE
1201 GFHLRTCYKR KVKTSKLDGL LERRVKQFTL EEKQRLERIK LKAASNSSDC KSLSPQRNTD ALPLVKTETD EKRAHSLGTK
1281 QSEDPVKDCL SPNNIQFNKN SESVQSLSCL NRLLDEESDL ISDQCSDTQC LIAANAHGSA SEPADTEKNK MLNKEDDNST
1361 MEHSQAIDNK ESTKCSVLED NEKSSEEITG PNCEDNIQPE GIEKNCEKDT ASLIKEENET LKTENQGRDS VEACNLEDHH
1441 AANKINVSLP KSSEDASEPK IALNPKPDLE GLKSLRTELI SEKAFEAQIP DIIGENIIDE ITHCKIAETN GEKLEQAKCD
1521 PPTSISKCID QIKSIADKNN NKNGEHDVQM DRGKSAPYQM NGKDADFKVL PNEECLKREA LAEAESNLDS KVNNINKNNL
1601 LGKTFPSNES VKPFMNGDIA VEDVDDRRNS DNEFSIGSSV DPNCDSRGGV LPQGDTSLSA QDSGRKQCSS ERSSPVDDAS
1681 ITAIHSCNEN SISSETECME IESSCVKKAA LSPVISCEES SLSNDFADQN GLQTHRIKNI NGENAVKAVV TEVTTTSTIS
1761 TESKTVRKMS ELSAVKEDTE RVMSSTENCS ISTITTTTTT TVTKLSTPTT NSSTDVISVK EHSKTVVTTT VTDSLTTPSG
1841 TLLTSMTVSK EYSTRNKVKL MKFTRPKKTR SGTALPSYRK FVTKSSKKSI FVLPSDDLKK LARRGGIREV PCFNYTAKPA
1921 LDIWPYPSPR PTFGITWRYR LQTVKSLAGV SLMLRLLWAC LKWDDMAAKP PPGGGTTRTE TSEMEITTTE IIKRRDVGPY
2001 GIRSEYCIRK IICPIGVPEA PKETPTPQRK GLRSSALRPK RPETPKQTGP VIIETWVAEE ELELWEIKAF SERVEKEKAQ
2081 AVEQQAKKRL EQQKQVPIVC GAATATTTSS TVTTVSTPQK VVVGQLPRSM PSGAKVVLAT KMGSPATVTF QQNKNFHQTF
2161 ATWVKQGQPS TGVVQVQQKV LGIIPSTTGA NQQTFTSFQP RTATVTIRPN NTGTVCTTSA SQVMPGTPLR PGMTVIRAPL
2241 QQSSLGKAII RTPVVVQQGV LPTSQTQQVV TQIIRGQPVS TAVSSPTTVS TSQKVATAPS QHMQPQAAPP HPPRPQQGQV
2321 KLTMAQLTQL TQGQGGSQGL TVVIQGQGQT TGQLQLIPQG VTVIPGPGQQ LMQAAMPNGT IQRFLFTPLS TAATAASTTS
2401 TTVSTSTSAA GEQKQTSLAQ TQTQPTPALP PAEPKPAPTS FSAPPPSSSA QTVMPAQTQT VQAPVPVEPH PFPEAQVPPS
2481 LPSPTVTSEA QPPRTQSTVT PTVAQPPPQS LAQGLPFPPV QNQAQPVMHL QTPPKGQTIQ PTQVQTAAPQ QVQIQPHPSV
2561 QIQTQQSQVP TSVPPLPPAS TVNQVTVLPP RPQLQIQPPQ TKVITMPQLQ QQVQVFSQLP SHMVAQIQAQ QSGLPQQIKL
2641 QLPIQIQQAS AVQTHQIQNV VTVQASVQEQ LQRVQQLREQ QQKKKQQHIE IKREHSLQAS NQSDIIQKQV VMKHNAVIEH
2721 LKQKKTLTPA EREENQRMIV CNQVMKYILD KIDKEEKQAA KKRKREESVE QKRSKQNASK LSALLFKHKE QLKAEILKKR
2801 ALLDKDLQIE VQEELKKDLT KIKREKEKAQ AAAAAAAAAA ALSPPPPPPP LPPPPPPPPP PPPPPLPPPA SSSSIPSTAS
2881 GTSYISSHKR KREEEKESAS SKSKKKKMIS TTSKETKKDT KLYCICKTPY DESKFYIGCD RCQNWYHGRC VGILQSEADL
2961 IDEYVCPQCQ STEDAMTVLS PLTDKDYEGL KRVLRSLQAH KMAWPFLEPV DPNDAPDYYG VIKEPMDLST MEERILKRYY
3041 KKVTEFVADM TKIFDNCRYY NPSDSPFYQC AEVLESFFVQ KLKGFKASRS HNNKLQSTAS
```

**Supporting Peptides:**

| Peptide | Uniq | -10lgP | Mass | Length | ppm | m/z | z | RT | Fraction | Scan | Source File | Area F1 | Area F2a | Area F3a | Area F4 | Area F5 | Area F6a | Area F7a | Area F7b | Area F8 | Area F9a | Area Sample 1F10a | Area F10b | Area F11a | Area F11b | Area F12a | Area F13 |
|---|---|---|---|---|---|---|---|---|---|---|---|---|---|---|---|---|---|---|---|---|---|---|---|---|---|---|---|
| R.AGAAAAAAGGGGGGGSRSSSSSQGRAAASSR.R | Y | 42.54 | 2534.1929 | 31 | -4.4 | 845.7345 | 3 | 68.02 | 6 | F6:46654 | BuCaTN01_F6a.raw | | | | | | 0 | | | | | | | | | | |
| total 1 peptides | | | | | | | | | | | | | | | | | | | | | | | | | | | |

| Peptide | #Feature | #Feature F1 | #Feature F2a | #Feature F3a | #Feature F4 | #Feature F5 | #Feature F6a | #Feature F7a | #Feature F7b | #Feature F8 | #Feature F9a | #Feature Sample 1F10a | #Feature F10b | #Feature F11a | #Feature F11b | #Feature F12a | #Feature F13 | Start | End | PTM | AScore | Found By |
|---|---|---|---|---|---|---|---|---|---|---|---|---|---|---|---|---|---|---|---|---|---|---|
| R.AGAAAAAAGGGGGGGSRSSSSSQGRAAASSR.R | 0 | 0 | 0 | 0 | 0 | 0 | 0 | 0 | 0 | 0 | 0 | 0 | 0 | 0 | 0 | 0 | 0 | 109 | 139 | | | PEAKS DB |
| total 1 peptides | | | | | | | | | | | | | | | | | | | | | | |

**Best Unique PSM (Scan F6:46654, m/z=845.7345, z=3, RT=68.02, ppm=-4.4):**

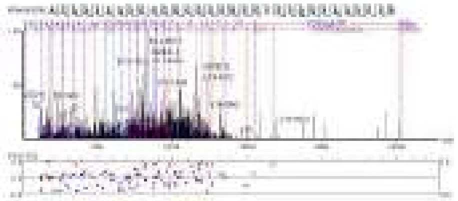

## XP_039182762.1

back to list

| Protein Coverage | Supporting Peptides | Best Unique PSM |

**Protein Coverage:**

1 MRGRRGRPPK HQQPAAAPAR ASTPAPPAPP GPIGGLRSRL RGSSRGRWAA SGQAAGAAGP RSRGSGAGQA STAAASSSSS
81 RGGGRRKGGG GSSSTPGGGG SSGRGRGR**AG AAAAAAGGGG GGGSRSSSSS QGRAAASSR**R SGGGKVVYDD HESDEEEEEE
161 SVGSDEDEED GEAEEKPDSQ EEEAVMEDDD DDDDSDYPEE MEDEDDASYC TESSFRSHST YSSTPGRRRQ RIHRPRSPIL
241 EEKDIPFLDL PKSSGDLLVP NEHIMNVIAI YEVLRNFGTV LRLSPFRFED FCAALVSQEQ CTLMAEMHIV LLKAVLREED
321 TSNTTFGPAD LKDSVNSTLY FIDGMTWPEV LRVYCESDKE YHHVLPFQEA EDYPYGPVKN KIQVLQFLVD QFLTTNIARE
401 ELMSEGVIQY DDHCRVCHKL GDLLCCETCS AVYHLECVKP PLEEVPEDEW QCEVCVAHKV PGVTDCIAEI QKNKPYIRHE
481 PIGYDRNRRK YWFLNRRIVI EEDSECEKHK RIWYYSTKVQ LAELTECLDK DYWEADICKT LEEMREEIHR HMDITEDLTN
561 KARGSNKSFL TAANEEILET IRGEKGELLE DRSLVADEEK TTVENKECTD TENGKEELSN QQMEVNKEIV AEESSEKGRK
641 EESADACNEN DSVLSLPGSS IPNVSAEAAD ASEGKTTVGC DSETPDDNKA DKNMASEDLK AISDETSKTI PNLNIDSSAE
721 THLSDQEHSS SKEPNSMQSE SVKNSDEVEI TEGDSQNSED PGDKSNGERS ESPNSGIGTP GSTRMVTRLR NPDSKLSQLK
801 IQQVAAAAHE ANKLFKEGKE VLVVNSQGEI SRMNTKKEIV MKGNISNYFK LGQEGKYRVY HNQYATNSFA LNKHQHREDH
881 DKRRHLSHKF CLSPAGEFKW NGSVHGSKVL TISTLRLTII QLENNIPSSF LHPNWASHRS NWIKAVQMCS KPREFALALA
961 ILECAIKPVV MLPIWRESLG HTRLRRMTAI EREEKEKVKK KERKQEEEET MQQATWVKYT FPIKHQVWKQ KGEEYRVTGY
1041 GGWIWISKTH VHRFVPRLPG NTNISYRKLL LAKNNTAAIT EQQYELDKRK NPAKVKVEHC SLKSRIKNFH EPHKKDQKET
1121 EHSETTKKSQ TKEENELNEE KAEIDGLFEK PDHAKEDEKG ICENDKDIKE EPMEIDEVKM DTSPKCEETP ISADLINVSE
1201 GFHLRTCYKR KVKTSKLDGL LERRVKQFTL EEKQRLERIK LKAASNSSDC KSLSPQRNTD ALPLVKTETD EKRAHSLGTK
1281 QSEDPVKDCL SPNNIQFNKN SESVQSLSCL NRLLDEESDL ISDQCSDTQC LIAANAHGSA SEPADTEKNK MLNKEDDNST
1361 MEHSQAIDNK ESTKCSVLED NEKSSEEITG PNCEDNIQPE GIEKNCEKDT ASLIKEENET LKTENQGRDS VEACNLEDHH
1441 AANKINVSLP KSSEDASEPK IALNPKPDLE GLKSLRTELI SEKAFEAQIP DIIGENIIDE ITHCKIAETN GEKLEQAKCD
1521 PPTSISKCID QIKSIADKNN NKNGEHDVQM DRGKSAPYQM NGKDADFKVL PNEECLKREA LAEAESNLDS KVNNINKNNL
1601 LGKTFPSNES VKPFMNGDIA VEDVDDRRNS DNEFSIGSSV DPNCDSRGGV LPQGDTSLSA QDSGRKQCSS ERSSPVDDAS
1681 ITAIHSCNEN SISSETECME IESSCVKKAA LSPVISCEES SLSNDFADQN GLQTHRIKNI NGENAVKAVV TEVTTTSTIS
1761 TESKTVRKMS ELSAVKEDTE RVMSSTENCS ISTITTTTTT TVTKLSTPTT NSSTDVISVK EHSKTVVTTT VTDSLTTPSG
1841 TLLTSMTVSK EYSTRNKVKL MKFTRPKKTR SGTALPSYRK FVTKSSKKSI FVLPSDDLKK LARRGGIREV PCFNYTAKPA
1921 LDIWPYPSPR PTFGITWRYR LQTVKSLAGV SLMLRLLWAC LKWDDMAAKP PPGGGTTRTE TSEMEITTTE IIKRRDVGPY
2001 GIRSEYCIRK IICPIGVPEA PKETPTPQRK GLRSSALRPK RPETPKQTGP VIIETWVAEE ELELWEIKAF SERVEKEKAQ
2081 AVEQQAKKRL EQQKQVPIVC GAATATTTSS TVTTVSTPQK VVVGQLPRSM PSGAKVVLAT KMGSPATVTF QQNKNFHQTF
2161 ATWVKQGQPS TGVVQVQQKV LGIIPSTTGA NQQTFTSFQP RTATVTIRPN NTGTVCTTSA SQVMPGTPLR PGMTVIRAPL
2241 QQSSLGKAII RTPVVVQQGV LPTSQTQQVV TQIIRGQPVS TAVSSPTTVS TSQKVATAPS QHMQPQAAPP HPPRPQQGQV
2321 KLTMAQLTQL TQGQGGSQGL TVVIQGQGQT TGQLQLIPQG VTVIPGPGQQ LMQAAMPNGT IQRFLFTPLS TAATAASTTS
2401 TTVSTSTSAA GEQKQTSLAQ TQTQPTPALP PAEPKPAPTS FSAPPPSSSA QTVMPAQTQT VQAPVPVEPH PFPEAQVPPS
2481 LPSPTVTSEA QPPRTQSTVT PTVAQPPPQS LAQGLPFPPV QNQAQPVMHL QTPPKGQTIQ PTQVQTAAPQ QVQIQPHPSV
2561 QIQTQQSQVP TSVPPLPPAS TVNQVTVLPP RPQLQIQPPQ TKVITMPQLQ QQVQVFSQLP SHMVAQIQAQ QSGLPQQIKL
2641 QLPIQIQQAS AVQTHQIQNV VTVQASVQEQ LQRVQQLREQ QQKKKQQHIE IKREHSLQAS NQSDIIQKQV VMKHNAVIEH
2721 LKQKKTLTPA EREENQRMIV CNQVMKYILD KIDKEEKQAA KKRKREESVE QKRSKQNASK LSALLFKHKE QLKAEILKKR
2801 ALLDKDLQIE VQEELKKDLT KIKREKEKAQ AAAAAAAAAA ALSPPPPPPP LPPPPPPPPP PPPPPLPPPA SSSSIPSTAS
2881 GTSYISSHKR KREEEKESAS SKSKKKKMIS TTSKETKKDT KLYCICKTPY DESKFYIGCD LCTNWYHGEC VGITEKEAKK
2961 MDVYICNECK RAQEGSSEEL YCICRTPYDE SQFYIGCDRC QNWYHGRCVG ILQSEADLID EYVCPQCQST EDAMTVLSPL
3041 TDKDYEGLKR VLRSLQAHKM AWPFLEPVDP NDAPDYYGVI KEPMDLSTME ERILKRYYKK VTEFVADMTK IFDNCRYYNP
3121 SDSPFYQCAE VLESFFVQKL KGFKASRSHN NKLQSTAS

**Supporting Peptides:**

| Peptide | Uniq | -10lgP | Mass | Length | ppm | m/z | z | RT | Fraction | Scan | Source File | Area F1 | Area F2a | Area F3a | Area F4 | Area F5 | Area F6a | Area F7a | Area F7b | Area F8 | Area F9a | Area Sample 1F10a | Area F10b | Area F11a | Area F11b | Area F12a | Area F13 |
|---|---|---|---|---|---|---|---|---|---|---|---|---|---|---|---|---|---|---|---|---|---|---|---|---|---|---|---|
| R.AGAAAAAAGGGGGGGSRSSSSSQGRAAASSR.R | Y | 42.54 | 2534.1929 | 31 | -4.4 | 845.7345 | 3 | 68.02 | 6 | F6:46654 | BuCaTN01_F6a.raw | | | | | | 0 | | | | | | | | | | |
| total 1 peptides | | | | | | | | | | | | | | | | | | | | | | | | | | | |

| Peptide | #Feature | #Feature F1 | #Feature F2a | #Feature F3a | #Feature F4 | #Feature F5 | #Feature F6a | #Feature F7a | #Feature F7b | #Feature F8 | #Feature F9a | #Feature Sample 1F10a | #Feature F10b | #Feature F11a | #Feature F11b | #Feature F12a | #Feature F13 | Start | End | PTM | AScore | Found By |
|---|---|---|---|---|---|---|---|---|---|---|---|---|---|---|---|---|---|---|---|---|---|---|
| R.AGAAAAAAGGGGGGGSRSSSSSQGRAAASSR.R | 0 | 0 | 0 | 0 | 0 | 0 | 0 | 0 | 0 | 0 | 0 | 0 | 0 | 0 | 0 | 0 | 0 | 109 | 139 | | | PEAKS DB |
| total 1 peptides | | | | | | | | | | | | | | | | | | | | | | |

**Best Unique PSM (Scan F6:46654, m/z=845.7345, z=3, RT=68.02, ppm=-4.4):**

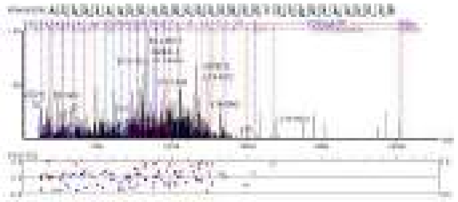

## XP_039182759.1

back to list

| Protein Coverage | Supporting Peptides | Best Unique PSM |

**Protein Coverage:**

1 MRGRRGRPPK HQQPAAAPAR ASTPAPPAPP GPIGGLRSRL RGSSRGRWAA SGQAAGAAGP RSRGSGAGQA STAAASSSSS
81 RGGGRRKGGG GSSSTPGGGG SSGRGRGR**AG AAAAAAGGGG GGGSRSSSSS QGRAAASSR**R SGGGKVVYDD HESDEEEEEE
161 SVGSDEDEED GEAEEKPDSQ EEEAVMEDDD DDDDSDYPEE MEDEDDASYC TESSFRSHST YSSTPGRRRQ RIHRPRSPIL
241 EEKDIPFLDL PKSSGDLLVP NEHIMNVIAI YEVLRNFGTV LRLSPFRFED FCAALVSQEQ CTLMAEMHIV LLKAVLREED
321 TSNTTFGPAD LKDSVNSTLY FIDGMTWPEV LRVYCESDKE YHHVLPFQEA EDYPYGPVKN KIQVLQFLVD QFLTTNIARE
401 ELMSEGVIQY DDHCRVCHKL GDLLCCETCS AVYHLECVKP PLEEVPEDEW QCEVCVAHKV PGVTDCIAEI QKNKPYIRHE
481 PIGYDRNRRK YWFLNRRIVI EEDSECEKHK RIWYYSTKVQ LAELTECLDK DYWEADICKT LEEMREEIHR HMDITEDLTN
561 KARGSNKSFL TAANEEILET IRGEKGELLE DRSLVADEEK TTVENKECTD TENGKEELSN QQMEVNKEIV AEESSEKGRK
641 EESADACNEN DSVLSLPGSS IPNVSAEAAD ASEGKTTVGC DSETPDDNKA DKNMASEDLK AISDETSKTI PNLNIDSSAE
721 THLSDQEHSS SKEPNSMQSE SVKNSDEVEI TEGDSQNSED PGDKSNGERS ESPNSGIGTP GSTRMVTRLR NPDSKLSQLK
801 IQQVAAAAHE ANKLFKEGKE VLVVNSQGEI SRMNTKKEIV MKGNISNYFK LGQEGKYRVY HNQYATNSFA LNKHQHREDH
881 DKRRHLSHKF CLSPAGEFKW NGSVHGSKVL TISTLRLTII QLENNIPSSF LHPNWASHRS NWIKAVQMCS KPREFALALA
961 ILECAIKPVV MLPIWRESLG HTRLRRMTAI EREEKEKVKK KERKQEEEET MQQATWVKYT FPIKHQVWKQ KGEEYRVTGY
1041 GGWIWISKTH VHRFVPRLPG NTNISYRKLL LAKNNTAAIT EQQYELDKRK NPAKVKVEHC SLKSRIKNFH EPHKKDQKET
1121 EHSETTKKSQ TKEENELNEE KAEIDGLFEK PDHAKEDEKG ICENDKDIKE EPMEIDEVKM DTSPKCEETP ISADLINVSE
1201 GFHLRTCYKR KVKTSKLDGL LERRVKQFTL EEKQRLERIK LKAASNSSDC KSLSPQRNTD ALPLVKTETD EKRAHSLGTK
1281 QSEDPVKDCL SPNNIQFNKN SESVQSLSCL NRLLDEESDL ISDQCSDTQC LIAANAHGSA SEPADTEKNK MLNKEDDNST
1361 MEHSQAIDNK ESTKCSVLED NEKSSEEITG PNCEDNIQPE GIEKNCEKDT ASLIKEENET LKTENQGRDS VEACNLEDHH
1441 AANKINVSLP KSSEDASEPK IALNPKPDLE GLKSLRTELI SEKAFEAQIP DIIGENIIDE ITHCKIAETN GEKLEQAKCD
1521 PPTSISKCID QIKSIADKNN NKNGEHDVQM DRGKSAPYQM NGKDADFKVL PNEECLKREA LAEAESNLDS KVNNINKNNL
1601 LGKTFPSNES VKPFMNGDIA VEDVDDRRNS DNEFSIGSSV DPNCDSRGGV LPQGDTSLSA QDSGRKQCSS ERSSPVDDAS
1681 ITAIHSCNEN SISSETECME IESSCVKKAA LSPVISCEES SLSNDFADQN GLQTHRIKNI NGENAVKAVV TEVTTTSTIS
1761 TESKTVRKMS ELSAVKEDTE RVMSSTENCS ISTITTTTTT TVTKLSTPTT NSSTDVISVK EHSKTVVTTT VTDSLTTPSG
1841 TLLTSMTVSK EYSTRNKVKL MKFTRPKKTR SGTALPSYRK FVTKSSKKSI FVLPSDDLKK LARRGGIREV PCFNYTAKPA
1921 LDIWPYPSPR PTFGITWRYR LQTVKSLAGV SLMLRLLWAC LKWDDMAAKP PPGGGTTRTE TSEMEITTTE IIKRRDVGPY
2001 GIRSEYCIRK IICPIGVPEA PKETPTPQRK GLRSSALRPK RPETPKQTGP VIIETWVAEE ELELWEIKAF SERVEKEKAQ
2081 AVEQQAKVSE QKKAEEFKAQ METQLKQQRL AAQQKRLEQQ KQVPIVCGAA TATTTSSTVT TVSTPQKVVV GQLPRSMPSG
2161 AKVVLATKMG SPATVTFQQN KNFHQTFATW VKQGQPSTGV VQVQQKVLGI IPSTTGANQQ TFTSFQPRTA TVTIRPNNTG
2241 TVCTTSASQV MPGTPLRPGM TVIRAPLQQS SLGKAIIRTP VVVQQGVLPT SQTQQVVTQI IRGQPVSTAV SSPTTVSTSQ
2321 KVATAPSQHM QPQAAPPHPP RPQQGQVKLT MAQLTQLTQG QGGSQGLTVV IQGQGQTTGQ LQLIPQGVTV IPGPGQQLMQ
2401 AAMPNGTIQR FLFTPLSTAA TAASTTSTTV STSTSAAGEQ KQTSLAQTQT QPTPALPPAE PKPAPTSFSA PPPSSSAQTV
2481 MPAQTQTVQA PVPVEPHPFP EAQVPPSLPS PTVTSEAQPP RTQSTVTPTV AQPPPQSLAQ GLPFPPVQNQ AQPVMHLQTP
2561 PKGQTIQPTQ VQTAAPQQVQ IQPHPSVQIQ TQQSQVPTSV PPLPPASTVN QVTVLPPRPQ LQIQPPQTKV ITMPQLQQQV
2641 QVFSQLPSHM VAQIQAQQSG LPQQIKLQLP IQIQQASAVQ THQIQNVVTV QASVQEQLQR VQQLREQQQK KKQQHIEIKR
2721 EHSLQASNQS DIIQKQVVMK HNAVIEHLKQ KKTLTPAERE ENQRMIVCNQ VMKYILDKID KEEKQAAKKR KREESVEQKR
2801 SKQNASKLSA LLFKHKEQLK AEILKKRALL DKDLQIEVQE ELKKDLTKIK REKEKAQAAA AAAAAAAALS PPPPPPPLPP
2881 PPPPPPPPPP PPLPPPASSS SIPSTASGTS YISSHKRKRE EEKESASSKS KKKKMISTTS KETKKDTKLY CICKTPYDES
2961 KFYIGCDLCT NWYHGECVGI TEKEAKKMDV YICNECKRAQ EGSSEELYCI CRTPYDESQF YIGCDRCQNW YHGRCVGILQ
3041 SEADLIDEYV CPQCQSTEDA MTVLSPLTDK DYEGLKRVLR SLQAHKMAWP FLEPVDPNDA PDYYGVIKEP MDLSTMEERI
3121 LKRYYKKVTE FVADMTKIFD NCRYYNPSDS PFYQCAEVLE SFFVQKLKGF KASRSHNNKL QSTAS

**Supporting Peptides:**

| Peptide | Uniq | -10lgP | Mass | Length | ppm | m/z | z | RT | Fraction | Scan | Source File | Area F1 | Area F2a | Area F3a | Area F4 | Area F5 | Area F6a | Area F7a | Area F7b | Area F8 | Area F9a | Area Sample 1F10a | Area F10b | Area F11a | Area F11b | Area F12a | Area F13 | #Feature | #Feature F1 | #Feature F2a | #Feature F3a | #Feature F4 | #Feature F5 | #Feature F6a | #Feature F7a | #Feature F7b | #Feature F8 | #Feature F9a | #Feature Sample 1F10a | #Feature F10b | #Feature F11a | #Feature F11b | #Feature F12a | #Feature F13 | Start | End | PTM | AScore | Found By |
|---|---|---|---|---|---|---|---|---|---|---|---|---|---|---|---|---|---|---|---|---|---|---|---|---|---|---|---|---|---|---|---|---|---|---|---|---|---|---|---|---|---|---|---|---|---|---|---|---|---|
| R.AGAAAAAAGGGGGGGSRSSSSSQGRAAASSR.R | Y | 42.54 | 2534.1929 | 31 | -4.4 | 845.7345 | 3 | 68.02 | 6 | F6:46654 | BuCaTN01_F6a.raw | | | | | | 0 | | | | | | | | | | | 0 | 0 | 0 | 0 | 0 | 0 | 0 | 0 | 0 | 0 | 0 | 0 | 0 | 0 | 0 | 0 | 0 | 109 | 139 | | | PEAKS DB |
| total 1 peptides | | | | | | | | | | | | | | | | | | | | | | | | | | | | | | | | | | | | | | | | | | | | | | | | | |

**Best Unique PSM (Scan F6:46654, m/z=845.7345, z=3, RT=68.02, ppm=-4.4):**

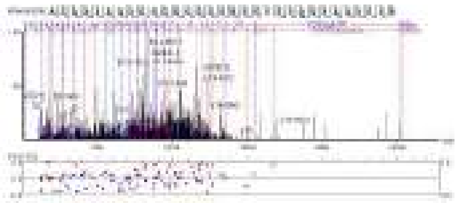

## XP_034278588.1

back to list

| Protein Coverage | Supporting Peptides | Best Unique PSM |

**Protein Coverage:**

1 MAEEAQGPAG PVGEDEAAVA AATTAAGLAA PSAGGSGTTP AASSTPRRRL SDLRVIDLRA ELKRRNLDTG GNKSVLLERL
81 QSAIEEEGGN PDEVPLASDN TSKKTPKRSL KGRKSEEEGG EDNGLEEENE KMLDILGDTC KSEFLKEDAS ESEQAQEANE
161 PSLGKKEEEE DALAAGTQSD EDLVDTDSQS GPAEAKNQED EGKLLVVEKG EPSEQTVEED KPDSDSLAVE SRSGGGGGGG
241 ESSKEAQTLE ACSEETAESD TGPQVVSPEA EKMEVKANVE EEPSATR**ESS APEGGDQNTS SEESKDPK**QP SKEEKGHSST
321 STGRNFWVSG LASSTRATDL KNLFSKYGKV VGAKVVTNAR SPGARCYGFV TMSTAEETTK CIAHCHKTEL HGKIISVERA
401 KNEPPTRKLL DKKDGEGKTE TSSGDRTSSA KKNEKADSKE DSNKADEKDE KGKEEQKSGS AEKSKTSKSG SKGTERTVIM
481 DKSKGEPVIR VKTTSRSKER SEKSQDRKSE SREKSRERQG SVPFNKTRDK RPNEDQHRSG DRRERQRLRL QAIWEQEERK
561 RLEITREKLE FERARLEREQ MERERLERER MYIEHERRRE QERIQREREE LRRQHEQLRY DKERRVALRR PYDMDGRRDD
641 PYWPEAKRMP MDDRHRSDFG RQDRFHDFDH RERGRYQGHP LDRREGSRGM MGDRERQHYA EERHRDPDRH SRDGWGGYGS
721 DRRMNEGRRI PPQSRDGRDW GDHIRKFEGH PDRSWPSNDD GGPPGREHER WRGGGRERRG HIMPRGGMSG QGFIPGGRQE
801 QTAQGEGFAG QERGNRPNDP RFSCHY

**Supporting Peptides:**

| Peptide | Uniq | -10lgP | Mass | Length | ppm | m/z | z | RT | Fraction | Scan | Source File | Area F1 | Area F2a | Area F3a | Area F4 | Area F5 | Area F6a | Area F7a | Area F7b | Area F8 | Area F9a | Area Sample 1F10a | Area F10b | Area F11a | Area F11b | Area F12a | Area F13 | #Feature | #Feature F1 | #Feature F2a | #Feature F3a | #Feature F4 | #Feature F5 | #Feature F6a | #Feature F7a | #Feature F7b | #Feature F8 | #Feature F9a | #Feature Sample 1F10a | #Feature F10b | #Feature F11a | #Feature F11b | #Feature F12a | #Feature F13 | Start | End | PTM | AScore | Found By |
|---|---|---|---|---|---|---|---|---|---|---|---|---|---|---|---|---|---|---|---|---|---|---|---|---|---|---|---|---|---|---|---|---|---|---|---|---|---|---|---|---|---|---|---|---|---|---|---|---|---|
| R.ESSAPEGGDQNTSSEESKDPK.Q | Y | 42.47 | 2177.9197 | 21 | 0.0 | 726.9775 | 3 | 40.77 | 14 | F14:18781 | BuCaTN01_F11b.raw | | | | | | | | | | | | | | 0 | | | 0 | 0 | 0 | 0 | 0 | 0 | 0 | 0 | 0 | 0 | 0 | 0 | 0 | 0 | 0 | 0 | 0 | 288 | 308 | | | PEAKS DB |
| total 1 peptides | | | | | | | | | | | | | | | | | | | | | | | | | | | | | | | | | | | | | | | | | | | | | | | | | |

**Best Unique PSM (Scan F14:18781, m/z=726.9775, z=3, RT=40.77, ppm=0.0):**

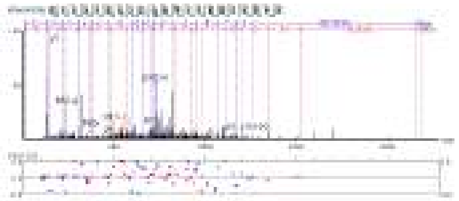

## P82464.1

back to list

| Protein Coverage | Supporting Peptides | Best Unique PSM |

**Protein Coverage:**

3 C

1 **TICYNHLTR**T SETTEICPDS WYFCYKISLA DGNDVRIKRG CTFTCPELRP TGIYVYCCRR DKCNQ

C Carbamidomethylation (+57.02)

**Supporting Peptides:**

| Peptide | Uniq | -10lgP | Mass | Length | ppm | m/z | z | RT | Fraction | Scan | Source File | Area F1 | Area F2a | Area F3a | Area F4 | Area F5 | Area F6a | Area F7a | Area F7b | Area F8 | Area F9a | Area Sample 1F10a | Area F10b | Area F11a | Area F11b | Area F12a | Area F13 | #Feature | #Feature F1 | #Feature F2a | #Feature F3a | #Feature F4 | #Feature F5 | #Feature F6a | #Feature F7a | #Feature F7b | #Feature F8 | #Feature F9a | #Feature Sample 1F10a | #Feature F10b | #Feature F11a | #Feature F11b | #Feature F12a | #Feature F13 | Start | End | PTM | AScore | Found By |
|---|---|---|---|---|---|---|---|---|---|---|---|---|---|---|---|---|---|---|---|---|---|---|---|---|---|---|---|---|---|---|---|---|---|---|---|---|---|---|---|---|---|---|---|---|---|---|---|---|---|
| TIC(+57.02)YNHLTR.T | Y | 42.32 | 1176.5709 | 9 | 0.0 | 393.1976 | 3 | 35.20 | 9 | F9:15736 | BuCaTN01_F8.raw | | | | | | | | | 3.152E5 | | | | | | | | 1 | 0 | 0 | 0 | 0 | 0 | 0 | 0 | 0 | 1 | 0 | 0 | 0 | 0 | 0 | 0 | 0 | 1 | 9 | Carbamidomethylation | C3:Carbamidomethylation :1000.00 | PEAKS DB |
| total 1 peptides | | | | | | | | | | | | | | | | | | | | | | | | | | | | | | | | | | | | | | | | | | | | | | | | | |

**Best Unique PSM (Scan F9:15736, m/z=393.1976, z=3, RT=35.20, ppm=0.0):**

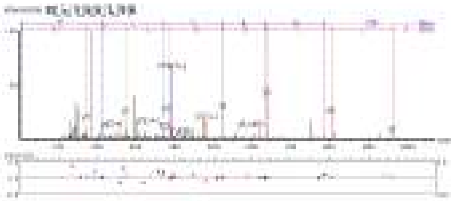

## XP_058025957.1

back to list

| Protein Coverage | Supporting Peptides | Best Unique PSM |

**Protein Coverage:**

```
   1 MDHCSEEAVL GKETDGSLGN IDDLAQQYAD YYNTCFTDVC ERMEELRKRR VSQDLDMEKS DASSTSLQLQ SQIEESLGFS
  81 SVASTPEIER KFSLHKSSSE ESAVGKVDWK KKNKYFWQNF RKNQKGLMRQ SSKGEDVGYV ASEITMSDEE RIQLMMMVKE
 161 KMITIEEALA RLKEYEAQHR QSGNMDASEW SDSPYPTFDV SSNCNSREQS DDESEESVKF KRLHKLVNST RRVRKKLIRV
 241 EEIKKPSTEG VDDHSFDTPA GPDGRPALYS GIHKKQFVLD HSSEKQPDNE NDSLTLSPSS SSLDTWGTNR KLLKTFSKSD
 321 SRGLIKPPKK LGTFFSYPEE DKIPKVCRSL TDGEMKNLGS LSHGVSKESI CFYDSRCKHH VLVSLEEIQS VRKQIRLKKA
 401 DPNYSCSRAF LCQRPARKGV SPSICDLQLL HHKPKRLGRG GSCGFSNRRL RGCNSVSEFN ITYVVERSLY SHLNLSHLVR
 481 PVTDRTLSKS EEQELRRCLL EEEAAAKRKW ASTVDRCTKR VLLRIHQKSR TCSFGGFDLA NRSLHIGNSS DQIGKDGDFV
 561 YKEVIKSPST SRISLGKKVK SVKETMKKRM SKKYSSSNLS EQEPSLDVRP ESLQAPQPDT DTLDKPKLKA GGSVESLRSS
 641 LSGQSSMSGQ TVSTTDSSTS NRESVKSEDG DDEELAYRGP FCGRARVHTD FTPSPYDTDS LKLKKGDIID IISKPPMGTW
 721 MGLLNNKVGT FKFIYVDVLN EEEEKPKKPT RRRRKGRLQQ PKSVEDLLDR INLKEYMPTF LFNGYEDLDT FKLLEEEDLD
 801 ELNIRDPEHR AVLLTAVELL QEYDSNSDQS GSQEKLLVDG QDLSGCSPRD SGCYESSENL ENAKIRQPCL LPSKLASEYH
 881 FKDFHRNPLA NFPNLPLIKS TEAYQQEKKT QFSCVHPISK NRLKLPAAMD LKKKGRSLPV GVCRSWEVLE RAPNMPTWPR
 961 SHSLGDLHGN IGTGSIKSVD TSPLDSSKRS TESEGGGQMI KLLLANQRKE VVDALKGKRK EFHLSATMPS TKMFPQRLPE
1041 NCDARTSSLS YGMTWTPFES CKKSTHDLGR ANISISKENV ETDQKDMSEV KMLPKVSSQP PPVPAKKNRE RISNGHCHLP
1121 STLSCSDVPS LPAKKVTLQS SSSSHELTAC RTFPDQEPKS PSSIRPPWLS DLPEIASVQQ HVFKLDPSSV RKISCNRGLD
1201 LEMLIENKLQ SEDIDLTEEP YSDKHGRCGI PEPLVQRYAE DLDHPEKDVA TNLDQVRVKQ LRKQHRMAIP SGGLTEICRK
1281 PASPGNISSI TDWLVSVGLP MYSVSLRAAG INDLSKVPSL SQTSLHEAGI TEERHISKLL TAARLLNPET I
```

**Supporting Peptides:**

| Peptide | Uniq | -10lgP | Mass | Length | ppm | m/z | z | RT | Fraction | Scan | Source File | Area F1 | Area F2a | Area F3a | Area F4 | Area F5 | Area F6a | Area F7a | Area F7b | Area F8 | Area F9a | Area Sample 1F10a | Area F10b | Area F11a | Area F11b | Area F12a | Area F13 | #Feature | #Feature F1 | #Feature F2a | #Feature F3a | #Feature F4 | #Feature F5 | #Feature F6a | #Feature F7a | #Feature F7b | #Feature F8 | #Feature F9a | #Feature Sample 1F10a | #Feature F10b | #Feature F11a | #Feature F11b | #Feature F12a | #Feature F13 | Start | End | PTM | AScore | Found By |
|---|---|---|---|---|---|---|---|---|---|---|---|---|---|---|---|---|---|---|---|---|---|---|---|---|---|---|---|---|---|---|---|---|---|---|---|---|---|---|---|---|---|---|---|---|---|---|---|---|---|
| K.RSTESEGGGQMIK.L | Y | 42.03 | 1378.6510 | 13 | 4.7 | 690.3360 | 2 | 46.98 | 10 | F10:24385 | BuCaTN01_F9a.raw | | | | | | | | | | 0 | | | | | | | 0 | 0 | 0 | 0 | 0 | 0 | 0 | 0 | 0 | 0 | 0 | 0 | 0 | 0 | 0 | 0 | 0 | 989 | 1001 | | | PEAKS DB |
| total 1 peptides | | | | | | | | | | | | | | | | | | | | | | | | | | | | | | | | | | | | | | | | | | | | | | | | | |

**Best Unique PSM (Scan F10:24385, m/z=690.3360, z=2, RT=46.98, ppm=4.7):**

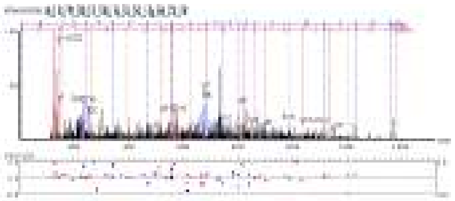

## Q8AY45.2

back to list

| Protein Coverage | Supporting Peptides | Best Unique PSM |

**Protein Coverage:**

```
 1 MSSGGLLLLL GLLTLWAELT PVSSRKRHPD CDKPPDTKIC QTVVRAFYYK PSAKRCVQFR YGGCNGNGNH FKSDHLCRCE
81 CLEYP
```

**Supporting Peptides:**

| Peptide | Uniq | -10lgP | Mass | Length | ppm | m/z | z | RT | Fraction | Scan | Source File | Area F1 | Area F2a | Area F3a | Area F4 | Area F5 | Area F6a | Area F7a | Area F7b | Area F8 | Area F9a | Area Sample 1F10a | Area F10b | Area F11a | Area F11b | Area F12a | Area F13 | #Feature | #Feature F1 | #Feature F2a | #Feature F3a | #Feature F4 | #Feature F5 | #Feature F6a | #Feature F7a | #Feature F7b | #Feature F8 | #Feature F9a | #Feature Sample 1F10a | #Feature F10b | #Feature F11a | #Feature F11b | #Feature F12a | #Feature F13 | Start | End | PTM | AScore | Found By |
|---|---|---|---|---|---|---|---|---|---|---|---|---|---|---|---|---|---|---|---|---|---|---|---|---|---|---|---|---|---|---|---|---|---|---|---|---|---|---|---|---|---|---|---|---|---|---|---|---|---|
| R.AFYYKPSAK.R | Y | 41.37 | 1073.5546 | 9 | 1.2 | 358.8592 | 3 | 33.53 | 5 | F5:16472 | BuCaTN01_F5.raw | | | | | 3.1771E6 | | | | | | | | | | | | 1 | 0 | 0 | 0 | 0 | 1 | 0 | 0 | 0 | 0 | 0 | 0 | 0 | 0 | 0 | 0 | 0 | 46 | 54 | | | PEAKS DB |
| total 1 peptides | | | | | | | | | | | | | | | | | | | | | | | | | | | | | | | | | | | | | | | | | | | | | | | | | |

**Best Unique PSM (Scan F5:16472, m/z=358.8592, z=3, RT=33.53, ppm=1.2):**

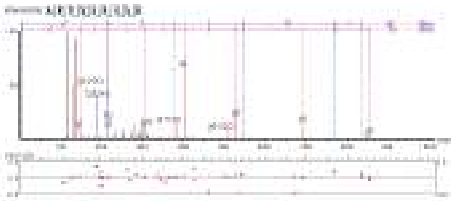

## AAL30066.1

back to list

| Protein Coverage | Supporting Peptides | Best Unique PSM |

**Protein Coverage:**

```
 1 PDGFIMSSGG LLLLLGLLTL WAELTPVSSR KRHPDCDKPP DTKICQTVVR AFYYKPSAKR CVQFRYGGCN GNGNHFKSDH
81 LCRCECLEYP
```

**Supporting Peptides:**

| Peptide | Uniq | -10lgP | Mass | Length | ppm | m/z | z | RT | Fraction | Scan | Source File | Area F1 | Area F2a | Area F3a | Area F4 | Area F5 | Area F6a | Area F7a | Area F7b | Area F8 | Area F9a | Area Sample 1F10a | Area F10b | Area F11a | Area F11b | Area F12a | Area F13 | #Feature | #Feature F1 | #Feature F2a | #Feature F3a | #Feature F4 | #Feature F5 | #Feature F6a | #Feature F7a | #Feature F7b | #Feature F8 | #Feature F9a | #Feature Sample 1F10a | #Feature F10b | #Feature F11a | #Feature F11b | #Feature F12a | #Feature F13 | Start | End | PTM | AScore | Found By |
|---|---|---|---|---|---|---|---|---|---|---|---|---|---|---|---|---|---|---|---|---|---|---|---|---|---|---|---|---|---|---|---|---|---|---|---|---|---|---|---|---|---|---|---|---|---|---|---|---|---|
| R.AFYYKPSAK.R | Y | 41.37 | 1073.5546 | 9 | 1.2 | 358.8592 | 3 | 33.53 | 5 | F5:16472 | BuCaTN01_F5.raw | | | | | 3.1771E6 | | | | | | | | | | | | 1 | 0 | 0 | 0 | 0 | 1 | 0 | 0 | 0 | 0 | 0 | 0 | 0 | 0 | 0 | 0 | 0 | 51 | 59 | | | PEAKS DB |
| total 1 peptides | | | | | | | | | | | | | | | | | | | | | | | | | | | | | | | | | | | | | | | | | | | | | | | | | |

**Best Unique PSM (Scan F5:16472, m/z=358.8592, z=3, RT=33.53, ppm=1.2):**

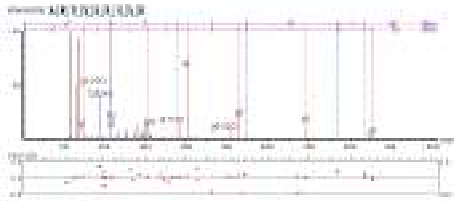

## AAB32179.1

back to list

| Protein Coverage | Supporting Peptides | Best Unique PSM |

**Protein Coverage:**

1 RKRHPDCDKP PDTKICQTVV R**AFYYKPSAK** RCVQFRYGG

**Supporting Peptides:**

| Peptide | Uniq | -10lgP | Mass | Length | ppm | m/z | z | RT | Fraction | Scan | Source File | Area F1 | Area F2a | Area F3a | Area F4 | Area F5 | Area F6a | Area F7a | Area F7b | Area F8 | Area F9a | Area Sample 1F10a | Area F10b | Area F11a | Area F11b | Area F12a | Area F13 | #Feature | #Feature F1 | #Feature F2a | #Feature F3a | #Feature F4 | #Feature F5 | #Feature F6a | #Feature F7a | #Feature F7b | #Feature F8 | #Feature F9a | #Feature Sample 1F10a | #Feature F10b | #Feature F11a | #Feature F11b | #Feature F12a | #Feature F13 | Start | End | PTM | AScore | Found By |
|---|---|---|---|---|---|---|---|---|---|---|---|---|---|---|---|---|---|---|---|---|---|---|---|---|---|---|---|---|---|---|---|---|---|---|---|---|---|---|---|---|---|---|---|---|---|---|---|---|---|
| R.AFYYKPSAK.R | Y | 41.37 | 1073.5546 | 9 | 1.2 | 358.8592 | 3 | 33.53 | 5 | F5:16472 | BuCaTN01_F5.raw | | | | | 3.1771E6 | | | | | | | | | | | | 1 | 0 | 0 | 0 | 0 | 1 | 0 | 0 | 0 | 0 | 0 | 0 | 0 | 0 | 0 | 0 | 0 | 22 | 30 | | | PEAKS DB |
| total 1 peptides | | | | | | | | | | | | | | | | | | | | | | | | | | | | | | | | | | | | | | | | | | | | | | | | | |

**Best Unique PSM (Scan F5:16472, m/z=358.8592, z=3, RT=33.53, ppm=1.2):**

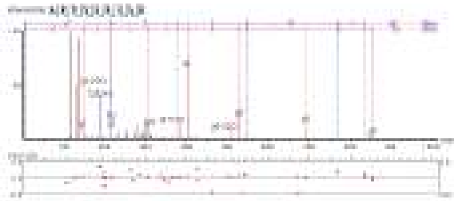

## 1BUN

back to list

| Protein Coverage | Supporting Peptides | Best Unique PSM |

**Protein Coverage:**

1 RKRHPDCDKP PDTKICQTVV R**AFYYKPSAK** RCVQFRYGGC NGNGNHFKSD HLCRCECLEY R

**Supporting Peptides:**

| Peptide | Uniq | -10lgP | Mass | Length | ppm | m/z | z | RT | Fraction | Scan | Source File | Area F1 | Area F2a | Area F3a | Area F4 | Area F5 | Area F6a | Area F7a | Area F7b | Area F8 | Area F9a | Area Sample 1F10a | Area F10b | Area F11a | Area F11b | Area F12a | Area F13 | #Feature | #Feature F1 | #Feature F2a | #Feature F3a | #Feature F4 | #Feature F5 | #Feature F6a | #Feature F7a | #Feature F7b | #Feature F8 | #Feature F9a | #Feature Sample 1F10a | #Feature F10b | #Feature F11a | #Feature F11b | #Feature F12a | #Feature F13 | Start | End | PTM | AScore | Found By |
|---|---|---|---|---|---|---|---|---|---|---|---|---|---|---|---|---|---|---|---|---|---|---|---|---|---|---|---|---|---|---|---|---|---|---|---|---|---|---|---|---|---|---|---|---|---|---|---|---|---|
| R.AFYYKPSAK.R | Y | 41.37 | 1073.5546 | 9 | 1.2 | 358.8592 | 3 | 33.53 | 5 | F5:16472 | BuCaTN01_F5.raw | | | | | 3.1771E6 | | | | | | | | | | | | 1 | 0 | 0 | 0 | 0 | 1 | 0 | 0 | 0 | 0 | 0 | 0 | 0 | 0 | 0 | 0 | 0 | 22 | 30 | | | PEAKS DB |
| total 1 peptides | | | | | | | | | | | | | | | | | | | | | | | | | | | | | | | | | | | | | | | | | | | | | | | | | |

**Best Unique PSM (Scan F5:16472, m/z=358.8592, z=3, RT=33.53, ppm=1.2):**

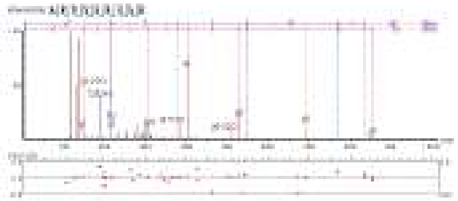

## CAB62504.1

back to list

| Protein Coverage | Supporting Peptides | Best Unique PSM |

**Protein Coverage:**

1 MSSGGLLLLL GLLTLCAELT PVSSRKRHPD CDKPPDTKIC QTVVR**AFYYK PSAK**RCVQFR YGGCNGNGNH FKSDHLCRCE

81 CL

**Supporting Peptides:**

| Peptide | Uniq | -10lgP | Mass | Length | ppm | m/z | z | RT | Fraction | Scan | Source File | Area F1 | Area F2a | Area F3a | Area F4 | Area F5 | Area F6a | Area F7a | Area F7b | Area F8 | Area F9a | Area Sample 1F10a | Area F10b | Area F11a | Area F11b | Area F12a | Area F13 | #Feature | #Feature F1 | #Feature F2a | #Feature F3a | #Feature F4 | #Feature F5 | #Feature F6a | #Feature F7a | #Feature F7b | #Feature F8 | #Feature F9a | #Feature Sample 1F10a | #Feature F10b | #Feature F11a | #Feature F11b | #Feature F12a | #Feature F13 | Start | End | PTM | AScore | Found By |
|---|---|---|---|---|---|---|---|---|---|---|---|---|---|---|---|---|---|---|---|---|---|---|---|---|---|---|---|---|---|---|---|---|---|---|---|---|---|---|---|---|---|---|---|---|---|---|---|---|---|
| R.AFYYKPSAK.R | Y | 41.37 | 1073.5546 | 9 | 1.2 | 358.8592 | 3 | 33.53 | 5 | F5:16472 | BuCaTN01_F5.raw | | | | | 3.1771E6 | | | | | | | | | | | | 1 | 0 | 0 | 0 | 0 | 1 | 0 | 0 | 0 | 0 | 0 | 0 | 0 | 0 | 0 | 0 | 0 | 46 | 54 | | | PEAKS DB |
| total 1 peptides | | | | | | | | | | | | | | | | | | | | | | | | | | | | | | | | | | | | | | | | | | | | | | | | | |

**Best Unique PSM (Scan F5:16472, m/z=358.8592, z=3, RT=33.53, ppm=1.2):**

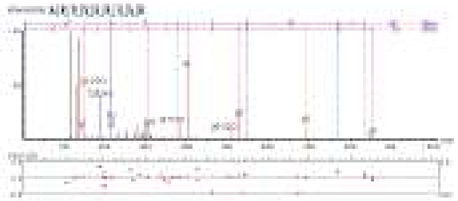

## CAJ18318.1

back to list

| Protein Coverage | Supporting Peptides | Best Unique PSM |

**Protein Coverage:**

1 MSSGGLLLLL GLLTLCAELT PVSSRKRHPD CDKPPDTKIC QTVVR**AFYYK PSAK**RCVQFR YGGCNGNGNH FKSDHLCRCE

81 CLEYR

**Supporting Peptides:**

| Peptide | Uniq | -10lgP | Mass | Length | ppm | m/z | z | RT | Fraction | Scan | Source File | Area F1 | Area F2a | Area F3a | Area F4 | Area F5 | Area F6a | Area F7a | Area F7b | Area F8 | Area F9a | Area Sample 1F10a | Area F10b | Area F11a | Area F11b | Area F12a | Area F13 | #Feature | #Feature F1 | #Feature F2a | #Feature F3a | #Feature F4 | #Feature F5 | #Feature F6a | #Feature F7a | #Feature F7b | #Feature F8 | #Feature F9a | #Feature Sample 1F10a | #Feature F10b | #Feature F11a | #Feature F11b | #Feature F12a | #Feature F13 | Start | End | PTM | AScore | Found By |
|---|---|---|---|---|---|---|---|---|---|---|---|---|---|---|---|---|---|---|---|---|---|---|---|---|---|---|---|---|---|---|---|---|---|---|---|---|---|---|---|---|---|---|---|---|---|---|---|---|---|
| R.AFYYKPSAK.R | Y | 41.37 | 1073.5546 | 9 | 1.2 | 358.8592 | 3 | 33.53 | 5 | F5:16472 | BuCaTN01_F5.raw | | | | | 3.1771E6 | | | | | | | | | | | | 1 | 0 | 0 | 0 | 0 | 1 | 0 | 0 | 0 | 0 | 0 | 0 | 0 | 0 | 0 | 0 | 0 | 46 | 54 | | | PEAKS DB |
| total 1 peptides | | | | | | | | | | | | | | | | | | | | | | | | | | | | | | | | | | | | | | | | | | | | | | | | | |

**Best Unique PSM (Scan F5:16472, m/z=358.8592, z=3, RT=33.53, ppm=1.2):**

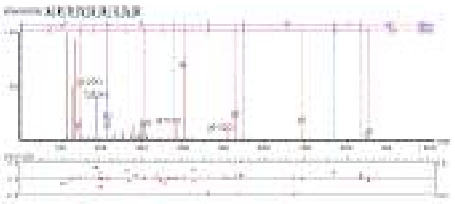

## CAJ18319.1

back to list

| Protein Coverage | Supporting Peptides | Best Unique PSM |

**Protein Coverage:**

1 MSSGGLLLLL GLLTLCAELT PVSSRKRHPD CDKPPDTKIC QTVVR**AFYYK PSAK**RCVQFR YGGCNGNGNH FKSDHLCRCE

81 CLEYS

**Supporting Peptides:**

| Peptide | Uniq | -10lgP | Mass | Length | ppm | m/z | z | RT | Fraction | Scan | Source File | Area F1 | Area F2a | Area F3a | Area F4 | Area F5 | Area F6a | Area F7a | Area F7b | Area F8 | Area F9a | Area Sample 1F10a | Area F10b | Area F11a | Area F11b | Area F12a | Area F13 | #Feature | #Feature F1 | #Feature F2a | #Feature F3a | #Feature F4 | #Feature F5 | #Feature F6a | #Feature F7a | #Feature F7b | #Feature F8 | #Feature F9a | #Feature Sample 1F10a | #Feature F10b | #Feature F11a | #Feature F11b | #Feature F12a | #Feature F13 | Start | End | PTM | AScore | Found By |
|---|---|---|---|---|---|---|---|---|---|---|---|---|---|---|---|---|---|---|---|---|---|---|---|---|---|---|---|---|---|---|---|---|---|---|---|---|---|---|---|---|---|---|---|---|---|---|---|---|---|
| R.AFYYKPSAK.R | Y | 41.37 | 1073.5546 | 9 | 1.2 | 358.8592 | 3 | 33.53 | 5 | F5:16472 | BuCaTN01_F5.raw | | | | | 3.1771E6 | | | | | | | | | | | | 1 | 0 | 0 | 0 | 0 | 1 | 0 | 0 | 0 | 0 | 0 | 0 | 0 | 0 | 0 | 0 | 0 | 46 | 54 | | | PEAKS DB |
| total 1 peptides | | | | | | | | | | | | | | | | | | | | | | | | | | | | | | | | | | | | | | | | | | | | | | | | | |

**Best Unique PSM (Scan F5:16472, m/z=358.8592, z=3, RT=33.53, ppm=1.2):**

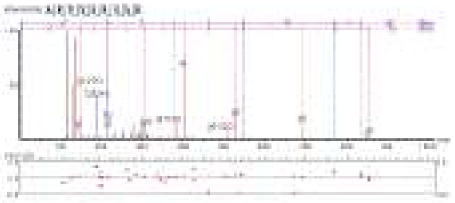

## Q1RPT0.1

back to list

| Protein Coverage | Supporting Peptides | Best Unique PSM |

**Protein Coverage:**

1 MSSGGLLLLL GLLTLCAELT PVSSRKRHPD CDKPPDTKIC QTVVR**AFYYK PSAK**RCVQFR YGGCNGNGNH FKSDHLCRCE

81 CLEYS

**Supporting Peptides:**

| Peptide | Uniq | -10lgP | Mass | Length | ppm | m/z | z | RT | Fraction | Scan | Source File | Area F1 | Area F2a | Area F3a | Area F4 | Area F5 | Area F6a | Area F7a | Area F7b | Area F8 | Area F9a | Area Sample 1F10a | Area F10b | Area F11a | Area F11b | Area F12a | Area F13 | #Feature | #Feature F1 | #Feature F2a | #Feature F3a | #Feature F4 | #Feature F5 | #Feature F6a | #Feature F7a | #Feature F7b | #Feature F8 | #Feature F9a | #Feature Sample 1F10a | #Feature F10b | #Feature F11a | #Feature F11b | #Feature F12a | #Feature F13 | Start | End | PTM | AScore | Found By |
|---|---|---|---|---|---|---|---|---|---|---|---|---|---|---|---|---|---|---|---|---|---|---|---|---|---|---|---|---|---|---|---|---|---|---|---|---|---|---|---|---|---|---|---|---|---|---|---|---|---|
| R.AFYYKPSAK.R | Y | 41.37 | 1073.5546 | 9 | 1.2 | 358.8592 | 3 | 33.53 | 5 | F5:16472 | BuCaTN01_F5.raw | | | | | 3.1771E6 | | | | | | | | | | | | 1 | 0 | 0 | 0 | 0 | 1 | 0 | 0 | 0 | 0 | 0 | 0 | 0 | 0 | 0 | 0 | 0 | 46 | 54 | | | PEAKS DB |
| total 1 peptides | | | | | | | | | | | | | | | | | | | | | | | | | | | | | | | | | | | | | | | | | | | | | | | | | |

**Best Unique PSM (Scan F5:16472, m/z=358.8592, z=3, RT=33.53, ppm=1.2):**

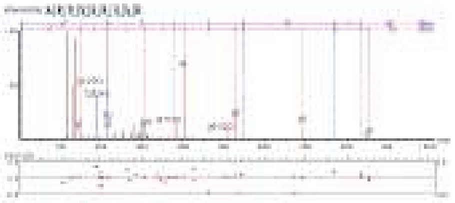

## P00989.2

back to list

| Protein Coverage | Supporting Peptides | Best Unique PSM |

**Protein Coverage:**

1 MSSGGLLLLL GLLTLCAELT PVSSRKRHPD CDKPPDTKIC QTVVR**AFYYK PSAK**RCVQFR YGGCNGNGNH FKSDHLCRCE

81 CLEYR

**Supporting Peptides:**

| Peptide | Uniq | -10lgP | Mass | Length | ppm | m/z | z | RT | Fraction | Scan | Source File | Area F1 | Area F2a | Area F3a | Area F4 | Area F5 | Area F6a | Area F7a | Area F7b | Area F8 | Area F9a | Area Sample 1F10a | Area F10b | Area F11a | Area F11b | Area F12a | Area F13 | #Feature | #Feature F1 | #Feature F2a | #Feature F3a | #Feature F4 | #Feature F5 | #Feature F6a | #Feature F7a | #Feature F7b | #Feature F8 | #Feature F9a | #Feature Sample 1F10a | #Feature F10b | #Feature F11a | #Feature F11b | #Feature F12a | #Feature F13 | Start | End | PTM | AScore | Found By |
|---|---|---|---|---|---|---|---|---|---|---|---|---|---|---|---|---|---|---|---|---|---|---|---|---|---|---|---|---|---|---|---|---|---|---|---|---|---|---|---|---|---|---|---|---|---|---|---|---|---|
| R.AFYYKPSAK.R | Y | 41.37 | 1073.5546 | 9 | 1.2 | 358.8592 | 3 | 33.53 | 5 | F5:16472 | BuCaTN01_F5.raw | | | | | 3.1771E6 | | | | | | | | | | | | 1 | 0 | 0 | 0 | 0 | 1 | 0 | 0 | 0 | 0 | 0 | 0 | 0 | 0 | 0 | 0 | 0 | 46 | 54 | | | PEAKS DB |
| total 1 peptides | | | | | | | | | | | | | | | | | | | | | | | | | | | | | | | | | | | | | | | | | | | | | | | | | |

**Best Unique PSM (Scan F5:16472, m/z=358.8592, z=3, RT=33.53, ppm=1.2):**

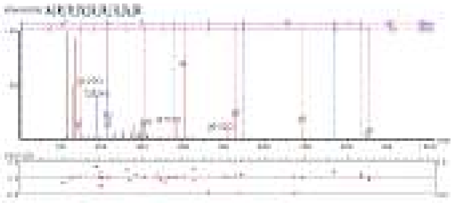

## CAA72810.1

back to list

| Protein Coverage | Supporting Peptides | Best Unique PSM |

**Protein Coverage:**

1 MSSGGLLLLL GLLTLCAELT PVSSRKRHPD CDKPPDTKIC QTVVR**AFYYK PSAK**RCVQFR YGGCNGNGNH FKSDHLCRCE

81 CLEYR

**Supporting Peptides:**

| Peptide | Uniq | -10lgP | Mass | Length | ppm | m/z | z | RT | Fraction | Scan | Source File | Area F1 | Area F2a | Area F3a | Area F4 | Area F5 | Area F6a | Area F7a | Area F7b | Area F8 | Area F9a | Area Sample 1F10a | Area F10b | Area F11a | Area F11b | Area F12a | Area F13 | #Feature | #Feature F1 | #Feature F2a | #Feature F3a | #Feature F4 | #Feature F5 | #Feature F6a | #Feature F7a | #Feature F7b | #Feature F8 | #Feature F9a | #Feature Sample 1F10a | #Feature F10b | #Feature F11a | #Feature F11b | #Feature F12a | #Feature F13 | Start | End | PTM | AScore | Found By |
|---|---|---|---|---|---|---|---|---|---|---|---|---|---|---|---|---|---|---|---|---|---|---|---|---|---|---|---|---|---|---|---|---|---|---|---|---|---|---|---|---|---|---|---|---|---|---|---|---|---|
| R.AFYYKPSAK.R | Y | 41.37 | 1073.5546 | 9 | 1.2 | 358.8592 | 3 | 33.53 | 5 | F5:16472 | BuCaTN01_F5.raw | | | | | 3.1771E6 | | | | | | | | | | | | 1 | 0 | 0 | 0 | 0 | 1 | 0 | 0 | 0 | 0 | 0 | 0 | 0 | 0 | 0 | 0 | 0 | 46 | 54 | | | PEAKS DB |
| total 1 peptides | | | | | | | | | | | | | | | | | | | | | | | | | | | | | | | | | | | | | | | | | | | | | | | | | |

**Best Unique PSM (Scan F5:16472, m/z=358.8592, z=3, RT=33.53, ppm=1.2):**

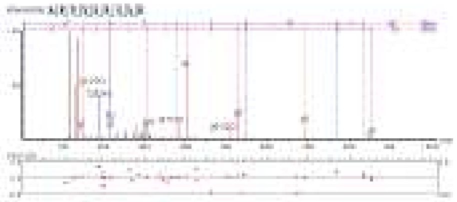

## ABY71037.1

back to list

| Protein Coverage | Supporting Peptides | Best Unique PSM |

**Protein Coverage:**

1 MSSGGLLLLL GLLTLWTELT PVSSRERHPD CDKPPDTGRC RKNVR**AFYYK PSAK**RCVQFI YGGCNANGNH FKSDHLCRCE

81 CLEYP

**Supporting Peptides:**

| Peptide | Uniq | -10lgP | Mass | Length | ppm | m/z | z | RT | Fraction | Scan | Source File | Area F1 | Area F2a | Area F3a | Area F4 | Area F5 | Area F6a | Area F7a | Area F7b | Area F8 | Area F9a | Area Sample 1F10a | Area F10b | Area F11a | Area F11b | Area F12a | Area F13 | #Feature | #Feature F1 | #Feature F2a | #Feature F3a | #Feature F4 | #Feature F5 | #Feature F6a | #Feature F7a | #Feature F7b | #Feature F8 | #Feature F9a | #Feature Sample 1F10a | #Feature F10b | #Feature F11a | #Feature F11b | #Feature F12a | #Feature F13 | Start | End | PTM | AScore | Found By |
|---|---|---|---|---|---|---|---|---|---|---|---|---|---|---|---|---|---|---|---|---|---|---|---|---|---|---|---|---|---|---|---|---|---|---|---|---|---|---|---|---|---|---|---|---|---|---|---|---|---|
| R.AFYYKPSAK.R | Y | 41.37 | 1073.5546 | 9 | 1.2 | 358.8592 | 3 | 33.53 | 5 | F5:16472 | BuCaTN01_F5.raw | | | | | 3.1771E6 | | | | | | | | | | | | 1 | 0 | 0 | 0 | 0 | 1 | 0 | 0 | 0 | 0 | 0 | 0 | 0 | 0 | 0 | 0 | 0 | 46 | 54 | | | PEAKS DB |
| total 1 peptides | | | | | | | | | | | | | | | | | | | | | | | | | | | | | | | | | | | | | | | | | | | | | | | | | |

**Best Unique PSM (Scan F5:16472, m/z=358.8592, z=3, RT=33.53, ppm=1.2):**

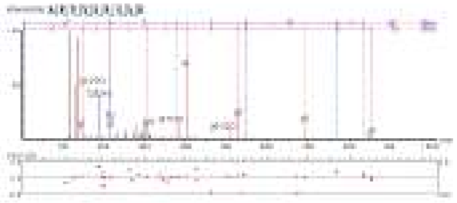

## B2KTG2.1

back to list

| Protein Coverage | Supporting Peptides | Best Unique PSM |

**Protein Coverage:**

1 MSSGGLLLLL GLLTLWTELT PVSSRERHPD CDKPPDTGRC RKNVR**AFYYK PSAK**RCVQFI YGGCNANGNH FKSDHLCRCE

81 CLEYP

**Supporting Peptides:**

| Peptide | Uniq | -10lgP | Mass | Length | ppm | m/z | z | RT | Fraction | Scan | Source File | Area F1 | Area F2a | Area F3a | Area F4 | Area F5 | Area F6a | Area F7a | Area F7b | Area F8 | Area F9a | Area Sample 1F10a | Area F10b | Area F11a | Area F11b | Area F12a | Area F13 | #Feature | #Feature F1 | #Feature F2a | #Feature F3a | #Feature F4 | #Feature F5 | #Feature F6a | #Feature F7a | #Feature F7b | #Feature F8 | #Feature F9a | #Feature Sample 1F10a | #Feature F10b | #Feature F11a | #Feature F11b | #Feature F12a | #Feature F13 | Start | End | PTM | AScore | Found By |
|---|---|---|---|---|---|---|---|---|---|---|---|---|---|---|---|---|---|---|---|---|---|---|---|---|---|---|---|---|---|---|---|---|---|---|---|---|---|---|---|---|---|---|---|---|---|---|---|---|---|
| R.AFYYKPSAK.R | Y | 41.37 | 1073.5546 | 9 | 1.2 | 358.8592 | 3 | 33.53 | 5 | F5:16472 | BuCaTN01_F5.raw | | | | | 3.1771E6 | | | | | | | | | | | | 1 | 0 | 0 | 0 | 0 | 1 | 0 | 0 | 0 | 0 | 0 | 0 | 0 | 0 | 0 | 0 | 0 | 46 | 54 | | | PEAKS DB |
| total 1 peptides | | | | | | | | | | | | | | | | | | | | | | | | | | | | | | | | | | | | | | | | | | | | | | | | | |

**Best Unique PSM (Scan F5:16472, m/z=358.8592, z=3, RT=33.53, ppm=1.2):**

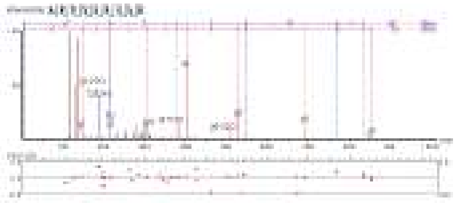

## 0805258B

back to list

| Protein Coverage | Supporting Peptides | Best Unique PSM |

**Protein Coverage:**

1 RKRHPDCDKP PDTKICQTVR **AFYYKPSAK**R CVQFRYGGCD GDHGNFKSDH LCRCECELYR

**Supporting Peptides:**

| Peptide | Uniq | -10lgP | Mass | Length | ppm | m/z | z | RT | Fraction | Scan | Source File | Area F1 | Area F2a | Area F3a | Area F4 | Area F5 | Area F6a | Area F7a | Area F7b | Area F8 | Area F9a | Area Sample 1F10a | Area F10b | Area F11a | Area F11b | Area F12a | Area F13 | #Feature | #Feature F1 | #Feature F2a | #Feature F3a | #Feature F4 | #Feature F5 | #Feature F6a | #Feature F7a | #Feature F7b | #Feature F8 | #Feature F9a | #Feature Sample 1F10a | #Feature F10b | #Feature F11a | #Feature F11b | #Feature F12a | #Feature F13 | Start | End | PTM | AScore | Found By |
|---|---|---|---|---|---|---|---|---|---|---|---|---|---|---|---|---|---|---|---|---|---|---|---|---|---|---|---|---|---|---|---|---|---|---|---|---|---|---|---|---|---|---|---|---|---|---|---|---|---|
| R.AFYYKPSAK.R | Y | 41.37 | 1073.5546 | 9 | 1.2 | 358.8592 | 3 | 33.53 | 5 | F5:16472 | BuCaTN01_F5.raw | | | | | 3.1771E6 | | | | | | | | | | | | 1 | 0 | 0 | 0 | 0 | 1 | 0 | 0 | 0 | 0 | 0 | 0 | 0 | 0 | 0 | 0 | 0 | 21 | 29 | | | PEAKS DB |
| total 1 peptides | | | | | | | | | | | | | | | | | | | | | | | | | | | | | | | | | | | | | | | | | | | | | | | | | |

**Best Unique PSM (Scan F5:16472, m/z=358.8592, z=3, RT=33.53, ppm=1.2):**

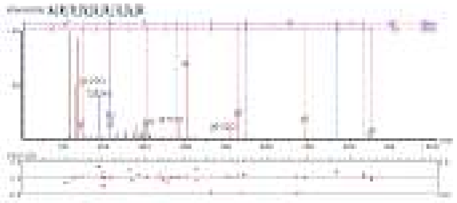

## XP_026523812.1

back to list

| Protein Coverage | Supporting Peptides | Best Unique PSM |

**Protein Coverage:**

1 MPGIRGRPPT SSLLGLAAAT FFLLLLLKSR PGSAAATSAV GLTRRPQQR**A GLSTPPSSMA APGPMFTLK**V QVNDIISHQY

Oxidation (M) (+15.99)

81 LYHAVVDIFV NYTKTNSTLT GKNGAVLIQV PYQLGLSLTI VSYKDGYMLT PLPWKTGRMP IYSSVTLSLF PQSRANIWLF
161 DDTVLITGKL SEAKSQPSVQ FPKYLIKLPV NHHITNVTAY LTVPQQFLKV EHFFYTTGIL LNKSGFKSIE LTPLAAICVN
241 IFSSGKELQV DGSIQITLPL LPTSAVKAGE PISAWTFDMK TGAWINRGLG MVKNMDDHLV WTYTAPSLGY WIAAPLSGAR
321 DSIITTVTKD ITAYHTVFLT AILGGTITII AGFFALLLCF CRDKCGRPKK RKKSPTKLDI LRKDQTTSTT HINHIGAAKE
401 SLKPEDKSRL YSPRVAFYSP QRRVSAEGEE RQVQDNFKVY TEGTPYQFSG KNLQCRSSPQ SLEAKTELRH LPPVKHLNGS
481 LPPSQNVQEQ NQYLPTTEEI YGLSSLPEQL MHLYSQPGAM LQTPELFHSQ EQLHATKSAT LPRKGQLVYT PVMESMNHEN
561 YTQTLPKMPM FAHLQEPTSR DETVAIDRQQ GLSPQTANWS RYTSSLLESV SVPGTLNEAV VMTPFSSELQ GISEQTLLEL
641 SKGKQCPNPR AWFVSLDGKP IAQVRHSFID LKKGRKTESN DTSLDSGVDM NEHNPSRKLE REKTFIKSTQ HSKTLYLEDL
721 DLNTSECGTM VSTPEDQAVK HVPEGSNDPI IEQESPEEMP RRKSIAKKHE ASPPPGKKQG RPRLTKTEGK TNIWKKRDER
801 PLIPKN

**Supporting Peptides:**

| Peptide | Uniq | -10lgP | Mass | Length | ppm | m/z | z | RT | Fraction | Scan | Source File | Area F1 | Area F2a | Area F3a | Area F4 | Area F5 | Area F6a | Area F7a | Area F7b | Area F8 | Area F9a | Area Sample 1F10a | Area F10b | Area F11a | Area F11b | Area F12a | Area F13 | #Feature | #Feature F1 | #Feature F2a | #Feature F3a | #Feature F4 | #Feature F5 | #Feature F6a | #Feature F7a | #Feature F7b | #Feature F8 | #Feature F9a | #Feature Sample 1F10a | #Feature F10b | #Feature F11a | #Feature F11b | #Feature F12a | #Feature F13 | Start | End | PTM | AScore | Found By |
|---|---|---|---|---|---|---|---|---|---|---|---|---|---|---|---|---|---|---|---|---|---|---|---|---|---|---|---|---|---|---|---|---|---|---|---|---|---|---|---|---|---|---|---|---|---|---|---|---|---|
| R.AGLSTPPSSM(+15.99)AAPGPMFTLK.V | Y | 41.12 | 1975.9747 | 20 | 3.2 | 495.0005 | 4 | 60.17 | 14 | F14:36220 | BuCaTN01_F11b.raw | | | | | | | | | | | | | | 4.8334E5 | | | 1 | 0 | 0 | 0 | 0 | 0 | 0 | 0 | 0 | 0 | 0 | 0 | 0 | 0 | 1 | 0 | 0 | 50 | 69 | Oxidation (M) | M10:Oxidation (M): 34.81 | PEAKS DB |
| total 1 peptides | | | | | | | | | | | | | | | | | | | | | | | | | | | | | | | | | | | | | | | | | | | | | | | | | |

**Best Unique PSM (Scan F14:36220, m/z=495.0005, z=4, RT=60.17, ppm=3.2):**

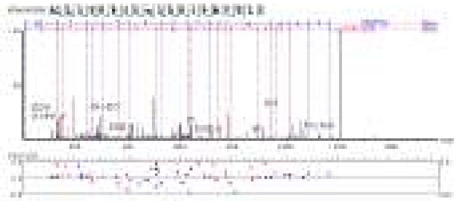

## XP_026556042.1

back to list

| Protein Coverage | Supporting Peptides | Best Unique PSM |

**Protein Coverage:**

1 MADPLRRTFS KLRSRRSQRG AGEAAAGDPS ERGGRGVGTL GGKTQQNLHF HHPCLENGVT TEACPGLGSQ VGGCEVLLVS
81 PLERPGLRES AAWSRWGKAE MPGERLPGAA VARSCPPRKG AEGRALQQPS PPELRAPPLA AELCFSSSSF FPRGSEPGPA
161 LECGGGSEED DYYDNQRLPG WDCQGTARTS EGAARGNK**EG SAAAVPGAGG GGGGGGGSSR AR**ARESTGKL RSQLQEAYYL
241 LIHAMHDLPP ASPTAGGPSP QPASHLPASE AAASDALRWS FSCPDGCSPL QRSDPCAQVS AKNPIRRQGI SKSLESLCFA
321 QPSASKLLLL PRCRSEGALQ PLPPEPLEPR GHLLLEPQEL GTSQETCFLS AATHGSGDSG CEKTLCSQGL GEAHPSGLQE
401 DMEELLAPEL YGAQGKMVDP ERATRGSHEG KPQHPPEDAS PGPFKPPAVT VRKLQKWMYK GRLLSLGMKG RGGTAGMSPP
481 SSRTKVTGSC RPLLGRGEGL TLSPPAHLNG PGPKARVSSS DQRILLTELI ENVYIPSVSR RELKNLKSSE VSECRPQIAN
561 ITVSKKRNWL HQSSLRVPSL EKGNSYKKFS GENYQVLKSS SPFFEPKMPQ TFSRFLGQES PRGRVSSAHA AGNGTRSLAL
641 QNCTLDFGED NDADDEGEIW YNPIPEDDEP EFPCFLSDHN NHLDPKALNY KIAPGNDLIK VIMHNEGLPQ FLNCSGNGQP
721 RETSQIDNVH SVEYLQTQKP KPVCNILTGS AVDEGLLKTT TGGPGIMSAN KAETGTAEYS PPSPSPLKKG GSINWPFPDK
801 IKSPRTVRKL SMKMKKLPEL SRKLSVKGNS SHNSSDNSLL LKNNCQEVGH APLASSGNVT AAASRNVISR YHLDSSVSSQ
881 HSYKKKNRGT SKSSSKGGYL SDGDSPELIA KSGKYAAESR VGKGKEVFAG NLGNKSEIDI DAFRHYTFVD QPKCSQYISG
961 LMSIHFYGAE DLKPPRMDSK DVFCAIQVDS VNKARTALLT CRTTFLDMDH TFNIEIENAQ HLKLVVFSWE PMPRKNRVCC
1041 HGTVVLPALF RVTKTHQLAV KLEPRGLIYV KLTLLEQWEN SFCGTDGERE PILFGVDARK VVEKENEGLM VPLLMKKCIT
1121 DIEKRGCQVV GLYRLCGSAA VKKELREAFE KNSKAVTLCE TQYPDINVIT GILKDYLREL PSPLITKQLY EAVLDAMVKR
1201 PLKMTANGCE NDCNDSEYTT ALLDCLPEVE KATLKMLLDH LKLVASYHEV NRMTCQNLAV CFGPVLLSQW QETTSHNNRV
1281 FTDSEELASA LDFKKHIEVL HYLLQLWPVR HPSTEESALL EQQSSVNYLR PKKQRPQMLN LSEVPGILRS RPARLDSSSS
1361 NRYAGDWTAC GENYFVGPKE ALSEADYDDV PSEEAESGDD GSKADASRTL LRRPICVSKE RAFQAYLKMQ TMDSAVNHQV
1441 NLKNLQESID TLIGNLEREL NKNKLNMSC

**Supporting Peptides:**

| Peptide | Uniq | -10lgP | Mass | Length | ppm | m/z | z | RT | Fraction | Scan | Source File | Area F1 | Area F2a | Area F3a | Area F4 | Area F5 | Area F6a | Area F7a | Area F7b | Area F8 | Area F9a | Area Sample 1F10a | Area F10b | Area F11a | Area F11b | Area F12a | Area F13 | #Feature | #Feature F1 | #Feature F2a | #Feature F3a | #Feature F4 | #Feature F5 | #Feature F6a | #Feature F7a | #Feature F7b | #Feature F8 | #Feature F9a | #Feature Sample 1F10a | #Feature F10b | #Feature F11a | #Feature F11b | #Feature F12a | #Feature F13 | Start | End | PTM | AScore | Found By |
|---|---|---|---|---|---|---|---|---|---|---|---|---|---|---|---|---|---|---|---|---|---|---|---|---|---|---|---|---|---|---|---|---|---|---|---|---|---|---|---|---|---|---|---|---|---|---|---|---|---|
| K.EGSAAAVPGAGGGGGGGGGSSRAR.A | Y | 41.02 | 1898.8943 | 24 | -2.2 | 633.9694 | 3 | 46.85 | 3 | F3:22983 | BuCaTN01_F3a.raw | | | 1.1974E6 | | | | | | | | | | | | | | 1 | 0 | 0 | 1 | 0 | 0 | 0 | 0 | 0 | 0 | 0 | 0 | 0 | 0 | 0 | 0 | 0 | 199 | 222 | | | PEAKS DB |
| total 1 peptides | | | | | | | | | | | | | | | | | | | | | | | | | | | | | | | | | | | | | | | | | | | | | | | | | |

**Best Unique PSM (Scan F3:22983, m/z=633.9694, z=3, RT=46.85, ppm=-2.2):**

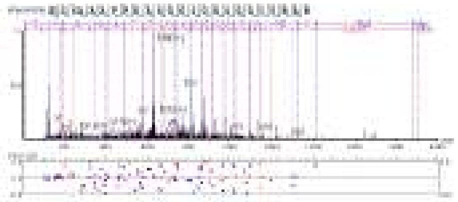

## Peptide List

Prepared with PEAKS ™ (bioinfor.com)

## Venom protein list- Western (MH) population of *B. caeruleus*

Protein Accession Contains:
Protein Description Contains:
Protein Sample Area >=
Protein PTM Contains:

| Protein Group | Protein ID | Accession | -10lgP | Coverage (%) | Coverage (%) F1 | Coverage (%) F2a | Coverage (%) F2b | Coverage (%) F3a | Coverage (%) F3b | Coverage (%) F4a | Coverage (%) F5a | Coverage (%) F5b | Coverage (%) F6a | Coverage (%) F6b | Coverage (%) F6c | Coverage (%) F7 | Coverage (%) F8a | Coverage (%) F8b | Coverage (%) F9a | Coverage (%) F9b | Coverage (%) F10a | Coverage (%) F11a | Coverage (%) F11b | Coverage (%) F12a | Coverage (%) F12b | Coverage (%) F12c | Coverage (%) F13a | Coverage (%) F13b | Coverage (%) F14 |
|---|---|---|---|---|---|---|---|---|---|---|---|---|---|---|---|---|---|---|---|---|---|---|---|---|---|---|---|---|---|
| 2 | 15 | AAL87004.1 | 443.94 | 76 | 0 | 24 | 11 | 24 | 0 | 0 | 20 | 42 | 8 | 33 | 34 | 18 | 12 | 45 | 60 | 70 | 59 | 53 | 64 | 47 | 28 | 50 | 44 | 58 | 0 |
| 2 | 16 | Q8QFW3.1 | 443.94 | 76 | 0 | 24 | 11 | 24 | 0 | 0 | 20 | 42 | 8 | 33 | 34 | 18 | 12 | 45 | 60 | 70 | 59 | 53 | 64 | 47 | 28 | 50 | 44 | 58 | 0 |
| 7 | 11 | Q8QFW4.1 | 408.72 | 73 | 0 | 21 | 0 | 10 | 0 | 0 | 0 | 10 | 0 | 20 | 23 | 0 | 12 | 42 | 48 | 63 | 65 | 48 | 48 | 48 | 25 | 48 | 26 | 48 | 0 |
| 7 | 12 | AAL87003.1 | 408.72 | 73 | 0 | 21 | 0 | 10 | 0 | 0 | 0 | 10 | 0 | 20 | 23 | 0 | 12 | 42 | 48 | 63 | 65 | 48 | 48 | 48 | 25 | 48 | 26 | 48 | 0 |
| 41 | 44 | 0702209A | 321.34 | 62 | 0 | 0 | 0 | 0 | 0 | 13 | 0 | 0 | 0 | 0 | 0 | 0 | 17 | 18 | 42 | 47 | 48 | 30 | 60 | 31 | 31 | 31 | 31 | 31 | 0 |
| 41 | 45 | P00606.2 | 321.34 | 50 | 0 | 0 | 0 | 0 | 0 | 10 | 0 | 0 | 0 | 0 | 0 | 0 | 14 | 14 | 34 | 39 | 39 | 24 | 49 | 26 | 26 | 26 | 26 | 26 | 0 |
| 41 | 46 | CAA37482.1 | 321.34 | 50 | 0 | 0 | 0 | 0 | 0 | 10 | 0 | 0 | 0 | 0 | 0 | 0 | 14 | 14 | 34 | 39 | 39 | 24 | 49 | 26 | 26 | 26 | 26 | 26 | 0 |
| 11 | 121 | 1G0Z | 318.42 | 61 | 0 | 16 | 0 | 0 | 0 | 0 | 0 | 0 | 0 | 0 | 15 | 0 | 12 | 17 | 28 | 41 | 38 | 35 | 35 | 25 | 43 | 47 | 35 | 36 | 0 |
| 11 | 122 | 1U4J | 318.42 | 61 | 0 | 16 | 0 | 0 | 0 | 0 | 0 | 0 | 0 | 0 | 15 | 0 | 12 | 17 | 28 | 41 | 38 | 35 | 35 | 25 | 43 | 47 | 35 | 36 | 0 |
| 11 | 123 | Q6SLM1.1 | 318.42 | 53 | 0 | 14 | 0 | 0 | 0 | 0 | 0 | 0 | 0 | 0 | 13 | 0 | 10 | 15 | 24 | 35 | 33 | 30 | 30 | 21 | 37 | 41 | 30 | 31 | 0 |
| 11 | 124 | AAR19228.1 | 318.42 | 53 | 0 | 14 | 0 | 0 | 0 | 0 | 0 | 0 | 0 | 0 | 13 | 0 | 10 | 15 | 24 | 35 | 33 | 30 | 30 | 21 | 37 | 41 | 30 | 31 | 0 |
| 40 | 258 | CAM11302.1 | 313.34 | 75 | 0 | 18 | 0 | 0 | 16 | 0 | 61 | 57 | 46 | 57 | 47 | 0 | 16 | 37 | 37 | 45 | 58 | 34 | 37 | 18 | 0 | 0 | 0 | 0 | 0 |
| 40 | 259 | D2N116.1 | 313.34 | 75 | 0 | 18 | 0 | 0 | 16 | 0 | 61 | 57 | 46 | 57 | 47 | 0 | 16 | 37 | 37 | 45 | 58 | 34 | 37 | 18 | 0 | 0 | 0 | 0 | 0 |
| 43 | 211 | JAA74765.1 | 313.10 | 26 | 0 | 0 | 0 | 0 | 0 | 0 | 0 | 0 | 0 | 0 | 0 | 0 | 0 | 0 | 6 | 0 | 0 | 18 | 9 | 18 | 26 | 18 | 18 | 9 | 0 |
| 43 | 212 | JAA74764.1 | 313.10 | 26 | 0 | 0 | 0 | 0 | 0 | 0 | 0 | 0 | 0 | 0 | 0 | 0 | 0 | 0 | 6 | 0 | 0 | 18 | 9 | 18 | 26 | 18 | 18 | 9 | 0 |
| 13 | 441 | ABU63166.1 | 308.92 | 29 | 0 | 13 | 0 | 0 | 0 | 0 | 0 | 0 | 0 | 0 | 14 | 0 | 10 | 15 | 15 | 20 | 22 | 15 | 29 | 15 | 13 | 15 | 13 | 13 | 0 |
| 13 | 449 | ABU63164.1 | 308.92 | 29 | 0 | 13 | 0 | 0 | 0 | 0 | 0 | 0 | 0 | 0 | 14 | 0 | 10 | 15 | 15 | 20 | 22 | 15 | 29 | 15 | 13 | 15 | 13 | 13 | 0 |
| 55 | 18 | ACE73577.1 | 303.68 | 42 | 0 | 0 | 0 | 0 | 0 | 0 | 0 | 0 | 0 | 0 | 0 | 0 | 0 | 0 | 0 | 0 | 0 | 19 | 0 | 22 | 31 | 26 | 13 | 0 | 0 |
| 22 | 48 | CAA37484.1 | 290.69 | 52 | 0 | 10 | 0 | 10 | 0 | 0 | 0 | 10 | 0 | 10 | 21 | 0 | 12 | 40 | 40 | 43 | 29 | 22 | 32 | 10 | 9 | 16 | 10 | 16 | 0 |
| 22 | 49 | P17934.1 | 290.69 | 52 | 0 | 10 | 0 | 10 | 0 | 0 | 0 | 10 | 0 | 10 | 21 | 0 | 12 | 40 | 40 | 43 | 29 | 22 | 32 | 10 | 9 | 16 | 10 | 16 | 0 |
| 20 | 108 | ABG90492.1 | 290.15 | 58 | 0 | 11 | 0 | 11 | 0 | 0 | 11 | 28 | 0 | 11 | 28 | 0 | 14 | 52 | 52 | 57 | 51 | 28 | 45 | 11 | 11 | 32 | 26 | 48 | 0 |
| 32 | 198 | Q90251.1 | 284.91 | 53 | 0 | 10 | 0 | 10 | 0 | 0 | 0 | 10 | 0 | 10 | 23 | 0 | 13 | 36 | 36 | 40 | 26 | 23 | 34 | 10 | 9 | 10 | 10 | 10 | 0 |
| 32 | 199 | CAA66869.1 | 284.91 | 53 | 0 | 10 | 0 | 10 | 0 | 0 | 0 | 10 | 0 | 10 | 23 | 0 | 13 | 36 | 36 | 40 | 26 | 23 | 34 | 10 | 9 | 10 | 10 | 10 | 0 |
| 46 | 161 | JAA74998.1 | 281.78 | 28 | 0 | 0 | 0 | 0 | 0 | 0 | 0 | 0 | 0 | 0 | 0 | 0 | 0 | 0 | 6 | 0 | 0 | 15 | 6 | 15 | 28 | 15 | 15 | 6 | 0 |
| 18 | 5193 | C0HLB8.1 | 280.76 | 62 | 0 | 59 | 0 | 0 | 0 | 0 | 0 | 0 | 0 | 0 | 56 | 0 | 44 | 59 | 59 | 59 | 59 | 59 | 62 | 59 | 59 | 62 | 59 | 59 | 0 |
| 14 | 228 | JAS05119.1 | 275.48 | 51 | 0 | 6 | 0 | 6 | 0 | 10 | 13 | 6 | 6 | 13 | 6 | 0 | 22 | 28 | 33 | 42 | 33 | 28 | 33 | 21 | 25 | 32 | 24 | 23 | 0 |
| 14 | 229 | JAS05120.1 | 275.48 | 51 | 0 | 6 | 0 | 6 | 0 | 10 | 13 | 6 | 6 | 13 | 6 | 0 | 22 | 28 | 33 | 42 | 33 | 28 | 33 | 21 | 25 | 32 | 24 | 23 | 0 |
| 14 | 230 | JAS05118.1 | 275.48 | 51 | 0 | 6 | 0 | 6 | 0 | 10 | 13 | 6 | 6 | 13 | 6 | 0 | 22 | 28 | 33 | 42 | 33 | 28 | 33 | 21 | 25 | 32 | 24 | 23 | 0 |
| 14 | 231 | JAS05121.1 | 275.48 | 51 | 0 | 6 | 0 | 6 | 0 | 10 | 13 | 6 | 6 | 13 | 6 | 0 | 22 | 28 | 33 | 42 | 33 | 28 | 33 | 21 | 25 | 32 | 24 | 23 | 0 |
| 26 | 71 | Q75S48.1 | 255.57 | 67 | 0 | 5 | 0 | 5 | 0 | 0 | 5 | 5 | 5 | 5 | 5 | 0 | 0 | 5 | 23 | 10 | 23 | 10 | 10 | 7 | 10 | 20 | 50 | 67 | 0 |
| 26 | 72 | BAD06270.1 | 255.57 | 67 | 0 | 5 | 0 | 5 | 0 | 0 | 5 | 5 | 5 | 5 | 5 | 0 | 0 | 5 | 23 | 10 | 23 | 10 | 10 | 7 | 10 | 20 | 50 | 67 | 0 |
| 8 | 366 | 1TC8 | 251.97 | 17 | 0 | 7 | 0 | 7 | 0 | 0 | 7 | 16 | 7 | 7 | 11 | 0 | 0 | 13 | 16 | 16 | 16 | 17 | 17 | 13 | 16 | 14 | 16 | 16 | 0 |
| 8 | 367 | 1PO8 | 251.97 | 17 | 0 | 7 | 0 | 7 | 0 | 0 | 7 | 16 | 7 | 7 | 11 | 0 | 0 | 13 | 16 | 16 | 16 | 17 | 17 | 13 | 16 | 14 | 16 | 16 | 0 |
| 8 | 369 | AAS20530.1 | 251.97 | 15 | 0 | 6 | 0 | 6 | 0 | 0 | 6 | 14 | 6 | 6 | 9 | 0 | 0 | 11 | 14 | 14 | 14 | 15 | 15 | 11 | 14 | 12 | 14 | 14 | 0 |
| 8 | 370 | Q6SLM2.1 | 251.97 | 15 | 0 | 6 | 0 | 6 | 0 | 0 | 6 | 14 | 6 | 6 | 9 | 0 | 0 | 11 | 14 | 14 | 14 | 15 | 15 | 11 | 14 | 12 | 14 | 14 | 0 |
| 16 | 74 | Q6YIJ3.1 | 250.52 | 39 | 0 | 22 | 0 | 10 | 0 | 0 | 0 | 10 | 0 | 22 | 9 | 7 | 0 | 29 | 29 | 39 | 39 | 29 | 22 | 22 | 9 | 29 | 10 | 36 | 0 |
| 16 | 75 | Q6YNQ0.1 | 250.52 | 39 | 0 | 22 | 0 | 10 | 0 | 0 | 0 | 10 | 0 | 22 | 9 | 7 | 0 | 29 | 29 | 39 | 39 | 29 | 22 | 22 | 9 | 29 | 10 | 36 | 0 |

| Accession | Area F1 | Area F2a | Area F2b | Area F3a | Area F3b | Area F4a | Area F5a | Area F5b | Area F6a | Area F6b | Area F6c | Area F7 | Area F8a | Area F8b | Area F9a | Area F9b | Area F10a | Area F11a | Area F11b | Area F12a | Area F12b | Area F12c | Area F13a | Area F13b | Area F14 |
|---|---|---|---|---|---|---|---|---|---|---|---|---|---|---|---|---|---|---|---|---|---|---|---|---|---|
| AAL87004.1 | | 1.5211E6 | 3.4058E4 | 3.8922E5 | | | 1.2367E6 | 2.1574E7 | 1.4378E5 | 6.4292E5 | 6.4738E6 | 1.4681E5 | | 1.0564E8 | 4.6038E8 | 1.4442E10 | 9.2415E7 | 2.3616E7 | 5.0157E8 | 7.5641E6 | 3.7077E6 | 9.2992E7 | 1.1642E7 | 2.549E8 | |
| Q8QFW3.1 | | 1.5211E6 | 3.4058E4 | 3.8922E5 | | | 1.2367E6 | 2.1574E7 | 1.4378E5 | 6.4292E5 | 6.4738E6 | 1.4681E5 | | 1.0564E8 | 4.6038E8 | 1.4442E10 | 9.2415E7 | 2.3616E7 | 5.0157E8 | 7.5641E6 | 3.7077E6 | 9.2992E7 | 1.1642E7 | 2.549E8 | |
| Q8QFW4.1 | | 6.2754E4 | | | | | | | | | 6.129E4 | | | 7.0959E6 | 6.7486E7 | 1.7056E9 | 4.4671E8 | 9.4393E6 | 2.9215E8 | 3.524E5 | 6.1513E4 | 1.1154E7 | 1.4294E6 | 7.2582E7 | |
| AAL87003.1 | | 6.2754E4 | | | | | | | | | 6.129E4 | | | 7.0959E6 | 6.7486E7 | 1.7056E9 | 4.4671E8 | 9.4393E6 | 2.9215E8 | 3.524E5 | 6.1513E4 | 1.1154E7 | 1.4294E6 | 7.2582E7 | |
| 0702209A | | | | | | | | | | | | | | 1.5644E5 | 5.3463E5 | 2.7048E5 | 2.0014E6 | 5.6378E6 | 1.1762E8 | 1.3616E6 | 3.5203E6 | 7.4853E7 | 1.3239E6 | 3.6884E6 | |
| P00606.2 | | | | | | | | | | | | | | 1.5644E5 | 5.3463E5 | 2.7048E5 | 2.0014E6 | 5.6378E6 | 1.1762E8 | 1.3616E6 | 3.5203E6 | 7.4853E7 | 1.3239E6 | 3.6884E6 | |
| CAA37482.1 | | | | | | | | | | | | | | 1.5644E5 | 5.3463E5 | 2.7048E5 | 2.0014E6 | 5.6378E6 | 1.1762E8 | 1.3616E6 | 3.5203E6 | 7.4853E7 | 1.3239E6 | 3.6884E6 | |
| 1G0Z | | | | | | | | | | | | | | 0 | 1.996E5 | 8.3363E4 | 8.7006E5 | 1.8987E7 | 5.2393E8 | 2.5248E7 | 1.0461E8 | 3.9237E9 | 5.9602E7 | 0 | |
| 1U4J | | | | | | | | | | | | | | 0 | 1.996E5 | 8.3363E4 | 8.7006E5 | 1.8987E7 | 5.2393E8 | 2.5248E7 | 1.0461E8 | 3.9237E9 | 5.9602E7 | 0 | |
| Q6SLM1.1 | | | | | | | | | | | | | | 0 | 1.996E5 | 8.3363E4 | 8.7006E5 | 1.8987E7 | 5.2393E8 | 2.5248E7 | 1.0461E8 | 3.9237E9 | 5.9602E7 | 0 | |
| AAR19228.1 | | | | | | | | | | | | | | 0 | 1.996E5 | 8.3363E4 | 8.7006E5 | 1.8987E7 | 5.2393E8 | 2.5248E7 | 1.0461E8 | 3.9237E9 | 5.9602E7 | 0 | |
| CAM11302.1 | | 0 | | | | | 1.2024E7 | 2.1463E7 | 4.5004E7 | 1.2831E8 | 8.9827E7 | | | 1.2798E5 | 7.8029E7 | 3.5561E8 | 9.1603E7 | 1.9299E6 | 3.9262E6 | 1.2584E5 | | | | | |
| D2N116.1 | | 0 | | | | | 1.2024E7 | 2.1463E7 | 4.5004E7 | 1.2831E8 | 8.9827E7 | | | 1.2798E5 | 7.8029E7 | 3.5561E8 | 9.1603E7 | 1.9299E6 | 3.9262E6 | 1.2584E5 | | | | | |
| JAA74765.1 | | | | | | | | | | | | | | | | | | | | | 1.3511E6 | | | | |
| JAA74764.1 | | | | | | | | | | | | | | | | | | | | | 1.3511E6 | | | | |
| ABU63166.1 | | | | | | | | | | | 2.8218E5 | | | 5.8693E6 | 4.1338E7 | 1.9224E8 | 2.5128E6 | 1.6455E5 | 7.7007E6 | 0 | | 6.6927E5 | | | |
| ABU63164.1 | | | | | | | | | | | 2.8218E5 | | | 5.8693E6 | 4.1338E7 | 1.9224E8 | 2.5128E6 | 1.6455E5 | 7.7007E6 | 0 | | 6.6927E5 | | | |
| ACE73577.1 | | | | | | | | | | | | | | | | | | 4.946E6 | | 3.0105E7 | 1.6276E8 | 0 | 5.8033E6 | | |
| CAA37484.1 | | | | | | | | | | | | | | | | 0 | | | | | | | | | |
| P17934.1 | | | | | | | | | | | | | | | | 0 | | | | | | | | | |
| ABG90492.1 | | | | | | | | | | | 0 | | | | | | | | | | | | | | |
| Q90251.1 | | | | | | | | | | | | | | | | | 0 | | | | | | | | |
| CAA66869.1 | | | | | | | | | | | | | | | | | 0 | | | | | | | | |
| JAA74998.1 | | | | | | | | | | | | | | | | | | | | | 1.9856E7 | | | | |
| C0HLB8.1 | | | | | | | | | | | | | | | | | | | 0 | | | 0 | | | |
| JAS05119.1 | | | | | | | | | | | | | | | | 3.6374E7 | | | | | | 0 | | | |
| JAS05120.1 | | | | | | | | | | | | | | | | 3.6374E7 | | | | | | 0 | | | |
| JAS05118.1 | | | | | | | | | | | | | | | | 3.6374E7 | | | | | | 0 | | | |
| JAS05121.1 | | | | | | | | | | | | | | | | 3.6374E7 | | | | | | 0 | | | |
| Q75S48.1 | | | | | | | | | | | | | | | 3.8693E5 | | 0 | | | | | 0 | 4.0081E6 | 2.1213E8 | |
| BAD06270.1 | | | | | | | | | | | | | | | 3.8693E5 | | 0 | | | | | 0 | 4.0081E6 | 2.1213E8 | |
| 1TC8 | | | | | | | | 1.1381E6 | | | 2.3688E6 | | | 6.1259E8 | 5.2828E8 | 1.3463E10 | 1.3991E8 | 5.3743E8 | 2.5391E10 | 2.5431E7 | 2.0998E8 | 1.1138E10 | 6.1353E8 | 1.9855E10 | |
| 1PO8 | | | | | | | | 1.1381E6 | | | 2.3688E6 | | | 6.1259E8 | 5.2828E8 | 1.3463E10 | 1.3991E8 | 5.3743E8 | 2.5391E10 | 2.5431E7 | 2.0998E8 | 1.1138E10 | 6.1353E8 | 1.9855E10 | |
| AAS20530.1 | | | | | | | | 1.1381E6 | | | 2.3688E6 | | | 6.1259E8 | 5.2828E8 | 1.3463E10 | 1.3991E8 | 5.3743E8 | 2.5391E10 | 2.5431E7 | 2.0998E8 | 1.1138E10 | 6.1353E8 | 1.9855E10 | |
| Q6SLM2.1 | | | | | | | | 1.1381E6 | | | 2.3688E6 | | | 6.1259E8 | 5.2828E8 | 1.3463E10 | 1.3991E8 | 5.3743E8 | 2.5391E10 | 2.5431E7 | 2.0998E8 | 1.1138E10 | 6.1353E8 | 1.9855E10 | |
| Q6YIJ3.1 | | | | | | | | | | | | | | | | 2.2947E7 | 0 | | | | | | | | |
| Q6YNQ0.1 | | | | | | | | | | | | | | | | 2.2947E7 | 0 | | | | | | | | |

| Accession | #Peptides | #Unique | #Spec F1 | #Spec F2a | #Spec F2b | #Spec F3a | #Spec F3b | #Spec F4a | #Spec F5a | #Spec F5b | #Spec F6a | #Spec F6b | #Spec F6c | #Spec F7 | #Spec F8a | #Spec F8b | #Spec F9a | #Spec F9b | #Spec F10a | #Spec F11a | #Spec F11b | #Spec F12a | #Spec F12b | #Spec F12c | #Spec F13a | #Spec F13b | #Spec F14 | PTM | Avg. Mass | Description |
|---|---|---|---|---|---|---|---|---|---|---|---|---|---|---|---|---|---|---|---|---|---|---|---|---|---|---|---|---|---|---|
| AAL87004.1 | 66 | 45 | 0 | 10 | 1 | 3 | 0 | 0 | 7 | 36 | 1 | 6 | 15 | 13 | 6 | 68 | 363 | 587 | 251 | 42 | 188 | 9 | 10 | 19 | 19 | 44 | 0 | Y | 16120 | beta-bungarotoxin A2 chain precursor [Bungarus caeruleus] |
| Q8QFW3.1 | 66 | 45 | 0 | 10 | 1 | 3 | 0 | 0 | 7 | 36 | 1 | 6 | 15 | 13 | 6 | 68 | 363 | 587 | 251 | 42 | 188 | 9 | 10 | 19 | 19 | 44 | 0 | Y | 16120 | RecName: Full=Basic phospholipase A2 beta-bungarotoxin A2 chain; Short=Beta-BuTX A2 chain; Short=svPLA2; AltName: Full=Phosphatidylcholine 2-acylhydrolase; Flags: Precursor |
| Q8QFW4.1 | 49 | 20 | 0 | 5 | 0 | 1 | 0 | 0 | 0 | 5 | 0 | 5 | 5 | 0 | 3 | 31 | 90 | 162 | 221 | 34 | 95 | 6 | 4 | 14 | 13 | 27 | 0 | Y | 16358 | RecName: Full=Basic phospholipase A2 beta-bungarotoxin A1 chain; Short=Beta-BuTX A1 chain; Short=svPLA2; AltName: Full=Phosphatidylcholine 2-acylhydrolase; Flags: Precursor |
| AAL87003.1 | 49 | 20 | 0 | 5 | 0 | 1 | 0 | 0 | 0 | 5 | 0 | 5 | 5 | 0 | 3 | 31 | 90 | 162 | 221 | 34 | 95 | 6 | 4 | 14 | 13 | 27 | 0 | Y | 16358 | beta-bungarotoxin A chain precursor [Bungarus caeruleus] |
| 0702209A | 25 | 7 | 0 | 0 | 0 | 0 | 0 | 3 | 0 | 0 | 0 | 0 | 0 | 0 | 6 | 3 | 18 | 8 | 24 | 20 | 49 | 9 | 19 | 26 | 9 | 10 | 0 | Y | 12832 | phospholipase A |
| P00606.2 | 25 | 7 | 0 | 0 | 0 | 0 | 0 | 3 | 0 | 0 | 0 | 0 | 0 | 0 | 6 | 3 | 18 | 8 | 24 | 20 | 49 | 9 | 19 | 26 | 9 | 10 | 0 | Y | 15593 | RecName: Full=Acidic phospholipase A2; Short=svPLA2; AltName: Full=Phosphatidylcholine 2-acylhydrolase; Flags: Precursor |
| CAA37482.1 | 25 | 7 | 0 | 0 | 0 | 0 | 0 | 3 | 0 | 0 | 0 | 0 | 0 | 0 | 6 | 3 | 18 | 8 | 24 | 20 | 49 | 9 | 19 | 26 | 9 | 10 | 0 | Y | 15593 | precursor polypeptide (AA-27 to 118) [Bungarus multicinctus] |
| 1G0Z | 19 | 3 | 0 | 3 | 0 | 0 | 0 | 0 | 0 | 0 | 0 | 0 | 9 | 0 | 1 | 22 | 25 | 18 | 32 | 29 | 60 | 26 | 47 | 106 | 34 | 17 | 0 | Y | 12970 | Chain B, PHOSPHOLIPASE A2 |
| 1U4J | 19 | 3 | 0 | 3 | 0 | 0 | 0 | 0 | 0 | 0 | 0 | 0 | 9 | 0 | 1 | 22 | 25 | 18 | 32 | 29 | 60 | 26 | 47 | 106 | 34 | 17 | 0 | Y | 12970 | Chain B, phospholipase A2 isoform 2 |
| Q6SLM1.1 | 19 | 3 | 0 | 3 | 0 | 0 | 0 | 0 | 0 | 0 | 0 | 0 | 9 | 0 | 1 | 22 | 25 | 18 | 32 | 29 | 60 | 26 | 47 | 106 | 34 | 17 | 0 | Y | 14827 | RecName: Full=Basic phospholipase A2 2; Short=svPLA2; AltName: Full=Phosphatidylcholine 2-acylhydrolase; Flags: Precursor |
| AAR19228.1 | 19 | 3 | 0 | 3 | 0 | 0 | 0 | 0 | 0 | 0 | 0 | 0 | 9 | 0 | 1 | 22 | 25 | 18 | 32 | 29 | 60 | 26 | 47 | 106 | 34 | 17 | 0 | Y | 14827 | phospholipase A2 isoform 2, partial [Bungarus caeruleus] |
| CAM11302.1 | 37 | 31 | 0 | 1 | 0 | 0 | 1 | 0 | 7 | 10 | 16 | 16 | 25 | 0 | 2 | 2 | 49 | 30 | 47 | 5 | 5 | 1 | 0 | 0 | 0 | 0 | 0 | Y | 8306 | alpha-delta-bungarotoxin-4, partial [Bungarus caeruleus] |
| D2N116.1 | 37 | 31 | 0 | 1 | 0 | 0 | 1 | 0 | 7 | 10 | 16 | 16 | 25 | 0 | 2 | 2 | 49 | 30 | 47 | 5 | 5 | 1 | 0 | 0 | 0 | 0 | 0 | Y | 8306 | RecName: Full=Alpha-delta-bungarotoxin-4; Short=Alpha-delta-Bgt-4; Flags: Precursor |
| JAA74765.1 | 21 | 1 | 0 | 0 | 0 | 0 | 0 | 0 | 0 | 0 | 0 | 0 | 0 | 0 | 0 | 0 | 1 | 0 | 0 | 20 | 3 | 49 | 103 | 11 | 31 | 2 | 0 | Y | 26376 | CRiSP-Ech-1 [Echiopsis curta] |
| JAA74764.1 | 21 | 1 | 0 | 0 | 0 | 0 | 0 | 0 | 0 | 0 | 0 | 0 | 0 | 0 | 0 | 0 | 1 | 0 | 0 | 20 | 3 | 49 | 103 | 11 | 31 | 2 | 0 | Y | 26376 | KP-Ech-8 [Echiopsis curta] |
| ABU63166.1 | 15 | 4 | 0 | 3 | 0 | 0 | 0 | 0 | 0 | 0 | 0 | 0 | 10 | 0 | 1 | 27 | 39 | 31 | 30 | 29 | 52 | 24 | 29 | 81 | 25 | 11 | 0 | Y | 16004 | phospholipase A2 precursor BF-42 [Bungarus fasciatus] |
| ABU63164.1 | 15 | 4 | 0 | 3 | 0 | 0 | 0 | 0 | 0 | 0 | 0 | 0 | 10 | 0 | 1 | 27 | 39 | 31 | 30 | 29 | 52 | 24 | 29 | 81 | 25 | 11 | 0 | Y | 15950 | phospholipase A2 precursor BF-40 [Bungarus fasciatus] |
| ACE73577.1 | 20 | 5 | 0 | 0 | 0 | 0 | 0 | 0 | 0 | 0 | 0 | 0 | 0 | 0 | 0 | 0 | 0 | 0 | 0 | 8 | 0 | 29 | 75 | 13 | 10 | 0 | 0 | Y | 26458 | cysteine-rich seceretory protein Bc-CRPb [Bungarus candidus] |
| CAA37484.1 | 18 | 1 | 0 | 3 | 0 | 1 | 0 | 0 | 0 | 5 | 0 | 4 | 4 | 0 | 3 | 21 | 35 | 65 | 66 | 14 | 34 | 2 | 1 | 3 | 10 | 12 | 0 | Y | 16177 | precursor polypeptide (AA-27 to 120) [Bungarus multicinctus] |
| P17934.1 | 18 | 1 | 0 | 3 | 0 | 1 | 0 | 0 | 0 | 5 | 0 | 4 | 4 | 0 | 3 | 21 | 35 | 65 | 66 | 14 | 34 | 2 | 1 | 3 | 10 | 12 | 0 | Y | 16177 | RecName: Full=Acidic phospholipase A2 beta-bungarotoxin A4 chain; Short=Beta-BuTX A4 chain; Short=svPLA2; AltName: Full=Phosphatidylcholine 2-acylhydrolase; Flags: Precursor |
| ABG90492.1 | 25 | 1 | 0 | 6 | 0 | 1 | 0 | 0 | 4 | 12 | 0 | 2 | 7 | 0 | 6 | 18 | 65 | 61 | 48 | 11 | 26 | 1 | 3 | 9 | 8 | 15 | 0 | Y | 13164 | beta-bungarotoxin A8 chain, partial [Bungarus multicinctus] |
| Q90251.1 | 16 | 1 | 0 | 3 | 0 | 1 | 0 | 0 | 0 | 5 | 0 | 4 | 4 | 0 | 3 | 19 | 31 | 48 | 58 | 14 | 34 | 2 | 1 | 2 | 10 | 11 | 0 | Y | 15103 | RecName: Full=Acidic phospholipase A2 beta-bungarotoxin A6 chain; Short=Beta-BuTX A6 chain; Short=svPLA2; AltName: Full=Phosphatidylcholine 2-acylhydrolase; Flags: Precursor |
| CAA66869.1 | 16 | 1 | 0 | 3 | 0 | 1 | 0 | 0 | 0 | 5 | 0 | 4 | 4 | 0 | 3 | 19 | 31 | 48 | 58 | 14 | 34 | 2 | 1 | 2 | 10 | 11 | 0 | Y | 15103 | beta-bungarotoxin A-chain precursor, partial [Bungarus multicinctus] |
| JAA74998.1 | 15 | 1 | 0 | 0 | 0 | 0 | 0 | 0 | 0 | 0 | 0 | 0 | 0 | 0 | 0 | 0 | 1 | 0 | 0 | 17 | 2 | 37 | 79 | 9 | 23 | 1 | 0 | Y | 26447 | CRiSP-Ver-3 [Vermicella annulata] |
| C0HLB8.1 | 10 | 1 | 0 | 3 | 0 | 0 | 0 | 0 | 0 | 0 | 0 | 0 | 9 | 0 | 1 | 21 | 20 | 14 | 24 | 26 | 47 | 23 | 29 | 80 | 25 | 11 | 0 | Y | 3728 | RecName: Full=Phospholipase A2; Short=MIPLA2-1; Short=svPLA2; AltName: Full=Phosphatidylcholine 2-acylhydrolase |
| JAS05119.1 | 22 | 2 | 0 | 1 | 0 | 1 | 0 | 3 | 3 | 5 | 1 | 3 | 3 | 0 | 23 | 16 | 22 | 40 | 32 | 26 | 54 | 13 | 16 | 31 | 16 | 33 | 0 | Y | 15836 | Phospholipase A2 6c [Micrurus tener] |
| JAS05120.1 | 22 | 2 | 0 | 1 | 0 | 1 | 0 | 3 | 3 | 5 | 1 | 3 | 3 | 0 | 23 | 16 | 22 | 40 | 32 | 26 | 54 | 13 | 16 | 31 | 16 | 33 | 0 | Y | 15836 | Phospholipase A2 6b [Micrurus tener] |
| JAS05118.1 | 22 | 2 | 0 | 1 | 0 | 1 | 0 | 3 | 3 | 5 | 1 | 3 | 3 | 0 | 23 | 16 | 22 | 40 | 32 | 26 | 54 | 13 | 16 | 31 | 16 | 33 | 0 | Y | 15835 | Phospholipase A2 6d [Micrurus tener] |
| JAS05121.1 | 22 | 2 | 0 | 1 | 0 | 1 | 0 | 3 | 3 | 5 | 1 | 3 | 3 | 0 | 23 | 16 | 22 | 40 | 32 | 26 | 54 | 13 | 16 | 31 | 16 | 33 | 0 | Y | 15836 | Phospholipase A2 6a [Micrurus tener] |
| Q75S48.1 | 15 | 7 | 0 | 1 | 0 | 1 | 0 | 0 | 2 | 5 | 1 | 1 | 3 | 0 | 0 | 7 | 11 | 30 | 18 | 12 | 28 | 8 | 9 | 23 | 18 | 58 | 0 | Y | 16855 | RecName: Full=Acidic phospholipase A2 1; Short=svPLA2; AltName: Full=PA2-I; AltName: Full=Phosphatidylcholine 2-acylhydrolase; Flags: Precursor |
| BAD06270.1 | 15 | 7 | 0 | 1 | 0 | 1 | 0 | 0 | 2 | 5 | 1 | 1 | 3 | 0 | 0 | 7 | 11 | 30 | 18 | 12 | 28 | 8 | 9 | 23 | 18 | 58 | 0 | Y | 16855 | phospholipase A2-I precursor [Bungarus candidus] |
| 1TC8 | 20 | 9 | 0 | 1 | 0 | 1 | 0 | 0 | 2 | 6 | 1 | 1 | 6 | 0 | 0 | 23 | 29 | 87 | 44 | 34 | 83 | 19 | 19 | 62 | 29 | 76 | 0 | Y | 13201 | Chain A, phospholipase A2 isoform 1 |
| 1PO8 | 20 | 9 | 0 | 1 | 0 | 1 | 0 | 0 | 2 | 6 | 1 | 1 | 6 | 0 | 0 | 23 | 29 | 87 | 44 | 34 | 83 | 19 | 19 | 62 | 29 | 76 | 0 | Y | 13201 | Chain A, Phospholipase A2 |
| AAS20530.1 | 20 | 9 | 0 | 1 | 0 | 1 | 0 | 0 | 2 | 6 | 1 | 1 | 6 | 0 | 0 | 23 | 29 | 87 | 44 | 34 | 83 | 19 | 19 | 62 | 29 | 76 | 0 | Y | 15058 | phospholipase A2 isoform 1, partial [Bungarus caeruleus] |
| Q6SLM2.1 | 20 | 9 | 0 | 1 | 0 | 1 | 0 | 0 | 2 | 6 | 1 | 1 | 6 | 0 | 0 | 23 | 29 | 87 | 44 | 34 | 83 | 19 | 19 | 62 | 29 | 76 | 0 | Y | 15058 | RecName: Full=Acidic phospholipase A2 1; Short=svPLA2; AltName: Full=Phosphatidylcholine 2-acylhydrolase; Flags: Precursor |
| Q6YIJ3.1 | 13 | 1 | 0 | 4 | 0 | 1 | 0 | 0 | 0 | 5 | 0 | 5 | 2 | 9 | 0 | 22 | 33 | 91 | 81 | 17 | 32 | 3 | 1 | 4 | 10 | 15 | 0 | Y | 15063 | RecName: Full=Acidic phospholipase A2 beta-bungarotoxin A chain; Short=Beta-BuTX A chain; Short=svPLA2; AltName: Full=Phosphatidylcholine 2-acylhydrolase; Flags: Precursor |
| Q6YNQ0.1 | 13 | 1 | 0 | 4 | 0 | 1 | 0 | 0 | 0 | 5 | 0 | 5 | 2 | 9 | 0 | 22 | 33 | 91 | 81 | 17 | 32 | 3 | 1 | 4 | 10 | 15 | 0 | Y | 15092 | RecName: Full=Acidic phospholipase A2 beta-bungarotoxin A3 chain; Short=Beta-BuTX A3 chain; Short=svPLA2; AltName: Full=Phosphatidylcholine 2-acylhydrolase; Flags: Precursor |

| | | | | | | | | | | | | | | | | | | | | | | | | | | | | | | | | | | | | | | | | | | | | | | | | | | | | | | | | | | | | | | | | | | | | | | | | | | | | | | | | | | | | |
|---|---|---|---|---|---|---|---|---|---|---|---|---|---|---|---|---|---|---|---|---|---|---|---|---|---|---|---|---|---|---|---|---|---|---|---|---|---|---|---|---|---|---|---|---|---|---|---|---|---|---|---|---|---|---|---|---|---|---|---|---|---|---|---|---|---|---|---|---|---|---|---|---|---|---|---|---|---|---|---|---|---|---|---|---|
| **16** | 76 | CAB62385.1 | 250.52 | 39 | 0 | 22 | 0 | 10 | 0 | 0 | 0 | 10 | 0 | 22 | 9 | 7 | 0 | 29 | 29 | 39 | 39 | 29 | 22 | 22 | 9 | 29 | 10 | 36 | 0 | | | | | | | | | | | | | | | | 2.2947E7 | 0 | | | | | | | | | 13 | 1 | 0 | 4 | 0 | 1 | 0 | 0 | 0 | 5 | 0 | 5 | 2 | 9 | 0 | 22 | 33 | 91 | 81 | 17 | 32 | 3 | 1 | 4 | 10 | 15 | 0 | Y | 15092 | beta-bungarotoxin A3 chain, partial [Bungarus multicinctus] |
| **16** | 77 | AAL30064.1 | 250.52 | 39 | 0 | 22 | 0 | 10 | 0 | 0 | 0 | 10 | 0 | 22 | 9 | 7 | 0 | 29 | 29 | 39 | 39 | 29 | 22 | 22 | 9 | 29 | 10 | 36 | 0 | | | | | | | | | | | | | | | | 2.2947E7 | 0 | | | | | | | | | 13 | 1 | 0 | 4 | 0 | 1 | 0 | 0 | 0 | 5 | 0 | 5 | 2 | 9 | 0 | 22 | 33 | 91 | 81 | 17 | 32 | 3 | 1 | 4 | 10 | 15 | 0 | Y | 15092 | beta bungarotoxin A3 chain, partial [Bungarus candidus] |
| **16** | 78 | AAN16113.1 | 250.52 | 39 | 0 | 22 | 0 | 10 | 0 | 0 | 0 | 10 | 0 | 22 | 9 | 7 | 0 | 29 | 29 | 39 | 39 | 29 | 22 | 22 | 9 | 29 | 10 | 36 | 0 | | | | | | | | | | | | | | | | 2.2947E7 | 0 | | | | | | | | | 13 | 1 | 0 | 4 | 0 | 1 | 0 | 0 | 0 | 5 | 0 | 5 | 2 | 9 | 0 | 22 | 33 | 91 | 81 | 17 | 32 | 3 | 1 | 4 | 10 | 15 | 0 | Y | 15063 | beta bungarotoxin A chain, partial [Bungarus candidus] |
| **16** | 79 | CAC08197.1 | 250.52 | 36 | 0 | 20 | 0 | 10 | 0 | 0 | 0 | 10 | 0 | 20 | 9 | 7 | 0 | 27 | 27 | 36 | 36 | 27 | 20 | 20 | 9 | 27 | 10 | 34 | 0 | | | | | | | | | | | | | | | | 2.2947E7 | 0 | | | | | | | | | 13 | 1 | 0 | 4 | 0 | 1 | 0 | 0 | 0 | 5 | 0 | 5 | 2 | 9 | 0 | 22 | 33 | 91 | 81 | 17 | 32 | 3 | 1 | 4 | 10 | 15 | 0 | Y | 16218 | beta-bungarotoxin A [Bungarus multicinctus multicinctus] |
| **16** | 80 | P00619.2 | 250.52 | 36 | 0 | 20 | 0 | 10 | 0 | 0 | 0 | 10 | 0 | 20 | 9 | 7 | 0 | 27 | 27 | 36 | 36 | 27 | 20 | 20 | 9 | 27 | 10 | 34 | 0 | | | | | | | | | | | | | | | | 2.2947E7 | 0 | | | | | | | | | 13 | 1 | 0 | 4 | 0 | 1 | 0 | 0 | 0 | 5 | 0 | 5 | 2 | 9 | 0 | 22 | 33 | 91 | 81 | 17 | 32 | 3 | 1 | 4 | 10 | 15 | 0 | Y | 16218 | RecName: Full=Acidic phospholipase A2 beta-bungarotoxin A3 chain; Short=Beta-BuTX A3 chain; Short=svPLA2; AltName: Full=Phosphatidylcholine 2-acylhydrolase; Flags: Precursor |
| **91** | 6 | 4QWW | 249.21 | 36 | 0 | 0 | 0 | 0 | 0 | 0 | 0 | 0 | 0 | 0 | 0 | 0 | 0 | 0 | 0 | 0 | 0 | 0 | 0 | 0 | 0 | 0 | 0 | 0 | 36 | | | | | | | | | | | | | | | | | | | | | | | | | 3.3595E5 | 13 | 3 | 0 | 0 | 0 | 0 | 0 | 0 | 0 | 0 | 0 | 0 | 0 | 0 | 0 | 0 | 0 | 0 | 0 | 0 | 0 | 0 | 0 | 0 | 0 | 0 | 63 | Y | 60267 | Chain B, Acetylcholinesterase |
| **91** | 7 | AAC59905.1 | 249.21 | 34 | 0 | 0 | 0 | 0 | 0 | 0 | 0 | 0 | 0 | 0 | 0 | 0 | 0 | 0 | 0 | 0 | 0 | 0 | 0 | 0 | 0 | 0 | 0 | 0 | 34 | | | | | | | | | | | | | | | | | | | | | | | | | 3.3595E5 | 13 | 3 | 0 | 0 | 0 | 0 | 0 | 0 | 0 | 0 | 0 | 0 | 0 | 0 | 0 | 0 | 0 | 0 | 0 | 0 | 0 | 0 | 0 | 0 | 0 | 0 | 63 | Y | 64723 | acetylcholinesterase [Bungarus fasciatus] |
| **91** | 8 | Q92035.2 | 249.21 | 33 | 0 | 0 | 0 | 0 | 0 | 0 | 0 | 0 | 0 | 0 | 0 | 0 | 0 | 0 | 0 | 0 | 0 | 0 | 0 | 0 | 0 | 0 | 0 | 0 | 33 | | | | | | | | | | | | | | | | | | | | | | | | | 3.3595E5 | 13 | 3 | 0 | 0 | 0 | 0 | 0 | 0 | 0 | 0 | 0 | 0 | 0 | 0 | 0 | 0 | 0 | 0 | 0 | 0 | 0 | 0 | 0 | 0 | 0 | 0 | 63 | Y | 68074 | RecName: Full=Acetylcholinesterase; Short=BfAChE; Flags: Precursor |
| **63** | 764 | JAS05108.1 | 246.17 | 24 | 0 | 0 | 0 | 0 | 0 | 10 | 0 | 0 | 0 | 0 | 0 | 0 | 14 | 14 | 14 | 24 | 14 | 18 | 23 | 13 | 14 | 13 | 13 | 12 | 0 | | | | | | | | | | | | | | | | 0 | | | 0 | | | | | | | 14 | 2 | 0 | 0 | 0 | 0 | 0 | 3 | 0 | 0 | 0 | 0 | 0 | 0 | 6 | 3 | 10 | 4 | 13 | 14 | 26 | 5 | 7 | 8 | 4 | 2 | 0 | Y | 15724 | Phospholipase A2 8d [Micrurus tener] |
| **63** | 765 | JAS05101.1 | 246.17 | 24 | 0 | 0 | 0 | 0 | 0 | 10 | 0 | 0 | 0 | 0 | 0 | 0 | 14 | 14 | 14 | 24 | 14 | 18 | 23 | 13 | 14 | 13 | 13 | 12 | 0 | | | | | | | | | | | | | | | | 0 | | | 0 | | | | | | | 14 | 2 | 0 | 0 | 0 | 0 | 0 | 3 | 0 | 0 | 0 | 0 | 0 | 0 | 6 | 3 | 10 | 4 | 13 | 14 | 26 | 5 | 7 | 8 | 4 | 2 | 0 | Y | 15739 | Phospholipase A2 8k [Micrurus tener] |
| **63** | 781 | JAS05106.1 | 246.17 | 24 | 0 | 0 | 0 | 0 | 0 | 10 | 0 | 0 | 0 | 0 | 0 | 0 | 14 | 14 | 14 | 24 | 14 | 18 | 23 | 13 | 14 | 13 | 13 | 12 | 0 | | | | | | | | | | | | | | | | 0 | | | 0 | | | | | | | 14 | 2 | 0 | 0 | 0 | 0 | 0 | 3 | 0 | 0 | 0 | 0 | 0 | 0 | 6 | 3 | 10 | 4 | 13 | 14 | 26 | 5 | 7 | 8 | 4 | 2 | 0 | Y | 15767 | Phospholipase A2 8f [Micrurus tener] |
| **63** | 782 | JAS05111.1 | 246.17 | 24 | 0 | 0 | 0 | 0 | 0 | 10 | 0 | 0 | 0 | 0 | 0 | 0 | 14 | 14 | 14 | 24 | 14 | 18 | 23 | 13 | 14 | 13 | 13 | 12 | 0 | | | | | | | | | | | | | | | | 0 | | | 0 | | | | | | | 14 | 2 | 0 | 0 | 0 | 0 | 0 | 3 | 0 | 0 | 0 | 0 | 0 | 0 | 6 | 3 | 10 | 4 | 13 | 14 | 26 | 5 | 7 | 8 | 4 | 2 | 0 | Y | 15766 | Phospholipase A2 8a [Micrurus tener] |
| **63** | 783 | JAS05104.1 | 246.17 | 24 | 0 | 0 | 0 | 0 | 0 | 10 | 0 | 0 | 0 | 0 | 0 | 0 | 14 | 14 | 14 | 24 | 14 | 18 | 23 | 13 | 14 | 13 | 13 | 12 | 0 | | | | | | | | | | | | | | | | 0 | | | 0 | | | | | | | 14 | 2 | 0 | 0 | 0 | 0 | 0 | 3 | 0 | 0 | 0 | 0 | 0 | 0 | 6 | 3 | 10 | 4 | 13 | 14 | 26 | 5 | 7 | 8 | 4 | 2 | 0 | Y | 15781 | Phospholipase A2 8h [Micrurus tener] |
| **63** | 784 | JAS05109.1 | 246.17 | 24 | 0 | 0 | 0 | 0 | 0 | 10 | 0 | 0 | 0 | 0 | 0 | 0 | 14 | 14 | 14 | 24 | 14 | 18 | 23 | 13 | 14 | 13 | 13 | 12 | 0 | | | | | | | | | | | | | | | | 0 | | | 0 | | | | | | | 14 | 2 | 0 | 0 | 0 | 0 | 0 | 3 | 0 | 0 | 0 | 0 | 0 | 0 | 6 | 3 | 10 | 4 | 13 | 14 | 26 | 5 | 7 | 8 | 4 | 2 | 0 | Y | 15865 | Phospholipase A2 8c [Micrurus tener] |
| **63** | 785 | JAS05107.1 | 246.17 | 24 | 0 | 0 | 0 | 0 | 0 | 10 | 0 | 0 | 0 | 0 | 0 | 0 | 14 | 14 | 14 | 24 | 14 | 18 | 23 | 13 | 14 | 13 | 13 | 12 | 0 | | | | | | | | | | | | | | | | 0 | | | 0 | | | | | | | 14 | 2 | 0 | 0 | 0 | 0 | 0 | 3 | 0 | 0 | 0 | 0 | 0 | 0 | 6 | 3 | 10 | 4 | 13 | 14 | 26 | 5 | 7 | 8 | 4 | 2 | 0 | Y | 15708 | Phospholipase A2 8e [Micrurus tener] |
| **63** | 786 | JAS05102.1 | 246.17 | 24 | 0 | 0 | 0 | 0 | 0 | 10 | 0 | 0 | 0 | 0 | 0 | 0 | 14 | 14 | 14 | 24 | 14 | 18 | 23 | 13 | 14 | 13 | 13 | 12 | 0 | | | | | | | | | | | | | | | | 0 | | | 0 | | | | | | | 14 | 2 | 0 | 0 | 0 | 0 | 0 | 3 | 0 | 0 | 0 | 0 | 0 | 0 | 6 | 3 | 10 | 4 | 13 | 14 | 26 | 5 | 7 | 8 | 4 | 2 | 0 | Y | 15781 | Phospholipase A2 8j [Micrurus tener] |
| **63** | 787 | JAS05110.1 | 246.17 | 24 | 0 | 0 | 0 | 0 | 0 | 10 | 0 | 0 | 0 | 0 | 0 | 0 | 14 | 14 | 14 | 24 | 14 | 18 | 23 | 13 | 14 | 13 | 13 | 12 | 0 | | | | | | | | | | | | | | | | 0 | | | 0 | | | | | | | 14 | 2 | 0 | 0 | 0 | 0 | 0 | 3 | 0 | 0 | 0 | 0 | 0 | 0 | 6 | 3 | 10 | 4 | 13 | 14 | 26 | 5 | 7 | 8 | 4 | 2 | 0 | Y | 15766 | Phospholipase A2 8b [Micrurus tener] |
| **63** | 808 | JAS05105.1 | 246.17 | 24 | 0 | 0 | 0 | 0 | 0 | 10 | 0 | 0 | 0 | 0 | 0 | 0 | 14 | 14 | 14 | 24 | 14 | 18 | 23 | 13 | 14 | 13 | 13 | 12 | 0 | | | | | | | | | | | | | | | | 0 | | | 0 | | | | | | | 14 | 2 | 0 | 0 | 0 | 0 | 0 | 3 | 0 | 0 | 0 | 0 | 0 | 0 | 6 | 3 | 10 | 4 | 13 | 14 | 26 | 5 | 7 | 8 | 4 | 2 | 0 | Y | 15952 | Phospholipase A2 8g [Micrurus tener] |
| **67** | 868 | AAB20783.1 | 242.11 | 36 | 0 | 0 | 0 | 0 | 0 | 13 | 0 | 0 | 0 | 0 | 0 | 0 | 14 | 15 | 16 | 36 | 16 | 16 | 30 | 16 | 16 | 16 | 16 | 15 | 0 | | | | | | | | | | | | | | | | 0 | | | 1.2181E6 | | | | | | | 12 | 3 | 0 | 0 | 0 | 0 | 0 | 3 | 0 | 0 | 0 | 0 | 0 | 0 | 5 | 2 | 8 | 4 | 10 | 10 | 22 | 5 | 6 | 8 | 4 | 2 | 0 | Y | 13014 | notechis 11'2=non-toxic phospholipase A2 [Notechis scutatus=Australian tiger snakes, ssp. scutatus, venom, Peptide, 118 aa] |
| **67** | 869 | P08873.1 | 242.11 | 30 | 0 | 0 | 0 | 0 | 0 | 10 | 0 | 0 | 0 | 0 | 0 | 0 | 12 | 12 | 13 | 30 | 13 | 13 | 24 | 13 | 13 | 13 | 13 | 12 | 0 | | | | | | | | | | | | | | | | 0 | | | 1.2181E6 | | | | | | | 12 | 3 | 0 | 0 | 0 | 0 | 0 | 3 | 0 | 0 | 0 | 0 | 0 | 0 | 5 | 2 | 8 | 4 | 10 | 10 | 22 | 5 | 6 | 8 | 4 | 2 | 0 | Y | 15903 | RecName: Full=Basic phospholipase A2 notechis 11'2; Short=svPLA2; AltName: Full=Phosphatidylcholine 2-acylhydrolase; Flags: Precursor |
| **67** | 870 | CAA31125.1 | 242.11 | 30 | 0 | 0 | 0 | 0 | 0 | 10 | 0 | 0 | 0 | 0 | 0 | 0 | 12 | 12 | 13 | 30 | 13 | 13 | 24 | 13 | 13 | 13 | 13 | 12 | 0 | | | | | | | | | | | | | | | | 0 | | | 1.2181E6 | | | | | | | 12 | 3 | 0 | 0 | 0 | 0 | 0 | 3 | 0 | 0 | 0 | 0 | 0 | 0 | 5 | 2 | 8 | 4 | 10 | 10 | 22 | 5 | 6 | 8 | 4 | 2 | 0 | Y | 15903 | PLA2 preprotein (AA-27 to 118) [Notechis scutatus] |
| **48** | 215 | Q3SB03.1 | 240.72 | 23 | 0 | 0 | 0 | 0 | 0 | 0 | 0 | 0 | 0 | 0 | 0 | 0 | 0 | 0 | 6 | 0 | 0 | 9 | 9 | 18 | 23 | 9 | 9 | 9 | 0 | | | | | | | | | | | | | | | | | | | | 0 | 3.6676E6 | | | | | 13 | 3 | 0 | 0 | 0 | 0 | 0 | 0 | 0 | 0 | 0 | 0 | 0 | 0 | 0 | 0 | 1 | 0 | 0 | 15 | 3 | 34 | 73 | 8 | 22 | 2 | 0 | Y | 26301 | RecName: Full=Cysteine-rich venom protein pseudechetoxin-like; Short=CRVP; Flags: Precursor |
| **48** | 216 | AAZ38984.1 | 240.72 | 23 | 0 | 0 | 0 | 0 | 0 | 0 | 0 | 0 | 0 | 0 | 0 | 0 | 0 | 0 | 6 | 0 | 0 | 9 | 9 | 18 | 23 | 9 | 9 | 9 | 0 | | | | | | | | | | | | | | | | | | | | 0 | 3.6676E6 | | | | | 13 | 3 | 0 | 0 | 0 | 0 | 0 | 0 | 0 | 0 | 0 | 0 | 0 | 0 | 0 | 0 | 1 | 0 | 0 | 15 | 3 | 34 | 73 | 8 | 22 | 2 | 0 | Y | 26301 | pseudechetoxin-like protein precursor [Hoplocephalus stephensii] |
| **112** | 431 | O12962.1 | 239.19 | 63 | 0 | 18 | 0 | 18 | 0 | 10 | 32 | 63 | 0 | 32 | 52 | 0 | 0 | 32 | 32 | 0 | 0 | 32 | 0 | 14 | 32 | 0 | 0 | 0 | 0 | | 0 | | 0 | | 3.7293E5 | 4.2638E6 | 9.3597E7 | | 0 | 0 | | | 1.1985E4 | 2.828E5 | | | 0 | | | 0 | | | | | 7 | 5 | 0 | 1 | 0 | 1 | 0 | 1 | 9 | 27 | 0 | 4 | 4 | 0 | 0 | 2 | 2 | 0 | 0 | 3 | 0 | 1 | 3 | 0 | 0 | 0 | 0 | Y | 9474 | RecName: Full=Kappa-5-bungarotoxin; Flags: Precursor |
| **112** | 432 | CAA72940.1 | 239.19 | 63 | 0 | 18 | 0 | 18 | 0 | 10 | 32 | 63 | 0 | 32 | 52 | 0 | 0 | 32 | 32 | 0 | 0 | 32 | 0 | 14 | 32 | 0 | 0 | 0 | 0 | | 0 | | 0 | | 3.7293E5 | 4.2638E6 | 9.3597E7 | | 0 | 0 | | | 1.1985E4 | 2.828E5 | | | 0 | | | 0 | | | | | 7 | 5 | 0 | 1 | 0 | 1 | 0 | 1 | 9 | 27 | 0 | 4 | 4 | 0 | 0 | 2 | 2 | 0 | 0 | 3 | 0 | 1 | 3 | 0 | 0 | 0 | 0 | Y | 9474 | kappa5 bungarotoxin [Bungarus multicinctus] |
| **64** | 566 | P00610.1 | 238.40 | 23 | 0 | 0 | 0 | 0 | 0 | 13 | 0 | 0 | 0 | 0 | 0 | 0 | 14 | 15 | 23 | 23 | 23 | 16 | 23 | 16 | 16 | 23 | 16 | 23 | 0 | | | | | | | | | | | | | | | 1.5239E6 | 2.9992E7 | 1.1481E6 | | 0 | | | 3.451E6 | | 7.4831E4 | | 10 | 1 | 0 | 0 | 0 | 0 | 0 | 3 | 0 | 0 | 0 | 0 | 0 | 0 | 5 | 2 | 10 | 9 | 13 | 10 | 21 | 5 | 6 | 11 | 4 | 7 | 0 | Y | 13446 | RecName: Full=Basic phospholipase A2; Short=svPLA2; AltName: Full=Myotoxin; AltName: Full=Phosphatidylcholine 2-acylhydrolase; AltName: Full=Toxin VI-5; AltName: Full=Toxin VI:5b |
| **64** | 602 | AAL55556.1 | 238.40 | 23 | 0 | 0 | 0 | 0 | 0 | 13 | 0 | 0 | 0 | 0 | 0 | 0 | 14 | 15 | 23 | 23 | 23 | 16 | 23 | 16 | 16 | 23 | 16 | 23 | 0 | | | | | | | | | | | | | | | 1.5239E6 | 2.9992E7 | 1.1481E6 | | 0 | | | 3.451E6 | | 7.4831E4 | | 10 | 1 | 0 | 0 | 0 | 0 | 0 | 3 | 0 | 0 | 0 | 0 | 0 | 0 | 5 | 2 | 10 | 9 | 13 | 10 | 21 | 5 | 6 | 11 | 4 | 7 | 0 | Y | 13210 | phospholipase A2, partial [Hydrophis hardwickii] |
| **64** | 603 | ABN54807.1 | 238.40 | 18 | 0 | 0 | 0 | 0 | 0 | 10 | 0 | 0 | 0 | 0 | 0 | 0 | 12 | 12 | 18 | 18 | 18 | 13 | 18 | 13 | 13 | 18 | 13 | 18 | 0 | | | | | | | | | | | | | | | 1.5239E6 | 2.9992E7 | 1.1481E6 | | 0 | | | 3.451E6 | | 7.4831E4 | | 10 | 1 | 0 | 0 | 0 | 0 | 0 | 3 | 0 | 0 | 0 | 0 | 0 | 0 | 5 | 2 | 10 | 9 | 13 | 10 | 21 | 5 | 6 | 11 | 4 | 7 | 0 | Y | 16078 | phospholipase A2 73 [Hydrophis hardwickii] |
| **64** | 604 | Q8UW30.2 | 238.40 | 18 | 0 | 0 | 0 | 0 | 0 | 10 | 0 | 0 | 0 | 0 | 0 | 0 | 12 | 12 | 18 | 18 | 18 | 13 | 18 | 13 | 13 | 18 | 13 | 18 | 0 | | | | | | | | | | | | | | | 1.5239E6 | 2.9992E7 | 1.1481E6 | | 0 | | | 3.451E6 | | 7.4831E4 | | 10 | 1 | 0 | 0 | 0 | 0 | 0 | 3 | 0 | 0 | 0 | 0 | 0 | 0 | 5 | 2 | 10 | 9 | 13 | 10 | 21 | 5 | 6 | 11 | 4 | 7 | 0 | Y | 16078 | RecName: Full=Basic phospholipase A2 73; Short=svPLA2; AltName: Full=Phosphatidylcholine 2-acylhydrolase; Flags: Precursor |
| **65** | 2354 | LAB37300.1 | 236.71 | 26 | 0 | 0 | 0 | 0 | 0 | 15 | 0 | 0 | 0 | 0 | 0 | 0 | 20 | 21 | 21 | 16 | 21 | 21 | 26 | 19 | 21 | 19 | 19 | 18 | 0 | | | | | | | | | | | | | | | | | | | 2.6873E5 | | | | | | | 12 | 1 | 0 | 0 | 0 | 0 | 0 | 3 | 0 | 0 | 0 | 0 | 0 | 0 | 6 | 3 | 10 | 2 | 13 | 13 | 25 | 5 | 7 | 8 | 4 | 2 | 0 | Y | 11154 | hypothetical protein, partial [Micrurus spixii] |
| **27** | 53 | Q9PU97.1 | 232.55 | 49 | 0 | 9 | 0 | 9 | 0 | 0 | 9 | 10 | 0 | 9 | 9 | 7 | 0 | 22 | 27 | 39 | 34 | 18 | 31 | 9 | 9 | 9 | 9 | 17 | 0 | | | | | | | | | | | | | | | | 7.5055E7 | | | | | | | | | | 15 | 1 | 0 | 6 | 0 | 1 | 0 | 0 | 4 | 10 | 0 | 2 | 3 | 9 | 0 | 11 | 47 | 67 | 31 | 12 | 19 | 1 | 3 | 5 | 7 | 11 | 0 | Y | 15120 | RecName: Full=Basic phospholipase A2 beta-bungarotoxin A7 chain; Short=Beta-BuTX A7 chain; Short=svPLA2; AltName: Full=Phosphatidylcholine 2-acylhydrolase; Flags: Precursor |
| **27** | 54 | CAB62384.1 | 232.55 | 49 | 0 | 9 | 0 | 9 | 0 | 0 | 9 | 10 | 0 | 9 | 9 | 7 | 0 | 22 | 27 | 39 | 34 | 18 | 31 | 9 | 9 | 9 | 9 | 17 | 0 | | | | | | | | | | | | | | | | 7.5055E7 | | | | | | | | | | 15 | 1 | 0 | 6 | 0 | 1 | 0 | 0 | 4 | 10 | 0 | 2 | 3 | 9 | 0 | 11 | 47 | 67 | 31 | 12 | 19 | 1 | 3 | 5 | 7 | 11 | 0 | Y | 15120 | beta-bungarotoxin A7 chain, partial [Bungarus multicinctus] |
| **69** | 2376 | 4E4C | 231.84 | 23 | 0 | 0 | 0 | 0 | 0 | 13 | 0 | 0 | 0 | 0 | 0 | 0 | 14 | 15 | 16 | 13 | 16 | 16 | 23 | 16 | 16 | 16 | 16 | 15 | 0 | | | | | | | | | | | | | | | | | | | 6.1143E5 | | | | | | | 10 | 1 | 0 | 0 | 0 | 0 | 0 | 3 | 0 | 0 | 0 | 0 | 0 | 0 | 5 | 2 | 8 | 2 | 10 | 10 | 25 | 5 | 6 | 8 | 4 | 2 | 0 | Y | 13593 | Chain A, Phospholipase A2 |
| **69** | 2377 | 1AE7 | 231.84 | 23 | 0 | 0 | 0 | 0 | 0 | 13 | 0 | 0 | 0 | 0 | 0 | 0 | 14 | 15 | 16 | 13 | 16 | 16 | 23 | 16 | 16 | 16 | 16 | 15 | 0 | | | | | | | | | | | | | | | | | | | 6.1143E5 | | | | | | | 10 | 1 | 0 | 0 | 0 | 0 | 0 | 3 | 0 | 0 | 0 | 0 | 0 | 0 | 5 | 2 | 8 | 2 | 10 | 10 | 25 | 5 | 6 | 8 | 4 | 2 | 0 | Y | 13593 | Chain A, Phospholipase A2 |
| **69** | 2378 | P00608.1 | 231.84 | 23 | 0 | 0 | 0 | 0 | 0 | 13 | 0 | 0 | 0 | 0 | 0 | 0 | 14 | 15 | 16 | 13 | 16 | 16 | 23 | 16 | 16 | 16 | 16 | 15 | 0 | | | | | | | | | | | | | | | | | | | 6.1143E5 | | | | | | | 10 | 1 | 0 | 0 | 0 | 0 | 0 | 3 | 0 | 0 | 0 | 0 | 0 | 0 | 5 | 2 | 8 | 2 | 10 | 10 | 25 | 5 | 6 | 8 | 4 | 2 | 0 | Y | 13593 | RecName: Full=Basic phospholipase A2 notexin; Short=svPLA2; AltName: Full=Phosphatidylcholine 2-acylhydrolase |
| **73** | 2169 | P00613.1 | 230.73 | 30 | 0 | 0 | 0 | 0 | 0 | 13 | 0 | 0 | 0 | 0 | 0 | 0 | 14 | 15 | 16 | 14 | 16 | 16 | 30 | 16 | 16 | 16 | 16 | 15 | 0 | | | | | | | | | | | | | | | | | | | 0 | | | | | | | 11 | 1 | 0 | 0 | 0 | 0 | 0 | 3 | 0 | 0 | 0 | 0 | 0 | 0 | 5 | 2 | 8 | 2 | 10 | 10 | 22 | 5 | 6 | 8 | 4 | 2 | 0 | Y | 13241 | RecName: Full=Basic phospholipase A2 4; Short=svPLA2; AltName: Full=Phosphatidylcholine 2-acylhydrolase; AltName: Full=Phospholipase A2 isozyme IV |
| **133** | 633 | 7ULR | 222.41 | 60 | 0 | 0 | 0 | 0 | 0 | 0 | 54 | 60 | 0 | 54 | 18 | 0 | 0 | 18 | 18 | 0 | 0 | 18 | 0 | 18 | 18 | 0 | 0 | 0 | 0 | | | | | | | 8.3295E5 | 1.8988E7 | | 1.5224E5 | | | | | | | | | | | | | | | | 5 | 3 | 0 | 0 | 0 | 0 | 0 | 0 | 5 | 20 | 0 | 3 | 2 | 0 | 0 | 1 | 1 | 0 | 0 | 2 | 0 | 1 | 2 | 0 | 0 | 0 | 0 | Y | 7407 | Chain B, Kappa-bungarotoxin |
| **133** | 653 | 1KBA | 222.41 | 61 | 0 | 0 | 0 | 0 | 0 | 0 | 55 | 61 | 0 | 55 | 18 | 0 | 0 | 18 | 18 | 0 | 0 | 18 | 0 | 18 | 18 | 0 | 0 | 0 | 0 | | | | | | | 8.3295E5 | 1.8988E7 | | 1.5224E5 | | | | | | | | | | | | | | | | 5 | 3 | 0 | 0 | 0 | 0 | 0 | 0 | 5 | 20 | 0 | 3 | 2 | 0 | 0 | 1 | 1 | 0 | 0 | 2 | 0 | 1 | 2 | 0 | 0 | 0 | 0 | Y | 7275 | Chain B, KAPPA-BUNGAROTOXIN |
| **133** | 654 | 2NBT | 222.41 | 61 | 0 | 0 | 0 | 0 | 0 | 0 | 55 | 61 | 0 | 55 | 18 | 0 | 0 | 18 | 18 | 0 | 0 | 18 | 0 | 18 | 18 | 0 | 0 | 0 | 0 | | | | | | | 8.3295E5 | 1.8988E7 | | 1.5224E5 | | | | | | | | | | | | | | | | 5 | 3 | 0 | 0 | 0 | 0 | 0 | 0 | 5 | 20 | 0 | 3 | 2 | 0 | 0 | 1 | 1 | 0 | 0 | 2 | 0 | 1 | 2 | 0 | 0 | 0 | 0 | Y | 7275 | Chain B, NEURONAL BUNGAROTOXIN |
| **95** | 19 | ABN72537.1 | 208.64 | 20 | 0 | 0 | 0 | 0 | 0 | 0 | 9 | 2 | 2 | 16 | 2 | 0 | 0 | 2 | 2 | 0 | 0 | 0 | 0 | 0 | 0 | 0 | 0 | 0 | 4 | | | | | | | 5.3207E6 | | | 4.365E7 | | | | | | | | | | | | | | | 0 | 15 | 6 | 0 | 0 | 0 | 0 | 0 | 0 | 0 | 13 | 1 | 1 | 36 | 1 | 0 | 0 | 1 | 1 | 0 | 0 | 0 | 0 | 0 | 0 | 0 | 0 | 3 | Y | 68988 | metalloproteinase [Bungarus multicinctus] |
| **95** | 20 | ABQL49.1 | 208.64 | 20 | 0 | 0 | 0 | 0 | 0 | 0 | 9 | 2 | 2 | 16 | 2 | 0 | 0 | 2 | 2 | 0 | 0 | 0 | 0 | 0 | 0 | 0 | 0 | 0 | 4 | | | | | | | 5.3207E6 | | | 4.365E7 | | | | | | | | | | | | | | | 0 | 15 | 6 | 0 | 0 | 0 | 0 | 0 | 0 | 0 | 13 | 1 | 1 | 36 | 1 | 0 | 0 | 1 | 1 | 0 | 0 | 0 | 0 | 0 | 0 | 0 | 0 | 3 | Y | 68988 | RecName: Full=Zinc metalloproteinase-disintegrin-like BmMP; AltName: Full=Snake venom metalloproteinase; Short=SVMP; Flags: Precursor |
| **23** | 265 | JAB52811.1 | 205.86 | 45 | 0 | 6 | 0 | 6 | 0 | 0 | 6 | 6 | 6 | 6 | 6 | 0 | 9 | 12 | 19 | 35 | 26 | 19 | 29 | 8 | 25 | 21 | 18 | 18 | 0 | | | | | | | | | | | | | | | | 1.2108E4 | | | 4.0558E5 | | | | | | | 17 | 2 | 0 | 1 | 0 | 1 | 0 | 0 | 2 | 5 | 1 | 1 | 3 | 0 | 17 | 8 | 14 | 32 | 20 | 14 | 32 | 8 | 12 | 25 | 13 | 32 | 0 | Y | 16160 | phospholipase A2 12 [Micrurus fulvius] |
| **62** | 116 | CAA06887.1 | 196.46 | 58 | 0 | 58 | 35 | 27 | 0 | 0 | 0 | 0 | 0 | 13 | 0 | 0 | 0 | 0 | 0 | 0 | 12 | 12 | 0 | 12 | 0 | 0 | 12 | 0 | 0 | | 1.2814E9 | 4.558E7 | 4.9638E6 | | | | | | 4.5406E5 | | | | | | | 0 | 0 | | 2.1317E5 | | | 8.301E4 | | | 13 | 13 | 0 | 77 | 12 | 3 | 0 | 0 | 0 | 0 | 0 | 2 | 0 | 0 | 0 | 0 | 0 | 0 | 1 | 1 | 0 | 1 | 0 | 0 | 1 | 0 | 0 | Y | 9519 | neurotoxin-like protein [Bungarus multicinctus multicinctus] |
| **62** | 117 | Q9YGI8.1 | 196.46 | 58 | 0 | 58 | 35 | 27 | 0 | 0 | 0 | 0 | 0 | 13 | 0 | 0 | 0 | 0 | 0 | 0 | 12 | 12 | 0 | 12 | 0 | 0 | 12 | 0 | 0 | | 1.2814E9 | 4.558E7 | 4.9638E6 | | | | | | 4.5406E5 | | | | | | | 0 | 0 | | 2.1317E5 | | | 8.301E4 | | | 13 | 13 | 0 | 77 | 12 | 3 | 0 | 0 | 0 | 0 | 0 | 2 | 0 | 0 | 0 | 0 | 0 | 0 | 1 | 1 | 0 | 1 | 0 | 0 | 1 | 0 | 0 | Y | 9519 | RecName: Full=Short neurotoxin homolog NTL4; Flags: Precursor |
| **49** | 734 | ABG90493.1 | 192.91 | 57 | 0 | 43 | 20 | 20 | 23 | 20 | 0 | 20 | 0 | 43 | 20 | 0 | 20 | 20 | 43 | 57 | 43 | 43 | 23 | 0 | 0 | 0 | 0 | 0 | 0 | | 3.8284E7 | 1.9034E6 | 5.8613E5 | 6.6621E7 | 3.0588E6 | | 5.6492E4 | | 4.1367E5 | 1.2184E7 | | 6.6756E5 | 7.0527E6 | 1.9911E7 | 1.2292E8 | 8.8584E6 | 2.8254E4 | 2.49E6 | | | | | | | 8 | 6 | 0 | 29 | 13 | 2 | 16 | 5 | 0 | 3 | 0 | 4 | 6 | 0 | 17 | 8 | 3 | 12 | 5 | 2 | 1 | 0 | 0 | 0 | 0 | 0 | 0 | Y | 7134 | beta-bungarotoxin B5 chain, partial [Bungarus multicinctus] |

| | | | | | | | | | | | | | | | | | | | | | | | | | | | | | | | | | | | | | | | | | | | | | | | | | | | | | | | | | | | | | | | | | | | | | | | | | | | | | | | | | | | | | |
|---|---|---|---|---|---|---|---|---|---|---|---|---|---|---|---|---|---|---|---|---|---|---|---|---|---|---|---|---|---|---|---|---|---|---|---|---|---|---|---|---|---|---|---|---|---|---|---|---|---|---|---|---|---|---|---|---|---|---|---|---|---|---|---|---|---|---|---|---|---|---|---|---|---|---|---|---|---|---|---|---|---|---|---|---|
| **49** | 735 | Q0PL65.1 | 192.91 | 57 | 0 | 43 | 20 | 20 | 23 | 20 | 0 | 20 | 0 | 43 | 20 | 0 | 20 | 20 | 43 | 57 | 43 | 43 | 23 | 0 | 0 | 0 | 0 | 0 | 0 | | 3.8284E7 | 1.9034E6 | 5.8613E5 | 6.6621E7 | 3.0588E6 | | 5.6492E4 | | 4.1367E5 | 1.2184E7 | | 6.6756E5 | 7.0527E6 | 1.9911E7 | 1.2292E8 | 8.8584E6 | 2.8254E4 | 2.49E6 | | | | | | | 8 | 6 | 0 | 29 | 13 | 2 | 16 | 5 | 0 | 3 | 0 | 4 | 6 | 0 | 17 | 8 | 3 | 12 | 5 | 2 | 1 | 0 | 0 | 0 | 0 | 0 | 0 | Y | 7134 | RecName: Full=Kunitz-type serine protease inhibitor homolog beta-bungarotoxin B5-B chain; AltName: Full=Beta-bungarotoxin B5 chain |
| **54** | 21 | ETE67131.1 | 187.81 | 4 | 2 | 2 | 0 | 0 | 0 | 1 | 1 | 1 | 0 | 1 | 0 | 0 | 0 | 1 | 1 | 0 | 1 | 2 | 1 | 3 | 1 | 1 | 2 | 0 | 0 | | 1.5725E4 | | | | 1.1777E4 | | | | 3.875E4 | | | | 0 | | | | 6.0727E4 | | | | | 4.565E4 | | | 11 | 1 | 19 | 5 | 0 | 1 | 0 | 2 | 8 | 2 | 1 | 2 | 1 | 0 | 0 | 1 | 2 | 0 | 4 | 5 | 3 | 8 | 2 | 7 | 4 | 1 | 0 | Y | 240642 | Keratin, type II cytoskeletal 1, partial [Ophiophagus hannah] |
| **30** | 483 | JAB52760.1 | 186.37 | 26 | 0 | 6 | 0 | 6 | 0 | 0 | 6 | 6 | 6 | 6 | 6 | 0 | 0 | 6 | 10 | 18 | 26 | 19 | 10 | 8 | 24 | 21 | 18 | 18 | 0 | | | | | | | | | | | | | | | | | 9.0694E4 | | | | | | | | | 11 | 1 | 0 | 1 | 0 | 1 | 0 | 0 | 2 | 5 | 1 | 1 | 3 | 0 | 0 | 7 | 10 | 31 | 20 | 14 | 28 | 8 | 11 | 25 | 13 | 32 | 0 | Y | 16202 | phospholipase A2 9 [Micrurus fulvius] |
| **109** | 106 | CAB62501.1 | 181.29 | 38 | 0 | 0 | 0 | 0 | 0 | 0 | 0 | 0 | 0 | 0 | 9 | 0 | 9 | 9 | 19 | 30 | 19 | 0 | 20 | 0 | 0 | 0 | 0 | 0 | 0 | | | | | | | | | | | | | | | | 5.3404E6 | | | 0 | | | | | | | 9 | 1 | 0 | 0 | 0 | 0 | 0 | 0 | 0 | 0 | 0 | 0 | 1 | 0 | 2 | 1 | 5 | 13 | 8 | 0 | 5 | 0 | 0 | 0 | 0 | 0 | 0 | Y | 15786 | beta-bungarotoxin A chain, partial [Bungarus multicinctus] |
| **109** | 107 | Q9PTA6.1 | 181.29 | 38 | 0 | 0 | 0 | 0 | 0 | 0 | 0 | 0 | 0 | 0 | 9 | 0 | 9 | 9 | 19 | 30 | 19 | 0 | 20 | 0 | 0 | 0 | 0 | 0 | 0 | | | | | | | | | | | | | | | | 5.3404E6 | | | 0 | | | | | | | 9 | 1 | 0 | 0 | 0 | 0 | 0 | 0 | 0 | 0 | 0 | 0 | 1 | 0 | 2 | 1 | 5 | 13 | 8 | 0 | 5 | 0 | 0 | 0 | 0 | 0 | 0 | Y | 15786 | RecName: Full=Basic phospholipase A2 beta-bungarotoxin A-AL3 chain; Short=Beta-BuTX A-AL3 chain; Short=svPLA2; AltName: Full=Phosphatidylcholine 2-acylhydrolase; Flags: Precursor |
| **120** | 1104 | 1DPY | 181.03 | 30 | 0 | 0 | 0 | 0 | 0 | 0 | 0 | 0 | 0 | 0 | 0 | 0 | 0 | 0 | 0 | 30 | 10 | 10 | 30 | 0 | 18 | 30 | 9 | 22 | 0 | | | | | | | | | | | | | | | | 0 | | | 1.0045E8 | | | 5.2339E6 | | 0 | | 9 | 6 | 0 | 0 | 0 | 0 | 0 | 0 | 0 | 0 | 0 | 0 | 0 | 0 | 0 | 0 | 0 | 2 | 2 | 2 | 6 | 0 | 2 | 5 | 1 | 2 | 0 | Y | 12936 | Chain A, PHOSPHOLIPASE A2 |
| **44** | 724 | XP_058026748.1 | 172.88 | 7 | 7 | 3 | 2 | 2 | 3 | 2 | 2 | 2 | 2 | 2 | 2 | 2 | 2 | 3 | 5 | 2 | 6 | 6 | 6 | 7 | 6 | 3 | 6 | 4 | 2 | 3.8925E5 | | | | | | | | | | | | | | | | | | | 8.8864E5 | 0 | | | 0 | | 6 | 2 | 37 | 9 | 3 | 5 | 4 | 7 | 8 | 2 | 3 | 3 | 7 | 2 | 5 | 7 | 3 | 4 | 12 | 11 | 7 | 11 | 7 | 3 | 10 | 3 | 2 | N | 53316 | keratin, type II cytoskeletal cochleal-like [Ahaetulla prasina] |
| **186** | 200 | LAA28499.1 | 172.34 | 14 | 2 | 0 | 0 | 0 | 0 | 0 | 0 | 0 | 0 | 0 | 0 | 2 | 0 | 0 | 0 | 0 | 0 | 0 | 0 | 0 | 0 | 0 | 12 | 0 | 0 | | | | | | | | | | | | | | | | | | | | | | | 4.7483E6 | | | 5 | 1 | 1 | 0 | 0 | 0 | 0 | 0 | 0 | 0 | 0 | 0 | 0 | 3 | 0 | 0 | 0 | 0 | 0 | 0 | 0 | 0 | 0 | 0 | 11 | 0 | 0 | Y | 39953 | hypothetical protein, partial [Micrurus lemniscatus carvalhoi] |
| **186** | 204 | XP_032079732.1 | 172.34 | 9 | 1 | 0 | 0 | 0 | 0 | 0 | 0 | 0 | 0 | 0 | 0 | 1 | 0 | 0 | 0 | 0 | 0 | 0 | 0 | 0 | 0 | 0 | 8 | 0 | 0 | | | | | | | | | | | | | | | | | | | | | | | 4.7483E6 | | | 5 | 1 | 1 | 0 | 0 | 0 | 0 | 0 | 0 | 0 | 0 | 0 | 0 | 3 | 0 | 0 | 0 | 0 | 0 | 0 | 0 | 0 | 0 | 0 | 11 | 0 | 0 | Y | 60758 | hepatocyte growth factor activator [Thamnophis elegans] |
| **57** | 70 | 0412250A | 169.88 | 48 | 0 | 0 | 0 | 0 | 0 | 11 | 0 | 11 | 0 | 11 | 11 | 8 | 0 | 11 | 30 | 38 | 38 | 8 | 19 | 11 | 0 | 11 | 11 | 19 | 0 | | | | | | 0 | | 1.3862E5 | | 6.0391E6 | 0 | | | 5.26E6 | 3.4642E5 | | 0 | | | 3.5744E6 | | 0 | 1.5719E5 | 0 | | 9 | 1 | 0 | 0 | 0 | 0 | 0 | 1 | 0 | 2 | 0 | 1 | 1 | 9 | 0 | 1 | 7 | 32 | 12 | 2 | 5 | 1 | 0 | 1 | 1 | 3 | 0 | Y | 13489 | toxin A,beta 1 bungaro |
| **119** | 209 | AAO84769.1 | 166.70 | 21 | 0 | 0 | 0 | 0 | 0 | 0 | 0 | 0 | 0 | 0 | 0 | 0 | 0 | 9 | 13 | 14 | 14 | 8 | 13 | 0 | 9 | 16 | 15 | 8 | 0 | | | | | | | | | | | | | | 9.3142E5 | 4.0342E5 | 8.3862E6 | 0 | 1.4979E5 | 4.8624E6 | | 9.8892E5 | 1.6673E8 | 1.4348E6 | 3.3887E6 | | 6 | 2 | 0 | 0 | 0 | 0 | 0 | 0 | 0 | 0 | 0 | 0 | 0 | 0 | 0 | 3 | 5 | 4 | 6 | 2 | 4 | 0 | 2 | 7 | 2 | 1 | 0 | Y | 15641 | phospholipase A2, partial [Bungarus candidus] |
| **119** | 210 | Q802I1.1 | 166.70 | 21 | 0 | 0 | 0 | 0 | 0 | 0 | 0 | 0 | 0 | 0 | 0 | 0 | 0 | 9 | 13 | 14 | 14 | 8 | 13 | 0 | 9 | 16 | 15 | 8 | 0 | | | | | | | | | | | | | | 9.3142E5 | 4.0342E5 | 8.3862E6 | 0 | 1.4979E5 | 4.8624E6 | | 9.8892E5 | 1.6673E8 | 1.4348E6 | 3.3887E6 | | 6 | 2 | 0 | 0 | 0 | 0 | 0 | 0 | 0 | 0 | 0 | 0 | 0 | 0 | 0 | 3 | 5 | 4 | 6 | 2 | 4 | 0 | 2 | 7 | 2 | 1 | 0 | Y | 15641 | RecName: Full=Acidic phospholipase A2 Bc-PL; Short=svPLA2; AltName: Full=Phosphatidylcholine 2-acylhydrolase; Flags: Precursor |
| **113** | 318 | CAJ77819.1 | 164.12 | 57 | 0 | 0 | 0 | 0 | 16 | 0 | 16 | 16 | 16 | 37 | 17 | 0 | 16 | 44 | 16 | 16 | 16 | 16 | 16 | 0 | 0 | 0 | 0 | 0 | 0 | | | | | | | | | | 0 | | | | | | | | | | | | | | | | 8 | 1 | 0 | 0 | 0 | 0 | 1 | 0 | 4 | 5 | 4 | 5 | 11 | 0 | 2 | 3 | 1 | 1 | 4 | 1 | 1 | 0 | 0 | 0 | 0 | 0 | 0 | Y | 8306 | alpha-delta-bungarotoxin-1, partial [Bungarus candidus] |
| **113** | 319 | A1IVR8.1 | 164.12 | 57 | 0 | 0 | 0 | 0 | 16 | 0 | 16 | 16 | 16 | 37 | 17 | 0 | 16 | 44 | 16 | 16 | 16 | 16 | 16 | 0 | 0 | 0 | 0 | 0 | 0 | | | | | | | | | | 0 | | | | | | | | | | | | | | | | 8 | 1 | 0 | 0 | 0 | 0 | 1 | 0 | 4 | 5 | 4 | 5 | 11 | 0 | 2 | 3 | 1 | 1 | 4 | 1 | 1 | 0 | 0 | 0 | 0 | 0 | 0 | Y | 8306 | RecName: Full=Alpha-elapitoxin-Bc2a; Short=Alpha-EPTX-Bc2a; AltName: Full=Alpha-delta-Bgt-1; AltName: Full=Alpha/delta-bungarotoxin-1; Short=Alpha/delta-BgTx-1; Flags: Precursor |
| **193** | 23 | ABN72539.1 | 161.10 | 15 | 0 | 0 | 0 | 0 | 0 | 0 | 0 | 0 | 0 | 0 | 0 | 0 | 0 | 0 | 0 | 0 | 0 | 0 | 0 | 0 | 0 | 0 | 0 | 0 | 15 | | | | | | | | | | | | | | | | | | | | | | | | | 1.5386E6 | 7 | 3 | 0 | 0 | 0 | 0 | 0 | 0 | 0 | 0 | 0 | 0 | 0 | 0 | 0 | 0 | 0 | 0 | 0 | 0 | 0 | 0 | 0 | 0 | 0 | 0 | 18 | N | 58811 | L-amino acid oxidase [Bungarus multicinctus] |
| **193** | 24 | A8QL51.1 | 161.10 | 15 | 0 | 0 | 0 | 0 | 0 | 0 | 0 | 0 | 0 | 0 | 0 | 0 | 0 | 0 | 0 | 0 | 0 | 0 | 0 | 0 | 0 | 0 | 0 | 0 | 15 | | | | | | | | | | | | | | | | | | | | | | | | | 1.5386E6 | 7 | 3 | 0 | 0 | 0 | 0 | 0 | 0 | 0 | 0 | 0 | 0 | 0 | 0 | 0 | 0 | 0 | 0 | 0 | 0 | 0 | 0 | 0 | 0 | 0 | 0 | 18 | N | 58811 | RecName: Full=L-amino-acid oxidase; Short=Bm-LAAO; Short=LAO; Flags: Precursor |
| **110** | 351 | Q9DF52.1 | 158.28 | 34 | 0 | 0 | 0 | 0 | 0 | 0 | 0 | 0 | 0 | 0 | 0 | 0 | 0 | 0 | 0 | 8 | 17 | 24 | 24 | 0 | 14 | 18 | 8 | 8 | 0 | | | | | | | | | | | | | | | | | | 1.0078E4 | 0 | | | | | | | 8 | 3 | 0 | 0 | 0 | 0 | 0 | 0 | 0 | 0 | 0 | 0 | 0 | 0 | 0 | 0 | 0 | 1 | 3 | 3 | 3 | 0 | 2 | 6 | 1 | 1 | 0 | Y | 15765 | RecName: Full=Basic phospholipase A2 KPA2; Short=svPLA2; AltName: Full=Phosphatidylcholine 2-acylhydrolase; Flags: Precursor |
| **110** | 352 | AAG13412.1 | 158.28 | 34 | 0 | 0 | 0 | 0 | 0 | 0 | 0 | 0 | 0 | 0 | 0 | 0 | 0 | 0 | 0 | 8 | 17 | 24 | 24 | 0 | 14 | 18 | 8 | 8 | 0 | | | | | | | | | | | | | | | | | | 1.0078E4 | 0 | | | | | | | 8 | 3 | 0 | 0 | 0 | 0 | 0 | 0 | 0 | 0 | 0 | 0 | 0 | 0 | 0 | 0 | 0 | 1 | 3 | 3 | 3 | 0 | 2 | 6 | 1 | 1 | 0 | Y | 15765 | phospholipase A2 [Bungarus caeruleus] |
| **98** | 706 | P20251.1 | 155.93 | 39 | 0 | 0 | 0 | 0 | 0 | 0 | 9 | 0 | 0 | 9 | 0 | 0 | 9 | 9 | 9 | 39 | 19 | 10 | 9 | 0 | 10 | 10 | 9 | 9 | 0 | | | | | | | | | | | | | | | | 1.0062E5 | | | | | | | | | | 5 | 1 | 0 | 0 | 0 | 0 | 0 | 0 | 1 | 0 | 0 | 2 | 0 | 0 | 18 | 6 | 2 | 3 | 4 | 2 | 2 | 0 | 1 | 2 | 1 | 1 | 0 | Y | 13152 | RecName: Full=Acidic phospholipase A2 PA-3; Short=svPLA2; AltName: Full=Phosphatidylcholine 2-acylhydrolase |
| **101** | 729 | AAZ22663.1 | 155.90 | 31 | 0 | 0 | 0 | 0 | 0 | 0 | 8 | 0 | 0 | 8 | 0 | 0 | 8 | 8 | 8 | 15 | 16 | 8 | 31 | 0 | 8 | 8 | 8 | 8 | 0 | | | | | | | | | | | | | | | | | | | 0 | | | | | | | 5 | 1 | 0 | 0 | 0 | 0 | 0 | 0 | 1 | 0 | 0 | 2 | 0 | 0 | 18 | 6 | 2 | 2 | 4 | 2 | 3 | 0 | 1 | 2 | 1 | 1 | 0 | Y | 15873 | Pa-18 precursor [Pseudechis australis] |
| **139** | 557 | AAL30054.1 | 155.57 | 46 | 0 | 0 | 0 | 0 | 0 | 0 | 40 | 46 | 0 | 14 | 39 | 0 | 0 | 14 | 14 | 0 | 0 | 41 | 0 | 14 | 14 | 0 | 0 | 0 | 0 | | | | | | | 5.7586E3 | 1.3951E7 | | | 1.9724E5 | | | | | | | 0 | | | | | | | | 5 | 3 | 0 | 0 | 0 | 0 | 0 | 0 | 4 | 17 | 0 | 1 | 3 | 0 | 0 | 1 | 1 | 0 | 0 | 3 | 0 | 1 | 2 | 0 | 0 | 0 | 0 | Y | 9580 | kappa 1a bungaratoxin [Bungarus candidus] |
| **139** | 558 | Q8AY56.1 | 155.57 | 46 | 0 | 0 | 0 | 0 | 0 | 0 | 40 | 46 | 0 | 14 | 39 | 0 | 0 | 14 | 14 | 0 | 0 | 41 | 0 | 14 | 14 | 0 | 0 | 0 | 0 | | | | | | | 5.7586E3 | 1.3951E7 | | | 1.9724E5 | | | | | | | 0 | | | | | | | | 5 | 3 | 0 | 0 | 0 | 0 | 0 | 0 | 4 | 17 | 0 | 1 | 3 | 0 | 0 | 1 | 1 | 0 | 0 | 3 | 0 | 1 | 2 | 0 | 0 | 0 | 0 | Y | 9580 | RecName: Full=Kappa 1a-bungarotoxin; Flags: Precursor |
| **134** | 4132 | Q8AY42.2 | 153.53 | 17 | 0 | 17 | 17 | 17 | 17 | 0 | 0 | 17 | 0 | 17 | 0 | 0 | 0 | 17 | 17 | 0 | 17 | 17 | 0 | 17 | 0 | 0 | 17 | 17 | 0 | | 1.1708E8 | 2.3805E7 | 2.9088E7 | 2.3708E7 | | | 2.7357E6 | | 8.1436E5 | | | | 0 | 1.0099E6 | | 0 | 1.1657E6 | | 5.5602E5 | | | 6.1651E5 | 2.3447E5 | | 5 | 2 | 0 | 6 | 10 | 2 | 9 | 0 | 0 | 1 | 0 | 1 | 0 | 0 | 0 | 1 | 1 | 0 | 1 | 1 | 0 | 1 | 0 | 0 | 1 | 1 | 0 | Y | 8919 | RecName: Full=Kunitz-type serine protease inhibitor B; AltName: Full=Kunitz inhibitor B; Flags: Precursor |
| **134** | 5197 | AAL30069.1 | 153.53 | 16 | 0 | 16 | 16 | 16 | 16 | 0 | 0 | 16 | 0 | 16 | 0 | 0 | 0 | 16 | 16 | 0 | 16 | 16 | 0 | 16 | 0 | 0 | 16 | 16 | 0 | | 1.1708E8 | 2.3805E7 | 2.9088E7 | 2.3708E7 | | | 2.7357E6 | | 8.1436E5 | | | | 0 | 1.0099E6 | | 0 | 1.1657E6 | | 5.5602E5 | | | 6.1651E5 | 2.3447E5 | | 5 | 2 | 0 | 6 | 10 | 2 | 9 | 0 | 0 | 1 | 0 | 1 | 0 | 0 | 0 | 1 | 1 | 0 | 1 | 1 | 0 | 1 | 0 | 0 | 1 | 1 | 0 | Y | 9391 | Kunitz inhibitor b, partial [Bungarus candidus] |
| **134** | 5198 | Q8AY43.2 | 153.53 | 17 | 0 | 17 | 17 | 17 | 17 | 0 | 0 | 17 | 0 | 17 | 0 | 0 | 0 | 17 | 17 | 0 | 17 | 17 | 0 | 17 | 0 | 0 | 17 | 17 | 0 | | 1.1708E8 | 2.3805E7 | 2.9088E7 | 2.3708E7 | | | 2.7357E6 | | 8.1436E5 | | | | 0 | 1.0099E6 | | 0 | 1.1657E6 | | 5.5602E5 | | | 6.1651E5 | 2.3447E5 | | 5 | 2 | 0 | 6 | 10 | 2 | 9 | 0 | 0 | 1 | 0 | 1 | 0 | 0 | 0 | 1 | 1 | 0 | 1 | 1 | 0 | 1 | 0 | 0 | 1 | 1 | 0 | Y | 9077 | RecName: Full=Kunitz-type serine protease inhibitor A; AltName: Full=Kunitz inhibitor A; Flags: Precursor |
| **134** | 5199 | AAL30068.1 | 153.53 | 16 | 0 | 16 | 16 | 16 | 16 | 0 | 0 | 16 | 0 | 16 | 0 | 0 | 0 | 16 | 16 | 0 | 16 | 16 | 0 | 16 | 0 | 0 | 16 | 16 | 0 | | 1.1708E8 | 2.3805E7 | 2.9088E7 | 2.3708E7 | | | 2.7357E6 | | 8.1436E5 | | | | 0 | 1.0099E6 | | 0 | 1.1657E6 | | 5.5602E5 | | | 6.1651E5 | 2.3447E5 | | 5 | 2 | 0 | 6 | 10 | 2 | 9 | 0 | 0 | 1 | 0 | 1 | 0 | 0 | 0 | 1 | 1 | 0 | 1 | 1 | 0 | 1 | 0 | 0 | 1 | 1 | 0 | Y | 9606 | Kunitz inhibitor a, partial [Bungarus candidus] |
| **173** | 1312 | O12963.1 | 148.71 | 21 | 0 | 21 | 0 | 16 | 21 | 0 | 0 | 0 | 0 | 0 | 15 | 0 | 0 | 16 | 0 | 16 | 16 | 16 | 0 | 16 | 0 | 0 | 16 | 0 | 0 | | 6.5111E7 | | 1.5108E8 | 8.0946E6 | | | | | | 0 | | | 3.0507E5 | | 0 | 1.0606E5 | 6.1383E4 | | 4.4738E4 | | | 1.1671E5 | | | 5 | 5 | 0 | 5 | 0 | 3 | 6 | 0 | 0 | 0 | 0 | 0 | 1 | 0 | 0 | 1 | 0 | 1 | 1 | 2 | 0 | 1 | 0 | 0 | 1 | 0 | 0 | Y | 9756 | RecName: Full=Long neurotoxin homolog TA-bm16; AltName: Full=Delta-protein; Flags: Precursor |
| **173** | 1313 | CAA72941.1 | 148.71 | 21 | 0 | 21 | 0 | 16 | 21 | 0 | 0 | 0 | 0 | 0 | 15 | 0 | 0 | 16 | 0 | 16 | 16 | 16 | 0 | 16 | 0 | 0 | 16 | 0 | 0 | | 6.5111E7 | | 1.5108E8 | 8.0946E6 | | | | | | 0 | | | 3.0507E5 | | 0 | 1.0606E5 | 6.1383E4 | | 4.4738E4 | | | 1.1671E5 | | | 5 | 5 | 0 | 5 | 0 | 3 | 6 | 0 | 0 | 0 | 0 | 0 | 1 | 0 | 0 | 1 | 0 | 1 | 1 | 2 | 0 | 1 | 0 | 0 | 1 | 0 | 0 | Y | 9756 | neurotoxin [Bungarus multicinctus] |
| **173** | 1392 | 1MR6 | 148.71 | 28 | 0 | 28 | 0 | 21 | 28 | 0 | 0 | 0 | 0 | 0 | 19 | 0 | 0 | 21 | 0 | 21 | 21 | 21 | 0 | 21 | 0 | 0 | 21 | 0 | 0 | | 6.5111E7 | | 1.5108E8 | 8.0946E6 | | | | | | 0 | | | 3.0507E5 | | 0 | 1.0606E5 | 6.1383E4 | | 4.4738E4 | | | 1.1671E5 | | | 5 | 5 | 0 | 5 | 0 | 3 | 6 | 0 | 0 | 0 | 0 | 0 | 1 | 0 | 0 | 1 | 0 | 1 | 1 | 2 | 0 | 1 | 0 | 0 | 1 | 0 | 0 | Y | 7535 | Chain A, neurotoxin |
| **173** | 1393 | CAA11214.1 | 148.71 | 21 | 0 | 21 | 0 | 16 | 21 | 0 | 0 | 0 | 0 | 0 | 15 | 0 | 0 | 16 | 0 | 16 | 16 | 16 | 0 | 16 | 0 | 0 | 16 | 0 | 0 | | 6.5111E7 | | 1.5108E8 | 8.0946E6 | | | | | | 0 | | | 3.0507E5 | | 0 | 1.0606E5 | 6.1383E4 | | 4.4738E4 | | | 1.1671E5 | | | 5 | 5 | 0 | 5 | 0 | 3 | 6 | 0 | 0 | 0 | 0 | 0 | 1 | 0 | 0 | 1 | 0 | 1 | 1 | 2 | 0 | 1 | 0 | 0 | 1 | 0 | 0 | Y | 9873 | neurotoxin-like protein [Bungarus multicinctus multicinctus] |
| **173** | 1394 | CAA06885.1 | 148.71 | 21 | 0 | 21 | 0 | 16 | 21 | 0 | 0 | 0 | 0 | 0 | 15 | 0 | 0 | 16 | 0 | 16 | 16 | 16 | 0 | 16 | 0 | 0 | 16 | 0 | 0 | | 6.5111E7 | | 1.5108E8 | 8.0946E6 | | | | | | 0 | | | 3.0507E5 | | 0 | 1.0606E5 | 6.1383E4 | | 4.4738E4 | | | 1.1671E5 | | | 5 | 5 | 0 | 5 | 0 | 3 | 6 | 0 | 0 | 0 | 0 | 0 | 1 | 0 | 0 | 1 | 0 | 1 | 1 | 2 | 0 | 1 | 0 | 0 | 1 | 0 | 0 | Y | 9826 | neurotoxin-like protein [Bungarus multicinctus multicinctus] |
| **173** | 1395 | Q9YGJ0.1 | 148.71 | 21 | 0 | 21 | 0 | 16 | 21 | 0 | 0 | 0 | 0 | 0 | 15 | 0 | 0 | 16 | 0 | 16 | 16 | 16 | 0 | 16 | 0 | 0 | 16 | 0 | 0 | | 6.5111E7 | | 1.5108E8 | 8.0946E6 | | | | | | 0 | | | 3.0507E5 | | 0 | 1.0606E5 | 6.1383E4 | | 4.4738E4 | | | 1.1671E5 | | | 5 | 5 | 0 | 5 | 0 | 3 | 6 | 0 | 0 | 0 | 0 | 0 | 1 | 0 | 0 | 1 | 0 | 1 | 1 | 2 | 0 | 1 | 0 | 0 | 1 | 0 | 0 | Y | 9826 | RecName: Full=Gamma-bungarotoxin; AltName: Full=Long neurotoxin homolog NTL2I; Flags: Precursor |
| **173** | 1396 | CAD01082.1 | 148.71 | 21 | 0 | 21 | 0 | 16 | 21 | 0 | 0 | 0 | 0 | 0 | 15 | 0 | 0 | 16 | 0 | 16 | 16 | 16 | 0 | 16 | 0 | 0 | 16 | 0 | 0 | | 6.5111E7 | | 1.5108E8 | 8.0946E6 | | | | | | 0 | | | 3.0507E5 | | 0 | 1.0606E5 | 6.1383E4 | | 4.4738E4 | | | 1.1671E5 | | | 5 | 5 | 0 | 5 | 0 | 3 | 6 | 0 | 0 | 0 | 0 | 0 | 1 | 0 | 0 | 1 | 0 | 1 | 1 | 2 | 0 | 1 | 0 | 0 | 1 | 0 | 0 | Y | 9826 | gamma-bungarotoxin [Bungarus multicinctus] |
| **173** | 1397 | Q9YGH9.1 | 148.71 | 21 | 0 | 21 | 0 | 16 | 21 | 0 | 0 | 0 | 0 | 0 | 15 | 0 | 0 | 16 | 0 | 16 | 16 | 16 | 0 | 16 | 0 | 0 | 16 | 0 | 0 | | 6.5111E7 | | 1.5108E8 | 8.0946E6 | | | | | | 0 | | | 3.0507E5 | | 0 | 1.0606E5 | 6.1383E4 | | 4.4738E4 | | | 1.1671E5 | | | 5 | 5 | 0 | 5 | 0 | 3 | 6 | 0 | 0 | 0 | 0 | 0 | 1 | 0 | 0 | 1 | 0 | 1 | 1 | 2 | 0 | 1 | 0 | 0 | 1 | 0 | 0 | Y | 9873 | RecName: Full=Long neurotoxin homolog NTL2; Flags: Precursor |
| **173** | 1398 | AAD41806.1 | 148.71 | 21 | 0 | 21 | 0 | 16 | 21 | 0 | 0 | 0 | 0 | 0 | 15 | 0 | 0 | 16 | 0 | 16 | 16 | 16 | 0 | 16 | 0 | 0 | 16 | 0 | 0 | | 6.5111E7 | | 1.5108E8 | 8.0946E6 | | | | | | 0 | | | 3.0507E5 | | 0 | 1.0606E5 | 6.1383E4 | | 4.4738E4 | | | 1.1671E5 | | | 5 | 5 | 0 | 5 | 0 | 3 | 6 | 0 | 0 | 0 | 0 | 0 | 1 | 0 | 0 | 1 | 0 | 1 | 1 | 2 | 0 | 1 | 0 | 0 | 1 | 0 | 0 | Y | 9826 | neurotoxin precursor [Bungarus multicinctus] |
| **83** | 93 | XP_026539668.1 | 143.53 | 15 | 5 | 3 | 0 | 0 | 0 | 0 | 3 | 2 | 0 | 2 | 0 | 0 | 0 | 0 | 4 | 0 | 12 | 6 | 5 | 7 | 4 | 3 | 5 | 2 | 0 | | | | | | | | | | | | | | | | | 0 | | | | | | | | | 6 | 1 | 16 | 2 | 0 | 0 | 0 | 0 | 4 | 1 | 0 | 1 | 0 | 0 | 0 | 0 | 2 | 0 | 5 | 4 | 3 | 5 | 2 | 6 | 3 | 1 | 0 | Y | 59563 | keratin, type II cytoskeletal 4-like [Notechis scutatus] |
| **135** | 835 | P00607.1 | 143.46 | 31 | 0 | 0 | 0 | 0 | 0 | 0 | 0 | 0 | 0 | 0 | 0 | 0 | 0 | 0 | 0 | 31 | 10 | 11 | 24 | 11 | 10 | 11 | 9 | 9 | 0 | | | | | | | | | | | | | | | | 0 | | | 3.3596E5 | | | | | | | 7 | 4 | 0 | 0 | 0 | 0 | 0 | 0 | 0 | 0 | 0 | 0 | 0 | 0 | 0 | 0 | 0 | 2 | 2 | 3 | 4 | 1 | 1 | 3 | 1 | 1 | 0 | Y | 13323 | RecName: Full=Basic phospholipase A2 homolog 1; Short=svPLA2 homolog; AltName: Full=Notechis II-1 |
| **107** | 605 | Q75S50.1 | 141.51 | 29 | 0 | 13 | 13 | 13 | 13 | 13 | 13 | 13 | 0 | 0 | 13 | 0 | 13 | 13 | 0 | 29 | 13 | 0 | 13 | 0 | 0 | 0 | 0 | 0 | 0 | | 6.4373E6 | 2.6768E6 | 5.734E5 | 4.9505E7 | 2.6154E7 | 6.2084E5 | 1.4464E7 | | | 2.7933E7 | | 1.7527E6 | 9.675E6 | | 4.5152E7 | 2.1544E6 | | 7.3861E5 | | | | | | | 5 | 4 | 0 | 3 | 2 | 1 | 7 | 5 | 1 | 6 | 0 | 0 | 2 | 0 | 1 | 2 | 0 | 9 | 2 | 0 | 1 | 0 | 0 | 0 | 0 | 0 | 0 | Y | 9751 | RecName: Full=Kunitz-type serine protease inhibitor homolog beta-bungarotoxin B3 chain; Flags: Precursor |
| **107** | 606 | BAD06268.1 | 141.51 | 29 | 0 | 13 | 13 | 13 | 13 | 13 | 13 | 13 | 0 | 0 | 13 | 0 | 13 | 13 | 0 | 29 | 13 | 0 | 13 | 0 | 0 | 0 | 0 | 0 | 0 | | 6.4373E6 | 2.6768E6 | 5.734E5 | 4.9505E7 | 2.6154E7 | 6.2084E5 | 1.4464E7 | | | 2.7933E7 | | 1.7527E6 | 9.675E6 | | 4.5152E7 | 2.1544E6 | | 7.3861E5 | | | | | | | 5 | 4 | 0 | 3 | 2 | 1 | 7 | 5 | 1 | 6 | 0 | 0 | 2 | 0 | 1 | 2 | 0 | 9 | 2 | 0 | 1 | 0 | 0 | 0 | 0 | 0 | 0 | Y | 9751 | beta-bungarotoxin B3 chain precursor [Bungarus candidus] |
| **107** | 607 | Q8AY44.2 | 141.51 | 29 | 0 | 13 | 13 | 13 | 13 | 13 | 13 | 13 | 0 | 0 | 13 | 0 | 13 | 13 | 0 | 29 | 13 | 0 | 13 | 0 | 0 | 0 | 0 | 0 | 0 | | 6.4373E6 | 2.6768E6 | 5.734E5 | 4.9505E7 | 2.6154E7 | 6.2084E5 | 1.4464E7 | | | 2.7933E7 | | 1.7527E6 | 9.675E6 | | 4.5152E7 | 2.1544E6 | | 7.3861E5 | | | | | | | 5 | 4 | 0 | 3 | 2 | 1 | 7 | 5 | 1 | 6 | 0 | 0 | 2 | 0 | 1 | 2 | 0 | 9 | 2 | 0 | 1 | 0 | 0 | 0 | 0 | 0 | 0 | Y | 9751 | RecName: Full=Kunitz-type serine protease inhibitor homolog beta-bungarotoxin B2b chain; Flags: Precursor |
| **107** | 608 | AAL30067.1 | 141.51 | 28 | 0 | 12 | 12 | 12 | 12 | 12 | 12 | 12 | 0 | 0 | 12 | 0 | 12 | 12 | 0 | 28 | 12 | 0 | 12 | 0 | 0 | 0 | 0 | 0 | 0 | | 6.4373E6 | 2.6768E6 | 5.734E5 | 4.9505E7 | 2.6154E7 | 6.2084E5 | 1.4464E7 | | | 2.7933E7 | | 1.7527E6 | 9.675E6 | | 4.5152E7 | 2.1544E6 | | 7.3861E5 | | | | | | | 5 | 4 | 0 | 3 | 2 | 1 | 7 | 5 | 1 | 6 | 0 | 0 | 2 | 0 | 1 | 2 | 0 | 9 | 2 | 0 | 1 | 0 | 0 | 0 | 0 | 0 | 0 | Y | 10281 | beta bungaratoxin B2b chain, partial [Bungarus candidus] |
| **82** | 91 | XP_032067281.1 | 140.30 | 10 | 5 | 3 | 0 | 0 | 0 | 0 | 3 | 2 | 0 | 2 | 0 | 0 | 0 | 0 | 4 | 0 | 5 | 6 | 7 | 7 | 4 | 3 | 5 | 2 | 0 | | | | | | | | | | | | | | | | | | | 1.6665E6 | | | | | | | 6 | 1 | 16 | 2 | 0 | 0 | 0 | 0 | 4 | 1 | 0 | 1 | 0 | 0 | 0 | 0 | 2 | 0 | 4 | 4 | 4 | 5 | 2 | 6 | 3 | 1 | 0 | N | 59706 | keratin, type II cytoskeletal 6A-like [Thamnophis elegans] |
| **82** | 92 | XP_013915209.1 | 140.30 | 10 | 5 | 3 | 0 | 0 | 0 | 0 | 3 | 2 | 0 | 2 | 0 | 0 | 0 | 0 | 4 | 0 | 5 | 6 | 7 | 7 | 4 | 3 | 5 | 2 | 0 | | | | | | | | | | | | | | | | | | | 1.6665E6 | | | | | | | 6 | 1 | 16 | 2 | 0 | 0 | 0 | 0 | 4 | 1 | 0 | 1 | 0 | 0 | 0 | 0 | 2 | 0 | 4 | 4 | 4 | 5 | 2 | 6 | 3 | 1 | 0 | N | 59764 | PREDICTED: keratin, type II cytoskeletal 6A-like [Thamnophis sirtalis] |

| | | | | | | | | | | | | | | | | | | | | | | | | | | | | | | | | | | | | | | | | | | | | | | | | | | | | | | | | | | | | | | | | | | | | | | | | | | | | | | | | | |
|---|---|---|---|---|---|---|---|---|---|---|---|---|---|---|---|---|---|---|---|---|---|---|---|---|---|---|---|---|---|---|---|---|---|---|---|---|---|---|---|---|---|---|---|---|---|---|---|---|---|---|---|---|---|---|---|---|---|---|---|---|---|---|---|---|---|---|---|---|---|---|---|---|---|---|---|---|---|---|---|---|---|---|---|---|
| 209 | 50 | ABN72540.1 | 138.02 | 12 | 0 | 0 | 0 | 0 | 0 | 0 | 0 | 0 | 0 | 0 | 0 | 0 | 0 | 0 | 0 | 0 | 0 | 0 | 0 | 0 | 0 | 0 | 0 | 0 | 12 |||||||||||||||||||||||||1.8424E5| 5 | 1 | 0 | 0 | 0 | 0 | 0 | 0 | 0 | 0 | 0 | 0 | 0 | 0 | 0 | 0 | 0 | 0 | 0 | 0 | 0 | 0 | 0 | 0 | 0 | 0 | 11 | N | 58764 | L-amino acid oxidase [Bungarus fasciatus] |
| 209 | 51 | A8QL52.1 | 138.02 | 12 | 0 | 0 | 0 | 0 | 0 | 0 | 0 | 0 | 0 | 0 | 0 | 0 | 0 | 0 | 0 | 0 | 0 | 0 | 0 | 0 | 0 | 0 | 0 | 0 | 12 |||||||||||||||||||||||||1.8424E5| 5 | 1 | 0 | 0 | 0 | 0 | 0 | 0 | 0 | 0 | 0 | 0 | 0 | 0 | 0 | 0 | 0 | 0 | 0 | 0 | 0 | 0 | 0 | 0 | 0 | 0 | 11 | N | 58764 | RecName: Full=L-amino-acid oxidase; Short=Bf-LAAO; Short=LAO; Flags: Precursor |
| 149 | 357 | Q8UW31.1 | 130.42 | 33 | 0 | 0 | 0 | 0 | 0 | 0 | 0 | 0 | 0 | 0 | 0 | 0 | 0 | 9 | 0 | 33 | 8 | 8 | 0 | 0 | 8 | 16 | 7 | 16 | 0 ||||||||||||||||0||||||||||| 4 | 1 | 0 | 0 | 0 | 0 | 0 | 0 | 0 | 0 | 0 | 0 | 0 | 0 | 0 | 1 | 0 | 3 | 2 | 2 | 0 | 0 | 1 | 3 | 1 | 2 | 0 | Y | 16943 | RecName: Full=Acidic phospholipase A2 57; Short=svPLA2; AltName: Full=Phosphatidylcholine 2-acylhydrolase; Flags: Precursor |
| 149 | 358 | AAL55555.1 | 130.42 | 33 | 0 | 0 | 0 | 0 | 0 | 0 | 0 | 0 | 0 | 0 | 0 | 0 | 0 | 9 | 0 | 33 | 8 | 8 | 0 | 0 | 8 | 16 | 7 | 16 | 0 ||||||||||||||||0||||||||||| 4 | 1 | 0 | 0 | 0 | 0 | 0 | 0 | 0 | 0 | 0 | 0 | 0 | 0 | 0 | 1 | 0 | 3 | 2 | 2 | 0 | 0 | 1 | 3 | 1 | 2 | 0 | Y | 16943 | phospholipase A2 [Hydrophis hardwickii] |
| 143 | 851 | P14615.2 | 129.26 | 23 | 0 | 0 | 0 | 0 | 0 | 0 | 0 | 0 | 0 | 0 | 0 | 0 | 11 | 8 | 11 | 0 | 8 | 0 | 0 | 0 | 8 | 0 | 0 | 12 | 0 ||||||||||||||||||||||||0|| 6 | 1 | 0 | 0 | 0 | 0 | 0 | 0 | 0 | 0 | 0 | 0 | 0 | 0 | 17 | 1 | 4 | 0 | 1 | 0 | 0 | 0 | 1 | 0 | 0 | 1 | 0 | Y | 13425 | RecName: Full=Neutral phospholipase A2 3; Short=svPLA2; AltName: Full=Kbf III; AltName: Full=Kbf-3; AltName: Full=Phosphatidylcholine 2-acylhydrolase |
| 143 | 852 | ABG75909.1 | 129.26 | 23 | 0 | 0 | 0 | 0 | 0 | 0 | 0 | 0 | 0 | 0 | 0 | 0 | 11 | 8 | 11 | 0 | 8 | 0 | 0 | 0 | 8 | 0 | 0 | 12 | 0 ||||||||||||||||||||||||0|| 6 | 1 | 0 | 0 | 0 | 0 | 0 | 0 | 0 | 0 | 0 | 0 | 0 | 0 | 17 | 1 | 4 | 0 | 1 | 0 | 0 | 0 | 1 | 0 | 0 | 1 | 0 | Y | 13425 | phospholipase A2 Kbf-III, partial [Bungarus fasciatus] |
| 148 | 1432 | Q45Z42.1 | 127.02 | 25 | 0 | 0 | 0 | 0 | 0 | 0 | 0 | 0 | 0 | 0 | 0 | 0 | 0 | 0 | 0 | 25 | 8 | 8 | 23 | 0 | 8 | 8 | 8 | 8 | 0 ||||||||||||||||0|||0||||||| 5 | 3 | 0 | 0 | 0 | 0 | 0 | 0 | 0 | 0 | 0 | 0 | 0 | 0 | 0 | 0 | 0 | 3 | 2 | 2 | 1 | 0 | 1 | 2 | 1 | 1 | 0 | Y | 16362 | RecName: Full=Basic phospholipase A2 paradoxin-like alpha chain; Short=svPLA2; AltName: Full=PLA-4; AltName: Full=Phosphatidylcholine 2-acylhydrolase; Flags: Precursor |
| 148 | 1433 | AAZ22642.1 | 127.02 | 25 | 0 | 0 | 0 | 0 | 0 | 0 | 0 | 0 | 0 | 0 | 0 | 0 | 0 | 0 | 0 | 25 | 8 | 8 | 23 | 0 | 8 | 8 | 8 | 8 | 0 ||||||||||||||||0|||0||||||| 5 | 3 | 0 | 0 | 0 | 0 | 0 | 0 | 0 | 0 | 0 | 0 | 0 | 0 | 0 | 0 | 0 | 3 | 2 | 2 | 1 | 0 | 1 | 2 | 1 | 1 | 0 | Y | 16362 | PLA-4 precursor [Oxyuranus microlepidotus] |
| 130 | 1211 | AAZ22652.1 | 126.81 | 23 | 0 | 0 | 0 | 0 | 0 | 0 | 0 | 0 | 0 | 0 | 0 | 0 | 0 | 0 | 0 | 23 | 8 | 8 | 23 | 0 | 8 | 23 | 7 | 7 | 0 ||||||||||||||||0|||1.3138E6|||0|||| 4 | 2 | 0 | 0 | 0 | 0 | 0 | 0 | 0 | 0 | 0 | 0 | 0 | 0 | 0 | 0 | 0 | 2 | 2 | 2 | 3 | 0 | 1 | 4 | 1 | 1 | 0 | Y | 16857 | PLA-2 precursor [Notechis scutatus] |
| 150 | 621 | Q9PUI0.1 | 123.90 | 23 | 0 | 0 | 0 | 0 | 0 | 0 | 0 | 0 | 0 | 0 | 0 | 0 | 0 | 0 | 0 | 23 | 8 | 8 | 0 | 0 | 8 | 8 | 7 | 7 | 0 ||||||||||||||||0||||||||||| 4 | 1 | 0 | 0 | 0 | 0 | 0 | 0 | 0 | 0 | 0 | 0 | 0 | 0 | 0 | 0 | 0 | 3 | 2 | 2 | 0 | 0 | 1 | 2 | 1 | 1 | 0 | Y | 16384 | RecName: Full=Acidic phospholipase A2 S7-48J; Short=svPLA2; AltName: Full=ASPLA4; AltName: Full=Phosphatidylcholine 2-acylhydrolase; Flags: Precursor |
| 150 | 622 | AAD56553.1 | 123.90 | 23 | 0 | 0 | 0 | 0 | 0 | 0 | 0 | 0 | 0 | 0 | 0 | 0 | 0 | 0 | 0 | 23 | 8 | 8 | 0 | 0 | 8 | 8 | 7 | 7 | 0 ||||||||||||||||0||||||||||| 4 | 1 | 0 | 0 | 0 | 0 | 0 | 0 | 0 | 0 | 0 | 0 | 0 | 0 | 0 | 0 | 0 | 3 | 2 | 2 | 0 | 0 | 1 | 2 | 1 | 1 | 0 | Y | 16384 | phospholipase A2 [Austrelaps superbus] |
| 100 | 437 | ADF50040.1 | 123.07 | 18 | 0 | 0 | 0 | 0 | 0 | 0 | 0 | 0 | 0 | 0 | 0 | 0 | 0 | 9 | 9 | 16 | 9 | 9 | 10 | 0 | 9 | 9 | 8 | 9 | 0 |||||||||||||||||||6.7331E5||||||| 6 | 1 | 0 | 0 | 0 | 0 | 0 | 0 | 0 | 0 | 0 | 0 | 0 | 0 | 0 | 1 | 2 | 5 | 5 | 5 | 10 | 0 | 2 | 5 | 1 | 4 | 0 | Y | 16208 | beta-bungarotoxin a chain isoform 3 [Bungarus flaviceps] |
| 100 | 438 | BAC77652.1 | 123.07 | 18 | 0 | 0 | 0 | 0 | 0 | 0 | 0 | 0 | 0 | 0 | 0 | 0 | 0 | 9 | 9 | 16 | 9 | 9 | 10 | 0 | 9 | 9 | 8 | 9 | 0 |||||||||||||||||||6.7331E5||||||| 6 | 1 | 0 | 0 | 0 | 0 | 0 | 0 | 0 | 0 | 0 | 0 | 0 | 0 | 0 | 1 | 2 | 5 | 5 | 5 | 10 | 0 | 2 | 5 | 1 | 4 | 0 | Y | 16208 | beta-bungarotoxin A2 chain precursor [Bungarus flaviceps] |
| 100 | 439 | Q7T1R1.1 | 123.07 | 18 | 0 | 0 | 0 | 0 | 0 | 0 | 0 | 0 | 0 | 0 | 0 | 0 | 0 | 9 | 9 | 16 | 9 | 9 | 10 | 0 | 9 | 9 | 8 | 9 | 0 |||||||||||||||||||6.7331E5||||||| 6 | 1 | 0 | 0 | 0 | 0 | 0 | 0 | 0 | 0 | 0 | 0 | 0 | 0 | 0 | 1 | 2 | 5 | 5 | 5 | 10 | 0 | 2 | 5 | 1 | 4 | 0 | Y | 16208 | RecName: Full=Basic phospholipase A2 beta-bungarotoxin A2 chain; Short=Beta-BuTX A2 chain; Short=svPLA2; AltName: Full=Phosphatidylcholine 2-acylhydrolase; Flags: Precursor |
| 141 | 856 | P59068.1 | 122.95 | 44 | 0 | 0 | 0 | 0 | 0 | 0 | 0 | 0 | 0 | 0 | 0 | 0 | 0 | 0 | 0 | 19 | 44 | 21 | 0 | 0 | 21 | 21 | 19 | 19 | 0 |||||||||||||||||9.127E4||||||||| 3 | 1 | 0 | 0 | 0 | 0 | 0 | 0 | 0 | 0 | 0 | 0 | 0 | 0 | 0 | 0 | 0 | 1 | 3 | 2 | 0 | 0 | 1 | 2 | 1 | 1 | 0 | Y | 6473 | RecName: Full=Phospholipase A2 superbin b; Short=svPLA2; AltName: Full=Phosphatidylcholine 2-acylhydrolase |
| 189 | 201 | ABK63559.1 | 122.90 | 9 | 0 | 0 | 0 | 0 | 0 | 0 | 4 | 2 | 2 | 7 | 0 | 0 | 0 | 2 | 0 | 0 | 0 | 0 | 0 | 0 | 0 | 0 | 0 | 0 | 0 |||||||4.9927E4|||6.704E6|||||||||||||||| 5 | 2 | 0 | 0 | 0 | 0 | 0 | 0 | 4 | 1 | 2 | 11 | 0 | 0 | 0 | 1 | 0 | 0 | 0 | 0 | 0 | 0 | 0 | 0 | 0 | 0 | 0 | Y | 68312 | metalloproteinase precursor [Demansia vestigiata] |
| 92 | 691 | 1F94 | 122.33 | 33 | 0 | 0 | 0 | 0 | 0 | 0 | 19 | 33 | 0 | 19 | 0 | 0 | 0 | 0 | 19 | 0 | 0 | 19 | 0 | 0 | 0 | 0 | 0 | 0 | 0 |||||||1.2401E7|4.2803E8||0|||||4.3597E5|||2.7947E5|||||||| 2 | 1 | 0 | 0 | 0 | 0 | 0 | 0 | 5 | 34 | 0 | 3 | 0 | 0 | 0 | 0 | 1 | 0 | 0 | 1 | 0 | 0 | 0 | 0 | 0 | 0 | 0 | Y | 7285 | Chain A, BUCANDIN |
| 92 | 692 | 1IJC | 122.33 | 33 | 0 | 0 | 0 | 0 | 0 | 0 | 19 | 33 | 0 | 19 | 0 | 0 | 0 | 0 | 19 | 0 | 0 | 19 | 0 | 0 | 0 | 0 | 0 | 0 | 0 |||||||1.2401E7|4.2803E8||0|||||4.3597E5|||2.7947E5|||||||| 2 | 1 | 0 | 0 | 0 | 0 | 0 | 0 | 5 | 34 | 0 | 3 | 0 | 0 | 0 | 0 | 1 | 0 | 0 | 1 | 0 | 0 | 0 | 0 | 0 | 0 | 0 | Y | 7285 | Chain A, bucandin |
| 92 | 693 | P81782.1 | 122.33 | 33 | 0 | 0 | 0 | 0 | 0 | 0 | 19 | 33 | 0 | 19 | 0 | 0 | 0 | 0 | 19 | 0 | 0 | 19 | 0 | 0 | 0 | 0 | 0 | 0 | 0 |||||||1.2401E7|4.2803E8||0|||||4.3597E5|||2.7947E5|||||||| 2 | 1 | 0 | 0 | 0 | 0 | 0 | 0 | 5 | 34 | 0 | 3 | 0 | 0 | 0 | 0 | 1 | 0 | 0 | 1 | 0 | 0 | 0 | 0 | 0 | 0 | 0 | Y | 7285 | RecName: Full=Bucandin |
| 137 | 1127 | Q45Z28.1 | 122.23 | 23 | 0 | 0 | 0 | 0 | 0 | 0 | 0 | 0 | 0 | 0 | 0 | 0 | 0 | 0 | 0 | 23 | 8 | 8 | 23 | 0 | 8 | 8 | 7 | 7 | 0 ||||||||||||||||0|||2.8252E6||||||| 4 | 2 | 0 | 0 | 0 | 0 | 0 | 0 | 0 | 0 | 0 | 0 | 0 | 0 | 0 | 0 | 0 | 3 | 2 | 2 | 2 | 0 | 1 | 2 | 1 | 1 | 0 | Y | 16932 | RecName: Full=Acidic phospholipase A2 3; Short=svPLA2; AltName: Full=Phosphatidylcholine 2-acylhydrolase 3; Short=PLA-3; Flags: Precursor |
| 137 | 1128 | AAZ22656.1 | 122.23 | 23 | 0 | 0 | 0 | 0 | 0 | 0 | 0 | 0 | 0 | 0 | 0 | 0 | 0 | 0 | 0 | 23 | 8 | 8 | 23 | 0 | 8 | 8 | 7 | 7 | 0 ||||||||||||||||0|||2.8252E6||||||| 4 | 2 | 0 | 0 | 0 | 0 | 0 | 0 | 0 | 0 | 0 | 0 | 0 | 0 | 0 | 0 | 0 | 3 | 2 | 2 | 2 | 0 | 1 | 2 | 1 | 1 | 0 | Y | 16932 | PLA-3 precursor [Tropidechis carinatus] |
| 182 | 213 | AAB25729.1 | 121.57 | 16 | 0 | 0 | 0 | 0 | 0 | 0 | 0 | 0 | 0 | 14 | 5 | 4 | 9 | 0 | 0 | 0 | 0 | 6 | 0 | 0 | 0 | 0 | 0 | 0 | 0 ||||||||||1.7106E6||1.1609E5|8.8399E6||||||||||||| 4 | 2 | 0 | 0 | 0 | 0 | 0 | 0 | 0 | 0 | 0 | 5 | 1 | 1 | 5 | 0 | 0 | 0 | 0 | 2 | 0 | 0 | 0 | 0 | 0 | 0 | 0 | Y | 27514 | nerve growth factor precursor [Bungarus multicinctus] |
| 182 | 214 | P34128.1 | 121.57 | 16 | 0 | 0 | 0 | 0 | 0 | 0 | 0 | 0 | 0 | 14 | 5 | 4 | 9 | 0 | 0 | 0 | 0 | 6 | 0 | 0 | 0 | 0 | 0 | 0 | 0 ||||||||||1.7106E6||1.1609E5|8.8399E6||||||||||||| 4 | 2 | 0 | 0 | 0 | 0 | 0 | 0 | 0 | 0 | 0 | 5 | 1 | 1 | 5 | 0 | 0 | 0 | 0 | 2 | 0 | 0 | 0 | 0 | 0 | 0 | 0 | Y | 27514 | RecName: Full=Venom nerve growth factor; Short=v-NGF; Short=vNGF; Flags: Precursor |
| 164 | 1418 | AAZ22640.1 | 120.76 | 23 | 0 | 0 | 0 | 0 | 0 | 0 | 0 | 0 | 0 | 0 | 0 | 0 | 0 | 0 | 0 | 23 | 8 | 8 | 0 | 0 | 8 | 8 | 8 | 8 | 0 ||||||||||||||||0||||||||||| 3 | 1 | 0 | 0 | 0 | 0 | 0 | 0 | 0 | 0 | 0 | 0 | 0 | 0 | 0 | 0 | 0 | 2 | 2 | 2 | 0 | 0 | 1 | 2 | 1 | 1 | 0 | Y | 16183 | PLA-2 precursor [Oxyuranus microlepidotus] |
| 164 | 2503 | AAZ22643.1 | 120.76 | 23 | 0 | 0 | 0 | 0 | 0 | 0 | 0 | 0 | 0 | 0 | 0 | 0 | 0 | 0 | 0 | 23 | 8 | 8 | 0 | 0 | 8 | 8 | 8 | 8 | 0 ||||||||||||||||0||||||||||| 3 | 1 | 0 | 0 | 0 | 0 | 0 | 0 | 0 | 0 | 0 | 0 | 0 | 0 | 0 | 0 | 0 | 2 | 2 | 2 | 0 | 0 | 1 | 2 | 1 | 1 | 0 | Y | 16162 | PLA-5 precursor [Oxyuranus microlepidotus] |
| 164 | 2504 | AAY47071.1 | 120.76 | 23 | 0 | 0 | 0 | 0 | 0 | 0 | 0 | 0 | 0 | 0 | 0 | 0 | 0 | 0 | 0 | 23 | 8 | 8 | 0 | 0 | 8 | 8 | 8 | 8 | 0 ||||||||||||||||0||||||||||| 3 | 1 | 0 | 0 | 0 | 0 | 0 | 0 | 0 | 0 | 0 | 0 | 0 | 0 | 0 | 0 | 0 | 2 | 2 | 2 | 0 | 0 | 1 | 2 | 1 | 1 | 0 | Y | 16087 | OS7 precursor [Oxyuranus scutellatus scutellatus] |
| 164 | 2505 | AAY47070.1 | 120.76 | 23 | 0 | 0 | 0 | 0 | 0 | 0 | 0 | 0 | 0 | 0 | 0 | 0 | 0 | 0 | 0 | 23 | 8 | 8 | 0 | 0 | 8 | 8 | 8 | 8 | 0 ||||||||||||||||0||||||||||| 3 | 1 | 0 | 0 | 0 | 0 | 0 | 0 | 0 | 0 | 0 | 0 | 0 | 0 | 0 | 0 | 0 | 2 | 2 | 2 | 0 | 0 | 1 | 2 | 1 | 1 | 0 | Y | 16145 | OS6 precursor [Oxyuranus scutellatus scutellatus] |
| 117 | 168 | XP_039209904.1 | 120.22 | 4 | 2 | 2 | 0 | 0 | 0 | 0 | 0 | 0 | 0 | 0 | 0 | 0 | 0 | 0 | 0 | 1 | 1 | 0 | 2 | 1 | 2 | 0 | 0 | 1 | 0 |1.0046E6||||||||||||||||||0|2.4897E4|4.5304E5|||8.321E5|| 5 | 2 | 12 | 2 | 0 | 0 | 0 | 0 | 0 | 0 | 0 | 0 | 0 | 0 | 0 | 0 | 0 | 1 | 1 | 0 | 2 | 1 | 2 | 0 | 0 | 1 | 0 | Y | 101157 | keratin, type I cytoskeletal 10-like [Crotalus tigris] |
| 117 | 176 | XP_034272439.1 | 120.22 | 4 | 2 | 2 | 0 | 0 | 0 | 0 | 0 | 0 | 0 | 0 | 0 | 0 | 0 | 0 | 0 | 1 | 1 | 0 | 2 | 1 | 2 | 0 | 0 | 1 | 0 |1.0046E6||||||||||||||||||0|2.4897E4|4.5304E5|||8.321E5|| 5 | 2 | 12 | 2 | 0 | 0 | 0 | 0 | 0 | 0 | 0 | 0 | 0 | 0 | 0 | 0 | 0 | 1 | 1 | 0 | 2 | 1 | 2 | 0 | 0 | 1 | 0 | Y | 112679 | keratin, type I cytoskeletal 9-like [Pantherophis guttatus] |
| 88 | 1619 | CAB53358.1 | 119.93 | 12 | 0 | 12 | 0 | 12 | 12 | 12 | 12 | 12 | 0 | 12 | 0 | 0 | 0 | 0 | 0 | 12 | 0 | 12 | 12 | 12 | 0 | 0 | 12 | 12 | 0 ||6.292E7||1.9102E8|1.6409E8|1.1264E6|8.8877E6|3.0947E7||1.9467E6||||||0||2.9368E6|0|3.3916E6|||1.9377E6|0|| 2 | 1 | 0 | 11 | 0 | 10 | 14 | 3 | 4 | 10 | 0 | 2 | 0 | 0 | 0 | 0 | 0 | 2 | 0 | 1 | 1 | 1 | 0 | 0 | 1 | 1 | 0 | N | 11334 | cardiotoxin-like protein [Bungarus multicinctus multicinctus] |
| 88 | 1620 | Q9PW19.1 | 119.93 | 12 | 0 | 12 | 0 | 12 | 12 | 12 | 12 | 12 | 0 | 12 | 0 | 0 | 0 | 0 | 0 | 12 | 0 | 12 | 12 | 12 | 0 | 0 | 12 | 12 | 0 ||6.292E7||1.9102E8|1.6409E8|1.1264E6|8.8877E6|3.0947E7||1.9467E6||||||0||2.9368E6|0|3.3916E6|||1.9377E6|0|| 2 | 1 | 0 | 11 | 0 | 10 | 14 | 3 | 4 | 10 | 0 | 2 | 0 | 0 | 0 | 0 | 0 | 2 | 0 | 1 | 1 | 1 | 0 | 0 | 1 | 1 | 0 | N | 11334 | RecName: Full=Toxin BMLCL; AltName: Full=BM8; Flags: Precursor |
| 88 | 1682 | Q9YGH0.1 | 119.93 | 12 | 0 | 12 | 0 | 12 | 12 | 12 | 12 | 12 | 0 | 12 | 0 | 0 | 0 | 0 | 0 | 12 | 0 | 12 | 12 | 12 | 0 | 0 | 12 | 12 | 0 ||6.292E7||1.9102E8|1.6409E8|1.1264E6|8.8877E6|3.0947E7||1.9467E6||||||0||2.9368E6|0|3.3916E6|||1.9377E6|0|| 2 | 1 | 0 | 11 | 0 | 10 | 14 | 3 | 4 | 10 | 0 | 2 | 0 | 0 | 0 | 0 | 0 | 2 | 0 | 1 | 1 | 1 | 0 | 0 | 1 | 1 | 0 | N | 11433 | RecName: Full=Cytotoxin-like protein TA-BMBGT3; Flags: Precursor |
| 88 | 1683 | CAA76607.1 | 119.93 | 12 | 0 | 12 | 0 | 12 | 12 | 12 | 12 | 12 | 0 | 12 | 0 | 0 | 0 | 0 | 0 | 12 | 0 | 12 | 12 | 12 | 0 | 0 | 12 | 12 | 0 ||6.292E7||1.9102E8|1.6409E8|1.1264E6|8.8877E6|3.0947E7||1.9467E6||||||0||2.9368E6|0|3.3916E6|||1.9377E6|0|| 2 | 1 | 0 | 11 | 0 | 10 | 14 | 3 | 4 | 10 | 0 | 2 | 0 | 0 | 0 | 0 | 0 | 2 | 0 | 1 | 1 | 1 | 0 | 0 | 1 | 1 | 0 | N | 11433 | cytotoxin-like protein [Bungarus multicinctus] |
| 142 | 628 | ABW24182.1 | 119.70 | 23 | 0 | 0 | 0 | 0 | 0 | 0 | 0 | 0 | 0 | 0 | 0 | 0 | 0 | 0 | 0 | 23 | 8 | 8 | 23 | 0 | 8 | 8 | 7 | 7 | 0 ||||||||||||||||0|||0||||||| 3 | 1 | 0 | 0 | 0 | 0 | 0 | 0 | 0 | 0 | 0 | 0 | 0 | 0 | 0 | 0 | 0 | 4 | 2 | 2 | 1 | 0 | 1 | 2 | 1 | 1 | 0 | Y | 16409 | PLA-20 precursor [Austrelaps superbus] |
| 206 | 128 | JAA97901.1 | 119.31 | 15 | 10 | 4 | 0 | 0 | 0 | 0 | 0 | 0 | 0 | 0 | 0 | 0 | 0 | 0 | 4 | 0 | 0 | 4 | 0 | 4 | 9 | 0 | 4 | 0 | 5 |3.9017E3|0|||||||||||||0|||0||0|0||0||0| 3 | 3 | 5 | 1 | 0 | 0 | 0 | 0 | 0 | 0 | 0 | 0 | 0 | 0 | 0 | 0 | 1 | 0 | 0 | 1 | 0 | 1 | 2 | 0 | 1 | 0 | 1 | Y | 41737 | Actin, cytoplasmic 1 [Crotalus horridus] |
| 206 | 129 | JAG47580.1 | 119.31 | 15 | 10 | 4 | 0 | 0 | 0 | 0 | 0 | 0 | 0 | 0 | 0 | 0 | 0 | 0 | 4 | 0 | 0 | 4 | 0 | 4 | 9 | 0 | 4 | 0 | 5 |3.9017E3|0|||||||||||||0|||0||0|0||0||0| 3 | 3 | 5 | 1 | 0 | 0 | 0 | 0 | 0 | 0 | 0 | 0 | 0 | 0 | 0 | 0 | 1 | 0 | 0 | 1 | 0 | 1 | 2 | 0 | 1 | 0 | 1 | Y | 41737 | Actin, cytoplasmic 1 [Crotalus horridus] |
| 206 | 130 | JAI14496.1 | 119.31 | 15 | 10 | 4 | 0 | 0 | 0 | 0 | 0 | 0 | 0 | 0 | 0 | 0 | 0 | 0 | 4 | 0 | 0 | 4 | 0 | 4 | 9 | 0 | 4 | 0 | 5 |3.9017E3|0|||||||||||||0|||0||0|0||0||0| 3 | 3 | 5 | 1 | 0 | 0 | 0 | 0 | 0 | 0 | 0 | 0 | 0 | 0 | 0 | 0 | 1 | 0 | 0 | 1 | 0 | 1 | 2 | 0 | 1 | 0 | 1 | Y | 41737 | Actin, cytoplasmic 1 [Crotalus adamanteus] |
| 206 | 131 | JAC96364.1 | 119.31 | 15 | 10 | 4 | 0 | 0 | 0 | 0 | 0 | 0 | 0 | 0 | 0 | 0 | 0 | 0 | 4 | 0 | 0 | 4 | 0 | 4 | 9 | 0 | 4 | 0 | 5 |3.9017E3|0|||||||||||||0|||0||0|0||0||0| 3 | 3 | 5 | 1 | 0 | 0 | 0 | 0 | 0 | 0 | 0 | 0 | 0 | 0 | 0 | 0 | 1 | 0 | 0 | 1 | 0 | 1 | 2 | 0 | 1 | 0 | 1 | Y | 41737 | Actin, cytoplasmic 1 [Hypsiglena sp. JMG-2014] |
| 206 | 132 | BAQ22177.1 | 119.31 | 15 | 10 | 4 | 0 | 0 | 0 | 0 | 0 | 0 | 0 | 0 | 0 | 0 | 0 | 0 | 4 | 0 | 0 | 4 | 0 | 4 | 9 | 0 | 4 | 0 | 5 |3.9017E3|0|||||||||||||0|||0||0|0||0||0| 3 | 3 | 5 | 1 | 0 | 0 | 0 | 0 | 0 | 0 | 0 | 0 | 0 | 0 | 0 | 0 | 1 | 0 | 0 | 1 | 0 | 1 | 2 | 0 | 1 | 0 | 1 | Y | 41737 | beta-actin [Protobothrops flavoviridis] |
| 206 | 133 | JAG45999.1 | 119.31 | 15 | 10 | 4 | 0 | 0 | 0 | 0 | 0 | 0 | 0 | 0 | 0 | 0 | 0 | 0 | 4 | 0 | 0 | 4 | 0 | 4 | 9 | 0 | 4 | 0 | 5 |3.9017E3|0|||||||||||||0|||0||0|0||0||0| 3 | 3 | 5 | 1 | 0 | 0 | 0 | 0 | 0 | 0 | 0 | 0 | 0 | 0 | 0 | 0 | 1 | 0 | 0 | 1 | 0 | 1 | 2 | 0 | 1 | 0 | 1 | Y | 41737 | actin, cytoplasmic 1 [Crotalus horridus] |
| 206 | 134 | JAI10342.1 | 119.31 | 15 | 10 | 4 | 0 | 0 | 0 | 0 | 0 | 0 | 0 | 0 | 0 | 0 | 0 | 0 | 4 | 0 | 0 | 4 | 0 | 4 | 9 | 0 | 4 | 0 | 5 |3.9017E3|0|||||||||||||0|||0||0|0||0||0| 3 | 3 | 5 | 1 | 0 | 0 | 0 | 0 | 0 | 0 | 0 | 0 | 0 | 0 | 0 | 0 | 1 | 0 | 0 | 1 | 0 | 1 | 2 | 0 | 1 | 0 | 1 | Y | 41737 | Actin, cytoplasmic 1 [Micrurus fulvius] |
| 206 | 135 | JAB54771.1 | 119.31 | 15 | 10 | 4 | 0 | 0 | 0 | 0 | 0 | 0 | 0 | 0 | 0 | 0 | 0 | 0 | 4 | 0 | 0 | 4 | 0 | 4 | 9 | 0 | 4 | 0 | 5 |3.9017E3|0|||||||||||||0|||0||0|0||0||0| 3 | 3 | 5 | 1 | 0 | 0 | 0 | 0 | 0 | 0 | 0 | 0 | 0 | 0 | 0 | 0 | 1 | 0 | 0 | 1 | 0 | 1 | 2 | 0 | 1 | 0 | 1 | Y | 41737 | Actin [Micrurus fulvius] |
| 206 | 136 | JAG68844.1 | 119.31 | 15 | 10 | 4 | 0 | 0 | 0 | 0 | 0 | 0 | 0 | 0 | 0 | 0 | 0 | 0 | 4 | 0 | 0 | 4 | 0 | 4 | 9 | 0 | 4 | 0 | 5 |3.9017E3|0|||||||||||||0|||0||0|0||0||0| 3 | 3 | 5 | 1 | 0 | 0 | 0 | 0 | 0 | 0 | 0 | 0 | 0 | 0 | 0 | 0 | 1 | 0 | 0 | 1 | 0 | 1 | 2 | 0 | 1 | 0 | 1 | Y | 41737 | Actin, cytoplasmic 1 [Boiga irregularis] |
| 206 | 137 | JAV51374.1 | 119.31 | 15 | 10 | 4 | 0 | 0 | 0 | 0 | 0 | 0 | 0 | 0 | 0 | 0 | 0 | 0 | 4 | 0 | 0 | 4 | 0 | 4 | 9 | 0 | 4 | 0 | 5 |3.9017E3|0|||||||||||||0|||0||0|0||0||0| 3 | 3 | 5 | 1 | 0 | 0 | 0 | 0 | 0 | 0 | 0 | 0 | 0 | 0 | 0 | 0 | 1 | 0 | 0 | 1 | 0 | 1 | 2 | 0 | 1 | 0 | 1 | Y | 41737 | Actin, cytoplasmic 1 [Agkistrodon contortrix contortrix] |
| 206 | 138 | AFJ49302.1 | 119.31 | 15 | 10 | 4 | 0 | 0 | 0 | 0 | 0 | 0 | 0 | 0 | 0 | 0 | 0 | 0 | 4 | 0 | 0 | 4 | 0 | 4 | 9 | 0 | 4 | 0 | 5 |3.9017E3|0|||||||||||||0|||0||0|0||0||0| 3 | 3 | 5 | 1 | 0 | 0 | 0 | 0 | 0 | 0 | 0 | 0 | 0 | 0 | 0 | 0 | 1 | 0 | 0 | 1 | 0 | 1 | 2 | 0 | 1 | 0 | 1 | Y | 41737 | Actin, cytoplasmic 1 [Crotalus adamanteus] |
| 206 | 139 | JAG68843.1 | 119.31 | 15 | 10 | 4 | 0 | 0 | 0 | 0 | 0 | 0 | 0 | 0 | 0 | 0 | 0 | 0 | 4 | 0 | 0 | 4 | 0 | 4 | 9 | 0 | 4 | 0 | 5 |3.9017E3|0|||||||||||||0|||0||0|0||0||0| 3 | 3 | 5 | 1 | 0 | 0 | 0 | 0 | 0 | 0 | 0 | 0 | 0 | 0 | 0 | 0 | 1 | 0 | 0 | 1 | 0 | 1 | 2 | 0 | 1 | 0 | 1 | Y | 41793 | Actin, cytoplasmic 2 [Boiga irregularis] |
| 206 | 140 | XP_013921350.1 | 119.31 | 15 | 10 | 4 | 0 | 0 | 0 | 0 | 0 | 0 | 0 | 0 | 0 | 0 | 0 | 0 | 4 | 0 | 0 | 4 | 0 | 4 | 9 | 0 | 4 | 0 | 5 |3.9017E3|0|||||||||||||0|||0||0|0||0||0| 3 | 3 | 5 | 1 | 0 | 0 | 0 | 0 | 0 | 0 | 0 | 0 | 0 | 0 | 0 | 0 | 1 | 0 | 0 | 1 | 0 | 1 | 2 | 0 | 1 | 0 | 1 | Y | 41793 | PREDICTED: actin, cytoplasmic 2 [Thamnophis sirtalis] |
| 206 | 141 | JAV51372.1 | 119.31 | 15 | 10 | 4 | 0 | 0 | 0 | 0 | 0 | 0 | 0 | 0 | 0 | 0 | 0 | 0 | 4 | 0 | 0 | 4 | 0 | 4 | 9 | 0 | 4 | 0 | 5 |3.9017E3|0|||||||||||||0|||0||0|0||0||0| 3 | 3 | 5 | 1 | 0 | 0 | 0 | 0 | 0 | 0 | 0 | 0 | 0 | 0 | 0 | 0 | 1 | 0 | 0 | 1 | 0 | 1 | 2 | 0 | 1 | 0 | 1 | Y | 41793 | Actin, cytoplasmic 2 [Agkistrodon contortrix contortrix] |
| 206 | 142 | JAB54395.1 | 119.31 | 15 | 10 | 4 | 0 | 0 | 0 | 0 | 0 | 0 | 0 | 0 | 0 | 0 | 0 | 0 | 4 | 0 | 0 | 4 | 0 | 4 | 9 | 0 | 4 | 0 | 5 |3.9017E3|0|||||||||||||0|||0||0|0||0||0| 3 | 3 | 5 | 1 | 0 | 0 | 0 | 0 | 0 | 0 | 0 | 0 | 0 | 0 | 0 | 0 | 1 | 0 | 0 | 1 | 0 | 1 | 2 | 0 | 1 | 0 | 1 | Y | 41793 | cytoplasmic 2 [Micrurus f |

| | | | | | | | | | | | | | | | | | | | | | | | | | | | | | | | | | | | | | | | | | | | | | | | | | | | | | | | | | | | | | | | | | | | | | | | | | | | | | | | | | | | | |
|---|---|---|---|---|---|---|---|---|---|---|---|---|---|---|---|---|---|---|---|---|---|---|---|---|---|---|---|---|---|---|---|---|---|---|---|---|---|---|---|---|---|---|---|---|---|---|---|---|---|---|---|---|---|---|---|---|---|---|---|---|---|---|---|---|---|---|---|---|---|---|---|---|---|---|---|---|---|---|---|---|---|---|---|---|
| | | | | | | | | | | | | | | | | | | | | | | | | | | | | | | | | | | | | | | | | | | | | | | | | | | | | | | | | | | | | | | | | | | | | | | | | | | | | | | | | | ulvius] |
| 206 | 143 | JAC96363.1 | 119.31 | 15 | 10 | 4 | 0 | 0 | 0 | 0 | 0 | 0 | 0 | 0 | 0 | 0 | 0 | 0 | 4 | 0 | 0 | 4 | 0 | 4 | 9 | 0 | 4 | 0 | 5 | 3.9017E3 | 0 | | | | | | | | | | | | | 0 | | | 0 | | 0 | 0 | | 0 | | 0 | 3 | 3 | 5 | 1 | 0 | 0 | 0 | 0 | 0 | 0 | 0 | 0 | 0 | 0 | 0 | 0 | 1 | 0 | 0 | 1 | 0 | 1 | 2 | 0 | 1 | 0 | 1 | Y | 41793 | Actin, cytoplasmic 2 [Hypsiglena sp. JMG-2014] |
| 206 | 144 | JAA97900.1 | 119.31 | 15 | 10 | 4 | 0 | 0 | 0 | 0 | 0 | 0 | 0 | 0 | 0 | 0 | 0 | 0 | 4 | 0 | 0 | 4 | 0 | 4 | 9 | 0 | 4 | 0 | 5 | 3.9017E3 | 0 | | | | | | | | | | | | | 0 | | | 0 | | 0 | 0 | | 0 | | 0 | 3 | 3 | 5 | 1 | 0 | 0 | 0 | 0 | 0 | 0 | 0 | 0 | 0 | 0 | 0 | 0 | 1 | 0 | 0 | 1 | 0 | 1 | 2 | 0 | 1 | 0 | 1 | Y | 41793 | Actin, cytoplasmic 2 [Crotalus horridus] |
| 206 | 145 | LAA21099.1 | 119.31 | 15 | 10 | 4 | 0 | 0 | 0 | 0 | 0 | 0 | 0 | 0 | 0 | 0 | 0 | 0 | 4 | 0 | 0 | 4 | 0 | 4 | 9 | 0 | 4 | 0 | 5 | 3.9017E3 | 0 | | | | | | | | | | | | | 0 | | | 0 | | 0 | 0 | | 0 | | 0 | 3 | 3 | 5 | 1 | 0 | 0 | 0 | 0 | 0 | 0 | 0 | 0 | 0 | 0 | 0 | 0 | 1 | 0 | 0 | 1 | 0 | 1 | 2 | 0 | 1 | 0 | 1 | Y | 41793 | hypothetical protein [Micrurus lemniscatus carvalhoi] |
[truncated: 4,919,147 more chars]
